# Directed Differentiation of Embryonic Stem Cells Using a Bead-Based Combinatorial Screening Method

**DOI:** 10.1371/journal.pone.0104301

**Published:** 2014-09-24

**Authors:** Marina Tarunina, Diana Hernandez, Christopher J. Johnson, Stanislav Rybtsov, Vidya Ramathas, Mylvaganam Jeyakumar, Thomas Watson, Lilian Hook, Alexander Medvinsky, Chris Mason, Yen Choo

**Affiliations:** 1 Plasticell Ltd, Stevenage Bioscience Catalyst, Stevenage, United Kingdom; 2 Advanced Centre for Biochemical Engineering, University College London, London, United Kingdom; 3 MRC Centre for Regenerative Medicine/Institute of Stem cell Research, University of Edinburgh, Edinburgh, United Kingdom; 4 Progenitor Labs Ltd, Stevenage Bioscience Catalyst, Stevenage, United Kingdom; Baylor College of Medicine, United States of America

## Abstract

We have developed a rapid, bead-based combinatorial screening method to determine optimal combinations of variables that direct stem cell differentiation to produce known or novel cell types having pre-determined characteristics. Here we describe three experiments comprising stepwise exposure of mouse or human embryonic cells to 10,000 combinations of serum-free differentiation media, through which we discovered multiple novel, efficient and robust protocols to generate a number of specific hematopoietic and neural lineages. We further demonstrate that the technology can be used to optimize existing protocols in order to substitute costly growth factors with bioactive small molecules and/or increase cell yield, and to identify in vitro conditions for the production of rare developmental intermediates such as an embryonic lymphoid progenitor cell that has not previously been reported.

## Introduction

As embryonic development is mediated by a succession of signals that bring about key cell fate decisions, differentiation of pluripotent stem cells in vitro is directed by recapitulating stages of the developmental process using a series of cell culture steps. Examples of such stepwise protocols include the differentiation of embryonic stem (ES) cells into motor neurons [1], oligodendrocytes [2], dopaminergic neurons [3], red blood cells [4], macrophages [5], hepatocytes [6], islet cells [7], germ cells [8] and many others [9]. Typically, such stem cell differentiation protocols are derived empirically and their development involves much futile effort. Therefore screening technologies capable of testing large numbers of protocols in parallel are required for systematic searches of the experimental space.

We have developed ‘Combinatorial Cell Culture (CombiCult) [10], [11], a bead-based screening technology that allows miniaturisation and multiplexing of large numbers of stepwise cell culture experiments, increasing throughput by orders of magnitude (for an overview see Fig S1; movie S1 or http://www.plasticell.co.uk/combicult/technology). Briefly, beads seeded with stem cells are shuffled randomly through multiple, predetermined combinations of cell culture media using a split-pool process analogous to that used in combinatorial chemistry. Each cell culture medium is spiked with a distinctive fluorescent tag that attaches to the bead substrate, allowing us to track the history of each bead. Following the split-pool process, beads are assayed to identify those on which stem cells have differentiated to a specific cell type (‘hits’). Hits are isolated using a large particle flow sorter and the beads are digested to release the fluorescent tags accumulated during the course of the experiment. These are analysed using a flow cytometer to deconvolute the cell culture history of the beads and thereby deduce differentiation protocols. A customised bioinformatics program (Ariadne) is used to collate data and perform statistical analysis to predict the most robust and effective protocols. Finally, a subset of candidate protocols is validated to quantitate cell yield, and study lineage markers and functional attributes of the resulting cells (for detailed protocols see Materials and Methods).

**Figure 1:**
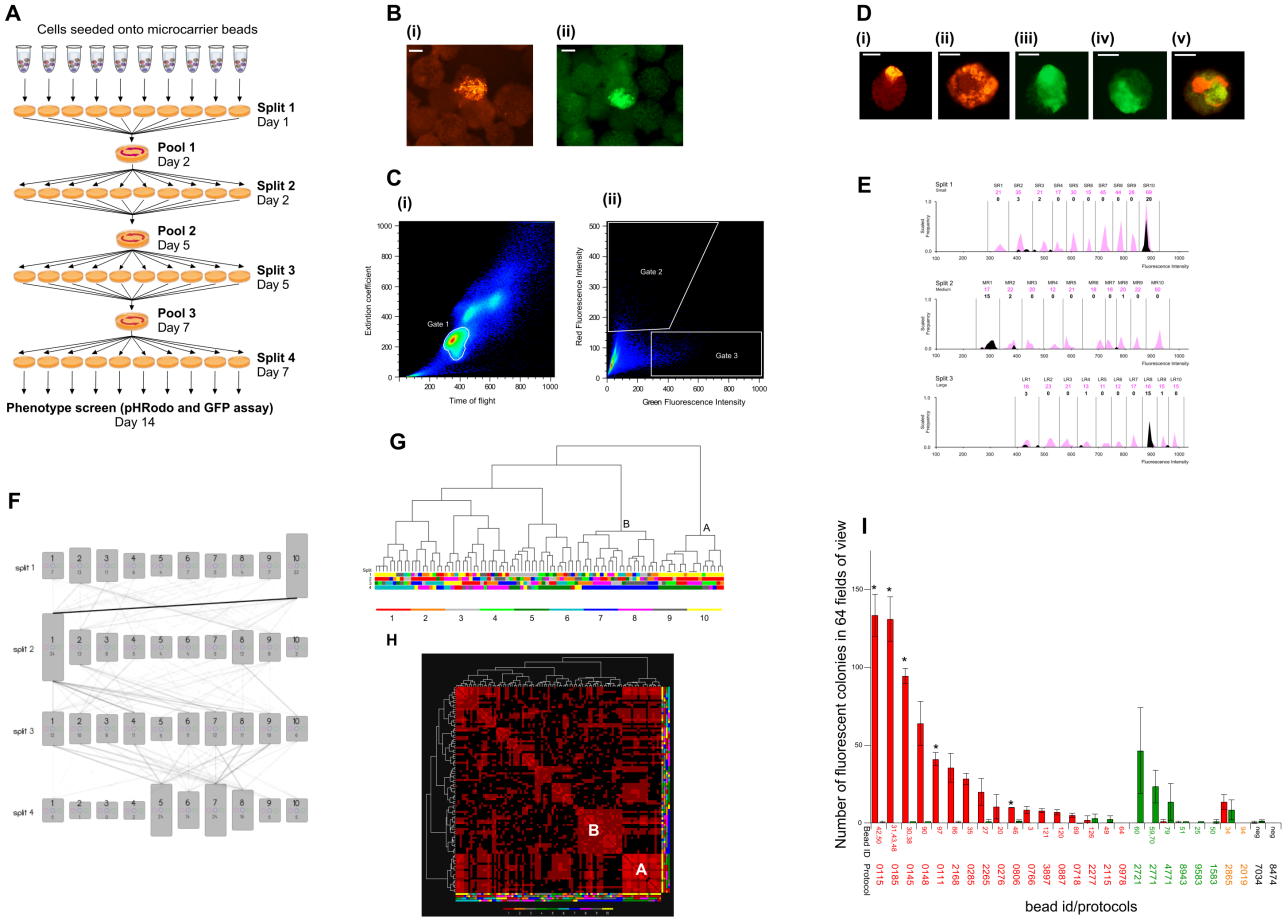
Mouse phagocytic screen. **(a)** Schematic diagram illustrating design of the CombiCult screen. Ten different cell culture media were tested in each of four stages of differentiation by split-pool passaging cells seeded onto beads in differentiation media spiked with tags, on day 1 (D1), D2, D5 and D7. On D14 beads were assayed to identify beads bearing phagocytes or neuroectodermal precursors (‘hits’). A total of 300,000 beads were used in the experiment to test 10,000 protocols so that on average each protocol was sampled by 30 beads. **(b)** Fluorescence micrographs showing hits bearing (i) red fluorescent phagocytic cells or (ii) GFP-positive neuroectoderm cells amongst negative beads. Scale bars  =  100 µm. **(c)** Cummulative large particle flow sorter dot plots, showing parameters used to sort hits. (i) Gating of monomeric beads (gate 1) from higher order agreggates. (ii) Sorting of monomeric beads with high red (gate 2) and green (gate 3) fluorescent signal. **(d)** Micrograph images of sorted hits: (i) bead 31 (phagocyte), (ii) bead 131 (phagocyte), (iii) bead 76 (neuroectoderm), (iv) bead 34 (neuroectoderm) and (v) bead 34 (phagocyte and neuroectoderm). Scale bars  =  100 µm. **(e)** Deconvolution of cell culture history of bead 31 (pictured in d(i) above; one of a triple hit from the phagocyte screen) by FACS analysis of bound tags. A set of 30 unique tags distinguishable by size (small, medium, large) and fluorescence intensity (ten levels for each size set) was used to spike cell culture media in the first three stages of differentiation. The figure shows magenta peaks corresponding to quantitation of a reference sample of the 30 tags used to calibrate the flow cytometer (histograms and tag numbers shown in magenta) in order to set gates. Quantitation of tags released from bead 31 is superimposed in black, showing detection of tags SR10, MR1 and LR8, corresponding to media 1.10, 2.1 and 3.8. **(f)** Schematic diagram illustrating an overlay of all protocols deconvoluted from phagocyte hits. The height of boxes representing each cell culture medium is proportional to the number of hits generated by that medium (written at the bottom of each box). The opacity of the linkage lines is proportional to the number of hits generated by specific media combinations - the darkest line corresponds to 21 hits. **(g)** Hierarchical clustering analysis of 92 unique protocols derived from the phagocyte screen, showing protocol clusters A and B. Each node at the bottom of the dendrogram (leaf node) corresponds to a hit bead. The associated protocol is denoted by the column of four colours directly below the node, specifying the media sampled in splits 1–4. The legend at the bottom of the figure specifies the colour used to denote the cell culture media in each split. **(h)** Similarity matrix comprising a pair-wise comparison of all protocols. Each column and each row corresponds to a protocol. The brightness of each cell in the matrix is proportional to the number of identical cell culture media shared by the two protocols. The brightest cell corresponds to identical protocols, while a black cell corresponds to two protocols with no common media. The diagonal row of cells (from the top left to bottom right) corresponds to protocols being compared to themselves. Protocol families with high internal homology appear as bright red squares, e.g. Clusters A and B marked on the diagram, with Cluster A protocols being more conserved. **(i)** Efficiency of phagocyte and/or neuroectoderm cell generation from mES cells on PTC5000 beads using a selection of protocols discovered by CombiCult. Coloured bars represent the number of fluorescent colonies per square centimeter (64 fields of view). Red bars correspond to phagocytic colonies, green bars correspond to GFP-expressing (neuroectodermal) colonies, orange bars correspond to beads with both phagocytic and neuroectodermal colonies, black bars correspond to negative protocols. Hit bead numbers and protocols listed below the histograms are coloured to show whether they were derived from phagocyte- (red), neuroectoderm- (green) or phagocyte/neuroectoderm- (orange) bearing hits, whereas black corresponds to two negative protocols – i.e. protocols which did not return any hits, these represent a range of 12 protocols tested, all but one (shown) had 0 colonies. Each bar represents the average of 2 wells (each one containing 4000 beads) and the graph is representative of 3 separate experiments. Protocols with a statistically significant difference (p>0.05) to the negative control are marked with an asterisk (*).

We have used this technology to derive differentiation protocols for the generation of various developmental intermediates and terminally differentiated cells. Here we report three experiments comprising combinatorial screening of ten different media in each of four stages of differentiation (Fig 1a, Fig S12), equal to 10×10×10×10 unique combinations or 10,000 putative differentiation protocols. In the first experiment we used mouse embryonic stem (mES) cells and screened simultaneously for two diverse phenotypic endpoints (phagocytes and neuroectodermal cells), while in the following pair of experiments we used either mES or human embryonic stem (hES) cells and screened for a common phenotypic endpoint (dopaminergic neurons).

## Materials and Methods

### mES cell culture

ES cell lines 46C Sox1-GFP and Oct4GiP cells (Stem Cell Sciences) were grown on 0.1% gelatin-coated plastic dishes in mES cell growth medium (KO-DMEM containing 1X non-essential amino acids (NEAA),1X GlutaMax, 0.5X penicillin/streptomycin (Life Technologies), 15% FBS (ES qualified, ATCC), 0.1 mM β-mercaptoethanol (Sigma) and 1000 U/ml Leukemia Inhibitory Factor (LIF) (Millipore)), in a humidified incubator at 37°C and 5% CO_2_.

### hES cell culture

Shef6 hES cells (UK Stem Cell Bank) were grown on mitomycin C inactivated mouse embryonic fibroblasts in hES cell growth medium (KO-DMEM supplemented with 20% KSR (Life Technologies), 1X GlutaMAX, 1X NEAA, 0.1 mM β-mercaptoethanol and 4 ng/mL bFGF(R&D)). For seeding, cells were harvested by washing in PBS and then incubated with TryplExpress (Life Technologies) for 5 min at 37°C. Following incubation, cells were dislodged by pipetting to obtain single cell suspensions, and centrifuged before being resuspended in complete media and counted.

### Combinatorial Cell Culture

#### (a) Screen for mouse macrophages

During the complete differentiation process cells on beads are maintained in a 37°C humidified incubator with 5% CO_2_.

On day 1 (d1) of the experiment ∼3×10^5^ PTC5000 beads (Plasticell) were equilibrated in each of ten media for split 1 (Table S1 in File S1), transferred into 8 wells of a 100 mm square Petri dish (25 wells; Bibby Sterilin) in 3 ml/well containing ∼4000 beads, and seeded by adding 4×10^5^ mES cells in 0.1 mL of DMEM to each well and mixing thoroughly. Beads in different media were coded by adding adherent tags with a bead to tag ratio of 1∶500 (see below). On d2, d5 and d7 of the experiment, beads from the different media groups were transferred into separate 70 µm cell strainers (Falcon), washed with DMEM to remove residual medium and unbound tags, then beads were pooled, mixed thoroughly and split equally into 10 new sets. Each set was resuspended in one of the media for split 2, spiked with an appropriate tag and plated in 8 wells of a 100 mm square Petri dish (as above). Following d7, the medium in each group was replaced with fresh medium (not containing tags) on d9 and d12. During the last split no tags were added to the media.

For the chemical screen on d1 of the experiment 8.1×10^5^ 46C cells were seeded on 9×10^4^ PTC5000 beads at 90 cells per bead in complete mES medium supplemented with FCS and LIF and next day (d2) beads were washed with DMEM and transferred into serum-free, LIF-free neuro-ectodermal medium containing ITS and RA (media 2.10Table S1 in File S1). On day 5 beads were split equally into 30 chemical mixtures (Table S2 in File S1) and each one was individually tagged (bead to tag ratio 1∶200). On day 7 beads were washed, pooled and split into 30 further conditions. On day 9 beads were washed and transferred into DMEM containing 1% ITS and 0.1% BSA. Media were refreshed on days 12 and 15. A phagocytosis assay was performed on d15 and d22.

#### (b) Screen for mouse dopaminergic neurons

On the day preceding the start of a split-pool experiment (d0), 4.8×10^6^ PTC5000 beads were equilibrated in standard mES growth medium and transferred to 12 well suspension culture plates (Greiner Bio) at a concentration of 4000 beads/well in 2 mL of media. mES cells were seeded onto beads by adding 1 ml of media containing 1.2×10^5^ cells to each well, then left to attach overnight. On d1 all beads were pooled, washed in serum free media and split equally into 10 tubes. The beads in each tube were resuspended in the media and tags required for the first split (Table S3 in File S1), and transferred into 12 wells of a 12 well suspension plate (Greiner Bio). On d7, 15 and 21, beads from different media groups were transferred into separate 70 µm cell strainers (Falcon), washed with DMEM to remove residual medium and unbound tags; then beads were pooled, mixed thoroughly and split equally into 10 sets which were incubated in media and tags as required for the next stage of the experiment, except the last split were no tags were added (Table S3 in File S1). Fresh media (not containing tags) were added on d18 and d24.

#### (c) Screen for human dopaminergic neurons

On day 0 (d0), 4.8×10^6^ PTC5000 beads were equilibrated in standard hES growth medium and plated into 12 well suspension culture plates (Greiner Bio) at a concentration of 4000 beads/well in 2 mL of media. hES cells were seeded onto beads by adding 1 ml of media containing 3.6×10^5^ cells to each well, then left to attach for 48 hours. On d2 all beads were pooled, washed in serum free media and split equally into 10 tubes. The beads in each tube were then resuspended into each one of the 10 media and tags required for the first split (Table S4 in File S1) and transferred into 12 wells of a 12 well suspension plate. On d8, 16 and 22 of the experiment, beads from the different media groups were transferred into separate cell strainers, washed with DMEM to remove residual medium and unbound tags, then beads were pooled, mixed thoroughly and split equally into 10 sets which were incubated in media and tags as required for the next stage of the experiment, except the last split were no tags were added (Table S4 in File S1). Fresh media (not containing tags) were added on d19 and d25.

### Phagocytosis assay

Beads were washed in DPBS and incubated with 1 mg/mL pHrodo *E.coli* BioParticles conjugate (Molecular Probes) in HBSS/HEPES buffer (pH 7.4) at 37°C for 2 hrs. The supernatant was removed and the beads were washed with HBSS/HEPES buffer (pH 7.4) once and then resuspended in the same buffer before being either sorted using the large particle sorter (COPAS PLUS, Union Biometrica), or photographed using a Nikon Eclipse 2000 microscope. An aliquot from the pool of beads was counterstained with calcein green to assess cell coverage and viability (Fig S3A).

For comparison, cells differentiated with either a positive control media or a negative control media (Stemline alone) were subjected to the pHrodo assay (Fig S3B).

### Immunostaining of mouse and human dopaminergic neurons

Cells on PTC5000 beads were washed in DPBS (Mg+ Ca+) twice and fixed in 4% paraformaldehyde for 20 min at room temperature. Following another wash in DPBS (Mg+ Ca+), cells were permeabilised using 0.25% Triton X-100 in PBS for 20 min at 25°C. Cells were then incubated in blocking solution (0.25% Triton, 1% BSA in PBS) for 30 min at 25°C and then incubated in the appropriate primary antibody diluted in blocking solution at 4°C overnight. Following primary antibody incubation, beads were washed 3 times in DPBS (Mg+ Ca+) incubated in secondary antibody solution for 2 hours at 25°C, then washed 3 times in DPBS (Mg+ Ca+) and resuspended in DPBS (Mg+ Ca+).

Antibodies used: primary: Rb anti-tyrosine hydroxylase (Millipore-AB152), Ms anti-βIII tubulin (Sigma clone 2G10), Goat anti-FOXA2 (Abcam) Secondary: Alexa Fluor 594 goat anti-rabbit IgG, Alexa Fluor 488 goat anti-rabbit IgG, Alexa Fluor 488 goat anti-mouse IgG, Alexa-Fluor 350 donkey anti-goat IgG (Life Technologies).

### Hit sorting

Hits were sorted using a COPAS PLUS (Union Biometrica) large particle flow sorter equipped with 488 nm and 561 solid state lasers and Green PMT 514/23 nm, Yellow PMT 585/20 nm, Red PMT 615/45 nm optical emission filters. The instrument was calibrated using a reference sample of beads. Sorting gates for size (TOF), optical density (EXT) and fluorescence parameters for each experiment were set using representative samples of beads that contained both cells and tags and were labelled with secondary antibody only. For the phagocyte screen the gates were set using a sample of tagged beads bearing cells grown under non-differentiating conditions, subjected to pHrodo assay. Beads were sorted at a speed of 50/sec and those decorated with red or green fluorescent cell clusters were dispensed automatically into separate wells of a 96-well plate. As this sorter can only sort on one channel at a time, in screen 1, the same sample of beads was run twice; first to sort for phagocytosis positive beads (red) and then neuroectodermal (green) positive beads. Fig S4 shows the two single colour controls samples run on the COPAS for gate setting. The sorting gate for the phagocytic (red) beads spilled across the double positive quadrant to sort any beads containing both phagocytic and neuroectodermal cells.

Wells were subsequently checked using a Nikon Eclipse 2000-S inverted epifluorescent microscope equipped with filter sets for visualization of TRITC, DAPI, GFP-B (all from Nikon) and Cy5.5 (Chroma Technology).

### Tags and FACS analysis

Tags used in the experiment comprised thirty unique populations of inert fluorescent microspheres (Plasticell) that adhere strongly to PTC5000 beads under cell culture conditions. Discrete populations in the size range of 1–10 microns were assigned according to diameter (three size groups: S, M, L) and fluorescence intensity (ten gradations for each size group). The binding efficiency of all tag species to the beads was very similar, thus there was no significant bias towards a specific interaction (Fig. S2c). Tags were analyzed using FACSCanto II equipped with 488, 635 and 405 nm lasers (Red PMT 710/50 nm) [Becton Dickinson] flow cytometer. Prior to tag analysis, a reference tag set was used to establish side and forward scattering gates and to calibrate fluorescence intensity of each tag set. Digestion of beads with tags did not affect the position of the tags in the flow cytometry plots (Fig S2a–b). Tag identification was performed using Ariadne bioinformatics software (Plasticell). Fluorescence histograms of each scattering gate and FSC/Red fluorescence plots were used to identify clusters of events with correlated scattering and fluorescence parameters, which are mapped to cell culture media as detailed in Tables S1–S4 in File S1. Clusters are identified when they contain ≥ 3 events with near identical scattering and fluorescence values. If two or more clusters of events were identified which mapped to cell media from the same split and/or the signal to noise ratio was too low, an accurate identification was not recorded.

### Probability simulations

Probability values for the occurrence of given events by chance were obtained from computer simulation experiments. A Mersenne Twister [12] random number generator was used to output uniformly distributed 32-bit integers which were scaled to cover 10,000, 1,000 or 100 possible pathways, when simulating common cell culture media on four out of four splits, three out of four splits or two out of four splits. Simulation begins by setting tally counters associated with each pathway to zero. To simulate probabilities for phagocyte screen, 96 paths were chosen at random and their counters incremented. Should any such counter exceed the specified threshold (2, 3, 4, 5, …, n beads per pathway), a positive result was recorded. Event probabilities were computed by repeating the process 100 million times and dividing the number of positive results by the total number of simulations, resulting in probability values accurate to eight decimal places. To obtain probabilities for events in the neuroectoderm screen, and the mouse dopaminergic neuron screen, 87 and 378 paths respectively were randomly chosen.

### Differentiation protocols for mouse phagocytes and neural progenitors validated on beads

For protocol validation studies, beads were transferred between media without splitting and pooling and without including fluorescent labels. On d1 of the experiment PCT5000 (Plasticell) or FACTIII (Solohill) beads were equilibrated in media and mES cells were seeded onto beads by adding 4×10^5^ cells in 0.1 mL of DMEM to each well containing 4000 beads in 3 mL of media (∼100 cells/bead). At each stage of differentiation media were decanted, beads washed twice in DMEM and the next media in the series were added. On d9 and d12 media were refreshed without washing. For each protocol validated, two independent wells were prepared. On d15, duplicate samples for each protocol were analyzed for phagocytic activity or GFP fluorescence (neural progenitors) and levels quantified from images obtained by epifluorescence microscopy using a Nikon Eclipse 2000 inverted microscope, and NIS-elements software.

### Hematopoietic colony forming assays

Cells were differentiated on FACTIII (Solohill) beads until d9, then harvested with trypsin/EDTA (Sigma), washed once with 10% FBS in DMEM, sieved through a 70 µm strainer and resuspended in IMDM media supplemented with 2% FCS. Cell suspensions were mixed with MethoCult semisolid media (M3434, StemCell Technologies) at a final concentration of 1×10^5^ cells/ml according to manufacturer's instructions. Colonies were scored after 2 weeks of incubation at 37°C in a humidified incubator with 5% CO_2_. MethoCult media containing Il-7 (M3630, Stem Cell Technology) was used to study B-lymphoid progenitors.

### FACS analysis of hematopoietic cells

Cells differentiated on FACTIII (Solohill) beads were harvested with Accutase solution (1X Accutase in DPBS, Sigma A6964). To analyze cells from colony forming units, semisolid media were diluted with staining/blocking solution (3% FBS in DPBS) and treated with Accutase to disrupt colonies. Prior to staining with specific antibody or the corresponding isotype control, cells were incubated with mouse BD Fc block (#553142, BD Biosciences). Antibodies and corresponding isotype controls used: anti-mouse CD11b-APC (clone M1/70), anti-mouse CD45-PE (clone 30-F11), anti-mouse CD3e-APC (clone145-2C11), anti-mouse CD45R/B220-APC or PE (clone RA3-6B2), anti-mouse CD5-APC (clone 53-7.3), anti-mouse CD43-PE (clone S7), anti-mouse CD19-PE (clone 1D3) and anti-mouse IgM-APC (clone 11/41) all purchased from BD Biosciences. A FACS Canto II flow cytometer was used for analysis.

### FACS sorting of hematopoietic cells

D15 cells differentiated on beads according to cluster B protocols were harvested with 0.25% trypsin/EDTA (Life technologies) and diluted with staining/blocking solution (3% FBS in DPBS). Prior to staining with specific antibody or the corresponding isotype control, cells were incubated with mouse BD Fc block (#553142, BD Biosciences). The antibodies and corresponding isotype controls used were anti-mouse CD11b-APC (clone M1/70) and APC-Rat IgG2a isotype control, both purchased from BD Biosciences. Cells were incubated with antibody for 1 hour on ice, washed twice with 3% FBS in PBS, and then sorted using a FACS Aria flow sorter (BD Bioscience). The live cells were gated using a propidium iodide (PI) stained control and the sorting gates were set using the isotype control stained cells. Both the CD11b positive and the CD11b negative cells were collected. The CD11b positive cells were plated into 2 wells of a 96 well tissue culture plate in Stemline medium (Sigma) containing TPO, IL3 and IL6 (All from R&D) and incubated at 37°C for 1 h to allow the cells to attach. After the incubation period the media were removed and the cells subjected to the pHrodo assay (Life Technologies) as above, then counterstained with calcein green. Cells were subsequently photographed using a Nikon Eclipse 2000 epifluorescence microscope.

### Maturation of cluster A-derived cells

Cells were harvested from beads at d15 of differentiation using Accutase, and cultured on OP9 feeder layers in MEM alpha, 10% FBS, 1X NEAA, 1X GlutaMax, 10 ng/mL IL7 and 20 ng/mL SDF1. Cells were re-plated onto fresh OP9 feeder layers once a week. Flow cytometry analyses were carried out after 2 and 3 weeks of co-culture. In parallel d15 differentiated cells were plated onto MethoCult supplemented with IL7 (Stem Cell Technologies) as per manufacturer's instructions and analyzed by flow cytometry 2 and 3 weeks after seeding.

### Mice and embryos

Embryos were obtained from C57BL/6 timed pregnant female mice (2–3 months old). Yolk sac (YS), aorta-gonad-mesonephros (AGM) region and when available foetal livers were isolated from E9 (20–28 somite pairs, s.p.), E10 (32–37s.p.) and E11 (40–47 s.p.) embryos and single cell suspensions obtained following collagenase treatment [13]. Mice were housed and bred in compliance with Home Office regulations and with approval of The University of Edinburgh Ethical Review Committee. Two separate experiments were performed and an average of 8 pregnant females were used per experiment.

### Mouse embryo cell isolation and analysis

Multicolour flow cytometry analysis of embryonic tissues was performed using LSR Fortessa (BD). Cell populations were isolated using a FACSAria II cell sorter (BD). The following anti-mouse antibodies (all from BD Pharmingen) were used for staining: anti-CD45 BD V500 or BD V450 (clone 30-F11); anti-CD5 BD V450 or PE clone (53-7.3); anti-CD43 FITC (clone S7); anti-B220-PE-cy7 (clone RA3-6B2); anti-CD3e-APC (clone 145-2C11); anti-CD19-PE or PerCP-cy5.5 (clone 1D3); anti-Ter119-V500 or PerCPcy5.5. Gating strategy was defined based on fluorescence minus one (FMO) control staining where one of each antibody was substituted by isotype control (IC) conjugated with corresponding fluorochrome. All analyses and sorting were done on the basis of 7AAD dead cell and Ter119+ cell exclusion.

### Differentiation protocols for dopaminergic neurons validated on beads

Equilibrated PTC5000 beads were plated onto 12 well suspension plates (Greiner Bio) at 4000 beads/well in 2 mL of complete growth media. To each well 1 mL of cells were added at a concentration of 1.2×10^5^ cells/mL (mES 46C Sox-1 GFP) or 3.6×10^5^ cells/mL (hES Shef6) and left to attach for either 24 (mES) or 48 (hES) hours. For each protocol validated, two independent wells were prepared.

After the attachment period, media were decanted; beads washed twice in DMEM (in situ) and appropriate media for each stage of differentiation added. After 1 week the washing process was repeated and the media for the next stage of differentiation added. In between stages, media were refreshed by exchanging half the volume with fresh media. At the end of the differentiation period beads were washed, fixed, immunostained and analysed using COPAS (Union Biometrica Inc.)

### Differentiation protocols for dopaminergic neurons validated by EB formation

Cells were harvested enzymatically, counted and transferred to bacteriological grade 10 cm dishes in mES cell growth medium. After cell aggregation, which usually required 24–48 hours, EBs were cultured in stage 1 media for seven days, then plated on adherent six well plates previously treated with 0.1% gelatin and cultured in stage 2 media, with media replacement every 2–3 days. Every seven days appropriate media for each stage of differentiation were introduced and refreshed every 2–3 days. Cells were passaged onto new laminin-coated wells on becoming confluent. Finally cells were fixed in 4% paraformaldehyde, immunostained as described and analysed by epifluorescence microscopy using a Nikon Eclipse 2000 inverted microscope, and NIS-elements software.

### Differentiation protocols for dopaminergic neurons validated on monolayer culture

mES and hES cells were harvested enzymatically and plated on either 0.1% gelatine (mouse) or CELLstart (Life Technologies) (human) at pre-defined densities and allowed to attach overnight in standard mES or hES growth media. After 24 h (or 48 h), the media were changed as directed by the differentiation protocols, with media refreshment every 2–3 days. Cells were passaged onto new plates pre-coated with laminin when they became confluent. At the end of the differentiation period the cells were fixed in 4% paraformaldehyde, immunostained as described and analysed by epifluorescence microscopy using a Nikon Eclipse 2000 inverted microscope, and the NIS-elements software.

## Results

### Differentiation of mES cells to haematopoietic phagocytes and/or neuroectoderm

In a first experiment we asked whether our system could identify mES cell differentiation protocols from combinations of media previously reported to direct ES cell differentiation towards ectodermal, mesodermal and endodermal lineages (Fig 1a,Table S1 in File S1). We hypothesised this extremely diverse screening matrix would contain media combinations that direct stem cell differentiation to a variety of cell types from all germ layers. At the end of the screen we assayed beads using red fluorescent bacterial particles to detect those bearing phagocytic cells (Fig 1b (i)). The rationale of using such a broad functional assay was to determine whether we could identify different types of phagocytic cells – e.g. macrophages [14], dendritic cells or B1 lymphocytes [15]–[18] – and if so to compare the protocols that generated each cell type.

Additionally, in this experiment we used an mES cell line that contains a GFP reporter of Sox-1 expression, which marks early neuroectoderm [19], [20], hence green fluorescence was used to detect differentiation towards a neural fate (Fig 1b (ii)). We expected to find that neuroectodermal cells and phagocytes are generated by very different protocols.

From over 250,000 monomeric beads screened at the end of our experiment (see Fig 1c, Gate 1), we identified and sorted a total of 137 red fluorescent hits (0.05%) (Fig 1 c–d). The rare incidence of fluorescent beads implies that only few combinations of culture conditions successfully directed differentiation towards phagocytic lineages. Fewer than 10 of these red fluorescent beads also contained traces of green fluorescence (Fig 1d (v)), indicating that the majority of phagocytes had appeared owing to directed, not spontaneous differentiation. Ninety-six hits (70%) carried sufficient tags to deduce the identity and sequence of media to which they had been exposed - i.e. the differentiation protocol. The remaining beads lacked sufficient tags from at least one split, or failed owing to total loss of the tag sample during handling (for a breakdown of causes see Data S1, Table 6).

Analysis of tags from each bead (Fig 1e), as well as comparison of the differentiation protocols deduced from all hits (Fig 1f), was performed using Ariadne (Data S1 for all hits). Since similarity amongst the putative differentiation protocols discovered by combinatorial cell culture likely indicates protocol efficacy, we first used hierarchical clustering analysis to group protocols (Fig 1g) and then scored a pairwise comparison of each (Fig 1h). In this way we identified two interesting protocol clusters with substantial internal homology: one group of 18 protocols was characterized predominantly by common media in the first two stages of differentiation (i.e. media 1.10 and 2.1, termed Cluster A), while a second group of 23 protocols featured common media in the final two stages (i.e. media 3.8/3.9 and 4.7, termed Cluster B).

Each of the media combinations tested by CombiCult is sampled by multiple beads, allowing statistical analysis of results. In the present experiment, 300,000 beads were used to test 10,000 protocols, therefore maximally efficient protocols should in theory return on average 30 hits (although taking into account factors such as bead loss during the experiment, in practice we would expect many fewer). We found three protocols within Cluster A that returned multiple hit beads: in particular, protocol 0185 (i.e. media combination 1.10→2.1→3.8→4.5) returned three hits, while protocols 0115 and 0145 each returned pairs of hits. We used a simulation algorithm to estimate the probability that a protocol would return multiple hits by chance, given the number of protocols screened and the number of hit beads obtained. In our dataset - in which 96 hits were derived from a screen of 10,000 protocols - a triple hit from the same protocol would occur randomly once in every thousand repetitions of the experiment and therefore its occurrence in our screen is likely to be significant (i.e. indicate a highly effective protocol).

We selected 17 protocols (including multiple hit protocols) for further study from the two protocol clusters (the relationship of these protocols is analysed in detail in Fig.S5). We tested these by differentiating mES cells on PTC5000 beads used in the screening experiment and determined that 14 protocols consistently directed differentiation to phagocytes in this system (Fig 1i). By quantitating green fluorescence from the Sox1-GFP transgene present in the mES cell line, we determined that none of the 17 phagocyte protocols produced significant amounts of neuroectoderm, confirming that stem cell differentiation was likely directed as opposed to stochastic. Interestingly, directed differentiation was even more efficient using a microcarrier bead (FACTIII) made of a different material, indicating that the protocols are adaptable to other culture systems and may be further optimised by altering factors such as cell substrate (Fig.S7). Consistent with our expectation that the protocols that generated multiple hits were likely the most productive, these performed best in validation experiments using either bead type.

We then tested 12 protocols that did not return hits, for the ability to generate phagocytes or neuroectoderm (two of which are shown in Fig 1i): five protocols were selected arbitrarily and seven on the basis that they comprised media that rarely produced hits. None generated appreciable amounts of either cell lineage, confirming that only specific combinations of media could efficiently direct terminal differentiation of mES cells.

We also sorted 395 green fluorescent beads (i.e. bearing Sox1-GFP-positive cells) from the CombiCult screen (Fig 1c (ii) – Gate 3), of which 106 were submitted to tag analysis, revealing 87 differentiation protocols (Data S2 and Fig.S6). As might be expected, there was little, if any, similarity between protocols that produced phagocytes and those that produced Sox1-positive neuroectoderm – demonstrating that different combinations of media are required to direct differentiation to divergent lineages. Nevertheless, seven beads with both green and red fluorescence were isolated from the phagocyte screen (representing seven different protocols) and by validating a small subset of these we determined that at least one protocol (2865) reproducibly resulted in mES cell differentiation into both lineages (Fig. 1i). Such protocols that efficiently generate mixtures of cell types may be useful in certain cell biology applications, for example the construction of more realistic tissue models.

### Cluster A protocols direct mES differentiation to a new lymphoid progenitor cell

**Figure 2:**
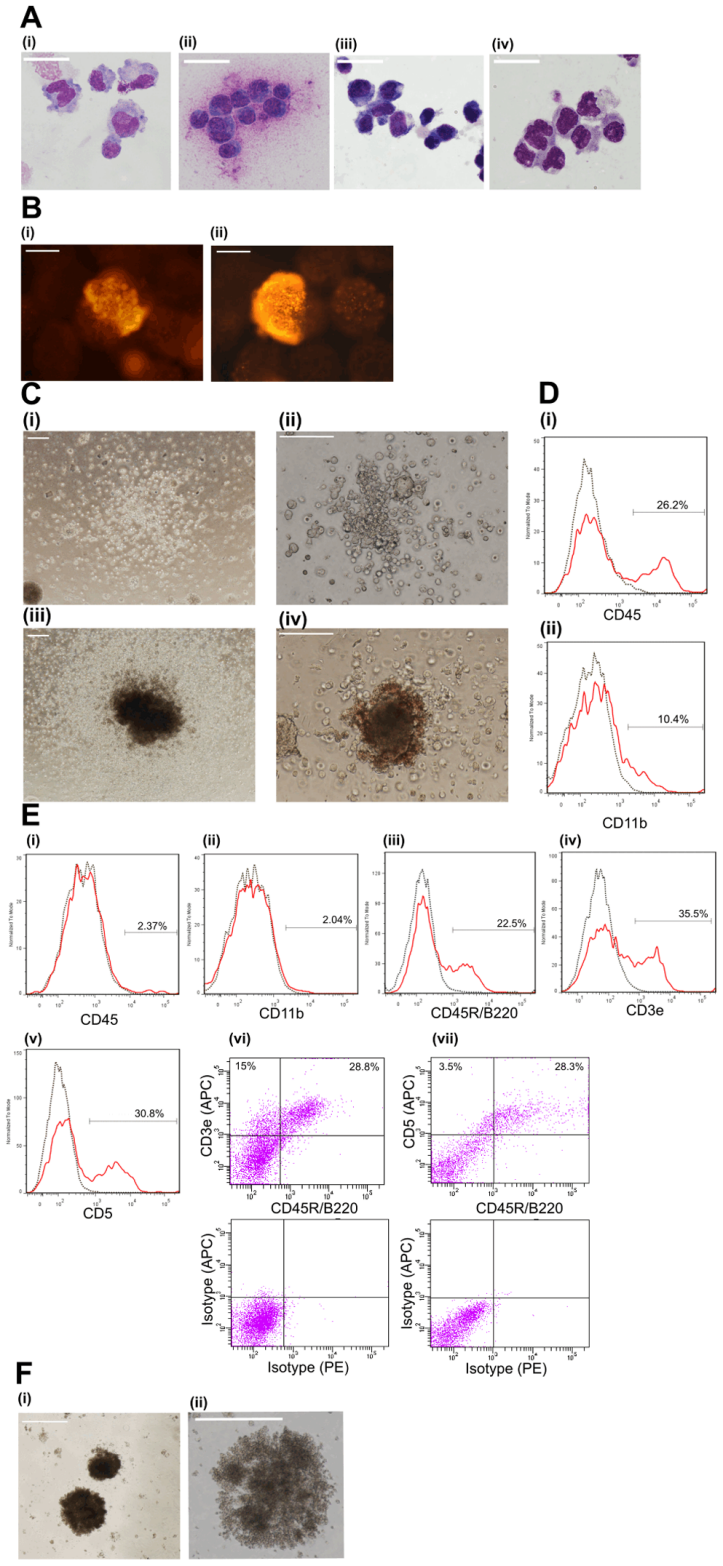
Analysis of phagocytes derived from mES cells using validated protocols discovered by CombiCult. Two protocol clusters generated two different types of phagocytes: Cluster B protocols gave rise to typical monocytes and macrophages while Cluster A protocols generated phagocytic cells that resemble B1a lymphocytes. Each panel is representative of at least 3 separate experiments. **(a)** Wright-Giemsa staining of cells generated on beads by protocol: (i) 0777 (Cluster B), (ii) 3897 (Cluster B), (iii and iv) 0185 (Cluster A), Scale bar =  5 µm. **(b)** Phagocytic cells on beads identified by pHrodo assay: (i) protocol 2277 (Cluster B) produced large vacuolarised phagocytes that attach loosely to beads. (ii) protocol 0185 (Cluster A) produced colonies of tightly adherent cells. Scale bars  =  100 µm. **(c)** Colony formation by mES-derived hematopoietic precursor cells in MethoCult. Precursors generated by Cluster B protocols gave rise to a range of monocytic, granulocytic or mixed type colonies; average colony formation efficiency was 3×10^−4^. (i) CFU-G (granulocytic) colonies and (ii) CFU-M (monocytic) colonies formed using protocol 2277, (iii) CFU-M colonies formed using protocol 0887, (iv) mixed type (CFU-GEMM) colonies formed using protocol 3897. Scale bars  =  100 µm. **(d)** Flow cytometry analysis of cells isolated from MethoCult following differentiation according to Cluster B protocols: (i) CD45 and (ii) CD11b staining of cells derived using protocol 3897. Black dotted lines represent the populations stained with a corresponding isotype control overlaid with the experimental population in red. **(e)** Flow cytometry analysis of cells differentiated for 15 days on microcarriers using protocol 0185 (Cluster A). In each histogram the black dotted line represents the population stained with a corresponding isotype control overlaid with the experimental population in red. The percentage of positively stained cells is shown in each panel. (i) CD45, (ii) CD11b, (iii) CD45R/B220, (iv) CD3e and (v) CD5 (vi) CD45R/B220 vs. CD3e with corresponding isotype control dot plot and (vii) CD45R/B220 vs. CD5 with corresponding isotype control dot plot. **(f)** Colony formation by mES derived progenitors in MethoCult supplemented with IIL7. Precursors generated by protocol 0185 (day 9) gave rise to round compact colonies. Average colony formation efficiency was 2×10^-3^ (i) 10X magnification and (ii) 20X magnification. Scale bar is 100 µm.

Next we investigated the phenotype of the phagocytic cells produced by Cluster A and B protocols by histology (Giemsa staining), analysis of cell surface antigens and colony-forming assays. Remarkably, we discovered that the two protocol clusters produce very different phenotypes. Cluster B protocols generate haematopoietic monocytes/macrophages (Fig 2a (i) and (ii)) that efficiently engulf bacterial particles (Fig 2b (i)), attach loosely to beads, and express the macrophage marker CD11b (Fig 2d and Fig S8,). Hematopoietic progenitors generated by these protocols on day 9 give rise to a variety of myeloid lineages when plated in MethoCult semisolid media containing SCF, IL3, IL6 and EPO (Stem Cell Technologies) (Fig 2c). Conventional feeder free protocols for macrophage differentiation from ES or iPS cells employ embryoid body formation using pre-selected fetal calf sera that support hematopoietic differentiation [14], [21]. Here we searched for combinations of cytokines and growth factors that direct macrophage differentiation in microculture under serum- and feeder- free conditions. Cluster B protocols feature various media combinations in the initial stages of differentiation, but later converge on common media that are well known to promote hematopoietic development. On day 5 these protocols feature exposure to BMP2/TGFβ1 or PDGF-BB (media 3.7, 3.8 and 3.9) - high concentrations of BMPs (BMP2 and BMP4) induce hematopoietic differentiation of ES cells [22], PDGF-BB regulates differentiation of hemangio-precursor cells in embryoid bodies [23] while TGFβ1 promotes maturation of pro-monocytic cells into macrophages, stimulates adherence to culture surfaces and enhances phagocytosis [24]. By day 7, all but two Cluster B protocols (Fig. 1g) require a cytokine mix containing IL3, IL6 and TPO at d7 (medium 4.7) in order to sustain and expand myeloid cells [25], [26].

In contrast, Cluster A protocols give rise to large cells with an ovoid or kidney shaped nucleus (Fig 2a (iii) and (iv)), that adhere firmly to beads (Fig 2b (ii)) and do not express the macrophage marker CD11b and the common leucocyte marker CD45 (Fig 2ei,ii ). Instead, these cells express the lymphocyte specific markers B220, CD5 and CD3e (Fig 2e (iii–vii)) but not CD19 or CD43. That these cells express B220 but not CD45 is unusual since B220 is an isoform of CD45. It would seem that the specific B220 epitope expressed in our cells is not recognised by the pan-CD45 antibodies used. In contrast to the findings with Cluster B protocols, progenitor cells generated by Cluster A protocols (on day 9) fail to generate myeloid colonies in MethoCult supplemented with SCF, IL3, IL6 and EPO, but do form colonies in IL7 containing MethoCult (Fig 2f).

To our knowledge the cell phenotype generated by Cluster A protocols has not been previously described, but resembles that of B1a cells; a lymphocyte that co-expresses B220 and CD5 and has been shown to adhere to plastic and be capable of phagocytosis in vitro [27]–[29] (but which has not been shown previously to express CD3 or lack CD45 expression). B1a progenitors are relatively abundant in foetus and neonates, however their repopulating ability declines during adulthood, with adult B1a cells mainly localized in the peritoneal cavity and to a lesser extent in spleen [30], [31]. Recent reports suggest that B1a progenitors emerge at different stages of embryonic development and are characterized by the expression of CD19 and B220 (low) but upon in vitro culture become CD19/B220 (high) [32], [33]. It has also been suggested that the precursor of the earliest B1a progenitors is yet to be described, and it remains possible that these cells represent a vestigial wave of B cell development similar to one seen during foetal erythropoiesis [34]. Adult B1a cells were recently shown to give rise to a further lineage, ‘immune response activator’ (IRA) cells, that is the source of granulocyte-colony stimulating factor (GM-CSF) and important in protecting against sepsis [16].

**Figure 3:**
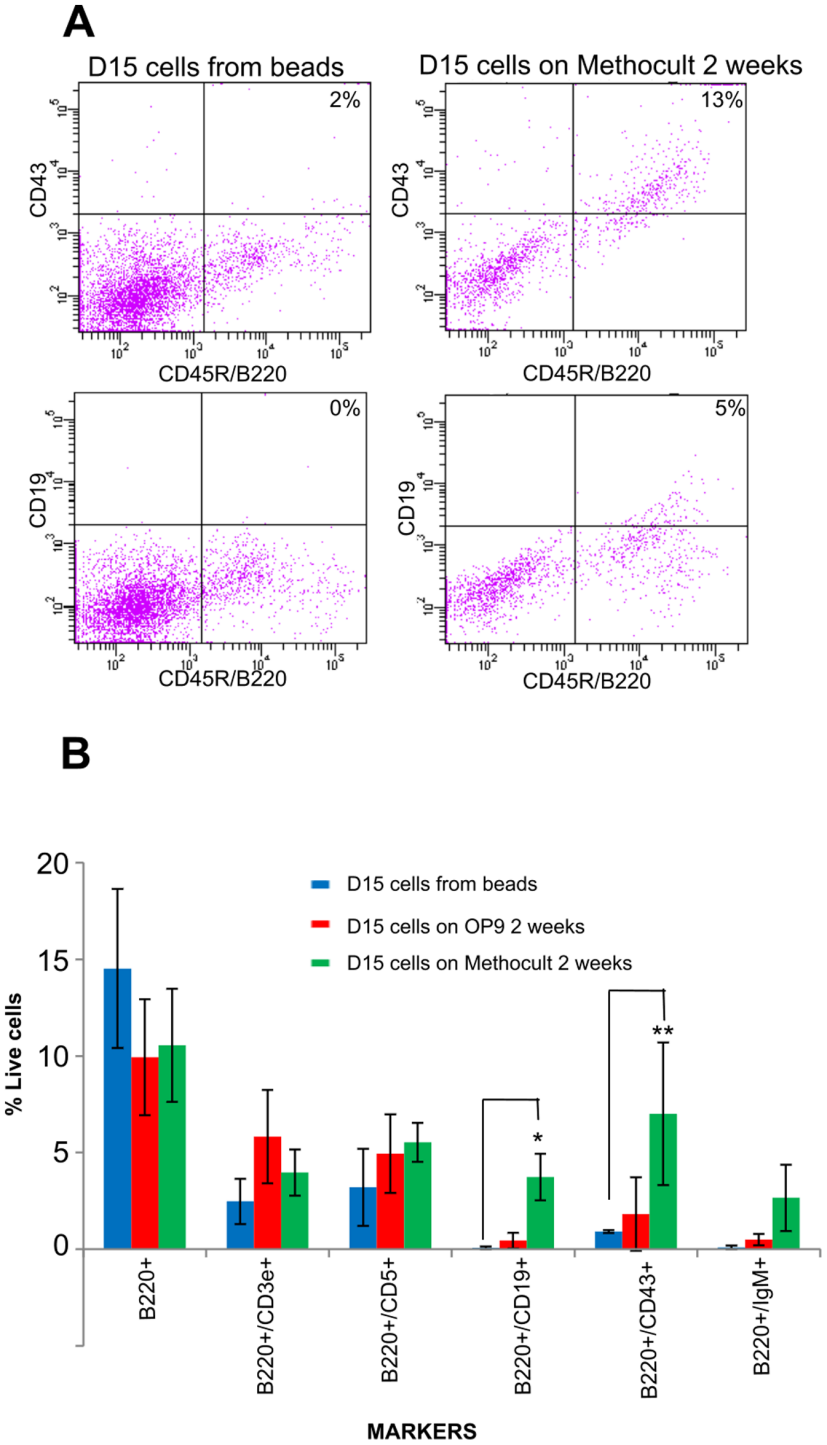
Analysis of Cluster A protocol derived phagocytes following further maturation in MethoCult or on OP9 cells supplemented with IL7. **(a)** Dot plots illustrating flow cytometry analysis of cells obtained after maturation in IL7 supplemented MethoCult for 2 weeks, showing the appearance of cells co-expressing B220/CD43 and B220/CD19; **(b)** Histogram showing the proportions of different cell populations present in MethoCult or OP9 derived cultures, measured by flow cytometry analysis. Blue bars represent cells taken off beads on D15 (prior to maturation), red bars represent cell isolated on D15 and cultured for a further 2 weeks on OP9 feeder layers and green bars represent cells isolated on D15 and cultured in MethoCult for 2 weeks. The experiment was carried out twice and in duplicate the bars show the average percentage of cells, the error bars show the standard deviation. There is a statistically significant difference between the cells taken from the beads at D15 and those cultured on MethoCult for 2 weeks for markers CD43 and CD19 *p<0.05.

Since Cluster A cells do not express markers of mature B1a cells such as IgM or CD43 [35] we hypothesised that they may be a type of B-lymphoid progenitor. To test this, we plated cells generated by Cluster A protocols, either at d9 or d15 of differentiation, into MethoCult semisolid medium supplemented with IL7 (Stem Cell Technologies), or onto OP9 feeder layers [36] to ascertain their ability to develop into more mature cells. In both systems, cells proliferated and gave rise to colonies of more mature cells that express B220, CD5 and CD43, although CD19 and IgM expression was only observed at very low levels (Fig 3 a,b) and CD45 continued to be absent. We further purified the B220+/CD5+/CD3e+ population by FACS and showed that they are capable of forming colonies in methylcellulose supplemented with IL7 (data not shown).

**Figure 4:**
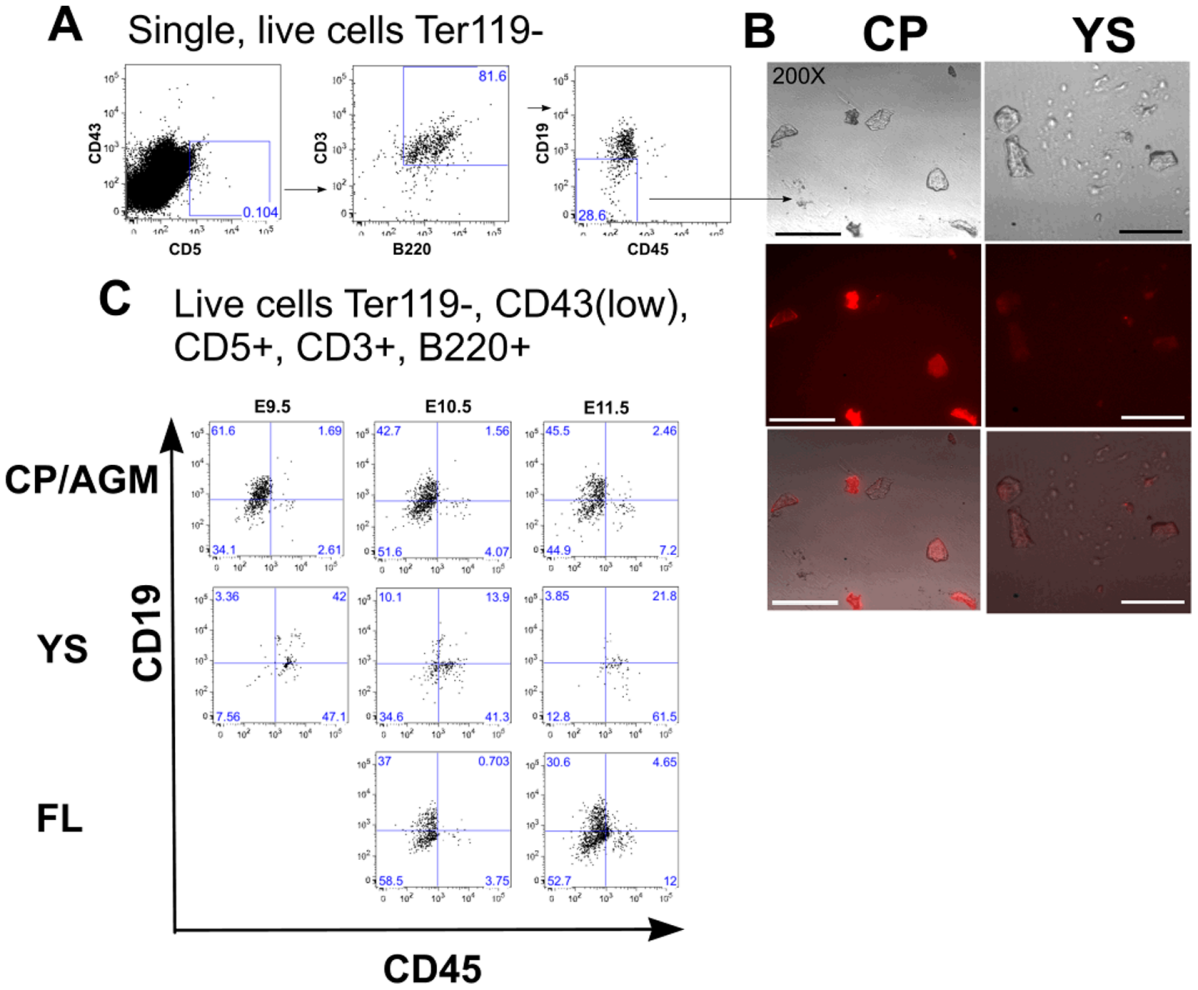
Characterisation of lymphoid progenitor cells in the early embryo. **(a)** Sorting strategy: Live (7AAD-), Ter119-single cells, were sorted for CD43^(low/-)^CD5^+^CD3^+^B220^+^CD45^-^CD19^-^ expression. **(b)** Cells sorted from yolk sac (YS) or caudal region/AGM (CP) were plated for pHrodo assay to assess phagocytosis. Upper panel, bright field; middle panel, fluorescent image of phagocytic cells; lower panel, merged image. Scale bar corresponds to 20 µm. **(c)** Lymphoid progenitor cells (Ter119^-^,CD43^(low/-)^CD5^+^CD3^+^B220^+^CD45^-^CD19^-^) are present on day 9 in the caudal part of the embryo and later in AGM and foetal liver (FL). The majority of CD43^(low/-)^CD5^+^CD3^+^B220^+^ are CD45^-^. The yolk sac (day 9–11) contains a small number of these CD45^-^ lymphoid progenitor cells. This is a representative analysis of two separate experiments each one performed using embryos from 8 pregnant females (average 6–8 embryos/female).

These assays confirm that Cluster A protocols result in mES differentiation to a lymphoid progenitor cell whose phenotype resembles but does not perfectly correlate with that of previously described B1a progenitor cells. In order to determine whether we had detected a previously undefined, naturally-occurring cell type, or had generated an *in vitro* artifact, we sought to prospectively identify a cell with the same phenotype *in vivo*. Since differentiation of mES cells is known to generate predominately primitive, rather than definitive, haemopoietic cell types (e.g. RBCs [37]) and because a subset of B1a progenitor cells are known to appear early in development in the yolk sac and para-aortic splanopleural region (producing a first primitive wave of B1a cell production [32]) we focused on D9.5-11.5 embryonic, rather than adult tissues. We were able to identify a minor population of cells in D9.5, 10.5 and 11.5 embryos that co-express CD5, B220 and CD3, are CD19 and CD45 negative, can adhere to tissue culture plastic and phagocytose (Fig. 4, see Fig S9 for all relevant flow cytometry controls). They are present in the caudal region/AGM, foetal liver and yolk sac, although interestingly phagocytosis is much less efficient in cells derived from the yolk sac (Fig. 4b). Here we show that a majority of these hematopoietic progenitors (7AAD^-^Ter119^-^CD45^-^CD43^(low/-)^CD19^-^CD5^+^CD3^+^B220^+^) from day 10.5 caudal region also express both CD135 (Flt3) and CD127 (IL7 receptor) indicating the lymphoid nature of these cells (Fig S10). The role of these novel lymphoid progenitor cells and their precise place in the hematopoietic lineage will require further investigation. The use of our method to produce a previously unknown progenitor cell illustrates the ability of combinatorial screening to generate rare intermediates for developmental biology and fate mapping studies, as well as for future use in small molecule screening to discover regenerative drugs.

### Use of CombiCult to substitute growth factors with small molecules

**Figure 5:**
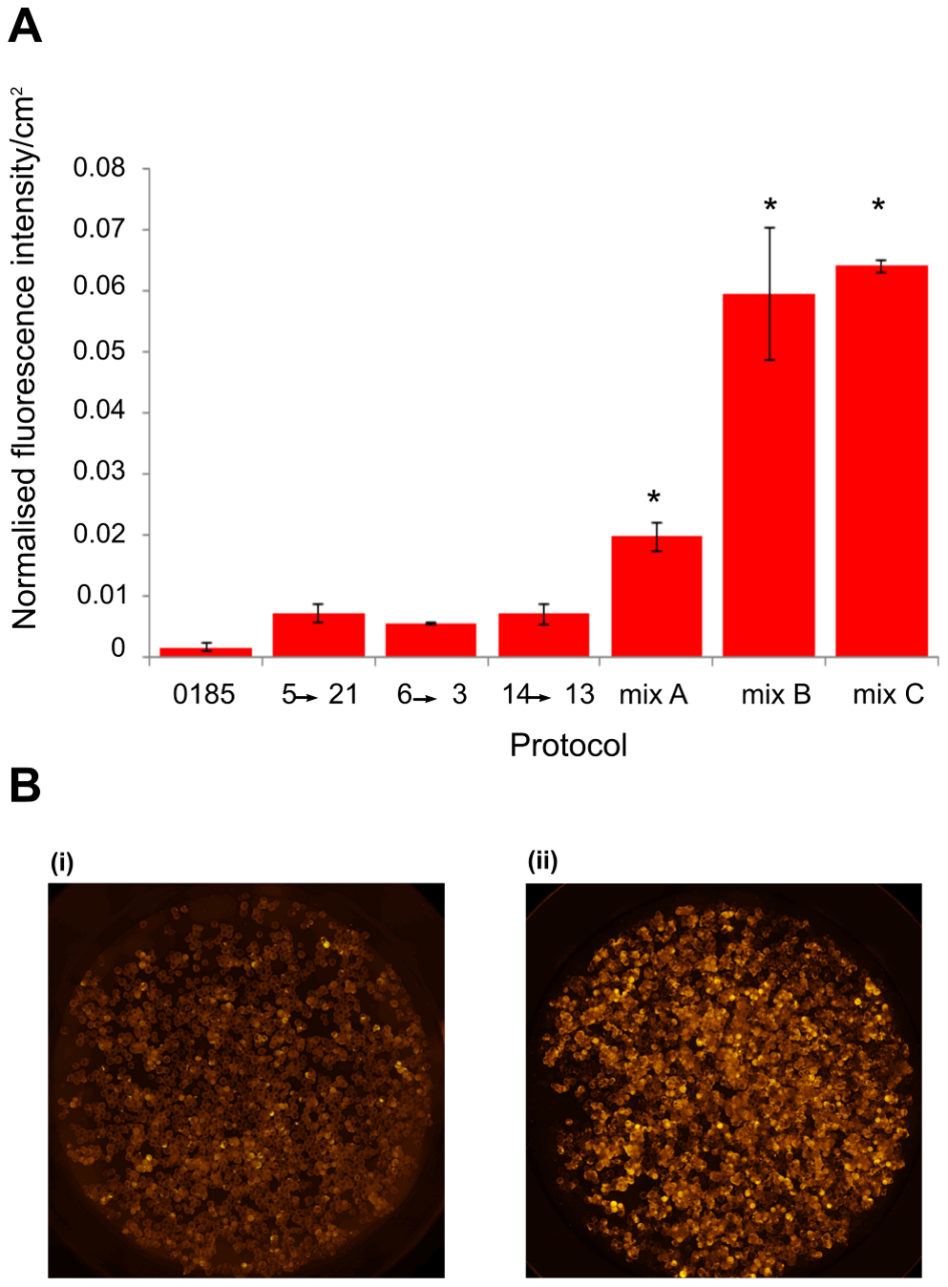
Combinatorial screening discovers small molecule-driven differentiation protocols for the generation of lymphoid progenitor cells. **(a)** Histogram showing efficiency of the most frequently occurring protocols derived from the CombiCult screen, as well as novel combinations of frequently occurring chemicals, compared to the most efficient cytokine-driven protocol (0185). Phagocytosis activity was measured by pHrodo assay. Mix A: metformin, sis3, kenpaullone and GPR-40 agonist on day 5, followed by pifithrin, capsaicin and DITPA on day 7. Mix B: metformin, sis3, kenpaullone, GPR-40 agonist on day 5, followed by pifithrin, capsaicin, DITPA, transRA, and VEGF inducer on day 7. Mix C: metformin, sis3, kenpaullone, GPR-40 agonist, TEA on day 5, followed by pifithrin, capsaicin, DITPA, transRA, and VEGF inducer on day 7. Each bar represents the average efficiency from 2 wells (each one containing 4000 beads) and the graph is representative of 3 separate experiments. There is a statistically significant difference between the cytokine protocol and the individual mixes *p<0.05. **(b)** Representative images of lymphoid progenitor cells generated on beads and treated with pHrodo particles (red fluorescence): (i) cytokine-driven protocol 0185 and (ii) chemically-driven protocol (Mix B).

An important application of combinatorial screening is to discover combinations of small molecules that drive stem cell differentiation. The B1a-like lymphoid progenitor cells described above arise from mES cells when these are transferred from standard mES cell expansion medium containing serum (medium 1.10) into a fully defined medium previously reported to induce neurogenesis (medium 2.1), followed by culture in media containing such growth factors as PDGF-AA, PDGF-BB, FGFa or bFGF. Both PDGFs and FGFs exert mitogenic and anti-apoptotic activities through tyrosine kinase receptors, MEK and PI3K signaling [38], [39]. PDGFs and FGFs are also implicated in proliferation and survival through interaction with other signaling pathways including Wnt and Shh [40], [41]. We therefore attempted a combinatorial screen to replace these growth factors with small molecules that target the same signaling pathways or chemicals shown to promote hematopoietic differentiation and/or prevent apoptosis. We seeded beads with mES cells in medium 1.10, transferred these to medium 2.1 for three days (D2-D4), then performed a combinatorial screen of (30×30  = ) 900 cell culture conditions containing 49 different bioactive compounds applied on D5 and D7 (Table S2 in File S1), followed by a phagocytosis assay. We obtained 146 hits of which 98 (67%) were deconvoluted to determine new, chemically-driven protocols. Remarkably, some combinations of small molecule mixes (including 5→11, 6→3 and 14→13) were able to generate Cluster A-like phagocytes with an equal or greater efficiency compared to Cluster A protocols (Fig 5a). Furthermore, by analysing the representation of individual chemicals in highly efficient protocols we developed novel combinations of small molecules that even further increased the yield of phagocytic cells. The most efficient protocol used two chemical cocktails: first a mix of four chemicals (SIS3, metformin, kenpaullone, GPR-40) on D5, followed by a mix of five chemicals (trans RA, VEGF inducer, DITPA, capsaicin, pifithrin A) on D7, which, in combination, increased phagocyte cell yield by up to 50-fold (Fig 5b). The first cocktail contains compounds that activate Wnt, AMPK and PKB/AKT signaling to stimulate proliferation, survival and the insulin response, while the second cocktail includes small molecules that prevent apoptosis and promote hematopoietic differentiation (i.e. DITPA or VEGF inducer). The increase in yield may be due to more efficient stimulation of the biochemical differentiation pathways, the preferential amplification of the desired cell type, or selective cell toxicity of contaminating cells. We further demonstrated that the cell phenotype generated by the growth factor- and small molecule- driven protocols was the same (Fig.S11). Our combinatorial screening method can therefore be used to develop novel protocols to generate cell types of a given phenotype using small molecules instead of growth factors, providing considerable advantages in process cost, yield and reproducibility.

### Comparison of the differentiation of human and mouse ES cells to a dopaminergic neuron fate

**Figure 6:**
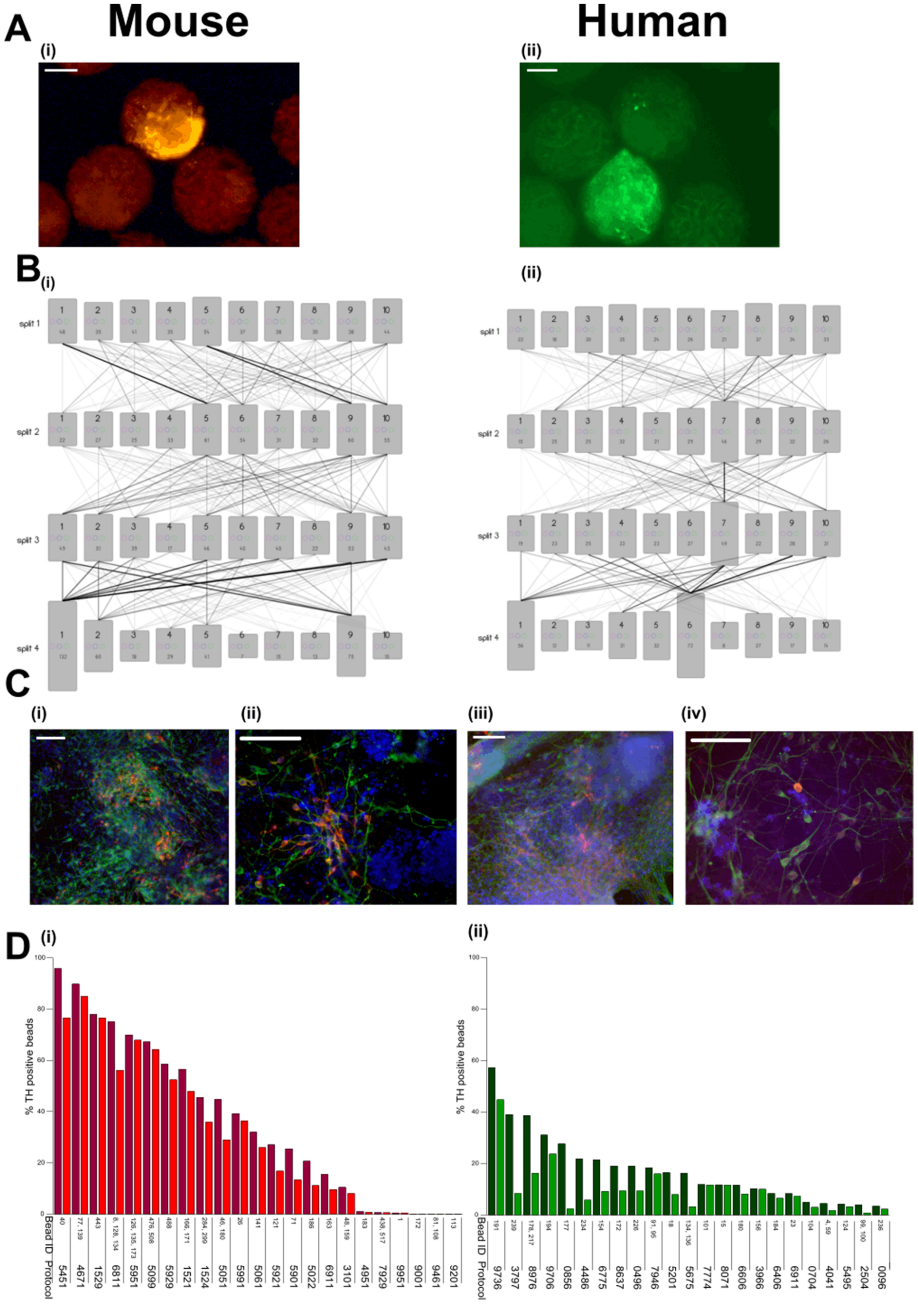
Mouse and human TH positive neuronal screens. **(a)** Fluorescence micrographs showing hits bearing (i) mouse and (ii) human TH+ cells amongst negative beads. Scale bars  =  100 µm. **(b)** Schematic diagram illustrating an overlay of all protocols deconvoluted from screens for TH+ neurons generated using (i) mES and (ii) hES cells. Histograms representing each cell culture medium are proportional to the number of hits generated (written at the bottom of each bar). The opacity of the linkage lines is proportional to the number of hits generated by specific media combinations - the darkest line in each plot corresponds to (i) 21 and (ii) 12 beads. **(c)** Microgrographs showing immunofluorescence images of differentiated ES cells in monolayer cultures. Differentiation protocols shown for mES cells are **(i)** 5929 and **(ii)** 4671, stained for TH (red), β-III tubulin (green) and DAPI (blue). Differentiation protocol shown for hES cells is 8976 (iii and iv) stained for TH (red), β-III tubulin (green) and DAPI (blue); except in (iv) where FOXA2 is stained blue. Scale bars  =  100 µm. Representative photographs from a field of view are shown. Monolayer differentiations were repeated at least twice for the protocols shown. **(d)** Efficiency of (i) mES (ii) hES differentiation to TH+ neurons on PTC5000 beads measured by COPAS. Experiments were performed in duplicate wells (containing 4000 beads/well) and the proportion of beads bearing TH+ neurons in each well is plotted separately (light and dark histograms) and ranked according to the average proportion of positive beads. The bead number(s) and the corresponding deconvoluted protocol are listed below the histogram.

The foregoing experiments demonstrate that combinatorial cell culture can be used to discover protocols that generate diverse mouse cell types. Next, we asked if the technology can be applied to hES cells, and to this end devised an experiment to compare the differentiation of mouse and human ES cells towards a common endpoint: we performed a pair of more focused screens to compare known protocols and discover new methods to make dopaminergic neurons. These are lost in Parkinson's disease and their differentiation from ES cells has been studied intensively [3], [42]–[46]. We devised two 10,000-plex screening matrices comprising published neurogenic media (some known to promote generic neural differentiation, others more directed dopaminergic specification) and new formulations based on subtle variations thereof. The two matrices shared 4,536 common protocols (compare matrices in Tables S3 and S4 in File S1). Following combinatorial cell culture, beads were fixed and stained using fluorescent antibodies against the intracellular enzyme tyrosine hydroxylase (TH), a marker of dopaminergic neurons (Fig 6 a (i) and (ii)).

From the mES screen, a total of 622 hits (0.36% of monomeric beads) were sorted by COPAS, of which 399 (64% of hits) were deconvoluted. From the hES screen a total of 367 hits (0.13% of monomeric beads) were processed, from which we obtained complete tagging data for 279 (76%), Ariadne analysis identified 378 unique protocols for mES cell differentiation and 274 unique protocols for hES cell differentiation towards a TH+ phenotype (Fig. 6 b (i) and (ii); Data S3 and S4). Hierarchical clustering of the mES dataset identified several protocol clusters (Fig S13): two related clusters (59XX and 50XX) each comprised of 17 and 16 beads respectively and containing the triple hit (5951) [conditions 9 and 10 in split 2 both contain Sonic hedgehog (SHH) or its agonist purmorphamine], the cluster (15XX) comprised of 20 beads and the cluster (XX01) also comprised of 20 beads (Data S3). Analysis of the hES dataset also identified several clusters (Fig S14): cluster 9XX6 comprised of 12 beads and the related clusters 6XX6 and 8XX6 comprised of 10 beads each (media 6, 8 and 9 in the first split all contain TGF-β inhibitors) as well as cluster X77X comprised of 11 beads (condition 7 in both split 2 and 3 is DKK1). For each cell type we chose around 20 protocols for further study based on number of hits per protocol and/or clustering analysis. The protocols were validated using one or more of microculture (bead), embryoid body (EB) or monolayer culture systems, and a number were found to generate neurons expressing β-III tubulin and TH, though with varying efficiency in different cell culture systems (Fig 6c–d). These protocols were sensitive to cell density, which might explain the variability in performance across different systems. hES differentiation using the most efficient and consistent protocol in monolayer and EB cell culture systems (8976) was equivalent to the most efficient published method [44] and in both protocols differentiation is induced by TGF-β inhibitors and subsequent patterning is achieved using SHH. As in previous screens, the top protocol returned multiple (two) hits and included commonly occurring media combinations (the 8XX6 linkage present in ten hits (P_cluster_ = 0.06) and the XX76 linkage present in 11 hits (P_cluster_ = 0.014)).

Of the 4,536 identical protocols tested in the mES and hES cell screens, eight gave rise to hits in both screens, though none generated more than one hit per screen, nor was any particularly effective on either ES cell type when tested. Therefore the finding that mouse and human ES cells largely favor distinct media combinations for efficient generation of TH+ neurons implies differences in the optimal differentiation mechanism. For example, efficient ectodermal differentiation in both hES and mouse epiblast (mEPi) cells requires TGFβsignalling inhibitors, whereas mES cells form ectoderm simply upon LIF withdrawal [47]. Accordingly, we found the most efficient hES cell differentiation protocols (9736, 8976, 9706),contain the TGFβ inhibitors Noggin and SB431542 in stage 1 of differentiation, followed by SHH/FGF8b or DKK1 in stage 2, while the most efficient mES differentiation protocols (5951, 5099, 5929) feature RHB-A medium (media 5) [48], [49] in stage 1 and SHH (or purmorphamine) and FGF8b in stage 2. Protocols featuring TGFβ inhibitors followed by SHH have been previously shown to be a particularly effective in producing TH+ neurons from hES cells [44], demonstrating that combinatorial cell culture identifies optimal media combinations. The results also suggest that DKK1 [conditions 7 in splits 2 and 3] (a repressor of Wnt signalling that facilitates the activity of SHH [50]) can be used as an efficient inducer of TH+ cell differentiation, though its effects in mouse development are known [51] it is seldom used to differentiate hES cells into DA neurons *in vitro*.

## Conclusion

In this paper we describe a new cell-based, high throughput combinatorial screening technology and apply it to discover stem cell differentiation protocols to generate pre-determined cell types, as well as a novel developmental intermediate cell type. The protocols we discovered are robust and efficient, result in relatively high yields of differentiated cells with predetermined characteristics and are readily adapted to various cell culture systems (microculture, monolayer, aggregates, semisolid media). Our method is simple and convenient, does not require specialised equipment and benefits from significant savings in time, labor and reagent costs.

Combinatorial cell culture is a powerful empirical ‘search engine’ that can be used to discover completely new methods for stem cell differentiation, as well as to optimize or further develop existing protocols, for example by varying the input cell type (e.g. iPS [52], [53] cells, adult stem cells, stable mesenchymal [54], [55] or neuroepithelial [56], [57] intermediates, or differentiated cultures derived by previously determined protocols comprising growth factors or small molecules [19], [58]), the cell culture media components or their concentration, the timing of media changes and many other factors important in cell culture. It can be used to devise protocols that are faster, more productive or cost-effective; protocols that generate higher quality cells, eliminate undefined components such as serum or substitute growth factors with small molecules. In addition, the method can be used to investigate developmental biology, provide in vitro models for the differentiation of human embryos, study species- or cell line- specific differences in differentiation, or identify and generate rare or transient developmental intermediates for further applications.

Here we have applied the technology exclusively to stem cell differentiation, however we expect it will find numerous other applications in diverse areas of cell biology where a more dynamic cell culture approach would better mimic the physiological environment of cells.

## Supporting Information

Figure S1 **Schematic diagram illustrating CombiCult technology.**
(TIF)

Figure S2 **Tag binding is not biased to any specific species within a size group.** **a.** Flow cytometry plot of a mixture of 10 different tag types within a group as a control. **b.** Flow cytometry plot of a digested bead tagged with 5 different tag types of the same size. **c.** Bar chart showing the average proportion of each tag type found in a group of 20 random beads.
(TIF)

Figure S3 **Controls for cell survival and efficiency of differentiation for the macrophage screen. a**. Cell survival. (i) Aliquot of pool of beads subjected to pHrodo assay showing one positive bead in a background of negative beads. (ii). The exact same field of view as (i) stained with calcein green to show that most beads have live cells, but are not positive for pHrodo. Scale bar 100 µm. **b.** Photomicrographs showing stitched images from a whole 48 well scan performed with the Nikon Eclipse 2000 with a 4X objective. (i). Beads seeded with mES cells (46C-Sox1-GFP) and cultured for 2 weeks in negative control media then subjected to pHrodo assay. (ii). Beads seeded with mES cells (46C-Sox1-GFP) and cultured for 2 weeks in positive control media then subjected to pHrodo assay. (iii) Beads seeded with mES cells (46C-Sox1-GFP) and cultured for 2 weeks in media for protocol (0145) then subjected to pHrodo assay.
(TIF)

Figure S4 **Large particle sorter COPAS, single colour positive control plots for gating strategy. a.** GFP (green) single colour positive control cells on beads. (i) Dot plot of the GFP positive cells on beads gated for bead size on single events. (ii) Dot plot of the population gated on size separated by red vs. green fluorescence intensity showing the position of the GFP (green) positive events. **b.** pHrodo (red) single colour positive control cells on beads. (i) Dot plot of the pHrodo positive cells beads gated for bead size on single events. (ii) Dot plot of the population gated on size separated by red vs. green fluorescence intensity showing the position of the pHrodo (red) positive events.
(TIF)

Figure S5 **Dendrograms illustrating validated protocols (magenta) and related protocols or media combinations.** (grey). The probability of an event occurring by chance is noted when probability (P)≤0.5. Protocols were scored qualitatively (−, +, ++, +++) to indicate efficiency of differentiation during validation experiments relative to other protocols tested in the same cell culture system. **(a)**–**(c)** Dendrograms derived from the mES/phagocytes screen showing protocols for differentiation to phagocytes validated in beads, CFCs MethoCult and preB MethoCult culture systems.
(PDF)

Figure S6 **Dendrograms illustrating validated protocols (magenta) and related protocols or media combinations (grey).** The probability of an event occurring by chance is noted when probability (P)≤0.5. Protocols were scored qualitatively (−, +, ++, +++) to indicate efficiency of differentiation during validation experiments relative to other protocols tested in the same cell culture system. **(a)**–**(c)** Dendrograms from mES/neuroectoderm screen showing protocols for differentiation to neuroectoderm validated on beads.
(PDF)

Figure S7 **Efficiency of mES cell differentiation to phagocytes on FACTIII beads. Histograms show the number of red fluorescent colonies per cm^2^ generated by each protocol as calculated by image analysis.** Experiments were performed in duplicate wells and results are plotted in the same order as in Fig. 1h which shows differentiation on PTC5000 beads. Hit/bead numbers and protocols below the histograms show whether they were deconvoluted from phagocyte- (red), neuroectoderm- (green) or phagocyte/neuroectoderm- (orange) bearing hits, or represent the two negative control protocols with the highest and lowest efficiency for phagocyte differentiation (black). Generation of phagocytes on. FACT III beads is generally more efficient and the ranking of individual protocols differs, nevertheless protocols that produced double and triple hits are the most efficient in both systems.
(TIF)

Figure S8 **mES cells differentiated with cluster B protocols, were stained with CD11b and flow sorted into positive and negative populations.** Both populations were then plated and subjected to a pHrodo phagocytosis assay (red) and subsequently stained with calcein green. **a**. Flow plots of the (i) isotype control and (ii) the sorted populations. **b**. Photomicrographs of CD11b positive cells stained for pHrodo (red) and calcein (green)- 20X magnification scale bars correspond to 20 µm. **c**. Photomicrographs of CD11b positive cells stained for pHrodo (red) and calcein (green)- 40X magnification scale bars correspond to 20 µm.
(TIF)

Figure S9 **Gating strategy and isotype controls for Ter119-single cells sorted for CD43^(low/-)^ CD5^+^CD3^+^B220^+^CD45^-^CD19^-^** **expression.**
(TIF)

Figure S10 **Characterisation of early B1 cells in day 10 embryo caudal part for expression of Flt3 (CD135) and IL7Ra (CD127).** Majority of live B1a cells (7AAD-Ter119-CD45-CD43-CD19-CD5+CD3+B220+)  express Flt3 and IL7ra.
(TIF)

Figure S11 **Phagocyte maturation in OP9 and Methocult after differentiation using cluster A protocols with chemical substitution. a.** Dot plots illustrating flow cytometry analysis of cells obtained after maturation in IL7 supplemented Methocult for 2 weeks, showing the appearance of cells co-expressing B220/CD43 and B220/CD19. **b.** Bar chart showing the proportions of different cell populations present in Methocult or OP9 derived cultures, measured by flow cytometry analysis. Blue bars represent cells taken off the beads at day 15 (prior to maturation), red bars represent cells isolated at D15 and cultured for a further 2 weeks on OP9 feeder layers and green bars represent cells cultured in Methocult for 2 weeks. Error bars depict the standard deviation of 3 biological repeats. * There is a statistically significant difference between the D15 cells and those cultured in Methocult for 2 weeks.
(TIF)

Figure S12 **Schematic diagram illustrating design of the tyrosine hydroxylase (TH) neuron CombiCult screen.** Ten different cell culture media were tested in each of 4 stages of differentiation, by split-pool passaging, cells seeded onto beads sample differentiation media spiked with tags on days D1, D7, D14 and D21. On day 28 beads were assayed to identify those bearing tyrosine hydroxylase (TH) positive cells. A total of 300,000 beads were used in the experiment to test 10,000 protocols so that on average each protocol is sampled by 30 beads.
(TIF)

Figure S13 **Dendrograms illustrating validated protocols (magenta) and related protocols or media combinations.** (grey) for the mES TH screen. The probability of an event occurring by chance is noted when P≤0.5. Protocols were scored qualitatively (−, +, ++, +++) to indicate efficiency of differentiation during validation experiments relative to other protocols tested in the same cell culture system. **(a)**–**(d)** Dendrograms from Experiment 3 (mES/TH+) showing protocols for differentiation to TH+ neurons validated using bead, monolayer culture and EB culture systems.
(PDF)

Figure S14 **Dendrograms illustrating validated protocols (magenta) and related protocols or media combinations (grey) for the hES TH screen.** The probability of an event occurring by chance is noted when P≤0.5. Protocols were scored qualitatively (−, +, ++, +++) to indicate efficiency of differentiation during validation experiments relative to other protocols tested in the same cell culture system. **(a)**–**(d)** Dendrograms from Experiment 4 (hES/TH+) showing protocols for differentiation to TH+ neurons validated using bead and monolayer culture systems.
(PDF)

File S1 **Tables S1–S4.** Table S1. List of components for media used in experiments 1 and 2: Screen for hematopoietic phagocytes and neural precursors. Table S2. List of components for media used in experiment 3: Chemical screen for hematopoietic phagocytes. Table S3. List of components for media used in experiment 4: Screen for TH positive neurons from mES cells. Table S4. List of components for media used in experiment 5: Screen for TH positive neurons from hES cells. List of references from which media recipes were derived.
(PDF)

Data S1 **Ariadne report for CombiCult screen 1: hematopoietic phagocytes from mES cells.**
(PDF)

Data S2 **Ariadne report for CombiCult screen 2: neuroectodermal precursors from mES cells.**
(PDF)

Data S3 **Ariadne report for CombiCult screen 3: TH positive neurons from mES cells.**
(PDF)

Data S4 **Ariadne report for CombiCult screen 4: TH positive neurons from hES cells.**
(PDF)

Movie S1 **This movie is an animation explaining CombiCult technology. (Quicktime).**
(MOV)
