# Supplementary figures and images for: Directed Differentiation of Embryonic Stem Cells Using a Bead-Based Combinatorial Screening Method
Source: PLoS One. 2014 Sep 24;9(9):e104301. doi: 10.1371/journal.pone.0104301 (PMC4174505; doi:10.1371/journal.pone.0104301)

### Figure S5

(a) mES/phagocytes - Dendrogram 1

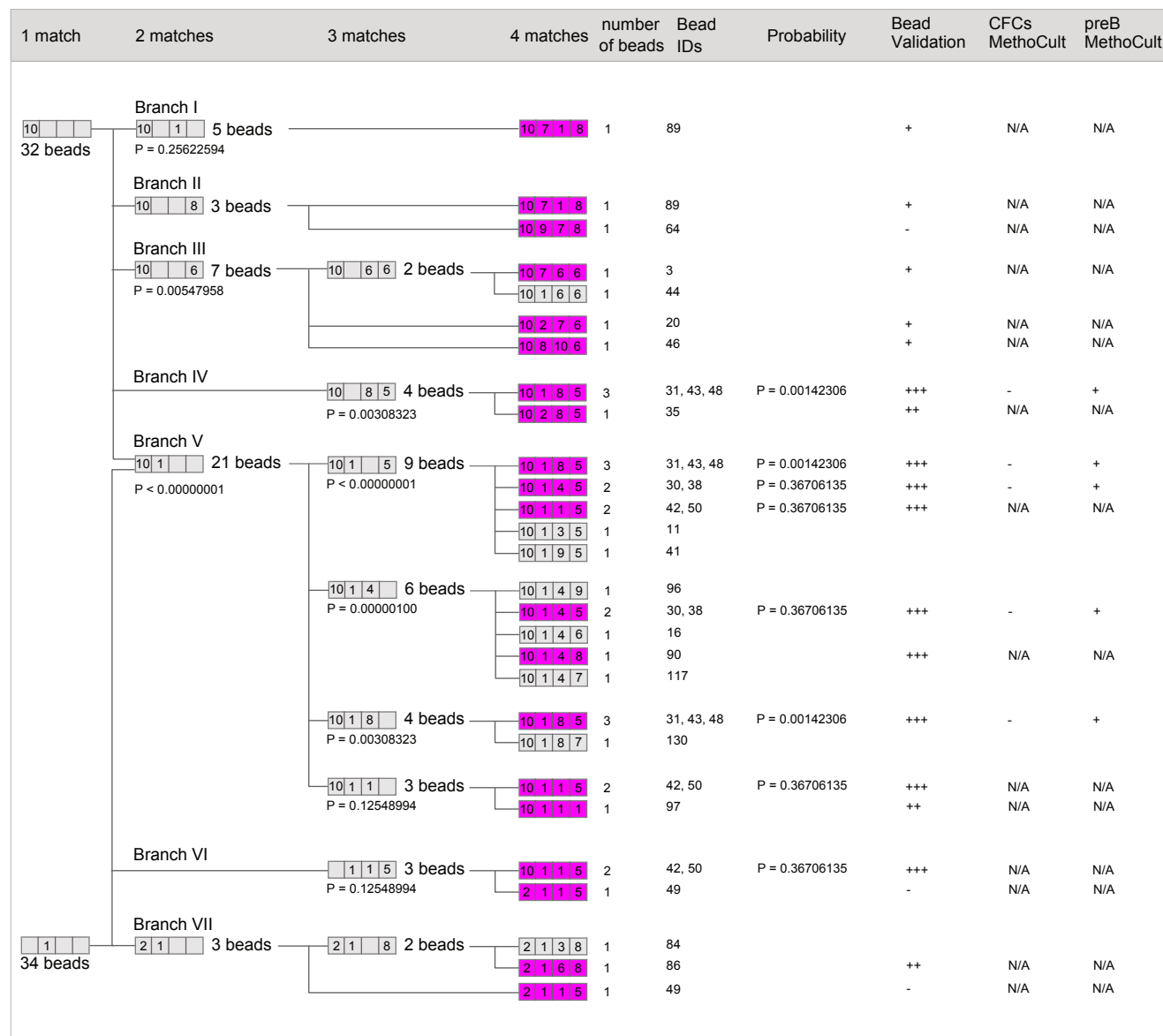

b) mES/phagocytes - Dendrogram 2

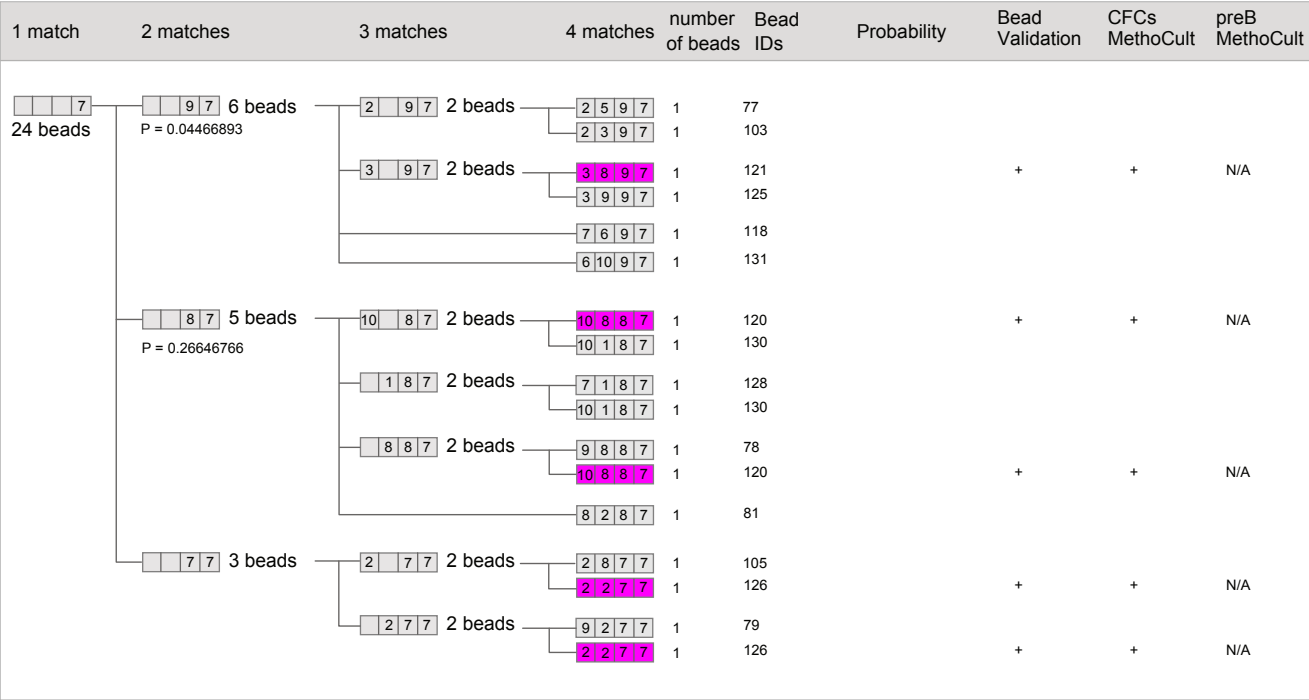

c) mES/phagocytes - Dendrogram 3

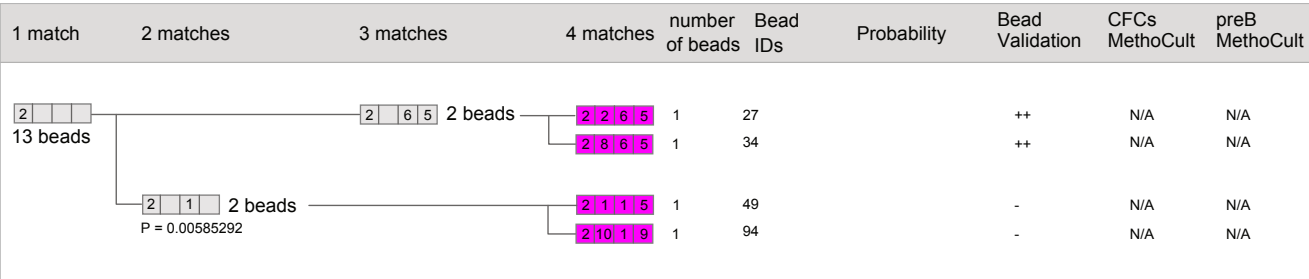

Supplement: Figure S5 — Dendrograms illustrating validated protocols (magenta) and related protocols or media combinations. (grey). The probability of an event occurring by chance is noted when probability (P)≤0.5. Protocols were scored qualitatively (−, +, ++, +++) to indicate efficiency of differentiation during validation experiments relative to other protocols tested in the same cell culture system. (a)–(c) Dendrograms derived from the mES/phagocytes screen showing protocols for differentiation to phagocytes validated in beads, CFCs MethoCult and preB MethoCult culture systems. (PDF) [file pone.0104301.s005.pdf]
