# Supplementary material for: Directed Differentiation of Embryonic Stem Cells Using a Bead-Based Combinatorial Screening Method
Source: PLoS One. 2014 Sep 24;9(9):e104301. doi: 10.1371/journal.pone.0104301 (PMC4174505; doi:10.1371/journal.pone.0104301)

Figure S6.

a) mES/neuroectoderm - Dendrogram 1

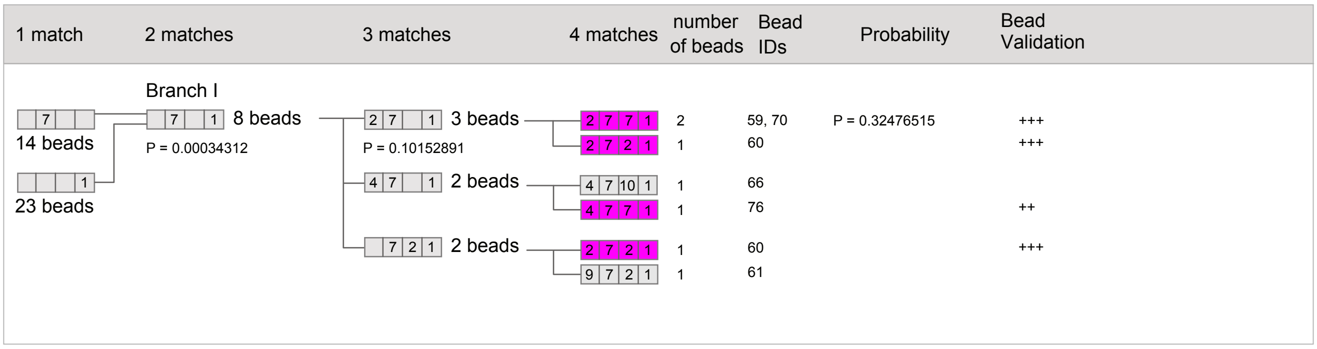

b) mES/neuroectoderm - Dendrogram 2

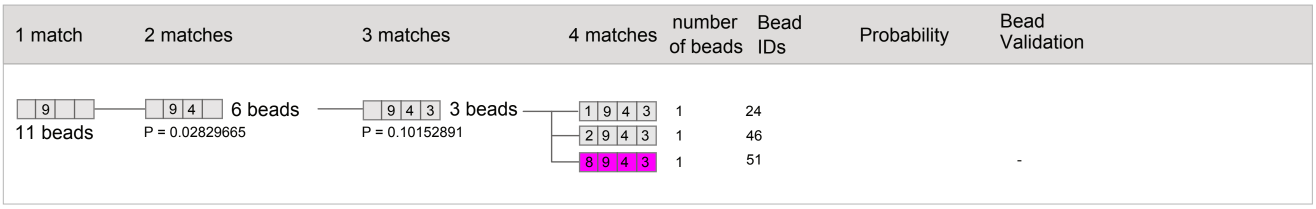

c) mES/neuroectoderm - Dendrogram 3

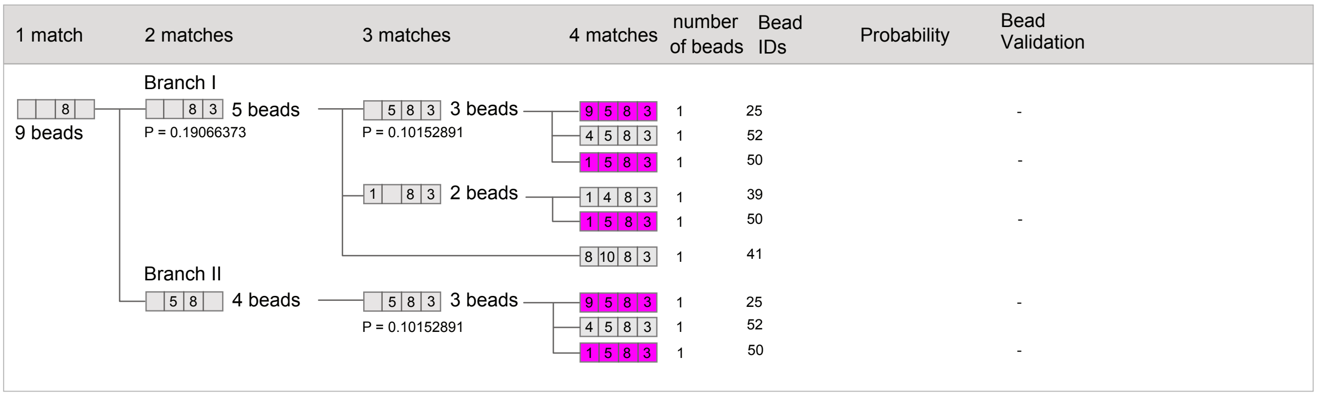

Supplement: Figure S6 — Dendrograms illustrating validated protocols (magenta) and related protocols or media combinations (grey). The probability of an event occurring by chance is noted when probability (P)≤0.5. Protocols were scored qualitatively (−, +, ++, +++) to indicate efficiency of differentiation during validation experiments relative to other protocols tested in the same cell culture system. (a)–(c) Dendrograms from mES/neuroectoderm screen showing protocols for differentiation to neuroectoderm validated on beads. (PDF) [file pone.0104301.s006.pdf]
