# Supplementary material for: Directed Differentiation of Embryonic Stem Cells Using a Bead-Based Combinatorial Screening Method
Source: PLoS One. 2014 Sep 24;9(9):e104301. doi: 10.1371/journal.pone.0104301 (PMC4174505; doi:10.1371/journal.pone.0104301)
Supplement: Figure S13 — Dendrograms illustrating validated protocols (magenta) and related protocols or media combinations. (grey) for the mES TH screen. The probability of an event occurring by chance is noted when P≤0.5. Protocols were scored qualitatively (−, +, ++, +++) to indicate efficiency of differentiation during validation experiments relative to other protocols tested in the same cell culture system. (a)–(d) Dendrograms from Experiment 3 (mES/TH+) showing protocols for differentiation to TH+ neurons validated using bead, monolayer culture and EB culture systems. (PDF) [file pone.0104301.s013.pdf]

Figure S13

a) Experiment 3 (mES/TH+) - Dendrogram 1

1 match	2 matches	3 matches	4 matches	number of beads	Bead IDs	Probability	Bead Validation	2D-Culture Validation	EB Validation
– – – 1 131 beads	Branch I – – 10 1 20 beads P = 0.00000079	– 1 10 1 3 beads	**3 1 10 1**	2	48, 159		+	N/A	N/A
			5 1 10 1	1	140				
		9 – 10 1 3 beads	9 3 10 1	1	78				
			**9 2 10 1**	1	113		-	N/A	N/A
			**9 10 10 1**	1	172		-	N/A	N/A
		5 – 10 1 3 beads	5 7 10 1	1	65				
			**5 9 10 1**	1	71		+	+	+
			5 1 10 1	1	140				
	Branch II 9 – – 1 20 beads P = 0.00000079	9 4 – 1 3 beads	**9 4 6 1**	2	81, 108		-	N/A	N/A
			9 4 2 1	1	21				
		9 9 – 1 3 beads	**9 9 5 1**	1	1		-	+	++
			9 9 4 1	1	70				
			9 9 9 1	1	75				
	Branch III – 6 – 1 18 beads P = 0.00001877	4 6 – 1 7 beads P = 0.00021298	**4 6 7 1**	2	77, 139		+++	+	+++
			4 6 9 1	1	18				
			4 6 2 1	1	37				
			4 6 6 1	1	83				
			4 6 5 1	1	175				
			4 6 1 1	1	178				
		– 6 7 1 6 beads P = 0.00385010	**4 6 7 1**	2	77,139		+++	+	+++
			7 6 7 1	1	5				
			10 6 7 1	1	32				
			9 6 7 1	1	55				
			1 6 7 1	1	136				
	Branch IV – – 5 1 18 beads P = 0.00001877	5 – 5 1 7 beads P = 0.0002198	**5 9 5 1**	3	126, 135, 173	P = 0.09710944	+++	+++	++
			**5 10 5 1**	2	46,180		++	N/A	N/A
			**5 4 5 1**	1	40		+++	+++	-
			5 5 5 1	1	167				
		– 9 5 1 5 beads	**5 9 5 1**	3	126, 135, 173	P = 0.09710944	+++	+++	++
			**9 9 5 1**	1	1		-	++	+
			**4 9 5 1**	1	183		-	N/A	N/A
	Branch V – 8 – 1 12 beads P = 0.08034176	– 8 1 1 4 beads	**6 8 1 1**	3	8, 128, 134	P = 0.09710944	+++	+++	+++
			10 8 1 1	1	97				
	Branch VI – 9 – 1 17 beads P = 0.00008932	5 9 – 1 6 beads P = 0.00385010	**5 9 5 1**	3	126, 135, 173	P = 0.09710944	+++	+++	++
			**5 9 9 1**	1	26		++	N/A	N/A
			**5 9 10 1**	1	71		+	+	+
			**5 9 2 1**	1	121		+	++	+++
		– 9 5 1 5 beads	**5 9 5 1**	3	126, 135, 173	P = 0.09710944	+++	+++	++
			**9 9 5 1**	1	1		-	+	++
			**4 9 5 1**	1	183		-	N/A	N/A
		– 9 9 1 3 beads	4 9 9 1	1	4				
			**5 9 9 1**	1	26		++	N/A	N/A
			9 9 9 1	1	75				
		– 9 1 1 2 beads	3 9 1 1	1	68				
			**6 9 1 1**	1	163		+	+	+
	Branch VII – 10 – 1 18 beads P = 0.00001877	5 10 – 1 3 beads	**5 10 5 1**	2	46, 180		++	N/A	N/A
			**5 10 6 1**	1	141		++	N/A	+
	Branch VIII – 5 – 1 16 beads P = 0.0039853	1 5 – 1 5 beads	**1 5 2 1**	2	166, 171		++	N/A	N/A
			1 5 1 1	1	66				
			1 5 9 1	1	101				
			1 5 7 1	1	174				

b) Experiment 3 (mES/TH+) - Dendrogram 2

1 match	2 matches	3 matches	4 matches	number of beads	Bead IDs	Probability	Bead Validation	2D-Culture Validation	EB Validation
_ _ _ 9 75 beads	Branch I _ 9 _ 9 14 beads P = 0.00658674	_ 9 2 9 6 beads P = 0.00385010	**7 9 2 9**	2	438, 517		-	N/A	+
			8 9 2 9	1	430				
			6 9 2 9	1	481				
			**5 9 2 9**	1	488		++	+	+++
			3 9 2 9	1	497				
	Branch II _ 5 _ 9 12 beads P = 0.08034176		**1 5 2 9**	1	443		+++	N/A	-
			1 5 1 9	1	430				
	Branch III _ _ 9 9 11 beads P = 0.23544554	_ 10 9 9 3 beads	**5 10 9 9**	2	476, 508		+++	+	++
			3 10 9 9	1	441				

c) Experiment 3 (mES/TH+) - Dendrogram 3

1 match	2 matches	3 matches	4 matches	number of beads	Bead IDs	Probability	Bead Validation	2D-Culture Validation	EB Validation
	Branch I 1 5 _ _ 21 beads P = 0.00000018	1 5 2 _ 7 beads P = 0.00021298	**1 5 2 1**	2	166, 171		++	N/A	N/A
			**1 5 2 4**	2	284, 299		++	N/A	N/A
			1 5 2 2	1	204				
			1 5 2 5	1	372				
			**1 5 2 9**	1	443		+++	N/A	-
		1 5 _ 1 5 beads	**1 5 2 1**	2	166, 171		++	N/A	N/A
			1 5 1 1	1	66				
			1 5 9 1	1	101				
			1 5 7 1	1	174				

d) Experiment 3 (mES/TH+) - Dendrogram 4

1 match	2 matches	3 matches	4 matches	number of beads	Bead IDs	Probability	Bead Validation	2D-Culture Validation	EB Validation
5 _ _ _ 54 beads	Branch I 5 9 _ _ 19 beads P = 0.00000373	5 9 2 _ 4 beads	**5 9 2 9**	1	488		++	+	+++
			5 9 2 3	1	271				
			5 9 2 2	1	198				
			**5 9 2 1**	1	121		+	++	+++
	Branch II 5 10 _ _ 16 beads P = 0.00039853		**5 10 5 1**	2	46, 180		++	N/A	N/A
		5 10 6 _ 3 beads	**5 10 6 1**	1	141		++	N/A	+
			5 10 6 4	1	294				
			5 10 6 5	1	373				
		5 10 9 _ 4 beads	5 10 9 3	2	262, 278				
			**5 10 9 9**	2	476, 508		+++	+	++
		5 10 _ 2 4 beads	**5 10 2 2**	1	186		+	N/A	+
			5 10 3 2	1	208				
			5 10 7 2	1	212				
			5 10 10 2	1	252				
