# Supplementary material for: Directed Differentiation of Embryonic Stem Cells Using a Bead-Based Combinatorial Screening Method
Source: PLoS One. 2014 Sep 24;9(9):e104301. doi: 10.1371/journal.pone.0104301 (PMC4174505; doi:10.1371/journal.pone.0104301)
Supplement: Figure S14 — Dendrograms illustrating validated protocols (magenta) and related protocols or media combinations (grey) for the hES TH screen. The probability of an event occurring by chance is noted when P≤0.5. Protocols were scored qualitatively (−, +, ++, +++) to indicate efficiency of differentiation during validation experiments relative to other protocols tested in the same cell culture system. (a)–(d) Dendrograms from Experiment 4 (hES/TH+) showing protocols for differentiation to TH+ neurons validated using bead and monolayer culture systems. (PDF) [file pone.0104301.s014.pdf]

Figure S14

a) Experiment 4 (hES/TH+) - Dendrogram 1

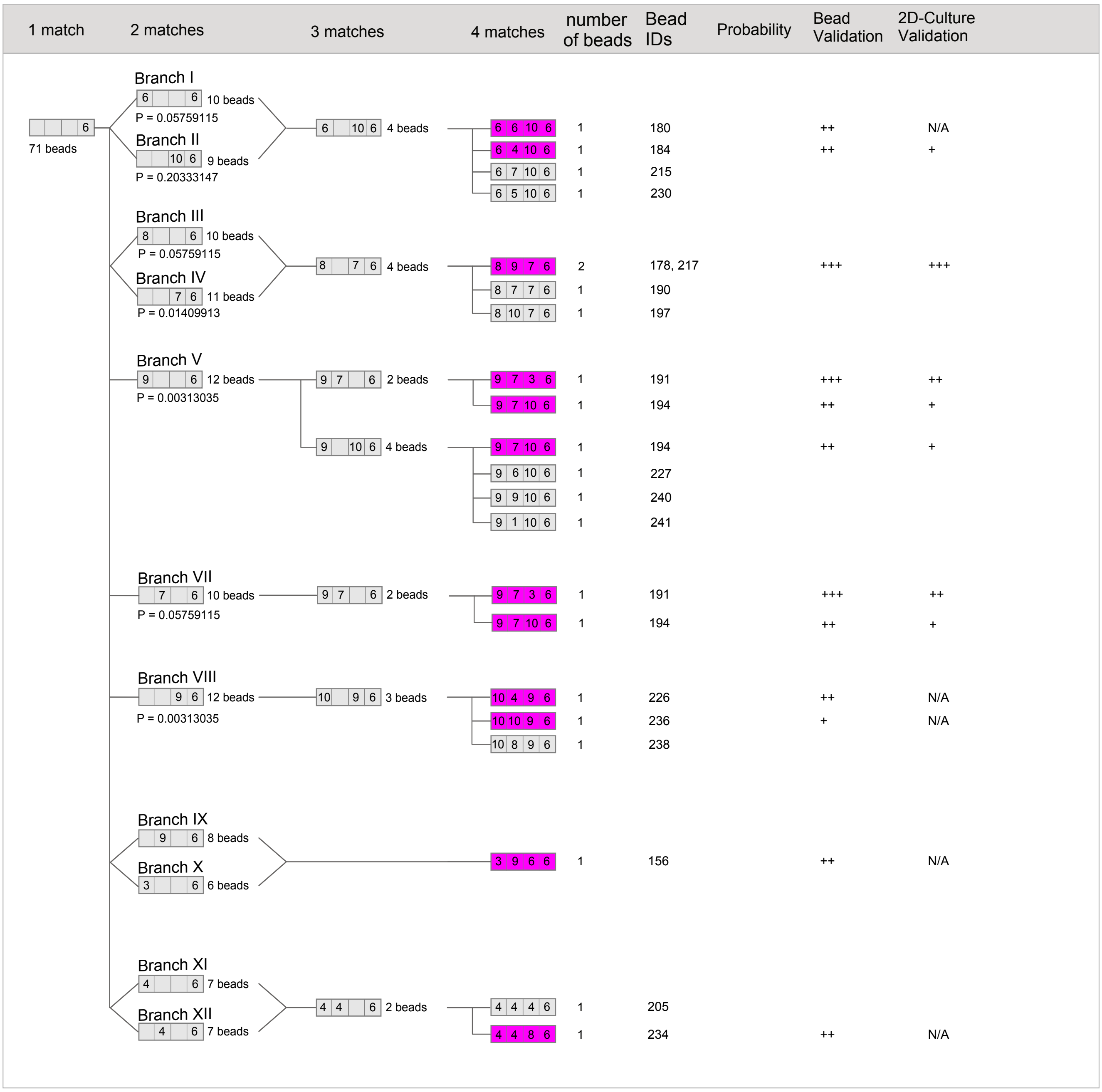

b) Experiment 4 (hES/TH+) - Dendrogram 2

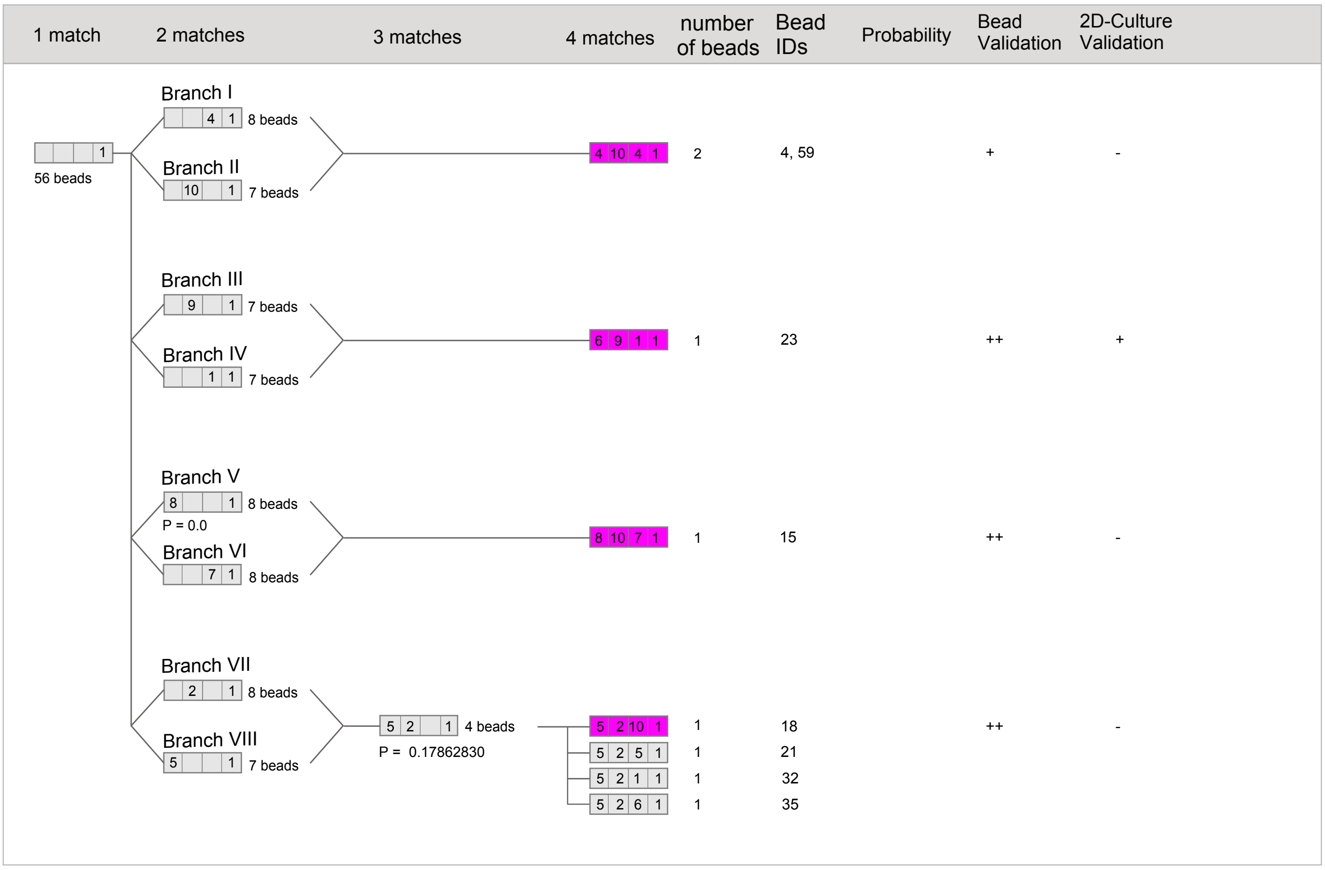

c) Experiment 4 (hES/TH+) - Dendrogram 3

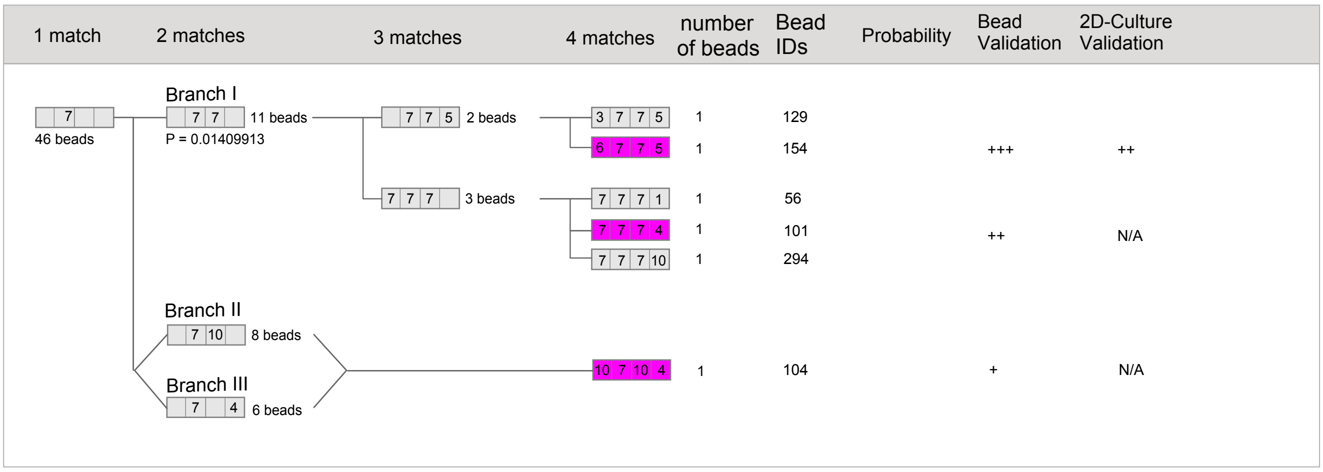

d) Experiment 4 (hES/TH+) - Dendrogram 4

1 match	2 matches	3 matches	4 matches	number of beads	Bead IDs	Probability	Bead Validation	2D-Culture Validation
	Branch I: – – 6 4 (6 beads)	– 9 6 4 (3 beads)	**7 9 6 4**	2	91, 95		++	+
			5 9 6 4	1	121			
	Branch II: 8 6 – – (6 beads); Branch III: 8 – 3 – (6 beads)	8 6 3 – (2 beads)	**8 6 3 7**	1	172		++	n/a
			8 6 3 1	1	24			
	Branch IV: – 6 7 – (7 beads); Branch V: – – 7 5 (7 beads)	– 6 7 5 (3 beads)	**5 6 7 5**	2	134, 136		++	-
			1 6 7 5	1	144			
	Branch VI: 2 – – 4 (5 beads)	2 5 – 4 (3 beads)	**2 5 10 4**	2	99, 100		+	-
			2 5 7 4	1	112			
	Branch VII: – 4 9 – (8 beads); Branch VIII: – 4 – 5 (7 beads)	– 4 9 5 (3 beads)	**5 4 9 5**	1	124		+	n/a
			3 4 9 5	1	135			
			10 4 9 5	1	149			
