# Supplementary material for: Directed Differentiation of Embryonic Stem Cells Using a Bead-Based Combinatorial Screening Method
Source: PLoS One. 2014 Sep 24;9(9):e104301. doi: 10.1371/journal.pone.0104301 (PMC4174505; doi:10.1371/journal.pone.0104301)
Supplement: File S1 — Tables S1–S4. Table S1. List of components for media used in experiments 1 and 2: Screen for hematopoietic phagocytes and neural precursors. Table S2. List of components for media used in experiment 3: Chemical screen for hematopoietic phagocytes. Table S3. List of components for media used in experiment 4: Screen for TH positive neurons from mES cells. Table S4. List of components for media used in experiment 5: Screen for TH positive neurons from hES cells. List of references from which media recipes were derived. (PDF) [file pone.0104301.s015.pdf]

Table S1

Experiments 1 and 2: Screen for hematopoietic phagocytes and neural precursors					
**Tag**	**Stage**	**Media**	**Basal Media**	**Supplements**	**Reference**
SR1	1	1	DMEM	1X N2	[1]
SR2	1	2	DMEM/F12 (50%)<br>Neurobasal (50%)	0.5X N2, 0.5X B27	[2]
SR3	1	3	Advanced DMEM/F12		[3]
SR4	1	4	RPMI	1X B27, 50ng/mL Activin A , 1 mM Sodium Butyrate	[4]
SR5	1	5	RPMI	100ng/mL Activin A , 25ng/mL Wnt 3A	[5]
SR6	1	6	RPMI	0.5mg/mL Albumun, 100 ng/mL Activin A	[6]
SR7	1	7	STEMLINE	50 ng/mL BMP4, 50ng/ml VEGF	[7]
SR8	1	8	IMDM (75%)<br>HAM F12 (25%)	1X N2 , 1X B27, 0.05% BSA, 0.5mM ascorbic acid	[8]
SR9	1	9	GMEM	5% KSR, 1ng/mL BMP4	[9]
SR10	1	10	KO-DMEM	15% FCS, 0.1% LIF	
MR1	2	1	DMEM/F12	1% ITS, 0.05µM RA	[10]
MR2	2	2	DMEM/F12 (50%)<br>Neurobasal (50%)	0.5X N2, 0.5X B27, 2ng/mL FGF4	[11]
MR3	2	3	DMEM/F12	1X ITS, 1X B27, 4ng/mL FGF2, 50ng/mL DKK1	
MR4	2	4	RPMI	1X B27, 50ng/mL Activin A, 0.5mM Sodium Butyrate	[4]
MR5	2	5	RPMI	100ng/mL Activin A, 25ng/mL Wnt 3A	[5]
MR6	2	6	RPMI	1X ITS, 0.5mg/mL BSA, 100 ng/mL Activin A	[6]
MR7	2	7	STEMLINE	20ng/mL SCF	
MR8	2	8	IMDM (75%)<br>HAM F12 (25%)	1% N2, 1% B27, 0.05% BSA, 0.5 mM ascorbic acid	[8]
MR9	2	9	GMEM	5% KSR, 2ng/mL TGFb , 10ng/mL BMP2	[12]
MR10	2	10	Advanced DMEM/F12		[3]
LR1	3	1	DMEM/F12	1X B27, 20ng/mL EGF, 20ng/mL bFGF, 20ng/mL PDGF AA	[13]
LR2	3	2	DMEM/F12 (50%)<br>Neurobasal (50%)	0.5X N2, 0.5X B27, 20ng/mL bFGF, 100ng/mL FGF8, 400ng/mL SSH	[11]
LR3	3	3	DMEM/F12	1X ITS, 1X B27, 4ng/mL FGF2, 50ng/mL DKK1	
LR4	3	4	RPMI	1X B27, 20ng/mL EGF, 2ng/mL bFGF , 100ng/mL noggin	[4]
LR5	3	5	DMEM	1X B27, 2 µM RA, 50ng/mL FGF10, 0.2µM CYC	[5]
LR6	3	6	HCM	30ng/mL FGF4, 20ng/mL BMP2	[6]
LR7	3	7	STEMLINE	20ng/mL SCF, 5ng/mL BMP2, 5ng/mL TGFb , 20ng/mL TPO	[14]
LR8	3	8	DMEM	1XITS, 1mg/mL BSA, 50ng/mL PDGF BB	[15]
LR9	3	9	GMEM	5% KSR, 2ng/mL TGFb, 10ng/mL BMP2, 1µg/mL Insulin, 50µg/mL Ascorbic acid	[12]
LR10	3	10	DMEM Low Glucose	1XITS, 5µg/mL Fibronectin	[16]
	4	1	DMEM/F12	1X B27, 20ng/mL PDGF AA	[13]
	4	2	DMEM/F12 (50%)<br>Neurobasal (50%)	0.5X N2, 0.5X B27, 20ng/mL bFGF, 100ng/mL FGF8, 400ng/mL SSH	[11]
	4	3	DMEM/F12	1X N2, 1X B27, 5ng/mL FGF2, 10ng/mL DKK1, 10ng/mL noggin, 10ng/mL IGF1	[17]
	4	4	DMEM	1X B27, 2 µM RA, 50ng/mL FGF10, 0.2µM CYC	[5]
	4	5	DMEM	1X B27, 100ng/mL FGFa	[18]
	4	6	HCM	20ng/ml HGF	[6]
	4	7	STEMLINE	30ng/mL IL3, 20ng/mL IL6, 20ng/mL TPO	[7]
	4	8	DMEM	1X ITS, 1mg/mL BSA , 50ng/mL PDGF BB	[15]
	4	9	GMEM	5% KSR, 2ng/mL TGFb, 10ng/mL BMP2, 1µg/mL Insulin, 50µg/mL Ascorbic acid	[12]
	4	10	Advanced DMEM/F12		[5]

Table S2

Experiment 3: Chemical screen				
**Tag**	**Stage**	**Media**	**Basal Media**	**Supplements**
SR1	3	1	DMEM, 1 % ITS	1μM Wnt agonist, 1μM Purmorphamine, 1nM FTY720-1, 0.05μM *trans*-retinoic acid
SR2	3	2	DMEM, 1 % ITS	250μM 8-Bromo-cAMP, 120nM GPR30 agonist, 6μM Scriptaid, 0.2μM Progesterone
SR3	3	3	DMEM, 1 % ITS	1μM TWS119, 100μM 8-pCPT-cGMP (TEA), 500nM DITPA
SR4	3	4	DMEM, 1 % ITS	20mM LiCl, 1μM HDAC inhibitor III, 1nM FTY720-1, 200μM ascorbic acid
SR5	3	5	DMEM, 1 % ITS	2μM SIS3, 100μM 8-pCPT-cGMP (TEA), 2mM Metformin, 500nM T3
SR6	3	6	DMEM, 1 % ITS	15μM Kenpaullone, 50nM GPR40 agonist, 10μM Y-27632
SR7	3	7	DMEM, 1 % ITS	5μM I3M, 2nM γ-Secretase inhibitor XX, 500nM Compound P
SR8	3	8	DMEM, 1 % ITS	1μM TWS119, 1μM SAG, 50nM TTNPB (RAR agonist), 500nM Compound P, 500nM T3
SR9	3	9	DMEM, 1 % ITS	1μM Wnt agonist
SR10	3	10	DMEM, 1 % ITS	1μM TWS1119
MR1	3	11	DMEM, 1 % ITS	1μM Wnt agonist, 1μM Purmorphamine
MR2	3	12	DMEM, 1 % ITS	1μM Purmorphamine
MR3	3	13	DMEM, 1 % ITS	1μM SAG
MR4	3	14	DMEM, 1 % ITS	2mM Metformin
MR5	3	15	DMEM, 1 % ITS	20nM PMA
MR6	3	16	DMEM, 1 % ITS	1μM TWS1119, 1μM SAG
MR7	3	17	DMEM, 1 % ITS	50nM Caspase-3 inhibitor VII
MR8	3	18	DMEM, 1 % ITS	1μM Asiaticoside
MR9	3	19	DMEM, 1 % ITS	6μM Scriptaid
MR10	3	20	DMEM, 1 % ITS	1μM Asiaticoside, 50nM TTNPB (RAR agonist)
LR1	3	21	DMEM, 1 % ITS	6μM Scriptaid, 50nM TTNPB (RAR agonist), 10μM Y-27632
LR2	3	22	DMEM, 1 % ITS	10nM VEGF inducer GS4012
LR3	3	23	DMEM, 1 % ITS	1μM PD 168393, 10μM Isoproterenol, 0.2μM Progesterone
LR4	3	24	DMEM, 1 % ITS	1mM Sodium Orthovanadate, 5μM SU5402, 10μM Pifithrin-a
LR5	3	25	DMEM, 1 % ITS	500nM Compound P, 20mM Inosine
LR6	3	26	DMEM, 1 % ITS	20μM 15d-PGJ2, 10μM DAQ B1
LR7	3	27	DMEM, 1 % ITS	10μM Prostaglandin D2
LR8	3	28	DMEM, 1 % ITS	1nM FTY20-1
LR9	3	29	DMEM, 1 % ITS	500nM DITPA, 50μM Capsaicin, 200μM Ascorbic acid
LR10	3	30	DMEM, 1 % ITS	
	4	1	DMEM, 1 % ITS	500nM Compound P
	4	2	DMEM, 1 % ITS	500nM Compound P, 0.05μM *trans*-retinoic acid, 25μM Compound C
	4	3	DMEM, 1 % ITS	0.05μM *trans*-retinoic acid, 10nM VEGF inducer GS4012
	4	4	DMEM, 1 % ITS	2nM γ-Secretase inhibitor XX
	4	5	DMEM, 1 % ITS	2nM γ-Secretase inhibitor XX, 1μM Wnt agonist
	4	6	DMEM, 1 % ITS	50nM TTNPB (RAR agonist)
	4	7	DMEM, 1 % ITS	5μM I3M, 2nM γ-Secretase inhibitor XX, 500nM Compound P
	4	8	DMEM, 1 % ITS	5μM Ciglitazone
	4	9	DMEM, 1 % ITS	10μM Hydroquinone
	4	10	DMEM, 1 % ITS	10μM Hydroquinone, 1nM FTY720-1
	4	11	DMEM, 1 % ITS	3μM G-CSF agonist
	4	12	DMEM, 1 % ITS	2mM Metformin
	4	13	DMEM, 1 % ITS	500nM DITPA, 50μM Capsaicin
	4	14	DMEM, 1 % ITS	50μM Capsaicin, 10μM Pifithrin-a

	4	15	DMEM, 1 % ITS	10µM Prostaglandin D2
	4	16	DMEM, 1 % ITS	10µM DAQ B1, 1nM FTY720-1
	4	17	DMEM, 1 % ITS	50nM Caspase-3 inhibitor VII
	4	18	DMEM, 1 % ITS	500nM Compound P, 2nM γ-Secretase inhibitor XX, 10nM Pifithrin-a
	4	19	DMEM, 1 % ITS	500nM Compound P, 20nM PMA
	4	20	DMEM, 1 % ITS	100nM Dexamethasone, 20mM Inosine, 100µM 8-pCPT-cGMP (TEA)
	4	21	DMEM, 1 % ITS	5µM PD98059, 50µg/ml Folic acid
	4	22	DMEM, 1 % ITS	50nM U0126, 1µM HDAC inhibitor
	4	23	DMEM, 1 % ITS	10µM Isoproterenol, 0.2µM Progesterone
	4	24	DMEM, 1 % ITS	1mM Sodium Orthovanadate, 10µM Pifirithrin-a
	4	25	DMEM, 1 % ITS	500nM Compound P, 20mM Inosine, 1µM Asiaticoside
	4	26	DMEM, 1 % ITS	20µM 15d-PGJ2, 10µM DAQ B1, 10µM LY294002
	4	27	DMEM, 1 % ITS	200nM Gö 6976
	4	28	DMEM, 1 % ITS	6µM Scriptaid
	4	29	DMEM, 1 % ITS	500nM DITPA, 50µM Capsaicin, 200µM Ascorbic acid
	4	30	DMEM, 1 % ITS	

Table S3

**Experiment 4: CombiCult screen for TH positive mouse neurons**					
**Tag**	**Stage**	**Media No.**	**Media composition**		**Reference**
			**Basal media**	**Supplements**	
MR1	1	1	KO DMEM	15% FBS, 2mM Glutamine, 1X NEAA, 0.1mM β-mercaptoethanol, 1000 Units/mL LIF(standard growth media for mES cells)	[19]
MR2	1	2	DMEM	10% FBS, 2mM Glutamine, 1X NEAA	
MR3	1	3	Advanced DMEM	2mM Glutamine	[3]
MR4	1	4	DMEM/F12	2mM Glutamine, 1% BSA, 1X N2 suppl., 1X B27 neuromix	[11]
MR5	1	5	RHB-A		[20]
MR6	1	6	KO DMEM	10% KSR, 2mM Glutamine	
MR7	1	7	DMEM/F12	2mM Glutamine,1% BSA, 1X ITS suppl., 20ng/mL bFGF.	[3]
MR8	1	8	DMEM/F12	2mM Glutamine,1% BSA, 1X N2 suppl. 200ng/mL mr Noggin.	[21]
MR9	1	9	DMEM/F12	2mM Glutamine,1% BSA, 1X N2suppl. 20µM SB431542.	[22]
MR10	1	10	DMEM/F12	2mM Glutamine,1% BSA, 1X ITS suppl., 0.5µM retinoic acid.	[23]
SR1	2	1	DMEM/F12	2mM Glutamine, 1X ITS suppl., 5µg/mL fibronectin.	[24]
SR2	2	2	DMEM/F12	2mM Glutamine, 1X ITS suppl., 200ng/mL mr Noggin.	[21]
SR3	2	3	DMEM/F12	2mM Glutamine, 1X ITS suppl., 20ng/mL bFGF.	
SR4	2	4	DMEM/F12	2mM Glutamine, 1X ITS suppl., 0.5µM retinoic acid.	[23]
SR5	2	5	RHB-A		[20]
SR6	2	6	KO DMEM,	10% KSR, 2mM Glutamine	
SR7	2	7	DMEM/F12	2mM Glutamine, 1X ITS suppl., 1X B27 neuromix, 20ng/mL bFGF, 50ng/mL mr Dkk1	[25]
SR8	2	8	Advanced DMEM	2mM Glutamine	[3]
SR9	2	9	DMEM/F12	2mM Glutamine, 1X ITS suppl., 100ng/mL mrFGF8b, 500ng/mL mrShh.	[24]
SR10	2	10	DMEM/F12	2mM Glutamine, 1X ITS suppl., 200µM ascorbic acid, 20ng/mL bFGF, 10ng/mL hrBDNF, 100ng/mL mrFGF8b, 1µM puromorphamine	[26]
LR1	3	1	DMEM/F12	2mM Glutamine, 1X ITS suppl., 20ng/mL bFGF.	[24]

LR2	3	2	DMEM/F12	2mM Glutamine, 1X ITS suppl., 20ng/mL bFGF, 1µg/mL laminin	
LR3	3	3	DMEM/F12	2mM Glutamine, 1X N2 suppl., 1µg/mL laminin, 100ng/mL FGF8b, 500ng/mL mrShh,	
LR4	3	4	Neurobasal media	2mM Glutamine, 1 X ITS suppl., 200 µM ascorbic Acid, 20ng/mL BDNF, 10ng/mL GDNF, 1µg/mL laminin.	[24]
LR5	3	5	RHB-A		[20]
LR6	3	6	KO DMEM	10% KSR, 2mM Glutamine	
LR7	3	7	DMEM/F12	2mM Glutamine, 1X ITS suppl., 1X B27 neuromix, 20ng/mL bFGF, 50ng/mL mr Dkk1	[25]
LR8	3	8	Neurobasal media	2mM Glutamine, 1X B27 neuromix., 20ng/mL bFGF	
LR9	3	9	Advanced DMEM	2mM Glutamine	[3]
LR10	3	10	DMEM/F12	2mM Glutamine, 1X N2 suppl., 20ng/mL bFGF, 20ng/mL EGF, 2ng/mL LIF.	[27]
	4	1	DMEM/F12	2mM Glutamine, 1X N2 suppl. , 200µM ascorbic acid, 1µg/mL laminin	[24]
	4	2	DMEM/F12	2mM Glutamine, 1X N2 suppl , 0.5% FBS, 20ng/mL hr GDNF, 20ng/mL hr BDNF	[28]
	4	3	DMEM/F12	2mM Glutamine, 1X N2 suppl., 200µM ascorbic acid, 20ng/mL hr BDNF	[29]
	4	4	DMEM/F12	2mM Glutamine, 1X N2 suppl., 0.5µM cAMP, 10ng/mL hrBDNF	
	4	5	RHB-A		[20]
	4	6	KO DMEM	10% KSR, 2mM Glutamine	
	4	7	Neurobasal media	2mM Glutamine, 1XB27 neuromix.	
	4	8	DMEM/F12	2mM Glutamine, 1X ITS suppl., 20ng/mL bFGF.	
	4	9	Advanced DMEM	2mM Glutamine	[3]
	4	10	DMEM/F12	2mM Glutamine, 1X ITS suppl., 10ng/mL NT3	[30]

Table S4

**Experiment 5: CombiCult screen for TH positive human neurons**					
**Tag**	**Stage**	**Media No.**	**Media composition**		
			**Basal media**	**Supplements**	
LR1	1	1	KO DMEM	20% KSR, 2mM Glutamine, 1X NEAA, 0.1mM β-mercaptoethanol, 4ng/mL bFGF (standard growth media for hES cells)	[31]
LR2	1	2	DMEM	10% FBS, 2mM Glutamine, 1X NEAA	
LR3	1	3	Advanced DMEM	2mM Glutamine	[3]
LR4	1	4	DMEM/F12	2mM Glutamine, 1% BSA, 1X N2 suppl., 1X B27 neuromix,	[11]
LR5	1	5	RHB-A		[20]
LR6	1	6	DMEM/F12,	2mM Glutamine, 1% BSA, 1X N2 suppl., 200ng/mL hr Noggin, 20µM SB431542	[22]
LR7	1	7	DMEM/F12	2mM Glutamine, 1% BSA , 1X ITS suppl., 20ng/mL bFGF	[3]
LR8	1	8	DMEM/F12	2mM Glutamine, 1% BSA, 1X N2 suppl., 200ng/mL hr Noggin	[21]
LR9	1	9	DMEM/F12	2mM Glutamine, 1% BSA, 1X N2 suppl. 20µM SB431542	[22]
LR10	1	10	DMEM/F12	2mM Glutamine, 1% BSA, 1X ITS suppl 0.5µM retinoic acid	[23]
MR1	2	1	DMEM/F12	2mM Glutamine, 1X ITS suppl. 5µg/mL fibronectin.	[24]
MR2	2	2	DMEM/F12	2mM Glutamine, 1X ITS suppl., 200ng/mL hr Noggin.	[21]
MR3	2	3	DMEM/F12	2mM Glutamine, 1X ITS suppl., 20ng/mL bFGF	[3]
MR4	2	4	DMEM/F12	2mM Glutamine, 1X ITS suppl 0.5µM retinoic acid.	[23]

MR5	2	5	RHB-A		[20]
MR6	2	6	DMEM/F12	2mM Glutamine ,1X N2 suppl.	
MR7	2	7	DMEM/F12	2mM Glutamine, 1X ITS suppl., 1X B27 neuromix, 20ng/mL bFGF, 50ng/mL hr DKK1	[25]
MR8	2	8	Advanced DMEM	2mM Glutamine.	[3]
MR9	2	9	DMEM/F12	2mM Glutamine, 1X ITS suppl., 100ng/mL mrFGF8b, 500ng/mL hr Shh.	[24]
MR10	2	10	DMEM/F12	2mM Glutamine, 1X ITS suppl., 200µM ascorbic acid, , 20ng/mL bFGF, 10ng/mL hrBDNF, 100ng/mL mrFGF8b, 1µM puromorphamine	[26]
SR1	3	1	DMEM/F12	2mM Glutamine, 1X ITS suppl., 20ng/mL bFGF	
SR2	3	2	DMEM/F12	2mM Glutamine, 1X N2 suppl., 1µg/mL laminin, , 10ng/mL hrBDNF, 200µM ascorbic acid	[23]
SR3	3	3	DMEM/F12	2mM Glutamine, 1X N2 suppl., 1µg/mL laminin, 100ng/mL mrFGF8b, 500ng/mL hr SHH.	[24]
SR4	3	4	Neurobasal media	2mM Glutamine, 1X ITS suppl., 200µM ascorbic acid, 20ng/mL hrBDNF, 10ng/mL hrGDNF, 0.5µM cAMP , 1ng/mL hrTGFβ	[32]
SR5	3	5	RHB-A		[20]
SR6	3	6	DMEM/F12	2mM Glutamine, 1X N2 suppl.,	
SR7	3	7	DMEM/F12	2mM Glutamine, 1X ITS suppl., 1X B27 neuromix, 20ng/mL bFGF, 50ng/mL hr DKK1	[25]
SR8	3	8	Neurobasal media	2mM Glutamine, 1X B27 neuromix., 20ng/mL bFGF	
SR9	3	9	Advanced DMEM,	2mM Glutamine.	[3]
SR10	3	10	DMEM/F12	2mM Glutamine, 1X N2 supp., l 20ng/mL bFGF, 20ng/mL hrEGF, 2ng/mL LIF	[27]
	4	1	DMEM/F12	2mM Glutamine, 1X N2 suppl., 200µM ascorbic acid 1µg/mL laminin	[24]
	4	2	DMEM/F12	2mM Glutamine, 1X N2 suppl., 0.5% FBS, 20ng/mL hrGDNF, 20ng/mL hrBDNF.	[28]
	4	3	DMEM/F12	2mM Glutamine, 1X N2 suppl., 200µM ascorbic acid 10ng/mL hrBDNF.	[29]
	4	4	DMEM/F12	2mM Glutamine, 1X N2 suppl., 0.5µM cAMP, 10ng/mL hrBDNF.	
	4	5	RHB-A		[20]
	4	6	DMEM/F12	2mM Glutamine, 1X N2 suppl.,	
	4	7	Neurobasal media	2mM Glutamine, 1X B27 neuromix	
	4	8	DMEM/F12	2mM Glutamine, 1X ITS suppl., 20ng/mL bFGF	
	4	9	Advanced DMEM	2mM Glutamine	[3]
	4	10	DMEM/F12	2mM Glutamine, 1X ITS suppl., 10ng/mL hrNT3	[30]

REFERENCES

1. Schulz TC, Noggle SA, Palmarini GM, Weiler DA, Lyons IG, et al. (2004) Differentiation of human embryonic stem cells to dopaminergic neurons in serum-free suspension culture. Stem Cells 22: 1218–1238.

2. Lowell S, Benchoua A, Heavey B, Smith AG (2006) Notch promotes neural lineage entry by pluripotent embryonic stem cells. PLoS Biol 4: e121.

3. Bouhon IA, Kato H, Chandran S, Allen ND (2005) Neural differentiation of mouse embryonic stem cells in chemically defined medium. Brain Res Bull 68: 62–75.

4. Jiang W, Shi Y, Zhao D, Chen S, Yong J, et al. (2007) In vitro derivation of functional insulin-producing cells from human embryonic stem cells. Cell Res 17: 333–344.

5. D'Amour KA, Bang AG, Eliazer S, Kelly OG, Agulnick AD, et al. (2006) Production of pancreatic hormone-expressing endocrine cells from human embryonic stem cells. Nat Biotechnol 24: 1392–1401.

6. Cai J, Zhao Y, Liu Y, Ye F, Song Z, et al. (2007) Directed differentiation of human embryonic stem cells into functional hepatic cells. Hepatology 45: 1229–1239.

7. Lu SJ, Feng Q, Caballero S, Chen Y, Moore MA, et al. (2007) Generation of functional hemangioblasts from human embryonic stem cells. Nat Methods 4: 501–509..

8. Gadue P, Huber TL, Paddison PJ, Keller GM (2006) Wnt and TGF-beta signaling are required for the induction of an in vitro model of primitive streak formation using embryonic stem cells. Proc Natl Acad Sci U S A 103: 16806–16811..

9. Honda M, Kurisaki A, Ohnuma K, Okochi H, Hamazaki TS, et al. (2006) N-cadherin is a useful marker for the progenitor of cardiomyocytes differentiated from mouse ES cells in serum-free condition. Biochem Biophys Res Commun 351: 877–882.

10. Pachernik J, Bryja V, Esner M, Hampl A, Dvorak P (2005) Retinoic acid-induced neural differentiation of P19 embryonal carcinoma cells is potentiated by leukemia inhibitory factor. Physiol Res 54: 257–262.

11. Ying QL, Stavridis M, Griffiths D, Li M, Smith A (2003) Conversion of embryonic stem cells into neuroectodermal precursors in adherent monoculture. Nat Biotechnol 21: 183–186.

12. Zur Nieden NI, Kempka G, Rancourt DE, Ahr HJ (2005) Induction of chondro-, osteo- and adipogenesis in embryonic stem cells by bone morphogenetic protein-2: effect of cofactors on differentiating lineages. BMC Dev Biol 5: 1.

13. Reubinoff BE, Itsykson P, Turetsky T, Pera MF, Reinhartz E, et al. (2001) Neural progenitors from human embryonic stem cells. Nat Biotechnol 19: 1134–1140.

14. Tian X, Morris JK, Linehan JL, Kaufman DS (2004) Cytokine requirements differ for stroma and embryoid body-mediated hematopoiesis from human embryonic stem cells. Exp Hematol 32: 1000–1009.

15. Sachinidis A, Fleischmann BK, Kolossov E, Wartenberg M, Sauer H, et al. (2003) Cardiac specific differentiation of mouse embryonic stem cells. Cardiovasc Res 58: 278–291.

16. Chang KH, Zandstra PW (2004) Quantitative screening of embryonic stem cell differentiation: endoderm formation as a model. Biotechnol Bioeng 88: 287–298.

17. Lamba DA, Karl MO, Ware CB, Reh TA (2006) Efficient generation of retinal progenitor cells from human embryonic stem cells. Proc Natl Acad Sci U S A 103: 12769–12774.

18. Lavon N, Yanuka O, Benvenisty N (2004) Differentiation and isolation of hepatic-like cells from human embryonic stem cells. Differentiation 72: 230–238.

19. Smith A (1991) Culture of embryonic stem cells. J Tiss Cult Meth 13: 89–94.

20. Diogo MM, Henrique D, Cabral JM (2008) Optimization and integration of expansion and neural commitment of mouse embryonic stem cells. Biotechnol Appl Biochem 49: 105–112.

21. Iacovitti L, Donaldson AE, Marshall CE, Suon S, Yang M (2007) A protocol for the differentiation of human embryonic stem cells into dopaminergic neurons using only chemically defined human additives: Studies in vitro and in vivo. Brain Res 1127: 19–25.

22. Chambers SM, Fasano C a, Papapetrou EP, Tomishima M, Sadelain M, et al. (2009) Highly efficient neural conversion of human ES and iPS cells by dual inhibition of SMAD signaling. Nat Biotechnol 27: 275–280.

23. Kumar M, Bagchi B, Gupta SK, Meena a S, Gressens P, et al. (2007) Neurospheres derived from human embryoid bodies treated with retinoic Acid show an increase in nestin and ngn2 expression that correlates with the proportion of tyrosine hydroxylase-positive cells. Stem Cells Dev 16: 667–681.

24. Lee SH, Lumelsky N, Studer L, Auerbach JM, McKay RD (2000) Efficient generation of midbrain and hindbrain neurons from mouse embryonic stem cells. Nat Biotechnol 18: 675–679.

25. Cajanek L, Ribeiro D, Liste I, Parish CL, Bryja V, et al. (2009) Wnt/beta-catenin signaling blockade promotes neuronal induction and dopaminergic differentiation in embryonic stem cells. Stem Cells 27: 2917–2927.

26. Hu BY, Zhang SC (2009) Differentiation of spinal motor neurons from pluripotent human stem cells. Nat Protoc 4: 1295–1304.

27. Andersen RK, Widmer HR, Zimmer J, Wahlberg LU, Meyer M (2009) Leukemia inhibitory factor favours neurogenic differentiation of long-term propagated human midbrain precursor cells. Neurosci Lett 464: 203–208.

28. Roy NS, Cleren C, Singh SK, Yang L, Beal MF, et al. (2006) Functional engraftment of human ES cell-derived dopaminergic neurons enriched by coculture with telomerase-immortalized midbrain astrocytes. Nat Med 12: 1259–1268.

29. Kim JY, Koh HC, Lee JY, Chang MY, Kim YC, et al. (2003) Dopaminergic neuronal differentiation from rat embryonic neural precursors by Nurr1 overexpression. J Neurochem 85: 1443–1454.

30. Wada T, Honda M, Minami I, Tooi N, Amagai Y, et al. (2009) Highly efficient differentiation and enrichment of spinal motor neurons derived from human and monkey embryonic stem cells. PLoS One 4: e6722.

31. Xu C, Inokuma MS, Denham J, Golds K, Kundu P, et al. (2001) Feeder-free growth of undifferentiated human embryonic stem cells. Nat Biotechnol 19: 971–974.

32. Swistowski A, Peng J, Han Y, Swistowska AM, Rao MS, et al. (2009) Xeno-free defined conditions for culture of human embryonic stem cells, neural stem cells and dopaminergic neurons derived from them. PLoS One 4: e6233.
