# Supplementary material for: Directed Differentiation of Embryonic Stem Cells Using a Bead-Based Combinatorial Screening Method
Source: PLoS One. 2014 Sep 24;9(9):e104301. doi: 10.1371/journal.pone.0104301 (PMC4174505; doi:10.1371/journal.pone.0104301)

Ariadne™

Experiment 1 - CombiCult™ screen for hematopoietic phagocytes using mES

Plasticell

EXECUTIVE SUMMARY

OBJECTIVE

- The objective of this CombiCult™ study was to identify novel serum-free protocols for the differentiation of mouse embryonic stem cells (mESC) to hematopoietic lineages.

COMBICULT™ EXPERIMENT

- 40 cell culture media were tested in 10,000 combinations.
- Approximately 300,000 beads were seeded with mES 46C Sox1-GFP cells (Stem Cell Sciences).
- The experimental matrix comprised four split-pool cycles, performed on days 1, 2, 5 and 7.
- 10 media were assayed in each cycle, resulting in an experimental complexity of 10x10x10x10 = 10,000 media combinations.
- On completion of cell culture (day 14) all beads were screened using a functional phagocytosis assay.

RESULTS

- 726 beads were isolated by COPAS
- 137 hits (0.053 % of monomeric beads) were verified following inspection by microscopy.
- The cell culture history of 96 (70.1 % of hits) could be deduced unambiguously.
- The 96 hits were derived from 92 distinct putative differentiation protocols.
- Protocols were ranked and selected for validation using bespoke bioinformatics software, Ariadne™ (v1.0).
- The selected 19 validation protocols are listed in Table 1.

 Confidential

Table 1: The 19 protocols selected for validation as by Ariadne™ (v1.0). Protocols are identified by the series of cell culture media that resulted in differentiation.

Validation #	Protocol	Bead Ids		Basal Medium	Supplements
1	10-1-8-5	31, 43, 48	Split 1 (Day 1)	KO-DMEM	FCS (15%), LIF (0.1%)
			Split 2 (Day 2)	DMEM/F12	ITS (1%), RA (0.05 μM)
			Split 3 (Day 5)	DMEM	ITS (1%), BSA (1mg/mL), PDGF BB (50ng/mL)
			Split 4 (Day 7)	DMEM	B27 (1%), FGF a (100ng/mL)
2	10-1-1-5	42, 50	Split 1 (Day 1)	KO-DMEM	FCS (15%), LIF (0.1%)
			Split 2 (Day 2)	DMEM/F12	ITS (1%), RA (0.05 μM)
			Split 3 (Day 5)	DMEM/F12	B27 (1%), EGF (20ng/mL),FGF b (20ng/mL), PDGF-AA (20ng/mL)
			Split 4 (Day 7)	DMEM	B27 (1%), FGF a (100ng/mL)
3	10-1-4-5	30, 38	Split 1 (Day 1)	KO-DMEM	FCS (15%), LIF (0.1%)
			Split 2 (Day 2)	DMEM/F12	ITS (1%), RA (0.05 μM)
			Split 3 (Day 5)	RPMI	B27 (1%), EGF (20ng/mL), FGF b (2ng/mL), Noggin (100ng/mL)
			Split 4 (Day 7)	DMEM	B27 (1%), FGF a (100ng/mL)
4	10-1-4-8	90	Split 1 (Day 1)	KO-DMEM	FCS (15%), LIF (0.1%)
			Split 2 (Day 2)	DMEM/F12	ITS (1%), RA (0.05 μM)
			Split 3 (Day 5)	RPMI	B27 (1%), EGF (20ng/mL), FGF b (2ng/mL), Noggin (100ng/mL)
			Split 4 (Day 7)	DMEM	ITS (1%), BSA (1mg/mL), PDGF-BB (50ng/mL)
5	10-1-1-1	97	Split 1 (Day 1)	KO-DMEM	FCS (15%), LIF (0.1%)
			Split 2 (Day 2)	DMEM/F12	ITS (1%), RA (0.05 μM)
			Split 3 (Day 5)	DMEM/F12	B27 (1%), EGF (20ng/mL),FGF b (20ng/mL), PDGF-AA (20ng/mL)
			Split 4 (Day 7)	DMEM/F12	B27 (1%), PDGF-AA (20ng/mL)
6	10-2-8-5	35	Split 1 (Day 1)	KO-DMEM	FCS (15%), LIF (0.1%)
			Split 2 (Day 2)	DMEM/F12/Neurobasal	N2 (0.5%), B27 (0.5%), FGF-4 (2ng/mL)
			Split 3 (Day 5)	DMEM	ITS (1%), BSA (1mg/mL), PDGF BB (50ng/mL)
			Split 4 (Day 7)	DMEM	B27 (1%), FGF a (100ng/mL)
7	2-1-6-8	86	Split 1 (Day 1)	DMEM/F12/Neurobasal	N2 (0.5%), B27 (0.5%)
			Split 2 (Day 2)	DMEM/F12	ITS (1%), RA (0.05 μM)
			Split 3 (Day 5)	HCM	FGF-4 (30ng/mL), BMP-2 (20ng/mL)
			Split 4 (Day 7)	DMEM	ITS (1%), BSA (1mg/mL), PDGF-BB (50ng/mL)
8	2-1-1-5	49	Split 1 (Day 1)	DMEM/F12/Neurobasal	N2 (0.5%), B27 (0.5%)
			Split 2 (Day 2)	DMEM/F12	ITS (1%), RA (0.05 μM)
			Split 3 (Day 5)	DMEM/F12	B27 (1%), EGF (20ng/mL),FGF b (20ng/mL), PDGF-AA (20ng/mL)
			Split 4 (Day 7)	DMEM	B27 (1%), FGF a (100ng/mL)
9	2-2-6-5	27	Split 1 (Day 1)	DMEM/F12/Neurobasal	N2 (0.5%), B27 (0.5%)
			Split 2 (Day 2)	DMEM/F12/Neurobasal	N2 (0.5%), B27 (0.5%), FGF-4 (2ng/mL)
			Split 3 (Day 5)	HCM	FGF-4 (30ng/mL), BMP-2 (20ng/mL)
			Split 4 (Day 7)	DMEM	B27 (1%), FGF a (100ng/mL)
10	10-2-7-6	20	Split 1 (Day 1)	KO-DMEM	FCS (15%), LIF (0.1%)
			Split 2 (Day 2)	DMEM/F12/Neurobasal	N2 (0.5%), B27 (0.5%), FGF-4 (2ng/mL)

Confidential

Validation #	Protocol	Bead Ids		Basal Medium	Supplements
			Split 3 (Day 5)	STEMLINE	SCF (20ng/mL), BMP-2 (5ng/mL), TGF-β1 (5ng/mL), TPO (20ng/mL)
			Split 4 (Day 7)	HCM	HGF (20 ng/mL)
11	10-8-10-6	46	Split 1 (Day 1)	KO-DMEM	FCS (15%), LIF (0.1%)
			Split 2 (Day 2)	IMDM (75 %)/HAM F12 (25 %)	N2 (1%), B27 (1%), BSA (0.5 mg/mL), Ascorbic Acid (0.5 mM)
			Split 3 (Day 5)	DMEM Low Glucose	ITS (1%), Fibronectin (5μg/mL)
			Split 4 (Day 7)	HCM	HGF (20 ng/mL)
12	10-7-6-6	3	Split 1 (Day 1)	KO-DMEM	FCS (15%), LIF (0.1%)
			Split 2 (Day 2)	STEMLINE	SCF (20ng/mL)
			Split 3 (Day 5)	HCM	FGF-4 (30ng/mL), BMP-2 (20ng/mL)
			Split 4 (Day 7)	HCM	HGF (20 ng/mL)
13	10-7-1-8	89	Split 1 (Day 1)	KO-DMEM	FCS (15%), LIF (0.1%)
			Split 2 (Day 2)	STEMLINE	SCF (20ng/mL)
			Split 3 (Day 5)	DMEM/F12	B27 (1%), EGF (20ng/mL),FGF b (20ng/mL), PDGF-AA (20ng/mL)
			Split 4 (Day 7)	DMEM	ITS (1%), BSA (1mg/mL), PDGF-BB (50ng/mL)
14	10-9-7-8	64	Split 1 (Day 1)	KO-DMEM	FCS (15%), LIF (0.1%)
			Split 2 (Day 2)	GMEM	KSR (5%), TGF-β1 (2ng/mL), BMP-2 (10ng/mL)
			Split 3 (Day 5)	STEMLINE	SCF (20ng/mL), BMP-2 (5ng/mL), TGF-β1 (5ng/mL), TPO (20ng/mL)
			Split 4 (Day 7)	DMEM	ITS (1%), BSA (1mg/mL), PDGF-BB (50ng/mL)
15	2-2-7-7	126	Split 1 (Day 1)	DMEM/F12/Neurobasal	N2 (0.5%), B27 (0.5%)
			Split 2 (Day 2)	DMEM/F12/Neurobasal	N2 (0.5%), B27 (0.5%), FGF-4 (2ng/mL)
			Split 3 (Day 5)	STEMLINE	SCF (20ng/mL), BMP-2 (5ng/mL), TGF-β1 (5ng/mL), TPO (20ng/mL)
			Split 4 (Day 7)	STEMLINE	IL-3 (30ng/mL), IL-6 (20ng/mL), TPO (20ng/mL)
16	10-8-8-7	120	Split 1 (Day 1)	KO-DMEM	FCS (15%), LIF (0.1%)
			Split 2 (Day 2)	IMDM (75 %)/HAM F12 (25 %)	N2 (1%), B27 (1%), BSA (0.5 mg/mL), Ascorbic Acid (0.5 mM)
			Split 3 (Day 5)	DMEM	ITS (1%), BSA (1mg/mL), PDGF BB (50ng/mL)
			Split 4 (Day 7)	STEMLINE	IL-3 (30ng/mL), IL-6 (20ng/mL), TPO (20ng/mL)
17	3-8-9-7	121	Split 1 (Day 1)	Advanced DMEM (CDM)	-
			Split 2 (Day 2)	IMDM (75 %)/HAM F12 (25 %)	N2 (1%), B27 (1%), BSA (0.5 mg/mL), Ascorbic Acid (0.5 mM)
			Split 3 (Day 5)	GMEM	KSR (5%), TGF-β1 (2ng/mL), BMP-2 (10ng/mL), Insulin (1μg/mL), Ascorbic Acid (50μg/mL)
			Split 4 (Day 7)	STEMLINE	IL-3 (30ng/mL), IL-6 (20ng/mL), TPO (20ng/mL)
18	2-8-6-5	34	Split 1 (Day 1)	DMEM/F12/Neurobasal	N2 (0.5%), B27 (0.5%)
			Split 2 (Day 2)	IMDM (75 %)/HAM F12 (25 %)	N2 (1%), B27 (1%), BSA (0.5 mg/mL), Ascorbic Acid (0.5 mM)
			Split 3 (Day 5)	HCM	FGF-4 (30ng/mL), BMP-2 (20ng/mL)
			Split 4 (Day 7)	DMEM	B27 (1%), FGF a (100ng/mL)
19	2-10-1-9	94	Split 1 (Day 1)	DMEM/F12/Neurobasal	N2 (0.5%), B27 (0.5%)
			Split 2 (Day 2)	Advanced DMEM	-
			Split 3 (Day 5)	DMEM/F12	B27 (1%), EGF (20ng/mL),FGF b (20ng/mL), PDGF-AA (20ng/mL)
			Split 4 (Day 7)	GMEM	KSR (5%), TGF-β1 (2ng/mL), BMP-2 (10ng/mL), Insulin (1μg/mL), Ascorbic Acid (50μg/mL)

Confidential

CONTENTS

Executive Summary

Report Nomenclature

1. Introduction

2. Study Setup

3. Split-Pool Experiment
3.1 Cell Culture Media
3.2 Tag Assignment

4. Bead Screening
4.1 Screening Assay
4.2 Flow Sorting of 'Hit' Beads

5. Tag Deconvolution

6. Protocol Analysis
6.1 Dataset Review
6.2 Linkage Analysis
6.3 Fingerprint Analysis
6.4 Hierarchical Clustering Dendrogram
6.5 Similarity Matrix

7. Summary and Conclusions

Annex 1: Flow sorting scatter plots

Annex 2: Tag reference set and calibrated selection gates for each tag identification session

Annex 3: Tag identification scatter plots and histograms for each hit

GLOSSARY

Screening matrix refers to the arrangement and identity of media in the split-pool experiment

Experimental complexity is the total number of combinations of media tested, or the number of different protocols tested

Hit is a bead which scores positive in the screening assay and is sorted by COPAS

COPAS is a large particle flow sorter manufactured by Union Bioimetrica Inc.

Deconvolution is the inference of cell culture history by tag analysis

Bin refers to the sum of beads in each final cell culture medium

Session refers to the FACS analysis of tags from a group of hits. Prior to each session a reference tag set was run to calibrate side and forward scatter, and fluorescence intensity gates

Fingerprint anaylsis is a method of finding and comparing hits derived from identical protocols and groups of beads with similar protocols

Probability (of occurrence of a group in the fingerprint analysis) is the probability of that cluster of protocols occurring by chance, assuming beads sample all protocols randomly

Hierarchical clustering is a hierarchy of protocol clusters, represented in a dendrogram

Similarity matrix is a diagrammatical representation of a pair-wise comparison of all protocols

1. INTRODUCTION

COMBICULT™ TECHNOLOGY

CombiCult™ is a proprietary bead-based combinatorial technology specifically developed for discovery of novel stem cell differentiation protocols. Stem cells on beads are exposed to multiple combinations of media, containing active agents such as growth factors or small molecules. The optimal combinations for effective differentiation can be deduced reliably, rapidly and cost effectively.

Figure 1: CombiCult™ technology.

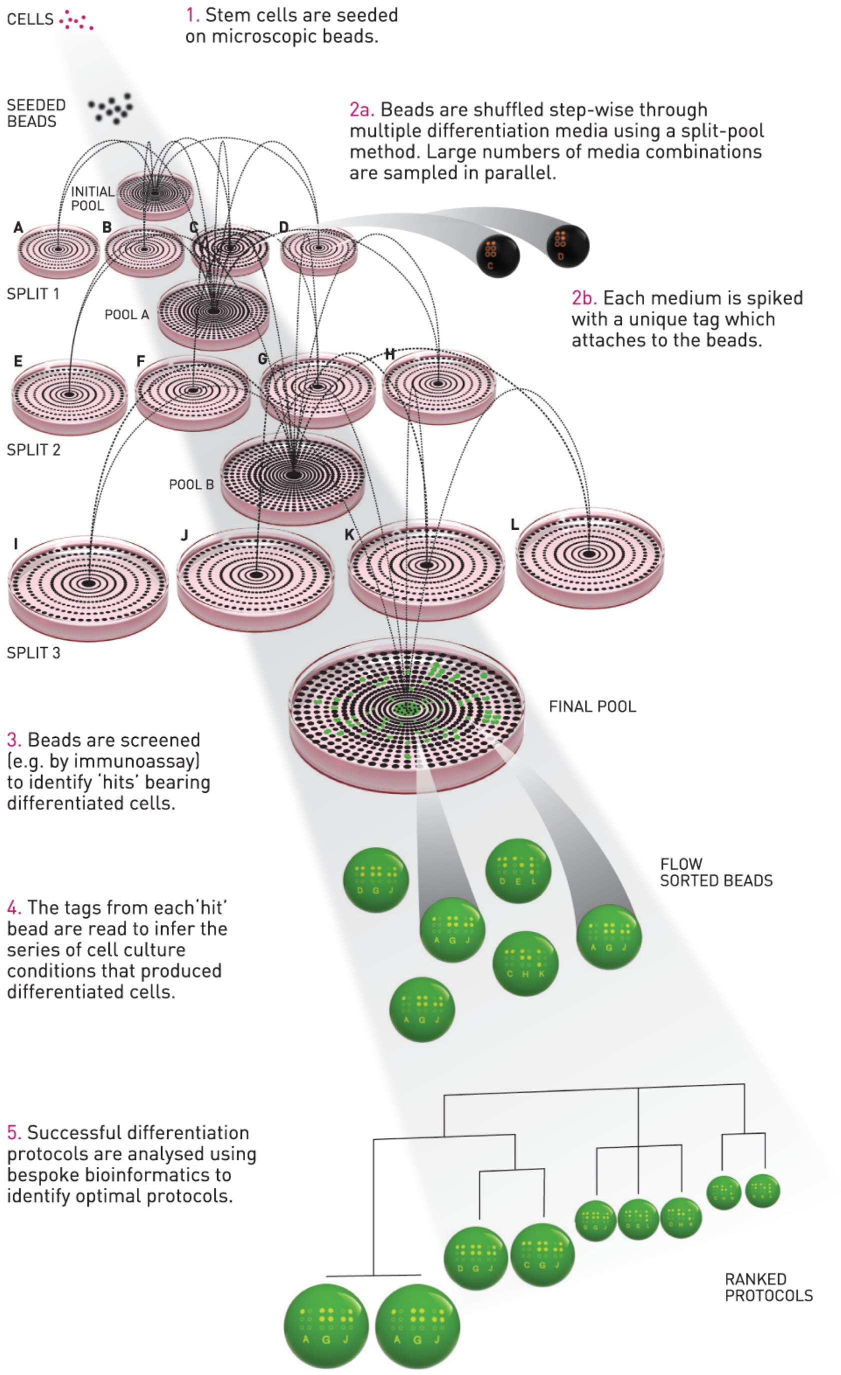

1. INTRODUCTION CONTINUED

OBJECTIVE

The objective of this CombiCult™ study was to identify novel serum-free protocols for the differentiation of mouse embryonic stem cells (mESC) to hematopoietic lineages.

STUDY

The experimental matrix comprised 40 cell culture media distributed over four split-pool cycles (splits) such that a total of 10,000 media combinations (protocols) were tested. Below is a depiction of the experimental design (Figure 2) showing the timing and number of media in each split. On completion of the cell culture phase, on day 14, beads were screened using a functional phagocytosis assay to identify differentiated cells.

Figure 2: Schematic diagram of the experimental matrix.

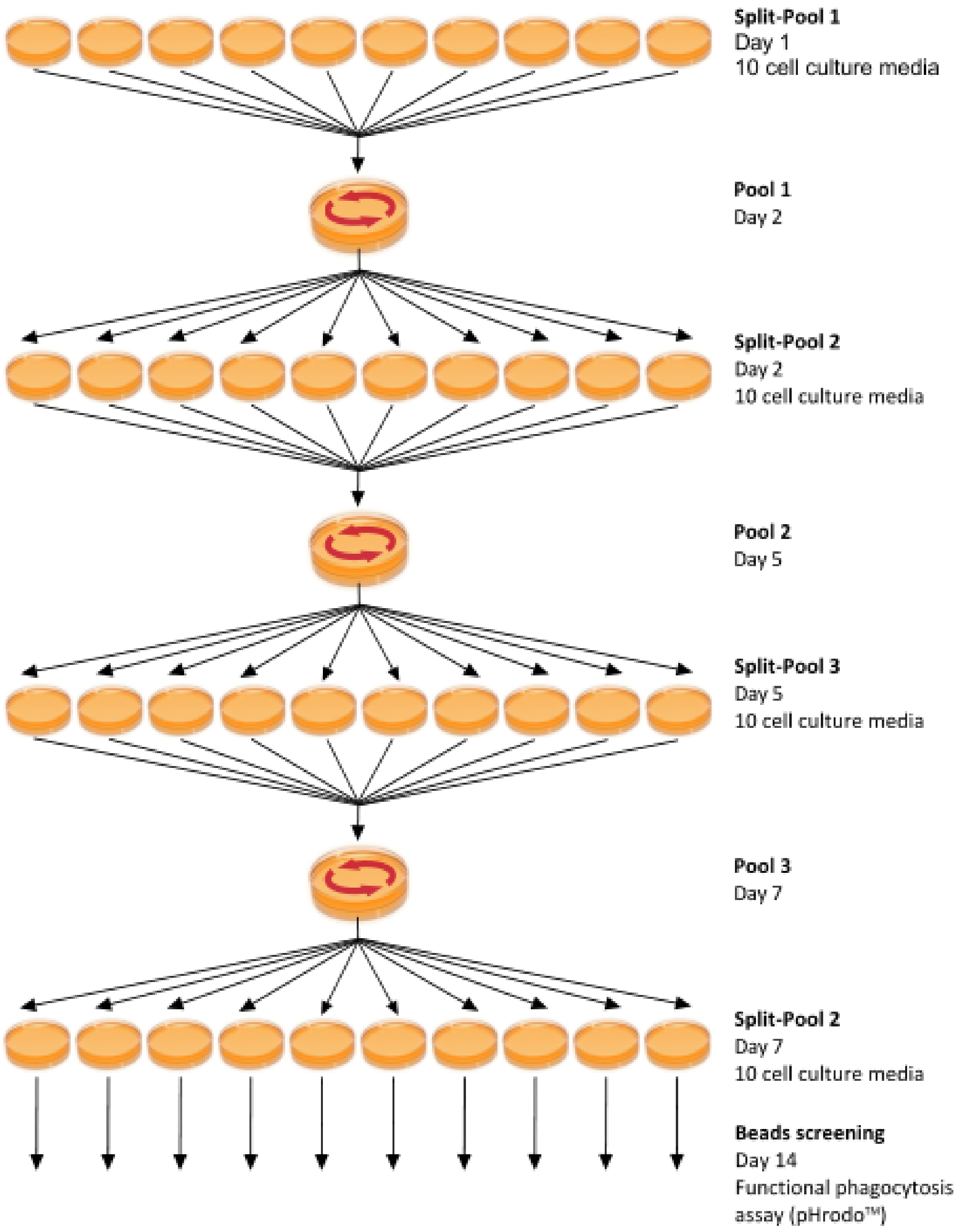

 Confidential

2. STUDY SETUP

OVERVIEW

Study title:	mES Macrophage experiment #1
Study start/finish dates:	24-Apr-08/01-Oct-08

SPLIT-POOL EXPERIMENT

Scientist Name:	Dr Marina Tarunina
Start/Finish Dates:	24-Apr-08/14-Jun-08
Cell Line:	mESC 46C Sox1-GFP cells (Stem Cell Sciences)
Split 1 Date:	24-April-08 (Day1)
Split 2 Date:	25-April-08 (Day 2)
Split 3 Date:	28-April-08 (Day 5)
Split 4 Date:	30-April-08 (Day 7)
Differentiation end Date:	08-May-08 (Day 14)
Tagging Date - Split 1:	24-April-08
Tagging Date - Split 2:	25-April-08
Tagging Date - Split 3:	28-April-08
Tagging Date - Split 4:	30-April-08
Number of cell culture media combinations tested:	10,000
Ratio of beads to complexity:	30:1
Total number of beads:	300,000
Ratio of cells to beads during seeding:	90:1
Additional notes:	None

BEAD SCREENING & SORTING

Scientist name:	Dr Marina Tarunina
Start/finish dates:	08 -May-11-May-08
Screening assay:	pHrodo E.Coli BioParticles (Molecular Probes)
Screening date:	08-May-08
Flow sorting instrument:	COPAS PLUS, Union Biometrica
Sorting PMT channel(s):	Red (615/45 nm) optical emission filters
Positive setup control:	N/A
Total number of sorted beads	726
Number of verified hits	137
Additional notes:	None

TAG DECONVOLUTION

**Bead Digestion**	
Scientist name:	Dr Christopher Johnson
Start/finish dates:	18-Jun-08/21-Jun-08
Additional notes:	None
**Tag Analysis**	
Scientist name:	Dr Christopher Johnson
Start/finish dates:	21-Jun-08/31-Jul-08
Tag analysis flow cytometry instrument:	BD FACSCalibur (sessions 1-6 & 8-12) & Beckman Coulter FC500 (session 7)
Analysis PMT channel(s) optical filter:	BD FACSCalibur FL3 (670 nm long pass). Beckman Coulter FC500 FL4 (675nm long pass).
Number of analysis sessions:	12
Additional notes:	None

PROTOCOL ANALYSIS & REPORT GENERATION

Software:	Ariadne™ version 1.0
User name:	Dr Christopher Johnson
Date, Time:	22-Oct-10, 16.00

3. SPLIT-POOL EXPERIMENT
3.1 CELL CULTURE MEDIA

Table 2: Compositions of the cell culture media tested in this study.

Split	Medium ID	Basal Medium	Supplements
1	1	DMEM	N2 (1%)
1	2	DMEM/F12/Neurobasal	N2 (0.5%), B27 (0.5%)
1	3	Advanced DMEM (CDM)	-
1	4	RPMI	B27 (1%), Activin A (50ng/mL), Sodium Butyrate (1 mM)
1	5	RPMI	Activin A (100ng/mL), Wnt-3A (25ng/mL)
1	6	RPMI	BSA (0.5mg/mL), Activin A (100 ng/mL)
1	7	STEMLINE	BMP-4 (50 ng/mL), VEGF (50ng/mL)
1	8	IMDM (75 %)/HAM F12 (25 %)	N2 (1%), B27 (1%), BSA (0.5 mg/mL)
1	9	GMEM	KSR (5%), BMP-4 (1ng/mL)
1	10	KO-DMEM	FCS (15%), LIF (0.1%)
2	1	DMEM/F12	ITS (1%), RA (0.05 µM)
2	2	DMEM/F12/Neurobasal	N2 (0.5%), B27 (0.5%), FGF-4 (2ng/mL)
2	3	DMEM/F12	ITS (1%), B27 (1%), FGF b (4ng/mL), DKK-1 (50ng/mL)
2	4	RPMI	B27(1%), Activin A (50ng/mL), Sodium Butyrate (0.5mM)
2	5	RPMI	Activin A (100ng/mL), Wnt-3A (25ng/mL)
2	6	RPMI	ITS (1%), BSA (0.5mg/mL), Activin A (100 ng/mL)
2	7	STEMLINE	SCF (20ng/mL)
2	8	IMDM (75 %)/HAM F12 (25 %)	N2 (1%), B27 (1%), BSA (0.5 mg/mL), Ascorbic Acid (0.5 mM)
2	9	GMEM	KSR (5%), TGF-β1 (2ng/mL), BMP-2 (10ng/mL)
2	10	Advanced DMEM	-
3	1	DMEM/F12	B27 (1%), EGF (20ng/mL),FGF b (20ng/mL), PDGF-AA (20ng/mL)
3	2	DMEM/F12/Neurobasal	N2 (0.5%), B27 (0.5%), FGF b (20ng/mL), FGF-8 (100ng/mL), SHH (400ng/mL)
3	3	DMEM/F12	ITS (1%), B27 (1%), FGF b (4ng/mL), DKK-1 (50ng/mL)
3	4	RPMI	B27 (1%), EGF (20ng/mL), FGF b (2ng/mL), Noggin (100ng/mL)
3	5	DMEM	B27 (1%), RA (2 µM), FGF-10 (50ng/mL), CYC (0.2µM)
3	6	HCM	FGF-4 (30ng/mL), BMP-2 (20ng/mL)
3	7	STEMLINE	SCF (20ng/mL), BMP-2 (5ng/mL), TGF-β1 (5ng/mL), TPO (20ng/mL)
3	8	DMEM	ITS (1%), BSA (1mg/mL), PDGF BB (50ng/mL)
3	9	GMEM	KSR (5%), TGF-β1 (2ng/mL), BMP-2 (10ng/mL), Insulin (1µg/mL), Ascorbic Acid (50µg/mL)
3	10	DMEM Low Glucose	ITS (1%), Fibronectin (5µg/mL)
4	1	DMEM/F12	B27 (1%), PDGF-AA (20ng/mL)
4	2	DMEM/F12/Neurobasal	N2 (0.5%), B27 (0.5%), FGF b (20ng/mL), FGF-8 (100ng/mL), SHH (400ng/mL)
4	3	DMEM/F12	N2 (1%), B27 (1%), FGF b (5ng/mL), DKK-1 (10ng/mL), Noggin (10ng/mL), IGF-1 (10ng/mL)
4	4	DMEM	B27 (1%), RA (2 µM), FGF-10 (50ng/mL), CYC (0.2µM)
4	5	DMEM	B27 (1%), FGF a (100ng/mL)
4	6	HCM	HGF (20 ng/mL)
4	7	STEMLINE	IL-3 (30ng/mL), IL-6 (20ng/mL), TPO (20ng/mL)
4	8	DMEM	ITS (1%), BSA (1mg/mL), PDGF-BB (50ng/mL)
4	9	GMEM	KSR (5%), TGF-β1 (2ng/mL), BMP-2 (10ng/mL), Insulin (1µg/mL), Ascorbic Acid (50µg/mL)
4	10	CMRL	B27 (1%), IGF-1 (50ng/mL), HGF (50ng/mL)

Confidential

3. SPLIT-POOL
3.1 CELL CULTURE MEDIA CONTINUED

Table 3: Suppliers of basal media and supplements.

Basal Media/Supplement	Supplier	Product Reference
Advanced DMEM	Fisher Scientific	VX12491015
CMRL	Fisher Scientific	VX21530027
DMEM/F12	Fisher Scientific	VX31331028
DMEM High Glucose	Fisher Scientific	VX31966047
DMEM Low Glucose	Fisher Scientific	VX21885108
FCS	Fischer Scientific	VX12662011
GMEM	Fisher Scientific	VX21710025
Ham F12	Fisher Scientific	VX31765027
HCM	Lonza	CC-3198
IMDM	Fisher Scientific	VX31980022
KO DMEM	Fisher Scientific	VX10829018
KSR	Fisher Scientific	VX10828028
Neurobasal	Fisher Scientific	VX21103049
RPMI	Fisher Scientific	VX21875034
Stemline	Sigma Aldrich	S0192
Activin A	R and D Systems	338-AC-005
Ascorbic Acid	Sigma Aldrich	A4403
B27 Supplement	Fisher Scientific	VX17504044
BMP-2	R and D Systems	355-BM-010
BMP-4	R and D Systems	314-BP-010
BSA	Sigma Aldrich	A1595
Cyclopamine KAAD	Toronto Research Chemicals	K171000
DKK-1	R and D Systems	1765-DK-010
EGF	R and D Systems	2028-EG-200
FGF-4	R and D Systems	235-F4-025
FGF-8b	R and D Systems	423-F8-025
FGF-10	R and D Systems	345-FG-025
FGF a	R and D Systems	232-FA-025
FGF b	R and D Systems	233-FB-025
Fibronectin	Fisher Scientific	VX33010018
HGF	R and D Systems	2207-HG-025
IGF I	R and D Systems	791-MG-050
IL-3	R and D Systems	403-ML-010
IL-6	R and D Systems	206-IL-010
Insulin	Sigma Aldrich	I0516
ITS	Sigma Aldrich	I3146
LIF	Millipore	ESG1106
N2 Supplement	Fisher Scientific	VX17502048
Noggin	R and D Systems	719-NG-050
PDGF-BB	R and D Systems	520-BB-050

Basal Media/Supplement	Supplier	Product Reference
Retinoic Acid	Merck	US1554720
SCF	R and D Systems	455-MC-010
SHH	R and D Systems	461-SH-025
Sodium Butyrate	Sigma Aldrich	B5887
TGF-β1	R and D Systems	4114-TC-01M
Tpo	R and D Systems	288-TP-005
VEGF	R and D Systems	293-VE-010
Wnt-3A	R and D Systems	1324-WN-002

3. SPLIT-POOL

3.2. TAG ASSIGNMENT

Each cell culture medium (except media in the last split-pool cycle) was spiked with a unique fluorescent tag that attaches to PTC5000 beads. Tag readout from individual beads enables determination of the series of cell culture media to which the bead was exposed. Thirty unique populations of tags were used, which differ in diameter, fluorescence colour and fluorescence intensity (ten gradations). Table 4 shows the tag code, size, fluorescence colour, fluorescence intensity, tag stock solution concentration and volume used to spike each cell culture medium.

Table 4: The tags used to spike different cell culture media.

Split	Medium ID	Tag code	Tag size	Tag fluorescence colour	Tag fluorescence intensity level	Stock solution concentration (% w/v)	V of stock solution added to media (μL)
1	1	SR01	S	Red	1	0.5	96
1	2	SR02	S	Red	2	0.5	96
1	3	SR03	S	Red	3	0.5	96
1	4	SR04	S	Red	4	0.5	96
1	5	SR05	S	Red	5	0.5	96
1	6	SR06	S	Red	6	0.5	96
1	7	SR07	S	Red	7	0.5	96
1	8	SR08	S	Red	8	0.5	96
1	9	SR09	S	Red	9	0.5	96
1	10	SR10	S	Red	10	0.5	96
2	1	MR01	M	Red	1	0.5	184
2	2	MR02	M	Red	2	0.5	184
2	3	MR03	M	Red	3	0.5	184
2	4	MR04	M	Red	4	0.5	184
2	5	MR05	M	Red	5	0.5	184
2	6	MR07	M	Red	7	0.5	184
2	7	MR06	M	Red	6	0.5	184
2	8	MR08	M	Red	8	0.5	184
2	9	MR09	M	Red	9	0.5	184
2	10	MR10	M	Red	10	0.5	184
3	1	LR01	L	Red	1	0.5	364
3	2	LR02	L	Red	2	0.5	364
3	3	LR03	L	Red	3	0.5	364
3	4	LR04	L	Red	4	0.5	364
3	5	LR05	L	Red	5	0.5	364
3	6	LR06	L	Red	6	0.5	364
3	7	LR07	L	Red	7	0.5	364
3	8	LR08	L	Red	8	0.5	364
3	9	LR09	L	Red	9	0.5	364
3	10	LR10	L	Red	10	0.5	364
4	1	No tag	-	-	-	-	-
4	2	No tag	-	-	-	-	-
4	3	No tag	-	-	-	-	-
4	4	No tag	-	-	-	-	-
4	5	No tag	-	-	-	-	-
4	6	No tag	-	-	-	-	-
4	7	No tag	-	-	-	-	-
4	8	No tag	-	-	-	-	-
4	9	No tag	-	-	-	-	-
4	10	No tag	-	-	-	-	-

4. BEAD SCREENING

4.1. SCREENING ASSAY

Following completion of the split-pool experiment at day 14, beads were screened using a functional phagocytosis assay to identify hits populated with 46C mES cells which had differentiated into phagocytic cells.

In brief, beads were washed in DPBS and incubated in Hanks balanced buffered solution (HBSS) with a red fluorescent pHrodo E.Coli BioParticles conjugate (Molecular Probes) at 37°C for 2 hrs. Beads were then washed twice with HBSS/HEPES buffer.

Figure 3 shows a 'hit' with a phagocytic cell colony in a background of negative beads (x10 objective lens). Images were obtained using a Nikon Eclipse TE2000-S fluorecent microscope with a TRITC filter (excitation λ = 540/25 nm, emmission λ = 605/55 nm). Scale bars = 100 μm.

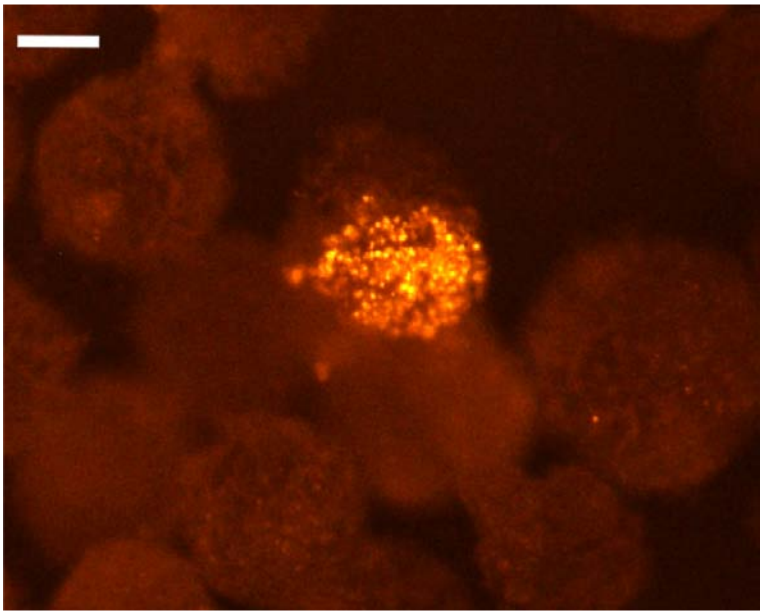

4. BEAD SCREENING

4.2. FLOW SORTING OF 'HIT' BEADS

Following the screening assay, beads were sorted using a large particle flow sorter (COPAS PLUS, Union Biometrica; Green PMT 514/23 nm and Red PMT 615/45 nm optical emission filters). Beads from each final cell culture medium were labeled as bin 1-10. The data from each bin is contained in one or more flow sorting data files. Annex 1 shows dot plots from each flow sorting data file.

Beads were gated according to size (time of flight [TOF] and optical extinction [ext] values) to exclude bead aggregates that had formed during cell culture. Gated beads were sorted based on their fluorescence properties, as defined by the screening assay, and hits individually dispensed into wells of a 96-well plate.

In figure 4 COPAS data from all bins have been combined on two dot-plots to provide an overview of bead sorting. Figure 4(a) is a dot plot of optical extinction (ext) vs. time of Flight (TOF). Events falling within the gate defining monomeric beads are represented in green: in this experiment 256,516 beads (85.5 % of the starting number) were sorted. Figure 4(b) is a dot plot of green vs. red fluorescence intensity for all sorted beads. Beads with a red fluorescence intensity value above the threshold value (depicted as red dots in Figure 4b) were individually dispensed into 96 well plates.

Once dispensed, hits were verified by fluorescence microscopy. Table 5 lists the number of positive beads sorted and verified as 'hit' beads from each bin. A total of 726 positive beads were isolated out of which 137 were verified as 'hit' beads (Table 5). This large discrepancy is due to the relatively low setting of the threshold value, resulting in sorting of beads with background fluorescence. Tags from the verified hits were subsequently analysed to determine the cell culture history (i.e. differentiation protocol) of each 'hit' bead, as detailed in the next section of the report.

Figure 4(a) and (b): COPAS dot plots, showing (a) the gate used to select monomeric beads (green dots) and (b) the threshold fluorescence intensity used to select positive 'hit' beads (red dots). The dot plots contain combined data recorded from all bins.

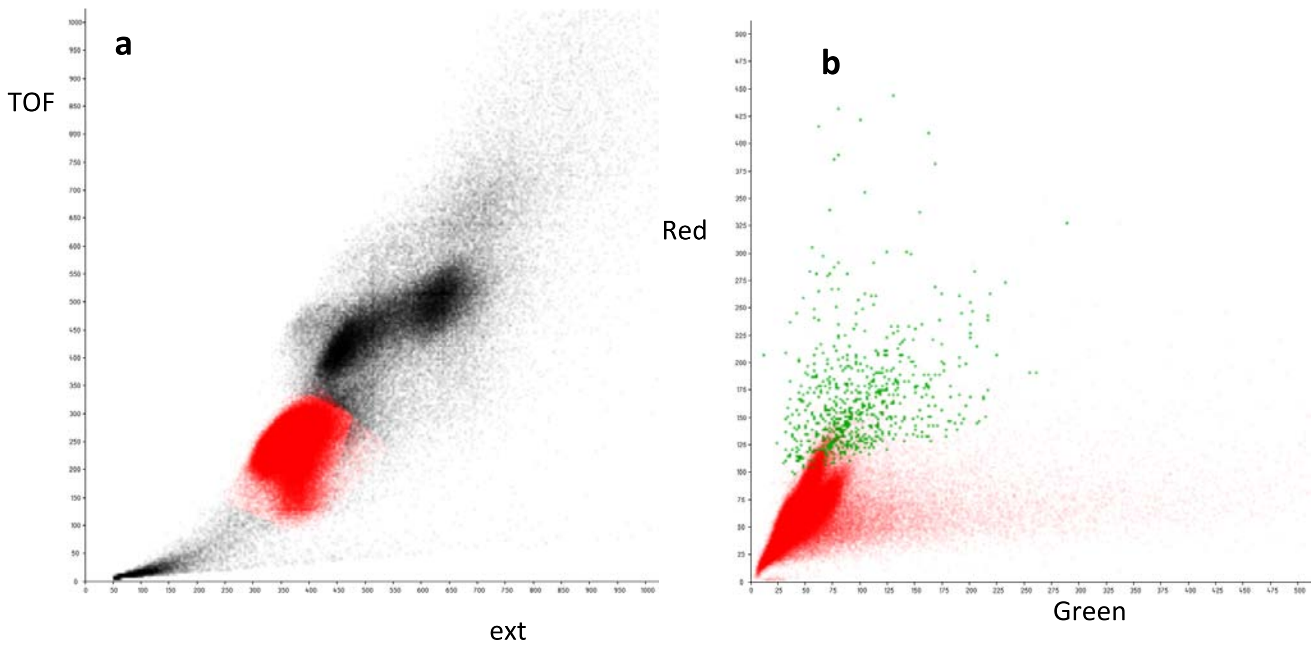

4.2. FLOW SORTING OF 'HITS' CONTINUED

Table 5: Number of positive beads sorted and number of verified 'hits' from each bin.

Bin Number	1	2	3	4	5	6	7	8	9	10	Total
Number of positive beads sorted	13	2	3	28	67	179	220	61	61	92	726
Number of verified hits	13	1	1	2	30	22	35	18	9	6	137

5. TAG DECONVOLUTION

Following bead sorting, tags from each hit are analysed by flow cytometry and related to the cell culture history. During this process, data is lost or excluded owing to various factors and this is summarized in Table 6.

The flow cytometry data acquisition was performed in a series of sessions and prior to each session a reference tag set was run to calibrate side/forward scatter and fluorescence intensity gates. Dot plots and histograms for each session are shown in Annex 2. The data was loaded into Ariadne™ which automatically identifies tags based on four unique parameter values (forward and side scatter and fluorescence colour and intensity).

Determination of tag identity is based on the number of events (≥ 3) within a gate, and the cluster tightness (Table 7). If two or more clusters of events are identified which mapped to cell media from the same split and/or the signal to noise ratio was too low, an accurate identification is not recorded.

Ariadne™ relates the tag identity to a cell culture medium using the tag assignment (Table 4), allowing it to log the cell culture history of each hit (Table 8).

Table 6. Summary of data attrition owing to various factors.

Bin Number	1	2	3	4	5	6	7	8	9	10	Total
Number of verified ‘hits’	13	1	1	2	30	22	35	18	9	6	137
Number of beads lost during digestion and sample preparation	(3)	0	(1)	0	(2)	0	0	0	0	0	(6)
Number of beads analysed by flow cytometry	10	1	0	2	28	22	35	18	9	6	131
Number of beads with no tagging data	0	0	0	0	0	0	(2)	0	0	0	(2)
Number of beads with incomplete tagging data	(5)	0	0	0	(4)	(8)	(9)	(2)	(4)	(1)	(33)
Number of beads containing too many tag clusters	0	0	0	0	0	0	0	0	0	0	0
Number of hits with conclusive tagging data	5	1	0	2	24	14	24	16	5	5	96

Table 7: Summary of the minimum, maximum and average number of tags derived from beads in each of the cell culture media. This data demonstrates that the average number of tags for all media is well above the minimum required to link a cell culture medium to a bead (3).

Split	Medium ID	hits	Minimum	Average	Maximum
1	1	7	41	63	103
1	2	13	46	57	82
1	3	11	26	82	148
1	4	6	29	41	77
1	5	4	24	45	73
1	6	7	41	57	86
1	7	5	26	33	48
1	8	4	25	36	56
1	9	7	5	10	16
1	10	32	16	35	65
2	1	34	5	18	62
2	2	13	14	34	83
2	3	8	8	21	34
2	4	5	9	22	38
2	5	4	13	28	48
2	6	4	25	40	54
2	7	5	8	24	44
2	8	12	4	45	127
2	9	8	13	40	69
2	10	3	47	67	83
3	1	12	10	29	56
3	2	10	22	52	98
3	3	8	9	28	64
3	4	11	6	40	74
3	5	5	11	34	48
3	6	11	7	38	104
3	7	12	5	27	45
3	8	11	4	27	64
3	9	10	7	25	48
3	10	6	11	36	75
4	1	5	-	-	-
4	2	1	-	-	-
4	3	0	-	-	-
4	4	2	-	-	-
4	5	24	-	-	-
4	6	14	-	-	-
4	7	24	-	-	-
4	8	16	-	-	-
4	9	5	-	-	-
4	10	5	-	-	-

Table 8: Information for each 'hit': i) the bead ID; ii) tag acquisition session number; iii) flow cytometry tag data file name; iv) series of cell culture media, i.e. protocol (number of tags upon which assignment of each cell culture media is based are in parentheses, "–" represents unreadable tag data) and v) whether the bead passes the tag deconvolution.

Bead ID	Tag acquisition session #	Tag data filename	Split 1 media (# of tags)		Split 2 media (# of tags)		Split 3 media (# of tags)		Split 4 media	Pass tag deconvolution criteria
1	1	Bin6_34.fcs	10	(31)	-		-		6	fail
2	1	Bin6_35.fcs	10	(39)	1	(12)	5	(11)	6	pass
3	1	Bin6_36.fcs	10	(37)	7	(8)	6	(9)	6	pass
4	1	Bin6_37.fcs	1	(39)	-		-		6	fail
5	1	Bin6_38.fcs	9	(15)	10	(72)	5	(39)	6	pass
6	1	Bin6_39.fcs	-		-		1	(57)	6	fail
7	1	Bin6_40.fcs	6	(41)	3	(22)	4	(23)	6	pass
8	1	Bin6_41.fcs	9	(5)	1	(15)	9	(48)	6	pass
9	1	Bin6_42.fcs	10	(37)	-		-		6	fail
10	1	Bin6_43.fcs	5	(23)	-		-		6	fail
11	2	Bin5_1	10	(24)	1	(1)	3	(22)	5	pass
12	2	Bin5_2	3	(60)	4	(9)	7	(16)	5	pass
13	2	Bin6_44	10	(24)	-		1	(12)	6	fail
14	2	Bin6_45	10	(25)	-		-		6	fail
15	2	Bin6_46	-		1	(39)	1	(37)	6	fail
16	2	Bin6_47	10	(29)	1	(25)	4	(55)	6	pass
17	2	Bin6_48	2	(59)	8	(41)	10	(75)	6	pass
18	2	Bin6_49	8	(34)	1	(8)	7	(45)	6	pass
19	2	Bin6_50	10	(55)	1	(25)	9	(8)	6	pass
20	2	Bin6_51	10	(24)	2	(23)	7	(36)	6	pass
21	3	Bin5_3	4	(77)	4	(22)	2	(22)	5	pass
22	3	Bin5_4	10	(17)	-		-		5	fail
23	3	Bin5_5	10	(38)	-		3	(23)	5	fail
24	3	Bin5_6	3	(60)	2	(19)	7	(25)	5	pass
25	3	Bin5_7	10	(33)	-		-		5	fail
26	3	Bin5_8	3	(71)	5	(29)	9	(19)	5	pass
27	3	Bin5_9	2	(50)	2	(58)	6	(14)	5	pass
28	3	Bin5_10	5	(46)	8	(23)	3	(26)	5	pass
29	3	Bin5_11	4	(36)	9	(59)	5	(48)	5	pass
30	3	Bin5_12	10	(24)	1	(5)	4	(6)	5	pass
31	3	Bin5_13	10	(20)	1	(16)	8	(15)	5	pass
32	3	Bin5_14	4	(34)	3	(15)	1	(30)	5	pass
33	4	Bin5_15	9	(16)	2	(34)	4	(74)	5	pass
34	4	Bin5_16	2	(70)	8	(46)	6	(26)	5	pass
35	4	Bin5_17	10	(45)	2	(19)	8	(4)	5	pass
36	4	Bin5_18	10	(42)	3	(8)	4	(24)	5	pass
37	4	Bin5_19	1	(59)	2	(83)	6	(53)	5	pass
38	4	Bin5_20	10	(61)	1	(22)	4	(28)	5	pass
39	4	Bin5_21	5	(73)	3	(26)	1	(11)	5	pass
40	4	Bin5_22	-		1	(26)	8	(31)	5	fail

 Confidential

Bead ID	Tag acquisition session #	Tag data filename	Split 1 media (# of tags)		Split 2 media (# of tags)		Split 3 media (# of tags)		Split 4 media	Pass tag deconvolution criteria
41	4	Bin5_23	10	(28)	1	(9)	9	(7)	5	pass
42	4	Bin5_24	10	(51)	1	(7)	1	(15)	5	pass
43	4	Bin5_25	10	(43)	1	(8)	8	(20)	5	pass
44	4	Bin6_6F1	10	(34)	1	(21)	6	(7)	6	pass
45	4	Bin6_6F6	3	(26)	7	(19)	8	(26)	6	pass
46	4	Bin6_6F7	10	(26)	8	(19)	10	(11)	6	pass
47	4	Bin6_6G11	4	(34)	3	(34)	2	(98)	6	pass
48	5	Bin5_26	10	(29)	1	(30)	8	(7)	5	pass
49	5	Bin5_27	2	(48)	1	(33)	1	(52)	5	pass
50	5	Bin5_28	10	(28)	1	(19)	1	(22)	5	pass
51	5	Bin9_92	10	(36)	8	(40)	1	(33)	9	pass
52	5	Bin9_93	4	(29)	1	(24)	4	(38)	9	pass
53	5	Bin9_94	-		-		2	(5)	9	fail
54	5	Bin9_96	5	(30)	-		8	(6)	9	fail
55	6	Bin1_1RA2	7	(32)	2	(21)	4	(37)	1	pass
56	6	Bin1_1RA3	10	(20)	-		-		1	fail
57	6	Bin1_1RA4	10	(20)	-		5	(55)	1	fail
58	7	Bin2_2R.LMD	10	(25)	1	(11)	6	(18)	2	pass
59	7	Bin8_80.LMD	1	(76)	1	(14)	2	(65)	8	pass
60	7	Bin8_81.LMD	-		-		-		8	fail
61	7	Bin8_82a.LMD	-		-		-		8	fail
62	7	Bin8_84.LMD	3	(148)	4	(31)	10	(44)	8	pass
63	7	Bin8_085a.LMD	1	(46)	9	(20)	7	(34)	8	pass
64	7	Bin8_86a.LMD	10	(24)	9	(69)	7	(26)	8	pass
65	7	Bin8_87.LMD	1	(41)	8	(127)	1	(30)	8	pass
66	7	Bin8_88.LMD	9	(14)	6	(25)	3	(27)	8	pass
67	7	Bin8_89.LMD	7	(48)	3	(21)	2	(49)	8	pass
68	7	Bin8_90.LMD	1	(44)	3	(24)	7	(10)	8	pass
69	7	Bin8_91.LMD	2	(57)	8	(84)	10	(46)	8	pass
70	7	Bin10_100.LMD	3	(99)	1	(36)	7	(21)	10	pass
71	7	Bin10_103.LMD	2	(46)	9	(51)	5	(36)	10	pass
72	7	Bin10_104.LMD	3	(115)	2	(56)	6	(75)	10	pass
73	7	Bin10_105.LMD	4	(34)	1	(5)	6	(19)	10	pass
74	7	Bin10_106.LMD	10	(51)	-		2	(32)	10	fail
75	7	Bin10_107.LMD	6	(51)	9	(31)	2	(52)	10	pass
76	7	Bin8_82b.LMD	-		-		-		8	fail
77	8	Bin7_7R1	2	(46)	5	(20)	9	(33)	7	pass
78	8	Bin7_7R2	9	(8)	8	(4)	8	(34)	7	pass
79	8	Bin7_7R3	9	(6)	2	(50)	7	(38)	7	pass
80	8	Bin7_7R4	1	(74)	5	(13)	6	(44)	7	pass
81	8	Bin7_7R5	8	(25)	2	(23)	8	(47)	7	pass
82	8	Bin7_7R6	5	(36)	4	(38)	2	(43)	7	pass
83	8	Bin7_7R7	1	(103)	1	(62)	6	(104)	7	pass

Confidential

Bead ID	Tag acquisition session #	Tag data filename	Split 1 media (# of tags)		Split 2 media (# of tags)		Split 3 media (# of tags)		Split 4 media	Pass tag deconvolution criteria
84	9	Bin8_72	2	(50)	1	(35)	3	(40)	8	pass
85	9	Bin8_73	8	(30)	7	(44)	1	(47)	8	pass
86	9	Bin8_74	2	(51)	1	(9)	6	(47)	8	pass
87	9	Bin8_75	7	(26)	1	(13)	7	(38)	8	pass
88	9	Bin8_76	5	(24)	9	(53)	2	(39)	8	pass
89	9	Bin8_77	10	(33)	7	(24)	1	(10)	8	pass
90	9	Bin8_78	10	(46)	1	(6)	4	(49)	8	pass
91	9	Bin8_79	10	(17)	-		2	(26)	8	fail
92	9	Bin9_97	10	(21)	-		4	(32)	9	fail
93	9	Bin9_98	3	(52)	-		3	(3)	9	fail
94	9	Bin9_99	2	(57)	10	(47)	1	(32)	9	pass
95	9	Bin9_101	3	(81)	2	(14)	3	(15)	9	pass
96	9	Bin9_102	10	(43)	1	(32)	4	(46)	9	pass
97	10	Bin1_1RA6	10	(65)	1	(13)	1	(12)	1	pass
98	10	Bin1_1RA7	6	(76)	8	(21)	10	(29)	1	pass
99	10	Bin1_1RA8	10	(42)	1	(5)	2	(33)	1	pass
100	10	Bin1_1RA9	10	(18)	2	(15)	10	(13)	1	pass
101	10	Bin1_1RA11	-		-		5	(42)	1	fail
102	10	Bin1_1RA12	-		-		-		1	fail
103	10	Bin7_7R8	2	(77)	3	(20)	9	(15)	7	pass
104	10	Bin7_7R9	10	(16)	9	(27)	3	(9)	7	pass
105	10	Bin7_70	2	(49)	8	(11)	7	(31)	7	pass
106	10	Bin7_110	6	(86)	1	(22)	3	(23)	7	pass
107	11	Bin1_1RB12	-		-		-		1	fail
108	11	Bin4_4R1	3	(82)	6	(29)	8	(64)	4	pass
109	11	Bin4_4R2	9	(9)	6	(54)	1	(56)	4	pass
110	11	Bin7_69	-		-		2	(5)	7	fail
111	11	Bin7_71	-		9	(48)	-		7	fail
112	11	Bin7_109	-		9	(37)	-		7	fail
113	11	Bin7_111	-		-		-		7	fail
114	11	Bin7_112	-		-		-		7	fail
115	11	Bin7_113	-		-		-		7	fail
116	11	Bin7_115	-		-		-		7	fail
117	12	Bin7_52	10	(37)	1	(11)	4	(61)	7	pass
118	12	Bin7_53	7	(29)	6	(51)	9	(42)	7	pass
119	12	Bin7_54	2	(28)	-		9	(34)	7	fail
120	12	Bin7_55	10	(16)	8	(59)	8	(59)	7	pass
121	12	Bin7_57	3	(102)	8	(66)	9	(38)	7	pass
122	12	Bin7_58	8	(56)	5	(48)	3	(64)	7	pass
123	12	Bin7_59	10	(33)	4	(11)	2	(38)	7	pass
124	12	Bin7_60	6	(43)	1	(12)	5	(34)	7	pass
125	12	Bin7_61	3	(63)	9	(13)	9	(9)	7	pass
126	12	Bin7_62	2	(82)	2	(22)	7	(5)	7	pass

Confidential

Bead ID	Tag acquisition session #	Tag data filename	Split 1 media (# of tags)	Split 2 media (# of tags)	Split 3 media (# of tags)	Split 4 media	Pass tag deconvolution criteria
127	12	Bin7_63	1 (37)	1 (15)	-	7	fail
128	12	Bin7_64	7 (31)	1 (20)	1 (7)	7	pass
129	12	Bin7_65	6 (53)	7 (23)	2 (80)	7	pass
130	12	Bin7_66	10 (31)	1 (14)	8 (11)	7	pass
131	12	Bin7_67	6 (50)	10 (83)	9 (31)	7	pass

6. PROTOCOL ANALYSIS

6.1 DATASET REVIEW

Once a dataset of protocols has been established, Ariadne™ allows post-acquisition resetting of gates on the COPAS sorting plot to specify a subset of hits for further analysis. For example, if 'hits were sorted using two antibodies, the user can select those which are positive for both antibodies. No post acquisition flow sort criterion was applied in this study.

Table 9 lists the bead IDs included in the dataset analyzed by Ariadne™ and presented in the following sections of the report. The dataset only includes beads which fall within the (reset) COPAS gates and can be assigned a full cell culture history. Ariadne™ allows a range of analyses to be performed, to select protocols for validation. Analysis methodologies include linkage analysis which identifies frequently occurring media combinations; fingerprint analysis which clusters groups of beads with identical or similar protocols (i.e. protocols with common cell culture media on the same split) and calculates the probability of these clusters occurring randomly; and methods of comparing entire protocols such as hierarchical clustering and a similarity matrix. These results are presented and discussed in the following sections.

Table 9: Dataset Review. The following parameters are shown for each hit: Bead ID, protocol ("–" represents unreadable tag data), whether the bead passed the tag deconvolution, whether it falls within the (reset) COPAS gate and whether it is included in the protocol analysis dataset.

	Protocol				Inclusion Criteria		
Bead ID	Split 1 media	Split 2 media	Split 3 media	Split 4 media	Tag deconvolution	Flow sort criteria	Included in analysis
1	10	-	-	6	fail	pass	No
2	10	1	5	6	pass	pass	Yes
3	10	7	6	6	pass	pass	Yes
4	1	-	-	6	fail	pass	No
5	9	10	5	6	pass	pass	Yes
6	-	-	1	6	fail	pass	No
7	6	3	4	6	pass	pass	Yes
8	9	1	9	6	pass	pass	Yes
9	10	-	-	6	fail	pass	No
10	5	-	-	6	fail	pass	No
11	10	1	3	5	pass	pass	Yes
12	3	4	7	5	pass	pass	Yes
13	10	-	1	6	fail	pass	No
14	10	-	-	6	fail	pass	No
15	-	1	1	6	fail	pass	No
16	10	1	4	6	pass	pass	Yes
17	2	8	10	6	pass	pass	Yes
18	8	1	7	6	pass	pass	Yes
19	10	1	9	6	pass	pass	Yes
20	10	2	7	6	pass	pass	Yes
21	4	4	2	5	pass	pass	Yes
22	10	-	-	5	fail	pass	No
23	10	-	3	5	fail	pass	No
24	3	2	7	5	pass	pass	Yes

	Protocol				Inclusion Criteria		
Bead ID	Split 1 media	Split 2 media	Split 3 media	Split 4 media	Tag deconvolution	Flow sort criteria	Included in analysis
25	10	-	-	5	fail	pass	No
26	3	5	9	5	pass	pass	Yes
27	2	2	6	5	pass	pass	Yes
28	5	8	3	5	pass	pass	Yes
29	4	9	5	5	pass	pass	Yes
30	10	1	4	5	pass	pass	Yes
31	10	1	8	5	pass	pass	Yes
32	4	3	1	5	pass	pass	Yes
33	9	2	4	5	pass	pass	Yes
34	2	8	6	5	pass	pass	Yes
35	10	2	8	5	pass	pass	Yes
36	10	3	4	5	pass	pass	Yes
37	1	2	6	5	pass	pass	Yes
38	10	1	4	5	pass	pass	Yes
39	5	3	1	5	pass	pass	Yes
40	-	1	8	5	fail	pass	No
41	10	1	9	5	pass	pass	Yes
42	10	1	1	5	pass	pass	Yes
43	10	1	8	5	pass	pass	Yes
44	10	1	6	6	pass	pass	Yes
45	3	7	8	6	pass	pass	Yes
46	10	8	10	6	pass	pass	Yes
47	4	3	2	6	pass	pass	Yes
48	10	1	8	5	pass	pass	Yes
49	2	1	1	5	pass	pass	Yes
50	10	1	1	5	pass	pass	Yes
51	10	8	1	9	pass	pass	Yes
52	4	1	4	9	pass	pass	Yes
53	-	-	2	9	fail	pass	No
54	5	-	8	9	fail	pass	No
55	7	2	4	1	pass	pass	Yes
56	10	-	-	1	fail	pass	No
57	10	-	5	1	fail	pass	No
58	10	1	6	2	pass	pass	Yes
59	1	1	2	8	pass	pass	Yes
60	-	-	-	8	fail	pass	No
61	-	-	-	8	fail	pass	No
62	3	4	10	8	pass	pass	Yes
63	1	9	7	8	pass	pass	Yes
64	10	9	7	8	pass	pass	Yes
65	1	8	1	8	pass	pass	Yes
66	9	6	3	8	pass	pass	Yes
67	7	3	2	8	pass	pass	Yes

	Protocol				Inclusion Criteria		
Bead ID	Split 1 media	Split 2 media	Split 3 media	Split 4 media	Tag deconvolution	Flow sort criteria	Included in analysis
68	1	3	7	8	pass	pass	Yes
69	2	8	10	8	pass	pass	Yes
70	3	1	7	10	pass	pass	Yes
71	2	9	5	10	pass	pass	Yes
72	3	2	6	10	pass	pass	Yes
73	4	1	6	10	pass	pass	Yes
74	10	-	2	10	fail	pass	No
75	6	9	2	10	pass	pass	Yes
76	-	-	-	8	fail	pass	No
77	2	5	9	7	pass	pass	Yes
78	9	8	8	7	pass	pass	Yes
79	9	2	7	7	pass	pass	Yes
80	1	5	6	7	pass	pass	Yes
81	8	2	8	7	pass	pass	Yes
82	5	4	2	7	pass	pass	Yes
83	1	1	6	7	pass	pass	Yes
84	2	1	3	8	pass	pass	Yes
85	8	7	1	8	pass	pass	Yes
86	2	1	6	8	pass	pass	Yes
87	7	1	7	8	pass	pass	Yes
88	5	9	2	8	pass	pass	Yes
89	10	7	1	8	pass	pass	Yes
90	10	1	4	8	pass	pass	Yes
91	10	-	2	8	fail	pass	No
92	10	-	4	9	fail	pass	No
93	3	-	3	9	fail	pass	No
94	2	10	1	9	pass	pass	Yes
95	3	2	3	9	pass	pass	Yes
96	10	1	4	9	pass	pass	Yes
97	10	1	1	1	fail	pass	No
98	6	8	10	1	pass	pass	Yes
99	10	1	2	1	pass	pass	Yes
100	10	2	10	1	pass	pass	Yes
101	-	-	5	1	fail	pass	No
102	-	-	-	1	fail	pass	No
103	2	3	9	7	pass	pass	Yes
104	10	9	3	7	pass	pass	Yes
105	2	8	7	7	pass	pass	Yes
106	6	1	3	7	pass	pass	Yes
107	-	-	-	1	fail	pass	No
108	3	6	8	4	pass	pass	Yes
109	9	6	1	4	pass	pass	Yes
110	-	-	2	7	fail	pass	No

Bead ID	Protocol<br>Split 1 media	Split 2 media	Split 3 media	Split 4 media	Inclusion Criteria<br>Tag deconvolution	Flow sort criteria	Included in analysis
111	-	9	-	7	fail	pass	No
112	-	9	-	7	fail	pass	No
113	-	-	-	7	fail	pass	No
114	-	-	-	7	fail	pass	No
115	-	-	-	7	fail	pass	No
116	-	-	-	7	fail	pass	No
117	10	1	4	7	pass	pass	Yes
118	7	6	9	7	pass	pass	Yes
119	2	-	9	7	fail	pass	No
120	10	8	8	7	pass	pass	Yes
121	3	8	9	7	pass	pass	Yes
122	8	5	3	7	pass	pass	Yes
123	10	4	2	7	pass	pass	Yes
124	6	1	5	7	pass	pass	Yes
125	3	9	9	7	pass	pass	Yes
126	2	2	7	7	pass	pass	Yes
127	1	1	-	7	fail	pass	No
128	7	1	1	7	pass	pass	Yes
129	6	7	2	7	pass	pass	Yes
130	10	1	8	7	pass	pass	Yes
131	6	10	9	7	pass	pass	Yes

6. PROTOCOL ANALYSIS

6.2. LINKAGE ANALYSIS

A schematic diagram of the split-pool experiment is shown in Figure 5. Each rectangle corresponds to a cell culture medium and each row of rectangles corresponds to a split. The black upper number within each rectangle is the medium number and the grey number below is the number of beads within the analysis dataset that passed through that medium (the height of each rectangle is proportional to this number). The linkage lines between rectangles depict frequently occurring combinations of media resulting in an overlay of all protocols in the dataset. The opacity of the linkage lines is proportional to the number of protocols that feature a certain media combination (in this dataset, the darkest and lightest lines correspond to 20 and 2 beads, respectively). An example of the information the linkage analysis provides is the highly represented transition between cell culture medium 10 (split 1) and medium 1 (split 2), 21 out of the 33 beads which start in medium 10 on split 1 transition to medium 1 on split 2. The high representation of this transition indicates that these media may be relevant for the differentiation of phagocytic cells from mES cells.

Figure 5: Schematic diagram showing an overlay of all protocols in the analysis dataset. The darkest line corresponds to 20 beads passing between the 2 connected cell culture media.

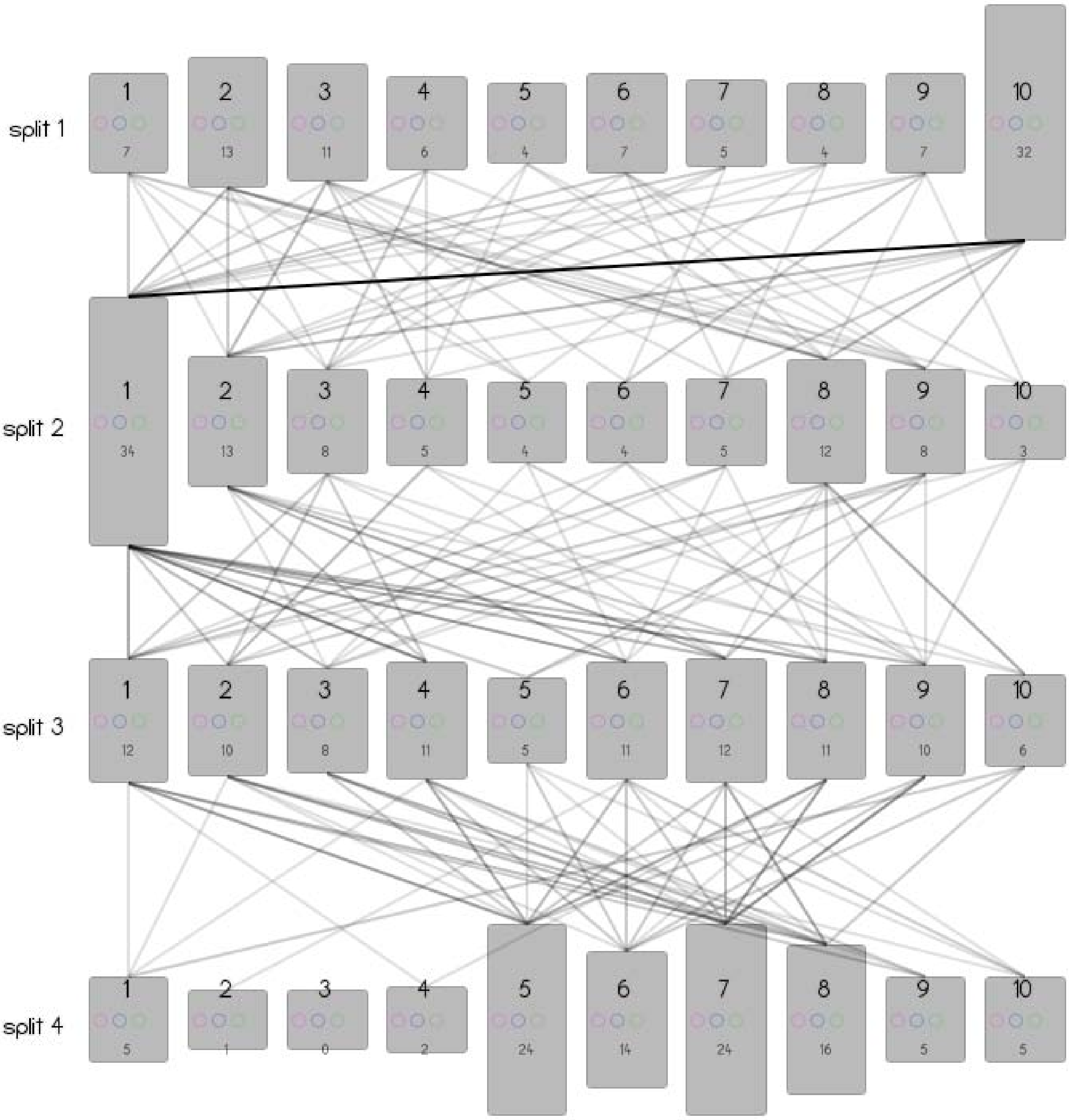

6. PROTOCOL ANALYSIS
6.3. FINGERPRINT ANALYSIS

Fingerprint analysis is a method of finding and comparing hits derived from identical protocols (i.e. four out of four matching cell culture media) and/or groups of beads with similar protocols (i.e. two or three matching media). This analysis method is complementary to linkage analysis which is only considers linkages between adjacent splits.

Table 10 details a fingerprint analysis of the dataset. Each row of the table represents a group of hits clustered according to protocol similarity. Column 2 identifies how many cell culture media the group has in common;, column 3 how many hits are included in the group; and column 4 the probability of the group occurring by chance. Columns 5 - 8 identify the common media in the groups ("-" represents divergence of media), and column 9 lists the bead IDs included in the group. The table is ordered first by probability value and then by number of media matches.

The two groups which have the lowest probability of occurring by chance are firstly, the 20 beads sharing cell culture medium 10 (split 1) and medium 1 (split 2) and secondly, a subset of 9 beads from this group which also share medium 5 on the final split (protocol = 10, 1, -, 5). The probability of either of these groups occurring by chance is extremely small ($p < 0.00000001$). Furthermore, both of these groups contain a triplet and two doublets of beads which have identical protocols (four out of four matching cell culture media). The probability of the dataset containing an identical triplet is low ($p = 0.00142306$) while the occurrence of a doublet is higher ($p = 0.36706135$).

In contrast to the multiple groups clustered around medium 10 (split 1) and medium 1 (split 2) two groups of beads also exist which feature medium 7 on the final split and either medium 9 or 8 on split 3 (groups 16 and 28, respectively).

Therefore two main clusters, or types, of protocols for the differentiation of phagocytic cells from mES cells have been identified. One is dominated by common media on split 1 (medium 10), 2 (medium 1) and to some degree split 4 (medium 5). A second protocol type is dominated by common media on splits 3 (media 8 or 9) and 4 (medium 7).

Table 10: Fingerprint analysis of protocols. Each row of the table represents a cluster of similar protocols . The second column identifies the number of common cell culture media in a group, the third column the number of hits included in the group, the fourth column the probability of the group occurring by chance, columns 5 – 8 the identity of the common media ("-" represents divergence of media), and the final column lists the beads which yielded the protocols included in the group. The data is sorted first by probability value then by number of media match.

Group	Matches (out of 4)	# of Beads	Probability (1= 100%)	Split 1 media #	Split 2 media #	Split 3 media #	Split 4 media #	Bead IDs
1	2	20	0.00000001	10	1	-	-	2,11,16,19,30,31,38,41,42,43,44,48,50,58,90,96,97,99,117,130
2	3	9	0.00000001	10	1	-	5	11,30,31,38,41,42,43,48,50
3	2	11	0.00000027	10	-	-	5	11,30,31,35,36,38,41,42,43,48,50
4	3	6	0.000001	10	1	4	-	16,30,38,90,96,117
5	2	10	0.00000481	-	1	-	5	11,30,31,38,41,42,43,48,49,50
6	4	3	0.00142306	10	1	8	5	31,43,48
7	3	4	0.00308323	10	1	8	-	31,43,48,130
8	3	4	0.00308323	10	1	-	6	2,16,19,44
9	3	4	0.00308323	10	-	8	5	31,35,43,48
10	2	7	0.00547958	10	-	4	-	16,30,36,38,90,96,117
11	2	7	0.00547958	10	-	-	6	2,3,16,19,20,44,46
12	2	7	0.00547958	-	1	4	-	16,30,38,52,90,96,117
13	2	6	0.04230879	10	-	8	-	31,35,43,48,120,130
14	2	6	0.04230879	-	1	-	6	2,8,16,18,19,44
15	2	6	0.04230879	-	1	-	7	83,106,117,124,128,130

Group	Matches (out of 4)	# of Beads	Probability (1= 100%)	Split 1 media #	Split 2 media #	Split 3 media #	Split 4 media #	Bead IDs
16	2	6	0.04230879	-	-	9	7	77,103,118,121,125,131
17	3	3	0.12548994	10	1	1	-	42,50,97
18	3	3	0.12548994	10	-	4	5	30,36,38
19	3	3	0.12548994	-	1	1	5	42,49,50
20	3	3	0.12548994	-	1	8	5	31,43,48
21	2	5	0.25622594	10	-	1	-	42,50,51,89,97
22	2	5	0.25622594	10	-	-	7	104,117,120,123,130
23	2	5	0.25622594	-	1	6	-	44,58,73,83,86
24	2	5	0.25622594	-	1	8	-	31,43,48,128,130
25	2	5	0.25622594	-	1	-	8	59,84,86,87,90
26	2	5	0.25622594	-	2	-	5	24,27,33,35,37
27	2	5	0.25622594	-	-	1	5	32,39,42,49,50
28	2	5	0.25622594	-	-	8	7	78,81,120,128,130
29	4	2	0.36706135	10	1	1	5	42,50
30	4	2	0.36706135	10	1	4	5	30,38
31	2	4	0.83296508	1	-	-	8	59,63,65,68
32	2	4	0.83296508	2	8	-	-	17,34,69,105
33	2	4	0.83296508	2	-	-	7	77,103,105,126
34	2	4	0.83296508	6	-	-	7	106,124,129,131
35	2	4	0.83296508	-	1	1	-	42,49,50,97
36	2	4	0.83296508	-	2	7	-	20,24,79,126
37	2	4	0.83296508	-	8	10	-	17,46,69,98
38	2	4	0.83296508	-	8	-	7	78,105,120,121
39	2	4	0.83296508	-	-	4	5	30,33,36,38
40	2	4	0.83296508	-	-	7	8	63,64,68,87
41	2	4	0.83296508	-	-	8	5	31,35,43,48
42	3	2	0.99101871	1	-	6	7	80,83
43	3	2	0.99101871	1	-	7	8	63,68
44	3	2	0.99101871	2	1	-	8	84,86
45	3	2	0.99101871	2	8	10	-	17,69
46	3	2	0.99101871	2	-	6	5	27,34
47	3	2	0.99101871	2	-	7	7	105,126
48	3	2	0.99101871	2	-	9	7	77,103
49	3	2	0.99101871	3	-	7	5	12,24
50	3	2	0.99101871	3	-	9	7	121,125
51	3	2	0.99101871	6	1	-	7	106,124
52	3	2	0.99101871	10	1	6	-	44,58
53	3	2	0.99101871	10	1	9	-	19,41
54	3	2	0.99101871	10	1	-	1	97,99
55	3	2	0.99101871	10	1	-	7	117,130
56	3	2	0.99101871	10	-	1	5	42,50
57	3	2	0.99101871	10	-	6	6	3,44
58	3	2	0.99101871	10	-	8	7	120,130
59	3	2	0.99101871	-	1	4	5	30,38
60	3	2	0.99101871	-	1	4	9	52,96
61	3	2	0.99101871	-	1	8	7	128,130
62	3	2	0.99101871	-	1	9	6	8,19
63	3	2	0.99101871	-	2	6	5	27,37
64	3	2	0.99101871	-	2	7	7	79,126
65	3	2	0.99101871	-	3	1	5	32,39
66	3	2	0.99101871	-	4	2	7	82,123
67	3	2	0.99101871	-	7	1	8	85,89
68	3	2	0.99101871	-	8	8	7	78,120
69	3	2	0.99101871	-	8	10	6	17,46
70	3	2	0.99101871	-	9	7	8	63,64
71	2	3	0.99996585	1	-	6	-	37,80,83

Confidential

Group	Matches (out of 4)	# of Beads	Probability (1= 100%)	Split 1 media #	Split 2 media #	Split 3 media #	Split 4 media #	Bead IDs
72	2	3	0.99996585	2	1	-	-	49,84,86
73	2	3	0.99996585	2	-	6	-	27,34,86
74	2	3	0.99996585	2	-	-	5	27,34,49
75	2	3	0.99996585	2	-	-	8	69,84,86
76	2	3	0.99996585	3	2	-	-	24,72,95
77	2	3	0.99996585	3	-	7	-	12,24,70
78	2	3	0.99996585	3	-	9	-	26,121,125
79	2	3	0.99996585	3	-	-	5	12,24,26
80	2	3	0.99996585	4	-	-	5	21,29,32
81	2	3	0.99996585	10	2	-	-	20,35,100
82	2	3	0.99996585	10	8	-	-	46,51,120
83	2	3	0.99996585	10	-	6	-	3,44,58
84	2	3	0.99996585	10	-	-	1	97,99,100
85	2	3	0.99996585	10	-	-	8	64,89,90
86	2	3	0.99996585	-	1	3	-	11,84,106
87	2	3	0.99996585	-	1	7	-	18,70,87
88	2	3	0.99996585	-	1	9	-	8,19,41
89	2	3	0.99996585	-	2	6	-	27,37,72
90	2	3	0.99996585	-	2	-	7	79,81,126
91	2	3	0.99996585	-	3	-	5	32,36,39
92	2	3	0.99996585	-	4	2	-	21,82,123
93	2	3	0.99996585	-	5	-	7	77,80,122
94	2	3	0.99996585	-	9	-	8	63,64,88
95	2	3	0.99996585	-	-	1	8	65,85,89
96	2	3	0.99996585	-	-	2	7	82,123,129
97	2	3	0.99996585	-	-	2	8	59,67,88
98	2	3	0.99996585	-	-	3	7	104,106,122
99	2	3	0.99996585	-	-	6	5	27,34,37
100	2	3	0.99996585	-	-	7	7	79,105,126

Confidential

6. PROTOCOL ANALYSIS
6.4. HIERACHICAL CLUSTERING DENDROGRAM

Hierarchical clustering is a method of grouping protocols according to similarity.

The hierarchical clustering illustrated in figure 6 has two sections. The upper dendrogram depicts the clustering structure and order. The lower coloured array is a graphical representation of the media combinations that comprise each protocol. The y-axis of the dendrogram measures intra-cluster similarity, i.e. horizontal bars closest to the bottom of the dendrogram link identical protocols whilst a horizontal bar half way up the y-axis represents a larger cluster containing beads whose protocols are not identical but do share some similarity. The hierarchical nature of the clustering means that beads are first clustered into small highly similar clusters, which are in turn included in larger clusters. This is repeated until the final cluster is reached (uppermost horizontal bar) which includes all beads in the dataset. Each node at the bottom of the dendrogram (leaf node) corresponds to a protocol. Each row of the coloured array corresponds to a split and each column of the array a protocol, with the columns aligned to the leaf nodes of the dendrogram. The horizontal coloured legend below the array specifies the colour associated with each media number. The two largest clusters are labeled A and B. Cluster A contains 18 beads, 14 of which share medium 10 (split 1) and medium 1 (split 2) and are a subset of the 20 beads in group 1 of the fingerprint analysis (table 10) and the strongest linkage line in figure 5. 12 of the beads in cluster A also share medium 5 (split 4) and include a triplet and two doublets of beads which have identical protocols (four out of four matching cell culture media). The probabilities of these groups occurring by chance are discussed in section 6.3. Cluster B includes beads which predominantly share medium 7 on the final split and media 8 or 9 on the third split.

Figure 6: Dendrogram showing the hierarchical clustering of similar protocols.

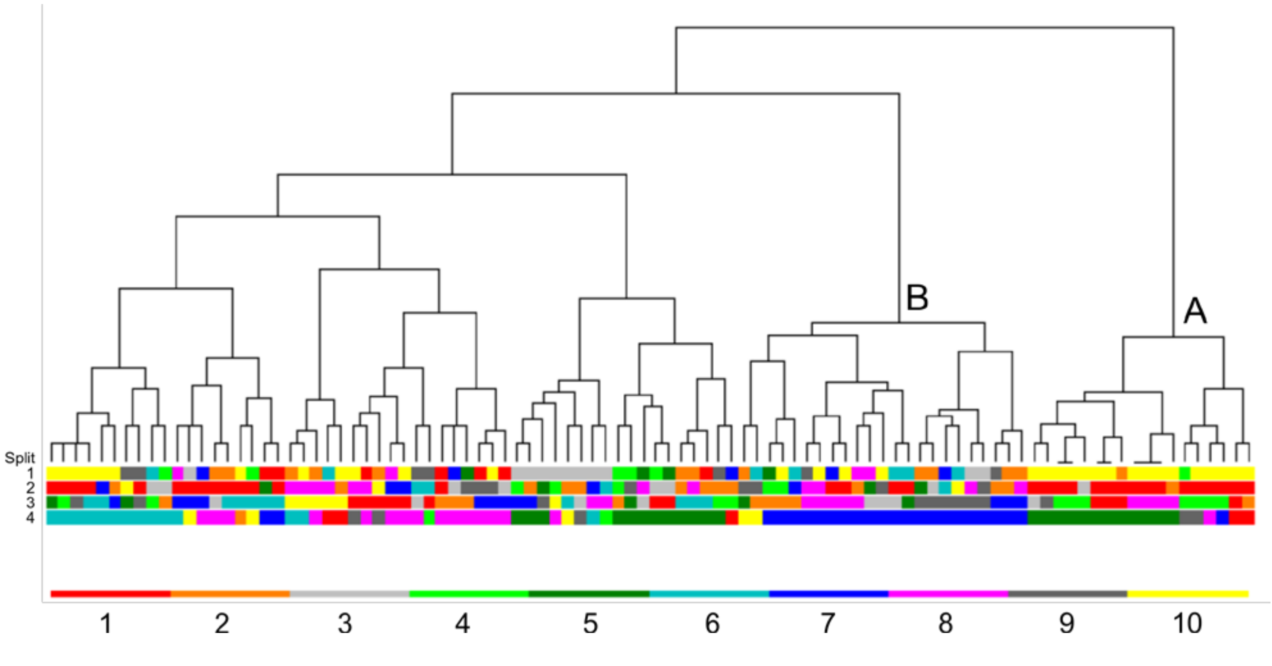

6. PROTOCOL ANALYSIS
6.5. SIMILARITY MATRIX

The similarity matrix is a diagrammatic representation of a pair-wise comparison of all protocols. Each column and each row corresponds to a protocol. The brightness of each cell in the matrix is proportional to the number of cell culture media shared by the two protocols. The brightest cell corresponds to protocols which have common media (i.e. identical protocols) in common, while a black cell corresponds to two protocols with no common media. The diagonal row of cells (from the top left to bottom right) corresponds to beads being compared to themselves. Beads are ordered along both the x- and y-axis according to the hierarchical clustering dendrogram illustrated in figure 7. The coloured arrays on the bottom and right of the matrix illustrate the protocol of each bead. The horizontal coloured legend below the matrix specifies the colour associated with each medium number.

The similarity matrix clearly displays clusters of similar protocols as square regions comprising brightly coloured cells. The size and overall brightness of the square regions are measures of the cluster size and intra-cluster protocol similarity, respectively. Additionally, the similarity matrix provides a global overview of the protocol similarity of beads or clusters to the remaining dataset. For example the brightness of the cells in the columns above cluster A indicate that the beads contained within cluster A also have similarity with many other beads in the dataset, especially to the beads contained in a smaller cluster labeled C. Interestingly, some beads in cluster B have similarity to beads of cluster A, but little similarity to the remaining dataset.

Figure 7: Similarity matrix comprising a pair-wise comparison of all protocols.

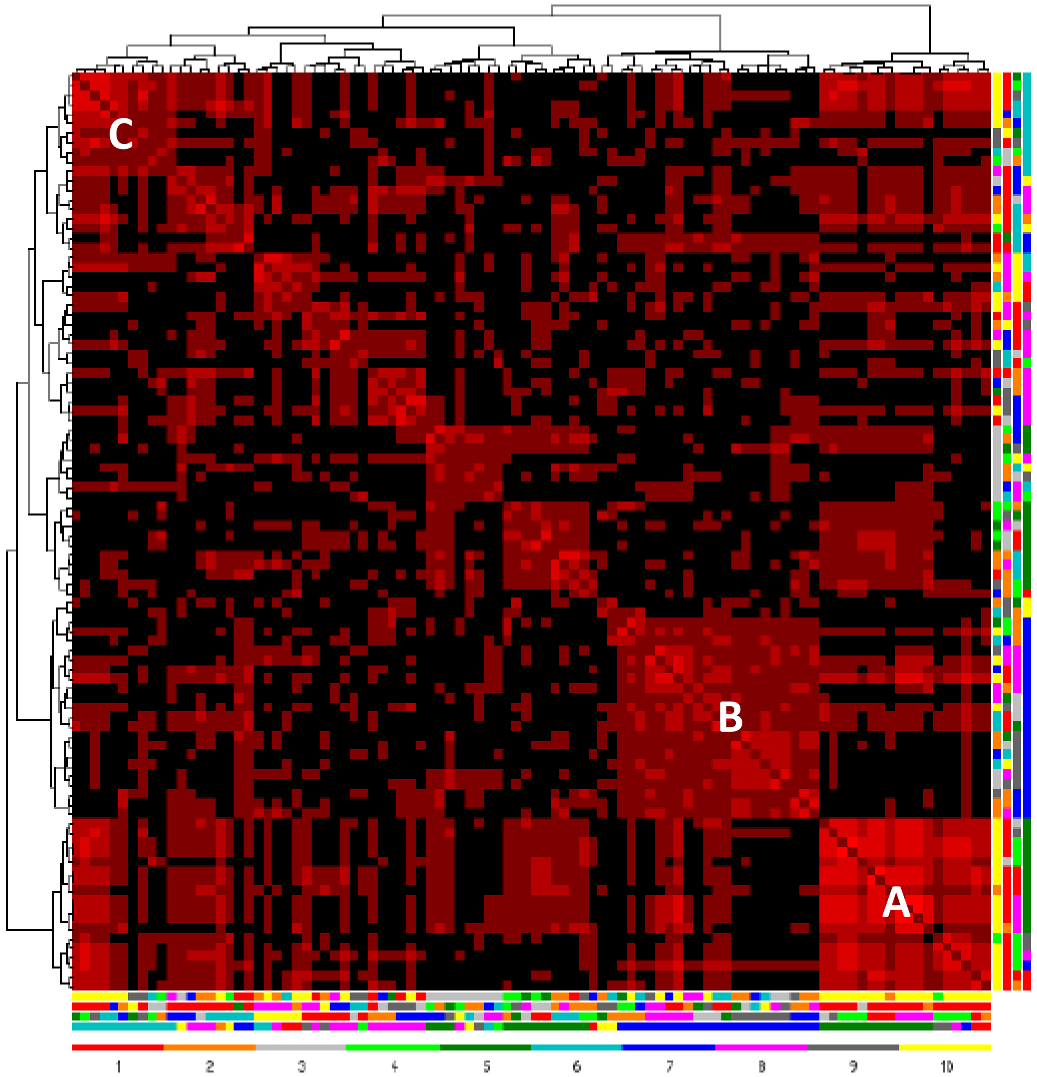

 Confidential

7. SUMMARY AND CONCLUSIONS

A CombiCult™ experiment was performed in which 40 serum-free cell culture media were screened in 10,000 combinations. The 40 media were tested over four split-pool cycles (performed on day 1, day 2, day 5 and day 7, respectively) with 10 media included in each cycle, such that 10,000 media combinations (protocols) are tested. A total of 300,000 beads were used so that on average each protocol was sampled by 30 beads. Following completion of the split-pool experiment (day 14) beads were screened using a functional phagocytosis assay and individual positive beads isolated.

Of the total number of beads screened, 137 (0.053 %) were sorted and verified as 'hits'. The tags from each hit were read to infer the series of cell culture media (protocol) that produced differentiated cells. Of the 137 'hit' beads, tags could be conclusively deconvoluted from 96 (70.1 %). Putative differentiation protocols were analysed using bespoke bioinformatics software Ariadne™ (v1.0) allowing protocols to be ranked and a subset to be selected for further validation and study. The 19 selected protocols are listed below in Table 11 as well as in the executive summary (Table 1).

Validated protocols 1-5 form part of cluster A shown in the hierarchical clustering dendrogram and similarity matrix and share cell culture medium 10 on split 1 and medium 1 on split 2. The first validated protocol is represented by a triplet of beads with identical protocols and has a low probability of occurring randomly ($p = 0.00142306$). The second and third validated protocols are represented by doublets of beads with identical protocols. Protocols 15-17 form part of cluster B of the hierarchical clustering dendrogram and similarity matrix and have the general protocol structure of X-Y-9/8-7 and protocols 18 and 19 are hit beads which had colonies of both neural precursors and phagocytes.

The 19 validated protocols therefore include two general types of protocol for the differentiation of phagocytic cells from mES cells. One is dominated by media 10 on split 1, media 1 on split 2 and to some degree media 5 on split 4. The second protocol type is dominated by media 8 or 9 on split 3 and media 7 on split 4.

Table 11: Validated 19 protocols determined by Ariadne™ (v1.0). The protocols are identified by the series of cell culture media on each split-pool cycle (split 1 – split 4), e.g. 10-1-8-5.

Validation #	Protocol	Bead Ids		Basal Medium	Supplements
1	10-1-8-5	31, 43, 48	Split 1 (Day 1)	KO-DMEM	FCS (15%), LIF (0.1%)
			Split 2 (Day 2)	DMEM/F12	ITS (1%), RA (0.05 µM)
			Split 3 (Day 5)	DMEM	ITS (1%), BSA (1mg/mL), PDGF BB (50ng/mL)
			Split 4 (Day 7)	DMEM	B27 (1%), FGF a (100ng/mL)
2	10-1-1-5	42, 50	Split 1 (Day 1)	KO-DMEM	FCS (15%), LIF (0.1%)
			Split 2 (Day 2)	DMEM/F12	ITS (1%), RA (0.05 µM)
			Split 3 (Day 5)	DMEM/F12	B27 (1%), EGF (20ng/mL),FGF b (20ng/mL), PDGF-AA (20ng/mL)
			Split 4 (Day 7)	DMEM	B27 (1%), FGF a (100ng/mL)
3	10-1-4-5	30, 38	Split 1 (Day 1)	KO-DMEM	FCS (15%), LIF (0.1%)
			Split 2 (Day 2)	DMEM/F12	ITS (1%), RA (0.05 µM)
			Split 3 (Day 5)	RPMI	B27 (1%), EGF (20ng/mL), FGF b (2ng/mL), Noggin (100ng/mL)
			Split 4 (Day 7)	DMEM	B27 (1%), FGF a (100ng/mL)
4	10-1-4-8	90	Split 1 (Day 1)	KO-DMEM	FCS (15%), LIF (0.1%)
			Split 2 (Day 2)	DMEM/F12	ITS (1%), RA (0.05 µM)
			Split 3 (Day 5)	RPMI	B27 (1%), EGF (20ng/mL), FGF b (2ng/mL), Noggin

Validation #	Protocol	Bead Ids		Basal Medium	Supplements
					(100ng/mL)
			Split 4 (Day 7)	DMEM	ITS (1%), BSA (1mg/mL), PDGF-BB (50ng/mL)
5	10-1-1-1	97	Split 1 (Day 1)	KO-DMEM	FCS (15%), LIF (0.1%)
			Split 2 (Day 2)	DMEM/F12	ITS (1%), RA (0.05 µM)
			Split 3 (Day 5)	DMEM/F12	B27 (1%), EGF (20ng/mL),FGF b (20ng/mL), PDGF-AA (20ng/mL)
			Split 4 (Day 7)	DMEM/F12	B27 (1%), PDGF-AA (20ng/mL)
6	10-2-8-5	35	Split 1 (Day 1)	KO-DMEM	FCS (15%), LIF (0.1%)
			Split 2 (Day 2)	DMEM/F12/Neurobasal	N2 (0.5%), B27 (0.5%), FGF-4 (2ng/mL)
			Split 3 (Day 5)	DMEM	ITS (1%), BSA (1mg/mL), PDGF BB (50ng/mL)
			Split 4 (Day 7)	DMEM	B27 (1%), FGF a (100ng/mL)
7	2-1-6-8	86	Split 1 (Day 1)	DMEM/F12/Neurobasal	N2 (0.5%), B27 (0.5%)
			Split 2 (Day 2)	DMEM/F12	ITS (1%), RA (0.05 µM)
			Split 3 (Day 5)	HCM	FGF-4 (30ng/mL), BMP-2 (20ng/mL)
			Split 4 (Day 7)	DMEM	ITS (1%), BSA (1mg/mL), PDGF-BB (50ng/mL)
8	2-1-1-5	49	Split 1 (Day 1)	DMEM/F12/Neurobasal	N2 (0.5%), B27 (0.5%)
			Split 2 (Day 2)	DMEM/F12	ITS (1%), RA (0.05 µM)
			Split 3 (Day 5)	DMEM/F12	B27 (1%), EGF (20ng/mL),FGF b (20ng/mL), PDGF-AA (20ng/mL)
			Split 4 (Day 7)	DMEM	B27 (1%), FGF a (100ng/mL)
9	2-2-6-5	27	Split 1 (Day 1)	DMEM/F12/Neurobasal	N2 (0.5%), B27 (0.5%)
			Split 2 (Day 2)	DMEM/F12/Neurobasal	N2 (0.5%), B27 (0.5%), FGF-4 (2ng/mL)
			Split 3 (Day 5)	HCM	FGF-4 (30ng/mL), BMP-2 (20ng/mL)
			Split 4 (Day 7)	DMEM	B27 (1%), FGF a (100ng/mL)
10	10-2-7-6	20	Split 1 (Day 1)	KO-DMEM	FCS (15%), LIF (0.1%)
			Split 2 (Day 2)	DMEM/F12/Neurobasal	N2 (0.5%), B27 (0.5%), FGF-4 (2ng/mL)
			Split 3 (Day 5)	STEMLINE	SCF (20ng/mL), BMP-2 (5ng/mL), TGF-β1 (5ng/mL), TPO (20ng/mL)
			Split 4 (Day 7)	HCM	HGF (20 ng/mL)
11	10-8-10-6	46	Split 1 (Day 1)	KO-DMEM	FCS (15%), LIF (0.1%)
			Split 2 (Day 2)	IMDM (75 %)/HAM F12 (25 %)	N2 (1%), B27 (1%), BSA (0.5 mg/mL), Ascorbic Acid (0.5 mM)
			Split 3 (Day 5)	DMEM Low Glucose	ITS (1%), Fibronectin (5µg/mL)
			Split 4 (Day 7)	HCM	HGF (20 ng/mL)
12	10-7-6-6	3	Split 1 (Day 1)	KO-DMEM	FCS (15%), LIF (0.1%)
			Split 2 (Day 2)	STEMLINE	SCF (20ng/mL)
			Split 3 (Day 5)	HCM	FGF-4 (30ng/mL), BMP-2 (20ng/mL)
			Split 4 (Day 7)	HCM	HGF (20 ng/mL)
13	10-7-1-8	89	Split 1 (Day 1)	KO-DMEM	FCS (15%), LIF (0.1%)
			Split 2 (Day 2)	STEMLINE	SCF (20ng/mL)
			Split 3 (Day 5)	DMEM/F12	B27 (1%), EGF (20ng/mL),FGF b (20ng/mL), PDGF-AA (20ng/mL)
			Split 4 (Day 7)	DMEM	ITS (1%), BSA (1mg/mL), PDGF-BB (50ng/mL)
14	10-9-7-8	64	Split 1 (Day 1)	KO-DMEM	FCS (15%), LIF (0.1%)
			Split 2 (Day 2)	GMEM	KSR (5%), TGF-β1 (2ng/mL), BMP-2 (10ng/mL)
			Split 3 (Day 5)	STEMLINE	SCF (20ng/mL), BMP-2 (5ng/mL), TGF-β1 (5ng/mL), TPO (20ng/mL)
			Split 4 (Day 7)	DMEM	ITS (1%), BSA (1mg/mL), PDGF-BB (50ng/mL)

Confidential

Validation #	Protocol	Bead Ids		Basal Medium	Supplements
15	2-2-7-7	126	Split 1 (Day 1)	DMEM/F12/Neurobasal	N2 (0.5%), B27 (0.5%)
			Split 2 (Day 2)	DMEM/F12/Neurobasal	N2 (0.5%), B27 (0.5%), FGF-4 (2ng/mL)
			Split 3 (Day 5)	STEMLINE	SCF (20ng/mL), BMP-2 (5ng/mL), TGF-β1 (5ng/mL), TPO (20ng/mL)
			Split 4 (Day 7)	STEMLINE	IL-3 (30ng/mL), IL-6 (20ng/mL), TPO (20ng/mL)
16	10-8-8-7	120	Split 1 (Day 1)	KO-DMEM	FCS (15%), LIF (0.1%)
			Split 2 (Day 2)	IMDM (75 %)/HAM F12 (25 %)	N2 (1%), B27 (1%), BSA (0.5 mg/mL), Ascorbic Acid (0.5 mM)
			Split 3 (Day 5)	DMEM	ITS (1%), BSA (1mg/mL), PDGF BB (50ng/mL)
			Split 4 (Day 7)	STEMLINE	IL-3 (30ng/mL), IL-6 (20ng/mL), TPO (20ng/mL)
17	3-8-9-7	121	Split 1 (Day 1)	Advanced DMEM (CDM)	-
			Split 2 (Day 2)	IMDM (75 %)/HAM F12 (25 %)	N2 (1%), B27 (1%), BSA (0.5 mg/mL), Ascorbic Acid (0.5 mM)
			Split 3 (Day 5)	GMEM	KSR (5%), TGF-β1 (2ng/mL), BMP-2 (10ng/mL), Insulin (1µg/mL), Ascorbic Acid (50µg/mL)
			Split 4 (Day 7)	STEMLINE	IL-3 (30ng/mL), IL-6 (20ng/mL), TPO (20ng/mL)
18	2-8-6-5	34	Split 1 (Day 1)	DMEM/F12/Neurobasal	N2 (0.5%), B27 (0.5%)
			Split 2 (Day 2)	IMDM (75 %)/HAM F12 (25 %)	N2 (1%), B27 (1%), BSA (0.5 mg/mL), Ascorbic Acid (0.5 mM)
			Split 3 (Day 5)	HCM	FGF-4 (30ng/mL), BMP-2 (20ng/mL)
			Split 4 (Day 7)	DMEM	B27 (1%), FGF a (100ng/mL)
19	2-10-1-9	94	Split 1 (Day 1)	DMEM/F12/Neurobasal	N2 (0.5%), B27 (0.5%)
			Split 2 (Day 2)	Advanced DMEM	-
			Split 3 (Day 5)	DMEM/F12	B27 (1%), EGF (20ng/mL),FGF b (20ng/mL), PDGF-AA (20ng/mL)
			Split 4 (Day 7)	GMEM	KSR (5%), TGF-β1 (2ng/mL), BMP-2 (10ng/mL), Insulin (1µg/mL), Ascorbic Acid (50µg/mL)

Confidential

ANNEX 1: FLOW SORTING BIN 1 – File 1 of 1

Bin 1: COPAS scatter plots showing events for medium 1 of the final split-pool cycle (bin 1). In the first scatter plot (optical extinction [ext] vs. time of flight [TOF]), events depicted by a red dot fall within the gate defining monomeric beads and were sorted. The second scatter plot displays the fluorescent values of the sorted beads (green vs. red fluorescence intensity). Beads with a red fluorescent intensity above the threshold value are depicted as green dots and were individually dispensed into 96 well plates.

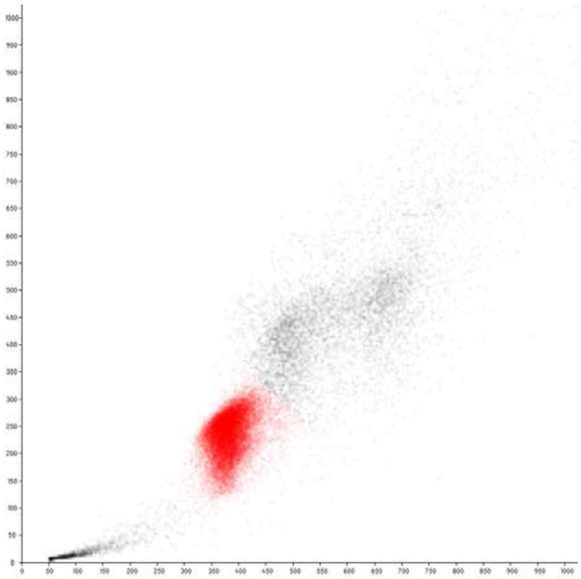

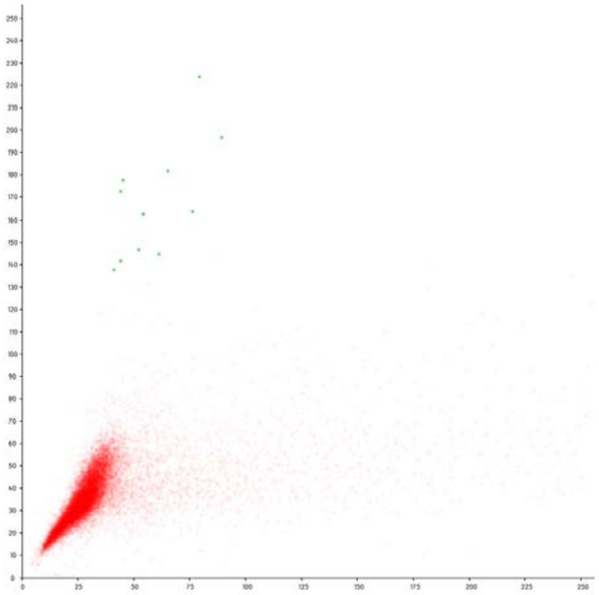

Number of beads individually dispensed: 13

ANNEX 1: FLOW SORTING BIN 2 – File 1 of 1

Bin 2: COPAS scatter plots showing events for medium 2 of the final split-pool cycle (bin 2). In the first scatter plot (optical extinction [ext] vs. time of flight [TOF]), events depicted by a red dot fall within the gate defining monomeric beads and were sorted. The second scatter plot displays the fluorescent values of the sorted beads (green vs. red fluorescence intensity). Beads with a red fluorescent intensity above the threshold value are depicted as green dots and were individually dispensed into 96 well plates.

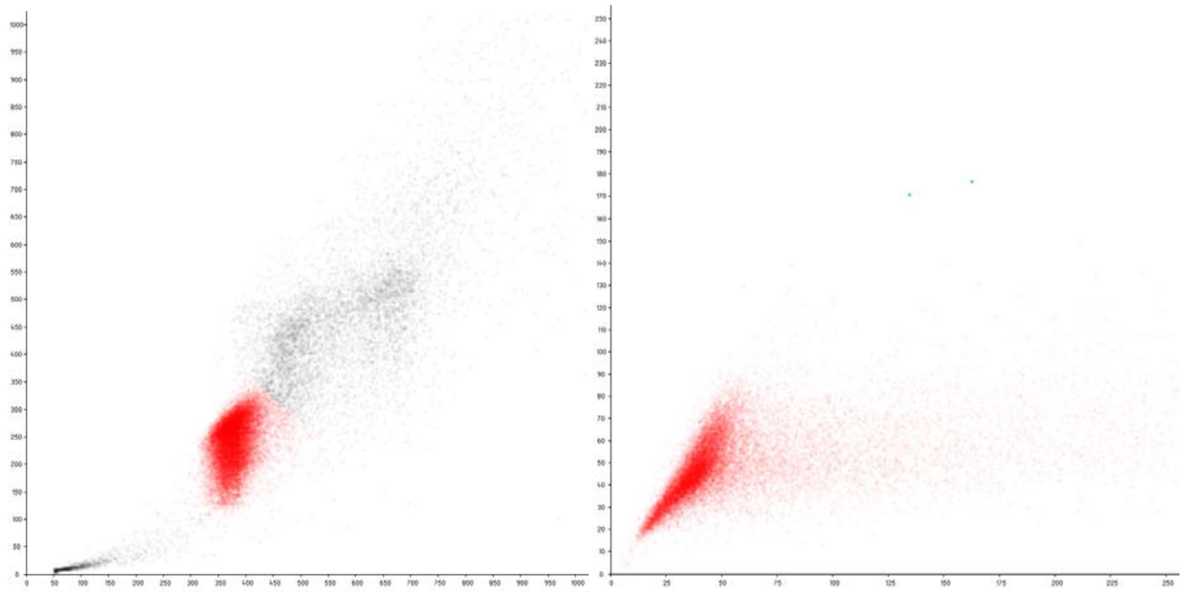

Number of beads individually dispensed: 2

ANNEX 1: FLOW SORTING BIN 3 – File 1 of 1

Bin 3: COPAS scatter plots showing events for medium 3 of the final split-pool cycle (bin 3). In the first scatter plot (optical extinction [ext] vs. time of flight [TOF]), events depicted by a red dot fall within the gate defining monomeric beads and were sorted. The second scatter plot displays the fluorescent values of the sorted beads (green vs. red fluorescence intensity). Beads with a red fluorescent intensity above the threshold value are depicted as green dots and were individually dispensed into 96 well plates.

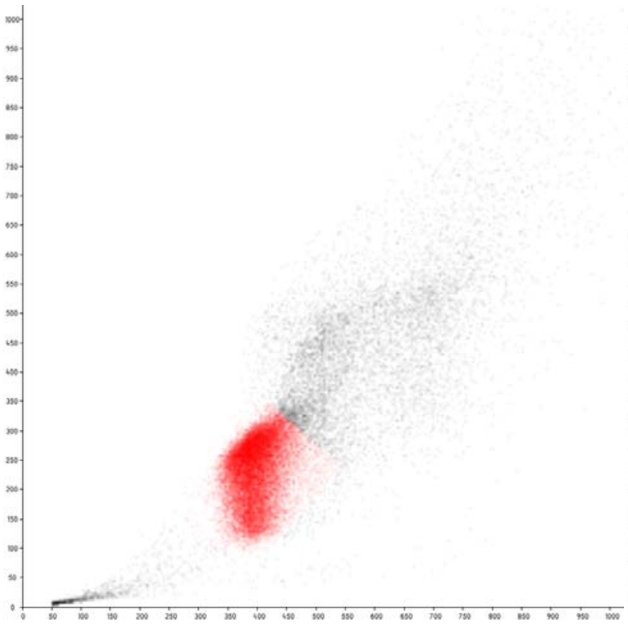

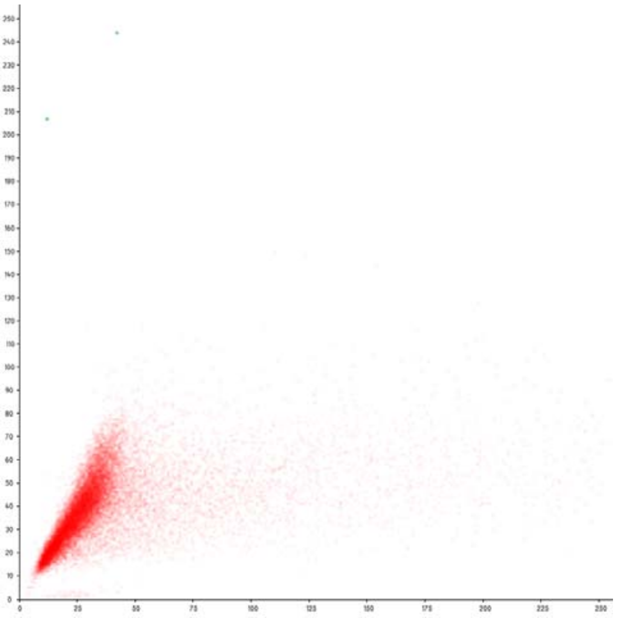

Number of beads individually dispensed: 3

ANNEX 1: FLOW SORTING BIN 4 – File 1 of 4

Bin 4: COPAS scatter plots showing events for medium 4 of the final split-pool cycle (bin 4). In the first scatter plot (optical extinction [ext] vs. time of flight [TOF]), events depicted by a red dot fall within the gate defining monomeric beads and were sorted. The second scatter plot displays the fluorescent values of the sorted beads (green vs. red fluorescence intensity). Beads with a red fluorescent intensity above the threshold value are depicted as green dots and were individually dispensed into 96 well plates.

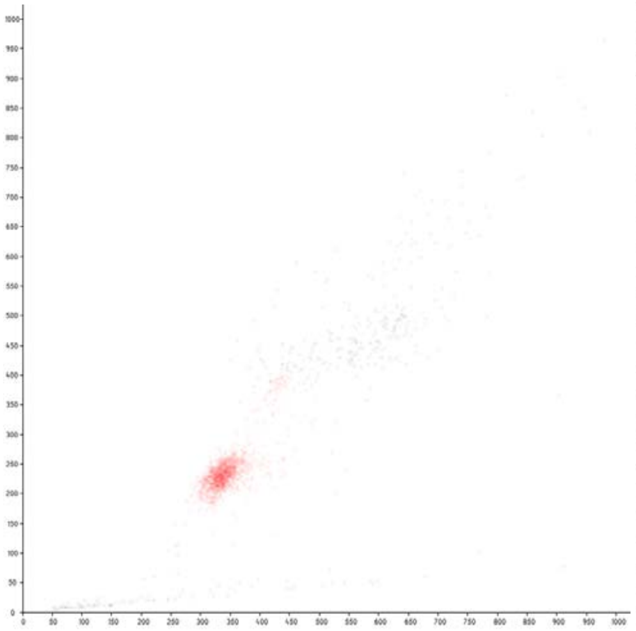

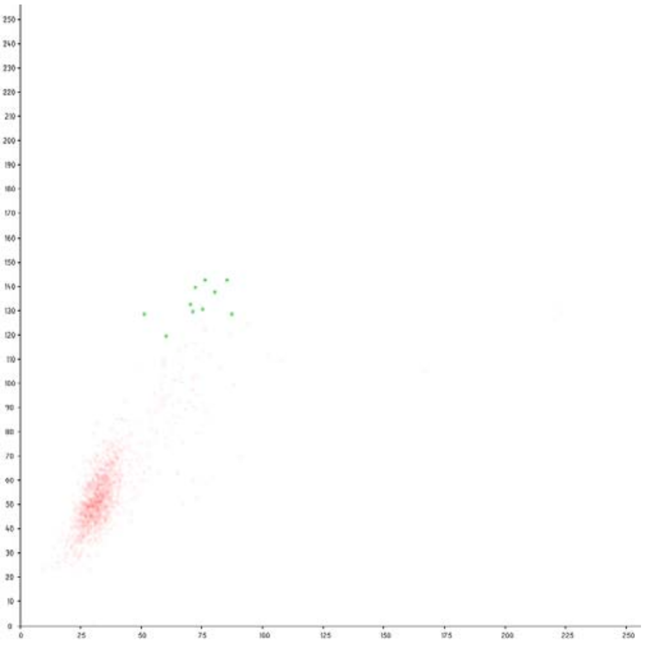

Number of beads individually dispensed: 10

ANNEX 1: FLOW SORTING BIN 4 – File 2 of 4

Bin 4: COPAS scatter plots showing events for medium 4 of the final split-pool cycle (bin 4). In the first scatter plot (optical extinction [ext] vs. time of flight [TOF]), events depicted by a red dot fall within the gate defining monomeric beads and were sorted. The second scatter plot displays the fluorescent values of the sorted beads (green vs. red fluorescence intensity). Beads with a red fluorescent intensity above the threshold value are depicted as green dots and were individually dispensed into 96 well plates.

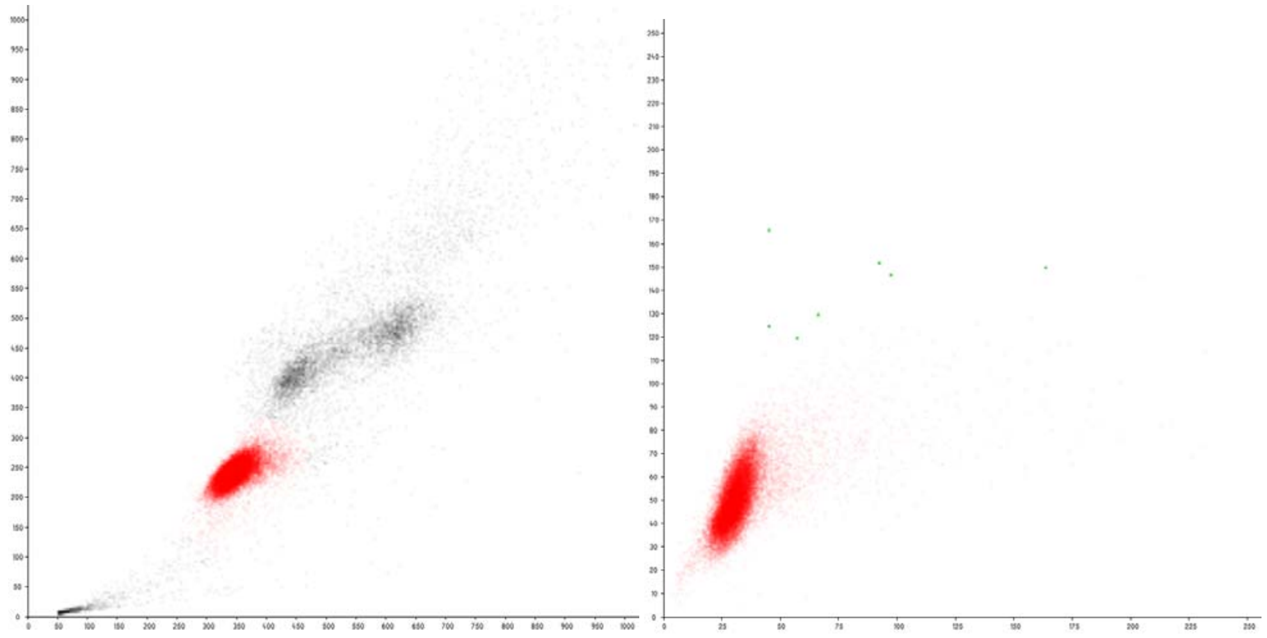

Number of beads individually dispensed: 7

ANNEX 1: FLOW SORTING BIN 4 – File 3 of 4

Bin 4: COPAS scatter plots showing events for medium 4 of the final split-pool cycle (bin 4). In the first scatter plot (optical extinction [ext] vs. time of flight [TOF]), events depicted by a red dot fall within the gate defining monomeric beads and were sorted. The second scatter plot displays the fluorescent values of the sorted beads (green vs. red fluorescence intensity). Beads with a red fluorescent intensity above the threshold value are depicted as green dots and were individually dispensed into 96 well plates.

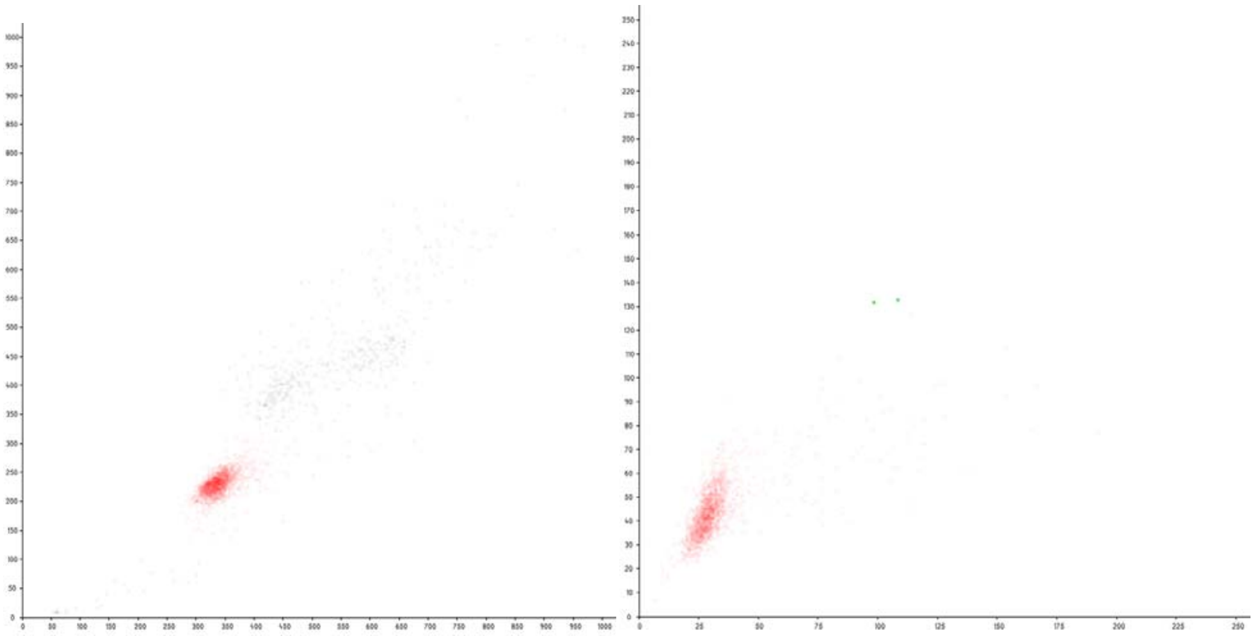

Number of beads individually dispensed: 2

ANNEX 1: FLOW SORTING BIN 4 – File 4 of 4

Bin 4: COPAS scatter plots showing events for medium 4 of the final split-pool cycle (bin 4). In the first scatter plot (optical extinction [ext] vs. time of flight [TOF]), events depicted by a red dot fall within the gate defining monomeric beads and were sorted. The second scatter plot displays the fluorescent values of the sorted beads (green vs. red fluorescence intensity). Beads with a red fluorescent intensity above the threshold value are depicted as green dots and were individually dispensed into 96 well plates.

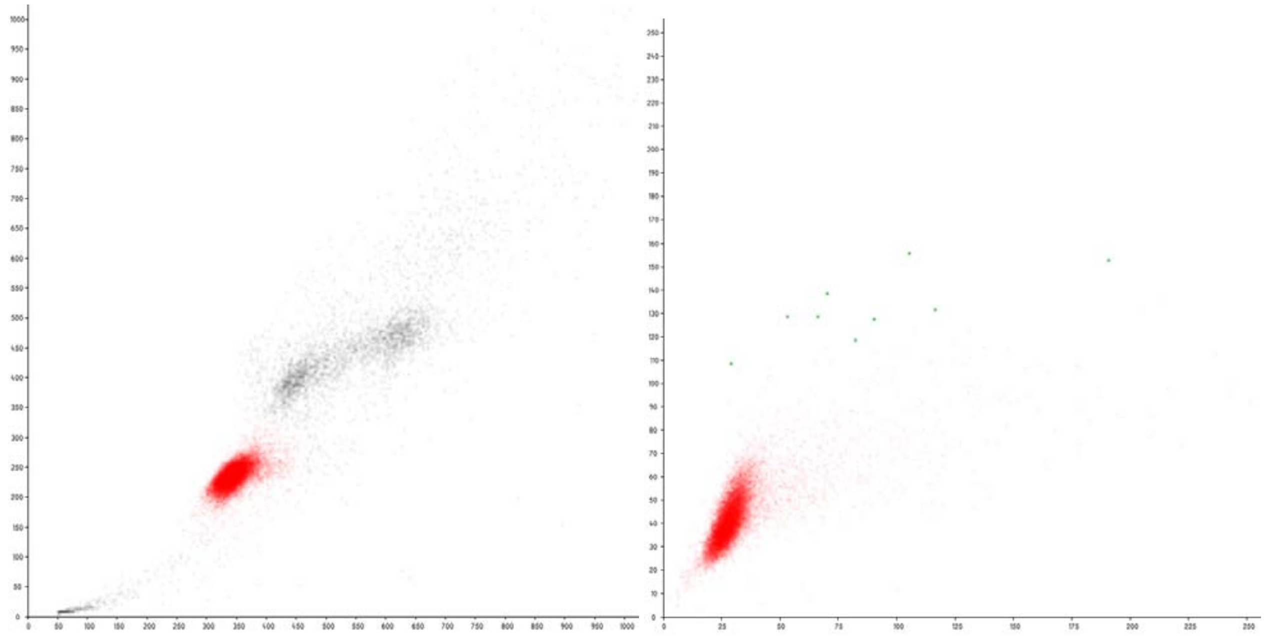

Number of beads individually dispensed: 9

ANNEX 1: FLOW SORTING BIN 5 – File 1 of 2

Bin 5: COPAS scatter plots showing events for medium 5 of the final split-pool cycle (bin 5). In the first scatter plot (optical extinction [ext] vs. time of flight [TOF]), events depicted by a red dot fall within the gate defining monomeric beads and were sorted. The second scatter plot displays the fluorescent values of the sorted beads (green vs. red fluorescence intensity). Beads with a red fluorescent intensity above the threshold value are depicted as green dots and were individually dispensed into 96 well plates.

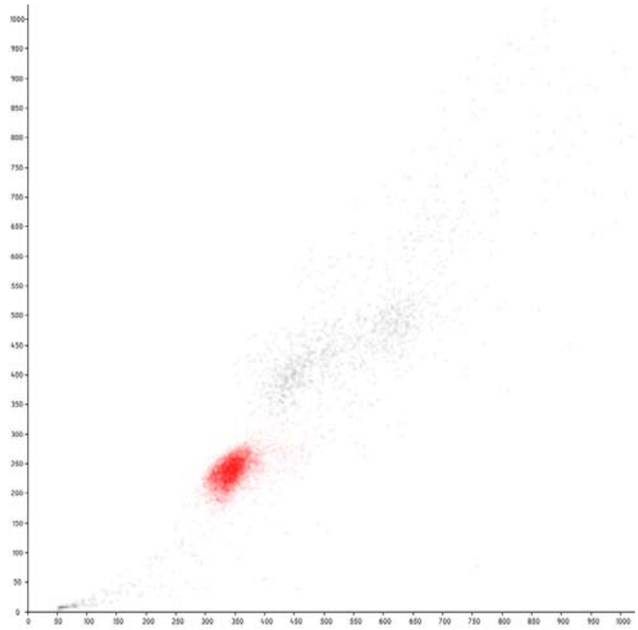

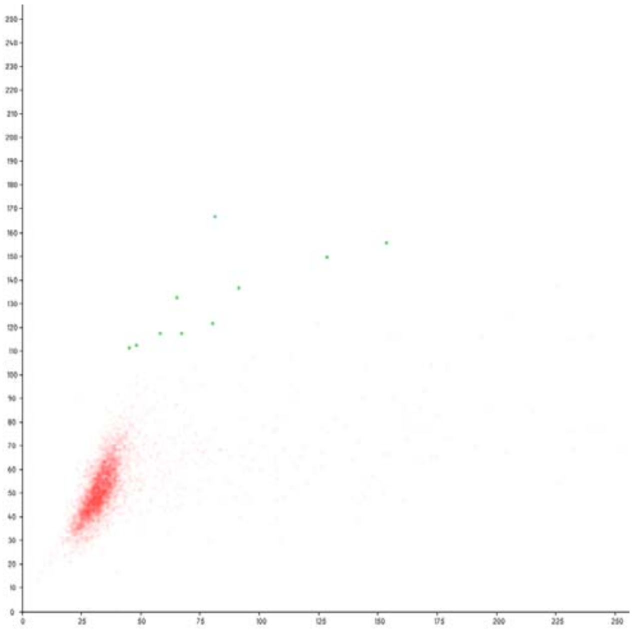

Number of beads individually dispensed: 11

ANNEX 1: FLOW SORTING BIN 5 – File 2 of 2

Bin 5: COPAS scatter plots showing events for medium 5 of the final split-pool cycle (bin 5). In the first scatter plot (optical extinction [ext] vs. time of flight [TOF]), events depicted by a red dot fall within the gate defining monomeric beads and were sorted. The second scatter plot displays the fluorescent values of the sorted beads (green vs. red fluorescence intensity). Beads with a red fluorescent intensity above the threshold value are depicted as green dots and were individually dispensed into 96 well plates.

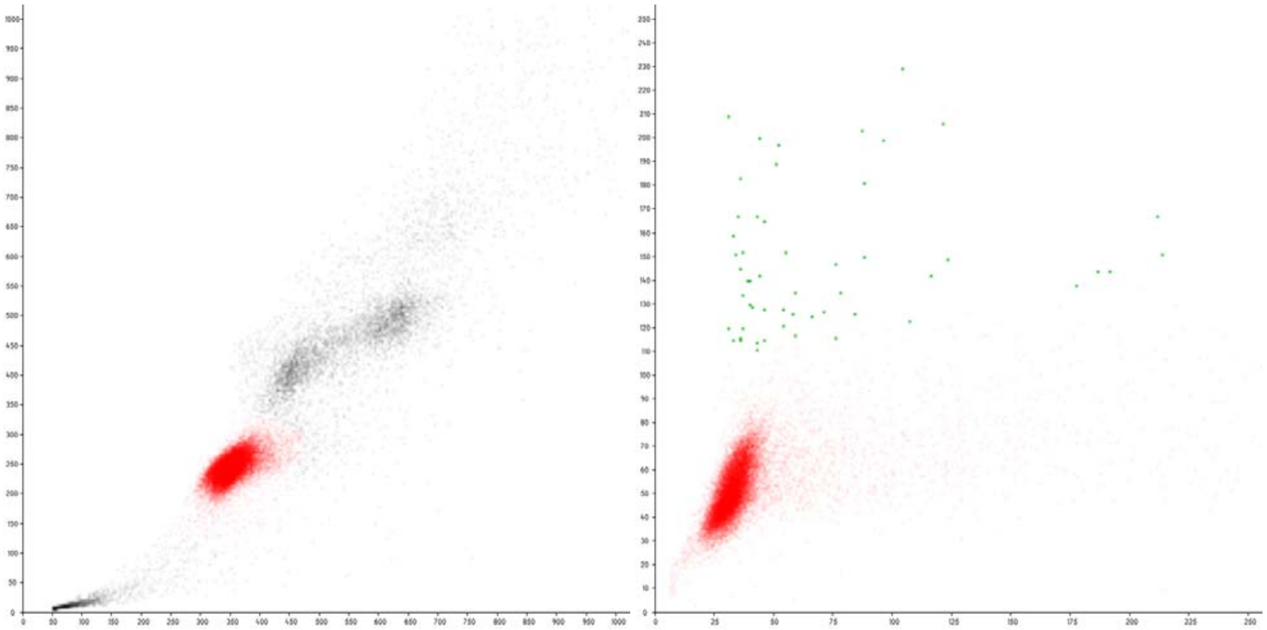

Number of beads individually dispensed: 56

ANNEX 1: FLOW SORTING BIN 6 – File 1 of 2

Bin 6: COPAS scatter plots showing events for medium 6 of the final split-pool cycle (bin 6). In the first scatter plot (optical extinction [ext] vs. time of flight [TOF]), events depicted by a red dot fall within the gate defining monomeric beads and were sorted. The second scatter plot displays the fluorescent values of the sorted beads (green vs. red fluorescence intensity). Beads with a red fluorescent intensity above the threshold value are depicted as green dots and were individually dispensed into 96 well plates.

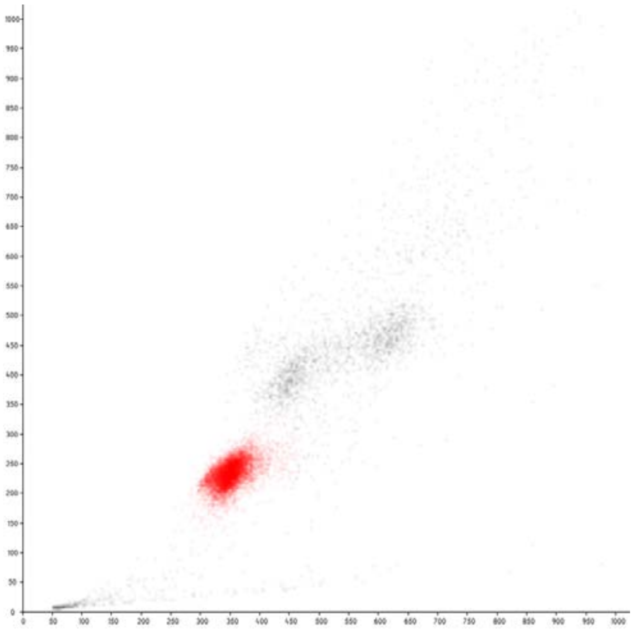

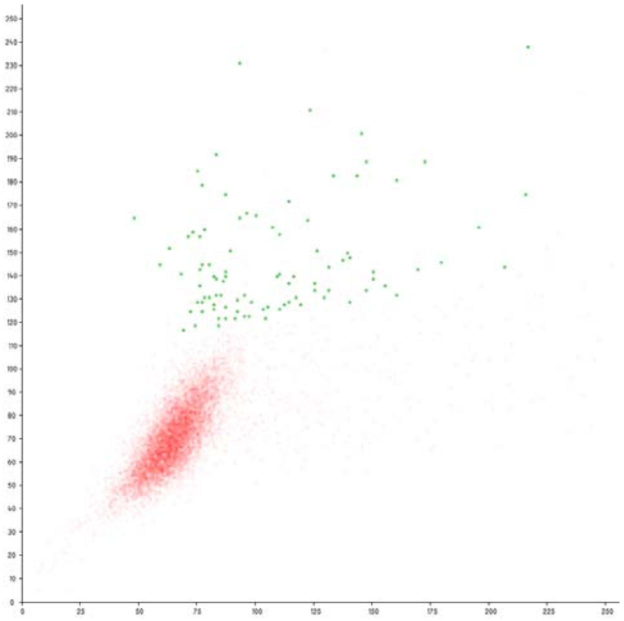

Number of beads individually dispensed: 96

ANNEX 1: FLOW SORTING BIN 6 – File 2 of 2

Bin 6: COPAS scatter plots showing events for medium 6 of the final split-pool cycle (bin 6). In the first scatter plot (optical extinction [ext] vs. time of flight [TOF]), events depicted by a red dot fall within the gate defining monomeric beads and were sorted. The second scatter plot displays the fluorescent values of the sorted beads (green vs. red fluorescence intensity). Beads with a red fluorescent intensity above the threshold value are depicted as green dots and were individually dispensed into 96 well plates.

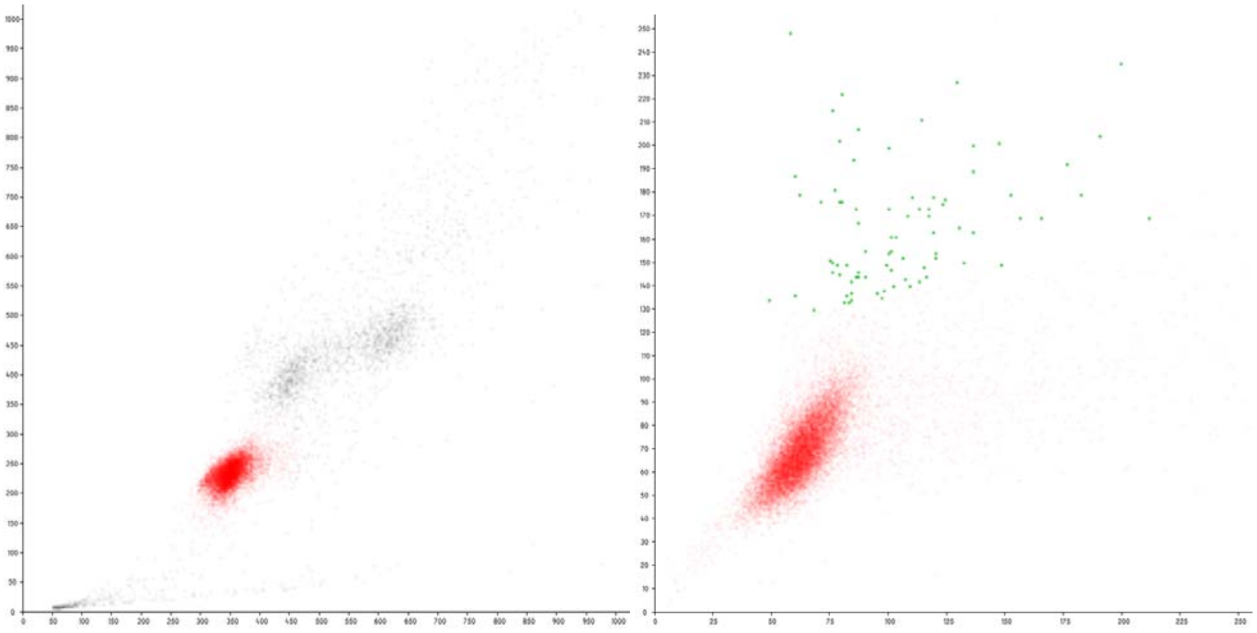

Number of beads individually dispensed: 83

ANNEX 1: FLOW SORTING BIN 7 – File 1 of 3

Bin 7: COPAS scatter plots showing events for medium 7 of the final split-pool cycle (bin 7). In the first scatter plot (optical extinction [ext] vs. time of flight [TOF]), events depicted by a red dot fall within the gate defining monomeric beads and were sorted. The second scatter plot displays the fluorescent values of the sorted beads (green vs. red fluorescence intensity). Beads with a red fluorescent intensity above the threshold value are depicted as green dots and were individually dispensed into 96 well plates.

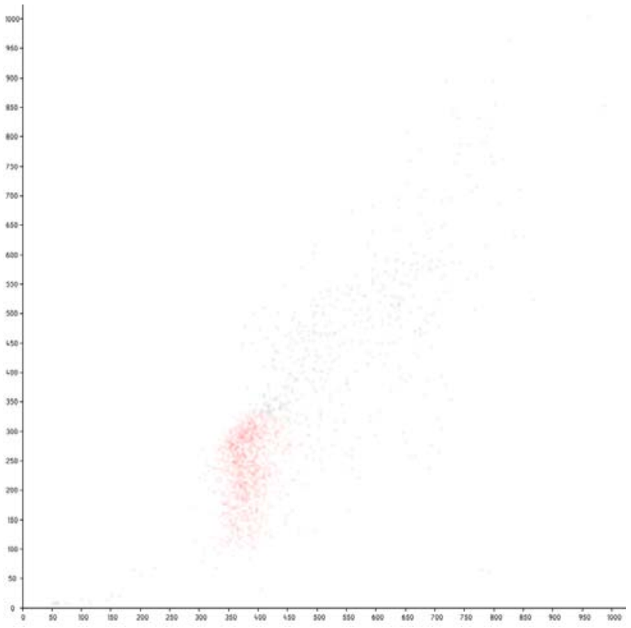

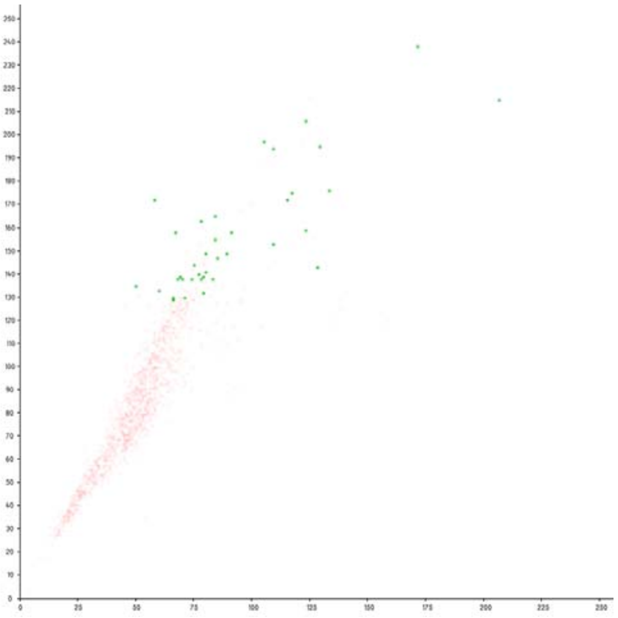

Number of beads individually dispensed: 37

ANNEX 1: FLOW SORTING BIN 7 – File 2 of 3

Bin 7: COPAS scatter plots showing events for medium 7 of the final split-pool cycle (bin 7). In the first scatter plot (optical extinction [ext] vs. time of flight [TOF]), events depicted by a red dot fall within the gate defining monomeric beads and were sorted. The second scatter plot displays the fluorescent values of the sorted beads (green vs. red fluorescence intensity). Beads with a red fluorescent intensity above the threshold value are depicted as green dots and were individually dispensed into 96 well plates.

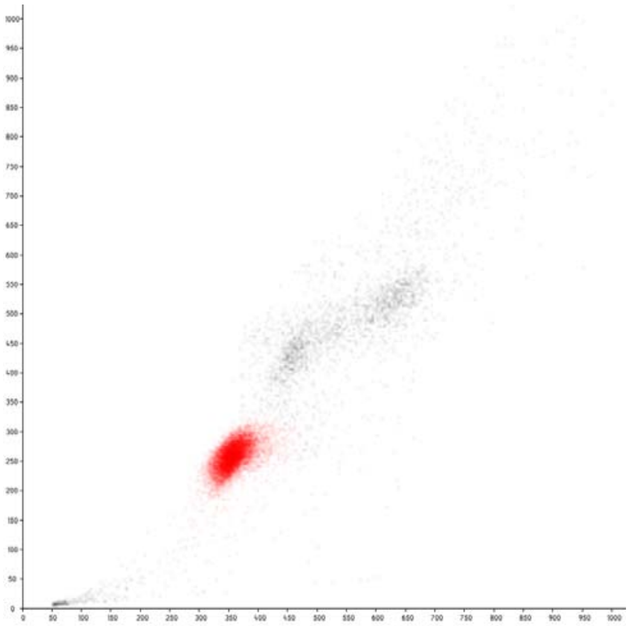

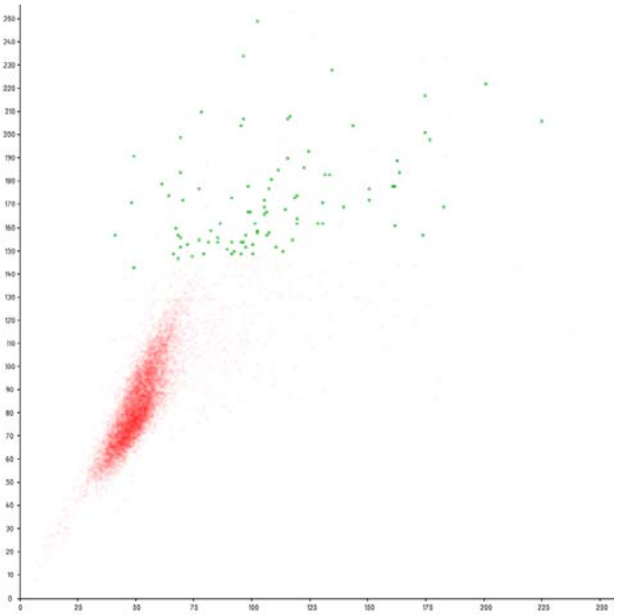

Number of beads individually dispensed: 96

ANNEX 1: FLOW SORTING BIN 7 – File 3 of 3

Bin 7: COPAS scatter plots showing events for medium 7 of the final split-pool cycle (bin 7). In the first scatter plot (optical extinction [ext] vs. time of flight [TOF]), events depicted by a red dot fall within the gate defining monomeric beads and were sorted. The second scatter plot displays the fluorescent values of the sorted beads (green vs. red fluorescence intensity). Beads with a red fluorescent intensity above the threshold value are depicted as green dots and were individually dispensed into 96 well plates.

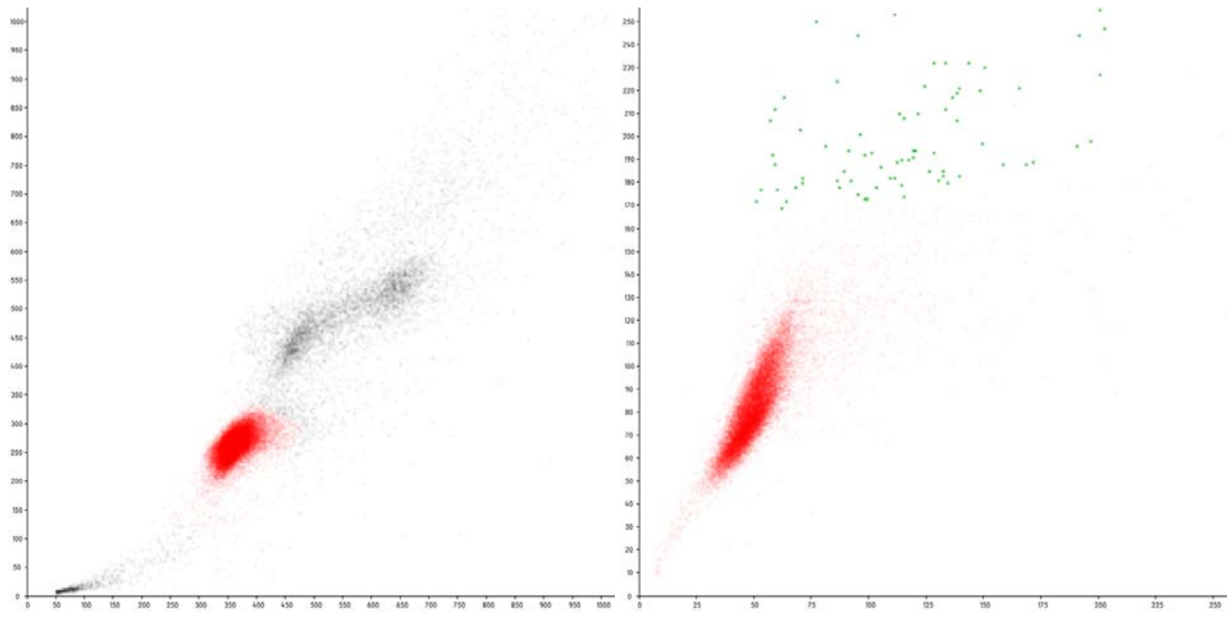

Number of beads individually dispensed: 87

ANNEX 1: FLOW SORTING BIN 8 – File 1 of 1

Bin 8: COPAS scatter plots showing events for medium 8 of the final split-pool cycle (bin 8). In the first scatter plot (optical extinction [ext] vs. time of flight [TOF]), events depicted by a red dot fall within the gate defining monomeric beads and were sorted. The second scatter plot displays the fluorescent values of the sorted beads (green vs. red fluorescence intensity). Beads with a red fluorescent intensity above the threshold value are depicted as green dots and were individually dispensed into 96 well plates.

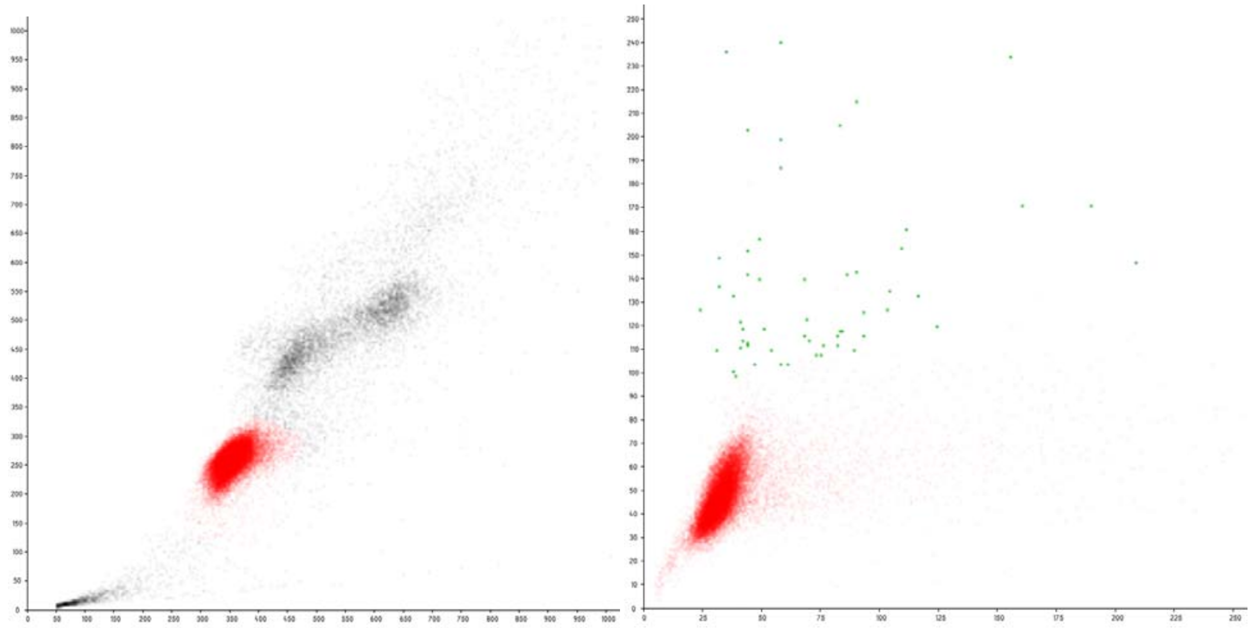

Number of beads individually dispensed: 61

ANNEX 1: FLOW SORTING BIN 9 – File 1 of 2

Bin 9: COPAS scatter plots showing events for medium 9 of the final split-pool cycle (bin 9). In the first scatter plot (optical extinction [ext] vs. time of flight [TOF]), events depicted by a red dot fall within the gate defining monomeric beads and were sorted. The second scatter plot displays the fluorescent values of the sorted beads (green vs. red fluorescence intensity). Beads with a red fluorescent intensity above the threshold value are depicted as green dots and were individually dispensed into 96 well plates.

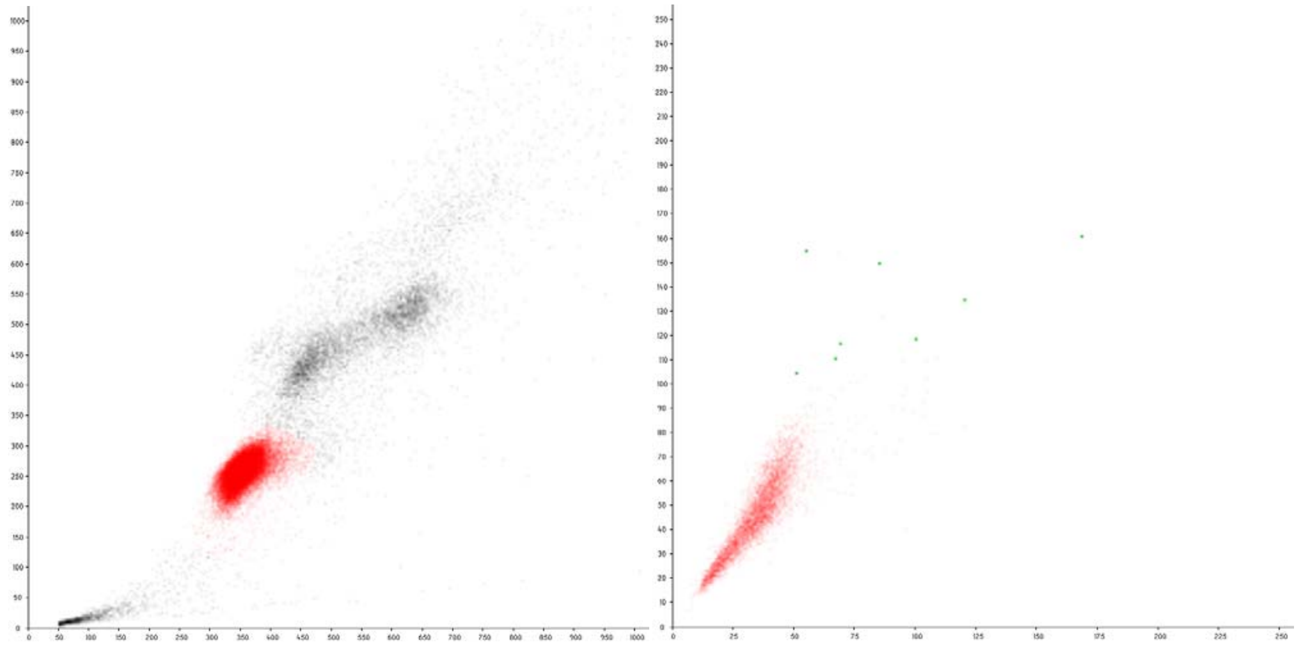

Number of beads individually dispensed: 8

ANNEX 1: FLOW SORTING BIN 9 – File 2 of 2

Bin 9: COPAS scatter plots showing events for medium 9 of the final split-pool cycle (bin 9). In the first scatter plot (optical extinction [ext] vs. time of flight [TOF]), events depicted by a red dot fall within the gate defining monomeric beads and were sorted. The second scatter plot displays the fluorescent values of the sorted beads (green vs. red fluorescence intensity). Beads with a red fluorescent intensity above the threshold value are depicted as green dots and were individually dispensed into 96 well plates.

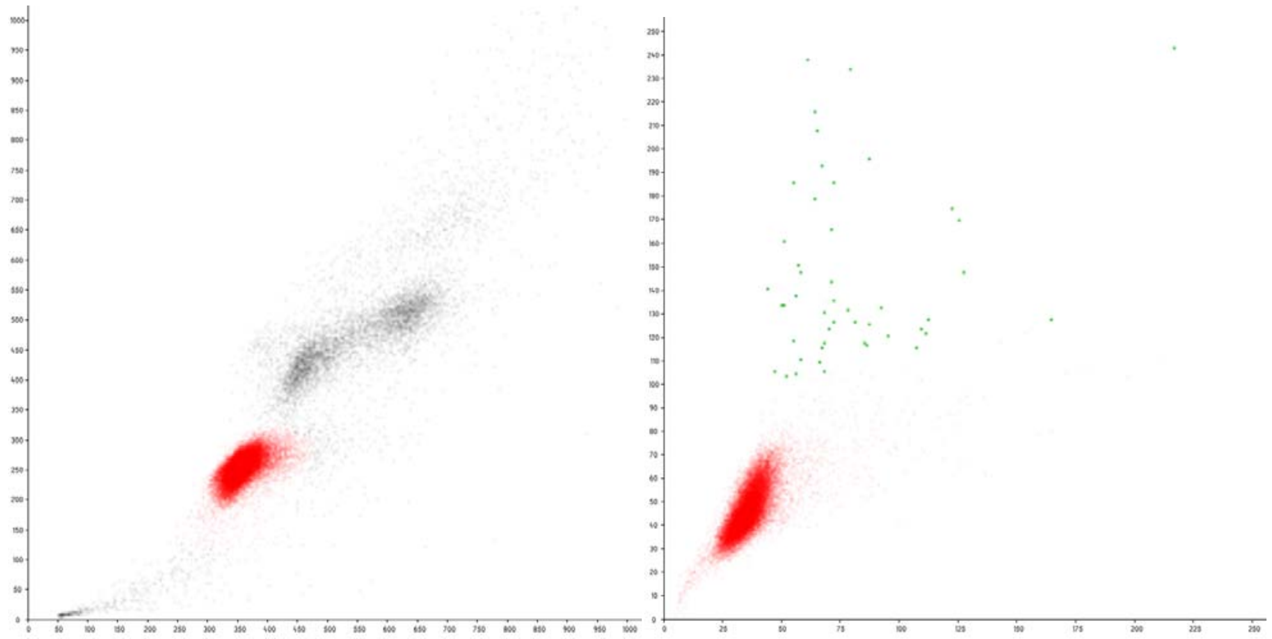

Number of beads individually dispensed: 53

ANNEX 1: FLOW SORTING BIN 10 – File 1 of 1

Bin 10: COPAS scatter plots showing events for medium 10 of the final split-pool cycle (bin 10). In the first scatter plot (optical extinction [ext] vs. time of flight [TOF]), events depicted by a red dot fall within the gate defining monomeric beads and were sorted. The second scatter plot displays the fluorescent values of the sorted beads (green vs. red fluorescence intensity). Beads with a red fluorescent intensity above the threshold value are depicted as green dots and were individually dispensed into 96 well plates.

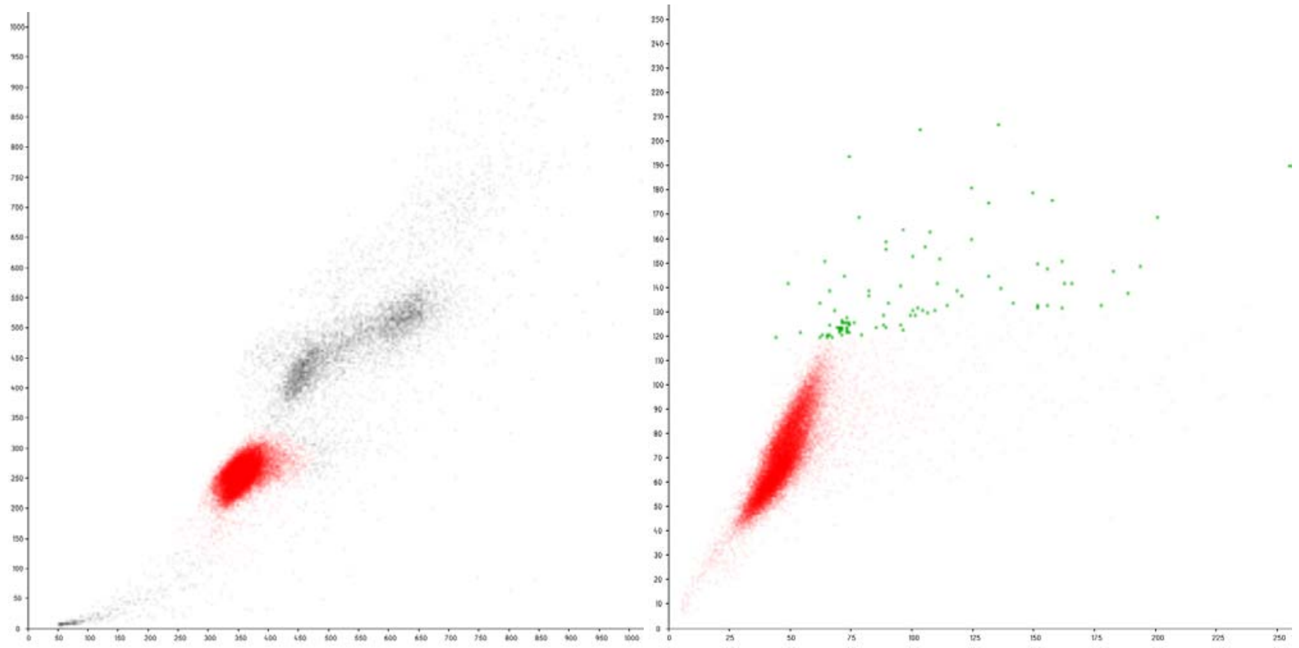

Number of beads individually dispensed: 92

ANNEX 2: TAG REFERENCE SET - TAG DECONVOLUTION SESSION 1

Tag analysis flow cytometry instrument: BD FACSCalibur

Number of gates: 30

Number of beads whose tags were deconvoluted: 10

Number of beads with conclusive tagging data: 5

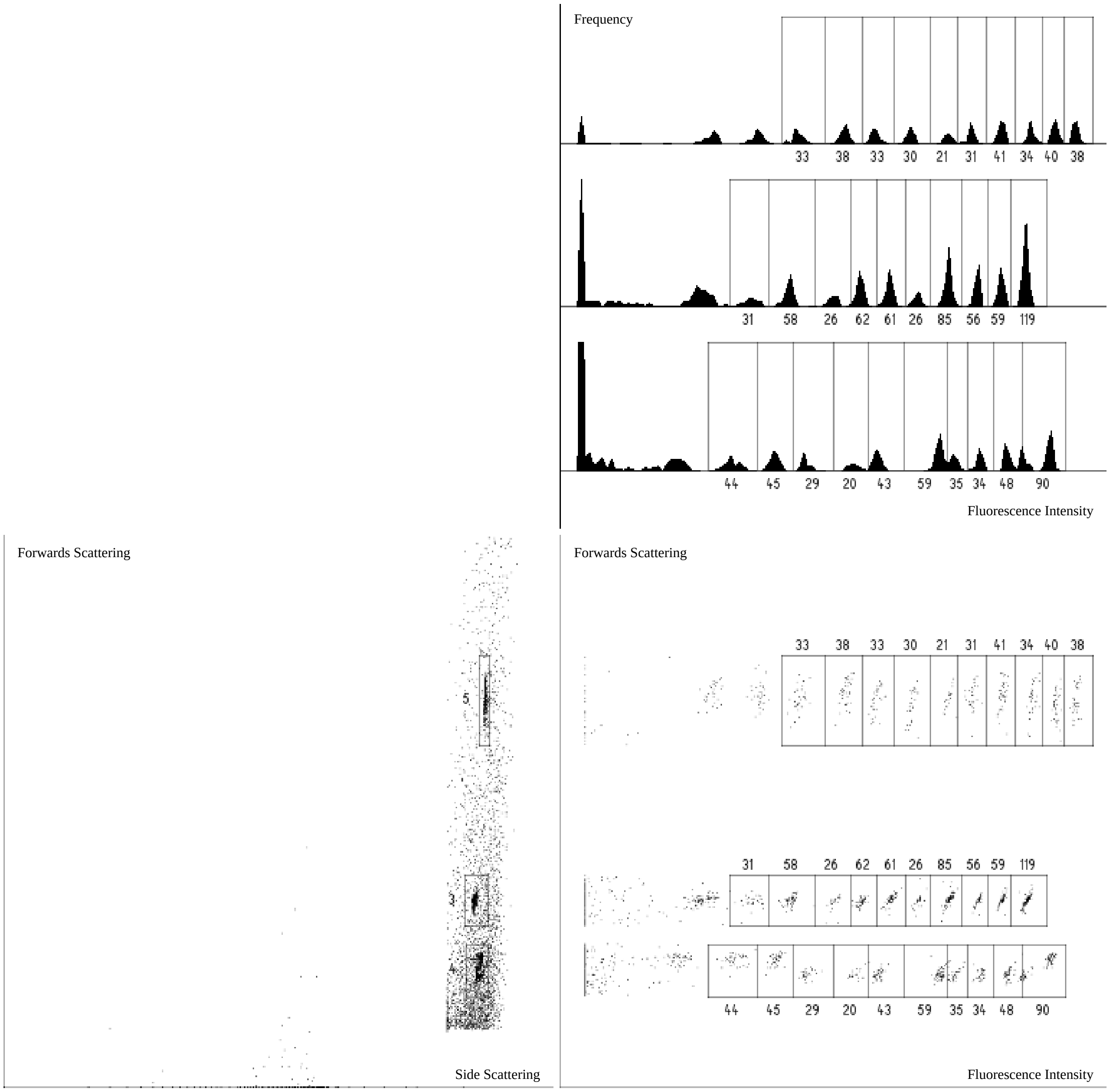

ANNEX 2: TAG REFERENCE SET - TAG DECONVOLUTION SESSION 2

Tag analysis flow cytometry instrument: BD FACSCalibur

Number of gates: 30

Number of beads whose tags were deconvoluted: 10

Number of beads with conclusive tagging data: 7

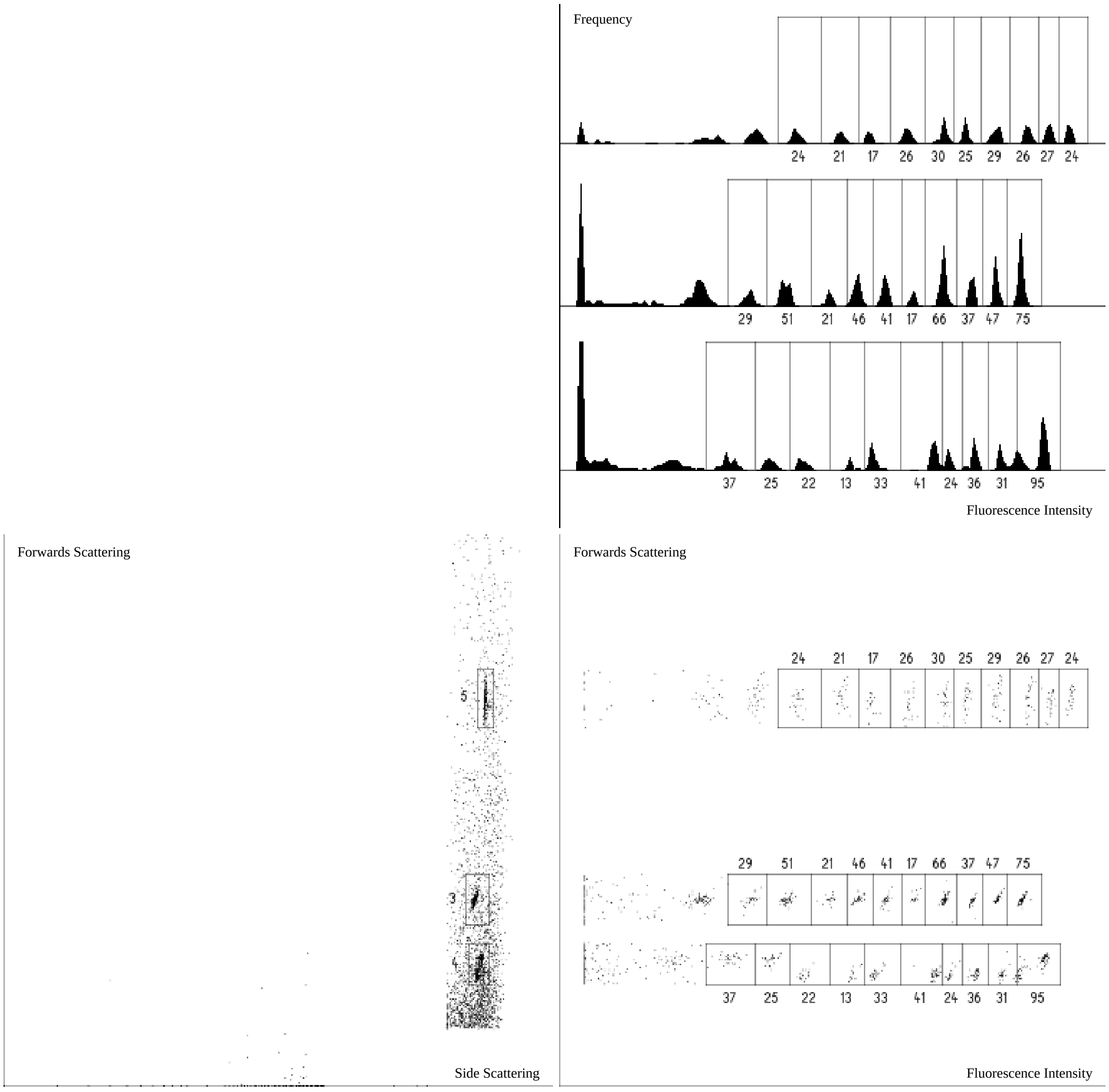

ANNEX 2: TAG REFERENCE SET - TAG DECONVOLUTION SESSION 3

Tag analysis flow cytometry instrument: BD FACSCalibur

Number of gates: 30

Number of beads whose tags were deconvoluted: 12

Number of beads with conclusive tagging data: 9

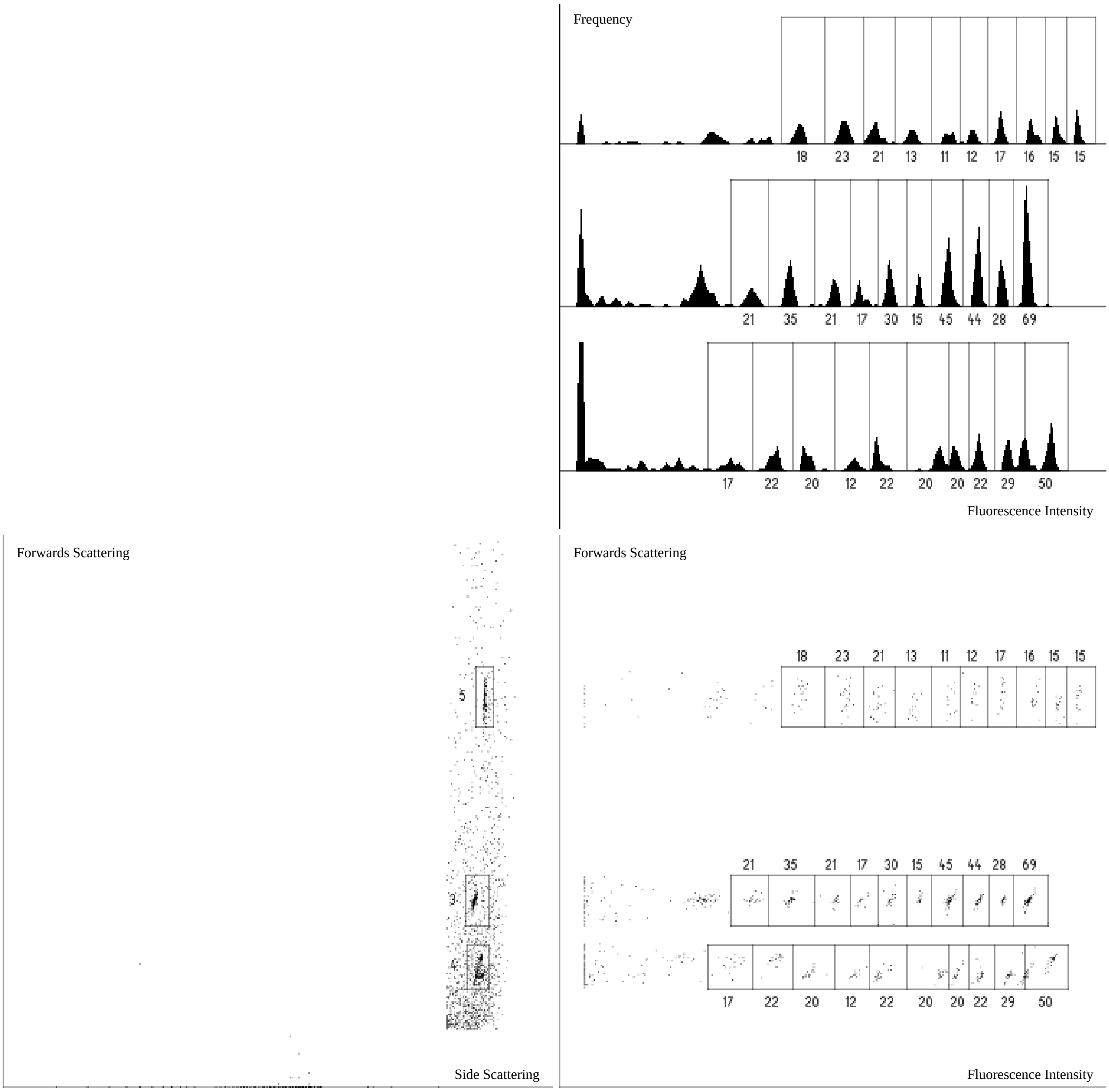

ANNEX 2: TAG REFERENCE SET - TAG DECONVOLUTION SESSION 4

Tag analysis flow cytometry instrument: BD FACSCalibur

Number of gates: 30

Number of beads whose tags were deconvoluted: 15

Number of beads with conclusive tagging data: 14

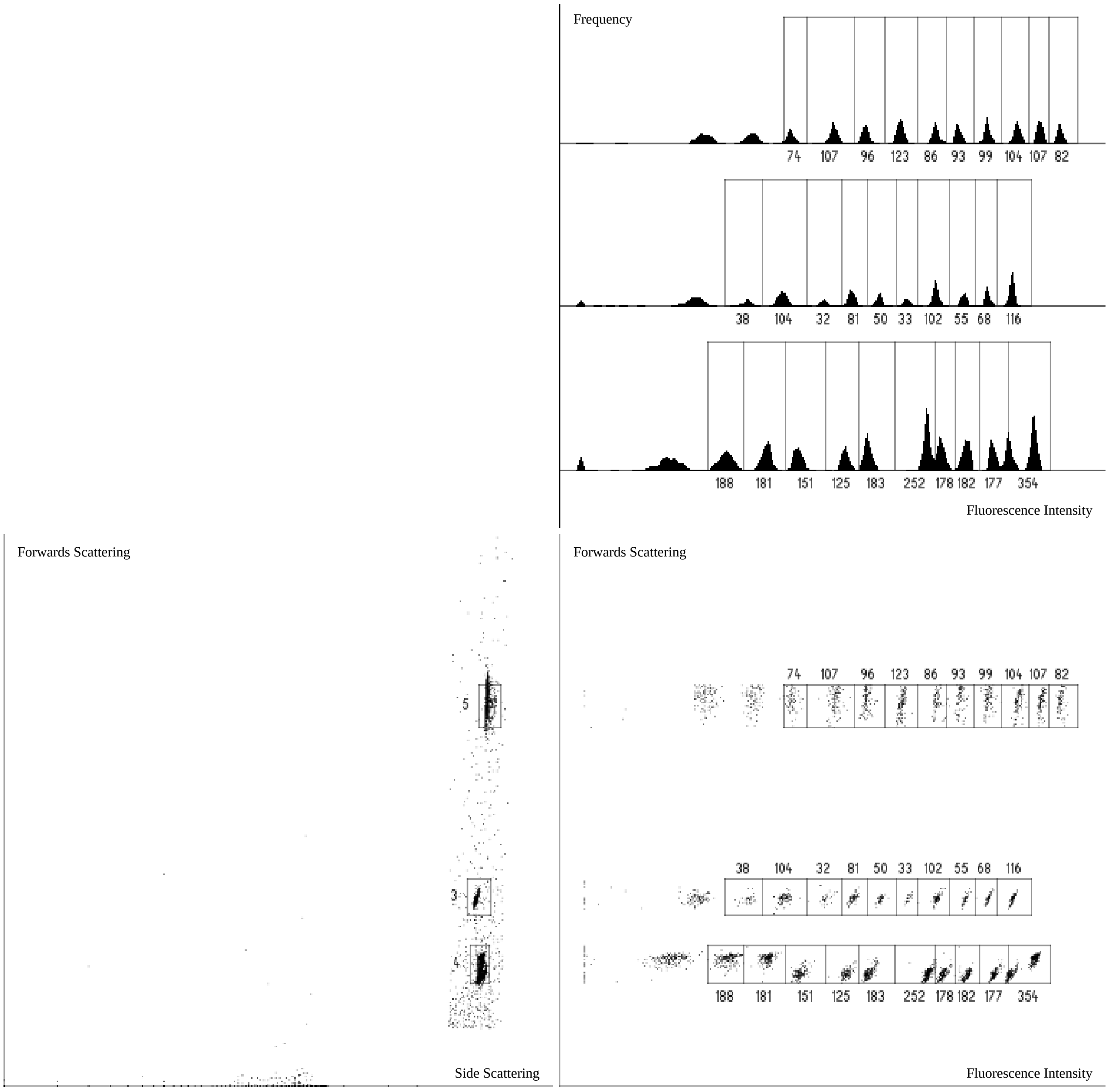

ANNEX 2: TAG REFERENCE SET - TAG DECONVOLUTION SESSION 5

Tag analysis flow cytometry instrument: BD FACSCalibur

Number of gates: 30

Number of beads whose tags were deconvoluted: 7

Number of beads with conclusive tagging data: 5

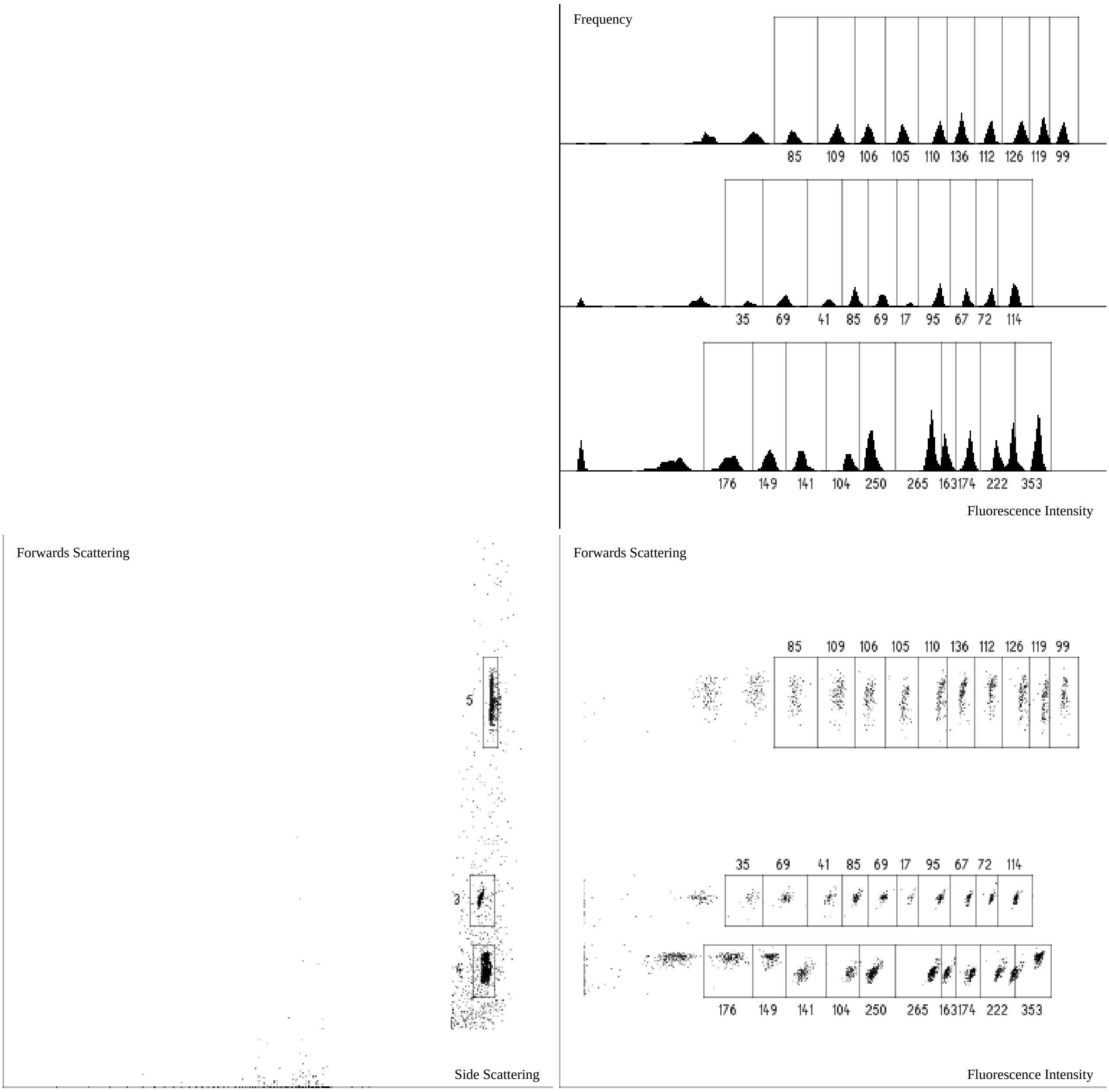

ANNEX 2: TAG REFERENCE SET - TAG DECONVOLUTION SESSION 6

Tag analysis flow cytometry instrument: BD FACSCalibur

Number of gates: 30

Number of beads whose tags were deconvoluted: 3

Number of beads with conclusive tagging data: 1

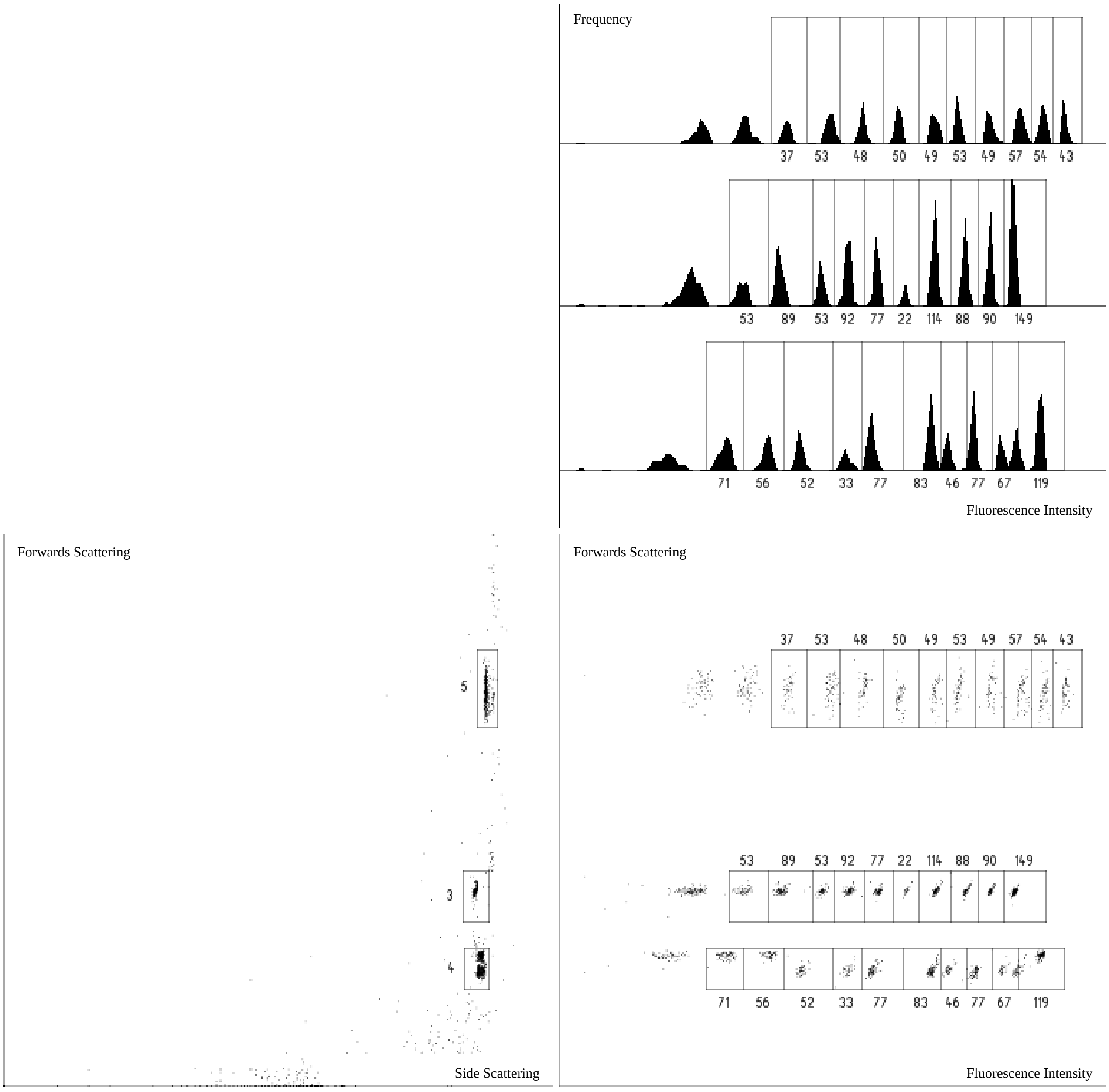

ANNEX 2: TAG REFERENCE SET - TAG DECONVOLUTION SESSION 7

Tag analysis flow cytometry instrument: BD FACSCalibur

Number of gates: 30

Number of beads whose tags were deconvoluted: 19

Number of beads with conclusive tagging data: 15

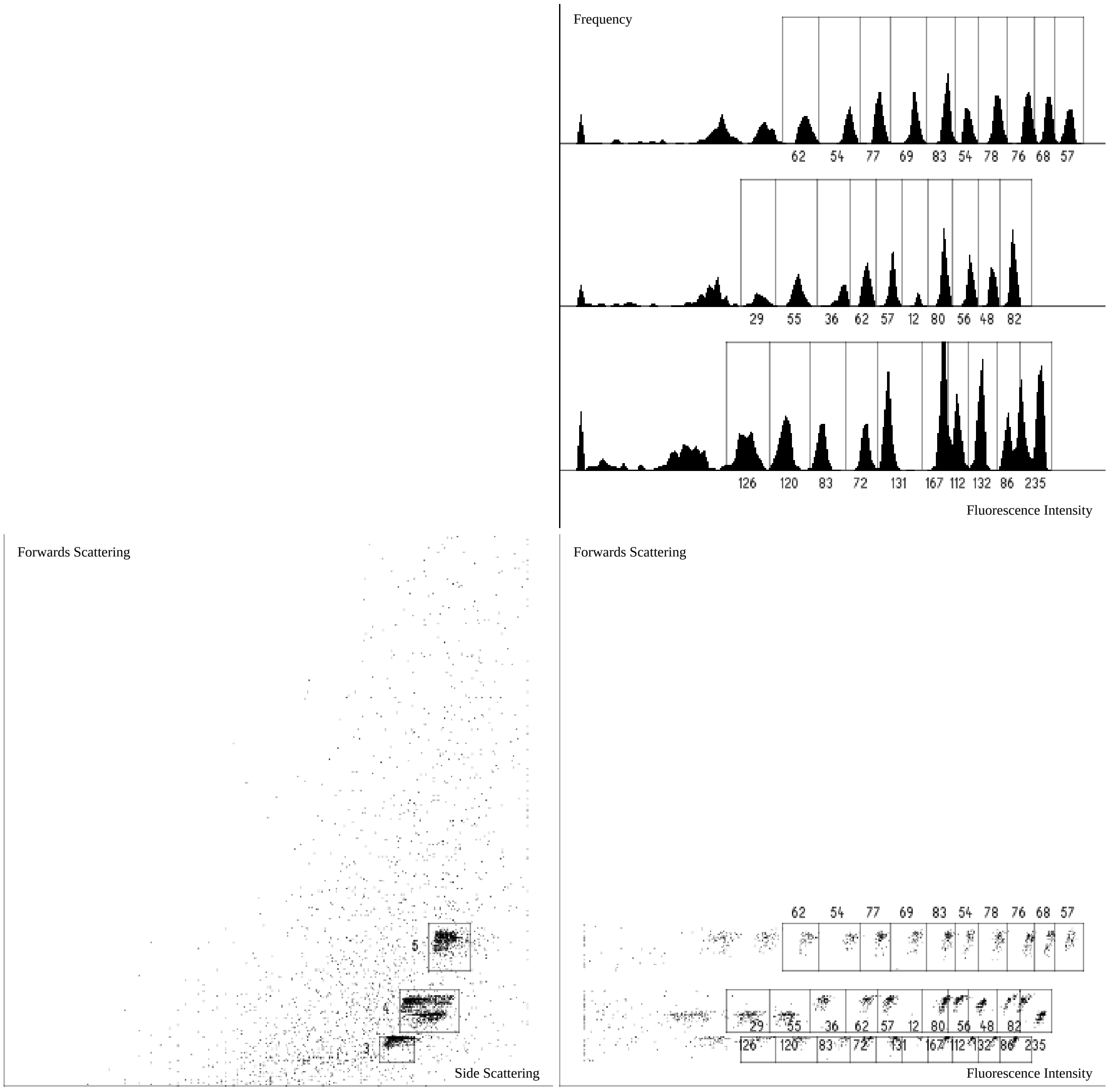

ANNEX 2: TAG REFERENCE SET - TAG DECONVOLUTION SESSION 8

Tag analysis flow cytometry instrument: BD FACSCalibur

Number of gates: 30

Number of beads whose tags were deconvoluted: 7

Number of beads with conclusive tagging data: 7

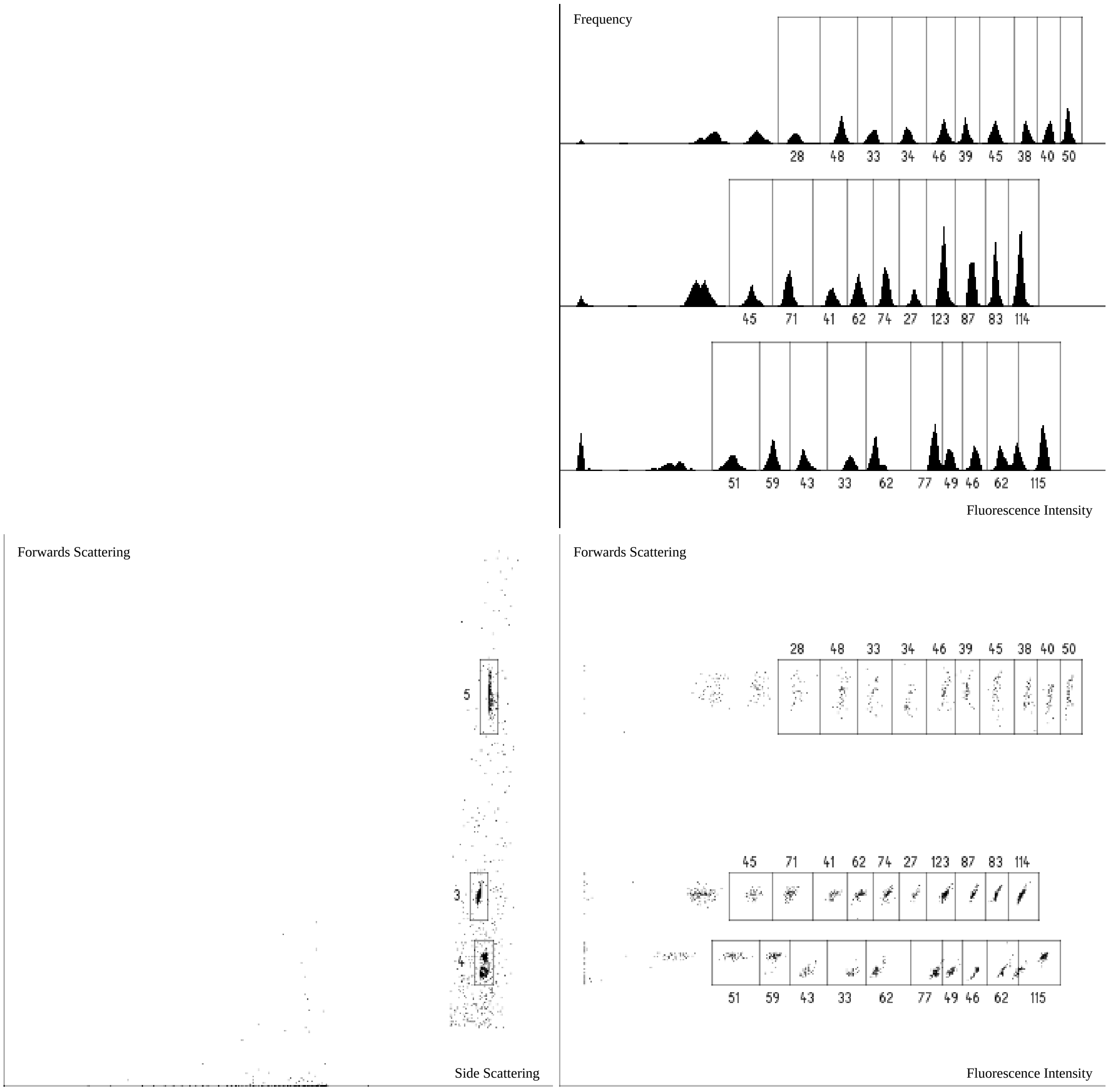

ANNEX 2: TAG REFERENCE SET - TAG DECONVOLUTION SESSION 9

Tag analysis flow cytometry instrument: BD FACSCalibur

Number of gates: 30

Number of beads whose tags were deconvoluted: 13

Number of beads with conclusive tagging data: 10

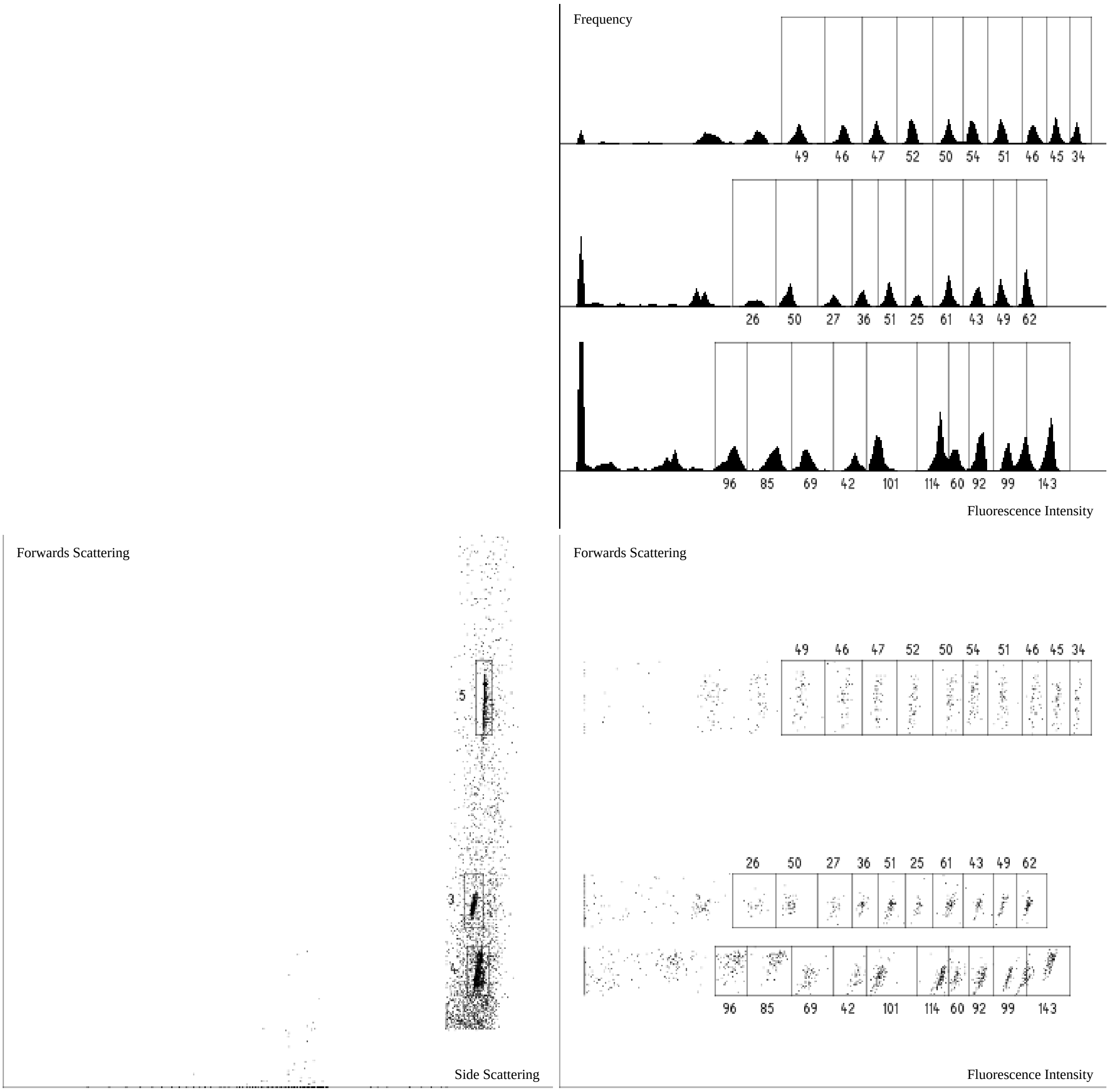

ANNEX 2: TAG REFERENCE SET - TAG DECONVOLUTION SESSION 10

Tag analysis flow cytometry instrument: BD FACSCalibur

Number of gates: 30

Number of beads whose tags were deconvoluted: 10

Number of beads with conclusive tagging data: 8

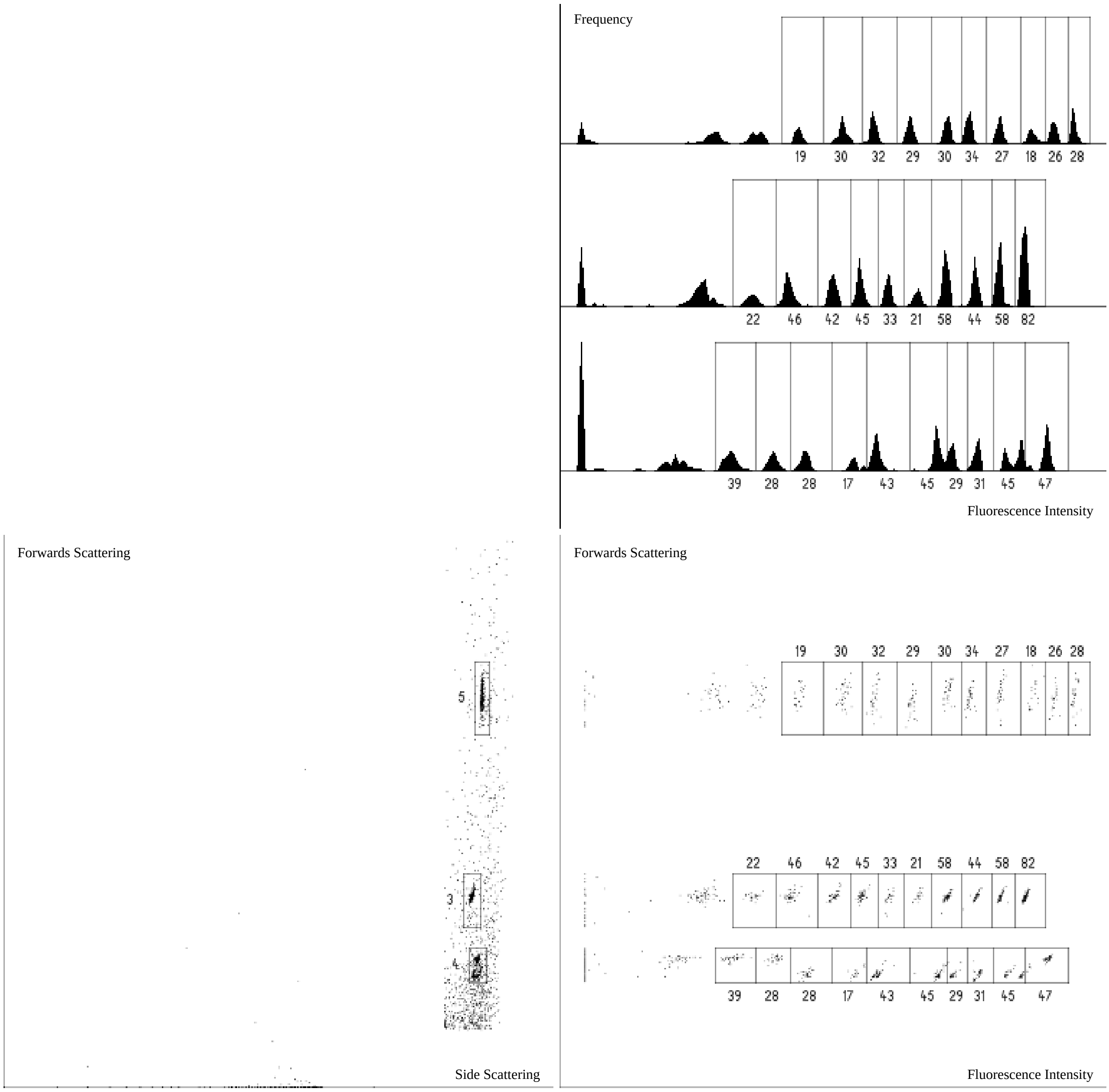

ANNEX 2: TAG REFERENCE SET - TAG DECONVOLUTION SESSION 11

Tag analysis flow cytometry instrument: BD FACSCalibur

Number of gates: 30

Number of beads whose tags were deconvoluted: 10

Number of beads with conclusive tagging data: 2

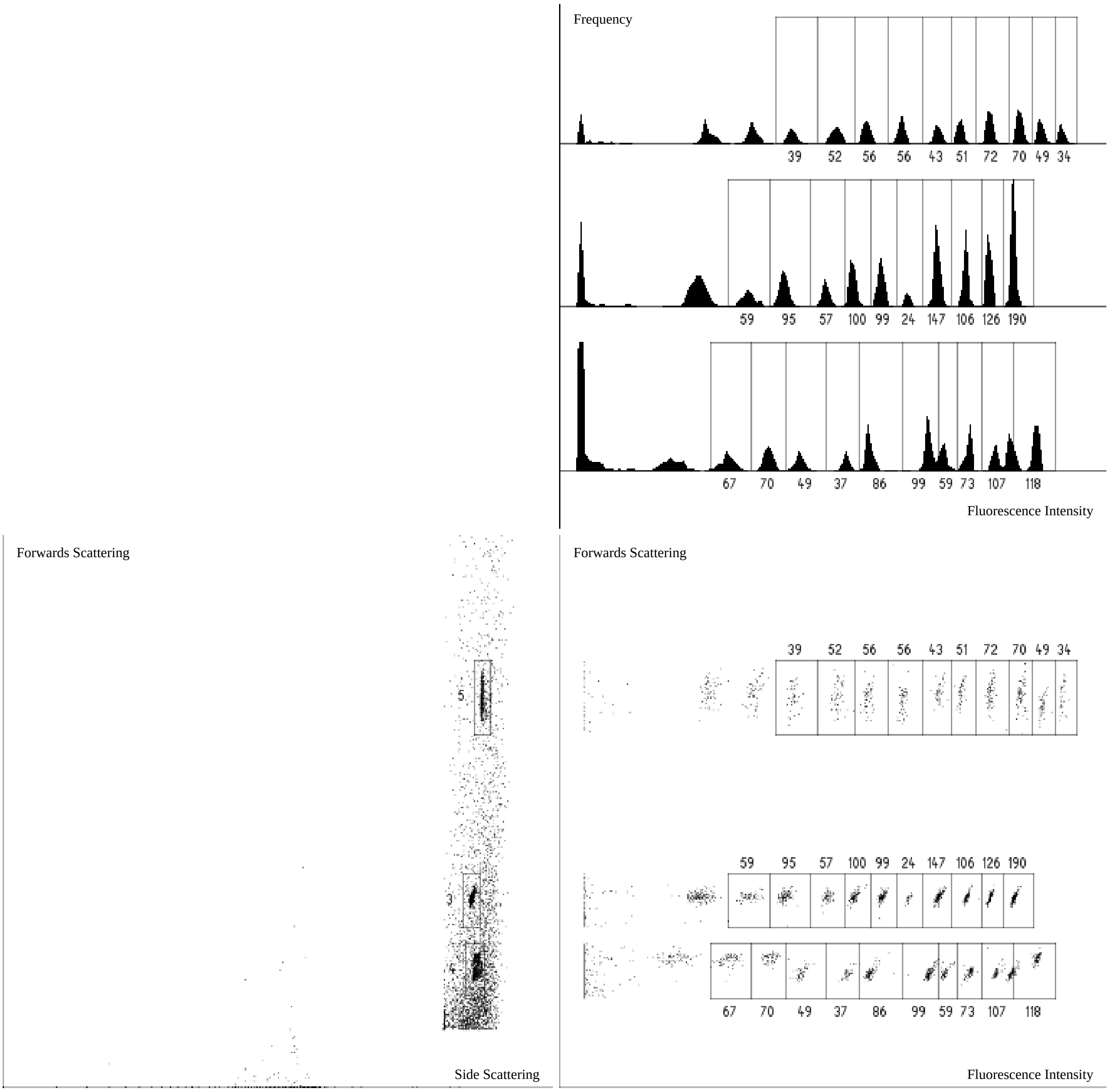

ANNEX 2: TAG REFERENCE SET - TAG DECONVOLUTION SESSION 12

Tag analysis flow cytometry instrument: BD FACSCalibur

Number of gates: 30

Number of beads whose tags were deconvoluted: 15

Number of beads with conclusive tagging data: 13

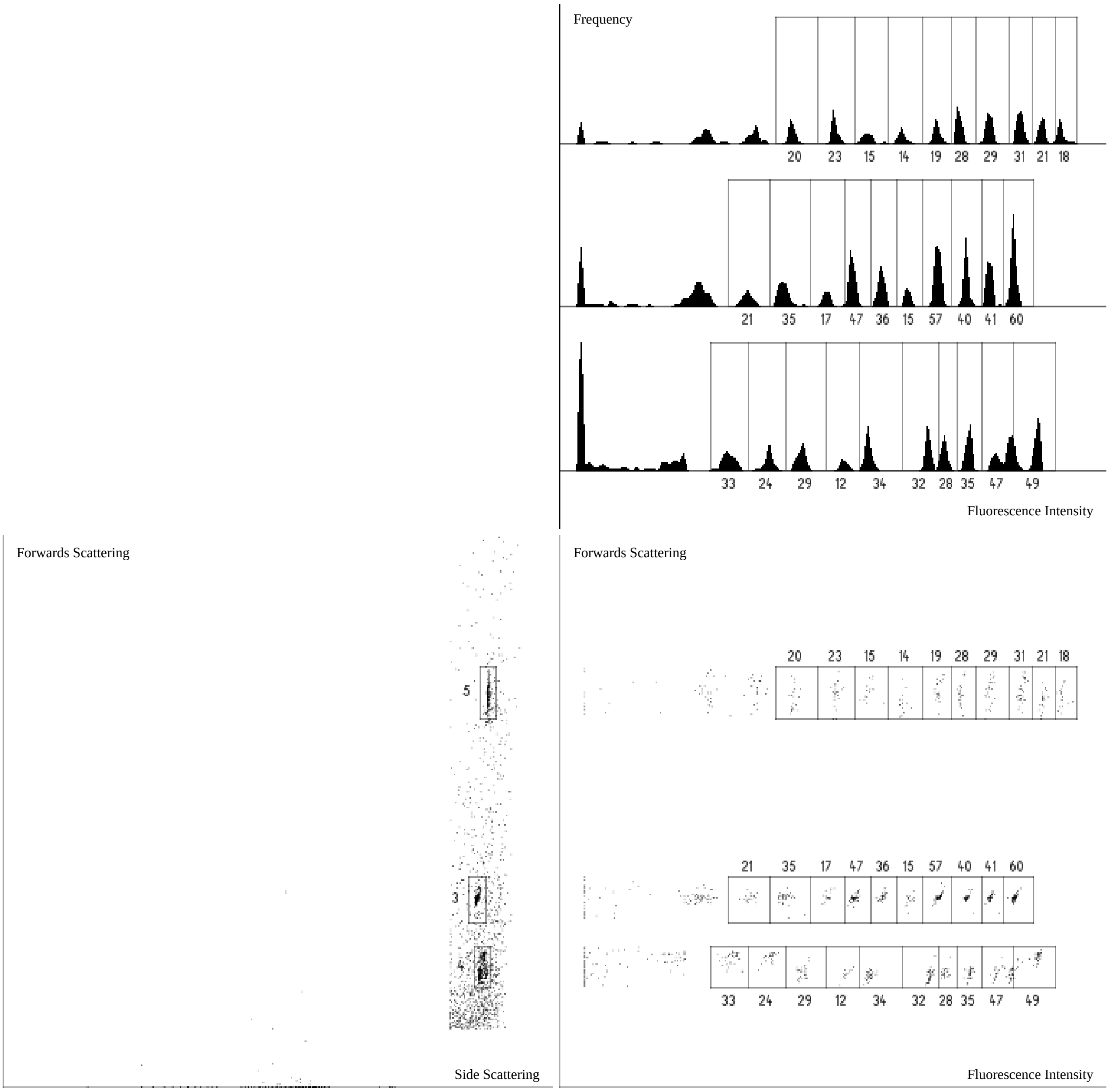

ANNEX 3: TAG DECONVOLUTION - BEAD 1

Passes flow sorting criteria: Yes

Passes tag deconvolution criteria: No

Included in protocol analysis: No

Protocol: N/A

Filename: Bin6_34.fcs

Split 1: Petrol shading

Split 2: Green shading

Split 3: Violet shading

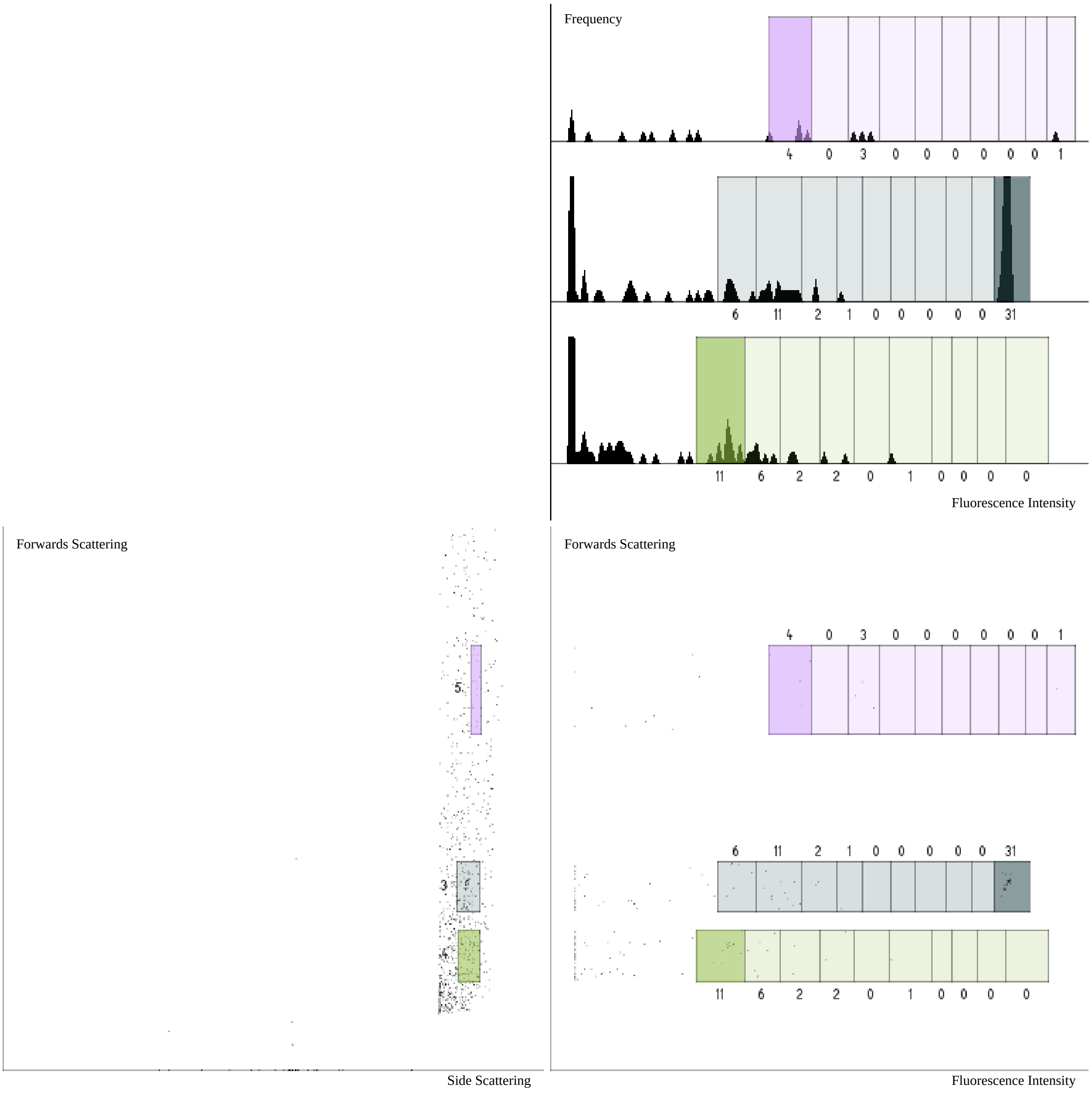

ANNEX 3: TAG DECONVOLUTION - BEAD 2

Passes flow sorting criteria: Yes

Passes tag deconvolution criteria: Yes

Included in protocol analysis: Yes

Protocol: 10, 1, 5, 6

Filename: Bin6_35.fcs

Split 1: Petrol shading

Split 2: Green shading

Split 3: Violet shading

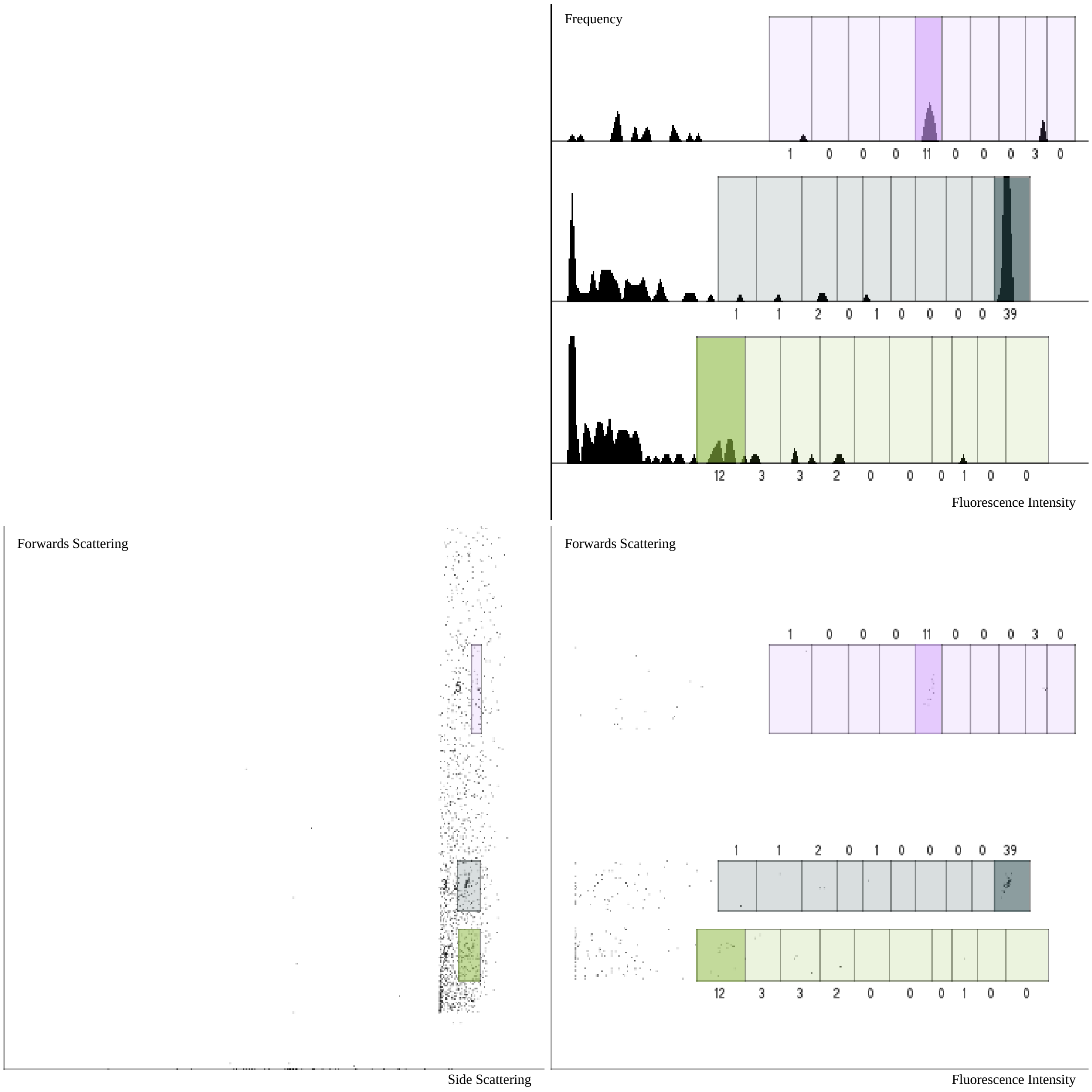

ANNEX 3: TAG DECONVOLUTION - BEAD 3

Passes flow sorting criteria: Yes

Passes tag deconvolution criteria: Yes

Included in protocol analysis: Yes

Protocol: 10, 7, 6, 6

Filename: Bin6_36.fcs

Split 1: Petrol shading

Split 2: Green shading

Split 3: Violet shading

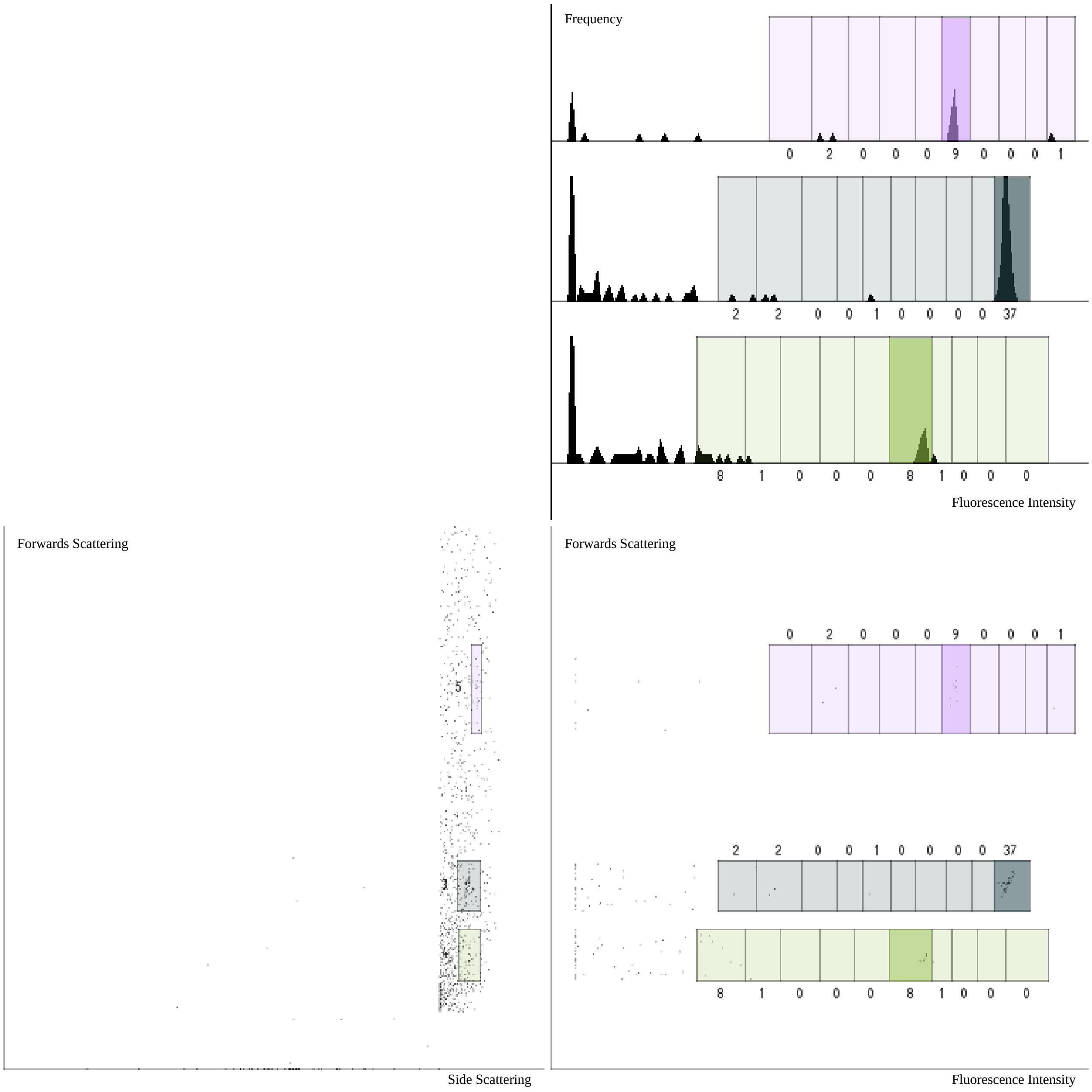

ANNEX 3: TAG DECONVOLUTION - BEAD 4

Passes flow sorting criteria: Yes

Passes tag deconvolution criteria: No

Included in protocol analysis: No

Protocol: N/A

Filename: Bin6_37.fcs

Split 1: Petrol shading

Split 2: Green shading

Split 3: Violet shading

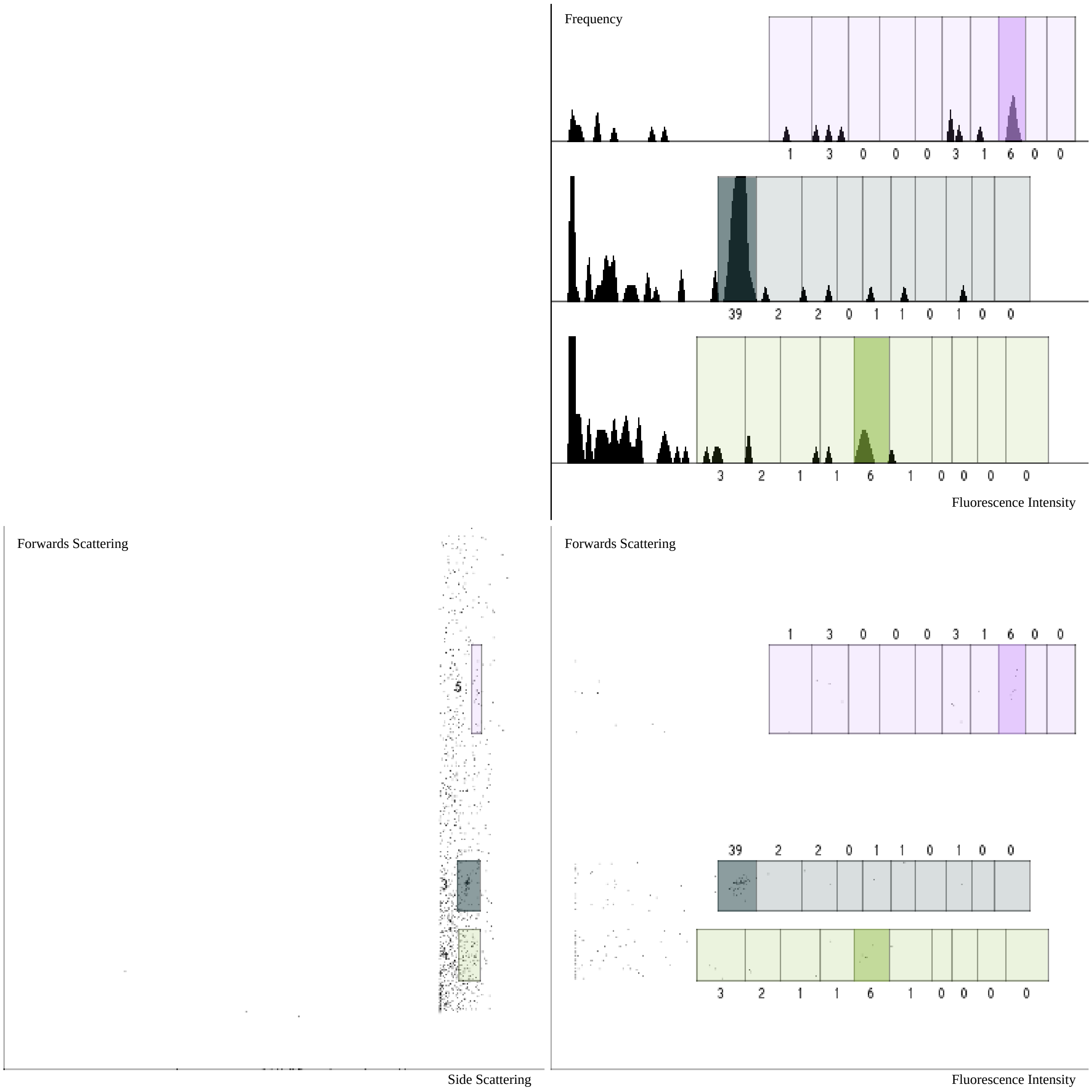

ANNEX 3: TAG DECONVOLUTION - BEAD 5

Passes flow sorting criteria: Yes

Passes tag deconvolution criteria: Yes

Included in protocol analysis: Yes

Protocol: 9, 10, 5, 6

Filename: Bin6_38.fcs

Split 1: Petrol shading

Split 2: Green shading

Split 3: Violet shading

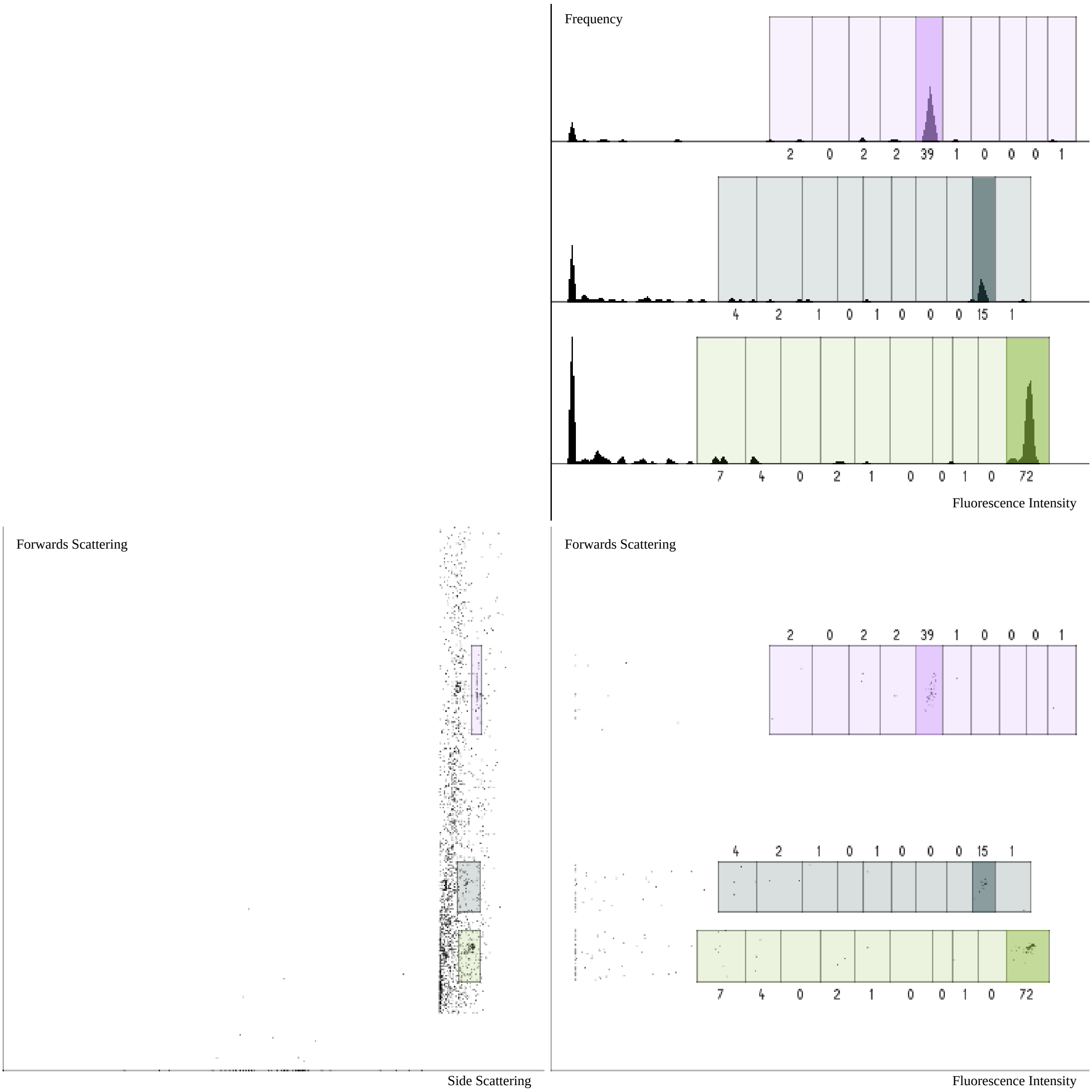

ANNEX 3: TAG DECONVOLUTION - BEAD 6

Passes flow sorting criteria: Yes

Passes tag deconvolution criteria: No

Included in protocol analysis: No

Protocol: N/A

Filename: Bin6_39.fcs

Split 1: Petrol shading

Split 2: Green shading

Split 3: Violet shading

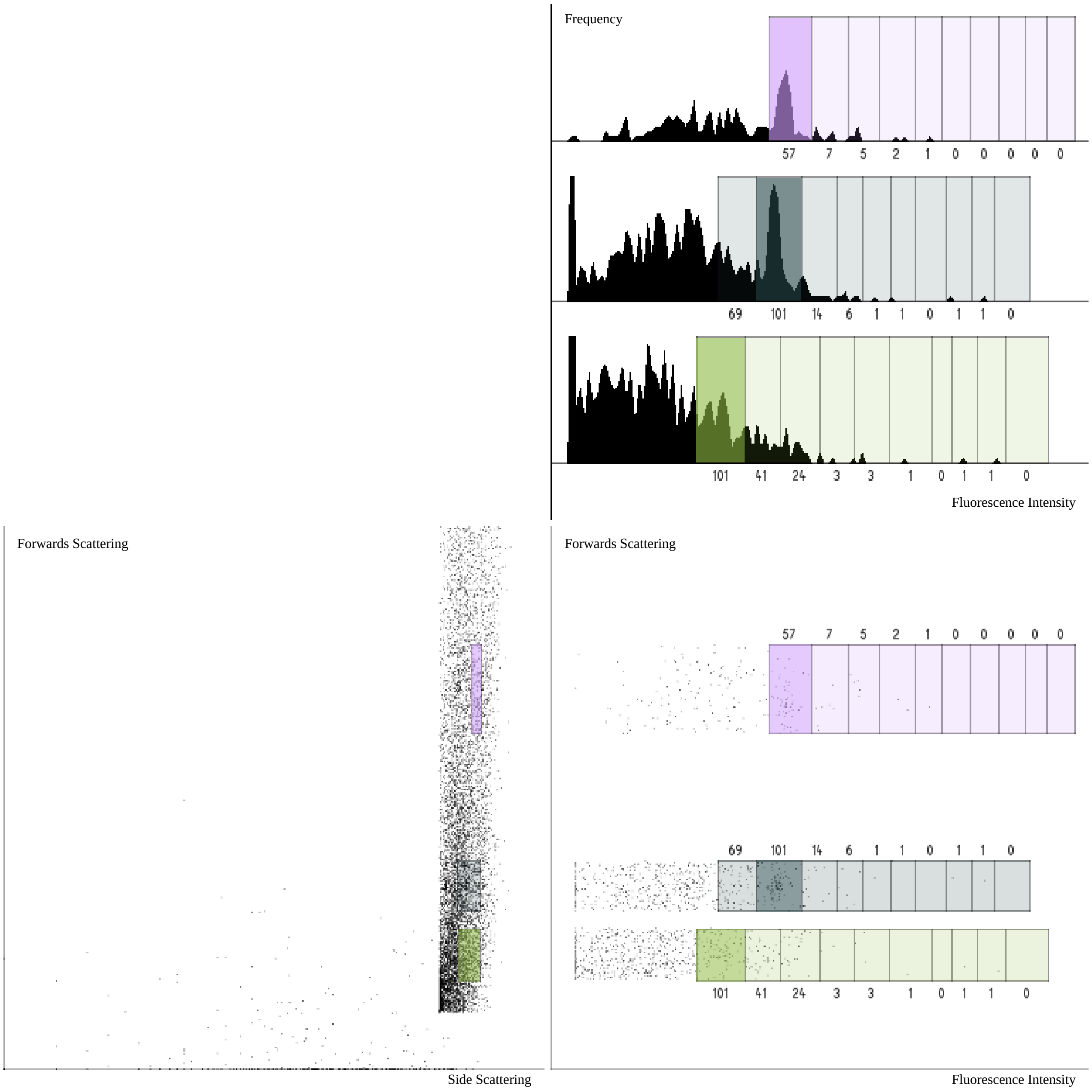

ANNEX 3: TAG DECONVOLUTION - BEAD 7

Passes flow sorting criteria: Yes

Passes tag deconvolution criteria: Yes

Included in protocol analysis: Yes

Protocol: 6, 3, 4, 6

Filename: Bin6_40.fcs

Split 1: Petrol shading

Split 2: Green shading

Split 3: Violet shading

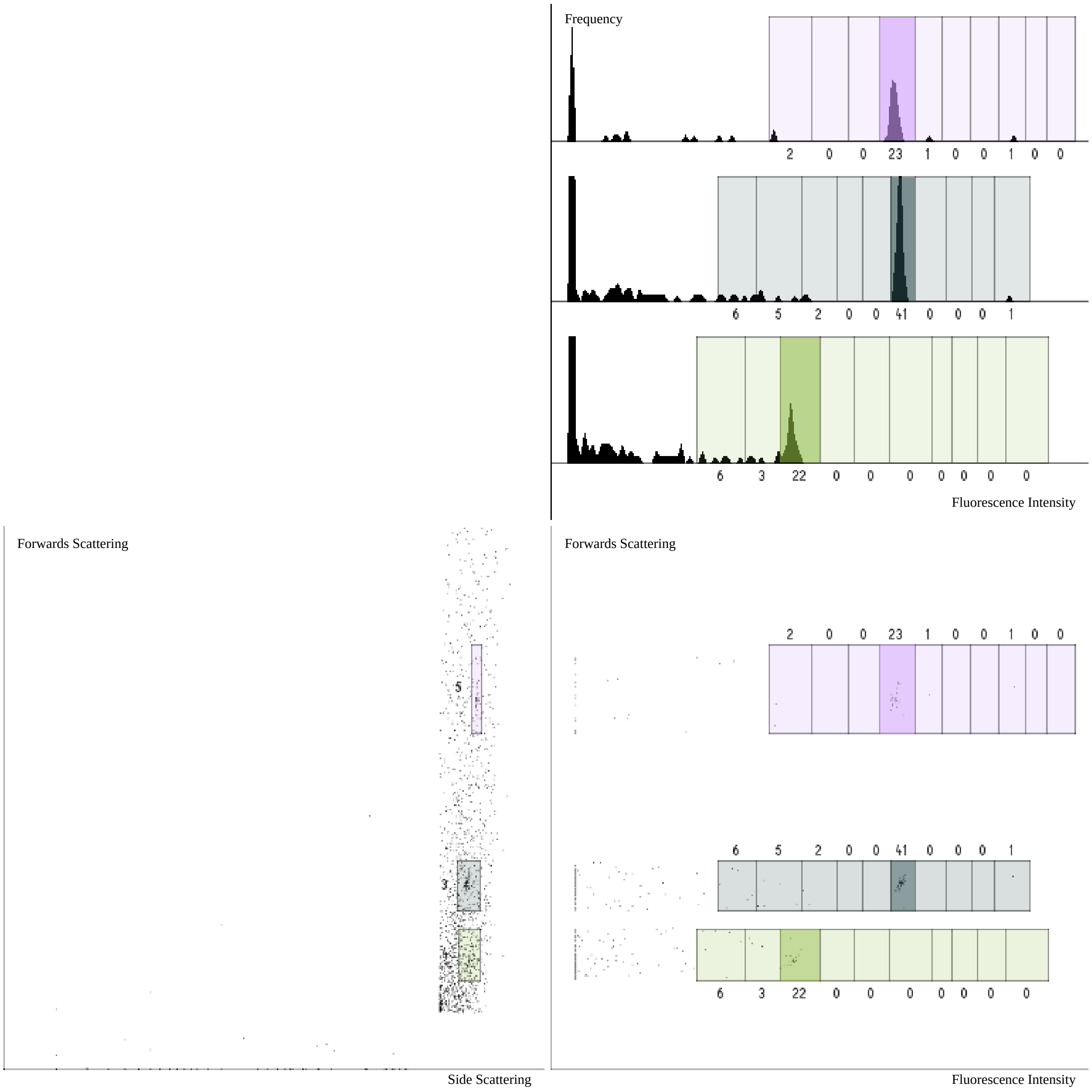

ANNEX 3: TAG DECONVOLUTION - BEAD 8

Passes flow sorting criteria: Yes

Passes tag deconvolution criteria: Yes

Included in protocol analysis: Yes

Protocol: 9, 1, 9, 6

Filename: Bin6_41.fcs

Split 1: Petrol shading

Split 2: Green shading

Split 3: Violet shading

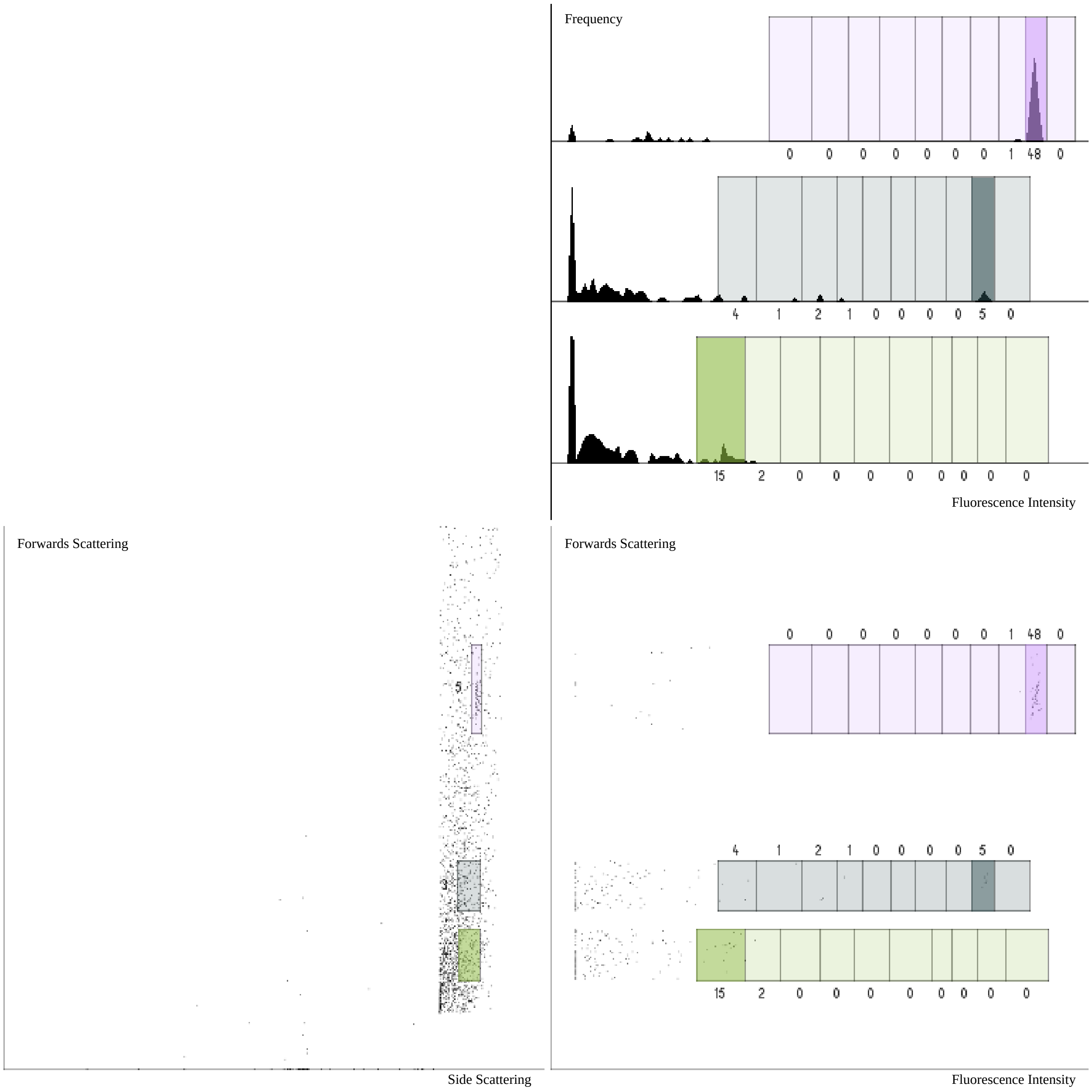

ANNEX 3: TAG DECONVOLUTION - BEAD 9

Passes flow sorting criteria: Yes

Passes tag deconvolution criteria: No

Included in protocol analysis: No

Protocol: N/A

Filename: Bin6_42.fcs

Split 1: Petrol shading

Split 2: Green shading

Split 3: Violet shading

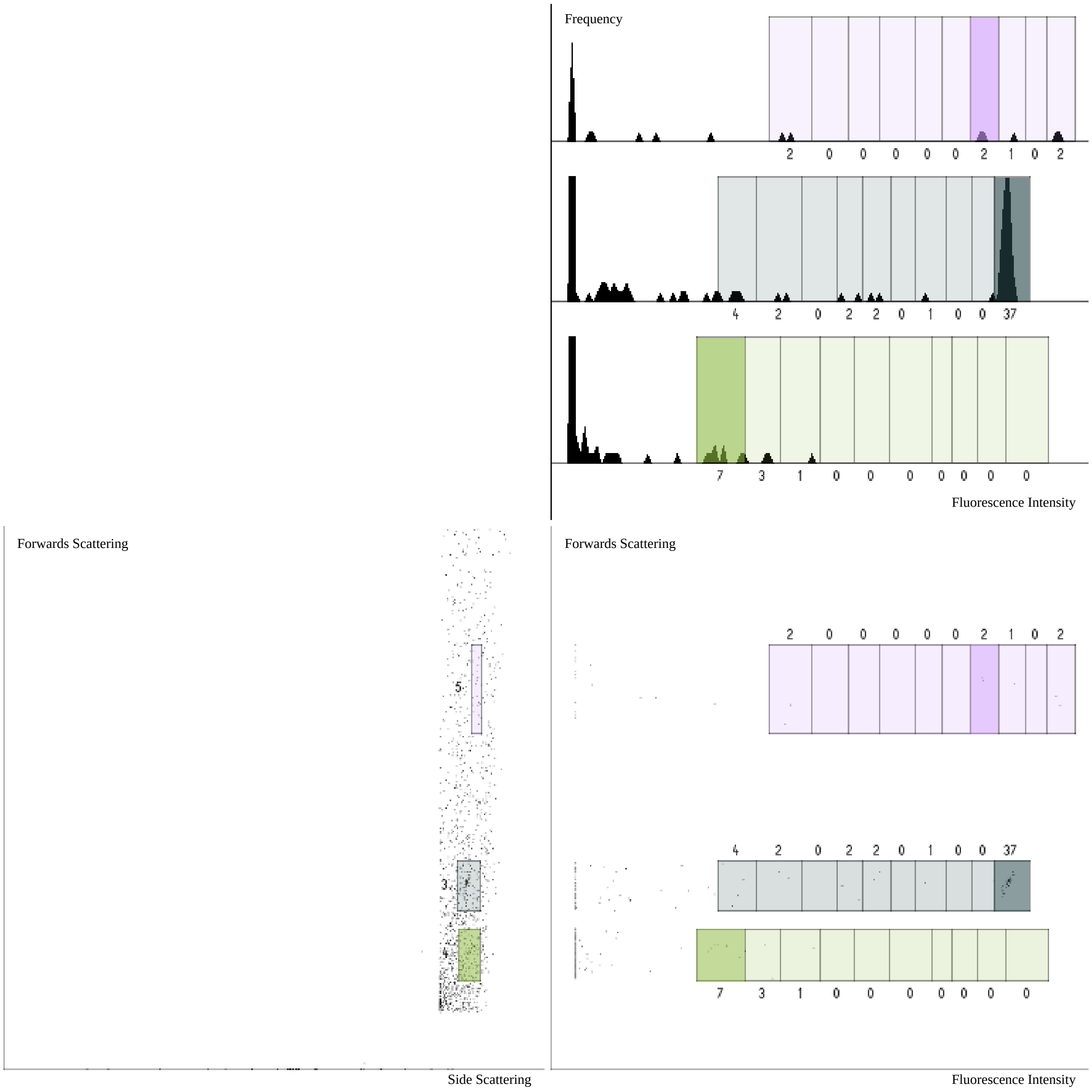

ANNEX 3: TAG DECONVOLUTION - BEAD 10

Passes flow sorting criteria: Yes

Passes tag deconvolution criteria: No

Included in protocol analysis: No

Protocol: N/A

Filename: Bin6_43.fcs

Split 1: Petrol shading

Split 2: Green shading

Split 3: Violet shading

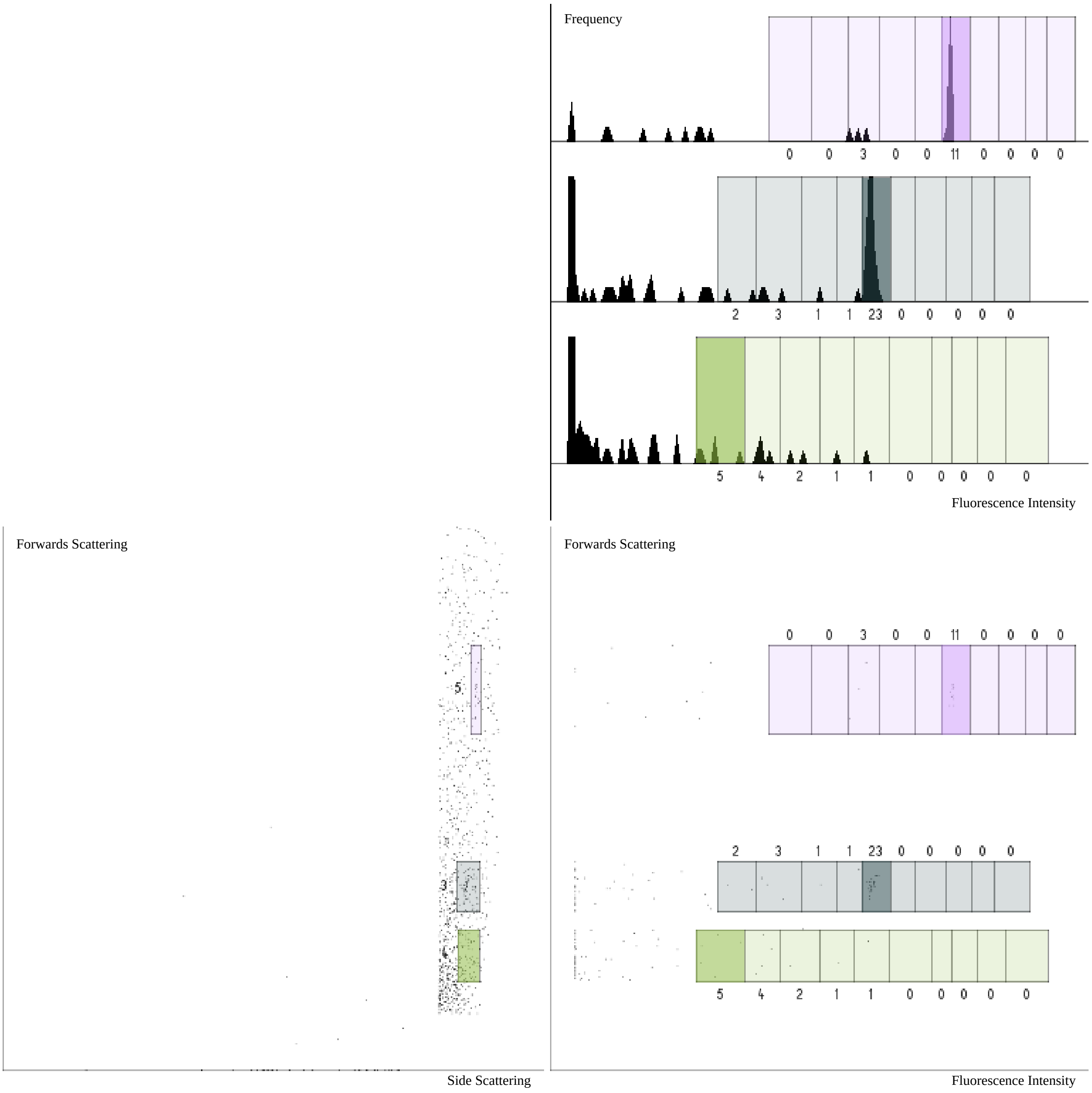

ANNEX 3: TAG DECONVOLUTION - BEAD 11

Passes flow sorting criteria: Yes

Passes tag deconvolution criteria: Yes

Included in protocol analysis: Yes

Protocol: 10, 1, 3, 5

Filename: Bin5_1

Split 1: Petrol shading

Split 2: Green shading

Split 3: Violet shading

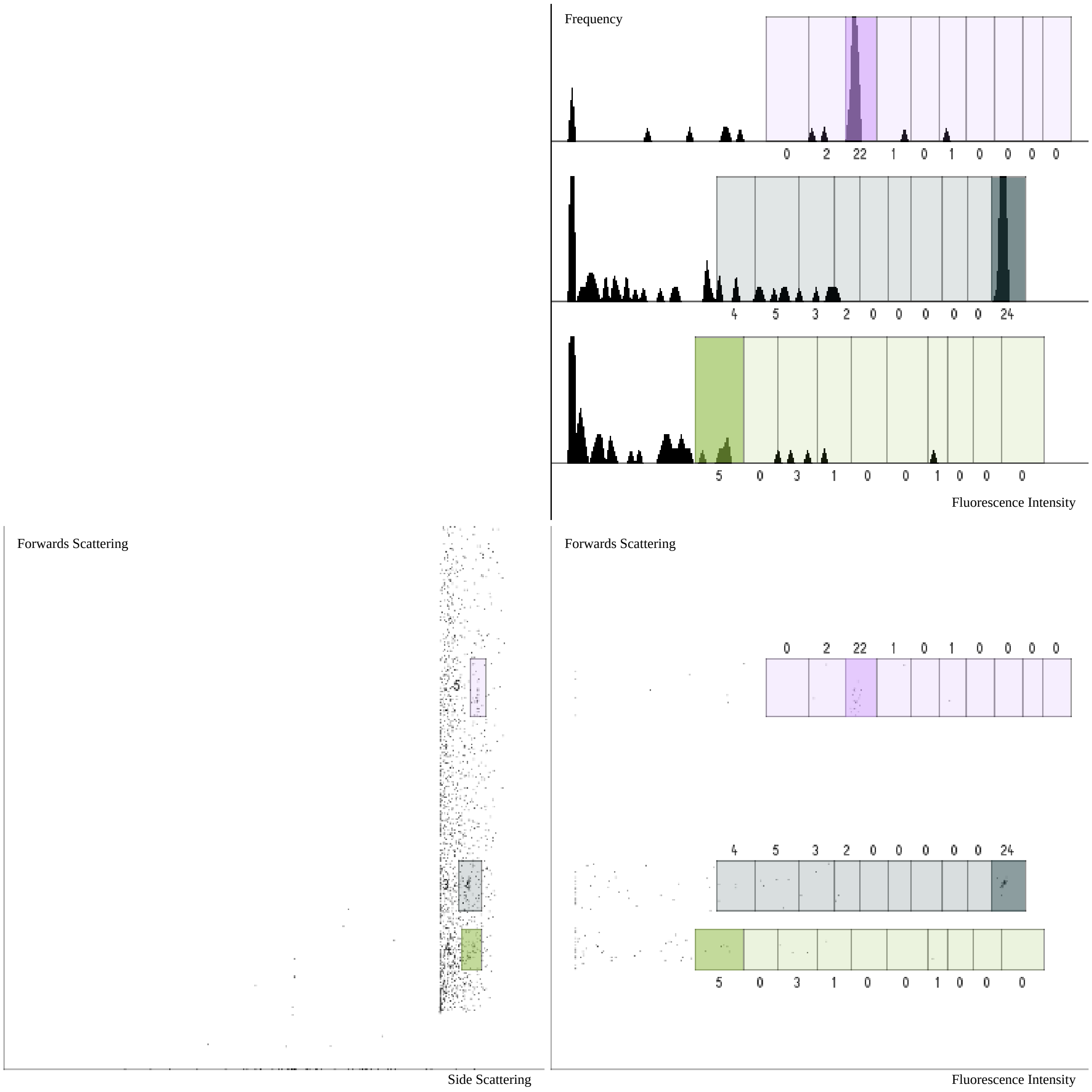

ANNEX 3: TAG DECONVOLUTION - BEAD 12

Passes flow sorting criteria: Yes

Passes tag deconvolution criteria: Yes

Included in protocol analysis: Yes

Protocol: 3, 4, 7, 5

Filename: Bin5_2

Split 1: Petrol shading

Split 2: Green shading

Split 3: Violet shading

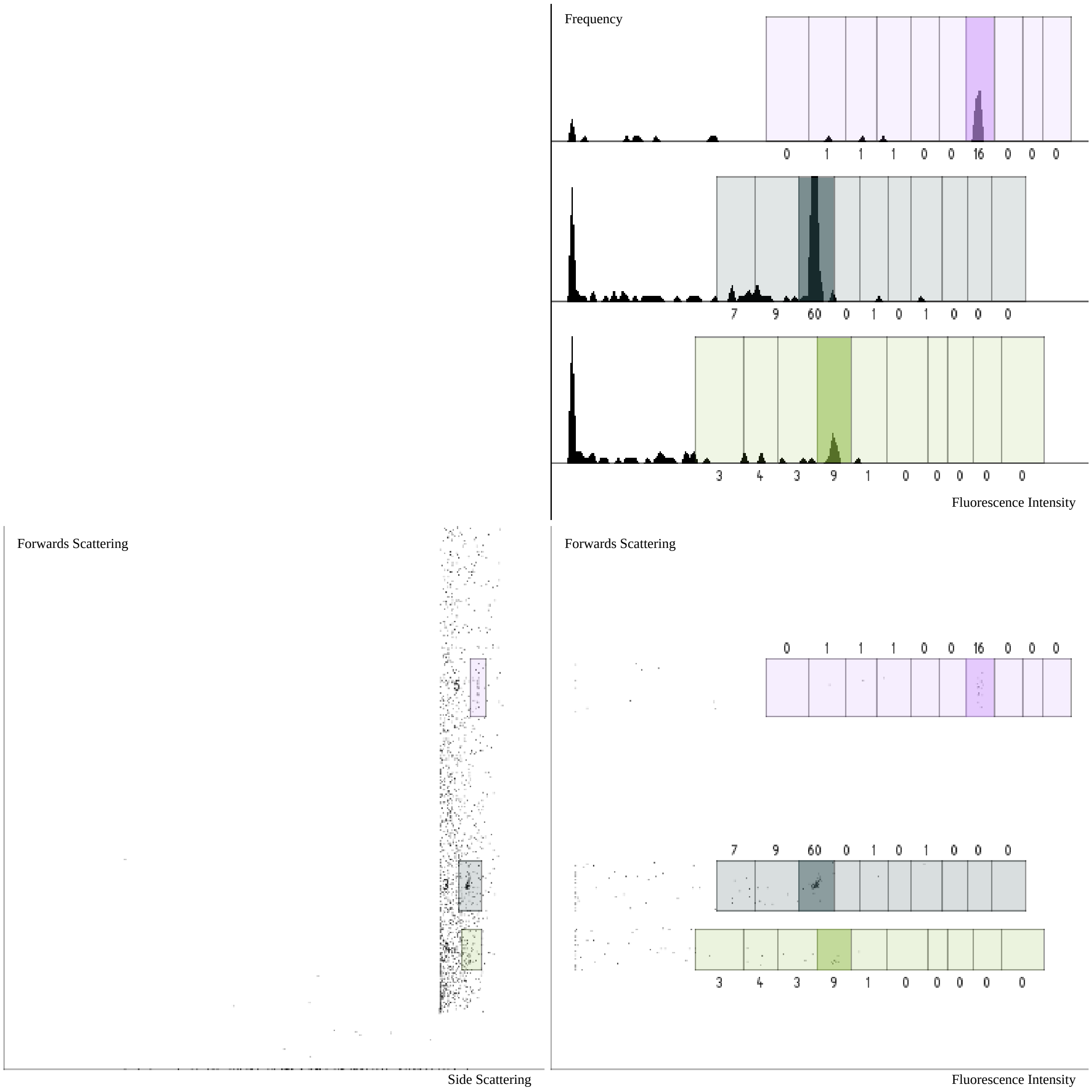

ANNEX 3: TAG DECONVOLUTION - BEAD 13

Passes flow sorting criteria: Yes

Passes tag deconvolution criteria: No

Included in protocol analysis: No

Protocol: N/A

Filename: Bin6_44

Split 1: Petrol shading

Split 2: Green shading

Split 3: Violet shading

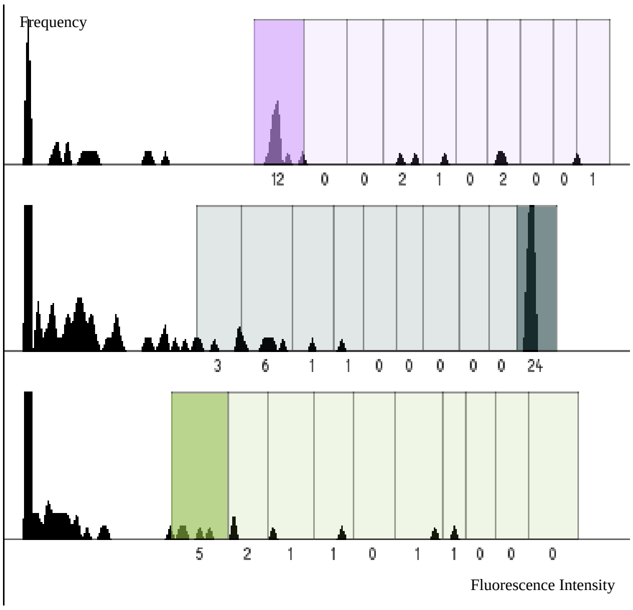

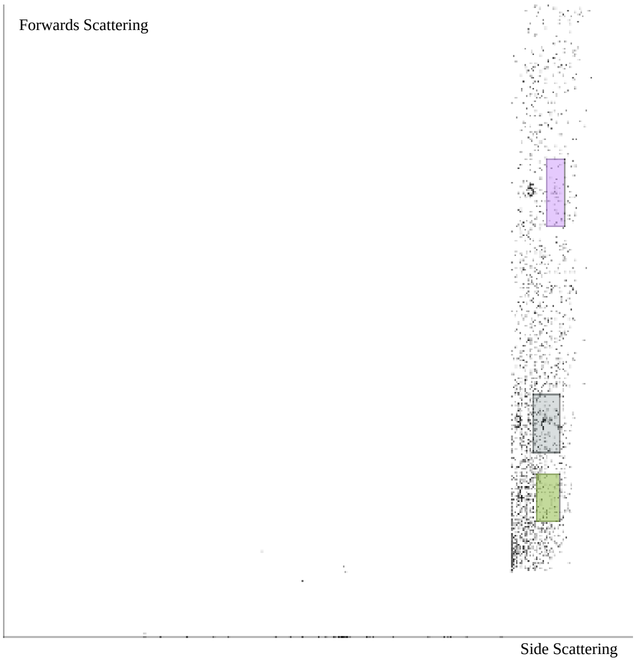

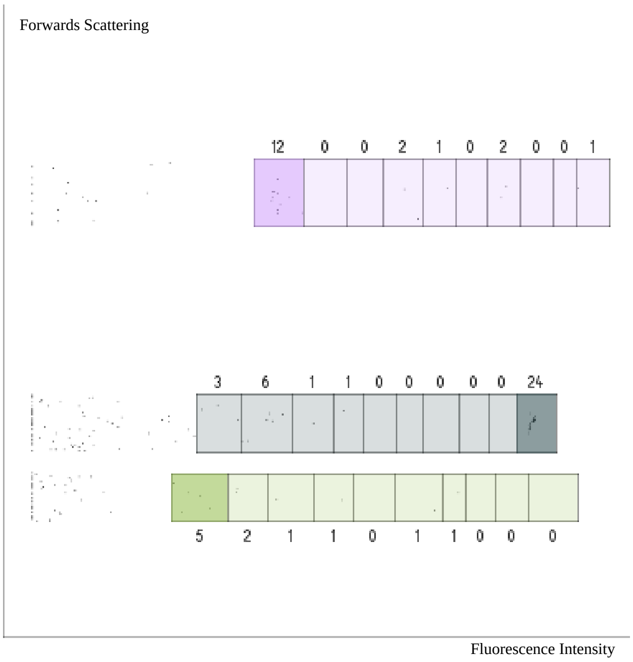

ANNEX 3: TAG DECONVOLUTION - BEAD 14

Passes flow sorting criteria: Yes

Passes tag deconvolution criteria: No

Included in protocol analysis: No

Protocol: N/A

Filename: Bin6_45

Split 1: Petrol shading

Split 2: Green shading

Split 3: Violet shading

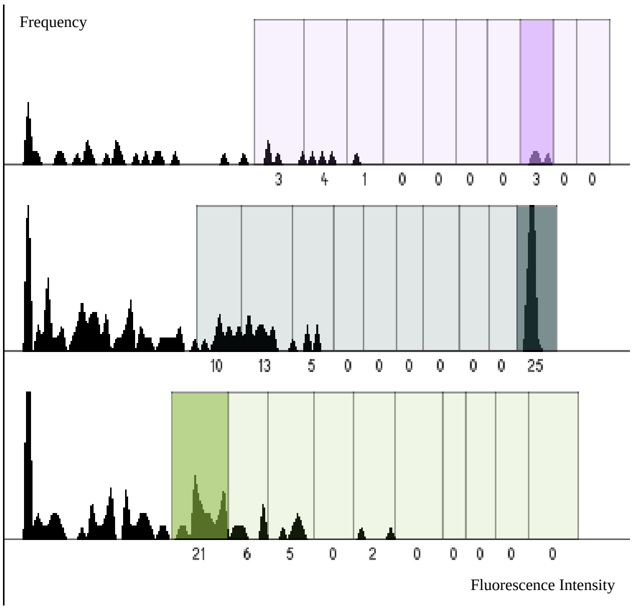

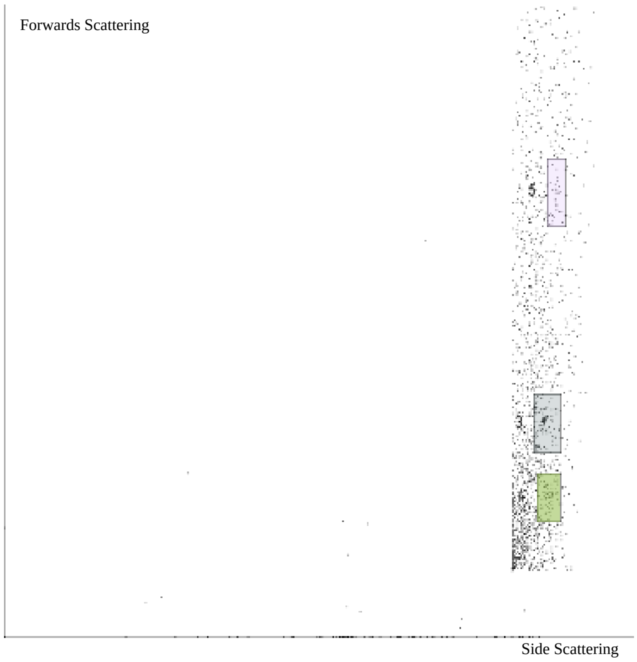

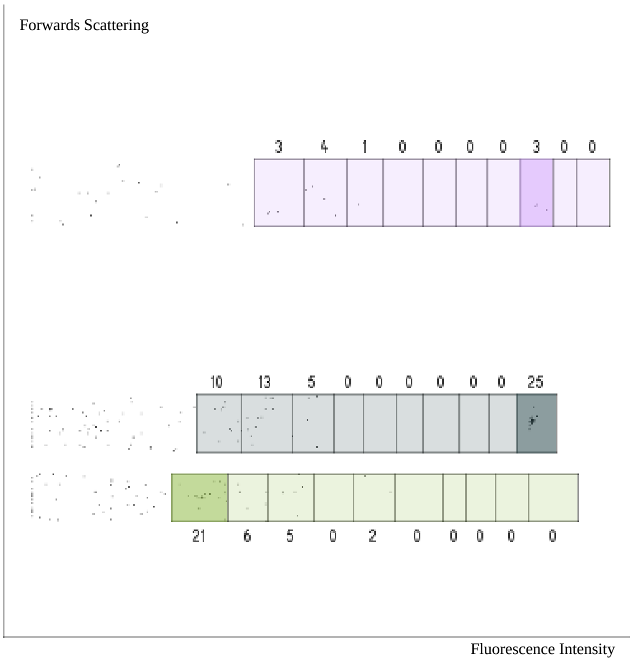

ANNEX 3: TAG DECONVOLUTION - BEAD 15

Passes flow sorting criteria: Yes

Passes tag deconvolution criteria: No

Included in protocol analysis: No

Protocol: N/A

Filename: Bin6_46

Split 1: Petrol shading

Split 2: Green shading

Split 3: Violet shading

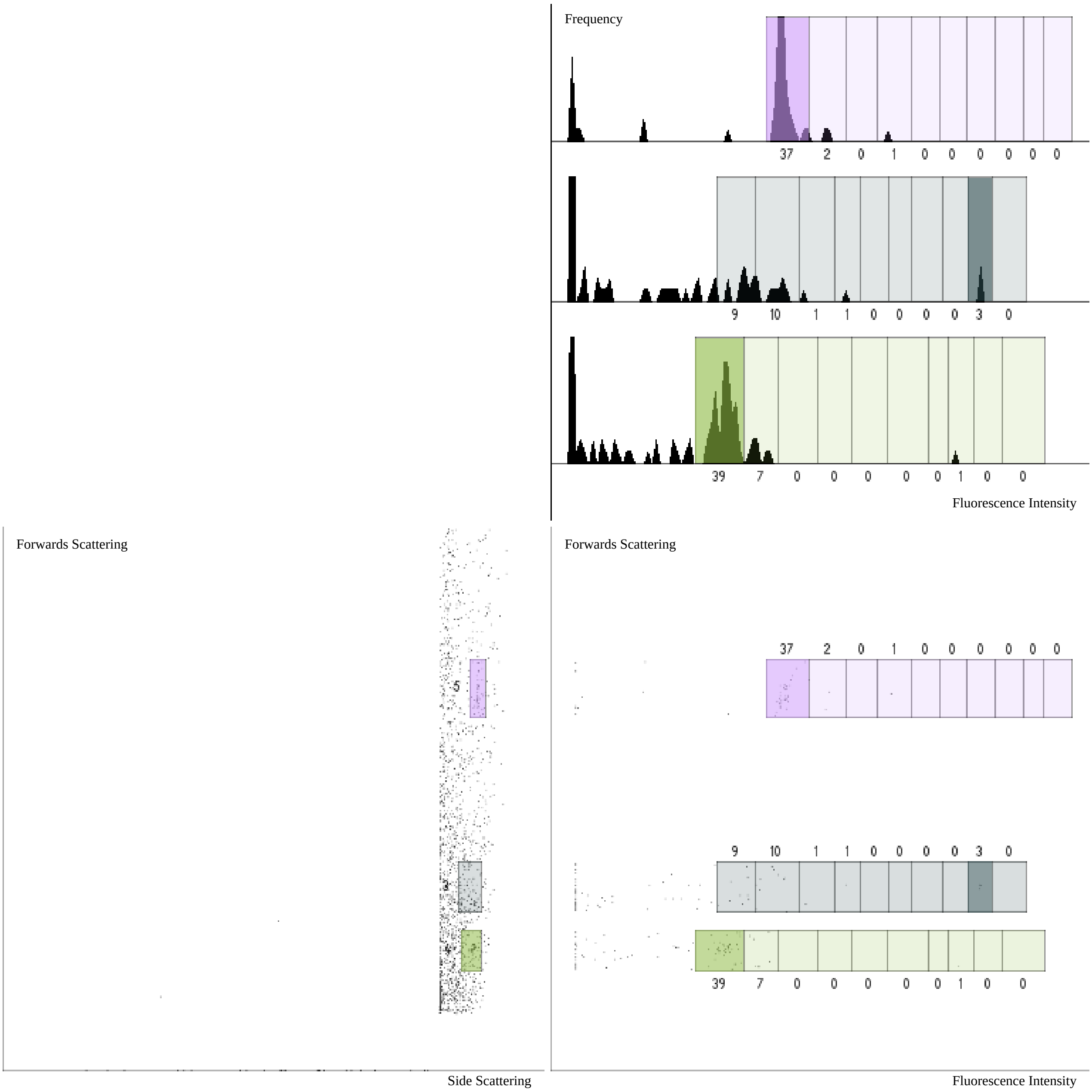

ANNEX 3: TAG DECONVOLUTION - BEAD 16

Passes flow sorting criteria: Yes

Passes tag deconvolution criteria: Yes

Included in protocol analysis: Yes

Protocol: 10, 1, 4, 6

Filename: Bin6_47

Split 1: Petrol shading

Split 2: Green shading

Split 3: Violet shading

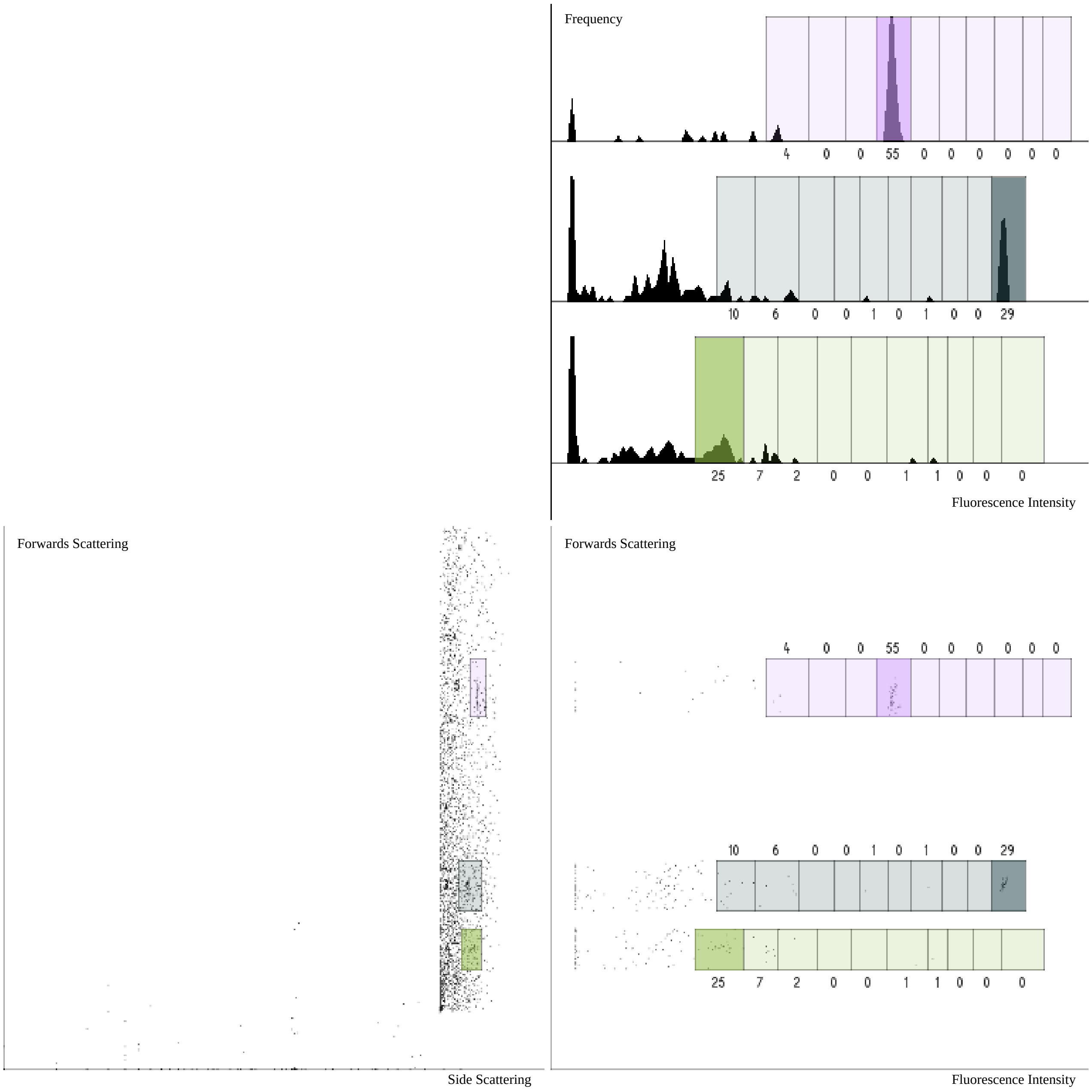

ANNEX 3: TAG DECONVOLUTION - BEAD 17

Passes flow sorting criteria: Yes

Passes tag deconvolution criteria: Yes

Included in protocol analysis: Yes

Protocol: 2, 8, 10, 6

Filename: Bin6_48

Split 1: Petrol shading

Split 2: Green shading

Split 3: Violet shading

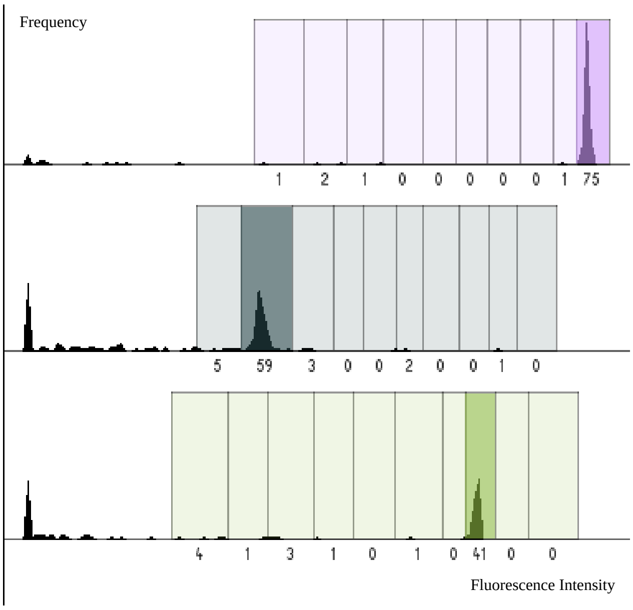

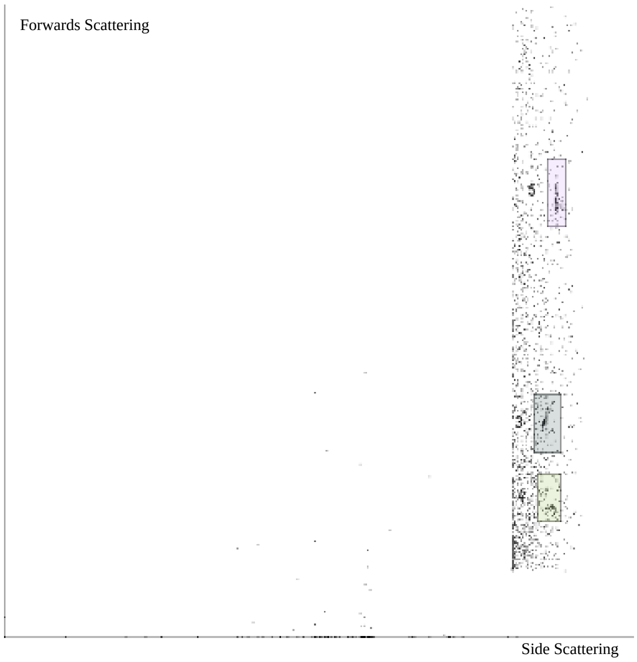

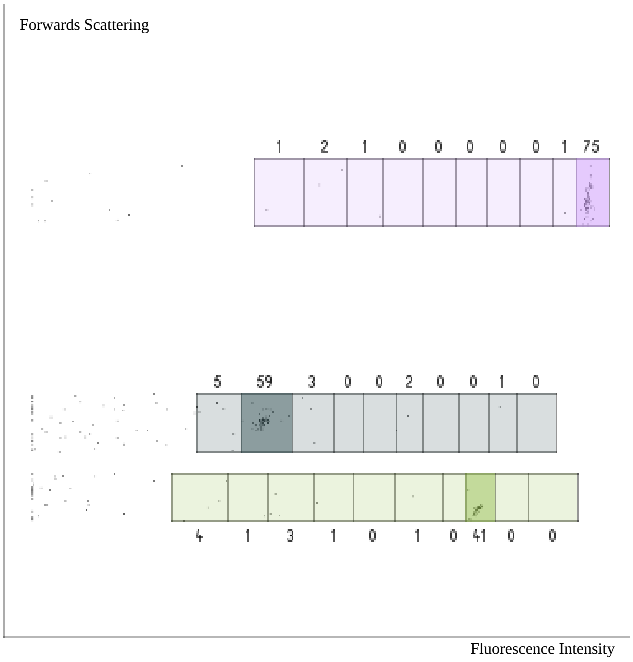

ANNEX 3: TAG DECONVOLUTION - BEAD 18

Passes flow sorting criteria: Yes

Passes tag deconvolution criteria: Yes

Included in protocol analysis: Yes

Protocol: 8, 1, 7, 6

Filename: Bin6_49

Split 1: Petrol shading

Split 2: Green shading

Split 3: Violet shading

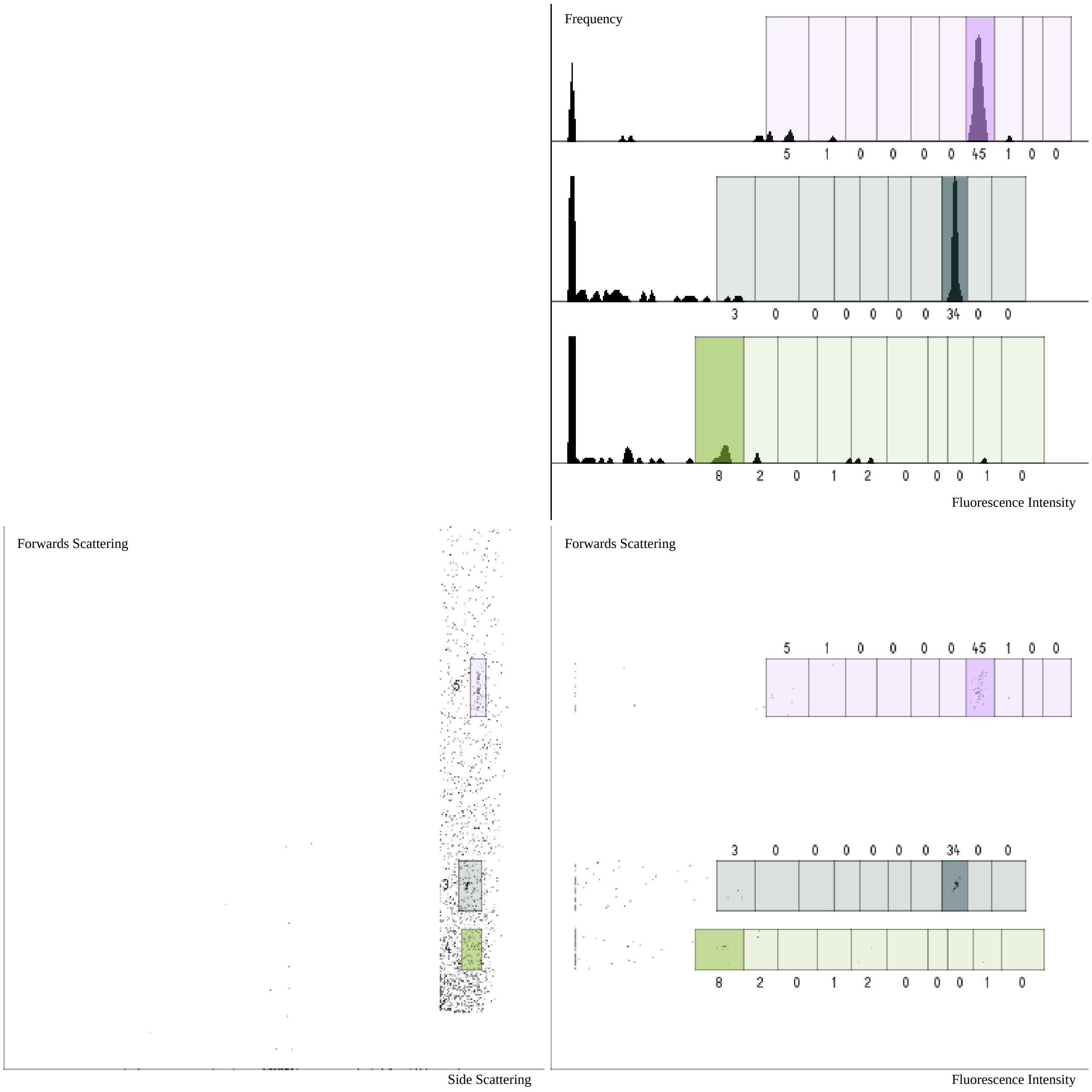

ANNEX 3: TAG DECONVOLUTION - BEAD 19

Passes flow sorting criteria: Yes

Passes tag deconvolution criteria: Yes

Included in protocol analysis: Yes

Protocol: 10, 1, 9, 6

Filename: Bin6_50

Split 1: Petrol shading

Split 2: Green shading

Split 3: Violet shading

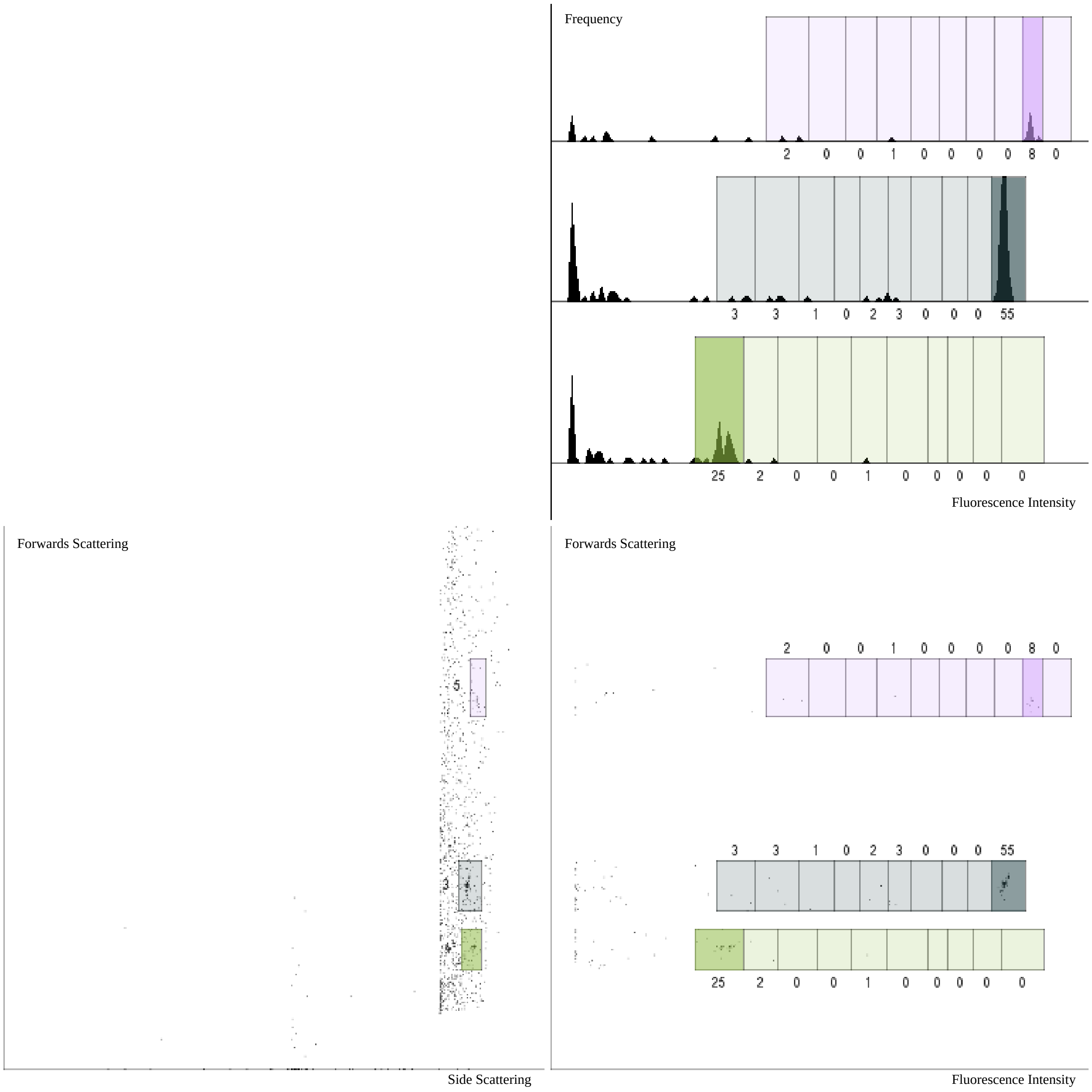

ANNEX 3: TAG DECONVOLUTION - BEAD 20

Passes flow sorting criteria: Yes

Passes tag deconvolution criteria: Yes

Included in protocol analysis: Yes

Protocol: 10, 2, 7, 6

Filename: Bin6_51

Split 1: Petrol shading

Split 2: Green shading

Split 3: Violet shading

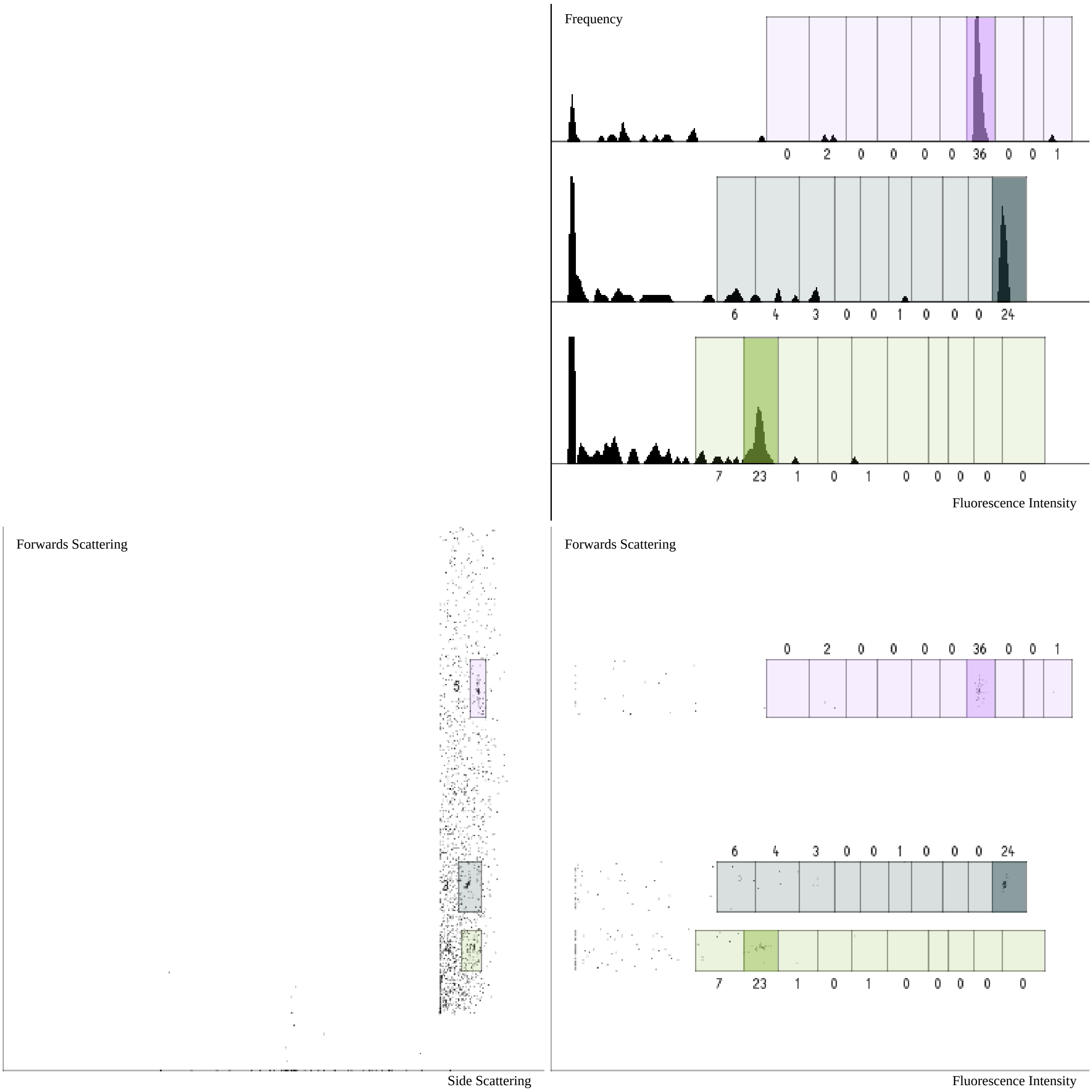

ANNEX 3: TAG DECONVOLUTION - BEAD 21

Passes flow sorting criteria: Yes

Passes tag deconvolution criteria: Yes

Included in protocol analysis: Yes

Protocol: 4, 4, 2, 5

Filename: Bin5_3

Split 1: Petrol shading

Split 2: Green shading

Split 3: Violet shading

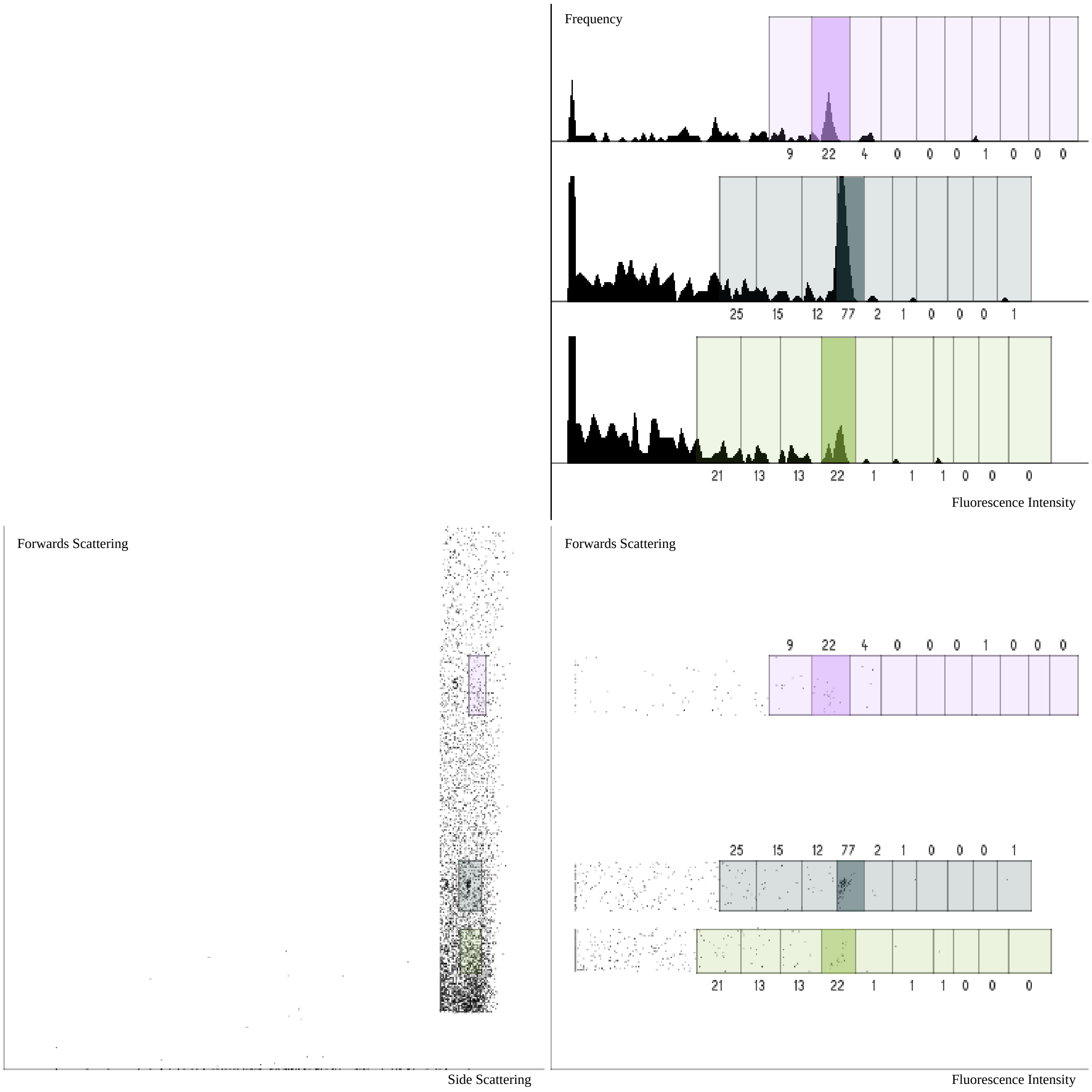

ANNEX 3: TAG DECONVOLUTION - BEAD 22

Passes flow sorting criteria: Yes

Passes tag deconvolution criteria: No

Included in protocol analysis: No

Protocol: N/A

Filename: Bin5_4

Split 1: Petrol shading

Split 2: Green shading

Split 3: Violet shading

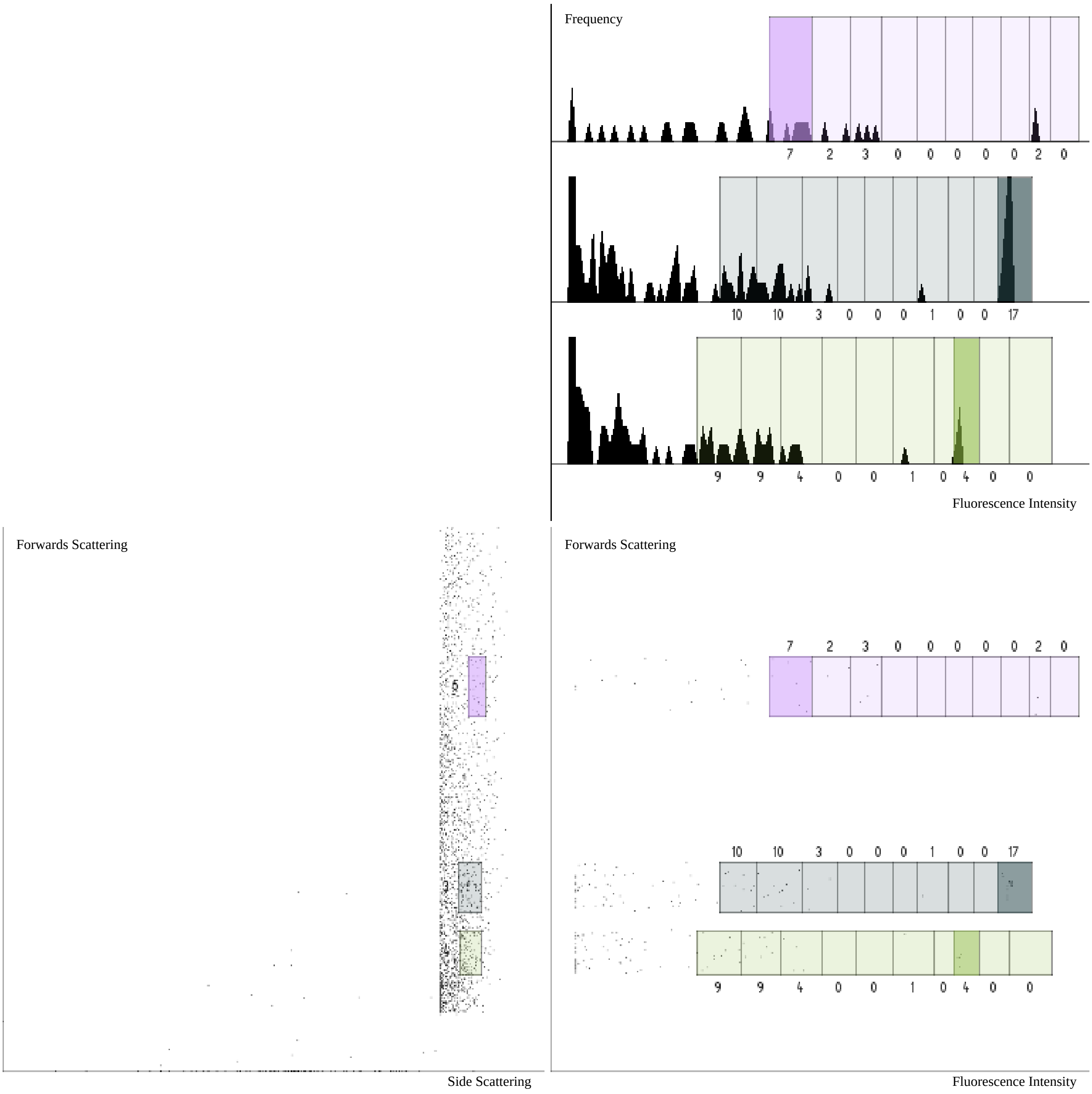

ANNEX 3: TAG DECONVOLUTION - BEAD 23

Passes flow sorting criteria: Yes

Passes tag deconvolution criteria: No

Included in protocol analysis: No

Protocol: N/A

Filename: Bin5_5

Split 1: Petrol shading

Split 2: Green shading

Split 3: Violet shading

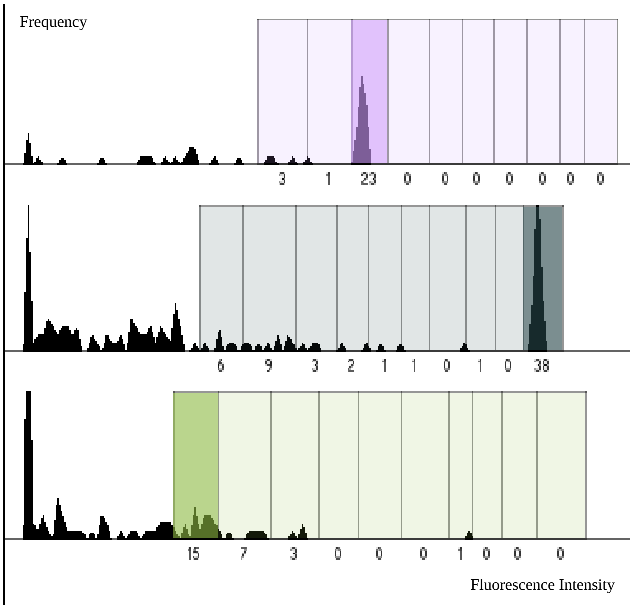

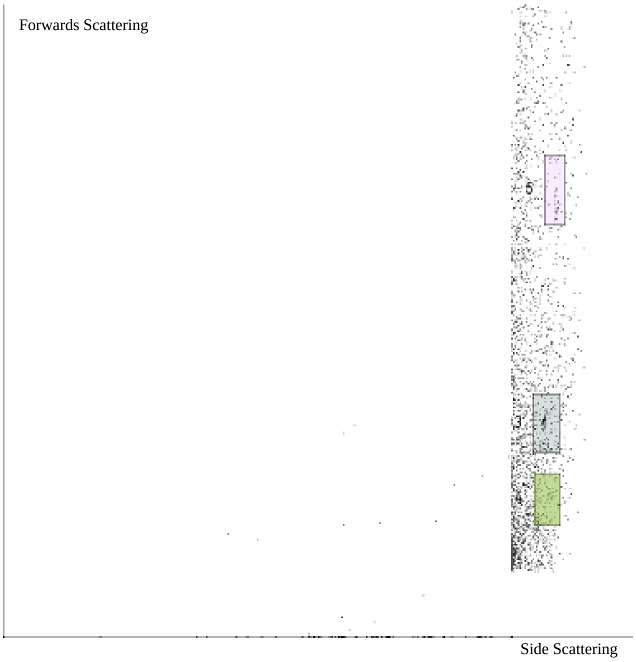

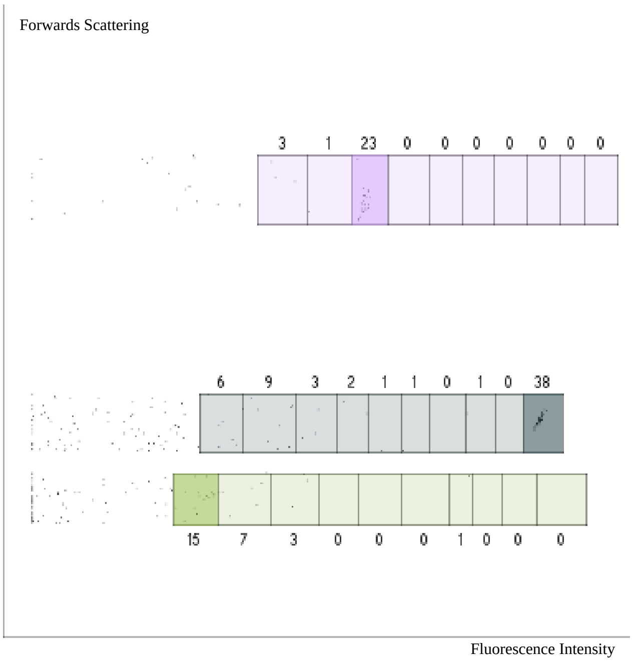

ANNEX 3: TAG DECONVOLUTION - BEAD 24

Passes flow sorting criteria: Yes

Passes tag deconvolution criteria: Yes

Included in protocol analysis: Yes

Protocol: 3, 2, 7, 5

Filename: Bin5_6

Split 1: Petrol shading

Split 2: Green shading

Split 3: Violet shading

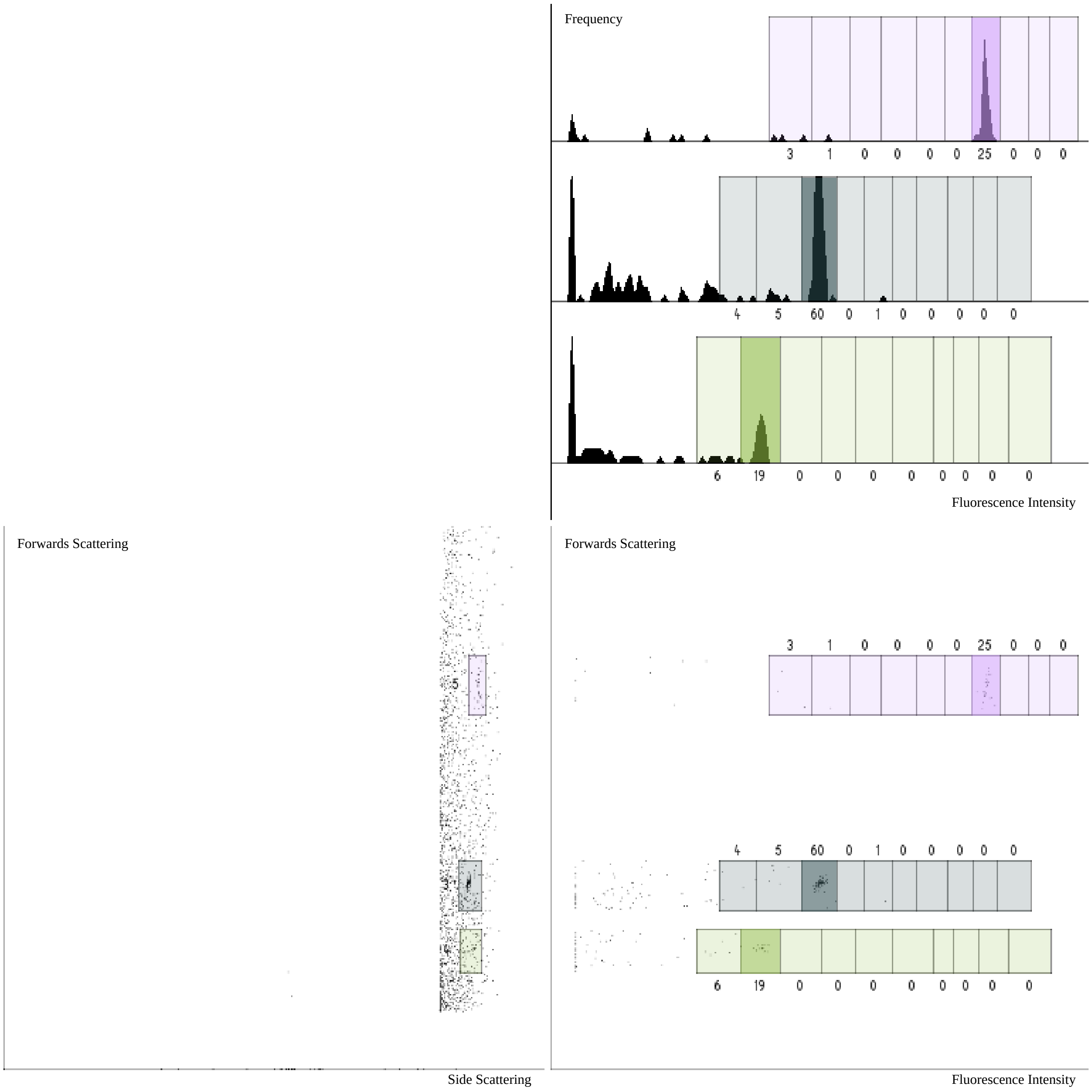

ANNEX 3: TAG DECONVOLUTION - BEAD 25

Passes flow sorting criteria: Yes

Passes tag deconvolution criteria: No

Included in protocol analysis: No

Protocol: N/A

Filename: Bin5_7

Split 1: Petrol shading

Split 2: Green shading

Split 3: Violet shading

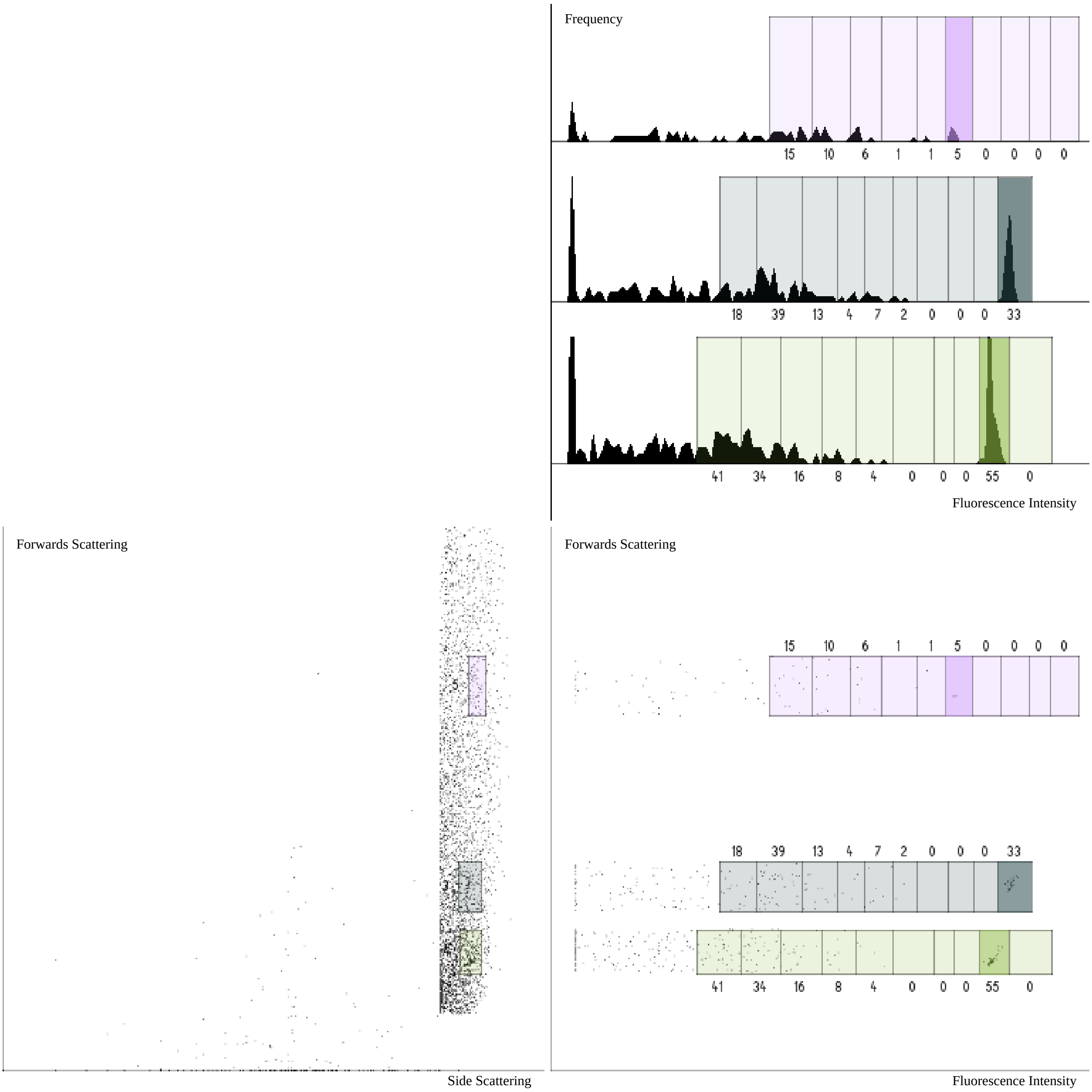

ANNEX 3: TAG DECONVOLUTION - BEAD 26

Passes flow sorting criteria: Yes

Passes tag deconvolution criteria: Yes

Included in protocol analysis: Yes

Protocol: 3, 5, 9, 5

Filename: Bin5_8

Split 1: Petrol shading

Split 2: Green shading

Split 3: Violet shading

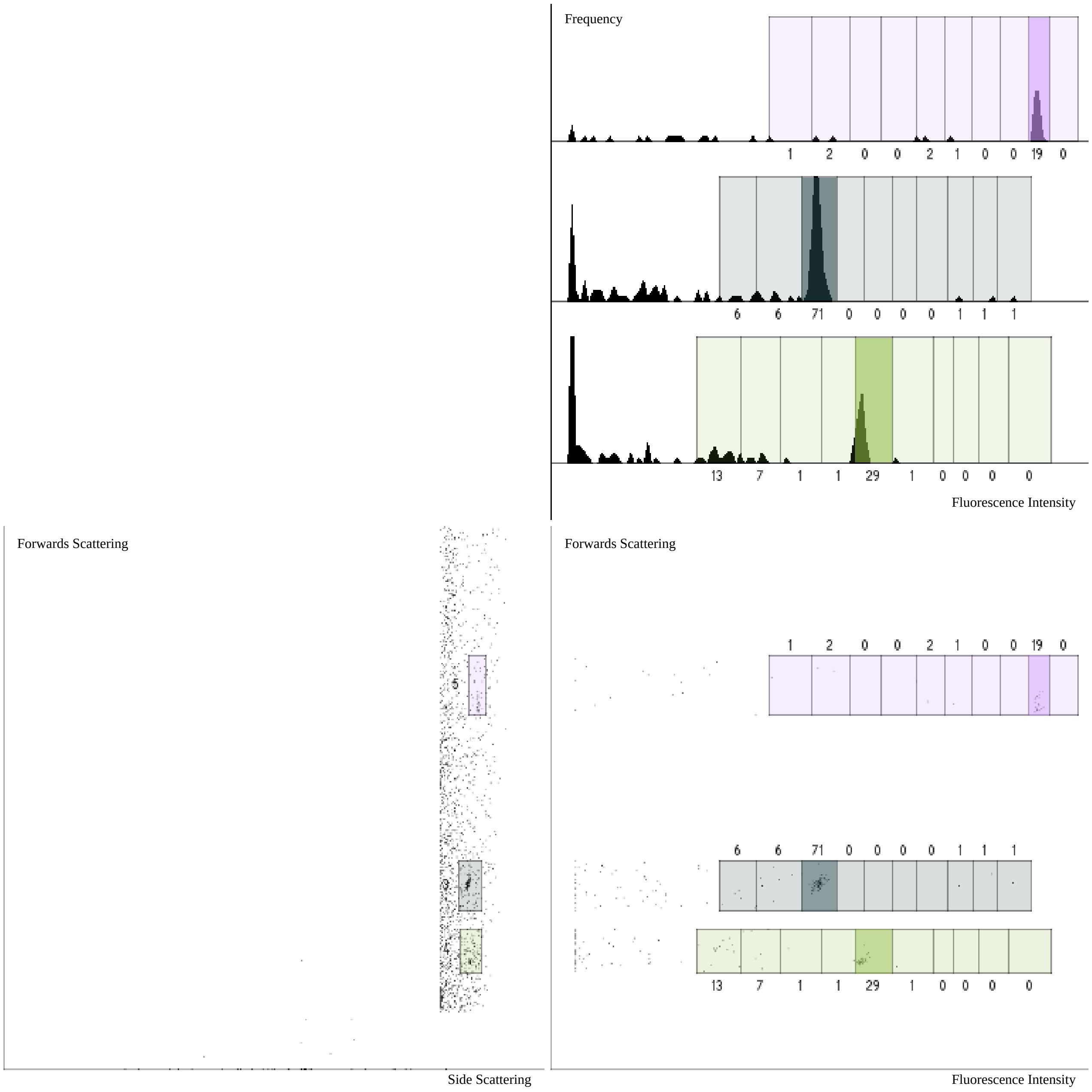

ANNEX 3: TAG DECONVOLUTION - BEAD 27

Passes flow sorting criteria: Yes

Passes tag deconvolution criteria: Yes

Included in protocol analysis: Yes

Protocol: 2, 2, 6, 5

Filename: Bin5_9

Split 1: Petrol shading

Split 2: Green shading

Split 3: Violet shading

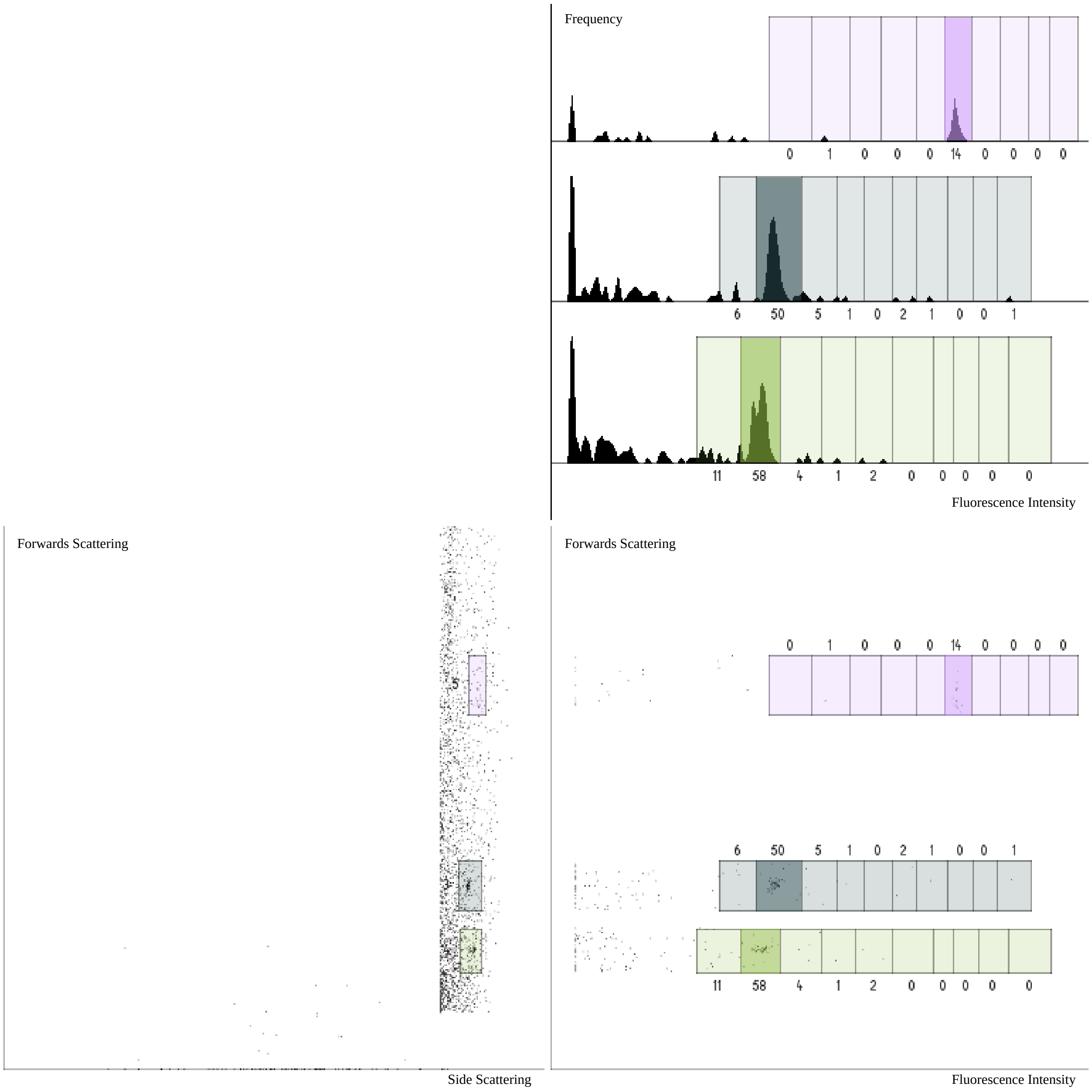

ANNEX 3: TAG DECONVOLUTION - BEAD 28

Passes flow sorting criteria: Yes

Passes tag deconvolution criteria: Yes

Included in protocol analysis: Yes

Protocol: 5, 8, 3, 5

Filename: Bin5_10

Split 1: Petrol shading

Split 2: Green shading

Split 3: Violet shading

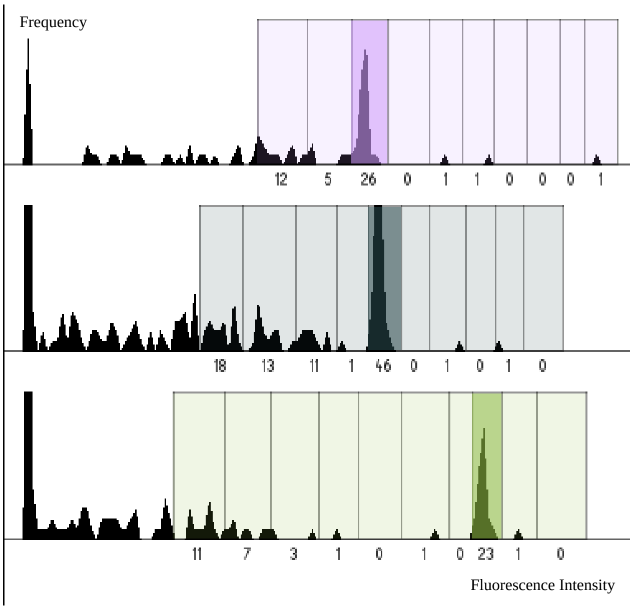

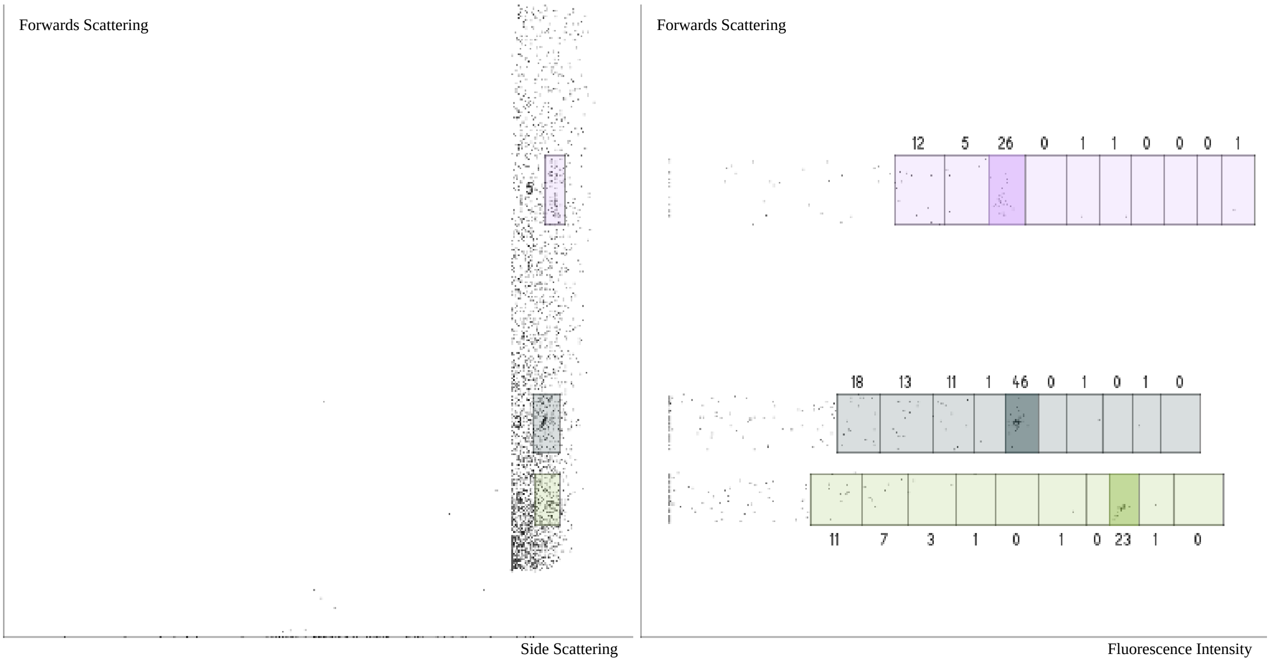

ANNEX 3: TAG DECONVOLUTION - BEAD 29

Passes flow sorting criteria: Yes

Passes tag deconvolution criteria: Yes

Included in protocol analysis: Yes

Protocol: 4, 9, 5, 5

Filename: Bin5_11

Split 1: Petrol shading

Split 2: Green shading

Split 3: Violet shading

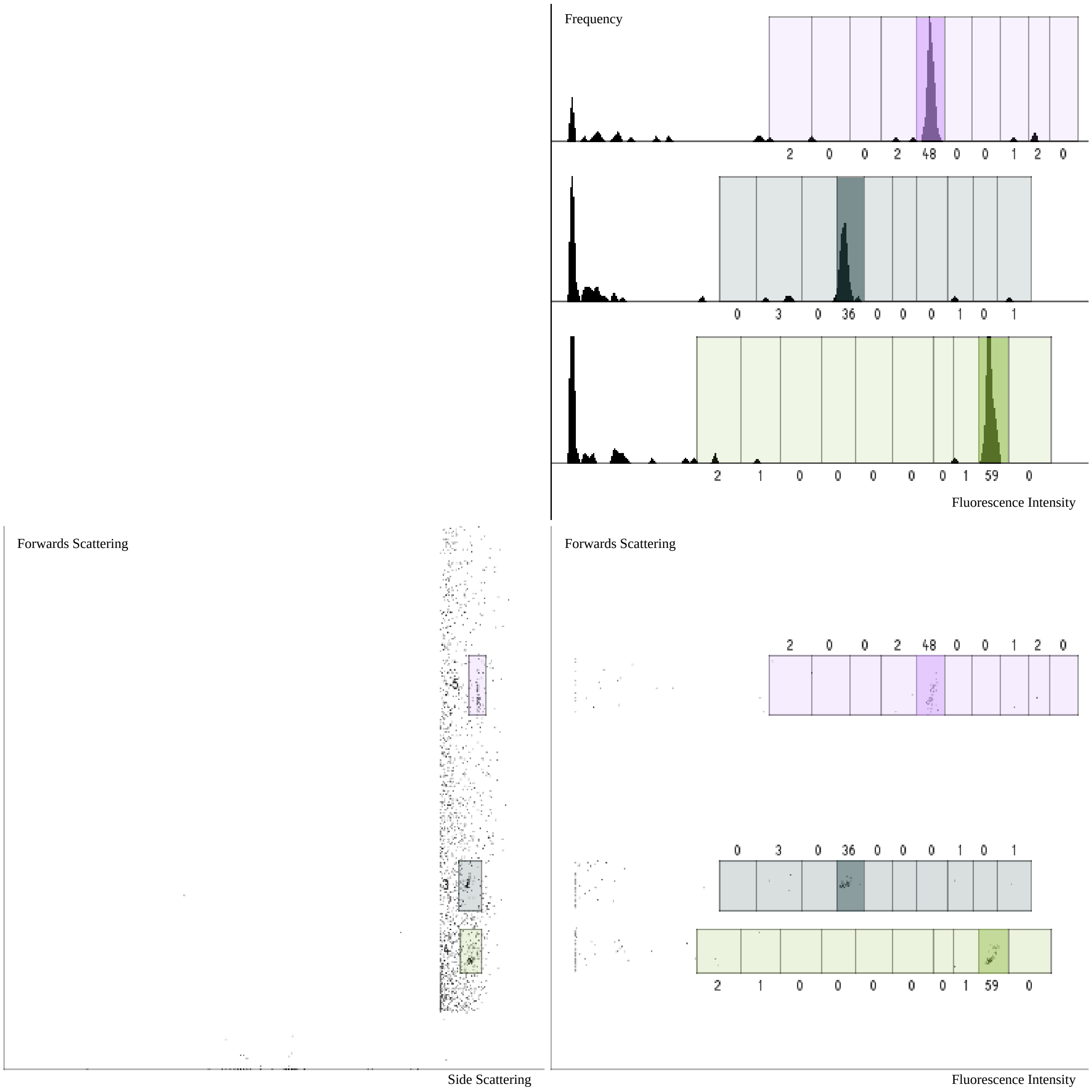

ANNEX 3: TAG DECONVOLUTION - BEAD 30

Passes flow sorting criteria: Yes

Passes tag deconvolution criteria: Yes

Included in protocol analysis: Yes

Protocol: 10, 1, 4, 5

Filename: Bin5_12

Split 1: Petrol shading

Split 2: Green shading

Split 3: Violet shading

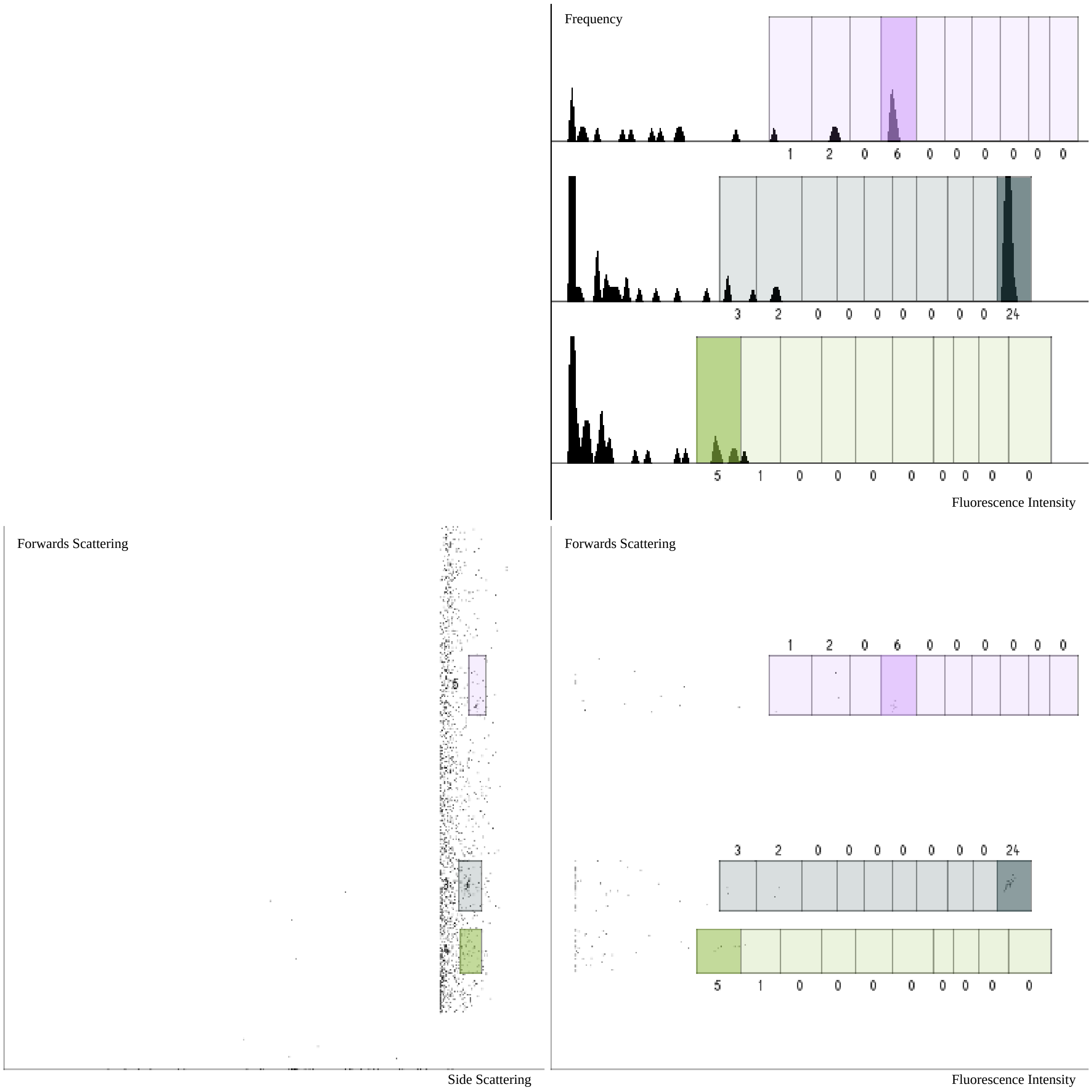

ANNEX 3: TAG DECONVOLUTION - BEAD 31

Passes flow sorting criteria: Yes

Passes tag deconvolution criteria: Yes

Included in protocol analysis: Yes

Protocol: 10, 1, 8, 5

Filename: Bin5_13

Split 1: Petrol shading

Split 2: Green shading

Split 3: Violet shading

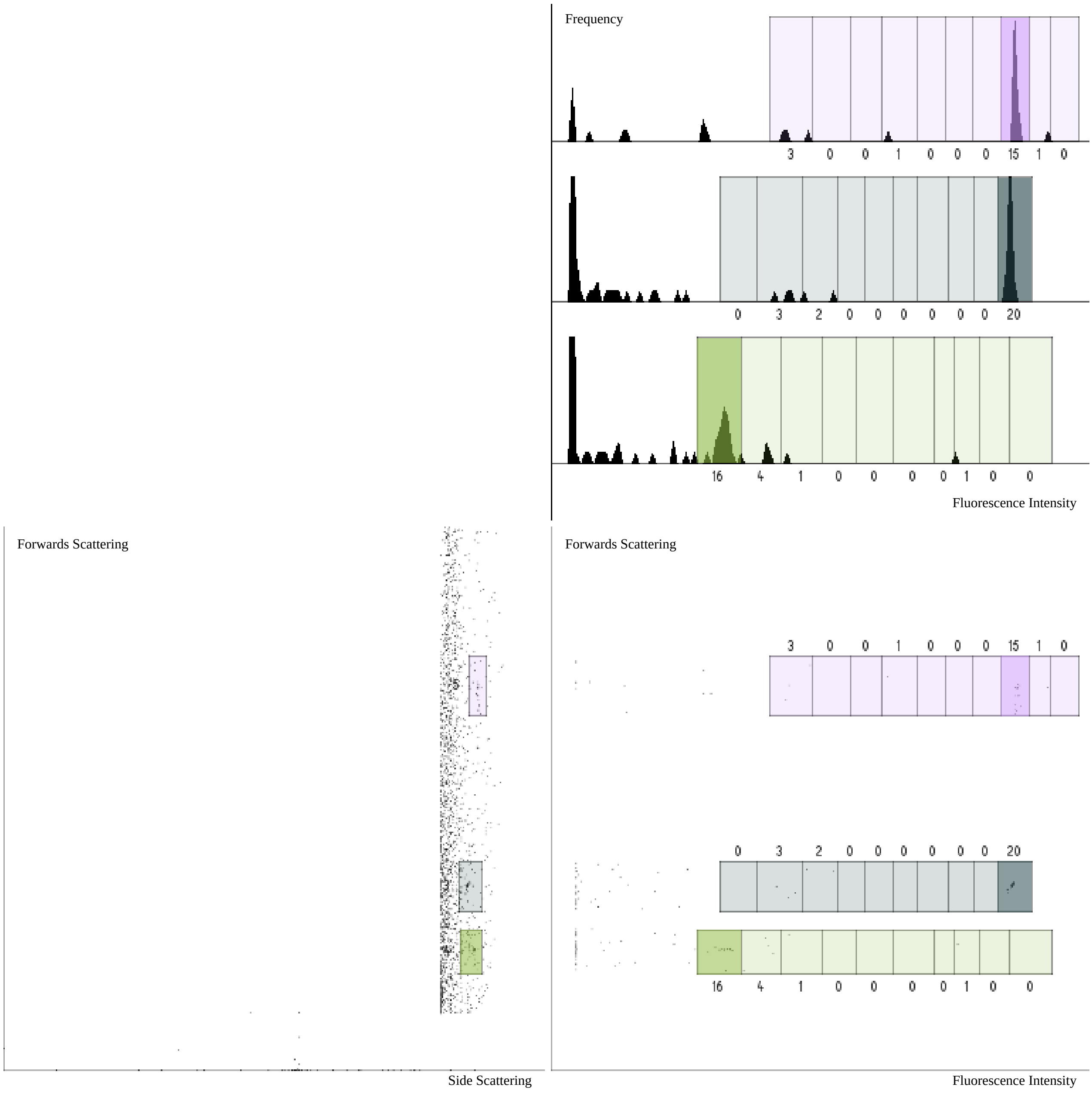

ANNEX 3: TAG DECONVOLUTION - BEAD 32

Passes flow sorting criteria: Yes

Passes tag deconvolution criteria: Yes

Included in protocol analysis: Yes

Protocol: 4, 3, 1, 5

Filename: Bin5_14

Split 1: Petrol shading

Split 2: Green shading

Split 3: Violet shading

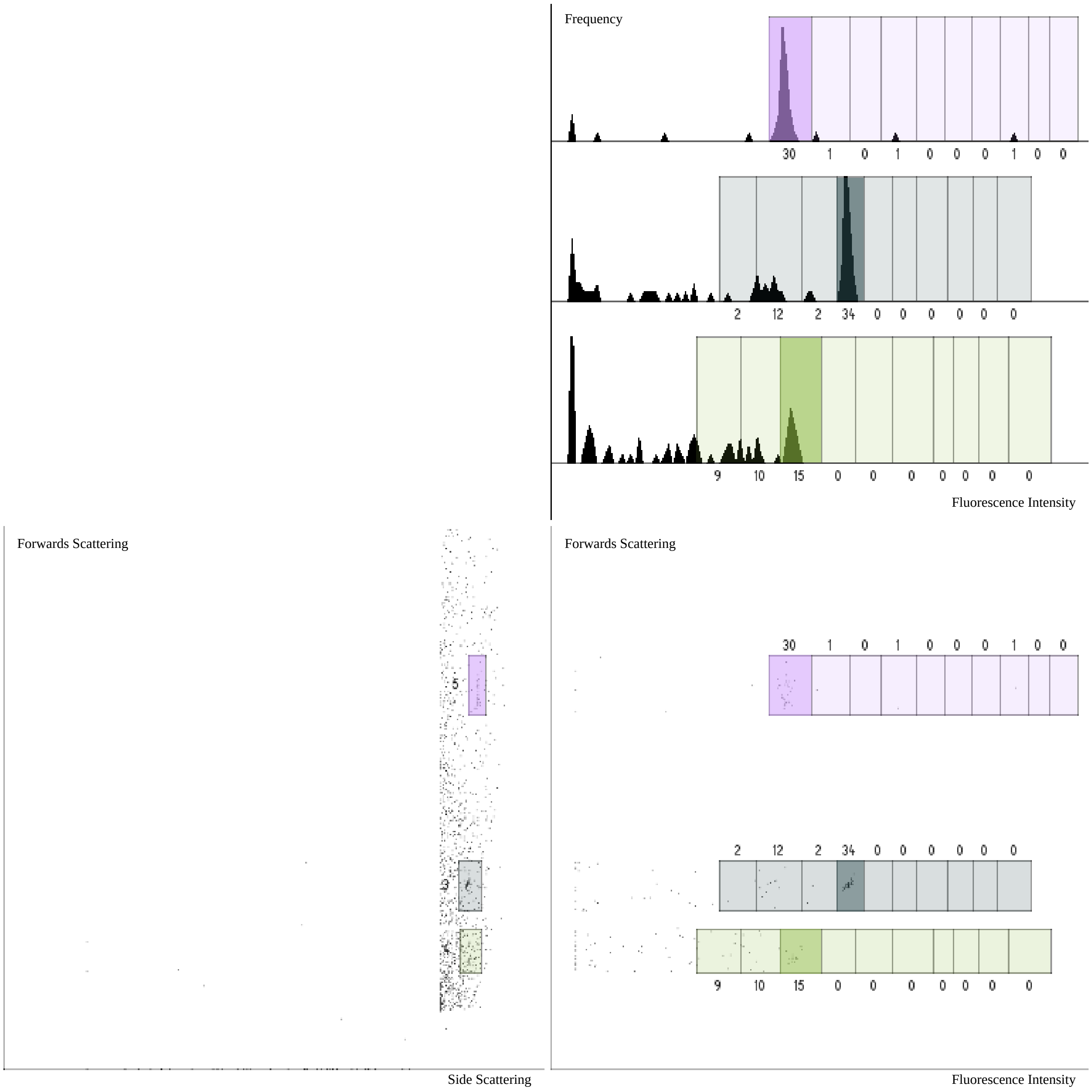

ANNEX 3: TAG DECONVOLUTION - BEAD 33

Passes flow sorting criteria: Yes

Passes tag deconvolution criteria: Yes

Included in protocol analysis: Yes

Protocol: 9, 2, 4, 5

Filename: Bin5_15

Split 1: Petrol shading

Split 2: Green shading

Split 3: Violet shading

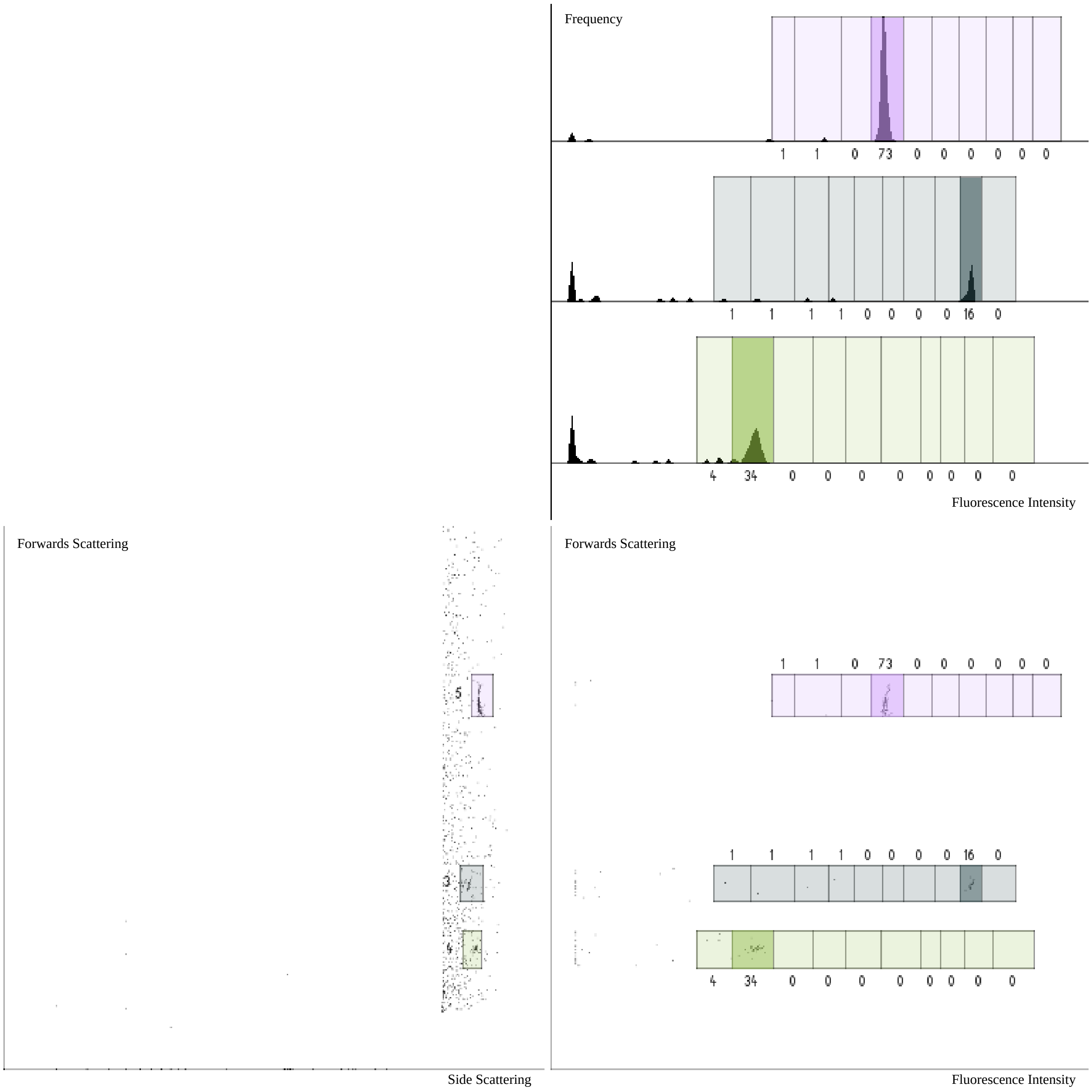

ANNEX 3: TAG DECONVOLUTION - BEAD 34

Passes flow sorting criteria: Yes

Passes tag deconvolution criteria: Yes

Included in protocol analysis: Yes

Protocol: 2, 8, 6, 5

Filename: Bin5_16

Split 1: Petrol shading

Split 2: Green shading

Split 3: Violet shading

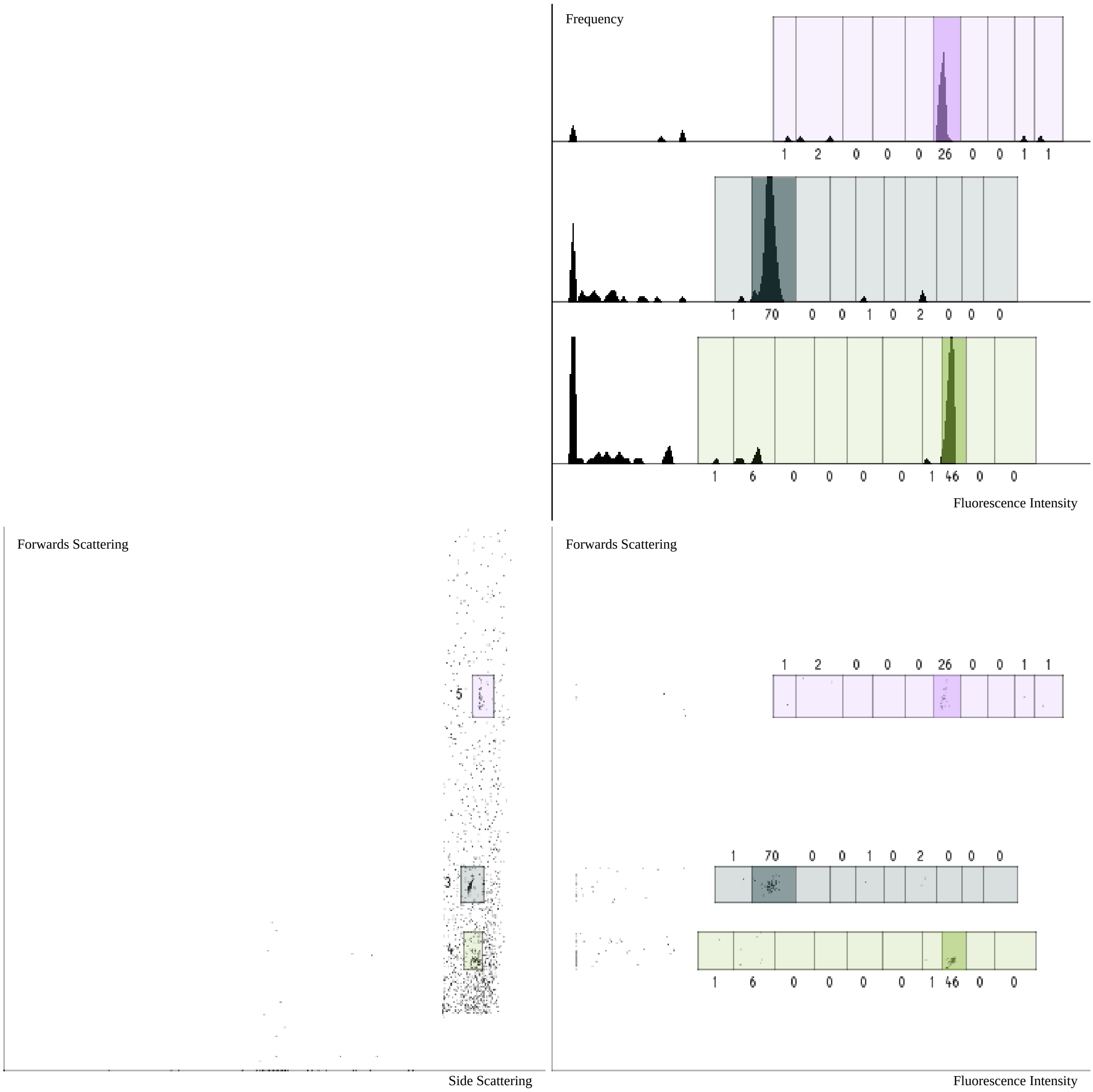

ANNEX 3: TAG DECONVOLUTION - BEAD 35

Passes flow sorting criteria: Yes

Passes tag deconvolution criteria: Yes

Included in protocol analysis: Yes

Protocol: 10, 2, 8, 5

Filename: Bin5_17

Split 1: Petrol shading

Split 2: Green shading

Split 3: Violet shading

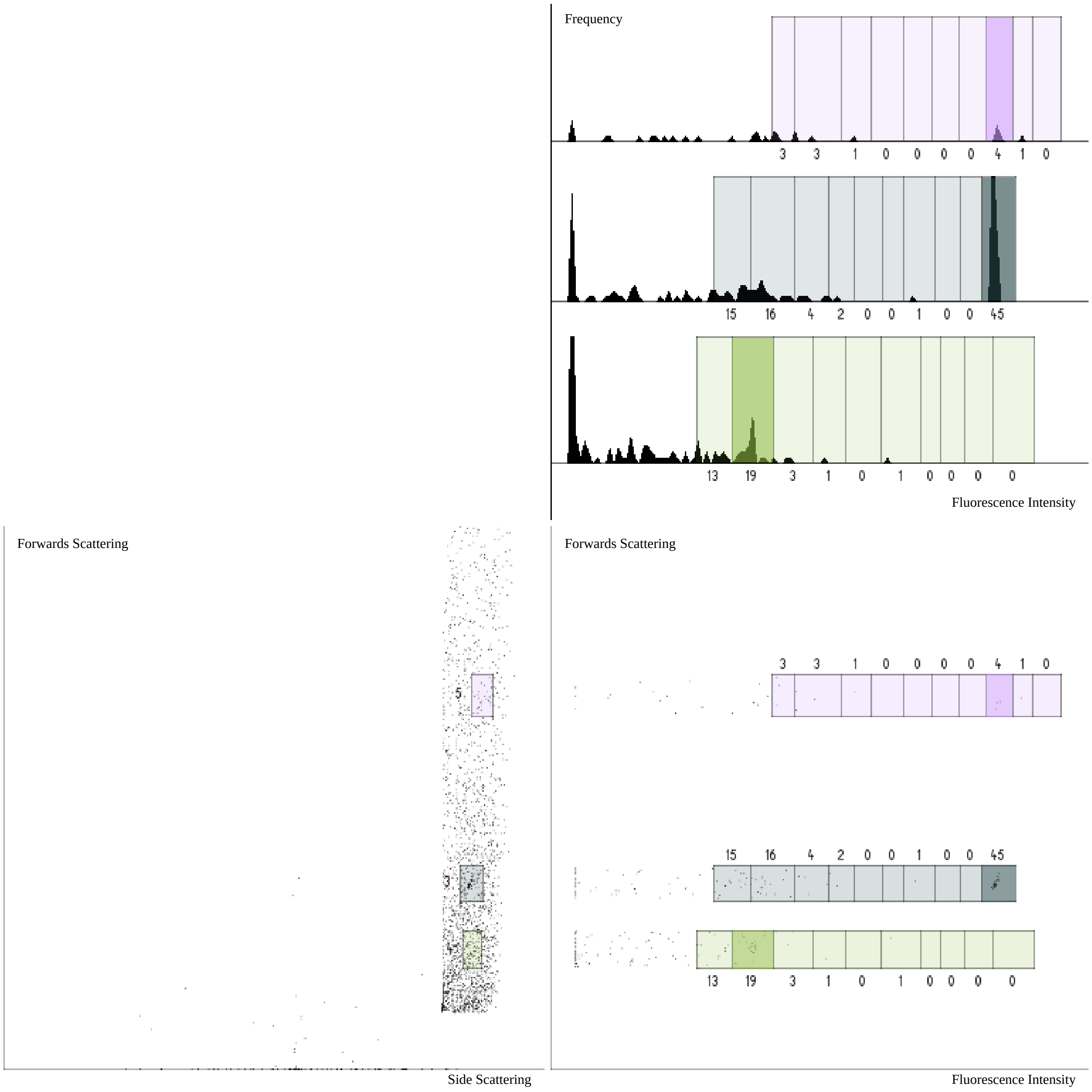

ANNEX 3: TAG DECONVOLUTION - BEAD 36

Passes flow sorting criteria: Yes

Passes tag deconvolution criteria: Yes

Included in protocol analysis: Yes

Protocol: 10, 3, 4, 5

Filename: Bin5_18

Split 1: Petrol shading

Split 2: Green shading

Split 3: Violet shading

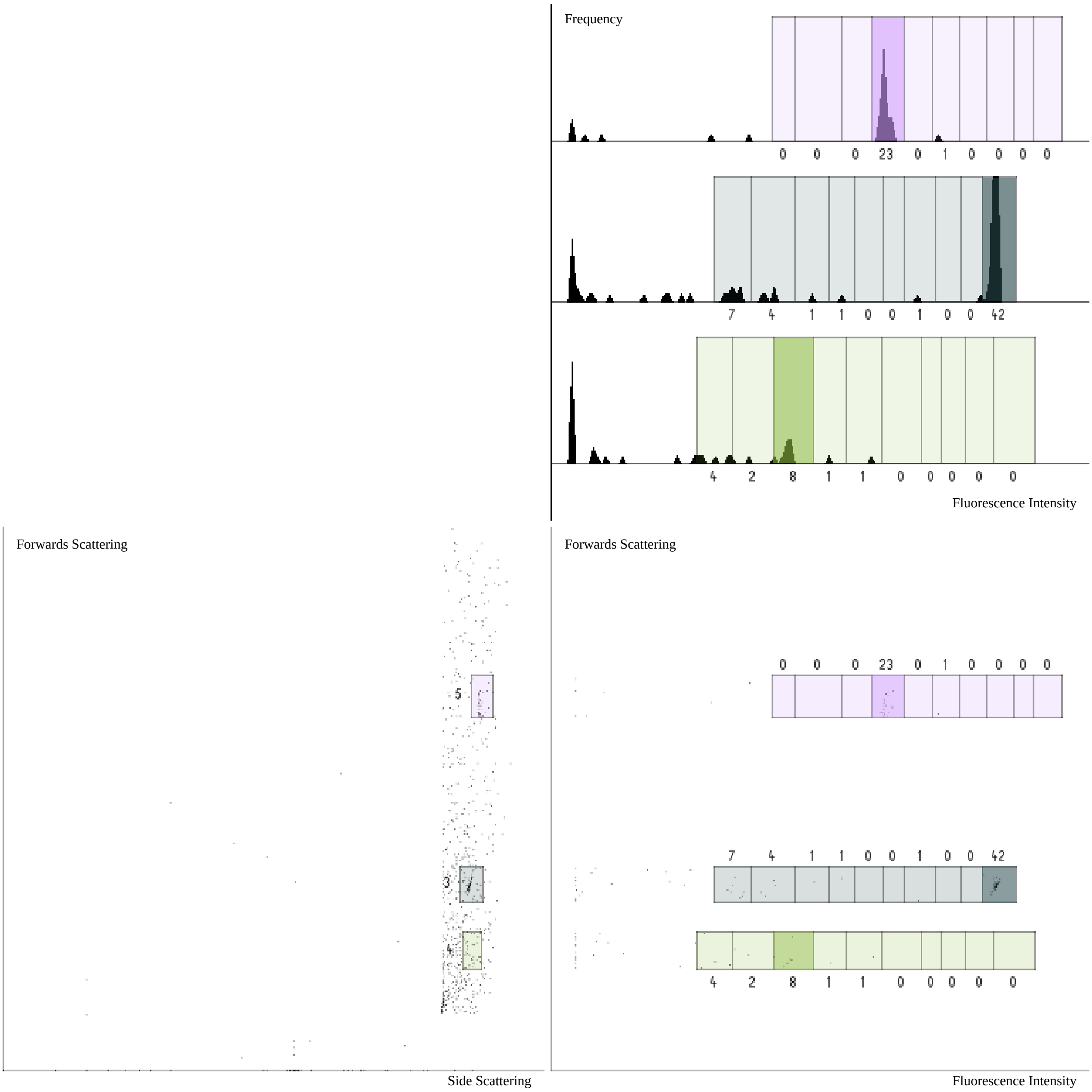

ANNEX 3: TAG DECONVOLUTION - BEAD 37

Passes flow sorting criteria: Yes

Passes tag deconvolution criteria: Yes

Included in protocol analysis: Yes

Protocol: 1, 2, 6, 5

Filename: Bin5_19

Split 1: Petrol shading

Split 2: Green shading

Split 3: Violet shading

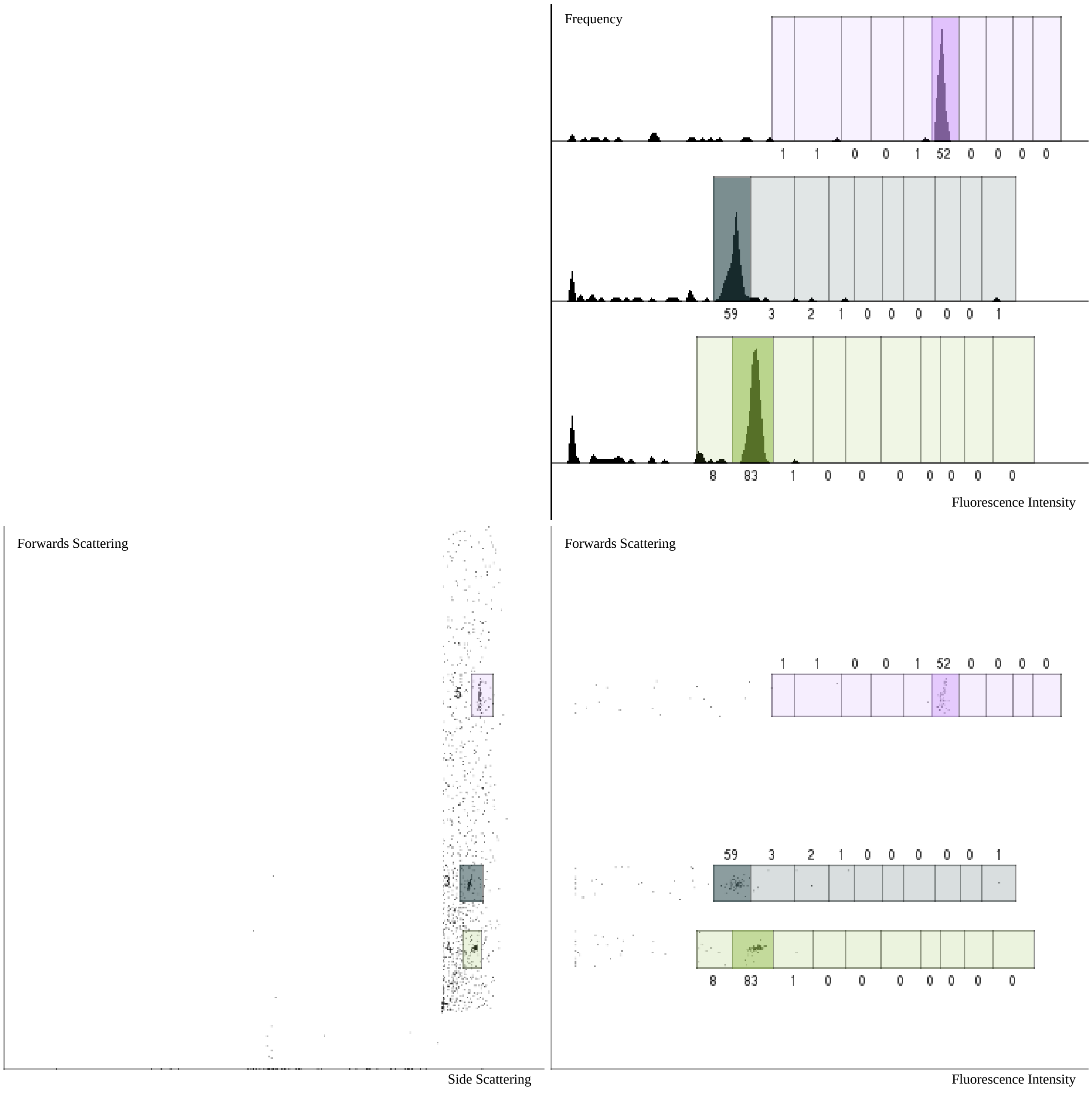

ANNEX 3: TAG DECONVOLUTION - BEAD 38

Passes flow sorting criteria: Yes

Passes tag deconvolution criteria: Yes

Included in protocol analysis: Yes

Protocol: 10, 1, 4, 5

Filename: Bin5_20

Split 1: Petrol shading

Split 2: Green shading

Split 3: Violet shading

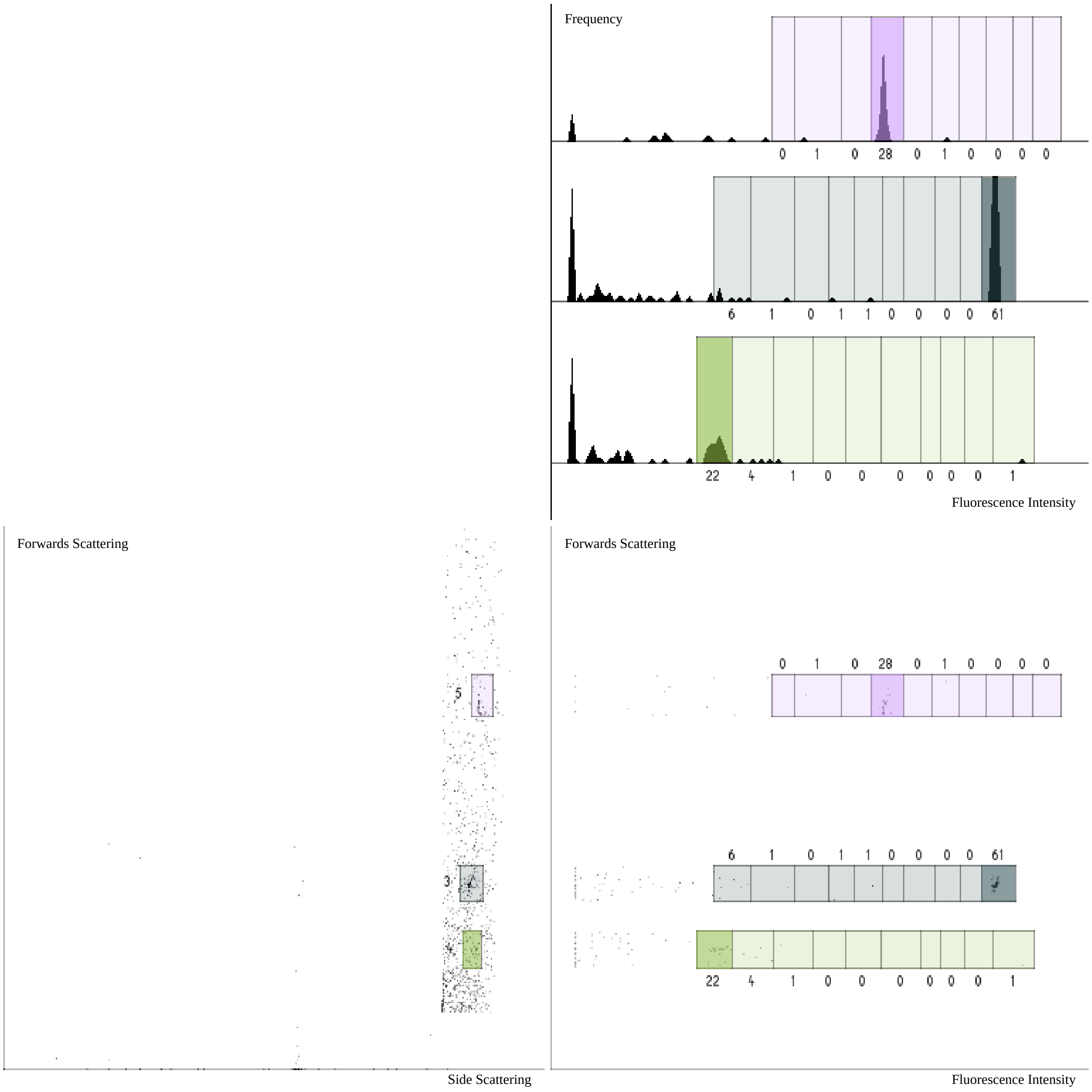

ANNEX 3: TAG DECONVOLUTION - BEAD 39

Passes flow sorting criteria: Yes

Passes tag deconvolution criteria: Yes

Included in protocol analysis: Yes

Protocol: 5, 3, 1, 5

Filename: Bin5_21

Split 1: Petrol shading

Split 2: Green shading

Split 3: Violet shading

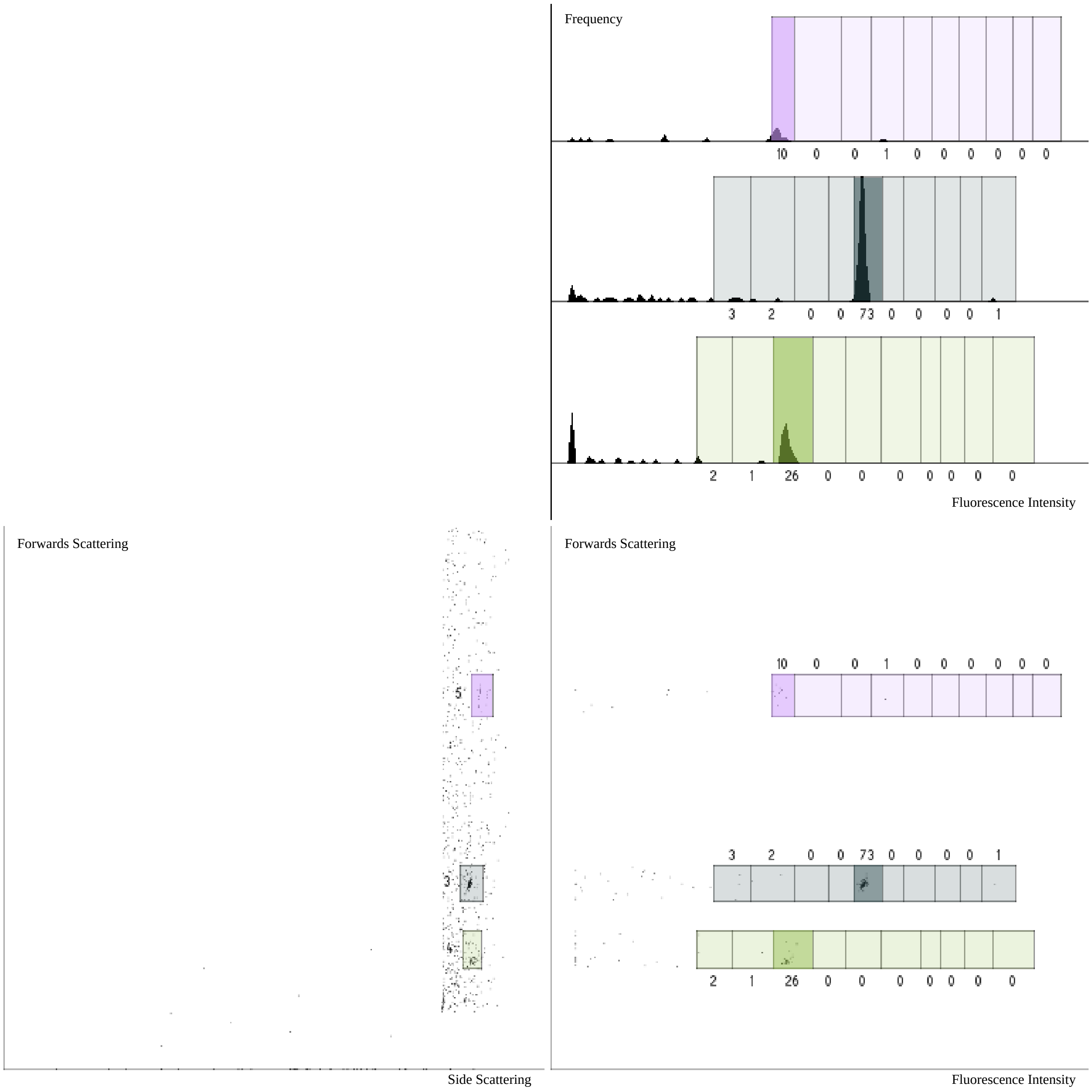

ANNEX 3: TAG DECONVOLUTION - BEAD 40

Passes flow sorting criteria: Yes

Passes tag deconvolution criteria: No

Included in protocol analysis: No

Protocol: N/A

Filename: Bin5_22

Split 1: Petrol shading

Split 2: Green shading

Split 3: Violet shading

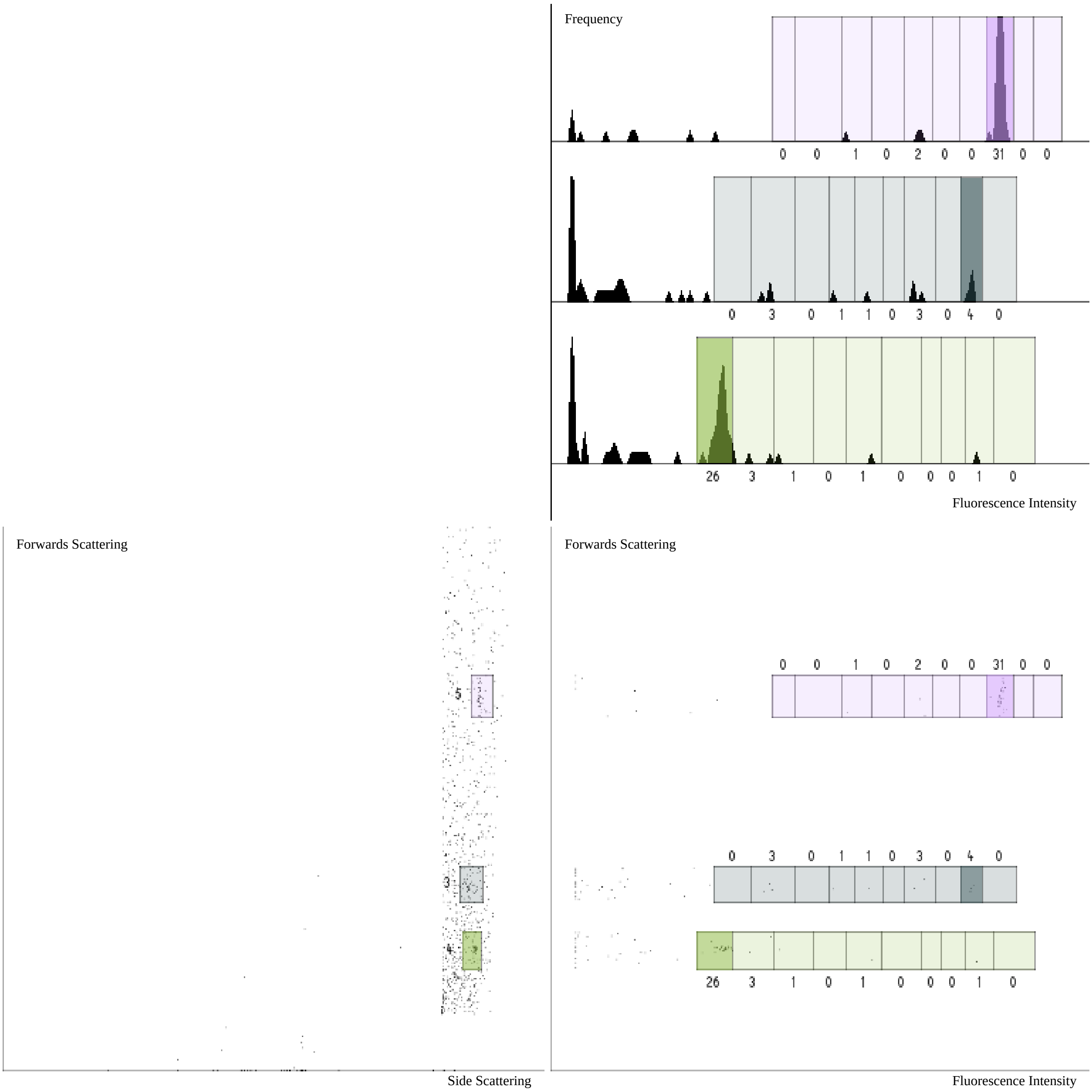

ANNEX 3: TAG DECONVOLUTION - BEAD 41

Passes flow sorting criteria: Yes

Passes tag deconvolution criteria: Yes

Included in protocol analysis: Yes

Protocol: 10, 1, 9, 5

Filename: Bin5_23

Split 1: Petrol shading

Split 2: Green shading

Split 3: Violet shading

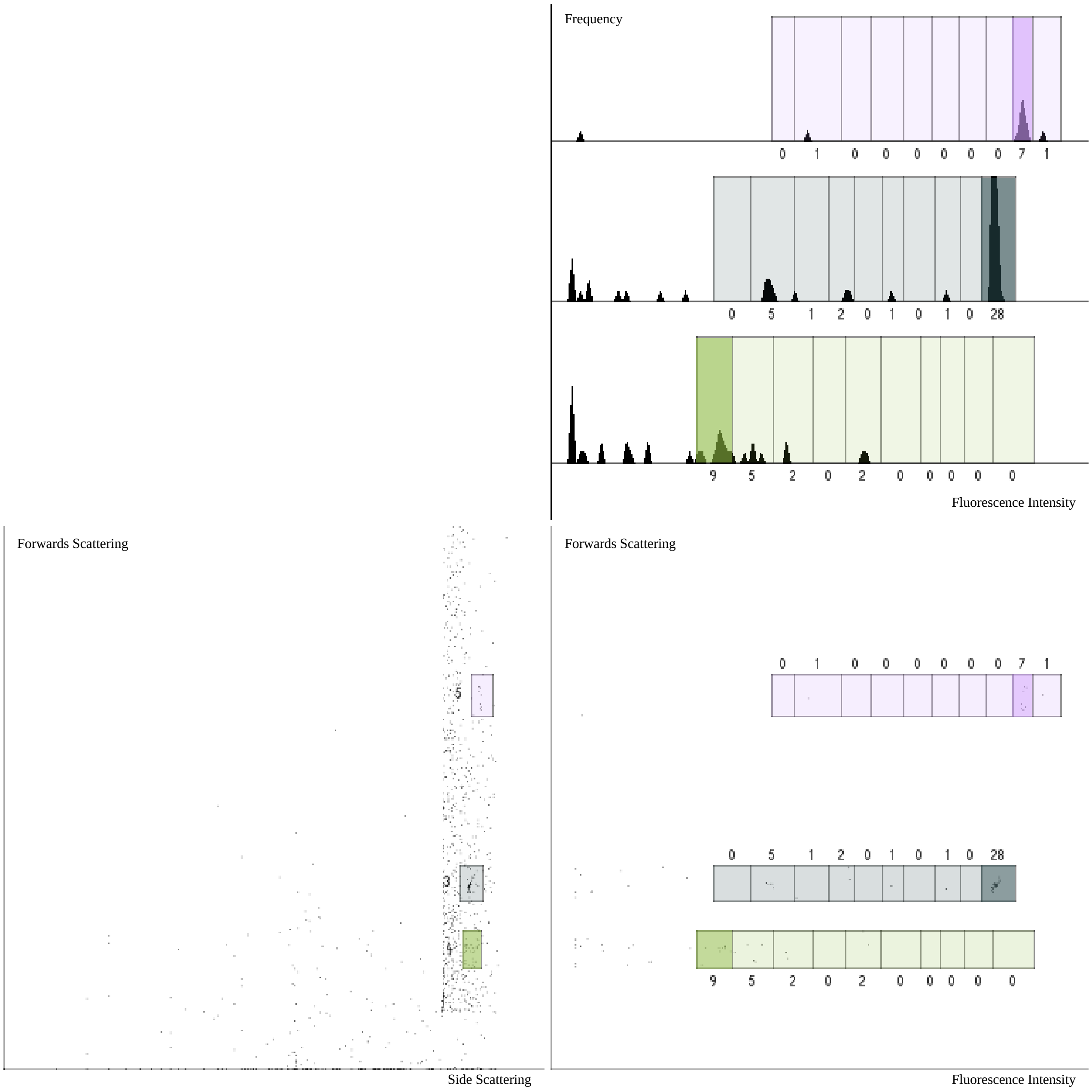

ANNEX 3: TAG DECONVOLUTION - BEAD 42

Passes flow sorting criteria: Yes

Passes tag deconvolution criteria: Yes

Included in protocol analysis: Yes

Protocol: 10, 1, 1, 5

Filename: Bin5_24

Split 1: Petrol shading

Split 2: Green shading

Split 3: Violet shading

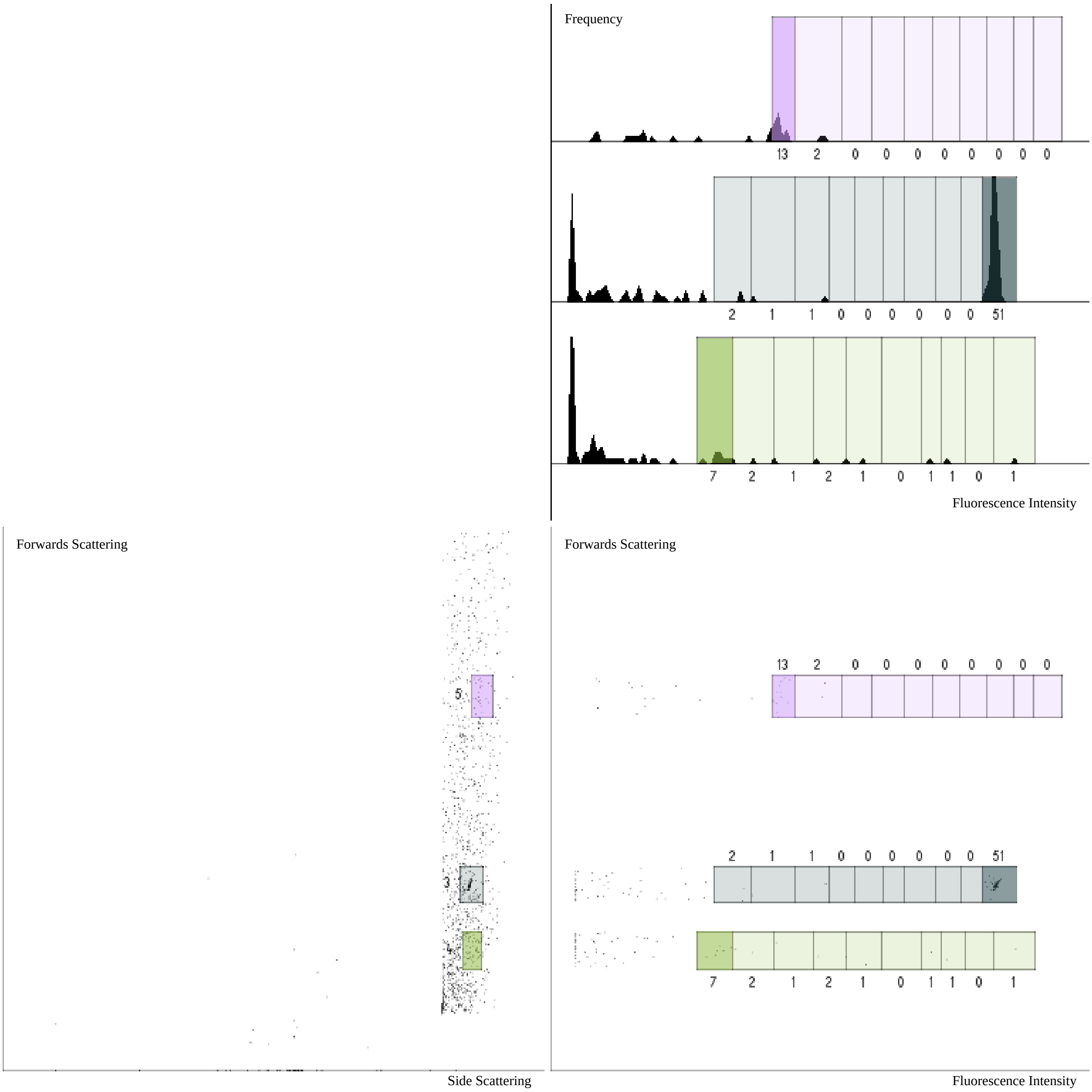

ANNEX 3: TAG DECONVOLUTION - BEAD 43

Passes flow sorting criteria: Yes

Passes tag deconvolution criteria: Yes

Included in protocol analysis: Yes

Protocol: 10, 1, 8, 5

Filename: Bin5_25

Split 1: Petrol shading

Split 2: Green shading

Split 3: Violet shading

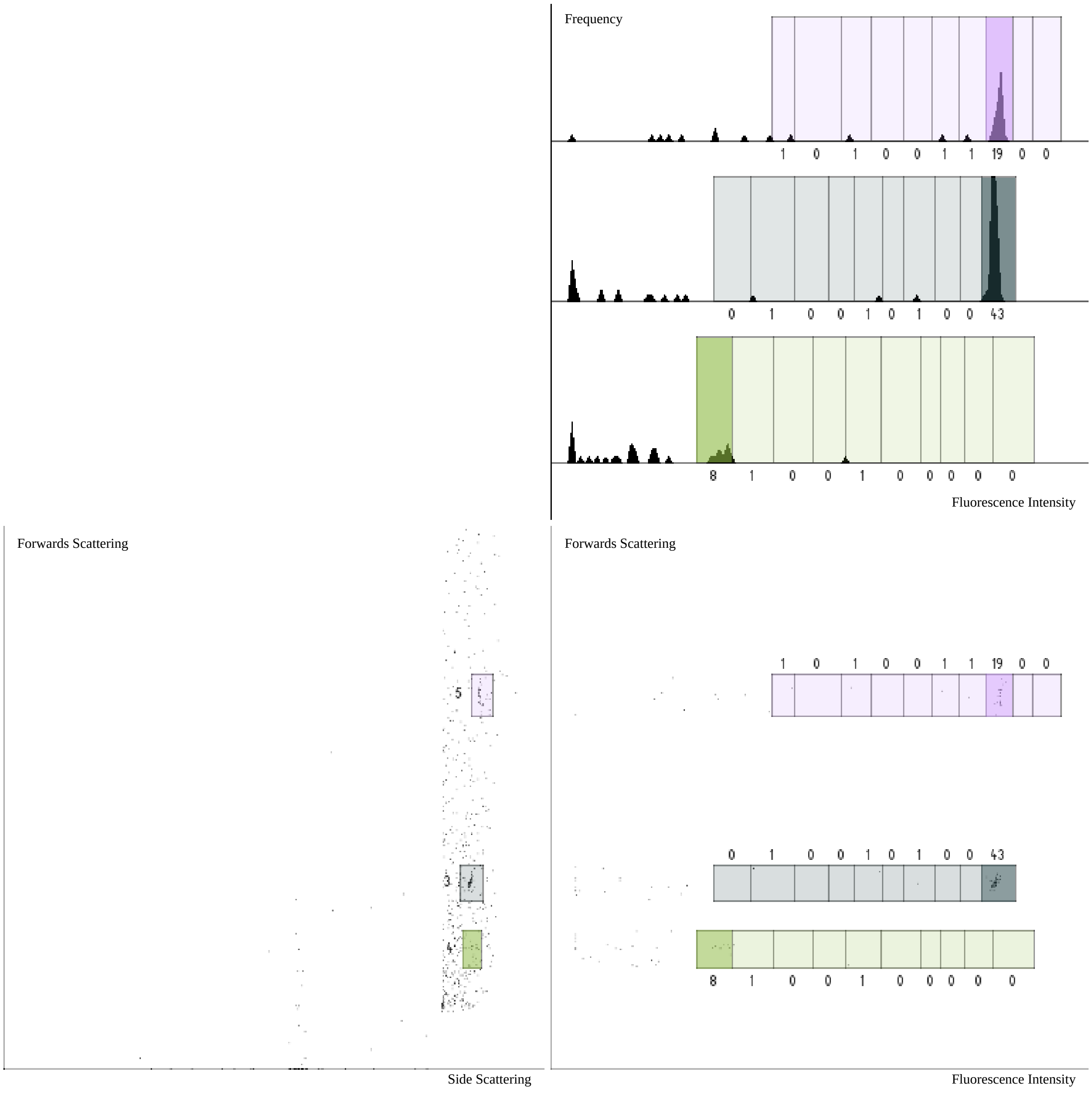

ANNEX 3: TAG DECONVOLUTION - BEAD 44

Passes flow sorting criteria: Yes

Passes tag deconvolution criteria: Yes

Included in protocol analysis: Yes

Protocol: 10, 1, 6, 6

Filename: Bin6_6F1

Split 1: Petrol shading

Split 2: Green shading

Split 3: Violet shading

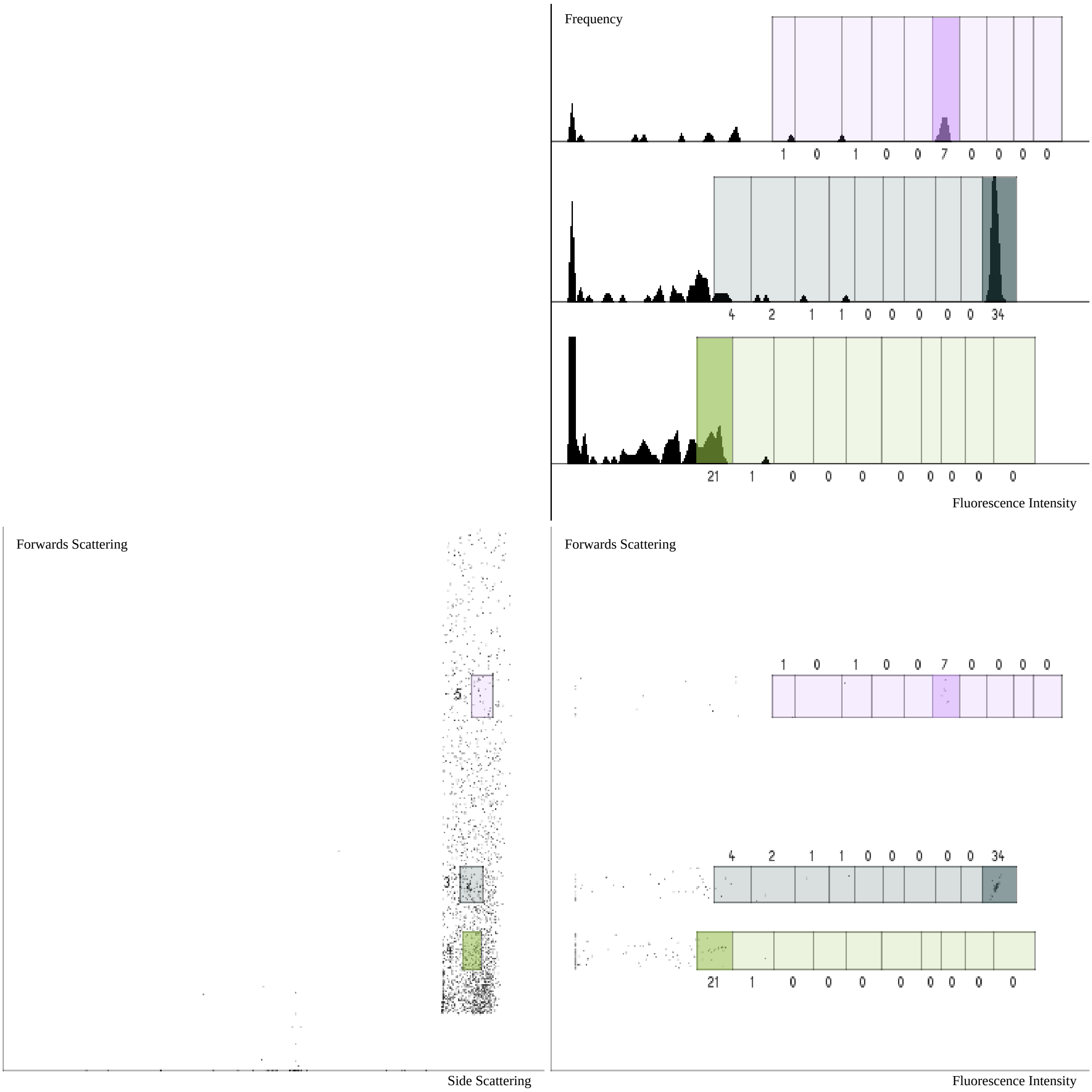

ANNEX 3: TAG DECONVOLUTION - BEAD 45

Passes flow sorting criteria: Yes

Passes tag deconvolution criteria: Yes

Included in protocol analysis: Yes

Protocol: 3, 7, 8, 6

Filename: Bin6_6F6

Split 1: Petrol shading

Split 2: Green shading

Split 3: Violet shading

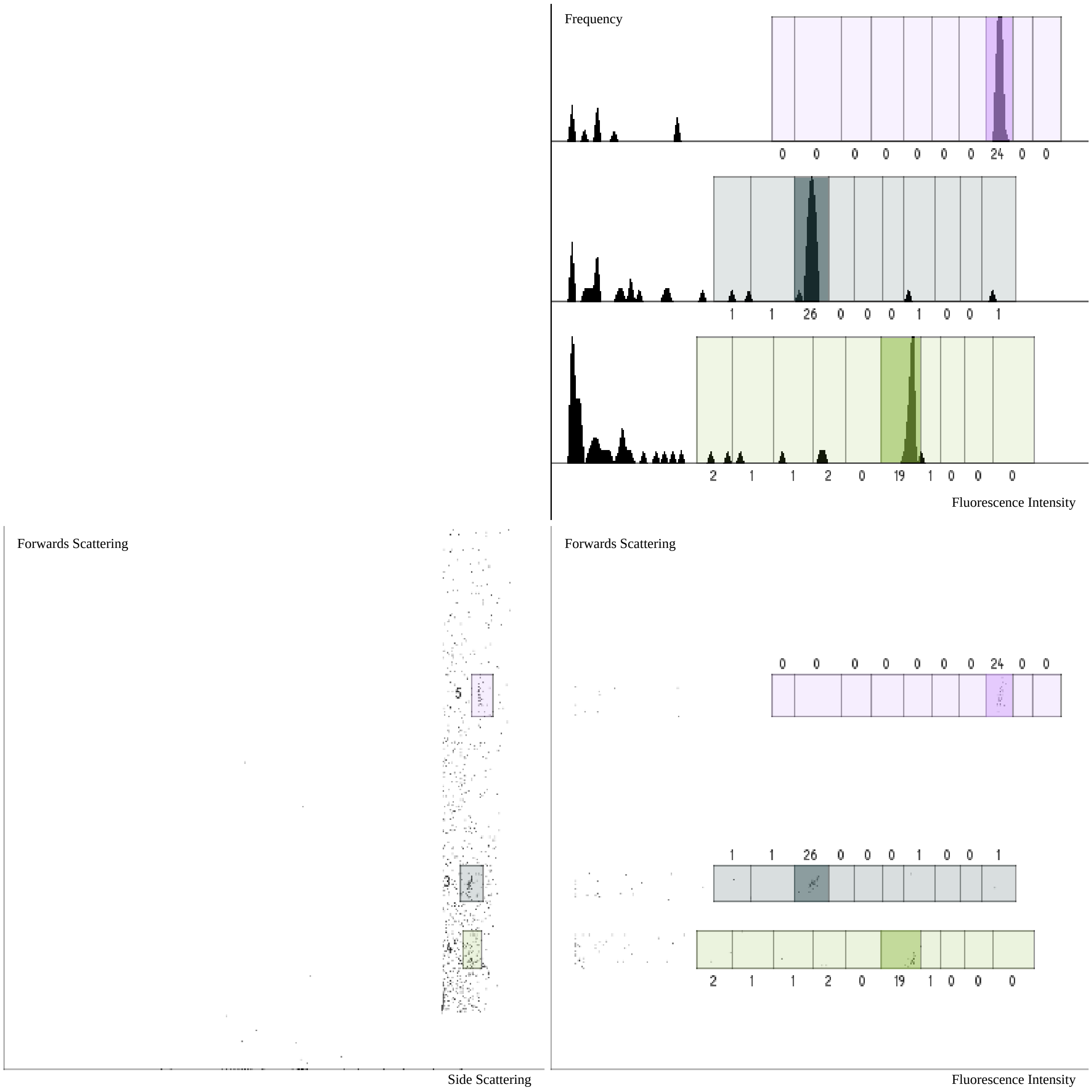

ANNEX 3: TAG DECONVOLUTION - BEAD 46

Passes flow sorting criteria: Yes

Passes tag deconvolution criteria: Yes

Included in protocol analysis: Yes

Protocol: 10, 8, 10, 6

Filename: Bin6_6F7

Split 1: Petrol shading

Split 2: Green shading

Split 3: Violet shading

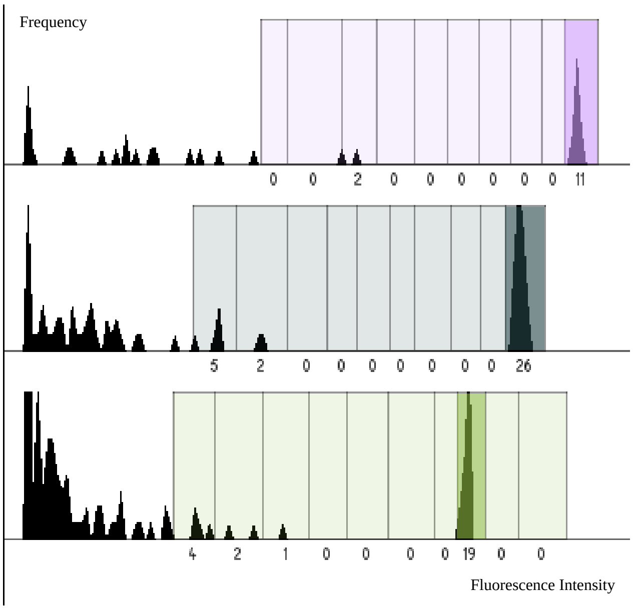

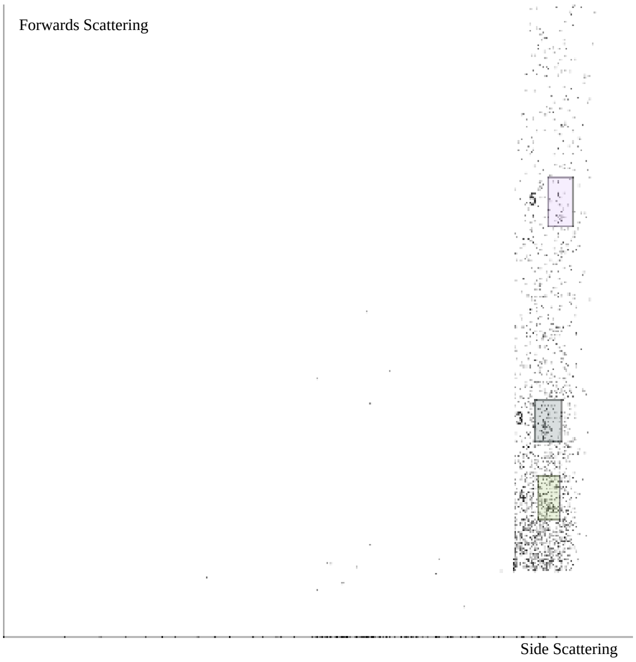

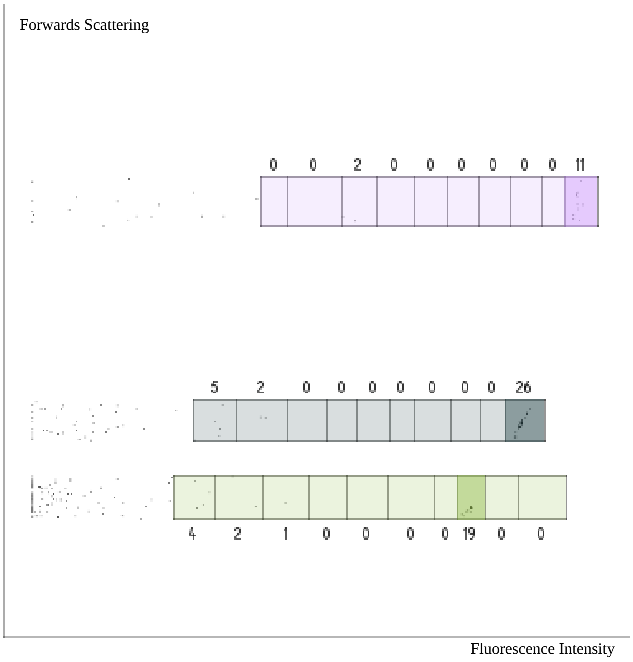

ANNEX 3: TAG DECONVOLUTION - BEAD 47

Passes flow sorting criteria: Yes

Passes tag deconvolution criteria: Yes

Included in protocol analysis: Yes

Protocol: 4, 3, 2, 6

Filename: Bin6_6G11

Split 1: Petrol shading

Split 2: Green shading

Split 3: Violet shading

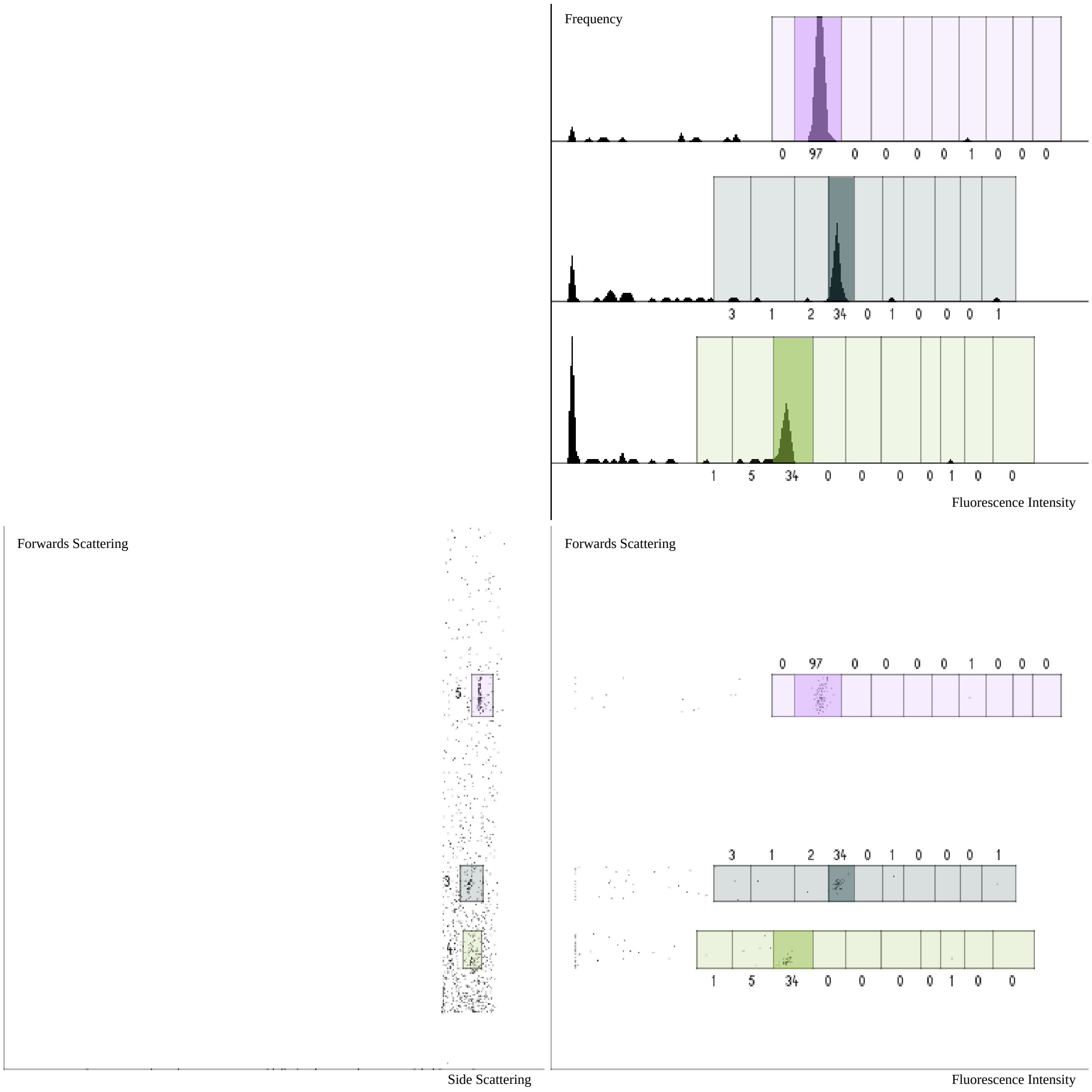

ANNEX 3: TAG DECONVOLUTION - BEAD 48

Passes flow sorting criteria: Yes

Passes tag deconvolution criteria: Yes

Included in protocol analysis: Yes

Protocol: 10, 1, 8, 5

Filename: Bin5_26

Split 1: Petrol shading

Split 2: Green shading

Split 3: Violet shading

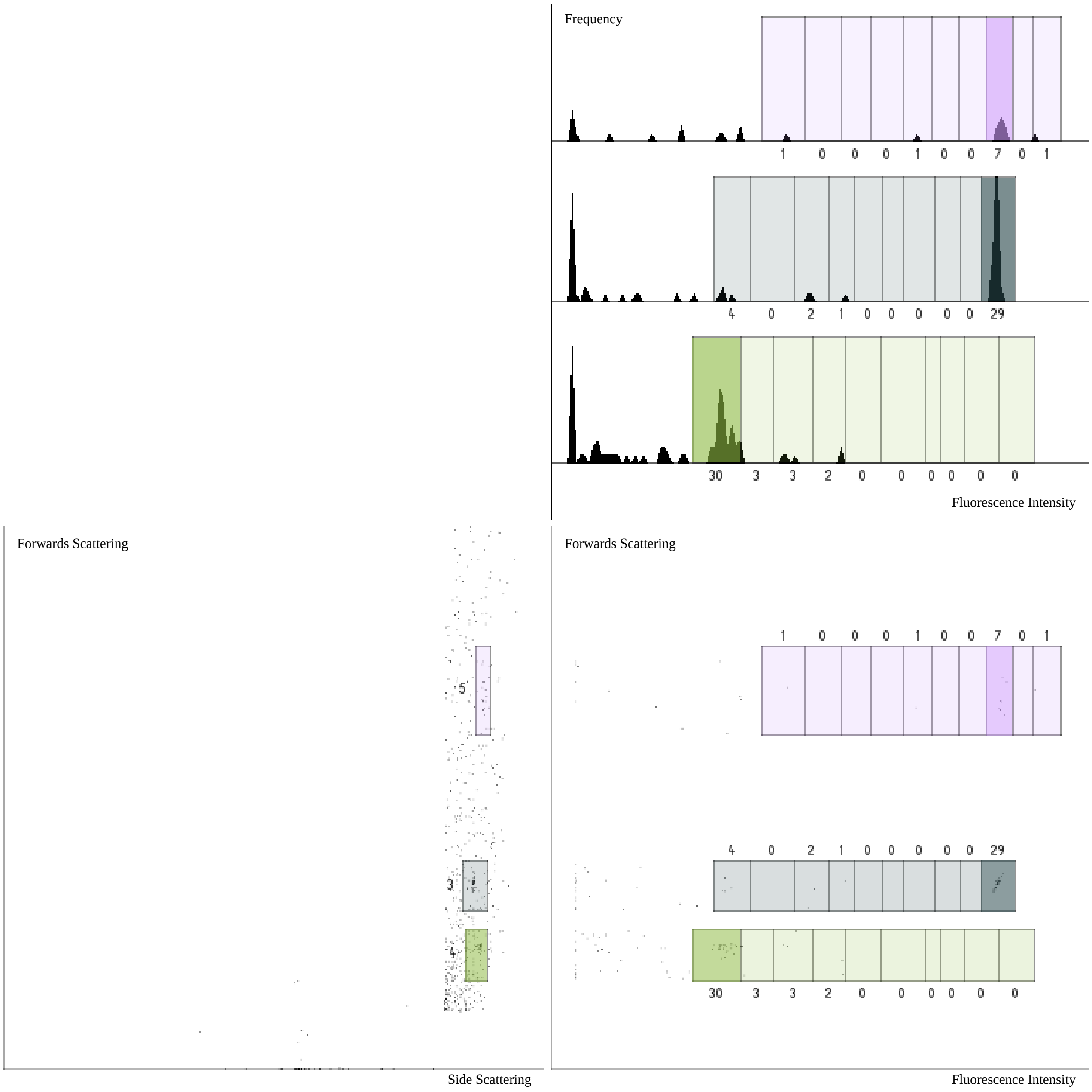

ANNEX 3: TAG DECONVOLUTION - BEAD 49

Passes flow sorting criteria: Yes

Passes tag deconvolution criteria: Yes

Included in protocol analysis: Yes

Protocol: 2, 1, 1, 5

Filename: Bin5_27

Split 1: Petrol shading

Split 2: Green shading

Split 3: Violet shading

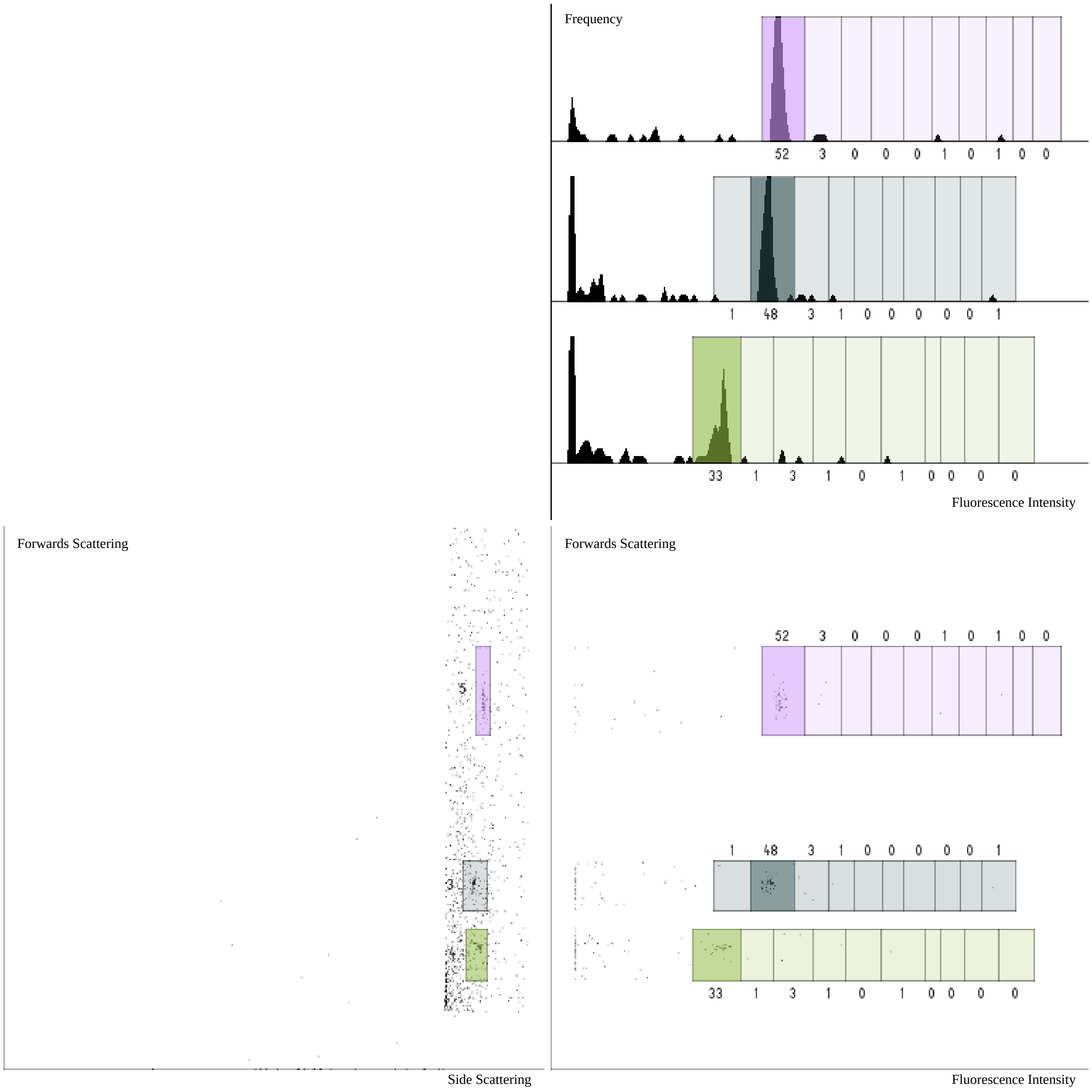

ANNEX 3: TAG DECONVOLUTION - BEAD 50

Passes flow sorting criteria: Yes

Passes tag deconvolution criteria: Yes

Included in protocol analysis: Yes

Protocol: 10, 1, 1, 5

Filename: Bin5_28

Split 1: Petrol shading

Split 2: Green shading

Split 3: Violet shading

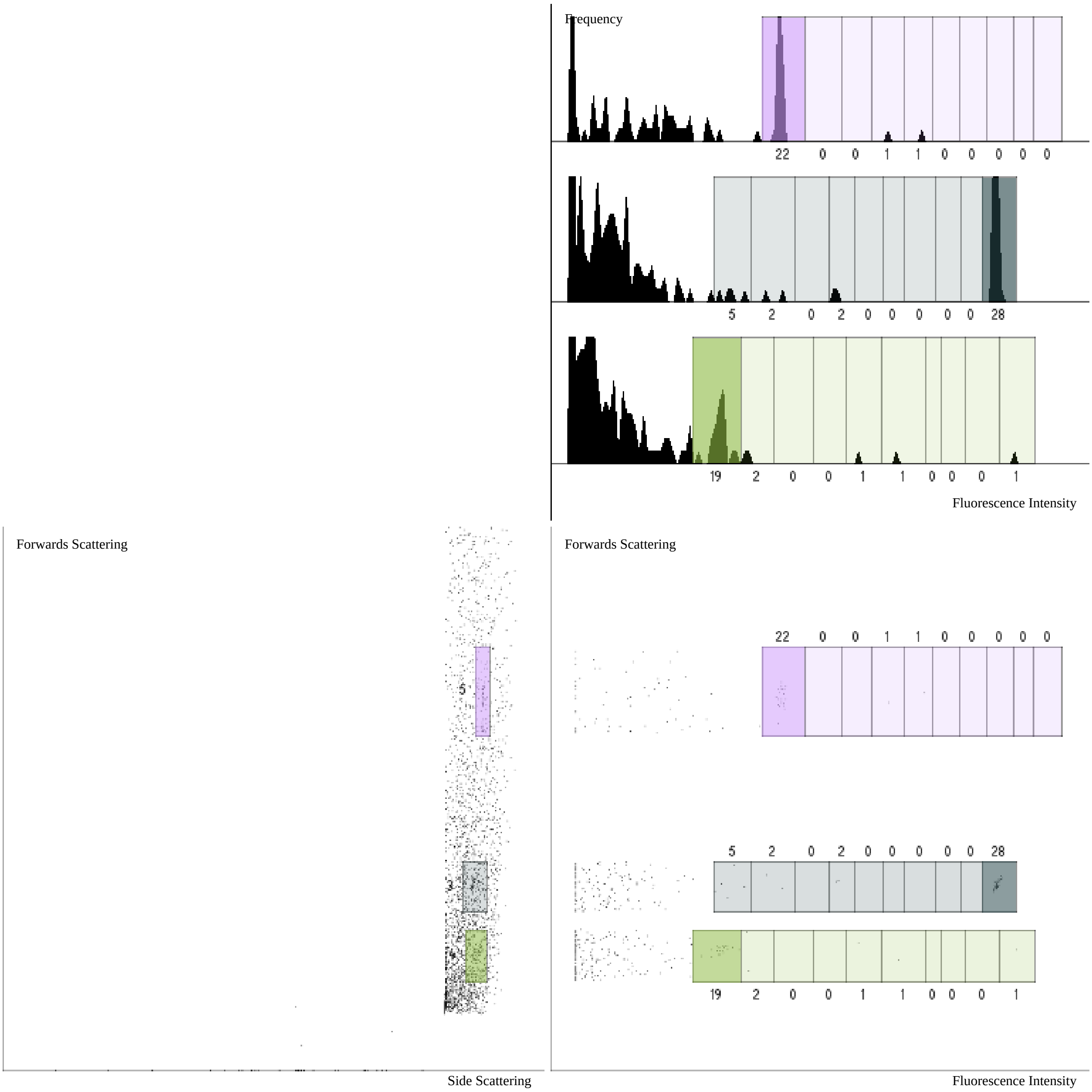

ANNEX 3: TAG DECONVOLUTION - BEAD 51

Passes flow sorting criteria: Yes

Passes tag deconvolution criteria: Yes

Included in protocol analysis: Yes

Protocol: 10, 8, 1, 9

Filename: Bin9_92

Split 1: Petrol shading

Split 2: Green shading

Split 3: Violet shading

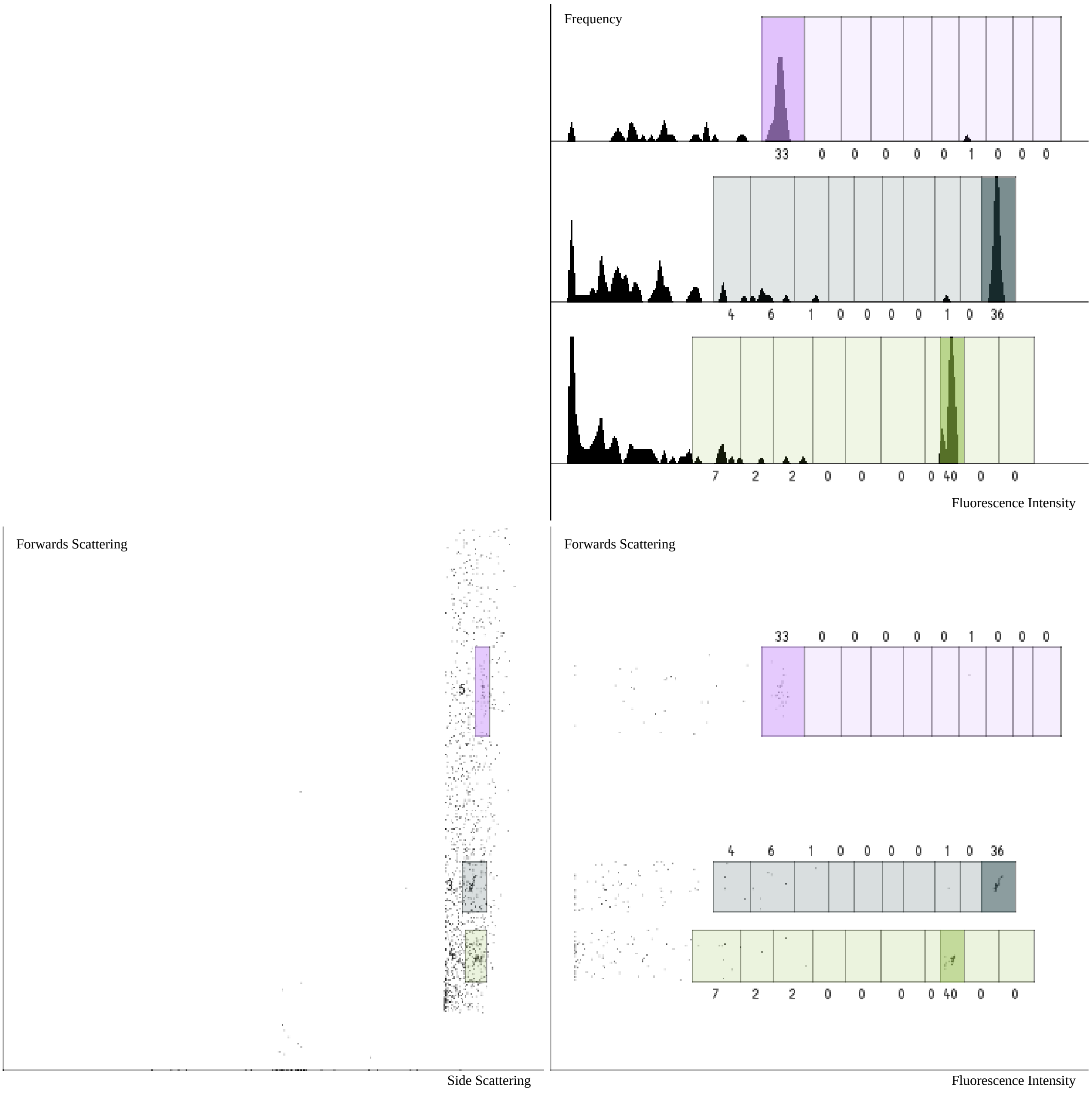

ANNEX 3: TAG DECONVOLUTION - BEAD 52

Passes flow sorting criteria: Yes

Passes tag deconvolution criteria: Yes

Included in protocol analysis: Yes

Protocol: 4, 1, 4, 9

Filename: Bin9_93

Split 1: Petrol shading

Split 2: Green shading

Split 3: Violet shading

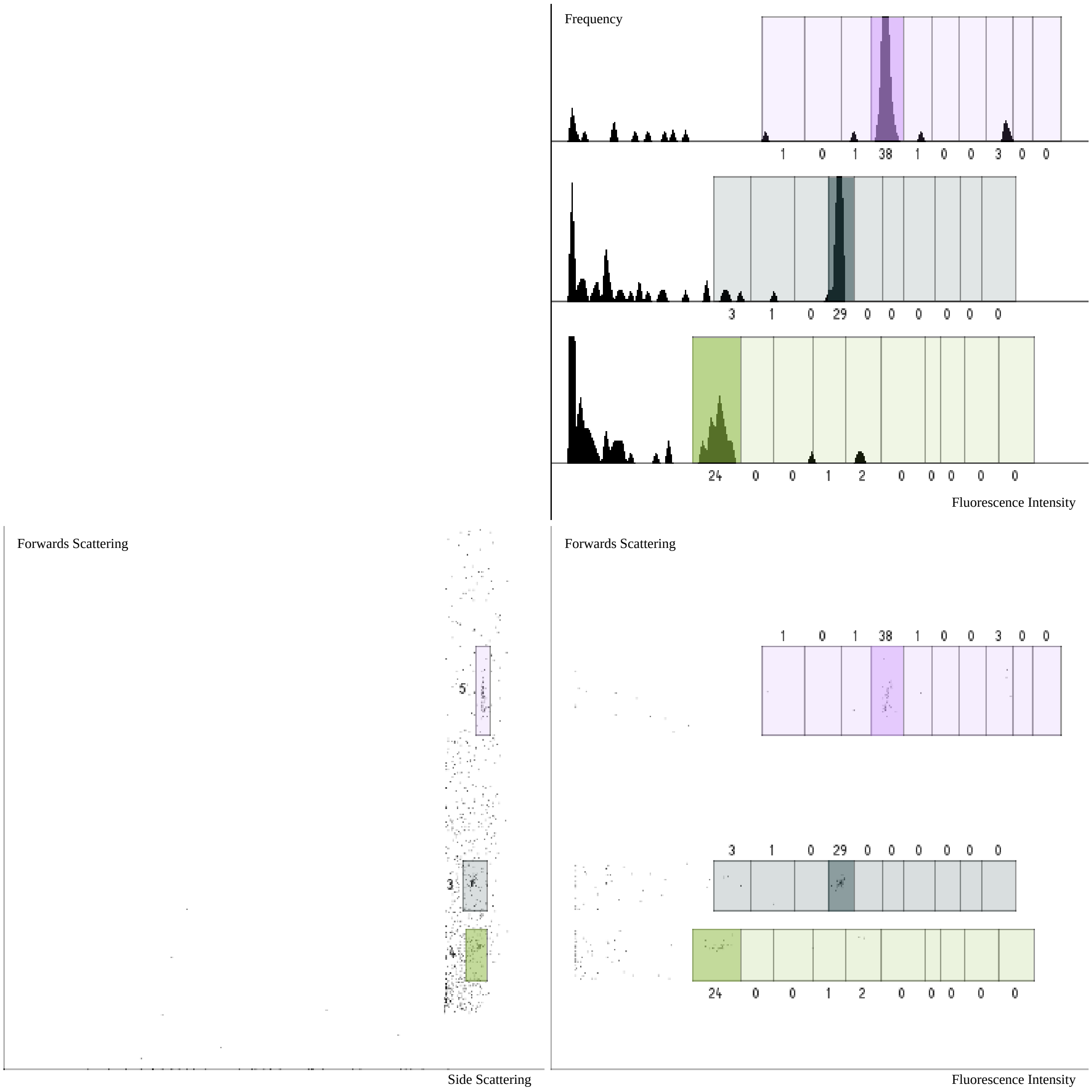

ANNEX 3: TAG DECONVOLUTION - BEAD 53

Passes flow sorting criteria: Yes

Passes tag deconvolution criteria: No

Included in protocol analysis: No

Protocol: N/A

Filename: Bin9_94

Split 1: Petrol shading

Split 2: Green shading

Split 3: Violet shading

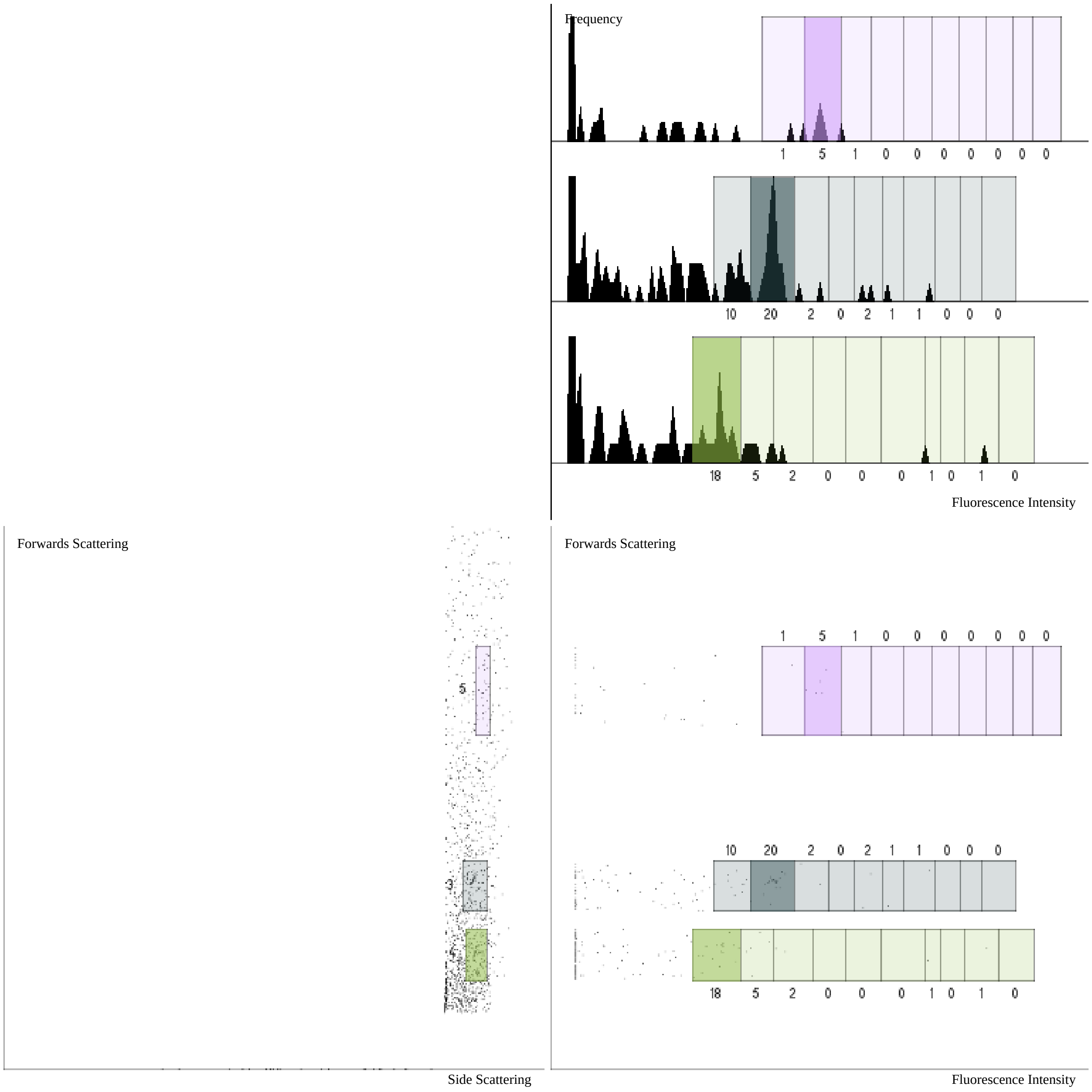

ANNEX 3: TAG DECONVOLUTION - BEAD 54

Passes flow sorting criteria: Yes

Passes tag deconvolution criteria: Yes

Included in protocol analysis: No

Protocol: N/A

Filename: Bin9_96

Split 1: Petrol shading

Split 2: Green shading

Split 3: Violet shading

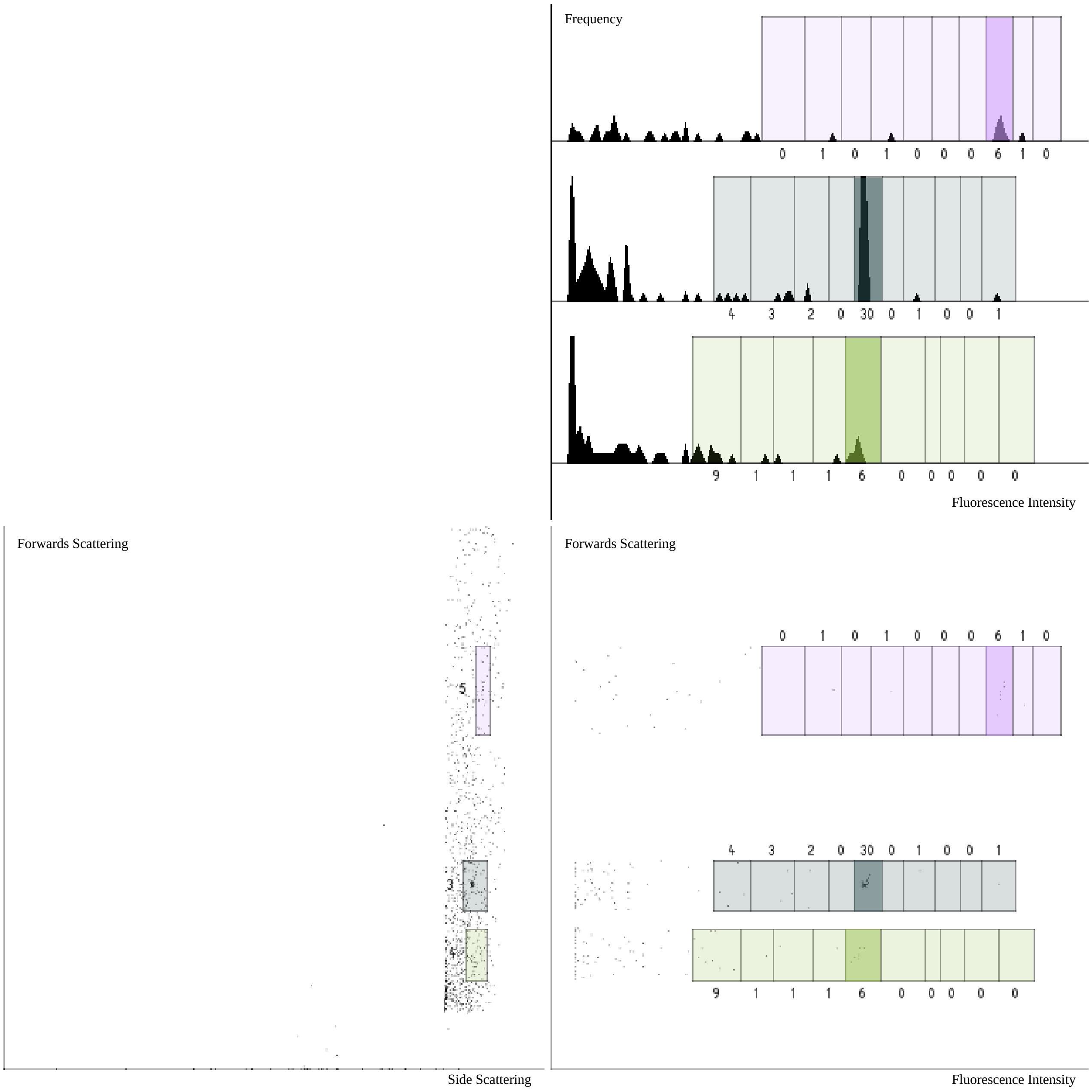

ANNEX 3: TAG DECONVOLUTION - BEAD 55

Passes flow sorting criteria: Yes

Passes tag deconvolution criteria: Yes

Included in protocol analysis: Yes

Protocol: 7, 2, 4, 1

Filename: Bin1_1RA2

Split 1: Petrol shading

Split 2: Green shading

Split 3: Violet shading

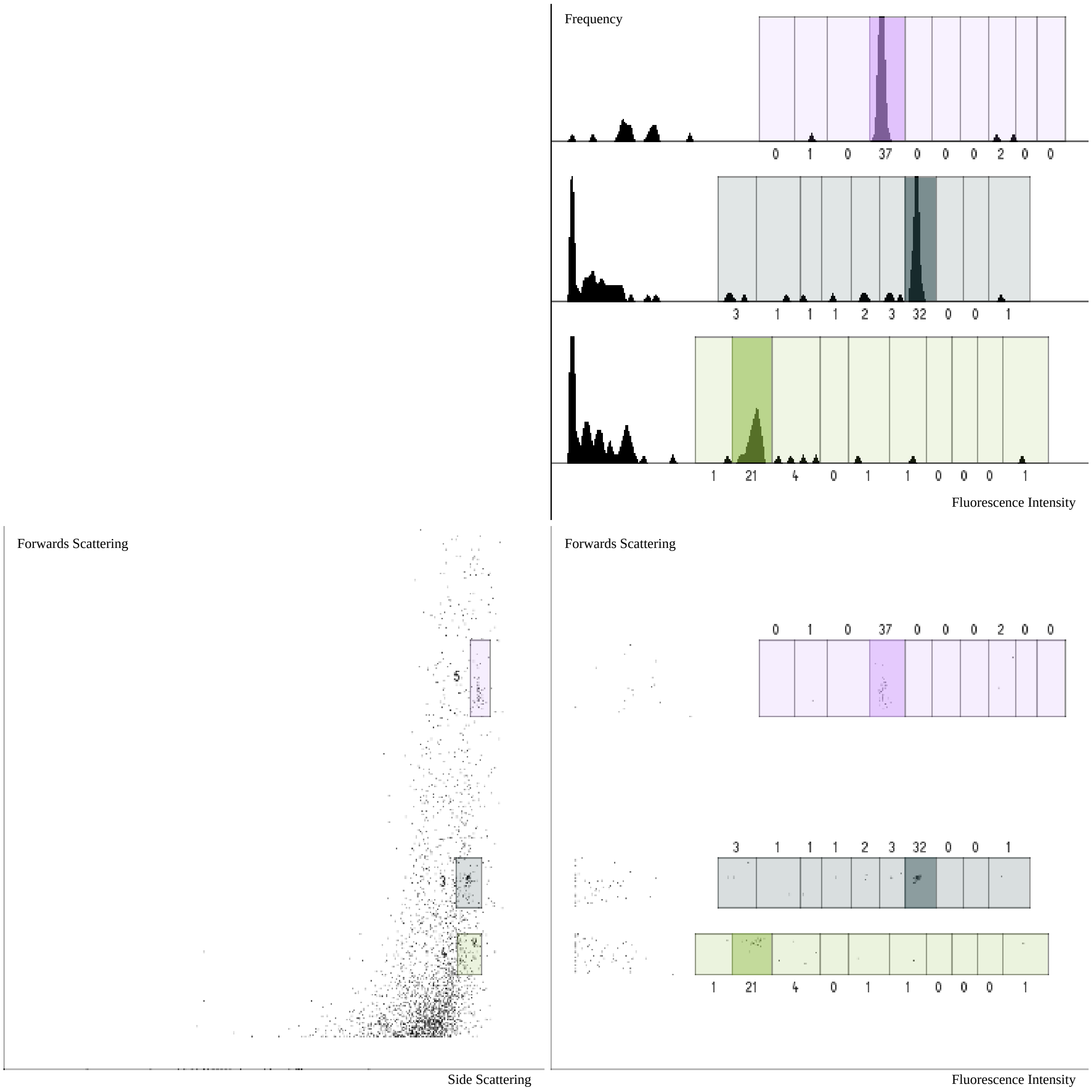

ANNEX 3: TAG DECONVOLUTION - BEAD 56

Passes flow sorting criteria: Yes

Passes tag deconvolution criteria: No

Included in protocol analysis: No

Protocol: N/A

Filename: Bin1_1RA3

Split 1: Petrol shading

Split 2: Green shading

Split 3: Violet shading

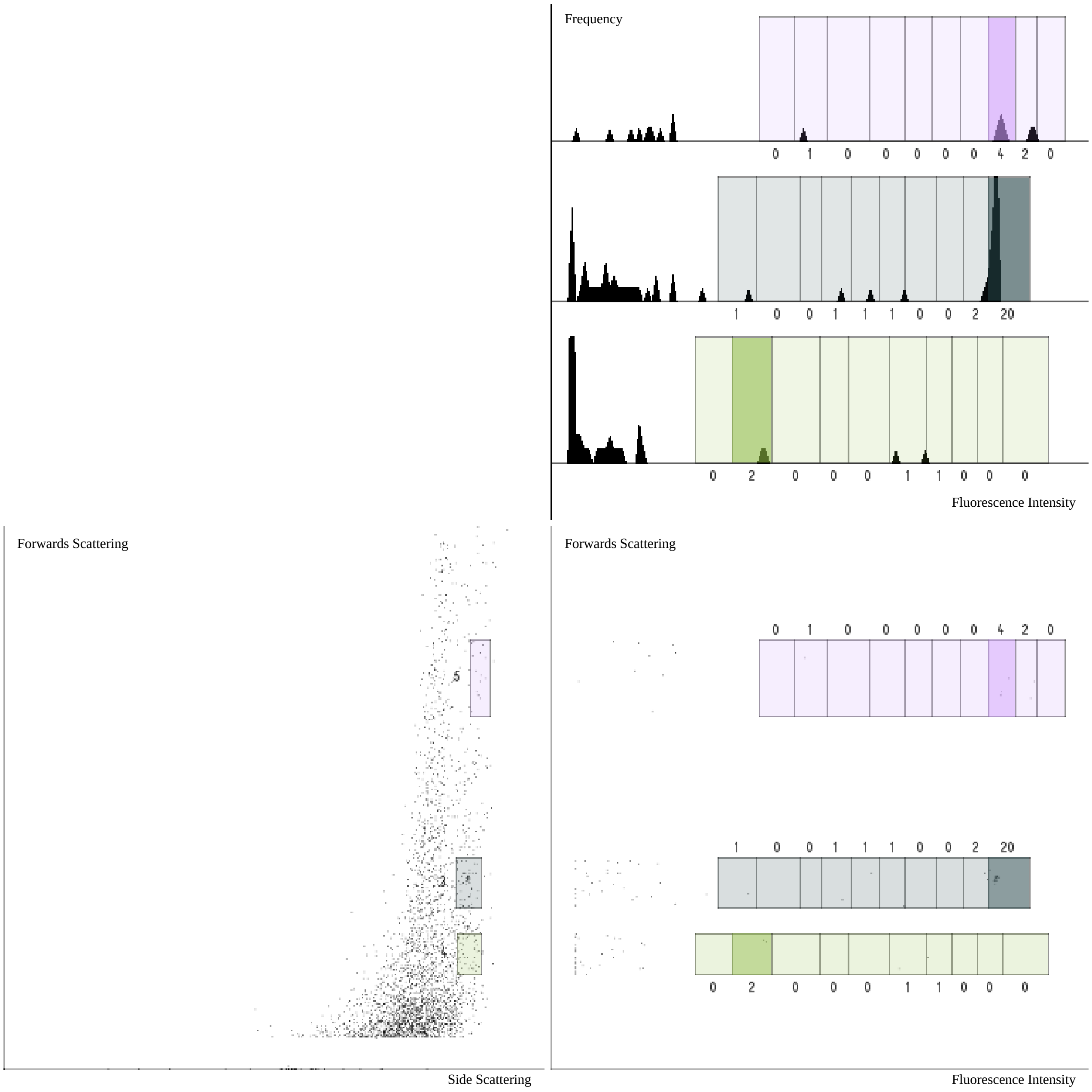

ANNEX 3: TAG DECONVOLUTION - BEAD 57

Passes flow sorting criteria: Yes

Passes tag deconvolution criteria: No

Included in protocol analysis: No

Protocol: N/A

Filename: Bin1_1RA4

Split 1: Petrol shading

Split 2: Green shading

Split 3: Violet shading

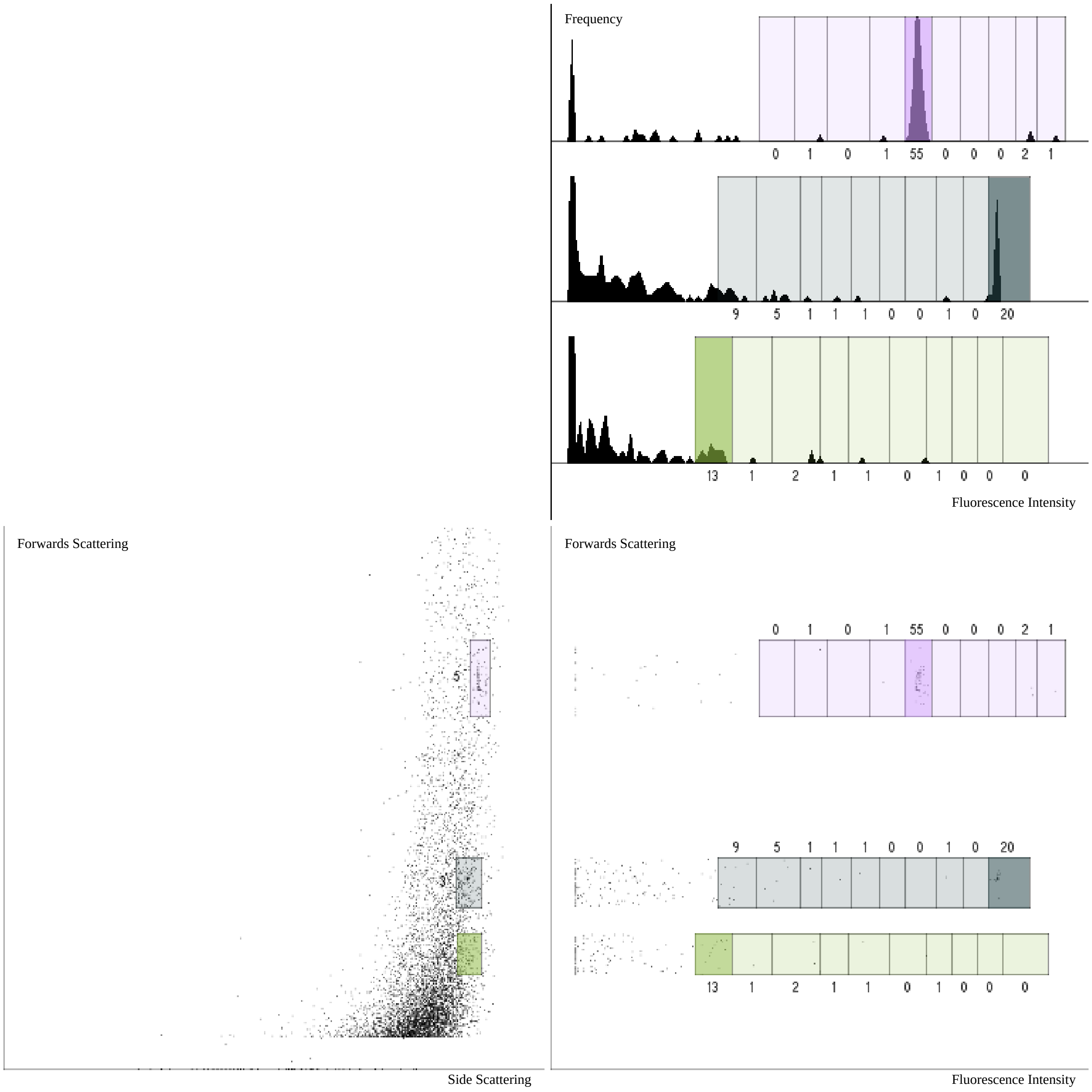

ANNEX 3: TAG DECONVOLUTION - BEAD 58

Passes flow sorting criteria: Yes

Passes tag deconvolution criteria: Yes

Included in protocol analysis: Yes

Protocol: 10, 1, 6, 2

Filename: Bin2_2R.LMD

Split 1: Petrol shading

Split 2: Green shading

Split 3: Violet shading

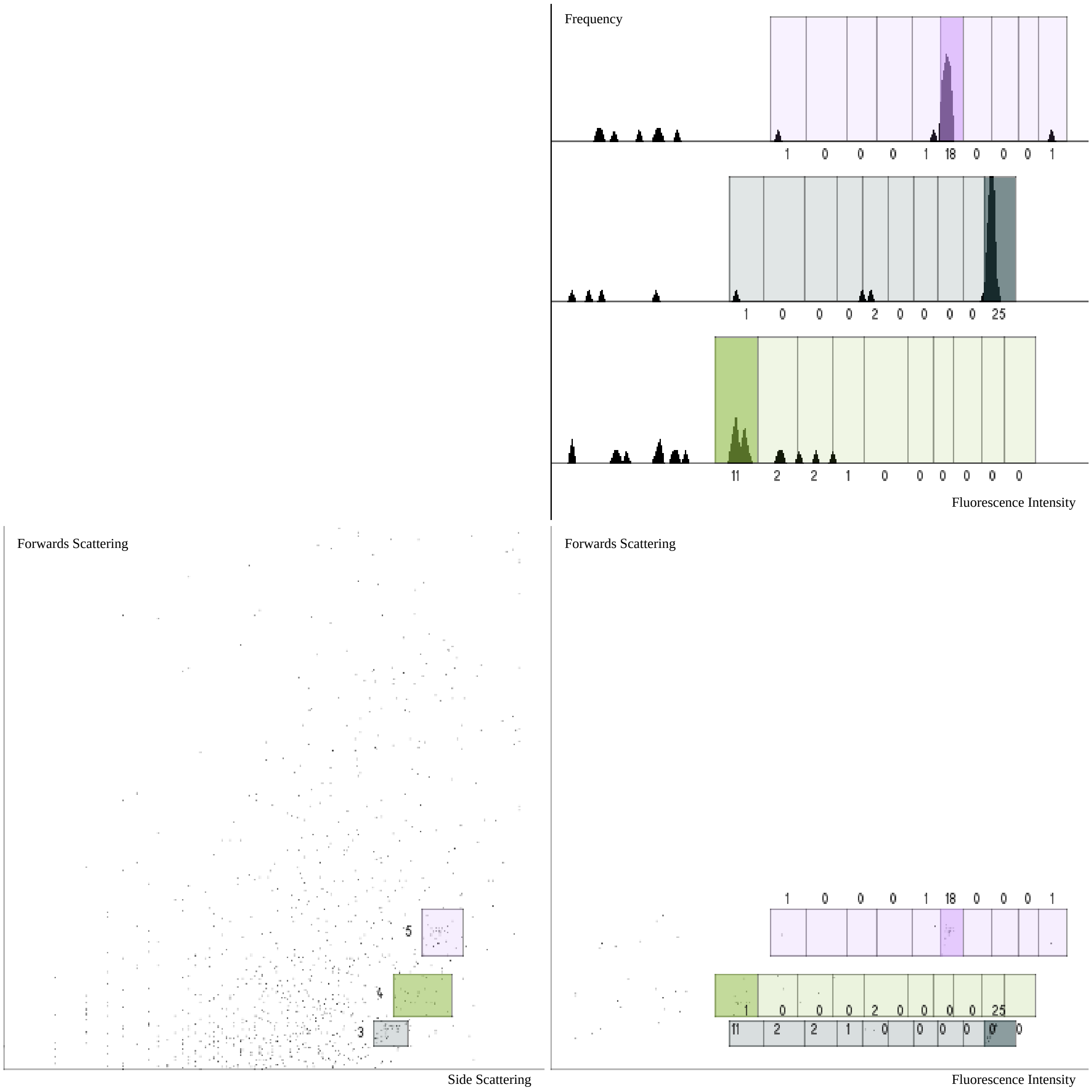

ANNEX 3: TAG DECONVOLUTION - BEAD 59

Passes flow sorting criteria: Yes

Passes tag deconvolution criteria: Yes

Included in protocol analysis: Yes

Protocol: 1, 1, 2, 8

Filename: Bin8_80.LMD

Split 1: Petrol shading

Split 2: Green shading

Split 3: Violet shading

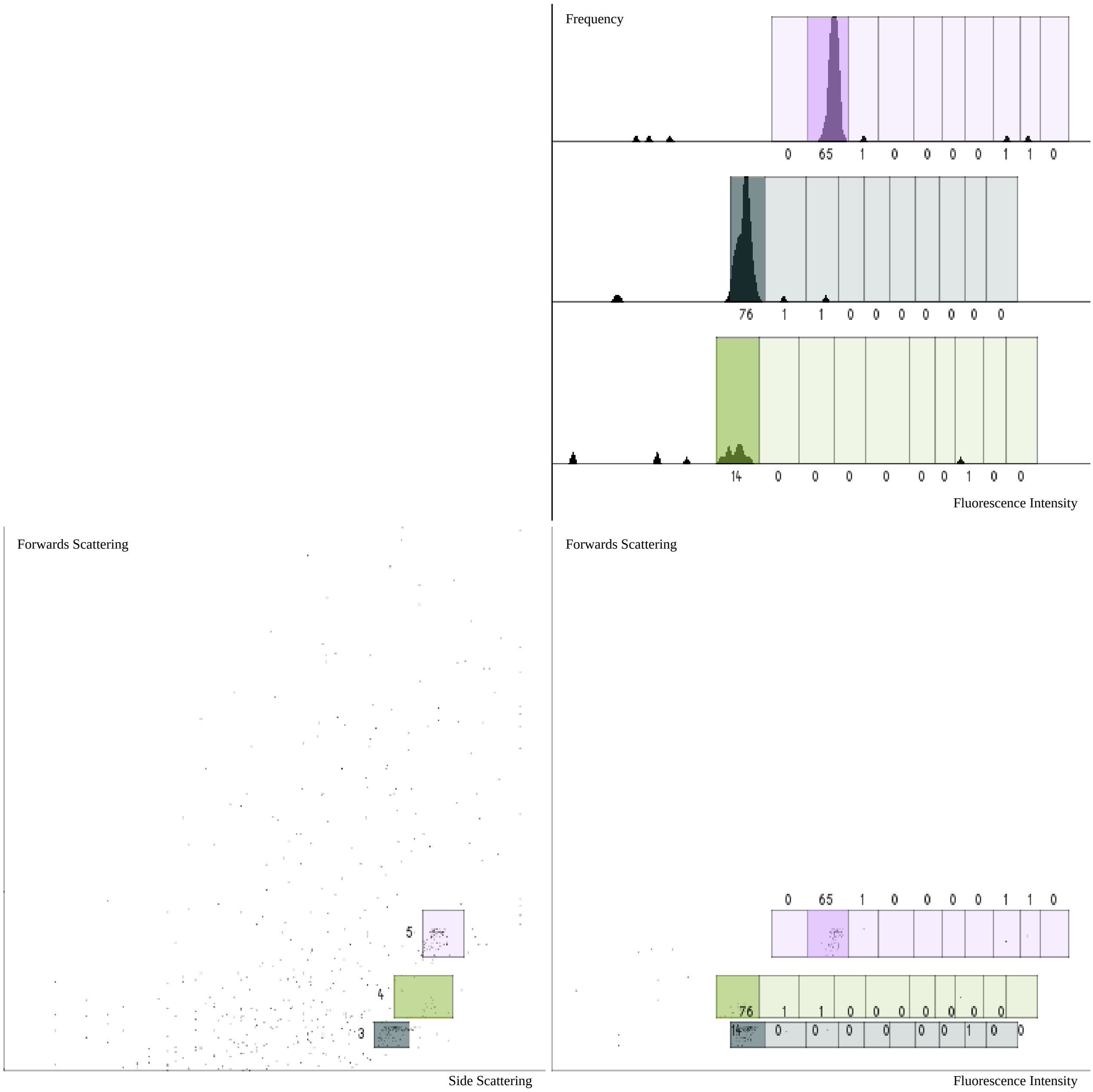

ANNEX 3: TAG DECONVOLUTION - BEAD 60

Passes flow sorting criteria: Yes

Passes tag deconvolution criteria: No

Included in protocol analysis: No

Protocol: N/A

Filename: Bin8_81.LMD

Split 1: Petrol shading

Split 2: Green shading

Split 3: Violet shading

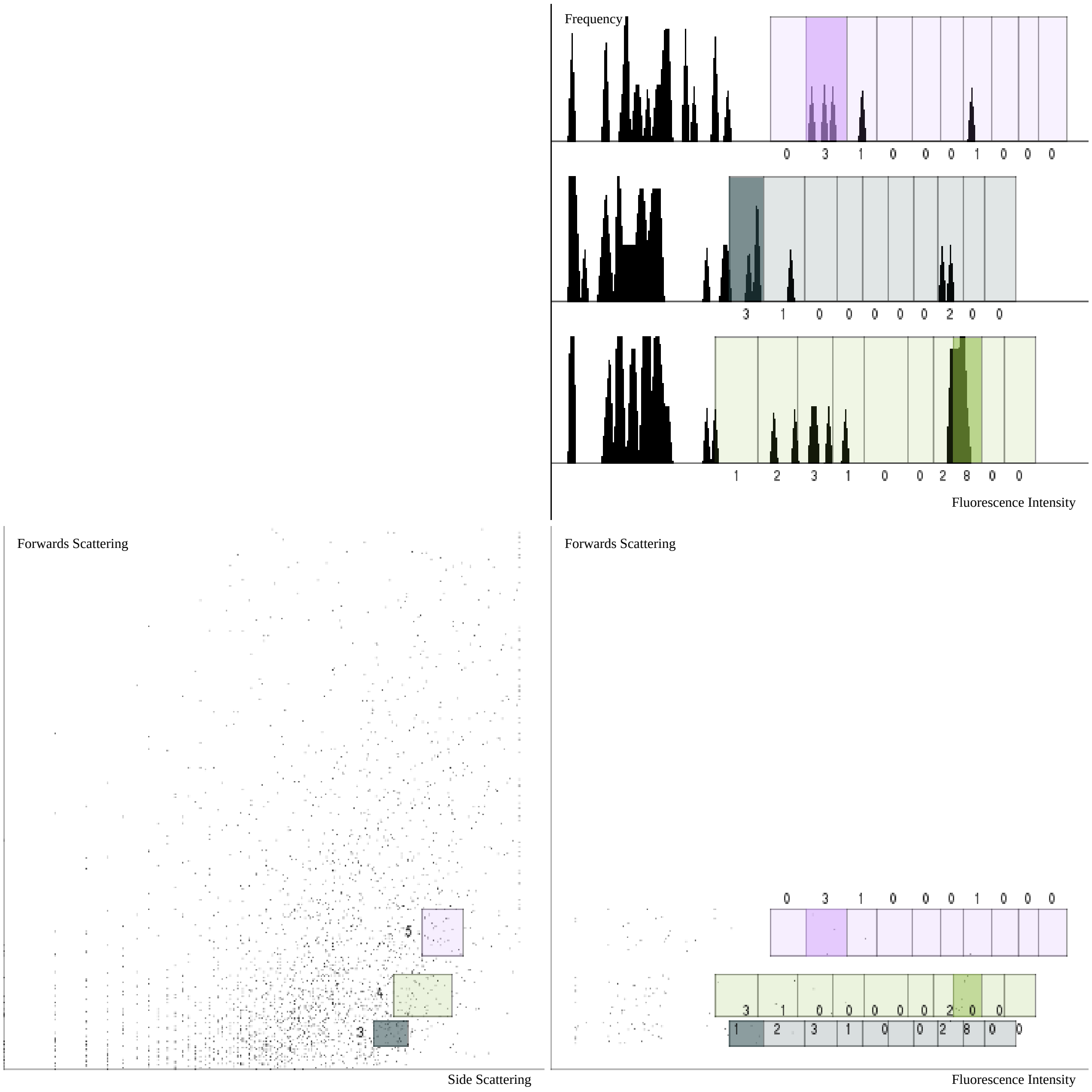

ANNEX 3: TAG DECONVOLUTION - BEAD 61

Passes flow sorting criteria: Yes

Passes tag deconvolution criteria: No

Included in protocol analysis: No

Protocol: N/A

Filename: Bin8_82a.LMD

Split 1: Petrol shading

Split 2: Green shading

Split 3: Violet shading

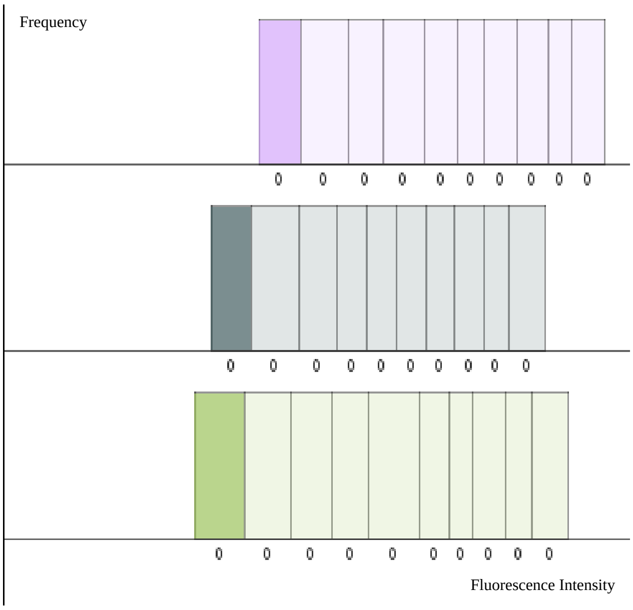

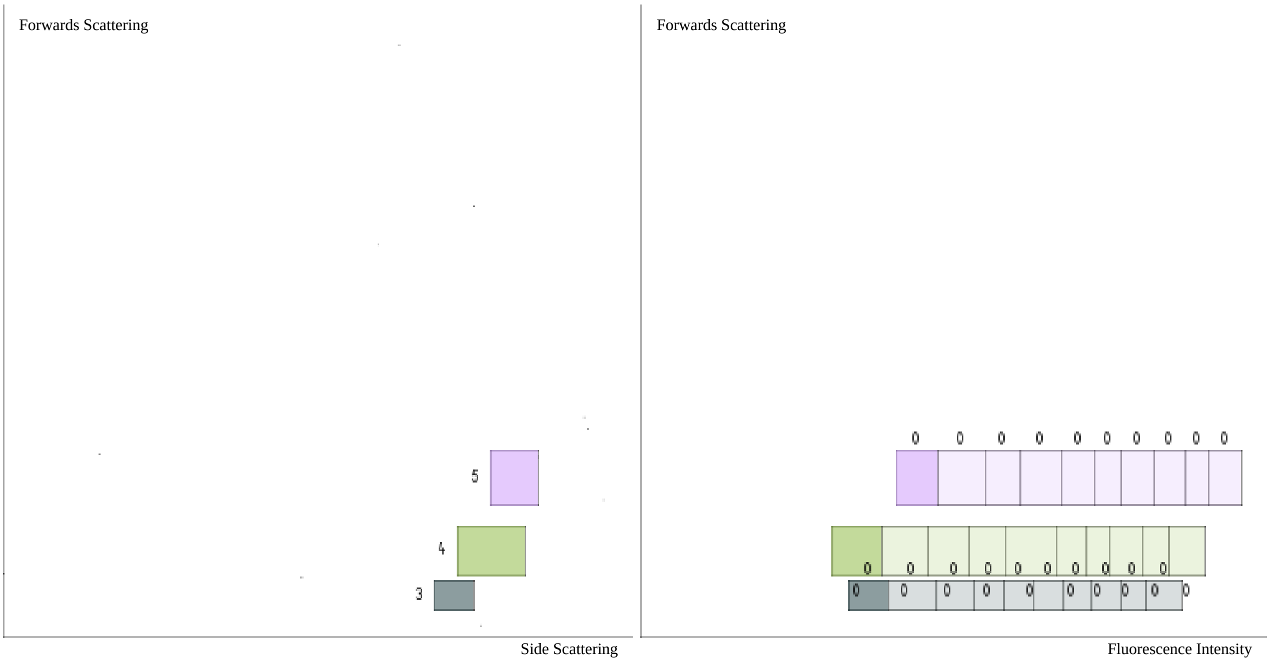

ANNEX 3: TAG DECONVOLUTION - BEAD 62

Passes flow sorting criteria: Yes

Passes tag deconvolution criteria: Yes

Included in protocol analysis: Yes

Protocol: 3, 4, 10, 8

Filename: Bin8_84.LMD

Split 1: Petrol shading

Split 2: Green shading

Split 3: Violet shading

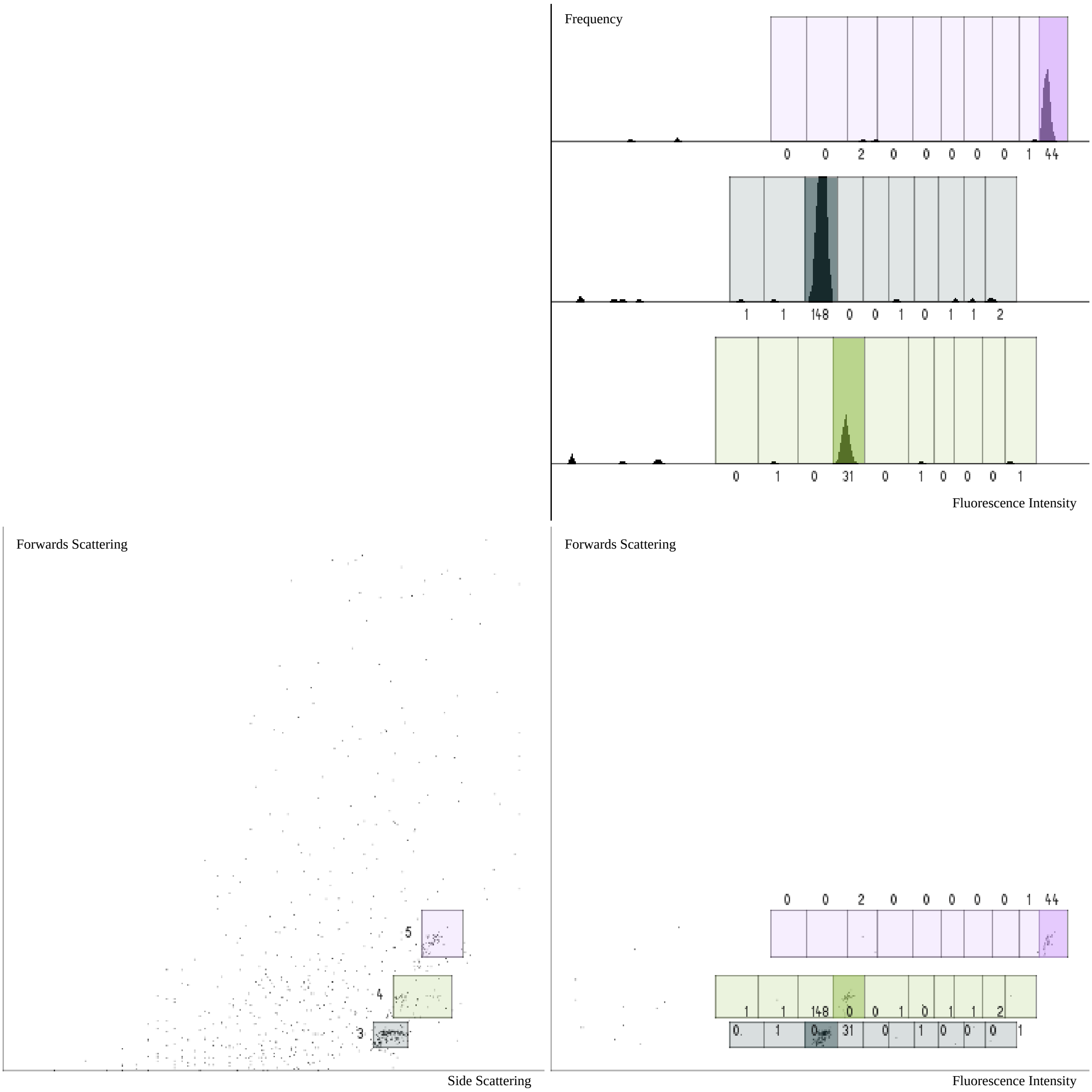

ANNEX 3: TAG DECONVOLUTION - BEAD 63

Passes flow sorting criteria: Yes

Passes tag deconvolution criteria: Yes

Included in protocol analysis: Yes

Protocol: 1, 9, 7, 8

Filename: Bin8_085a.LMD

Split 1: Petrol shading

Split 2: Green shading

Split 3: Violet shading

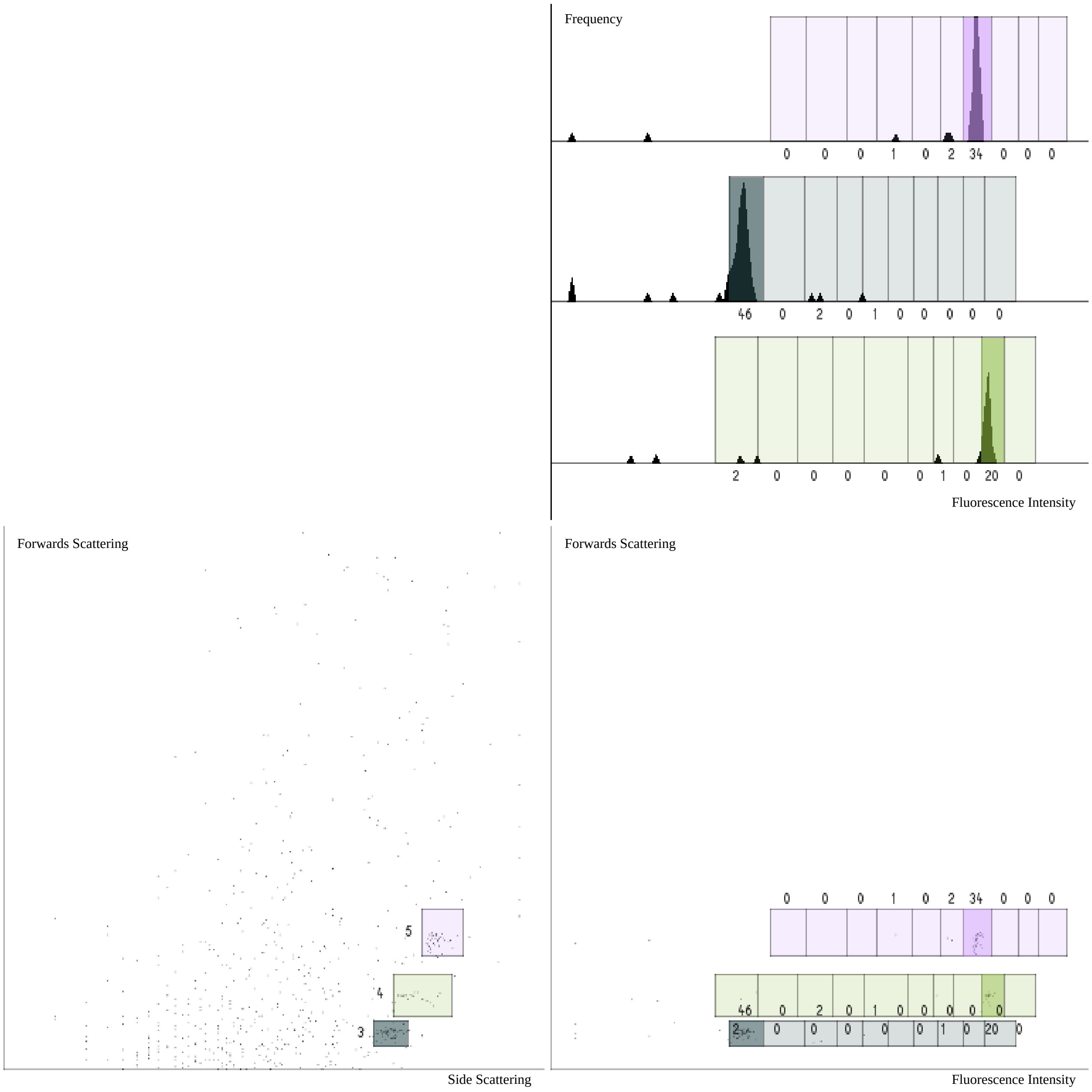

ANNEX 3: TAG DECONVOLUTION - BEAD 64

Passes flow sorting criteria: Yes

Passes tag deconvolution criteria: Yes

Included in protocol analysis: Yes

Protocol: 10, 9, 7, 8

Filename: Bin8_86a.LMD

Split 1: Petrol shading

Split 2: Green shading

Split 3: Violet shading

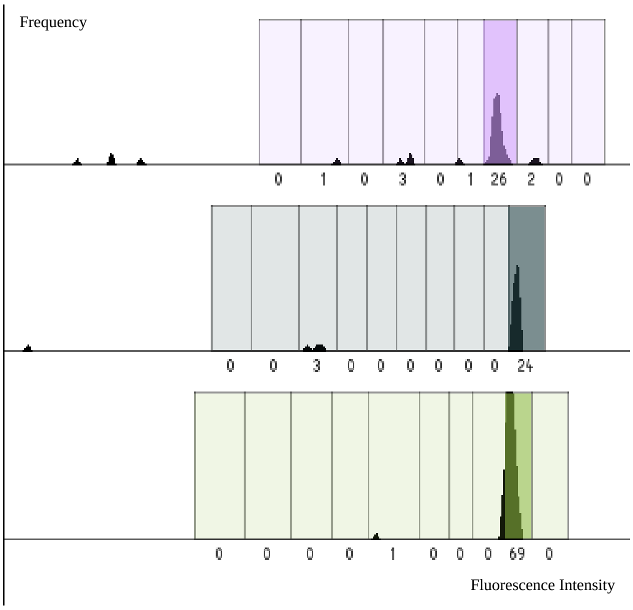

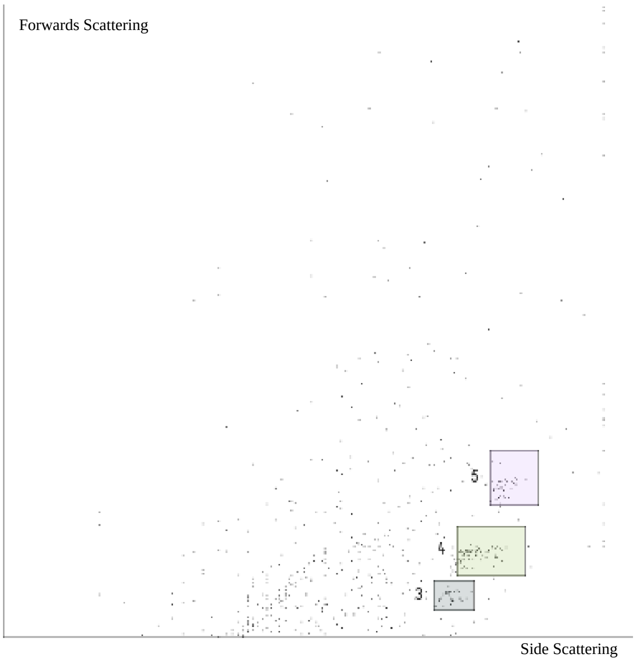

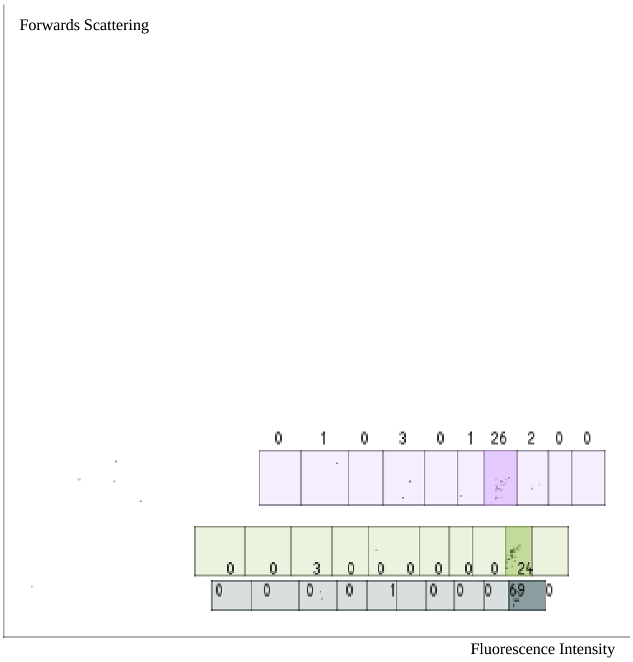

ANNEX 3: TAG DECONVOLUTION - BEAD 65

Passes flow sorting criteria: Yes

Passes tag deconvolution criteria: Yes

Included in protocol analysis: Yes

Protocol: 1, 8, 1, 8

Filename: Bin8_87.LMD

Split 1: Petrol shading

Split 2: Green shading

Split 3: Violet shading

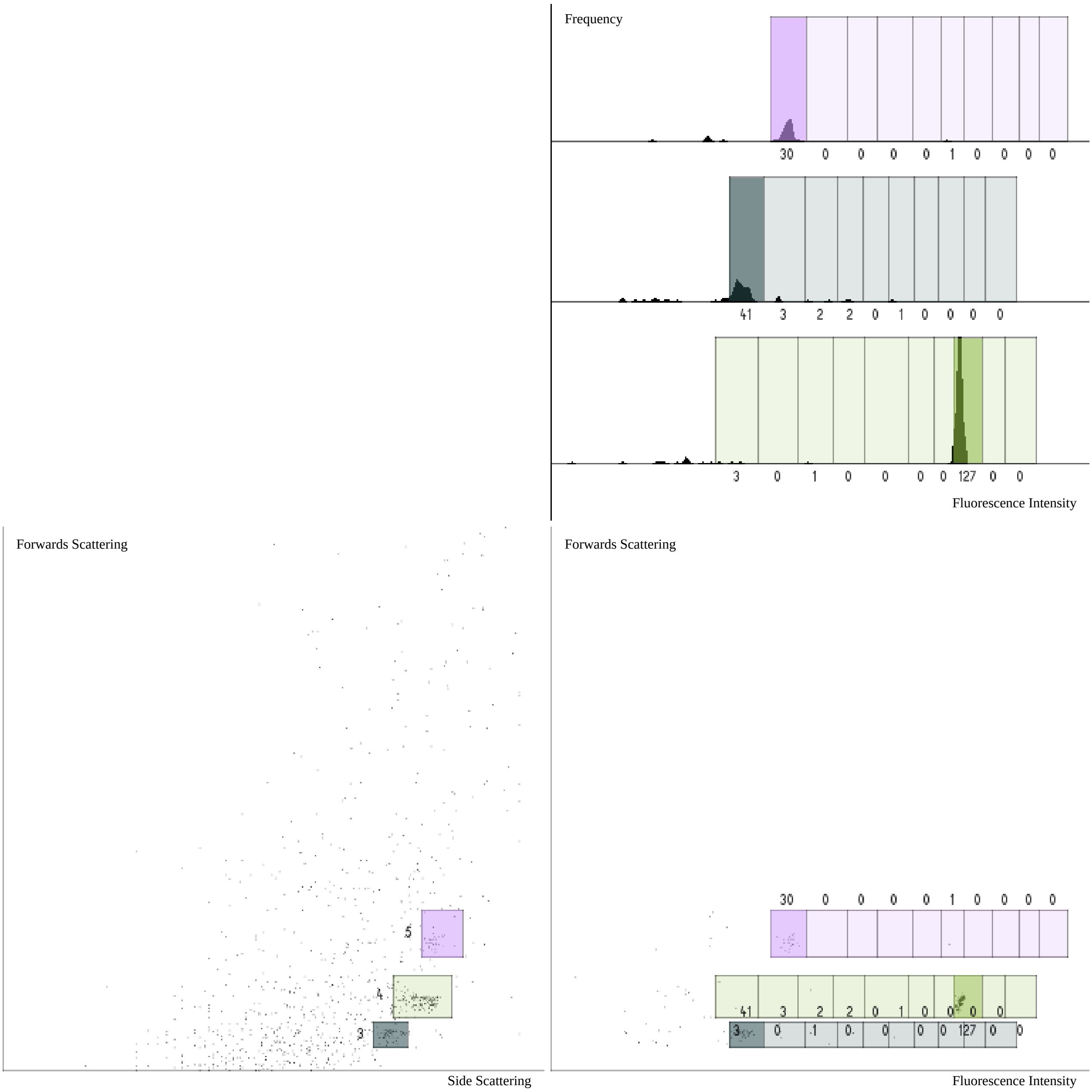

ANNEX 3: TAG DECONVOLUTION - BEAD 66

Passes flow sorting criteria: Yes

Passes tag deconvolution criteria: Yes

Included in protocol analysis: Yes

Protocol: 9, 6, 3, 8

Filename: Bin8_88.LMD

Split 1: Petrol shading

Split 2: Green shading

Split 3: Violet shading

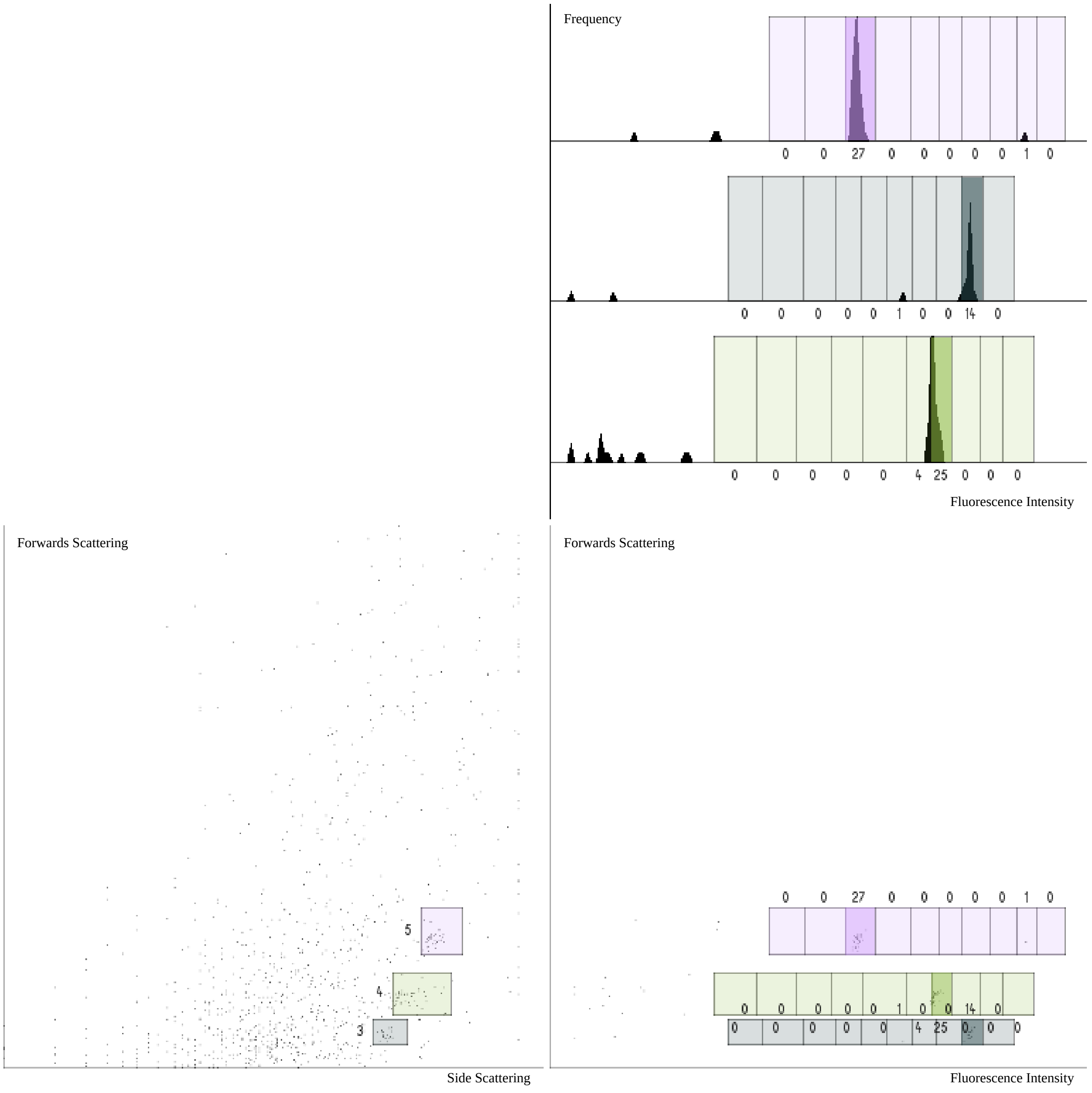

ANNEX 3: TAG DECONVOLUTION - BEAD 67

Passes flow sorting criteria: Yes

Passes tag deconvolution criteria: Yes

Included in protocol analysis: Yes

Protocol: 7, 3, 2, 8

Filename: Bin8_89.LMD

Split 1: Petrol shading

Split 2: Green shading

Split 3: Violet shading

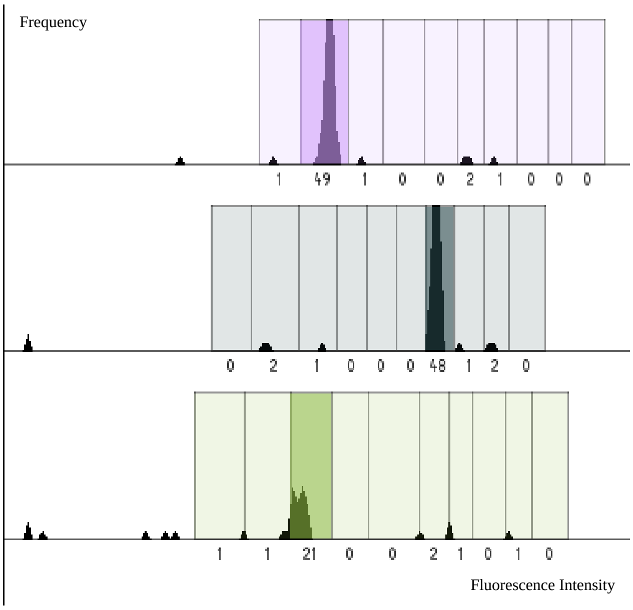

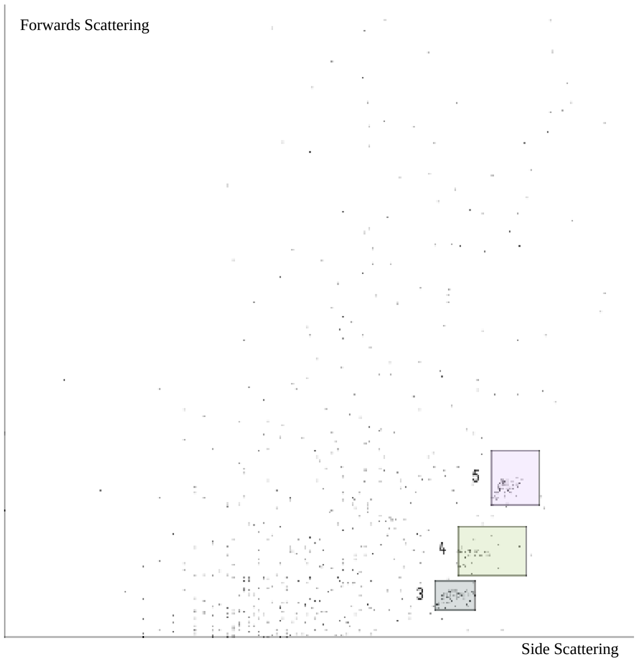

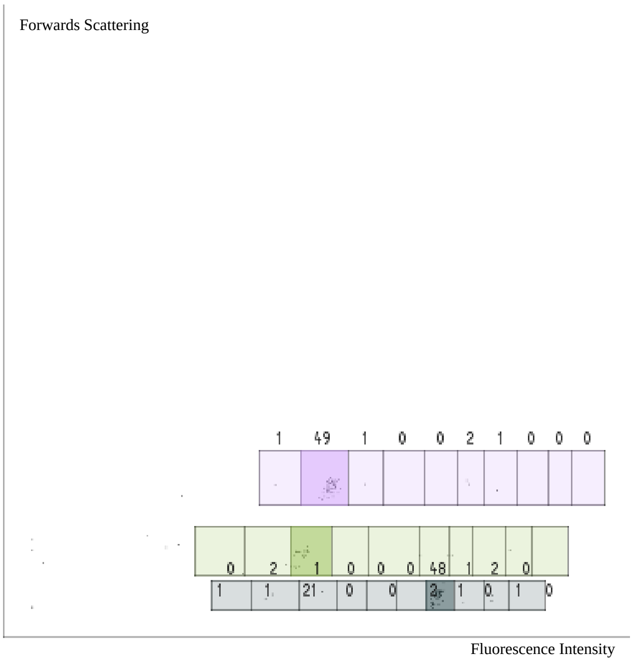

ANNEX 3: TAG DECONVOLUTION - BEAD 68

Passes flow sorting criteria: Yes

Passes tag deconvolution criteria: Yes

Included in protocol analysis: Yes

Protocol: 1, 3, 7, 8

Filename: Bin8_90.LMD

Split 1: Petrol shading

Split 2: Green shading

Split 3: Violet shading

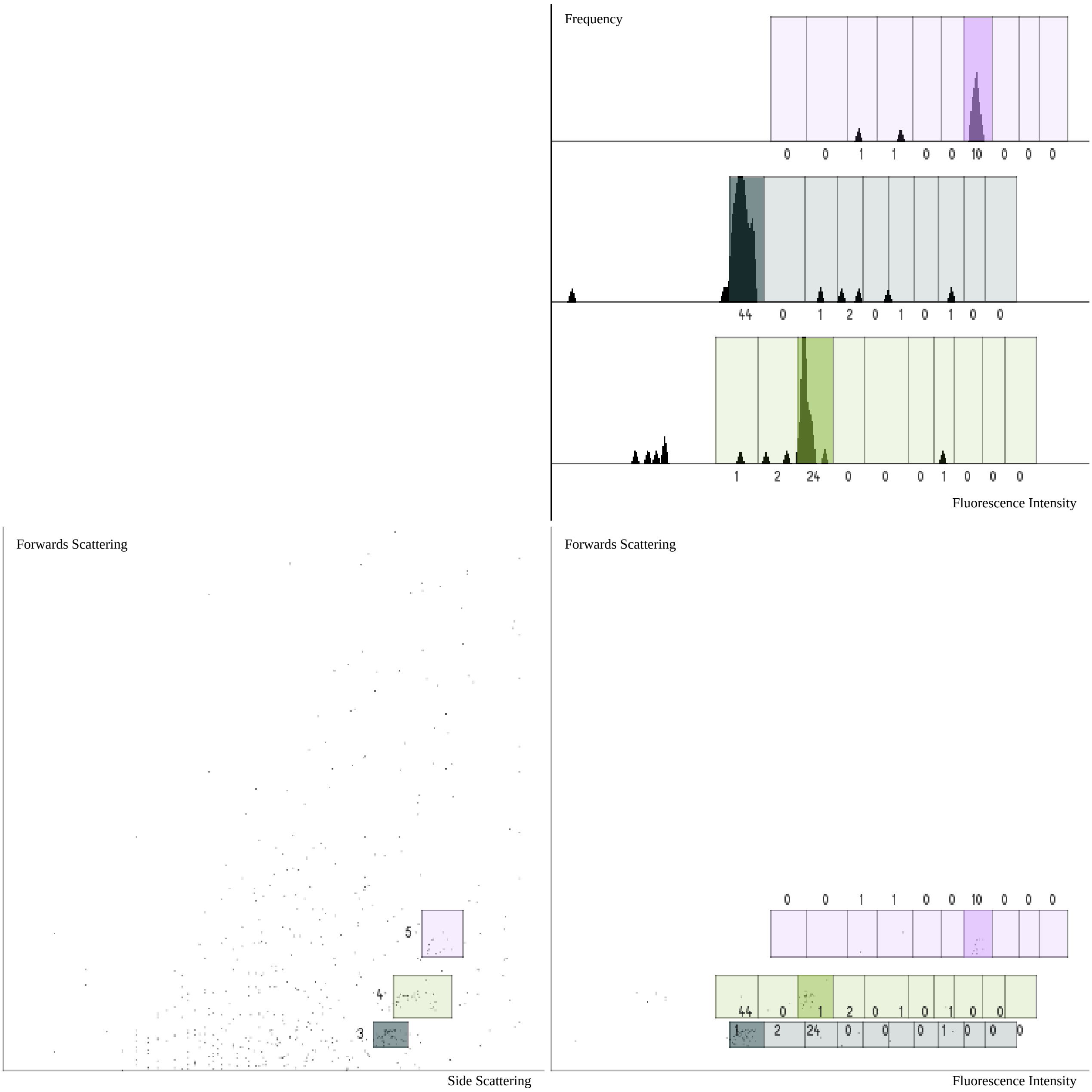

ANNEX 3: TAG DECONVOLUTION - BEAD 69

Passes flow sorting criteria: Yes

Passes tag deconvolution criteria: Yes

Included in protocol analysis: Yes

Protocol: 2, 8, 10, 8

Filename: Bin8_91.LMD

Split 1: Petrol shading

Split 2: Green shading

Split 3: Violet shading

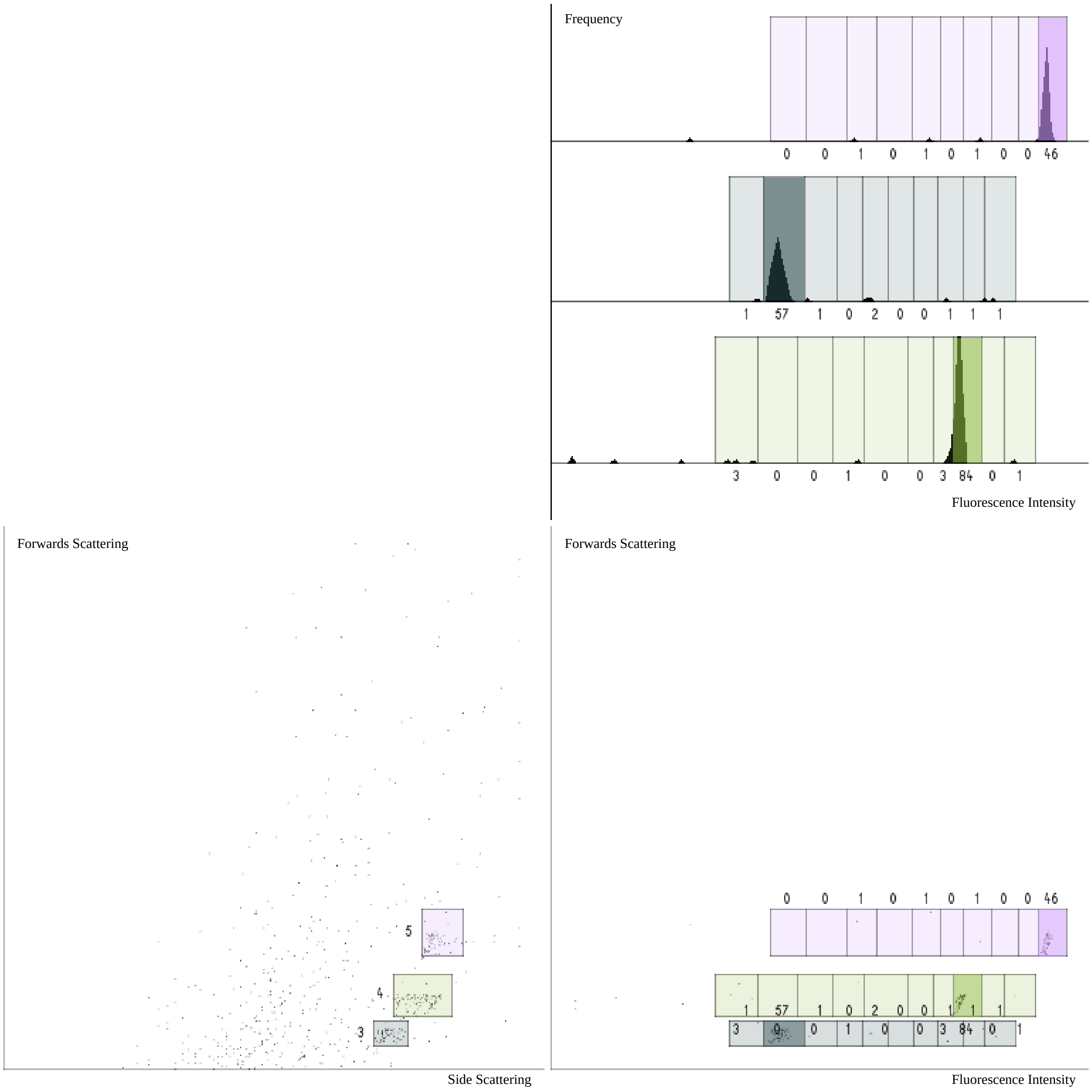

ANNEX 3: TAG DECONVOLUTION - BEAD 70

Passes flow sorting criteria: Yes

Passes tag deconvolution criteria: Yes

Included in protocol analysis: Yes

Protocol: 3, 1, 7, 10

Filename: Bin10_100.LMD

Split 1: Petrol shading

Split 2: Green shading

Split 3: Violet shading

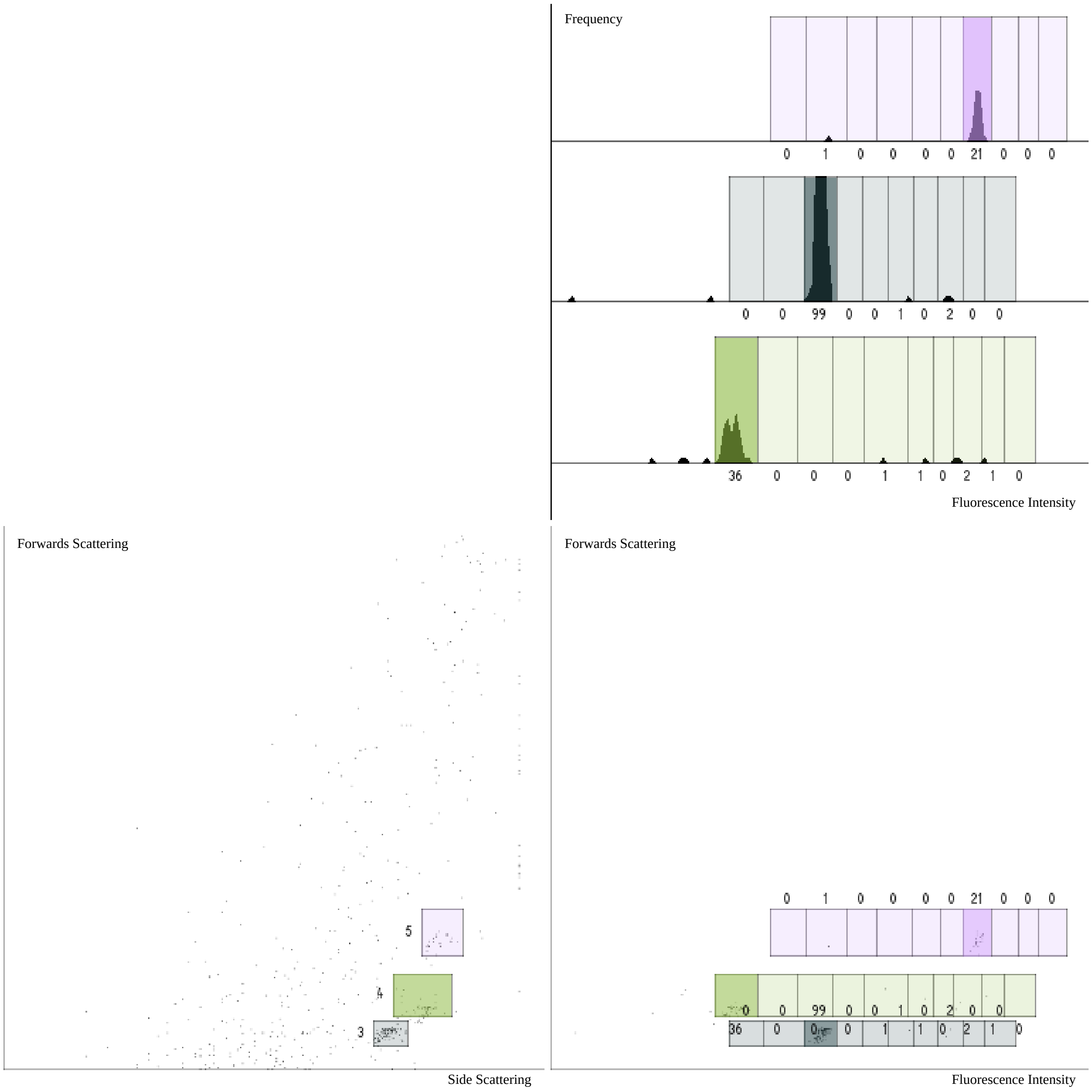

ANNEX 3: TAG DECONVOLUTION - BEAD 71

Passes flow sorting criteria: Yes

Passes tag deconvolution criteria: Yes

Included in protocol analysis: Yes

Protocol: 2, 9, 5, 10

Filename: Bin10_103.LMD

Split 1: Petrol shading

Split 2: Green shading

Split 3: Violet shading

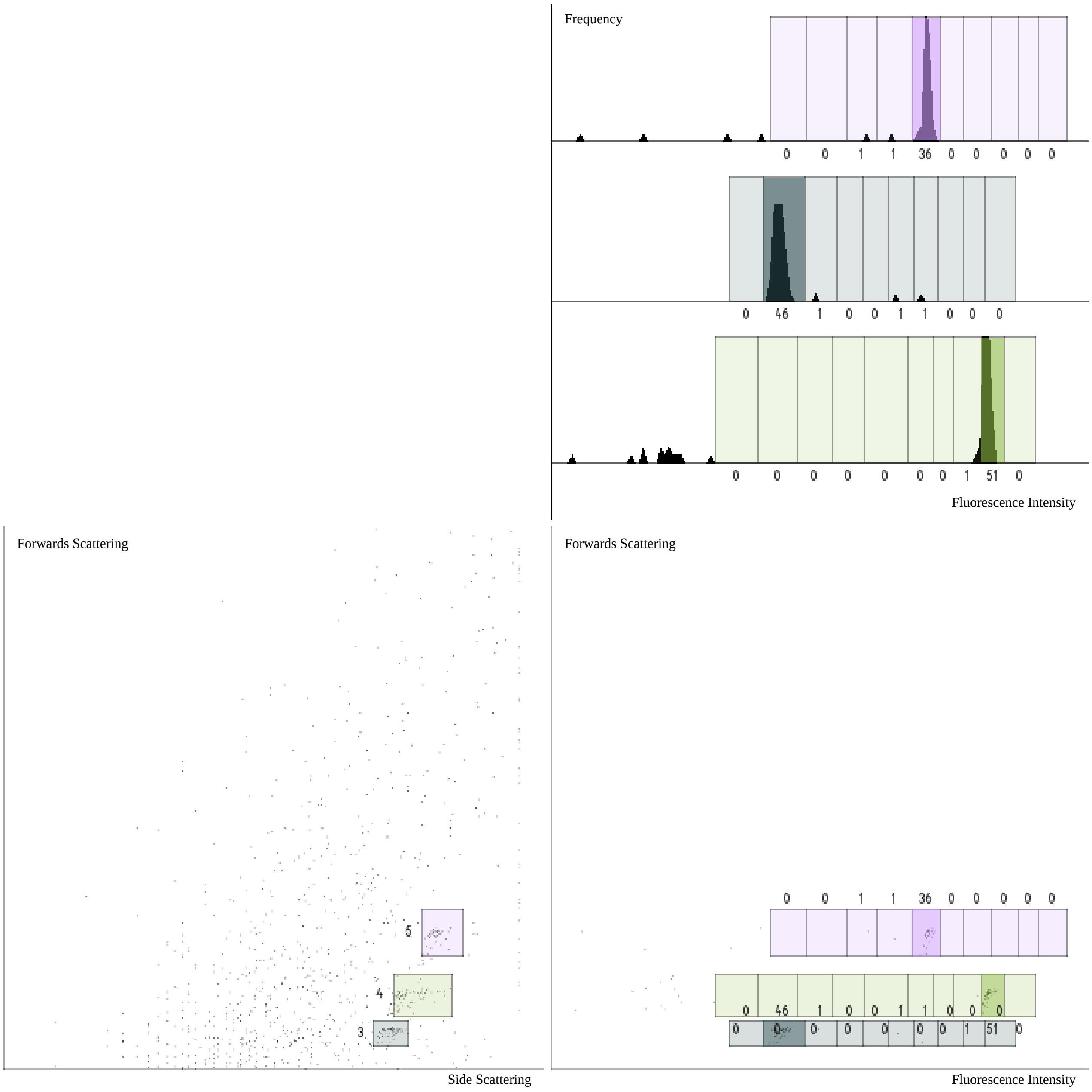

ANNEX 3: TAG DECONVOLUTION - BEAD 72

Passes flow sorting criteria: Yes

Passes tag deconvolution criteria: Yes

Included in protocol analysis: Yes

Protocol: 3, 2, 6, 10

Filename: Bin10_104.LMD

Split 1: Petrol shading

Split 2: Green shading

Split 3: Violet shading

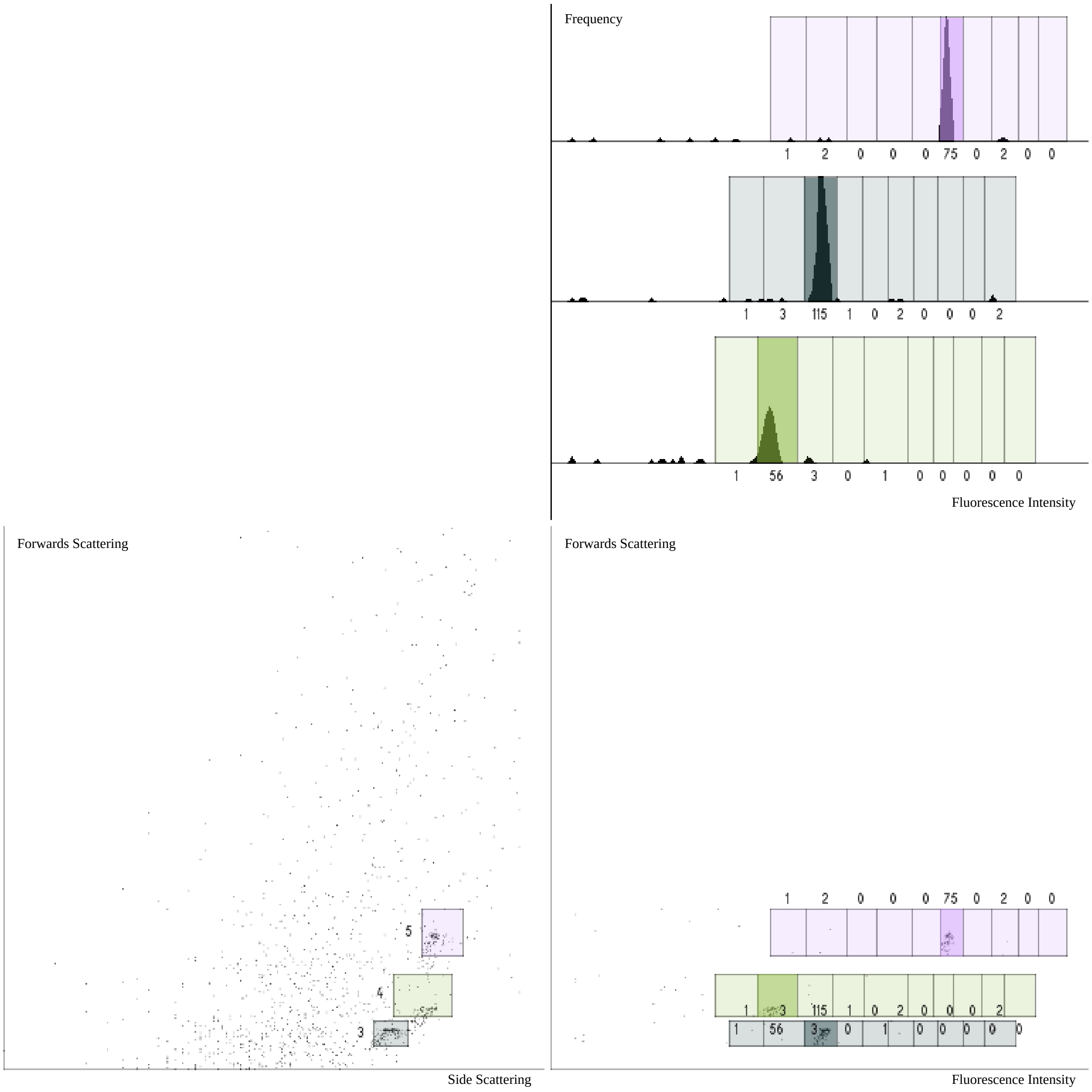

ANNEX 3: TAG DECONVOLUTION - BEAD 73

Passes flow sorting criteria: Yes

Passes tag deconvolution criteria: Yes

Included in protocol analysis: Yes

Protocol: 4, 1, 6, 10

Filename: Bin10_105.LMD

Split 1: Petrol shading

Split 2: Green shading

Split 3: Violet shading

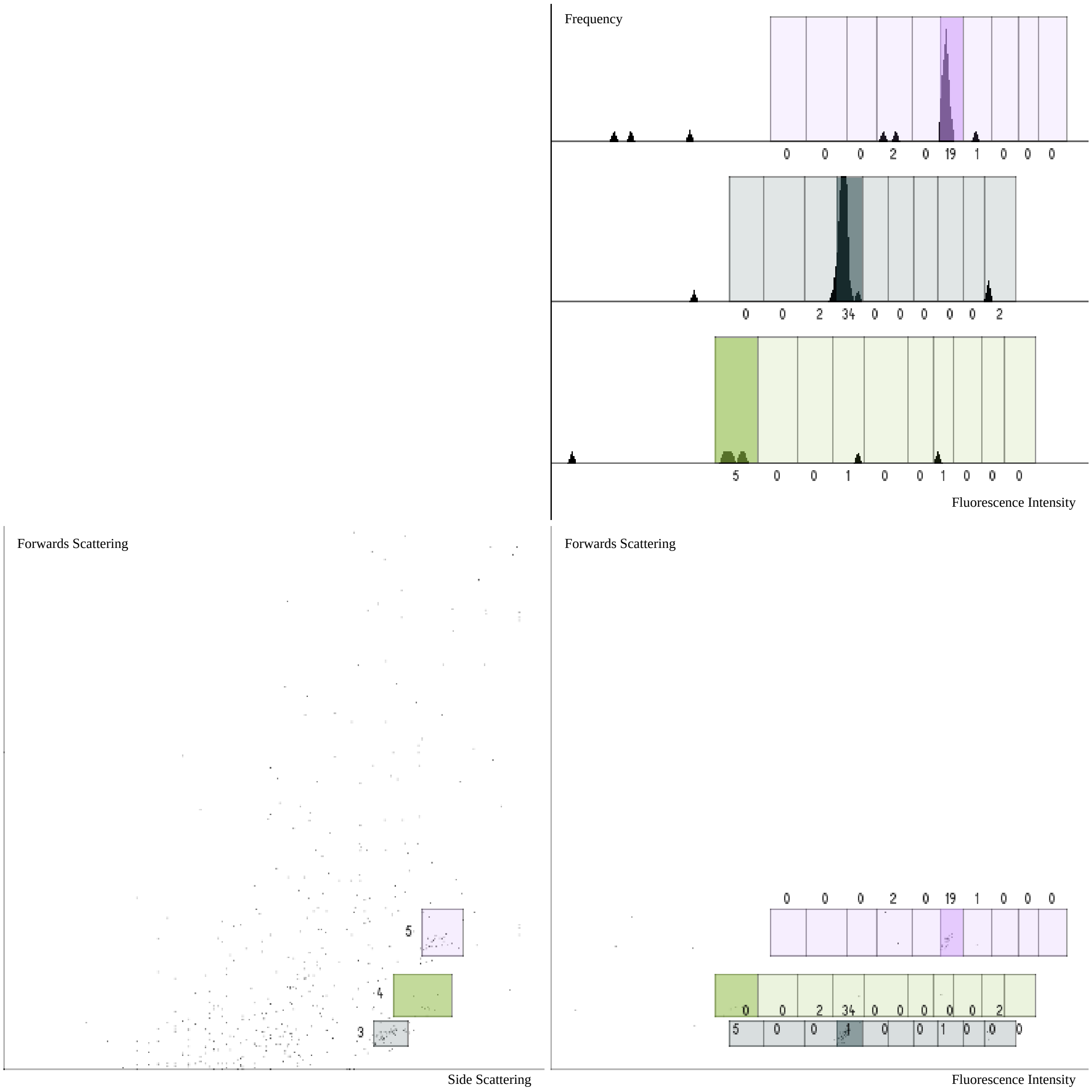

ANNEX 3: TAG DECONVOLUTION - BEAD 74

Passes flow sorting criteria: Yes

Passes tag deconvolution criteria: No

Included in protocol analysis: No

Protocol: N/A

Filename: Bin10_106.LMD

Split 1: Petrol shading

Split 2: Green shading

Split 3: Violet shading

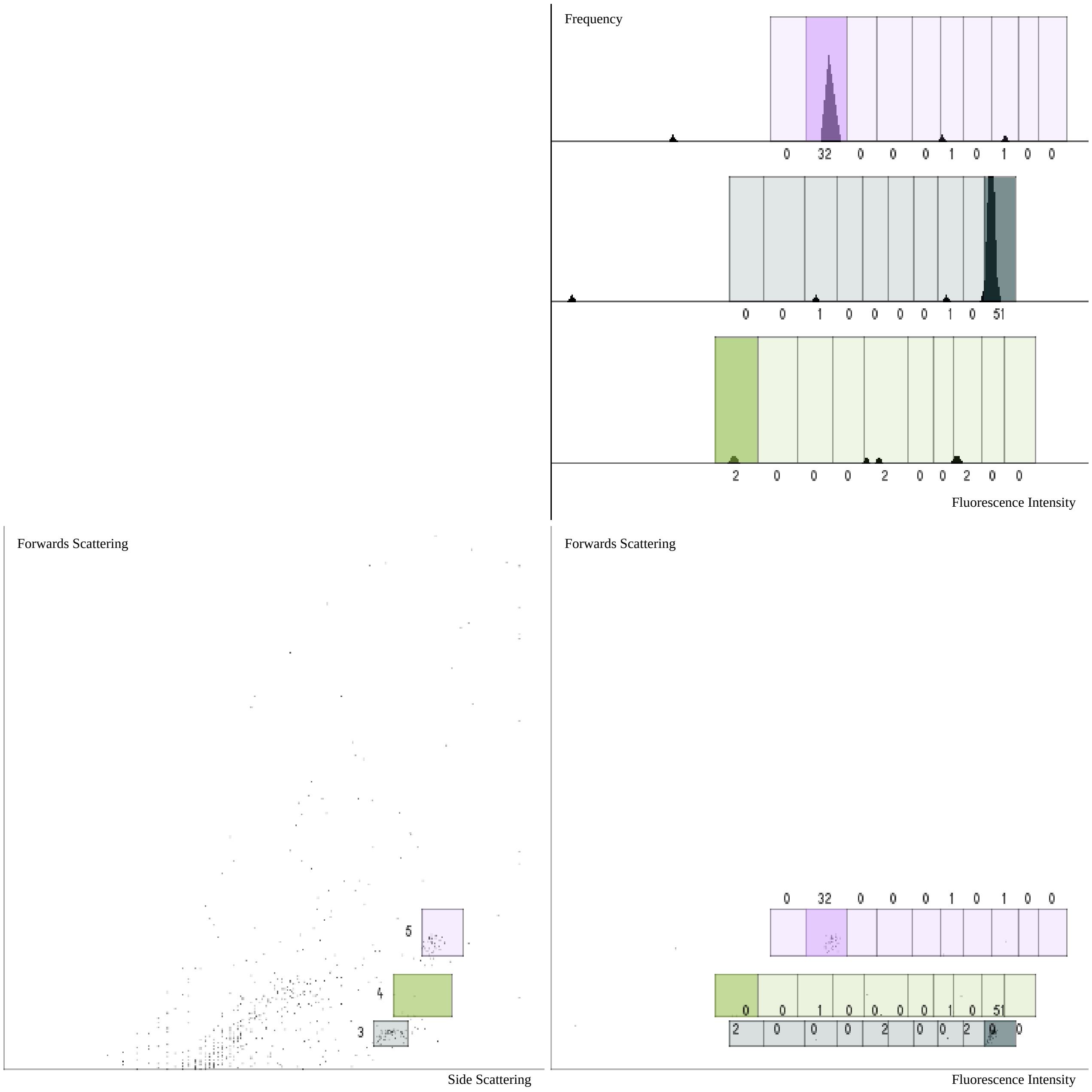

ANNEX 3: TAG DECONVOLUTION - BEAD 75

Passes flow sorting criteria: Yes

Passes tag deconvolution criteria: Yes

Included in protocol analysis: Yes

Protocol: 6, 9, 2, 10

Filename: Bin10_107.LMD

Split 1: Petrol shading

Split 2: Green shading

Split 3: Violet shading

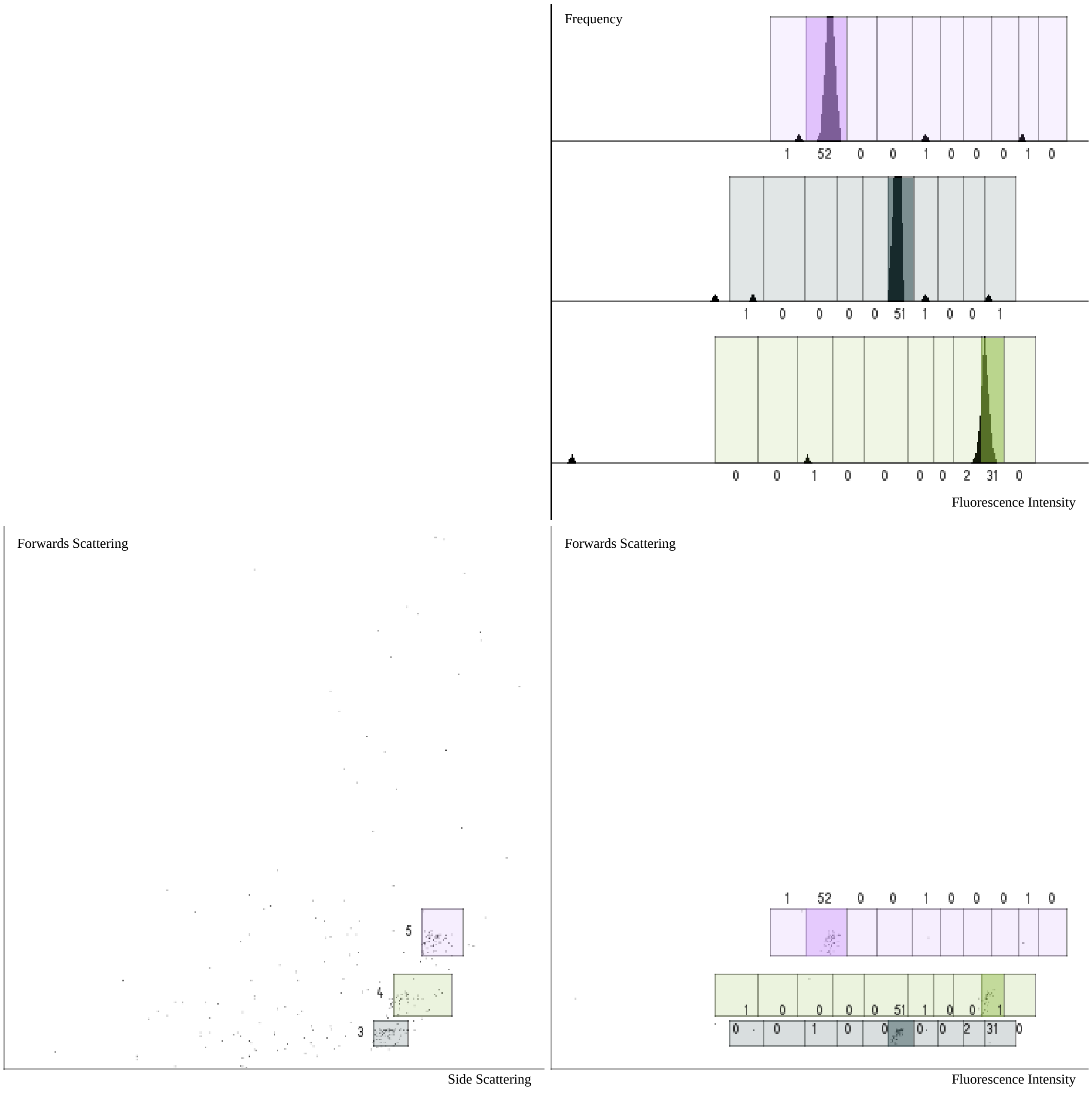

ANNEX 3: TAG DECONVOLUTION - BEAD 76

Passes flow sorting criteria: Yes

Passes tag deconvolution criteria: No

Included in protocol analysis: No

Protocol: N/A

Filename: Bin8_82b.LMD

Split 1: Petrol shading

Split 2: Green shading

Split 3: Violet shading

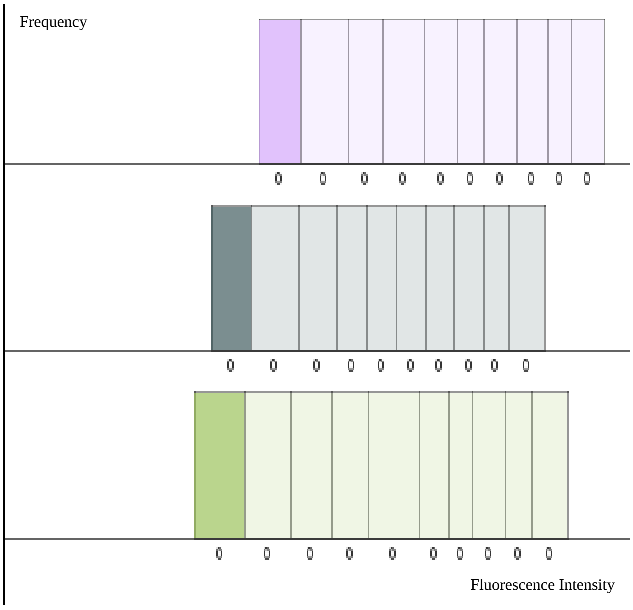

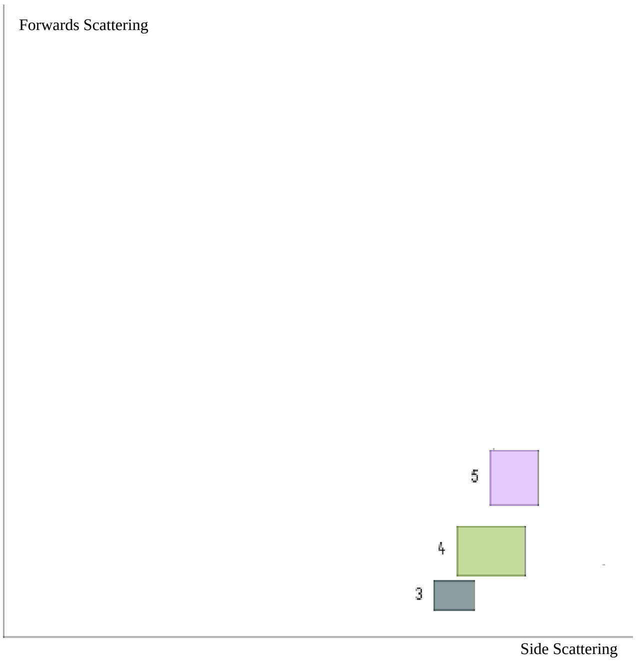

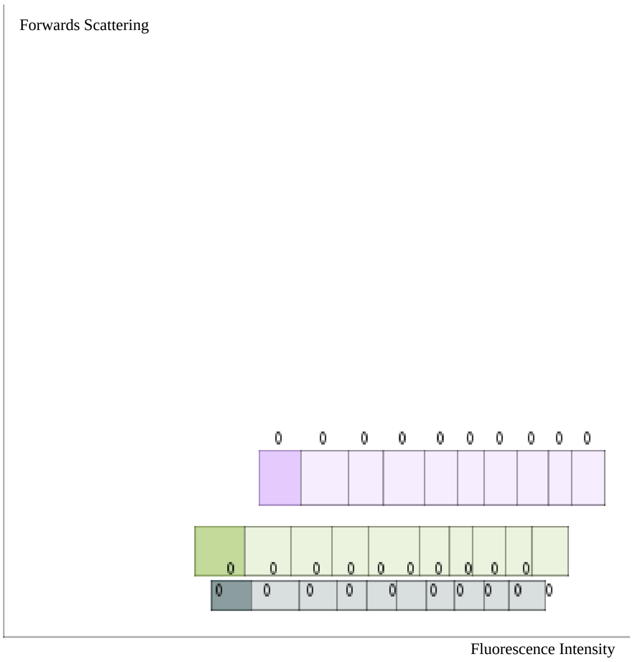

ANNEX 3: TAG DECONVOLUTION - BEAD 77

Passes flow sorting criteria: Yes

Passes tag deconvolution criteria: Yes

Included in protocol analysis: Yes

Protocol: 2, 5, 9, 7

Filename: Bin7_7R1

Split 1: Petrol shading

Split 2: Green shading

Split 3: Violet shading

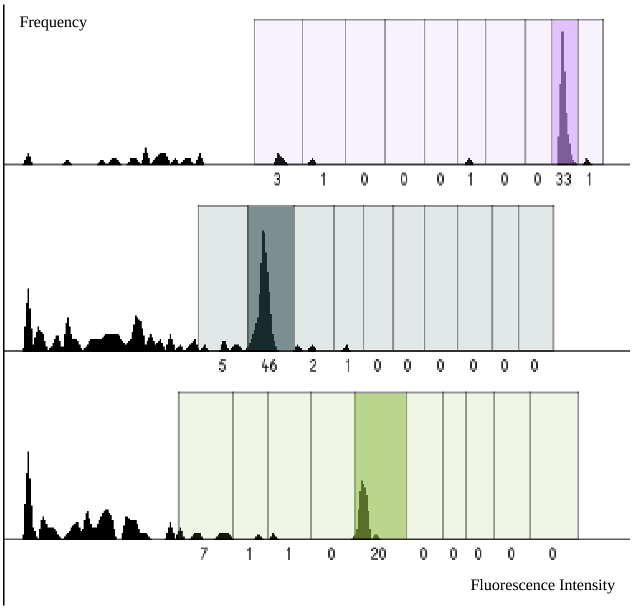

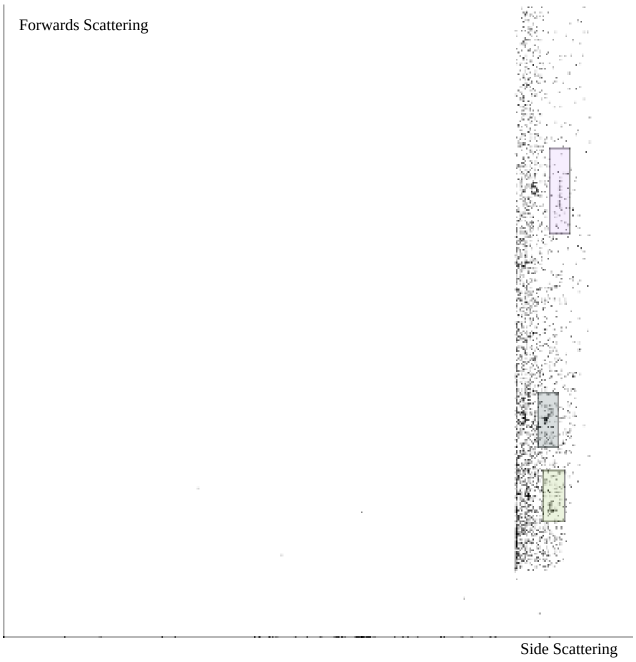

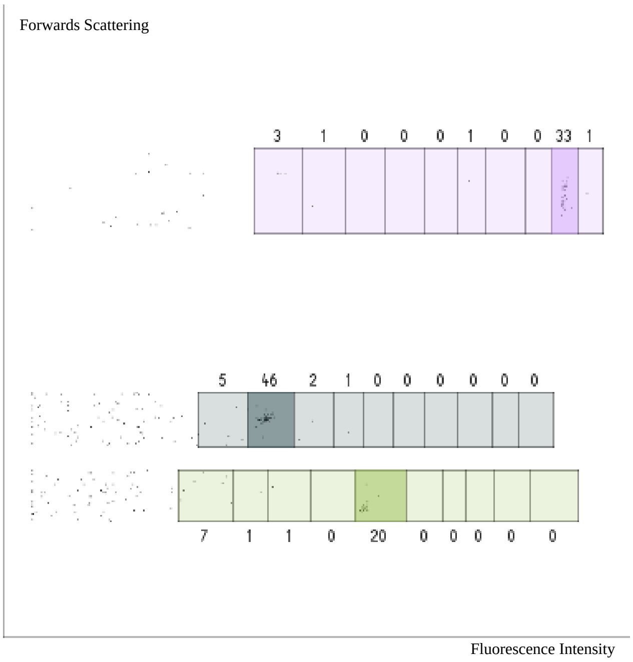

ANNEX 3: TAG DECONVOLUTION - BEAD 78

Passes flow sorting criteria: Yes

Passes tag deconvolution criteria: Yes

Included in protocol analysis: Yes

Protocol: 9, 8, 8, 7

Filename: Bin7_7R2

Split 1: Petrol shading

Split 2: Green shading

Split 3: Violet shading

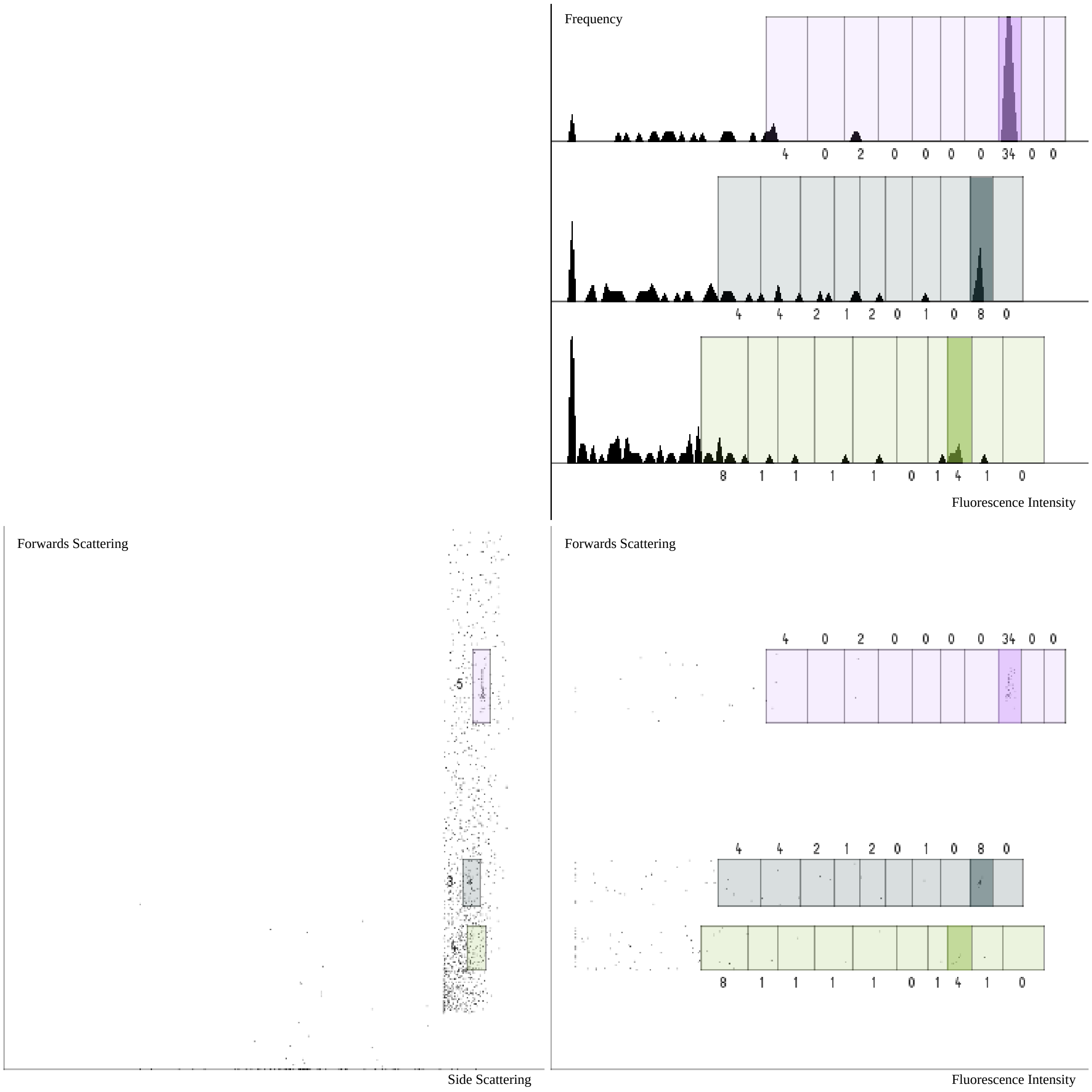

ANNEX 3: TAG DECONVOLUTION - BEAD 79

Passes flow sorting criteria: Yes

Passes tag deconvolution criteria: Yes

Included in protocol analysis: Yes

Protocol: 9, 2, 7, 7

Filename: Bin7_7R3

Split 1: Petrol shading

Split 2: Green shading

Split 3: Violet shading

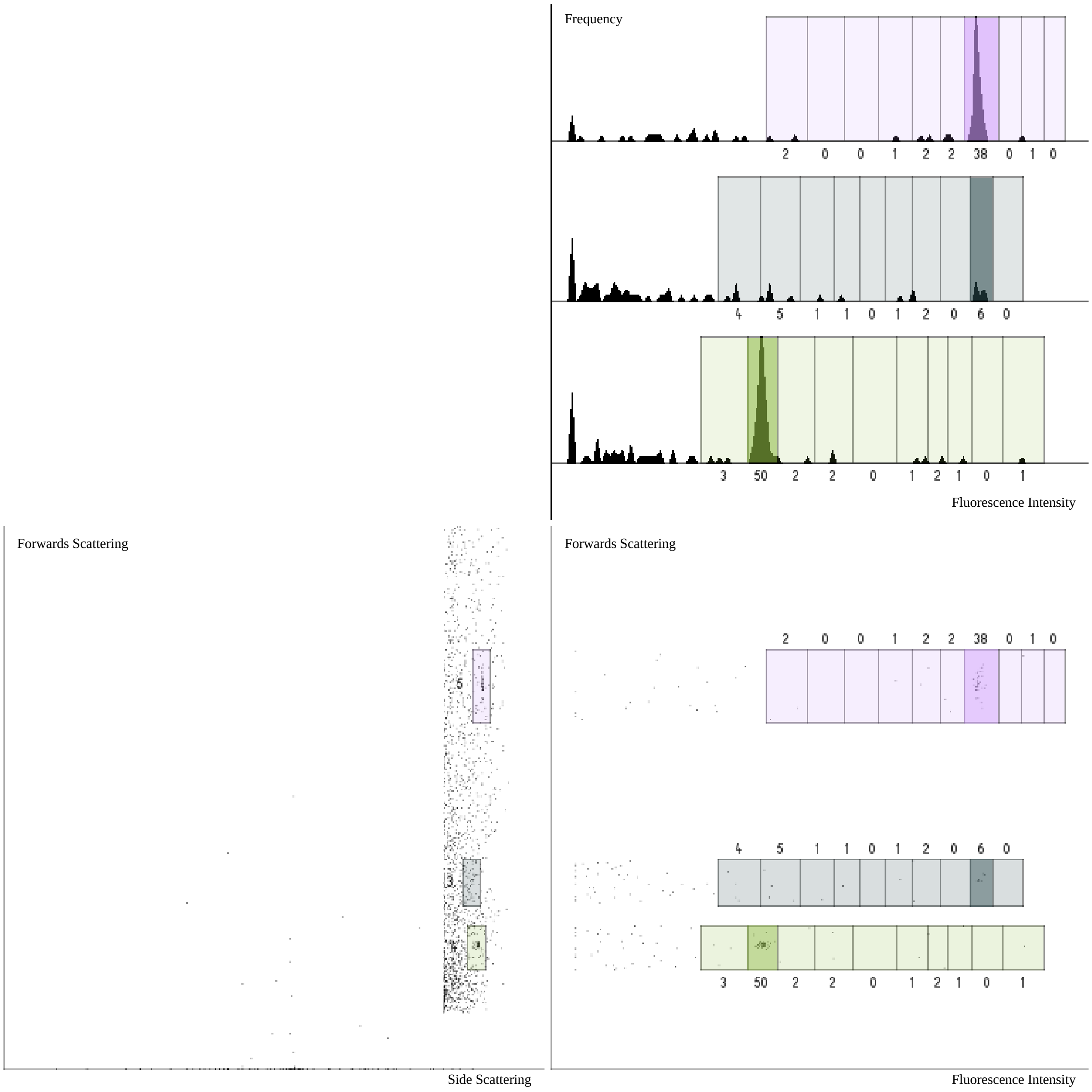

ANNEX 3: TAG DECONVOLUTION - BEAD 80

Passes flow sorting criteria: Yes

Passes tag deconvolution criteria: Yes

Included in protocol analysis: Yes

Protocol: 1, 5, 6, 7

Filename: Bin7_7R4

Split 1: Petrol shading

Split 2: Green shading

Split 3: Violet shading

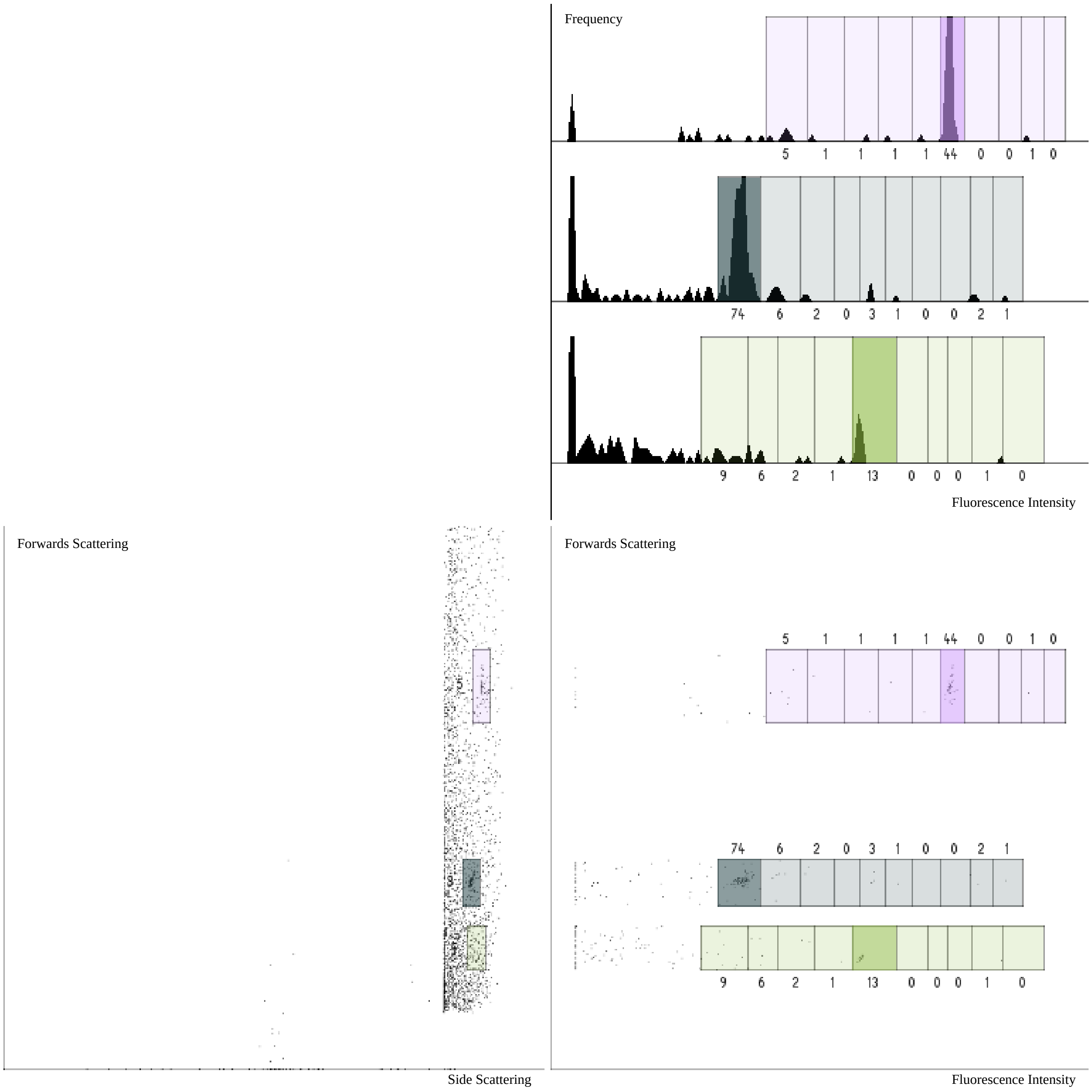

ANNEX 3: TAG DECONVOLUTION - BEAD 81

Passes flow sorting criteria: Yes

Passes tag deconvolution criteria: Yes

Included in protocol analysis: Yes

Protocol: 8, 2, 8, 7

Filename: Bin7_7R5

Split 1: Petrol shading

Split 2: Green shading

Split 3: Violet shading

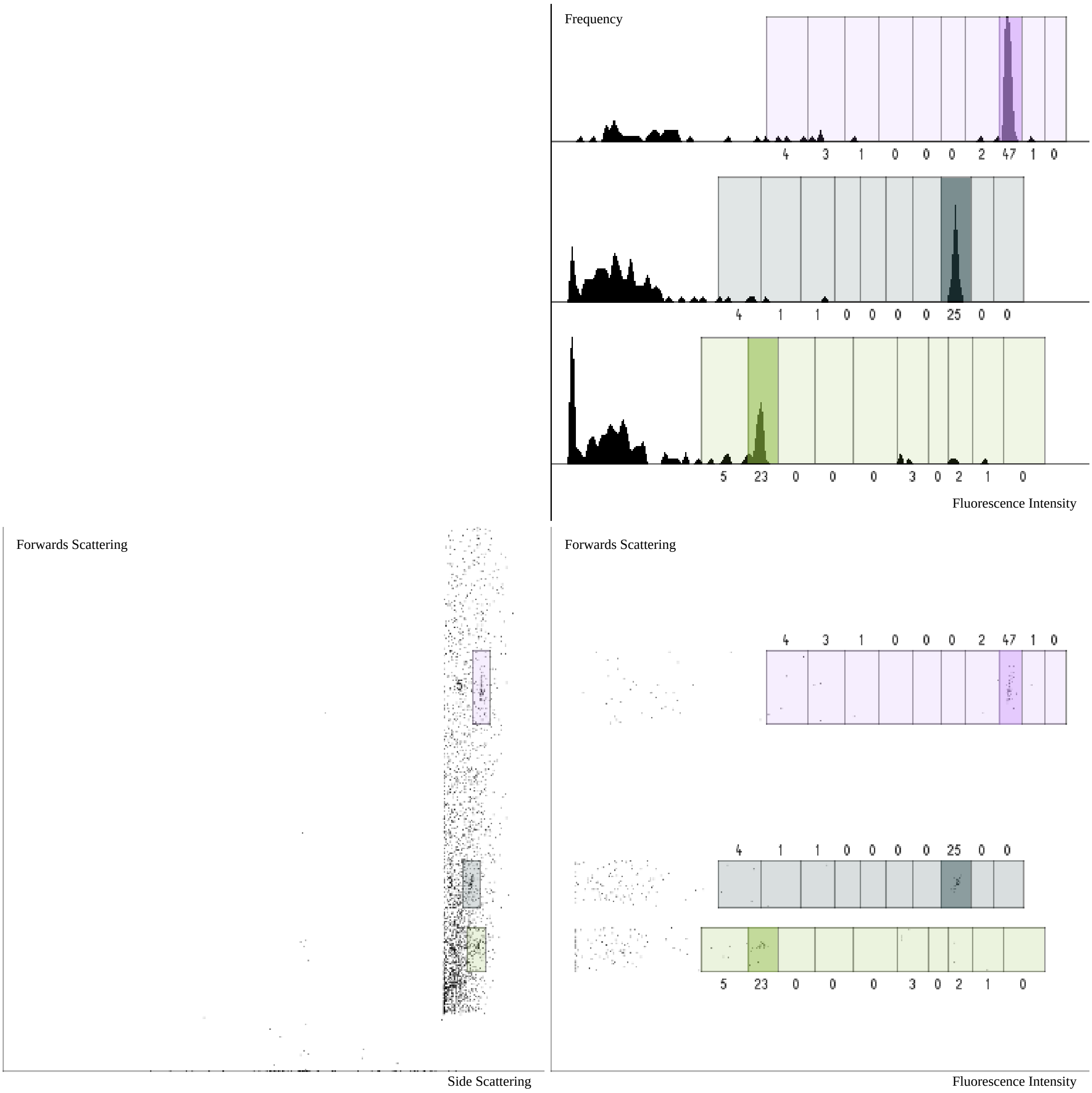

ANNEX 3: TAG DECONVOLUTION - BEAD 82

Passes flow sorting criteria: Yes

Passes tag deconvolution criteria: Yes

Included in protocol analysis: Yes

Protocol: 5, 4, 2, 7

Filename: Bin7_7R6

Split 1: Petrol shading

Split 2: Green shading

Split 3: Violet shading

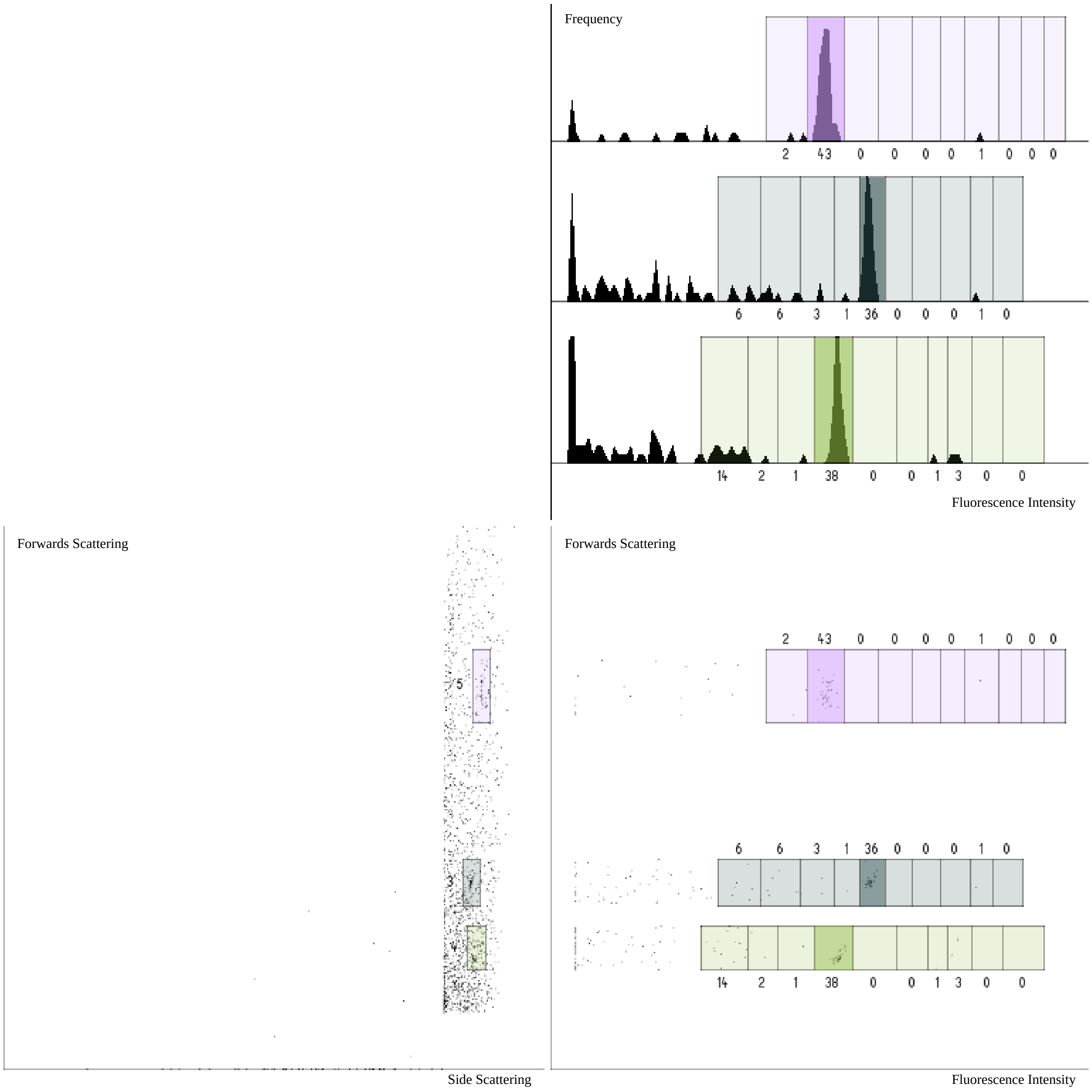

ANNEX 3: TAG DECONVOLUTION - BEAD 83

Passes flow sorting criteria: Yes

Passes tag deconvolution criteria: Yes

Included in protocol analysis: Yes

Protocol: 1, 1, 6, 7

Filename: Bin7_7R7

Split 1: Petrol shading

Split 2: Green shading

Split 3: Violet shading

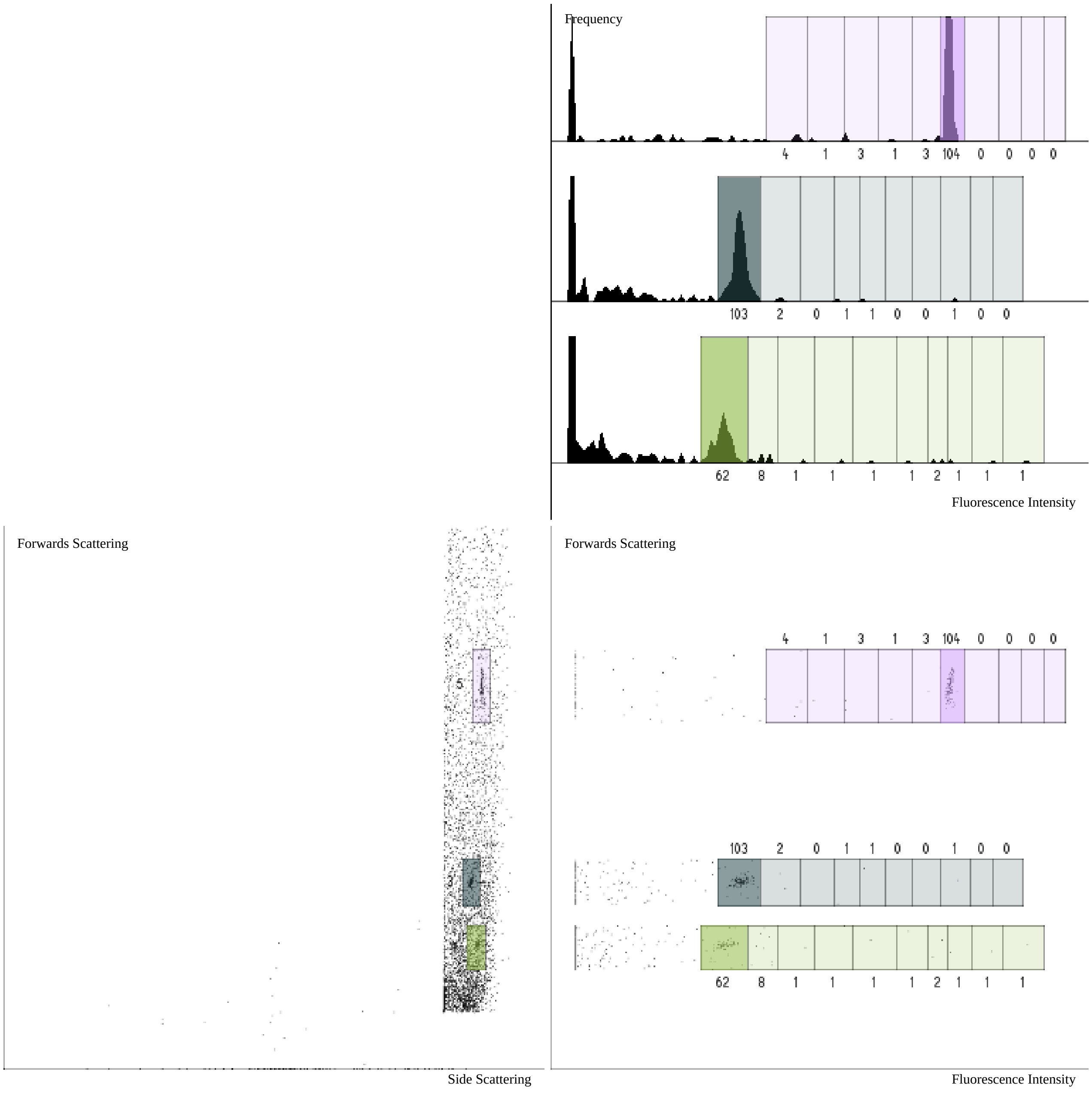

ANNEX 3: TAG DECONVOLUTION - BEAD 84

Passes flow sorting criteria: Yes

Passes tag deconvolution criteria: Yes

Included in protocol analysis: Yes

Protocol: 2, 1, 3, 8

Filename: Bin8_72

Split 1: Petrol shading

Split 2: Green shading

Split 3: Violet shading

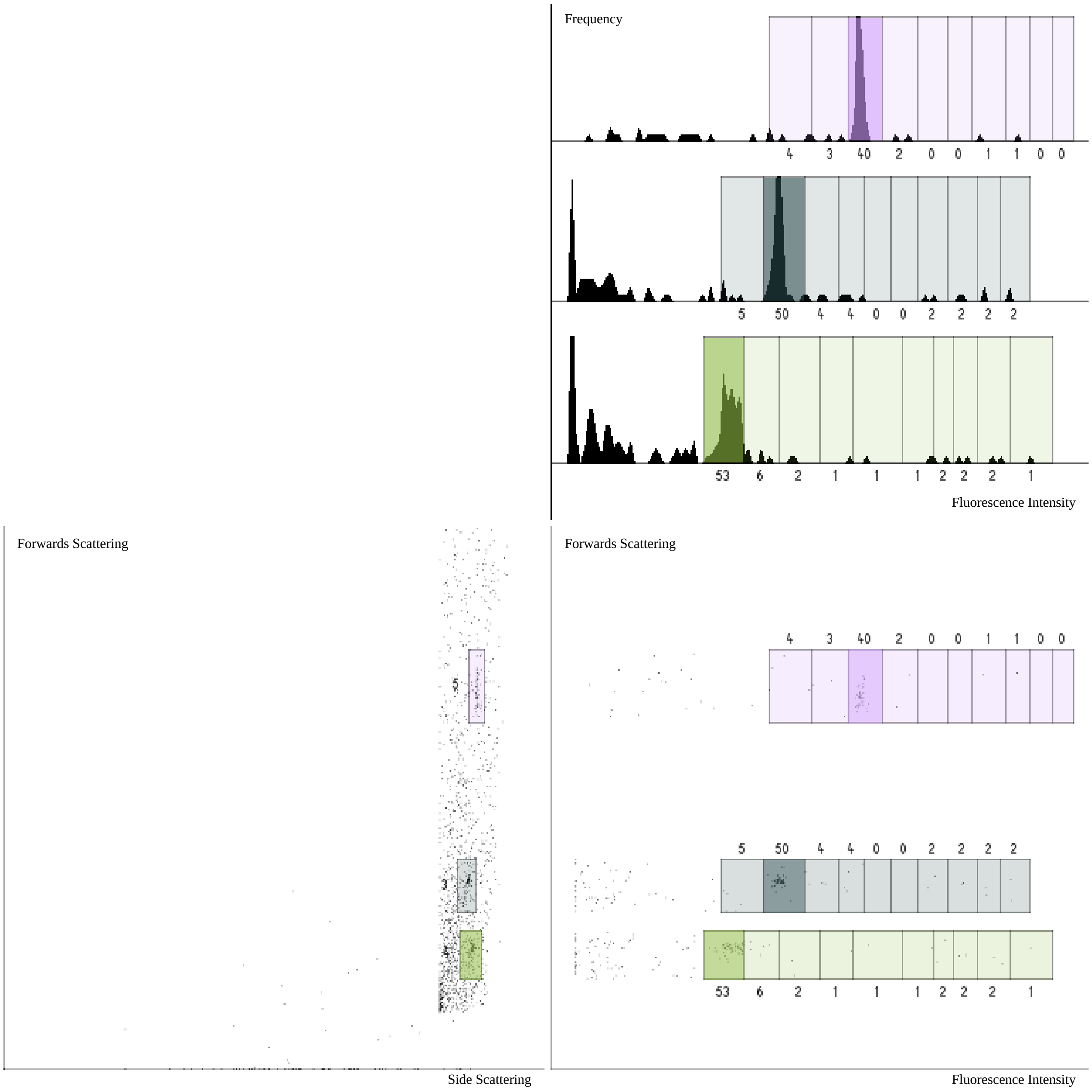

ANNEX 3: TAG DECONVOLUTION - BEAD 85

Passes flow sorting criteria: Yes

Passes tag deconvolution criteria: Yes

Included in protocol analysis: Yes

Protocol: 8, 7, 1, 8

Filename: Bin8_73

Split 1: Petrol shading

Split 2: Green shading

Split 3: Violet shading

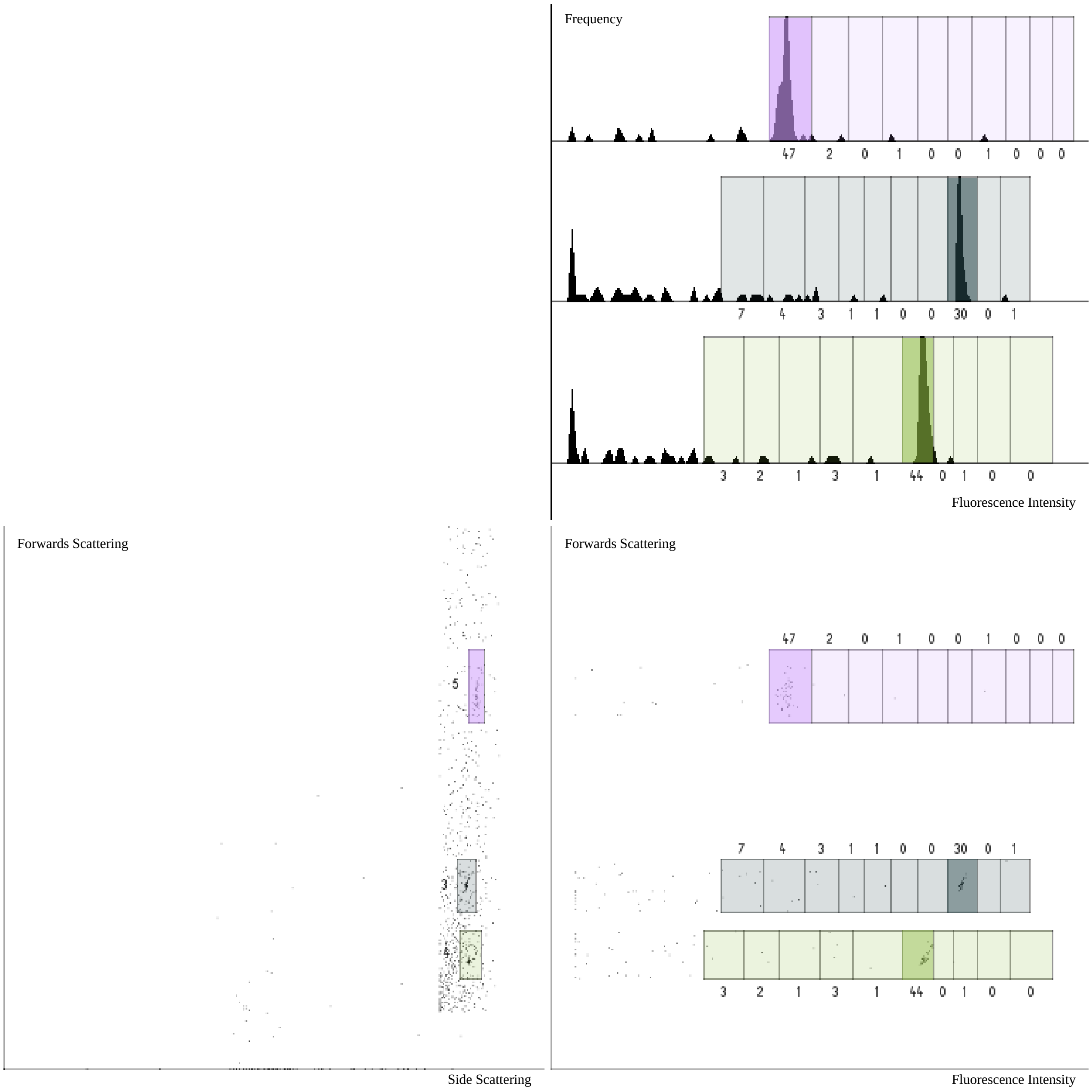

ANNEX 3: TAG DECONVOLUTION - BEAD 86

Passes flow sorting criteria: Yes

Passes tag deconvolution criteria: Yes

Included in protocol analysis: Yes

Protocol: 2, 1, 6, 8

Filename: Bin8_74

Split 1: Petrol shading

Split 2: Green shading

Split 3: Violet shading

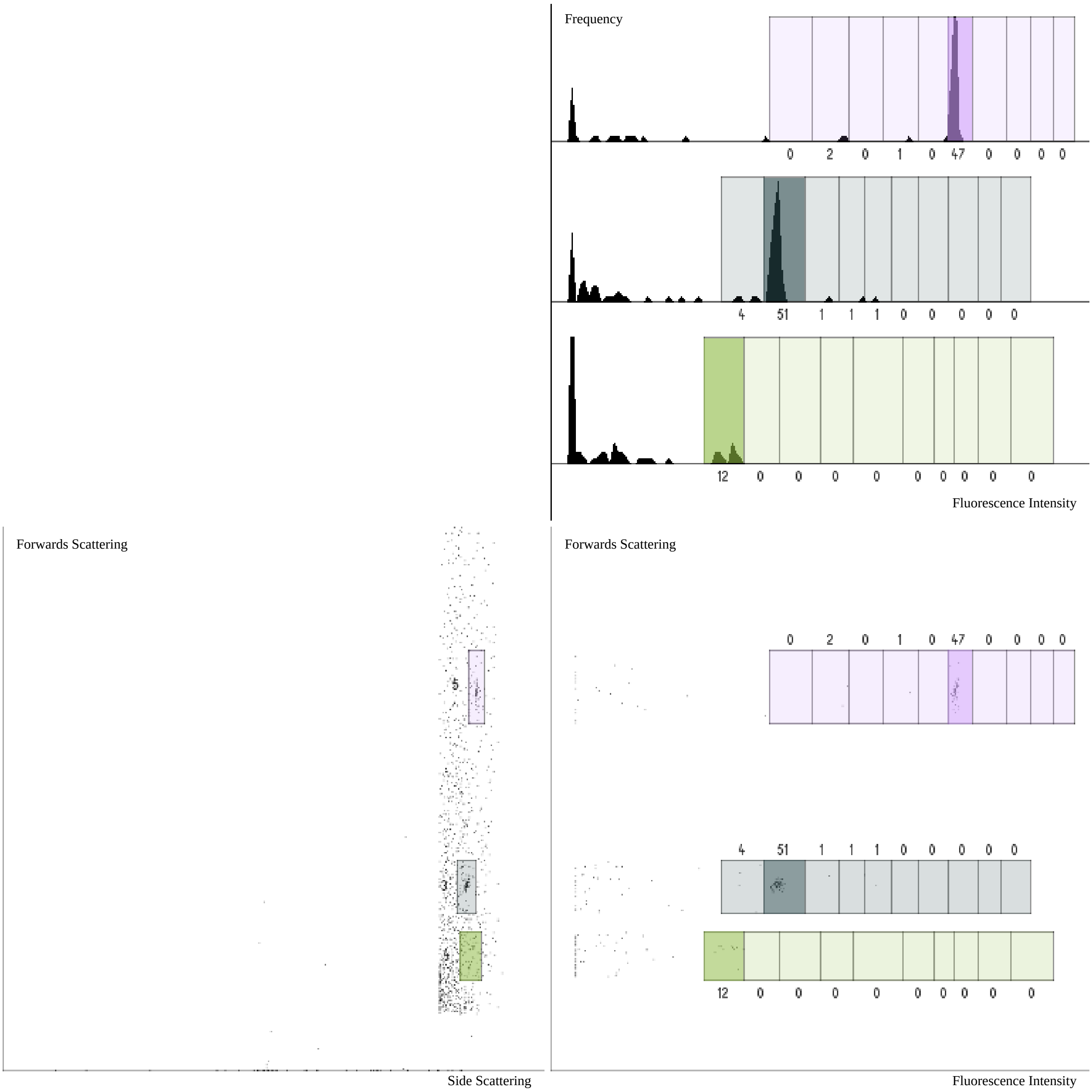

ANNEX 3: TAG DECONVOLUTION - BEAD 87

Passes flow sorting criteria: Yes

Passes tag deconvolution criteria: Yes

Included in protocol analysis: Yes

Protocol: 7, 1, 7, 8

Filename: Bin8_75

Split 1: Petrol shading

Split 2: Green shading

Split 3: Violet shading

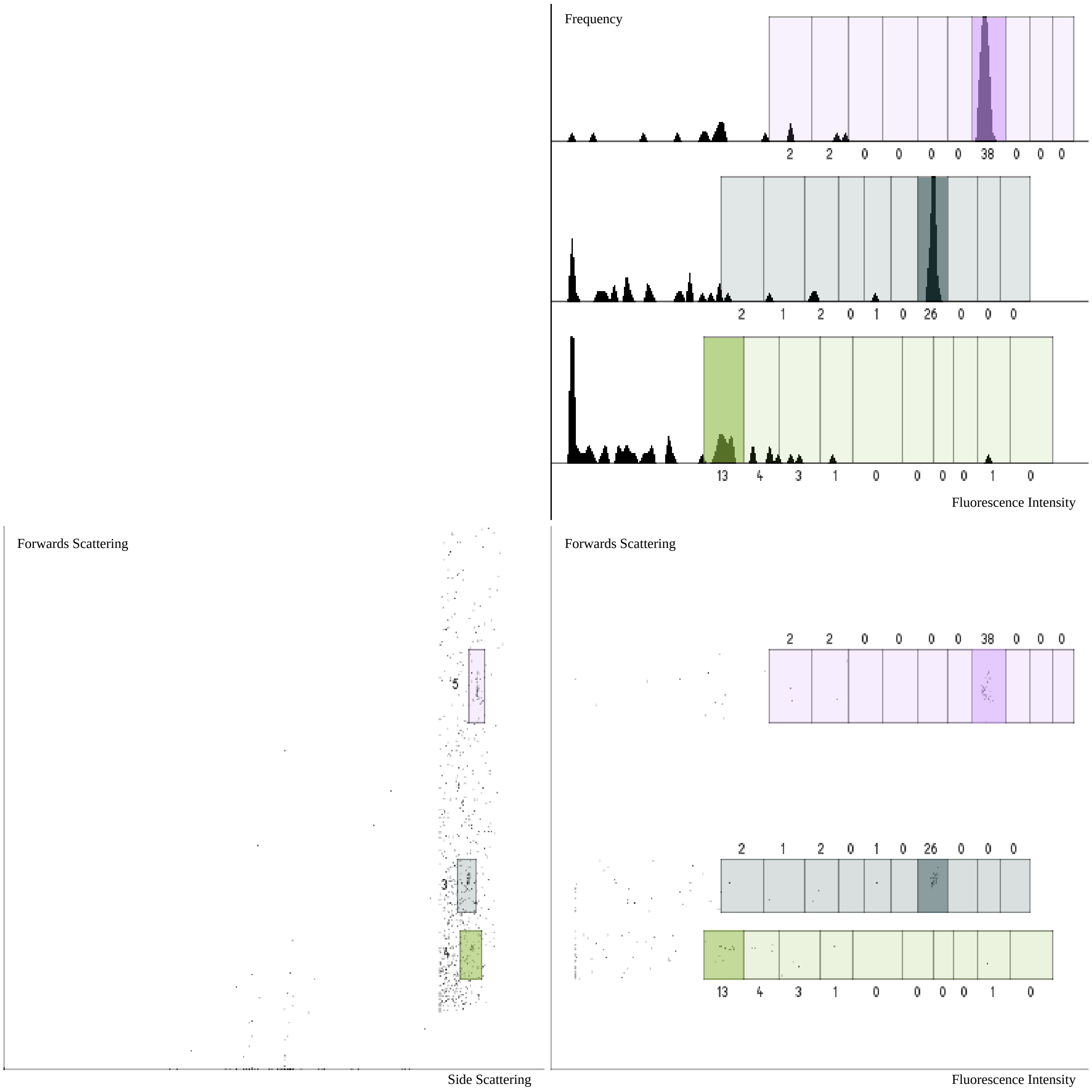

ANNEX 3: TAG DECONVOLUTION - BEAD 88

Passes flow sorting criteria: Yes

Passes tag deconvolution criteria: Yes

Included in protocol analysis: Yes

Protocol: 5, 9, 2, 8

Filename: Bin8_76

Split 1: Petrol shading

Split 2: Green shading

Split 3: Violet shading

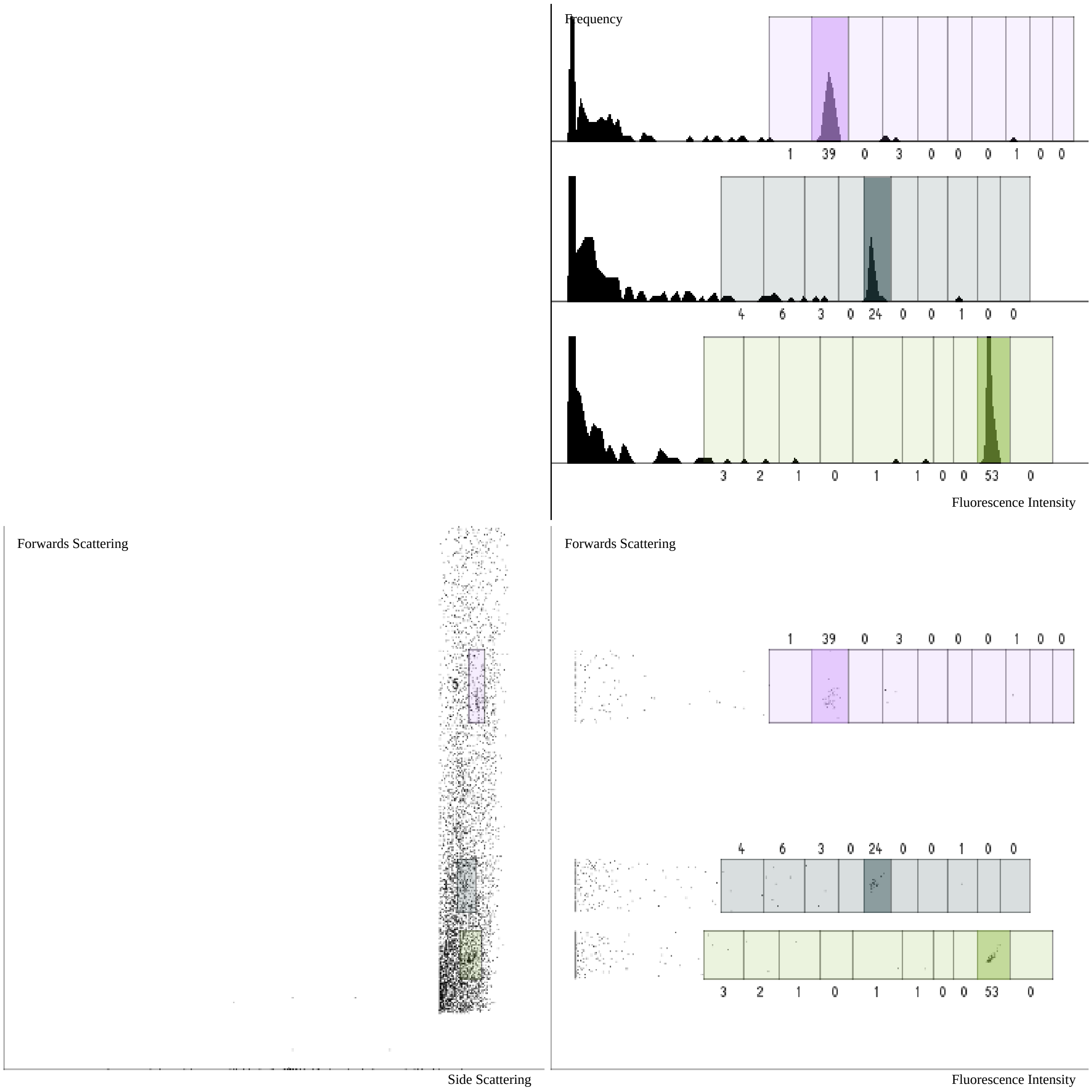

ANNEX 3: TAG DECONVOLUTION - BEAD 89

Passes flow sorting criteria: Yes

Passes tag deconvolution criteria: Yes

Included in protocol analysis: Yes

Protocol: 10, 7, 1, 8

Filename: Bin8_77

Split 1: Petrol shading

Split 2: Green shading

Split 3: Violet shading

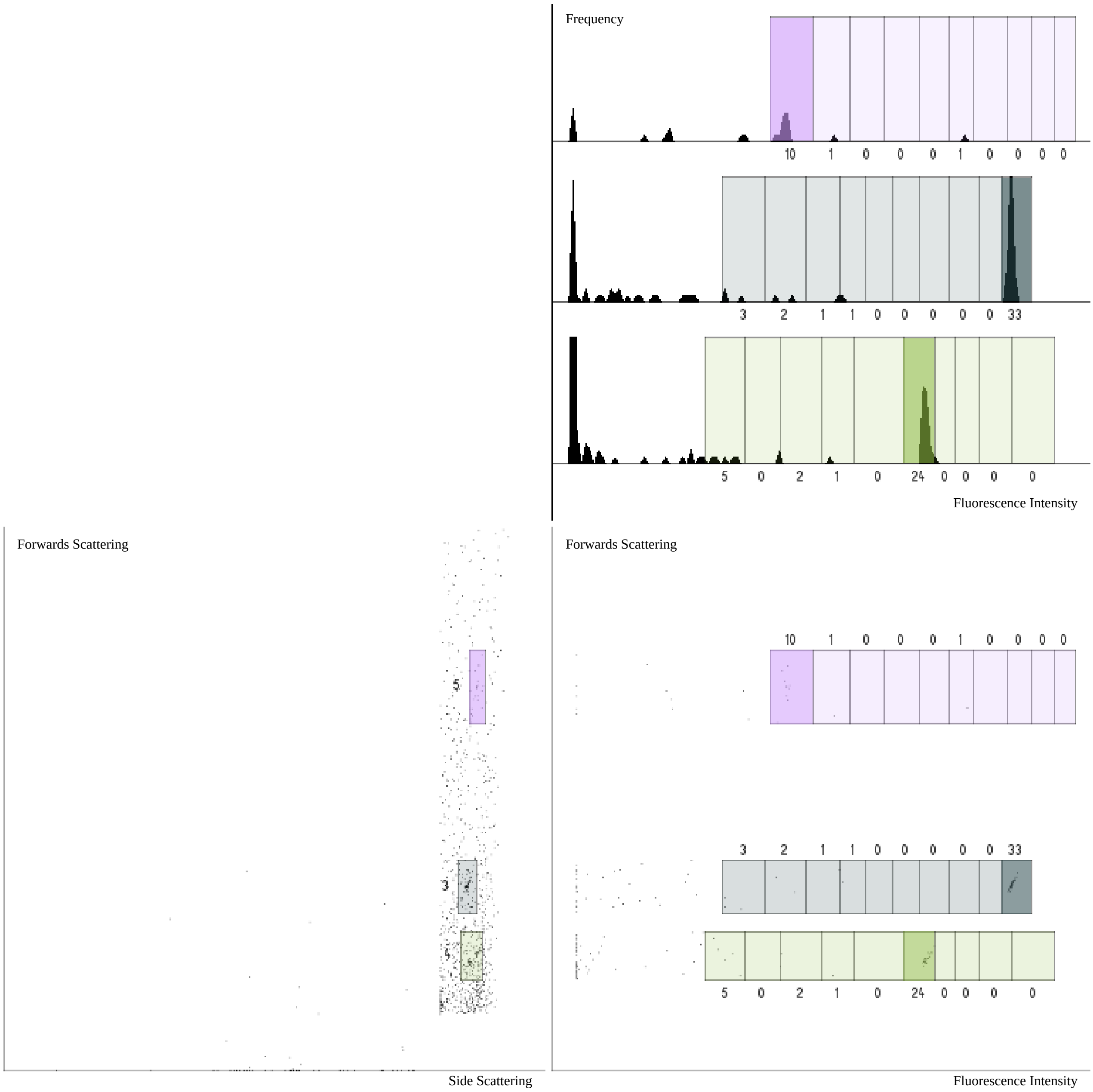

ANNEX 3: TAG DECONVOLUTION - BEAD 90

Passes flow sorting criteria: Yes

Passes tag deconvolution criteria: Yes

Included in protocol analysis: Yes

Protocol: 10, 1, 4, 8

Filename: Bin8_78

Split 1: Petrol shading

Split 2: Green shading

Split 3: Violet shading

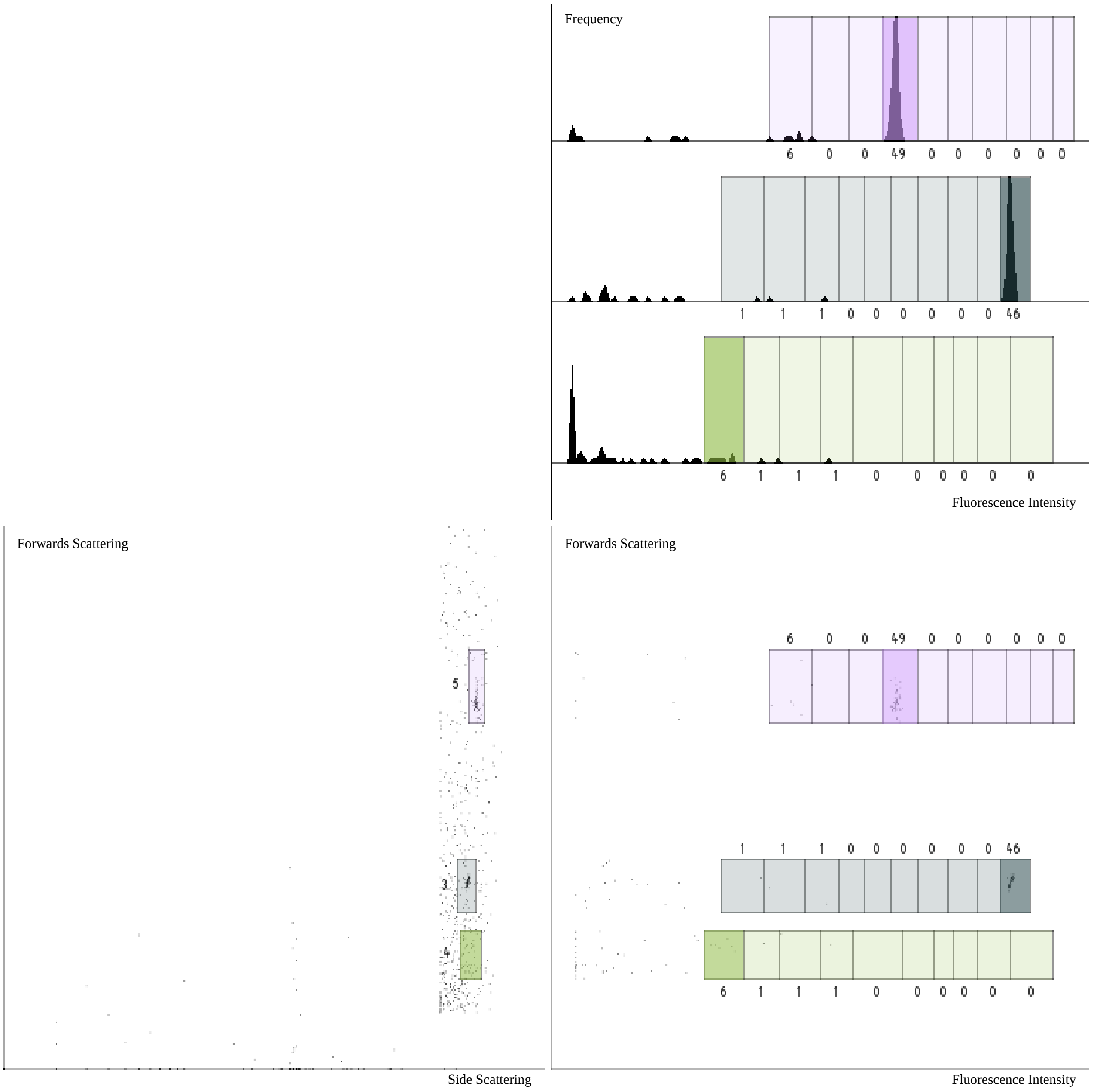

ANNEX 3: TAG DECONVOLUTION - BEAD 91

Passes flow sorting criteria: Yes

Passes tag deconvolution criteria: Yes

Included in protocol analysis: No

Protocol: N/A

Filename: Bin8_79

Split 1: Petrol shading

Split 2: Green shading

Split 3: Violet shading

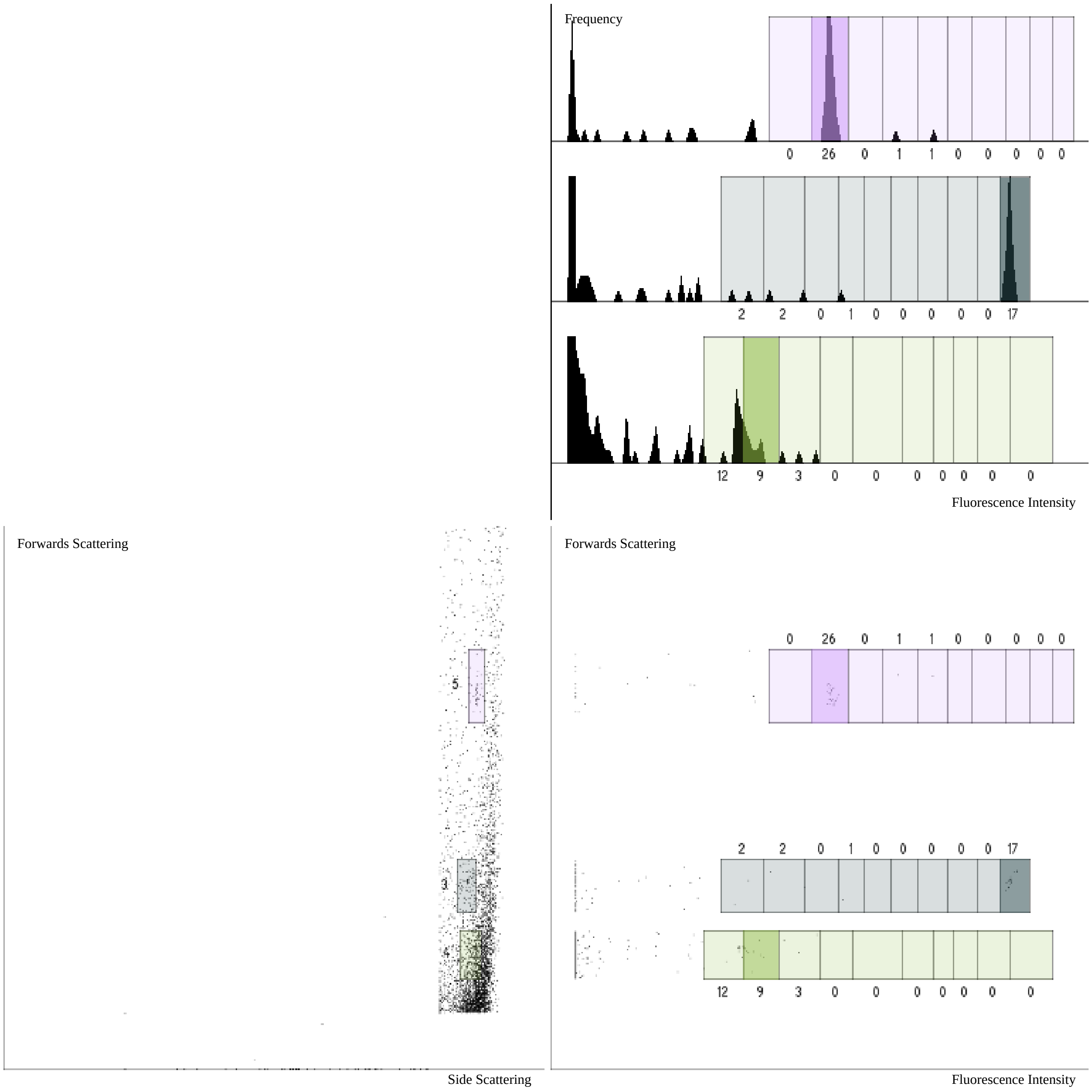

ANNEX 3: TAG DECONVOLUTION - BEAD 92

Passes flow sorting criteria: Yes

Passes tag deconvolution criteria: No

Included in protocol analysis: No

Protocol: N/A

Filename: Bin9_97

Split 1: Petrol shading

Split 2: Green shading

Split 3: Violet shading

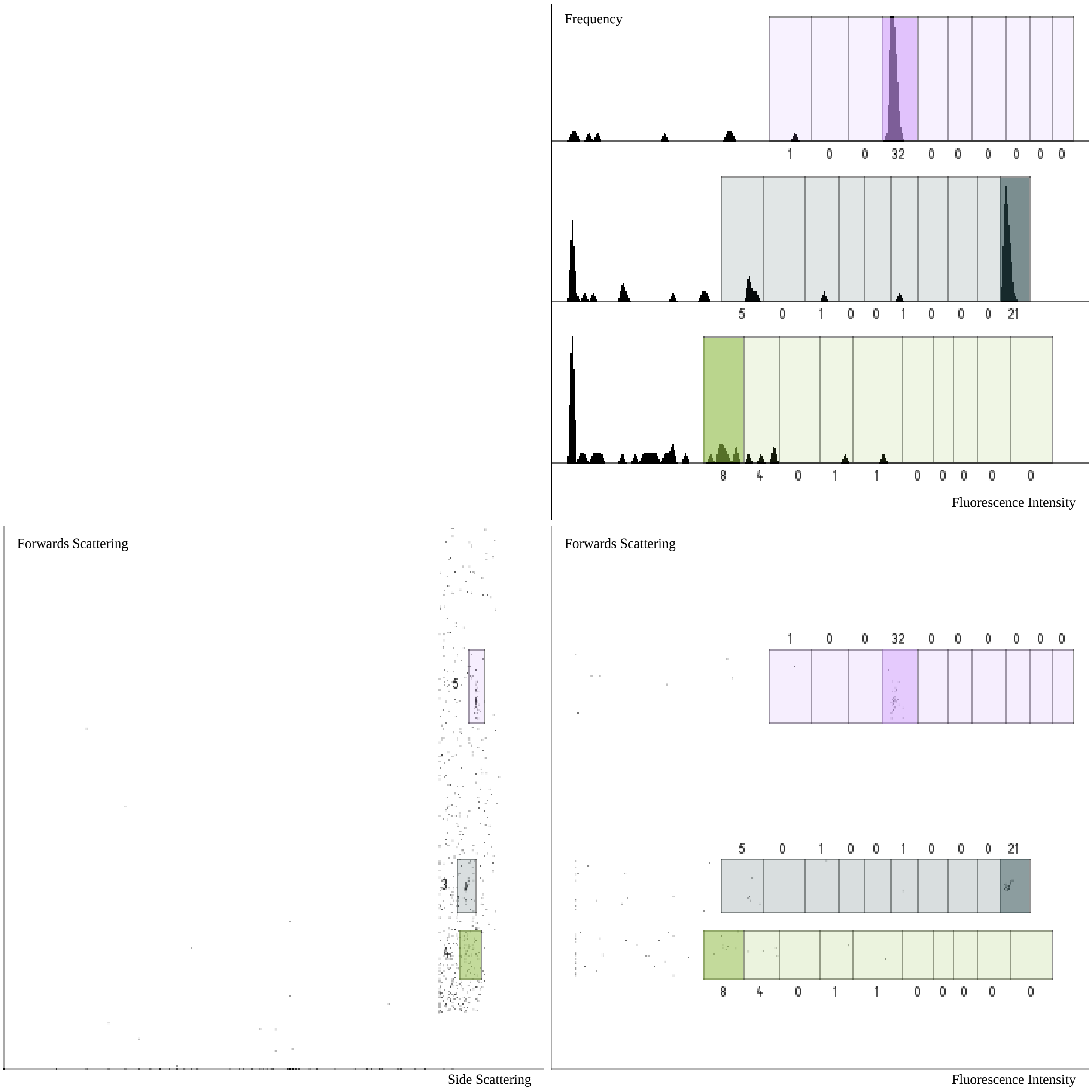

ANNEX 3: TAG DECONVOLUTION - BEAD 93

Passes flow sorting criteria: Yes

Passes tag deconvolution criteria: No

Included in protocol analysis: No

Protocol: N/A

Filename: Bin9_98

Split 1: Petrol shading

Split 2: Green shading

Split 3: Violet shading

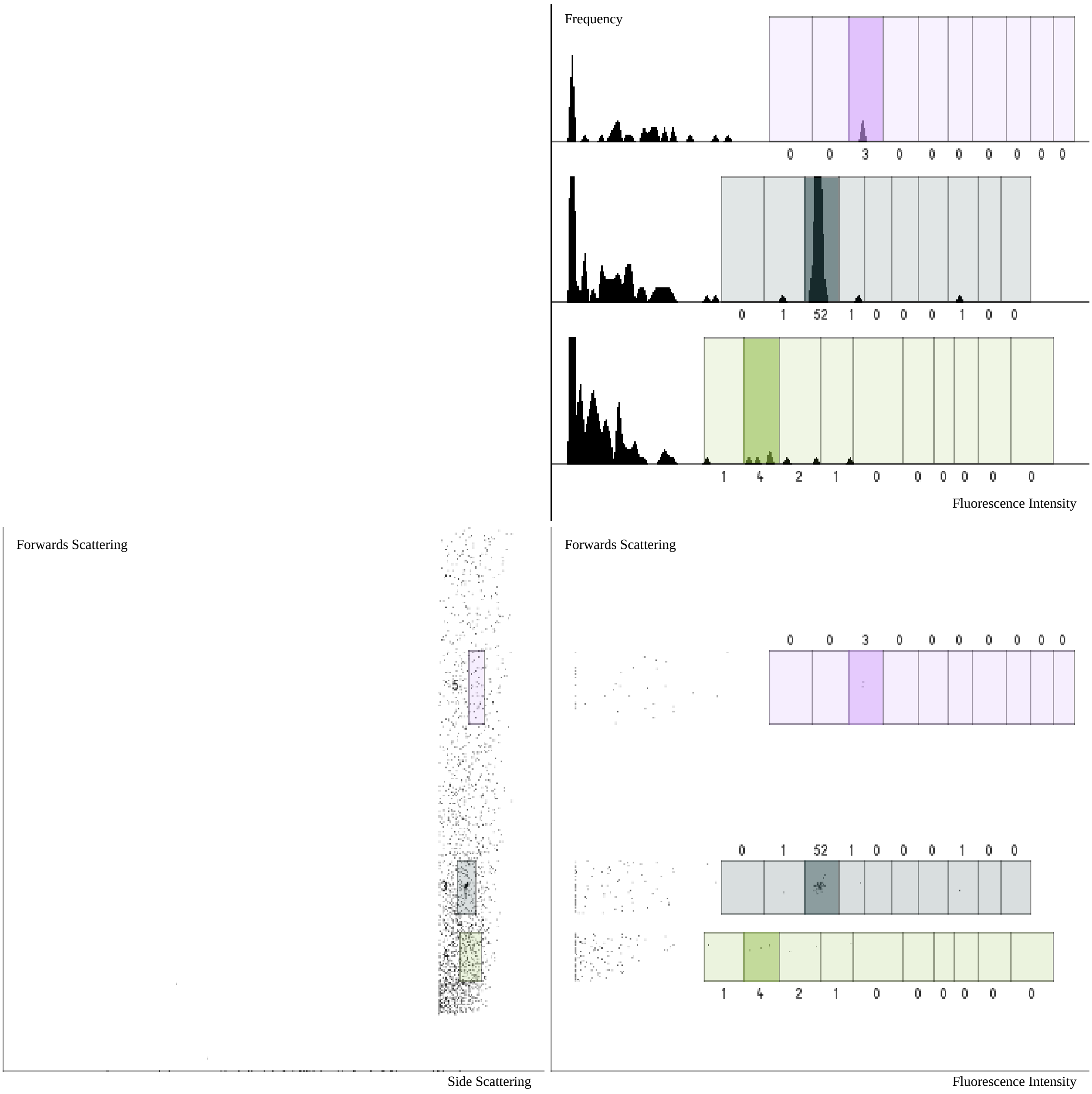

ANNEX 3: TAG DECONVOLUTION - BEAD 94

Passes flow sorting criteria: Yes

Passes tag deconvolution criteria: Yes

Included in protocol analysis: Yes

Protocol: 2, 10, 1, 9

Filename: Bin9_99

Split 1: Petrol shading

Split 2: Green shading

Split 3: Violet shading

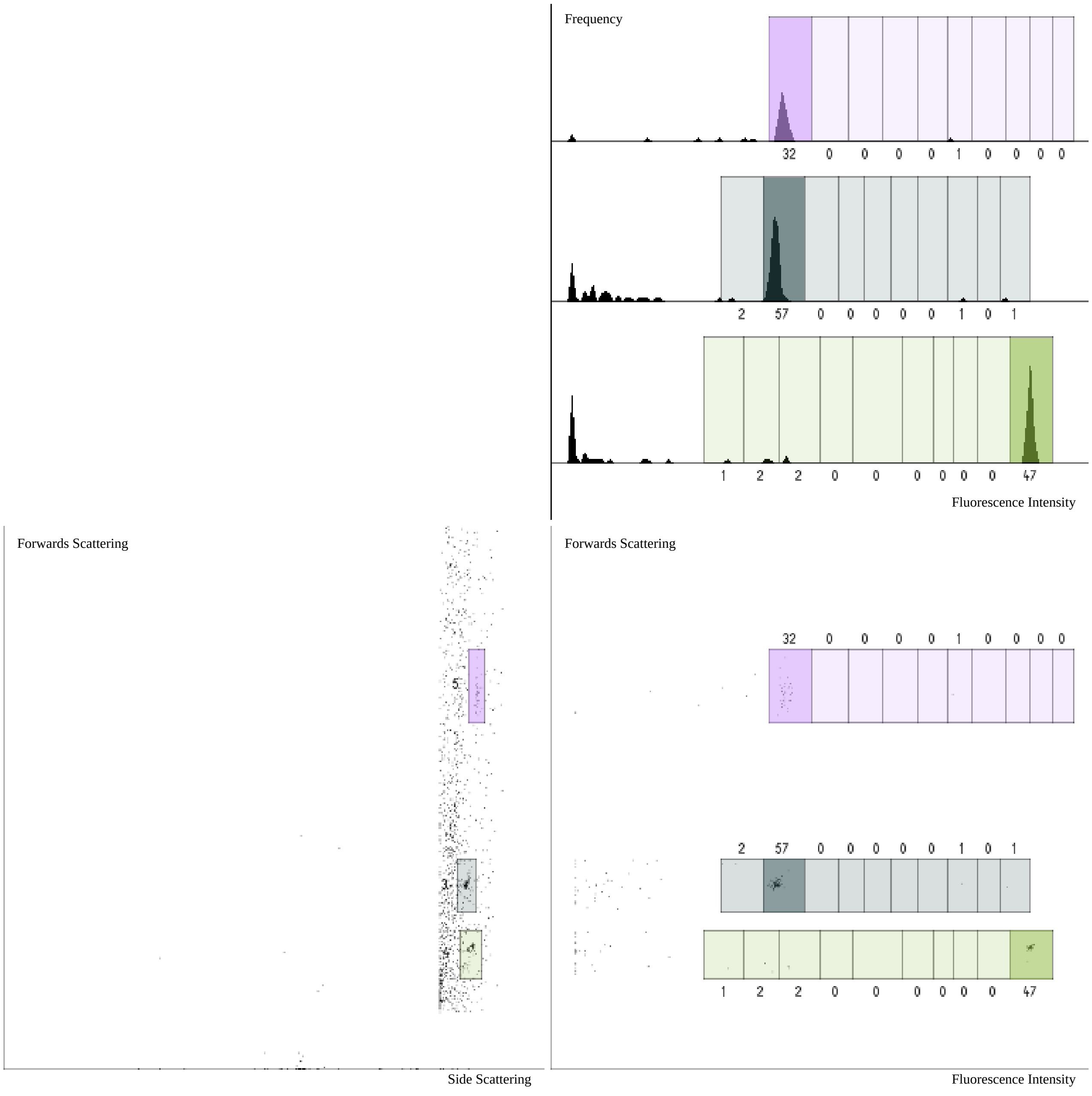

ANNEX 3: TAG DECONVOLUTION - BEAD 95

Passes flow sorting criteria: Yes

Passes tag deconvolution criteria: Yes

Included in protocol analysis: Yes

Protocol: 3, 2, 3, 9

Filename: Bin9_101

Split 1: Petrol shading

Split 2: Green shading

Split 3: Violet shading

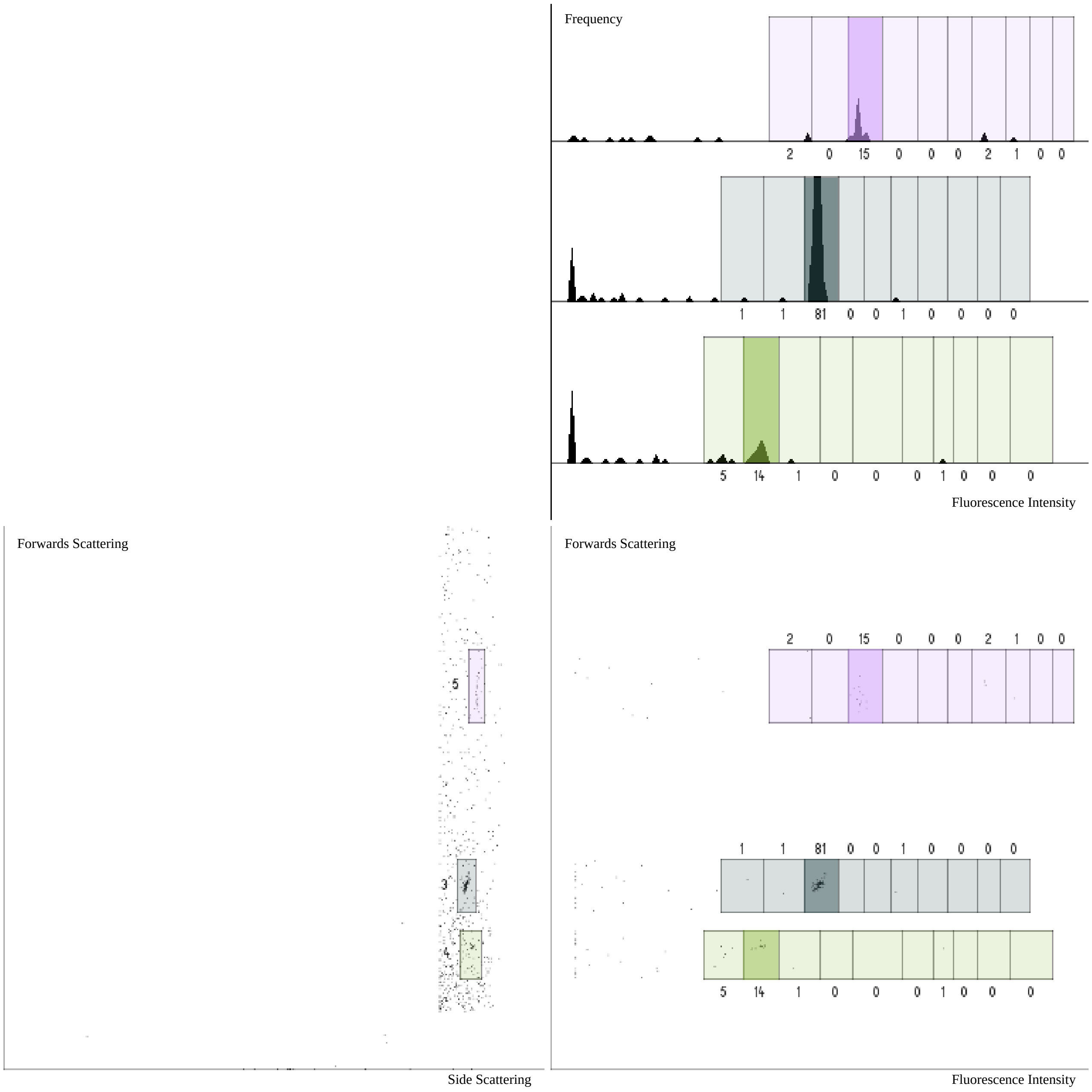

ANNEX 3: TAG DECONVOLUTION - BEAD 96

Passes flow sorting criteria: Yes

Passes tag deconvolution criteria: Yes

Included in protocol analysis: Yes

Protocol: 10, 1, 4, 9

Filename: Bin9_102

Split 1: Petrol shading

Split 2: Green shading

Split 3: Violet shading

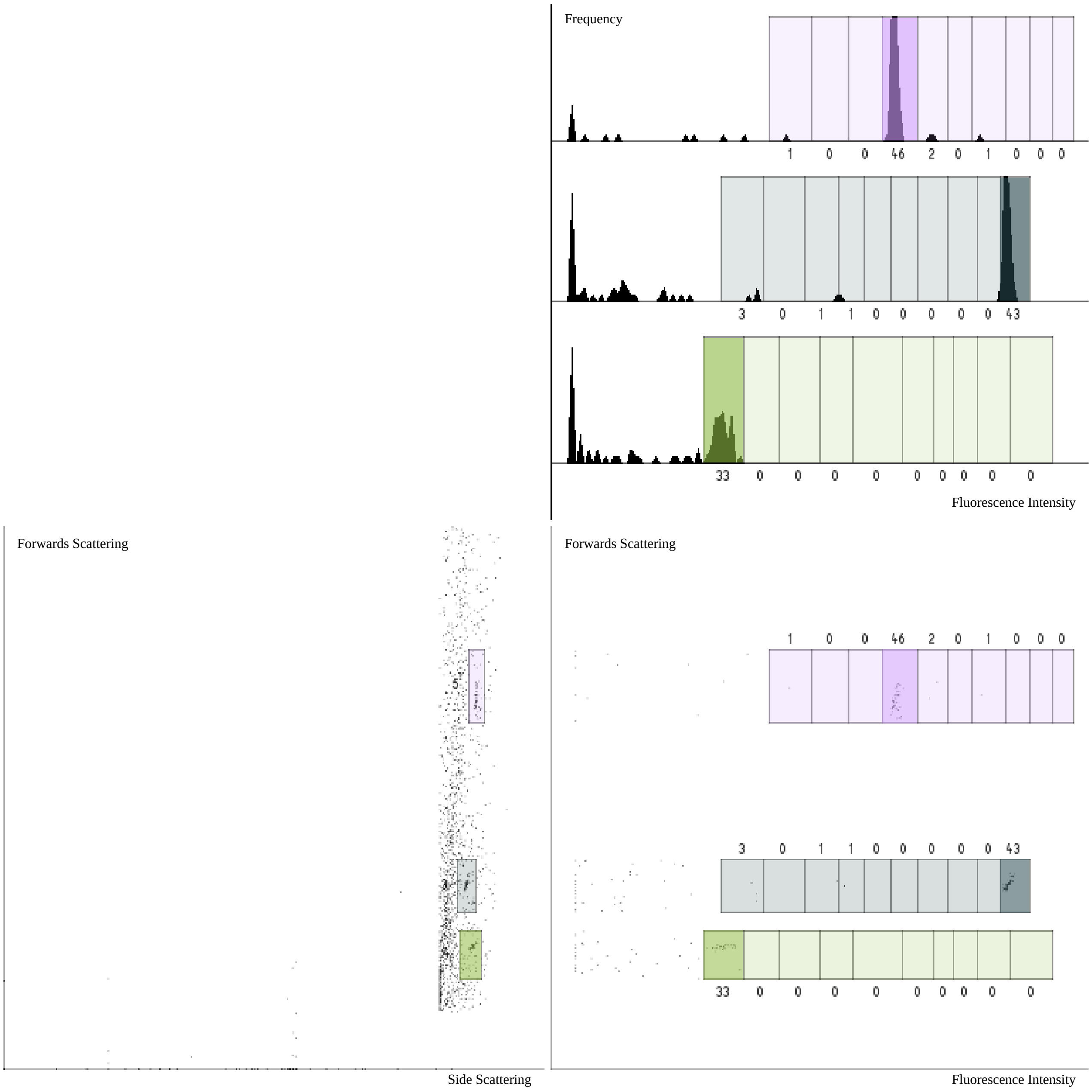

ANNEX 3: TAG DECONVOLUTION - BEAD 97

Passes flow sorting criteria: Yes

Passes tag deconvolution criteria: Yes

Included in protocol analysis: Yes

Protocol: 10, 1, 1, 1

Filename: Bin1_1RA6

Split 1: Petrol shading

Split 2: Green shading

Split 3: Violet shading

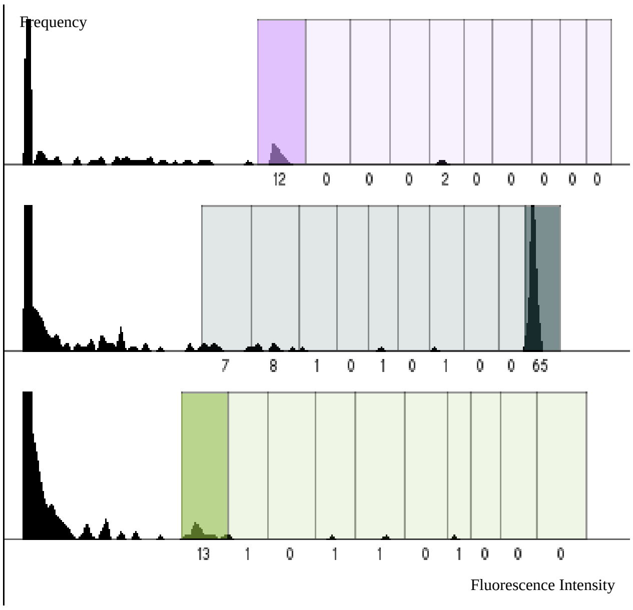

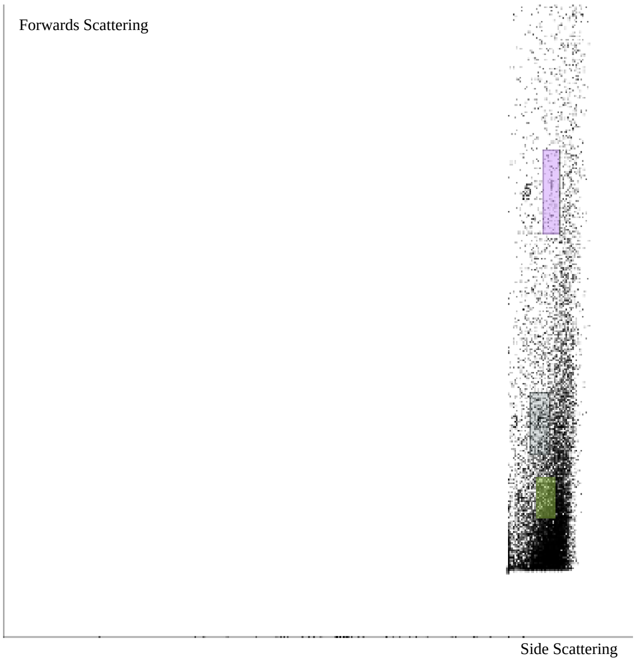

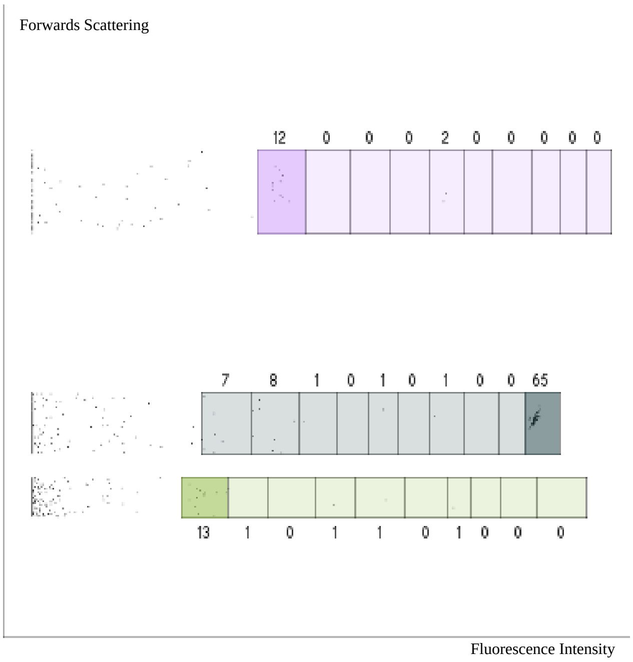

ANNEX 3: TAG DECONVOLUTION - BEAD 98

Passes flow sorting criteria: Yes

Passes tag deconvolution criteria: Yes

Included in protocol analysis: Yes

Protocol: 6, 8, 10, 1

Filename: Bin1_1RA7

Split 1: Petrol shading

Split 2: Green shading

Split 3: Violet shading

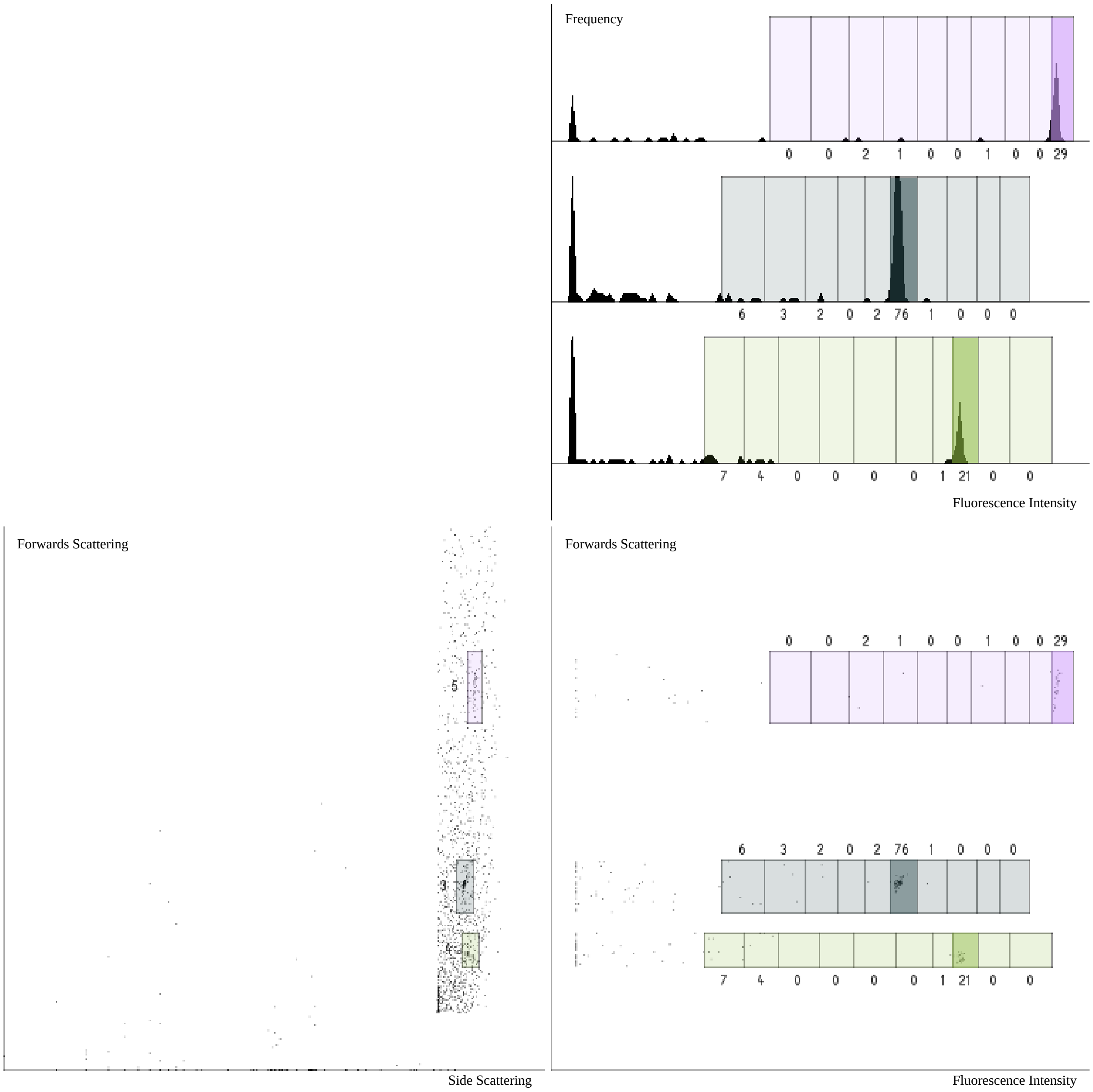

ANNEX 3: TAG DECONVOLUTION - BEAD 99

Passes flow sorting criteria: Yes

Passes tag deconvolution criteria: Yes

Included in protocol analysis: Yes

Protocol: 10, 1, 2, 1

Filename: Bin1_1RA8

Split 1: Petrol shading

Split 2: Green shading

Split 3: Violet shading

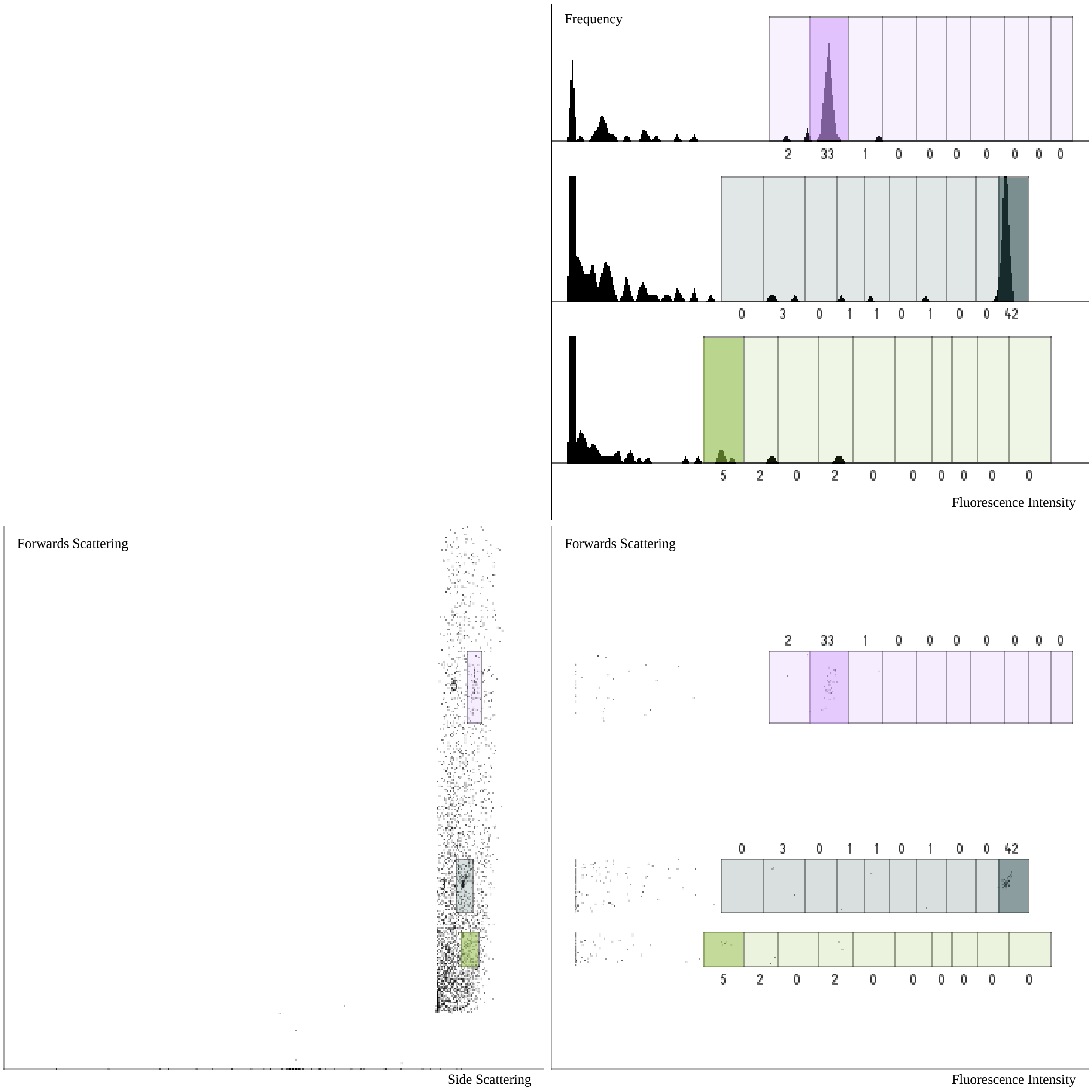

ANNEX 3: TAG DECONVOLUTION - BEAD 100

Passes flow sorting criteria: Yes

Passes tag deconvolution criteria: Yes

Included in protocol analysis: Yes

Protocol: 10, 2, 10, 1

Filename: Bin1_1RA9

Split 1: Petrol shading

Split 2: Green shading

Split 3: Violet shading

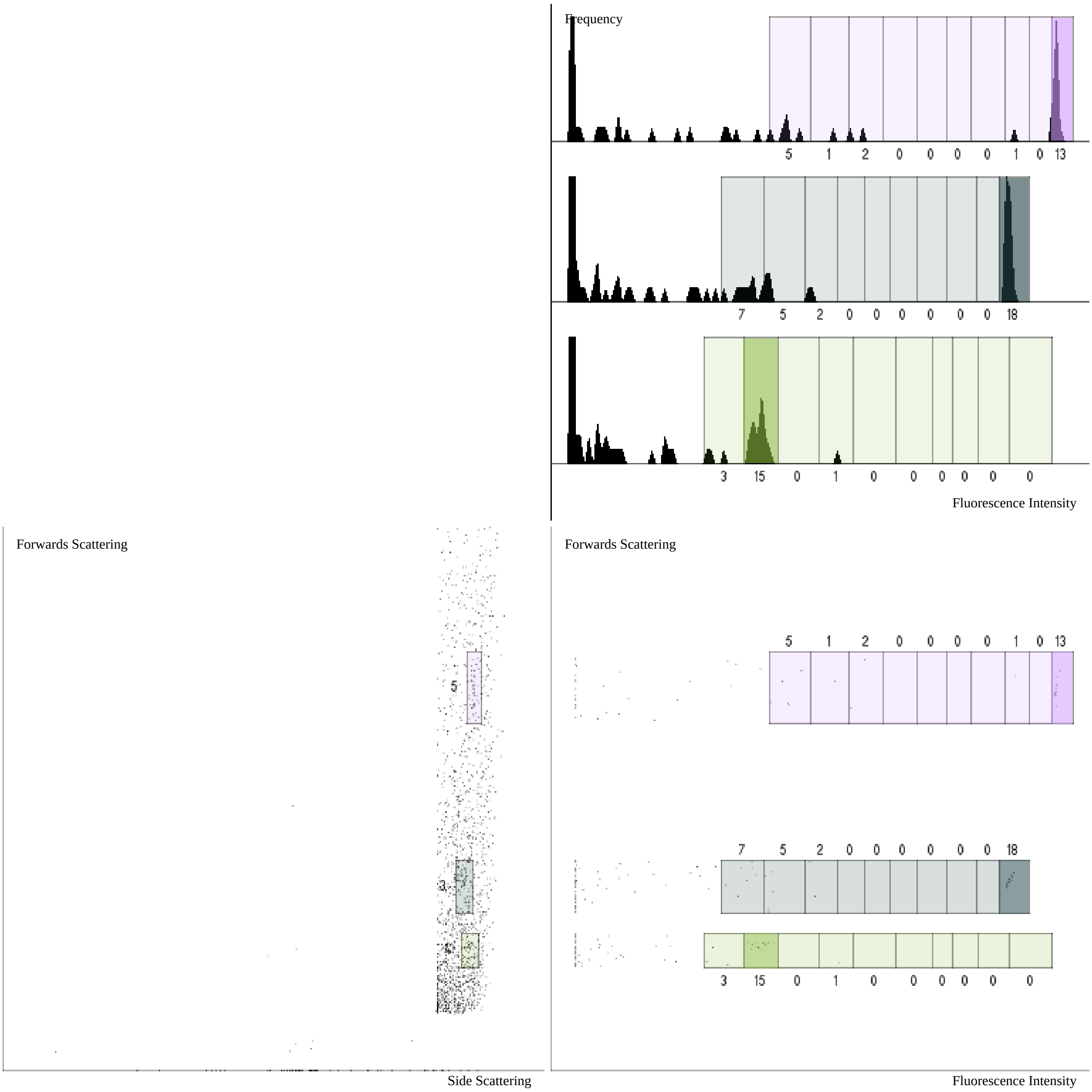

ANNEX 3: TAG DECONVOLUTION - BEAD 101

Passes flow sorting criteria: Yes

Passes tag deconvolution criteria: No

Included in protocol analysis: No

Protocol: N/A

Filename: Bin1_1RA11

Split 1: Petrol shading

Split 2: Green shading

Split 3: Violet shading

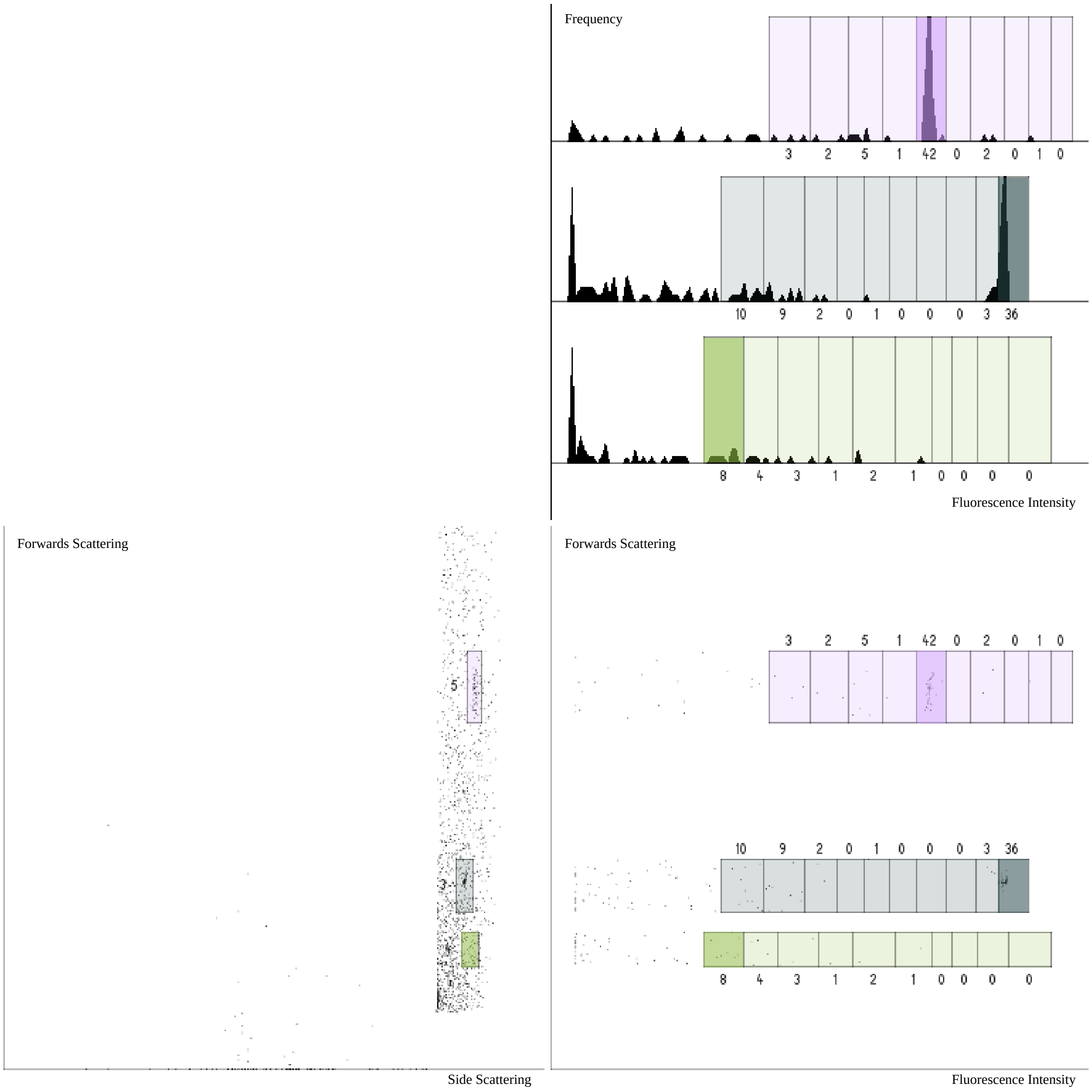

ANNEX 3: TAG DECONVOLUTION - BEAD 102

Passes flow sorting criteria: Yes

Passes tag deconvolution criteria: No

Included in protocol analysis: No

Protocol: N/A

Filename: Bin1_1RA12

Split 1: Petrol shading

Split 2: Green shading

Split 3: Violet shading

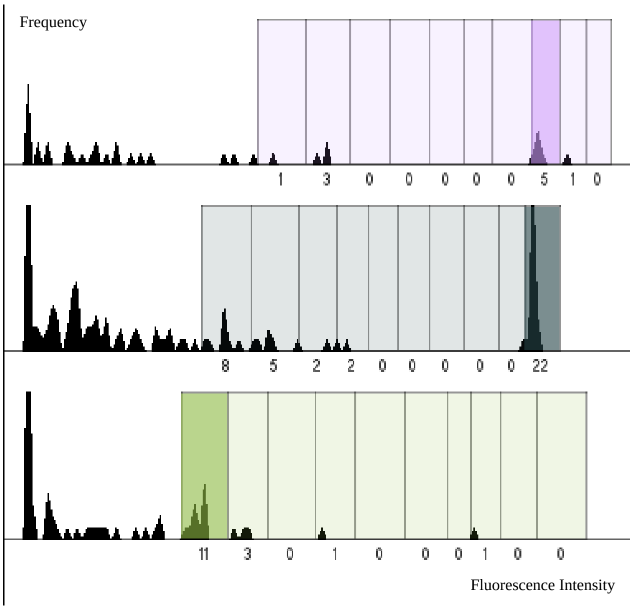

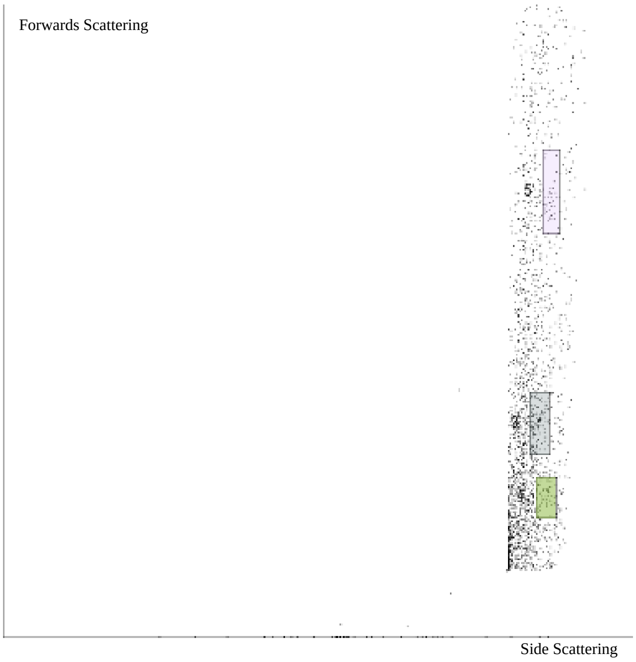

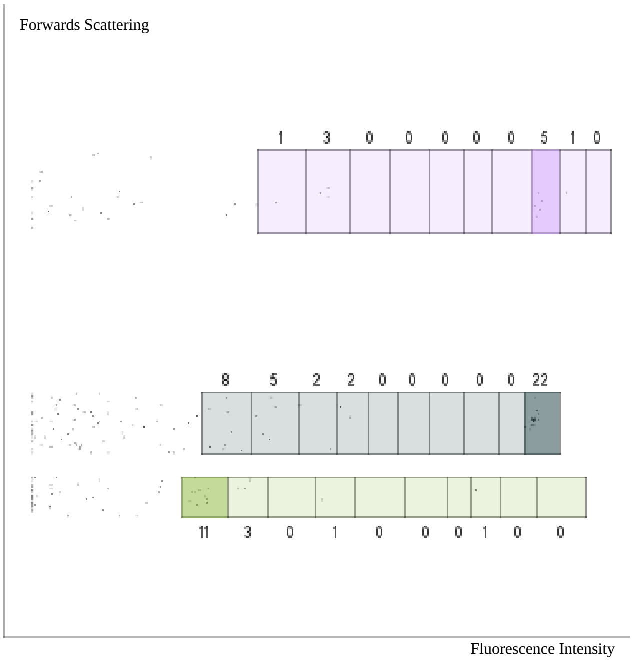

ANNEX 3: TAG DECONVOLUTION - BEAD 103

Passes flow sorting criteria: Yes

Passes tag deconvolution criteria: Yes

Included in protocol analysis: Yes

Protocol: 2, 3, 9, 7

Filename: Bin7_7R8

Split 1: Petrol shading

Split 2: Green shading

Split 3: Violet shading

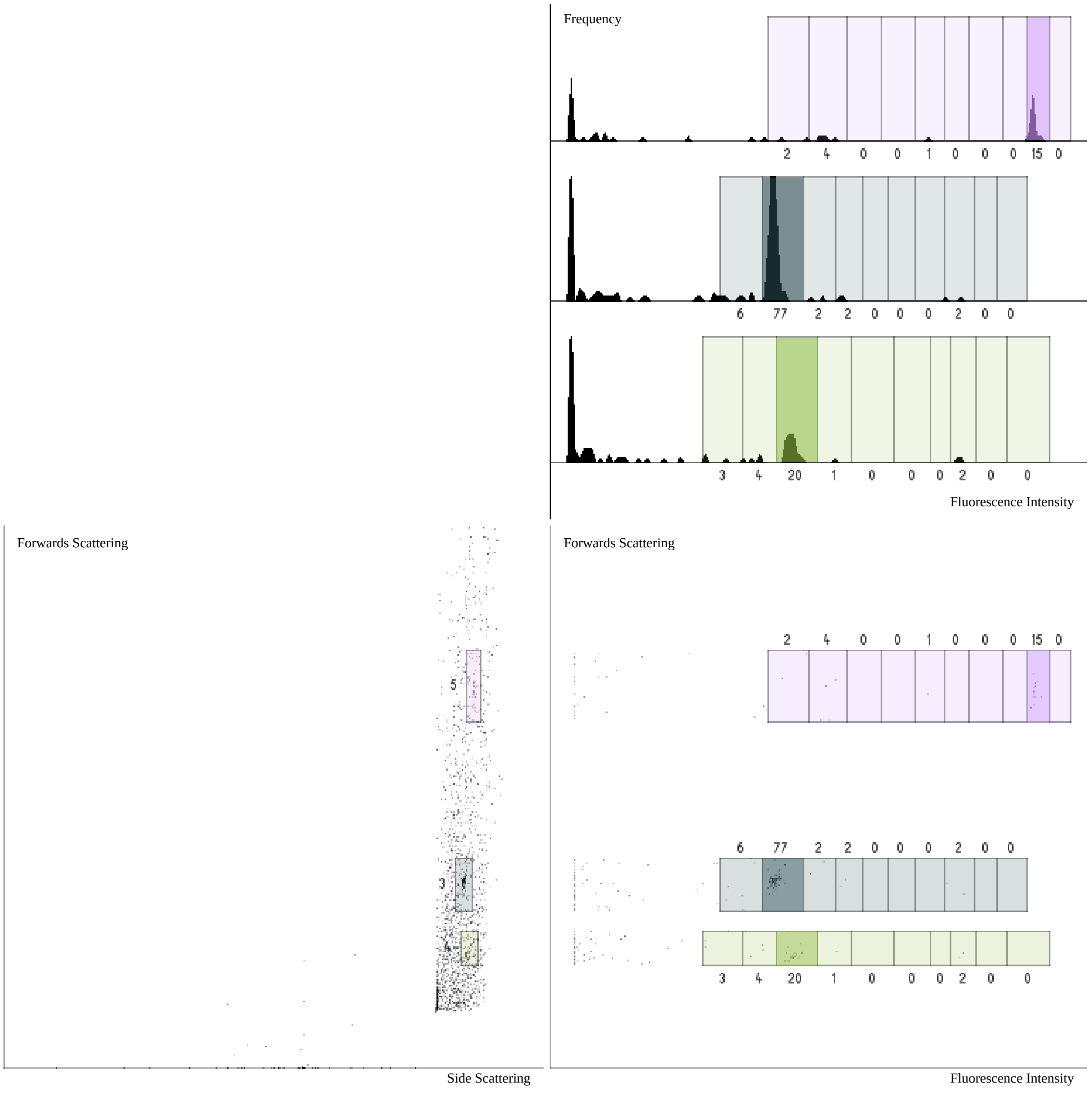

ANNEX 3: TAG DECONVOLUTION - BEAD 104

Passes flow sorting criteria: Yes

Passes tag deconvolution criteria: Yes

Included in protocol analysis: Yes

Protocol: 10, 9, 3, 7

Filename: Bin7_7R9

Split 1: Petrol shading

Split 2: Green shading

Split 3: Violet shading

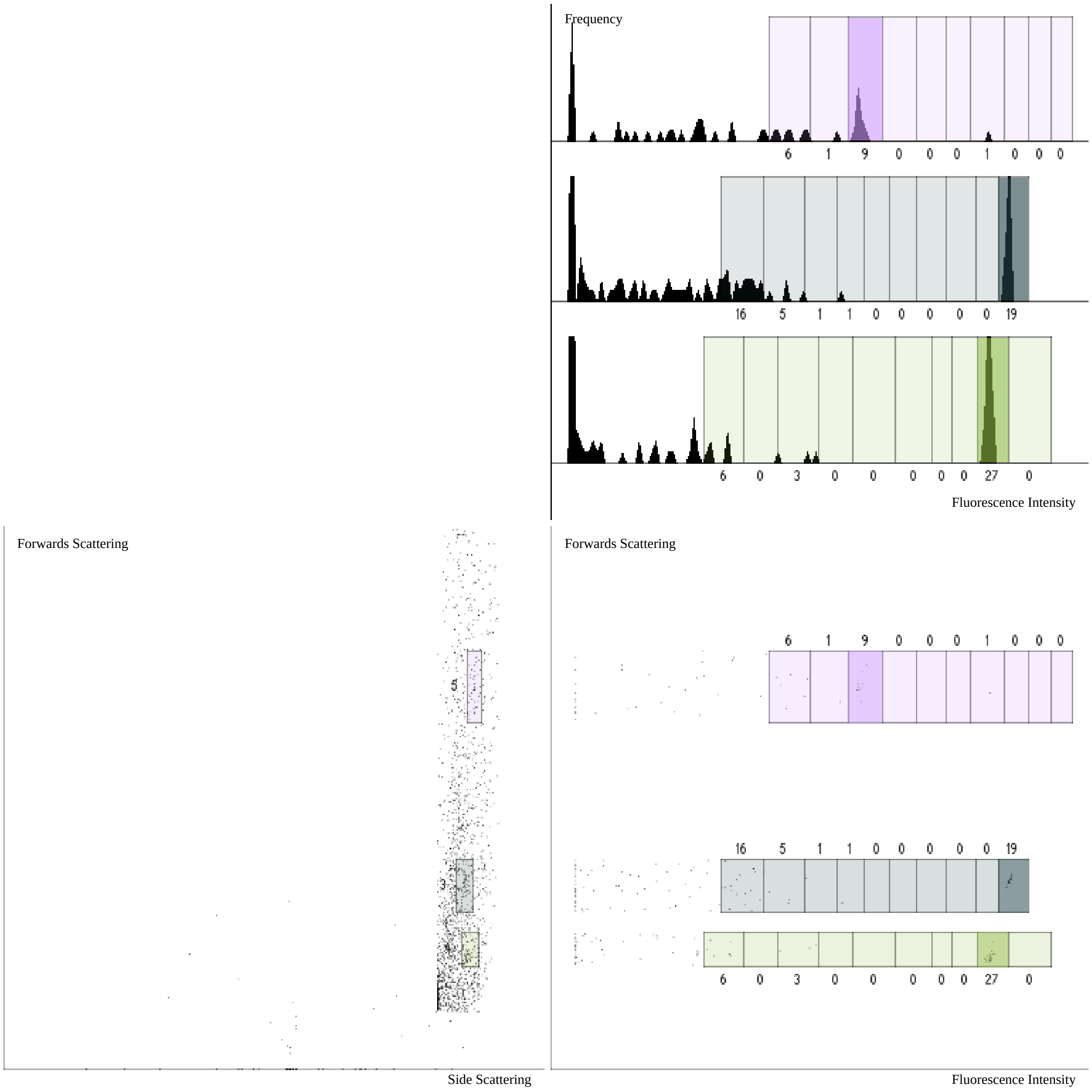

ANNEX 3: TAG DECONVOLUTION - BEAD 105

Passes flow sorting criteria: Yes

Passes tag deconvolution criteria: Yes

Included in protocol analysis: Yes

Protocol: 2, 8, 7, 7

Filename: Bin7_70

Split 1: Petrol shading

Split 2: Green shading

Split 3: Violet shading

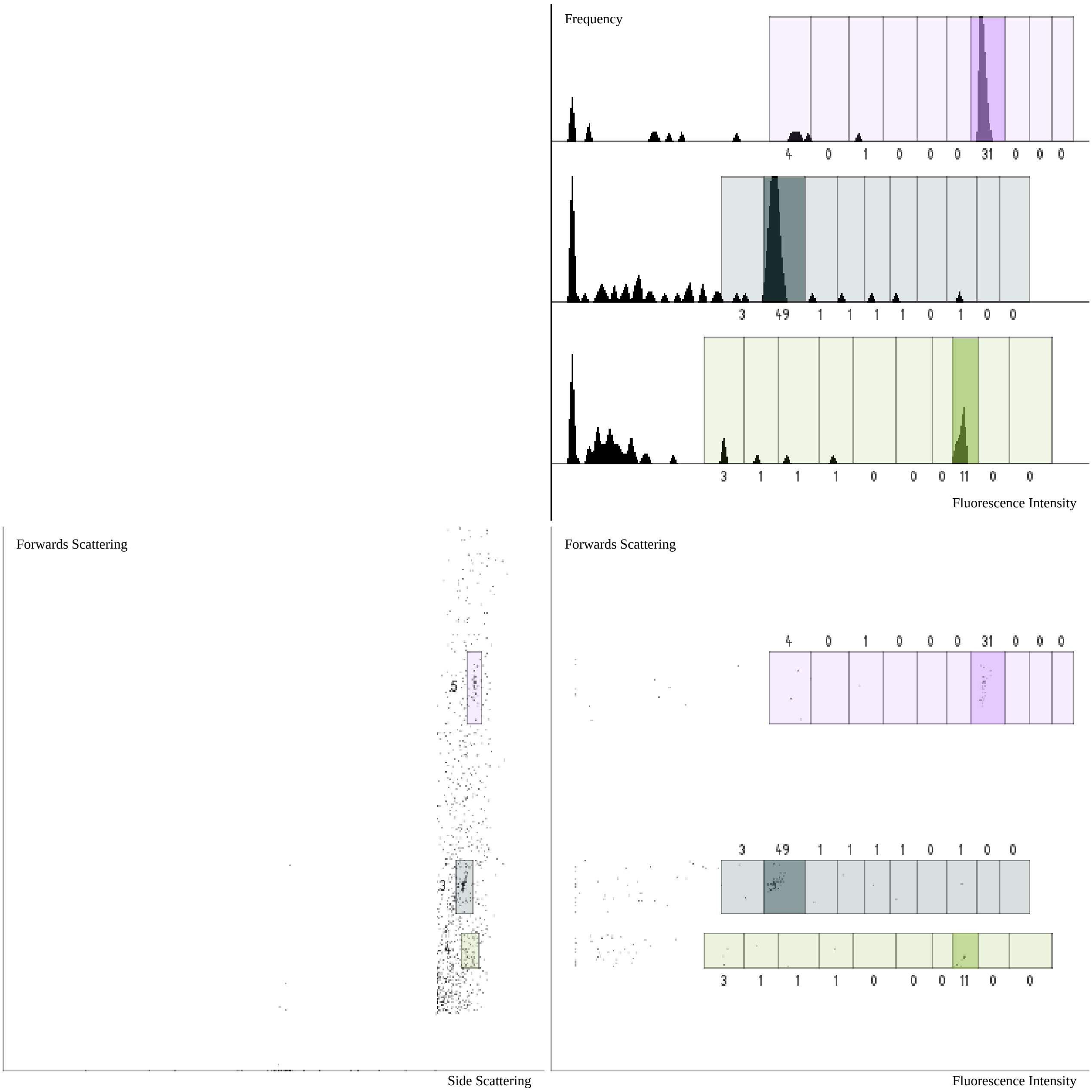

ANNEX 3: TAG DECONVOLUTION - BEAD 106

Passes flow sorting criteria: Yes

Passes tag deconvolution criteria: Yes

Included in protocol analysis: Yes

Protocol: 6, 1, 3, 7

Filename: Bin7_110

Split 1: Petrol shading

Split 2: Green shading

Split 3: Violet shading

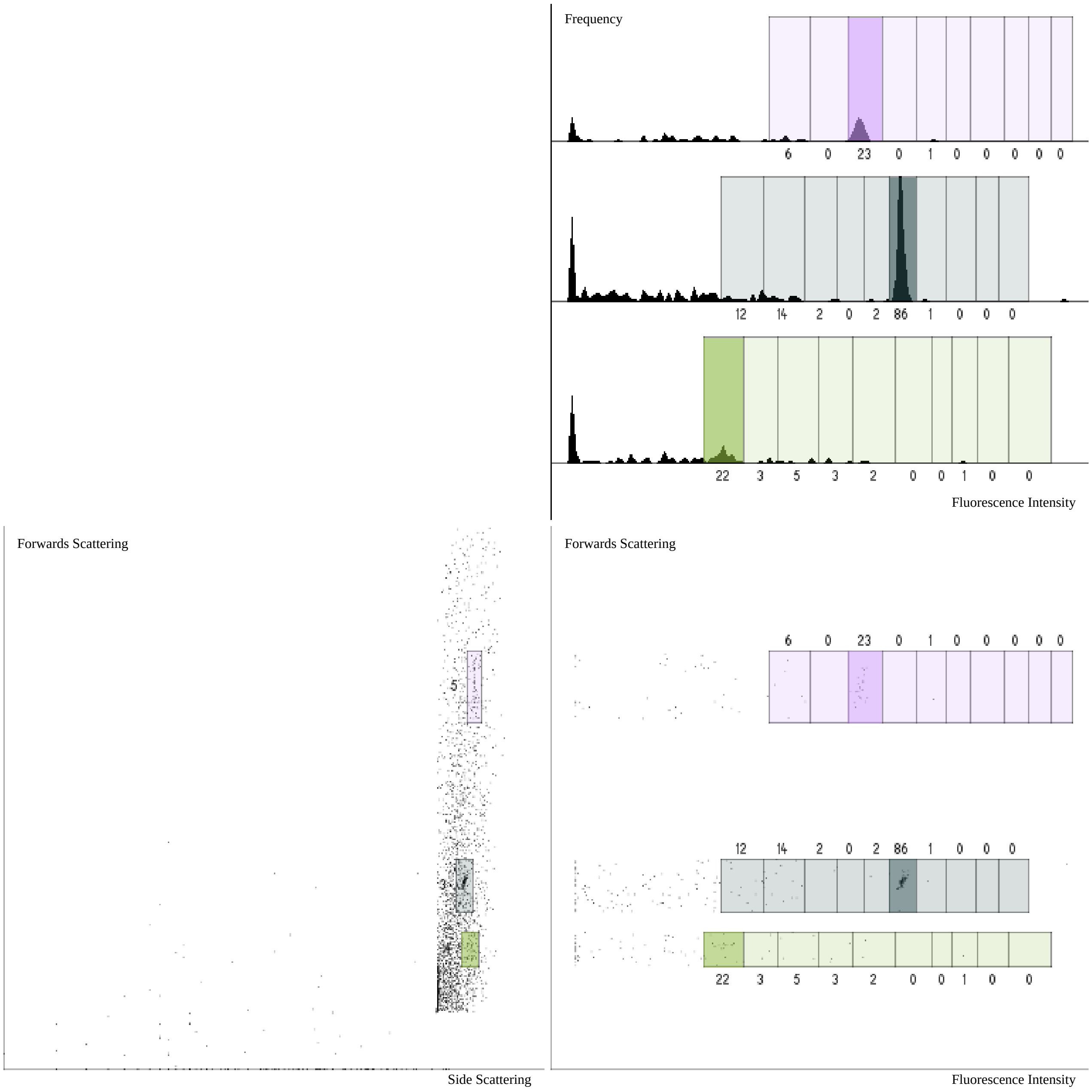

ANNEX 3: TAG DECONVOLUTION - BEAD 107

Passes flow sorting criteria: Yes

Passes tag deconvolution criteria: No

Included in protocol analysis: No

Protocol: N/A

Filename: Bin1_1RB12

Split 1: Petrol shading

Split 2: Green shading

Split 3: Violet shading

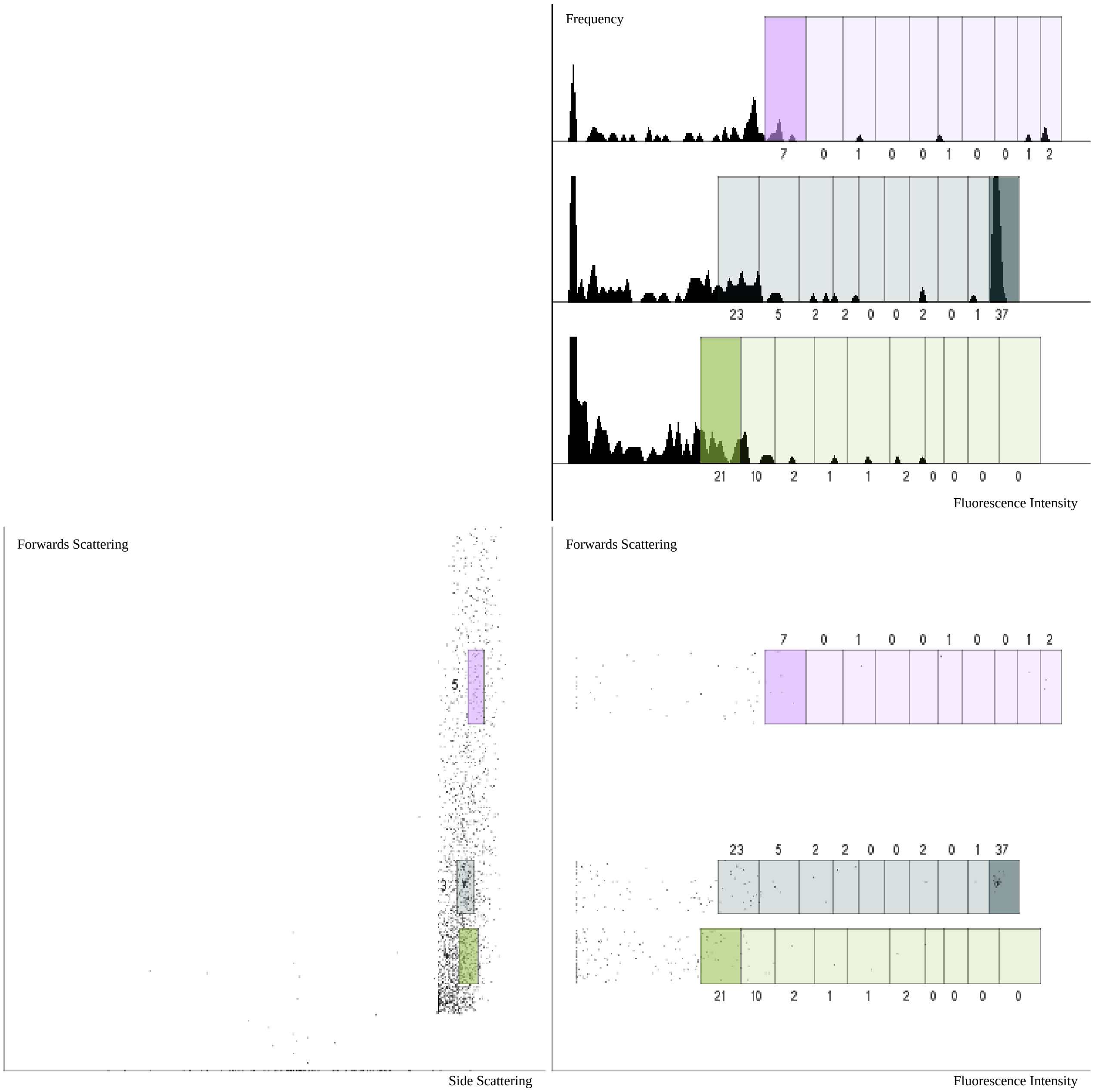

ANNEX 3: TAG DECONVOLUTION - BEAD 108

Passes flow sorting criteria: Yes

Passes tag deconvolution criteria: Yes

Included in protocol analysis: Yes

Protocol: 3, 6, 8, 4

Filename: Bin4_4R1

Split 1: Petrol shading

Split 2: Green shading

Split 3: Violet shading

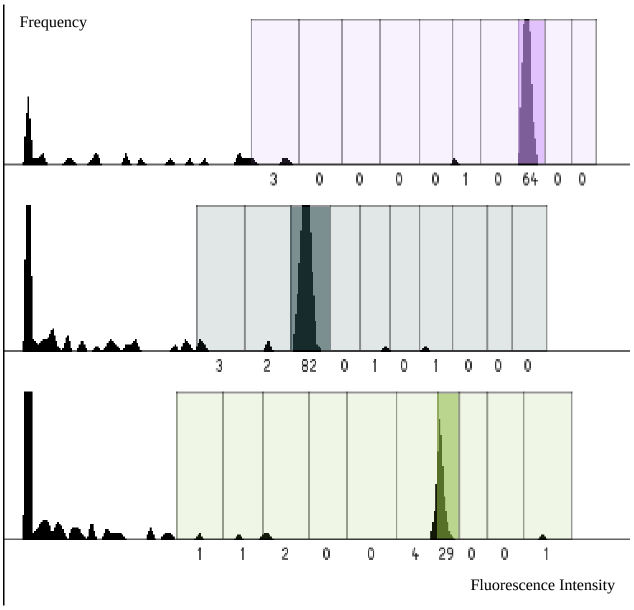

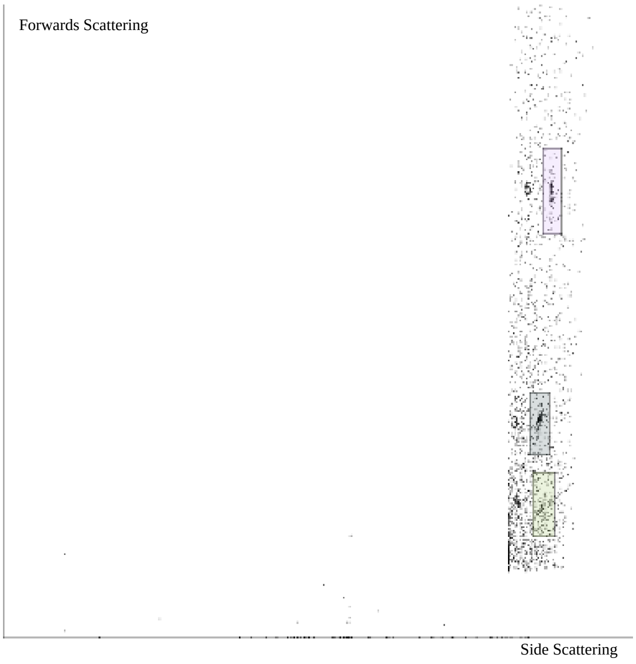

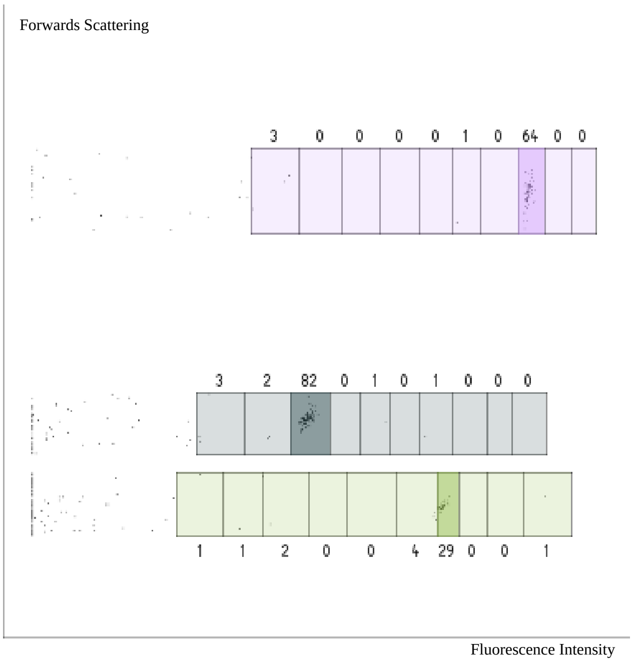

ANNEX 3: TAG DECONVOLUTION - BEAD 109

Passes flow sorting criteria: Yes

Passes tag deconvolution criteria: Yes

Included in protocol analysis: Yes

Protocol: 9, 6, 1, 4

Filename: Bin4_4R2

Split 1: Petrol shading

Split 2: Green shading

Split 3: Violet shading

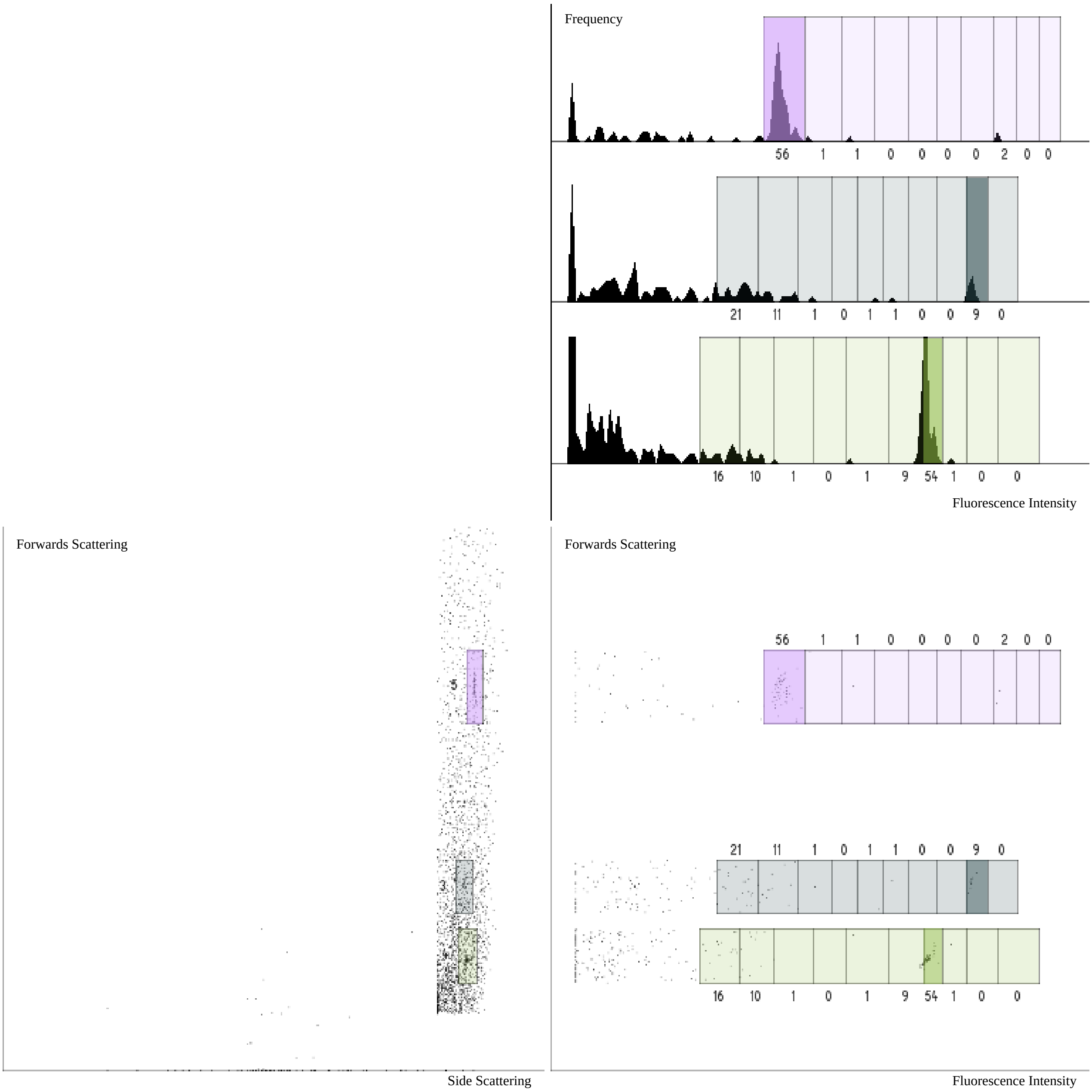

ANNEX 3: TAG DECONVOLUTION - BEAD 110

Passes flow sorting criteria: Yes

Passes tag deconvolution criteria: No

Included in protocol analysis: No

Protocol: N/A

Filename: Bin7_69

Split 1: Petrol shading

Split 2: Green shading

Split 3: Violet shading

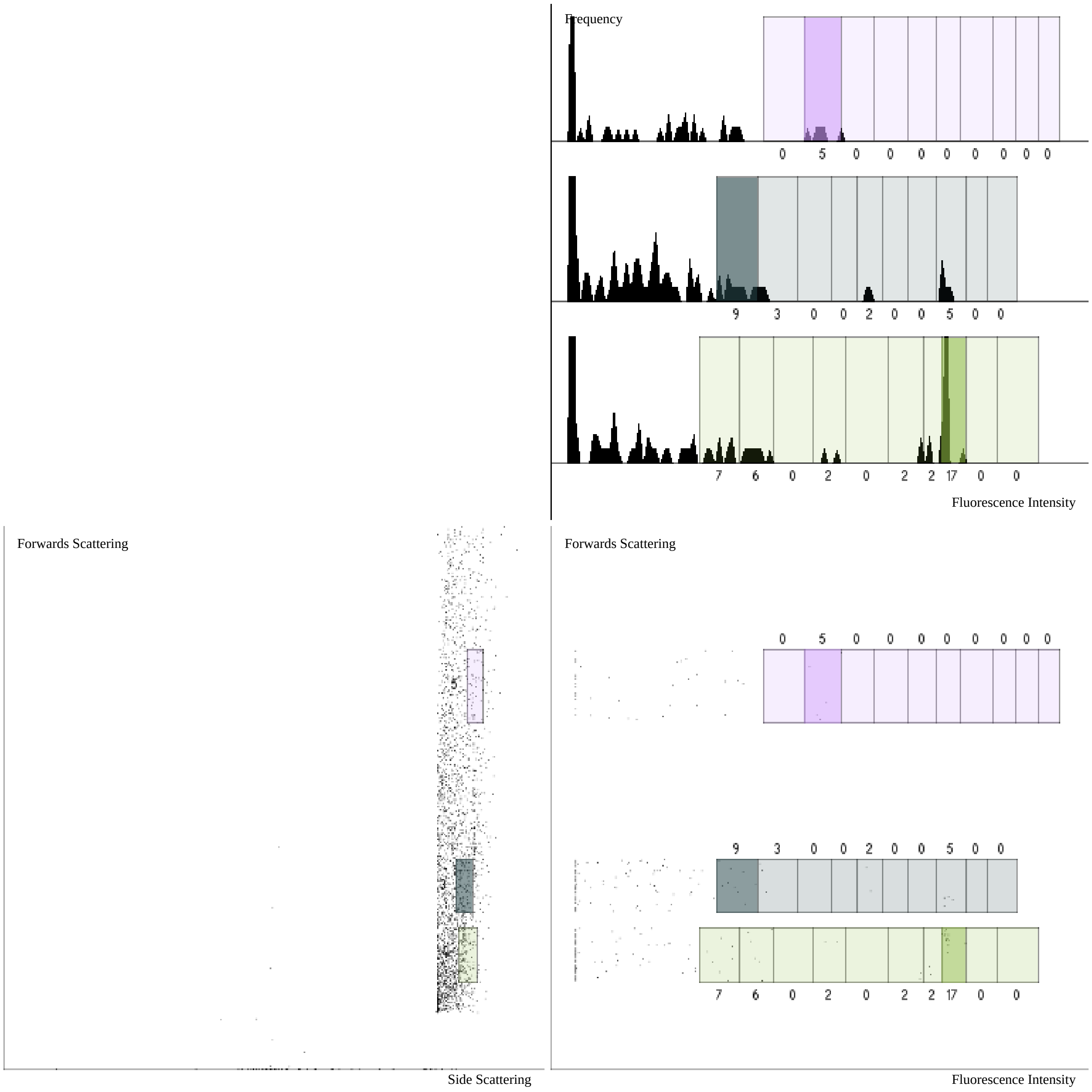

ANNEX 3: TAG DECONVOLUTION - BEAD 111

Passes flow sorting criteria: Yes

Passes tag deconvolution criteria: No

Included in protocol analysis: No

Protocol: N/A

Filename: Bin7_71

Split 1: Petrol shading

Split 2: Green shading

Split 3: Violet shading

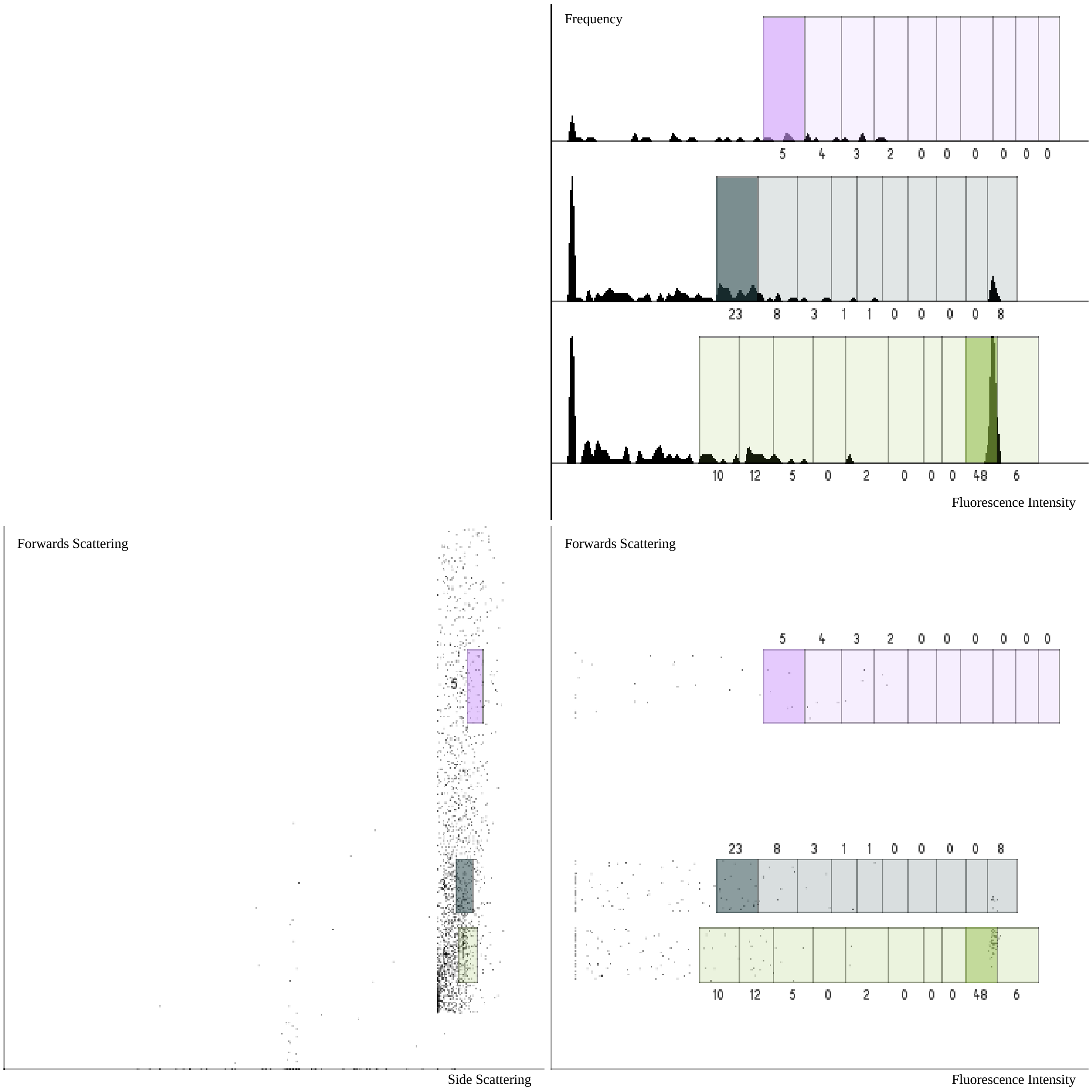

ANNEX 3: TAG DECONVOLUTION - BEAD 112

Passes flow sorting criteria: Yes

Passes tag deconvolution criteria: No

Included in protocol analysis: No

Protocol: N/A

Filename: Bin7_109

Split 1: Petrol shading

Split 2: Green shading

Split 3: Violet shading

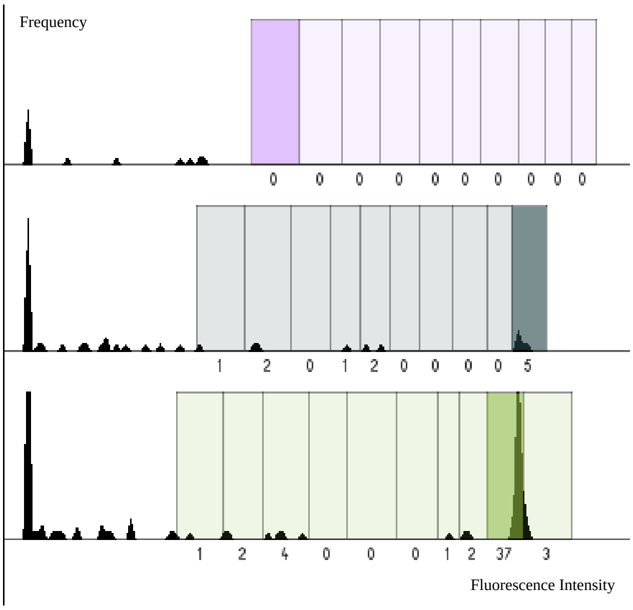

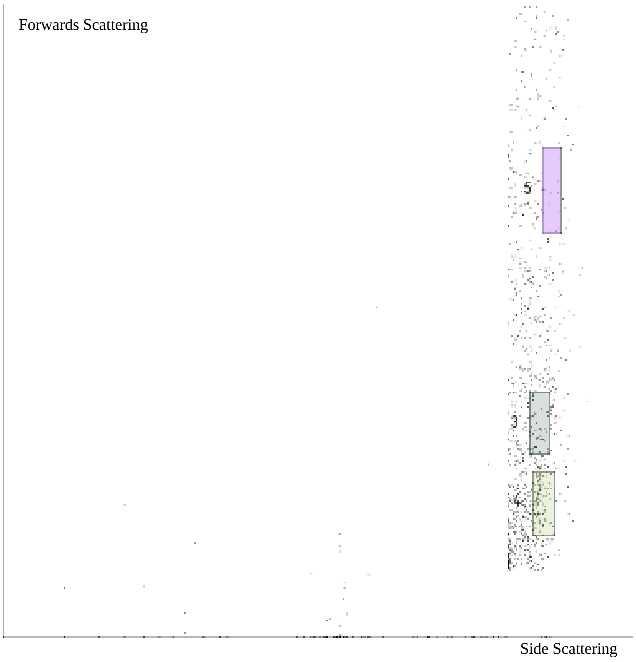

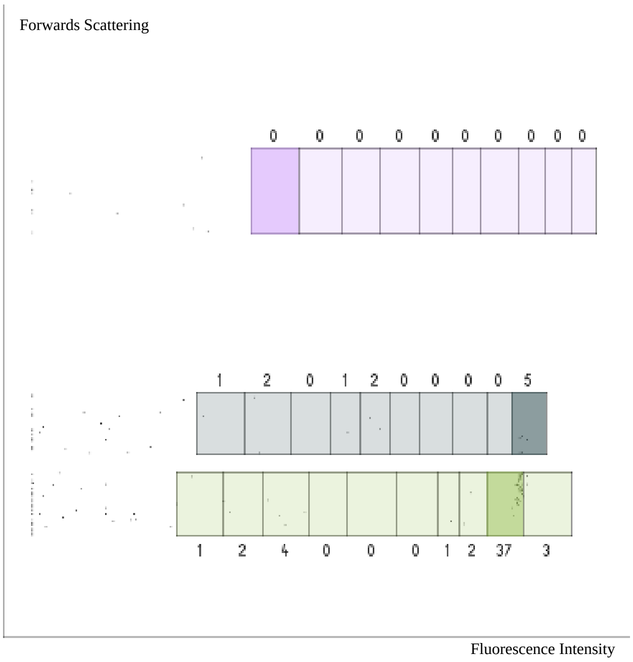

ANNEX 3: TAG DECONVOLUTION - BEAD 113

Passes flow sorting criteria: Yes

Passes tag deconvolution criteria: No

Included in protocol analysis: No

Protocol: N/A

Filename: Bin7_111

Split 1: Petrol shading

Split 2: Green shading

Split 3: Violet shading

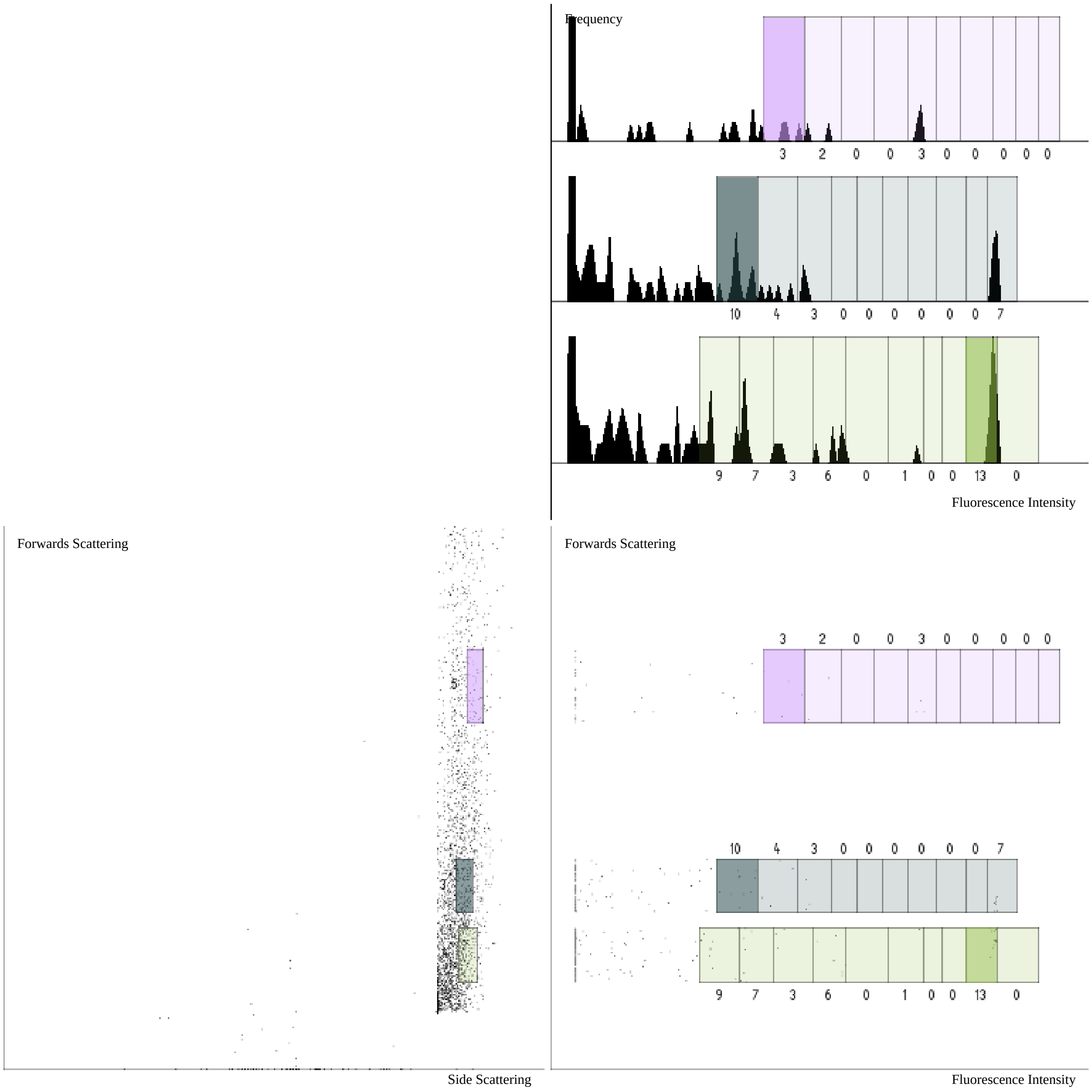

ANNEX 3: TAG DECONVOLUTION - BEAD 114

Passes flow sorting criteria: Yes

Passes tag deconvolution criteria: No

Included in protocol analysis: No

Protocol: N/A

Filename: Bin7_112

Split 1: Petrol shading

Split 2: Green shading

Split 3: Violet shading

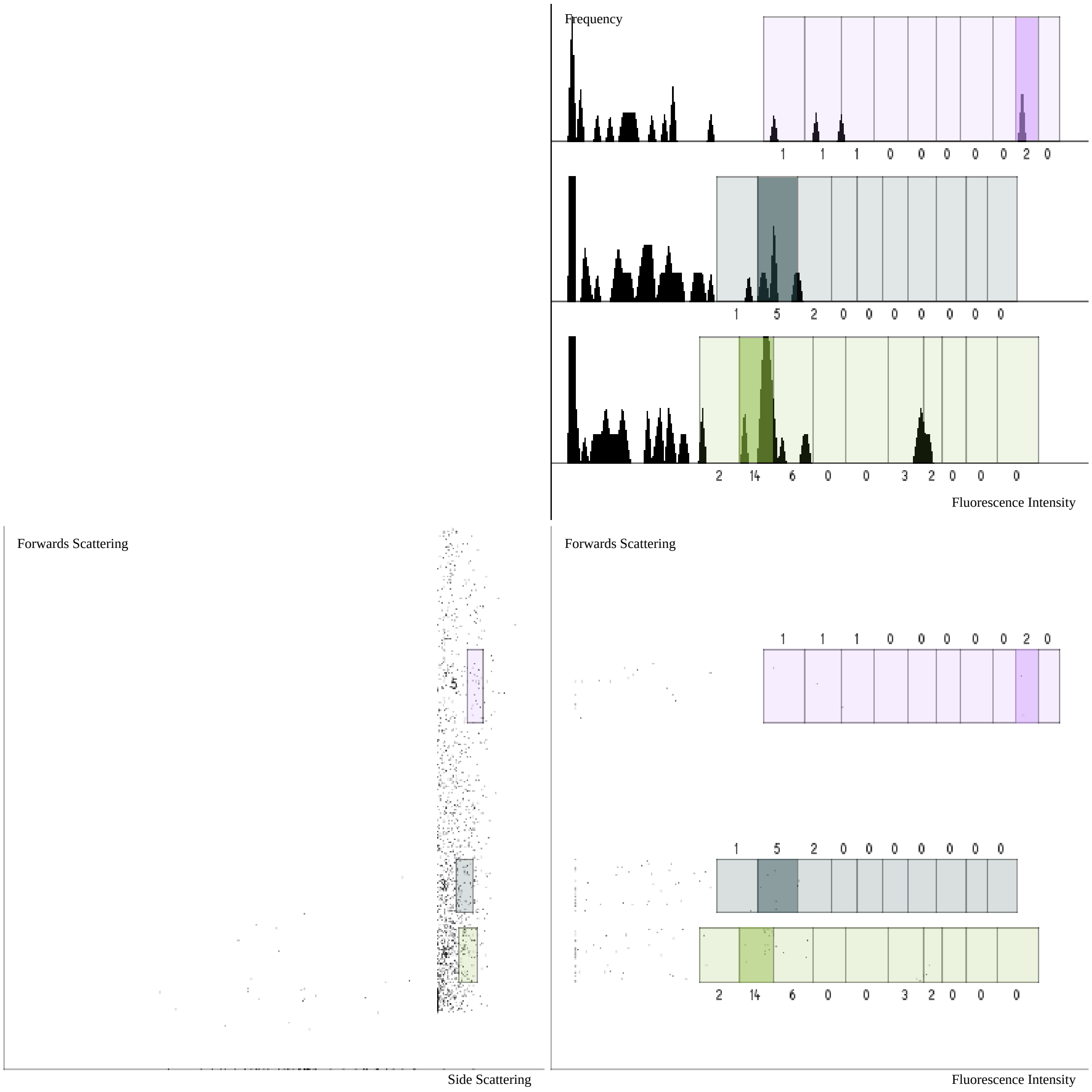

ANNEX 3: TAG DECONVOLUTION - BEAD 115

Passes flow sorting criteria: Yes

Passes tag deconvolution criteria: No

Included in protocol analysis: No

Protocol: N/A

Filename: Bin7_113

Split 1: Petrol shading

Split 2: Green shading

Split 3: Violet shading

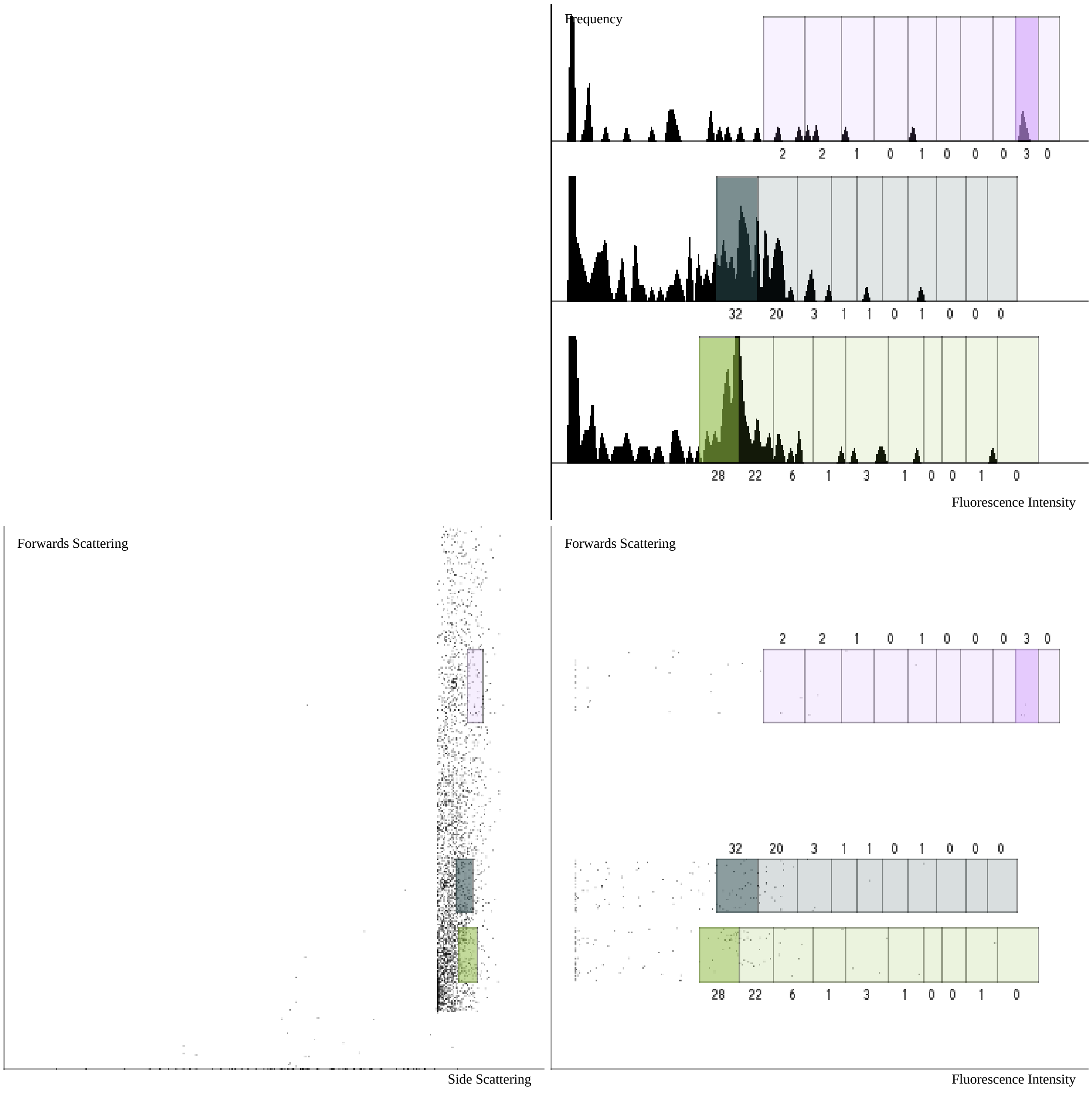

ANNEX 3: TAG DECONVOLUTION - BEAD 116

Passes flow sorting criteria: Yes

Passes tag deconvolution criteria: No

Included in protocol analysis: No

Protocol: N/A

Filename: Bin7_115

Split 1: Petrol shading

Split 2: Green shading

Split 3: Violet shading

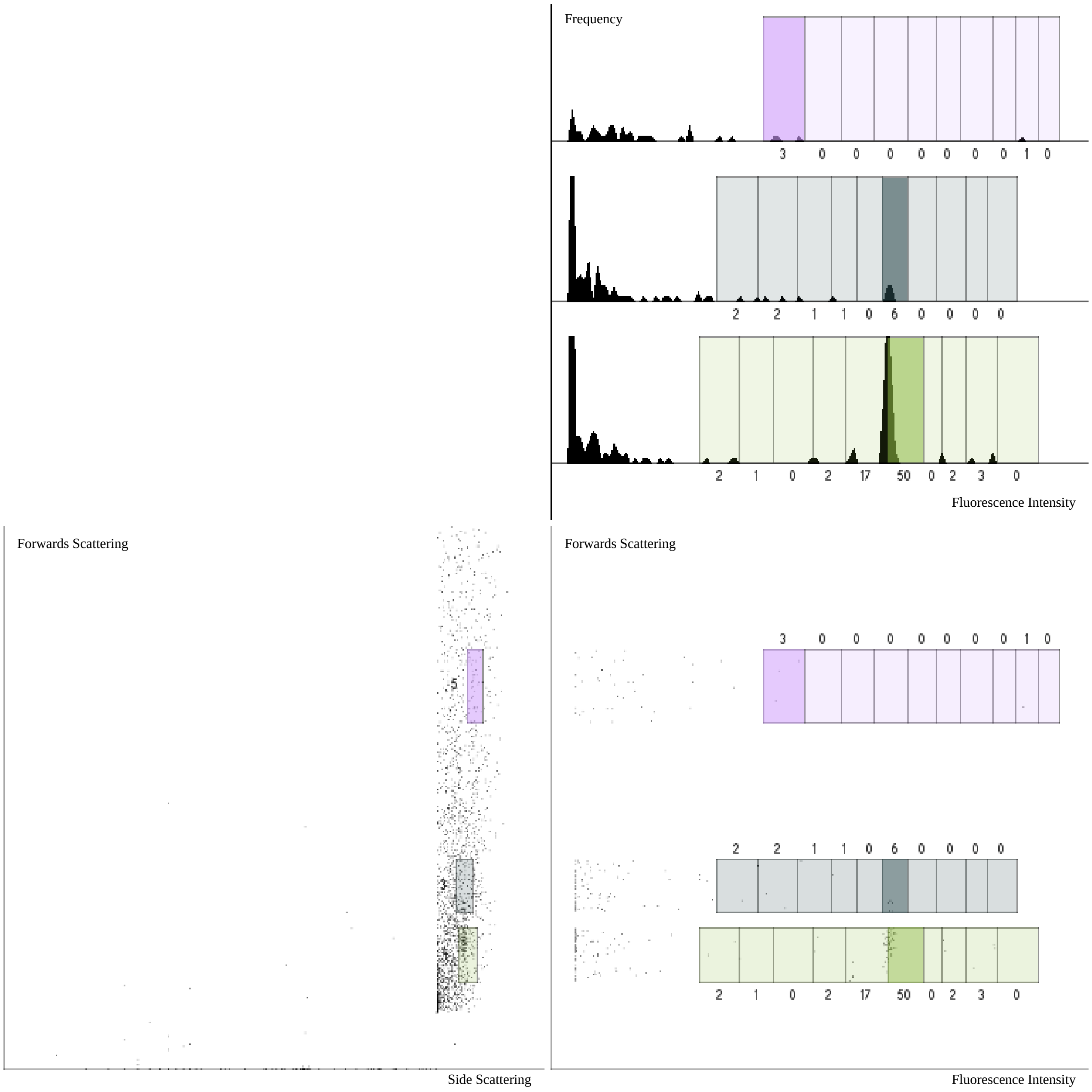

ANNEX 3: TAG DECONVOLUTION - BEAD 117

Passes flow sorting criteria: Yes

Passes tag deconvolution criteria: Yes

Included in protocol analysis: Yes

Protocol: 10, 1, 4, 7

Filename: Bin7_52

Split 1: Petrol shading

Split 2: Green shading

Split 3: Violet shading

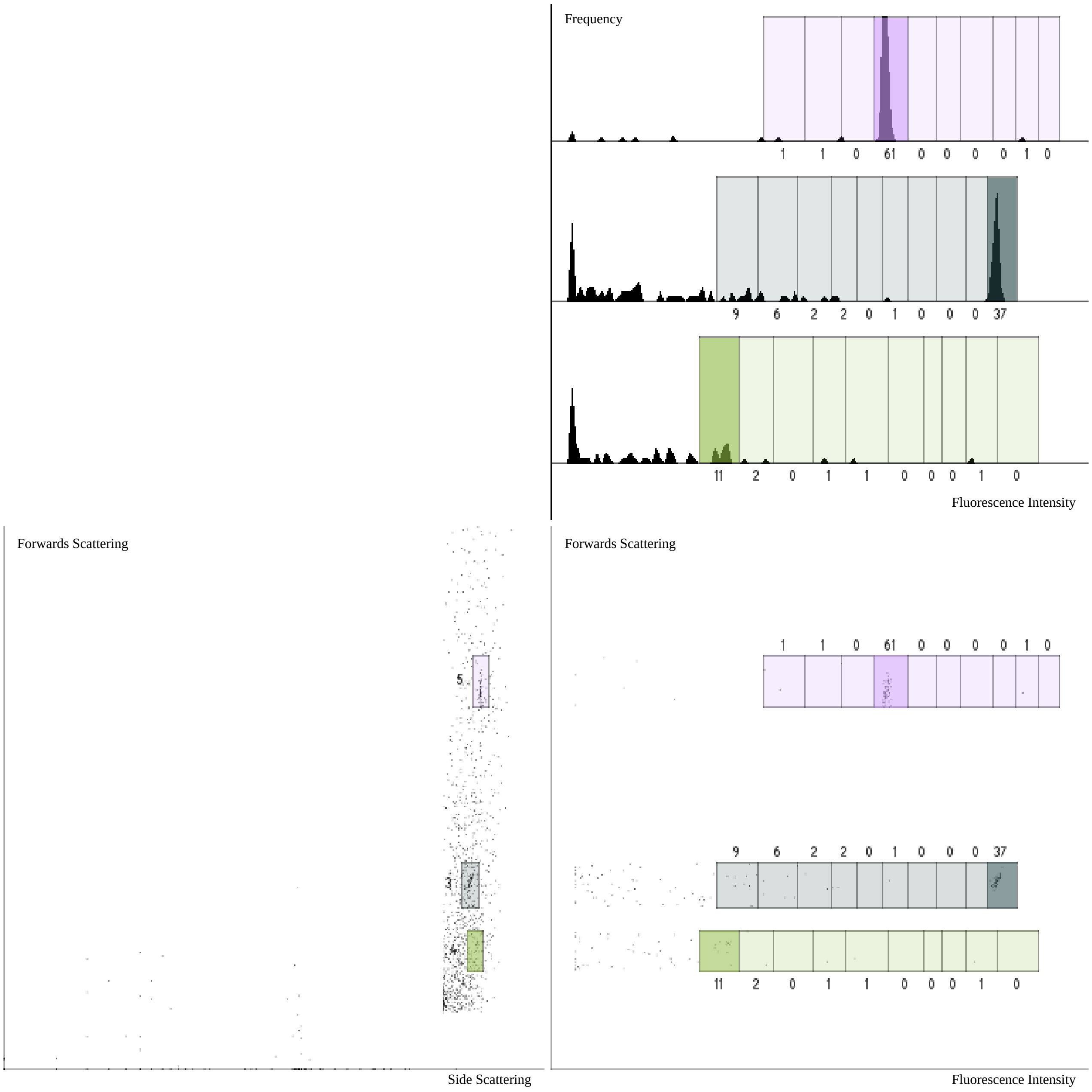

ANNEX 3: TAG DECONVOLUTION - BEAD 118

Passes flow sorting criteria: Yes

Passes tag deconvolution criteria: Yes

Included in protocol analysis: Yes

Protocol: 7, 6, 9, 7

Filename: Bin7_53

Split 1: Petrol shading

Split 2: Green shading

Split 3: Violet shading

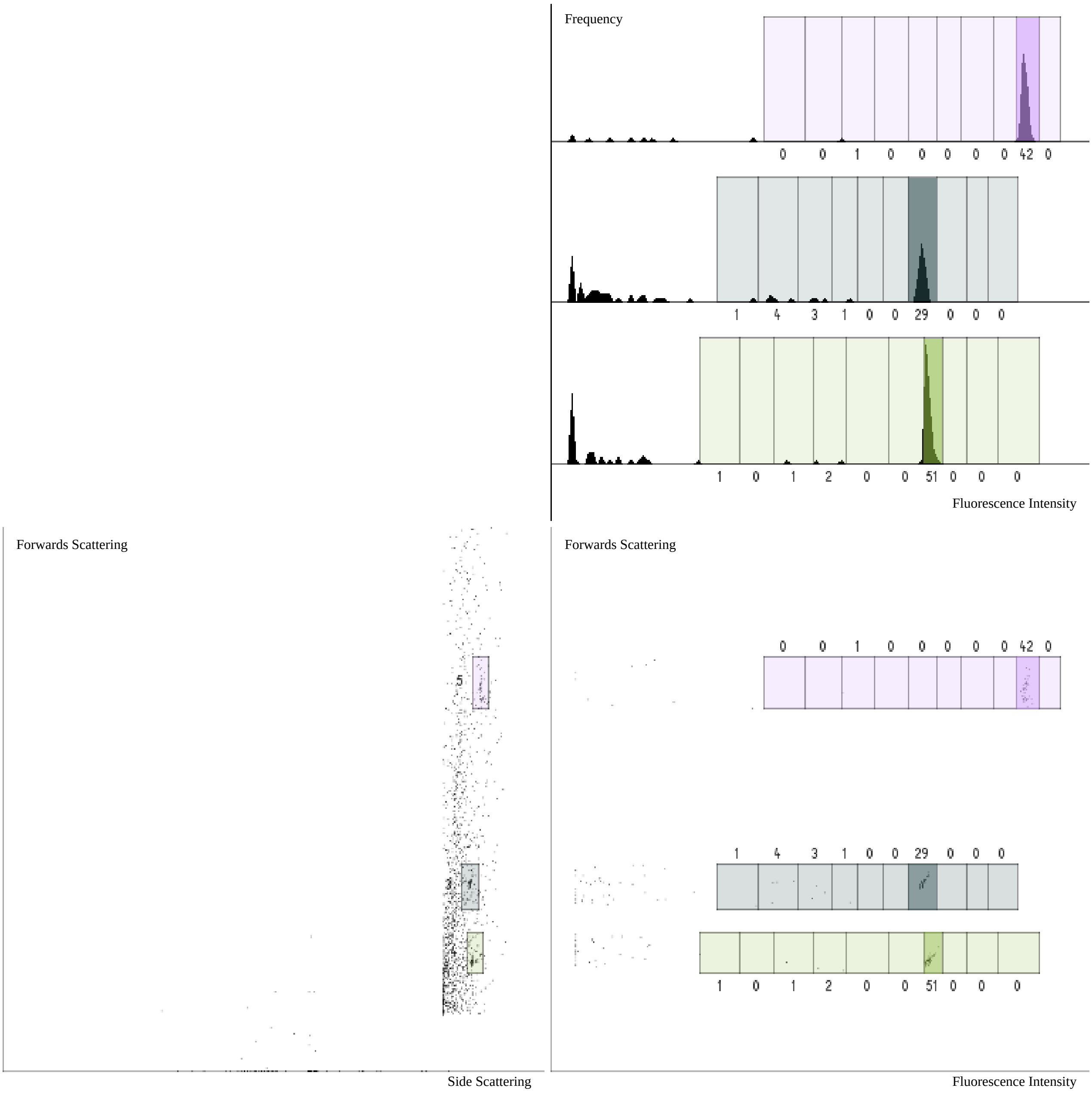

ANNEX 3: TAG DECONVOLUTION - BEAD 119

Passes flow sorting criteria: Yes

Passes tag deconvolution criteria: No

Included in protocol analysis: No

Protocol: N/A

Filename: Bin7_54

Split 1: Petrol shading

Split 2: Green shading

Split 3: Violet shading

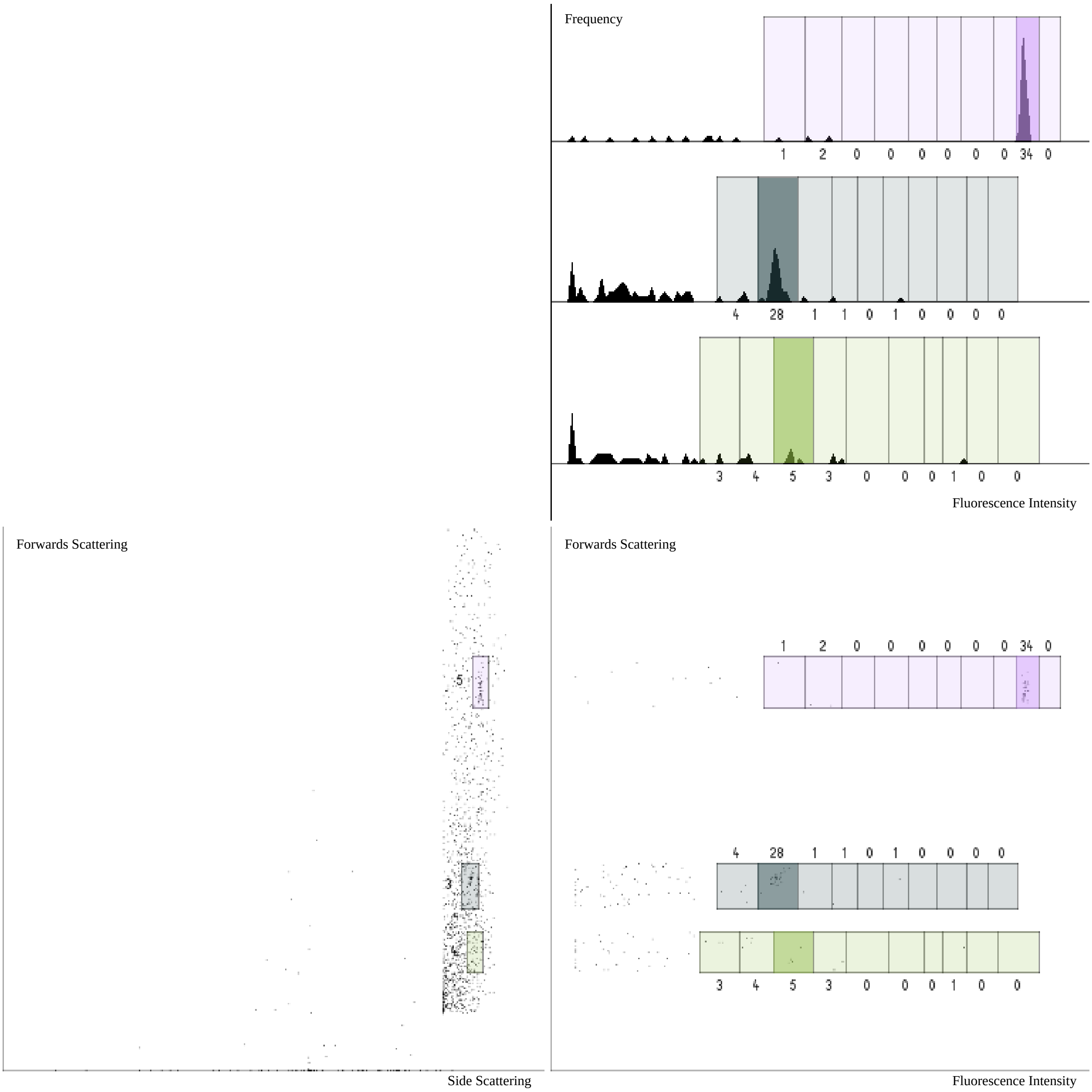

ANNEX 3: TAG DECONVOLUTION - BEAD 120

Passes flow sorting criteria: Yes

Passes tag deconvolution criteria: Yes

Included in protocol analysis: Yes

Protocol: 10, 8, 8, 7

Filename: Bin7_55

Split 1: Petrol shading

Split 2: Green shading

Split 3: Violet shading

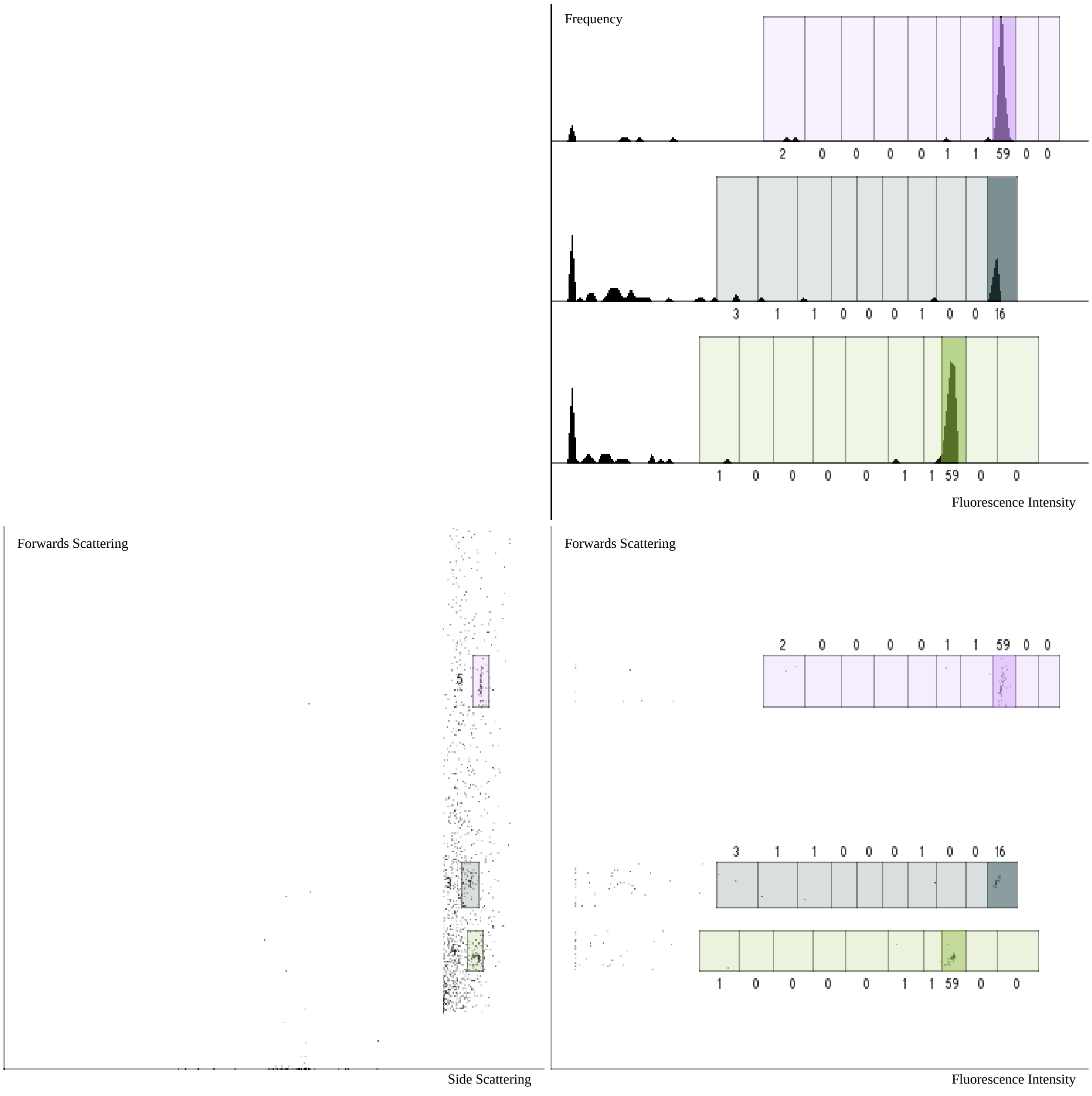

ANNEX 3: TAG DECONVOLUTION - BEAD 121

Passes flow sorting criteria: Yes

Passes tag deconvolution criteria: Yes

Included in protocol analysis: Yes

Protocol: 3, 8, 9, 7

Filename: Bin7_57

Split 1: Petrol shading

Split 2: Green shading

Split 3: Violet shading

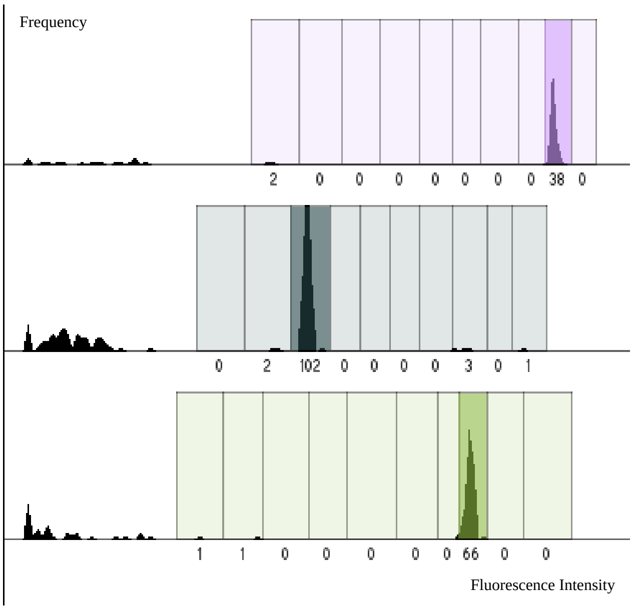

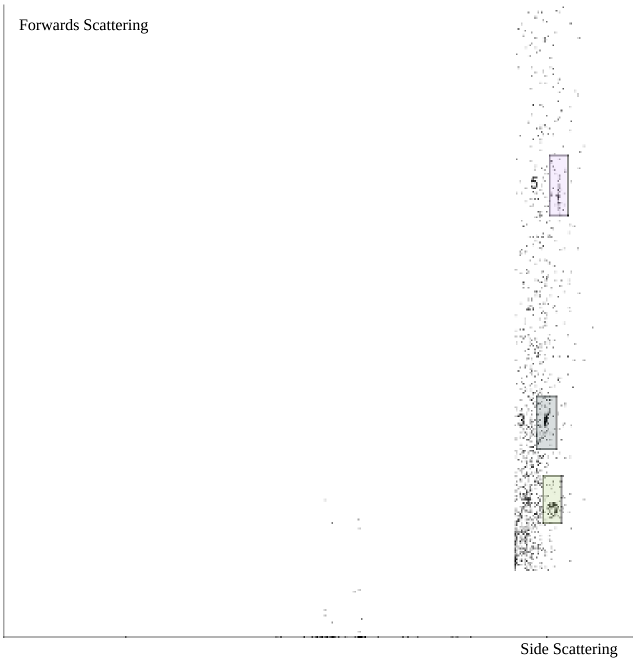

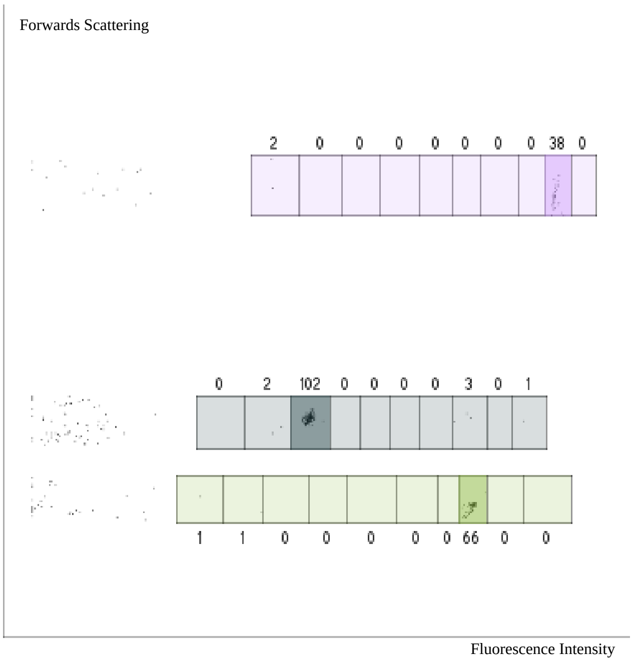

ANNEX 3: TAG DECONVOLUTION - BEAD 122

Passes flow sorting criteria: Yes

Passes tag deconvolution criteria: Yes

Included in protocol analysis: Yes

Protocol: 8, 5, 3, 7

Filename: Bin7_58

Split 1: Petrol shading

Split 2: Green shading

Split 3: Violet shading

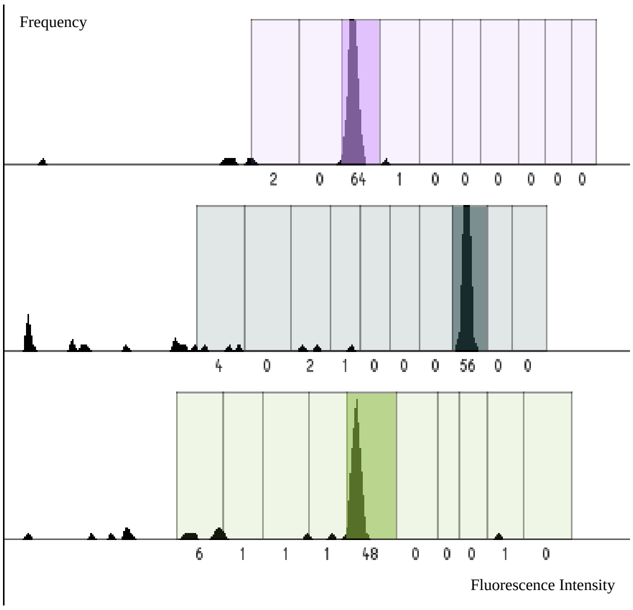

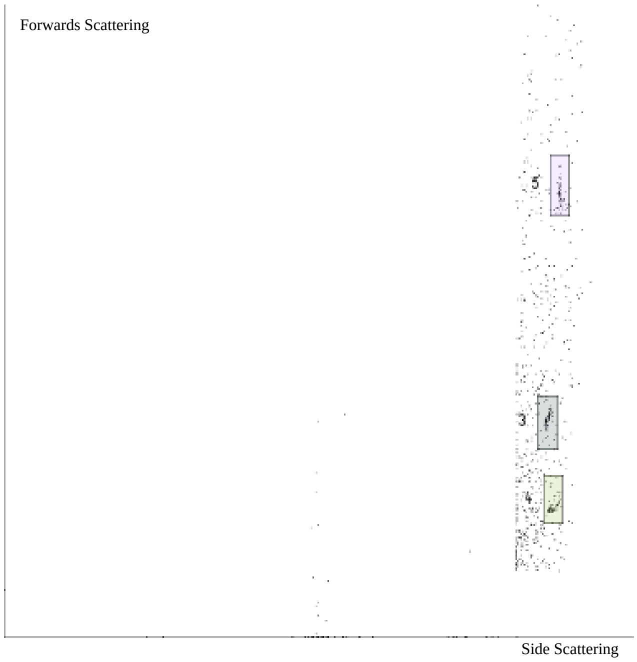

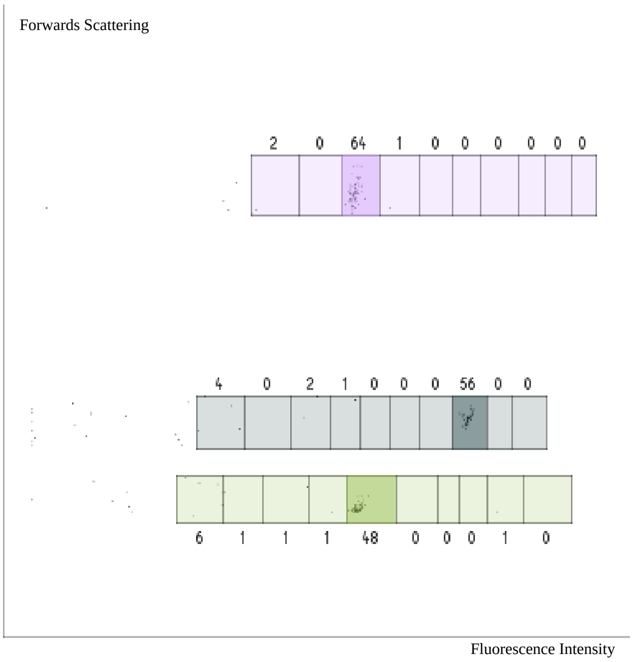

ANNEX 3: TAG DECONVOLUTION - BEAD 123

Passes flow sorting criteria: Yes

Passes tag deconvolution criteria: Yes

Included in protocol analysis: Yes

Protocol: 10, 4, 2, 7

Filename: Bin7_59

Split 1: Petrol shading

Split 2: Green shading

Split 3: Violet shading

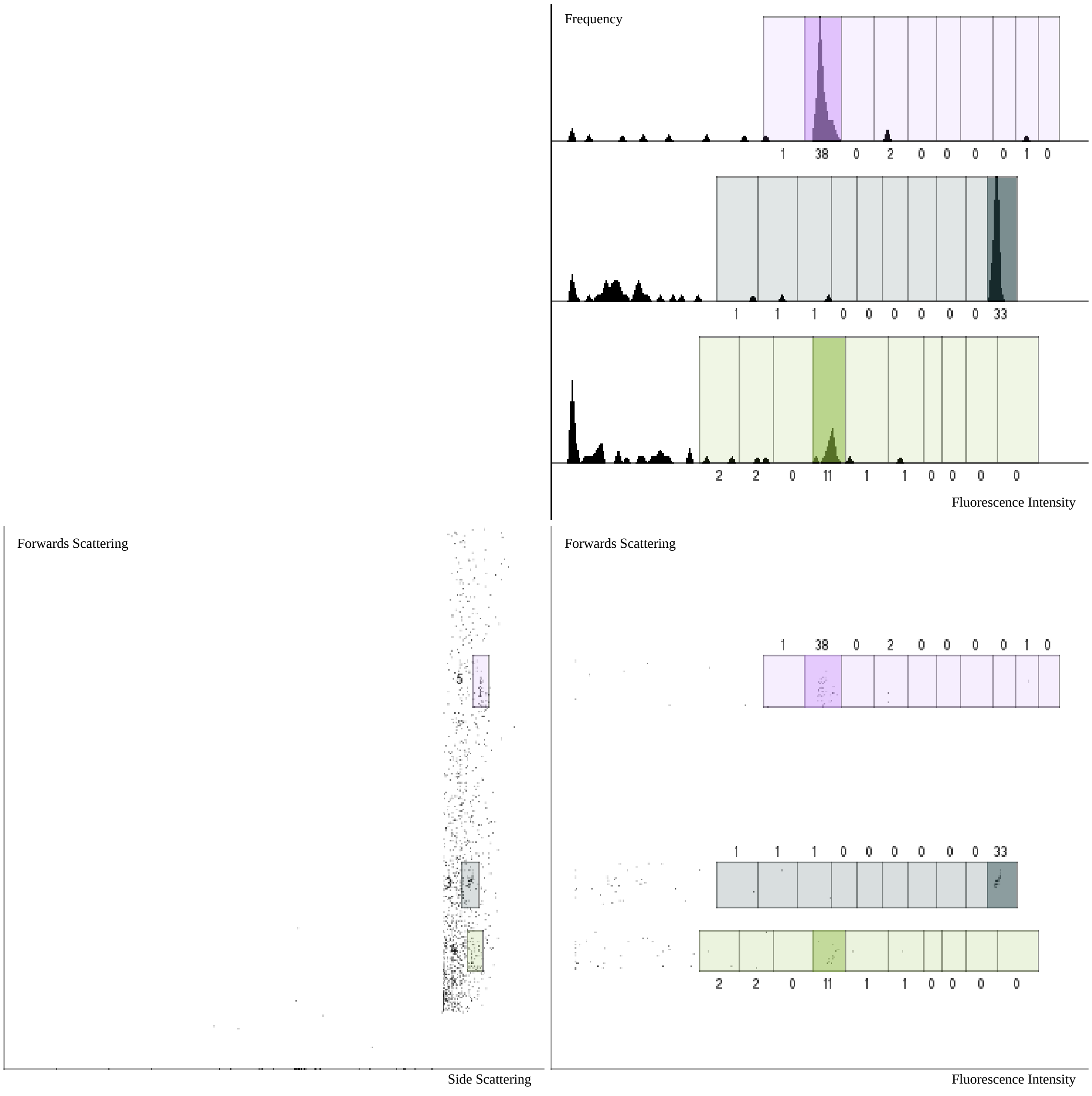

ANNEX 3: TAG DECONVOLUTION - BEAD 124

Passes flow sorting criteria: Yes

Passes tag deconvolution criteria: Yes

Included in protocol analysis: Yes

Protocol: 6, 1, 5, 7

Filename: Bin7_60

Split 1: Petrol shading

Split 2: Green shading

Split 3: Violet shading

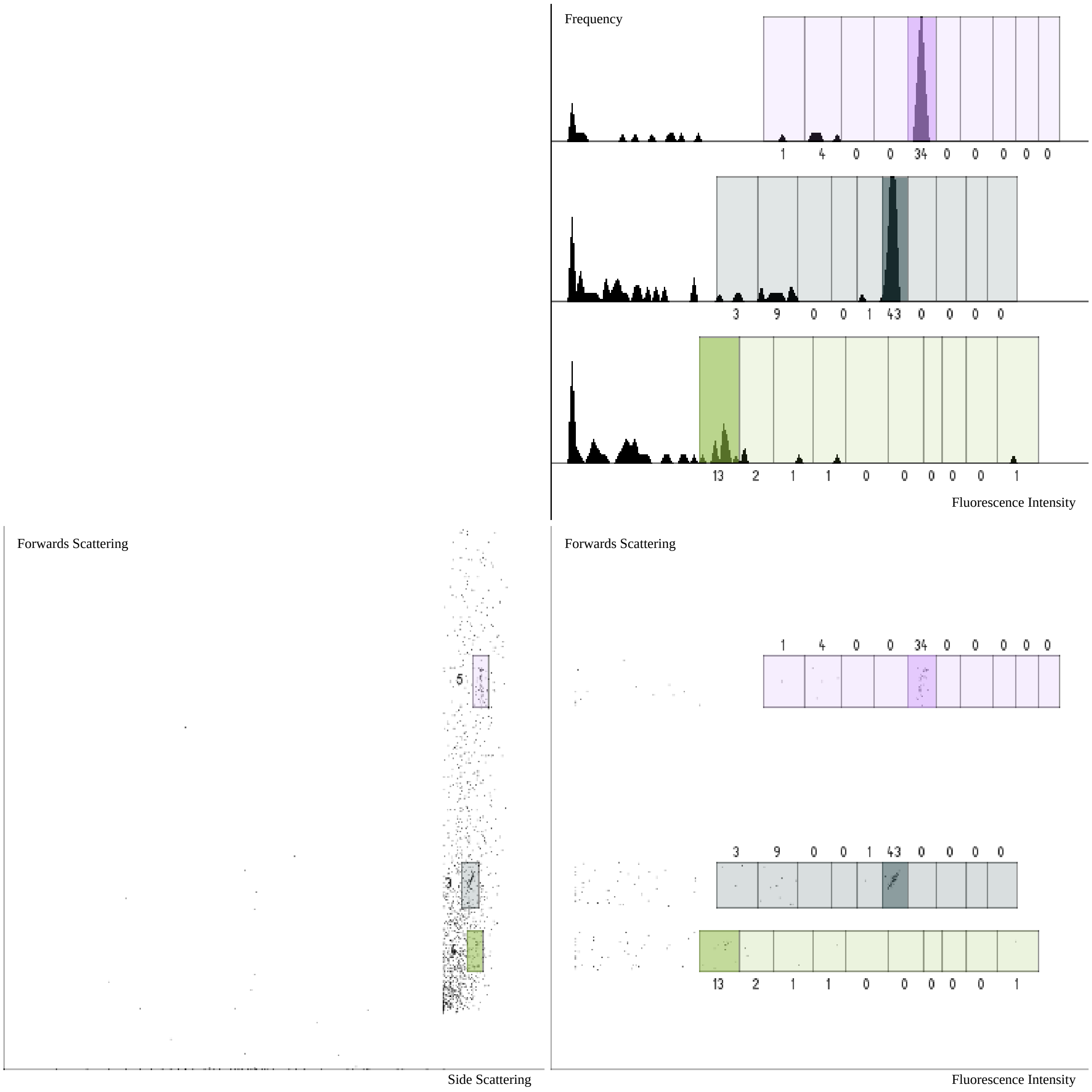

ANNEX 3: TAG DECONVOLUTION - BEAD 125

Passes flow sorting criteria: Yes

Passes tag deconvolution criteria: Yes

Included in protocol analysis: Yes

Protocol: 3, 9, 9, 7

Filename: Bin7_61

Split 1: Petrol shading

Split 2: Green shading

Split 3: Violet shading

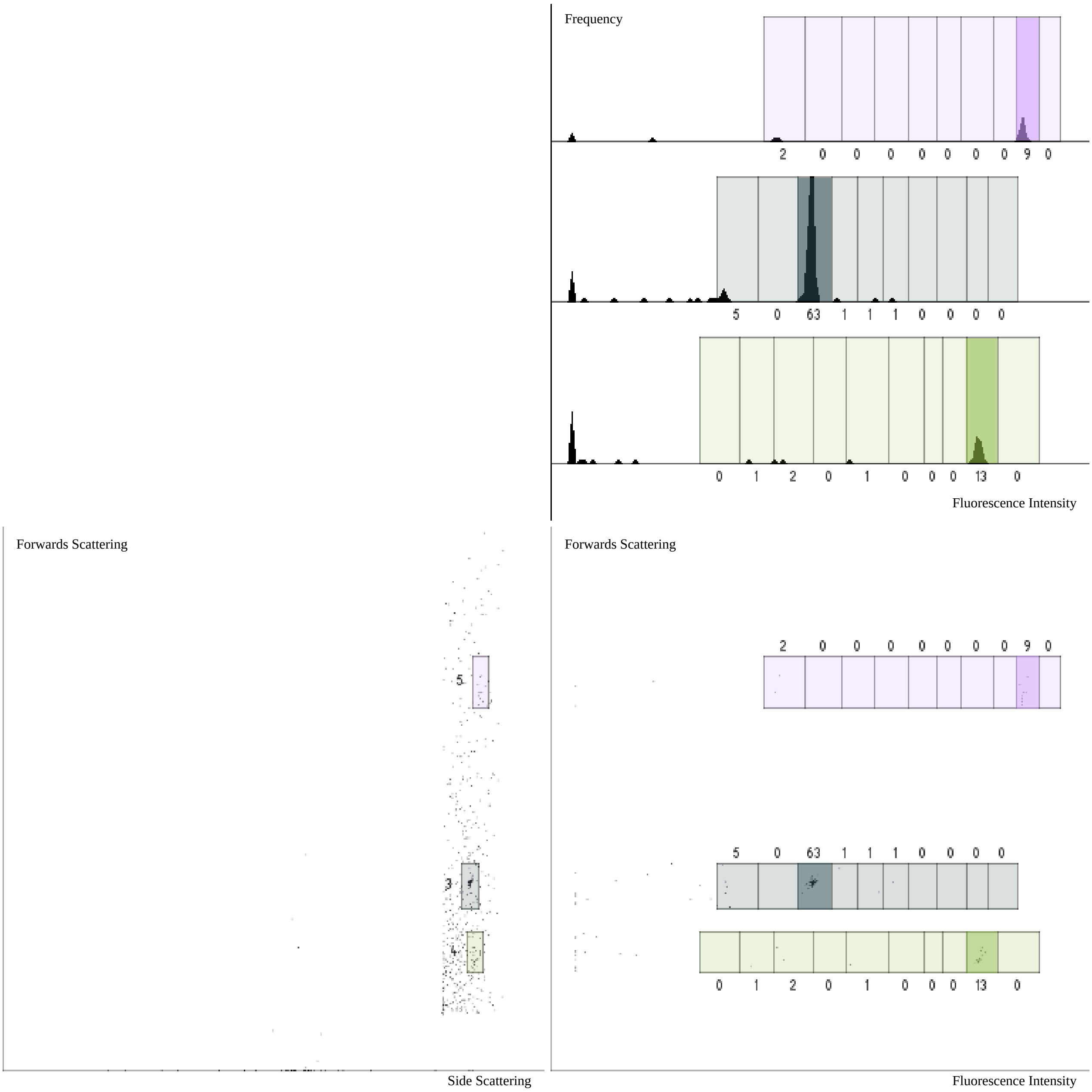

ANNEX 3: TAG DECONVOLUTION - BEAD 126

Passes flow sorting criteria: Yes

Passes tag deconvolution criteria: Yes

Included in protocol analysis: Yes

Protocol: 2, 2, 7, 7

Filename: Bin7_62

Split 1: Petrol shading

Split 2: Green shading

Split 3: Violet shading

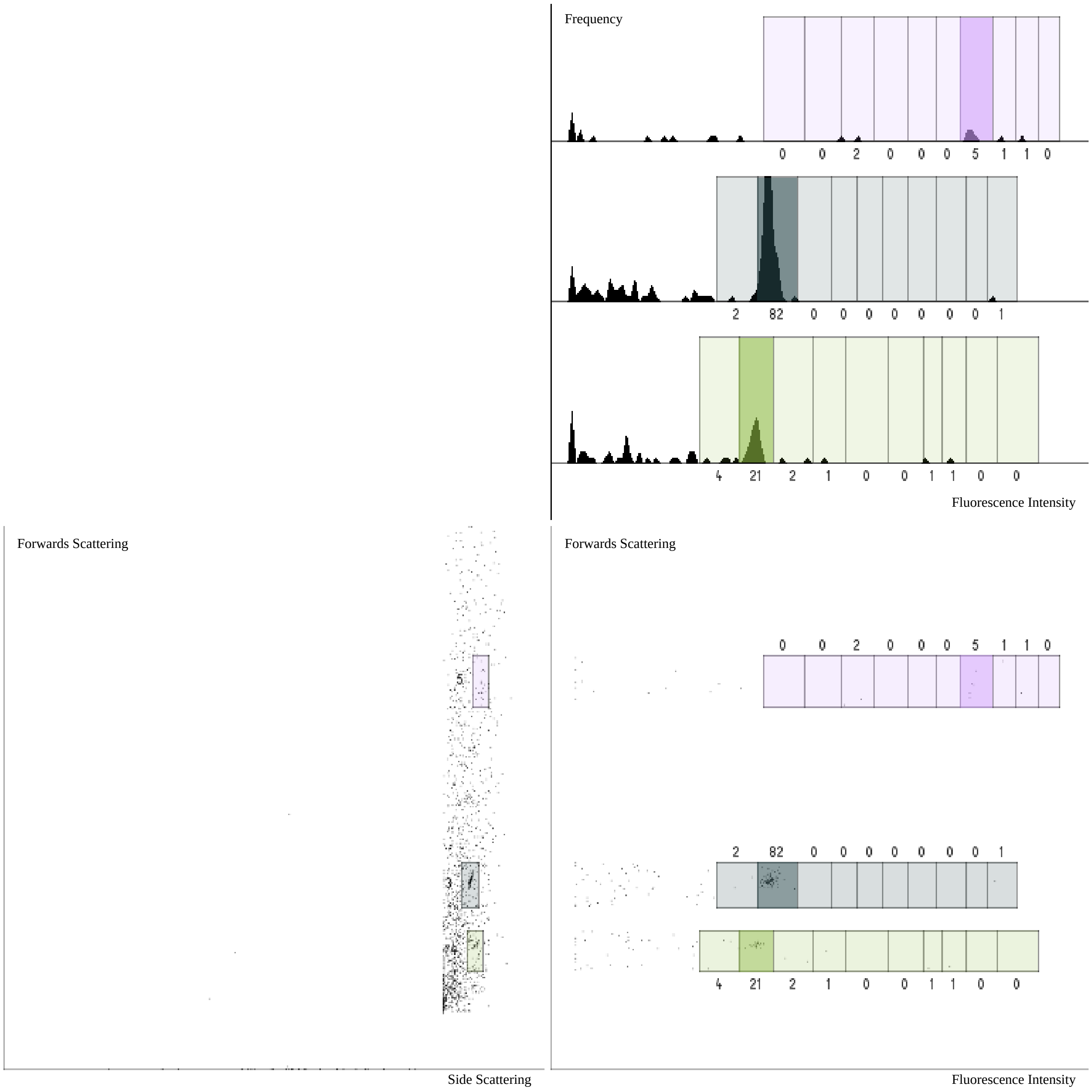

ANNEX 3: TAG DECONVOLUTION - BEAD 127

Passes flow sorting criteria: Yes

Passes tag deconvolution criteria: No

Included in protocol analysis: No

Protocol: N/A

Filename: Bin7_63

Split 1: Petrol shading

Split 2: Green shading

Split 3: Violet shading

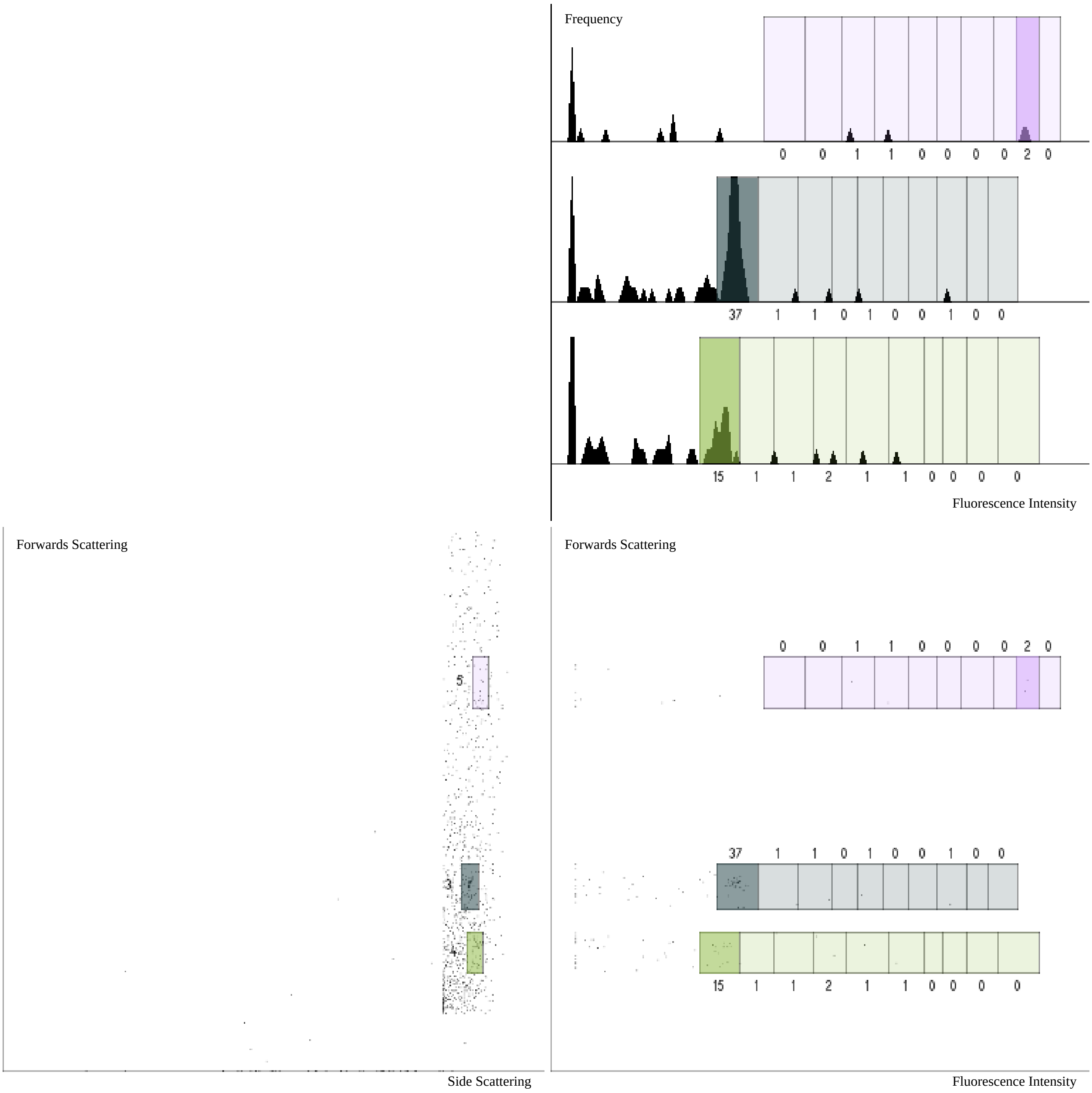

ANNEX 3: TAG DECONVOLUTION - BEAD 128

Passes flow sorting criteria: Yes

Passes tag deconvolution criteria: Yes

Included in protocol analysis: Yes

Protocol: 7, 1, 8, 7

Filename: Bin7_64

Split 1: Petrol shading

Split 2: Green shading

Split 3: Violet shading

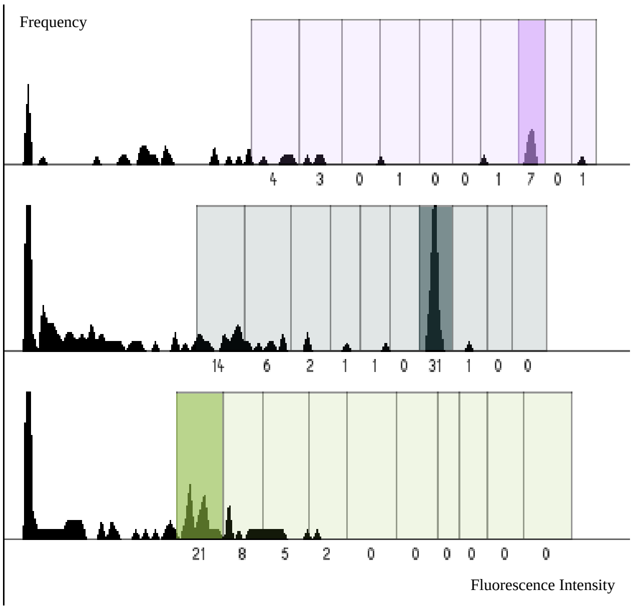

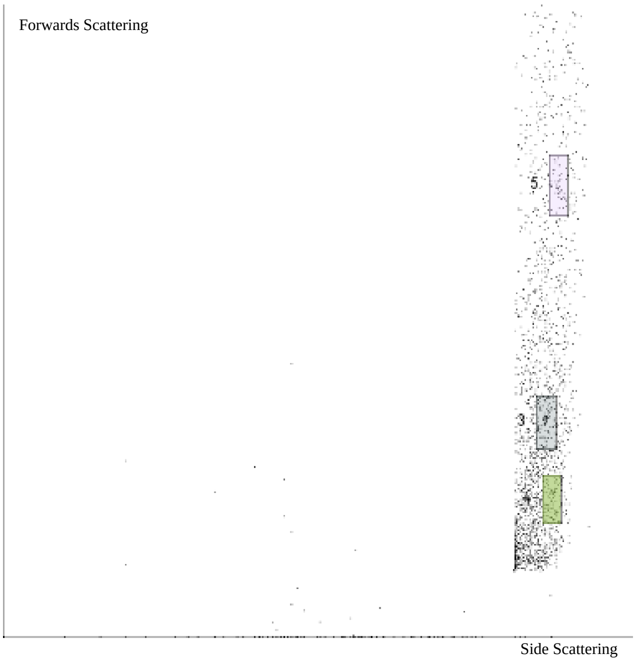

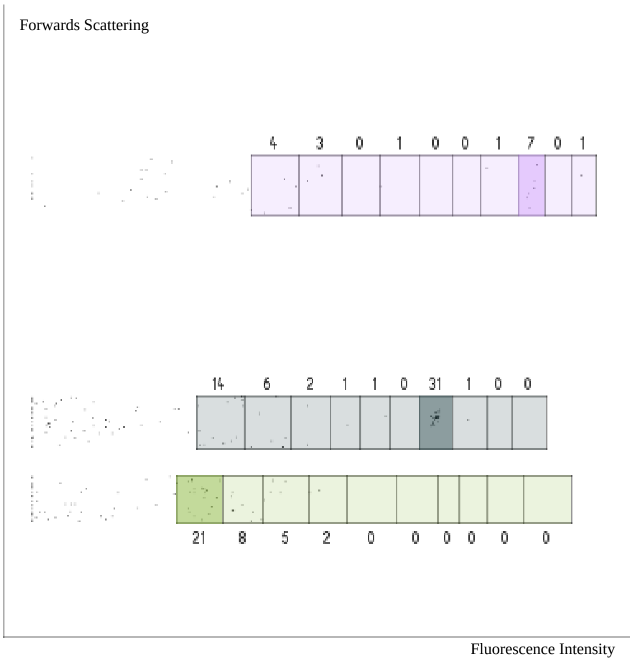

ANNEX 3: TAG DECONVOLUTION - BEAD 129

Passes flow sorting criteria: Yes

Passes tag deconvolution criteria: Yes

Included in protocol analysis: Yes

Protocol: 6, 7, 2, 7

Filename: Bin7_65

Split 1: Petrol shading

Split 2: Green shading

Split 3: Violet shading

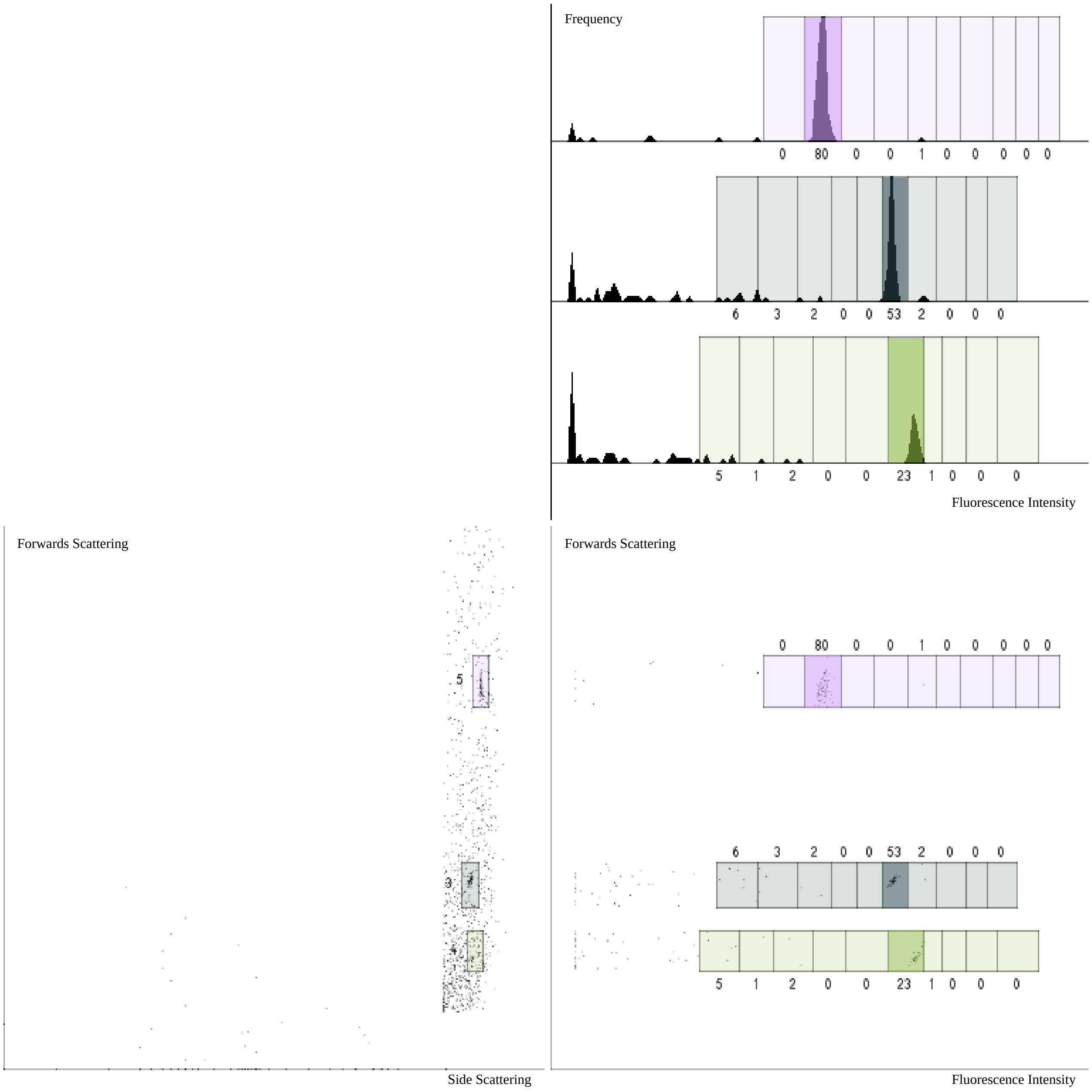

ANNEX 3: TAG DECONVOLUTION - BEAD 130

Passes flow sorting criteria: Yes

Passes tag deconvolution criteria: Yes

Included in protocol analysis: Yes

Protocol: 10, 1, 8, 7

Filename: Bin7_66

Split 1: Petrol shading

Split 2: Green shading

Split 3: Violet shading

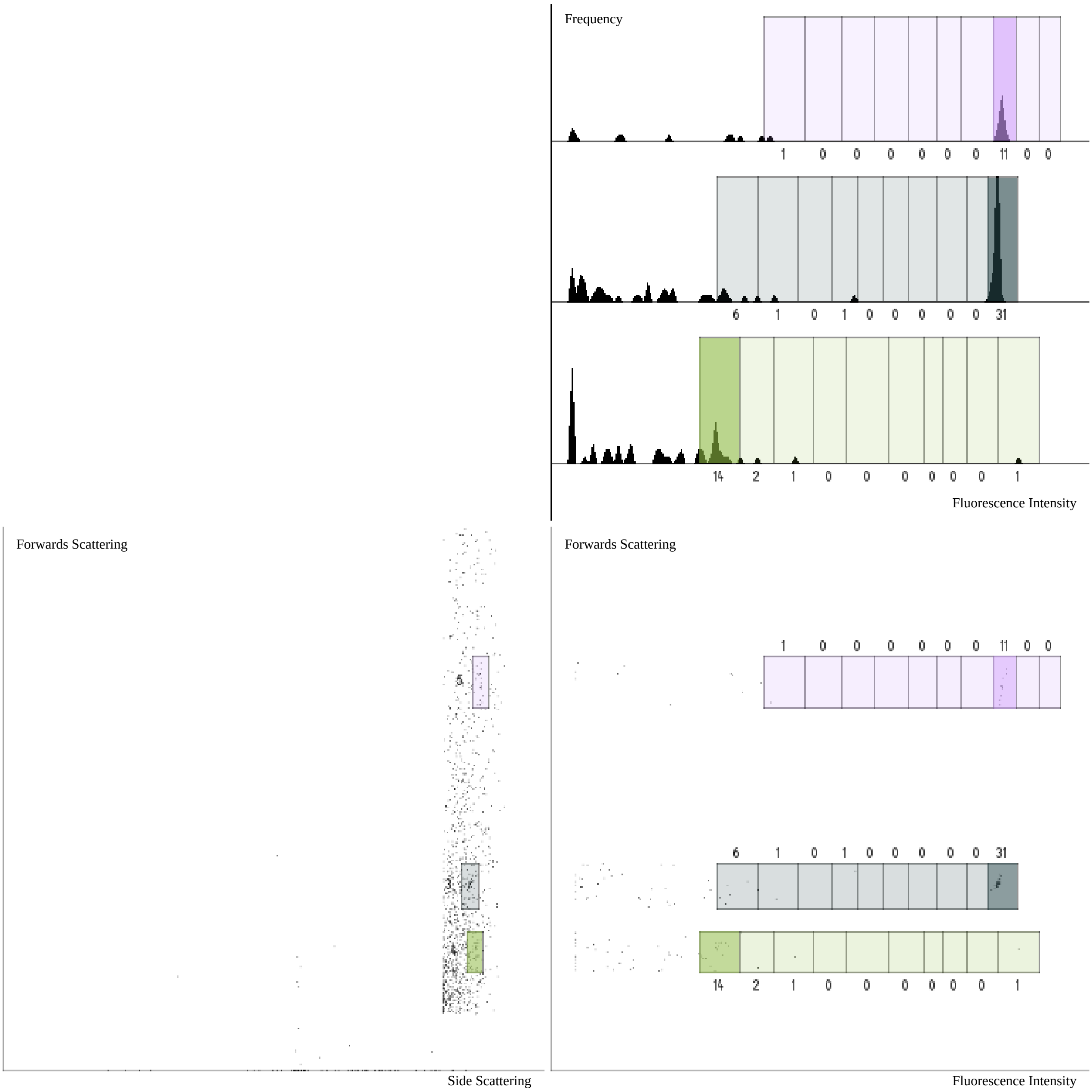

ANNEX 3: TAG DECONVOLUTION - BEAD 131

Passes flow sorting criteria: Yes

Passes tag deconvolution criteria: Yes

Included in protocol analysis: Yes

Protocol: 6, 10, 9, 7

Filename: Bin7_67

Split 1: Petrol shading

Split 2: Green shading

Split 3: Violet shading

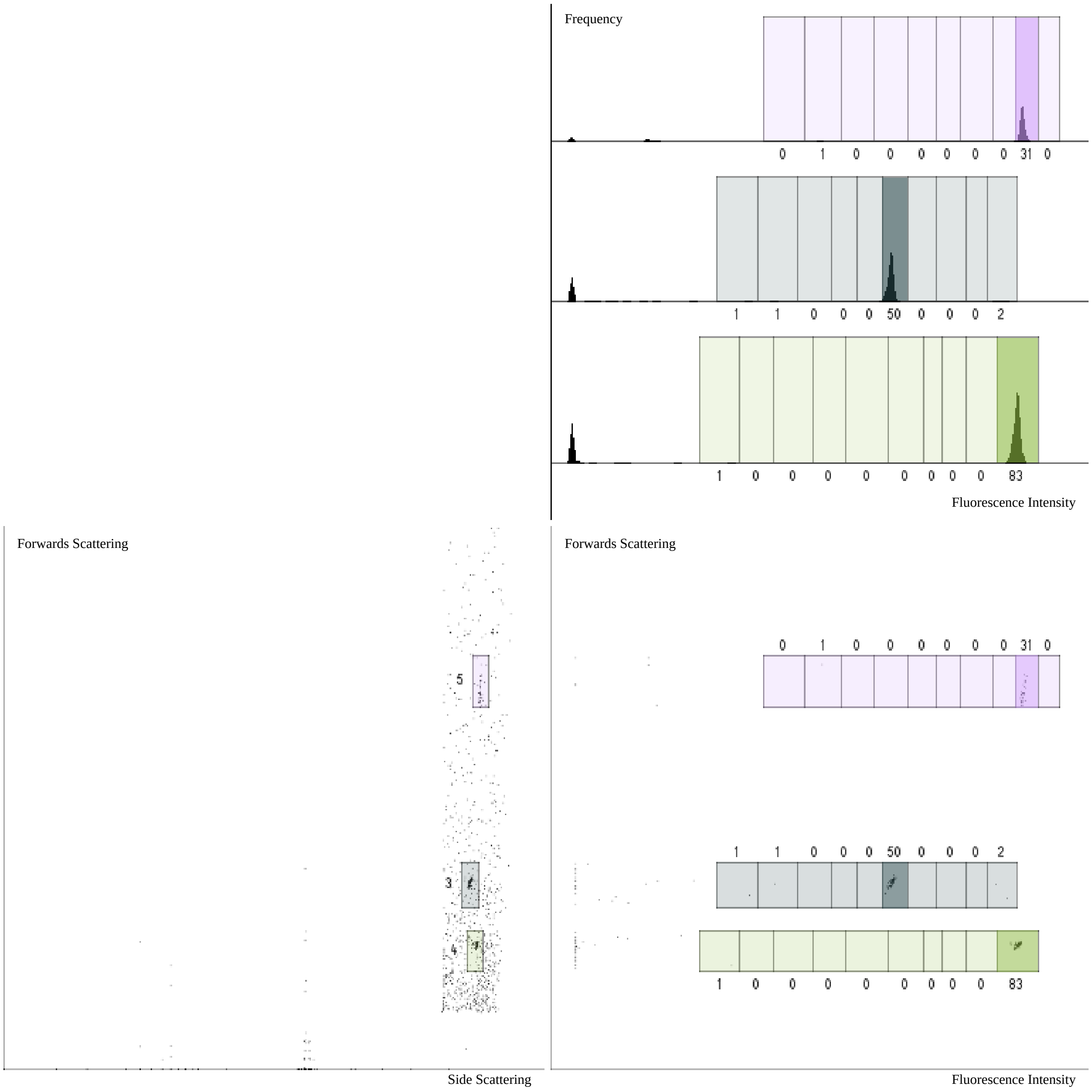

Supplement: Data S1 — Ariadne report for CombiCult screen 1: hematopoietic phagocytes from mES cells. (PDF) [file pone.0104301.s016.pdf]
