# Supplementary material for: Directed Differentiation of Embryonic Stem Cells Using a Bead-Based Combinatorial Screening Method
Source: PLoS One. 2014 Sep 24;9(9):e104301. doi: 10.1371/journal.pone.0104301 (PMC4174505; doi:10.1371/journal.pone.0104301)

Ariadne™

Experiment 1 - CombiCult™ screen for neural precursors using mES

Plasticell

EXECUTIVE SUMMARY

OBJECTIVE

- The objective of this CombiCult^{TM} study was to identify novel protocols for the differentiation of mouse embryonic stem cells (mESC) to neuronal (Sox1 positive) lineages using a diverse screening matrix comprising of media previously reported to direct ES cell differentiation towards ectodermal, mesodermal and endodermal lineages.

COMBICULT^{TM} EXPERIMENT

- 40 cell culture media were tested in 10,000 combinations.
- Approximately 300,000 beads were seeded with mES 46C Sox1-GFP cells (Stem Cell Sciences).
- The experimental matrix comprised four split-pool cycles, performed on days 1, 2, 5 and 7.
- 10 media were assayed in each cycle, resulting in an experimental complexity of 10x10x10x10 = 10,000 media combinations.
- On completion of cell culture (day 14) all beads were screened using Sox1-GFP expression.
- 580 beads were isolated by COPAS.

RESULTS

- 395 hits (0.21 % of monomeric beads) were verified following inspection by microscopy.
- 106 verified hits from cell culture media 1-3 on the final split were chosen for tag deconvolution
- The cell culture history of 87 (82 % of chosen hits) were deduced unambiguously.
- The 87 hits were derived from 86 distinct putative differentiation protocols.
- Protocols were ranked and chosen for validation using bespoke bioinformatics software, Ariadne^{TM} (v1.0).
- The 6 validated protocols are listed in Table 1.

EXECUTIVE SUMMARY CONTINUED

Table 1: The 6 protocols selected for validation as by Ariadne™ (v1.0). Protocols are identified by the series of cell culture media that resulted in differentiation.

Validation #	Protocol	Bead Ids		Basal Medium	Supplements
1	2-7-7-1	59, 70	Split 1 (Day 1)	DMEM/F12/Neurobasal	N2 (0.5%), B27 (0.5%)
			Split 2 (Day 2)	STEMLINE	SCF (20ng/mL)
			Split 3 (Day 5)	STEMLINE	SCF (20ng/mL), BMP-2 (5ng/mL), TGF-β1 (5ng/mL), TPO (20ng/mL)
			Split 4 (Day 7)	DMEM/F12	B27 (1%), PDGF-AA (20ng/mL)
2	4-7-7-1	76	Split 1 (Day 1)	RPMI	B27 (1%), Activin A (50ng/mL), Sodium Butyrate (1 mM)
			Split 2 (Day 2)	STEMLINE	SCF (20ng/mL)
			Split 3 (Day 5)	STEMLINE	SCF (20ng/mL), BMP-2 (5ng/mL), TGF-β1 (5ng/mL), TPO (20ng/mL)
			Split 4 (Day 7)	DMEM/F12	B27 (1%), PDGF-AA (20ng/mL)
3	2-7-2-1	60	Split 1 (Day 1)	DMEM/F12/Neurobasal	N2 (0.5%), B27 (0.5%)
			Split 2 (Day 2)	STEMLINE	SCF (20ng/mL)
			Split 3 (Day 5)	DMEM/F12/Neurobasal	N2 (0.5%), B27 (0.5%), FGF b (20ng/mL), FGF-8 (100ng/mL), SHH (400ng/mL)
			Split 4 (Day 7)	DMEM/F12	B27 (1%), PDGF-AA (20ng/mL)
4	8-9-4-3	51	Split 1 (Day 1)	IMDM (75 %)/HAM F12 (25 %)	N2 (1%), B27 (1%), BSA (0.5 mg/mL)
			Split 2 (Day 2)	GMEM	KSR (5%), TGF-β1 (2ng/mL), BMP-2 (10ng/mL)
			Split 3 (Day 5)	RPMI	B27 (1%), EGF (20ng/mL), FGF b (2ng/mL), Noggin (100ng/mL)
			Split 4 (Day 7)	DMEM/F12	N2 (1%), B27 (1%), FGF b (5ng/mL), DKK-1 (10ng/mL), Noggin (10ng/mL), IGF-1 (10ng/mL)
5	9-5-8-3	25	Split 1 (Day 1)	GMEM	KSR (5%), BMP-4 (1ng/mL)
			Split 2 (Day 2)	RPMI	Activin A (100ng/mL), Wnt-3A (25ng/mL)
			Split 3 (Day 5)	DMEM	ITS (1%), BSA (1mg/mL), PDGF BB (50ng/mL)
			Split 4 (Day 7)	DMEM/F12	N2 (1%), B27 (1%), FGF b (5ng/mL), DKK-1 (10ng/mL), Noggin (10ng/mL), IGF-1 (10ng/mL)
6	1-5-8-3	50	Split 1 (Day 1)	DMEM	N2 (1%)
			Split 2 (Day 2)	RPMI	Activin A (100ng/mL), Wnt-3A (25ng/mL)
			Split 3 (Day 5)	DMEM	ITS (1%), BSA (1mg/mL), PDGF BB (50ng/mL)
			Split 4 (Day 7)	DMEM/F12	N2 (1%), B27 (1%), FGF b (5ng/mL), DKK-1 (10ng/mL), Noggin (10ng/mL), IGF-1 (10ng/mL)

CONTENTS

Executive Summary

Report Nomenclature

1. Introduction

2. Study Setup

3. Split-Pool Experiment
3.1 Cell Culture Media
3.2 Tag Assignment

4. Bead Screening
4.1 Screening Assay
4.2 Flow Sorting of 'Hit' Beads

5. Tag Deconvolution

6. Protocol Analysis
6.1 Dataset Review
6.2 Linkage Analysis
6.3 Fingerprint Analysis
6.4 Hierachical Clustering Dendrogram
6.5 Similarity Matrix

7. Summary and Conclusions

Annex 1: Flow sorting scatter plots

Annex 2: Tag reference set and calibrated selection gates for each tag identification session

Annex 3: Tag identification scatter plots and histograms for each hit

GLOSSARY

Screening matrix refers to the arrangement and identity of media in the split-pool experiment

Experimental complexity is the total number of combinations of media tested, or the number of different protocols tested

Hit is a bead which scores positive in the screening assay and is sorted by COPAS

COPAS is a large particle flow sorter manufactured by Union Bioimetrica Inc.

Deconvolution is the inference of cell culture history by tag analysis

Bin refers to the sum of beads in each final cell culture medium

Session refers to the FACS analysis of tags from a group of hits. Prior to each session a reference tag set was run to calibrate side and forward scatter, and fluorescence intensity gates

Fingerprint anaylsis is a method of finding and comparing hits derived from identical protocols and groups of beads with similar protocols

Probability (of occurrence of a group in the fingerprint analysis) is the probability of that cluster of protocols occurring by chance, assuming beads sample all protocols randomly

Hierarchical clustering is a hierarchy of protocol clusters, represented in a dendrogram

Similarity matrix is a diagrammatical representation of a pair-wise comparison of all protocols

1. INTRODUCTION

COMBICULT™ TECHNOLOGY

CombiCult™ is a proprietary bead-based combinatorial technology specifically developed for discovery of novel stem cell differentiation protocols. Stem cells on beads are exposed to multiple combinations of media, containing active agents such as growth factors or small molecules. The optimal combinations for effective differentiation can be deduced reliably, rapidly and cost effectively.

Figure 1: CombiCult^{TM} technology

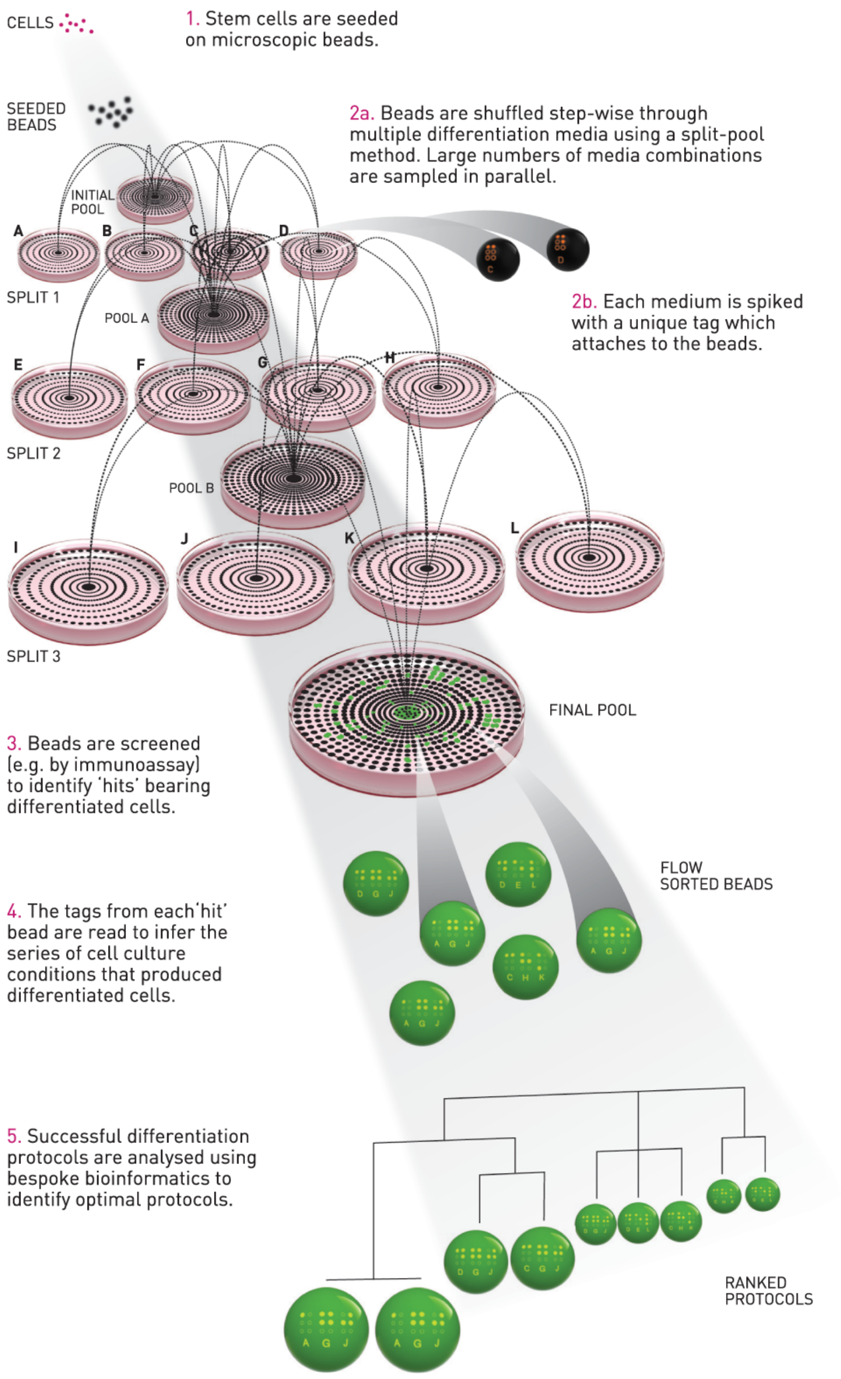

1. INTRODUCTION CONTINUED

OBJECTIVE

The objective of this CombiCult™ study was to identify novel serum-free protocols for the differentiation of mouse embryonic stem cells (mESC) to neuronal (Sox1 positive) lineages.

STUDY

The experimental matrix comprised 40 cell culture media distributed over four split-pool cycles (splits) such that a total of 10,000 media combinations (protocols) were tested. Below is a depiction of the experimental design (Figure 2) showing the timing and number of media in each split. On completion of the cell culture phase, on day 14, beads were screened using Sox1-GFP expression to identify differentiated cells.

Figure 2: Schematic diagram of the experimental matrix.

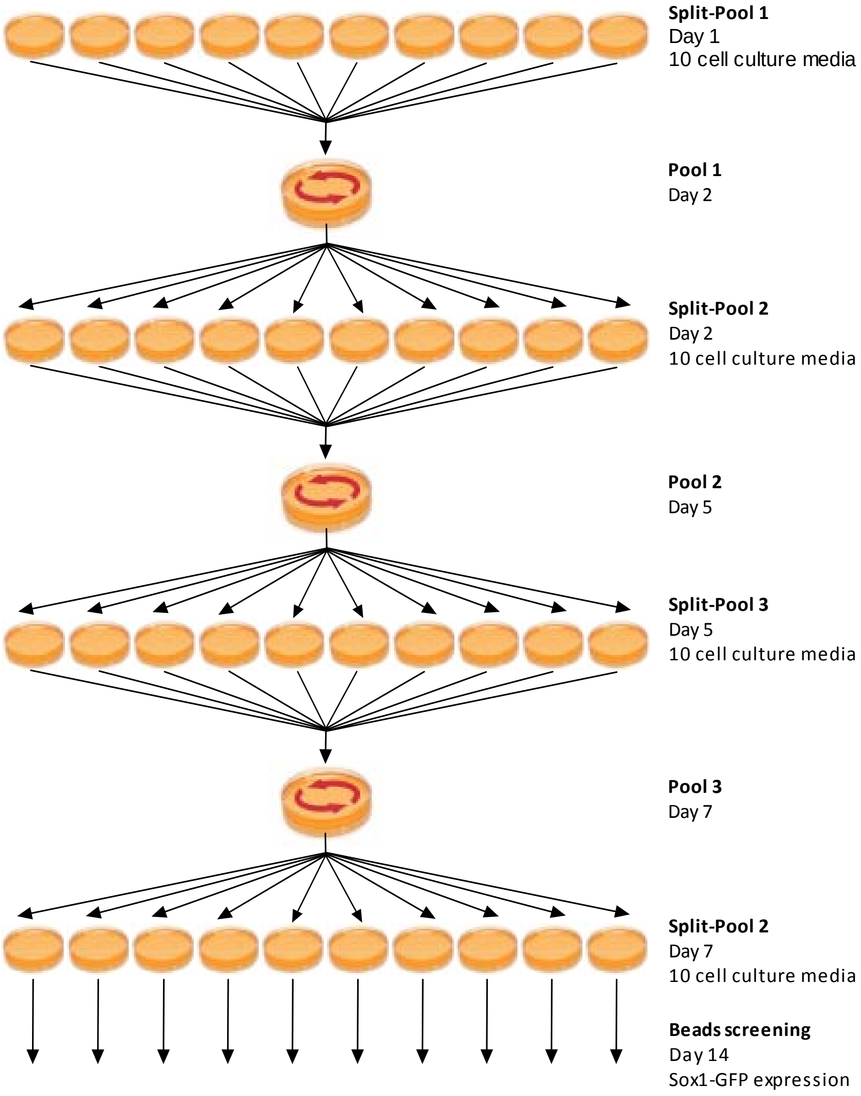

2. STUDY SETUP

OVERVIEW

Study title:	mES Macrophage experiment #1 (GFP Screen)
Study start/finish dates:	24-Apr-08/31-Oct-08

SPLIT-POOL EXPERIMENT

Scientist Name:	Dr Marina Tarunina
Start/Finish Dates:	24-Apr-08/14-Jun-08
Cell Line:	mESC 46C Sox1-GFP cells (Stem Cell Sciences)
Split 1 Date:	24-Apr-08 (Day1)
Split 2 Date:	25-Apr-08 (Day 2)
Split 3 Date:	28-Apr-08 (Day 5)
Split 4 Date:	30-Apr-08 (Day 7)
Differentiation end Date:	08-May-08 (Day 14)
Tagging Date - Split 1:	24-Apr-08
Tagging Date - Split 2:	25-Apr-08
Tagging Date - Split 3:	28-Apr-08
Tagging Date - Split 4:	30-Apr-08
Number of cell culture media combinations tested:	10,000
Ratio of beads to complexity:	30:1
Total number of beads:	300,000
Ratio of cells to beads during seeding:	90:1
Additional notes:	None

BEAD SCREENING & SORTING

Scientist name:	Dr Marina Tarunina
Start/finish dates:	8-May-08/11-May-08
Screening assay:	Sox1-GFP expression
Screening date:	08-May-08
Flow sorting instrument:	COPAS PLUS, Union Biometrica
Sorting PMT channel(s):	Green (514/23 nm) optical emission filters
Positive setup control:	N/A
Total number of sorted beads	580
Number of verified hits	395
Additional notes:	106 verified hits from cell culture media 1-3 on the final split were chosen for tag deconvolution.

TAG DECONVOLUTION

**Bead Digestion**	
Scientist name:	Dr Christopher Johnson
Start/finish dates:	18-Jun-08/21-Jun-08
Additional notes:	None
**Tag Analysis**	
Scientist name:	Dr Christopher Johnson
Start/finish dates:	21-Jun-08/31-Jul-08
Tag analysis flow cytometry instrument:	Beckman Coulter FC500
Analysis PMT channel(s) optical filter:	Beckman Coulter FC500 FL4 (675nm long pass).
Number of analysis sessions:	3
Additional notes:	None

PROTOCOL ANALYSIS & REPORT GENERATION

Software:	Ariadne™ version 1.0
User name:	Dr Christopher Johnson
Date, Time:	22-Oct-10, 16.00

Confidential

3. SPLIT-POOL EXPERIMENT

3.1 Cell Culture Media

Table 2: Compositions of the cell culture media tested in this study.

Split	Medium ID	Basal Medium	Supplements
1	1	DMEM	N2 (1%)
1	2	DMEM/F12/Neurobasal	N2 (0.5%), B27 (0.5%)
1	3	Advanced DMEM (CDM)	-
1	4	RPMI	B27 (1%), Activin A (50ng/mL), Sodium Butyrate (1 mM)
1	5	RPMI	Activin A (100ng/mL), Wnt-3A (25ng/mL)
1	6	RPMI	BSA (0.5mg/mL), Activin A (100 ng/mL)
1	7	STEMLINE	BMP-4 (50 ng/mL), VEGF (50ng/mL)
1	8	IMDM (75 %)/HAM F12 (25 %)	N2 (1%), B27 (1%), BSA (0.5 mg/mL)
1	9	GMEM	KSR (5%), BMP-4 (1ng/mL)
1	10	KO-DMEM	FCS (15%), LIF (0.1%)
2	1	DMEM/F12	ITS (1%), RA (0.05 µM)
2	2	DMEM/F12/Neurobasal	N2 (0.5%), B27 (0.5%), FGF-4 (2ng/mL)
2	3	DMEM/F12	ITS (1%), B27 (1%), FGF b (4ng/mL), DKK-1 (50ng/mL)
2	4	RPMI	B27(1%), Activin A (50ng/mL), Sodium Butyrate (0.5mM)
2	5	RPMI	Activin A (100ng/mL), Wnt-3A (25ng/mL)
2	6	RPMI	ITS (1%), BSA (0.5mg/mL), Activin A (100 ng/mL)
2	7	STEMLINE	SCF (20ng/mL)
2	8	IMDM (75 %)/HAM F12 (25 %)	N2 (1%), B27 (1%), BSA (0.5 mg/mL), Ascorbic Acid (0.5 mM)
2	9	GMEM	KSR (5%), TGF-β1 (2ng/mL), BMP-2 (10ng/mL)
2	10	Advanced DMEM	-
3	1	DMEM/F12	B27 (1%), EGF (20ng/mL),FGF b (20ng/mL), PDGF-AA (20ng/mL)
3	2	DMEM/F12/Neurobasal	N2 (0.5%), B27 (0.5%), FGF b (20ng/mL), FGF-8 (100ng/mL), SHH (400ng/mL)
3	3	DMEM/F12	ITS (1%), B27 (1%), FGF b (4ng/mL), DKK-1 (50ng/mL)
3	4	RPMI	B27 (1%), EGF (20ng/mL), FGF b (2ng/mL), Noggin (100ng/mL)
3	5	DMEM	B27 (1%), RA (2 µM), FGF-10 (50ng/mL), CYC (0.2µM)
3	6	HCM	FGF-4 (30ng/mL), BMP-2 (20ng/mL)
3	7	STEMLINE	SCF (20ng/mL), BMP-2 (5ng/mL), TGF-β1 (5ng/mL), TPO (20ng/mL)
3	8	DMEM	ITS (1%), BSA (1mg/mL), PDGF BB (50ng/mL)
3	9	GMEM	KSR (5%), TGF-β1 (2ng/mL), BMP-2 (10ng/mL), Insulin (1µg/mL), Ascorbic Acid (50µg/mL)
3	10	DMEM Low Glucose	ITS (1%), Fibronectin (5µg/mL)
4	1	DMEM/F12	B27 (1%), PDGF-AA (20ng/mL)
4	2	DMEM/F12/Neurobasal	N2 (0.5%), B27 (0.5%), FGF b (20ng/mL), FGF-8 (100ng/mL), SHH (400ng/mL)
4	3	DMEM/F12	N2 (1%), B27 (1%), FGF b (5ng/mL), DKK-1 (10ng/mL), Noggin (10ng/mL), IGF-1 (10ng/mL)
4	4	DMEM	B27 (1%), RA (2 µM), FGF-10 (50ng/mL), CYC (0.2µM)
4	5	DMEM	B27 (1%), FGF a (100ng/mL)
4	6	HCM	HGF (20 ng/mL)
4	7	STEMLINE	IL-3 (30ng/mL), IL-6 (20ng/mL), TPO (20ng/mL)
4	8	DMEM	ITS (1%), BSA (1mg/mL), PDGF-BB (50ng/mL)
4	9	GMEM	KSR (5%), TGF-β1 (2ng/mL), BMP-2 (10ng/mL), Insulin (1µg/mL), Ascorbic Acid (50µg/mL)
4	10	CMRL	B27 (1%), IGF-1 (50ng/mL), HGF (50ng/mL)

Confidential

3. SPLIT-POOL

3.1 CELL CULTURE MEDIA CONTINUED

Table 3: Suppliers of basal media and supplements.

Basal Media/Supplement	Supplier	Product Reference
Advanced DMEM	Fisher Scientific	VX12491015
CMRL	Fisher Scientific	VX21530027
DMEM/F12	Fisher Scientific	VX31331028
DMEM High Glucose	Fisher Scientific	VX31966047
DMEM Low Glucose	Fisher Scientific	VX21885108
FCS	Fischer Scientific	VX12662011
GMEM	Fisher Scientific	VX21710025
Ham F12	Fisher Scientific	VX31765027
HCM	Lonza	CC-3198
IMDM	Fisher Scientific	VX31980022
KO DMEM	Fisher Scientific	VX10829018
KSR	Fisher Scientific	VX10828028
Neurobasal	Fisher Scientific	VX21103049
RPMI	Fisher Scientific	VX21875034
Stemline	Sigma Aldrich	S0192
Activin A	R and D Systems	338-AC-005
Ascorbic Acid	Sigma Aldrich	A4403
B27 Supplement	Fisher Scientific	VX17504044
BMP-2	R and D Systems	355-BM-010
BMP-4	R and D Systems	314-BP-010
BSA	Sigma Aldrich	A1595
Cyclopamine KAAD	Toronto Research Chemicals	K171000
DKK-1	R and D Systems	1765-DK-010
EGF	R and D Systems	2028-EG-200
FGF-4	R and D Systems	235-F4-025
FGF-8b	R and D Systems	423-F8-025
FGF-10	R and D Systems	345-FG-025
FGF a	R and D Systems	232-FA-025
FGF b	R and D Systems	233-FB-025
Fibronectin	Fisher Scientific	VX33010018
HGF	R and D Systems	2207-HG-025
IGF I	R and D Systems	791-MG-050
IL-3	R and D Systems	403-ML-010
IL-6	R and D Systems	206-IL-010
Insulin	Sigma Aldrich	I0516
ITS	Sigma Aldrich	I3146
LIF	Millipore	ESG1106
N2 Supplement	Fisher Scientific	VX17502048
Noggin	R and D Systems	719-NG-050
PDGF-BB	R and D Systems	520-BB-050

Basal Media/Supplement	Supplier	Product Reference
Retinoic Acid	Merck	US1554720
SCF	R and D Systems	455-MC-010
SHH	R and D Systems	461-SH-025
Sodium Butyrate	Sigma Aldrich	B5887
TGF-β1	R and D Systems	4114-TC-01M
Tpo	R and D Systems	288-TP-005
VEGF	R and D Systems	293-VE-010
Wnt-3A	R and D Systems	1324-WN-002

3. SPLIT-POOL
3.2. TAG ASSIGNMENT

Each cell culture medium (except media in the last split-pool cycle) was spiked with a unique fluorescent tag that attaches to PTC5000 beads. Tag readout from individual beads enables determination of the series of cell culture media to which the bead was exposed. Thirty unique populations of tags were used, which differ in diameter, fluorescence colour and fluorescence intensity (ten gradations). Table 4 shows the tag code, size, fluorescence colour, fluorescence intensity, sample lot number, tag stock solution concentration and volume used, to spike each cell culture medium.

Table 4: The tags used to spike different cell culture media.

Split	Medium ID	Tag code	Tag size	Tag fluorescence colour	Tag fluorescence intensity level	Stock solution concentration (% w/v)	V of stock solution added to media (μL)
1	1	SR01	S	Red	1	0.5	96
1	2	SR02	S	Red	2	0.5	96
1	3	SR03	S	Red	3	0.5	96
1	4	SR04	S	Red	4	0.5	96
1	5	SR05	S	Red	5	0.5	96
1	6	SR06	S	Red	6	0.5	96
1	7	SR07	S	Red	7	0.5	96
1	8	SR08	S	Red	8	0.5	96
1	9	SR09	S	Red	9	0.5	96
1	10	SR10	S	Red	10	0.5	96
2	1	MR01	M	Red	1	0.5	184
2	2	MR02	M	Red	2	0.5	184
2	3	MR03	M	Red	3	0.5	184
2	4	MR04	M	Red	4	0.5	184
2	5	MR05	M	Red	5	0.5	184
2	6	MR07	M	Red	7	0.5	184
2	7	MR06	M	Red	6	0.5	184
2	8	MR08	M	Red	8	0.5	184
2	9	MR09	M	Red	9	0.5	184
2	10	MR10	M	Red	10	0.5	184
3	1	LR01	L	Red	1	0.5	364
3	2	LR02	L	Red	2	0.5	364
3	3	LR03	L	Red	3	0.5	364
3	4	LR04	L	Red	4	0.5	364
3	5	LR05	L	Red	5	0.5	364
3	6	LR06	L	Red	6	0.5	364
3	7	LR07	L	Red	7	0.5	364
3	8	LR08	L	Red	8	0.5	364
3	9	LR09	L	Red	9	0.5	364
3	10	LR10	L	Red	10	0.5	364
4	1	No tag	-	-	-	-	-
4	2	No tag	-	-	-	-	-
4	3	No tag	-	-	-	-	-
4	4	No tag	-	-	-	-	-
4	5	No tag	-	-	-	-	-
4	6	No tag	-	-	-	-	-
4	7	No tag	-	-	-	-	-
4	8	No tag	-	-	-	-	-
4	9	No tag	-	-	-	-	-
4	10	No tag	-	-	-	-	-

4. BEAD SCREENING

4.1. SCREENING ASSAY

Following completion of the split-pool experiment at day 14, beads were screened using Sox1-GFP expression to identify hits populated with 46C mES cells which had differentiated into SOX1 expressing neurons.

Figure 3: Shows an image of a single 'hit' decorated with Sox1 positive cell colonies amongst negative beads (x4 objective lens, scale bar = 100 μm). The image was obtained using a Nikon Eclipse TE2000-S fluorecent microscope with a FITC filter (λex = 485-495 nm, λem = 515-555 nm).

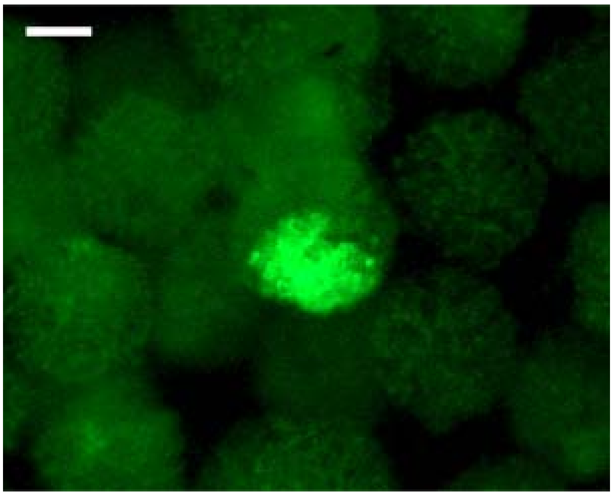

4. BEAD SCREENING

4.2. FLOW SORTING OF 'HIT' BEADS

Following the screening assay, beads were sorted using a large particle flow sorter (COPAS PLUS, Union Biometrica; Green PMT 514/23 nm optical emission filters). Beads from each final cell culture medium were labeled as bin 1-10. The data from each bin is contained in one or more flow sorting data files. Annex 1 shows dot plots from each flow sorting data file.

Beads were gated according to size (time of flight [TOF] and optical extinction [ext] values) to exclude bead aggregates that had formed during cell culture. Gated beads were sorted based on their fluorescence properties, as defined by the screening assay, and hits individually dispensed into wells of a 96-well plate.

In figure 4 COPAS data from all bins have been combined on two dot-plots to provide an overview of bead sorting. Figure 4(a) is a dot plot of optical extinction (ext) vs. time of Flight (TOF). Events falling within the gate defining monomeric beads are represented in red: in this experiment 185,628 beads (62 % of the starting number) were sorted. Figure 4(b) is a dot plot of green vs. red fluorescence intensity for all sorted beads. Beads with a green fluoresence intensity value above the threshold value (depicted as green dots in Figure 4b) were individually dispensed into 96 well plates.

Once dispensed, hits were verified by fluorescence microscopy. Table 5 lists the number of positive beads sorted and verified as 'hit' beads from each bin. A total of 580 positive beads were isolated out of which 395 were verified as 'hit' beads (Table 5). This large discrepancy is due to the relatively low setting of the threshold value, resulting in sorting of beads with background fluorescence. Tags from 106 verified hits chosen from cell culture media 1-3 on the final split were subsequently analysed to determine their cell culture history (i.e. differentiation protocol), as detailed in the next section of the report.

Figure 4(a) and (b): COPAS dot plots, showing (a) the gate used to select monomeric beads (red dots) and (b) the threshold fluorescence intensity used to select positive 'hit' beads (green dots). The dot plots contain combined data recorded from all bins.

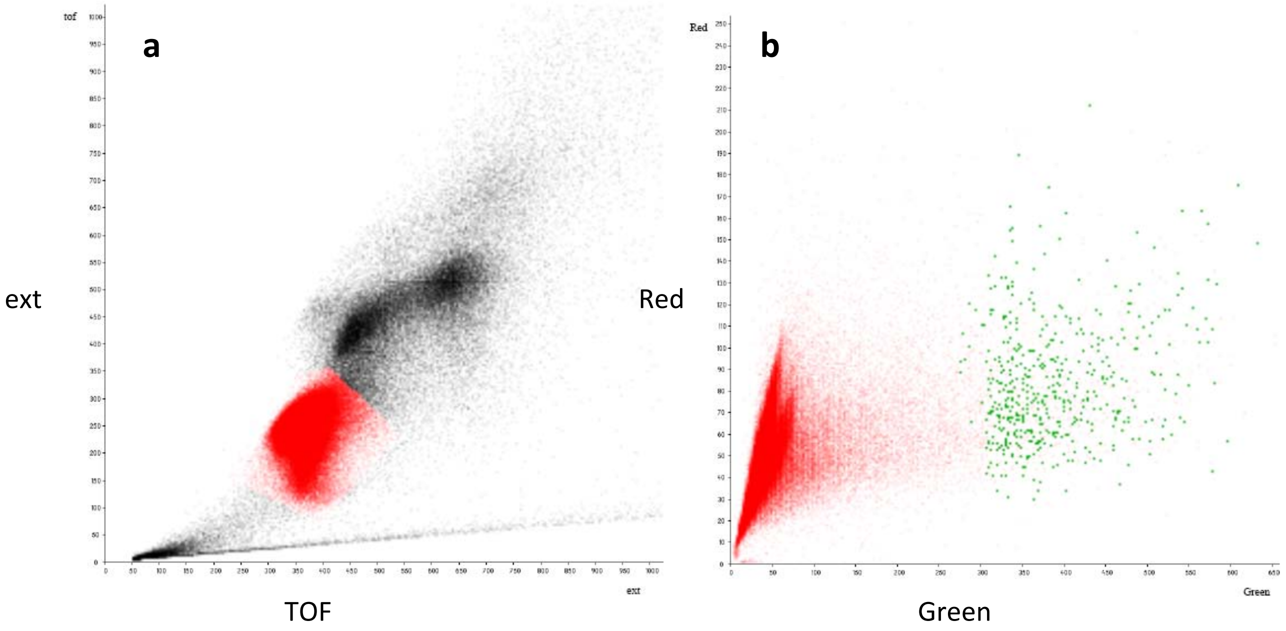

4. BEAD SCREENING

4.2. FLOW SORTING OF 'HITS' CONTINUED

Table 5: Number of positive beads sorted, number of verified 'hits' from each bin.

Bin Number	1	2	3	4	5	6	7	8	9	10	Total
Number of positive beads sorted	56	290	63	0	41	50	0	58	0	22	580
Number of verified hits	47	179	46	0	19	38	0	57	0	9	395

5. TAG DECONVOLUTION

Following bead sorting, tags from each hit within the chosen subset of 106 validated hits from cell culture media 1-3 on the final split, are analysed by flow cytometry and related to the cell culture history. During this process, data is lost or excluded owing to various factors and this is summarized in Table 6.

The flow cytometry data acquisition was performed in a series of sessions and prior to each session a reference tag set was run to calibrate side/forward scatter and fluorescence intensity gates. Dot plots and histograms for each session are shown in Annex 3. The data was loaded into Ariadne™ which automatically identifies tags based on four unique parameter values (forward and side scatter and fluorescence colour and intensity).

Determination of tag identity is performed by an algorithm that measures the number of events (minimum 3) within a gate, and the cluster tightness (Table 7). If two or more tight clusters are present, the ratio of tag numbers between the largest and second largest cluster must be greater or equal to three.

Ariadne™ relates the tag identity to a cell culture medium using the tag assignment (Table 4), allowing it to log the cell culture history of each hit (Table 8).

Table 6: Summary of data attrition owing to various factors

Bin Number	1	2	3	4	5	6	7	8	9	10	Total
Number of verified 'hits'	47	179	46	0	19	38	0	57	0	9	395
Number of beads chosen for tag analysis	33	37	36	0	0	0	0	0	0	0	106
Number of beads lost during digestion and sample preparation	(1)	(3)	(2)	0	0	0	0	0	0	0	(6)
Number of beads analysed by flow cytometry	32	34	34	0	0	0	0	0	0	0	100
Number of beads with no tagging data	(5)	0	0	0	0	0	0	0	0	0	(5)
Number of beads with incomplete tagging data	(4)	(1)	(3)	0	0	0	0	0	0	0	(8)
Number of beads containing too many tag clusters	(0)	0	0	0	0	0	0	0	0	0	(0)
Number of hits with conclusive tagging data	23	33	31	0	0	0	0	0	0	0	87

Table 7: Summary of the minimum, maximum and average number of tags derived from beads in each of the cell culture media. This data demonstrates that the average number of tags for all media is well above the minimum required to link a cell culture medium to a bead (3).

Split	Medium ID	# of beads	Minimum	Average	Maximum
1	1	9	20	40	66
1	2	10	31	56	91
1	3	6	54	71	103
1	4	12	8	37	78
1	5	9	23	43	73
1	6	6	48	71	113
1	7	5	30	41	54
1	8	10	18	40	61
1	9	10	4	9	23
1	10	10	14	31	56
2	1	4	6	11	15
2	2	15	4	25	50
2	3	8	13	25	38
2	4	7	8	17	36
2	5	8	11	44	98
2	6	6	6	19	35
2	7	14	8	43	99
2	8	11	6	49	95
2	9	10	15	45	96
2	10	4	23	31	41
3	1	11	6	24	61
3	2	11	8	28	70
3	3	6	11	23	37
3	4	16	8	46	96
3	5	5	20	44	83
3	6	8	12	36	78
3	7	9	7	30	56
3	8	7	18	25	32
3	9	7	3	43	78
3	10	7	11	26	55
4	1	23	-	-	-
4	2	33	-	-	-
4	3	31	-	-	-
4	4	0	-	-	-
4	5	0	-	-	-
4	6	0	-	-	-
4	7	0	-	-	-
4	8	0	-	-	-
4	9	0	-	-	-
4	10	0	-	-	-

Table 8: Information for each 'hit': i) the bead ID; ii) tag acquisition session number; iii) flow cytometry tag data file name; iv) series of cell culture media, i.e. protocol (number of tags upon which assignment of each cell culture media is based are in parentheses, "–" represents unreadable tag data) and v) whether the bead passes the tag deconvolution.

Bead ID	Tag acquisition session #	Tag data filename	Split 1 media (# of tags)	Split 2 media (# of tags)	Split 3 media (# of tags)	Split 4 media	Pass tag deconvolution criteria
1	1	Bin3_plateA1_C10.LMD	8 (48)	4 (37)	1 (12)	3	pass
2	1	Bin2_plateA1_A1.LMD	7 (30)	8 (62)	7 (56)	2	pass
3	1	Bin2_plateA1_A2.LMD	10 (14)	5 (61)	8 (29)	2	pass
4	1	Bin2_plateA1_A3.LMD	6 (105)	7 (52)	9 (43)	2	pass
5	1	Bin2_plateA1_A4.LMD	1 (59)	2 (44)	2 (50)	2	pass
6	1	Bin2_plateA1_A5.LMD	8 (49)	8 (95)	2 (54)	2	pass
7	1	Bin2_plateA1_A6.LMD	5 (41)	2 (47)	10 (55)	2	pass
8	1	Bin2_plateA1_A7.LMD	4 (8)	2 (7)	6 (31)	2	pass
9	1	Bin2_plateA1_A8.LMD	5 (37)	2 (22)	1 (6)	2	pass
10	1	Bin2_plateA1_A9.LMD	4 (49)	7 (63)	10 (23)	2	pass
11	1	Bin2_plateA1_A10.LMD	3 (57)	8 (60)	4 (54)	2	pass
12	1	Bin2_plateA1_A11.LMD	2 (31)	8 (27)	5 (83)	2	pass
13	1	Bin2_plateA1_A12.LMD	10 (44)	8 (6)	3 (37)	2	pass
14	1	Bin2_plateA1_B1.LMD	8 (48)	3 (24)	6 (41)	2	pass
15	1	Bin2_plateA1_B2.LMD	9 (12)	3 (31)	9 (31)	2	pass
16	1	Bin2_plateA1_B3.LMD	2 (76)	5 (98)	4 (69)	2	pass
17	1	Bin2_plateA1_B4.LMD	9 (23)	4 (9)	10 (36)	2	pass
18	1	Bin2_plateA1_B5.LMD	9 (9)	1 (11)	6 (23)	2	pass
19	1	Bin2_plateA1_B6.LMD	10 (29)	2 (4)	10 (12)	2	pass
20	1	Bin2_plateA1_B7.LMD	4 (23)	9 (28)	3 (11)	2	pass
21	1	Bin2_plateA1_B8.LMD	5 (23)	6 (6)	4 (25)	2	pass
22	1	Bin3_plateA1_A1.LMD	7 (54)	4 (13)	1 (61)	3	pass
23	1	Bin3_plateA1_A2.LMD	8 (61)	2 (50)	9 (48)	3	pass
24	1	Bin3_plateA1_A3.LMD	1 (57)	9 (41)	4 (75)	3	pass
25	1	Bin3_plateA1_A4.LMD	9 (10)	5 (63)	8 (18)	3	pass
26	1	Bin3_plateA1_A5.LMD	10 (56)	8 (56)	7 (39)	3	pass
27	1	Bin3_plateA1_A6.LMD	1 (24)	6 (23)	1 (15)	3	pass
28	1	Bin3_plateA1_A7.LMD	10 (62)	-	7 (19)	3	fail
29	1	Bin3_plateA1_A8.LMD	4 (33)	1 (15)	1 (44)	3	pass
30	1	Bin3_plateA1_A9.LMD	6 (113)	10 (27)	10 (13)	3	pass
31	1	Bin3_plateA1_A10.LMD	4 (78)	3 (38)	10 (30)	3	pass
32	1	Bin3_plateA1_A11.LMD	8 (34)	7 (15)	6 (78)	3	pass
33	1	Bin3_plateA1_A12.LMD	5 (64)	3 (20)	1 (7)	3	pass
34	1	Bin3_plateA1_B1.LMD	8 (41)	8 (47)	5 (30)	3	pass
35	1	Bin3_plateA1_B2.LMD	4 (46)	7 (52)	6 (16)	3	pass
36	1	Bin3_plateA1_B3.LMD	2 (43)	7 (46)	2 (31)	3	pass
37	1	Bin3_plateA1_B4.LMD	9 (4)	2 (22)	3 (32)	3	pass
38	1	Bin3_plateA1_B5.LMD	-	-	-	3	fail
39	1	Bin3_plateA1_B6.LMD	1 (66)	4 (8)	8 (32)	3	pass
40	1	Bin3_plateA1_B7.LMD	3 (103)	9 (96)	5 (53)	3	pass
41	1	Bin3_plateA1_B8.LMD	8 (44)	10 (41)	8 (20)	3	pass
42	1	Bin3_plateA1_B9.LMD	5 (34)	2 (15)	9 (78)	3	pass
43	1	Bin3_plateA1_B10.LMD	6 (50)	2 (31)	2 (8)	3	pass
44	1	Bin3_plateA1_B11.LMD	3 (57)	4 (17)	9 (3)	3	pass
45	1	Bin3_plateA1_B12.LMD	2 (77)	8 (53)	2 (10)	3	pass
46	1	Bin3_plateA1_C1.LMD	2 (39)	9 (42)	4 (29)	3	pass
47	1	Bin3_plateA1_C2.LMD	5 (42)	9 (17)	3 (28)	3	pass
48	1	Bin3_plateA1_C3.LMD	4 (36)	10 (23)	7 (30)	3	pass

Bead ID	Tag acquisition session #	Tag data filename	Split 1 media (# of tags)	Split 2 media (# of tags)	Split 3 media (# of tags)	Split 4 media	Pass tag deconvolution criteria
49	1	Bin3_plateA1_C4.LMD	10 (26)	2 (28)	1 (19)	3	pass
50	1	Bin3_plateA1_C5.LMD	1 (27)	5 (30)	8 (25)	3	pass
51	1	Bin3_plateA1_C6.LMD	8 (18)	9 (54)	4 (20)	3	pass
52	1	Bin3_plateA1_C7.LMD	4 (55)	5 (43)	8 (18)	3	pass
53	1	Bin3_plateA1_C8.LMD	10 (7)	-	-	3	fail
54	1	Bin3_plateA1_C9.LMD	9 (8)	7 (59)	6 (12)	3	pass
55	2	Bin2_plateA1_C10.LMD	2 (54)	3 (15)	6 (38)	2	pass
56	2	Bin1_plateA1_A1.LMD	9 (5)	2 (10)	7 (36)	1	pass
57	2	Bin1_plateA1_A2.LMD	1 (32)	4 (23)	2 (8)	1	pass
58	2	Bin1_plateA1_A3.LMD	1 (20)	3 (14)	1 (15)	1	pass
59	2	Bin1_plateA1_A4.LMD	2 (49)	7 (46)	7 (23)	1	pass
60	2	Bin1_plateA1_A5.LMD	2 (52)	7 (35)	2 (26)	1	pass
61	2	Bin1_plateA1_A6.LMD	9 (8)	7 (101)	2 (70)	1	pass
62	2	Bin1_plateA1_A7.LMD	6 (50)	8 (43)	4 (8)	1	pass
63	2	Bin1_plateA1_A8.LMD	5 (73)	7 (56)	9 (64)	1	pass
64	2	Bin1_plateA1_A9.LMD	10 (23)	-	4 (31)	1	fail
65	2	Bin1_plateA1_A10.LMD	8 (25)	5 (24)	4 (66)	1	pass
66	2	Bin1_plateA1_A11.LMD	4 (30)	7 (17)	10 (11)	1	pass
67	2	Bin1_plateA1_A12.LMD	5 (53)	9 (53)	4 (73)	1	pass
68	2	Bin1_plateA1_B1.LMD	4 (25)	10 (29)	-	1	fail
69	2	Bin1_plateA1_B2.LMD	-	-	-	1	fail
70	2	Bin1_plateA1_B3.LMD	2 (52)	7 (35)	7 (35)	1	pass
71	2	Bin1_plateA1_B4.LMD	3 (54)	8 (26)	3 (13)	1	pass
72	2	Bin1_plateA1_B5.LMD	3 (104)	-	6 (43)	1	fail
73	2	Bin1_plateA1_B6.LMD	1 (39)	5 (23)	7 (7)	1	pass
74	2	Bin1_plateA1_B7.LMD	3 (97)	10 (41)	1 (35)	1	pass
75	2	Bin1_plateA1_B8.LMD	9 (4)	4 (15)	6 (46)	1	pass
76	2	Bin1_plateA1_B9.LMD	4 (39)	7 (32)	7 (25)	1	pass
77	2	Bin1_plateA1_B10.LMD	10 (29)	2 (16)	7 (20)	1	pass
78	2	Bin1_plateA1_B11.LMD	5 (11)	-	-	1	fail
79	2	Bin1_plateA1_B12.LMD	4 (10)	6 (15)	2 (16)	1	pass
80	2	Bin1_plateA1_C1.LMD	-	-	-	1	fail
81	2	Bin1_plateA1_C2.LMD	10 (19)	7 (8)	4 (34)	1	pass
82	2	Bin1_plateA1_C3.LMD	6 (60)	6 (12)	5 (35)	1	pass
83	2	Bin1_plateA1_C4.LMD	9 (7)	3 (32)	4 (17)	1	pass
84	2	Bin1_plateA1_C5.LMD	6 (48)	1 (16)	2 (20)	1	pass
85	2	Bin1_plateA1_C6.LMD	-	-	-	1	fail
86	2	Bin1_plateA1_C7.LMD	-	-	-	1	fail
87	2	Bin1_plateA1_C8.LMD	-	-	-	1	fail
88	2	Bin2_plateA1_B9.LMD	4 (36)	9 (71)	4 (96)	2	pass
89	2	Bin2_plateA1_B10.LMD	10 (39)	8 (68)	4 (21)	2	pass
90	2	Bin2_plateA1_B11.LMD	1 (34)	2 (25)	4 (17)	2	pass
91	2	Bin2_plateA1_B12.LMD	5 (23)	5 (12)	1 (29)	2	pass
92	2	Bin2_plateA1_C1.LMD	10 (29)	9 (37)	8 (32)	2	pass
93	2	Bin2_plateA1_C2.LMD	7 (39)	6 (24)	9 (34)	2	pass
94	2	Bin2_plateA1_C3.LMD	3 (57)	1 (6)	2 (18)	2	pass
95	2	Bin2_plateA1_C4.LMD	2 (53)	-	6 (35)	2	fail
96	2	Bin2_plateA1_C5.LMD	2 (91)	9 (15)	4 (72)	2	pass
97	2	Bin2_plateA1_C6.LMD	10 (24)	6 (35)	5 (20)	2	pass
98	2	Bin2_plateA1_C7.LMD	8 (28)	2 (34)	4 (58)	2	pass
99	2	Bin2_plateA1_C8.LMD	7 (38)	2 (21)	3 (16)	2	pass
100	2	Bin2_plateA1_C9.LMD	7 (46)	3 (25)	1 (24)	2	pass

6. PROTOCOL ANALYSIS

6.1 DATASET REVIEW

Once a dataset of protocols has been established, Ariadne^{TM} allows post-acquisition resetting of gates on the COPAS sorting plot to specify a subset of hits for further analysis. For example, if 'hits were sorted using two antibodies, the user can select those which are positive for both antibodies. No post acquisition flow sort criterion was applied in this study.

Table 9 lists the bead IDs included in the dataset analyzed by Ariadne^{TM} and presented in the following sections of the report. The dataset only includes beads which fall within the (reset) COPAS gates and can be assigned a full cell culture history. Ariadne^{TM} allows a range of analyses to be performed, to select protocols for validation. Analysis methodologies include linkage analysis which identifies frequently occurring media combinations; fingerprint analysis which clusters groups of beads with identical or similar protocols (i.e. protocols with common cell culture media on the same split) and calculates the probability of these clusters occurring randomly; and methods of comparing entire protocols such as hierarchical clustering and a similarity matrix. These results are presented and discussed in the following sections.

Table 9: Dataset Review. The following parameters are shown for each hit: Bead ID, protocol ("–" represents unreadable tag data), whether the bead passed the tag deconvolution, whether it falls within the (reset) COPAS gate and whether it is included in the protocol analysis dataset.

	Protocol				Inclusion Criteria		
Bead ID	Split 1 media	Split 2 media	Split 3 media	Split 4 media	Tag deconvolution	Flow sort criteria	Included in analysis
1	8	4	1	3	pass	pass	Yes
2	7	8	7	2	pass	pass	Yes
3	10	5	8	2	pass	pass	Yes
4	6	7	9	2	pass	pass	Yes
5	1	2	2	2	pass	pass	Yes
6	8	8	2	2	pass	pass	Yes
7	5	2	10	2	pass	pass	Yes
8	4	2	6	2	pass	pass	Yes
9	5	2	1	2	pass	pass	Yes
10	4	7	10	2	pass	pass	Yes
11	3	8	4	2	pass	pass	Yes
12	2	8	5	2	pass	pass	Yes
13	10	8	3	2	pass	pass	Yes
14	8	3	6	2	pass	pass	Yes
15	9	3	9	2	pass	pass	Yes
16	2	5	4	2	pass	pass	Yes
17	9	4	10	2	pass	pass	Yes
18	9	1	6	2	pass	pass	Yes
19	10	2	10	2	pass	pass	Yes
20	4	9	3	2	pass	pass	Yes
21	5	6	4	2	pass	pass	Yes
22	7	4	1	3	pass	pass	Yes
23	8	2	9	3	pass	pass	Yes
24	1	9	4	3	pass	pass	Yes
25	9	5	8	3	pass	pass	Yes
26	10	8	7	3	pass	pass	Yes
27	1	6	1	3	pass	pass	Yes
28	10	-	7	3	fail	pass	No
29	4	1	1	3	pass	pass	Yes
30	6	10	10	3	pass	pass	Yes
31	4	3	10	3	pass	pass	Yes

	Protocol				Inclusion Criteria		
Bead ID	Split 1 media	Split 2 media	Split 3 media	Split 4 media	Tag deconvolution	Flow sort criteria	Included in analysis
32	8	7	6	3	pass	pass	Yes
33	5	3	1	3	pass	pass	Yes
34	8	8	5	3	pass	pass	Yes
35	4	7	6	3	pass	pass	Yes
36	2	7	2	3	pass	pass	Yes
37	9	2	3	3	pass	pass	Yes
38	-	-	-	3	fail	pass	No
39	1	4	8	3	pass	pass	Yes
40	3	9	5	3	pass	pass	Yes
41	8	10	8	3	pass	pass	Yes
42	5	2	9	3	pass	pass	Yes
43	6	2	2	3	pass	pass	Yes
44	3	4	9	3	pass	pass	Yes
45	2	8	2	3	pass	pass	Yes
46	2	9	4	3	pass	pass	Yes
47	5	9	3	3	pass	pass	Yes
48	4	10	7	3	pass	pass	Yes
49	10	2	1	3	pass	pass	Yes
50	1	5	8	3	pass	pass	Yes
51	8	9	4	3	pass	pass	Yes
52	4	5	8	3	pass	pass	Yes
53	10	-	-	3	fail	pass	No
54	9	7	6	3	pass	pass	Yes
55	2	3	6	2	pass	pass	Yes
56	9	2	7	1	pass	pass	Yes
57	1	4	2	1	pass	pass	Yes
58	1	3	1	1	pass	pass	Yes
59	2	7	7	1	pass	pass	Yes
60	2	7	2	1	pass	pass	Yes
61	9	7	2	1	pass	pass	Yes
62	6	8	4	1	pass	pass	Yes
63	5	7	9	1	pass	pass	Yes
64	10	-	4	1	fail	pass	No
65	8	5	4	1	pass	pass	Yes
66	4	7	10	1	pass	pass	Yes
67	5	9	4	1	pass	pass	Yes
68	4	10	-	1	fail	pass	No
69	-	-	-	1	fail	pass	No
70	2	7	7	1	pass	pass	Yes
71	3	8	3	1	pass	pass	Yes
72	3	-	6	1	fail	pass	No
73	1	5	7	1	pass	pass	Yes
74	3	10	1	1	pass	pass	Yes
75	9	4	6	1	pass	pass	Yes
76	4	7	7	1	pass	pass	Yes
77	10	2	7	1	pass	pass	Yes
78	5	-	-	1	fail	pass	No
79	4	6	2	1	pass	pass	Yes
80	-	-	-	1	fail	pass	No
81	10	7	4	1	pass	pass	Yes
82	6	6	5	1	pass	pass	Yes
83	9	3	4	1	pass	pass	Yes
84	6	1	2	1	pass	pass	Yes
85	-	-	-	1	fail	pass	No
86	-	-	-	1	fail	pass	No
87	-	-	-	1	fail	pass	No
88	4	9	4	2	pass	pass	Yes
89	10	8	4	2	pass	pass	Yes

	Protocol				Inclusion Criteria		
Bead ID	Split 1 media	Split 2 media	Split 3 media	Split 4 media	Tag deconvolution	Flow sort criteria	Included in analysis
90	1	2	4	2	pass	pass	Yes
91	5	5	1	2	pass	pass	Yes
92	10	9	8	2	pass	pass	Yes
93	7	6	9	2	pass	pass	Yes
94	3	1	2	2	pass	pass	Yes
95	2	-	6	2	fail	pass	No
96	2	9	4	2	pass	pass	Yes
97	10	6	5	2	pass	pass	Yes
98	8	2	4	2	pass	pass	Yes
99	7	2	3	2	pass	pass	Yes
100	7	3	1	2	pass	pass	Yes

6.2. LINKAGE ANALYSIS

A schematic diagram of the split-pool experiment is shown in Figure 5. Each rectangle corresponds to a cell culture medium and each row of rectangles corresponds to a split. The black upper number within each rectangle is the medium number and the grey number below is the number of beads within the analysis dataset that passed through that medium (the height of each rectangle is proportional to this number). The linkage lines between rectangles depict frequently occurring combinations of media resulting in an overlay of all protocols in the dataset. The opacity of the linkage lines is proportional to the number of protocols that feature a certain media combination (in this dataset, the darkest and lightest lines correspond to 8 and 1 beads respectively). An example of the information the linkage analysis provides is the highly represented transition between cell culture medium 4 (split 3) and medium 2 (split 4), 8 out of the 16 beads which start in medium 4 on split 3 transition to medium 4 on the final split. The high representation of this transition indicates that these media may be relevant for the differentiation of neuronal (Sox1 positive) cells from mES cells.

Figure 5: Schematic diagram showing an overlay of all protocols in the analysis dataset. The darkest line corresponds to 8 beads passing between the 2 connected cell culture media.

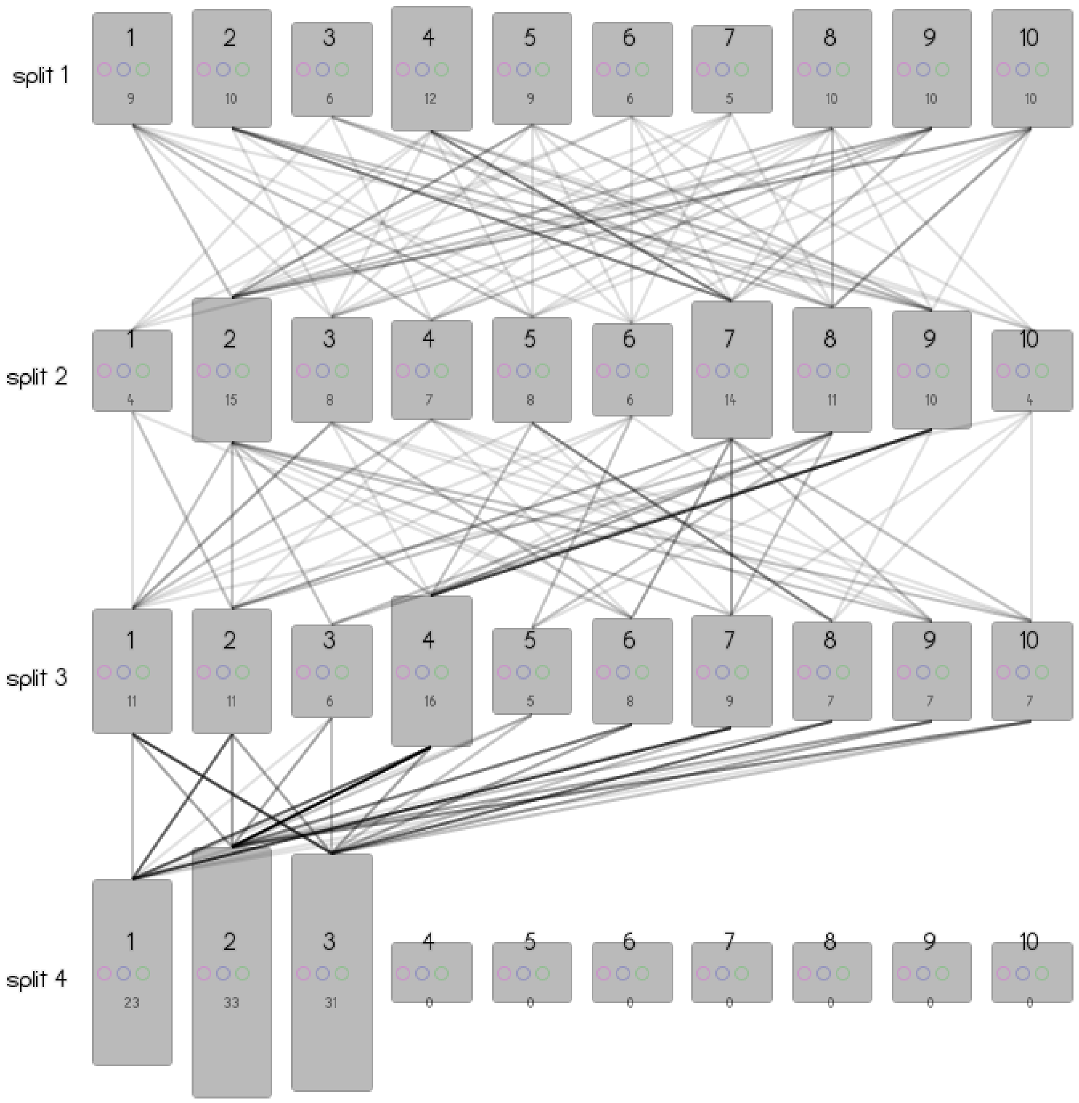

6. PROTOCOL ANALYSIS

6.3. FINGERPRINT ANALYSIS

Fingerprint analysis is a method of finding and comparing hits derived from identical protocols (i.e. four out of four matching cell culture media) and/or groups of beads with similar protocols (i.e. two or three matching media). This analysis method is complementary to linkage analysis which only considers linkages between adjacent splits.

Table 10 details a fingerprint analysis of the dataset. Each row of the table represents a group of hits clustered according to protocol similarity. Column 2 identifies how many cell culture media the group has in common;, column 3 how many hits are included in the group; and column 4 the probability of the group occurring by chance. Columns 5 - 8 identify the common media in the groups ("-" represents divergence of media), and column 9 lists the bead IDs included in the group. The table is ordered first by probability value and then by number of media matches.

The groups which have the lowest probability of occurring by chance (p = 0.00028859) are three groups of 8 beads. The beads within the second group share cell culture medium 7 (split 2) and medium 1 (split 4). A subset of 3 beads from this group also share medium 2 (split 1). Furthermore, this subset contains a doublet of beads which have identical protocols (four out of four matching cell culture media) (protocol = 2,7,7,1).

Table 10: Fingerprint analysis of protocols. Each row of the table represents a cluster of similar protocols. The second column identifies the number of common cell culture media in a group, the third column the number of hits included in the group, the fourth column the probability of the group occurring by chance, columns 5 - 8 the identity of the common media ("-" represents divergence of media), and the final column lists the beads which yielded the protocols included in the group. The data is sorted first by probability value then by number of media matches.

Group #	Matches (out of 4)	# of beads	Probability (1 = 100%)	Split 1 media #	Split 2 media #	Split 3 media #	Split 4 media #	Bead ID's
1	2	8	0.00028859	-	2	-	2	5,7,8,9,19,90,98,99
2	2	8	0.00028859	-	7	-	1	59,60,61,63,66,70,76,81
3	2	8	0.00028859	-	-	4	2	11,16,21,88,89,90,96,98
4	2	6	0.02504662	8	-	-	3	1,23,32,34,41,51
5	2	6	0.02504662	10	-	-	2	3,13,19,89,92,97
6	2	6	0.02504662	-	8	-	2	2,6,11,12,13,89
7	2	6	0.02504662	-	9	4	-	24,46,51,67,88,96
8	2	6	0.02504662	-	-	1	3	1,22,27,29,33,49
9	2	6	0.02504662	-	-	7	1	56,59,70,73,76,77
10	3	3	0.09522599	2	7	-	1	59,60,70
11	3	3	0.09522599	-	5	8	3	25,50,52
12	3	3	0.09522599	-	7	6	3	32,35,54
13	3	3	0.09522599	-	7	7	1	59,70,76
14	3	3	0.09522599	-	9	4	3	24,46,51
15	2	5	0.17397404	4	-	-	3	29,31,35,48,52
16	2	5	0.17397404	-	2	-	3	23,37,42,43,49
17	2	5	0.17397404	-	9	-	3	24,40,46,47,51
18	2	5	0.17397404	-	-	2	1	57,60,61,79,84
19	2	5	0.17397404	-	-	4	1	62,65,67,81,83
20	2	5	0.17397404	-	-	8	3	25,39,41,50,52
21	4	2	0.31279248	2	7	7	1	59,70
22	2	4	0.71559697	1	-	-	3	24,27,39,50
23	2	4	0.71559697	2	7	-	-	36,59,60,70
24	2	4	0.71559697	2	-	-	2	12,16,55,96
25	2	4	0.71559697	4	7	-	-	10,35,66,76
26	2	4	0.71559697	4	-	-	2	8,10,20,88
27	2	4	0.71559697	5	-	-	2	7,9,21,91
28	2	4	0.71559697	7	-	-	2	2,93,99,100
29	2	4	0.71559697	9	-	-	1	56,61,75,83

Group #	Matches (out of 4)	# of beads	Probability (1 = 100%)	Split 1 media #	Split 2 media #	Split 3 media #	Split 4 media #	Bead ID's
30	2	4	0.71559697	-	3	-	2	14,15,55,100
31	2	4	0.71559697	-	4	-	3	1,22,39,44
32	2	4	0.71559697	-	5	8	-	3,25,50,52
33	2	4	0.71559697	-	7	-	3	32,35,36,54
34	2	4	0.71559697	-	9	-	2	20,88,92,96
35	2	4	0.71559697	-	-	6	2	8,14,18,55
36	2	4	0.71559697	-	-	10	2	7,10,17,19
37	3	2	0.97882658	1	2	-	2	5,90
38	3	2	0.97882658	1	-	8	3	39,50
39	3	2	0.97882658	2	7	2	-	36,60
40	3	2	0.97882658	2	7	7	-	59,70
41	3	2	0.97882658	2	9	4	-	46,96
42	3	2	0.97882658	2	-	2	3	36,45
43	3	2	0.97882658	2	-	4	2	16,96
44	3	2	0.97882658	2	-	7	1	59,70
45	3	2	0.97882658	4	7	10	-	10,66
46	3	2	0.97882658	4	7	-	1	66,76
47	3	2	0.97882658	4	9	-	2	20,88
48	3	2	0.97882658	5	2	-	2	7,9
49	3	2	0.97882658	5	-	1	2	9,91
50	3	2	0.97882658	10	8	-	2	13,89
51	3	2	0.97882658	10	-	8	2	3,92
52	3	2	0.97882658	-	2	4	2	90,98
53	3	2	0.97882658	-	2	7	1	56,77
54	3	2	0.97882658	-	2	9	3	23,42
55	3	2	0.97882658	-	2	10	2	7,19
56	3	2	0.97882658	-	3	6	2	14,55
57	3	2	0.97882658	-	4	1	3	1,22
58	3	2	0.97882658	-	7	2	1	60,61
59	3	2	0.97882658	-	8	4	2	11,89
60	3	2	0.97882658	-	9	4	2	88,96
61	2	3	0.99955547	1	-	-	1	57,58,73
62	2	3	0.99955547	2	-	2	-	36,45,60
63	2	3	0.99955547	2	-	4	-	16,46,96
64	2	3	0.99955547	2	-	-	1	59,60,70
65	2	3	0.99955547	2	-	-	3	36,45,46
66	2	3	0.99955547	4	-	10	-	10,31,66
67	2	3	0.99955547	4	-	-	1	66,76,79
68	2	3	0.99955547	5	2	-	-	7,9,42
69	2	3	0.99955547	5	-	1	-	9,33,91
70	2	3	0.99955547	5	-	-	3	33,42,47
71	2	3	0.99955547	6	-	-	1	62,82,84
72	2	3	0.99955547	8	-	4	-	51,65,98
73	2	3	0.99955547	8	-	-	2	6,14,98
74	2	3	0.99955547	9	-	6	-	18,54,75
75	2	3	0.99955547	9	-	-	2	15,17,18
76	2	3	0.99955547	9	-	-	3	25,37,54
77	2	3	0.99955547	10	2	-	-	19,49,77
78	2	3	0.99955547	10	8	-	-	13,26,89
79	2	3	0.99955547	-	3	1	-	33,58,100
80	2	3	0.99955547	-	5	-	2	3,16,91
81	2	3	0.99955547	-	5	-	3	25,50,52
82	2	3	0.99955547	-	6	-	2	21,93,97
83	2	3	0.99955547	-	7	2	-	36,60,61
84	2	3	0.99955547	-	7	6	-	32,35,54
85	2	3	0.99955547	-	7	7	-	59,70,76
86	2	3	0.99955547	-	8	4	-	11,62,89
87	2	3	0.99955547	-	8	-	3	26,34,45
88	2	3	0.99955547	-	10	-	3	30,41,48

Group #	Matches (out of 4)	# of beads	Probability (1 = 100%)	Split 1 media #	Split 2 media #	Split 3 media #	Split 4 media #	Bead ID's
89	2	3	0.99955547	-	-	1	2	9,91,100
90	2	3	0.99955547	-	-	2	2	5,6,94
91	2	3	0.99955547	-	-	2	3	36,43,45
92	2	3	0.99955547	-	-	3	2	13,20,99
93	2	3	0.99955547	-	-	4	3	24,46,51
94	2	3	0.99955547	-	-	6	3	32,35,54
95	2	3	0.99955547	-	-	9	2	4,15,93
96	2	3	0.99955547	-	-	9	3	23,42,44

6. PROTOCOL ANALYSIS
6.4. HIERACHICAL CLUSTERING DENDROGRAM

Hierarchical clustering is a method of grouping protocols according to similarity.

The hierarchical clustering illustrated in figure 6 has two sections. The upper dendrogram depicts the clustering structure and order. The lower coloured array is a graphical representation of the media combinations that comprise each protocol. The y-axis of the dendrogram measures intra-cluster similarity, i.e. horizontal bars closest to the bottom of the dendrogram link identical protocols whilst a horizontal bar half way up the y-axis represents a larger cluster containing beads whose protocols are not identical but do share some similarity. The hierarchical nature of the clustering means that beads are first clustered into small highly similar clusters, which are in turn included in larger clusters. This is repeated until the final cluster is reached (uppermost horizontal bar) which includes all beads in the dataset. Each node at the bottom of the dendrogram (leaf node) corresponds to a protocol. Each row of the coloured array corresponds to a split and each column of the array a protocol, with the columns aligned to the leaf nodes of the dendrogram. The horizontal coloured legend below the array specifies the colour associated with each media number. The dataset contains three clusters labeled A, B and C, which arise from the three cell culture media on the final split-pool cycle, from which the beads selected for deconvolution were chosen from.

Figure 6: Dendrogram showing the hierarchical clustering of similar protocols.

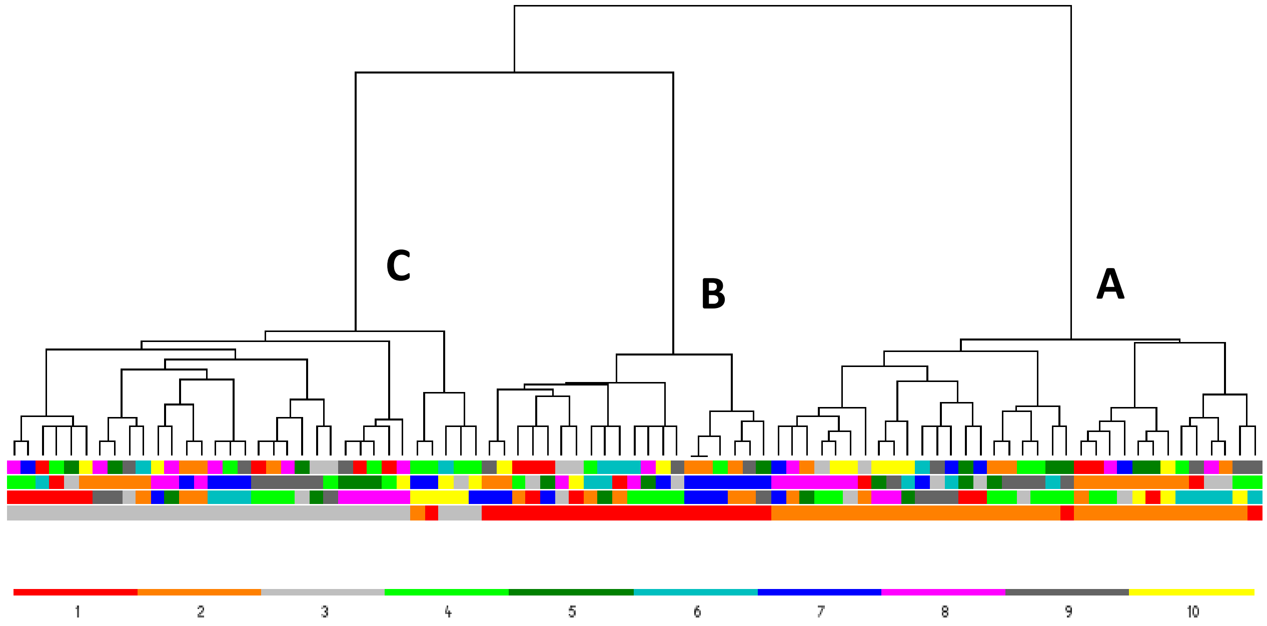

6. PROTOCOL ANALYSIS

6.5. SIMILARITY MATRIX

The similarity matrix is a diagrammatic representation of a pair-wise comparison of all protocols. Each column and each row corresponds to a protocol. The brightness of each cell in the matrix is proportional to the number of cell culture media shared by the two protocols. The brightest cell corresponds to protocols which have common media (i.e. identical protocols) in common, while a black cell corresponds to two protocols with no common media. The diagonal row of cells (from the top left to bottom right) corresponds to beads being compared to themselves. Beads are ordered along both the x- and y-axis according to the hierarchical clustering dendrogram illustrated in figure 6. The coloured arrays on the bottom and right of the matrix illustrate the protocol of each bead. The horizontal coloured legend below the matrix specifies the colour associated with each medium number.

The similarity matrix clearly displays clusters of similar protocols as square regions comprising brightly coloured cells. The size and overall brightness of the square regions are measures of the cluster size and intra-cluster protocol similarity, respectively. Additionally, the similarity matrix provides a global overview of the protocol similarity of beads or clusters to the remaining dataset. For example the darkness of the cells in the columns above cluster A indicate that the beads contained within cluster A have little similarity with the other beads in the dataset.

Figure 7: Similarity matrix comprising a pair-wise comparison of all protocols

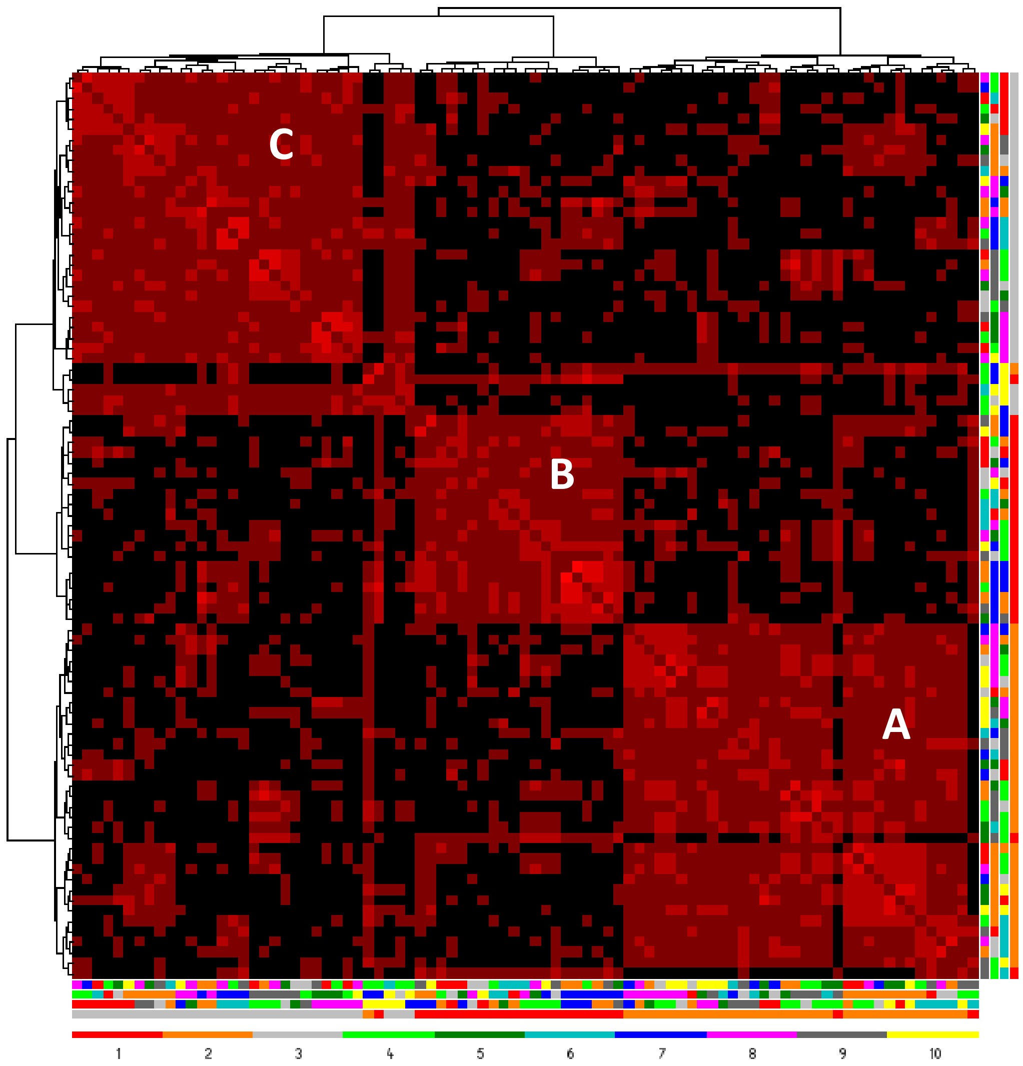

7. SUMMARY AND CONCLUSIONS

A CombiCult™ experiment was performed in which 40 serum-free cell culture media were screened in 10,000 combinations. The 40 media were tested over four split-pool cycles (performed on day 1, day 2, day 5 and day 7 respectively) with 10 media included in each cycle, such that 10,000 media combinations (protocols) are tested. A total of 300,000 beads were used so that on average each protocol was sampled by 30 beads. Following completion of the split-pool experiment (day 14) beads were screened using the expression of Sox1-GFP reporter gene and individual positive beads isolated.

Of the total number of monomeric beads sorted by COPAS (185,628), 395 (0.21 %) were sorted and verified as 'hits'. The tags from a sub-set of 106 beads were read to infer the series of cell culture media (protocol) that produced differentiated cells. Of the 106 'hit' beads selected for tag deconvolution, tags could be conclusively deconvoluted from 87 (82 %). Putative differentiation protocols were analysed using bespoke bioinformatics software Ariadne™ (v1.0) allowing protocols to be ranked and a subset to be selected for further validation and study. The 6 selected protocols are listed below in table 11 as well as in the executive summary (table 1).

The validated protocols numbered 1-3 form part of cluster B shown in the hierarchical clustering dendrogram and similarity matrix and share cell culture medium 7 on split 2 and medium 1 on the final split. The first protocol is represented by the only doublet beads sharing four out of four common cell culture media. Protocols numbered 4 - 6 form part of cluster C of the hierarchical clustering dendrogram and similarity matrix and have the general protocol structure of X-Y-4/8-3 and are present in groups 11 and 14 of the fingerprint analysis.

The 6 validated protocols therefore include two general types of protocol for the differentiation of Sox1 expressing neuronal cells from mES cells. One is dominated by media 7 on split 2 and media 1 on split 4. The second protocol type is dominated by media 4 or 8 on split 3 and media 3 on split 4.

Table 11: Top 6 Protocols determined by Ariadne™ (v1.0). The protocols are identified by the series of cell culture media on each split-pool cycle (split 1 - split 4), e.g. 2-7-7-1.

Validation #	Protocol	Bead Ids		Basal Medium	Supplements
1	2-7-7-1	59, 70	Split 1 (Day 1)	DMEM/F12/Neurobasal	N2 (0.5%), B27 (0.5%)
			Split 2 (Day 2)	STEMLINE	SCF (20ng/mL)
			Split 3 (Day 5)	STEMLINE	SCF (20ng/mL), BMP-2 (5ng/mL), TGF-β1 (5ng/mL), TPO (20ng/mL)
			Split 4 (Day 7)	DMEM/F12	B27 (1%), PDGF-AA (20ng/mL)
2	4-7-7-1	76	Split 1 (Day 1)	RPMI	B27 (1%), Activin A (50ng/mL), Sodium Butyrate (1 mM)
			Split 2 (Day 2)	STEMLINE	SCF (20ng/mL)
			Split 3 (Day 5)	STEMLINE	SCF (20ng/mL), BMP-2 (5ng/mL), TGF-β1 (5ng/mL), TPO (20ng/mL)
			Split 4 (Day 7)	DMEM/F12	B27 (1%), PDGF-AA (20ng/mL)
3	2-7-2-1	60	Split 1 (Day 1)	DMEM/F12/Neurobasal	N2 (0.5%), B27 (0.5%)
			Split 2 (Day 2)	STEMLINE	SCF (20ng/mL)
			Split 3 (Day 5)	DMEM/F12/Neurobasal	N2 (0.5%), B27 (0.5%), FGF b (20ng/mL), FGF-8 (100ng/mL), SHH (400ng/mL)
			Split 4 (Day 7)	DMEM/F12	B27 (1%), PDGF-AA (20ng/mL)
4	8-9-4-3	51	Split 1 (Day 1)	IMDM (75 %)/HAM F12 (25 %)	N2 (1%), B27 (1%), BSA (0.5 mg/mL)
			Split 2 (Day 2)	GMEM	KSR (5%), TGF-β1 (2ng/mL), BMP-2 (10ng/mL)
			Split 3 (Day 5)	RPMI	B27 (1%), EGF (20ng/mL), FGF b (2ng/mL), Noggin (100ng/mL)

Validation #	Protocol	Bead Ids		Basal Medium	Supplements
			Split 4 (Day 7)	DMEM/F12	N2 (1%), B27 (1%), FGF b (5ng/mL), DKK-1 (10ng/mL), Noggin (10ng/mL), IGF-1 (10ng/mL)
5	9-5-8-3	25	Split 1 (Day 1)	GMEM	KSR (5%), BMP-4 (1ng/mL)
			Split 2 (Day 2)	RPMI	Activin A (100ng/mL), Wnt-3A (25ng/mL)
			Split 3 (Day 5)	DMEM	ITS (1x), BSA (1mg/mL), PDGF BB (50ng/mL)
			Split 4 (Day 7)	DMEM/F12	N2 (1%), B27 (1%), FGF b (5ng/mL), DKK-1 (10ng/mL), Noggin (10ng/mL), IGF-1 (10ng/mL)
6	1-5-8-3	50	Split 1 (Day 1)	DMEM	N2 (1%)
			Split 2 (Day 2)	RPMI	Activin A (100ng/mL), Wnt-3A (25ng/mL)
			Split 3 (Day 5)	DMEM	ITS (1x), BSA (1mg/mL), PDGF BB (50ng/mL)
			Split 4 (Day 7)	DMEM/F12	N2 (1%), B27 (1%), FGF b (5ng/mL), DKK-1 (10ng/mL), Noggin (10ng/mL), IGF-1 (10ng/mL)

ANNEX 1: FLOW SORTING BIN 1 – File 1 of 1

Bin 1: COPAS scatter plots showing events for medium 1 of the final split-pool cycle (bin 1). In the first scatter plot (optical extinction [ext] vs. time of flight [TOF]), events depicted by a red dot fall within the gate defining monomeric beads and were sorted. The second scatter plot displays the fluorescent values of the sorted beads (green vs. red fluorescence intensity). Beads with a red fluorescent intensity above the threshold value are depicted as green dots and were individually dispensed into 96 well plates.

Number of beads individually dispensed: 56

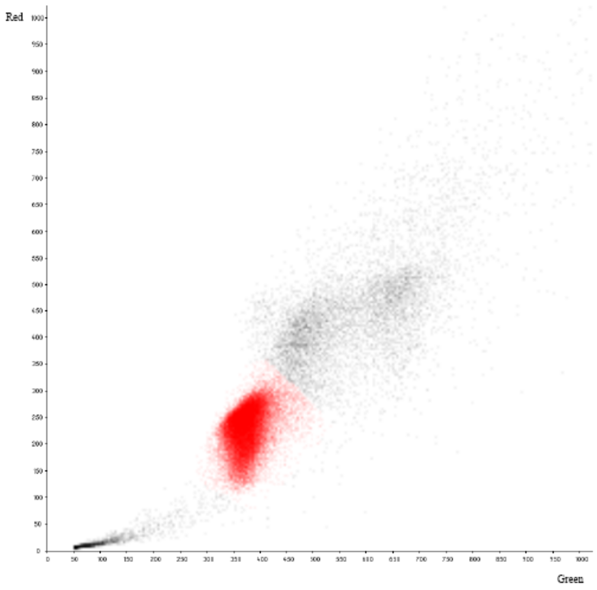

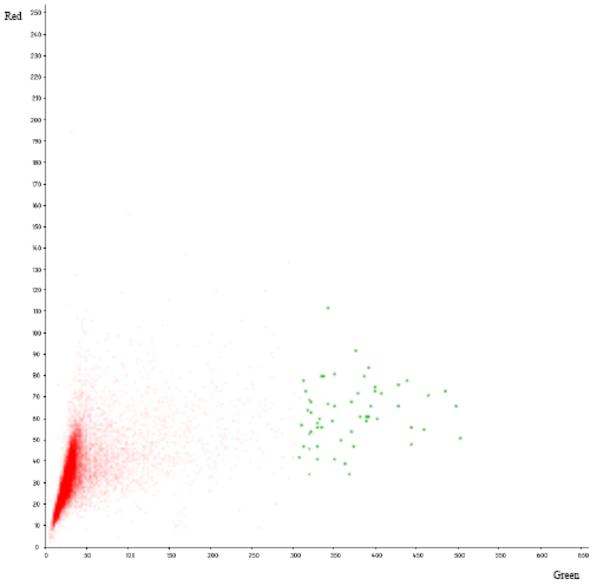

 Confidential

ANNEX 1: FLOW SORTING BIN 2 – File 1 of 3

Bin 2: COPAS scatter plots showing events for medium 2 of the final split-pool cycle (bin 2). In the first scatter plot (optical extinction [ext] vs. time of flight [TOF]), events depicted by a red dot fall within the gate defining monomeric beads and were sorted. The second scatter plot displays the fluorescent values of the sorted beads (green vs. red fluorescence intensity). Beads with a red fluorescent intensity above the threshold value are depicted as green dots and were individually dispensed into 96 well plates.

Number of beads individually dispensed: 2

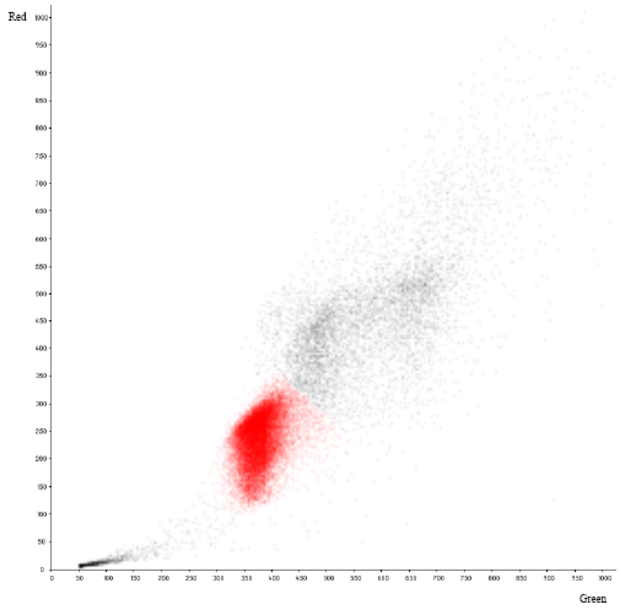

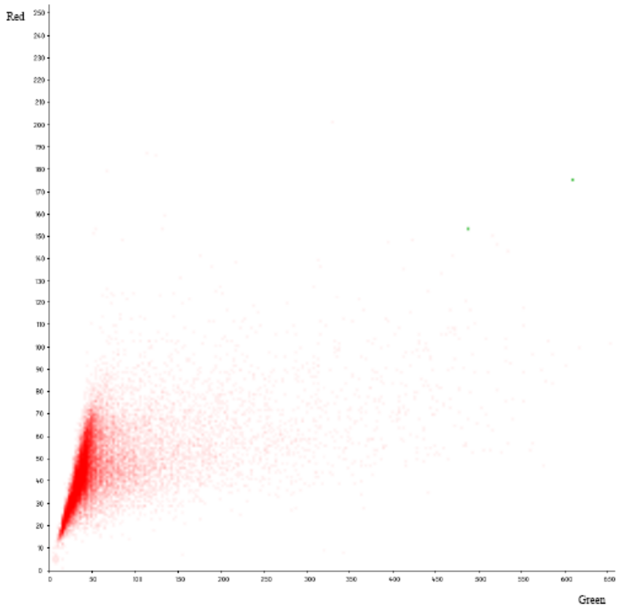

ANNEX 1: FLOW SORTING BIN 2 – File 2 of 3

Bin 2: COPAS scatter plots showing events for medium 2 of the final split-pool cycle (bin 2). In the first scatter plot (optical extinction [ext] vs. time of flight [TOF]), events depicted by a red dot fall within the gate defining monomeric beads and were sorted. The second scatter plot displays the fluorescent values of the sorted beads (green vs. red fluorescence intensity). Beads with a red fluorescent intensity above the threshold value are depicted as green dots and were individually dispensed into 96 well plates.

Number of beads individually dispensed: 96

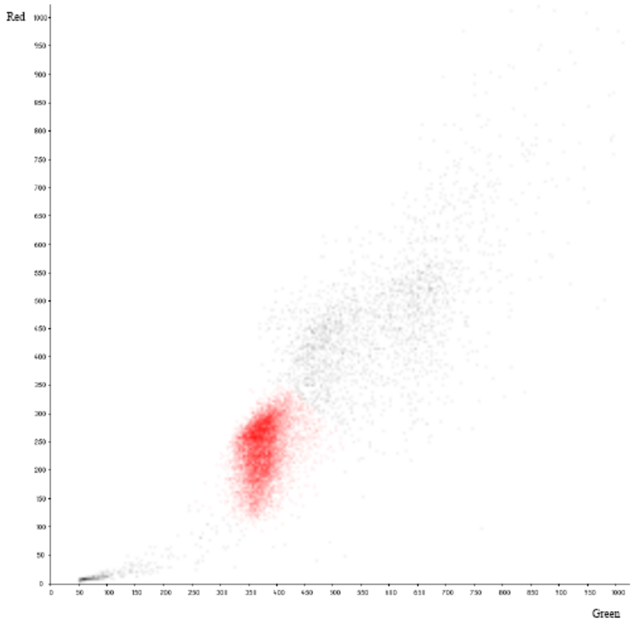

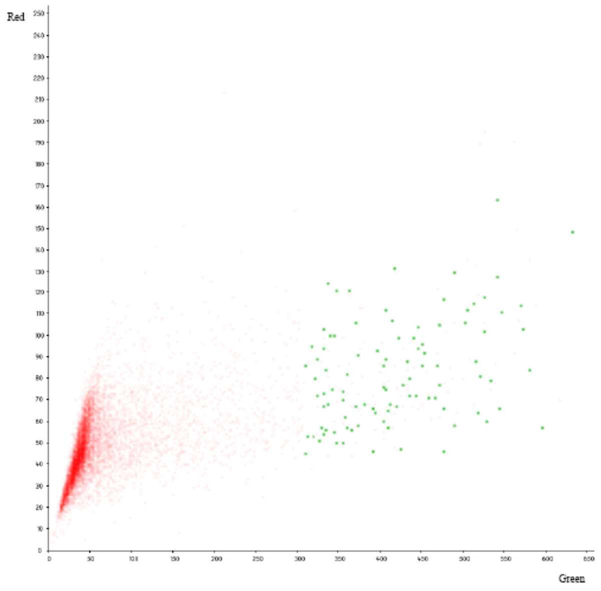

ANNEX 1: FLOW SORTING BIN 2 – File 3 of 3

Bin 2: COPAS scatter plots showing events for medium 2 of the final split-pool cycle (bin 2). In the first scatter plot (optical extinction [ext] vs. time of flight [TOF]), events depicted by a red dot fall within the gate defining monomeric beads and were sorted. The second scatter plot displays the fluorescent values of the sorted beads (green vs. red fluorescence intensity). Beads with a red fluorescent intensity above the threshold value are depicted as green dots and were individually dispensed into 96 well plates.

Number of beads individually dispensed: 192

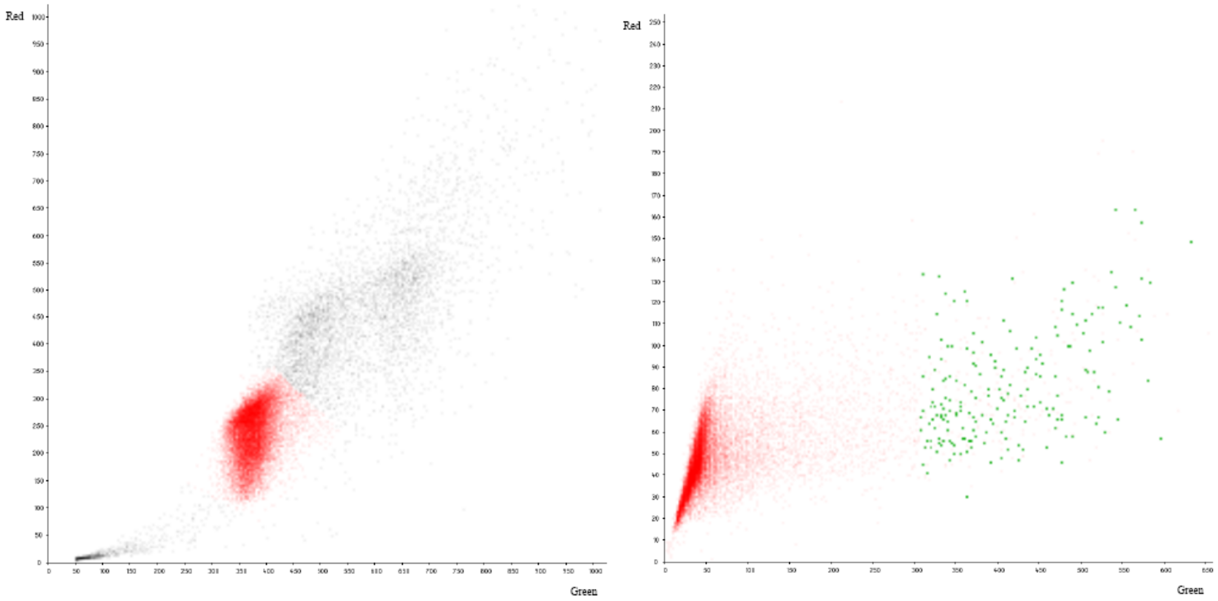

ANNEX 1: FLOW SORTING BIN 3 – File 1 of 2

Bin 3: COPAS scatter plots showing events for medium 3 of the final split-pool cycle (bin 3). In the first scatter plot (optical extinction [ext] vs. time of flight [TOF]), events depicted by a red dot fall within the gate defining monomeric beads and were sorted. The second scatter plot displays the fluorescent values of the sorted beads (green vs. red fluorescence intensity). Beads with a red fluorescent intensity above the threshold value are depicted as green dots and were individually dispensed into 96 well plates.

Number of beads individually dispensed: 46

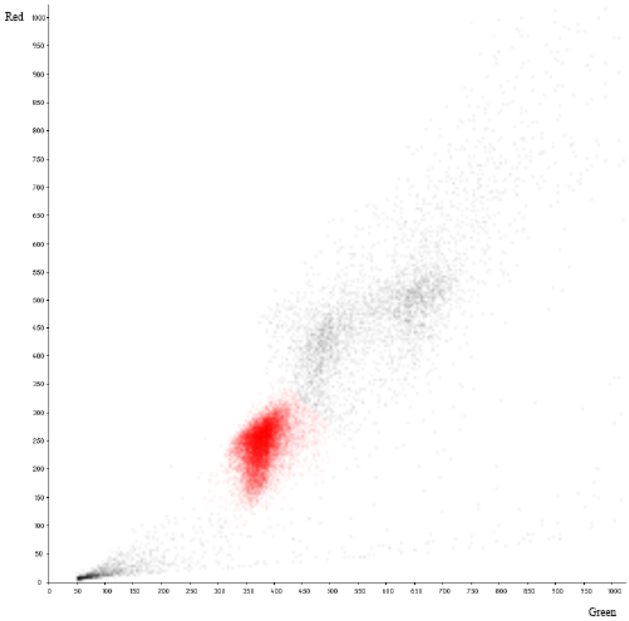

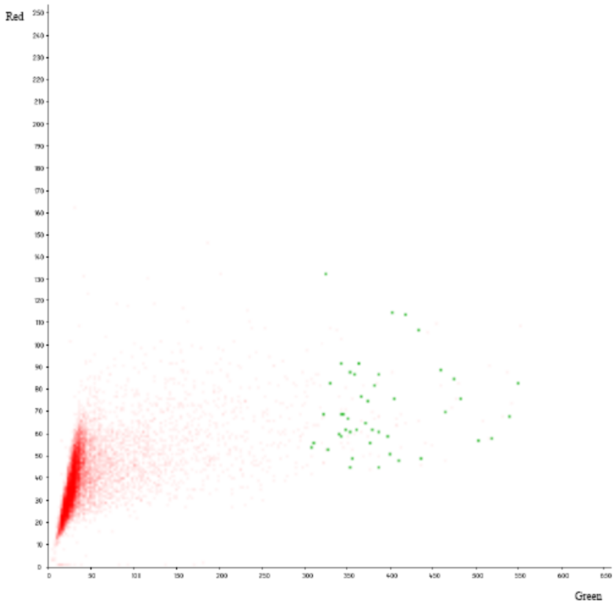

ANNEX 1: FLOW SORTING BIN 3 – File 2 of 2

Bin 3: COPAS scatter plots showing events for medium 3 of the final split-pool cycle (bin 3). In the first scatter plot (optical extinction [ext] vs. time of flight [TOF]), events depicted by a red dot fall within the gate defining monomeric beads and were sorted. The second scatter plot displays the fluorescent values of the sorted beads (green vs. red fluorescence intensity). Beads with a red fluorescent intensity above the threshold value are depicted as green dots and were individually dispensed into 96 well plates.

Number of beads individually dispensed: 17

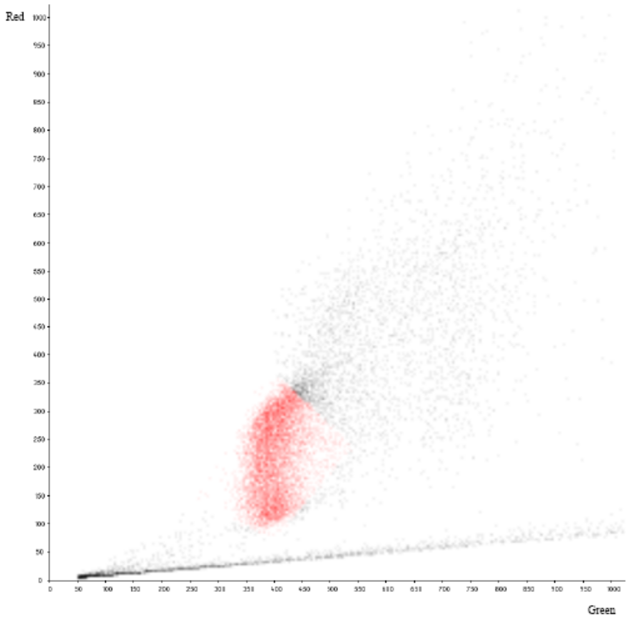

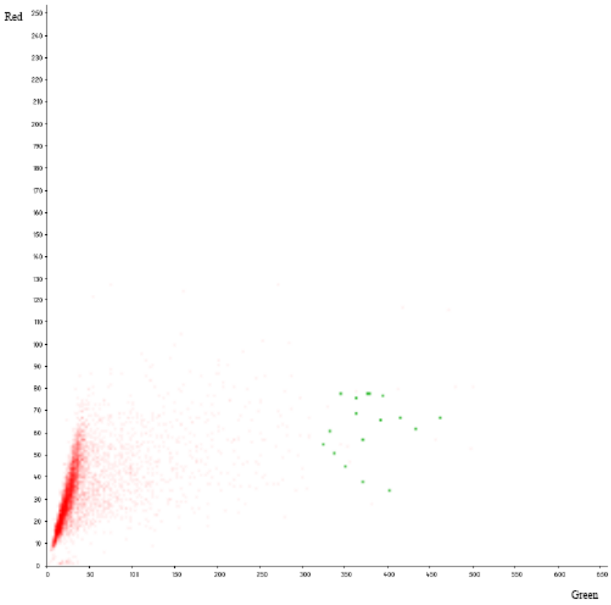

ANNEX 1: FLOW SORTING BIN 5 – File 1 of 1

Bin 5: COPAS scatter plots showing events for medium 5 of the final split-pool cycle (bin 5). In the first scatter plot (optical extinction [ext] vs. time of flight [TOF]), events depicted by a red dot fall within the gate defining monomeric beads and were sorted. The second scatter plot displays the fluorescent values of the sorted beads (green vs. red fluorescence intensity). Beads with a red fluorescent intensity above the threshold value are depicted as green dots and were individually dispensed into 96 well plates.

Number of beads individually dispensed: 41

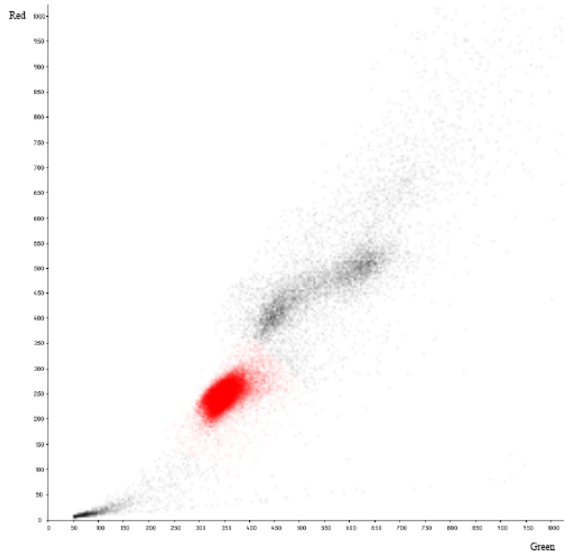

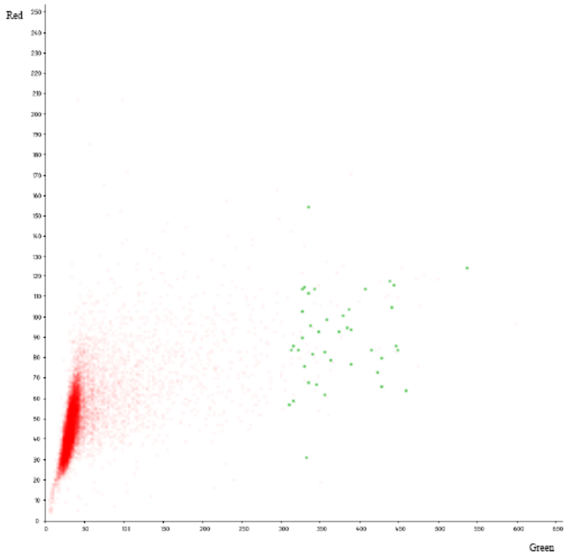

ANNEX 1: FLOW SORTING BIN 6 – File 1 of 1

Bin 6: COPAS scatter plots showing events for medium 6 of the final split-pool cycle (bin 6). In the first scatter plot (optical extinction [ext] vs. time of flight [TOF]), events depicted by a red dot fall within the gate defining monomeric beads and were sorted. The second scatter plot displays the fluorescent values of the sorted beads (green vs. red fluorescence intensity). Beads with a red fluorescent intensity above the threshold value are depicted as green dots and were individually dispensed into 96 well plates.

Number of beads individually dispensed: 50

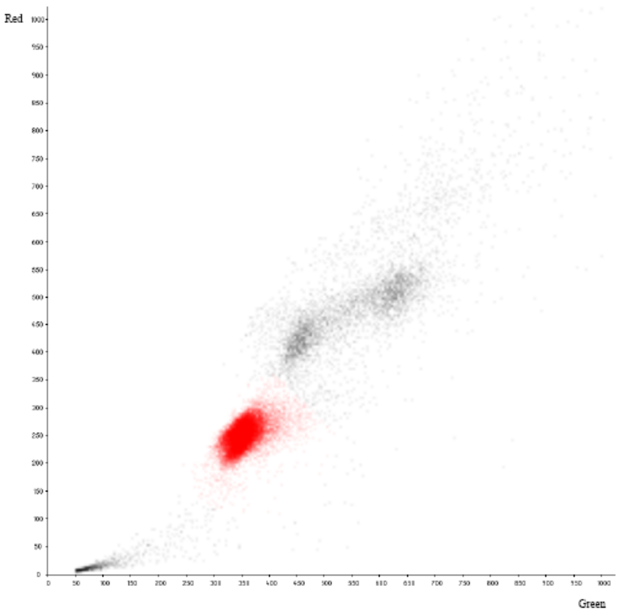

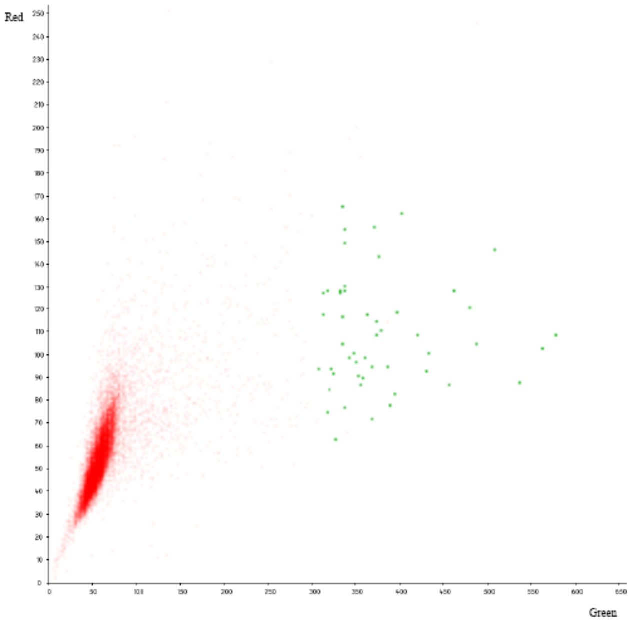

ANNEX 1: FLOW SORTING BIN 8 – File 1 of 1

Bin 8: COPAS scatter plots showing events for medium 8 of the final split-pool cycle (bin 8). In the first scatter plot (optical extinction [ext] vs. time of flight [TOF]), events depicted by a red dot fall within the gate defining monomeric beads and were sorted. The second scatter plot displays the fluorescent values of the sorted beads (green vs. red fluorescence intensity). Beads with a red fluorescent intensity above the threshold value are depicted as green dots and were individually dispensed into 96 well plates.

Number of beads individually dispensed: 58

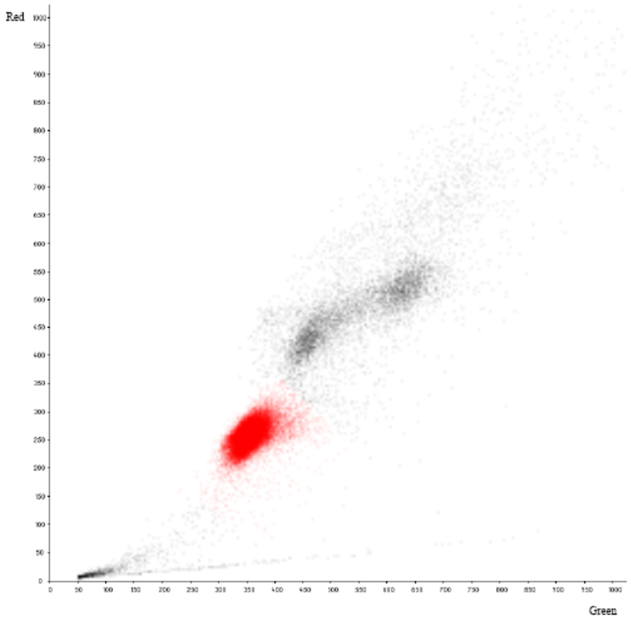

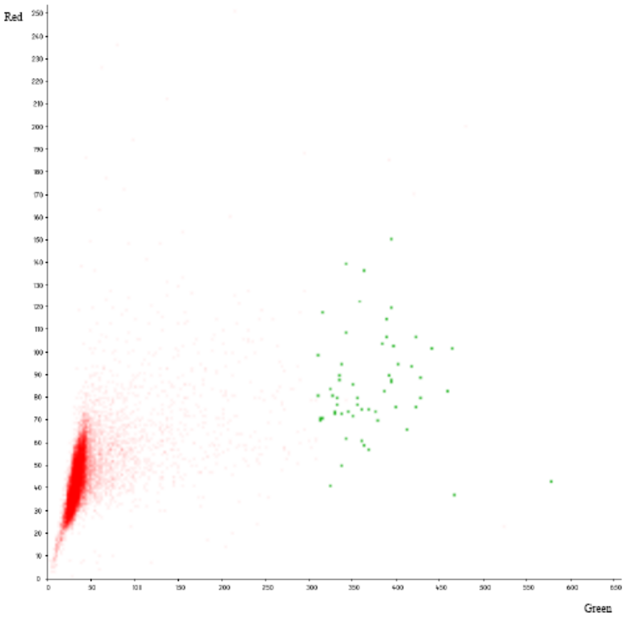

ANNEX 1: FLOW SORTING BIN 10 – File 1 of 1

Bin 8: COPAS scatter plots showing events for medium 8 of the final split-pool cycle (bin 8). In the first scatter plot (optical extinction [ext] vs. time of flight [TOF]), events depicted by a red dot fall within the gate defining monomeric beads and were sorted. The second scatter plot displays the fluorescent values of the sorted beads (green vs. red fluorescence intensity). Beads with a red fluorescent intensity above the threshold value are depicted as green dots and were individually dispensed into 96 well plates.

Number of beads individually dispensed: 22

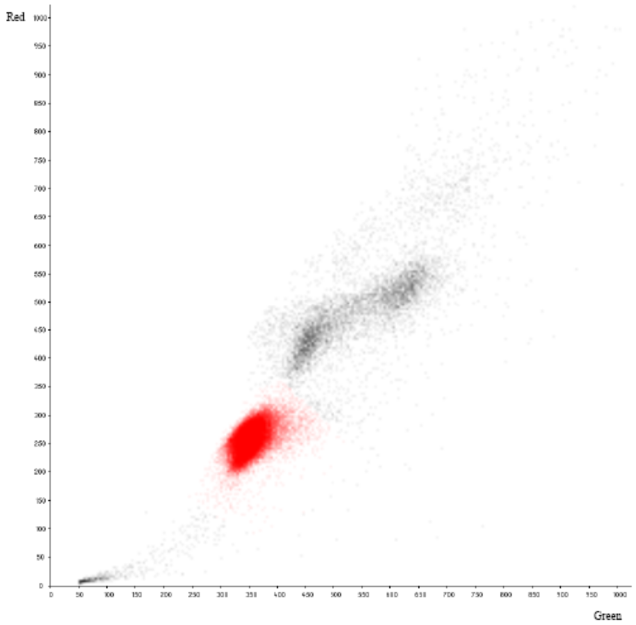

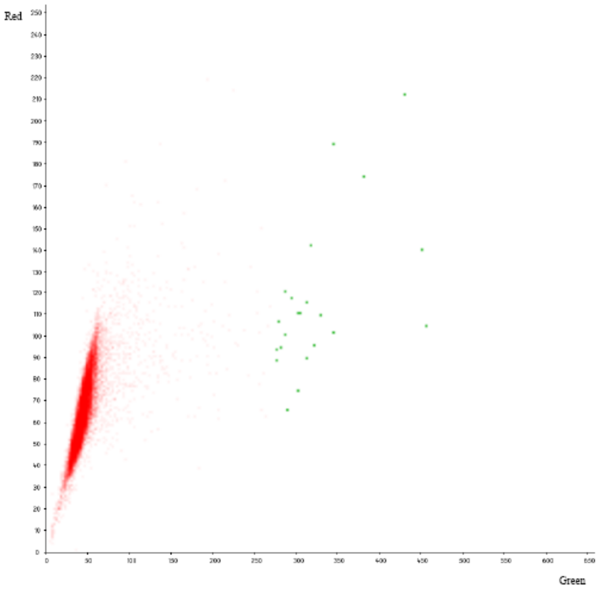

ANNEX 2: TAG REFERENCE SET - TAG DECONVOLUTION SESSION 1

Tag analysis flow cytometry instrument: BD FACSCalibur

Number of gates: 30

Number of beads whose tags were deconvoluted: 54

Number of beads with conclusive tagging data: 51

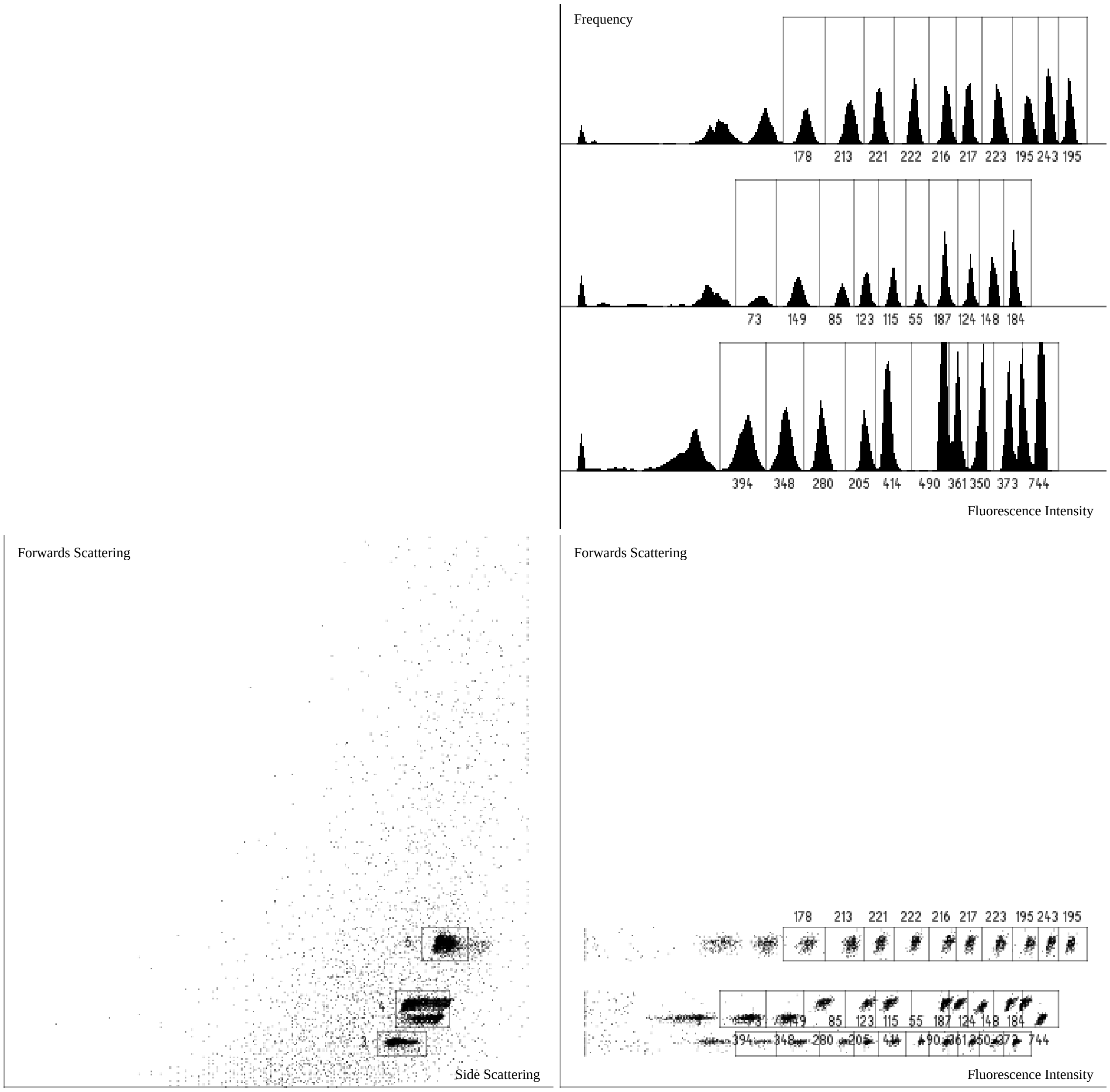

ANNEX 2: TAG REFERENCE SET - TAG DECONVOLUTION SESSION 2

Tag analysis flow cytometry instrument: BD FACSCalibur

Number of gates: 30

Number of beads whose tags were deconvoluted: 46

Number of beads with conclusive tagging data: 36

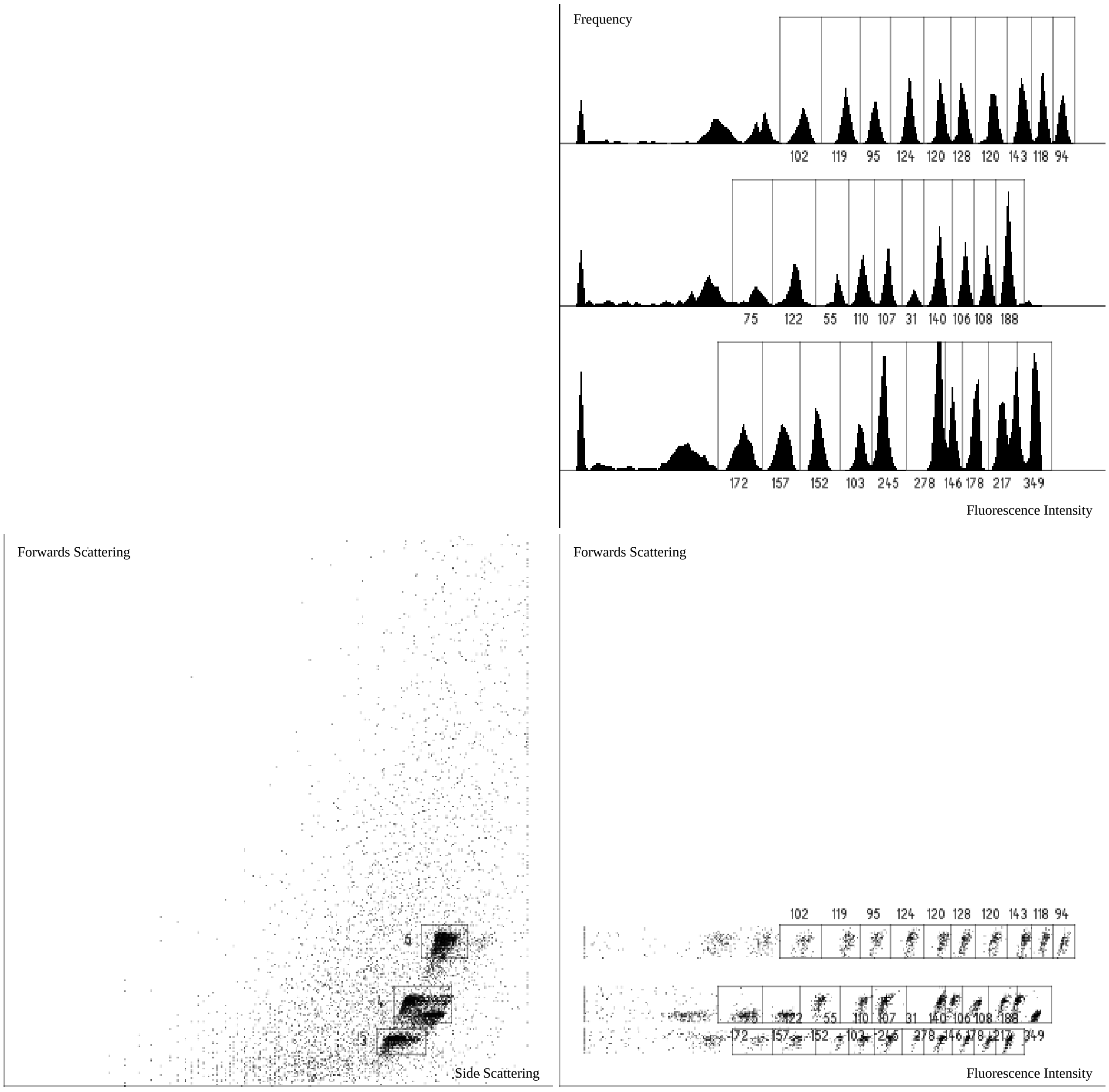

ANNEX 3: TAG DECONVOLUTION - BEAD 1

Passes flow sorting criteria: Yes

Passes tag deconvolution criteria: Yes

Included in protocol analysis: Yes

Protocol: 8, 4, 1, 3

Filename: Bin3_plateA1_C10.LMD

Split 1: Petrol shading

Split 2: Green shading

Split 3: Violet shading

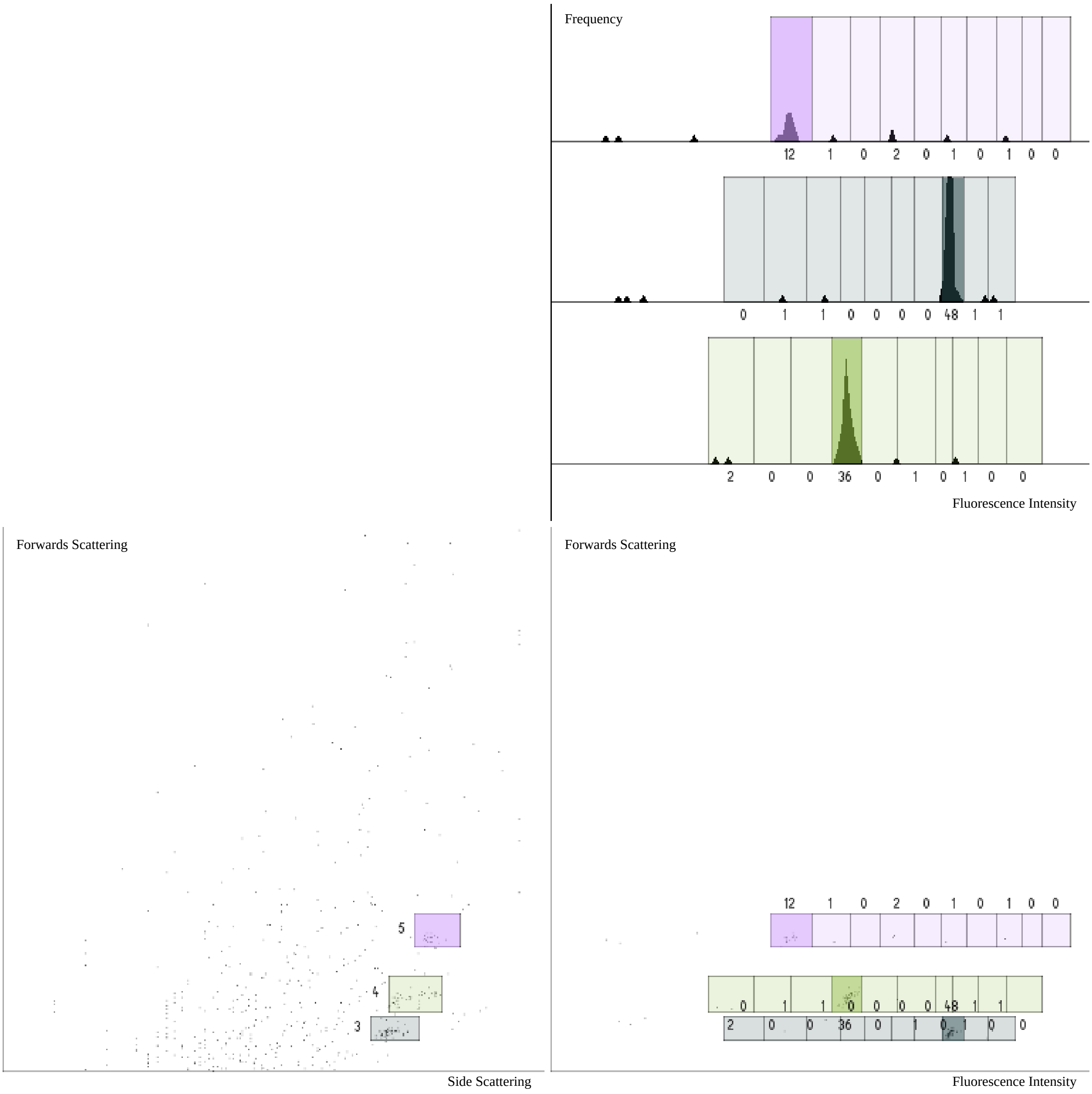

ANNEX 3: TAG DECONVOLUTION - BEAD 2

Passes flow sorting criteria: Yes

Passes tag deconvolution criteria: Yes

Included in protocol analysis: Yes

Protocol: 7, 8, 7, 2

Filename: Bin2_plateA1_A1.LMD

Split 1: Petrol shading

Split 2: Green shading

Split 3: Violet shading

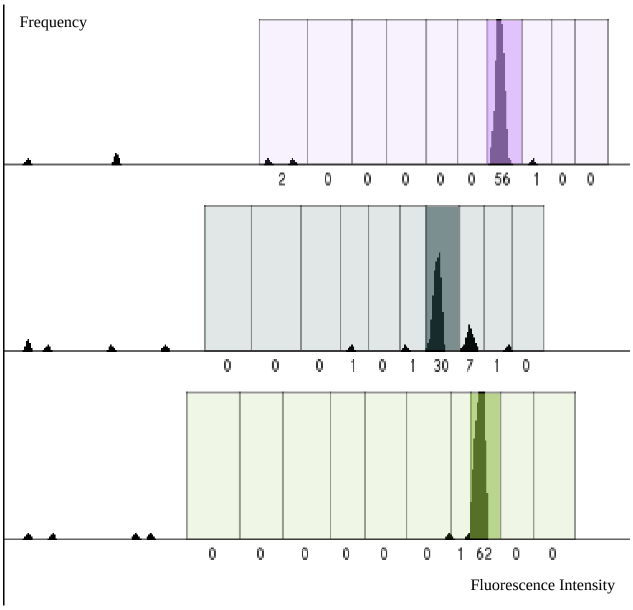

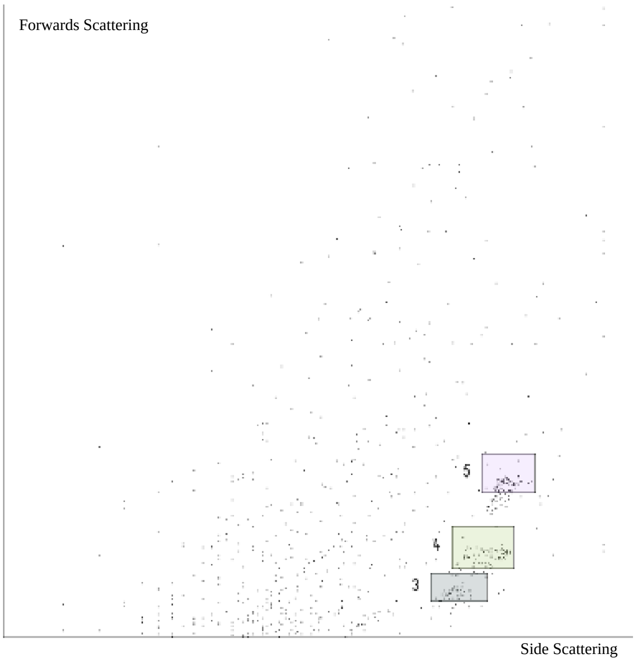

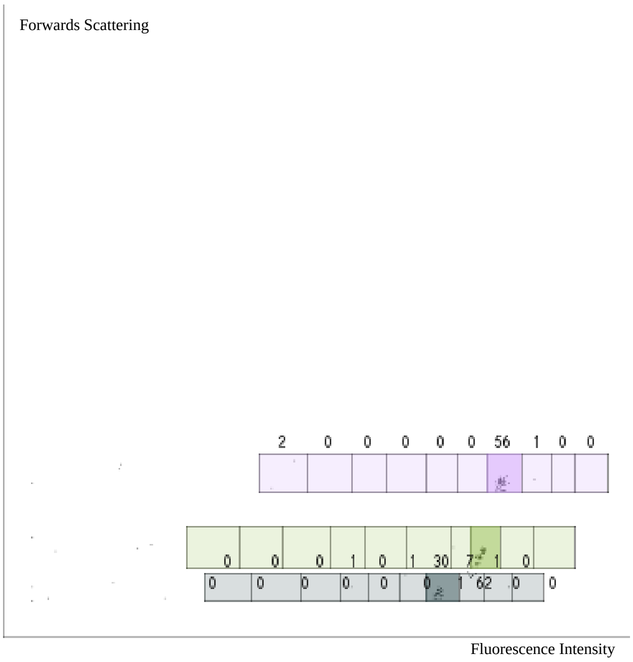

ANNEX 3: TAG DECONVOLUTION - BEAD 3

Passes flow sorting criteria: Yes

Passes tag deconvolution criteria: Yes

Included in protocol analysis: Yes

Protocol: 10, 5, 8, 2

Filename: Bin2_plateA1_A2.LMD

Split 1: Petrol shading

Split 2: Green shading

Split 3: Violet shading

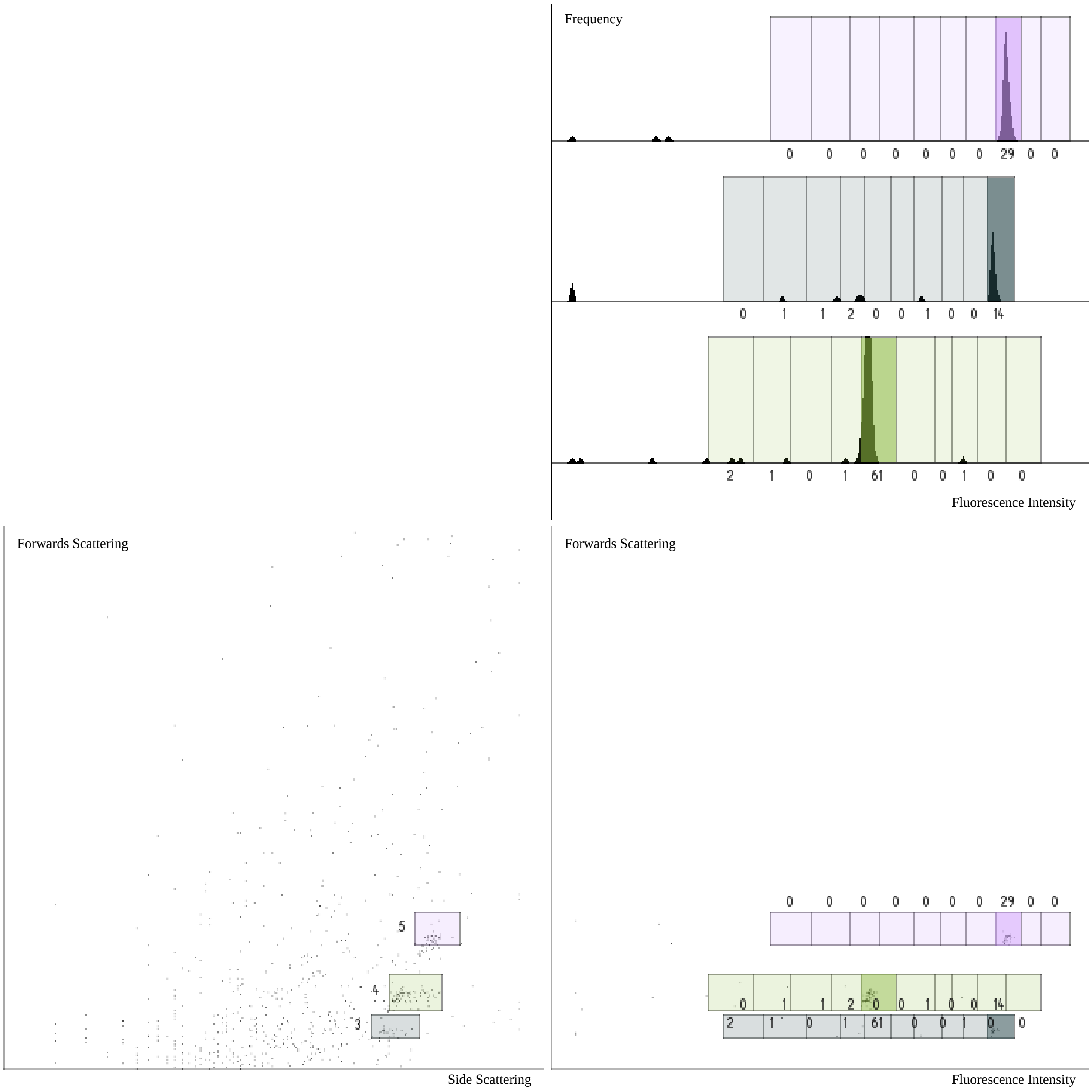

ANNEX 3: TAG DECONVOLUTION - BEAD 4

Passes flow sorting criteria: Yes

Passes tag deconvolution criteria: Yes

Included in protocol analysis: Yes

Protocol: 6, 7, 9, 2

Filename: Bin2_plateA1_A3.LMD

Split 1: Petrol shading

Split 2: Green shading

Split 3: Violet shading

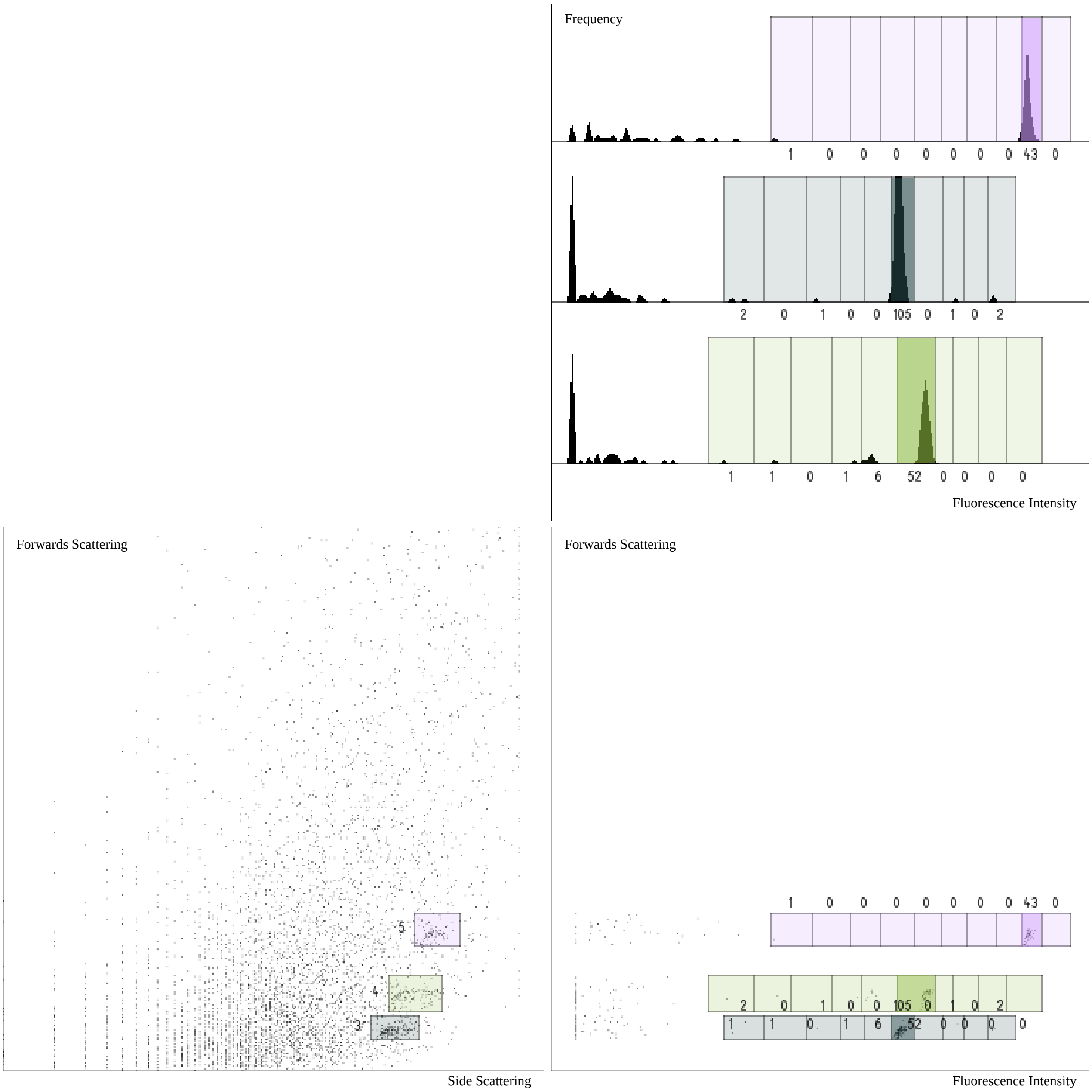

ANNEX 3: TAG DECONVOLUTION - BEAD 5

Passes flow sorting criteria: Yes

Passes tag deconvolution criteria: Yes

Included in protocol analysis: Yes

Protocol: 1, 2, 2, 2

Filename: Bin2_plateA1_A4.LMD

Split 1: Petrol shading

Split 2: Green shading

Split 3: Violet shading

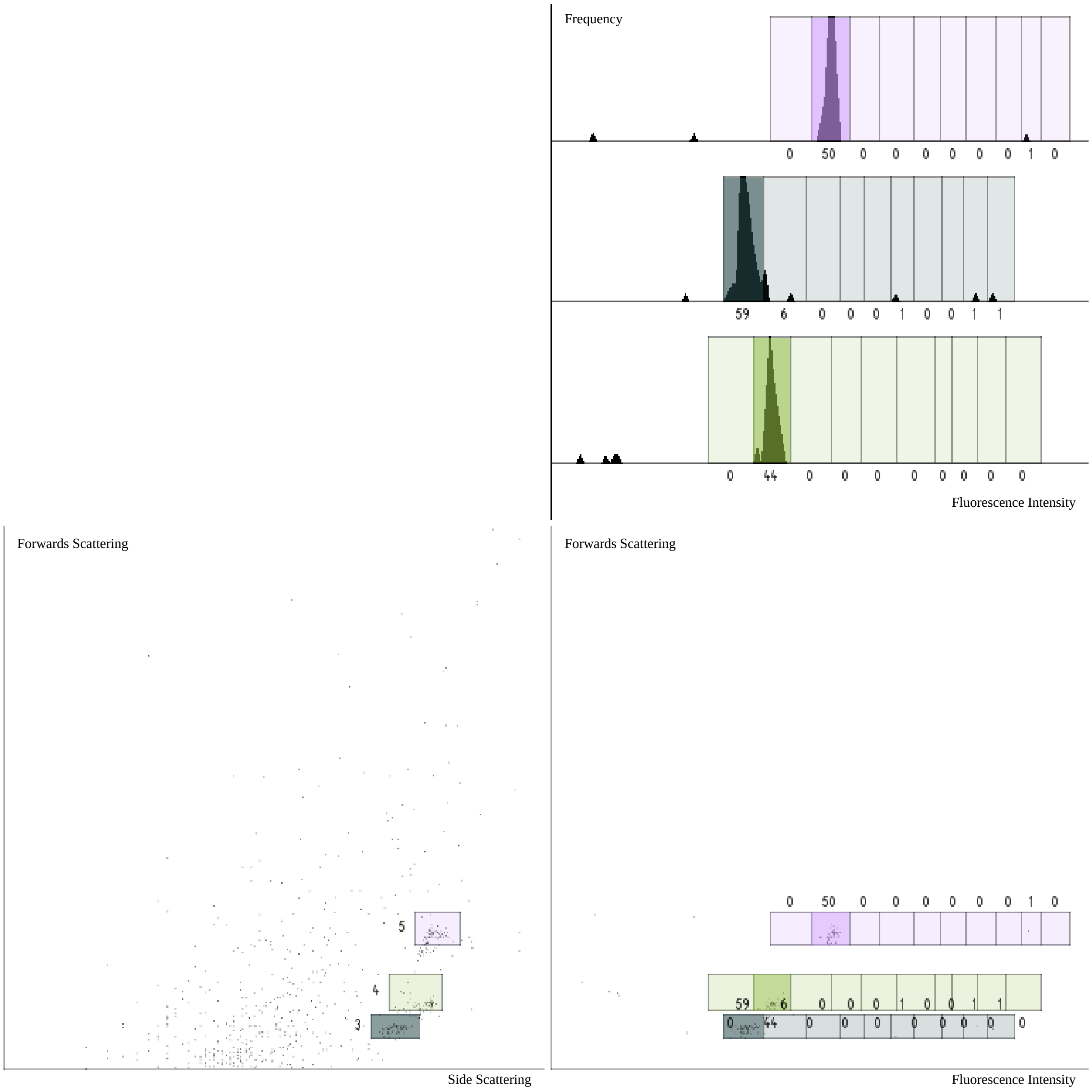

ANNEX 3: TAG DECONVOLUTION - BEAD 6

Passes flow sorting criteria: Yes

Passes tag deconvolution criteria: Yes

Included in protocol analysis: Yes

Protocol: 8, 8, 2, 2

Filename: Bin2_plateA1_A5.LMD

Split 1: Petrol shading

Split 2: Green shading

Split 3: Violet shading

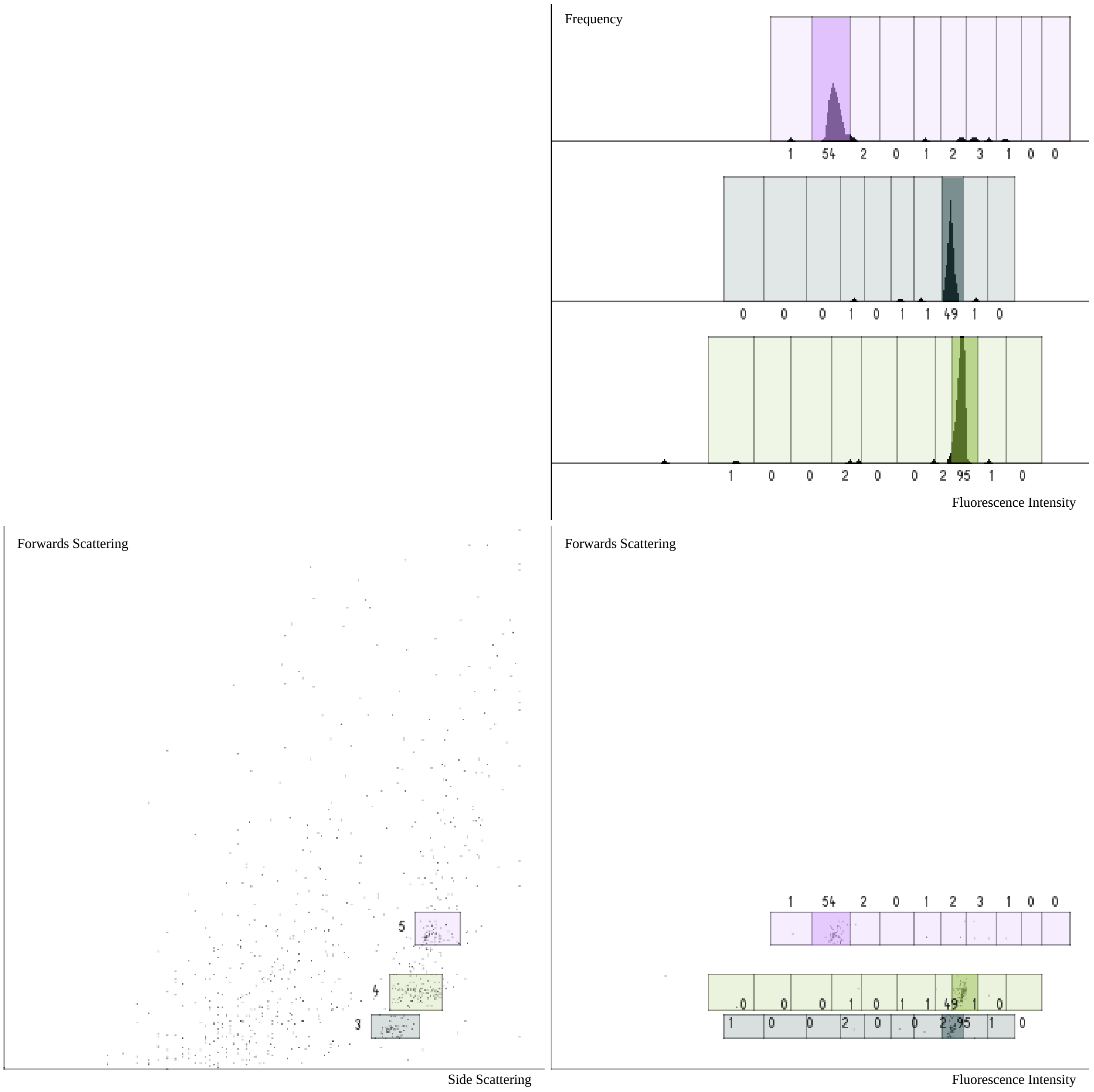

ANNEX 3: TAG DECONVOLUTION - BEAD 7

Passes flow sorting criteria: Yes

Passes tag deconvolution criteria: Yes

Included in protocol analysis: Yes

Protocol: 5, 2, 10, 2

Filename: Bin2_plateA1_A6.LMD

Split 1: Petrol shading

Split 2: Green shading

Split 3: Violet shading

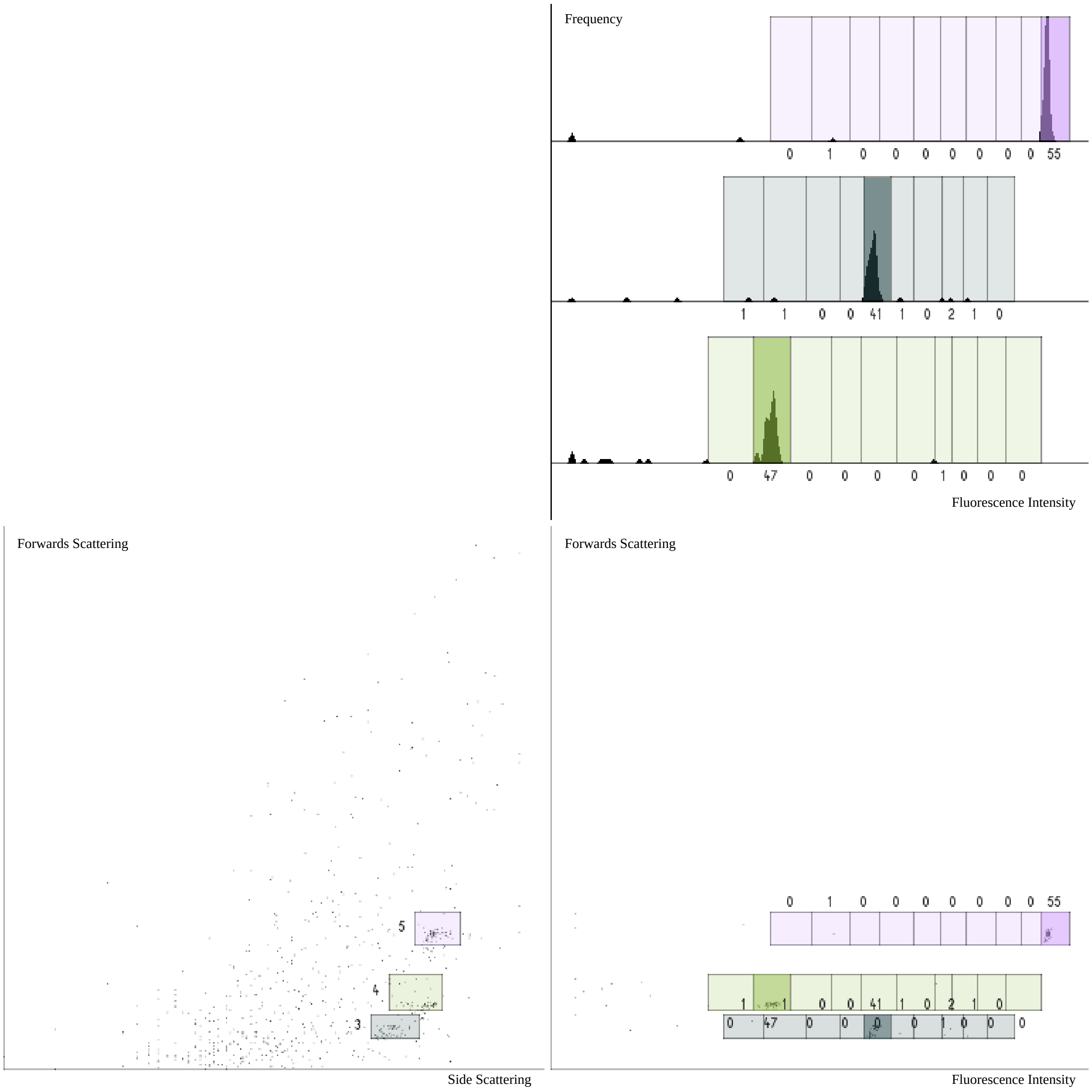

ANNEX 3: TAG DECONVOLUTION - BEAD 8

Passes flow sorting criteria: Yes

Passes tag deconvolution criteria: Yes

Included in protocol analysis: Yes

Protocol: 4, 2, 6, 2

Filename: Bin2_plateA1_A7.LMD

Split 1: Petrol shading

Split 2: Green shading

Split 3: Violet shading

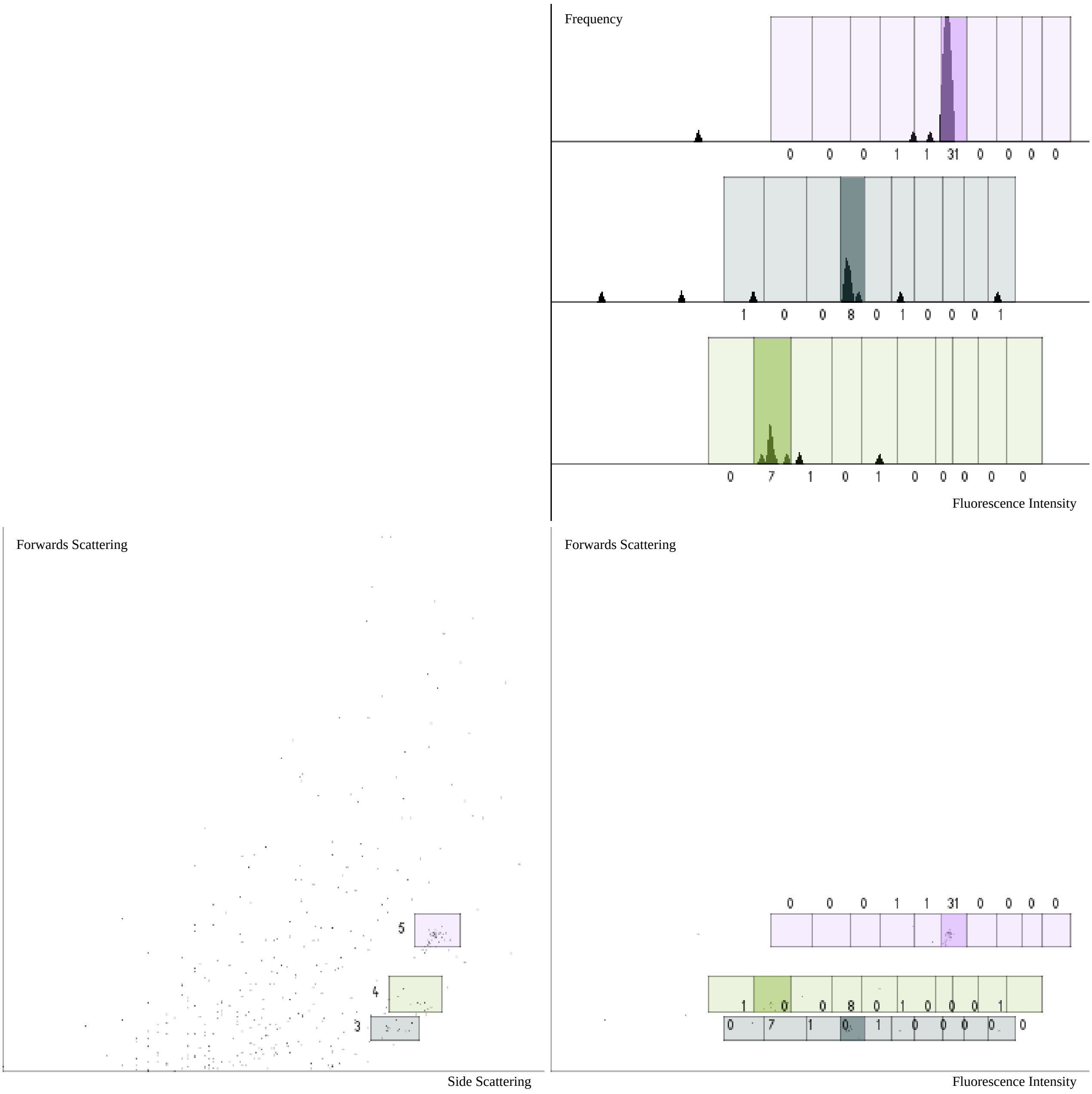

ANNEX 3: TAG DECONVOLUTION - BEAD 9

Passes flow sorting criteria: Yes

Passes tag deconvolution criteria: Yes

Included in protocol analysis: Yes

Protocol: 5, 2, 1, 2

Filename: Bin2_plateA1_A8.LMD

Split 1: Petrol shading

Split 2: Green shading

Split 3: Violet shading

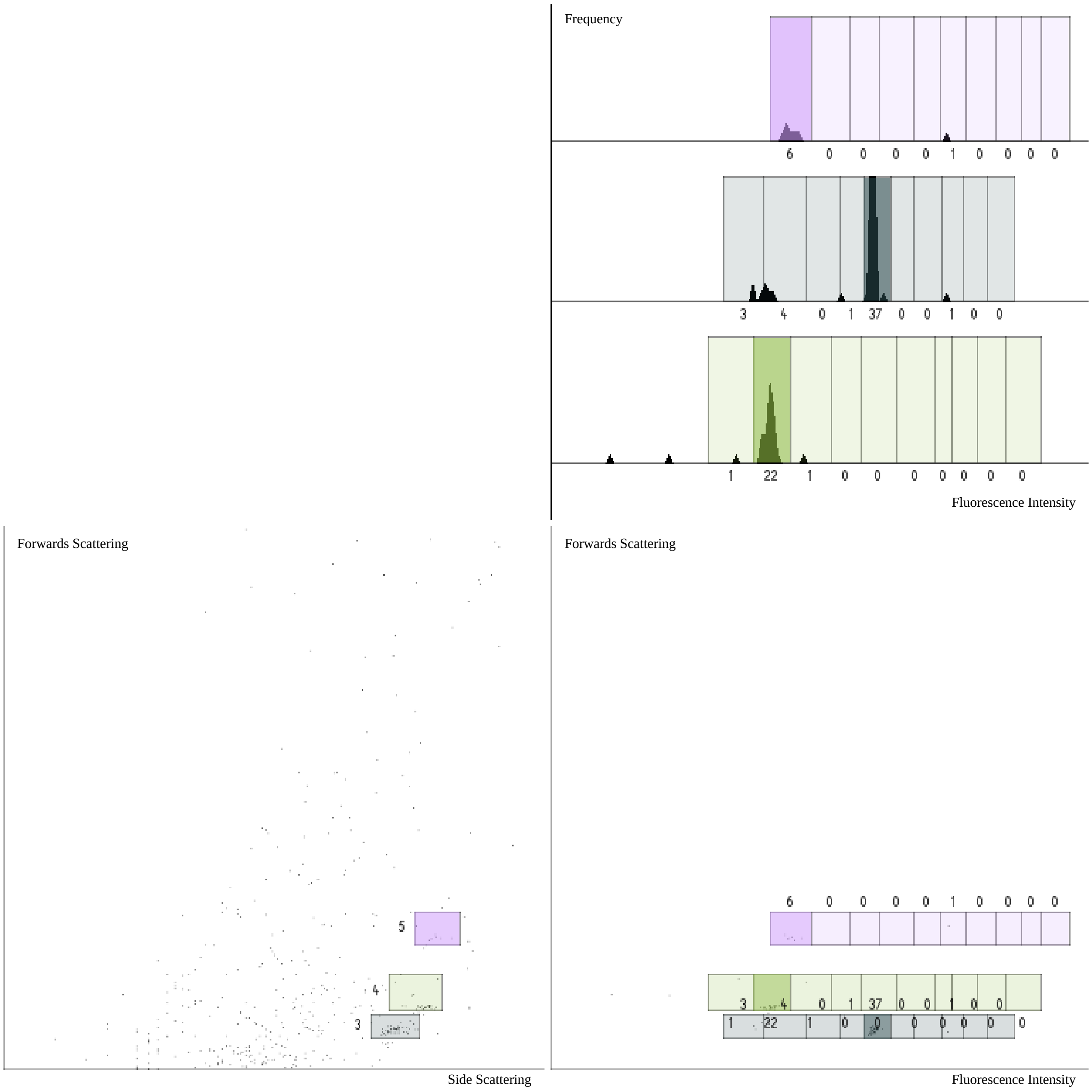

ANNEX 3: TAG DECONVOLUTION - BEAD 10

Passes flow sorting criteria: Yes

Passes tag deconvolution criteria: Yes

Included in protocol analysis: Yes

Protocol: 4, 7, 10, 2

Filename: Bin2_plateA1_A9.LMD

Split 1: Petrol shading

Split 2: Green shading

Split 3: Violet shading

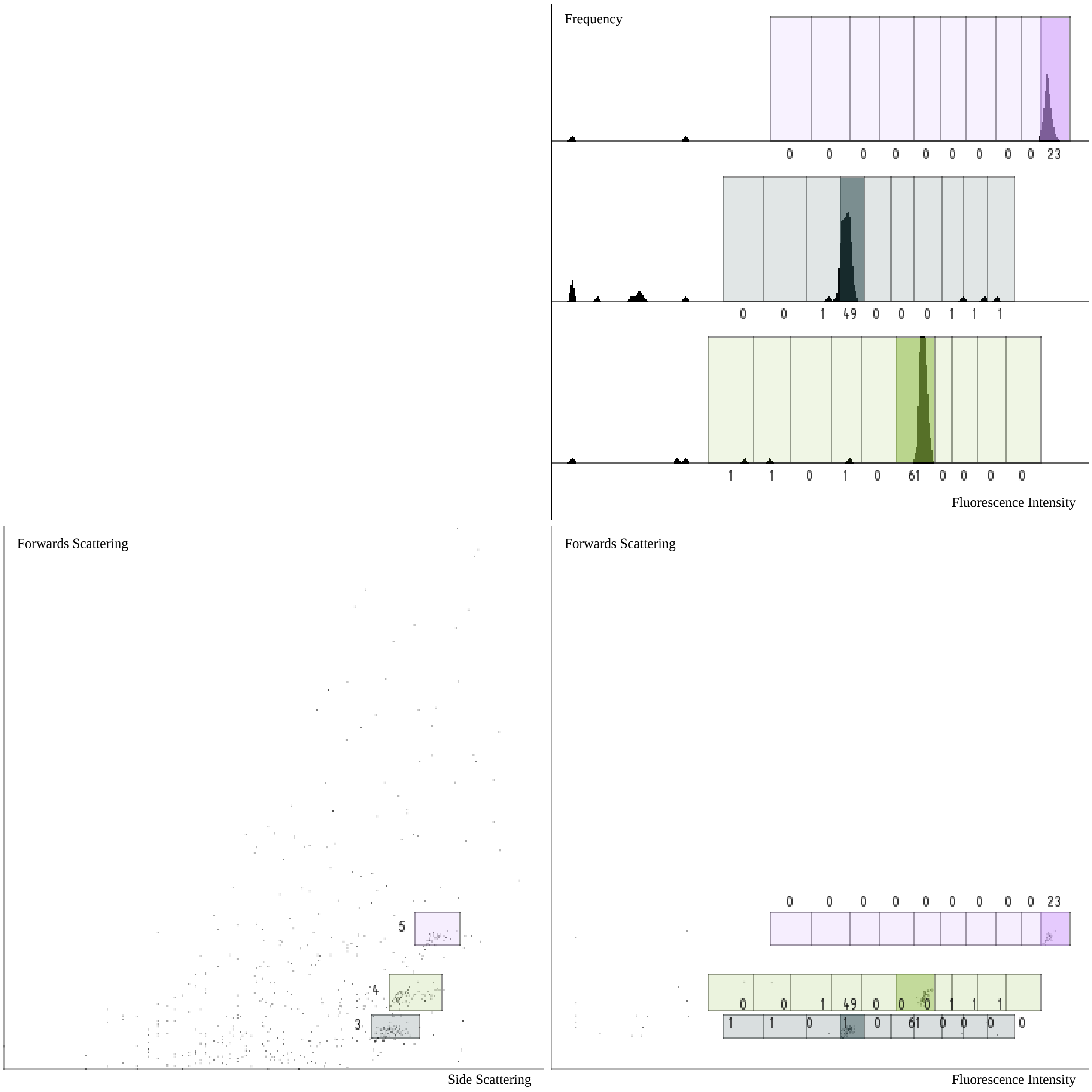

ANNEX 3: TAG DECONVOLUTION - BEAD 11

Passes flow sorting criteria: Yes

Passes tag deconvolution criteria: Yes

Included in protocol analysis: Yes

Protocol: 3, 8, 4, 2

Filename: Bin2_plateA1_A10.LMD

Split 1: Petrol shading

Split 2: Green shading

Split 3: Violet shading

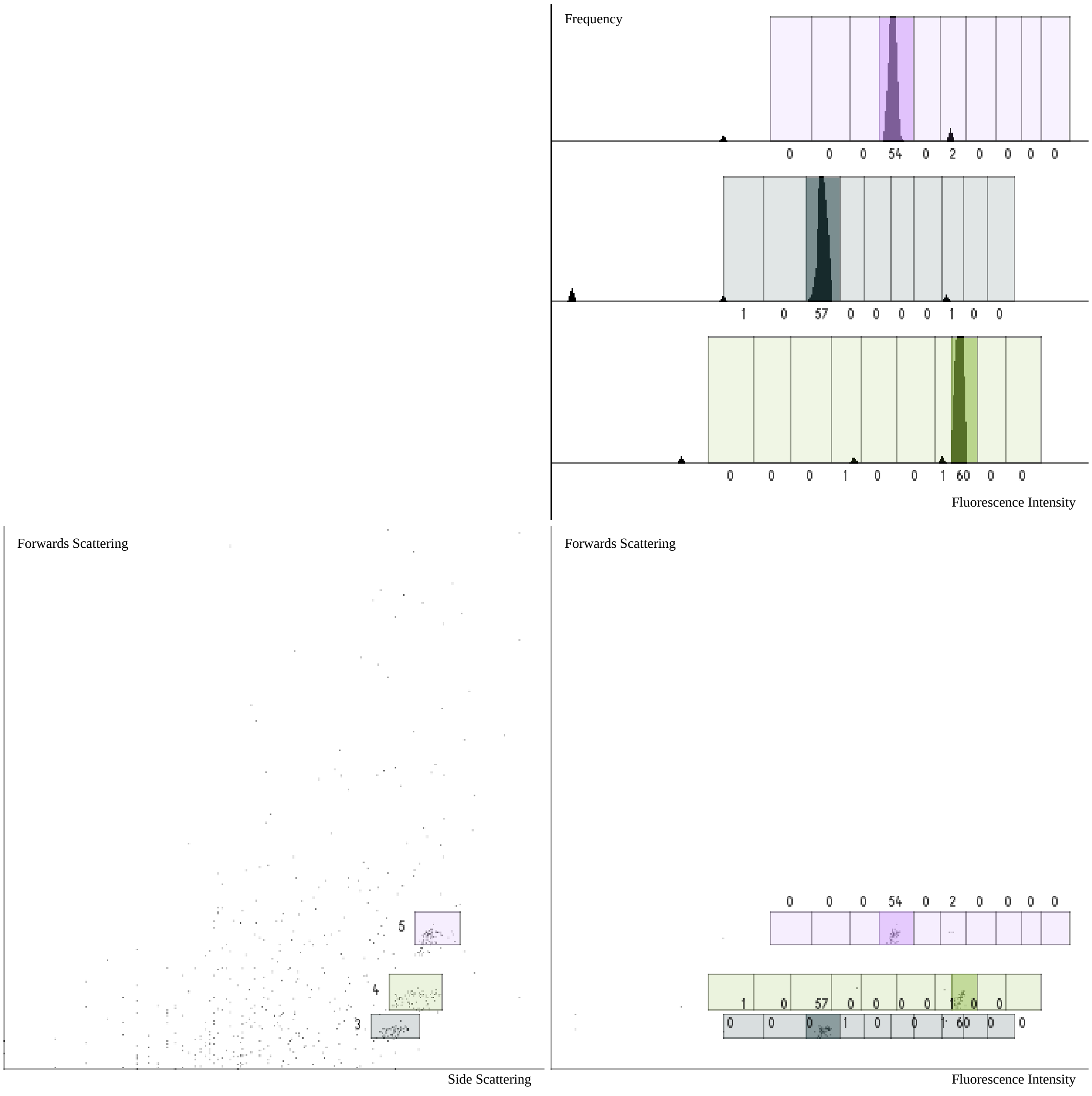

ANNEX 3: TAG DECONVOLUTION - BEAD 12

Passes flow sorting criteria: Yes

Passes tag deconvolution criteria: Yes

Included in protocol analysis: Yes

Protocol: 2, 8, 5, 2

Filename: Bin2_plateA1_A11.LMD

Split 1: Petrol shading

Split 2: Green shading

Split 3: Violet shading

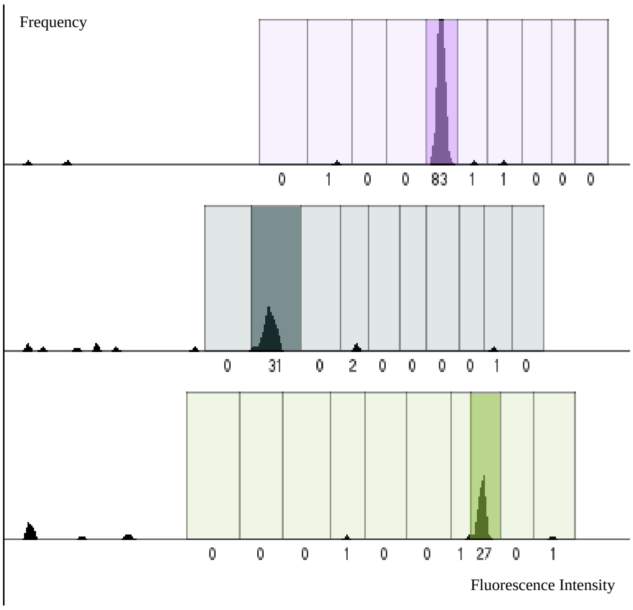

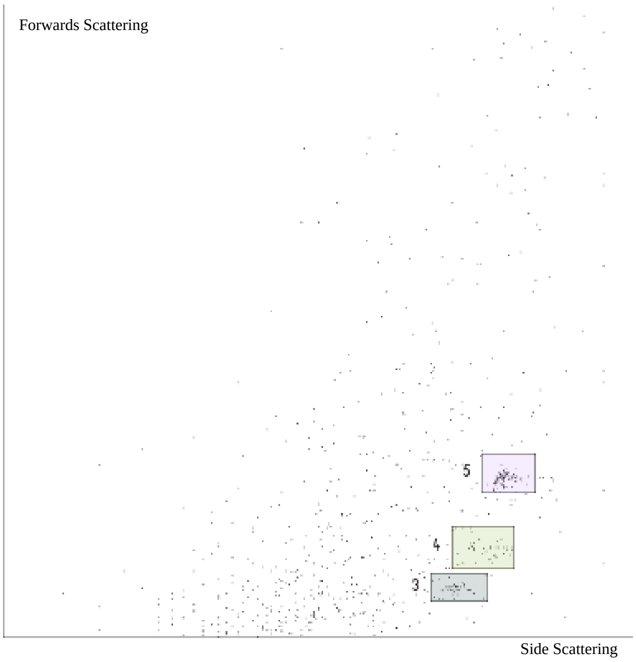

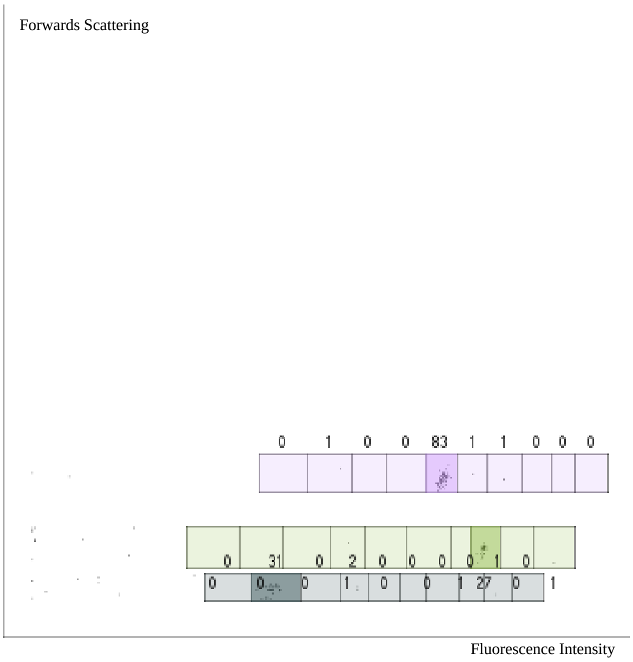

ANNEX 3: TAG DECONVOLUTION - BEAD 13

Passes flow sorting criteria: Yes

Passes tag deconvolution criteria: Yes

Included in protocol analysis: Yes

Protocol: 10, 8, 3, 2

Filename: Bin2_plateA1_A12.LMD

Split 1: Petrol shading

Split 2: Green shading

Split 3: Violet shading

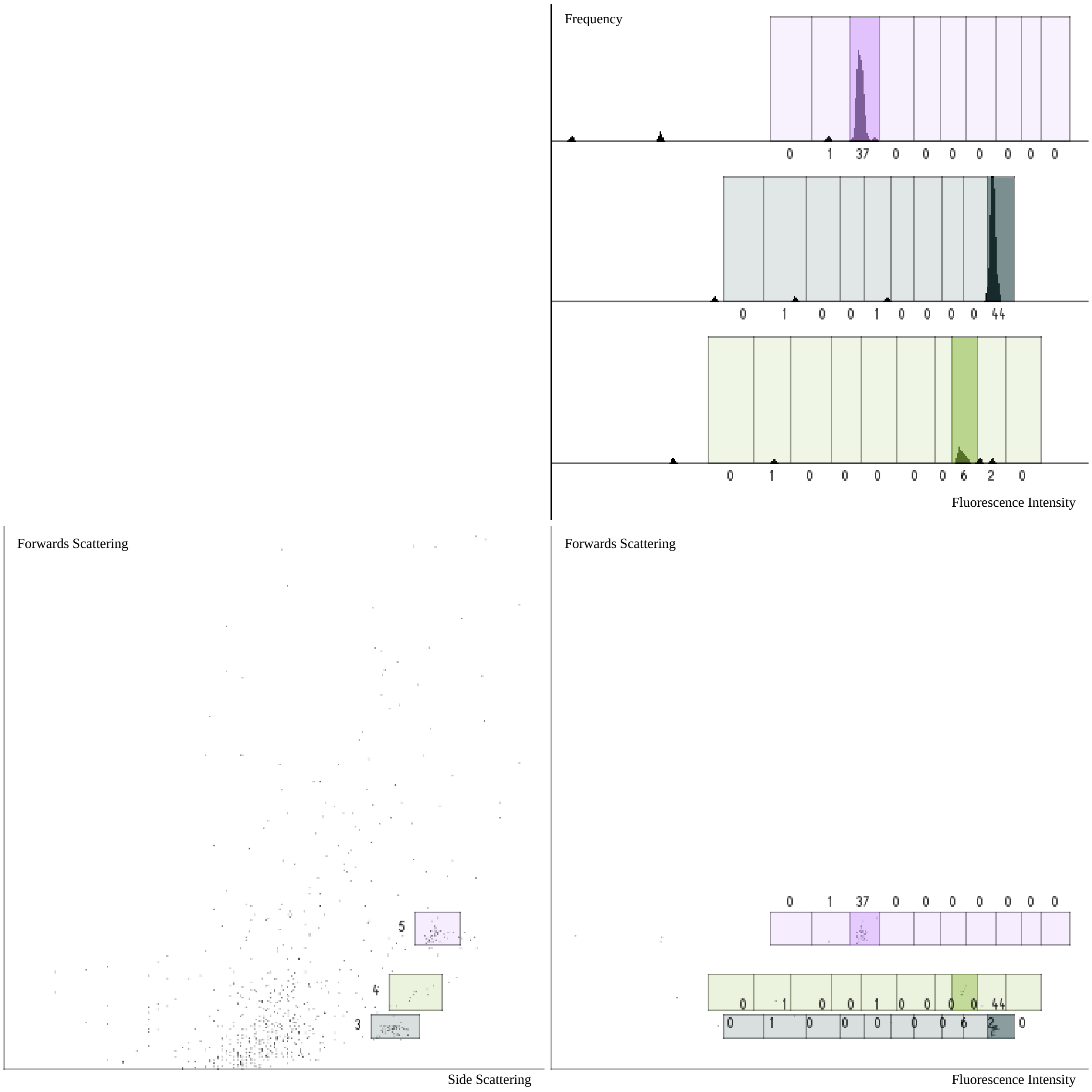

ANNEX 3: TAG DECONVOLUTION - BEAD 14

Passes flow sorting criteria: Yes

Passes tag deconvolution criteria: Yes

Included in protocol analysis: Yes

Protocol: 8, 3, 6, 2

Filename: Bin2_plateA1_B1.LMD

Split 1: Petrol shading

Split 2: Green shading

Split 3: Violet shading

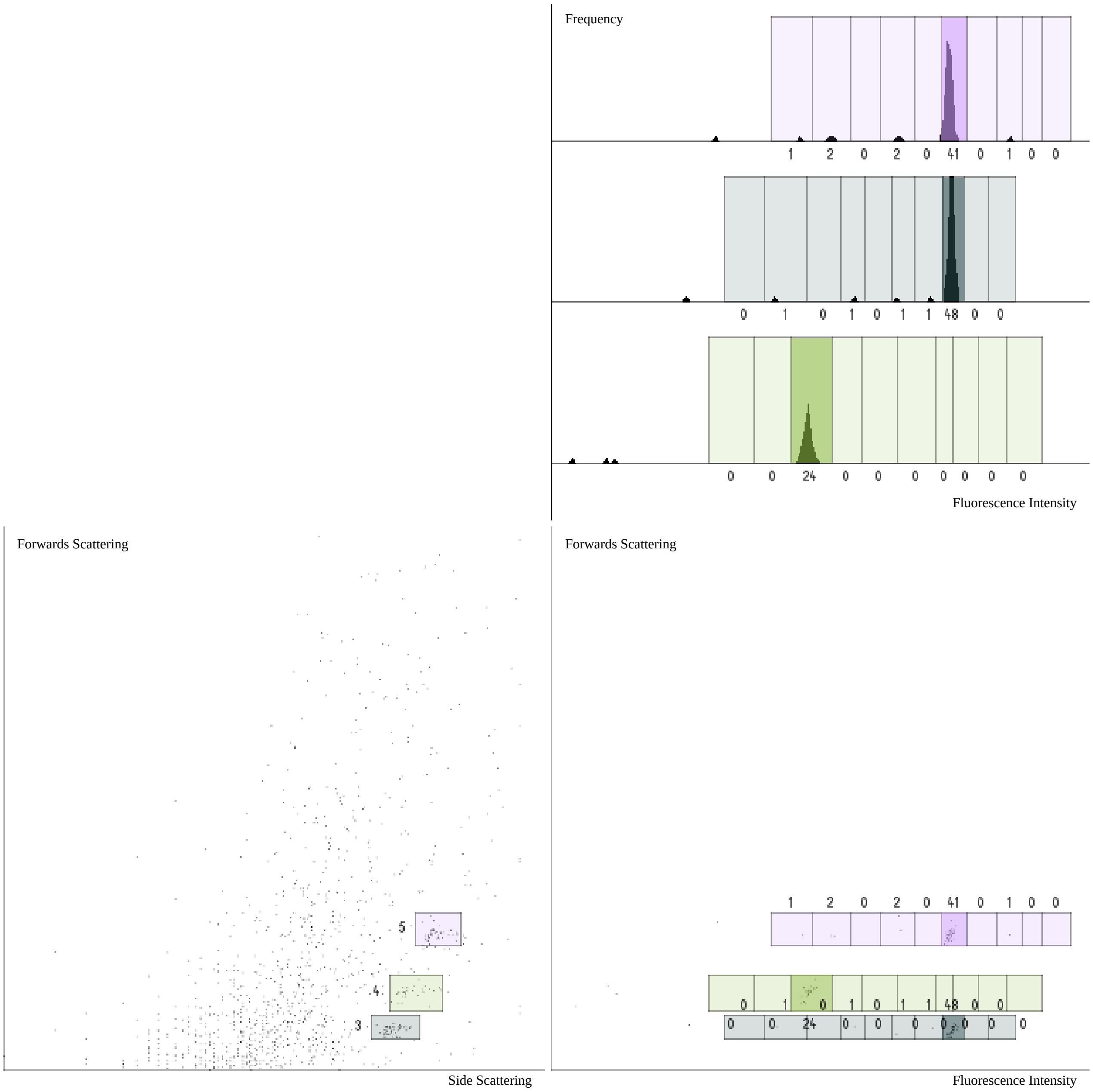

ANNEX 3: TAG DECONVOLUTION - BEAD 15

Passes flow sorting criteria: Yes

Passes tag deconvolution criteria: Yes

Included in protocol analysis: Yes

Protocol: 9, 3, 9, 2

Filename: Bin2_plateA1_B2.LMD

Split 1: Petrol shading

Split 2: Green shading

Split 3: Violet shading

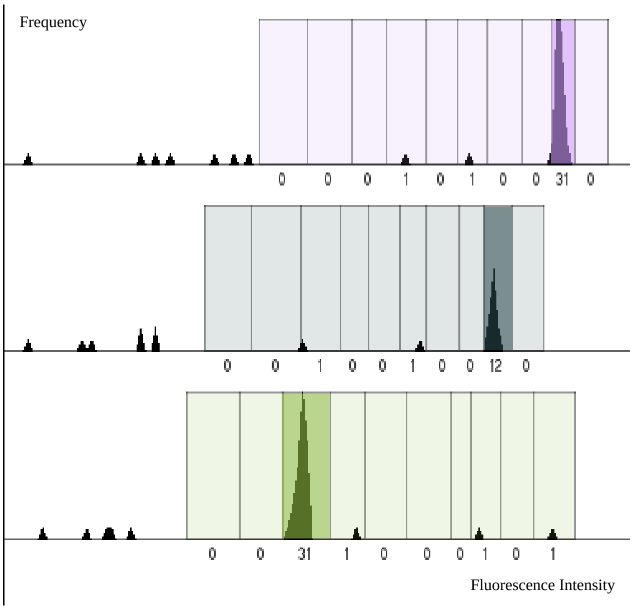

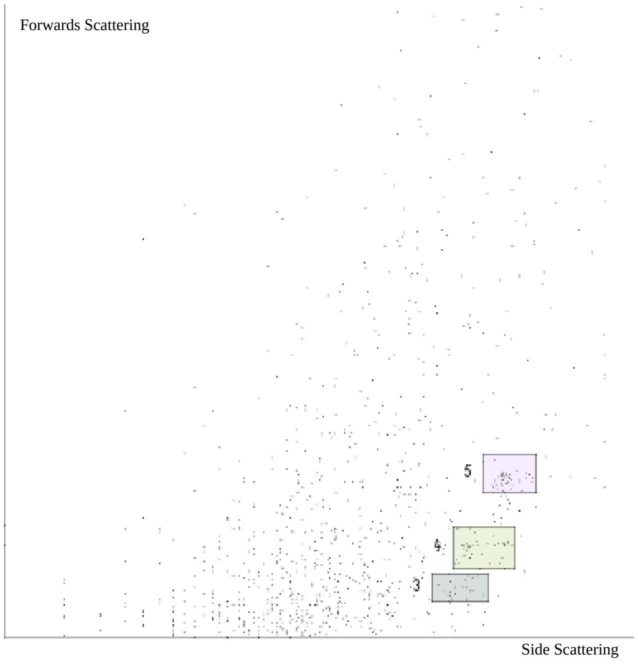

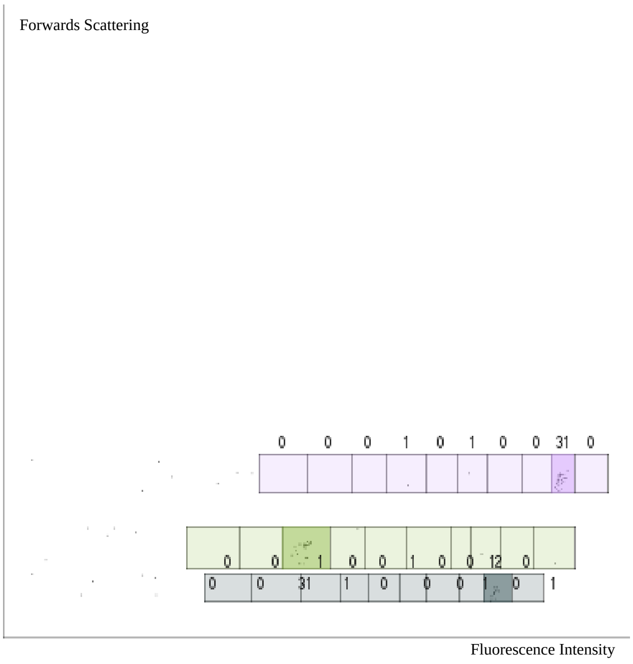

ANNEX 3: TAG DECONVOLUTION - BEAD 16

Passes flow sorting criteria: Yes

Passes tag deconvolution criteria: Yes

Included in protocol analysis: Yes

Protocol: 2, 5, 4, 2

Filename: Bin2_plateA1_B3.LMD

Split 1: Petrol shading

Split 2: Green shading

Split 3: Violet shading

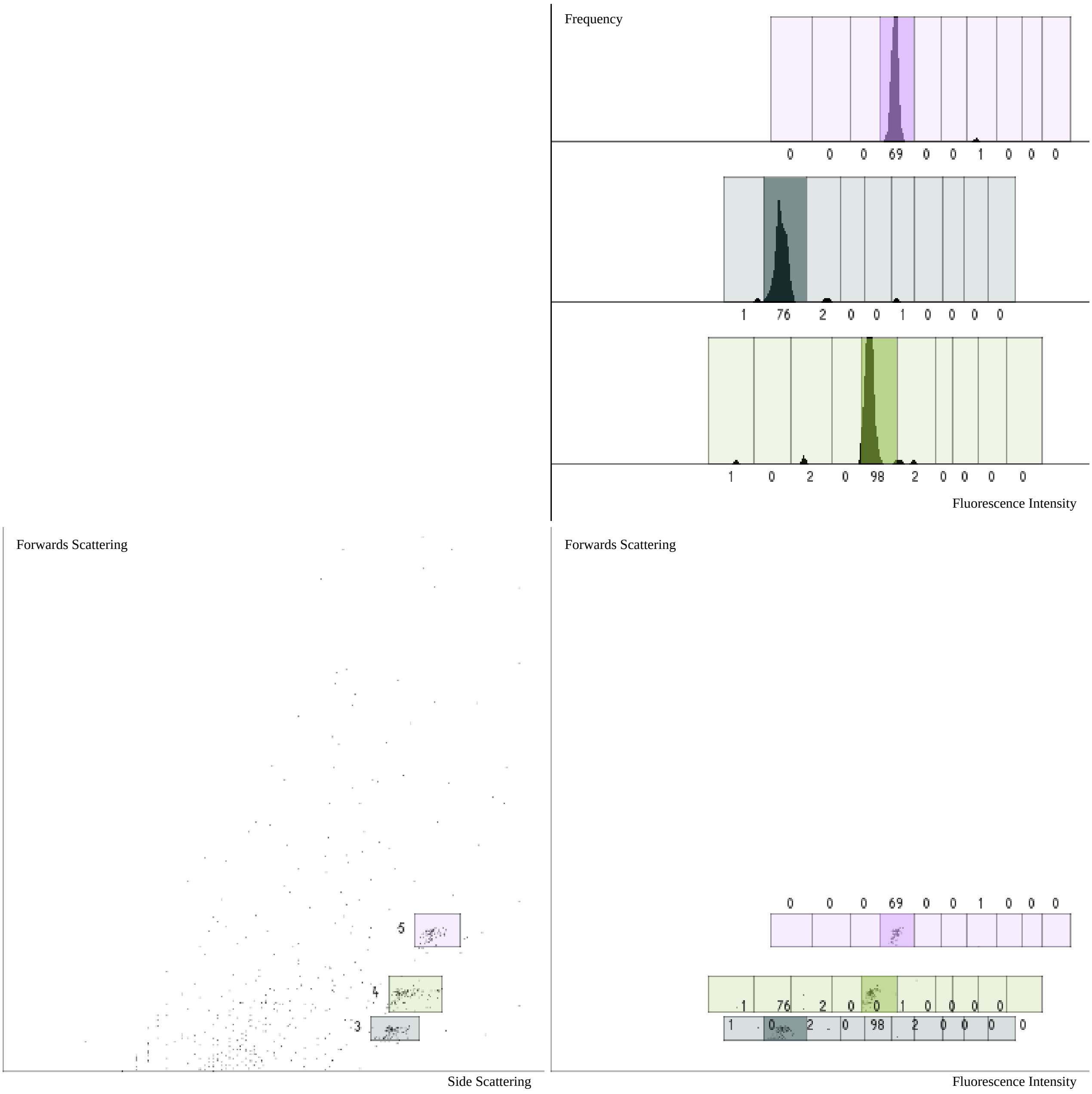

ANNEX 3: TAG DECONVOLUTION - BEAD 17

Passes flow sorting criteria: Yes

Passes tag deconvolution criteria: Yes

Included in protocol analysis: Yes

Protocol: 9, 4, 10, 2

Filename: Bin2_plateA1_B4.LMD

Split 1: Petrol shading

Split 2: Green shading

Split 3: Violet shading

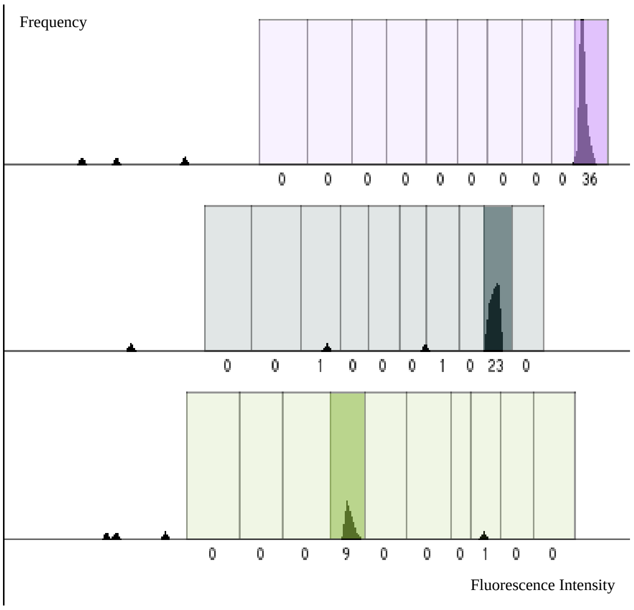

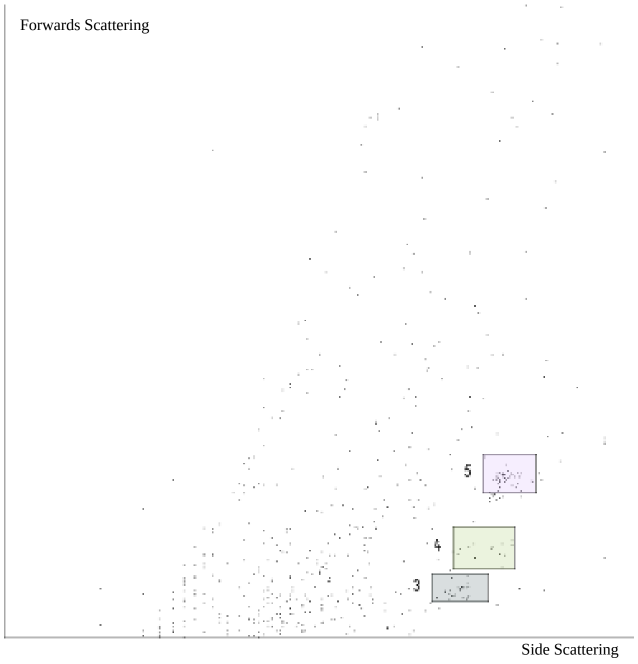

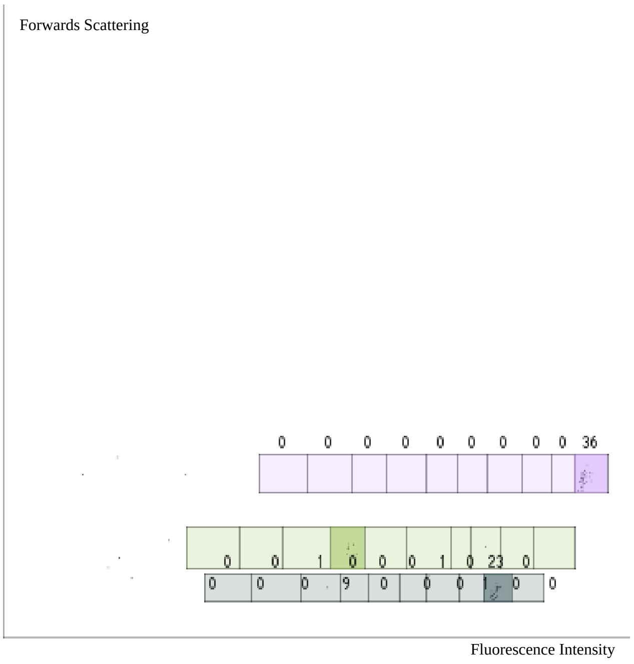

ANNEX 3: TAG DECONVOLUTION - BEAD 18

Passes flow sorting criteria: Yes

Passes tag deconvolution criteria: Yes

Included in protocol analysis: Yes

Protocol: 9, 1, 6, 2

Filename: Bin2_plateA1_B5.LMD

Split 1: Petrol shading

Split 2: Green shading

Split 3: Violet shading

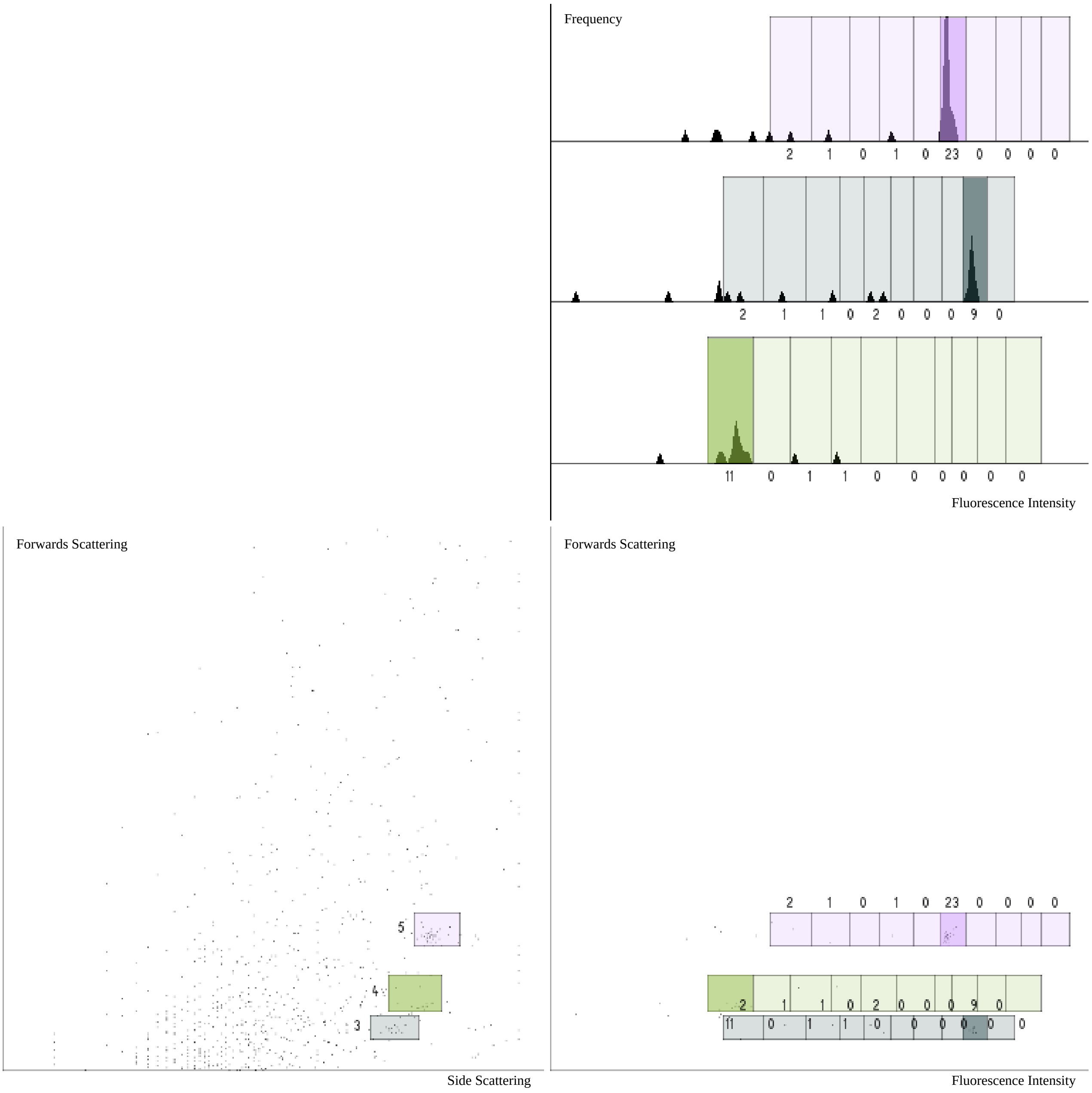

ANNEX 3: TAG DECONVOLUTION - BEAD 19

Passes flow sorting criteria: Yes

Passes tag deconvolution criteria: Yes

Included in protocol analysis: Yes

Protocol: 10, 2, 10, 2

Filename: Bin2_plateA1_B6.LMD

Split 1: Petrol shading

Split 2: Green shading

Split 3: Violet shading

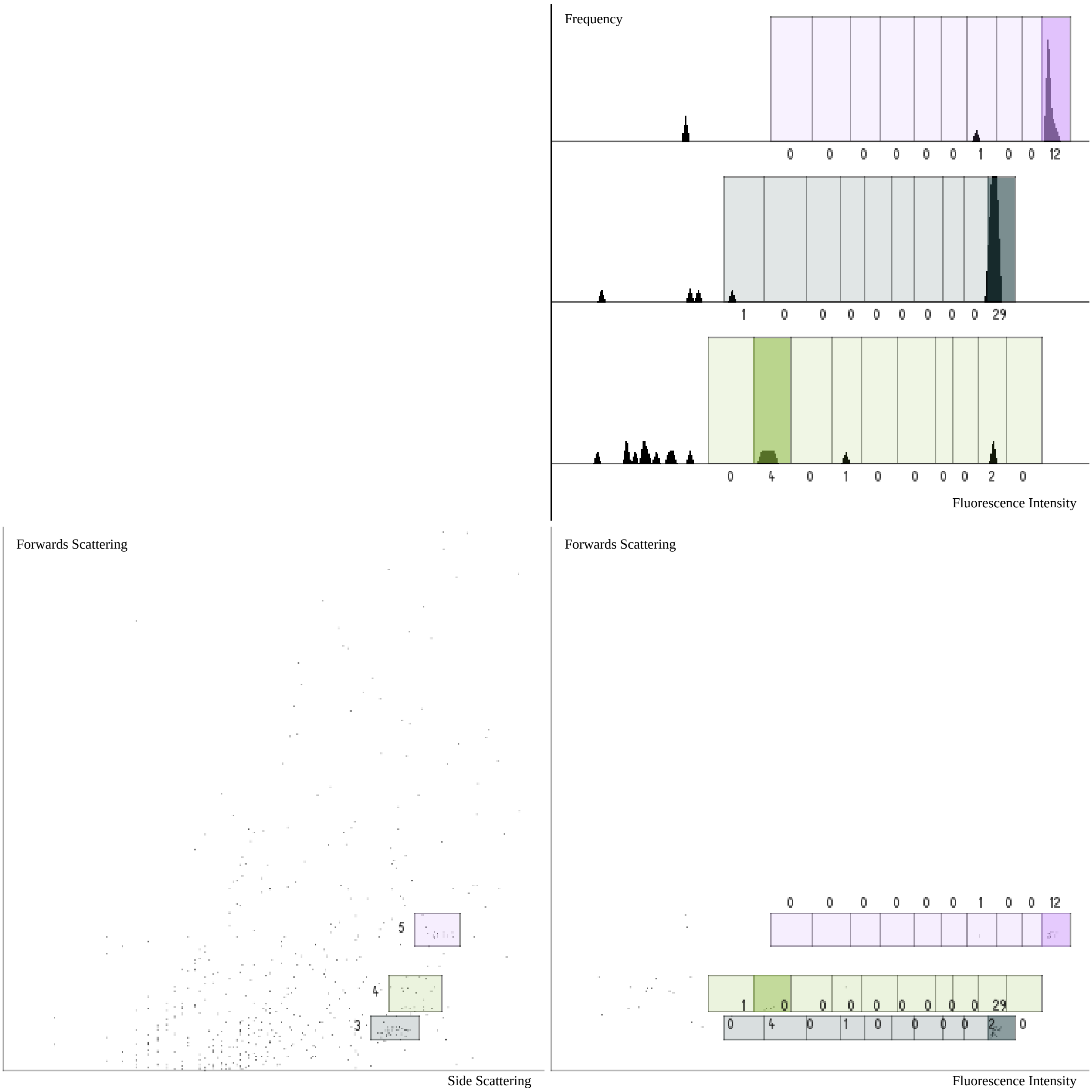

ANNEX 3: TAG DECONVOLUTION - BEAD 20

Passes flow sorting criteria: Yes

Passes tag deconvolution criteria: Yes

Included in protocol analysis: Yes

Protocol: 4, 9, 3, 2

Filename: Bin2_plateA1_B7.LMD

Split 1: Petrol shading

Split 2: Green shading

Split 3: Violet shading

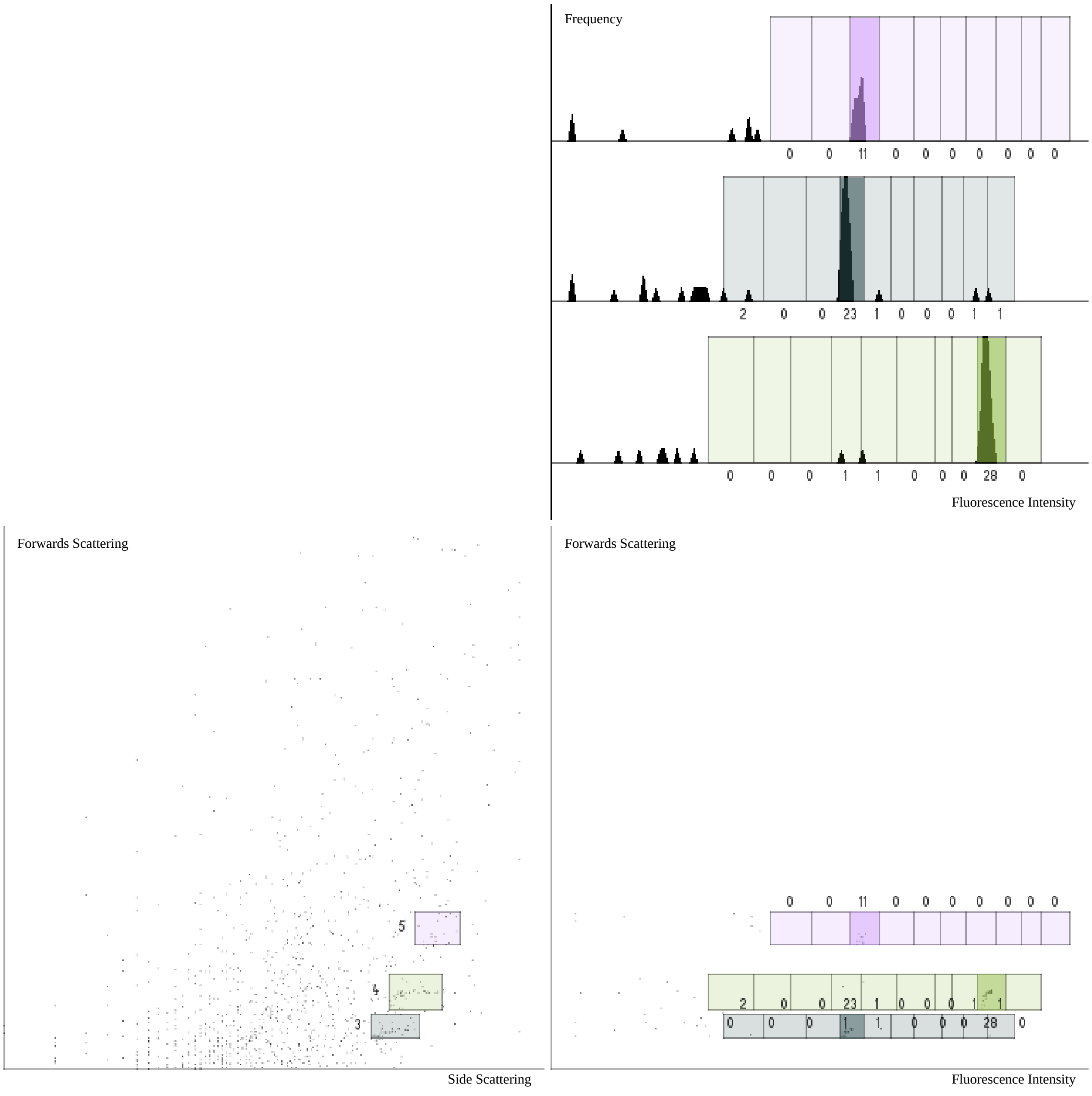

ANNEX 3: TAG DECONVOLUTION - BEAD 21

Passes flow sorting criteria: Yes

Passes tag deconvolution criteria: Yes

Included in protocol analysis: Yes

Protocol: 5, 6, 4, 2

Filename: Bin2_plateA1_B8.LMD

Split 1: Petrol shading

Split 2: Green shading

Split 3: Violet shading

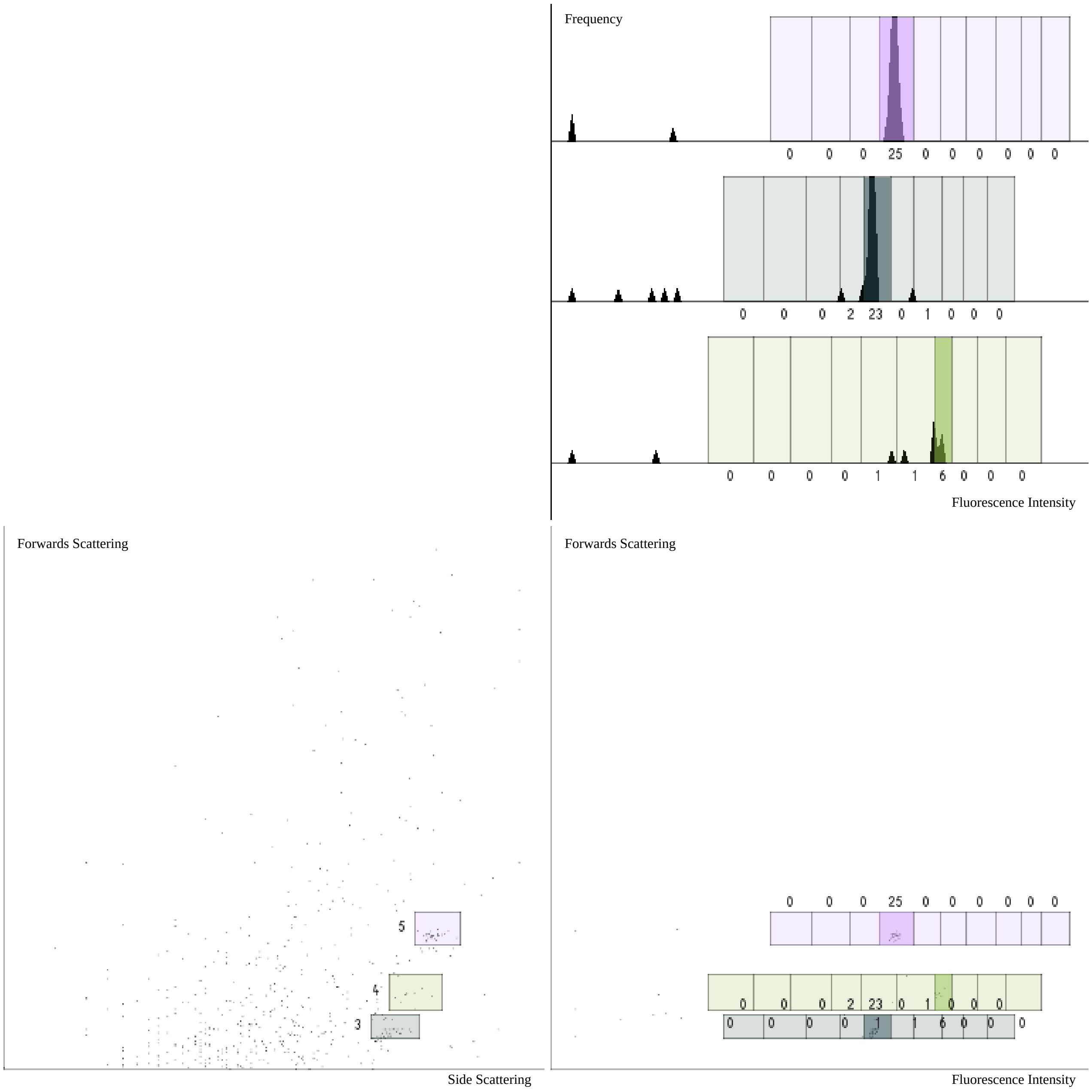

ANNEX 3: TAG DECONVOLUTION - BEAD 22

Passes flow sorting criteria: Yes

Passes tag deconvolution criteria: Yes

Included in protocol analysis: Yes

Protocol: 7, 4, 1, 3

Filename: Bin3_plateA1_A1.LMD

Split 1: Petrol shading

Split 2: Green shading

Split 3: Violet shading

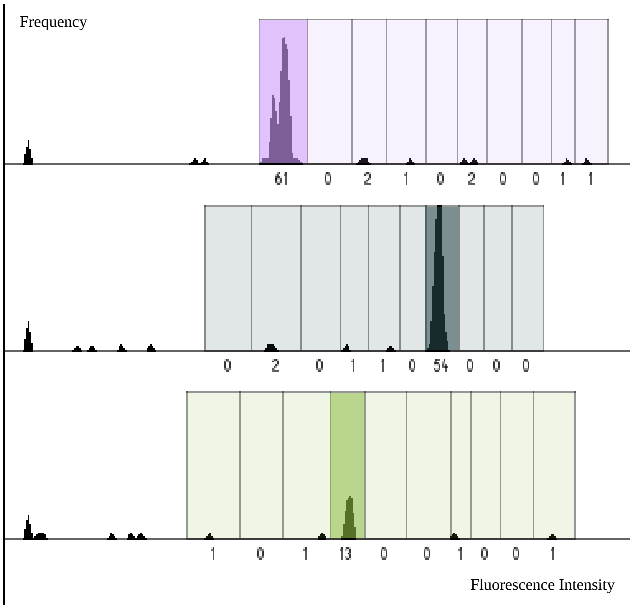

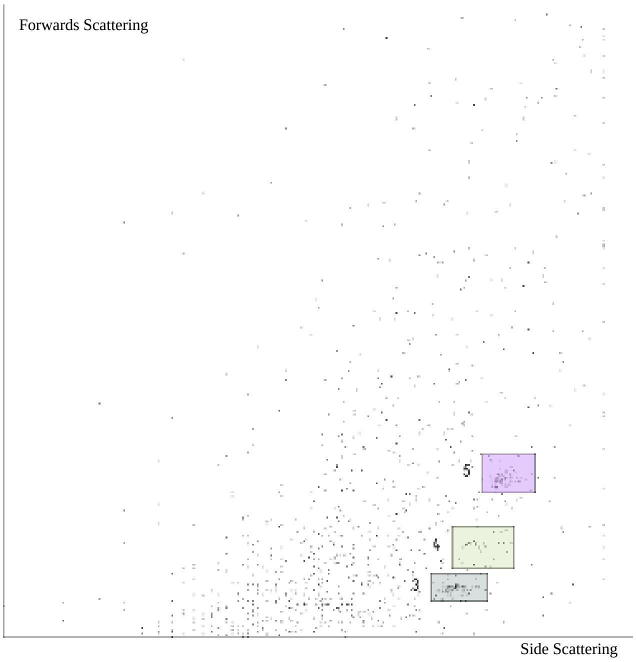

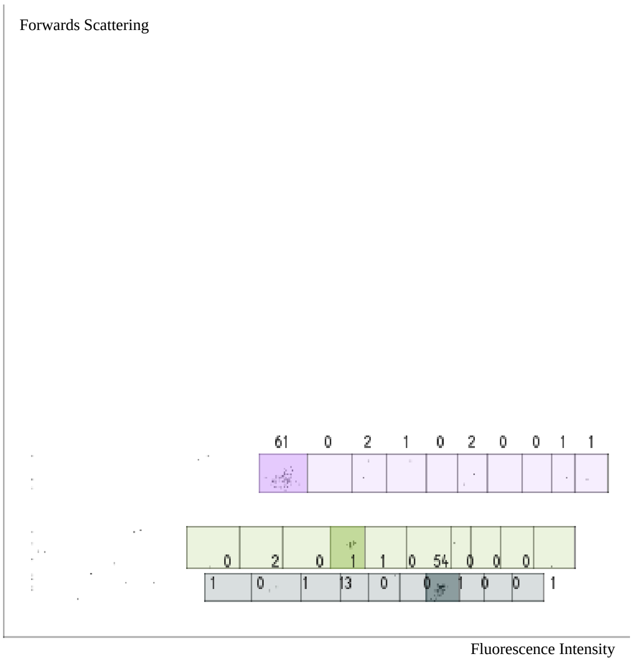

ANNEX 3: TAG DECONVOLUTION - BEAD 23

Passes flow sorting criteria: Yes

Passes tag deconvolution criteria: Yes

Included in protocol analysis: Yes

Protocol: 8, 2, 9, 3

Filename: Bin3_plateA1_A2.LMD

Split 1: Petrol shading

Split 2: Green shading

Split 3: Violet shading

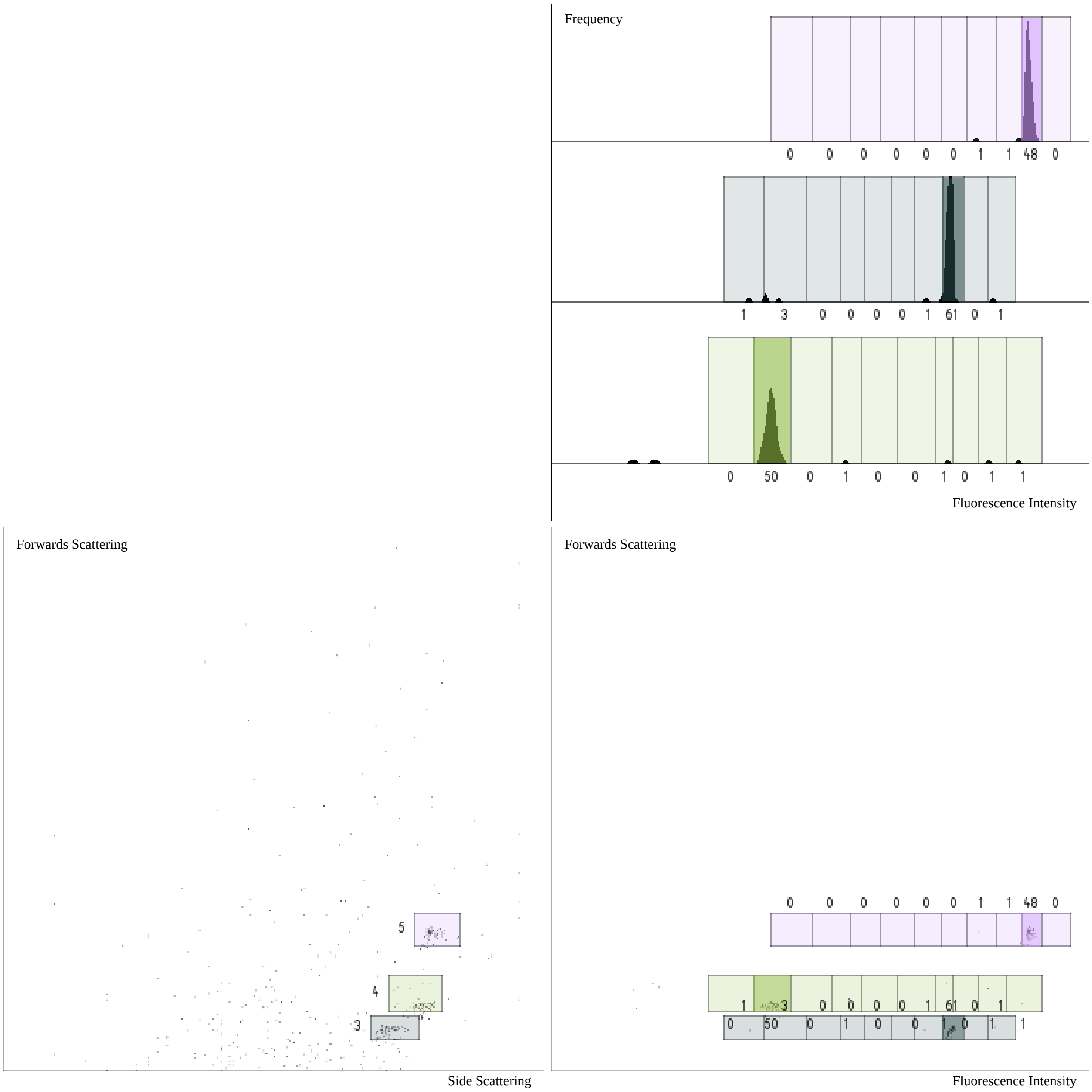

ANNEX 3: TAG DECONVOLUTION - BEAD 24

Passes flow sorting criteria: Yes

Passes tag deconvolution criteria: Yes

Included in protocol analysis: Yes

Protocol: 1, 9, 4, 3

Filename: Bin3_plateA1_A3.LMD

Split 1: Petrol shading

Split 2: Green shading

Split 3: Violet shading

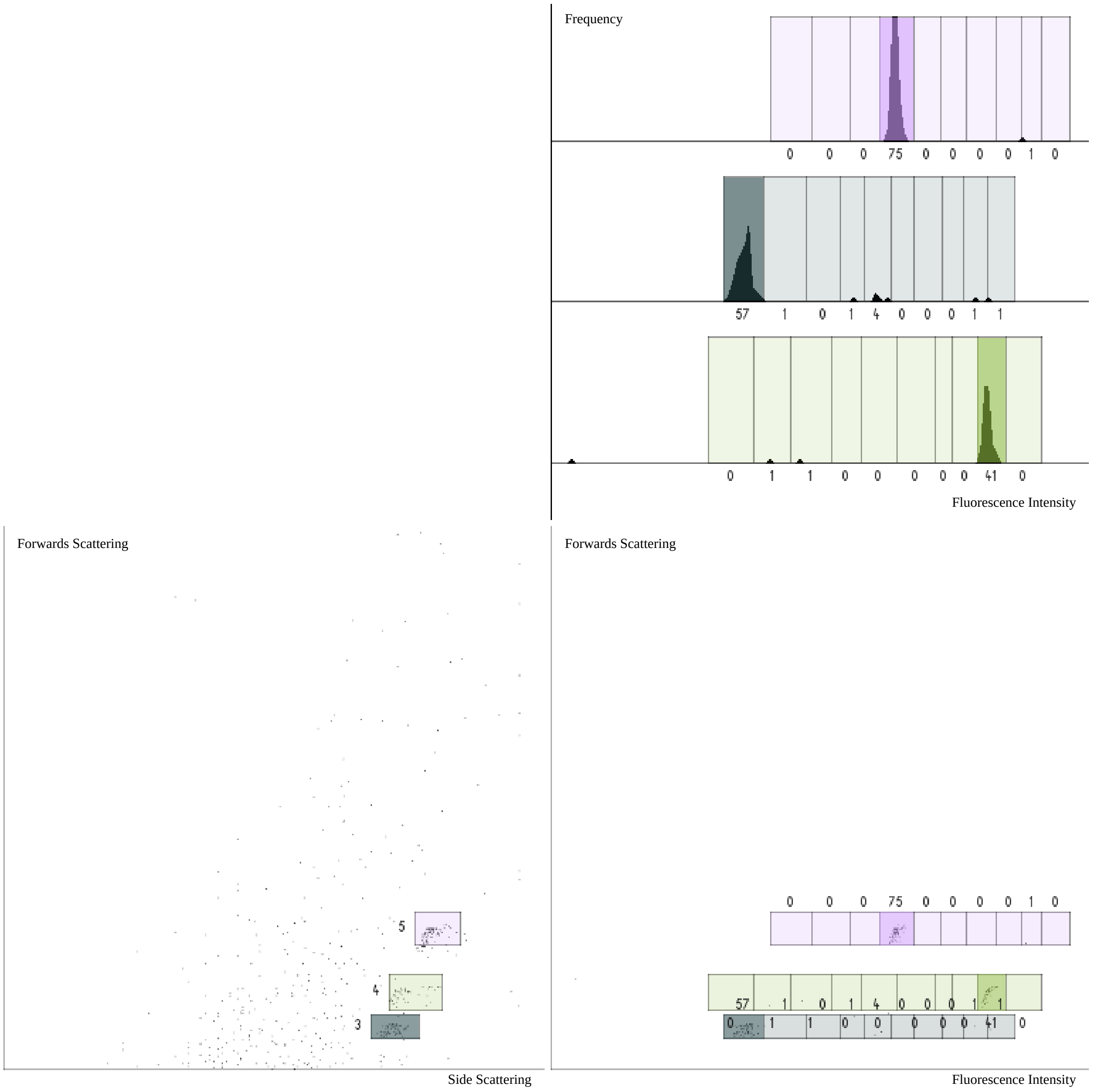

ANNEX 3: TAG DECONVOLUTION - BEAD 25

Passes flow sorting criteria: Yes

Passes tag deconvolution criteria: Yes

Included in protocol analysis: Yes

Protocol: 9, 5, 8, 3

Filename: Bin3_plateA1_A4.LMD

Split 1: Petrol shading

Split 2: Green shading

Split 3: Violet shading

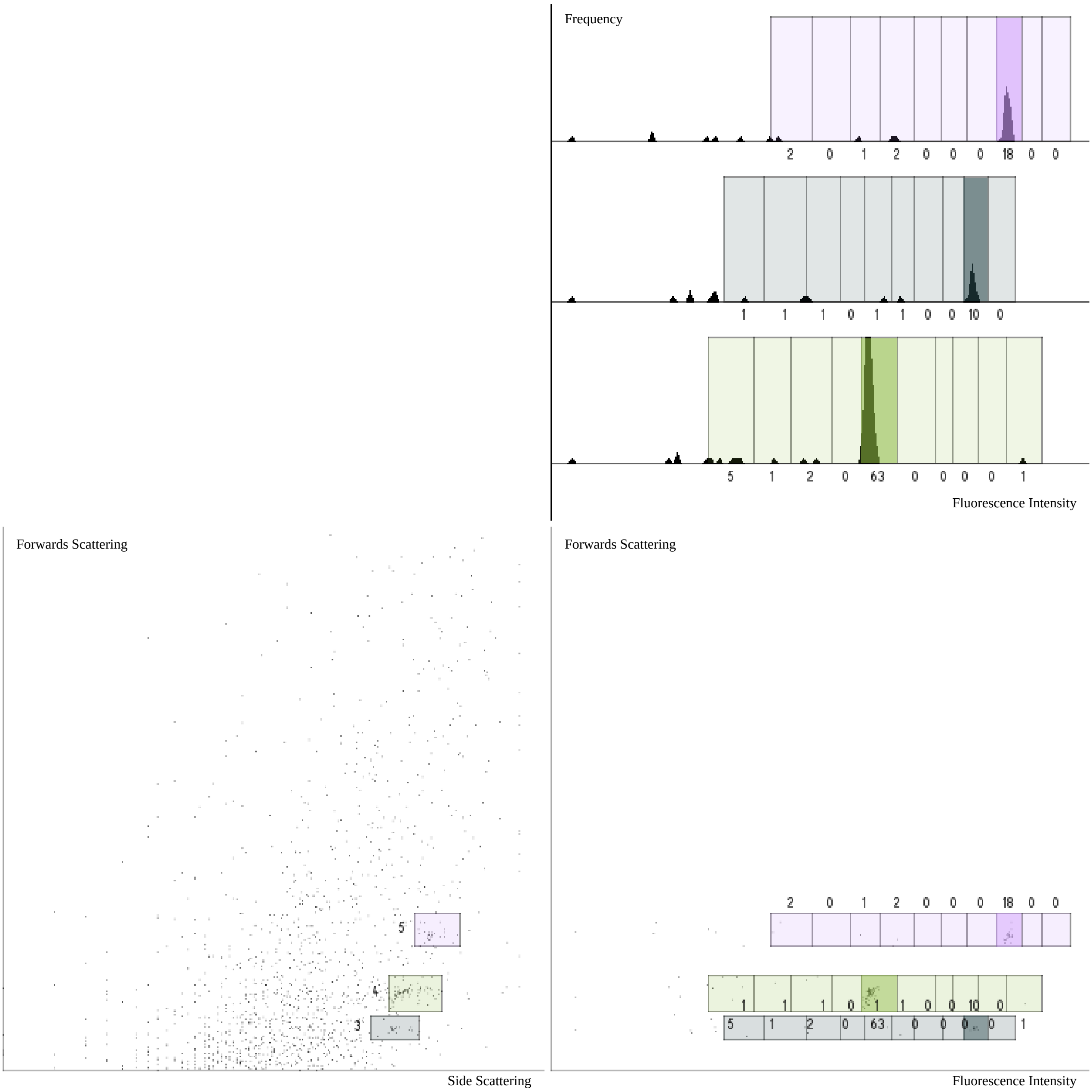

ANNEX 3: TAG DECONVOLUTION - BEAD 26

Passes flow sorting criteria: Yes

Passes tag deconvolution criteria: Yes

Included in protocol analysis: Yes

Protocol: 10, 8, 7, 3

Filename: Bin3_plateA1_A5.LMD

Split 1: Petrol shading

Split 2: Green shading

Split 3: Violet shading

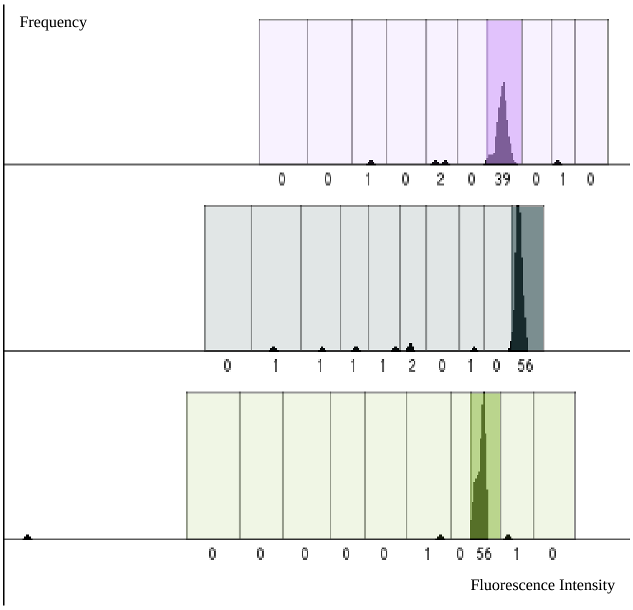

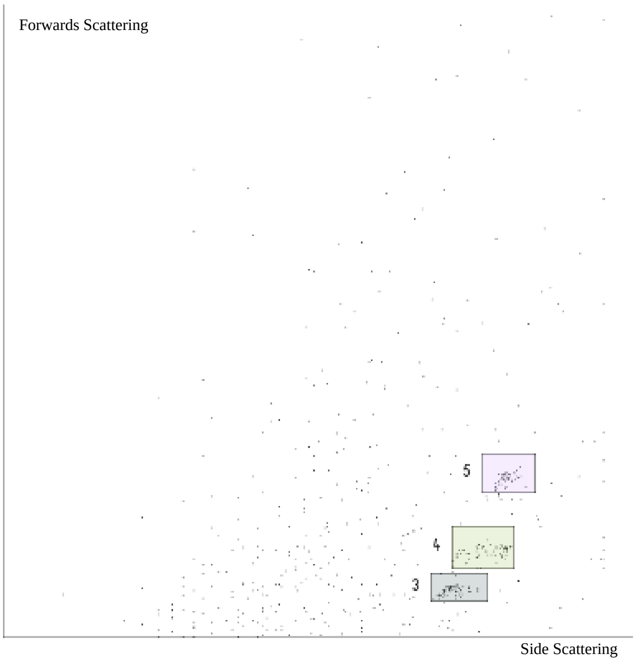

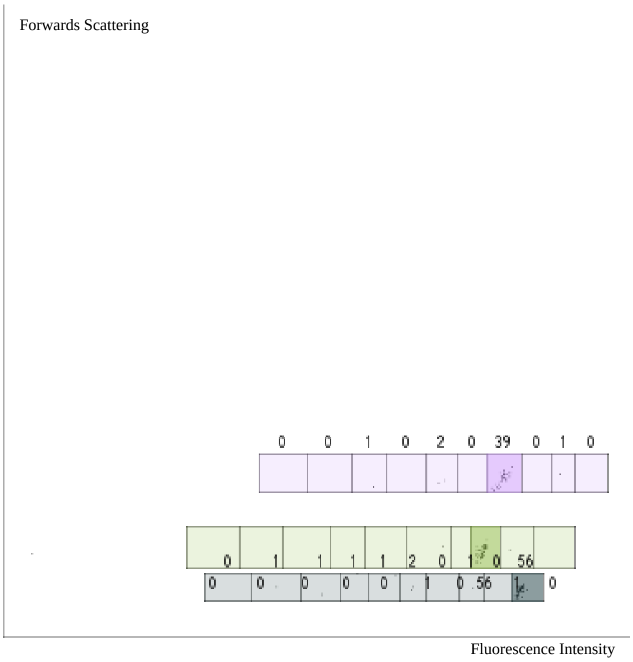

ANNEX 3: TAG DECONVOLUTION - BEAD 27

Passes flow sorting criteria: Yes

Passes tag deconvolution criteria: Yes

Included in protocol analysis: Yes

Protocol: 1, 6, 1, 3

Filename: Bin3_plateA1_A6.LMD

Split 1: Petrol shading

Split 2: Green shading

Split 3: Violet shading

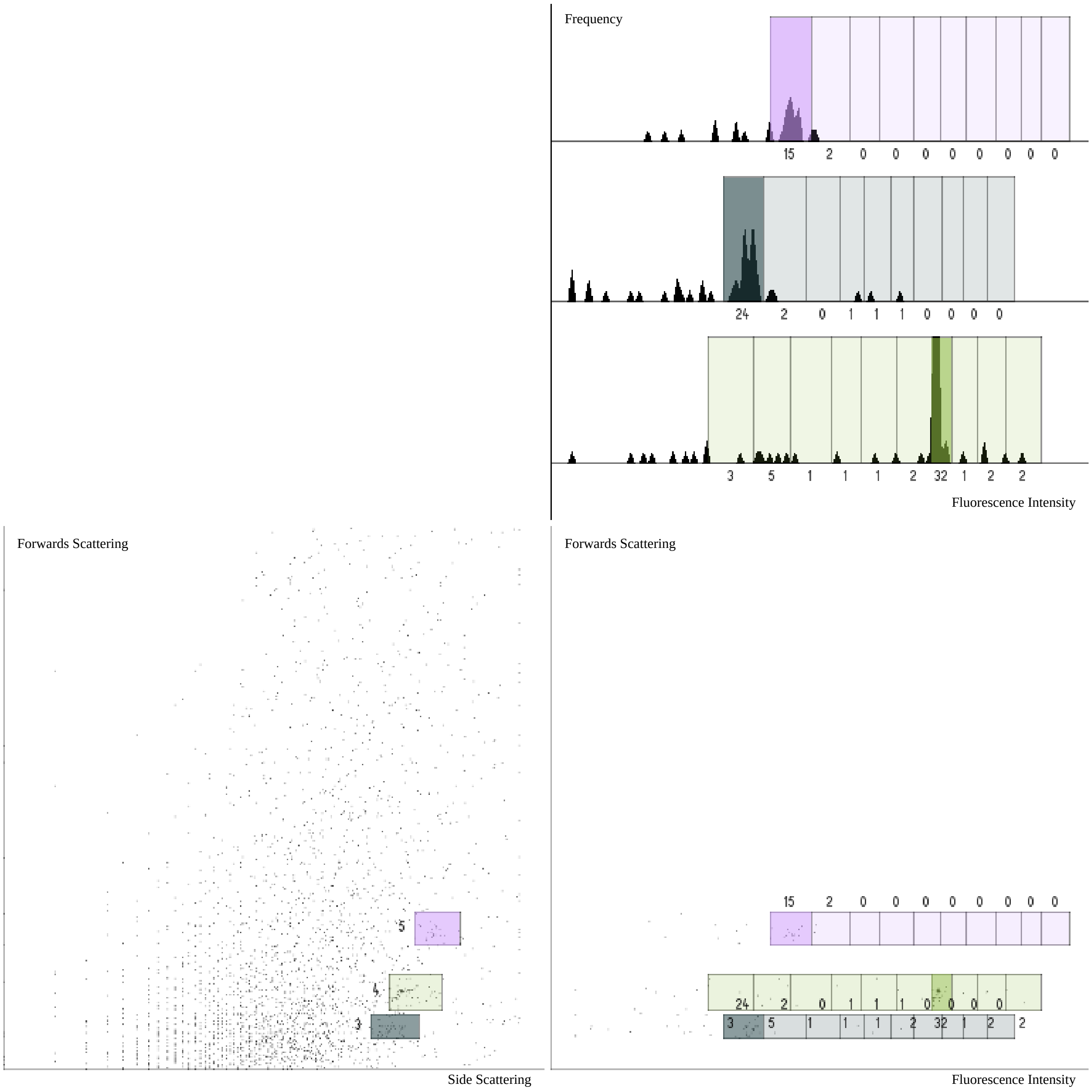

ANNEX 3: TAG DECONVOLUTION - BEAD 28

Passes flow sorting criteria: Yes

Passes tag deconvolution criteria: No

Included in protocol analysis: No

Protocol: N/A

Filename: Bin3_plateA1_A7.LMD

Split 1: Petrol shading

Split 2: Green shading

Split 3: Violet shading

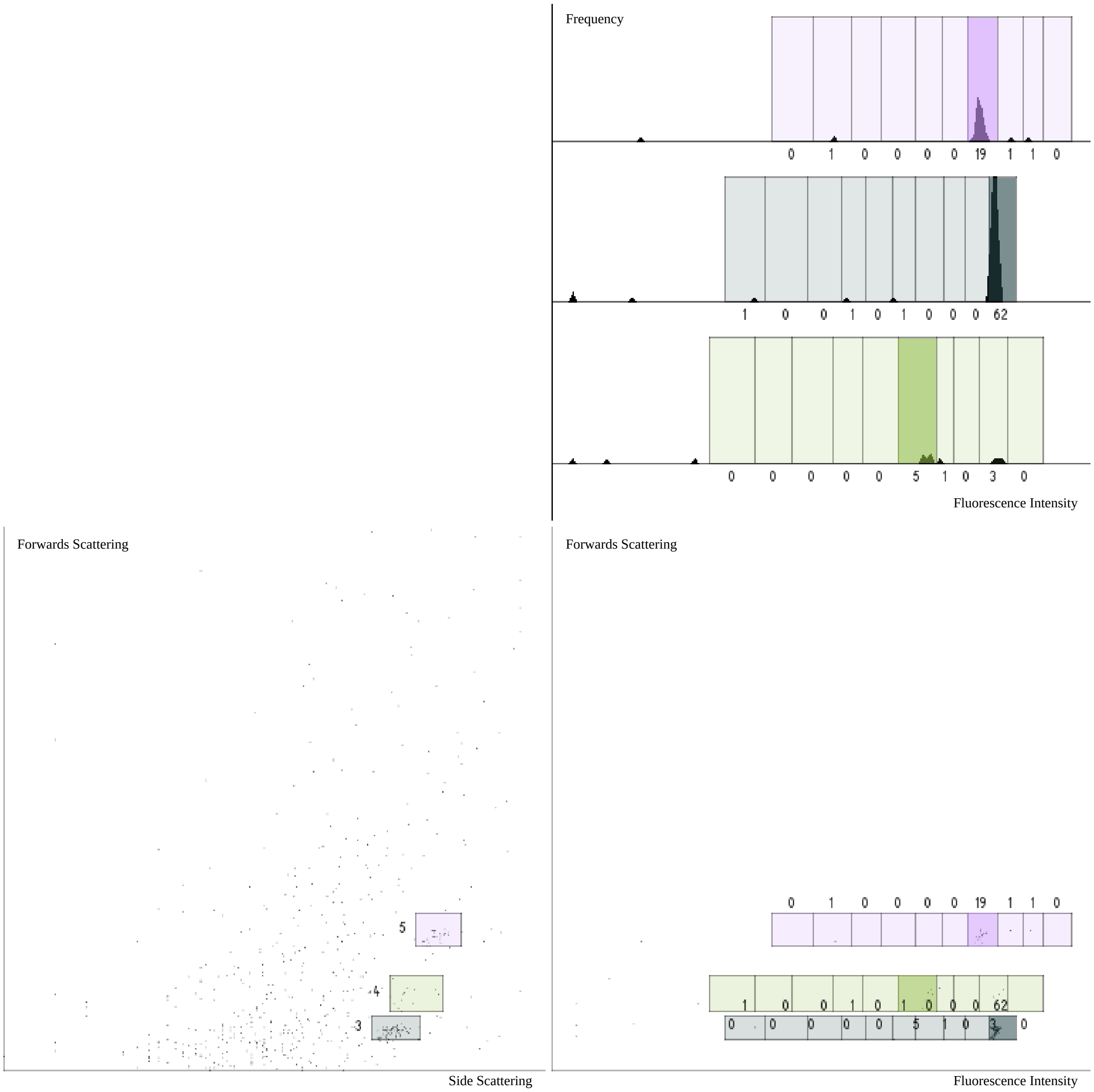

ANNEX 3: TAG DECONVOLUTION - BEAD 29

Passes flow sorting criteria: Yes

Passes tag deconvolution criteria: Yes

Included in protocol analysis: Yes

Protocol: 4, 1, 1, 3

Filename: Bin3_plateA1_A8.LMD

Split 1: Petrol shading

Split 2: Green shading

Split 3: Violet shading

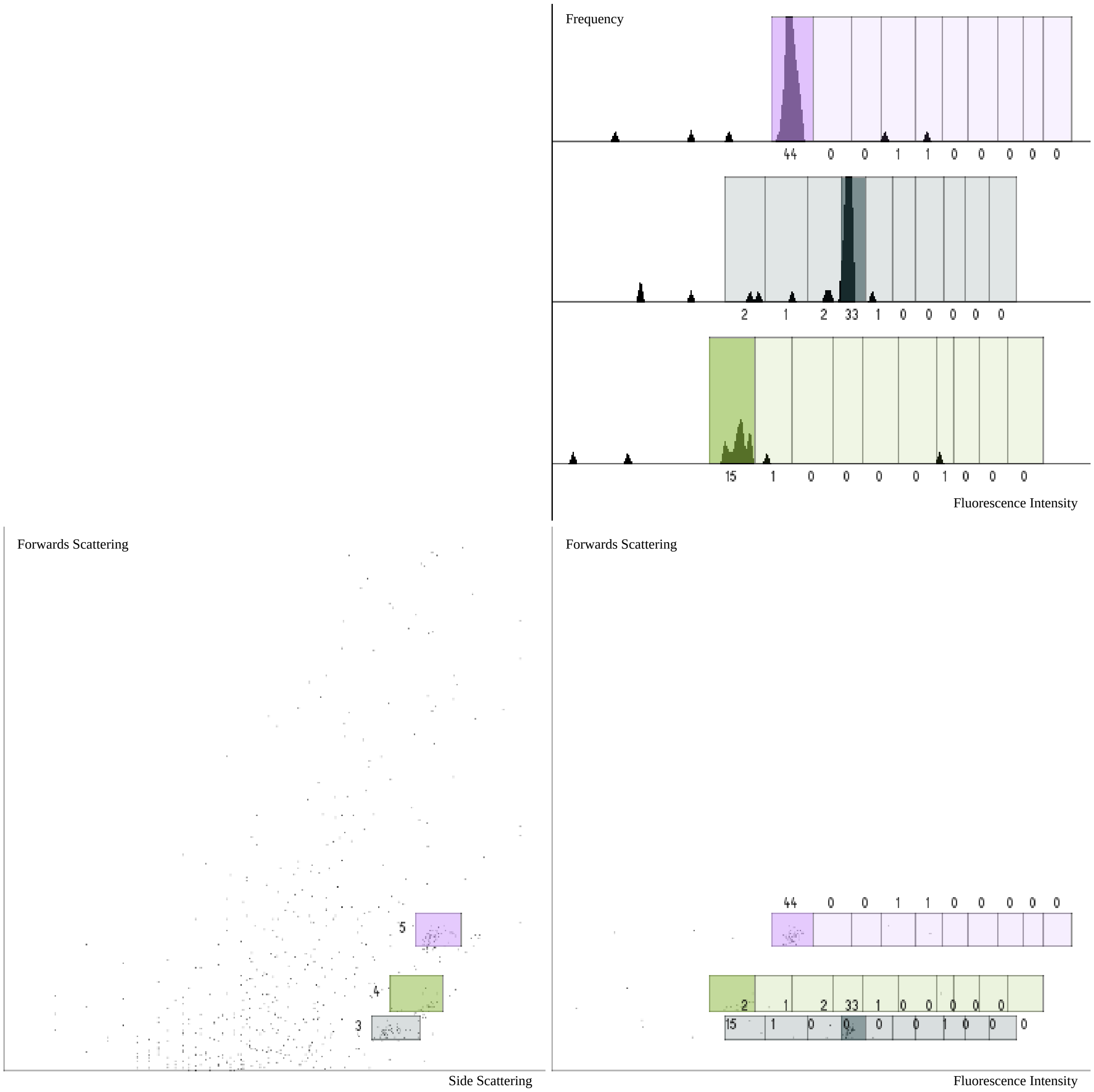

ANNEX 3: TAG DECONVOLUTION - BEAD 30

Passes flow sorting criteria: Yes

Passes tag deconvolution criteria: Yes

Included in protocol analysis: Yes

Protocol: 6, 10, 10, 3

Filename: Bin3_plateA1_A9.LMD

Split 1: Petrol shading

Split 2: Green shading

Split 3: Violet shading

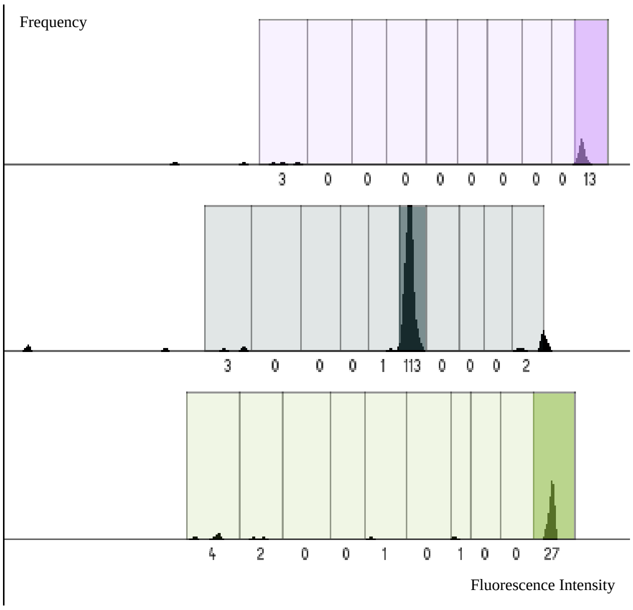

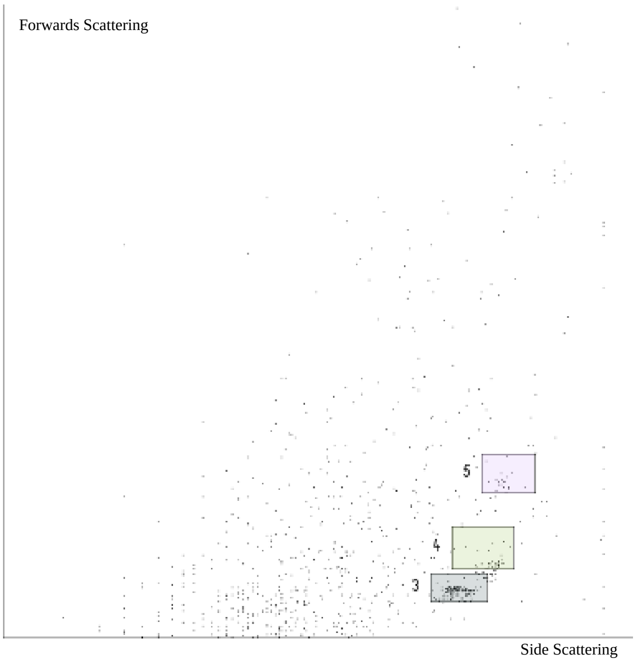

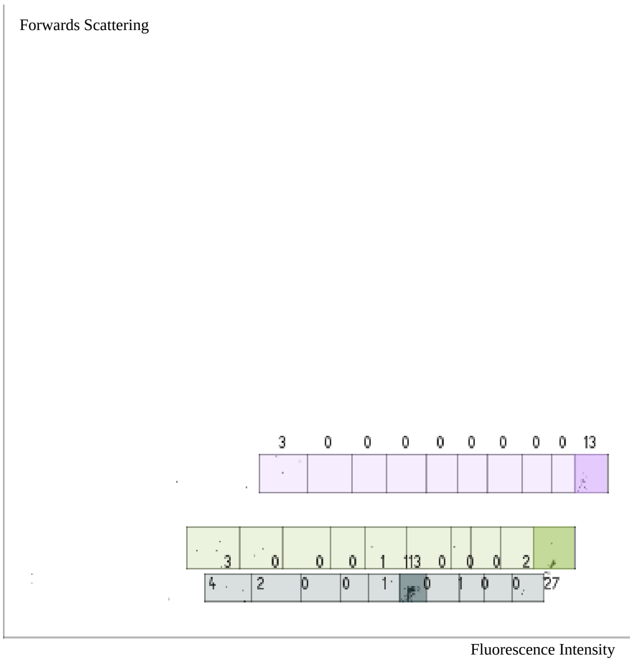

ANNEX 3: TAG DECONVOLUTION - BEAD 31

Passes flow sorting criteria: Yes

Passes tag deconvolution criteria: Yes

Included in protocol analysis: Yes

Protocol: 4, 3, 10, 3

Filename: Bin3_plateA1_A10.LMD

Split 1: Petrol shading

Split 2: Green shading

Split 3: Violet shading

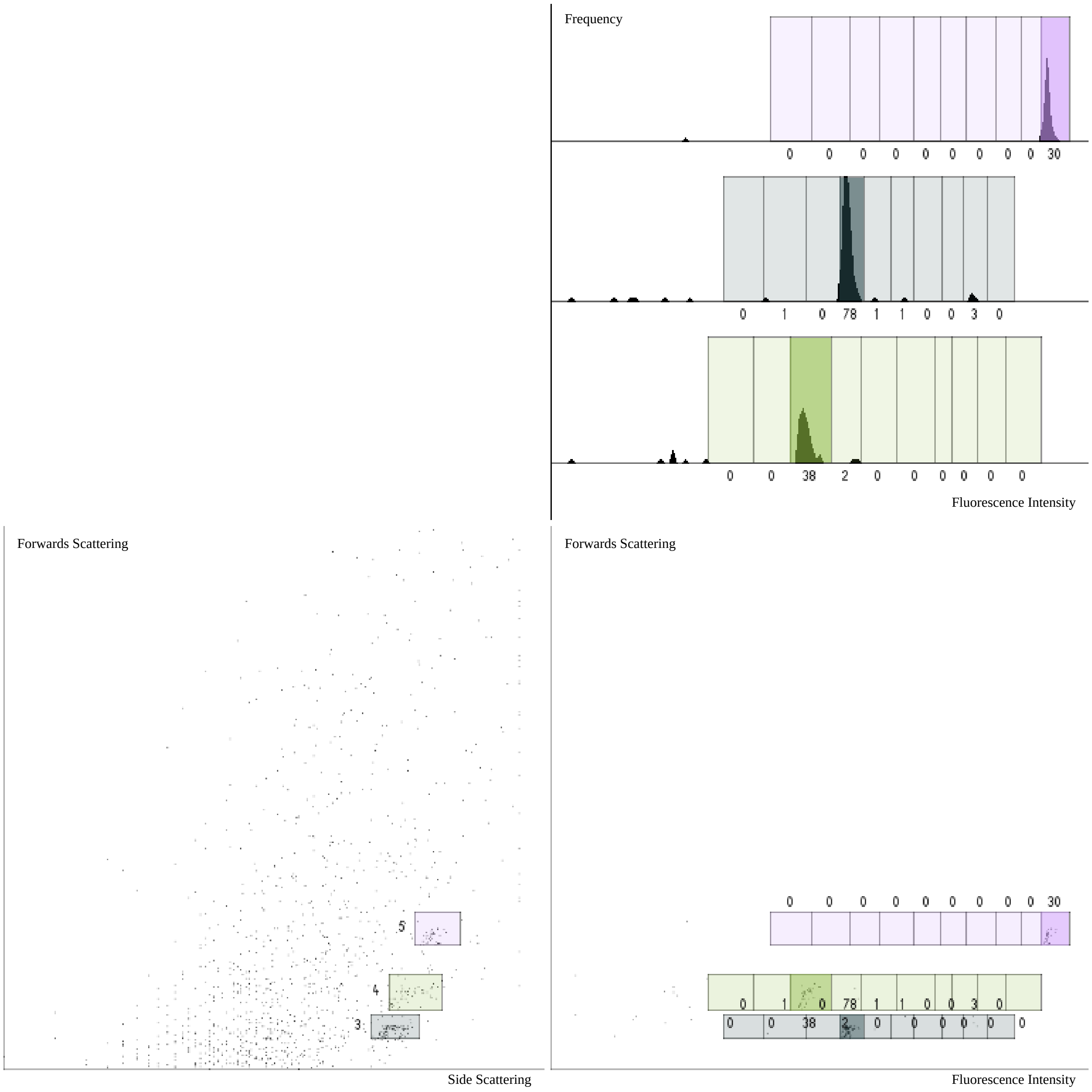

ANNEX 3: TAG DECONVOLUTION - BEAD 32

Passes flow sorting criteria: Yes

Passes tag deconvolution criteria: Yes

Included in protocol analysis: Yes

Protocol: 8, 7, 6, 3

Filename: Bin3_plateA1_A11.LMD

Split 1: Petrol shading

Split 2: Green shading

Split 3: Violet shading

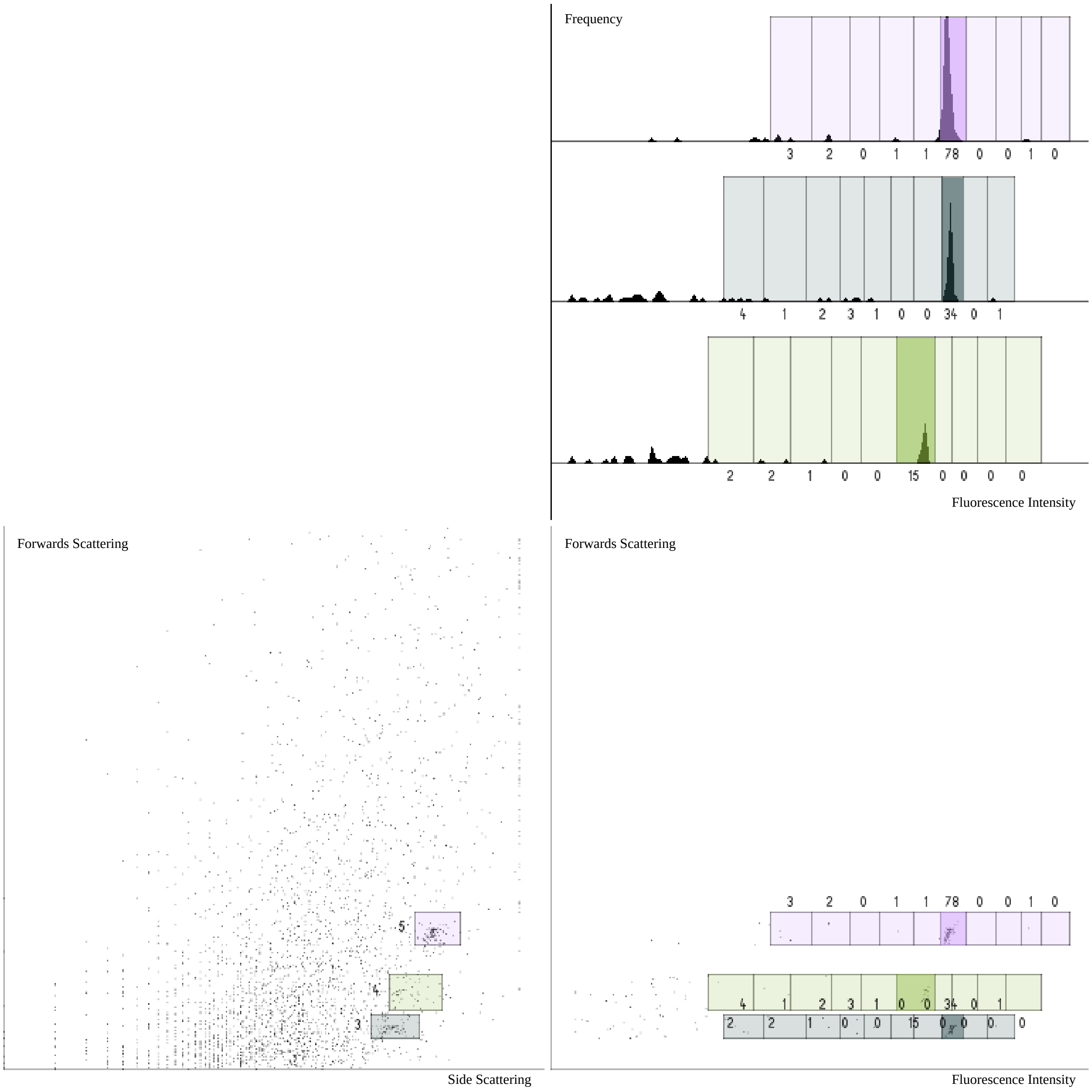

ANNEX 3: TAG DECONVOLUTION - BEAD 33

Passes flow sorting criteria: Yes

Passes tag deconvolution criteria: Yes

Included in protocol analysis: Yes

Protocol: 5, 3, 1, 3

Filename: Bin3_plateA1_A12.LMD

Split 1: Petrol shading

Split 2: Green shading

Split 3: Violet shading

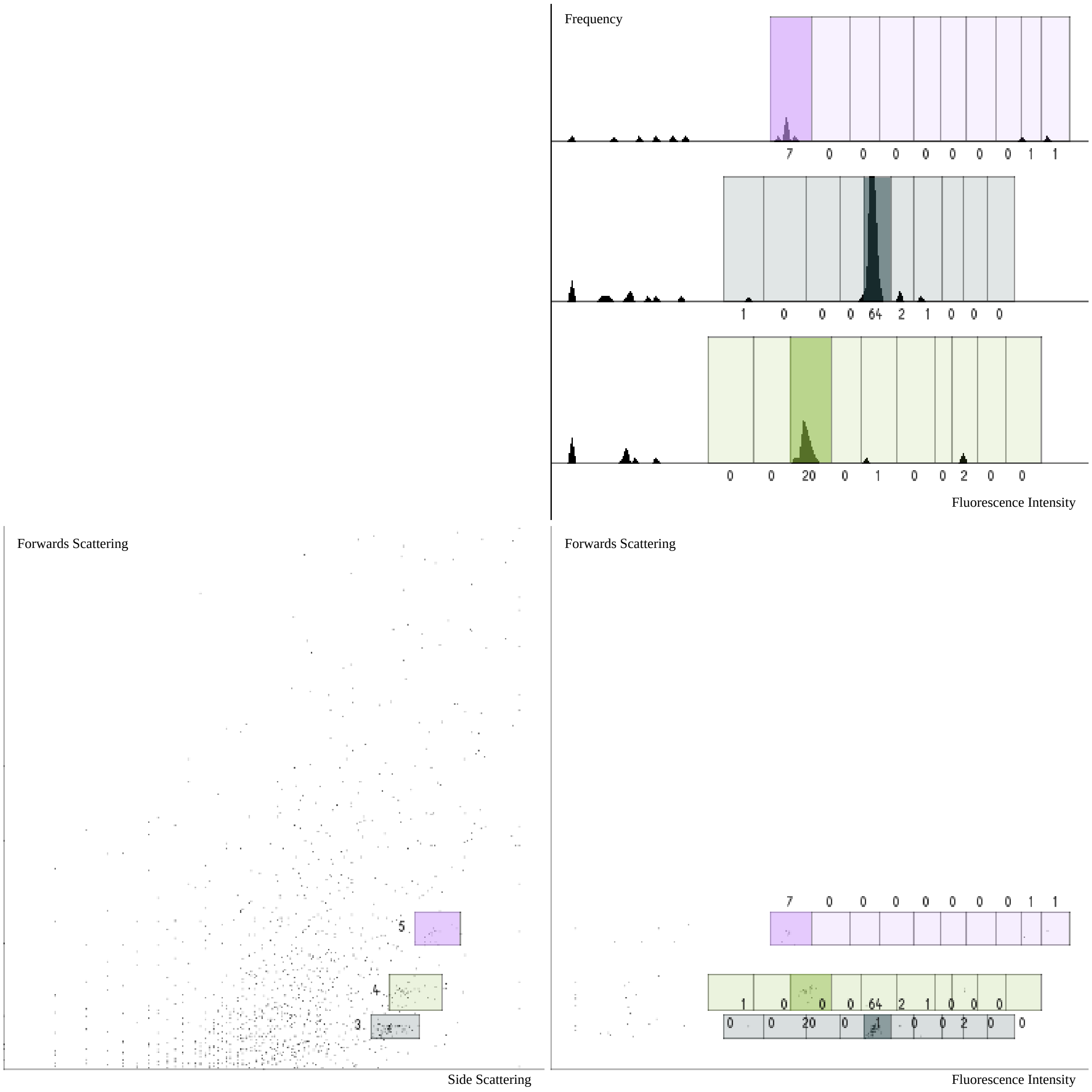

ANNEX 3: TAG DECONVOLUTION - BEAD 34

Passes flow sorting criteria: Yes

Passes tag deconvolution criteria: Yes

Included in protocol analysis: Yes

Protocol: 8, 8, 5, 3

Filename: Bin3_plateA1_B1.LMD

Split 1: Petrol shading

Split 2: Green shading

Split 3: Violet shading

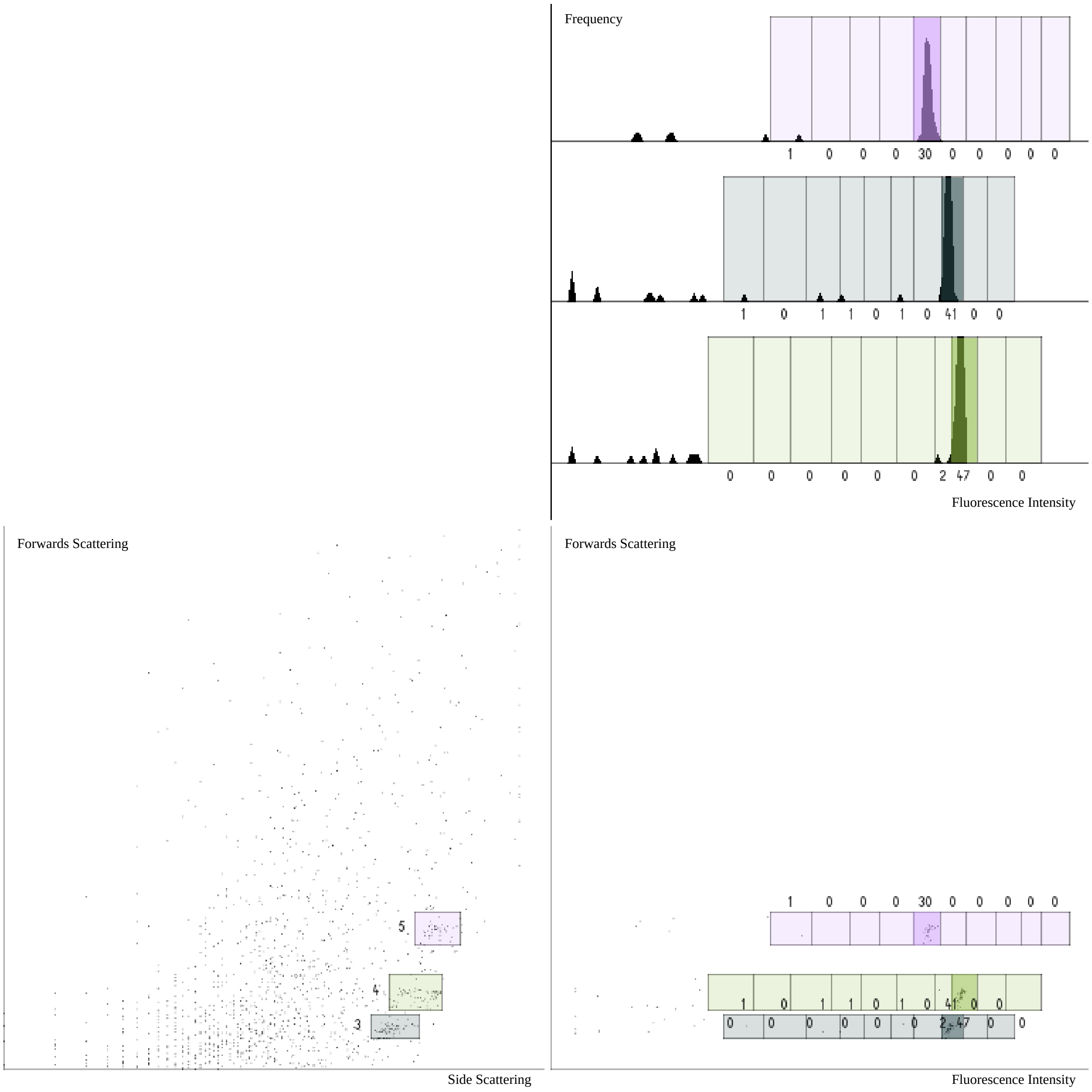

ANNEX 3: TAG DECONVOLUTION - BEAD 35

Passes flow sorting criteria: Yes

Passes tag deconvolution criteria: Yes

Included in protocol analysis: Yes

Protocol: 4, 7, 6, 3

Filename: Bin3_plateA1_B2.LMD

Split 1: Petrol shading

Split 2: Green shading

Split 3: Violet shading

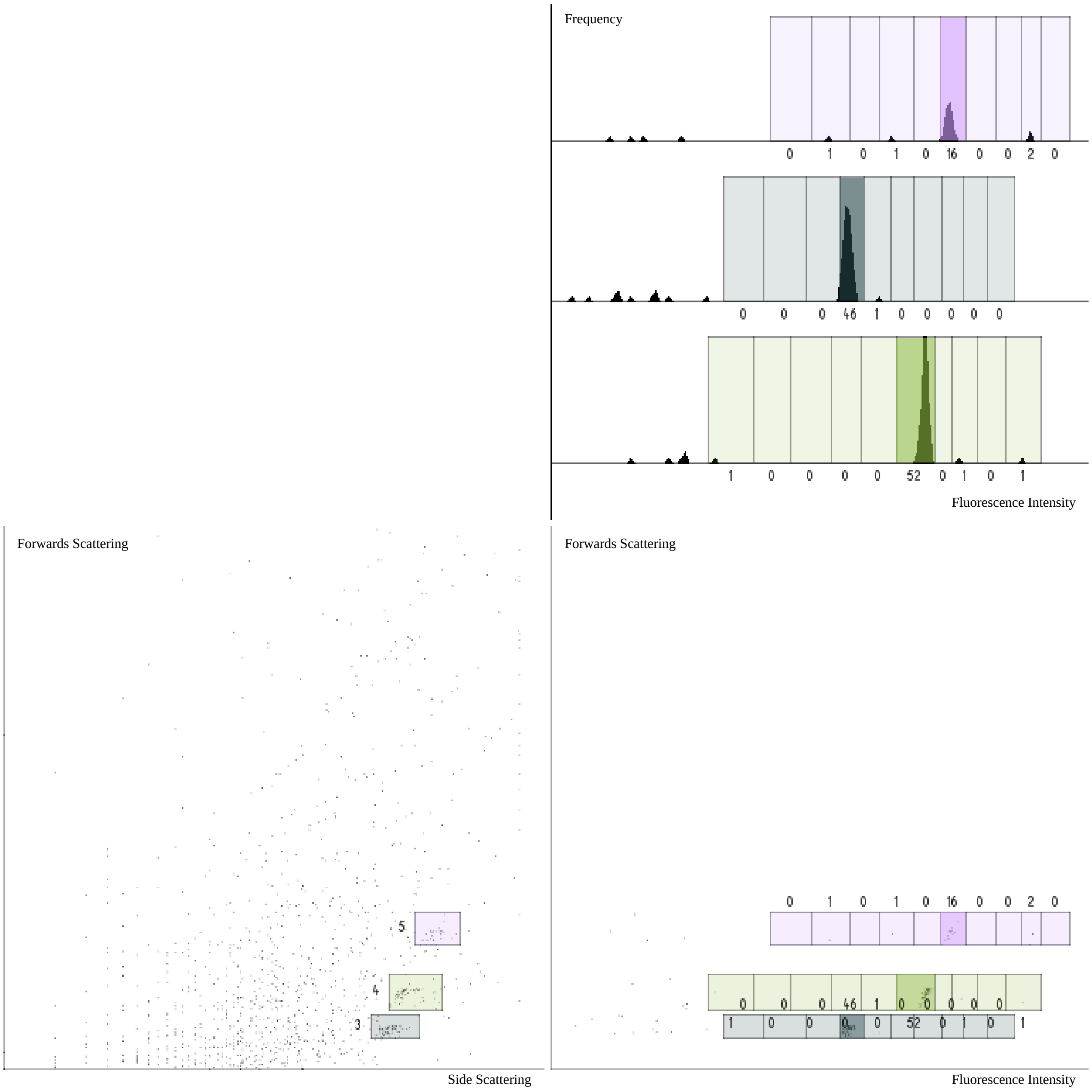

ANNEX 3: TAG DECONVOLUTION - BEAD 36

Passes flow sorting criteria: Yes

Passes tag deconvolution criteria: Yes

Included in protocol analysis: Yes

Protocol: 2, 7, 2, 3

Filename: Bin3_plateA1_B3.LMD

Split 1: Petrol shading

Split 2: Green shading

Split 3: Violet shading

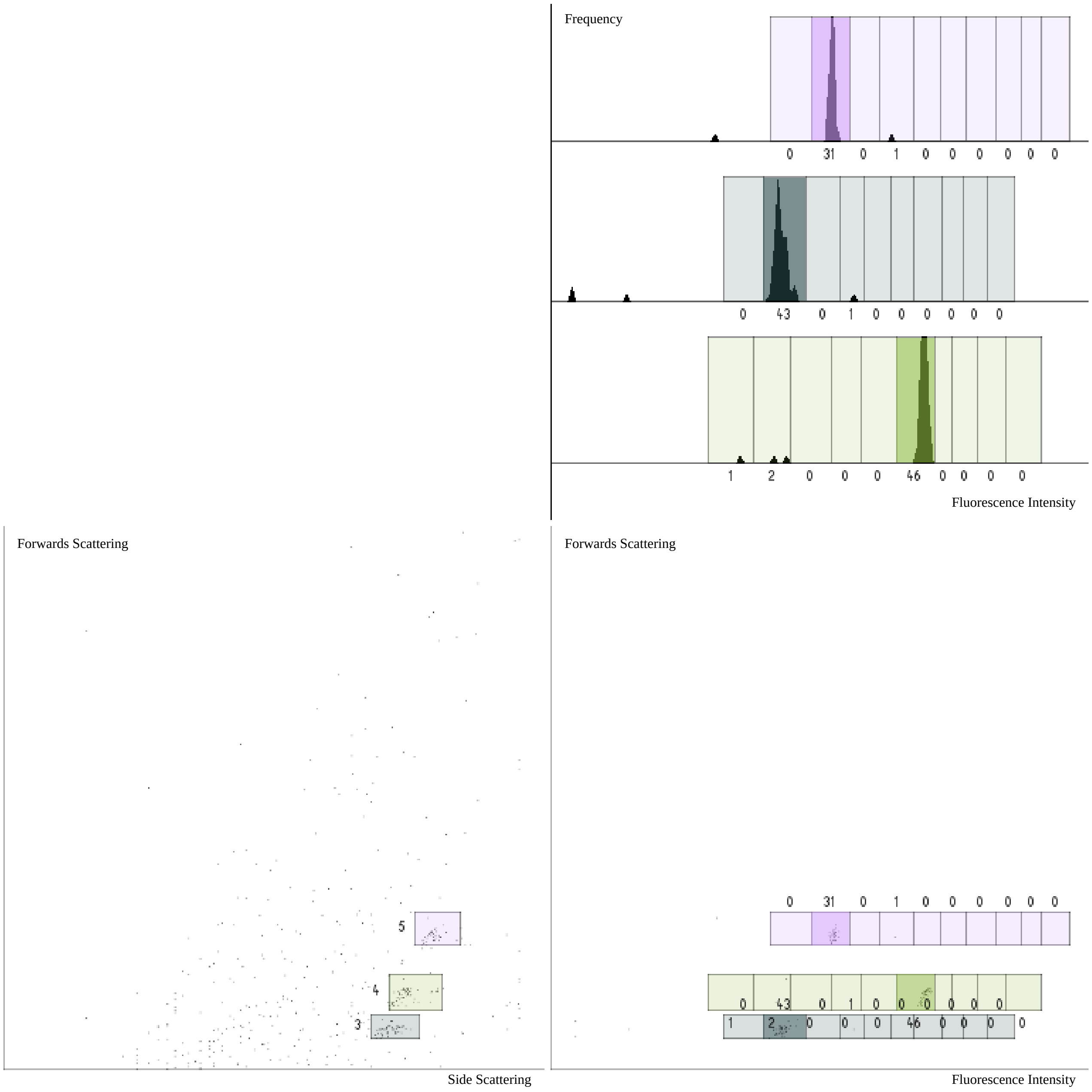

ANNEX 3: TAG DECONVOLUTION - BEAD 37

Passes flow sorting criteria: Yes

Passes tag deconvolution criteria: Yes

Included in protocol analysis: Yes

Protocol: 9, 2, 3, 3

Filename: Bin3_plateA1_B4.LMD

Split 1: Petrol shading

Split 2: Green shading

Split 3: Violet shading

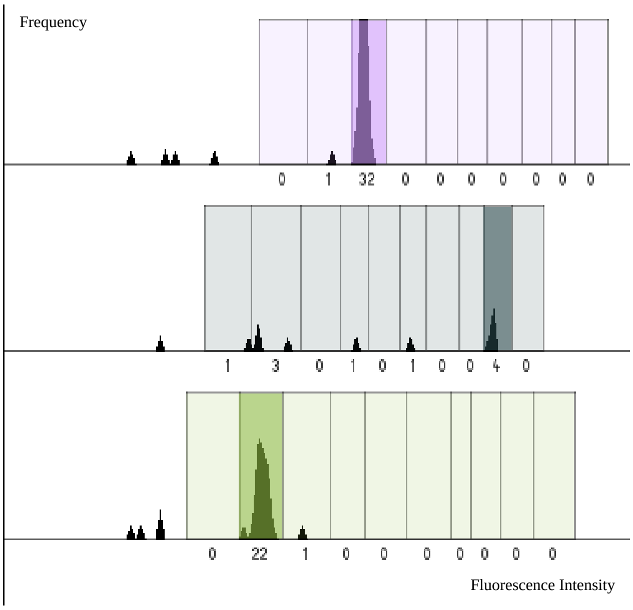

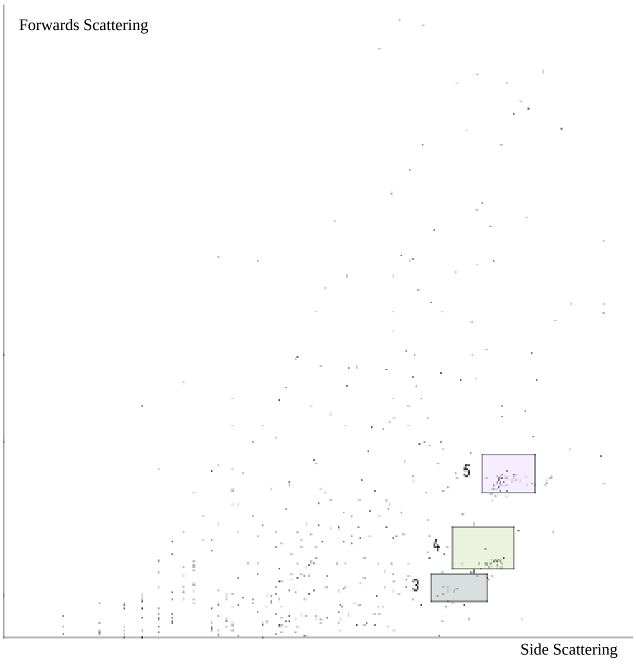

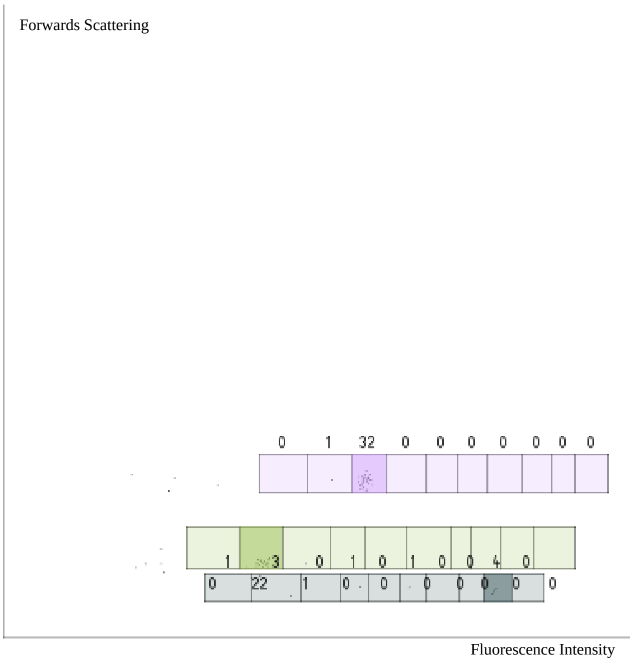

ANNEX 3: TAG DECONVOLUTION - BEAD 38

Passes flow sorting criteria: Yes

Passes tag deconvolution criteria: No

Included in protocol analysis: No

Protocol: N/A

Filename: Bin3_plateA1_B5.LMD

Split 1: Petrol shading

Split 2: Green shading

Split 3: Violet shading

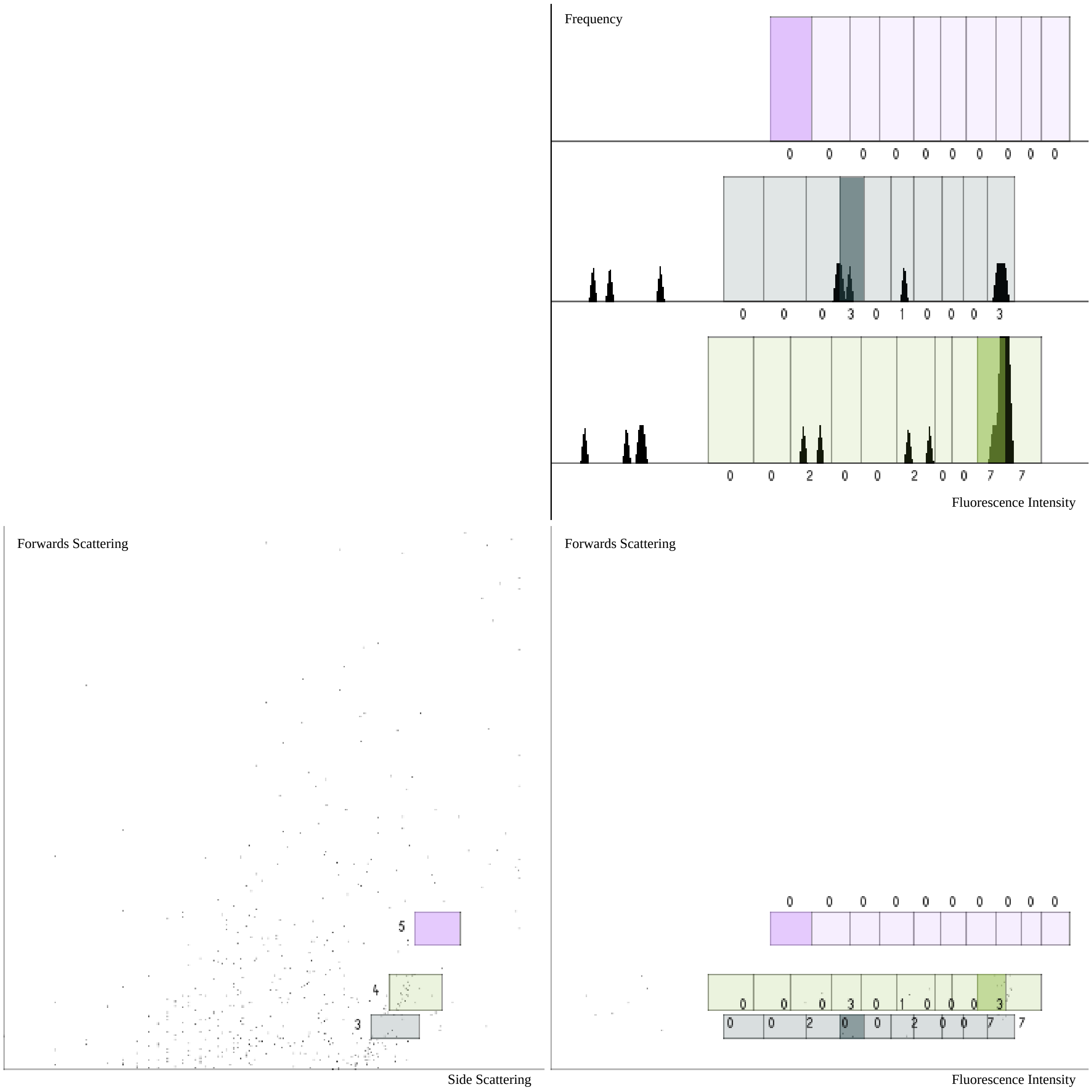

ANNEX 3: TAG DECONVOLUTION - BEAD 39

Passes flow sorting criteria: Yes

Passes tag deconvolution criteria: Yes

Included in protocol analysis: Yes

Protocol: 1, 4, 8, 3

Filename: Bin3_plateA1_B6.LMD

Split 1: Petrol shading

Split 2: Green shading

Split 3: Violet shading

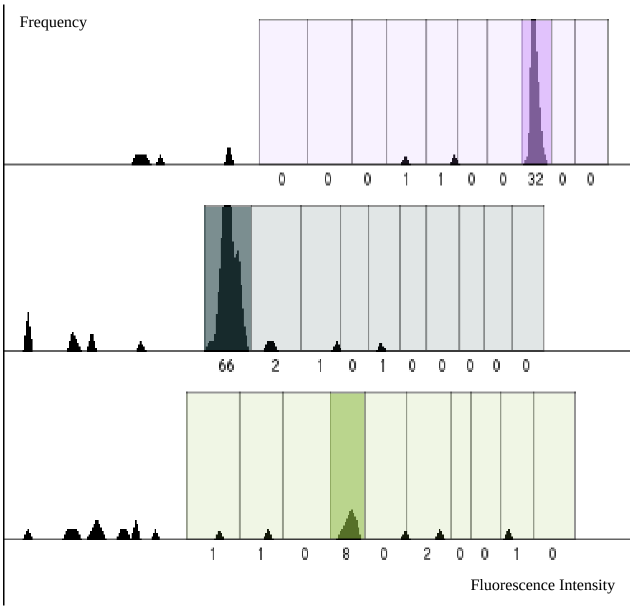

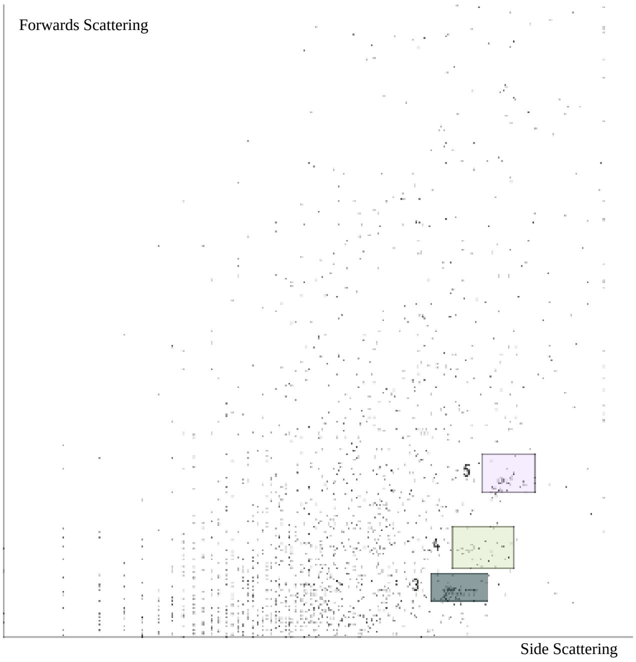

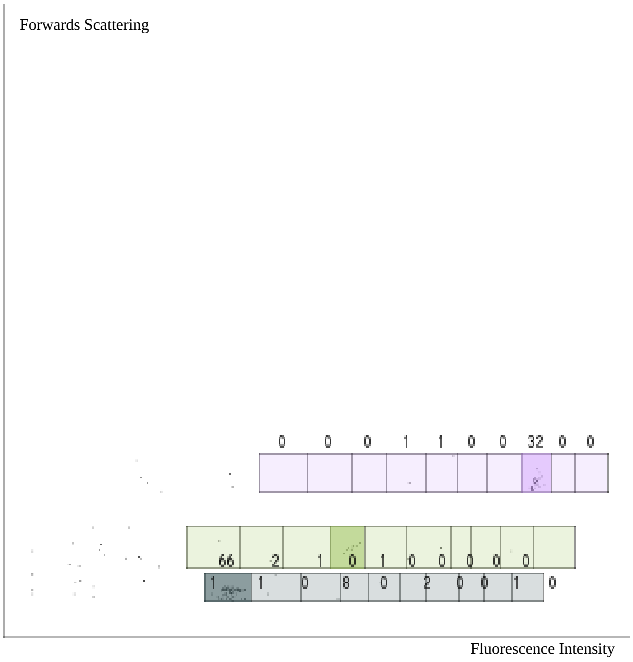

ANNEX 3: TAG DECONVOLUTION - BEAD 40

Passes flow sorting criteria: Yes

Passes tag deconvolution criteria: Yes

Included in protocol analysis: Yes

Protocol: 3, 9, 5, 3

Filename: Bin3_plateA1_B7.LMD

Split 1: Petrol shading

Split 2: Green shading

Split 3: Violet shading

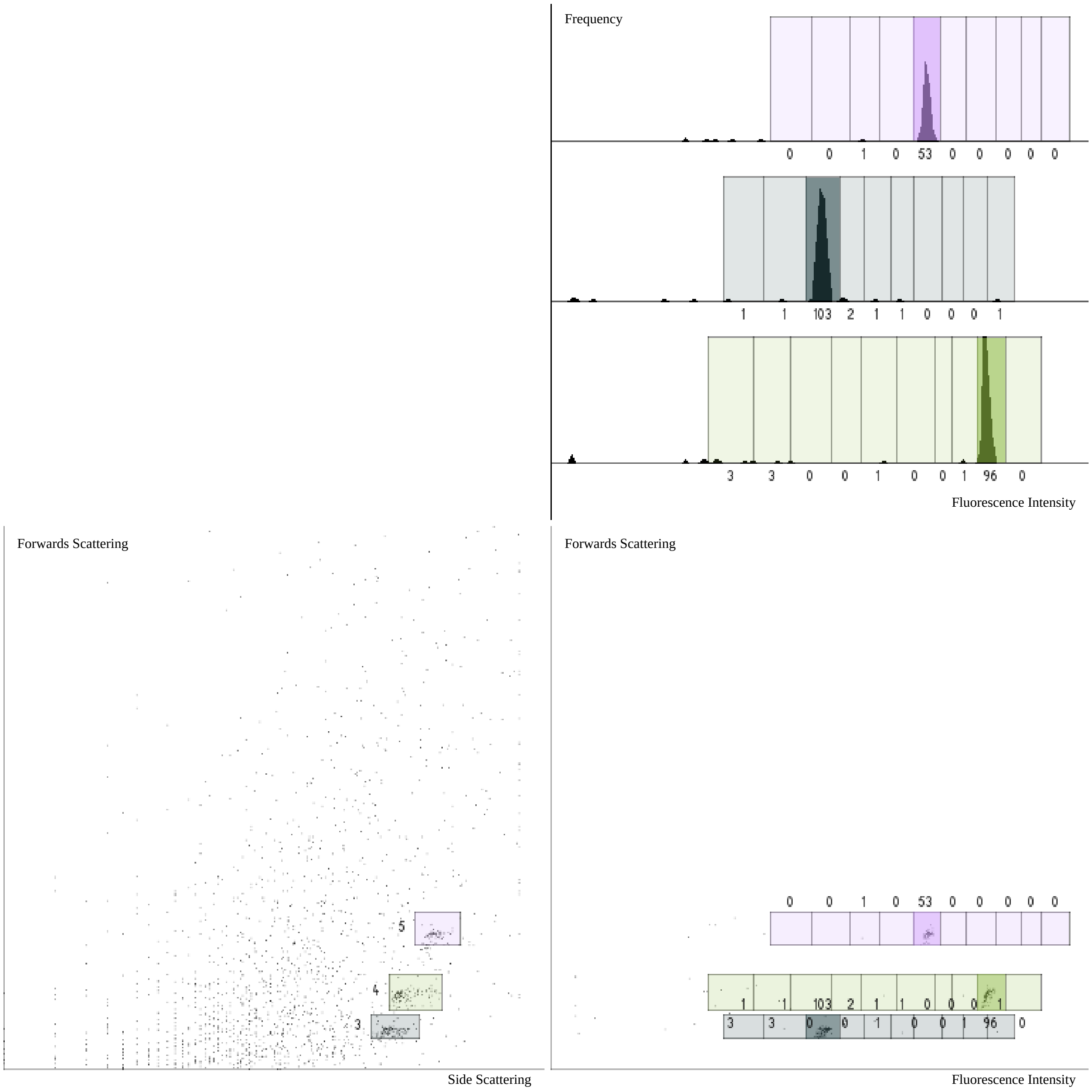

ANNEX 3: TAG DECONVOLUTION - BEAD 41

Passes flow sorting criteria: Yes

Passes tag deconvolution criteria: Yes

Included in protocol analysis: Yes

Protocol: 8, 10, 8, 3

Filename: Bin3_plateA1_B8.LMD

Split 1: Petrol shading

Split 2: Green shading

Split 3: Violet shading

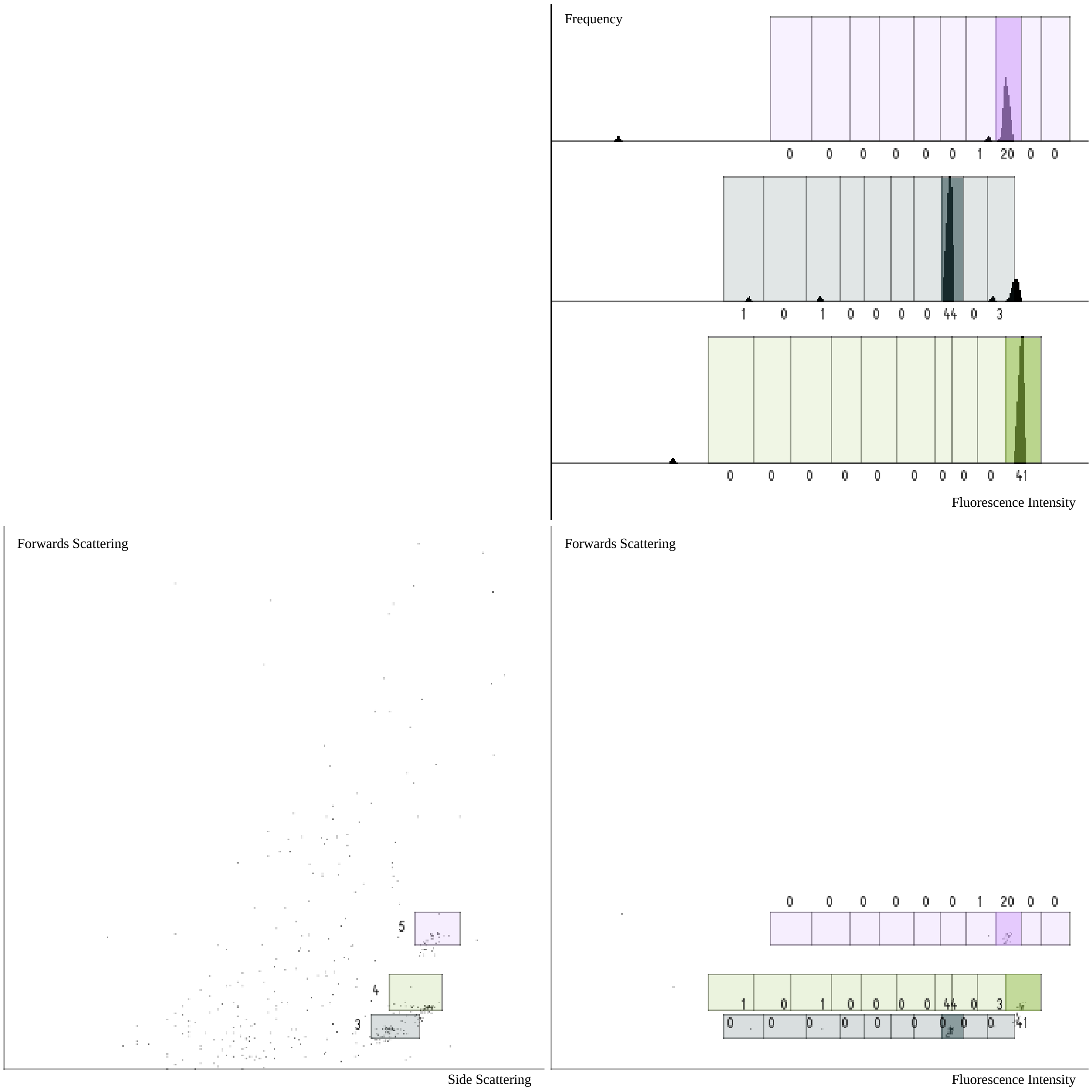

ANNEX 3: TAG DECONVOLUTION - BEAD 42

Passes flow sorting criteria: Yes

Passes tag deconvolution criteria: Yes

Included in protocol analysis: Yes

Protocol: 5, 2, 9, 3

Filename: Bin3_plateA1_B9.LMD

Split 1: Petrol shading

Split 2: Green shading

Split 3: Violet shading

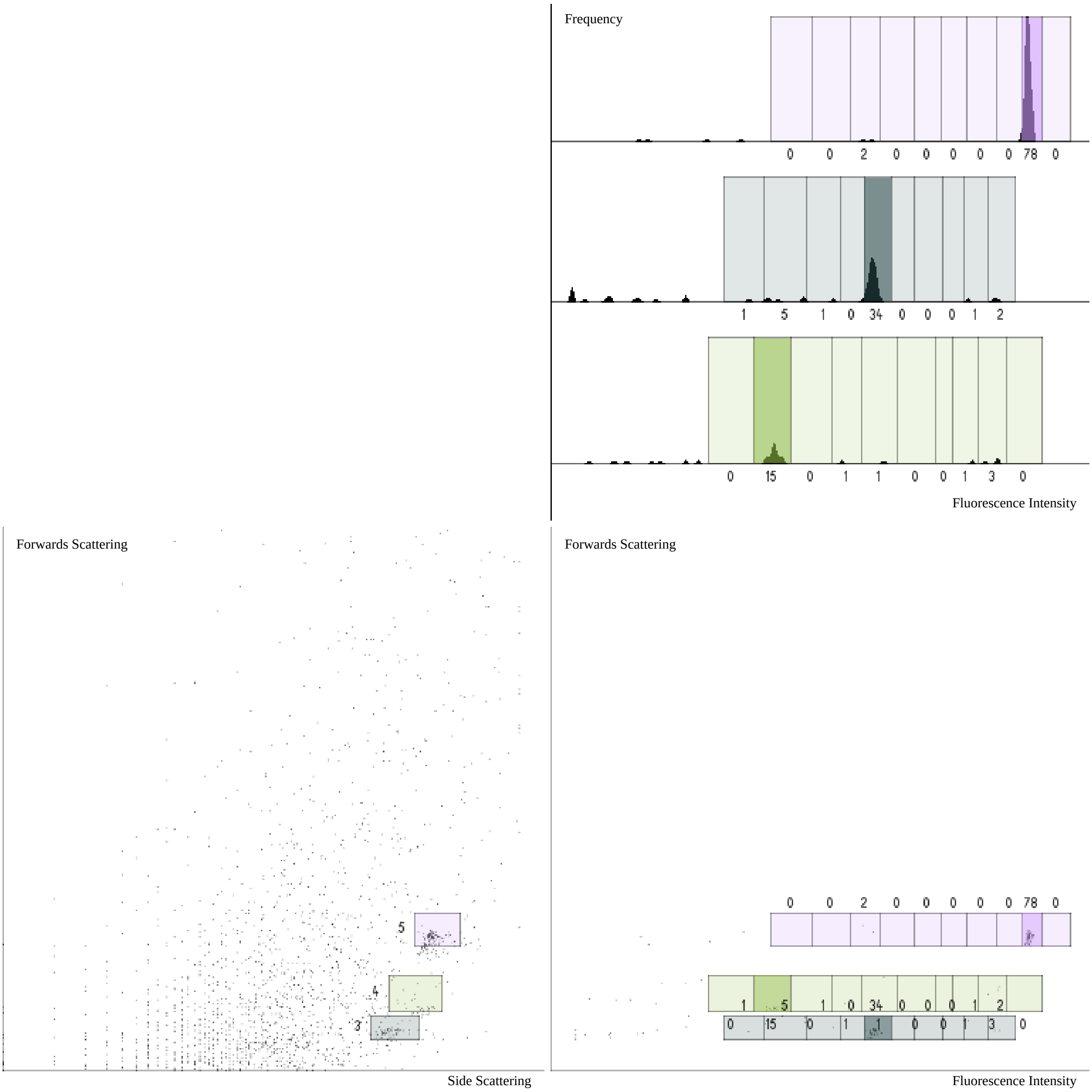

ANNEX 3: TAG DECONVOLUTION - BEAD 43

Passes flow sorting criteria: Yes

Passes tag deconvolution criteria: Yes

Included in protocol analysis: Yes

Protocol: 6, 2, 2, 3

Filename: Bin3_plateA1_B10.LMD

Split 1: Petrol shading

Split 2: Green shading

Split 3: Violet shading

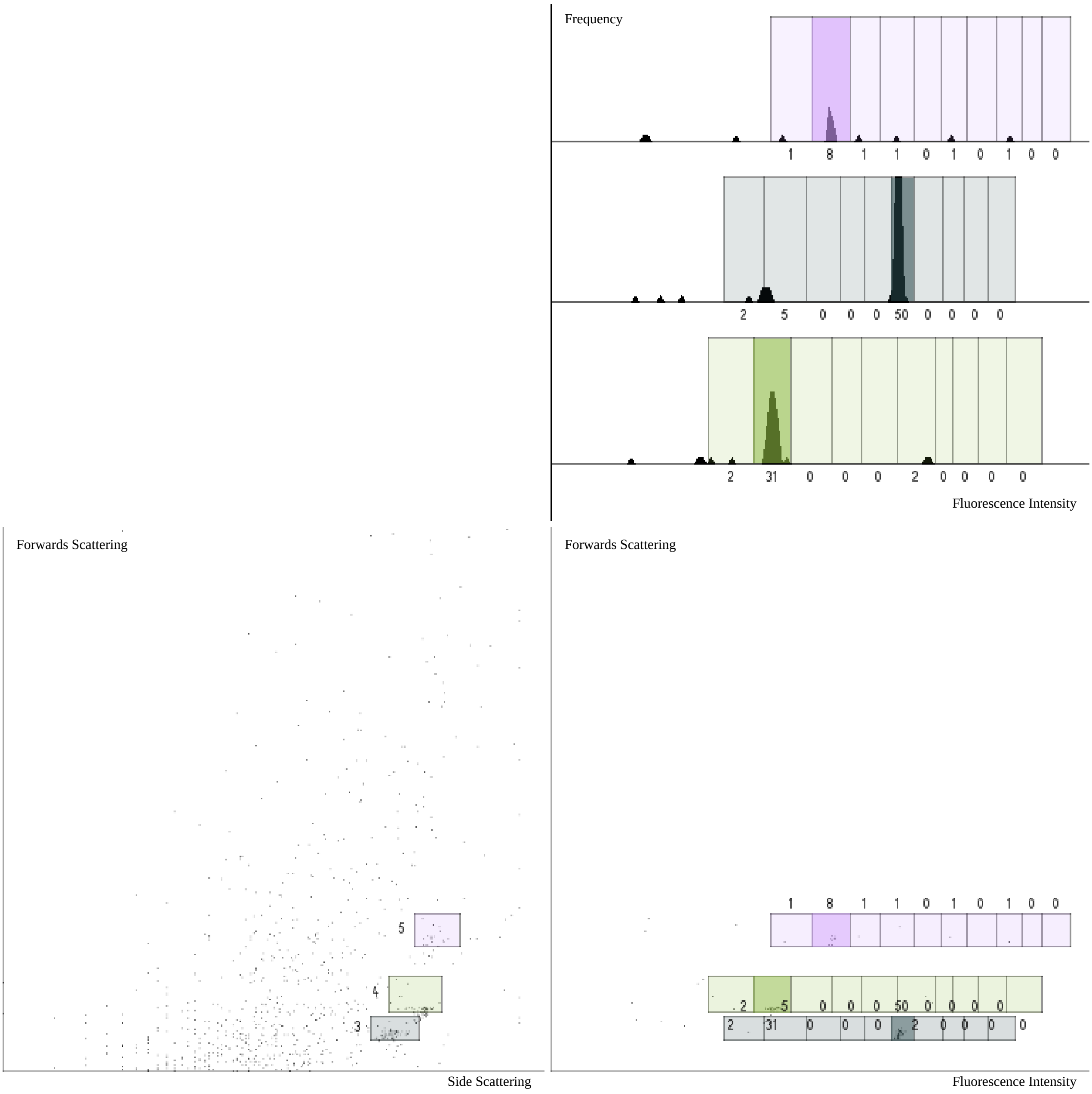

ANNEX 3: TAG DECONVOLUTION - BEAD 44

Passes flow sorting criteria: Yes

Passes tag deconvolution criteria: Yes

Included in protocol analysis: Yes

Protocol: 3, 4, 9, 3

Filename: Bin3_plateA1_B11.LMD

Split 1: Petrol shading

Split 2: Green shading

Split 3: Violet shading

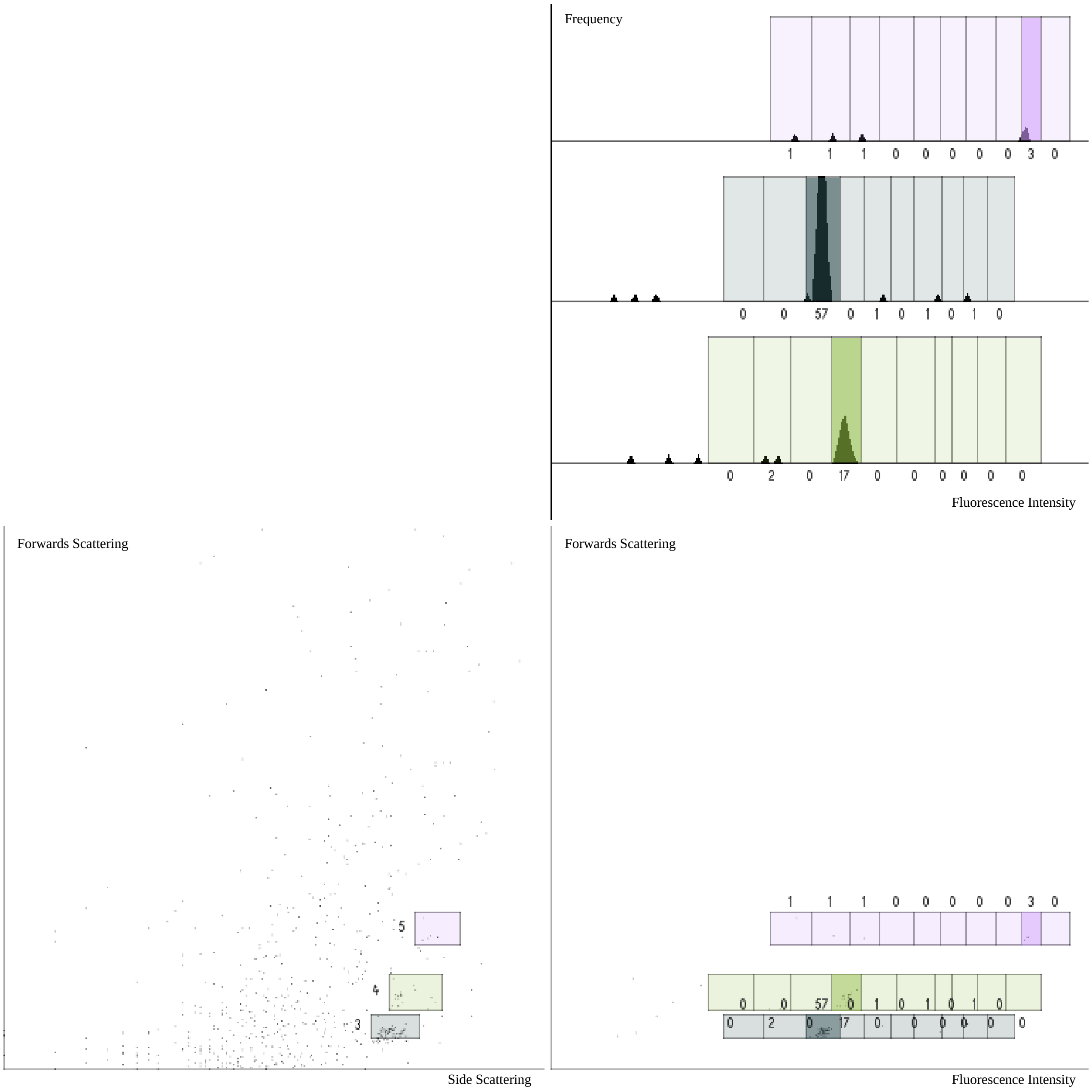

ANNEX 3: TAG DECONVOLUTION - BEAD 45

Passes flow sorting criteria: Yes

Passes tag deconvolution criteria: Yes

Included in protocol analysis: Yes

Protocol: 2, 8, 2, 3

Filename: Bin3_plateA1_B12.LMD

Split 1: Petrol shading

Split 2: Green shading

Split 3: Violet shading

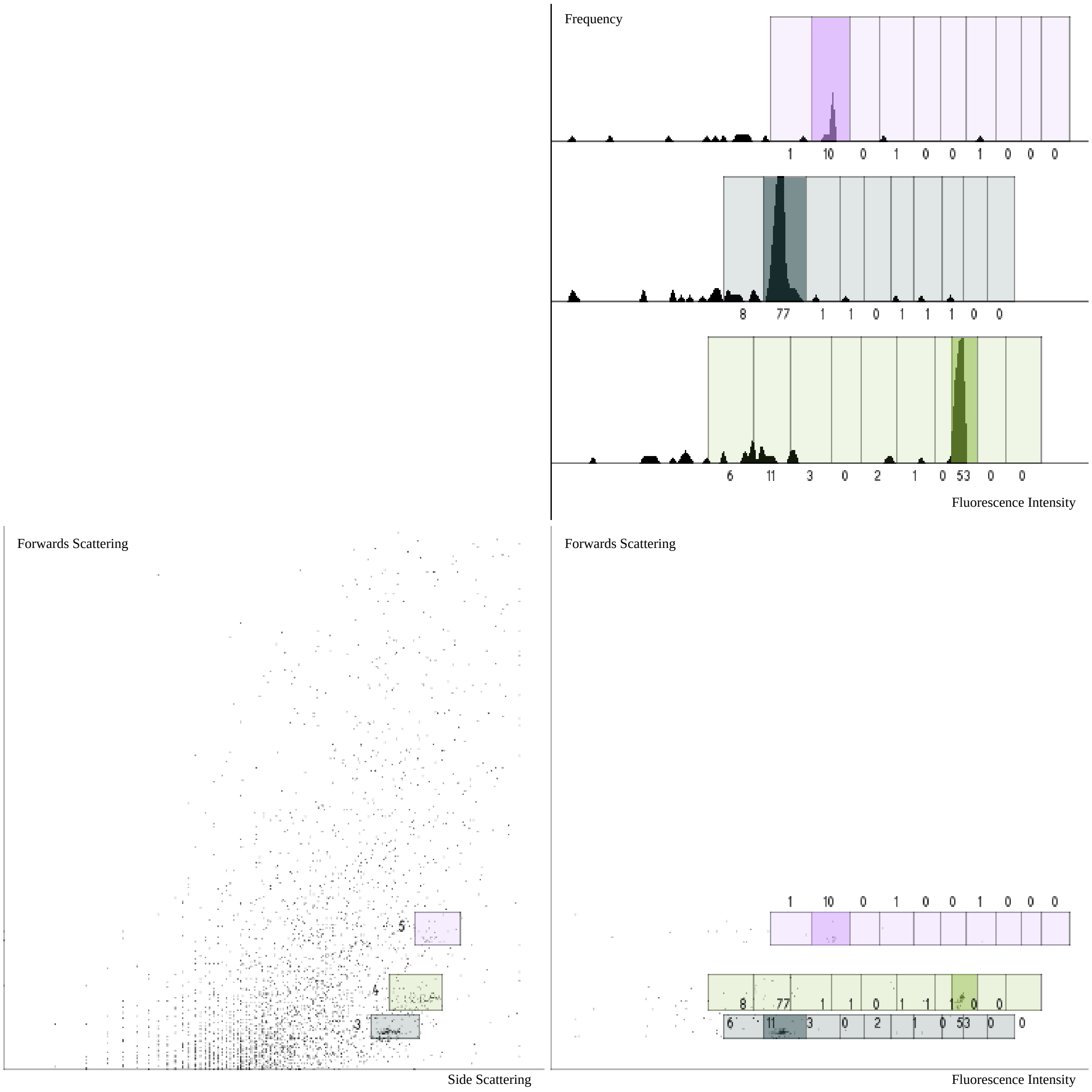

ANNEX 3: TAG DECONVOLUTION - BEAD 46

Passes flow sorting criteria: Yes

Passes tag deconvolution criteria: Yes

Included in protocol analysis: Yes

Protocol: 2, 9, 4, 3

Filename: Bin3_plateA1_C1.LMD

Split 1: Petrol shading

Split 2: Green shading

Split 3: Violet shading

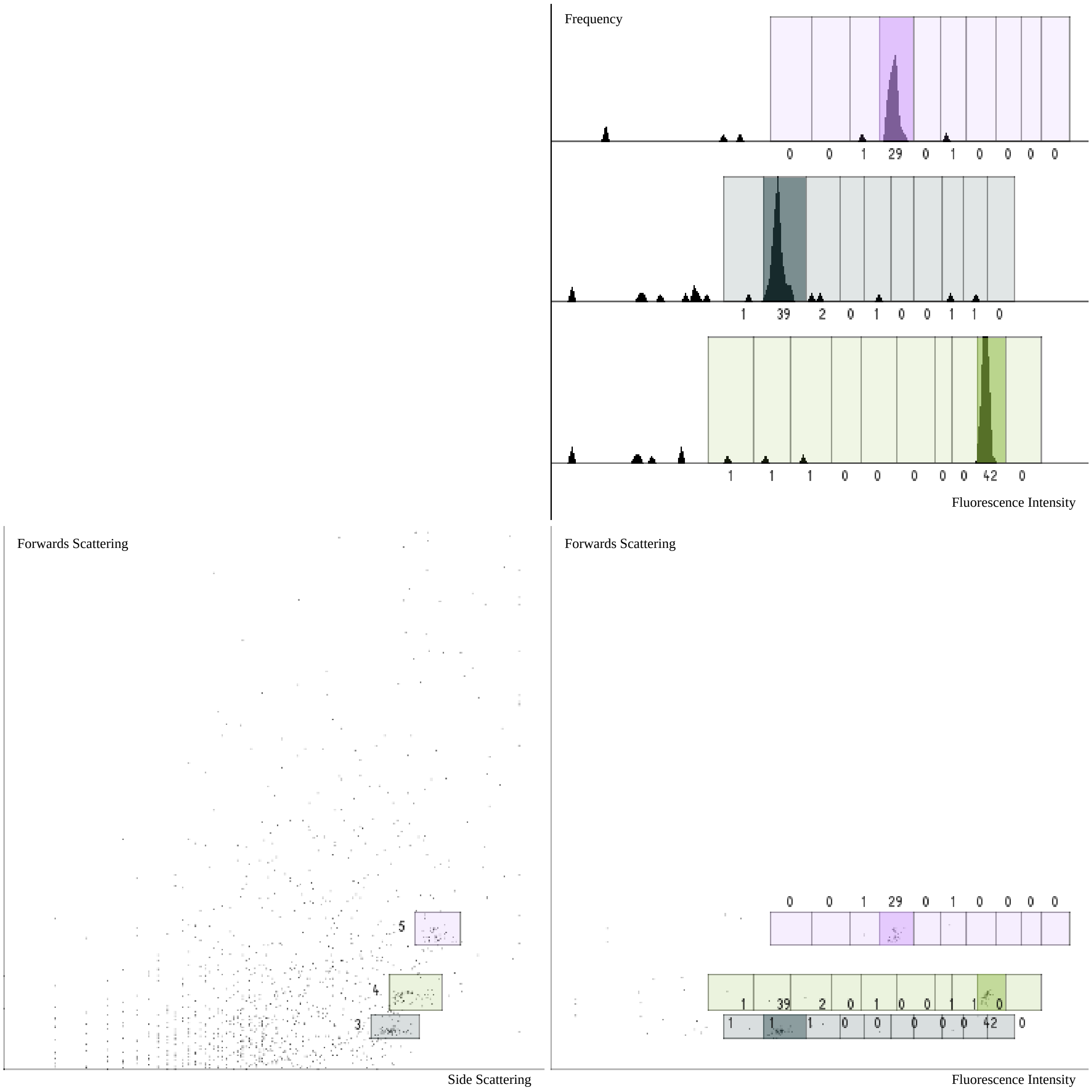

ANNEX 3: TAG DECONVOLUTION - BEAD 47

Passes flow sorting criteria: Yes

Passes tag deconvolution criteria: Yes

Included in protocol analysis: Yes

Protocol: 5, 9, 3, 3

Filename: Bin3_plateA1_C2.LMD

Split 1: Petrol shading

Split 2: Green shading

Split 3: Violet shading

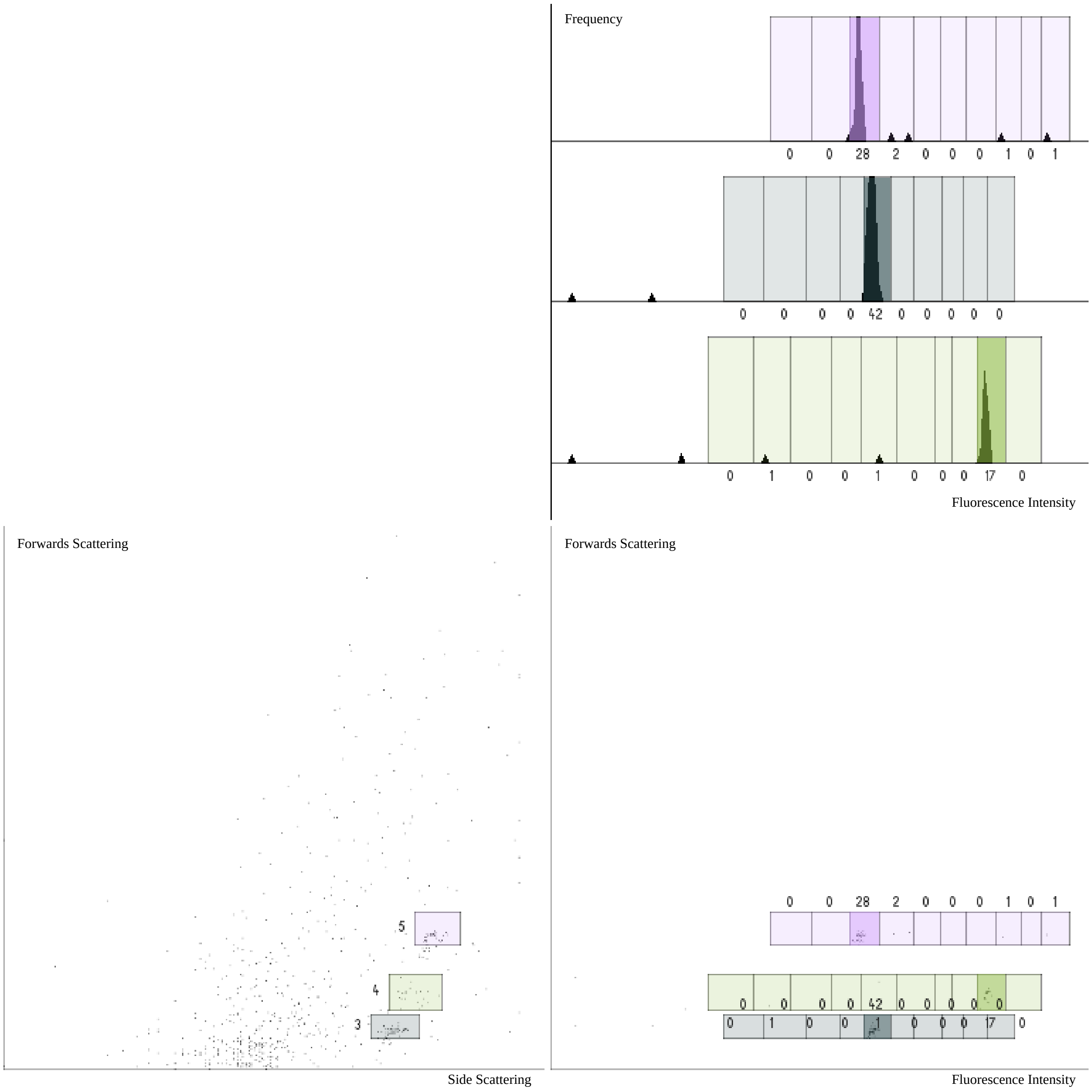

ANNEX 3: TAG DECONVOLUTION - BEAD 48

Passes flow sorting criteria: Yes

Passes tag deconvolution criteria: Yes

Included in protocol analysis: Yes

Protocol: 4, 10, 7, 3

Filename: Bin3_plateA1_C3.LMD

Split 1: Petrol shading

Split 2: Green shading

Split 3: Violet shading

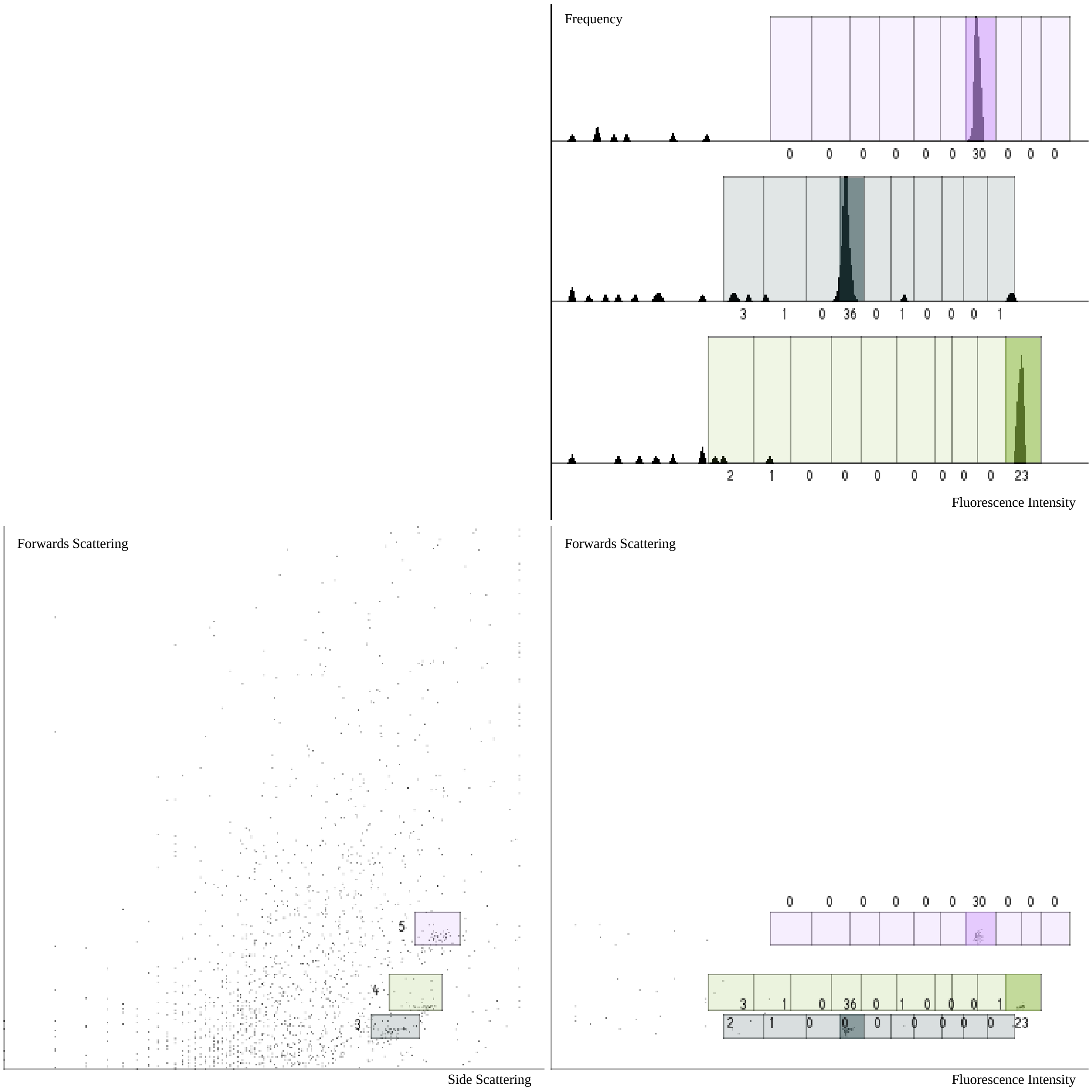

ANNEX 3: TAG DECONVOLUTION - BEAD 49

Passes flow sorting criteria: Yes

Passes tag deconvolution criteria: Yes

Included in protocol analysis: Yes

Protocol: 10, 2, 1, 3

Filename: Bin3_plateA1_C4.LMD

Split 1: Petrol shading

Split 2: Green shading

Split 3: Violet shading

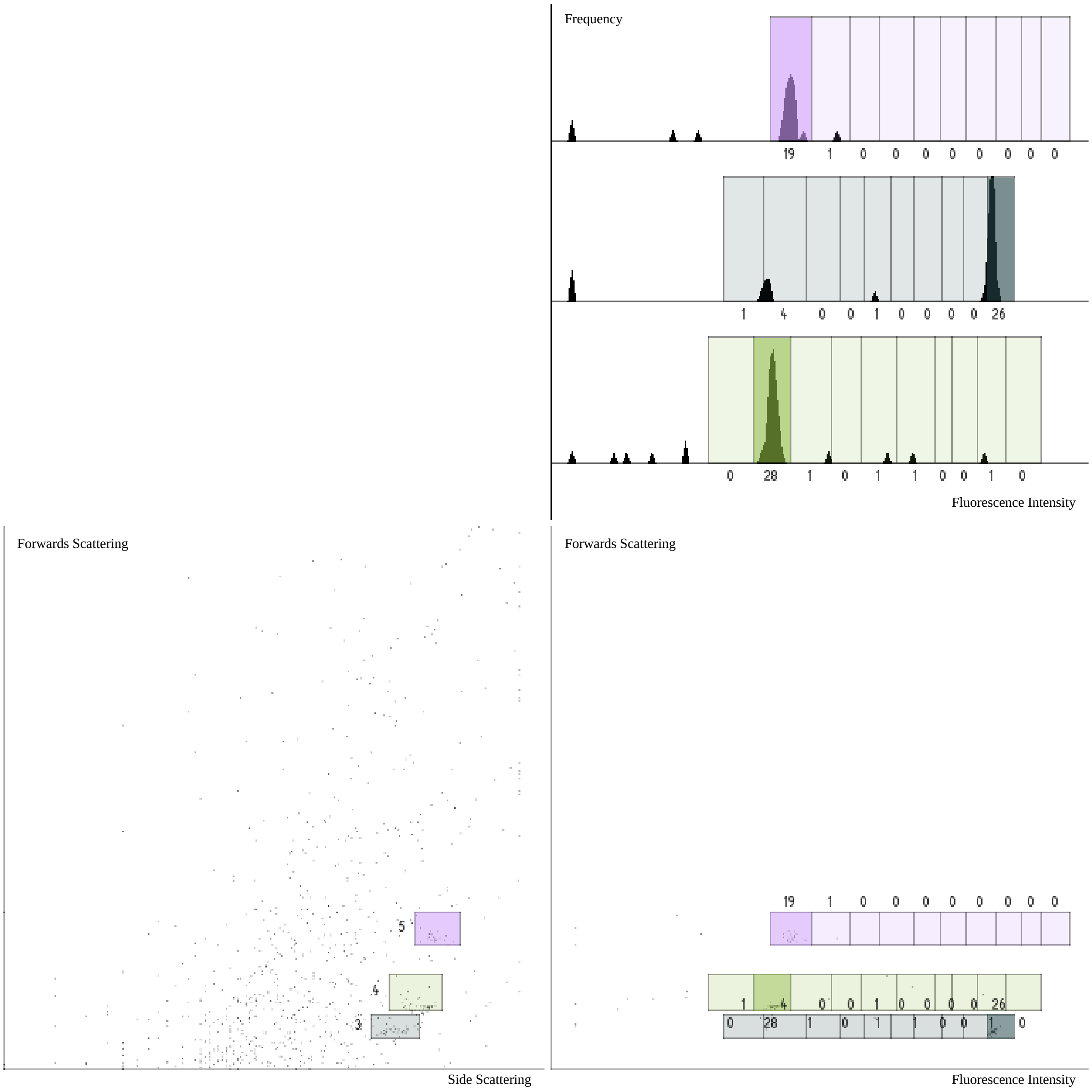

ANNEX 3: TAG DECONVOLUTION - BEAD 50

Passes flow sorting criteria: Yes

Passes tag deconvolution criteria: Yes

Included in protocol analysis: Yes

Protocol: 1, 5, 8, 3

Filename: Bin3_plateA1_C5.LMD

Split 1: Petrol shading

Split 2: Green shading

Split 3: Violet shading

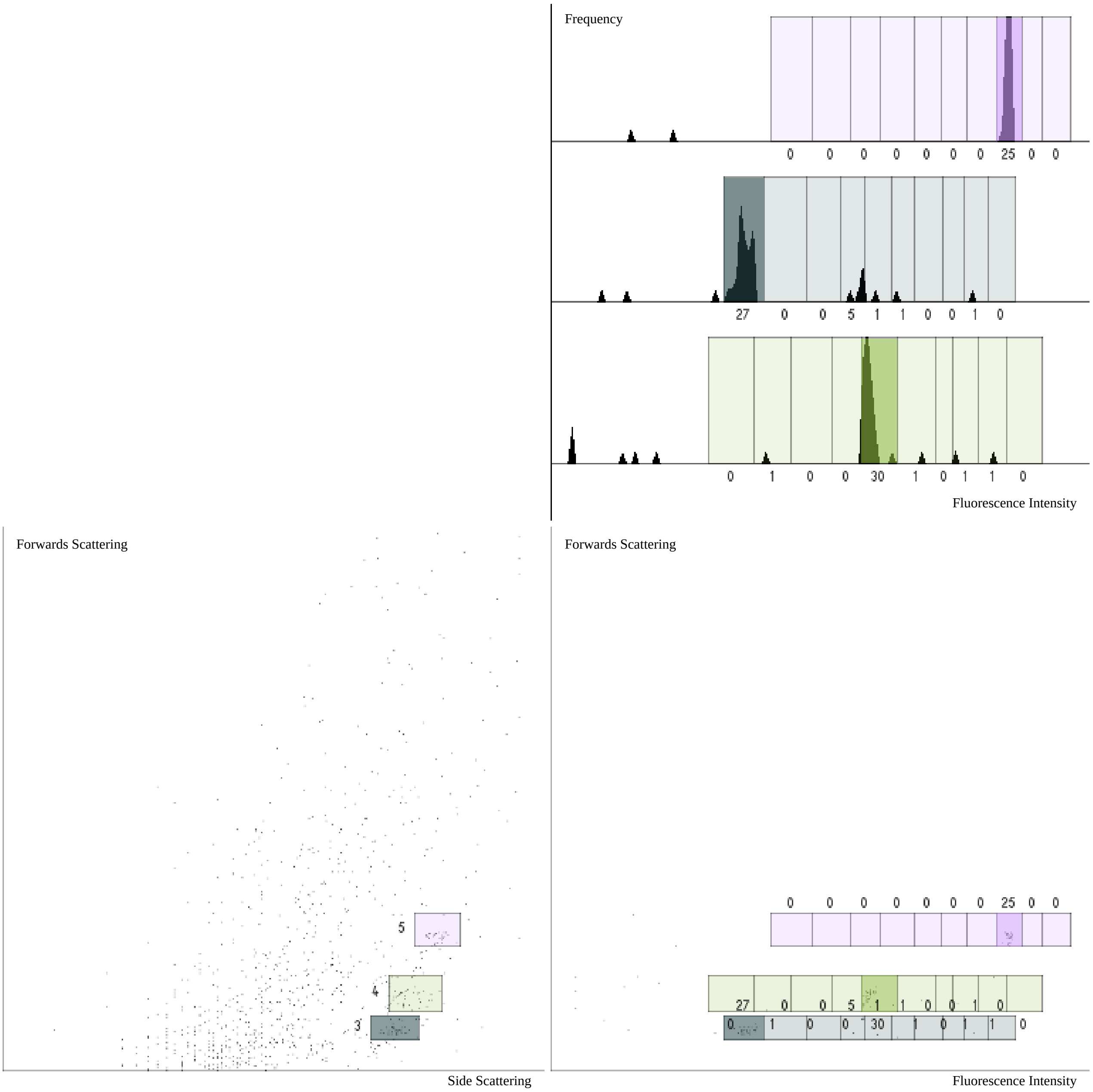

ANNEX 3: TAG DECONVOLUTION - BEAD 51

Passes flow sorting criteria: Yes

Passes tag deconvolution criteria: Yes

Included in protocol analysis: Yes

Protocol: 8, 9, 4, 3

Filename: Bin3_plateA1_C6.LMD

Split 1: Petrol shading

Split 2: Green shading

Split 3: Violet shading

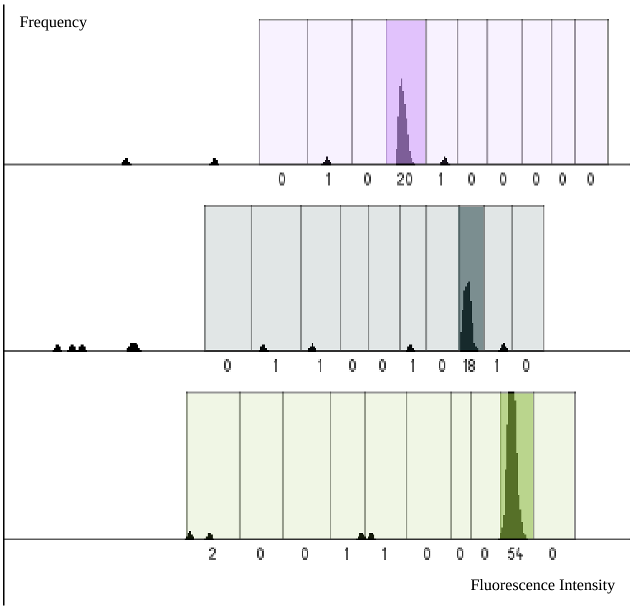

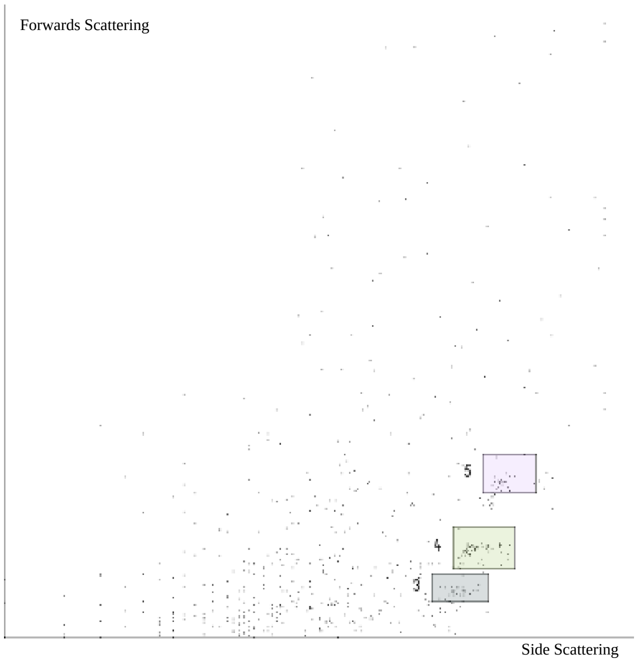

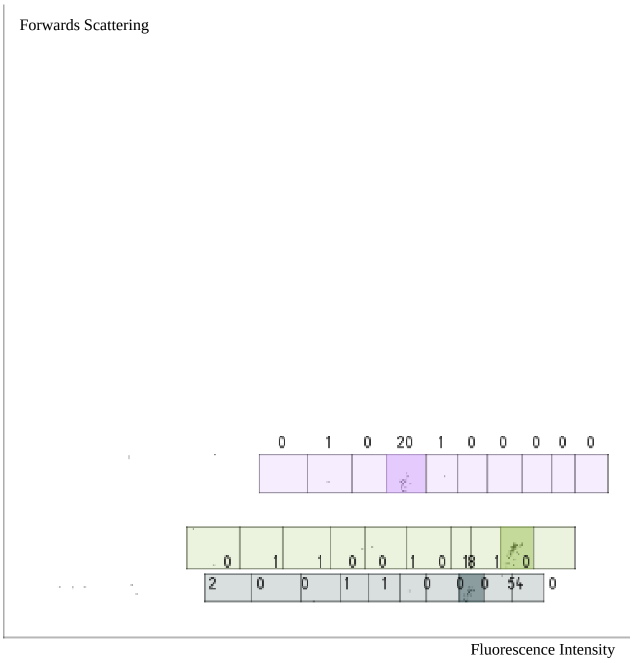

ANNEX 3: TAG DECONVOLUTION - BEAD 52

Passes flow sorting criteria: Yes

Passes tag deconvolution criteria: Yes

Included in protocol analysis: Yes

Protocol: 4, 5, 8, 3

Filename: Bin3_plateA1_C7.LMD

Split 1: Petrol shading

Split 2: Green shading

Split 3: Violet shading

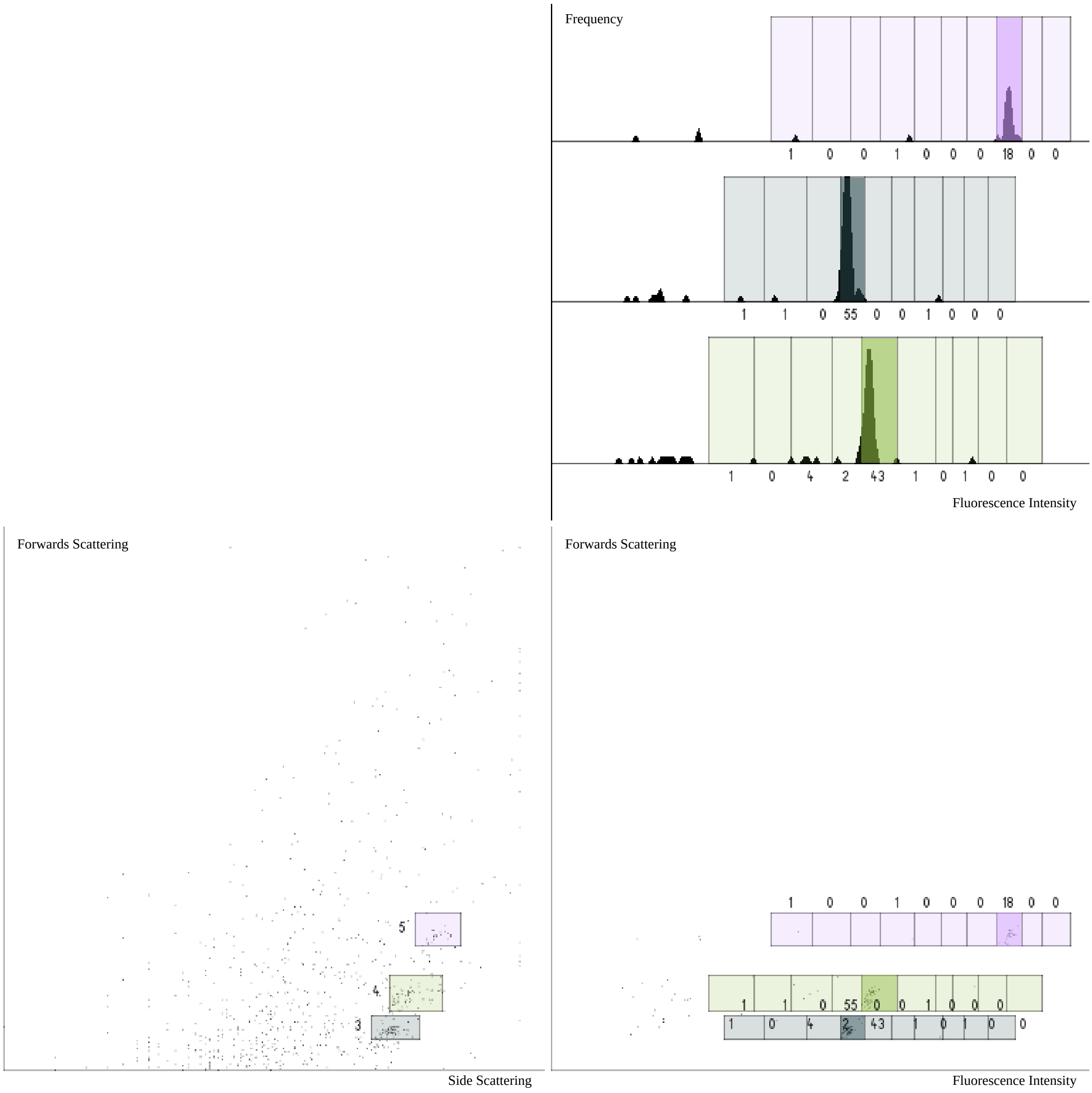

ANNEX 3: TAG DECONVOLUTION - BEAD 53

Passes flow sorting criteria: Yes

Passes tag deconvolution criteria: No

Included in protocol analysis: No

Protocol: N/A

Filename: Bin3_plateA1_C8.LMD

Split 1: Petrol shading

Split 2: Green shading

Split 3: Violet shading

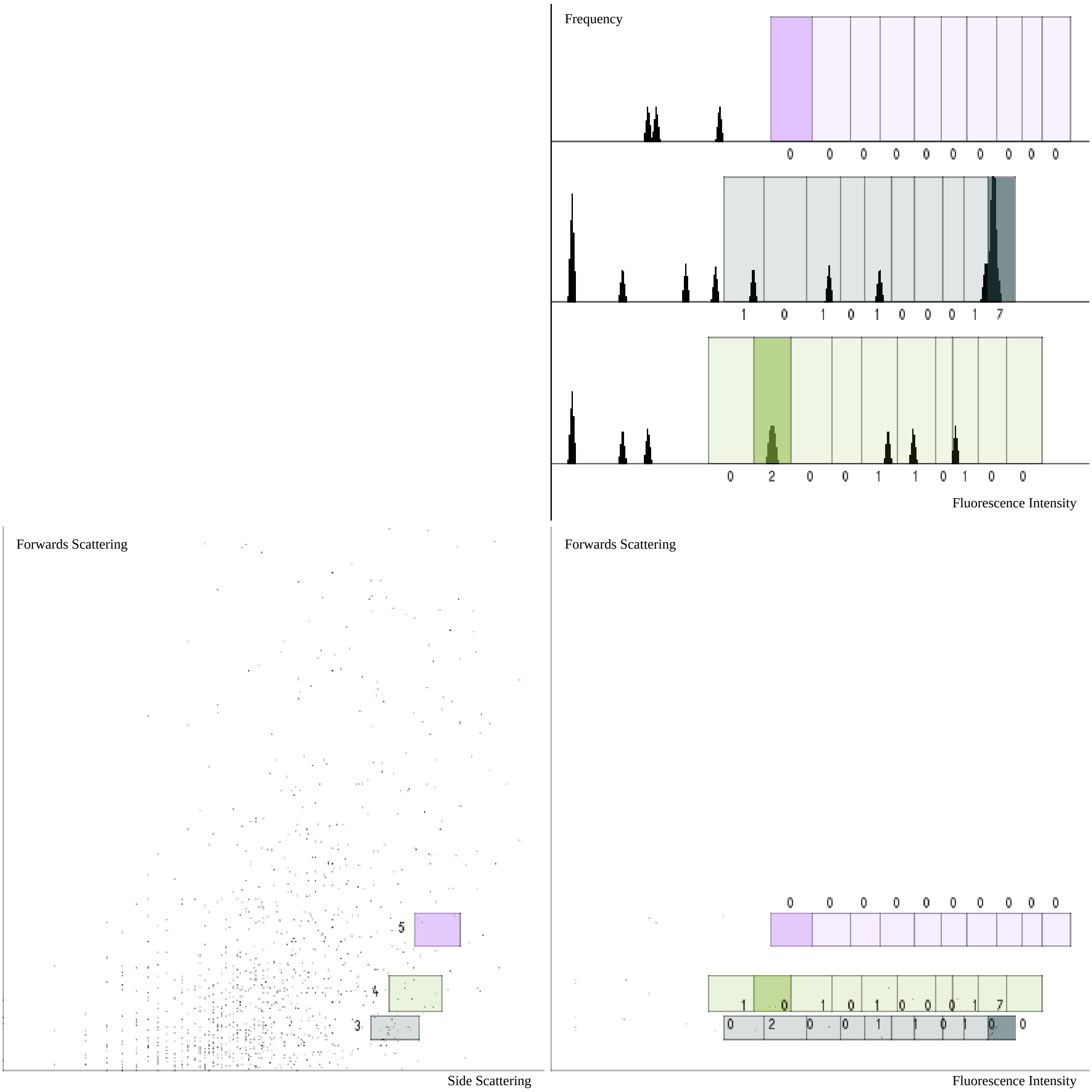

ANNEX 3: TAG DECONVOLUTION - BEAD 54

Passes flow sorting criteria: Yes

Passes tag deconvolution criteria: Yes

Included in protocol analysis: Yes

Protocol: 9, 7, 6, 3

Filename: Bin3_plateA1_C9.LMD

Split 1: Petrol shading

Split 2: Green shading

Split 3: Violet shading

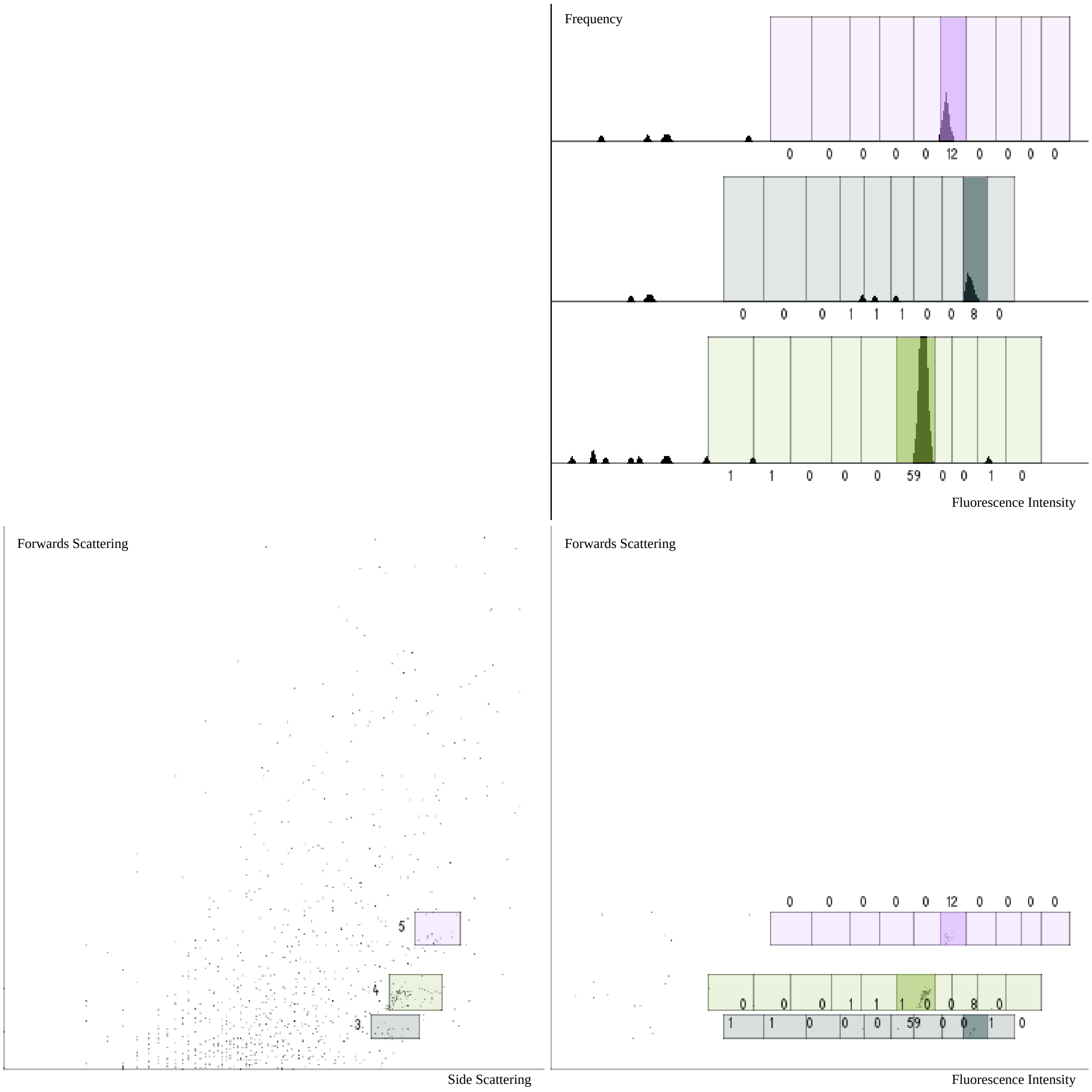

ANNEX 3: TAG DECONVOLUTION - BEAD 55

Passes flow sorting criteria: Yes

Passes tag deconvolution criteria: Yes

Included in protocol analysis: Yes

Protocol: 2, 3, 6, 2

Filename: Bin2_plateA1_C10.LMD

Split 1: Petrol shading

Split 2: Green shading

Split 3: Violet shading

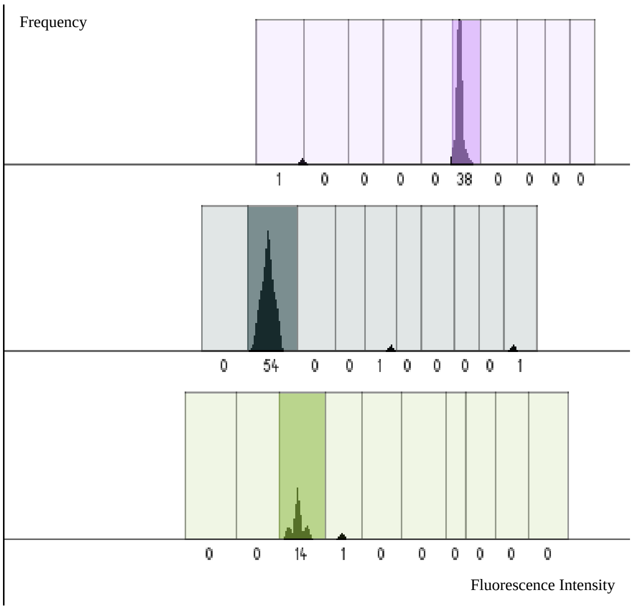

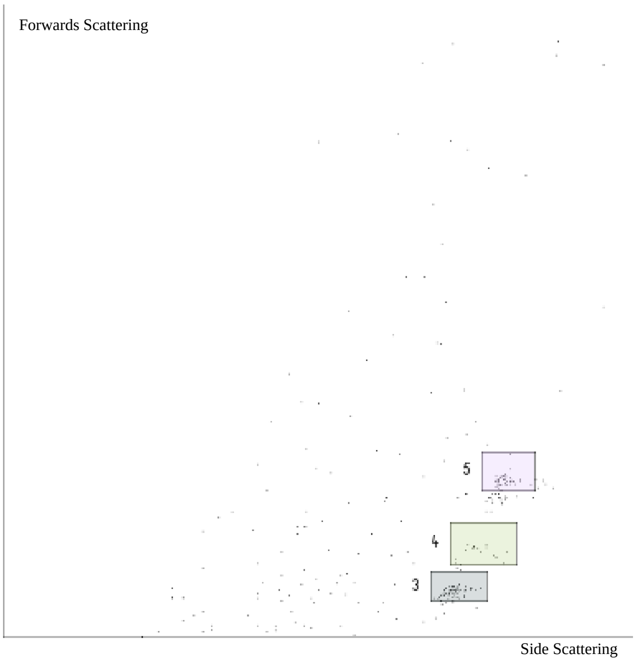

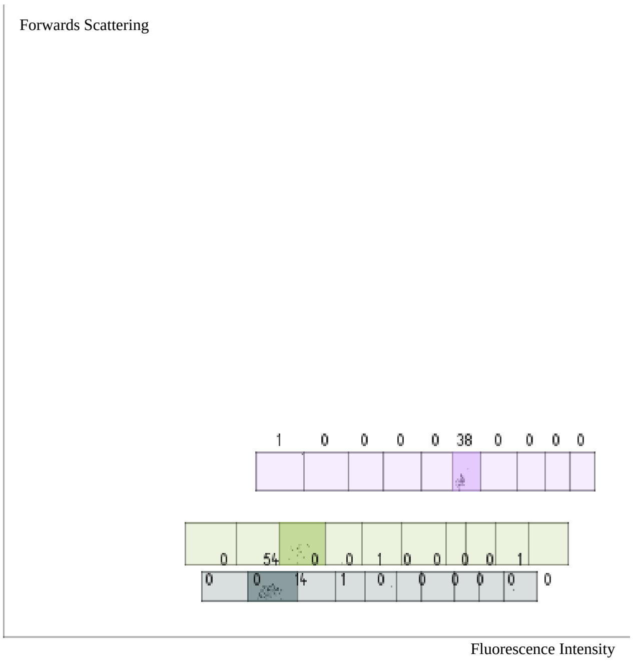

ANNEX 3: TAG DECONVOLUTION - BEAD 56

Passes flow sorting criteria: Yes

Passes tag deconvolution criteria: Yes

Included in protocol analysis: Yes

Protocol: 9, 2, 7, 1

Filename: Bin1_plateA1_A1.LMD

Split 1: Petrol shading

Split 2: Green shading

Split 3: Violet shading

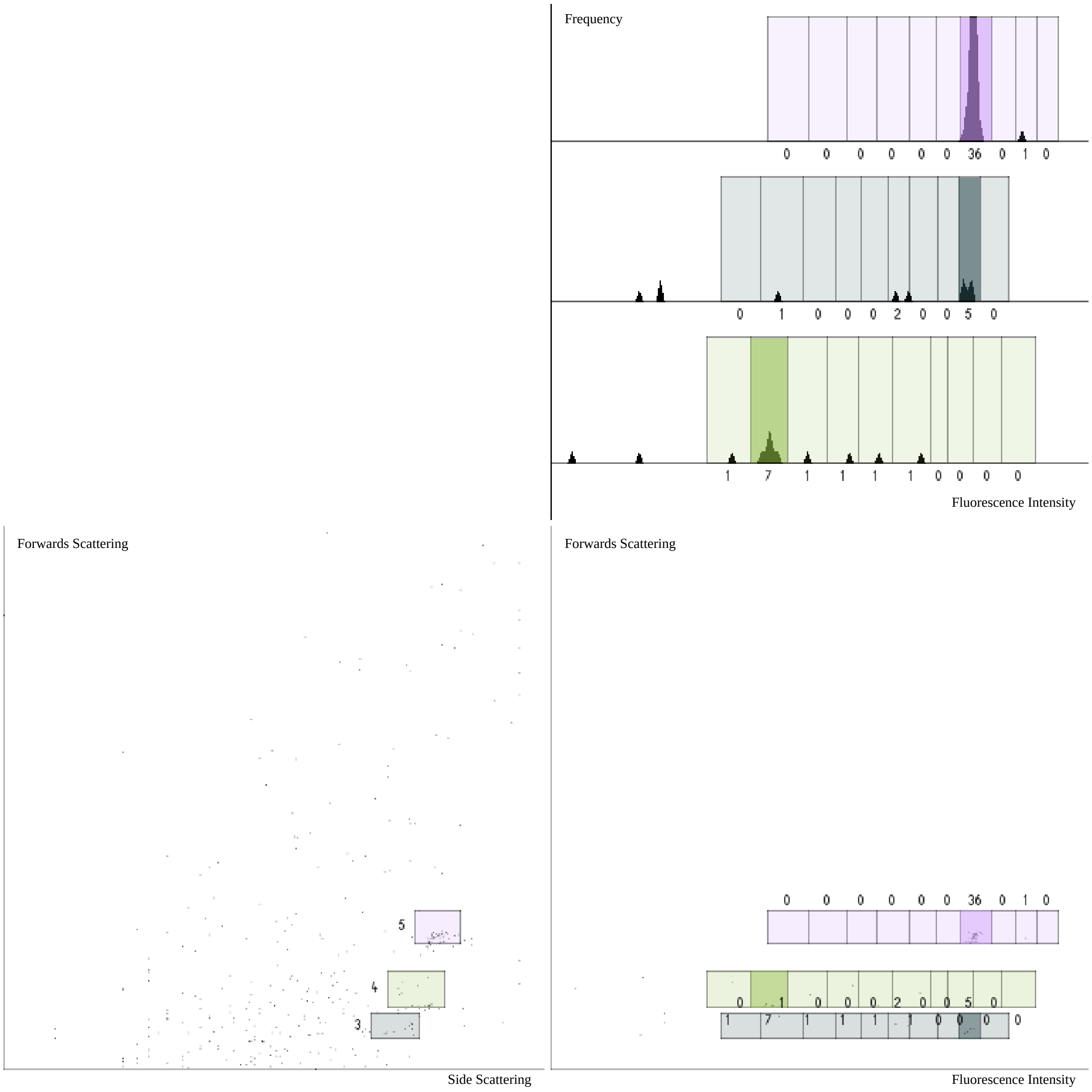

ANNEX 3: TAG DECONVOLUTION - BEAD 57

Passes flow sorting criteria: Yes

Passes tag deconvolution criteria: Yes

Included in protocol analysis: Yes

Protocol: 1, 4, 2, 1

Filename: Bin1_plateA1_A2.LMD

Split 1: Petrol shading

Split 2: Green shading

Split 3: Violet shading

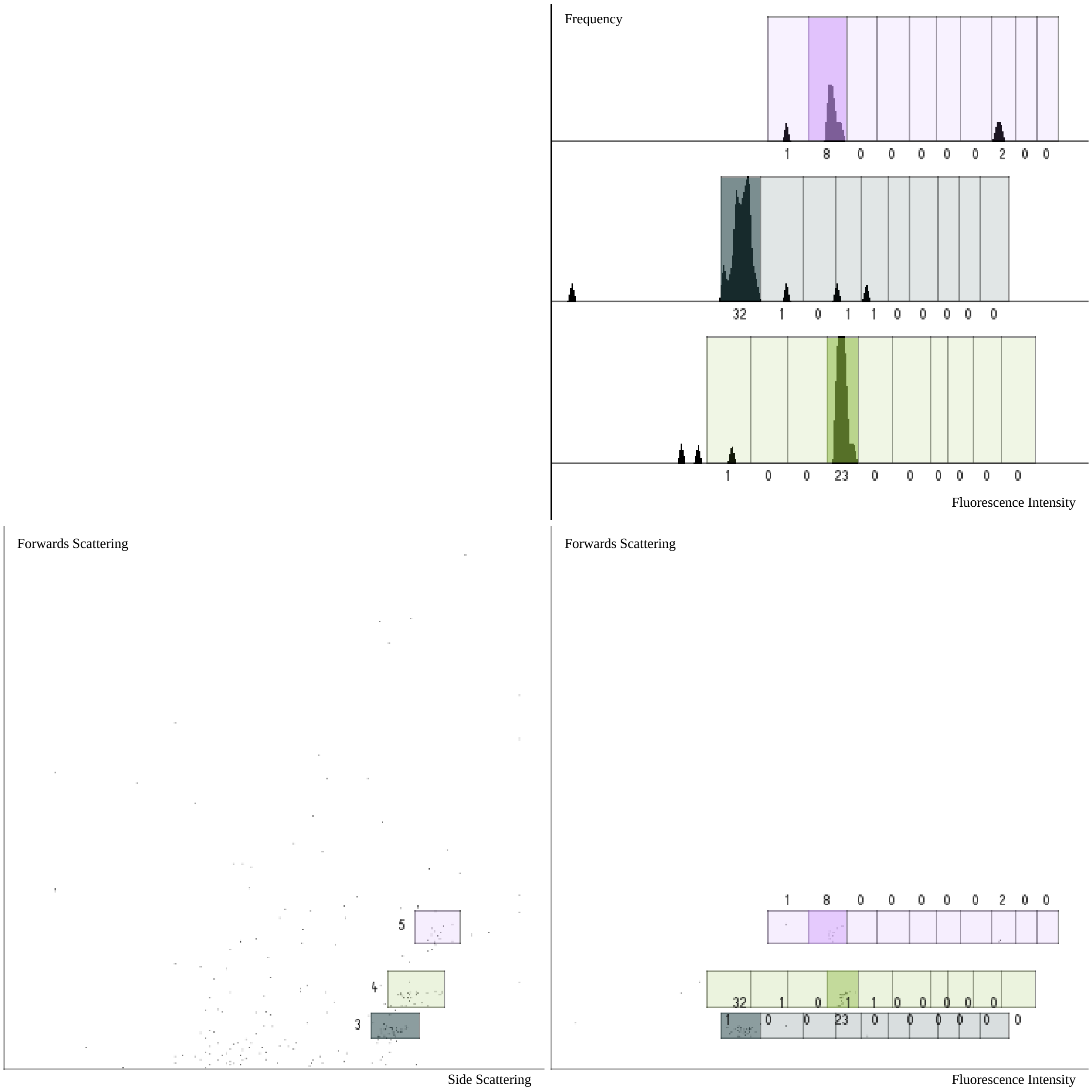

ANNEX 3: TAG DECONVOLUTION - BEAD 58

Passes flow sorting criteria: Yes

Passes tag deconvolution criteria: Yes

Included in protocol analysis: Yes

Protocol: 1, 3, 1, 1

Filename: Bin1_plateA1_A3.LMD

Split 1: Petrol shading

Split 2: Green shading

Split 3: Violet shading

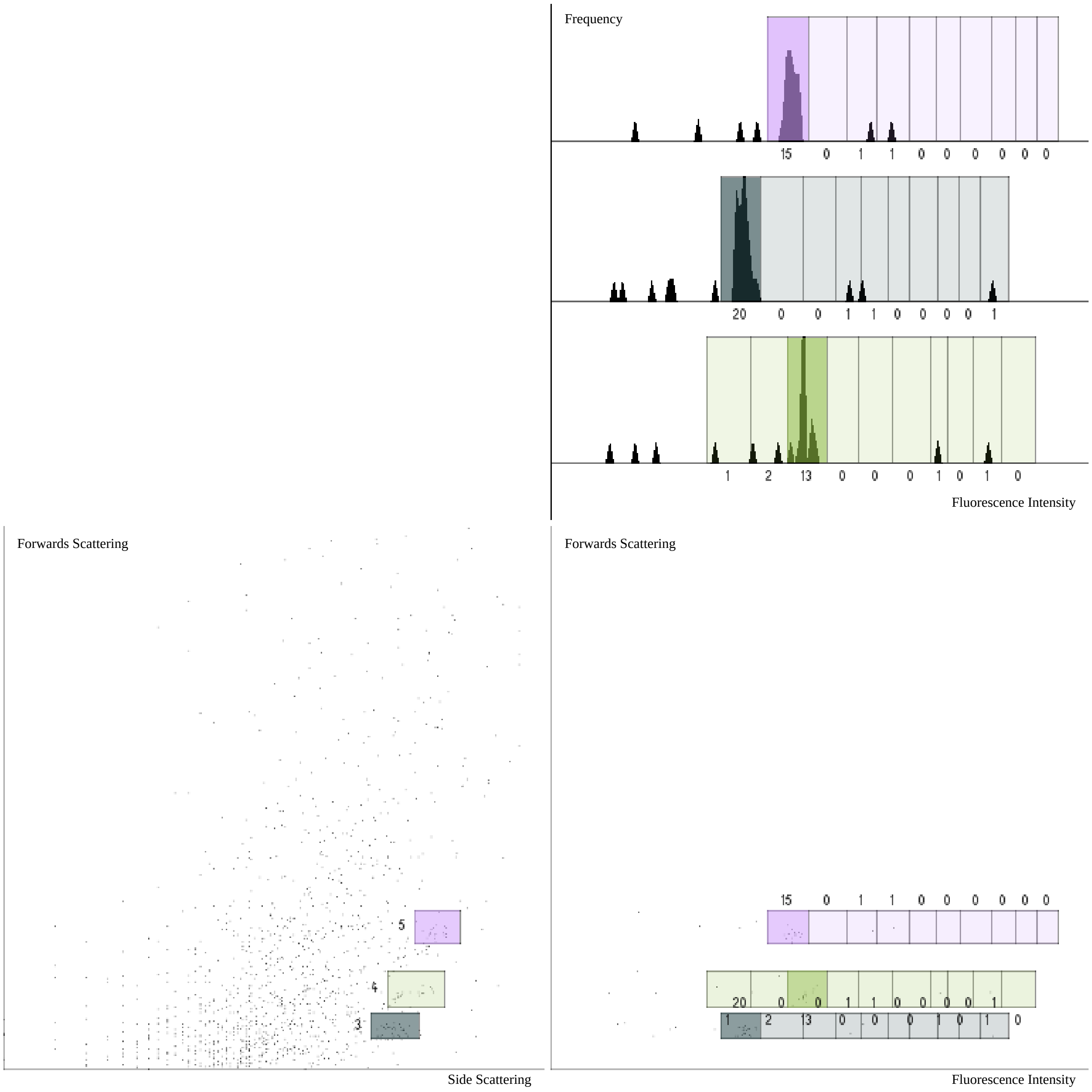

ANNEX 3: TAG DECONVOLUTION - BEAD 59

Passes flow sorting criteria: Yes

Passes tag deconvolution criteria: Yes

Included in protocol analysis: Yes

Protocol: 2, 7, 7, 1

Filename: Bin1_plateA1_A4.LMD

Split 1: Petrol shading

Split 2: Green shading

Split 3: Violet shading

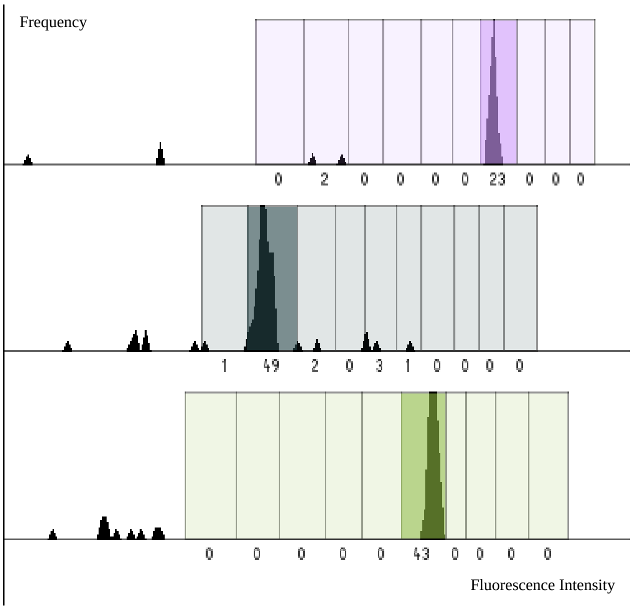

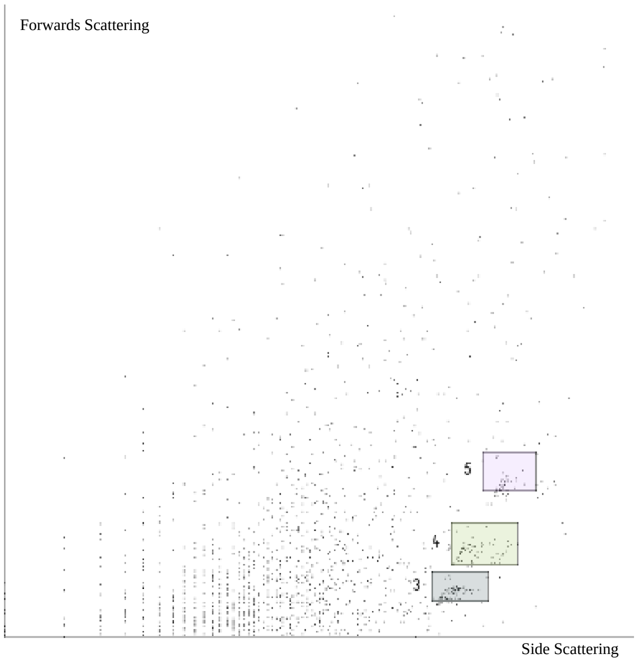

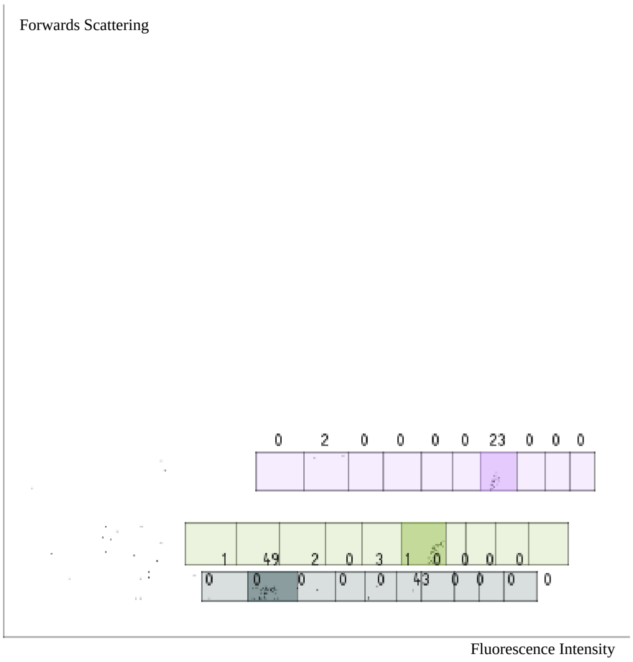

ANNEX 3: TAG DECONVOLUTION - BEAD 60

Passes flow sorting criteria: Yes

Passes tag deconvolution criteria: Yes

Included in protocol analysis: Yes

Protocol: 2, 7, 2, 1

Filename: Bin1_plateA1_A5.LMD

Split 1: Petrol shading

Split 2: Green shading

Split 3: Violet shading

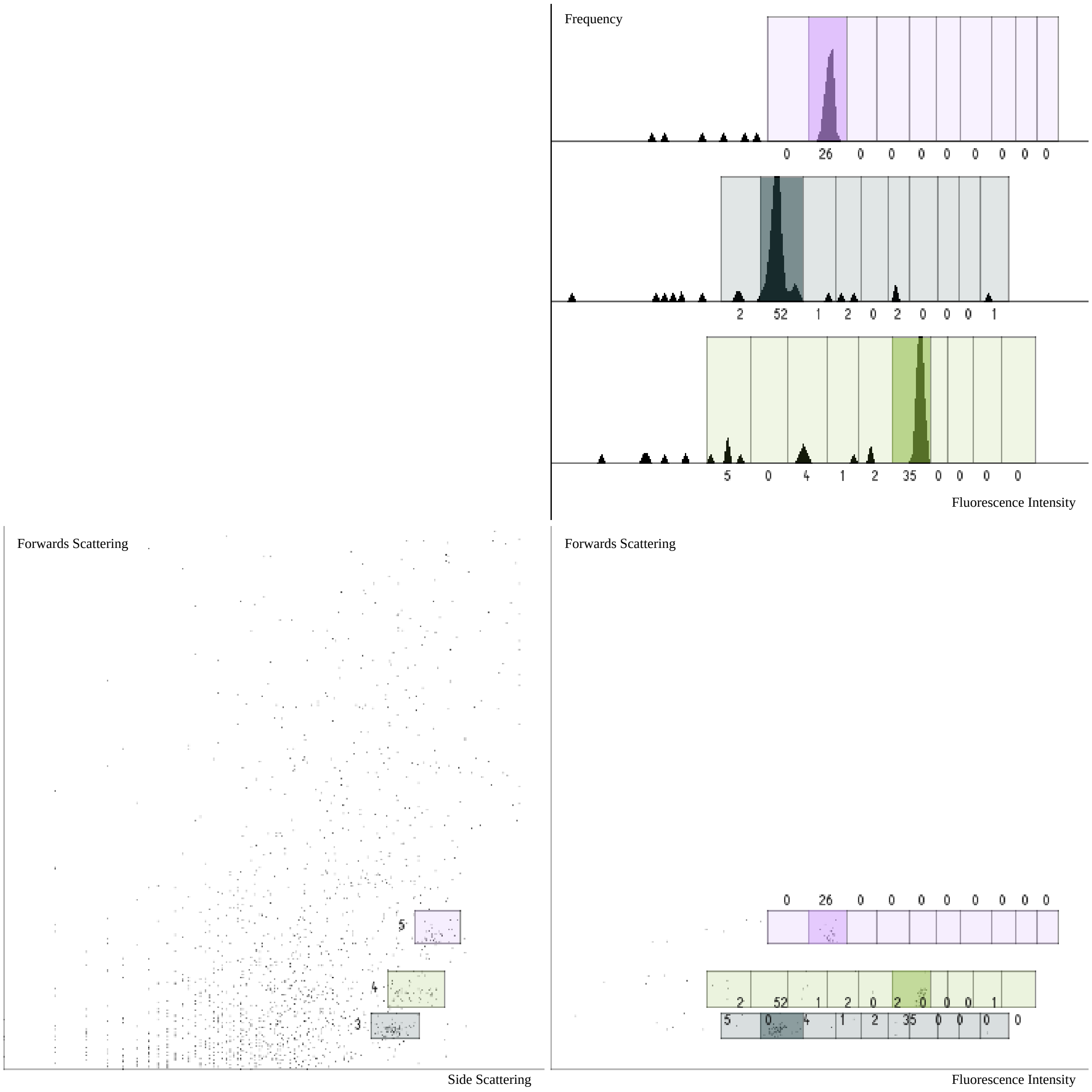

ANNEX 3: TAG DECONVOLUTION - BEAD 61

Passes flow sorting criteria: Yes

Passes tag deconvolution criteria: Yes

Included in protocol analysis: Yes

Protocol: 9, 7, 2, 1

Filename: Bin1_plateA1_A6.LMD

Split 1: Petrol shading

Split 2: Green shading

Split 3: Violet shading

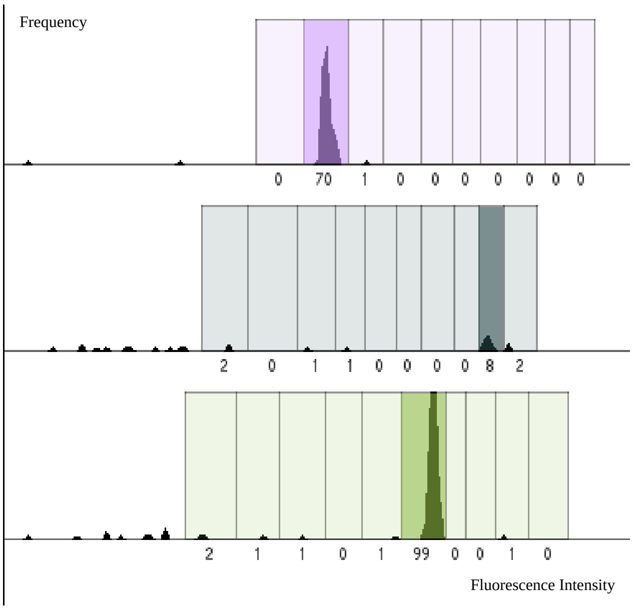

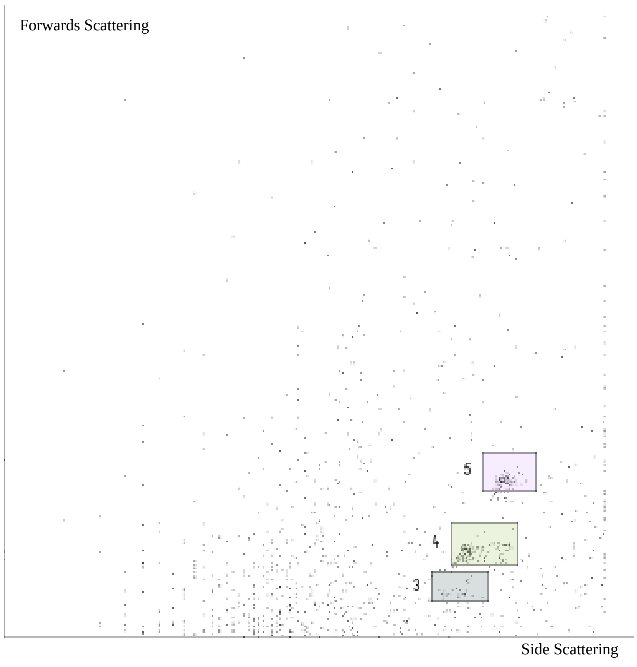

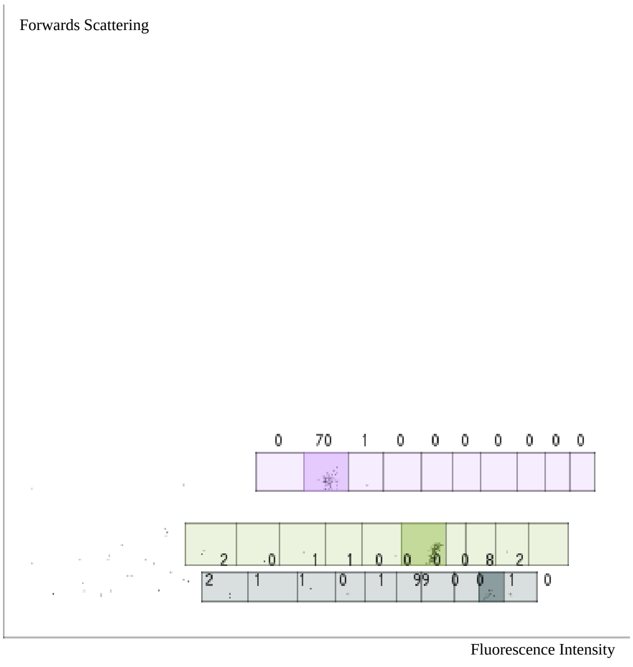

ANNEX 3: TAG DECONVOLUTION - BEAD 62

Passes flow sorting criteria: Yes

Passes tag deconvolution criteria: Yes

Included in protocol analysis: Yes

Protocol: 6, 8, 4, 1

Filename: Bin1_plateA1_A7.LMD

Split 1: Petrol shading

Split 2: Green shading

Split 3: Violet shading

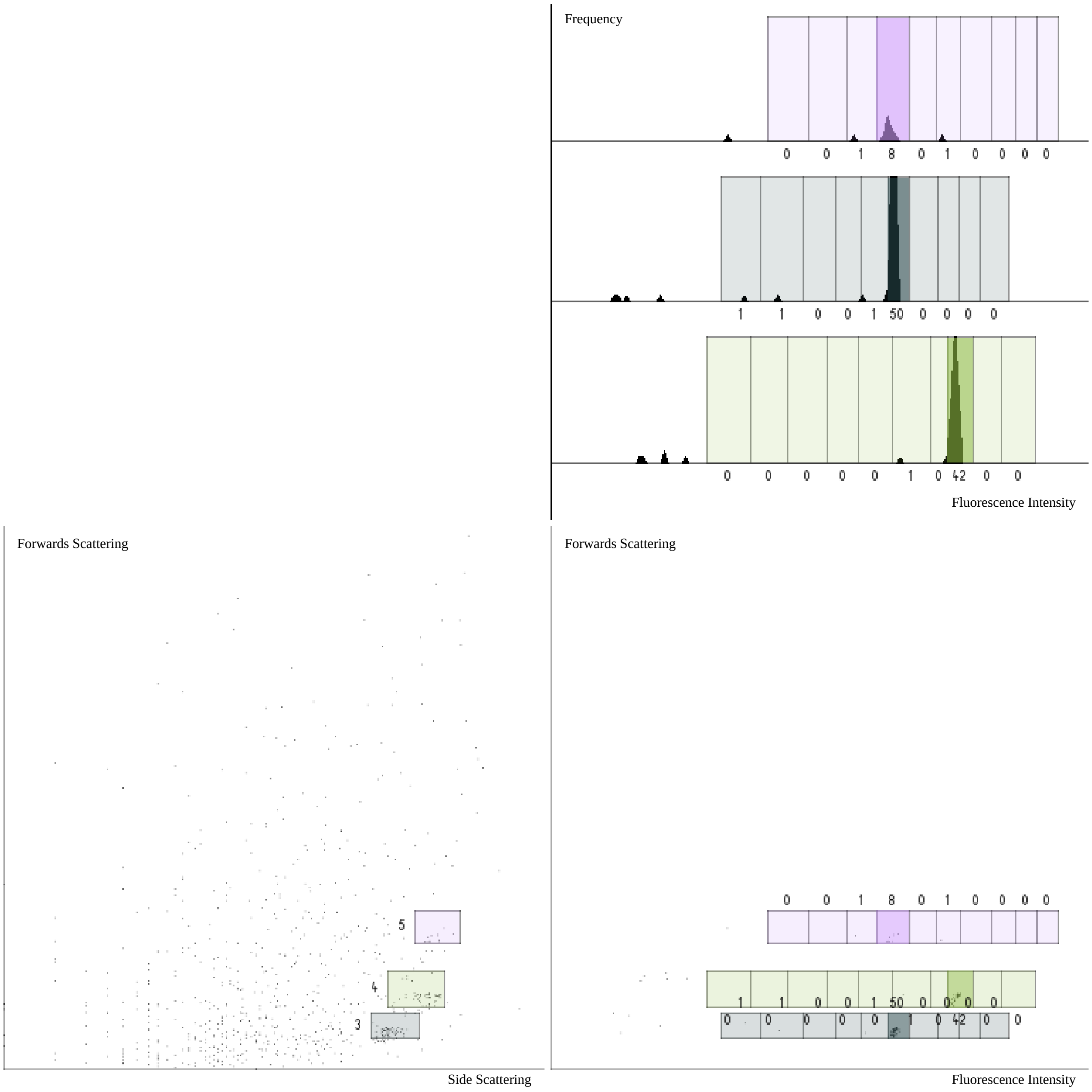

ANNEX 3: TAG DECONVOLUTION - BEAD 63

Passes flow sorting criteria: Yes

Passes tag deconvolution criteria: Yes

Included in protocol analysis: Yes

Protocol: 5, 7, 9, 1

Filename: Bin1_plateA1_A8.LMD

Split 1: Petrol shading

Split 2: Green shading

Split 3: Violet shading

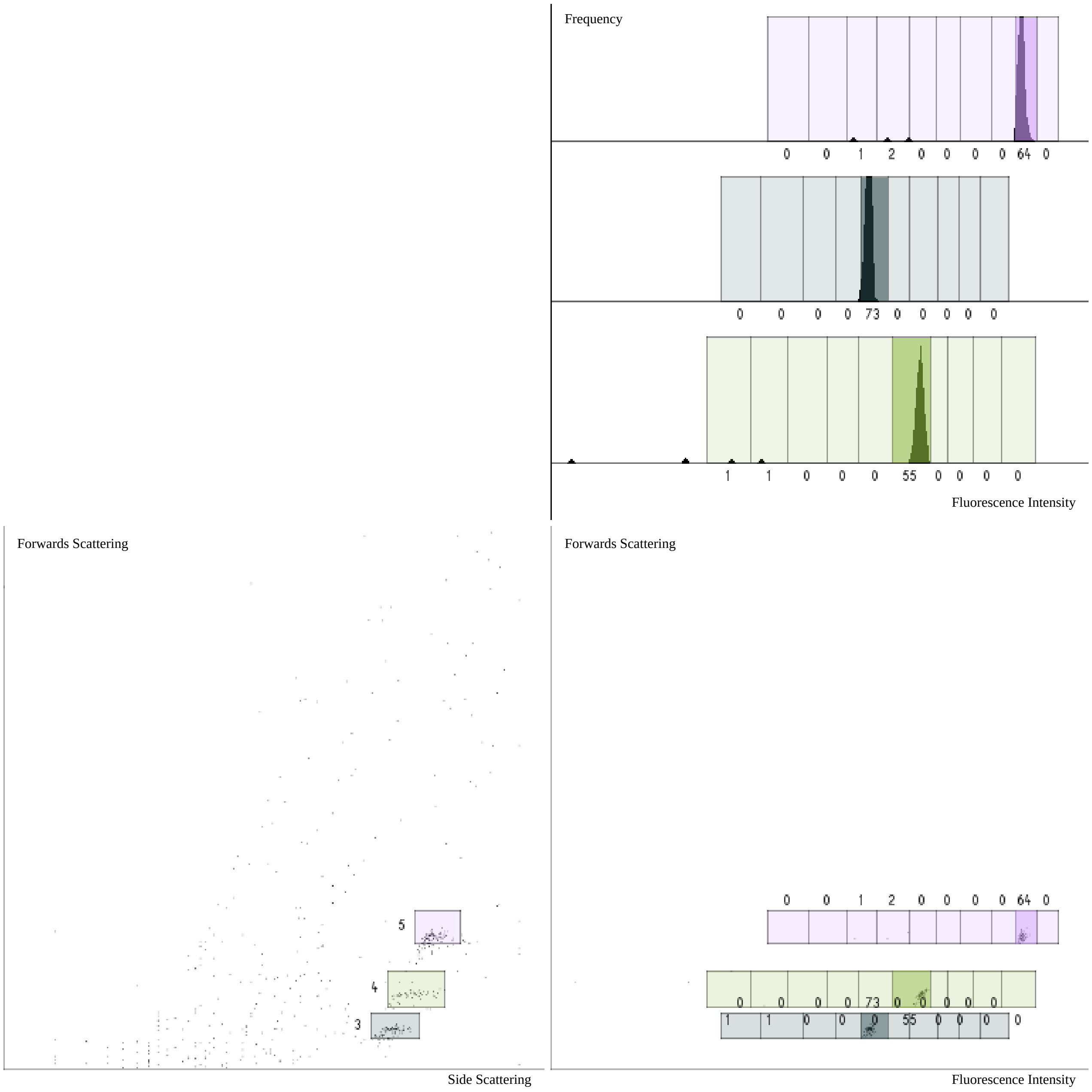

ANNEX 3: TAG DECONVOLUTION - BEAD 64

Passes flow sorting criteria: Yes

Passes tag deconvolution criteria: No

Included in protocol analysis: No

Protocol: N/A

Filename: Bin1_plateA1_A9.LMD

Split 1: Petrol shading

Split 2: Green shading

Split 3: Violet shading

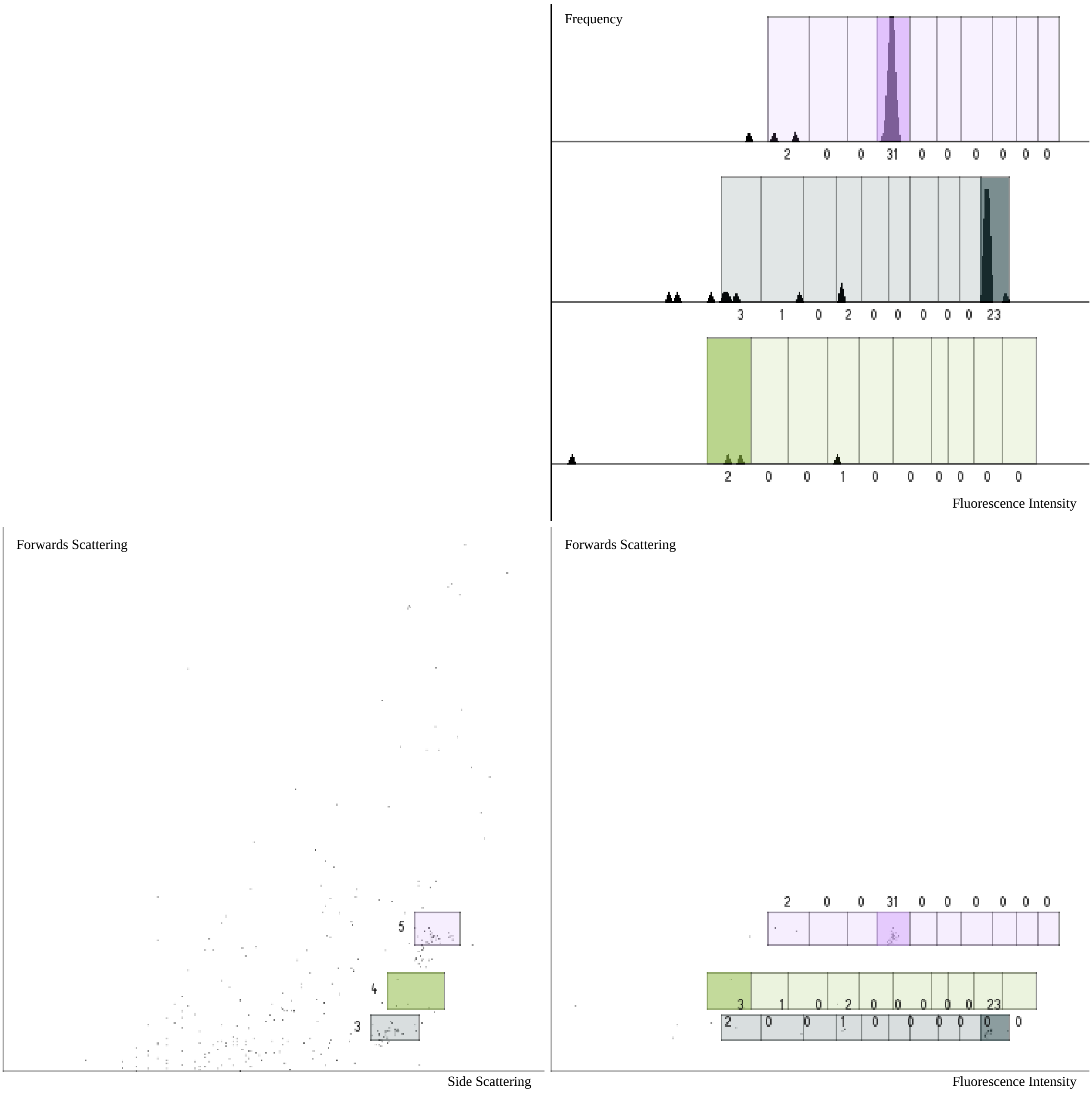

ANNEX 3: TAG DECONVOLUTION - BEAD 65

Passes flow sorting criteria: Yes

Passes tag deconvolution criteria: Yes

Included in protocol analysis: Yes

Protocol: 8, 5, 4, 1

Filename: Bin1_plateA1_A10.LMD

Split 1: Petrol shading

Split 2: Green shading

Split 3: Violet shading

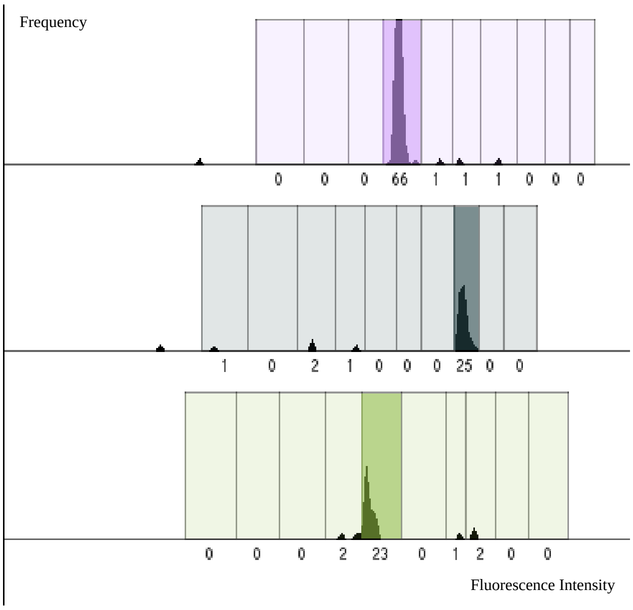

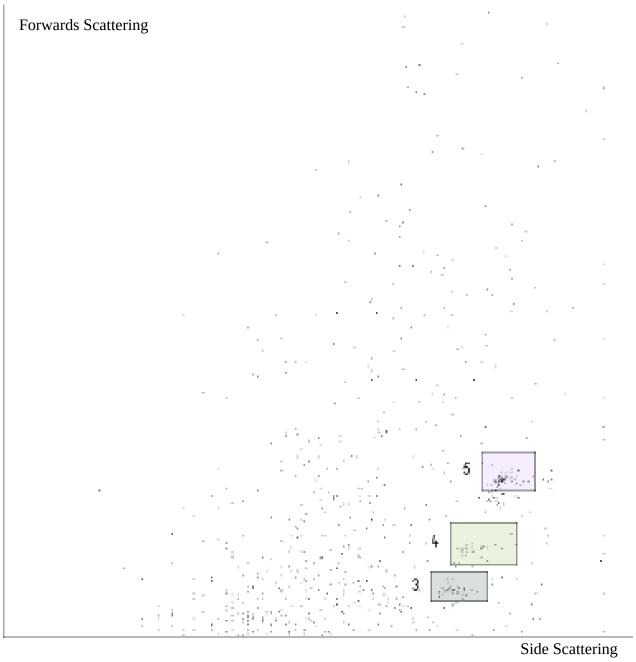

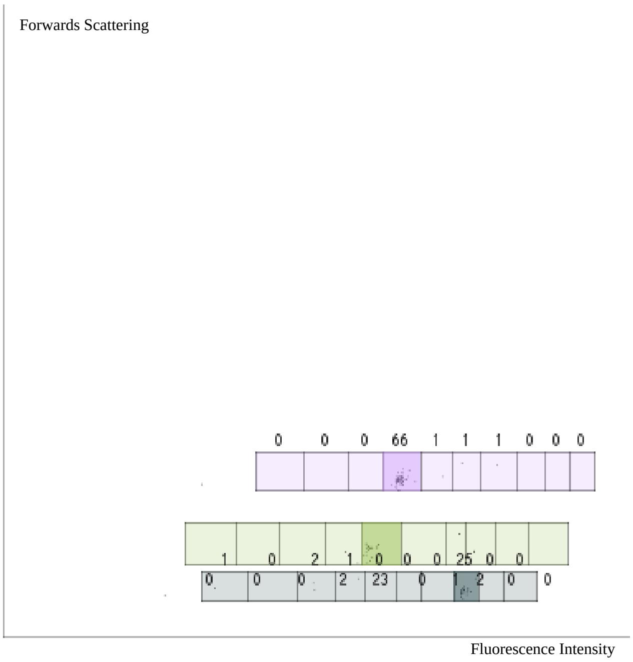

ANNEX 3: TAG DECONVOLUTION - BEAD 66

Passes flow sorting criteria: Yes

Passes tag deconvolution criteria: Yes

Included in protocol analysis: Yes

Protocol: 4, 7, 10, 1

Filename: Bin1_plateA1_A11.LMD

Split 1: Petrol shading

Split 2: Green shading

Split 3: Violet shading

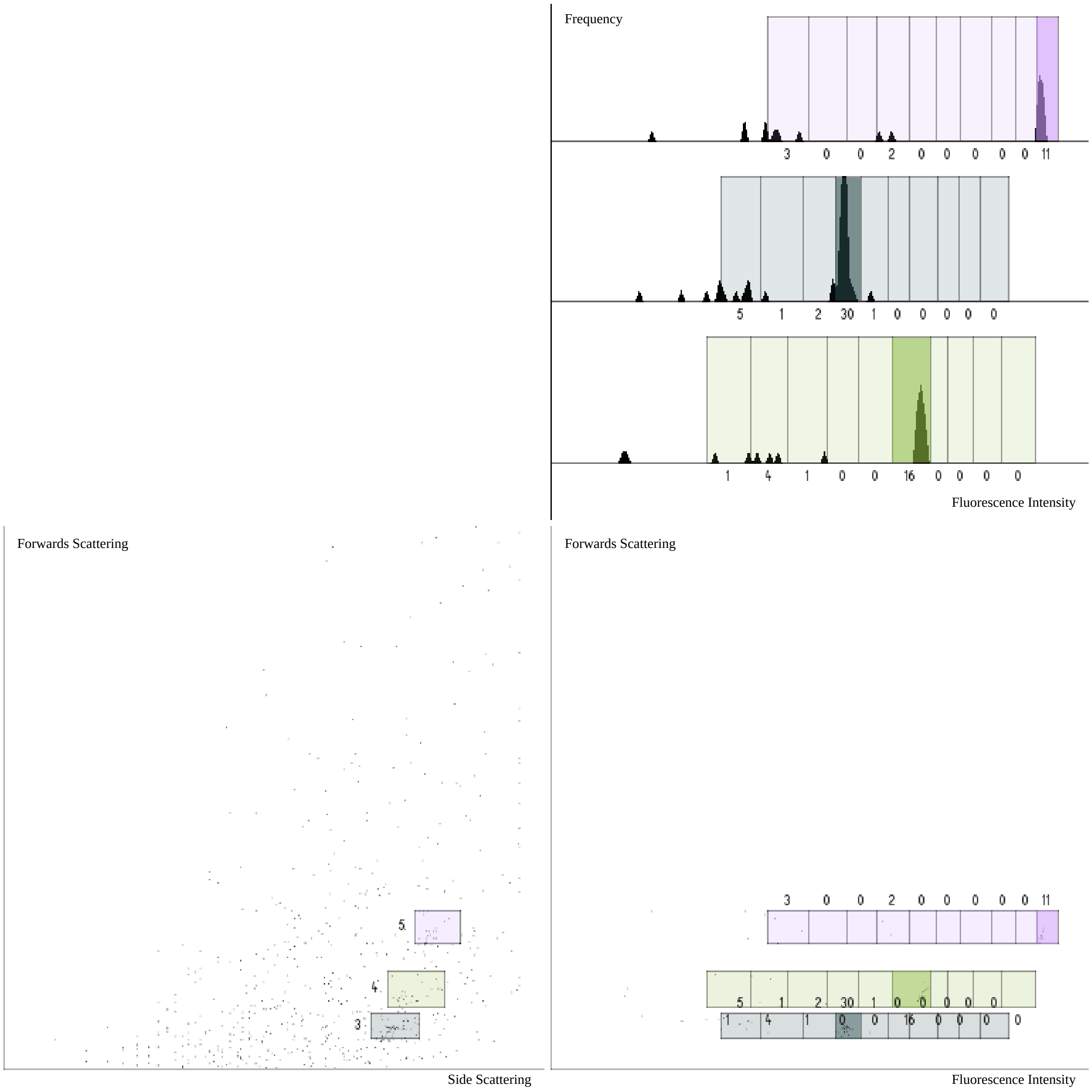

ANNEX 3: TAG DECONVOLUTION - BEAD 67

Passes flow sorting criteria: Yes

Passes tag deconvolution criteria: Yes

Included in protocol analysis: Yes

Protocol: 5, 9, 4, 1

Filename: Bin1_plateA1_A12.LMD

Split 1: Petrol shading

Split 2: Green shading

Split 3: Violet shading

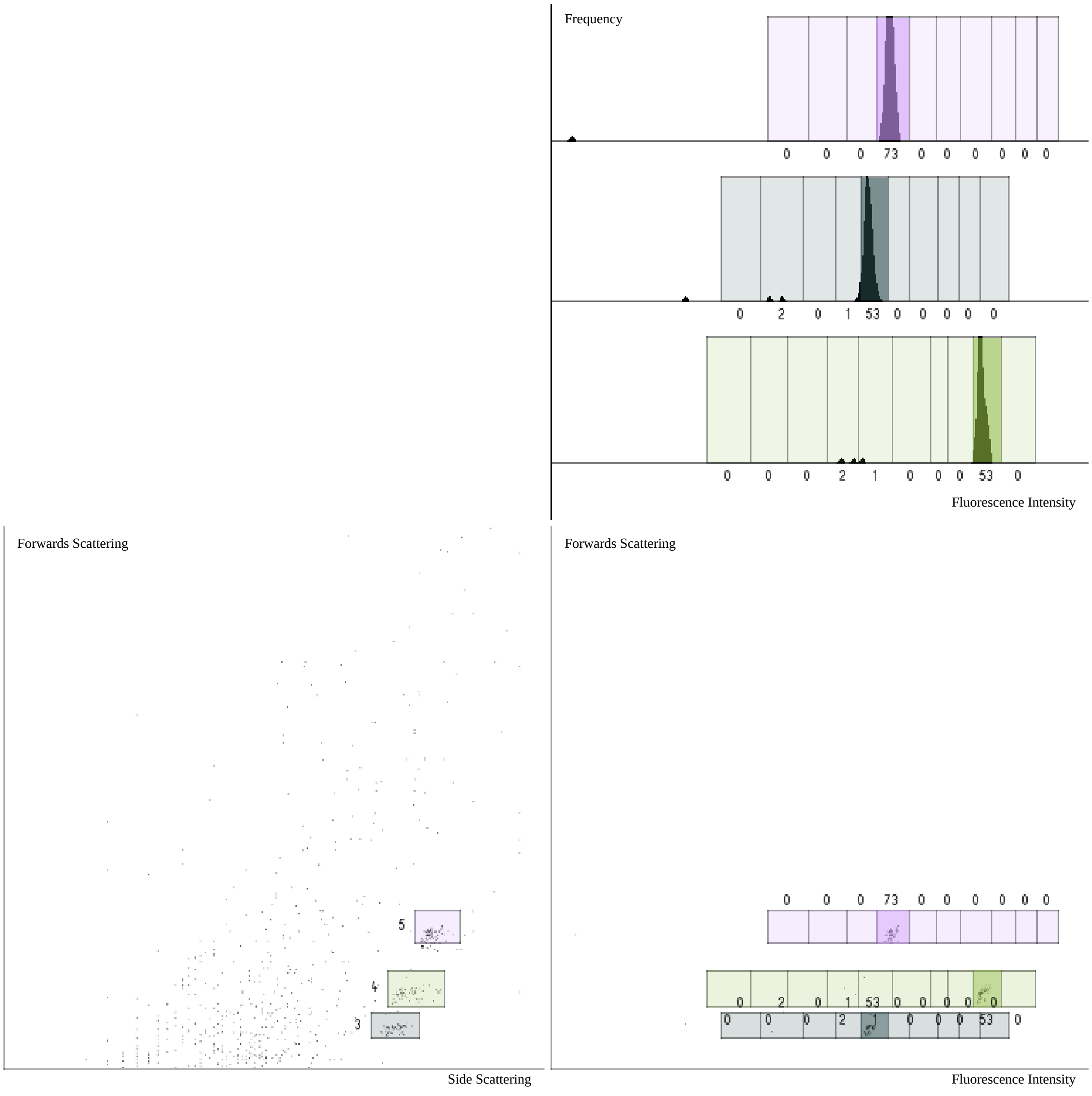

ANNEX 3: TAG DECONVOLUTION - BEAD 68

Passes flow sorting criteria: Yes

Passes tag deconvolution criteria: No

Included in protocol analysis: No

Protocol: N/A

Filename: Bin1_plateA1_B1.LMD

Split 1: Petrol shading

Split 2: Green shading

Split 3: Violet shading

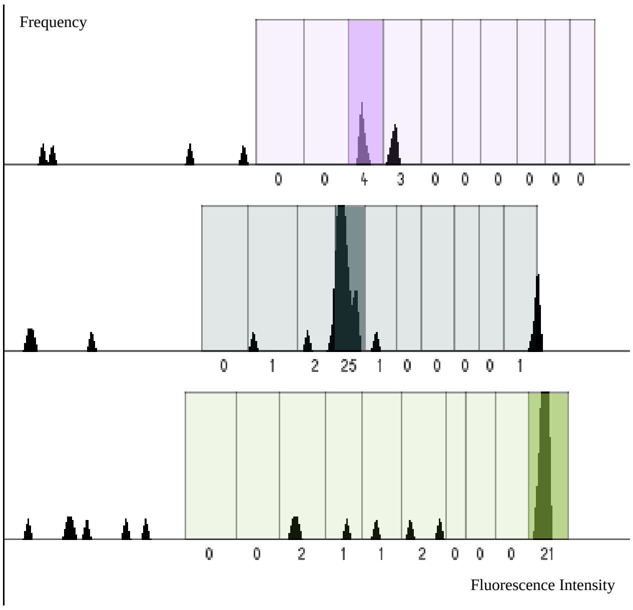

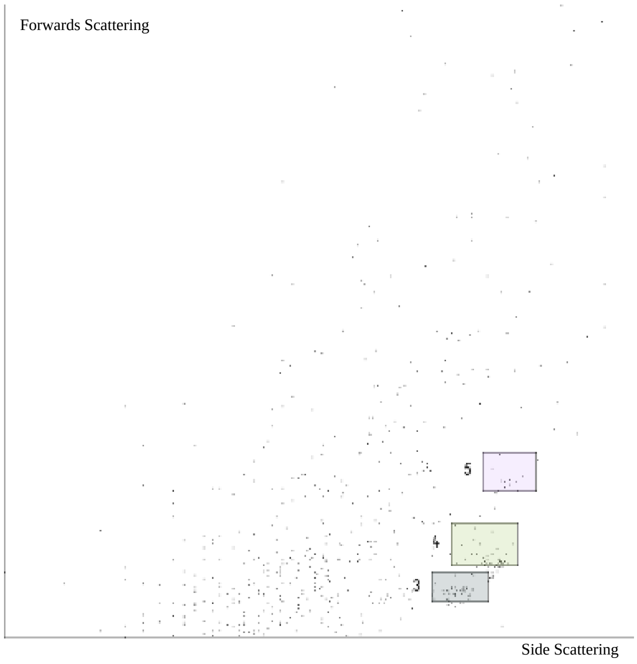

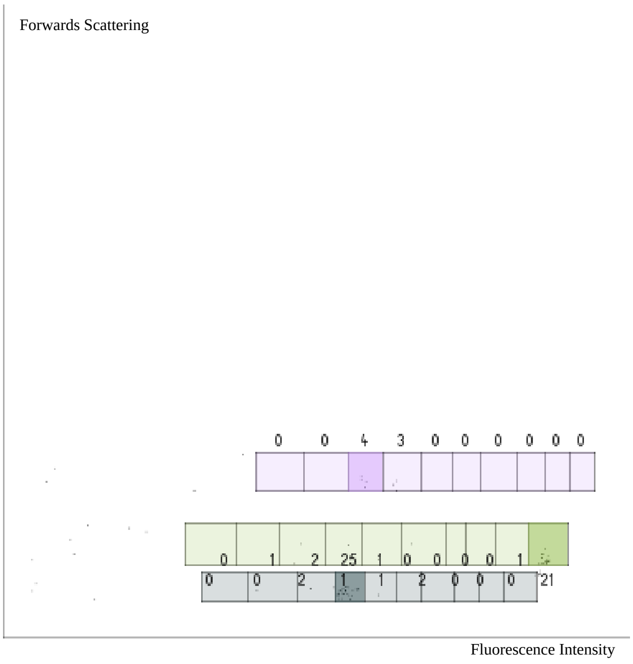

ANNEX 3: TAG DECONVOLUTION - BEAD 69

Passes flow sorting criteria: Yes

Passes tag deconvolution criteria: No

Included in protocol analysis: No

Protocol: N/A

Filename: Bin1_plateA1_B2.LMD

Split 1: Petrol shading

Split 2: Green shading

Split 3: Violet shading

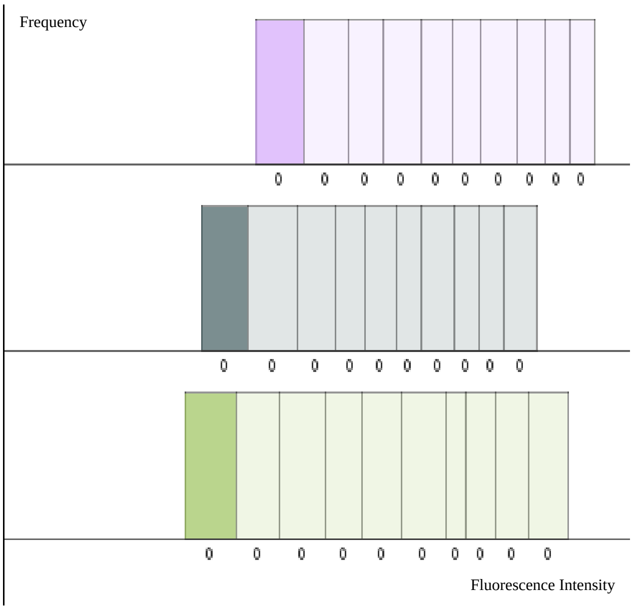

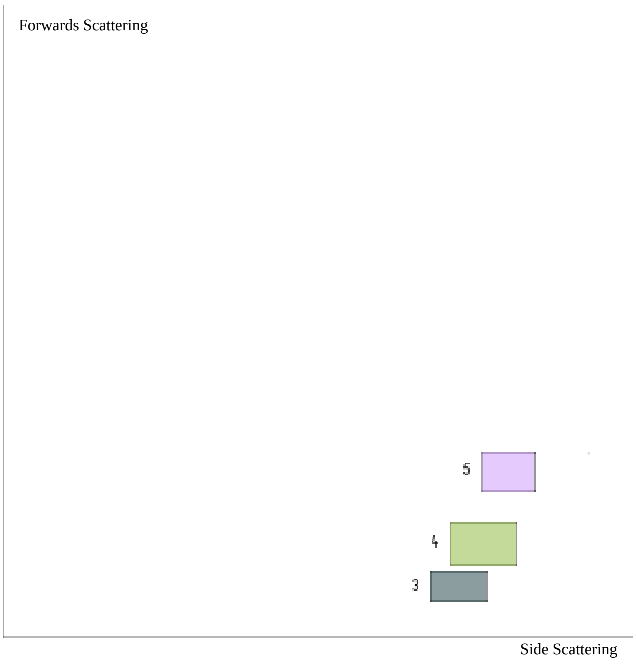

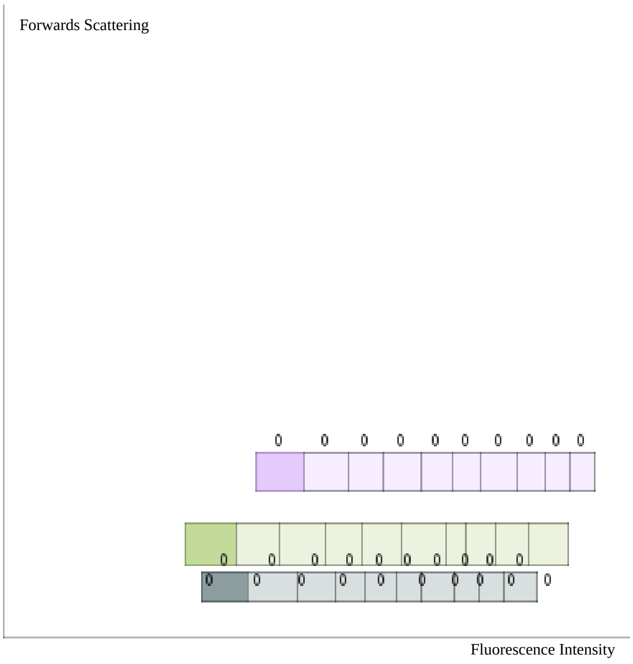

ANNEX 3: TAG DECONVOLUTION - BEAD 70

Passes flow sorting criteria: Yes

Passes tag deconvolution criteria: Yes

Included in protocol analysis: Yes

Protocol: 2, 7, 7, 1

Filename: Bin1_plateA1_B3.LMD

Split 1: Petrol shading

Split 2: Green shading

Split 3: Violet shading

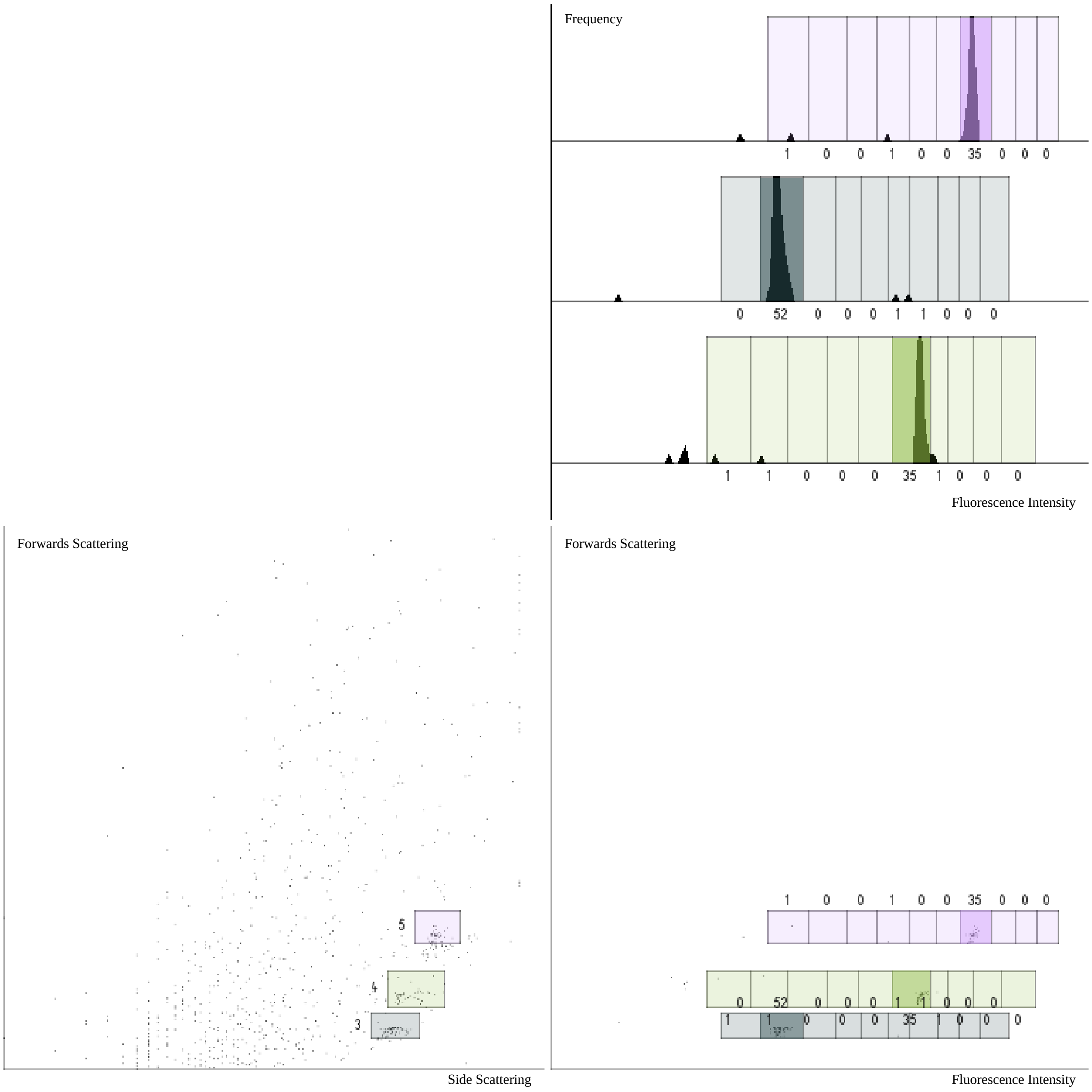

ANNEX 3: TAG DECONVOLUTION - BEAD 71

Passes flow sorting criteria: Yes

Passes tag deconvolution criteria: Yes

Included in protocol analysis: Yes

Protocol: 3, 8, 3, 1

Filename: Bin1_plateA1_B4.LMD

Split 1: Petrol shading

Split 2: Green shading

Split 3: Violet shading

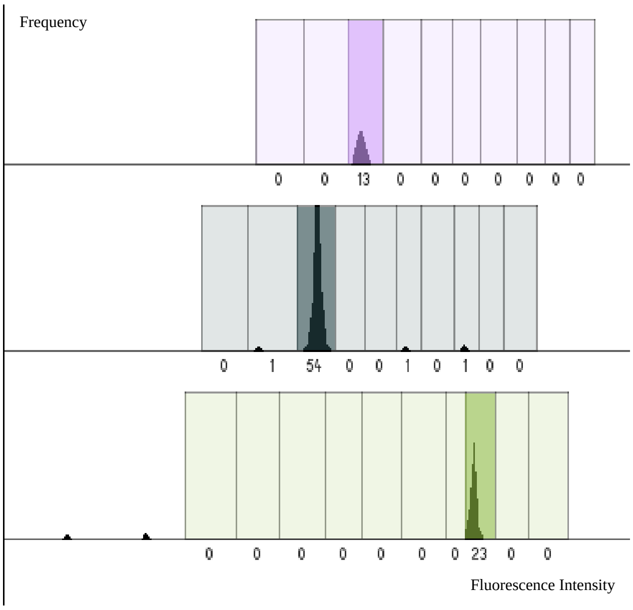

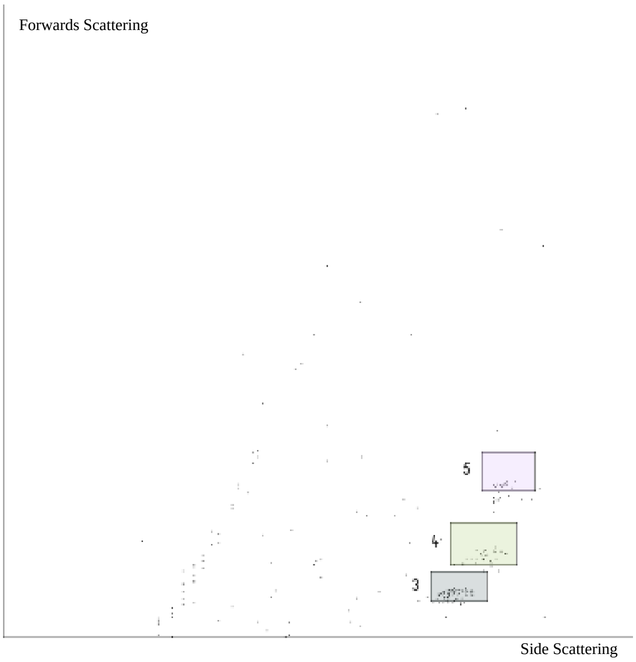

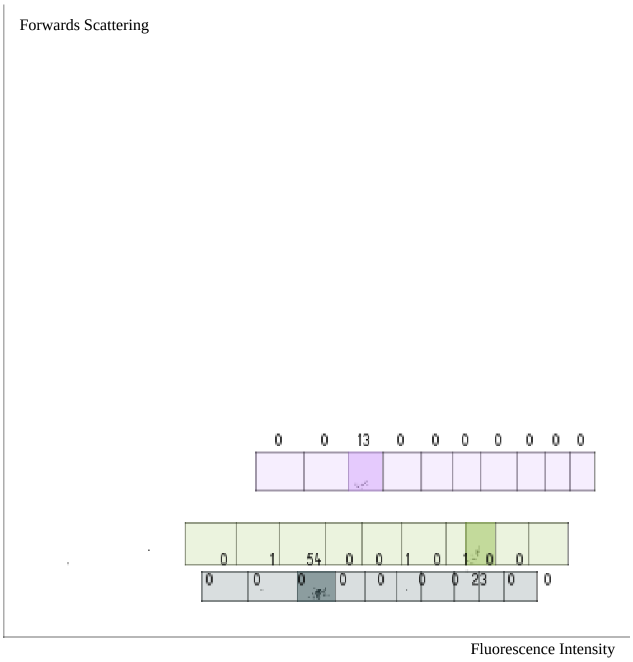

ANNEX 3: TAG DECONVOLUTION - BEAD 72

Passes flow sorting criteria: Yes

Passes tag deconvolution criteria: No

Included in protocol analysis: No

Protocol: N/A

Filename: Bin1_plateA1_B5.LMD

Split 1: Petrol shading

Split 2: Green shading

Split 3: Violet shading

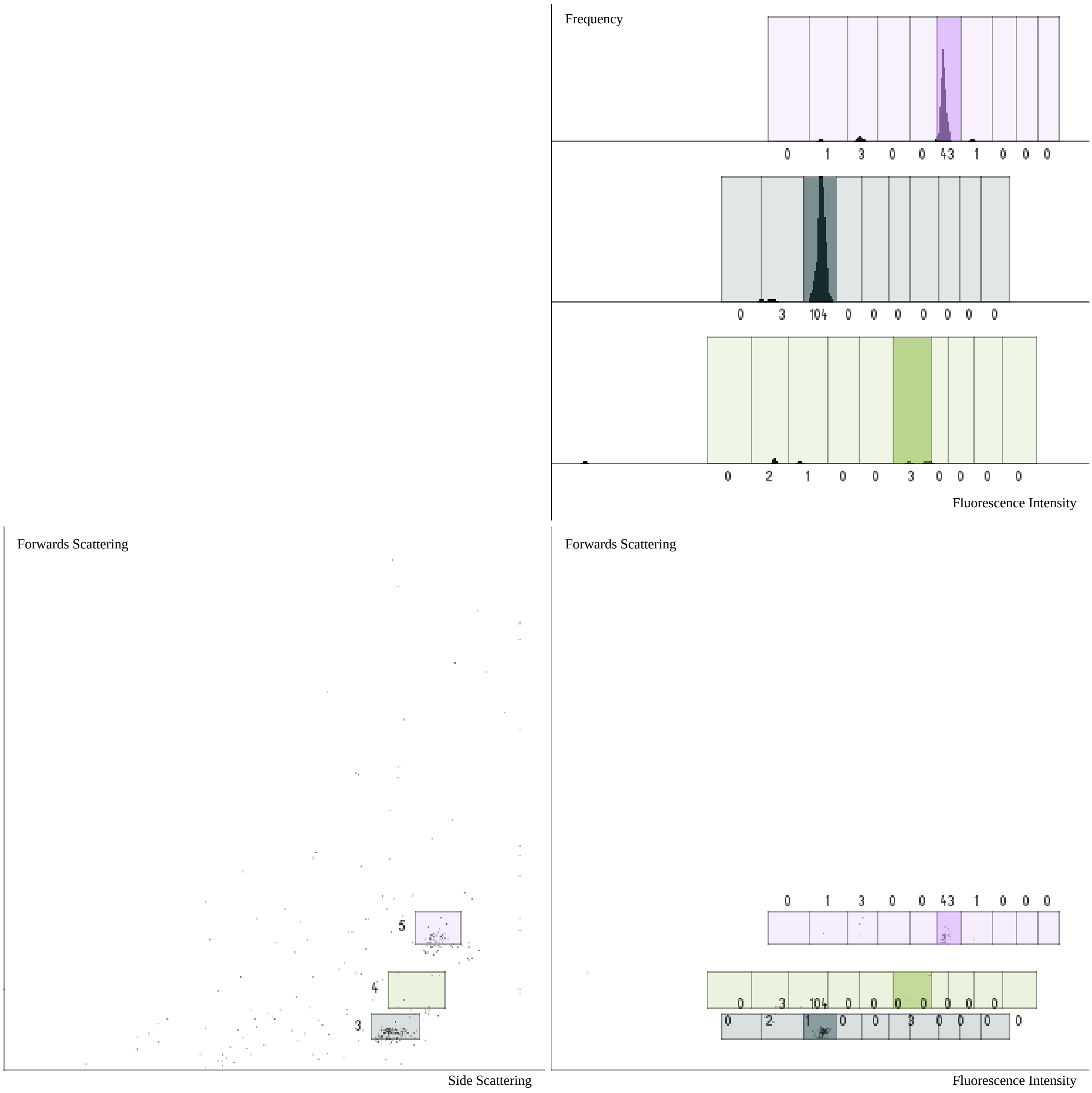

ANNEX 3: TAG DECONVOLUTION - BEAD 73

Passes flow sorting criteria: Yes

Passes tag deconvolution criteria: Yes

Included in protocol analysis: Yes

Protocol: 1, 5, 7, 1

Filename: Bin1_plateA1_B6.LMD

Split 1: Petrol shading

Split 2: Green shading

Split 3: Violet shading

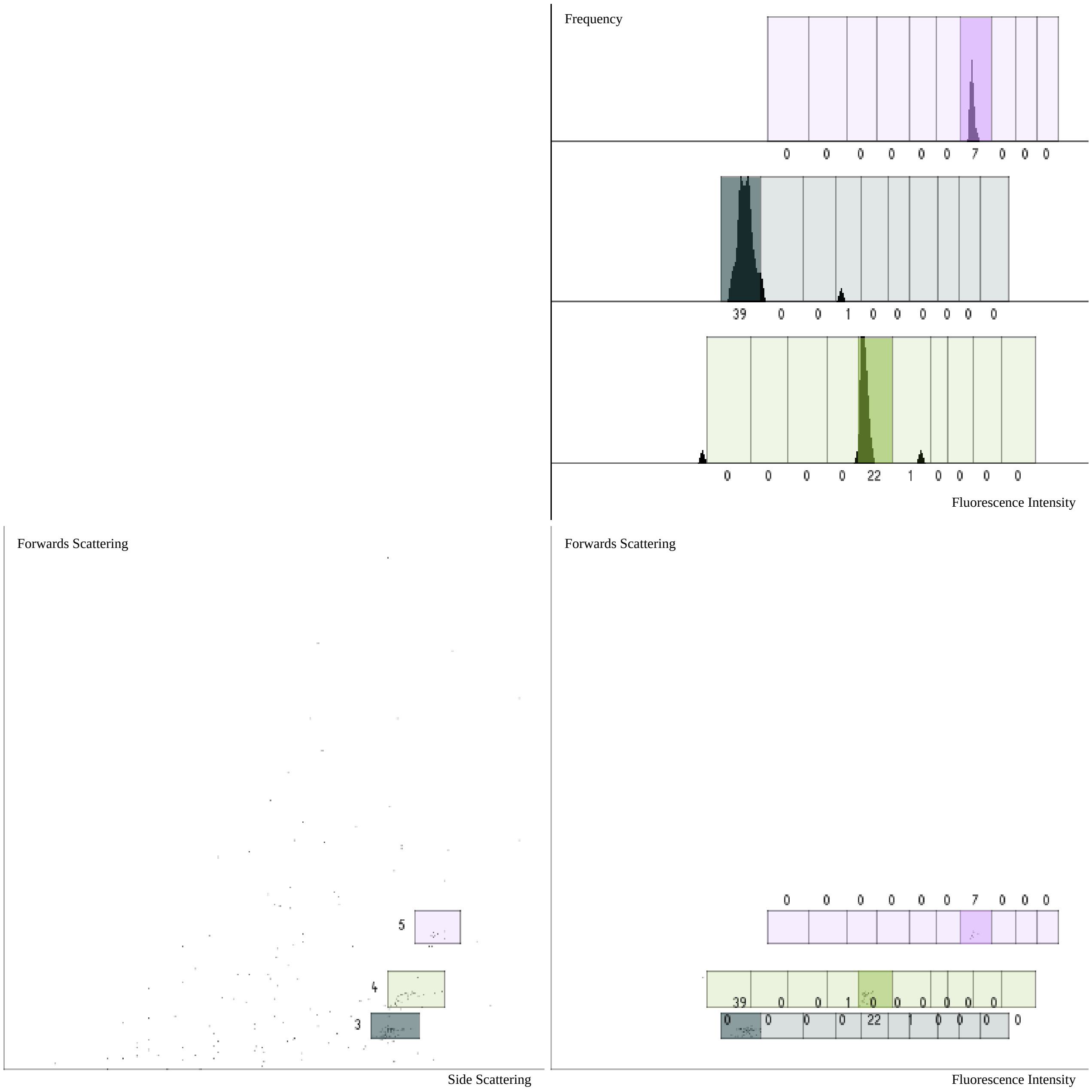

ANNEX 3: TAG DECONVOLUTION - BEAD 74

Passes flow sorting criteria: Yes

Passes tag deconvolution criteria: Yes

Included in protocol analysis: Yes

Protocol: 3, 10, 1, 1

Filename: Bin1_plateA1_B7.LMD

Split 1: Petrol shading

Split 2: Green shading

Split 3: Violet shading

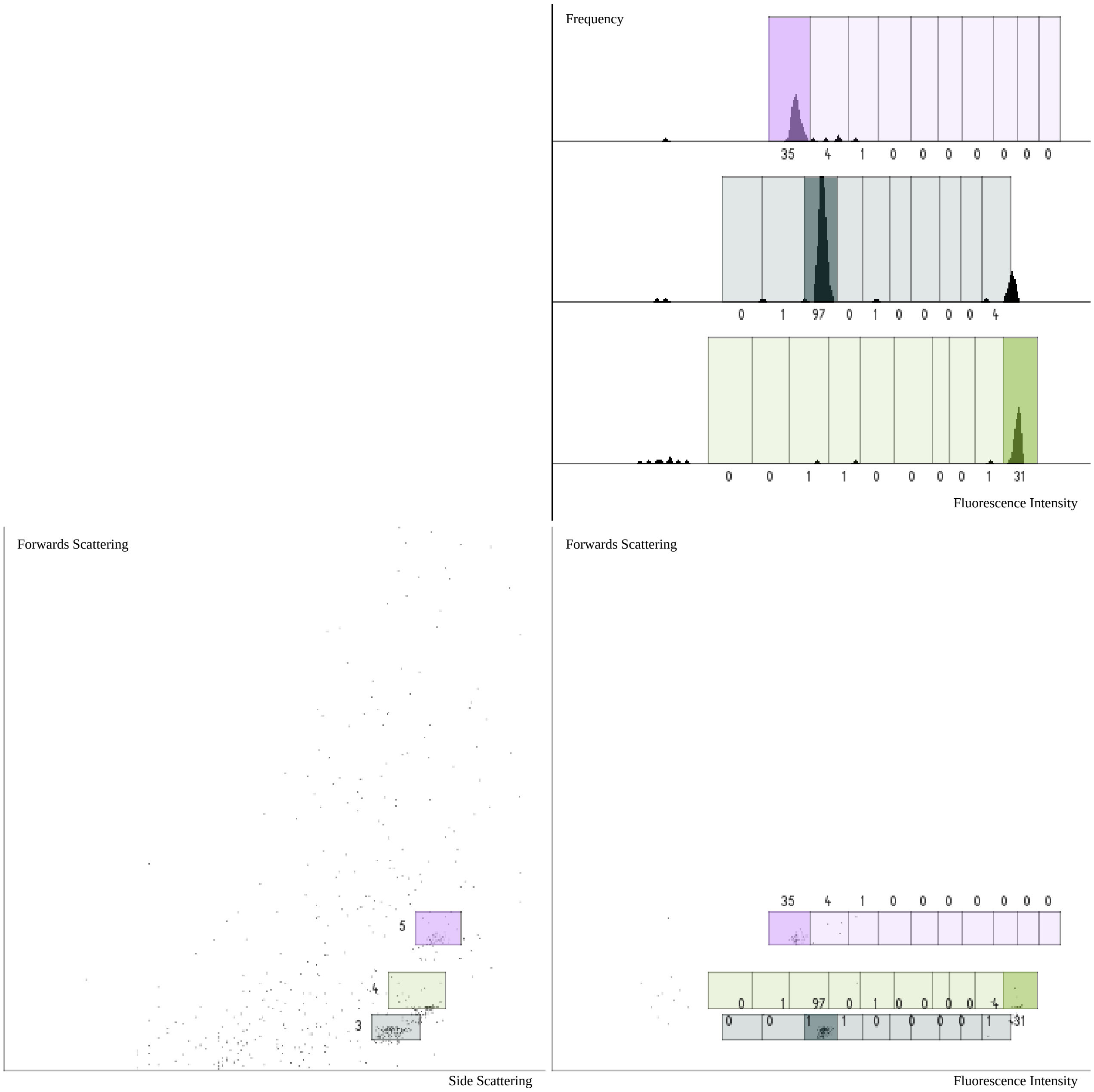

ANNEX 3: TAG DECONVOLUTION - BEAD 75

Passes flow sorting criteria: Yes

Passes tag deconvolution criteria: Yes

Included in protocol analysis: Yes

Protocol: 9, 4, 6, 1

Filename: Bin1_plateA1_B8.LMD

Split 1: Petrol shading

Split 2: Green shading

Split 3: Violet shading

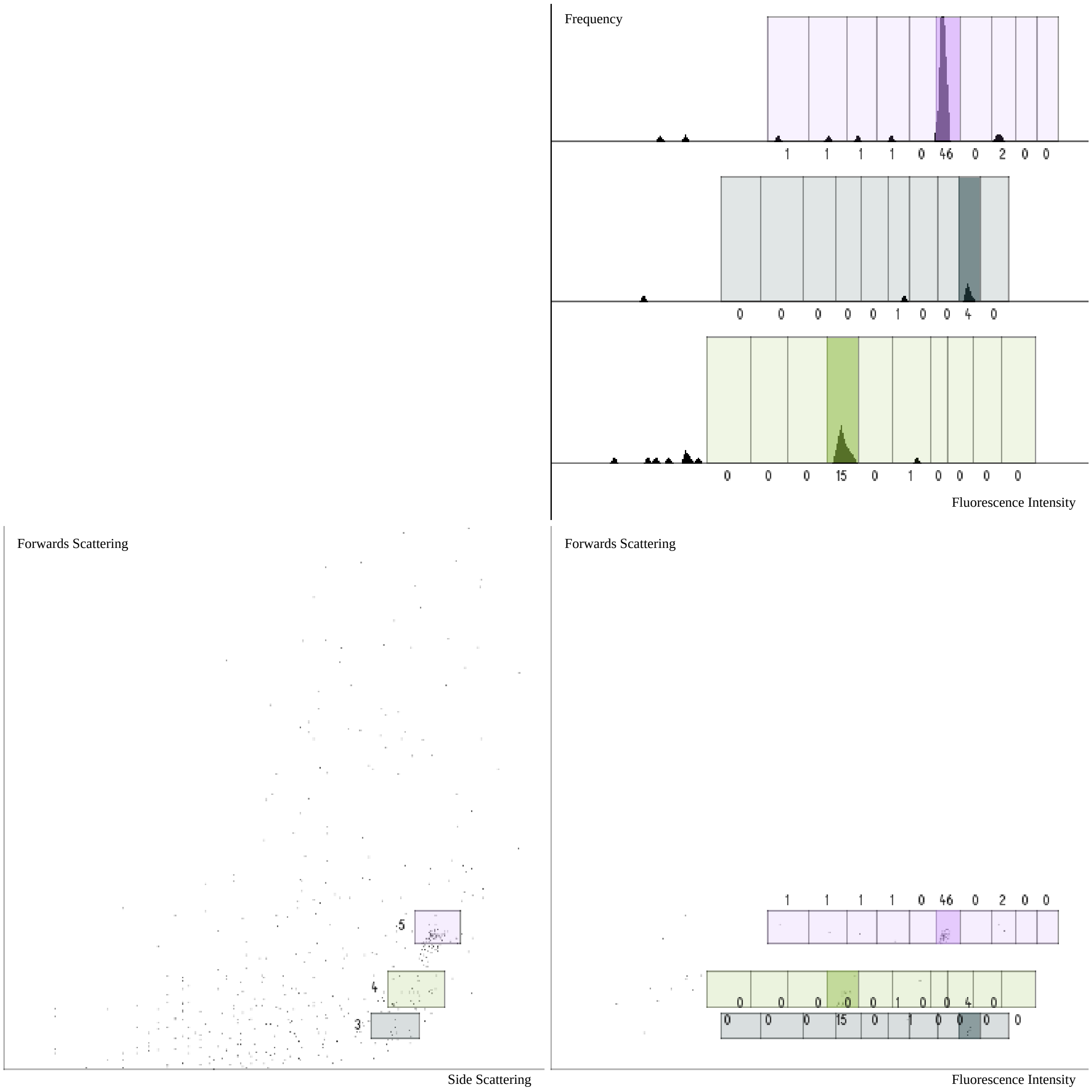

ANNEX 3: TAG DECONVOLUTION - BEAD 76

Passes flow sorting criteria: Yes

Passes tag deconvolution criteria: Yes

Included in protocol analysis: Yes

Protocol: 4, 7, 7, 1

Filename: Bin1_plateA1_B9.LMD

Split 1: Petrol shading

Split 2: Green shading

Split 3: Violet shading

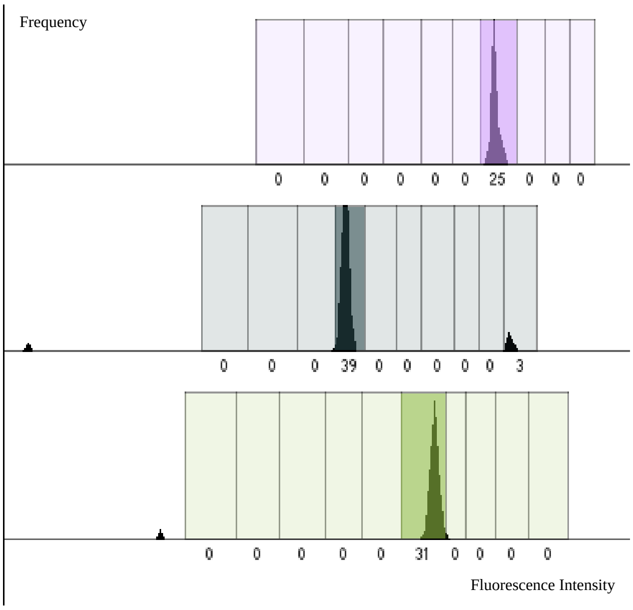

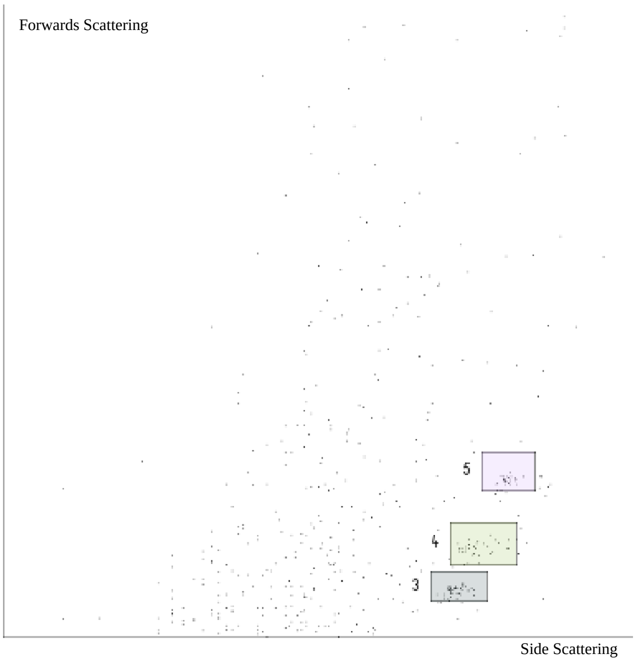

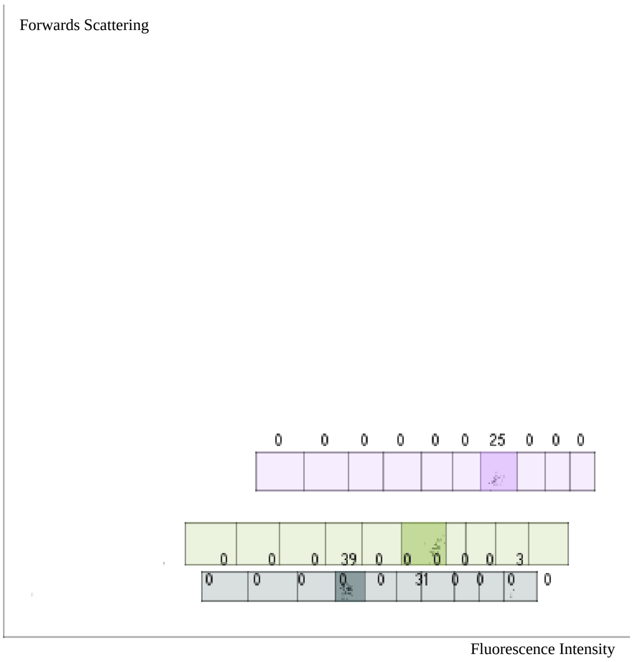

ANNEX 3: TAG DECONVOLUTION - BEAD 77

Passes flow sorting criteria: Yes

Passes tag deconvolution criteria: Yes

Included in protocol analysis: Yes

Protocol: 10, 2, 7, 1

Filename: Bin1_plateA1_B10.LMD

Split 1: Petrol shading

Split 2: Green shading

Split 3: Violet shading

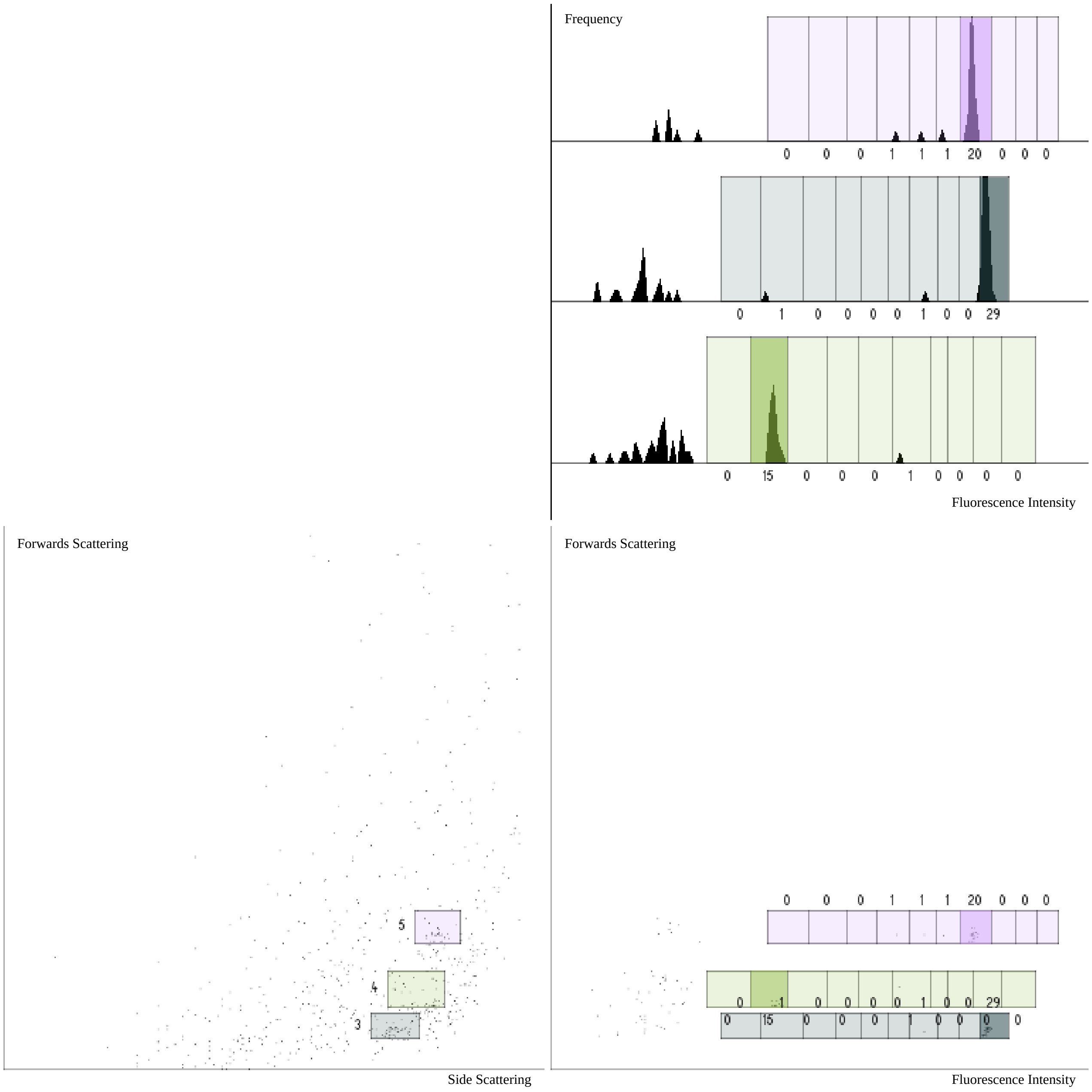

ANNEX 3: TAG DECONVOLUTION - BEAD 78

Passes flow sorting criteria: Yes

Passes tag deconvolution criteria: No

Included in protocol analysis: No

Protocol: N/A

Filename: Bin1_plateA1_B11.LMD

Split 1: Petrol shading

Split 2: Green shading

Split 3: Violet shading

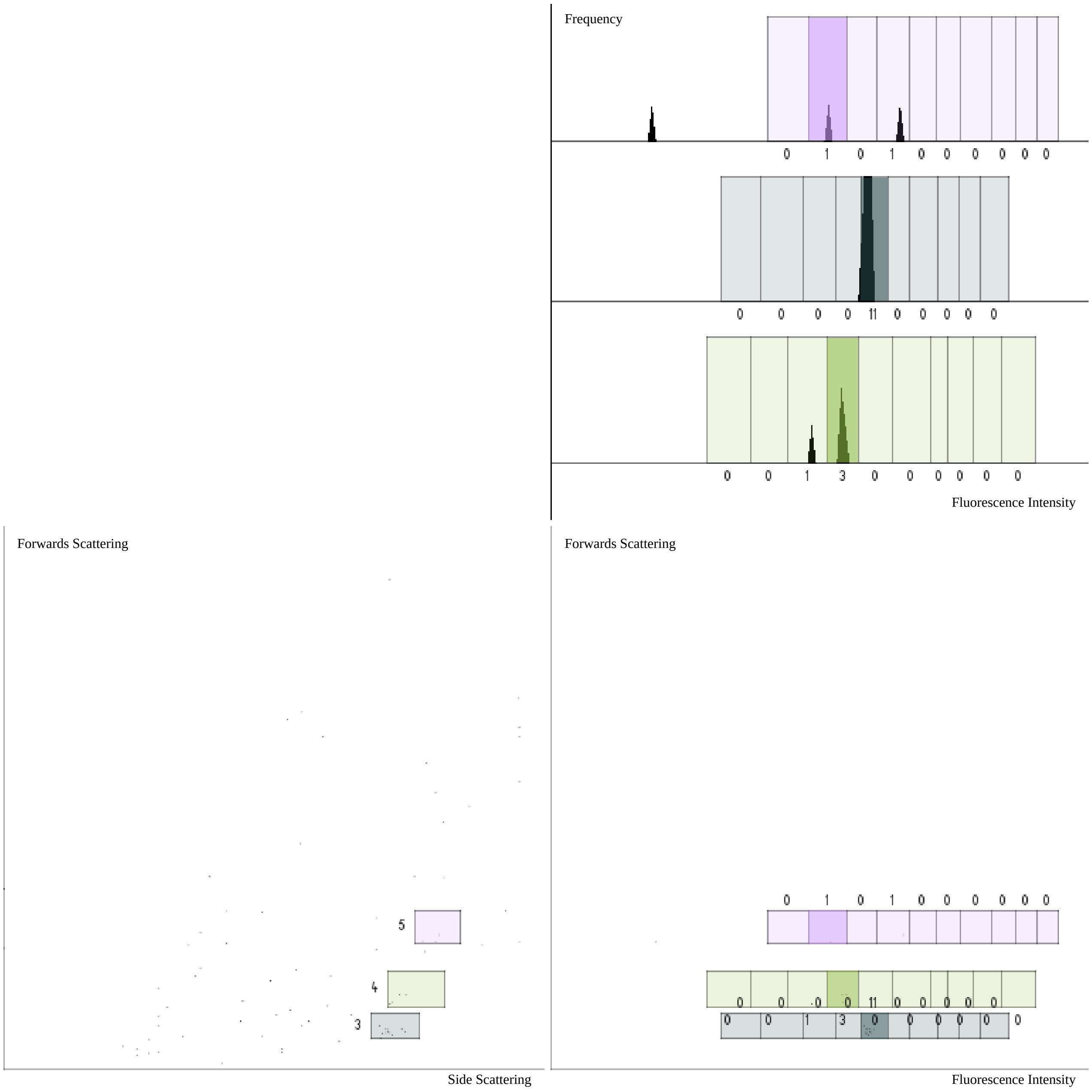

ANNEX 3: TAG DECONVOLUTION - BEAD 79

Passes flow sorting criteria: Yes

Passes tag deconvolution criteria: Yes

Included in protocol analysis: Yes

Protocol: 4, 6, 2, 1

Filename: Bin1_plateA1_B12.LMD

Split 1: Petrol shading

Split 2: Green shading

Split 3: Violet shading

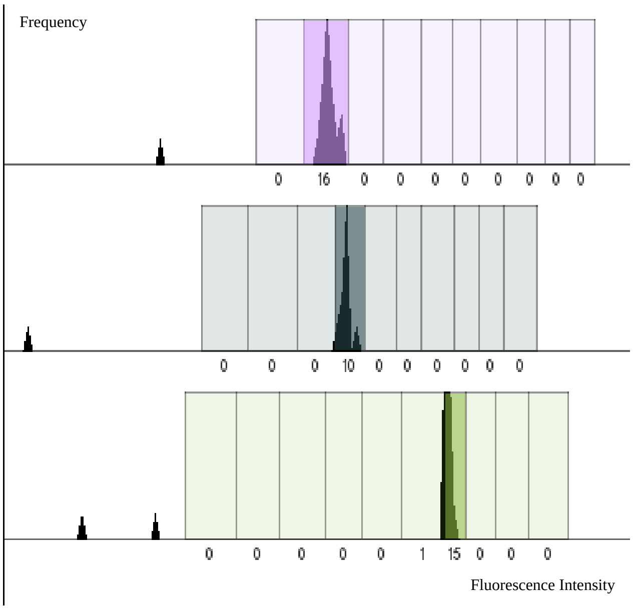

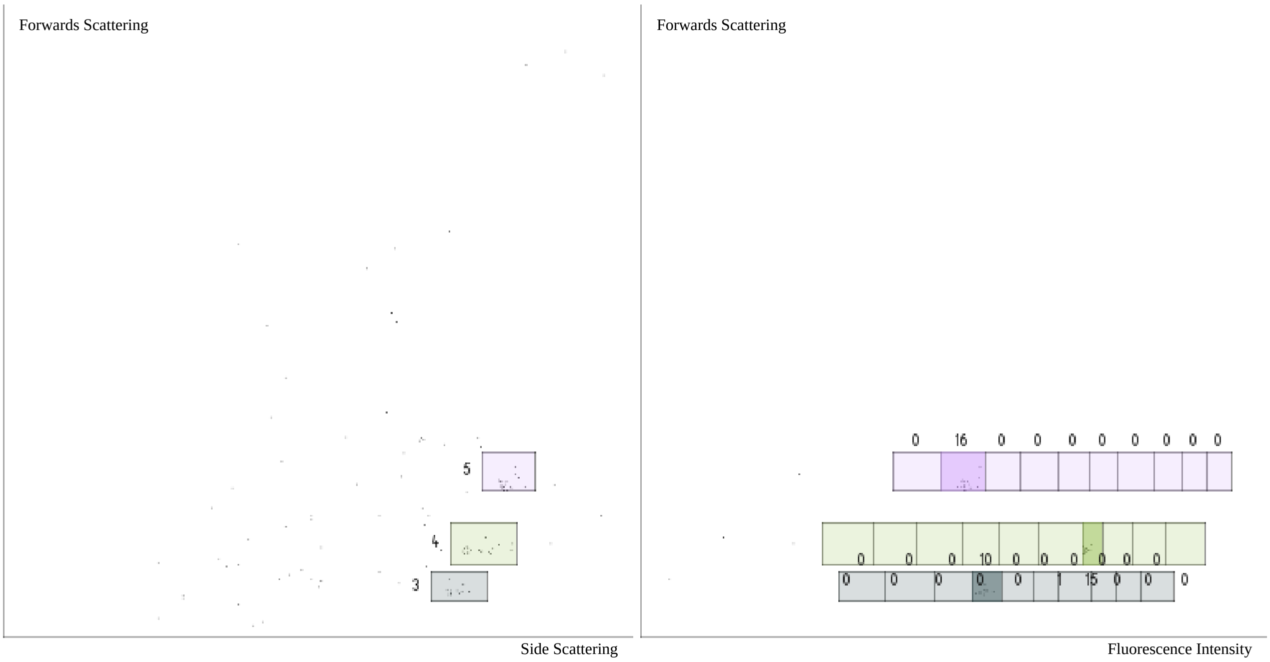

ANNEX 3: TAG DECONVOLUTION - BEAD 80

Passes flow sorting criteria: Yes

Passes tag deconvolution criteria: No

Included in protocol analysis: No

Protocol: N/A

Filename: Bin1_plateA1_C1.LMD

Split 1: Petrol shading

Split 2: Green shading

Split 3: Violet shading

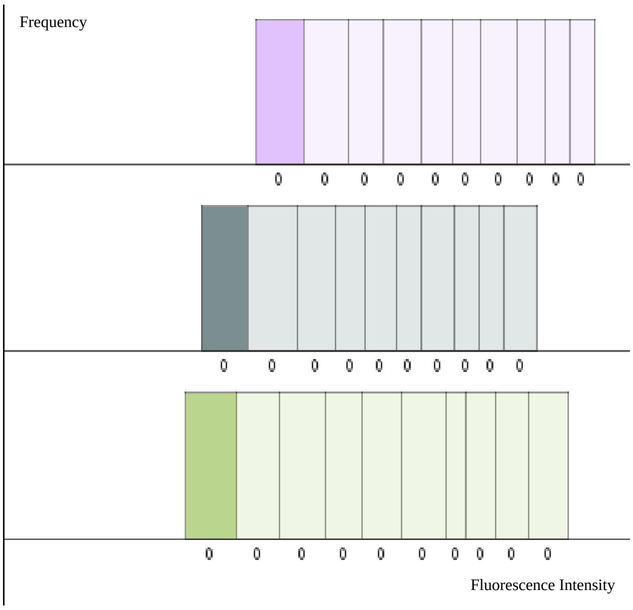

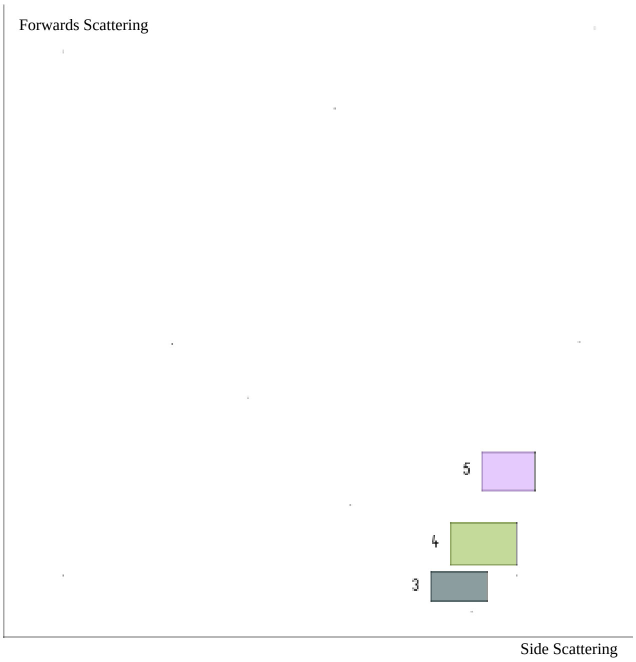

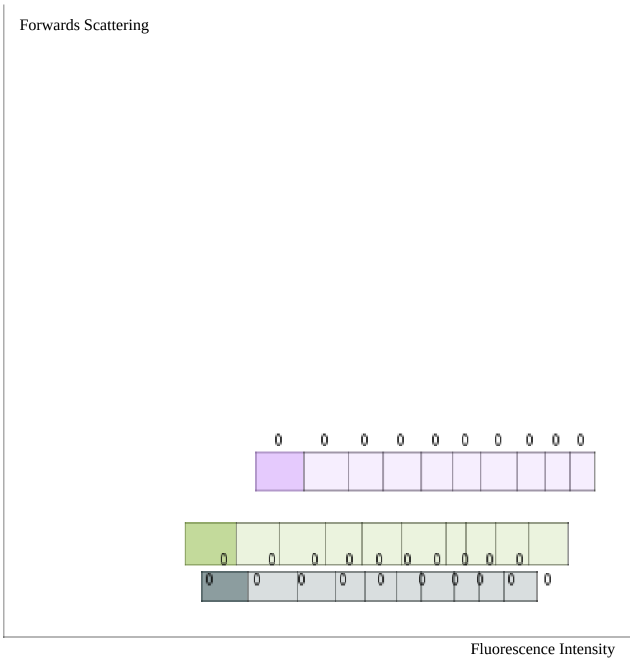

ANNEX 3: TAG DECONVOLUTION - BEAD 81

Passes flow sorting criteria: Yes

Passes tag deconvolution criteria: Yes

Included in protocol analysis: Yes

Protocol: 10, 7, 4, 1

Filename: Bin1_plateA1_C2.LMD

Split 1: Petrol shading

Split 2: Green shading

Split 3: Violet shading

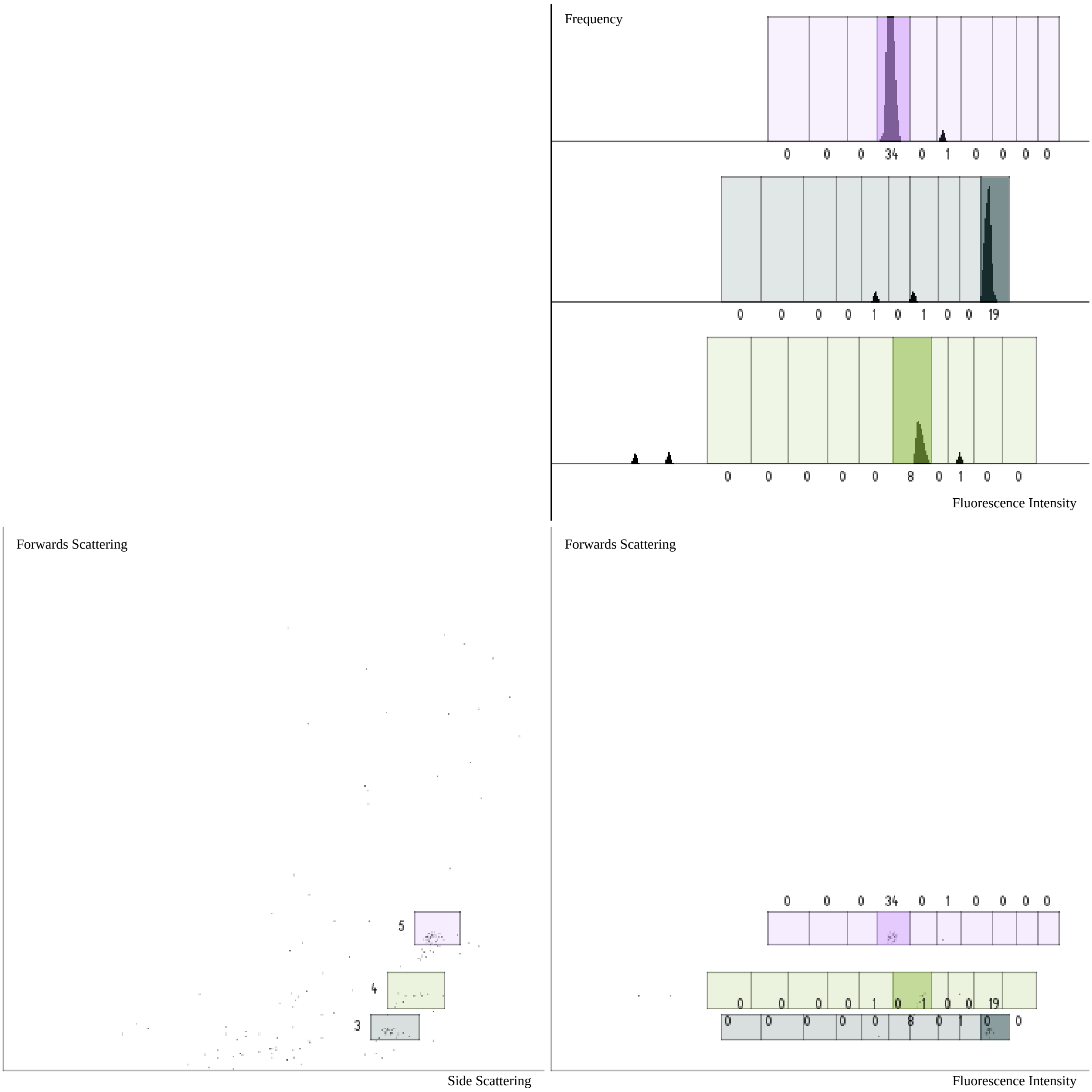

ANNEX 3: TAG DECONVOLUTION - BEAD 82

Passes flow sorting criteria: Yes

Passes tag deconvolution criteria: Yes

Included in protocol analysis: Yes

Protocol: 6, 6, 5, 1

Filename: Bin1_plateA1_C3.LMD

Split 1: Petrol shading

Split 2: Green shading

Split 3: Violet shading

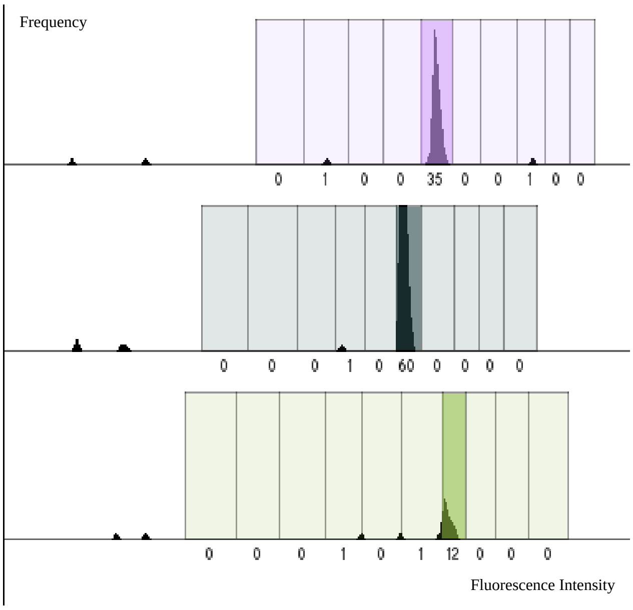

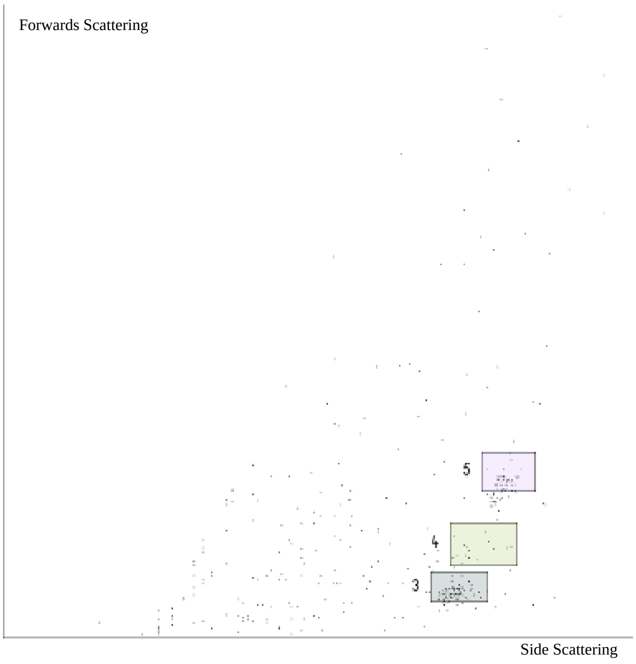

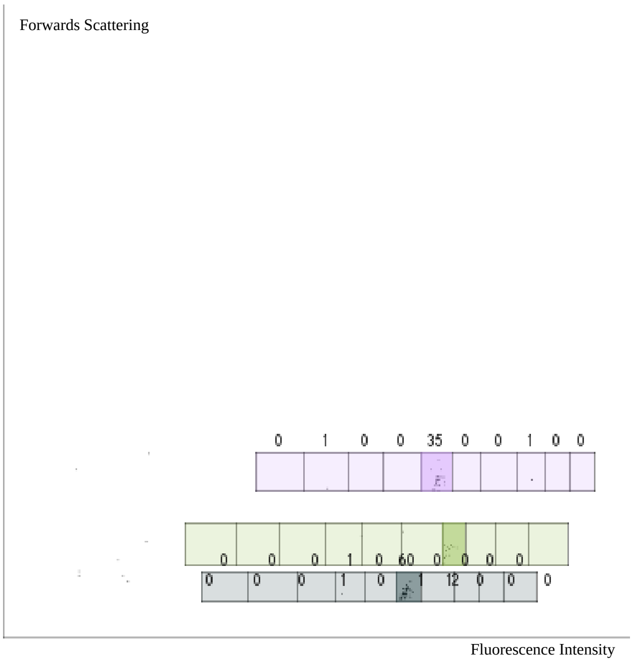

ANNEX 3: TAG DECONVOLUTION - BEAD 83

Passes flow sorting criteria: Yes

Passes tag deconvolution criteria: Yes

Included in protocol analysis: Yes

Protocol: 9, 3, 4, 1

Filename: Bin1_plateA1_C4.LMD

Split 1: Petrol shading

Split 2: Green shading

Split 3: Violet shading

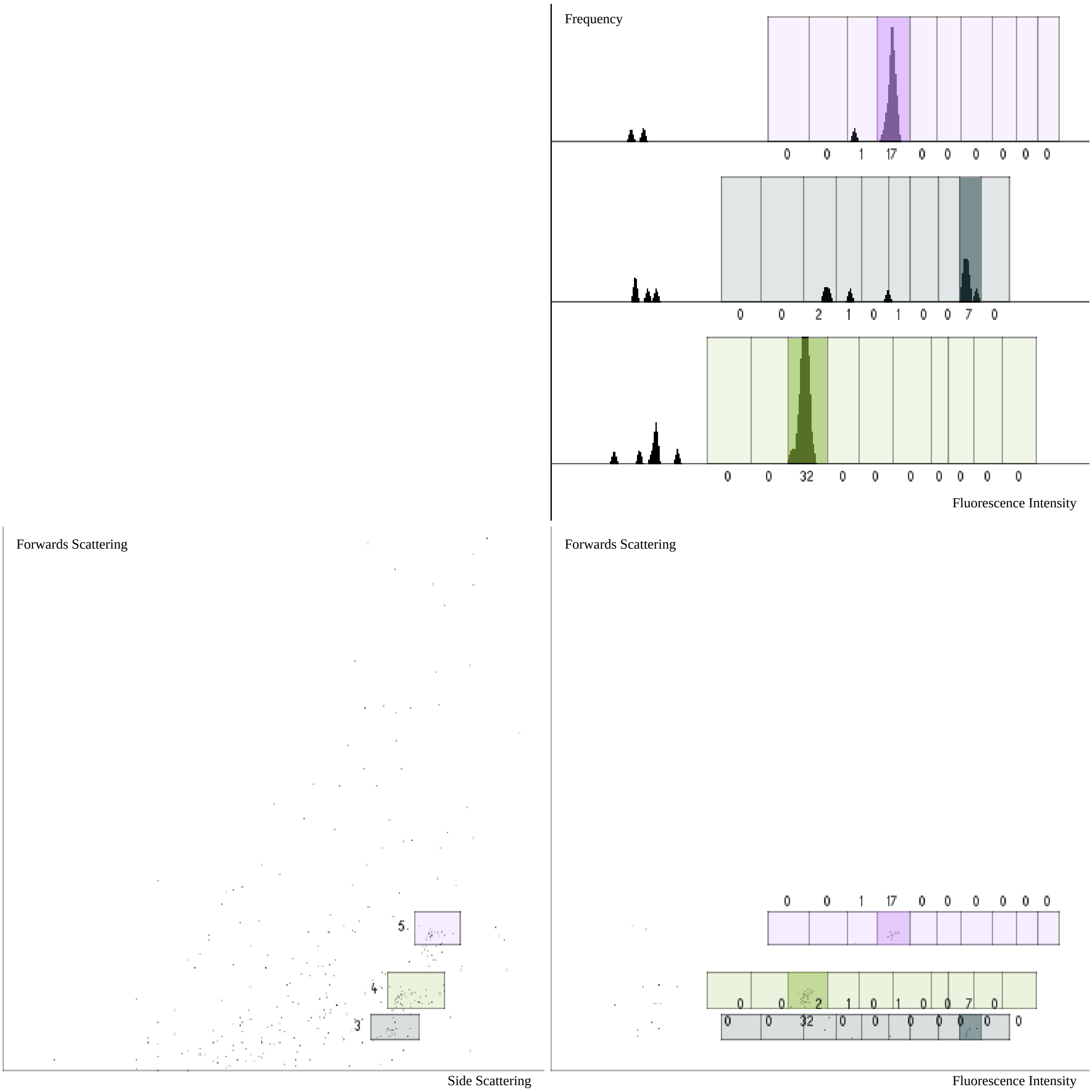

ANNEX 3: TAG DECONVOLUTION - BEAD 84

Passes flow sorting criteria: Yes

Passes tag deconvolution criteria: Yes

Included in protocol analysis: Yes

Protocol: 6, 1, 2, 1

Filename: Bin1_plateA1_C5.LMD

Split 1: Petrol shading

Split 2: Green shading

Split 3: Violet shading

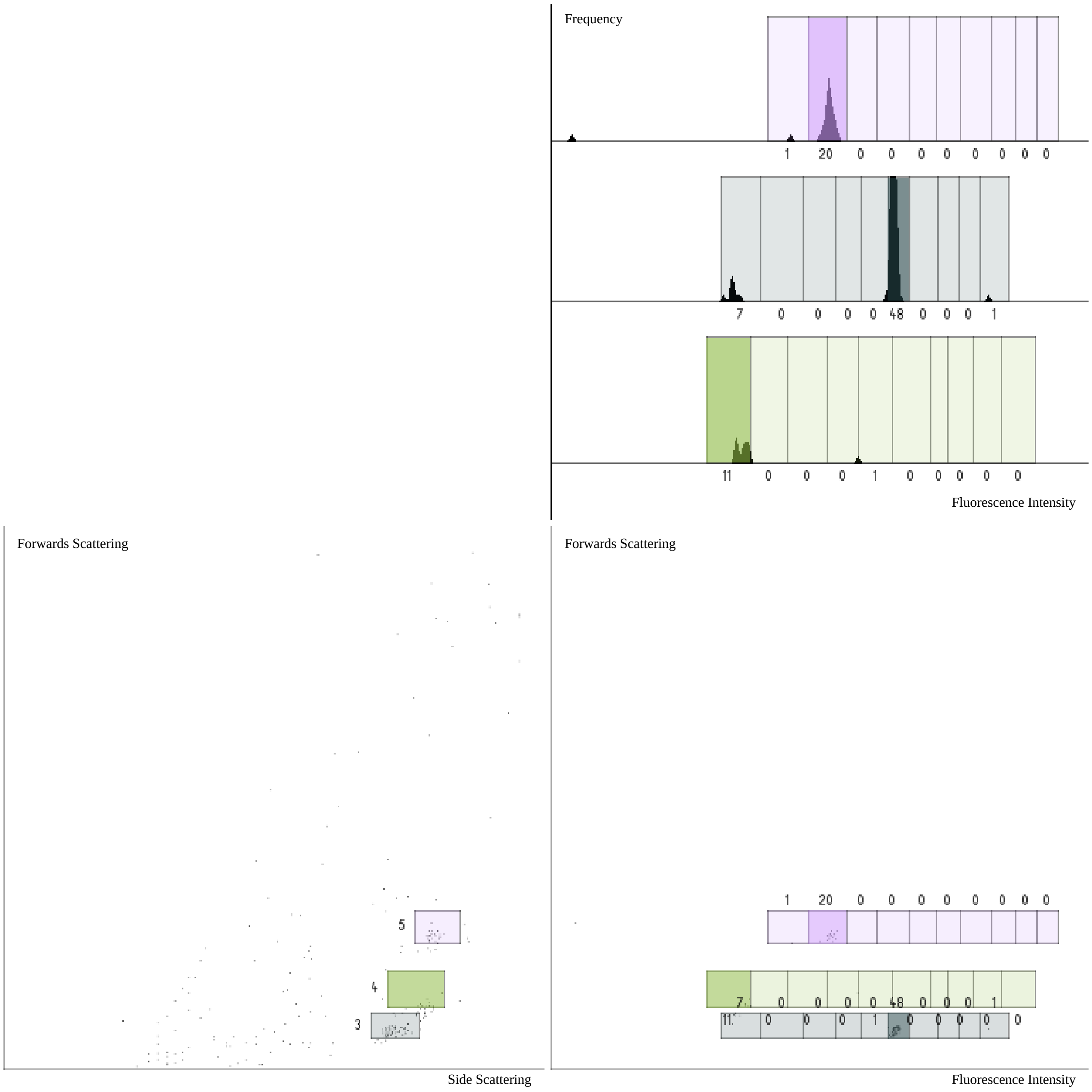

ANNEX 3: TAG DECONVOLUTION - BEAD 85

Passes flow sorting criteria: Yes

Passes tag deconvolution criteria: No

Included in protocol analysis: No

Protocol: N/A

Filename: Bin1_plateA1_C6.LMD

Split 1: Petrol shading

Split 2: Green shading

Split 3: Violet shading

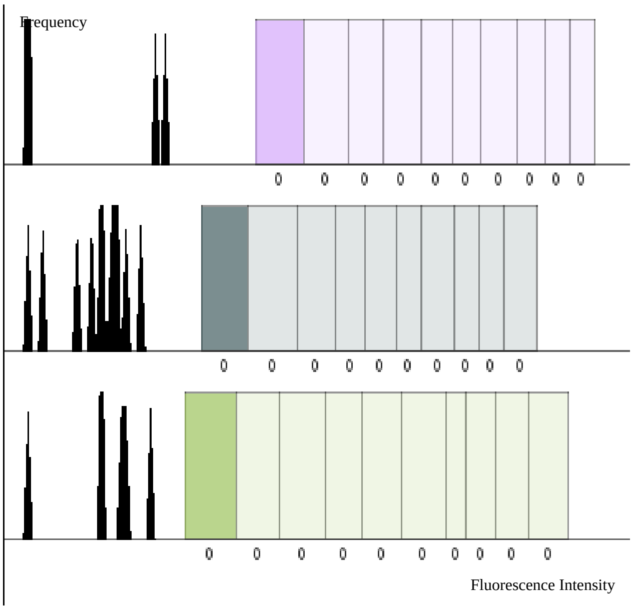

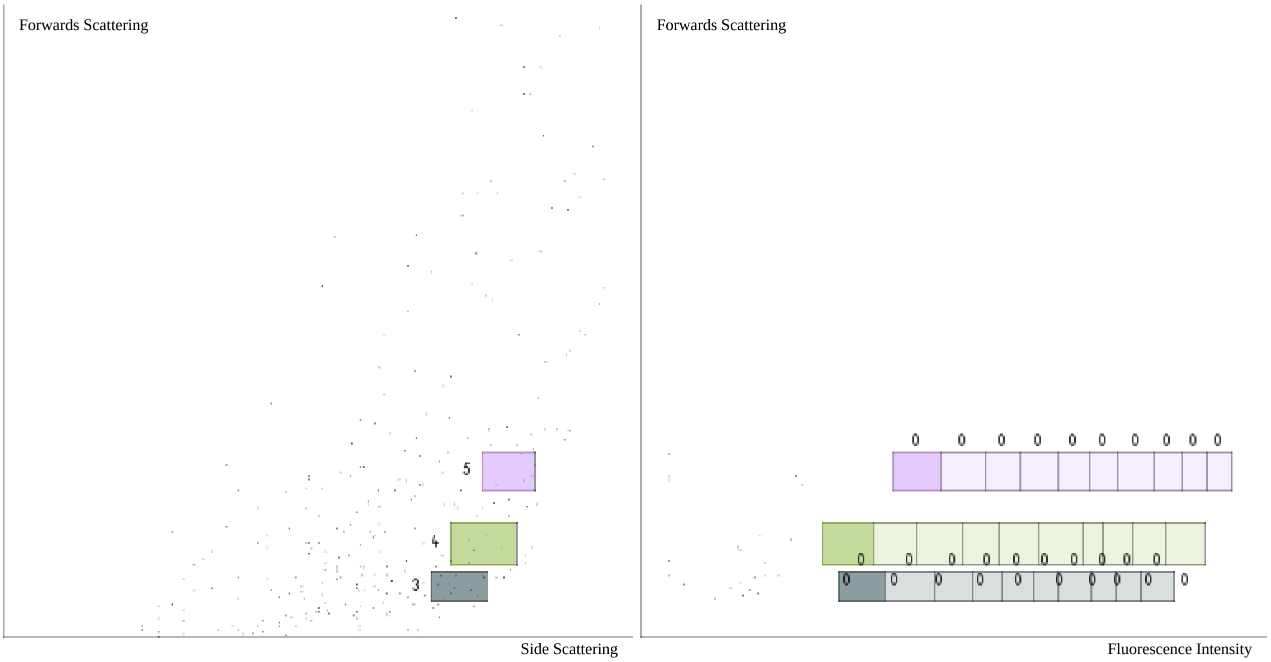

ANNEX 3: TAG DECONVOLUTION - BEAD 86

Passes flow sorting criteria: Yes

Passes tag deconvolution criteria: No

Included in protocol analysis: No

Protocol: N/A

Filename: Bin1_plateA1_C7.LMD

Split 1: Petrol shading

Split 2: Green shading

Split 3: Violet shading

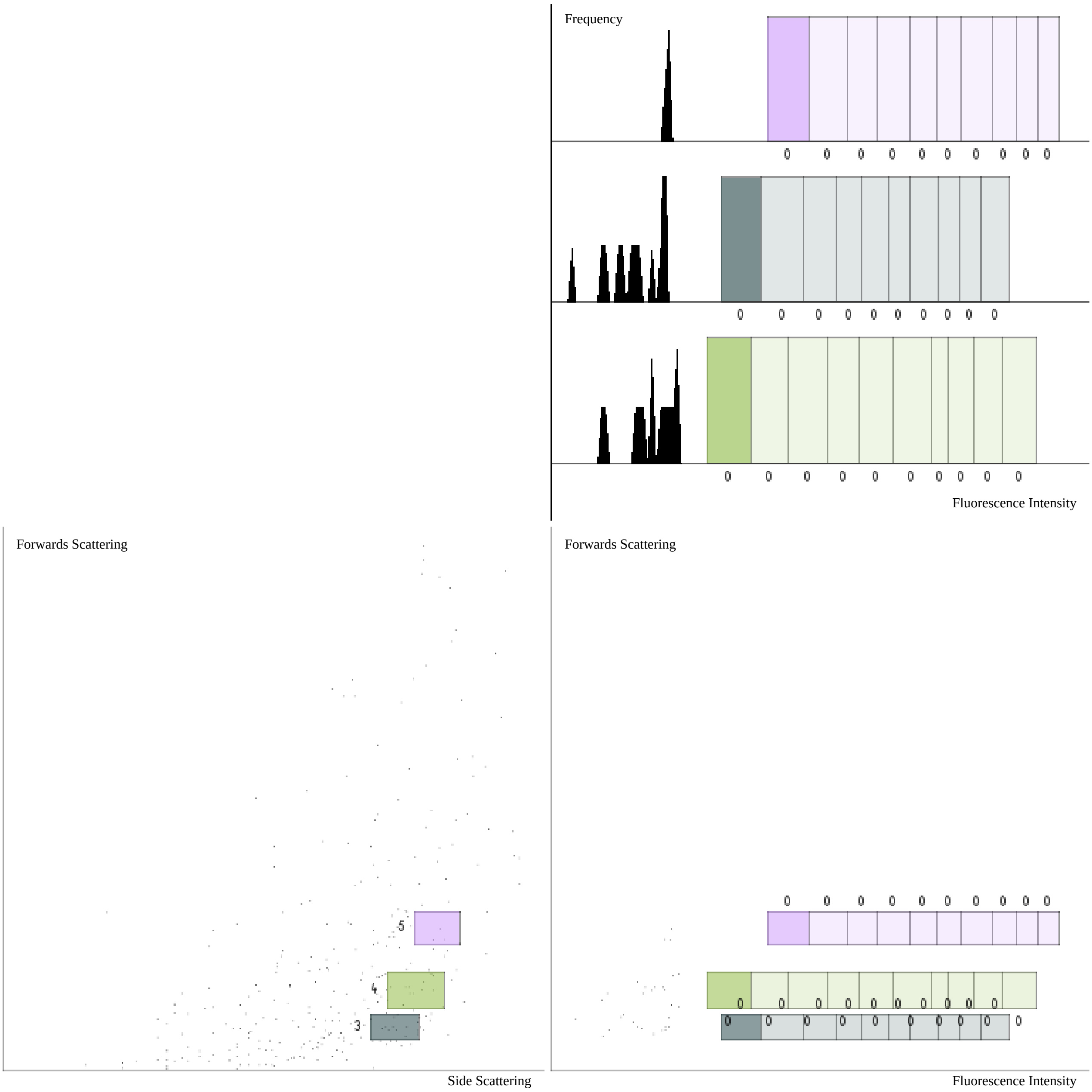

ANNEX 3: TAG DECONVOLUTION - BEAD 87

Passes flow sorting criteria: Yes

Passes tag deconvolution criteria: No

Included in protocol analysis: No

Protocol: N/A

Filename: Bin1_plateA1_C8.LMD

Split 1: Petrol shading

Split 2: Green shading

Split 3: Violet shading

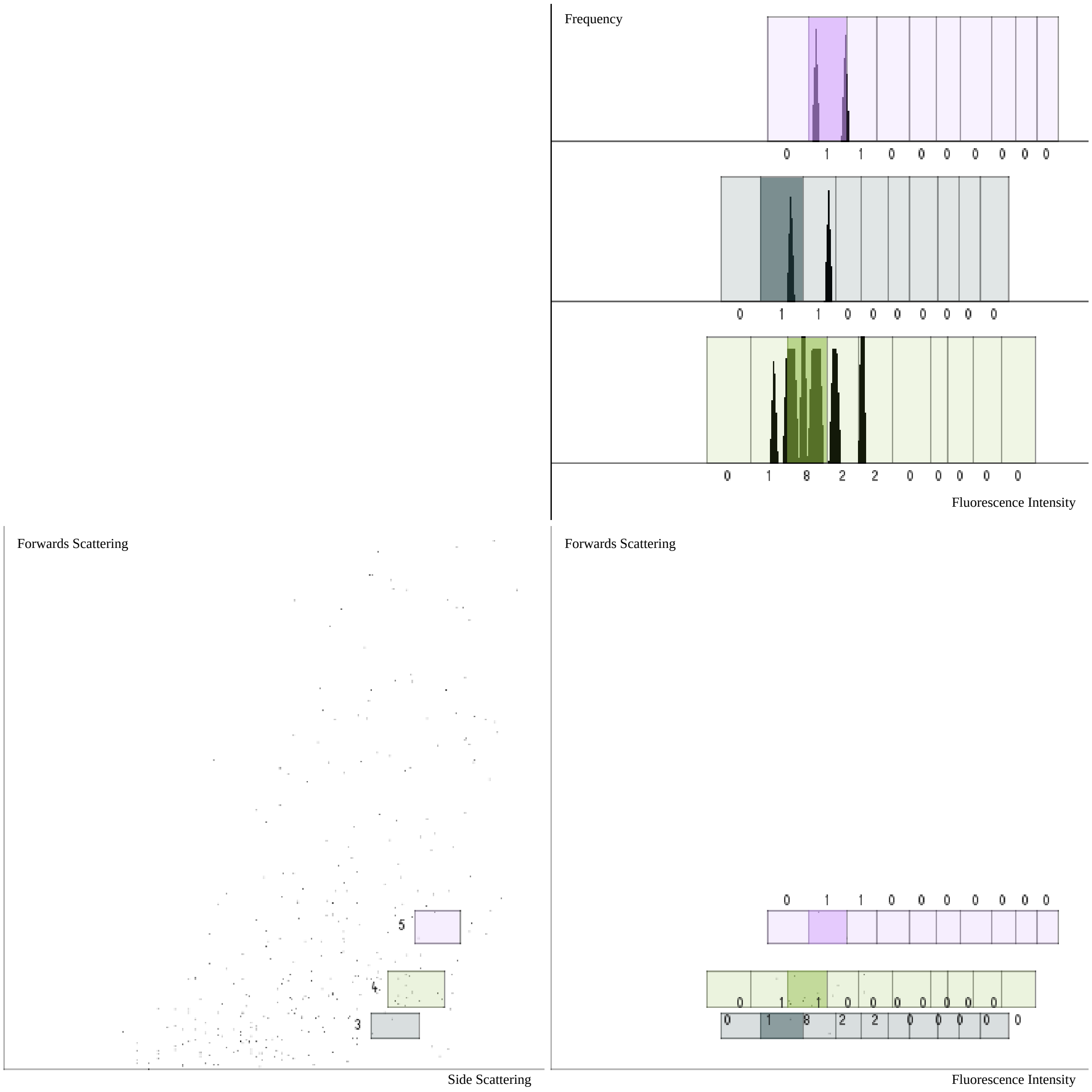

ANNEX 3: TAG DECONVOLUTION - BEAD 88

Passes flow sorting criteria: Yes

Passes tag deconvolution criteria: Yes

Included in protocol analysis: Yes

Protocol: 4, 9, 4, 2

Filename: Bin2_plateA1_B9.LMD

Split 1: Petrol shading

Split 2: Green shading

Split 3: Violet shading

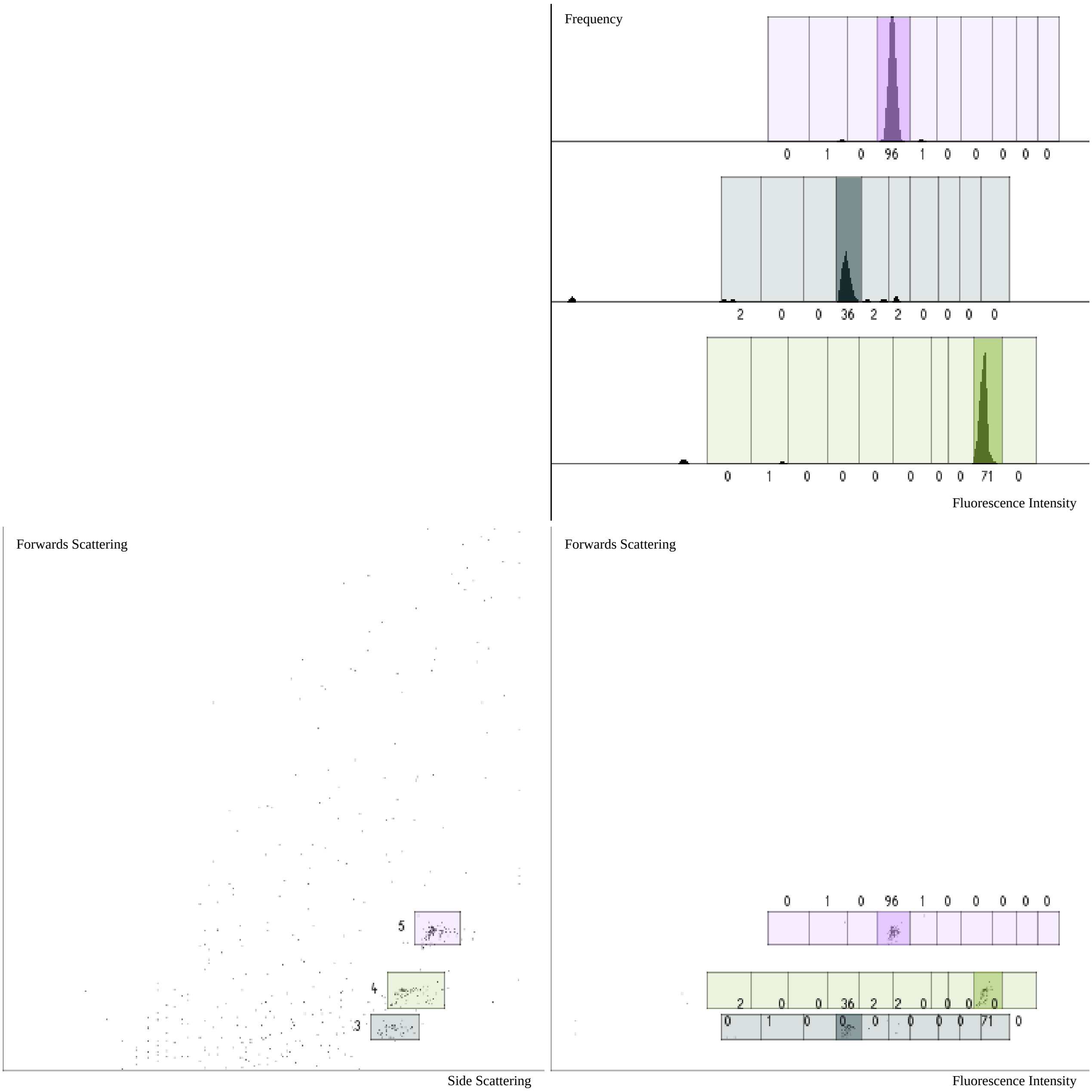

ANNEX 3: TAG DECONVOLUTION - BEAD 89

Passes flow sorting criteria: Yes

Passes tag deconvolution criteria: Yes

Included in protocol analysis: Yes

Protocol: 10, 8, 4, 2

Filename: Bin2_plateA1_B10.LMD

Split 1: Petrol shading

Split 2: Green shading

Split 3: Violet shading

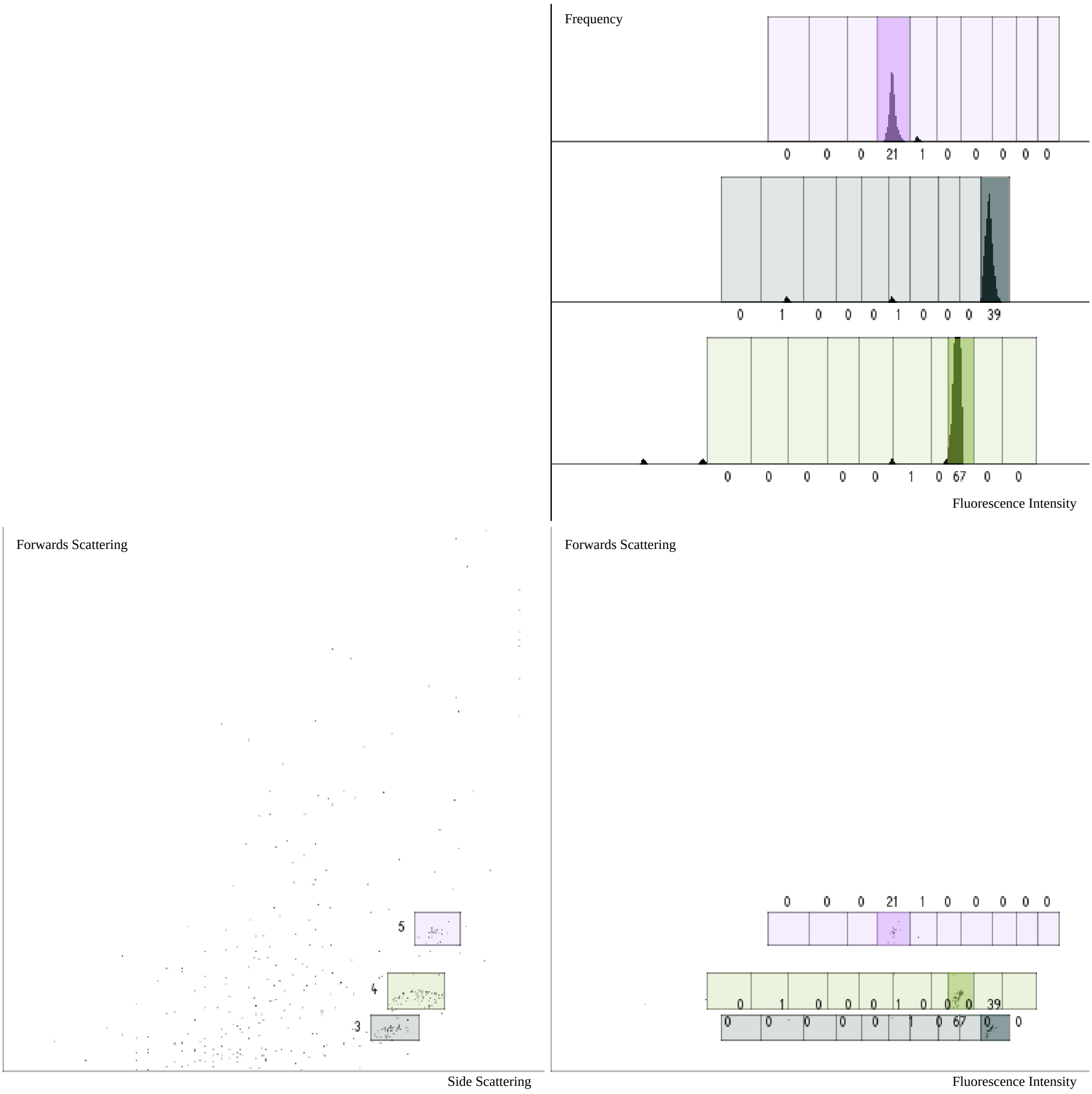

ANNEX 3: TAG DECONVOLUTION - BEAD 90

Passes flow sorting criteria: Yes

Passes tag deconvolution criteria: Yes

Included in protocol analysis: Yes

Protocol: 1, 2, 4, 2

Filename: Bin2_plateA1_B11.LMD

Split 1: Petrol shading

Split 2: Green shading

Split 3: Violet shading

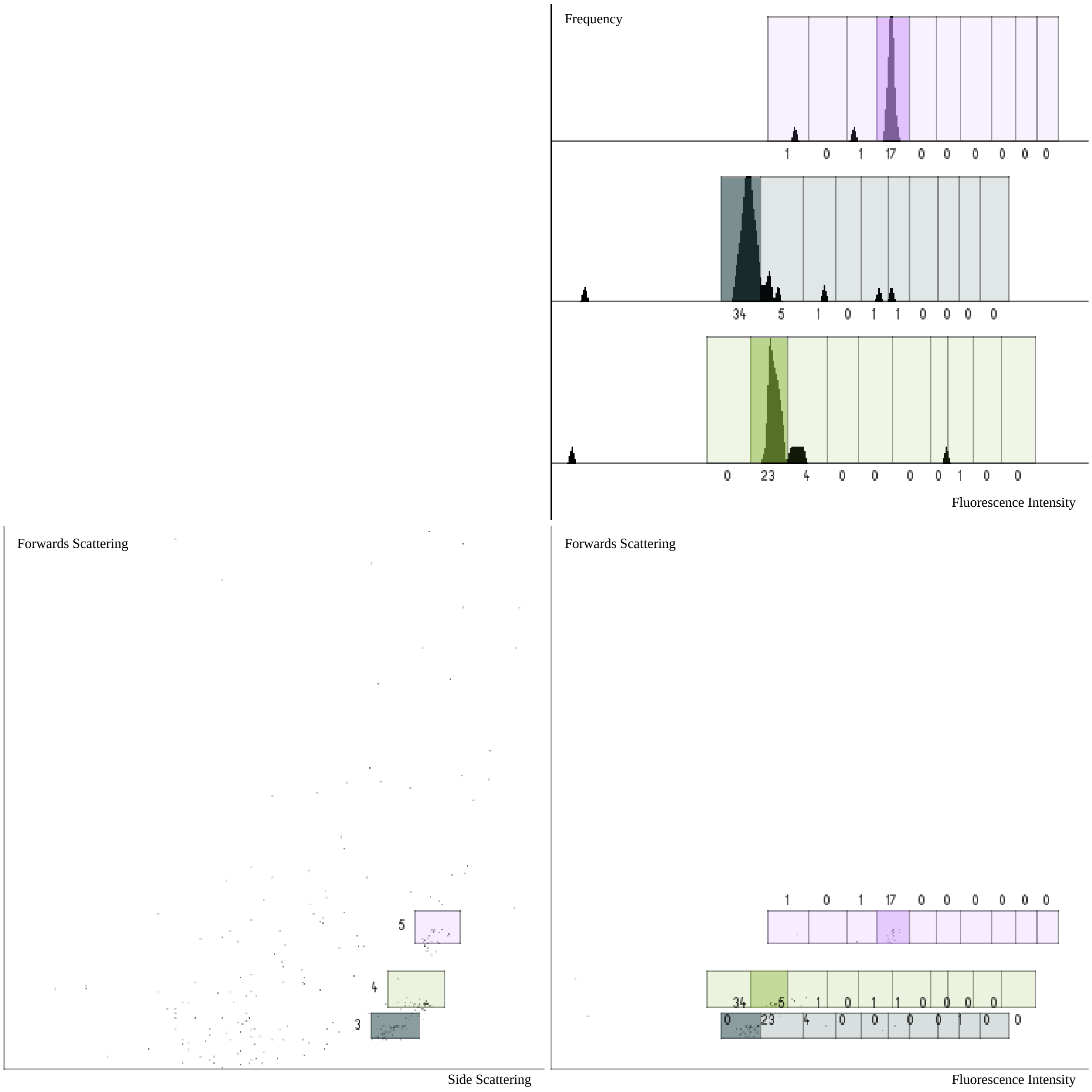

ANNEX 3: TAG DECONVOLUTION - BEAD 91

Passes flow sorting criteria: Yes

Passes tag deconvolution criteria: Yes

Included in protocol analysis: Yes

Protocol: 5, 5, 1, 2

Filename: Bin2_plateA1_B12.LMD

Split 1: Petrol shading

Split 2: Green shading

Split 3: Violet shading

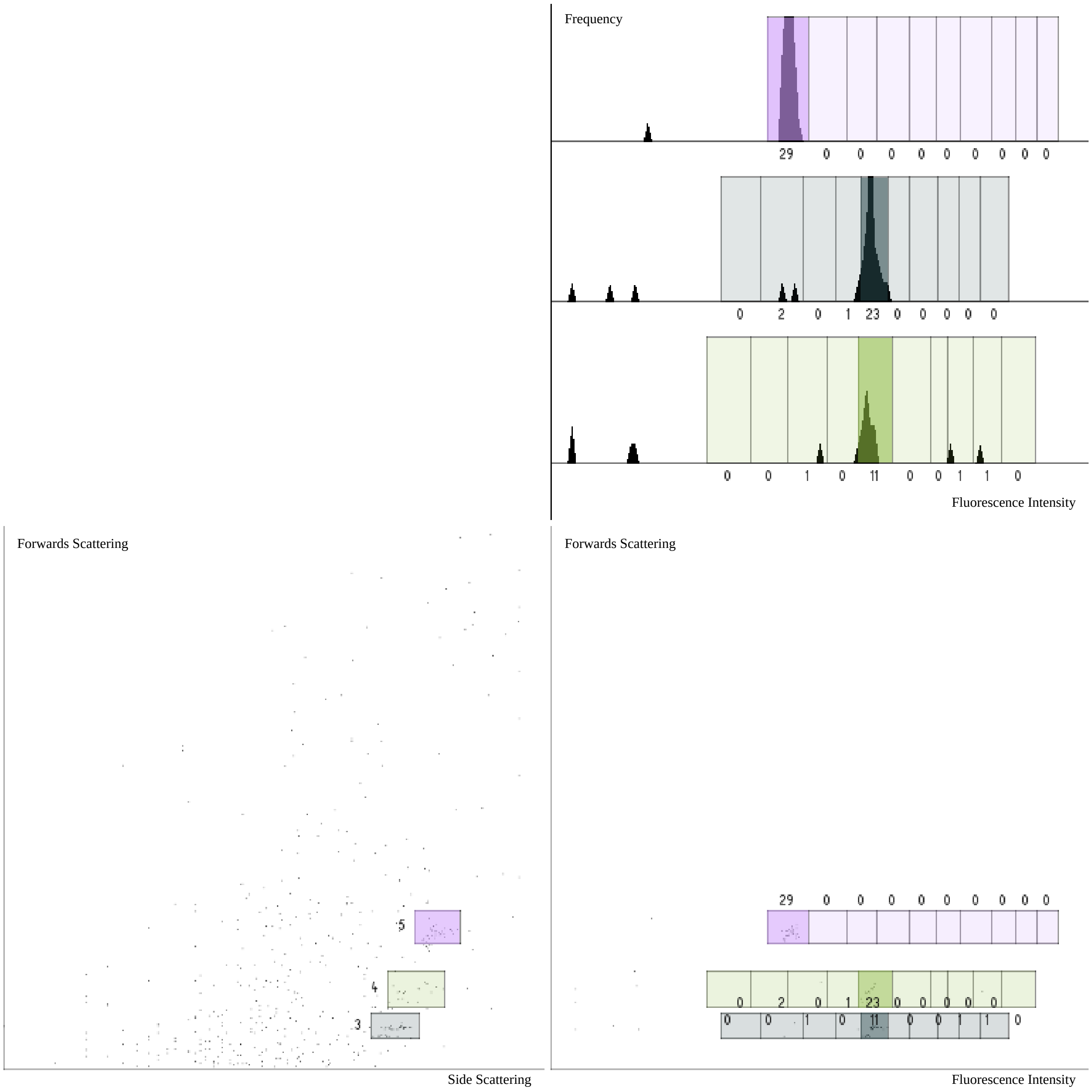

ANNEX 3: TAG DECONVOLUTION - BEAD 92

Passes flow sorting criteria: Yes

Passes tag deconvolution criteria: Yes

Included in protocol analysis: Yes

Protocol: 10, 9, 8, 2

Filename: Bin2_plateA1_C1.LMD

Split 1: Petrol shading

Split 2: Green shading

Split 3: Violet shading

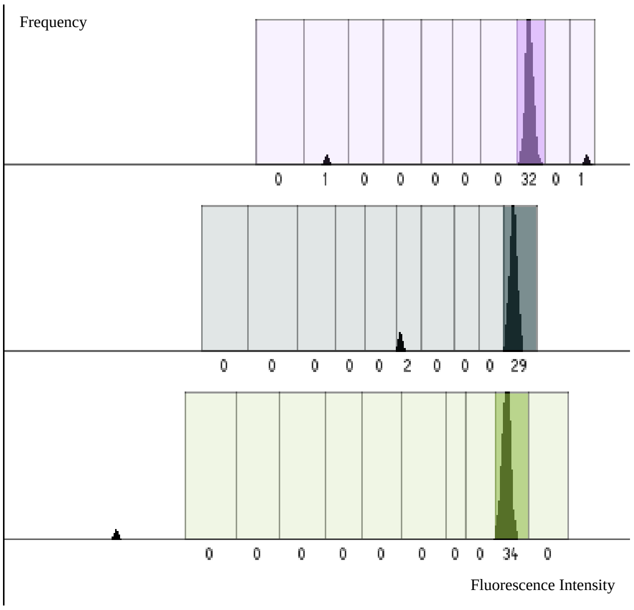

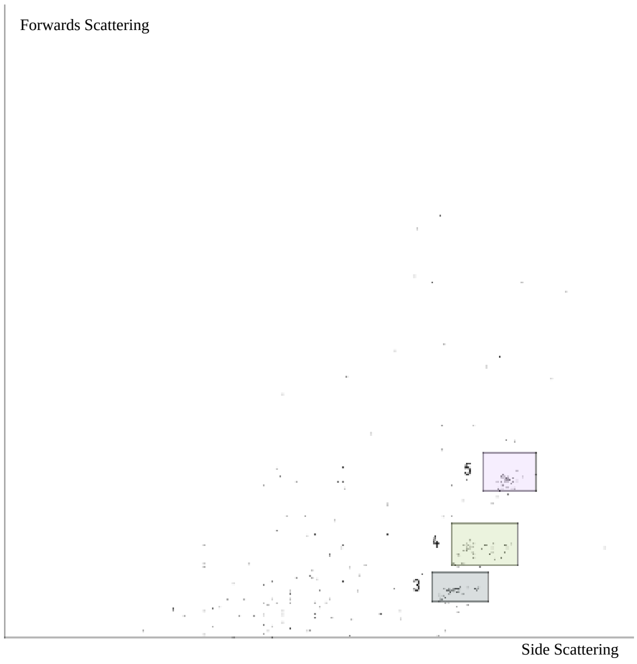

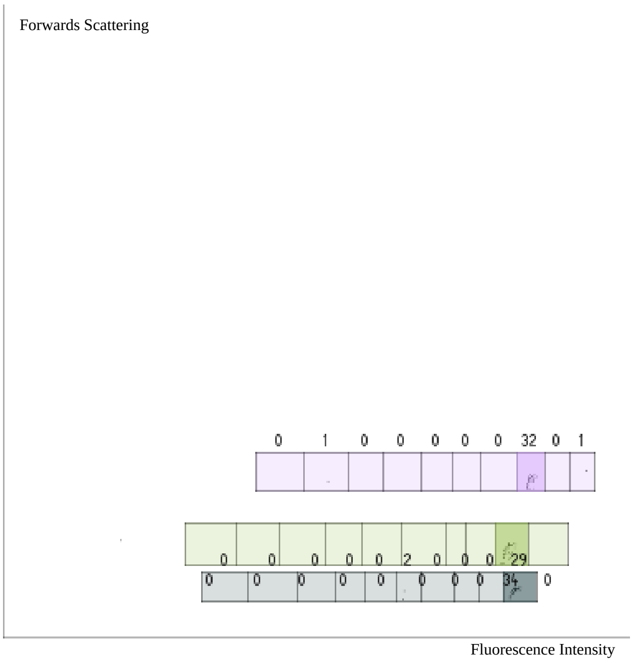

ANNEX 3: TAG DECONVOLUTION - BEAD 93

Passes flow sorting criteria: Yes

Passes tag deconvolution criteria: Yes

Included in protocol analysis: Yes

Protocol: 7, 6, 9, 2

Filename: Bin2_plateA1_C2.LMD

Split 1: Petrol shading

Split 2: Green shading

Split 3: Violet shading

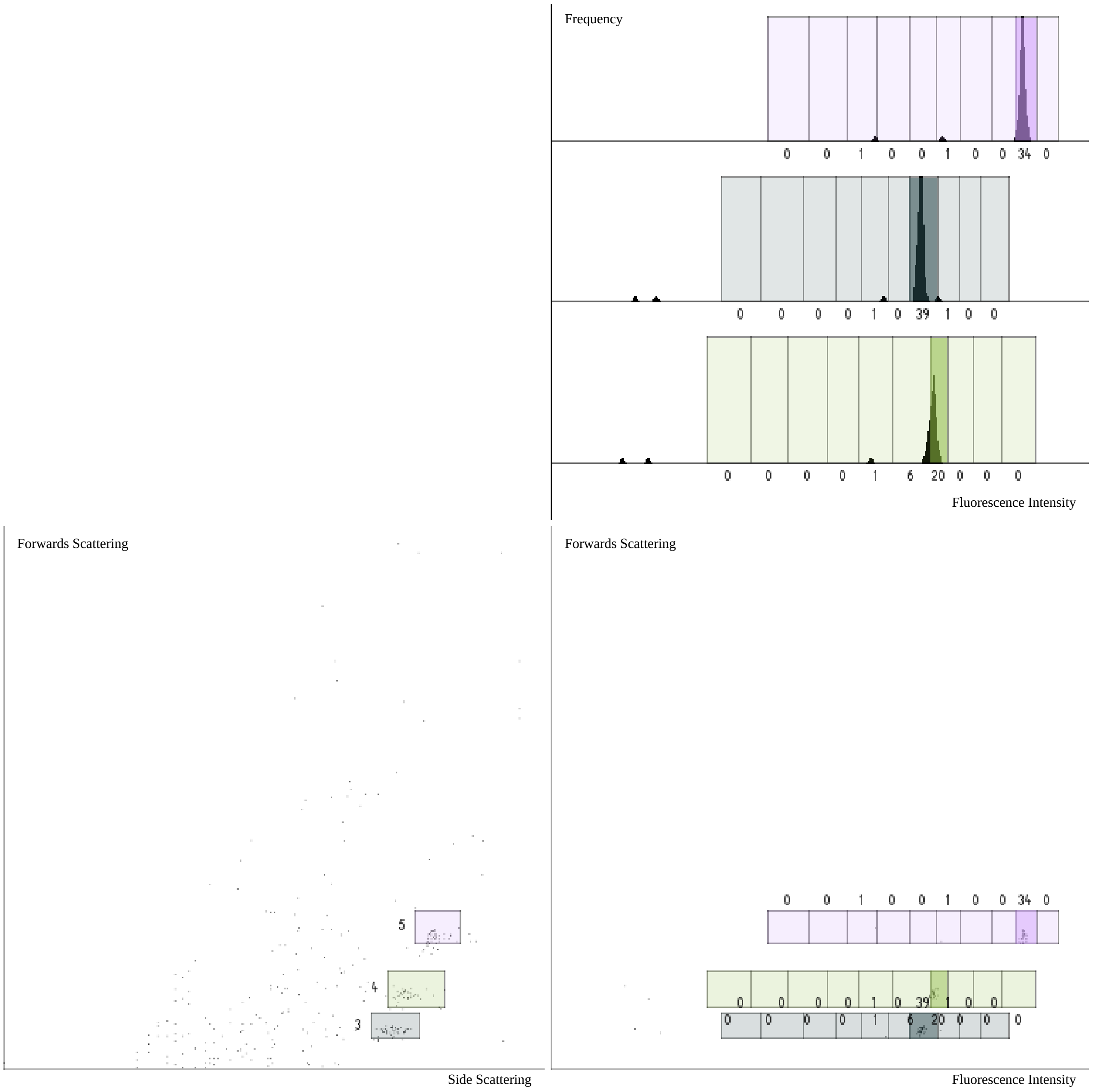

ANNEX 3: TAG DECONVOLUTION - BEAD 94

Passes flow sorting criteria: Yes

Passes tag deconvolution criteria: Yes

Included in protocol analysis: Yes

Protocol: 3, 1, 2, 2

Filename: Bin2_plateA1_C3.LMD

Split 1: Petrol shading

Split 2: Green shading

Split 3: Violet shading

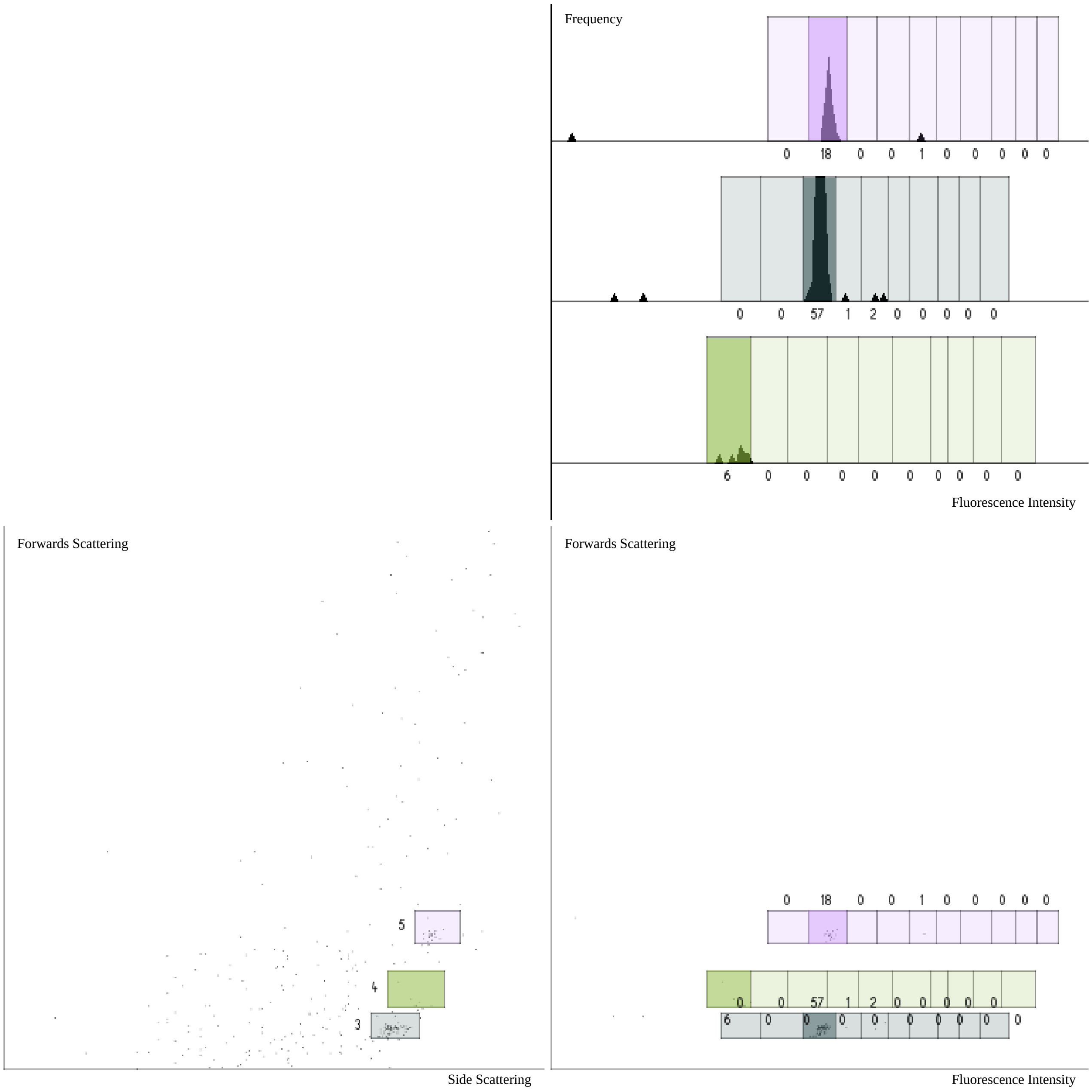

ANNEX 3: TAG DECONVOLUTION - BEAD 95

Passes flow sorting criteria: Yes

Passes tag deconvolution criteria: No

Included in protocol analysis: No

Protocol: N/A

Filename: Bin2_plateA1_C4.LMD

Split 1: Petrol shading

Split 2: Green shading

Split 3: Violet shading

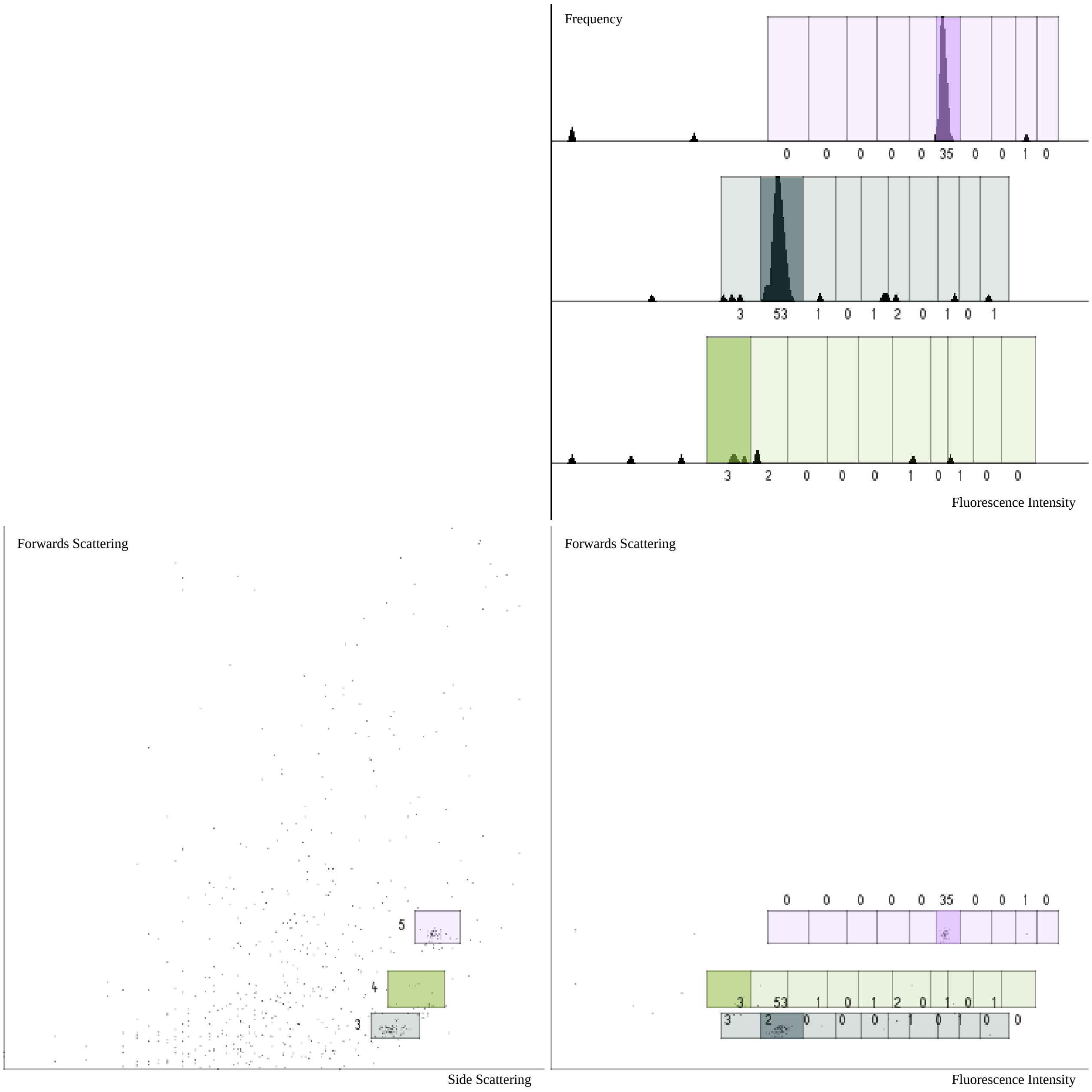

ANNEX 3: TAG DECONVOLUTION - BEAD 96

Passes flow sorting criteria: Yes

Passes tag deconvolution criteria: Yes

Included in protocol analysis: Yes

Protocol: 2, 9, 4, 2

Filename: Bin2_plateA1_C5.LMD

Split 1: Petrol shading

Split 2: Green shading

Split 3: Violet shading

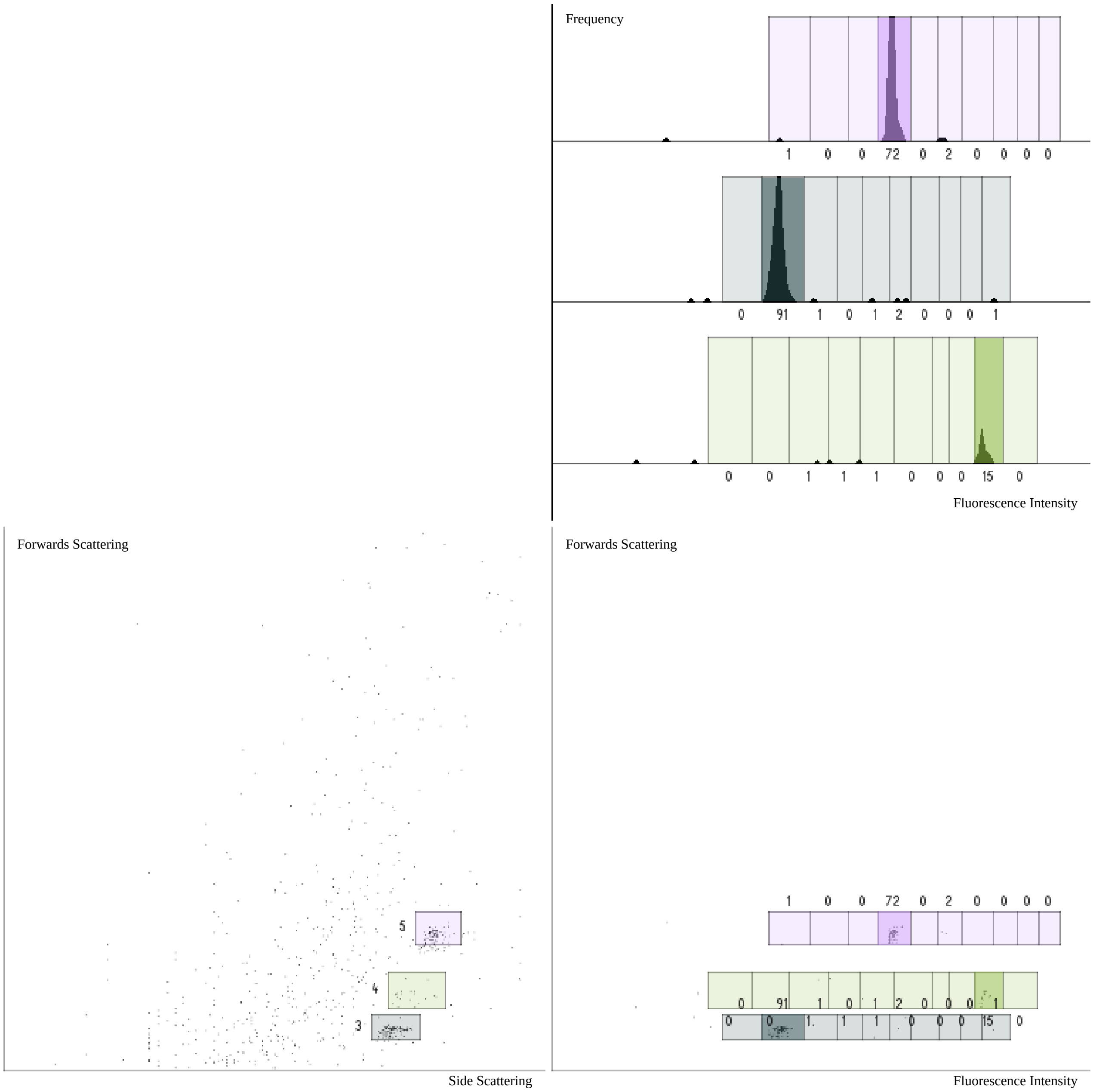

ANNEX 3: TAG DECONVOLUTION - BEAD 97

Passes flow sorting criteria: Yes

Passes tag deconvolution criteria: Yes

Included in protocol analysis: Yes

Protocol: 10, 6, 5, 2

Filename: Bin2_plateA1_C6.LMD

Split 1: Petrol shading

Split 2: Green shading

Split 3: Violet shading

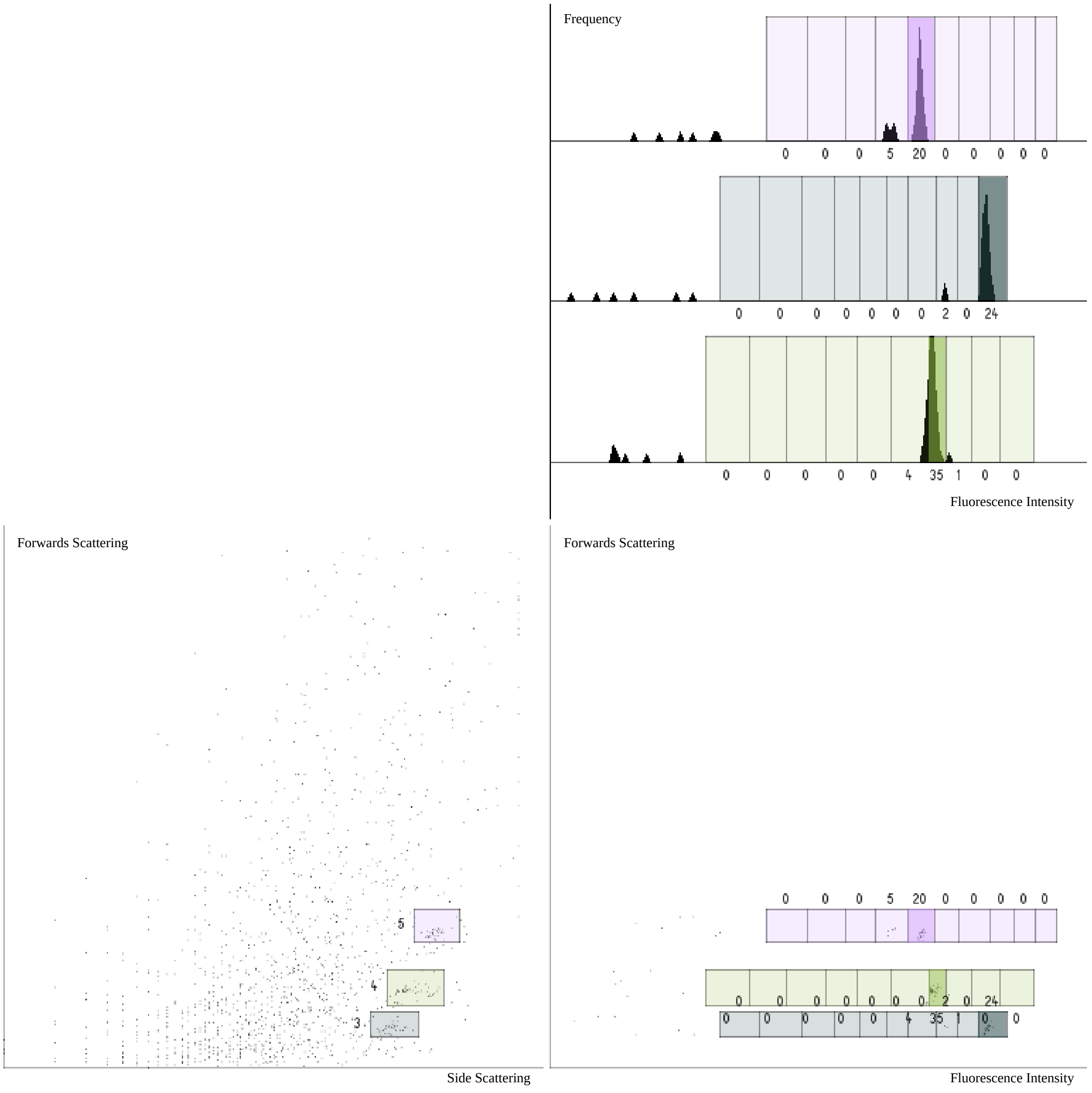

ANNEX 3: TAG DECONVOLUTION - BEAD 98

Passes flow sorting criteria: Yes

Passes tag deconvolution criteria: Yes

Included in protocol analysis: Yes

Protocol: 8, 2, 4, 2

Filename: Bin2_plateA1_C7.LMD

Split 1: Petrol shading

Split 2: Green shading

Split 3: Violet shading

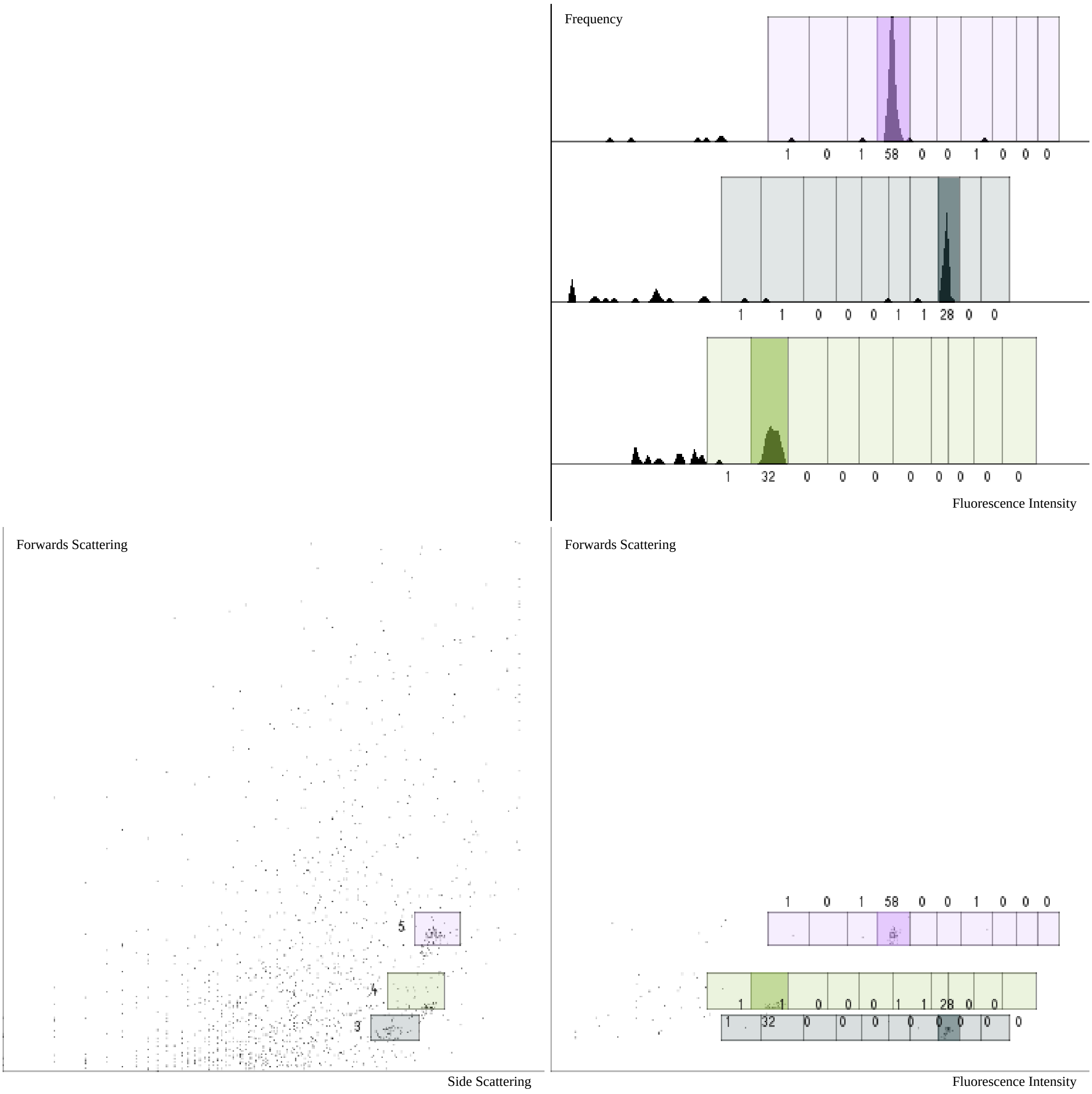

ANNEX 3: TAG DECONVOLUTION - BEAD 99

Passes flow sorting criteria: Yes

Passes tag deconvolution criteria: Yes

Included in protocol analysis: Yes

Protocol: 7, 2, 3, 2

Filename: Bin2_plateA1_C8.LMD

Split 1: Petrol shading

Split 2: Green shading

Split 3: Violet shading

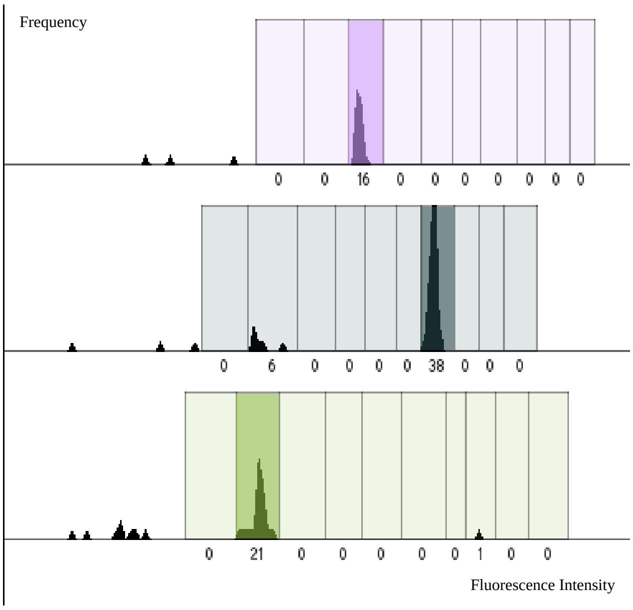

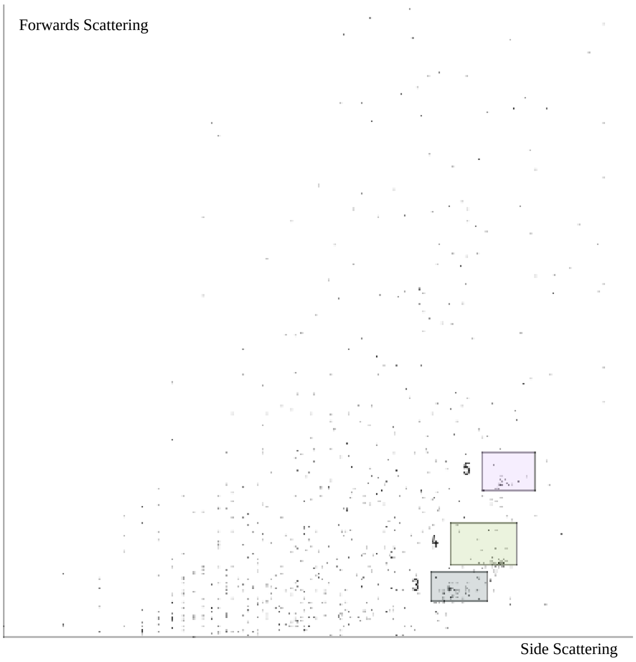

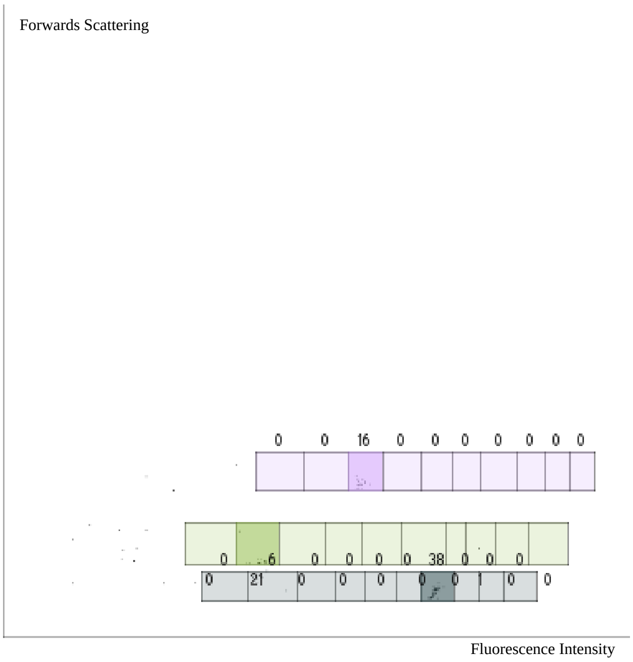

ANNEX 3: TAG DECONVOLUTION - BEAD 100

Passes flow sorting criteria: Yes

Passes tag deconvolution criteria: Yes

Included in protocol analysis: Yes

Protocol: 7, 3, 1, 2

Filename: Bin2_plateA1_C9.LMD

Split 1: Petrol shading

Split 2: Green shading

Split 3: Violet shading

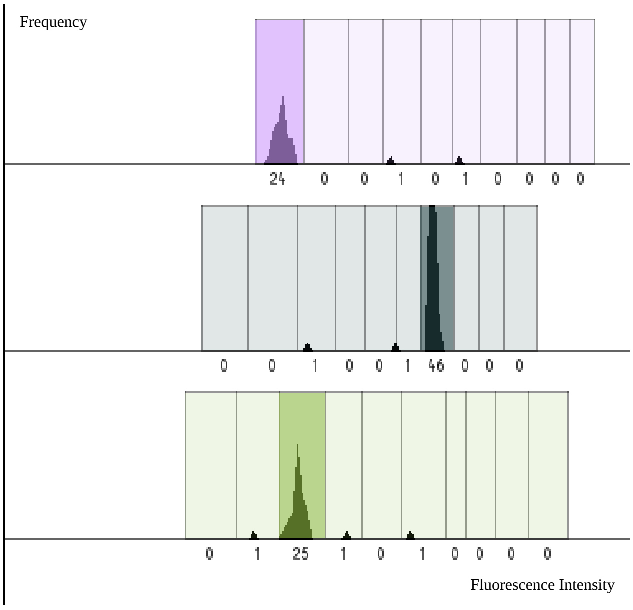

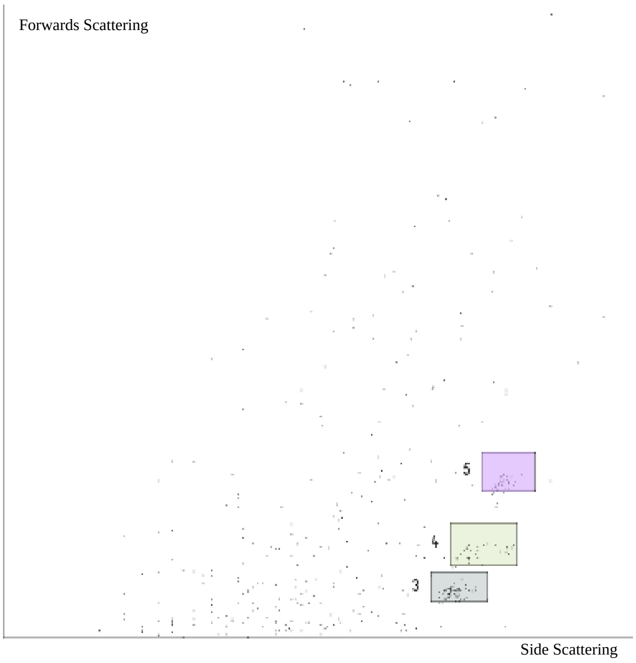

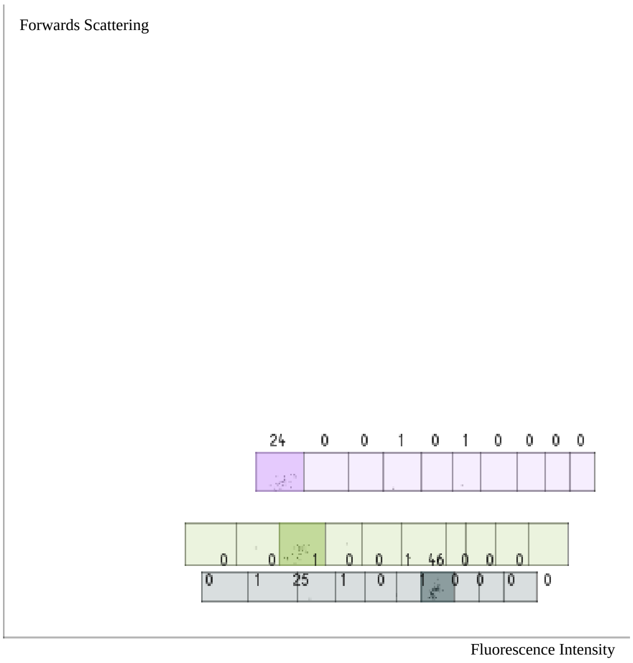

Supplement: Data S2 — Ariadne report for CombiCult screen 2: neuroectodermal precursors from mES cells. (PDF) [file pone.0104301.s017.pdf]
