# Supplementary material for: Directed Differentiation of Embryonic Stem Cells Using a Bead-Based Combinatorial Screening Method
Source: PLoS One. 2014 Sep 24;9(9):e104301. doi: 10.1371/journal.pone.0104301 (PMC4174505; doi:10.1371/journal.pone.0104301)

Ariadne™

Experiment 2 - CombiCult™ screen for Tyrosine Hydroxylase positive neurons using mES

Plasticell

EXECUTIVE SUMMARY

OBJECTIVE

- The objective of this CombiCult^{TM} study was to identify novel and/or efficient serum-free protocols for the differentiation of mouse embryonic stem cells (mESC) to dopaminergic neurons.

COMBICULT^{TM} EXPERIMENT

- 40 cell culture media were tested in 10,000 combinations.
- Approximately 480,000 beads were seeded with mES46C Sox1-GFP cells (Stem Cell Sciences).
- The experimental matrix comprised four split-pool cycles, performed on days 1, 7,14 and 21.
- 10 media were assayed in each cycle, resulting in an experimental complexity of 10x10x10x10 = 10,000 media combinations.
- On completion of cell culture (day 28) all beads were screened using an immunocytochemistry assay for the enzyme tyrosine hydroxylase, beads were isolated by COPAS.

RESULTS

- 622 hits (0.36% of monomeric beads) were verified following inspection by microscopy.
- The cell culture history of 399 (64 % of hits) could be deduced unambiguously.
- The 399 hits were derived from 378 distinct putative differentiation protocols.
- Protocols were ranked and selected for validation using bespoke bioinformatics software, Ariadne^{TM} (v1.0).
- The selected 23 validation protocols are listed in Table 1.

Table 1: The23 protocols selected for validation as by Ariadne™ (v1.0). Protocols are identified by the series of cell culture media that resulted in differentiation.

Validation Number	Protocol	Bead Ids		Basal Medium	Supplements
1	5-9-5-1	126, 135, 173	Split 1 (Day 1)	RHB-A	-
			Split 2 (Day 7)	DMEM/F12	2mM Glutamine, 1X ITS suppl., 100ng/mL FGF8b, 500ng/mL mrShh.
			Split 3 (Day 15)	RHB-A	-
			Split 4 (Day 21)	DMEM/F12	2mM Glutamine, 1X N2 suppl. , 200µM ascorbic acid, 1mg/mL laminin
2	9-9-5-1	1	Split 1 (Day 1)	DMEM/F12	2mM Glutamine,1% BSA, 1X N2suppl., 20µM SB431542.
			Split 2 (Day 7)	DMEM/F12	2mM Glutamine, 1X ITS suppl., 100ng/mL FGF8b, 500ng/mL mrShh.
			Split 3 (Day 15)	RHB-A	-
			Split 4 (Day 21)	DMEM/F12	2mM Glutamine, 1X N2 suppl. , 200µM ascorbic acid, 1mg/mL laminin
3	5-9-10-1	71	Split 1 (Day 1)	RHB-A	-
			Split 2 (Day 7)	DMEM/F12	2mM Glutamine, 1X ITS suppl., 100ng/mL FGF8b, 500ng/mL mrShh.
			Split 3 (Day 15)	DMEM/F12	2mM Glutamine, 1X N2 suppl., 20ng/mL bFGF, 20ng/mL EGF, 2ng/mL LIF.
			Split 4 (Day 21)	DMEM/F12	2mM Glutamine, 1X N2 suppl. , 200µM ascorbic acid, 1mg/mL laminin
4	5-10-5-1	46, 180	Split 1 (Day 1)	RHB-A	-
			Split 2 (Day 7)	DMEM/F12	2mM Glutamine, 1X ITS suppl., 200µM ascorbic acid, 20ng/mL bFGF, 10ng/mL BDNF 100ng/mL FGF8b, 1µM purmorphamine
			Split 3 (Day 15)	RHB-A	-
			Split 4 (Day 21)	DMEM/F12	2mM Glutamine, 1X N2 suppl. , 200µM ascorbic acid, 1mg/mL laminin
5	5-9-9-1	26	Split 1 (Day 1)	RHB-A	-
			Split 2 (Day 7)	DMEM/F12	2mM Glutamine, 1X ITS suppl., 100ng/mL FGF8b, 500ng/mL mrShh.
			Split 3 (Day 15)	Advanced DMEM	2mM Glutamine
			Split 4 (Day 21)	DMEM/F12	2mM Glutamine, 1X N2 suppl. , 200µM ascorbic acid, 1mg/mL laminin
6	5-4-5-1	40	Split 1 (Day 1)	RHB-A	-
			Split 2 (Day 7)	DMEM/F12	2mM Glutamine, 1X ITS suppl., 0.5µM retinoic acid.
			Split 3 (Day 15)	RHB-A	-
			Split 4 (Day 21)	DMEM/F12	2mM Glutamine, 1X N2 suppl., 200µM ascorbic acid, 1mg/mL laminin.
7	4-9-5-1	183	Split 1 (Day 1)	DMEM/F12	2mM Glutamine,1% BSA, 1X N2 suppl., 1X B27 neuromix.
			Split 2 (Day 7)	DMEM/F12	2mM Glutamine, 1X ITS suppl., 100ng/mL FGF8b, 500ng/mL mrShh.
			Split 3 (Day 15)	RHB-A	-
			Split 4 (Day 21)	DMEM/F12	2mM Glutamine, 1X N2 suppl. , 200µM ascorbic acid, 1mg/mL laminin
8	6-8-1-1	8, 128, 134	Split 1 (Day 1)	KO DMEM	10% KSR, 1mM Glutamine
			Split 2 (Day 7)	Advanced DMEM	2mM Glutamine
			Split 3 (Day 15)	DMEM/F12	2mM Glutamine, 1X ITS suppl., 20ng/mL bFGF.

Confidential

Validation Number	Protocol	Bead Ids		Basal Medium	Supplements
			Split 4 (Day 21)	DMEM/F12	2mM Glutamine, 1X N2 suppl. , 200µM ascorbic acid, 1mg/mL laminin
9	6-9-1-1	163	Split 1 (Day 1)	KO DMEM	10% KSR, 1mM Glutamine
			Split 2 (Day 7)	DMEM/F12	2mM Glutamine, 1X ITS suppl., 100ng/mL FGF8b, 500ng/mL mrShh.
			Split 3 (Day 15)	DMEM/F12	2mM Glutamine, 1X ITS suppl., 20ng/mL bFGF.
			Split 4 (Day 21)	DMEM/F12	2mM Glutamine, 1X N2 suppl., 200µM ascorbic acid, 1mg/mL laminin
10	1-5-2-1	166, 171	Split 1 (Day 1)	KO DMEM	15% FBS, 2mM Glutamine, 0.1mM β-mercaptoethanol, 1X NEAA, 1000 Units/mL LIF (standard growth media for mES cells).
			Split 2 (Day 7)	RHB-A	-
			Split 3 (Day 15)	DMEM/F12	2mM Glutamine, 1X ITS suppl., 20ng/mL bFGF, 1mg/mL laminin
			Split 4 (Day 21)	DMEM/F12	2mM Glutamine, 1X N2 suppl. , 200µM ascorbic acid, 1mg/mL laminin
11	1-5-2-4	284, 299	Split 1 (Day 1)	KO DMEM	15% FBS, 2mM Glutamine, 0.1mM β-mercaptoethanol, 1X NEAA, 1000 Units/mL LIF (standard growth media for mES cells).
			Split 2 (Day 7)	RHB-A	-
			Split 3 (Day 15)	DMEM/F12	2mM Glutamine, 1X ITS suppl., 20ng/mL bFGF, 1mg/mL laminin
			Split 4 (Day 21)	DMEM/F12	2mM Glutamine, 1X ITS suppl., 1µM cAMP, 10ng/mL hrBDNF
12	1-5-2-9	443	Split 1 (Day 1)	KO DMEM	15% FBS, 2mM Glutamine, 0.1mM β-mercaptoethanol, 1X NEAA, (standard growth media for mES cells), 1000 Units/mL LIF
			Split 2 (Day 7)	RHB-A	-
			Split 3 (Day 15)	DMEM/F12	2mM Glutamine, 1X ITS suppl., 20ng/mL bFGF, 1mg/mL laminin
			Split 4 (Day 21)	Advanced DMEM	2mM Glutamine
13	4-6-7-1	77, 139	Split 1 (Day 1)	DMEM/F12	2mM Glutamine, 1% BSA, 1X N2 suppl., 1X B27 neuromix.
			Split 2 (Day 7)	KO DMEM	10% KSR, 2mM Glutamine.
			Split 3 (Day 15)	DMEM/F12	2mM Glutamine, 1X ITS suppl., 1X B27 suppl., 50ng/mL mr Dkk1
			Split 4 (Day 21)	DMEM/F12	2mM Glutamine, 1X N2 suppl. , 200µM ascorbic acid, 1mg/mL laminin
14	7-9-2-9	438, 517	Split 1 (Day 1)	DMEM/F12	2mM Glutamine, 1% BSA, 1X ITS suppl., 20ng/mL bFGF
			Split 2 (Day 7)	DMEM/F12	2mM Glutamine, 1X ITS suppl., 100ng/mL FGF8b, 500ng/mL mrShh.
			Split 3 (Day 15)	DMEM/F12	2mM Glutamine, 1X ITS suppl., 20ng/mL bFGF, 1mg/mL laminin
			Split 4 (Day 21)	Advanced DMEM	2mM Glutamine
15	5-9-2-9	488	Split 1 (Day 1)	RHB-A	-
			Split 2 (Day 7)	DMEM/F12	2mM Glutamine, 1X ITS suppl., 100ng/mL FGF8b, 500ng/mL mrShh.
			Split 3 (Day 15)	DMEM/F12	2mM Glutamine, 1X N2 suppl., 20ng/mL bFGF, 1mg/mL laminin
			Split 4 (Day 21)	Advanced DMEM	2mM Glutamine
16	5-9-2-1	121	Split 1 (Day 1)	RHB-A	-
			Split 2 (Day 7)	DMEM/F12	2mM Glutamine, 1X ITS suppl., 100ng/mL FGF8b, 500ng/mL mrShh.
			Split 3 (Day 15)	DMEM/F12	2mM Glutamine, 1X N2 suppl., 20ng/mL bFGF, 1mg/mL laminin
			Split 4 (Day 21)	DMEM/F12	2mM Glutamine, 1X N2 suppl. , 200µM ascorbic acid, 1mg/mL laminin

Confidential

Validation Number	Protocol	Bead Ids		Basal Medium	Supplements
17	3-1-10-1	48, 159	Split 1 (Day 1)	Advanced DMEM	2mM Glutamine
			Split 2 (Day 7)	DMEM/F12	2mM Glutamine, 1X ITS suppl., 5mg/mL fibronectin.
			Split 3 (Day 15)	DMEM/F12	2mM Glutamine, 1X N2 suppl., 20ng/mL bFGF, 20ng/mL EGF, 2ng/mL LIF.
			Split 4 (Day 21)	DMEM/F12	2mM Glutamine, 1X N2 suppl. , 200µM ascorbic acid, 1mg/mL laminin
18	9-10-10-1	172	Split 1 (Day 1)	DMEM/F12	2mM Glutamine,1% BSA, 1X N2suppl., 20mM SB431542.
			Split 2 (Day 7)	DMEM/F12	2mM Glutamine, 1X ITS suppl., 200mM ascorbic acid, 20ng/mL bFGF, 10ng/mL BDNF 100ng/mL FGF8b, 1µM purmorphamine
			Split 3 (Day 15)	DMEM/F12	2mM Glutamine, 1X N2 suppl., 20ng/mL bFGF, 20ng/mL EGF, 2ng/mL LIF.
			Split 4 (Day 21)	DMEM/F12	2mM Glutamine, 1X N2 suppl. , 200mM ascorbic acid, 1mg/mL laminin
19	9-2-10-1	113	Split 1 (Day 1)	DMEM/F12	2mM Glutamine,1% BSA, 1X N2suppl., 20µM SB431542.
			Split 2 (Day 7)	DMEM/F12	2mM Glutamine, 1X ITS suppl., 100ng/mL mr Noggin.
			Split 3 (Day 15)	DMEM/F12	2mM Glutamine, 1X N2 suppl., 20ng/mL bFGF, 20ng/mL EGF, 2ng/mL LIF.
			Split 4 (Day 21)	DMEM/F12	2mM Glutamine, 1X N2 suppl. , 200mM ascorbic acid, 1mg/mL laminin
20	9-4-6-1	81, 108	Split 1 (Day 1)	DMEM/F12	2mM Glutamine,1% BSA, 1X N2suppl., 20µM SB431542.
			Split 2 (Day 7)	DMEM/F12	2mM Glutamine, 1X ITS suppl., 0.5µM retinoic acid.
			Split 3 (Day 15)	KO DMEM	10% KSR, 2mM Glutamine
			Split 4 (Day 21)	DMEM/F12	2mM Glutamine, 1X N2 suppl. , 200µM ascorbic acid, 1mg/mL laminin
21	5-10-2-2	186	Split 1 (Day 1)	RHB-A	-
			Split 2 (Day 7)	DMEM/F12	2mM Glutamine, 1X ITS suppl., 200µM ascorbic acid, 20ng/mL bFGF, 10ng/mL BDNF 100ng/mL FGF8b, 1µM purmorphamine
			Split 3 (Day 15)	DMEM/F12	2mM Glutamine, 1X ITS suppl., 20ng/mL bFGF, 1mg/mL laminin
			Split 4 (Day 21)	DMEM/F12	2mM Glutamine, 0.5% FBS, 1X N2 suppl., 20ng/mL hr GDNF, 20ng/mL hr BDNF
22	5-10-9-9	476, 508	Split 1 (Day 1)	RHB-A	-
			Split 2 (Day 7)	DMEM/F12	2mM Glutamine, 1X ITS suppl., 200µM ascorbic acid, 20ng/mL bFGF, 10ng/mL BDNF 100ng/mL FGF8b, 1µM purmorphamine
			Split 3 (Day 15)	Advanced DMEM	2mM Glutamine
			Split 4 (Day 21)	Advanced DMEM	2mM Glutamine
23	5-10-6-1	141	Split 1 (Day 1)	RHB-A	-
			Split 2 (Day 7)	DMEM/F12	2mM Glutamine, 1X ITS suppl., 200µM ascorbic acid, 20ng/mL bFGF, 10ng/mL BDNF 100ng/mL FGF8b, 1µM purmorphamine
			Split 3 (Day 15)	KO DMEM	10% KSR, 2mM Glutamine
			Split 4 (Day 21)	DMEM/F12	2mM Glutamine, 1X N2 suppl. , 200µM ascorbic acid, 1mg/mL laminin

CONTENTS

Executive Summary

Report Nomenclature

1. Introduction

2. Study Setup

3. Split-Pool Experiment
3.1 Cell Culture Media
3.2 Tag Assignment

4. Bead Screening
4.1 Screening Assay
4.2 Flow Sorting of ‘Hit’ Beads

5. Tag Deconvolution

6. Protocol Analysis
6.1 Dataset Review
6.2 Linkage Analysis
6.3 Fingerprint Analysis
6.4 Hierarchical Clustering Dendrogram
6.5 Similarity Matrix

7. Summary and Conclusions

Annex 1: Flow sorting scatter plots

Annex 2: Tag reference set and calibrated selection gates for each tag identification session

Annex 3: Tag identification scatter plots and histograms for each hit

GLOSSARY

Screening matrix refers to the arrangement and identity of media in the split-pool experiment

Experimental complexity is the total number of combinations of media tested, or the number of different protocols tested

Hit is a bead which scores positive in the screening assay and is sorted by COPAS

COPAS is a large particle flow sorter manufactured by Union Bioimetrica Inc.

Deconvolution is the inference of cell culture history by tag analysis

Bin refers to the sum of beads in each final cell culture medium

Session refers to the FACS analysis of tags from a group of hits. Prior to each session a reference tag set was run to calibrate side and forward scatter, and fluorescence intensity gates

Fingerprint analysis is a method of finding and comparing hits derived from identical protocols and groups of beads with similar protocols

Probability (of occurrence of a group in the fingerprint analysis) is the probability of that cluster of protocols occurring by chance, assuming beads sample all protocols randomly

Hierarchical clustering is a hierarchy of protocol clusters, represented in a dendrogram

Similarity matrix is a diagrammatical representation of a pair-wise comparison of all protocols

DPBS ++ is Dulbecco's modified phosphate buffer saline with calcium chloride and magnesium chloride.

Confidential

1. INTRODUCTION

COMBICULT™ TECHNOLOGY

CombiCult™ is a proprietary bead-based combinatorial technology specifically developed for discovery of novel stem cell differentiation protocols. Stem cells on beads are exposed to multiple combinations of media, containing active agents such as growth factors or small molecules. The optimal combinations for effective differentiation can be deduced reliably, rapidly and cost effectively.

Figure 1: CombiCult^{TM} technology

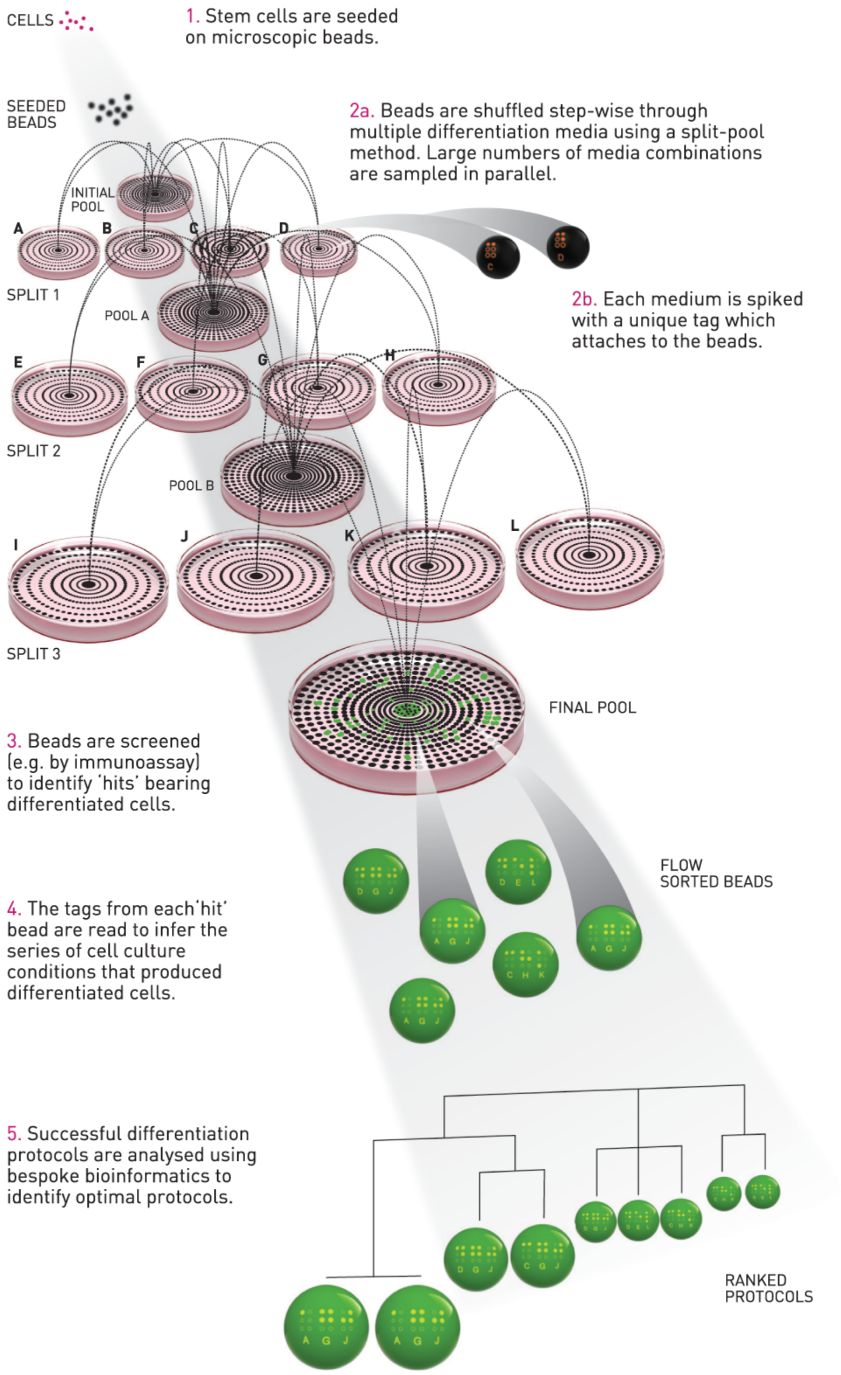

1. INTRODUCTION CONTINUED

OBJECTIVE

The objective of this CombiCult^{TM} study was to identify efficient and/or novel protocols for the differentiation of mouse embryonic stem cells (mESC) to dopaminergic neurons.

STUDY

The experimental matrix comprised 40 cell culture media distributed over four split-pool cycles (splits) such that a total of 10,000 media combinations (protocols) were tested. Below is a depiction of the experimental design (Figure 2) showing the timing and number of media in each split.On completion of the cell culture phase, on day 28, beads were screened using immunocytochemistry assay to identify differentiated cells.

Figure 2: Schematic diagram of the experimental matrix.

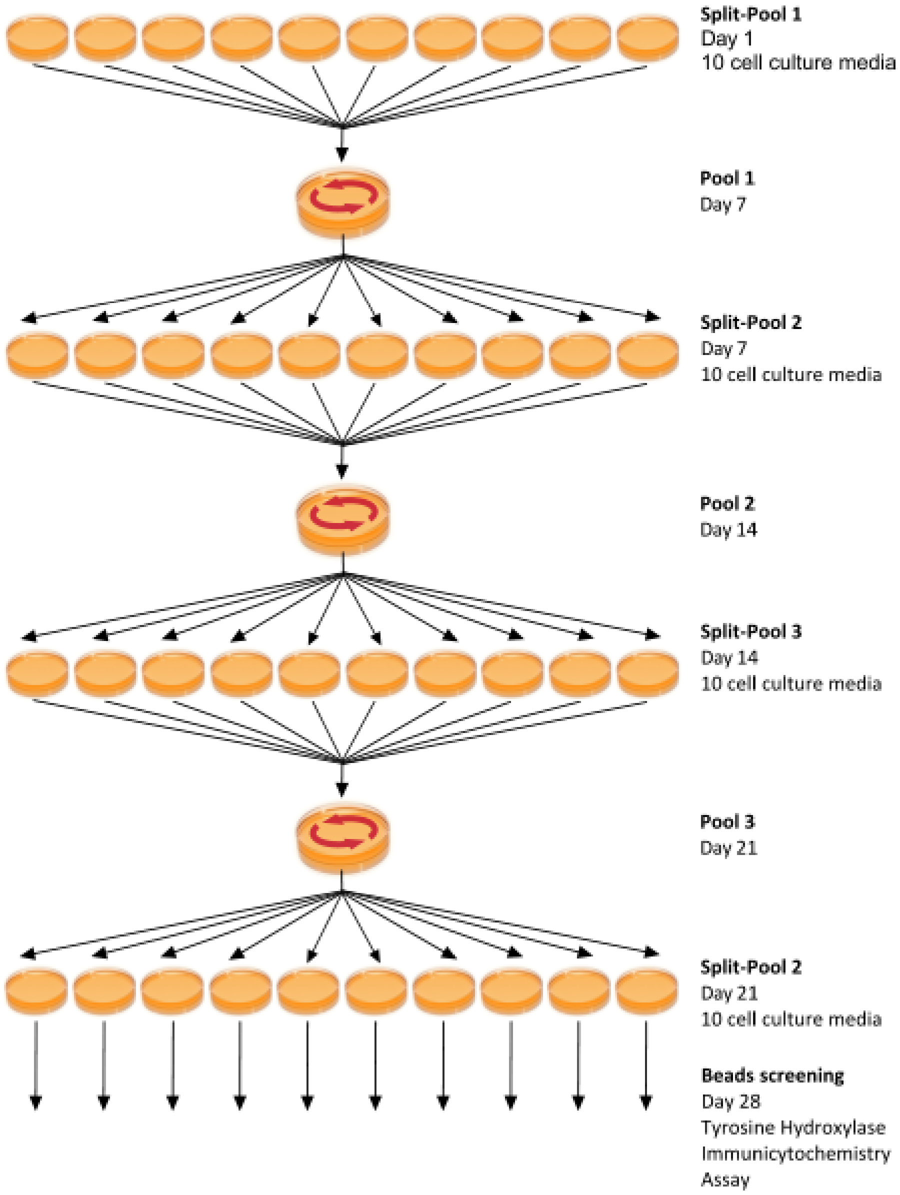

 Confidential

2. STUDY SETUP

OVERVIEW

Study title:	mES neuronal experiment #1
Study start/finish dates:	03-March-10/20-April-10

SPLIT-POOL EXPERIMENT

Scientist Name:	Dr Diana Hernandez
Start/Finish Dates:	03-Mar-10/31-Mar-10
Cell Line:	mESC 46C Sox1-GFP cells (Stem Cell Sciences)
Split 1 Date:	04-Mar-10 (Day1)
Split 2 Date:	10-Mar-10 (Day 7)
Split 3 Date:	17-Mar-10 (Day 14)
Split 4 Date:	24-Mar -10 (Day 21)
Differentiation end Date:	31-Mar-10 (Day 28)
Tagging Date - Split 1:	09-Mar-10
Tagging Date - Split 2:	12-Mar-10
Tagging Date - Split 3:	17-Mar-10
Tagging Date - Split 4:	N/A
Number of cell culture media combinations tested:	10,000
Ratio of beads to complexity:	48:1
Total number of beads:	480,000
Ratio of cells to beads during seeding:	30:1
Additional notes:	None

BEAD SCREENING & SORTING

Scientist name:	Dr Diana Hernandez
Start/finish dates:	31-Mar-10/20-Apr-10
Screening assay:	Rb Anti-TH antibody (Millipore) + Alexa Fluor 594 anti-Rb (Invitrogen)
Screening date:	20-Apr-10
Flow sorting instrument:	COPAS PLUS, Union Biometrica
Sorting PMT channel(s):	Red (615/45 nm) optical emission filters
Positive setup control:	N/A
Total number of sorted beads	622
Number of verified hits	622
Additional notes:	None

Confidential

TAG DECONVOLUTION

**Bead Digestion**	
Scientist name:	Dr Christopher Johnson
Start/finish dates:	21-May-10/18-Jul-11
Additional notes:	None
**Tag Analysis**	
Scientist name:	Dr Christopher Johnson
Start/finish dates:	26-May-10/22-Jul-10
Tag analysis flow cytometry instrument:	BD FACSCanto II
Analysis PMT channel(s) optical filter:	710/50 nm band pass
Number of analysis sessions:	21
Additional notes:	None

PROTOCOL ANALYSIS & REPORT GENERATION

Software:	Ariadne™ version 1.0
User name:	Dr Christopher Johnson
Date, Time:	24-Jun-11, 16.00

3. SPLIT-POOL EXPERIMENT

3.1 Cell Culture Media

Table 2: Compositions of the cell culture media tested in this study.

Split	Medium ID	Basal Media	Supplements
1	1	KO DMEM	15% FBS, 2mM Glutamine, 0.1mM β-mercaptoethanol, 1X NEAA, 1000 Units/mL LIF(standard growth media for mES cells),
1	2	DMEM	10% FBS, 2mM Glutamine, 1X NEAA
1	3	Advanced DMEM	2mM Glutamine
1	4	DMEM/F12	2mM Glutamine,1% BSA, 1X N2 suppl., 1X B27 neuromix.
1	5	RHB-A	
1	6	KO DMEM	10% KSR, 2mM Glutamine
1	7	DMEM/F12	2mM Glutamine, 1% BSA, 1X ITS suppl., 20ng/mL bFGF
1	8	DMEM/F12	2mM Glutamine,1% BSA, 1X N2 suppl., 100ng/mL mr Noggin.
1	9	DMEM/F12	2mM Glutamine, 1% BSA, 1X N2 suppl., 20μM SB431542.
1	10	DMEM/F12	2mM Glutamine, 1% BSA, 1X ITS suppl., 0.5μM retinoic acid.
2	1	DMEM/F12	2mM Glutamine, 1X ITS suppl., 5μg/mL fibronectin.
2	2	DMEM/F12	2mM Glutamine, 1X ITS suppl., 100ng/mL mr Noggin.
2	3	DMEM/F12	2mM Glutamine, 1X ITS suppl., 20ng/mL bFGF.
2	4	DMEM/F12	2mM Glutamine, 1X ITS suppl., 0.5μM retinoic acid.
2	5	RHB-A	
2	6	KO DMEM	10% KSR, 2mM Glutamine
2	7	DMEM/F12	2mM Glutamine, 1X ITS suppl., 1X B27 neuromix, 20ng/mL bFGF, 50ng/mL mr Dkk1
2	8	Advanced DMEM	2mM Glutamine
2	9	DMEM/F12	2mM Glutamine, 1X ITS suppl., 100ng/mL FGF8b, 500ng/mL mrShh.
2	10	DMEM/F12	2mM Glutamine, 1X ITS suppl., 200μM ascorbic acid, 20ng/mL bFGF, 10ng/mL BDNF 100ng/mL FGF8b, 1μM purmorphamine
3	1	DMEM/F12	2mM Glutamine, 1X ITS suppl., 20ng/mL bFGF.
3	2	DMEM/F12	2mM Glutamine, 1X ITS suppl., 20ng/mL bFGF, 1μg/mL laminin
3	3	DMEM/F12	2mM Glutamine, 1X N2 suppl., 1mg/mL laminin, 100ng/mL FGF8b, 500ng/mL mrShh,
3	4	Neurobasal media	2mM Glutamine, 1 X ITS suppl., 1mg/mL laminin, 200 μM ascorbic acid, 10ng/mL BDNF, 10ng/mL GDNF,
3	5	RHB-A	
3	6	KO DMEM	10% KSR, 2mM Glutamine
3	7	DMEM/F12	2mM Glutamine, 1X ITS suppl., 1X B27 suppl., 50ng/mL mr Dkk1
3	8	Neurobasal media	2mM Glutamine, 1X B27 neuromix., 20ng/mL bFGF
3	9	Advanced DMEM	2mM Glutamine
3	10	DMEM/F12	2mM Glutamine, 1X N2 suppl., 20ng/mL bFGF, 20ng/mL EGF, 2ng/mL LIF.
4	1	DMEM/F12	2mM Glutamine, 1X N2 suppl. , 200μM ascorbic acid, 1mg/mL laminin
4	2	DMEM/F12	2mM Glutamine, 0.5% FBS, 1X N2 suppl., 20ng/mL hr GDNF, 20ng/mL hr BDNF

Confidential

Split	Medium ID	Basal Media	Supplements
4	3	DMEM/F12	2mM Glutamine, 1X N2 suppl., 200μM ascorbic acid, 20ng/mL hr BDNF
4	4	DMEM/F12	2mM Glutamine, 1X ITS suppl., 0.5μM cAMP, 10ng/mL hrBDNF
4	5	RHB-A	
4	6	KO DMEM	10% KSR, 2mM Glutamine
4	7	Neurobasal media	2mM Glutamine, 1XB27 neuromix.
4	8	DMEM/F12	2mM Glutamine, 1X ITS suppl., 20ng/mL bFGF.
4	9	Advanced DMEM	2mM Glutamine
4	10	DMEM/F12	2mM Glutamine, 1X ITS suppl., 10ng/mL NT3

3. SPLIT-POOL

3.1 CELL CULTURE MEDIA CONTINUED

Table 3: Suppliers of basal media and supplements.

Basal Media/Supplement	Supplier	Product Reference
Advanced DMEM	Life Technologies	12491-015
DMEM/F12	Life Technologies	21041-025
FBS	SLI	EU-000-F
Glutamine	Life Technologies	25030-024
NEAA	Life Technologies	11140035
β-mercaptoethanol	Sigma Aldrich	M7522
KO DMEM	Life Technologies	10829-018
KSR	Life Technologies	10828-028
Neurobasal	Life Technologies	21103-049
RHB-A	Stem Cells Inc.	SCS-SF-NB-01
Ascorbic Acid	Sigma Aldrich	A4403
B27 Neuromix	PAA	F01-002
BSA	Life Technologies	A10008-01
DKK-1	R and D Systems	1765-DK-010
EGF	R and D Systems	2028-EG-200
FGF-8b	R and D Systems	423-F8-025
bFGF	R and D Systems	233-FB-025
Fibronectin	R and D Systems	1918-FN-02M
ITS	Sigma Aldrich	I3146
LIF-ESGRO	Millipore	ESG1106
N2 Supplement	PAA	F005-004
Noggin	R and D Systems	719-NG-050
SB431542	Sigma Aldrich	S4317
Retinoic Acid	Sigma Aldrich	R2625
SHH	R and D Systems	461-SH-025
TGF-β1	R and D Systems	4114-TC-01M
Purmorphamine	Calbiochem	540220
BDNF	R and D Systems	248-BD-005
GDNF	R and D Systems	212-GD-010
Laminin	Sigma Aldrich	L2020
cAMP	Sigma Aldrich	A9501
NT3	R and D Systems	267-N3-005

Confidential

3. SPLIT-POOL

3.2. TAG ASSIGNMENT

Each cell culture medium (except media in the last split-pool cycle) was spiked with a unique fluorescent tag that attaches to PTC5000 beads. Tag readout from individual beads enables determination of the series of cell culture media to which the bead was exposed. Thirty unique populations of tags were used, which differ in diameter and fluorescence intensity (ten gradations). Table 4 shows the tag code, size, fluorescence colour, fluorescence intensity, sample lot number, tag stock solution concentration and volume used, to spike each cell culture medium.

Table 4: The tags used to spike different cell culture media.

Split	Medium ID	Tag code	Tag size	Tag fluorescence colour	Tag fluorescence intensity level	Stock solution concentration (% w/v)	V of stock solution added to media (µL)
1	1	MR01	Medium	Red	1	0.5	220
1	2	MR02	Medium	Red	2	0.5	220
1	3	MR03	Medium	Red	3	0.5	220
1	4	MR04	Medium	Red	4	0.5	220
1	5	MR05	Medium	Red	5	0.5	220
1	6	MR06	Medium	Red	6	0.5	220
1	7	MR07	Medium	Red	7	0.5	220
1	8	MR08	Medium	Red	8	0.5	220
1	9	MR09	Medium	Red	9	0.5	220
1	10	MR10	Medium	Red	10	0.5	220
2	1	SR01	Small	Red	1	0.5	111
2	2	SR02	Small	Red	2	0.5	111
2	3	SR03	Small	Red	3	0.5	111
2	4	SR04	Small	Red	4	0.5	111
2	5	SR05	Small	Red	5	0.5	111
2	6	SR06	Small	Red	6	0.5	111
2	7	SR07	Small	Red	7	0.5	111
2	8	SR08	Small	Red	8	0.5	111
2	9	SR09	Small	Red	9	0.5	111
2	10	SR10	Small	Red	10	0.5	111
3	1	LR01	Large	Red	1	0.5	440
3	2	LR02	Large	Red	2	0.5	440
3	3	LR03	Large	Red	3	0.5	440
3	4	LR04	Large	Red	4	0.5	440
3	5	LR05	Large	Red	5	0.5	440
3	6	LR06	Large	Red	6	0.5	440
3	7	LR07	Large	Red	7	0.5	440
3	8	LR08	Large	Red	8	0.5	440
3	9	LR09	Large	Red	9	0.5	440
3	10	LR10	Large	Red	10	0.5	440
4	1	No tag	-	-	-	-	-
4	2	No tag	-	-	-	-	-
4	3	No tag	-	-	-	-	-
4	4	No tag	-	-	-	-	-
4	5	No tag	-	-	-	-	-
4	6	No tag	-	-	-	-	-
4	7	No tag	-	-	-	-	-
4	8	No tag	-	-	-	-	-
4	9	No tag	-	-	-	-	-
4	10	No tag	-	-	-	-	-

4. BEAD SCREENING
4.1. SCREENING ASSAY

Following completion of the split-pool experiment at day 28, beads were screened using an immunocytochemistry assay for the enzyme tyrosine hydroxylase to identify hits populated with 46C mES cells which had differentiated into dopaminergic neurons.
In brief, beads were washed in DPBS , fixed in 4% paraformaldehyde, permeabilised with 0.25% Triton X-100 in DPBS for 20min at 25°C. Following permeabilisation, cells were incubated in blocking solution (0.25% Triton, 1% BSA in DPBS) for 30 min at 25°C and then incubated in the appropriate primary antibody diluted in blocking solution at 4°C overnight. Following primary antibody incubation, beads were washed three times in DPBS++, incubated in secondary antibody solution for two hours at 25°C, then washed three times in DPBS++ and resuspended in PBS before being sorted. Antibodies used: primary: Rb anti-tyrosine hydroxylase (Millipore), Secondary: Alexa Fluor 594 goat anti-rabbit IgG.

Figure 3: Shows a 'hit' in a background of negative beads (10x objective lens, scale bar = 100 μm). The image was obtained using a Nikon Eclipse TE2000-S fluorescent microscope with a TRITC filter (excitation 540/25 nm, emission 605/55 nm).

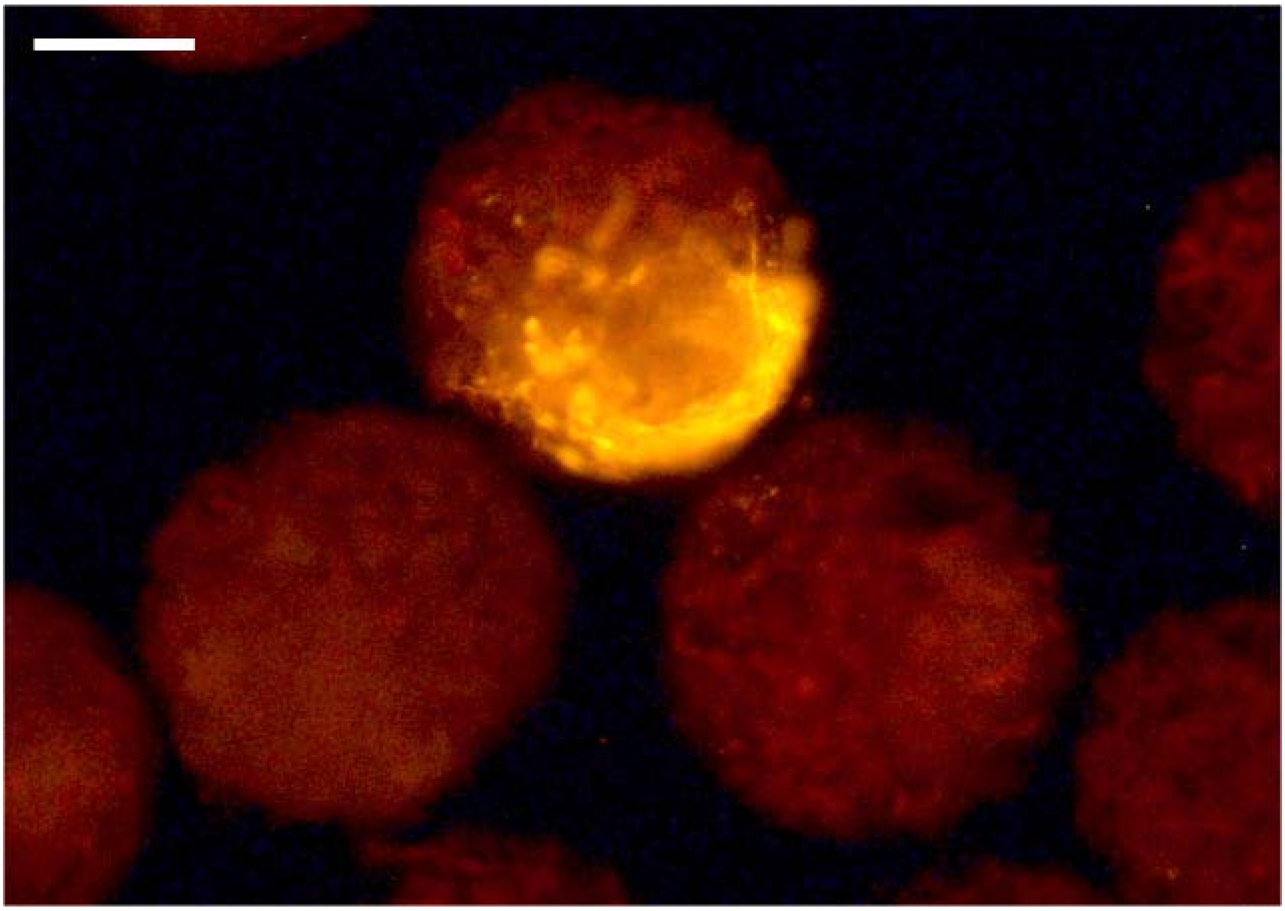

4. BEAD SCREENING

4.2. FLOW SORTING OF 'HIT' BEADS

Following the screening assay, beads were sorted using a large particle flow sorter (COPAS PLUS, Union Biometrica; Red PMT 615/45 nm optical emission filters). Beads from each final cell culture medium were labeled as bin 1-10. The data from each bin is contained in one or more flow sorting data files. Annex 1 shows dot plots from each flow sorting data file.
Beads were gated according to size (time of flight [TOF] and optical extinction [ext] values) to exclude bead aggregates that had formed during cell culture. Gated beads were sorted based on their fluorescence properties, as defined by the screening assay, and hits individually dispensed into wells of a 96-well plate.

In figure 4 COPAS data from all bins have been combined on two dot-plots to provide an overview of bead sorting. Figure 4(a) is a dot plot of time of Flight (TOF) vs. optical extinction (ext). Events falling within the gate defining monomeric beads are represented in red: in this experiment 175,062 beads (36.5 % of the starting number) were sorted. Figure 4(b) is a dot plot of TOF vs. red fluorescence intensity for all sorted beads. Beads with a red fluorescence intensity value above the threshold value (depicted as green dots in Figure 4b) were individually dispensed into 96 well plates.

Once dispensed, hits were verified by fluorescence microscopy. Table 5 lists the number of positive beads sorted and verified as 'hit' beads from each bin. A total of 622 positive beads were isolated all of which were verified as 'hit' beads (Table 5). Tags from the verified 'hits' were subsequently analysed to determine the cell culture history (i.e. differentiation protocol) of each 'hit' bead, as detailed in the next section of the report.

Figure 4(a) and (b): COPAS dot plots, showing (a) the gate used to select monomeric beads (red dots) and (b) the threshold fluorescence intensity used to select positive 'hit' beads (green dots). The dot plots contain combined data recorded from all bins.

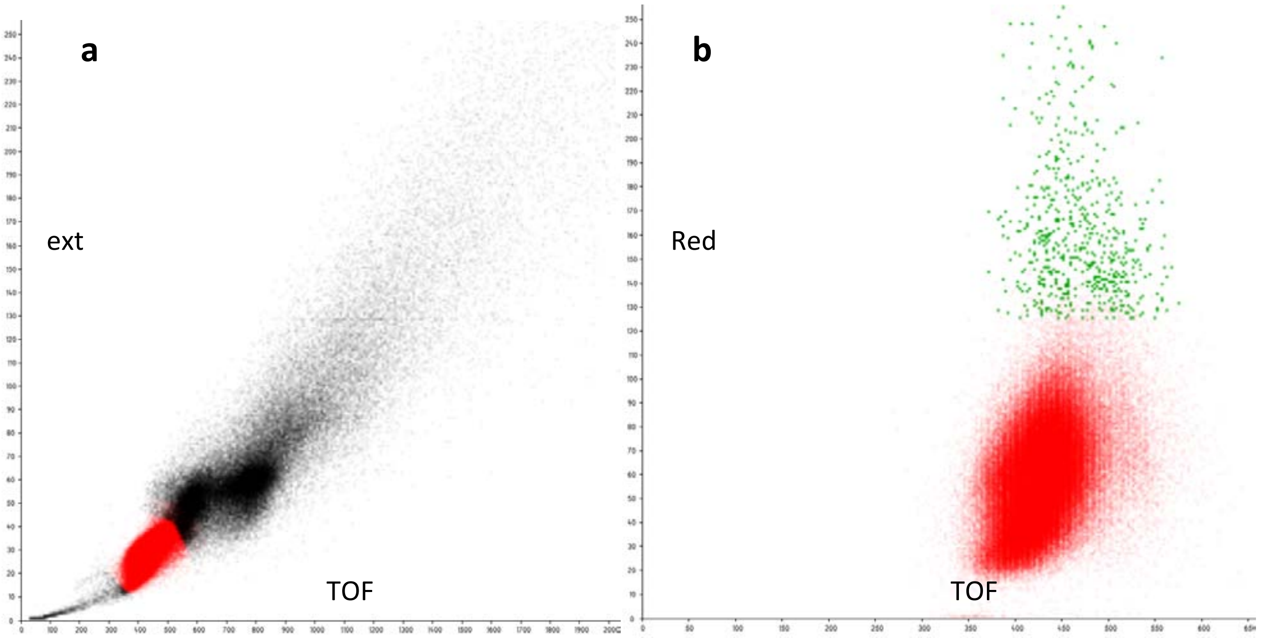

4. BEAD SCREENING

4.2. FLOW SORTING OF 'HITS' CONTINUED

Table 5: Number of positive beads sorted, number of verified 'hits' from each bin.

Bin Number	1	2	3	4	5	6	7	8	9	10	Total
Number of positive beads sorted	222	80	23	45	77	11	20	21	106	17	622
Number of verified 'hits'	222	80	23	45	77	11	20	21	106	17	622

5. TAG DECONVOLUTION

Following bead sorting, tags from each hit are analysed by flow cytometry and related to the cell culture history. During this process, data is lost or excluded owing to various factors and this is summarized in Table 6.

The flow cytometry data acquisition was performed in a series of sessions and prior to each session a reference tag set was run to calibrate side/forward scatter and fluorescence intensity gates. Dot plots and histograms for each session are shown in Annex 2. The data was loaded into Ariadne™ which automatically identifies tags based on four unique parameter values (forward and side scatter and fluorescence colour and intensity).

Determination of tag identity is based on the number of events (≥ 3) within a gate, and the cluster tightness (Table 7). If two or more clusters of events are identified which mapped to cell media from the same split and/or the signal to noise ratio was too low, an accurate identification is not recorded.

Ariadne™ relates the tag identity to a cell culture medium using the tag assignment (Table 4), allowing it to log the cell culture history of each hit (Table 8).

Table 6: Summary of data attrition owing to various factors.

Bin Number	1	2	3	4	5	6	7	8	9	10	Total
Number of verified 'hits'	222	80	23	45	77	11	20	21	106	17	622
Number of beads lost during digestion and sample preparation	(38)	(5)	(1)	(1)	(24)	(2)	(2)	0	(8)	(2)	(83)
Number of beads analysed by flow cytometry	184	75	22	44	53	9	18	21	98	15	539
Number of beads with no tagging data	(6)	0	0	0	(1)	(2)	(1)	(3)	(8)	(1)	(22)
Number of beads with incomplete tagging data	(17)	(4)	(3)	(7)	(3)	0	0	0	(1)	(4)	(39)
Number of beads containing too many tag clusters	(30)	(11)	(1)	(8)	(8)	0	(2)	(5)	(14)	0	(79)
Number of hits with conclusive tagging data	131	60	18	29	41	7	15	13	75	10	399

Confidential

Table 7: Summary of the minimum, maximum and average number of tags derived from beads in each of the cell culture media. This data demonstrates that the average number of tags for all media is well above the minimum required to link a cell culture medium to a bead (3), and only seven media were identified using the minimum number of tags.

Split	Medium ID	# of 'hit' beads per media	Minimum # of tags	Average # of tags	Maximum # of tags
1	1	47	4	16	106
1	2	35	19	80	205
1	3	41	20	100	402
1	4	35	24	117	242
1	5	54	7	34	86
1	6	37	10	76	237
1	7	38	23	86	183
1	8	30	19	80	177
1	9	38	7	92	282
1	10	44	16	106	246
2	1	22	7	37	117
2	2	27	6	29	96
2	3	25	10	40	91
2	4	32	6	32	115
2	5	61	4	56	178
2	6	54	4	50	147
2	7	31	8	59	177
2	8	32	9	27	86
2	9	60	15	81	234
2	10	55	11	83	215
3	1	49	5	31	129
3	2	51	4	47	242
3	3	39	7	42	170
3	4	17	6	63	223
3	5	46	4	59	204
3	6	40	4	84	279
3	7	40	5	70	411
3	8	22	19	56	118
3	9	53	7	49	230
3	10	42	5	43	157
4	1	132	No tag	-	-
4	2	60	No tag	-	-
4	3	18	No tag	-	-
4	4	29	No tag	-	-
4	5	41	No tag	-	-
4	6	7	No tag	-	-
4	7	15	No tag	-	-
4	8	13	No tag	-	-
4	9	75	No tag	-	-
4	10	10	No tag	-	-

Table 8: Information for each 'hit': i) the bead ID; ii) tag acquisition session number; iii) flow cytometry tag data file name; iv) series of cell culture media, i.e. protocol (number of tags upon which assignment of each cell culture media is based are in parentheses, "–" represents unreadable tag data) and v) whether the bead passes the tag deconvolution.

Bead ID	Tag acquisition session #	Tag data filename	Split 1 media (# of tags)	Split 2 media (# of tags)	Split 3 media (# of tags)	Split 4 media	Pass tag deconvolution criteria
1	1	Bin1_plateA0_H3.fcs	9 (282)	9 (175)	5 (109)	1	pass
2	1	Bin1_plateA0_A2.fcs	8 (72)	-	10 (26)	1	fail
3	1	Bin1_plateA0_A3.fcs	9 (37)	-	6 (43)	1	fail
4	1	Bin1_plateA0_B1.fcs	4 (159)	9 (28)	9 (47)	1	pass
5	1	Bin1_plateA0_B2.fcs	7 (99)	6 (131)	7 (88)	1	pass
6	1	Bin1_plateA0_B3.fcs	10 (111)	4 (23)	4 (121)	1	pass
7	1	Bin1_plateA0_C1.fcs	7 (51)	10 (74)	2 (20)	1	pass
8	1	Bin1_plateA0_C2.fcs	6 (112)	8 (36)	1 (129)	1	pass
9	1	Bin1_plateA0_C3.fcs	3 (127)	6 (147)	3 (82)	1	pass
10	1	Bin1_plateA0_D1.fcs	8 (177)	2 (29)	2 (34)	1	pass
11	1	Bin1_plateA0_D2.fcs	1 (32)	-	2 (52)	1	fail
12	1	Bin1_plateA0_D3.fcs	2 (72)	5 (51)	4 (65)	1	pass
13	1	Bin1_plateA0_E1.fcs	10 (89)	8 (11)	2 (81)	1	pass
14	1	Bin1_plateA0_E2.fcs	3 (184)	7 (177)	3 (44)	1	pass
15	1	Bin1_plateA0_E3.fcs	1 (20)	-	4 (85)	1	fail
16	1	Bin1_plateA0_F1.fcs	5 (74)	-	5 (248)	1	fail
17	1	Bin1_plateA0_F2.fcs	10 (165)	10 (215)	5 (49)	1	pass
18	1	Bin1_plateA0_F3.fcs	4 (173)	6 (90)	9 (155)	1	pass
19	1	Bin1_plateA0_G1.fcs	7 (76)	2 (7)	10 (42)	1	pass
20	1	Bin1_plateA0_G2.fcs	-	-	1 (20)	1	fail
21	1	Bin1_plateA0_G3.fcs	9 (108)	4 (105)	2 (13)	1	pass
22	1	Bin1_plateA0_H1.fcs	1 (8)	-	8 (44)	1	fail
23	1	Bin1_plateA0_H2.fcs	1 (106)	8 (86)	9 (57)	1	pass
24	2	Bin1_plateA0_H6.fcs	6 (10)	7 (12)	6 (18)	1	pass
25	2	Bin1_plateA0_A4.fcs	9 (128)	7 (58)	5 (22)	1	pass
26	2	Bin1_plateA0_A5.fcs	5 (25)	9 (42)	9 (19)	1	pass
27	2	Bin1_plateA0_A6.fcs	10 (121)	7 (97)	5 (76)	1	pass
28	2	Bin1_plateA0_A7.fcs	6 (236)	1 (54)	9 (29)	1	pass
29	2	Bin1_plateA0_B4.fcs	10 (216)	7 (33)	9 (41)	1	pass
30	2	Bin1_plateA0_B5.fcs	-	-	1 (15)	1	fail
31	2	Bin1_plateA0_B6.fcs	9 (20)	-	-	1	fail
32	2	Bin1_plateA0_B7.fcs	10 (118)	6 (55)	7 (203)	1	pass
33	2	Bin1_plateA0_C4.fcs	1 (11)	3 (11)	-	1	fail
34	2	Bin1_plateA0_C5.fcs	6 (90)	10 (83)	6 (83)	1	pass
35	2	Bin1_plateA0_C6.fcs	5 (85)	2 (55)	7 (169)	1	pass
36	2	Bin1_plateA0_C7.fcs	1 (12)	10 (148)	5 (29)	1	pass
37	2	Bin1_plateA0_D4.fcs	4 (96)	6 (29)	2 (47)	1	pass
38	2	Bin1_plateA0_D5.fcs	8 (90)	10 (27)	8 (118)	1	pass
39	2	Bin1_plateA0_D6.fcs	-	-	2 (62)	1	fail
40	2	Bin1_plateA0_D7.fcs	5 (44)	4 (39)	5 (46)	1	pass

Bead ID	Tag acquisition session #	Tag data filename	Split 1 media (# of tags)	Split 2 media (# of tags)	Split 3 media (# of tags)	Split 4 media	Pass tag deconvolution criteria
41	2	Bin1_plateA0_E4.fcs	-	-	-	1	fail
42	2	Bin1_plateA0_E5.fcs	2 (115)	1 (42)	5 (132)	1	pass
43	2	Bin1_plateA0_E6.fcs	-	-	-	1	fail
44	2	Bin1_plateA0_F5.fcs	-	4 (31)	10 (53)	1	fail
45	2	Bin1_plateA0_F6.fcs	1 (15)	3 (13)	6 (67)	1	pass
46	2	Bin1_plateA0_G4.fcs	5 (50)	10 (33)	5 (74)	1	pass
47	2	Bin1_plateA0_G5.fcs	1 (41)	10 (188)	-	1	fail
48	2	Bin1_plateA0_G6.fcs	3 (94)	1 (22)	10 (22)	1	pass
49	2	Bin1_plateA0_H4.fcs	-	-	9 (338)	1	fail
50	2	Bin1_plateA0_H5.fcs	9 (48)	5 (9)	8 (72)	1	pass
51	3	Bin1_plateA0_H10.fcs	9 (87)	1 (23)	7 (74)	1	pass
52	3	Bin1_plateA0_A8.fcs	6 (28)	4 (36)	7 (30)	1	pass
53	3	Bin1_plateA0_A9.fcs	9 (82)	3 (40)	7 (56)	1	pass
54	3	Bin1_plateA0_A10.fcs	2 (142)	5 (89)	3 (15)	1	pass
55	3	Bin1_plateA0_B8.fcs	9 (143)	6 (63)	7 (63)	1	pass
56	3	Bin1_plateA0_B9.fcs	7 (70)	6 (24)	10 (36)	1	pass
57	3	Bin1_plateA0_B10.fcs	6 (29)	9 (19)	6 (120)	1	pass
58	3	Bin1_plateA0_C8.fcs	7 (183)	7 (98)	2 (29)	1	pass
59	3	Bin1_plateA0_C9.fcs	5 (51)	3 (44)	-	1	fail
60	3	Bin1_plateA0_C10.fcs	10 (39)	2 (27)	3 (20)	1	pass
61	3	Bin1_plateA0_D8.fcs	9 (108)	2 (40)	6 (26)	1	pass
62	3	Bin1_plateA0_D9.fcs	6 (60)	3 (83)	-	1	fail
63	3	Bin1_plateA0_D10.fcs	3 (128)	8 (23)	2 (40)	1	pass
64	3	Bin1_plateA0_E7.fcs	10 (158)	4 (40)	-	1	fail
65	3	Bin1_plateA0_E8.fcs	5 (71)	7 (17)	10 (42)	1	pass
66	3	Bin1_plateA0_E10.fcs	1 (13)	5 (28)	1 (5)	1	pass
67	3	Bin1_plateA0_F7.fcs	2 (146)	10 (65)	1 (29)	1	pass
68	3	Bin1_plateA0_F8.fcs	3 (92)	9 (53)	1 (22)	1	pass
69	3	Bin1_plateA0_F9.fcs	9 (42)	10 (44)	3 (28)	1	pass
70	3	Bin1_plateA0_F10.fcs	9 (46)	9 (30)	4 (23)	1	pass
71	3	Bin1_plateA0_G7.fcs	5 (35)	9 (130)	10 (54)	1	pass
72	3	Bin1_plateA0_G8.fcs	-	9 (21)	7 (82)	1	fail
73	3	Bin1_plateA0_G9.fcs	10 (114)	2 (25)	6 (161)	1	pass
74	3	Bin1_plateA0_G10.fcs	1 (11)	10 (157)	-	1	fail
75	3	Bin1_plateA0_H7.fcs	9 (139)	9 (77)	9 (71)	1	pass
76	3	Bin1_plateA0_H8.fcs	1 (16)	-	4 (40)	1	fail
77	3	Bin1_plateA0_H9.fcs	4 (93)	6 (43)	7 (79)	1	pass
78	4	Bin1_plateA0_H12.fcs	9 (211)	3 (57)	10 (32)	1	pass
79	4	Bin1_plateA0_A11.fcs	3 (402)	6 (120)	1 (29)	1	pass
80	4	Bin1_plateA0_A12.fcs	2 (205)	5 (113)	9 (65)	1	pass
81	4	Bin1_plateA0_B11.fcs	9 (116)	4 (52)	6 (55)	1	pass
82	4	Bin1_plateA0_B12.fcs	10 (235)	8 (41)	10 (34)	1	pass
83	4	Bin1_plateA0_C11.fcs	4 (129)	6 (36)	6 (115)	1	pass

Confidential

Bead ID	Tag acquisition session #	Tag data filename	Split 1 media (# of tags)	Split 2 media (# of tags)	Split 3 media (# of tags)	Split 4 media	Pass tag deconvolution criteria
84	4	Bin1_plateA0_C12.fcs	2 (90)	10 (56)	5 (50)	1	pass
85	4	Bin1_plateA0_D11.fcs	8 (117)	8 (47)	2 (47)	1	pass
86	4	Bin1_plateA0_D12.fcs	1 (27)	6 (7)	2 (6)	1	pass
87	4	Bin1_plateA0_E11.fcs	6 (74)	3 (66)	7 (55)	1	pass
88	4	Bin1_plateA0_E12.fcs	8 (153)	7 (156)	10 (71)	1	pass
89	4	Bin1_plateA0_F11.fcs	3 (173)	9 (86)	3 (38)	1	pass
90	4	Bin1_plateA0_F12.fcs	6 (103)	3 (54)	9 (46)	1	pass
91	4	Bin1_plateA0_G11.fcs	4 (133)	10 (59)	4 (81)	1	pass
92	4	Bin1_plateA0_G12.fcs	1 (27)	-	4 (227)	1	fail
93	4	Bin1_plateA0_H11.fcs	9 (94)	8 (66)	8 (106)	1	pass
94	5	Bin1_plateB1_H7.fcs	9 (52)	-	6 (13)	1	fail
95	5	Bin1_plateB1_A1.fcs	9 (119)	7 (55)	3 (41)	1	pass
96	5	Bin1_plateB1_A6.fcs	10 (47)	10 (39)	7 (25)	1	pass
97	5	Bin1_plateB1_A7.fcs	10 (133)	8 (28)	1 (16)	1	pass
98	5	Bin1_plateB1_B1.fcs	10 (83)	5 (31)	8 (49)	1	pass
99	5	Bin1_plateB1_B2.fcs	7 (100)	-	7 (92)	1	fail
100	5	Bin1_plateB1_B3.fcs	-	-	-	1	fail
101	5	Bin1_plateB1_B4.fcs	1 (11)	5 (22)	9 (15)	1	pass
102	5	Bin1_plateB1_C1.fcs	9 (3)	5 (11)	-	1	fail
103	5	Bin1_plateB1_C2.fcs	9 (5)	-	-	1	fail
104	5	Bin1_plateB1_C4.fcs	-	-	10 (10)	1	fail
105	5	Bin1_plateB1_C5.fcs	-	-	6 (57)	1	fail
106	5	Bin1_plateB1_C6.fcs	9 (79)	5 (20)	7 (34)	1	pass
107	5	Bin1_plateB1_C7.fcs	-	-	1 (4)	1	fail
108	5	Bin1_plateB1_D1.fcs	9 (7)	4 (11)	6 (5)	1	pass
109	5	Bin1_plateB1_D4.fcs	10 (31)	-	6 (6)	1	fail
110	5	Bin1_plateB1_D6.fcs	7 (76)	3 (10)	1 (67)	1	pass
111	5	Bin1_plateB1_D7.fcs	5 (8)	5 (12)	-	1	fail
112	5	Bin1_plateB1_E3.fcs	8 (25)	4 (7)	7 (15)	1	pass
113	5	Bin1_plateB1_E6.fcs	9 (23)	2 (7)	10 (13)	1	pass
114	5	Bin1_plateB1_E7.fcs	4 (45)	10 (70)	4 (42)	1	pass
115	5	Bin1_plateB1_F1.fcs	-	-	-	1	fail
116	5	Bin1_plateB1_F2.fcs	3 (83)	10 (152)	4 (32)	1	pass
117	5	Bin1_plateB1_F4.fcs	10 (24)	6 (15)	1 (7)	1	pass
118	5	Bin1_plateB1_F5.fcs	9 (28)	7 (16)	3 (26)	1	pass
119	5	Bin1_plateB1_F6.fcs	4 (24)	1 (28)	2 (13)	1	pass
120	5	Bin1_plateB1_F7.fcs	10 (64)	2 (27)	9 (46)	1	pass
121	5	Bin1_plateB1_G2.fcs	5 (10)	9 (34)	2 (5)	1	pass
122	5	Bin1_plateB1_G3.fcs	9 (90)	-	10 (32)	1	fail
123	5	Bin1_plateB1_G4.fcs	-	-	-	1	fail
124	5	Bin1_plateB1_G5.fcs	8 (63)	10 (24)	9 (7)	1	pass
125	5	Bin1_plateB1_H1.fcs	1 (13)	4 (10)	10 (118)	1	pass
126	5	Bin1_plateB1_H2.fcs	5 (16)	9 (20)	5 (65)	1	pass

 Confidential

Bead ID	Tag acquisition session #	Tag data filename	Split 1 media (# of tags)	Split 2 media (# of tags)	Split 3 media (# of tags)	Split 4 media	Pass tag deconvolution criteria
127	5	Bin1_plateB1_H3.fcs	2 (49)	-	-	1	fail
128	5	Bin1_plateB1_H4.fcs	6 (15)	8 (15)	1 (12)	1	pass
129	5	Bin1_plateB1_H5.fcs	2 (38)	4 (8)	3 (9)	1	pass
130	5	Bin1_plateB1_H6.fcs	6 (49)	6 (16)	10 (18)	1	pass
131	6	Bin1_plateB1_H10.fcs	-	-	-	1	fail
132	6	Bin1_plateB1_A10.fcs	2 (25)	7 (33)	4 (6)	1	pass
133	6	Bin1_plateB1_B8.fcs	10 (31)	-	-	1	fail
134	6	Bin1_plateB1_B10.fcs	6 (60)	8 (9)	1 (18)	1	pass
135	6	Bin1_plateB1_C8.fcs	5 (15)	9 (32)	5 (36)	1	pass
136	6	Bin1_plateB1_C9.fcs	1 (7)	6 (40)	7 (6)	1	pass
137	6	Bin1_plateB1_D8.fcs	8 (3)	-	8 (3)	1	fail
138	6	Bin1_plateB1_D9.fcs	-	3 (42)	-	1	fail
139	6	Bin1_plateB1_D10.fcs	4 (44)	6 (20)	7 (5)	1	pass
140	6	Bin1_plateB1_E8.fcs	5 (65)	1 (14)	10 (114)	1	pass
141	6	Bin1_plateB1_E9.fcs	5 (14)	10 (75)	6 (15)	1	pass
142	6	Bin1_plateB1_E10.fcs	9 (56)	2 (47)	-	1	fail
143	6	Bin1_plateB1_F8.fcs	-	10 (53)	6 (92)	1	fail
144	6	Bin1_plateB1_F9.fcs	-	-	-	1	fail
145	6	Bin1_plateB1_F10.fcs	1 (4)	2 (8)	10 (14)	1	pass
146	6	Bin1_plateB1_G8.fcs	9 (13)	-	6 (4)	1	fail
147	6	Bin1_plateB1_G9.fcs	2 (56)	3 (43)	8 (21)	1	pass
148	6	Bin1_plateB1_G10.fcs	2 (19)	4 (29)	2 (11)	1	pass
149	6	Bin1_plateB1_H8.fcs	-	4 (40)	5 (421)	1	fail
150	6	Bin1_plateB1_H9.fcs	4 (44)	7 (25)	10 (8)	1	pass
151	7	Bin1_plateB1_H11.fcs	4 (138)	4 (25)	5 (49)	1	pass
152	7	Bin1_plateB1_A12.fcs	7 (110)	9 (212)	7 (411)	1	pass
153	7	Bin1_plateB1_B11.fcs	-	6 (79)	1 (48)	1	fail
154	7	Bin1_plateB1_B12.fcs	-	-	-	1	fail
155	7	Bin1_plateB1_C12.fcs	8 (81)	8 (17)	9 (21)	1	pass
156	7	Bin1_plateB1_F12.fcs	-	3 (145)	1 (35)	1	fail
157	7	Bin1_plateB1_G11.fcs	7 (78)	-	4 (70)	1	fail
158	8	Bin1_plateC2_C6.fcs	10 (180)	5 (154)	10 (36)	1	pass
159	8	Bin1_plateC2_A1.fcs	3 (178)	1 (28)	10 (157)	1	pass
160	8	Bin1_plateC2_A2.fcs	8 (131)	6 (72)	9 (75)	1	pass
161	8	Bin1_plateC2_A4.fcs	8 (69)	4 (24)	8 (22)	1	pass
162	8	Bin1_plateC2_A5.fcs	8 (107)	10 (154)	3 (30)	1	pass
163	8	Bin1_plateC2_A6.fcs	6 (208)	9 (162)	1 (23)	1	pass
164	8	Bin1_plateC2_A7.fcs	8 (106)	5 (42)	10 (31)	1	pass
165	8	Bin1_plateC2_A8.fcs	-	-	-	1	fail
166	8	Bin1_plateC2_A9.fcs	1 (14)	5 (70)	2 (48)	1	pass
167	8	Bin1_plateC2_A10.fcs	5 (22)	5 (15)	5 (124)	1	pass
168	8	Bin1_plateC2_A11.fcs	-	-	-	1	fail
169	8	Bin1_plateC2_A12.fcs	7 (60)	8 (18)	10 (11)	1	pass

Confidential

Bead ID	Tag acquisition session #	Tag data filename	Split 1 media (# of tags)	Split 2 media (# of tags)	Split 3 media (# of tags)	Split 4 media	Pass tag deconvolution criteria
170	8	Bin1_plateC2_B1.fcs	8 (19)	7 (12)	9 (10)	1	pass
171	8	Bin1_plateC2_B2.fcs	1 (8)	5 (28)	2 (98)	1	pass
172	8	Bin1_plateC2_B3.fcs	9 (193)	10 (130)	10 (49)	1	pass
173	8	Bin1_plateC2_B4.fcs	5 (23)	9 (19)	5 (18)	1	pass
174	8	Bin1_plateC2_B5.fcs	1 (4)	5 (4)	7 (26)	1	pass
175	8	Bin1_plateC2_B6.fcs	4 (152)	6 (65)	5 (45)	1	pass
176	8	Bin1_plateC2_B7.fcs	6 (19)	7 (18)	5 (17)	1	pass
177	8	Bin1_plateC2_B8.fcs	-	5 (17)	2 (71)	1	fail
178	8	Bin1_plateC2_B9.fcs	4 (100)	6 (37)	1 (16)	1	pass
179	8	Bin1_plateC2_B10.fcs	6 (24)	5 (29)	6 (47)	1	pass
180	8	Bin1_plateC2_B11.fcs	5 (25)	10 (115)	5 (24)	1	pass
181	8	Bin1_plateC2_B12.fcs	1 (22)	3 (62)	10 (99)	1	pass
182	8	Bin1_plateC2_C1.fcs	6 (29)	5 (18)	3 (11)	1	pass
183	8	Bin1_plateC2_C4.fcs	4 (84)	9 (29)	5 (37)	1	pass
184	8	Bin1_plateC2_C5.fcs	2 (154)	9 (122)	6 (66)	1	pass
185	9	Bin2_plateA2_E8.fcs	3 (89)	4 (39)	1 (18)	2	pass
186	9	Bin2_plateA2_A1.fcs	5 (40)	10 (28)	2 (31)	2	pass
187	9	Bin2_plateA2_A3.fcs	8 (65)	6 (67)	5 (103)	2	pass
188	9	Bin2_plateA2_A4.fcs	9 (50)	4 (22)	9 (17)	2	pass
189	9	Bin2_plateA2_A5.fcs	4 (100)	9 (72)	3 (26)	2	pass
190	9	Bin2_plateA2_A6.fcs	3 (101)	6 (70)	10 (33)	2	pass
191	9	Bin2_plateA2_A7.fcs	5 (33)	9 (29)	-	2	fail
192	9	Bin2_plateA2_A8.fcs	10 (111)	-	1 (34)	2	fail
193	9	Bin2_plateA2_A9.fcs	10 (105)	4 (115)	4 (91)	2	pass
194	9	Bin2_plateA2_A10.fcs	10 (36)	6 (17)	9 (38)	2	pass
195	9	Bin2_plateA2_A11.fcs	5 (27)	8 (14)	9 (58)	2	pass
196	9	Bin2_plateA2_A12.fcs	4 (126)	1 (24)	2 (38)	2	pass
197	9	Bin2_plateA2_B1.fcs	2 (45)	3 (43)	10 (17)	2	pass
198	9	Bin2_plateA2_B3.fcs	5 (57)	9 (39)	2 (29)	2	pass
199	9	Bin2_plateA2_B5.fcs	3 (74)	5 (53)	9 (90)	2	pass
200	9	Bin2_plateA2_B6.fcs	2 (40)	9 (108)	3 (100)	2	pass
201	9	Bin2_plateA2_B7.fcs	3 (46)	6 (26)	9 (36)	2	pass
202	9	Bin2_plateA2_B8.fcs	5 (14)	10 (318)	-	2	fail
203	9	Bin2_plateA2_B9.fcs	10 (104)	6 (30)	7 (111)	2	pass
204	9	Bin2_plateA2_B10.fcs	1 (19)	5 (97)	2 (86)	2	pass
205	9	Bin2_plateA2_B11.fcs	5 (38)	10 (93)	-	2	fail
206	9	Bin2_plateA2_B12.fcs	2 (66)	6 (31)	3 (26)	2	pass
207	9	Bin2_plateA2_C1.fcs	9 (51)	10 (140)	5 (28)	2	pass
208	9	Bin2_plateA2_C2.fcs	5 (58)	10 (21)	3 (28)	2	pass
209	9	Bin2_plateA2_C3.fcs	8 (89)	6 (67)	10 (24)	2	pass
210	9	Bin2_plateA2_C4.fcs	10 (79)	10 (103)	5 (41)	2	pass
211	9	Bin2_plateA2_C5.fcs	10 (85)	6 (72)	9 (84)	2	pass
212	9	Bin2_plateA2_C6.fcs	5 (18)	10 (68)	7 (58)	2	pass

Bead ID	Tag acquisition session #	Tag data filename	Split 1 media (# of tags)	Split 2 media (# of tags)	Split 3 media (# of tags)	Split 4 media	Pass tag deconvolution criteria
213	9	Bin2_plateA2_C7.fcs	6 (63)	6 (52)	3 (30)	2	pass
214	9	Bin2_plateA2_C8.fcs	4 (84)	10 (63)	3 (7)	2	pass
215	9	Bin2_plateA2_C9.fcs	7 (85)	4 (27)	2 (24)	2	pass
216	9	Bin2_plateA2_C10.fcs	1 (19)	5 (160)	3 (131)	2	pass
217	9	Bin2_plateA2_C11.fcs	10 (81)	8 (23)	7 (97)	2	pass
218	9	Bin2_plateA2_C12.fcs	7 (84)	4 (21)	8 (30)	2	pass
219	9	Bin2_plateA2_D1.fcs	9 (44)	6 (31)	6 (63)	2	pass
220	9	Bin2_plateA2_D2.fcs	10 (50)	5 (81)	5 (112)	2	pass
221	9	Bin2_plateA2_D3.fcs	6 (32)	8 (24)	2 (72)	2	pass
222	9	Bin2_plateA2_D4.fcs	7 (104)	3 (37)	6 (111)	2	pass
223	9	Bin2_plateA2_D5.fcs	5 (41)	9 (62)	5 (35)	2	pass
224	9	Bin2_plateA2_D6.fcs	8 (99)	2 (13)	2 (83)	2	pass
225	9	Bin2_plateA2_D7.fcs	3 (102)	2 (37)	-	2	fail
226	9	Bin2_plateA2_D8.fcs	-	9 (10)	3 (32)	2	fail
227	9	Bin2_plateA2_D9.fcs	1 (16)	5 (27)	6 (80)	2	pass
228	9	Bin2_plateA2_D10.fcs	5 (43)	2 (25)	2 (72)	2	pass
229	9	Bin2_plateA2_D11.fcs	6 (100)	5 (52)	8 (27)	2	pass
230	9	Bin2_plateA2_D12.fcs	3 (36)	-	1 (13)	2	fail
231	9	Bin2_plateA2_E1.fcs	-	3 (62)	-	2	fail
232	9	Bin2_plateA2_E2.fcs	-	-	-	2	fail
233	9	Bin2_plateA2_E3.fcs	2 (66)	-	3 (33)	2	fail
234	9	Bin2_plateA2_E4.fcs	6 (97)	2 (55)	9 (147)	2	pass
235	9	Bin2_plateA2_E5.fcs	1 (8)	6 (39)	5 (173)	2	pass
236	9	Bin2_plateA2_E6.fcs	8 (119)	3 (47)	2 (27)	2	pass
237	9	Bin2_plateA2_E7.fcs	3 (117)	5 (66)	8 (55)	2	pass
238	10	Bin2_plateA2_G8.fcs	6 (26)	5 (24)	2 (4)	2	pass
239	10	Bin2_plateA2_E9.fcs	5 (12)	7 (38)	6 (34)	2	pass
240	10	Bin2_plateA2_E10.fcs	10 (89)	8 (28)	9 (74)	2	pass
241	10	Bin2_plateA2_E11.fcs	10 (107)	-	-	2	fail
242	10	Bin2_plateA2_E12.fcs	6 (66)	4 (26)	6 (68)	2	pass
243	10	Bin2_plateA2_F1.fcs	4 (85)	4 (34)	10 (80)	2	pass
244	10	Bin2_plateA2_F2.fcs	8 (129)	10 (34)	9 (81)	2	pass
245	10	Bin2_plateA2_F3.fcs	5 (42)	4 (19)	7 (114)	2	pass
246	10	Bin2_plateA2_F4.fcs	5 (18)	9 (97)	1 (13)	2	pass
247	10	Bin2_plateA2_F5.fcs	9 (78)	10 (42)	9 (69)	2	pass
248	10	Bin2_plateA2_F6.fcs	5 (38)	6 (27)	6 (155)	2	pass
249	10	Bin2_plateA2_F7.fcs	4 (193)	10 (116)	7 (28)	2	pass
250	10	Bin2_plateA2_F8.fcs	-	3 (8)	1 (15)	2	fail
251	10	Bin2_plateA2_F9.fcs	3 (52)	4 (32)	6 (78)	2	pass
252	10	Bin2_plateA2_F10.fcs	5 (24)	10 (45)	10 (33)	2	pass
253	10	Bin2_plateA2_F11.fcs	3 (96)	3 (91)	5 (89)	2	pass
254	10	Bin2_plateA2_F12.fcs	4 (52)	-	6 (93)	2	fail
255	10	Bin2_plateA2_G1.fcs	2 (54)	3 (31)	1 (10)	2	pass

Confidential

Bead ID	Tag acquisition session #	Tag data filename	Split 1 media (# of tags)	Split 2 media (# of tags)	Split 3 media (# of tags)	Split 4 media	Pass tag deconvolution criteria
256	10	Bin2_plateA2_G4.fcs	-	9 (50)	3 (11)	2	fail
257	10	Bin2_plateA2_G5.fcs	-	7 (45)	10 (16)	2	fail
258	10	Bin2_plateA2_G6.fcs	5 (54)	5 (20)	3 (17)	2	pass
259	10	Bin2_plateA2_G7.fcs	2 (89)	10 (100)	5 (9)	2	pass
260	11	Bin3_plateA2_B9.fcs	3 (121)	5 (129)	8 (78)	3	pass
261	11	Bin3_plateA2_A1.fcs	5 (8)	9 (195)	1 (46)	3	pass
262	11	Bin3_plateA2_A2.fcs	5 (9)	10 (59)	9 (27)	3	pass
263	11	Bin3_plateA2_A3.fcs	9 (87)	4 (9)	3 (14)	3	pass
264	11	Bin3_plateA2_A4.fcs	9 (44)	5 (143)	7 (25)	3	pass
265	11	Bin3_plateA2_A5.fcs	8 (111)	6 (75)	10 (52)	3	pass
266	11	Bin3_plateA2_A6.fcs	1 (5)	7 (85)	10 (5)	3	pass
267	11	Bin3_plateA2_A7.fcs	7 (106)	1 (117)	7 (64)	3	pass
268	11	Bin3_plateA2_A8.fcs	7 (41)	4 (12)	2 (6)	3	pass
269	11	Bin3_plateA2_A9.fcs	5 (15)	10 (105)	-	3	fail
270	11	Bin3_plateA2_A10.fcs	2 (77)	3 (33)	8 (29)	3	pass
271	11	Bin3_plateA2_A11.fcs	5 (34)	9 (66)	2 (7)	3	pass
272	11	Bin3_plateA2_A12.fcs	1 (15)	5 (17)	7 (50)	3	pass
273	11	Bin3_plateA2_B2.fcs	6 (72)	-	-	3	fail
274	11	Bin3_plateA2_B3.fcs	7 (35)	10 (124)	2 (14)	3	pass
275	11	Bin3_plateA2_B5.fcs	-	-	4 (32)	3	fail
276	11	Bin3_plateA2_B6.fcs	2 (51)	6 (101)	10 (22)	3	pass
277	11	Bin3_plateA2_B7.fcs	7 (48)	10 (61)	3 (20)	3	pass
278	11	Bin3_plateA2_B8.fcs	5 (22)	10 (81)	9 (13)	3	pass
279	12	Bin3_plateA2_B12.fcs	-	-	9 (99)	3	fail
280	12	Bin3_plateA2_B10.fcs	1 (16)	5 (74)	3 (57)	3	pass
281	12	Bin3_plateA2_B11.fcs	3 (66)	5 (40)	2 (61)	3	pass
282	13	Bin4_plateA2_E8.fcs	10 (246)	8 (20)	7 (104)	4	pass
283	13	Bin4_plateA2_D1.fcs	10 (131)	9 (137)	5 (134)	4	pass
284	13	Bin4_plateA2_D2.fcs	1 (21)	5 (37)	2 (242)	4	pass
285	13	Bin4_plateA2_D3.fcs	10 (140)	1 (40)	5 (98)	4	pass
286	13	Bin4_plateA2_D4.fcs	3 (59)	1 (51)	8 (95)	4	pass
287	13	Bin4_plateA2_D5.fcs	3 (76)	2 (13)	2 (60)	4	pass
288	13	Bin4_plateA2_D6.fcs	1 (8)	2 (23)	9 (37)	4	pass
289	13	Bin4_plateA2_D7.fcs	1 (8)	-	8 (23)	4	fail
290	13	Bin4_plateA2_D8.fcs	-	-	-	4	fail
291	13	Bin4_plateA2_D9.fcs	5 (50)	5 (37)	1 (57)	4	pass
292	13	Bin4_plateA2_D10.fcs	-	9 (46)	2 (9)	4	fail
293	13	Bin4_plateA2_D11.fcs	3 (21)	-	8 (153)	4	fail
294	13	Bin4_plateA2_D12.fcs	5 (12)	10 (75)	6 (116)	4	pass
295	13	Bin4_plateA2_E1.fcs	10 (113)	8 (17)	8 (86)	4	pass
296	13	Bin4_plateA2_E2.fcs	4 (90)	6 (8)	-	4	fail
297	13	Bin4_plateA2_E3.fcs	-	6 (7)	6 (11)	4	fail
298	13	Bin4_plateA2_E4.fcs	1 (6)	9 (61)	5 (7)	4	pass

 Confidential

Bead ID	Tag acquisition session #	Tag data filename	Split 1 media (# of tags)	Split 2 media (# of tags)	Split 3 media (# of tags)	Split 4 media	Pass tag deconvolution criteria
299	13	Bin4_plateA2_E5.fcs	1 (5)	5 (37)	2 (17)	4	pass
300	13	Bin4_plateA2_E6.fcs	-	5 (23)	9 (7)	4	fail
301	13	Bin4_plateA2_E7.fcs	2 (61)	6 (8)	7 (15)	4	pass
302	14	Bin4_plateA2_G12.fcs	5 (31)	-	2 (78)	4	fail
303	14	Bin4_plateA2_E9.fcs	6 (34)	10 (86)	10 (41)	4	pass
304	14	Bin4_plateA2_E10.fcs	4 (179)	5 (71)	6 (131)	4	pass
305	14	Bin4_plateA2_E11.fcs	1 (16)	8 (40)	6 (64)	4	pass
306	14	Bin4_plateA2_E12.fcs	7 (23)	6 (31)	6 (115)	4	pass
307	14	Bin4_plateA2_F1.fcs	9 (69)	9 (29)	10 (102)	4	pass
308	14	Bin4_plateA2_F2.fcs	10 (175)	5 (24)	9 (120)	4	pass
309	14	Bin4_plateA2_F3.fcs	3 (57)	10 (151)	10 (18)	4	pass
310	14	Bin4_plateA2_F4.fcs	1 (6)	5 (10)	9 (9)	4	pass
311	14	Bin4_plateA2_F5.fcs	-	5 (55)	3 (14)	4	fail
312	14	Bin4_plateA2_F6.fcs	1 (23)	10 (44)	7 (66)	4	pass
313	14	Bin4_plateA2_F7.fcs	-	-	1 (36)	4	fail
314	14	Bin4_plateA2_F8.fcs	7 (96)	3 (34)	-	4	fail
315	14	Bin4_plateA2_F9.fcs	2 (32)	5 (47)	9 (27)	4	pass
316	14	Bin4_plateA2_F10.fcs	5 (27)	3 (38)	9 (84)	4	pass
317	14	Bin4_plateA2_F11.fcs	6 (105)	8 (35)	4 (28)	4	pass
318	14	Bin4_plateA2_F12.fcs	5 (29)	9 (96)	3 (134)	4	pass
319	14	Bin4_plateA2_G4.fcs	7 (42)	2 (6)	3 (14)	4	pass
320	14	Bin4_plateA2_G6.fcs	5 (4)	10 (199)	-	4	fail
321	14	Bin4_plateA2_G7.fcs	4 (90)	8 (29)	3 (28)	4	pass
322	14	Bin4_plateA2_G8.fcs	-	9 (19)	10 (21)	4	fail
323	14	Bin4_plateA2_G9.fcs	1 (7)	1 (7)	6 (28)	4	pass
324	14	Bin4_plateA2_G10.fcs	1 (15)	-	-	4	fail
325	14	Bin4_plateA2_G11.fcs	1 (6)	-	2 (30)	4	fail
326	15	Bin5_plateA0_C8.fcs	3 (134)	1 (37)	5 (46)	5	pass
327	15	Bin5_plateA0_B7.fcs	6 (12)	2 (9)	5 (18)	5	pass
328	15	Bin5_plateA0_B8.fcs	5 (43)	9 (134)	6 (110)	5	pass
329	15	Bin5_plateA0_B9.fcs	6 (54)	6 (20)	10 (19)	5	pass
330	15	Bin5_plateA0_B10.fcs	4 (157)	6 (100)	5 (29)	5	pass
331	15	Bin5_plateA0_C1.fcs	4 (242)	2 (75)	3 (33)	5	pass
332	15	Bin5_plateA0_C2.fcs	2 (162)	10 (170)	-	5	fail
333	15	Bin5_plateA0_C3.fcs	1 (19)	-	6 (102)	5	fail
334	15	Bin5_plateA0_C4.fcs	4 (198)	5 (58)	1 (32)	5	pass
335	15	Bin5_plateA0_C5.fcs	-	5 (23)	3 (36)	5	fail
336	15	Bin5_plateA0_C6.fcs	4 (222)	-	4 (102)	5	fail
337	15	Bin5_plateA0_C7.fcs	10 (150)	10 (86)	1 (22)	5	pass
338	16	Bin5_plateA0_G5.fcs	7 (163)	2 (96)	7 (41)	5	pass
339	16	Bin5_plateA0_C9.fcs	7 (78)	9 (117)	6 (64)	5	pass
340	16	Bin5_plateA0_C10.fcs	5 (12)	9 (25)	5 (18)	5	pass
341	16	Bin5_plateA0_C11.fcs	2 (73)	7 (116)	7 (70)	5	pass

Bead ID	Tag acquisition session #	Tag data filename	Split 1 media (# of tags)	Split 2 media (# of tags)	Split 3 media (# of tags)	Split 4 media	Pass tag deconvolution criteria
342	16	Bin5_plateA0_D1.fcs	1 (11)	6 (47)	6 (117)	5	pass
343	16	Bin5_plateA0_D2.fcs	6 (53)	10 (31)	10 (5)	5	pass
344	16	Bin5_plateA0_D3.fcs	10 (101)	6 (76)	2 (49)	5	pass
345	16	Bin5_plateA0_D4.fcs	4 (178)	9 (44)	8 (48)	5	pass
346	16	Bin5_plateA0_D5.fcs	-	-	-	5	fail
347	16	Bin5_plateA0_D6.fcs	5 (30)	2 (32)	6 (130)	5	pass
348	16	Bin5_plateA0_D7.fcs	7 (153)	9 (159)	9 (22)	5	pass
349	16	Bin5_plateA0_D8.fcs	10 (123)	1 (42)	6 (101)	5	pass
350	16	Bin5_plateA0_D9.fcs	5 (42)	9 (25)	9 (19)	5	pass
351	16	Bin5_plateA0_D10.fcs	9 (110)	1 (16)	6 (279)	5	pass
352	16	Bin5_plateA0_D11.fcs	4 (169)	-	10 (238)	5	fail
353	16	Bin5_plateA0_D12.fcs	3 (120)	2 (50)	5 (116)	5	pass
354	16	Bin5_plateA0_E1.fcs	-	8 (45)	8 (179)	5	fail
355	16	Bin5_plateA0_E2.fcs	1 (26)	5 (42)	1 (22)	5	pass
356	16	Bin5_plateA0_E3.fcs	7 (127)	7 (31)	5 (204)	5	pass
357	16	Bin5_plateA0_E4.fcs	8 (85)	7 (70)	3 (43)	5	pass
358	16	Bin5_plateA0_E5.fcs	10 (58)	5 (89)	5 (28)	5	pass
359	16	Bin5_plateA0_E6.fcs	-	10 (22)	4 (22)	5	fail
360	16	Bin5_plateA0_E7.fcs	9 (110)	-	1 (142)	5	fail
361	16	Bin5_plateA0_E9.fcs	10 (47)	2 (12)	5 (31)	5	pass
362	16	Bin5_plateA0_E10.fcs	-	-	2 (4)	5	fail
363	16	Bin5_plateA0_E11.fcs	9 (76)	5 (57)	1 (72)	5	pass
364	16	Bin5_plateA0_E12.fcs	2 (21)	4 (6)	2 (52)	5	pass
365	16	Bin5_plateA0_F1.fcs	1 (23)	9 (99)	1 (78)	5	pass
366	16	Bin5_plateA0_F2.fcs	5 (11)	9 (153)	9 (11)	5	pass
367	16	Bin5_plateA0_F3.fcs	1 (35)	5 (39)	10 (41)	5	pass
368	16	Bin5_plateA0_F4.fcs	1 (17)	9 (234)	7 (33)	5	pass
369	16	Bin5_plateA0_F5.fcs	6 (21)	9 (9)	-	5	fail
370	16	Bin5_plateA0_F6.fcs	10 (44)	10 (34)	8 (90)	5	pass
371	16	Bin5_plateA0_F7.fcs	-	-	-	5	fail
372	16	Bin5_plateA0_F8.fcs	1 (25)	5 (28)	2 (45)	5	pass
373	16	Bin5_plateA0_F9.fcs	5 (8)	10 (11)	6 (4)	5	pass
374	16	Bin5_plateA0_F10.fcs	9 (89)	9 (83)	10 (30)	5	pass
375	16	Bin5_plateA0_F12.fcs	7 (26)	8 (10)	5 (8)	5	pass
376	16	Bin5_plateA0_G2.fcs	4 (44)	2 (22)	3 (15)	5	pass
377	16	Bin5_plateA0_G3.fcs	3 (20)	6 (14)	3 (15)	5	pass
378	16	Bin5_plateA0_G4.fcs	1 (6)	6 (87)	8 (60)	5	pass
379	17	Bin7_plateA0_C3.fcs	1 (13)	5 (88)	1 (25)	7	pass
380	17	Bin6_plateA2_A1.fcs	1 (14)	5 (52)	3 (56)	6	pass
381	17	Bin6_plateA2_A2.fcs	-	-	-	6	fail
382	17	Bin6_plateA2_A4.fcs	3 (131)	1 (70)	2 (135)	6	pass
383	17	Bin6_plateA2_A5.fcs	9 (221)	6 (144)	10 (50)	6	pass
384	17	Bin6_plateA2_A6.fcs	5 (86)	7 (77)	1 (35)	6	pass

Bead ID	Tag acquisition session #	Tag data filename	Split 1 media (# of tags)	Split 2 media (# of tags)	Split 3 media (# of tags)	Split 4 media	Pass tag deconvolution criteria
385	17	Bin6_plateA2_A7.fcs	-	-	-	6	fail
386	17	Bin6_plateA2_A9.fcs	7 (31)	9 (35)	6 (84)	6	pass
387	17	Bin6_plateA2_A10.fcs	10 (42)	3 (36)	9 (9)	6	pass
388	17	Bin6_plateA2_A11.fcs	5 (62)	10 (134)	1 (6)	6	pass
389	17	Bin7_plateA0_C1.fcs	1 (7)	10 (26)	5 (4)	7	pass
390	17	Bin7_plateA0_C2.fcs	2 (89)	3 (27)	2 (42)	7	pass
391	18	Bin8_plateA0_G12.fcs	10 (102)	9 (43)	4 (83)	8	pass
392	18	Bin7_plateA0_C4.fcs	8 (105)	-	7 (201)	7	fail
393	18	Bin7_plateA0_C5.fcs	7 (102)	6 (17)	1 (26)	7	pass
394	18	Bin7_plateA0_C7.fcs	8 (35)	10 (134)	6 (115)	7	pass
395	18	Bin7_plateA0_C8.fcs	-	-	9 (330)	7	fail
396	18	Bin7_plateA0_C9.fcs	4 (159)	1 (41)	3 (32)	7	pass
397	18	Bin7_plateA0_C11.fcs	3 (59)	2 (27)	3 (74)	7	pass
398	18	Bin7_plateA0_C12.fcs	6 (39)	6 (32)	6 (54)	7	pass
399	18	Bin7_plateA0_D5.fcs	5 (56)	10 (171)	1 (6)	7	pass
400	18	Bin7_plateA0_D6.fcs	8 (32)	8 (12)	4 (10)	7	pass
401	18	Bin7_plateA0_D7.fcs	-	-	-	7	fail
402	18	Bin7_plateA0_D8.fcs	5 (25)	3 (12)	2 (24)	7	pass
403	18	Bin7_plateA0_D9.fcs	7 (116)	3 (33)	6 (49)	7	pass
404	18	Bin7_plateA0_D10.fcs	8 (46)	2 (54)	10 (51)	7	pass
405	18	Bin7_plateA0_D11.fcs	3 (82)	3 (77)	4 (223)	7	pass
406	18	Bin7_plateA0_D12.fcs	10 (16)	4 (9)	9 (25)	7	pass
407	18	Bin8_plateA0_F1.fcs	-	-	-	8	fail
408	18	Bin8_plateA0_F2.fcs	1 (6)	5 (30)	1 (7)	8	pass
409	18	Bin8_plateA0_F3.fcs	8 (83)	5 (46)	9 (33)	8	pass
410	18	Bin8_plateA0_F4.fcs	7 (152)	9 (65)	10 (77)	8	pass
411	18	Bin8_plateA0_F5.fcs	7 (97)	6 (54)	1 (92)	8	pass
412	18	Bin8_plateA0_F6.fcs	6 (74)	6 (35)	6 (82)	8	pass
413	18	Bin8_plateA0_F7.fcs	2 (47)	3 (24)	5 (136)	8	pass
414	18	Bin8_plateA0_F8.fcs	-	-	1 (25)	8	fail
415	18	Bin8_plateA0_F9.fcs	-	-	-	8	fail
416	18	Bin8_plateA0_F10.fcs	8 (102)	-	1 (106)	8	fail
417	18	Bin8_plateA0_F11.fcs	10 (84)	9 (37)	-	8	fail
418	18	Bin8_plateA0_F12.fcs	2 (97)	5 (87)	7 (34)	8	pass
419	18	Bin8_plateA0_G4.fcs	-	5 (47)	2 (5)	8	fail
420	18	Bin8_plateA0_G5.fcs	1 (13)	8 (32)	1 (17)	8	pass
421	18	Bin8_plateA0_G6.fcs	-	-	-	8	fail
422	18	Bin8_plateA0_G7.fcs	9 (50)	9 (24)	9 (29)	8	pass
423	18	Bin8_plateA0_G8.fcs	5 (29)	9 (146)	9 (11)	8	pass
424	18	Bin8_plateA0_G9.fcs	3 (55)	3 (32)	10 (12)	8	pass
425	18	Bin8_plateA0_G10.fcs	7 (87)	6 (45)	8 (32)	8	pass
426	18	Bin8_plateA0_G11.fcs	-	6 (7)	2 (29)	8	fail
427	19	Bin9_plateA0_D7.fcs	4 (119)	7 (48)	7 (139)	9	pass

Bead ID	Tag acquisition session #	Tag data filename	Split 1 media (# of tags)	Split 2 media (# of tags)	Split 3 media (# of tags)	Split 4 media	Pass tag deconvolution criteria
428	19	Bin9_plateA0_A1.fcs	3 (90)	9 (191)	1 (27)	9	pass
429	19	Bin9_plateA0_A2.fcs	10 (173)	10 (106)	3 (170)	9	pass
430	19	Bin9_plateA0_A3.fcs	8 (52)	9 (103)	2 (63)	9	pass
431	19	Bin9_plateA0_A4.fcs	6 (33)	7 (30)	2 (66)	9	pass
432	19	Bin9_plateA0_A5.fcs	6 (85)	5 (36)	1 (18)	9	pass
433	19	Bin9_plateA0_A6.fcs	1 (9)	1 (53)	9 (230)	9	pass
434	19	Bin9_plateA0_A7.fcs	4 (188)	8 (22)	3 (74)	9	pass
435	19	Bin9_plateA0_A8.fcs	6 (149)	6 (34)	6 (203)	9	pass
436	19	Bin9_plateA0_A9.fcs	5 (58)	6 (4)	9 (89)	9	pass
437	19	Bin9_plateA0_A10.fcs	4 (38)	9 (31)	10 (12)	9	pass
438	19	Bin9_plateA0_A11.fcs	7 (58)	9 (48)	2 (38)	9	pass
439	19	Bin9_plateA0_B1.fcs	-	-	-	9	fail
440	19	Bin9_plateA0_B2.fcs	-	-	-	9	fail
441	19	Bin9_plateA0_B3.fcs	3 (120)	10 (94)	9 (39)	9	pass
442	19	Bin9_plateA0_B4.fcs	2 (198)	4 (49)	2 (83)	9	pass
443	19	Bin9_plateA0_B5.fcs	1 (28)	5 (78)	2 (27)	9	pass
444	19	Bin9_plateA0_B6.fcs	7 (121)	3 (20)	1 (53)	9	pass
445	19	Bin9_plateA0_B7.fcs	7 (125)	8 (29)	9 (57)	9	pass
446	19	Bin9_plateA0_B8.fcs	-	-	-	9	fail
447	19	Bin9_plateA0_B9.fcs	4 (62)	6 (53)	7 (7)	9	pass
448	19	Bin9_plateA0_B10.fcs	7 (146)	2 (51)	-	9	fail
449	19	Bin9_plateA0_B11.fcs	9 (76)	8 (19)	9 (37)	9	pass
450	19	Bin9_plateA0_B12.fcs	8 (87)	9 (97)	8 (29)	9	pass
451	19	Bin9_plateA0_C1.fcs	2 (69)	10 (66)	6 (40)	9	pass
452	19	Bin9_plateA0_C2.fcs	1 (8)	5 (84)	-	9	fail
453	19	Bin9_plateA0_C3.fcs	2 (160)	5 (178)	5 (15)	9	pass
454	19	Bin9_plateA0_C4.fcs	3 (114)	6 (52)	6 (30)	9	pass
455	19	Bin9_plateA0_C5.fcs	10 (116)	5 (19)	1 (17)	9	pass
456	19	Bin9_plateA0_C6.fcs	3 (57)	4 (89)	7 (132)	9	pass
457	19	Bin9_plateA0_C7.fcs	-	-	-	9	fail
458	19	Bin9_plateA0_C8.fcs	7 (126)	4 (18)	4 (53)	9	pass
459	19	Bin9_plateA0_C9.fcs	-	-	-	9	fail
460	19	Bin9_plateA0_C10.fcs	-	-	-	9	fail
461	19	Bin9_plateA0_C11.fcs	10 (162)	4 (33)	2 (24)	9	pass
462	19	Bin9_plateA0_C12.fcs	7 (66)	5 (77)	9 (32)	9	pass
463	19	Bin9_plateA0_D1.fcs	9 (83)	4 (12)	1 (35)	9	pass
464	19	Bin9_plateA0_D2.fcs	5 (12)	-	6 (177)	9	fail
465	19	Bin9_plateA0_D3.fcs	6 (123)	10 (129)	1 (44)	9	pass
466	19	Bin9_plateA0_D4.fcs	10 (159)	-	-	9	fail
467	19	Bin9_plateA0_D5.fcs	1 (17)	5 (131)	1 (7)	9	pass
468	19	Bin9_plateA0_D6.fcs	6 (43)	8 (21)	5 (33)	9	pass
469	20	Bin9_plateA0_H12.fcs	2 (103)	4 (41)	1 (16)	9	pass
470	20	Bin9_plateA0_D8.fcs	-	-	-	9	fail

Bead ID	Tag acquisition session #	Tag data filename	Split 1 media (# of tags)	Split 2 media (# of tags)	Split 3 media (# of tags)	Split 4 media	Pass tag deconvolution criteria
471	20	Bin9_plateA0_D9.fcs	-	-	-	9	fail
472	20	Bin9_plateA0_D10.fcs	5 (50)	2 (22)	8 (53)	9	pass
473	20	Bin9_plateA0_D11.fcs	7 (64)	7 (44)	3 (38)	9	pass
474	20	Bin9_plateA0_D12.fcs	10 (71)	5 (38)	5 (76)	9	pass
475	20	Bin9_plateA0_E1.fcs	2 (115)	5 (44)	1 (24)	9	pass
476	20	Bin9_plateA0_E2.fcs	5 (48)	10 (116)	9 (32)	9	pass
477	20	Bin9_plateA0_E3.fcs	5 (30)	10 (122)	7 (24)	9	pass
478	20	Bin9_plateA0_E4.fcs	7 (81)	6 (55)	1 (40)	9	pass
479	20	Bin9_plateA0_E5.fcs	-	-	-	9	fail
480	20	Bin9_plateA0_E6.fcs	6 (93)	8 (17)	2 (60)	9	pass
481	20	Bin9_plateA0_E7.fcs	6 (237)	9 (169)	2 (23)	9	pass
482	20	Bin9_plateA0_E8.fcs	10 (83)	4 (33)	2 (47)	9	pass
483	20	Bin9_plateA0_E9.fcs	7 (67)	-	-	9	fail
484	20	Bin9_plateA0_E10.fcs	10 (135)	7 (71)	1 (16)	9	pass
485	20	Bin9_plateA0_E11.fcs	3 (140)	8 (30)	1 (37)	9	pass
486	20	Bin9_plateA0_E12.fcs	-	-	-	9	fail
487	20	Bin9_plateA0_F1.fcs	2 (83)	9 (157)	7 (9)	9	pass
488	20	Bin9_plateA0_F2.fcs	5 (41)	9 (41)	2 (42)	9	pass
489	20	Bin9_plateA0_F3.fcs	5 (31)	9 (49)	7 (28)	9	pass
490	20	Bin9_plateA0_F4.fcs	7 (92)	6 (6)	1 (19)	9	pass
491	20	Bin9_plateA0_F6.fcs	9 (97)	5 (89)	6 (93)	9	pass
492	20	Bin9_plateA0_F7.fcs	5 (17)	2 (19)	7 (31)	9	pass
493	20	Bin9_plateA0_F8.fcs	4 (141)	7 (103)	5 (11)	9	pass
494	20	Bin9_plateA0_F9.fcs	3 (125)	3 (21)	10 (24)	9	pass
495	20	Bin9_plateA0_F10.fcs	8 (38)	7 (51)	4 (44)	9	pass
496	20	Bin9_plateA0_F11.fcs	8 (81)	7 (94)	7 (73)	9	pass
497	20	Bin9_plateA0_F12.fcs	3 (40)	9 (100)	2 (62)	9	pass
498	20	Bin9_plateA0_G1.fcs	3 (100)	7 (71)	5 (59)	9	pass
499	20	Bin9_plateA0_G2.fcs	3 (91)	5 (9)	9 (57)	9	pass
500	20	Bin9_plateA0_G3.fcs	9 (82)	1 (25)	1 (32)	9	pass
501	20	Bin9_plateA0_G4.fcs	7 (44)	9 (46)	1 (18)	9	pass
502	20	Bin9_plateA0_G6.fcs	2 (40)	6 (42)	9 (51)	9	pass
503	20	Bin9_plateA0_G7.fcs	3 (49)	5 (35)	5 (39)	9	pass
504	20	Bin9_plateA0_G8.fcs	-	-	-	9	fail
505	20	Bin9_plateA0_G9.fcs	2 (39)	9 (59)	3 (10)	9	pass
506	20	Bin9_plateA0_G10.fcs	-	9 (90)	8 (191)	9	fail
507	20	Bin9_plateA0_G11.fcs	-	-	-	9	fail
508	20	Bin9_plateA0_G12.fcs	5 (7)	10 (174)	9 (34)	9	pass
509	20	Bin9_plateA0_H1.fcs	2 (62)	3 (60)	8 (28)	9	pass
510	20	Bin9_plateA0_H2.fcs	6 (86)	10 (21)	1 (69)	9	pass
511	20	Bin9_plateA0_H3.fcs	1 (10)	9 (48)	9 (43)	9	pass
512	20	Bin9_plateA0_H4.fcs	9 (70)	5 (41)	4 (27)	9	pass
513	20	Bin9_plateA0_H5.fcs	1 (15)	10 (89)	1 (21)	9	pass

Bead ID	Tag acquisition session #	Tag data filename	Split 1 media (# of tags)	Split 2 media (# of tags)	Split 3 media (# of tags)	Split 4 media	Pass tag deconvolution criteria
514	20	Bin9_plateA0_H6.fcs	8 (48)	8 (25)	3 (23)	9	pass
515	20	Bin9_plateA0_H7.fcs	3 (137)	-	3 (136)	9	fail
516	20	Bin9_plateA0_H8.fcs	-	-	-	9	fail
517	20	Bin9_plateA0_H9.fcs	7 (53)	9 (62)	2 (39)	9	pass
518	20	Bin9_plateA0_H10.fcs	3 (90)	7 (35)	2 (89)	9	pass
519	20	Bin9_plateA0_H11.fcs	-	5 (111)	5 (40)	9	fail
520	21	Bin10_plateA1_D12.fcs	3 (36)	9 (15)	2 (32)	10	pass
521	21	Bin9_plateB1_A1.fcs	1 (18)	1 (22)	10 (37)	9	pass
522	21	Bin9_plateB1_A2.fcs	6 (121)	-	2 (206)	9	fail
523	21	Bin9_plateB1_A6.fcs	4 (87)	6 (39)	4 (108)	9	pass
524	21	Bin9_plateB1_A7.fcs	8 (31)	10 (28)	3 (71)	9	pass
525	21	Bin9_plateB1_A8.fcs	-	3 (9)	2 (51)	9	fail
526	21	Bin10_plateA1_C1.fcs	7 (10)	-	5 (20)	10	fail
527	21	Bin10_plateA1_C2.fcs	3 (46)	1 (26)	8 (87)	10	pass
528	21	Bin10_plateA1_C3.fcs	10 (50)	9 (16)	1 (19)	10	pass
529	21	Bin10_plateA1_C4.fcs	1 (15)	7 (50)	7 (111)	10	pass
530	21	Bin10_plateA1_C5.fcs	2 (32)	8 (14)	4 (33)	10	pass
531	21	Bin10_plateA1_C6.fcs	-	-	-	10	fail
532	21	Bin10_plateA1_C7.fcs	9 (21)	2 (7)	8 (19)	10	pass
533	21	Bin10_plateA1_C8.fcs	5 (15)	-	6 (32)	10	fail
534	21	Bin10_plateA1_C9.fcs	-	8 (26)	6 (11)	10	fail
535	21	Bin10_plateA1_C10.fcs	-	5 (65)	10 (10)	10	fail
536	21	Bin10_plateA1_D8.fcs	4 (114)	7 (8)	9 (33)	10	pass
537	21	Bin10_plateA1_D9.fcs	4 (93)	1 (39)	3 (18)	10	pass
538	21	Bin10_plateA1_D10.fcs	6 (108)	5 (72)	9 (12)	10	pass
539	21	Bin10_plateA1_D11.fcs	8 (46)	9 (25)	1 (39)	10	pass

Confidential

6. PROTOCOL ANALYSIS

6.1 DATASET REVIEW

Once a dataset of protocols has been established, Ariadne™ allows post-acquisition resetting of gates on the COPAS sorting plot to specify a subset of hits for further analysis. For example, if ‘hits were sorted using two antibodies, the user can select those which are positive for both antibodies. No post acquisition flow sort criterion was applied in this study.

Table 9 lists the bead IDs included in the dataset analyzed by Ariadne™ and presented in the following sections of the report. The dataset only includes beads which fall within the (reset) COPAS gates and can be assigned a full cell culture history. Ariadne™ allows a range of analyses to be performed, to select protocols for validation. Analysis methodologies include linkage analysis which identifies frequently occurring media combinations; fingerprint analysis which clusters groups of beads with identical or similar protocols (i.e. protocols with common cell culture media on the same split) and calculates the probability of these clusters occurring randomly; and methods of comparing entire protocols such as hierarchical clustering and a similarity matrix. These results are presented and discussed in the following sections.

Table 9: Dataset Review. The following parameters are shown for each hit: Bead ID, protocol (“–” represents unreadable tag data), whether the bead passed the tag deconvolution, whether it falls within the (reset) COPAS gate and whether it is included in the protocol analysis dataset.

	Protocol				Inclusion Criteria		
Bead ID	Split 1 media	Split 2 media	Split 3 media	Split 4 media	Tag deconvolution	Flow sort criteria	Included in analysis
1	9	9	5	1	pass	pass	Yes
2	8	-	10	1	fail	pass	No
3	9	-	6	1	fail	pass	No
4	4	9	9	1	pass	pass	Yes
5	7	6	7	1	pass	pass	Yes
6	10	4	4	1	pass	pass	Yes
7	7	10	2	1	pass	pass	Yes
8	6	8	1	1	pass	pass	Yes
9	3	6	3	1	pass	pass	Yes
10	8	2	2	1	pass	pass	Yes
11	1	-	2	1	fail	pass	No
12	2	5	4	1	pass	pass	Yes
13	10	8	2	1	pass	pass	Yes
14	3	7	3	1	pass	pass	Yes
15	1	-	4	1	fail	pass	No
16	5	-	5	1	fail	pass	No
17	10	10	5	1	pass	pass	Yes
18	4	6	9	1	pass	pass	Yes
19	7	2	10	1	pass	pass	Yes
20	-	-	1	1	fail	pass	No
21	9	4	2	1	pass	pass	Yes
22	1	-	8	1	fail	pass	No
23	1	8	9	1	pass	pass	Yes
24	6	7	6	1	pass	pass	Yes
25	9	7	5	1	pass	pass	Yes

Bead ID	Protocol				Inclusion Criteria		
	Split 1 media	Split 2 media	Split 3 media	Split 4 media	Tag deconvolution	Flow sort criteria	Included in analysis
26	5	9	9	1	pass	pass	Yes
27	10	7	5	1	pass	pass	Yes
28	6	1	9	1	pass	pass	Yes
29	10	7	9	1	pass	pass	Yes
30	-	-	1	1	fail	pass	No
31	9	-	-	1	fail	pass	No
32	10	6	7	1	pass	pass	Yes
33	1	3	-	1	fail	pass	No
34	6	10	6	1	pass	pass	Yes
35	5	2	7	1	pass	pass	Yes
36	1	10	5	1	pass	pass	Yes
37	4	6	2	1	pass	pass	Yes
38	8	10	8	1	pass	pass	Yes
39	-	-	2	1	fail	pass	No
40	5	4	5	1	pass	pass	Yes
41	-	-	-	1	fail	pass	No
42	2	1	5	1	pass	pass	Yes
43	-	-	-	1	fail	pass	No
44	-	4	10	1	fail	pass	No
45	1	3	6	1	pass	pass	Yes
46	5	10	5	1	pass	pass	Yes
47	1	10	-	1	fail	pass	No
48	3	1	10	1	pass	pass	Yes
49	-	-	9	1	fail	pass	No
50	9	5	8	1	pass	pass	Yes
51	9	1	7	1	pass	pass	Yes
52	6	4	7	1	pass	pass	Yes
53	9	3	7	1	pass	pass	Yes
54	2	5	3	1	pass	pass	Yes
55	9	6	7	1	pass	pass	Yes
56	7	6	10	1	pass	pass	Yes
57	6	9	6	1	pass	pass	Yes
58	7	7	2	1	pass	pass	Yes
59	5	3	-	1	fail	pass	No
60	10	2	3	1	pass	pass	Yes
61	9	2	6	1	pass	pass	Yes
62	6	3	-	1	fail	pass	No
63	3	8	2	1	pass	pass	Yes
64	10	4	-	1	fail	pass	No
65	5	7	10	1	pass	pass	Yes
66	1	5	1	1	pass	pass	Yes
67	2	10	1	1	pass	pass	Yes
68	3	9	1	1	pass	pass	Yes

Bead ID	Protocol				Inclusion Criteria		
	Split 1 media	Split 2 media	Split 3 media	Split 4 media	Tag deconvolution	Flow sort criteria	Included in analysis
69	9	10	3	1	pass	pass	Yes
70	9	9	4	1	pass	pass	Yes
71	5	9	10	1	pass	pass	Yes
72	-	9	7	1	fail	pass	No
73	10	2	6	1	pass	pass	Yes
74	1	10	-	1	fail	pass	No
75	9	9	9	1	pass	pass	Yes
76	1	-	4	1	fail	pass	No
77	4	6	7	1	pass	pass	Yes
78	9	3	10	1	pass	pass	Yes
79	3	6	1	1	pass	pass	Yes
80	2	5	9	1	pass	pass	Yes
81	9	4	6	1	pass	pass	Yes
82	10	8	10	1	pass	pass	Yes
83	4	6	6	1	pass	pass	Yes
84	2	10	5	1	pass	pass	Yes
85	8	8	2	1	pass	pass	Yes
86	1	6	2	1	pass	pass	Yes
87	6	3	7	1	pass	pass	Yes
88	8	7	10	1	pass	pass	Yes
89	3	9	3	1	pass	pass	Yes
90	6	3	9	1	pass	pass	Yes
91	4	10	4	1	pass	pass	Yes
92	1	-	4	1	fail	pass	No
93	9	8	8	1	pass	pass	Yes
94	9	-	6	1	fail	pass	No
95	9	7	3	1	pass	pass	Yes
96	10	10	7	1	pass	pass	Yes
97	10	8	1	1	pass	pass	Yes
98	10	5	8	1	pass	pass	Yes
99	7	-	7	1	fail	pass	No
100	-	-	-	1	fail	pass	No
101	1	5	9	1	pass	pass	Yes
102	9	5	-	1	fail	pass	No
103	9	-	-	1	fail	pass	No
104	-	-	10	1	fail	pass	No
105	-	-	6	1	fail	pass	No
106	9	5	7	1	pass	pass	Yes
107	-	-	1	1	fail	pass	No
108	9	4	6	1	pass	pass	Yes
109	10	-	6	1	fail	pass	No
110	7	3	1	1	pass	pass	Yes
111	5	5	-	1	fail	pass	No

Bead ID	Protocol				Inclusion Criteria		
	Split 1 media	Split 2 media	Split 3 media	Split 4 media	Tag deconvolution	Flow sort criteria	Included in analysis
112	8	4	7	1	pass	pass	Yes
113	9	2	10	1	pass	pass	Yes
114	4	10	4	1	pass	pass	Yes
115	-	-	-	1	fail	pass	No
116	3	10	4	1	pass	pass	Yes
117	10	6	1	1	pass	pass	Yes
118	9	7	3	1	pass	pass	Yes
119	4	1	2	1	pass	pass	Yes
120	10	2	9	1	pass	pass	Yes
121	5	9	2	1	pass	pass	Yes
122	9	-	10	1	fail	pass	No
123	-	-	-	1	fail	pass	No
124	8	10	9	1	pass	pass	Yes
125	1	4	10	1	pass	pass	Yes
126	5	9	5	1	pass	pass	Yes
127	2	-	-	1	fail	pass	No
128	6	8	1	1	pass	pass	Yes
129	2	4	3	1	pass	pass	Yes
130	6	6	10	1	pass	pass	Yes
131	-	-	-	1	fail	pass	No
132	2	7	4	1	pass	pass	Yes
133	10	-	-	1	fail	pass	No
134	6	8	1	1	pass	pass	Yes
135	5	9	5	1	pass	pass	Yes
136	1	6	7	1	pass	pass	Yes
137	8	-	8	1	fail	pass	No
138	-	3	-	1	fail	pass	No
139	4	6	7	1	pass	pass	Yes
140	5	1	10	1	pass	pass	Yes
141	5	10	6	1	pass	pass	Yes
142	9	2	-	1	fail	pass	No
143	-	10	6	1	fail	pass	No
144	-	-	-	1	fail	pass	No
145	1	2	10	1	pass	pass	Yes
146	9	-	6	1	fail	pass	No
147	2	3	8	1	pass	pass	Yes
148	2	4	2	1	pass	pass	Yes
149	-	4	5	1	fail	pass	No
150	4	7	10	1	pass	pass	Yes
151	4	4	5	1	pass	pass	Yes
152	7	9	7	1	pass	pass	Yes
153	-	6	1	1	fail	pass	No
154	-	-	-	1	fail	pass	No

Bead ID	Protocol				Inclusion Criteria		
	Split 1 media	Split 2 media	Split 3 media	Split 4 media	Tag deconvolution	Flow sort criteria	Included in analysis
155	8	8	9	1	pass	pass	Yes
156	-	3	1	1	fail	pass	No
157	7	-	4	1	fail	pass	No
158	10	5	10	1	pass	pass	Yes
159	3	1	10	1	pass	pass	Yes
160	8	6	9	1	pass	pass	Yes
161	8	4	8	1	pass	pass	Yes
162	8	10	3	1	pass	pass	Yes
163	6	9	1	1	pass	pass	Yes
164	8	5	10	1	pass	pass	Yes
165	-	-	-	1	fail	pass	No
166	1	5	2	1	pass	pass	Yes
167	5	5	5	1	pass	pass	Yes
168	-	-	-	1	fail	pass	No
169	7	8	10	1	pass	pass	Yes
170	8	7	9	1	pass	pass	Yes
171	1	5	2	1	pass	pass	Yes
172	9	10	10	1	pass	pass	Yes
173	5	9	5	1	pass	pass	Yes
174	1	5	7	1	pass	pass	Yes
175	4	6	5	1	pass	pass	Yes
176	6	7	5	1	pass	pass	Yes
177	-	5	2	1	fail	pass	No
178	4	6	1	1	pass	pass	Yes
179	6	5	6	1	pass	pass	Yes
180	5	10	5	1	pass	pass	Yes
181	1	3	10	1	pass	pass	Yes
182	6	5	3	1	pass	pass	Yes
183	4	9	5	1	pass	pass	Yes
184	2	9	6	1	pass	pass	Yes
185	3	4	1	2	pass	pass	Yes
186	5	10	2	2	pass	pass	Yes
187	8	6	5	2	pass	pass	Yes
188	9	4	9	2	pass	pass	Yes
189	4	9	3	2	pass	pass	Yes
190	3	6	10	2	pass	pass	Yes
191	5	9	-	2	fail	pass	No
192	10	-	1	2	fail	pass	No
193	10	4	4	2	pass	pass	Yes
194	10	6	9	2	pass	pass	Yes
195	5	8	9	2	pass	pass	Yes
196	4	1	2	2	pass	pass	Yes
197	2	3	10	2	pass	pass	Yes

	Protocol				Inclusion Criteria		
Bead ID	Split 1 media	Split 2 media	Split 3 media	Split 4 media	Tag deconvolution	Flow sort criteria	Included in analysis
198	5	9	2	2	pass	pass	Yes
199	3	5	9	2	pass	pass	Yes
200	2	9	3	2	pass	pass	Yes
201	3	6	9	2	pass	pass	Yes
202	5	10	-	2	fail	pass	No
203	10	6	7	2	pass	pass	Yes
204	1	5	2	2	pass	pass	Yes
205	5	10	-	2	fail	pass	No
206	2	6	3	2	pass	pass	Yes
207	9	10	5	2	pass	pass	Yes
208	5	10	3	2	pass	pass	Yes
209	8	6	10	2	pass	pass	Yes
210	10	10	5	2	pass	pass	Yes
211	10	6	9	2	pass	pass	Yes
212	5	10	7	2	pass	pass	Yes
213	6	6	3	2	pass	pass	Yes
214	4	10	3	2	pass	pass	Yes
215	7	4	2	2	pass	pass	Yes
216	1	5	3	2	pass	pass	Yes
217	10	8	7	2	pass	pass	Yes
218	7	4	8	2	pass	pass	Yes
219	9	6	6	2	pass	pass	Yes
220	10	5	5	2	pass	pass	Yes
221	6	8	2	2	pass	pass	Yes
222	7	3	6	2	pass	pass	Yes
223	5	9	5	2	pass	pass	Yes
224	8	2	2	2	pass	pass	Yes
225	3	2	-	2	fail	pass	No
226	-	9	3	2	fail	pass	No
227	1	5	6	2	pass	pass	Yes
228	5	2	2	2	pass	pass	Yes
229	6	5	8	2	pass	pass	Yes
230	3	-	1	2	fail	pass	No
231	-	3	-	2	fail	pass	No
232	-	-	-	2	fail	pass	No
233	2	-	3	2	fail	pass	No
234	6	2	9	2	pass	pass	Yes
235	1	6	5	2	pass	pass	Yes
236	8	3	2	2	pass	pass	Yes
237	3	5	8	2	pass	pass	Yes
238	6	5	2	2	pass	pass	Yes
239	5	7	6	2	pass	pass	Yes
240	10	8	9	2	pass	pass	Yes

	Protocol				Inclusion Criteria		
Bead ID	Split 1 media	Split 2 media	Split 3 media	Split 4 media	Tag deconvolution	Flow sort criteria	Included in analysis
241	10	-	-	2	fail	pass	No
242	6	4	6	2	pass	pass	Yes
243	4	4	10	2	pass	pass	Yes
244	8	10	9	2	pass	pass	Yes
245	5	4	7	2	pass	pass	Yes
246	5	9	1	2	pass	pass	Yes
247	9	10	9	2	pass	pass	Yes
248	5	6	6	2	pass	pass	Yes
249	4	10	7	2	pass	pass	Yes
250	-	3	1	2	fail	pass	No
251	3	4	6	2	pass	pass	Yes
252	5	10	10	2	pass	pass	Yes
253	3	3	5	2	pass	pass	Yes
254	4	-	6	2	fail	pass	No
255	2	3	1	2	pass	pass	Yes
256	-	9	3	2	fail	pass	No
257	-	7	10	2	fail	pass	No
258	5	5	3	2	pass	pass	Yes
259	2	10	5	2	pass	pass	Yes
260	3	5	8	3	pass	pass	Yes
261	5	9	1	3	pass	pass	Yes
262	5	10	9	3	pass	pass	Yes
263	9	4	3	3	pass	pass	Yes
264	9	5	7	3	pass	pass	Yes
265	8	6	10	3	pass	pass	Yes
266	1	7	10	3	pass	pass	Yes
267	7	1	7	3	pass	pass	Yes
268	7	4	2	3	pass	pass	Yes
269	5	10	-	3	fail	pass	No
270	2	3	8	3	pass	pass	Yes
271	5	9	2	3	pass	pass	Yes
272	1	5	7	3	pass	pass	Yes
273	6	-	-	3	fail	pass	No
274	7	10	2	3	pass	pass	Yes
275	-	-	4	3	fail	pass	No
276	2	6	10	3	pass	pass	Yes
277	7	10	3	3	pass	pass	Yes
278	5	10	9	3	pass	pass	Yes
279	-	-	9	3	fail	pass	No
280	1	5	3	3	pass	pass	Yes
281	3	5	2	3	pass	pass	Yes
282	10	8	7	4	pass	pass	Yes
283	10	9	5	4	pass	pass	Yes

Bead ID	Protocol				Inclusion Criteria		
	Split 1 media	Split 2 media	Split 3 media	Split 4 media	Tag deconvolution	Flow sort criteria	Included in analysis
284	1	5	2	4	pass	pass	Yes
285	10	1	5	4	pass	pass	Yes
286	3	1	8	4	pass	pass	Yes
287	3	2	2	4	pass	pass	Yes
288	1	2	9	4	pass	pass	Yes
289	1	-	8	4	fail	pass	No
290	-	-	-	4	fail	pass	No
291	5	5	1	4	pass	pass	Yes
292	-	9	2	4	fail	pass	No
293	3	-	8	4	fail	pass	No
294	5	10	6	4	pass	pass	Yes
295	10	8	8	4	pass	pass	Yes
296	4	6	-	4	fail	pass	No
297	-	6	6	4	fail	pass	No
298	1	9	5	4	pass	pass	Yes
299	1	5	2	4	pass	pass	Yes
300	-	5	9	4	fail	pass	No
301	2	6	7	4	pass	pass	Yes
302	5	-	2	4	fail	pass	No
303	6	10	10	4	pass	pass	Yes
304	4	5	6	4	pass	pass	Yes
305	1	8	6	4	pass	pass	Yes
306	7	6	6	4	pass	pass	Yes
307	9	9	10	4	pass	pass	Yes
308	10	5	9	4	pass	pass	Yes
309	3	10	10	4	pass	pass	Yes
310	1	5	9	4	pass	pass	Yes
311	-	5	3	4	fail	pass	No
312	1	10	7	4	pass	pass	Yes
313	-	-	1	4	fail	pass	No
314	7	3	-	4	fail	pass	No
315	2	5	9	4	pass	pass	Yes
316	5	3	9	4	pass	pass	Yes
317	6	8	4	4	pass	pass	Yes
318	5	9	3	4	pass	pass	Yes
319	7	2	3	4	pass	pass	Yes
320	5	10	-	4	fail	pass	No
321	4	8	3	4	pass	pass	Yes
322	-	9	10	4	fail	pass	No
323	1	1	6	4	pass	pass	Yes
324	1	-	-	4	fail	pass	No
325	1	-	2	4	fail	pass	No
326	3	1	5	5	pass	pass	Yes

Bead ID	Protocol Split 1 media	Split 2 media	Split 3 media	Split 4 media	Inclusion Criteria Tag deconvolution	Flow sort criteria	Included in analysis
327	6	2	5	5	pass	pass	Yes
328	5	9	6	5	pass	pass	Yes
329	6	6	10	5	pass	pass	Yes
330	4	6	5	5	pass	pass	Yes
331	4	2	3	5	pass	pass	Yes
332	2	10	-	5	fail	pass	No
333	1	-	6	5	fail	pass	No
334	4	5	1	5	pass	pass	Yes
335	-	5	3	5	fail	pass	No
336	4	-	4	5	fail	pass	No
337	10	10	1	5	pass	pass	Yes
338	7	2	7	5	pass	pass	Yes
339	7	9	6	5	pass	pass	Yes
340	5	9	5	5	pass	pass	Yes
341	2	7	7	5	pass	pass	Yes
342	1	6	6	5	pass	pass	Yes
343	6	10	10	5	pass	pass	Yes
344	10	6	2	5	pass	pass	Yes
345	4	9	8	5	pass	pass	Yes
346	-	-	-	5	fail	pass	No
347	5	2	6	5	pass	pass	Yes
348	7	9	9	5	pass	pass	Yes
349	10	1	6	5	pass	pass	Yes
350	5	9	9	5	pass	pass	Yes
351	9	1	6	5	pass	pass	Yes
352	4	-	10	5	fail	pass	No
353	3	2	5	5	pass	pass	Yes
354	-	8	8	5	fail	pass	No
355	1	5	1	5	pass	pass	Yes
356	7	7	5	5	pass	pass	Yes
357	8	7	3	5	pass	pass	Yes
358	10	5	5	5	pass	pass	Yes
359	-	10	4	5	fail	pass	No
360	9	-	1	5	fail	pass	No
361	10	2	5	5	pass	pass	Yes
362	-	-	2	5	fail	pass	No
363	9	5	1	5	pass	pass	Yes
364	2	4	2	5	pass	pass	Yes
365	1	9	1	5	pass	pass	Yes
366	5	9	9	5	pass	pass	Yes
367	1	5	10	5	pass	pass	Yes
368	1	9	7	5	pass	pass	Yes
369	6	9	-	5	fail	pass	No

Confidential

Bead ID	Protocol				Inclusion Criteria		
	Split 1 media	Split 2 media	Split 3 media	Split 4 media	Tag deconvolution	Flow sort criteria	Included in analysis
370	10	10	8	5	pass	pass	Yes
371	-	-	-	5	fail	pass	No
372	1	5	2	5	pass	pass	Yes
373	5	10	6	5	pass	pass	Yes
374	9	9	10	5	pass	pass	Yes
375	7	8	5	5	pass	pass	Yes
376	4	2	3	5	pass	pass	Yes
377	3	6	3	5	pass	pass	Yes
378	1	6	8	5	pass	pass	Yes
379	1	5	1	7	pass	pass	Yes
380	1	5	3	6	pass	pass	Yes
381	-	-	-	6	fail	pass	No
382	3	1	2	6	pass	pass	Yes
383	9	6	10	6	pass	pass	Yes
384	5	7	1	6	pass	pass	Yes
385	-	-	-	6	fail	pass	No
386	7	9	6	6	pass	pass	Yes
387	10	3	9	6	pass	pass	Yes
388	5	10	1	6	pass	pass	Yes
389	1	10	5	7	pass	pass	Yes
390	2	3	2	7	pass	pass	Yes
391	10	9	4	8	pass	pass	Yes
392	8	-	7	7	fail	pass	No
393	7	6	1	7	pass	pass	Yes
394	8	10	6	7	pass	pass	Yes
395	-	-	9	7	fail	pass	No
396	4	1	3	7	pass	pass	Yes
397	3	2	3	7	pass	pass	Yes
398	6	6	6	7	pass	pass	Yes
399	5	10	1	7	pass	pass	Yes
400	8	8	4	7	pass	pass	Yes
401	-	-	-	7	fail	pass	No
402	5	3	2	7	pass	pass	Yes
403	7	3	6	7	pass	pass	Yes
404	8	2	10	7	pass	pass	Yes
405	3	3	4	7	pass	pass	Yes
406	10	4	9	7	pass	pass	Yes
407	-	-	-	8	fail	pass	No
408	1	5	1	8	pass	pass	Yes
409	8	5	9	8	pass	pass	Yes
410	7	9	10	8	pass	pass	Yes
411	7	6	1	8	pass	pass	Yes
412	6	6	6	8	pass	pass	Yes

Confidential

Bead ID	Protocol				Inclusion Criteria		
	Split 1 media	Split 2 media	Split 3 media	Split 4 media	Tag deconvolution	Flow sort criteria	Included in analysis
413	2	3	5	8	pass	pass	Yes
414	-	-	1	8	fail	pass	No
415	-	-	-	8	fail	pass	No
416	8	-	1	8	fail	pass	No
417	10	9	-	8	fail	pass	No
418	2	5	7	8	pass	pass	Yes
419	-	5	2	8	fail	pass	No
420	1	8	1	8	pass	pass	Yes
421	-	-	-	8	fail	pass	No
422	9	9	9	8	pass	pass	Yes
423	5	9	9	8	pass	pass	Yes
424	3	3	10	8	pass	pass	Yes
425	7	6	8	8	pass	pass	Yes
426	-	6	2	8	fail	pass	No
427	4	7	7	9	pass	pass	Yes
428	3	9	1	9	pass	pass	Yes
429	10	10	3	9	pass	pass	Yes
430	8	9	2	9	pass	pass	Yes
431	6	7	2	9	pass	pass	Yes
432	6	5	1	9	pass	pass	Yes
433	1	1	9	9	pass	pass	Yes
434	4	8	3	9	pass	pass	Yes
435	6	6	6	9	pass	pass	Yes
436	5	6	9	9	pass	pass	Yes
437	4	9	10	9	pass	pass	Yes
438	7	9	2	9	pass	pass	Yes
439	-	-	-	9	fail	pass	No
440	-	-	-	9	fail	pass	No
441	3	10	9	9	pass	pass	Yes
442	2	4	2	9	pass	pass	Yes
443	1	5	2	9	pass	pass	Yes
444	7	3	1	9	pass	pass	Yes
445	7	8	9	9	pass	pass	Yes
446	-	-	-	9	fail	pass	No
447	4	6	7	9	pass	pass	Yes
448	7	2	-	9	fail	pass	No
449	9	8	9	9	pass	pass	Yes
450	8	9	8	9	pass	pass	Yes
451	2	10	6	9	pass	pass	Yes
452	1	5	-	9	fail	pass	No
453	2	5	5	9	pass	pass	Yes
454	3	6	6	9	pass	pass	Yes
455	10	5	1	9	pass	pass	Yes

Bead ID	Protocol				Inclusion Criteria		
	Split 1 media	Split 2 media	Split 3 media	Split 4 media	Tag deconvolution	Flow sort criteria	Included in analysis
456	3	4	7	9	pass	pass	Yes
457	-	-	-	9	fail	pass	No
458	7	4	4	9	pass	pass	Yes
459	-	-	-	9	fail	pass	No
460	-	-	-	9	fail	pass	No
461	10	4	2	9	pass	pass	Yes
462	7	5	9	9	pass	pass	Yes
463	9	4	1	9	pass	pass	Yes
464	5	-	6	9	fail	pass	No
465	6	10	1	9	pass	pass	Yes
466	10	-	-	9	fail	pass	No
467	1	5	1	9	pass	pass	Yes
468	6	8	5	9	pass	pass	Yes
469	2	4	1	9	pass	pass	Yes
470	-	-	-	9	fail	pass	No
471	-	-	-	9	fail	pass	No
472	5	2	8	9	pass	pass	Yes
473	7	7	3	9	pass	pass	Yes
474	10	5	5	9	pass	pass	Yes
475	2	5	1	9	pass	pass	Yes
476	5	10	9	9	pass	pass	Yes
477	5	10	7	9	pass	pass	Yes
478	7	6	1	9	pass	pass	Yes
479	-	-	-	9	fail	pass	No
480	6	8	2	9	pass	pass	Yes
481	6	9	2	9	pass	pass	Yes
482	10	4	2	9	pass	pass	Yes
483	7	-	-	9	fail	pass	No
484	10	7	1	9	pass	pass	Yes
485	3	8	1	9	pass	pass	Yes
486	-	-	-	9	fail	pass	No
487	2	9	7	9	pass	pass	Yes
488	5	9	2	9	pass	pass	Yes
489	5	9	7	9	pass	pass	Yes
490	7	6	1	9	pass	pass	Yes
491	9	5	6	9	pass	pass	Yes
492	5	2	7	9	pass	pass	Yes
493	4	7	5	9	pass	pass	Yes
494	3	3	10	9	pass	pass	Yes
495	8	7	4	9	pass	pass	Yes
496	8	7	7	9	pass	pass	Yes
497	3	9	2	9	pass	pass	Yes
498	3	7	5	9	pass	pass	Yes

Bead ID	Protocol				Inclusion Criteria		
	Split 1 media	Split 2 media	Split 3 media	Split 4 media	Tag deconvolution	Flow sort criteria	Included in analysis
499	3	5	9	9	pass	pass	Yes
500	9	1	1	9	pass	pass	Yes
501	7	9	1	9	pass	pass	Yes
502	2	6	9	9	pass	pass	Yes
503	3	5	5	9	pass	pass	Yes
504	-	-	-	9	fail	pass	No
505	2	9	3	9	pass	pass	Yes
506	-	9	8	9	fail	pass	No
507	-	-	-	9	fail	pass	No
508	5	10	9	9	pass	pass	Yes
509	2	3	8	9	pass	pass	Yes
510	6	10	1	9	pass	pass	Yes
511	1	9	9	9	pass	pass	Yes
512	9	5	4	9	pass	pass	Yes
513	1	10	1	9	pass	pass	Yes
514	8	8	3	9	pass	pass	Yes
515	3	-	3	9	fail	pass	No
516	-	-	-	9	fail	pass	No
517	7	9	2	9	pass	pass	Yes
518	3	7	2	9	pass	pass	Yes
519	-	5	5	9	fail	pass	No
520	3	9	2	10	pass	pass	Yes
521	1	1	10	9	pass	pass	Yes
522	6	-	2	9	fail	pass	No
523	4	6	4	9	pass	pass	Yes
524	8	10	3	9	pass	pass	Yes
525	-	3	2	9	fail	pass	No
526	7	-	5	10	fail	pass	No
527	3	1	8	10	pass	pass	Yes
528	10	9	1	10	pass	pass	Yes
529	1	7	7	10	pass	pass	Yes
530	2	8	4	10	pass	pass	Yes
531	-	-	-	10	fail	pass	No
532	9	2	8	10	pass	pass	Yes
533	5	-	6	10	fail	pass	No
534	-	8	6	10	fail	pass	No
535	-	5	10	10	fail	pass	No
536	4	7	9	10	pass	pass	Yes
537	4	1	3	10	pass	pass	Yes
538	6	5	9	10	pass	pass	Yes
539	8	9	1	10	pass	pass	Yes

Confidential

6. PROTOCOL ANALYSIS
6.2. LINKAGE ANALYSIS

A schematic diagram of the split-pool experiment is shown in Figure 5. Each rectangle corresponds to a cell culture medium and each row of rectangles corresponds to a split. The black upper number within each rectangle is the medium number and the grey number below is the number of beads within the analysis dataset that passed through that medium (the height of each rectangle is proportional to this number). The linkage lines between rectangles depict frequently occurring combinations of media resulting in an overlay of all protocols in the dataset. The opacity of the linkage lines is proportional to the number of protocols that feature a certain media combination (in this dataset, the darkest line corresponds to 21beads.

Figure 5: Schematic diagram showing an overlay of all protocols in the analysis dataset. The darkest line corresponds to 21 beads passing between the 2 connected conditions.

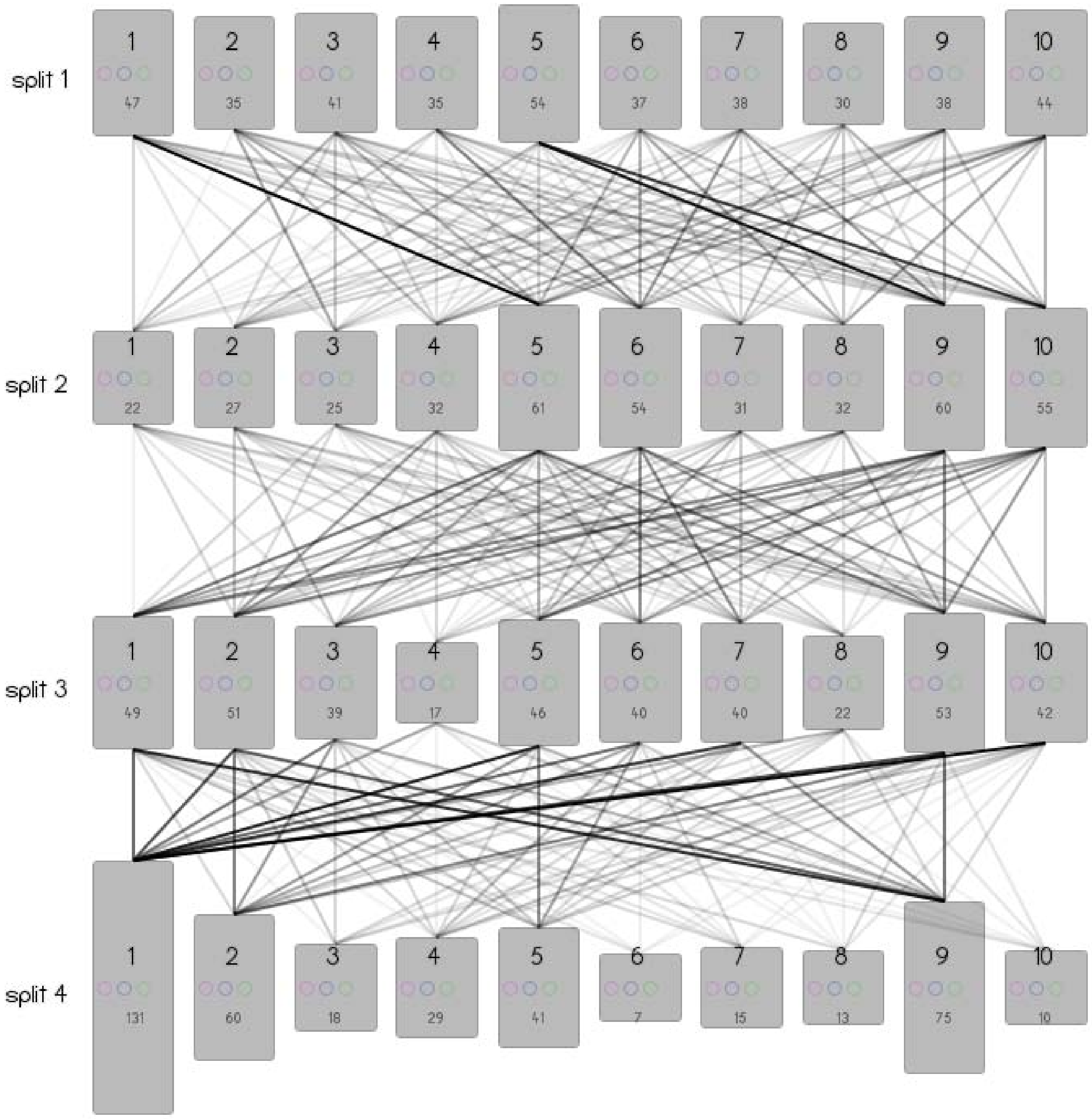

6.3. FINGERPRINT ANALYSIS

Fingerprint analysis is a method of finding and comparing hits derived from identical protocols (i.e. four out of four matching cell culture media) and/or groups of beads with similar protocols (i.e. two or three matching media). This analysis method is complementary to linkage analysis which only considers linkages between adjacent splits.

Table 10 details a fingerprint analysis of the dataset. Each row of the table represents a group of hits clustered according to protocol similarity. Column 2 identifies how many cell culture media the group has in common;, column 3 how many hits are included in the group; and column 4 the probability of the group occurring by chance. Columns 5 - 8 identify the common media in the groups ("-" represents divergence of media), and column 9 lists the bead IDs included in the group. The table is ordered first by probability value and then by number of media matches.

The group with the lowest probability of occurring by chance (p = 0.0000018) is a group of 21 beads which share cell culture medium 1 (split 1) and medium 5 (split 2). A subset of 7 beads from this group with p = 0.00021298 also shares medium 2 on the third split (group 10)and comprises two doublets of beads which have identical protocols (four out of four matching cell culture media). A second group of 7 beads which share media on three splits (split 1 = 4, split 2 = 6, split 4 = 1) is group 11. Group 11 is a subset of group 5 which share cell culture medium 6 (split 2) and medium 1 (split 4). These two groups contain a doublet of beads which have identical protocol 4-6-7-1.

Table 10: Fingerprint analysis of protocols. Each row of the table represents a cluster of similar protocols. The second column identifies the number of common cell culture media in a group, the third column the number of hits included in the group, the fourth column the probability of the group occurring by chance, columns 5-8 the identity of the common media ("-" represents divergence of media), and the final column lists the beads which yielded the protocols included in the group. The data is sorted first by probability value then by number of media matches.

Group	Matches	Beads	Probability	Split 1	Split 2	Split 3	Split 4	Bead Ids
1	2	21	0.00000018	1	5	-	-	66,101,166,171,174,204,216,227,272,280,284,299,310,355,367,372,379,380,408,443,467
2	2	20	0.00000079	9	-	-	1	1,21,25,50,51,53,55,61,69,70,75,78,81,93,95,106,108,113,118,172
3	2	20	0.00000079	-	-	10	1	19,48,56,65,71,78,82,88,113,125,130,140,145,150,158,159,164,169,172,181
4	2	19	0.00000373	5	9	-	-	26,71,121,126,135,173,198,223,246,261,271,318,328,340,350,366,423,488,489
5	2	18	0.00001877	-	6	-	1	5,9,18,32,37,55,56,77,79,83,86,117,130,136,139,160,175,178
6	2	18	0.00001877	-	10	-	1	7,17,34,36,38,46,67,69,84,91,96,114,116,124,141,162,172,180
7	2	18	0.00001877	-	-	5	1	1,17,25,27,36,40,42,46,84,126,135,151,167,173,175,176,180,183
8	2	17	0.00008932	-	9	-	1	1,4,26,57,68,70,71,75,89,121,126,135,152,163,173,183,184
9	2	17	0.00008932	-	-	1	9	428,432,444,455,463,465,467,469,475,478,484,485,490,500,501,510,513
10	3	7	0.00021298	1	5	2	-	166,171,204,284,299,372,443
11	3	7	0.00021298	4	6	-	1	18,37,77,83,139,175,178
12	3	7	0.00021298	5	-	5	1	40,46,126,135,167,173,180
13	2	16	0.00039853	5	10	-	-	46,141,180,186,208,212,252,262,278,294,373,388,399,476,477,508
14	2	16	0.00039853	-	5	-	1	12,50,54,66,80,98,101,106,158,164,166,167,171,174,179,182
15	2	16	0.00039853	-	-	7	1	5,32,35,51,52,53,55,77,87,96,106,112,136,139,152,174
16	2	15	0.00167372	6	-	-	1	8,24,28,34,52,57,87,90,128,130,134,163,176,179,182
17	2	15	0.00167372	10	-	-	1	6,13,17,27,29,32,60,73,82,96,97,98,117,120,158

Group	Matches	Beads	Probability	Split 1	Split 2	Split 3	Split 4	Bead Ids
18	2	15	0.00167372	-	-	9	1	4,18,23,26,28,29,75,80,90,101,120,124,155,160,170
19	3	6	0.0038501	5	9	-	1	26,71,121,126,135,173
20	3	6	0.0038501	-	6	7	1	5,32,55,77,136,139
21	3	6	0.0038501	-	9	2	9	430,438,481,488,497,517
22	2	14	0.00658674	4	-	-	1	4,18,37,77,83,91,114,119,139,150,151,175,178,183
23	2	14	0.00658674	5	-	-	1	26,35,40,46,65,71,121,126,135,140,141,167,173,180
24	2	14	0.00658674	-	7	-	1	14,24,25,27,29,58,65,88,95,118,132,150,170,176
25	2	14	0.00658674	-	9	-	9	428,430,437,438,450,481,487,488,489,497,501,505,511,517
26	2	14	0.00658674	-	-	2	1	7,10,13,21,37,58,63,85,86,119,121,148,166,171
27	2	13	0.02407892	1	-	-	1	23,36,45,66,86,101,125,136,145,166,171,174,181
28	2	13	0.02407892	5	-	-	2	186,195,198,208,212,223,228,239,245,246,248,252,258
29	2	13	0.02407892	-	-	2	9	430,431,438,442,443,461,480,481,482,488,497,517,518
30	3	5	0.05762738	1	5	1	-	66,355,379,408,467
31	3	5	0.05762738	1	5	-	1	66,101,166,171,174
32	3	5	0.05762738	5	9	5	-	126,135,173,223,340
33	3	5	0.05762738	-	9	5	1	1,126,135,173,183
34	3	5	0.05762738	-	10	5	1	17,36,46,84,180
35	2	12	0.08034176	8	-	-	1	10,38,85,88,112,124,155,160,161,162,164,170
36	2	12	0.08034176	-	4	-	1	6,21,40,52,81,108,112,125,129,148,151,161
37	2	12	0.08034176	-	5	-	9	432,443,453,455,462,467,474,475,491,499,503,512
38	2	12	0.08034176	-	6	-	2	187,190,194,201,203,206,209,211,213,219,235,248
39	2	12	0.08034176	-	8	-	1	8,13,23,63,82,85,93,97,128,134,155,169
40	2	12	0.08034176	-	-	1	1	8,66,67,68,79,97,110,117,128,134,163,178
41	2	12	0.08034176	-	-	6	1	24,34,45,57,61,73,81,83,108,141,179,184
42	4	3	0.09710944	5	9	5	1	126,135,173
43	4	3	0.09710944	6	8	1	1	8,128,134
44	2	11	0.23544554	2	-	-	1	12,42,54,67,80,84,129,132,147,148,184
45	2	11	0.23544554	3	-	-	9	428,441,454,456,485,494,497,498,499,503,518
46	2	11	0.23544554	5	-	9	-	26,195,262,278,316,350,366,423,436,476,508
47	2	11	0.23544554	-	5	1	-	66,291,334,355,363,379,408,432,455,467,475
48	2	11	0.23544554	-	10	-	2	186,207,208,210,212,214,244,247,249,252,259
49	2	11	0.23544554	-	-	3	1	9,14,54,60,69,89,95,118,129,162,182
50	2	11	0.23544554	-	-	9	9	433,436,441,445,449,462,476,499,502,508,511
51	3	4	0.53708327	5	9	2	-	121,198,271,488
52	3	4	0.53708327	5	9	9	-	26,350,366,423
53	3	4	0.53708327	5	9	-	5	328,340,350,366
54	3	4	0.53708327	5	10	9	-	262,278,476,508
55	3	4	0.53708327	5	10	-	2	186,208,212,252
56	3	4	0.53708327	6	-	1	1	8,128,134,163
57	3	4	0.53708327	6	-	6	1	24,34,57,179
58	3	4	0.53708327	7	6	1	-	393,411,478,490
59	3	4	0.53708327	7	-	1	9	444,478,490,501
60	3	4	0.53708327	8	-	9	1	124,155,160,170
61	3	4	0.53708327	9	-	7	1	51,53,55,106
62	3	4	0.53708327	-	5	1	9	432,455,467,475
63	3	4	0.53708327	-	8	1	1	8,97,128,134
64	2	10	0.55245245	4	6	-	-	18,37,77,83,139,175,178,330,447,523
65	2	10	0.55245245	7	-	-	9	438,444,445,458,462,473,478,490,501,517
66	2	10	0.55245245	-	5	9	-	80,101,199,308,310,315,409,462,499,538
67	2	10	0.55245245	-	9	2	-	121,198,271,430,438,481,488,497,517,520
68	2	10	0.55245245	-	9	-	5	328,339,340,345,348,350,365,366,368,374
69	2	10	0.55245245	-	10	-	9	429,441,451,465,476,477,508,510,513,524
70	2	10	0.55245245	-	-	2	2	186,196,198,204,215,221,224,228,236,238
71	2	10	0.55245245	-	-	9	2	188,194,195,199,201,211,234,240,244,247

Group	Matches	Beads	Probability	Split 1	Split 2	Split 3	Split 4	Bead Ids
72	2	9	0.89621097	2	-	-	9	442,451,453,469,475,487,502,505,509
73	2	9	0.89621097	3	-	-	1	9,14,48,63,68,79,89,116,159
74	2	9	0.89621097	5	-	5	-	40,46,126,135,167,173,180,223,340
75	2	9	0.89621097	10	-	5	-	17,27,210,220,283,285,358,361,474
76	2	9	0.89621097	-	2	-	1	10,19,35,60,61,73,113,120,145
77	2	9	0.89621097	-	4	-	2	185,188,193,215,218,242,243,245,251
78	2	9	0.89621097	-	5	2	-	166,171,204,238,281,284,299,372,443
79	2	9	0.89621097	-	5	-	2	199,204,216,220,227,229,237,238,258
80	2	9	0.89621097	-	6	6	-	83,219,248,306,342,398,412,435,454
81	2	9	0.89621097	-	6	7	-	5,32,55,77,136,139,203,301,447
82	2	9	0.89621097	-	7	-	9	427,431,473,484,493,495,496,498,518
83	2	9	0.89621097	-	9	1	-	68,163,246,261,365,428,501,528,539
84	2	9	0.89621097	-	9	5	-	1,126,135,173,183,223,283,298,340
85	2	9	0.89621097	-	9	9	-	4,26,75,348,350,366,422,423,511
86	2	9	0.89621097	-	10	5	-	17,36,46,84,180,207,210,259,389
87	2	9	0.89621097	-	-	5	5	326,327,330,340,353,356,358,361,375
88	2	8	0.99771118	1	-	1	-	66,355,365,379,408,420,467,513
89	2	8	0.99771118	1	-	2	-	86,166,171,204,284,299,372,443
90	2	8	0.99771118	1	-	-	4	284,288,298,299,305,310,312,323
91	2	8	0.99771118	4	-	3	-	189,214,321,331,376,396,434,537
92	2	8	0.99771118	5	-	-	9	436,472,476,477,488,489,492,508
93	2	8	0.99771118	6	-	6	-	24,34,57,179,242,398,412,435
94	2	8	0.99771118	6	-	-	9	431,432,435,465,468,480,481,510
95	2	8	0.99771118	7	6	-	-	5,56,306,393,411,425,478,490
96	2	8	0.99771118	7	9	-	-	152,339,348,386,410,438,501,517
97	2	8	0.99771118	7	-	-	1	5,7,19,56,58,110,152,169
98	2	8	0.99771118	10	-	9	-	29,120,194,211,240,308,387,406
99	2	8	0.99771118	10	-	-	2	193,194,203,210,211,217,220,240
100	2	8	0.99771118	-	3	-	1	45,53,78,87,90,110,147,181
101	2	8	0.99771118	-	4	2	-	21,148,215,268,364,442,461,482
102	2	8	0.99771118	-	6	10	-	56,130,190,209,265,276,329,383
103	2	8	0.99771118	-	6	-	9	435,436,447,454,478,490,502,523
104	2	8	0.99771118	-	10	9	-	124,244,247,262,278,441,476,508
105	2	8	0.99771118	-	-	3	2	189,200,206,208,213,214,216,258
106	2	8	0.99771118	-	-	5	2	187,207,210,220,223,235,253,259
107	2	8	0.99771118	-	-	7	9	427,447,456,477,487,489,492,496
108	4	2	0.99968117	1	5	2	1	166,171
109	4	2	0.99968117	1	5	2	4	284,299
110	4	2	0.99968117	3	1	10	1	48,159
111	4	2	0.99968117	4	2	3	5	331,376
112	4	2	0.99968117	4	6	7	1	77,139
113	4	2	0.99968117	4	10	4	1	91,114
114	4	2	0.99968117	5	9	9	5	350,366
115	4	2	0.99968117	5	10	5	1	46,180
116	4	2	0.99968117	5	10	9	3	262,278
117	4	2	0.99968117	5	10	9	9	476,508
118	4	2	0.99968117	6	10	1	9	465,510
119	4	2	0.99968117	7	6	1	9	478,490
120	4	2	0.99968117	7	9	2	9	438,517
121	4	2	0.99968117	9	4	6	1	81,108
122	4	2	0.99968117	9	7	3	1	95,118
123	4	2	0.99968117	10	4	2	9	461,482
124	4	2	0.99968117	10	6	9	2	194,211
125	3	3	0.99979776	1	5	3	-	216,280,380
126	3	3	0.99979776	1	5	-	2	204,216,227
127	3	3	0.99979776	1	5	-	4	284,299,310
128	3	3	0.99979776	1	5	-	5	355,367,372
129	3	3	0.99979776	1	-	2	1	86,166,171
130	3	3	0.99979776	1	-	10	1	125,145,181
131	3	3	0.99979776	2	3	8	-	147,270,509
132	3	3	0.99979776	2	4	2	-	148,364,442

Group	Matches	Beads	Probability	Split 1	Split 2	Split 3	Split 4	Bead Ids
133	3	3	0.99979776	2	5	-	1	12,54,80
134	3	3	0.99979776	3	-	3	1	9,14,89
135	3	3	0.99979776	4	6	7	-	77,139,447
136	3	3	0.99979776	4	-	5	1	151,175,183
137	3	3	0.99979776	5	9	-	2	198,223,246
138	3	3	0.99979776	5	10	6	-	141,294,373
139	3	3	0.99979776	5	10	-	1	46,141,180
140	3	3	0.99979776	5	10	-	9	476,477,508
141	3	3	0.99979776	5	-	2	2	186,198,228
142	3	3	0.99979776	5	-	6	5	328,347,373
143	3	3	0.99979776	5	-	7	9	477,489,492
144	3	3	0.99979776	5	-	9	9	436,476,508
145	3	3	0.99979776	5	-	10	1	65,71,140
146	3	3	0.99979776	6	6	6	-	398,412,435
147	3	3	0.99979776	6	8	1	-	8,128,134
148	3	3	0.99979776	6	8	-	1	8,128,134
149	3	3	0.99979776	6	-	1	9	432,465,510
150	3	3	0.99979776	6	-	2	9	431,480,481
151	3	3	0.99979776	7	9	-	9	438,501,517
152	3	3	0.99979776	7	-	10	1	19,56,169
153	3	3	0.99979776	8	10	-	1	38,124,162
154	3	3	0.99979776	9	4	-	1	21,81,108
155	3	3	0.99979776	9	7	-	1	25,95,118
156	3	3	0.99979776	9	9	-	1	1,70,75
157	3	3	0.99979776	9	-	3	1	69,95,118
158	3	3	0.99979776	9	-	6	1	61,81,108
159	3	3	0.99979776	9	-	10	1	78,113,172
160	3	3	0.99979776	10	2	-	1	60,73,120
161	3	3	0.99979776	10	5	5	-	220,358,474
162	3	3	0.99979776	10	6	-	2	194,203,211
163	3	3	0.99979776	10	8	-	1	13,82,97
164	3	3	0.99979776	10	-	9	2	194,211,240
165	3	3	0.99979776	-	1	10	1	48,140,159
166	3	3	0.99979776	-	2	5	5	327,353,361
167	3	3	0.99979776	-	2	10	1	19,113,145
168	3	3	0.99979776	-	4	2	9	442,461,482
169	3	3	0.99979776	-	5	1	5	334,355,363
170	3	3	0.99979776	-	5	5	9	453,474,503
171	3	3	0.99979776	-	5	9	4	308,310,315
172	3	3	0.99979776	-	6	1	1	79,117,178
173	3	3	0.99979776	-	6	9	2	194,201,211
174	3	3	0.99979776	-	7	3	1	14,95,118
175	3	3	0.99979776	-	7	5	1	25,27,176
176	3	3	0.99979776	-	7	10	1	65,88,150
177	3	3	0.99979776	-	8	2	1	13,63,85
178	3	3	0.99979776	-	9	9	1	4,26,75
179	3	3	0.99979776	-	9	9	5	348,350,366
180	3	3	0.99979776	-	10	1	9	465,510,513
181	3	3	0.99979776	-	10	4	1	91,114,116
182	3	3	0.99979776	-	10	5	2	207,210,259
183	3	3	0.99979776	-	10	9	9	441,476,508
184	2	7	0.99999988	1	-	-	5	342,355,365,367,368,372,378
185	2	7	0.99999988	2	3	-	-	147,197,255,270,390,413,509
186	2	7	0.99999988	2	5	-	-	12,54,80,315,418,453,475
187	2	7	0.99999988	3	-	2	-	63,281,287,382,497,518,520
188	2	7	0.99999988	3	-	-	2	185,190,199,201,237,251,253
189	2	7	0.99999988	5	-	2	-	121,186,198,228,271,402,488
190	2	7	0.99999988	5	-	6	-	141,239,248,294,328,347,373
191	2	7	0.99999988	6	8	-	-	8,128,134,221,317,468,480
192	2	7	0.99999988	6	-	1	-	8,128,134,163,432,465,510
193	2	7	0.99999988	7	-	1	-	110,393,411,444,478,490,501

Confidential

Group	Matches	Beads	Probability	Split 1	Split 2	Split 3	Split 4	Bead Ids
194	2	7	0.99999988	7	-	2	-	7,58,215,268,274,438,517
195	2	7	0.99999988	10	5	-	-	98,158,220,308,358,455,474
196	2	7	0.99999988	10	8	-	-	13,82,97,217,240,282,295
197	2	7	0.99999988	-	1	-	1	28,42,48,51,119,140,159
198	2	7	0.99999988	-	2	-	5	327,331,338,347,353,361,376
199	2	7	0.99999988	-	4	-	9	442,456,458,461,463,469,482
200	2	7	0.99999988	-	5	-	4	284,291,299,304,308,310,315
201	2	7	0.99999988	-	6	1	-	79,117,178,393,411,478,490
202	2	7	0.99999988	-	6	9	-	18,160,194,201,211,436,502
203	2	7	0.99999988	-	8	-	9	434,445,449,468,480,485,514
204	2	7	0.99999988	-	10	1	-	67,337,388,399,465,510,513
205	2	7	0.99999988	-	10	3	-	69,162,208,214,277,429,524
206	2	7	0.99999988	-	-	4	1	6,12,70,91,114,116,132
207	2	7	0.99999988	-	-	6	2	219,222,227,239,242,248,251
208	2	7	0.99999988	-	-	6	5	328,339,342,347,349,351,373
209	2	6	0.99999999	1	-	7	-	136,174,272,312,368,529
210	2	6	0.99999999	1	-	9	-	23,101,288,310,433,511
211	2	6	0.99999999	1	-	10	-	125,145,181,266,367,521
212	2	6	0.99999999	1	-	-	9	433,443,467,511,513,521
213	2	6	0.99999999	3	1	-	-	48,159,286,326,382,527
214	2	6	0.99999999	3	5	-	-	199,237,260,281,499,503
215	2	6	0.99999999	3	6	-	-	9,79,190,201,377,454
216	2	6	0.99999999	3	-	10	-	48,159,190,309,424,494
217	2	6	0.99999999	4	-	-	9	427,434,437,447,493,523
218	2	6	0.99999999	5	-	1	-	246,261,291,384,388,399
219	2	6	0.99999999	5	-	7	-	35,212,245,477,489,492
220	2	6	0.99999999	5	-	-	5	328,340,347,350,366,373
221	2	6	0.99999999	6	5	-	-	179,182,229,238,432,538
222	2	6	0.99999999	6	6	-	-	130,213,329,398,412,435
223	2	6	0.99999999	6	-	-	2	213,221,229,234,238,242
224	2	6	0.99999999	8	10	-	-	38,124,162,244,394,524
225	2	6	0.99999999	8	-	9	-	124,155,160,170,244,409
226	2	6	0.99999999	8	-	-	9	430,450,495,496,514,524
227	2	6	0.99999999	9	4	-	-	21,81,108,188,263,463
228	2	6	0.99999999	9	5	-	-	50,106,264,363,491,512
229	2	6	0.99999999	9	9	-	-	1,70,75,307,374,422
230	2	6	0.99999999	9	-	6	-	61,81,108,219,351,491
231	2	6	0.99999999	9	-	10	-	78,113,172,307,374,383
232	2	6	0.99999999	10	6	-	-	32,117,194,203,211,344
233	2	6	0.99999999	10	10	-	-	17,96,210,337,370,429
234	2	6	0.99999999	10	-	1	-	97,117,337,455,484,528
235	2	6	0.99999999	10	-	-	5	337,344,349,358,361,370
236	2	6	0.99999999	10	-	-	9	429,455,461,474,482,484
237	2	6	0.99999999	-	5	3	-	54,182,216,258,280,380
238	2	6	0.99999999	-	5	5	-	167,220,358,453,474,503
239	2	6	0.99999999	-	5	-	5	334,355,358,363,367,372
240	2	6	0.99999999	-	6	-	5	329,330,342,344,377,378
241	2	6	0.99999999	-	7	5	-	25,27,176,356,493,498
242	2	6	0.99999999	-	8	1	-	8,97,128,134,420,485
243	2	6	0.99999999	-	8	9	-	23,155,195,240,445,449
244	2	6	0.99999999	-	10	6	-	34,141,294,373,394,451
245	2	6	0.99999999	-	-	3	9	429,434,473,505,514,524
246	2	6	0.99999999	-	-	5	9	453,468,474,493,498,503
247	2	6	0.99999999	-	-	8	1	38,50,93,98,147,161
248	2	5	0.99999999	1	6	-	-	86,136,235,342,378
249	2	5	0.99999999	1	-	6	-	45,227,305,323,342
250	2	5	0.99999999	2	4	-	-	129,148,364,442,469
251	2	5	0.99999999	2	-	3	-	54,129,200,206,505
252	2	5	0.99999999	2	-	5	-	42,84,259,413,453
253	2	5	0.99999999	2	-	-	2	197,200,206,255,259
254	2	5	0.99999999	3	9	-	-	68,89,428,497,520

Group	Matches	Beads	Probability	Split 1	Split 2	Split 3	Split 4	Bead Ids
255	2	5	0.99999999	3	-	1	-	68,79,185,428,485
256	2	5	0.99999999	3	-	3	-	9,14,89,377,397
257	2	5	0.99999999	3	-	5	-	253,326,353,498,503
258	2	5	0.99999999	4	9	-	-	4,183,189,345,437
259	2	5	0.99999999	4	-	5	-	151,175,183,330,493
260	2	5	0.99999999	4	-	7	-	77,139,249,427,447
261	2	5	0.99999999	4	-	-	2	189,196,214,243,249
262	2	5	0.99999999	4	-	-	5	330,331,334,345,376
263	2	5	0.99999999	5	2	-	-	35,228,347,472,492
264	2	5	0.99999999	6	10	-	-	34,303,343,465,510
265	2	5	0.99999999	6	-	2	-	221,238,431,480,481
266	2	5	0.99999999	7	-	6	-	222,306,339,386,403
267	2	5	0.99999999	7	-	-	5	338,339,348,356,375
268	2	5	0.99999999	8	7	-	-	88,170,357,495,496
269	2	5	0.99999999	8	-	2	-	10,85,224,236,430
270	2	5	0.99999999	8	-	10	-	88,164,209,265,404
271	2	5	0.99999999	8	-	-	2	187,209,224,236,244
272	2	5	0.99999999	9	-	7	-	51,53,55,106,264
273	2	5	0.99999999	9	-	9	-	75,188,247,422,449
274	2	5	0.99999999	9	-	-	9	449,463,491,500,512
275	2	5	0.99999999	10	4	-	-	6,193,406,461,482
276	2	5	0.99999999	10	-	7	-	32,96,203,217,282
277	2	5	0.99999999	10	-	-	4	282,283,285,295,308
278	2	5	0.99999999	-	2	3	-	60,319,331,376,397
279	2	5	0.99999999	-	3	10	-	78,181,197,424,494
280	2	5	0.99999999	-	3	-	2	197,222,236,253,255
281	2	5	0.99999999	-	5	7	-	106,174,264,272,418
282	2	5	0.99999999	-	5	8	-	50,98,229,237,260
283	2	5	0.99999999	-	5	-	3	260,264,272,280,281
284	2	5	0.99999999	-	7	3	-	14,95,118,357,473
285	2	5	0.99999999	-	8	2	-	13,63,85,221,480
286	2	5	0.99999999	-	8	-	4	282,295,305,317,321
287	2	5	0.99999999	-	9	3	-	89,189,200,318,505
288	2	5	0.99999999	-	9	6	-	57,184,328,339,386
289	2	5	0.99999999	-	9	10	-	71,307,374,410,437
290	2	5	0.99999999	-	9	-	2	189,198,200,223,246
291	2	5	0.99999999	-	10	7	-	96,212,249,312,477
292	2	5	0.99999999	-	10	10	-	172,252,303,309,343
293	2	5	0.99999999	-	-	1	5	334,337,355,363,365
294	2	5	0.99999999	-	-	6	4	294,304,305,306,323
295	2	5	0.99999999	-	-	7	2	203,212,217,245,249
296	2	5	0.99999999	-	-	9	4	288,308,310,315,316
297	2	5	0.99999999	-	-	10	2	190,197,209,243,252
298	2	4	0.99999999	1	9	-	-	298,365,368,511
299	2	4	0.99999999	1	10	-	-	36,312,389,513
300	2	4	0.99999999	1	-	5	-	36,235,298,389
301	2	4	0.99999999	1	-	-	2	204,216,227,235
302	2	4	0.99999999	2	6	-	-	206,276,301,502
303	2	4	0.99999999	2	9	-	-	184,200,487,505
304	2	4	0.99999999	2	10	-	-	67,84,259,451
305	2	4	0.99999999	2	-	1	-	67,255,469,475
306	2	4	0.99999999	2	-	2	-	148,364,390,442
307	2	4	0.99999999	2	-	7	-	301,341,418,487
308	2	4	0.99999999	3	3	-	-	253,405,424,494
309	2	4	0.99999999	3	-	8	-	237,260,286,527
310	2	4	0.99999999	3	-	9	-	199,201,441,499
311	2	4	0.99999999	4	1	-	-	119,196,396,537
312	2	4	0.99999999	4	7	-	-	150,427,493,536
313	2	4	0.99999999	4	10	-	-	91,114,214,249
314	2	4	0.99999999	5	-	10	-	65,71,140,252
315	2	4	0.99999999	5	-	-	3	261,262,271,278

Group	Matches	Beads	Probability	Split 1	Split 2	Split 3	Split 4	Bead Ids
316	2	4	0.99999999	5	-	-	4	291,294,316,318
317	2	4	0.99999999	6	-	9	-	28,90,234,538
318	2	4	0.99999999	6	-	10	-	130,303,329,343
319	2	4	0.99999999	7	3	-	-	110,222,403,444
320	2	4	0.99999999	7	4	-	-	215,218,268,458
321	2	4	0.99999999	7	-	7	-	5,152,267,338
322	2	4	0.99999999	7	-	10	-	19,56,169,410
323	2	4	0.99999999	7	-	-	3	267,268,274,277
324	2	4	0.99999999	8	6	-	-	160,187,209,265
325	2	4	0.99999999	8	8	-	-	85,155,400,514
326	2	4	0.99999999	8	-	3	-	162,357,514,524
327	2	4	0.99999999	9	10	-	-	69,172,207,247
328	2	4	0.99999999	9	-	3	-	69,95,118,263
329	2	4	0.99999999	9	-	-	2	188,207,219,247
330	2	4	0.99999999	10	2	-	-	60,73,120,361
331	2	4	0.99999999	10	-	2	-	13,344,461,482
332	2	4	0.99999999	-	1	10	-	48,140,159,521
333	2	4	0.99999999	-	2	2	-	10,224,228,287
334	2	4	0.99999999	-	2	10	-	19,113,145,404
335	2	4	0.99999999	-	3	-	7	390,402,403,405
336	2	4	0.99999999	-	4	6	-	81,108,242,251
337	2	4	0.99999999	-	4	7	-	52,112,245,456
338	2	4	0.99999999	-	5	6	-	179,227,304,491
339	2	4	0.99999999	-	6	3	-	9,206,213,377
340	2	4	0.99999999	-	6	5	-	175,187,235,330
341	2	4	0.99999999	-	7	7	-	341,427,496,529
342	2	4	0.99999999	-	7	10	-	65,88,150,266
343	2	4	0.99999999	-	8	-	2	195,217,221,240
344	2	4	0.99999999	-	9	7	-	152,368,487,489
345	2	4	0.99999999	-	9	-	4	283,298,307,318
346	2	4	0.99999999	-	9	-	8	391,410,422,423
347	2	4	0.99999999	-	10	-	3	262,274,277,278
348	2	4	0.99999999	-	10	-	4	294,303,309,312
349	2	4	0.99999999	-	10	-	5	337,343,370,373
350	2	4	0.99999999	-	-	2	3	268,271,274,281
351	2	4	0.99999999	-	-	3	5	331,357,376,377
352	2	4	0.99999999	-	-	4	9	458,495,512,523
353	2	4	0.99999999	-	-	6	9	435,451,454,491
354	2	4	0.99999999	-	-	10	5	329,343,367,374

6. PROTOCOL ANALYSIS

6.4. HIERACHICAL CLUSTERING DENDROGRAM

Hierarchical clustering is a method of grouping protocols according to similarity.

The hierarchical clustering illustrated in figure 6 has two sections. The upper dendrogram depicts the clustering structure and order. The lower coloured array is a graphical representation of the media combinations that comprise each protocol. The y-axis of the dendrogram measures intra-cluster similarity, i.e. horizontal bars closest to the bottom of the dendrogram link identical protocols whilst a horizontal bar half way up the y-axis represents a larger cluster containing beads whose protocols are not identical but do share some similarity. The hierarchical nature of the clustering means that beads are first clustered into small highly similar clusters, which are in turn included in larger clusters. This is repeated until the final cluster is reached (uppermost horizontal bar) which includes all beads in the dataset. Each node at the bottom of the dendrogram (leaf node) corresponds to a protocol. Each row of the coloured array corresponds to a split and each column of the array a protocol, with the columns aligned to the leaf nodes of the dendrogram. The horizontal coloured legend below the array specifies the colour associated with each media number.

Three large clusters within the dataset are labeled A, B and C. Cluster A is the largest and includes 109 beads which predominantly share cell culture medium 1 on the final split and comprises one triplet and 6 doublet of beads which have identical protocols (four out of four matching cell culture media). Cluster B contains 69 beads, which predominately end in media 9 (split 4), and also contains two subset of beads, one with 14 beads also sharing media 9 (split 2), and second containing 11 beads sharing media 9 (split 3). Each subset also comprises a doublet of beads which have identical protocols (protocols 7-9-2-9 and 5-10-9-9). Cluster C contains 34 beads, 20 of which share cell culture medium 1 (split 1) and medium 5 (split 2). This cluster is the same as group 1 of the fingerprint analysis (table 9) and the strongest linkage line in figure 5. Cluster C also includes two doublets of beads which have identical protocols. The probabilities of these groups occurring by chance are discussed in section 6.3.

Figure 6: Dendrogram showing the hierarchical clustering of similar protocols.

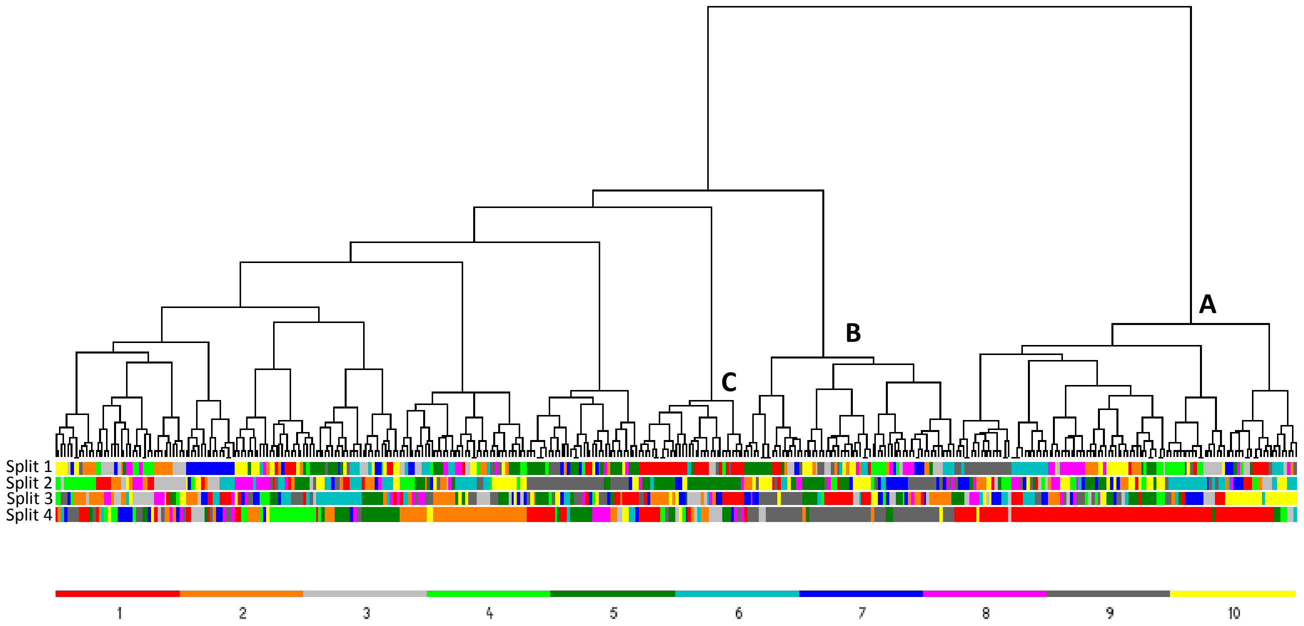

 Confidential

6. PROTOCOL ANALYSIS
6.5. SIMILARITY MATRIX

The similarity matrix is a diagrammatic representation of a pair-wise comparison of all protocols. Each column and each row corresponds to a protocol. The brightness of each cell in the matrix is proportional to the number of cell culture media shared by the two protocols. The brightest cell corresponds to protocols which have common media (i.e. identical protocols) in common, while a black cell corresponds to two protocols with no common media. The diagonal row of cells (from the top left to bottom right) corresponds to beads being compared to themselves. Beads are ordered along both the x- and y-axis according to the hierarchical clustering dendrogram illustrated in figure 6. The coloured arrays on the bottom and right of the matrix illustrate the protocol of each bead. The horizontal coloured legend below the matrix specifies the colour associated with each medium number.

The similarity matrix clearly displays clusters of similar protocols as square regions comprising brightly coloured cells. The size and overall brightness of the square regions are measures of the cluster size and intra-cluster protocol similarity, respectively. Additionally, the similarity matrix provides a global overview of the protocol similarity of beads or clusters to the remaining dataset. For example the brightness of the cells in the columns above cluster A indicate that the beads contained within cluster A have little similarity with the beads contained in cluster B and some similarity with the beads contained within cluster C.

Figure 7: Similarity matrix comprising a pair-wise comparison of all protocols

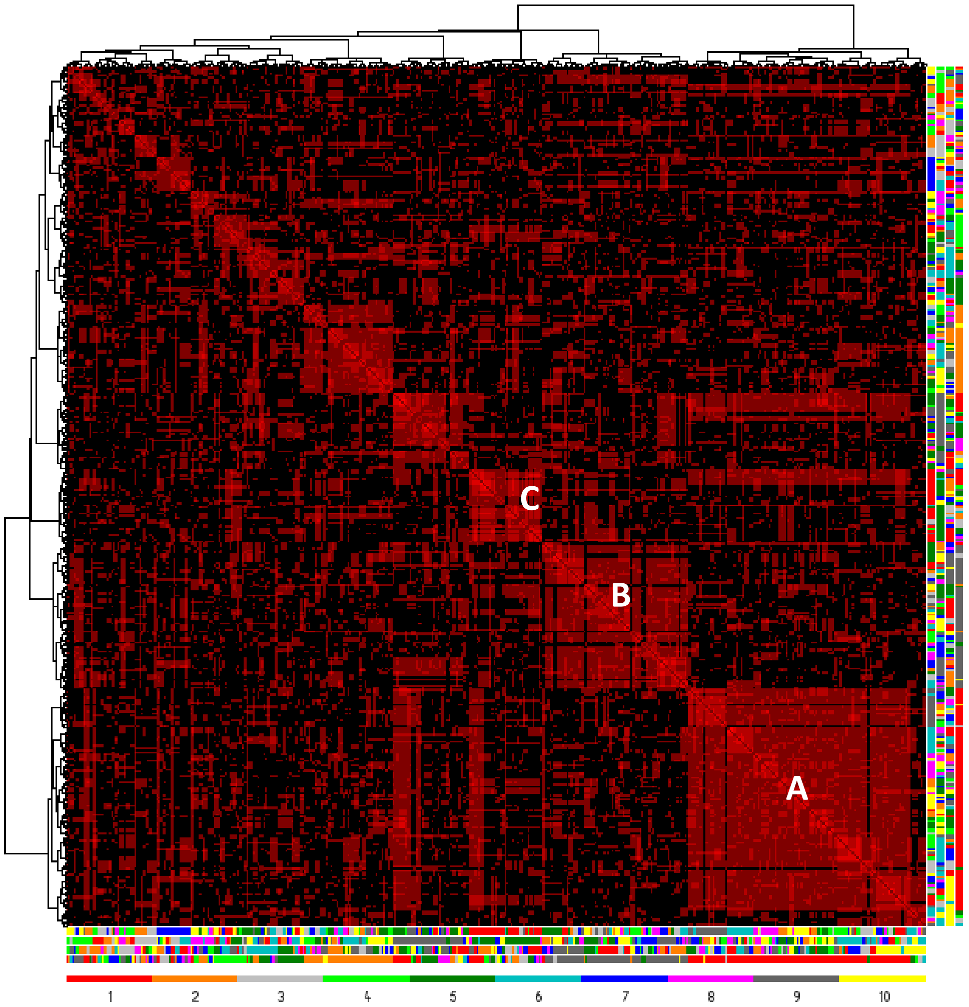

7. SUMMARY AND CONCLUSIONS

A CombiCult™ experiment was performed in which 40 serum-free cell culture media were screened in 10,000 combinations. The 40 media were tested over four split-pool cycles (performed on day 1, day 7, day 14 and day 21 respectively) with 10 media included in each cycle, such that 10,000 media combinations (protocols) are tested. A total of 480,000 beads were used so that on average each protocol was sampled by 48 beads. Following completion of the split-pool experiment (day 28) beads were screened using an immunocytochemistry assay and individual positive beads isolated.

Of the total number of monomeric beads screened, 622 (0.36 %) were sorted and verified as 'hits'. The tags from each hit were read to infer the series of cell culture media (protocol) that produced differentiated cells. Of the 622 'hit' beads, tags could be conclusively deconvoluted from 399 (64 %). Putative differentiation protocols were analysed using bespoke bioinformatics software Ariadne™ (v1.0) allowing protocols to be ranked and a subset to be selected for further validation and study. The 23 selected protocols are listed below in Table 11 as well as in the executive summary (Table 1). Validation numbers 1, 2, 4, 6 and 7 form part of group 7 in the fingerprint analysis and share cell culture medium 5 on split 3 and medium 1 on the final split. The first validated protocol is represented by a triplet of beads and has a one in ten probability of occurring randomly ($p = 0.0971$) and is also a subset of group 4 in the fingerprint analysis (19 beads sharing medium 5 on split 1 and medium 9 on split 2). Validation numbers 10-12 form part of cluster C shown in the hierarchical clustering dendrogram and similarity matrix and share cell culture medium 1 (split 1) and 5 (split 2). Both protocols 10 and 11 are represented by doublet of beads. Finally, protocols 21-23 share medium 5 on split 1 and medium 10 on split 2 and form part of group 13 in the fingerprint analysis.

Table 11: Selected23 validation protocols determined by Ariadne™ (v1.0). The protocols are identified by the series of cell culture media on each split-pool cycle (split 1 – split 4), e.g. 5-9-5-1.

Validation Number	Protocol	Bead Ids		Basal Medium	Supplements
1	5-9-5-1	126, 135, 173	Split 1 (Day 1)	RHB-A	-
			Split 2 (Day 7)	DMEM/F12	2mM Glutamine, 1X ITS suppl., 100ng/mL FGF8b, 500ng/mL mrShh.
			Split 3 (Day 15)	RHB-A	-
			Split 4 (Day 21)	DMEM/F12	2mM Glutamine, 1X N2 suppl., 200µM ascorbic acid, 1mg/mL laminin
2	9-9-5-1	1	Split 1 (Day 1)	DMEM/F12	2mM Glutamine,1% BSA, 1X N2suppl., 20µM SB431542.
			Split 2 (Day 7)	DMEM/F12	2mM Glutamine, 1X ITS suppl., 100ng/mL FGF8b, 500ng/mL mrShh.
			Split 3 (Day 15)	RHB-A	-
			Split 4 (Day 21)	DMEM/F12	2mM Glutamine, 1X N2 suppl. , 200µM ascorbic acid, 1mg/mL laminin
3	5-9-10-1	71	Split 1 (Day 1)	RHB-A	-
			Split 2 (Day 7)	DMEM/F12	2mM Glutamine, 1X ITS suppl., 100ng/mL FGF8b, 500ng/mL mrShh.
			Split 3 (Day 15)	DMEM/F12	2mM Glutamine, 1X N2 suppl., 20ng/mL bFGF, 20ng/mL EGF, 2ng/mL LIF.
			Split 4 (Day 21)	DMEM/F12	2mM Glutamine, 1X N2 suppl. , 200µM ascorbic acid, 1mg/mL laminin
4	5-10-5-1	46, 180	Split 1 (Day 1)	RHB-A	-
			Split 2 (Day 7)	DMEM/F12	2mM Glutamine, 1X ITS suppl., 200µM ascorbic acid, 20ng/mL bFGF, 10ng/mL BDNF 100ng/mL FGF8b, 1µM purmorphamine
			Split 3 (Day 15)	RHB-A	-

Validation Number	Protocol	Bead Ids		Basal Medium	Supplements
			Split 4 (Day 21)	DMEM/F12	2mM Glutamine, 1X N2 suppl. , 200μM ascorbic acid, 1mg/mL laminin
5	5-9-9-1	26	Split 1 (Day 1)	RHB-A	-
			Split 2 (Day 7)	DMEM/F12	2mM Glutamine, 1X ITS suppl., 100ng/mL FGF8b, 500ng/mL mrShh.
			Split 3 (Day 15)	Advanced DMEM	2mM Glutamine
			Split 4 (Day 21)	DMEM/F12	2mM Glutamine, 1X N2 suppl. , 200μM ascorbic acid, 1mg/mL laminin
6	5-4-5-1	40	Split 1 (Day 1)	RHB-A	-
			Split 2 (Day 7)	DMEM/F12	2mM Glutamine, 1X ITS suppl., 0.5μM retinoic acid.
			Split 3 (Day 15)	RHB-A	-
			Split 4 (Day 21)	DMEM/F12	2mM Glutamine, 1X N2 suppl. , 200μM ascorbic acid, 1mg/mL laminin.
7	4-9-5-1	183	Split 1 (Day 1)	DMEM/F12	2mM Glutamine 1% BSA, 1X N2 suppl., 1X B27 neuromix.
			Split 2 (Day 7)	DMEM/F12	2mM Glutamine, 1X ITS suppl., 100ng/mL FGF8b, 500ng/mL mrShh.
			Split 3 (Day 15)	RHB-A	-
			Split 4 (Day 21)	DMEM/F12	2mM Glutamine, 1X N2 suppl. , 200μM ascorbic acid, 1mg/mL laminin
8	6-8-1-1	8, 128, 134	Split 1 (Day 1)	KO DMEM	10% KSR, 2mM Glutamine
			Split 2 (Day 7)	Advanced DMEM	2mM Glutamine
			Split 3 (Day 15)	DMEM/F12	2mM Glutamine, 1X ITS suppl., 20ng/mL bFGF.
			Split 4 (Day 21)	DMEM/F12	2mM Glutamine, 1X N2 suppl. , 200μM ascorbic acid, 1mg/mL laminin
9	6-9-1-1	163	Split 1 (Day 1)	KO DMEM	10% KSR, 2mM Glutamine
			Split 2 (Day 7)	DMEM/F12	2mM Glutamine, 1X ITS suppl., 100ng/mL FGF8b, 500ng/mL mrShh.
			Split 3 (Day 15)	DMEM/F12	2mM Glutamine, 1X ITS suppl., 20ng/mL bFGF.
			Split 4 (Day 21)	DMEM/F12	2mM Glutamine, 1X N2 suppl. , 200μM ascorbic acid, 1mg/mL laminin
10	1-5-2-1	166, 171	Split 1 (Day 1)	KO DMEM	15% FBS, 2mM Glutamine, 0.1mM β-mercaptoethanol, 1X NEAA, 1000 Units/mL LIF (standard growth media for mES cells).
			Split 2 (Day 7)	RHB-A	-
			Split 3 (Day 15)	DMEM/F12	2mM Glutamine, 1X ITS suppl., 20ng/mL bFGF, 1mg/mL laminin
			Split 4 (Day 21)	DMEM/F12	2mM Glutamine, 1X N2 suppl. , 200μM ascorbic acid, 1mg/mL laminin
11	1-5-2-4	284, 299	Split 1 (Day 1)	KO DMEM	15% FBS, 1mM Glutamine, 0.1mM β-mercaptoethanol, 1X NEAA,), 1000 Units/mL LIF (standard growth media for mES cells)
			Split 2 (Day 7)	RHB-A	-
			Split 3 (Day 15)	DMEM/F12	2mM Glutamine, 1X ITS suppl., 20ng/mL bFGF, 1mg/mL laminin
			Split 4 (Day 21)	DMEM/F12	2mM Glutamine, 1X ITS suppl., 1μM cAMP, 10ng/mL hrBDNF

Confidential

Validation Number	Protocol	Bead Ids		Basal Medium	Supplements
12	1-5-2-9	443	Split 1 (Day 1)	KO DMEM	15% FBS, 1mM Glutamine, 0.1mM β-mercaptoethanol, 1X NEAA, (standard growth media for mES cells), 1000 Units/mL LIF
			Split 2 (Day 7)	RHB-A	-
			Split 3 (Day 15)	DMEM/F12	2mM Glutamine, 1X ITS suppl., 20ng/mL bFGF, 1mg/mL laminin
			Split 4 (Day 21)	Advanced DMEM	2mM Glutamine
13	4-6-7-1	77, 139	Split 1 (Day 1)	DMEM/F12	1% BSA, 1X N2 suppl., 1X B27 neuromix,. 1mM Glutamine
			Split 2 (Day 7)	KO DMEM	10% KSR, 2mM Glutamine
			Split 3 (Day 15)	DMEM/F12	2mM Glutamine, 1X ITS suppl., 1X B27 suppl., 50ng/mL mr Dkk1
			Split 4 (Day 21)	DMEM/F12	2mM Glutamine, 1X N2 suppl. , 200µM ascorbic acid., 1mg/mL laminin
14	7-9-2-9	438, 517	Split 1 (Day 1)	DMEM/F12	2mM Glutamine, 1% BSA, 1X ITS suppl., 20ng/mL bFGF
			Split 2 (Day 7)	DMEM/F12	2mM Glutamine, 1X ITS suppl., 100ng/mL FGF8b, 500ng/mL mrShh.
			Split 3 (Day 15)	DMEM/F12	2mM Glutamine, 1X ITS suppl., 20ng/mL bFGF, 1mg/mL laminin
			Split 4 (Day 21)	Advanced DMEM	2mM Glutamine
15	5-9-2-9	488	Split 1 (Day 1)	RHB-A	-
			Split 2 (Day 7)	DMEM/F12	2mM Glutamine, 1X ITS suppl., 100ng/mL FGF8b, 500ng/mL mrShh.
			Split 3 (Day 15)	DMEM/F12	2mM Glutamine, 1X N2 suppl., 20ng/mL bFGF, 1mg/mL laminin
			Split 4 (Day 21)	Advanced DMEM	2mM Glutamine
16	5-9-2-1	121	Split 1 (Day 1)	RHB-A	-
			Split 2 (Day 7)	DMEM/F12	2mM Glutamine, 1X ITS suppl., 100ng/mL FGF8b, 500ng/mL mrShh.
			Split 3 (Day 15)	DMEM/F12	2mM Glutamine, 1X N2 suppl., 20ng/mL bFGF, 1mg/mL laminin
			Split 4 (Day 21)	DMEM/F12	2mM Glutamine, 1X N2 suppl. , 200µM ascorbic acid, 1mg/mL laminin
17	3-1-10-1	48, 159	Split 1 (Day 1)	Advanced DMEM	1mM Glutamine
			Split 2 (Day 7)	DMEM/F12	2mM Glutamine, 1X ITS suppl., 5mg/mL fibronectin.
			Split 3 (Day 15)	DMEM/F12	2mM Glutamine, 1X N2 suppl., 20ng/mL bFGF, 20ng/mL EGF, 2ng/mL LIF.
			Split 4 (Day 21)	DMEM/F12	2mM Glutamine, 1X N2 suppl. , 200µM ascorbic acid, 1mg/mL laminin
18	9-10-10-1	172	Split 1 (Day 1)	DMEM/F12	2mM Glutamine,1% BSA, 1X N2suppl., 20µM SB431542.
			Split 2 (Day 7)	DMEM/F12	2mM Glutamine, 1X ITS suppl., 200µM ascorbic acid, 20ng/mL bFGF, 10ng/mL BDNF 100ng/mL FGF8b, 1µM purmorphamine
			Split 3 (Day 15)	DMEM/F12	2mM Glutamine, 1X N2 suppl., 20ng/mL bFGF, 20ng/mL EGF, 2ng/mL LIF.
			Split 4 (Day 21)	DMEM/F12	2mM Glutamine, 1X N2 suppl. , 200µM ascorbic acid, 1mg/mL laminin
19	9-2-10-1	113	Split 1 (Day 1)	DMEM/F12	2mM Glutamine,1% BSA, 1X N2suppl., 20µM SB431542.
			Split 2 (Day 7)	DMEM/F12	2mM Glutamine, 1X ITS suppl., 100ng/mL mr Noggin.
			Split 3 (Day 15)	DMEM/F12	2mM Glutamine, 1X N2 suppl., 20ng/mL bFGF, 20ng/mL EGF, 2ng/mL LIF.
			Split 4 (Day 21)	DMEM/F12	2mM Glutamine, 1X N2 suppl. , 200µM ascorbic acid, 1mg/mL laminin

Validation Number	Protocol	Bead Ids		Basal Medium	Supplements
20	9-4-6-1	81, 108	Split 1 (Day 1)	DMEM/F12	2mM Glutamine,1% BSA, 1X N2suppl., 20µM SB431542.
			Split 2 (Day 7)	DMEM/F12	2mM Glutamine, 1X ITS suppl., 0.5µM retinoic acid.
			Split 3 (Day 15)	KO DMEM	10% KSR, 2mM Glutamine
			Split 4 (Day 21)	DMEM/F12	2mM Glutamine, 1X N2 suppl. , 200µM ascorbic acid, 1mg/mL laminin
21	5-10-2-2	186	Split 1 (Day 1)	RHB-A	-
			Split 2 (Day 7)	DMEM/F12	2mM Glutamine, 1X ITS suppl., 200µM ascorbic acid, 20ng/mL bFGF, 10ng/mL BDNF 100ng/mL FGF8b, 1µM purmorphamine
			Split 3 (Day 15)	DMEM/F12	2mM Glutamine, 1X ITS suppl., 20ng/mL bFGF, 1mg/mL laminin
			Split 4 (Day 21)	DMEM/F12	2mM Glutamine, 0.5% FBS, 1X N2 suppl., 20ng/mL hr GDNF, 20ng/mL hr BDNF
22	5-10-9-9	476, 508	Split 1 (Day 1)	RHB-A	-
			Split 2 (Day 7)	DMEM/F12	2mM Glutamine, 1X ITS suppl., 200µM ascorbic acid, 20ng/mL bFGF, 10ng/mL BDNF 100ng/mL FGF8b, 1µM purmorphamine
			Split 3 (Day 15)	Advanced DMEM	2mM Glutamine
			Split 4 (Day 21)	Advanced DMEM	2mM Glutamine
23	5-10-6-1	141	Split 1 (Day 1)	RHB-A	-
			Split 2 (Day 7)	DMEM/F12	2mM Glutamine, 1X ITS suppl., 200µM ascorbic acid, 20ng/mL bFGF, 10ng/mL BDNF 100ng/mL FGF8b, 1µM purmorphamine
			Split 3 (Day 15)	KO DMEM	10% KSR, 2mM Glutamine
			Split 4 (Day 21)	DMEM/F12	2mM Glutamine, 1X N2 suppl. , 200µM ascorbic acid, 1mg/mL laminin

Confidential

ANNEX 1: FLOW SORTINGBIN 1 – File 1 of 3

Bin 1: COPAS scatter plots showing events for medium 1 of the final split-pool cycle (bin 1). In the first scatter plot (time of flight [TOF]vs. optical extinction [ext]), events depicted by a red dot fall within the gate defining monomeric beads and were sorted. The second scatter plot displays the fluorescent values of the sorted beads (TOF vs. red fluorescence intensity). Beads with a red fluorescent intensity above the threshold value are depicted as green dots and were individually dispensed into 96 well plates.

Number of beads individually dispensed: 30

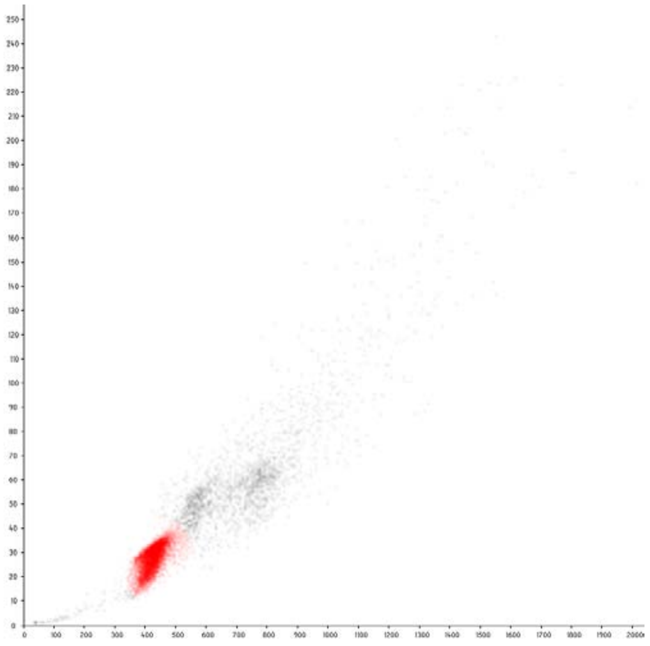

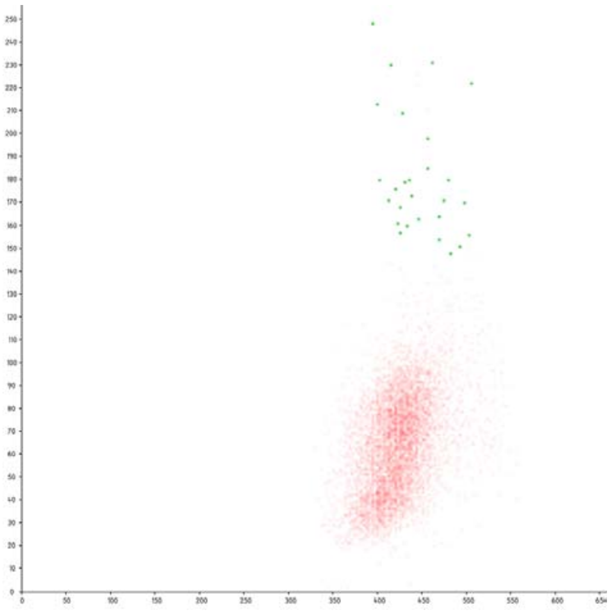

ANNEX 1: FLOW SORTINGBIN 1 – File 2 of 3

Bin 1: COPAS scatter plots showing events for medium 1 of the final split-pool cycle (bin 1). In the first scatter plot (time of flight [TOF] vs. optical extinction [ext]), events depicted by a red dot fall within the gate defining monomeric beads and were sorted. The second scatter plot displays the fluorescent values of the sorted beads (TOF vs. red fluorescence intensity). Beads with a red fluorescent intensity above the threshold value are depicted as green dots and were individually dispensed into 96 well plates.

Number of beads individually dispensed: 96

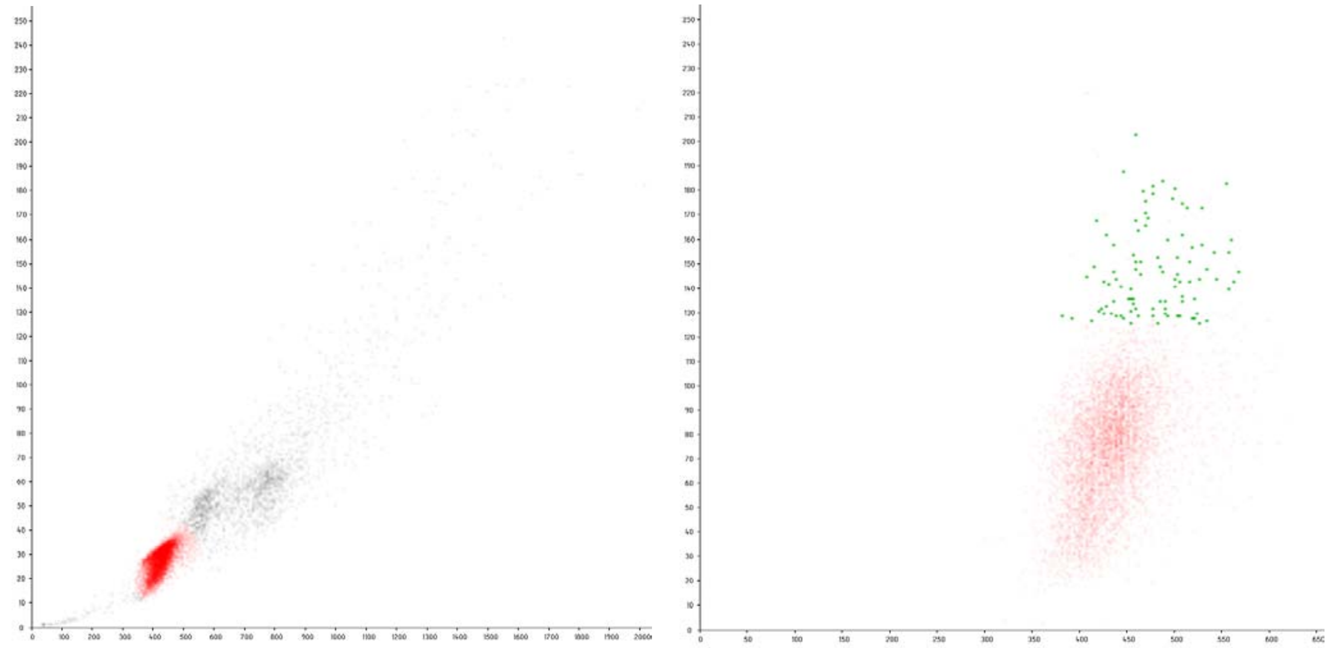

ANNEX 1: FLOW SORTINGBIN 1 – File 3 of 3

Bin 1: COPAS scatter plots showing events for medium 1 of the final split-pool cycle (bin 1). In the first scatter plot (time of flight [TOF] vs. optical extinction [ext]), events depicted by a red dot fall within the gate defining monomeric beads and were sorted. The second scatter plot displays the fluorescent values of the sorted beads (TOF vs. red fluorescence intensity). Beads with a red fluorescent intensity above the threshold value are depicted as green dots and were individually dispensed into 96 well plates.

Number of beads individually dispensed: 96

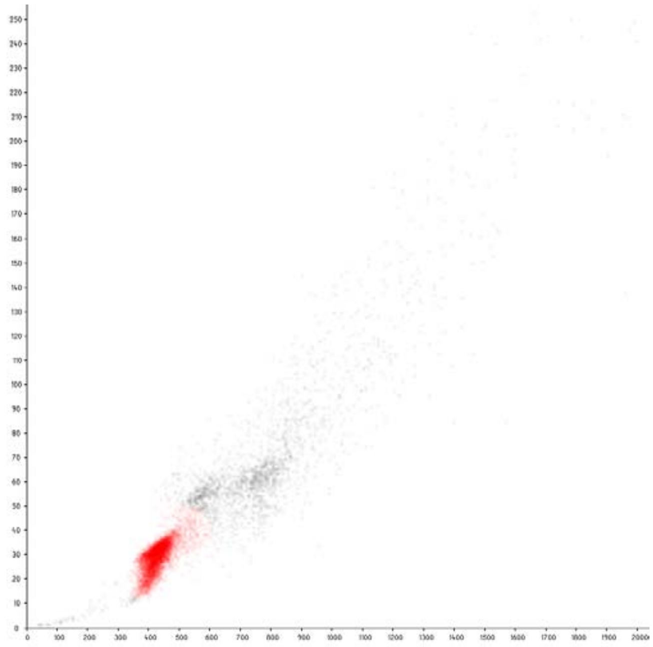

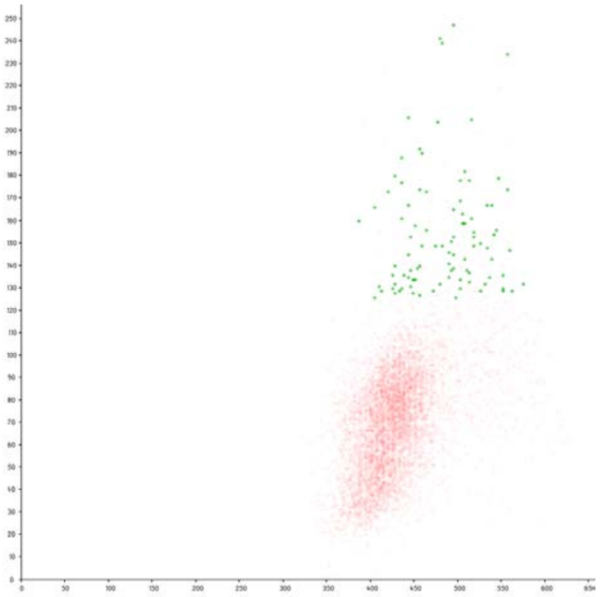

ANNEX 1: FLOW SORTINGBIN 2 – File 1 of 1

Bin 2: COPAS scatter plots showing events for medium 2 of the final split-pool cycle (bin 2). In the first scatter plot (time of flight [TOF] vs. optical extinction [ext]), events depicted by a red dot fall within the gate defining monomeric beads and were sorted. The second scatter plot displays the fluorescent values of the sorted beads (TOF vs. red fluorescence intensity). Beads with a red fluorescent intensity above the threshold value are depicted as green dots and were individually dispensed into 96 well plates.

Number of beads individually dispensed: 80

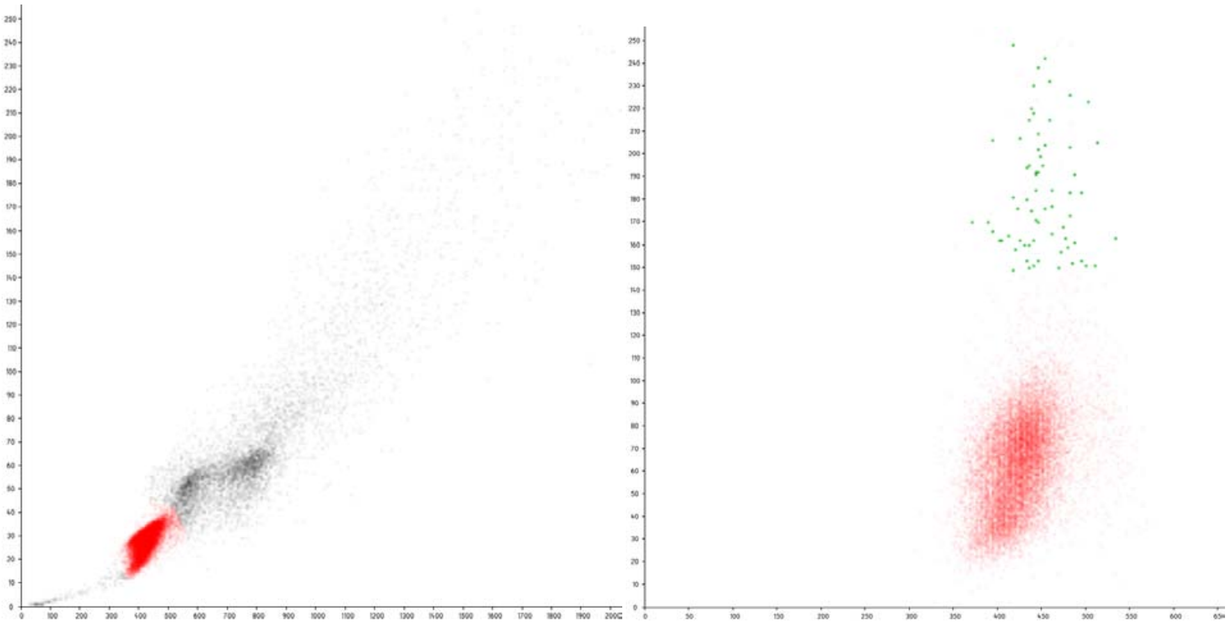

ANNEX 1: FLOW SORTINGBIN 3 – File 1 of 1

Bin 3: COPAS scatter plots showing events for medium 3 of the final split-pool cycle (bin 3). In the first scatter plot (time of flight [TOF] vs. optical extinction [ext]), events depicted by a red dot fall within the gate defining monomeric beads and were sorted. The second scatter plot displays the fluorescent values of the sorted beads (TOF vs. red fluorescence intensity). Beads with a red fluorescent intensity above the threshold value are depicted as green dots and were individually dispensed into 96 well plates.

Number of beads individually dispensed: 23

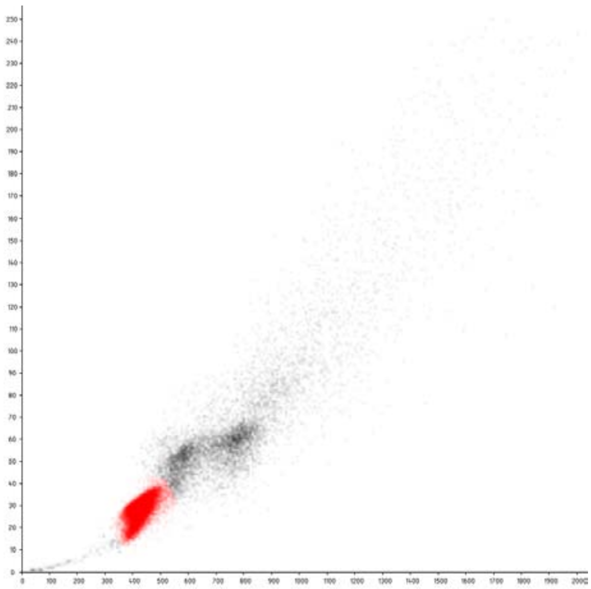

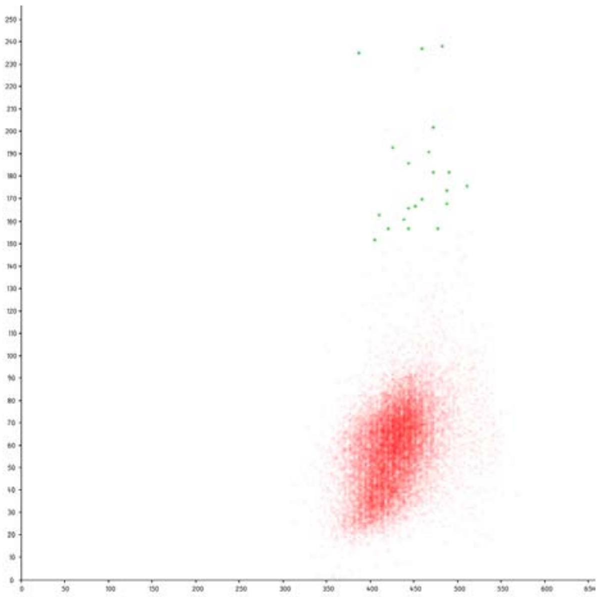

ANNEX 1: FLOW SORTINGBIN 4 – File 1 of 1

Bin 4: COPAS scatter plots showing events for medium 4 of the final split-pool cycle (bin 4). In the first scatter plot (time of flight [TOF] vs. optical extinction [ext]), events depicted by a red dot fall within the gate defining monomeric beads and were sorted. The second scatter plot displays the fluorescent values of the sorted beads (TOF vs. red fluorescence intensity). Beads with a red fluorescent intensity above the threshold value are depicted as green dots and were individually dispensed into 96 well plates.

Number of beads individually dispensed: 45

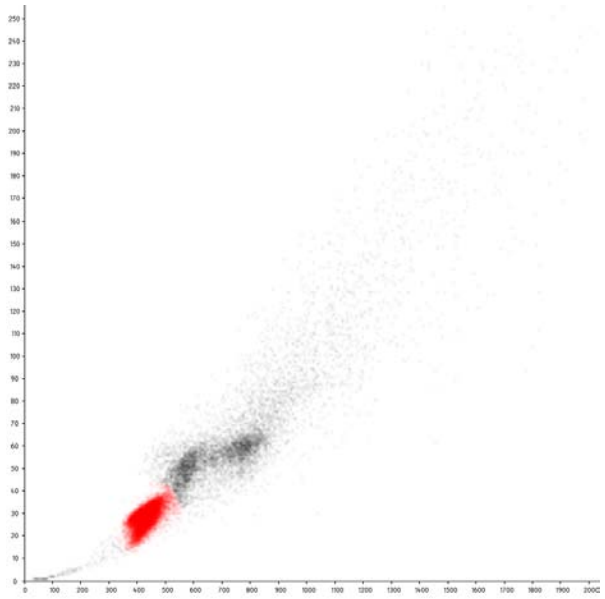

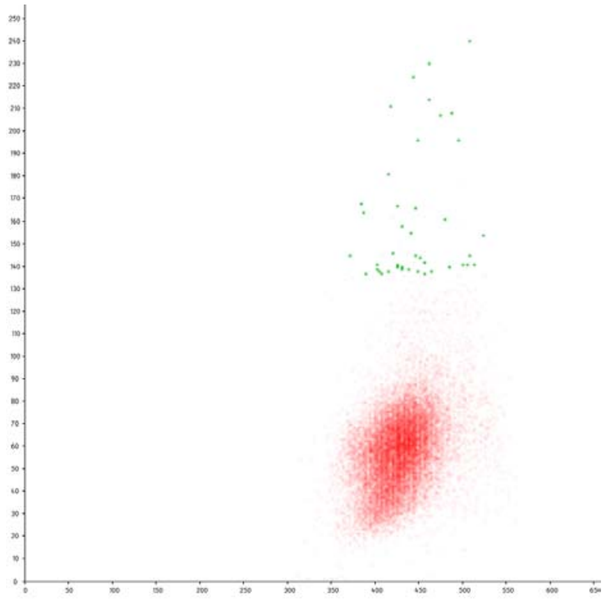

ANNEX 1: FLOW SORTINGBIN 5 – File 1 of 1

Bin 5: COPAS scatter plots showing events for medium 5 of the final split-pool cycle (bin 5). In the first scatter plot (time of flight [TOF] vs. optical extinction [ext]), events depicted by a red dot fall within the gate defining monomeric beads and were sorted. The second scatter plot displays the fluorescent values of the sorted beads (TOF vs. red fluorescence intensity). Beads with a red fluorescent intensity above the threshold value are depicted as green dots and were individually dispensed into 96 well plates.

Number of beads individually dispensed: 77

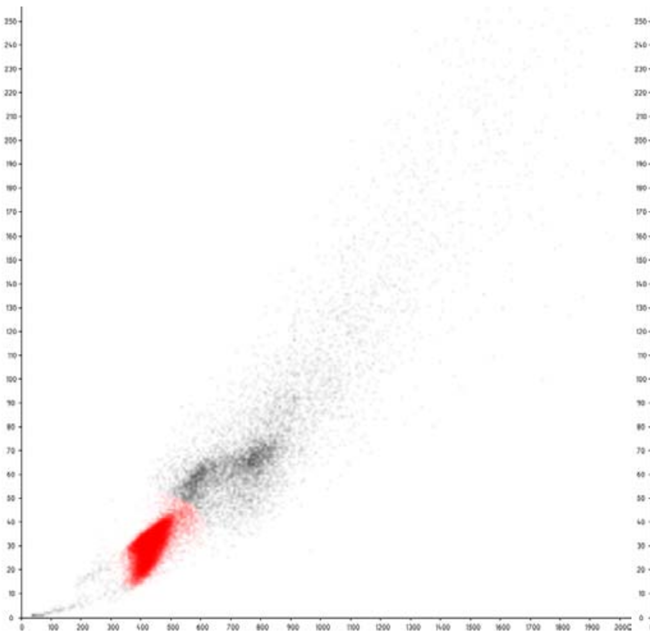

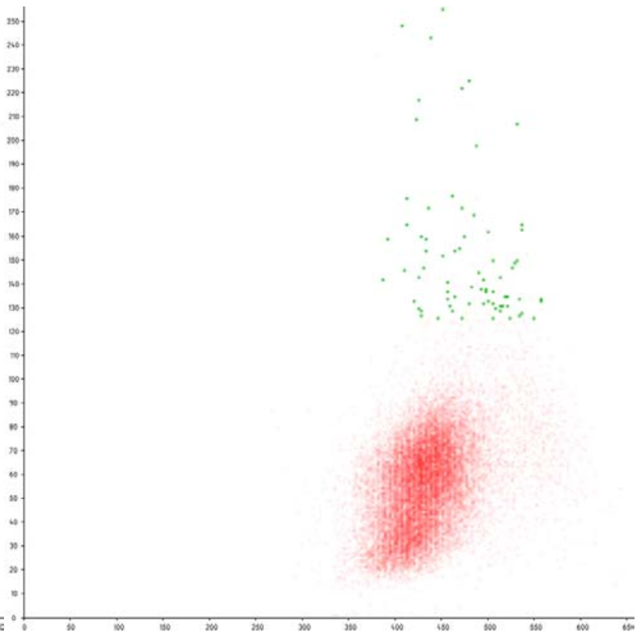

ANNEX 1: FLOW SORTINGBIN 6 – File 1 of 1

Bin 6: COPAS scatter plots showing events for medium 6 of the final split-pool cycle (bin 6). In the first scatter plot (time of flight [TOF] vs. optical extinction [ext]), events depicted by a red dot fall within the gate defining monomeric beads and were sorted. The second scatter plot displays the fluorescent values of the sorted beads (TOF vs. red fluorescence intensity). Beads with a red fluorescent intensity above the threshold value are depicted as green dots and were individually dispensed into 96 well plates.

Number of beads individually dispensed: 8

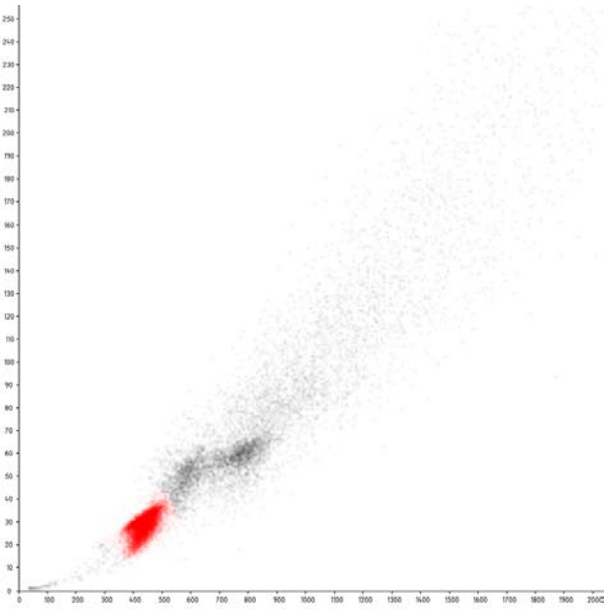

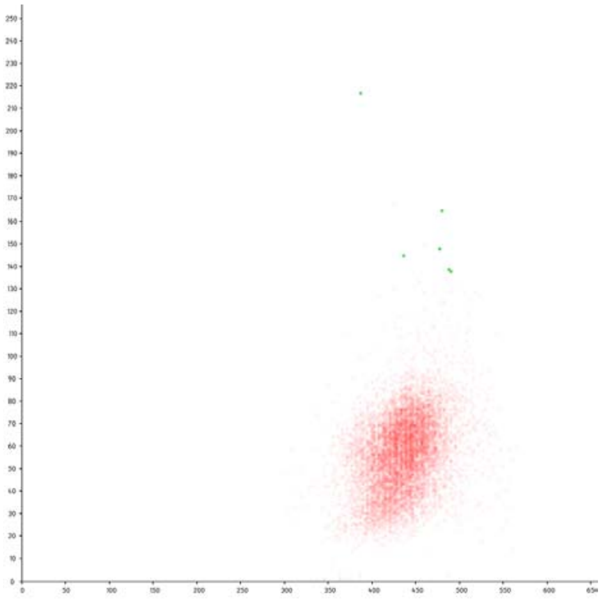

ANNEX 1: FLOW SORTINGBIN 7 – File 1 of 1

Bin 7: COPAS scatter plots showing events for medium 7 of the final split-pool cycle (bin 7). In the first scatter plot (time of flight [TOF] vs. optical extinction [ext]), events depicted by a red dot fall within the gate defining monomeric beads and were sorted. The second scatter plot displays the fluorescent values of the sorted beads (TOF vs. red fluorescence intensity). Beads with a red fluorescent intensity above the threshold value are depicted as green dots and were individually dispensed into 96 well plates.

Number of beads individually dispensed: 20

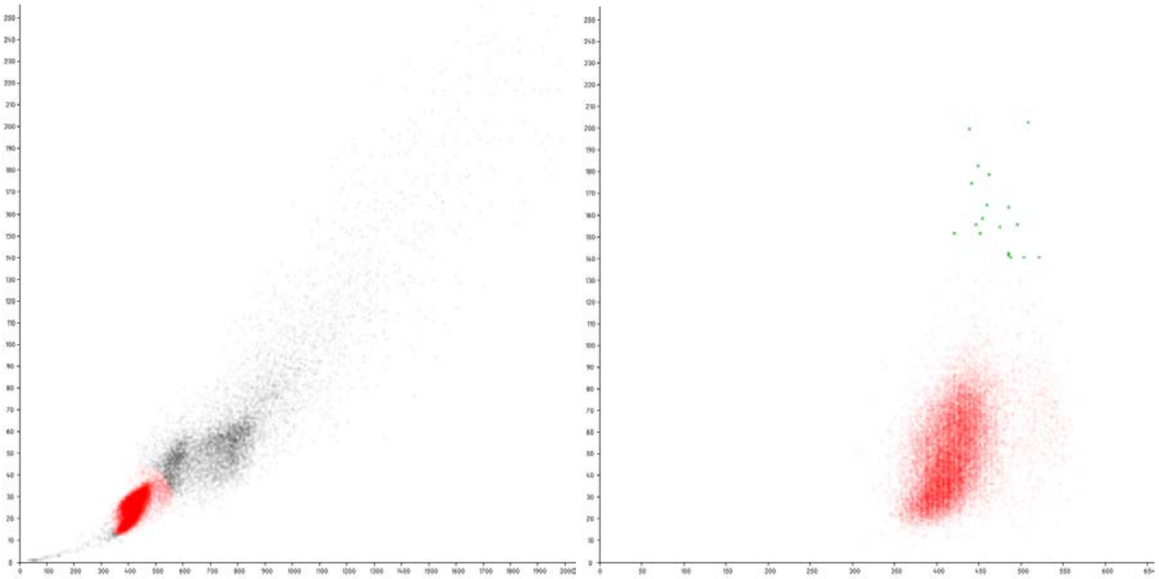

ANNEX 1: FLOW SORTINGBIN 8 – File 1 of 1

Bin 8: COPAS scatter plots showing events for medium 8 of the final split-pool cycle (bin 8). In the first scatter plot (time of flight [TOF] vs. optical extinction [ext]), events depicted by a red dot fall within the gate defining monomeric beads and were sorted. The second scatter plot displays the fluorescent values of the sorted beads (TOF vs. red fluorescence intensity). Beads with a red fluorescent intensity above the threshold value are depicted as green dots and were individually dispensed into 96 well plates.

Number of beads individually dispensed: 21

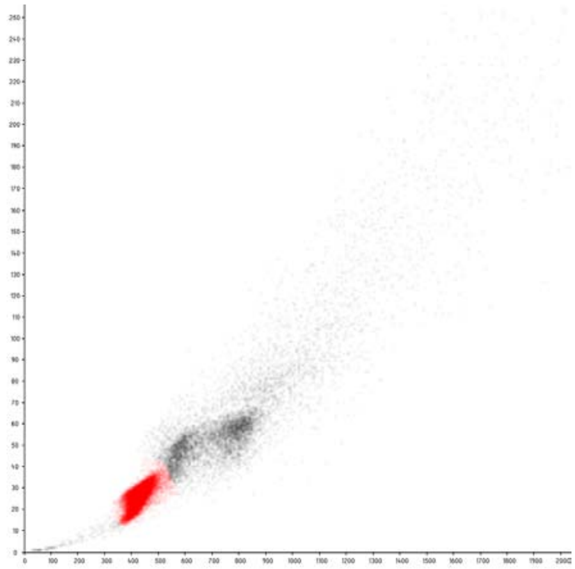

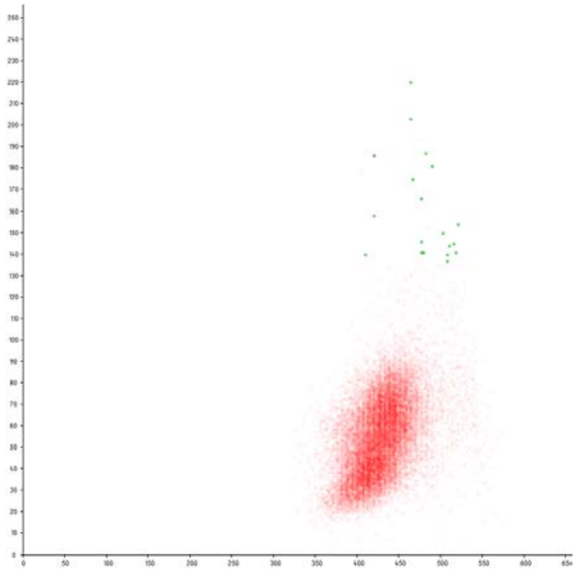

ANNEX 1: FLOW SORTINGBIN 9 – File 1 of 2

Bin 9: COPAS scatter plots showing events for medium 9 of the final split-pool cycle (bin 9). In the first scatter plot (time of flight [TOF] vs. optical extinction [ext]), events depicted by a red dot fall within the gate defining monomeric beads and were sorted. The second scatter plot displays the fluorescent values of the sorted beads (TOF vs. red fluorescence intensity). Beads with a red fluorescent intensity above the threshold value are depicted as green dots and were individually dispensed into 96 well plates.

Number of beads individually dispensed: 10

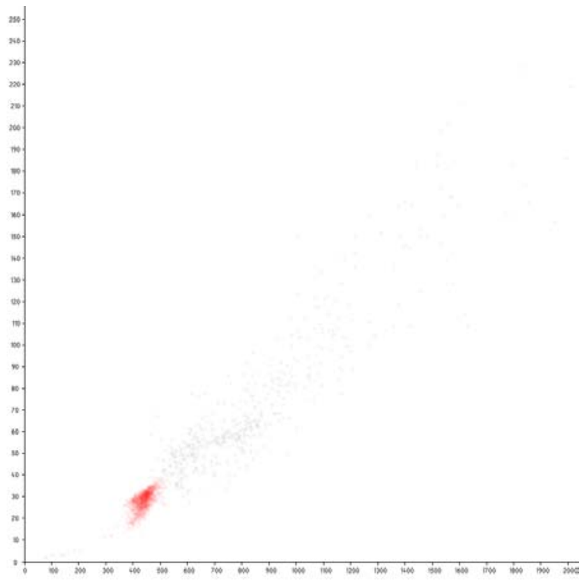

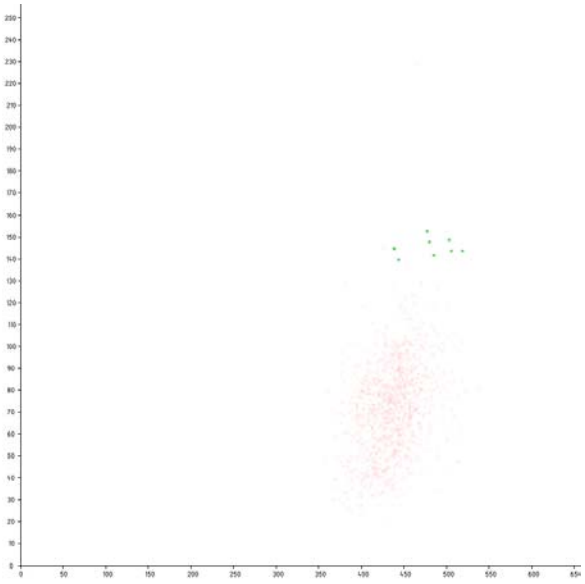

ANNEX 1: FLOW SORTINGBIN 9 – File 2 of 2

Bin 9: COPAS scatter plots showing events for medium 9 of the final split-pool cycle (bin 9). In the first scatter plot (time of flight [TOF] vs. optical extinction [ext]), events depicted by a red dot fall within the gate defining monomeric beads and were sorted. The second scatter plot displays the fluorescent values of the sorted beads (TOF vs. red fluorescence intensity). Beads with a red fluorescent intensity above the threshold value are depicted as green dots and were individually dispensed into 96 well plates.

Number of beads individually dispensed: 96

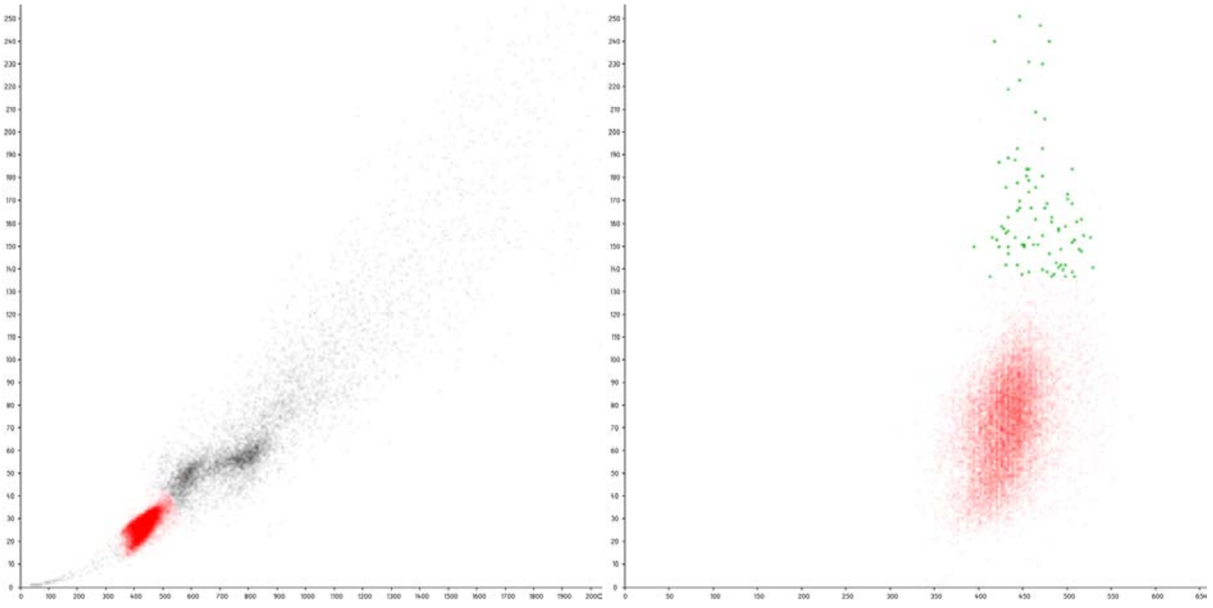

ANNEX 1: FLOW SORTINGBIN 10 – File 1 of 1

Bin 10: COPAS scatter plots showing events for medium 10 of the final split-pool cycle (bin 10). In the first scatter plot (time of flight [TOF] vs. optical extinction [ext]), events depicted by a red dot fall within the gate defining monomeric beads and were sorted. The second scatter plot displays the fluorescent values of the sorted beads (TOF vs. red fluorescence intensity). Beads with a red fluorescent intensity above the threshold value are depicted as green dots and were individually dispensed into 96 well plates.

Number of beads individually dispensed: 17

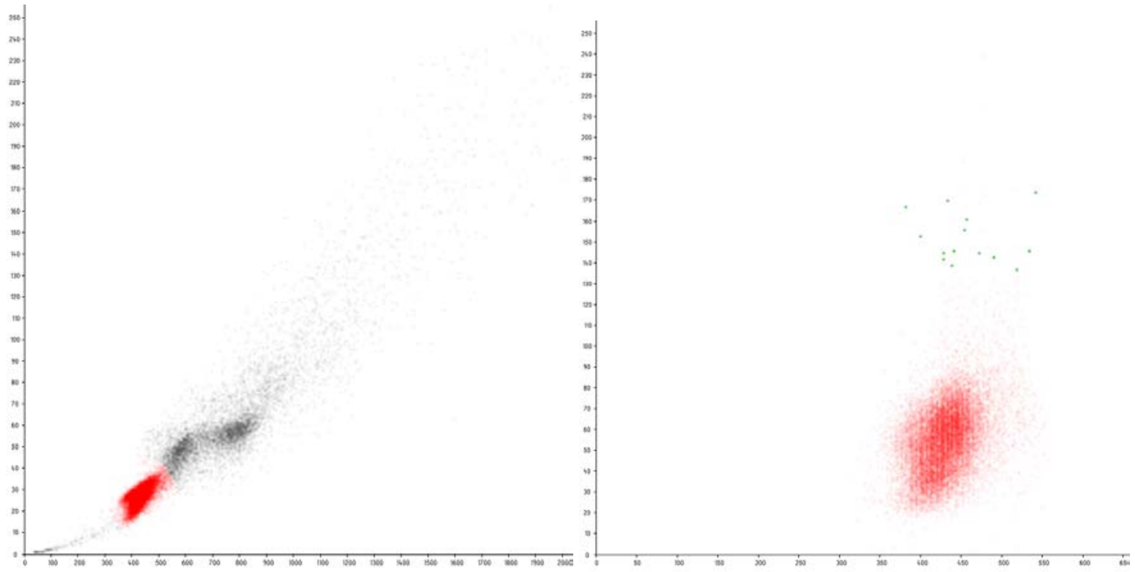

ANNEX 2: TAG REFERENCE SET - TAG DECONVOLUTION SESSION 1

Tag analysis flow cytometry instrument: BD FACSCalibur

Number of gates: 30

Number of beads whose tags were deconvoluted: 23

Number of beads with conclusive tagging data: 16

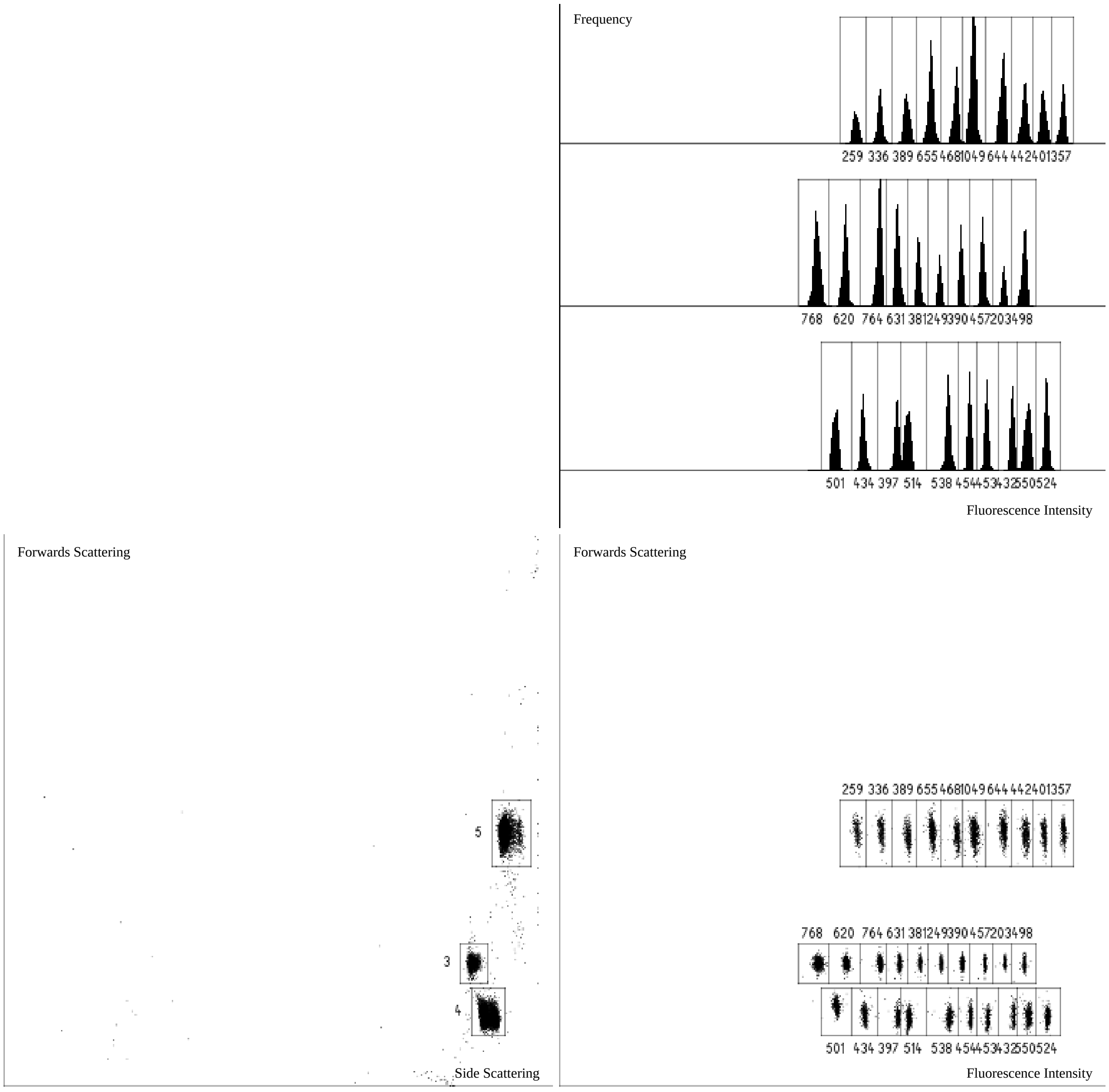

ANNEX 2: TAG REFERENCE SET - TAG DECONVOLUTION SESSION 2

Tag analysis flow cytometry instrument: BD FACSCalibur

Number of gates: 30

Number of beads whose tags were deconvoluted: 27

Number of beads with conclusive tagging data: 18

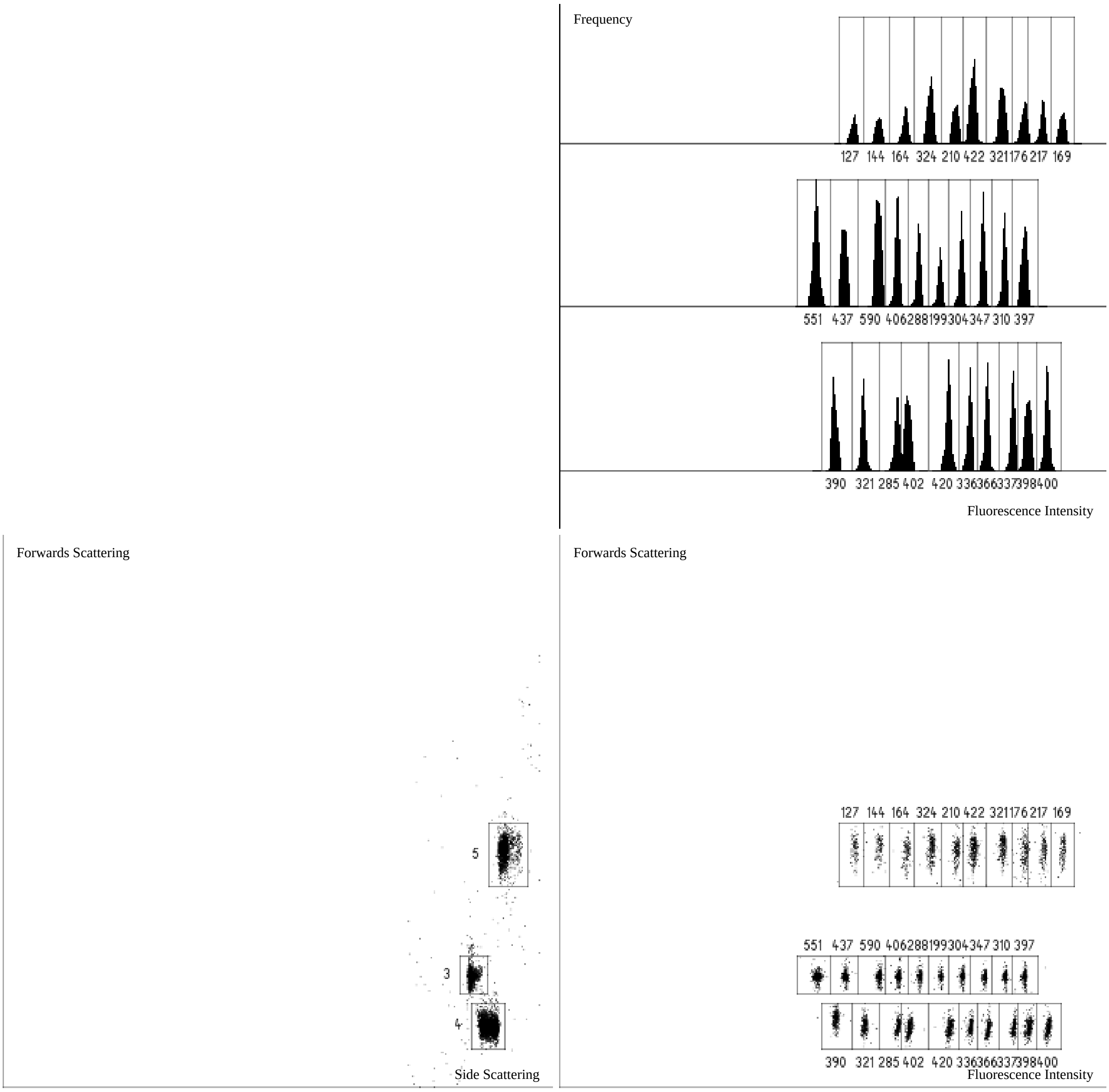

ANNEX 2: TAG REFERENCE SET - TAG DECONVOLUTION SESSION 3

Tag analysis flow cytometry instrument: BD FACSCalibur

Number of gates: 30

Number of beads whose tags were deconvoluted: 27

Number of beads with conclusive tagging data: 21

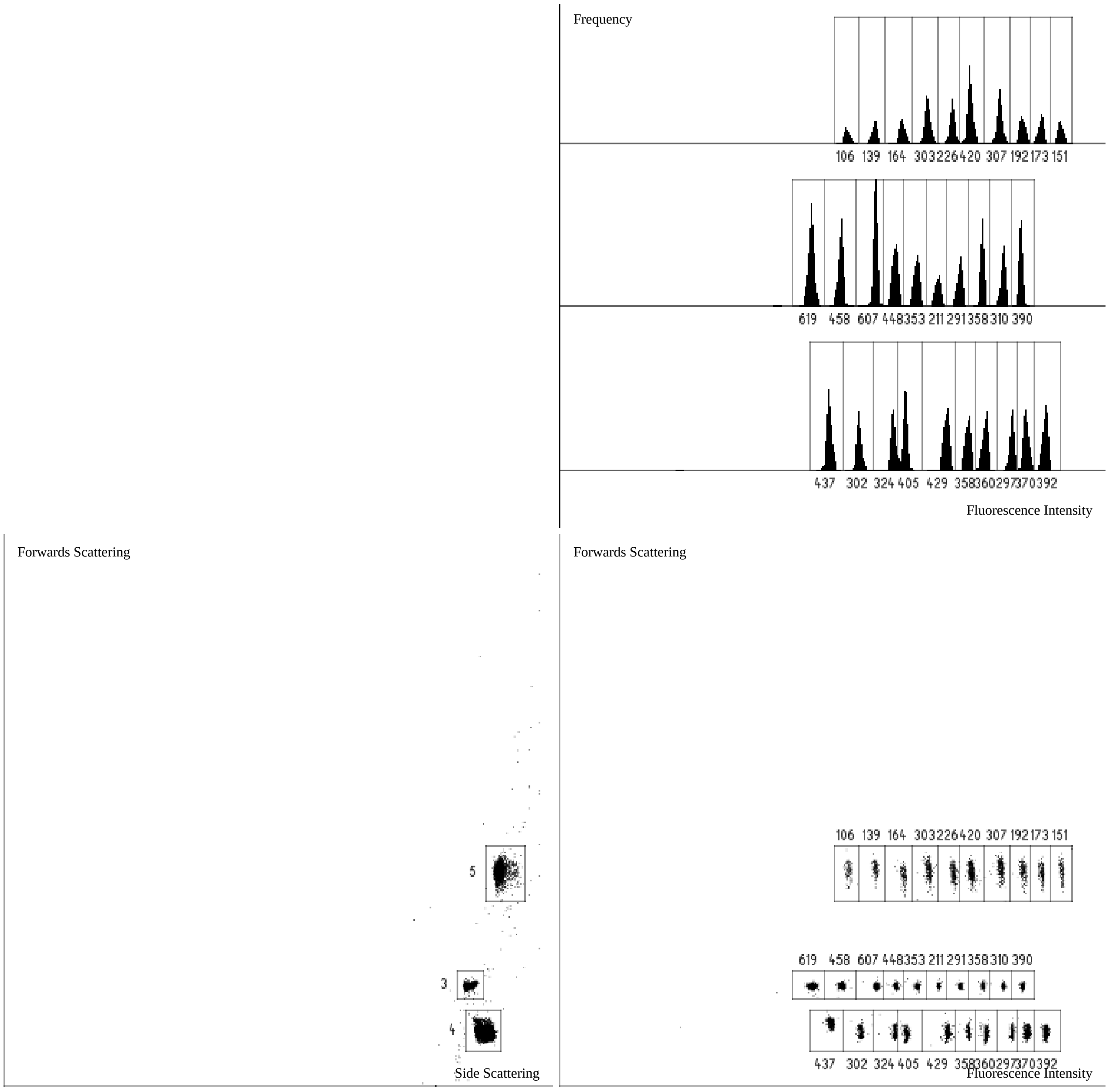

ANNEX 2: TAG REFERENCE SET - TAG DECONVOLUTION SESSION 4

Tag analysis flow cytometry instrument: BD FACSCalibur

Number of gates: 30

Number of beads whose tags were deconvoluted: 16

Number of beads with conclusive tagging data: 15

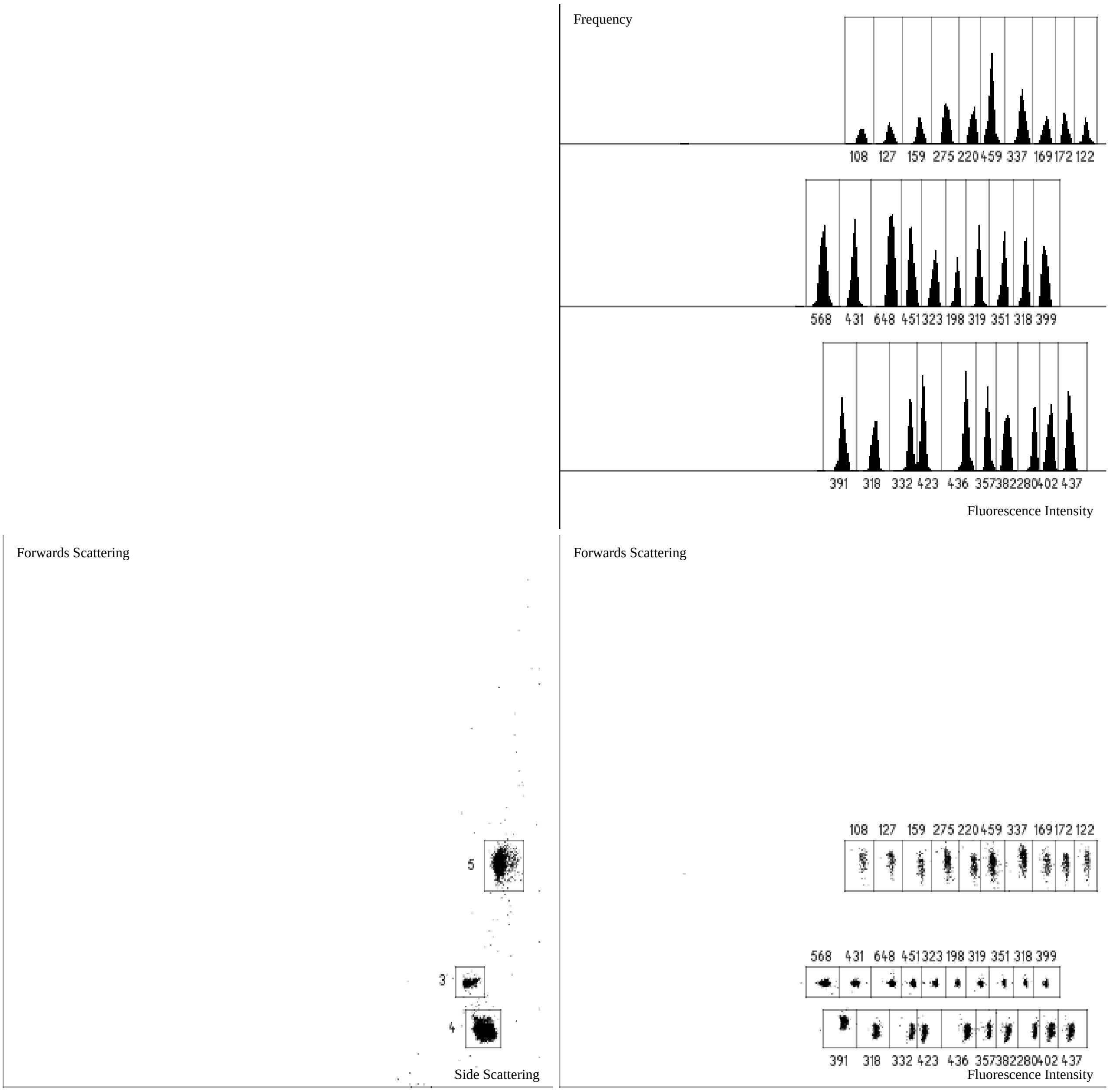

ANNEX 2: TAG REFERENCE SET - TAG DECONVOLUTION SESSION 5

Tag analysis flow cytometry instrument: BD FACSCalibur

Number of gates: 30

Number of beads whose tags were deconvoluted: 37

Number of beads with conclusive tagging data: 23

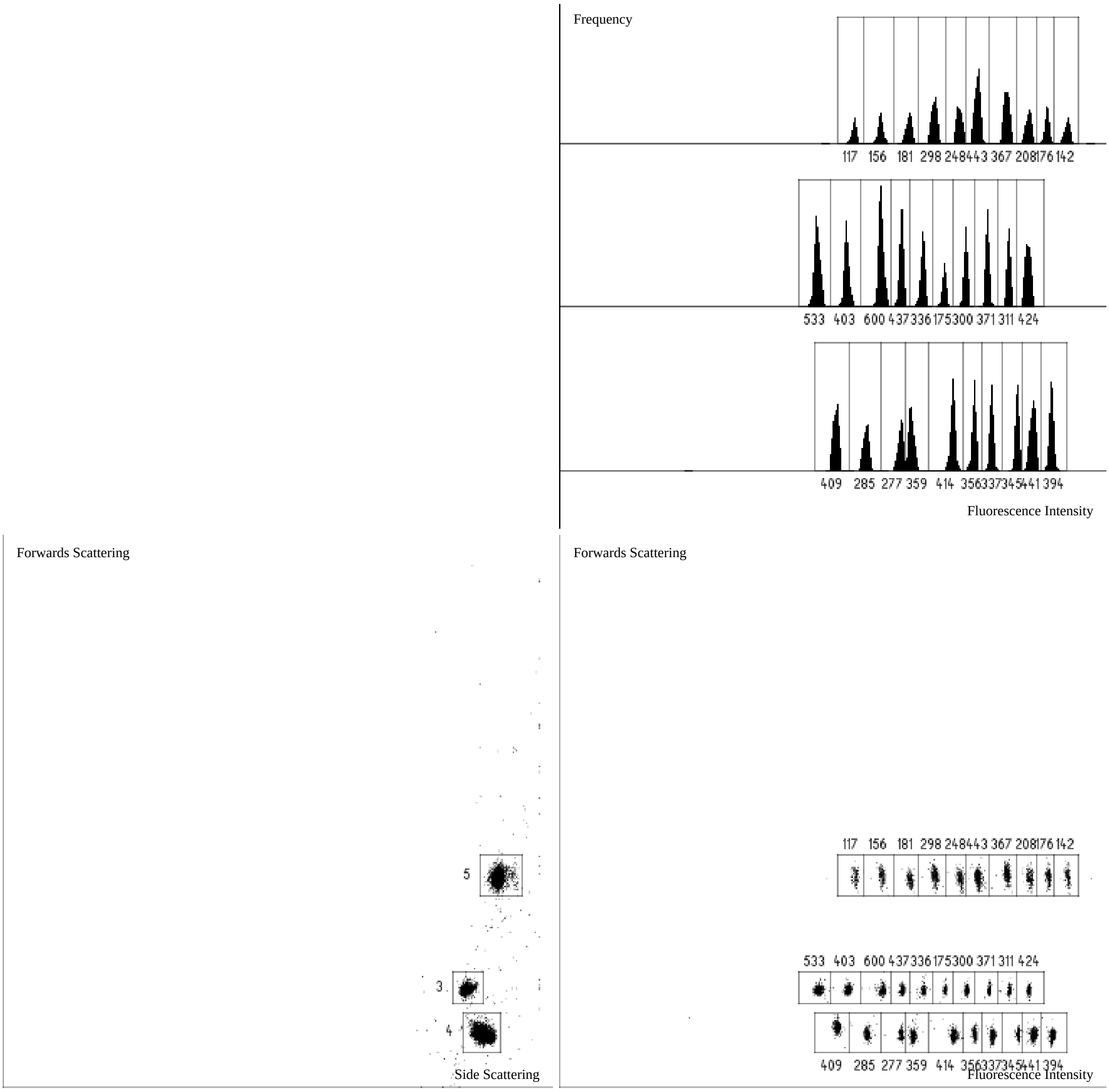

ANNEX 2: TAG REFERENCE SET - TAG DECONVOLUTION SESSION 6

Tag analysis flow cytometry instrument: BD FACSCalibur

Number of gates: 30

Number of beads whose tags were deconvoluted: 20

Number of beads with conclusive tagging data: 11

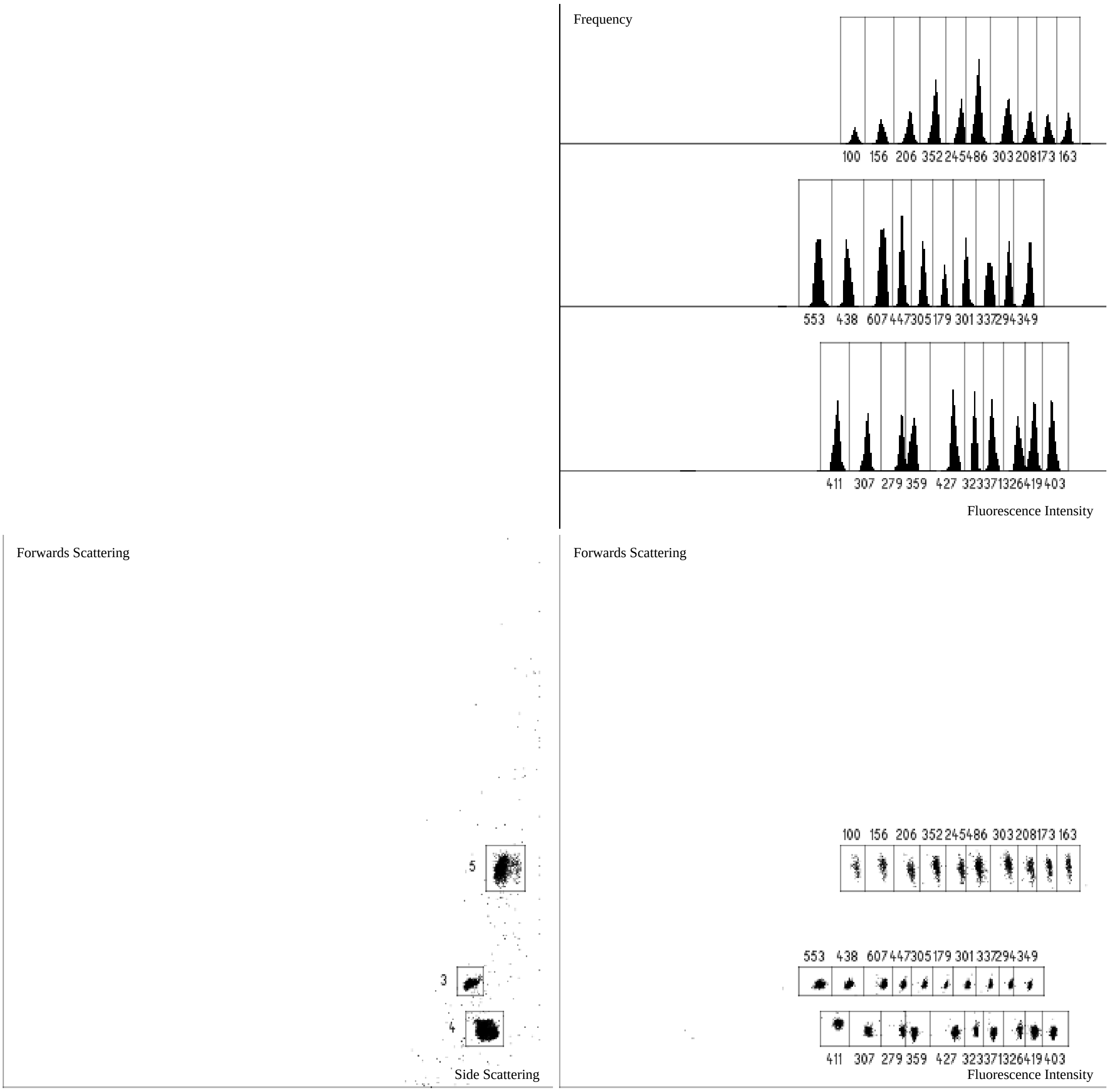

ANNEX 2: TAG REFERENCE SET - TAG DECONVOLUTION SESSION 7

Tag analysis flow cytometry instrument: BD FACSCalibur

Number of gates: 30

Number of beads whose tags were deconvoluted: 7

Number of beads with conclusive tagging data: 3

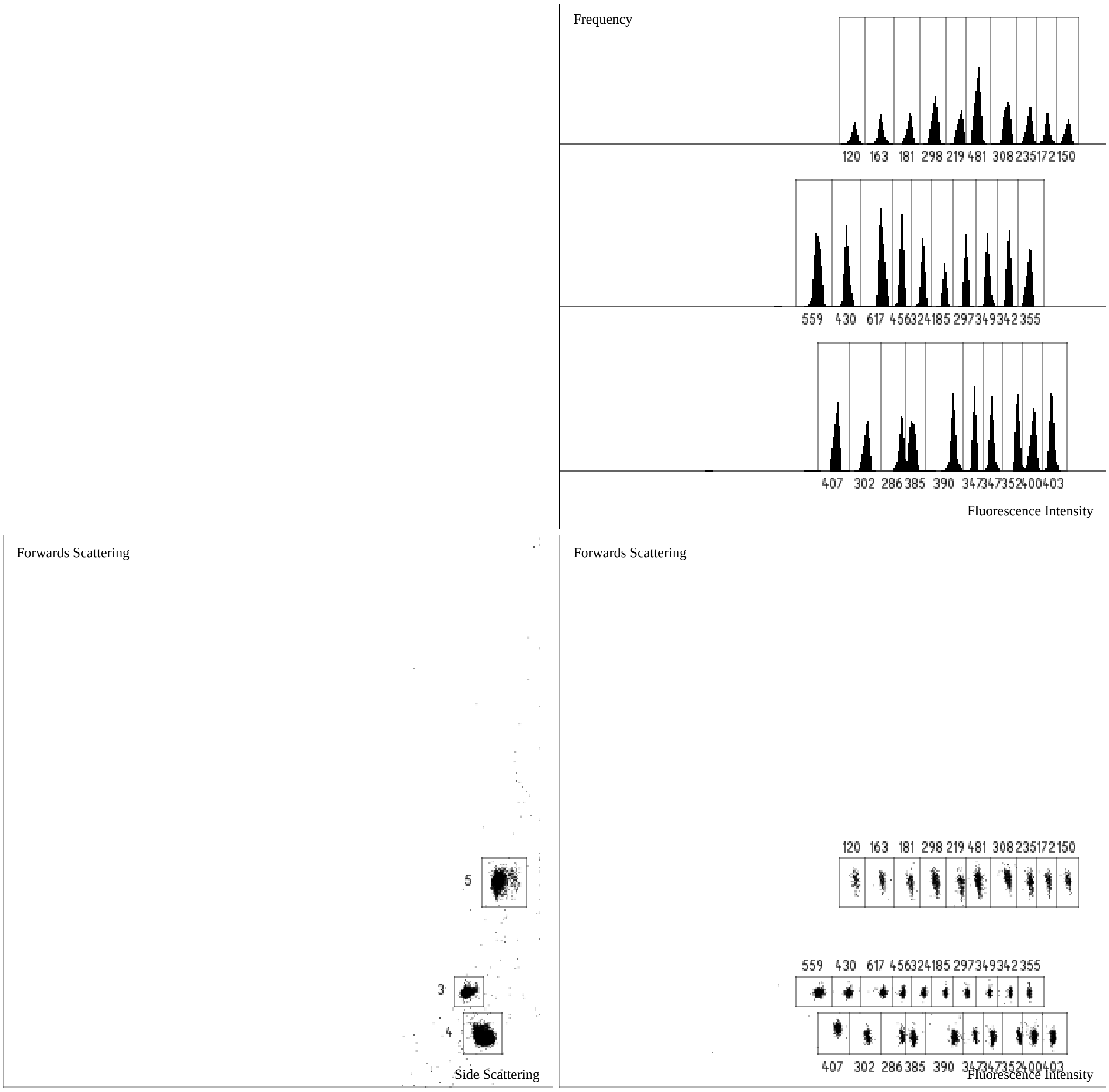

ANNEX 2: TAG REFERENCE SET - TAG DECONVOLUTION SESSION 8

Tag analysis flow cytometry instrument: BD FACSCalibur

Number of gates: 30

Number of beads whose tags were deconvoluted: 27

Number of beads with conclusive tagging data: 24

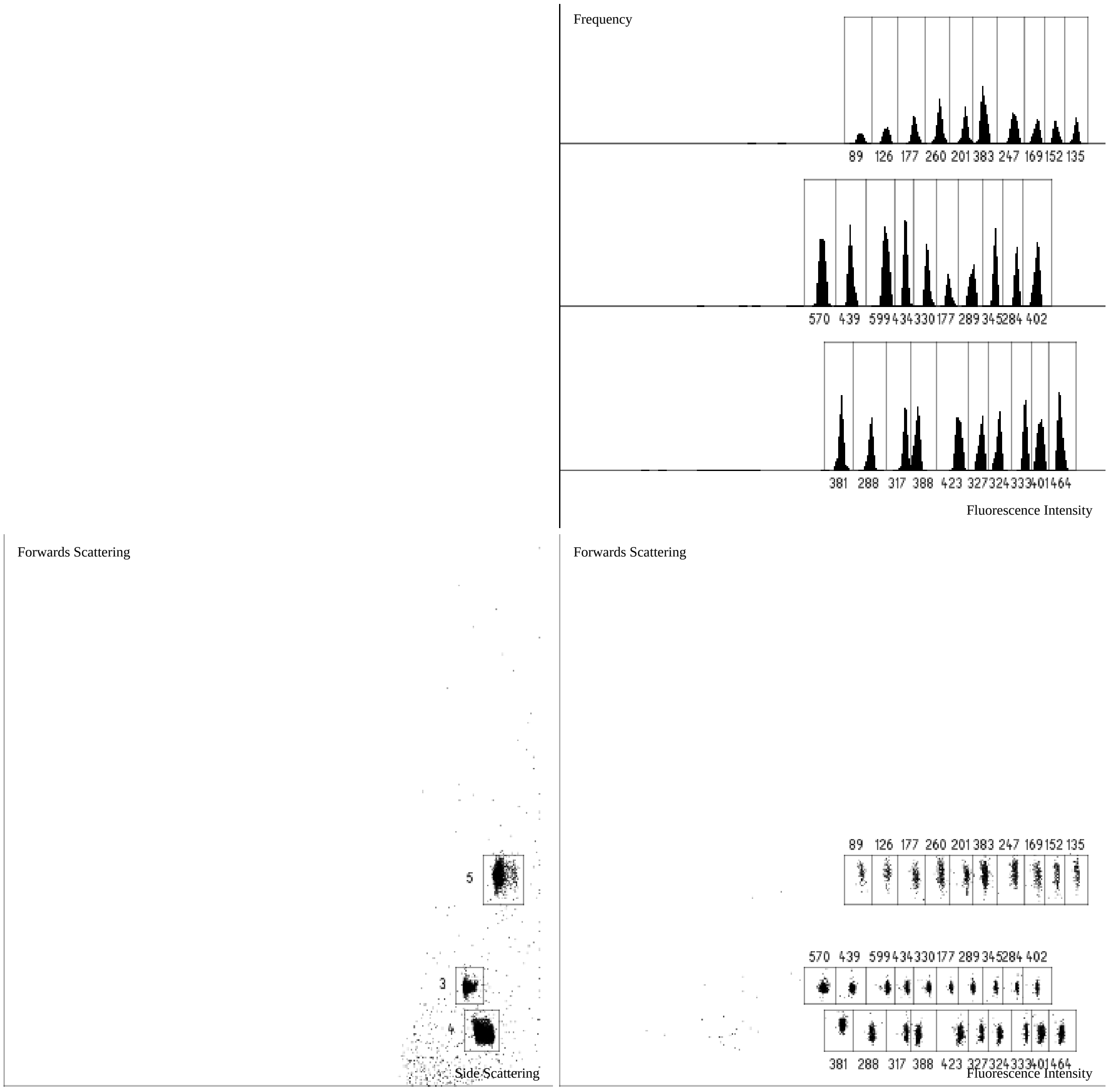

ANNEX 2: TAG REFERENCE SET - TAG DECONVOLUTION SESSION 9

Tag analysis flow cytometry instrument: BD FACSCalibur

Number of gates: 30

Number of beads whose tags were deconvoluted: 53

Number of beads with conclusive tagging data: 43

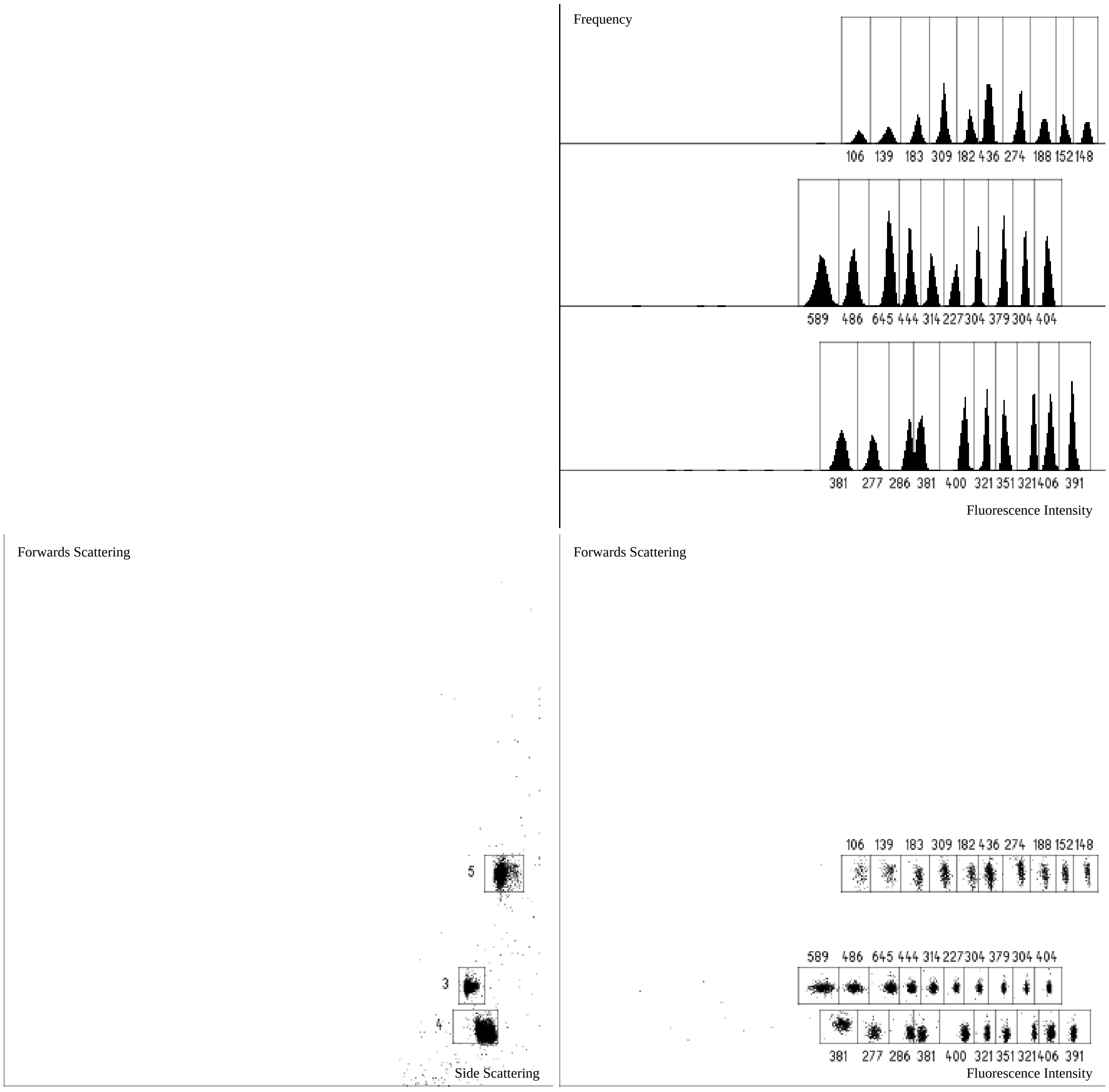

ANNEX 2: TAG REFERENCE SET - TAG DECONVOLUTION SESSION 10

Tag analysis flow cytometry instrument: BD FACSCalibur

Number of gates: 30

Number of beads whose tags were deconvoluted: 22

Number of beads with conclusive tagging data: 17

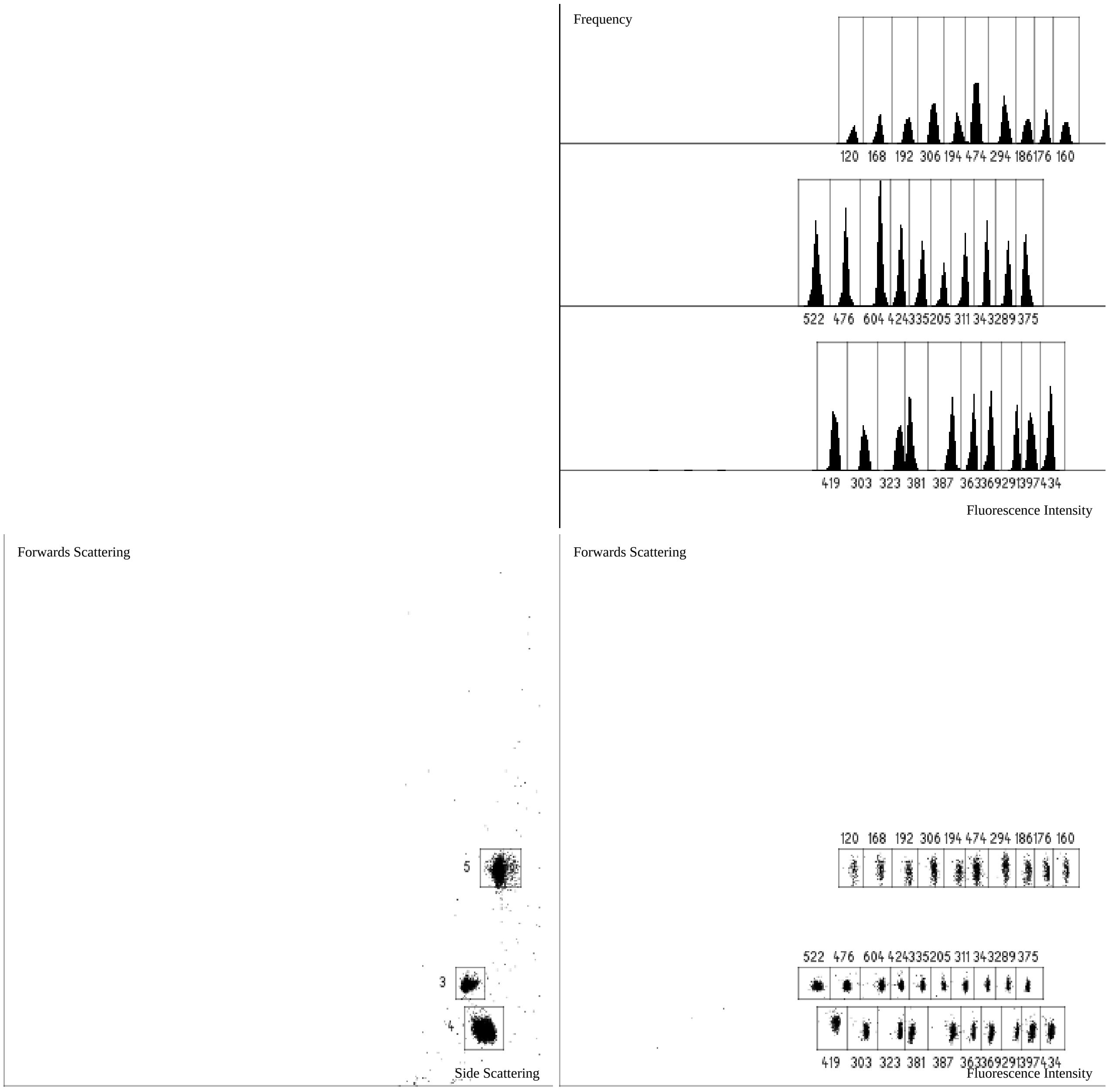

ANNEX 2: TAG REFERENCE SET - TAG DECONVOLUTION SESSION 11

Tag analysis flow cytometry instrument: BD FACSCalibur

Number of gates: 30

Number of beads whose tags were deconvoluted: 19

Number of beads with conclusive tagging data: 16

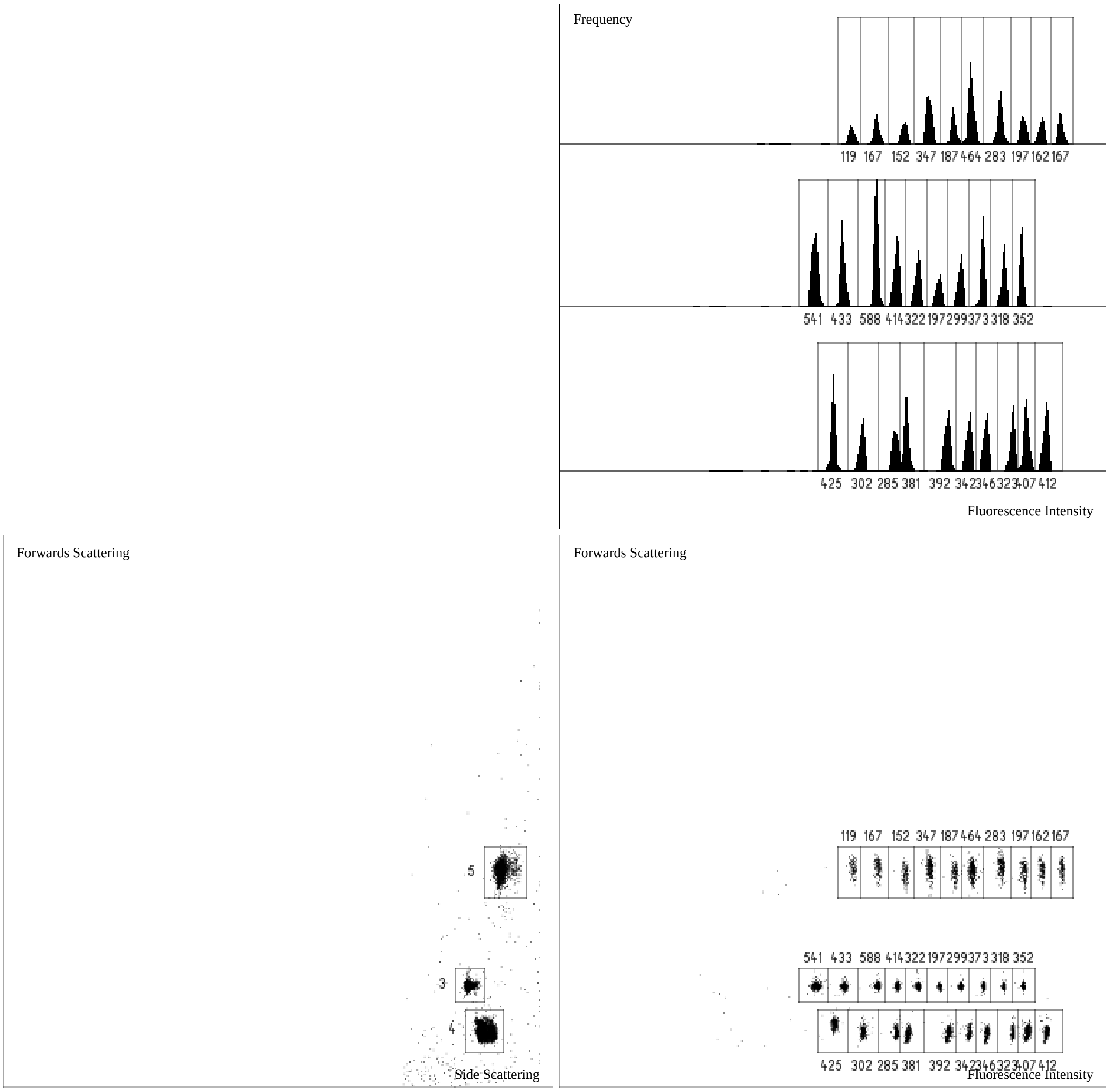

ANNEX 2: TAG REFERENCE SET - TAG DECONVOLUTION SESSION 12

Tag analysis flow cytometry instrument: BD FACSCalibur

Number of gates: 30

Number of beads whose tags were deconvoluted: 3

Number of beads with conclusive tagging data: 2

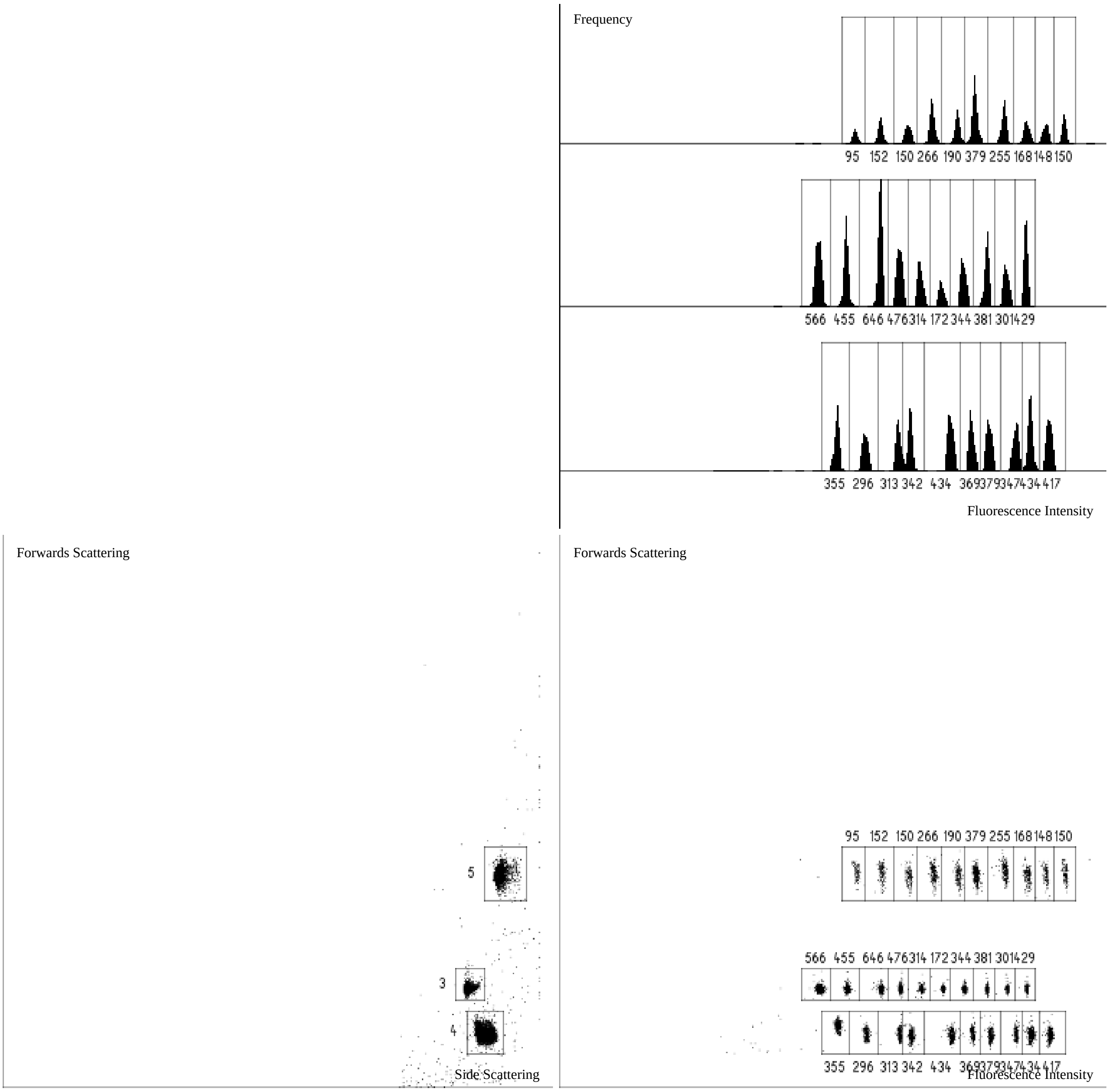

ANNEX 2: TAG REFERENCE SET - TAG DECONVOLUTION SESSION 13

Tag analysis flow cytometry instrument: BD FACSCalibur

Number of gates: 30

Number of beads whose tags were deconvoluted: 20

Number of beads with conclusive tagging data: 13

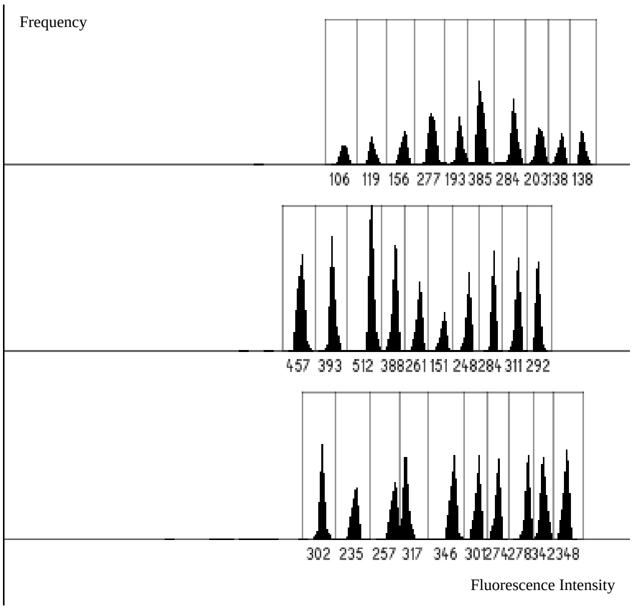

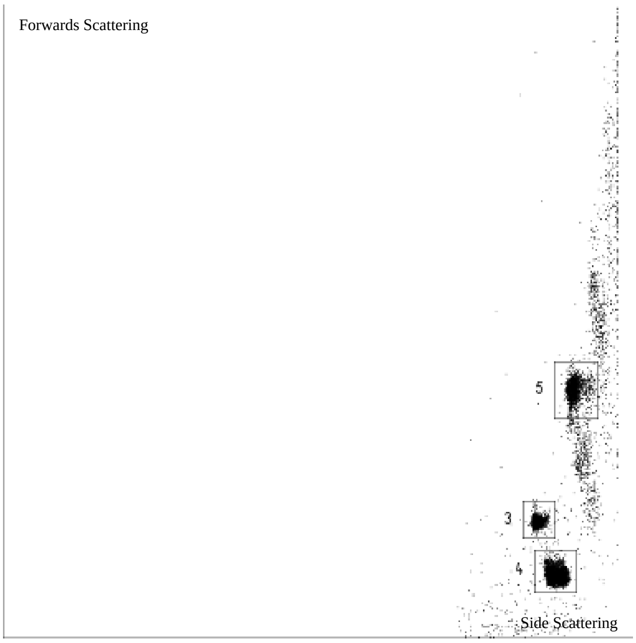

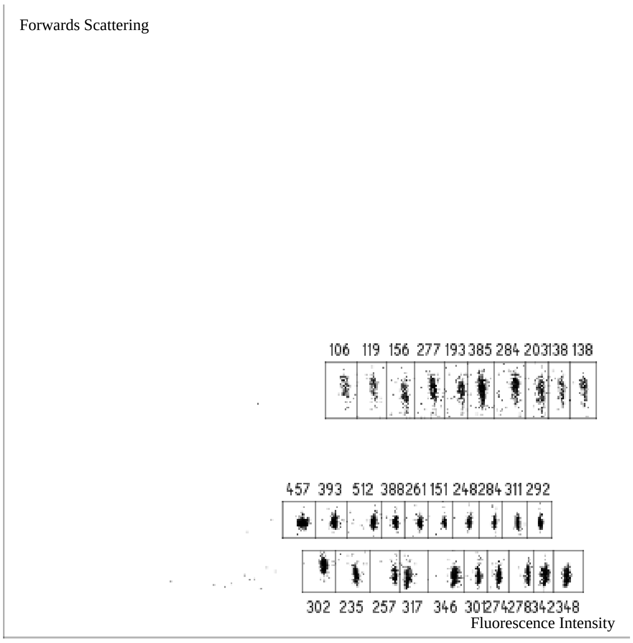

ANNEX 2: TAG REFERENCE SET - TAG DECONVOLUTION SESSION 14

Tag analysis flow cytometry instrument: BD FACSCalibur

Number of gates: 30

Number of beads whose tags were deconvoluted: 24

Number of beads with conclusive tagging data: 16

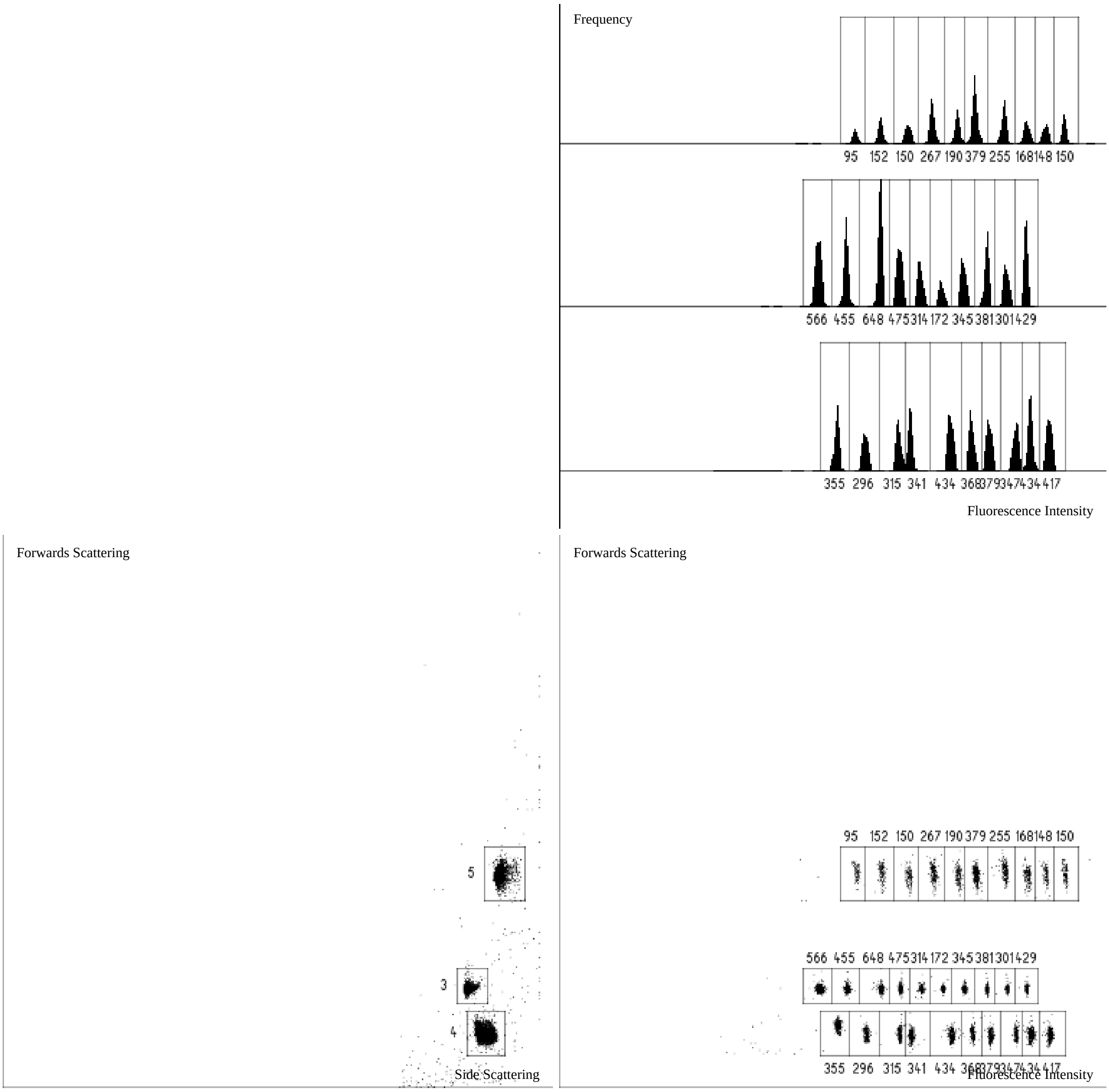

ANNEX 2: TAG REFERENCE SET - TAG DECONVOLUTION SESSION 15

Tag analysis flow cytometry instrument: BD FACSCalibur

Number of gates: 30

Number of beads whose tags were deconvoluted: 12

Number of beads with conclusive tagging data: 8

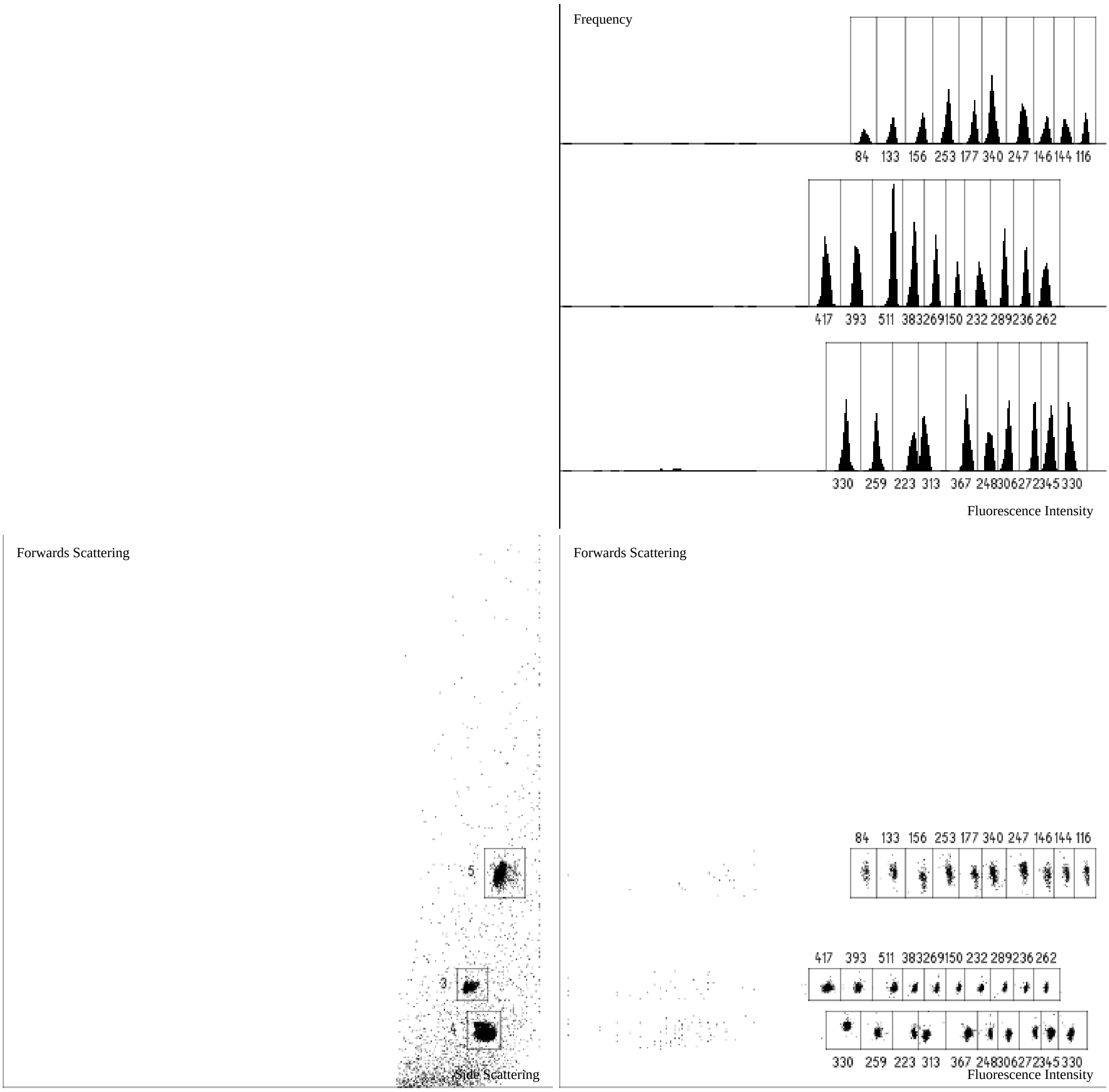

ANNEX 2: TAG REFERENCE SET - TAG DECONVOLUTION SESSION 16

Tag analysis flow cytometry instrument: BD FACSCalibur

Number of gates: 30

Number of beads whose tags were deconvoluted: 41

Number of beads with conclusive tagging data: 33

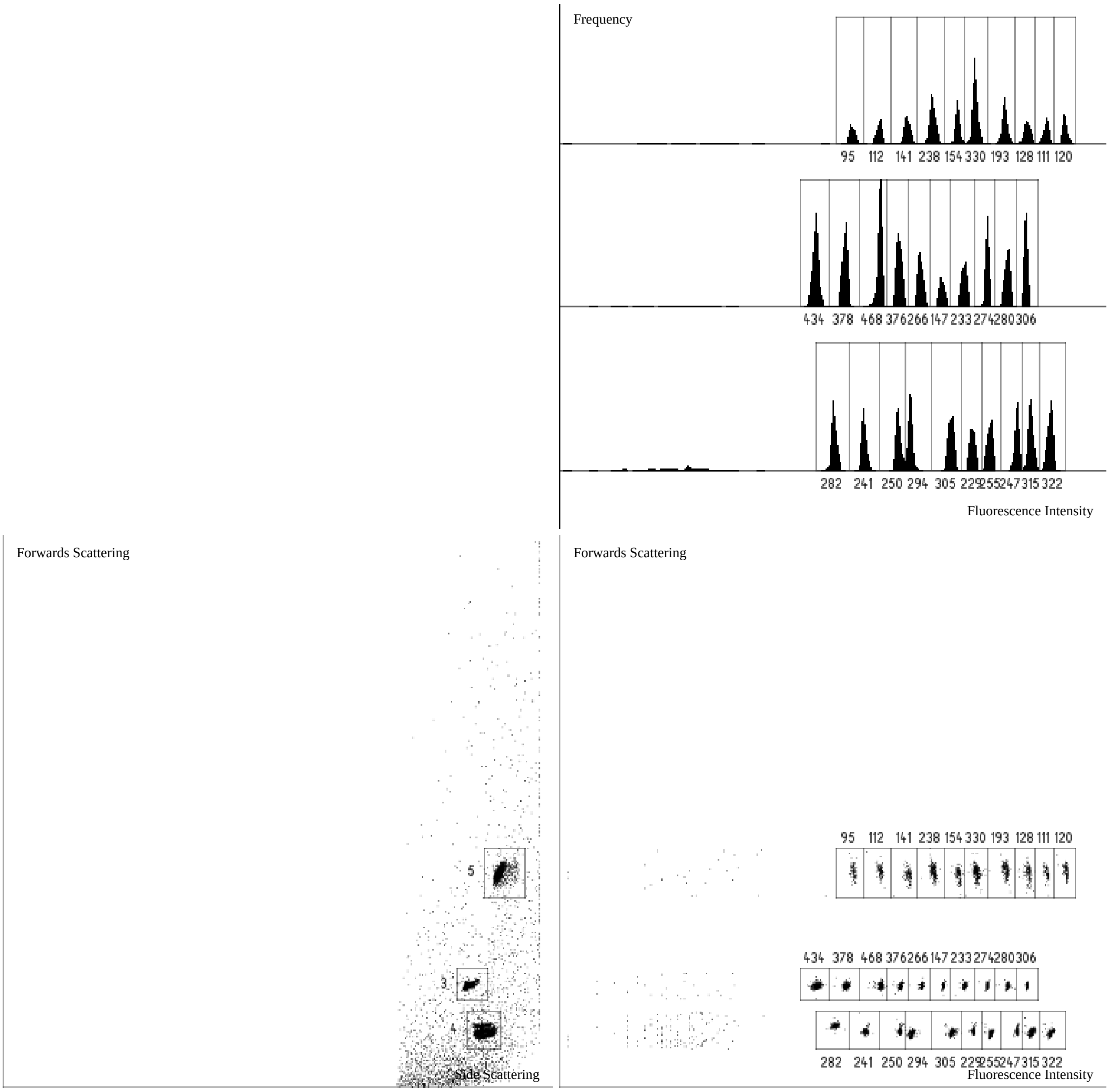

ANNEX 2: TAG REFERENCE SET - TAG DECONVOLUTION SESSION 17

Tag analysis flow cytometry instrument: BD FACSCalibur

Number of gates: 30

Number of beads whose tags were deconvoluted: 12

Number of beads with conclusive tagging data: 10

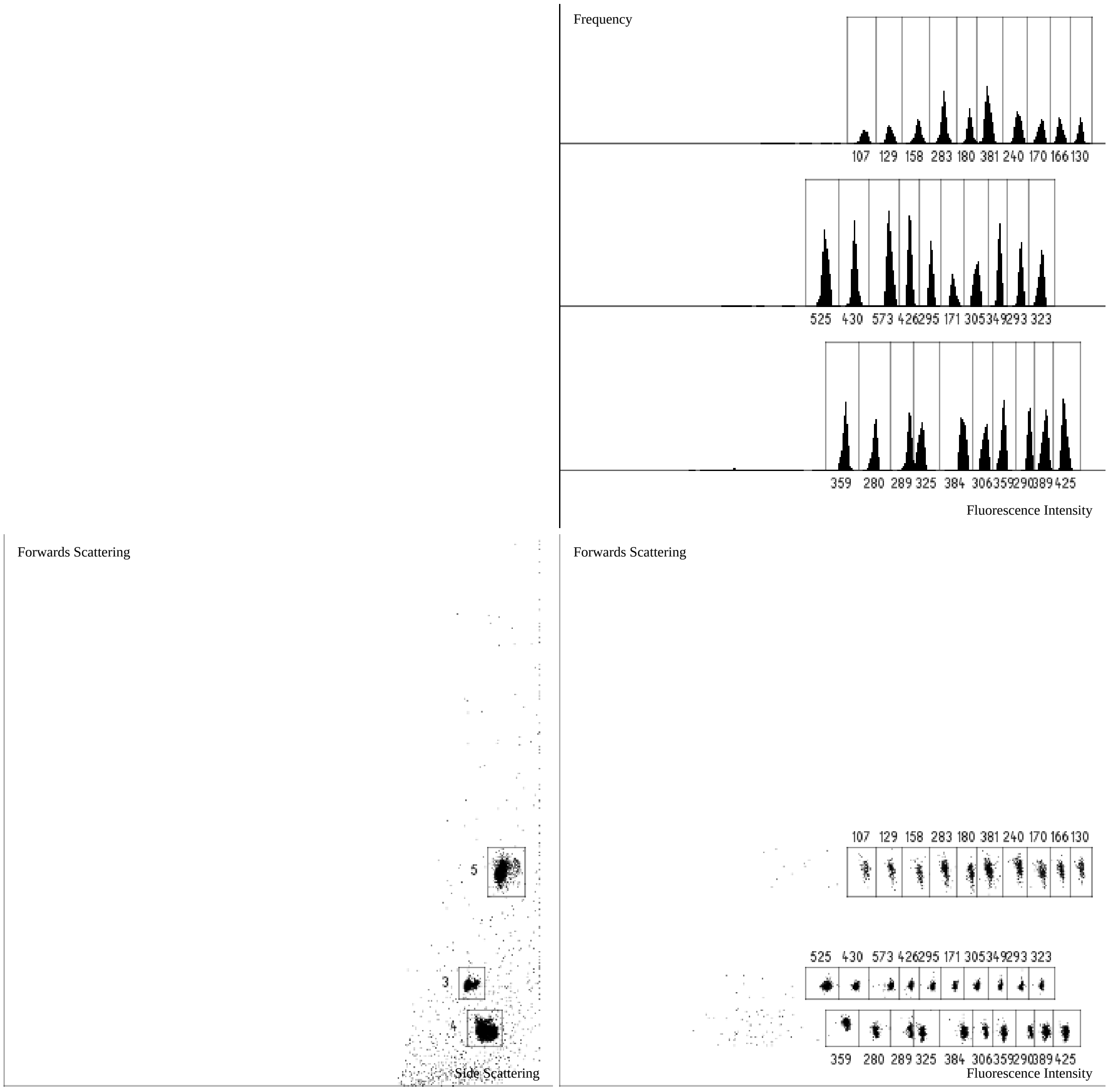

ANNEX 2: TAG REFERENCE SET - TAG DECONVOLUTION SESSION 18

Tag analysis flow cytometry instrument: BD FACSCalibur

Number of gates: 30

Number of beads whose tags were deconvoluted: 36

Number of beads with conclusive tagging data: 25

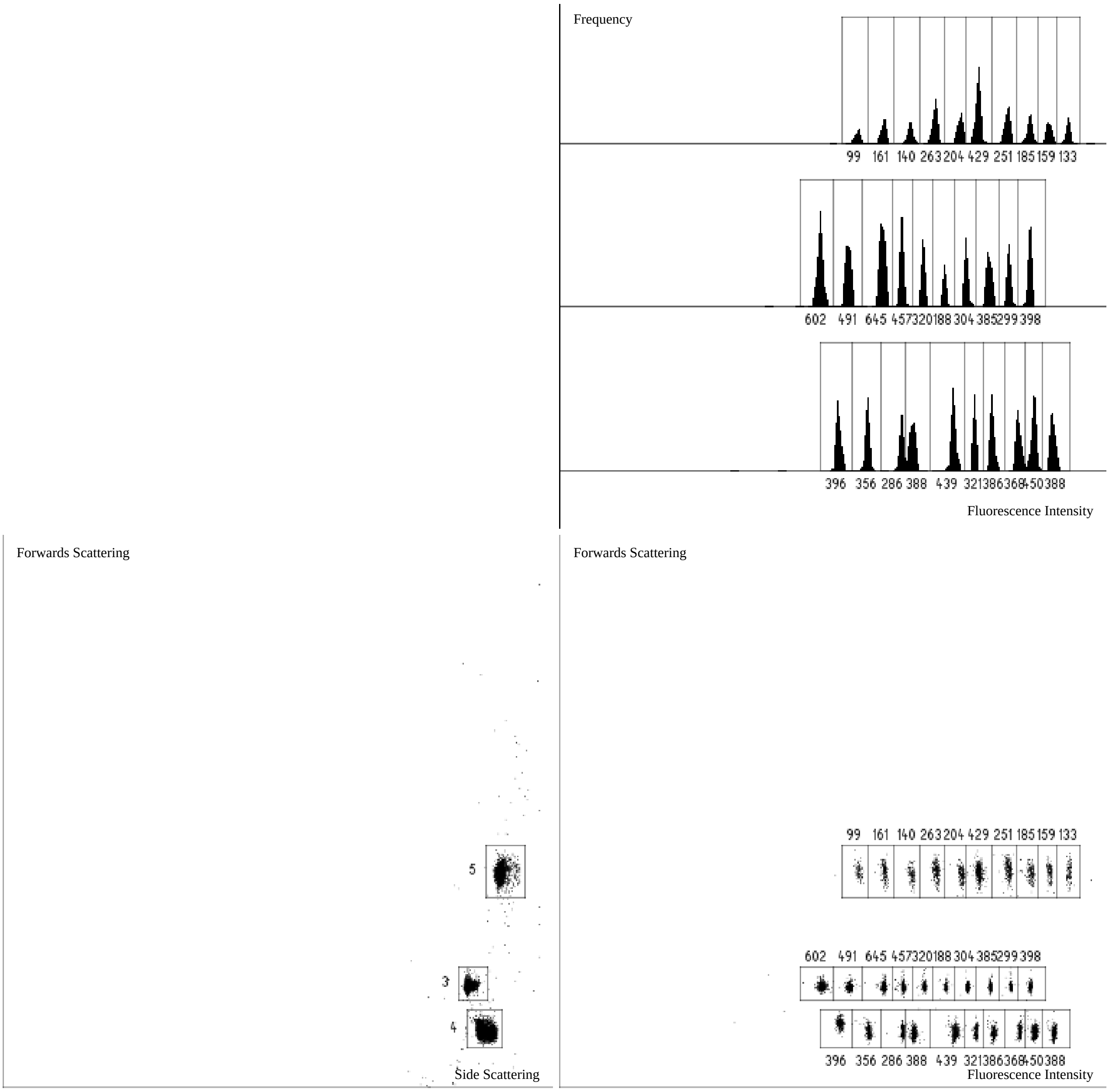

ANNEX 2: TAG REFERENCE SET - TAG DECONVOLUTION SESSION 19

Tag analysis flow cytometry instrument: BD FACSCalibur

Number of gates: 30

Number of beads whose tags were deconvoluted: 42

Number of beads with conclusive tagging data: 32

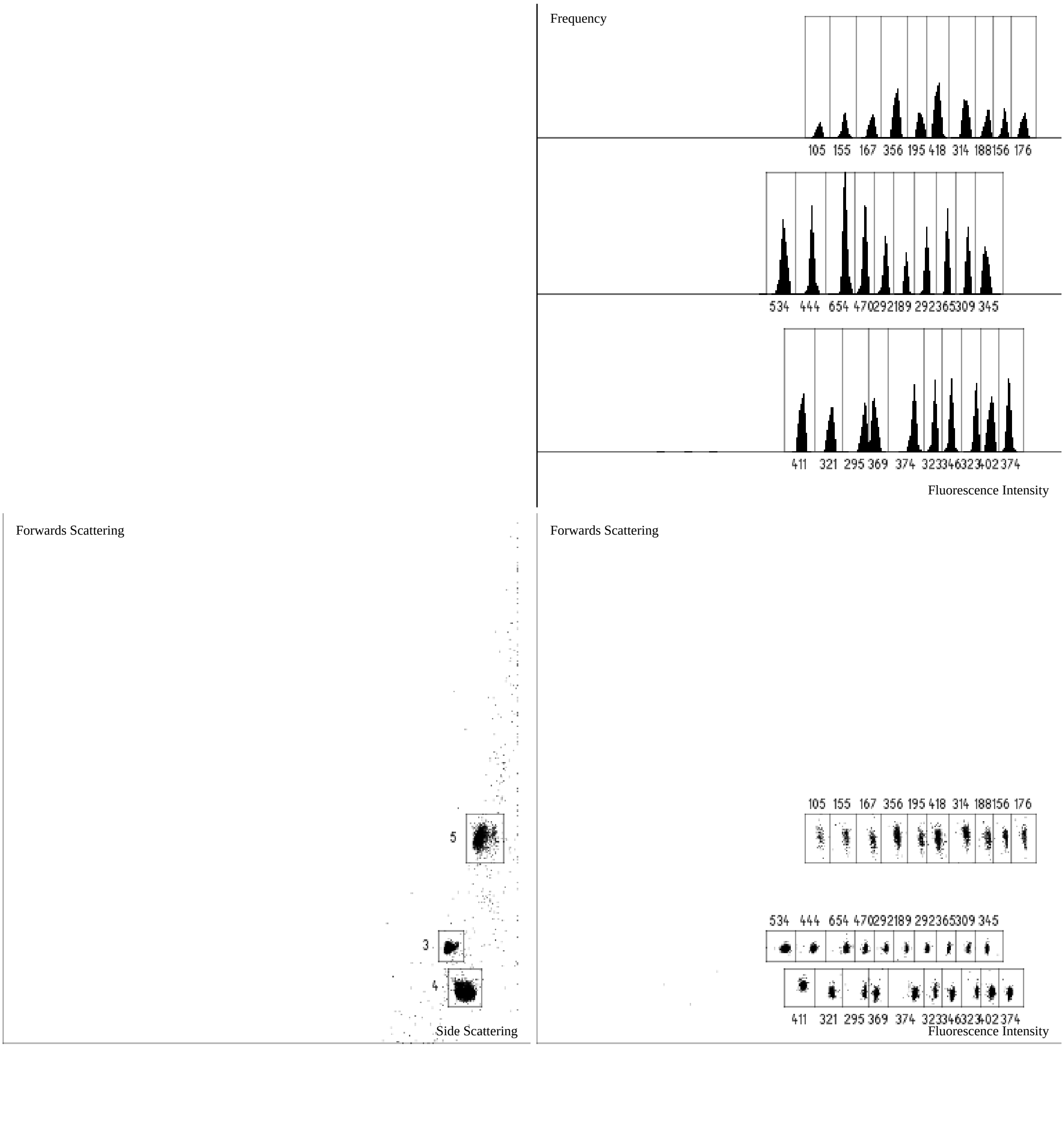

ANNEX 2: TAG REFERENCE SET - TAG DECONVOLUTION SESSION 20

Tag analysis flow cytometry instrument: BD FACSCalibur

Number of gates: 30

Number of beads whose tags were deconvoluted: 51

Number of beads with conclusive tagging data: 40

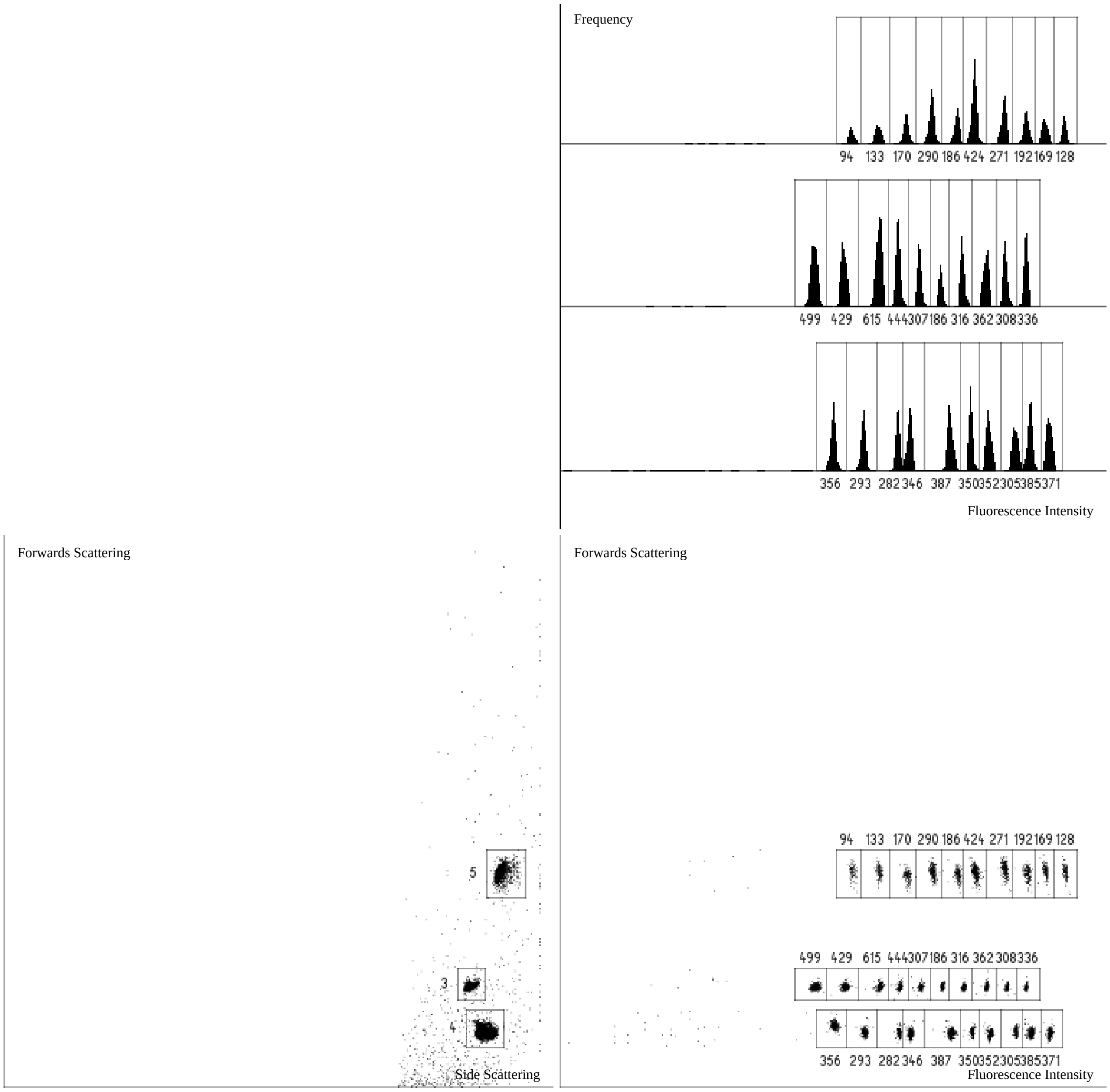

ANNEX 2: TAG REFERENCE SET - TAG DECONVOLUTION SESSION 21

Tag analysis flow cytometry instrument: BD FACSCalibur

Number of gates: 30

Number of beads whose tags were deconvoluted: 20

Number of beads with conclusive tagging data: 13

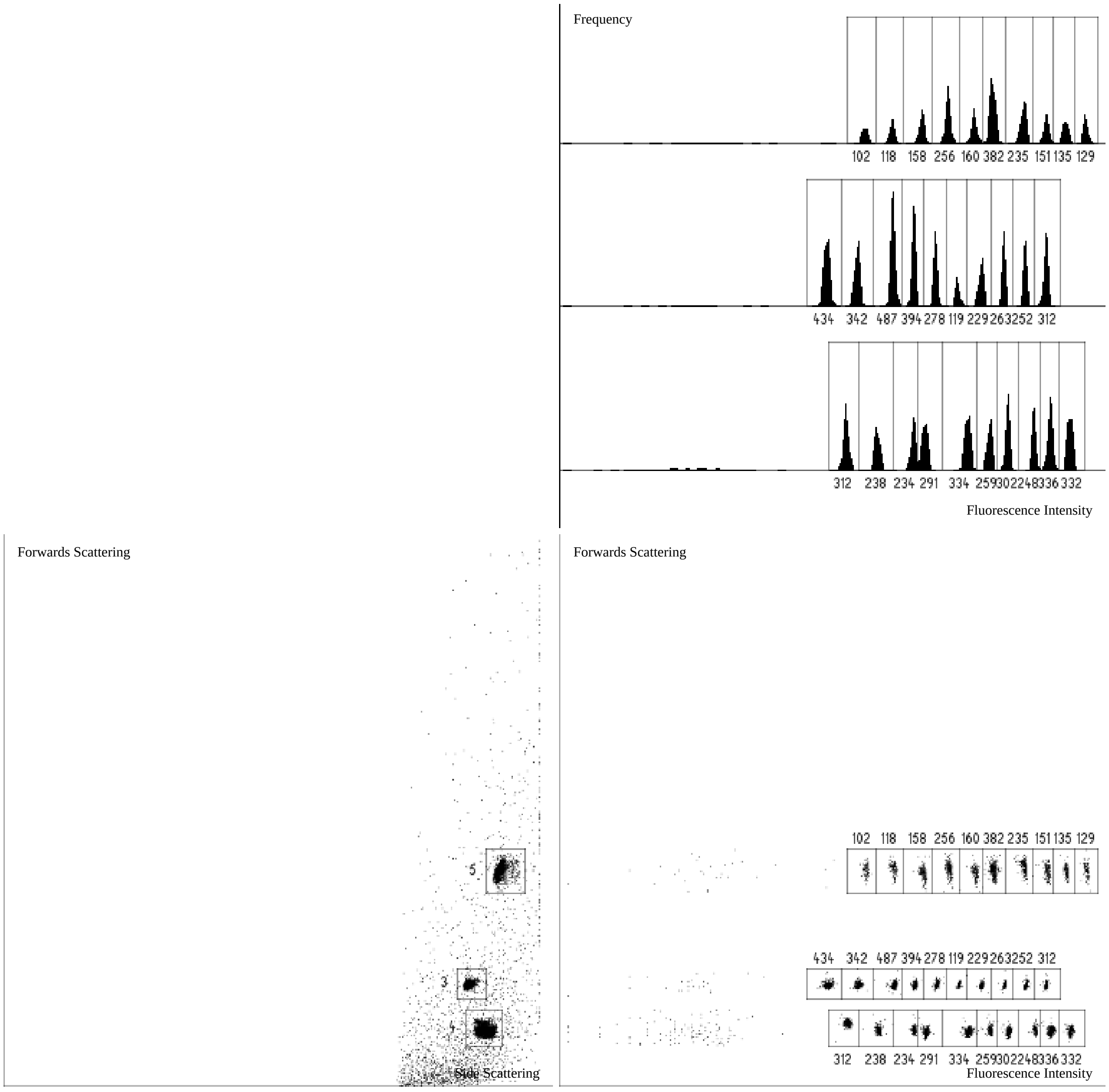

ANNEX 3: TAG DECONVOLUTION - BEAD 1

Passes flow sorting criteria: Yes

Passes tag deconvolution criteria: Yes

Included in protocol analysis: Yes

Protocol: 9, 9, 5, 1

Filename: Bin1_plateA0_H3.fcs

Split 1: Petrol shading

Split 2: Green shading

Split 3: Violet shading

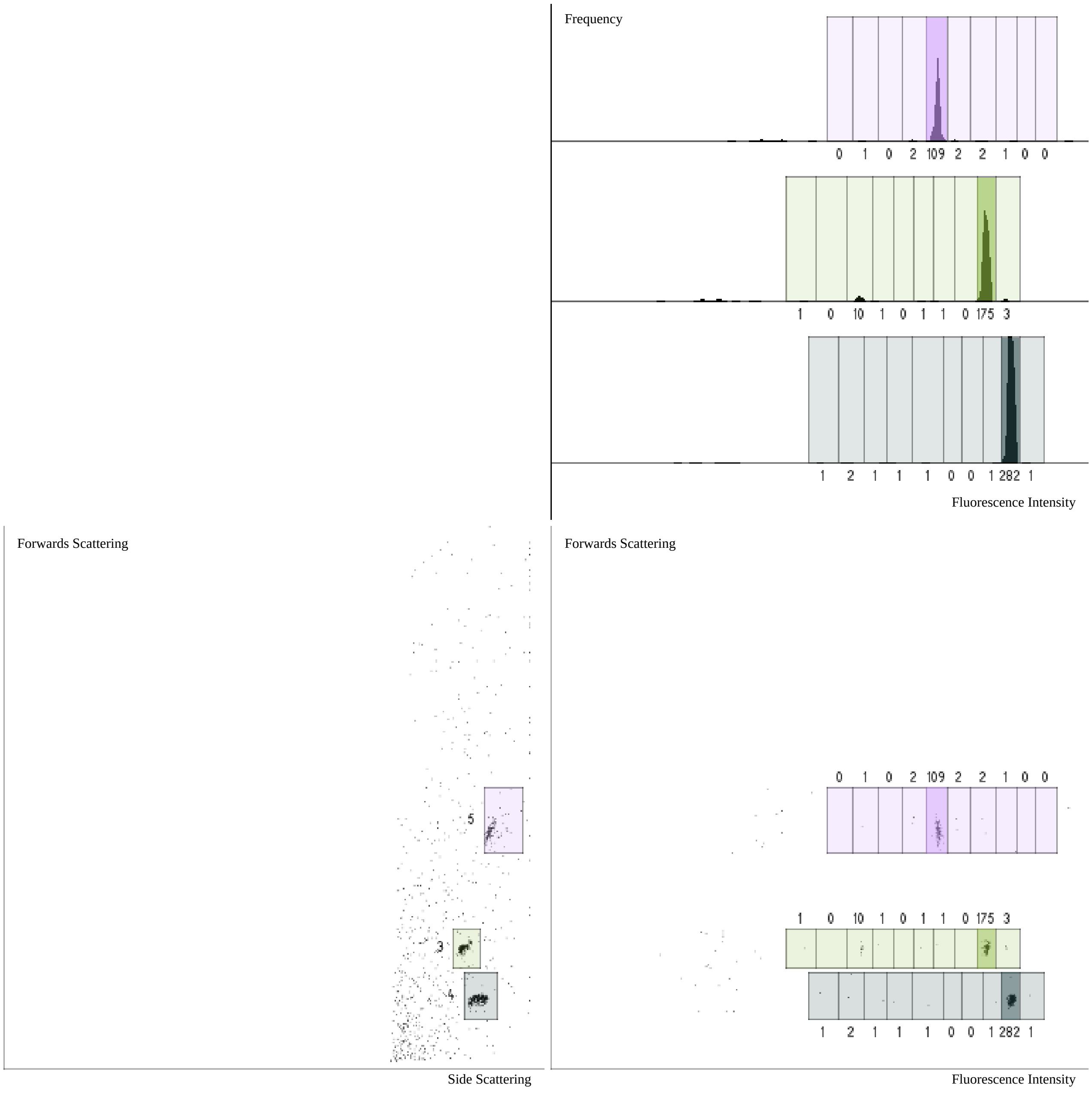

ANNEX 3: TAG DECONVOLUTION - BEAD 2

Passes flow sorting criteria: Yes

Passes tag deconvolution criteria: No

Included in protocol analysis: No

Protocol: N/A

Filename: Bin1_plateA0_A2.fcs

Split 1: Petrol shading

Split 2: Green shading

Split 3: Violet shading

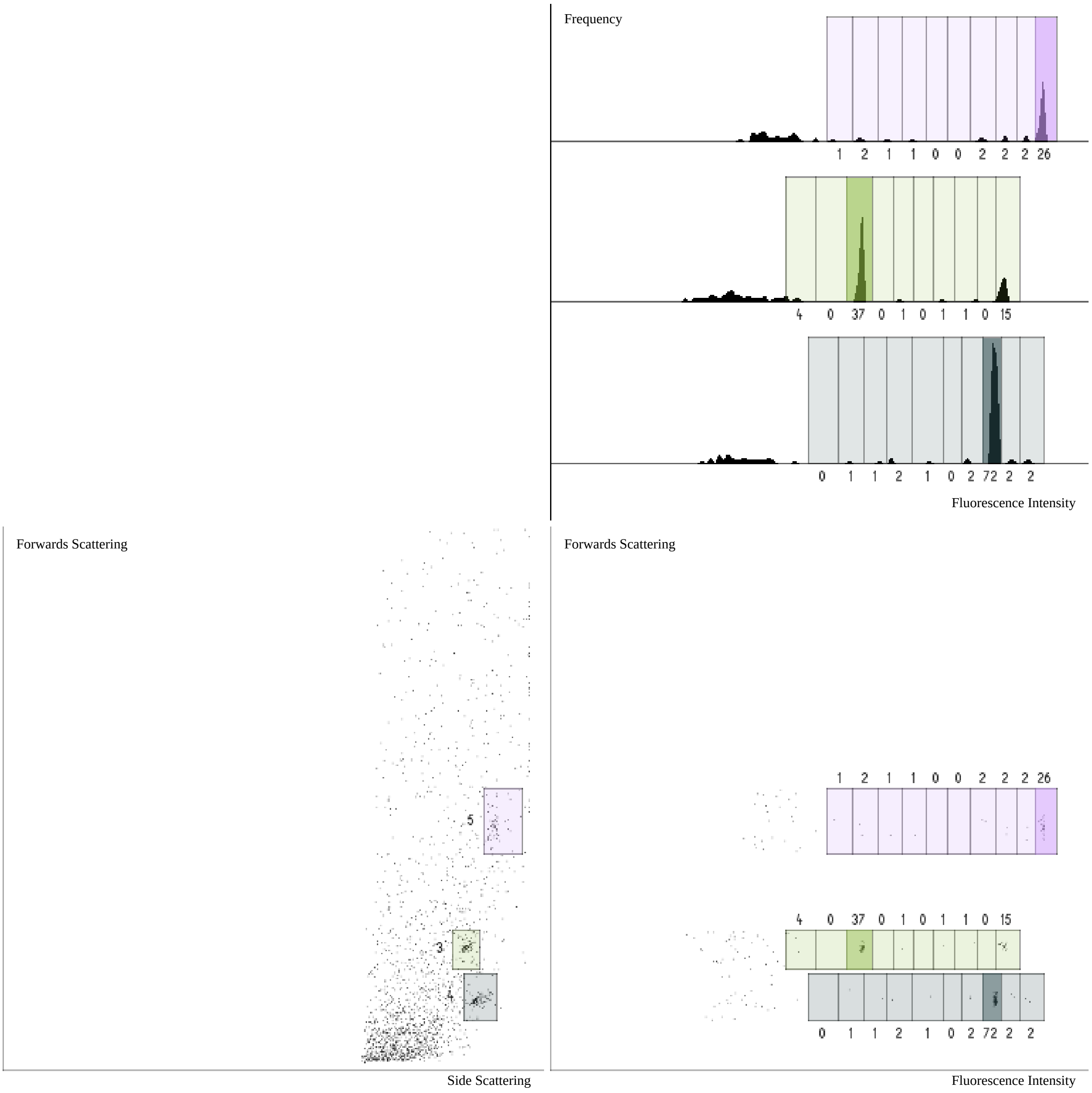

ANNEX 3: TAG DECONVOLUTION - BEAD 3

Passes flow sorting criteria: Yes

Passes tag deconvolution criteria: No

Included in protocol analysis: No

Protocol: N/A

Filename: Bin1_plateA0_A3.fcs

Split 1: Petrol shading

Split 2: Green shading

Split 3: Violet shading

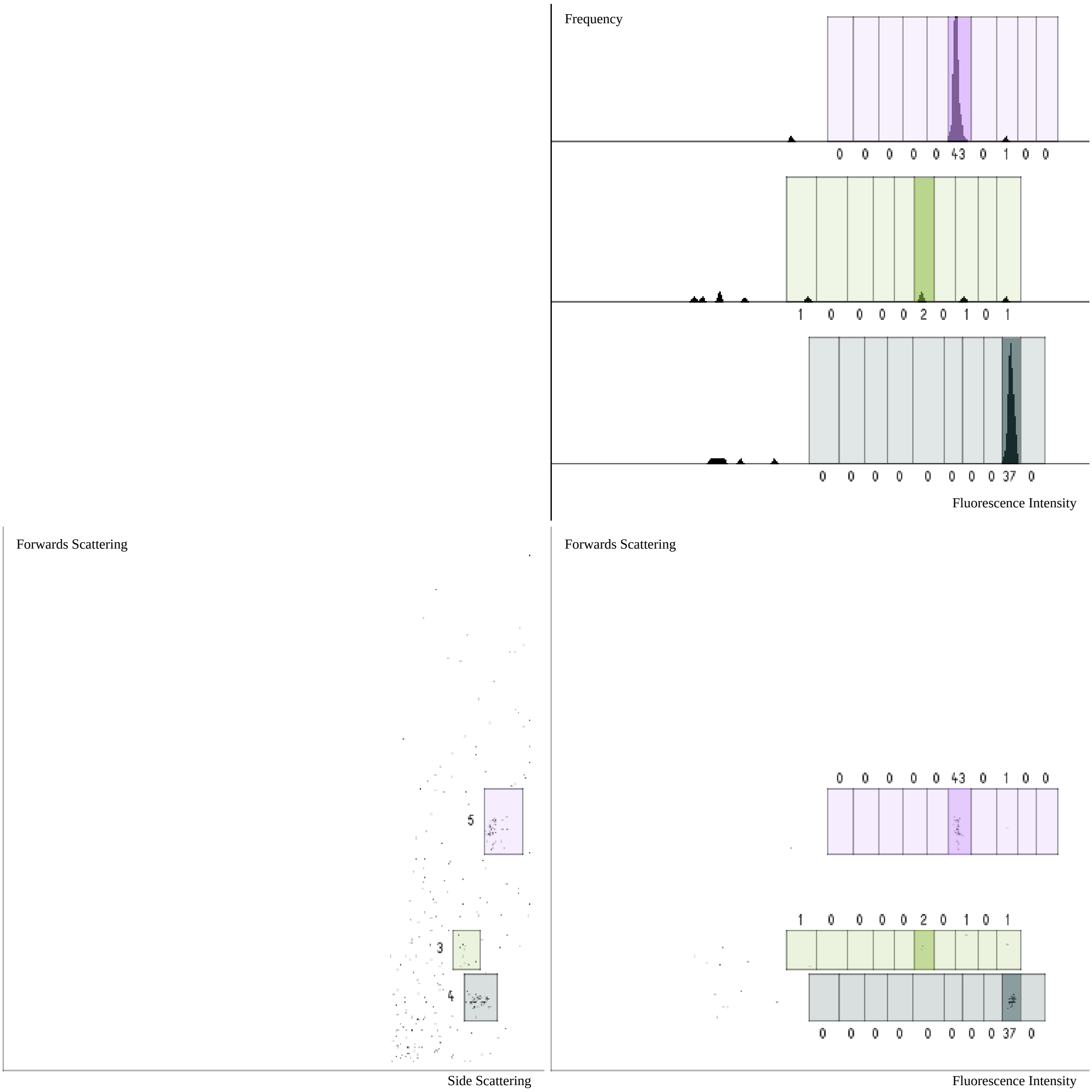

ANNEX 3: TAG DECONVOLUTION - BEAD 4

Passes flow sorting criteria: Yes

Passes tag deconvolution criteria: Yes

Included in protocol analysis: Yes

Protocol: 4, 9, 9, 1

Filename: Bin1_plateA0_B1.fcs

Split 1: Petrol shading

Split 2: Green shading

Split 3: Violet shading

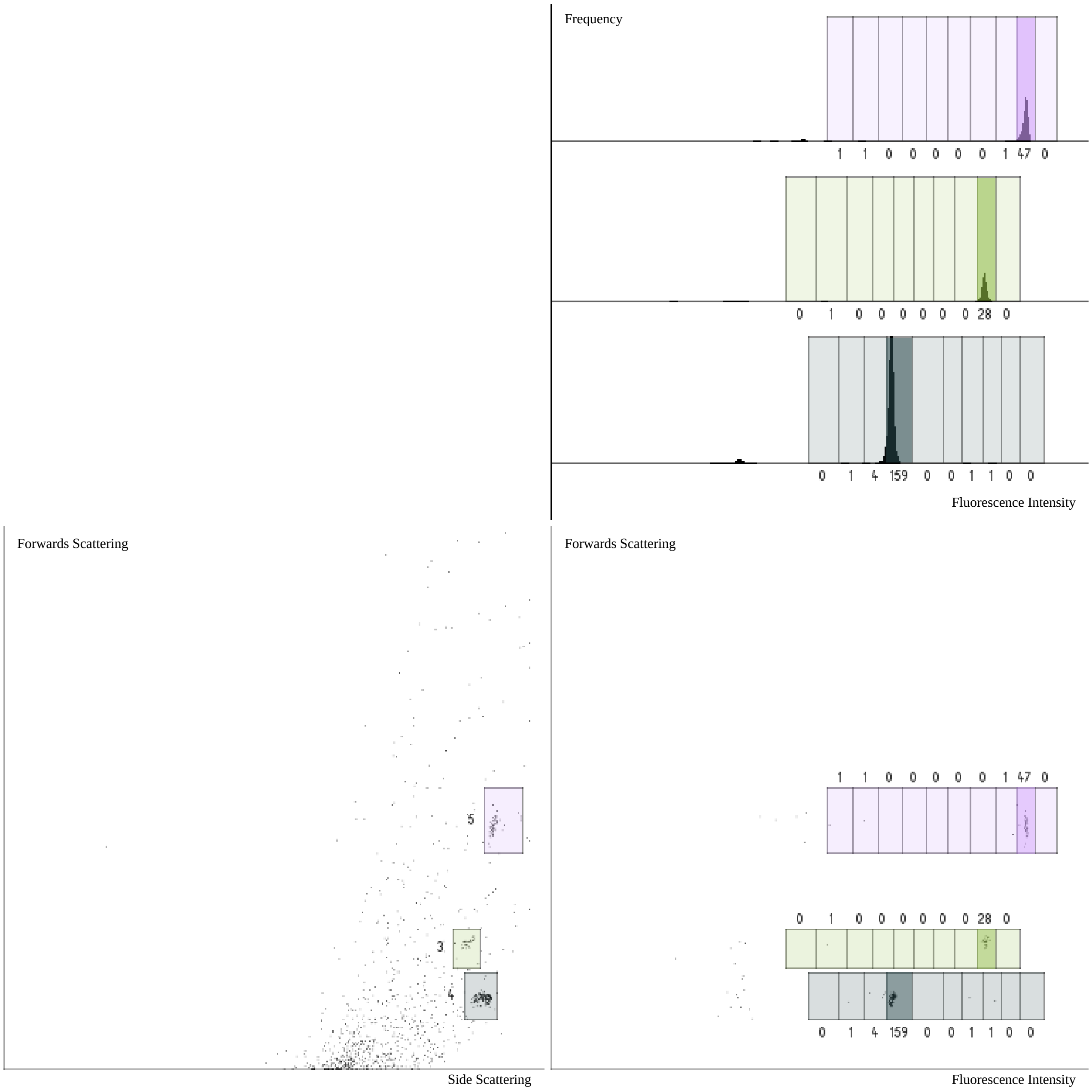

ANNEX 3: TAG DECONVOLUTION - BEAD 5

Passes flow sorting criteria: Yes

Passes tag deconvolution criteria: Yes

Included in protocol analysis: Yes

Protocol: 7, 6, 7, 1

Filename: Bin1_plateA0_B2.fcs

Split 1: Petrol shading

Split 2: Green shading

Split 3: Violet shading

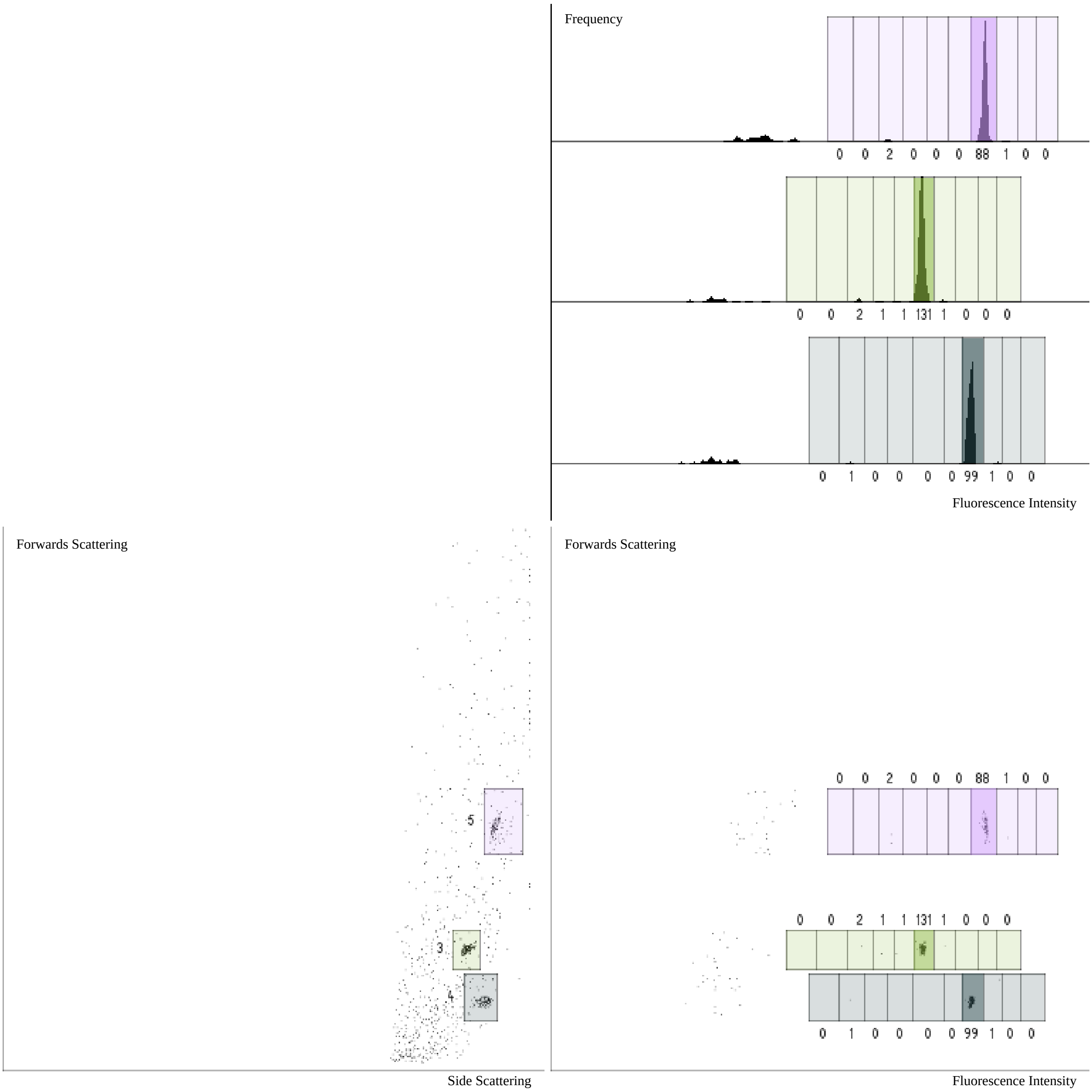

ANNEX 3: TAG DECONVOLUTION - BEAD 6

Passes flow sorting criteria: Yes

Passes tag deconvolution criteria: Yes

Included in protocol analysis: Yes

Protocol: 10, 4, 4, 1

Filename: Bin1_plateA0_B3.fcs

Split 1: Petrol shading

Split 2: Green shading

Split 3: Violet shading

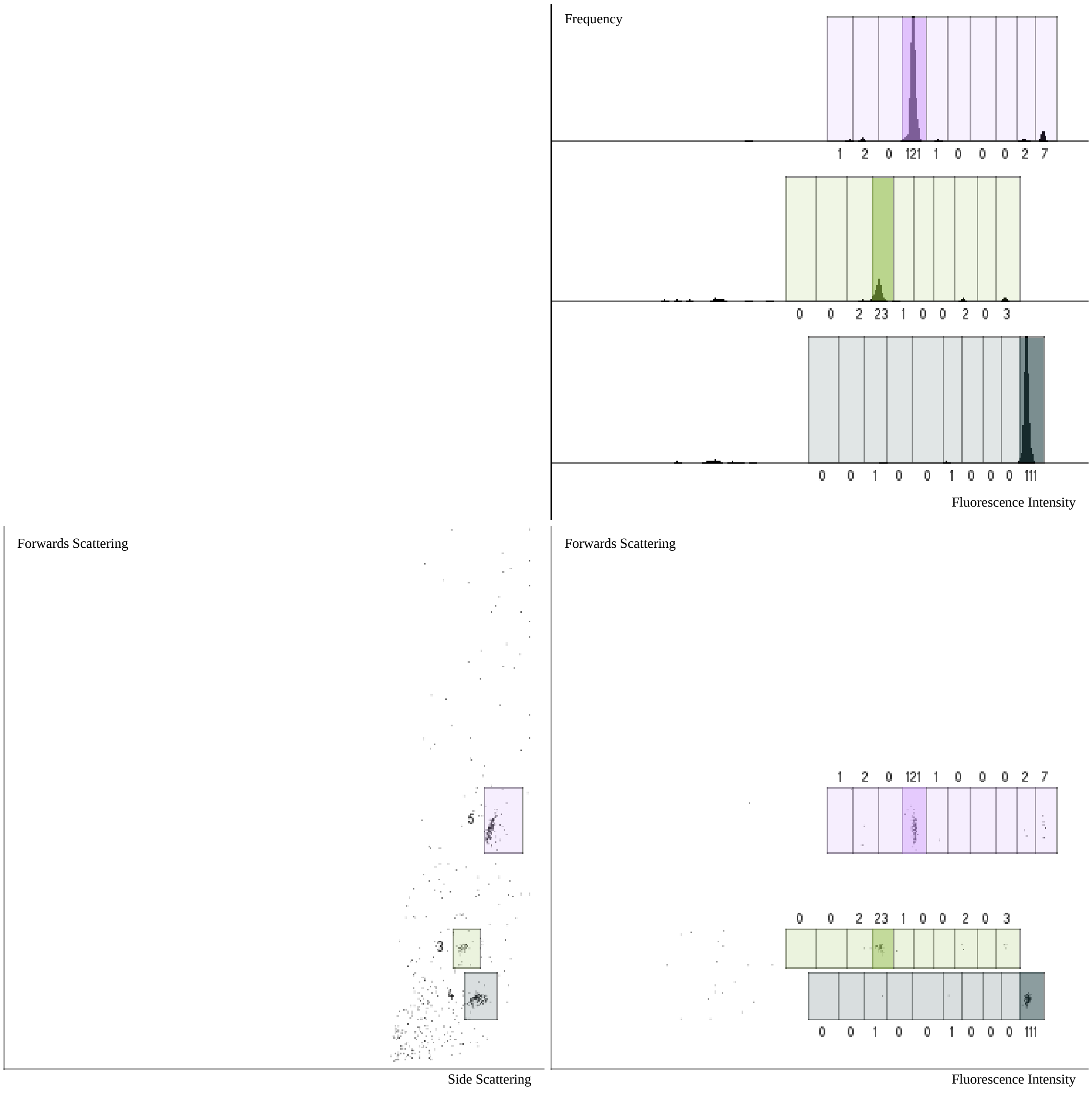

ANNEX 3: TAG DECONVOLUTION - BEAD 7

Passes flow sorting criteria: Yes

Passes tag deconvolution criteria: Yes

Included in protocol analysis: Yes

Protocol: 7, 10, 2, 1

Filename: Bin1_plateA0_C1.fcs

Split 1: Petrol shading

Split 2: Green shading

Split 3: Violet shading

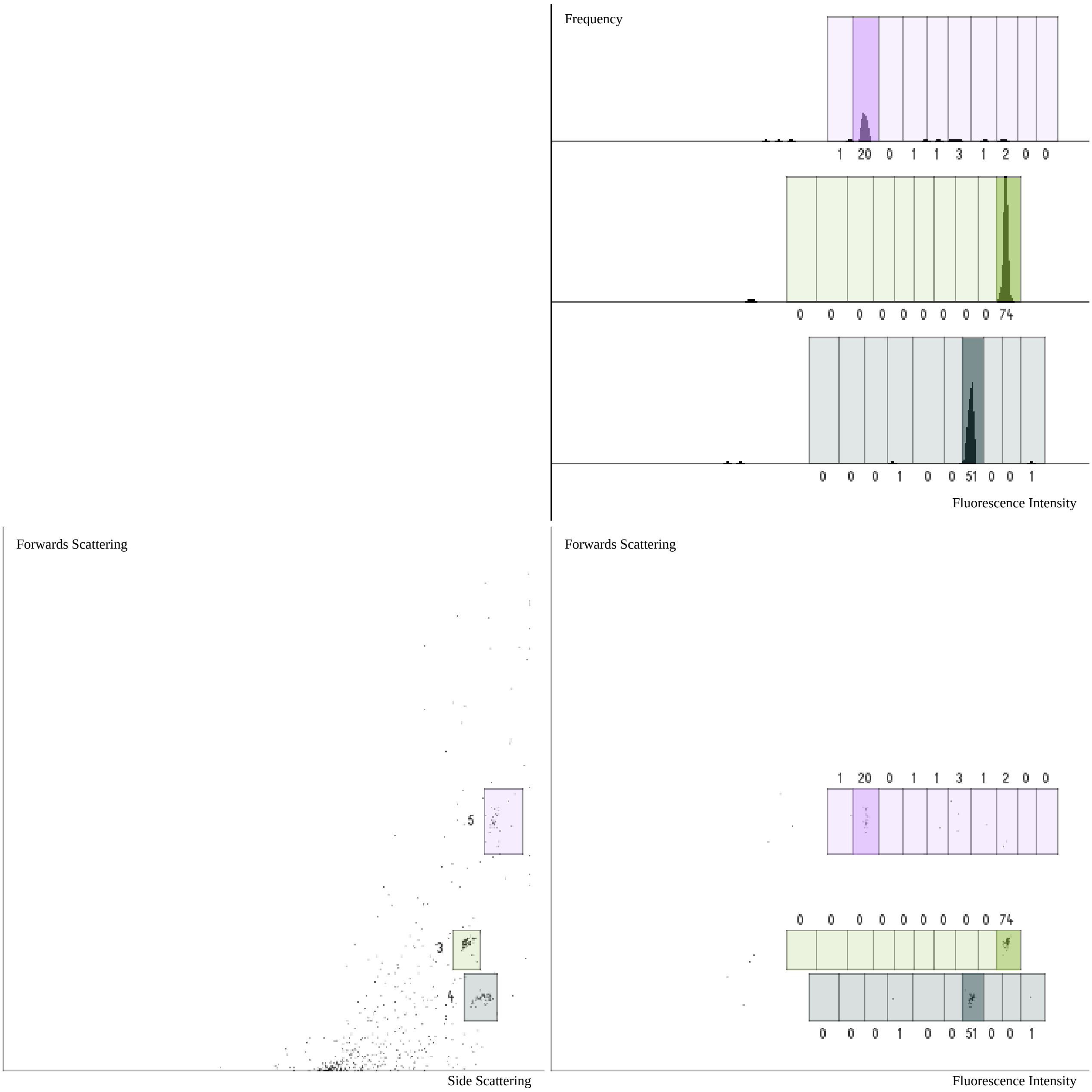

ANNEX 3: TAG DECONVOLUTION - BEAD 8

Passes flow sorting criteria: Yes

Passes tag deconvolution criteria: Yes

Included in protocol analysis: Yes

Protocol: 6, 8, 1, 1

Filename: Bin1_plateA0_C2.fcs

Split 1: Petrol shading

Split 2: Green shading

Split 3: Violet shading

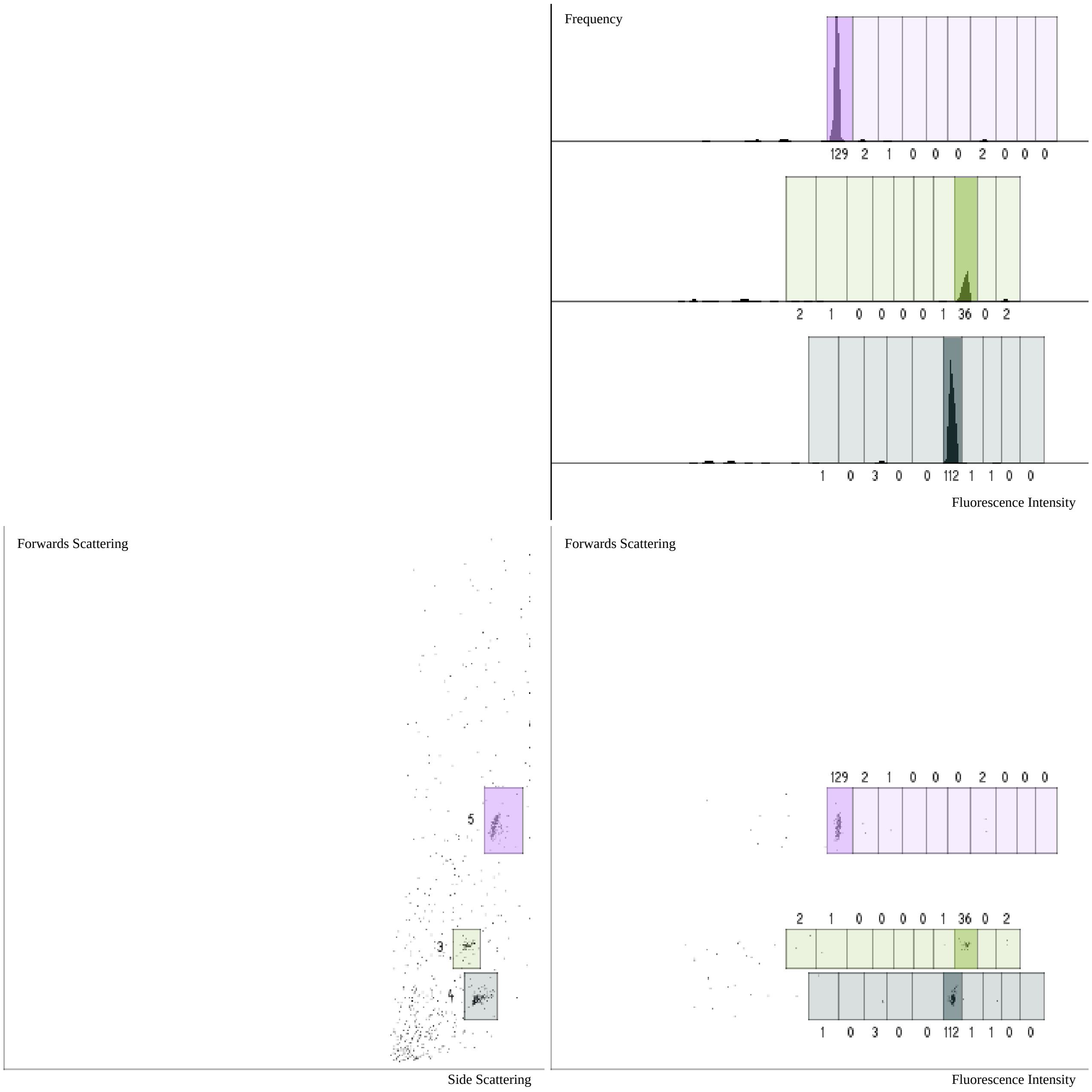

ANNEX 3: TAG DECONVOLUTION - BEAD 9

Passes flow sorting criteria: Yes

Passes tag deconvolution criteria: Yes

Included in protocol analysis: Yes

Protocol: 3, 6, 3, 1

Filename: Bin1_plateA0_C3.fcs

Split 1: Petrol shading

Split 2: Green shading

Split 3: Violet shading

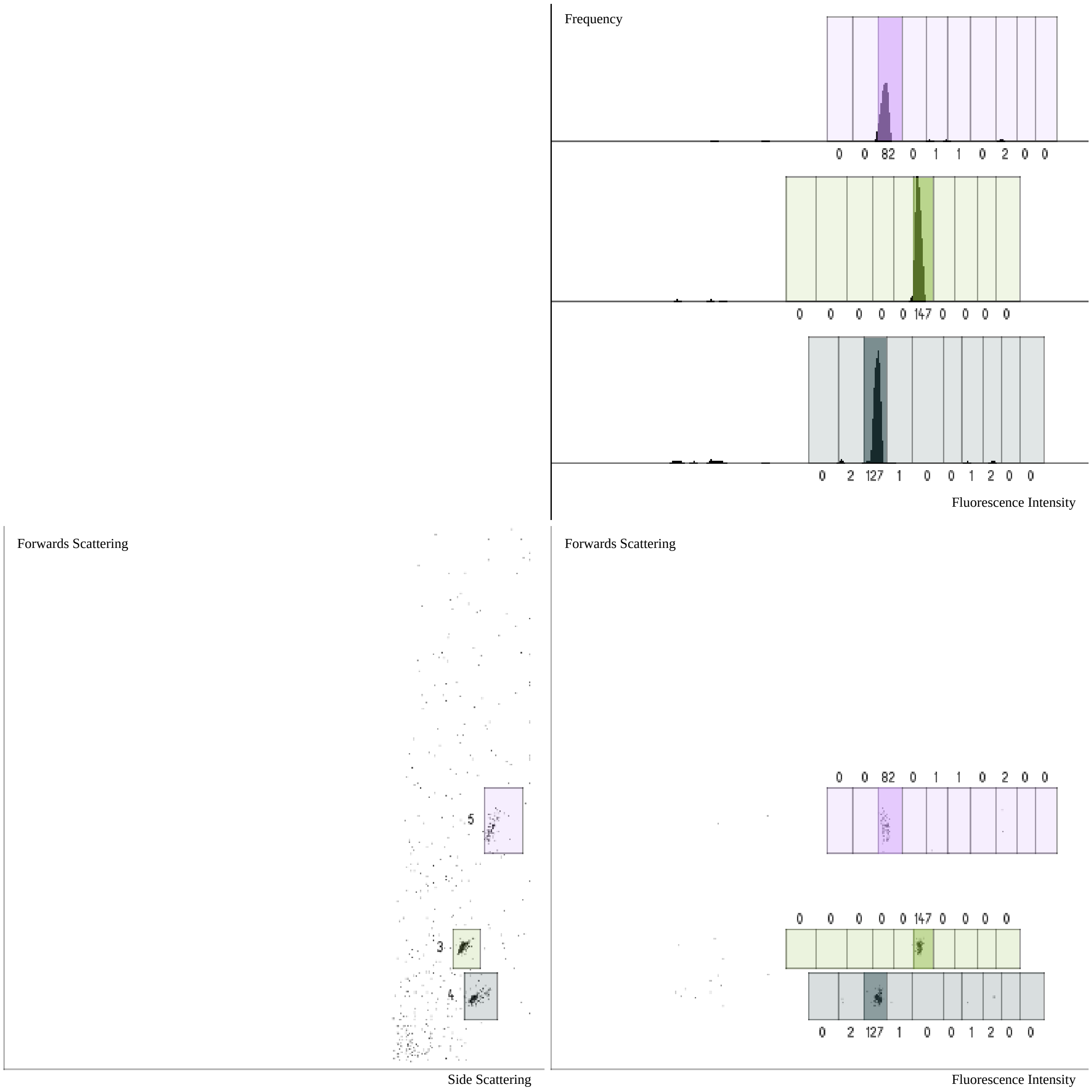

ANNEX 3: TAG DECONVOLUTION - BEAD 10

Passes flow sorting criteria: Yes

Passes tag deconvolution criteria: Yes

Included in protocol analysis: Yes

Protocol: 8, 2, 2, 1

Filename: Bin1_plateA0_D1.fcs

Split 1: Petrol shading

Split 2: Green shading

Split 3: Violet shading

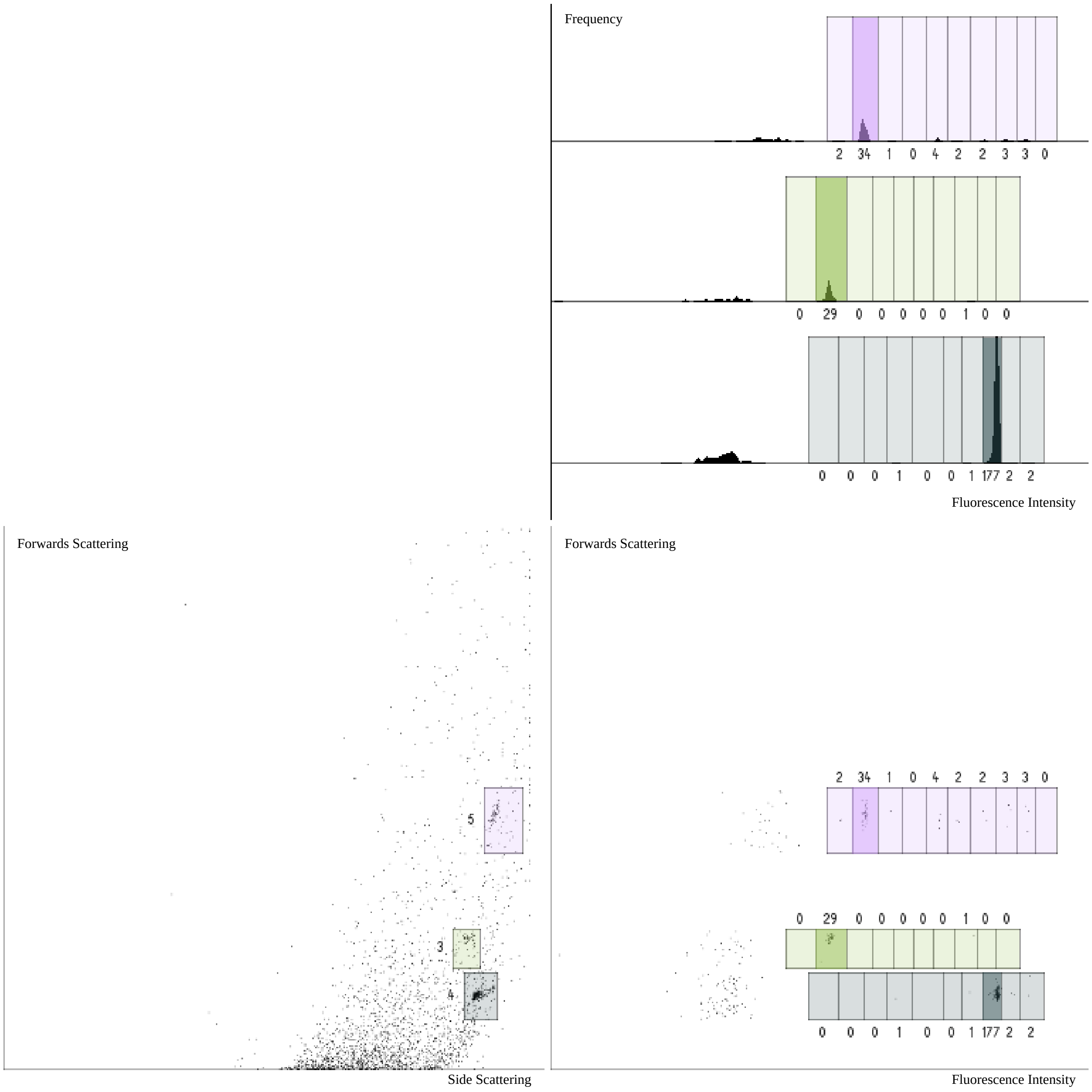

ANNEX 3: TAG DECONVOLUTION - BEAD 11

Passes flow sorting criteria: Yes

Passes tag deconvolution criteria: No

Included in protocol analysis: No

Protocol: N/A

Filename: Bin1_plateA0_D2.fcs

Split 1: Petrol shading

Split 2: Green shading

Split 3: Violet shading

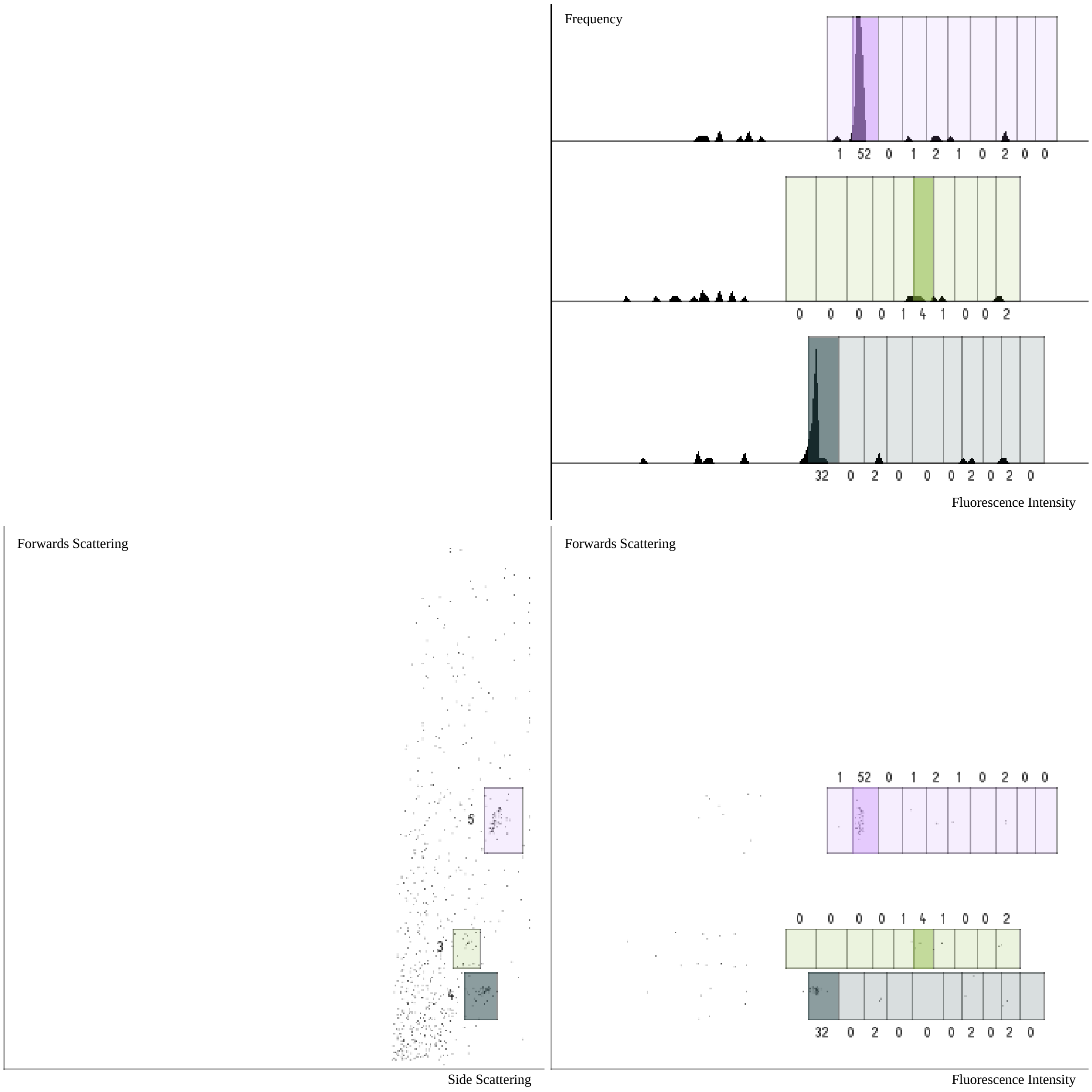

ANNEX 3: TAG DECONVOLUTION - BEAD 12

Passes flow sorting criteria: Yes

Passes tag deconvolution criteria: Yes

Included in protocol analysis: Yes

Protocol: 2, 5, 4, 1

Filename: Bin1_plateA0_D3.fcs

Split 1: Petrol shading

Split 2: Green shading

Split 3: Violet shading

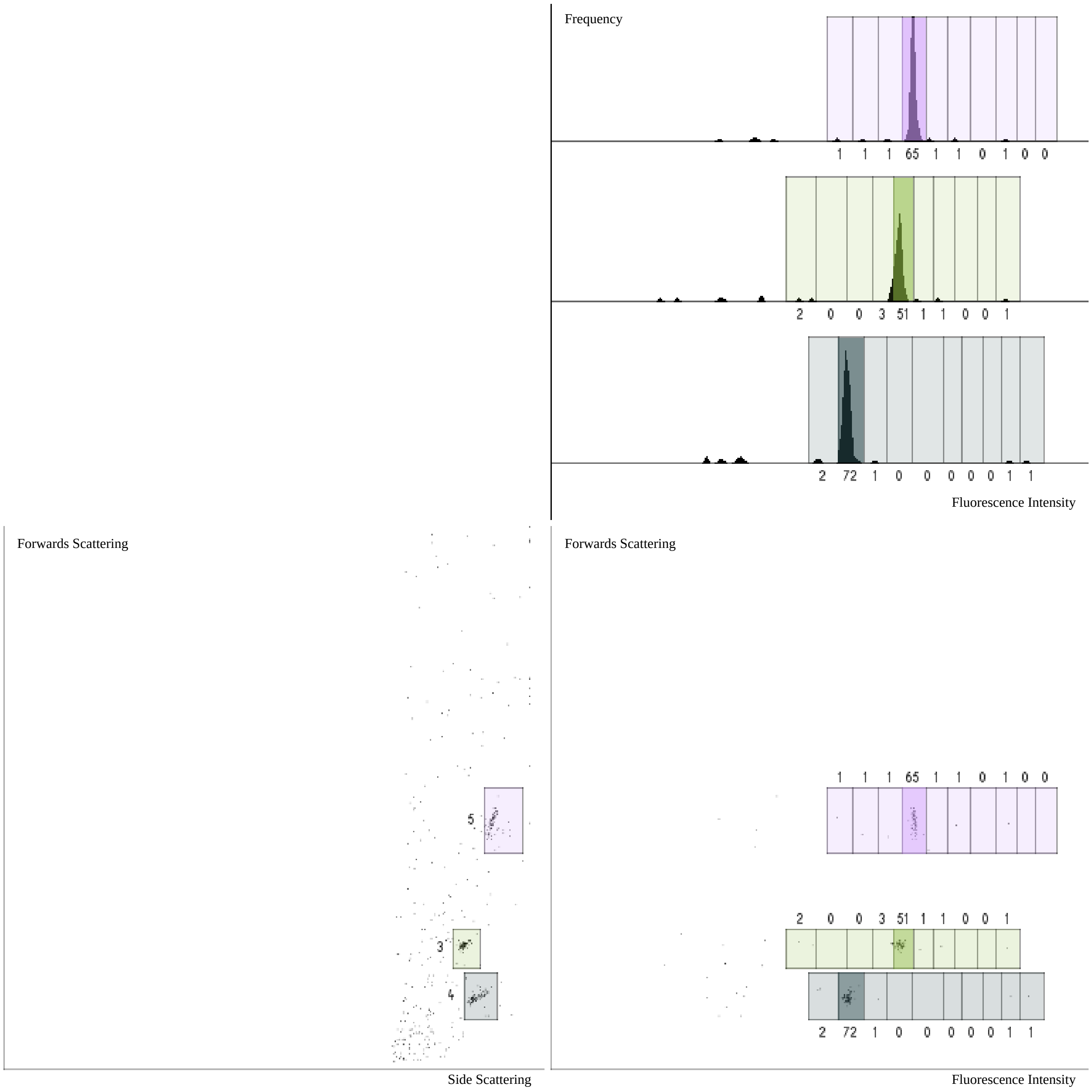

ANNEX 3: TAG DECONVOLUTION - BEAD 13

Passes flow sorting criteria: Yes

Passes tag deconvolution criteria: Yes

Included in protocol analysis: Yes

Protocol: 10, 8, 2, 1

Filename: Bin1_plateA0_E1.fcs

Split 1: Petrol shading

Split 2: Green shading

Split 3: Violet shading

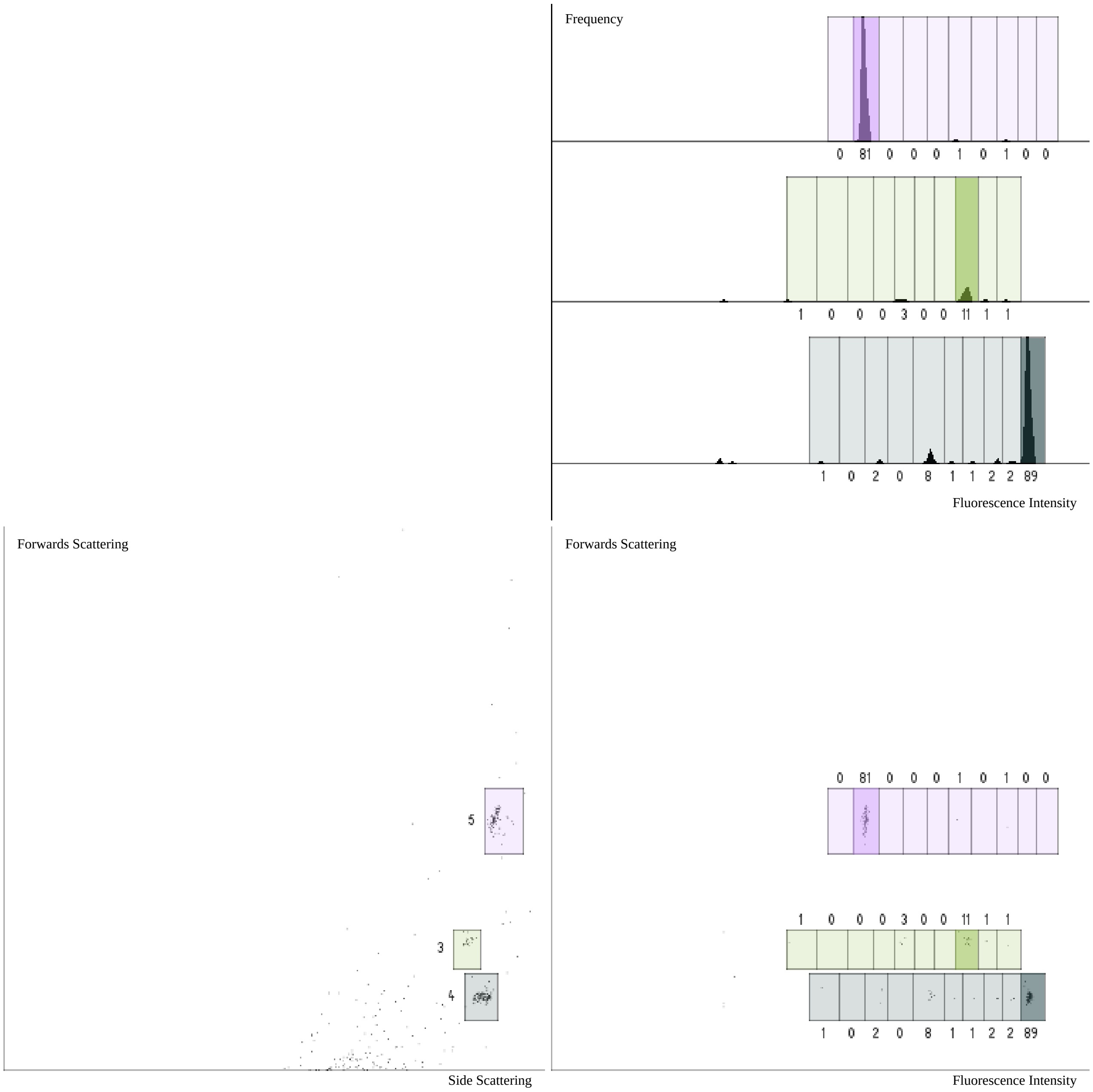

ANNEX 3: TAG DECONVOLUTION - BEAD 14

Passes flow sorting criteria: Yes

Passes tag deconvolution criteria: Yes

Included in protocol analysis: Yes

Protocol: 3, 7, 3, 1

Filename: Bin1_plateA0_E2.fcs

Split 1: Petrol shading

Split 2: Green shading

Split 3: Violet shading

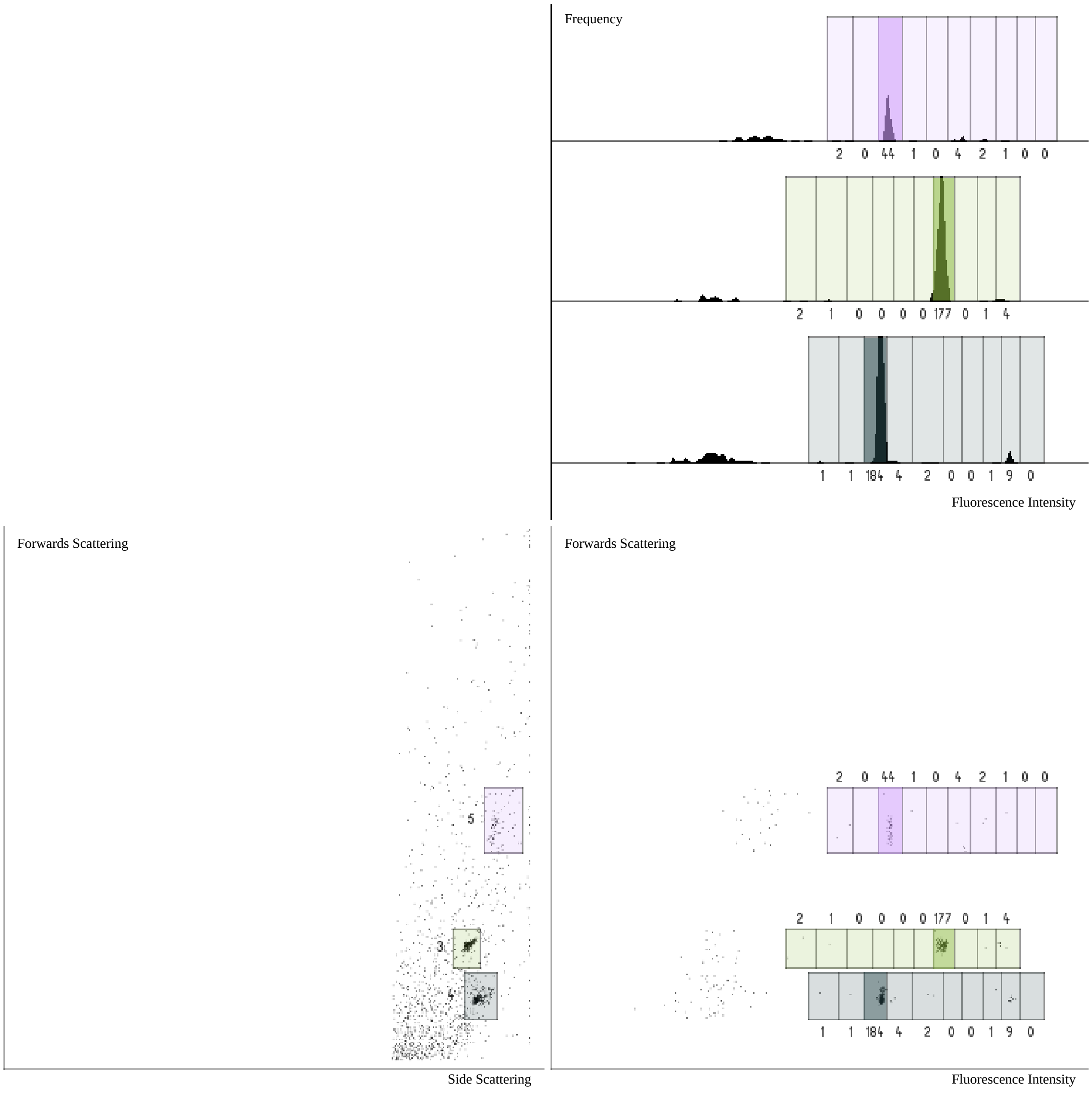

ANNEX 3: TAG DECONVOLUTION - BEAD 15

Passes flow sorting criteria: Yes

Passes tag deconvolution criteria: No

Included in protocol analysis: No

Protocol: N/A

Filename: Bin1_plateA0_E3.fcs

Split 1: Petrol shading

Split 2: Green shading

Split 3: Violet shading

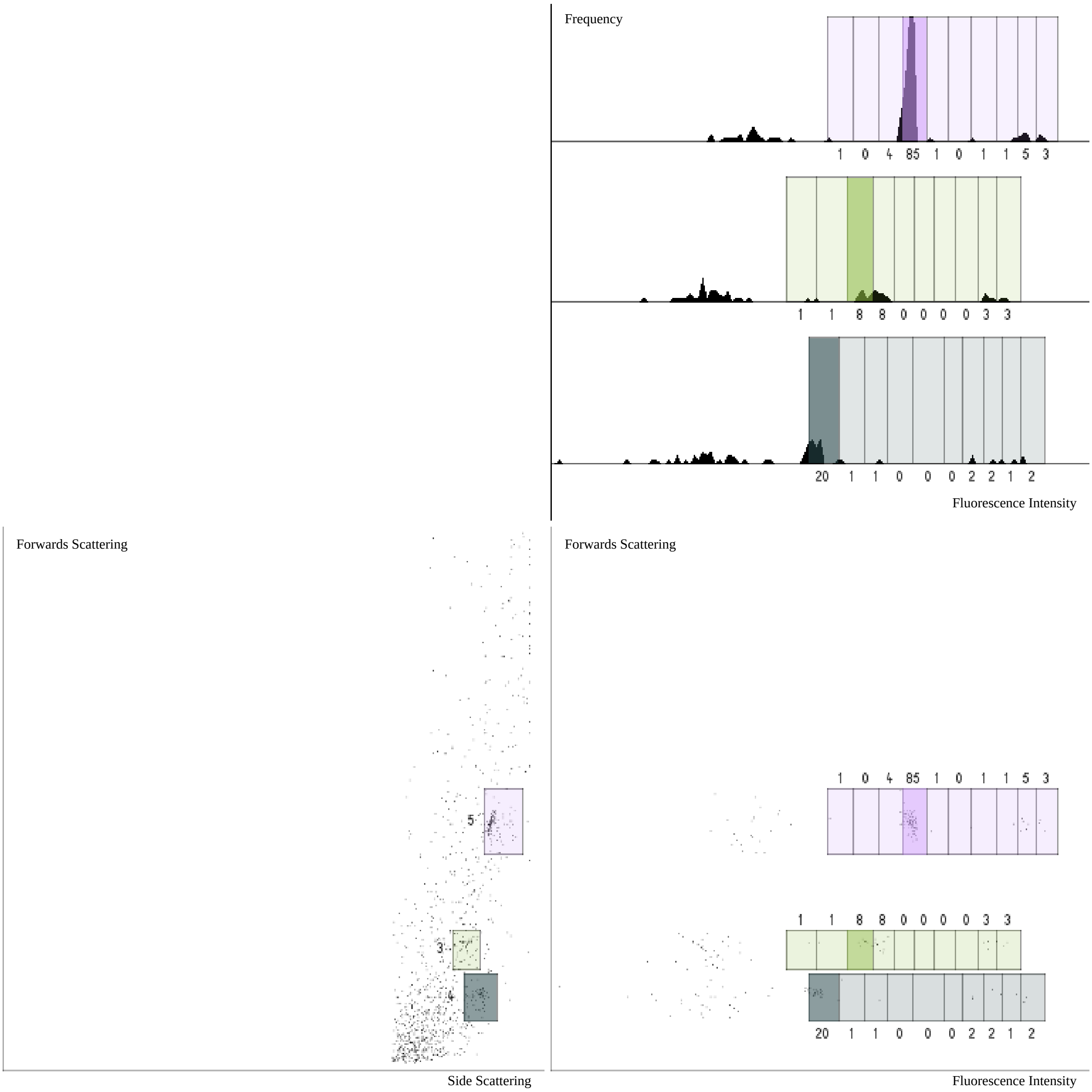

ANNEX 3: TAG DECONVOLUTION - BEAD 16

Passes flow sorting criteria: Yes

Passes tag deconvolution criteria: No

Included in protocol analysis: No

Protocol: N/A

Filename: Bin1_plateA0_F1.fcs

Split 1: Petrol shading

Split 2: Green shading

Split 3: Violet shading

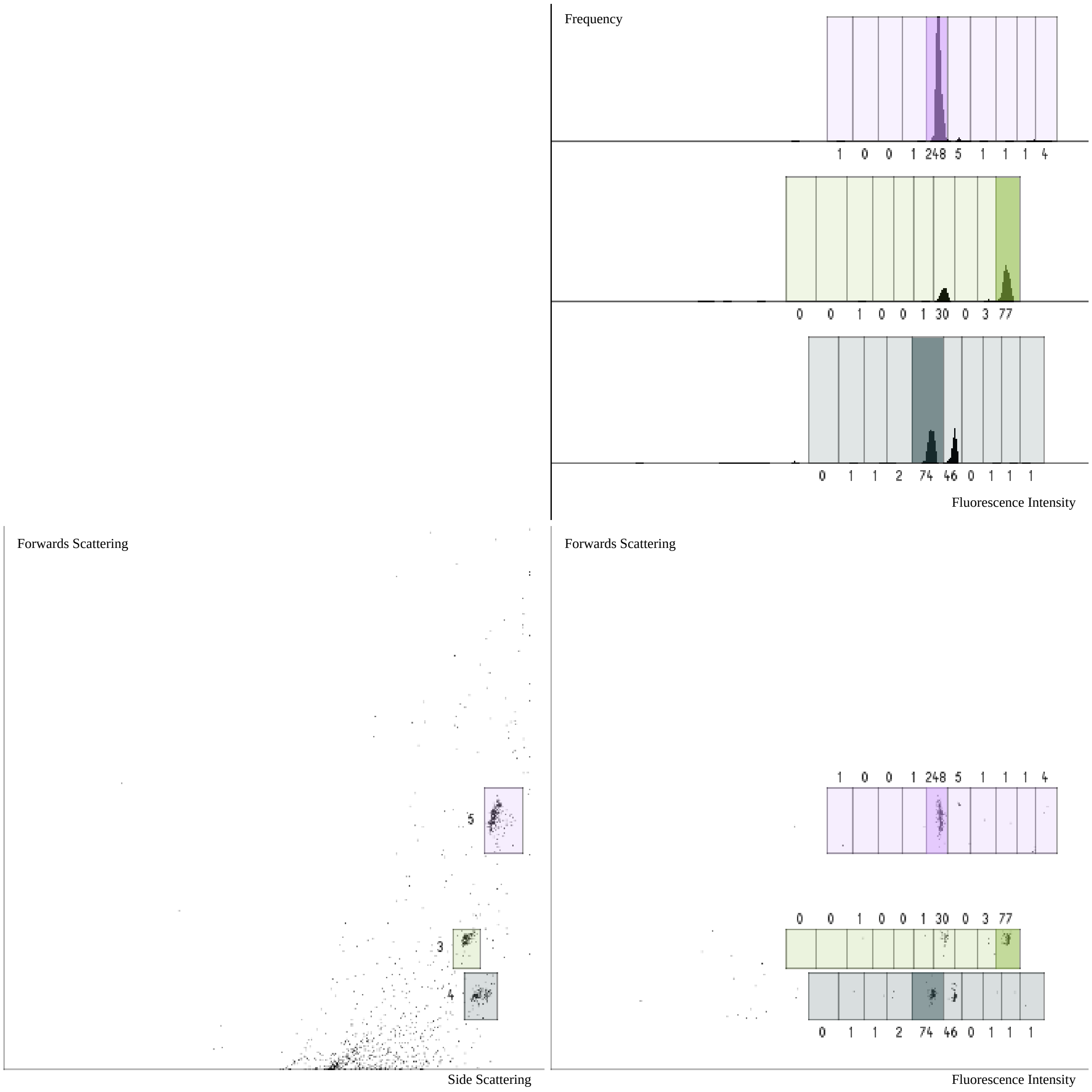

ANNEX 3: TAG DECONVOLUTION - BEAD 17

Passes flow sorting criteria: Yes

Passes tag deconvolution criteria: Yes

Included in protocol analysis: Yes

Protocol: 10, 10, 5, 1

Filename: Bin1_plateA0_F2.fcs

Split 1: Petrol shading

Split 2: Green shading

Split 3: Violet shading

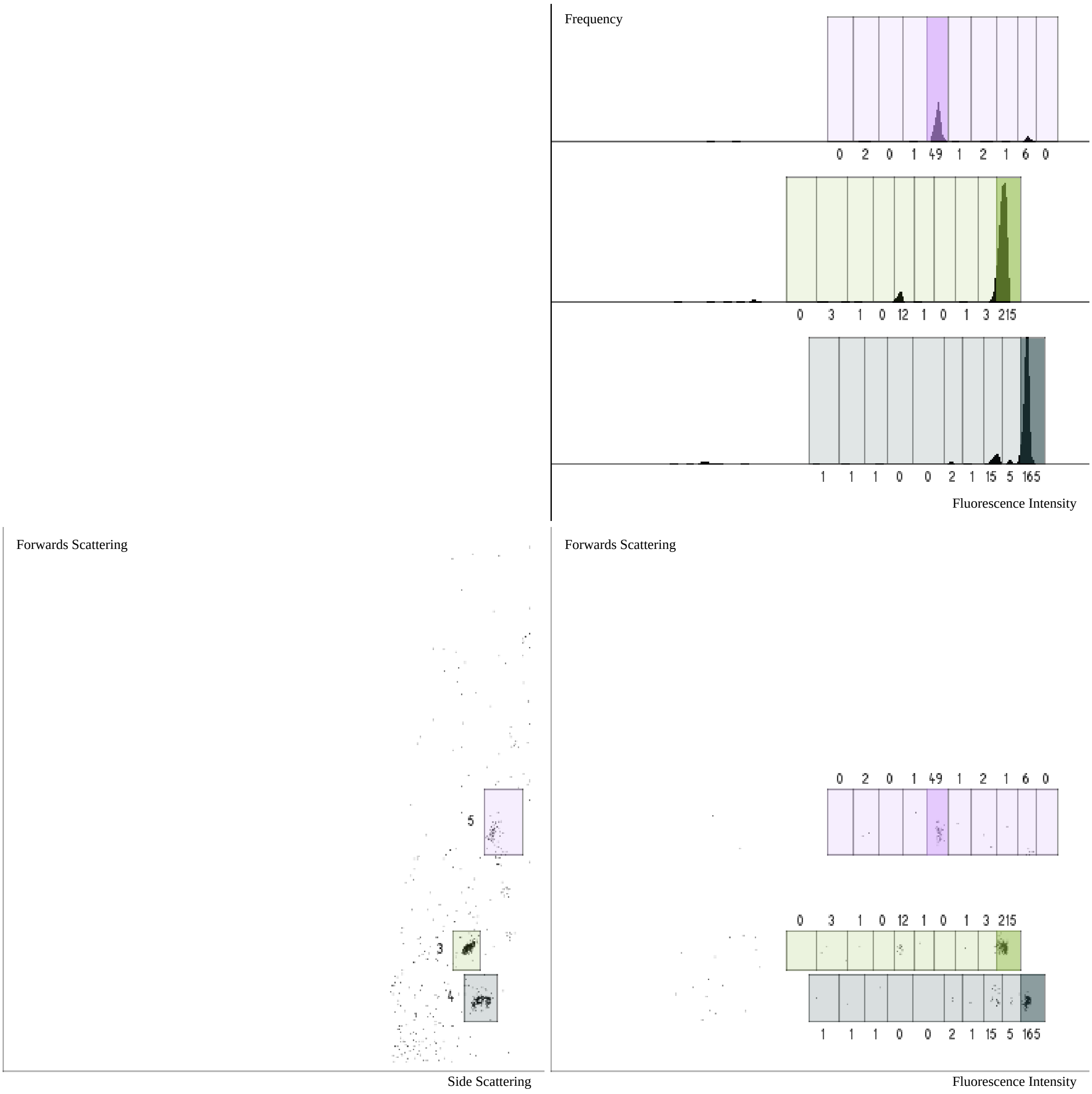

ANNEX 3: TAG DECONVOLUTION - BEAD 18

Passes flow sorting criteria: Yes

Passes tag deconvolution criteria: Yes

Included in protocol analysis: Yes

Protocol: 4, 6, 9, 1

Filename: Bin1_plateA0_F3.fcs

Split 1: Petrol shading

Split 2: Green shading

Split 3: Violet shading

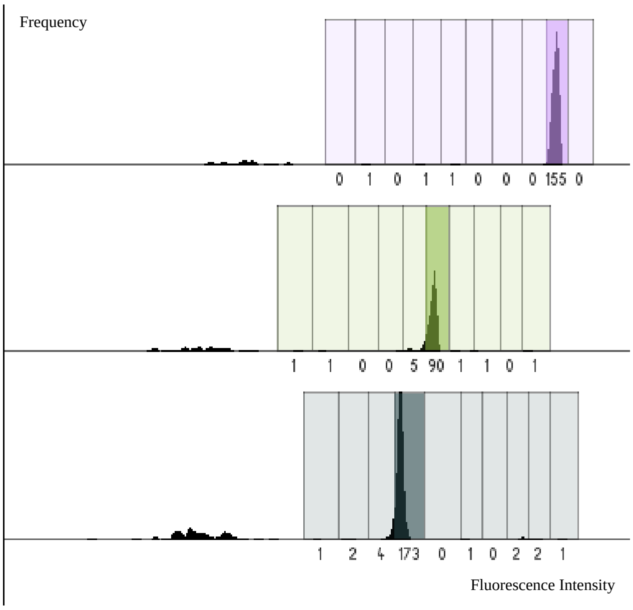

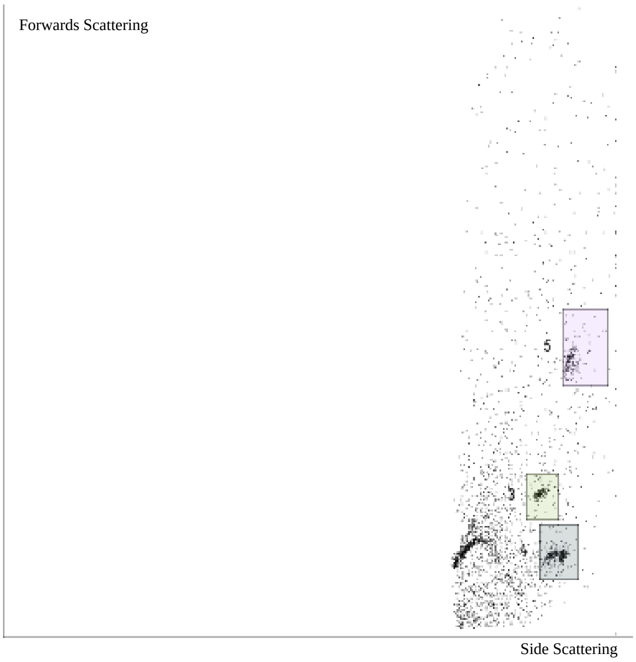

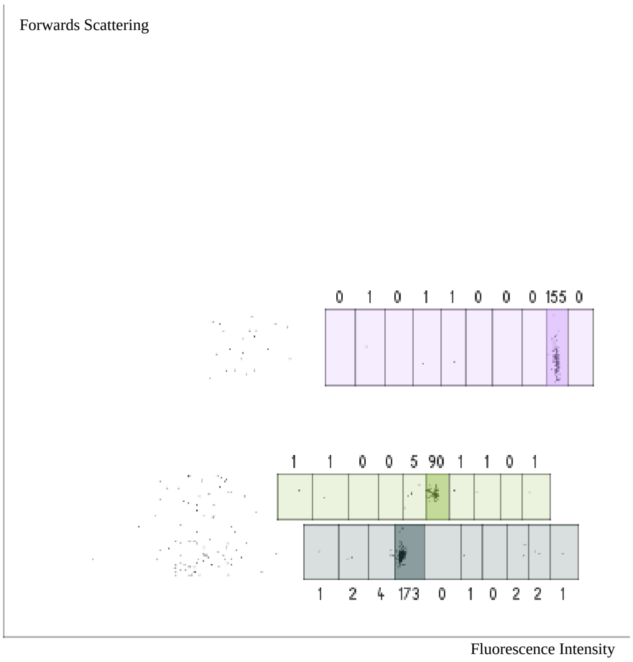

ANNEX 3: TAG DECONVOLUTION - BEAD 19

Passes flow sorting criteria: Yes

Passes tag deconvolution criteria: Yes

Included in protocol analysis: Yes

Protocol: 7, 2, 10, 1

Filename: Bin1_plateA0_G1.fcs

Split 1: Petrol shading

Split 2: Green shading

Split 3: Violet shading

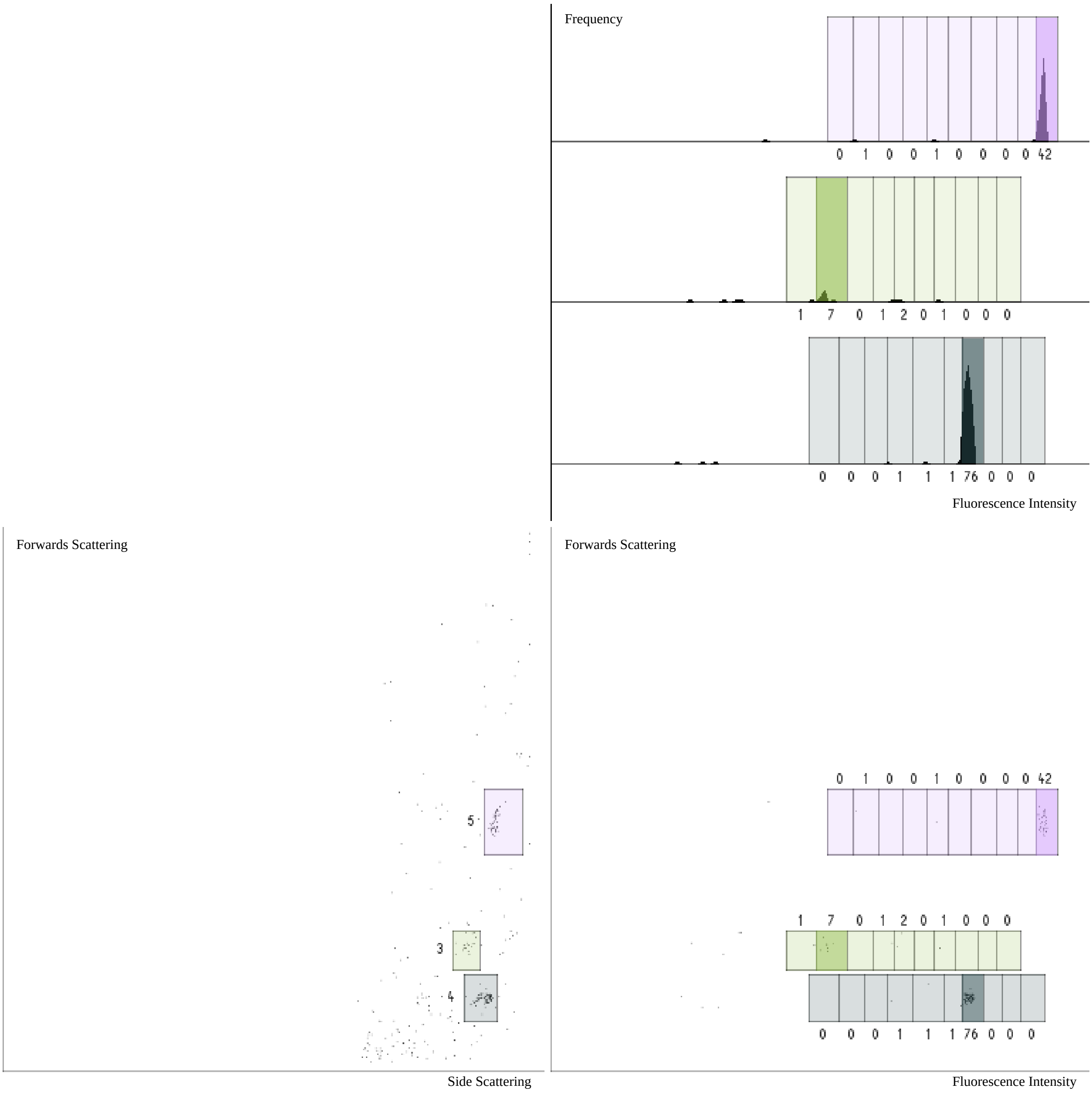

ANNEX 3: TAG DECONVOLUTION - BEAD 20

Passes flow sorting criteria: Yes

Passes tag deconvolution criteria: No

Included in protocol analysis: No

Protocol: N/A

Filename: Bin1_plateA0_G2.fcs

Split 1: Petrol shading

Split 2: Green shading

Split 3: Violet shading

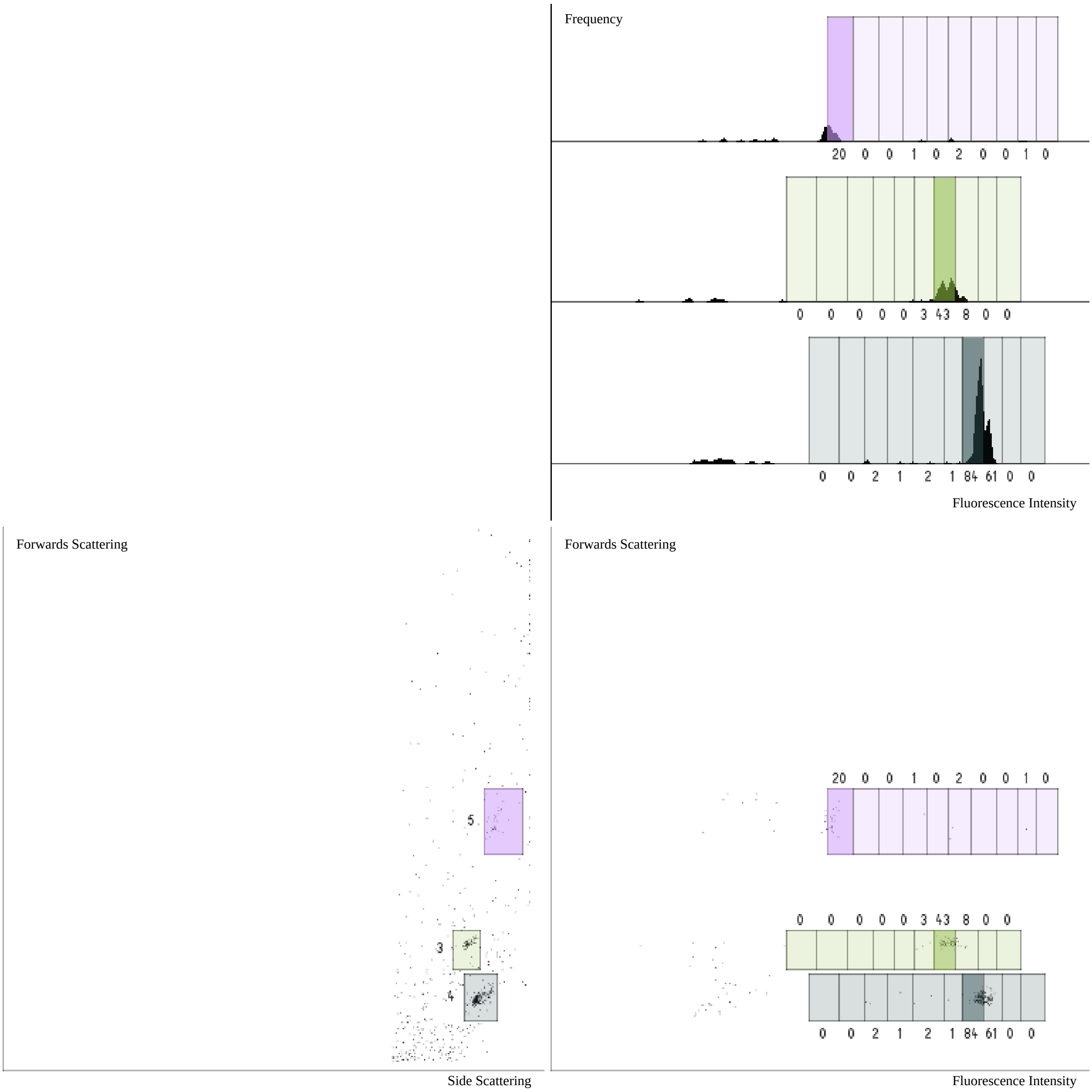

ANNEX 3: TAG DECONVOLUTION - BEAD 21

Passes flow sorting criteria: Yes

Passes tag deconvolution criteria: Yes

Included in protocol analysis: Yes

Protocol: 9, 4, 2, 1

Filename: Bin1_plateA0_G3.fcs

Split 1: Petrol shading

Split 2: Green shading

Split 3: Violet shading

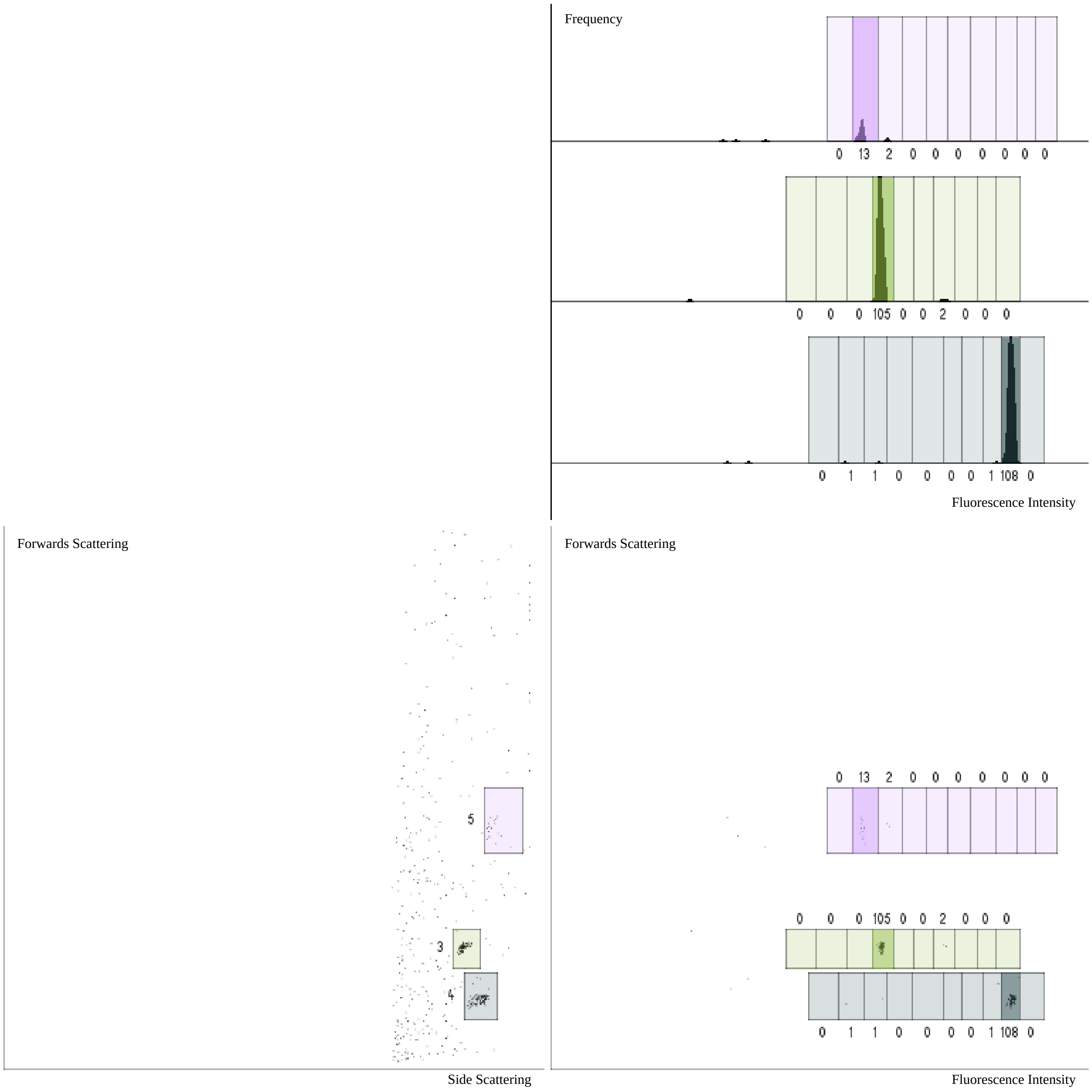

ANNEX 3: TAG DECONVOLUTION - BEAD 22

Passes flow sorting criteria: Yes

Passes tag deconvolution criteria: No

Included in protocol analysis: No

Protocol: N/A

Filename: Bin1_plateA0_H1.fcs

Split 1: Petrol shading

Split 2: Green shading

Split 3: Violet shading

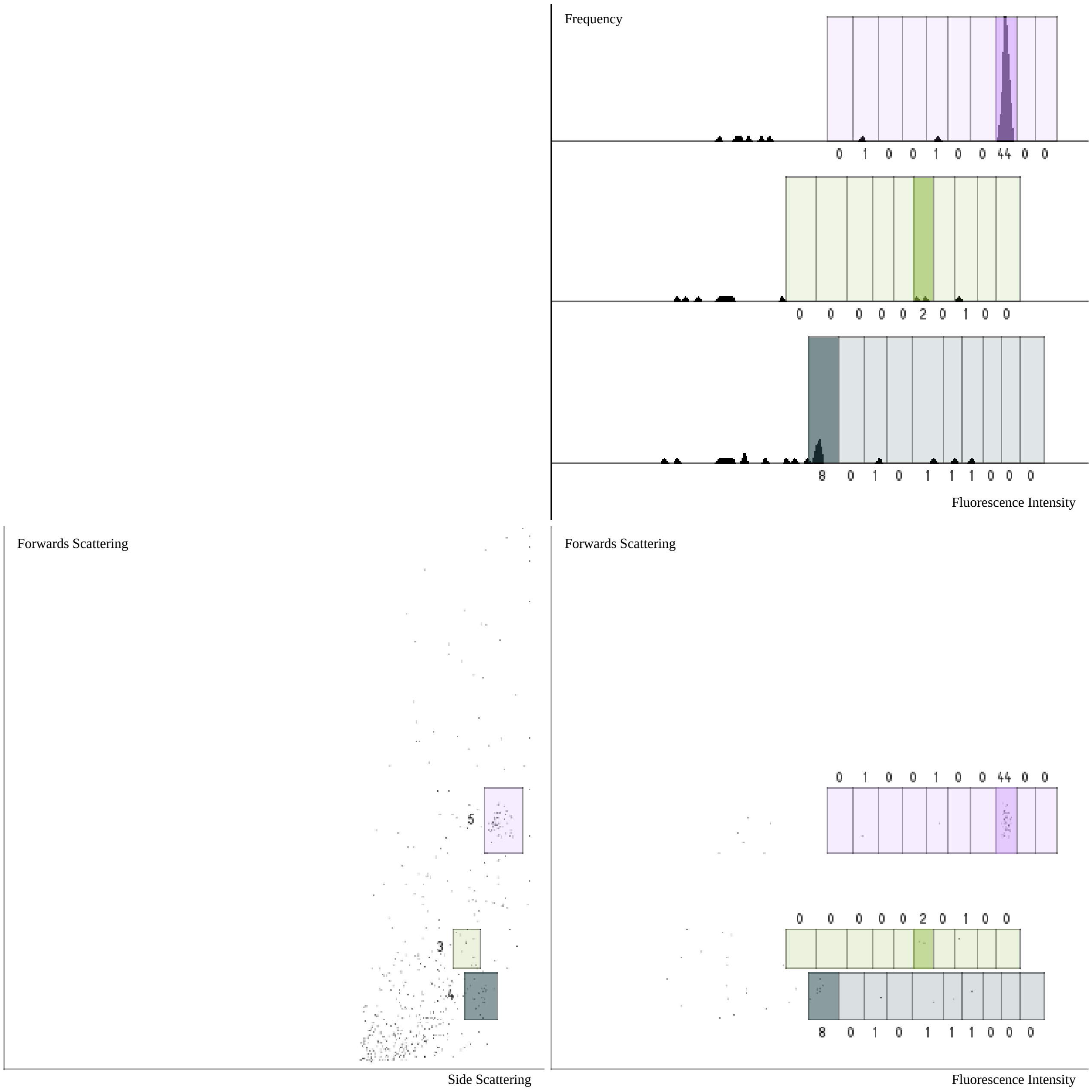

ANNEX 3: TAG DECONVOLUTION - BEAD 23

Passes flow sorting criteria: Yes

Passes tag deconvolution criteria: Yes

Included in protocol analysis: Yes

Protocol: 1, 8, 9, 1

Filename: Bin1_plateA0_H2.fcs

Split 1: Petrol shading

Split 2: Green shading

Split 3: Violet shading

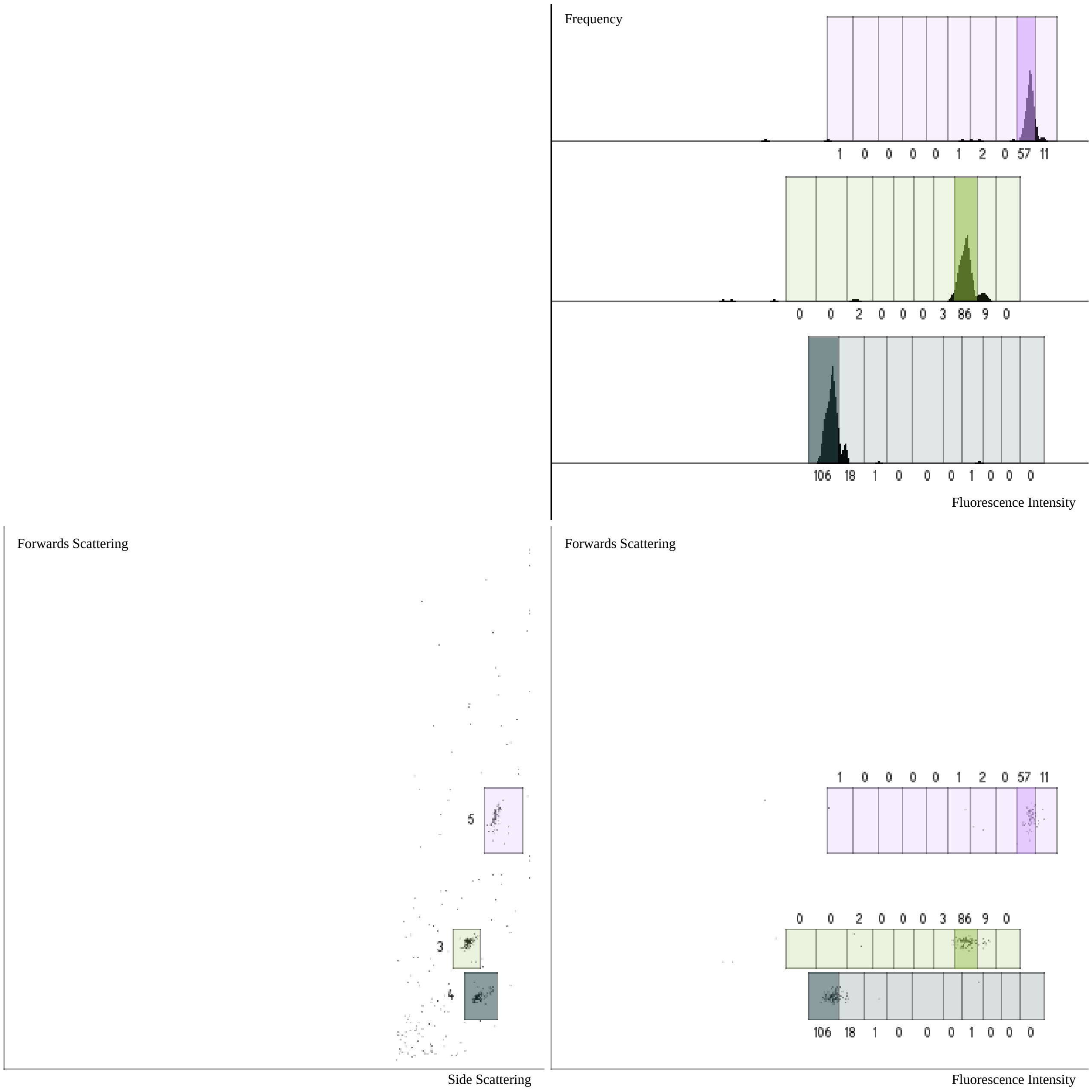

ANNEX 3: TAG DECONVOLUTION - BEAD 24

Passes flow sorting criteria: Yes

Passes tag deconvolution criteria: Yes

Included in protocol analysis: Yes

Protocol: 6, 7, 6, 1

Filename: Bin1_plateA0_H6.fcs

Split 1: Petrol shading

Split 2: Green shading

Split 3: Violet shading

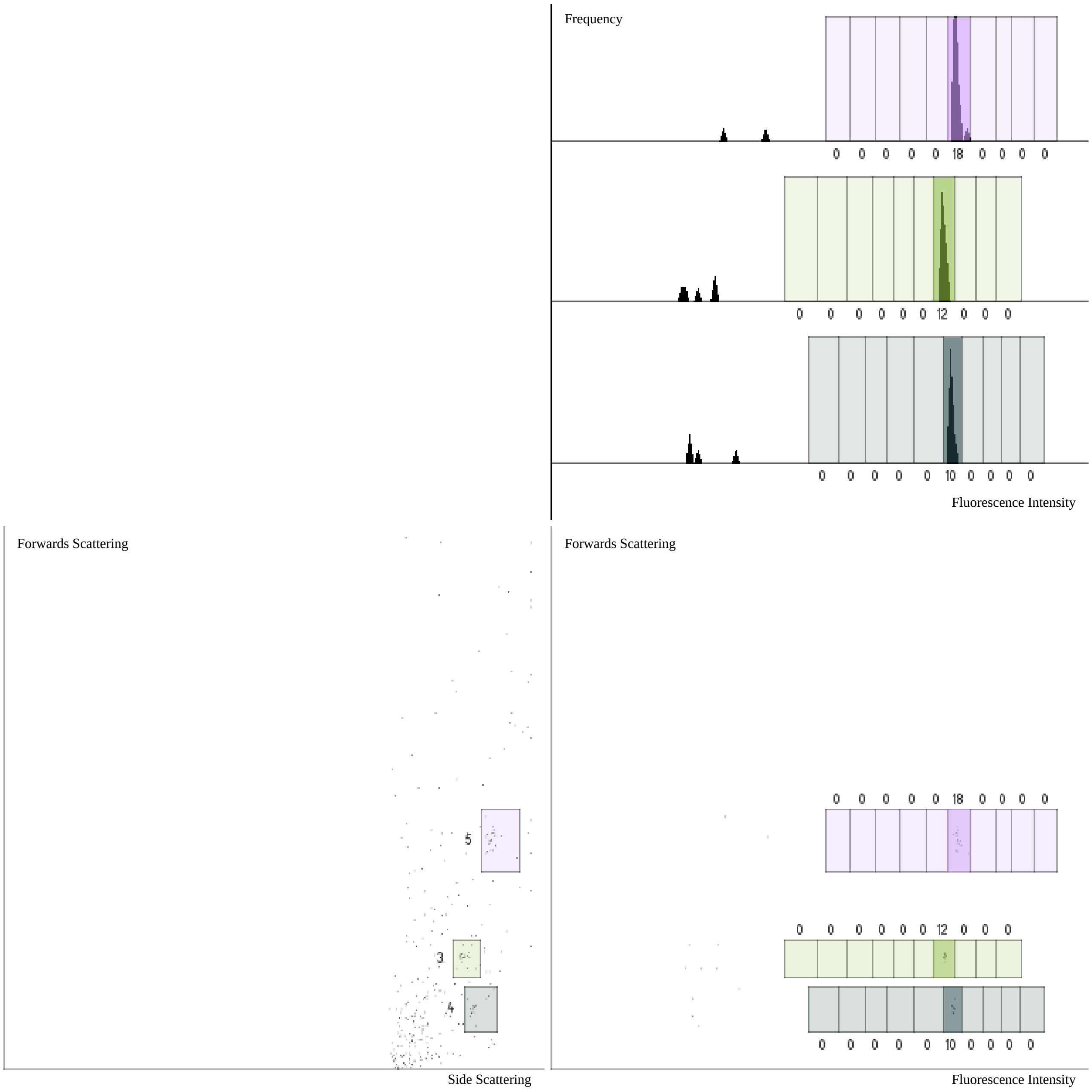

ANNEX 3: TAG DECONVOLUTION - BEAD 25

Passes flow sorting criteria: Yes

Passes tag deconvolution criteria: Yes

Included in protocol analysis: Yes

Protocol: 9, 7, 5, 1

Filename: Bin1_plateA0_A4.fcs

Split 1: Petrol shading

Split 2: Green shading

Split 3: Violet shading

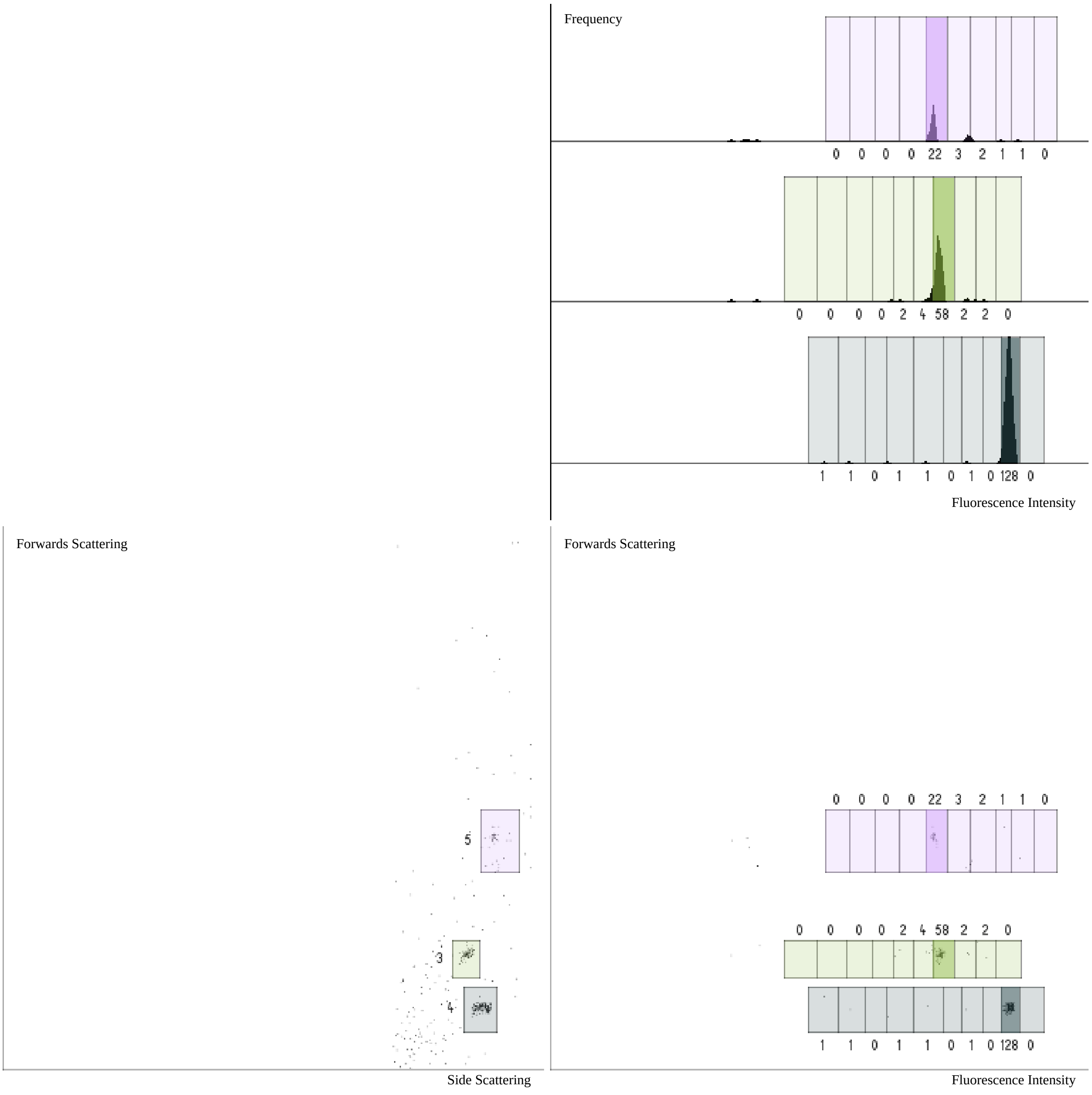

ANNEX 3: TAG DECONVOLUTION - BEAD 26

Passes flow sorting criteria: Yes

Passes tag deconvolution criteria: Yes

Included in protocol analysis: Yes

Protocol: 5, 9, 9, 1

Filename: Bin1_plateA0_A5.fcs

Split 1: Petrol shading

Split 2: Green shading

Split 3: Violet shading

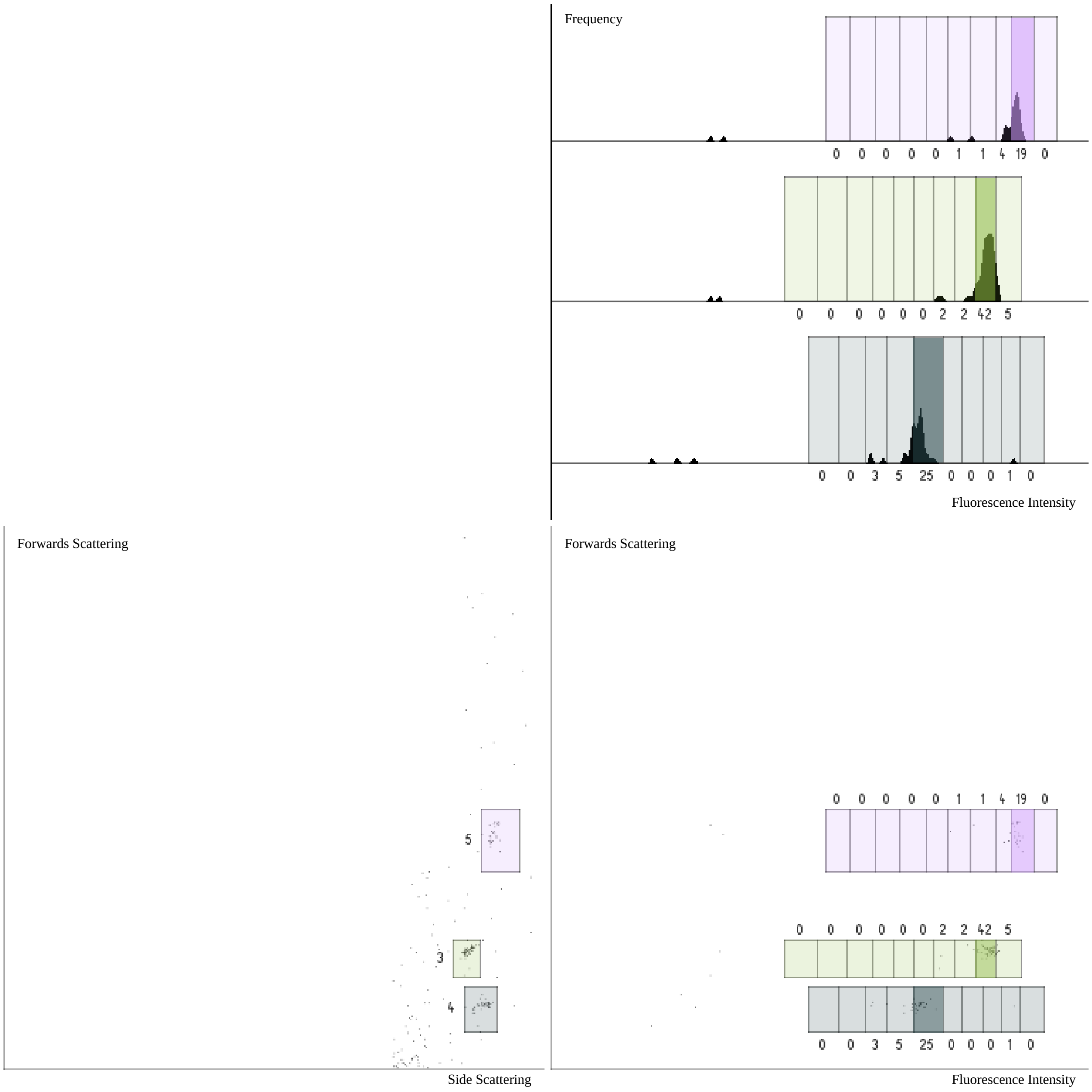

ANNEX 3: TAG DECONVOLUTION - BEAD 27

Passes flow sorting criteria: Yes

Passes tag deconvolution criteria: Yes

Included in protocol analysis: Yes

Protocol: 10, 7, 5, 1

Filename: Bin1_plateA0_A6.fcs

Split 1: Petrol shading

Split 2: Green shading

Split 3: Violet shading

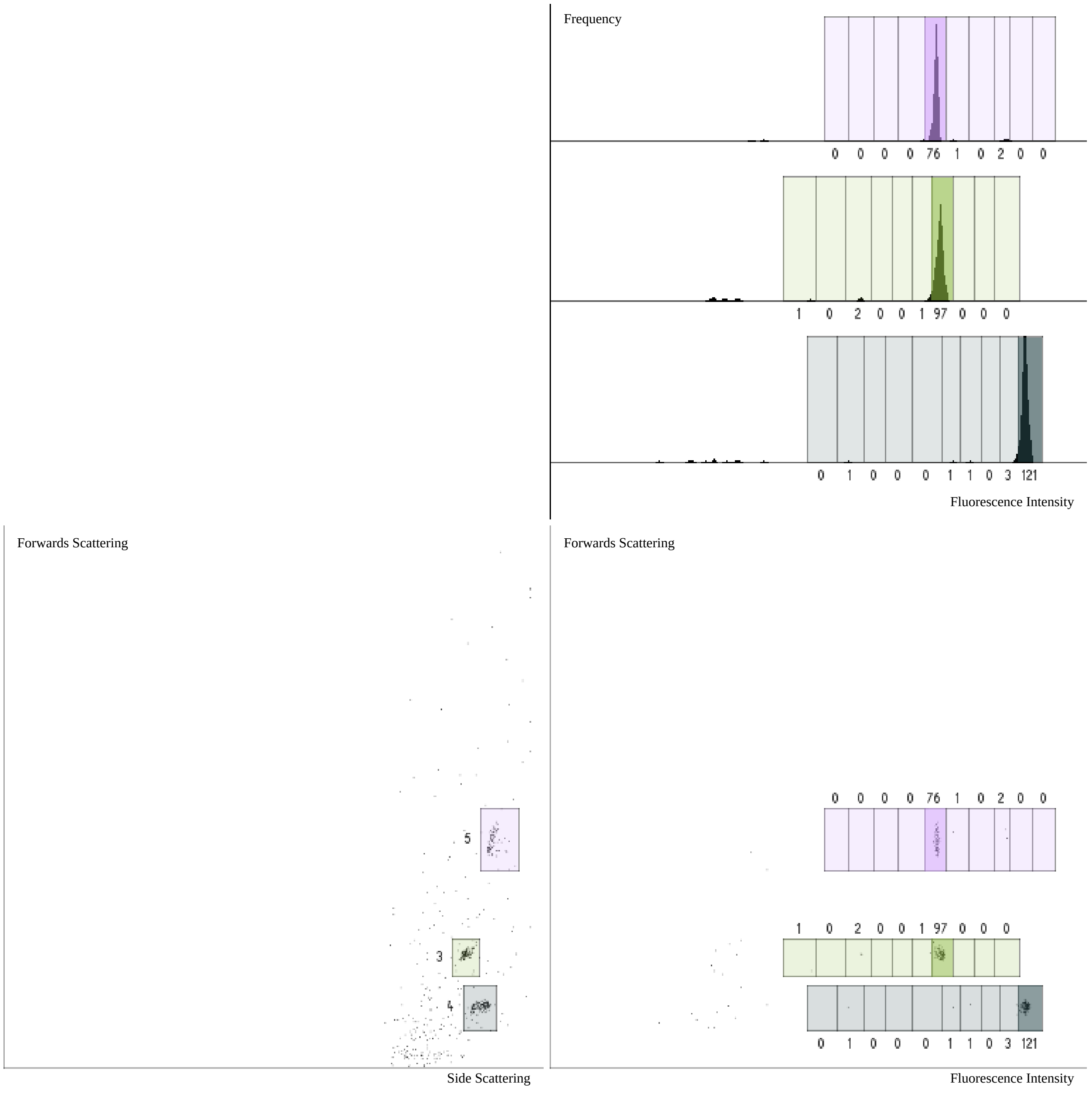

ANNEX 3: TAG DECONVOLUTION - BEAD 28

Passes flow sorting criteria: Yes

Passes tag deconvolution criteria: Yes

Included in protocol analysis: Yes

Protocol: 6, 1, 9, 1

Filename: Bin1_plateA0_A7.fcs

Split 1: Petrol shading

Split 2: Green shading

Split 3: Violet shading

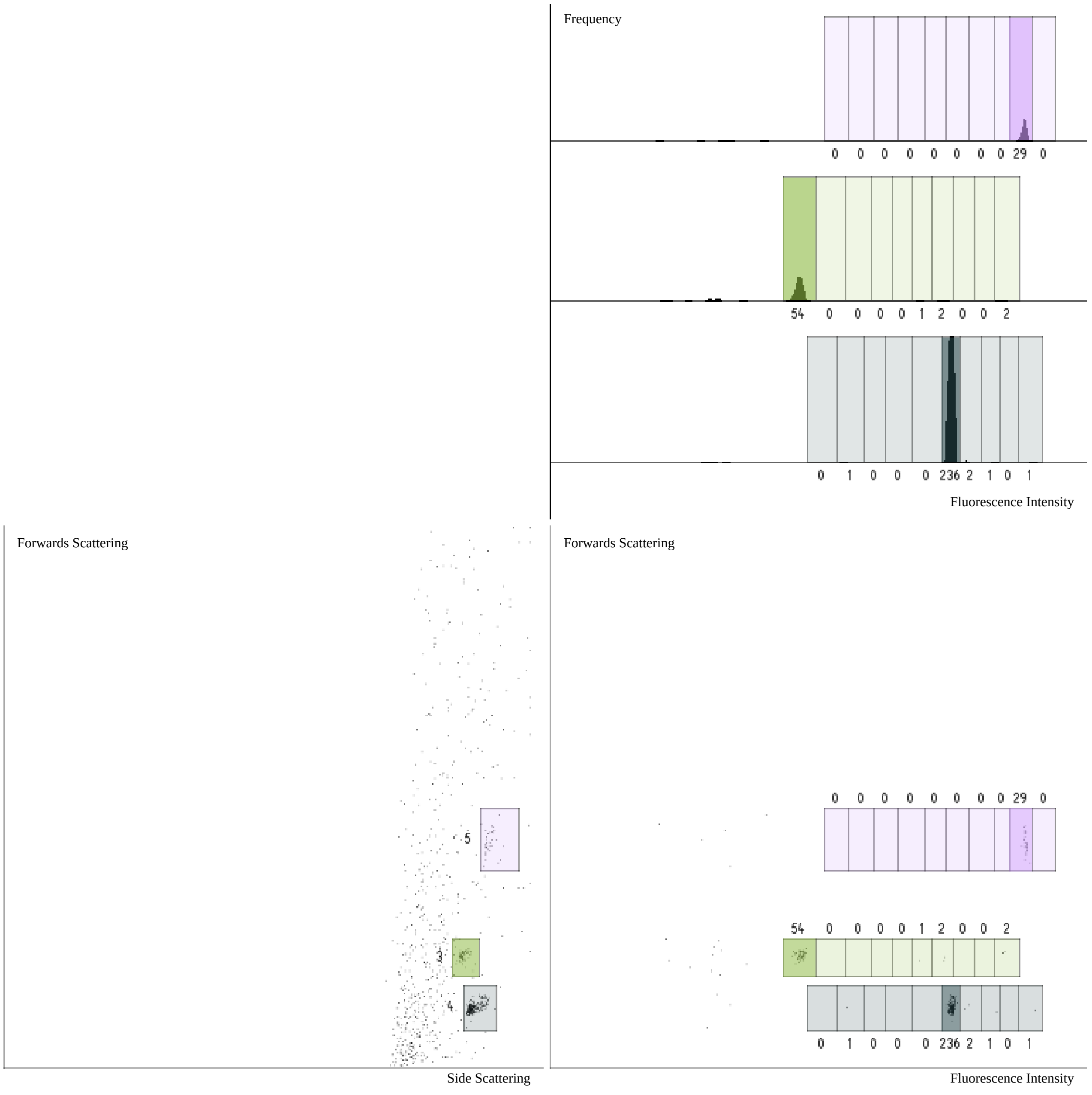

ANNEX 3: TAG DECONVOLUTION - BEAD 29

Passes flow sorting criteria: Yes

Passes tag deconvolution criteria: Yes

Included in protocol analysis: Yes

Protocol: 10, 7, 9, 1

Filename: Bin1_plateA0_B4.fcs

Split 1: Petrol shading

Split 2: Green shading

Split 3: Violet shading

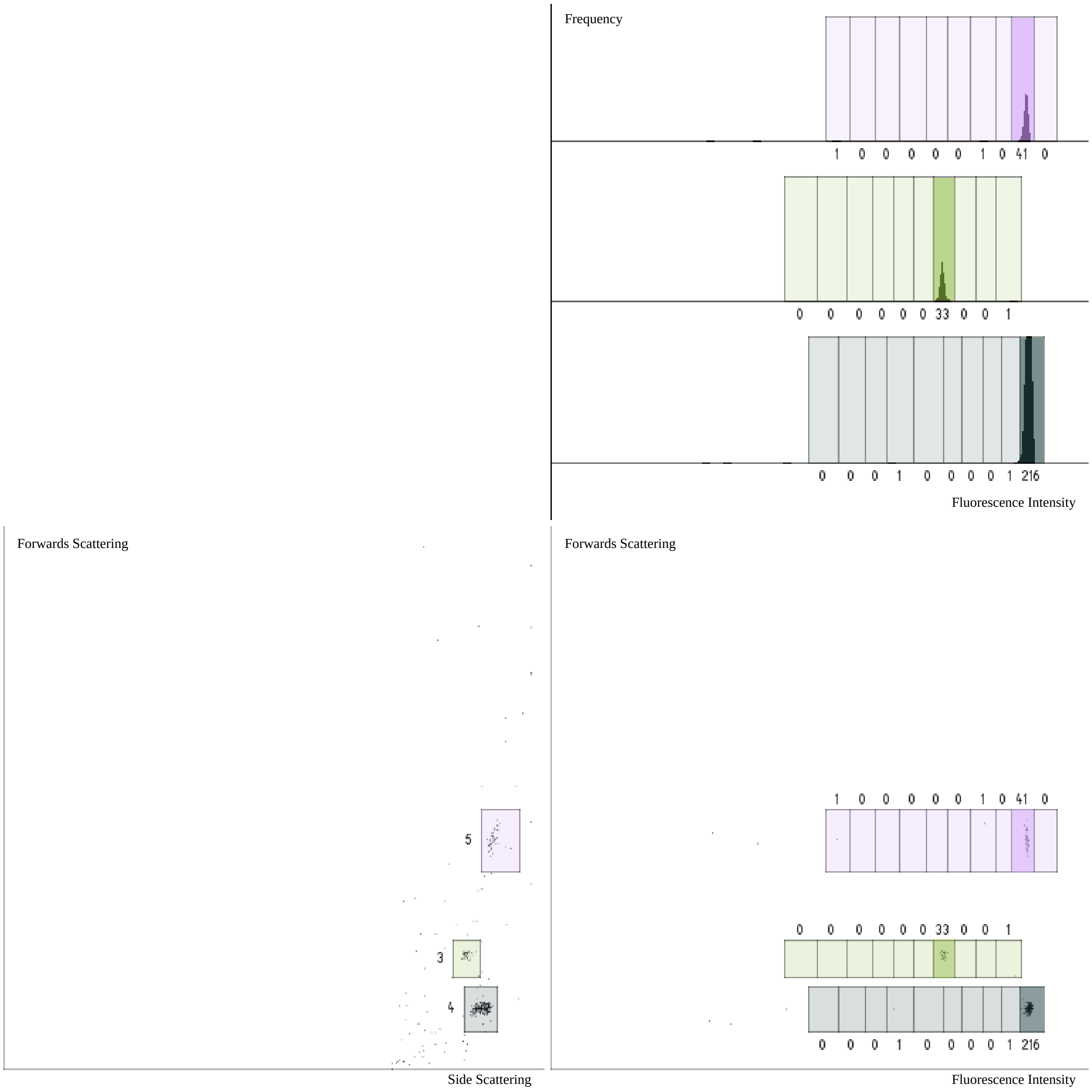

ANNEX 3: TAG DECONVOLUTION - BEAD 30

Passes flow sorting criteria: Yes

Passes tag deconvolution criteria: No

Included in protocol analysis: No

Protocol: N/A

Filename: Bin1_plateA0_B5.fcs

Split 1: Petrol shading

Split 2: Green shading

Split 3: Violet shading

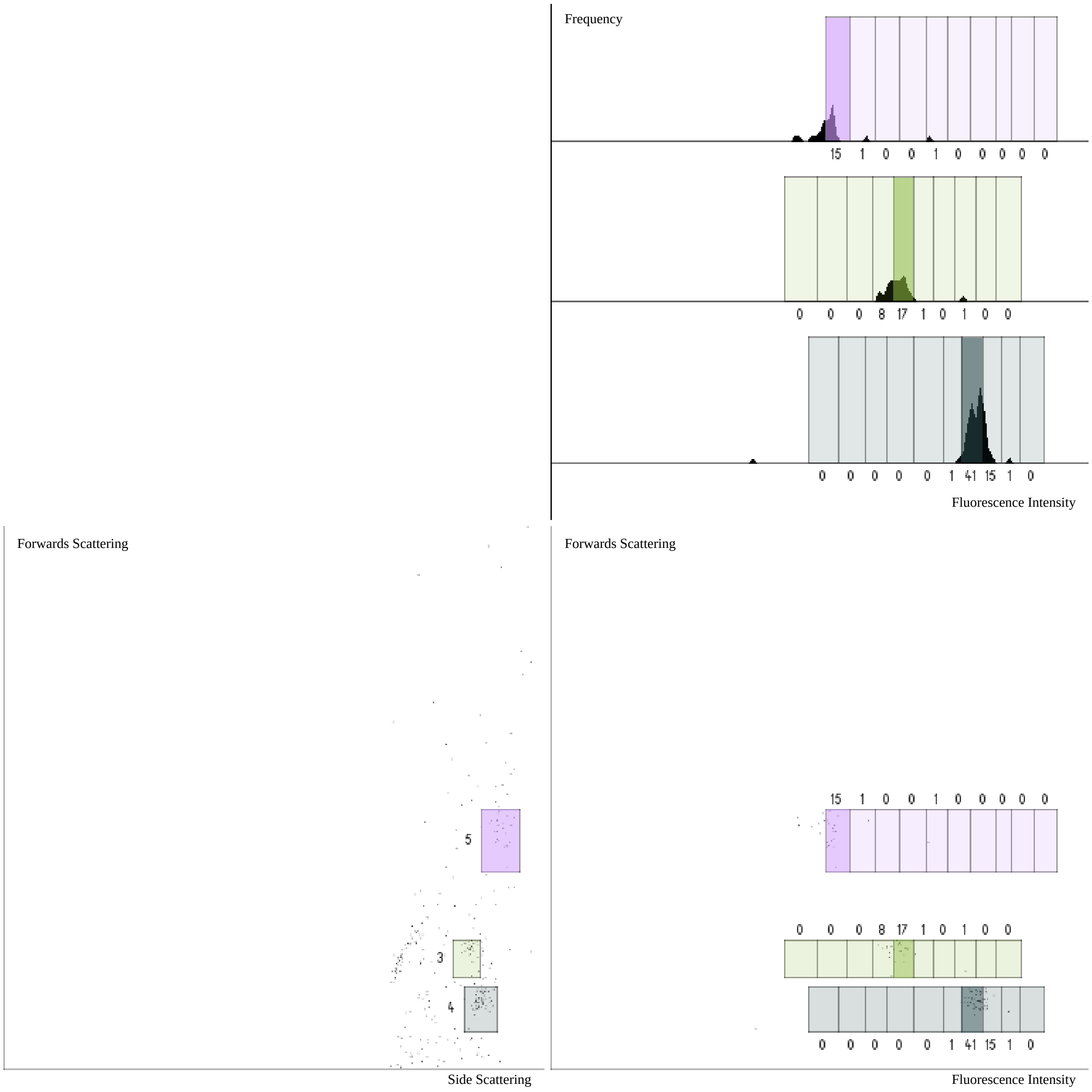

ANNEX 3: TAG DECONVOLUTION - BEAD 31

Passes flow sorting criteria: Yes

Passes tag deconvolution criteria: No

Included in protocol analysis: No

Protocol: N/A

Filename: Bin1_plateA0_B6.fcs

Split 1: Petrol shading

Split 2: Green shading

Split 3: Violet shading

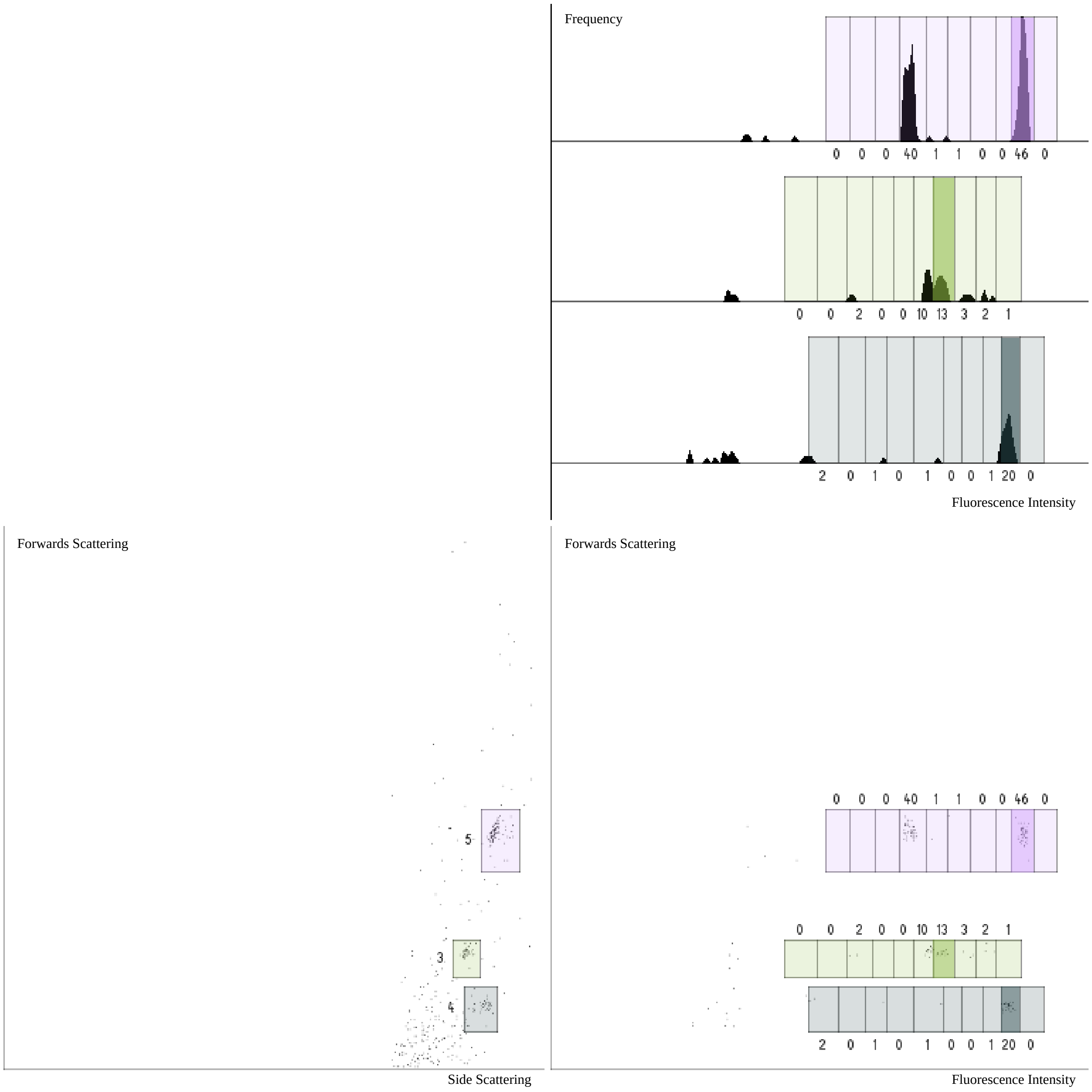

ANNEX 3: TAG DECONVOLUTION - BEAD 32

Passes flow sorting criteria: Yes

Passes tag deconvolution criteria: Yes

Included in protocol analysis: Yes

Protocol: 10, 6, 7, 1

Filename: Bin1_plateA0_B7.fcs

Split 1: Petrol shading

Split 2: Green shading

Split 3: Violet shading

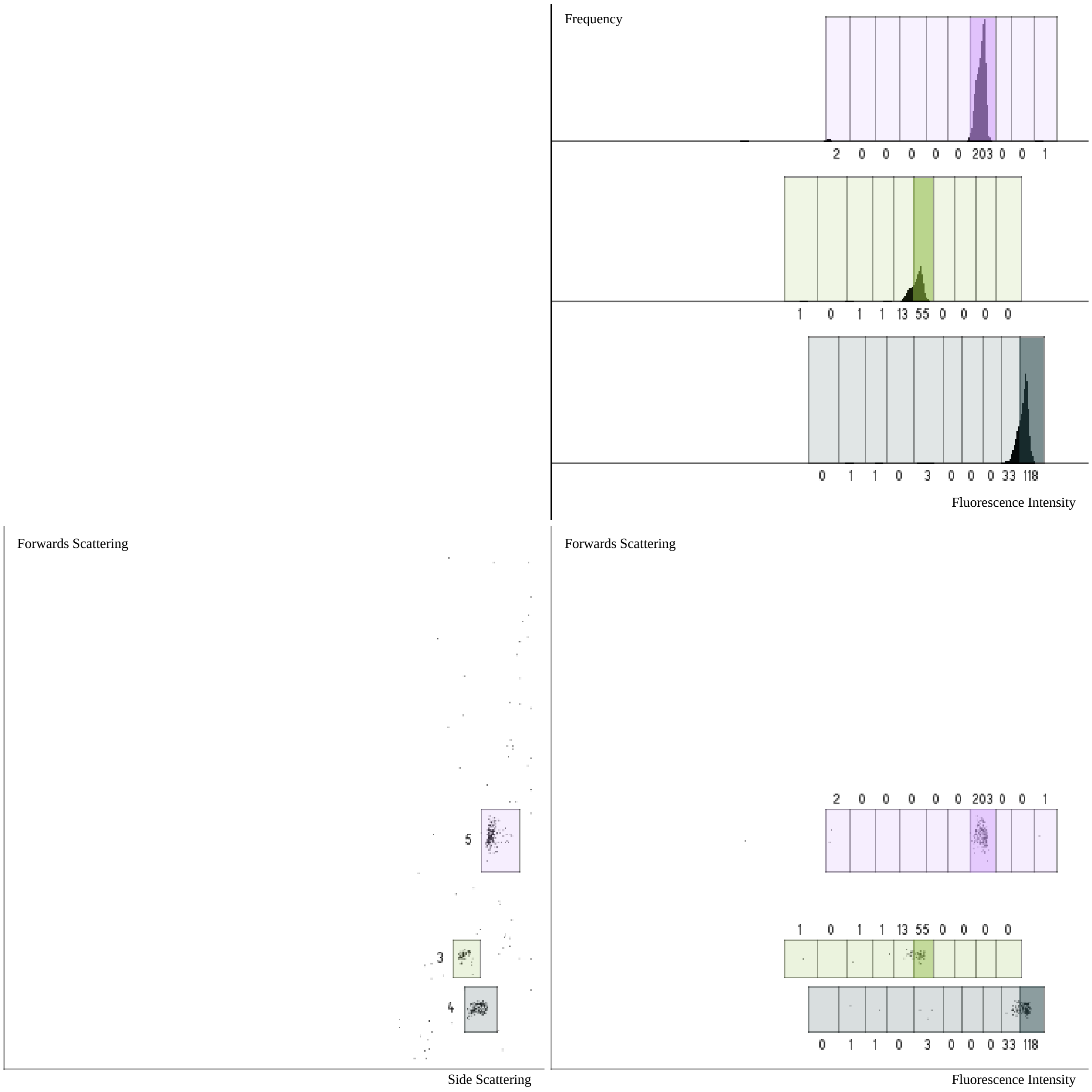

ANNEX 3: TAG DECONVOLUTION - BEAD 33

Passes flow sorting criteria: Yes

Passes tag deconvolution criteria: No

Included in protocol analysis: No

Protocol: N/A

Filename: Bin1_plateA0_C4.fcs

Split 1: Petrol shading

Split 2: Green shading

Split 3: Violet shading

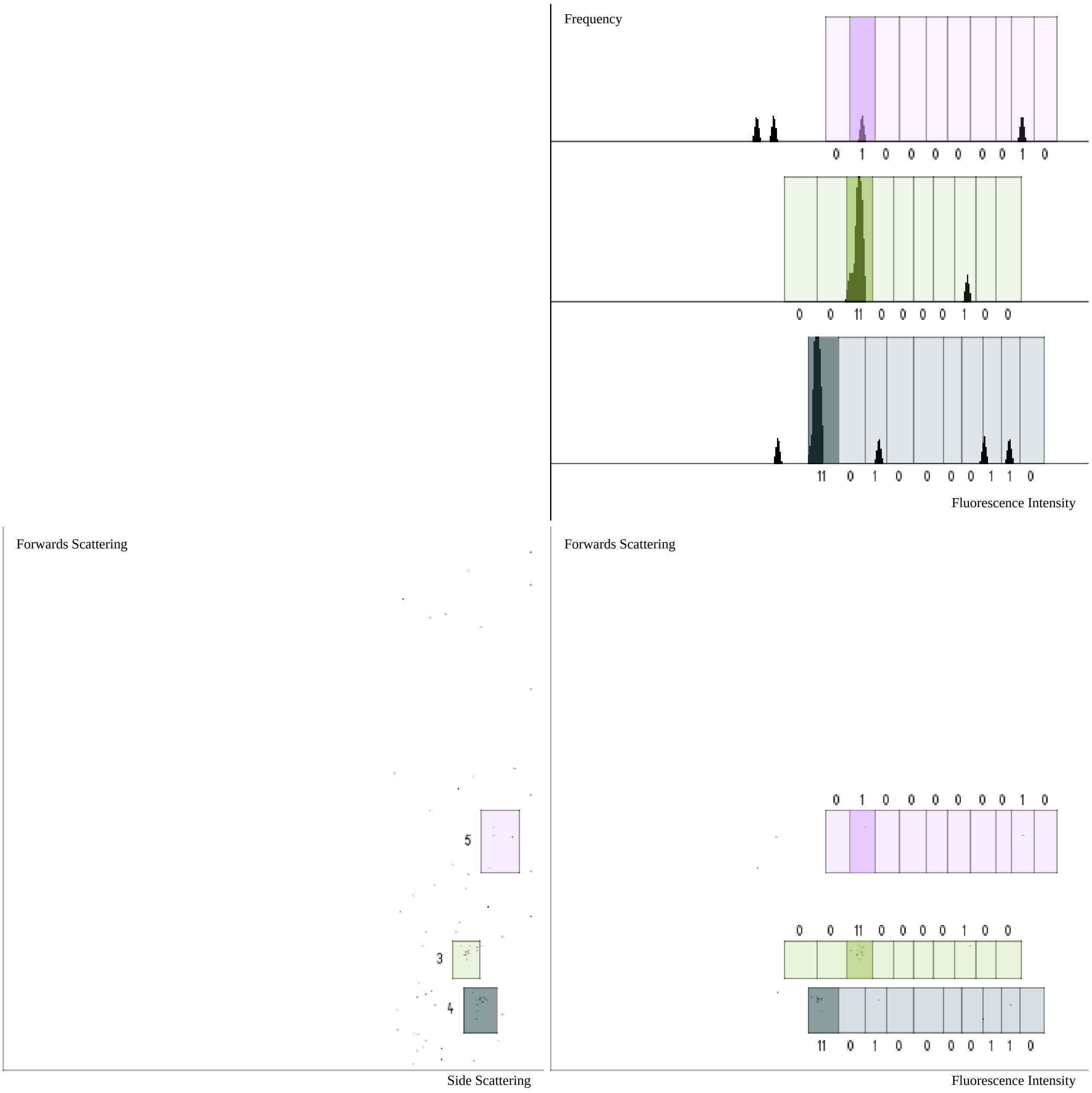

ANNEX 3: TAG DECONVOLUTION - BEAD 34

Passes flow sorting criteria: Yes

Passes tag deconvolution criteria: Yes

Included in protocol analysis: Yes

Protocol: 6, 10, 6, 1

Filename: Bin1_plateA0_C5.fcs

Split 1: Petrol shading

Split 2: Green shading

Split 3: Violet shading

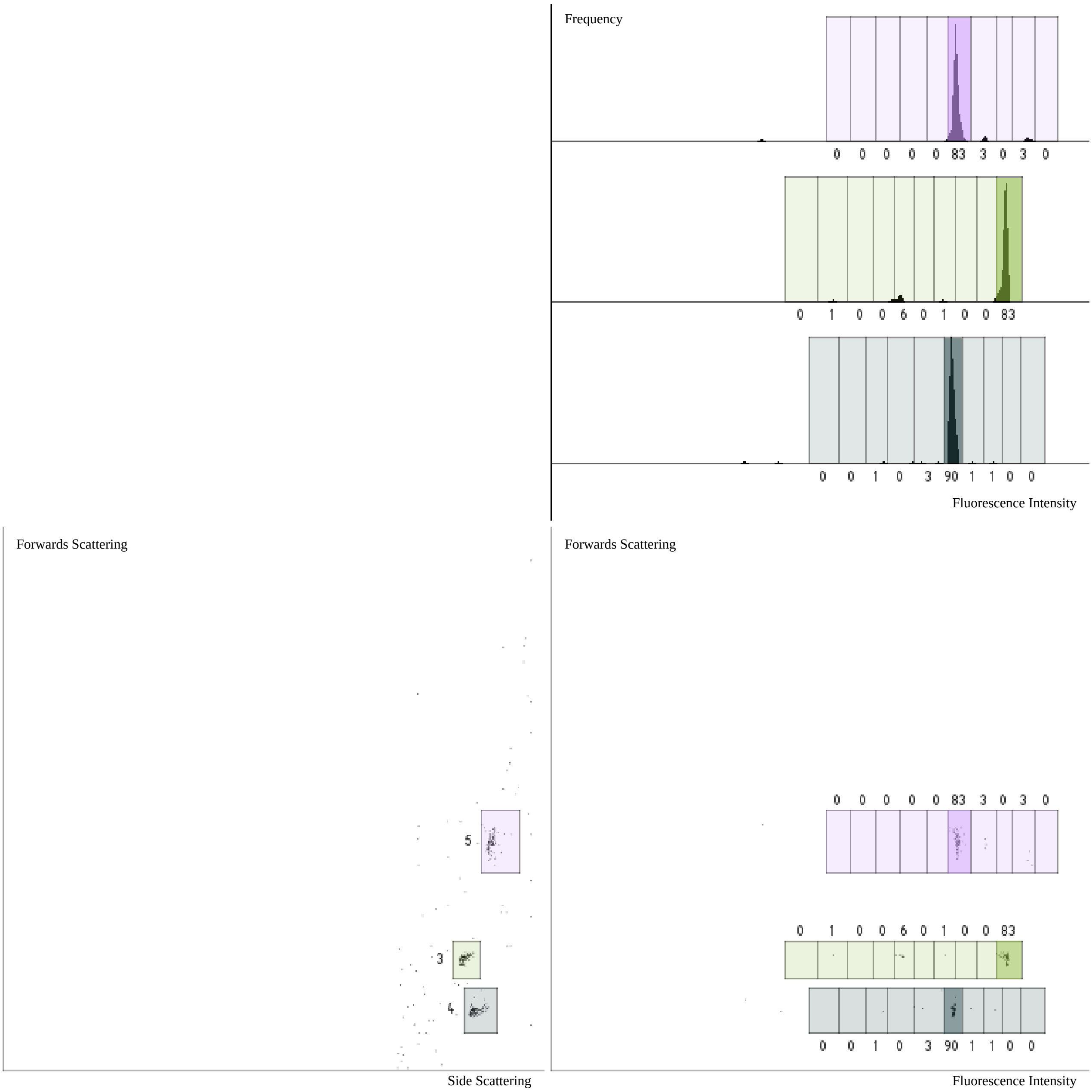

ANNEX 3: TAG DECONVOLUTION - BEAD 35

Passes flow sorting criteria: Yes

Passes tag deconvolution criteria: Yes

Included in protocol analysis: Yes

Protocol: 5, 2, 7, 1

Filename: Bin1_plateA0_C6.fcs

Split 1: Petrol shading

Split 2: Green shading

Split 3: Violet shading

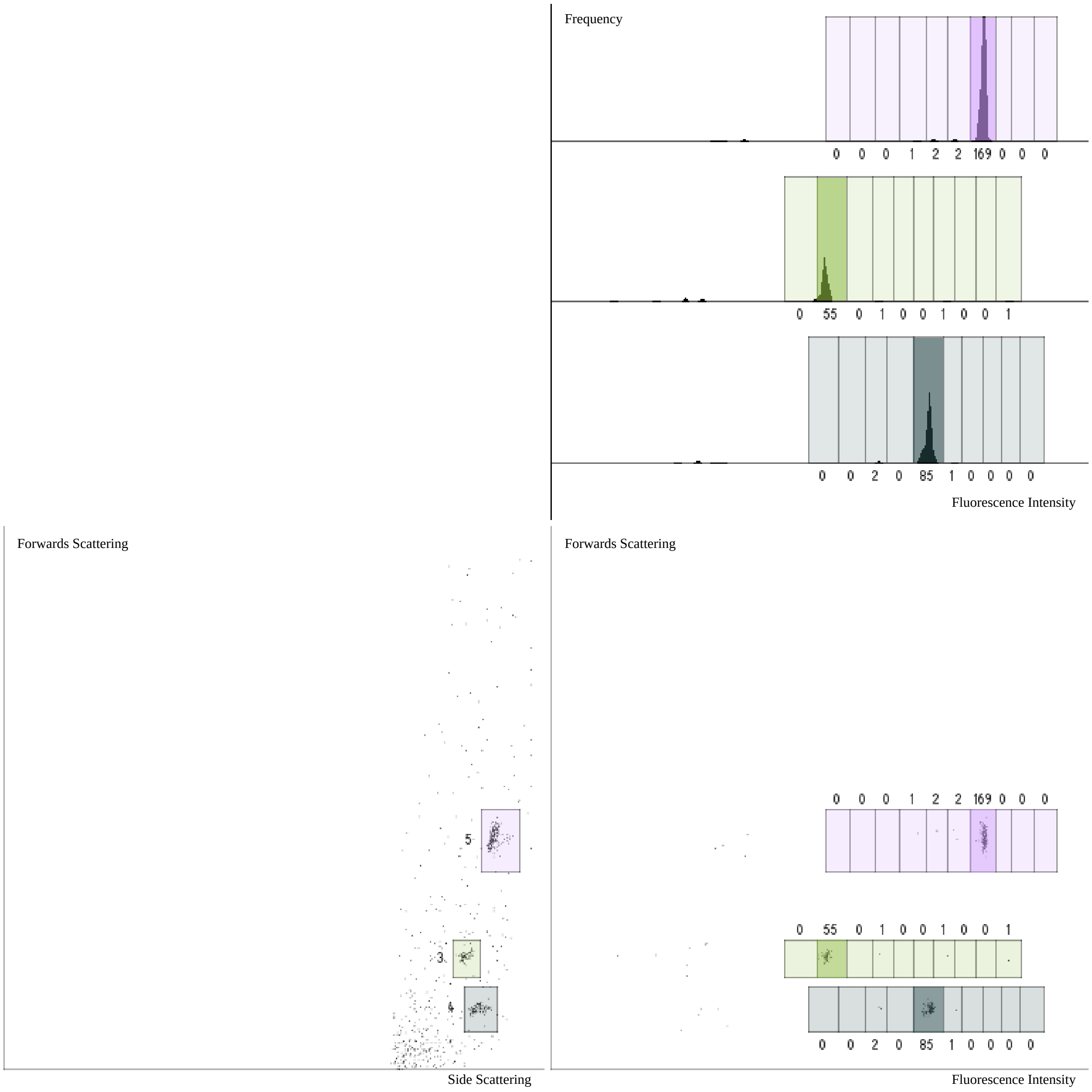

ANNEX 3: TAG DECONVOLUTION - BEAD 36

Passes flow sorting criteria: Yes

Passes tag deconvolution criteria: Yes

Included in protocol analysis: Yes

Protocol: 1, 10, 5, 1

Filename: Bin1_plateA0_C7.fcs

Split 1: Petrol shading

Split 2: Green shading

Split 3: Violet shading

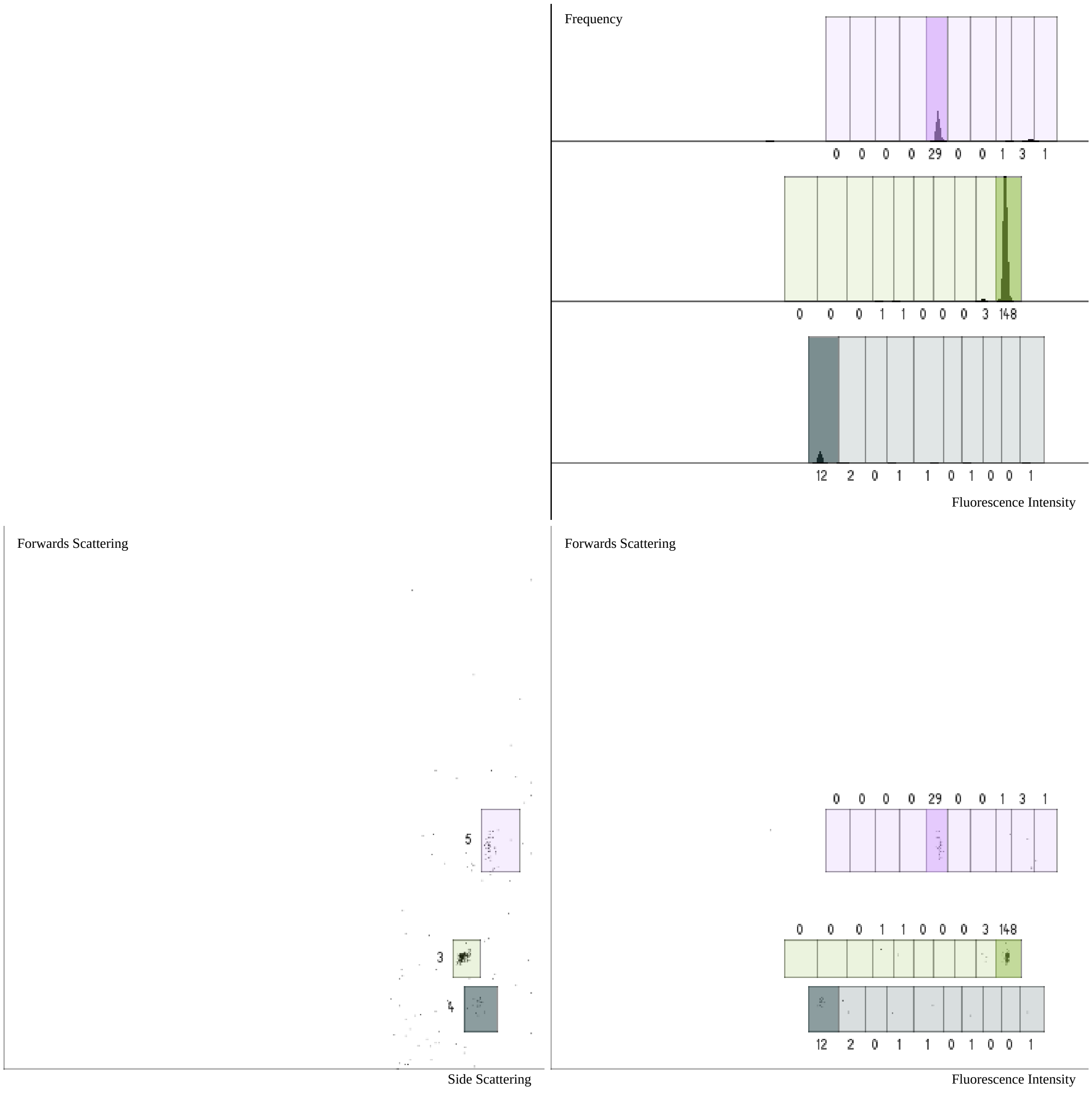

ANNEX 3: TAG DECONVOLUTION - BEAD 37

Passes flow sorting criteria: Yes

Passes tag deconvolution criteria: Yes

Included in protocol analysis: Yes

Protocol: 4, 6, 2, 1

Filename: Bin1_plateA0_D4.fcs

Split 1: Petrol shading

Split 2: Green shading

Split 3: Violet shading

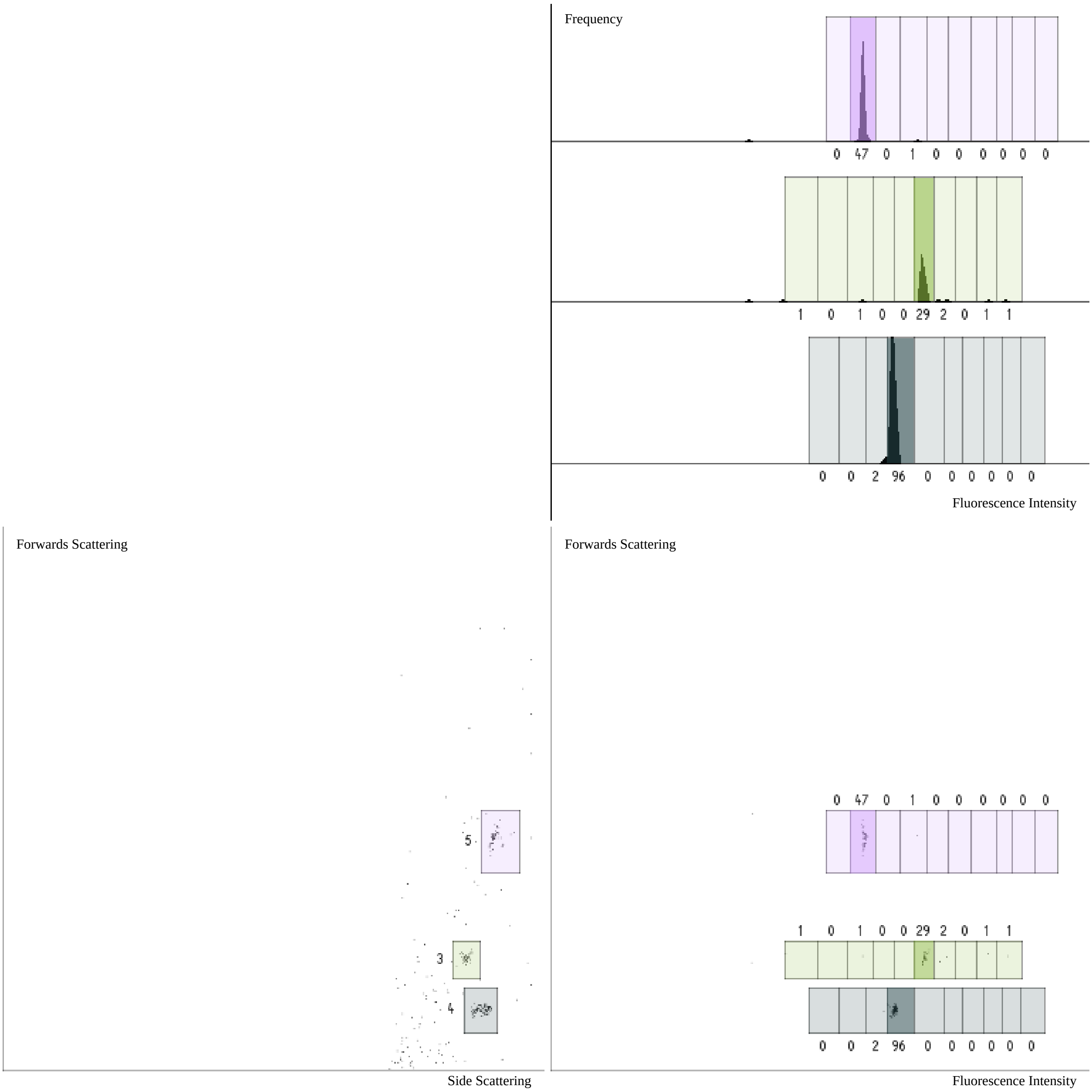

ANNEX 3: TAG DECONVOLUTION - BEAD 38

Passes flow sorting criteria: Yes

Passes tag deconvolution criteria: Yes

Included in protocol analysis: Yes

Protocol: 8, 10, 8, 1

Filename: Bin1_plateA0_D5.fcs

Split 1: Petrol shading

Split 2: Green shading

Split 3: Violet shading

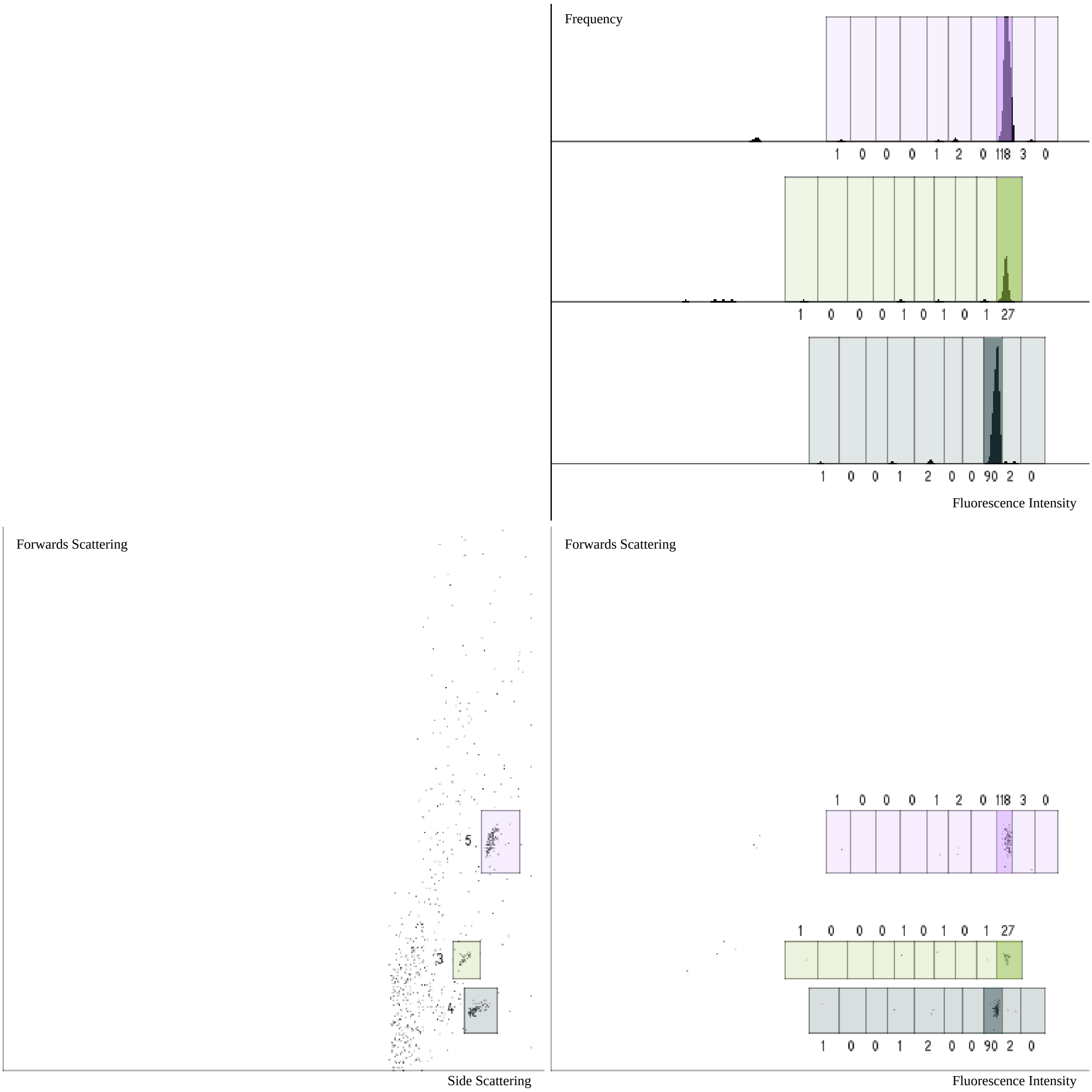

ANNEX 3: TAG DECONVOLUTION - BEAD 39

Passes flow sorting criteria: Yes

Passes tag deconvolution criteria: No

Included in protocol analysis: No

Protocol: N/A

Filename: Bin1_plateA0_D6.fcs

Split 1: Petrol shading

Split 2: Green shading

Split 3: Violet shading

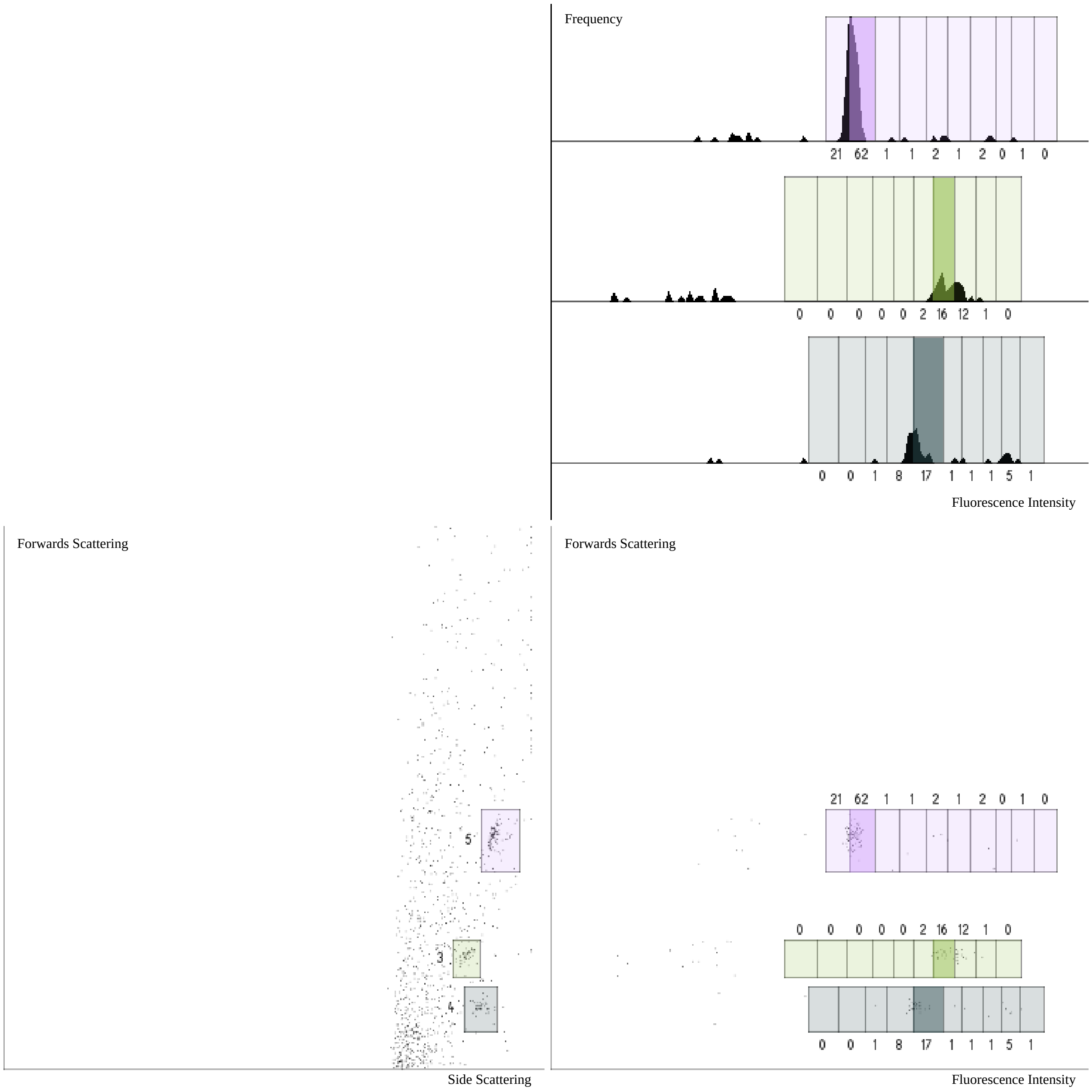

ANNEX 3: TAG DECONVOLUTION - BEAD 40

Passes flow sorting criteria: Yes

Passes tag deconvolution criteria: Yes

Included in protocol analysis: Yes

Protocol: 5, 4, 5, 1

Filename: Bin1_plateA0_D7.fcs

Split 1: Petrol shading

Split 2: Green shading

Split 3: Violet shading

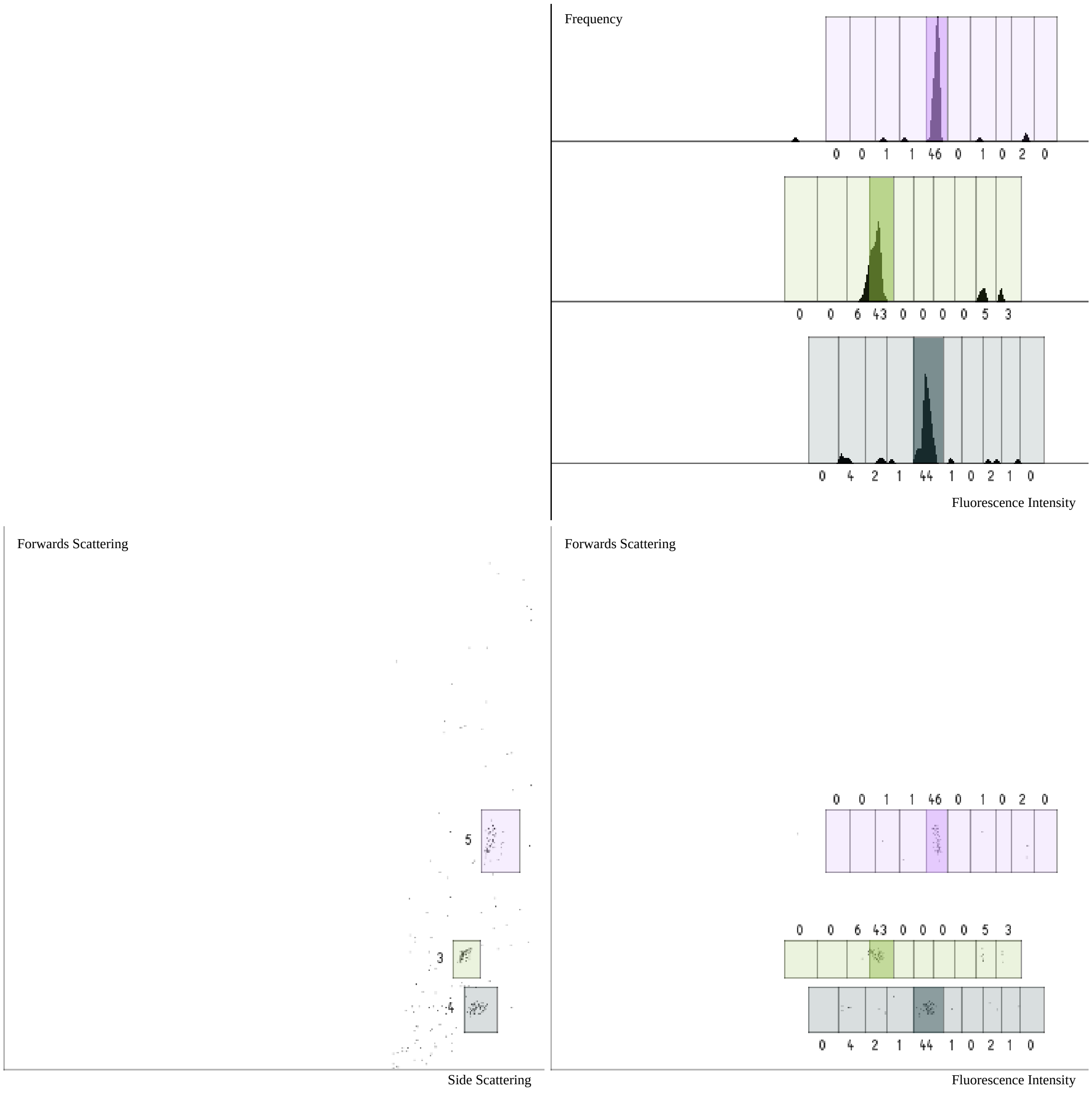

ANNEX 3: TAG DECONVOLUTION - BEAD 41

Passes flow sorting criteria: Yes

Passes tag deconvolution criteria: No

Included in protocol analysis: No

Protocol: N/A

Filename: Bin1_plateA0_E4.fcs

Split 1: Petrol shading

Split 2: Green shading

Split 3: Violet shading

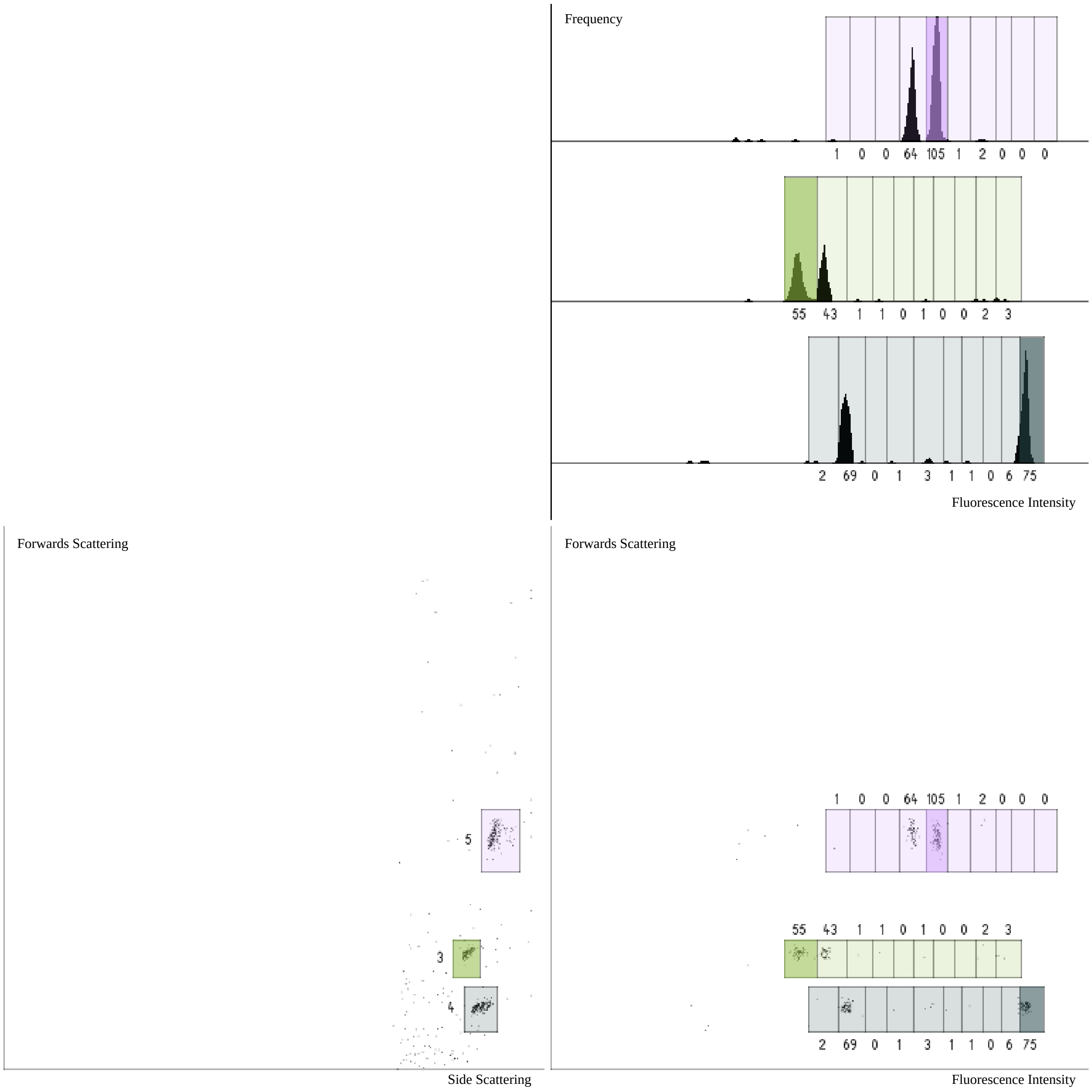

ANNEX 3: TAG DECONVOLUTION - BEAD 42

Passes flow sorting criteria: Yes

Passes tag deconvolution criteria: Yes

Included in protocol analysis: Yes

Protocol: 2, 1, 5, 1

Filename: Bin1_plateA0_E5.fcs

Split 1: Petrol shading

Split 2: Green shading

Split 3: Violet shading

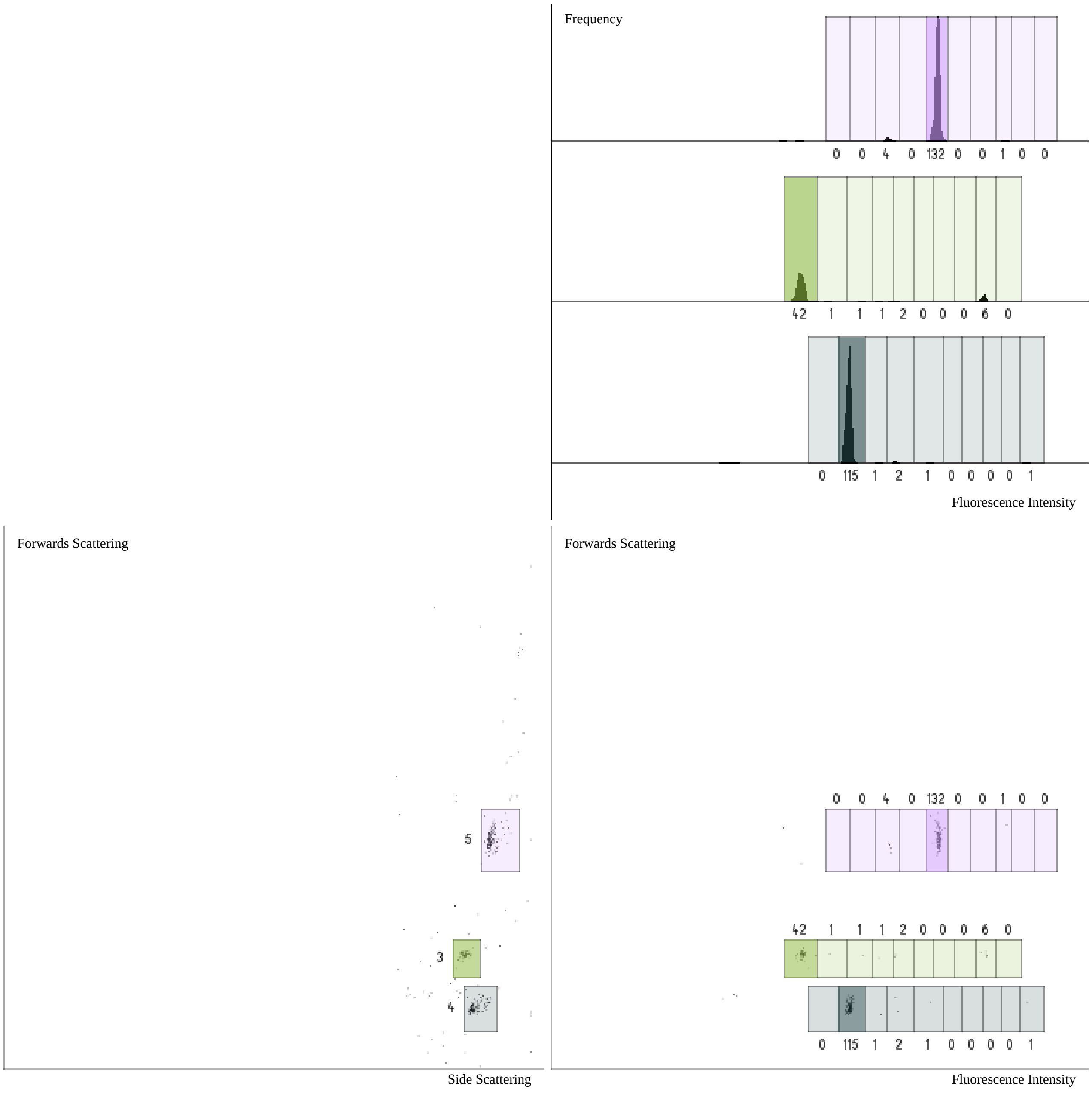

ANNEX 3: TAG DECONVOLUTION - BEAD 43

Passes flow sorting criteria: Yes

Passes tag deconvolution criteria: No

Included in protocol analysis: No

Protocol: N/A

Filename: Bin1_plateA0_E6.fcs

Split 1: Petrol shading

Split 2: Green shading

Split 3: Violet shading

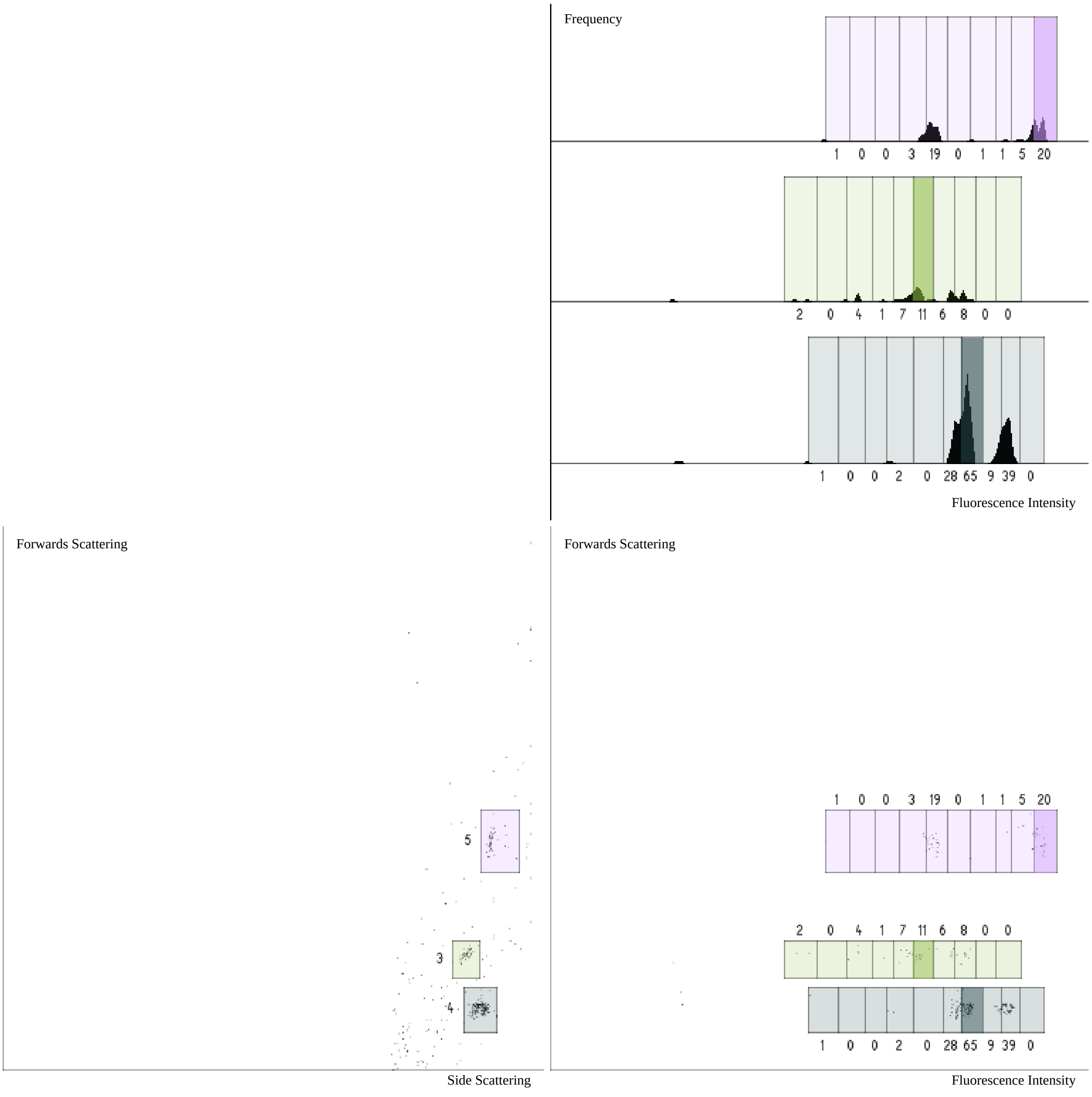

ANNEX 3: TAG DECONVOLUTION - BEAD 44

Passes flow sorting criteria: Yes

Passes tag deconvolution criteria: No

Included in protocol analysis: No

Protocol: N/A

Filename: Bin1_plateA0_F5.fcs

Split 1: Petrol shading

Split 2: Green shading

Split 3: Violet shading

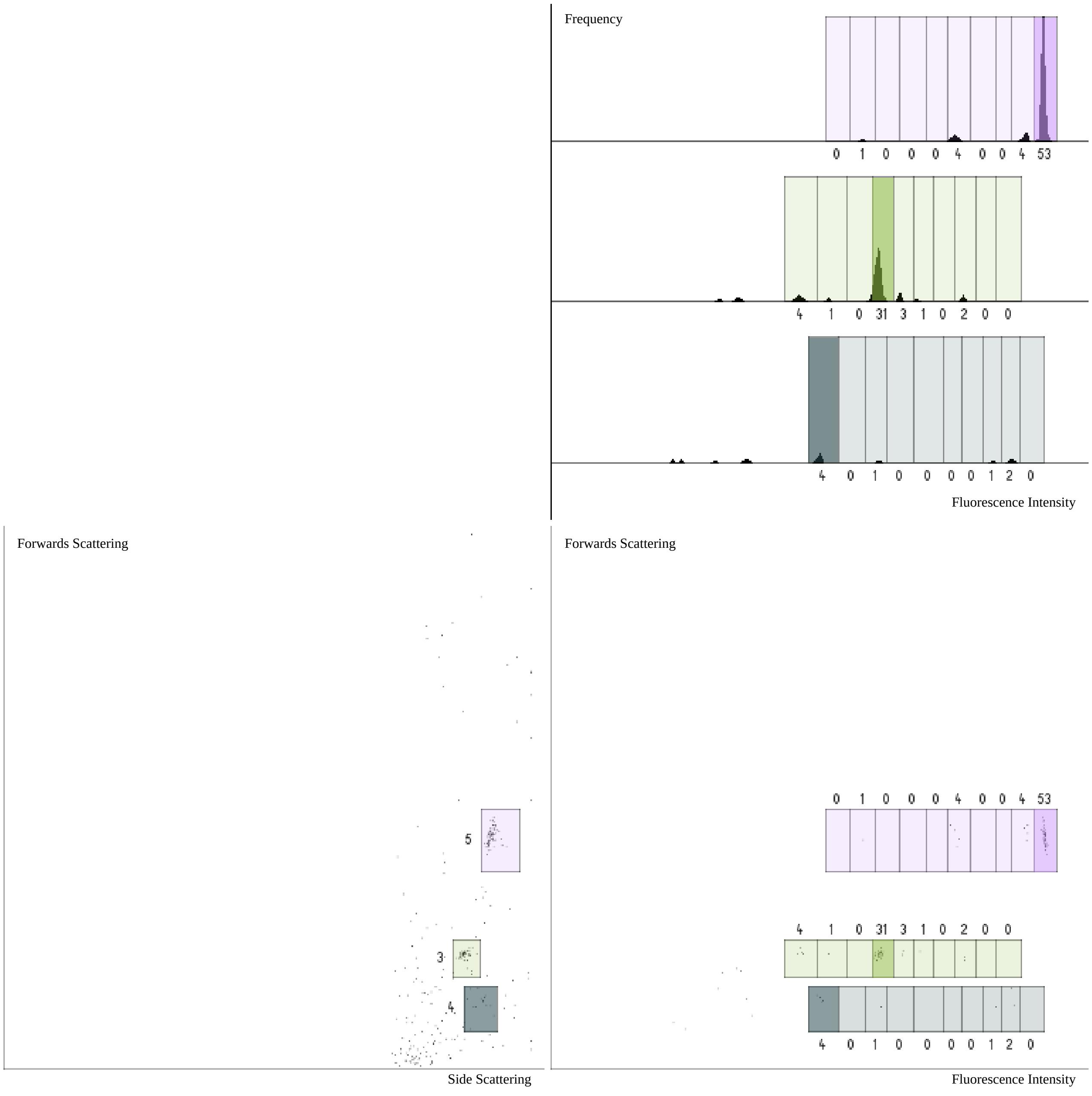

ANNEX 3: TAG DECONVOLUTION - BEAD 45

Passes flow sorting criteria: Yes

Passes tag deconvolution criteria: Yes

Included in protocol analysis: Yes

Protocol: 1, 3, 6, 1

Filename: Bin1_plateA0_F6.fcs

Split 1: Petrol shading

Split 2: Green shading

Split 3: Violet shading

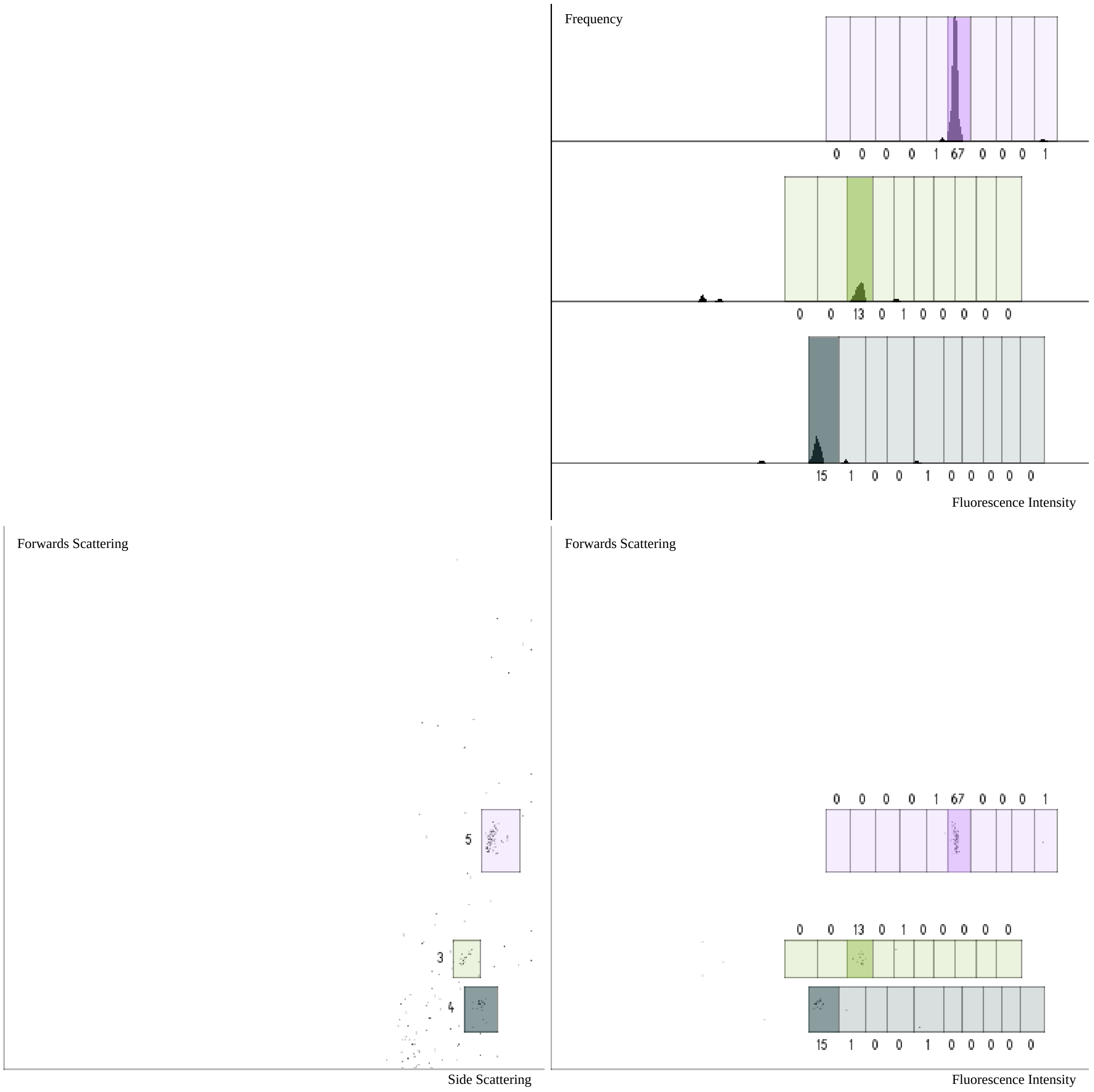

ANNEX 3: TAG DECONVOLUTION - BEAD 46

Passes flow sorting criteria: Yes

Passes tag deconvolution criteria: Yes

Included in protocol analysis: Yes

Protocol: 5, 10, 5, 1

Filename: Bin1_plateA0_G4.fcs

Split 1: Petrol shading

Split 2: Green shading

Split 3: Violet shading

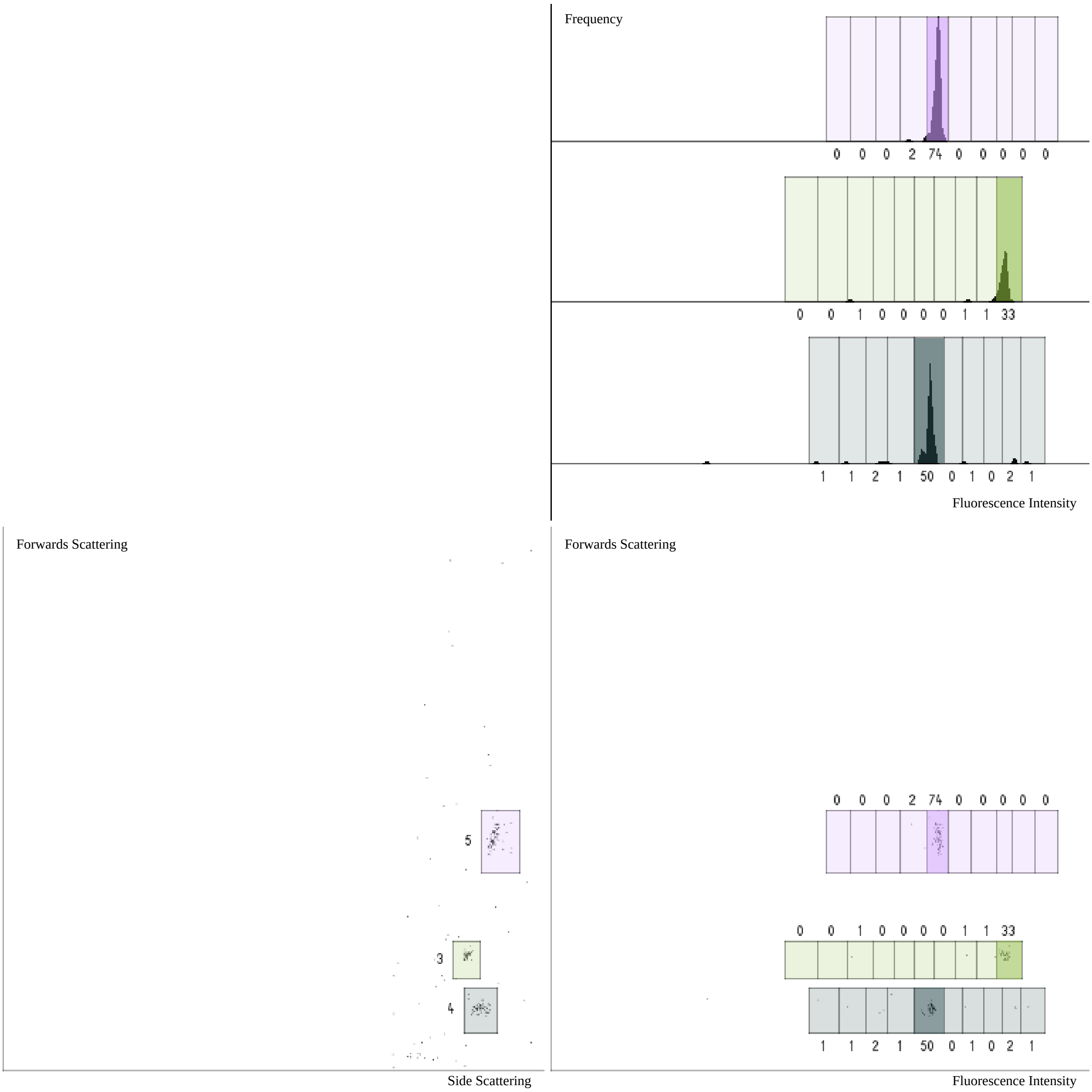

ANNEX 3: TAG DECONVOLUTION - BEAD 47

Passes flow sorting criteria: Yes

Passes tag deconvolution criteria: No

Included in protocol analysis: No

Protocol: N/A

Filename: Bin1_plateA0_G5.fcs

Split 1: Petrol shading

Split 2: Green shading

Split 3: Violet shading

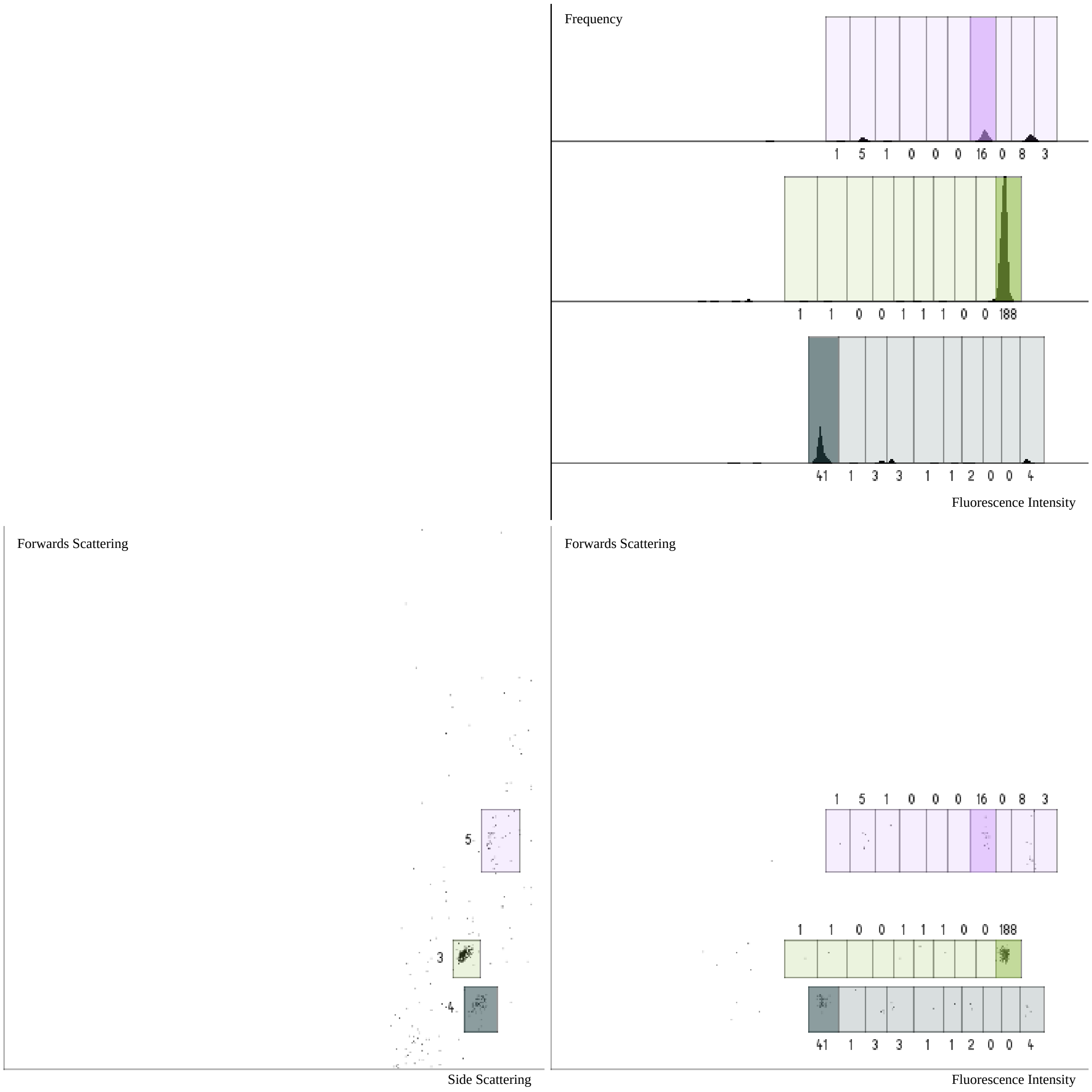

ANNEX 3: TAG DECONVOLUTION - BEAD 48

Passes flow sorting criteria: Yes

Passes tag deconvolution criteria: Yes

Included in protocol analysis: Yes

Protocol: 3, 1, 10, 1

Filename: Bin1_plateA0_G6.fcs

Split 1: Petrol shading

Split 2: Green shading

Split 3: Violet shading

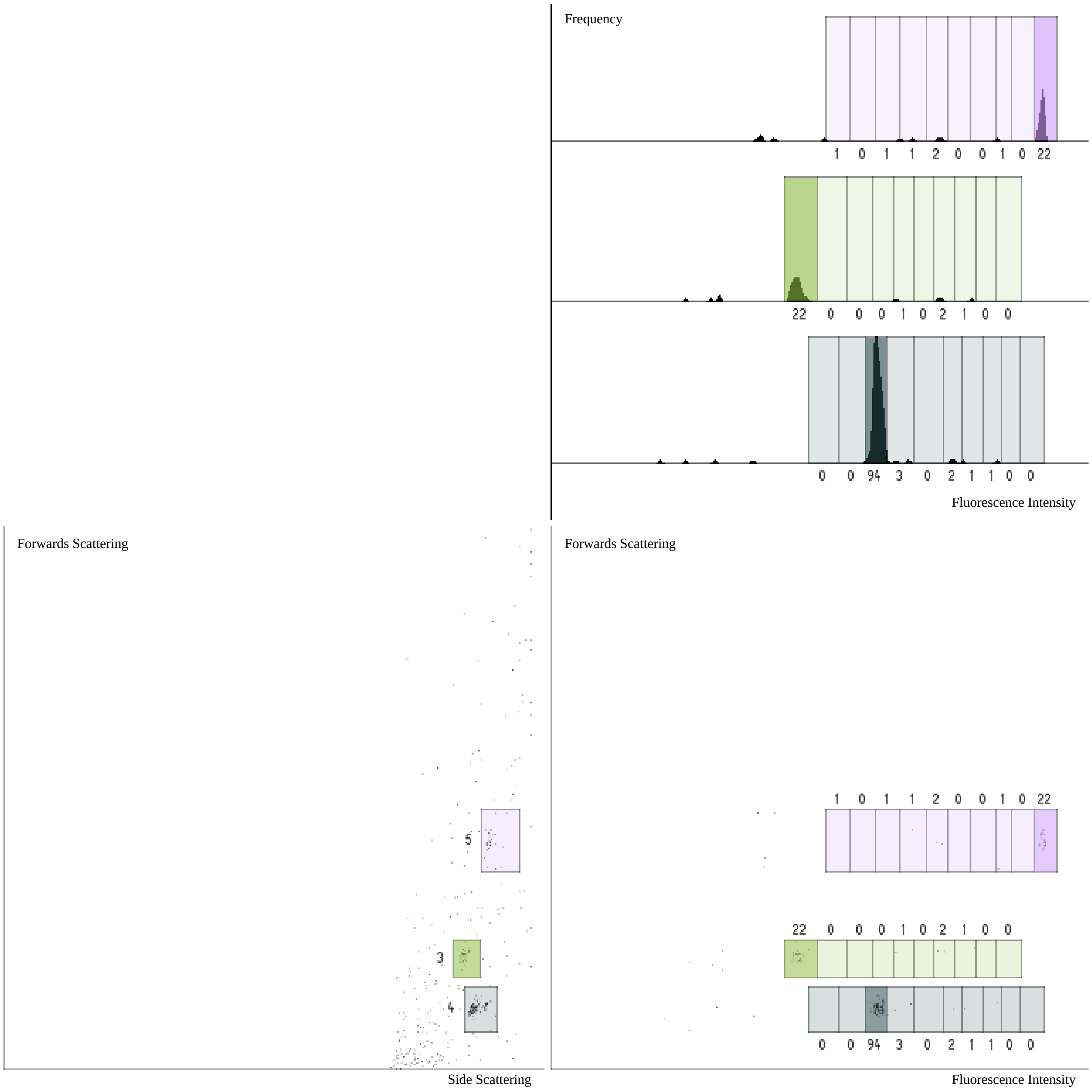

ANNEX 3: TAG DECONVOLUTION - BEAD 49

Passes flow sorting criteria: Yes

Passes tag deconvolution criteria: No

Included in protocol analysis: No

Protocol: N/A

Filename: Bin1_plateA0_H4.fcs

Split 1: Petrol shading

Split 2: Green shading

Split 3: Violet shading

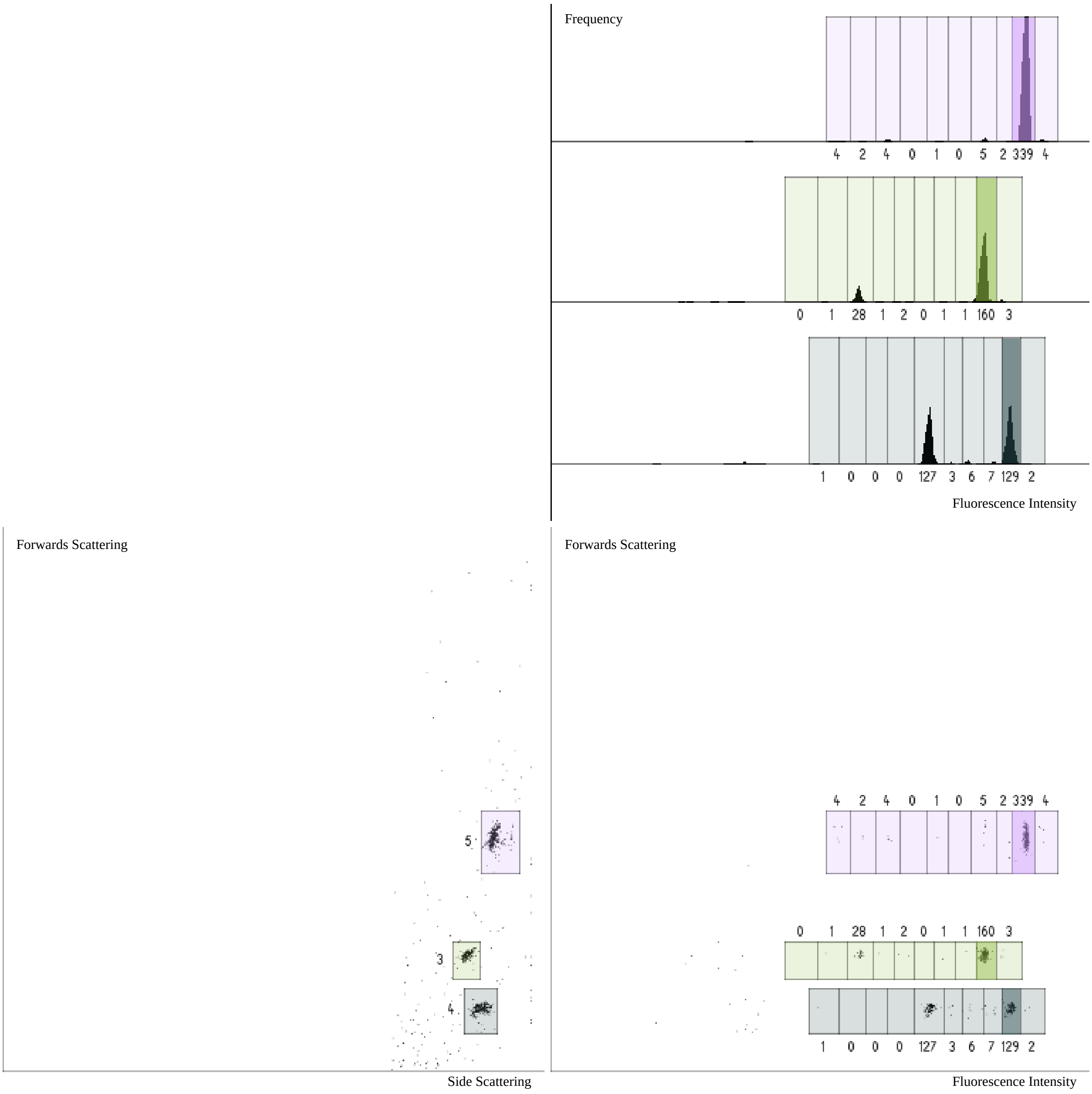

ANNEX 3: TAG DECONVOLUTION - BEAD 50

Passes flow sorting criteria: Yes

Passes tag deconvolution criteria: Yes

Included in protocol analysis: Yes

Protocol: 9, 5, 8, 1

Filename: Bin1_plateA0_H5.fcs

Split 1: Petrol shading

Split 2: Green shading

Split 3: Violet shading

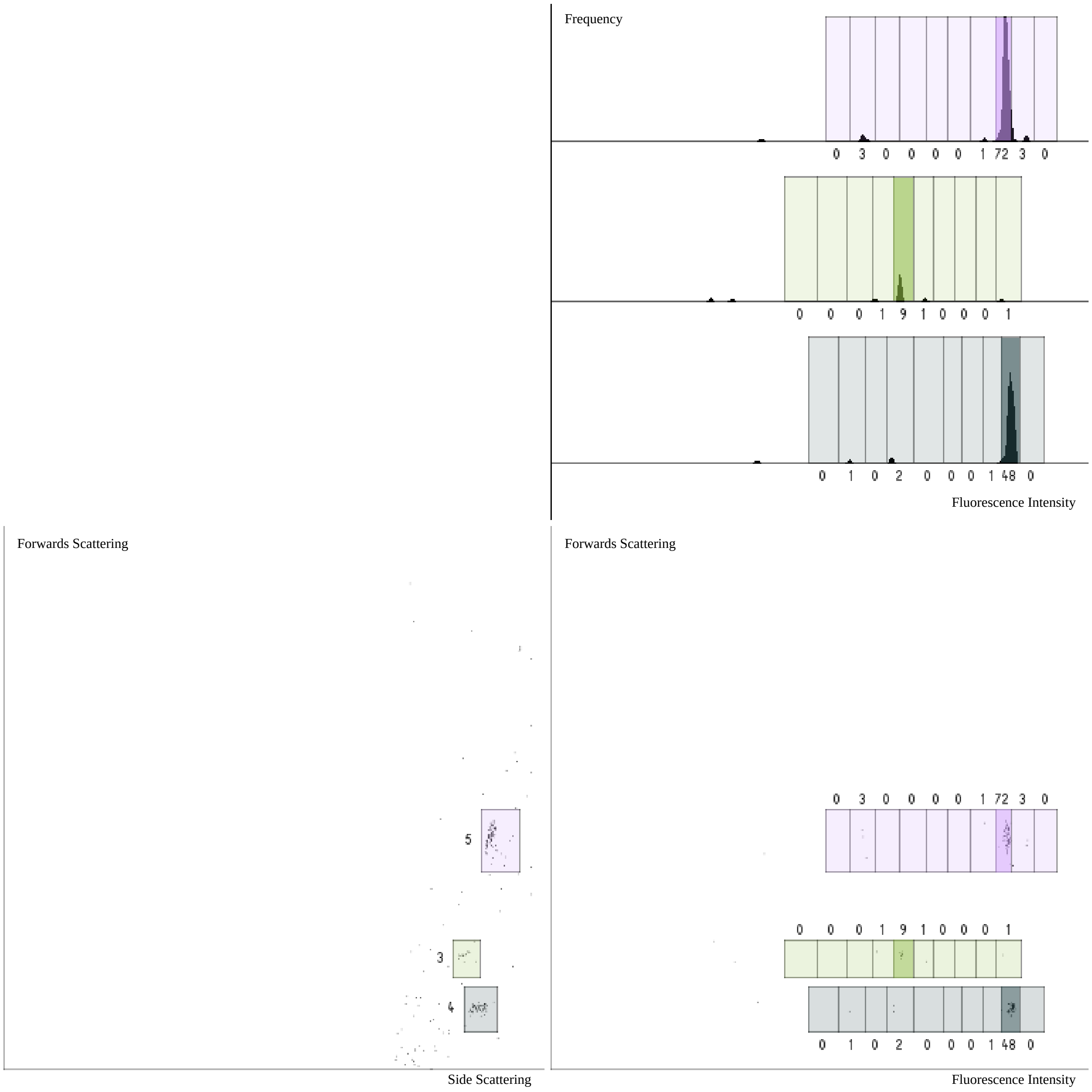

ANNEX 3: TAG DECONVOLUTION - BEAD 51

Passes flow sorting criteria: Yes

Passes tag deconvolution criteria: Yes

Included in protocol analysis: Yes

Protocol: 9, 1, 7, 1

Filename: Bin1_plateA0_H10.fcs

Split 1: Petrol shading

Split 2: Green shading

Split 3: Violet shading

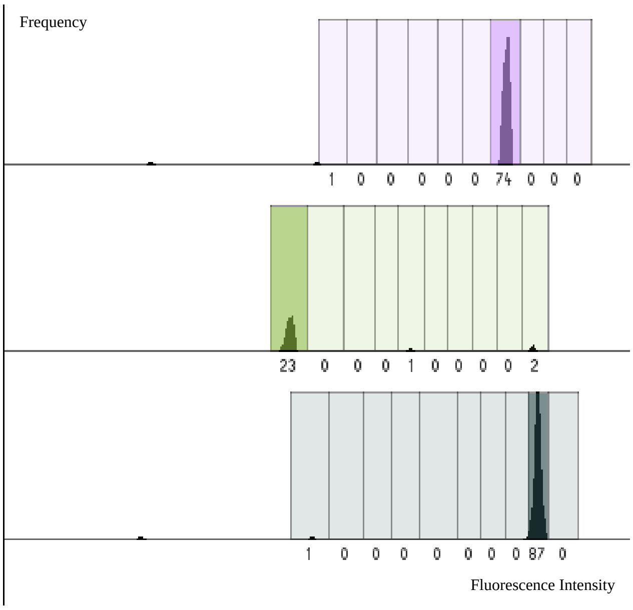

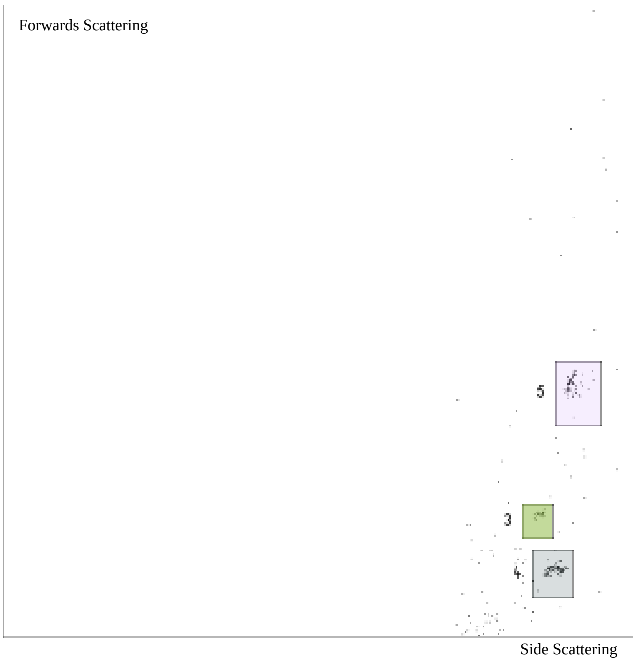

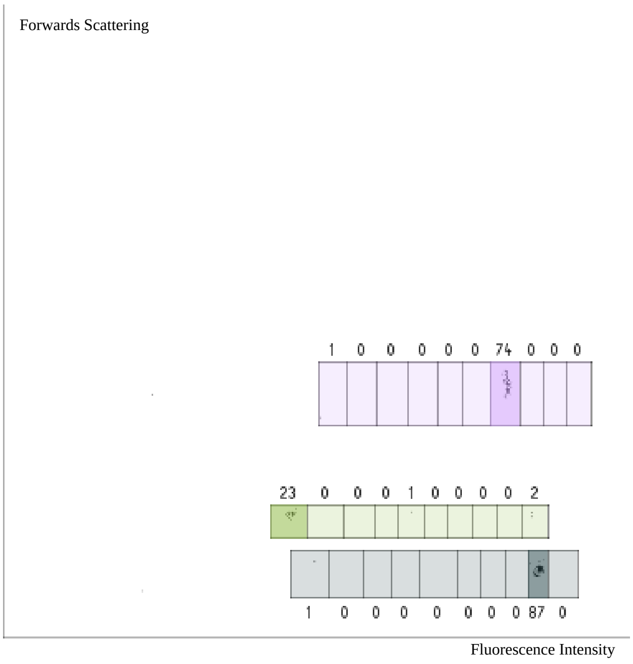

ANNEX 3: TAG DECONVOLUTION - BEAD 52

Passes flow sorting criteria: Yes

Passes tag deconvolution criteria: Yes

Included in protocol analysis: Yes

Protocol: 6, 4, 7, 1

Filename: Bin1_plateA0_A8.fcs

Split 1: Petrol shading

Split 2: Green shading

Split 3: Violet shading

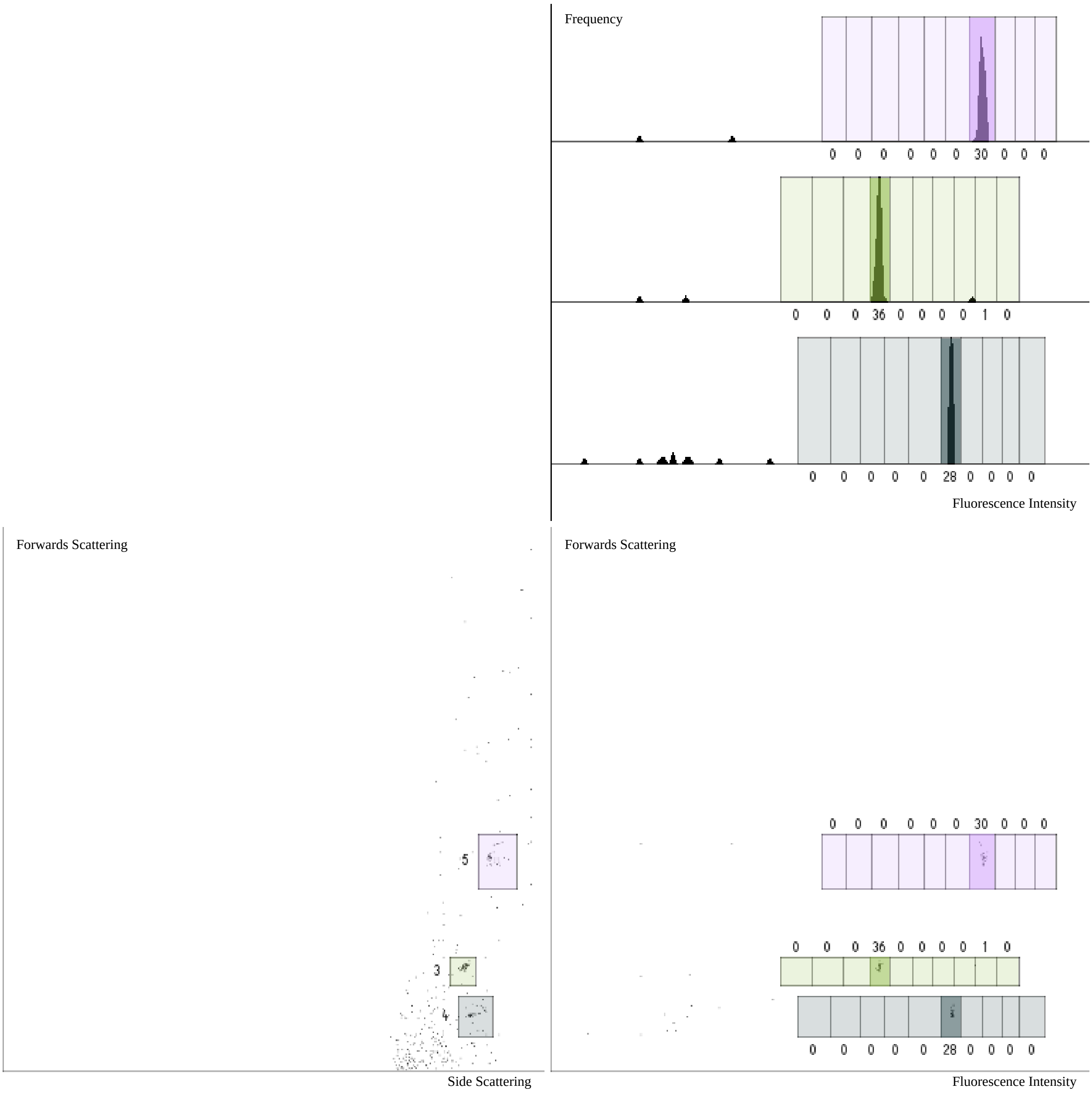

ANNEX 3: TAG DECONVOLUTION - BEAD 53

Passes flow sorting criteria: Yes

Passes tag deconvolution criteria: Yes

Included in protocol analysis: Yes

Protocol: 9, 3, 7, 1

Filename: Bin1_plateA0_A9.fcs

Split 1: Petrol shading

Split 2: Green shading

Split 3: Violet shading

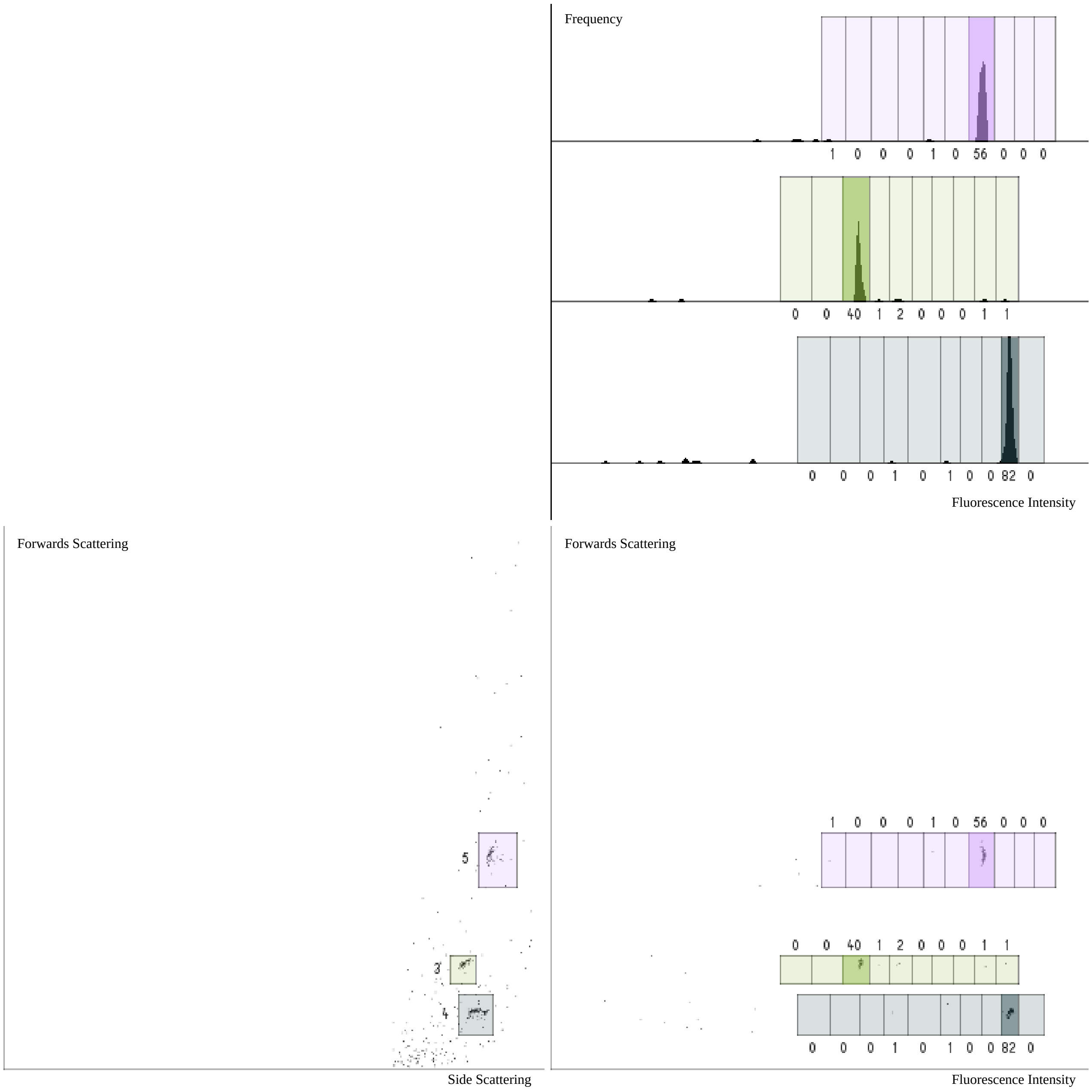

ANNEX 3: TAG DECONVOLUTION - BEAD 54

Passes flow sorting criteria: Yes

Passes tag deconvolution criteria: Yes

Included in protocol analysis: Yes

Protocol: 2, 5, 3, 1

Filename: Bin1_plateA0_A10.fcs

Split 1: Petrol shading

Split 2: Green shading

Split 3: Violet shading

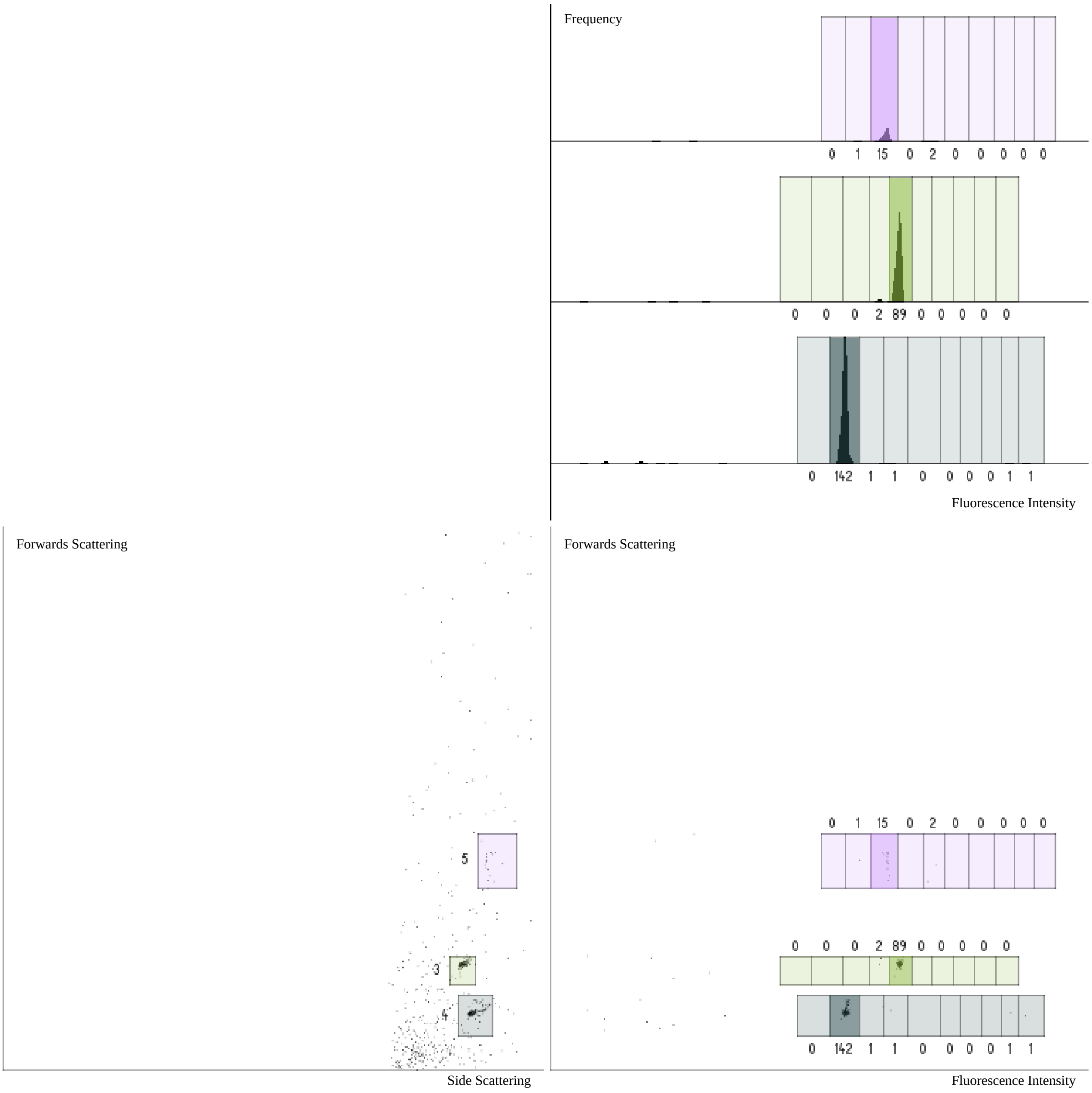

ANNEX 3: TAG DECONVOLUTION - BEAD 55

Passes flow sorting criteria: Yes

Passes tag deconvolution criteria: Yes

Included in protocol analysis: Yes

Protocol: 9, 6, 7, 1

Filename: Bin1_plateA0_B8.fcs

Split 1: Petrol shading

Split 2: Green shading

Split 3: Violet shading

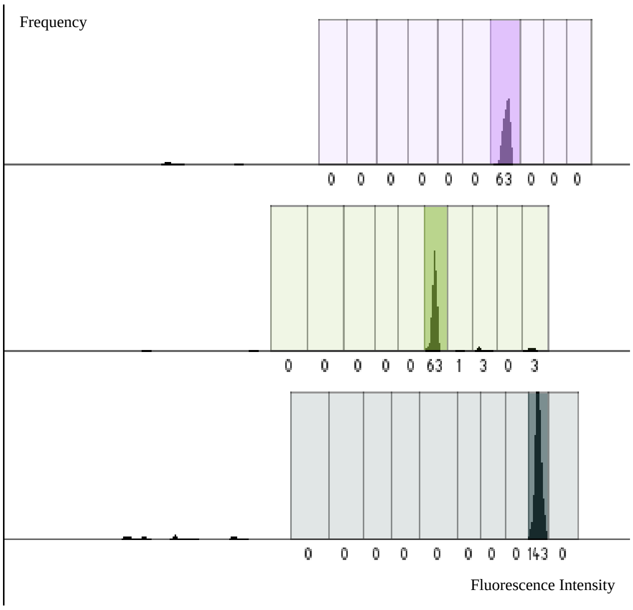

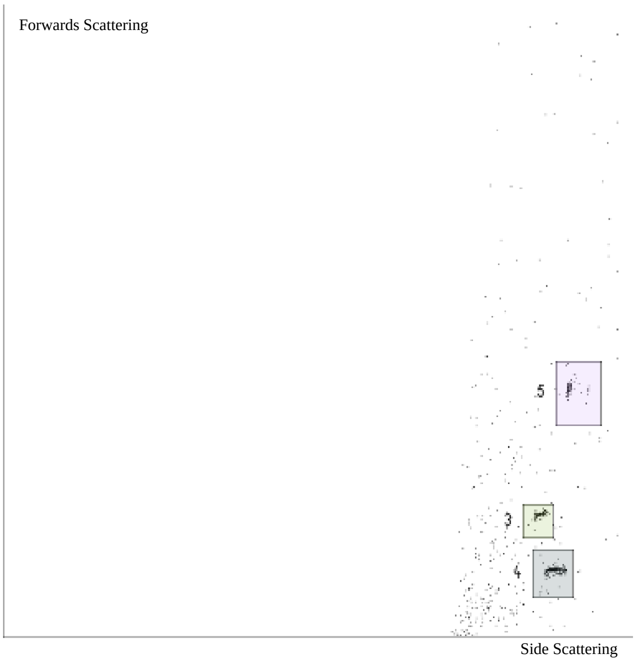

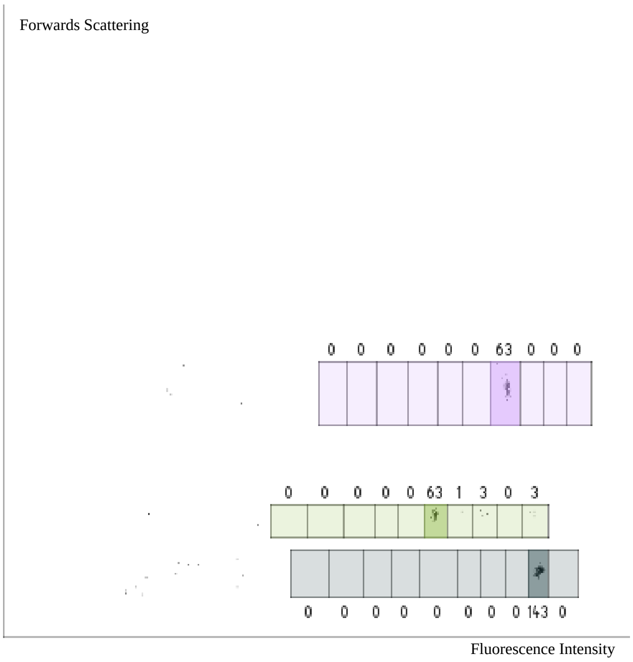

ANNEX 3: TAG DECONVOLUTION - BEAD 56

Passes flow sorting criteria: Yes

Passes tag deconvolution criteria: Yes

Included in protocol analysis: Yes

Protocol: 7, 6, 10, 1

Filename: Bin1_plateA0_B9.fcs

Split 1: Petrol shading

Split 2: Green shading

Split 3: Violet shading

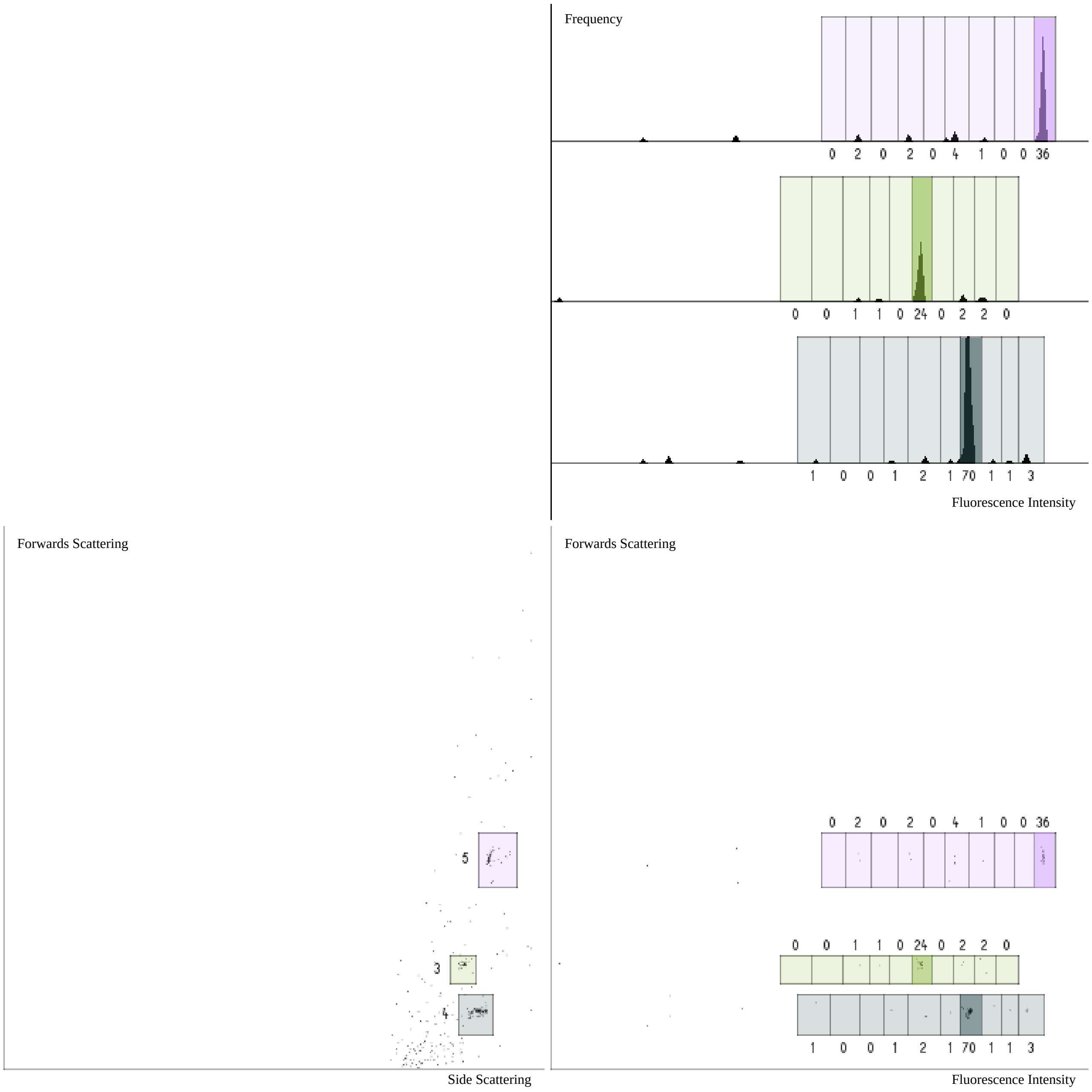

ANNEX 3: TAG DECONVOLUTION - BEAD 57

Passes flow sorting criteria: Yes

Passes tag deconvolution criteria: Yes

Included in protocol analysis: Yes

Protocol: 6, 9, 6, 1

Filename: Bin1_plateA0_B10.fcs

Split 1: Petrol shading

Split 2: Green shading

Split 3: Violet shading

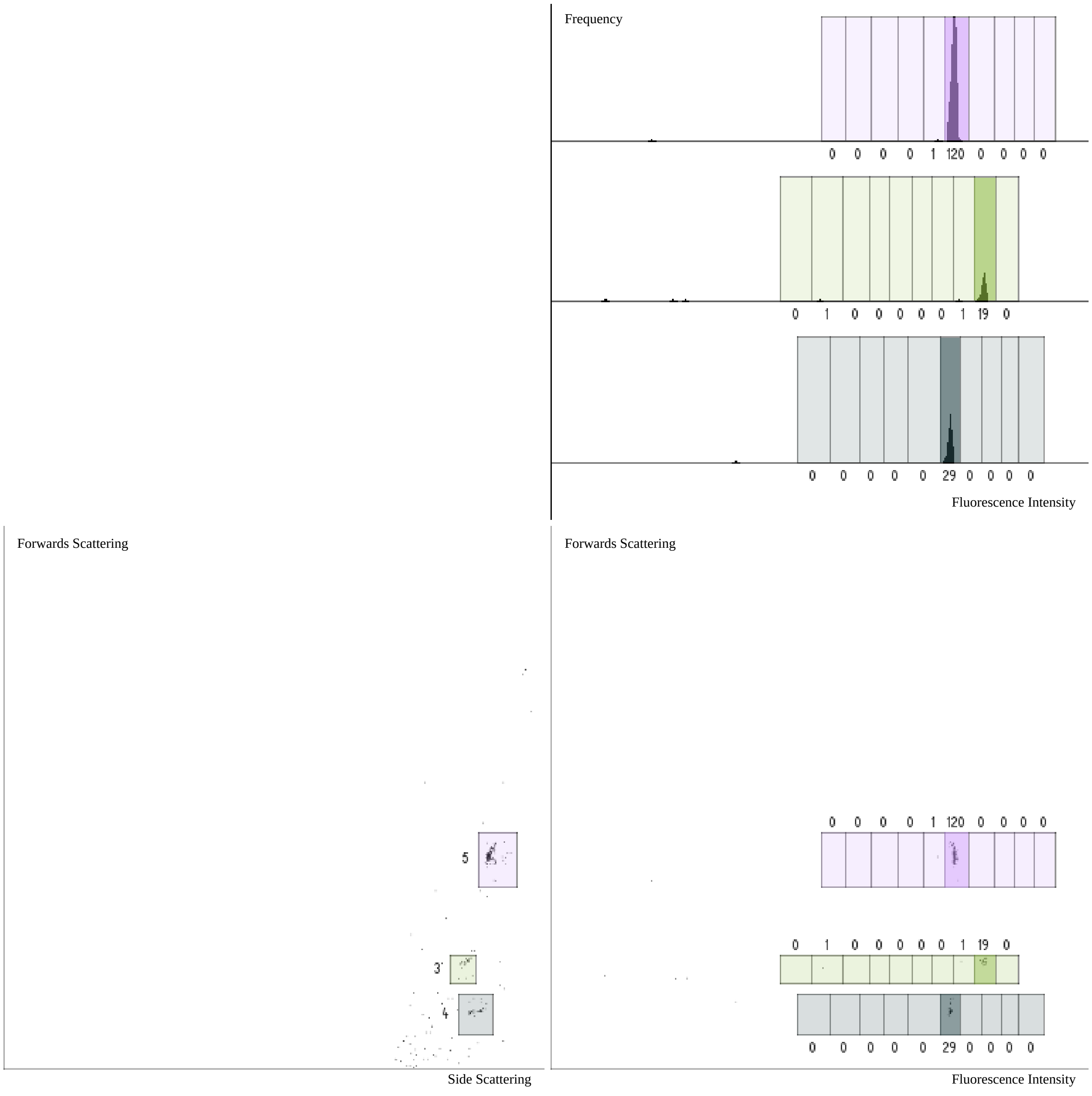

ANNEX 3: TAG DECONVOLUTION - BEAD 58

Passes flow sorting criteria: Yes

Passes tag deconvolution criteria: Yes

Included in protocol analysis: Yes

Protocol: 7, 7, 2, 1

Filename: Bin1_plateA0_C8.fcs

Split 1: Petrol shading

Split 2: Green shading

Split 3: Violet shading

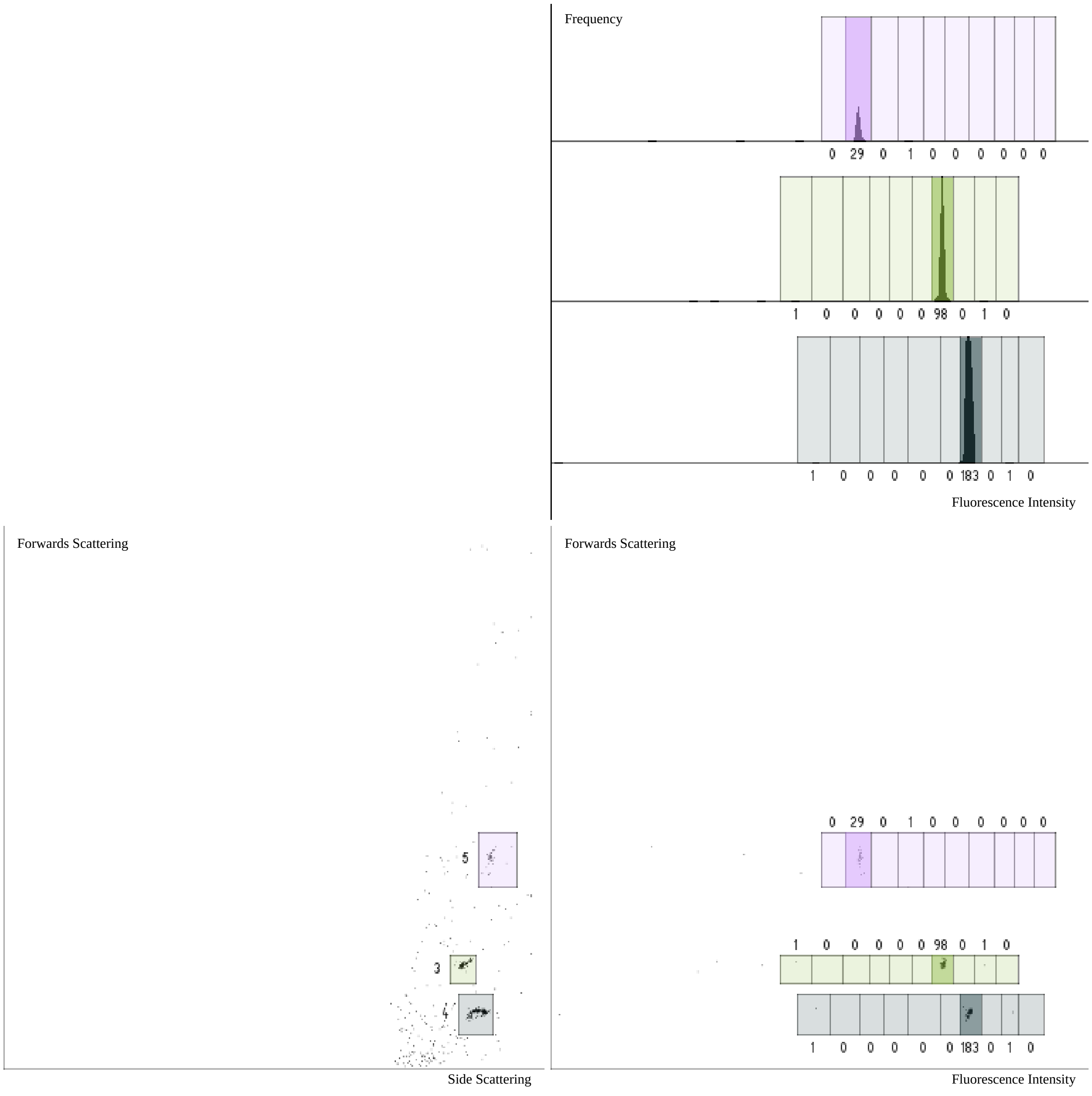

ANNEX 3: TAG DECONVOLUTION - BEAD 59

Passes flow sorting criteria: Yes

Passes tag deconvolution criteria: No

Included in protocol analysis: No

Protocol: N/A

Filename: Bin1_plateA0_C9.fcs

Split 1: Petrol shading

Split 2: Green shading

Split 3: Violet shading

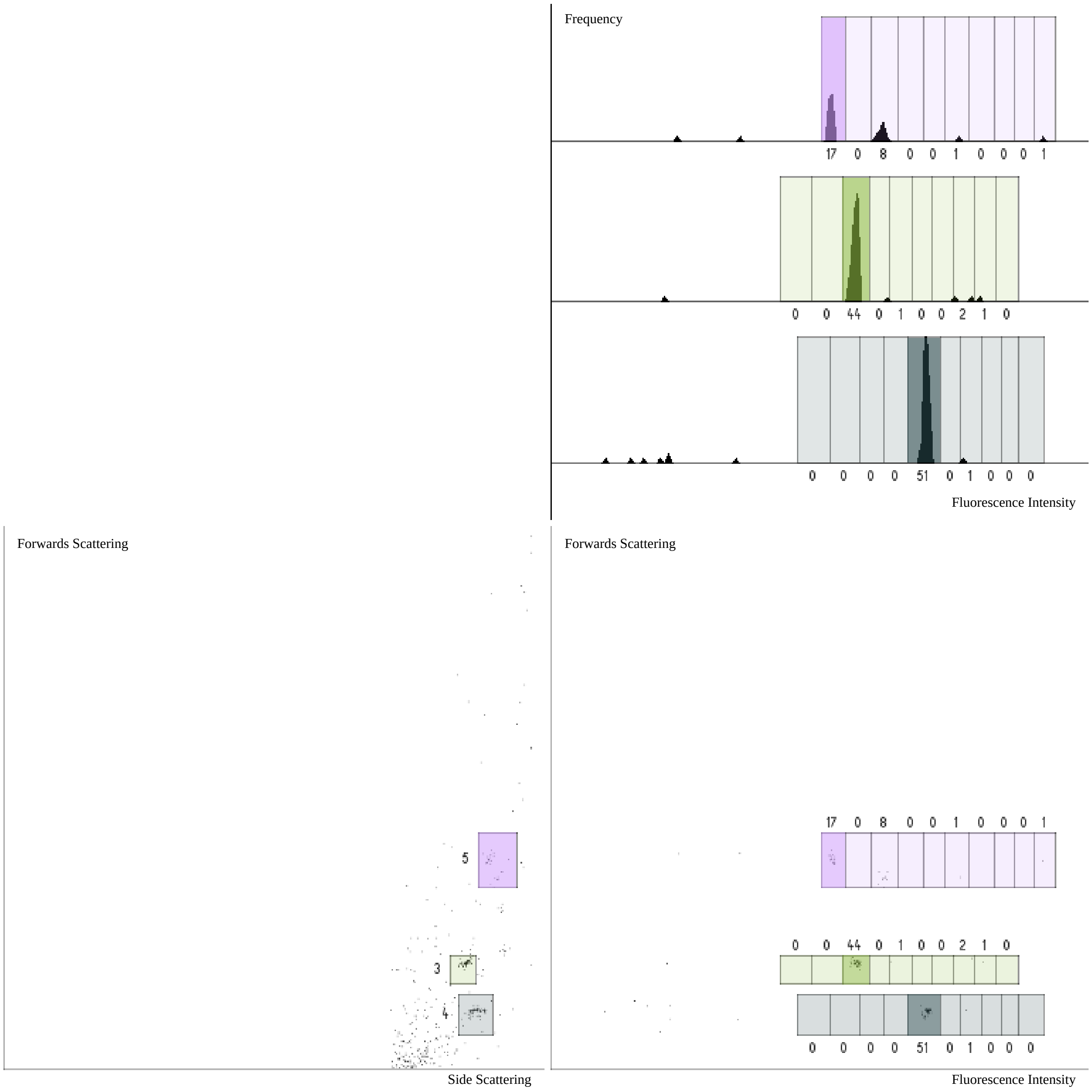

ANNEX 3: TAG DECONVOLUTION - BEAD 60

Passes flow sorting criteria: Yes

Passes tag deconvolution criteria: Yes

Included in protocol analysis: Yes

Protocol: 10, 2, 3, 1

Filename: Bin1_plateA0_C10.fcs

Split 1: Petrol shading

Split 2: Green shading

Split 3: Violet shading

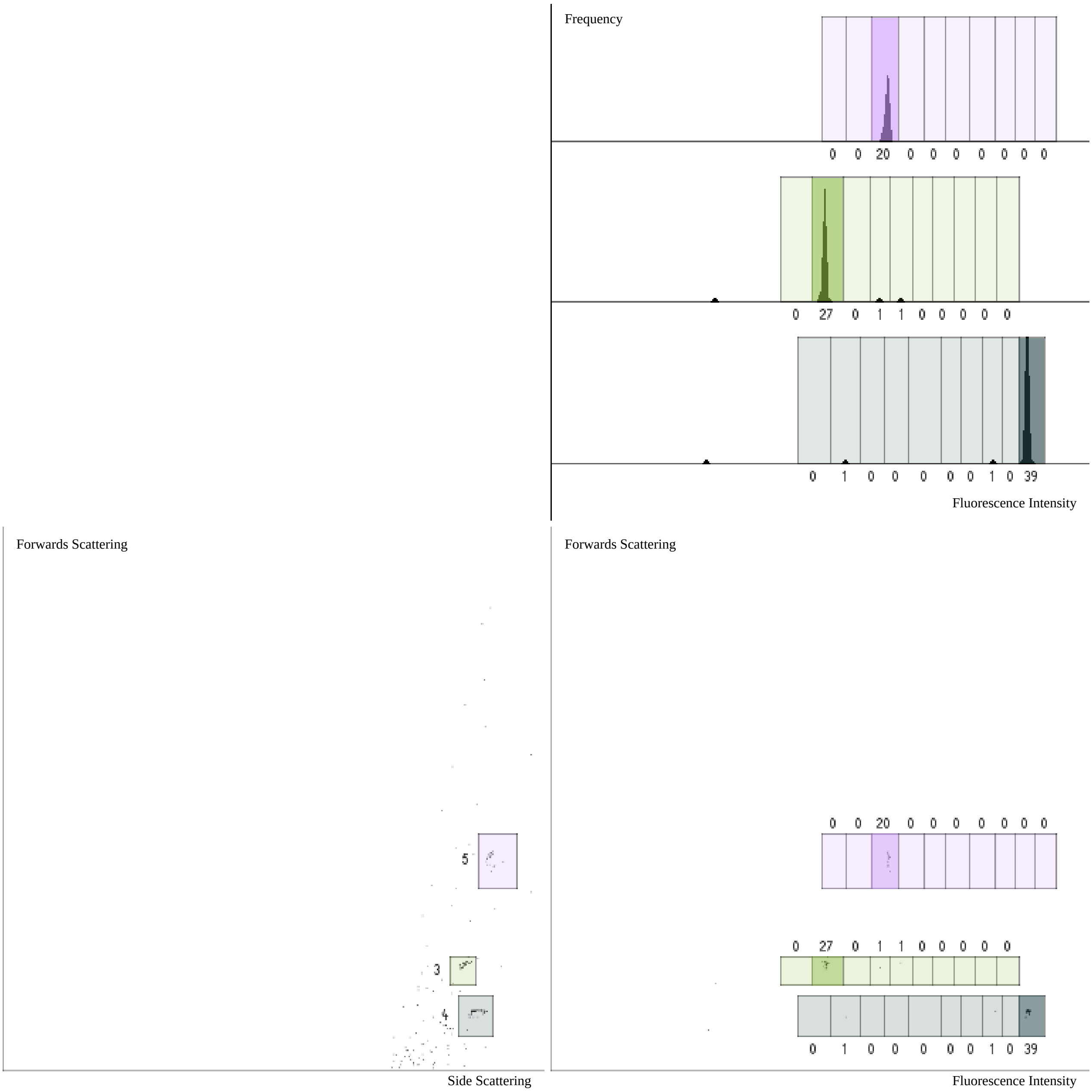

ANNEX 3: TAG DECONVOLUTION - BEAD 61

Passes flow sorting criteria: Yes

Passes tag deconvolution criteria: Yes

Included in protocol analysis: Yes

Protocol: 9, 2, 6, 1

Filename: Bin1_plateA0_D8.fcs

Split 1: Petrol shading

Split 2: Green shading

Split 3: Violet shading

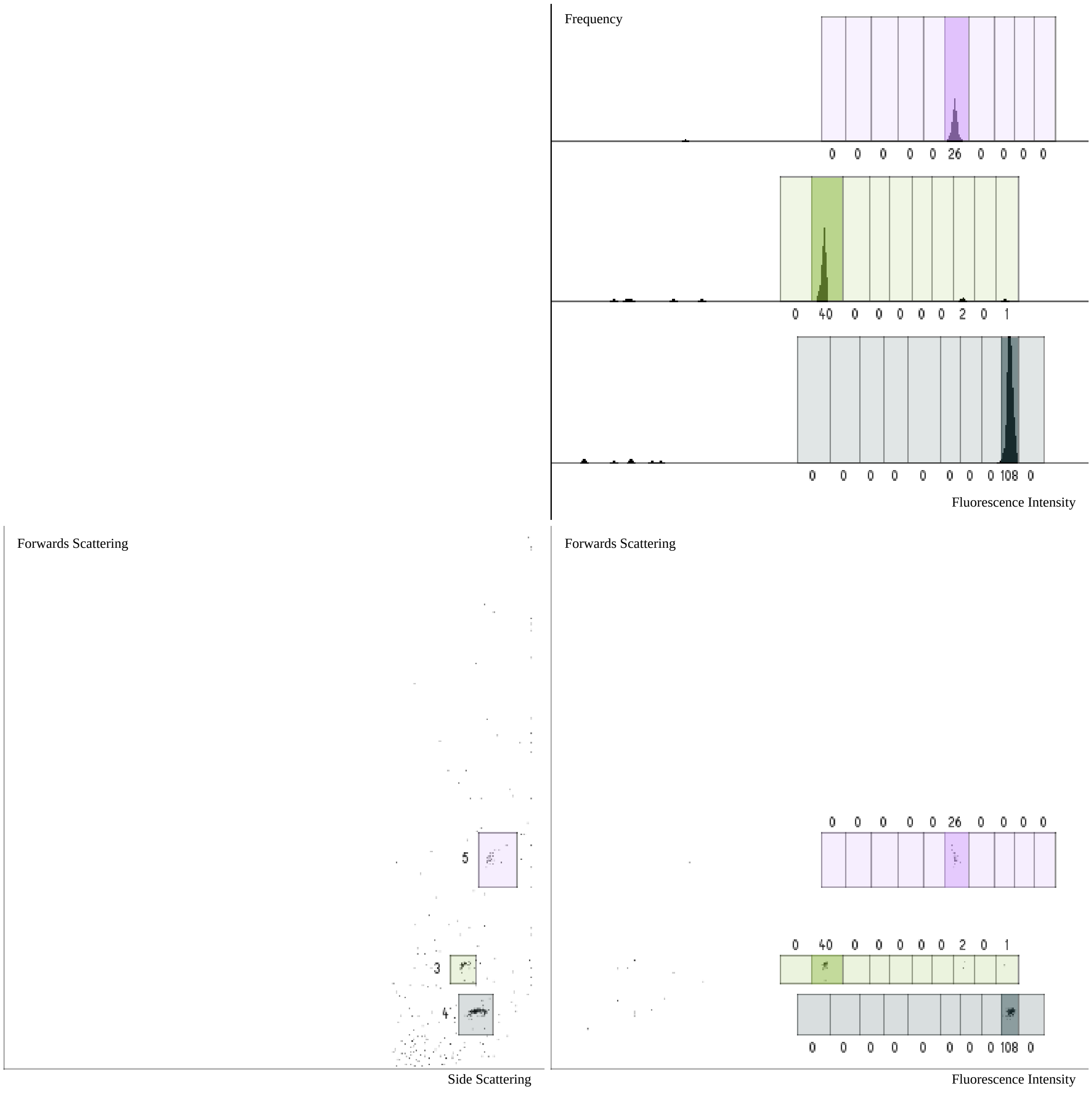

ANNEX 3: TAG DECONVOLUTION - BEAD 62

Passes flow sorting criteria: Yes

Passes tag deconvolution criteria: No

Included in protocol analysis: No

Protocol: N/A

Filename: Bin1_plateA0_D9.fcs

Split 1: Petrol shading

Split 2: Green shading

Split 3: Violet shading

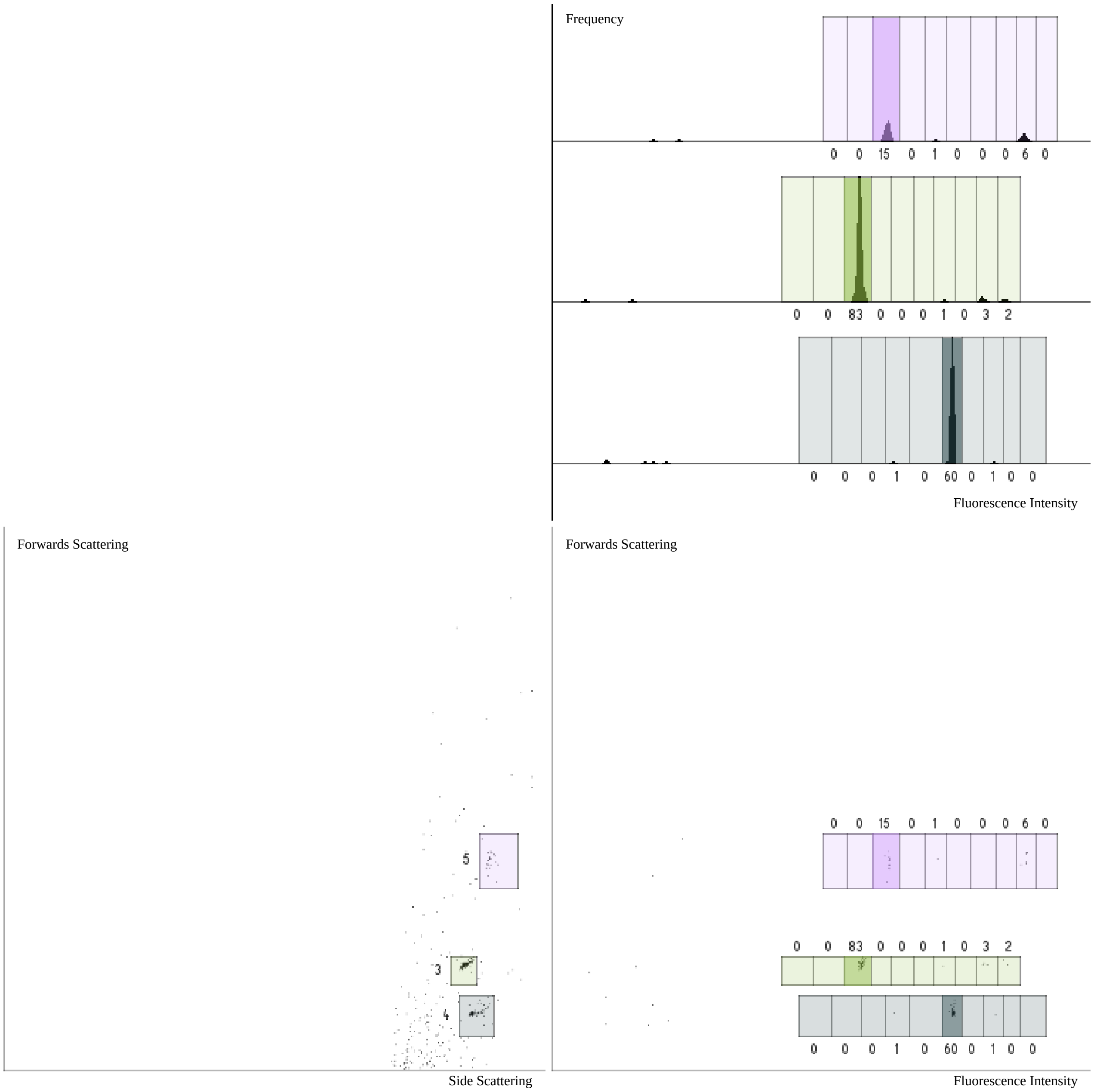

ANNEX 3: TAG DECONVOLUTION - BEAD 63

Passes flow sorting criteria: Yes

Passes tag deconvolution criteria: Yes

Included in protocol analysis: Yes

Protocol: 3, 8, 2, 1

Filename: Bin1_plateA0_D10.fcs

Split 1: Petrol shading

Split 2: Green shading

Split 3: Violet shading

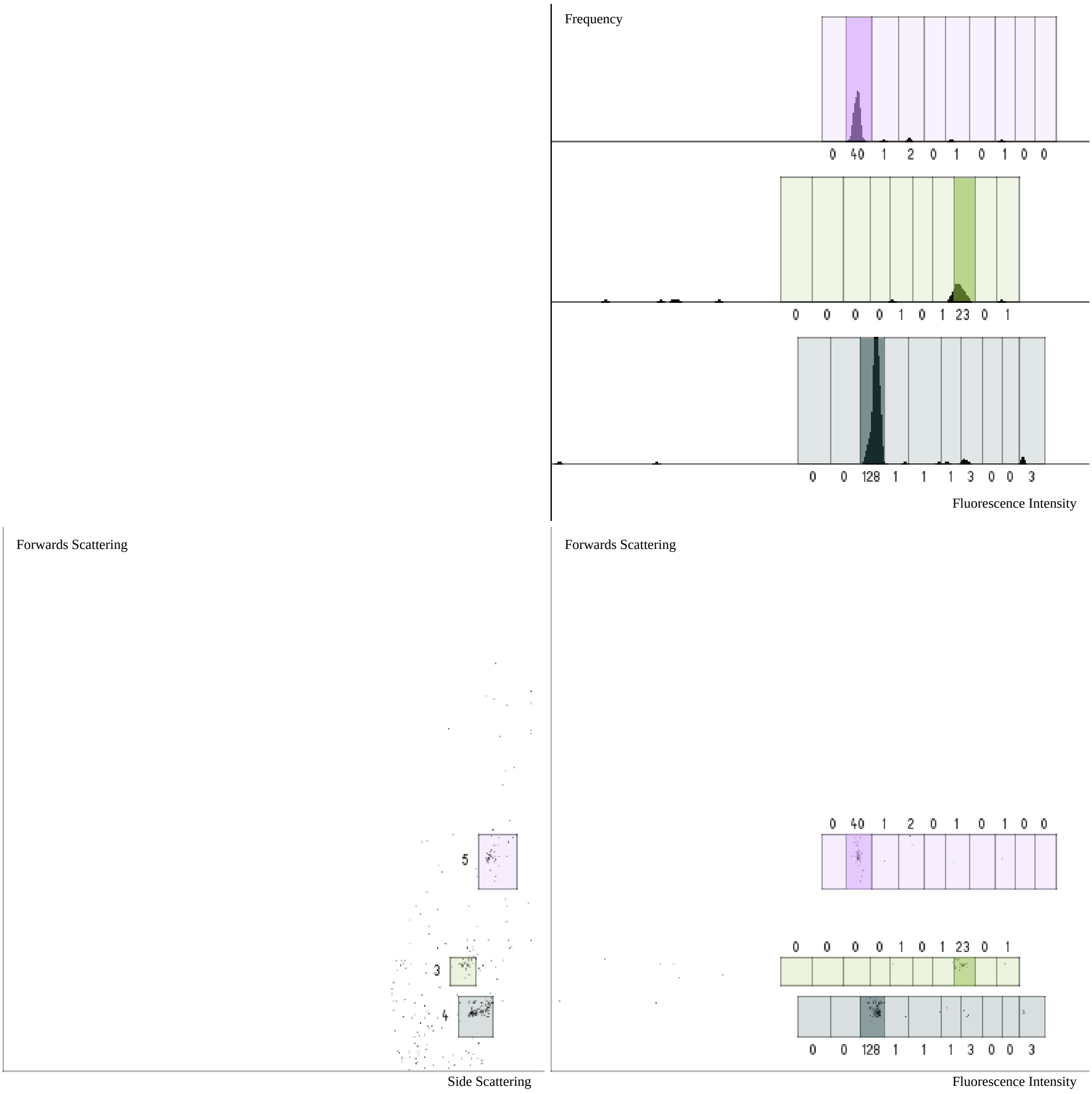

ANNEX 3: TAG DECONVOLUTION - BEAD 64

Passes flow sorting criteria: Yes

Passes tag deconvolution criteria: No

Included in protocol analysis: No

Protocol: N/A

Filename: Bin1_plateA0_E7.fcs

Split 1: Petrol shading

Split 2: Green shading

Split 3: Violet shading

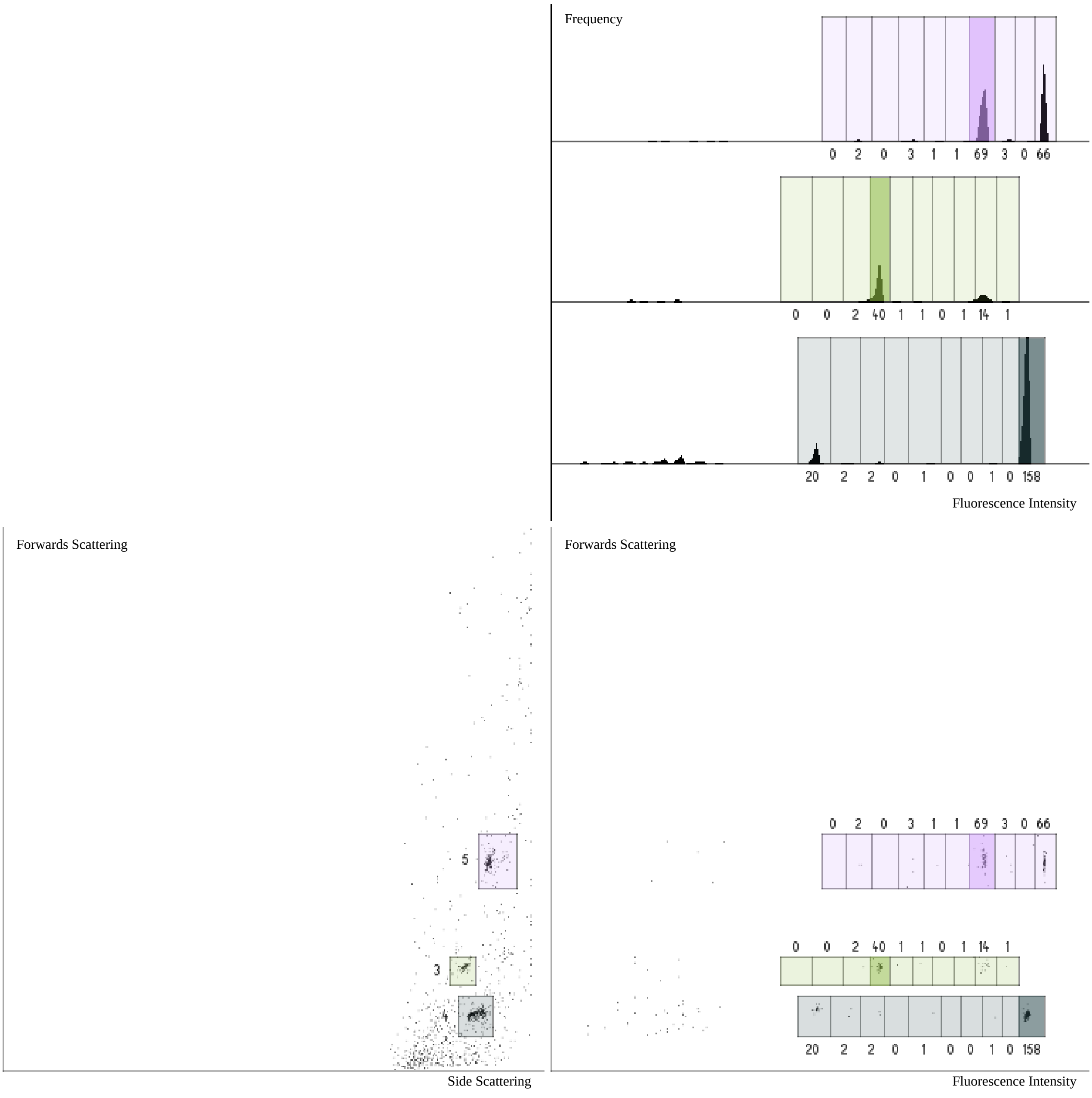

ANNEX 3: TAG DECONVOLUTION - BEAD 65

Passes flow sorting criteria: Yes

Passes tag deconvolution criteria: Yes

Included in protocol analysis: Yes

Protocol: 5, 7, 10, 1

Filename: Bin1_plateA0_E8.fcs

Split 1: Petrol shading

Split 2: Green shading

Split 3: Violet shading

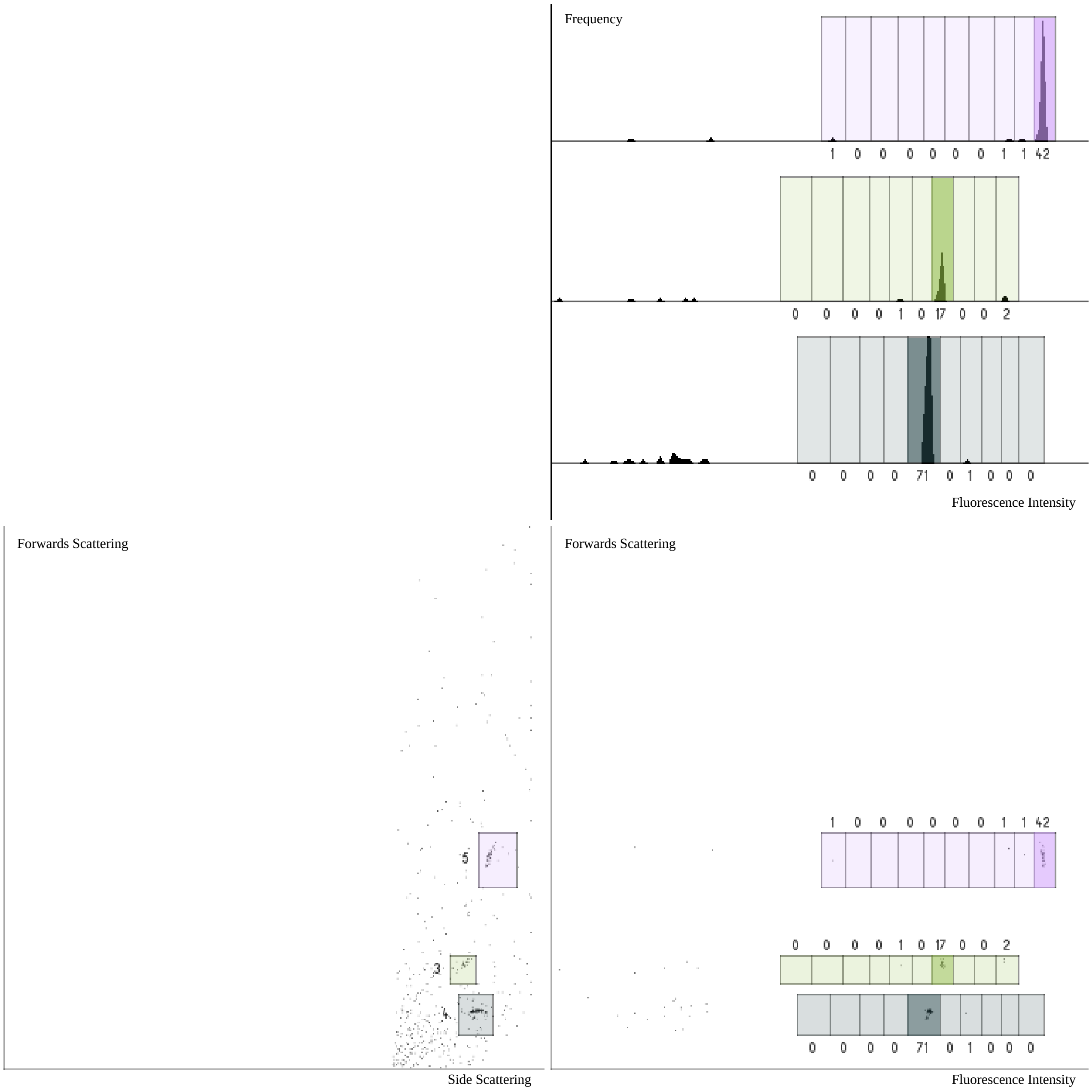

ANNEX 3: TAG DECONVOLUTION - BEAD 66

Passes flow sorting criteria: Yes

Passes tag deconvolution criteria: Yes

Included in protocol analysis: Yes

Protocol: 1, 5, 1, 1

Filename: Bin1_plateA0_E10.fcs

Split 1: Petrol shading

Split 2: Green shading

Split 3: Violet shading

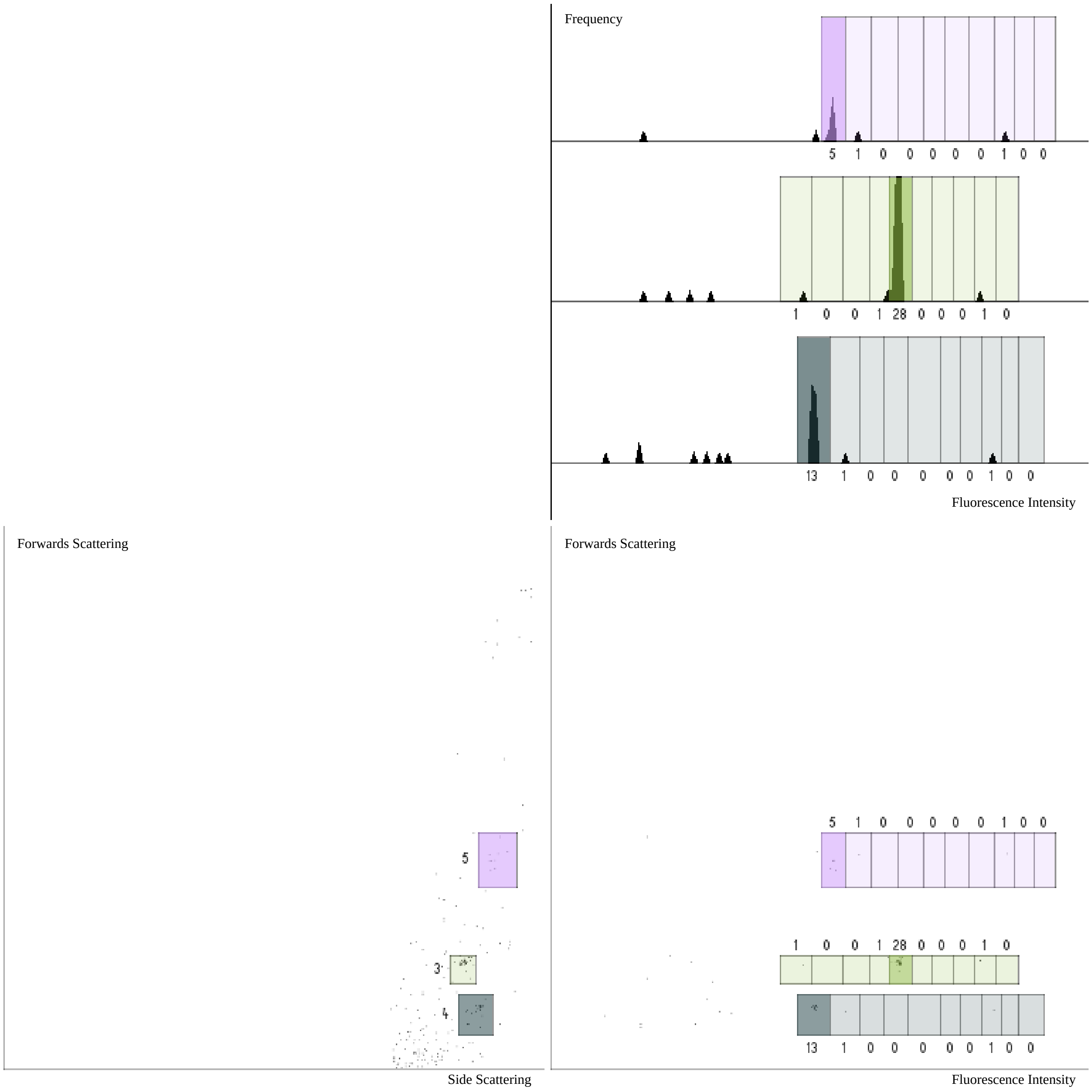

ANNEX 3: TAG DECONVOLUTION - BEAD 67

Passes flow sorting criteria: Yes

Passes tag deconvolution criteria: Yes

Included in protocol analysis: Yes

Protocol: 2, 10, 1, 1

Filename: Bin1_plateA0_F7.fcs

Split 1: Petrol shading

Split 2: Green shading

Split 3: Violet shading

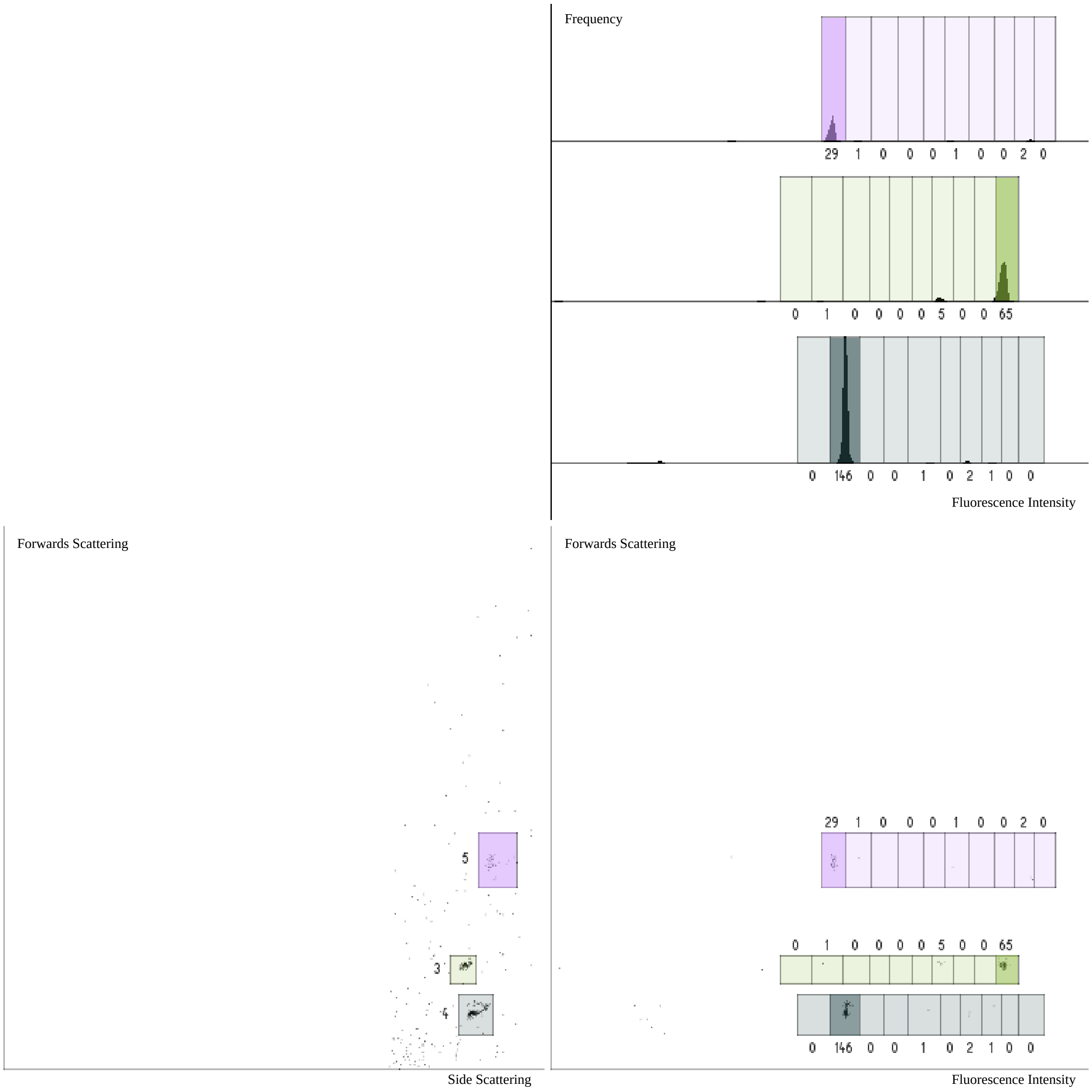

ANNEX 3: TAG DECONVOLUTION - BEAD 68

Passes flow sorting criteria: Yes

Passes tag deconvolution criteria: Yes

Included in protocol analysis: Yes

Protocol: 3, 9, 1, 1

Filename: Bin1_plateA0_F8.fcs

Split 1: Petrol shading

Split 2: Green shading

Split 3: Violet shading

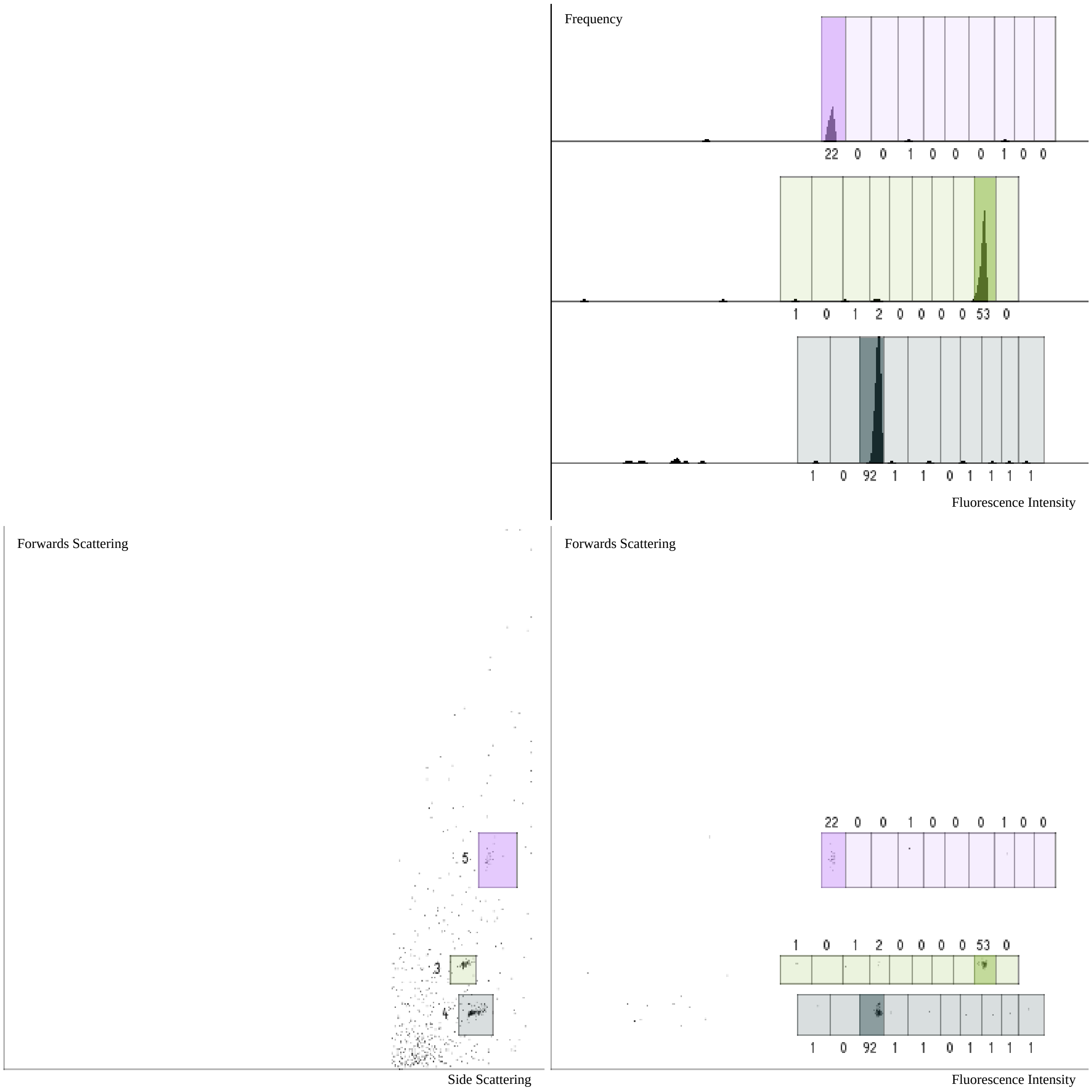

ANNEX 3: TAG DECONVOLUTION - BEAD 69

Passes flow sorting criteria: Yes

Passes tag deconvolution criteria: Yes

Included in protocol analysis: Yes

Protocol: 9, 10, 3, 1

Filename: Bin1_plateA0_F9.fcs

Split 1: Petrol shading

Split 2: Green shading

Split 3: Violet shading

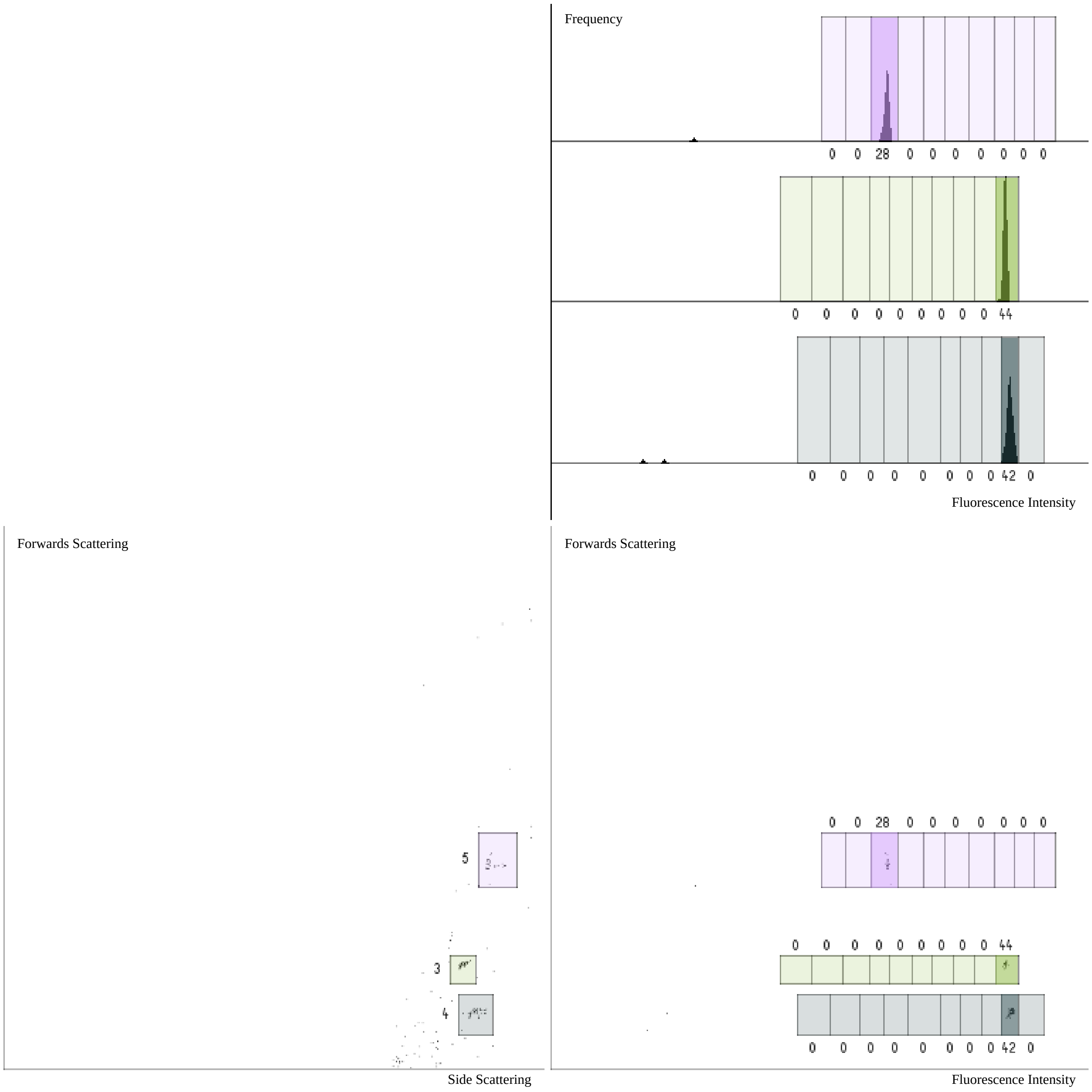

ANNEX 3: TAG DECONVOLUTION - BEAD 70

Passes flow sorting criteria: Yes

Passes tag deconvolution criteria: Yes

Included in protocol analysis: Yes

Protocol: 9, 9, 4, 1

Filename: Bin1_plateA0_F10.fcs

Split 1: Petrol shading

Split 2: Green shading

Split 3: Violet shading

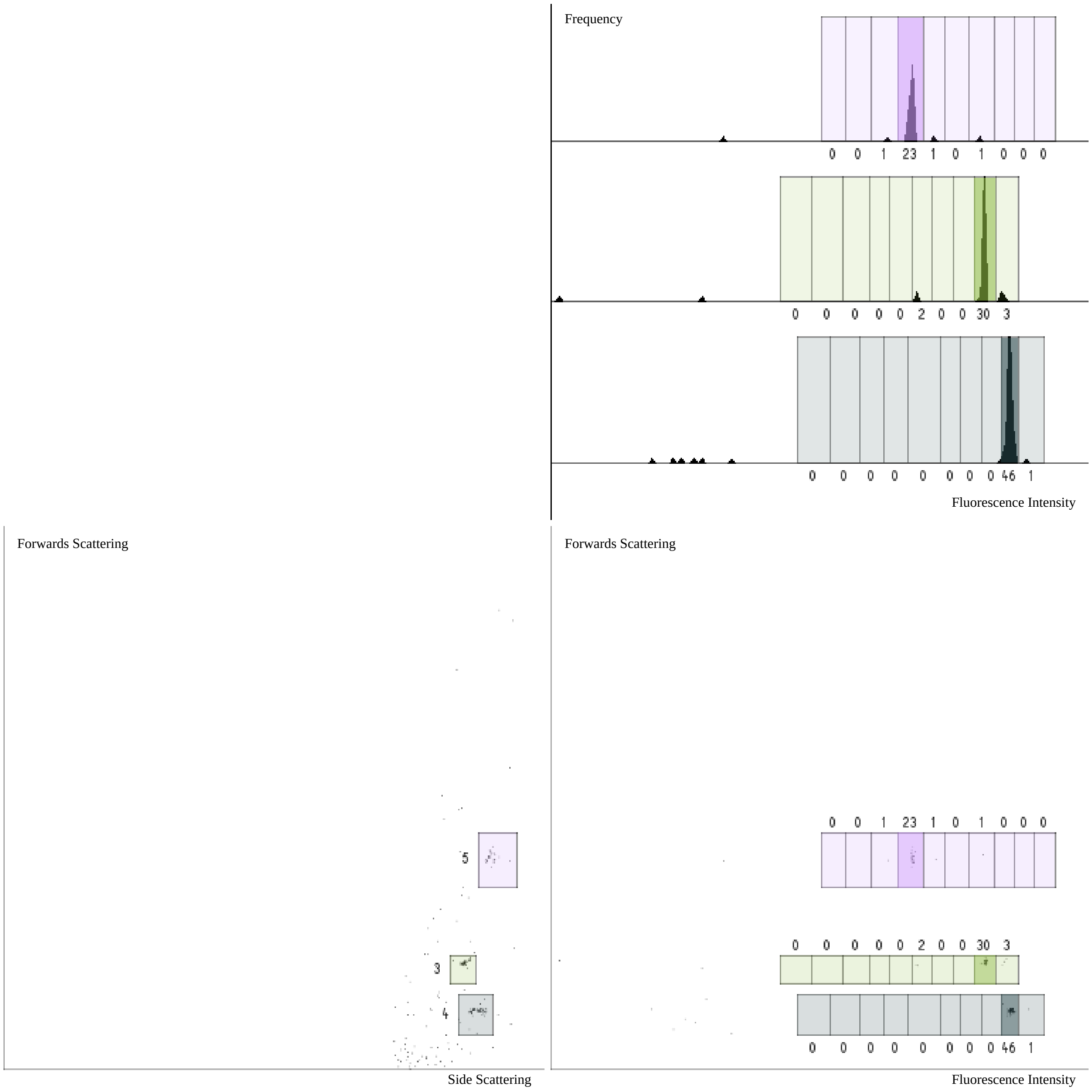

ANNEX 3: TAG DECONVOLUTION - BEAD 71

Passes flow sorting criteria: Yes

Passes tag deconvolution criteria: Yes

Included in protocol analysis: Yes

Protocol: 5, 9, 10, 1

Filename: Bin1_plateA0_G7.fcs

Split 1: Petrol shading

Split 2: Green shading

Split 3: Violet shading

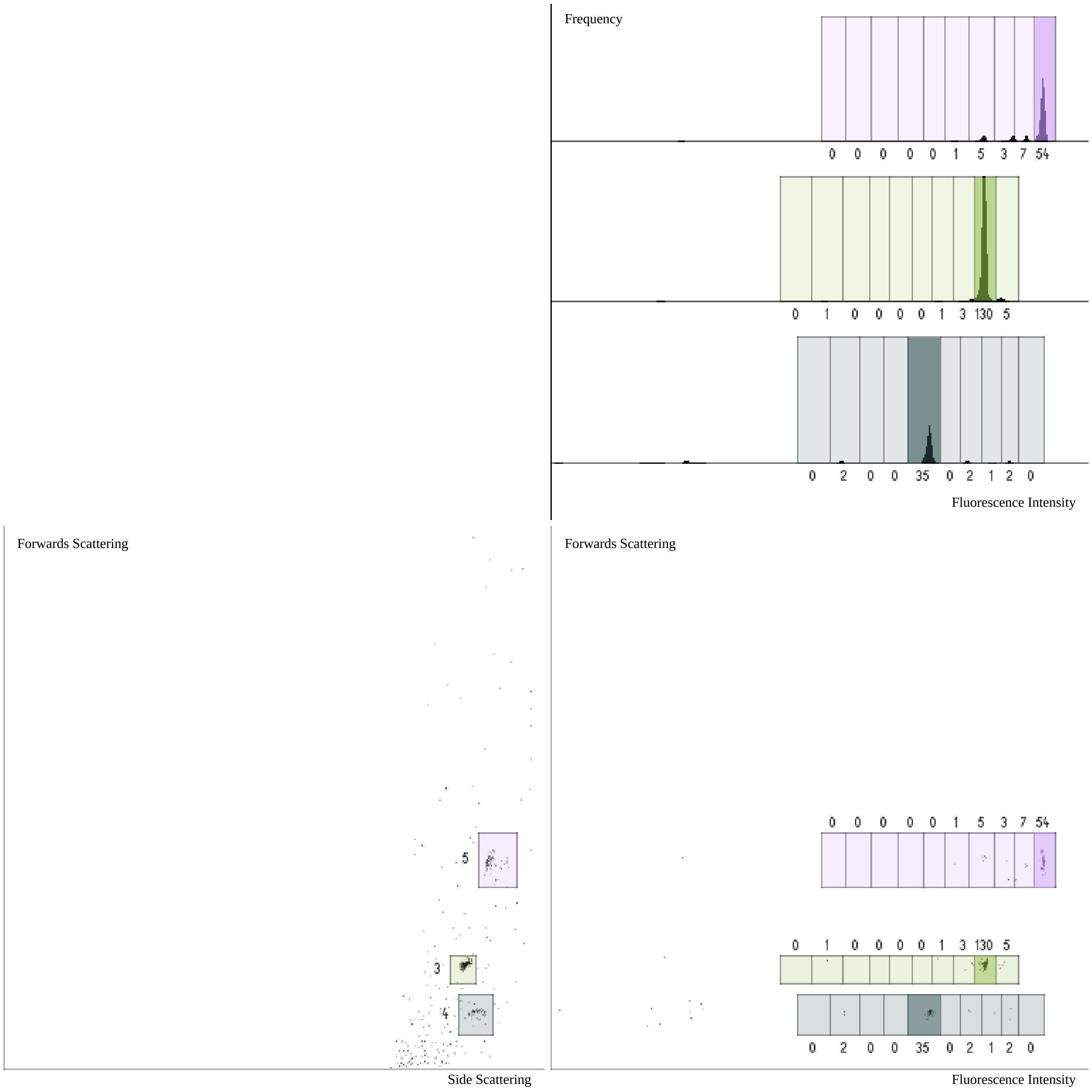

ANNEX 3: TAG DECONVOLUTION - BEAD 72

Passes flow sorting criteria: Yes

Passes tag deconvolution criteria: No

Included in protocol analysis: No

Protocol: N/A

Filename: Bin1_plateA0_G8.fcs

Split 1: Petrol shading

Split 2: Green shading

Split 3: Violet shading

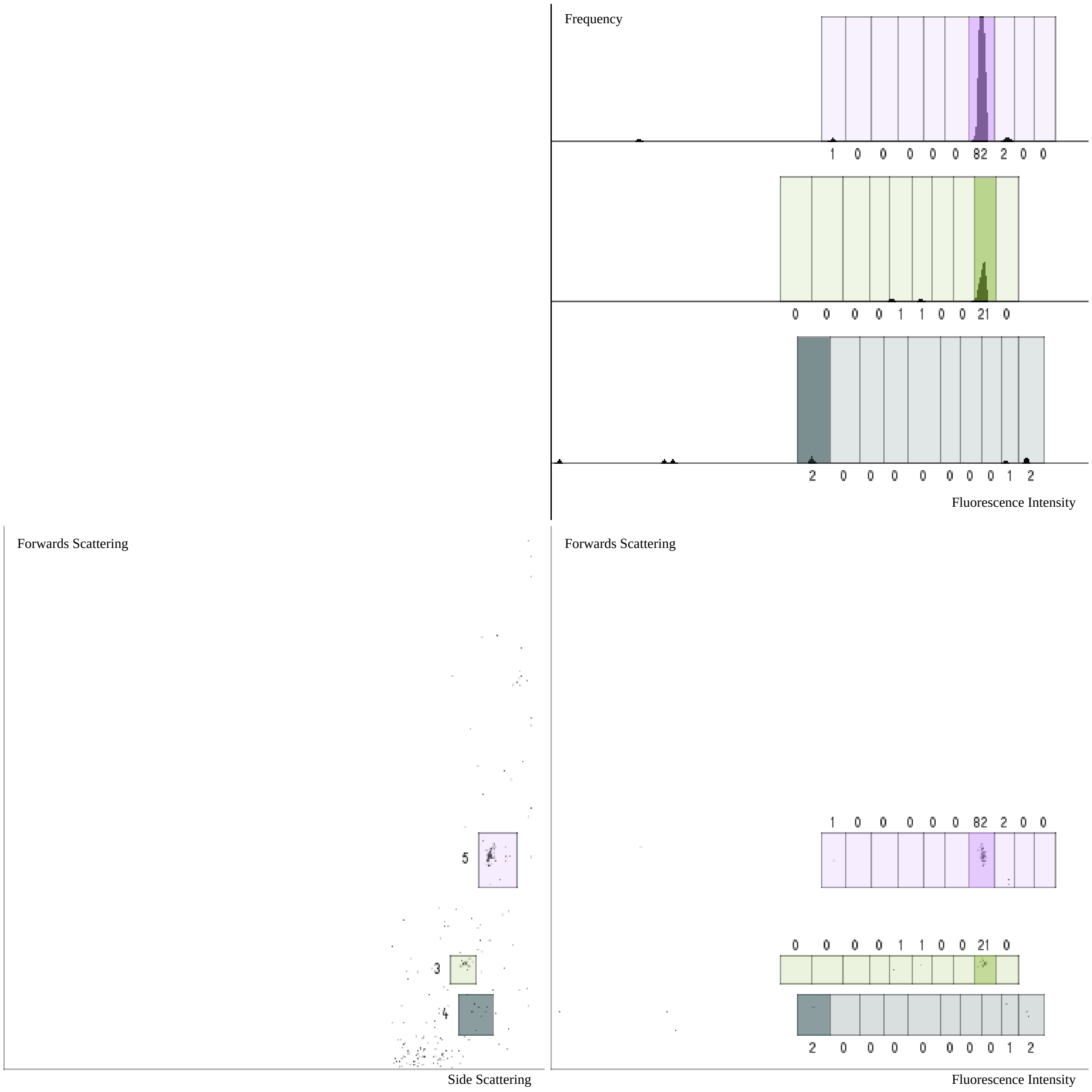

ANNEX 3: TAG DECONVOLUTION - BEAD 73

Passes flow sorting criteria: Yes

Passes tag deconvolution criteria: Yes

Included in protocol analysis: Yes

Protocol: 10, 2, 6, 1

Filename: Bin1_plateA0_G9.fcs

Split 1: Petrol shading

Split 2: Green shading

Split 3: Violet shading

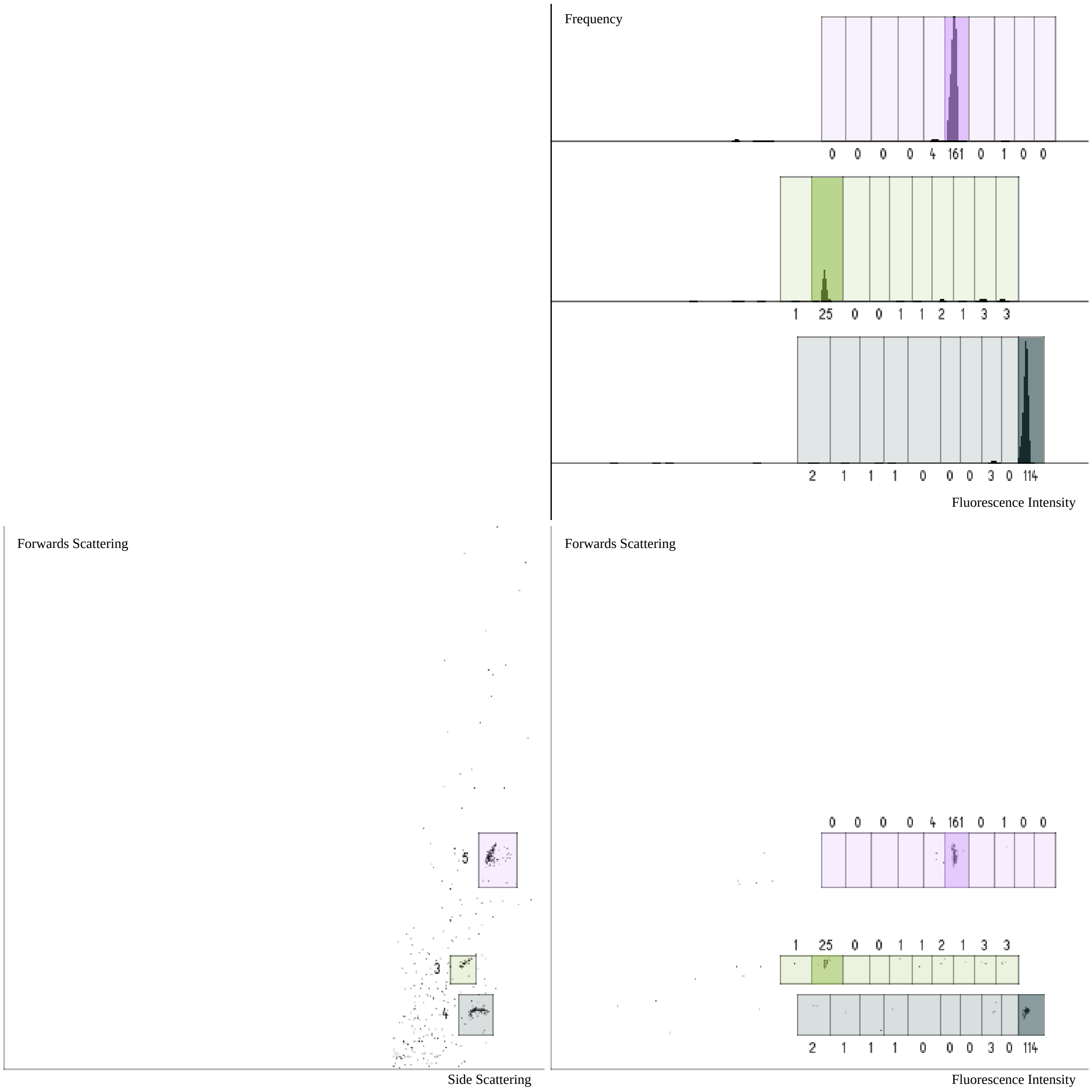

ANNEX 3: TAG DECONVOLUTION - BEAD 74

Passes flow sorting criteria: Yes

Passes tag deconvolution criteria: No

Included in protocol analysis: No

Protocol: N/A

Filename: Bin1_plateA0_G10.fcs

Split 1: Petrol shading

Split 2: Green shading

Split 3: Violet shading

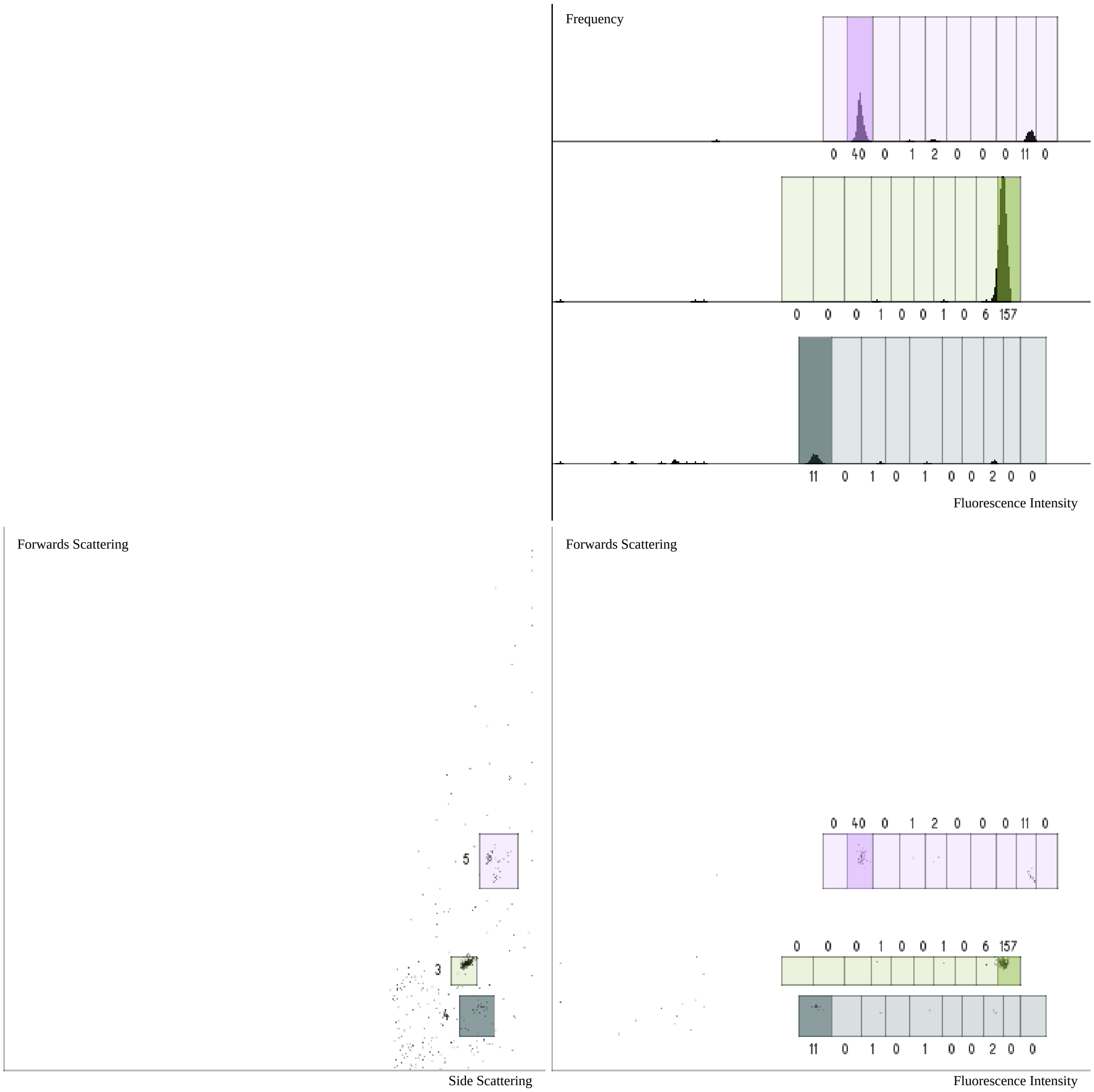

ANNEX 3: TAG DECONVOLUTION - BEAD 75

Passes flow sorting criteria: Yes

Passes tag deconvolution criteria: Yes

Included in protocol analysis: Yes

Protocol: 9, 9, 9, 1

Filename: Bin1_plateA0_H7.fcs

Split 1: Petrol shading

Split 2: Green shading

Split 3: Violet shading

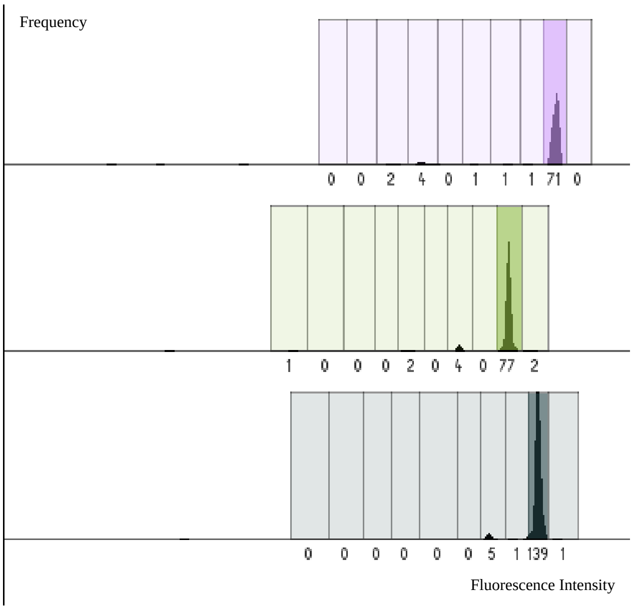

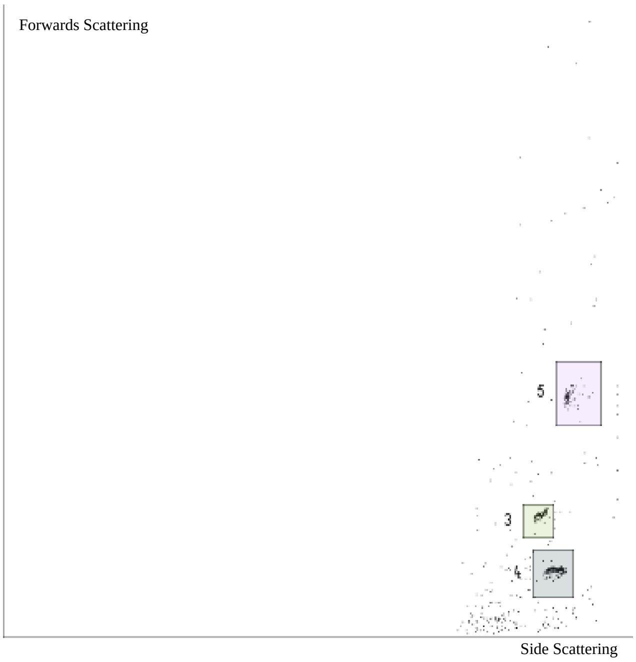

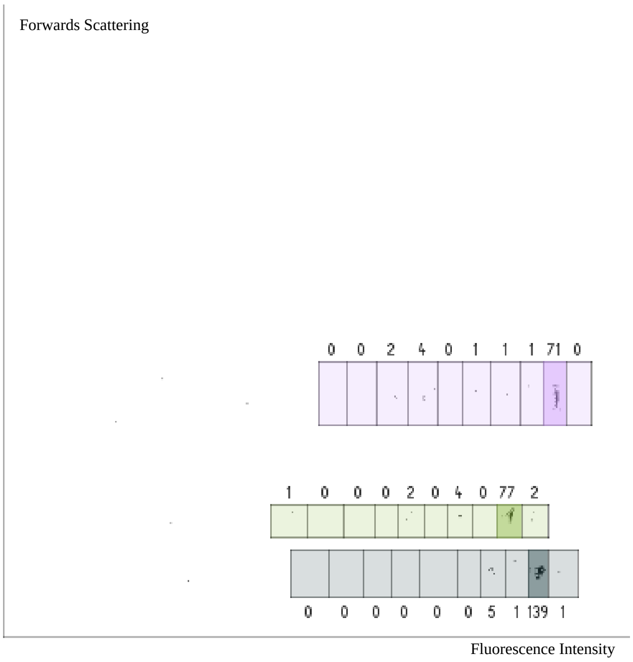

ANNEX 3: TAG DECONVOLUTION - BEAD 76

Passes flow sorting criteria: Yes

Passes tag deconvolution criteria: No

Included in protocol analysis: No

Protocol: N/A

Filename: Bin1_plateA0_H8.fcs

Split 1: Petrol shading

Split 2: Green shading

Split 3: Violet shading

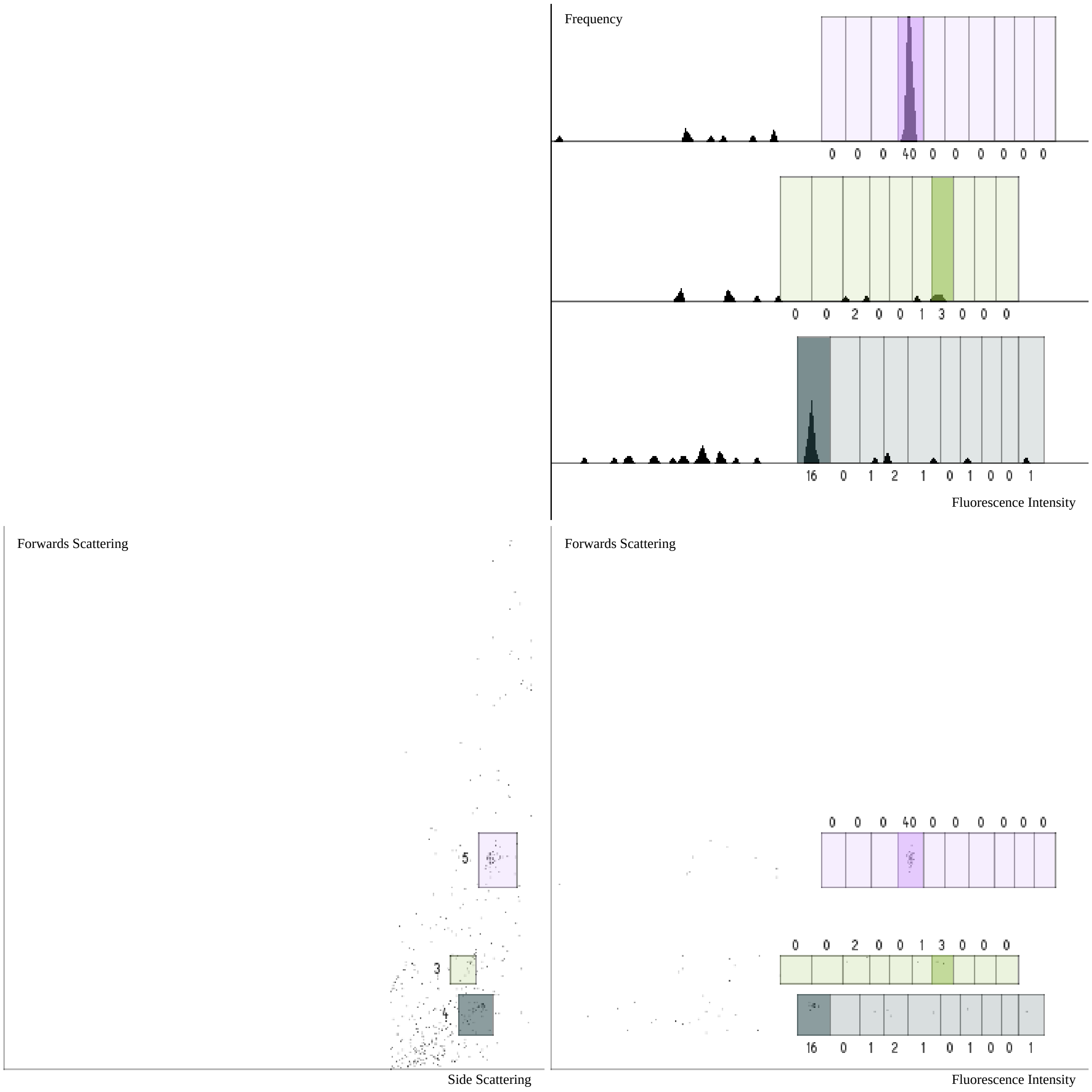

ANNEX 3: TAG DECONVOLUTION - BEAD 77

Passes flow sorting criteria: Yes

Passes tag deconvolution criteria: Yes

Included in protocol analysis: Yes

Protocol: 4, 6, 7, 1

Filename: Bin1_plateA0_H9.fcs

Split 1: Petrol shading

Split 2: Green shading

Split 3: Violet shading

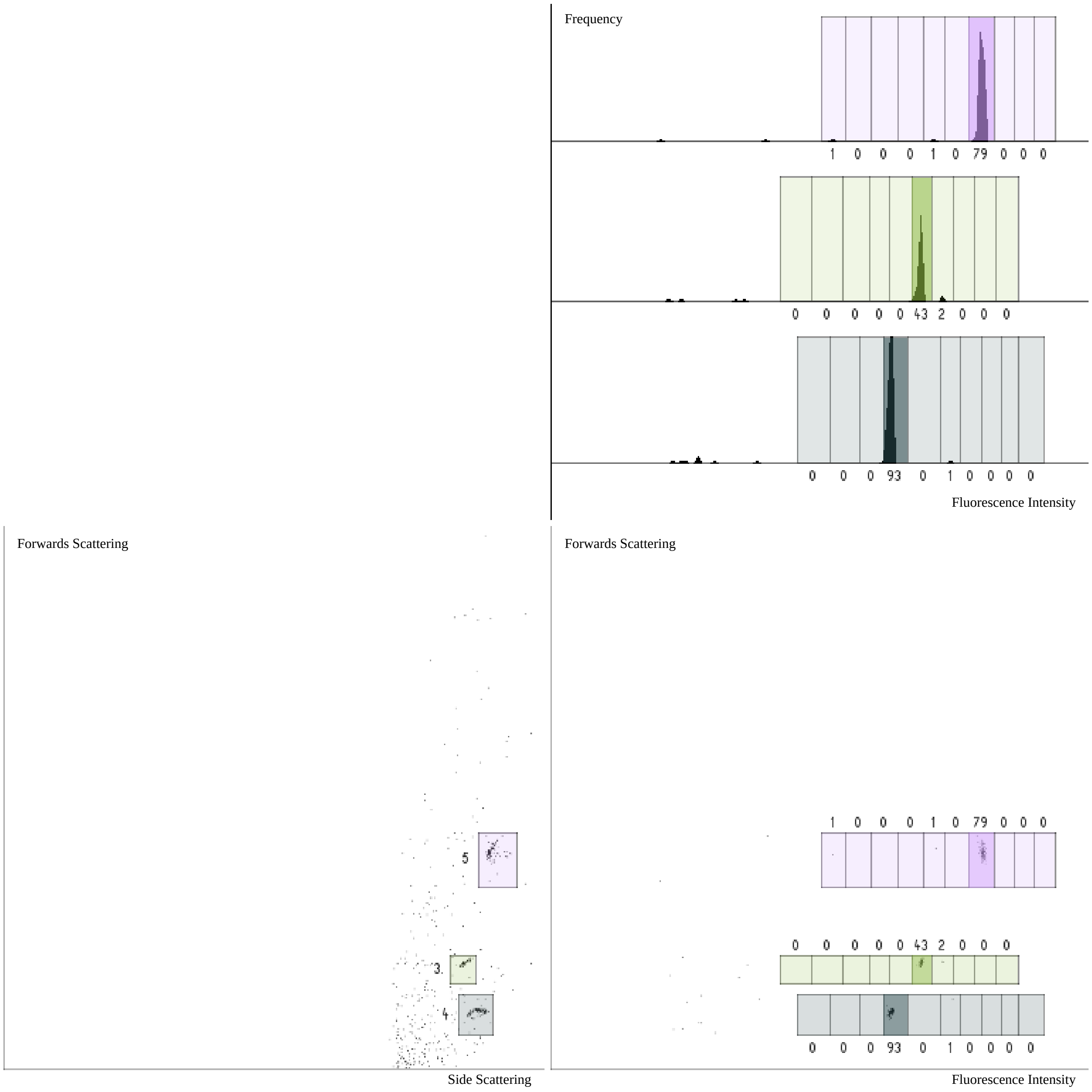

ANNEX 3: TAG DECONVOLUTION - BEAD 78

Passes flow sorting criteria: Yes

Passes tag deconvolution criteria: Yes

Included in protocol analysis: Yes

Protocol: 9, 3, 10, 1

Filename: Bin1_plateA0_H12.fcs

Split 1: Petrol shading

Split 2: Green shading

Split 3: Violet shading

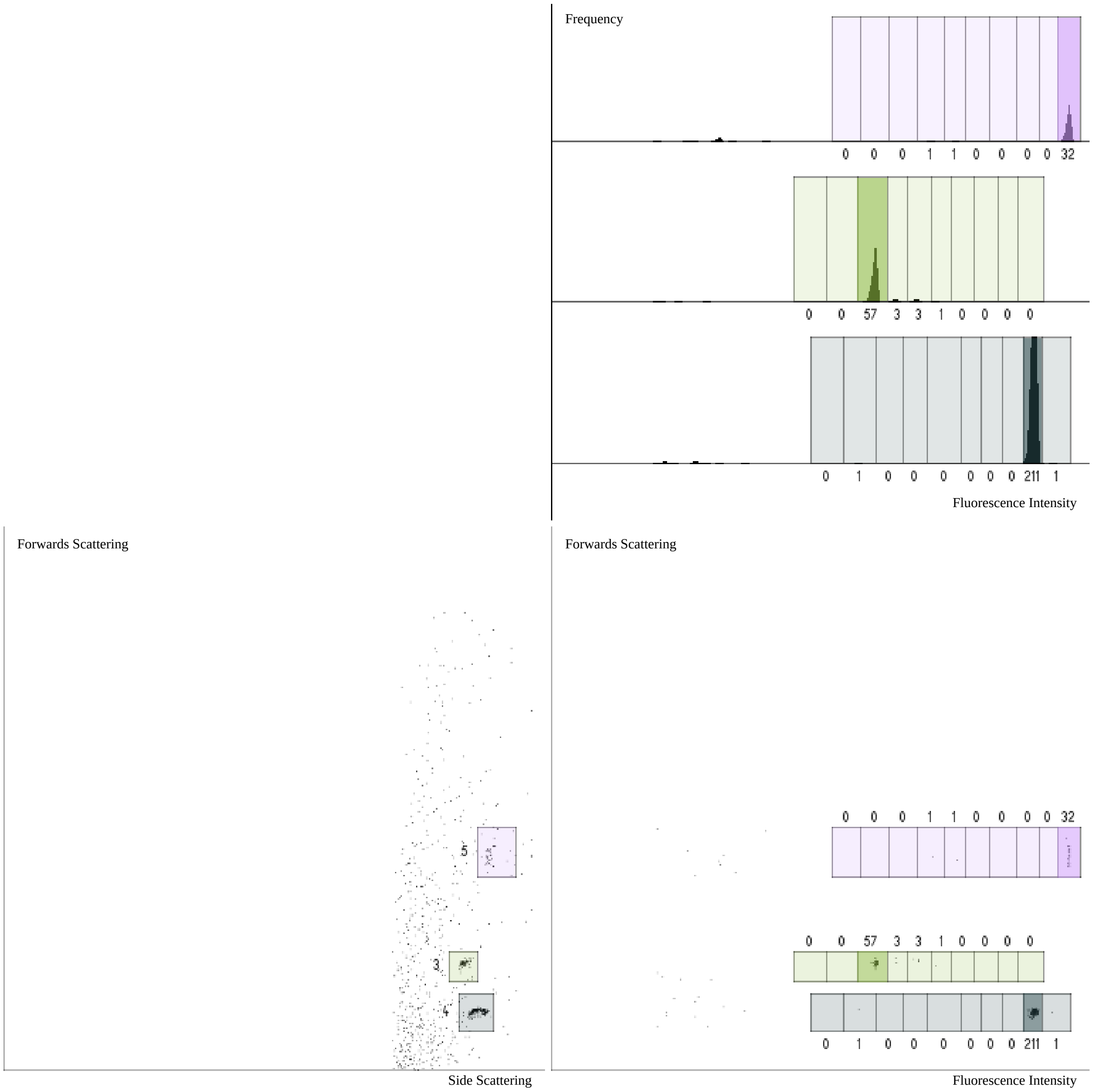

ANNEX 3: TAG DECONVOLUTION - BEAD 79

Passes flow sorting criteria: Yes

Passes tag deconvolution criteria: Yes

Included in protocol analysis: Yes

Protocol: 3, 6, 1, 1

Filename: Bin1_plateA0_A11.fcs

Split 1: Petrol shading

Split 2: Green shading

Split 3: Violet shading

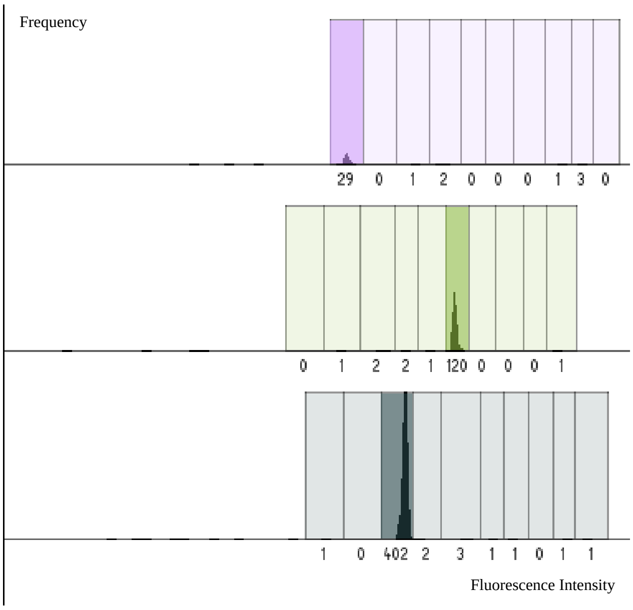

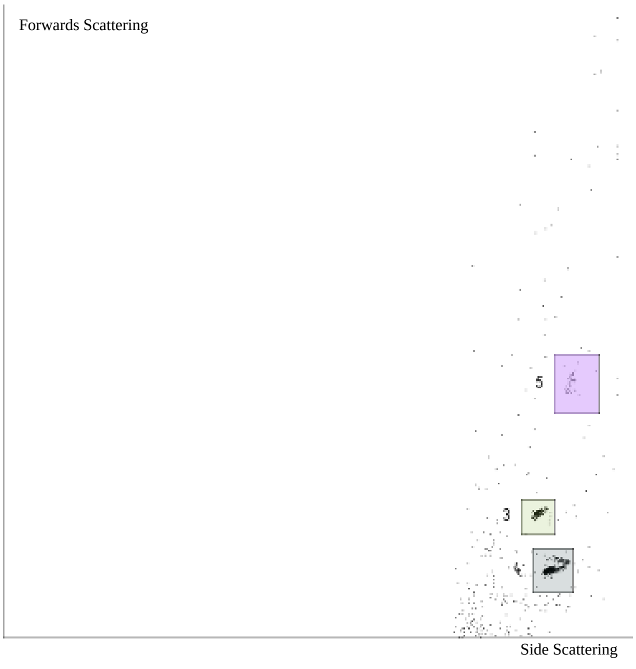

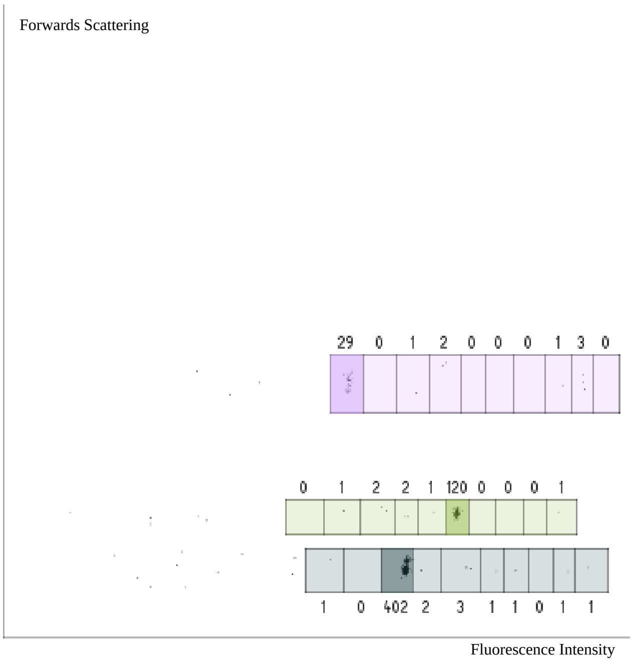

ANNEX 3: TAG DECONVOLUTION - BEAD 80

Passes flow sorting criteria: Yes

Passes tag deconvolution criteria: Yes

Included in protocol analysis: Yes

Protocol: 2, 5, 9, 1

Filename: Bin1_plateA0_A12.fcs

Split 1: Petrol shading

Split 2: Green shading

Split 3: Violet shading

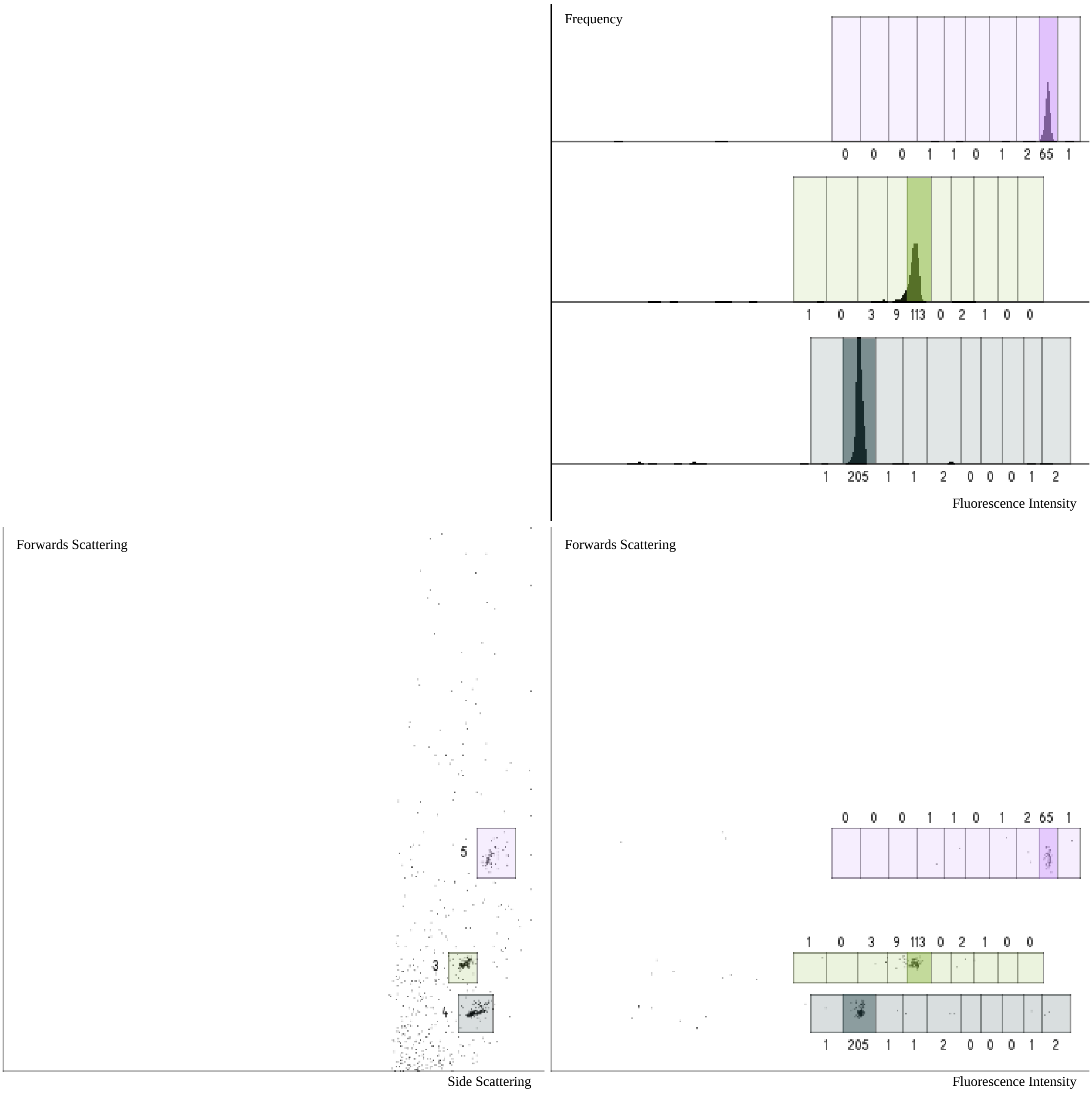

ANNEX 3: TAG DECONVOLUTION - BEAD 81

Passes flow sorting criteria: Yes

Passes tag deconvolution criteria: Yes

Included in protocol analysis: Yes

Protocol: 9, 4, 6, 1

Filename: Bin1_plateA0_B11.fcs

Split 1: Petrol shading

Split 2: Green shading

Split 3: Violet shading

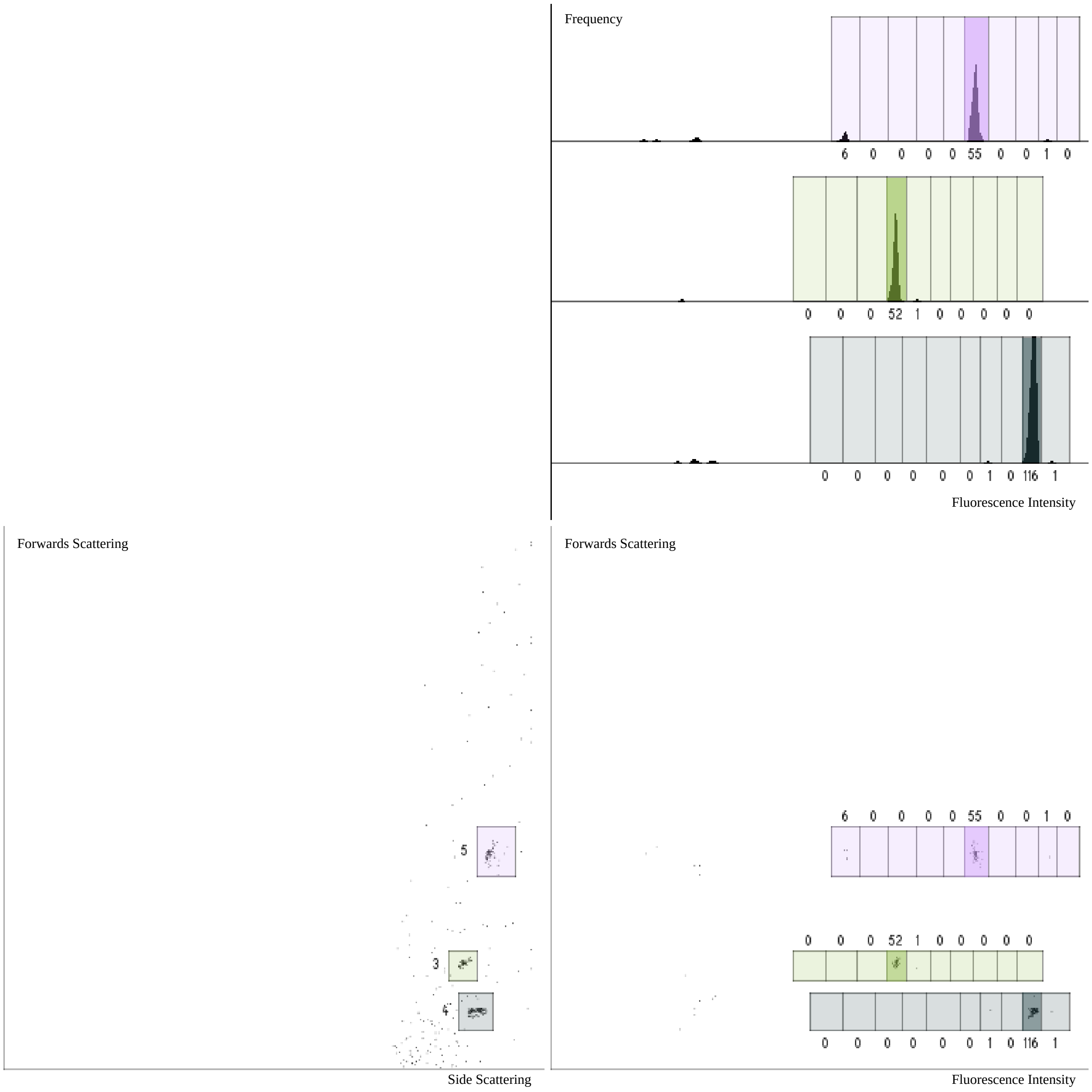

ANNEX 3: TAG DECONVOLUTION - BEAD 82

Passes flow sorting criteria: Yes

Passes tag deconvolution criteria: Yes

Included in protocol analysis: Yes

Protocol: 10, 8, 10, 1

Filename: Bin1_plateA0_B12.fcs

Split 1: Petrol shading

Split 2: Green shading

Split 3: Violet shading

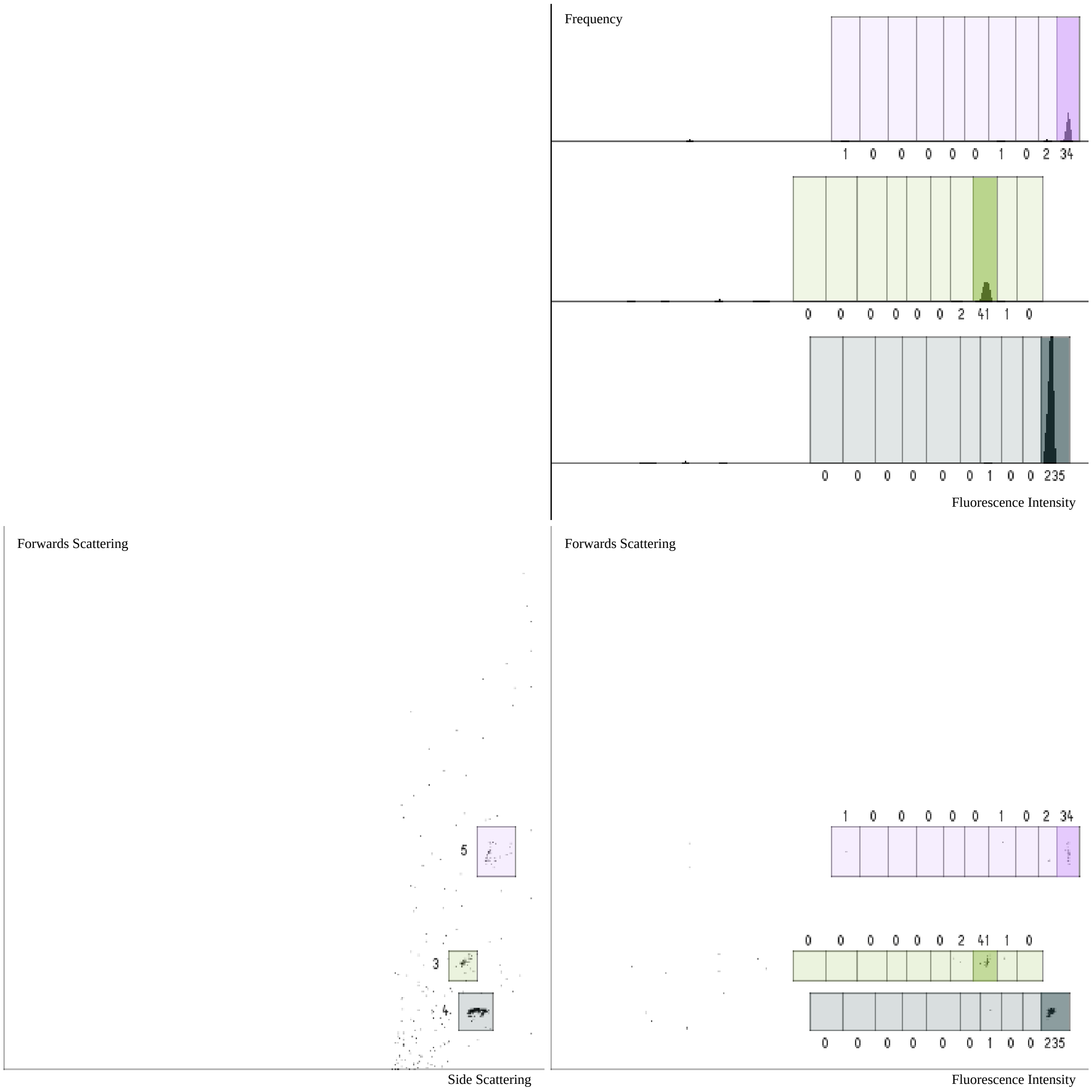

ANNEX 3: TAG DECONVOLUTION - BEAD 83

Passes flow sorting criteria: Yes

Passes tag deconvolution criteria: Yes

Included in protocol analysis: Yes

Protocol: 4, 6, 6, 1

Filename: Bin1_plateA0_C11.fcs

Split 1: Petrol shading

Split 2: Green shading

Split 3: Violet shading

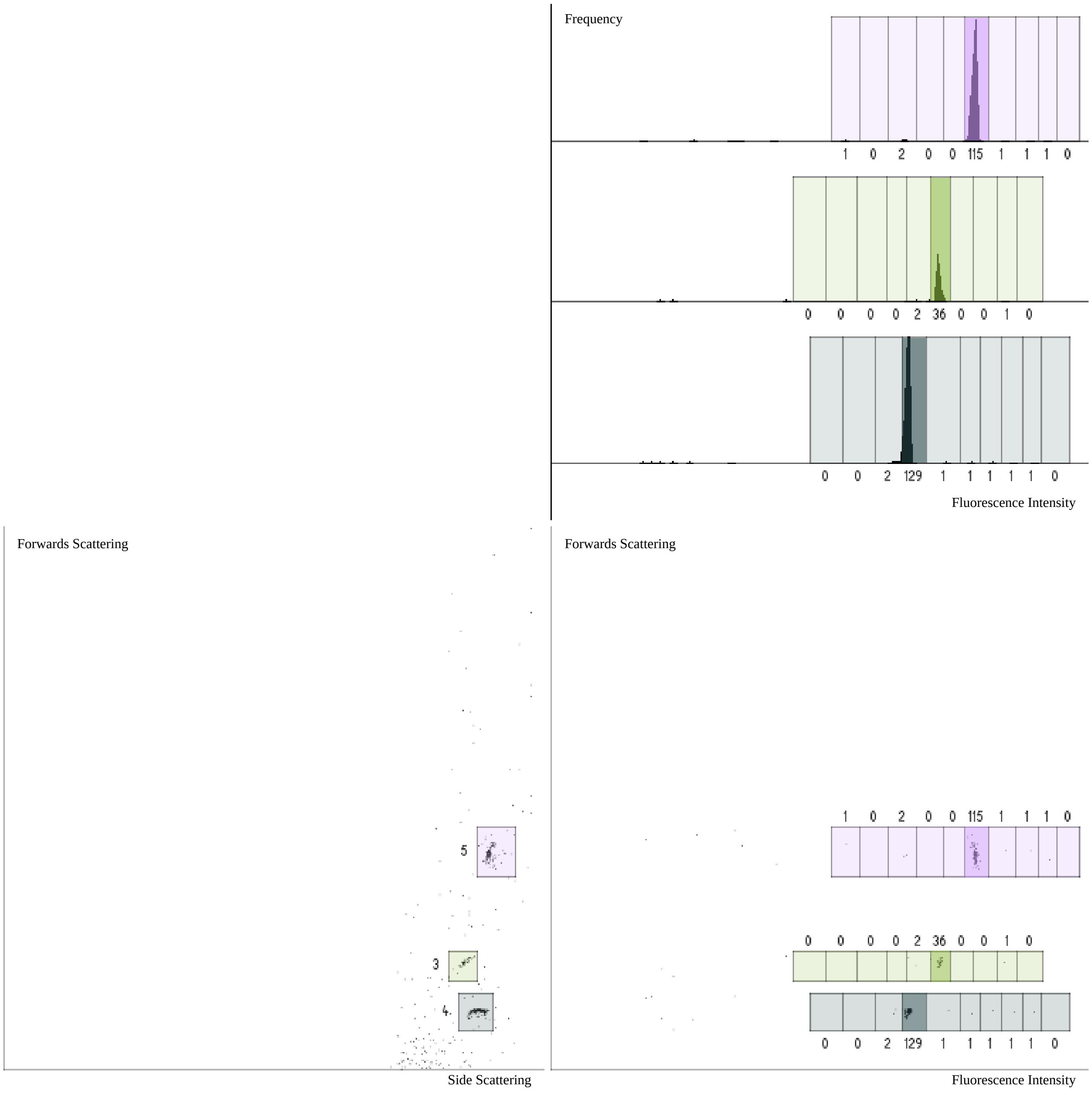

ANNEX 3: TAG DECONVOLUTION - BEAD 84

Passes flow sorting criteria: Yes

Passes tag deconvolution criteria: Yes

Included in protocol analysis: Yes

Protocol: 2, 10, 5, 1

Filename: Bin1_plateA0_C12.fcs

Split 1: Petrol shading

Split 2: Green shading

Split 3: Violet shading

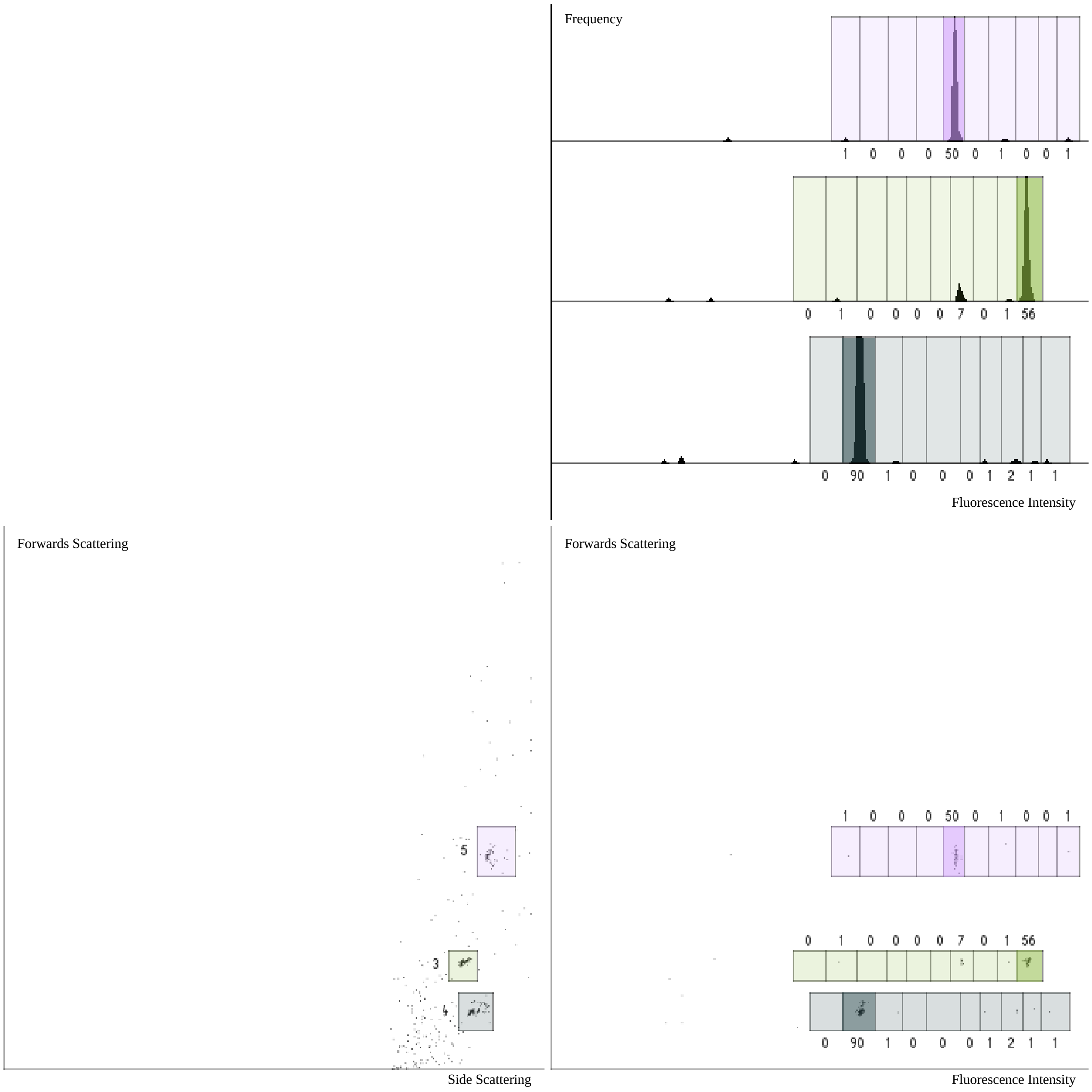

ANNEX 3: TAG DECONVOLUTION - BEAD 85

Passes flow sorting criteria: Yes

Passes tag deconvolution criteria: Yes

Included in protocol analysis: Yes

Protocol: 8, 8, 2, 1

Filename: Bin1_plateA0_D11.fcs

Split 1: Petrol shading

Split 2: Green shading

Split 3: Violet shading

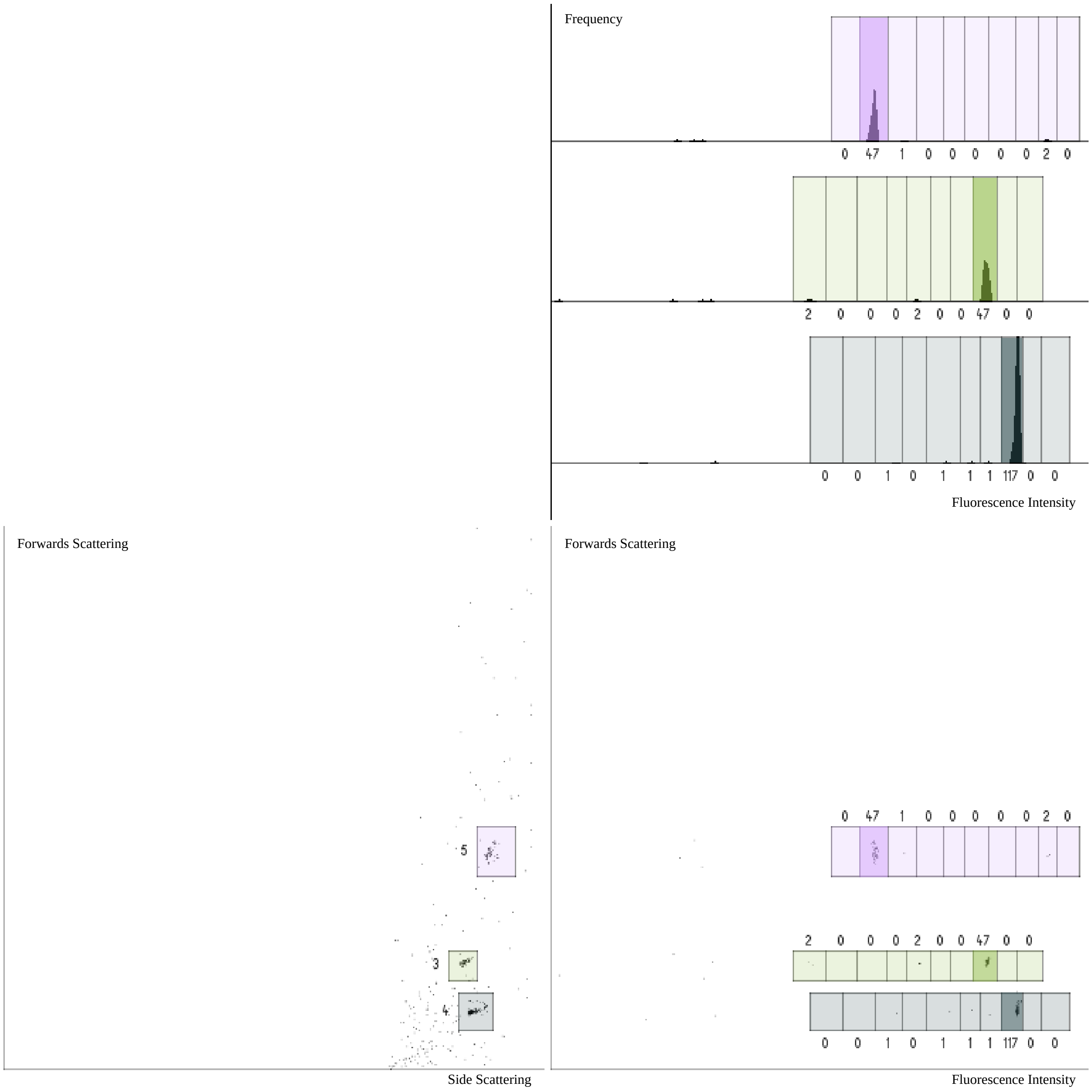

ANNEX 3: TAG DECONVOLUTION - BEAD 86

Passes flow sorting criteria: Yes

Passes tag deconvolution criteria: Yes

Included in protocol analysis: Yes

Protocol: 1, 6, 2, 1

Filename: Bin1_plateA0_D12.fcs

Split 1: Petrol shading

Split 2: Green shading

Split 3: Violet shading

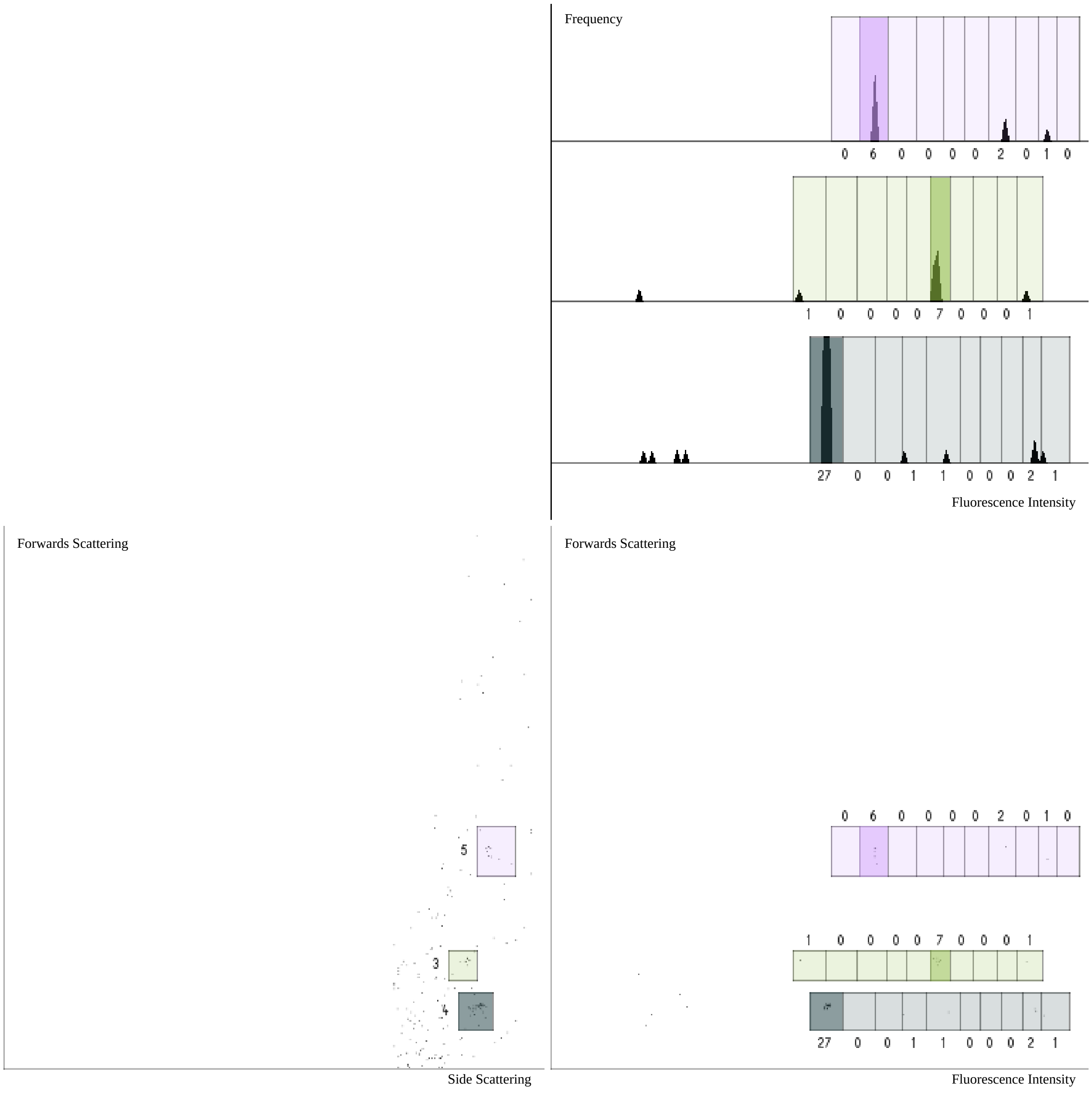

ANNEX 3: TAG DECONVOLUTION - BEAD 87

Passes flow sorting criteria: Yes

Passes tag deconvolution criteria: Yes

Included in protocol analysis: Yes

Protocol: 6, 3, 7, 1

Filename: Bin1_plateA0_E11.fcs

Split 1: Petrol shading

Split 2: Green shading

Split 3: Violet shading

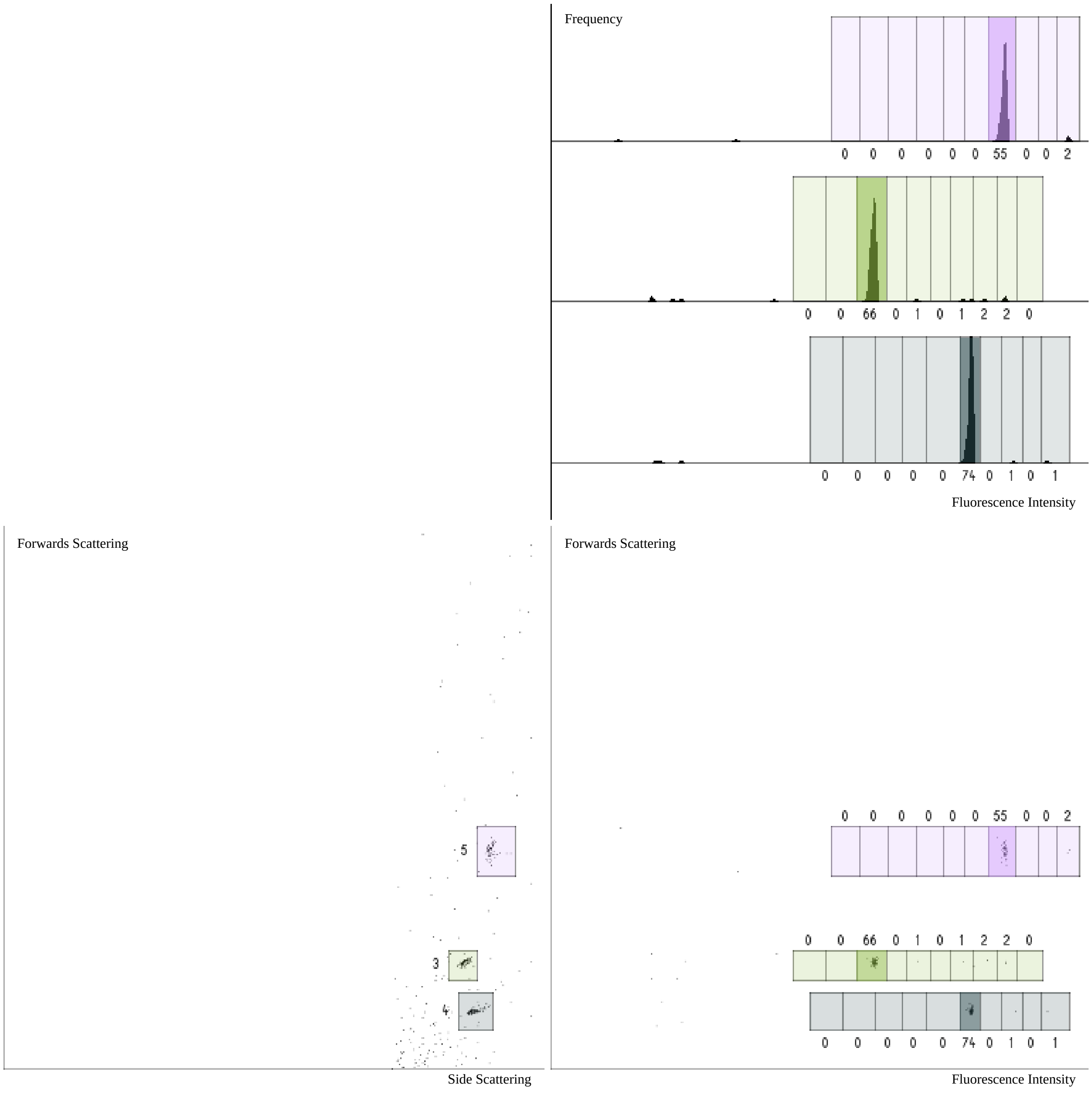

ANNEX 3: TAG DECONVOLUTION - BEAD 88

Passes flow sorting criteria: Yes

Passes tag deconvolution criteria: Yes

Included in protocol analysis: Yes

Protocol: 8, 7, 10, 1

Filename: Bin1_plateA0_E12.fcs

Split 1: Petrol shading

Split 2: Green shading

Split 3: Violet shading

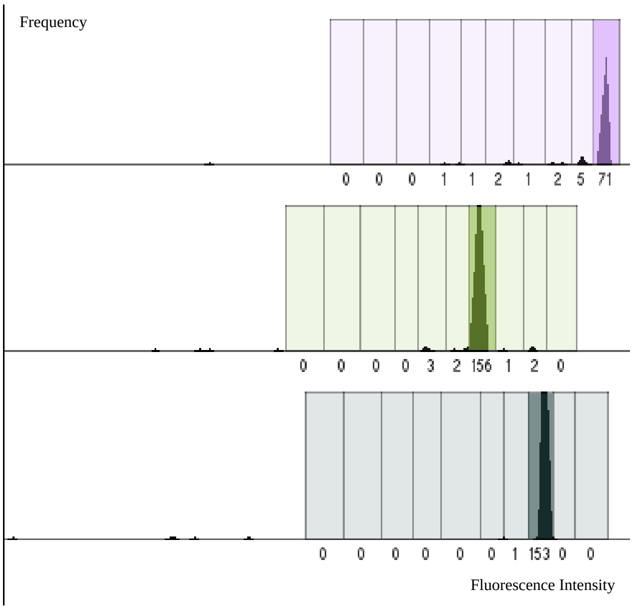

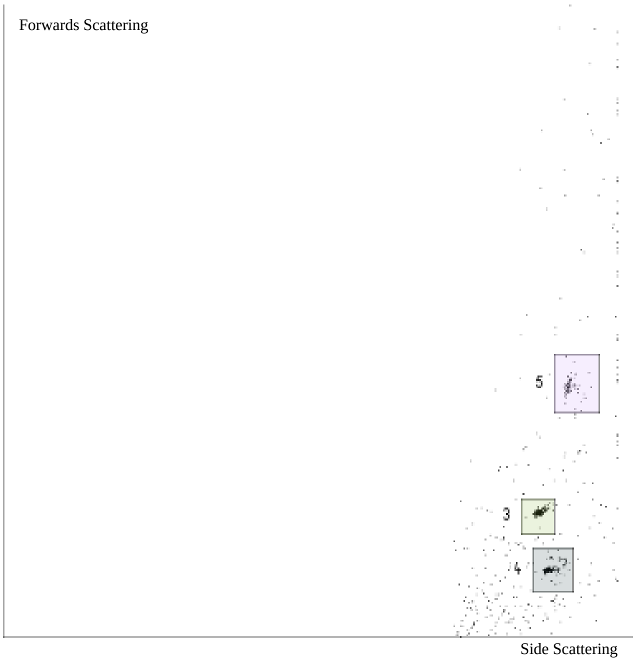

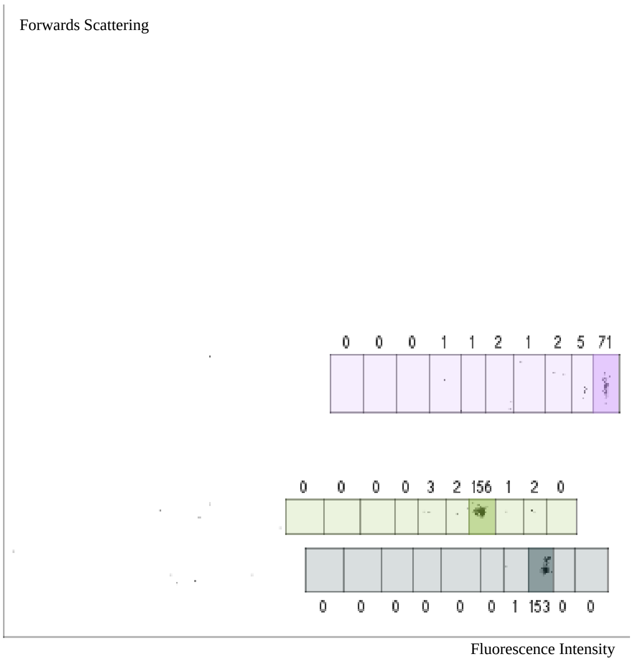

ANNEX 3: TAG DECONVOLUTION - BEAD 89

Passes flow sorting criteria: Yes

Passes tag deconvolution criteria: Yes

Included in protocol analysis: Yes

Protocol: 3, 9, 3, 1

Filename: Bin1_plateA0_F11.fcs

Split 1: Petrol shading

Split 2: Green shading

Split 3: Violet shading

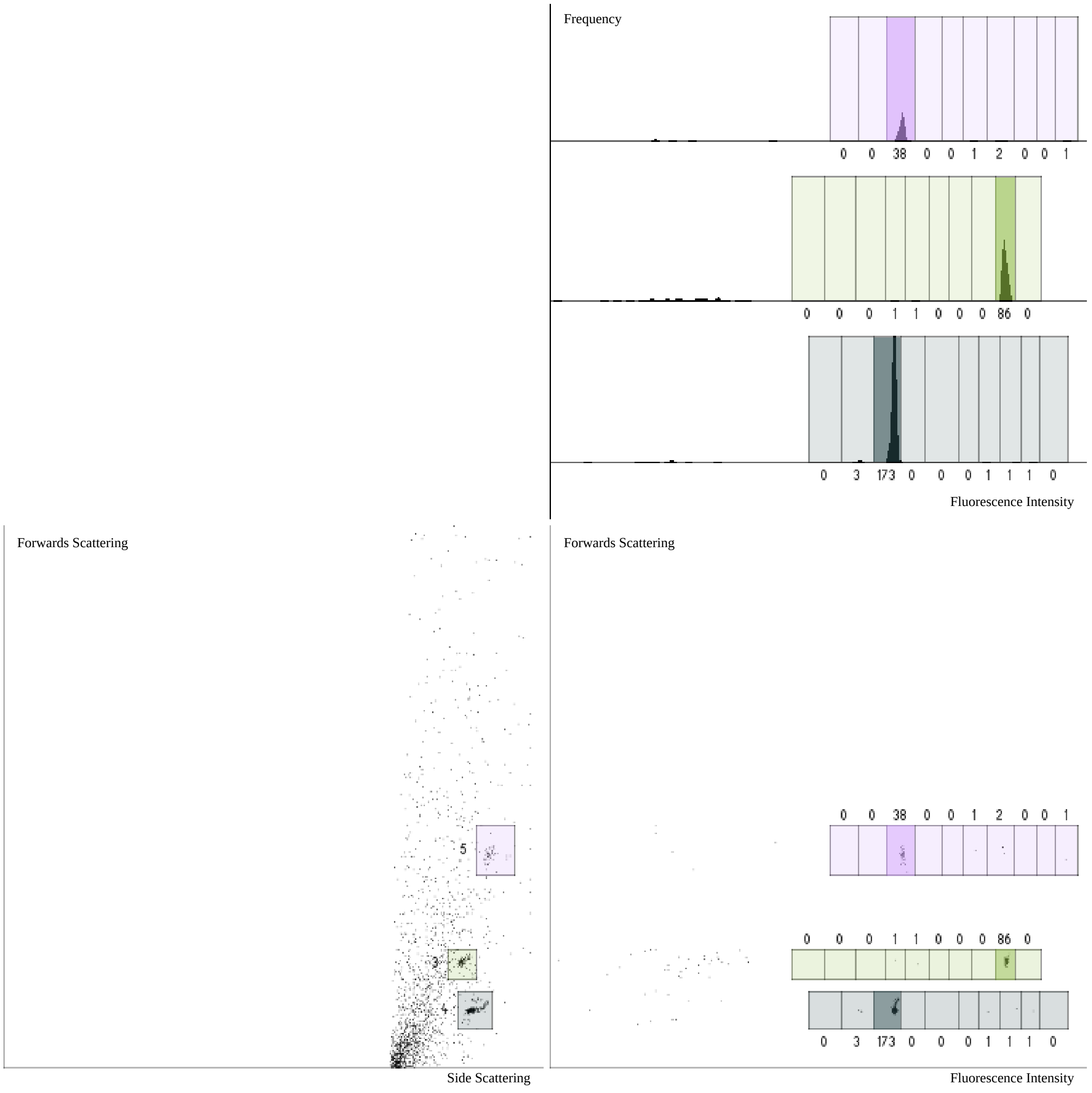

ANNEX 3: TAG DECONVOLUTION - BEAD 90

Passes flow sorting criteria: Yes

Passes tag deconvolution criteria: Yes

Included in protocol analysis: Yes

Protocol: 6, 3, 9, 1

Filename: Bin1_plateA0_F12.fcs

Split 1: Petrol shading

Split 2: Green shading

Split 3: Violet shading

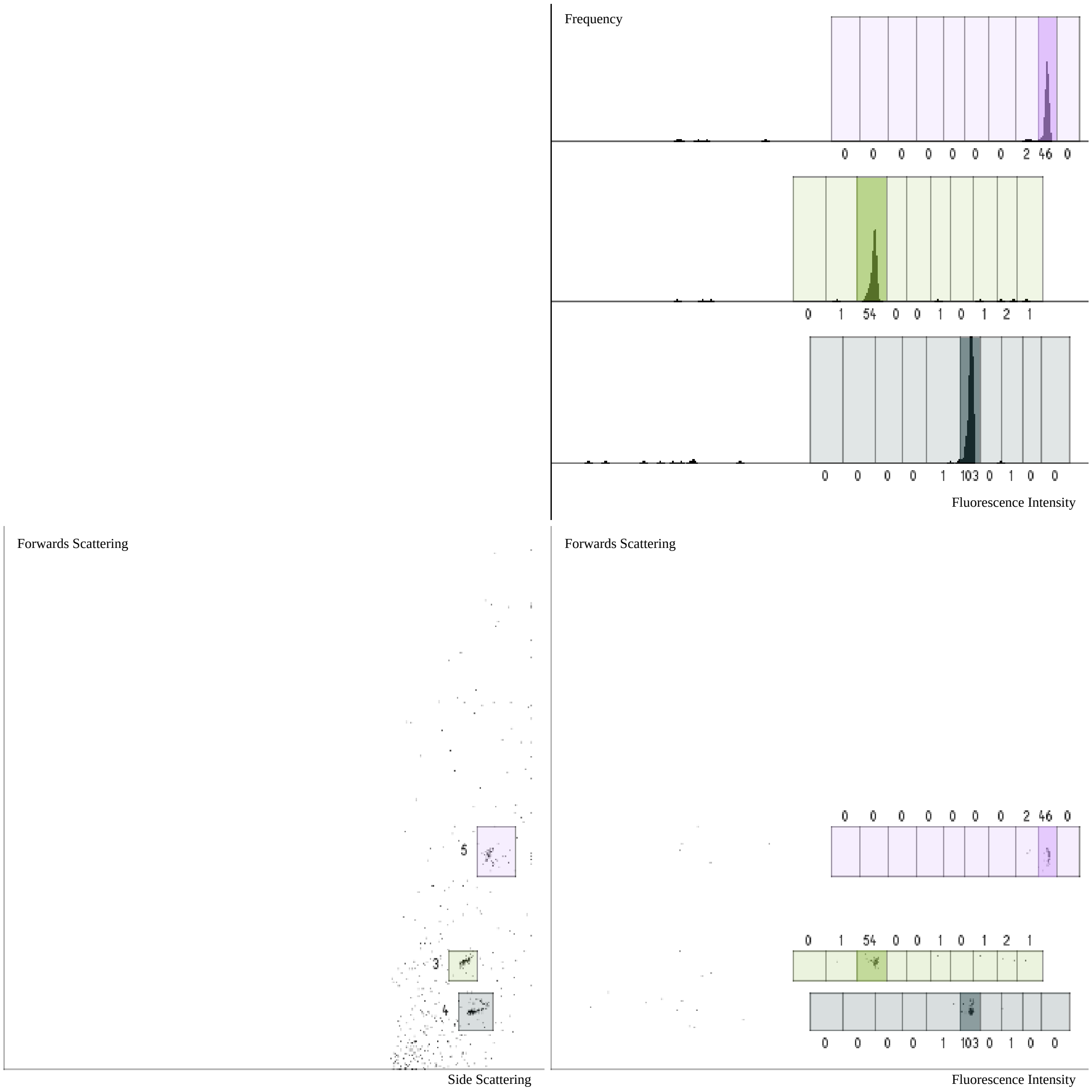

ANNEX 3: TAG DECONVOLUTION - BEAD 91

Passes flow sorting criteria: Yes

Passes tag deconvolution criteria: Yes

Included in protocol analysis: Yes

Protocol: 4, 10, 4, 1

Filename: Bin1_plateA0_G11.fcs

Split 1: Petrol shading

Split 2: Green shading

Split 3: Violet shading

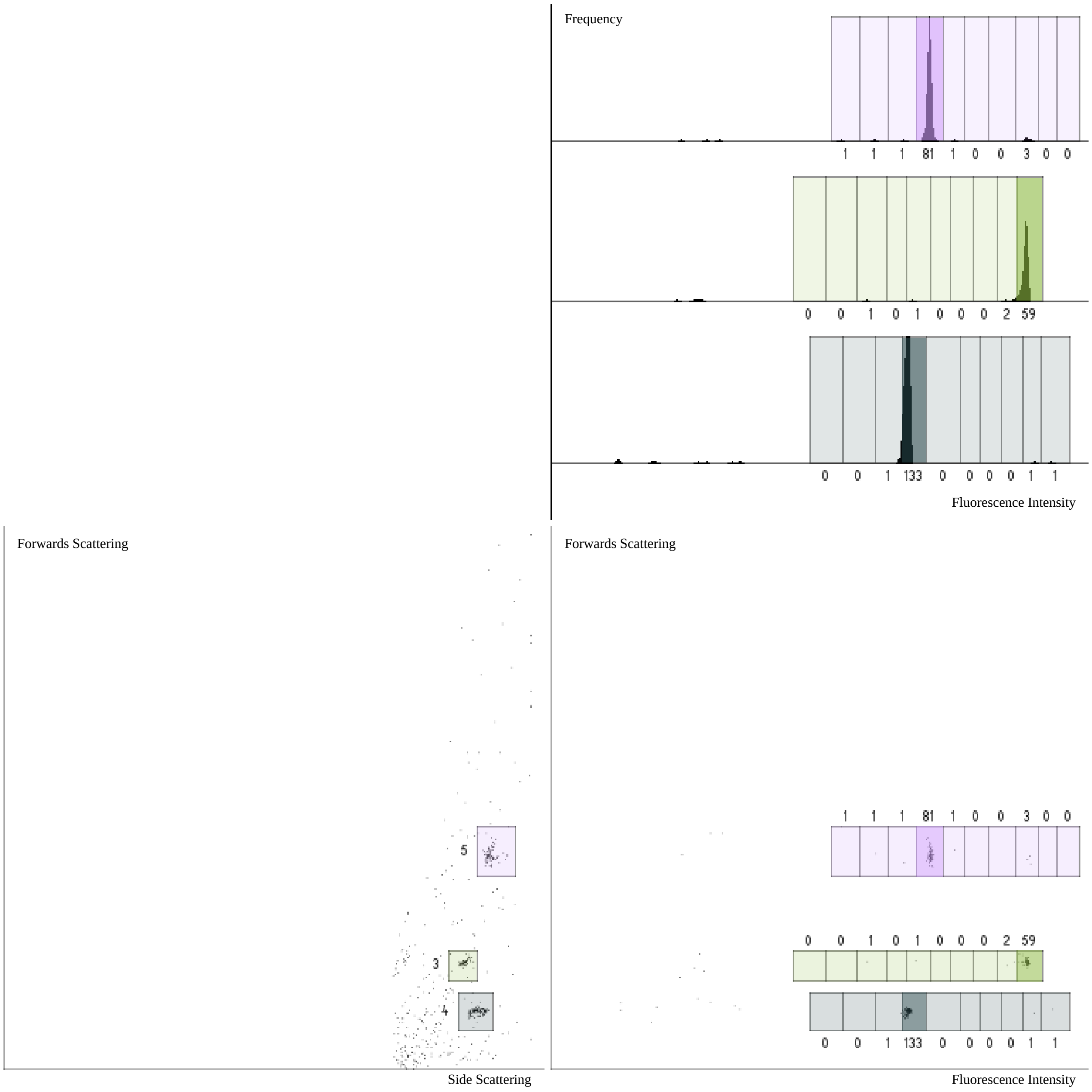

ANNEX 3: TAG DECONVOLUTION - BEAD 92

Passes flow sorting criteria: Yes

Passes tag deconvolution criteria: No

Included in protocol analysis: No

Protocol: N/A

Filename: Bin1_plateA0_G12.fcs

Split 1: Petrol shading

Split 2: Green shading

Split 3: Violet shading

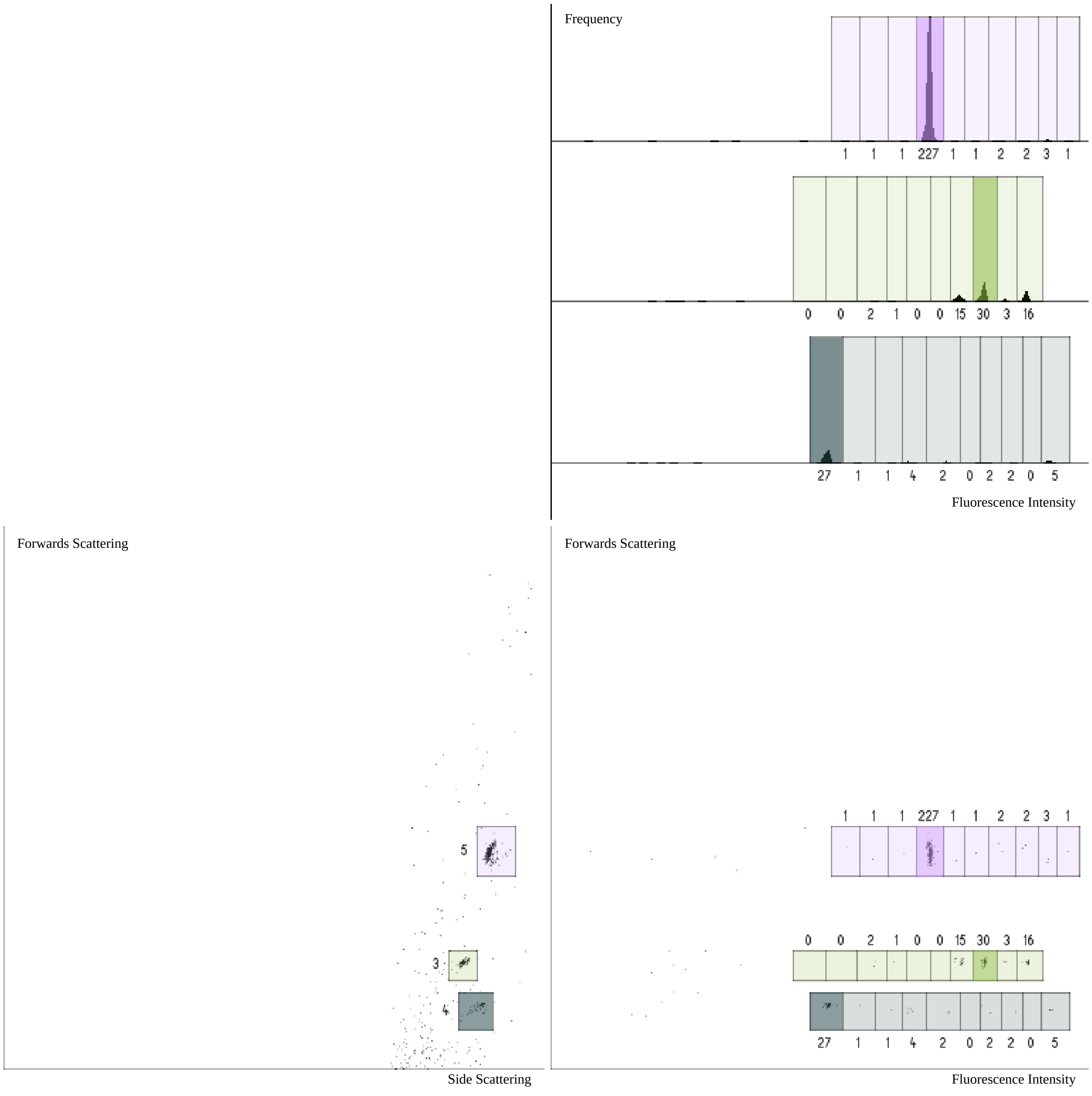

ANNEX 3: TAG DECONVOLUTION - BEAD 93

Passes flow sorting criteria: Yes

Passes tag deconvolution criteria: Yes

Included in protocol analysis: Yes

Protocol: 9, 8, 8, 1

Filename: Bin1_plateA0_H11.fcs

Split 1: Petrol shading

Split 2: Green shading

Split 3: Violet shading

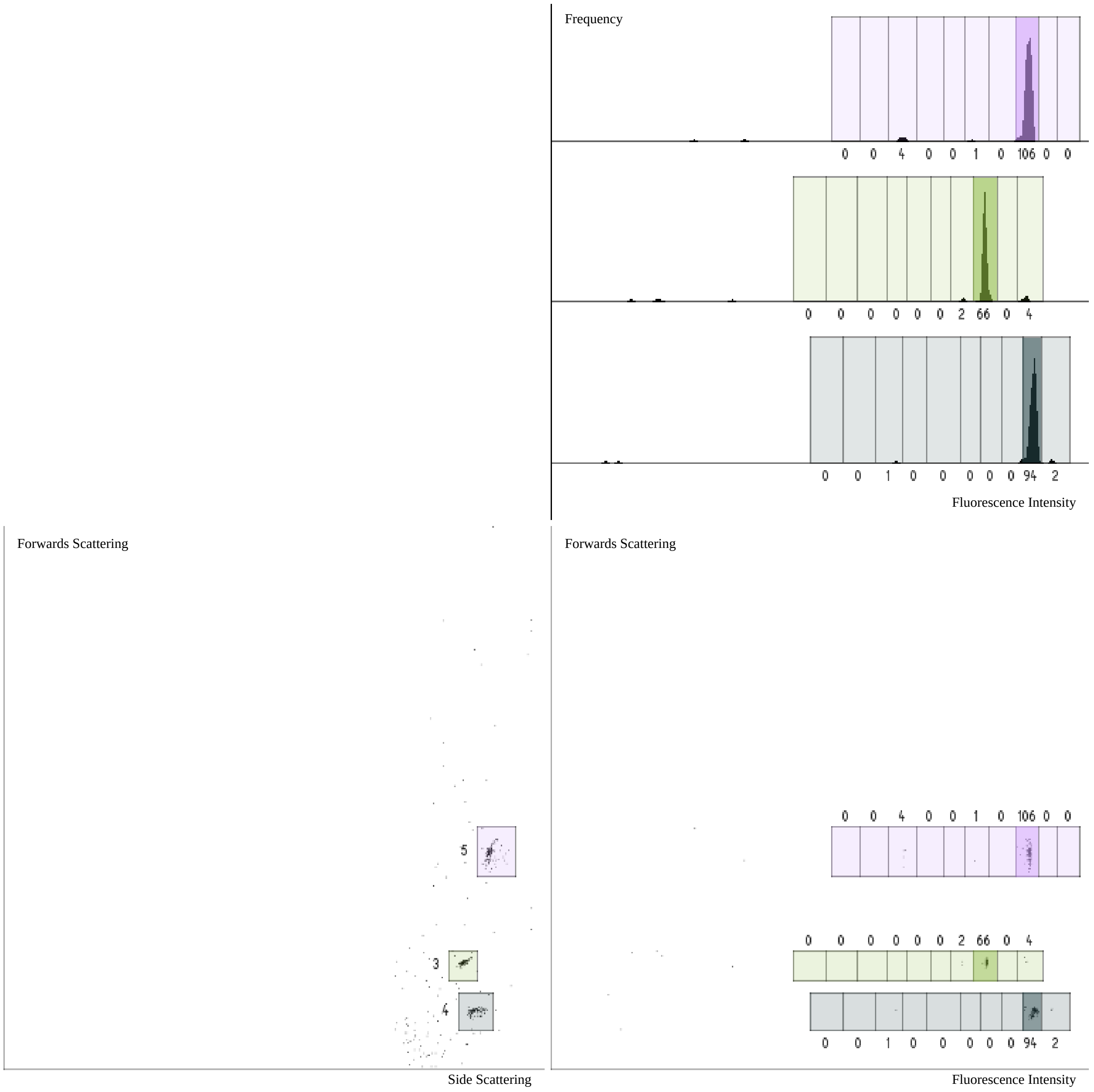

ANNEX 3: TAG DECONVOLUTION - BEAD 94

Passes flow sorting criteria: Yes

Passes tag deconvolution criteria: No

Included in protocol analysis: No

Protocol: N/A

Filename: Bin1_plateB1_H7.fcs

Split 1: Petrol shading

Split 2: Green shading

Split 3: Violet shading

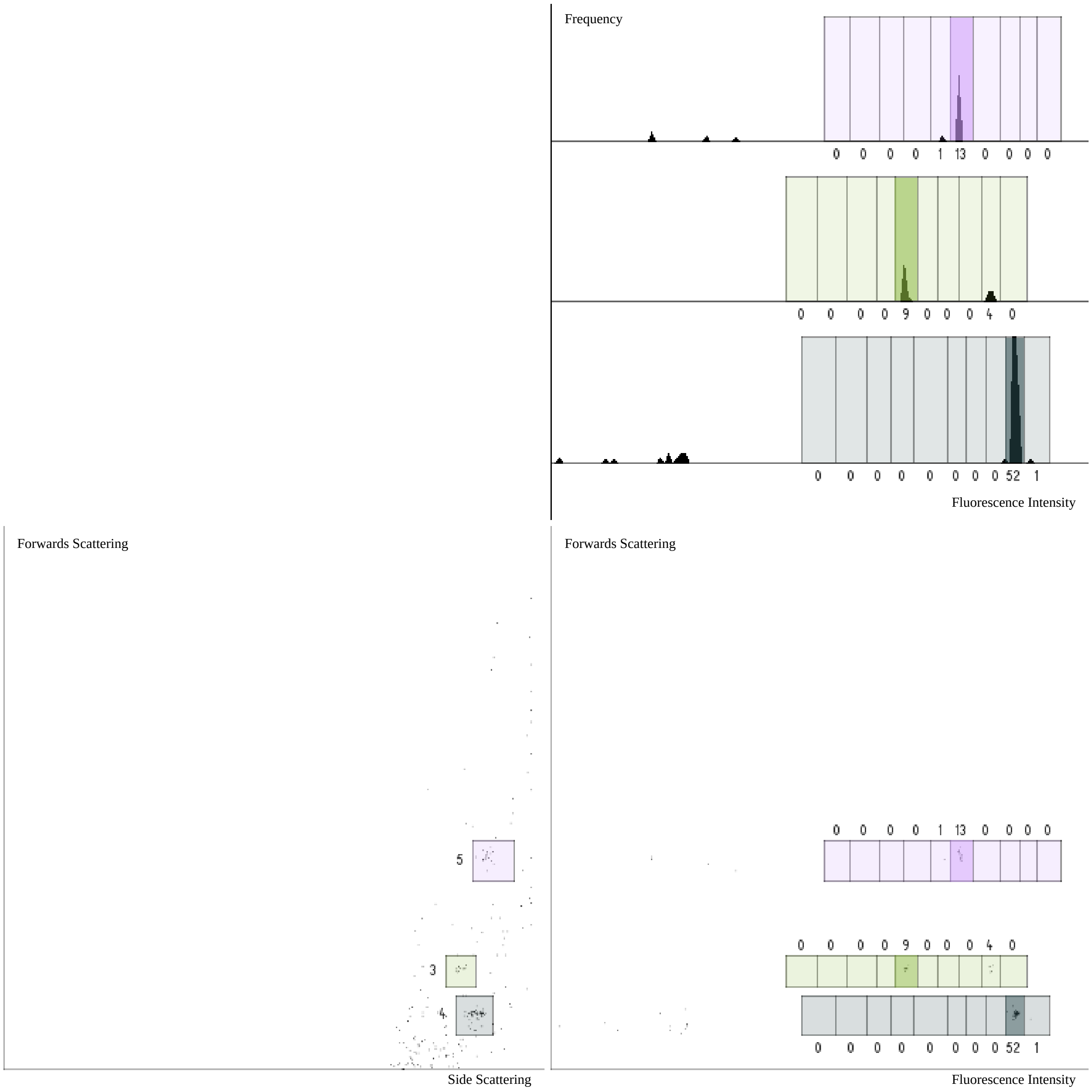

ANNEX 3: TAG DECONVOLUTION - BEAD 95

Passes flow sorting criteria: Yes

Passes tag deconvolution criteria: Yes

Included in protocol analysis: Yes

Protocol: 9, 7, 3, 1

Filename: Bin1_plateB1_A1.fcs

Split 1: Petrol shading

Split 2: Green shading

Split 3: Violet shading

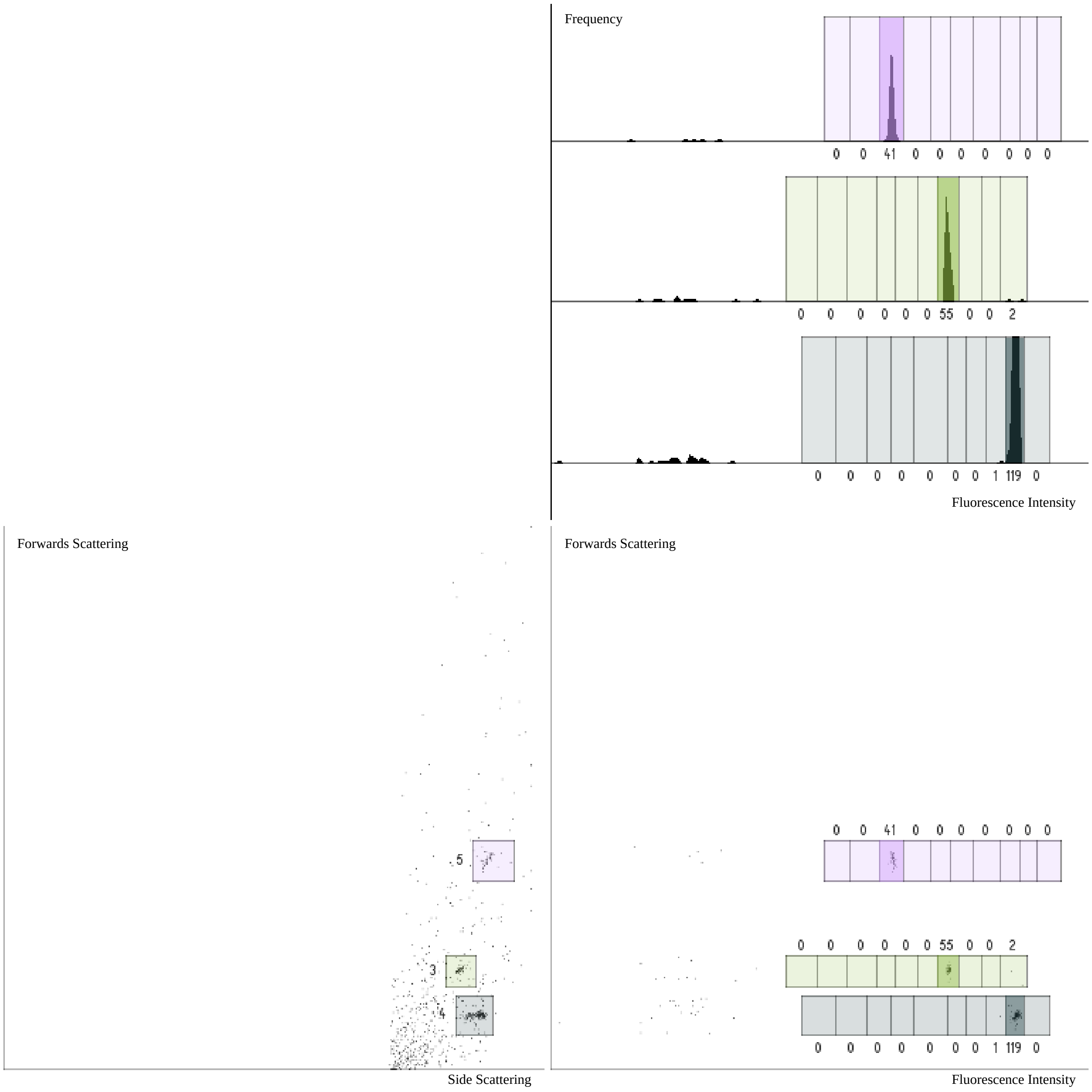

ANNEX 3: TAG DECONVOLUTION - BEAD 96

Passes flow sorting criteria: Yes

Passes tag deconvolution criteria: Yes

Included in protocol analysis: Yes

Protocol: 10, 10, 7, 1

Filename: Bin1_plateB1_A6.fcs

Split 1: Petrol shading

Split 2: Green shading

Split 3: Violet shading

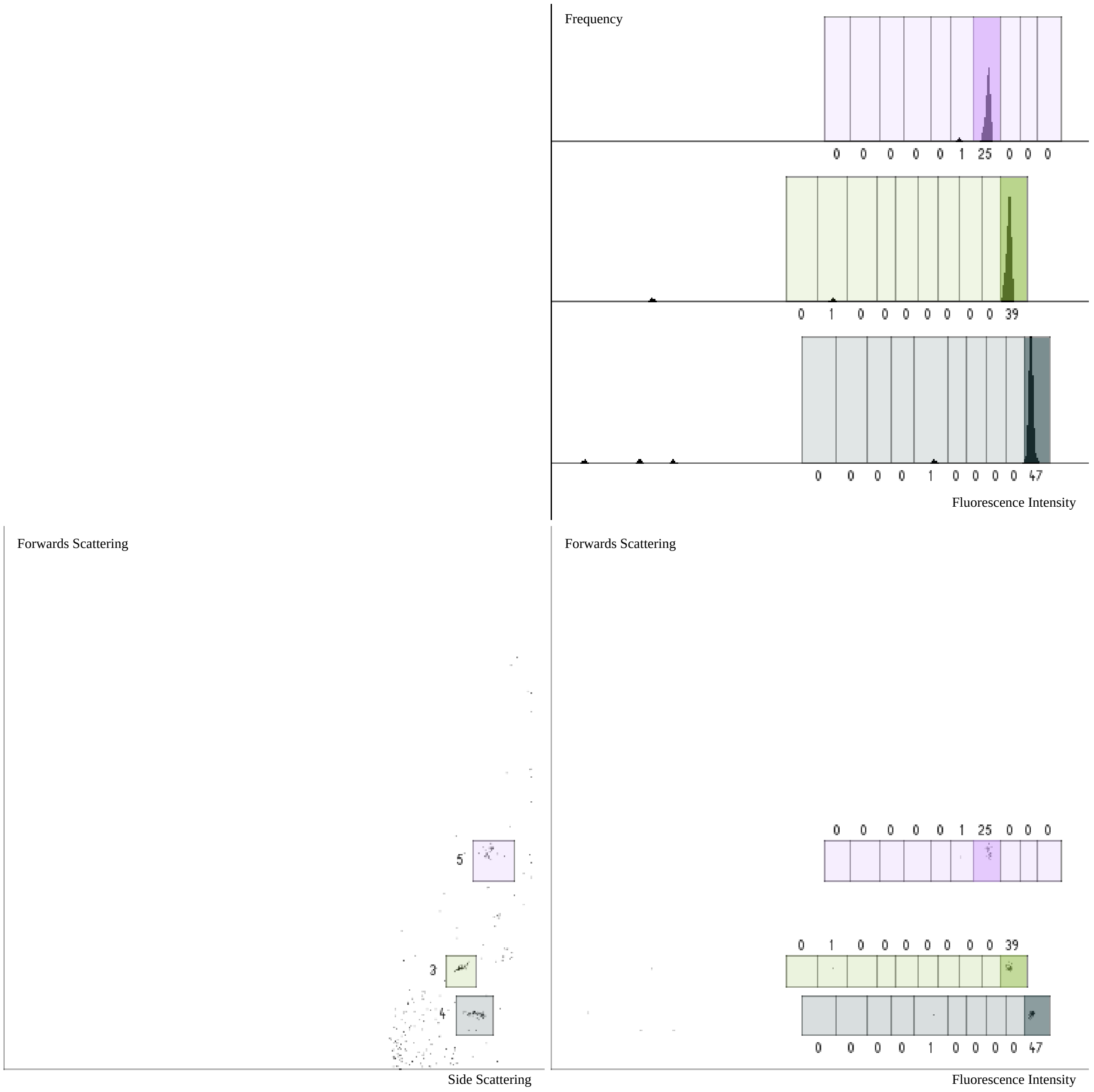

ANNEX 3: TAG DECONVOLUTION - BEAD 97

Passes flow sorting criteria: Yes

Passes tag deconvolution criteria: Yes

Included in protocol analysis: Yes

Protocol: 10, 8, 1, 1

Filename: Bin1_plateB1_A7.fcs

Split 1: Petrol shading

Split 2: Green shading

Split 3: Violet shading

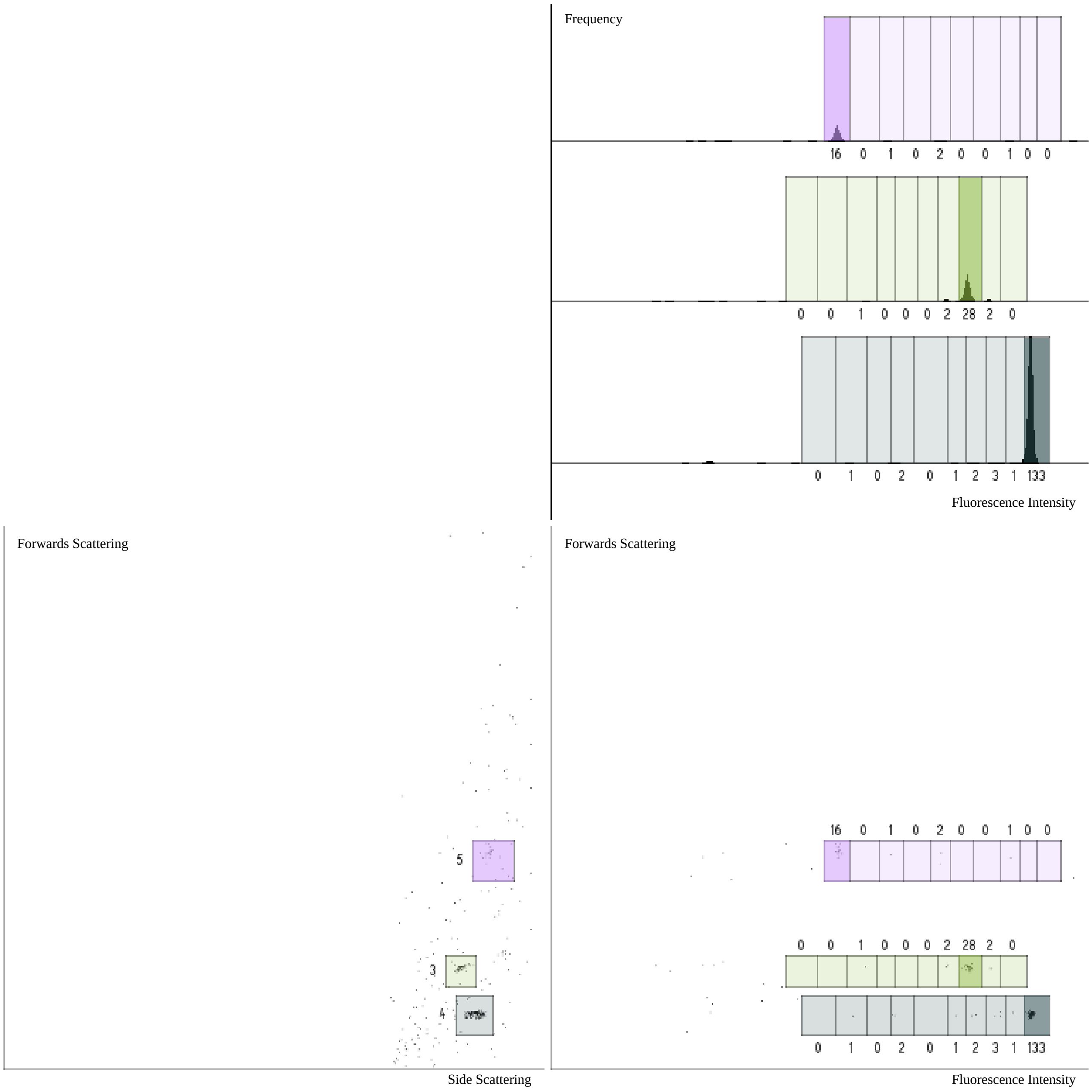

ANNEX 3: TAG DECONVOLUTION - BEAD 98

Passes flow sorting criteria: Yes

Passes tag deconvolution criteria: Yes

Included in protocol analysis: Yes

Protocol: 10, 5, 8, 1

Filename: Bin1_plateB1_B1.fcs

Split 1: Petrol shading

Split 2: Green shading

Split 3: Violet shading

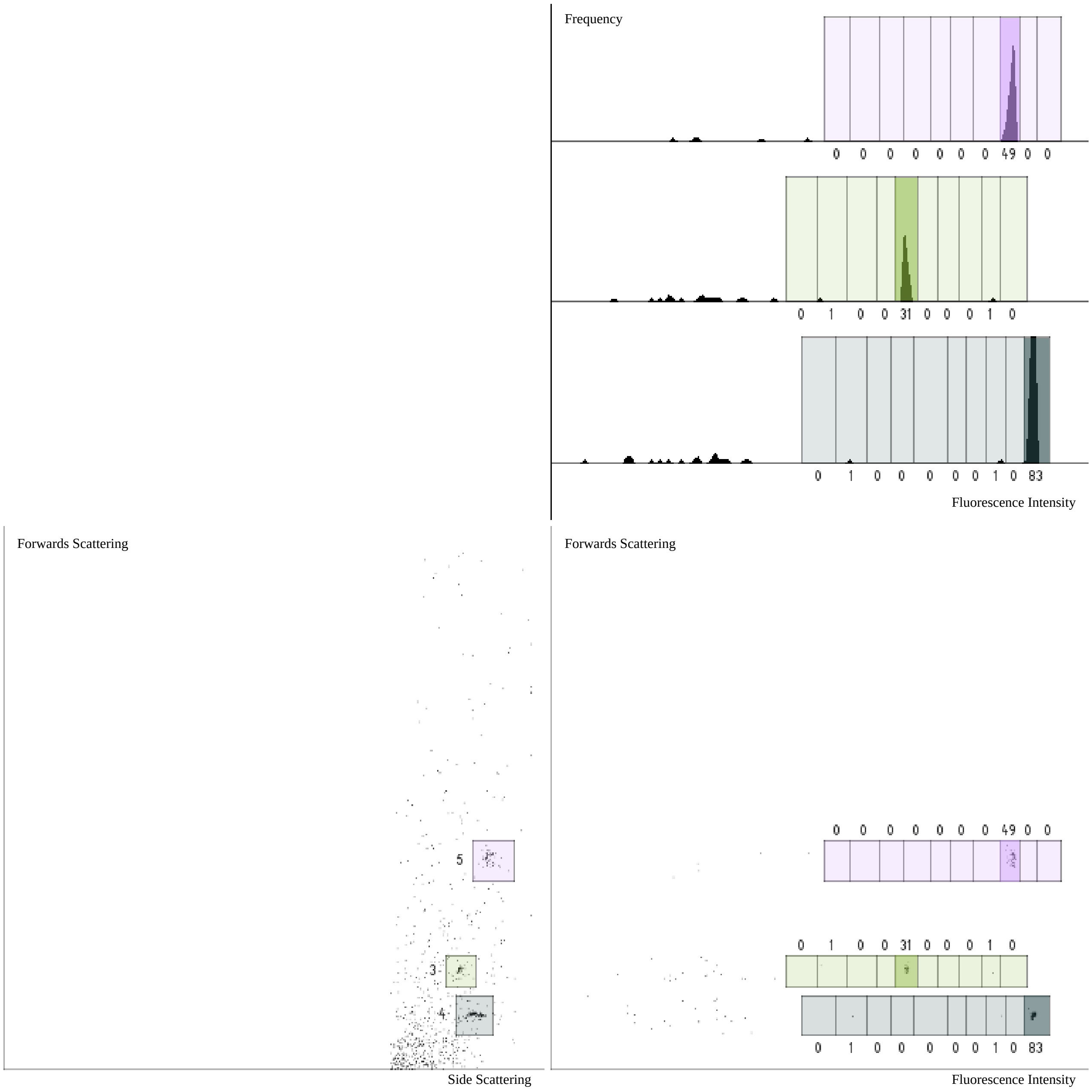

ANNEX 3: TAG DECONVOLUTION - BEAD 99

Passes flow sorting criteria: Yes

Passes tag deconvolution criteria: No

Included in protocol analysis: No

Protocol: N/A

Filename: Bin1_plateB1_B2.fcs

Split 1: Petrol shading

Split 2: Green shading

Split 3: Violet shading

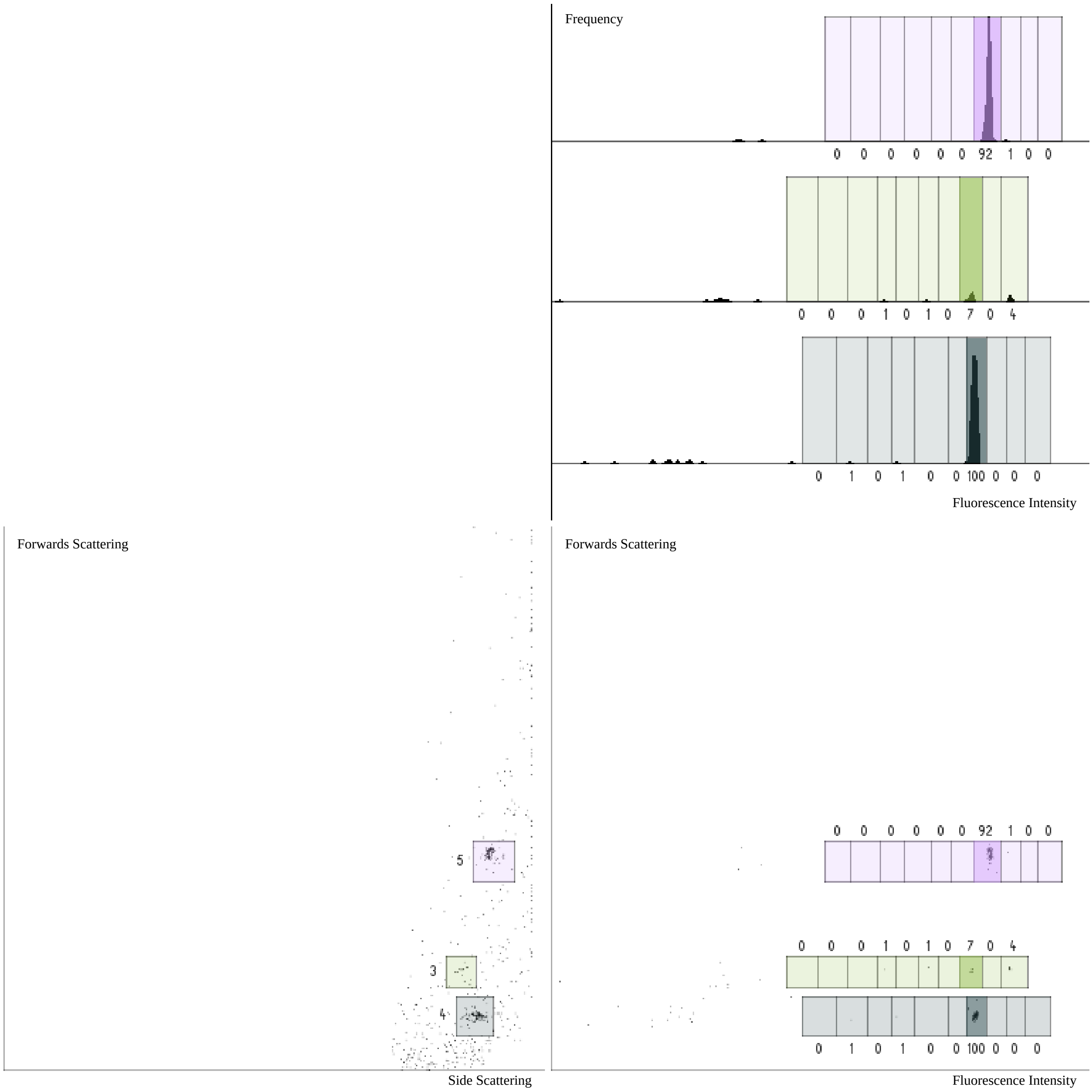

ANNEX 3: TAG DECONVOLUTION - BEAD 100

Passes flow sorting criteria: Yes

Passes tag deconvolution criteria: No

Included in protocol analysis: No

Protocol: N/A

Filename: Bin1_plateB1_B3.fcs

Split 1: Petrol shading

Split 2: Green shading

Split 3: Violet shading

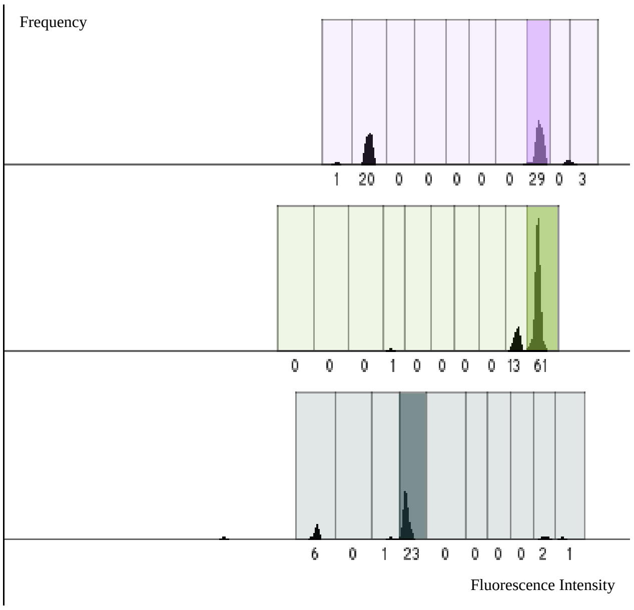

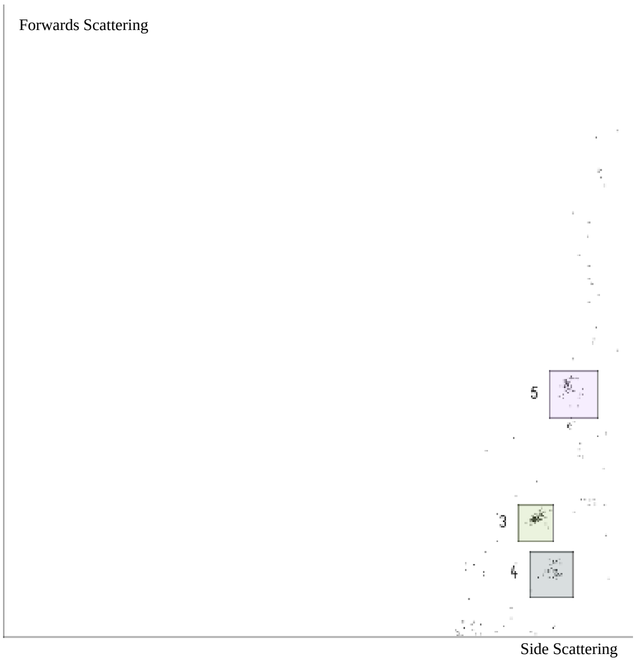

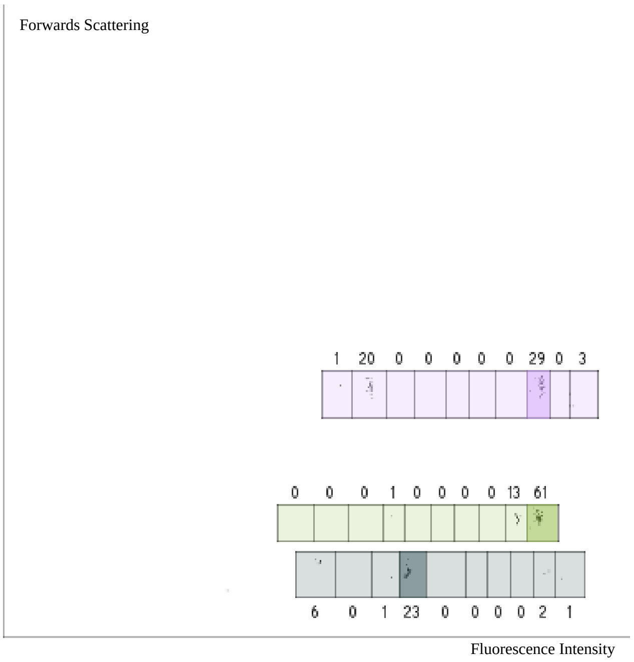

ANNEX 3: TAG DECONVOLUTION - BEAD 101

Passes flow sorting criteria: Yes

Passes tag deconvolution criteria: Yes

Included in protocol analysis: Yes

Protocol: 1, 5, 9, 1

Filename: Bin1_plateB1_B4.fcs

Split 1: Petrol shading

Split 2: Green shading

Split 3: Violet shading

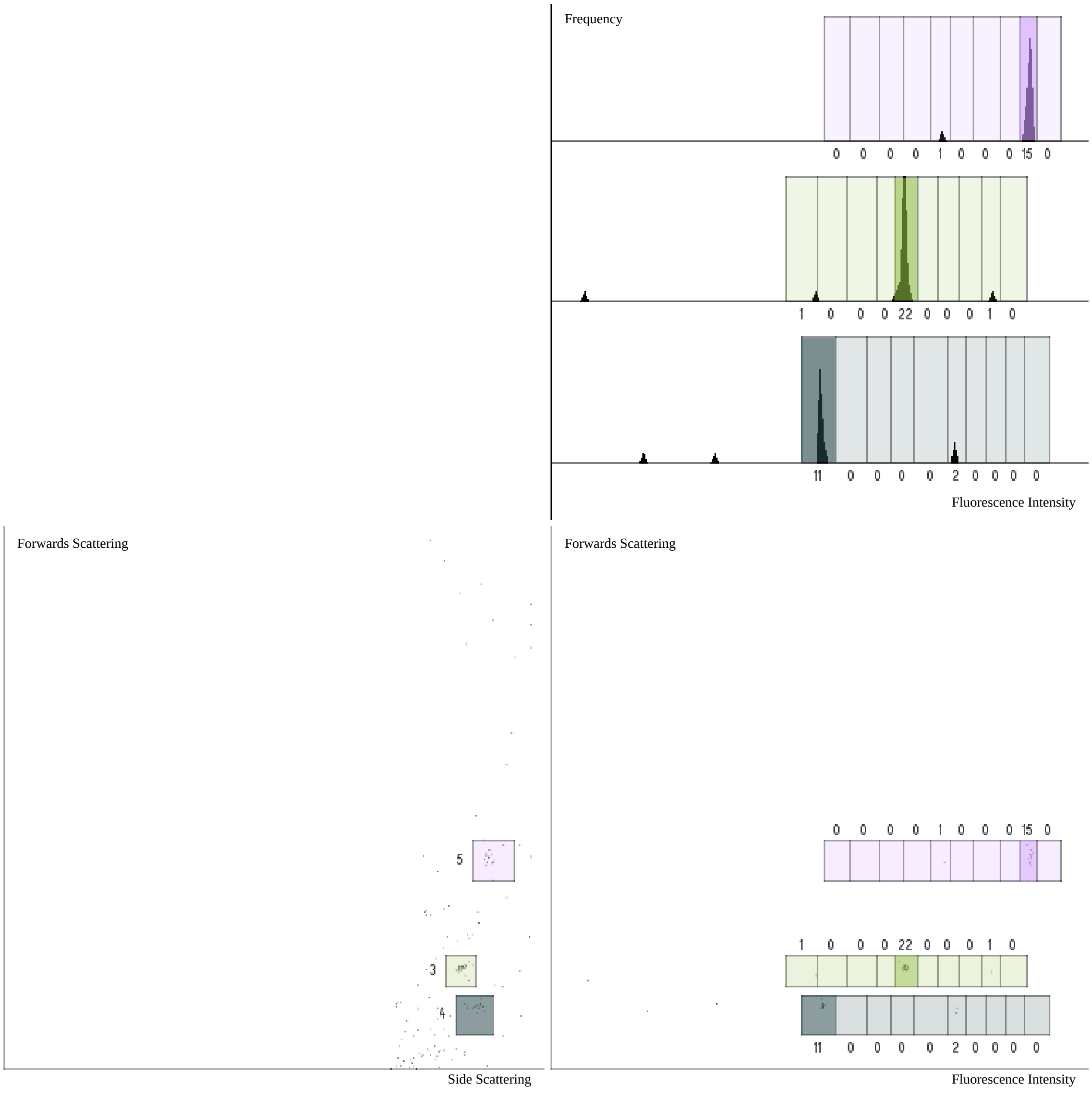

ANNEX 3: TAG DECONVOLUTION - BEAD 102

Passes flow sorting criteria: Yes

Passes tag deconvolution criteria: No

Included in protocol analysis: No

Protocol: N/A

Filename: Bin1_plateB1_C1.fcs

Split 1: Petrol shading

Split 2: Green shading

Split 3: Violet shading

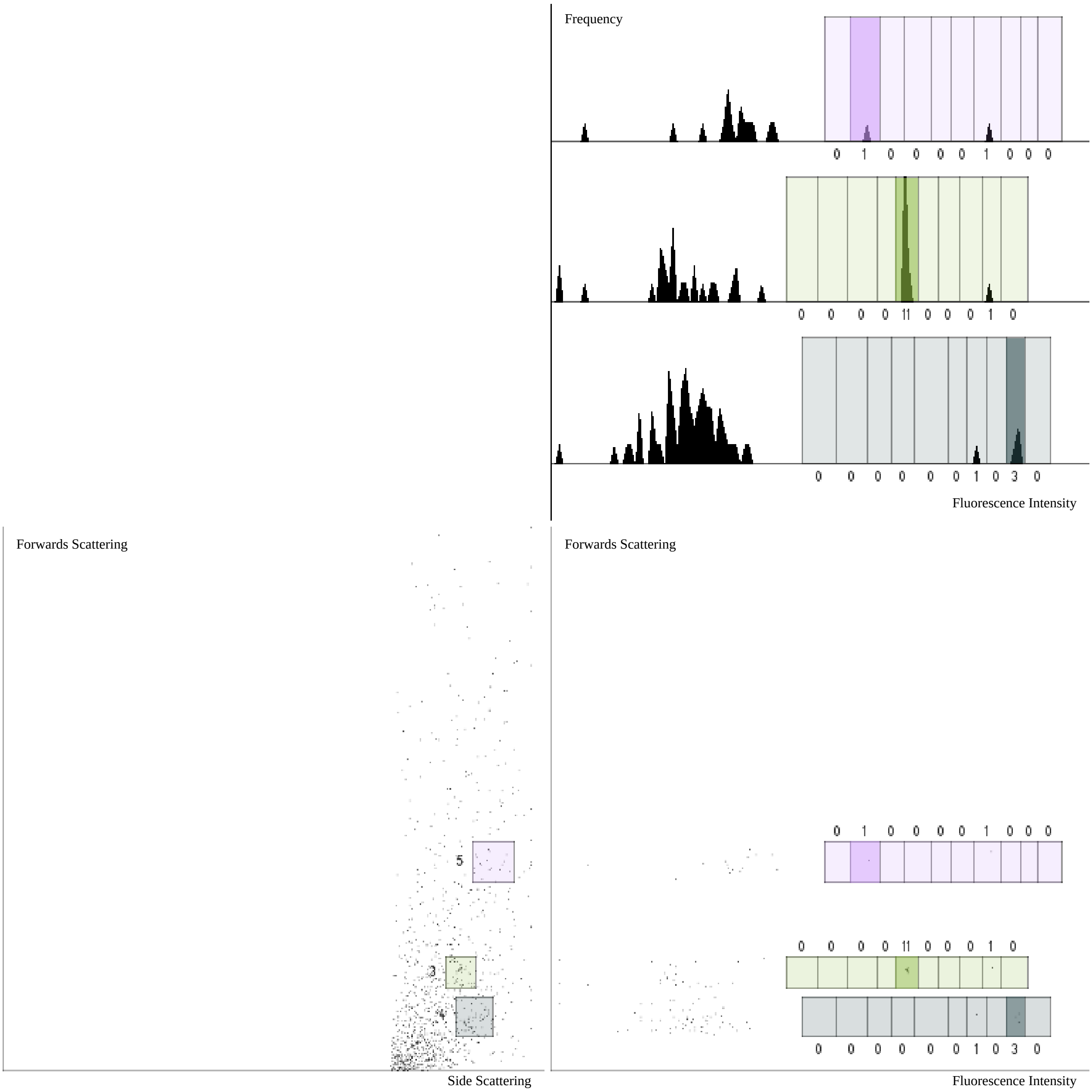

ANNEX 3: TAG DECONVOLUTION - BEAD 103

Passes flow sorting criteria: Yes

Passes tag deconvolution criteria: No

Included in protocol analysis: No

Protocol: N/A

Filename: Bin1_plateB1_C2.fcs

Split 1: Petrol shading

Split 2: Green shading

Split 3: Violet shading

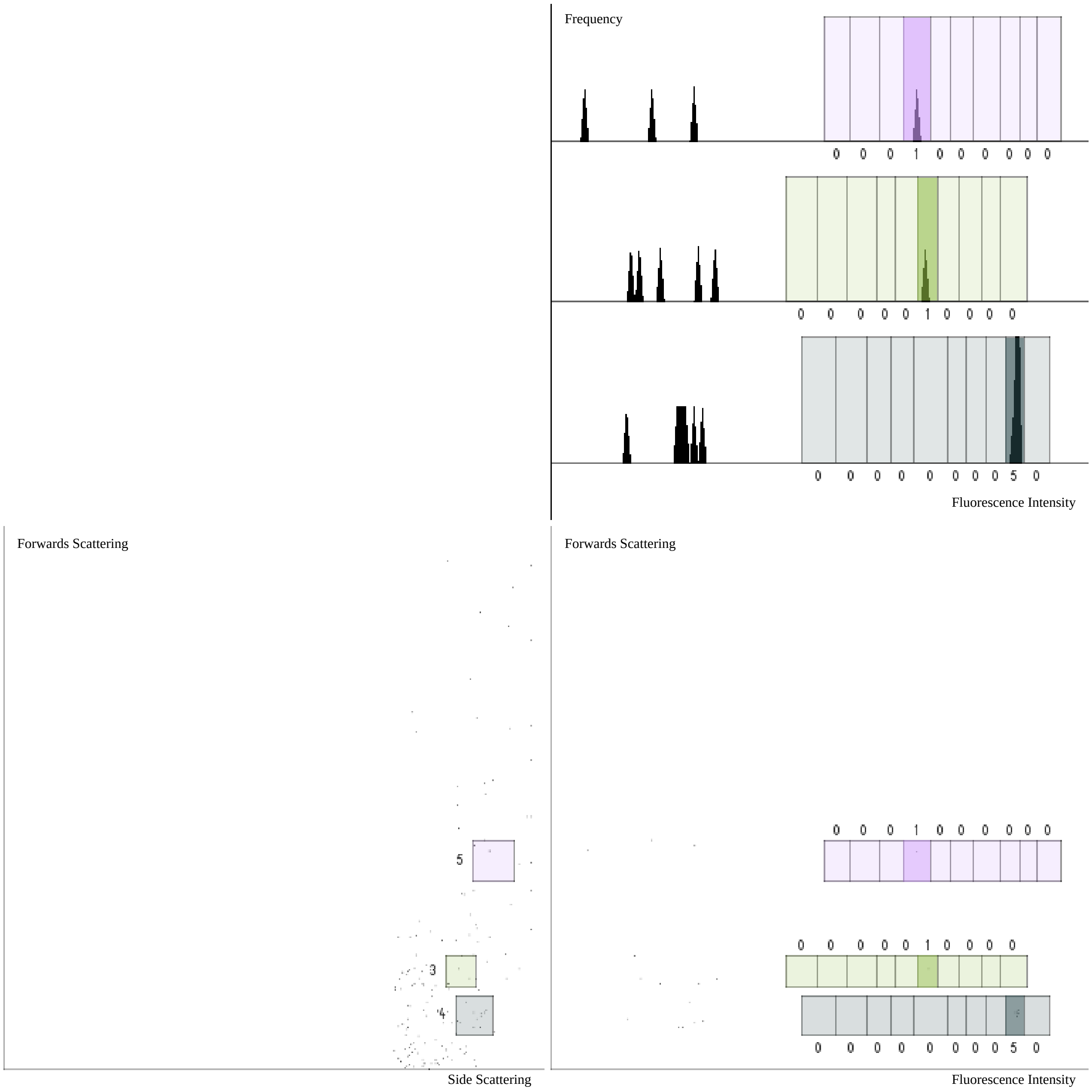

ANNEX 3: TAG DECONVOLUTION - BEAD 104

Passes flow sorting criteria: Yes

Passes tag deconvolution criteria: No

Included in protocol analysis: No

Protocol: N/A

Filename: Bin1_plateB1_C4.fcs

Split 1: Petrol shading

Split 2: Green shading

Split 3: Violet shading

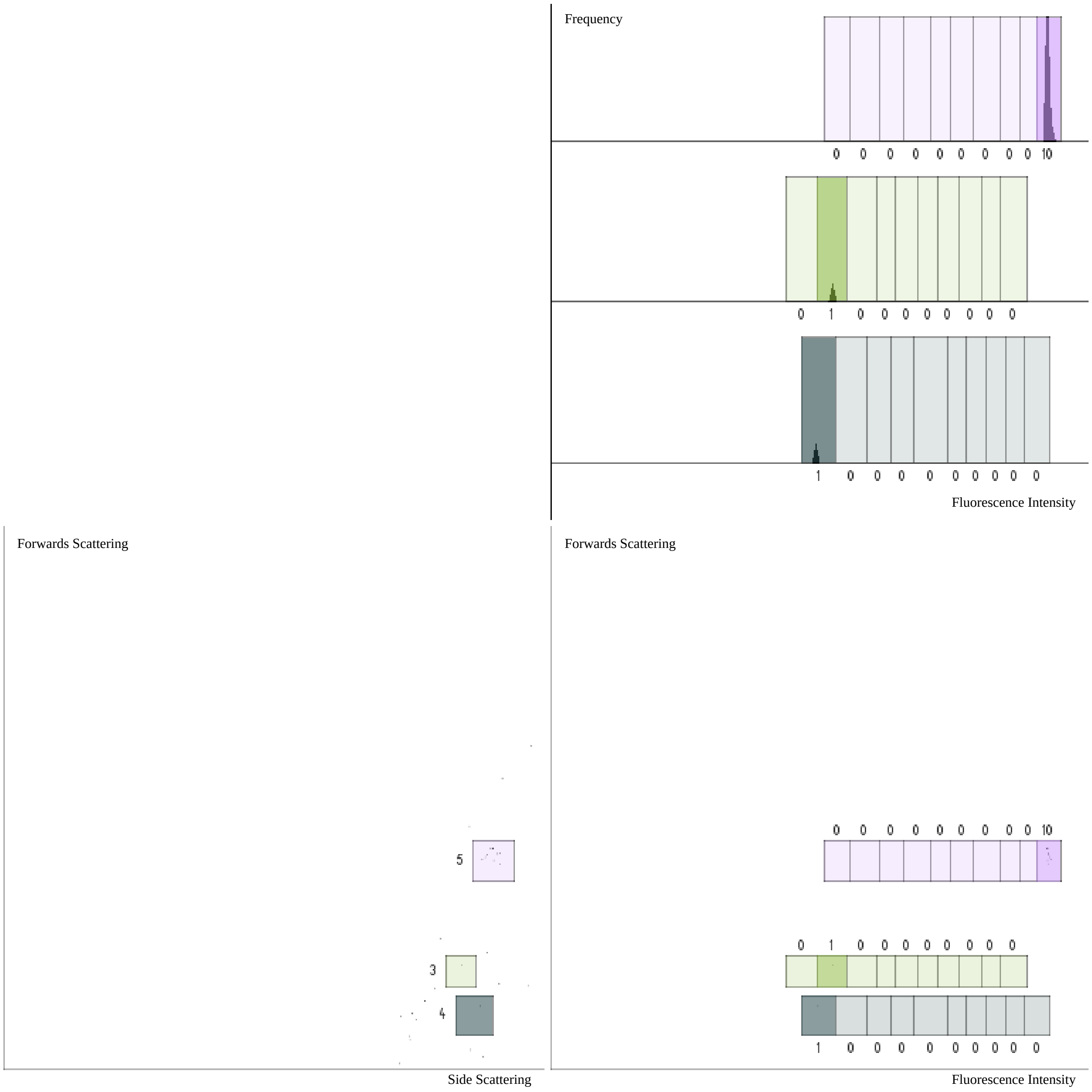

ANNEX 3: TAG DECONVOLUTION - BEAD 105

Passes flow sorting criteria: Yes

Passes tag deconvolution criteria: No

Included in protocol analysis: No

Protocol: N/A

Filename: Bin1_plateB1_C5.fcs

Split 1: Petrol shading

Split 2: Green shading

Split 3: Violet shading

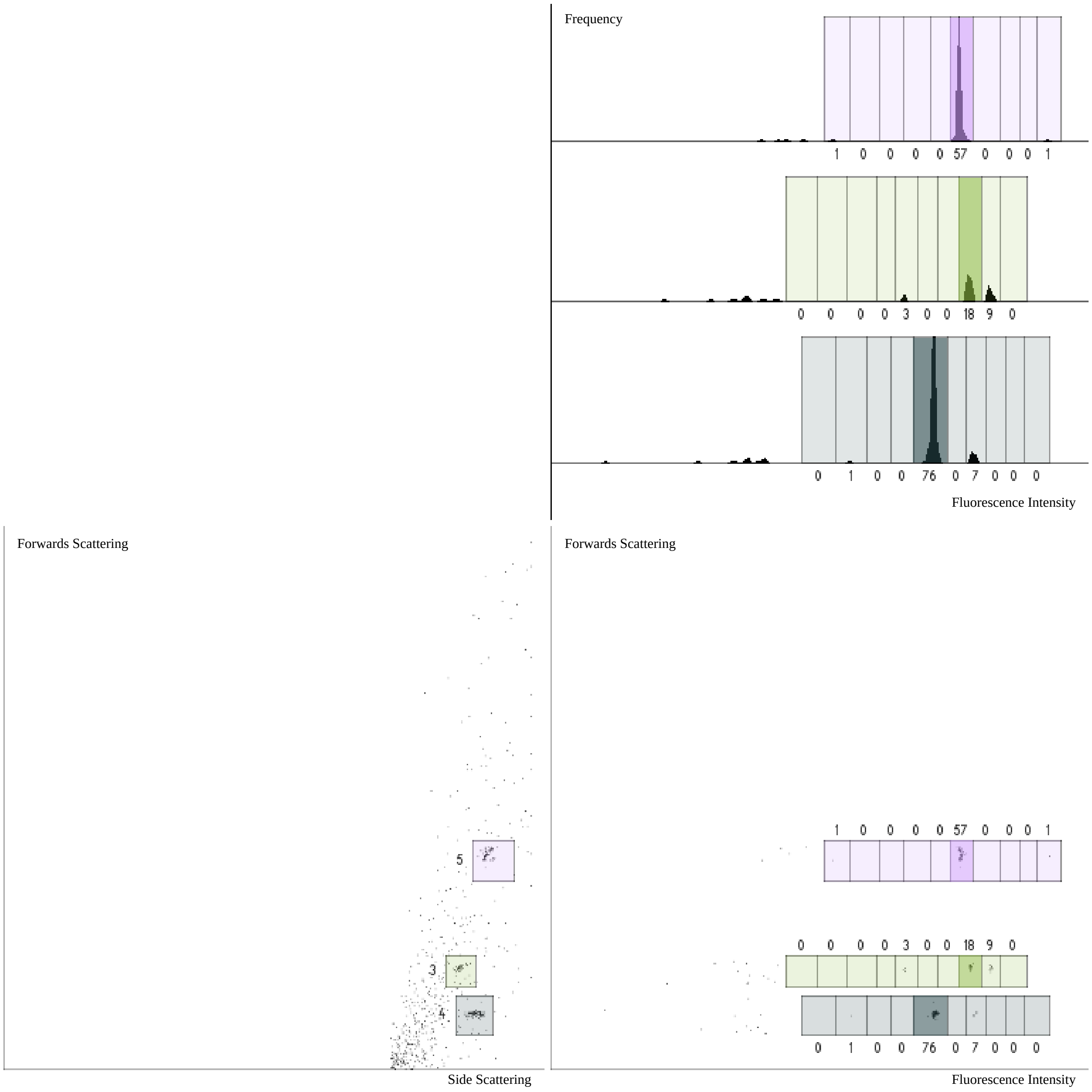

ANNEX 3: TAG DECONVOLUTION - BEAD 106

Passes flow sorting criteria: Yes

Passes tag deconvolution criteria: Yes

Included in protocol analysis: Yes

Protocol: 9, 5, 7, 1

Filename: Bin1_plateB1_C6.fcs

Split 1: Petrol shading

Split 2: Green shading

Split 3: Violet shading

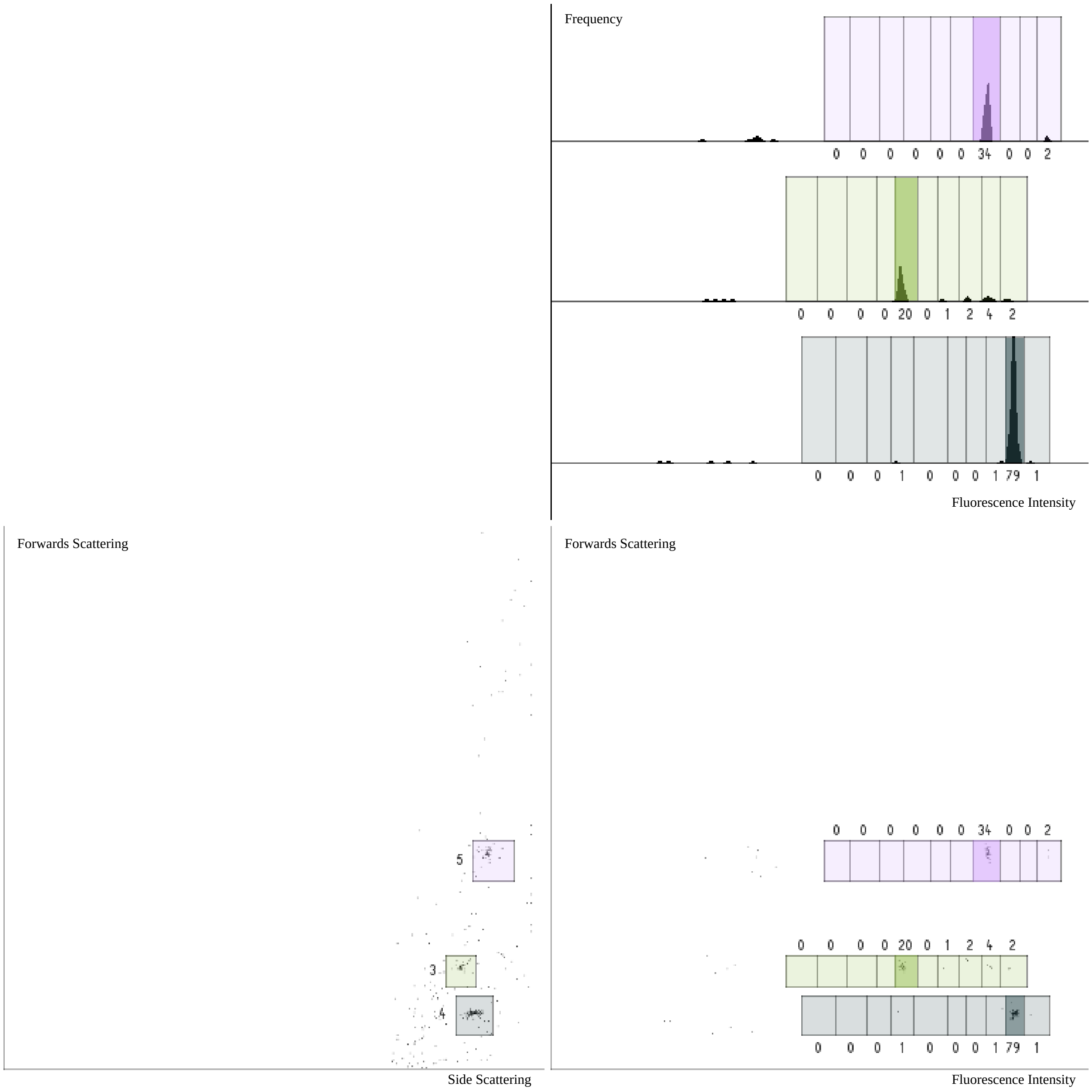

ANNEX 3: TAG DECONVOLUTION - BEAD 107

Passes flow sorting criteria: Yes

Passes tag deconvolution criteria: No

Included in protocol analysis: No

Protocol: N/A

Filename: Bin1_plateB1_C7.fcs

Split 1: Petrol shading

Split 2: Green shading

Split 3: Violet shading

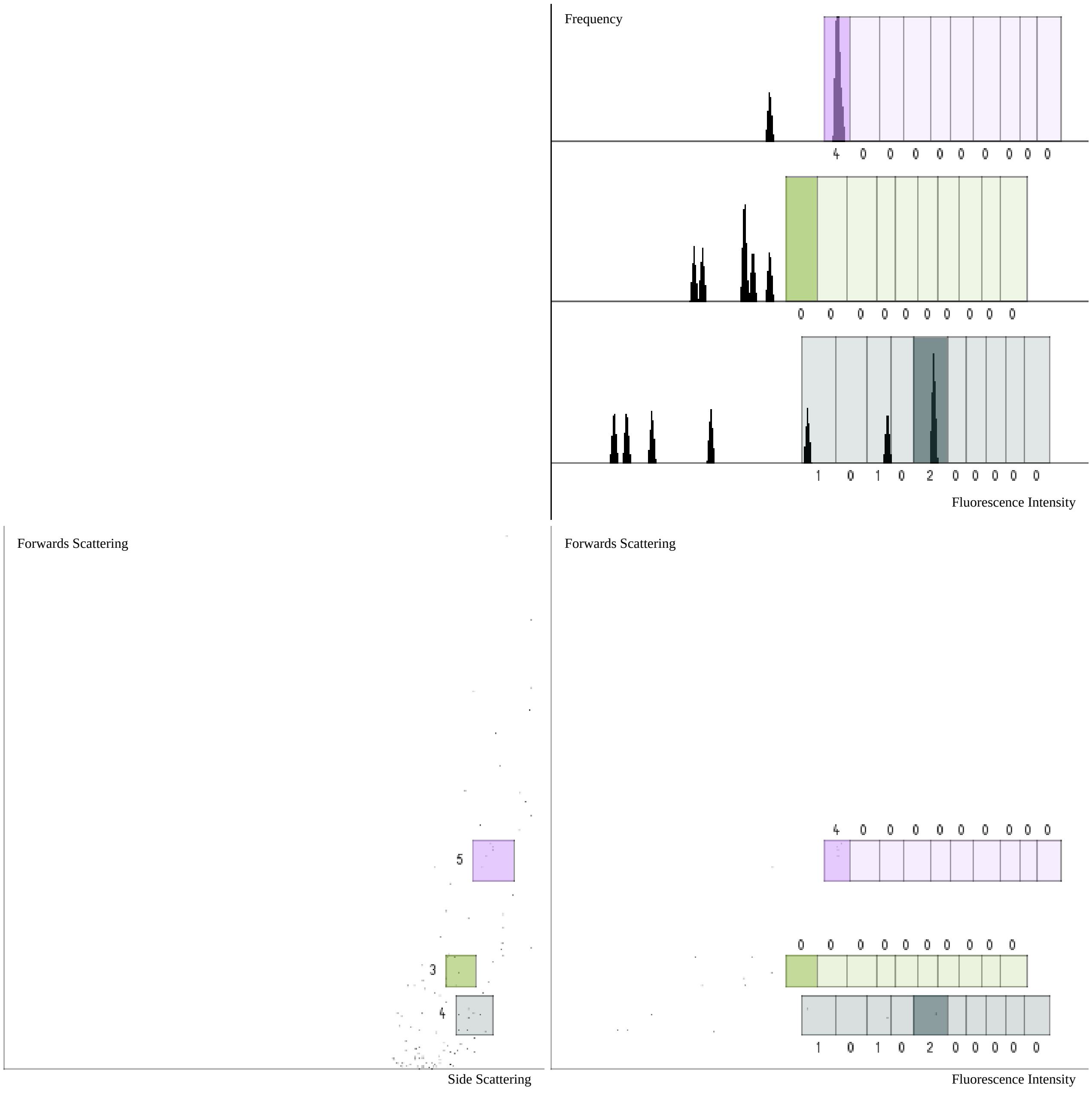

ANNEX 3: TAG DECONVOLUTION - BEAD 108

Passes flow sorting criteria: Yes

Passes tag deconvolution criteria: Yes

Included in protocol analysis: Yes

Protocol: 9, 4, 6, 1

Filename: Bin1_plateB1_D1.fcs

Split 1: Petrol shading

Split 2: Green shading

Split 3: Violet shading

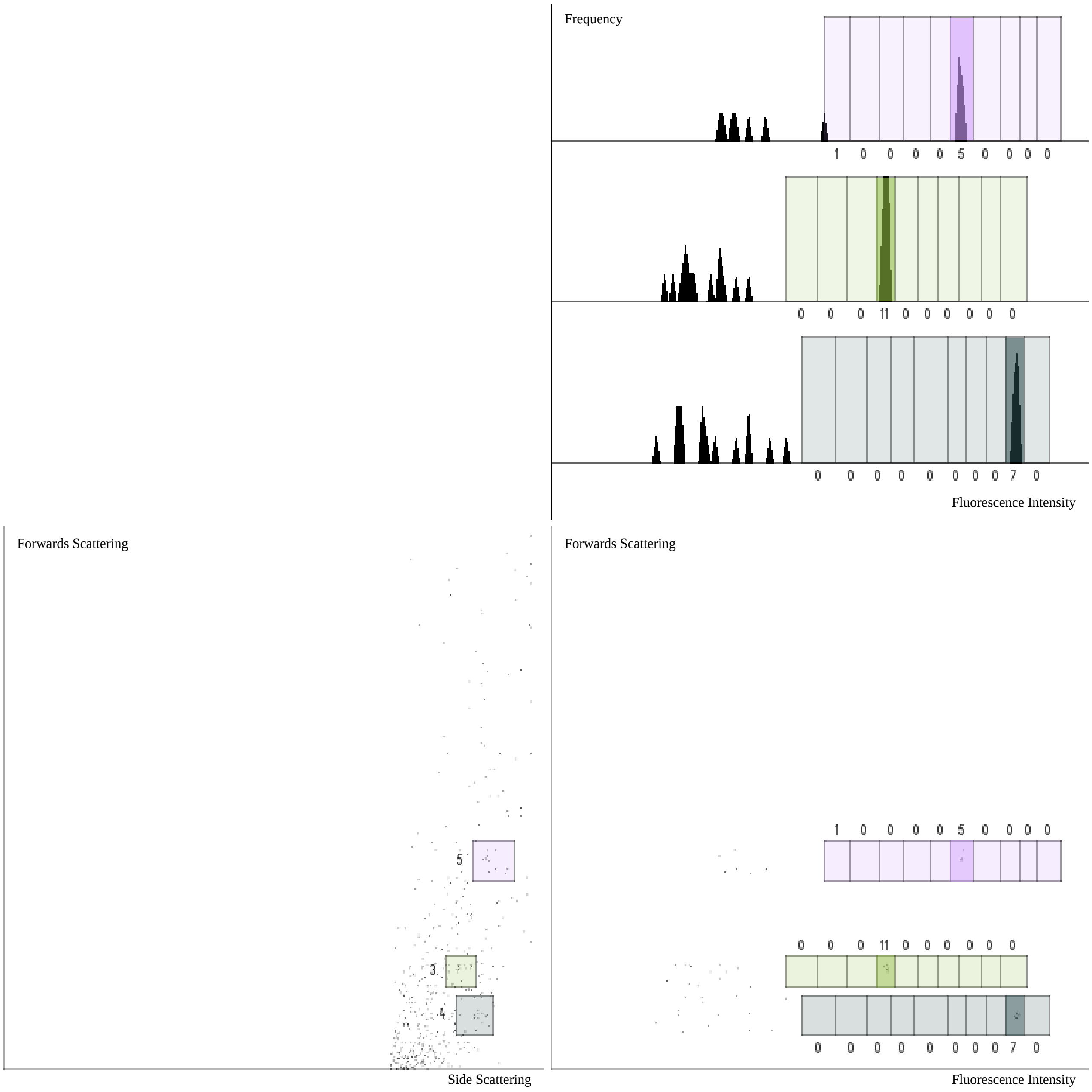

ANNEX 3: TAG DECONVOLUTION - BEAD 109

Passes flow sorting criteria: Yes

Passes tag deconvolution criteria: Yes

Included in protocol analysis: No

Protocol: N/A

Filename: Bin1_plateB1_D4.fcs

Split 1: Petrol shading

Split 2: Green shading

Split 3: Violet shading

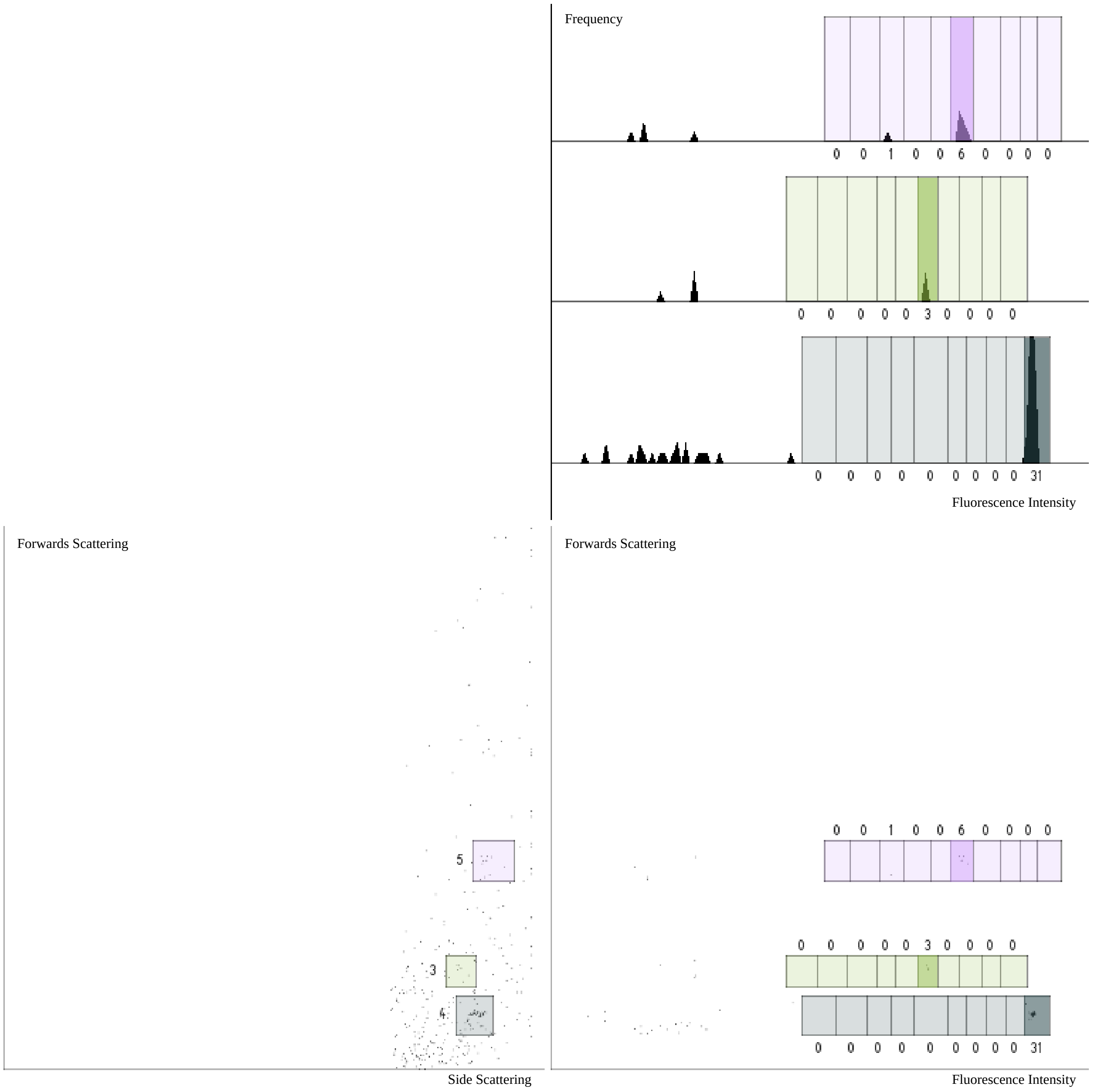

ANNEX 3: TAG DECONVOLUTION - BEAD 110

Passes flow sorting criteria: Yes

Passes tag deconvolution criteria: Yes

Included in protocol analysis: Yes

Protocol: 7, 3, 1, 1

Filename: Bin1_plateB1_D6.fcs

Split 1: Petrol shading

Split 2: Green shading

Split 3: Violet shading

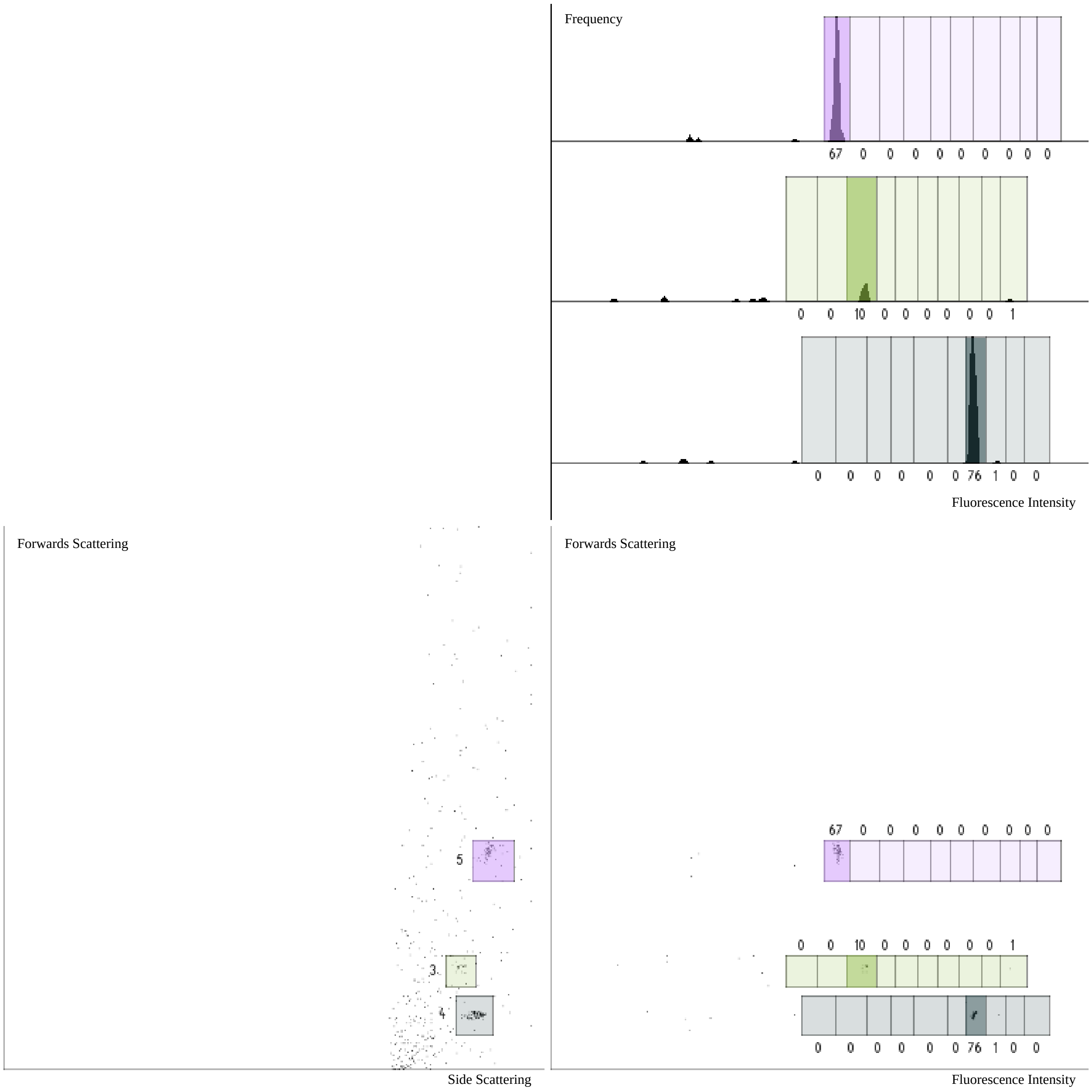

ANNEX 3: TAG DECONVOLUTION - BEAD 111

Passes flow sorting criteria: Yes

Passes tag deconvolution criteria: No

Included in protocol analysis: No

Protocol: N/A

Filename: Bin1_plateB1_D7.fcs

Split 1: Petrol shading

Split 2: Green shading

Split 3: Violet shading

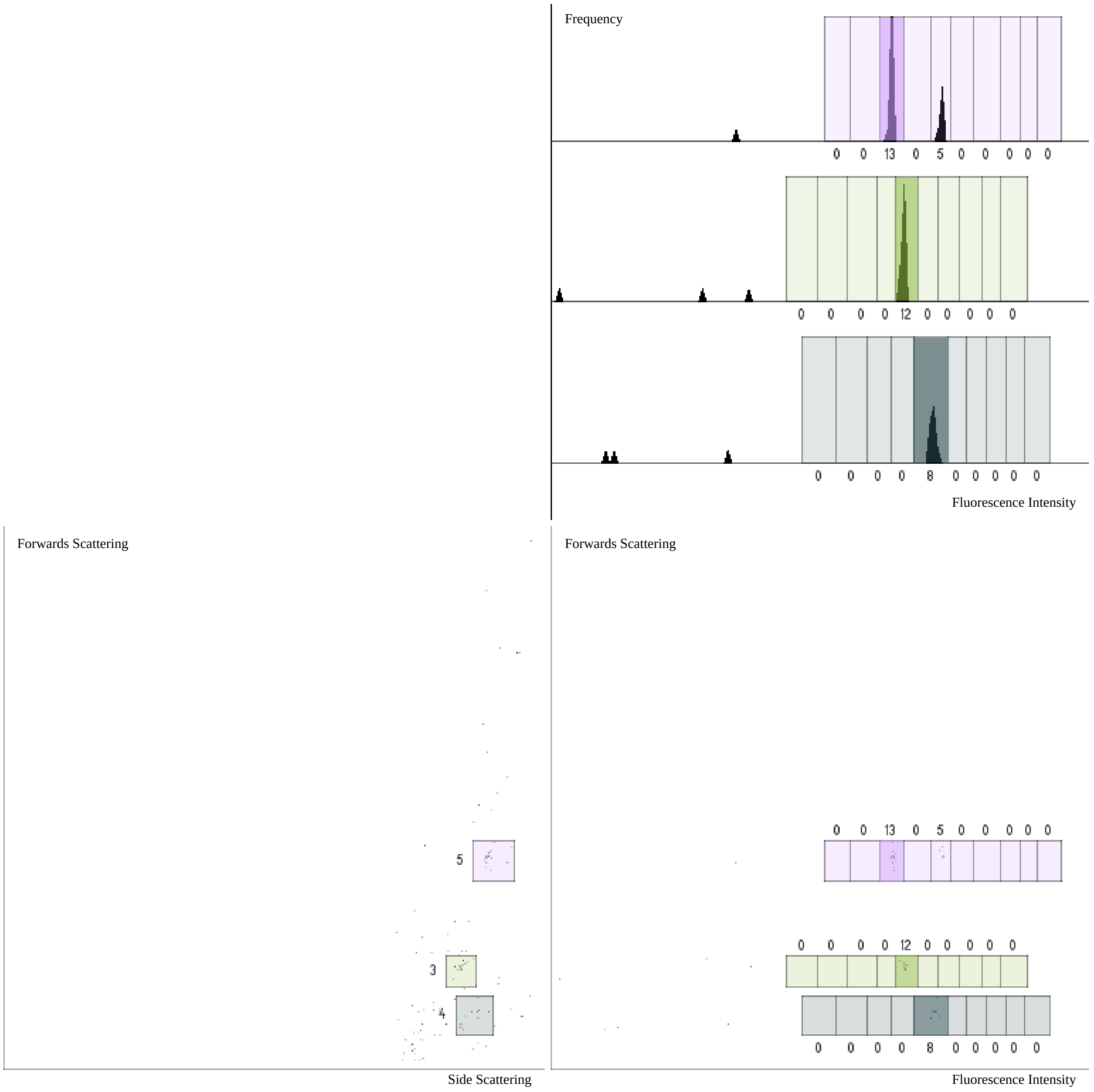

ANNEX 3: TAG DECONVOLUTION - BEAD 112

Passes flow sorting criteria: Yes

Passes tag deconvolution criteria: Yes

Included in protocol analysis: Yes

Protocol: 8, 4, 7, 1

Filename: Bin1_plateB1_E3.fcs

Split 1: Petrol shading

Split 2: Green shading

Split 3: Violet shading

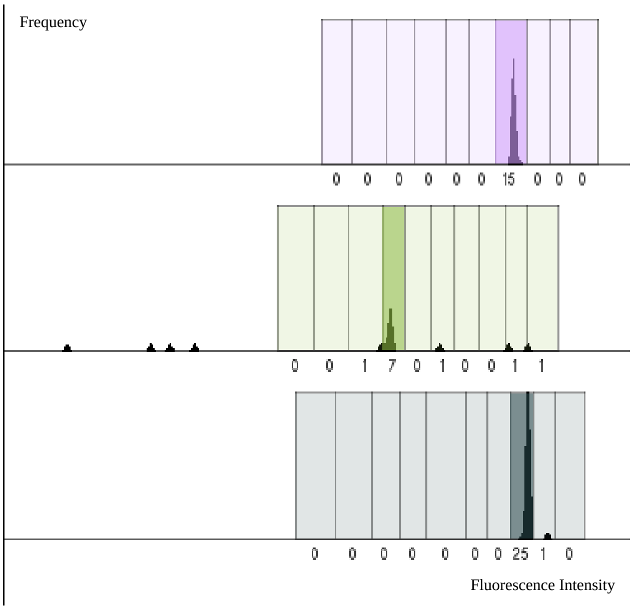

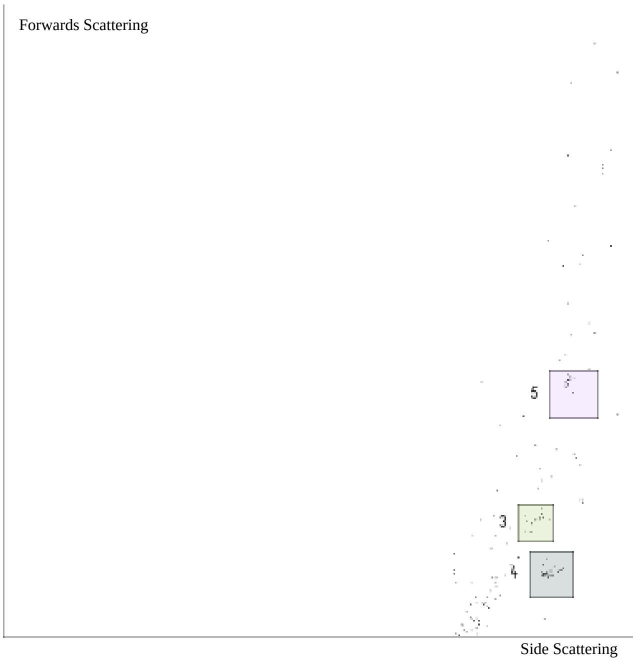

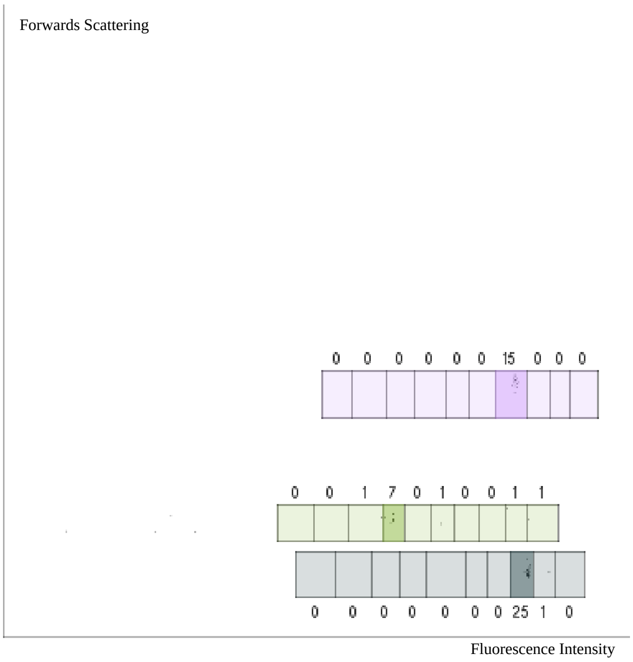

ANNEX 3: TAG DECONVOLUTION - BEAD 113

Passes flow sorting criteria: Yes

Passes tag deconvolution criteria: Yes

Included in protocol analysis: Yes

Protocol: 9, 2, 10, 1

Filename: Bin1_plateB1_E6.fcs

Split 1: Petrol shading

Split 2: Green shading

Split 3: Violet shading

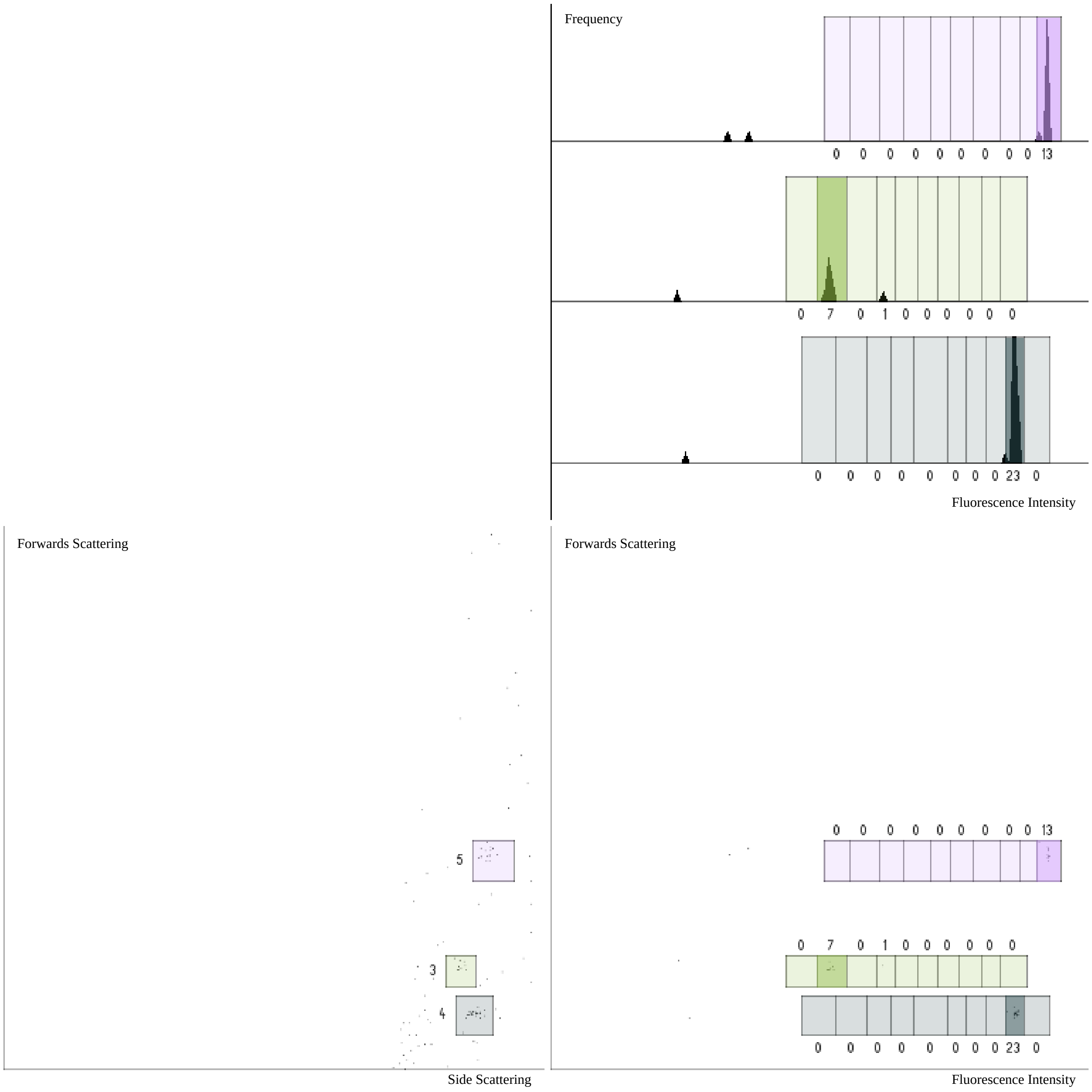

ANNEX 3: TAG DECONVOLUTION - BEAD 114

Passes flow sorting criteria: Yes

Passes tag deconvolution criteria: Yes

Included in protocol analysis: Yes

Protocol: 4, 10, 4, 1

Filename: Bin1_plateB1_E7.fcs

Split 1: Petrol shading

Split 2: Green shading

Split 3: Violet shading

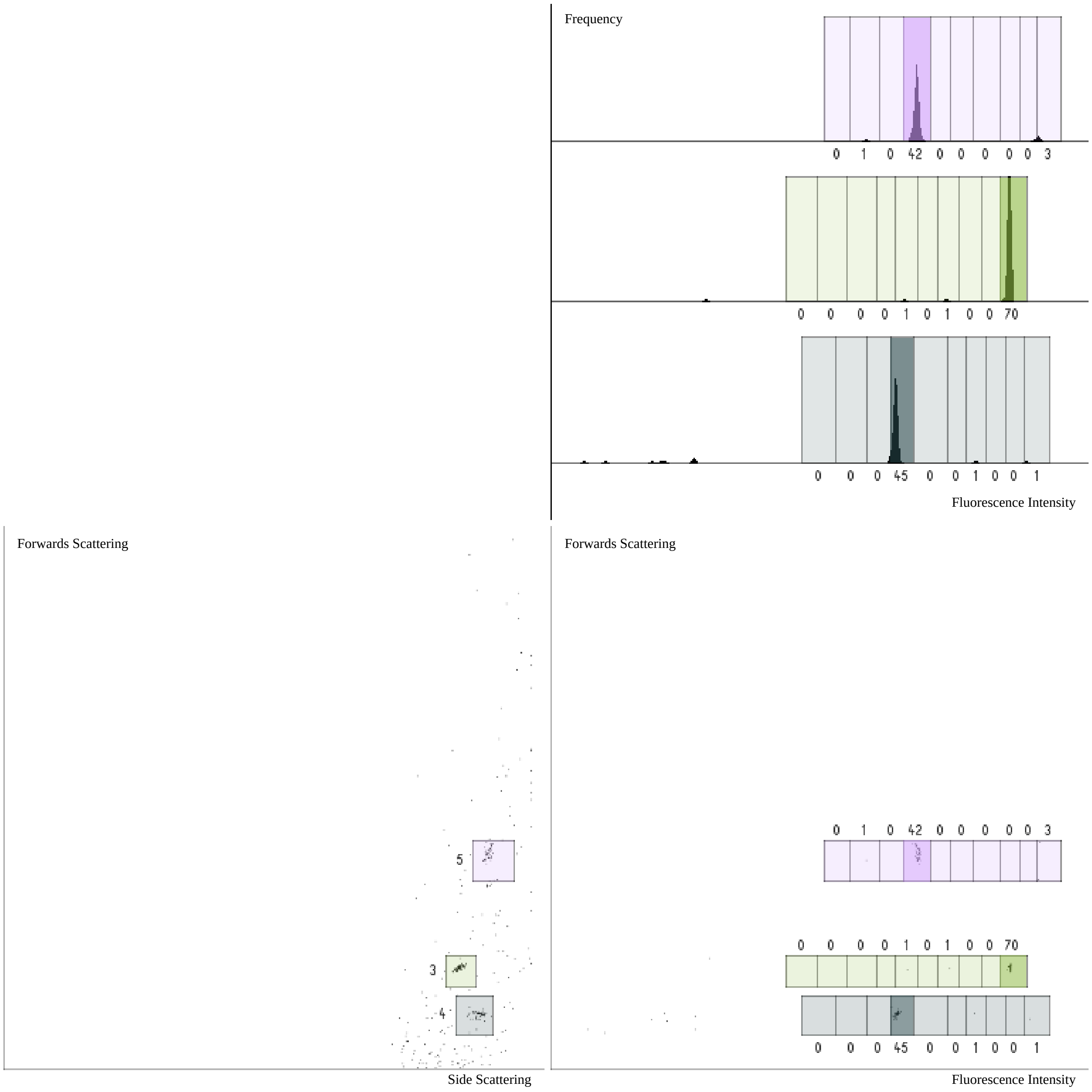

ANNEX 3: TAG DECONVOLUTION - BEAD 115

Passes flow sorting criteria: Yes

Passes tag deconvolution criteria: No

Included in protocol analysis: No

Protocol: N/A

Filename: Bin1_plateB1_F1.fcs

Split 1: Petrol shading

Split 2: Green shading

Split 3: Violet shading

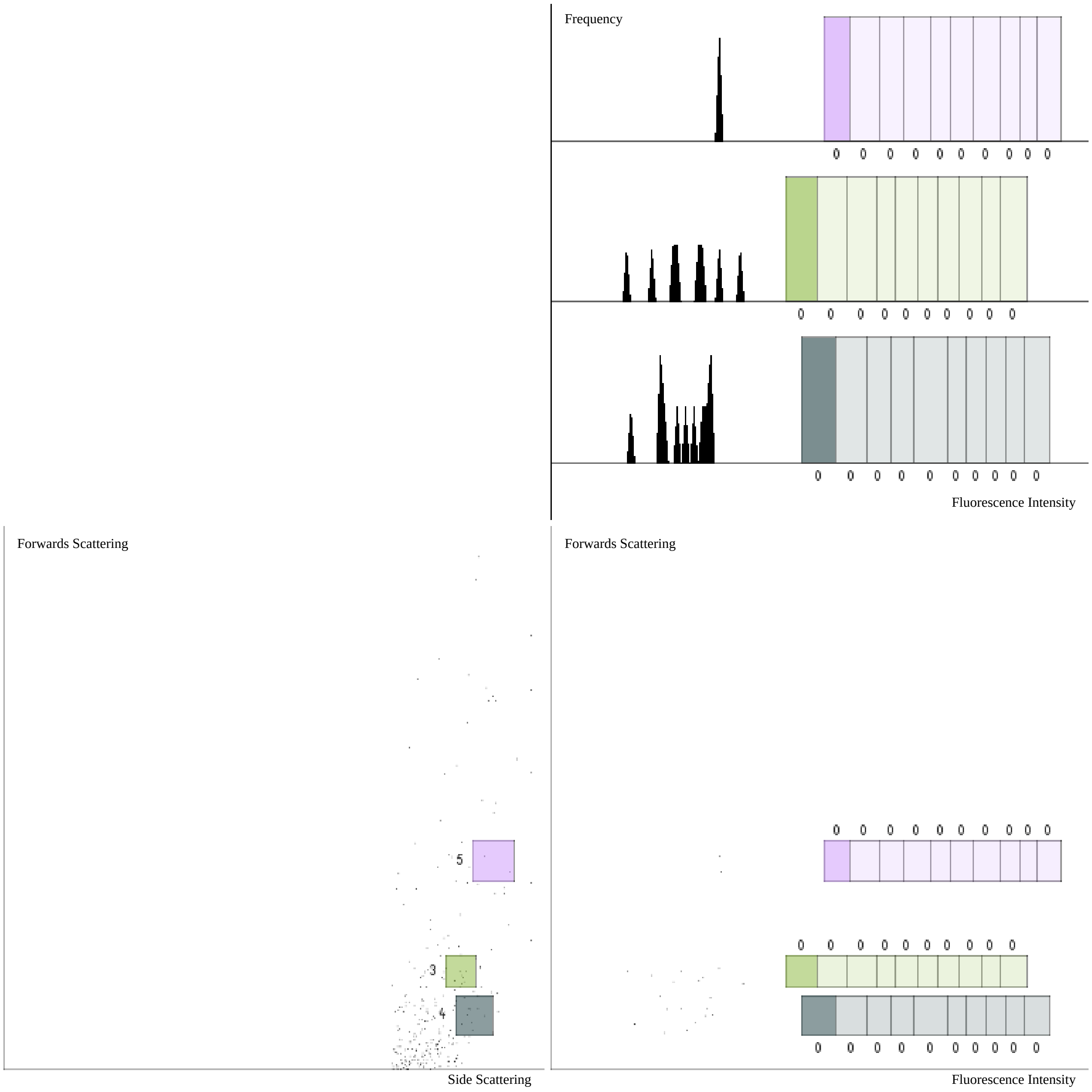

ANNEX 3: TAG DECONVOLUTION - BEAD 116

Passes flow sorting criteria: Yes

Passes tag deconvolution criteria: Yes

Included in protocol analysis: Yes

Protocol: 3, 10, 4, 1

Filename: Bin1_plateB1_F2.fcs

Split 1: Petrol shading

Split 2: Green shading

Split 3: Violet shading

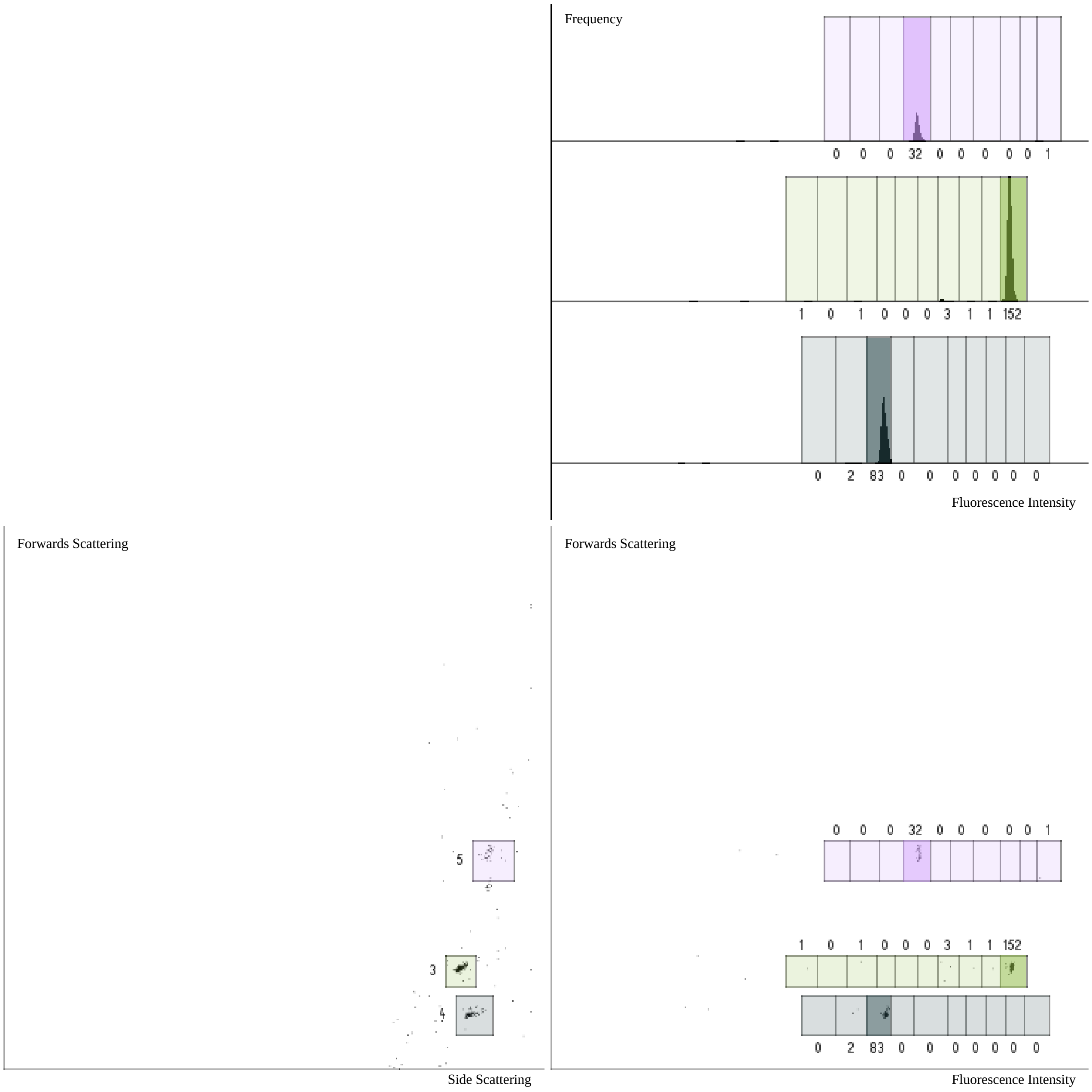

ANNEX 3: TAG DECONVOLUTION - BEAD 117

Passes flow sorting criteria: Yes

Passes tag deconvolution criteria: Yes

Included in protocol analysis: Yes

Protocol: 10, 6, 1, 1

Filename: Bin1_plateB1_F4.fcs

Split 1: Petrol shading

Split 2: Green shading

Split 3: Violet shading

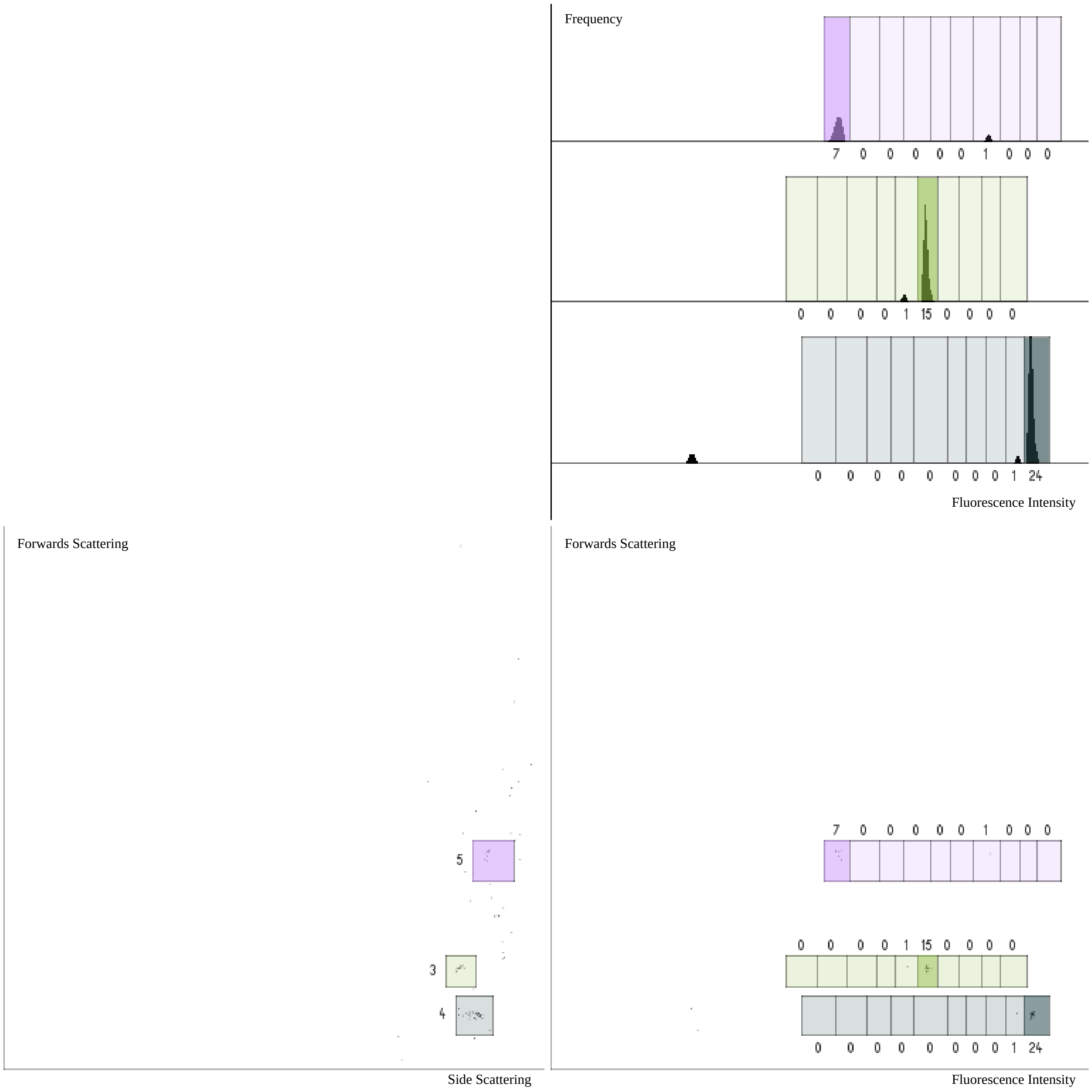

ANNEX 3: TAG DECONVOLUTION - BEAD 118

Passes flow sorting criteria: Yes

Passes tag deconvolution criteria: Yes

Included in protocol analysis: Yes

Protocol: 9, 7, 3, 1

Filename: Bin1_plateB1_F5.fcs

Split 1: Petrol shading

Split 2: Green shading

Split 3: Violet shading

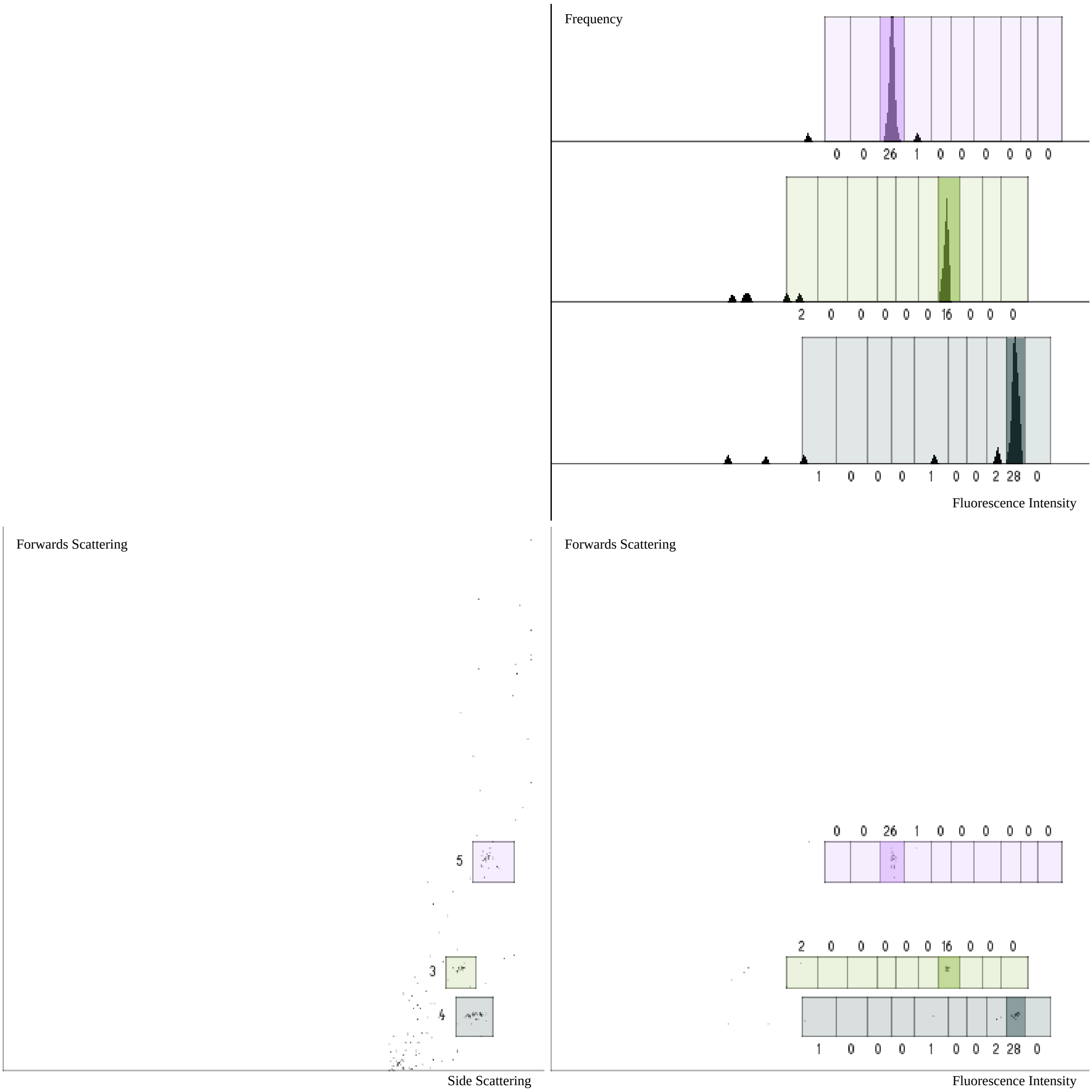

ANNEX 3: TAG DECONVOLUTION - BEAD 119

Passes flow sorting criteria: Yes

Passes tag deconvolution criteria: Yes

Included in protocol analysis: Yes

Protocol: 4, 1, 2, 1

Filename: Bin1_plateB1_F6.fcs

Split 1: Petrol shading

Split 2: Green shading

Split 3: Violet shading

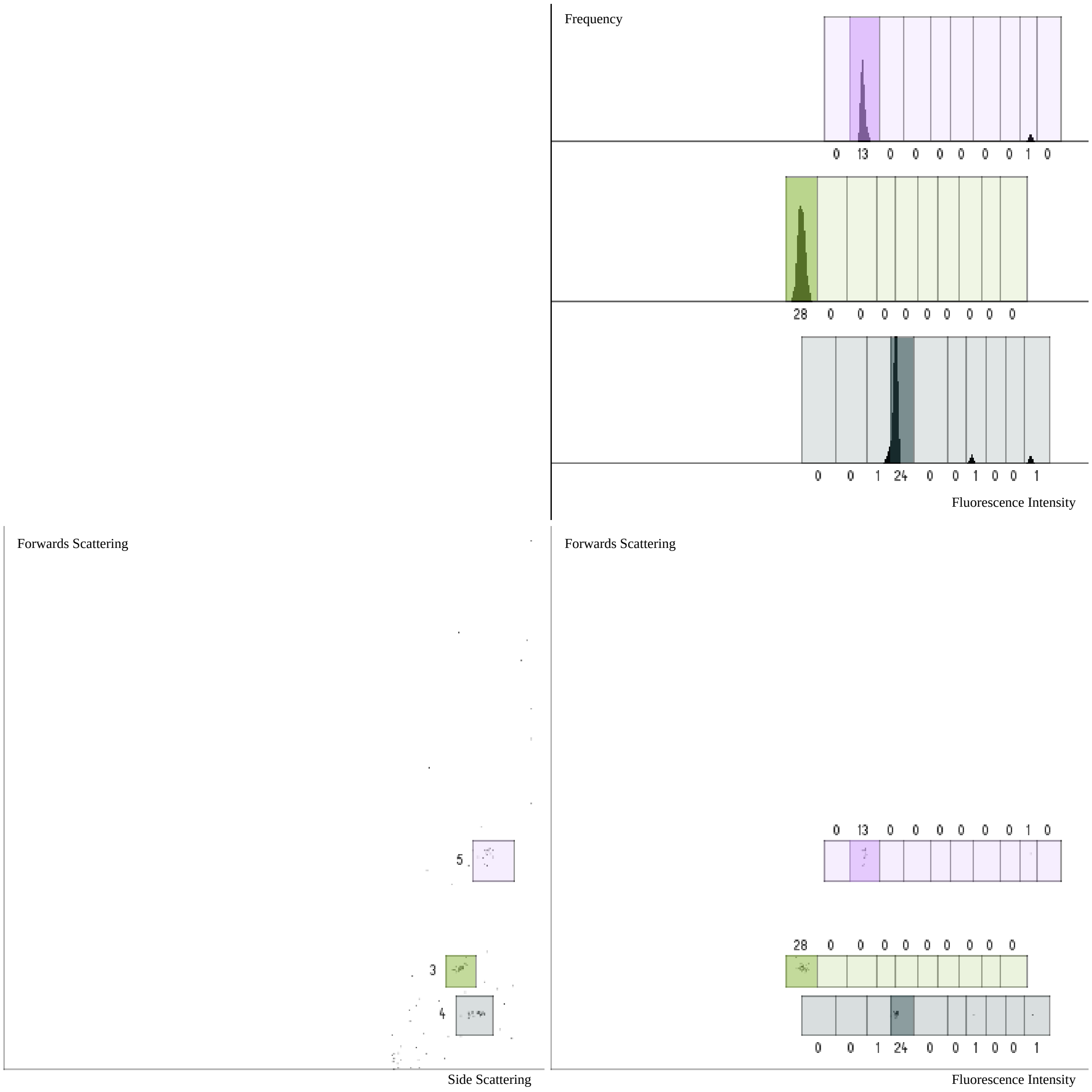

ANNEX 3: TAG DECONVOLUTION - BEAD 120

Passes flow sorting criteria: Yes

Passes tag deconvolution criteria: Yes

Included in protocol analysis: Yes

Protocol: 10, 2, 9, 1

Filename: Bin1_plateB1_F7.fcs

Split 1: Petrol shading

Split 2: Green shading

Split 3: Violet shading

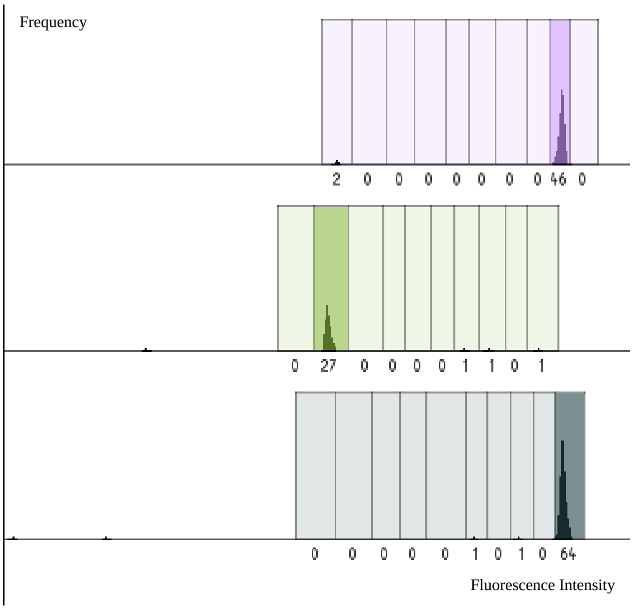

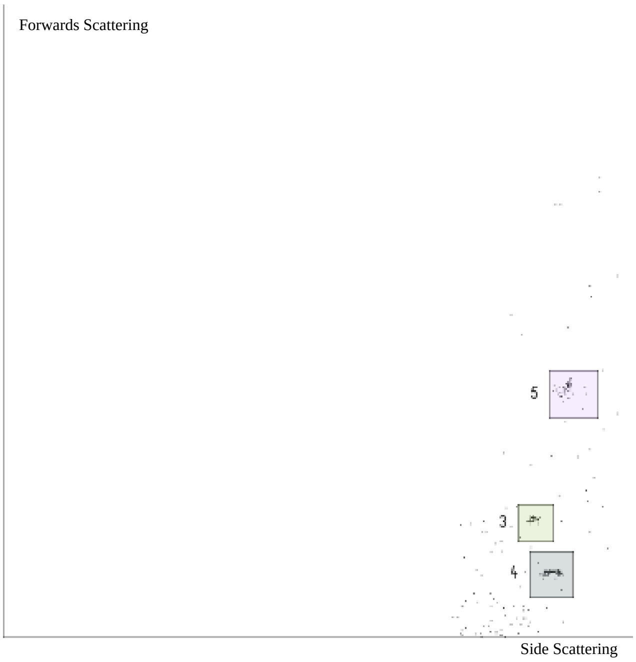

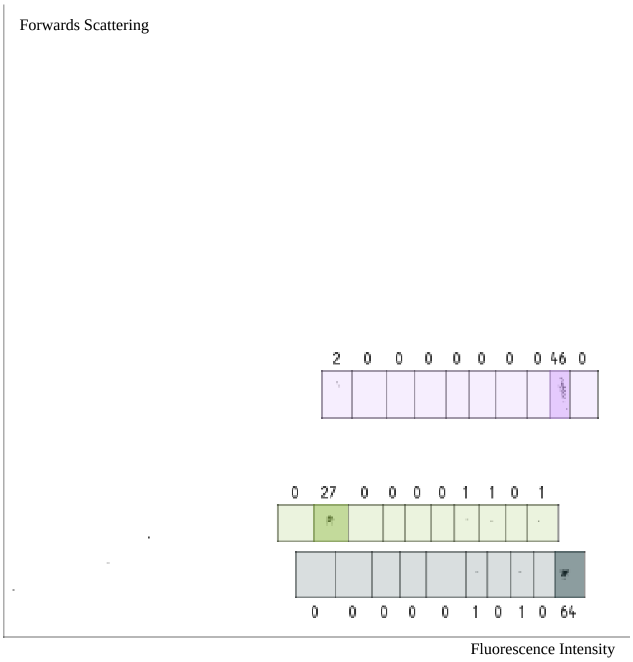

ANNEX 3: TAG DECONVOLUTION - BEAD 121

Passes flow sorting criteria: Yes

Passes tag deconvolution criteria: Yes

Included in protocol analysis: Yes

Protocol: 5, 9, 2, 1

Filename: Bin1_plateB1_G2.fcs

Split 1: Petrol shading

Split 2: Green shading

Split 3: Violet shading

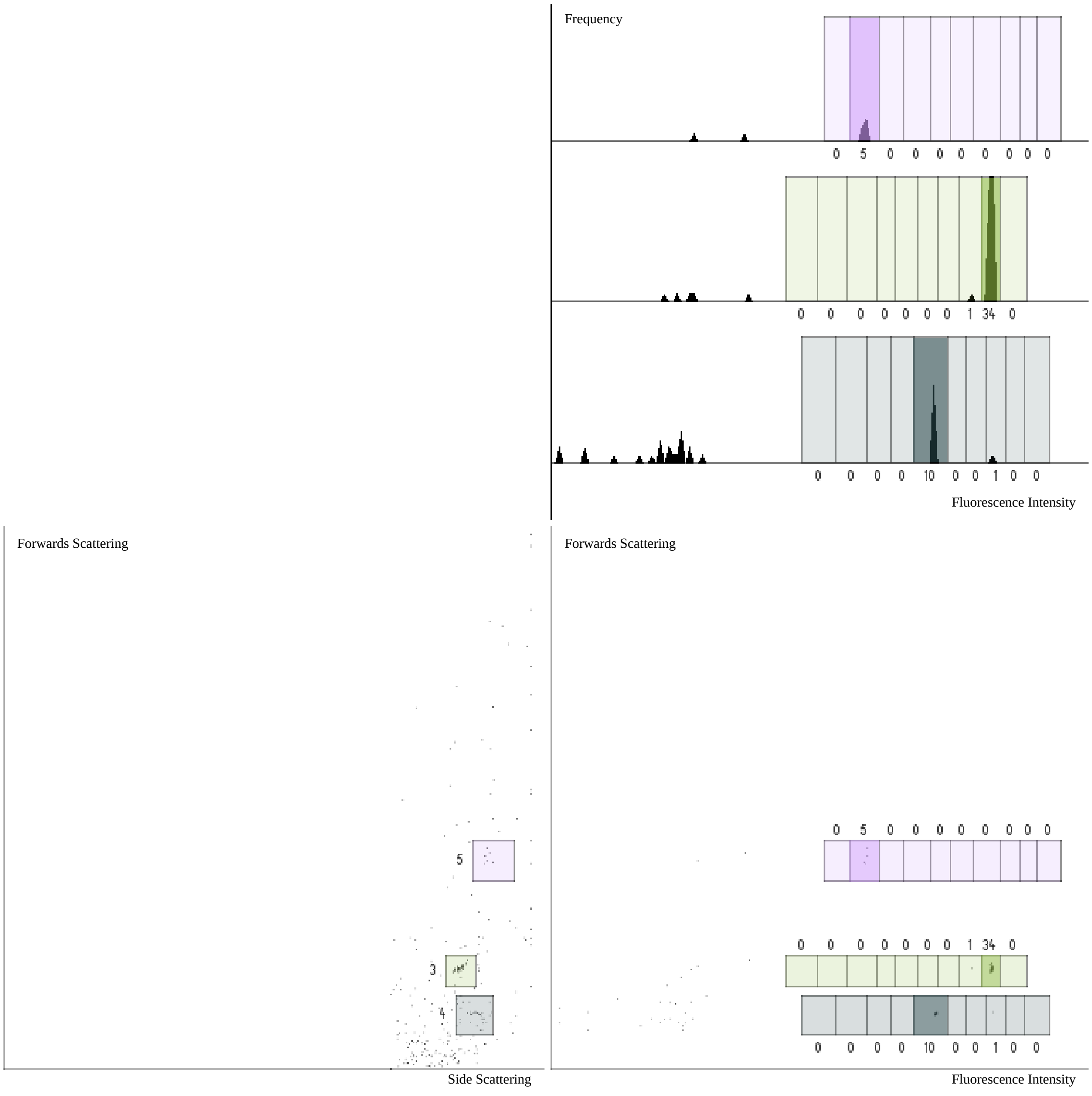

ANNEX 3: TAG DECONVOLUTION - BEAD 122

Passes flow sorting criteria: Yes

Passes tag deconvolution criteria: No

Included in protocol analysis: No

Protocol: N/A

Filename: Bin1_plateB1_G3.fcs

Split 1: Petrol shading

Split 2: Green shading

Split 3: Violet shading

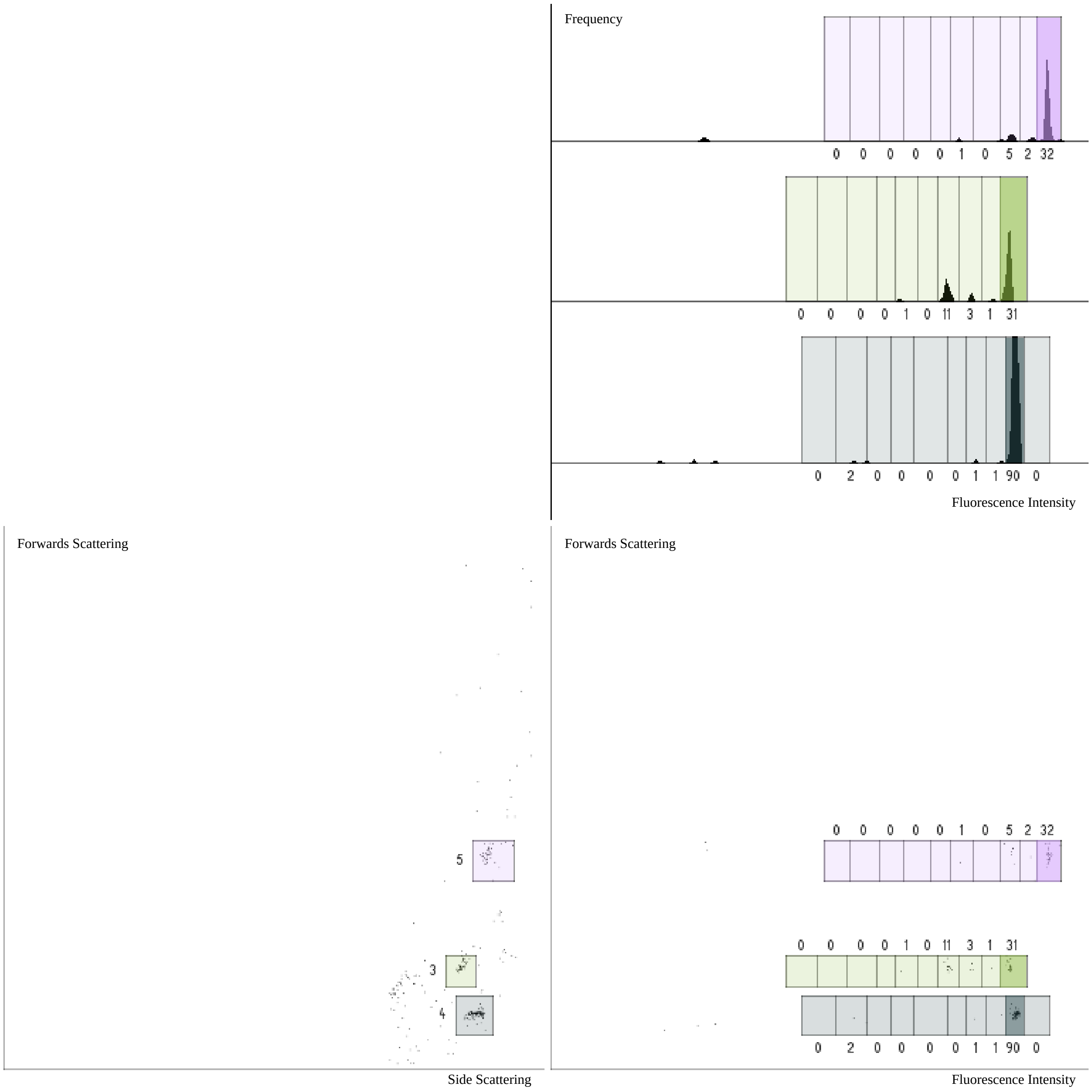

ANNEX 3: TAG DECONVOLUTION - BEAD 123

Passes flow sorting criteria: Yes

Passes tag deconvolution criteria: No

Included in protocol analysis: No

Protocol: N/A

Filename: Bin1_plateB1_G4.fcs

Split 1: Petrol shading

Split 2: Green shading

Split 3: Violet shading

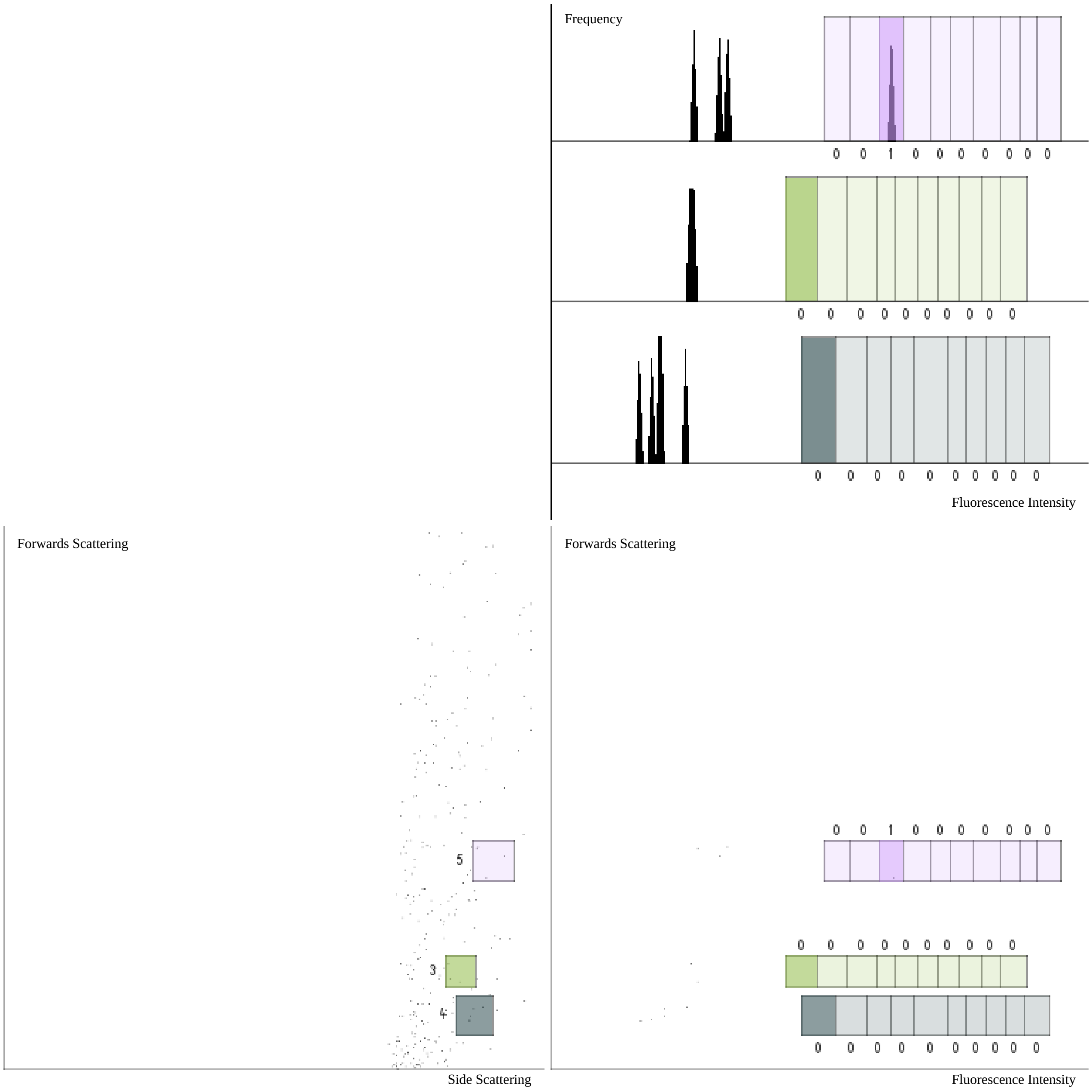

ANNEX 3: TAG DECONVOLUTION - BEAD 124

Passes flow sorting criteria: Yes

Passes tag deconvolution criteria: Yes

Included in protocol analysis: Yes

Protocol: 8, 10, 9, 1

Filename: Bin1_plateB1_G5.fcs

Split 1: Petrol shading

Split 2: Green shading

Split 3: Violet shading

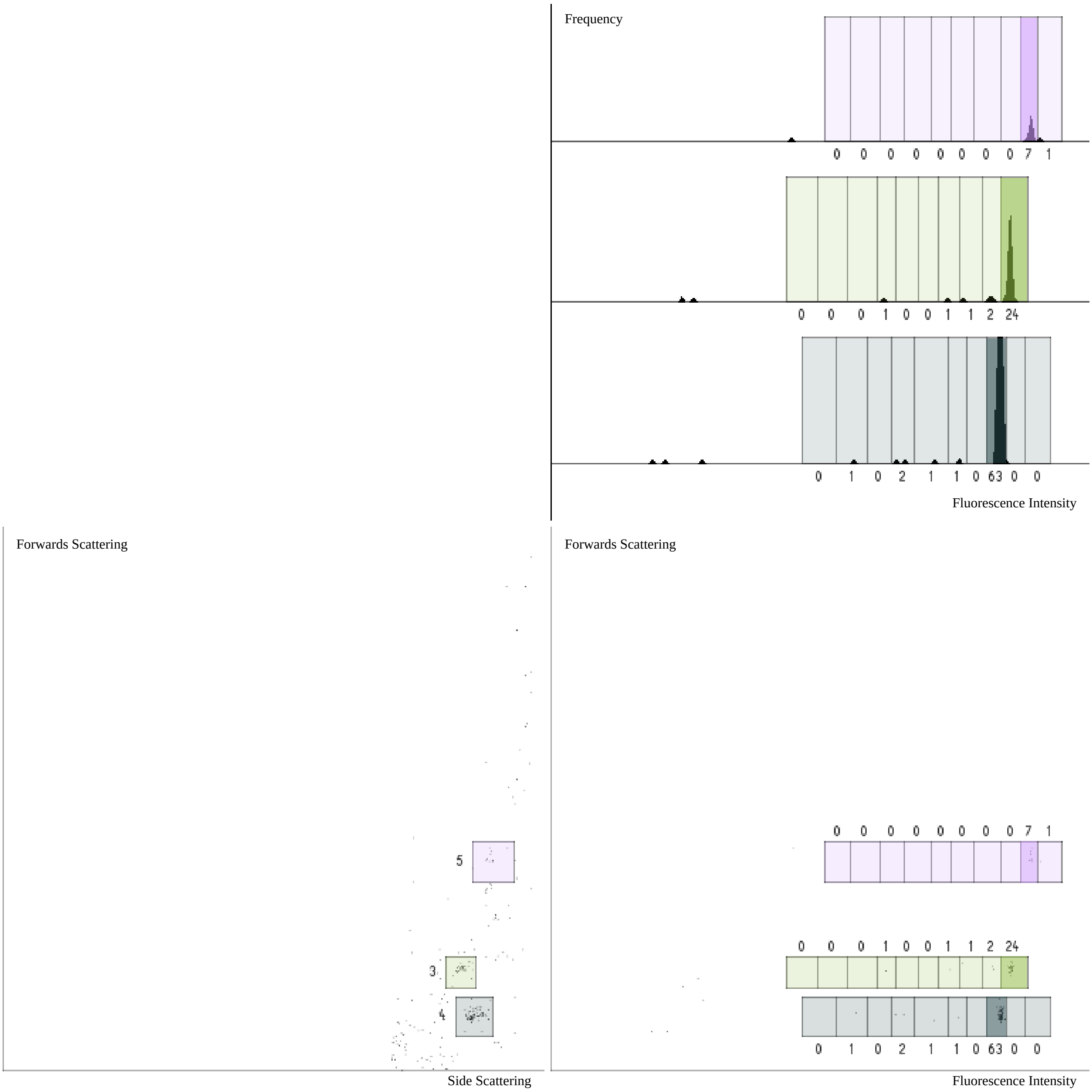

ANNEX 3: TAG DECONVOLUTION - BEAD 125

Passes flow sorting criteria: Yes

Passes tag deconvolution criteria: Yes

Included in protocol analysis: Yes

Protocol: 1, 4, 10, 1

Filename: Bin1_plateB1_H1.fcs

Split 1: Petrol shading

Split 2: Green shading

Split 3: Violet shading

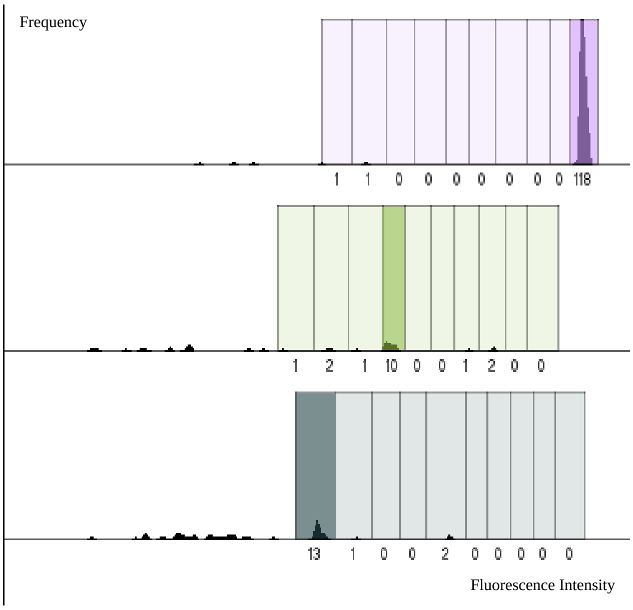

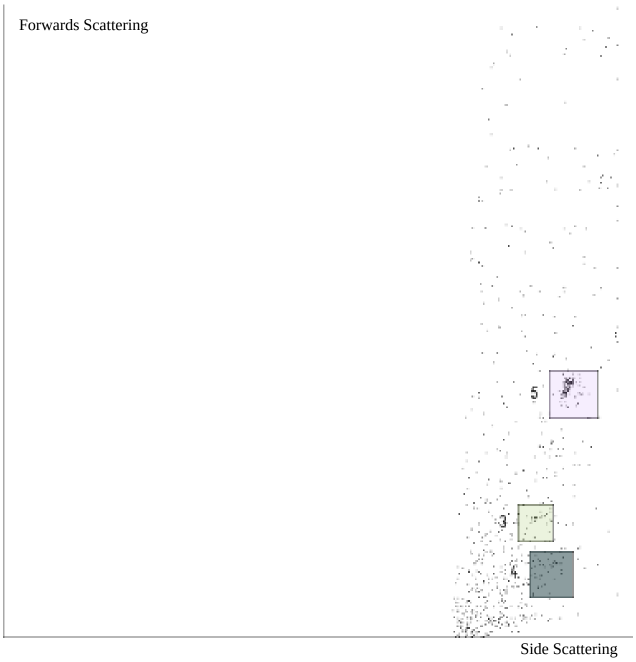

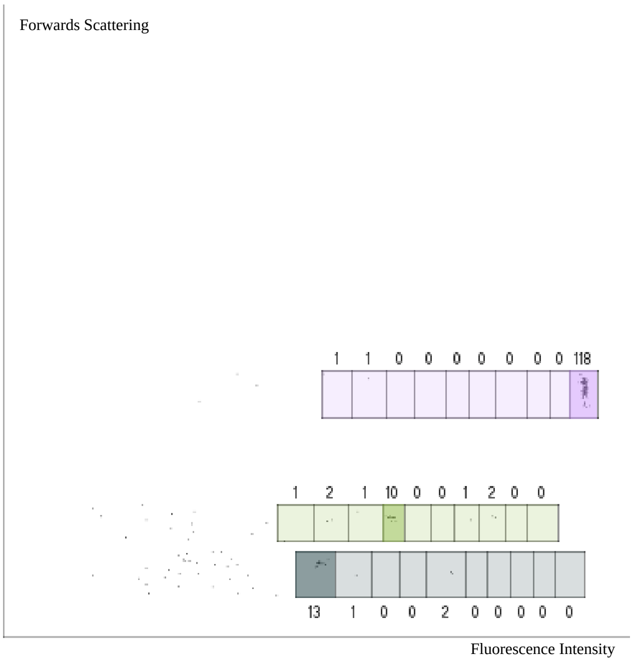

ANNEX 3: TAG DECONVOLUTION - BEAD 126

Passes flow sorting criteria: Yes

Passes tag deconvolution criteria: Yes

Included in protocol analysis: Yes

Protocol: 5, 9, 5, 1

Filename: Bin1_plateB1_H2.fcs

Split 1: Petrol shading

Split 2: Green shading

Split 3: Violet shading

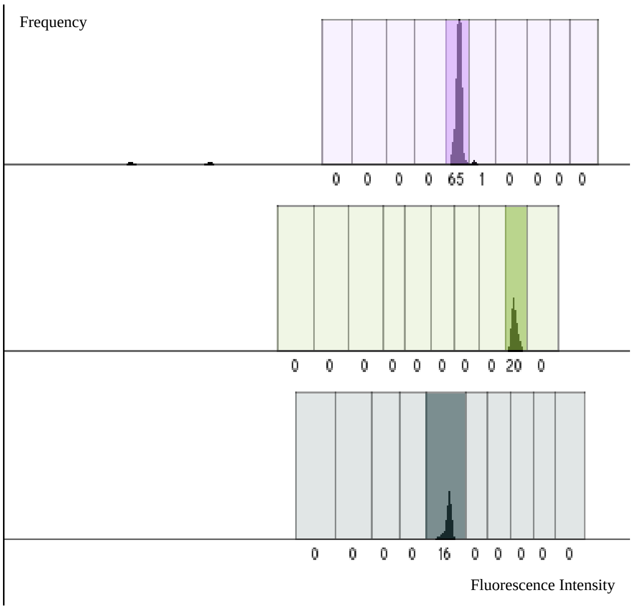

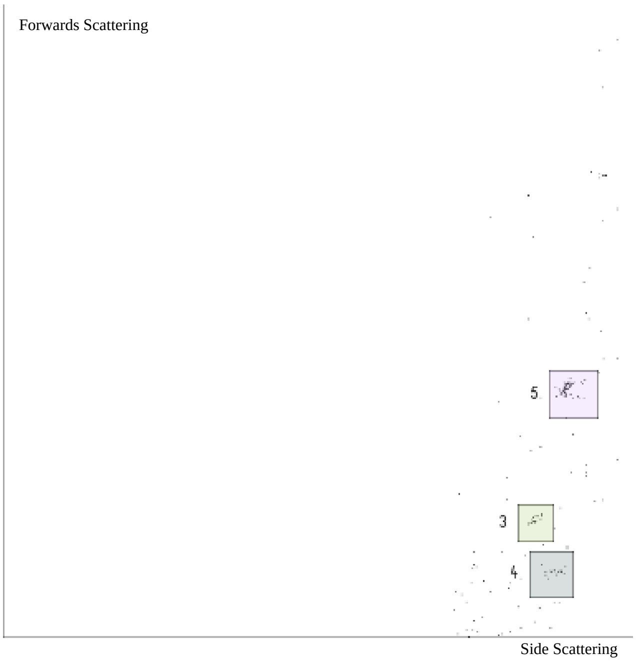

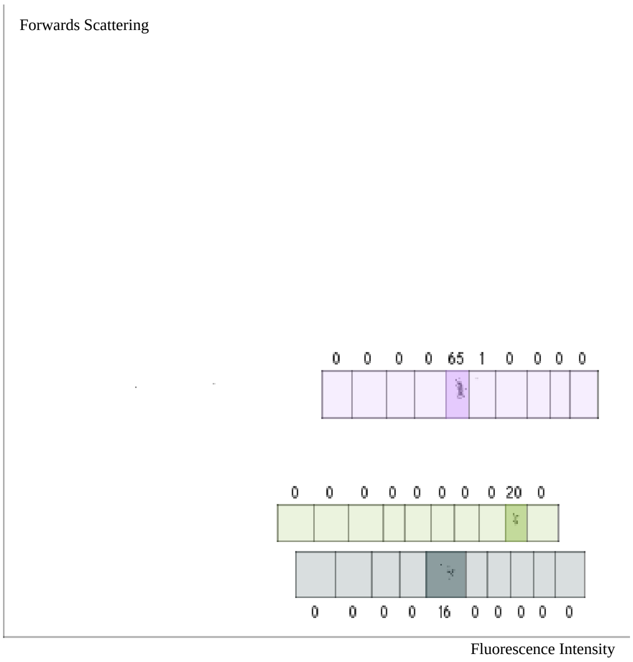

ANNEX 3: TAG DECONVOLUTION - BEAD 127

Passes flow sorting criteria: Yes

Passes tag deconvolution criteria: No

Included in protocol analysis: No

Protocol: N/A

Filename: Bin1_plateB1_H3.fcs

Split 1: Petrol shading

Split 2: Green shading

Split 3: Violet shading

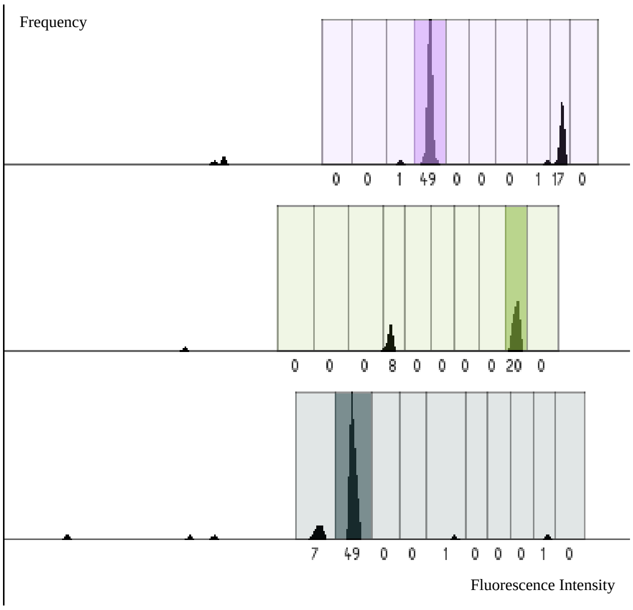

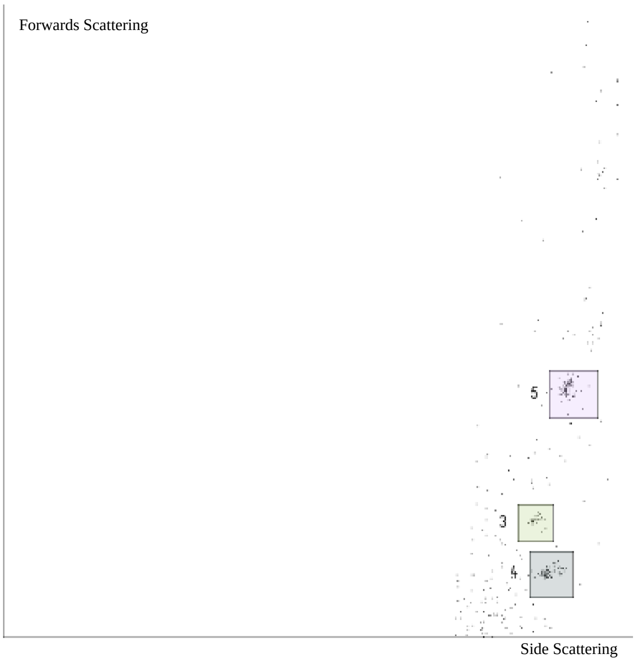

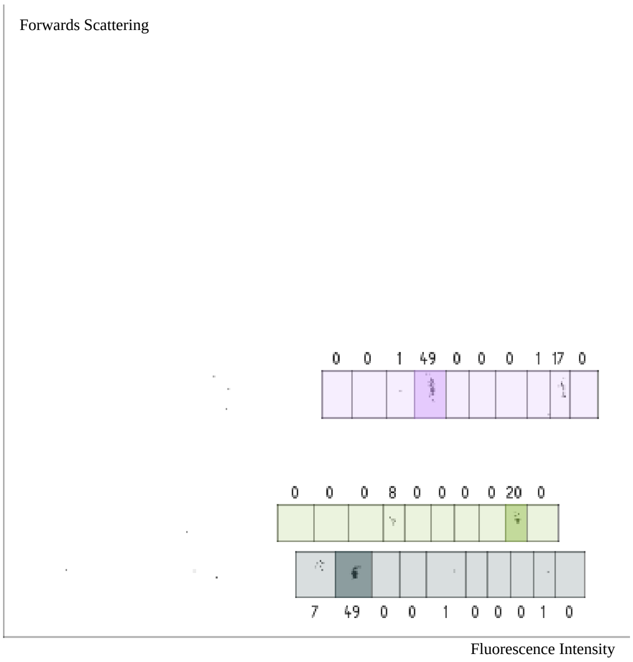

ANNEX 3: TAG DECONVOLUTION - BEAD 128

Passes flow sorting criteria: Yes

Passes tag deconvolution criteria: Yes

Included in protocol analysis: Yes

Protocol: 6, 8, 1, 1

Filename: Bin1_plateB1_H4.fcs

Split 1: Petrol shading

Split 2: Green shading

Split 3: Violet shading

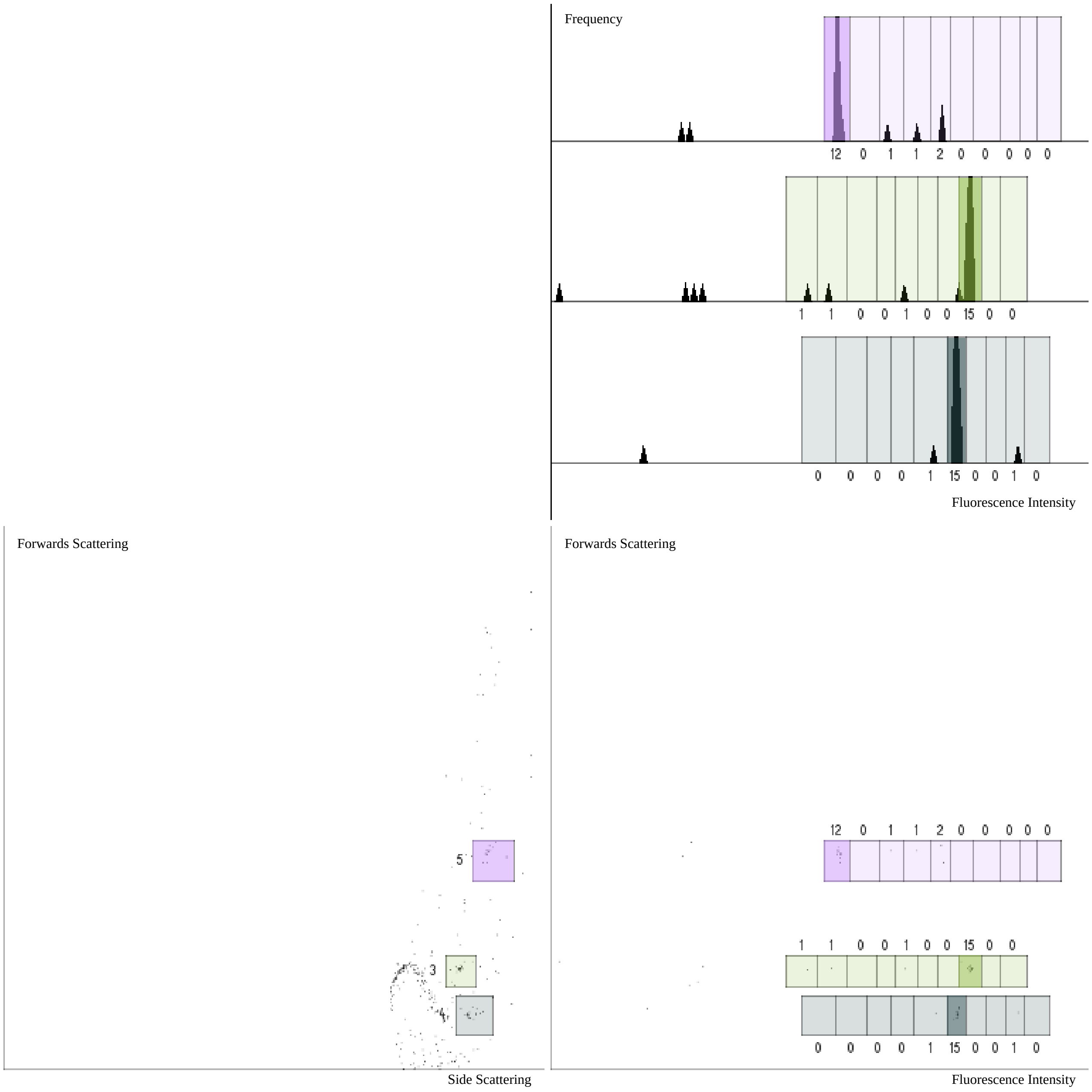

ANNEX 3: TAG DECONVOLUTION - BEAD 129

Passes flow sorting criteria: Yes

Passes tag deconvolution criteria: Yes

Included in protocol analysis: Yes

Protocol: 2, 4, 3, 1

Filename: Bin1_plateB1_H5.fcs

Split 1: Petrol shading

Split 2: Green shading

Split 3: Violet shading

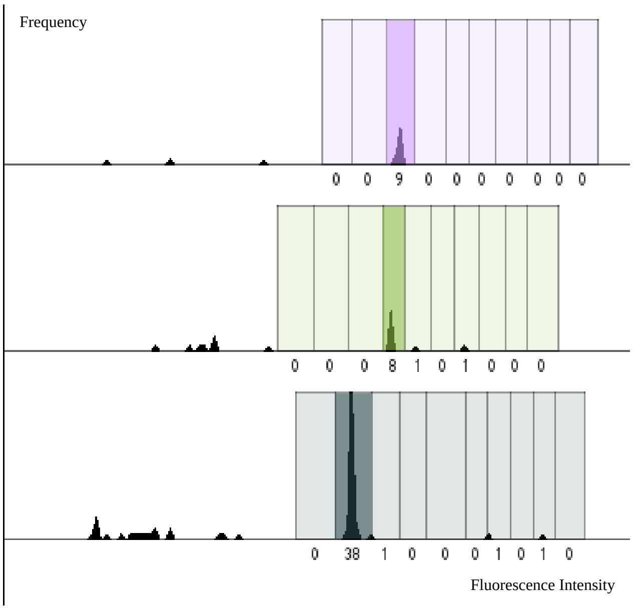

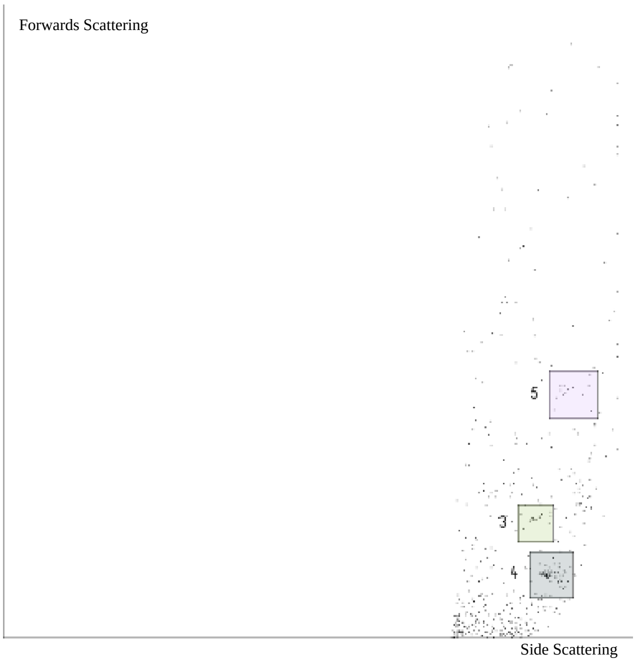

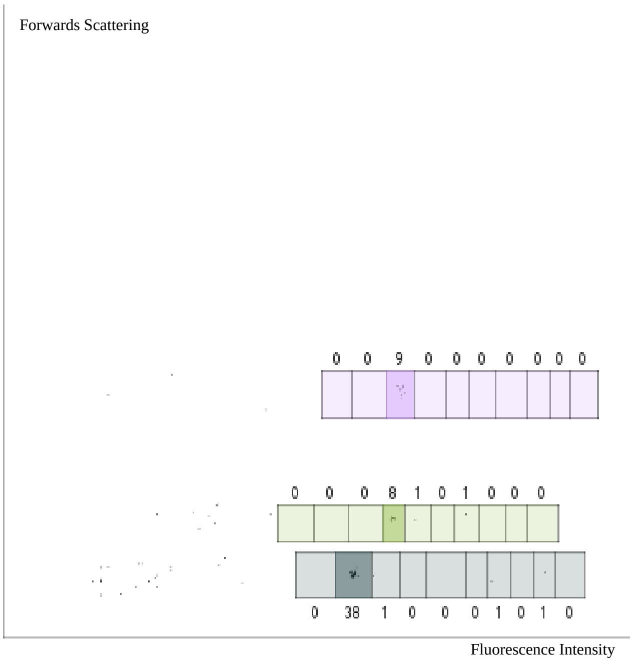

ANNEX 3: TAG DECONVOLUTION - BEAD 130

Passes flow sorting criteria: Yes

Passes tag deconvolution criteria: Yes

Included in protocol analysis: Yes

Protocol: 6, 6, 10, 1

Filename: Bin1_plateB1_H6.fcs

Split 1: Petrol shading

Split 2: Green shading

Split 3: Violet shading

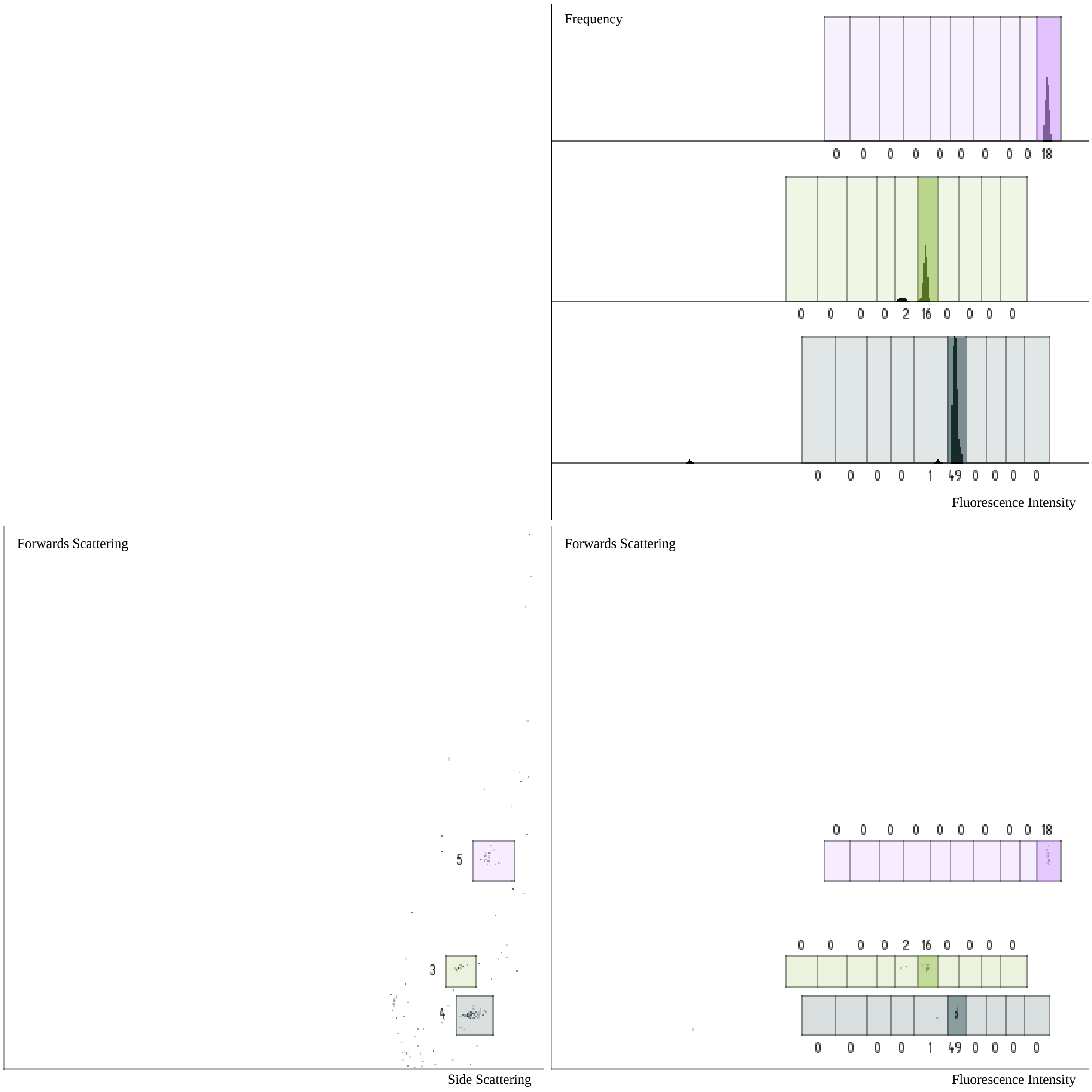

ANNEX 3: TAG DECONVOLUTION - BEAD 131

Passes flow sorting criteria: Yes

Passes tag deconvolution criteria: No

Included in protocol analysis: No

Protocol: N/A

Filename: Bin1_plateB1_H10.fcs

Split 1: Petrol shading

Split 2: Green shading

Split 3: Violet shading

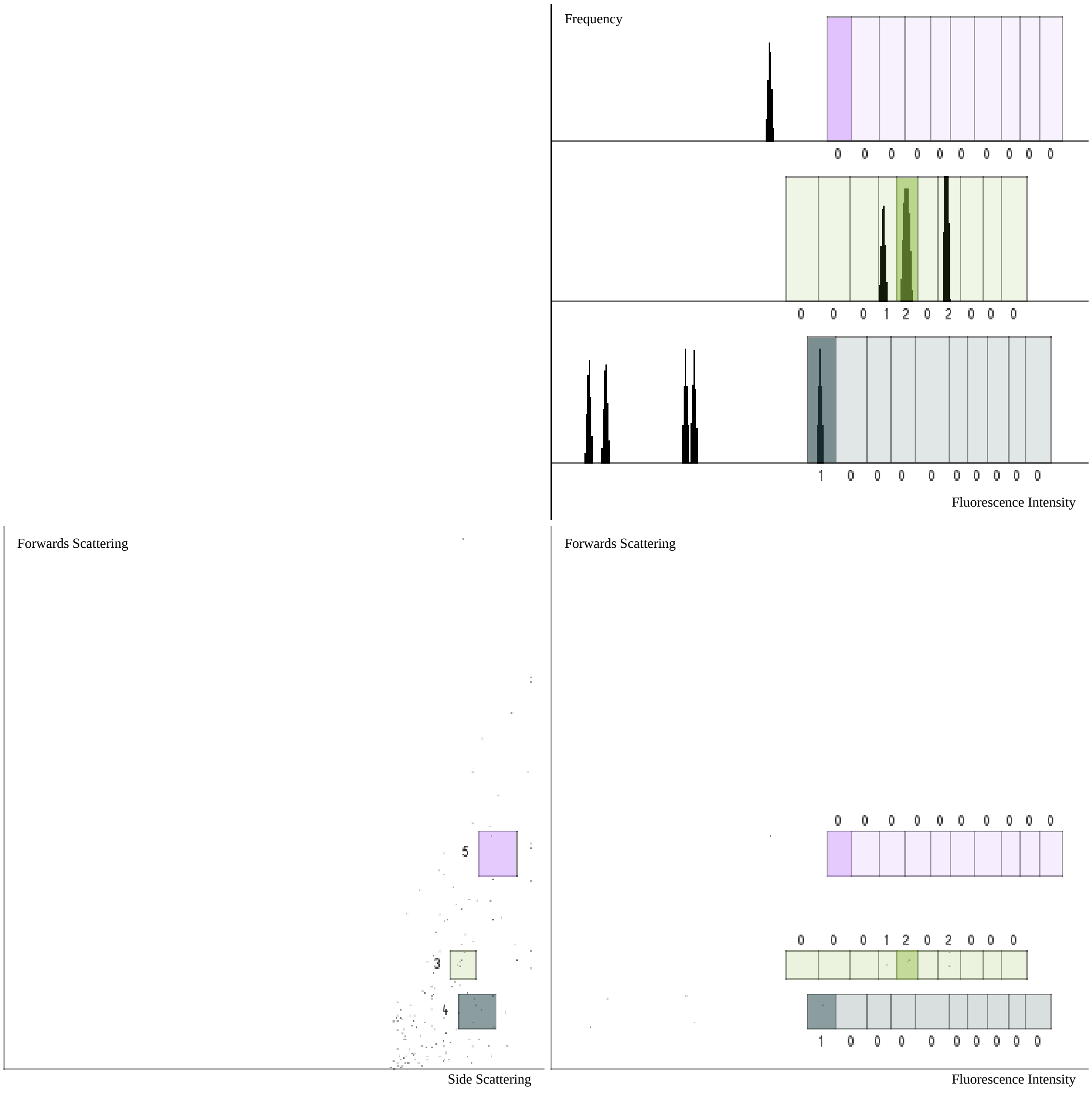

ANNEX 3: TAG DECONVOLUTION - BEAD 132

Passes flow sorting criteria: Yes

Passes tag deconvolution criteria: Yes

Included in protocol analysis: Yes

Protocol: 2, 7, 4, 1

Filename: Bin1_plateB1_A10.fcs

Split 1: Petrol shading

Split 2: Green shading

Split 3: Violet shading

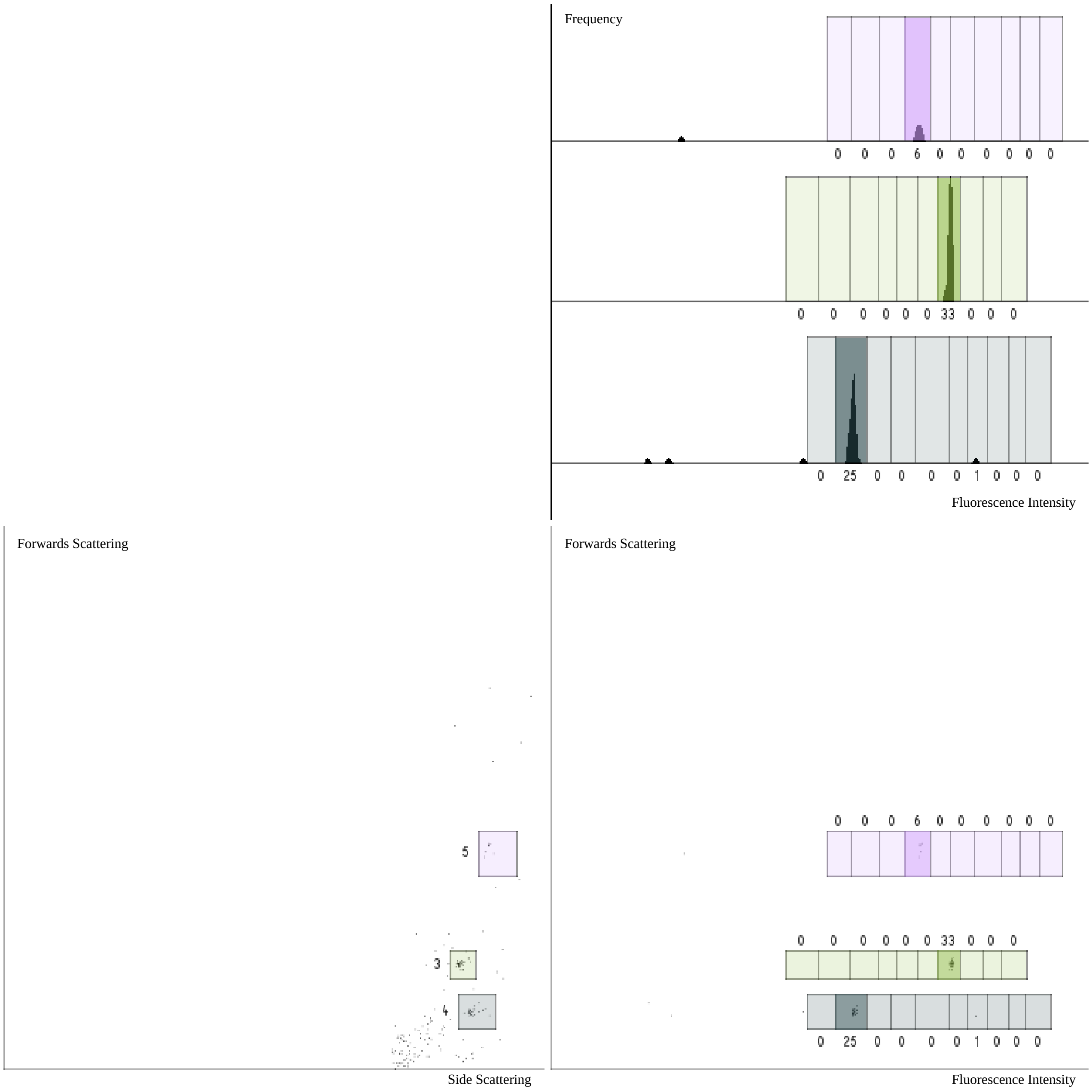

ANNEX 3: TAG DECONVOLUTION - BEAD 133

Passes flow sorting criteria: Yes

Passes tag deconvolution criteria: No

Included in protocol analysis: No

Protocol: N/A

Filename: Bin1_plateB1_B8.fcs

Split 1: Petrol shading

Split 2: Green shading

Split 3: Violet shading

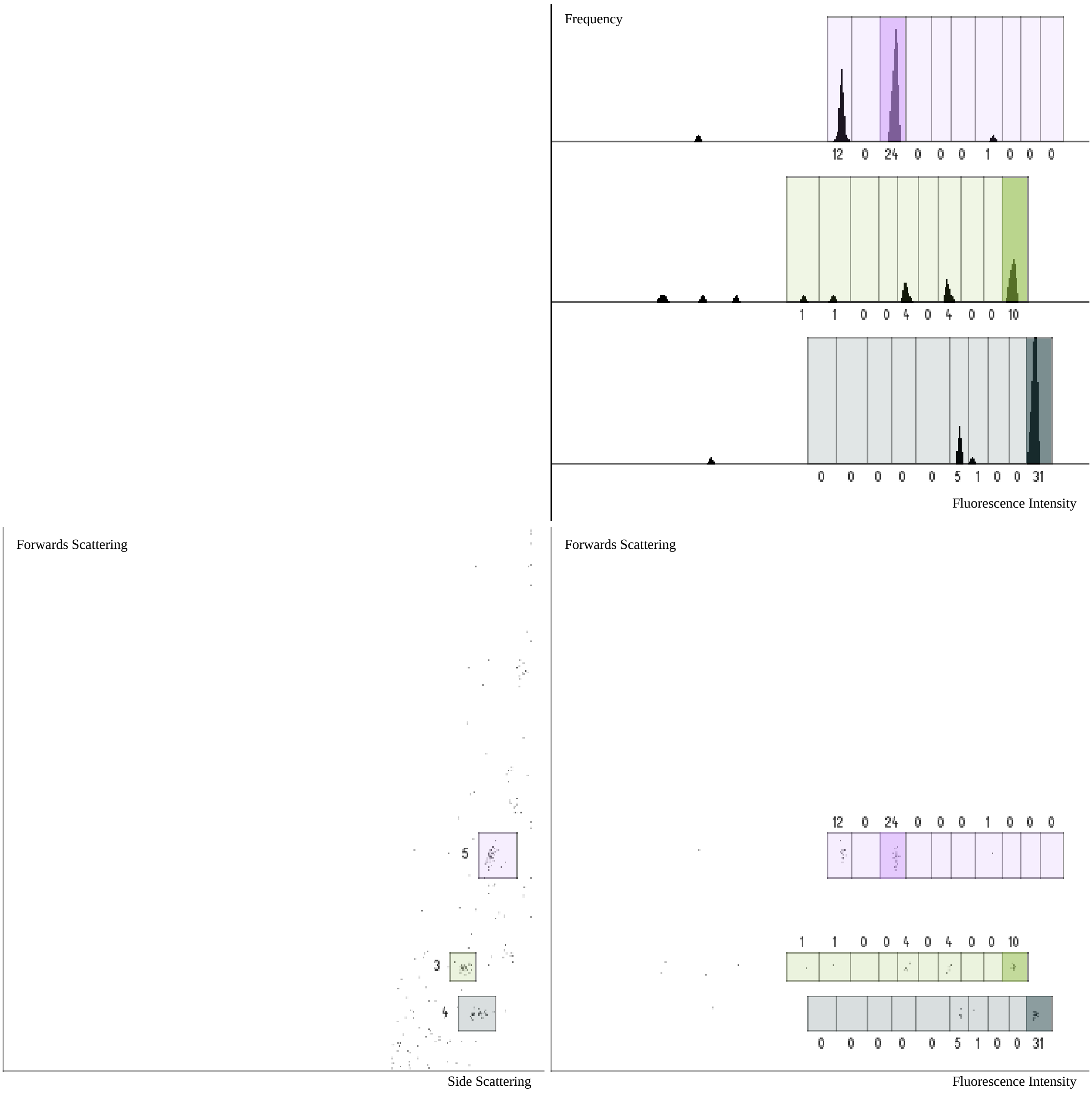

ANNEX 3: TAG DECONVOLUTION - BEAD 134

Passes flow sorting criteria: Yes

Passes tag deconvolution criteria: Yes

Included in protocol analysis: Yes

Protocol: 6, 8, 1, 1

Filename: Bin1_plateB1_B10.fcs

Split 1: Petrol shading

Split 2: Green shading

Split 3: Violet shading

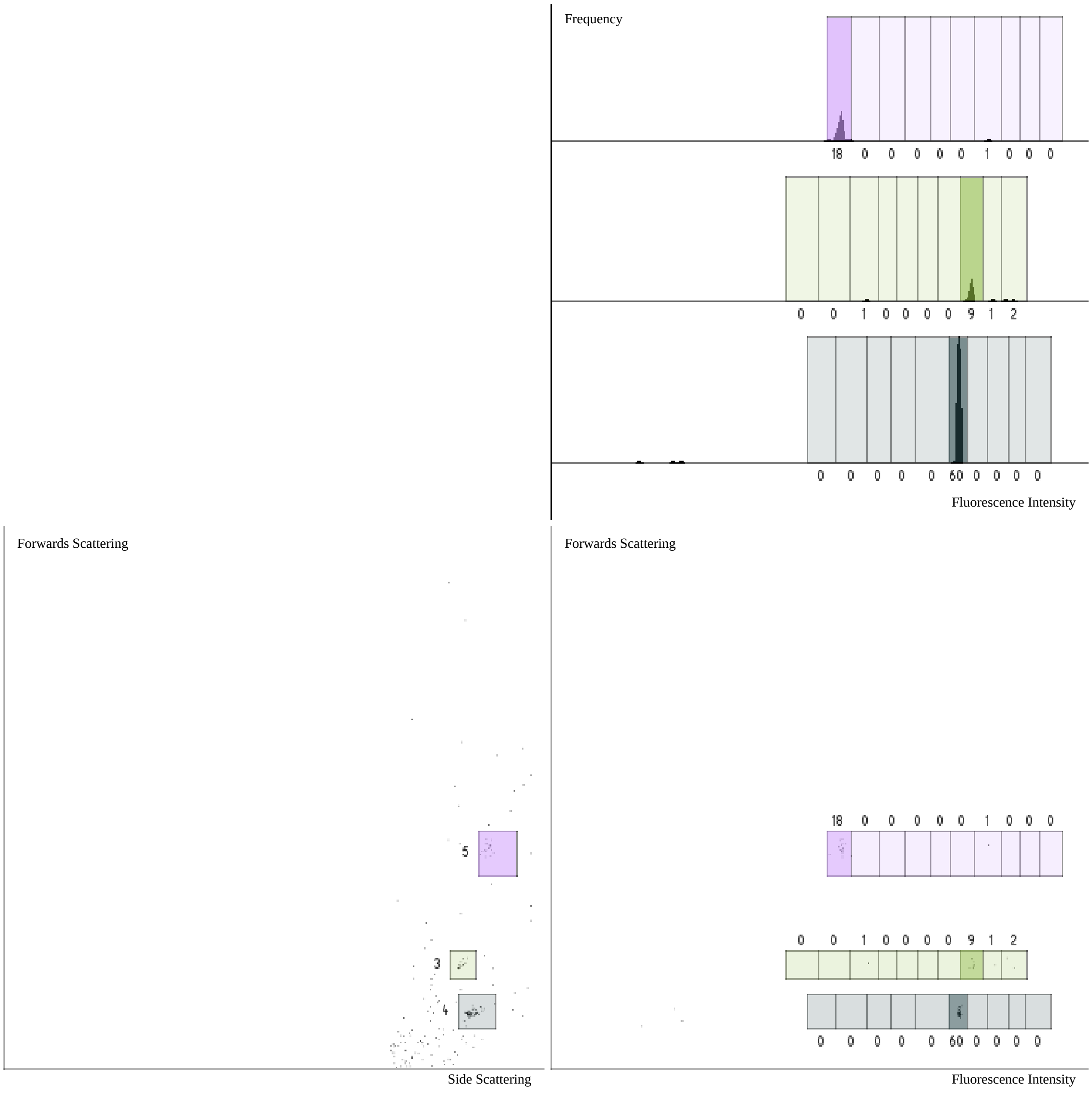

ANNEX 3: TAG DECONVOLUTION - BEAD 135

Passes flow sorting criteria: Yes

Passes tag deconvolution criteria: Yes

Included in protocol analysis: Yes

Protocol: 5, 9, 5, 1

Filename: Bin1_plateB1_C8.fcs

Split 1: Petrol shading

Split 2: Green shading

Split 3: Violet shading

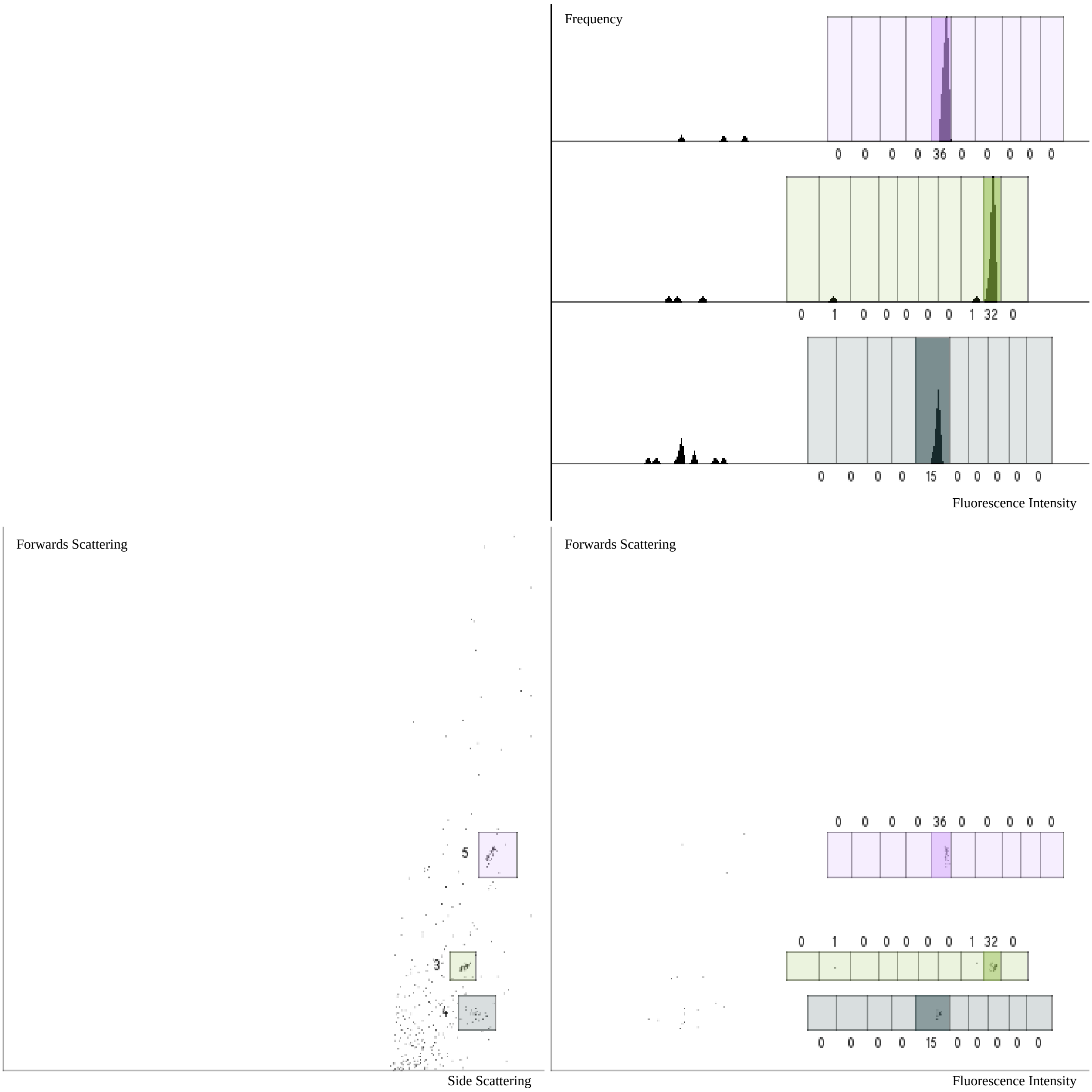

ANNEX 3: TAG DECONVOLUTION - BEAD 136

Passes flow sorting criteria: Yes

Passes tag deconvolution criteria: Yes

Included in protocol analysis: Yes

Protocol: 1, 6, 7, 1

Filename: Bin1_plateB1_C9.fcs

Split 1: Petrol shading

Split 2: Green shading

Split 3: Violet shading

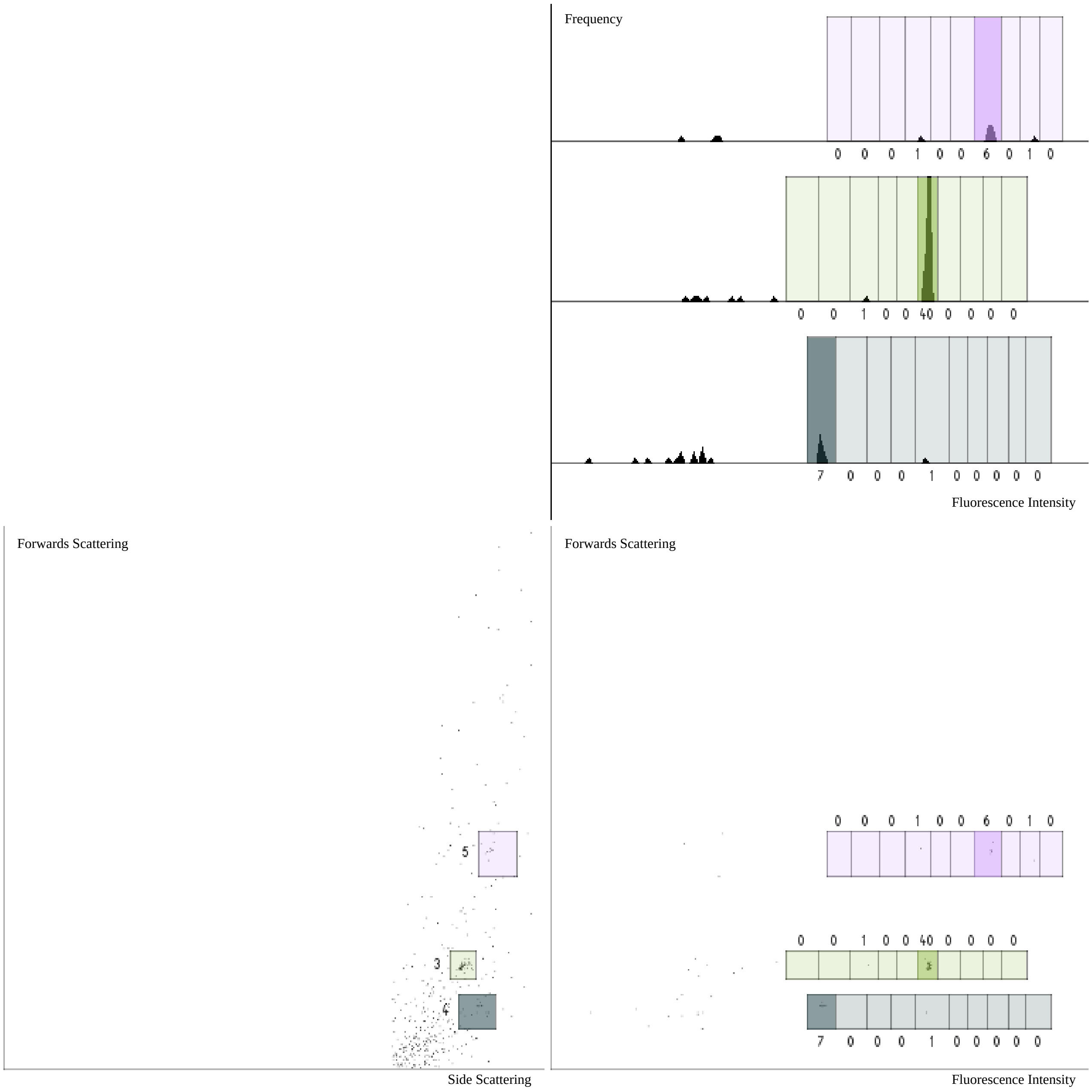

ANNEX 3: TAG DECONVOLUTION - BEAD 137

Passes flow sorting criteria: Yes

Passes tag deconvolution criteria: No

Included in protocol analysis: No

Protocol: N/A

Filename: Bin1_plateB1_D8.fcs

Split 1: Petrol shading

Split 2: Green shading

Split 3: Violet shading

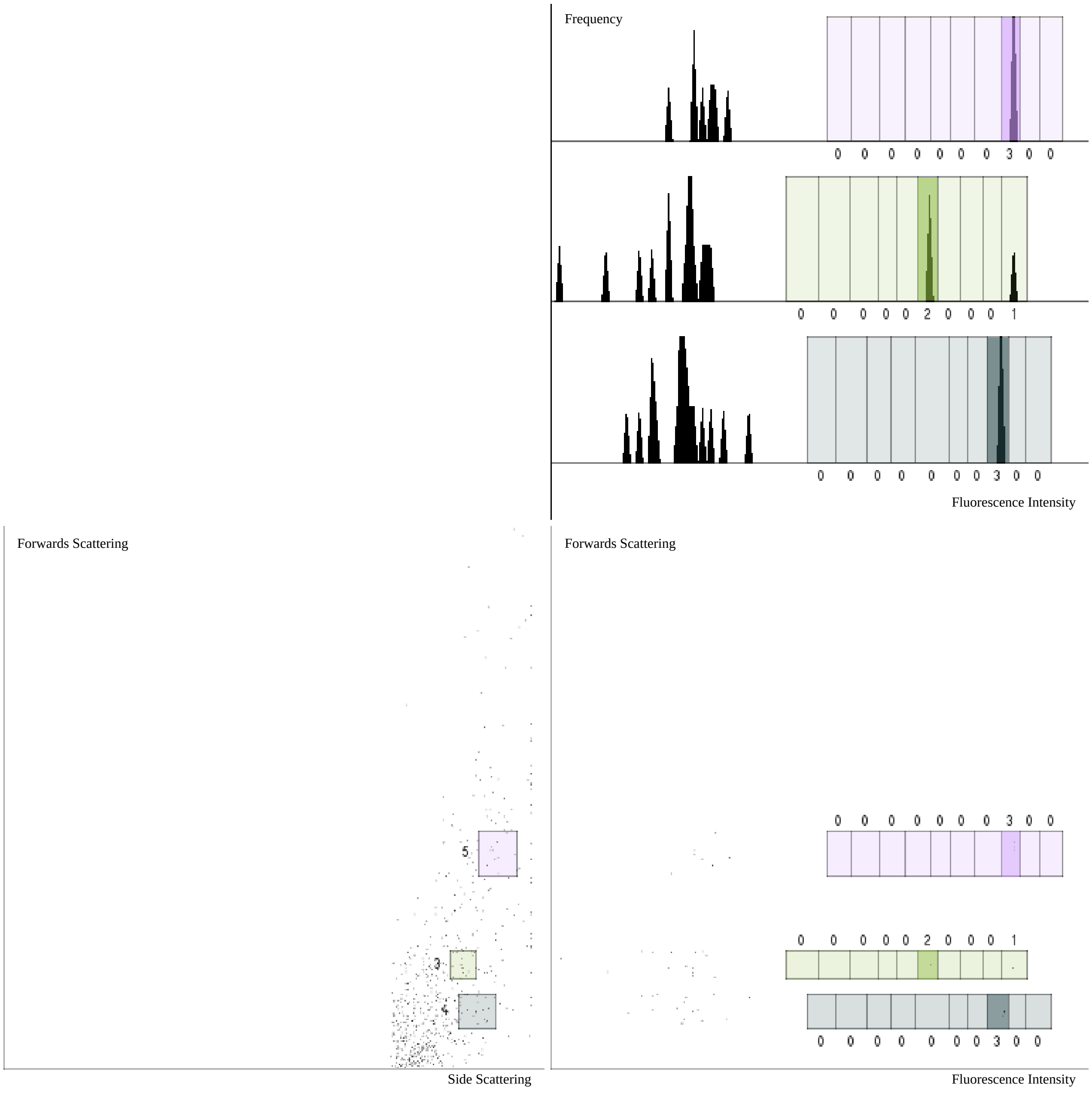

ANNEX 3: TAG DECONVOLUTION - BEAD 138

Passes flow sorting criteria: Yes

Passes tag deconvolution criteria: No

Included in protocol analysis: No

Protocol: N/A

Filename: Bin1_plateB1_D9.fcs

Split 1: Petrol shading

Split 2: Green shading

Split 3: Violet shading

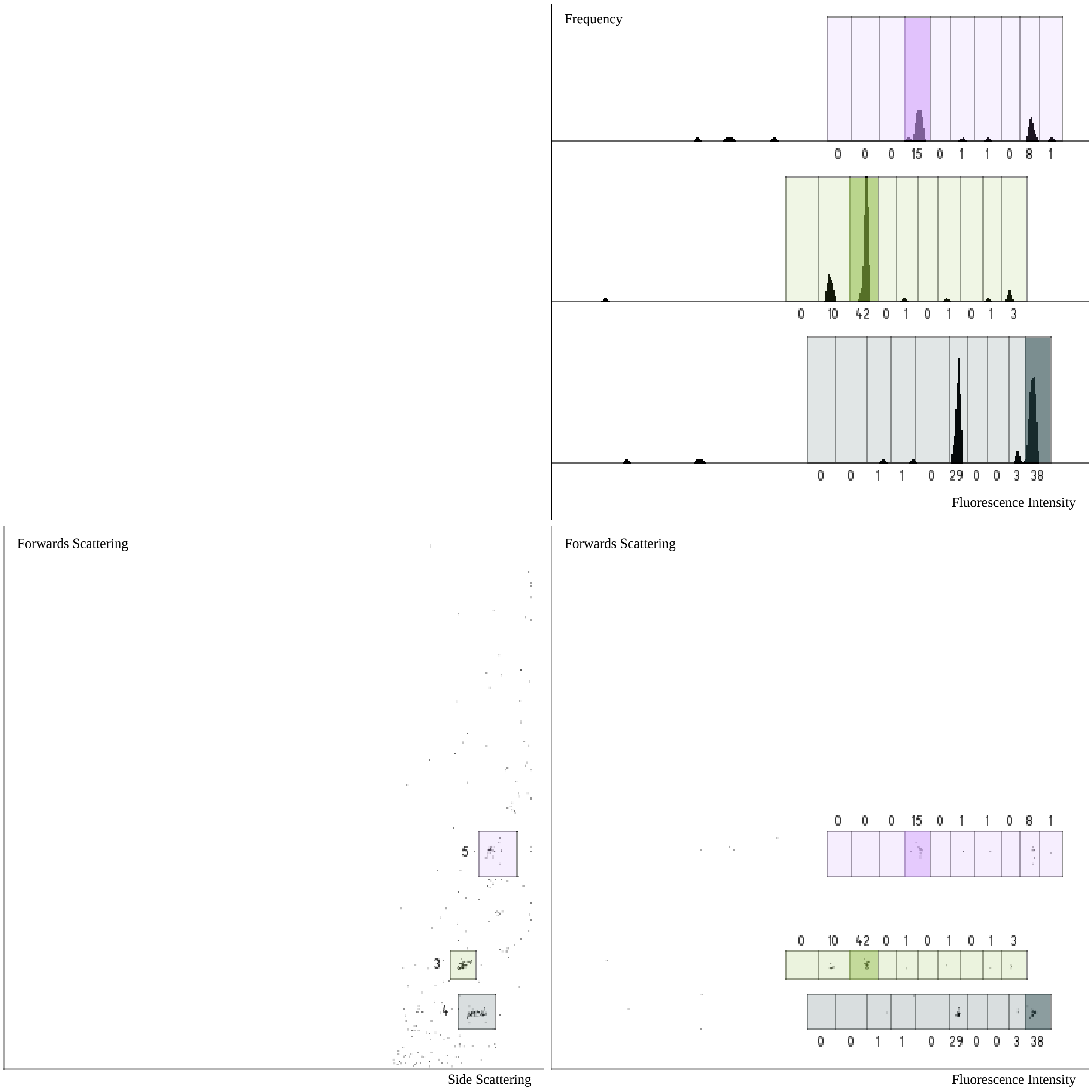

ANNEX 3: TAG DECONVOLUTION - BEAD 139

Passes flow sorting criteria: Yes

Passes tag deconvolution criteria: Yes

Included in protocol analysis: Yes

Protocol: 4, 6, 7, 1

Filename: Bin1_plateB1_D10.fcs

Split 1: Petrol shading

Split 2: Green shading

Split 3: Violet shading

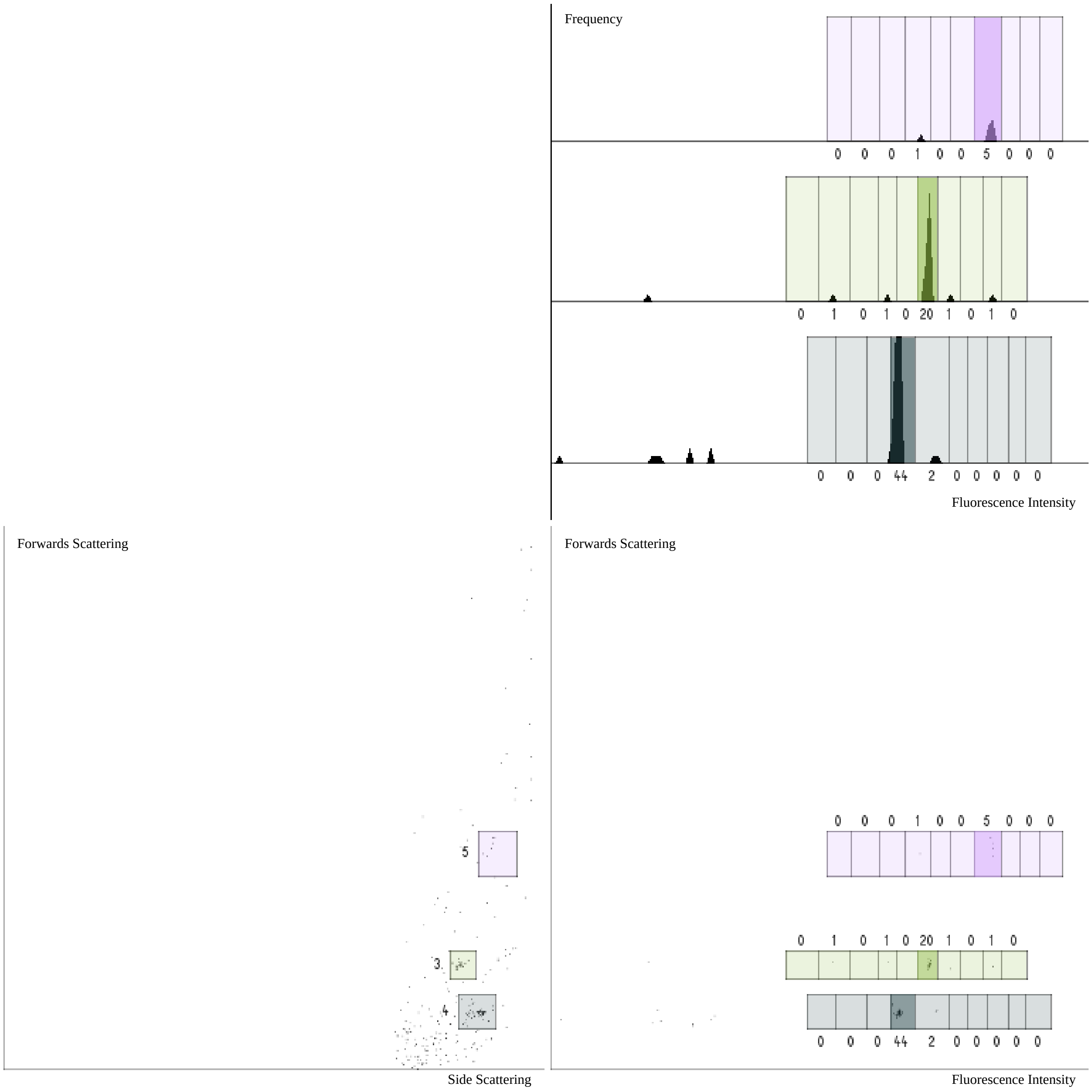

ANNEX 3: TAG DECONVOLUTION - BEAD 140

Passes flow sorting criteria: Yes

Passes tag deconvolution criteria: Yes

Included in protocol analysis: Yes

Protocol: 5, 1, 10, 1

Filename: Bin1_plateB1_E8.fcs

Split 1: Petrol shading

Split 2: Green shading

Split 3: Violet shading

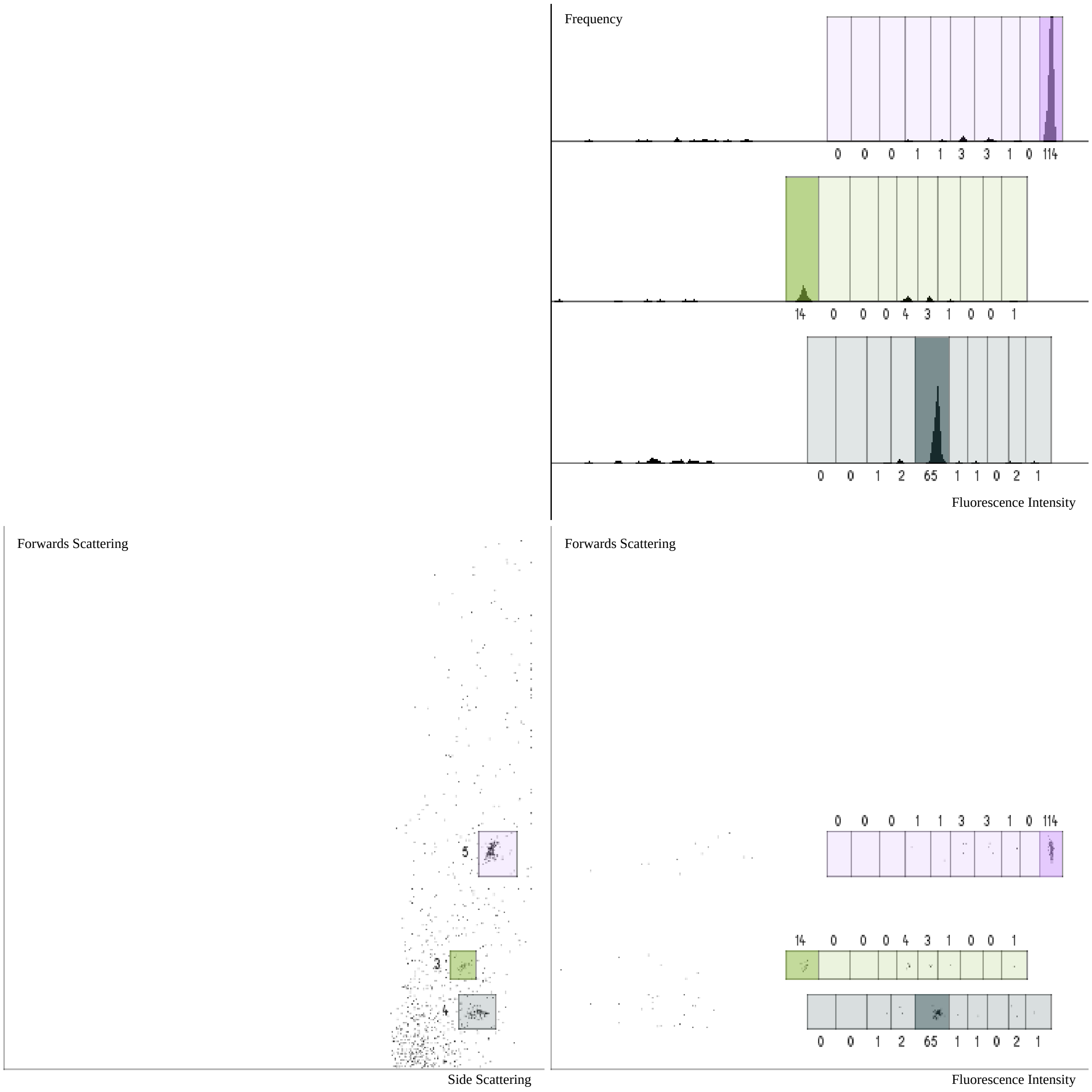

ANNEX 3: TAG DECONVOLUTION - BEAD 141

Passes flow sorting criteria: Yes

Passes tag deconvolution criteria: Yes

Included in protocol analysis: Yes

Protocol: 5, 10, 6, 1

Filename: Bin1_plateB1_E9.fcs

Split 1: Petrol shading

Split 2: Green shading

Split 3: Violet shading

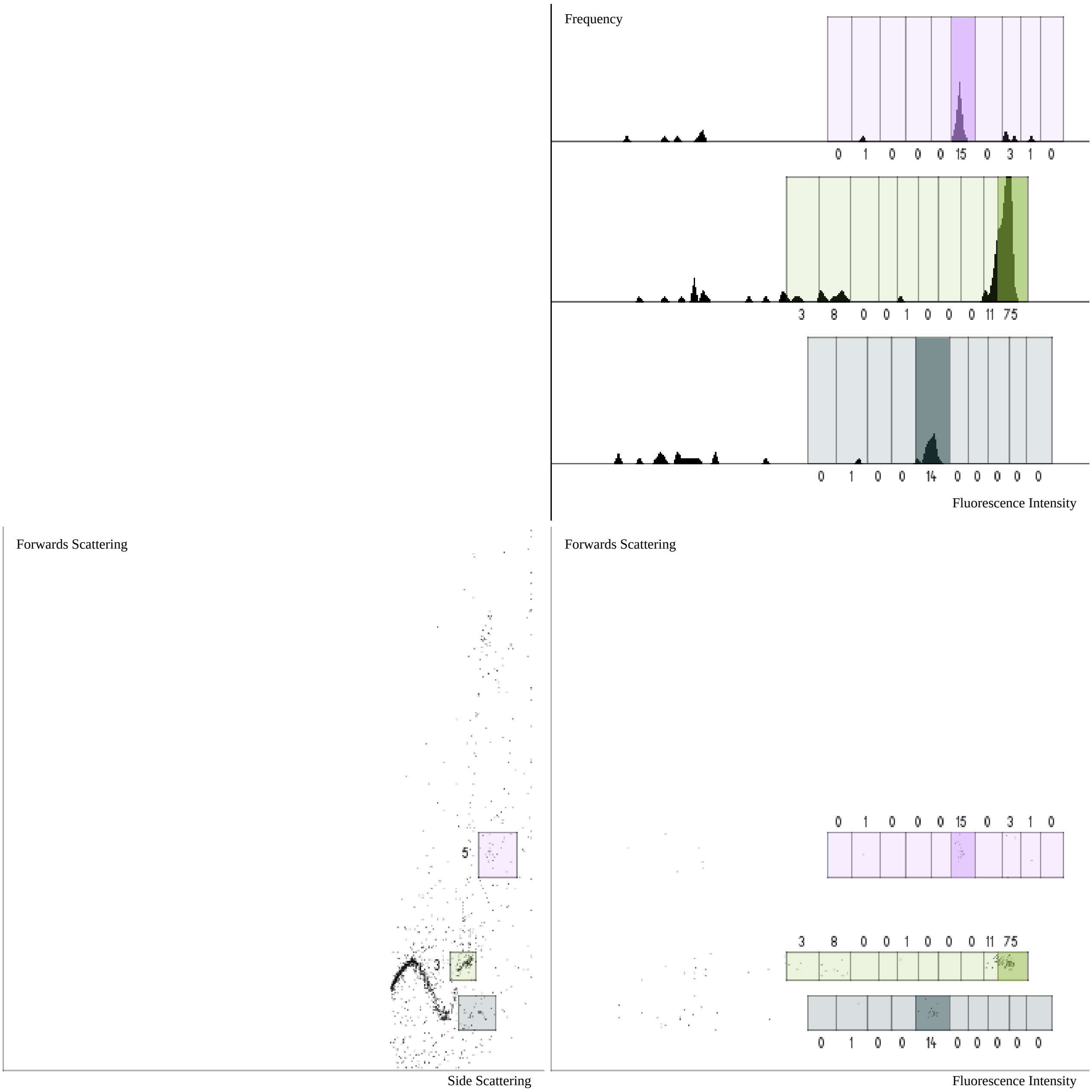

ANNEX 3: TAG DECONVOLUTION - BEAD 142

Passes flow sorting criteria: Yes

Passes tag deconvolution criteria: No

Included in protocol analysis: No

Protocol: N/A

Filename: Bin1_plateB1_E10.fcs

Split 1: Petrol shading

Split 2: Green shading

Split 3: Violet shading

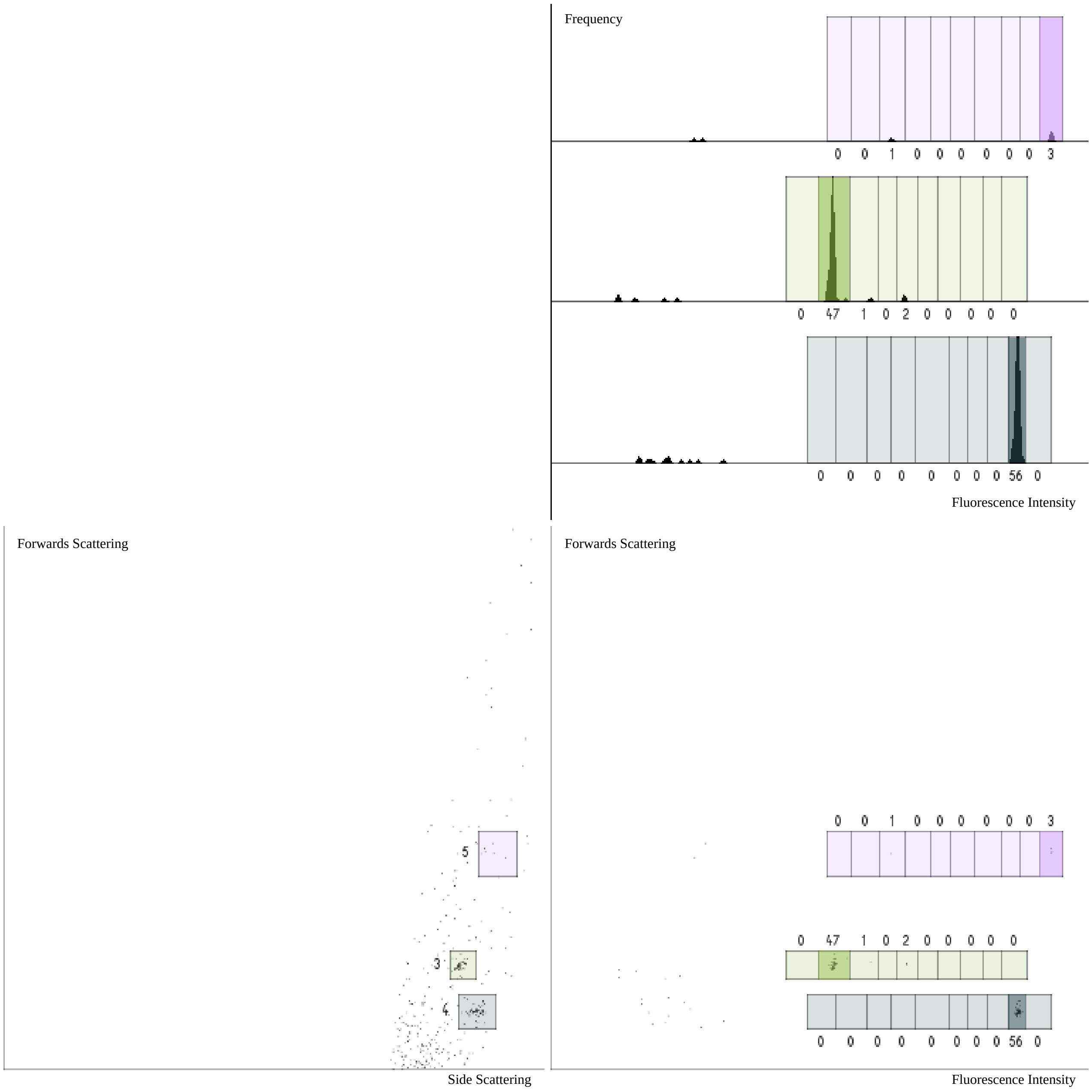

ANNEX 3: TAG DECONVOLUTION - BEAD 143

Passes flow sorting criteria: Yes

Passes tag deconvolution criteria: No

Included in protocol analysis: No

Protocol: N/A

Filename: Bin1_plateB1_F8.fcs

Split 1: Petrol shading

Split 2: Green shading

Split 3: Violet shading

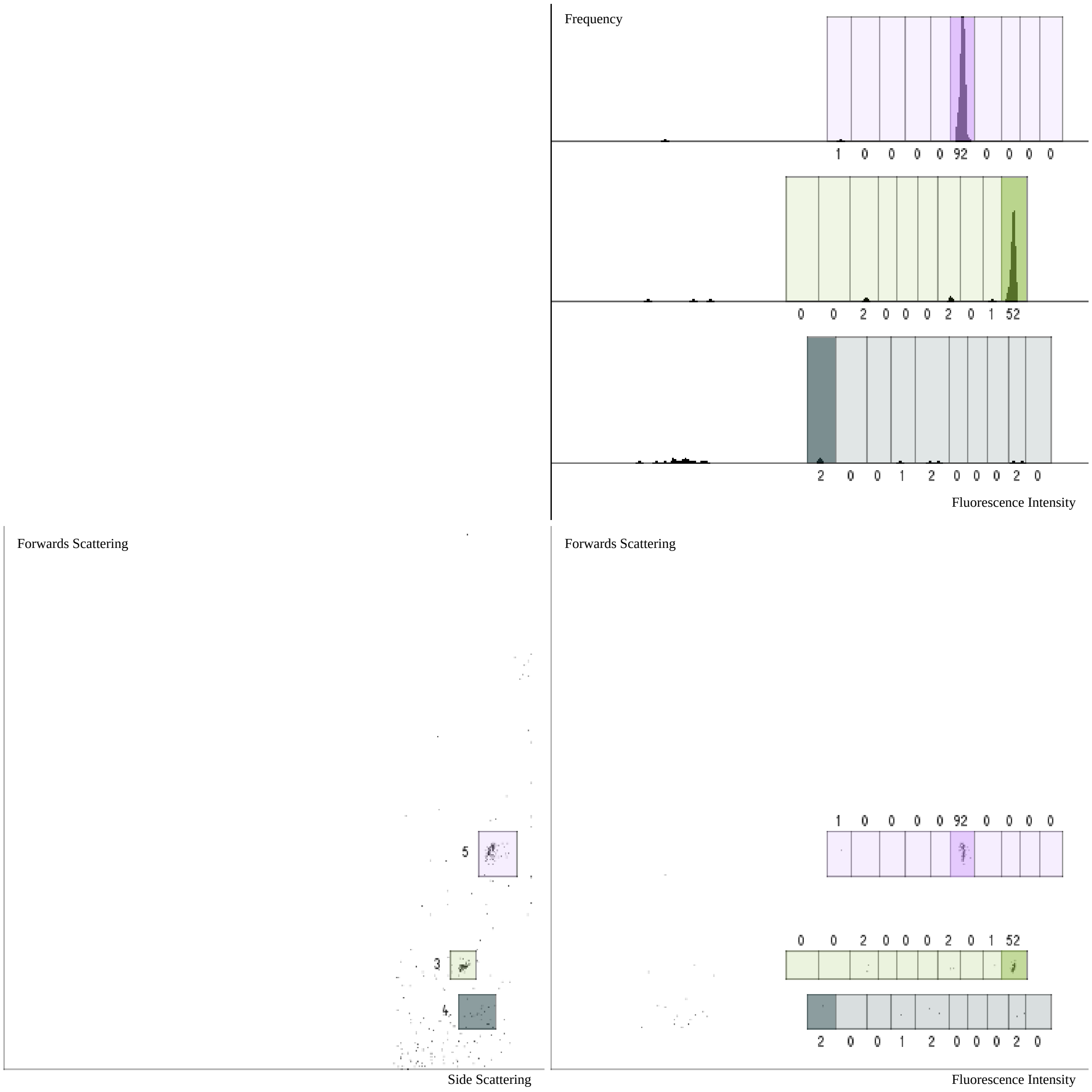

ANNEX 3: TAG DECONVOLUTION - BEAD 144

Passes flow sorting criteria: Yes

Passes tag deconvolution criteria: No

Included in protocol analysis: No

Protocol: N/A

Filename: Bin1_plateB1_F9.fcs

Split 1: Petrol shading

Split 2: Green shading

Split 3: Violet shading

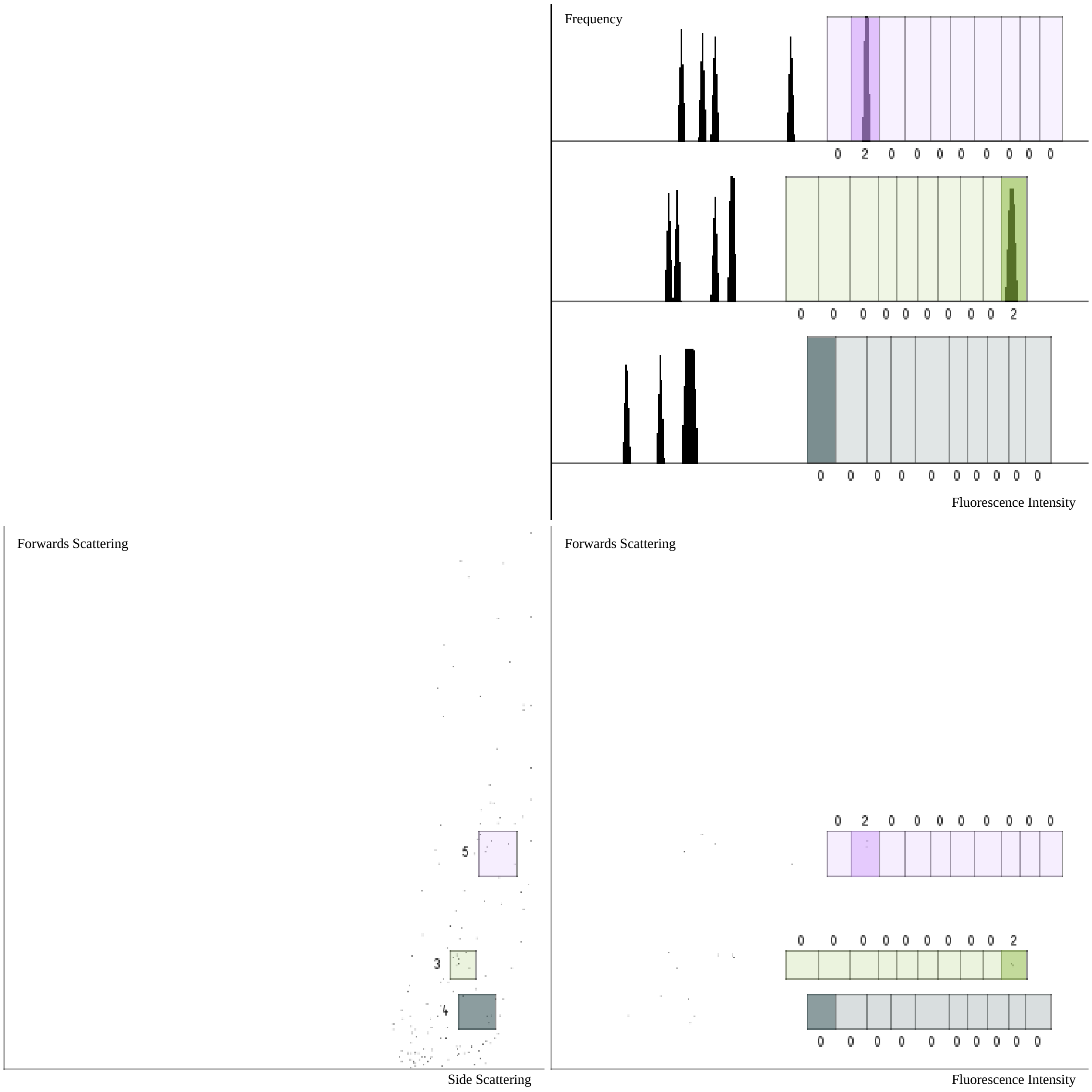

ANNEX 3: TAG DECONVOLUTION - BEAD 145

Passes flow sorting criteria: Yes

Passes tag deconvolution criteria: Yes

Included in protocol analysis: Yes

Protocol: 1, 2, 10, 1

Filename: Bin1_plateB1_F10.fcs

Split 1: Petrol shading

Split 2: Green shading

Split 3: Violet shading

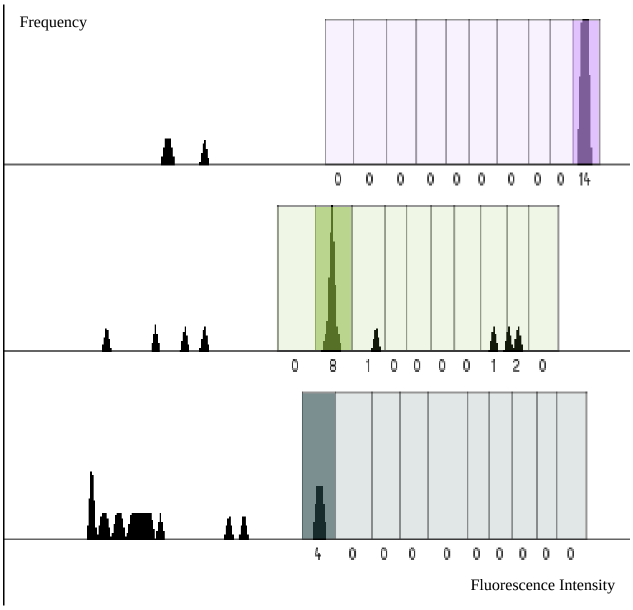

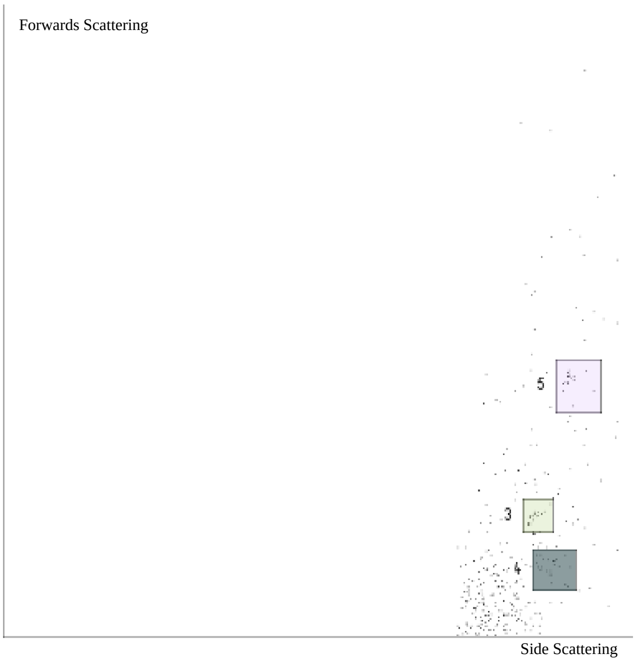

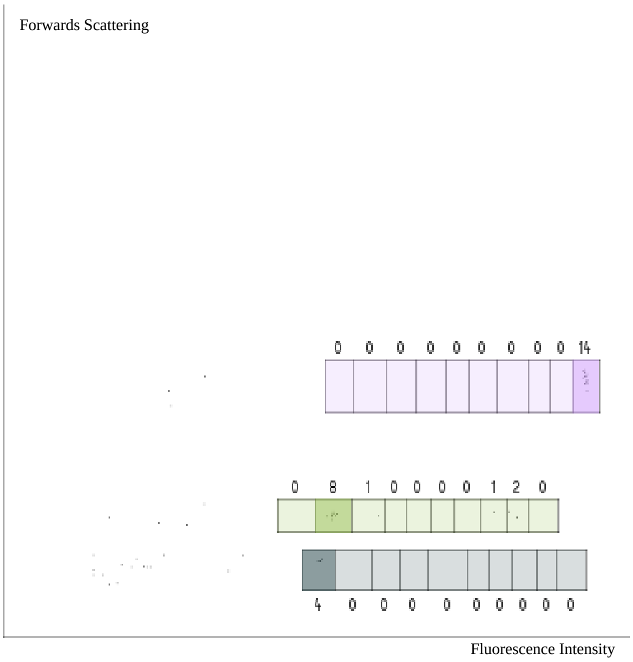

ANNEX 3: TAG DECONVOLUTION - BEAD 146

Passes flow sorting criteria: Yes

Passes tag deconvolution criteria: No

Included in protocol analysis: No

Protocol: N/A

Filename: Bin1_plateB1_G8.fcs

Split 1: Petrol shading

Split 2: Green shading

Split 3: Violet shading

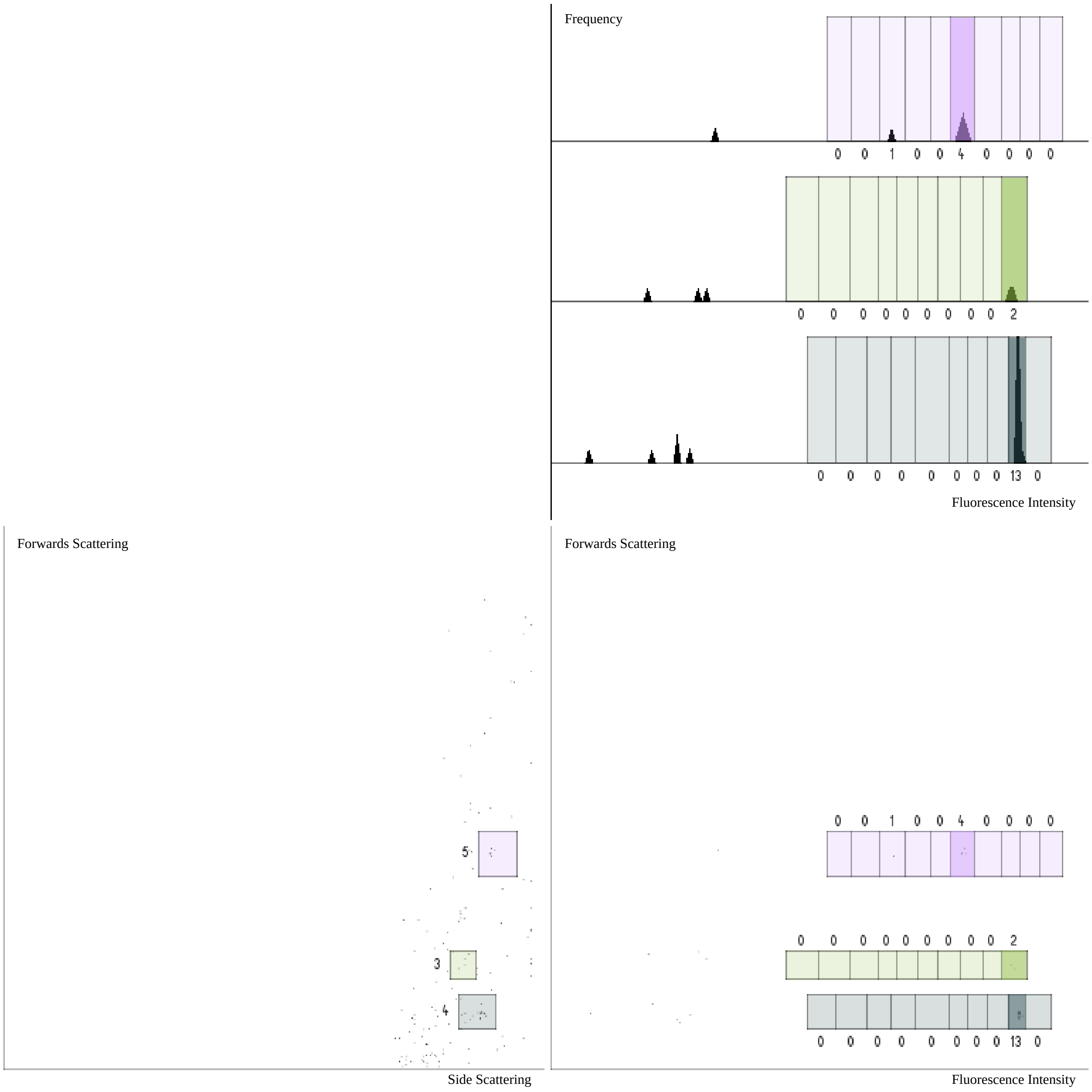

ANNEX 3: TAG DECONVOLUTION - BEAD 147

Passes flow sorting criteria: Yes

Passes tag deconvolution criteria: Yes

Included in protocol analysis: Yes

Protocol: 2, 3, 8, 1

Filename: Bin1_plateB1_G9.fcs

Split 1: Petrol shading

Split 2: Green shading

Split 3: Violet shading

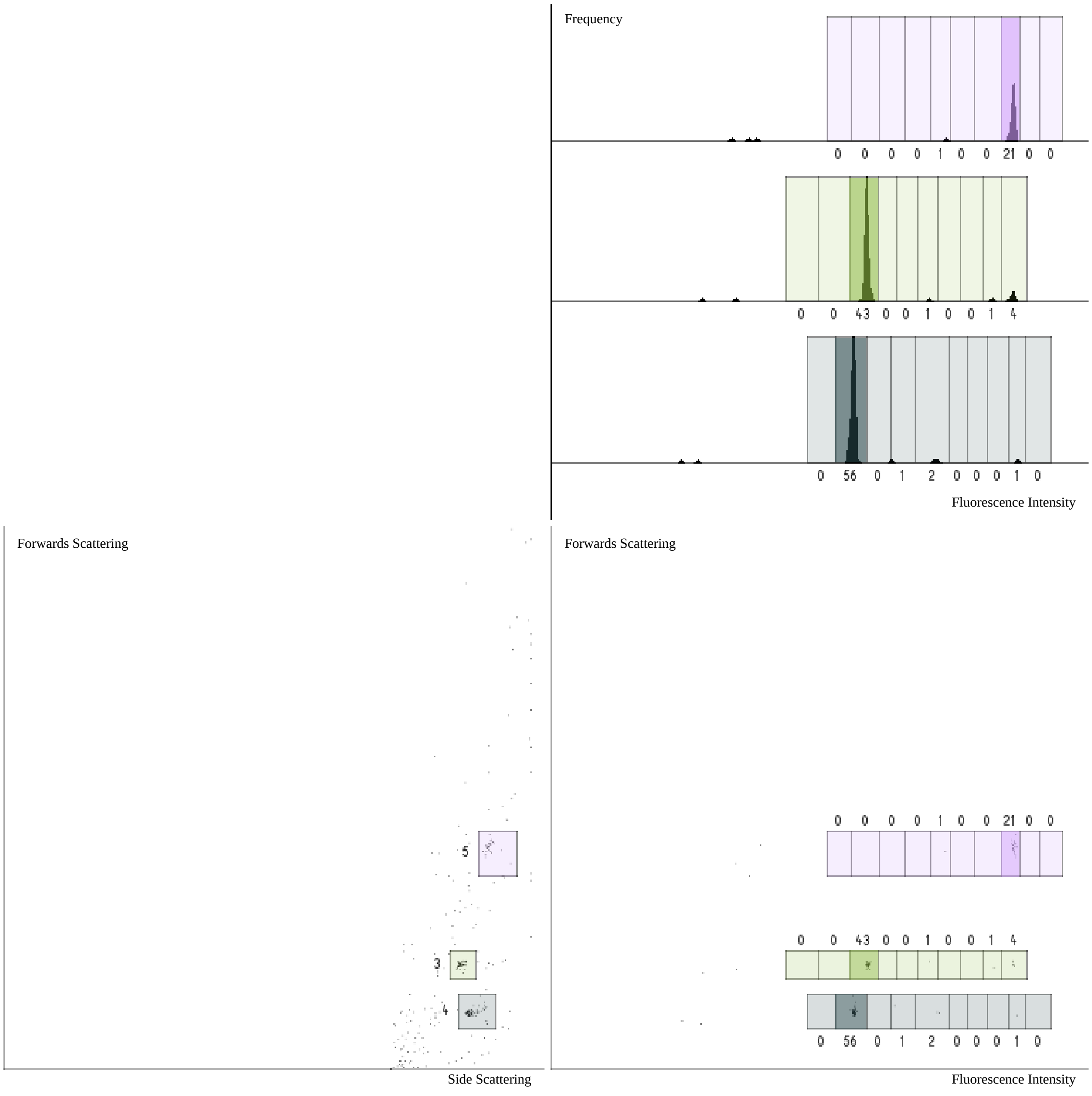

ANNEX 3: TAG DECONVOLUTION - BEAD 148

Passes flow sorting criteria: Yes

Passes tag deconvolution criteria: Yes

Included in protocol analysis: Yes

Protocol: 2, 4, 2, 1

Filename: Bin1_plateB1_G10.fcs

Split 1: Petrol shading

Split 2: Green shading

Split 3: Violet shading

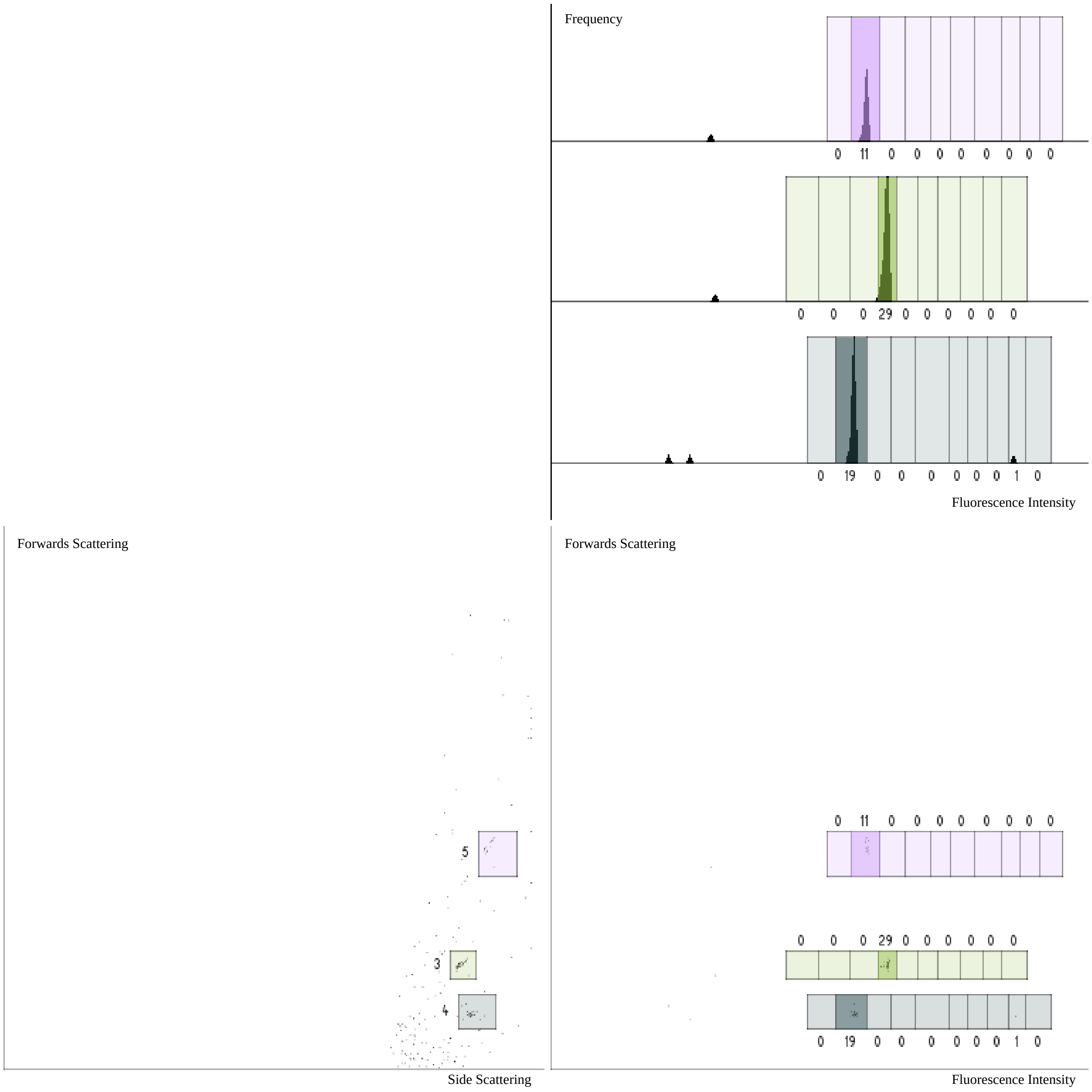

ANNEX 3: TAG DECONVOLUTION - BEAD 149

Passes flow sorting criteria: Yes

Passes tag deconvolution criteria: No

Included in protocol analysis: No

Protocol: N/A

Filename: Bin1_plateB1_H8.fcs

Split 1: Petrol shading

Split 2: Green shading

Split 3: Violet shading

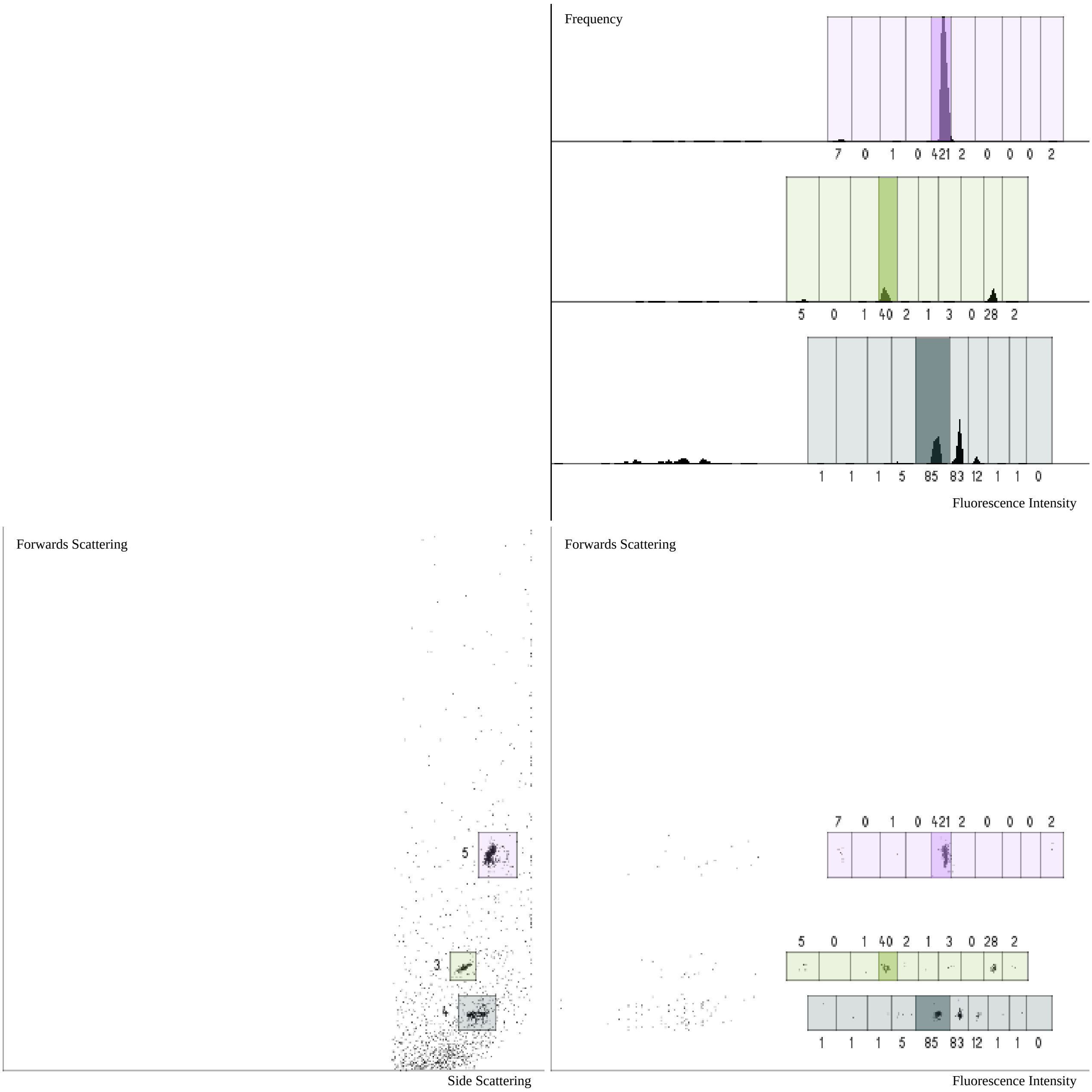

ANNEX 3: TAG DECONVOLUTION - BEAD 150

Passes flow sorting criteria: Yes

Passes tag deconvolution criteria: Yes

Included in protocol analysis: Yes

Protocol: 4, 7, 10, 1

Filename: Bin1_plateB1_H9.fcs

Split 1: Petrol shading

Split 2: Green shading

Split 3: Violet shading

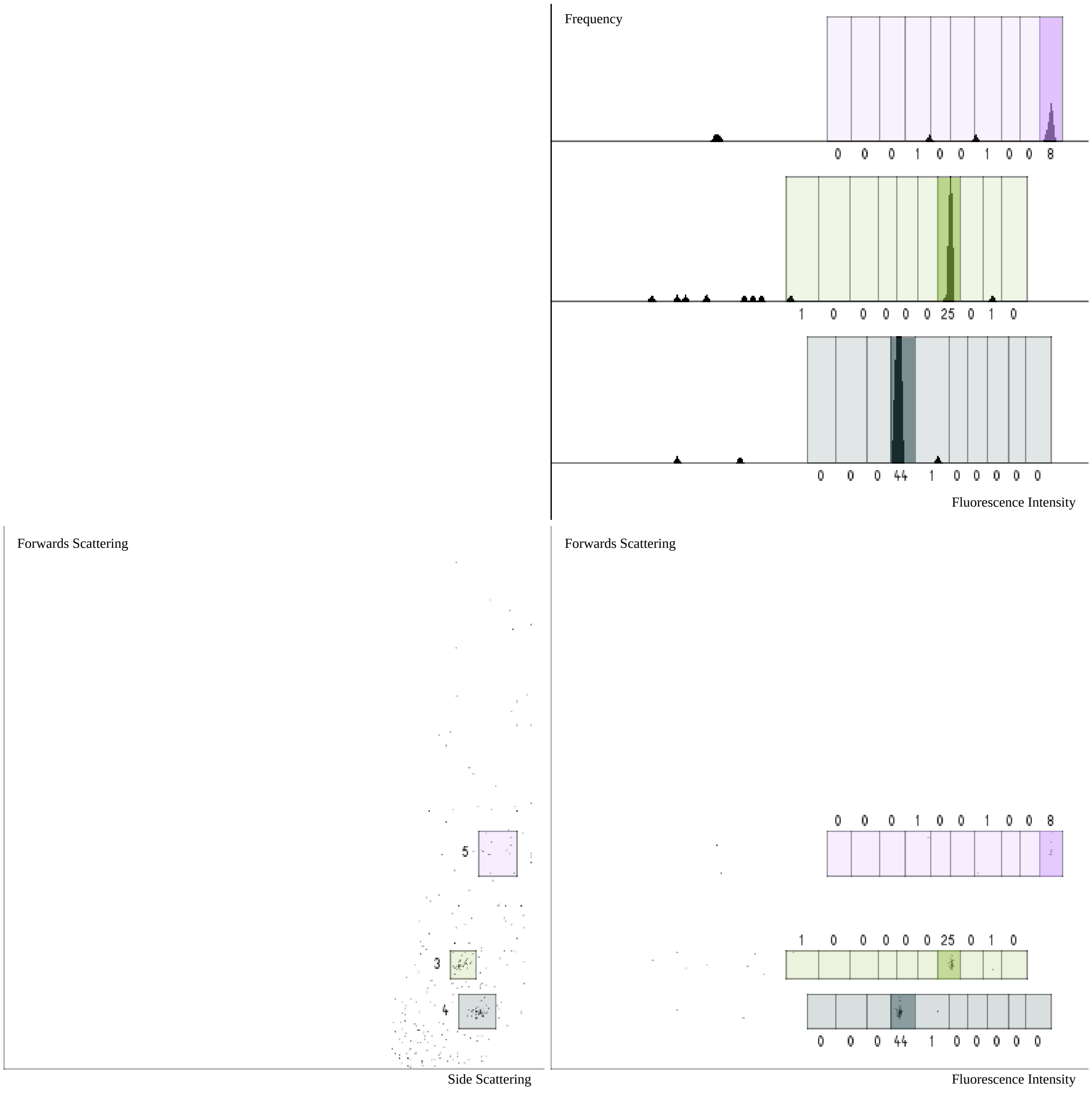

ANNEX 3: TAG DECONVOLUTION - BEAD 151

Passes flow sorting criteria: Yes

Passes tag deconvolution criteria: Yes

Included in protocol analysis: Yes

Protocol: 4, 4, 5, 1

Filename: Bin1_plateB1_H11.fcs

Split 1: Petrol shading

Split 2: Green shading

Split 3: Violet shading

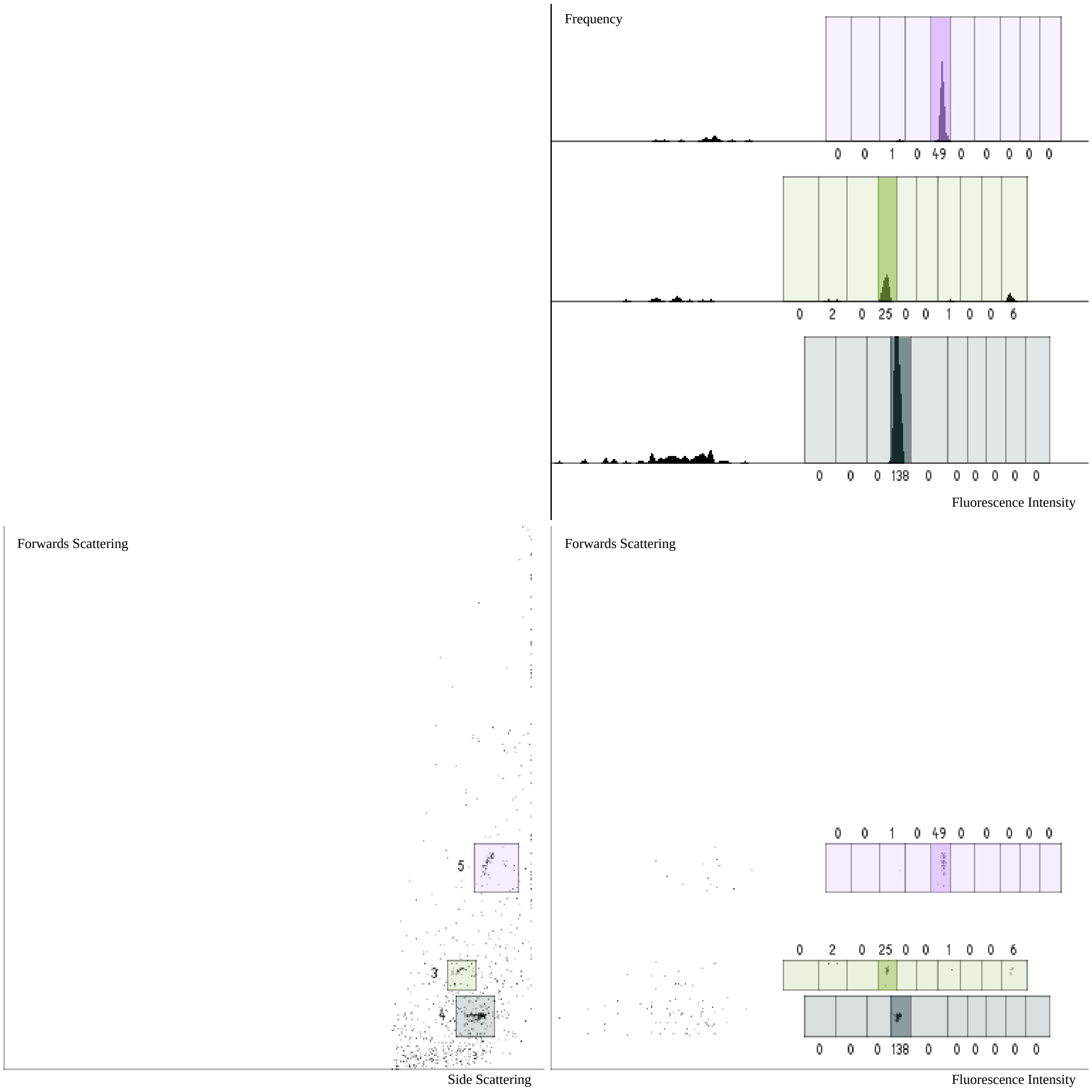

ANNEX 3: TAG DECONVOLUTION - BEAD 152

Passes flow sorting criteria: Yes

Passes tag deconvolution criteria: Yes

Included in protocol analysis: Yes

Protocol: 7, 9, 7, 1

Filename: Bin1_plateB1_A12.fcs

Split 1: Petrol shading

Split 2: Green shading

Split 3: Violet shading

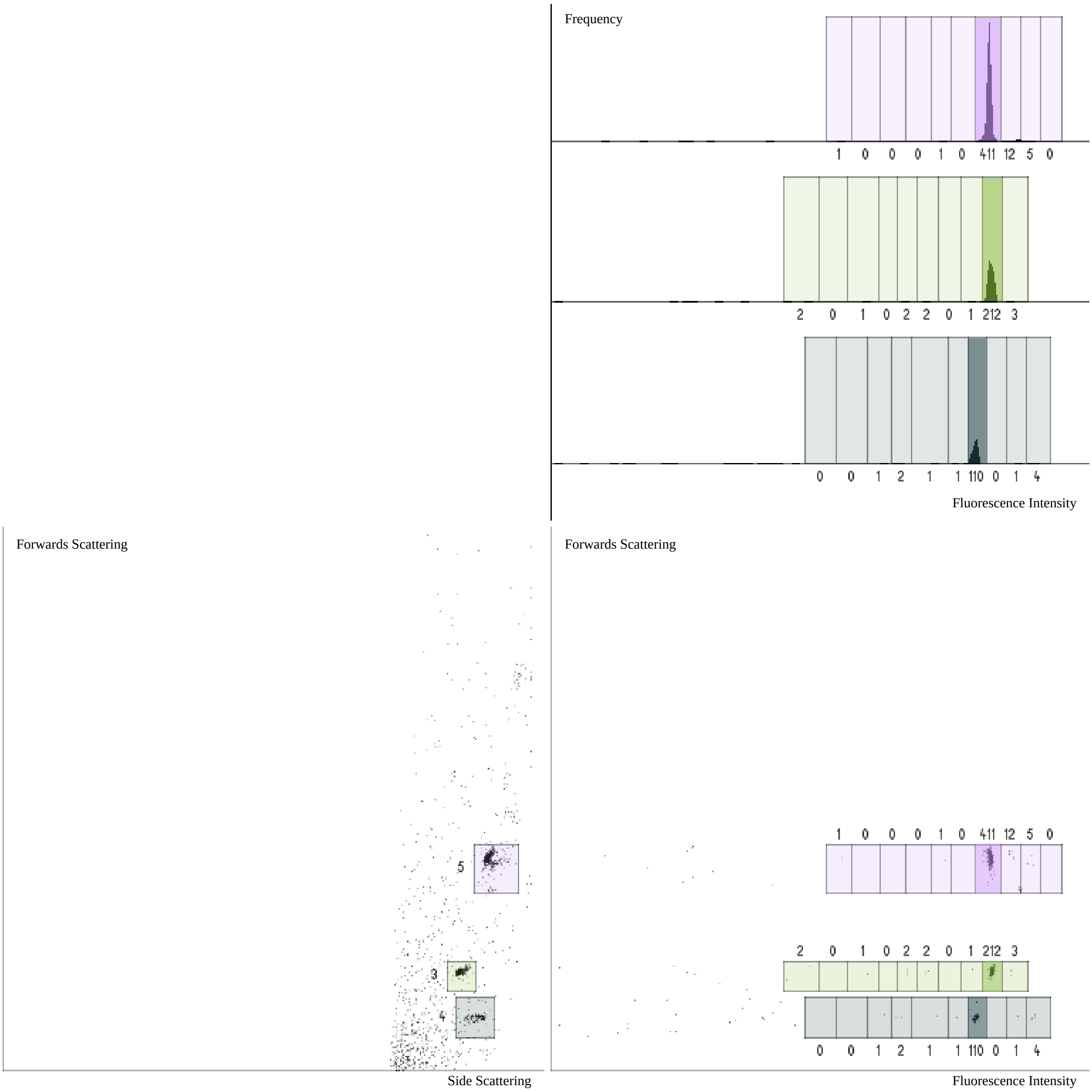

ANNEX 3: TAG DECONVOLUTION - BEAD 153

Passes flow sorting criteria: Yes

Passes tag deconvolution criteria: No

Included in protocol analysis: No

Protocol: N/A

Filename: Bin1_plateB1_B11.fcs

Split 1: Petrol shading

Split 2: Green shading

Split 3: Violet shading

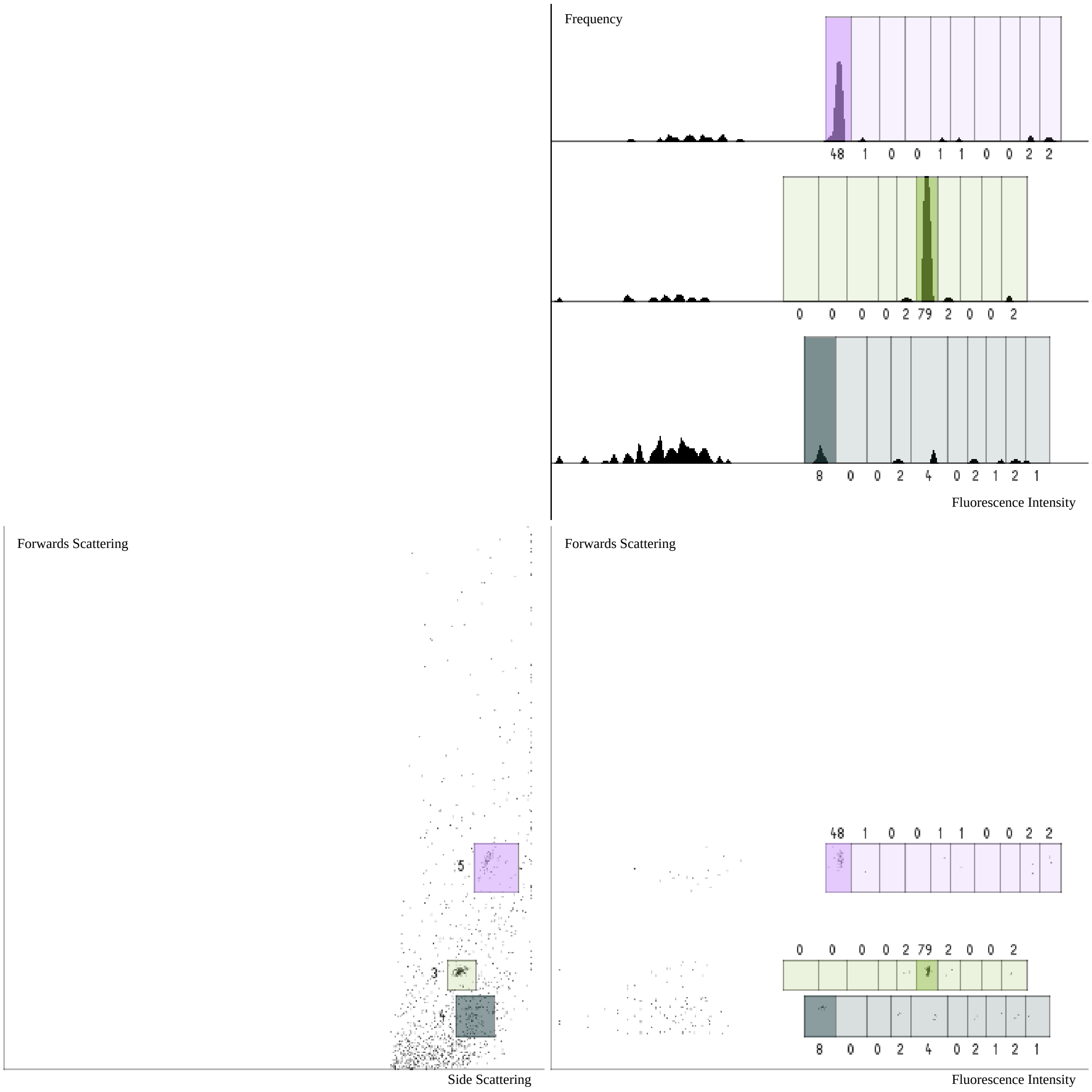

ANNEX 3: TAG DECONVOLUTION - BEAD 154

Passes flow sorting criteria: Yes

Passes tag deconvolution criteria: No

Included in protocol analysis: No

Protocol: N/A

Filename: Bin1_plateB1_B12.fcs

Split 1: Petrol shading

Split 2: Green shading

Split 3: Violet shading

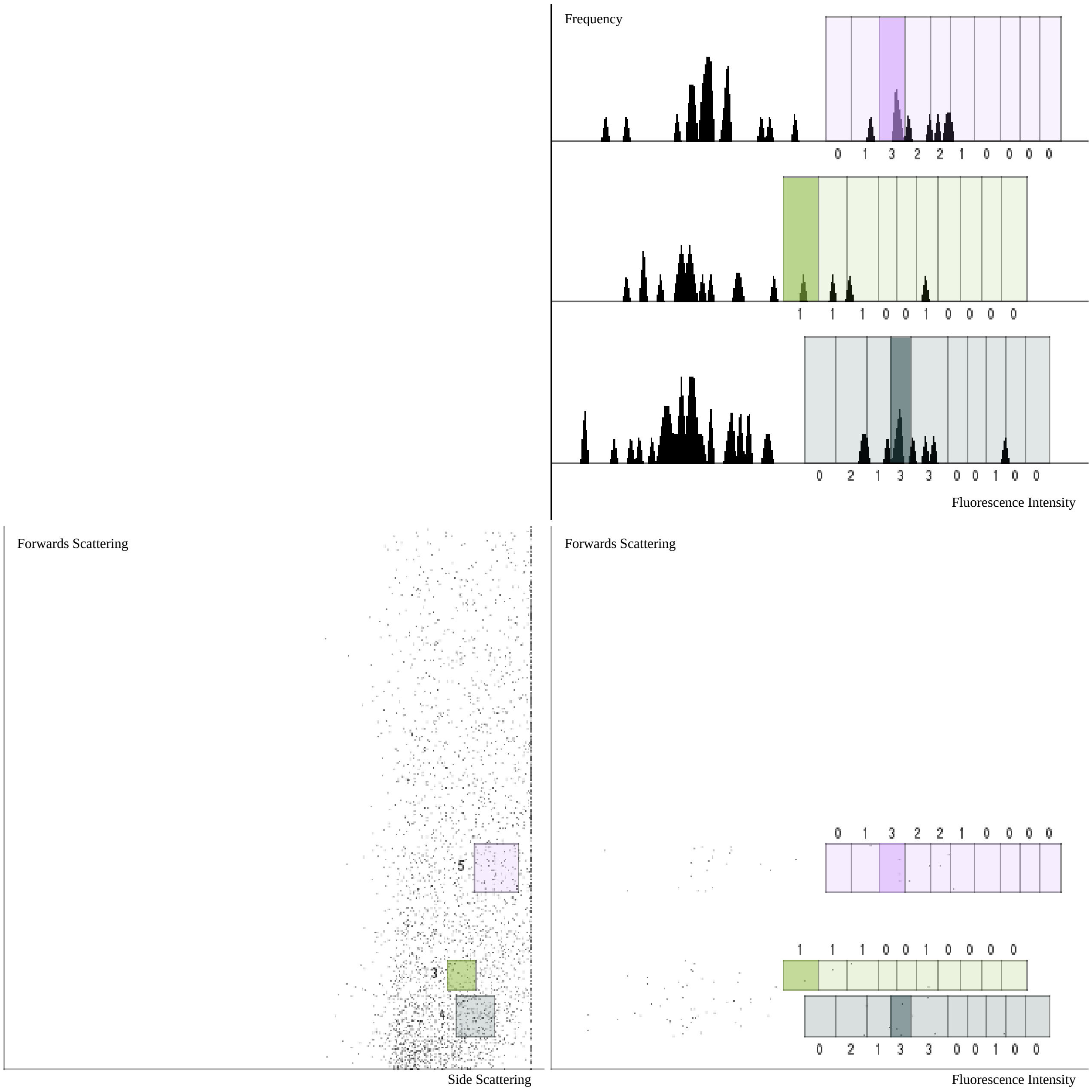

ANNEX 3: TAG DECONVOLUTION - BEAD 155

Passes flow sorting criteria: Yes

Passes tag deconvolution criteria: Yes

Included in protocol analysis: Yes

Protocol: 8, 8, 9, 1

Filename: Bin1_plateB1_C12.fcs

Split 1: Petrol shading

Split 2: Green shading

Split 3: Violet shading

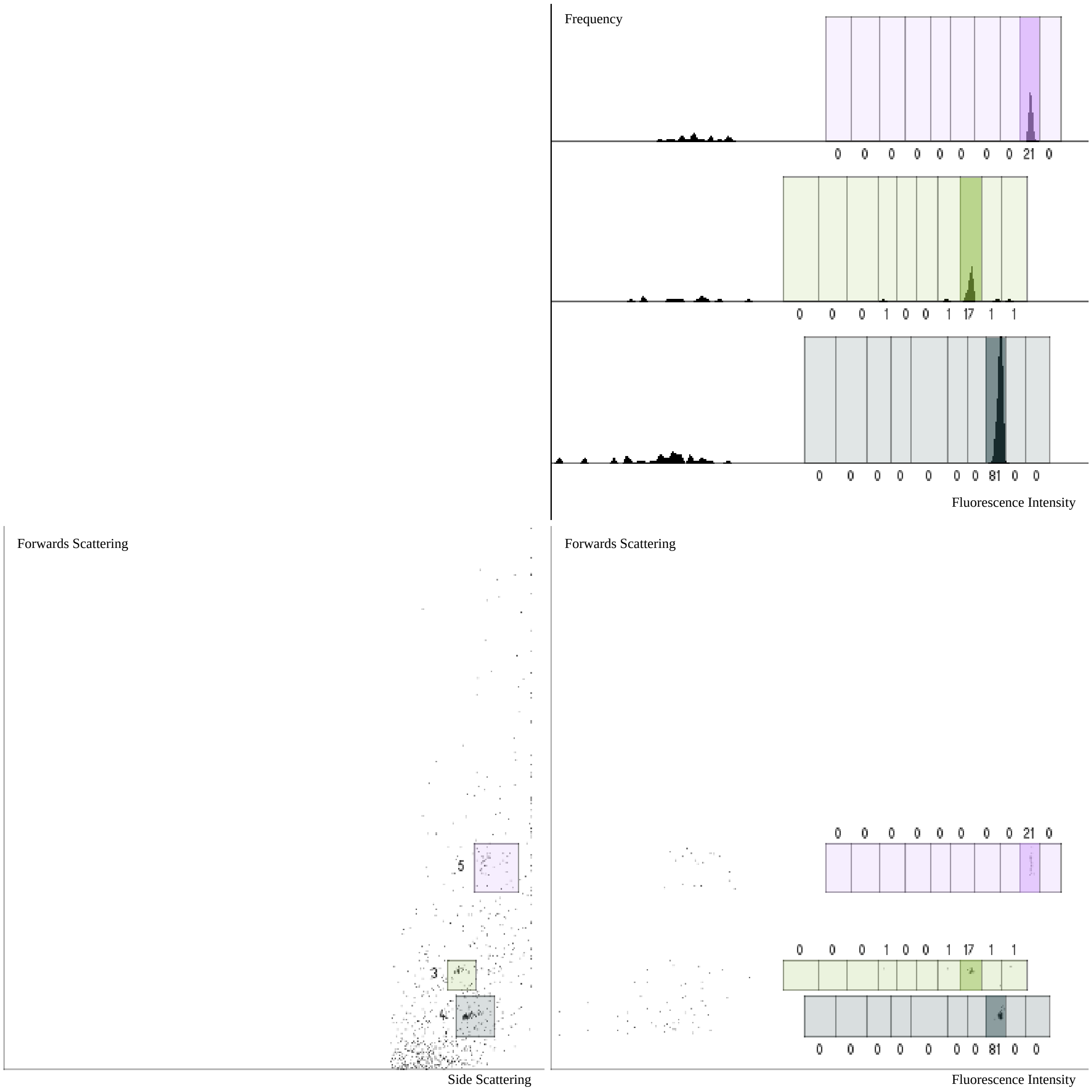

ANNEX 3: TAG DECONVOLUTION - BEAD 156

Passes flow sorting criteria: Yes

Passes tag deconvolution criteria: No

Included in protocol analysis: No

Protocol: N/A

Filename: Bin1_plateB1_F12.fcs

Split 1: Petrol shading

Split 2: Green shading

Split 3: Violet shading

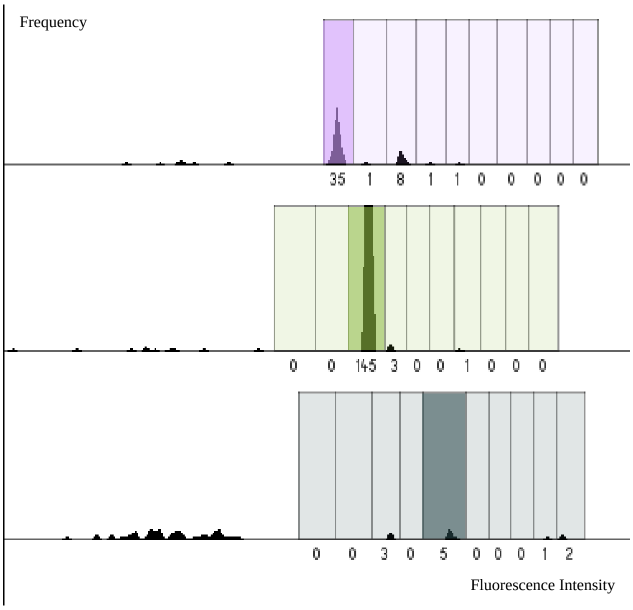

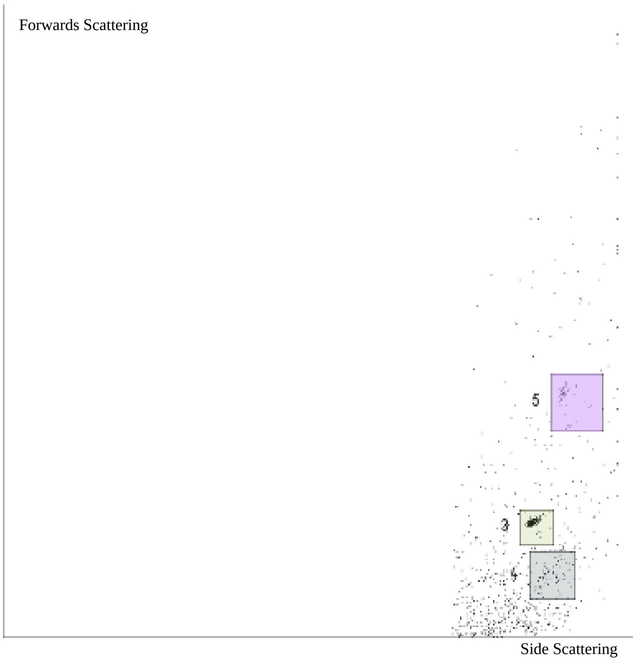

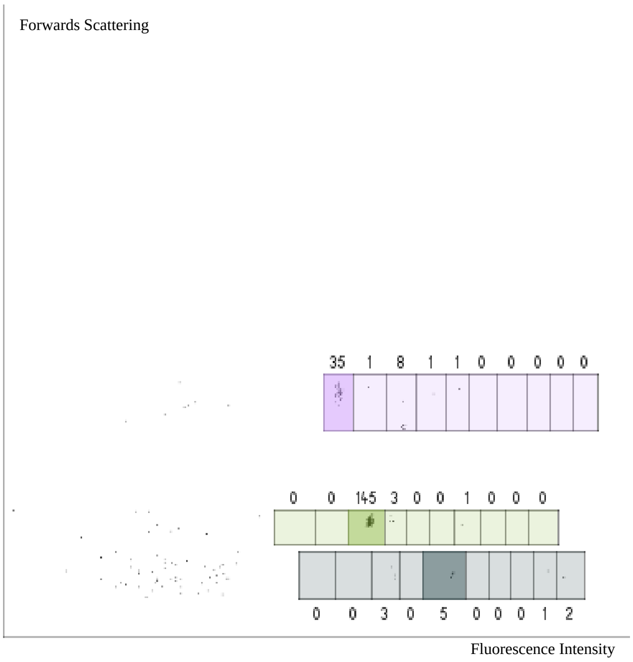

ANNEX 3: TAG DECONVOLUTION - BEAD 157

Passes flow sorting criteria: Yes

Passes tag deconvolution criteria: No

Included in protocol analysis: No

Protocol: N/A

Filename: Bin1_plateB1_G11.fcs

Split 1: Petrol shading

Split 2: Green shading

Split 3: Violet shading

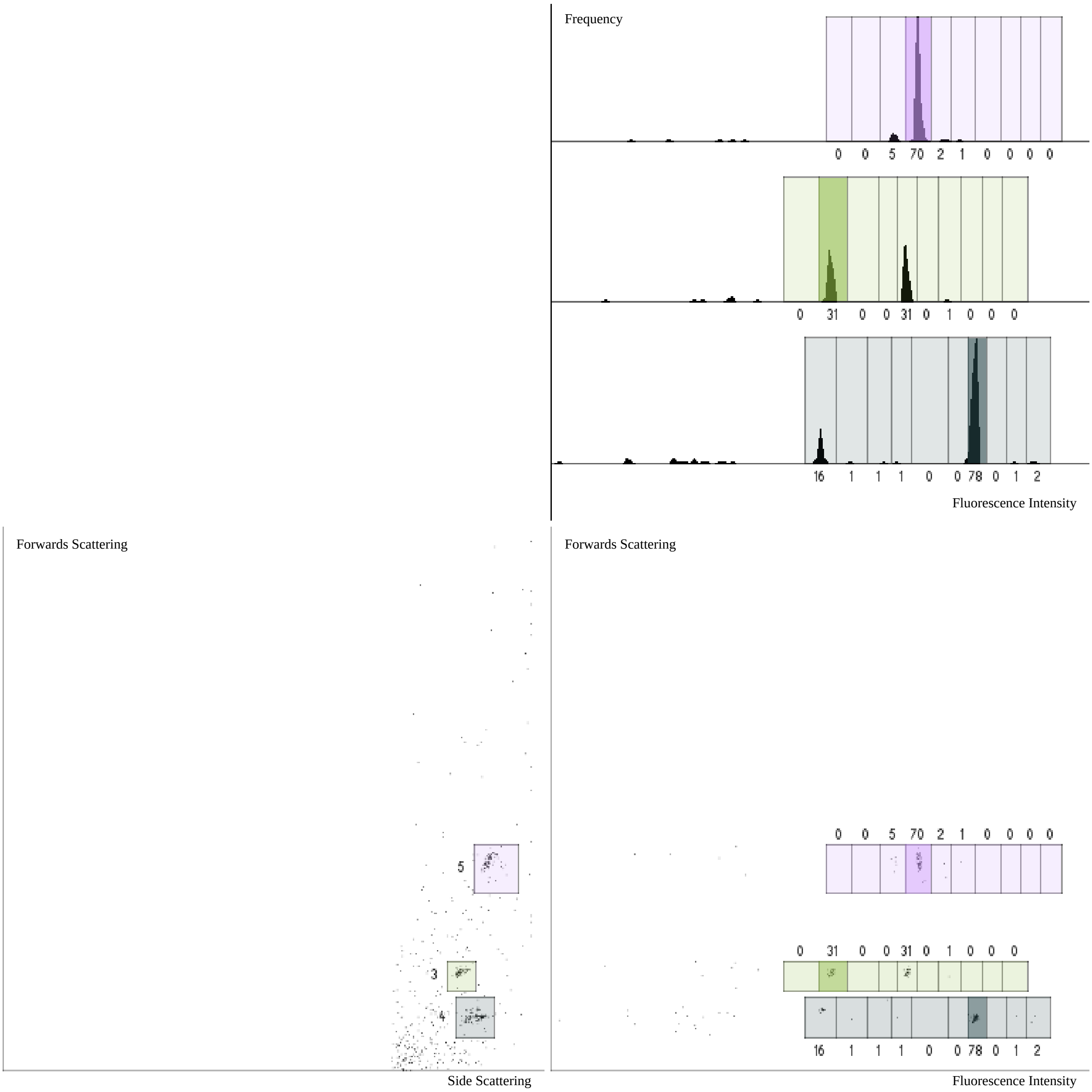

ANNEX 3: TAG DECONVOLUTION - BEAD 158

Passes flow sorting criteria: Yes

Passes tag deconvolution criteria: Yes

Included in protocol analysis: Yes

Protocol: 10, 5, 10, 1

Filename: Bin1_plateC2_C6.fcs

Split 1: Petrol shading

Split 2: Green shading

Split 3: Violet shading

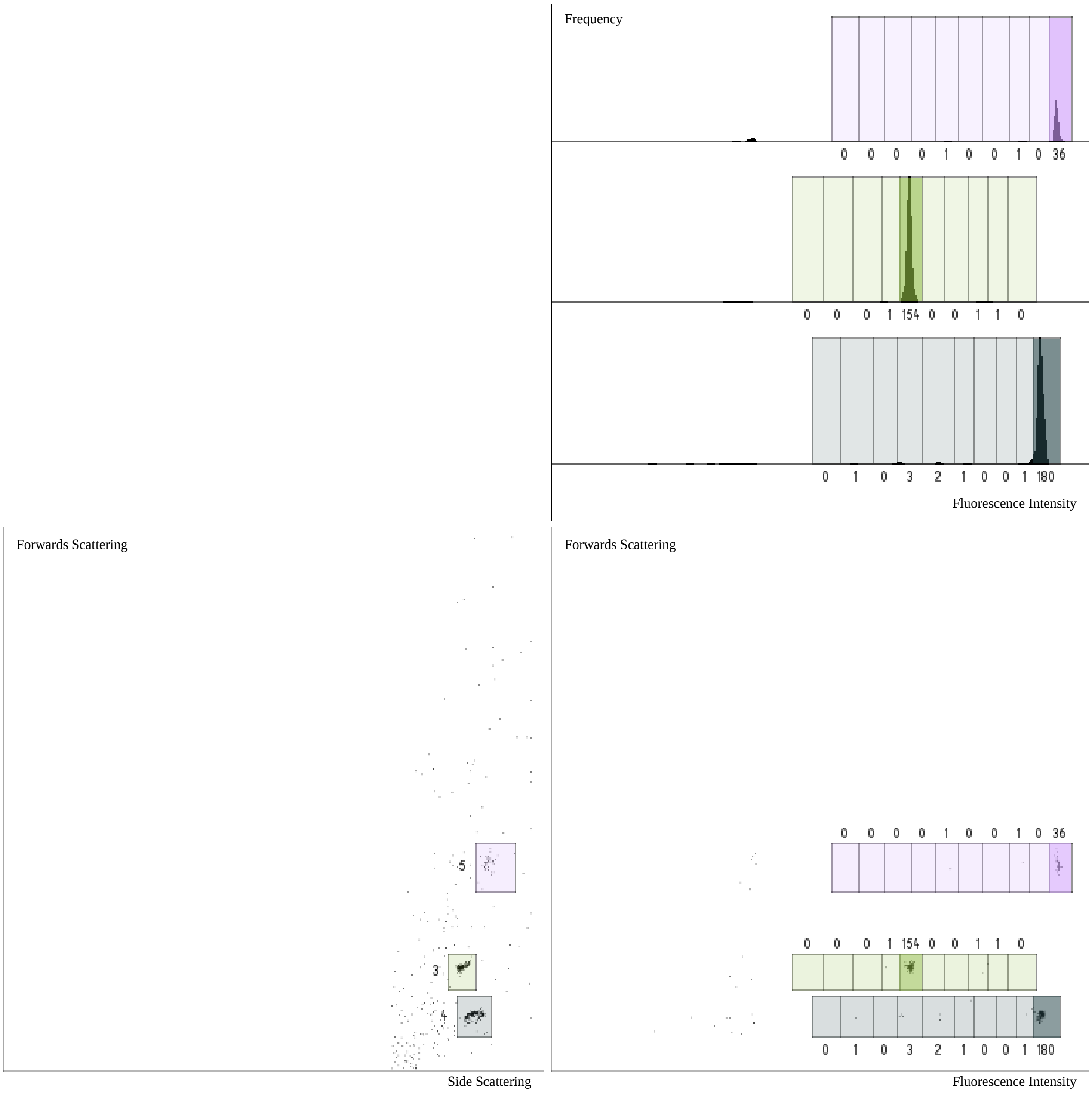

ANNEX 3: TAG DECONVOLUTION - BEAD 159

Passes flow sorting criteria: Yes

Passes tag deconvolution criteria: Yes

Included in protocol analysis: Yes

Protocol: 3, 1, 10, 1

Filename: Bin1_plateC2_A1.fcs

Split 1: Petrol shading

Split 2: Green shading

Split 3: Violet shading

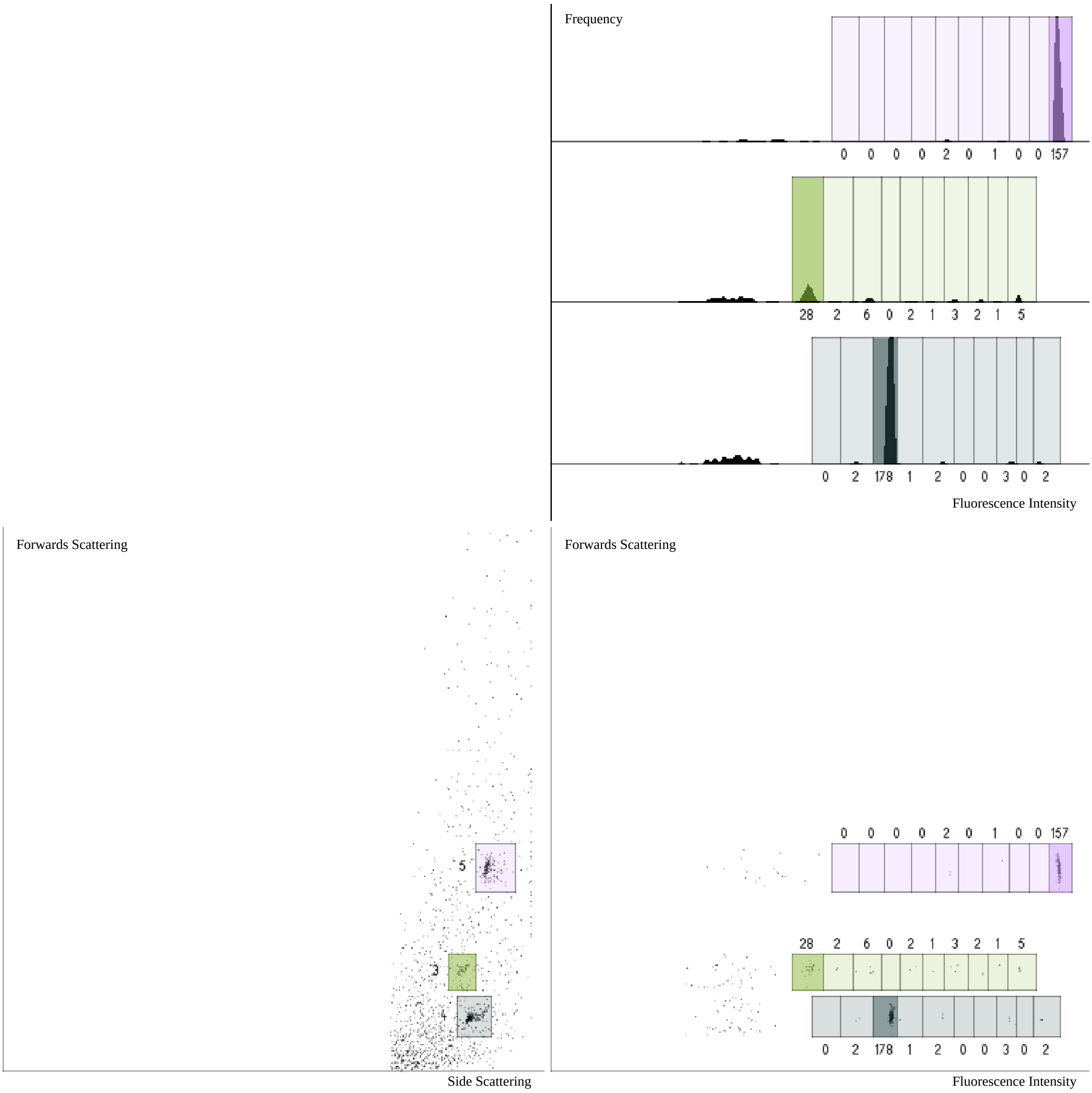

ANNEX 3: TAG DECONVOLUTION - BEAD 160

Passes flow sorting criteria: Yes

Passes tag deconvolution criteria: Yes

Included in protocol analysis: Yes

Protocol: 8, 6, 9, 1

Filename: Bin1_plateC2_A2.fcs

Split 1: Petrol shading

Split 2: Green shading

Split 3: Violet shading

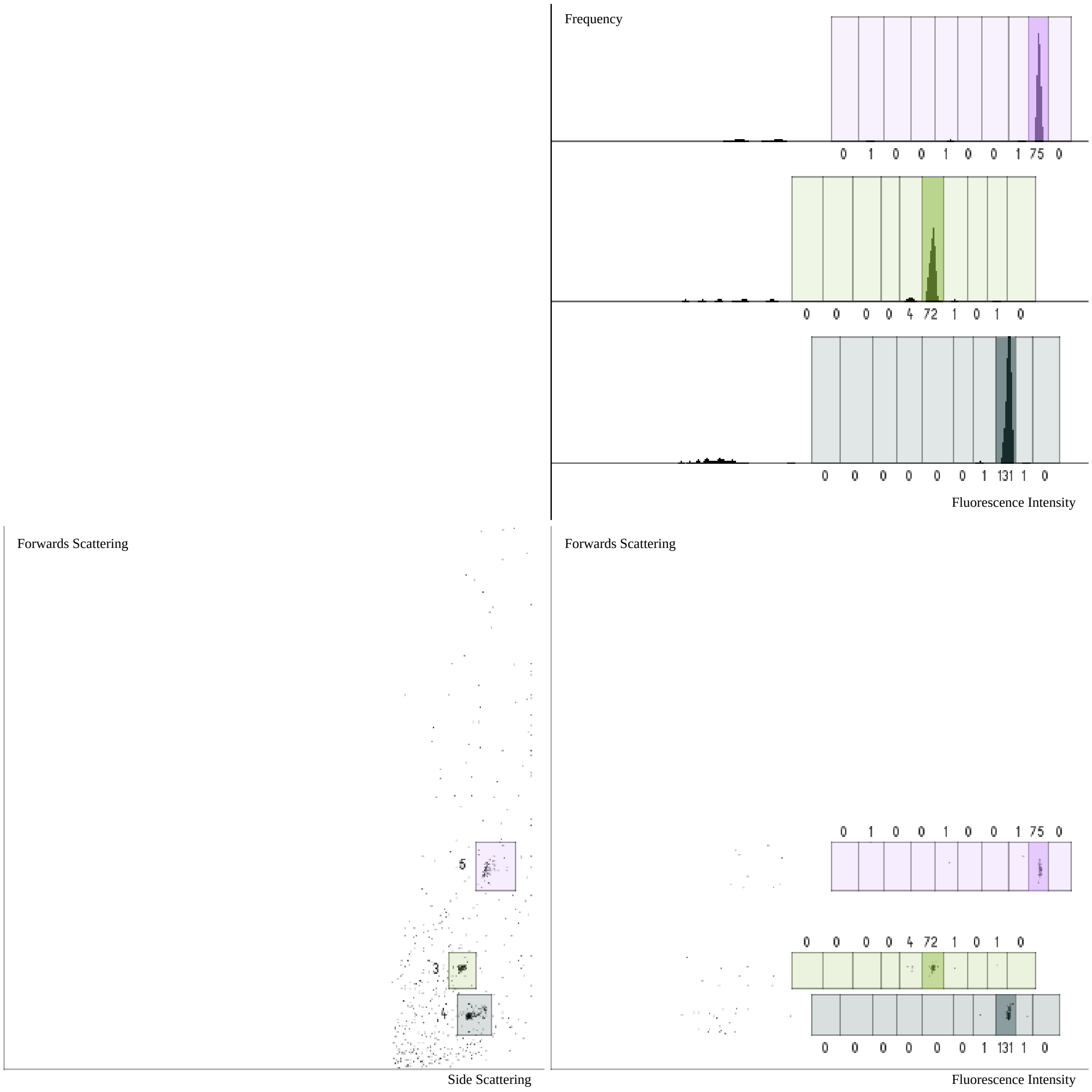

ANNEX 3: TAG DECONVOLUTION - BEAD 161

Passes flow sorting criteria: Yes

Passes tag deconvolution criteria: Yes

Included in protocol analysis: Yes

Protocol: 8, 4, 8, 1

Filename: Bin1_plateC2_A4.fcs

Split 1: Petrol shading

Split 2: Green shading

Split 3: Violet shading

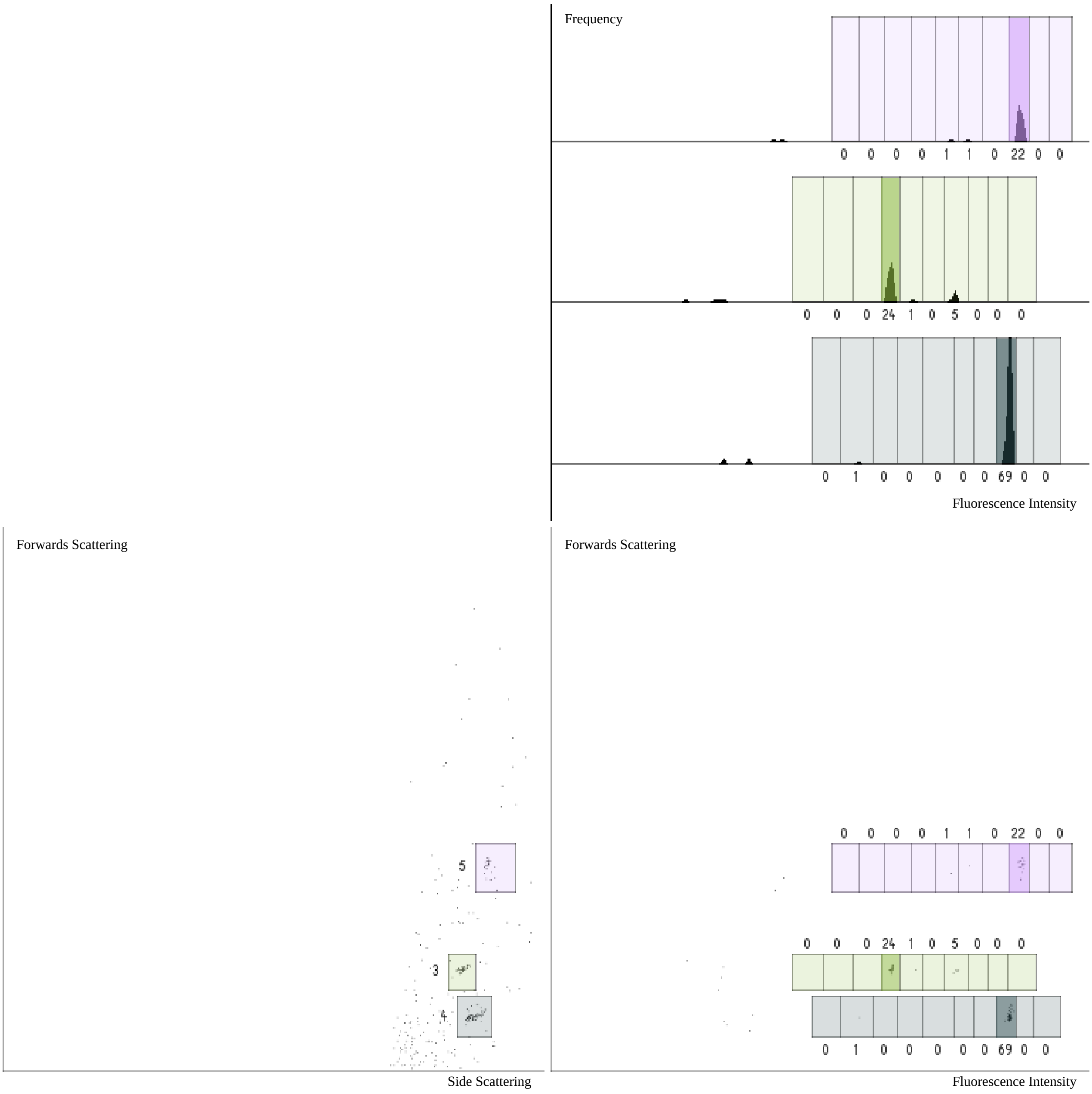

ANNEX 3: TAG DECONVOLUTION - BEAD 162

Passes flow sorting criteria: Yes

Passes tag deconvolution criteria: Yes

Included in protocol analysis: Yes

Protocol: 8, 10, 3, 1

Filename: Bin1_plateC2_A5.fcs

Split 1: Petrol shading

Split 2: Green shading

Split 3: Violet shading

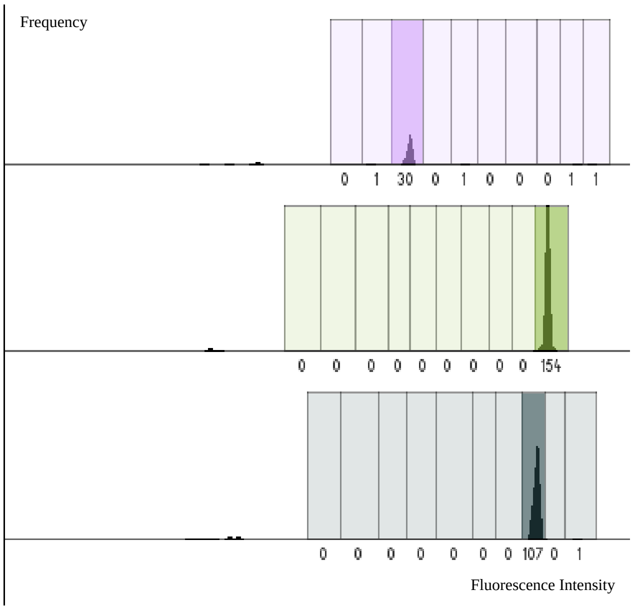

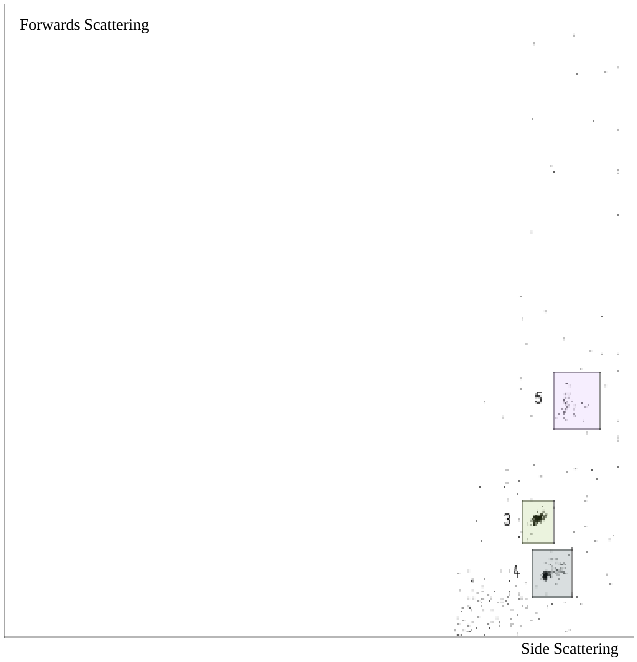

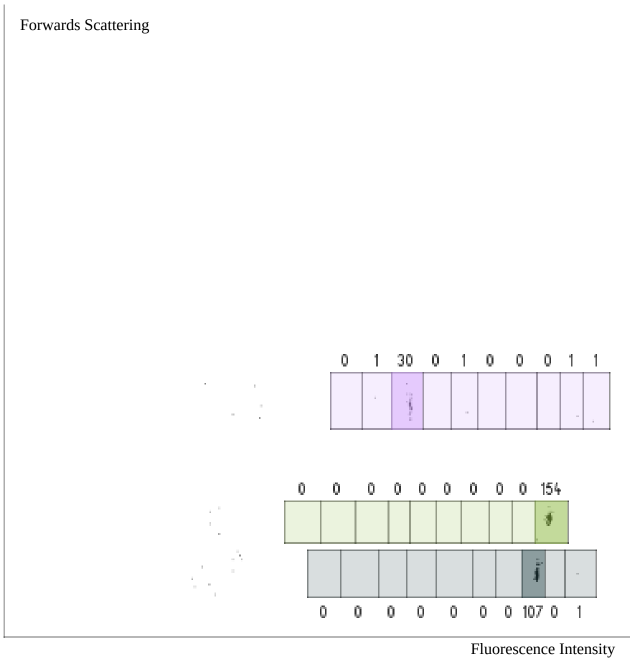

ANNEX 3: TAG DECONVOLUTION - BEAD 163

Passes flow sorting criteria: Yes

Passes tag deconvolution criteria: Yes

Included in protocol analysis: Yes

Protocol: 6, 9, 1, 1

Filename: Bin1_plateC2_A6.fcs

Split 1: Petrol shading

Split 2: Green shading

Split 3: Violet shading

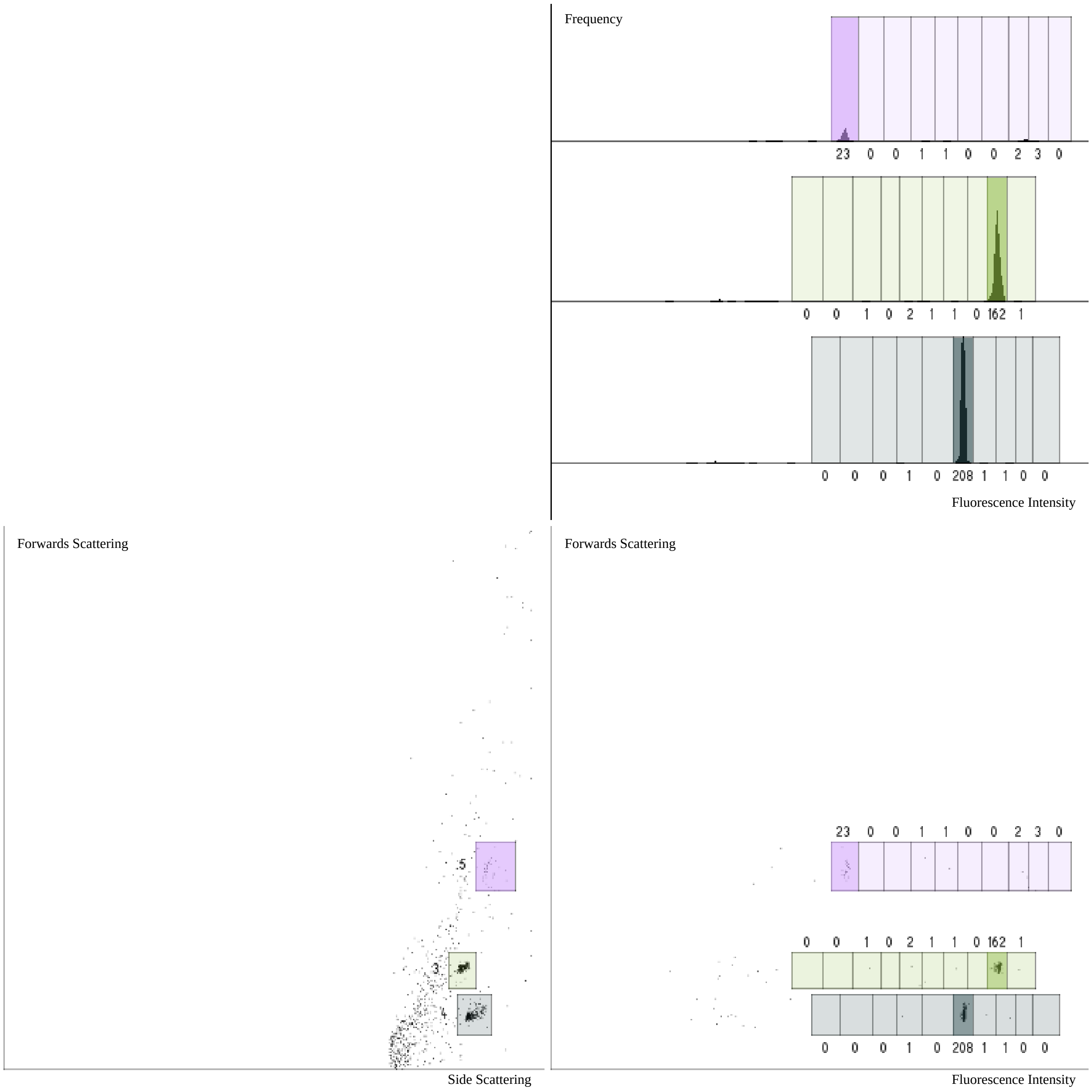

ANNEX 3: TAG DECONVOLUTION - BEAD 164

Passes flow sorting criteria: Yes

Passes tag deconvolution criteria: Yes

Included in protocol analysis: Yes

Protocol: 8, 5, 10, 1

Filename: Bin1_plateC2_A7.fcs

Split 1: Petrol shading

Split 2: Green shading

Split 3: Violet shading

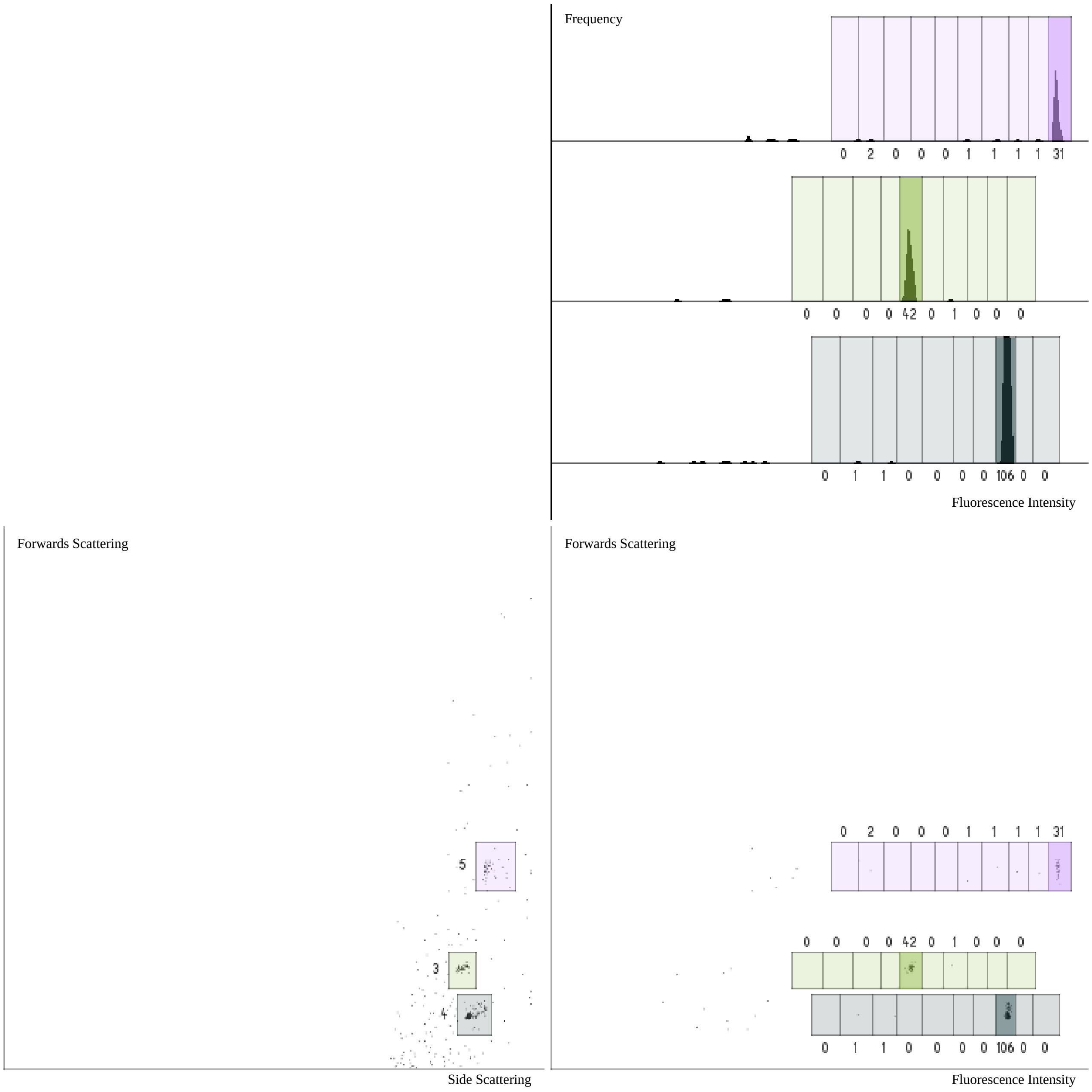

ANNEX 3: TAG DECONVOLUTION - BEAD 165

Passes flow sorting criteria: Yes

Passes tag deconvolution criteria: No

Included in protocol analysis: No

Protocol: N/A

Filename: Bin1_plateC2_A8.fcs

Split 1: Petrol shading

Split 2: Green shading

Split 3: Violet shading

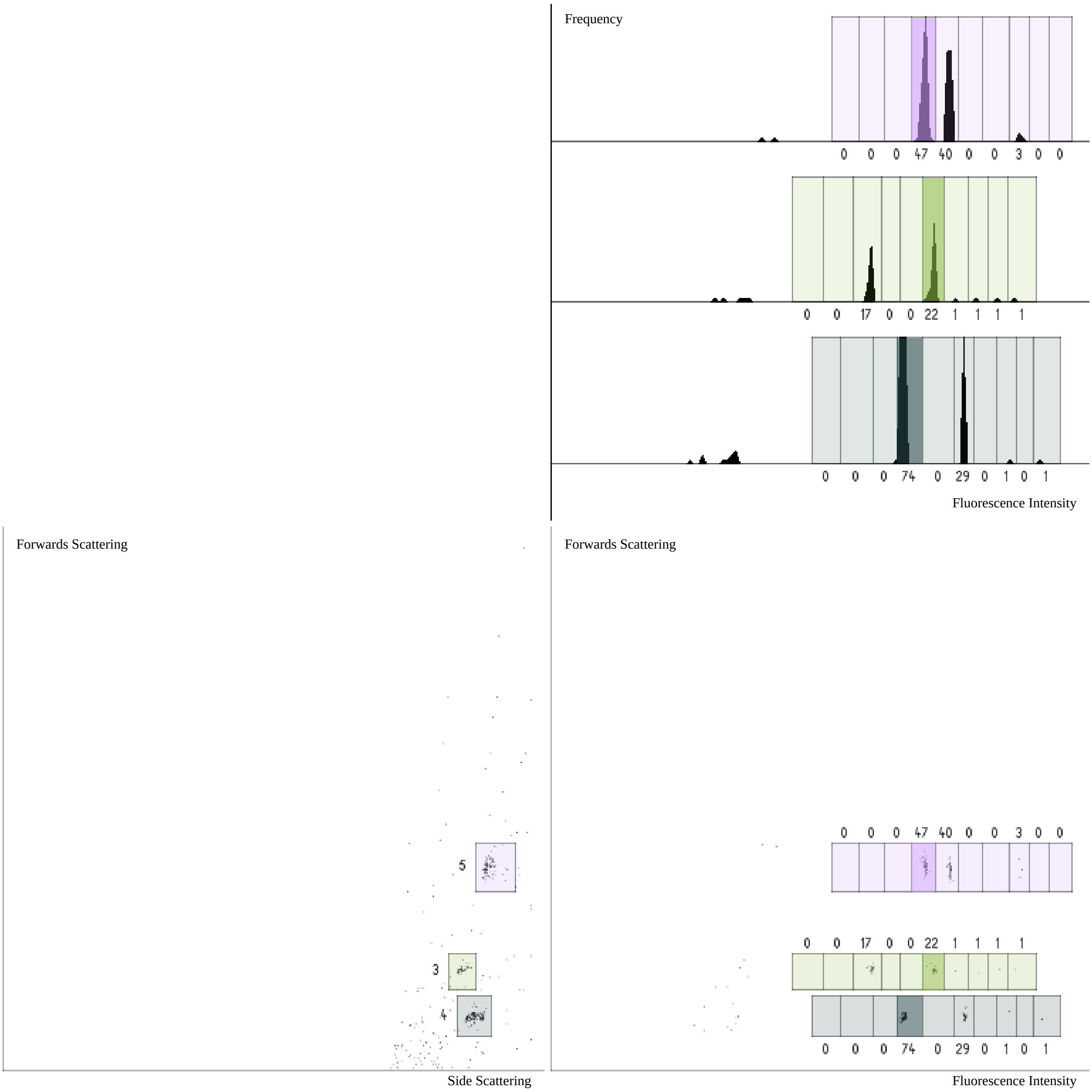

ANNEX 3: TAG DECONVOLUTION - BEAD 166

Passes flow sorting criteria: Yes

Passes tag deconvolution criteria: Yes

Included in protocol analysis: Yes

Protocol: 1, 5, 2, 1

Filename: Bin1_plateC2_A9.fcs

Split 1: Petrol shading

Split 2: Green shading

Split 3: Violet shading

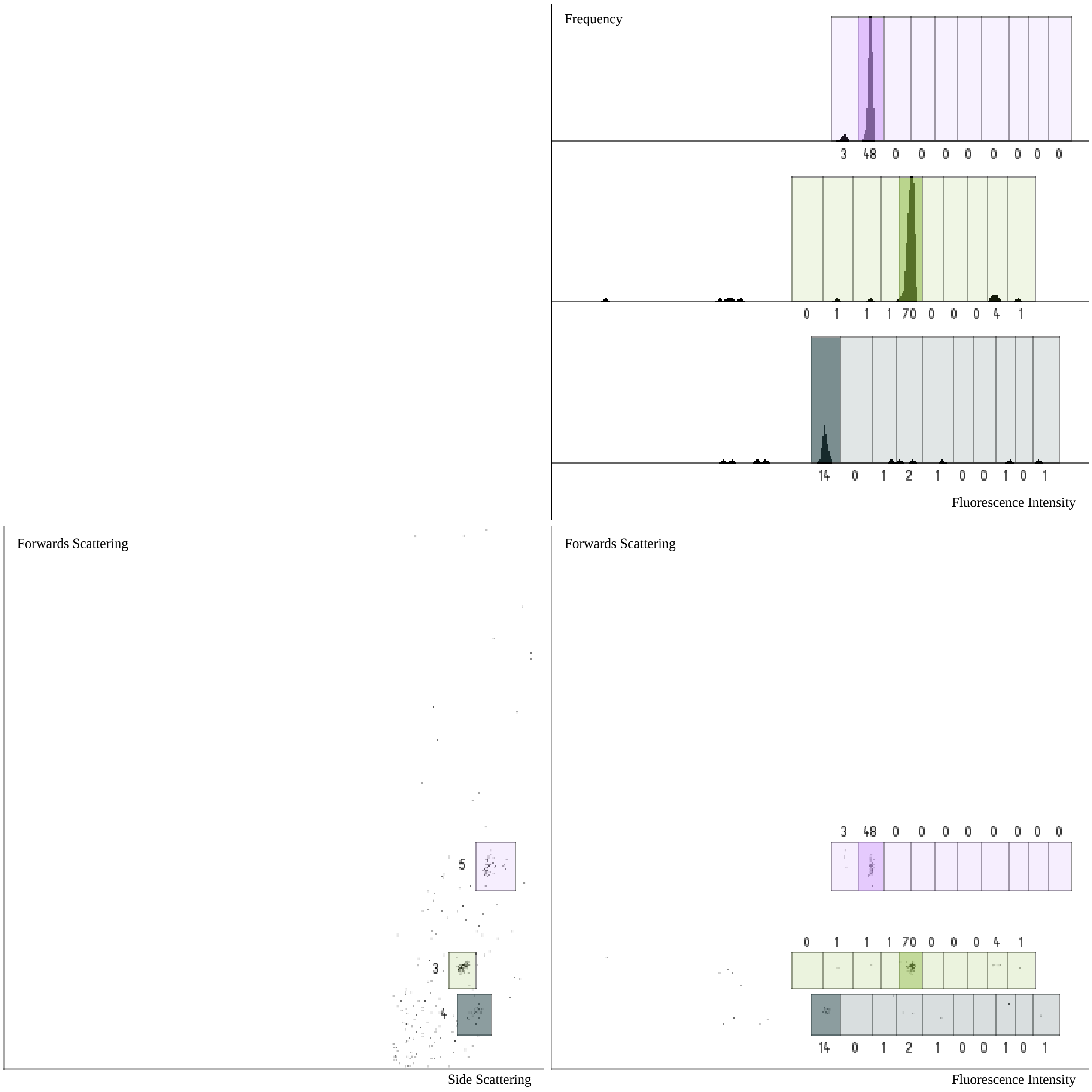

ANNEX 3: TAG DECONVOLUTION - BEAD 167

Passes flow sorting criteria: Yes

Passes tag deconvolution criteria: Yes

Included in protocol analysis: Yes

Protocol: 5, 5, 5, 1

Filename: Bin1_plateC2_A10.fcs

Split 1: Petrol shading

Split 2: Green shading

Split 3: Violet shading

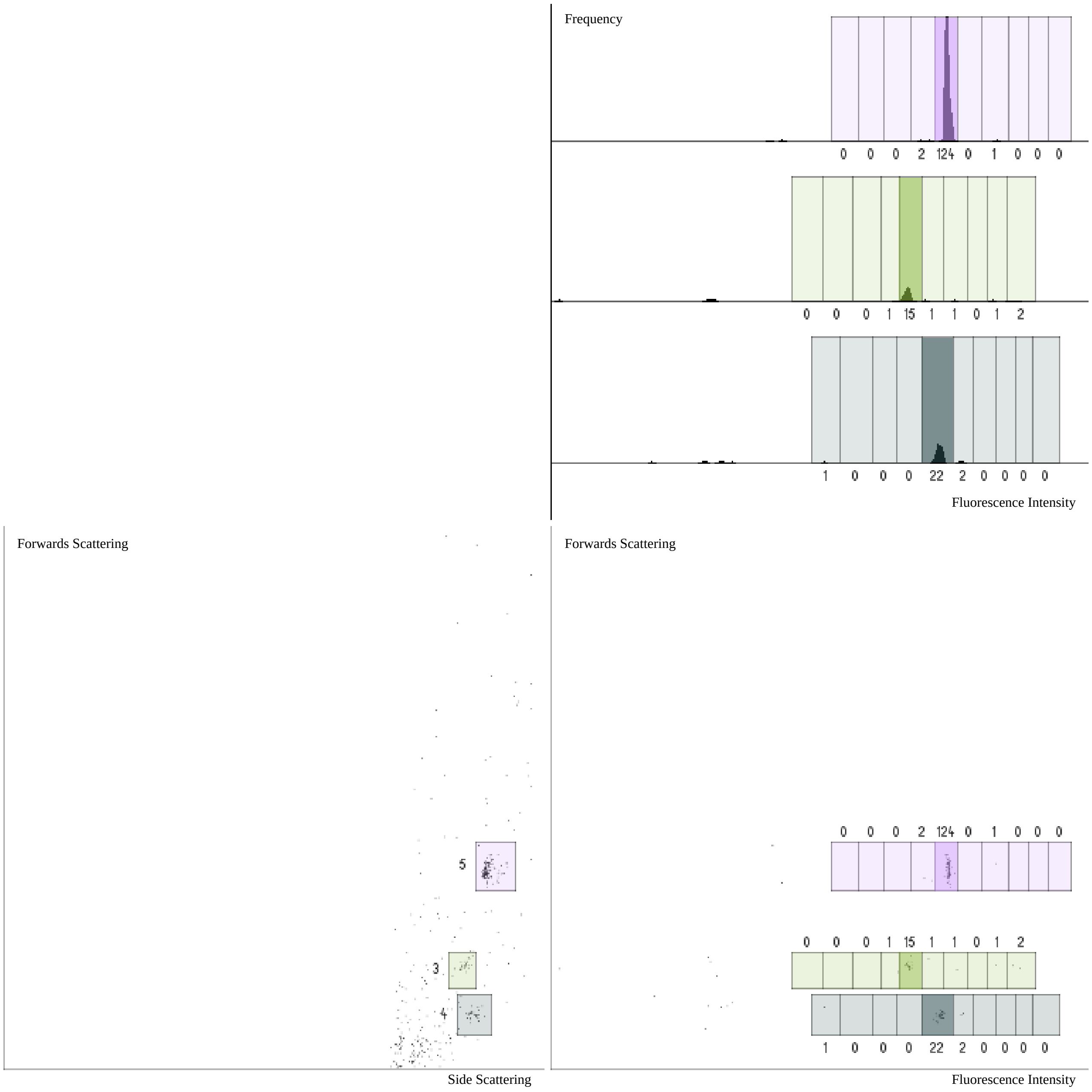

ANNEX 3: TAG DECONVOLUTION - BEAD 168

Passes flow sorting criteria: Yes

Passes tag deconvolution criteria: No

Included in protocol analysis: No

Protocol: N/A

Filename: Bin1_plateC2_A11.fcs

Split 1: Petrol shading

Split 2: Green shading

Split 3: Violet shading

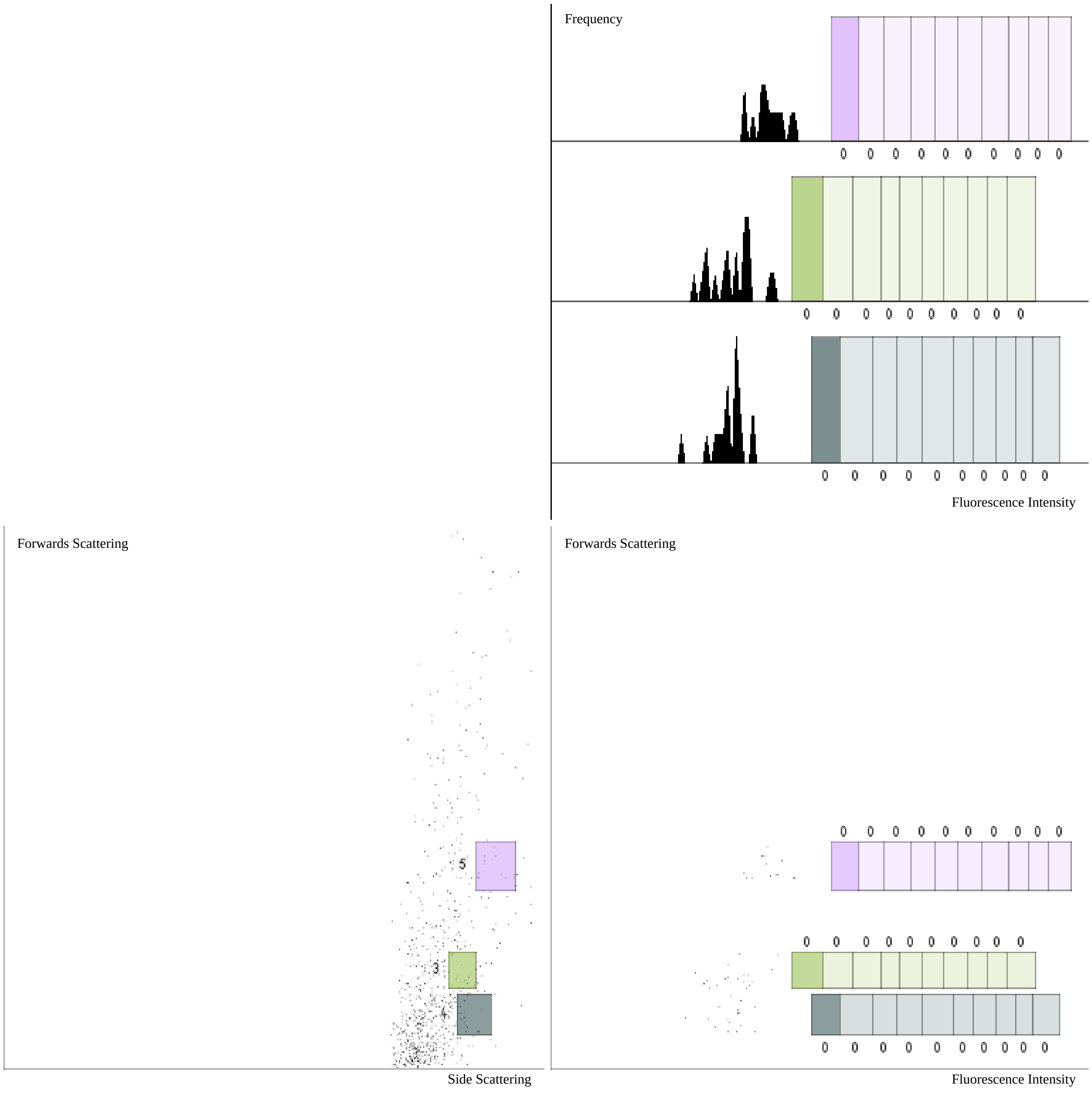

ANNEX 3: TAG DECONVOLUTION - BEAD 169

Passes flow sorting criteria: Yes

Passes tag deconvolution criteria: Yes

Included in protocol analysis: Yes

Protocol: 7, 8, 10, 1

Filename: Bin1_plateC2_A12.fcs

Split 1: Petrol shading

Split 2: Green shading

Split 3: Violet shading

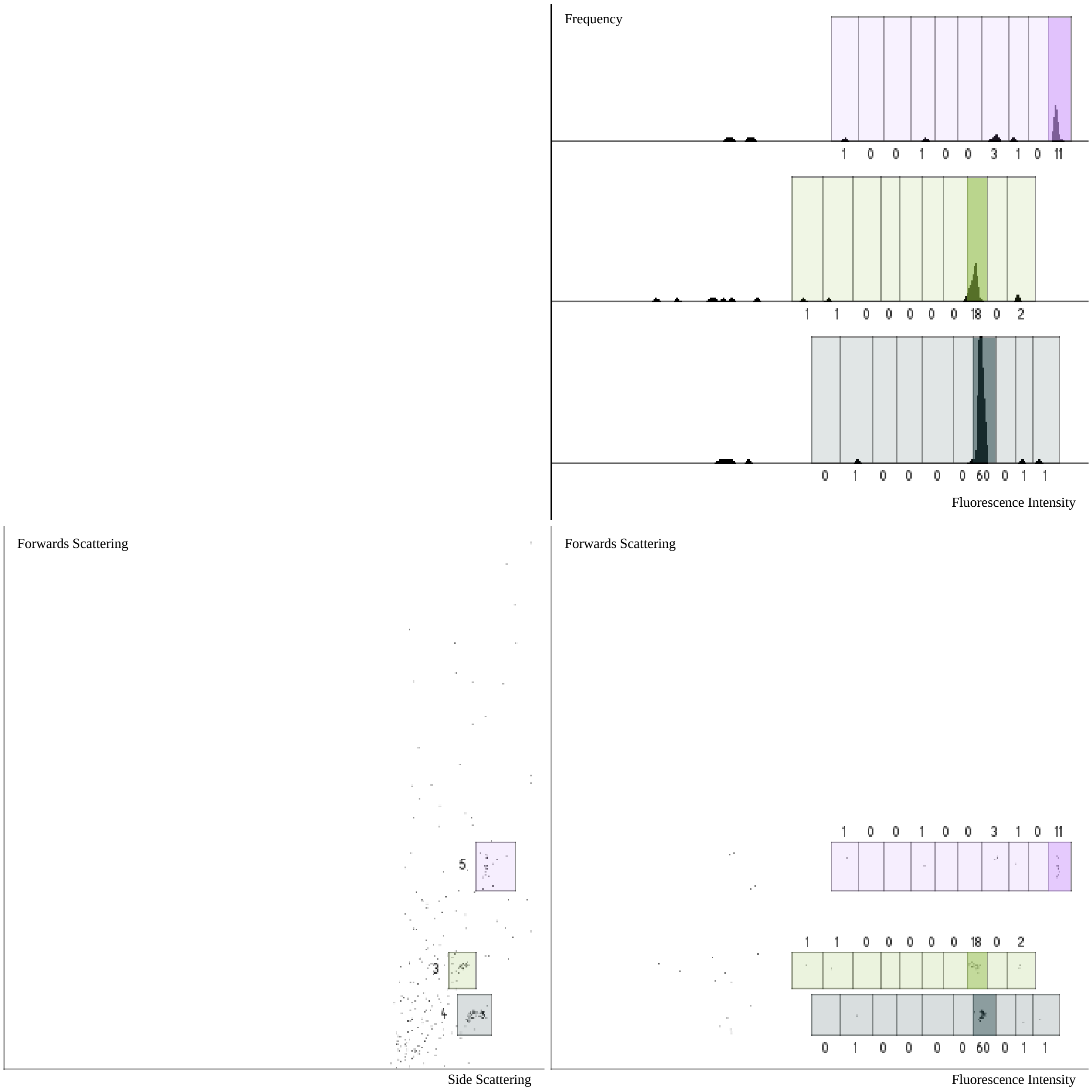

ANNEX 3: TAG DECONVOLUTION - BEAD 170

Passes flow sorting criteria: Yes

Passes tag deconvolution criteria: Yes

Included in protocol analysis: Yes

Protocol: 8, 7, 9, 1

Filename: Bin1_plateC2_B1.fcs

Split 1: Petrol shading

Split 2: Green shading

Split 3: Violet shading

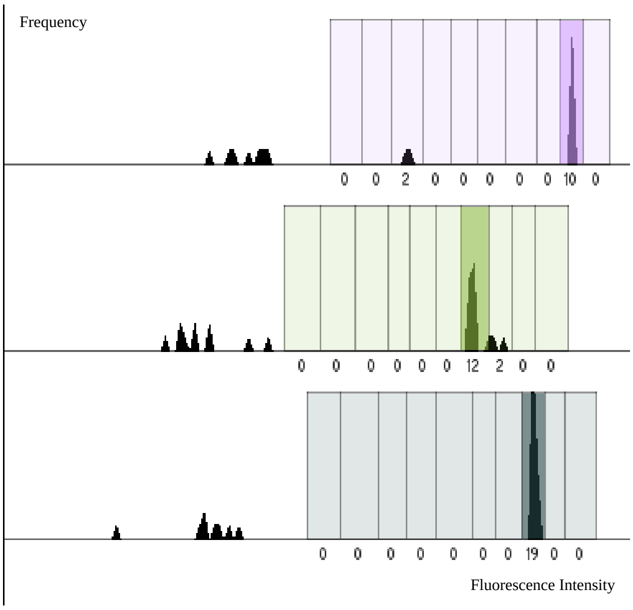

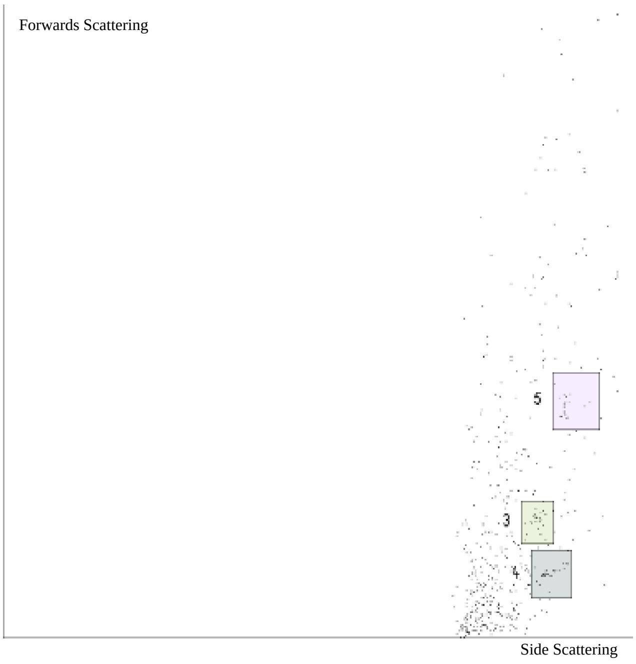

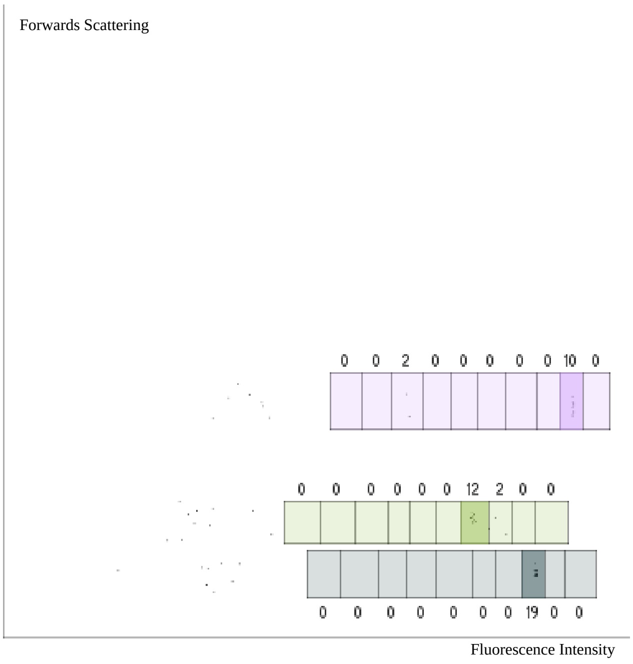

ANNEX 3: TAG DECONVOLUTION - BEAD 171

Passes flow sorting criteria: Yes

Passes tag deconvolution criteria: Yes

Included in protocol analysis: Yes

Protocol: 1, 5, 2, 1

Filename: Bin1_plateC2_B2.fcs

Split 1: Petrol shading

Split 2: Green shading

Split 3: Violet shading

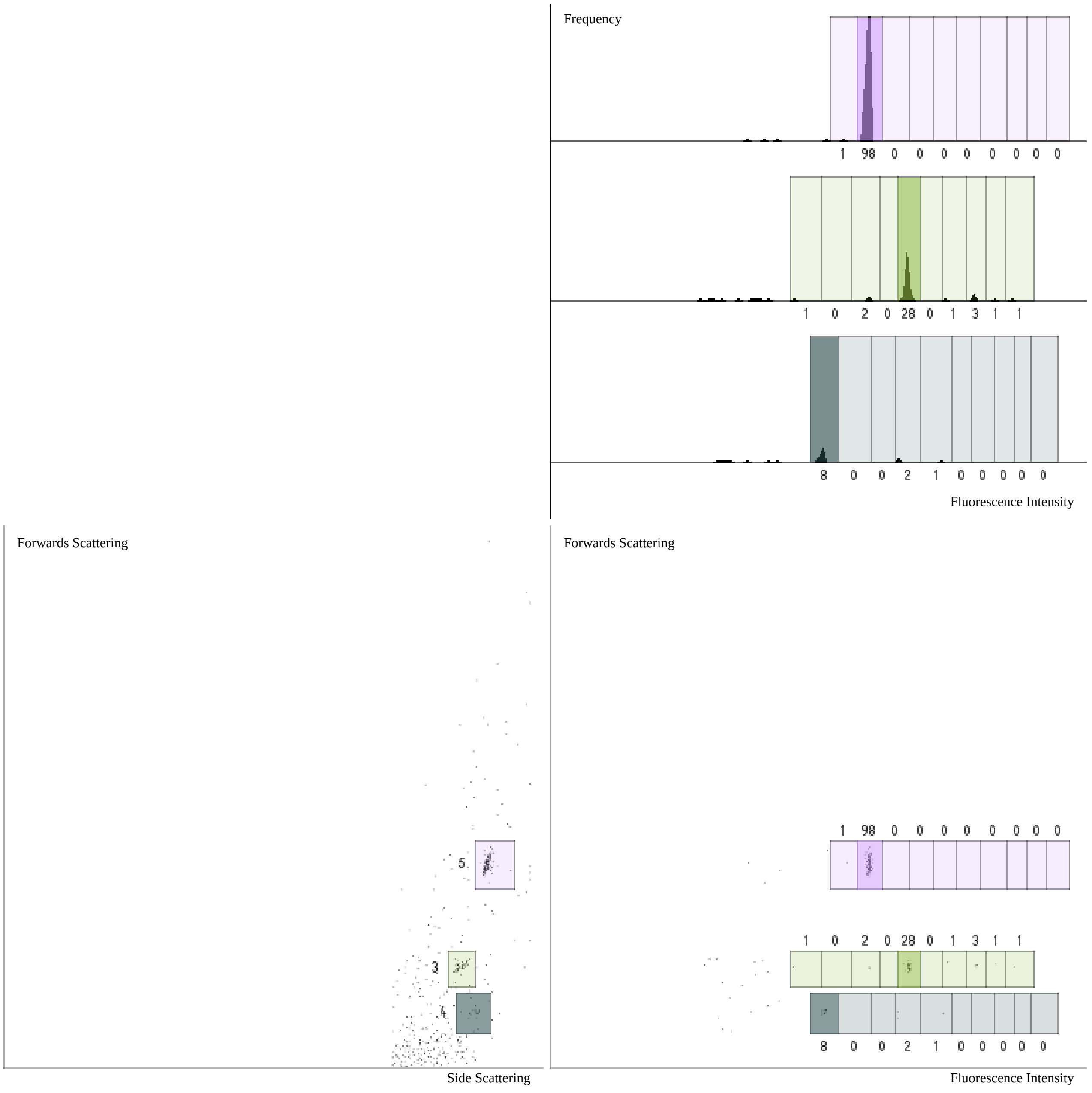

ANNEX 3: TAG DECONVOLUTION - BEAD 172

Passes flow sorting criteria: Yes

Passes tag deconvolution criteria: Yes

Included in protocol analysis: Yes

Protocol: 9, 10, 10, 1

Filename: Bin1_plateC2_B3.fcs

Split 1: Petrol shading

Split 2: Green shading

Split 3: Violet shading

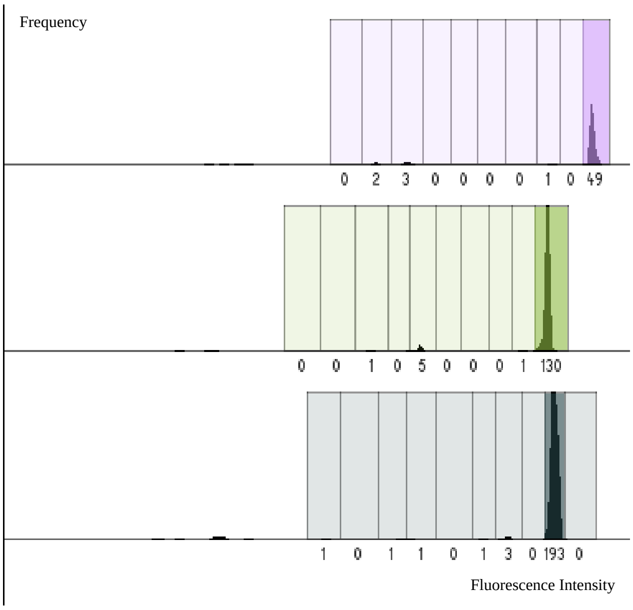

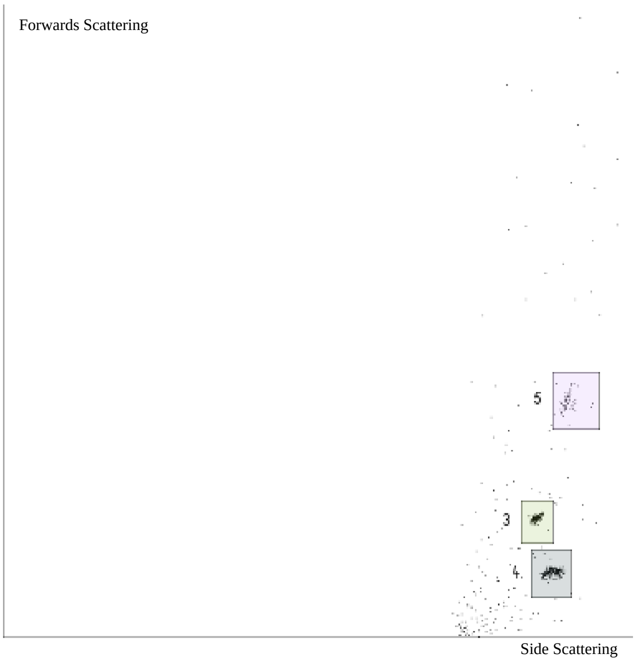

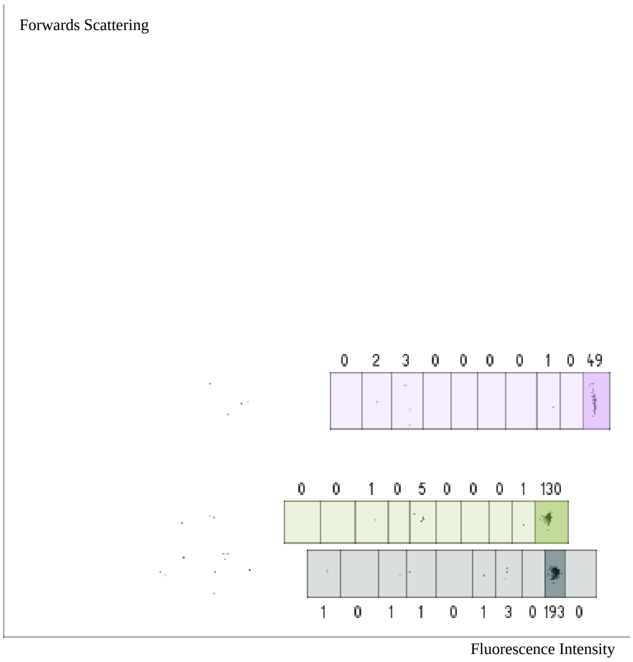

ANNEX 3: TAG DECONVOLUTION - BEAD 173

Passes flow sorting criteria: Yes

Passes tag deconvolution criteria: Yes

Included in protocol analysis: Yes

Protocol: 5, 9, 5, 1

Filename: Bin1_plateC2_B4.fcs

Split 1: Petrol shading

Split 2: Green shading

Split 3: Violet shading

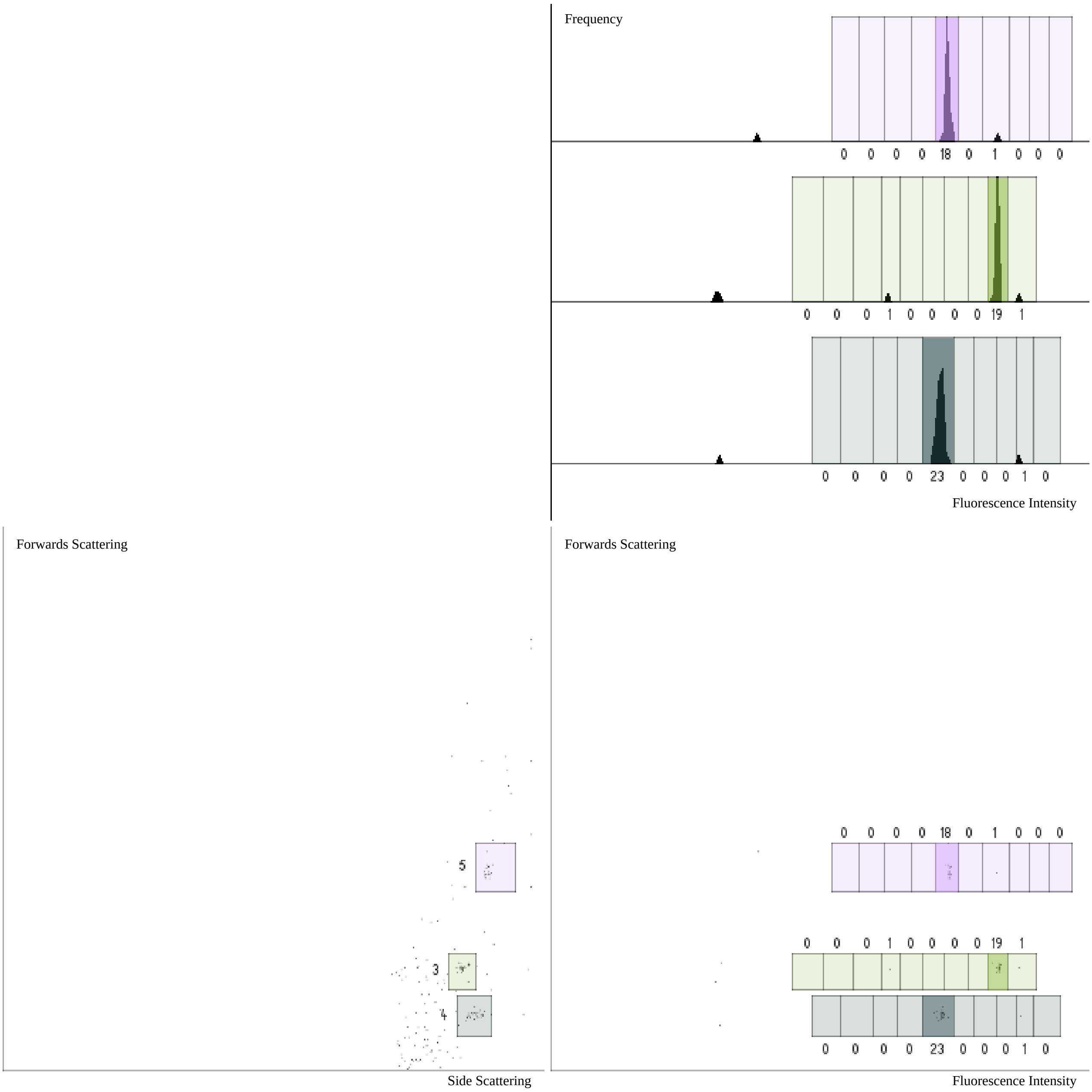

ANNEX 3: TAG DECONVOLUTION - BEAD 174

Passes flow sorting criteria: Yes

Passes tag deconvolution criteria: Yes

Included in protocol analysis: Yes

Protocol: 1, 5, 7, 1

Filename: Bin1_plateC2_B5.fcs

Split 1: Petrol shading

Split 2: Green shading

Split 3: Violet shading

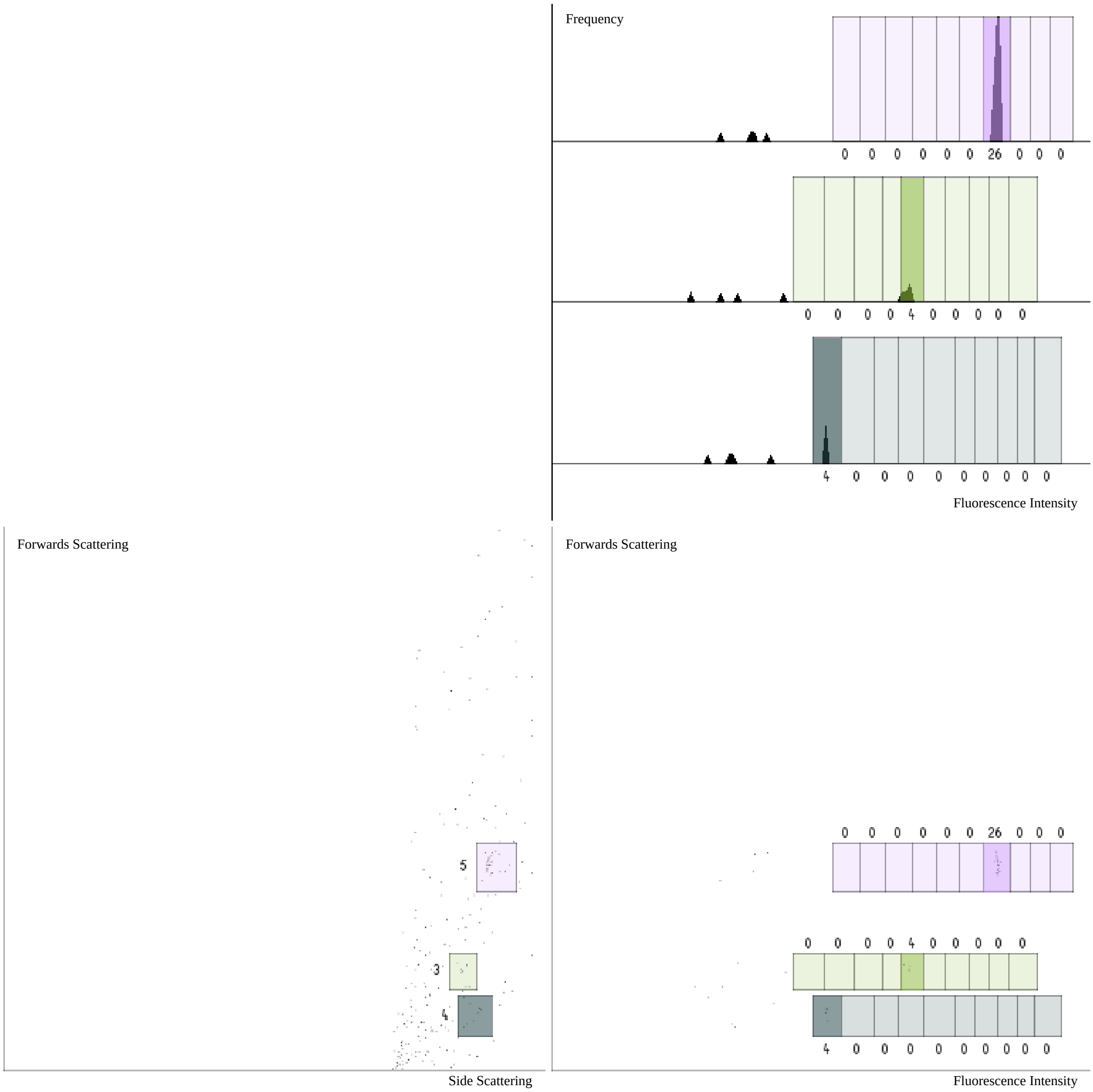

ANNEX 3: TAG DECONVOLUTION - BEAD 175

Passes flow sorting criteria: Yes

Passes tag deconvolution criteria: Yes

Included in protocol analysis: Yes

Protocol: 4, 6, 5, 1

Filename: Bin1_plateC2_B6.fcs

Split 1: Petrol shading

Split 2: Green shading

Split 3: Violet shading

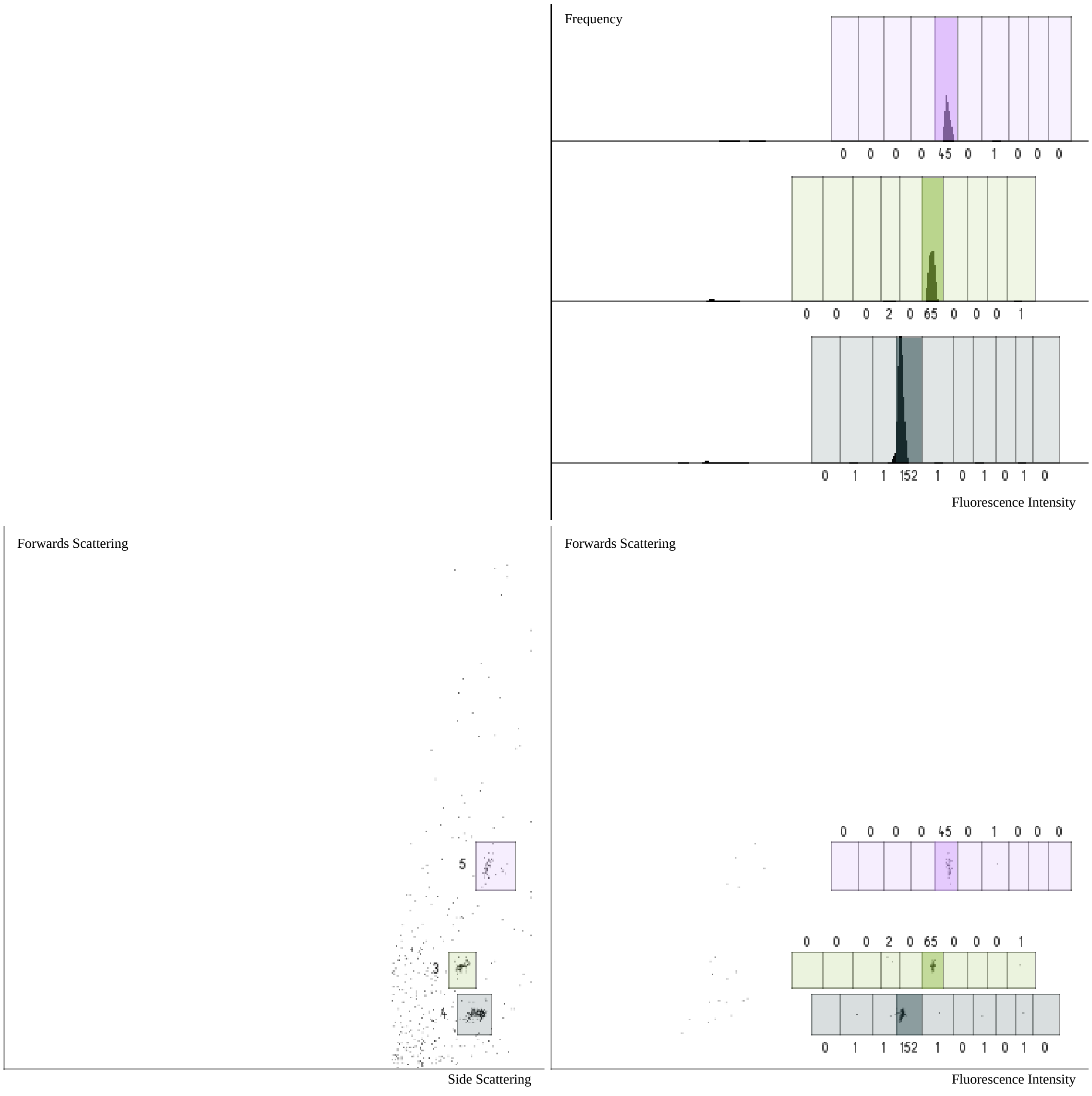

ANNEX 3: TAG DECONVOLUTION - BEAD 176

Passes flow sorting criteria: Yes

Passes tag deconvolution criteria: Yes

Included in protocol analysis: Yes

Protocol: 6, 7, 5, 1

Filename: Bin1_plateC2_B7.fcs

Split 1: Petrol shading

Split 2: Green shading

Split 3: Violet shading

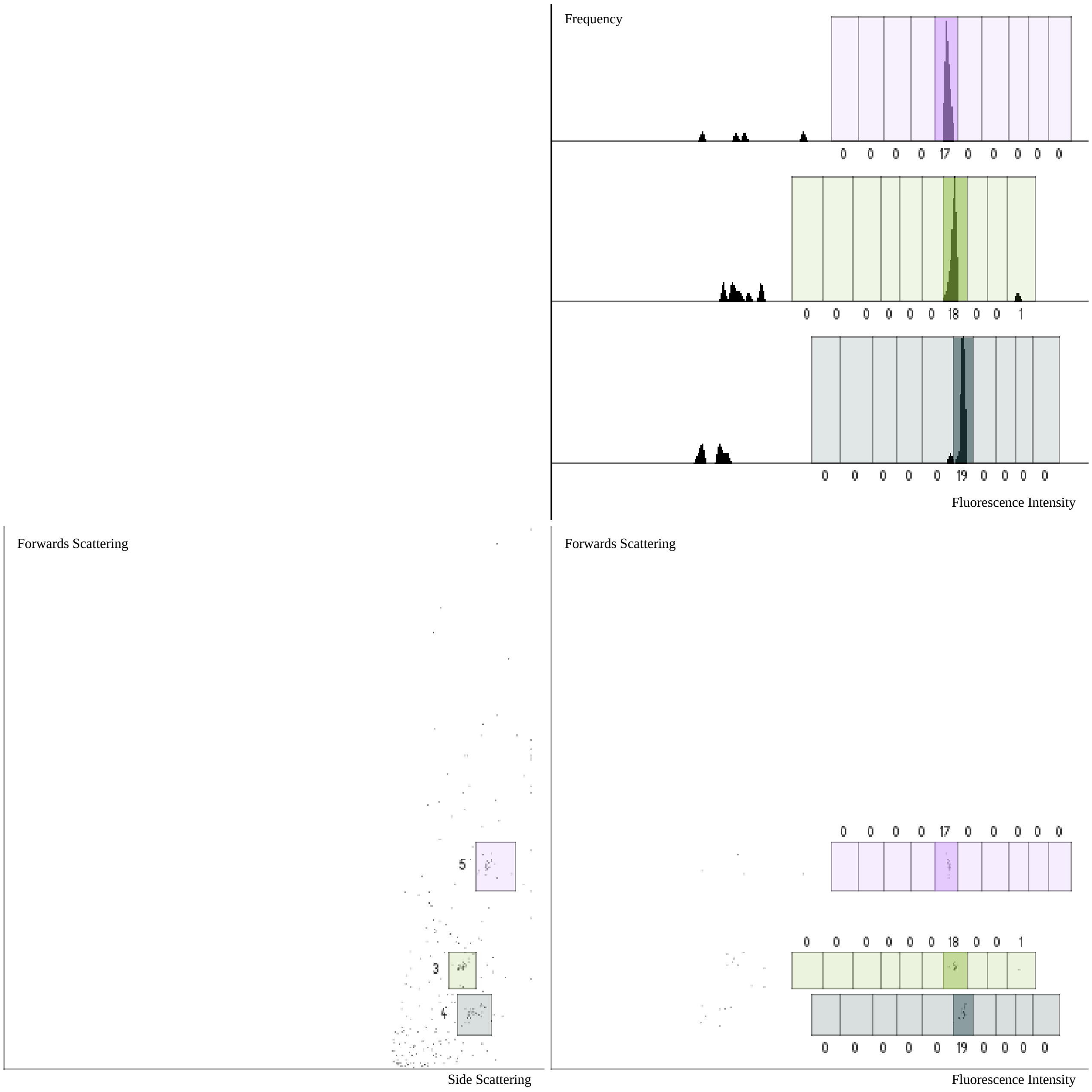

ANNEX 3: TAG DECONVOLUTION - BEAD 177

Passes flow sorting criteria: Yes

Passes tag deconvolution criteria: No

Included in protocol analysis: No

Protocol: N/A

Filename: Bin1_plateC2_B8.fcs

Split 1: Petrol shading

Split 2: Green shading

Split 3: Violet shading

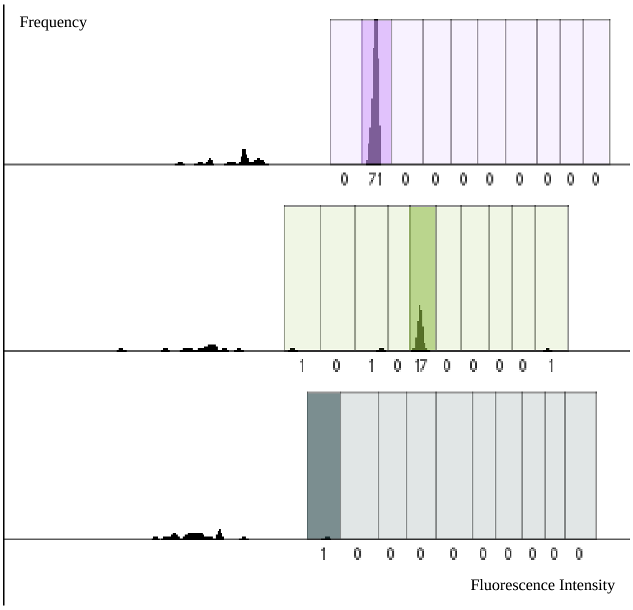

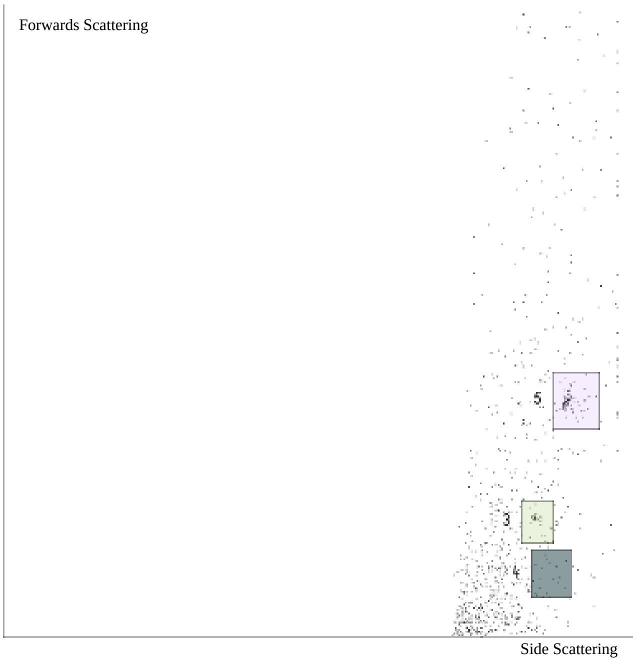

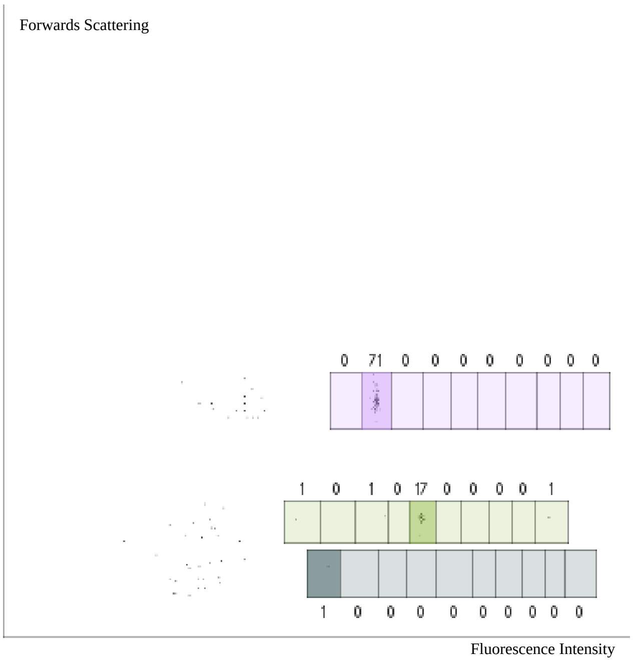

ANNEX 3: TAG DECONVOLUTION - BEAD 178

Passes flow sorting criteria: Yes

Passes tag deconvolution criteria: Yes

Included in protocol analysis: Yes

Protocol: 4, 6, 1, 1

Filename: Bin1_plateC2_B9.fcs

Split 1: Petrol shading

Split 2: Green shading

Split 3: Violet shading

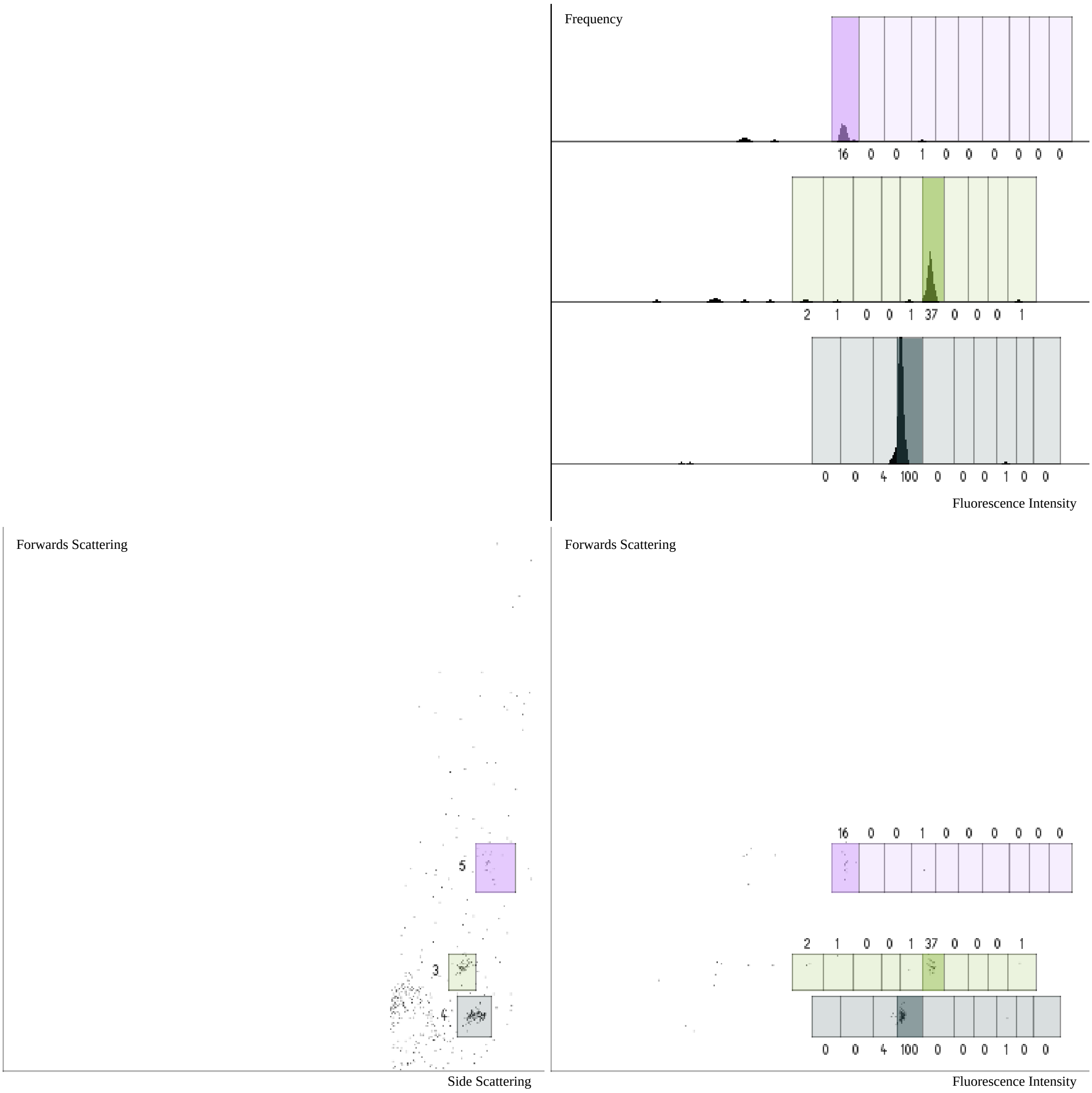

ANNEX 3: TAG DECONVOLUTION - BEAD 179

Passes flow sorting criteria: Yes

Passes tag deconvolution criteria: Yes

Included in protocol analysis: Yes

Protocol: 6, 5, 6, 1

Filename: Bin1_plateC2_B10.fcs

Split 1: Petrol shading

Split 2: Green shading

Split 3: Violet shading

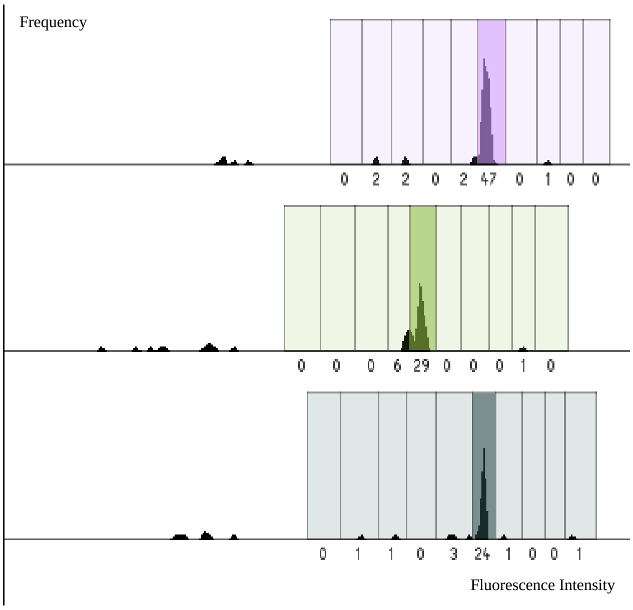

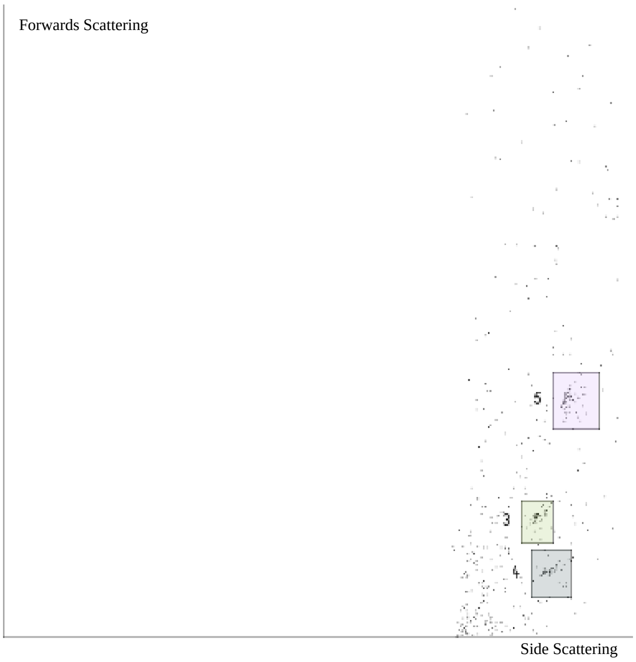

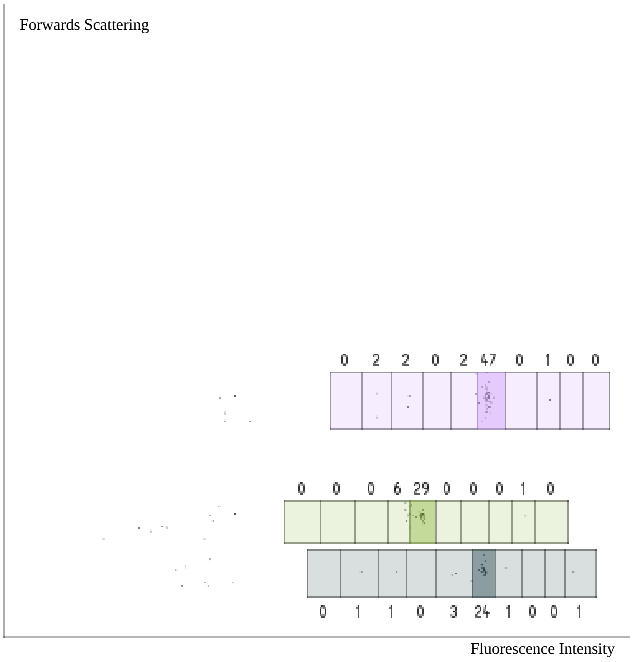

ANNEX 3: TAG DECONVOLUTION - BEAD 180

Passes flow sorting criteria: Yes

Passes tag deconvolution criteria: Yes

Included in protocol analysis: Yes

Protocol: 5, 10, 5, 1

Filename: Bin1_plateC2_B11.fcs

Split 1: Petrol shading

Split 2: Green shading

Split 3: Violet shading

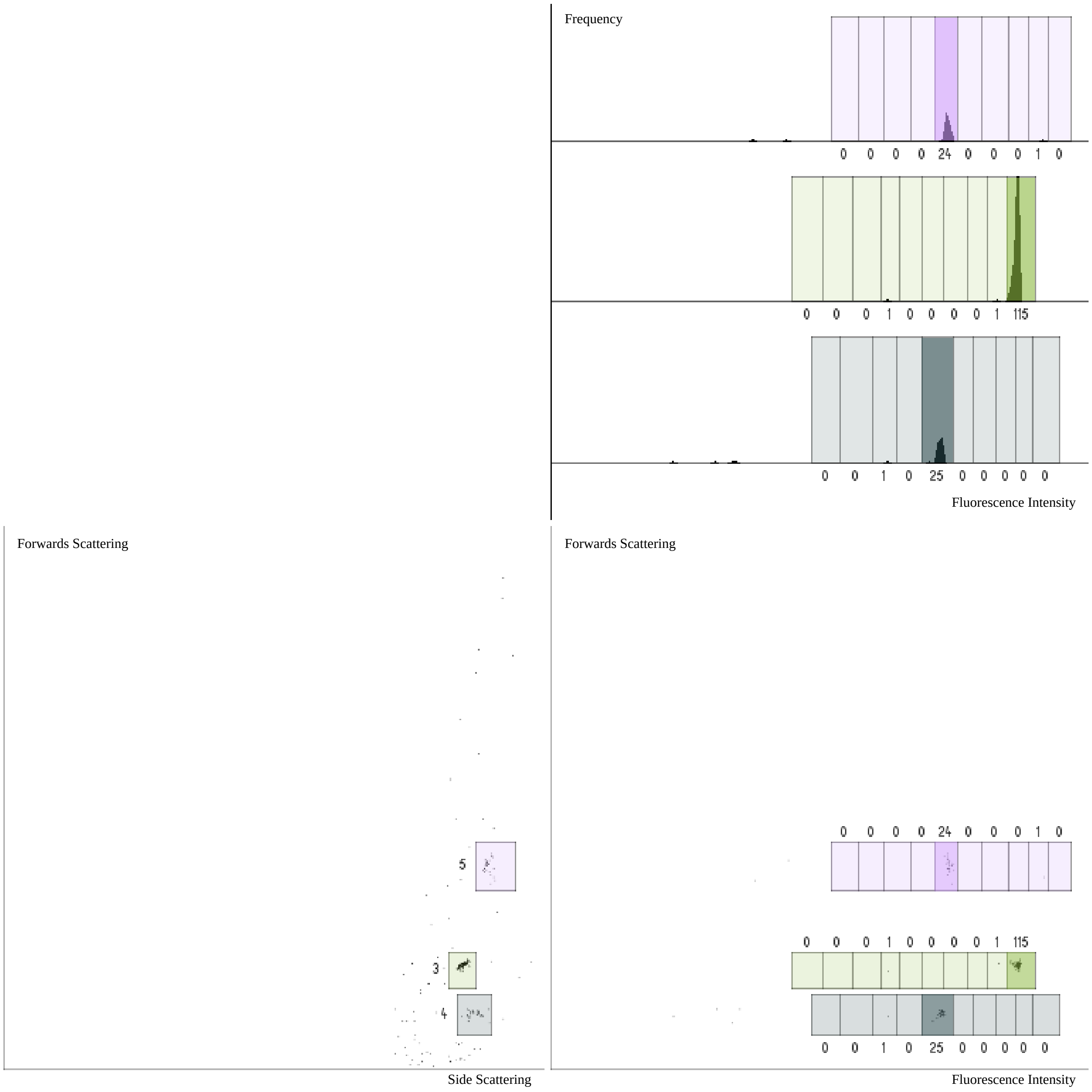

ANNEX 3: TAG DECONVOLUTION - BEAD 181

Passes flow sorting criteria: Yes

Passes tag deconvolution criteria: Yes

Included in protocol analysis: Yes

Protocol: 1, 3, 10, 1

Filename: Bin1_plateC2_B12.fcs

Split 1: Petrol shading

Split 2: Green shading

Split 3: Violet shading

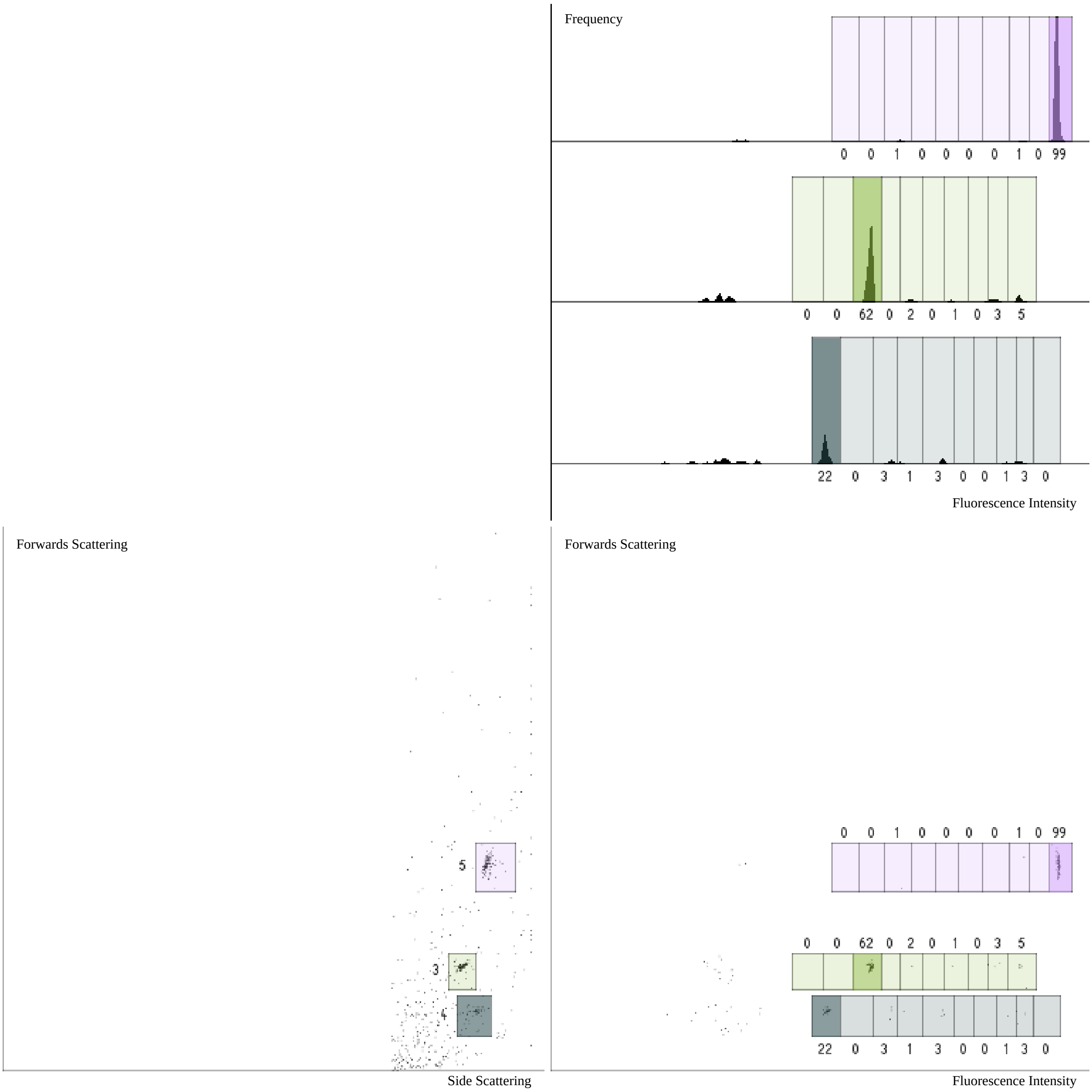

ANNEX 3: TAG DECONVOLUTION - BEAD 182

Passes flow sorting criteria: Yes

Passes tag deconvolution criteria: Yes

Included in protocol analysis: Yes

Protocol: 6, 5, 3, 1

Filename: Bin1_plateC2_C1.fcs

Split 1: Petrol shading

Split 2: Green shading

Split 3: Violet shading

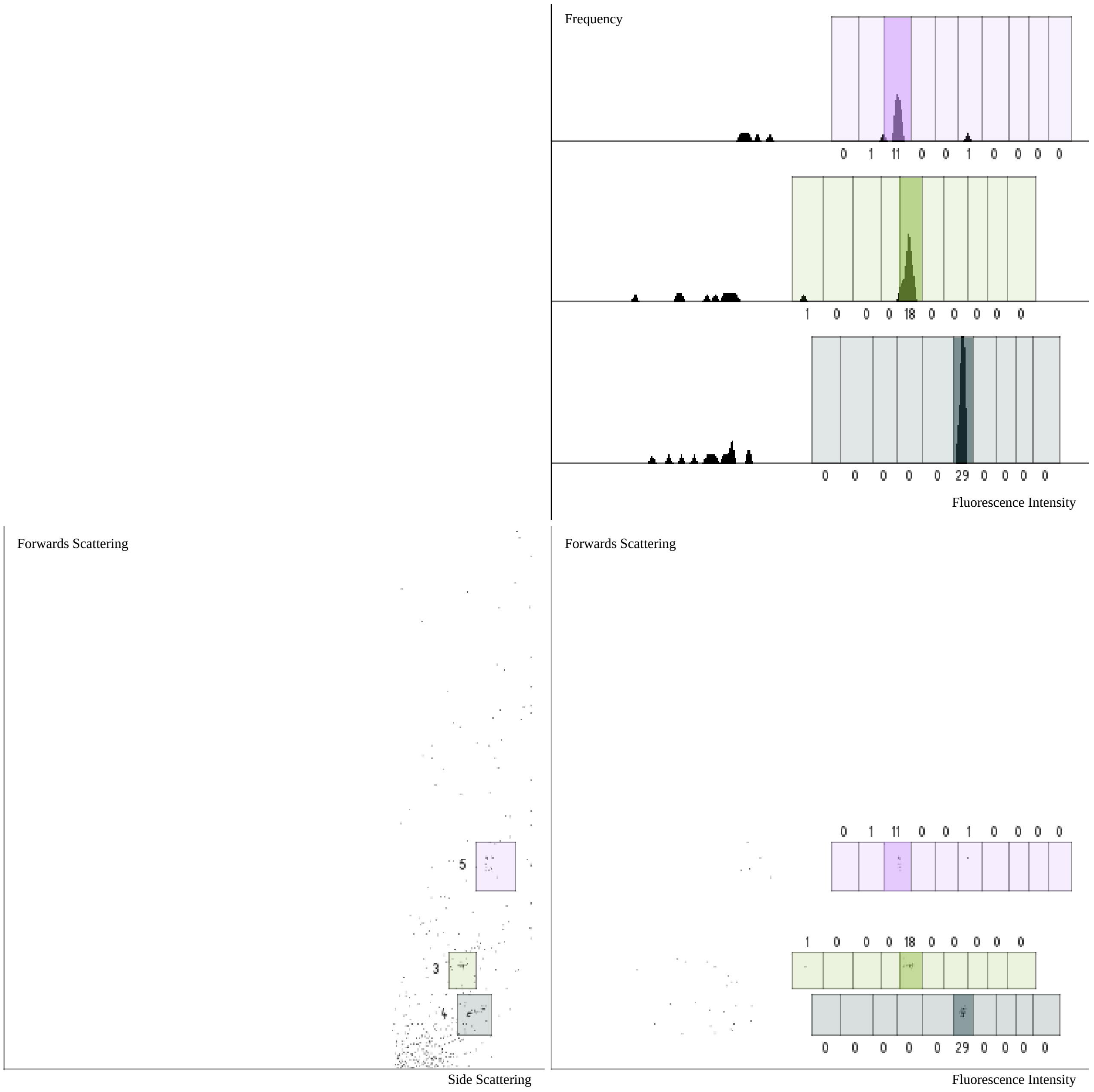

ANNEX 3: TAG DECONVOLUTION - BEAD 183

Passes flow sorting criteria: Yes

Passes tag deconvolution criteria: Yes

Included in protocol analysis: Yes

Protocol: 4, 9, 5, 1

Filename: Bin1_plateC2_C4.fcs

Split 1: Petrol shading

Split 2: Green shading

Split 3: Violet shading

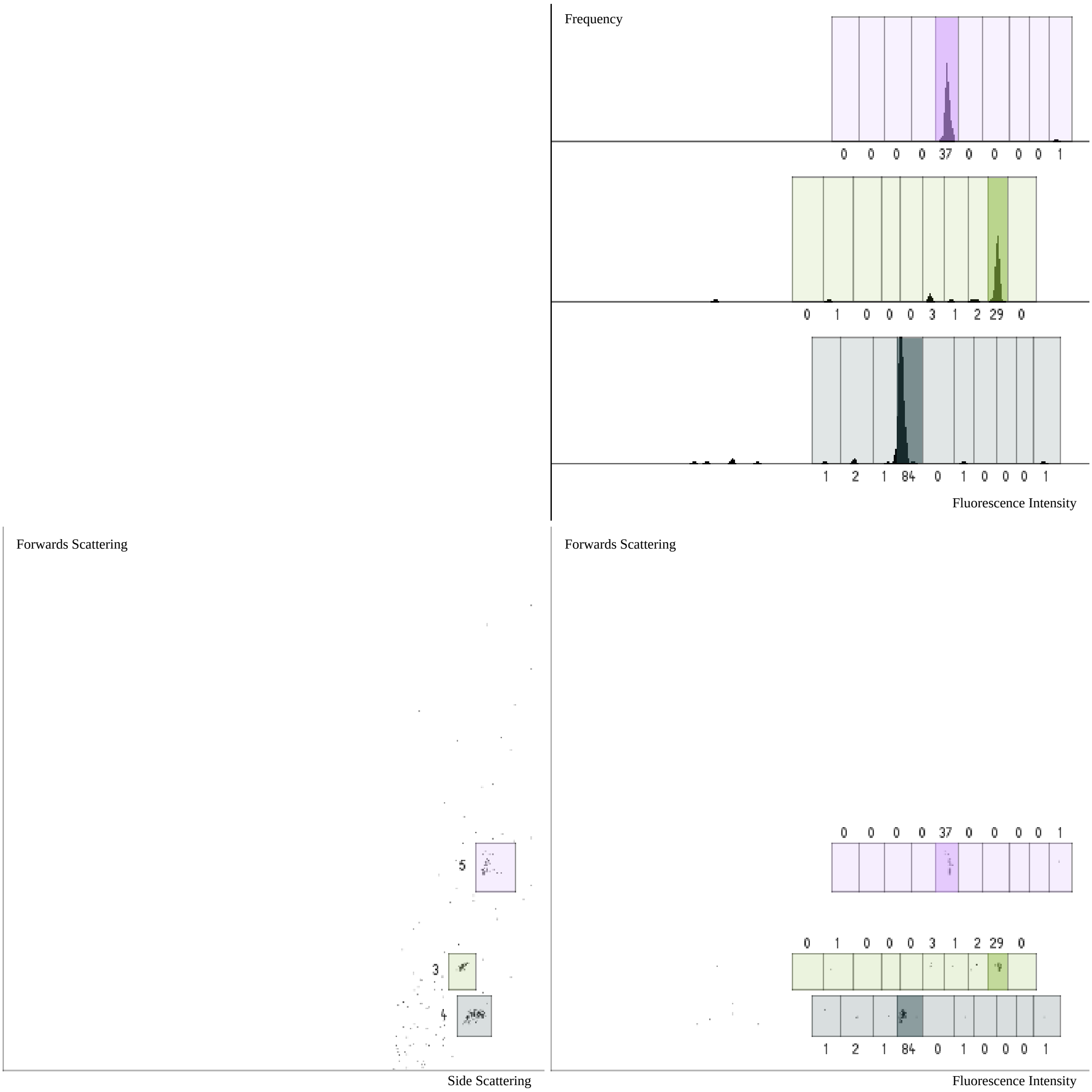

ANNEX 3: TAG DECONVOLUTION - BEAD 184

Passes flow sorting criteria: Yes

Passes tag deconvolution criteria: Yes

Included in protocol analysis: Yes

Protocol: 2, 9, 6, 1

Filename: Bin1_plateC2_C5.fcs

Split 1: Petrol shading

Split 2: Green shading

Split 3: Violet shading

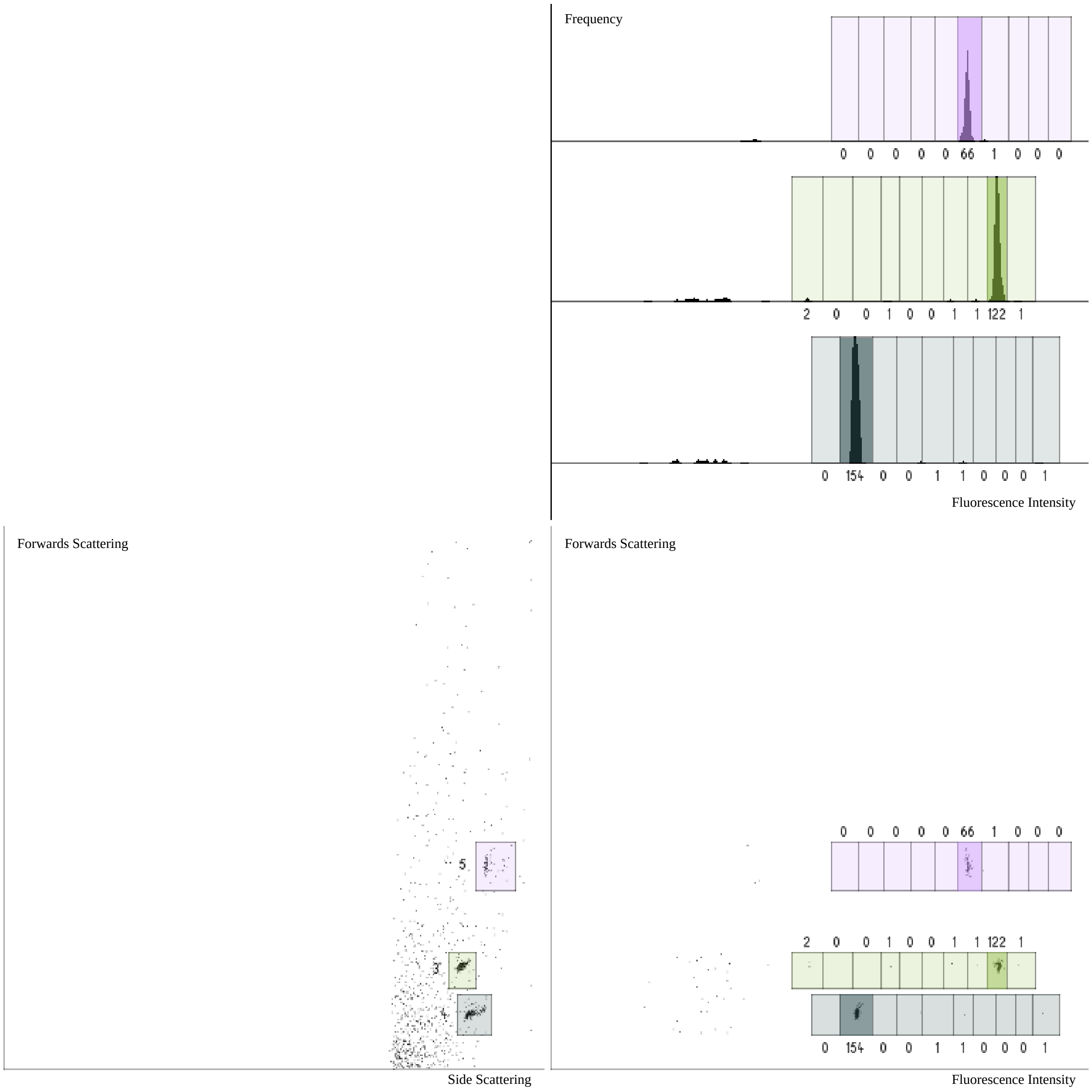

ANNEX 3: TAG DECONVOLUTION - BEAD 185

Passes flow sorting criteria: Yes

Passes tag deconvolution criteria: Yes

Included in protocol analysis: Yes

Protocol: 3, 4, 1, 2

Filename: Bin2_plateA2_E8.fcs

Split 1: Petrol shading

Split 2: Green shading

Split 3: Violet shading

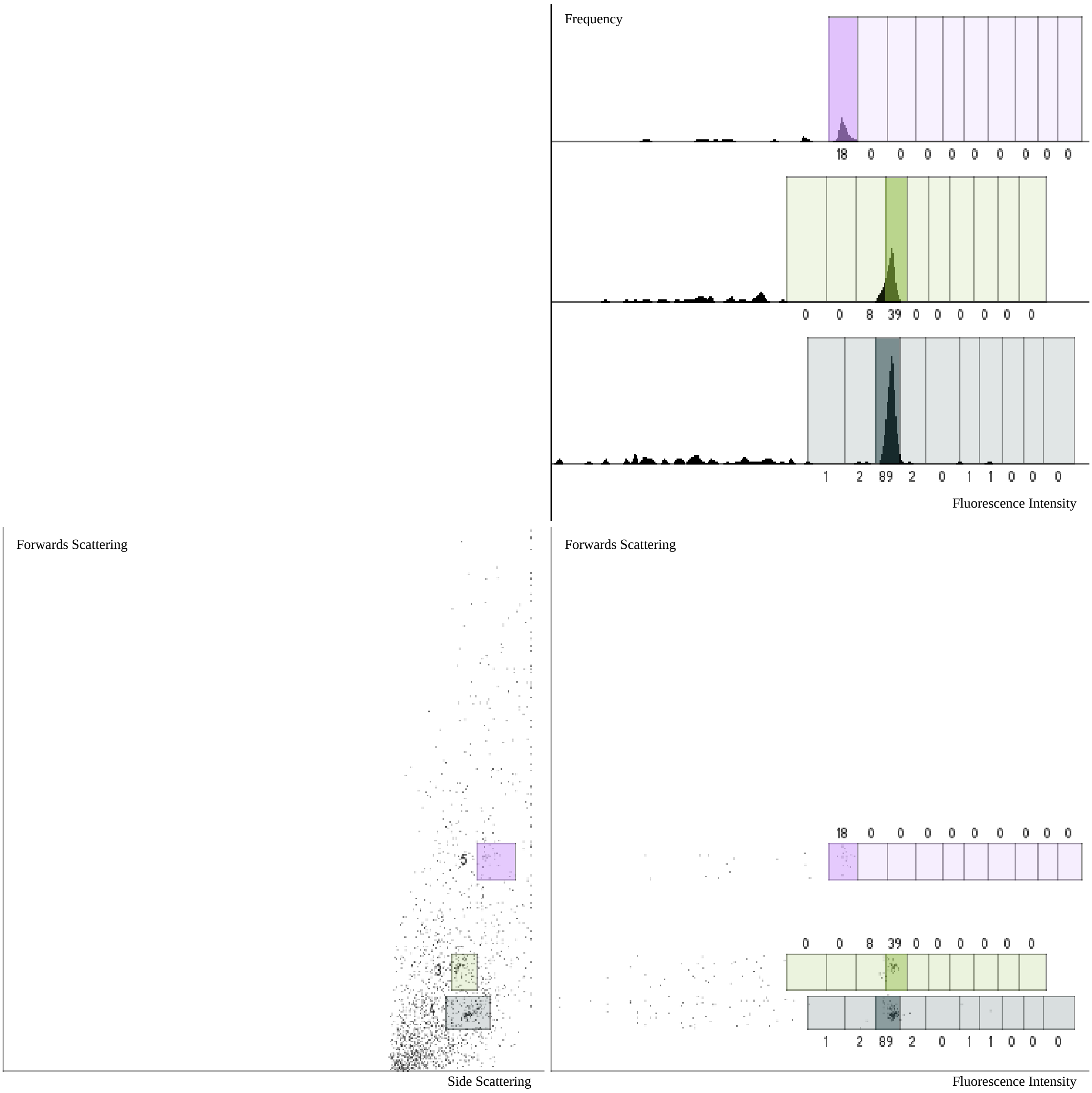

ANNEX 3: TAG DECONVOLUTION - BEAD 186

Passes flow sorting criteria: Yes

Passes tag deconvolution criteria: Yes

Included in protocol analysis: Yes

Protocol: 5, 10, 2, 2

Filename: Bin2_plateA2_A1.fcs

Split 1: Petrol shading

Split 2: Green shading

Split 3: Violet shading

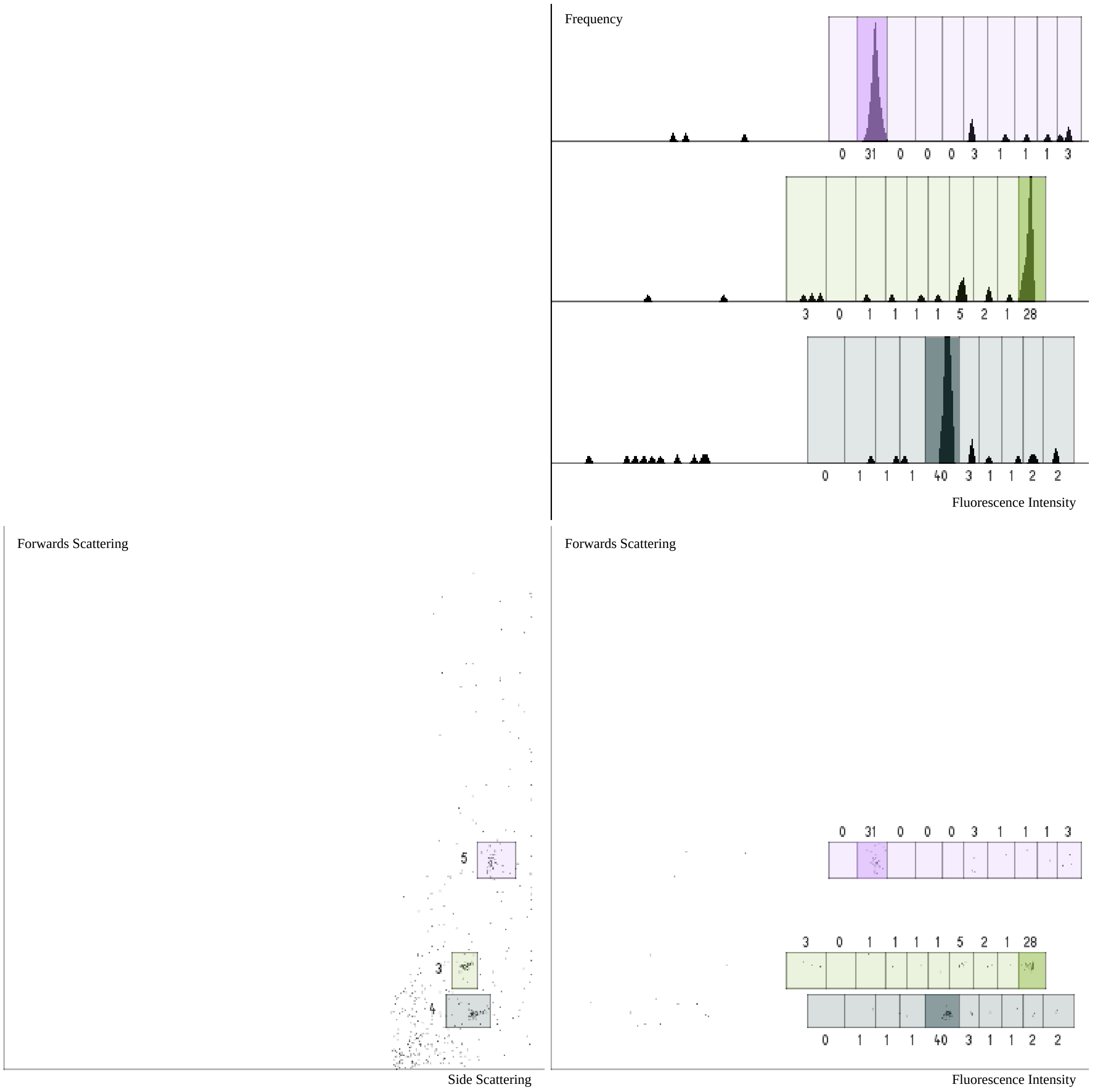

ANNEX 3: TAG DECONVOLUTION - BEAD 187

Passes flow sorting criteria: Yes

Passes tag deconvolution criteria: Yes

Included in protocol analysis: Yes

Protocol: 8, 6, 5, 2

Filename: Bin2_plateA2_A3.fcs

Split 1: Petrol shading

Split 2: Green shading

Split 3: Violet shading

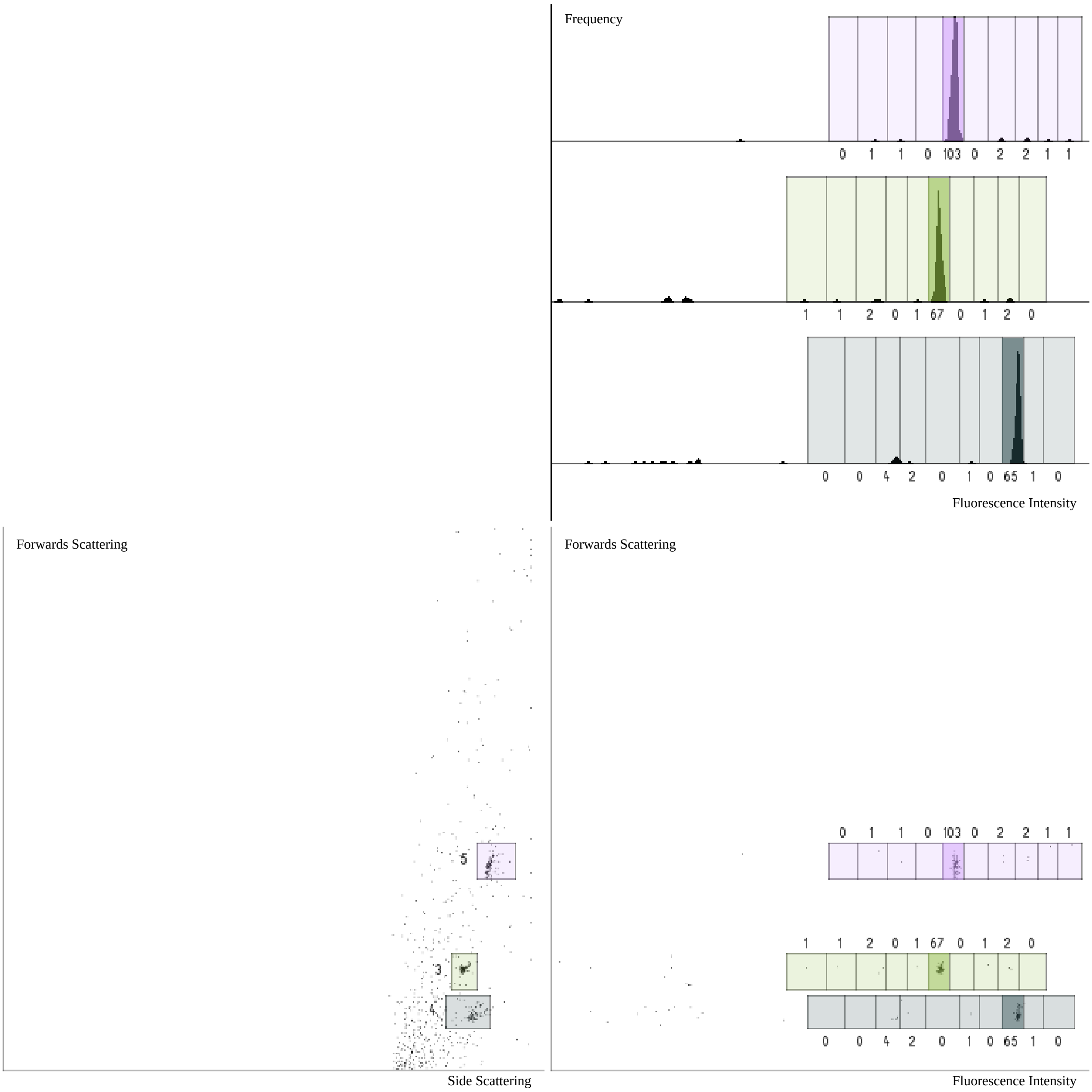

ANNEX 3: TAG DECONVOLUTION - BEAD 188

Passes flow sorting criteria: Yes

Passes tag deconvolution criteria: Yes

Included in protocol analysis: Yes

Protocol: 9, 4, 9, 2

Filename: Bin2_plateA2_A4.fcs

Split 1: Petrol shading

Split 2: Green shading

Split 3: Violet shading

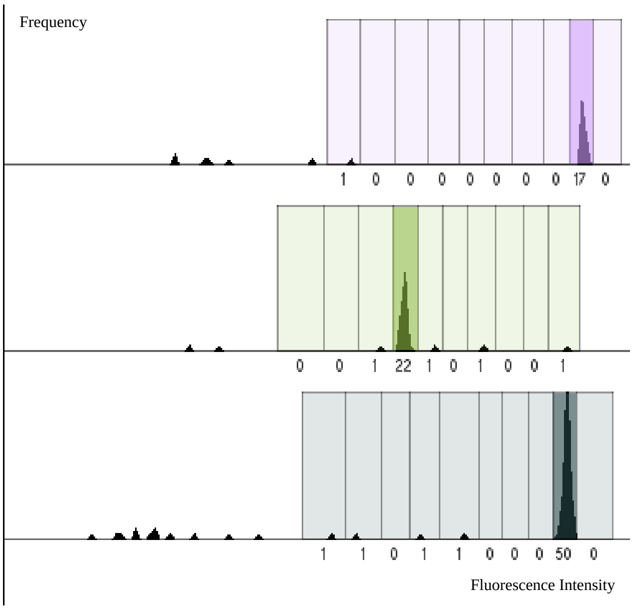

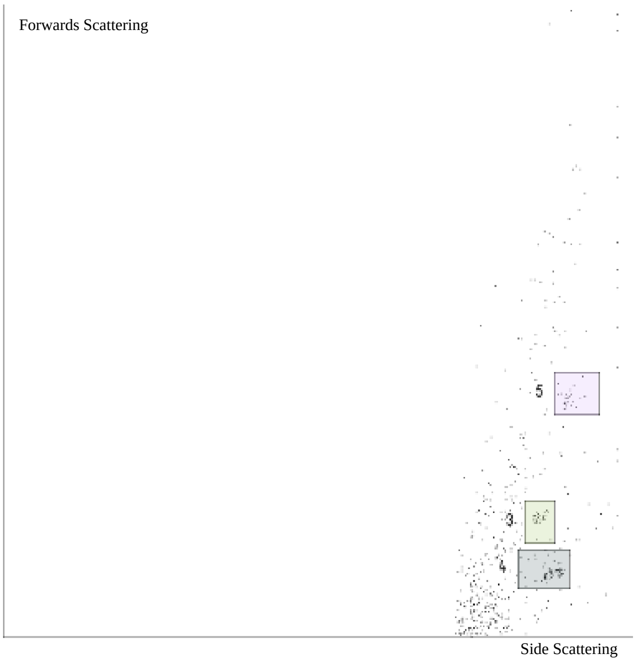

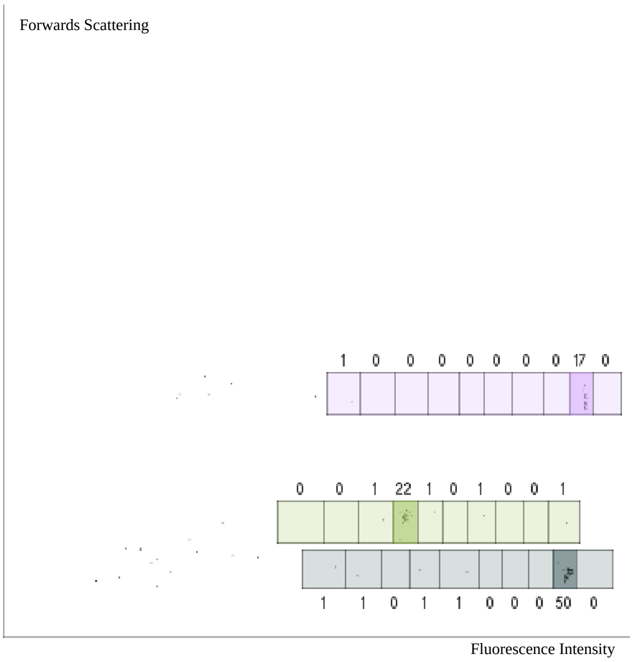

ANNEX 3: TAG DECONVOLUTION - BEAD 189

Passes flow sorting criteria: Yes

Passes tag deconvolution criteria: Yes

Included in protocol analysis: Yes

Protocol: 4, 9, 3, 2

Filename: Bin2_plateA2_A5.fcs

Split 1: Petrol shading

Split 2: Green shading

Split 3: Violet shading

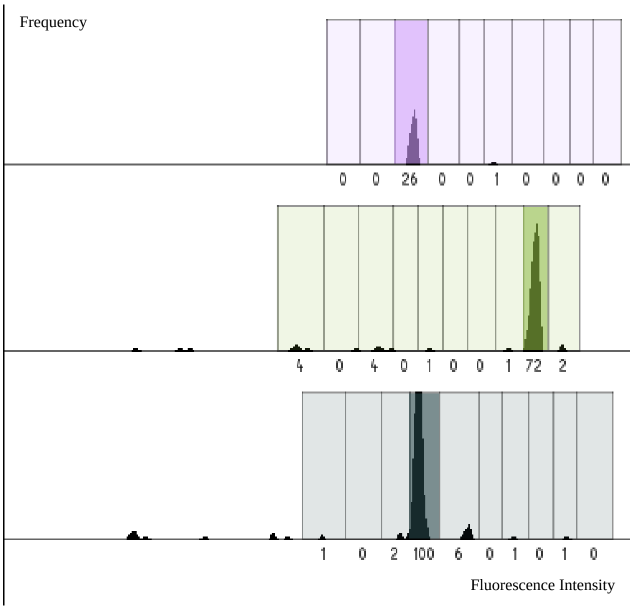

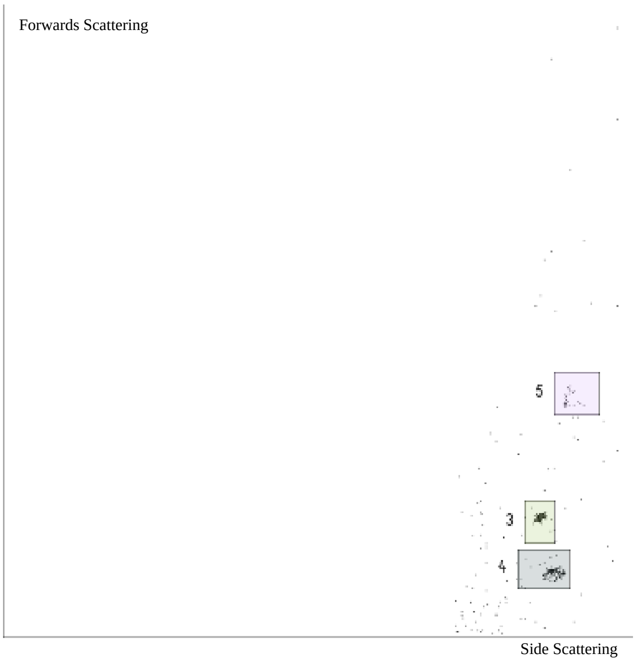

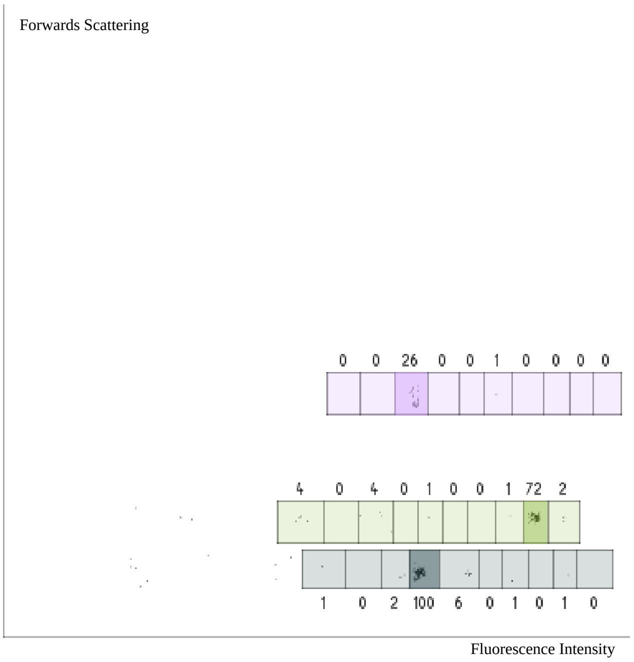

ANNEX 3: TAG DECONVOLUTION - BEAD 190

Passes flow sorting criteria: Yes

Passes tag deconvolution criteria: Yes

Included in protocol analysis: Yes

Protocol: 3, 6, 10, 2

Filename: Bin2_plateA2_A6.fcs

Split 1: Petrol shading

Split 2: Green shading

Split 3: Violet shading

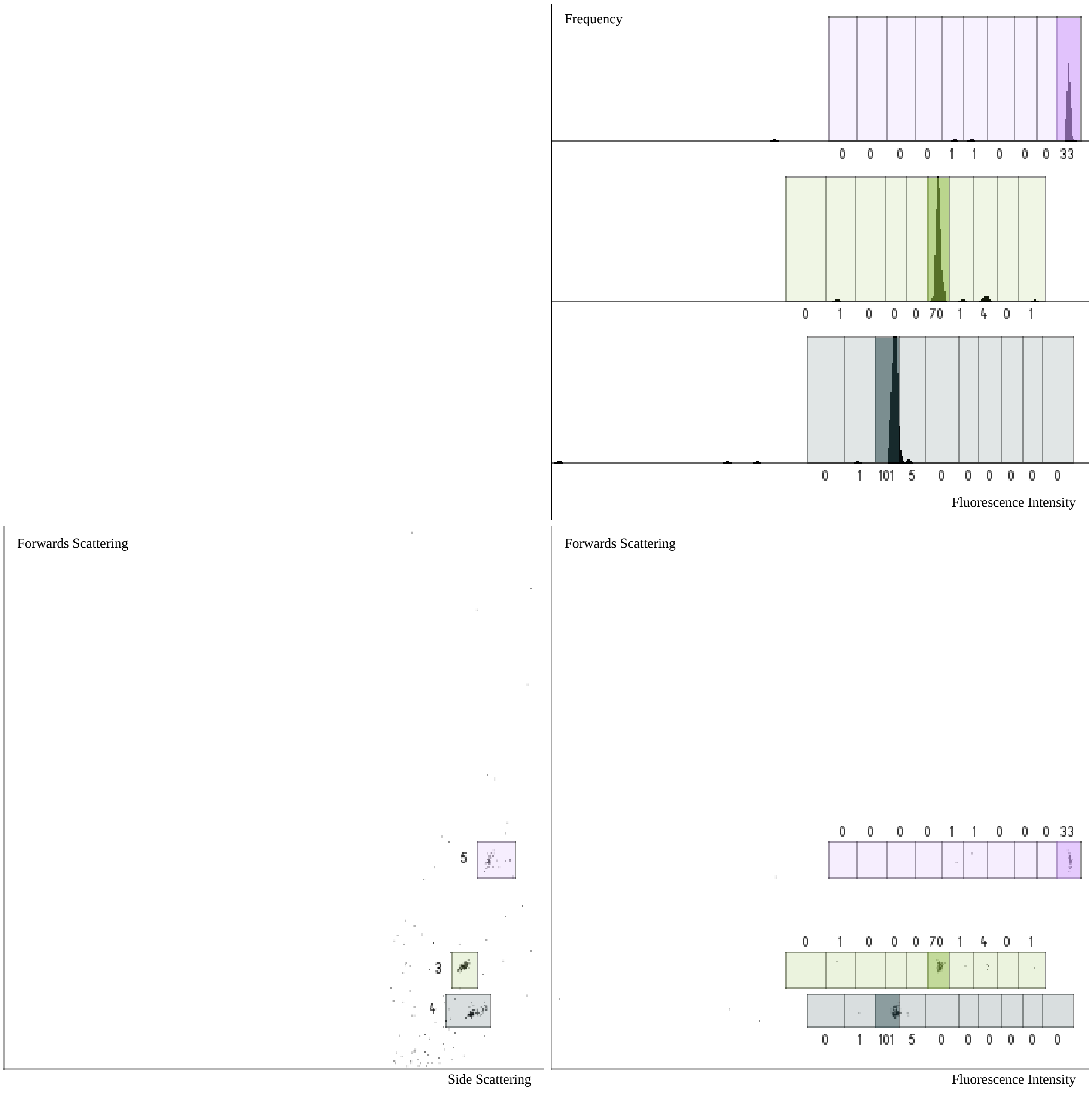

ANNEX 3: TAG DECONVOLUTION - BEAD 191

Passes flow sorting criteria: Yes

Passes tag deconvolution criteria: No

Included in protocol analysis: No

Protocol: N/A

Filename: Bin2_plateA2_A7.fcs

Split 1: Petrol shading

Split 2: Green shading

Split 3: Violet shading

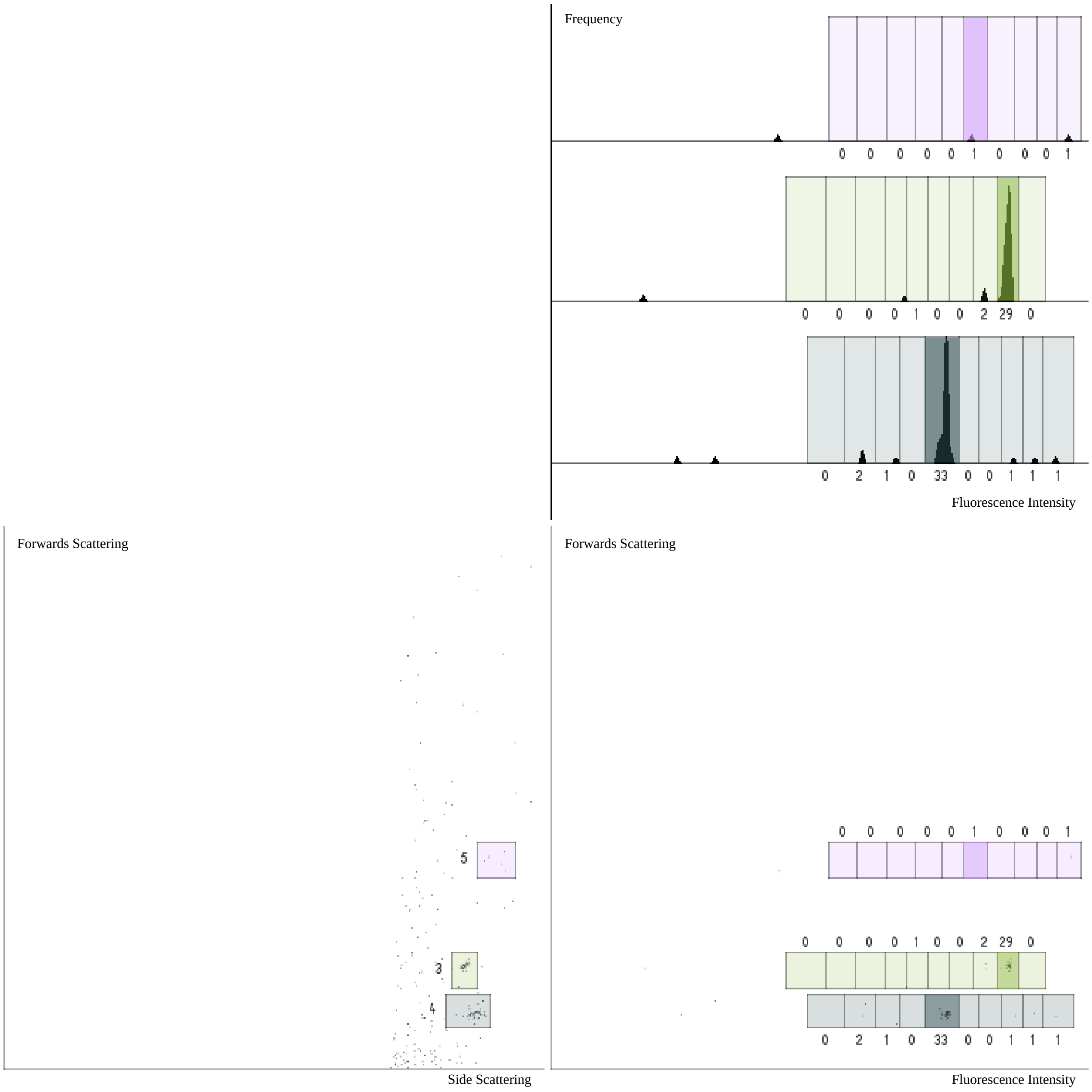

ANNEX 3: TAG DECONVOLUTION - BEAD 192

Passes flow sorting criteria: Yes

Passes tag deconvolution criteria: No

Included in protocol analysis: No

Protocol: N/A

Filename: Bin2_plateA2_A8.fcs

Split 1: Petrol shading

Split 2: Green shading

Split 3: Violet shading

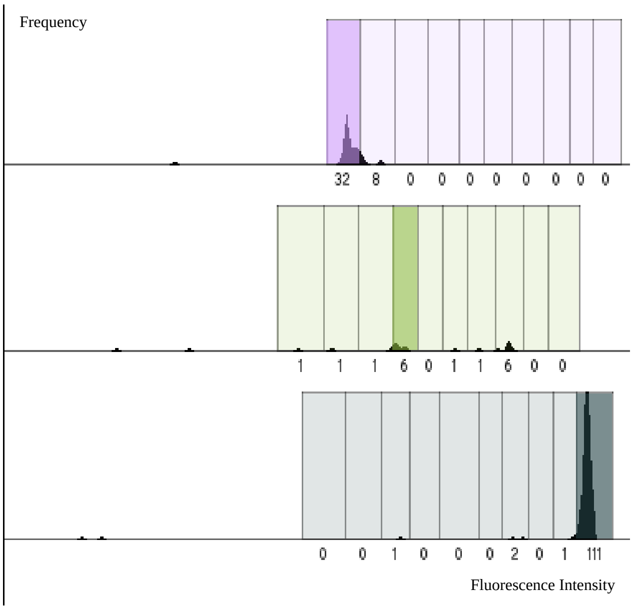

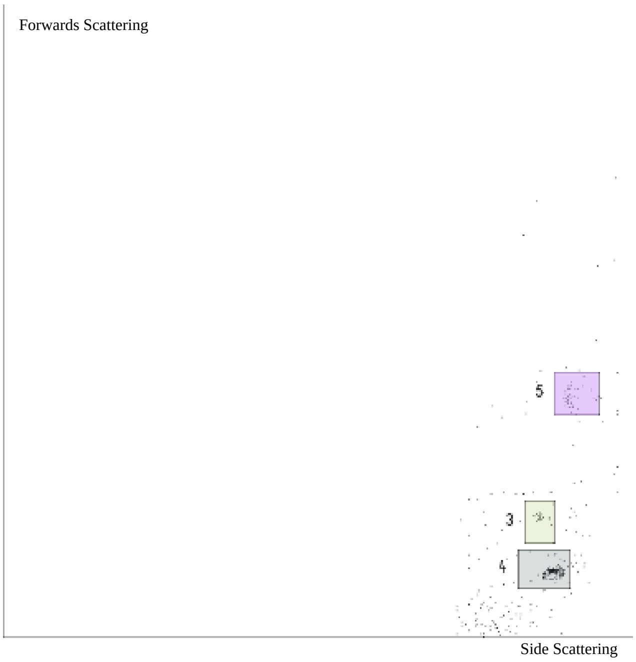

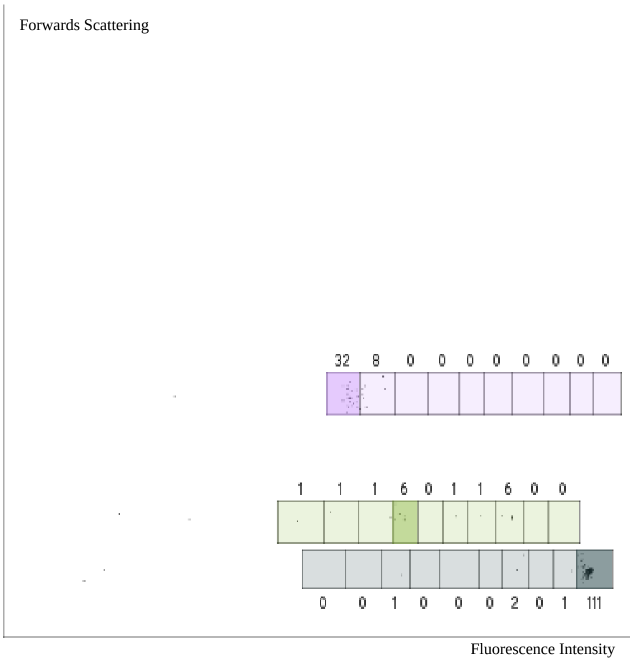

ANNEX 3: TAG DECONVOLUTION - BEAD 193

Passes flow sorting criteria: Yes

Passes tag deconvolution criteria: Yes

Included in protocol analysis: Yes

Protocol: 10, 4, 4, 2

Filename: Bin2_plateA2_A9.fcs

Split 1: Petrol shading

Split 2: Green shading

Split 3: Violet shading

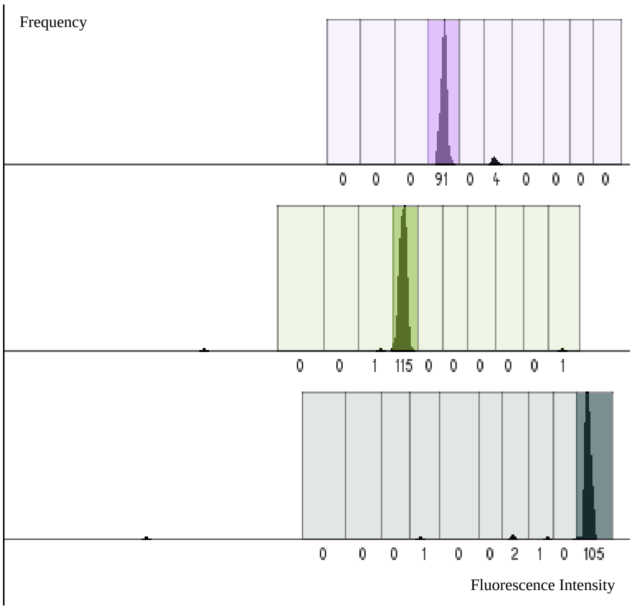

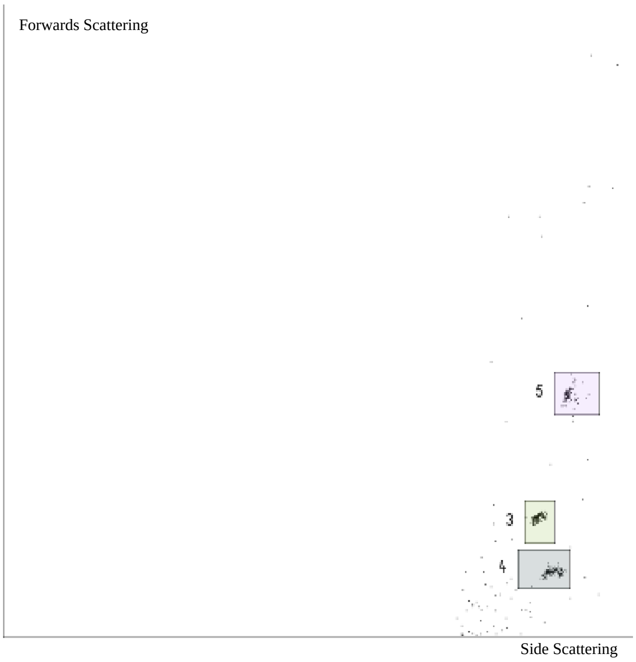

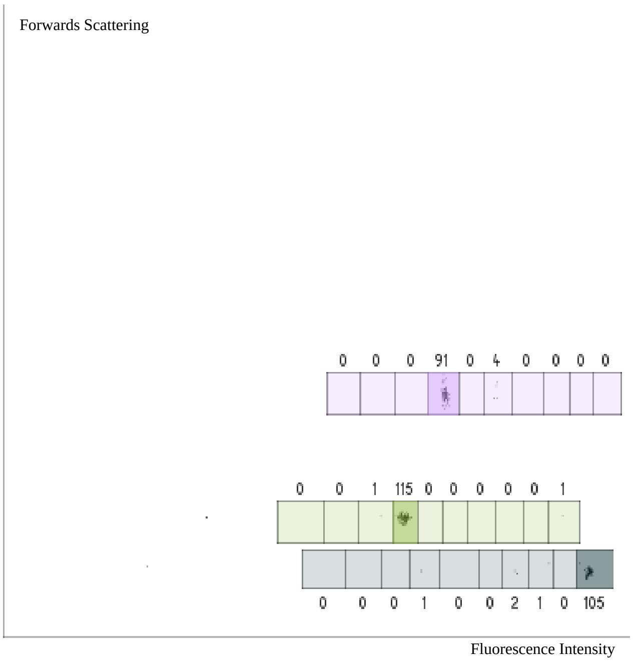

ANNEX 3: TAG DECONVOLUTION - BEAD 194

Passes flow sorting criteria: Yes

Passes tag deconvolution criteria: Yes

Included in protocol analysis: Yes

Protocol: 10, 6, 9, 2

Filename: Bin2_plateA2_A10.fcs

Split 1: Petrol shading

Split 2: Green shading

Split 3: Violet shading

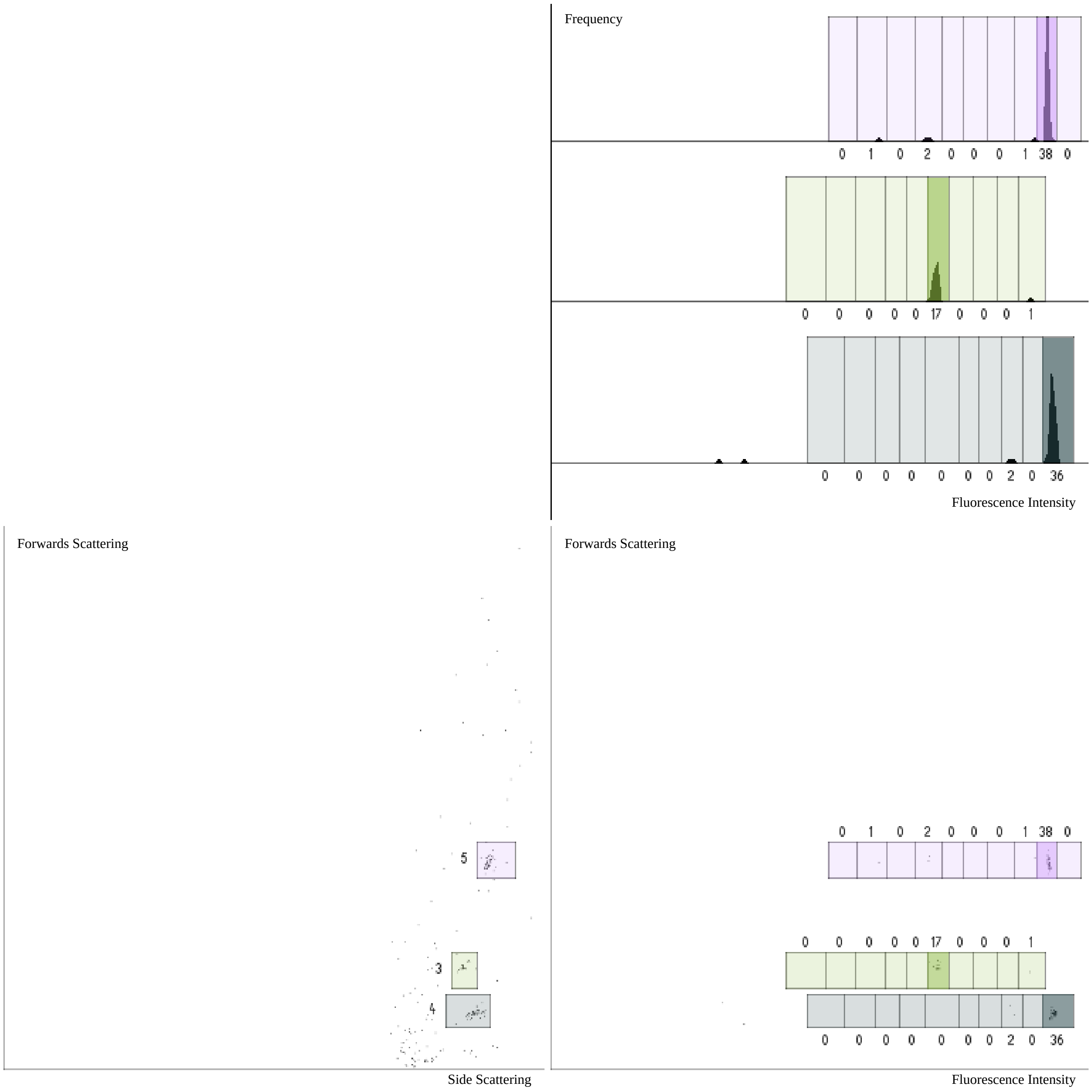

ANNEX 3: TAG DECONVOLUTION - BEAD 195

Passes flow sorting criteria: Yes

Passes tag deconvolution criteria: Yes

Included in protocol analysis: Yes

Protocol: 5, 8, 9, 2

Filename: Bin2_plateA2_A11.fcs

Split 1: Petrol shading

Split 2: Green shading

Split 3: Violet shading

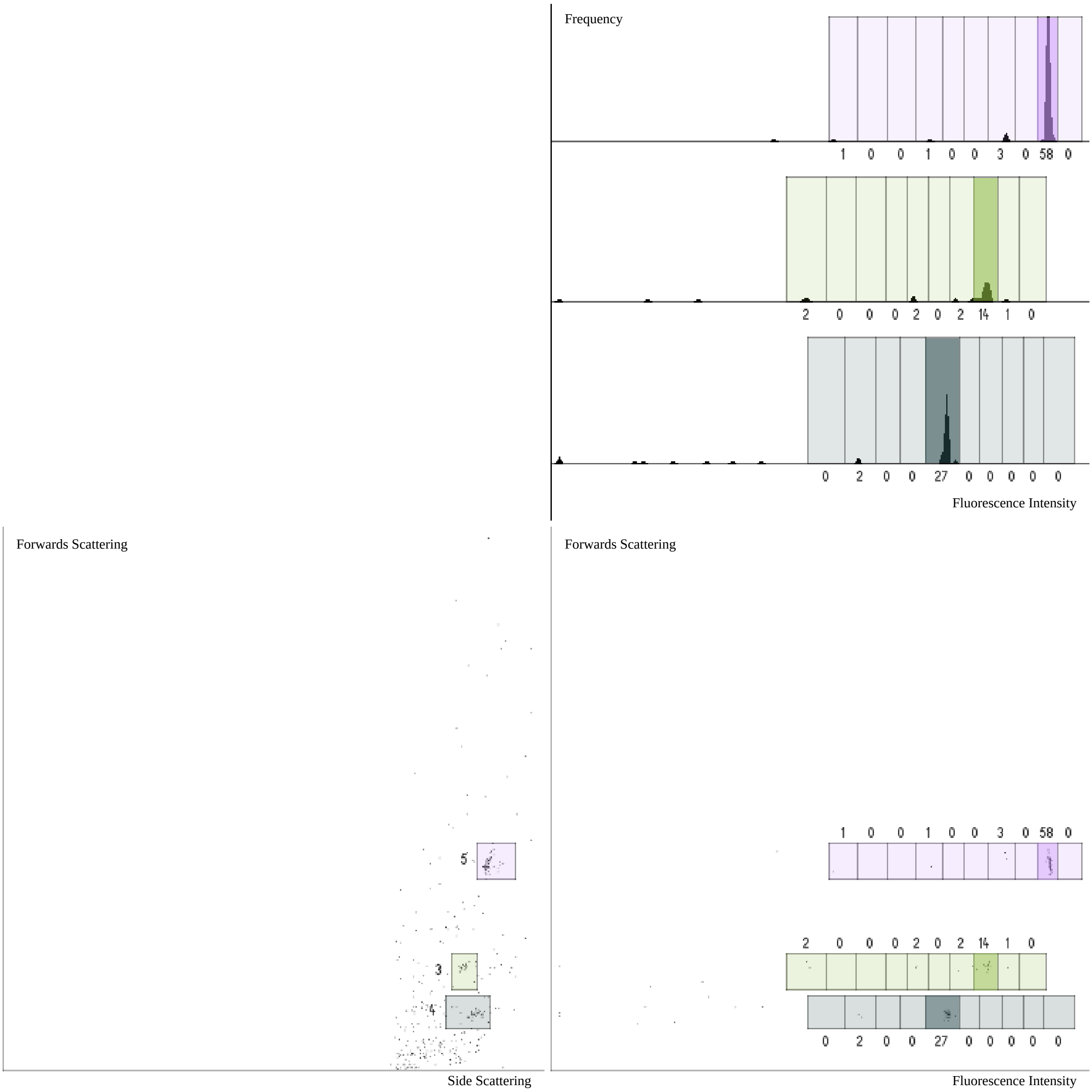

ANNEX 3: TAG DECONVOLUTION - BEAD 196

Passes flow sorting criteria: Yes

Passes tag deconvolution criteria: Yes

Included in protocol analysis: Yes

Protocol: 4, 1, 2, 2

Filename: Bin2_plateA2_A12.fcs

Split 1: Petrol shading

Split 2: Green shading

Split 3: Violet shading

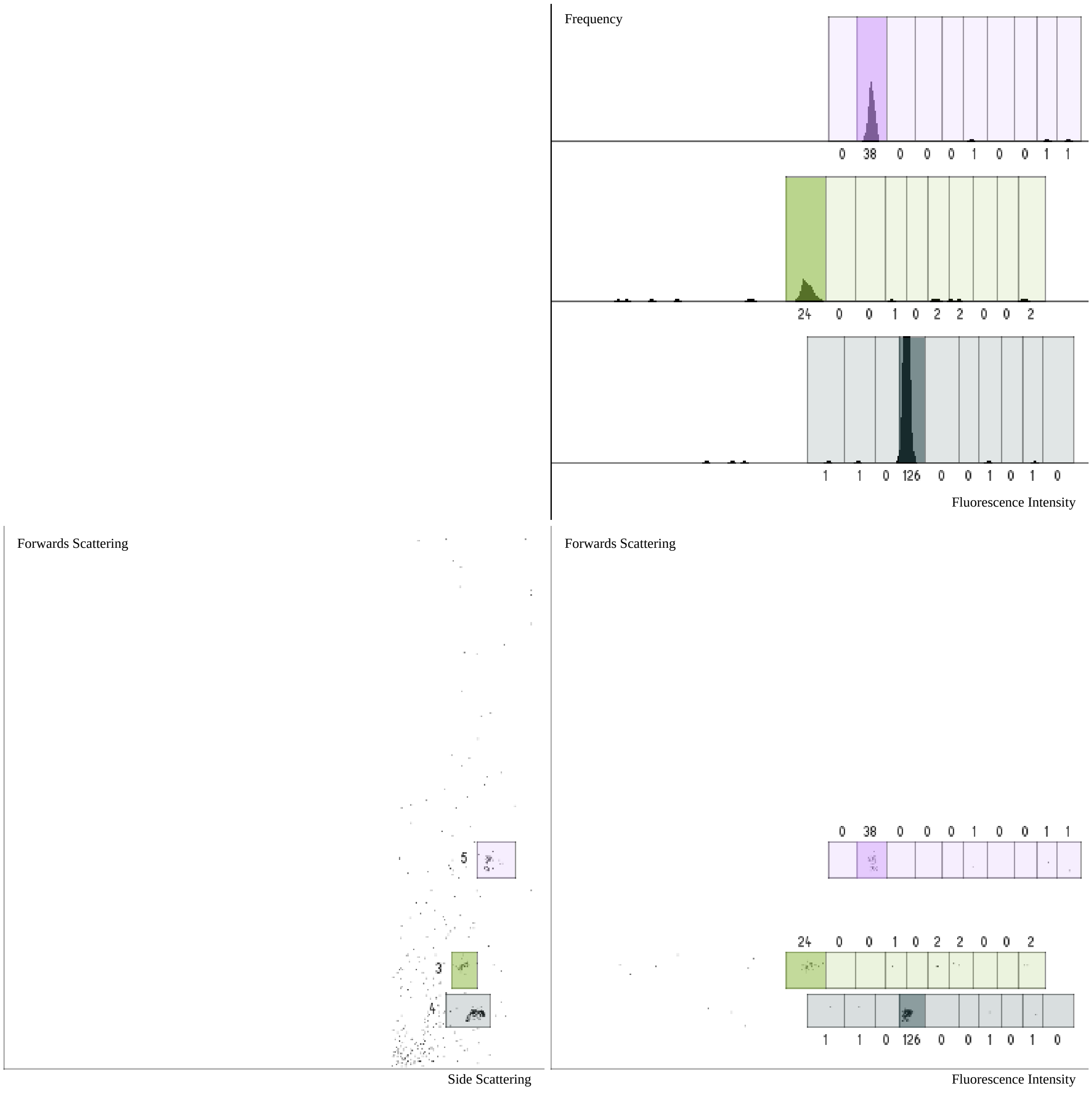

ANNEX 3: TAG DECONVOLUTION - BEAD 197

Passes flow sorting criteria: Yes

Passes tag deconvolution criteria: Yes

Included in protocol analysis: Yes

Protocol: 2, 3, 10, 2

Filename: Bin2_plateA2_B1.fcs

Split 1: Petrol shading

Split 2: Green shading

Split 3: Violet shading

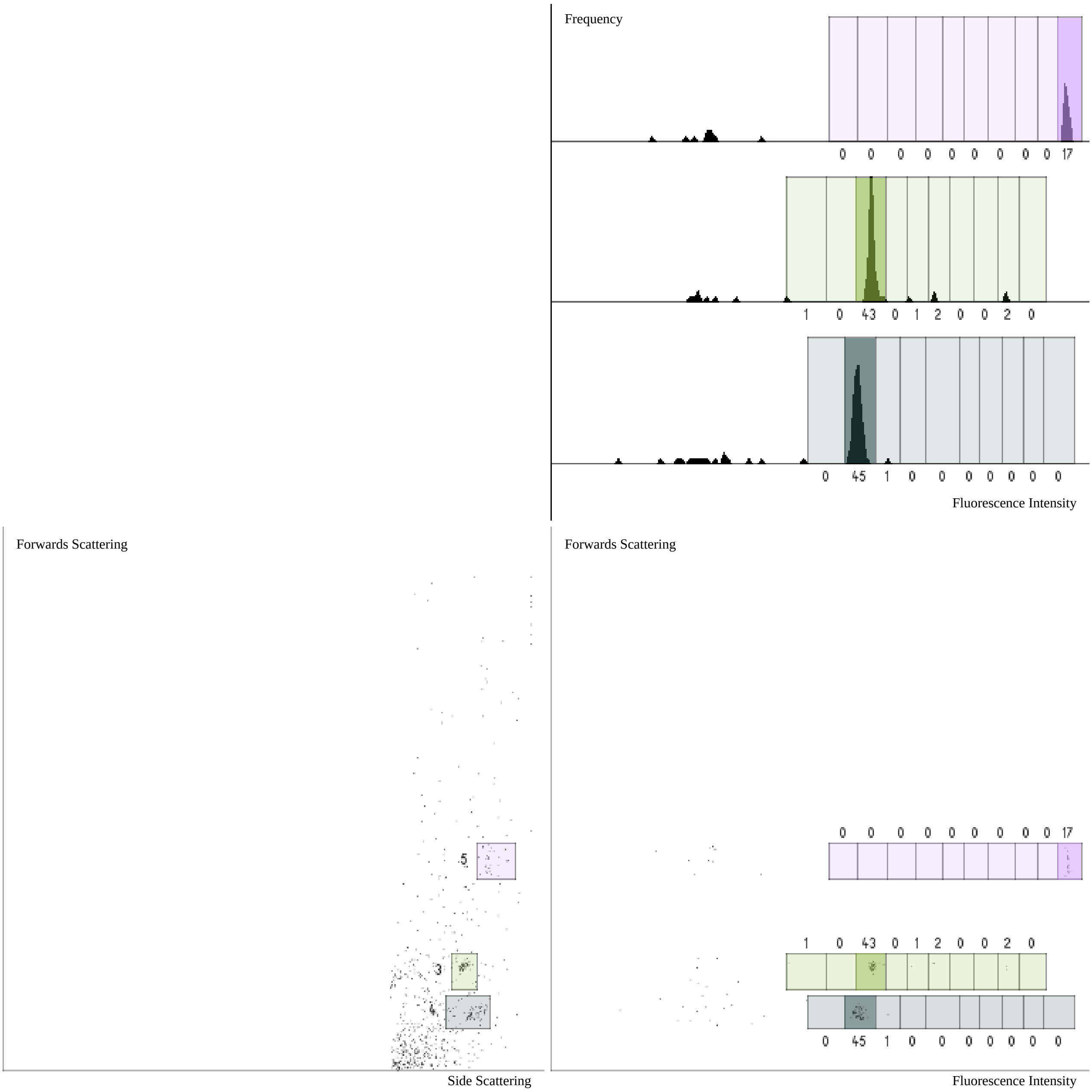

ANNEX 3: TAG DECONVOLUTION - BEAD 198

Passes flow sorting criteria: Yes

Passes tag deconvolution criteria: Yes

Included in protocol analysis: Yes

Protocol: 5, 9, 2, 2

Filename: Bin2_plateA2_B3.fcs

Split 1: Petrol shading

Split 2: Green shading

Split 3: Violet shading

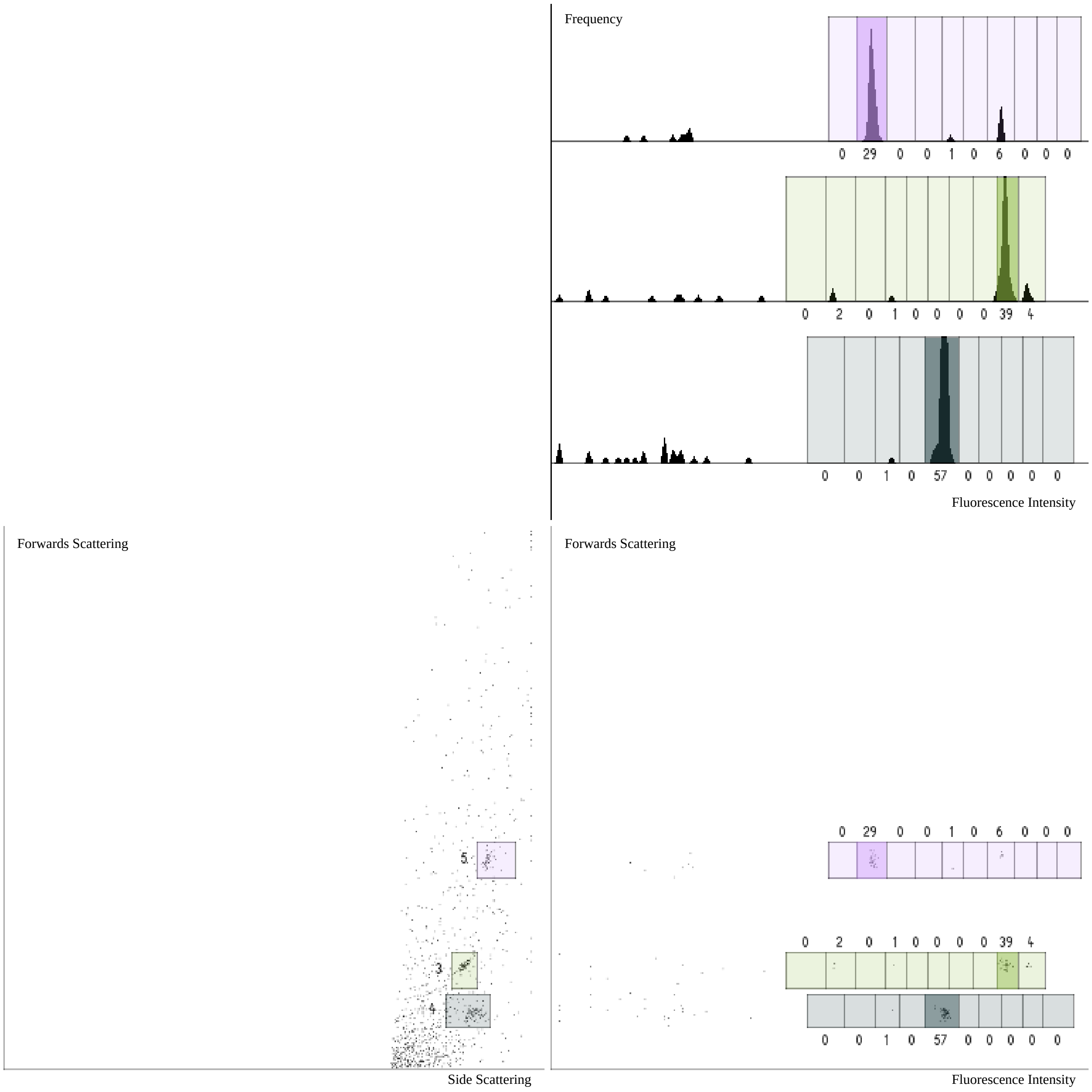

ANNEX 3: TAG DECONVOLUTION - BEAD 199

Passes flow sorting criteria: Yes

Passes tag deconvolution criteria: Yes

Included in protocol analysis: Yes

Protocol: 3, 5, 9, 2

Filename: Bin2_plateA2_B5.fcs

Split 1: Petrol shading

Split 2: Green shading

Split 3: Violet shading

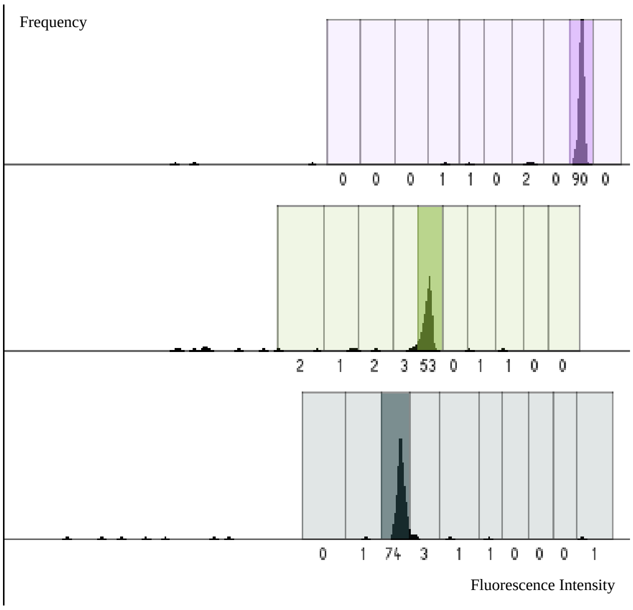

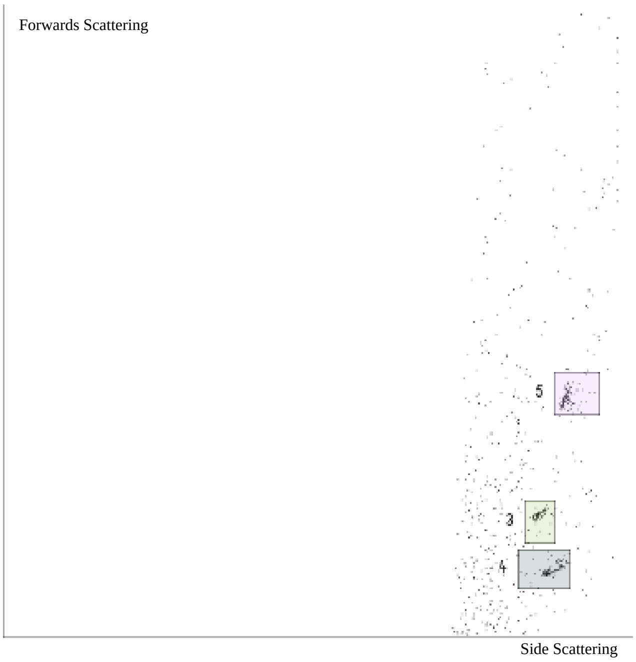

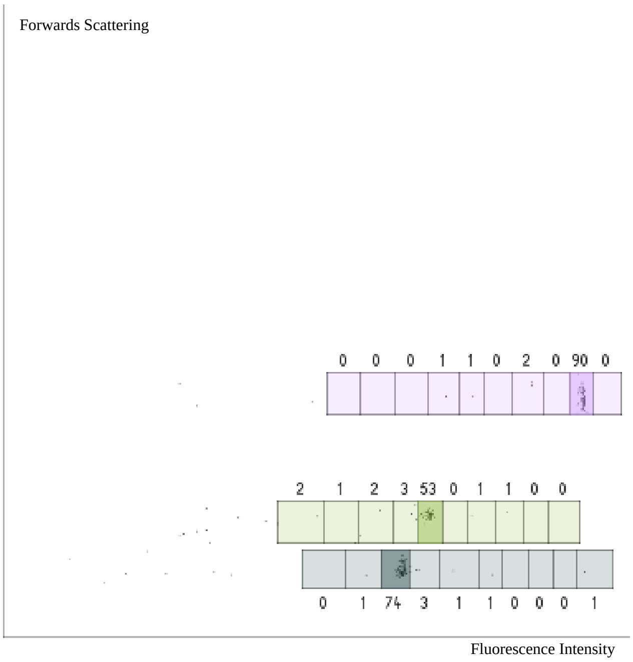

ANNEX 3: TAG DECONVOLUTION - BEAD 200

Passes flow sorting criteria: Yes

Passes tag deconvolution criteria: Yes

Included in protocol analysis: Yes

Protocol: 2, 9, 3, 2

Filename: Bin2_plateA2_B6.fcs

Split 1: Petrol shading

Split 2: Green shading

Split 3: Violet shading

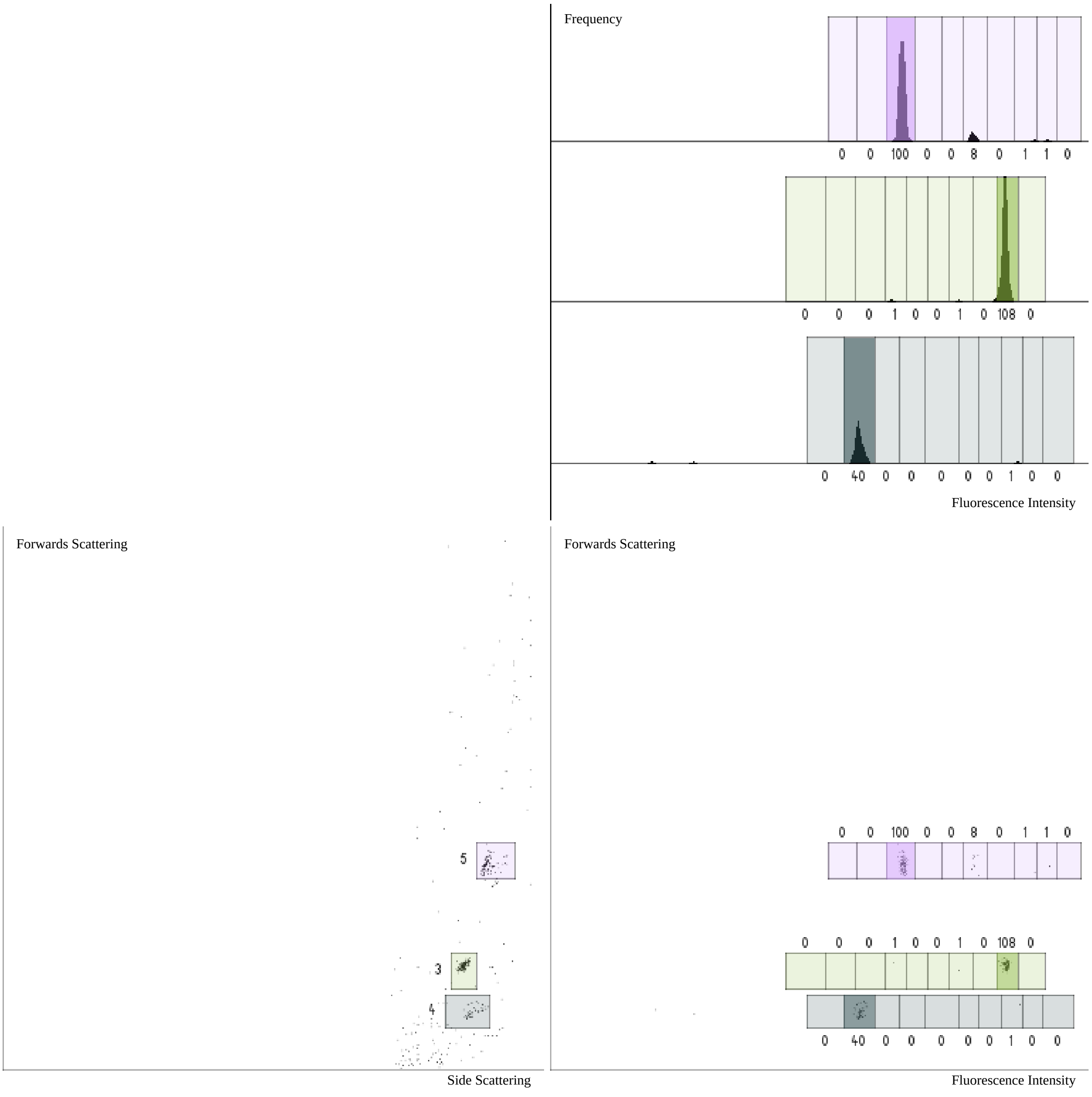

ANNEX 3: TAG DECONVOLUTION - BEAD 201

Passes flow sorting criteria: Yes

Passes tag deconvolution criteria: Yes

Included in protocol analysis: Yes

Protocol: 3, 6, 9, 2

Filename: Bin2_plateA2_B7.fcs

Split 1: Petrol shading

Split 2: Green shading

Split 3: Violet shading

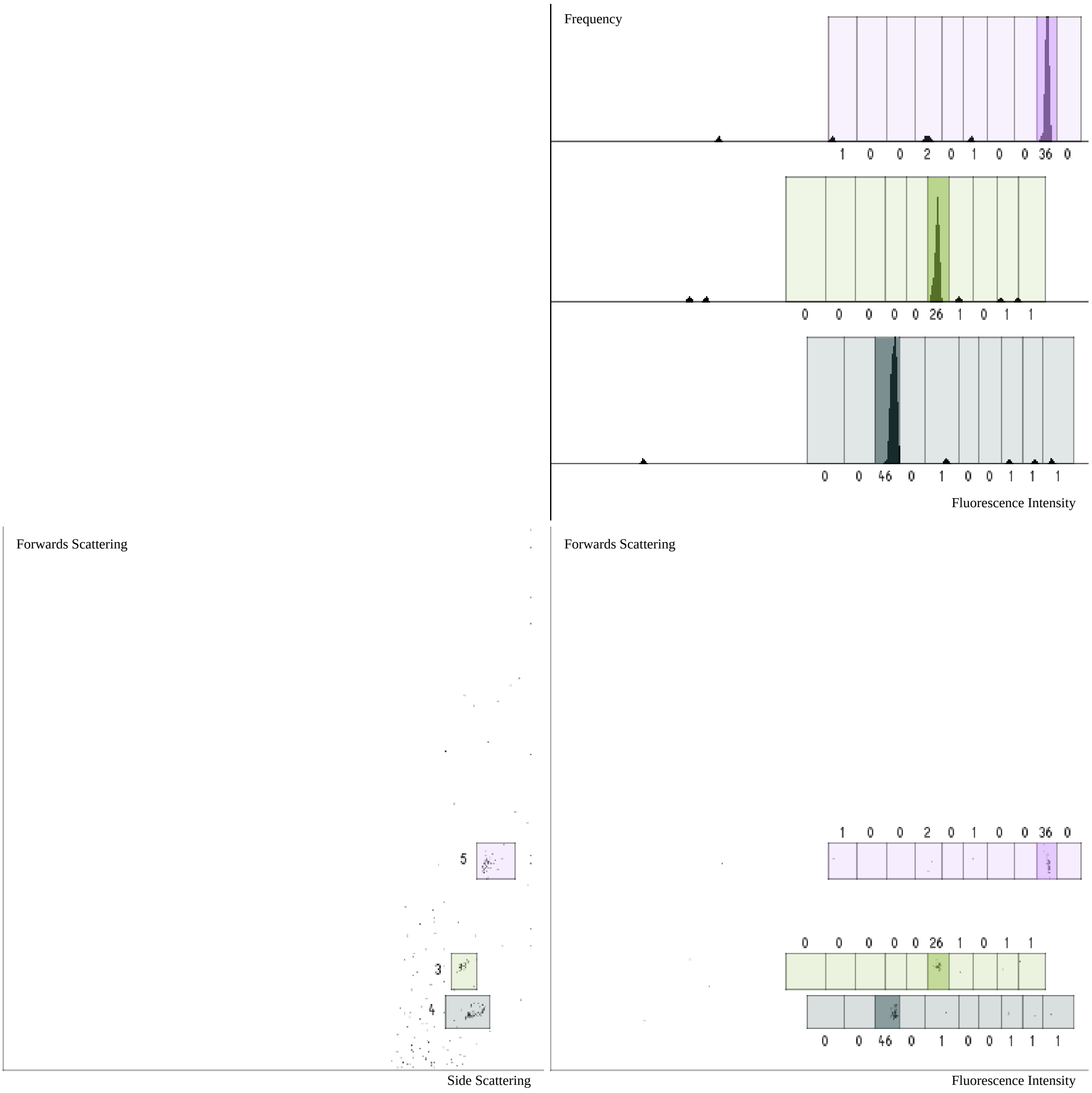

ANNEX 3: TAG DECONVOLUTION - BEAD 202

Passes flow sorting criteria: Yes

Passes tag deconvolution criteria: No

Included in protocol analysis: No

Protocol: N/A

Filename: Bin2_plateA2_B8.fcs

Split 1: Petrol shading

Split 2: Green shading

Split 3: Violet shading

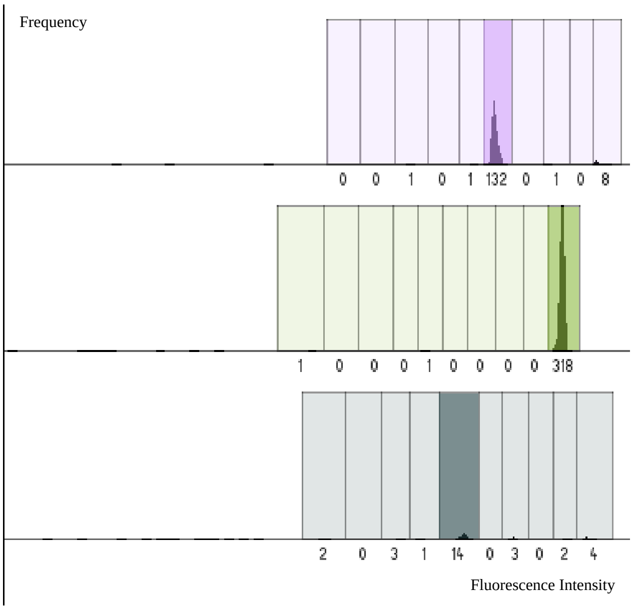

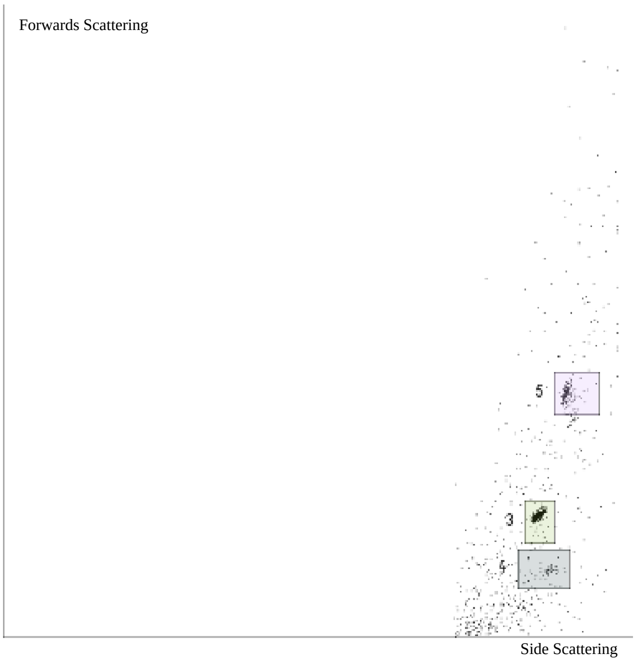

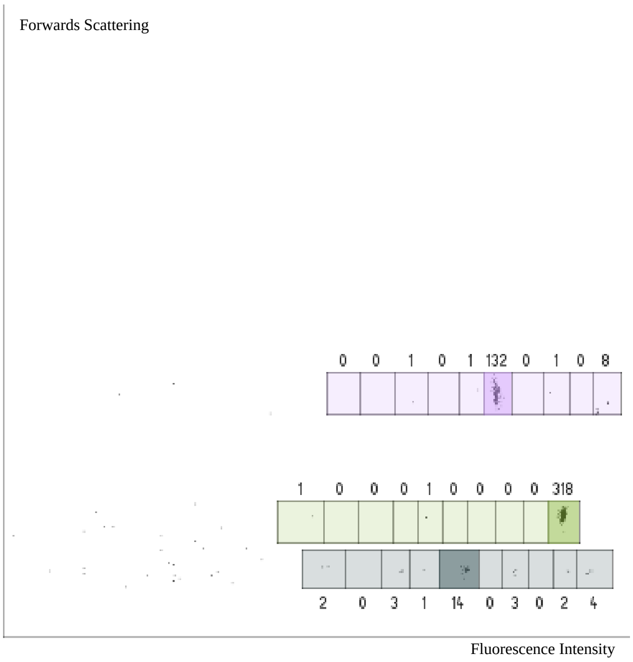

ANNEX 3: TAG DECONVOLUTION - BEAD 203

Passes flow sorting criteria: Yes

Passes tag deconvolution criteria: Yes

Included in protocol analysis: Yes

Protocol: 10, 6, 7, 2

Filename: Bin2_plateA2_B9.fcs

Split 1: Petrol shading

Split 2: Green shading

Split 3: Violet shading

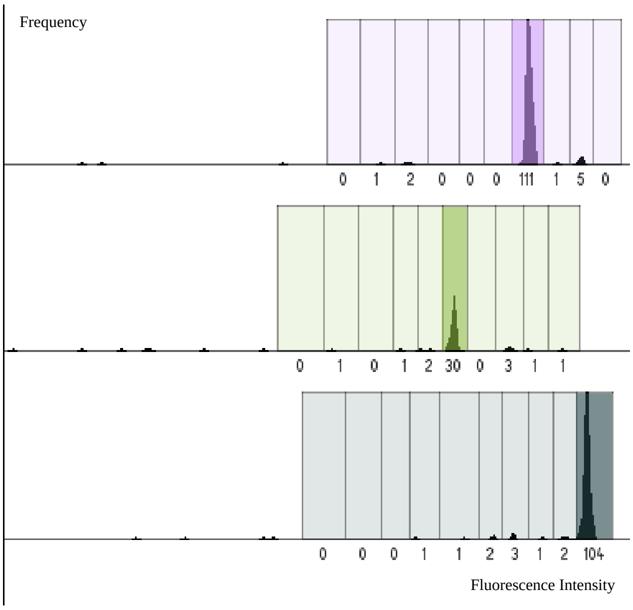

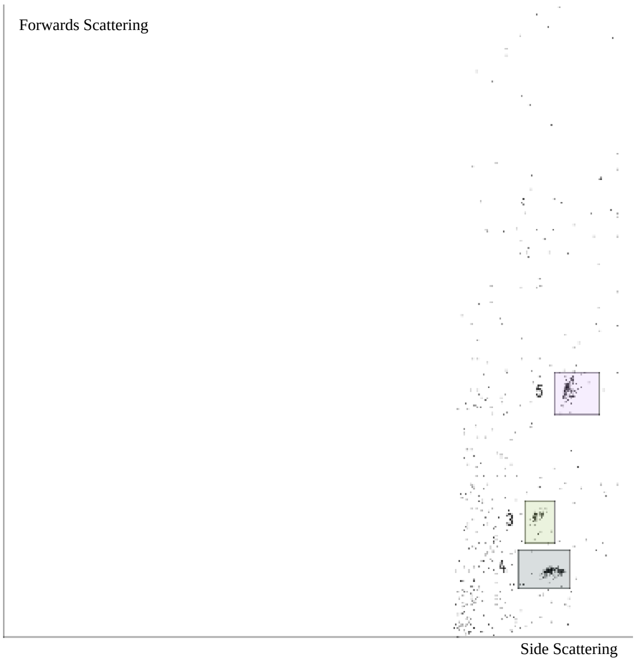

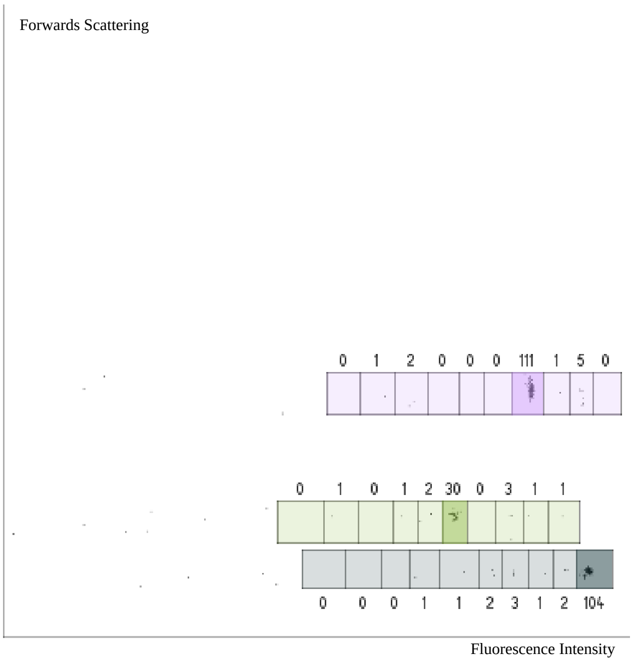

ANNEX 3: TAG DECONVOLUTION - BEAD 204

Passes flow sorting criteria: Yes

Passes tag deconvolution criteria: Yes

Included in protocol analysis: Yes

Protocol: 1, 5, 2, 2

Filename: Bin2_plateA2_B10.fcs

Split 1: Petrol shading

Split 2: Green shading

Split 3: Violet shading

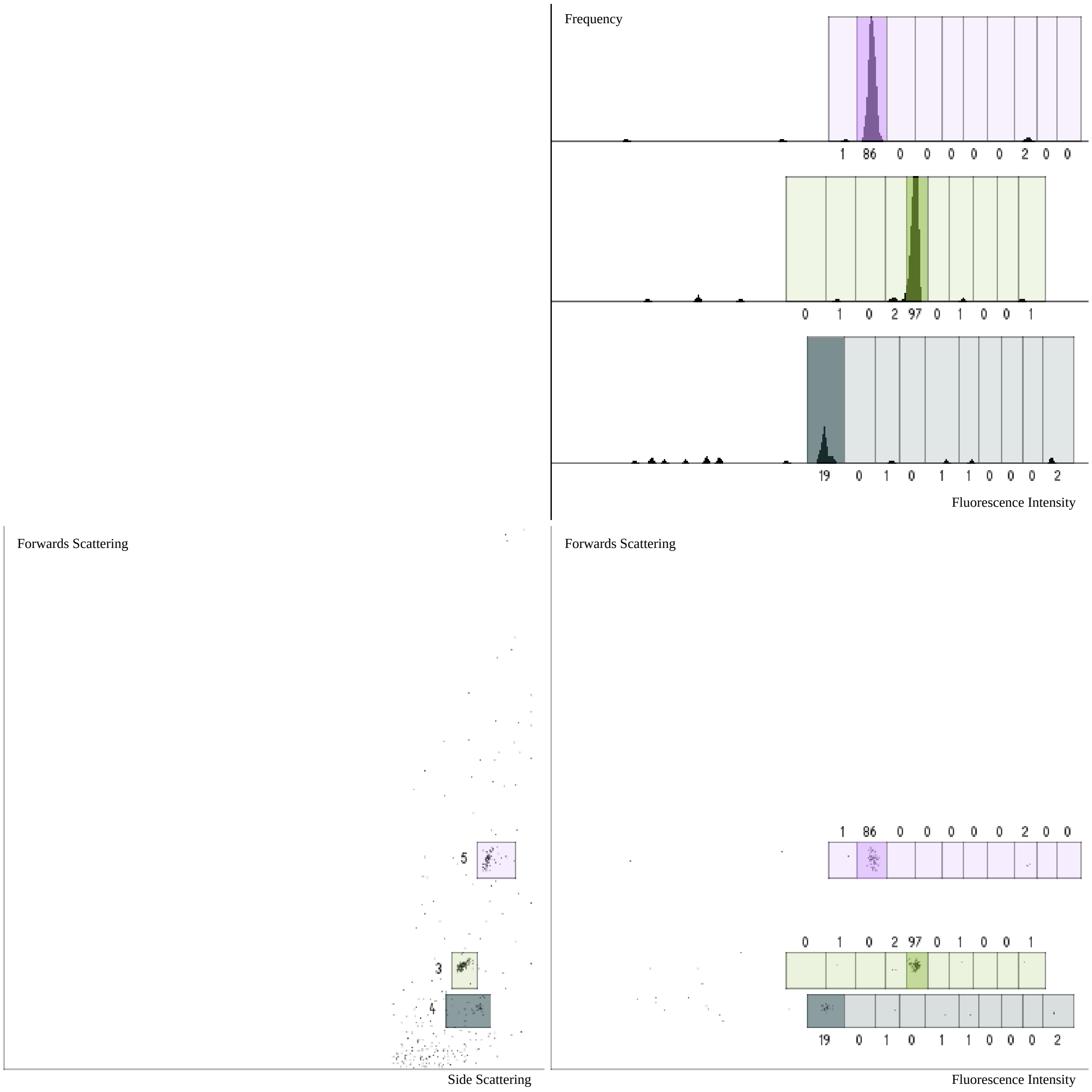

ANNEX 3: TAG DECONVOLUTION - BEAD 205

Passes flow sorting criteria: Yes

Passes tag deconvolution criteria: No

Included in protocol analysis: No

Protocol: N/A

Filename: Bin2_plateA2_B11.fcs

Split 1: Petrol shading

Split 2: Green shading

Split 3: Violet shading

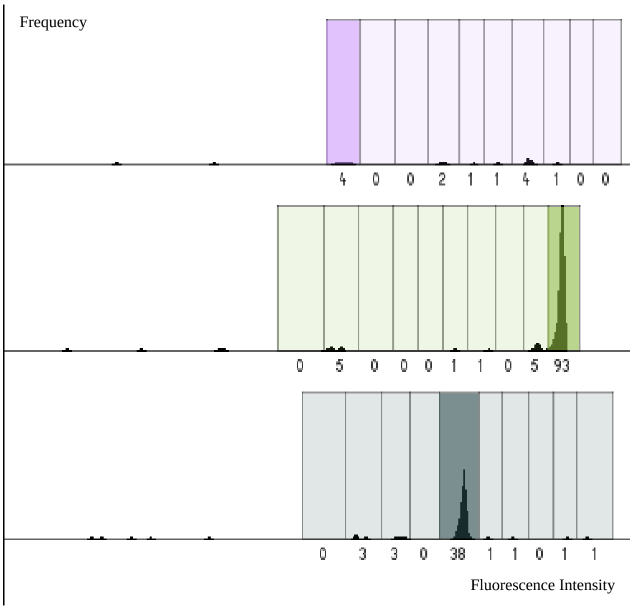

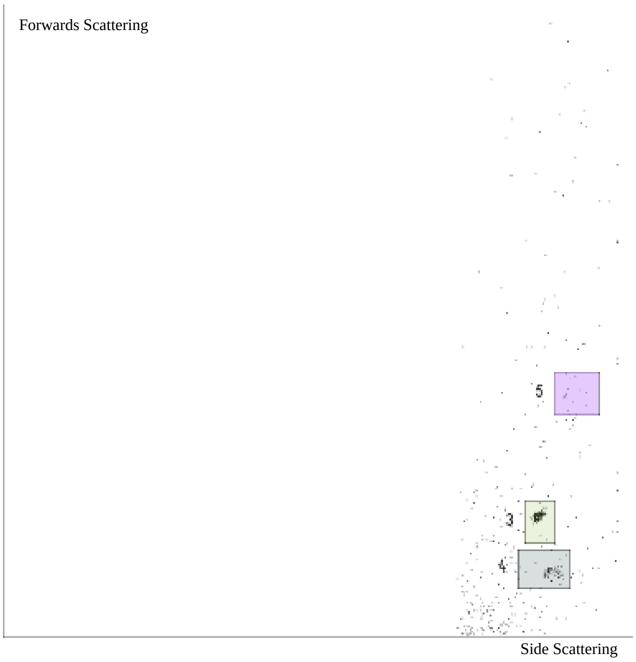

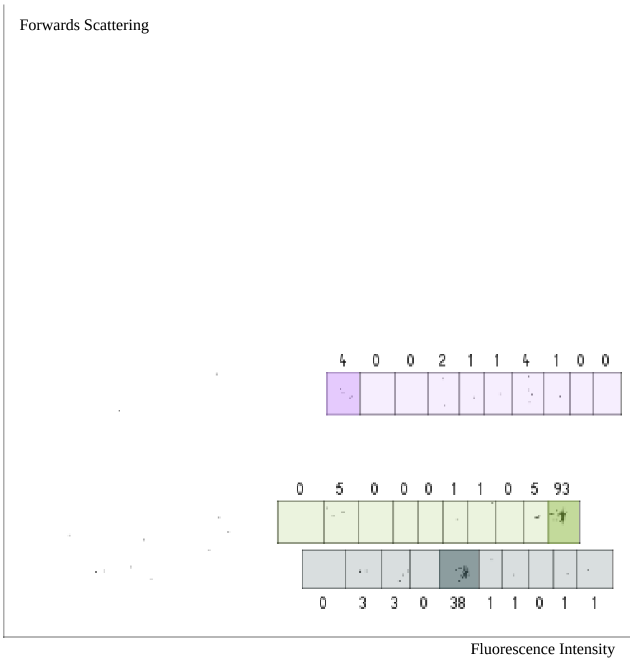

ANNEX 3: TAG DECONVOLUTION - BEAD 206

Passes flow sorting criteria: Yes

Passes tag deconvolution criteria: Yes

Included in protocol analysis: Yes

Protocol: 2, 6, 3, 2

Filename: Bin2_plateA2_B12.fcs

Split 1: Petrol shading

Split 2: Green shading

Split 3: Violet shading

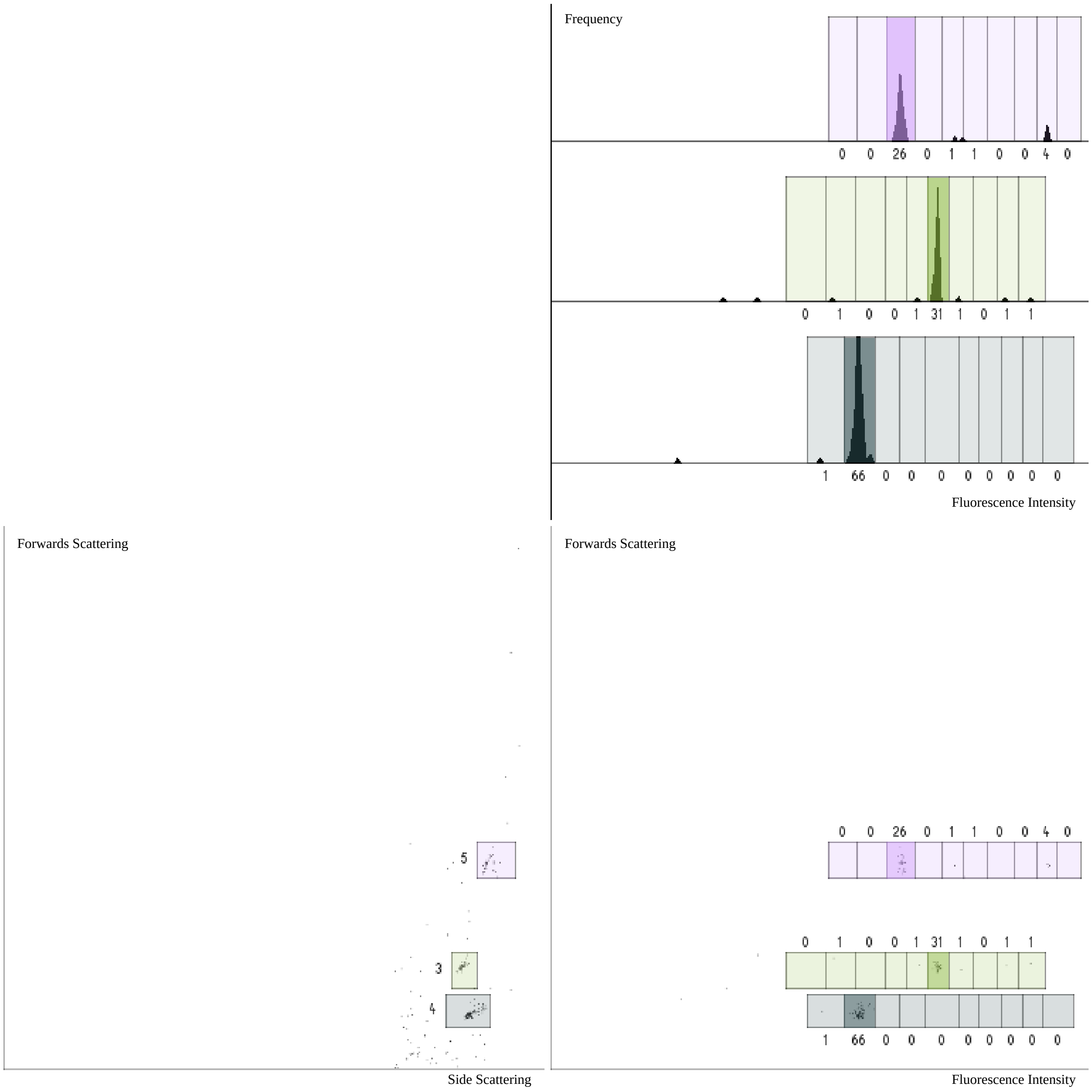

ANNEX 3: TAG DECONVOLUTION - BEAD 207

Passes flow sorting criteria: Yes

Passes tag deconvolution criteria: Yes

Included in protocol analysis: Yes

Protocol: 9, 10, 5, 2

Filename: Bin2_plateA2_C1.fcs

Split 1: Petrol shading

Split 2: Green shading

Split 3: Violet shading

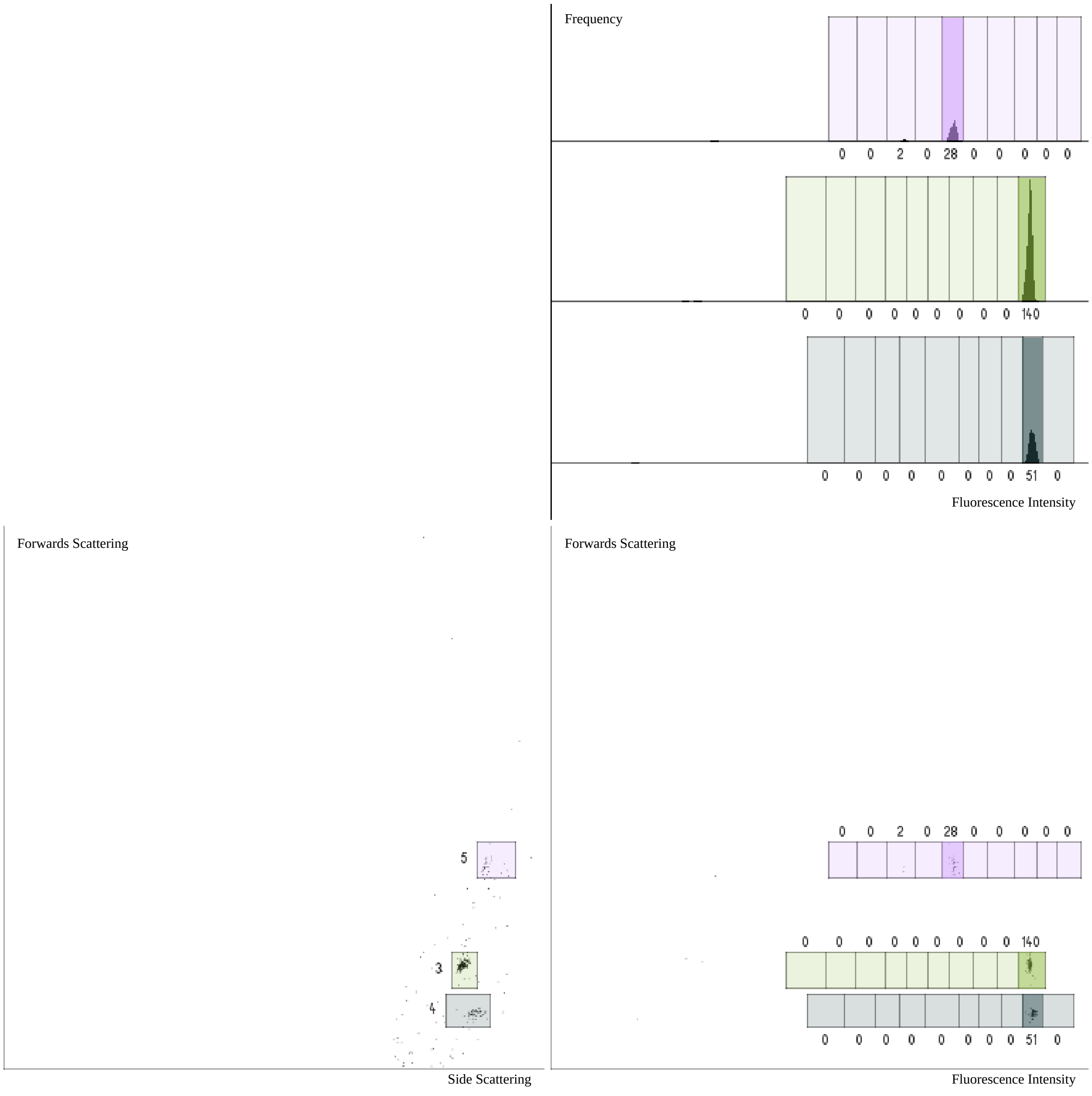

ANNEX 3: TAG DECONVOLUTION - BEAD 208

Passes flow sorting criteria: Yes

Passes tag deconvolution criteria: Yes

Included in protocol analysis: Yes

Protocol: 5, 10, 3, 2

Filename: Bin2_plateA2_C2.fcs

Split 1: Petrol shading

Split 2: Green shading

Split 3: Violet shading

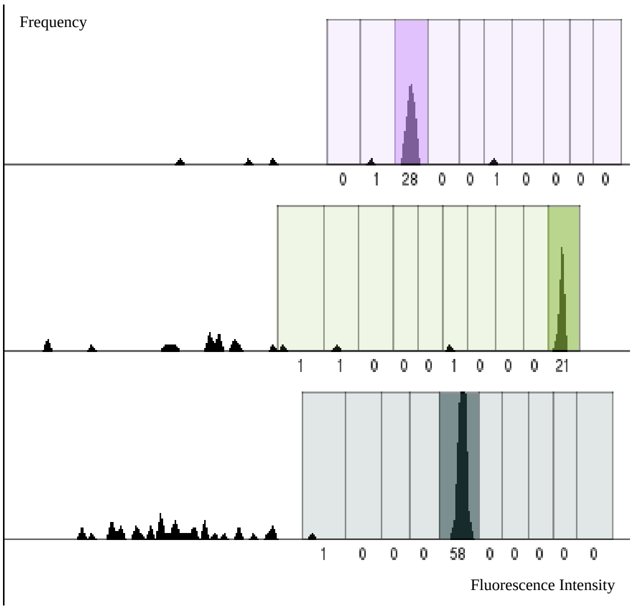

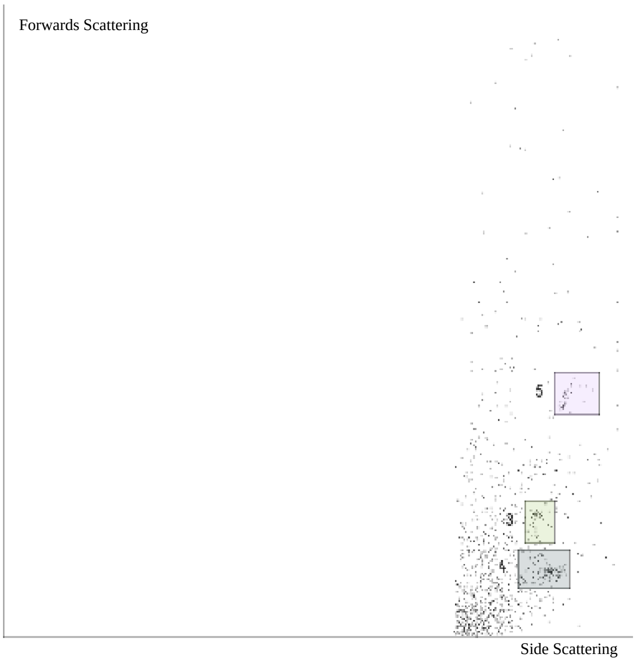

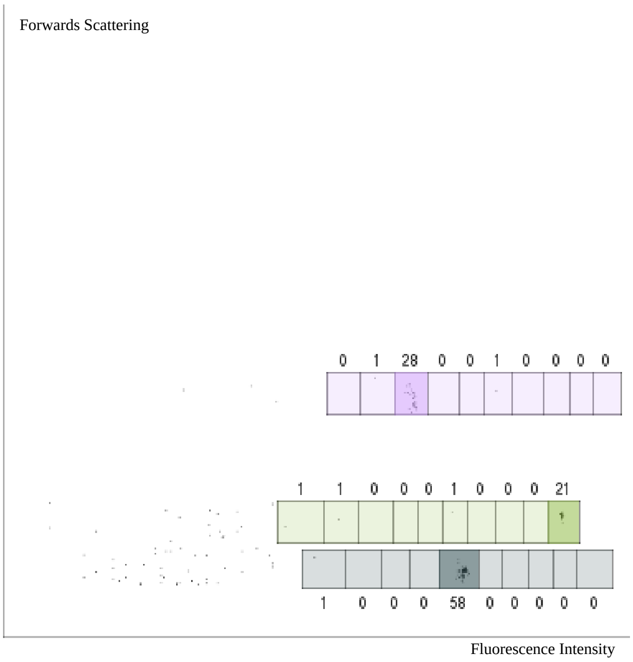

ANNEX 3: TAG DECONVOLUTION - BEAD 209

Passes flow sorting criteria: Yes

Passes tag deconvolution criteria: Yes

Included in protocol analysis: Yes

Protocol: 8, 6, 10, 2

Filename: Bin2_plateA2_C3.fcs

Split 1: Petrol shading

Split 2: Green shading

Split 3: Violet shading

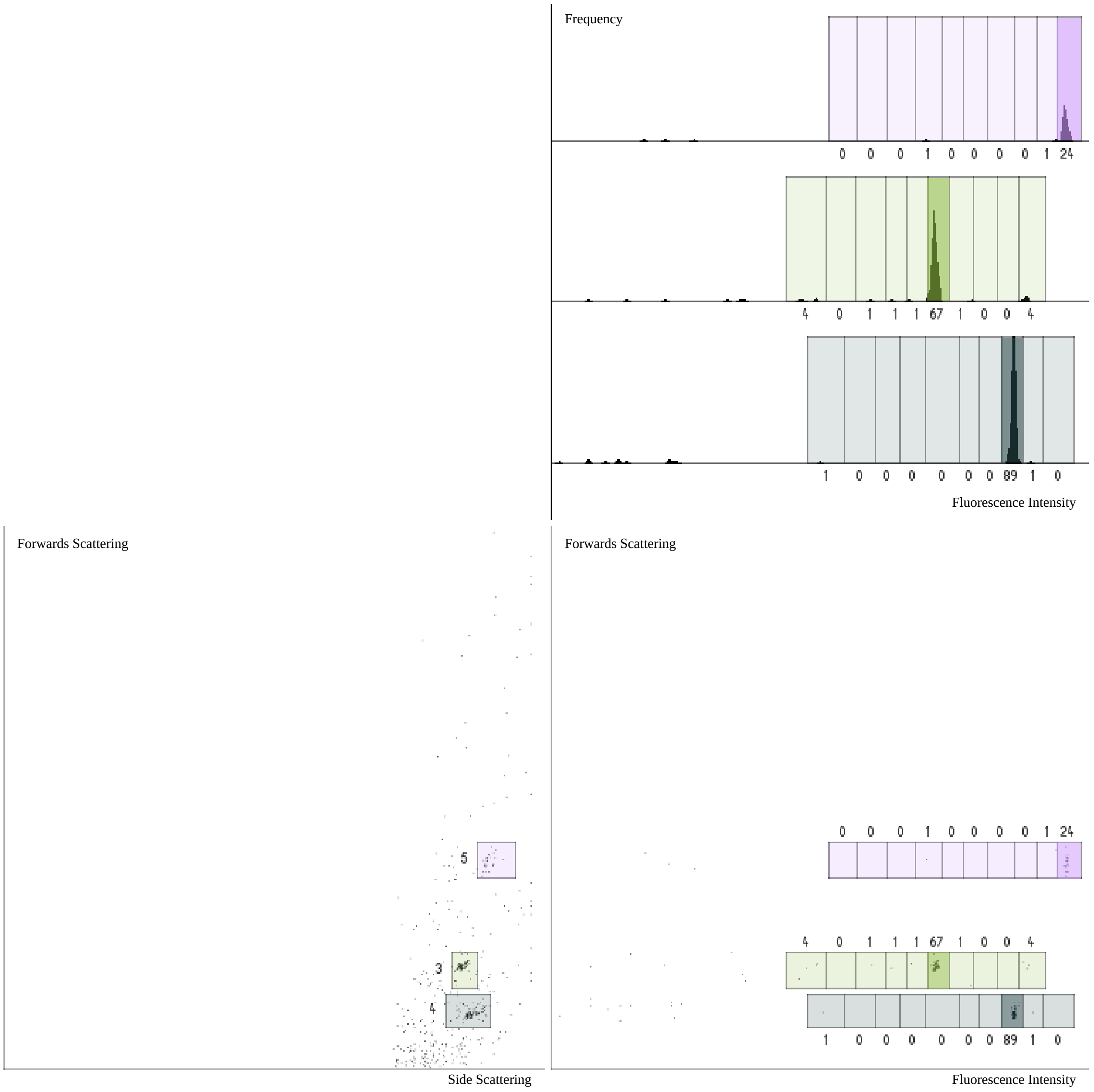

ANNEX 3: TAG DECONVOLUTION - BEAD 210

Passes flow sorting criteria: Yes

Passes tag deconvolution criteria: Yes

Included in protocol analysis: Yes

Protocol: 10, 10, 5, 2

Filename: Bin2_plateA2_C4.fcs

Split 1: Petrol shading

Split 2: Green shading

Split 3: Violet shading

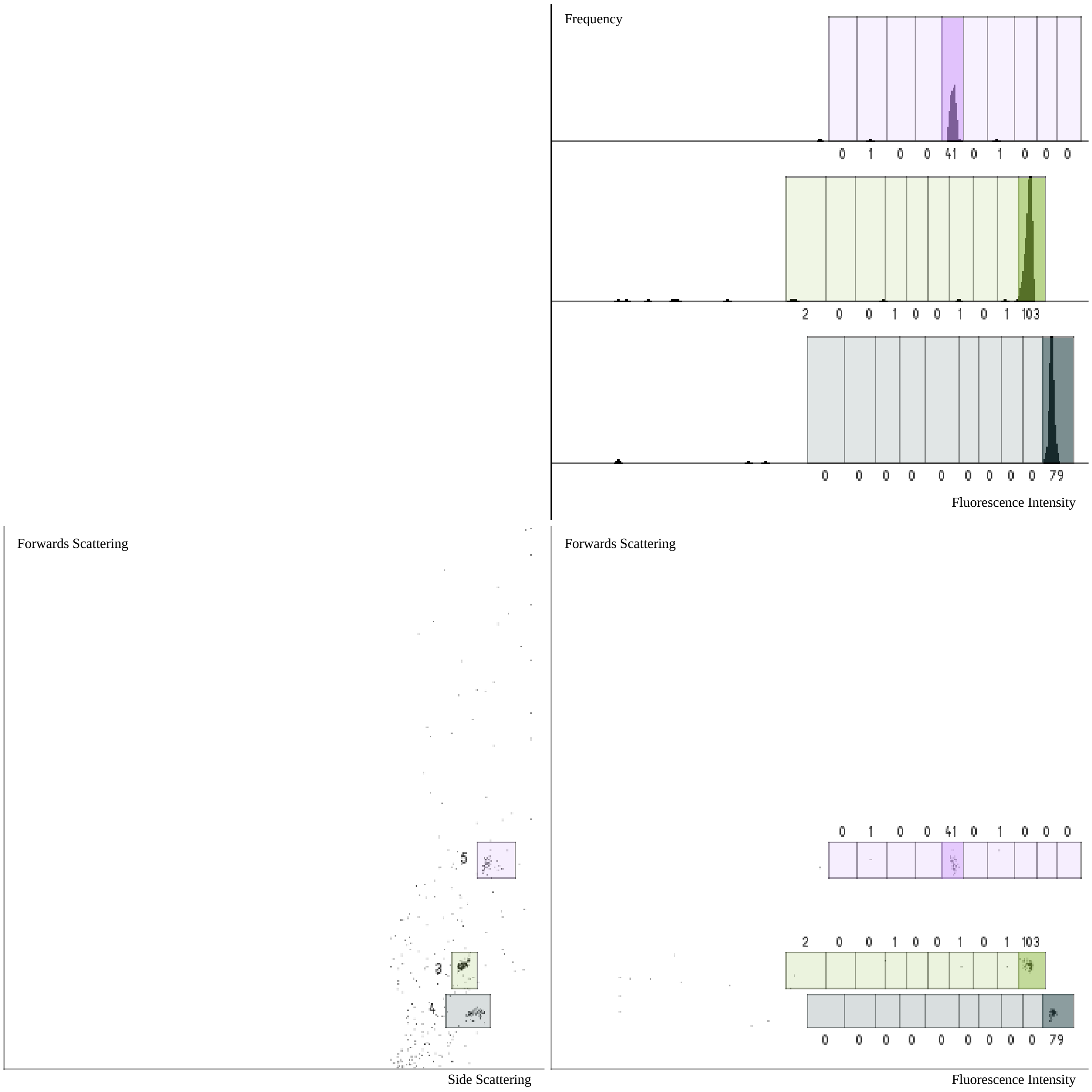

ANNEX 3: TAG DECONVOLUTION - BEAD 211

Passes flow sorting criteria: Yes

Passes tag deconvolution criteria: Yes

Included in protocol analysis: Yes

Protocol: 10, 6, 9, 2

Filename: Bin2_plateA2_C5.fcs

Split 1: Petrol shading

Split 2: Green shading

Split 3: Violet shading

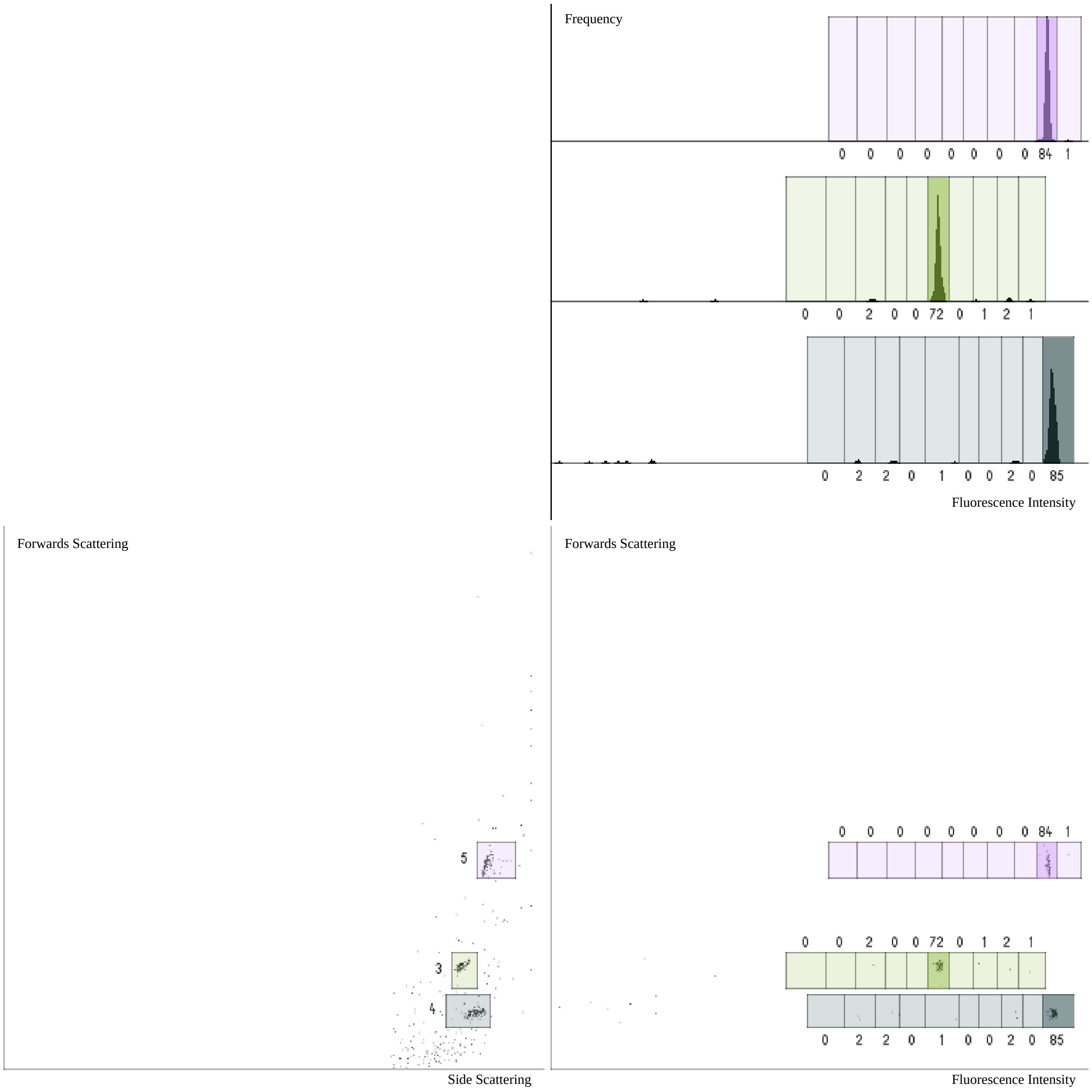

ANNEX 3: TAG DECONVOLUTION - BEAD 212

Passes flow sorting criteria: Yes

Passes tag deconvolution criteria: Yes

Included in protocol analysis: Yes

Protocol: 5, 10, 7, 2

Filename: Bin2_plateA2_C6.fcs

Split 1: Petrol shading

Split 2: Green shading

Split 3: Violet shading

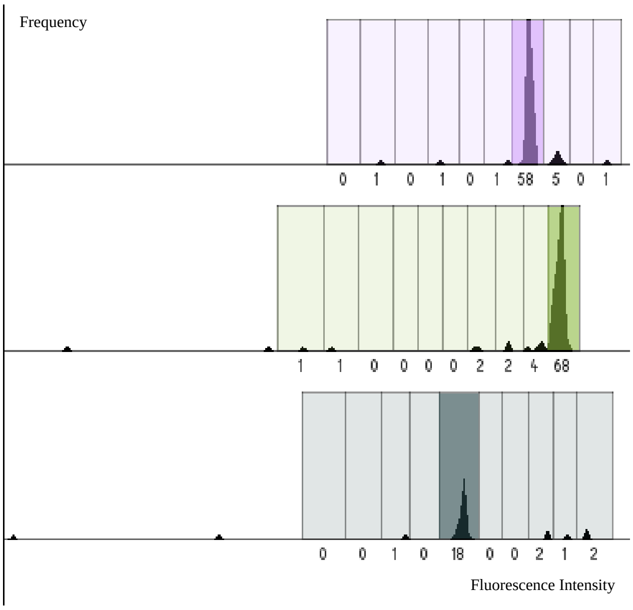

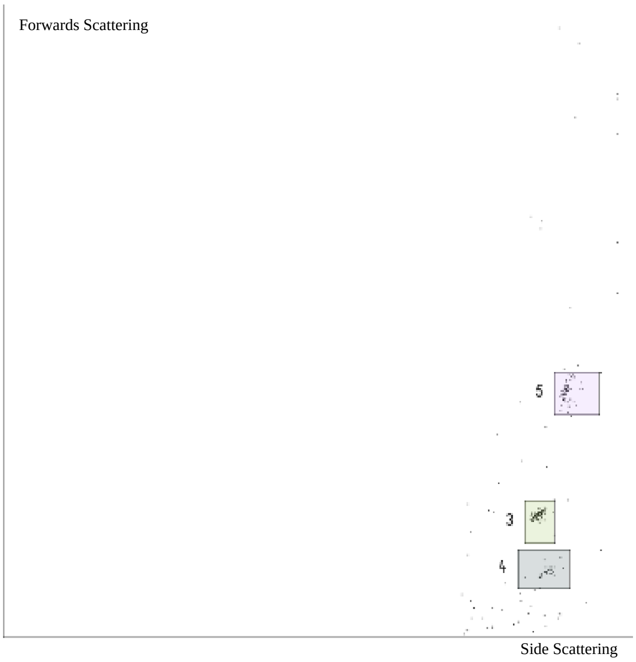

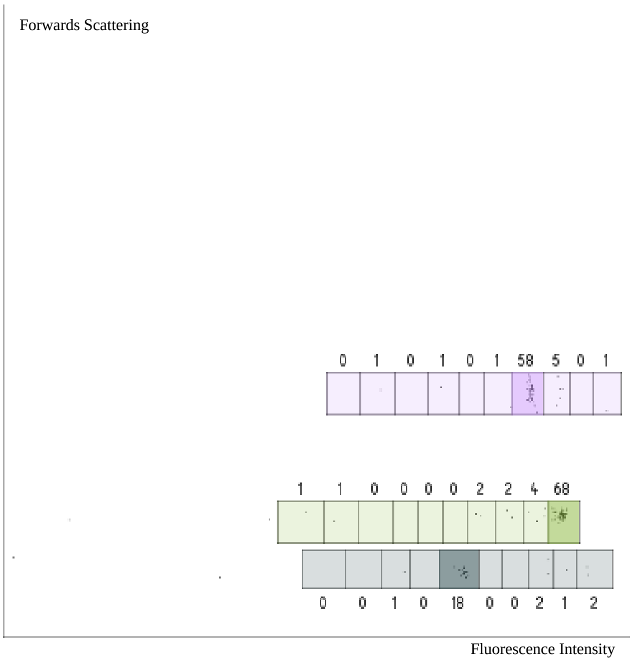

ANNEX 3: TAG DECONVOLUTION - BEAD 213

Passes flow sorting criteria: Yes

Passes tag deconvolution criteria: Yes

Included in protocol analysis: Yes

Protocol: 6, 6, 3, 2

Filename: Bin2_plateA2_C7.fcs

Split 1: Petrol shading

Split 2: Green shading

Split 3: Violet shading

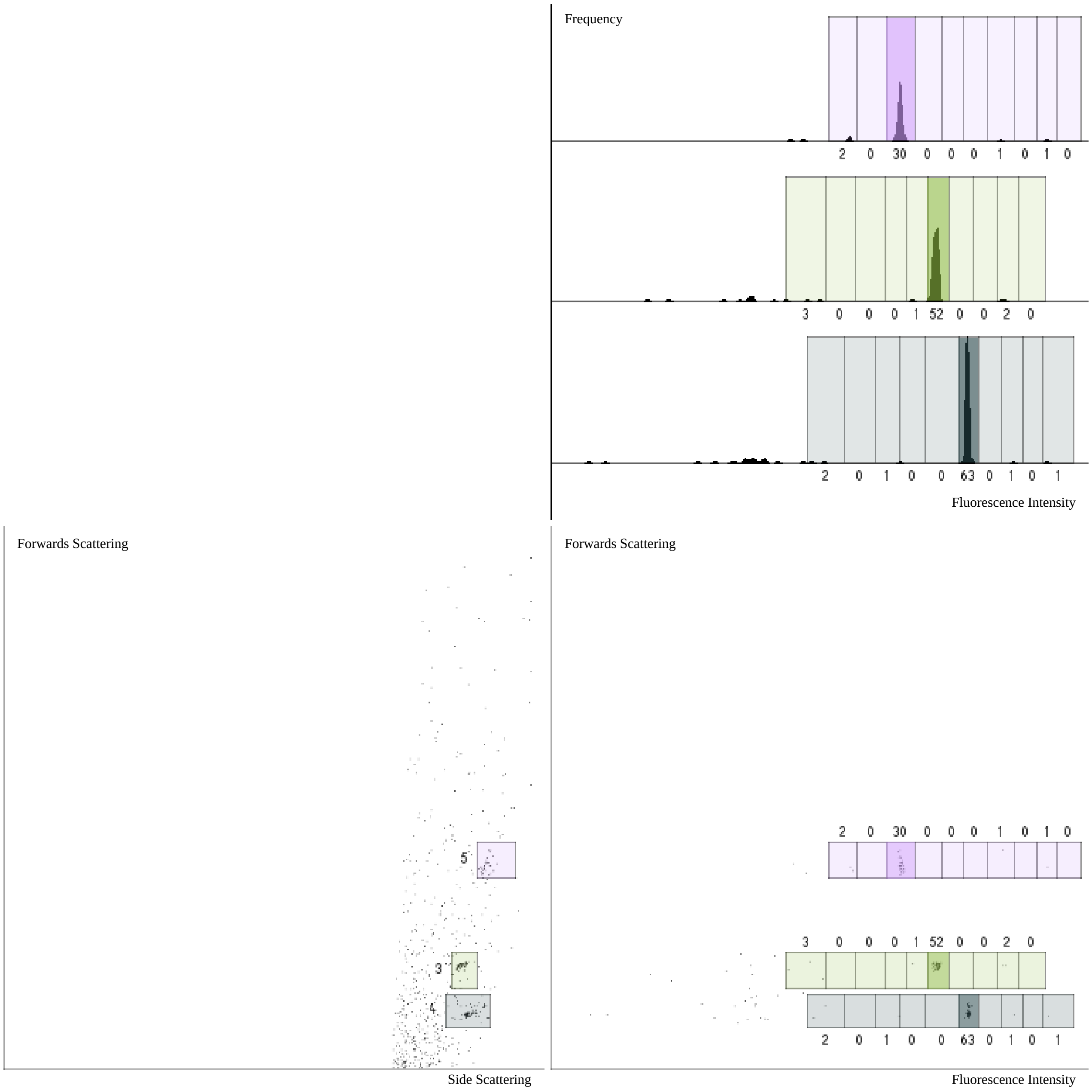

ANNEX 3: TAG DECONVOLUTION - BEAD 214

Passes flow sorting criteria: Yes

Passes tag deconvolution criteria: Yes

Included in protocol analysis: Yes

Protocol: 4, 10, 3, 2

Filename: Bin2_plateA2_C8.fcs

Split 1: Petrol shading

Split 2: Green shading

Split 3: Violet shading

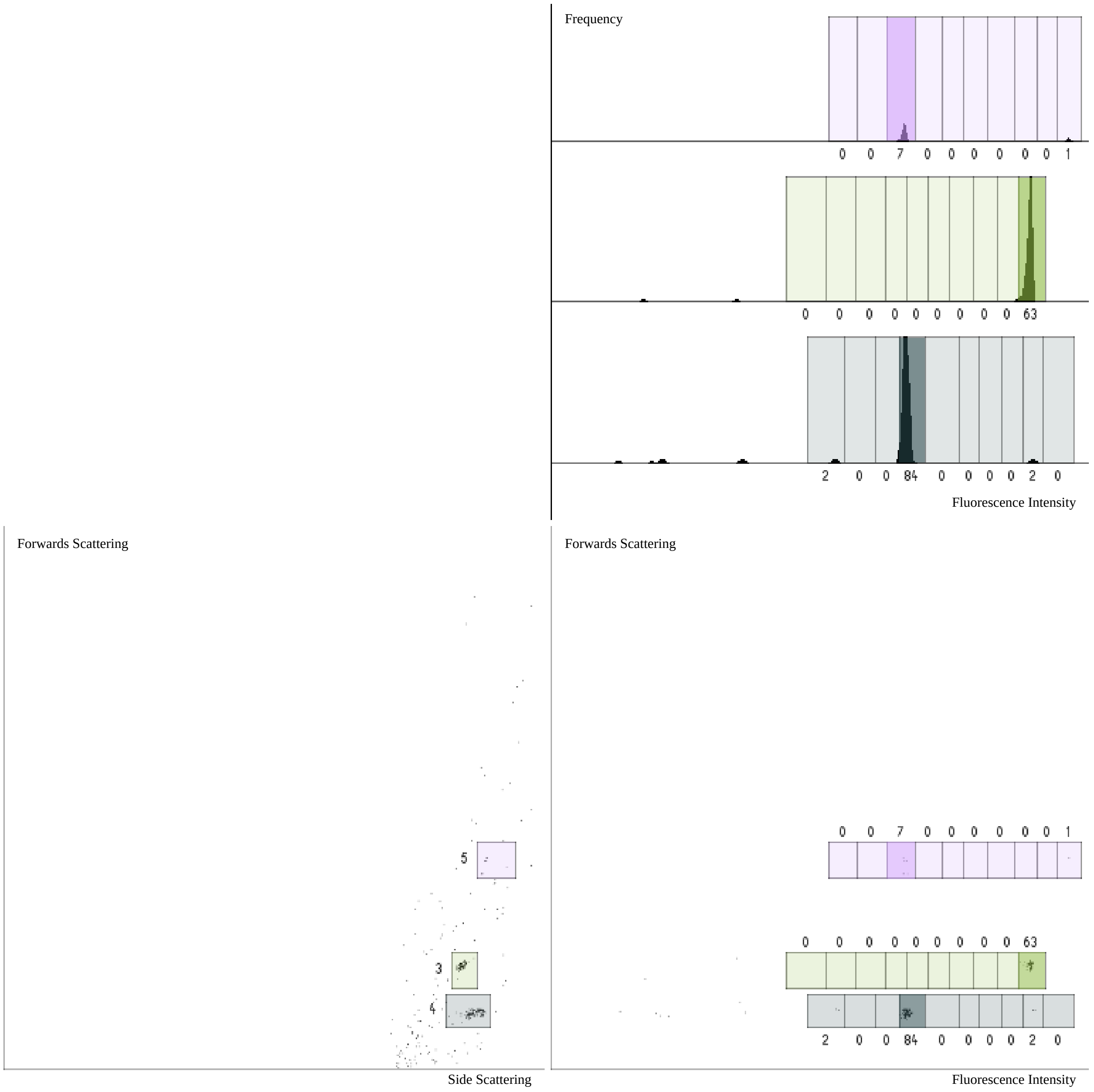

ANNEX 3: TAG DECONVOLUTION - BEAD 215

Passes flow sorting criteria: Yes

Passes tag deconvolution criteria: Yes

Included in protocol analysis: Yes

Protocol: 7, 4, 2, 2

Filename: Bin2_plateA2_C9.fcs

Split 1: Petrol shading

Split 2: Green shading

Split 3: Violet shading

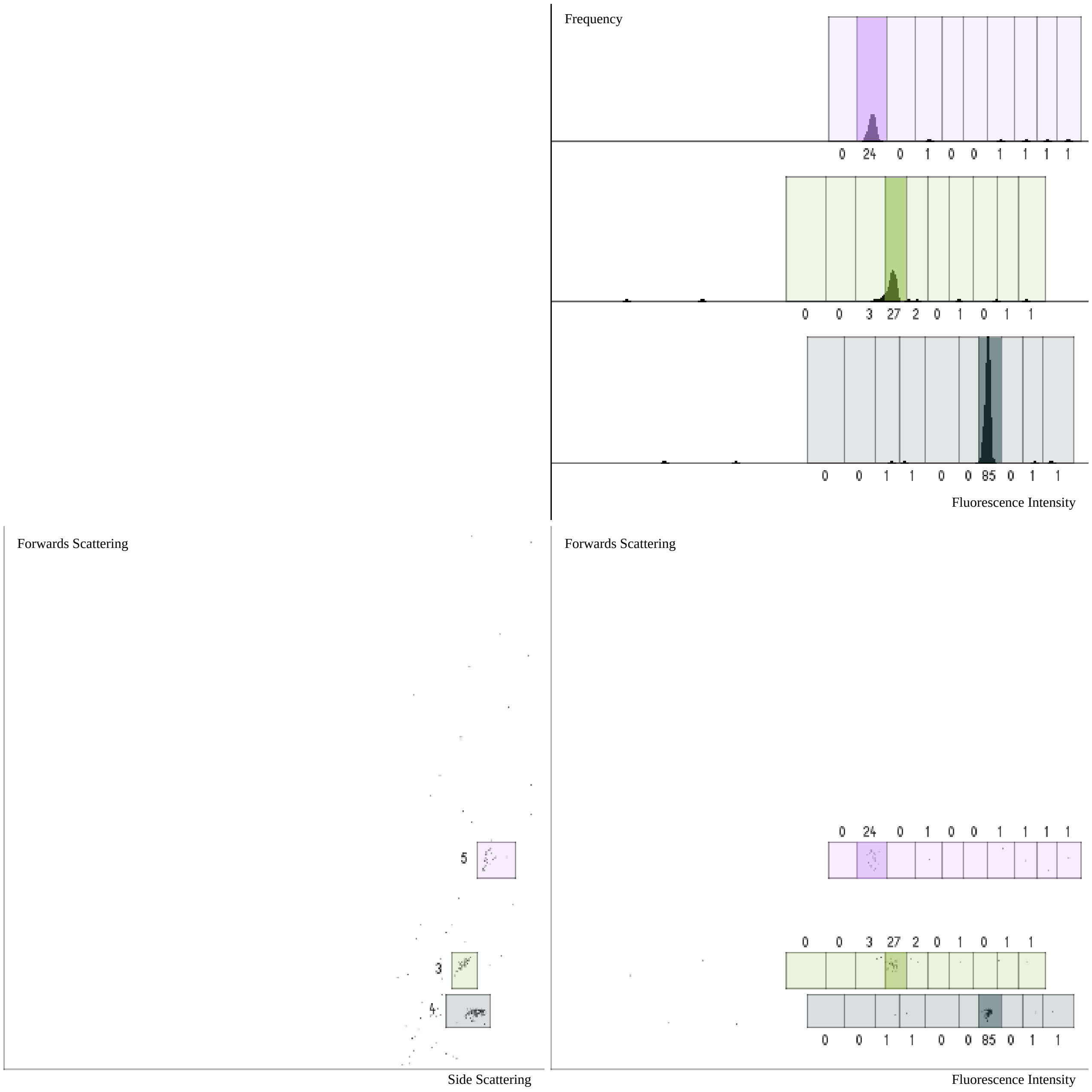

ANNEX 3: TAG DECONVOLUTION - BEAD 216

Passes flow sorting criteria: Yes

Passes tag deconvolution criteria: Yes

Included in protocol analysis: Yes

Protocol: 1, 5, 3, 2

Filename: Bin2_plateA2_C10.fcs

Split 1: Petrol shading

Split 2: Green shading

Split 3: Violet shading

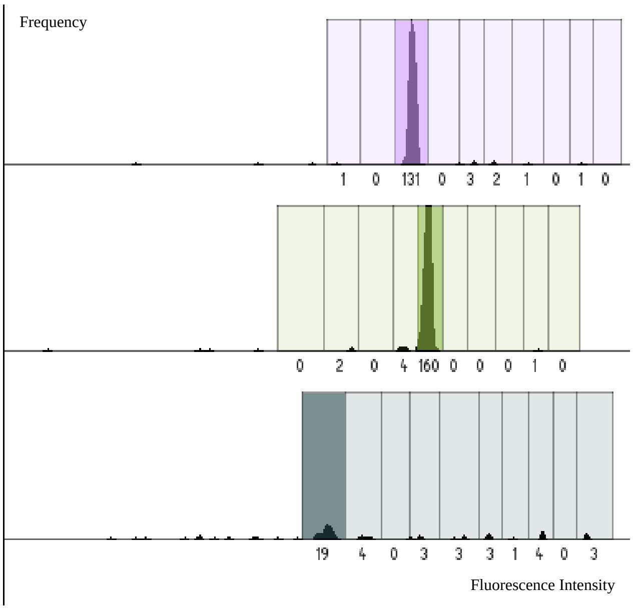

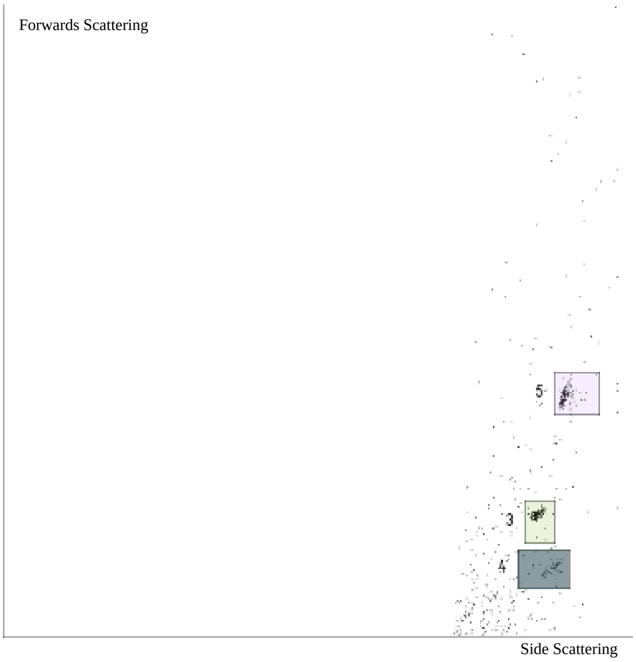

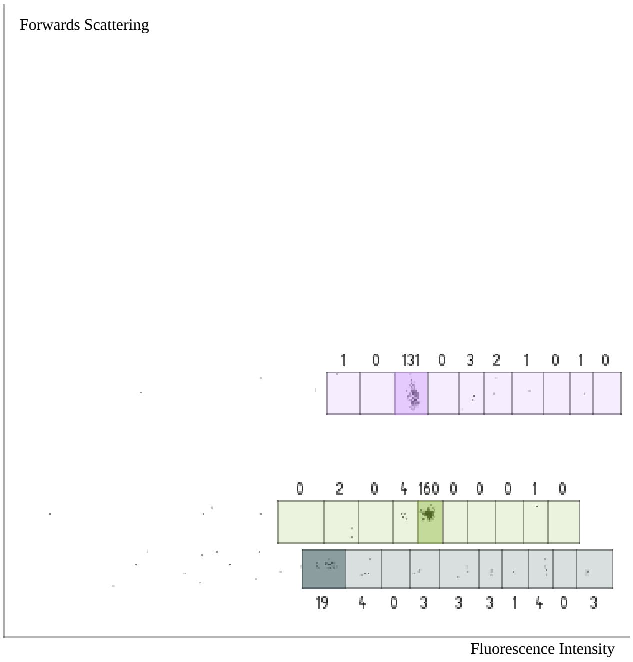

ANNEX 3: TAG DECONVOLUTION - BEAD 217

Passes flow sorting criteria: Yes

Passes tag deconvolution criteria: Yes

Included in protocol analysis: Yes

Protocol: 10, 8, 7, 2

Filename: Bin2_plateA2_C11.fcs

Split 1: Petrol shading

Split 2: Green shading

Split 3: Violet shading

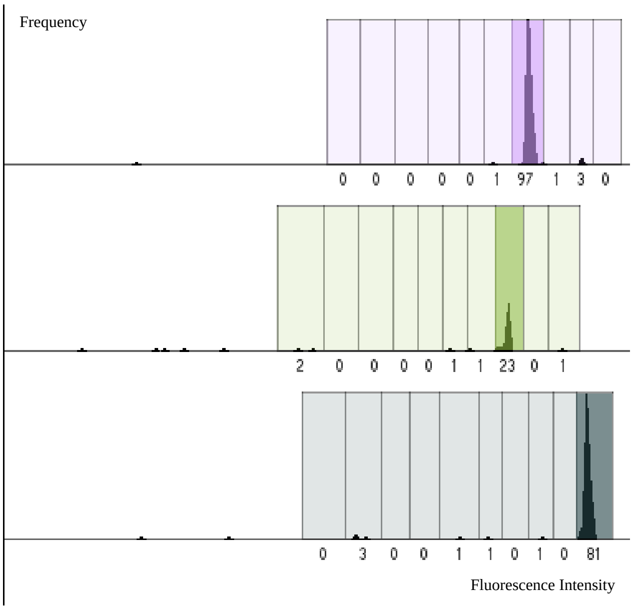

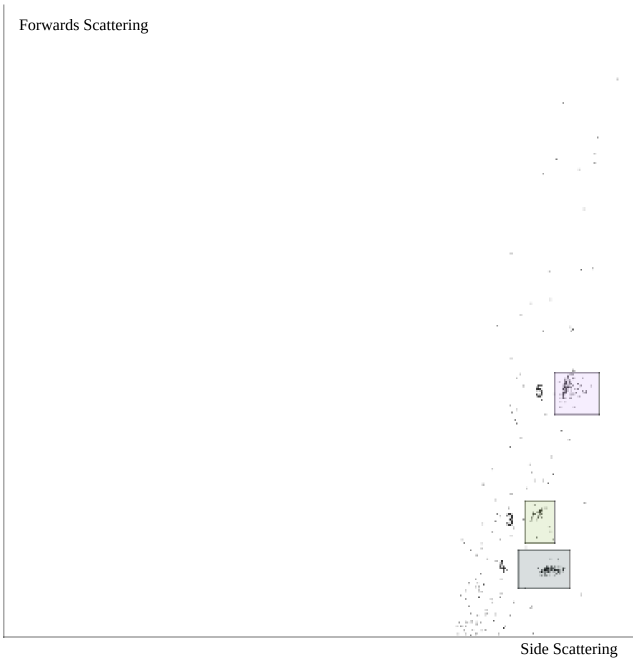

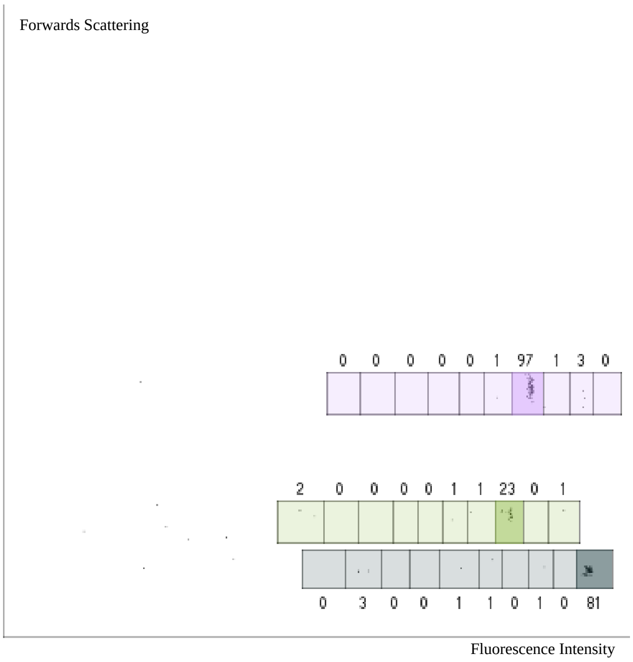

ANNEX 3: TAG DECONVOLUTION - BEAD 218

Passes flow sorting criteria: Yes

Passes tag deconvolution criteria: Yes

Included in protocol analysis: Yes

Protocol: 7, 4, 8, 2

Filename: Bin2_plateA2_C12.fcs

Split 1: Petrol shading

Split 2: Green shading

Split 3: Violet shading

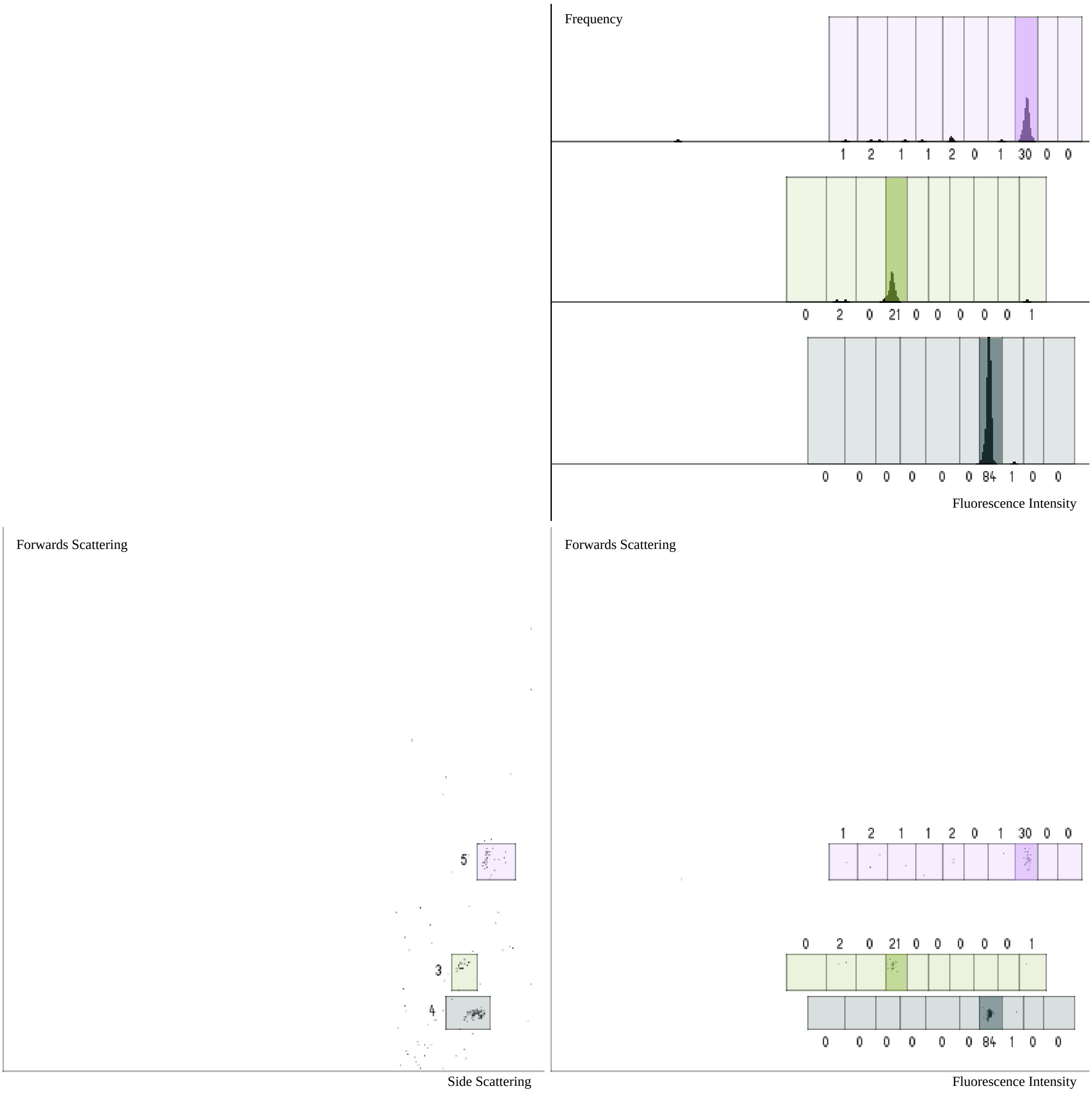

ANNEX 3: TAG DECONVOLUTION - BEAD 219

Passes flow sorting criteria: Yes

Passes tag deconvolution criteria: Yes

Included in protocol analysis: Yes

Protocol: 9, 6, 6, 2

Filename: Bin2_plateA2_D1.fcs

Split 1: Petrol shading

Split 2: Green shading

Split 3: Violet shading

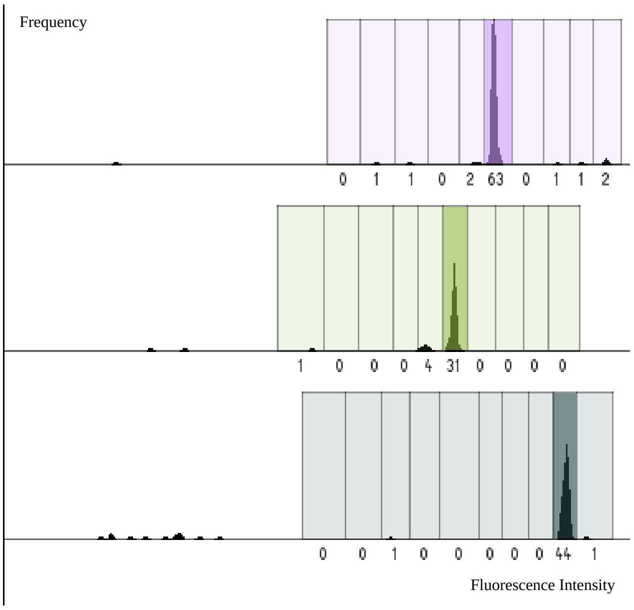

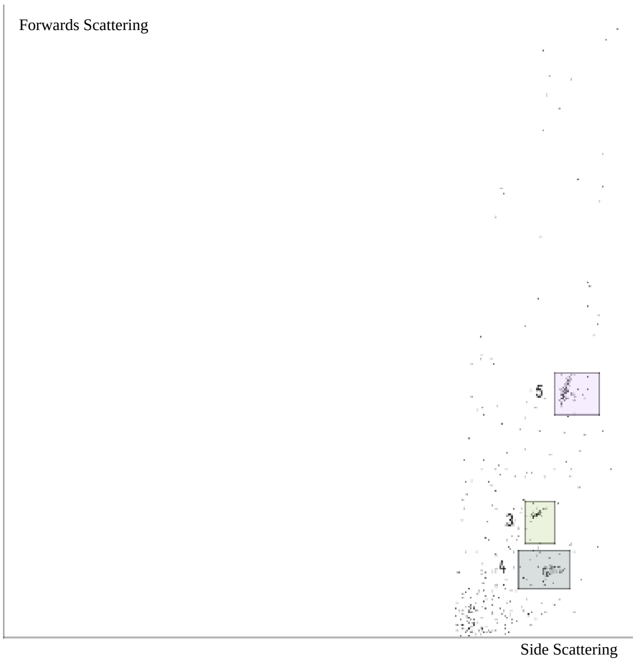

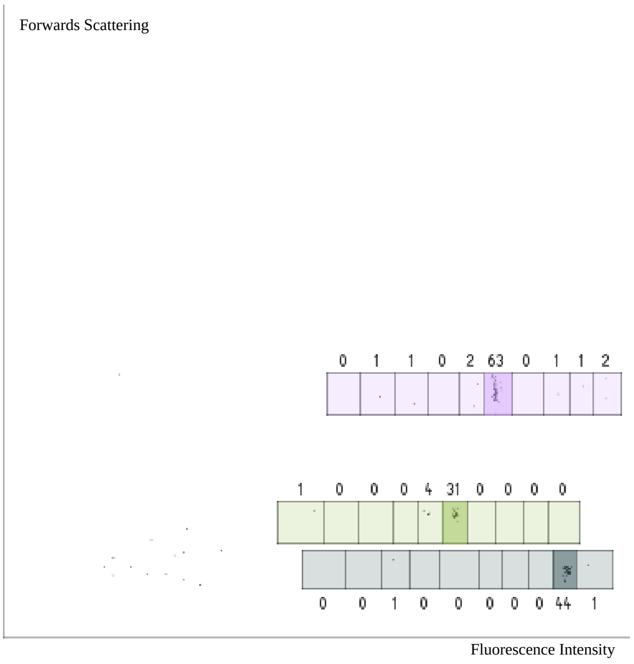

ANNEX 3: TAG DECONVOLUTION - BEAD 220

Passes flow sorting criteria: Yes

Passes tag deconvolution criteria: Yes

Included in protocol analysis: Yes

Protocol: 10, 5, 5, 2

Filename: Bin2_plateA2_D2.fcs

Split 1: Petrol shading

Split 2: Green shading

Split 3: Violet shading

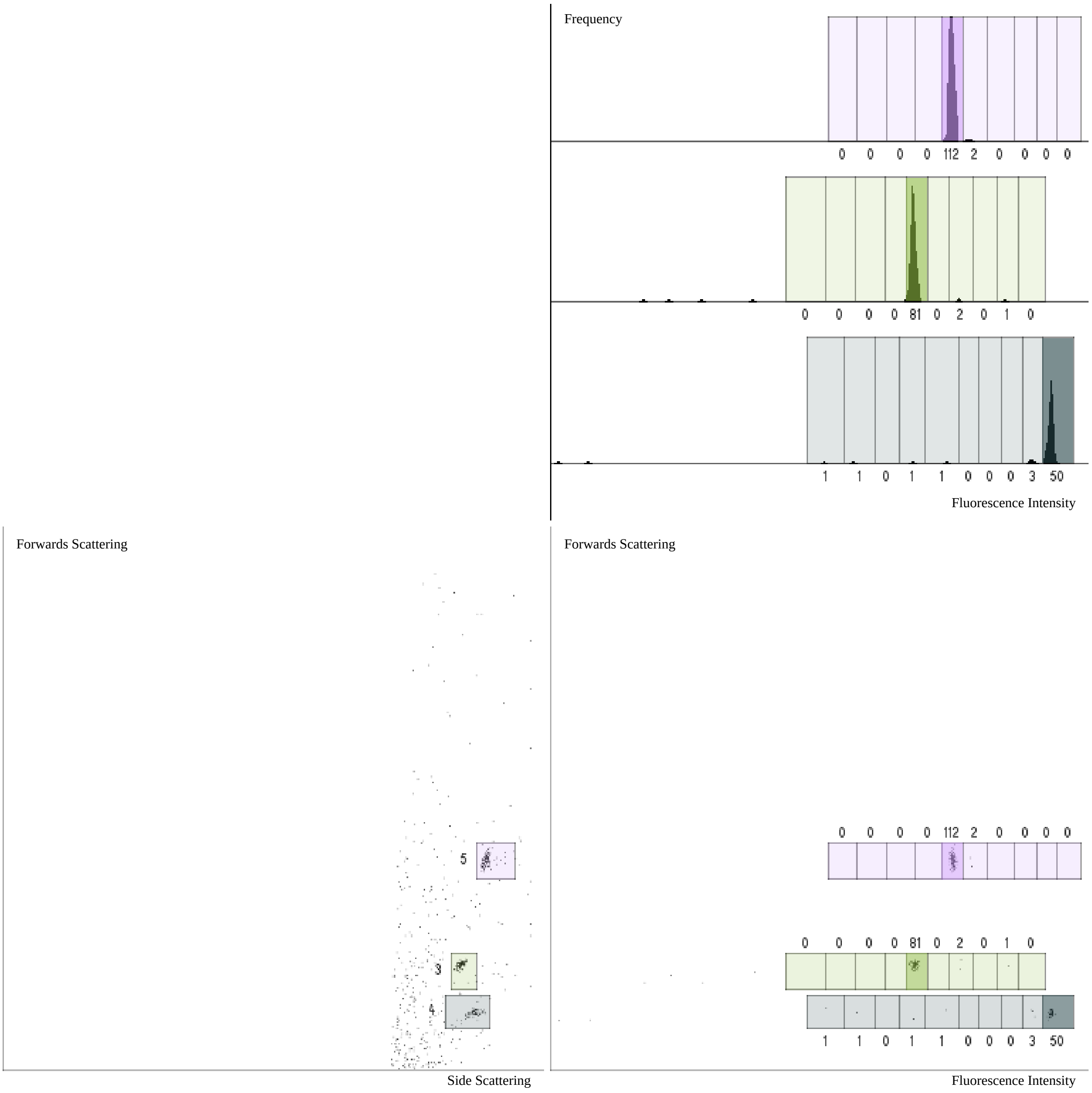

ANNEX 3: TAG DECONVOLUTION - BEAD 221

Passes flow sorting criteria: Yes

Passes tag deconvolution criteria: Yes

Included in protocol analysis: Yes

Protocol: 6, 8, 2, 2

Filename: Bin2_plateA2_D3.fcs

Split 1: Petrol shading

Split 2: Green shading

Split 3: Violet shading

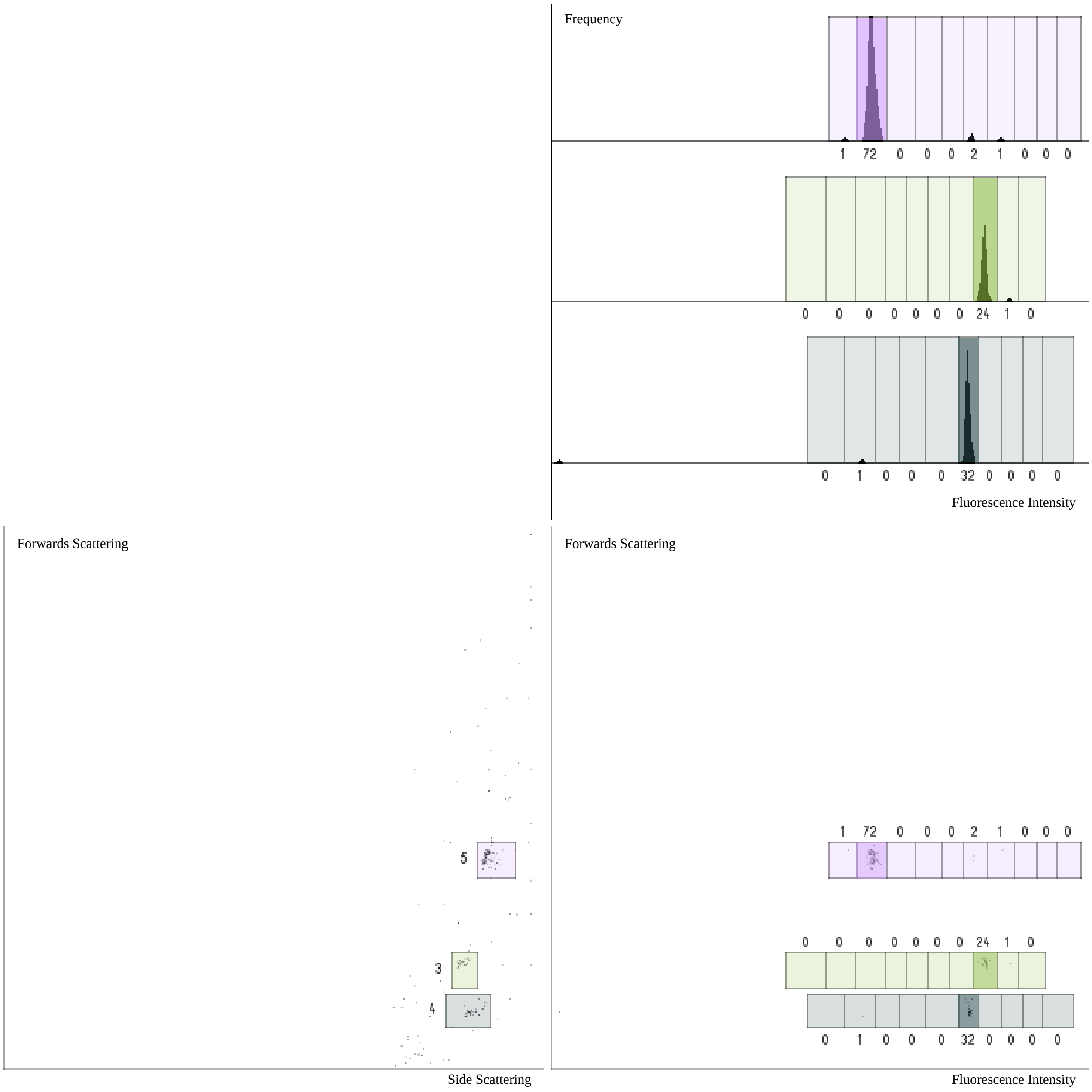

ANNEX 3: TAG DECONVOLUTION - BEAD 222

Passes flow sorting criteria: Yes

Passes tag deconvolution criteria: Yes

Included in protocol analysis: Yes

Protocol: 7, 3, 6, 2

Filename: Bin2_plateA2_D4.fcs

Split 1: Petrol shading

Split 2: Green shading

Split 3: Violet shading

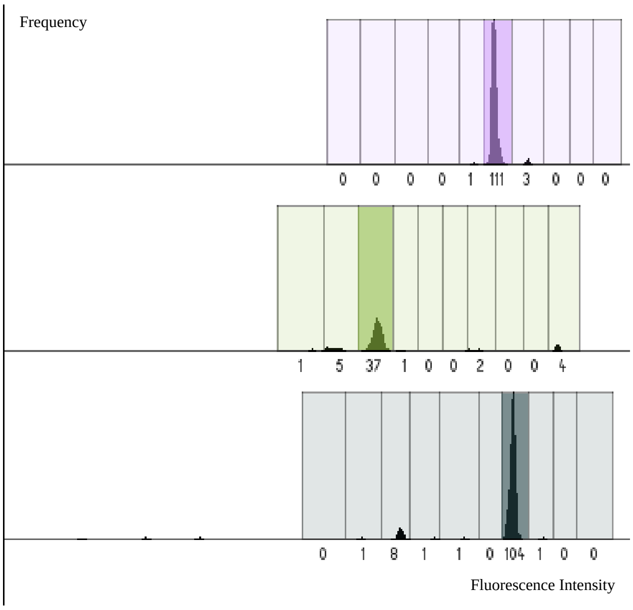

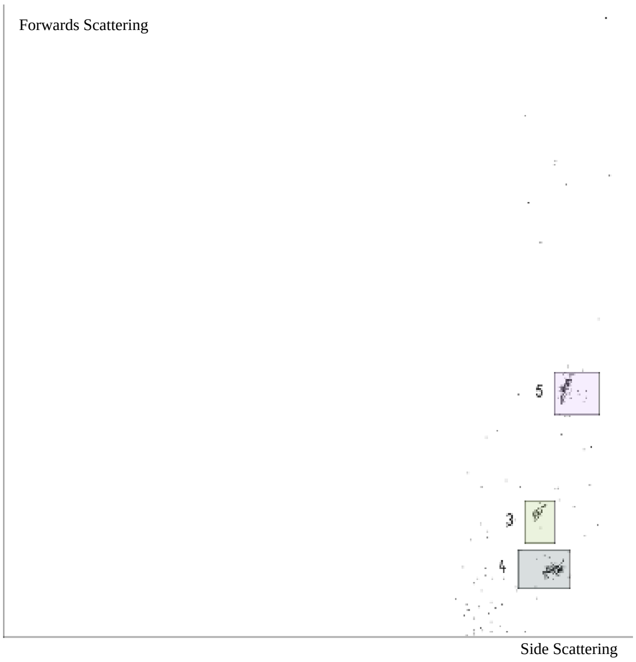

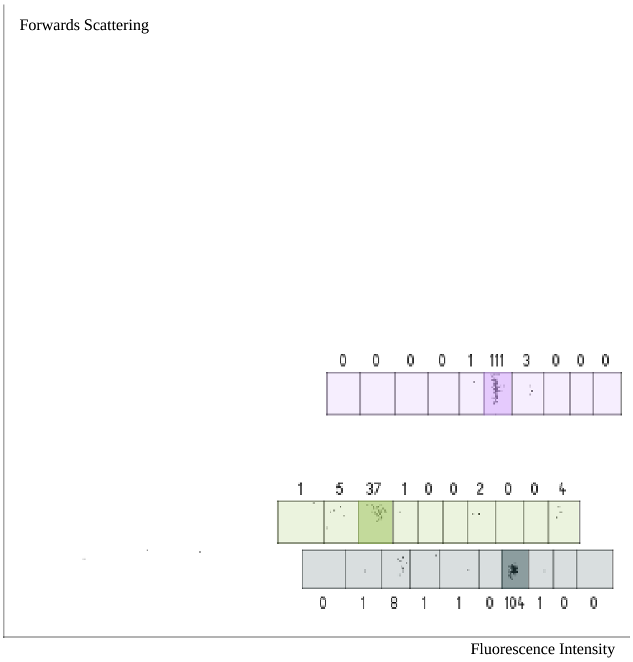

ANNEX 3: TAG DECONVOLUTION - BEAD 223

Passes flow sorting criteria: Yes

Passes tag deconvolution criteria: Yes

Included in protocol analysis: Yes

Protocol: 5, 9, 5, 2

Filename: Bin2_plateA2_D5.fcs

Split 1: Petrol shading

Split 2: Green shading

Split 3: Violet shading

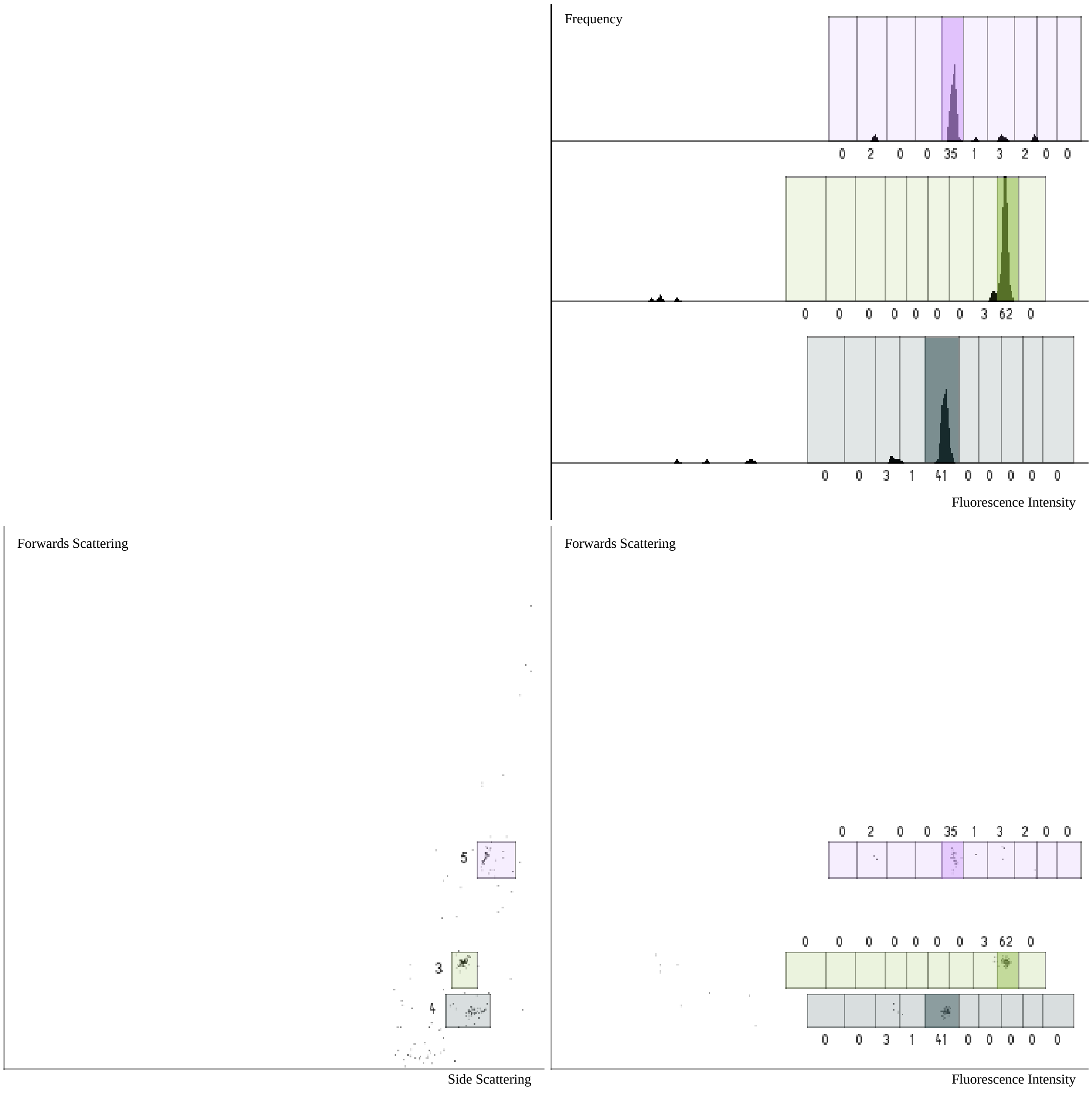

ANNEX 3: TAG DECONVOLUTION - BEAD 224

Passes flow sorting criteria: Yes

Passes tag deconvolution criteria: Yes

Included in protocol analysis: Yes

Protocol: 8, 2, 2, 2

Filename: Bin2_plateA2_D6.fcs

Split 1: Petrol shading

Split 2: Green shading

Split 3: Violet shading

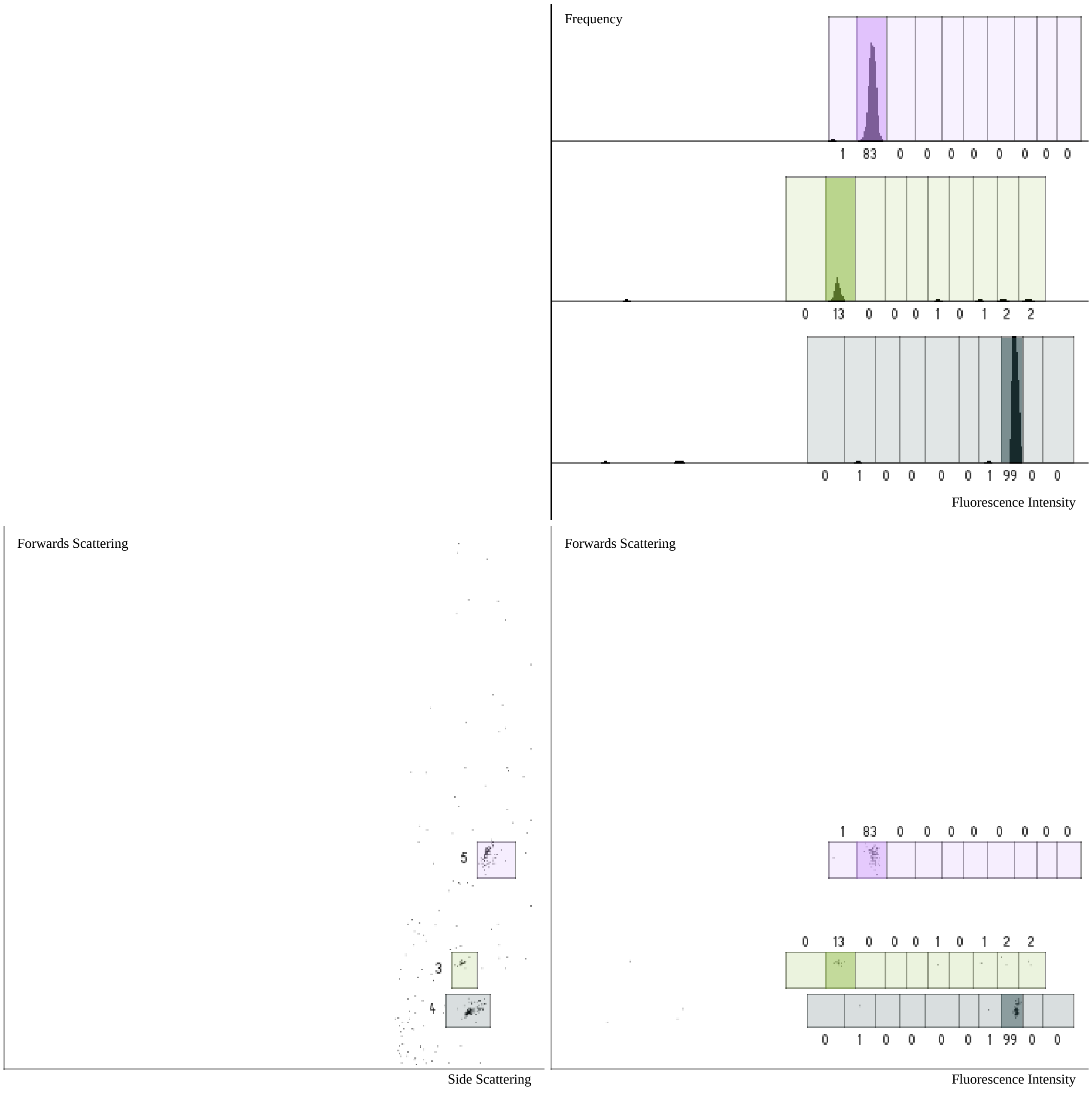

ANNEX 3: TAG DECONVOLUTION - BEAD 225

Passes flow sorting criteria: Yes

Passes tag deconvolution criteria: No

Included in protocol analysis: No

Protocol: N/A

Filename: Bin2_plateA2_D7.fcs

Split 1: Petrol shading

Split 2: Green shading

Split 3: Violet shading

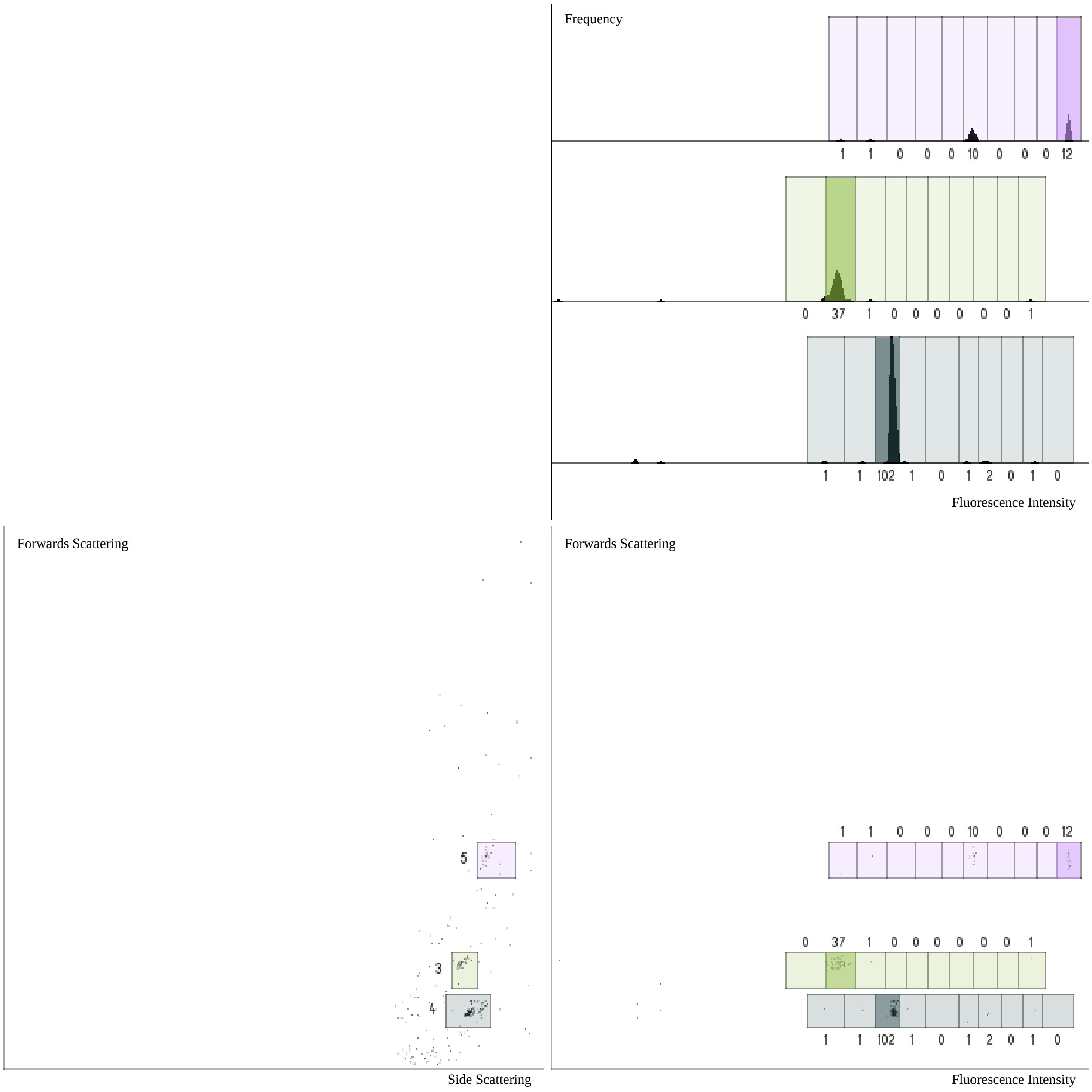

ANNEX 3: TAG DECONVOLUTION - BEAD 226

Passes flow sorting criteria: Yes

Passes tag deconvolution criteria: No

Included in protocol analysis: No

Protocol: N/A

Filename: Bin2_plateA2_D8.fcs

Split 1: Petrol shading

Split 2: Green shading

Split 3: Violet shading

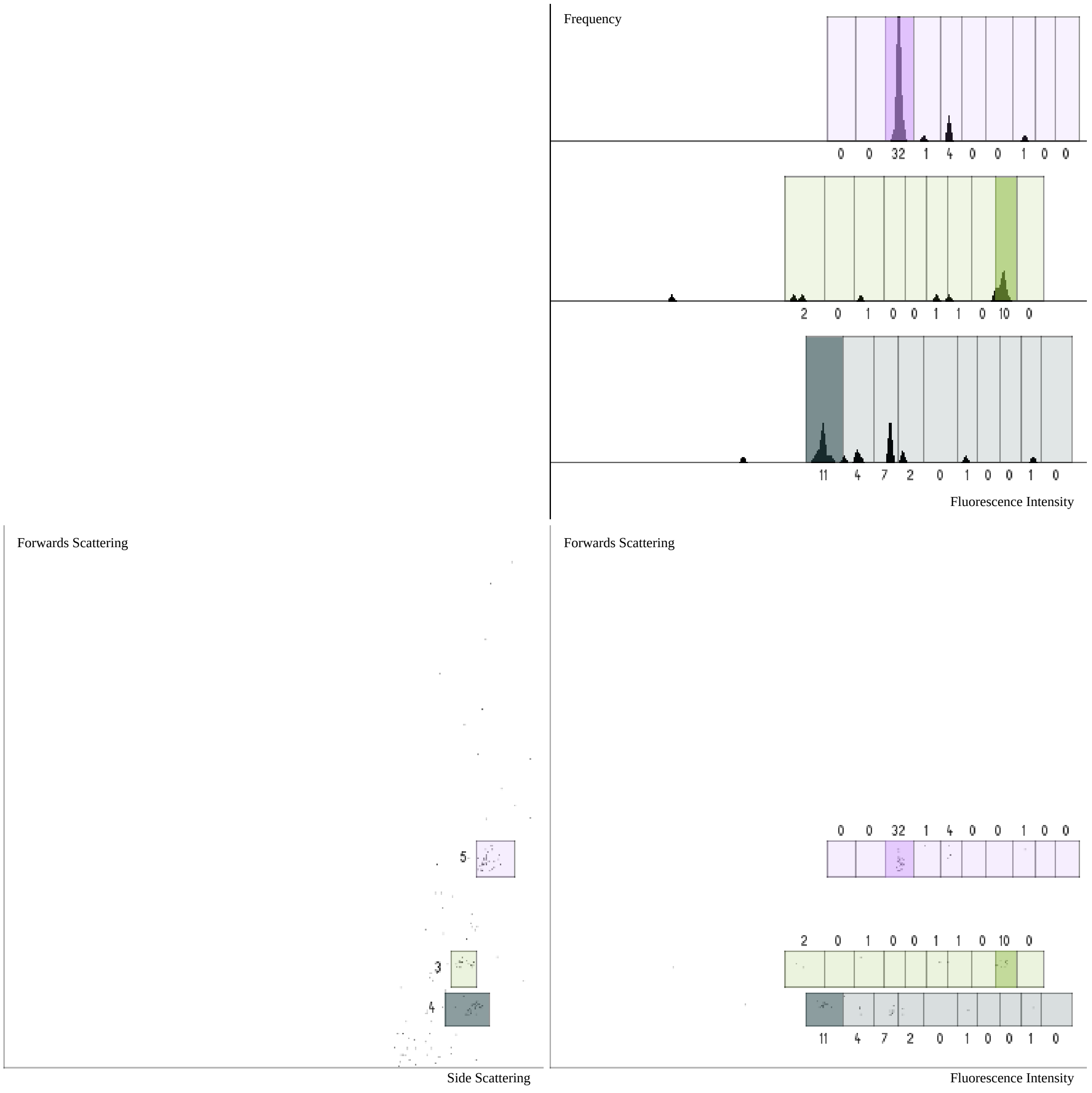

ANNEX 3: TAG DECONVOLUTION - BEAD 227

Passes flow sorting criteria: Yes

Passes tag deconvolution criteria: Yes

Included in protocol analysis: Yes

Protocol: 1, 5, 6, 2

Filename: Bin2_plateA2_D9.fcs

Split 1: Petrol shading

Split 2: Green shading

Split 3: Violet shading

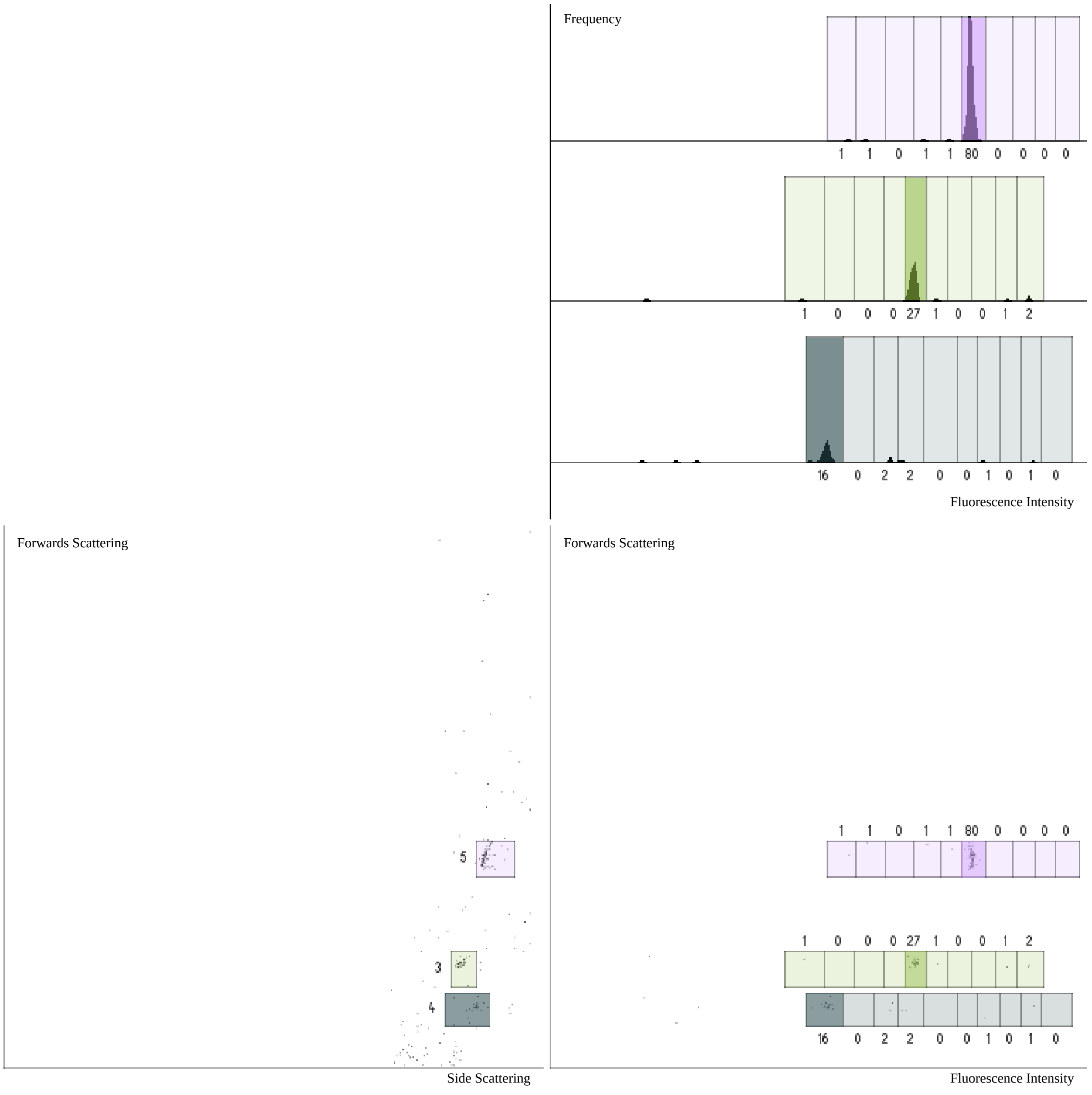

ANNEX 3: TAG DECONVOLUTION - BEAD 228

Passes flow sorting criteria: Yes

Passes tag deconvolution criteria: Yes

Included in protocol analysis: Yes

Protocol: 5, 2, 2, 2

Filename: Bin2_plateA2_D10.fcs

Split 1: Petrol shading

Split 2: Green shading

Split 3: Violet shading

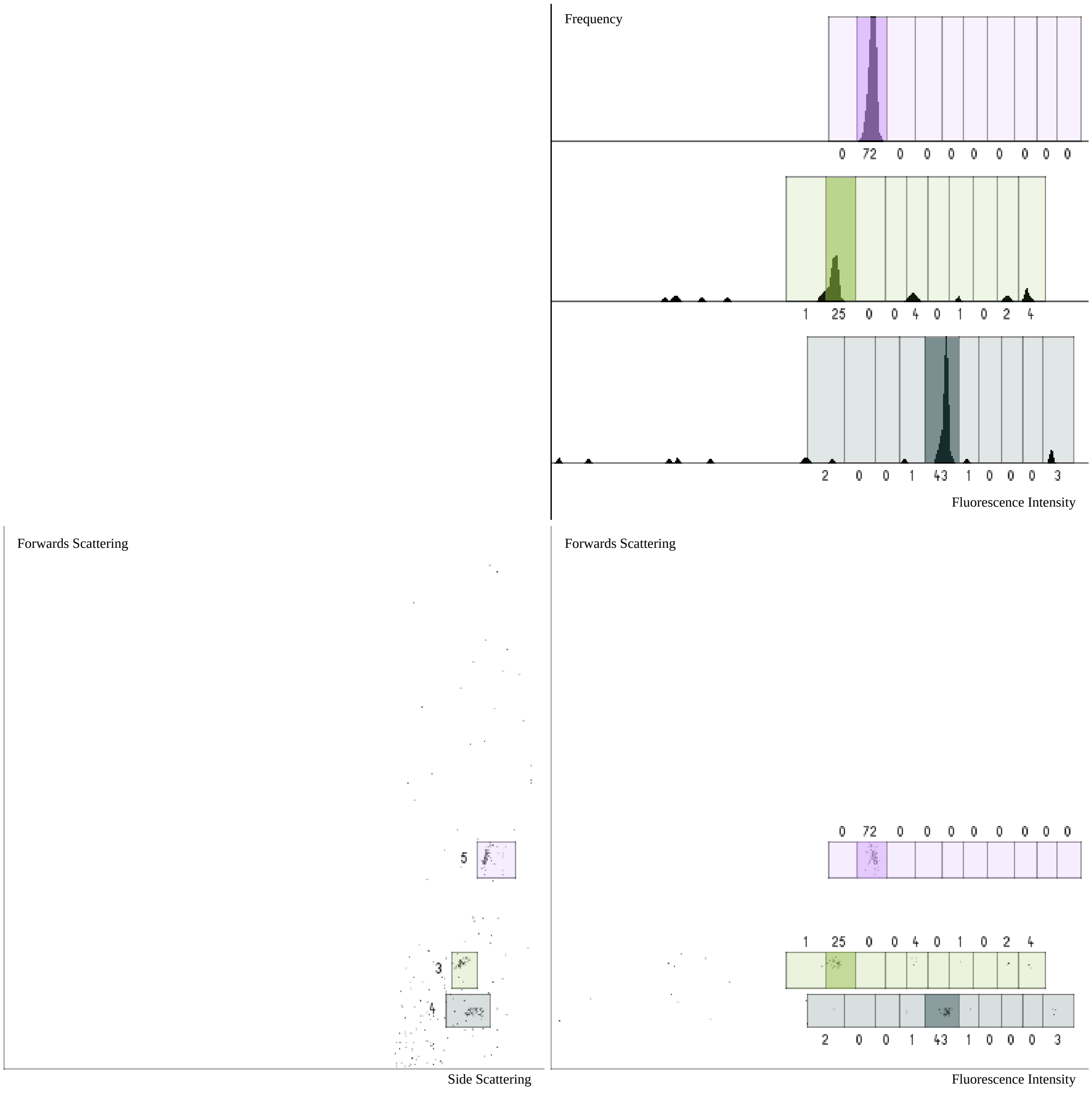

ANNEX 3: TAG DECONVOLUTION - BEAD 229

Passes flow sorting criteria: Yes

Passes tag deconvolution criteria: Yes

Included in protocol analysis: Yes

Protocol: 6, 5, 8, 2

Filename: Bin2_plateA2_D11.fcs

Split 1: Petrol shading

Split 2: Green shading

Split 3: Violet shading

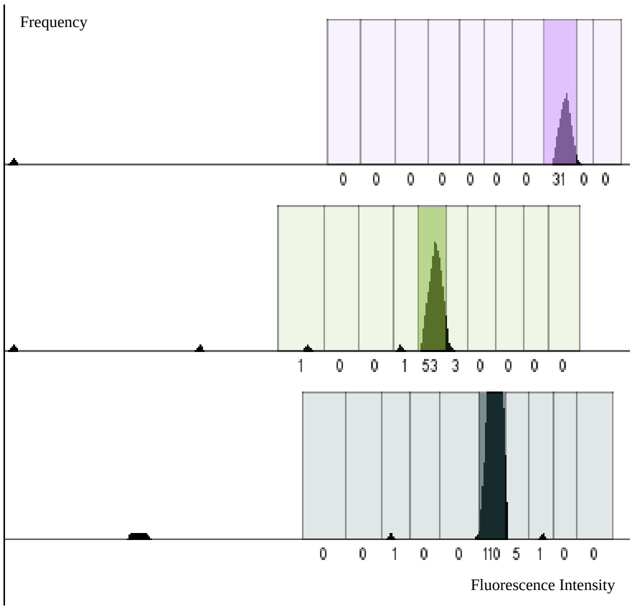

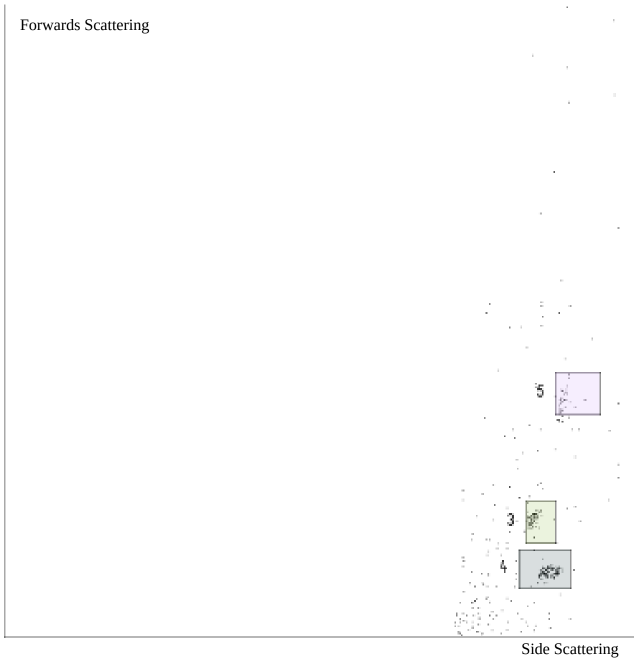

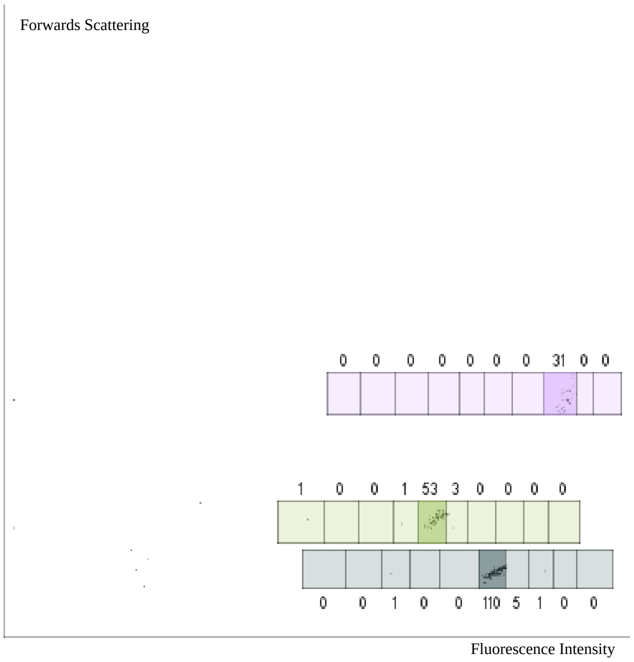

ANNEX 3: TAG DECONVOLUTION - BEAD 230

Passes flow sorting criteria: Yes

Passes tag deconvolution criteria: No

Included in protocol analysis: No

Protocol: N/A

Filename: Bin2_plateA2_D12.fcs

Split 1: Petrol shading

Split 2: Green shading

Split 3: Violet shading

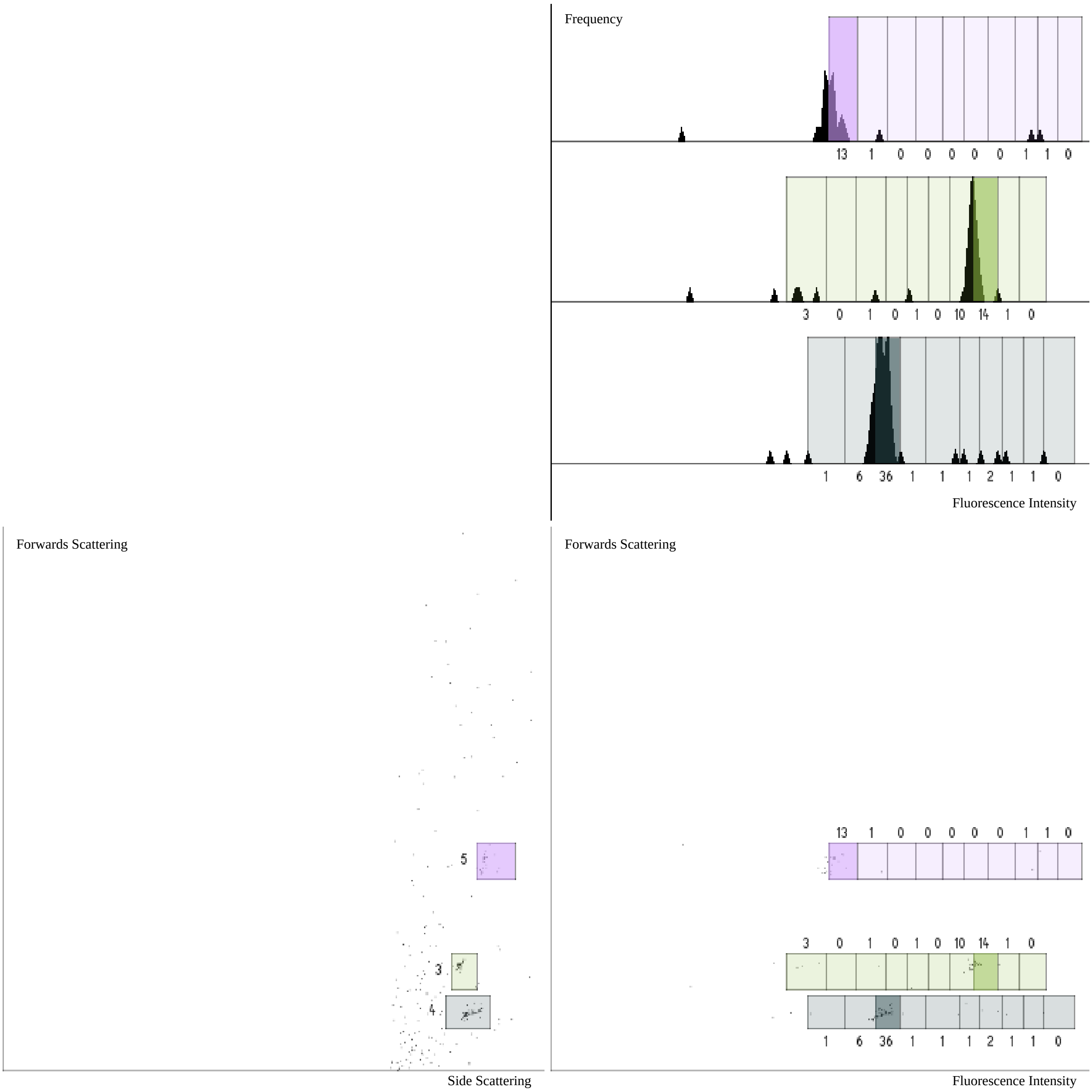

ANNEX 3: TAG DECONVOLUTION - BEAD 231

Passes flow sorting criteria: Yes

Passes tag deconvolution criteria: No

Included in protocol analysis: No

Protocol: N/A

Filename: Bin2_plateA2_E1.fcs

Split 1: Petrol shading

Split 2: Green shading

Split 3: Violet shading

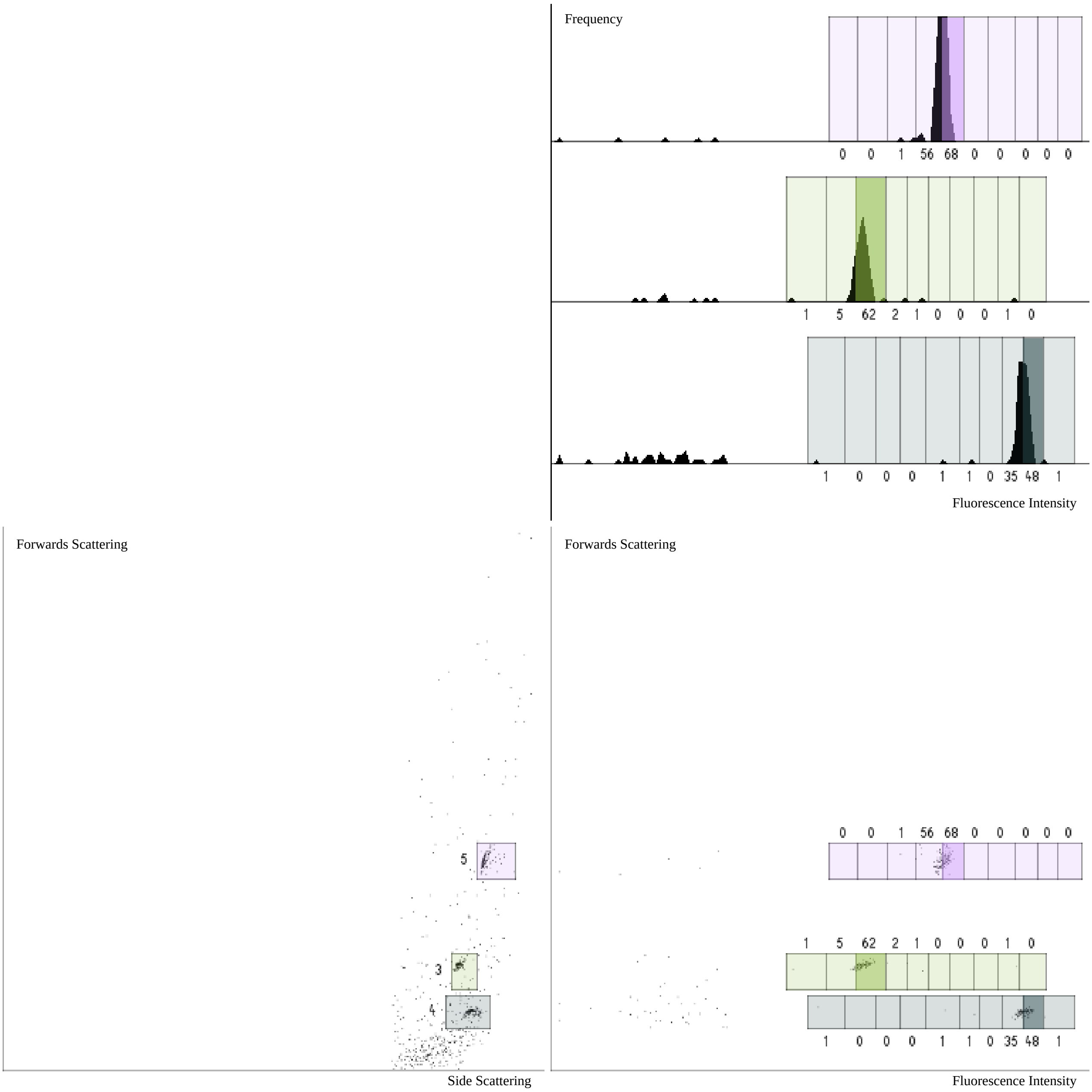

ANNEX 3: TAG DECONVOLUTION - BEAD 232

Passes flow sorting criteria: Yes

Passes tag deconvolution criteria: No

Included in protocol analysis: No

Protocol: N/A

Filename: Bin2_plateA2_E2.fcs

Split 1: Petrol shading

Split 2: Green shading

Split 3: Violet shading

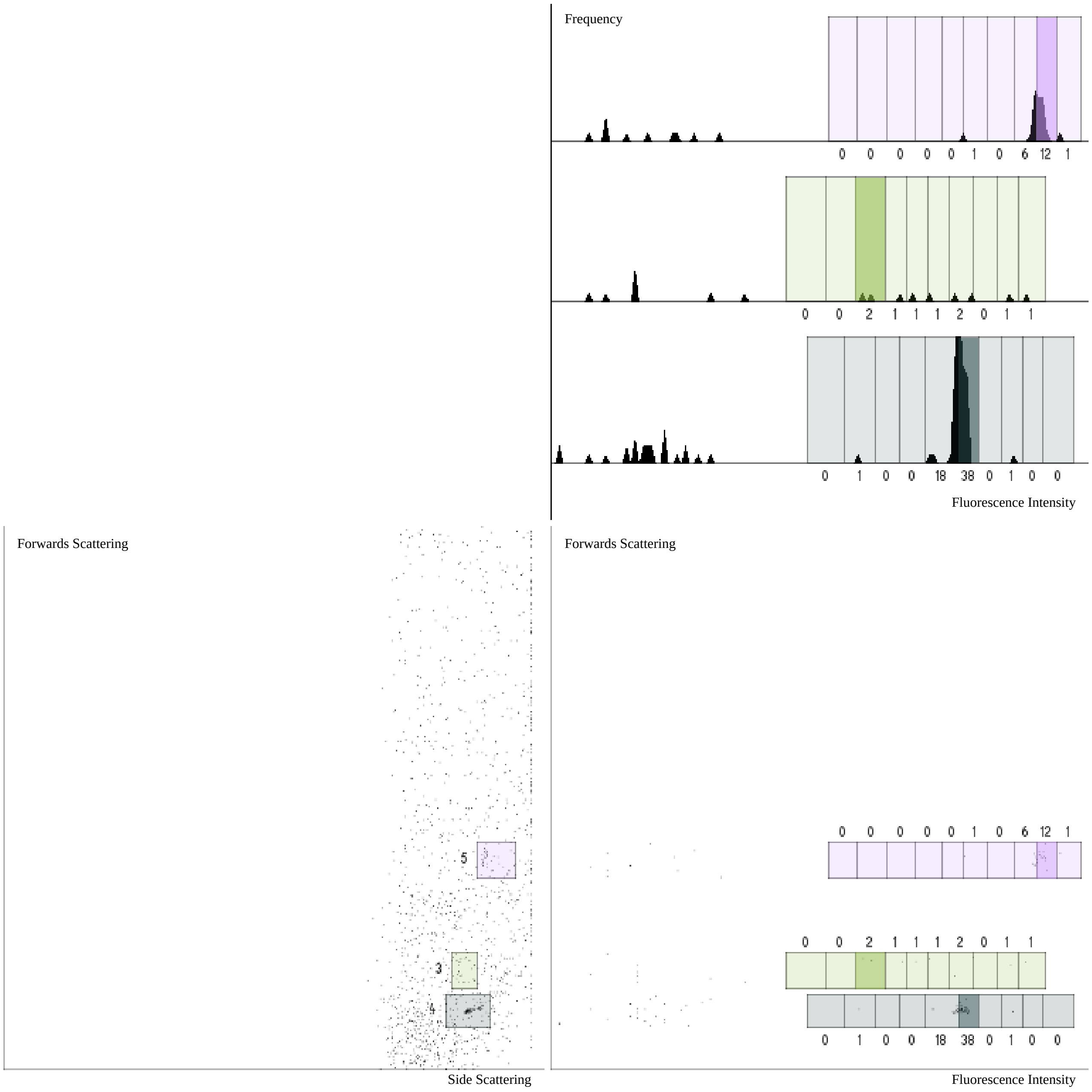

ANNEX 3: TAG DECONVOLUTION - BEAD 233

Passes flow sorting criteria: Yes

Passes tag deconvolution criteria: No

Included in protocol analysis: No

Protocol: N/A

Filename: Bin2_plateA2_E3.fcs

Split 1: Petrol shading

Split 2: Green shading

Split 3: Violet shading

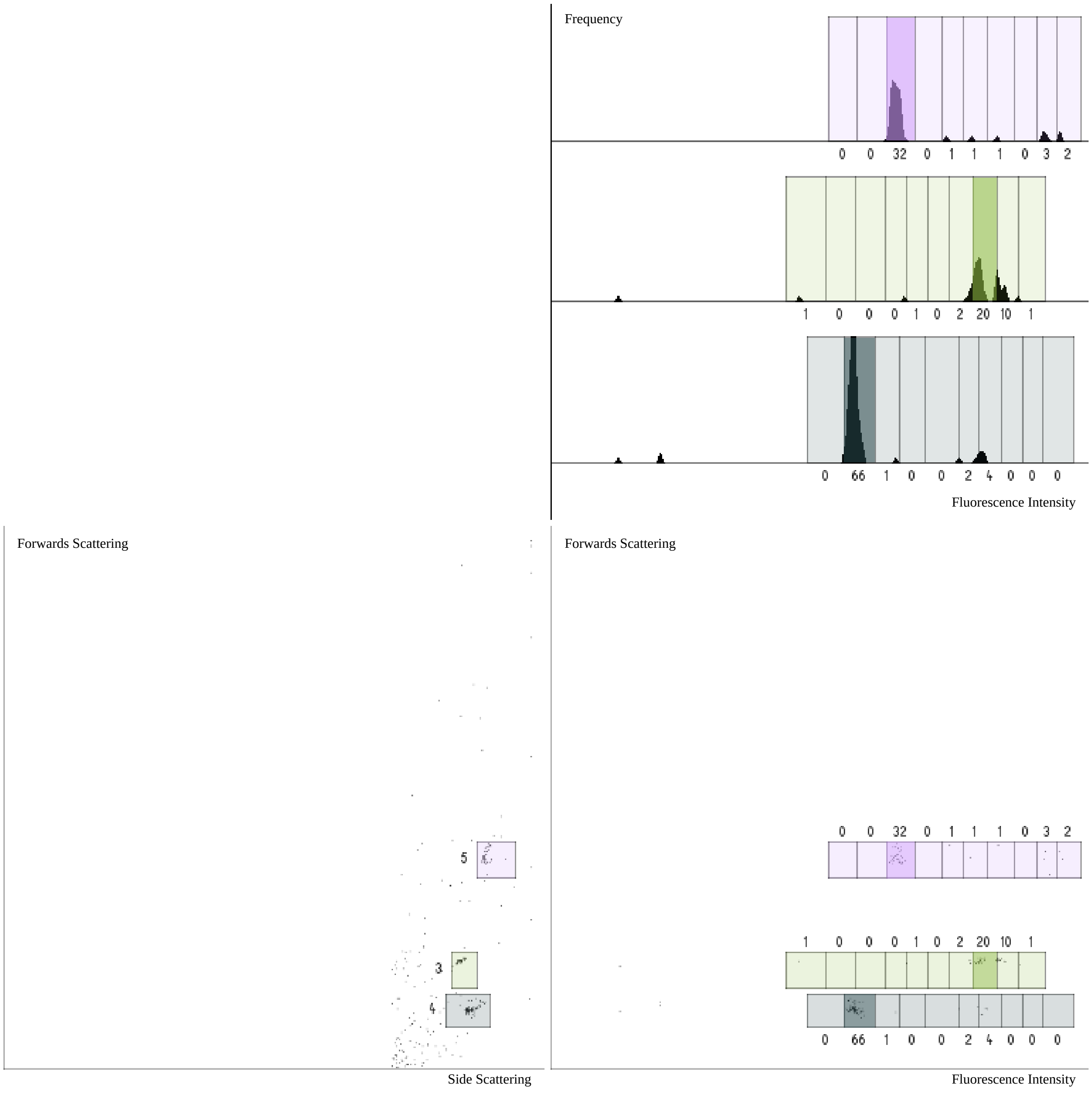

ANNEX 3: TAG DECONVOLUTION - BEAD 234

Passes flow sorting criteria: Yes

Passes tag deconvolution criteria: Yes

Included in protocol analysis: Yes

Protocol: 6, 2, 9, 2

Filename: Bin2_plateA2_E4.fcs

Split 1: Petrol shading

Split 2: Green shading

Split 3: Violet shading

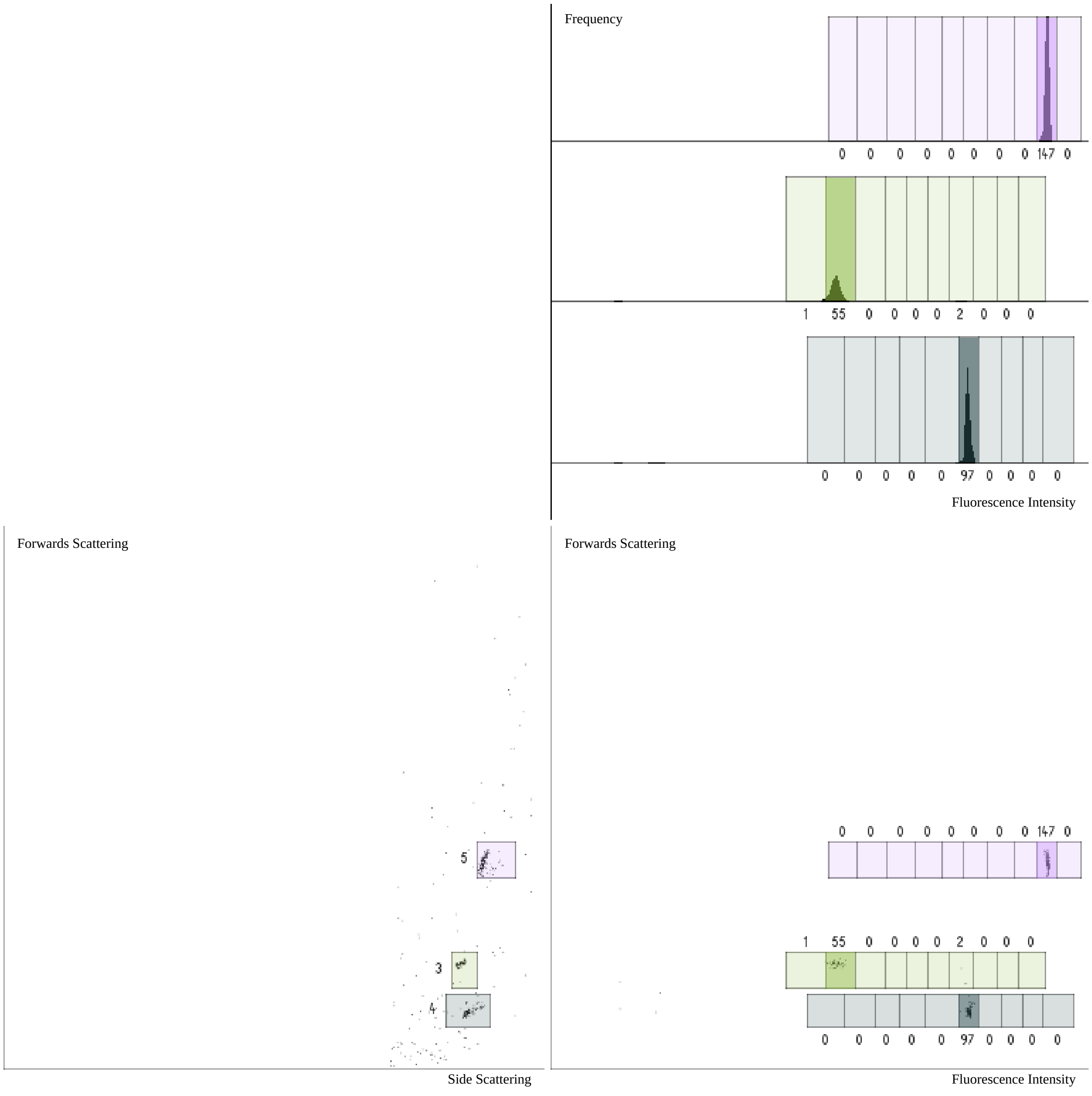

ANNEX 3: TAG DECONVOLUTION - BEAD 235

Passes flow sorting criteria: Yes

Passes tag deconvolution criteria: Yes

Included in protocol analysis: Yes

Protocol: 1, 6, 5, 2

Filename: Bin2_plateA2_E5.fcs

Split 1: Petrol shading

Split 2: Green shading

Split 3: Violet shading

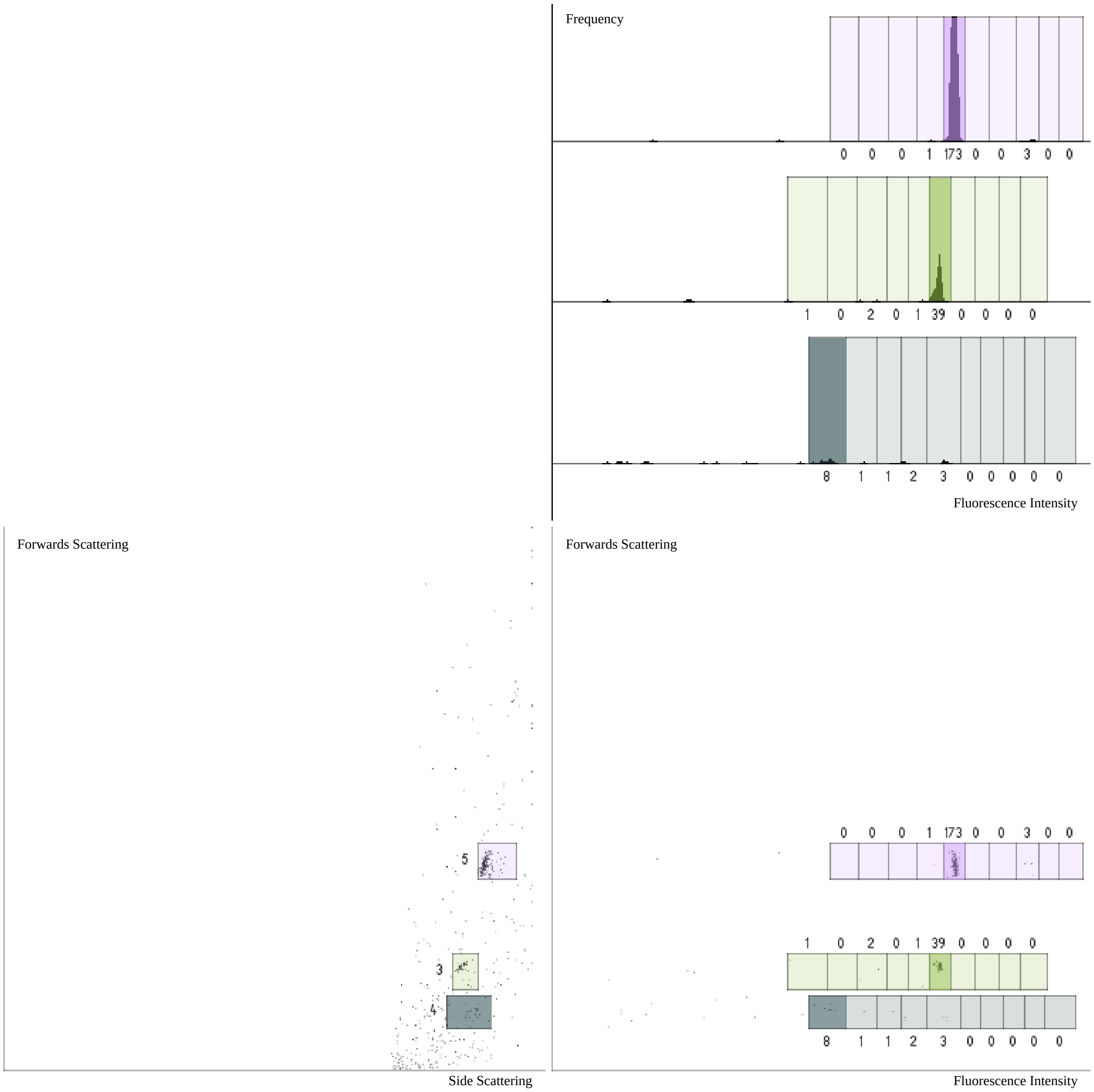

ANNEX 3: TAG DECONVOLUTION - BEAD 236

Passes flow sorting criteria: Yes

Passes tag deconvolution criteria: Yes

Included in protocol analysis: Yes

Protocol: 8, 3, 2, 2

Filename: Bin2_plateA2_E6.fcs

Split 1: Petrol shading

Split 2: Green shading

Split 3: Violet shading

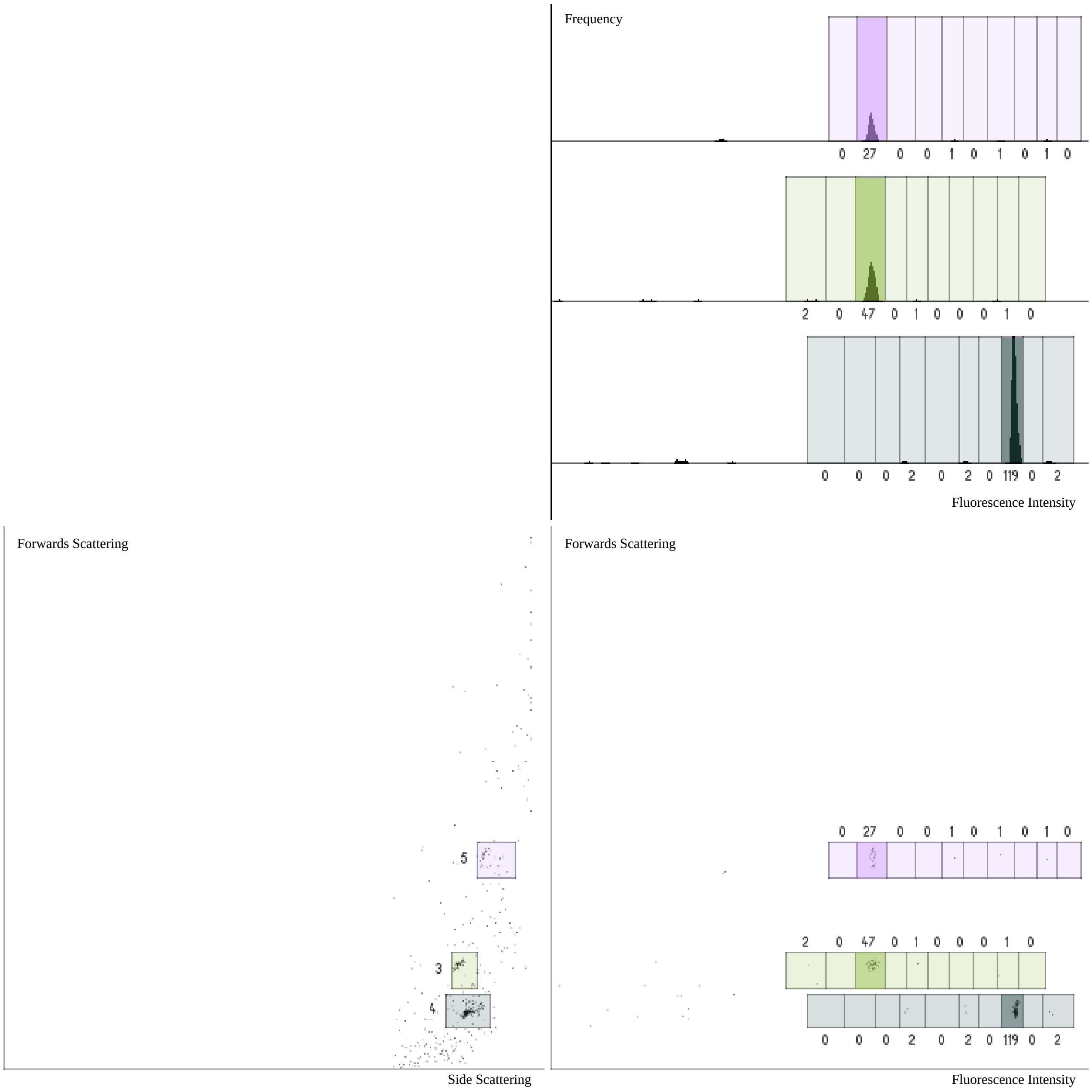

ANNEX 3: TAG DECONVOLUTION - BEAD 237

Passes flow sorting criteria: Yes

Passes tag deconvolution criteria: Yes

Included in protocol analysis: Yes

Protocol: 3, 5, 8, 2

Filename: Bin2_plateA2_E7.fcs

Split 1: Petrol shading

Split 2: Green shading

Split 3: Violet shading

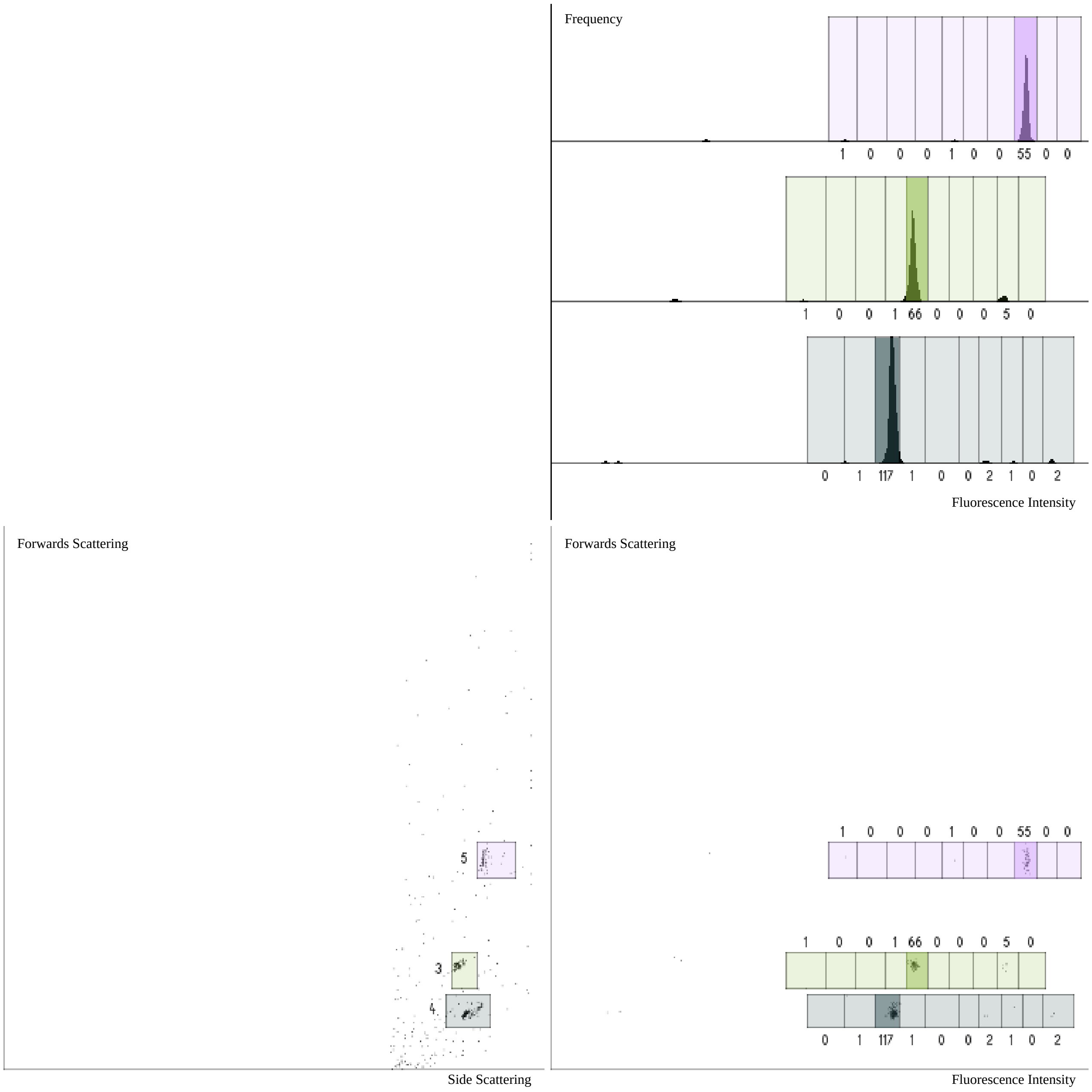

ANNEX 3: TAG DECONVOLUTION - BEAD 238

Passes flow sorting criteria: Yes

Passes tag deconvolution criteria: Yes

Included in protocol analysis: Yes

Protocol: 6, 5, 2, 2

Filename: Bin2_plateA2_G8.fcs

Split 1: Petrol shading

Split 2: Green shading

Split 3: Violet shading

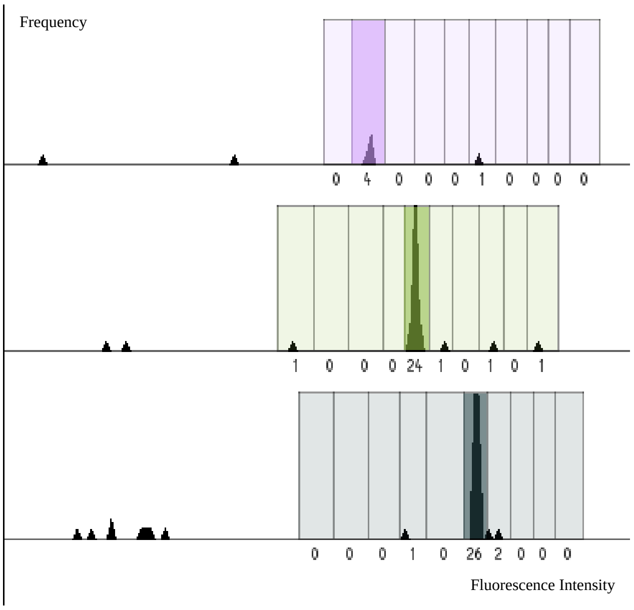

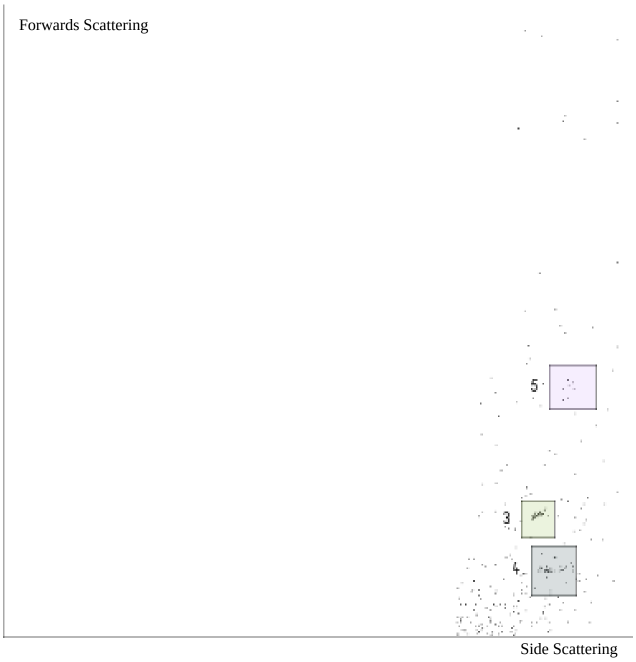

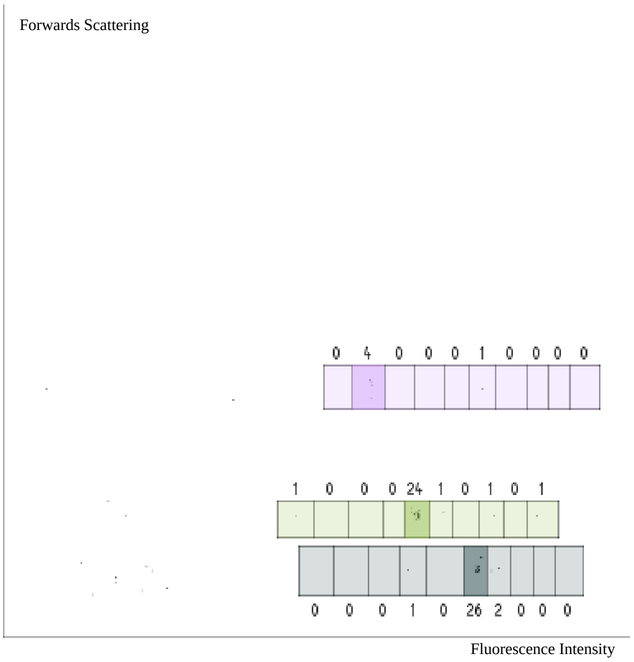

ANNEX 3: TAG DECONVOLUTION - BEAD 239

Passes flow sorting criteria: Yes

Passes tag deconvolution criteria: Yes

Included in protocol analysis: Yes

Protocol: 5, 7, 6, 2

Filename: Bin2_plateA2_E9.fcs

Split 1: Petrol shading

Split 2: Green shading

Split 3: Violet shading

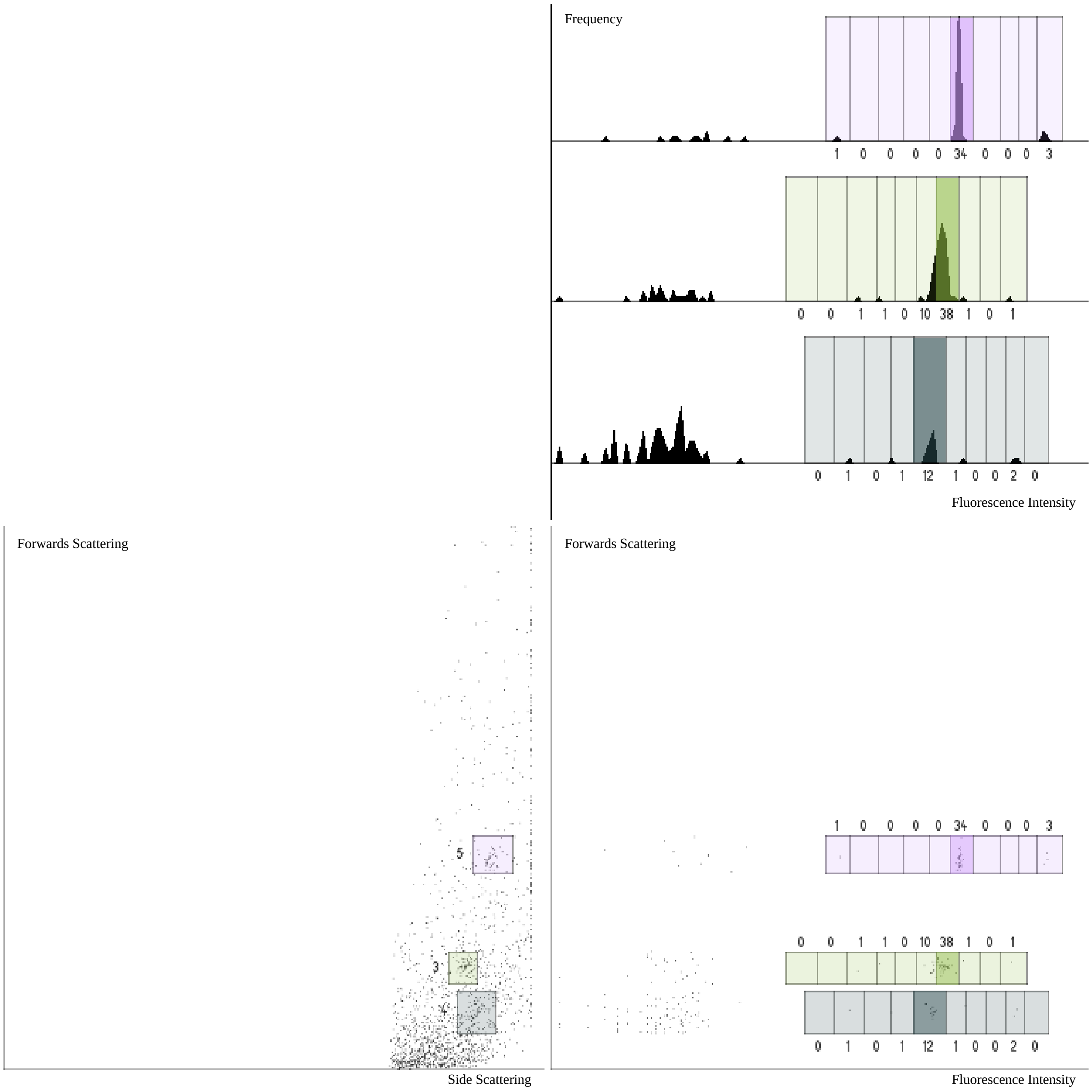

ANNEX 3: TAG DECONVOLUTION - BEAD 240

Passes flow sorting criteria: Yes

Passes tag deconvolution criteria: Yes

Included in protocol analysis: Yes

Protocol: 10, 8, 9, 2

Filename: Bin2_plateA2_E10.fcs

Split 1: Petrol shading

Split 2: Green shading

Split 3: Violet shading

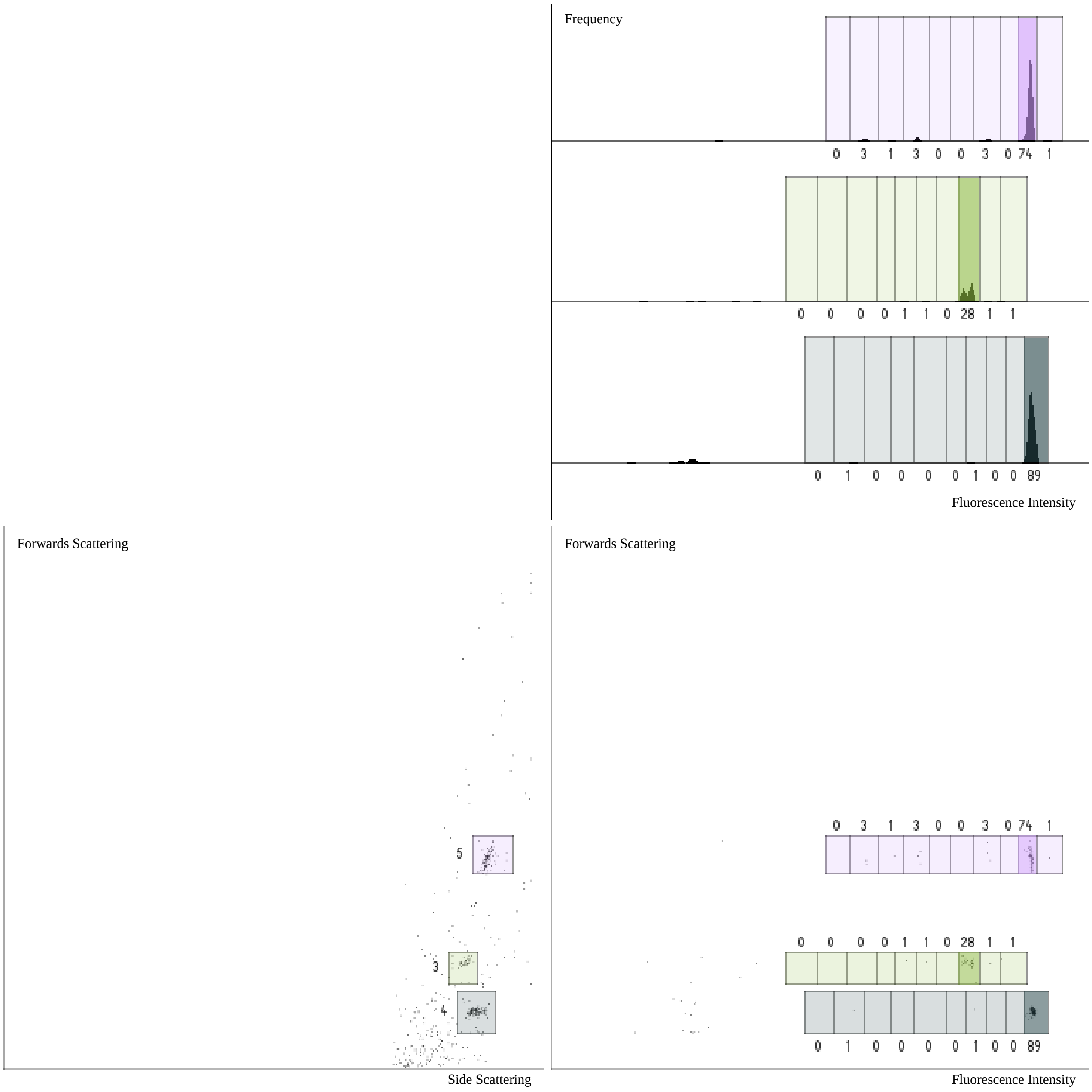

ANNEX 3: TAG DECONVOLUTION - BEAD 241

Passes flow sorting criteria: Yes

Passes tag deconvolution criteria: No

Included in protocol analysis: No

Protocol: N/A

Filename: Bin2_plateA2_E11.fcs

Split 1: Petrol shading

Split 2: Green shading

Split 3: Violet shading

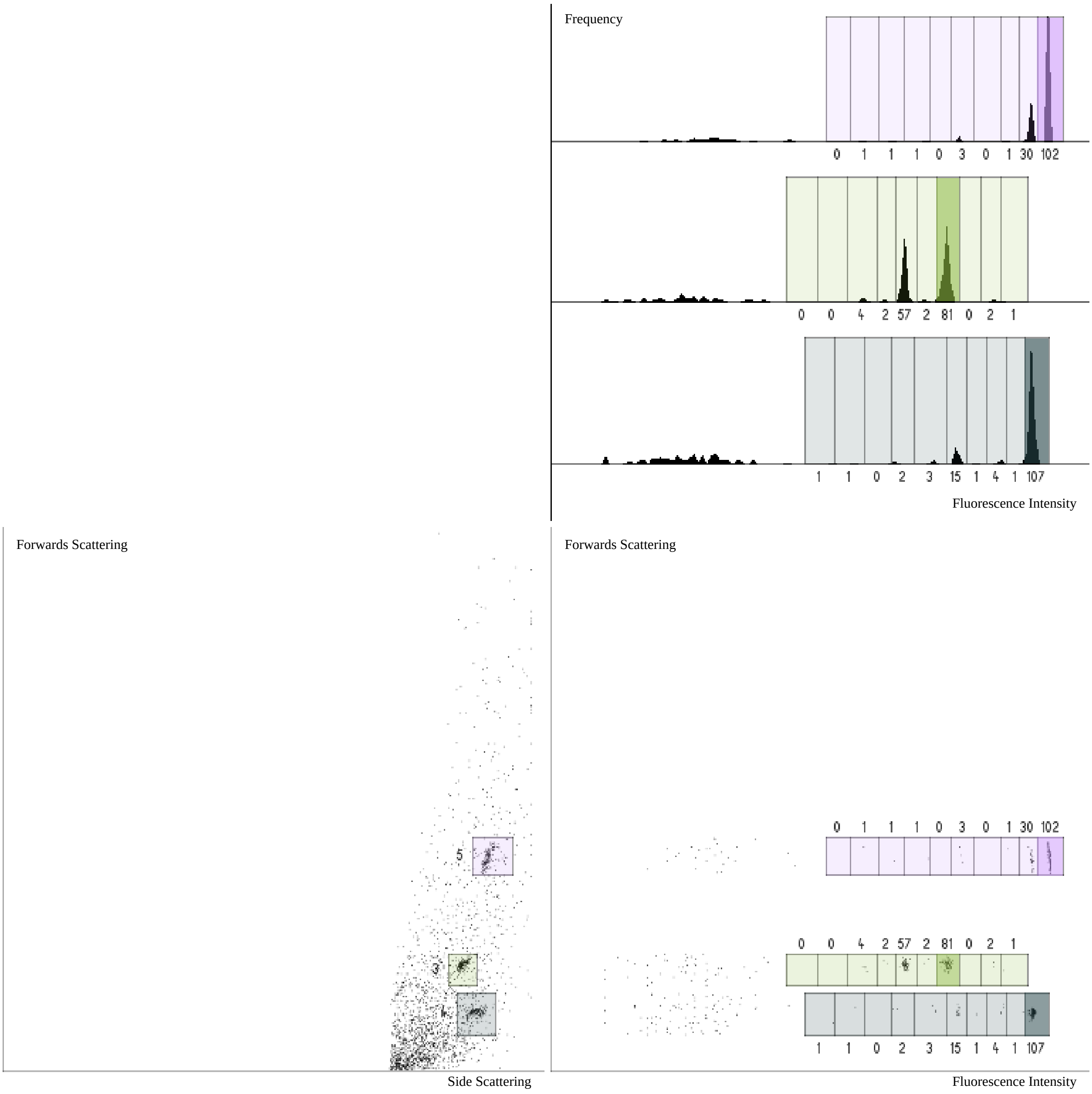

ANNEX 3: TAG DECONVOLUTION - BEAD 242

Passes flow sorting criteria: Yes

Passes tag deconvolution criteria: Yes

Included in protocol analysis: Yes

Protocol: 6, 4, 6, 2

Filename: Bin2_plateA2_E12.fcs

Split 1: Petrol shading

Split 2: Green shading

Split 3: Violet shading

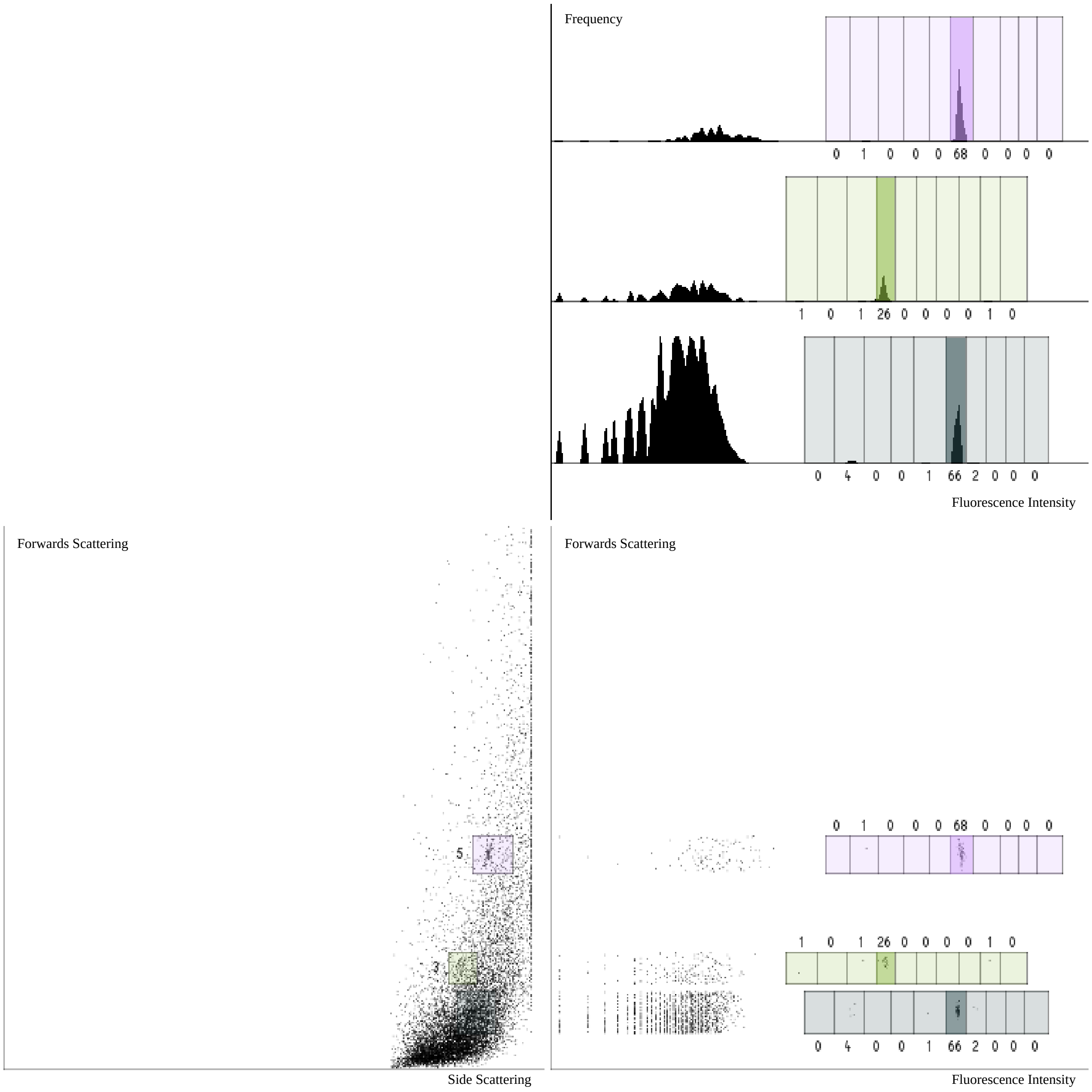

ANNEX 3: TAG DECONVOLUTION - BEAD 243

Passes flow sorting criteria: Yes

Passes tag deconvolution criteria: Yes

Included in protocol analysis: Yes

Protocol: 4, 4, 10, 2

Filename: Bin2_plateA2_F1.fcs

Split 1: Petrol shading

Split 2: Green shading

Split 3: Violet shading

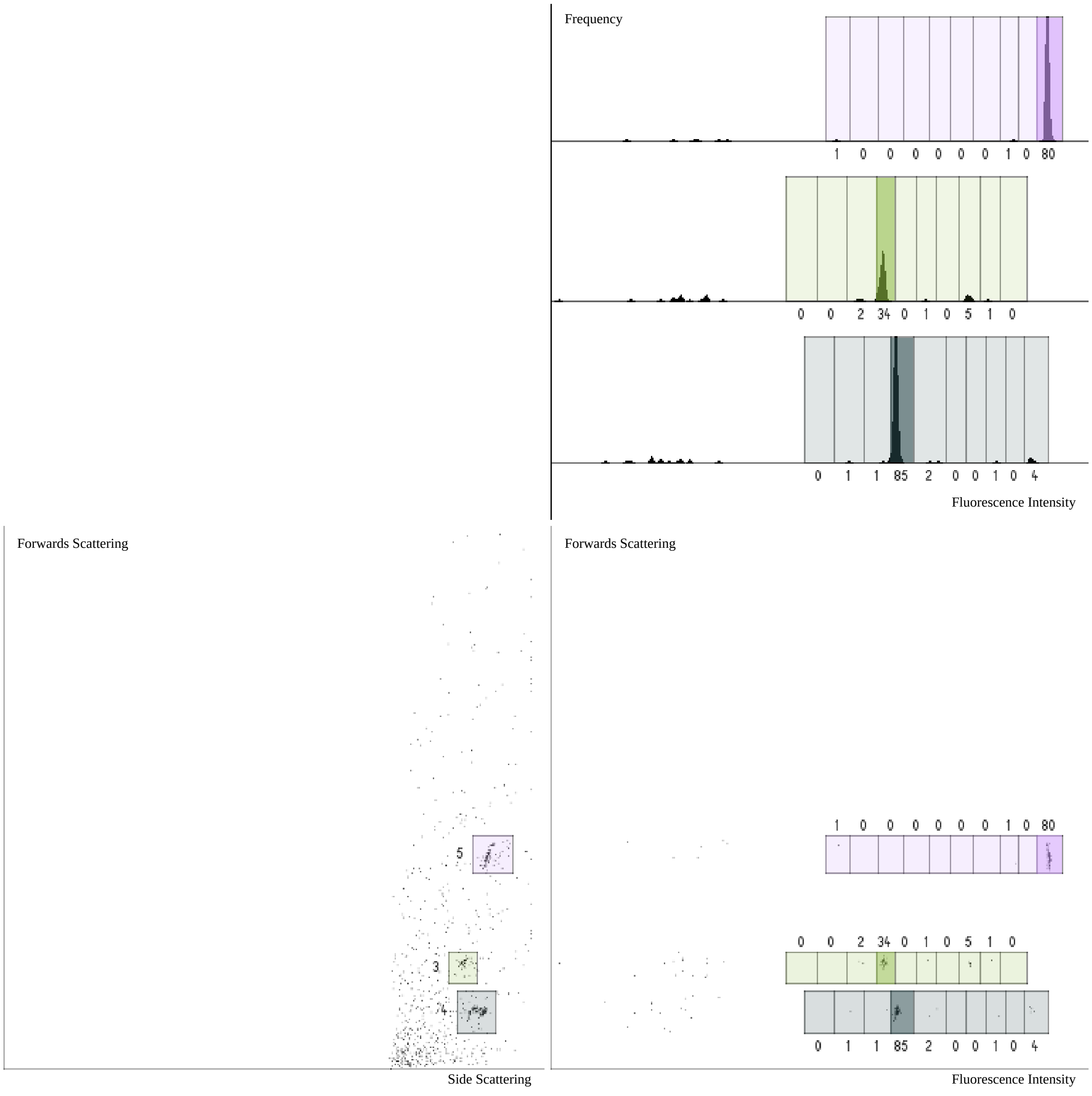

ANNEX 3: TAG DECONVOLUTION - BEAD 244

Passes flow sorting criteria: Yes

Passes tag deconvolution criteria: Yes

Included in protocol analysis: Yes

Protocol: 8, 10, 9, 2

Filename: Bin2_plateA2_F2.fcs

Split 1: Petrol shading

Split 2: Green shading

Split 3: Violet shading

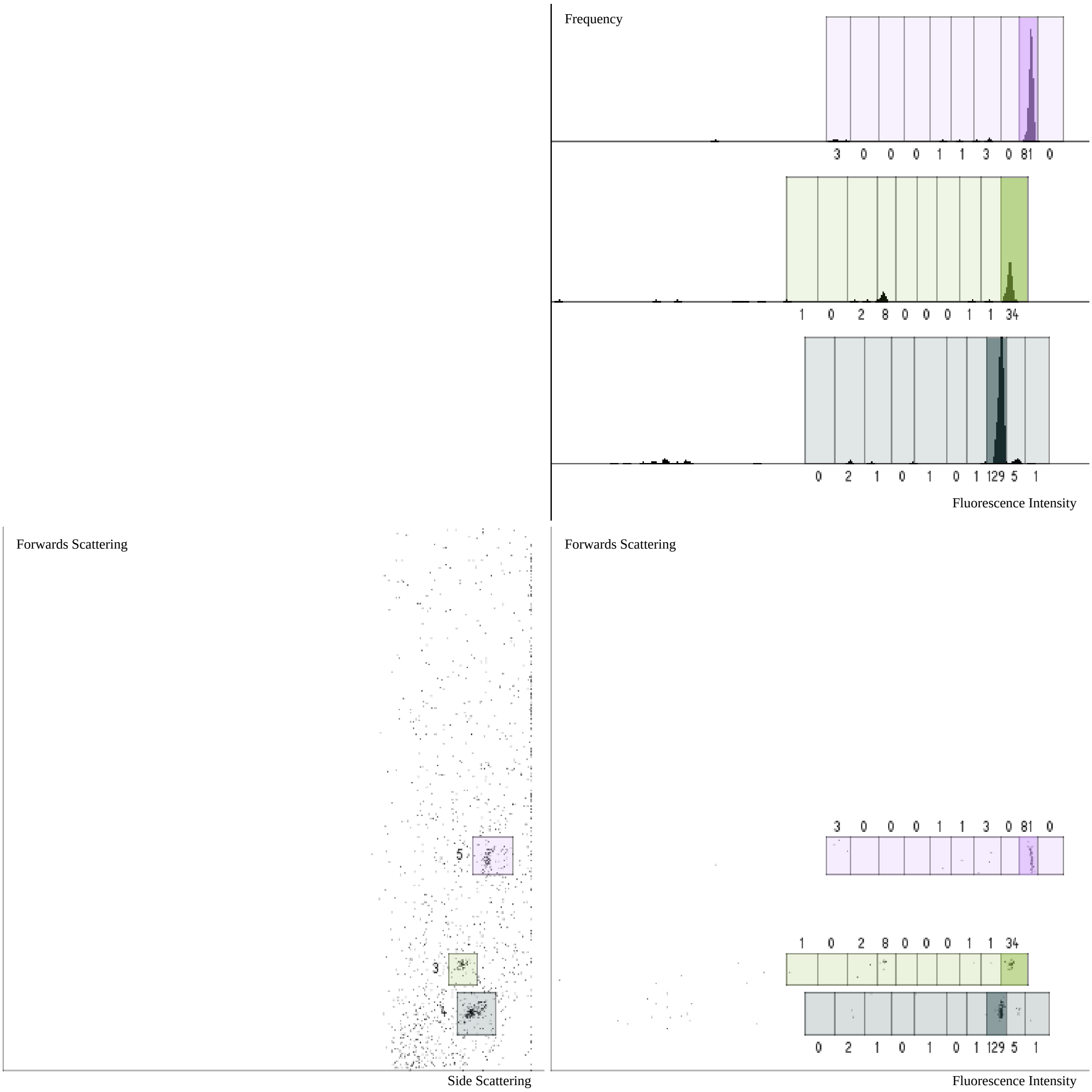

ANNEX 3: TAG DECONVOLUTION - BEAD 245

Passes flow sorting criteria: Yes

Passes tag deconvolution criteria: Yes

Included in protocol analysis: Yes

Protocol: 5, 4, 7, 2

Filename: Bin2_plateA2_F3.fcs

Split 1: Petrol shading

Split 2: Green shading

Split 3: Violet shading

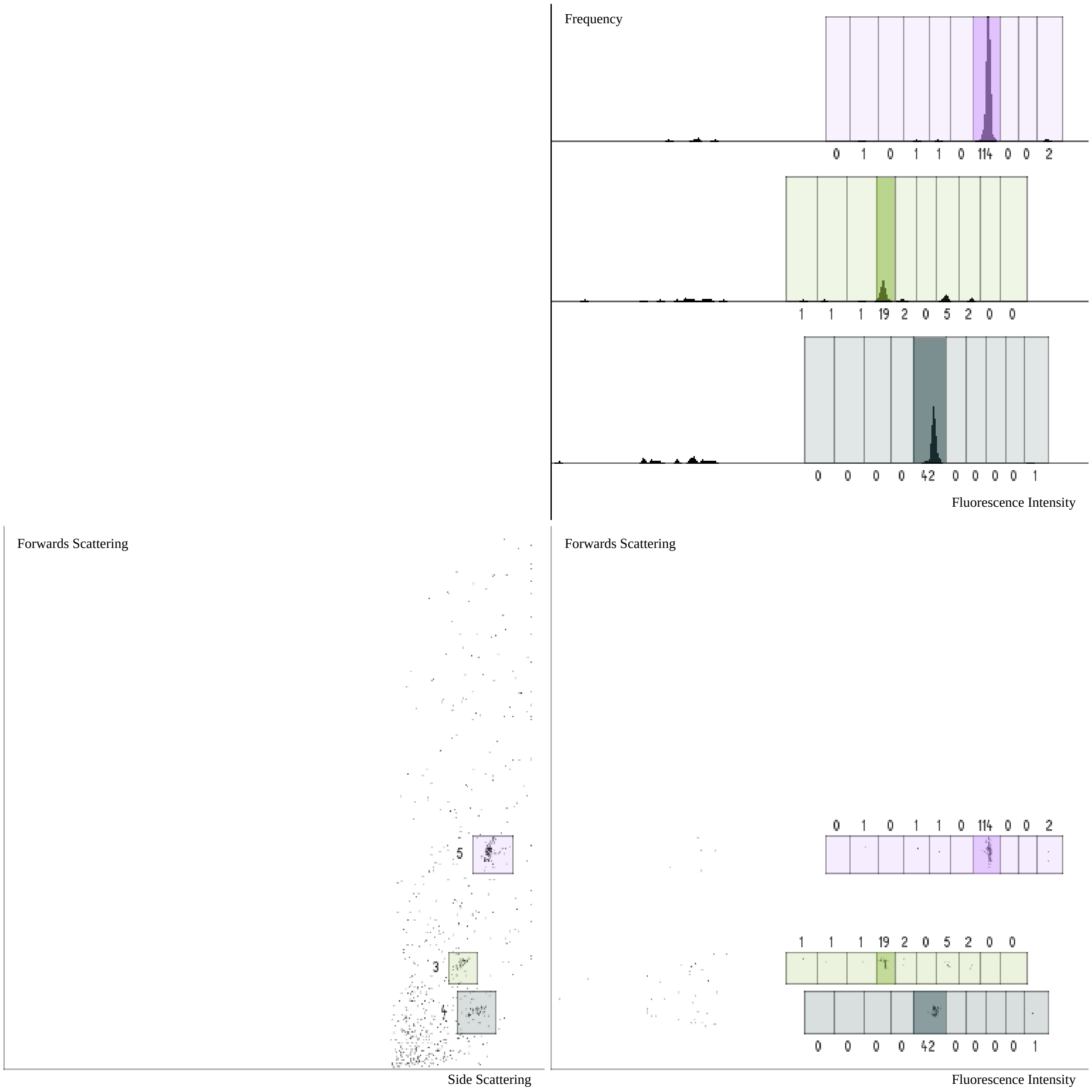

ANNEX 3: TAG DECONVOLUTION - BEAD 246

Passes flow sorting criteria: Yes

Passes tag deconvolution criteria: Yes

Included in protocol analysis: Yes

Protocol: 5, 9, 1, 2

Filename: Bin2_plateA2_F4.fcs

Split 1: Petrol shading

Split 2: Green shading

Split 3: Violet shading

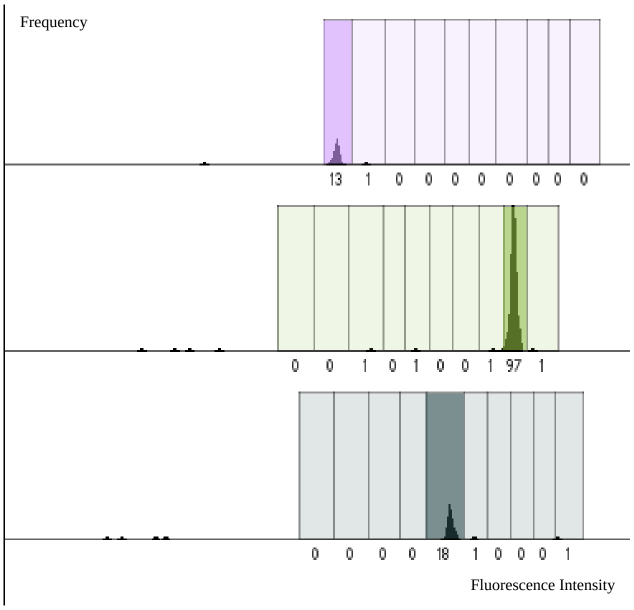

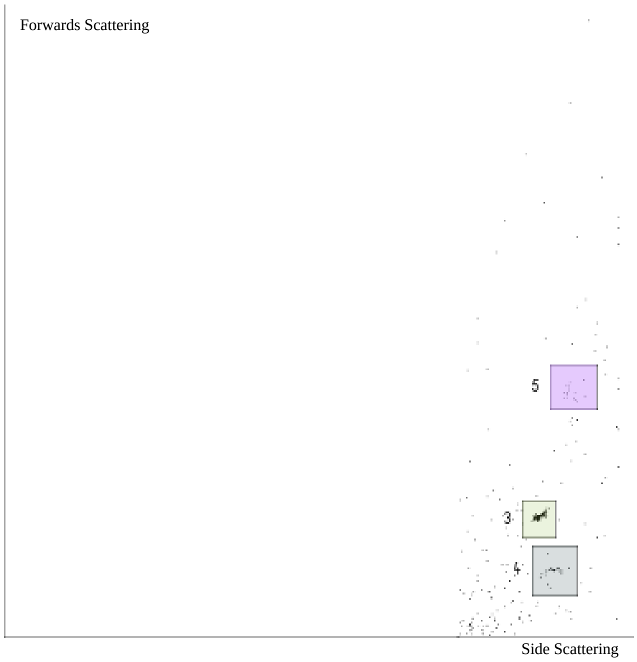

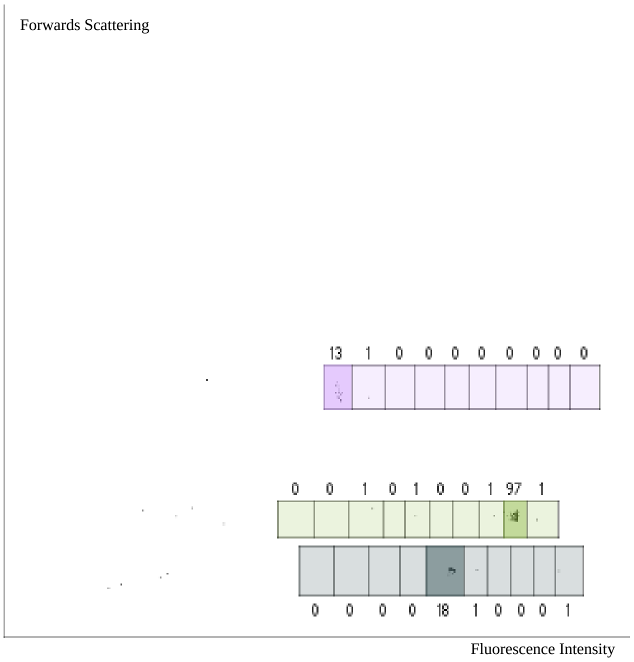

ANNEX 3: TAG DECONVOLUTION - BEAD 247

Passes flow sorting criteria: Yes

Passes tag deconvolution criteria: Yes

Included in protocol analysis: Yes

Protocol: 9, 10, 9, 2

Filename: Bin2_plateA2_F5.fcs

Split 1: Petrol shading

Split 2: Green shading

Split 3: Violet shading

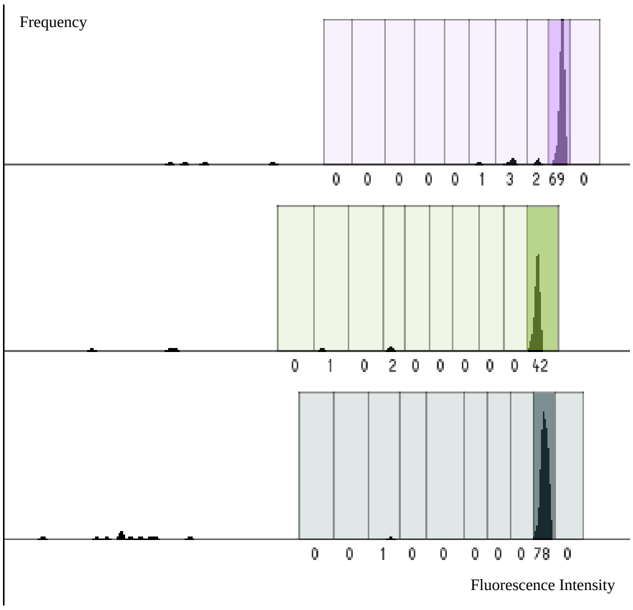

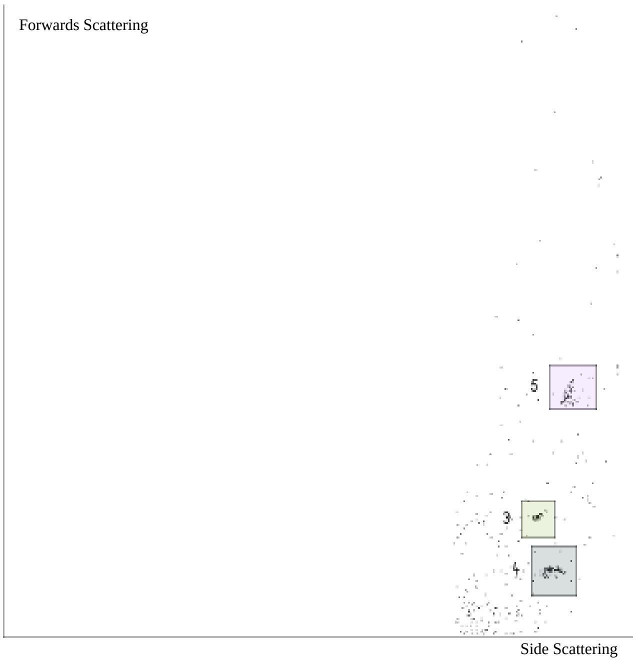

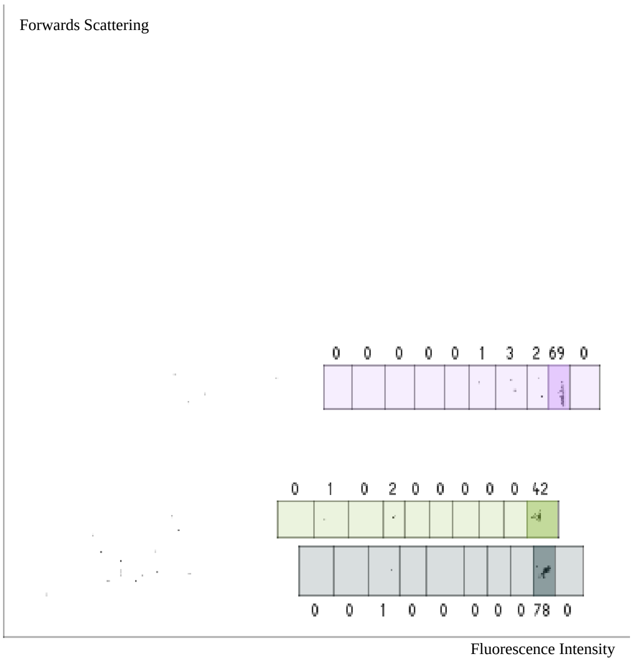

ANNEX 3: TAG DECONVOLUTION - BEAD 248

Passes flow sorting criteria: Yes

Passes tag deconvolution criteria: Yes

Included in protocol analysis: Yes

Protocol: 5, 6, 6, 2

Filename: Bin2_plateA2_F6.fcs

Split 1: Petrol shading

Split 2: Green shading

Split 3: Violet shading

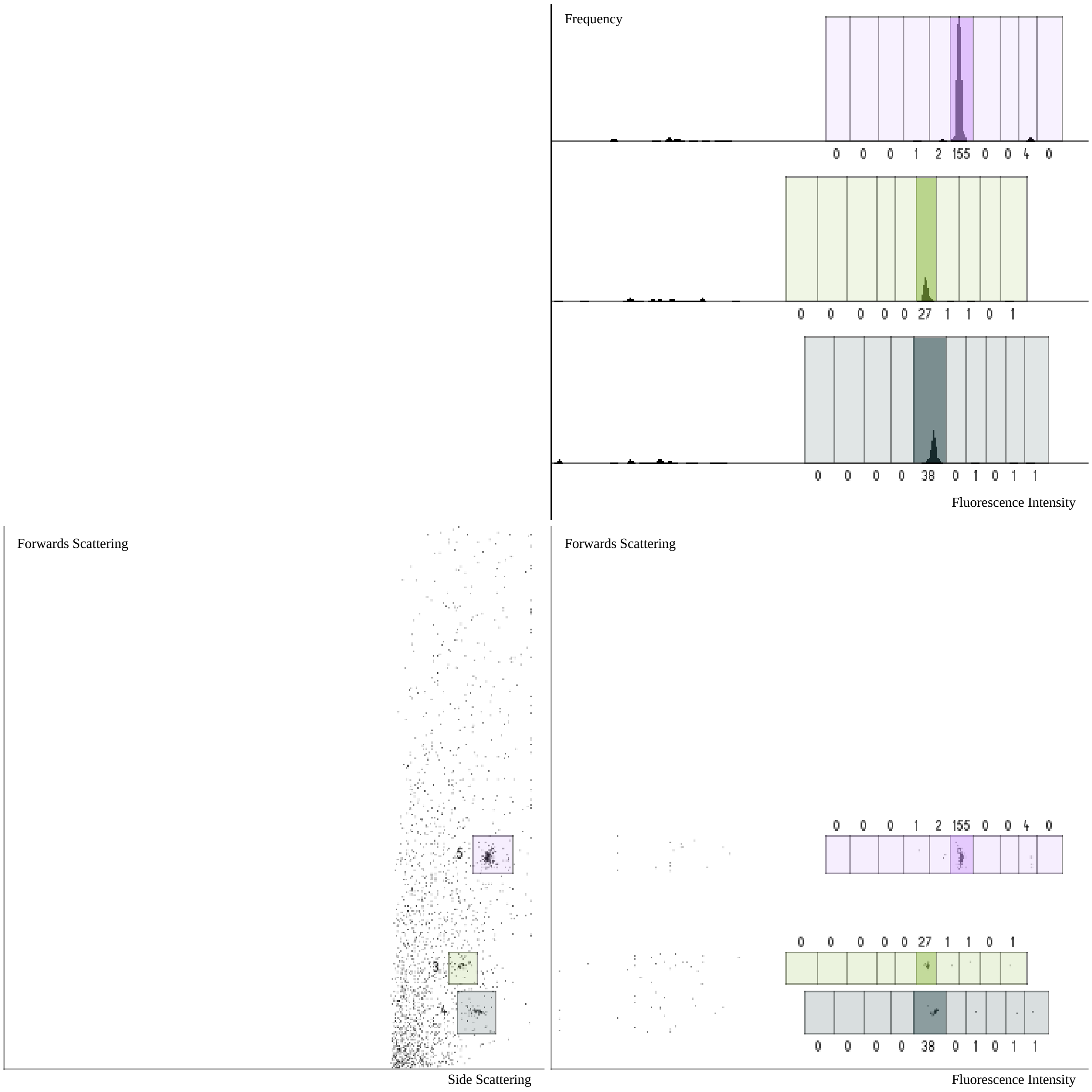

ANNEX 3: TAG DECONVOLUTION - BEAD 249

Passes flow sorting criteria: Yes

Passes tag deconvolution criteria: Yes

Included in protocol analysis: Yes

Protocol: 4, 10, 7, 2

Filename: Bin2_plateA2_F7.fcs

Split 1: Petrol shading

Split 2: Green shading

Split 3: Violet shading

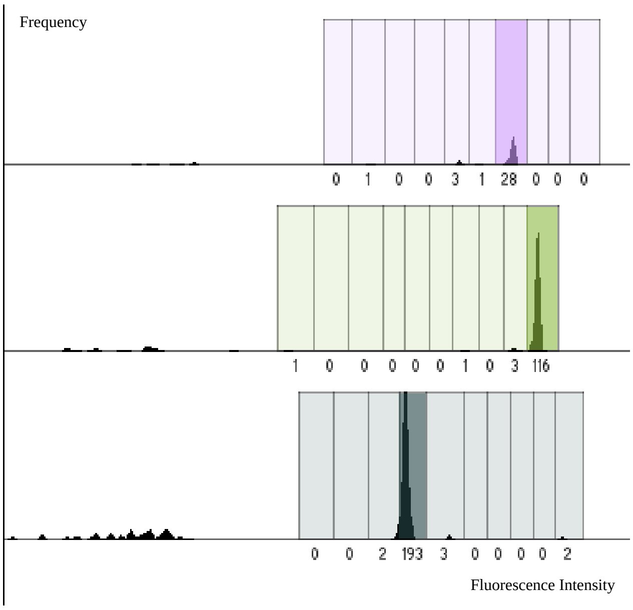

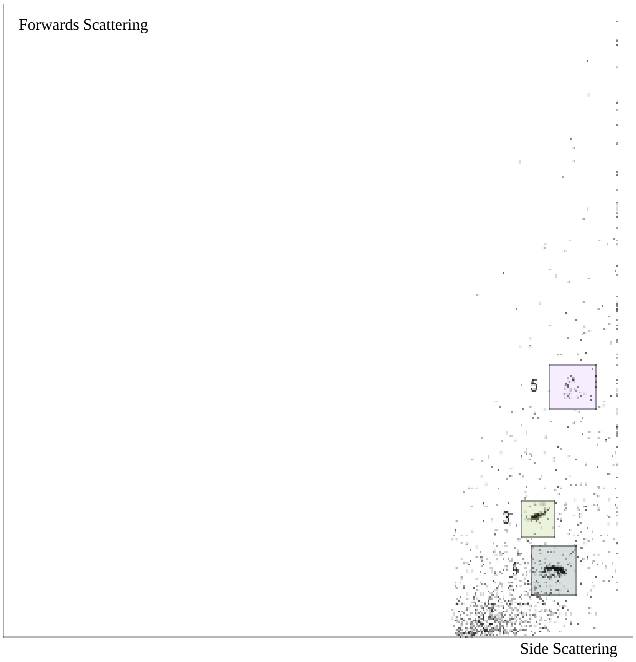

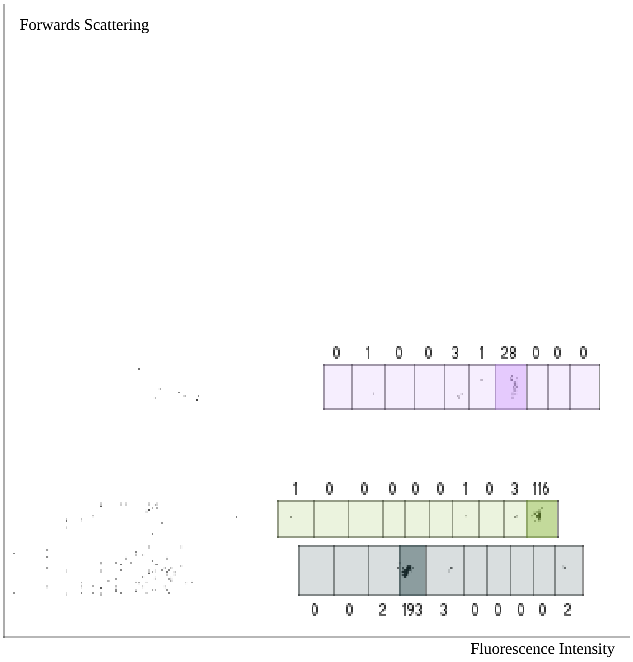

ANNEX 3: TAG DECONVOLUTION - BEAD 250

Passes flow sorting criteria: Yes

Passes tag deconvolution criteria: No

Included in protocol analysis: No

Protocol: N/A

Filename: Bin2_plateA2_F8.fcs

Split 1: Petrol shading

Split 2: Green shading

Split 3: Violet shading

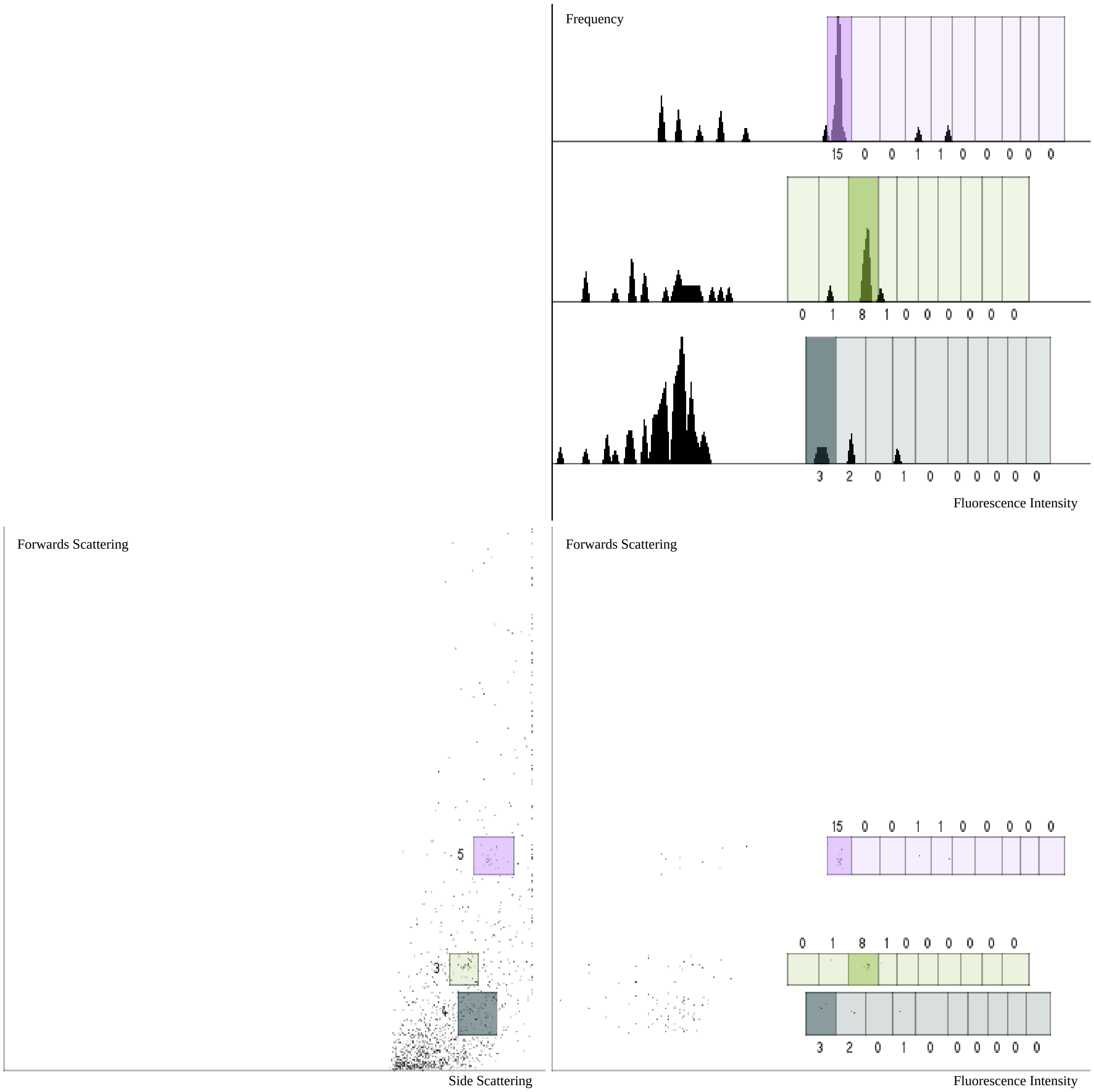

ANNEX 3: TAG DECONVOLUTION - BEAD 251

Passes flow sorting criteria: Yes

Passes tag deconvolution criteria: Yes

Included in protocol analysis: Yes

Protocol: 3, 4, 6, 2

Filename: Bin2_plateA2_F9.fcs

Split 1: Petrol shading

Split 2: Green shading

Split 3: Violet shading

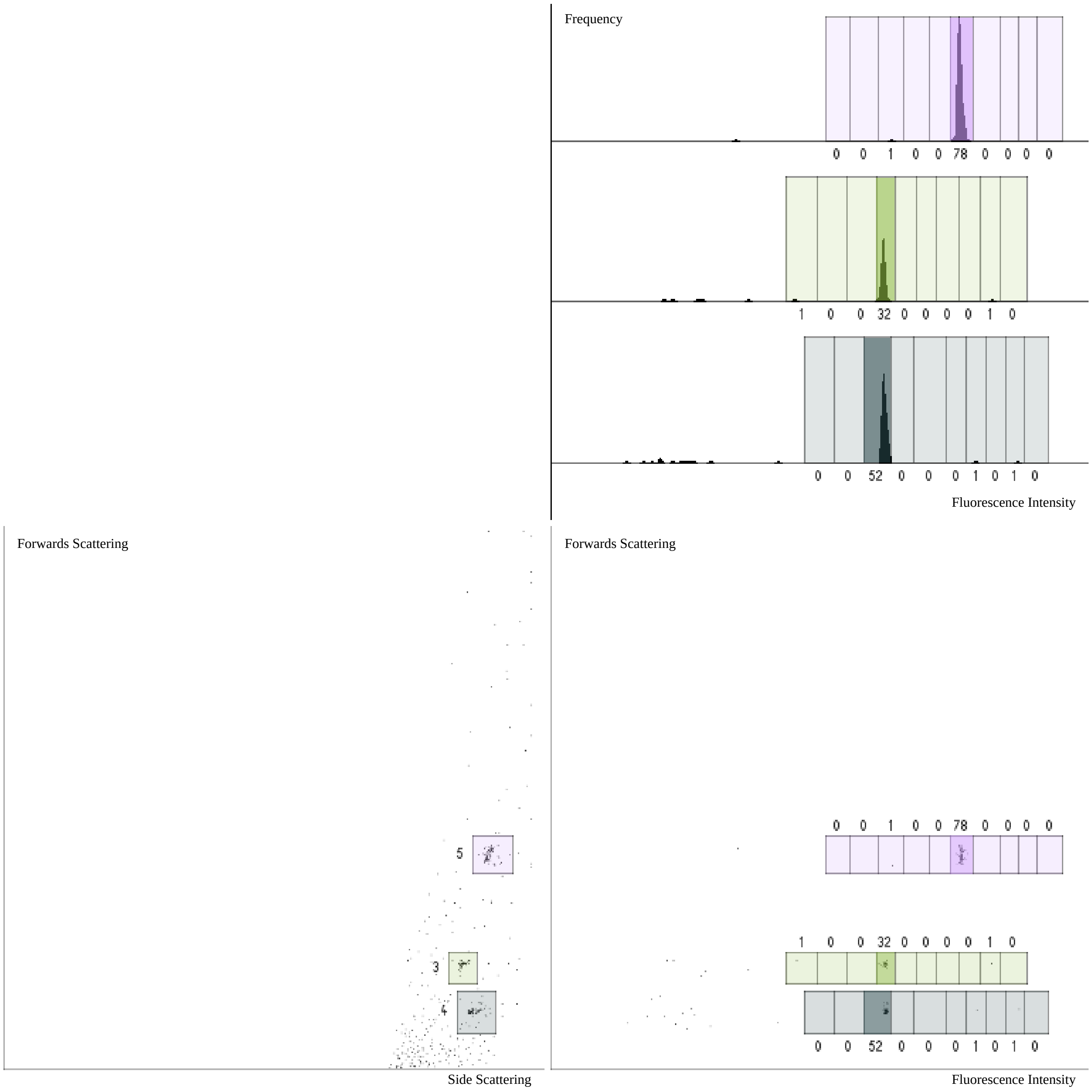

ANNEX 3: TAG DECONVOLUTION - BEAD 252

Passes flow sorting criteria: Yes

Passes tag deconvolution criteria: Yes

Included in protocol analysis: Yes

Protocol: 5, 10, 10, 2

Filename: Bin2_plateA2_F10.fcs

Split 1: Petrol shading

Split 2: Green shading

Split 3: Violet shading

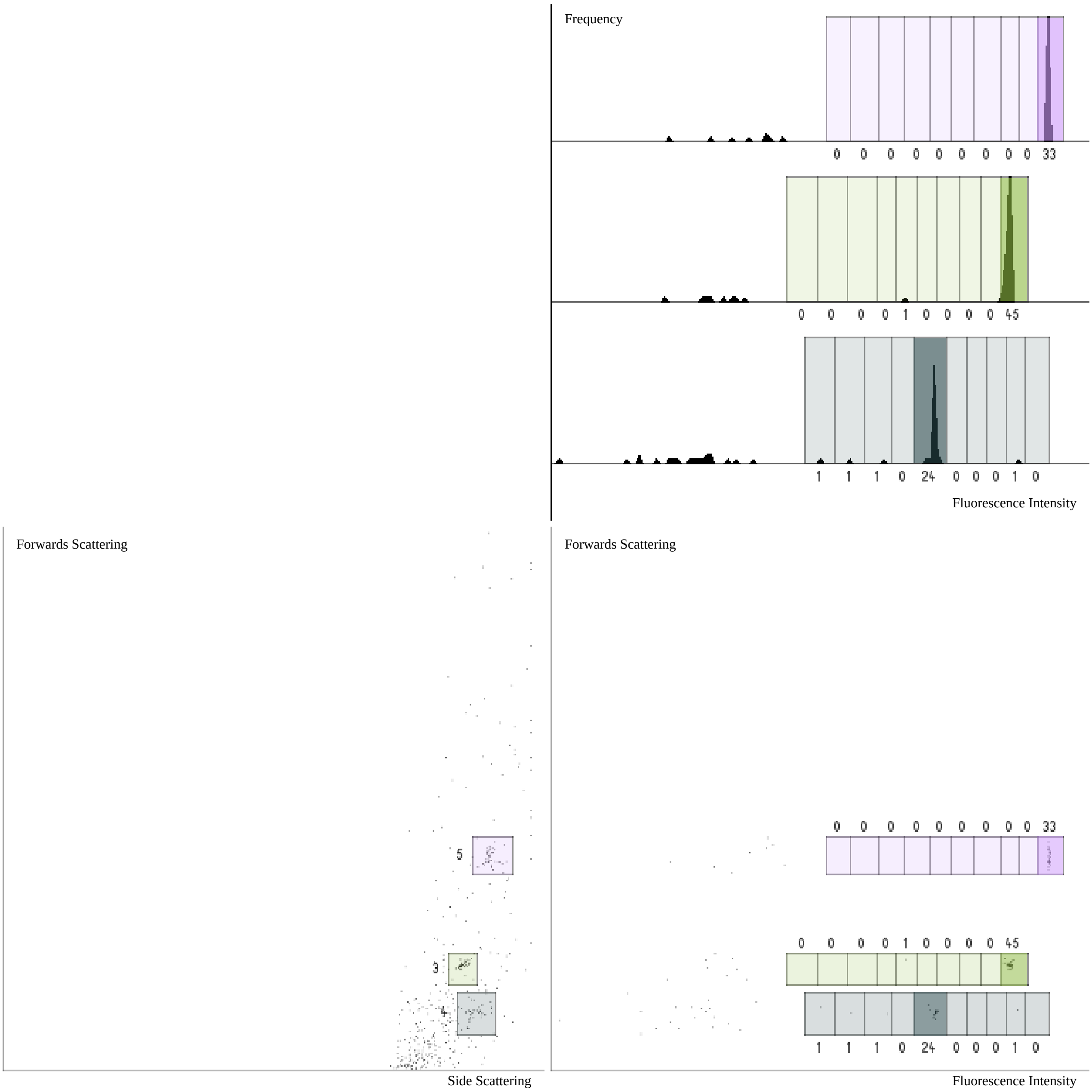

ANNEX 3: TAG DECONVOLUTION - BEAD 253

Passes flow sorting criteria: Yes

Passes tag deconvolution criteria: Yes

Included in protocol analysis: Yes

Protocol: 3, 3, 5, 2

Filename: Bin2_plateA2_F11.fcs

Split 1: Petrol shading

Split 2: Green shading

Split 3: Violet shading

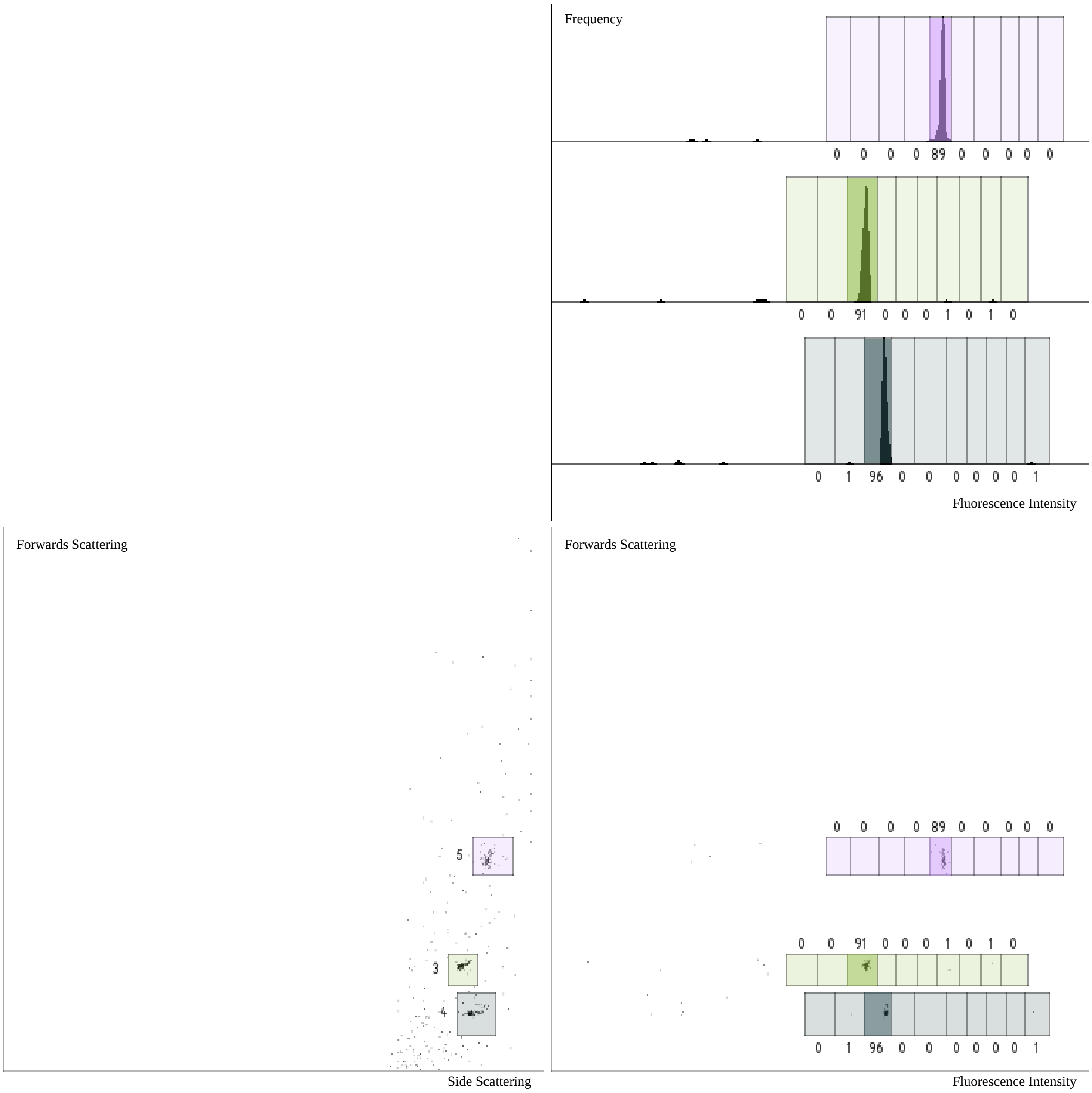

ANNEX 3: TAG DECONVOLUTION - BEAD 254

Passes flow sorting criteria: Yes

Passes tag deconvolution criteria: No

Included in protocol analysis: No

Protocol: N/A

Filename: Bin2_plateA2_F12.fcs

Split 1: Petrol shading

Split 2: Green shading

Split 3: Violet shading

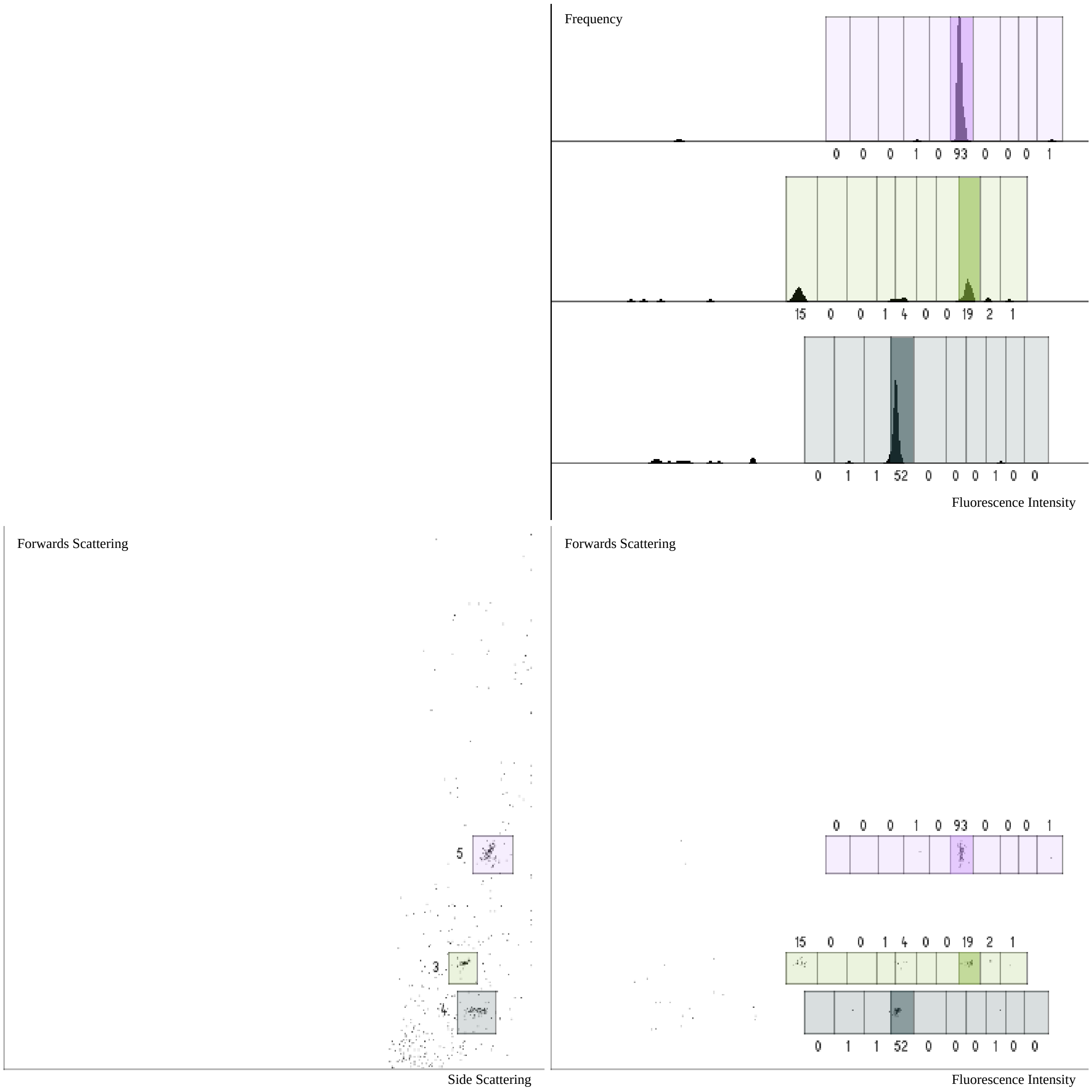

ANNEX 3: TAG DECONVOLUTION - BEAD 255

Passes flow sorting criteria: Yes

Passes tag deconvolution criteria: Yes

Included in protocol analysis: Yes

Protocol: 2, 3, 1, 2

Filename: Bin2_plateA2_G1.fcs

Split 1: Petrol shading

Split 2: Green shading

Split 3: Violet shading

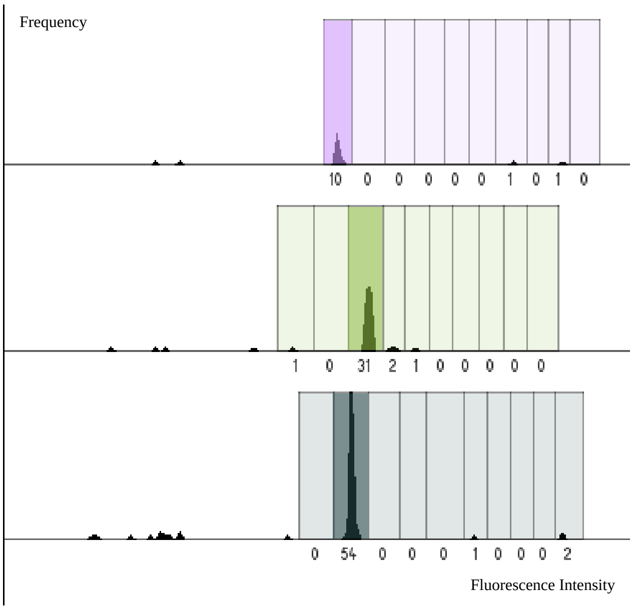

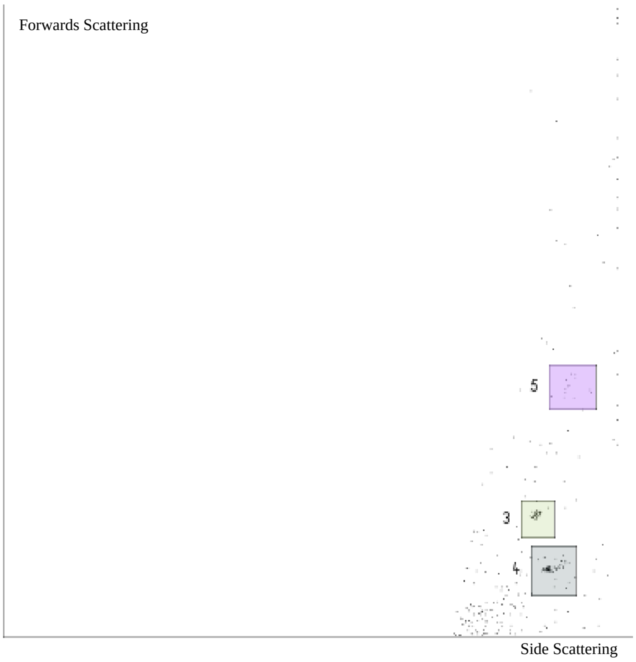

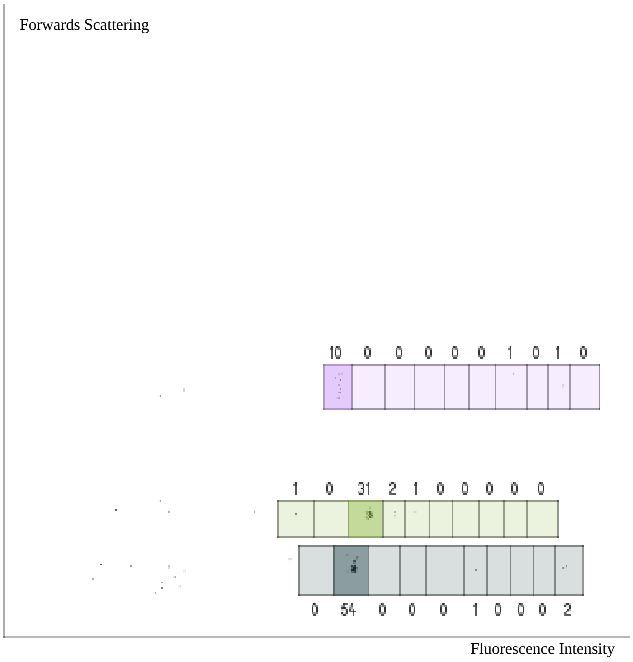

ANNEX 3: TAG DECONVOLUTION - BEAD 256

Passes flow sorting criteria: Yes

Passes tag deconvolution criteria: No

Included in protocol analysis: No

Protocol: N/A

Filename: Bin2_plateA2_G4.fcs

Split 1: Petrol shading

Split 2: Green shading

Split 3: Violet shading

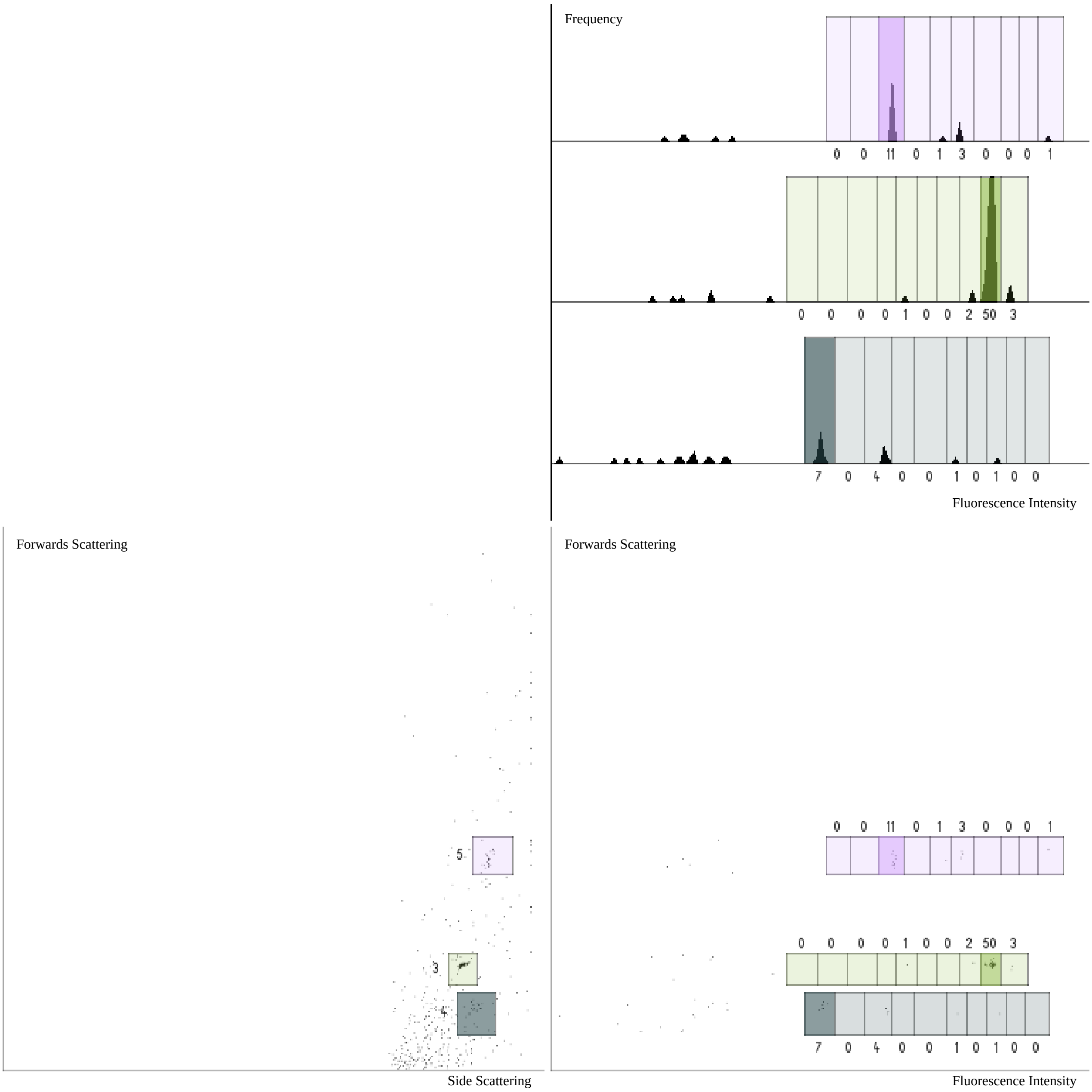

ANNEX 3: TAG DECONVOLUTION - BEAD 257

Passes flow sorting criteria: Yes

Passes tag deconvolution criteria: No

Included in protocol analysis: No

Protocol: N/A

Filename: Bin2_plateA2_G5.fcs

Split 1: Petrol shading

Split 2: Green shading

Split 3: Violet shading

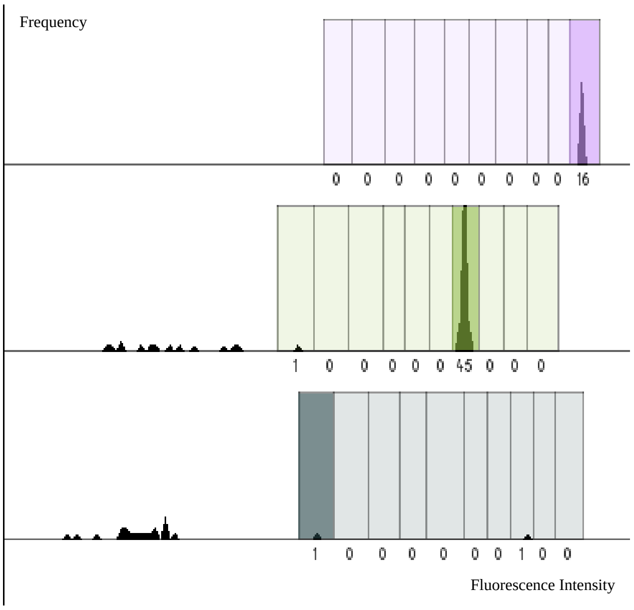

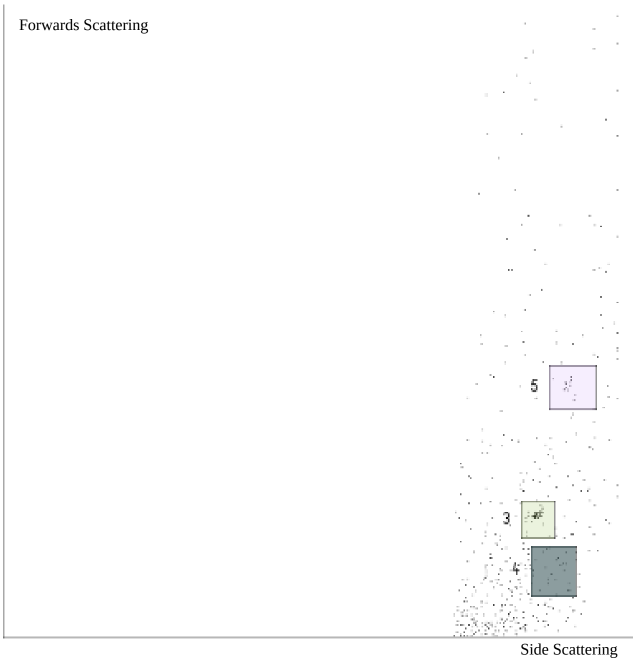

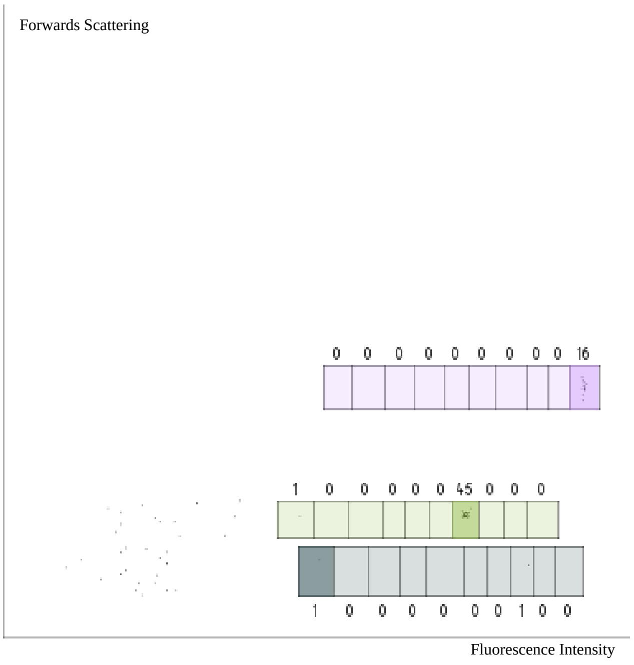

ANNEX 3: TAG DECONVOLUTION - BEAD 258

Passes flow sorting criteria: Yes

Passes tag deconvolution criteria: Yes

Included in protocol analysis: Yes

Protocol: 5, 5, 3, 2

Filename: Bin2_plateA2_G6.fcs

Split 1: Petrol shading

Split 2: Green shading

Split 3: Violet shading

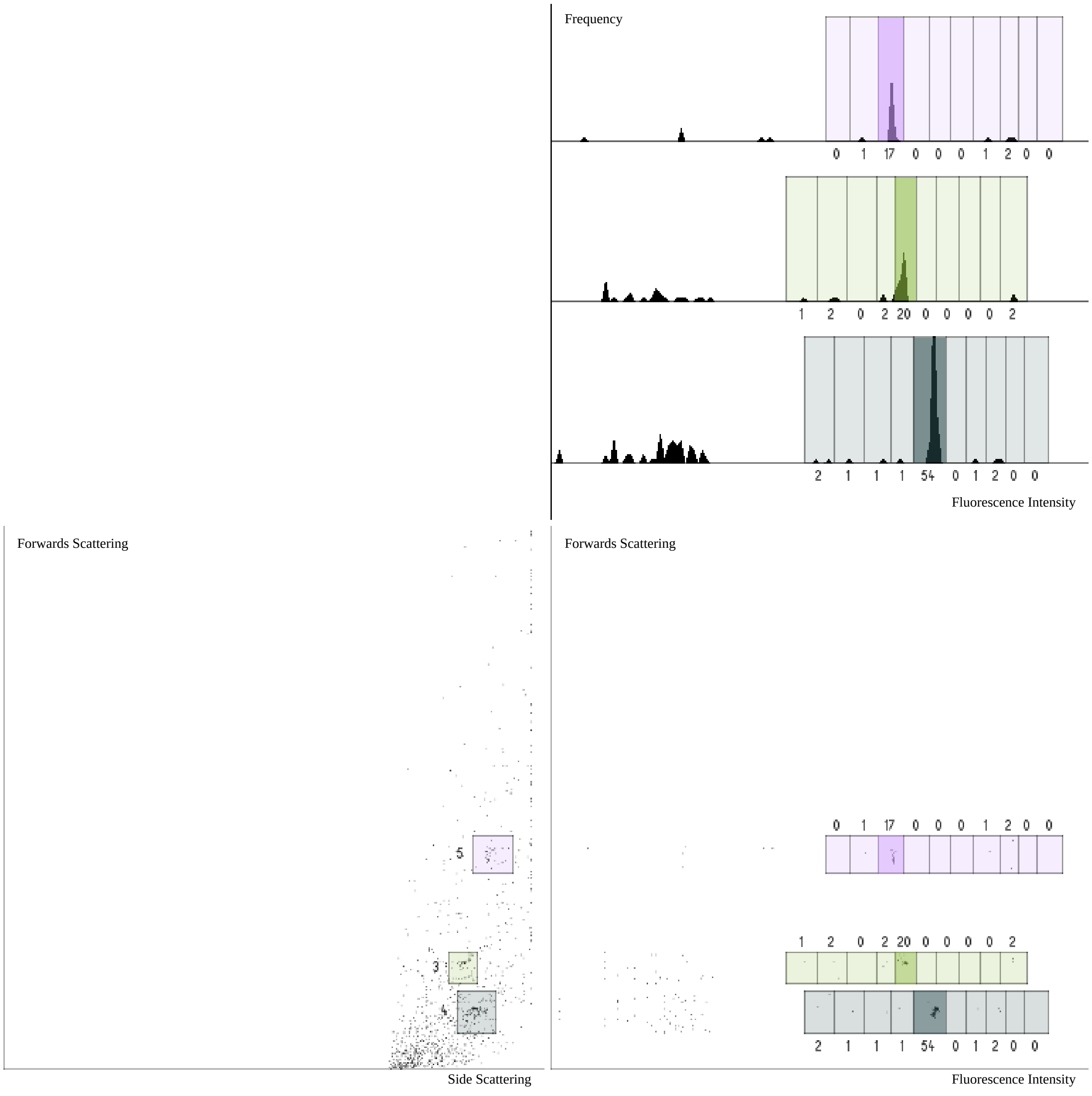

ANNEX 3: TAG DECONVOLUTION - BEAD 259

Passes flow sorting criteria: Yes

Passes tag deconvolution criteria: Yes

Included in protocol analysis: Yes

Protocol: 2, 10, 5, 2

Filename: Bin2_plateA2_G7.fcs

Split 1: Petrol shading

Split 2: Green shading

Split 3: Violet shading

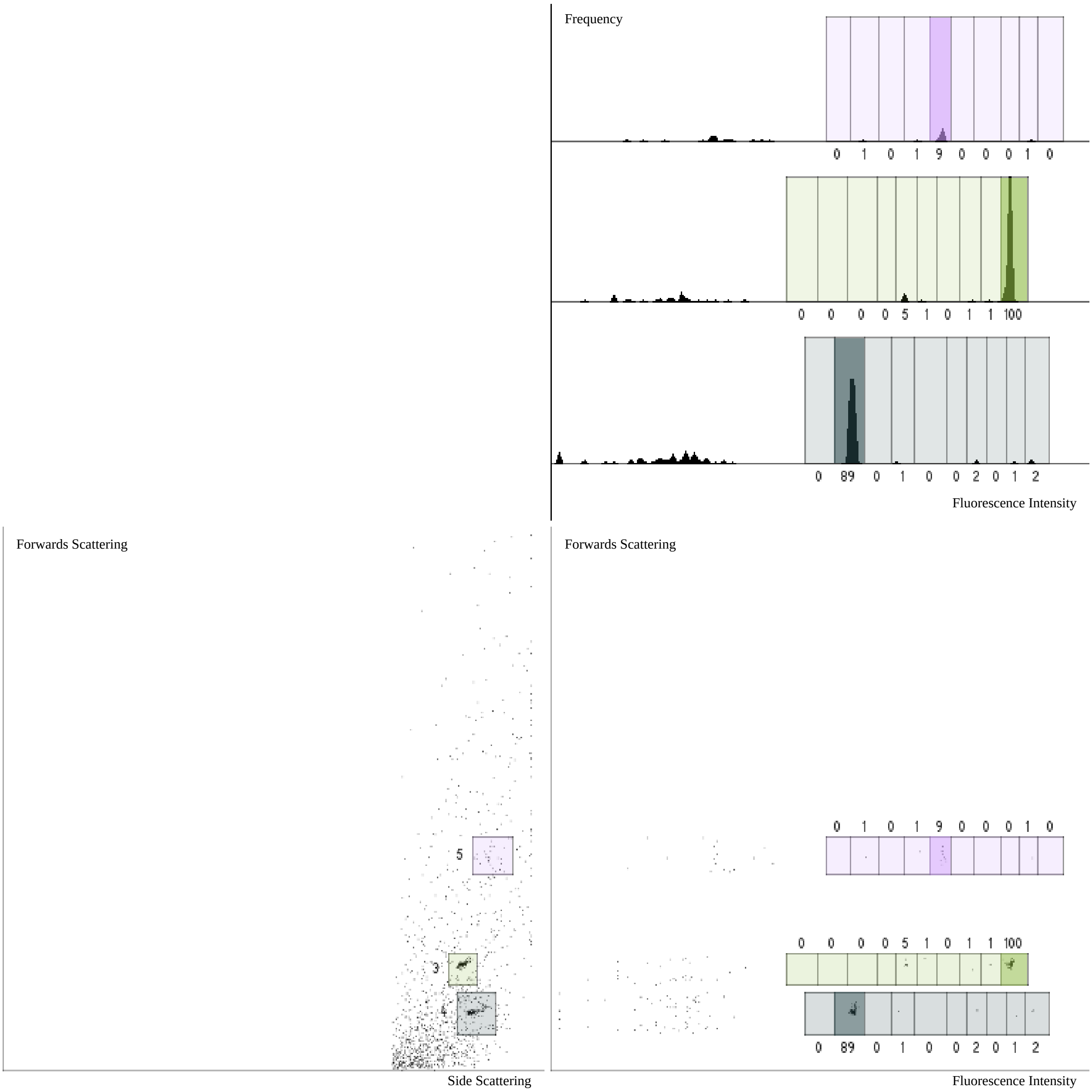

ANNEX 3: TAG DECONVOLUTION - BEAD 260

Passes flow sorting criteria: Yes

Passes tag deconvolution criteria: Yes

Included in protocol analysis: Yes

Protocol: 3, 5, 8, 3

Filename: Bin3_plateA2_B9.fcs

Split 1: Petrol shading

Split 2: Green shading

Split 3: Violet shading

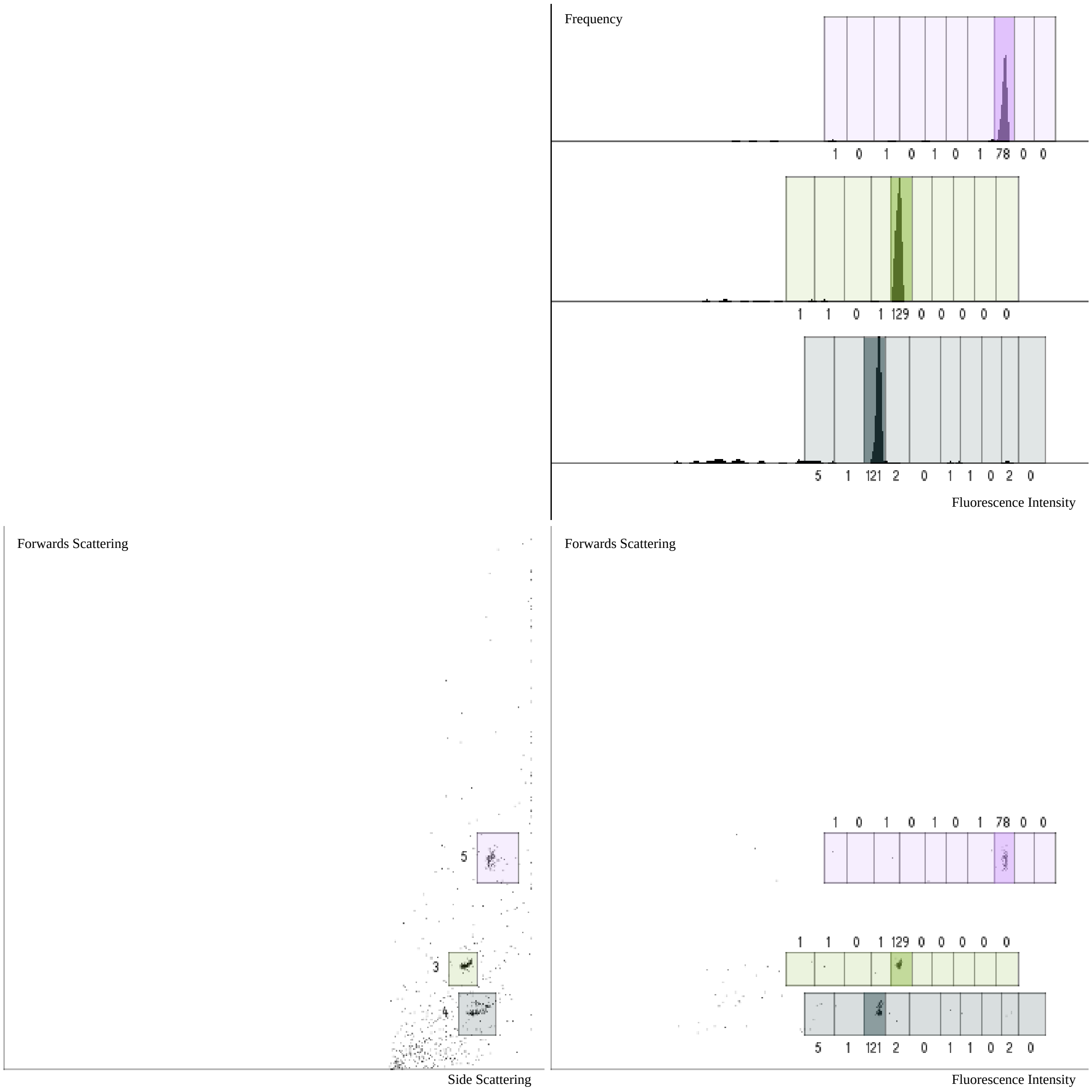

ANNEX 3: TAG DECONVOLUTION - BEAD 261

Passes flow sorting criteria: Yes

Passes tag deconvolution criteria: Yes

Included in protocol analysis: Yes

Protocol: 5, 9, 1, 3

Filename: Bin3_plateA2_A1.fcs

Split 1: Petrol shading

Split 2: Green shading

Split 3: Violet shading

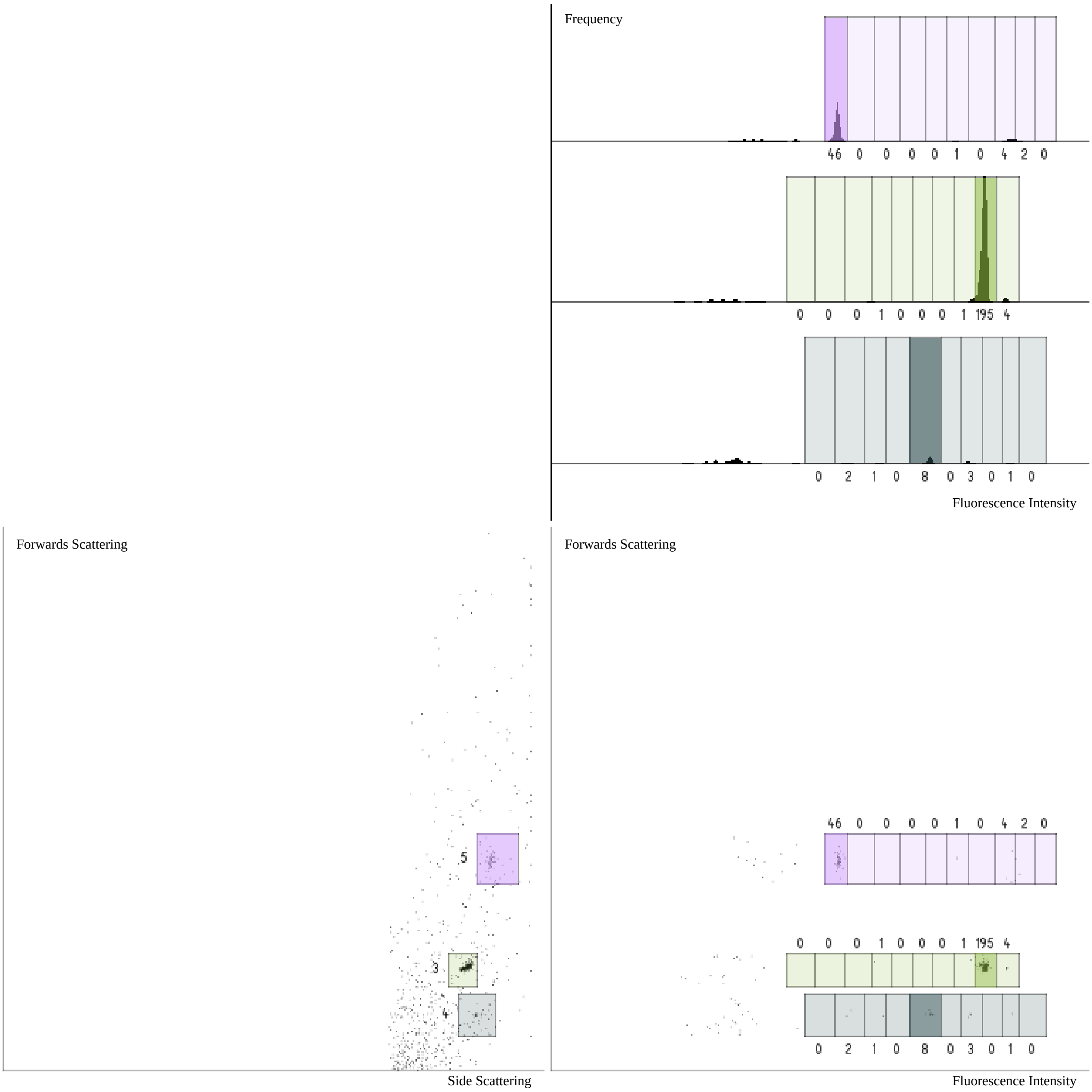

ANNEX 3: TAG DECONVOLUTION - BEAD 262

Passes flow sorting criteria: Yes

Passes tag deconvolution criteria: Yes

Included in protocol analysis: Yes

Protocol: 5, 10, 9, 3

Filename: Bin3_plateA2_A2.fcs

Split 1: Petrol shading

Split 2: Green shading

Split 3: Violet shading

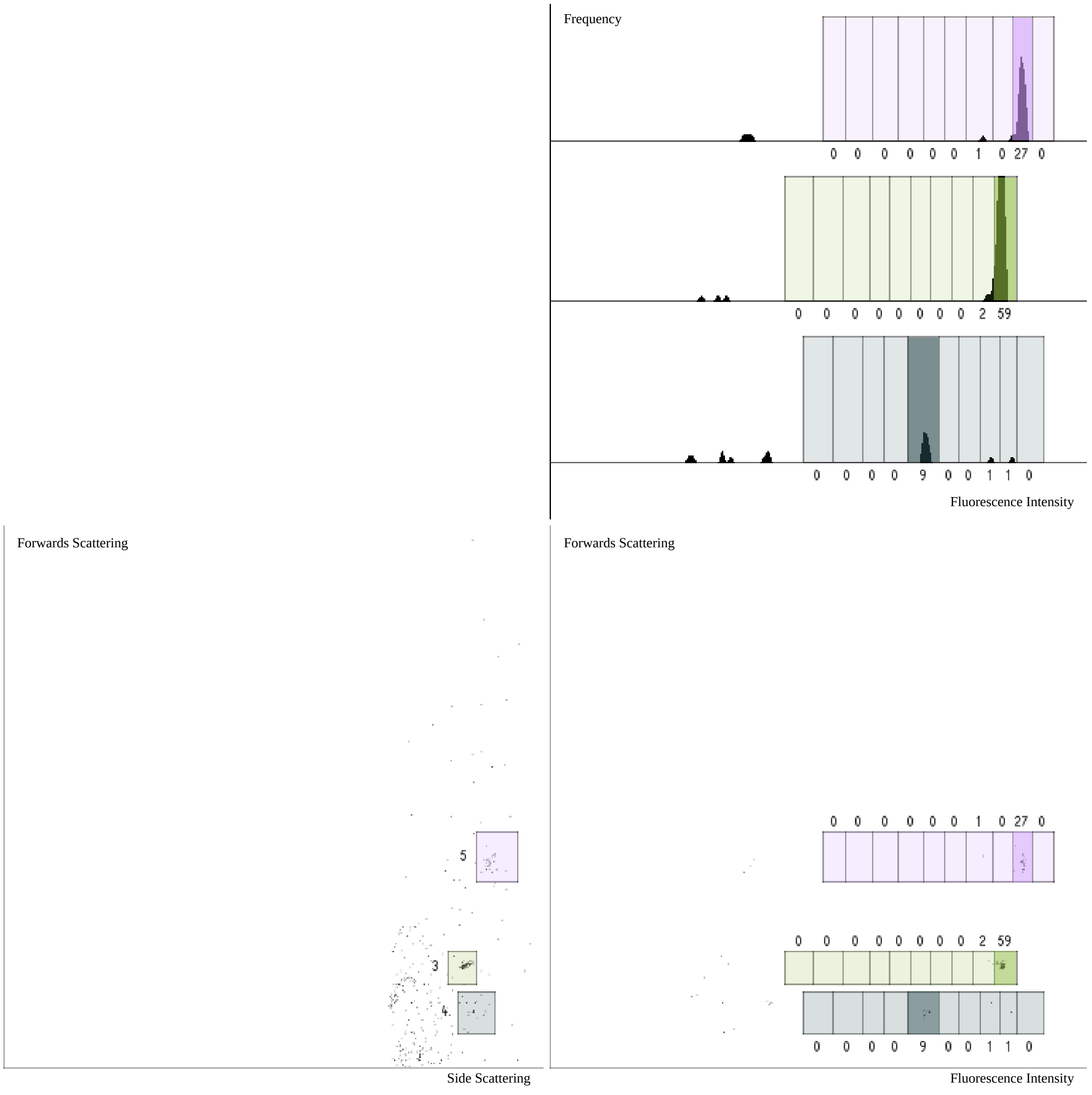

ANNEX 3: TAG DECONVOLUTION - BEAD 263

Passes flow sorting criteria: Yes

Passes tag deconvolution criteria: Yes

Included in protocol analysis: Yes

Protocol: 9, 4, 3, 3

Filename: Bin3_plateA2_A3.fcs

Split 1: Petrol shading

Split 2: Green shading

Split 3: Violet shading

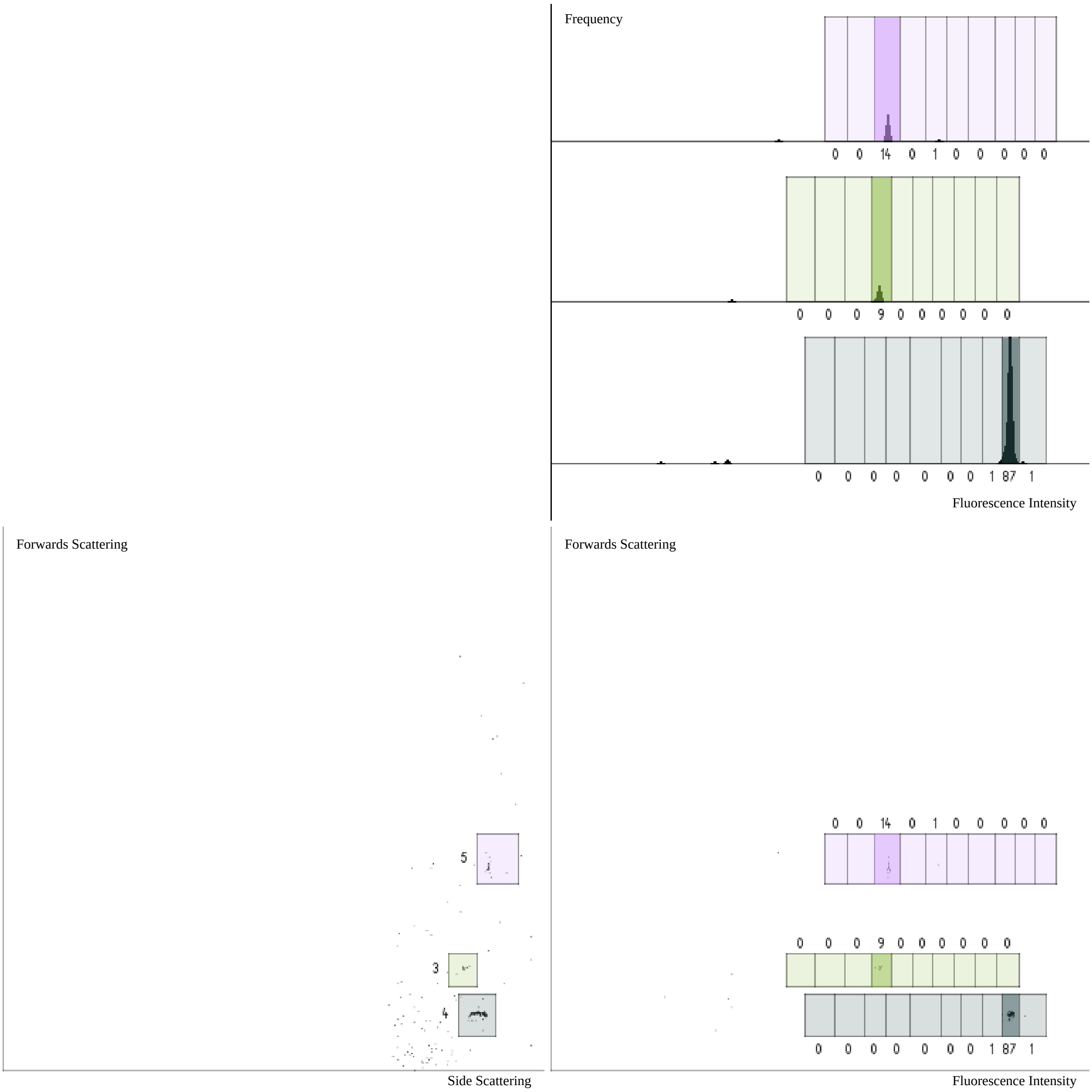

ANNEX 3: TAG DECONVOLUTION - BEAD 264

Passes flow sorting criteria: Yes

Passes tag deconvolution criteria: Yes

Included in protocol analysis: Yes

Protocol: 9, 5, 7, 3

Filename: Bin3_plateA2_A4.fcs

Split 1: Petrol shading

Split 2: Green shading

Split 3: Violet shading

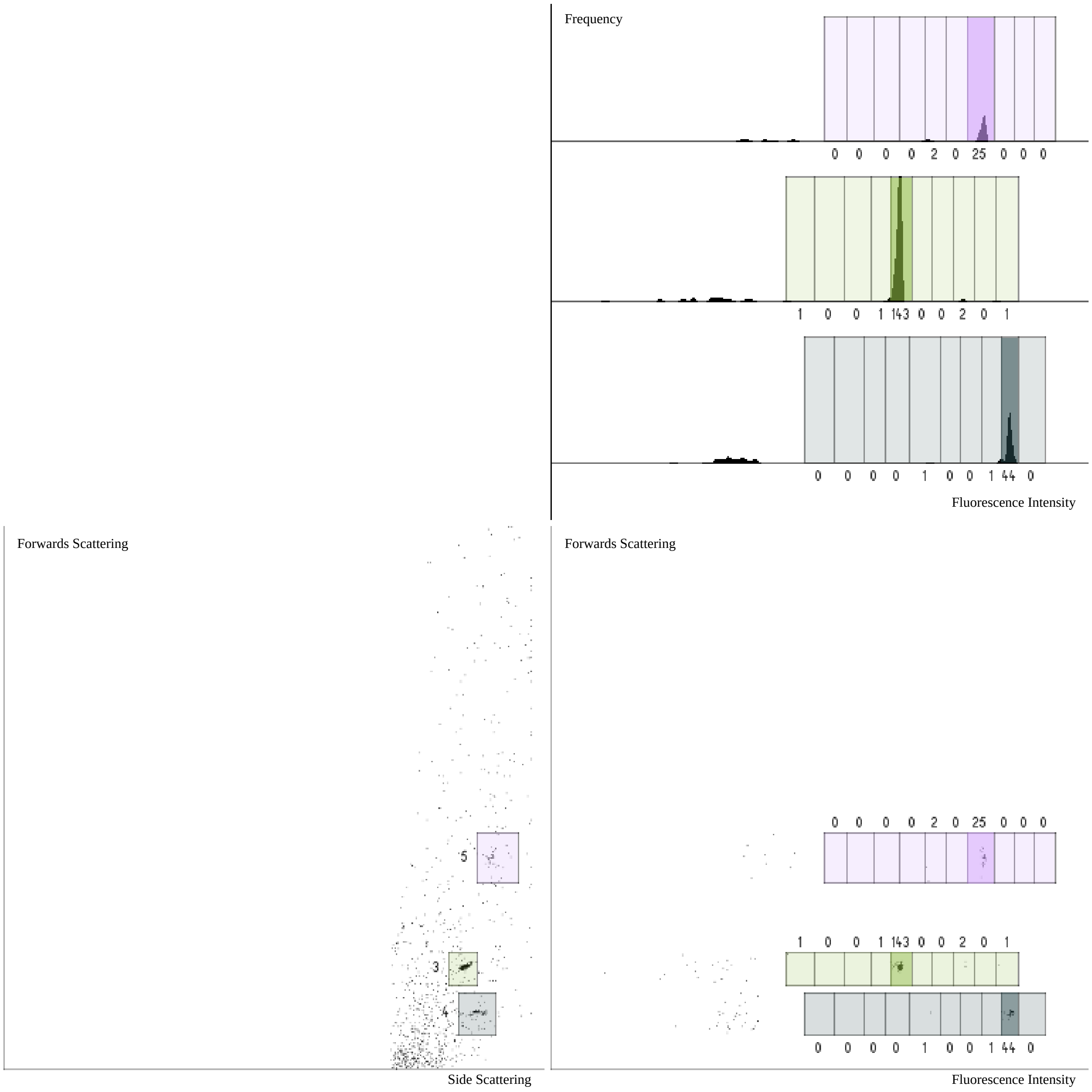

ANNEX 3: TAG DECONVOLUTION - BEAD 265

Passes flow sorting criteria: Yes

Passes tag deconvolution criteria: Yes

Included in protocol analysis: Yes

Protocol: 8, 6, 10, 3

Filename: Bin3_plateA2_A5.fcs

Split 1: Petrol shading

Split 2: Green shading

Split 3: Violet shading

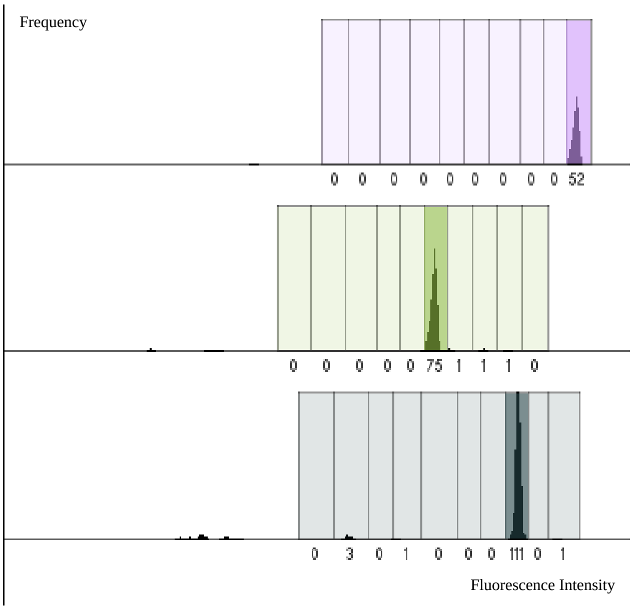

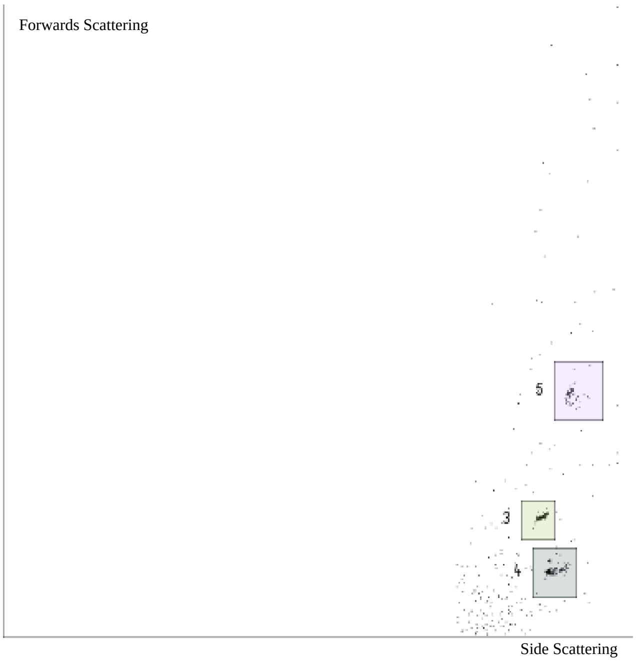

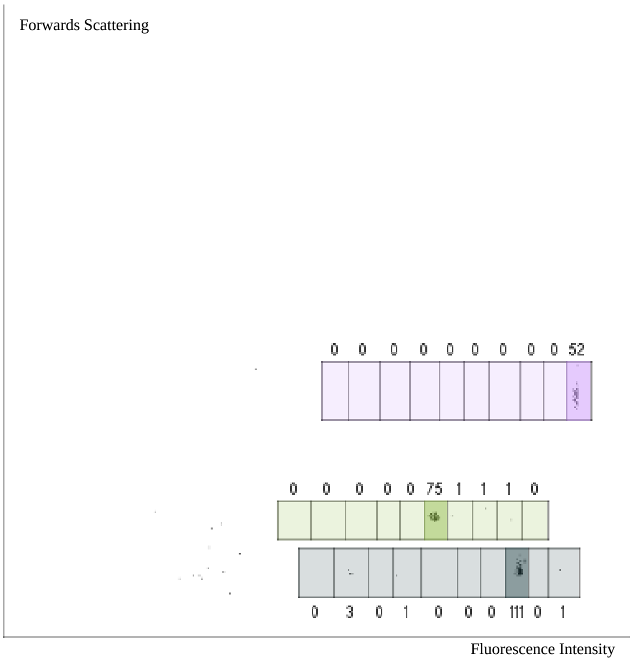

ANNEX 3: TAG DECONVOLUTION - BEAD 266

Passes flow sorting criteria: Yes

Passes tag deconvolution criteria: Yes

Included in protocol analysis: Yes

Protocol: 1, 7, 10, 3

Filename: Bin3_plateA2_A6.fcs

Split 1: Petrol shading

Split 2: Green shading

Split 3: Violet shading

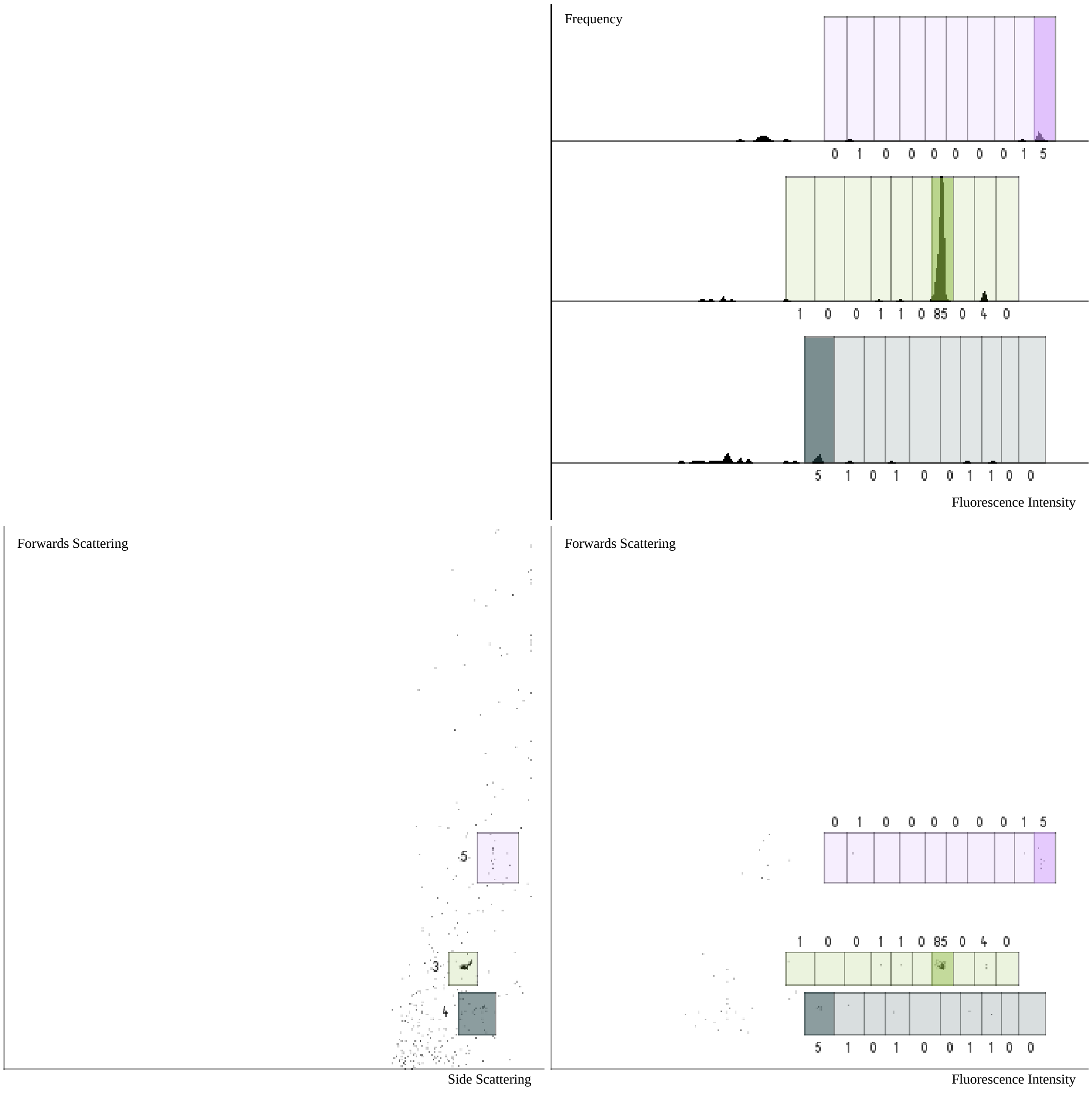

ANNEX 3: TAG DECONVOLUTION - BEAD 267

Passes flow sorting criteria: Yes

Passes tag deconvolution criteria: Yes

Included in protocol analysis: Yes

Protocol: 7, 1, 7, 3

Filename: Bin3_plateA2_A7.fcs

Split 1: Petrol shading

Split 2: Green shading

Split 3: Violet shading

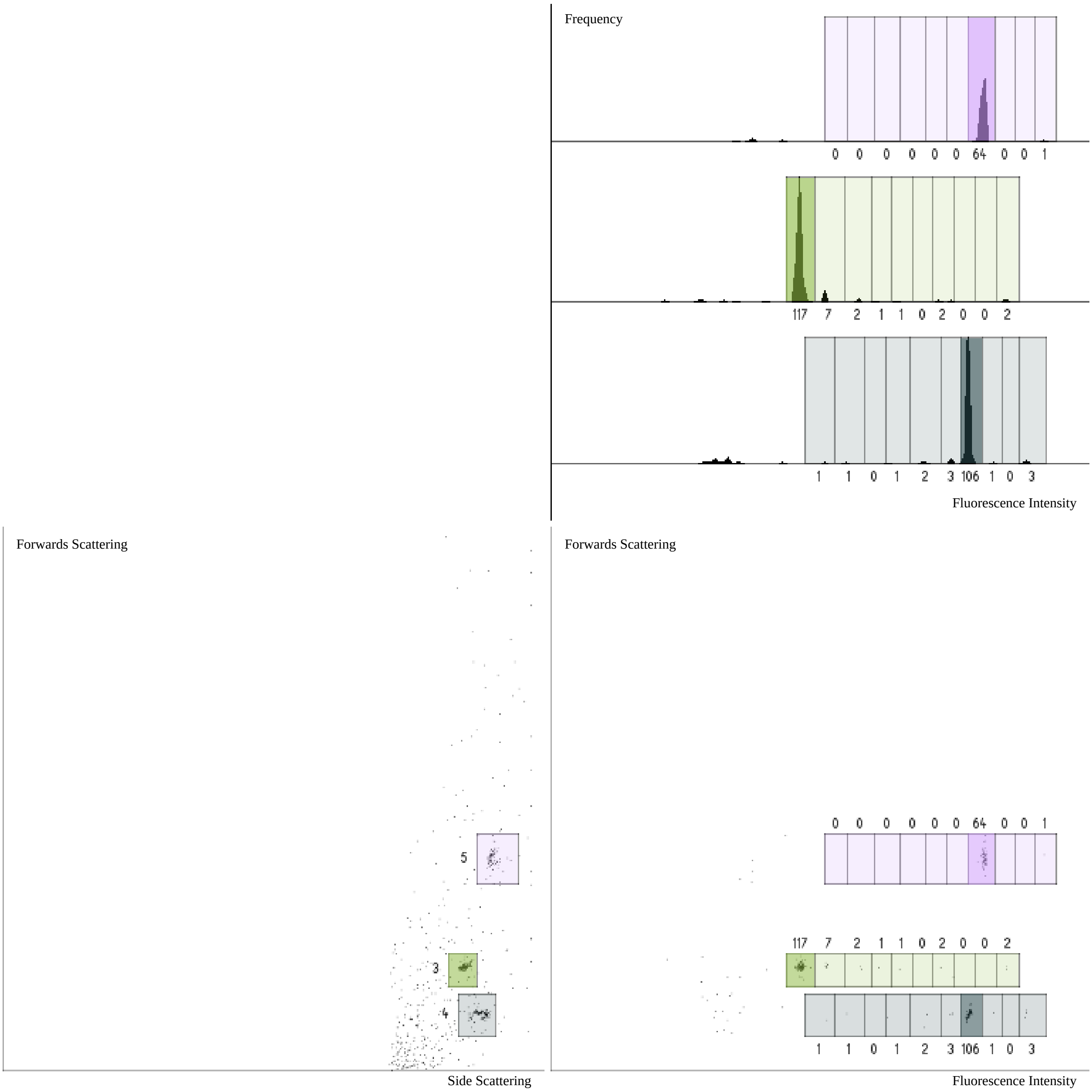

ANNEX 3: TAG DECONVOLUTION - BEAD 268

Passes flow sorting criteria: Yes

Passes tag deconvolution criteria: Yes

Included in protocol analysis: Yes

Protocol: 7, 4, 2, 3

Filename: Bin3_plateA2_A8.fcs

Split 1: Petrol shading

Split 2: Green shading

Split 3: Violet shading

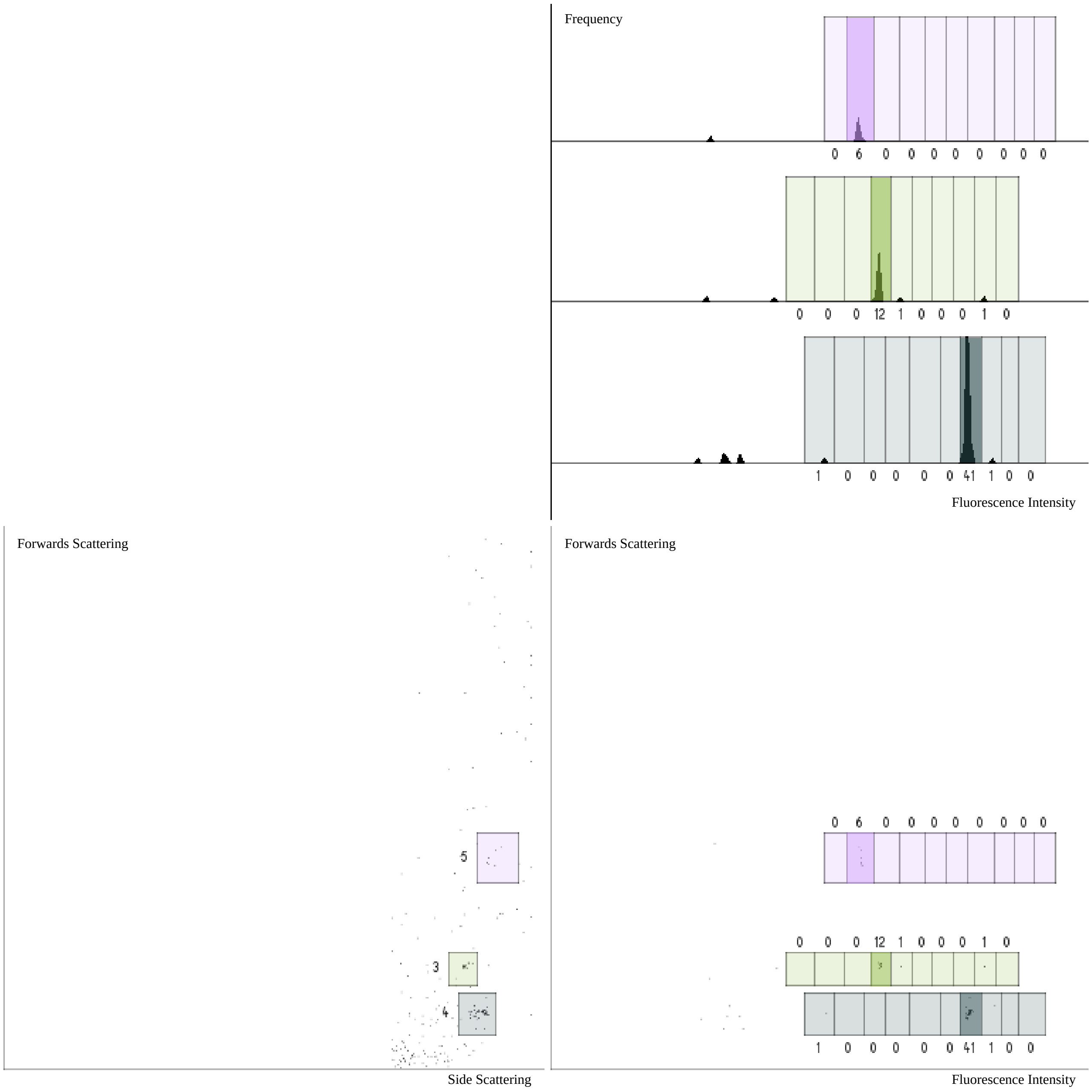

ANNEX 3: TAG DECONVOLUTION - BEAD 269

Passes flow sorting criteria: Yes

Passes tag deconvolution criteria: No

Included in protocol analysis: No

Protocol: N/A

Filename: Bin3_plateA2_A9.fcs

Split 1: Petrol shading

Split 2: Green shading

Split 3: Violet shading

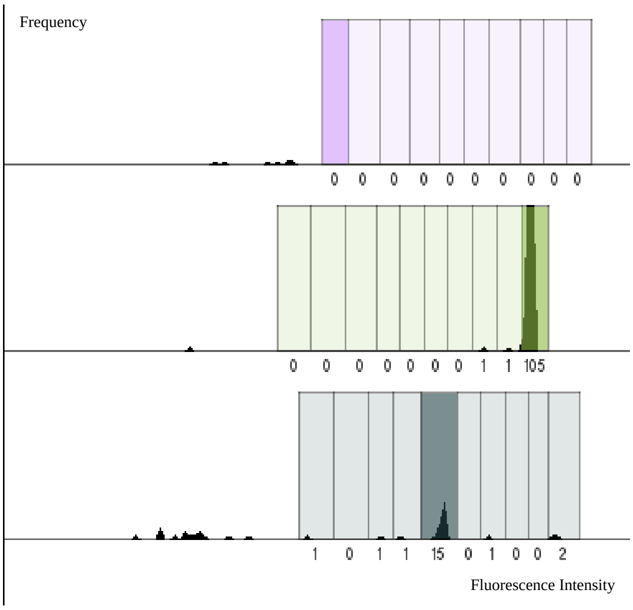

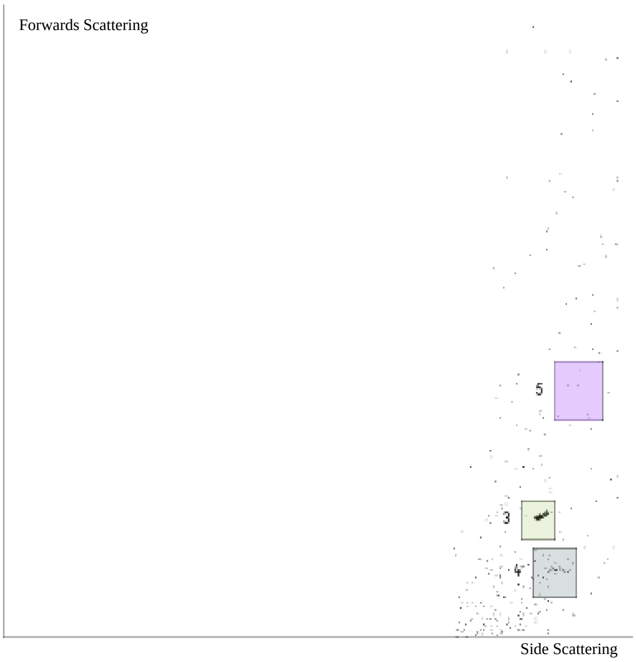

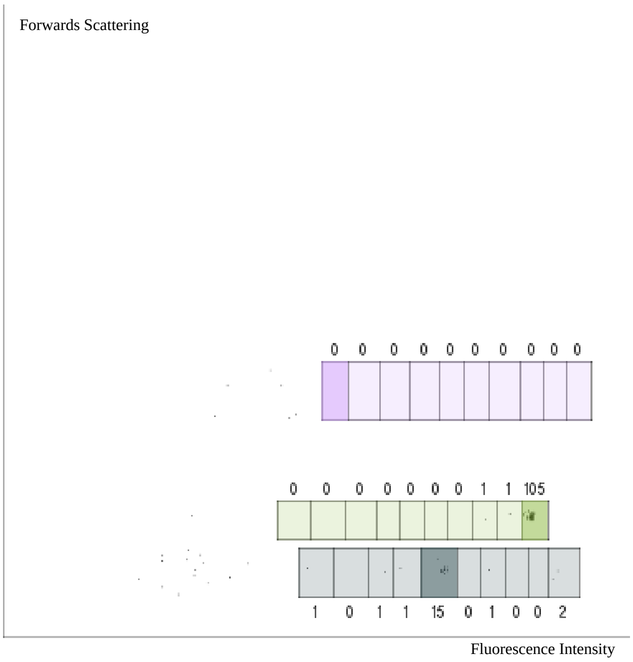

ANNEX 3: TAG DECONVOLUTION - BEAD 270

Passes flow sorting criteria: Yes

Passes tag deconvolution criteria: Yes

Included in protocol analysis: Yes

Protocol: 2, 3, 8, 3

Filename: Bin3_plateA2_A10.fcs

Split 1: Petrol shading

Split 2: Green shading

Split 3: Violet shading

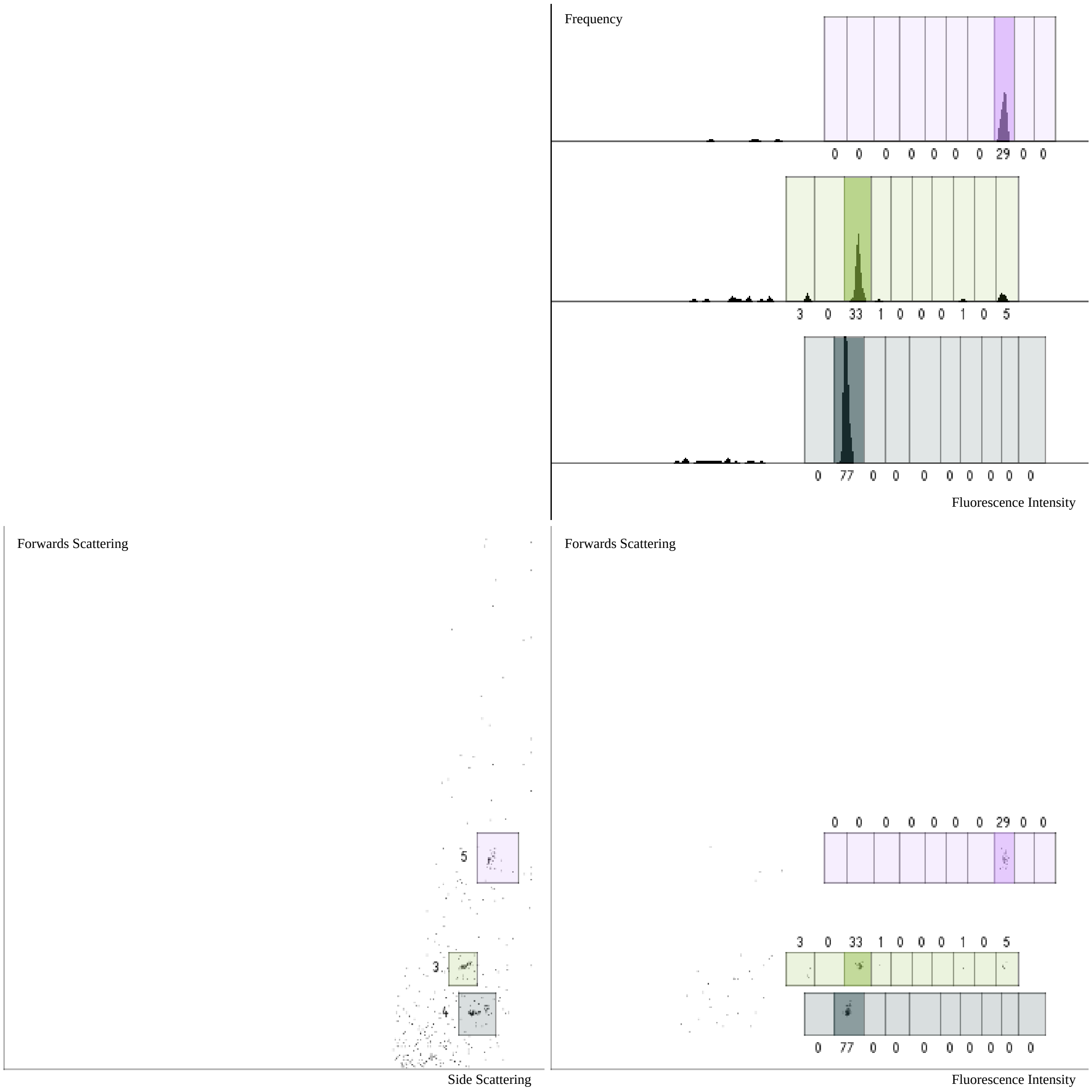

ANNEX 3: TAG DECONVOLUTION - BEAD 271

Passes flow sorting criteria: Yes

Passes tag deconvolution criteria: Yes

Included in protocol analysis: Yes

Protocol: 5, 9, 2, 3

Filename: Bin3_plateA2_A11.fcs

Split 1: Petrol shading

Split 2: Green shading

Split 3: Violet shading

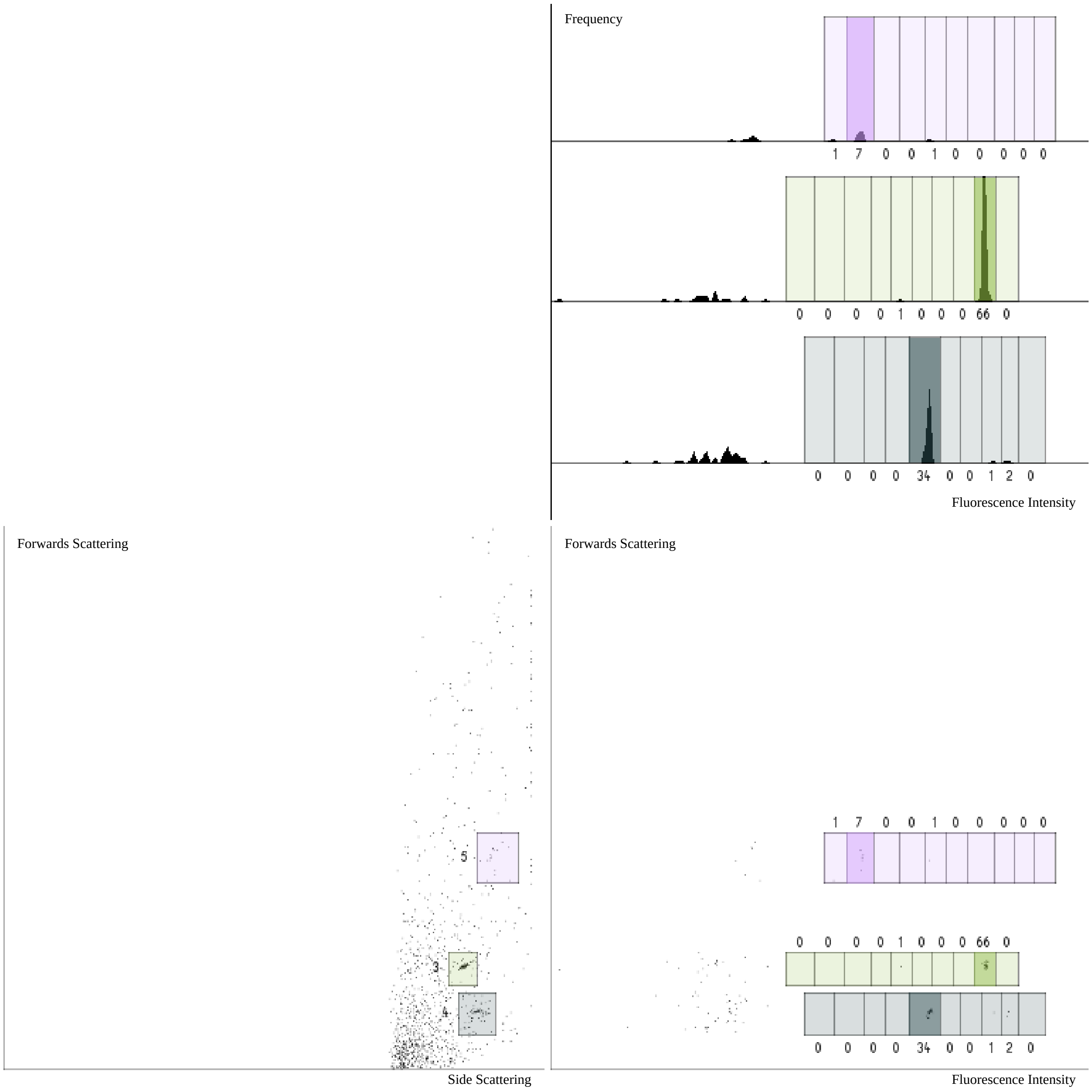

ANNEX 3: TAG DECONVOLUTION - BEAD 272

Passes flow sorting criteria: Yes

Passes tag deconvolution criteria: Yes

Included in protocol analysis: Yes

Protocol: 1, 5, 7, 3

Filename: Bin3_plateA2_A12.fcs

Split 1: Petrol shading

Split 2: Green shading

Split 3: Violet shading

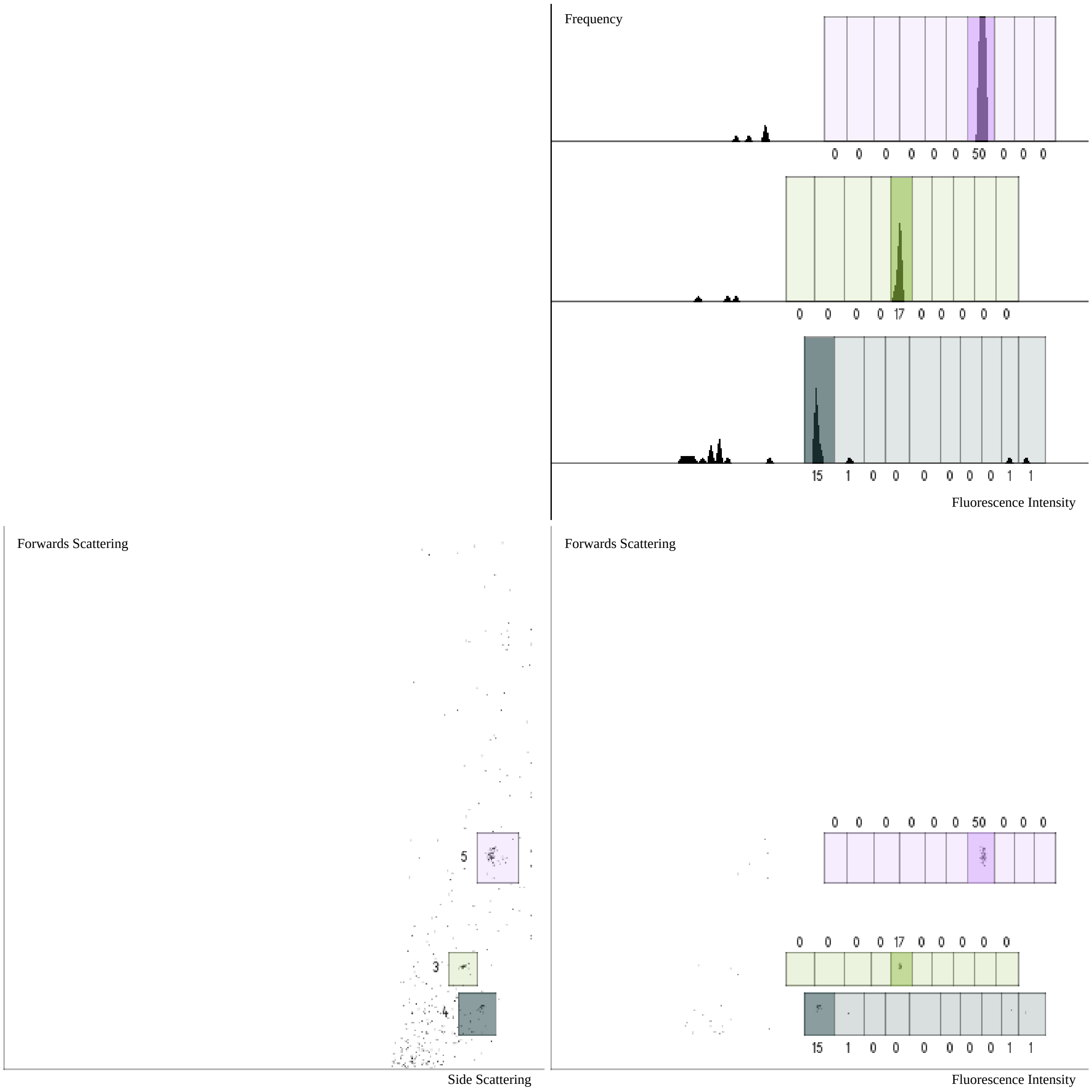

ANNEX 3: TAG DECONVOLUTION - BEAD 273

Passes flow sorting criteria: Yes

Passes tag deconvolution criteria: No

Included in protocol analysis: No

Protocol: N/A

Filename: Bin3_plateA2_B2.fcs

Split 1: Petrol shading

Split 2: Green shading

Split 3: Violet shading

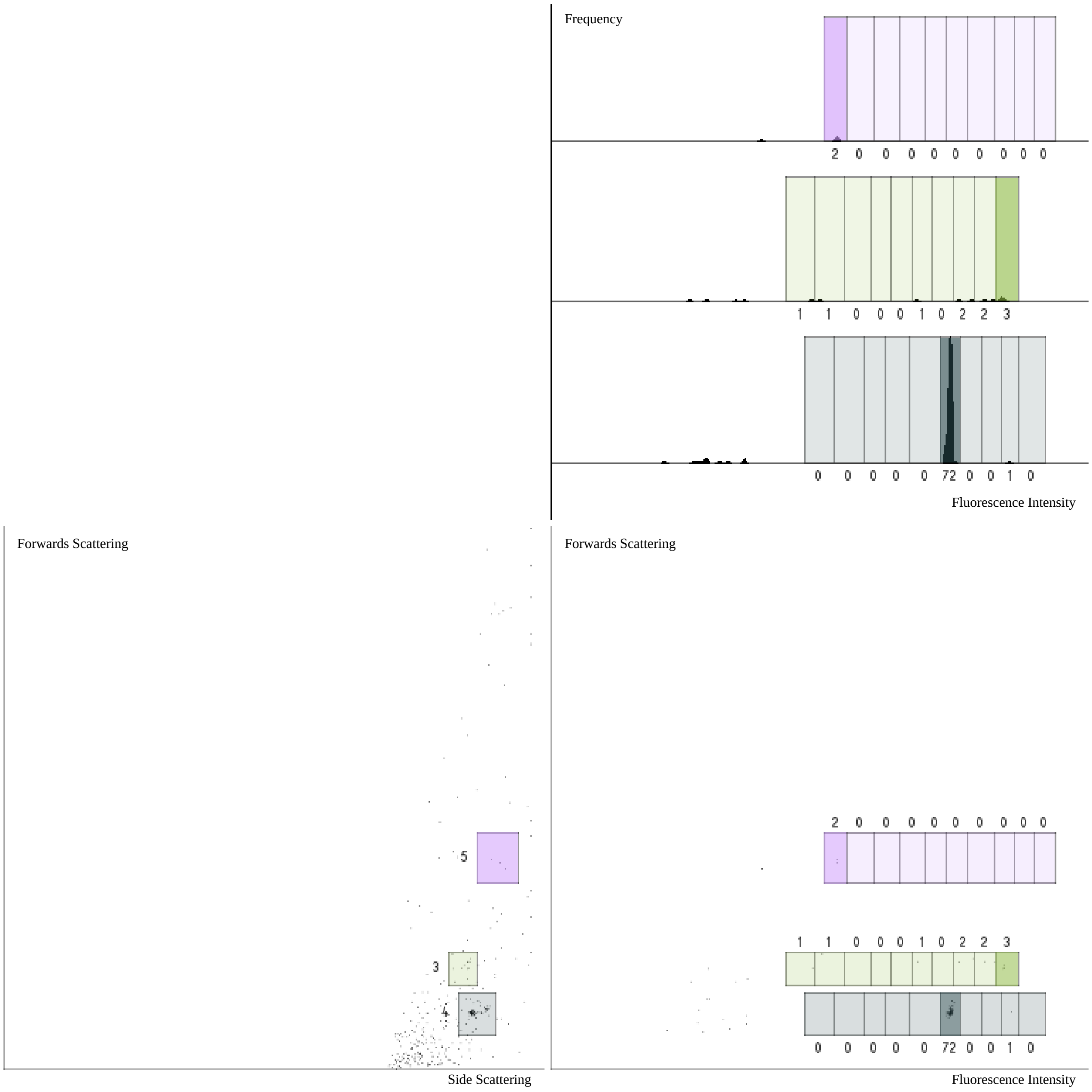

ANNEX 3: TAG DECONVOLUTION - BEAD 274

Passes flow sorting criteria: Yes

Passes tag deconvolution criteria: Yes

Included in protocol analysis: Yes

Protocol: 7, 10, 2, 3

Filename: Bin3_plateA2_B3.fcs

Split 1: Petrol shading

Split 2: Green shading

Split 3: Violet shading

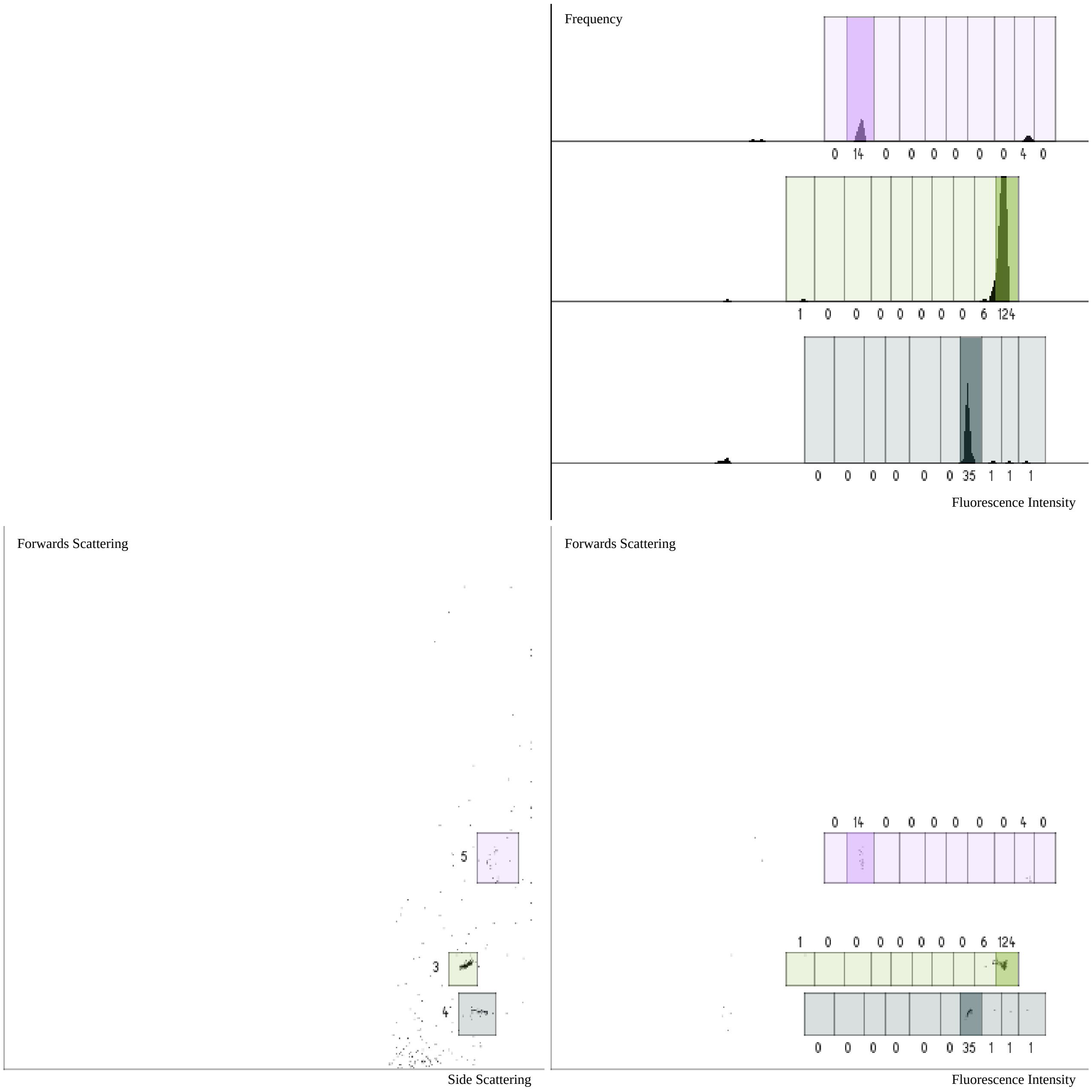

ANNEX 3: TAG DECONVOLUTION - BEAD 275

Passes flow sorting criteria: Yes

Passes tag deconvolution criteria: No

Included in protocol analysis: No

Protocol: N/A

Filename: Bin3_plateA2_B5.fcs

Split 1: Petrol shading

Split 2: Green shading

Split 3: Violet shading

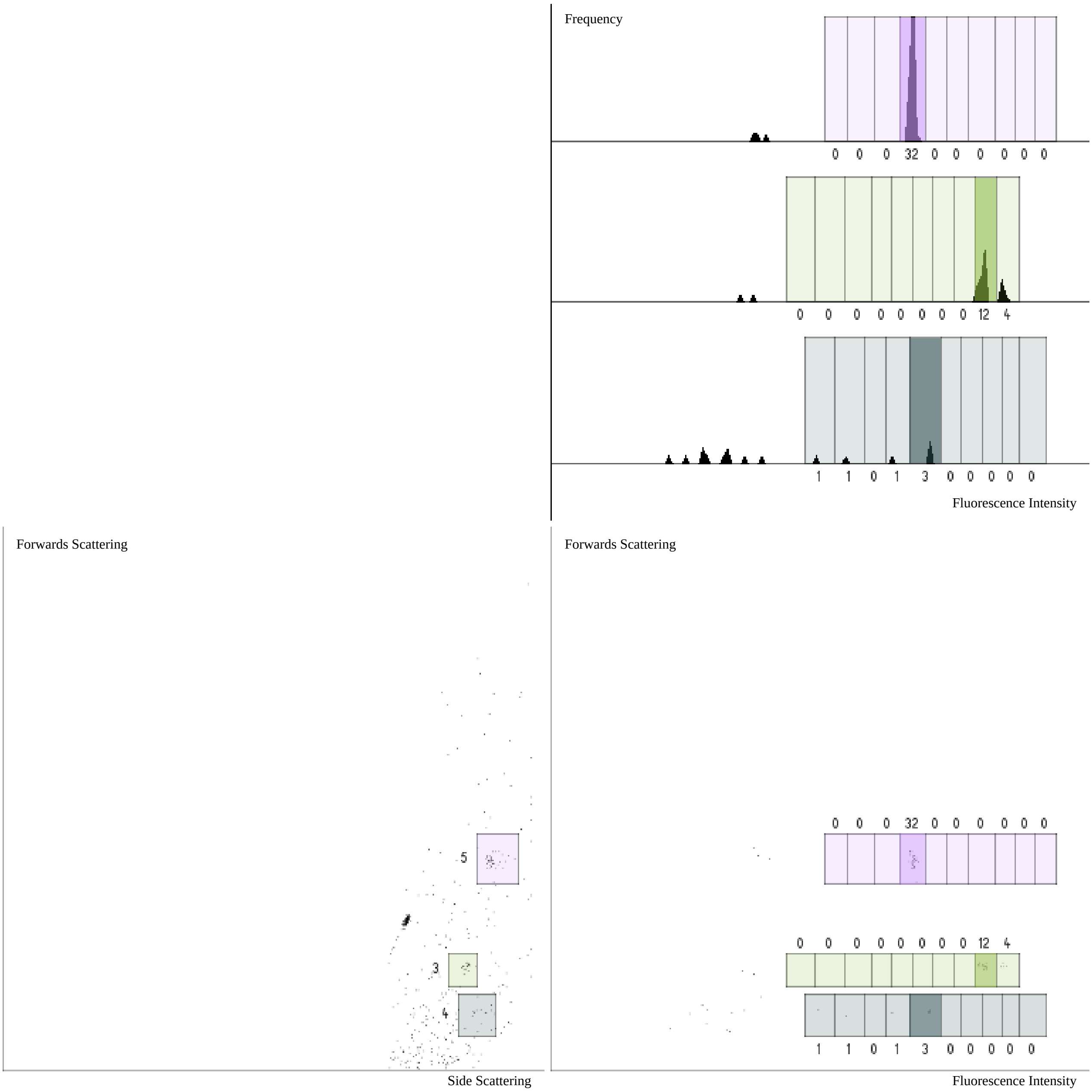

ANNEX 3: TAG DECONVOLUTION - BEAD 276

Passes flow sorting criteria: Yes

Passes tag deconvolution criteria: Yes

Included in protocol analysis: Yes

Protocol: 2, 6, 10, 3

Filename: Bin3_plateA2_B6.fcs

Split 1: Petrol shading

Split 2: Green shading

Split 3: Violet shading

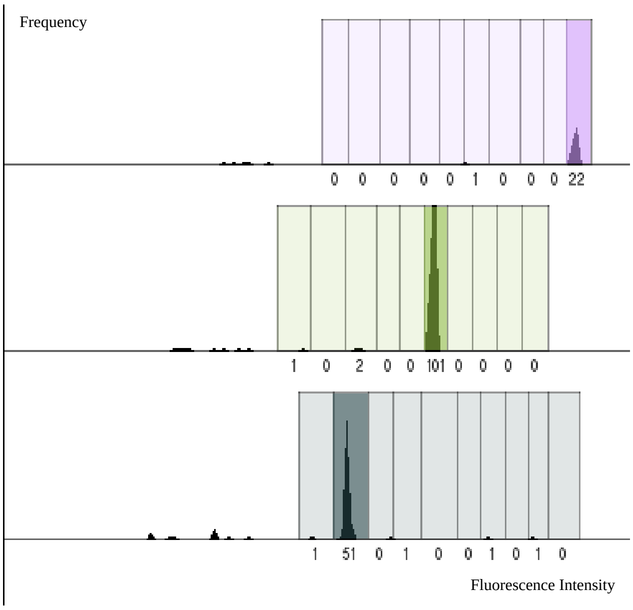

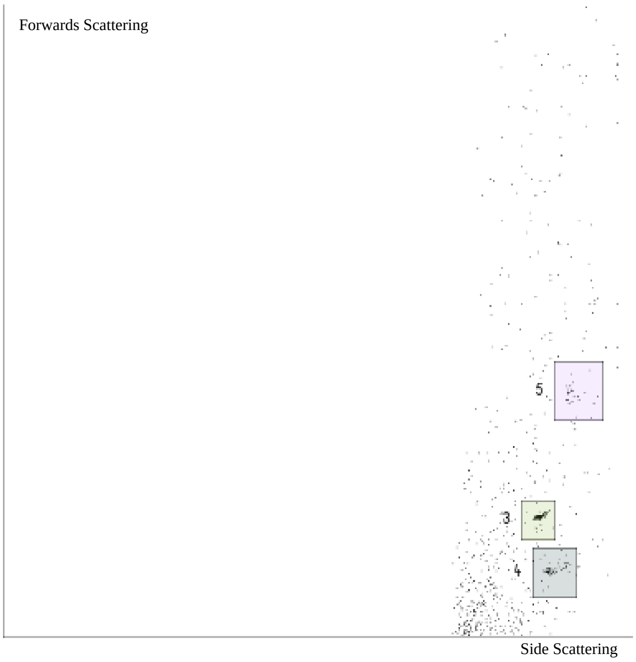

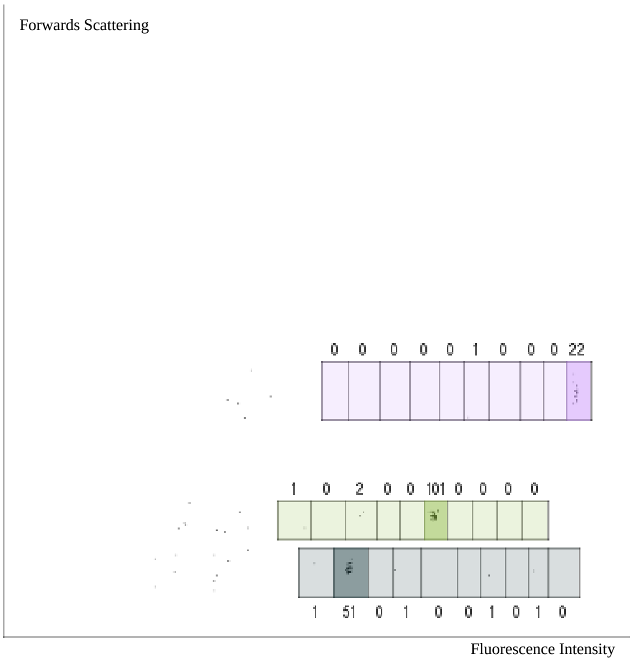

ANNEX 3: TAG DECONVOLUTION - BEAD 277

Passes flow sorting criteria: Yes

Passes tag deconvolution criteria: Yes

Included in protocol analysis: Yes

Protocol: 7, 10, 3, 3

Filename: Bin3_plateA2_B7.fcs

Split 1: Petrol shading

Split 2: Green shading

Split 3: Violet shading

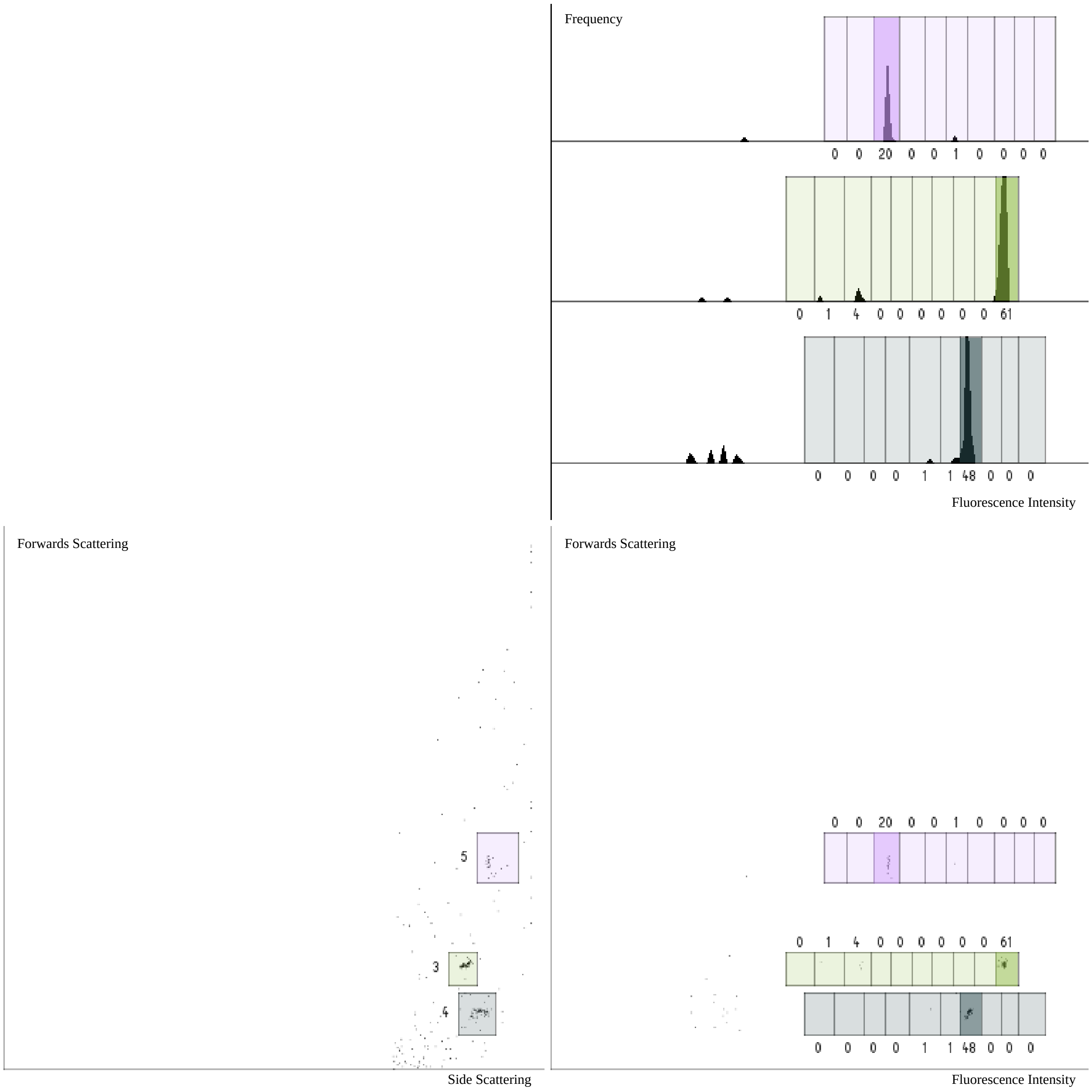

ANNEX 3: TAG DECONVOLUTION - BEAD 278

Passes flow sorting criteria: Yes

Passes tag deconvolution criteria: Yes

Included in protocol analysis: Yes

Protocol: 5, 10, 9, 3

Filename: Bin3_plateA2_B8.fcs

Split 1: Petrol shading

Split 2: Green shading

Split 3: Violet shading

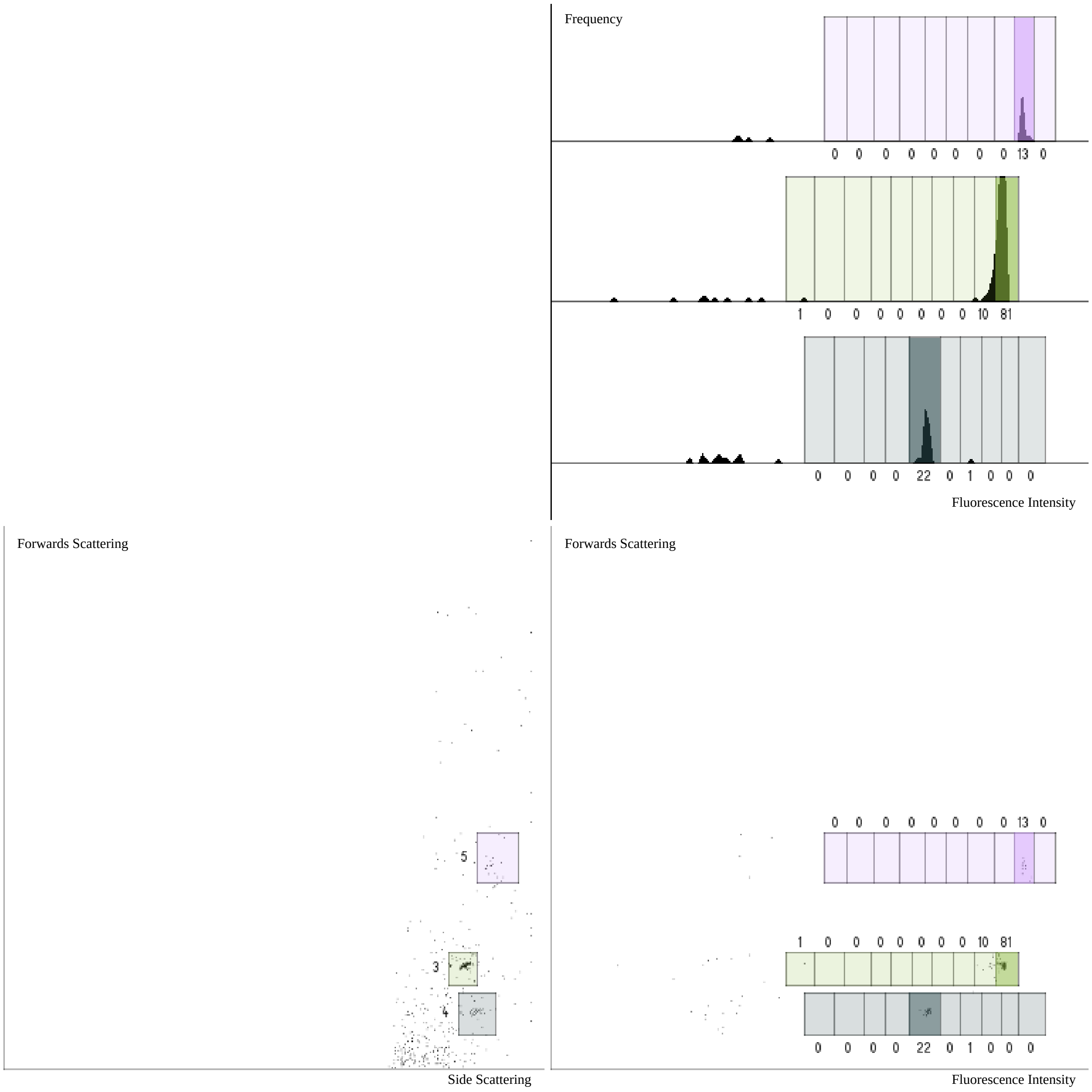

ANNEX 3: TAG DECONVOLUTION - BEAD 279

Passes flow sorting criteria: Yes

Passes tag deconvolution criteria: No

Included in protocol analysis: No

Protocol: N/A

Filename: Bin3_plateA2_B12.fcs

Split 1: Petrol shading

Split 2: Green shading

Split 3: Violet shading

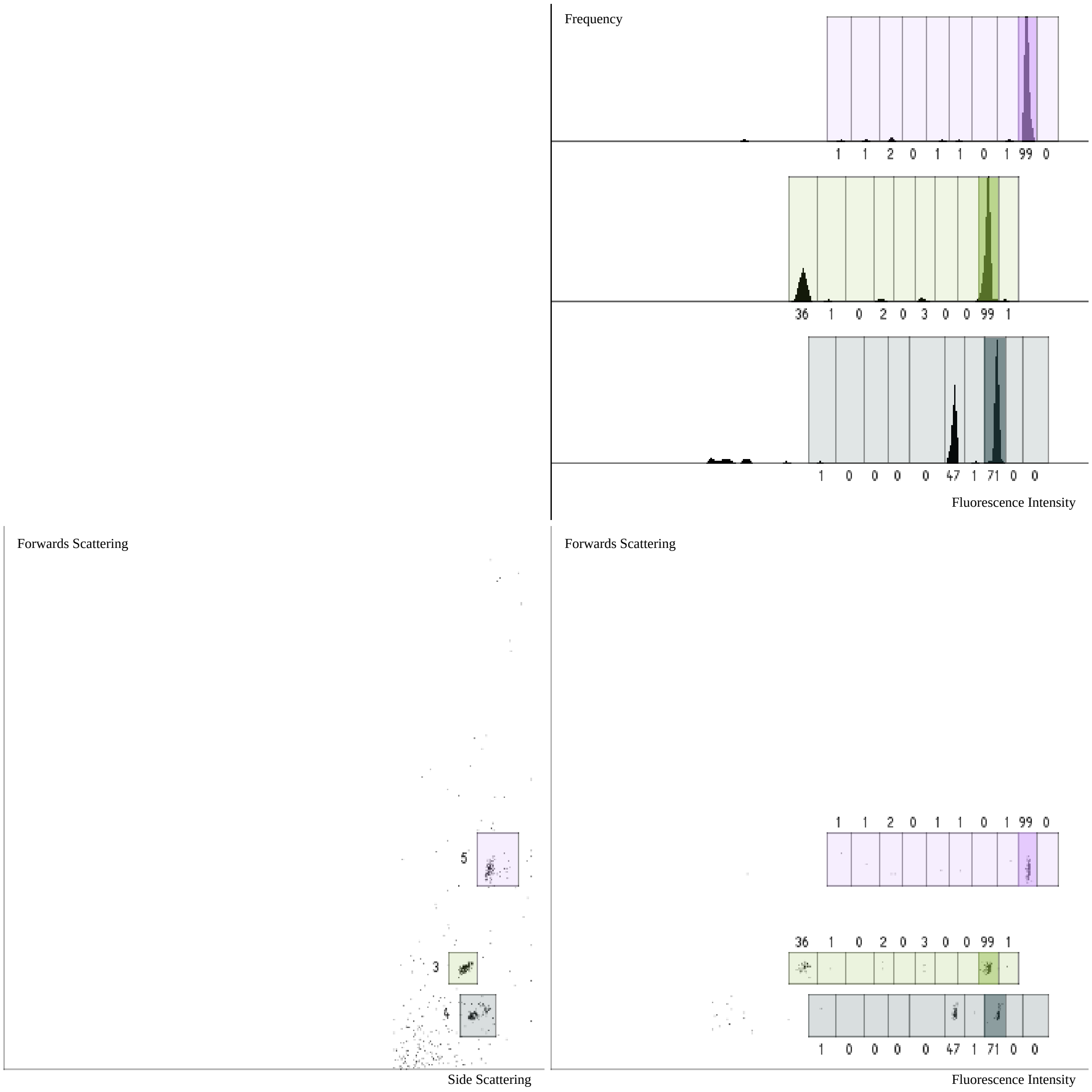

ANNEX 3: TAG DECONVOLUTION - BEAD 280

Passes flow sorting criteria: Yes

Passes tag deconvolution criteria: Yes

Included in protocol analysis: Yes

Protocol: 1, 5, 3, 3

Filename: Bin3_plateA2_B10.fcs

Split 1: Petrol shading

Split 2: Green shading

Split 3: Violet shading

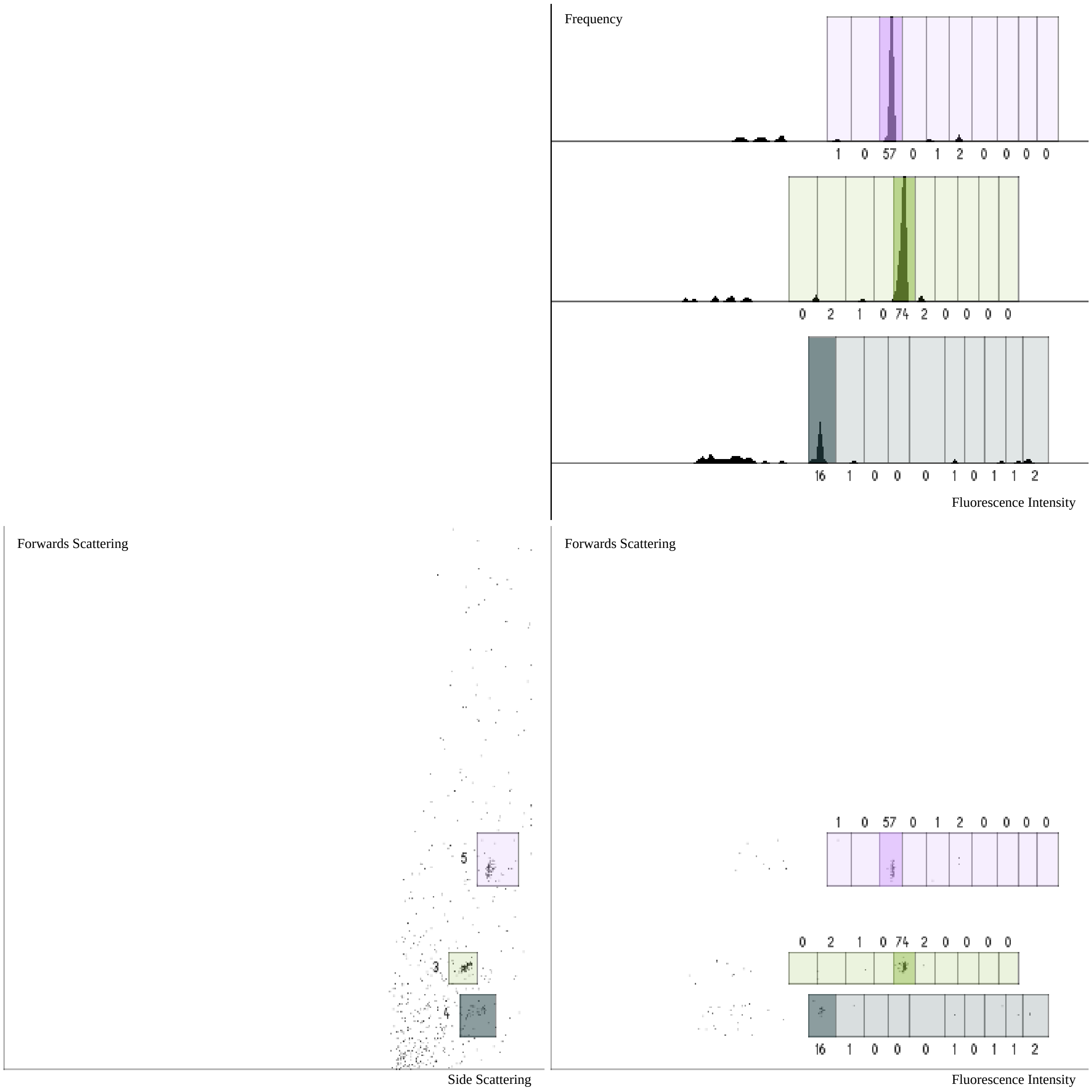

ANNEX 3: TAG DECONVOLUTION - BEAD 281

Passes flow sorting criteria: Yes

Passes tag deconvolution criteria: Yes

Included in protocol analysis: Yes

Protocol: 3, 5, 2, 3

Filename: Bin3_plateA2_B11.fcs

Split 1: Petrol shading

Split 2: Green shading

Split 3: Violet shading

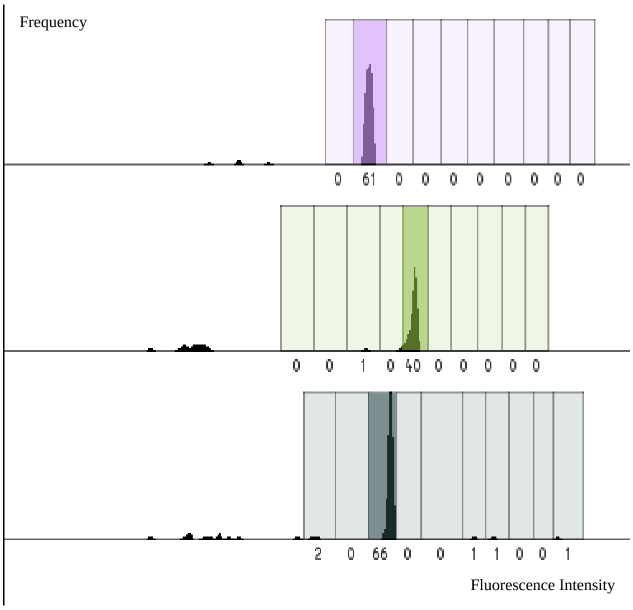

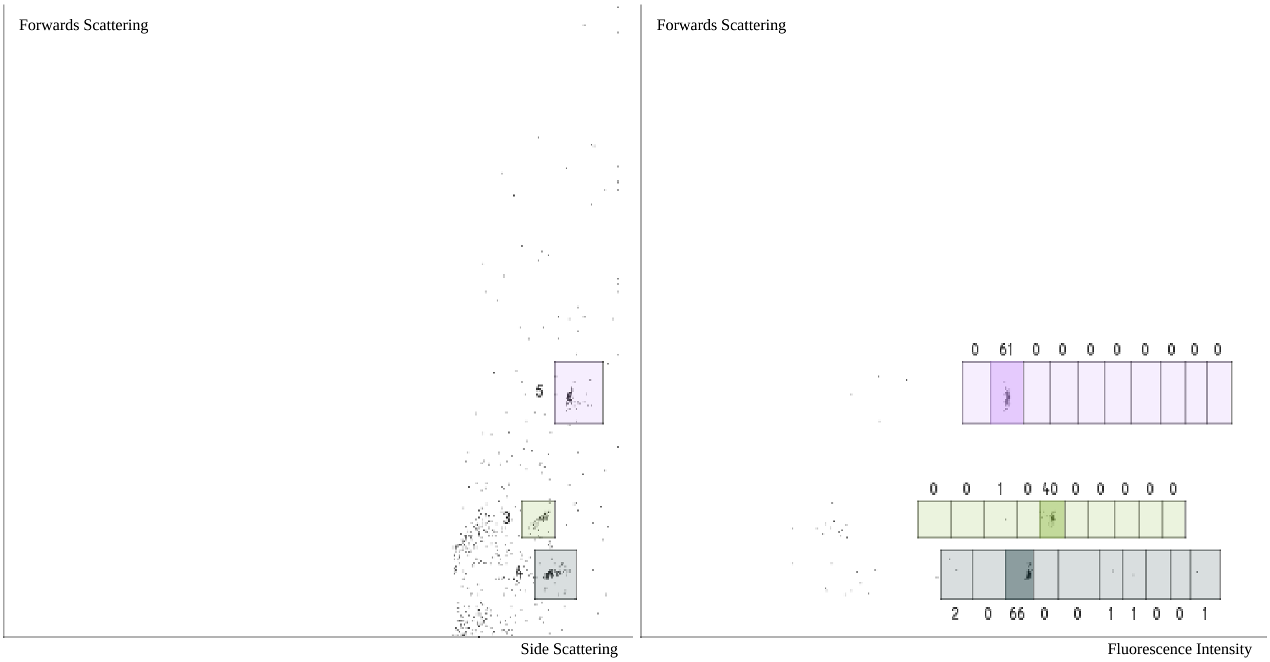

ANNEX 3: TAG DECONVOLUTION - BEAD 282

Passes flow sorting criteria: Yes

Passes tag deconvolution criteria: Yes

Included in protocol analysis: Yes

Protocol: 10, 8, 7, 4

Filename: Bin4_plateA2_E8.fcs

Split 1: Petrol shading

Split 2: Green shading

Split 3: Violet shading

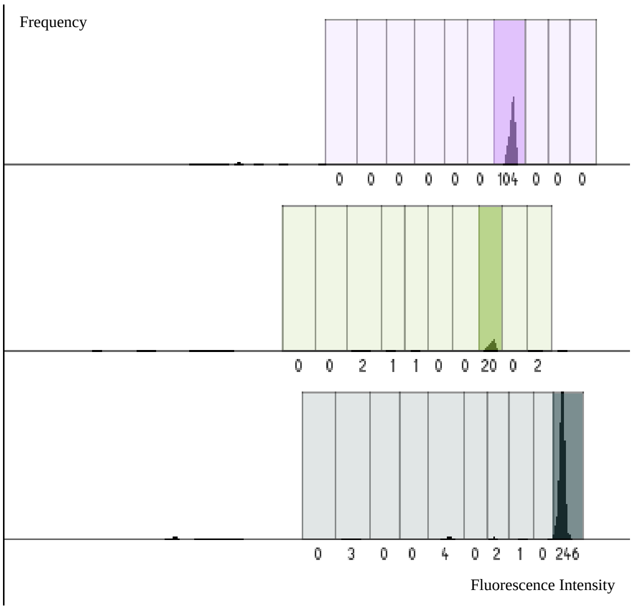

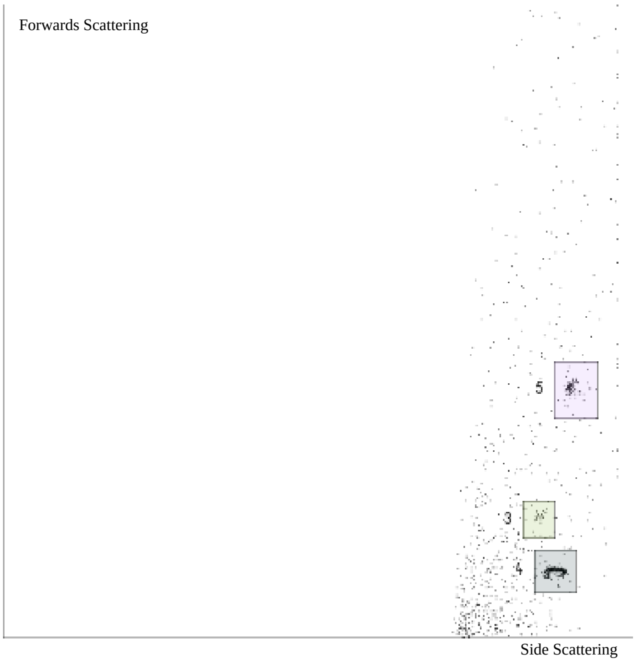

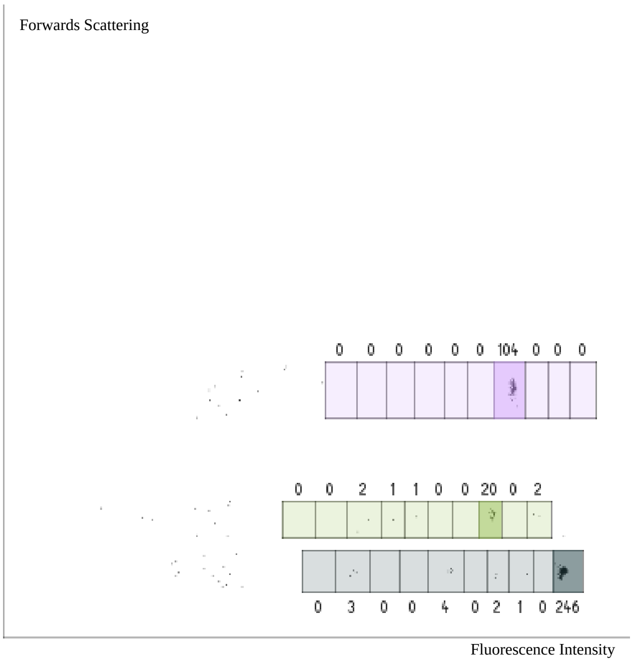

ANNEX 3: TAG DECONVOLUTION - BEAD 283

Passes flow sorting criteria: Yes

Passes tag deconvolution criteria: Yes

Included in protocol analysis: Yes

Protocol: 10, 9, 5, 4

Filename: Bin4_plateA2_D1.fcs

Split 1: Petrol shading

Split 2: Green shading

Split 3: Violet shading

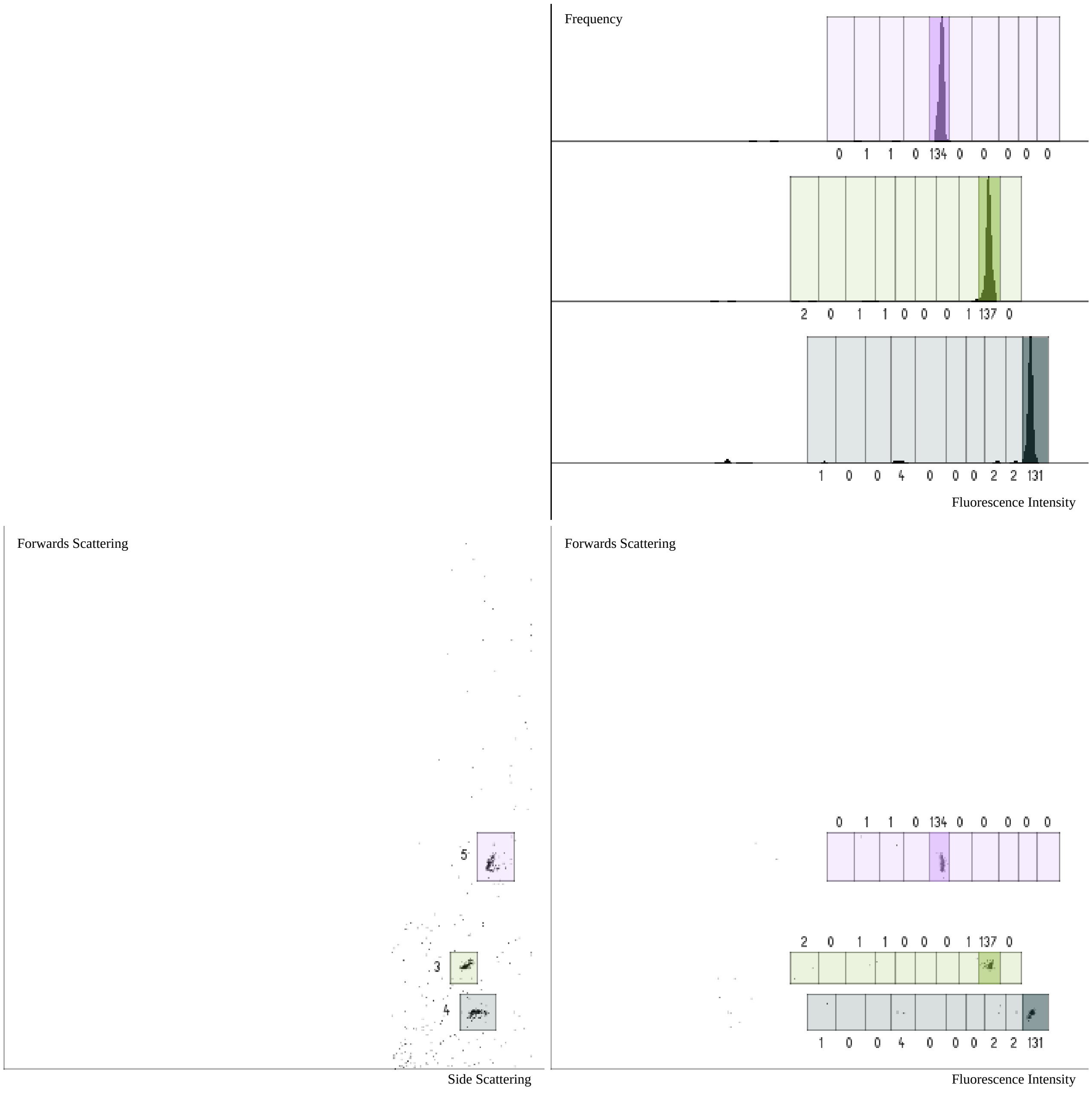

ANNEX 3: TAG DECONVOLUTION - BEAD 284

Passes flow sorting criteria: Yes

Passes tag deconvolution criteria: Yes

Included in protocol analysis: Yes

Protocol: 1, 5, 2, 4

Filename: Bin4_plateA2_D2.fcs

Split 1: Petrol shading

Split 2: Green shading

Split 3: Violet shading

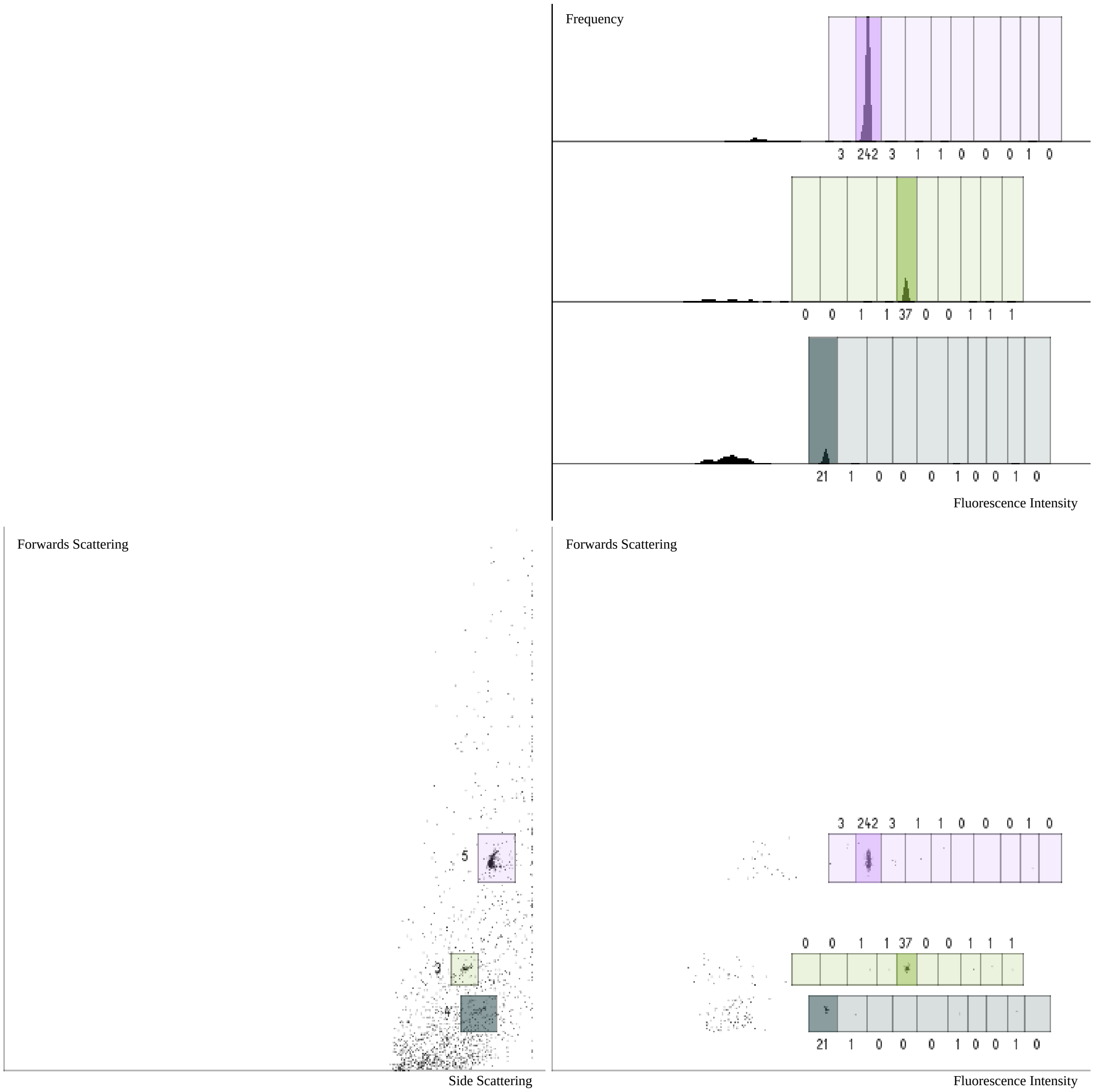

ANNEX 3: TAG DECONVOLUTION - BEAD 285

Passes flow sorting criteria: Yes

Passes tag deconvolution criteria: Yes

Included in protocol analysis: Yes

Protocol: 10, 1, 5, 4

Filename: Bin4_plateA2_D3.fcs

Split 1: Petrol shading

Split 2: Green shading

Split 3: Violet shading

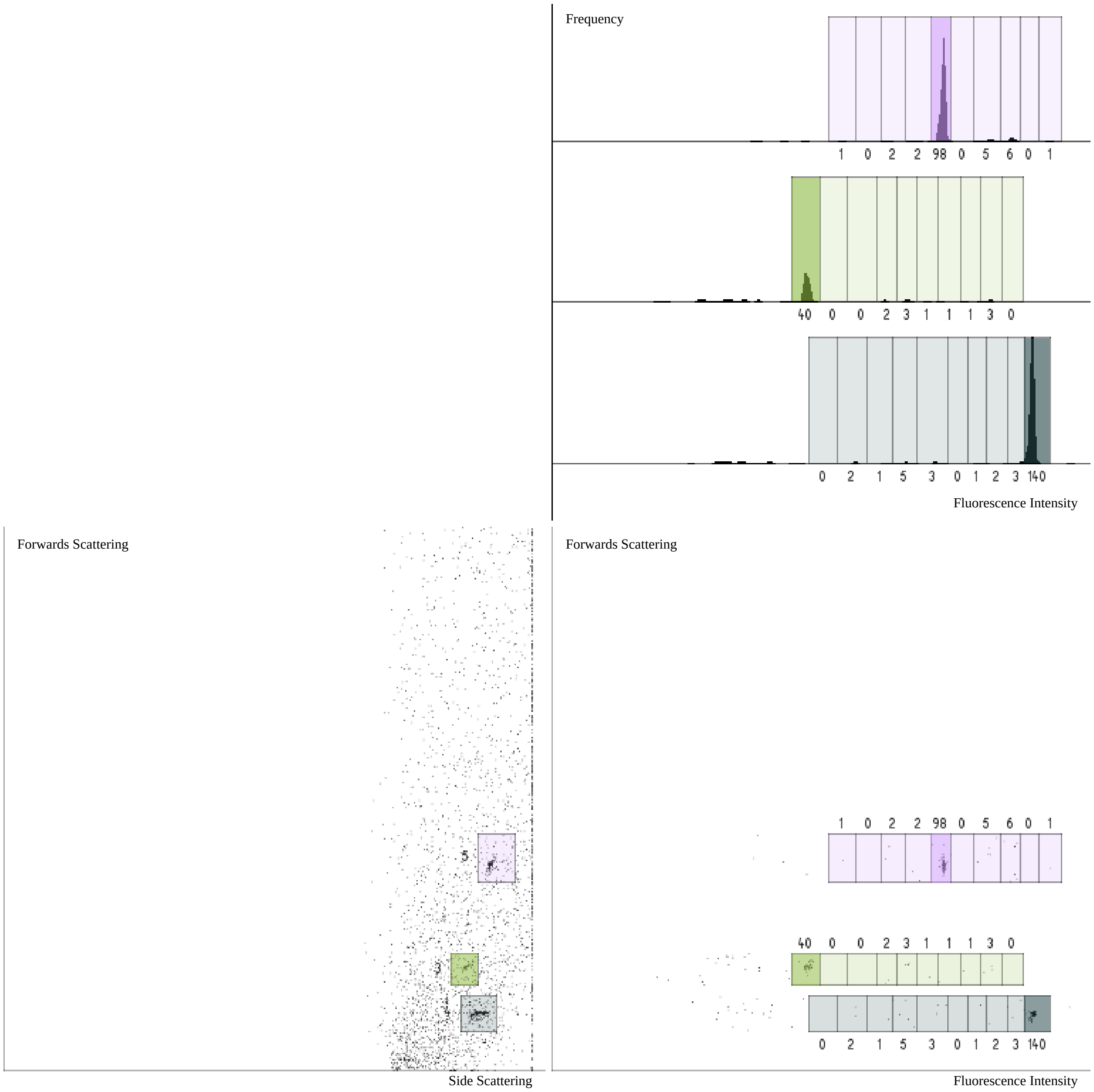

ANNEX 3: TAG DECONVOLUTION - BEAD 286

Passes flow sorting criteria: Yes

Passes tag deconvolution criteria: Yes

Included in protocol analysis: Yes

Protocol: 3, 1, 8, 4

Filename: Bin4_plateA2_D4.fcs

Split 1: Petrol shading

Split 2: Green shading

Split 3: Violet shading

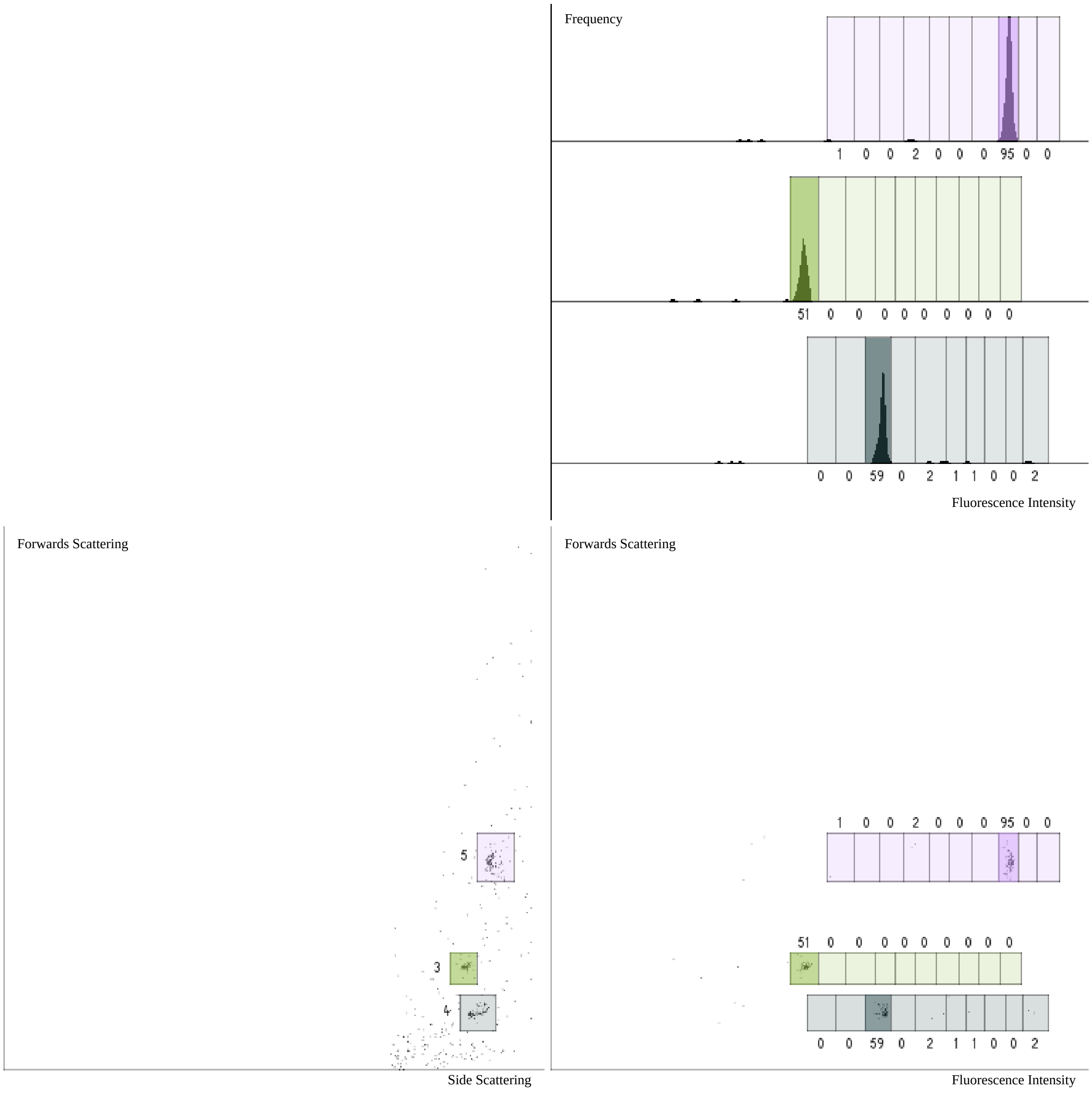

ANNEX 3: TAG DECONVOLUTION - BEAD 287

Passes flow sorting criteria: Yes

Passes tag deconvolution criteria: Yes

Included in protocol analysis: Yes

Protocol: 3, 2, 2, 4

Filename: Bin4_plateA2_D5.fcs

Split 1: Petrol shading

Split 2: Green shading

Split 3: Violet shading

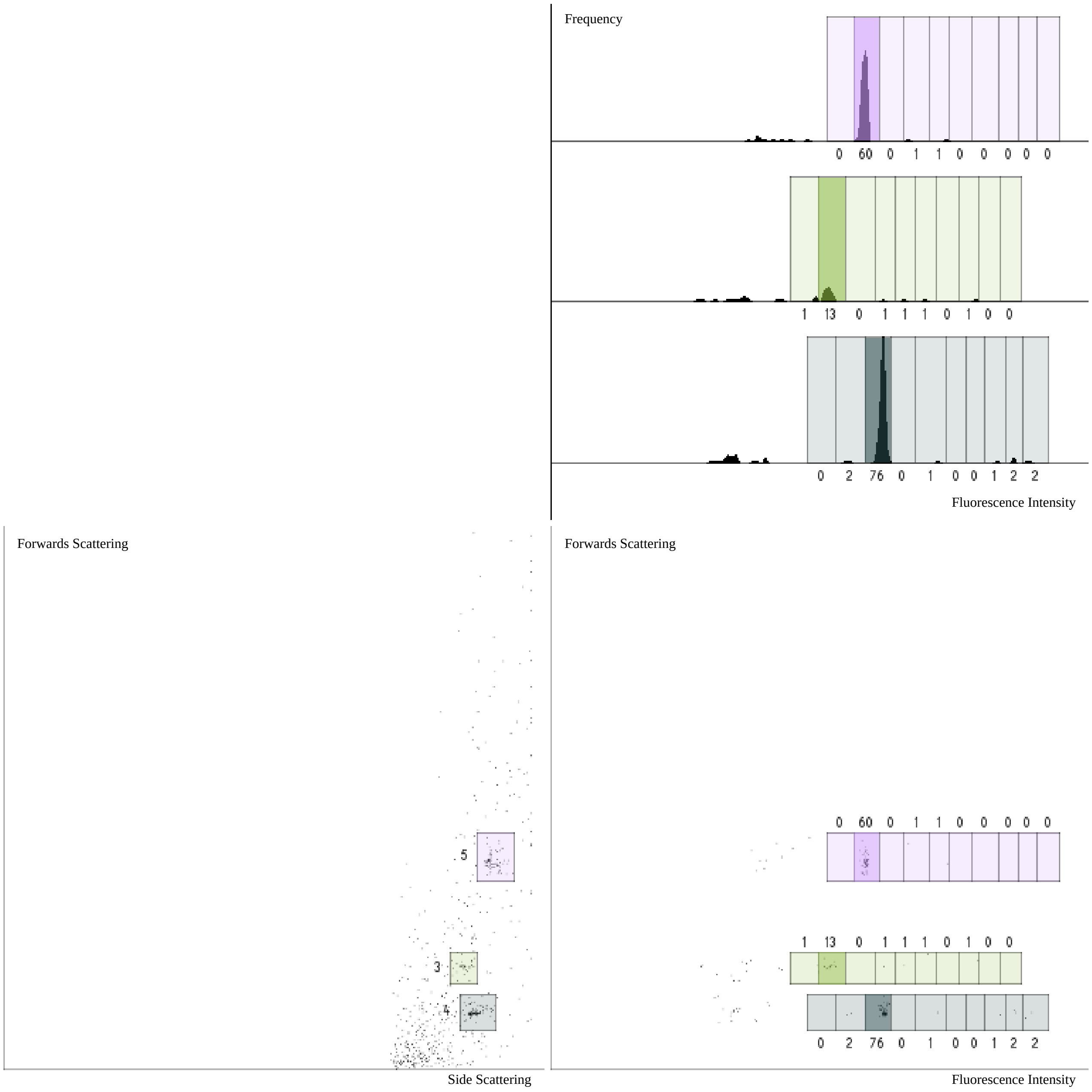

ANNEX 3: TAG DECONVOLUTION - BEAD 288

Passes flow sorting criteria: Yes

Passes tag deconvolution criteria: Yes

Included in protocol analysis: Yes

Protocol: 1, 2, 9, 4

Filename: Bin4_plateA2_D6.fcs

Split 1: Petrol shading

Split 2: Green shading

Split 3: Violet shading

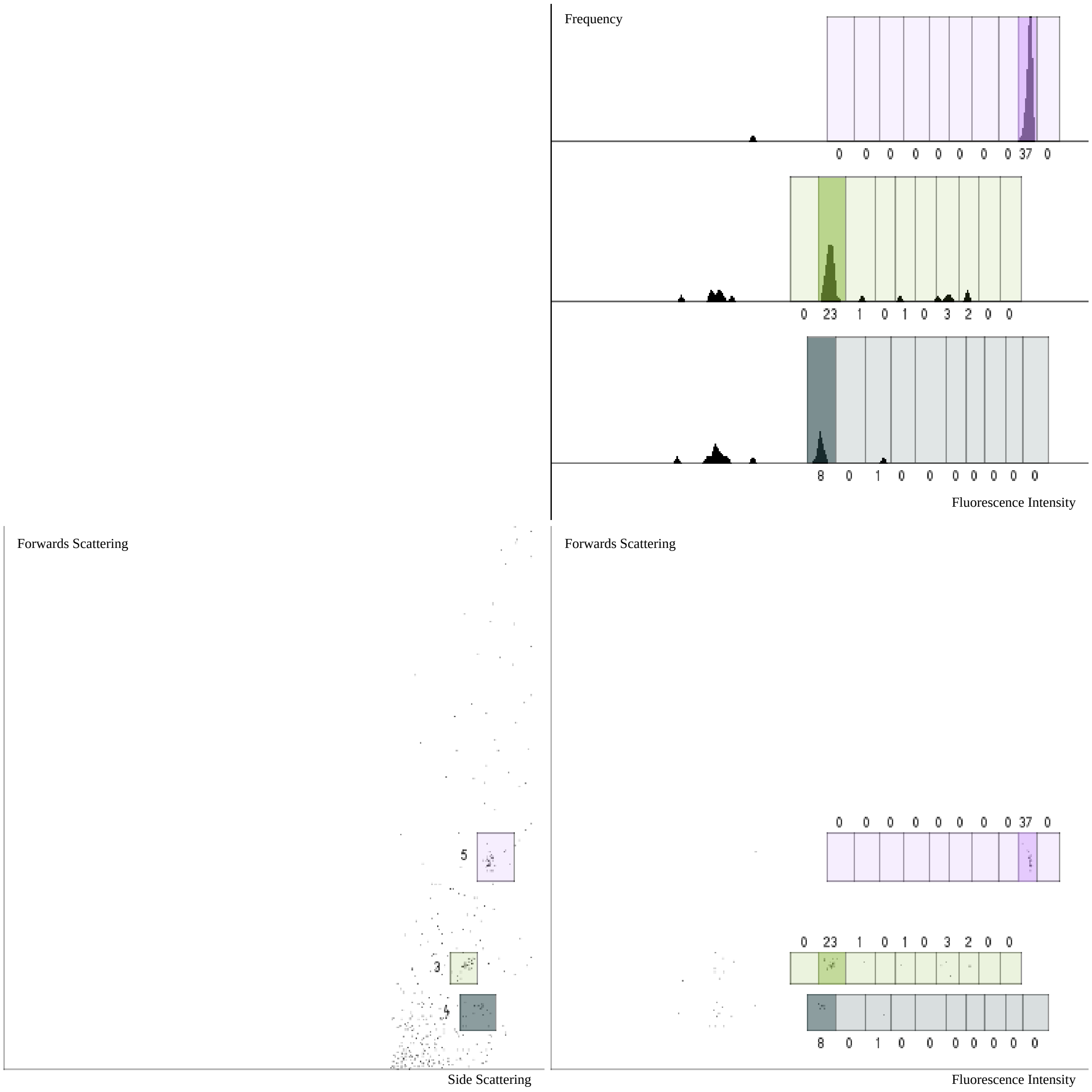

ANNEX 3: TAG DECONVOLUTION - BEAD 289

Passes flow sorting criteria: Yes

Passes tag deconvolution criteria: No

Included in protocol analysis: No

Protocol: N/A

Filename: Bin4_plateA2_D7.fcs

Split 1: Petrol shading

Split 2: Green shading

Split 3: Violet shading

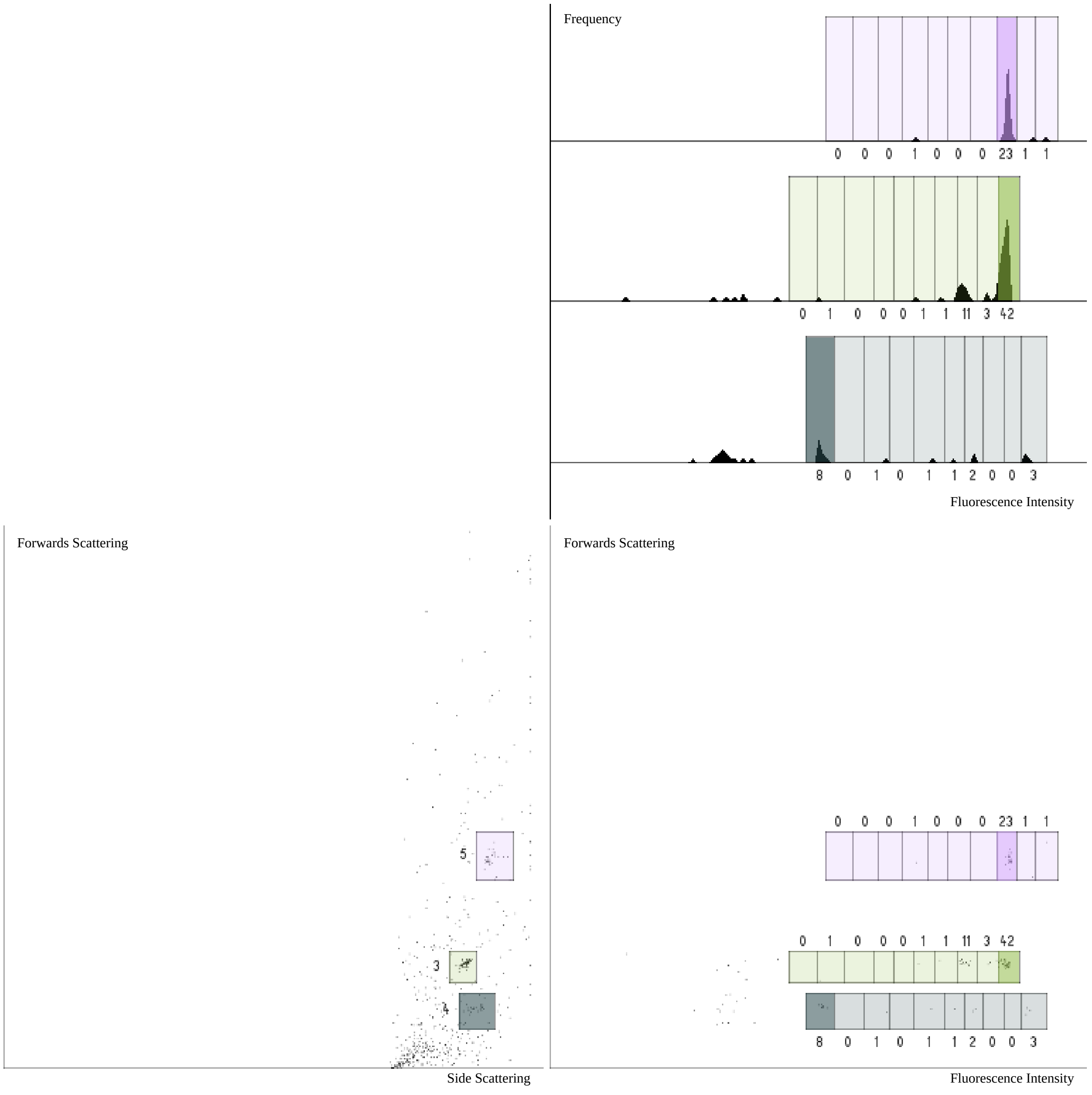

ANNEX 3: TAG DECONVOLUTION - BEAD 290

Passes flow sorting criteria: Yes

Passes tag deconvolution criteria: No

Included in protocol analysis: No

Protocol: N/A

Filename: Bin4_plateA2_D8.fcs

Split 1: Petrol shading

Split 2: Green shading

Split 3: Violet shading

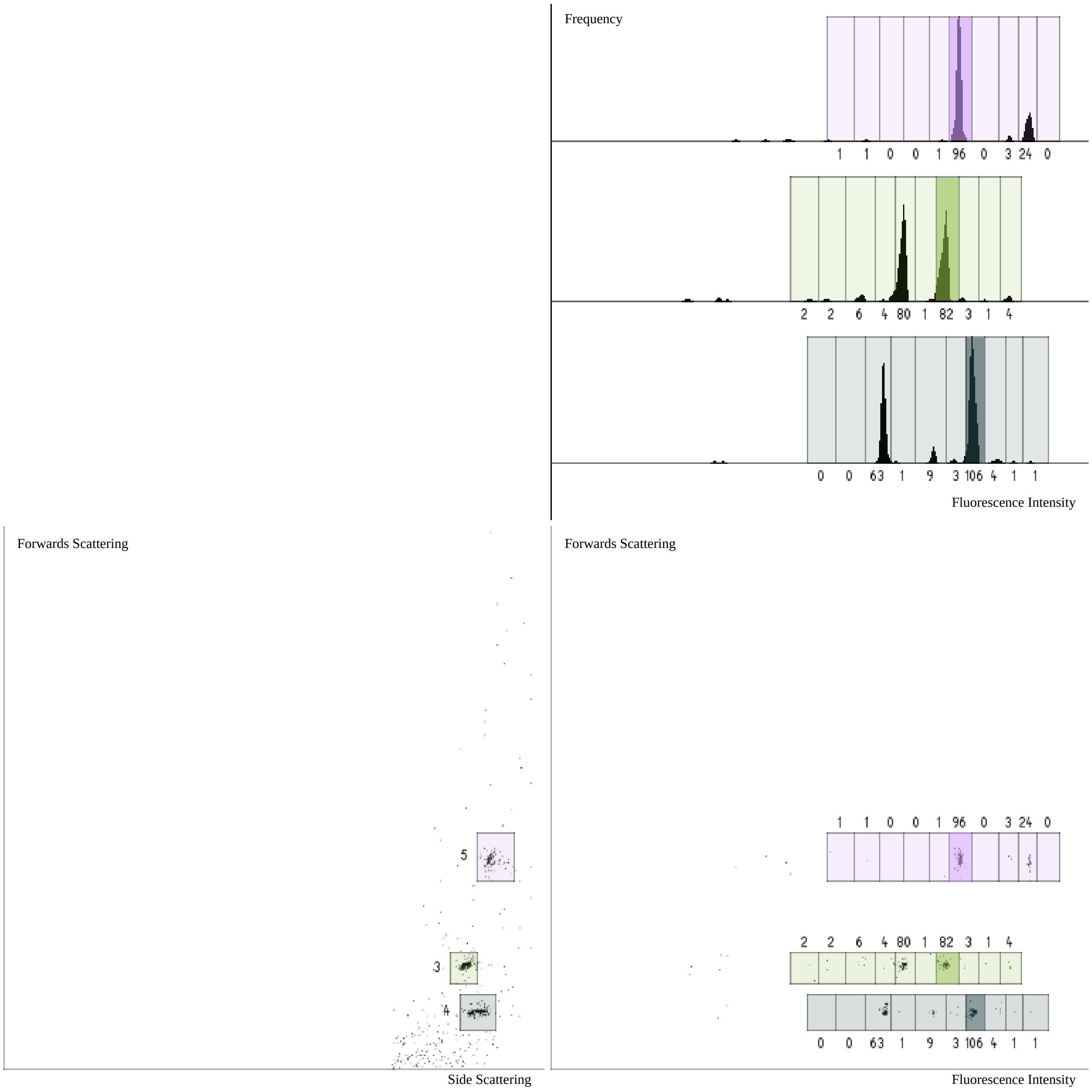

ANNEX 3: TAG DECONVOLUTION - BEAD 291

Passes flow sorting criteria: Yes

Passes tag deconvolution criteria: Yes

Included in protocol analysis: Yes

Protocol: 5, 5, 1, 4

Filename: Bin4_plateA2_D9.fcs

Split 1: Petrol shading

Split 2: Green shading

Split 3: Violet shading

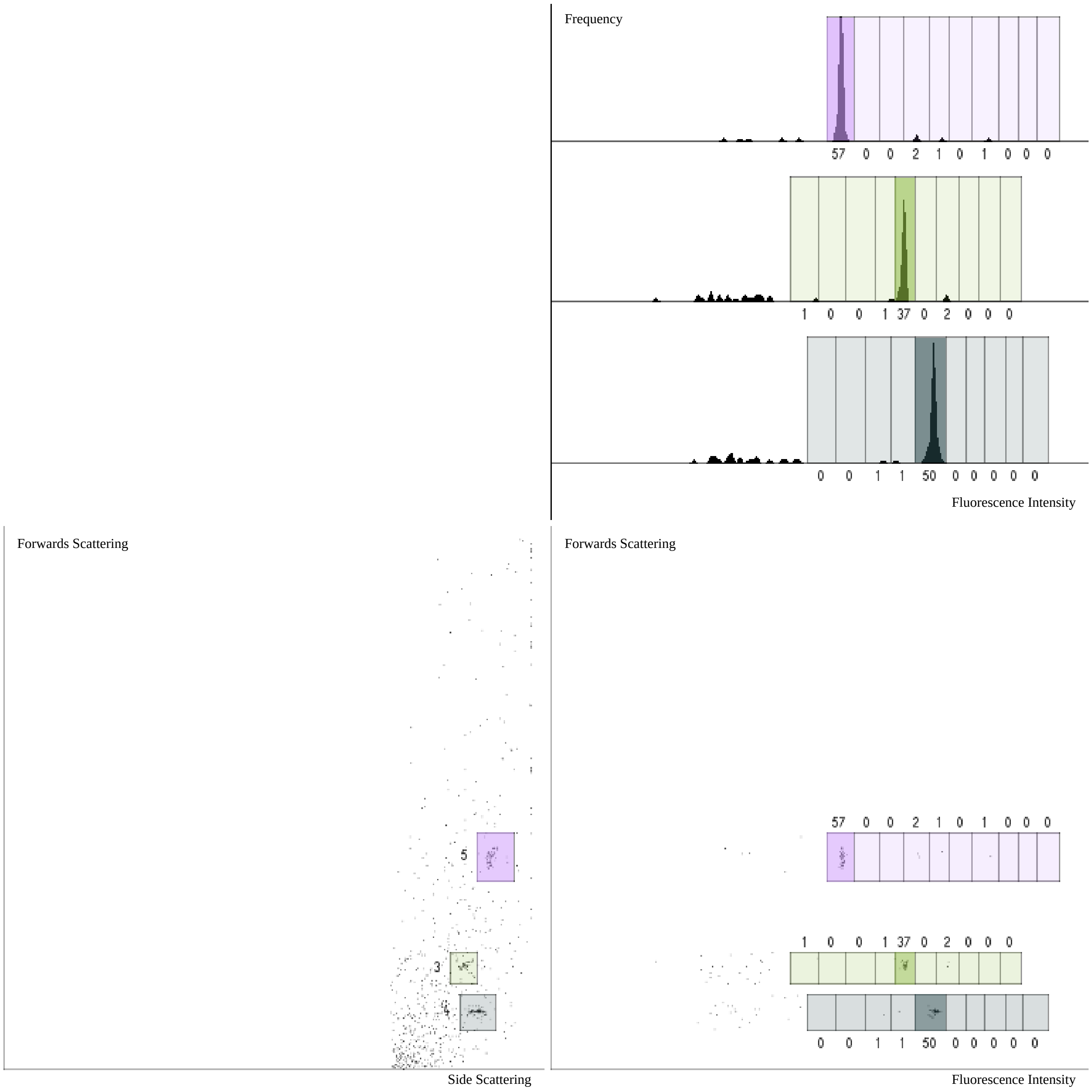

ANNEX 3: TAG DECONVOLUTION - BEAD 292

Passes flow sorting criteria: Yes

Passes tag deconvolution criteria: No

Included in protocol analysis: No

Protocol: N/A

Filename: Bin4_plateA2_D10.fcs

Split 1: Petrol shading

Split 2: Green shading

Split 3: Violet shading

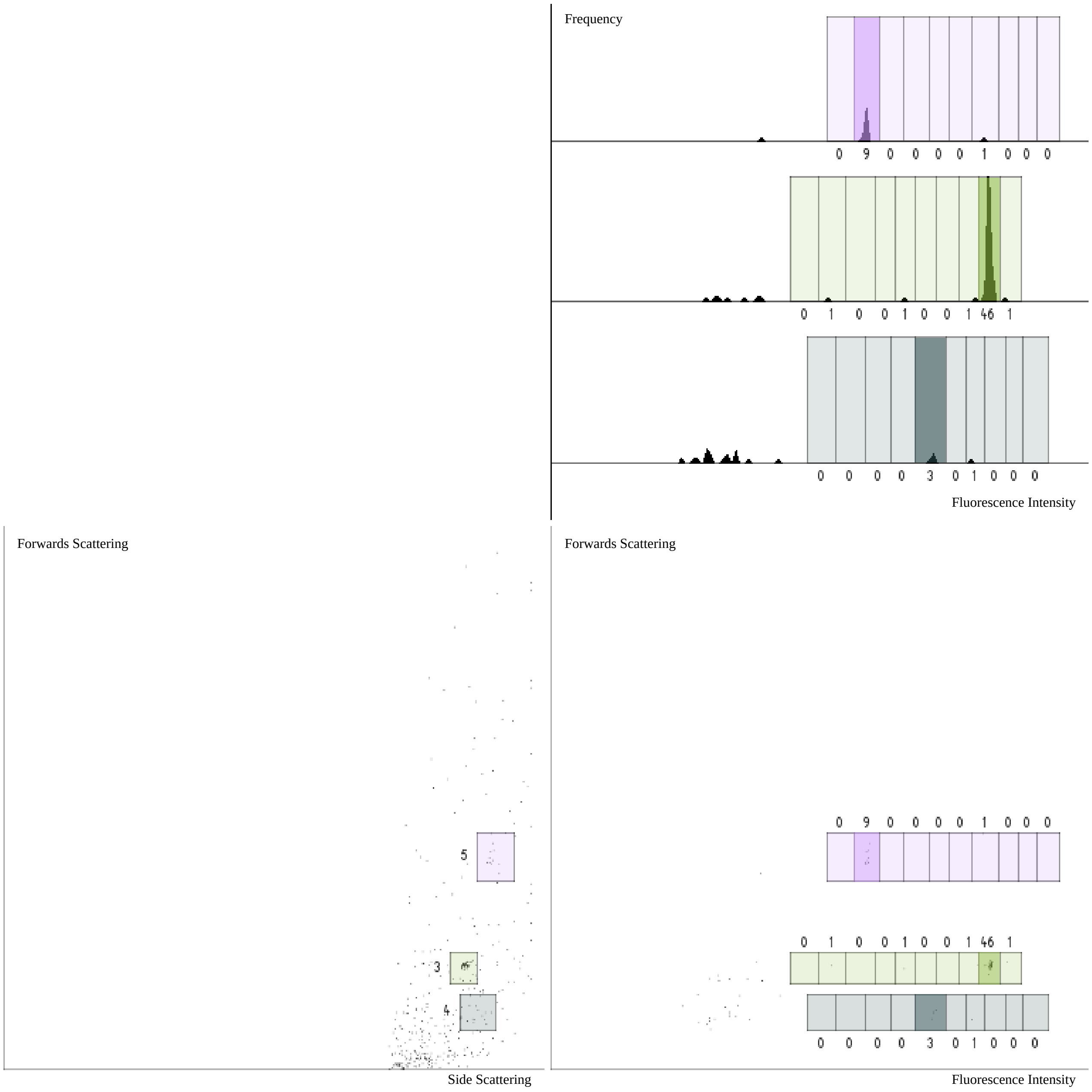

ANNEX 3: TAG DECONVOLUTION - BEAD 293

Passes flow sorting criteria: Yes

Passes tag deconvolution criteria: No

Included in protocol analysis: No

Protocol: N/A

Filename: Bin4_plateA2_D11.fcs

Split 1: Petrol shading

Split 2: Green shading

Split 3: Violet shading

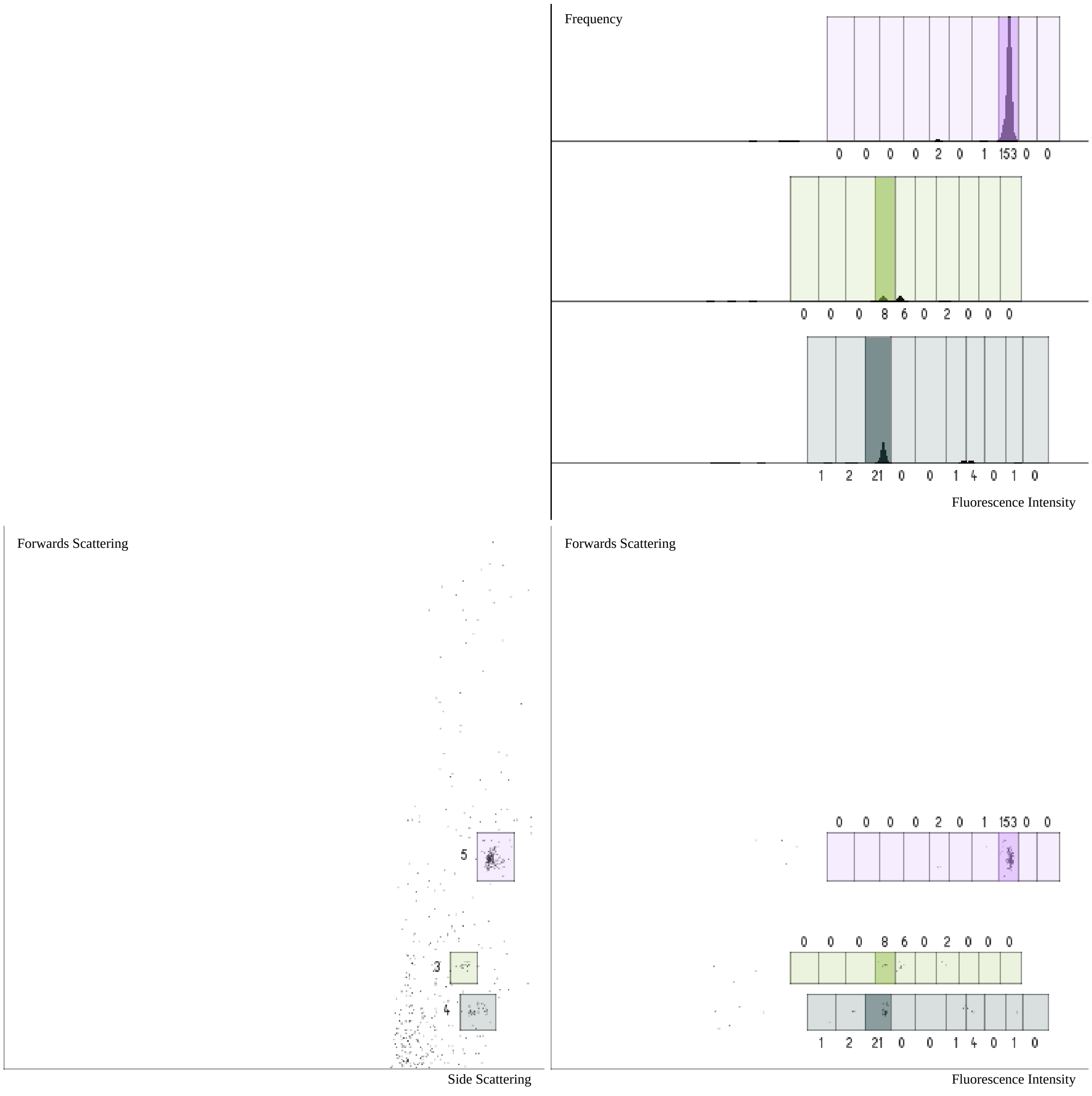

ANNEX 3: TAG DECONVOLUTION - BEAD 294

Passes flow sorting criteria: Yes

Passes tag deconvolution criteria: Yes

Included in protocol analysis: Yes

Protocol: 5, 10, 6, 4

Filename: Bin4_plateA2_D12.fcs

Split 1: Petrol shading

Split 2: Green shading

Split 3: Violet shading

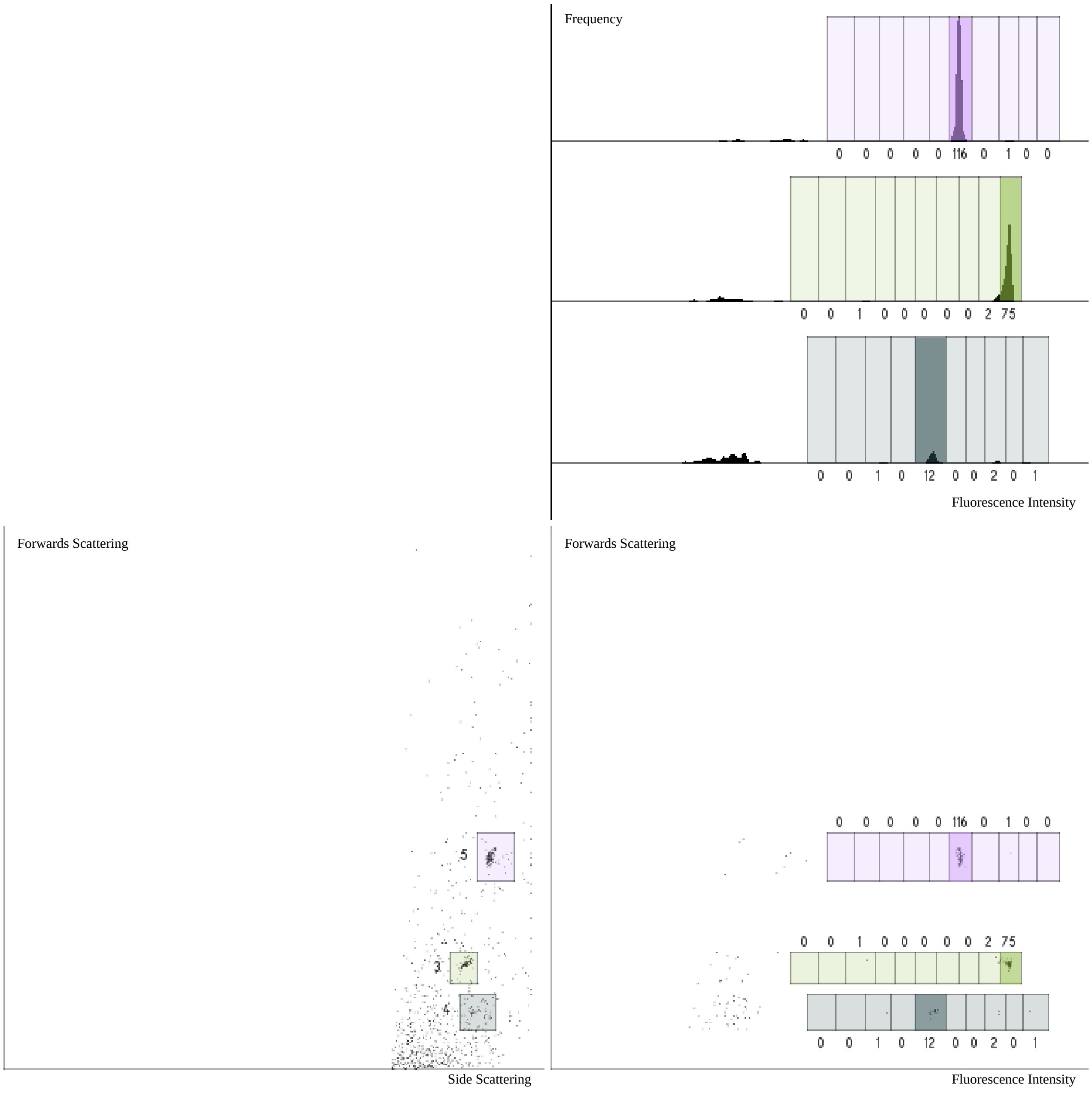

ANNEX 3: TAG DECONVOLUTION - BEAD 295

Passes flow sorting criteria: Yes

Passes tag deconvolution criteria: Yes

Included in protocol analysis: Yes

Protocol: 10, 8, 8, 4

Filename: Bin4_plateA2_E1.fcs

Split 1: Petrol shading

Split 2: Green shading

Split 3: Violet shading

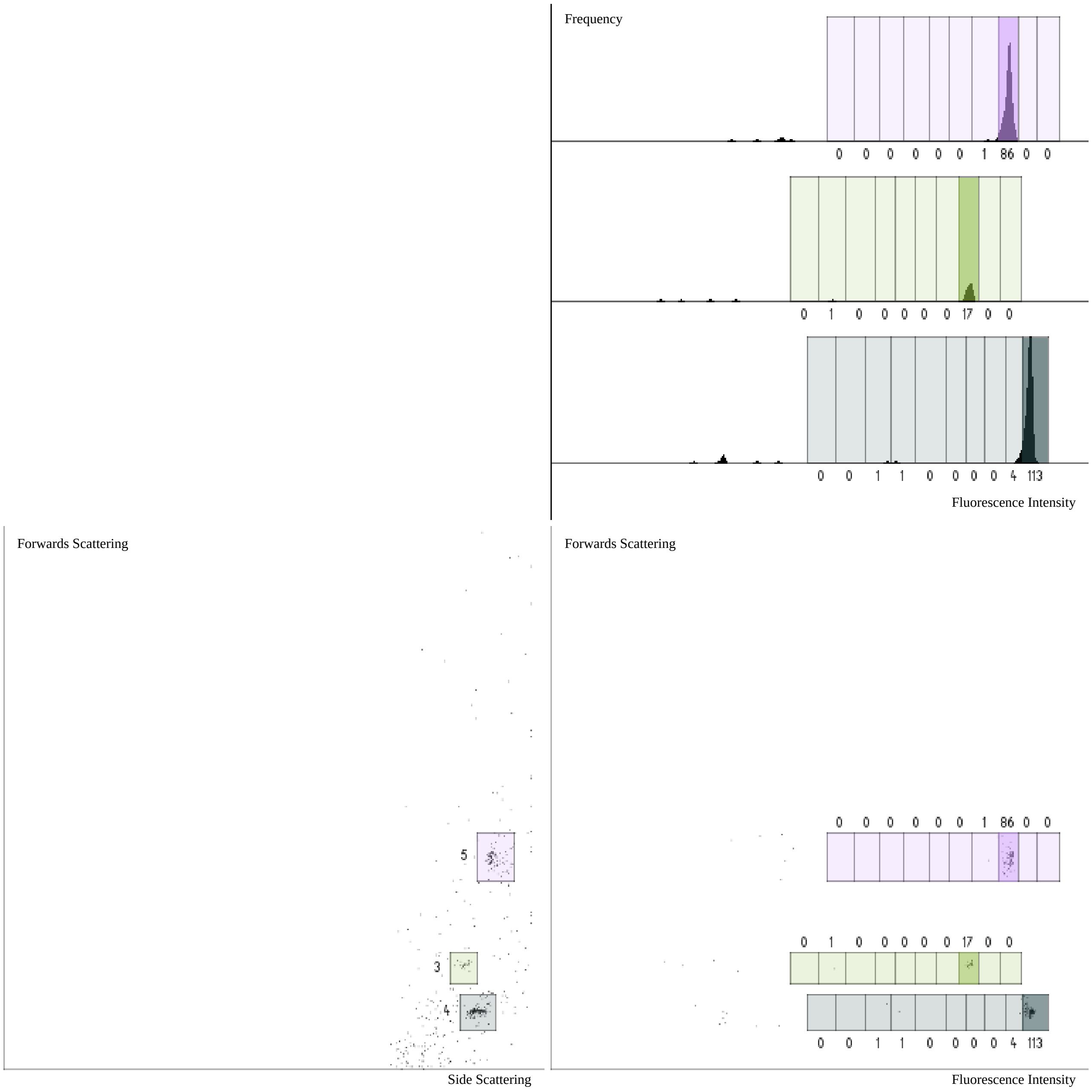

ANNEX 3: TAG DECONVOLUTION - BEAD 296

Passes flow sorting criteria: Yes

Passes tag deconvolution criteria: No

Included in protocol analysis: No

Protocol: N/A

Filename: Bin4_plateA2_E2.fcs

Split 1: Petrol shading

Split 2: Green shading

Split 3: Violet shading

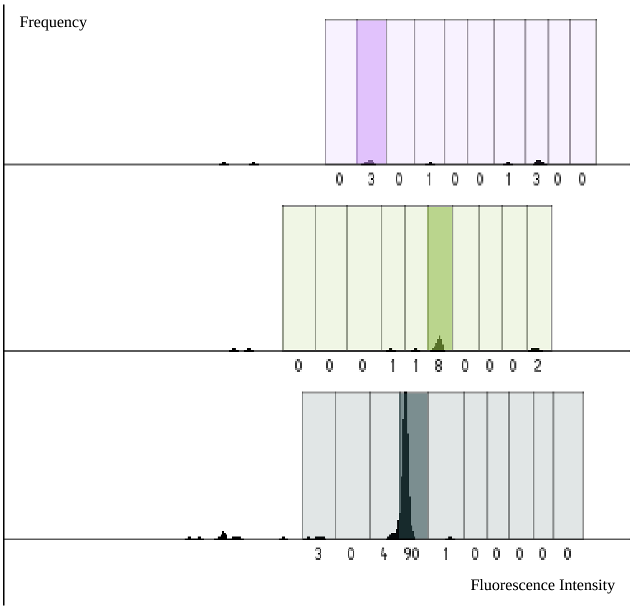

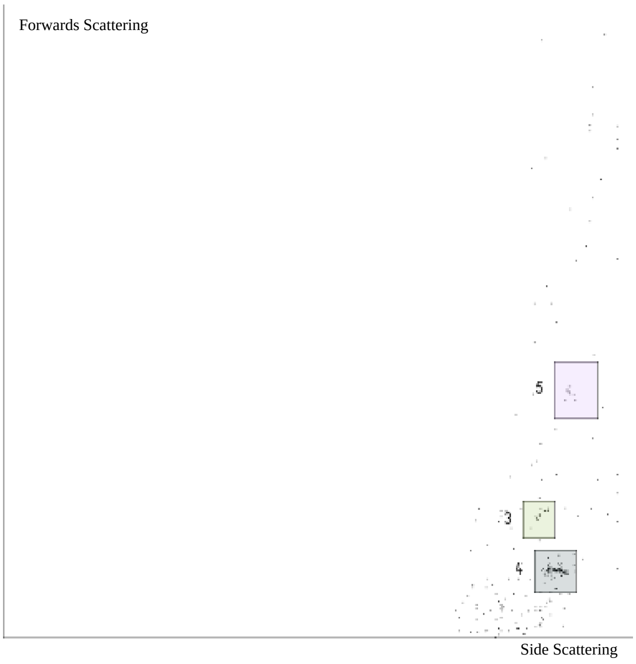

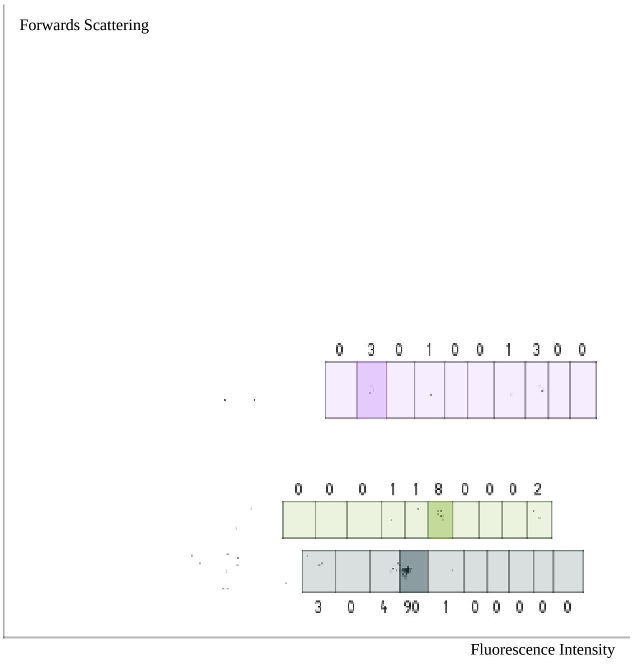

ANNEX 3: TAG DECONVOLUTION - BEAD 297

Passes flow sorting criteria: Yes

Passes tag deconvolution criteria: No

Included in protocol analysis: No

Protocol: N/A

Filename: Bin4_plateA2_E3.fcs

Split 1: Petrol shading

Split 2: Green shading

Split 3: Violet shading

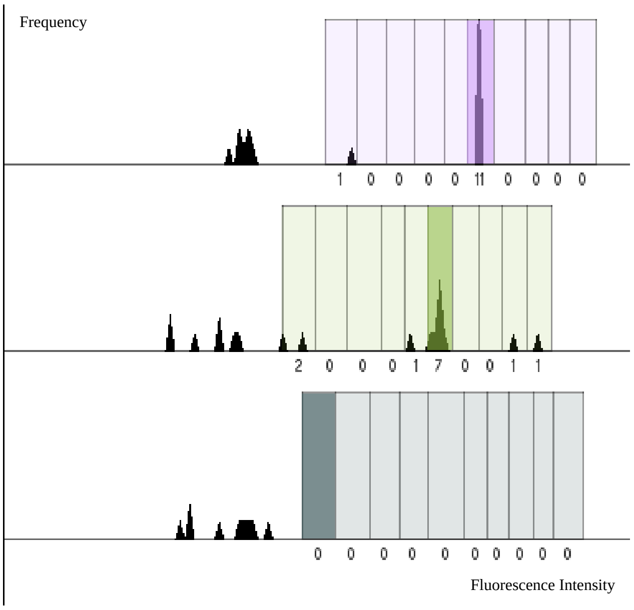

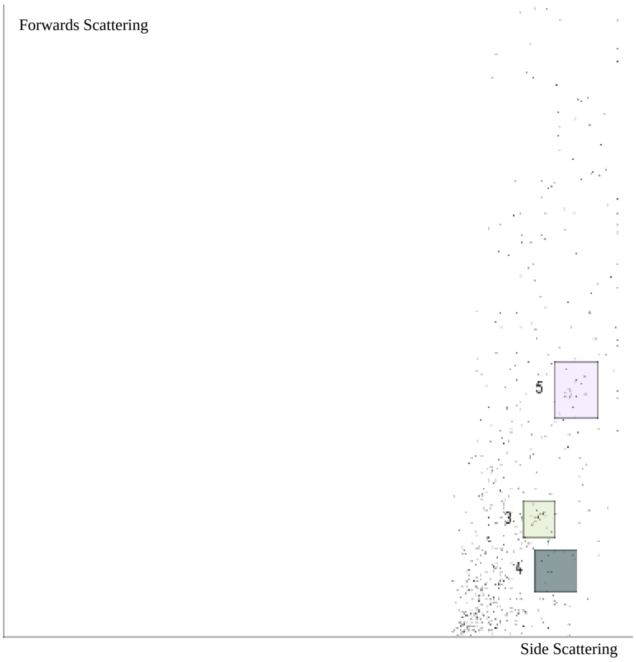

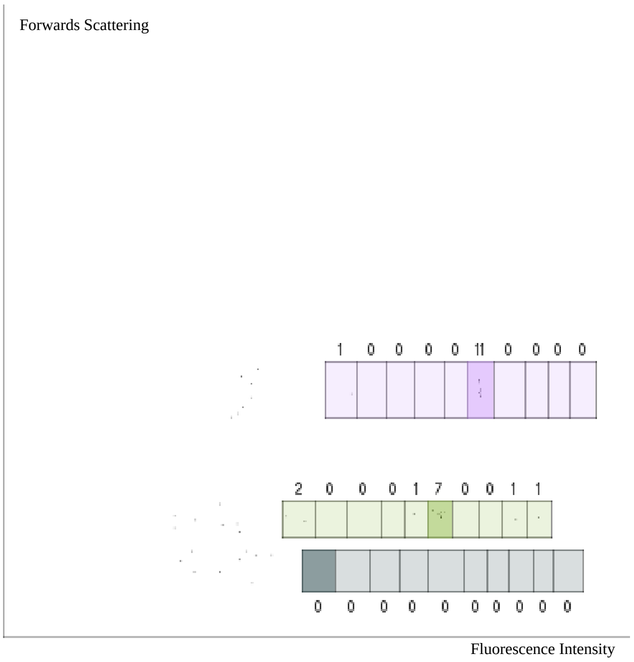

ANNEX 3: TAG DECONVOLUTION - BEAD 298

Passes flow sorting criteria: Yes

Passes tag deconvolution criteria: Yes

Included in protocol analysis: Yes

Protocol: 1, 9, 5, 4

Filename: Bin4_plateA2_E4.fcs

Split 1: Petrol shading

Split 2: Green shading

Split 3: Violet shading

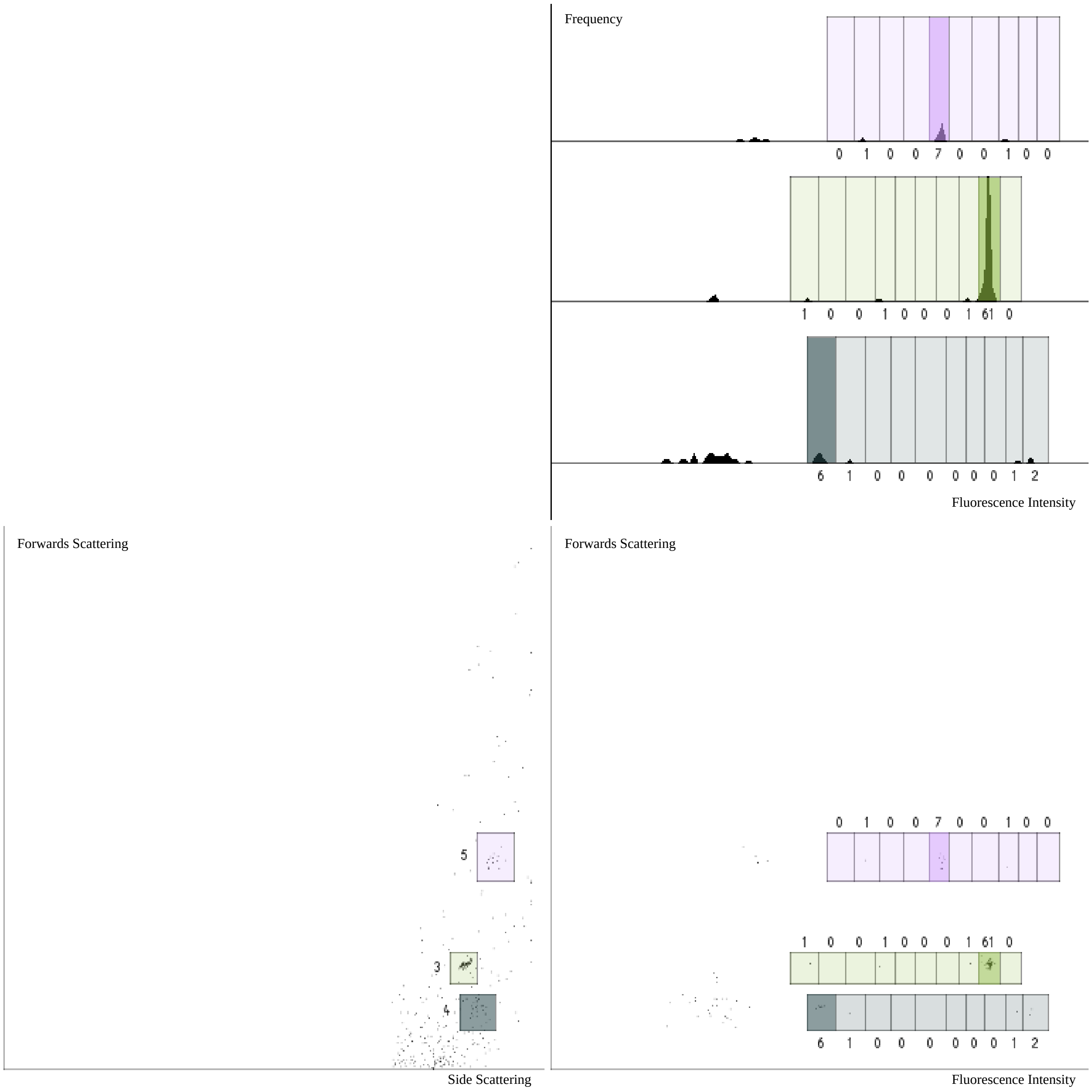

ANNEX 3: TAG DECONVOLUTION - BEAD 299

Passes flow sorting criteria: Yes

Passes tag deconvolution criteria: Yes

Included in protocol analysis: Yes

Protocol: 1, 5, 2, 4

Filename: Bin4_plateA2_E5.fcs

Split 1: Petrol shading

Split 2: Green shading

Split 3: Violet shading

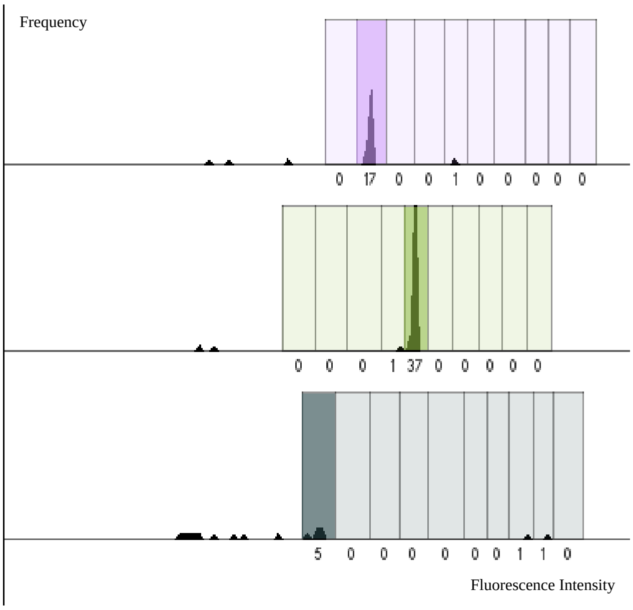

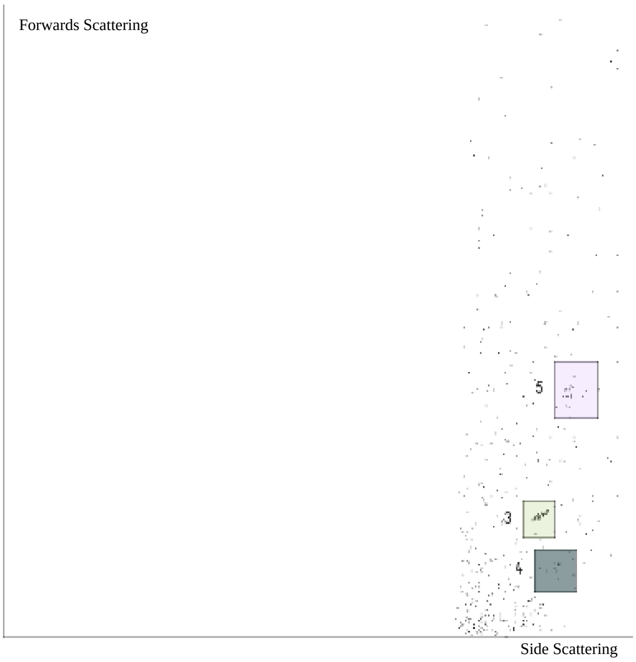

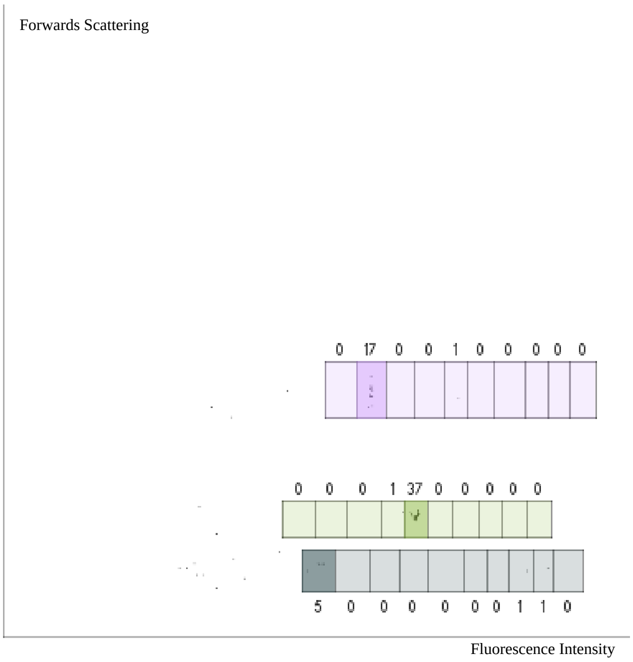

ANNEX 3: TAG DECONVOLUTION - BEAD 300

Passes flow sorting criteria: Yes

Passes tag deconvolution criteria: No

Included in protocol analysis: No

Protocol: N/A

Filename: Bin4_plateA2_E6.fcs

Split 1: Petrol shading

Split 2: Green shading

Split 3: Violet shading

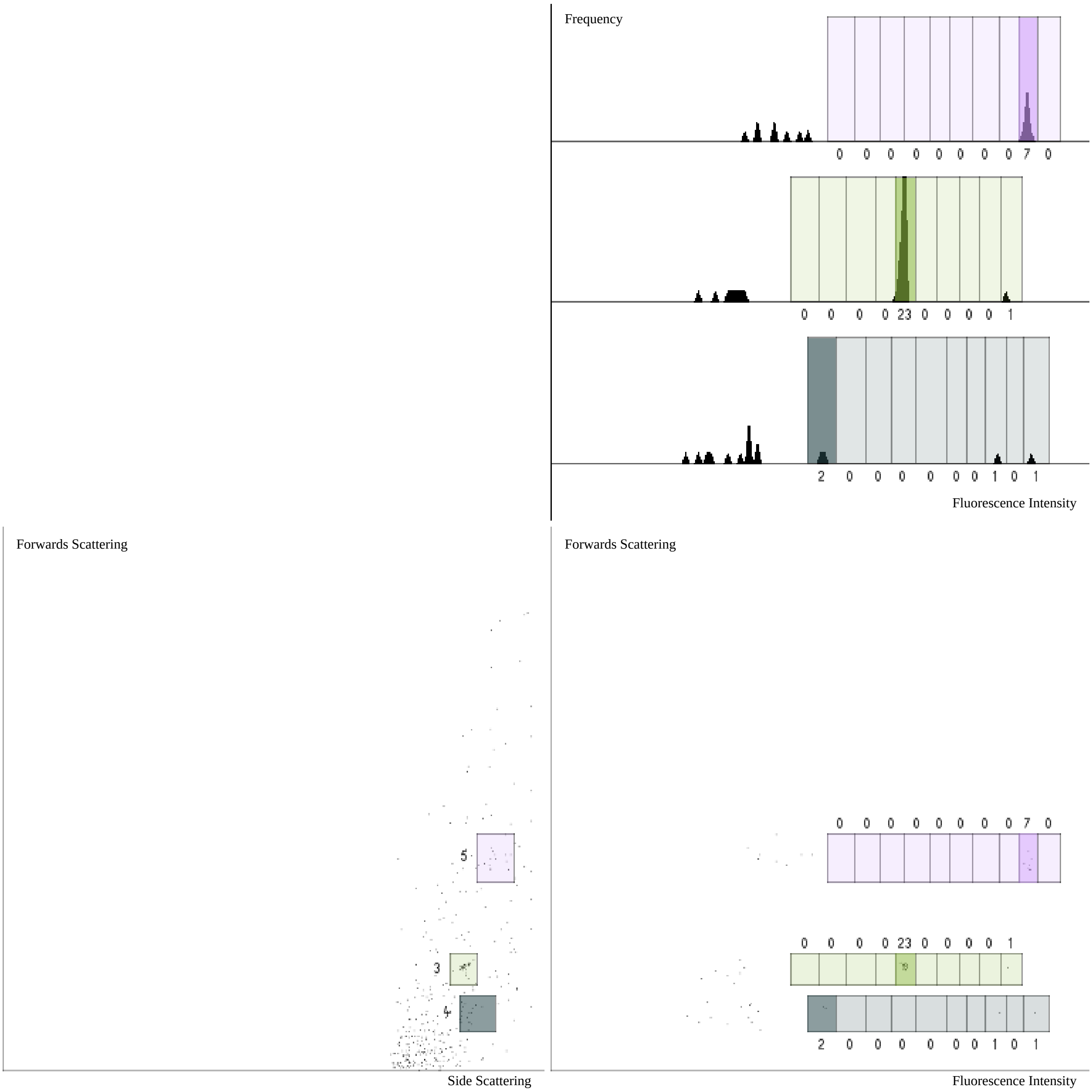

ANNEX 3: TAG DECONVOLUTION - BEAD 301

Passes flow sorting criteria: Yes

Passes tag deconvolution criteria: Yes

Included in protocol analysis: Yes

Protocol: 2, 6, 7, 4

Filename: Bin4_plateA2_E7.fcs

Split 1: Petrol shading

Split 2: Green shading

Split 3: Violet shading

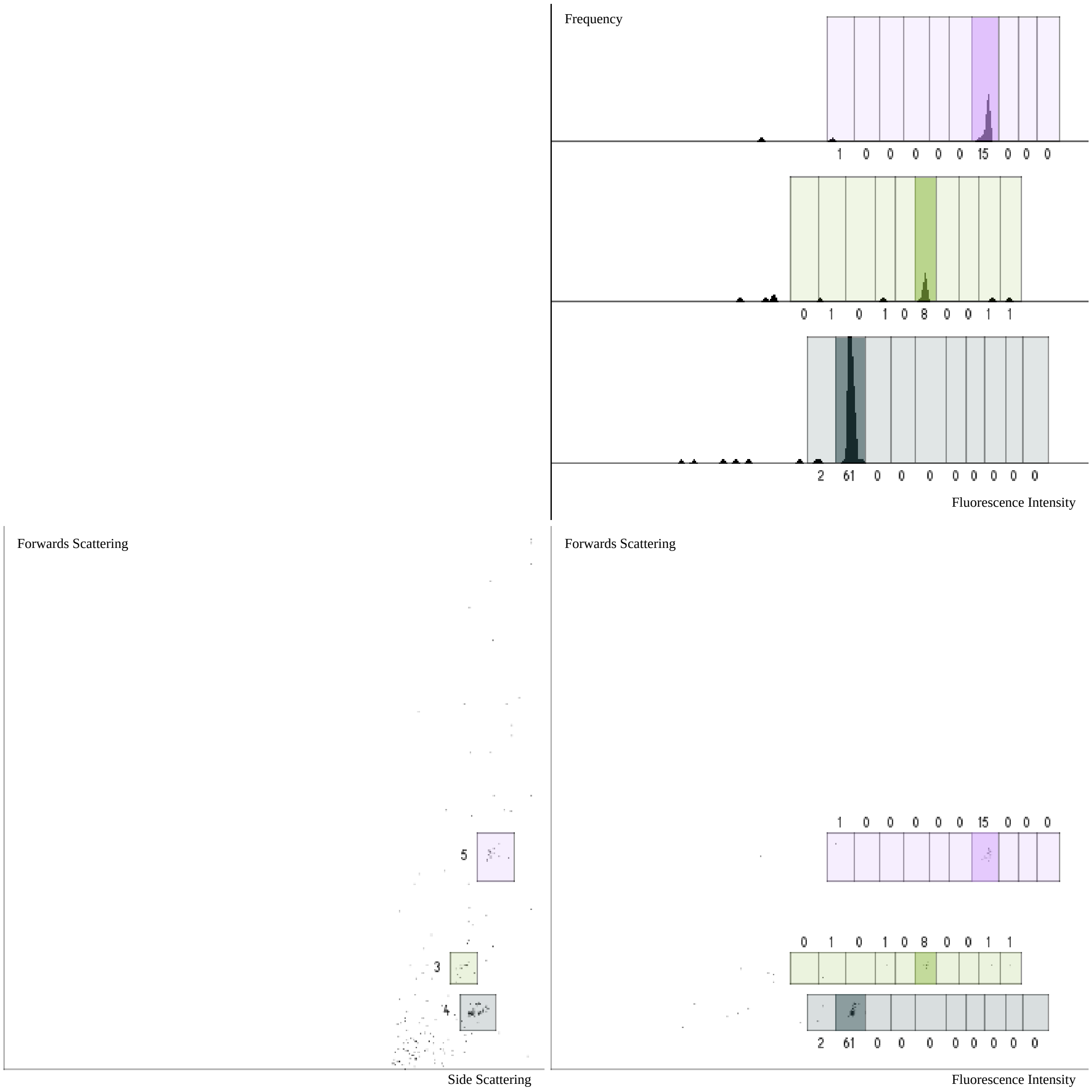

ANNEX 3: TAG DECONVOLUTION - BEAD 302

Passes flow sorting criteria: Yes

Passes tag deconvolution criteria: No

Included in protocol analysis: No

Protocol: N/A

Filename: Bin4_plateA2_G12.fcs

Split 1: Petrol shading

Split 2: Green shading

Split 3: Violet shading

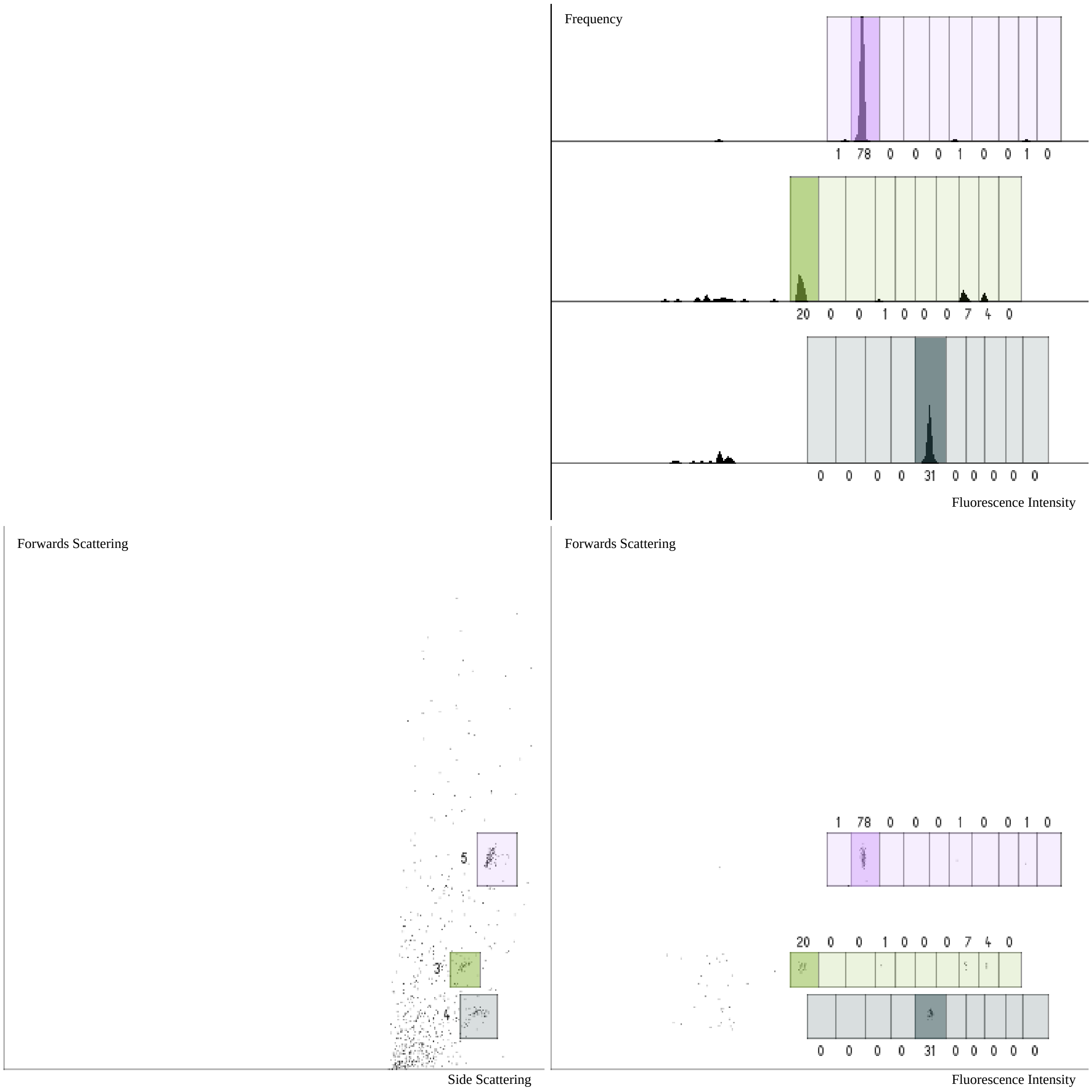

ANNEX 3: TAG DECONVOLUTION - BEAD 303

Passes flow sorting criteria: Yes

Passes tag deconvolution criteria: Yes

Included in protocol analysis: Yes

Protocol: 6, 10, 10, 4

Filename: Bin4_plateA2_E9.fcs

Split 1: Petrol shading

Split 2: Green shading

Split 3: Violet shading

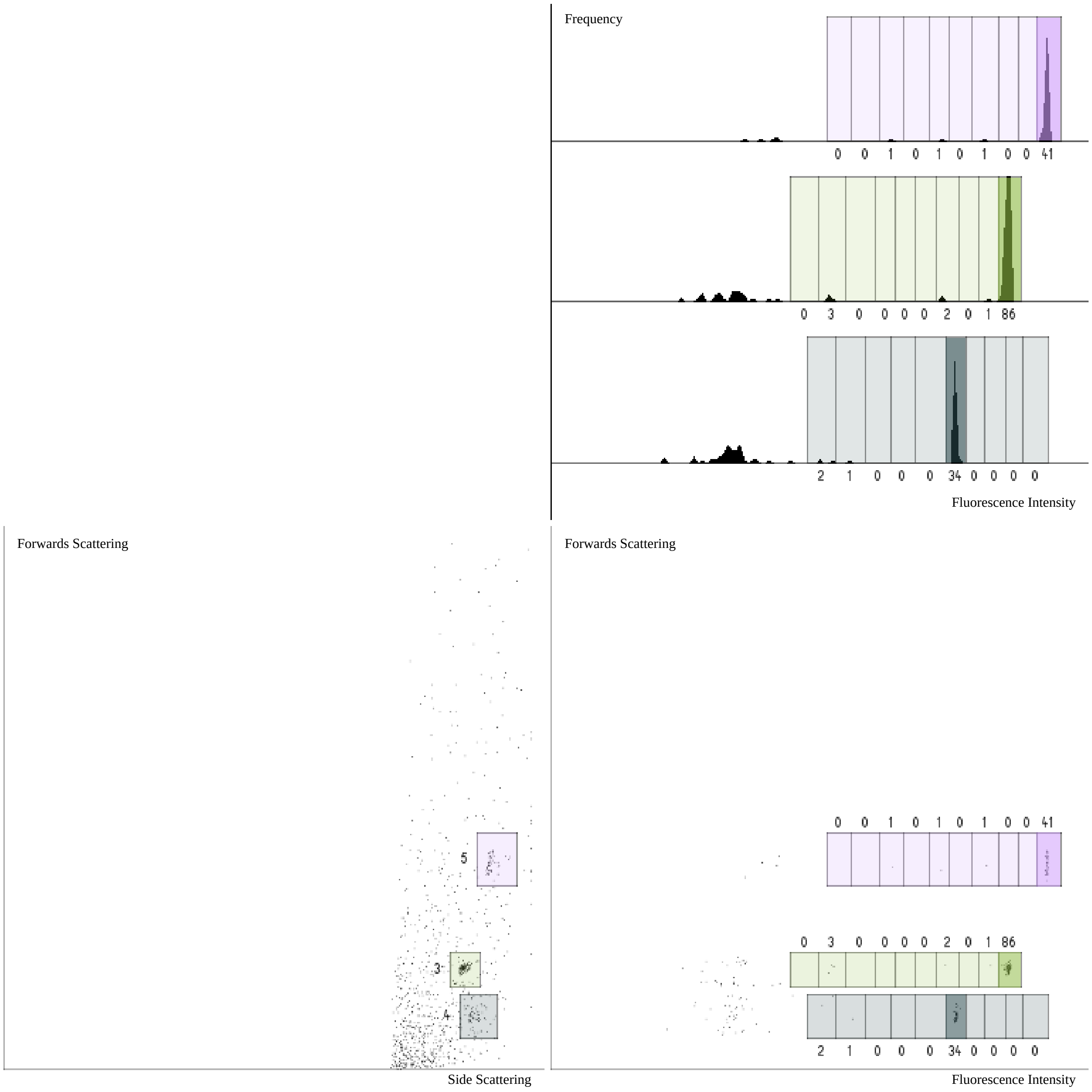

ANNEX 3: TAG DECONVOLUTION - BEAD 304

Passes flow sorting criteria: Yes

Passes tag deconvolution criteria: Yes

Included in protocol analysis: Yes

Protocol: 4, 5, 6, 4

Filename: Bin4_plateA2_E10.fcs

Split 1: Petrol shading

Split 2: Green shading

Split 3: Violet shading

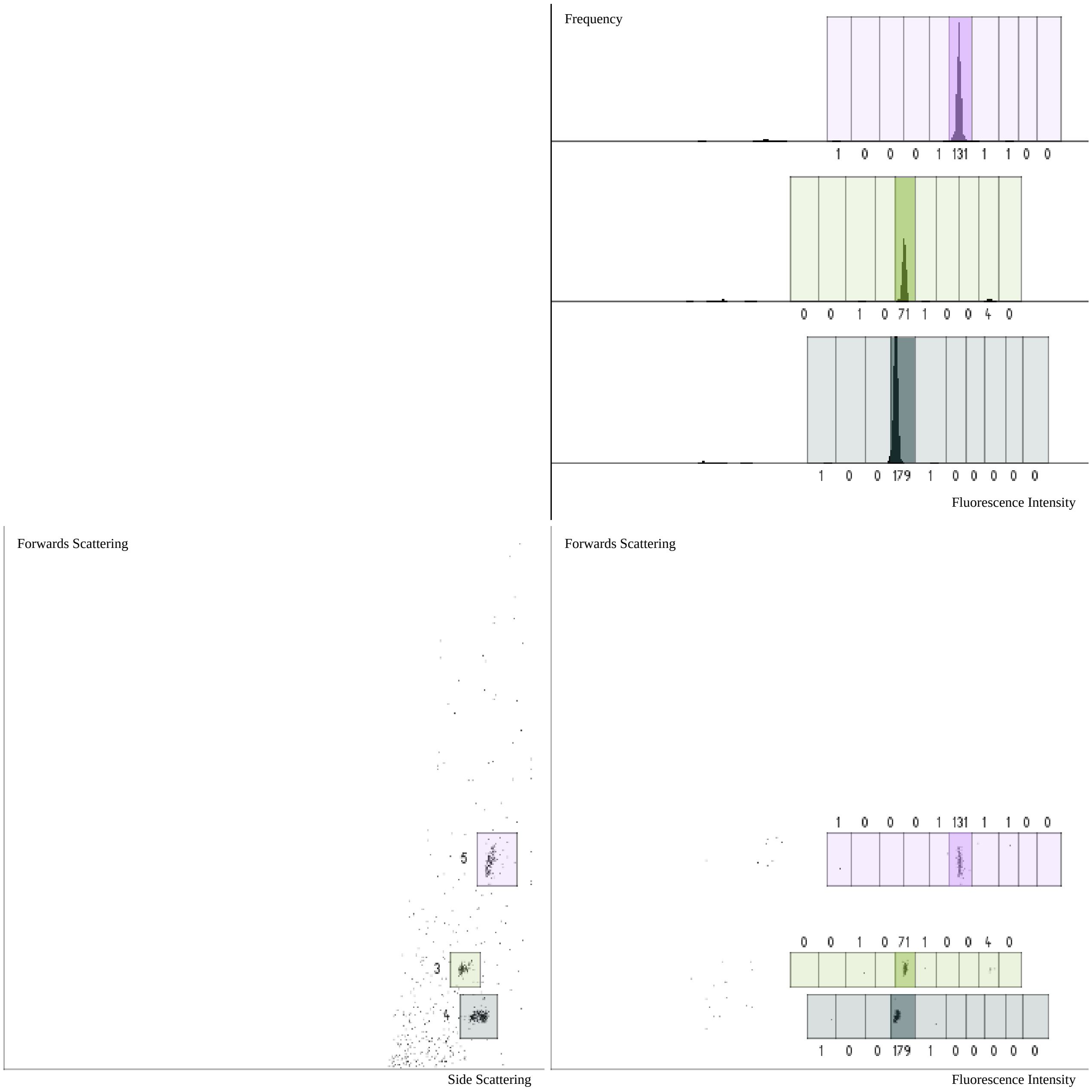

ANNEX 3: TAG DECONVOLUTION - BEAD 305

Passes flow sorting criteria: Yes

Passes tag deconvolution criteria: Yes

Included in protocol analysis: Yes

Protocol: 1, 8, 6, 4

Filename: Bin4_plateA2_E11.fcs

Split 1: Petrol shading

Split 2: Green shading

Split 3: Violet shading

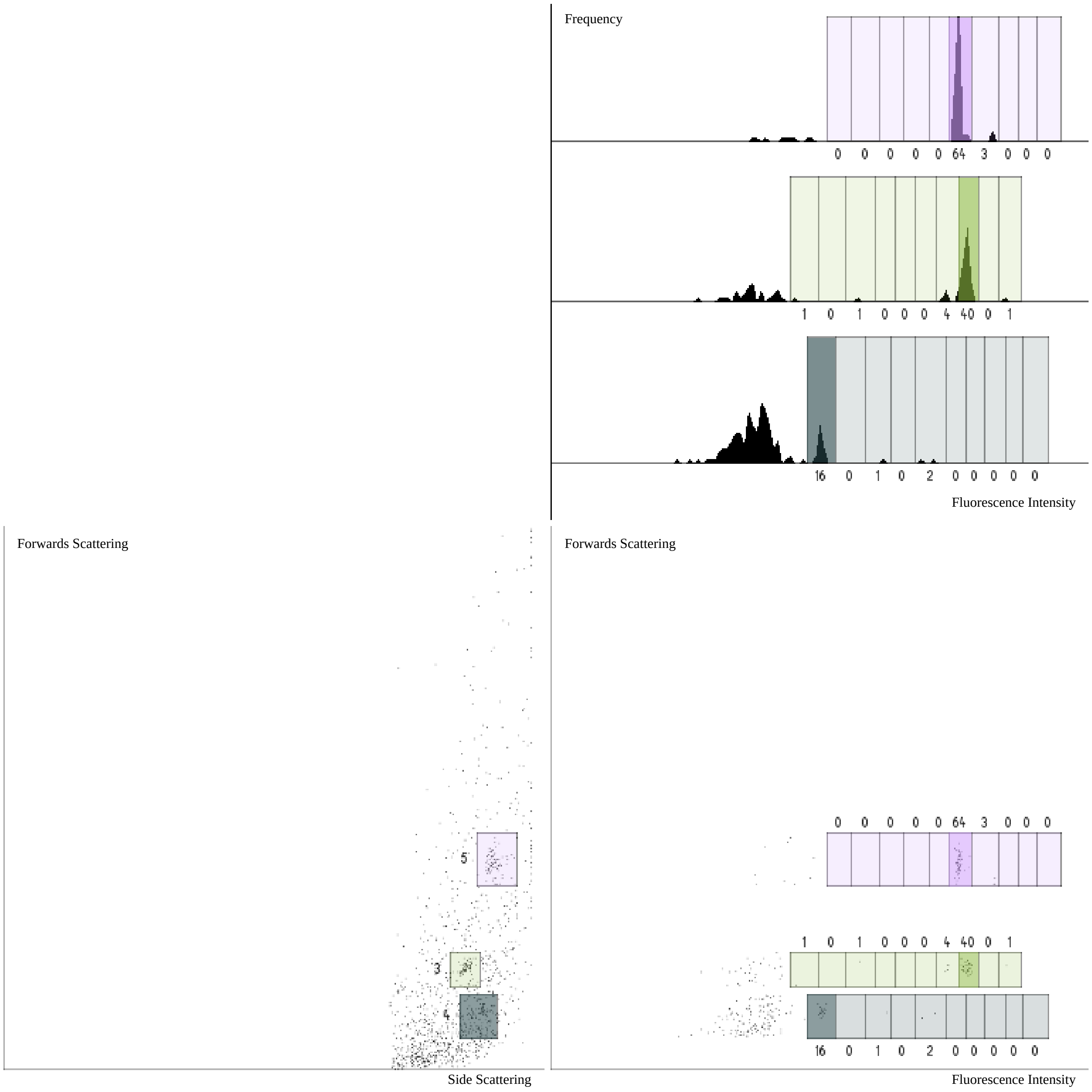

ANNEX 3: TAG DECONVOLUTION - BEAD 306

Passes flow sorting criteria: Yes

Passes tag deconvolution criteria: Yes

Included in protocol analysis: Yes

Protocol: 7, 6, 6, 4

Filename: Bin4_plateA2_E12.fcs

Split 1: Petrol shading

Split 2: Green shading

Split 3: Violet shading

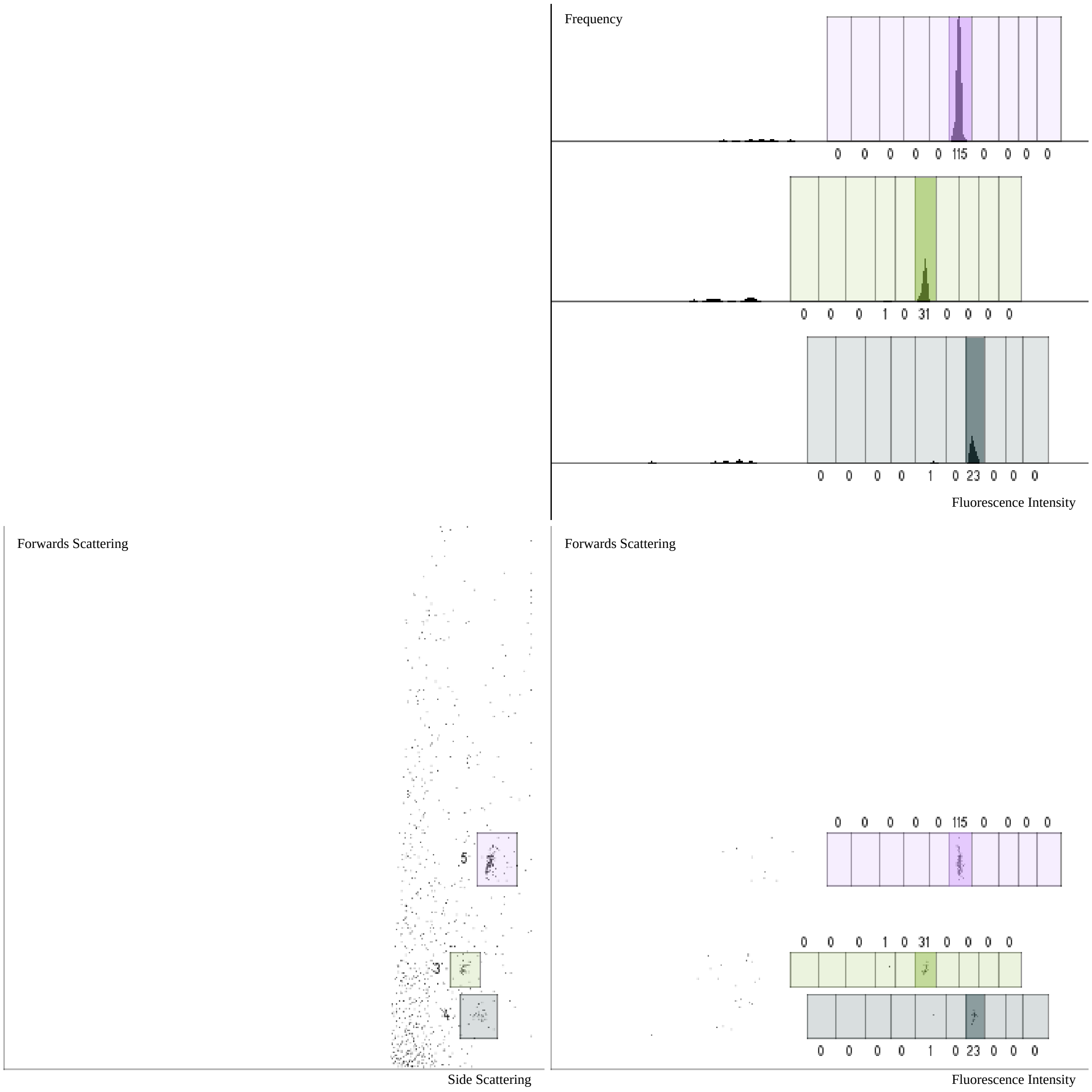

ANNEX 3: TAG DECONVOLUTION - BEAD 307

Passes flow sorting criteria: Yes

Passes tag deconvolution criteria: Yes

Included in protocol analysis: Yes

Protocol: 9, 9, 10, 4

Filename: Bin4_plateA2_F1.fcs

Split 1: Petrol shading

Split 2: Green shading

Split 3: Violet shading

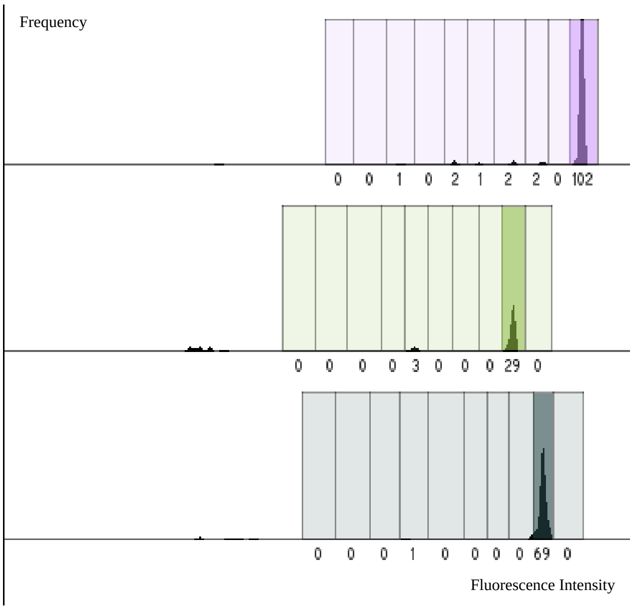

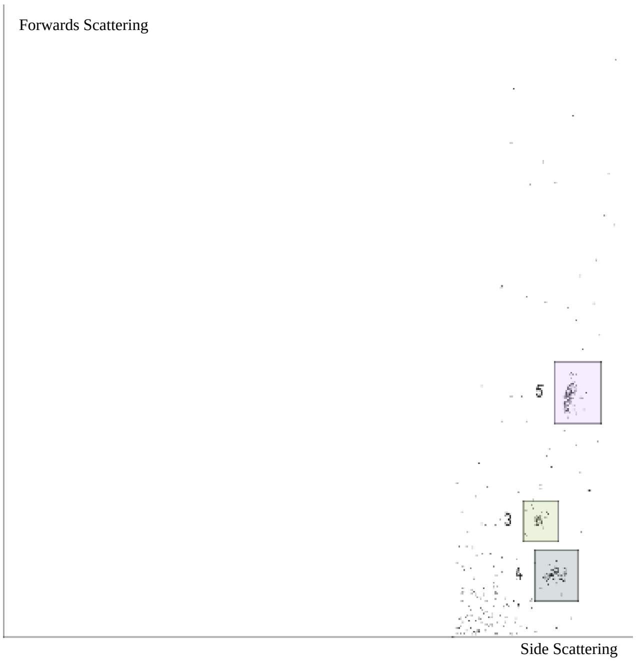

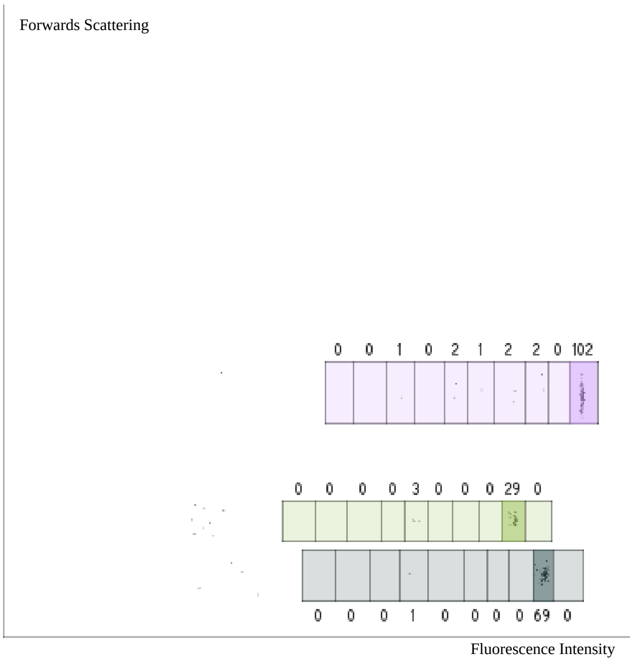

ANNEX 3: TAG DECONVOLUTION - BEAD 308

Passes flow sorting criteria: Yes

Passes tag deconvolution criteria: Yes

Included in protocol analysis: Yes

Protocol: 10, 5, 9, 4

Filename: Bin4_plateA2_F2.fcs

Split 1: Petrol shading

Split 2: Green shading

Split 3: Violet shading

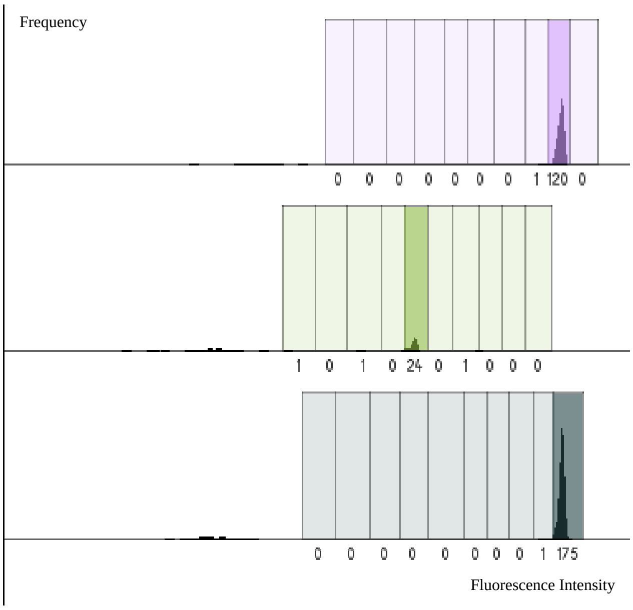

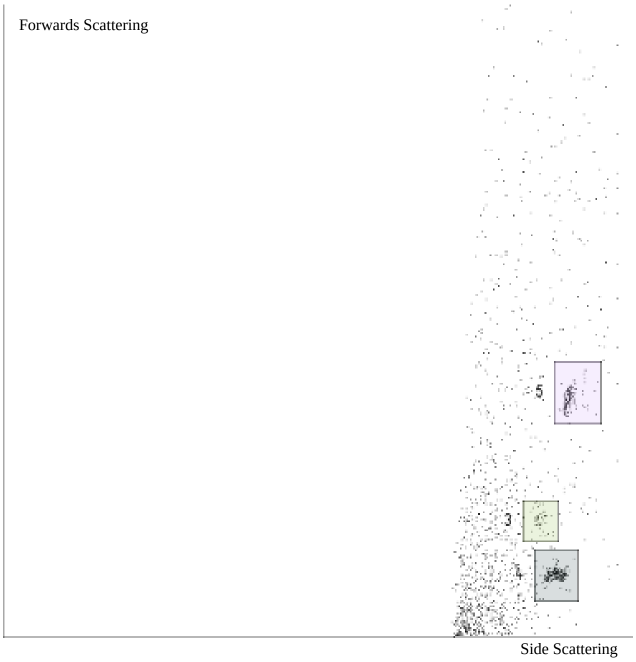

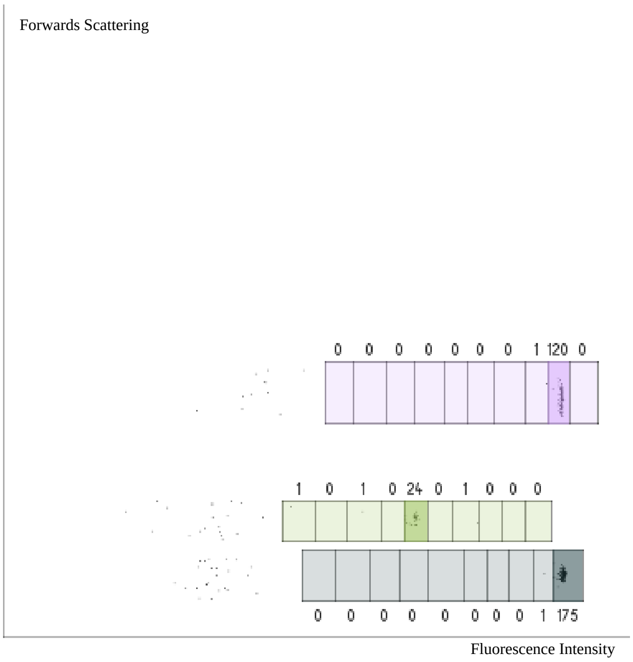

ANNEX 3: TAG DECONVOLUTION - BEAD 309

Passes flow sorting criteria: Yes

Passes tag deconvolution criteria: Yes

Included in protocol analysis: Yes

Protocol: 3, 10, 10, 4

Filename: Bin4_plateA2_F3.fcs

Split 1: Petrol shading

Split 2: Green shading

Split 3: Violet shading

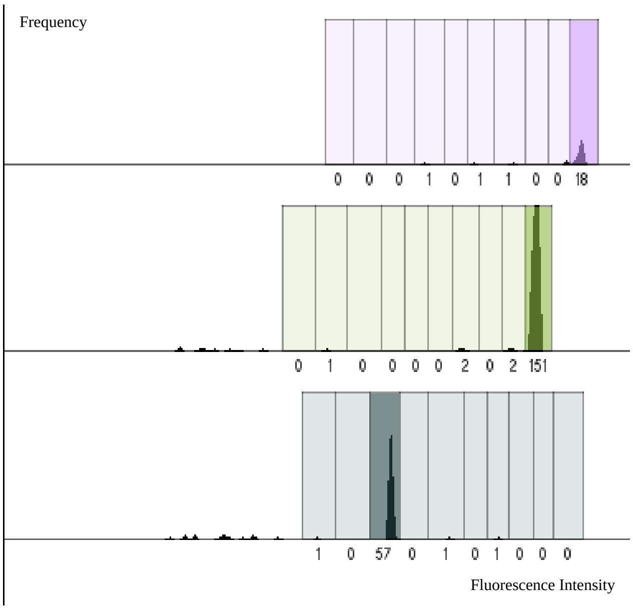

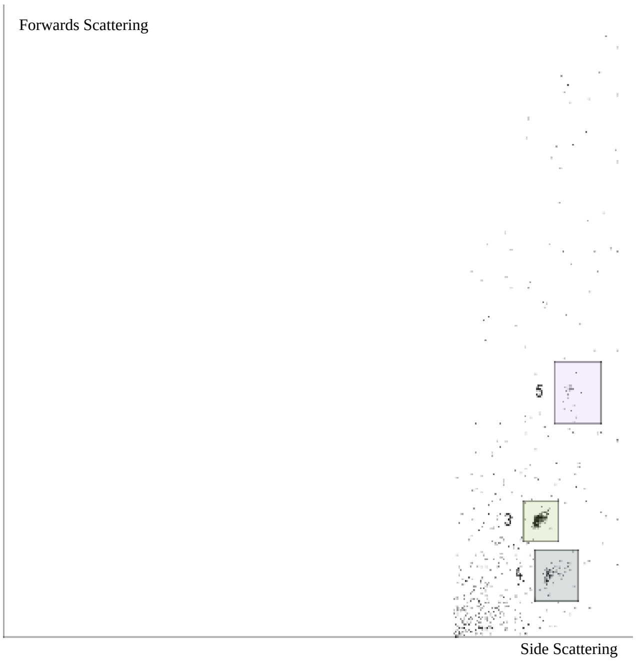

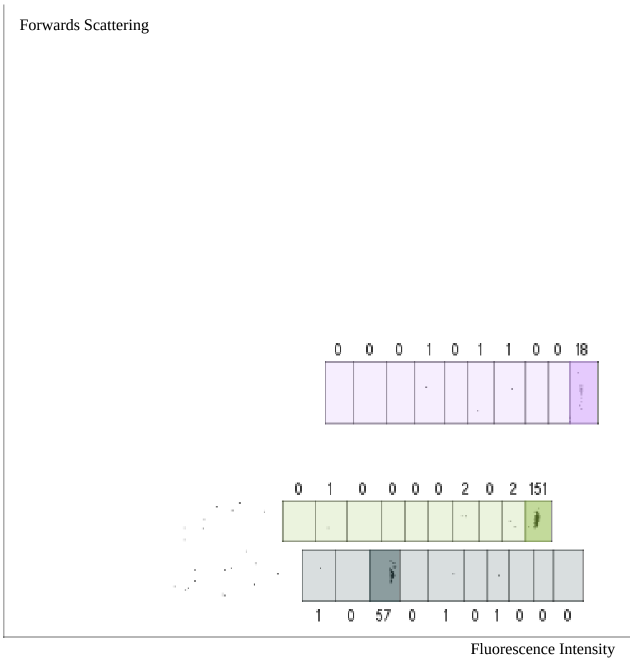

ANNEX 3: TAG DECONVOLUTION - BEAD 310

Passes flow sorting criteria: Yes

Passes tag deconvolution criteria: Yes

Included in protocol analysis: Yes

Protocol: 1, 5, 9, 4

Filename: Bin4_plateA2_F4.fcs

Split 1: Petrol shading

Split 2: Green shading

Split 3: Violet shading

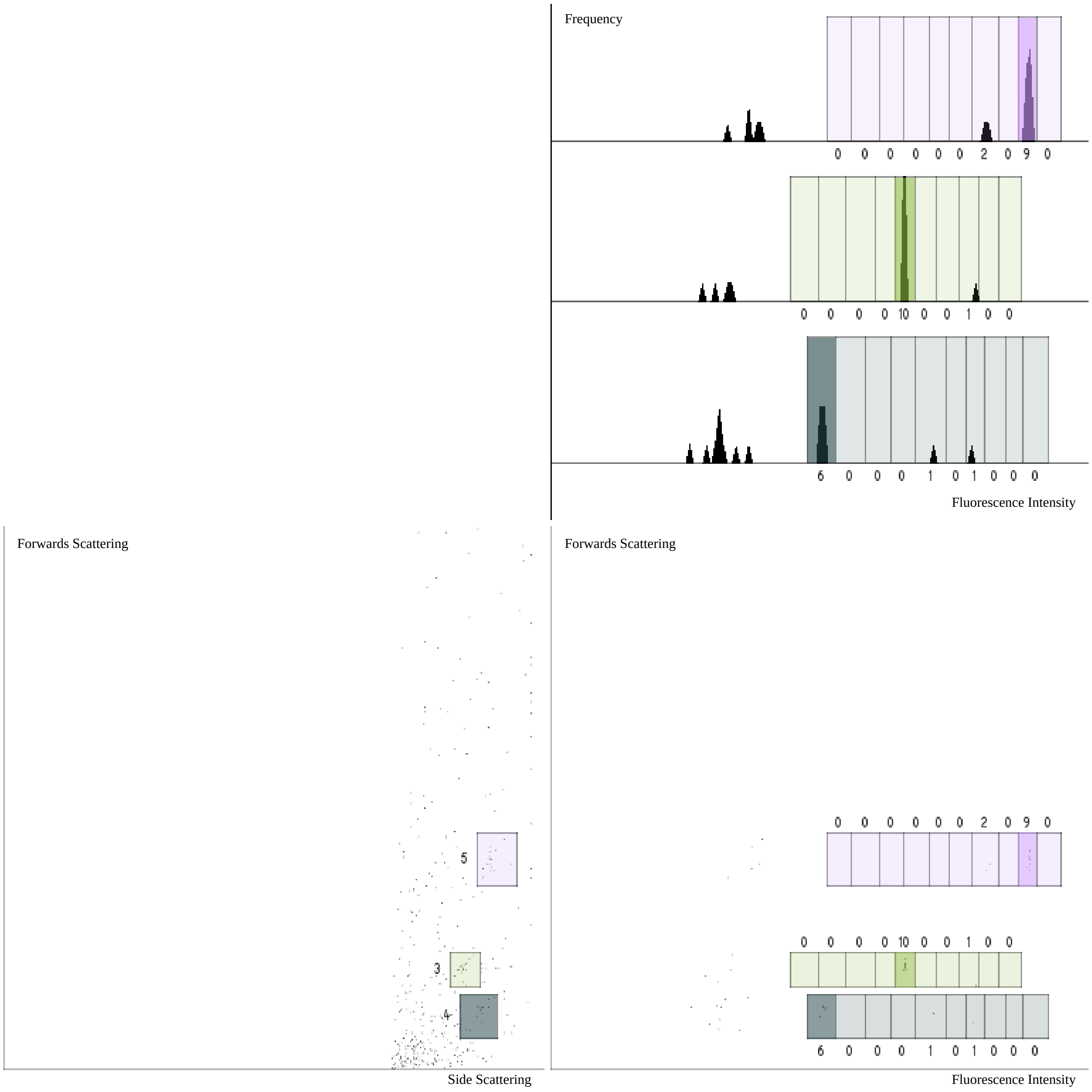

ANNEX 3: TAG DECONVOLUTION - BEAD 311

Passes flow sorting criteria: Yes

Passes tag deconvolution criteria: No

Included in protocol analysis: No

Protocol: N/A

Filename: Bin4_plateA2_F5.fcs

Split 1: Petrol shading

Split 2: Green shading

Split 3: Violet shading

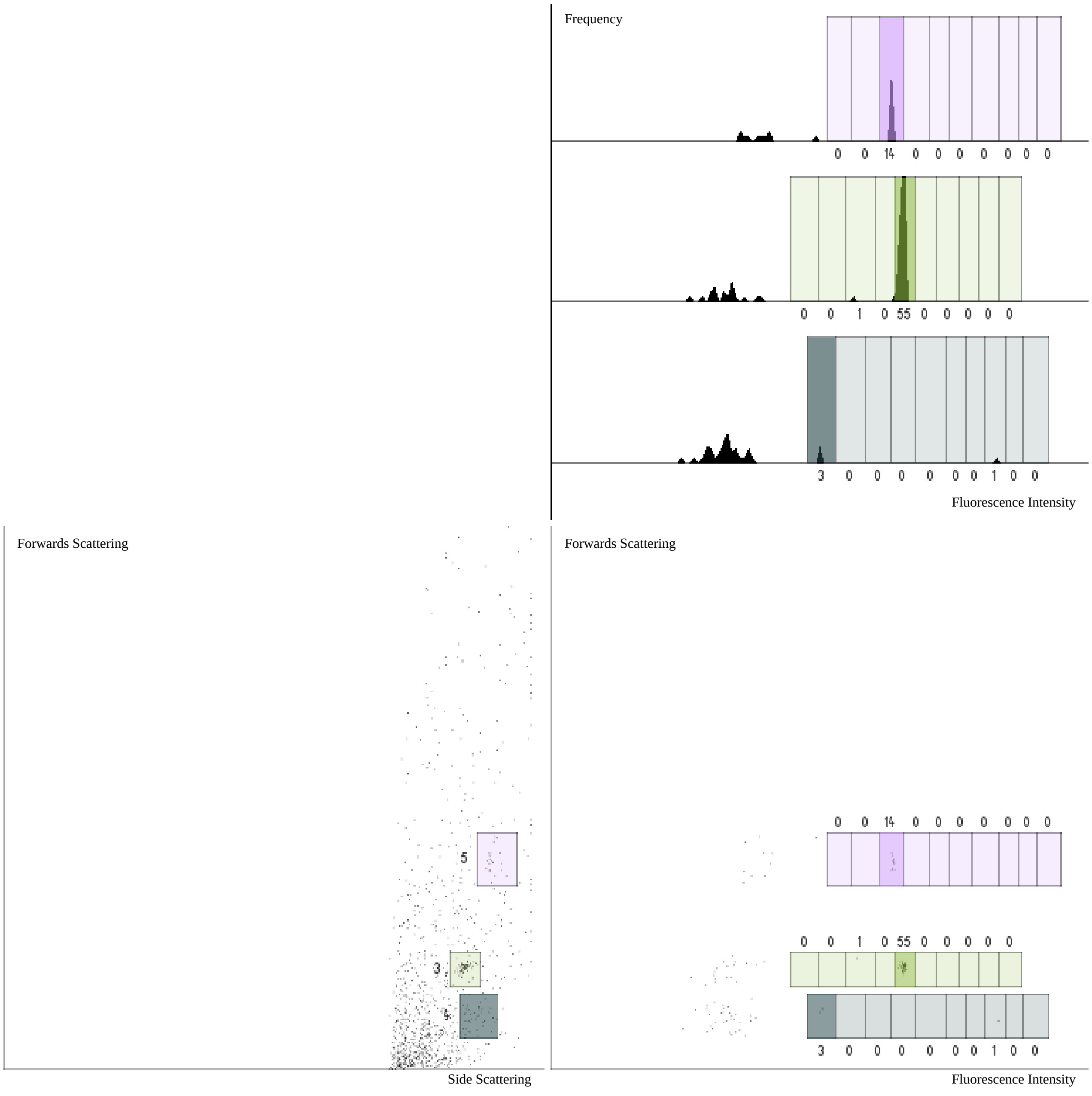

ANNEX 3: TAG DECONVOLUTION - BEAD 312

Passes flow sorting criteria: Yes

Passes tag deconvolution criteria: Yes

Included in protocol analysis: Yes

Protocol: 1, 10, 7, 4

Filename: Bin4_plateA2_F6.fcs

Split 1: Petrol shading

Split 2: Green shading

Split 3: Violet shading

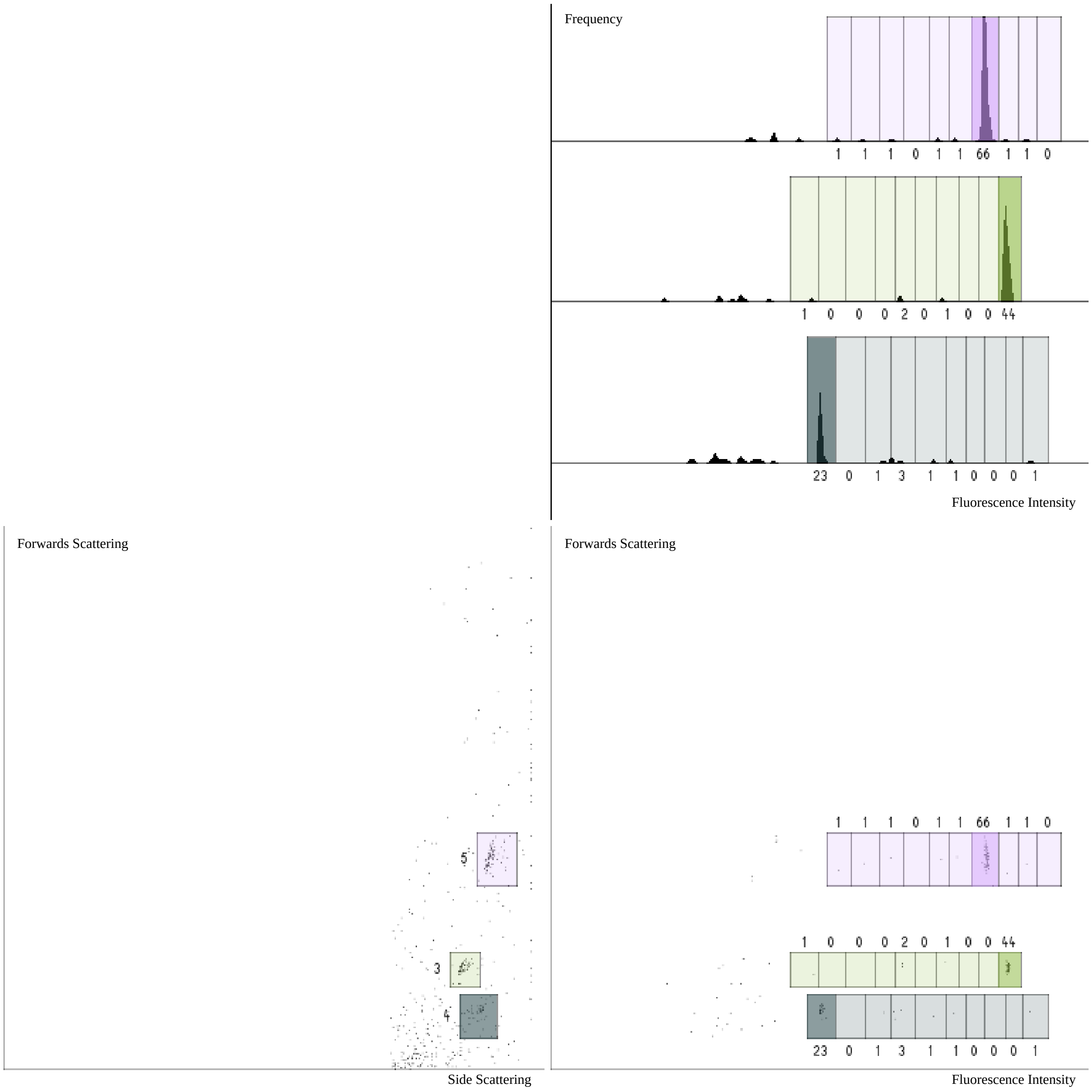

ANNEX 3: TAG DECONVOLUTION - BEAD 313

Passes flow sorting criteria: Yes

Passes tag deconvolution criteria: No

Included in protocol analysis: No

Protocol: N/A

Filename: Bin4_plateA2_F7.fcs

Split 1: Petrol shading

Split 2: Green shading

Split 3: Violet shading

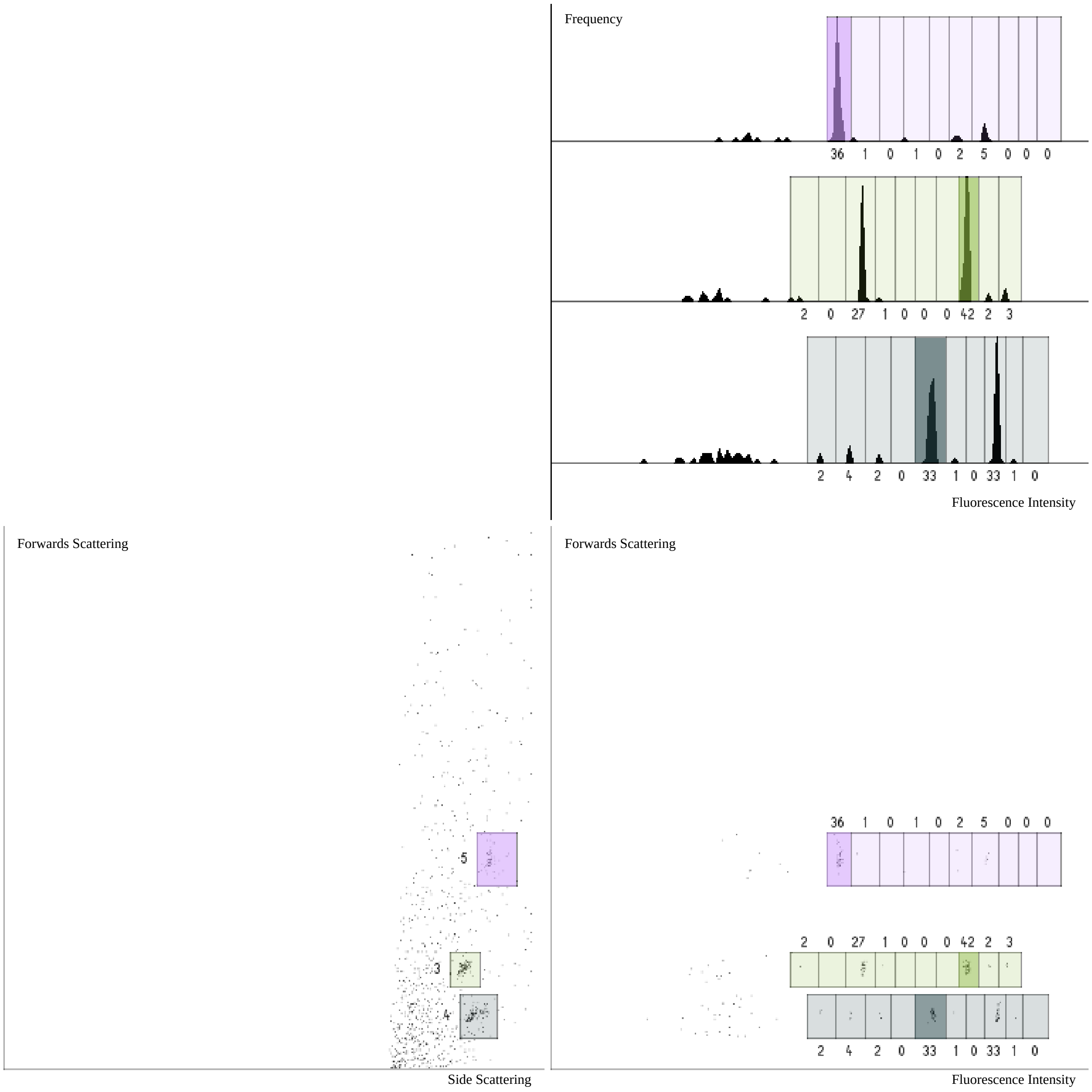

ANNEX 3: TAG DECONVOLUTION - BEAD 314

Passes flow sorting criteria: Yes

Passes tag deconvolution criteria: No

Included in protocol analysis: No

Protocol: N/A

Filename: Bin4_plateA2_F8.fcs

Split 1: Petrol shading

Split 2: Green shading

Split 3: Violet shading

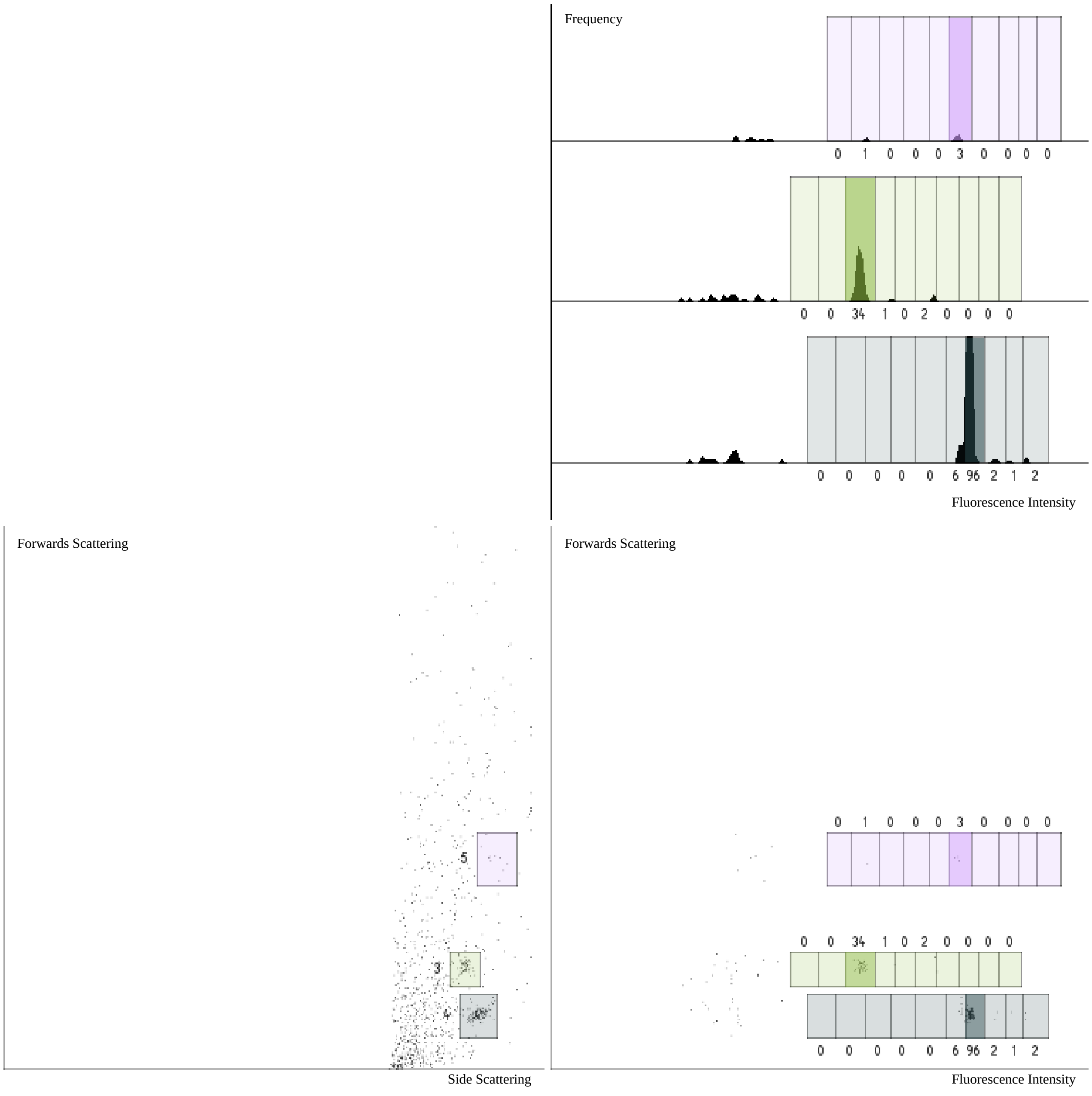

ANNEX 3: TAG DECONVOLUTION - BEAD 315

Passes flow sorting criteria: Yes

Passes tag deconvolution criteria: Yes

Included in protocol analysis: Yes

Protocol: 2, 5, 9, 4

Filename: Bin4_plateA2_F9.fcs

Split 1: Petrol shading

Split 2: Green shading

Split 3: Violet shading

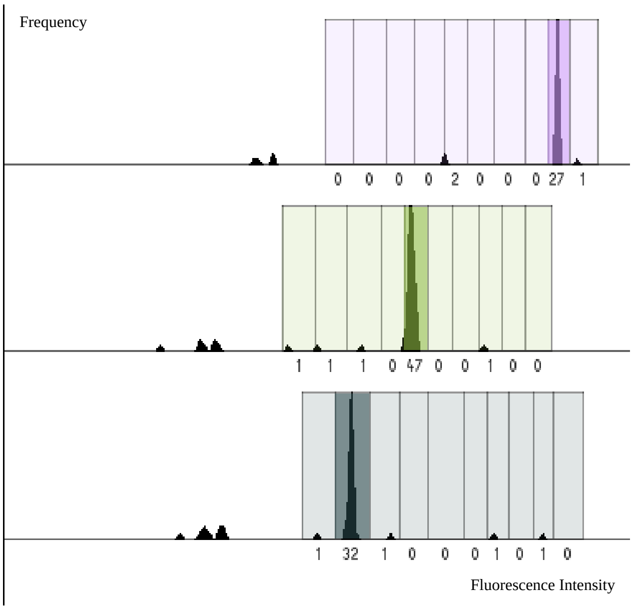

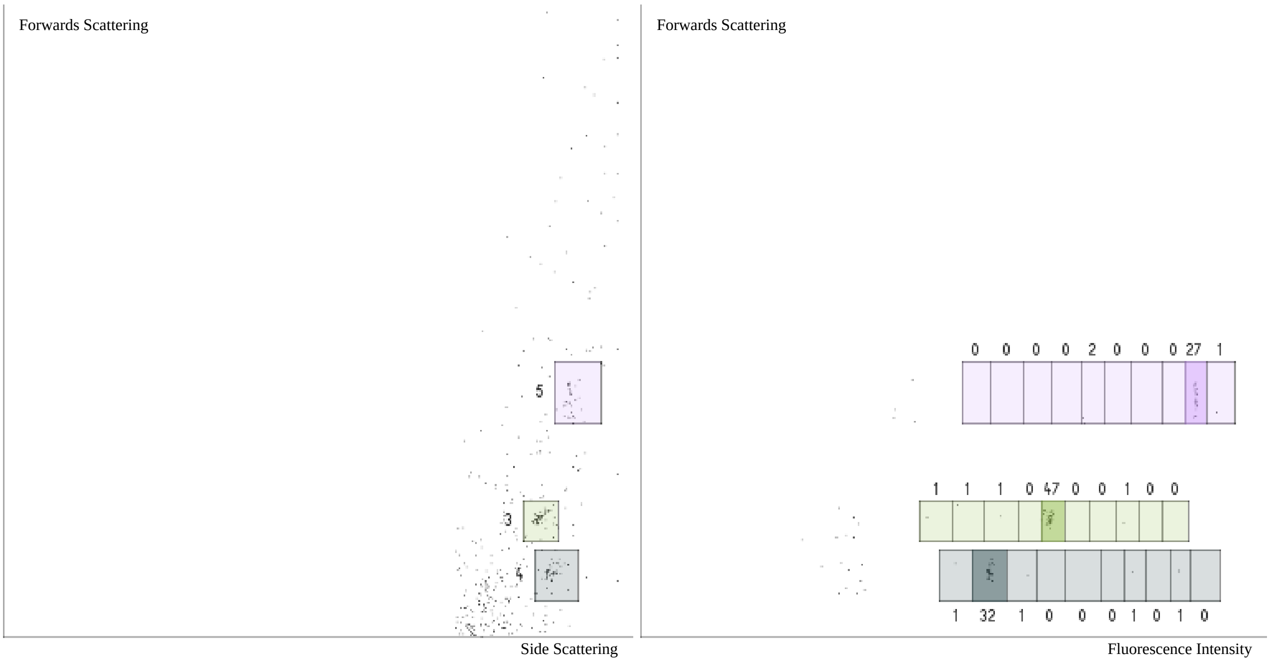

ANNEX 3: TAG DECONVOLUTION - BEAD 316

Passes flow sorting criteria: Yes

Passes tag deconvolution criteria: Yes

Included in protocol analysis: Yes

Protocol: 5, 3, 9, 4

Filename: Bin4_plateA2_F10.fcs

Split 1: Petrol shading

Split 2: Green shading

Split 3: Violet shading

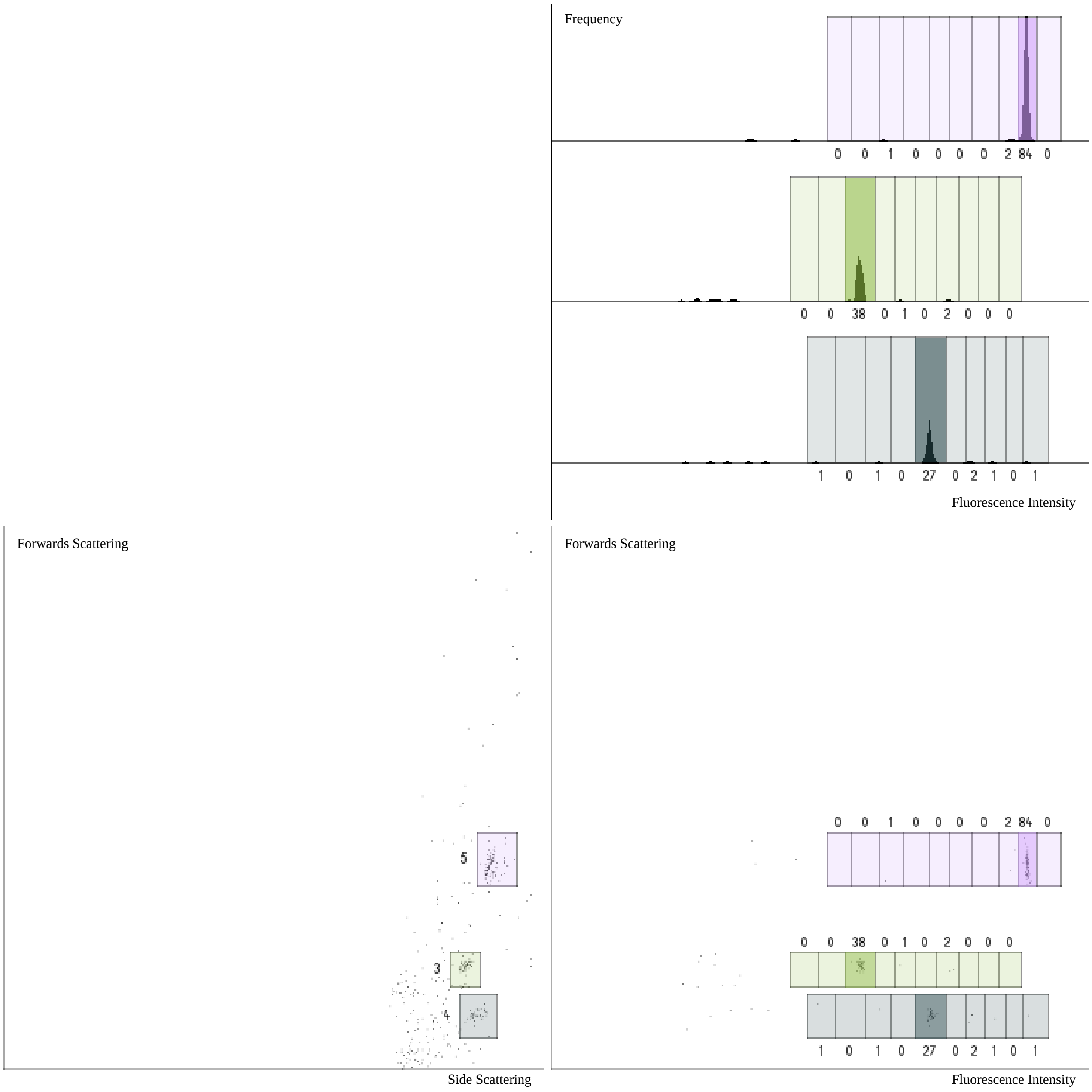

ANNEX 3: TAG DECONVOLUTION - BEAD 317

Passes flow sorting criteria: Yes

Passes tag deconvolution criteria: Yes

Included in protocol analysis: Yes

Protocol: 6, 8, 4, 4

Filename: Bin4_plateA2_F11.fcs

Split 1: Petrol shading

Split 2: Green shading

Split 3: Violet shading

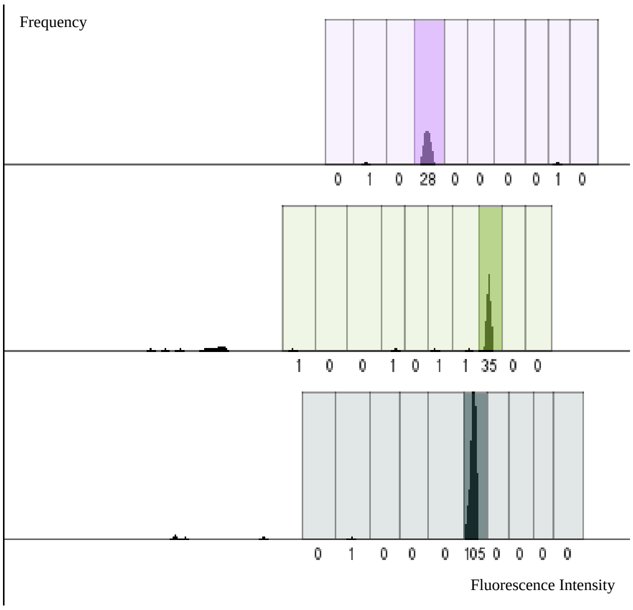

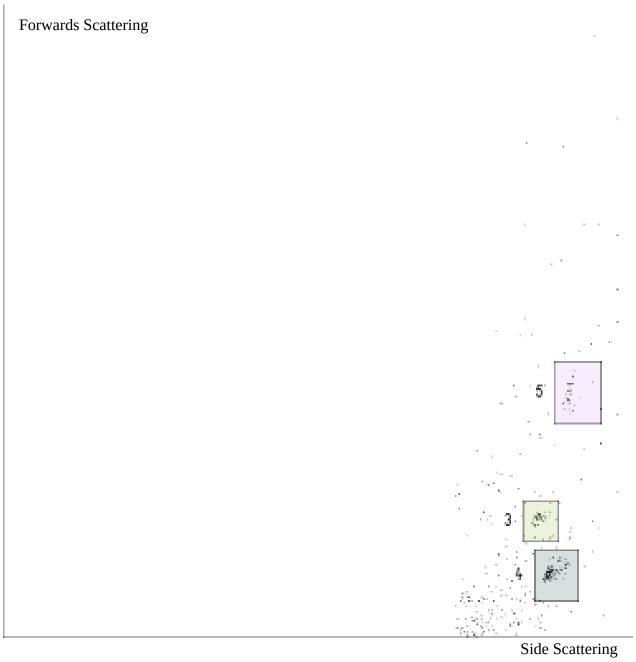

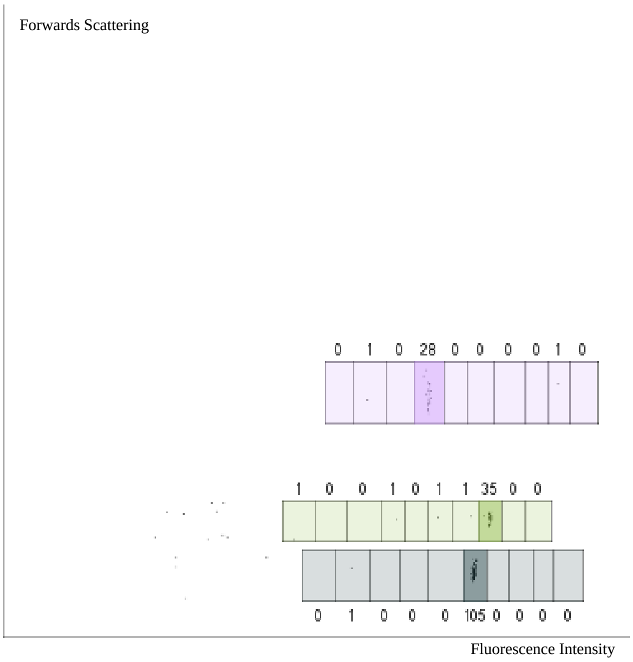

ANNEX 3: TAG DECONVOLUTION - BEAD 318

Passes flow sorting criteria: Yes

Passes tag deconvolution criteria: Yes

Included in protocol analysis: Yes

Protocol: 5, 9, 3, 4

Filename: Bin4_plateA2_F12.fcs

Split 1: Petrol shading

Split 2: Green shading

Split 3: Violet shading

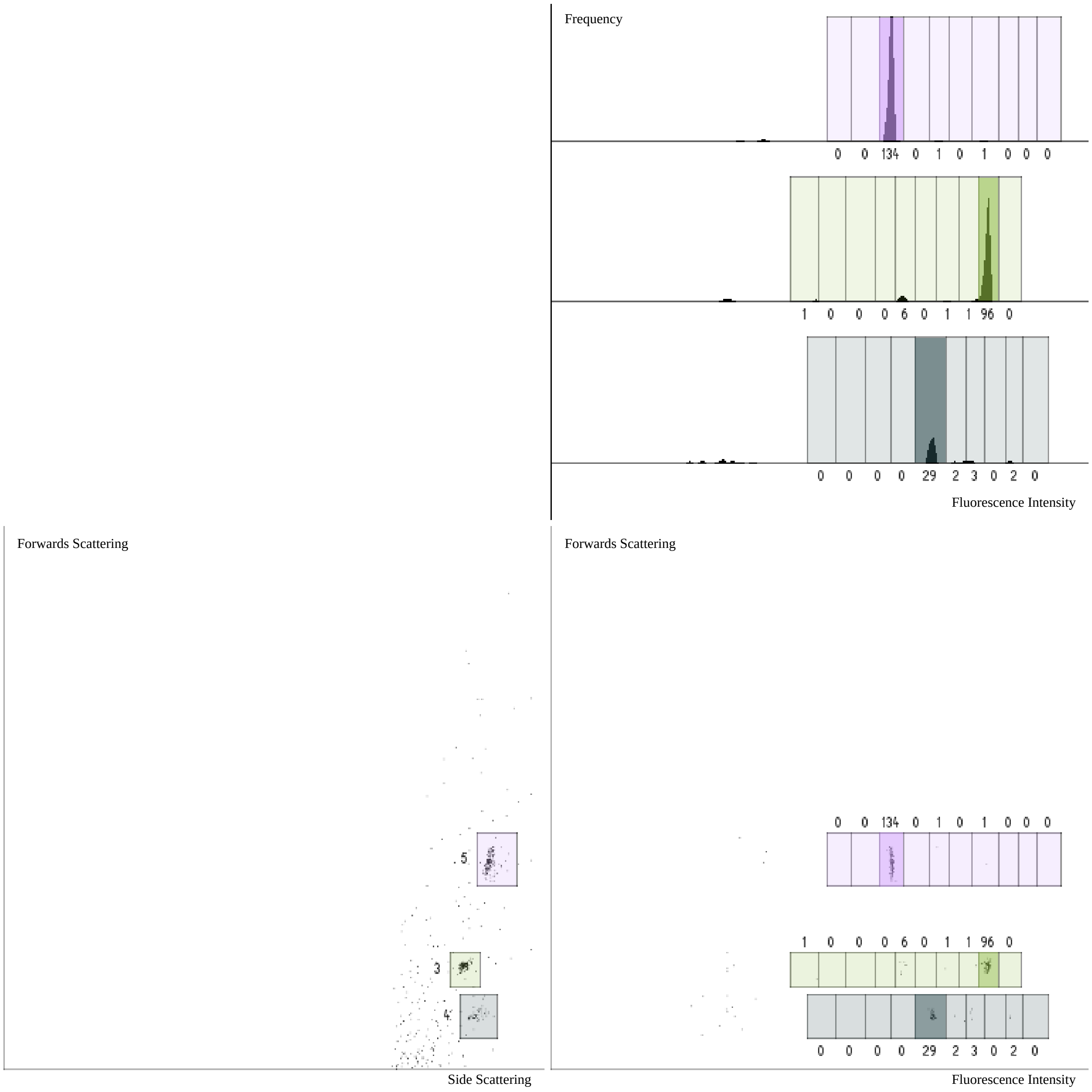

ANNEX 3: TAG DECONVOLUTION - BEAD 319

Passes flow sorting criteria: Yes

Passes tag deconvolution criteria: Yes

Included in protocol analysis: Yes

Protocol: 7, 2, 3, 4

Filename: Bin4_plateA2_G4.fcs

Split 1: Petrol shading

Split 2: Green shading

Split 3: Violet shading

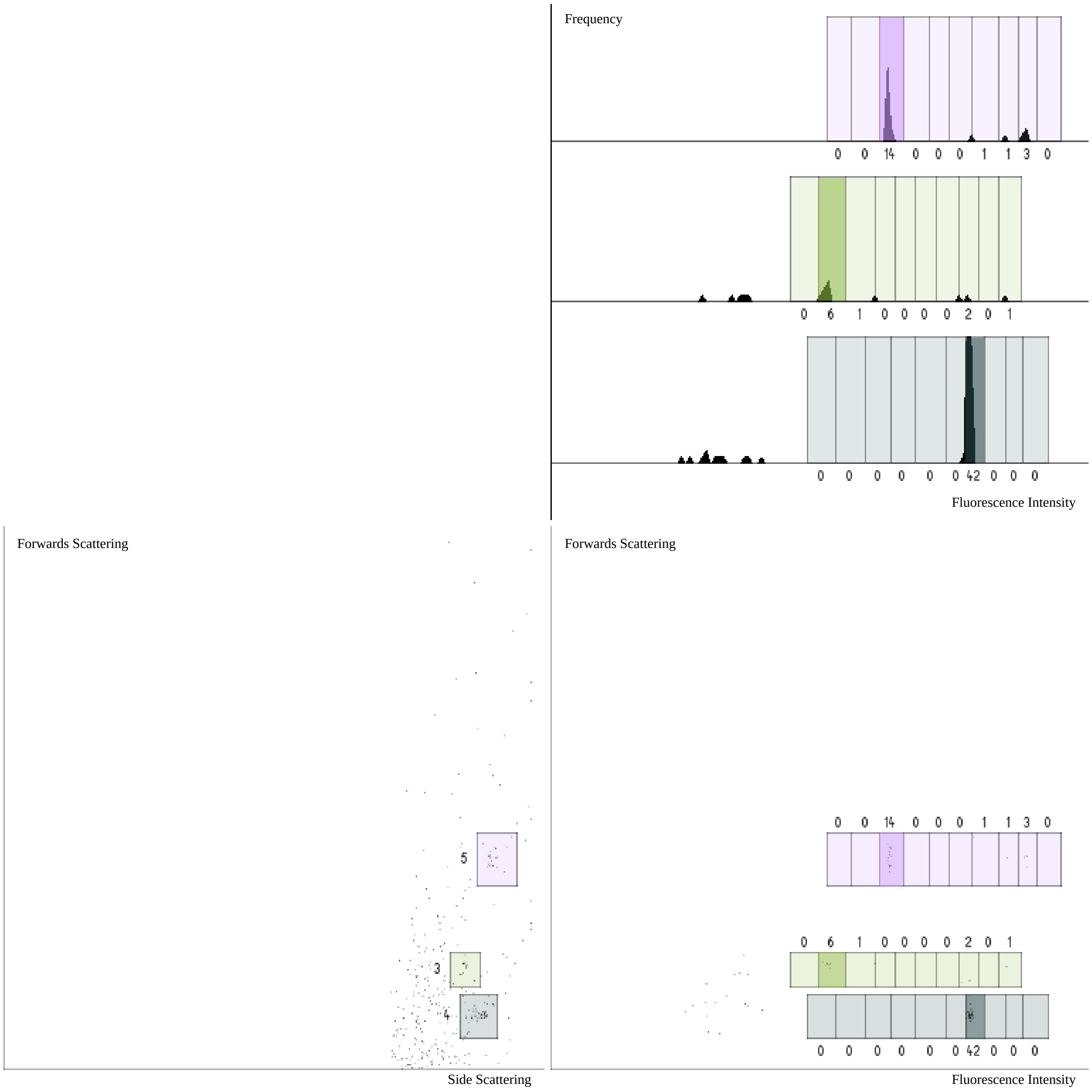

ANNEX 3: TAG DECONVOLUTION - BEAD 320

Passes flow sorting criteria: Yes

Passes tag deconvolution criteria: No

Included in protocol analysis: No

Protocol: N/A

Filename: Bin4_plateA2_G6.fcs

Split 1: Petrol shading

Split 2: Green shading

Split 3: Violet shading

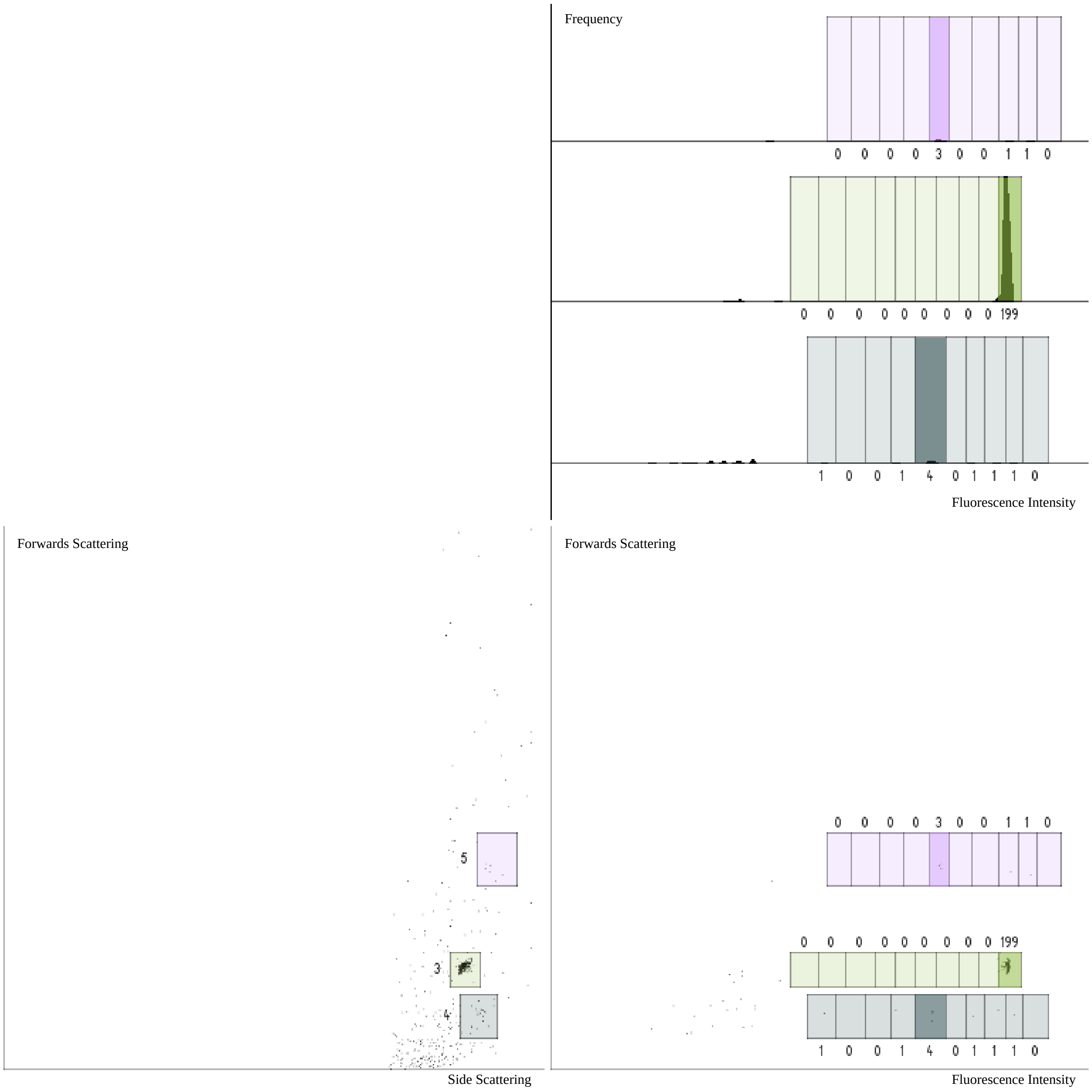

ANNEX 3: TAG DECONVOLUTION - BEAD 321

Passes flow sorting criteria: Yes

Passes tag deconvolution criteria: Yes

Included in protocol analysis: Yes

Protocol: 4, 8, 3, 4

Filename: Bin4_plateA2_G7.fcs

Split 1: Petrol shading

Split 2: Green shading

Split 3: Violet shading

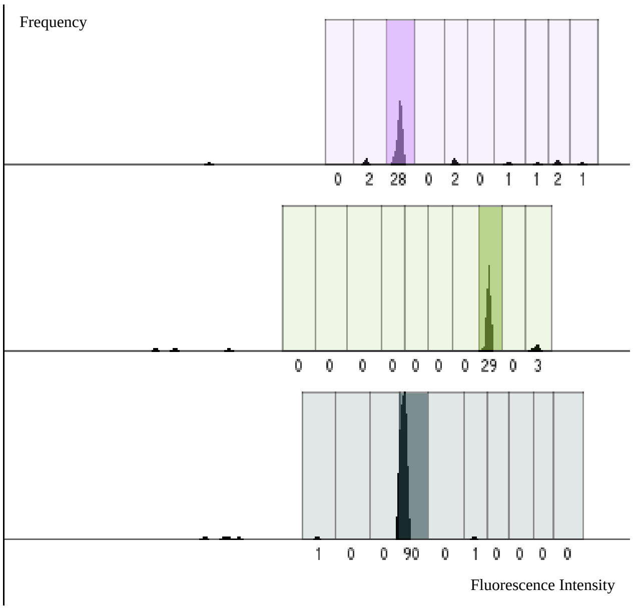

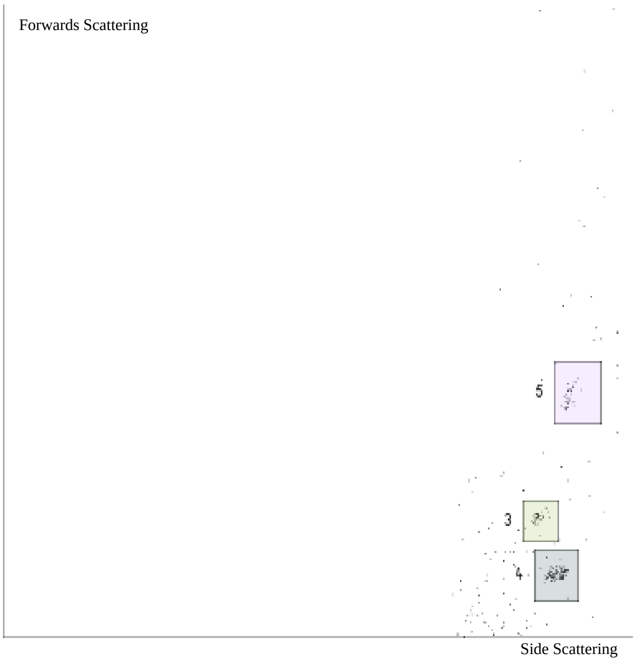

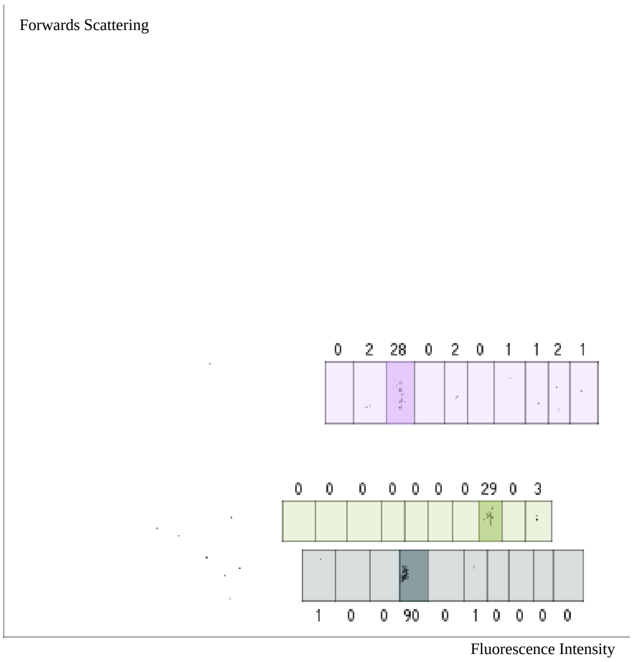

ANNEX 3: TAG DECONVOLUTION - BEAD 322

Passes flow sorting criteria: Yes

Passes tag deconvolution criteria: No

Included in protocol analysis: No

Protocol: N/A

Filename: Bin4_plateA2_G8.fcs

Split 1: Petrol shading

Split 2: Green shading

Split 3: Violet shading

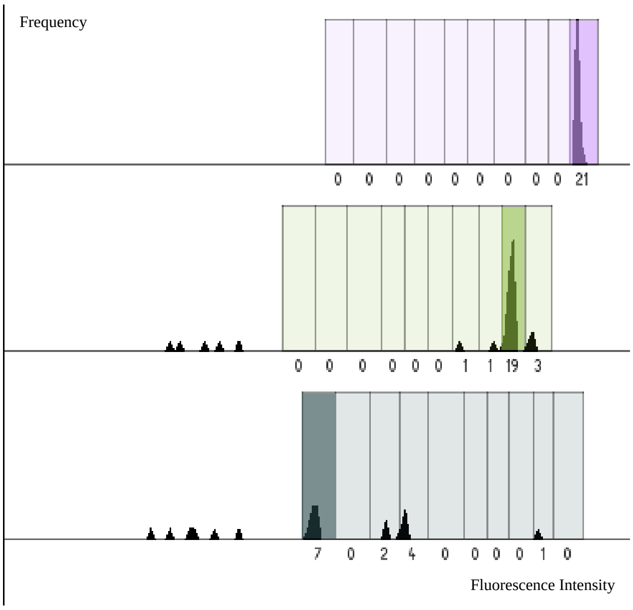

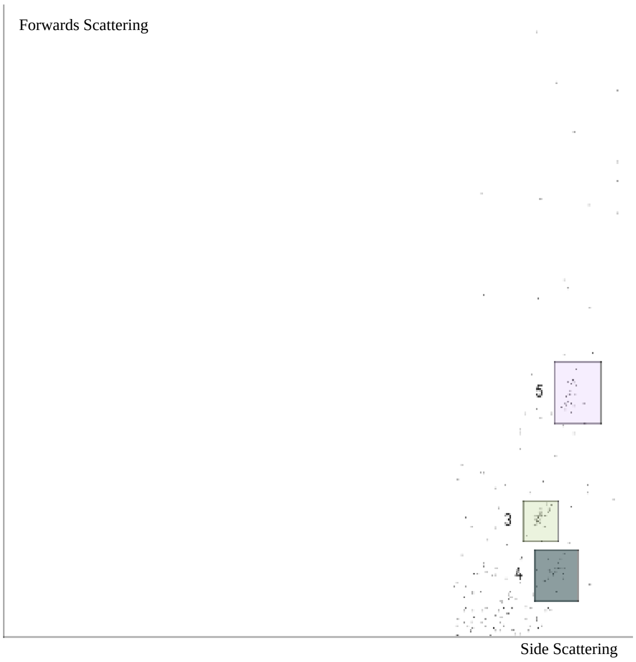

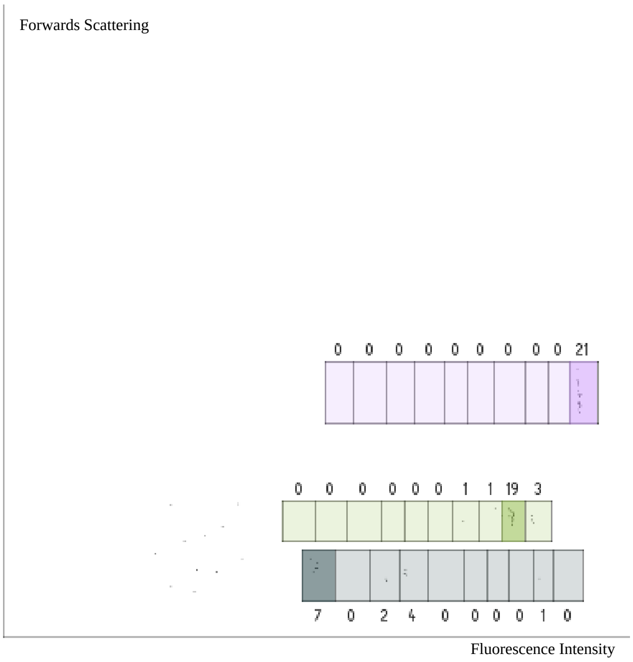

ANNEX 3: TAG DECONVOLUTION - BEAD 323

Passes flow sorting criteria: Yes

Passes tag deconvolution criteria: Yes

Included in protocol analysis: Yes

Protocol: 1, 1, 6, 4

Filename: Bin4_plateA2_G9.fcs

Split 1: Petrol shading

Split 2: Green shading

Split 3: Violet shading

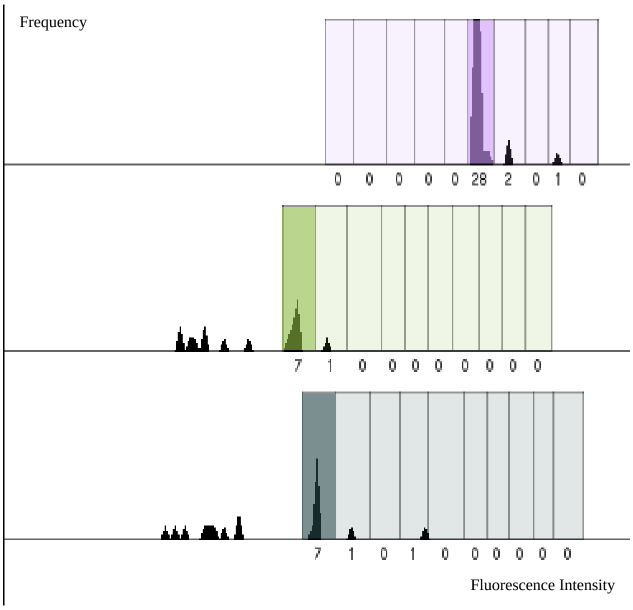

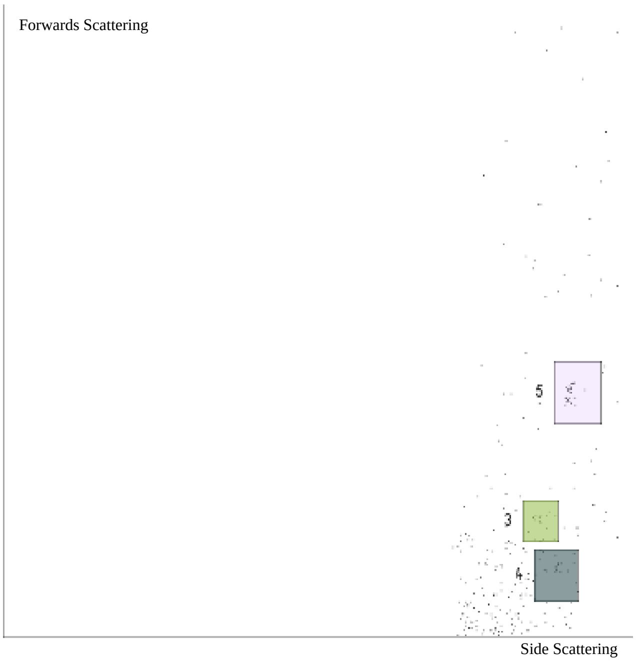

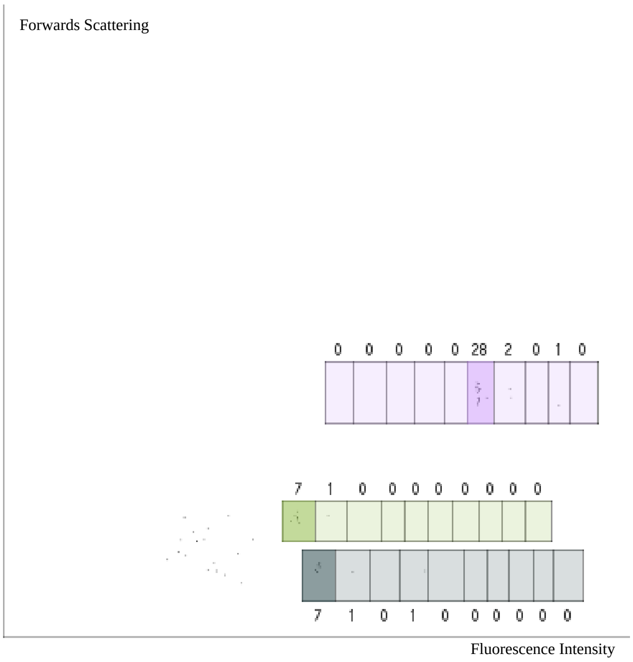

ANNEX 3: TAG DECONVOLUTION - BEAD 324

Passes flow sorting criteria: Yes

Passes tag deconvolution criteria: No

Included in protocol analysis: No

Protocol: N/A

Filename: Bin4_plateA2_G10.fcs

Split 1: Petrol shading

Split 2: Green shading

Split 3: Violet shading

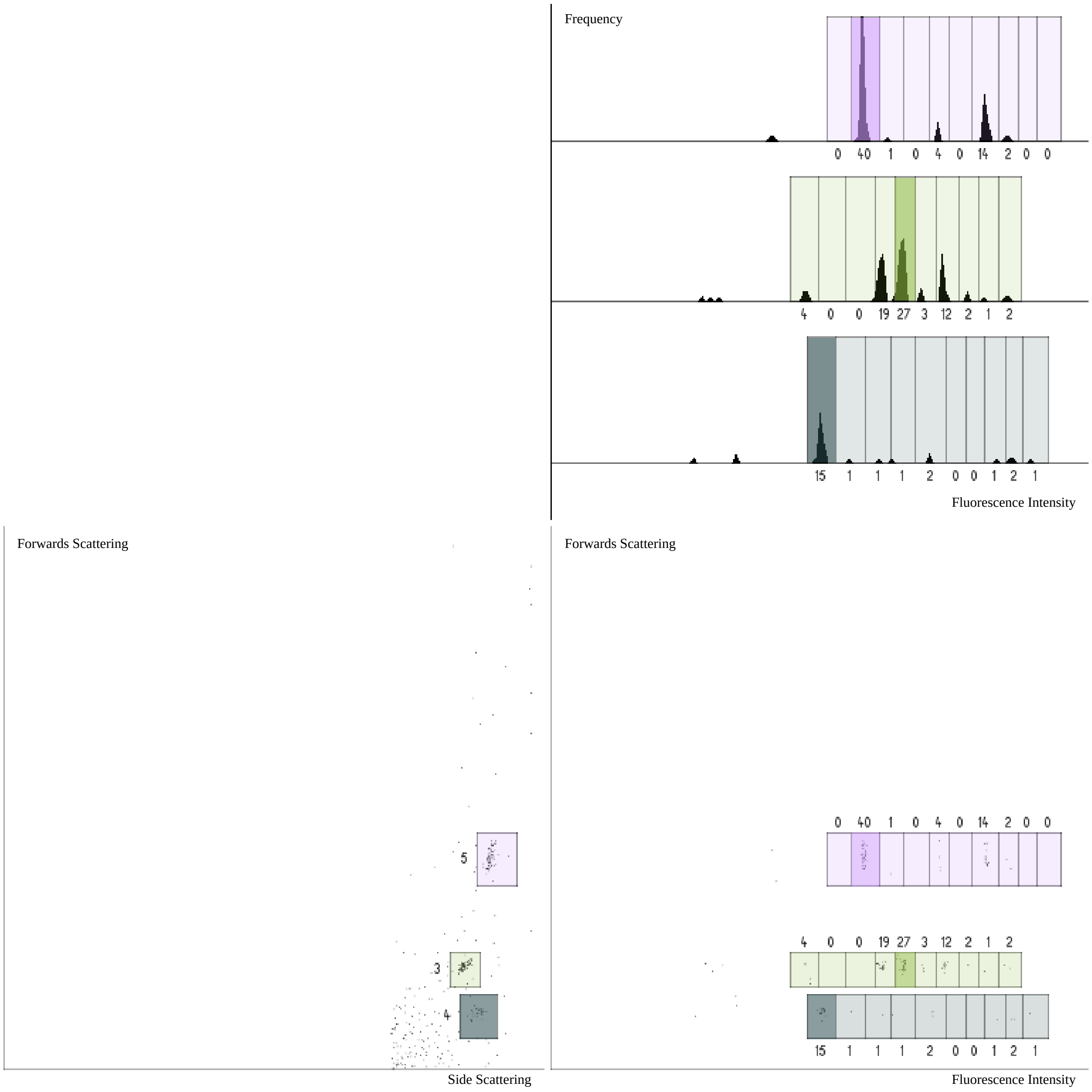

ANNEX 3: TAG DECONVOLUTION - BEAD 325

Passes flow sorting criteria: Yes

Passes tag deconvolution criteria: No

Included in protocol analysis: No

Protocol: N/A

Filename: Bin4_plateA2_G11.fcs

Split 1: Petrol shading

Split 2: Green shading

Split 3: Violet shading

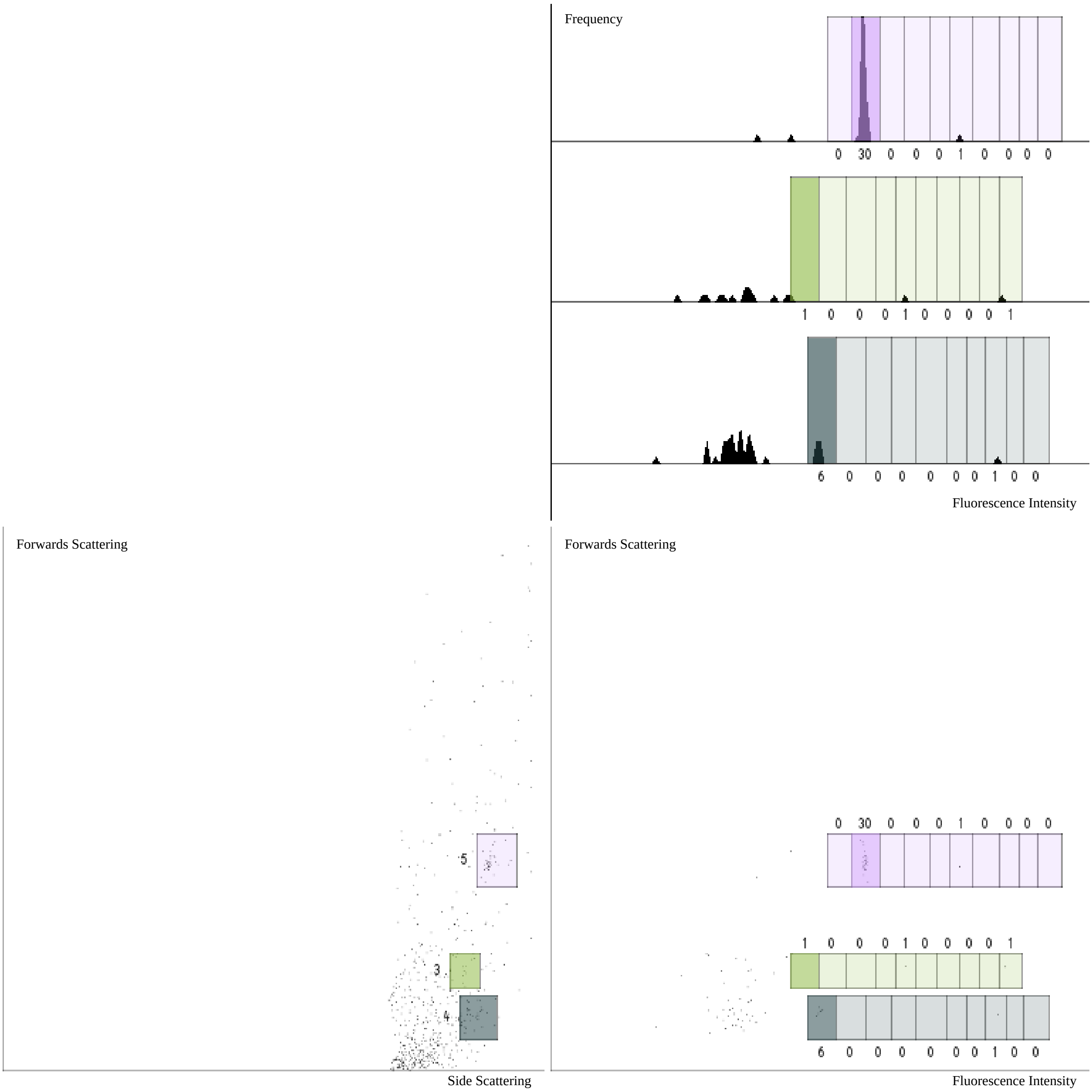

ANNEX 3: TAG DECONVOLUTION - BEAD 326

Passes flow sorting criteria: Yes

Passes tag deconvolution criteria: Yes

Included in protocol analysis: Yes

Protocol: 3, 1, 5, 5

Filename: Bin5_plateA0_C8.fcs

Split 1: Petrol shading

Split 2: Green shading

Split 3: Violet shading

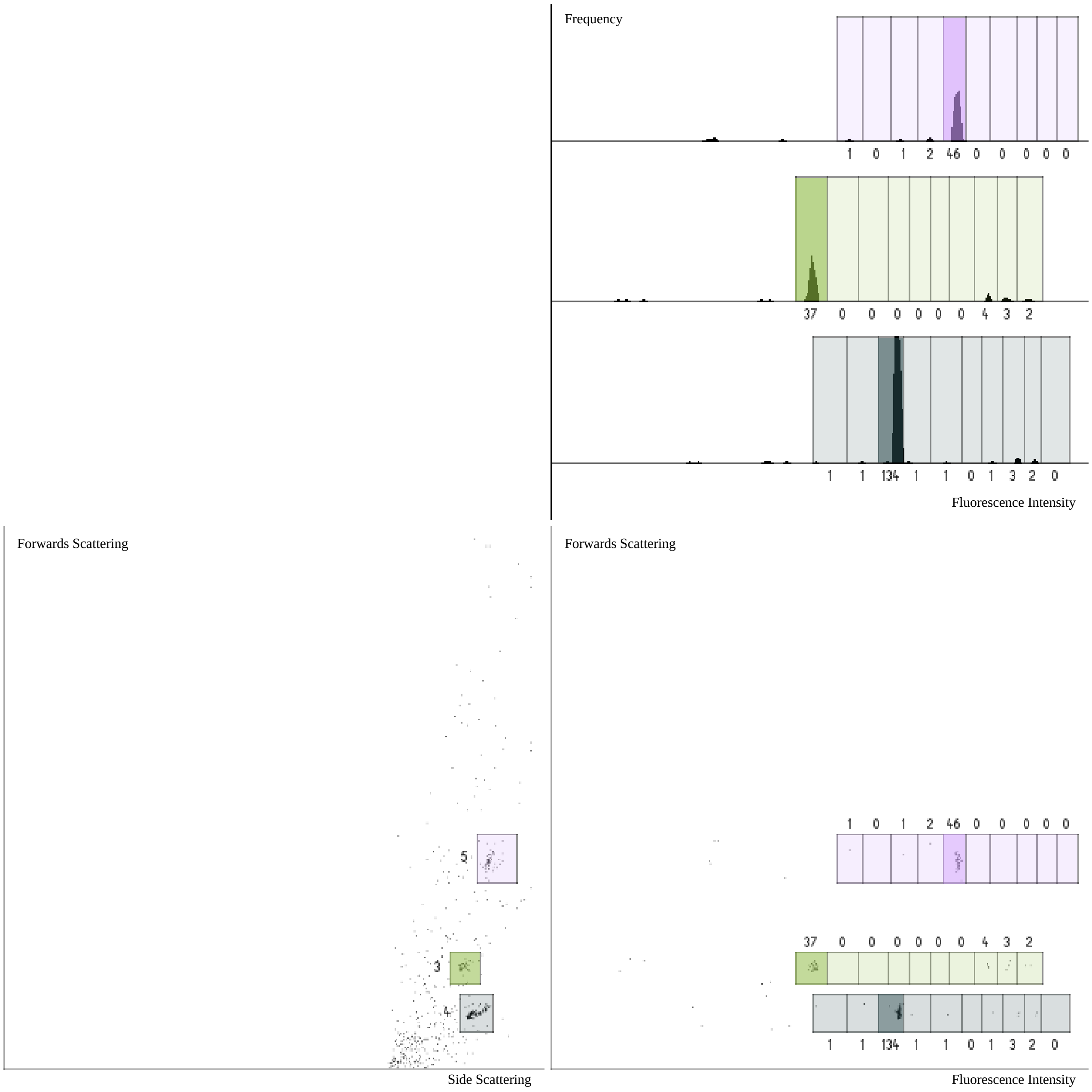

ANNEX 3: TAG DECONVOLUTION - BEAD 327

Passes flow sorting criteria: Yes

Passes tag deconvolution criteria: Yes

Included in protocol analysis: Yes

Protocol: 6, 2, 5, 5

Filename: Bin5_plateA0_B7.fcs

Split 1: Petrol shading

Split 2: Green shading

Split 3: Violet shading

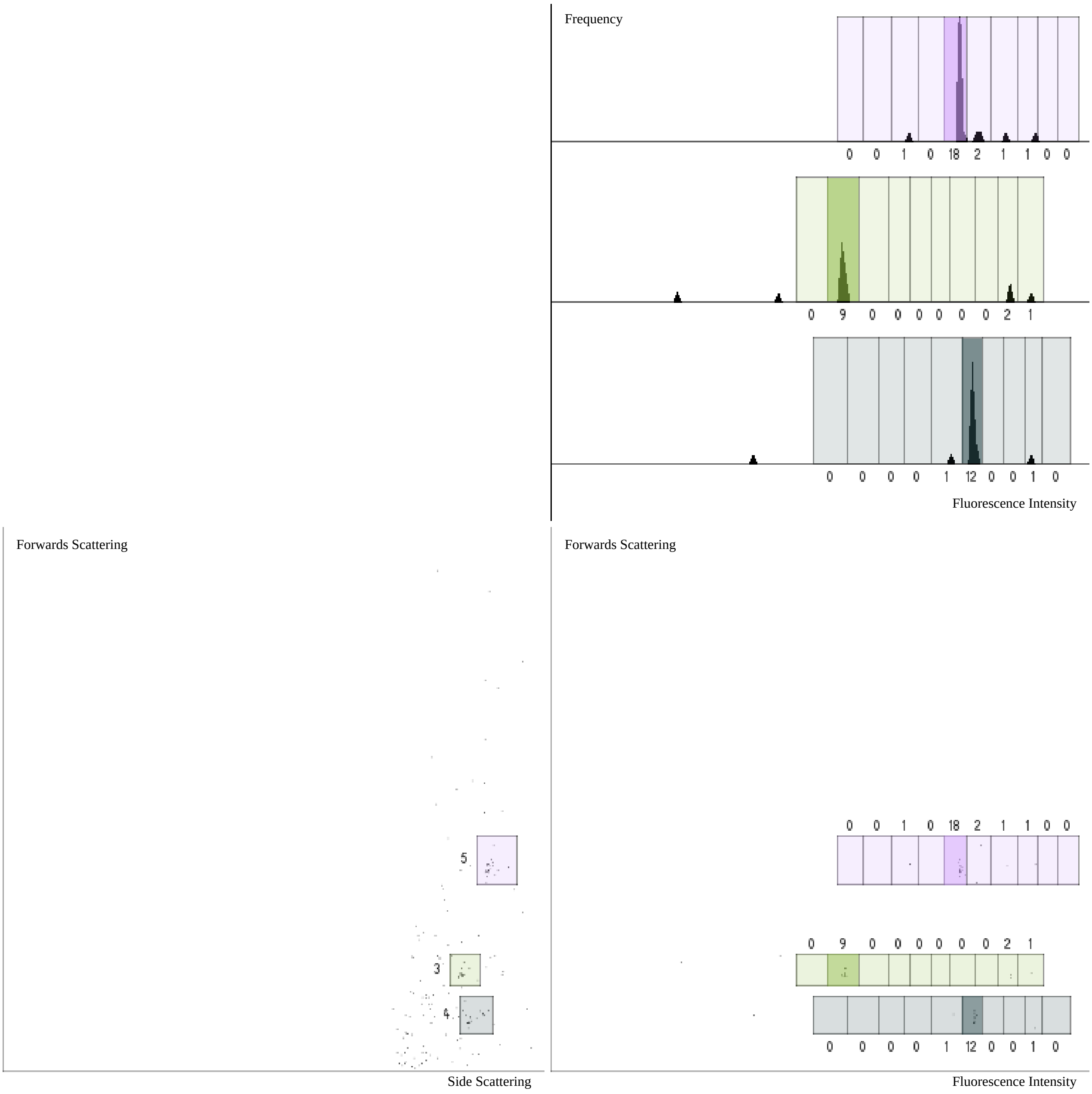

ANNEX 3: TAG DECONVOLUTION - BEAD 328

Passes flow sorting criteria: Yes

Passes tag deconvolution criteria: Yes

Included in protocol analysis: Yes

Protocol: 5, 9, 6, 5

Filename: Bin5_plateA0_B8.fcs

Split 1: Petrol shading

Split 2: Green shading

Split 3: Violet shading

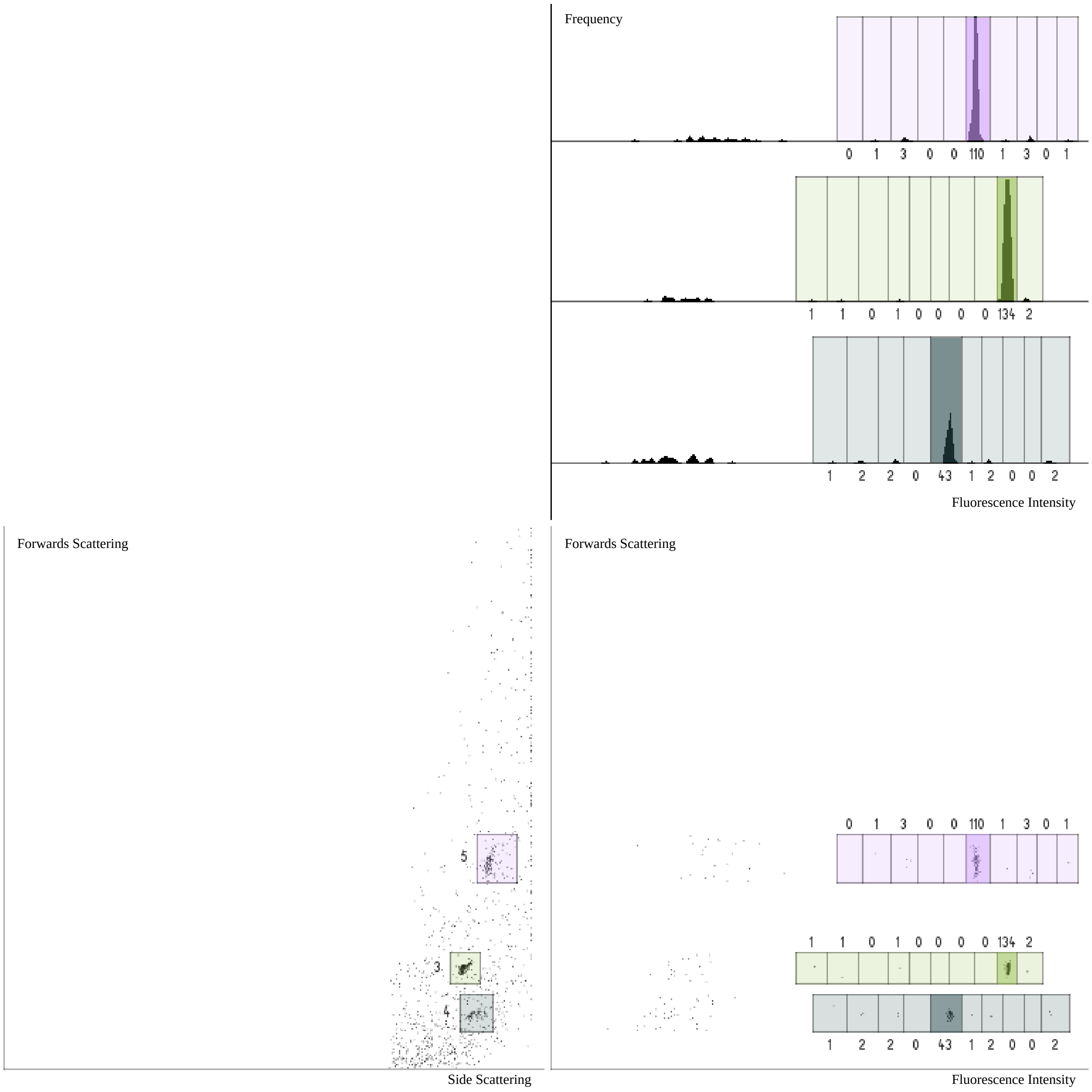

ANNEX 3: TAG DECONVOLUTION - BEAD 329

Passes flow sorting criteria: Yes

Passes tag deconvolution criteria: Yes

Included in protocol analysis: Yes

Protocol: 6, 6, 10, 5

Filename: Bin5_plateA0_B9.fcs

Split 1: Petrol shading

Split 2: Green shading

Split 3: Violet shading

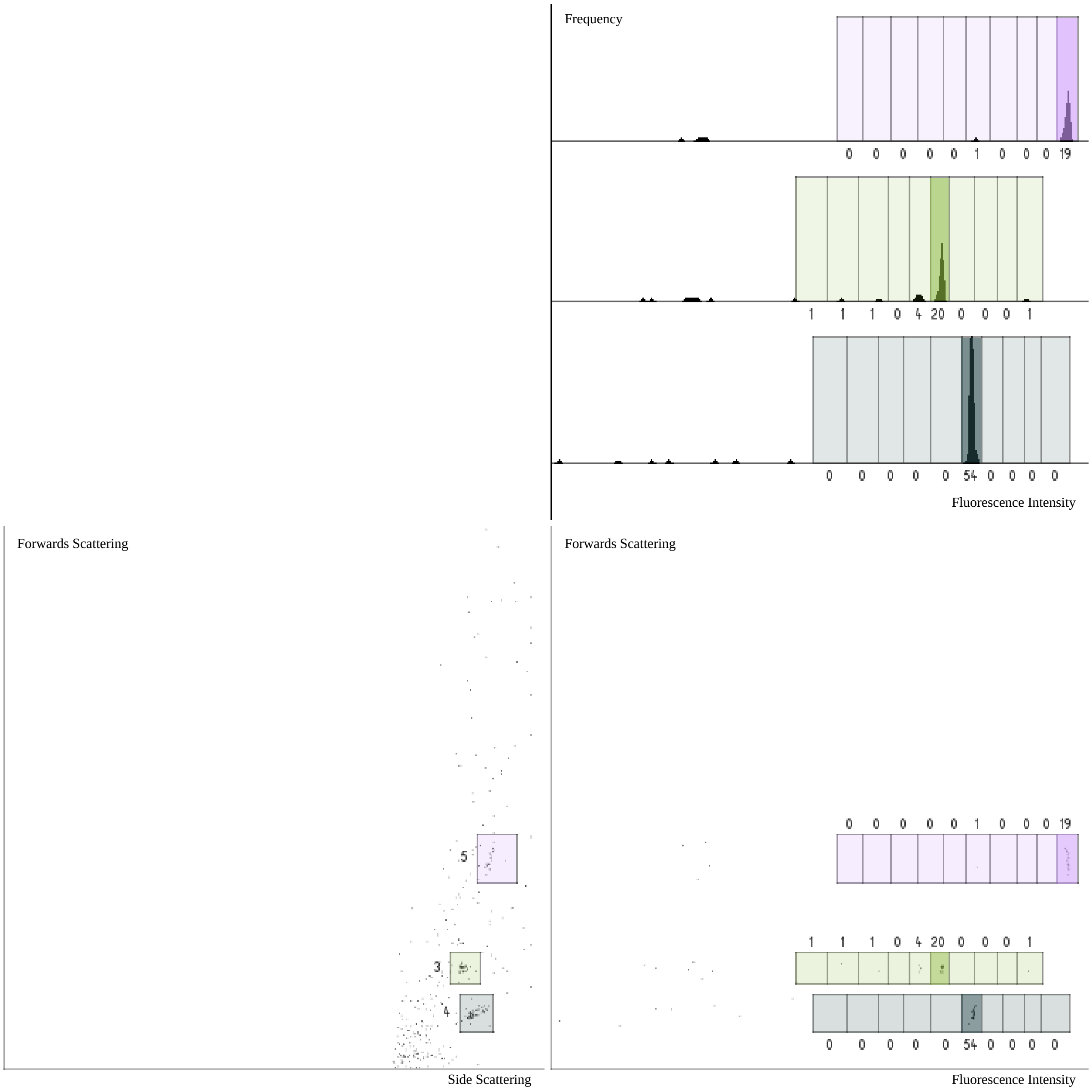

ANNEX 3: TAG DECONVOLUTION - BEAD 330

Passes flow sorting criteria: Yes

Passes tag deconvolution criteria: Yes

Included in protocol analysis: Yes

Protocol: 4, 6, 5, 5

Filename: Bin5_plateA0_B10.fcs

Split 1: Petrol shading

Split 2: Green shading

Split 3: Violet shading

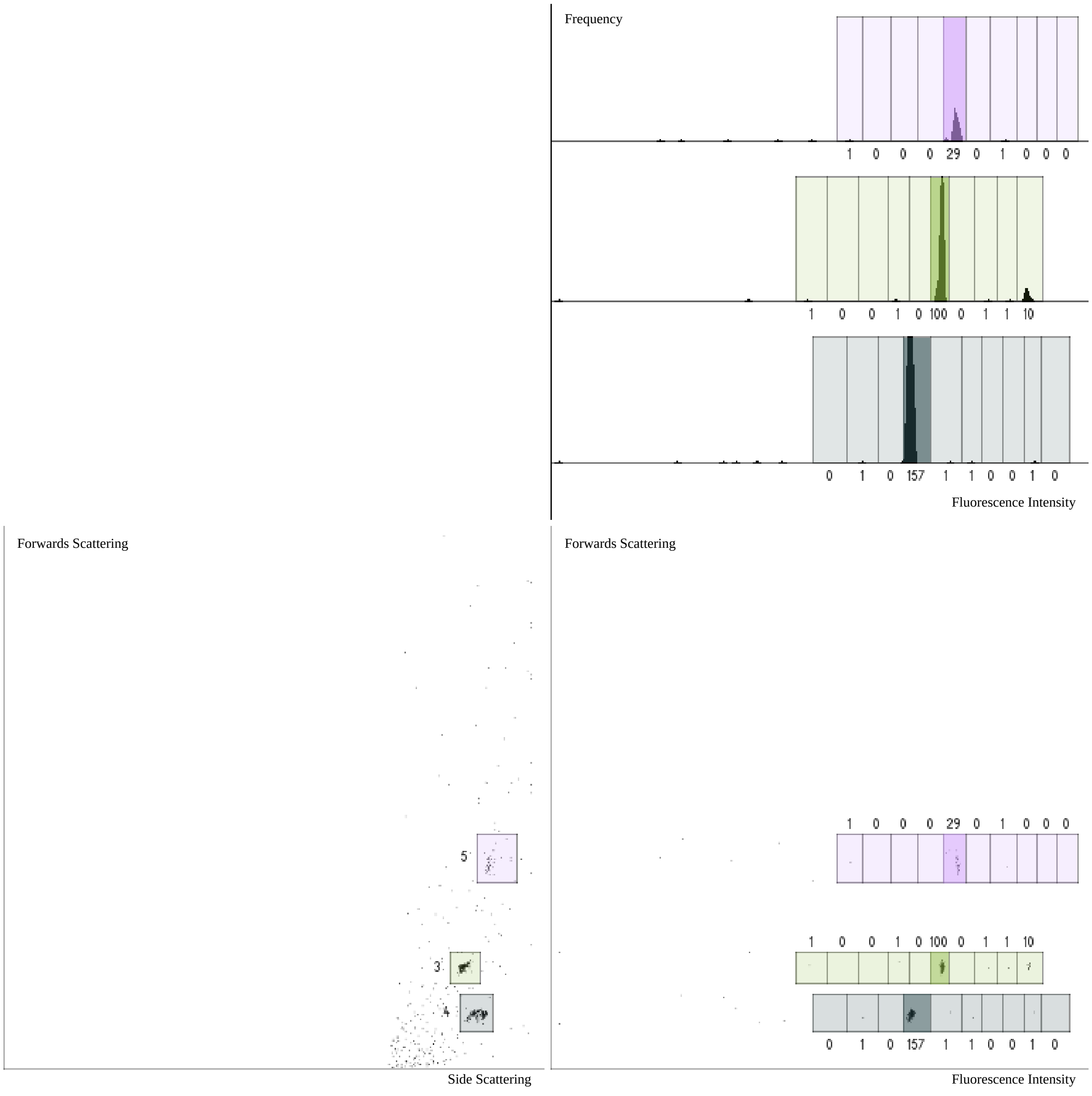

ANNEX 3: TAG DECONVOLUTION - BEAD 331

Passes flow sorting criteria: Yes

Passes tag deconvolution criteria: Yes

Included in protocol analysis: Yes

Protocol: 4, 2, 3, 5

Filename: Bin5_plateA0_C1.fcs

Split 1: Petrol shading

Split 2: Green shading

Split 3: Violet shading

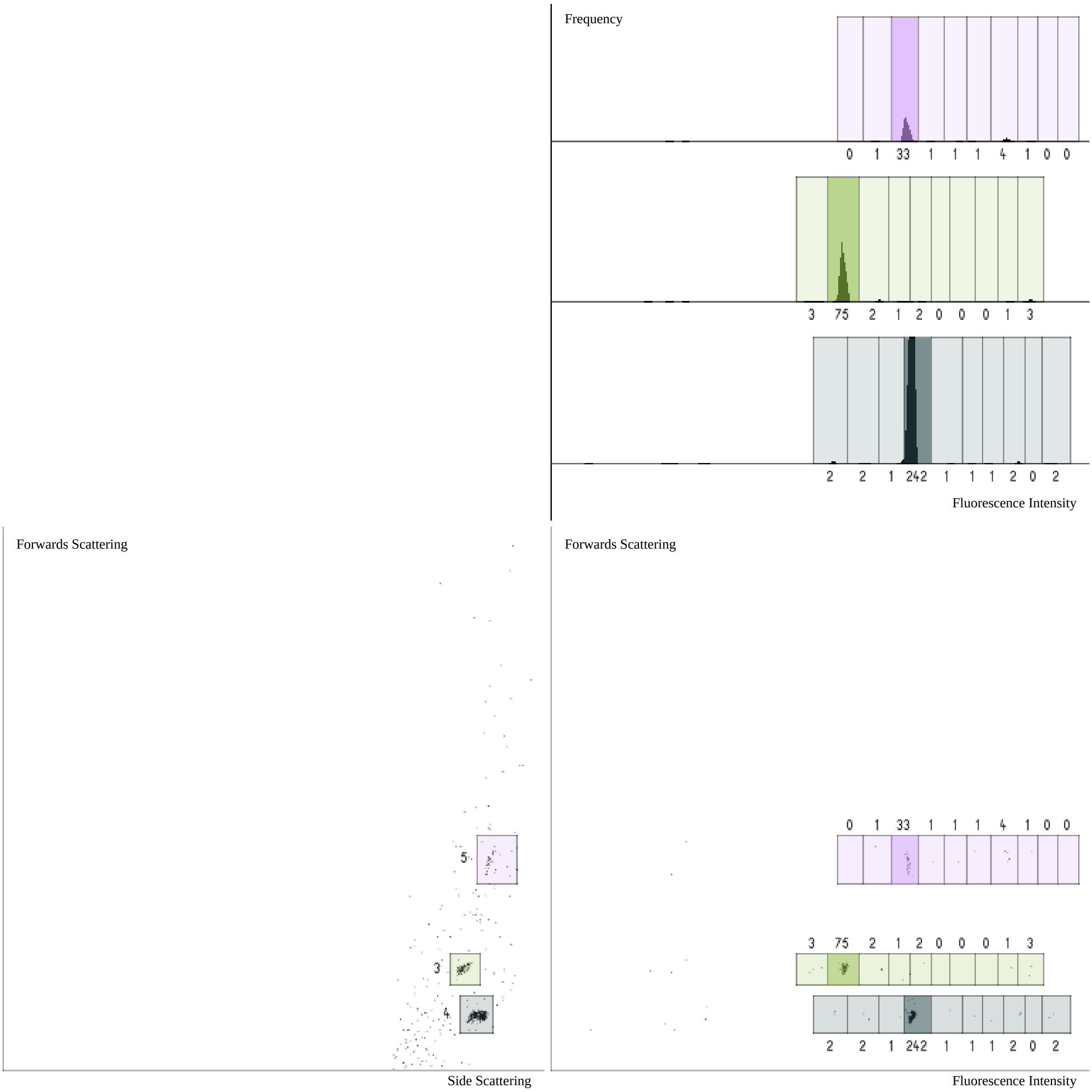

ANNEX 3: TAG DECONVOLUTION - BEAD 332

Passes flow sorting criteria: Yes

Passes tag deconvolution criteria: No

Included in protocol analysis: No

Protocol: N/A

Filename: Bin5_plateA0_C2.fcs

Split 1: Petrol shading

Split 2: Green shading

Split 3: Violet shading

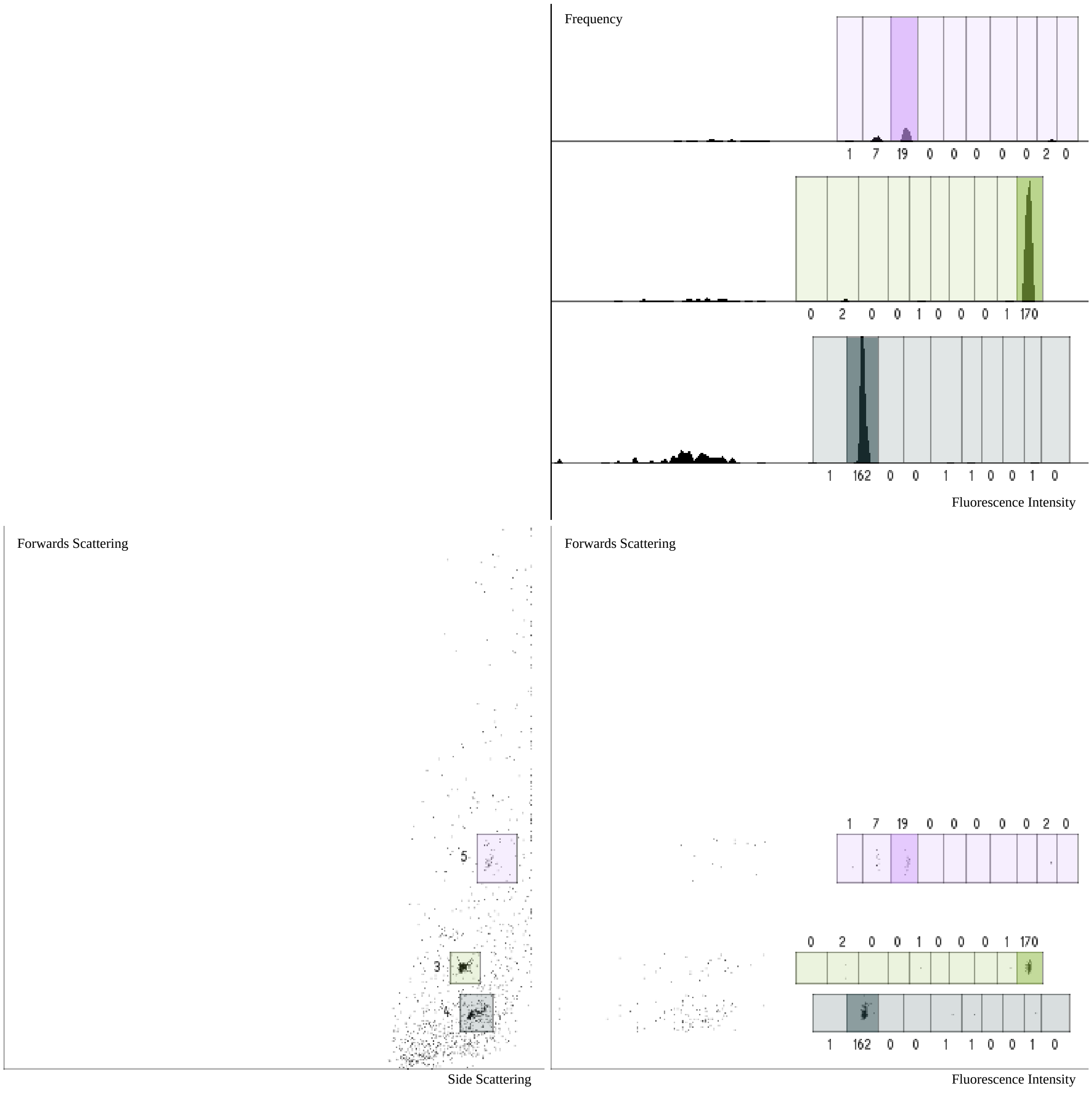

ANNEX 3: TAG DECONVOLUTION - BEAD 333

Passes flow sorting criteria: Yes

Passes tag deconvolution criteria: No

Included in protocol analysis: No

Protocol: N/A

Filename: Bin5_plateA0_C3.fcs

Split 1: Petrol shading

Split 2: Green shading

Split 3: Violet shading

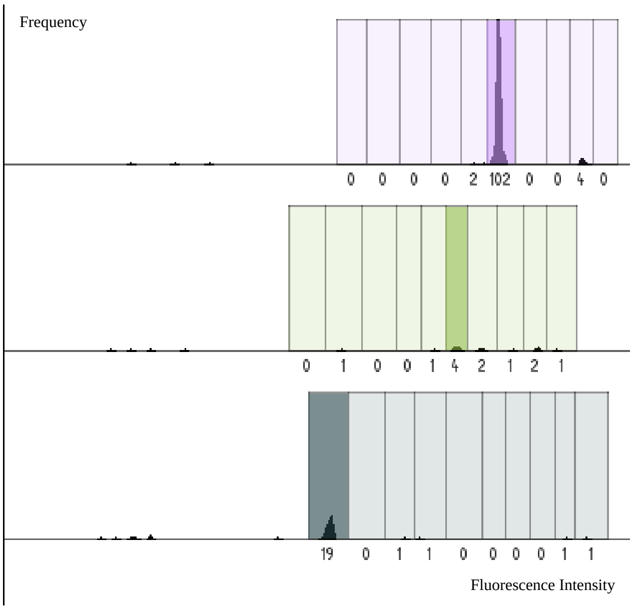

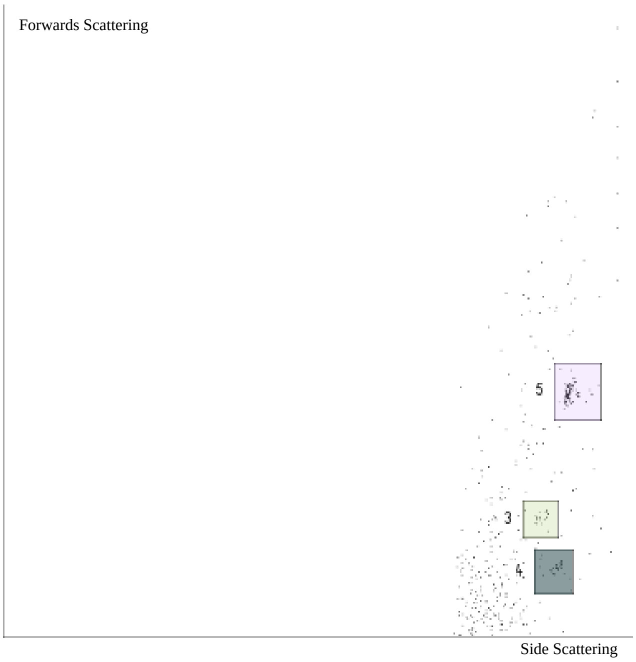

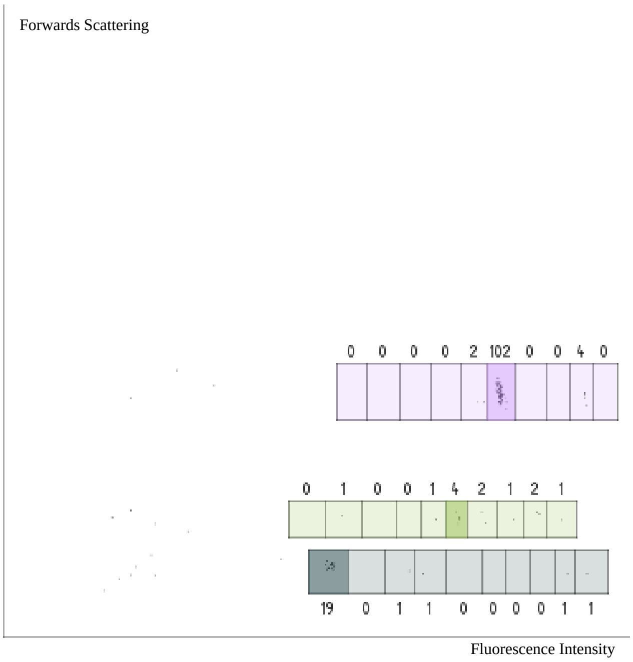

ANNEX 3: TAG DECONVOLUTION - BEAD 334

Passes flow sorting criteria: Yes

Passes tag deconvolution criteria: Yes

Included in protocol analysis: Yes

Protocol: 4, 5, 1, 5

Filename: Bin5_plateA0_C4.fcs

Split 1: Petrol shading

Split 2: Green shading

Split 3: Violet shading

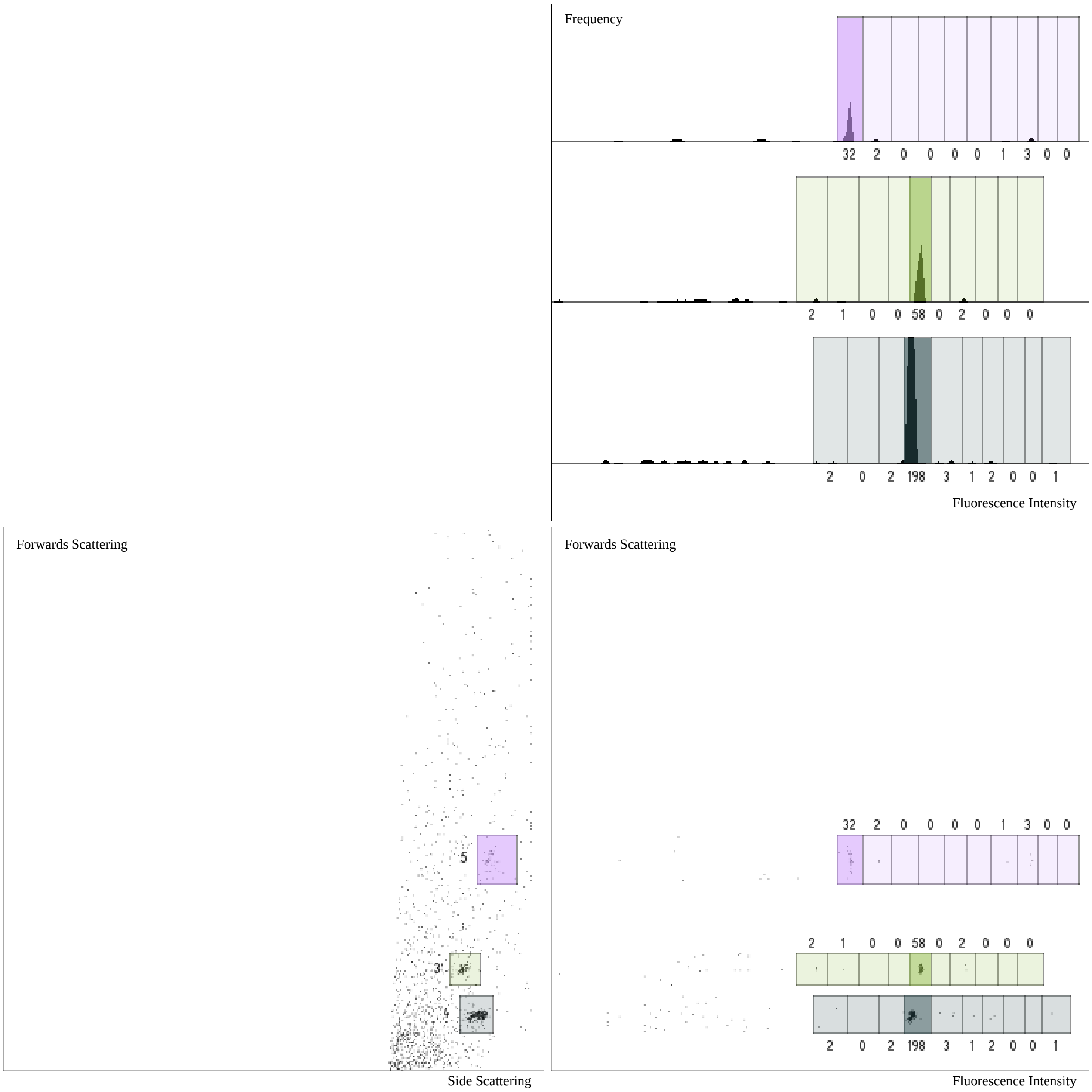

ANNEX 3: TAG DECONVOLUTION - BEAD 335

Passes flow sorting criteria: Yes

Passes tag deconvolution criteria: No

Included in protocol analysis: No

Protocol: N/A

Filename: Bin5_plateA0_C5.fcs

Split 1: Petrol shading

Split 2: Green shading

Split 3: Violet shading

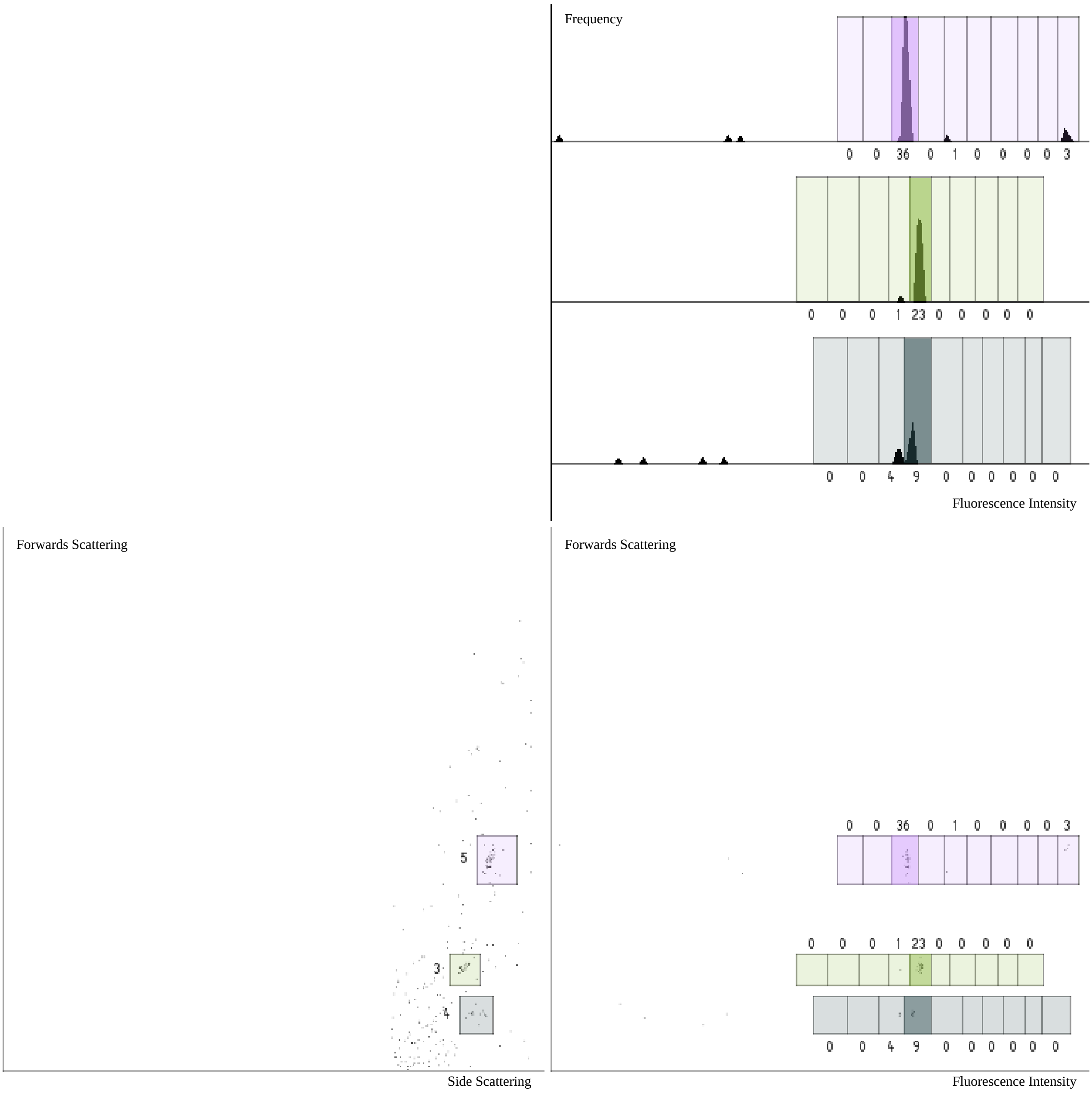

ANNEX 3: TAG DECONVOLUTION - BEAD 336

Passes flow sorting criteria: Yes

Passes tag deconvolution criteria: No

Included in protocol analysis: No

Protocol: N/A

Filename: Bin5_plateA0_C6.fcs

Split 1: Petrol shading

Split 2: Green shading

Split 3: Violet shading

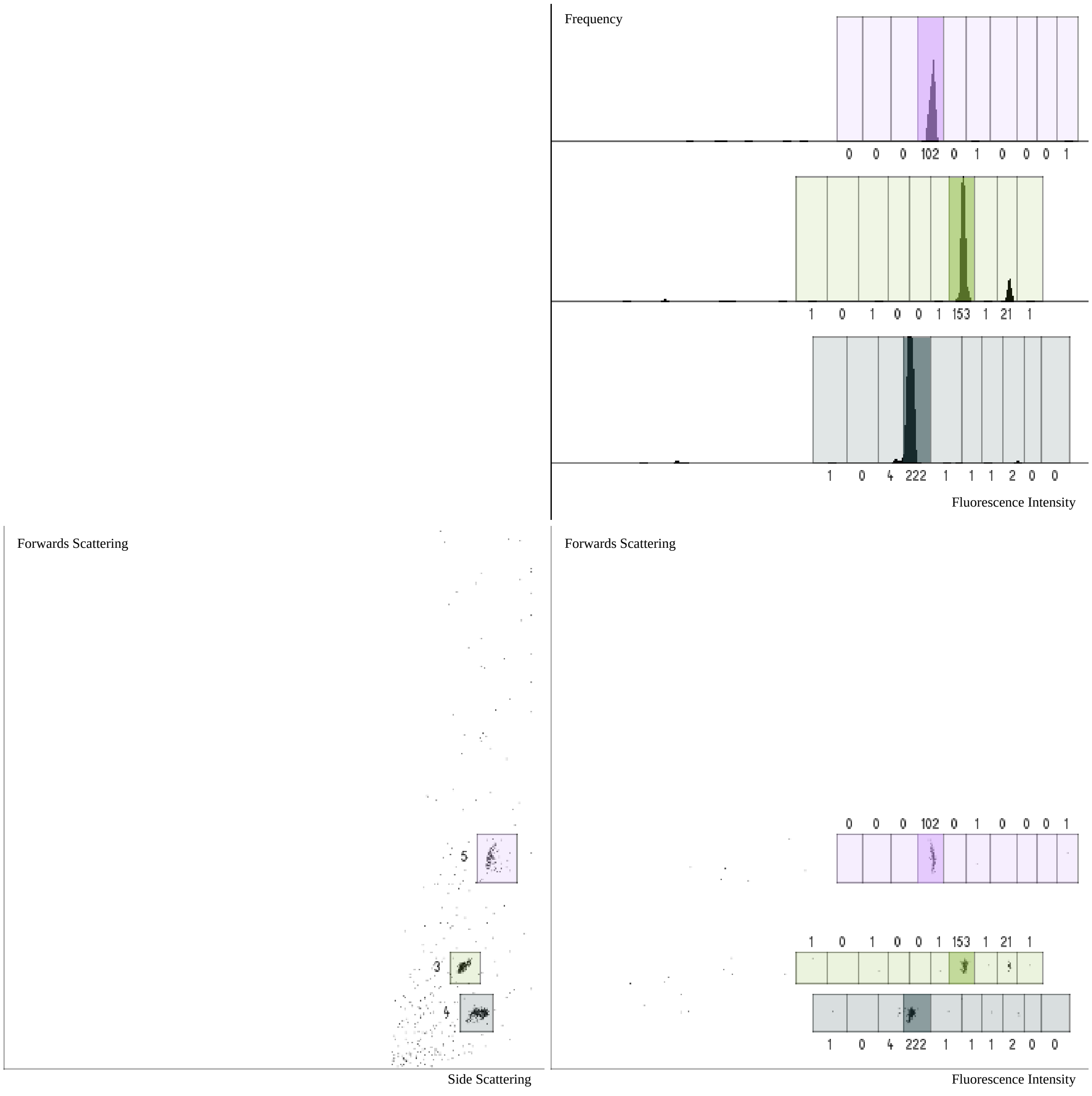

ANNEX 3: TAG DECONVOLUTION - BEAD 337

Passes flow sorting criteria: Yes

Passes tag deconvolution criteria: Yes

Included in protocol analysis: Yes

Protocol: 10, 10, 1, 5

Filename: Bin5_plateA0_C7.fcs

Split 1: Petrol shading

Split 2: Green shading

Split 3: Violet shading

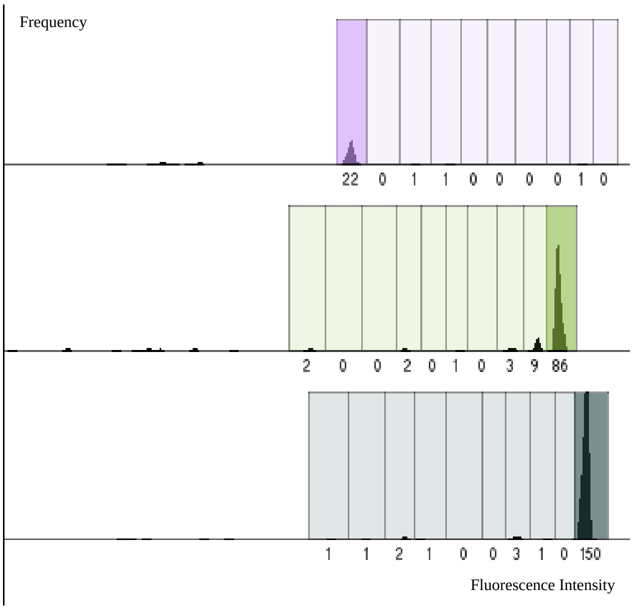

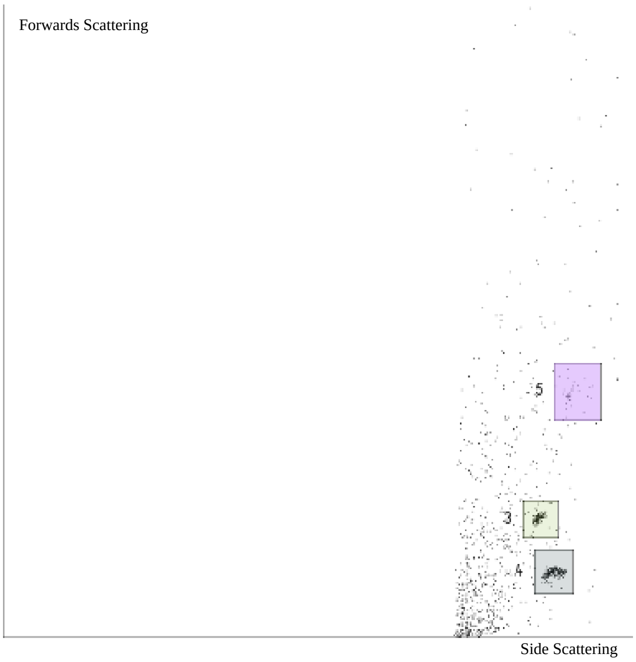

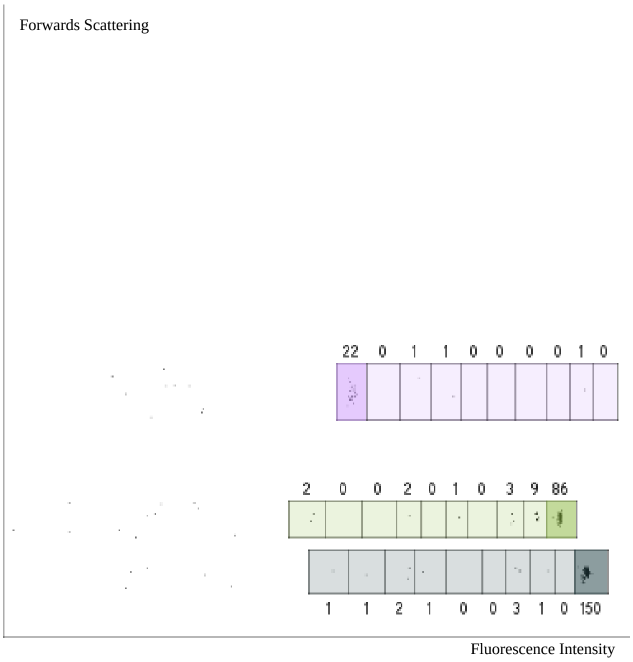

ANNEX 3: TAG DECONVOLUTION - BEAD 338

Passes flow sorting criteria: Yes

Passes tag deconvolution criteria: Yes

Included in protocol analysis: Yes

Protocol: 7, 2, 7, 5

Filename: Bin5_plateA0_G5.fcs

Split 1: Petrol shading

Split 2: Green shading

Split 3: Violet shading

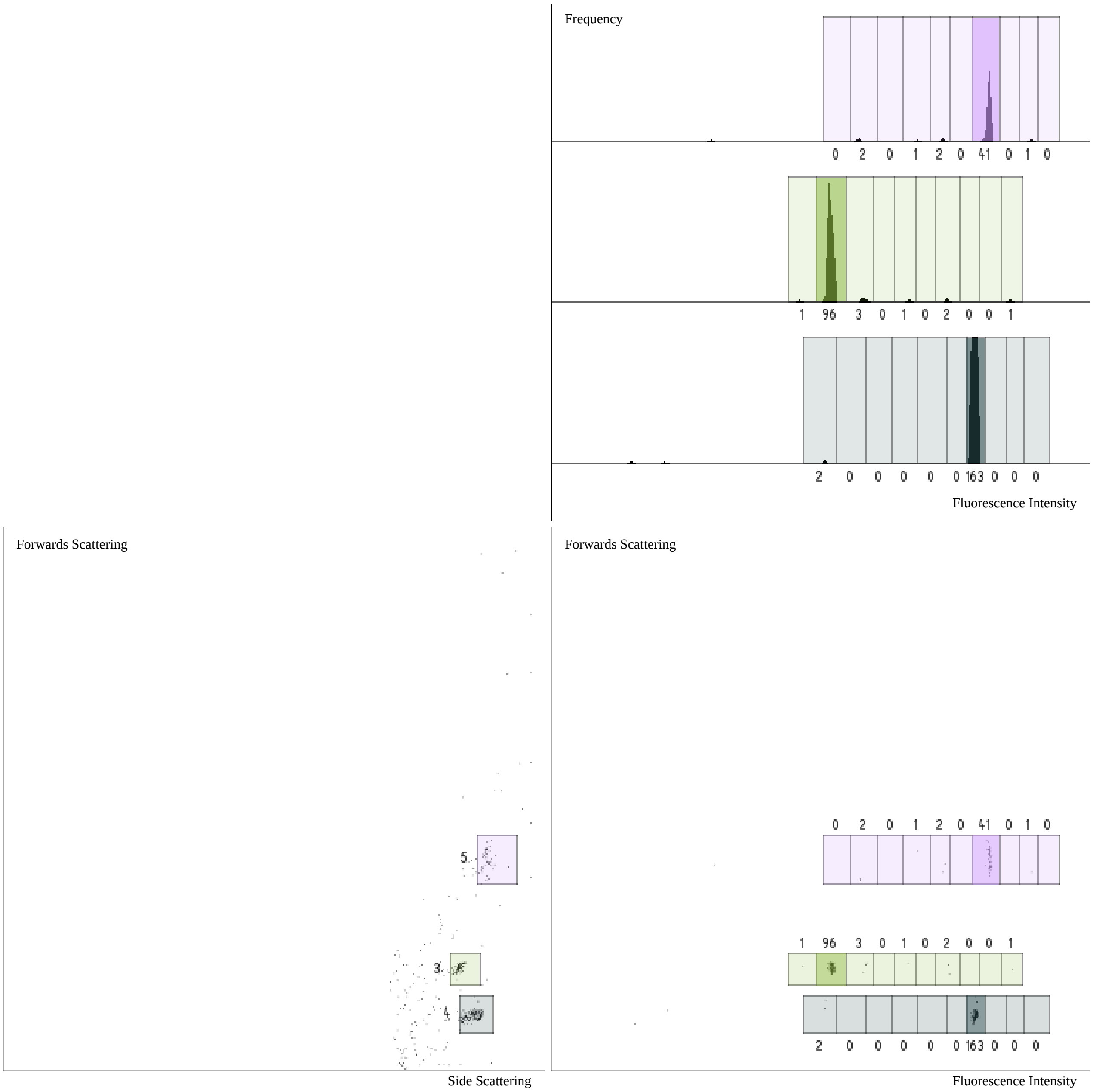

ANNEX 3: TAG DECONVOLUTION - BEAD 339

Passes flow sorting criteria: Yes

Passes tag deconvolution criteria: Yes

Included in protocol analysis: Yes

Protocol: 7, 9, 6, 5

Filename: Bin5_plateA0_C9.fcs

Split 1: Petrol shading

Split 2: Green shading

Split 3: Violet shading

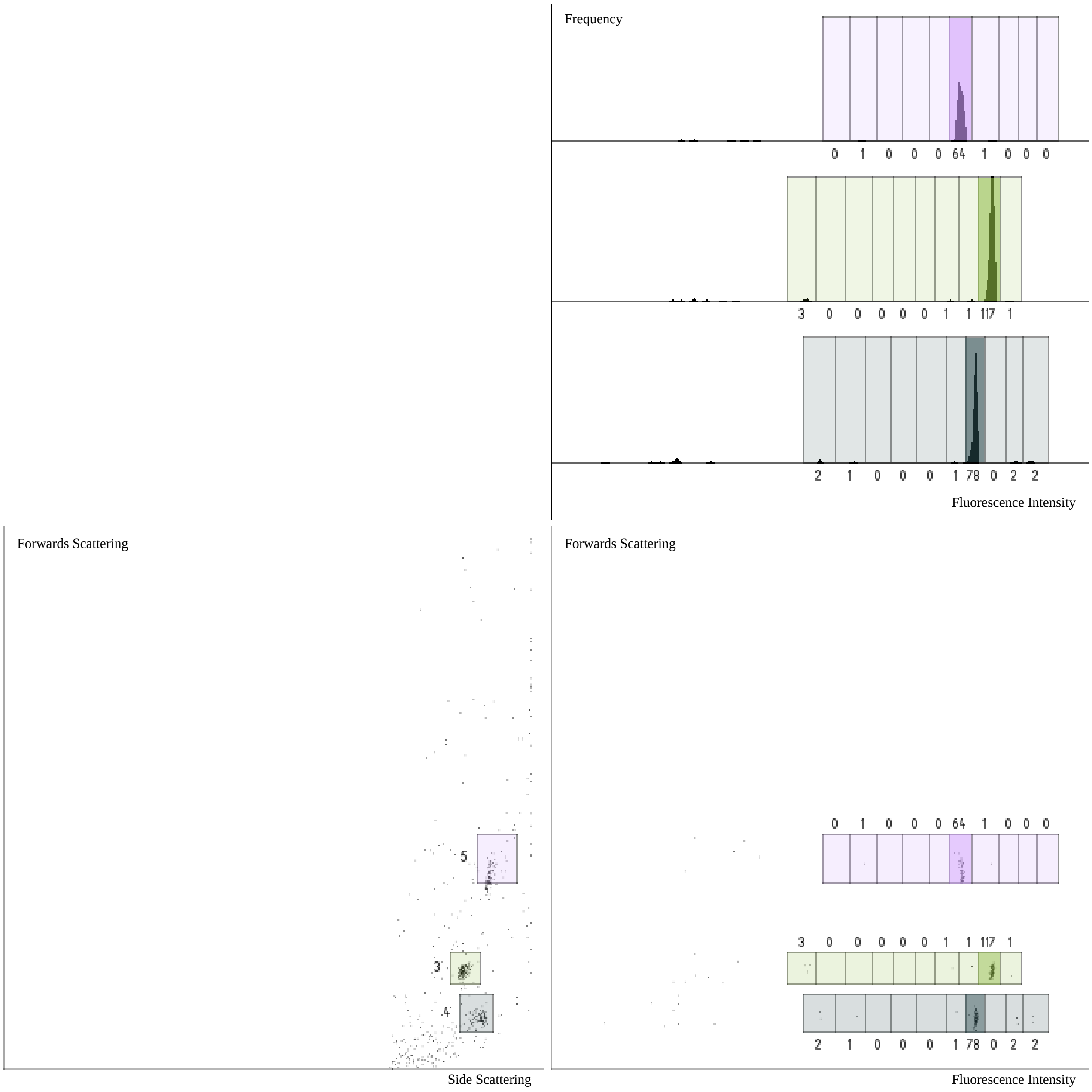

ANNEX 3: TAG DECONVOLUTION - BEAD 340

Passes flow sorting criteria: Yes

Passes tag deconvolution criteria: Yes

Included in protocol analysis: Yes

Protocol: 5, 9, 5, 5

Filename: Bin5_plateA0_C10.fcs

Split 1: Petrol shading

Split 2: Green shading

Split 3: Violet shading

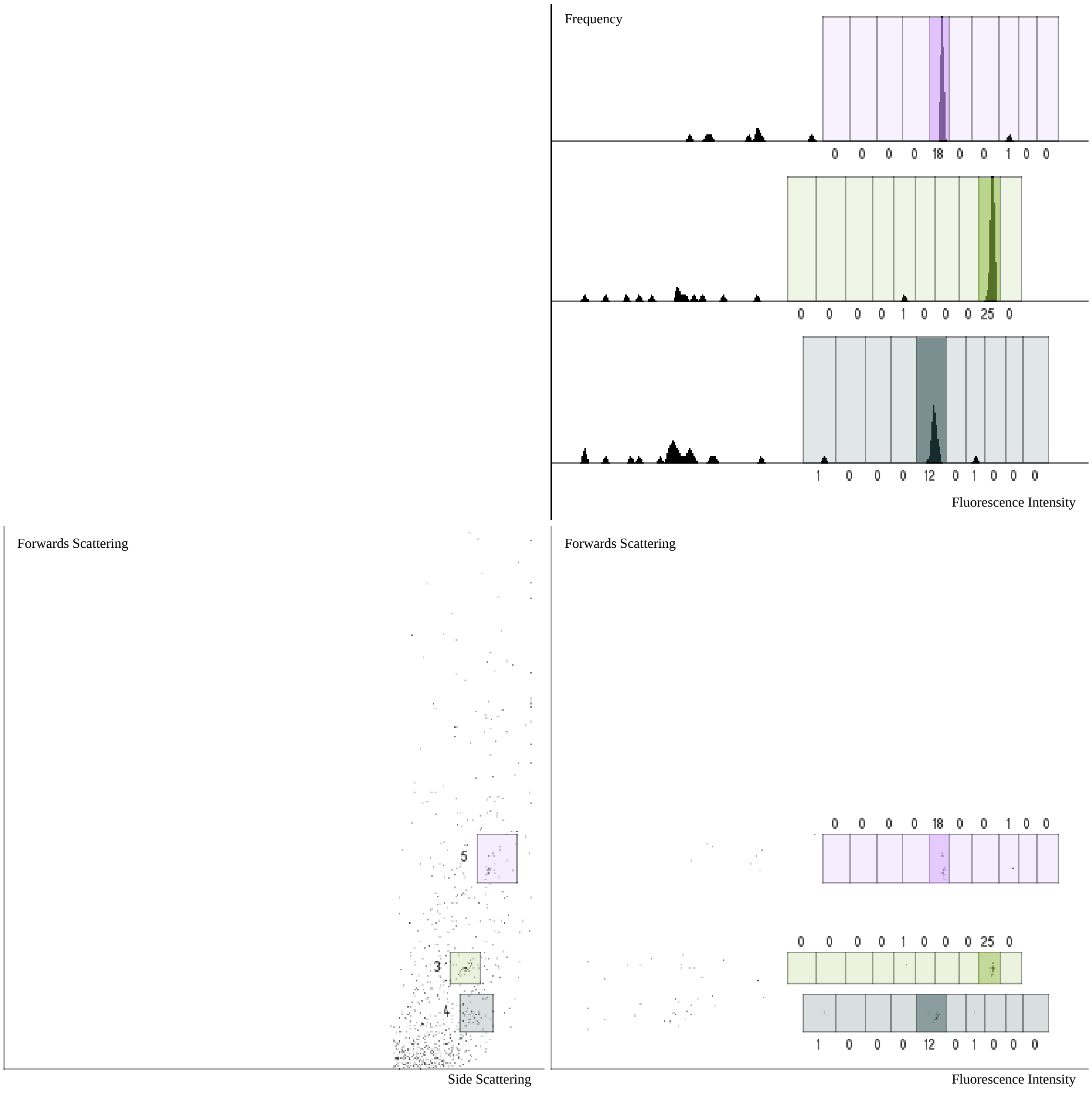

ANNEX 3: TAG DECONVOLUTION - BEAD 341

Passes flow sorting criteria: Yes

Passes tag deconvolution criteria: Yes

Included in protocol analysis: Yes

Protocol: 2, 7, 7, 5

Filename: Bin5_plateA0_C11.fcs

Split 1: Petrol shading

Split 2: Green shading

Split 3: Violet shading

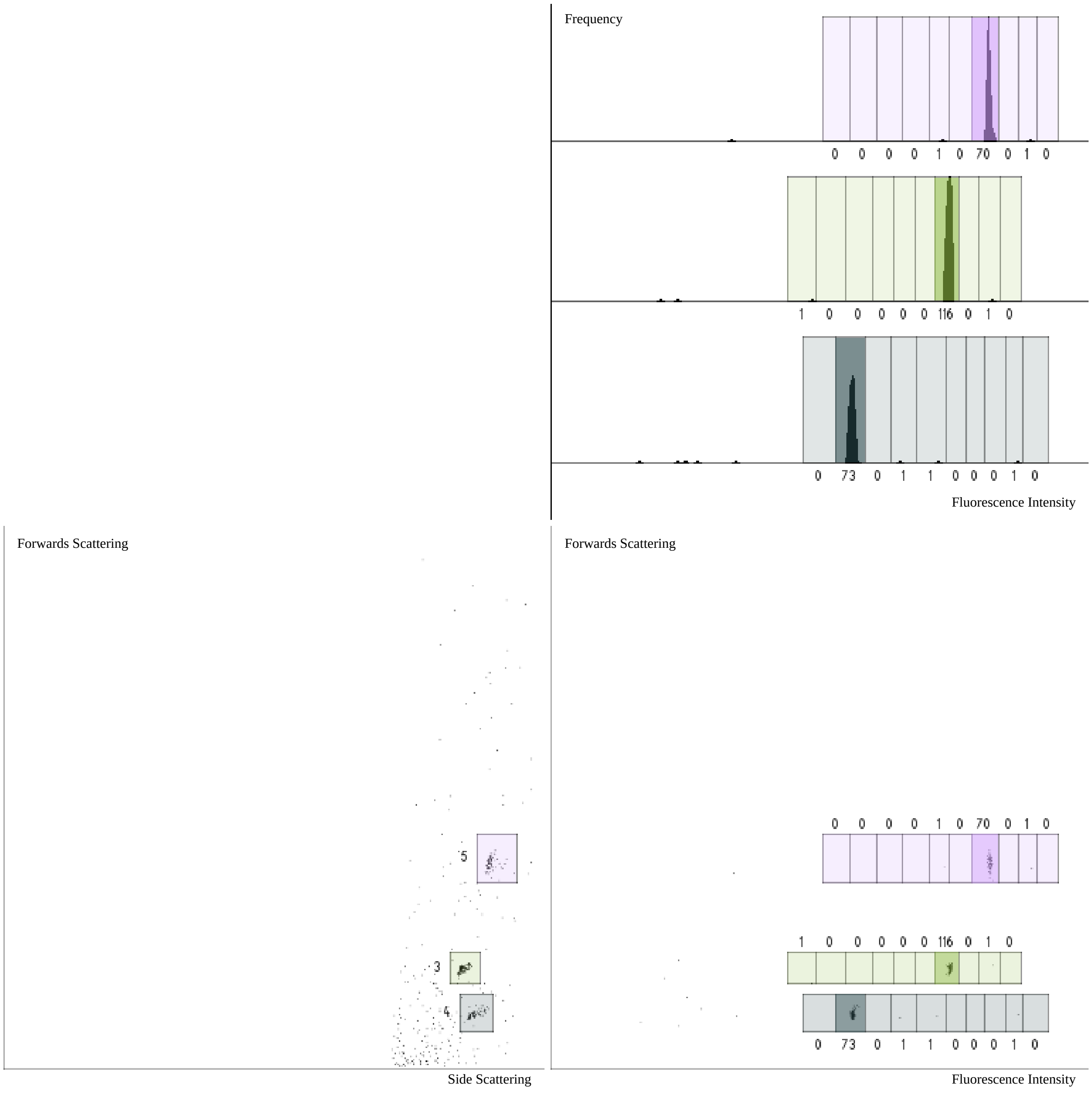

ANNEX 3: TAG DECONVOLUTION - BEAD 342

Passes flow sorting criteria: Yes

Passes tag deconvolution criteria: Yes

Included in protocol analysis: Yes

Protocol: 1, 6, 6, 5

Filename: Bin5_plateA0_D1.fcs

Split 1: Petrol shading

Split 2: Green shading

Split 3: Violet shading

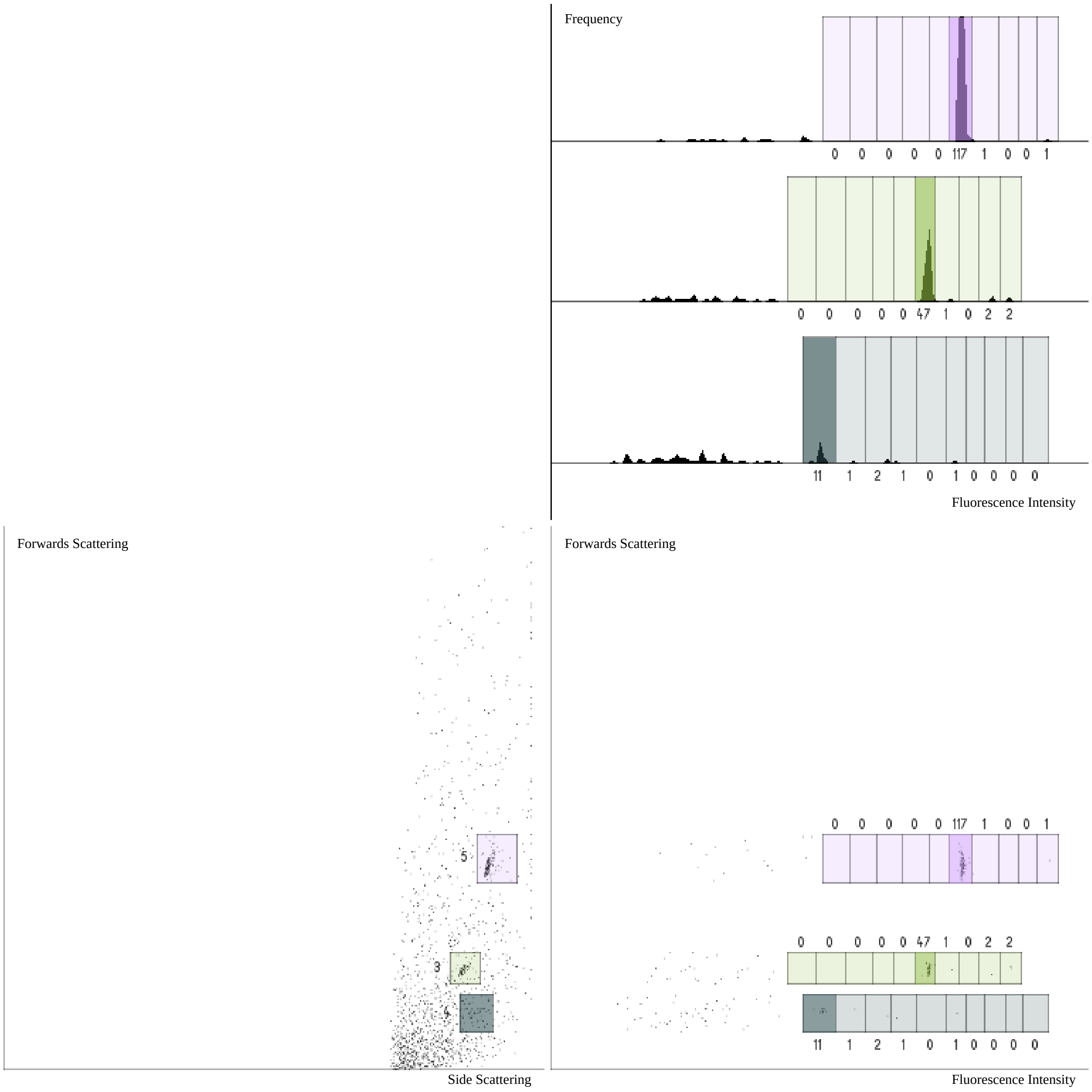

ANNEX 3: TAG DECONVOLUTION - BEAD 343

Passes flow sorting criteria: Yes

Passes tag deconvolution criteria: Yes

Included in protocol analysis: Yes

Protocol: 6, 10, 10, 5

Filename: Bin5_plateA0_D2.fcs

Split 1: Petrol shading

Split 2: Green shading

Split 3: Violet shading

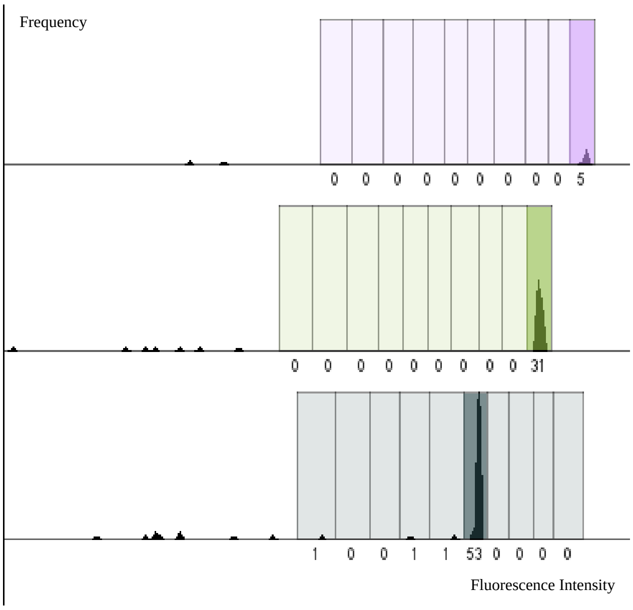

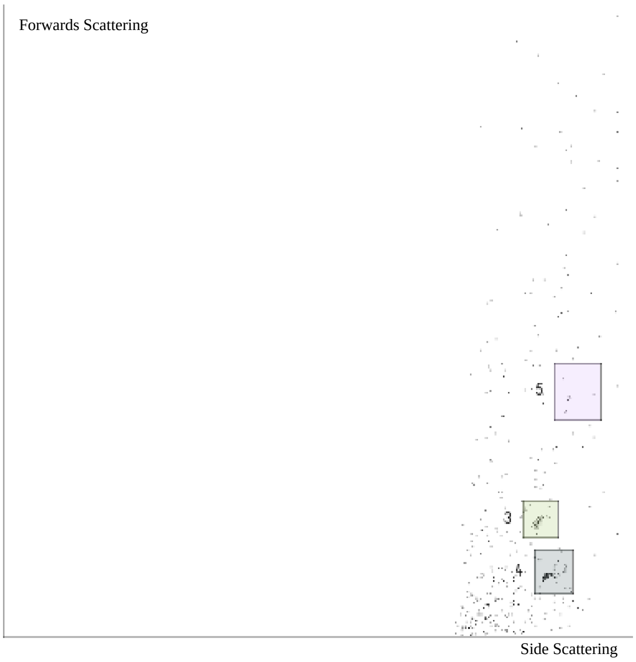

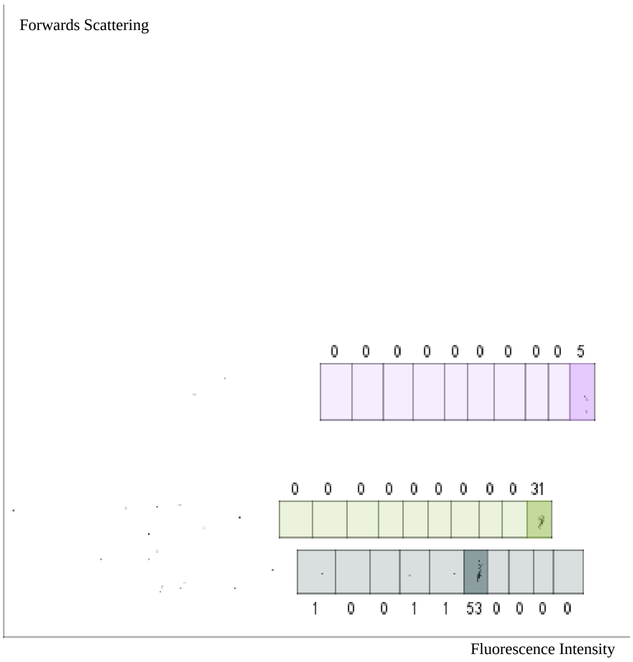

ANNEX 3: TAG DECONVOLUTION - BEAD 344

Passes flow sorting criteria: Yes

Passes tag deconvolution criteria: Yes

Included in protocol analysis: Yes

Protocol: 10, 6, 2, 5

Filename: Bin5_plateA0_D3.fcs

Split 1: Petrol shading

Split 2: Green shading

Split 3: Violet shading

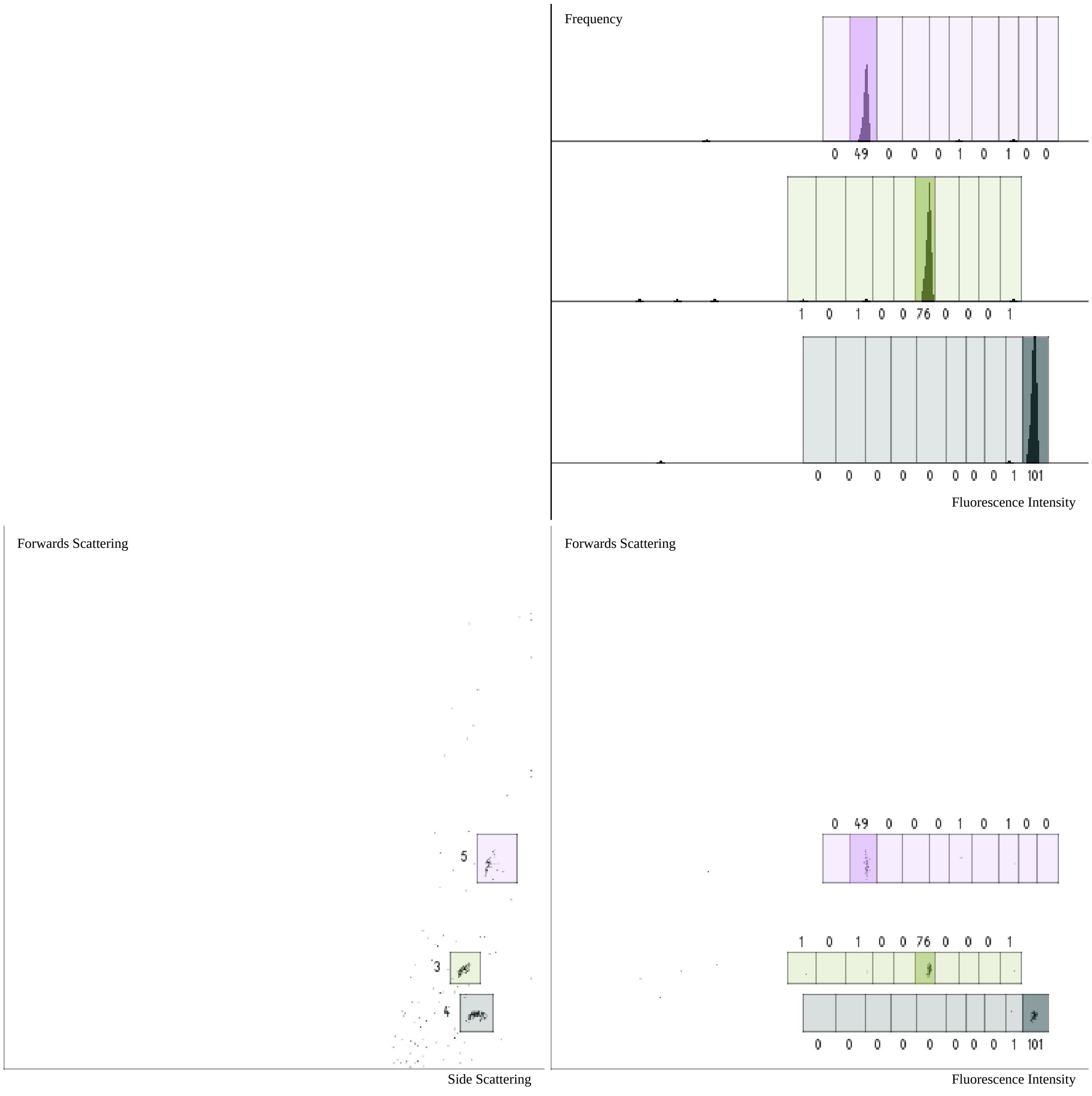

ANNEX 3: TAG DECONVOLUTION - BEAD 345

Passes flow sorting criteria: Yes

Passes tag deconvolution criteria: Yes

Included in protocol analysis: Yes

Protocol: 4, 9, 8, 5

Filename: Bin5_plateA0_D4.fcs

Split 1: Petrol shading

Split 2: Green shading

Split 3: Violet shading

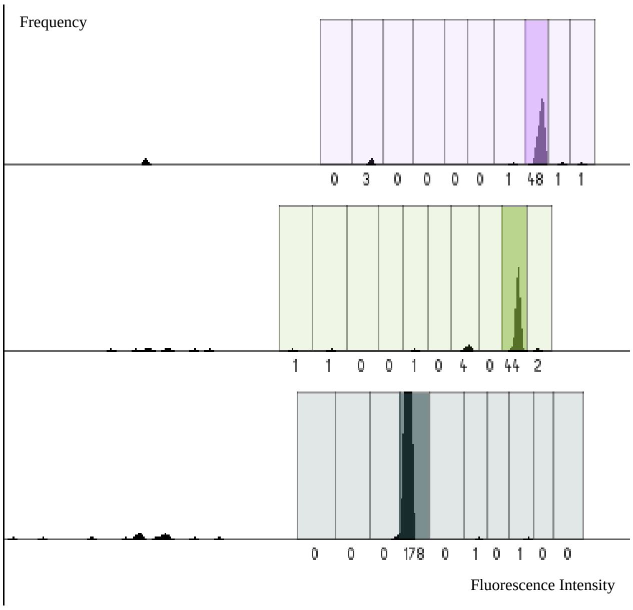

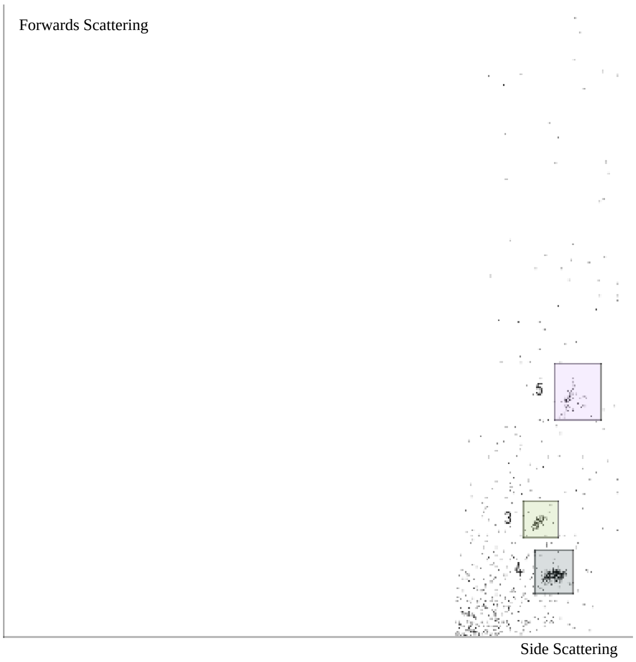

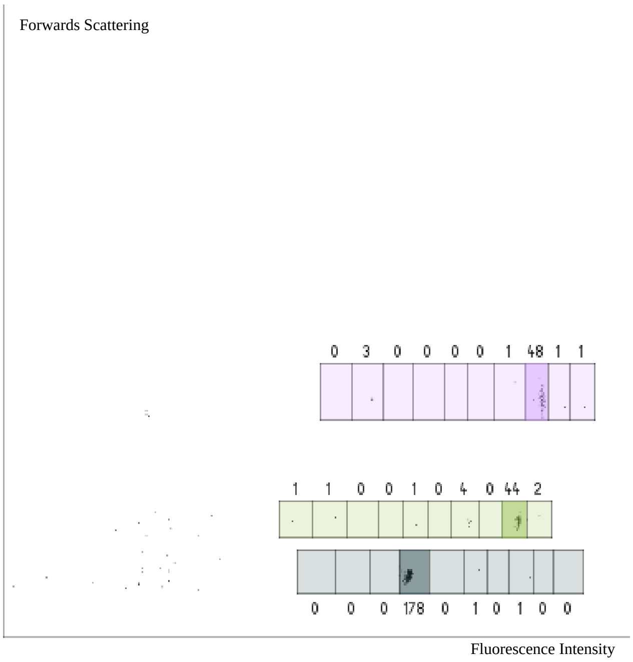

ANNEX 3: TAG DECONVOLUTION - BEAD 346

Passes flow sorting criteria: Yes

Passes tag deconvolution criteria: No

Included in protocol analysis: No

Protocol: N/A

Filename: Bin5_plateA0_D5.fcs

Split 1: Petrol shading

Split 2: Green shading

Split 3: Violet shading

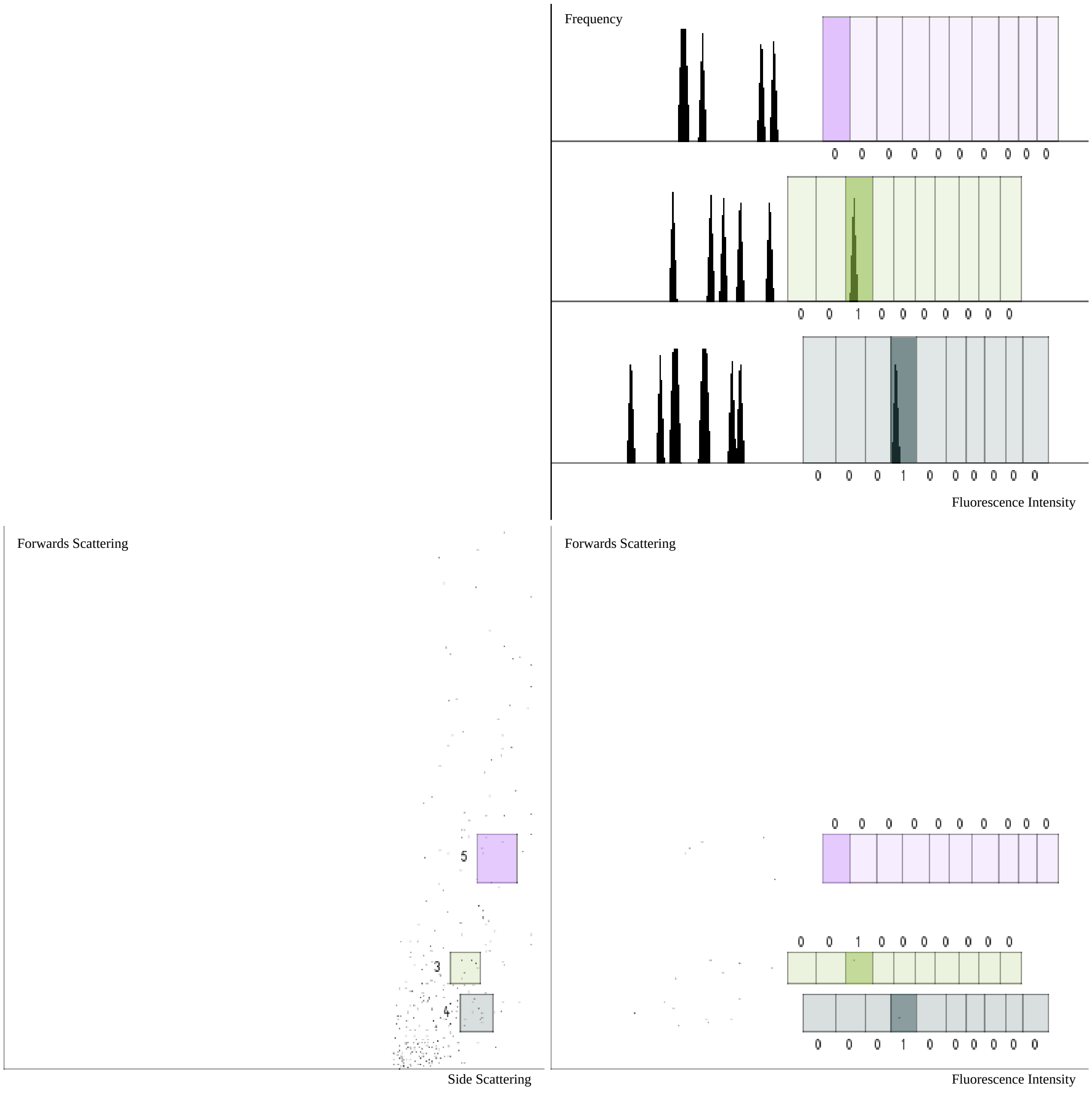

ANNEX 3: TAG DECONVOLUTION - BEAD 347

Passes flow sorting criteria: Yes

Passes tag deconvolution criteria: Yes

Included in protocol analysis: Yes

Protocol: 5, 2, 6, 5

Filename: Bin5_plateA0_D6.fcs

Split 1: Petrol shading

Split 2: Green shading

Split 3: Violet shading

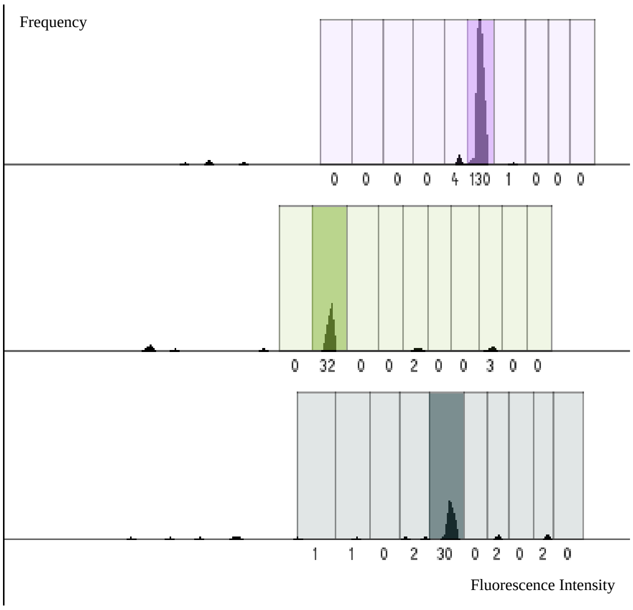

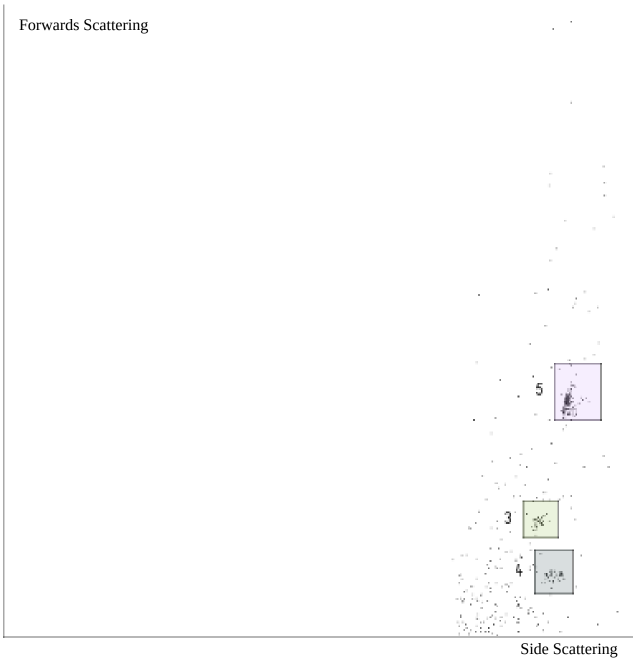

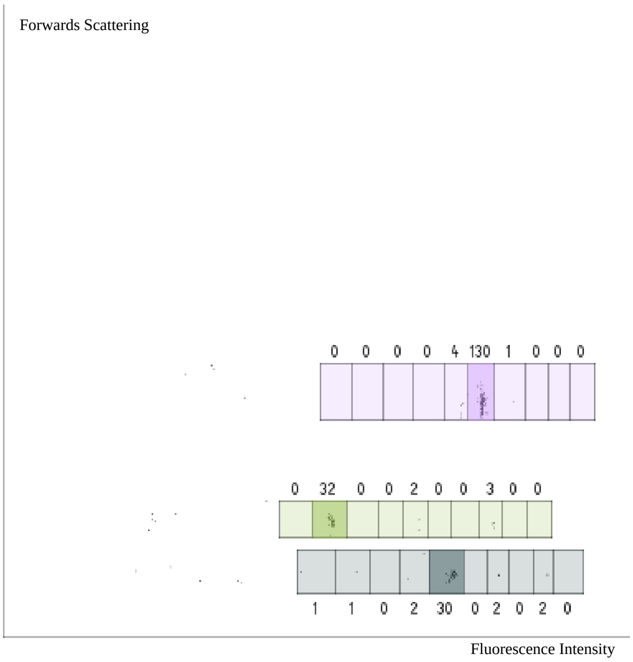

ANNEX 3: TAG DECONVOLUTION - BEAD 348

Passes flow sorting criteria: Yes

Passes tag deconvolution criteria: Yes

Included in protocol analysis: Yes

Protocol: 7, 9, 9, 5

Filename: Bin5_plateA0_D7.fcs

Split 1: Petrol shading

Split 2: Green shading

Split 3: Violet shading

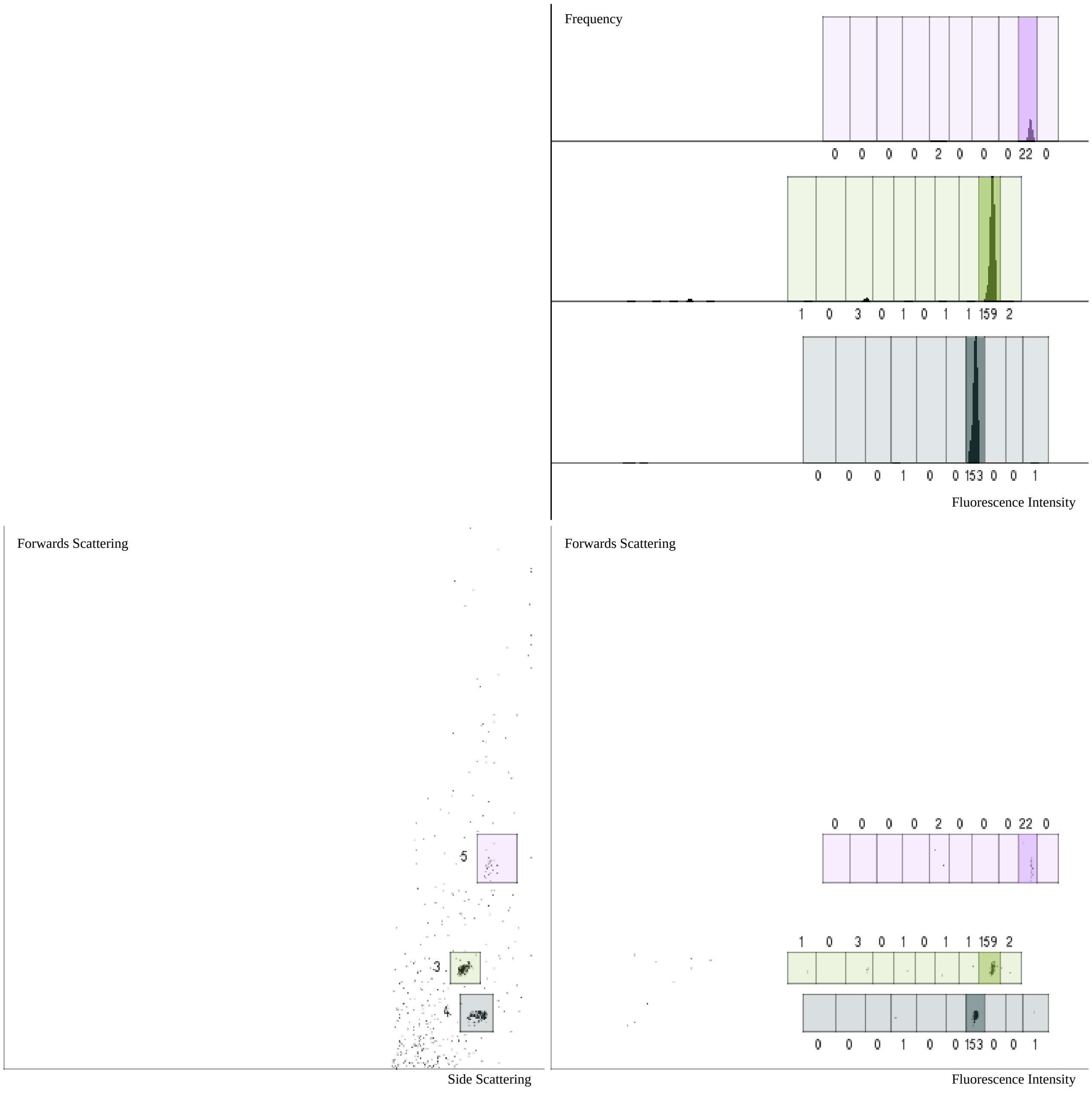

ANNEX 3: TAG DECONVOLUTION - BEAD 349

Passes flow sorting criteria: Yes

Passes tag deconvolution criteria: Yes

Included in protocol analysis: Yes

Protocol: 10, 1, 6, 5

Filename: Bin5_plateA0_D8.fcs

Split 1: Petrol shading

Split 2: Green shading

Split 3: Violet shading

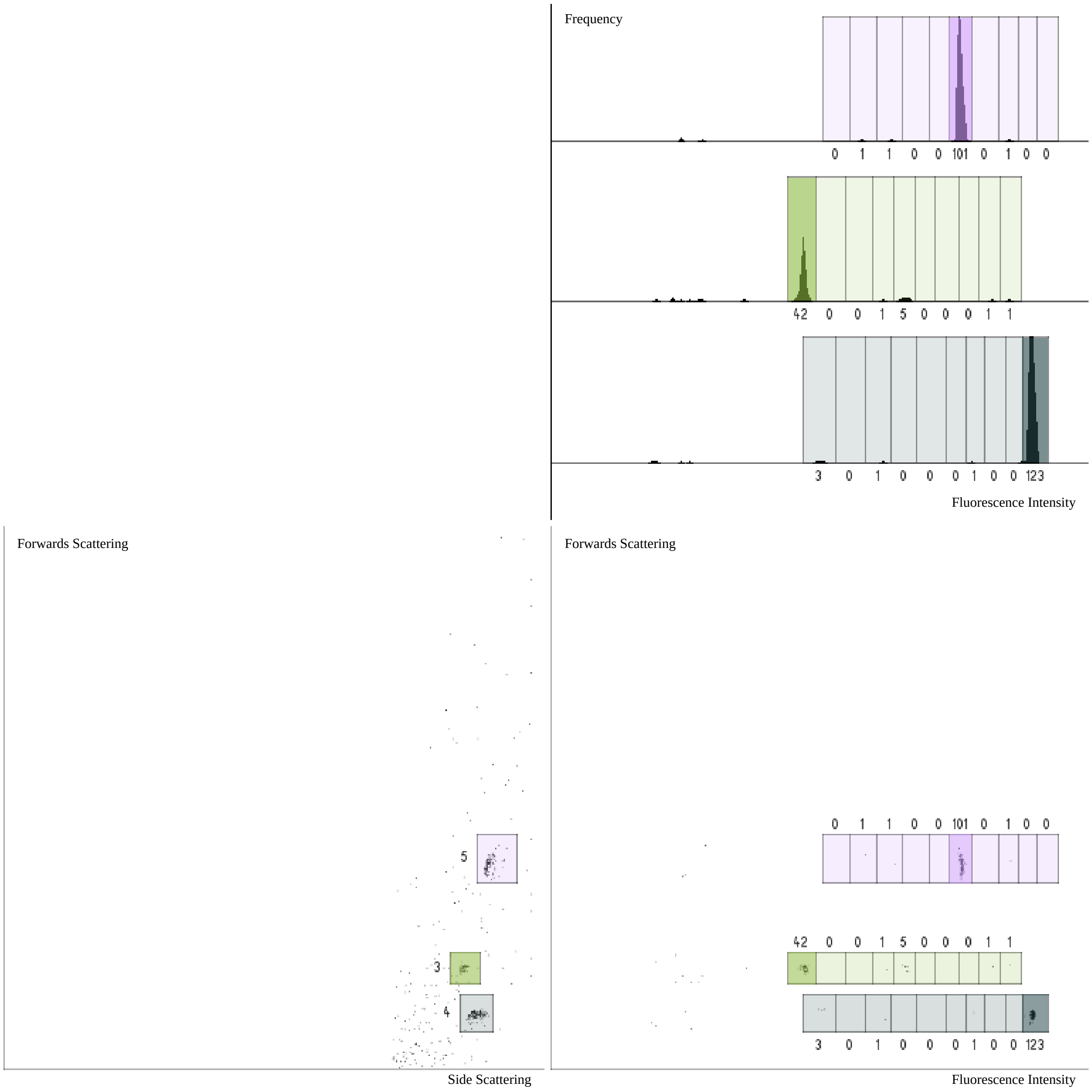

ANNEX 3: TAG DECONVOLUTION - BEAD 350

Passes flow sorting criteria: Yes

Passes tag deconvolution criteria: Yes

Included in protocol analysis: Yes

Protocol: 5, 9, 9, 5

Filename: Bin5_plateA0_D9.fcs

Split 1: Petrol shading

Split 2: Green shading

Split 3: Violet shading

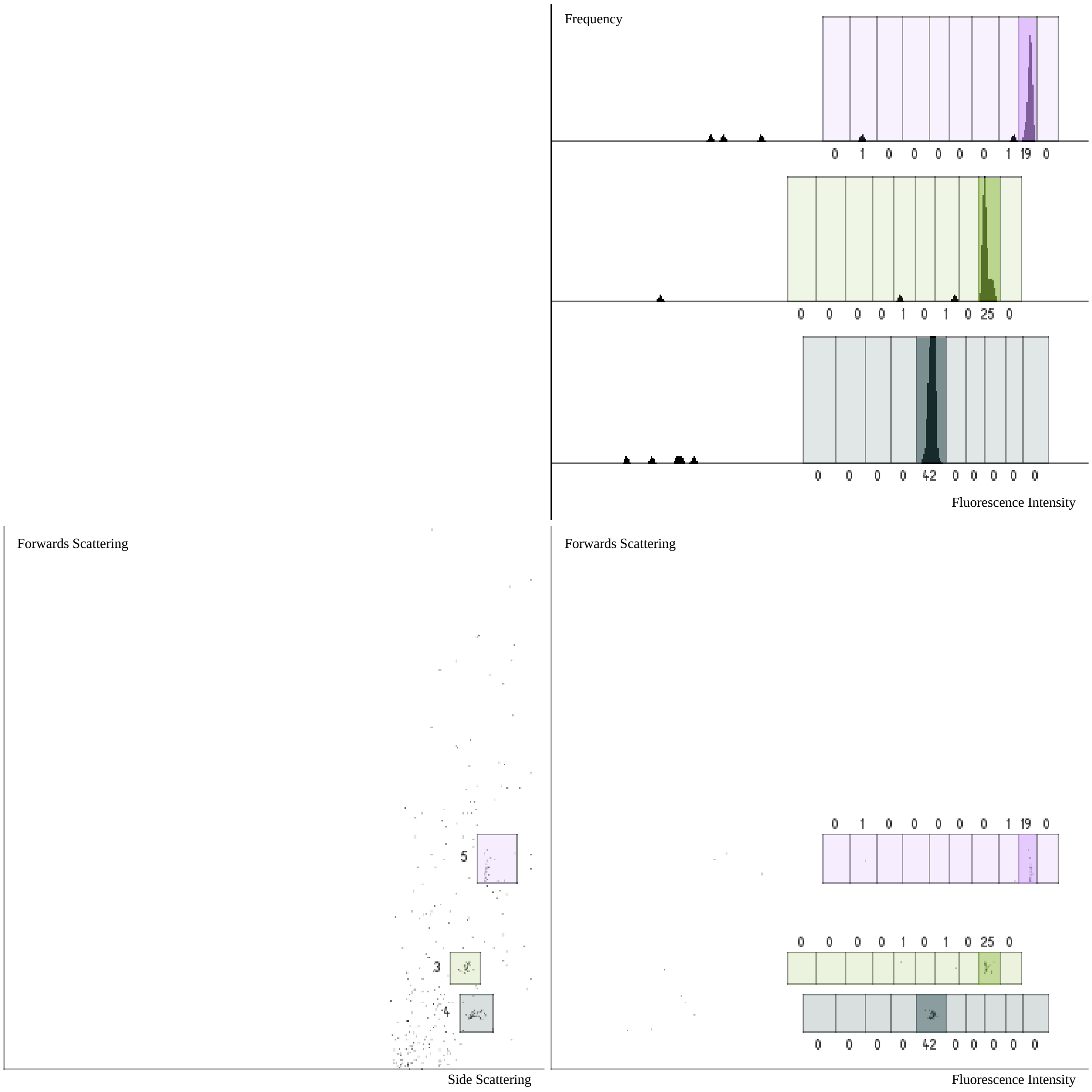

ANNEX 3: TAG DECONVOLUTION - BEAD 351

Passes flow sorting criteria: Yes

Passes tag deconvolution criteria: Yes

Included in protocol analysis: Yes

Protocol: 9, 1, 6, 5

Filename: Bin5_plateA0_D10.fcs

Split 1: Petrol shading

Split 2: Green shading

Split 3: Violet shading

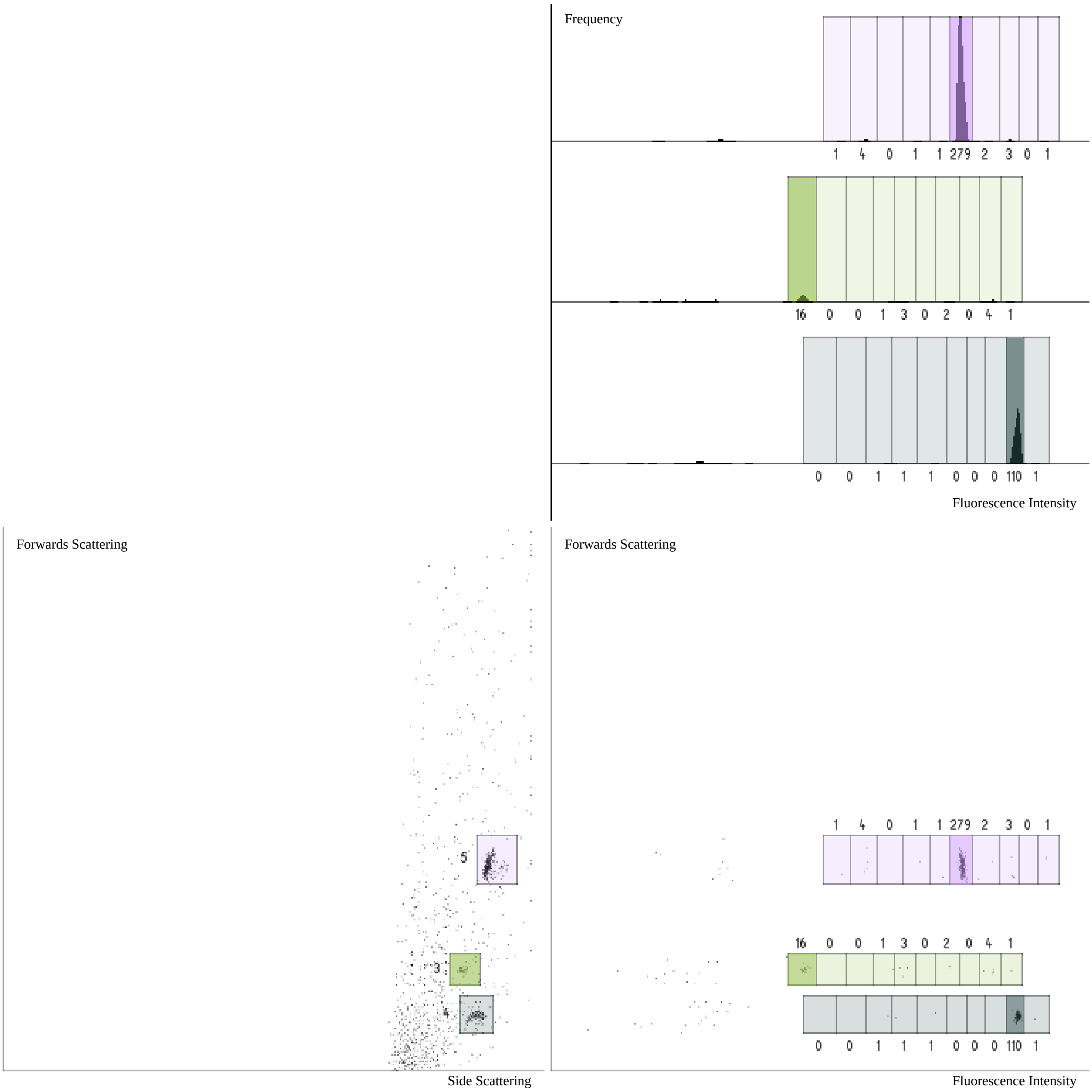

ANNEX 3: TAG DECONVOLUTION - BEAD 352

Passes flow sorting criteria: Yes

Passes tag deconvolution criteria: No

Included in protocol analysis: No

Protocol: N/A

Filename: Bin5_plateA0_D11.fcs

Split 1: Petrol shading

Split 2: Green shading

Split 3: Violet shading

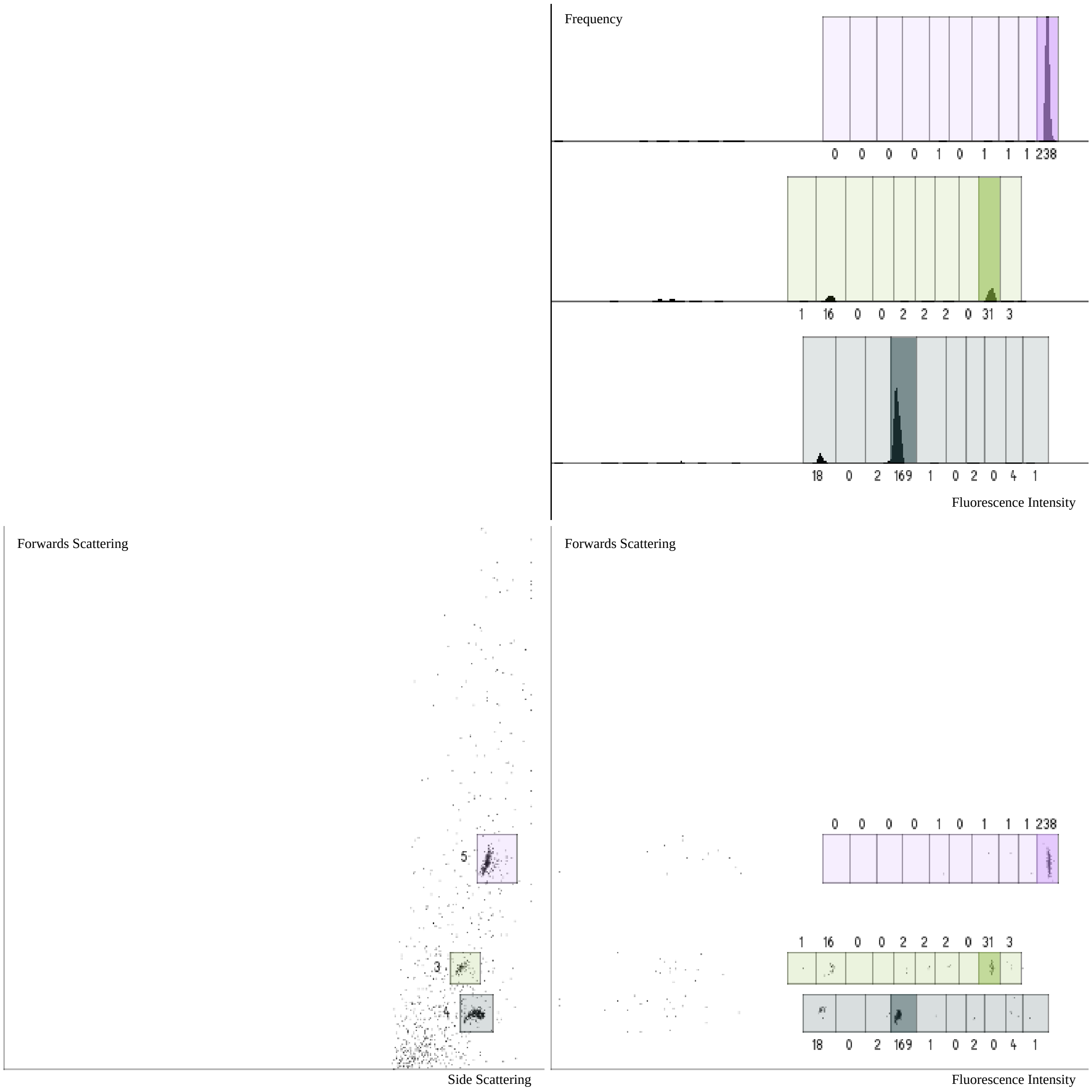

ANNEX 3: TAG DECONVOLUTION - BEAD 353

Passes flow sorting criteria: Yes

Passes tag deconvolution criteria: Yes

Included in protocol analysis: Yes

Protocol: 3, 2, 5, 5

Filename: Bin5_plateA0_D12.fcs

Split 1: Petrol shading

Split 2: Green shading

Split 3: Violet shading

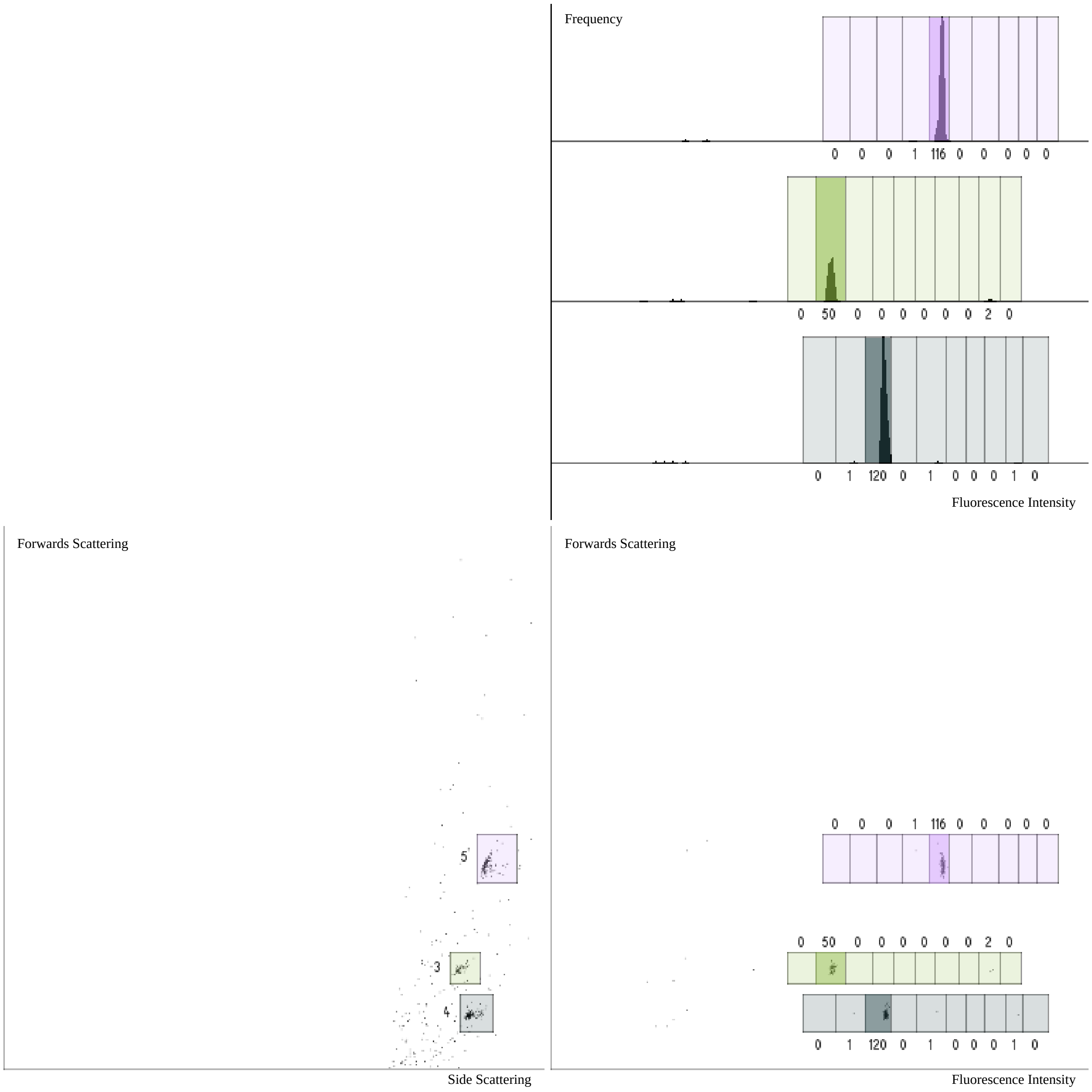

ANNEX 3: TAG DECONVOLUTION - BEAD 354

Passes flow sorting criteria: Yes

Passes tag deconvolution criteria: No

Included in protocol analysis: No

Protocol: N/A

Filename: Bin5_plateA0_E1.fcs

Split 1: Petrol shading

Split 2: Green shading

Split 3: Violet shading

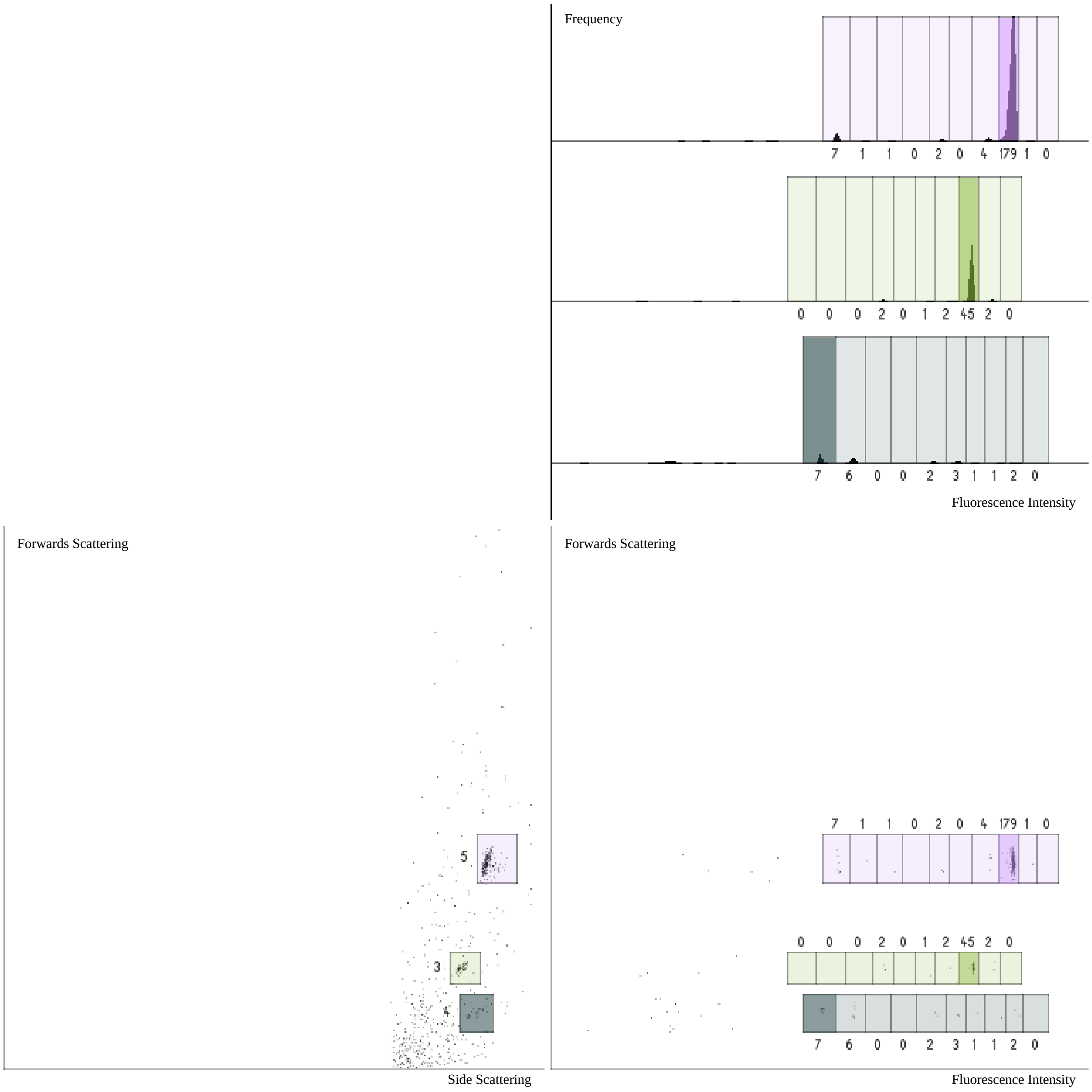

ANNEX 3: TAG DECONVOLUTION - BEAD 355

Passes flow sorting criteria: Yes

Passes tag deconvolution criteria: Yes

Included in protocol analysis: Yes

Protocol: 1, 5, 1, 5

Filename: Bin5_plateA0_E2.fcs

Split 1: Petrol shading

Split 2: Green shading

Split 3: Violet shading

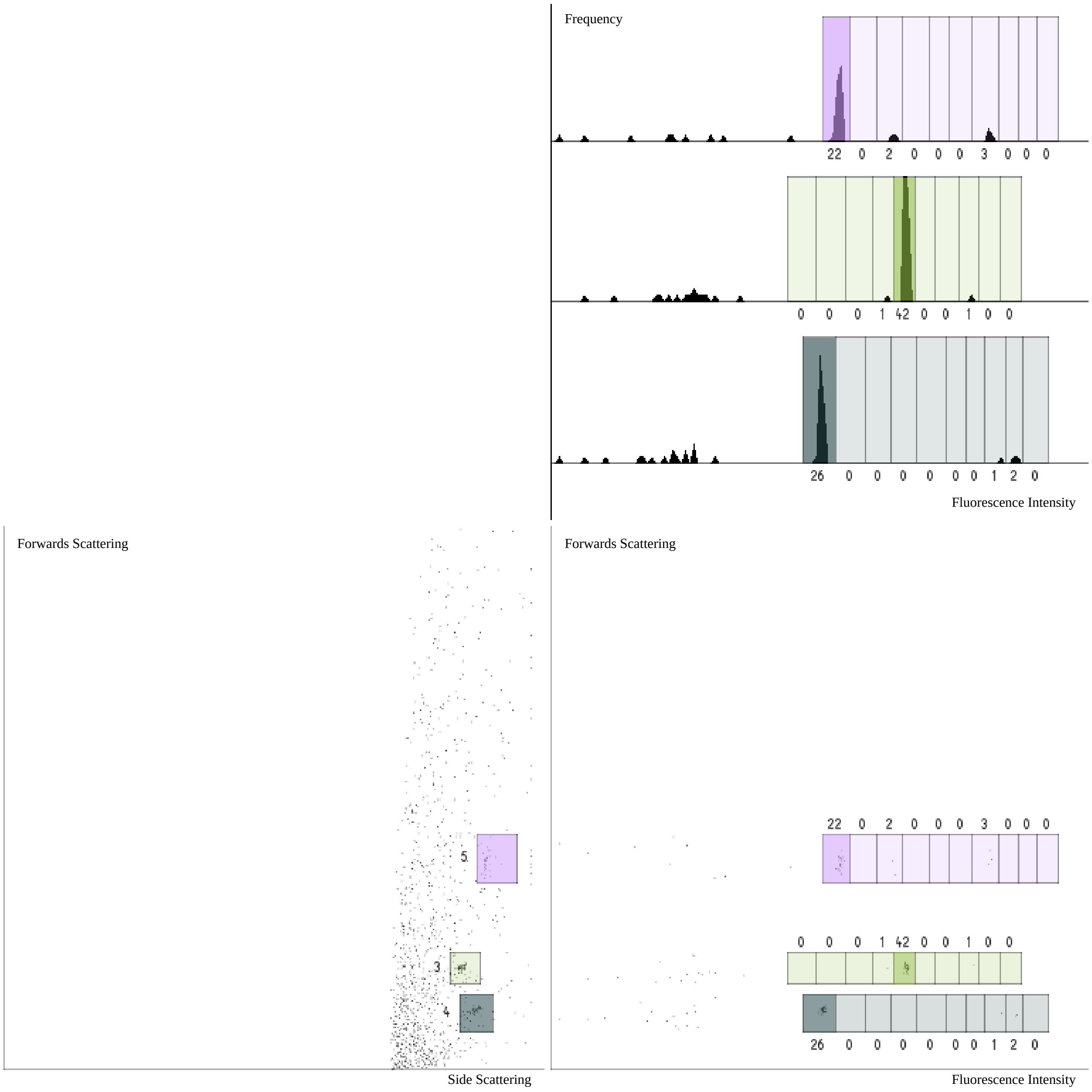

ANNEX 3: TAG DECONVOLUTION - BEAD 356

Passes flow sorting criteria: Yes

Passes tag deconvolution criteria: Yes

Included in protocol analysis: Yes

Protocol: 7, 7, 5, 5

Filename: Bin5_plateA0_E3.fcs

Split 1: Petrol shading

Split 2: Green shading

Split 3: Violet shading

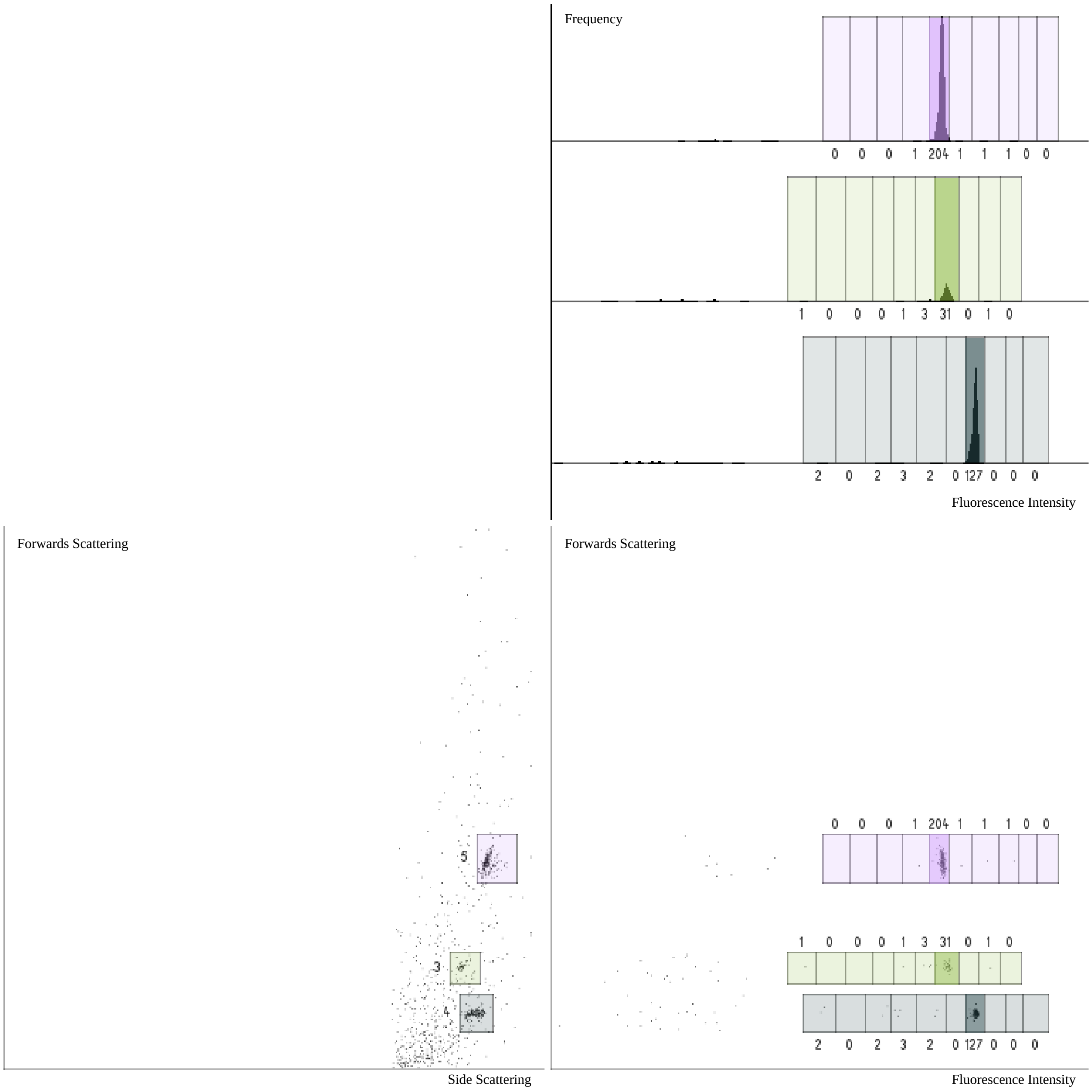

ANNEX 3: TAG DECONVOLUTION - BEAD 357

Passes flow sorting criteria: Yes

Passes tag deconvolution criteria: Yes

Included in protocol analysis: Yes

Protocol: 8, 7, 3, 5

Filename: Bin5_plateA0_E4.fcs

Split 1: Petrol shading

Split 2: Green shading

Split 3: Violet shading

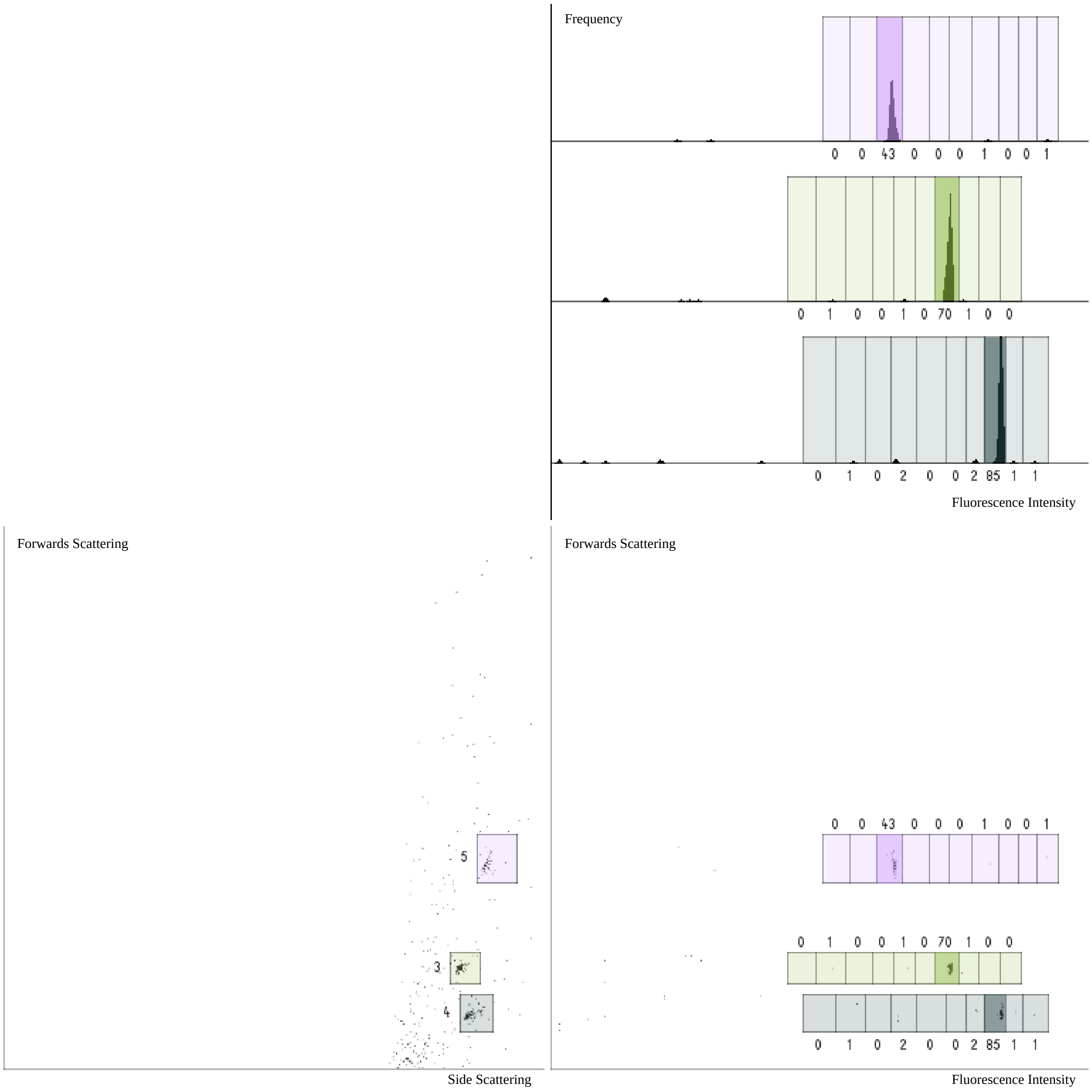

ANNEX 3: TAG DECONVOLUTION - BEAD 358

Passes flow sorting criteria: Yes

Passes tag deconvolution criteria: Yes

Included in protocol analysis: Yes

Protocol: 10, 5, 5, 5

Filename: Bin5_plateA0_E5.fcs

Split 1: Petrol shading

Split 2: Green shading

Split 3: Violet shading

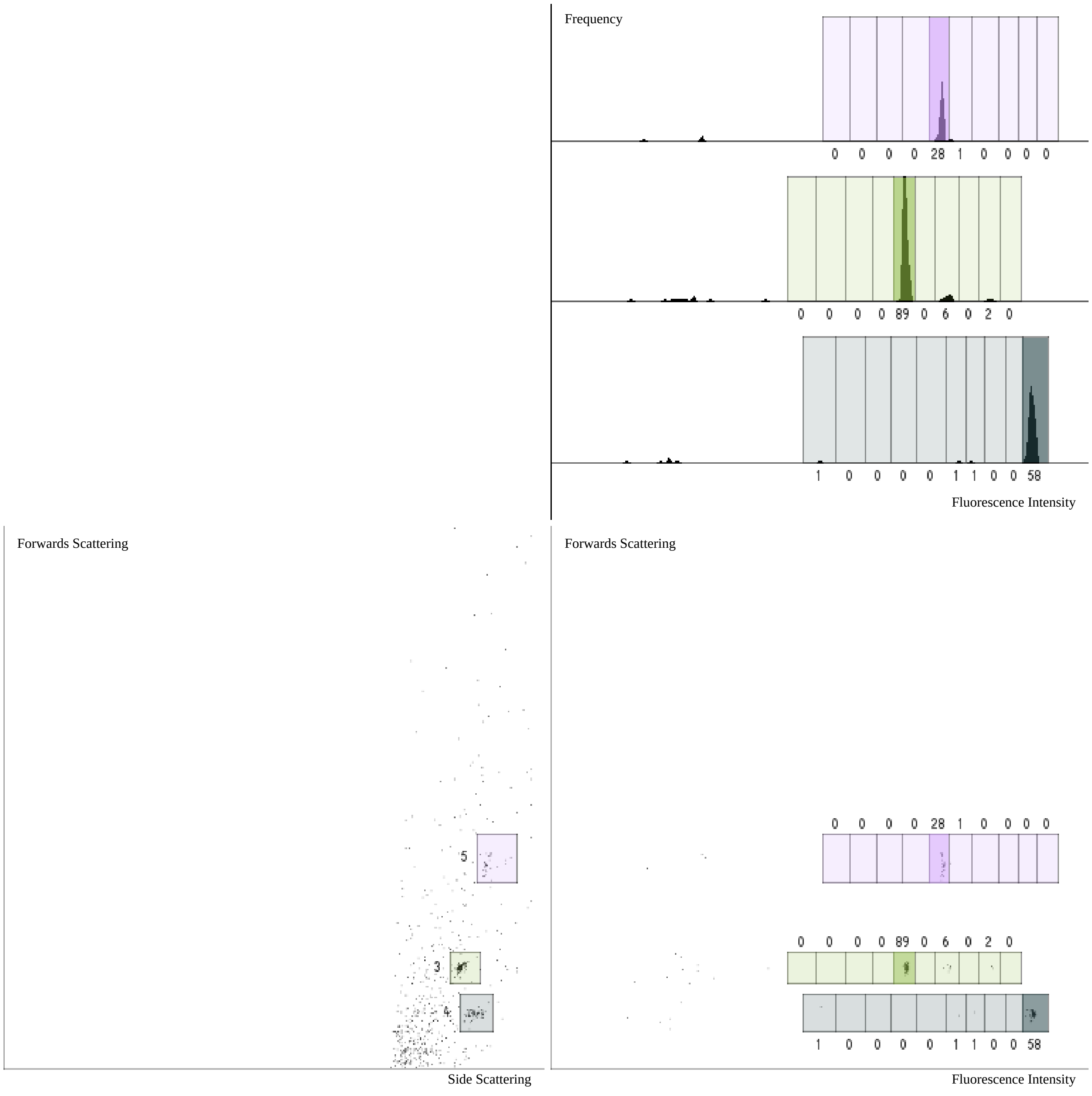

ANNEX 3: TAG DECONVOLUTION - BEAD 359

Passes flow sorting criteria: Yes

Passes tag deconvolution criteria: No

Included in protocol analysis: No

Protocol: N/A

Filename: Bin5_plateA0_E6.fcs

Split 1: Petrol shading

Split 2: Green shading

Split 3: Violet shading

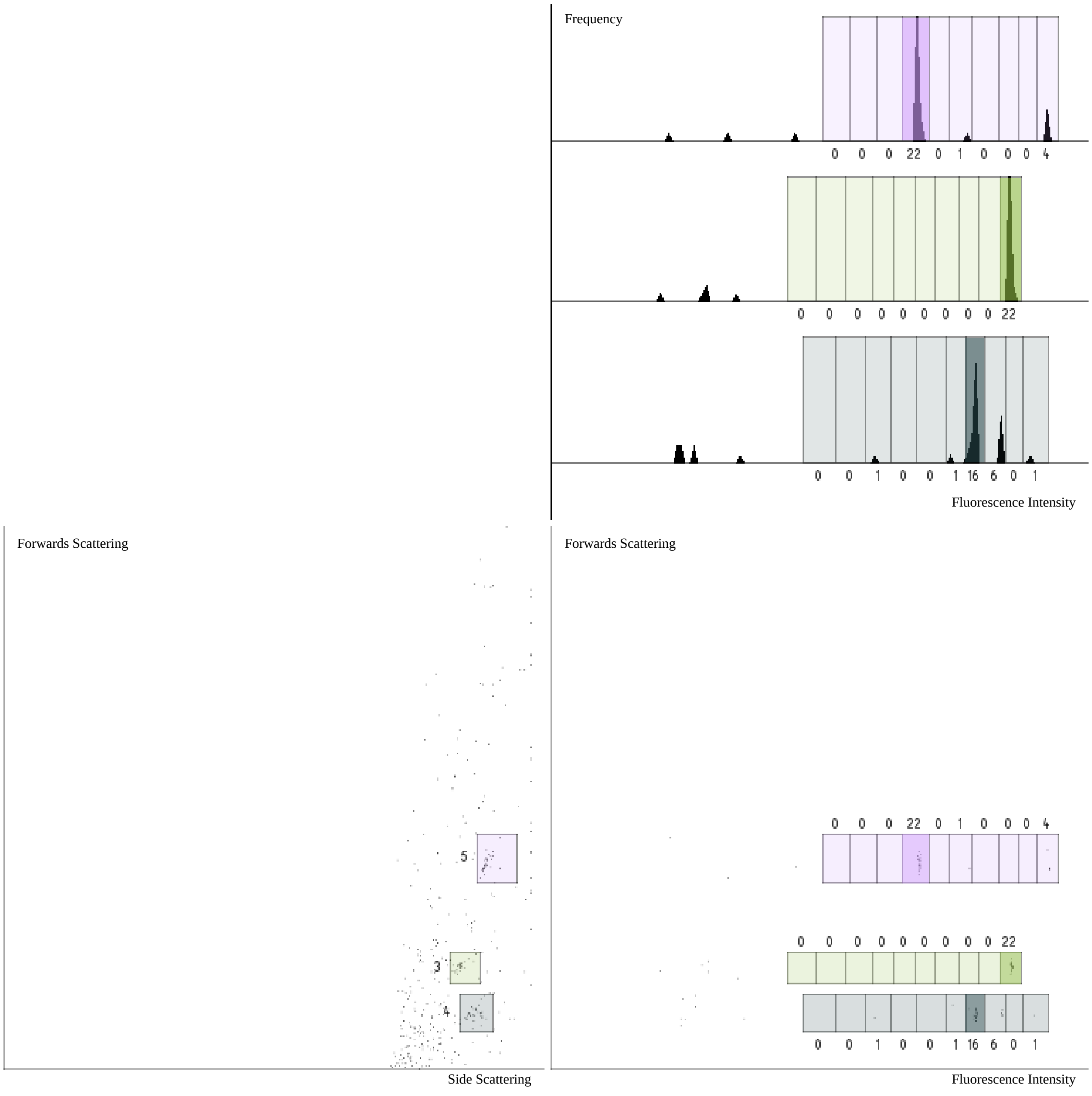

ANNEX 3: TAG DECONVOLUTION - BEAD 360

Passes flow sorting criteria: Yes

Passes tag deconvolution criteria: No

Included in protocol analysis: No

Protocol: N/A

Filename: Bin5_plateA0_E7.fcs

Split 1: Petrol shading

Split 2: Green shading

Split 3: Violet shading

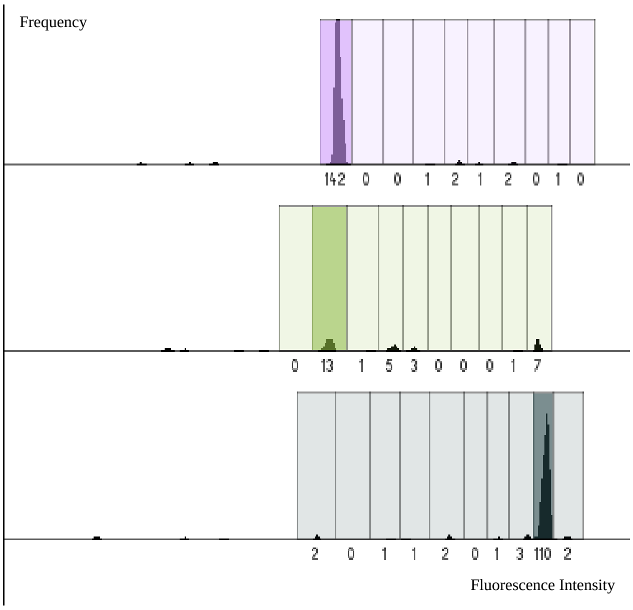

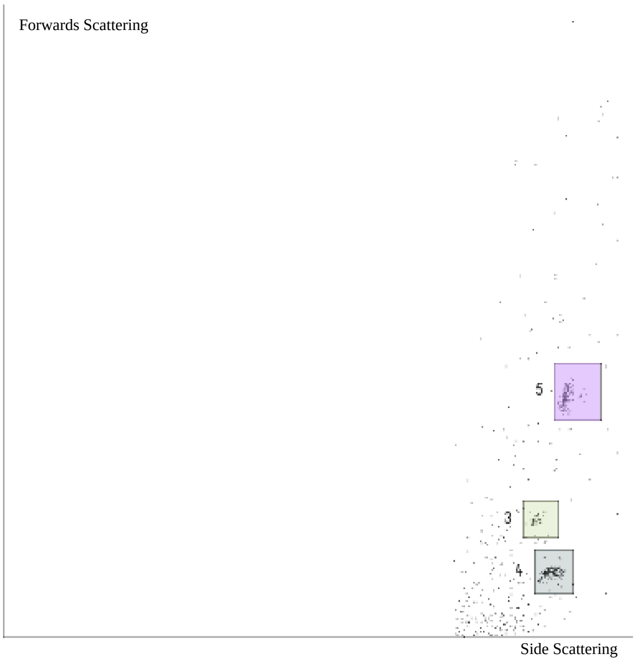

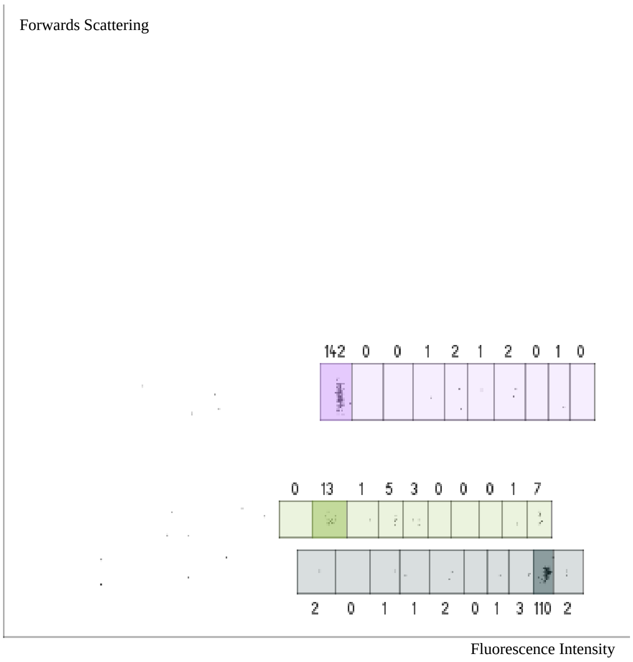

ANNEX 3: TAG DECONVOLUTION - BEAD 361

Passes flow sorting criteria: Yes

Passes tag deconvolution criteria: Yes

Included in protocol analysis: Yes

Protocol: 10, 2, 5, 5

Filename: Bin5_plateA0_E9.fcs

Split 1: Petrol shading

Split 2: Green shading

Split 3: Violet shading

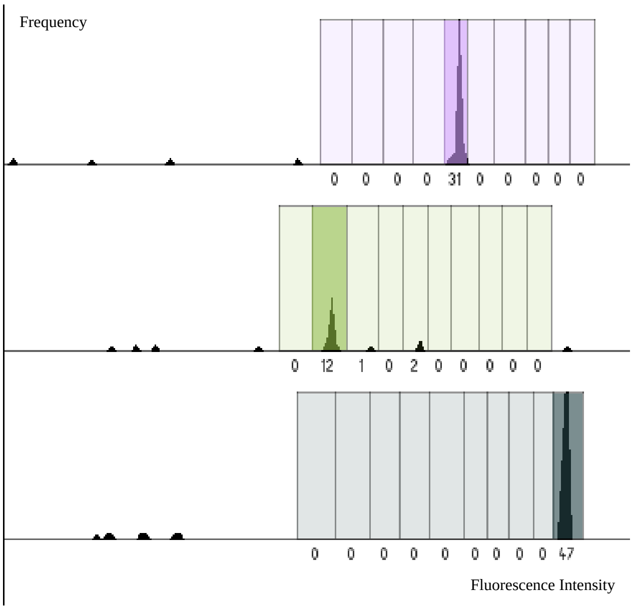

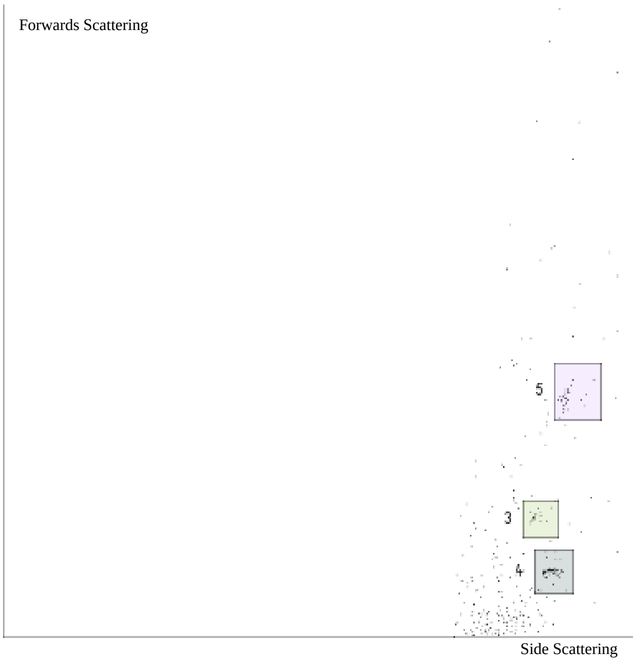

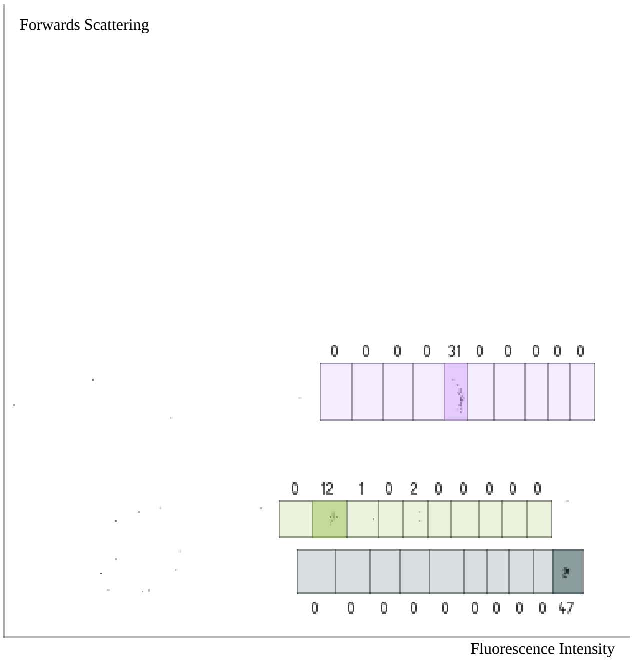

ANNEX 3: TAG DECONVOLUTION - BEAD 362

Passes flow sorting criteria: Yes

Passes tag deconvolution criteria: No

Included in protocol analysis: No

Protocol: N/A

Filename: Bin5_plateA0_E10.fcs

Split 1: Petrol shading

Split 2: Green shading

Split 3: Violet shading

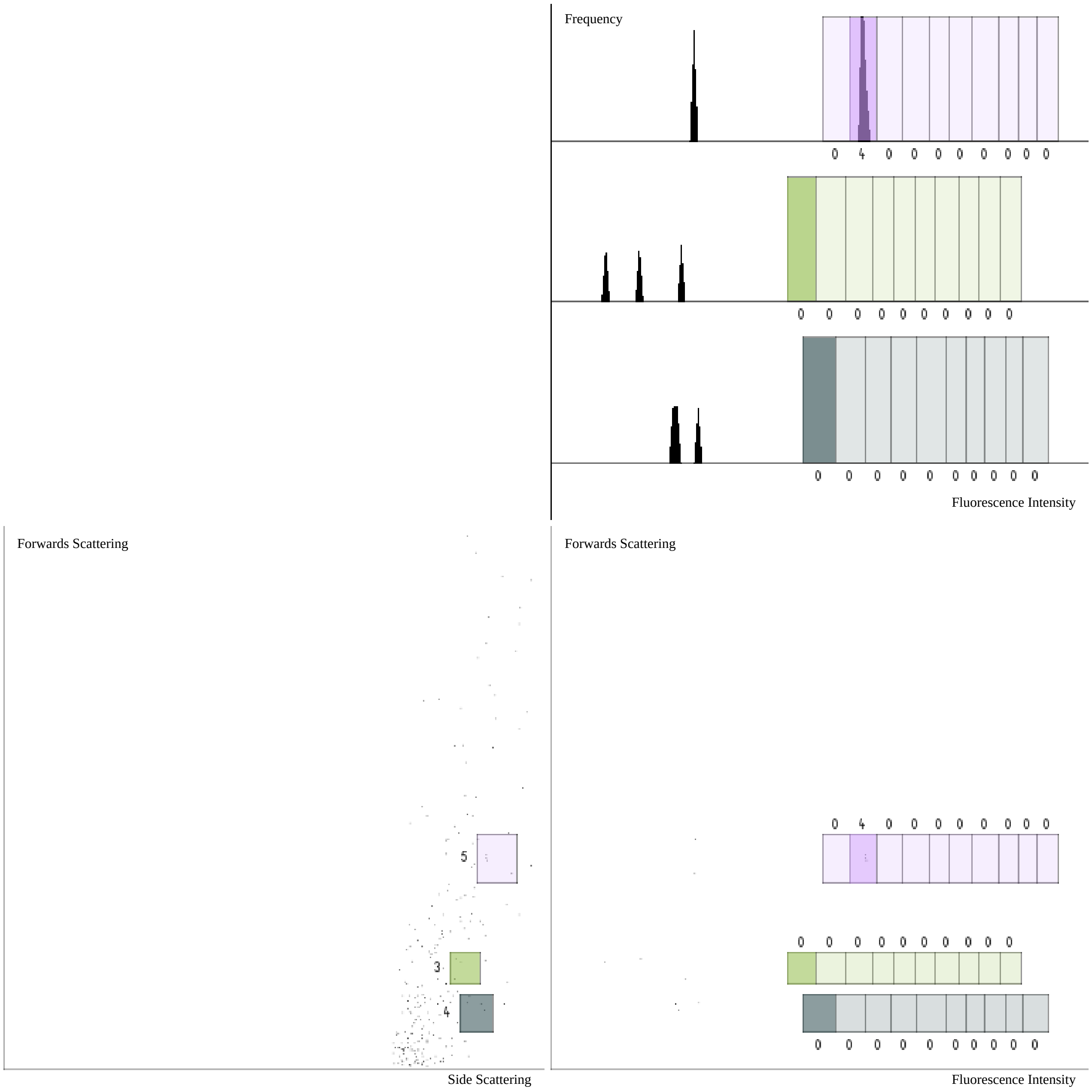

ANNEX 3: TAG DECONVOLUTION - BEAD 363

Passes flow sorting criteria: Yes

Passes tag deconvolution criteria: Yes

Included in protocol analysis: Yes

Protocol: 9, 5, 1, 5

Filename: Bin5_plateA0_E11.fcs

Split 1: Petrol shading

Split 2: Green shading

Split 3: Violet shading

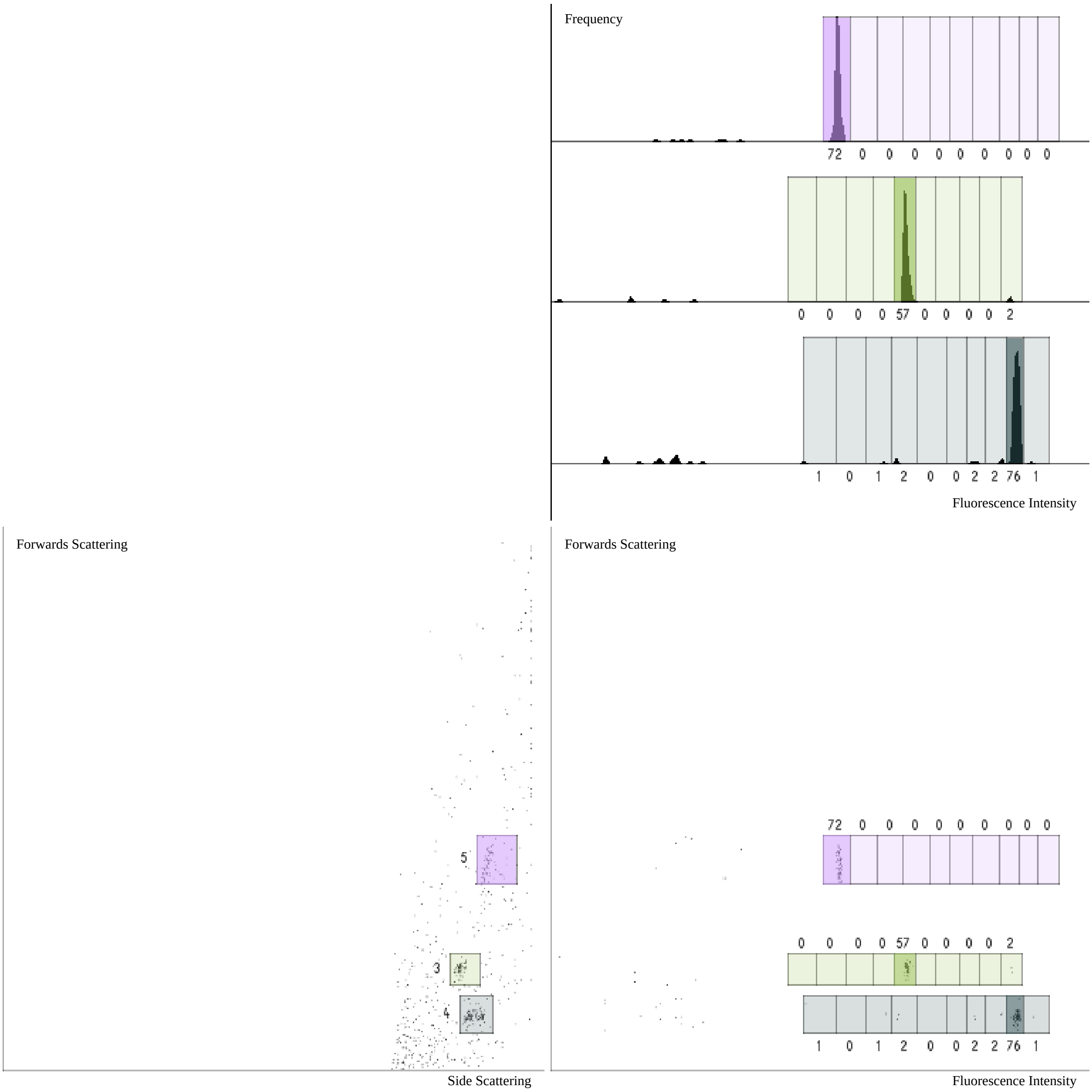

ANNEX 3: TAG DECONVOLUTION - BEAD 364

Passes flow sorting criteria: Yes

Passes tag deconvolution criteria: Yes

Included in protocol analysis: Yes

Protocol: 2, 4, 2, 5

Filename: Bin5_plateA0_E12.fcs

Split 1: Petrol shading

Split 2: Green shading

Split 3: Violet shading

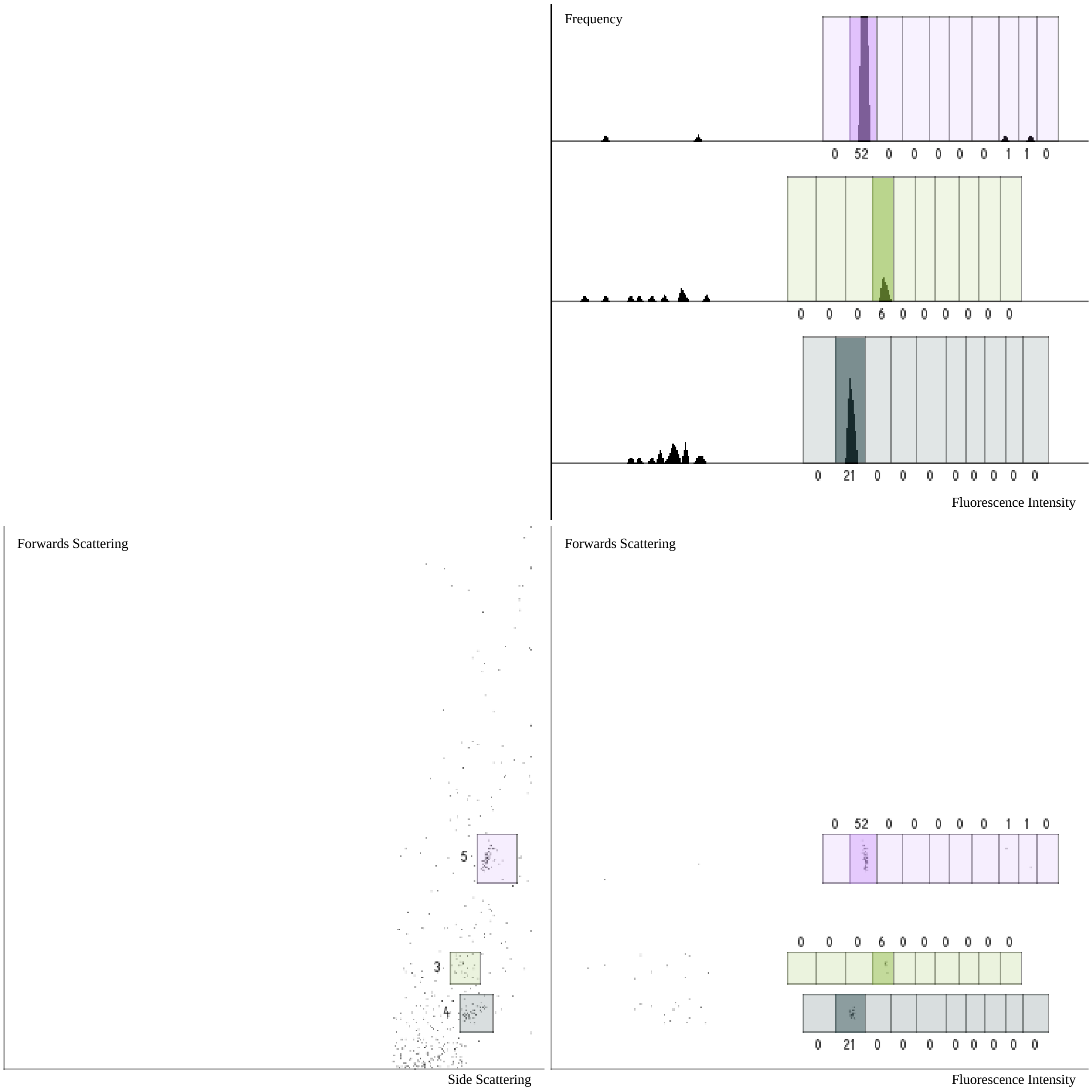

ANNEX 3: TAG DECONVOLUTION - BEAD 365

Passes flow sorting criteria: Yes

Passes tag deconvolution criteria: Yes

Included in protocol analysis: Yes

Protocol: 1, 9, 1, 5

Filename: Bin5_plateA0_F1.fcs

Split 1: Petrol shading

Split 2: Green shading

Split 3: Violet shading

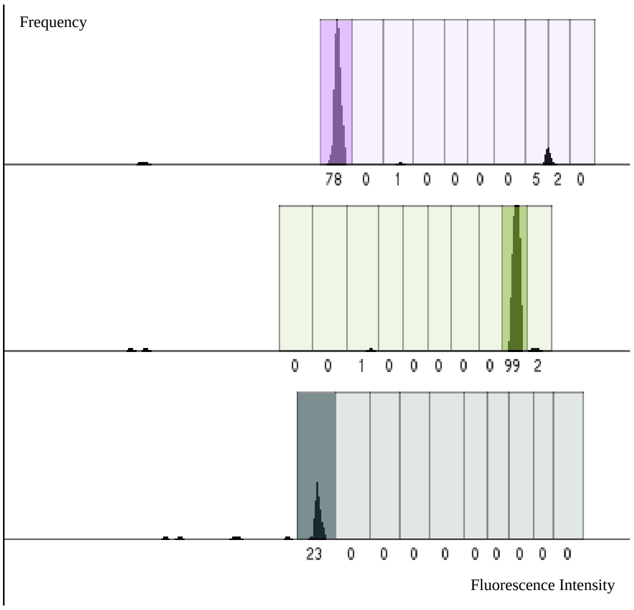

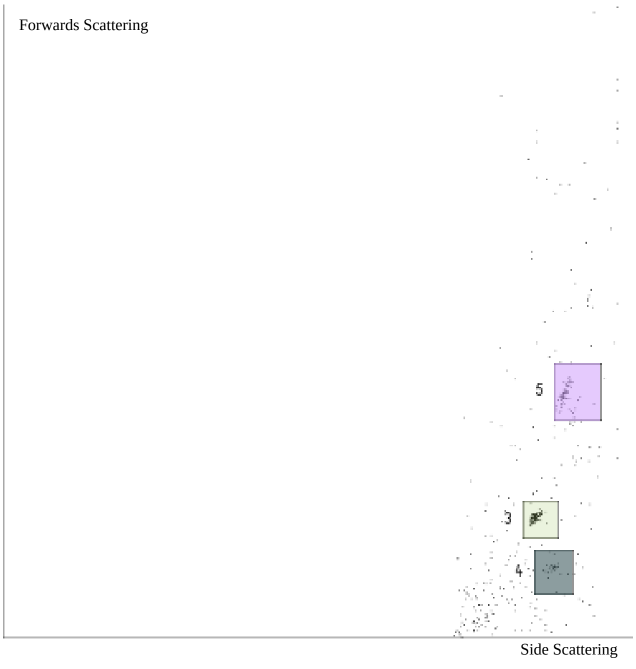

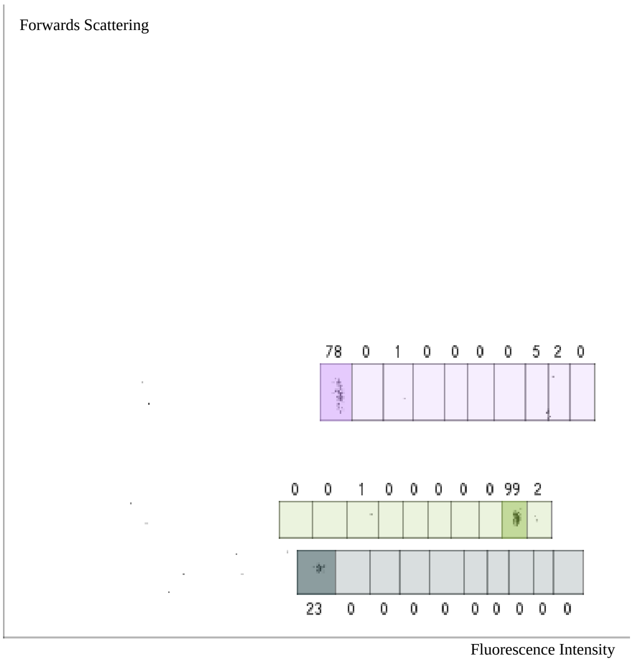

ANNEX 3: TAG DECONVOLUTION - BEAD 366

Passes flow sorting criteria: Yes

Passes tag deconvolution criteria: Yes

Included in protocol analysis: Yes

Protocol: 5, 9, 9, 5

Filename: Bin5_plateA0_F2.fcs

Split 1: Petrol shading

Split 2: Green shading

Split 3: Violet shading

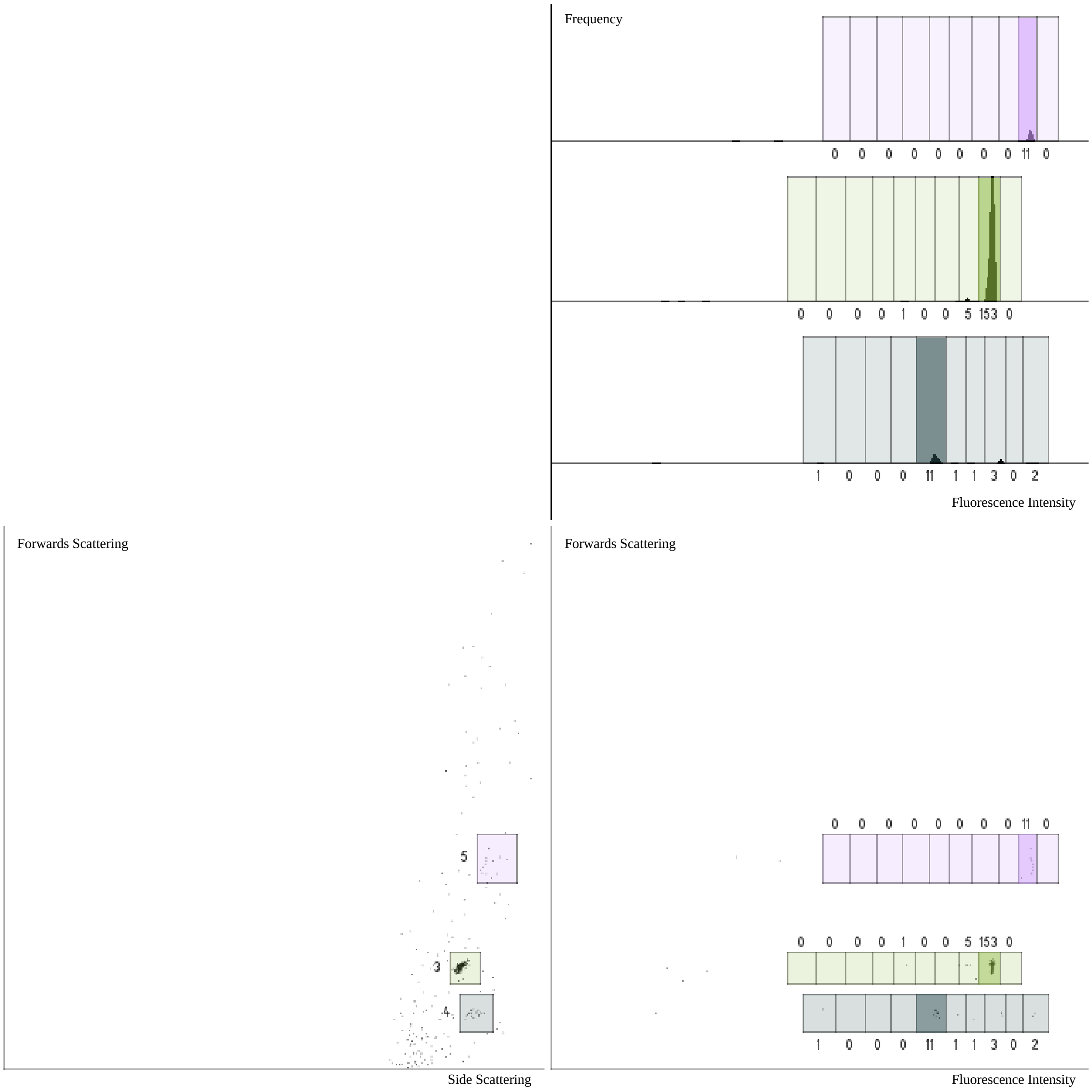

ANNEX 3: TAG DECONVOLUTION - BEAD 367

Passes flow sorting criteria: Yes

Passes tag deconvolution criteria: Yes

Included in protocol analysis: Yes

Protocol: 1, 5, 10, 5

Filename: Bin5_plateA0_F3.fcs

Split 1: Petrol shading

Split 2: Green shading

Split 3: Violet shading

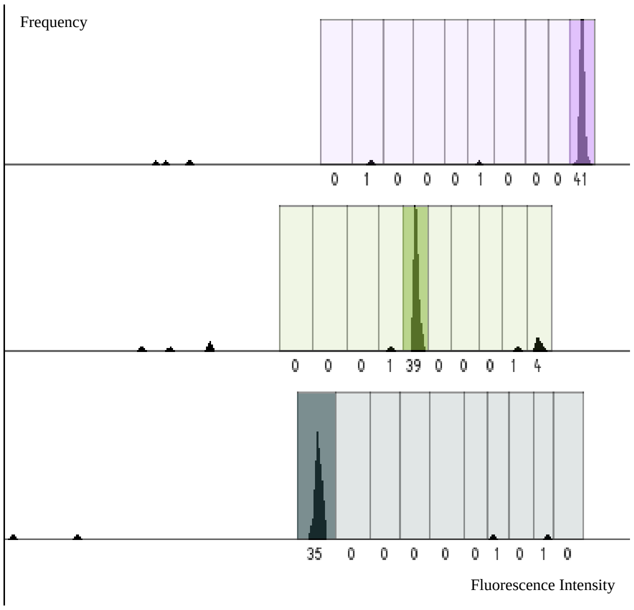

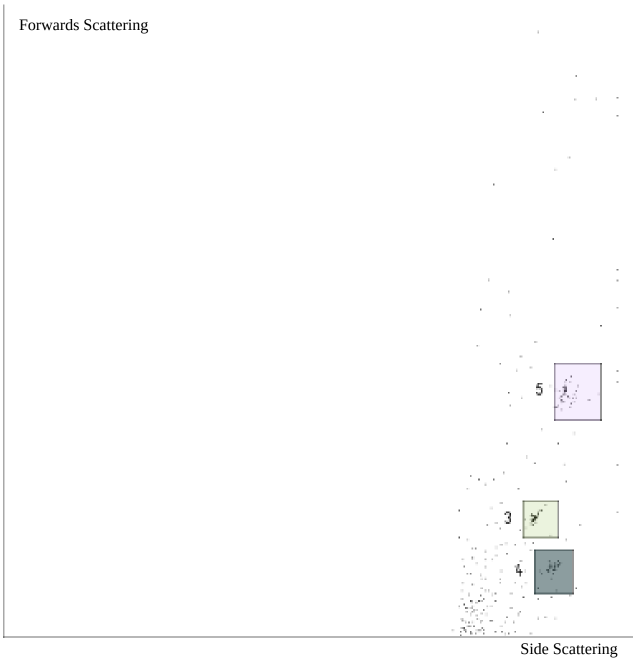

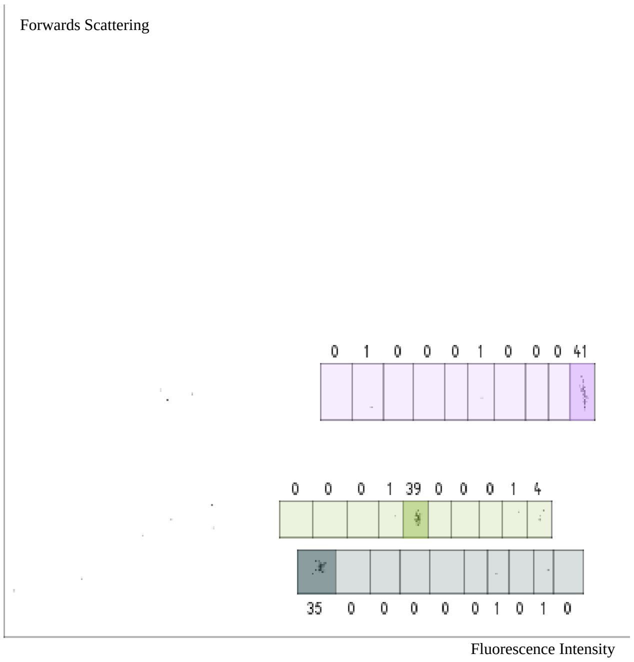

ANNEX 3: TAG DECONVOLUTION - BEAD 368

Passes flow sorting criteria: Yes

Passes tag deconvolution criteria: Yes

Included in protocol analysis: Yes

Protocol: 1, 9, 7, 5

Filename: Bin5_plateA0_F4.fcs

Split 1: Petrol shading

Split 2: Green shading

Split 3: Violet shading

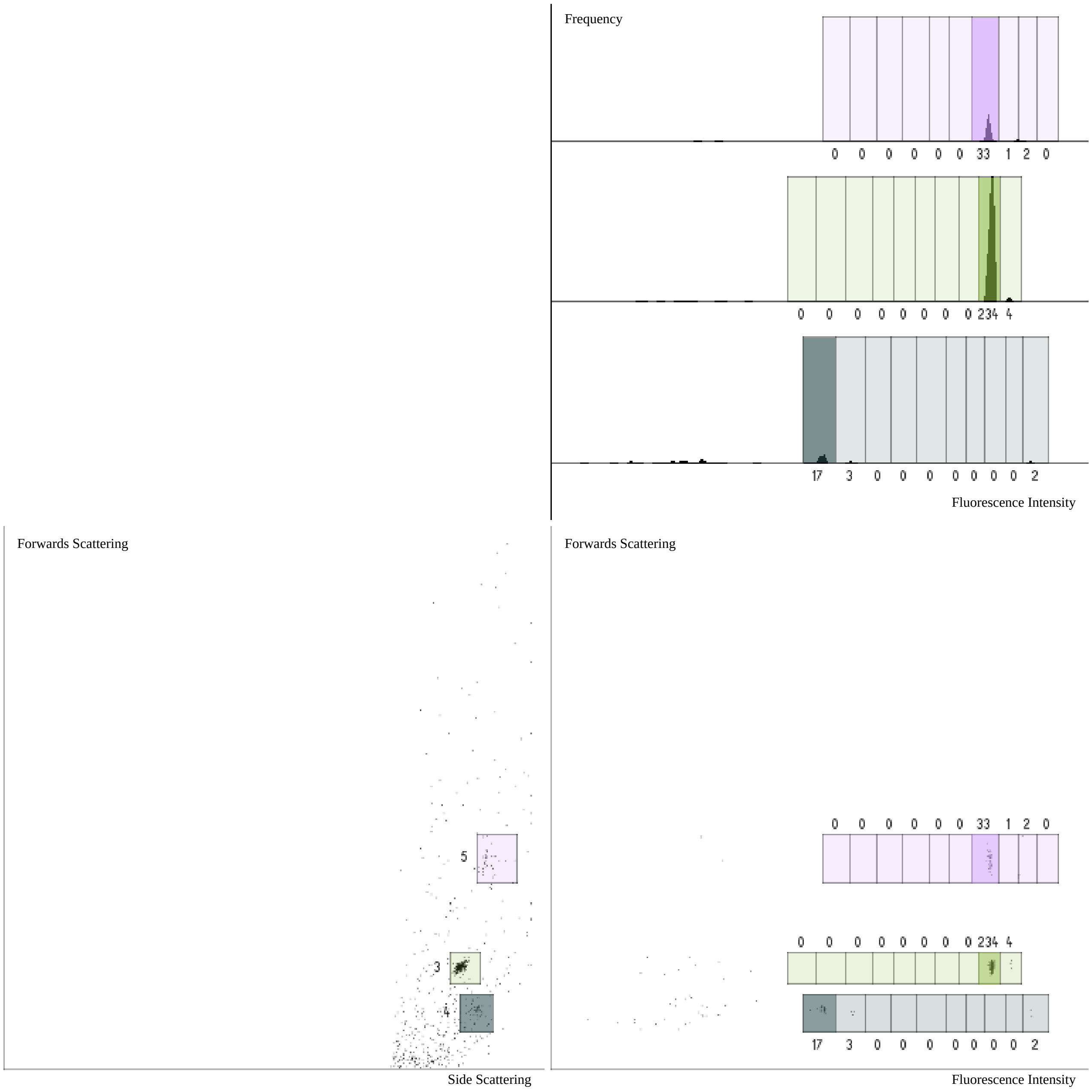

ANNEX 3: TAG DECONVOLUTION - BEAD 369

Passes flow sorting criteria: Yes

Passes tag deconvolution criteria: No

Included in protocol analysis: No

Protocol: N/A

Filename: Bin5_plateA0_F5.fcs

Split 1: Petrol shading

Split 2: Green shading

Split 3: Violet shading

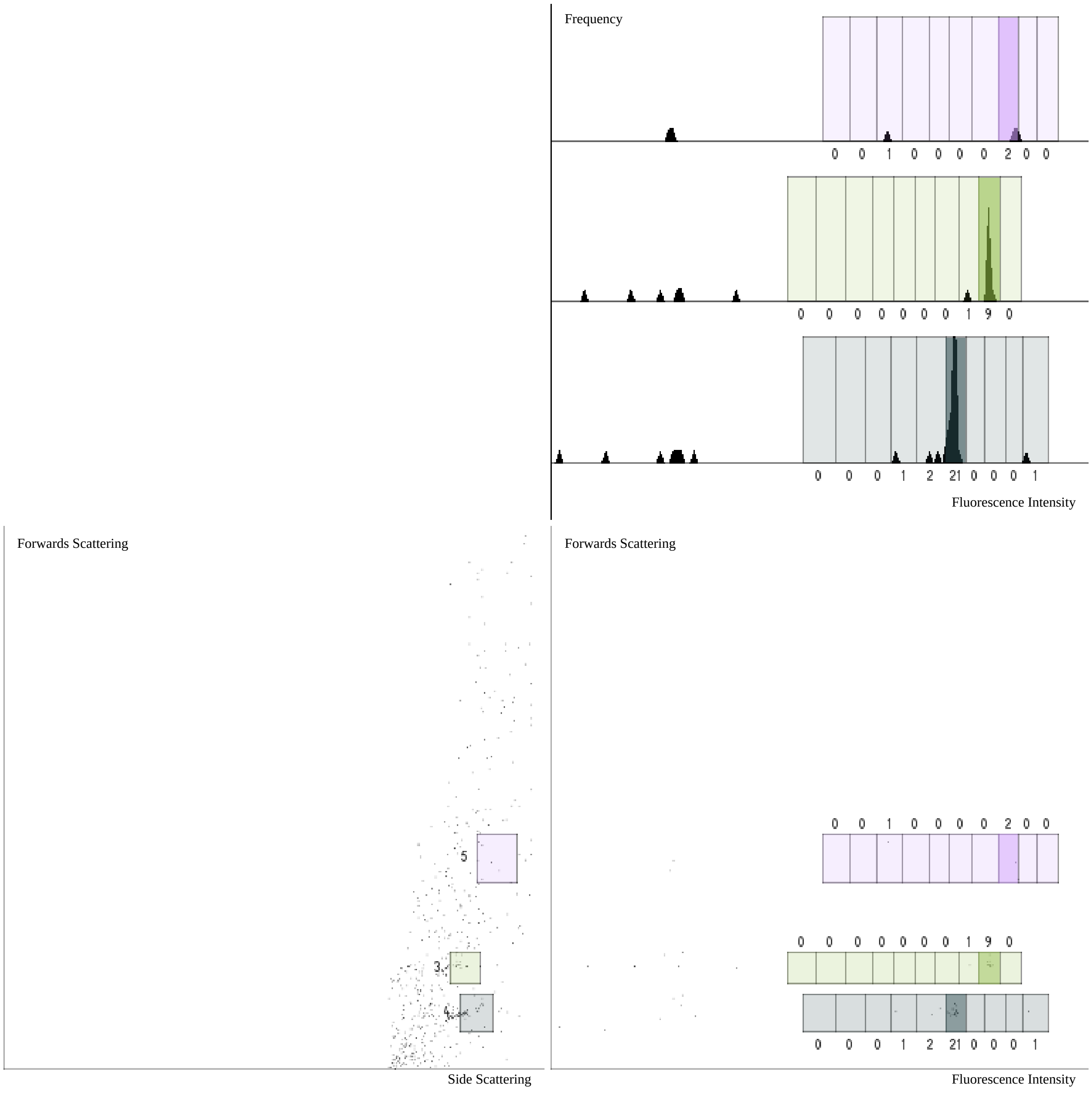

ANNEX 3: TAG DECONVOLUTION - BEAD 370

Passes flow sorting criteria: Yes

Passes tag deconvolution criteria: Yes

Included in protocol analysis: Yes

Protocol: 10, 10, 8, 5

Filename: Bin5_plateA0_F6.fcs

Split 1: Petrol shading

Split 2: Green shading

Split 3: Violet shading

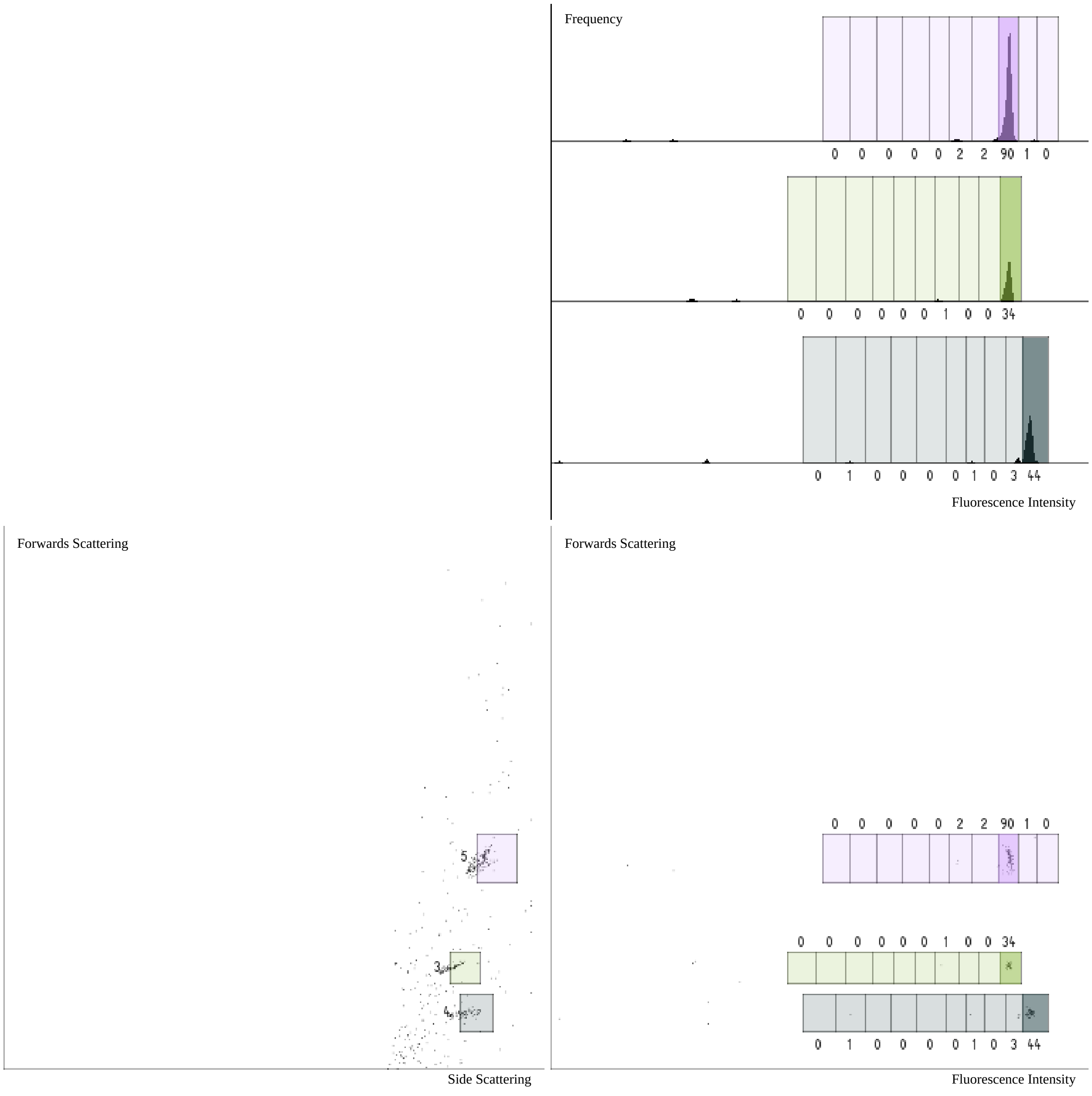

ANNEX 3: TAG DECONVOLUTION - BEAD 371

Passes flow sorting criteria: Yes

Passes tag deconvolution criteria: No

Included in protocol analysis: No

Protocol: N/A

Filename: Bin5_plateA0_F7.fcs

Split 1: Petrol shading

Split 2: Green shading

Split 3: Violet shading

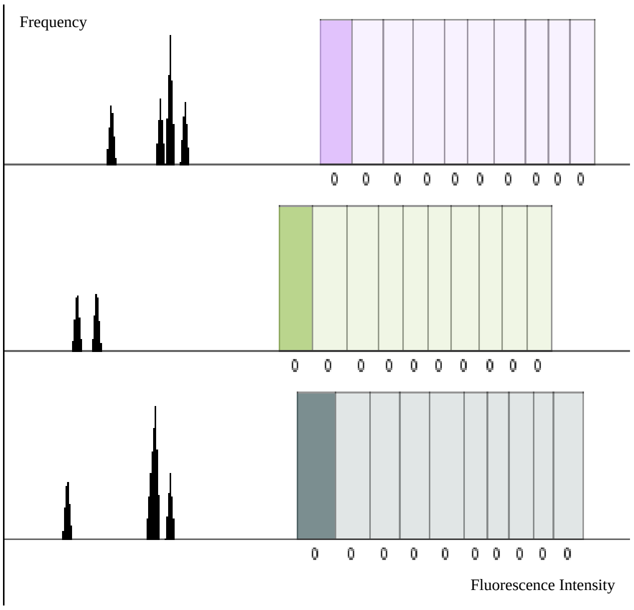

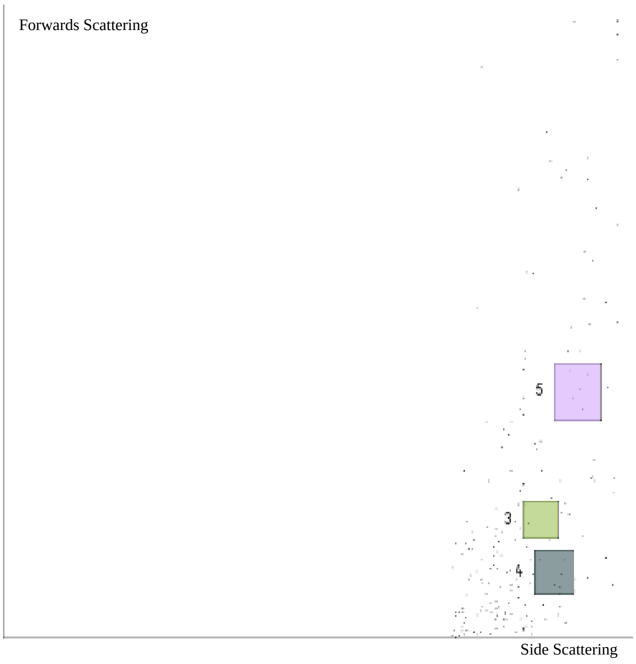

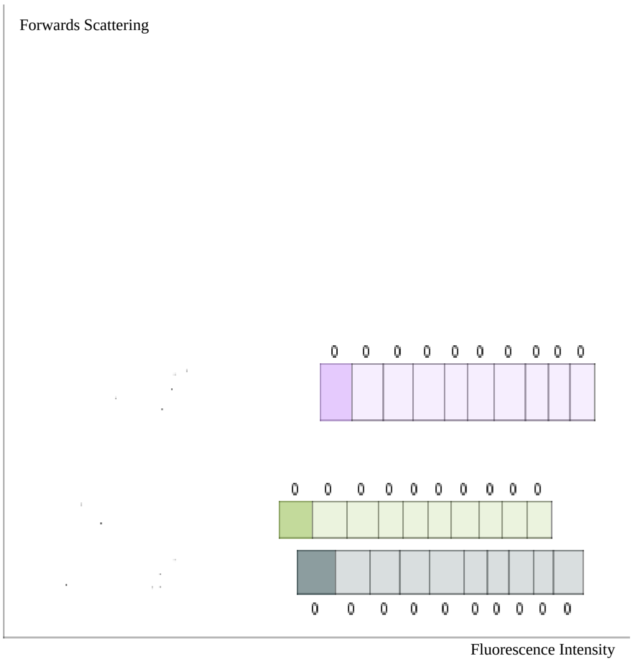

ANNEX 3: TAG DECONVOLUTION - BEAD 372

Passes flow sorting criteria: Yes

Passes tag deconvolution criteria: Yes

Included in protocol analysis: Yes

Protocol: 1, 5, 2, 5

Filename: Bin5_plateA0_F8.fcs

Split 1: Petrol shading

Split 2: Green shading

Split 3: Violet shading

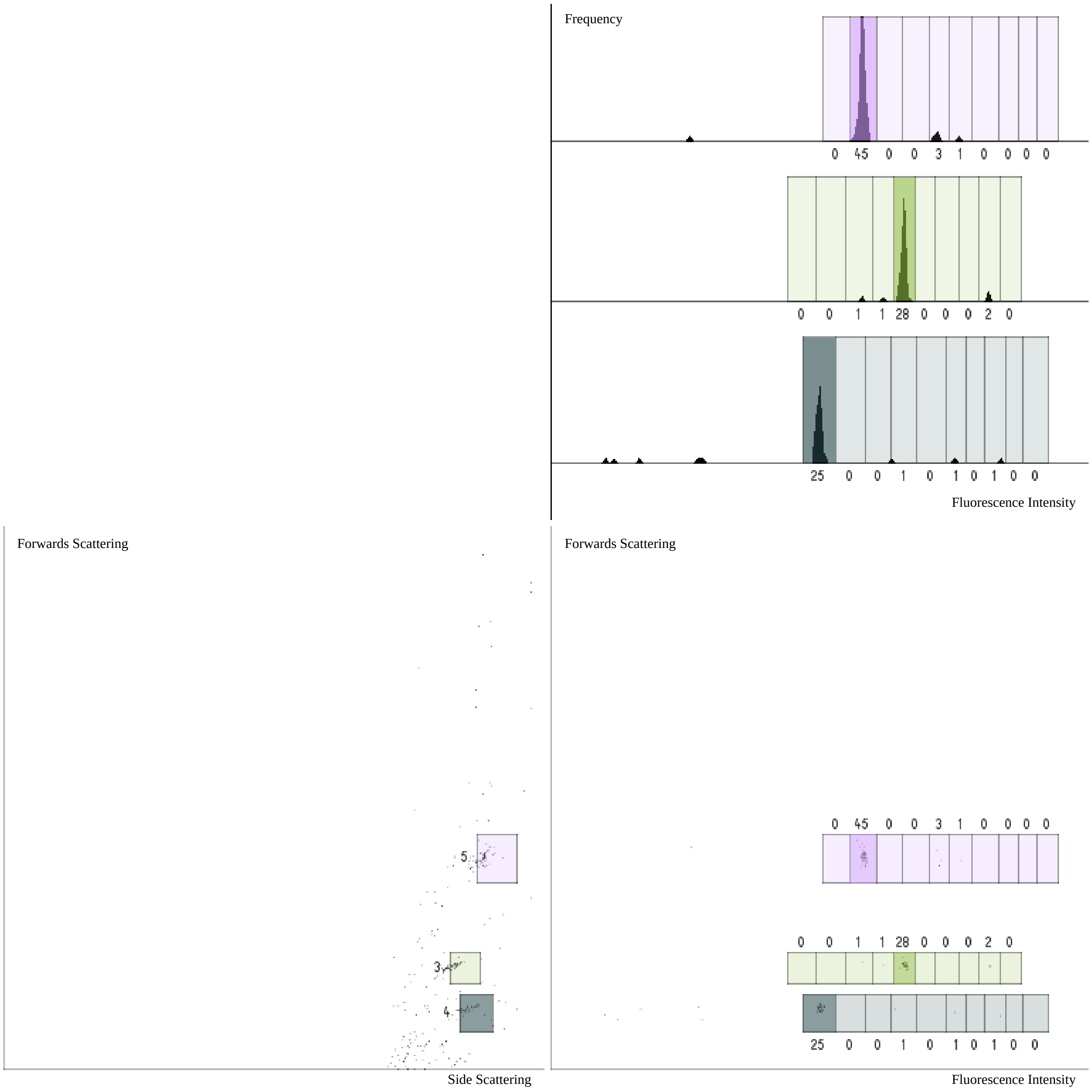

ANNEX 3: TAG DECONVOLUTION - BEAD 373

Passes flow sorting criteria: Yes

Passes tag deconvolution criteria: Yes

Included in protocol analysis: Yes

Protocol: 5, 10, 6, 5

Filename: Bin5_plateA0_F9.fcs

Split 1: Petrol shading

Split 2: Green shading

Split 3: Violet shading

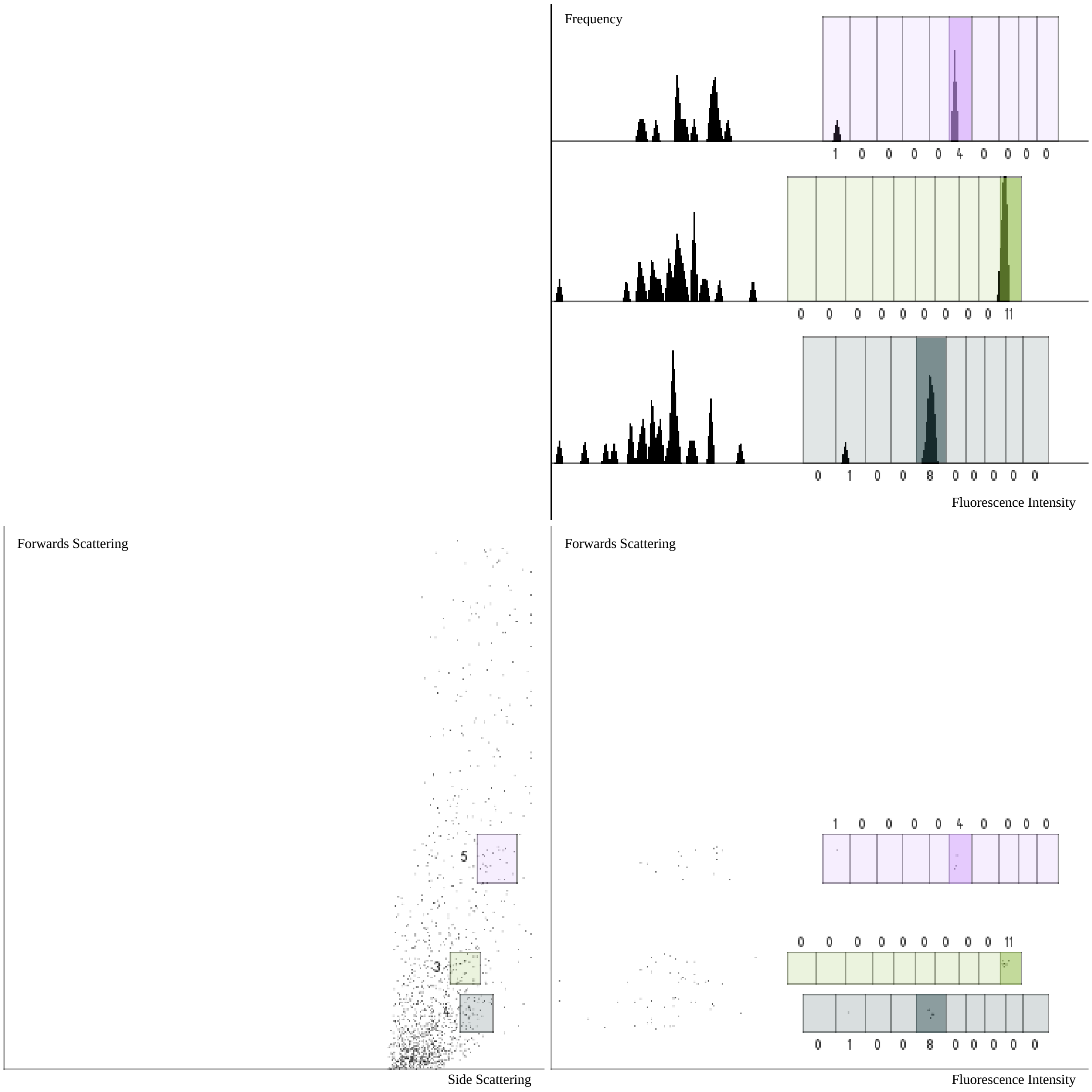

ANNEX 3: TAG DECONVOLUTION - BEAD 374

Passes flow sorting criteria: Yes

Passes tag deconvolution criteria: Yes

Included in protocol analysis: Yes

Protocol: 9, 9, 10, 5

Filename: Bin5_plateA0_F10.fcs

Split 1: Petrol shading

Split 2: Green shading

Split 3: Violet shading

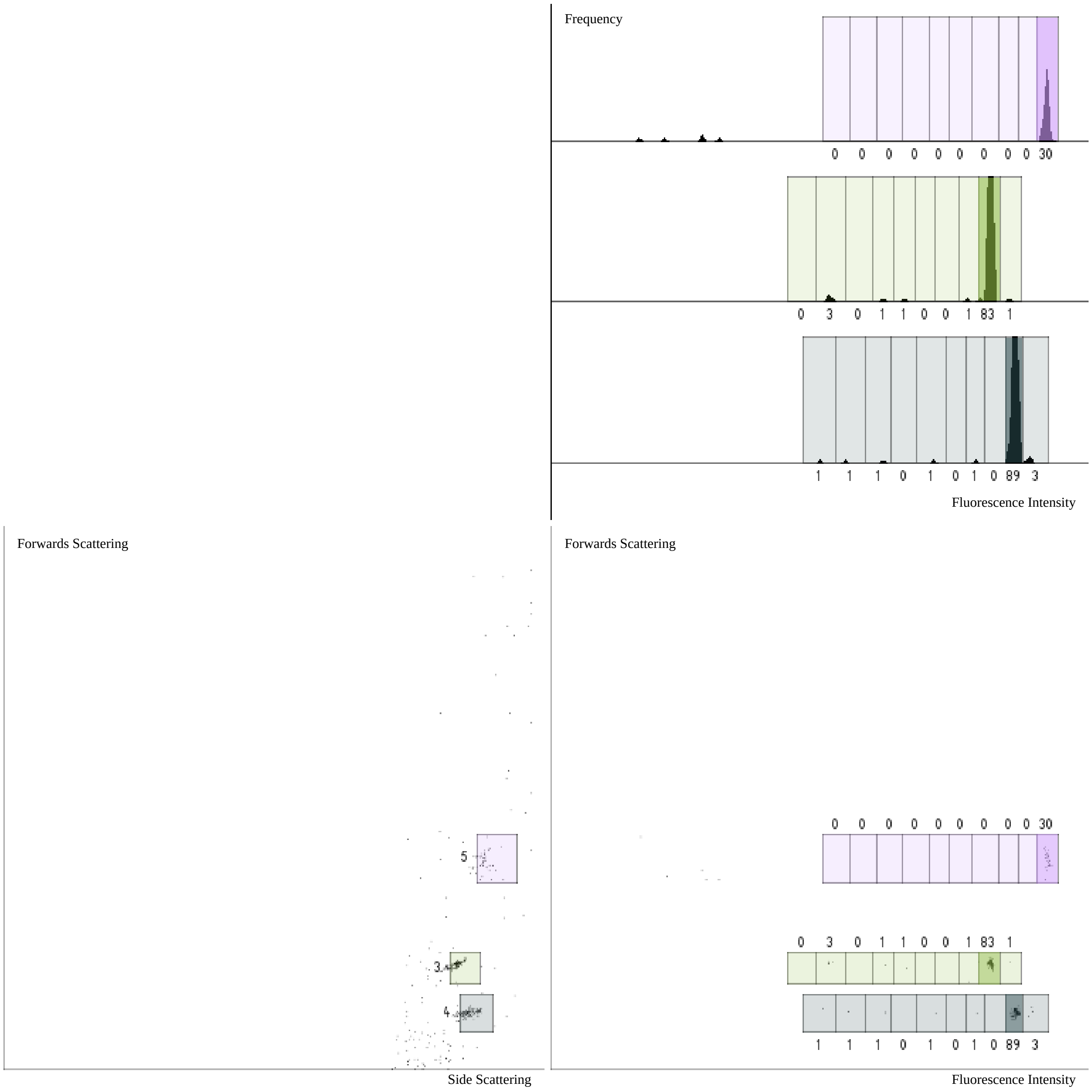

ANNEX 3: TAG DECONVOLUTION - BEAD 375

Passes flow sorting criteria: Yes

Passes tag deconvolution criteria: Yes

Included in protocol analysis: Yes

Protocol: 7, 8, 5, 5

Filename: Bin5_plateA0_F12.fcs

Split 1: Petrol shading

Split 2: Green shading

Split 3: Violet shading

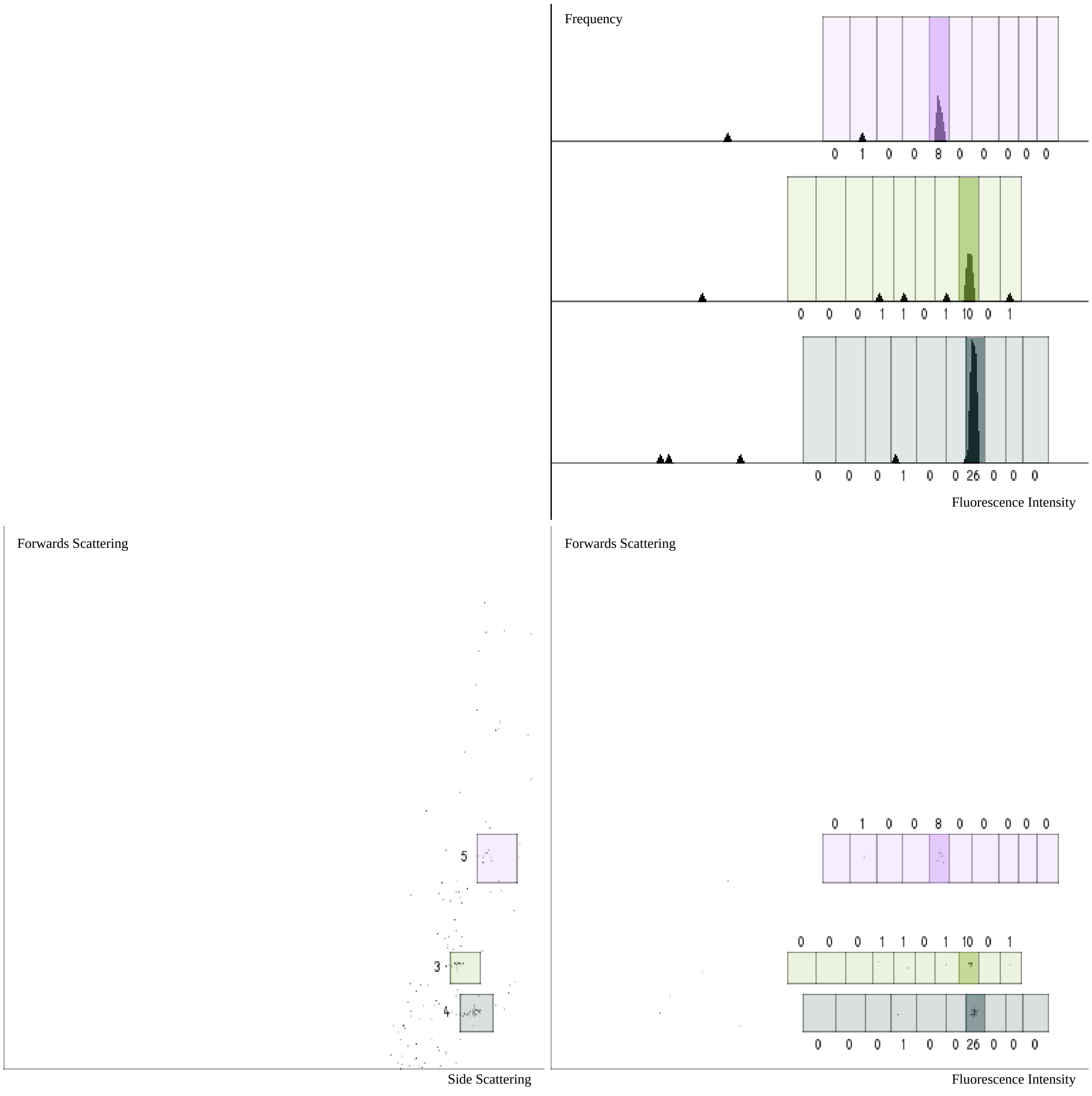

ANNEX 3: TAG DECONVOLUTION - BEAD 376

Passes flow sorting criteria: Yes

Passes tag deconvolution criteria: Yes

Included in protocol analysis: Yes

Protocol: 4, 2, 3, 5

Filename: Bin5_plateA0_G2.fcs

Split 1: Petrol shading

Split 2: Green shading

Split 3: Violet shading

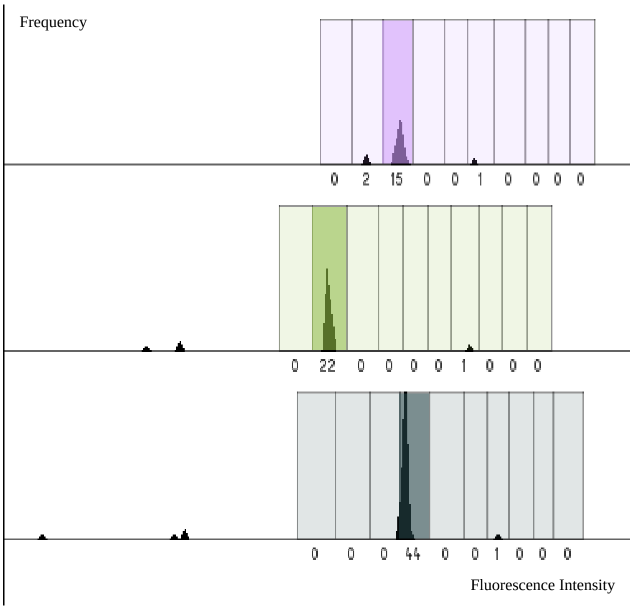

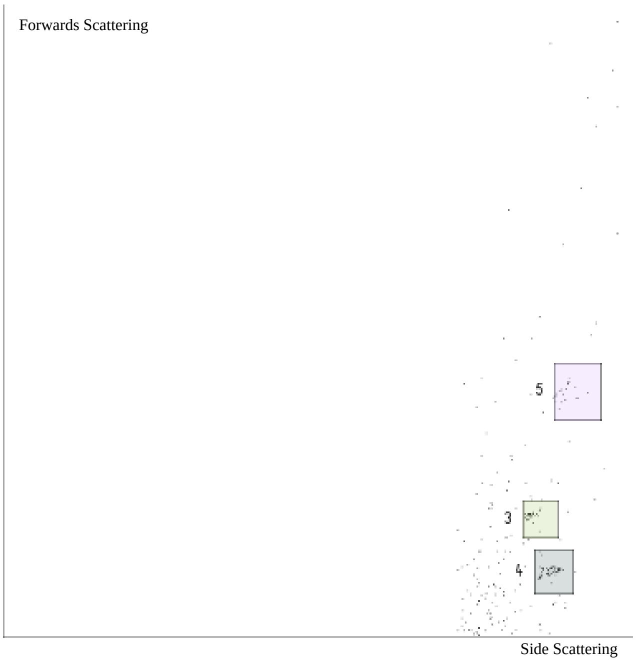

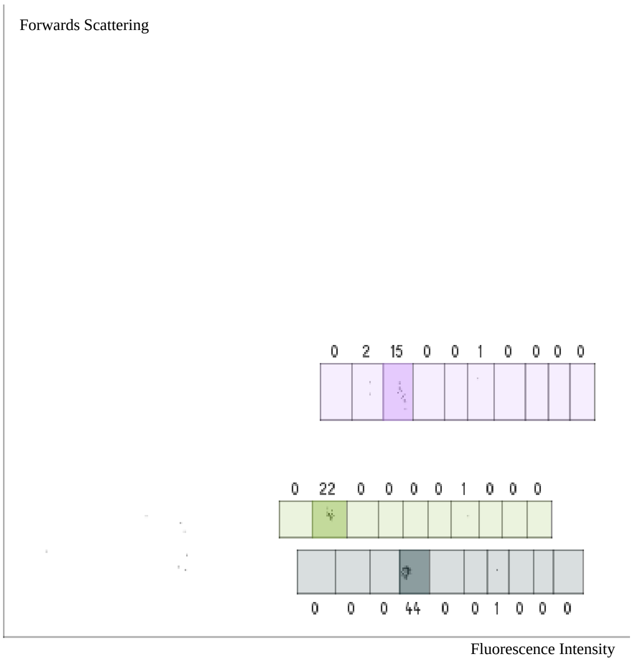

ANNEX 3: TAG DECONVOLUTION - BEAD 377

Passes flow sorting criteria: Yes

Passes tag deconvolution criteria: Yes

Included in protocol analysis: Yes

Protocol: 3, 6, 3, 5

Filename: Bin5_plateA0_G3.fcs

Split 1: Petrol shading

Split 2: Green shading

Split 3: Violet shading

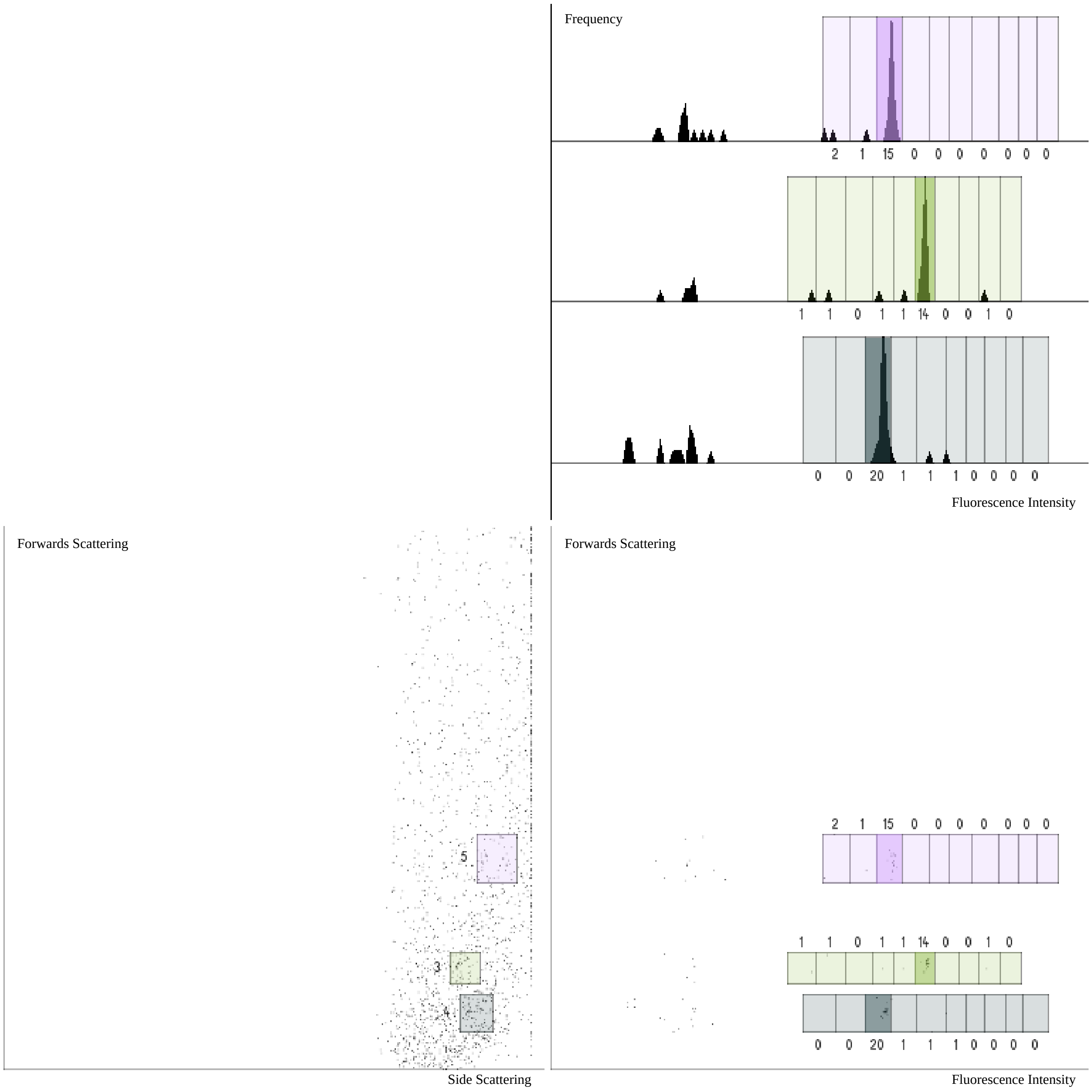

ANNEX 3: TAG DECONVOLUTION - BEAD 378

Passes flow sorting criteria: Yes

Passes tag deconvolution criteria: Yes

Included in protocol analysis: Yes

Protocol: 1, 6, 8, 5

Filename: Bin5_plateA0_G4.fcs

Split 1: Petrol shading

Split 2: Green shading

Split 3: Violet shading

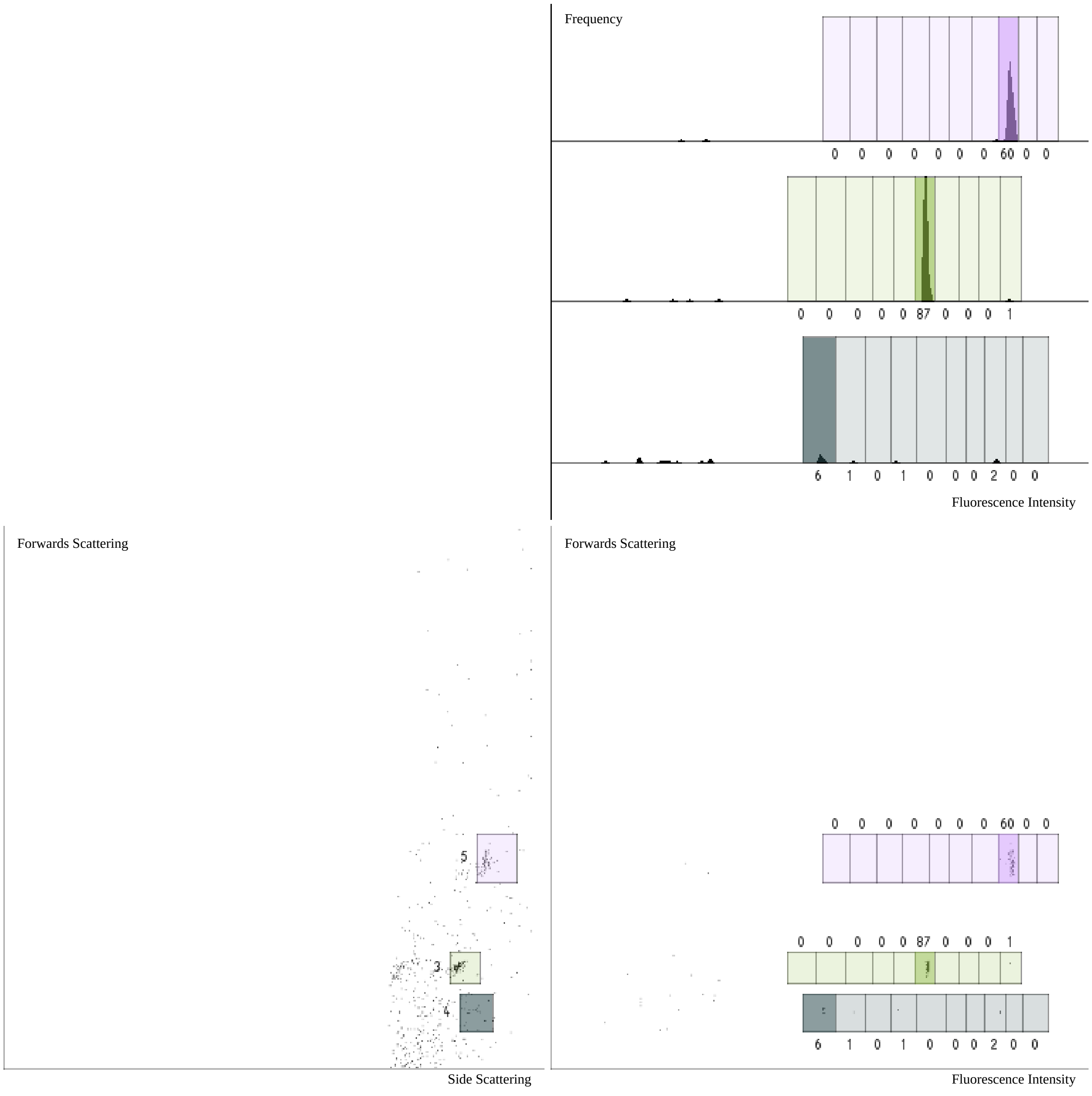

ANNEX 3: TAG DECONVOLUTION - BEAD 379

Passes flow sorting criteria: Yes

Passes tag deconvolution criteria: Yes

Included in protocol analysis: Yes

Protocol: 1, 5, 1, 7

Filename: Bin7_plateA0_C3.fcs

Split 1: Petrol shading

Split 2: Green shading

Split 3: Violet shading

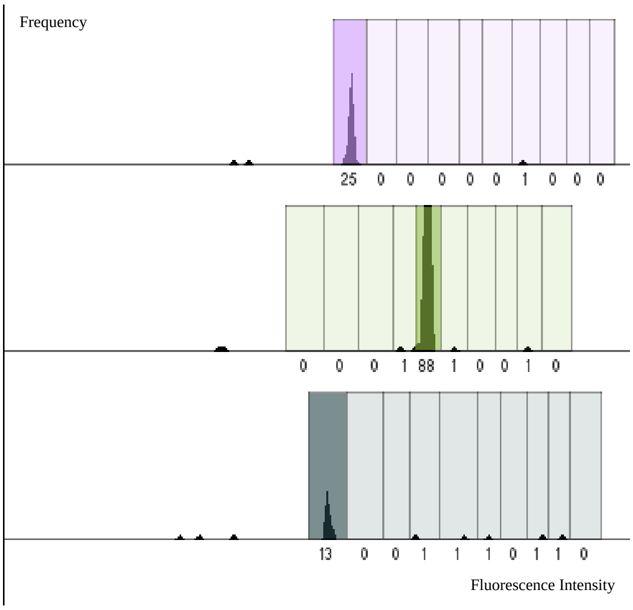

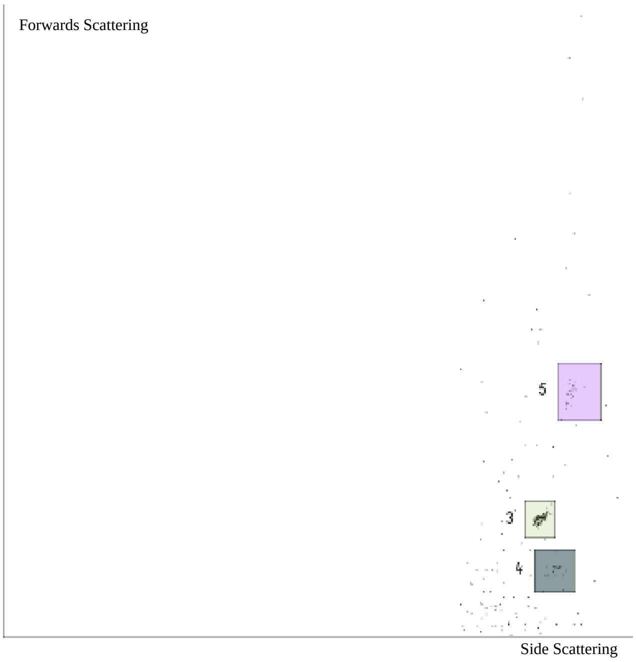

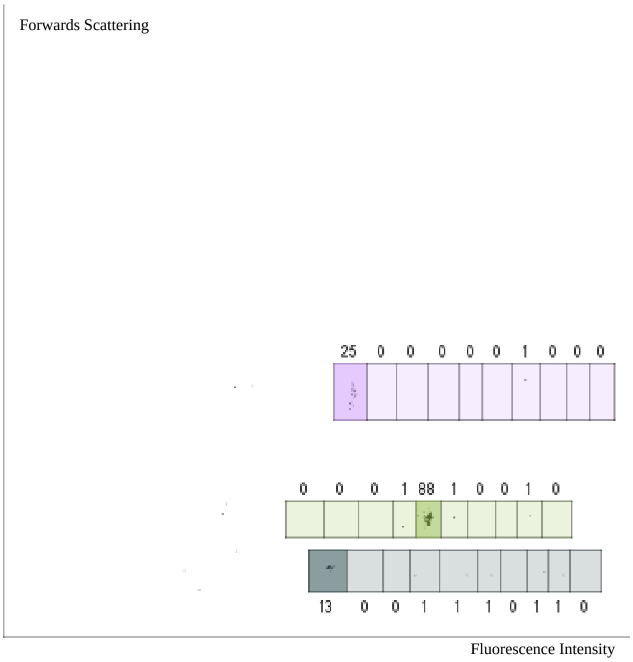

ANNEX 3: TAG DECONVOLUTION - BEAD 380

Passes flow sorting criteria: Yes

Passes tag deconvolution criteria: Yes

Included in protocol analysis: Yes

Protocol: 1, 5, 3, 6

Filename: Bin6_plateA2_A1.fcs

Split 1: Petrol shading

Split 2: Green shading

Split 3: Violet shading

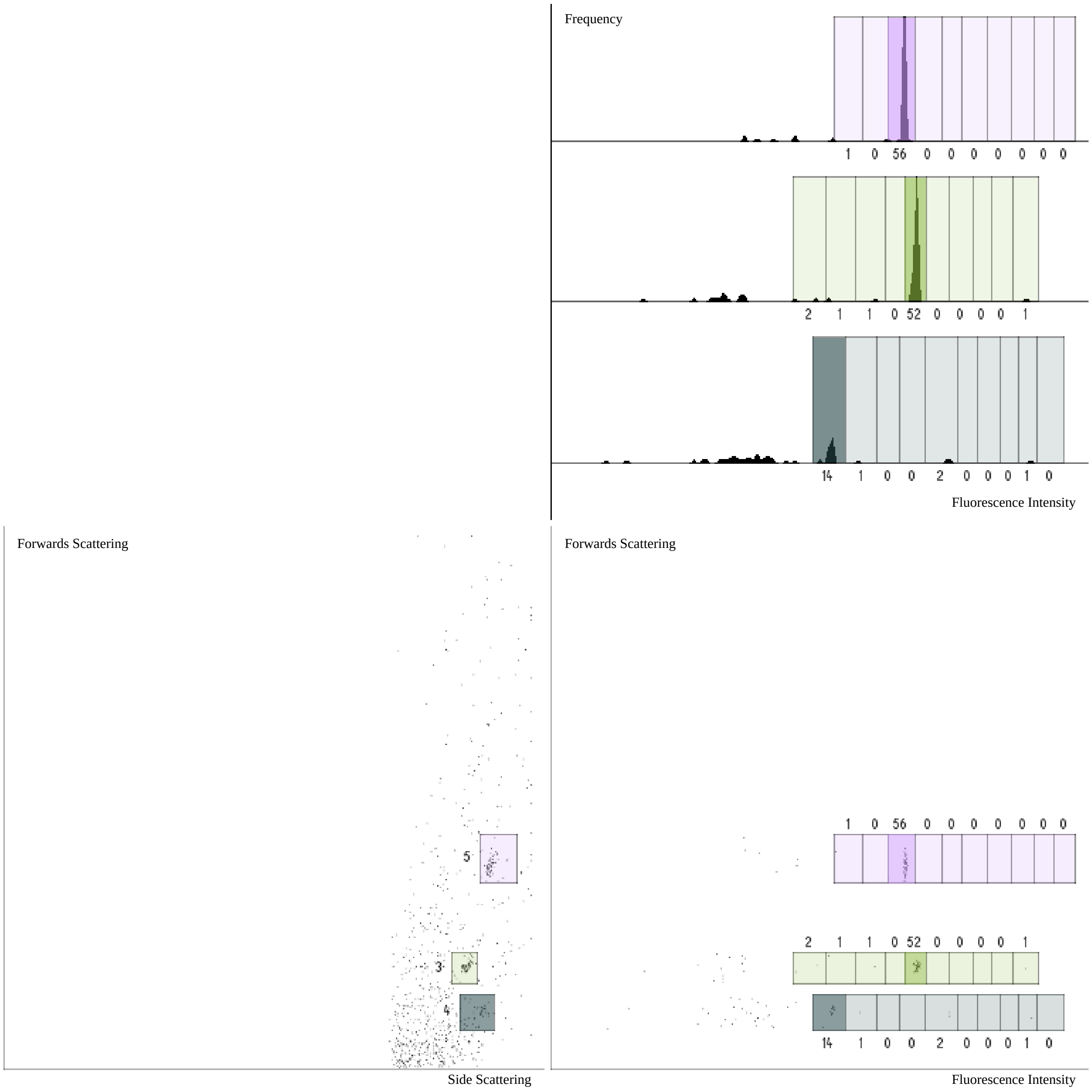

ANNEX 3: TAG DECONVOLUTION - BEAD 381

Passes flow sorting criteria: Yes

Passes tag deconvolution criteria: No

Included in protocol analysis: No

Protocol: N/A

Filename: Bin6_plateA2_A2.fcs

Split 1: Petrol shading

Split 2: Green shading

Split 3: Violet shading

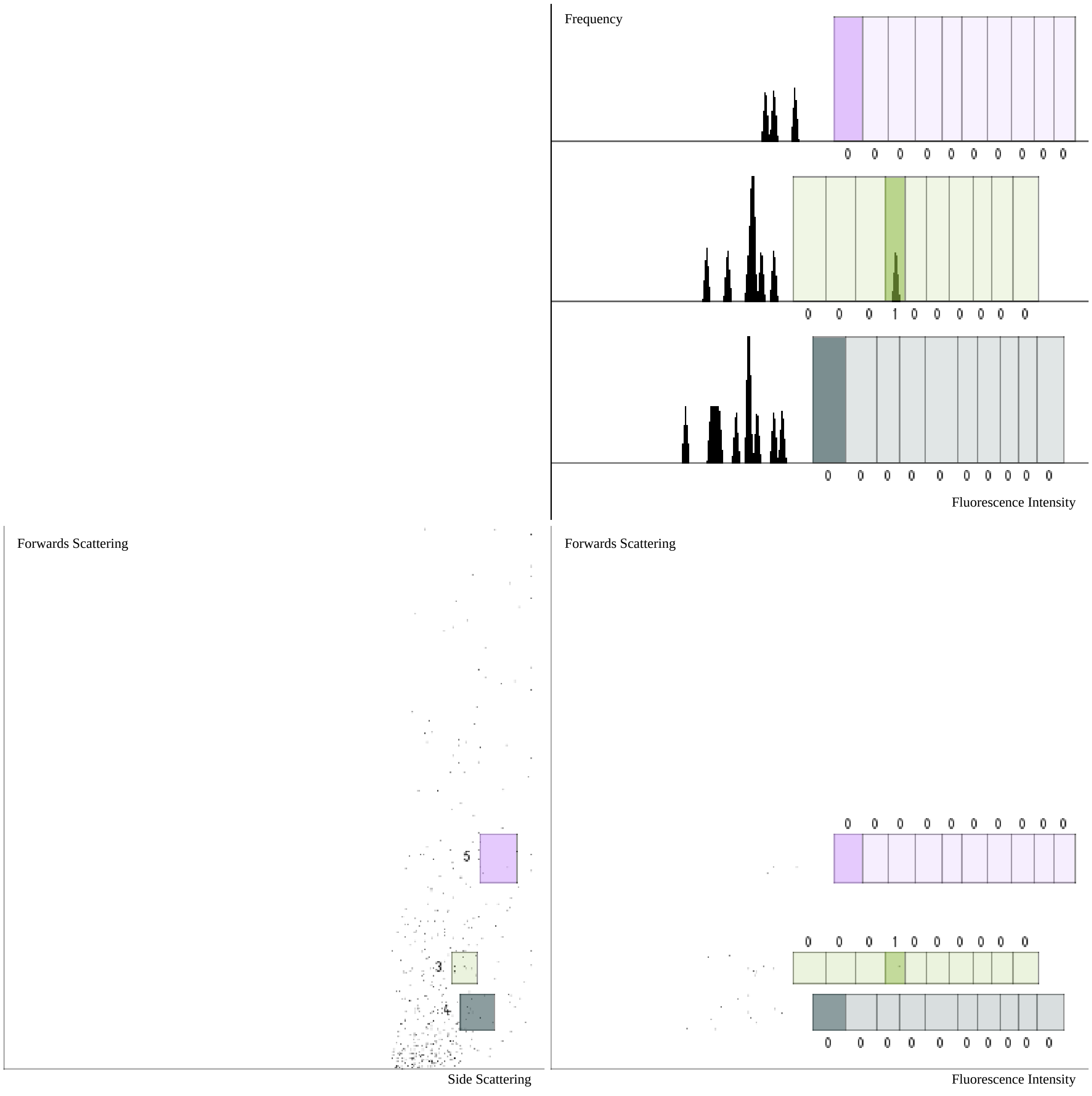

ANNEX 3: TAG DECONVOLUTION - BEAD 382

Passes flow sorting criteria: Yes

Passes tag deconvolution criteria: Yes

Included in protocol analysis: Yes

Protocol: 3, 1, 2, 6

Filename: Bin6_plateA2_A4.fcs

Split 1: Petrol shading

Split 2: Green shading

Split 3: Violet shading

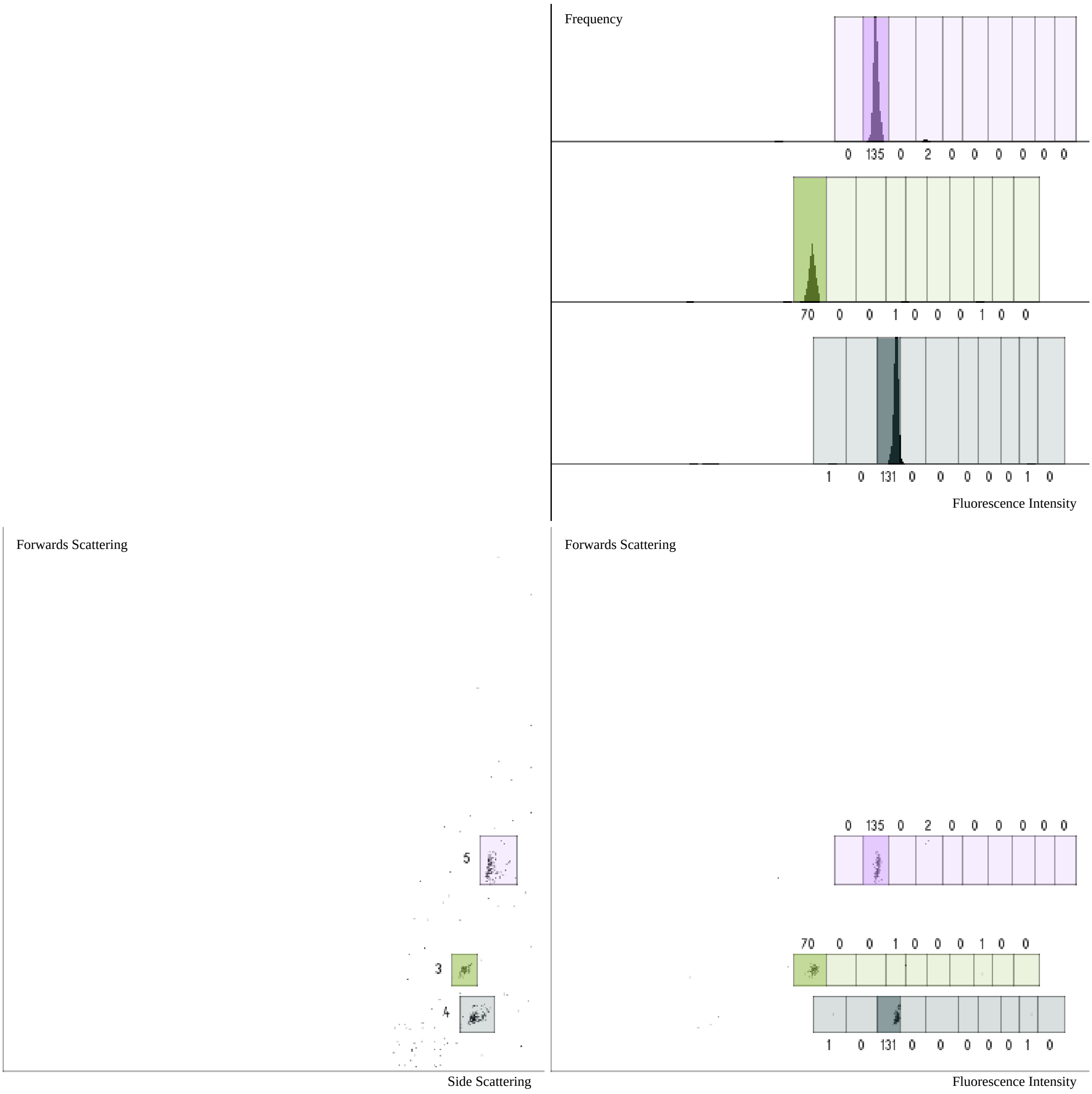

ANNEX 3: TAG DECONVOLUTION - BEAD 383

Passes flow sorting criteria: Yes

Passes tag deconvolution criteria: Yes

Included in protocol analysis: Yes

Protocol: 9, 6, 10, 6

Filename: Bin6_plateA2_A5.fcs

Split 1: Petrol shading

Split 2: Green shading

Split 3: Violet shading

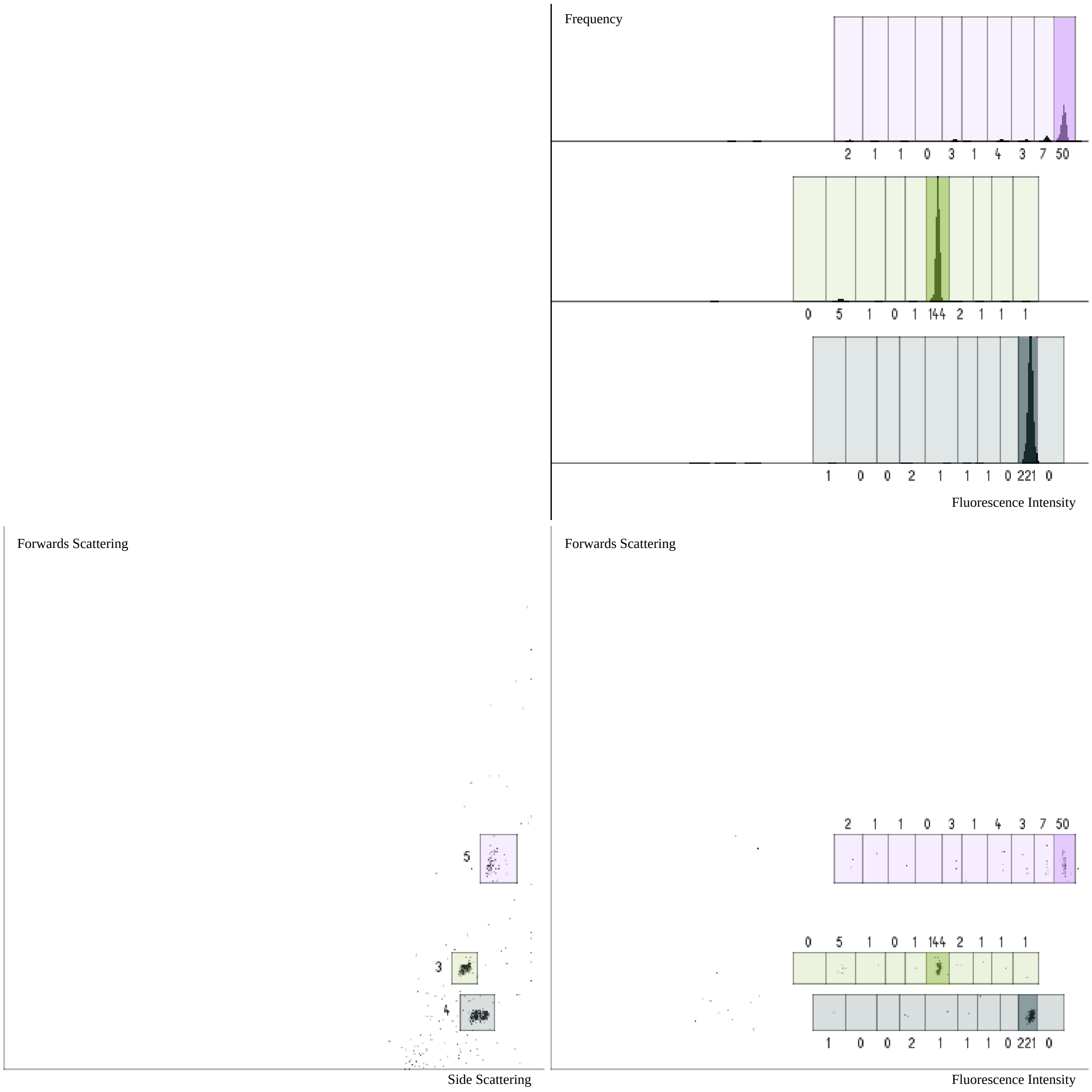

ANNEX 3: TAG DECONVOLUTION - BEAD 384

Passes flow sorting criteria: Yes

Passes tag deconvolution criteria: Yes

Included in protocol analysis: Yes

Protocol: 5, 7, 1, 6

Filename: Bin6_plateA2_A6.fcs

Split 1: Petrol shading

Split 2: Green shading

Split 3: Violet shading

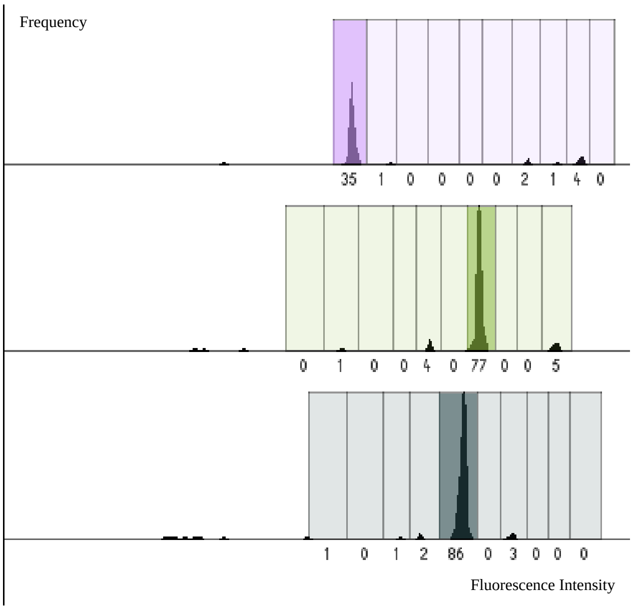

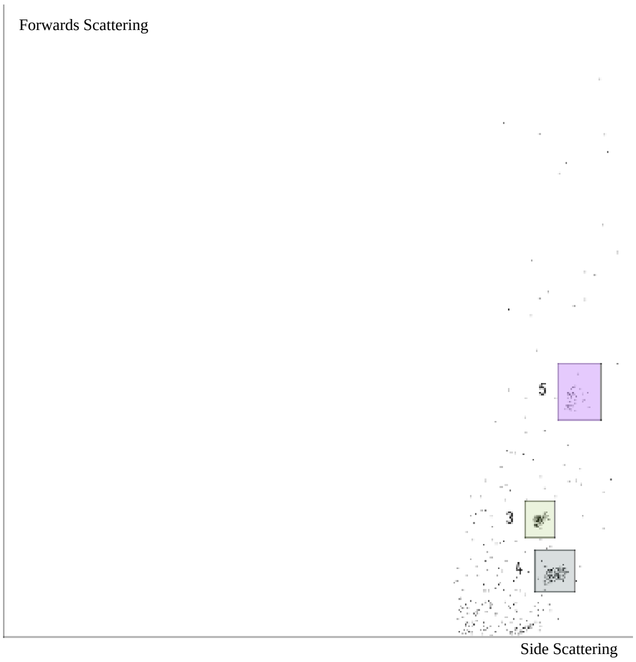

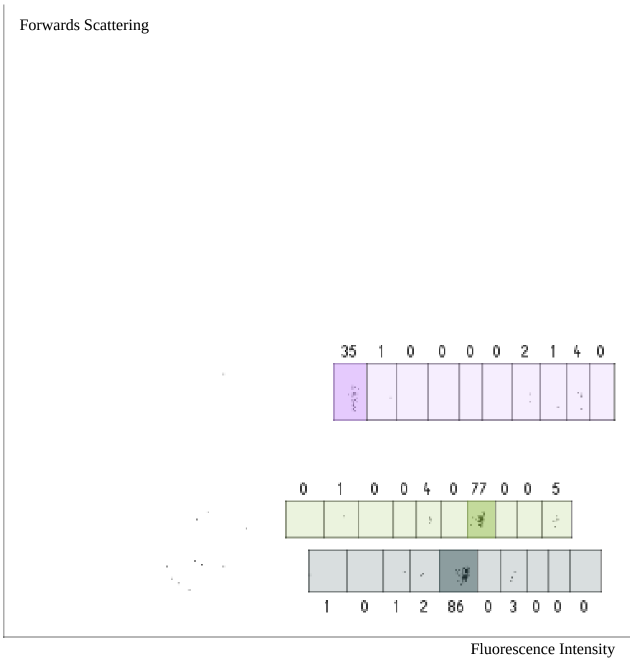

ANNEX 3: TAG DECONVOLUTION - BEAD 385

Passes flow sorting criteria: Yes

Passes tag deconvolution criteria: No

Included in protocol analysis: No

Protocol: N/A

Filename: Bin6_plateA2_A7.fcs

Split 1: Petrol shading

Split 2: Green shading

Split 3: Violet shading

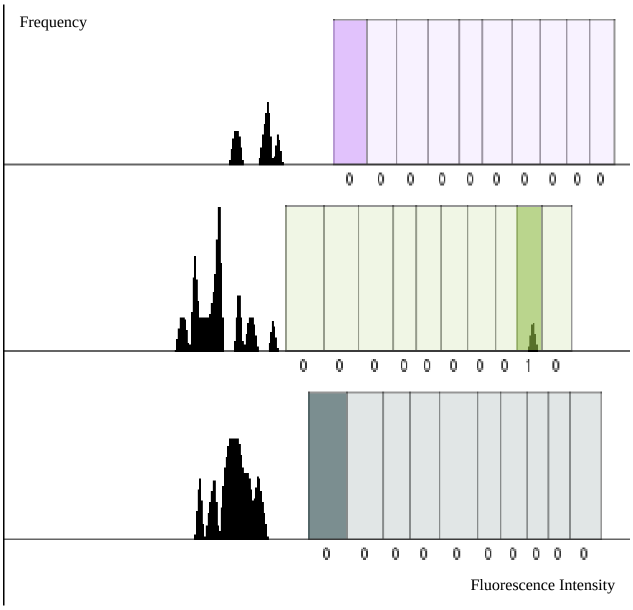

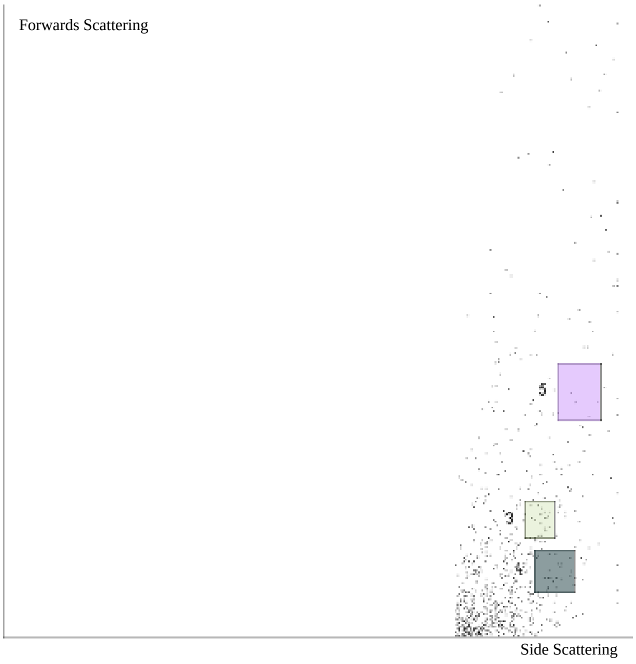

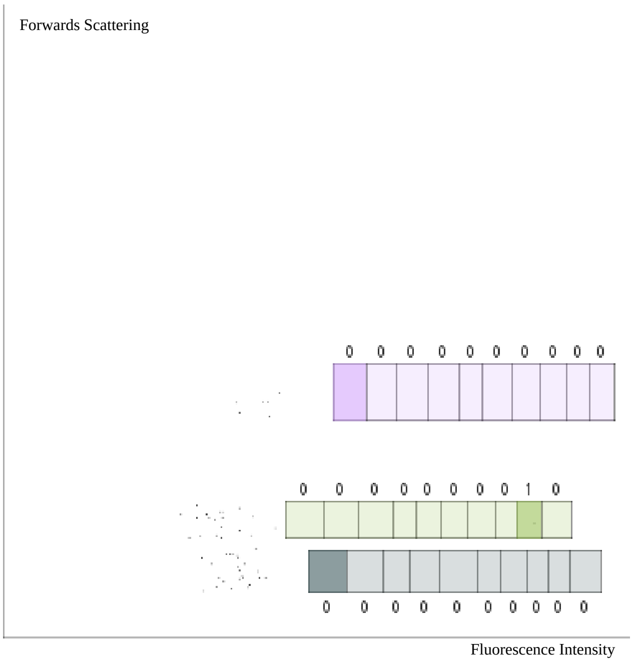

ANNEX 3: TAG DECONVOLUTION - BEAD 386

Passes flow sorting criteria: Yes

Passes tag deconvolution criteria: Yes

Included in protocol analysis: Yes

Protocol: 7, 9, 6, 6

Filename: Bin6_plateA2_A9.fcs

Split 1: Petrol shading

Split 2: Green shading

Split 3: Violet shading

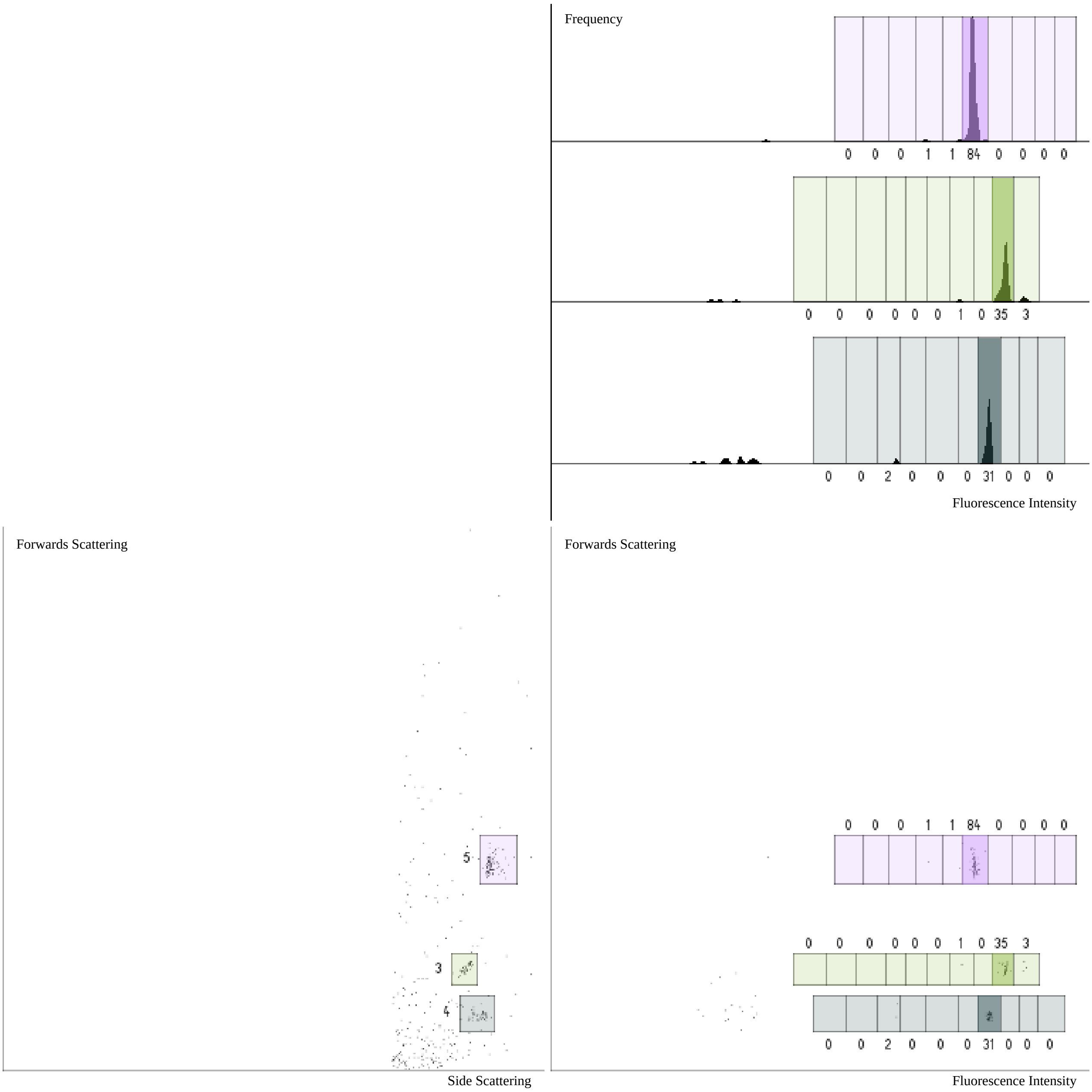

ANNEX 3: TAG DECONVOLUTION - BEAD 387

Passes flow sorting criteria: Yes

Passes tag deconvolution criteria: Yes

Included in protocol analysis: Yes

Protocol: 10, 3, 9, 6

Filename: Bin6_plateA2_A10.fcs

Split 1: Petrol shading

Split 2: Green shading

Split 3: Violet shading

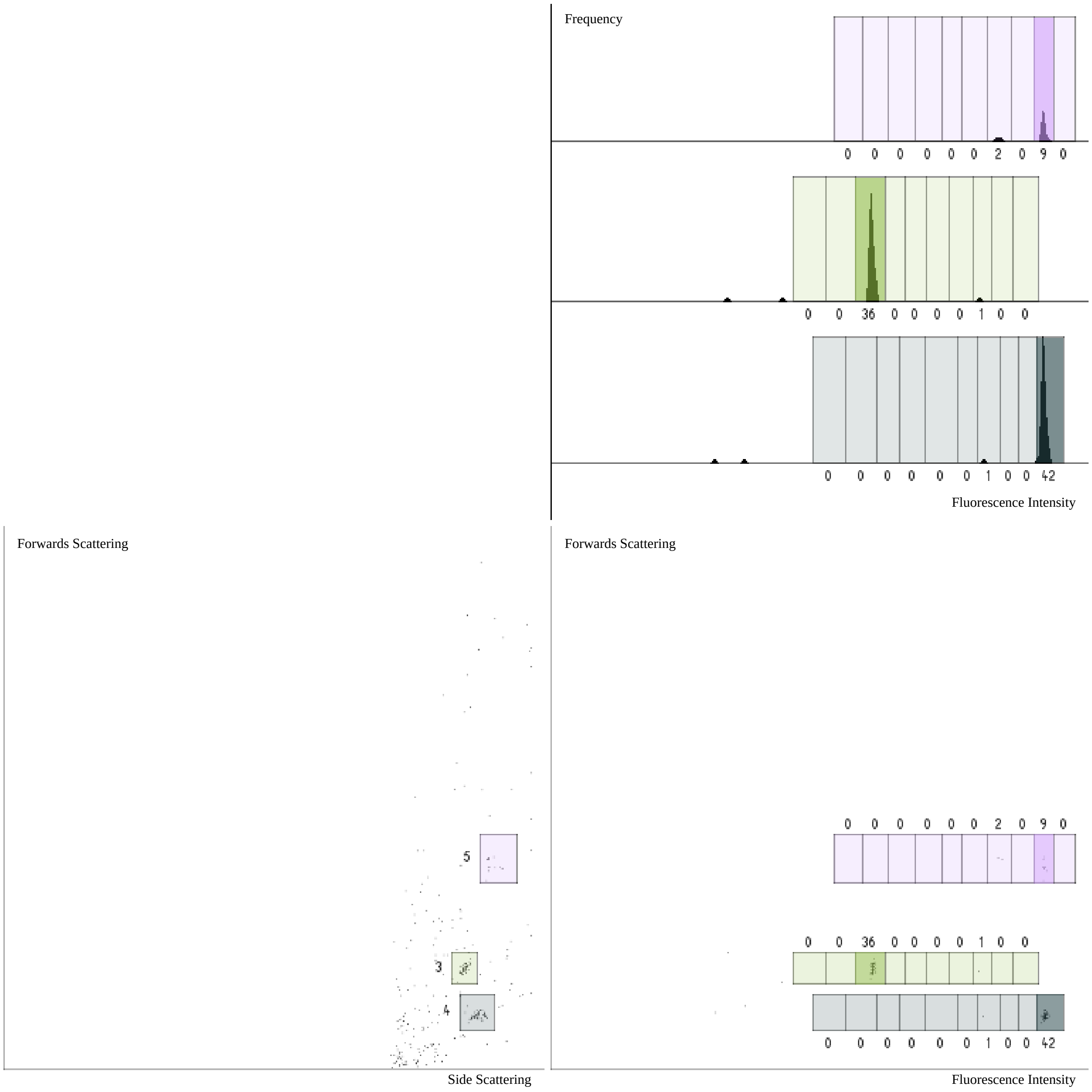

ANNEX 3: TAG DECONVOLUTION - BEAD 388

Passes flow sorting criteria: Yes

Passes tag deconvolution criteria: Yes

Included in protocol analysis: Yes

Protocol: 5, 10, 1, 6

Filename: Bin6_plateA2_A11.fcs

Split 1: Petrol shading

Split 2: Green shading

Split 3: Violet shading

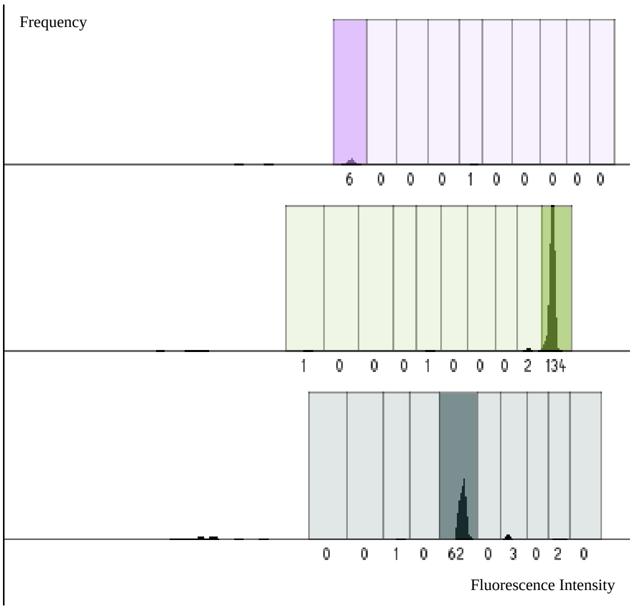

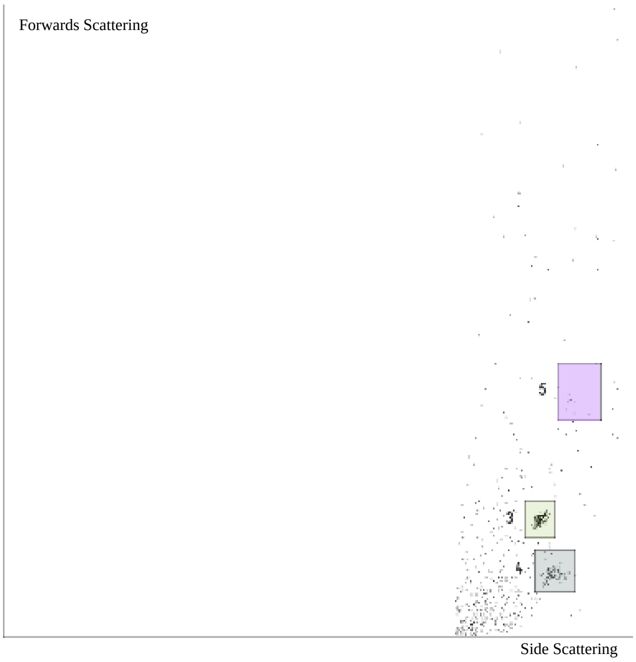

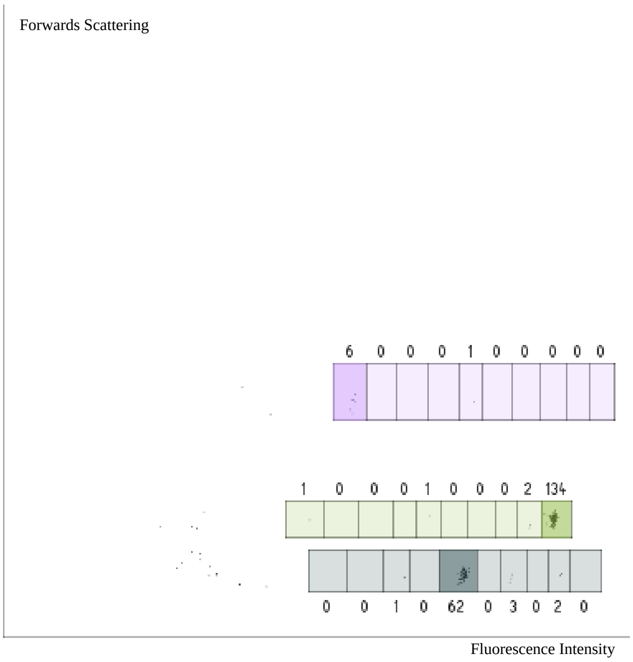

ANNEX 3: TAG DECONVOLUTION - BEAD 389

Passes flow sorting criteria: Yes

Passes tag deconvolution criteria: Yes

Included in protocol analysis: Yes

Protocol: 1, 10, 5, 7

Filename: Bin7_plateA0_C1.fcs

Split 1: Petrol shading

Split 2: Green shading

Split 3: Violet shading

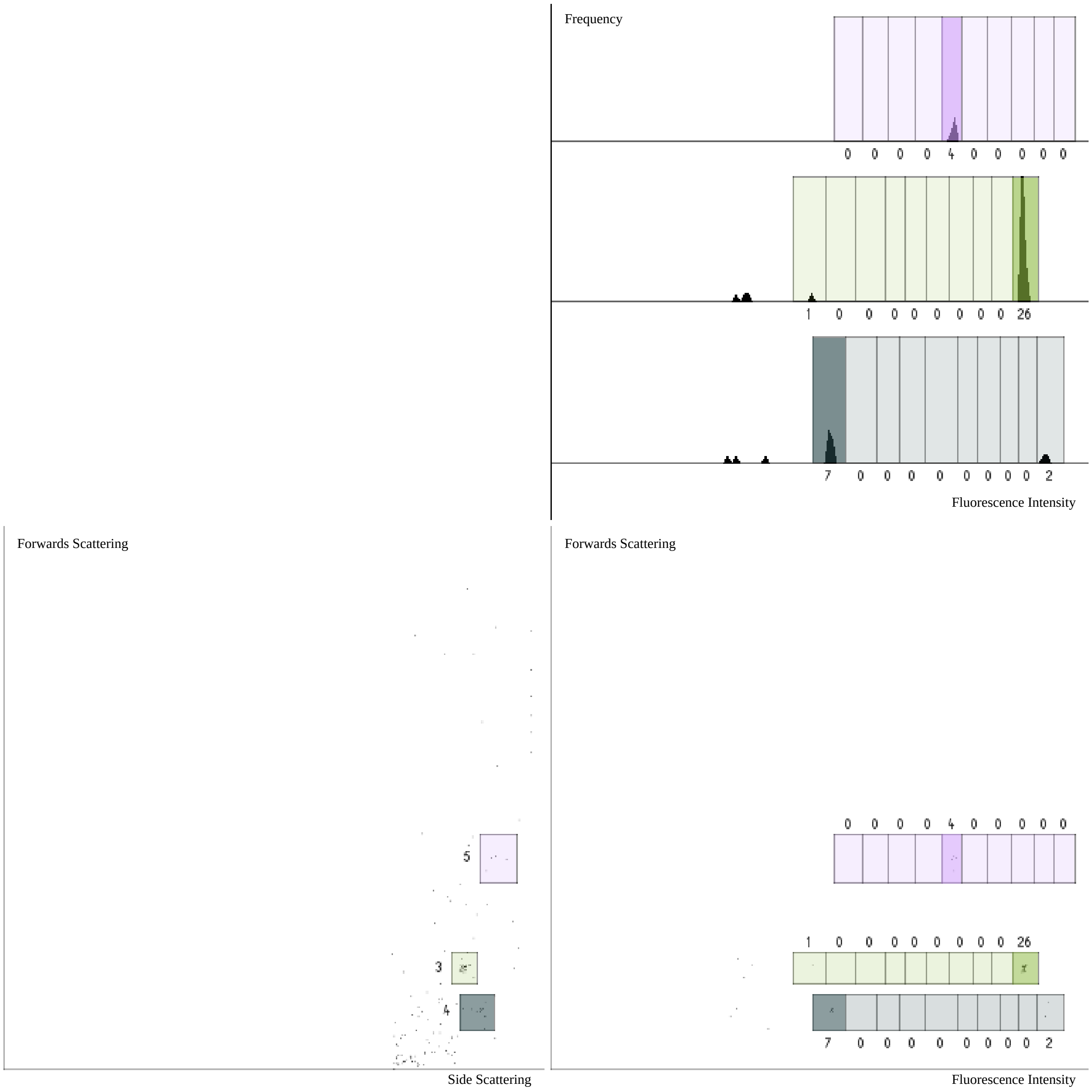

ANNEX 3: TAG DECONVOLUTION - BEAD 390

Passes flow sorting criteria: Yes

Passes tag deconvolution criteria: Yes

Included in protocol analysis: Yes

Protocol: 2, 3, 2, 7

Filename: Bin7_plateA0_C2.fcs

Split 1: Petrol shading

Split 2: Green shading

Split 3: Violet shading

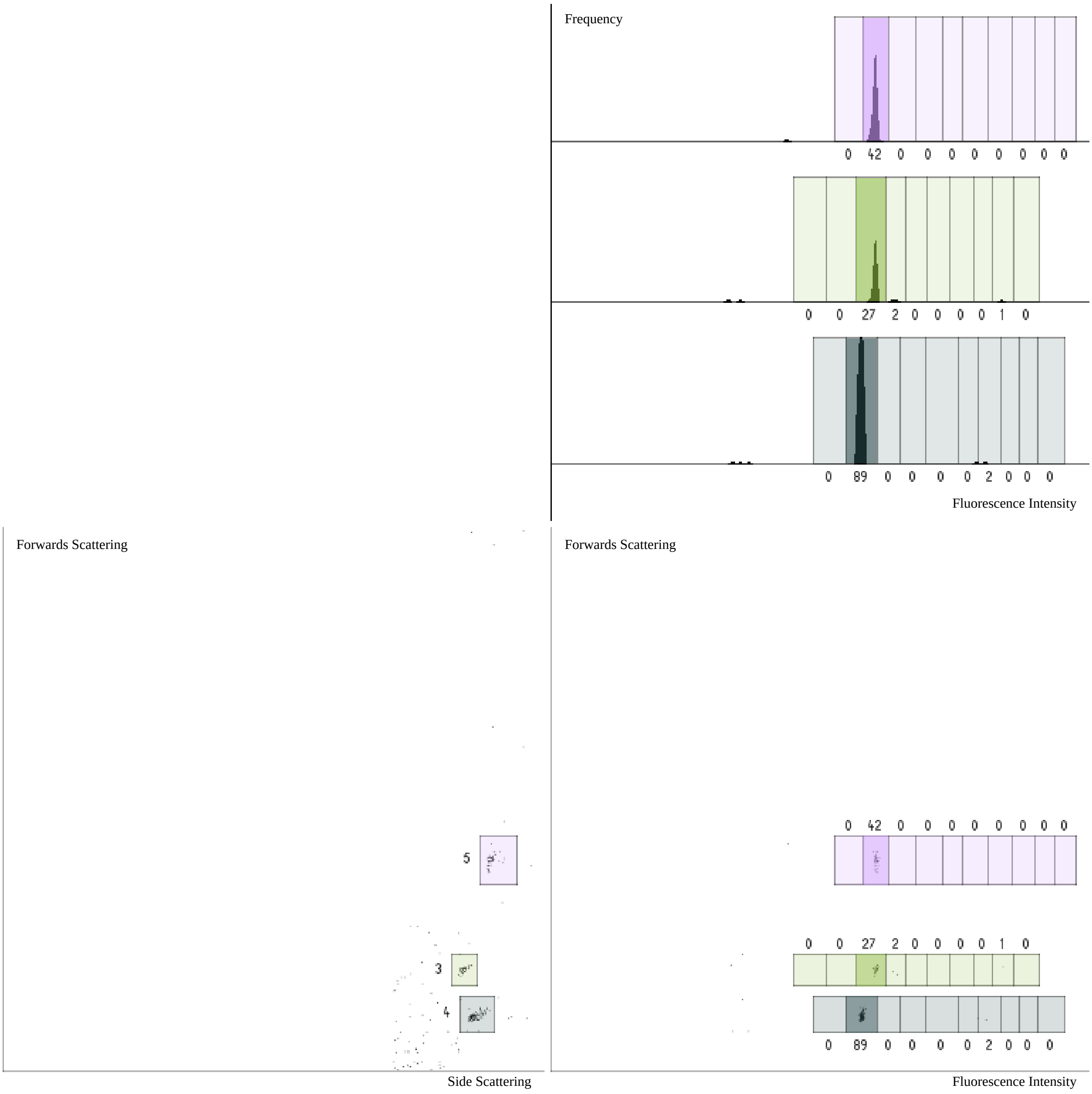

ANNEX 3: TAG DECONVOLUTION - BEAD 391

Passes flow sorting criteria: Yes

Passes tag deconvolution criteria: Yes

Included in protocol analysis: Yes

Protocol: 10, 9, 4, 8

Filename: Bin8_plateA0_G12.fcs

Split 1: Petrol shading

Split 2: Green shading

Split 3: Violet shading

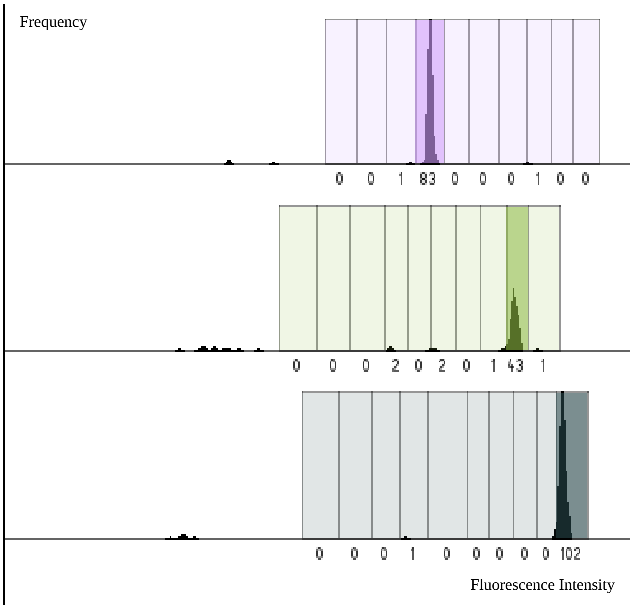

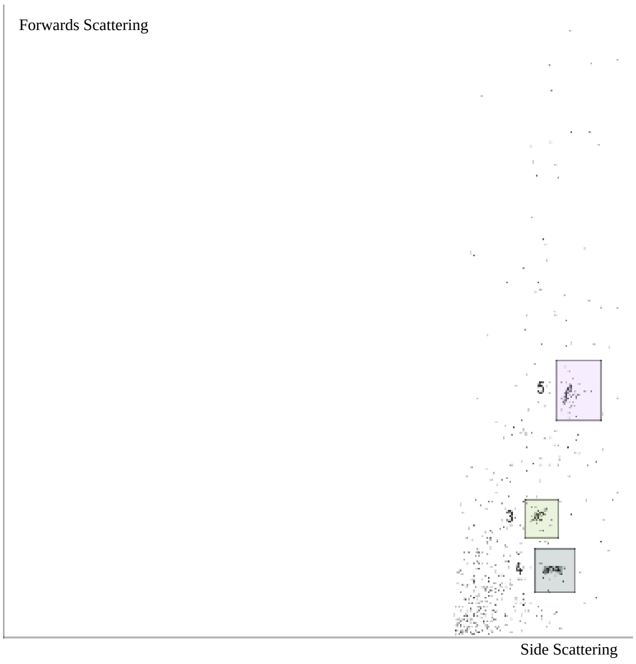

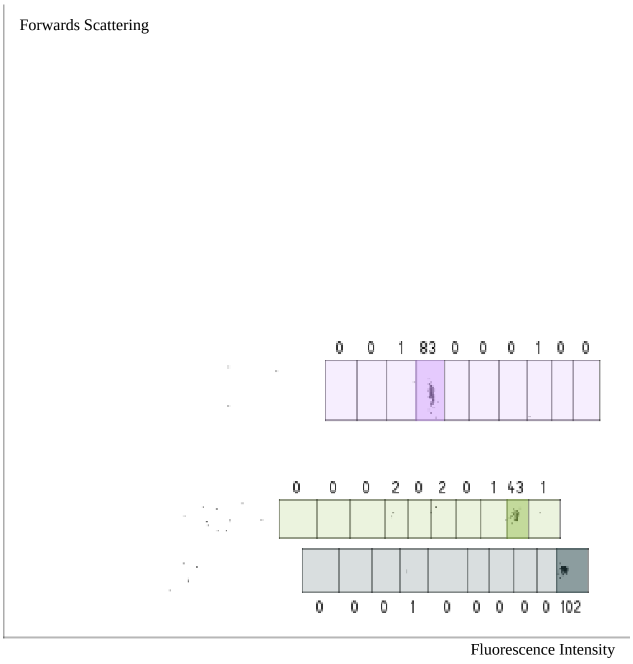

ANNEX 3: TAG DECONVOLUTION - BEAD 392

Passes flow sorting criteria: Yes

Passes tag deconvolution criteria: No

Included in protocol analysis: No

Protocol: N/A

Filename: Bin7_plateA0_C4.fcs

Split 1: Petrol shading

Split 2: Green shading

Split 3: Violet shading

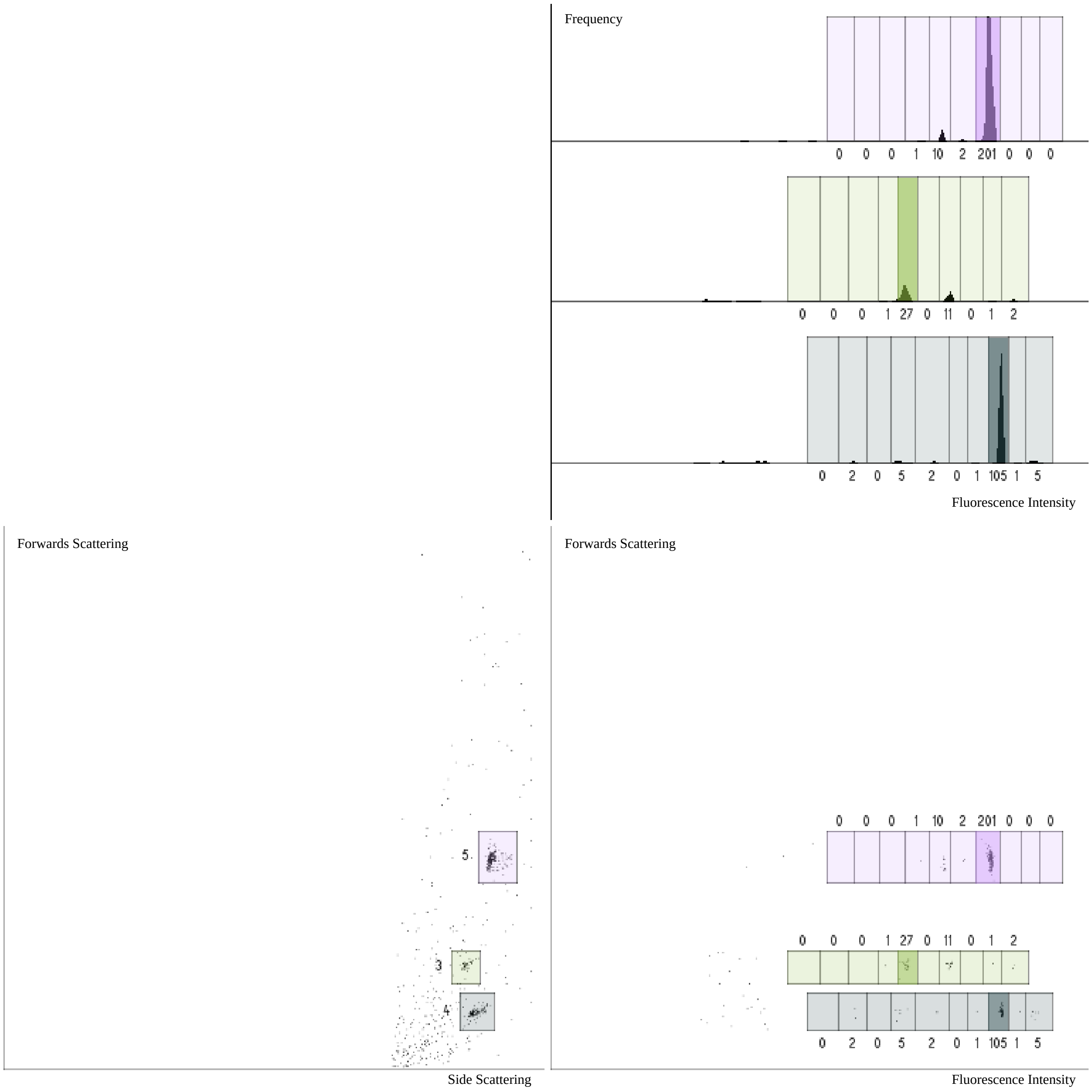

ANNEX 3: TAG DECONVOLUTION - BEAD 393

Passes flow sorting criteria: Yes

Passes tag deconvolution criteria: Yes

Included in protocol analysis: Yes

Protocol: 7, 6, 1, 7

Filename: Bin7_plateA0_C5.fcs

Split 1: Petrol shading

Split 2: Green shading

Split 3: Violet shading

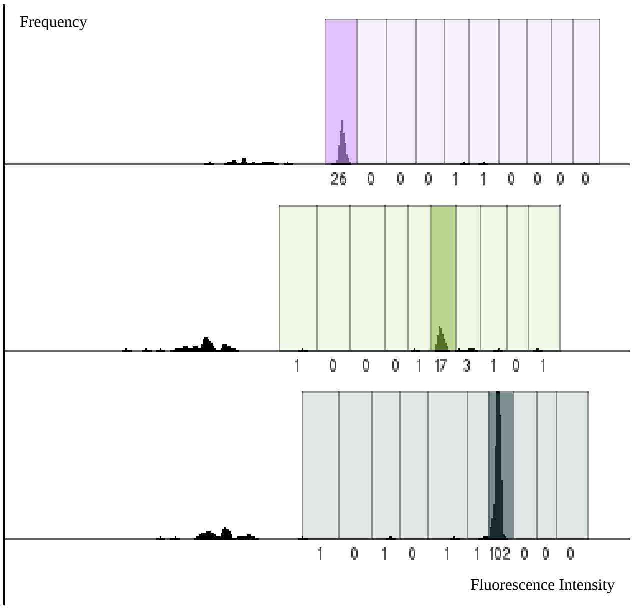

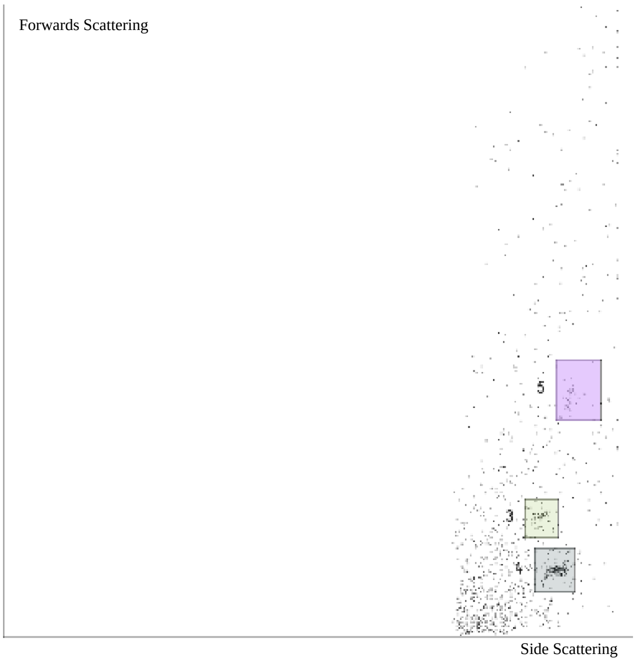

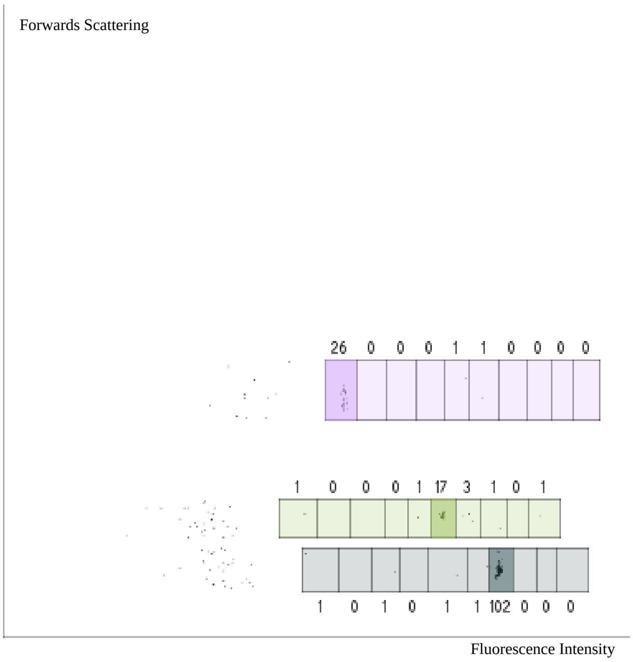

ANNEX 3: TAG DECONVOLUTION - BEAD 394

Passes flow sorting criteria: Yes

Passes tag deconvolution criteria: Yes

Included in protocol analysis: Yes

Protocol: 8, 10, 6, 7

Filename: Bin7_plateA0_C7.fcs

Split 1: Petrol shading

Split 2: Green shading

Split 3: Violet shading

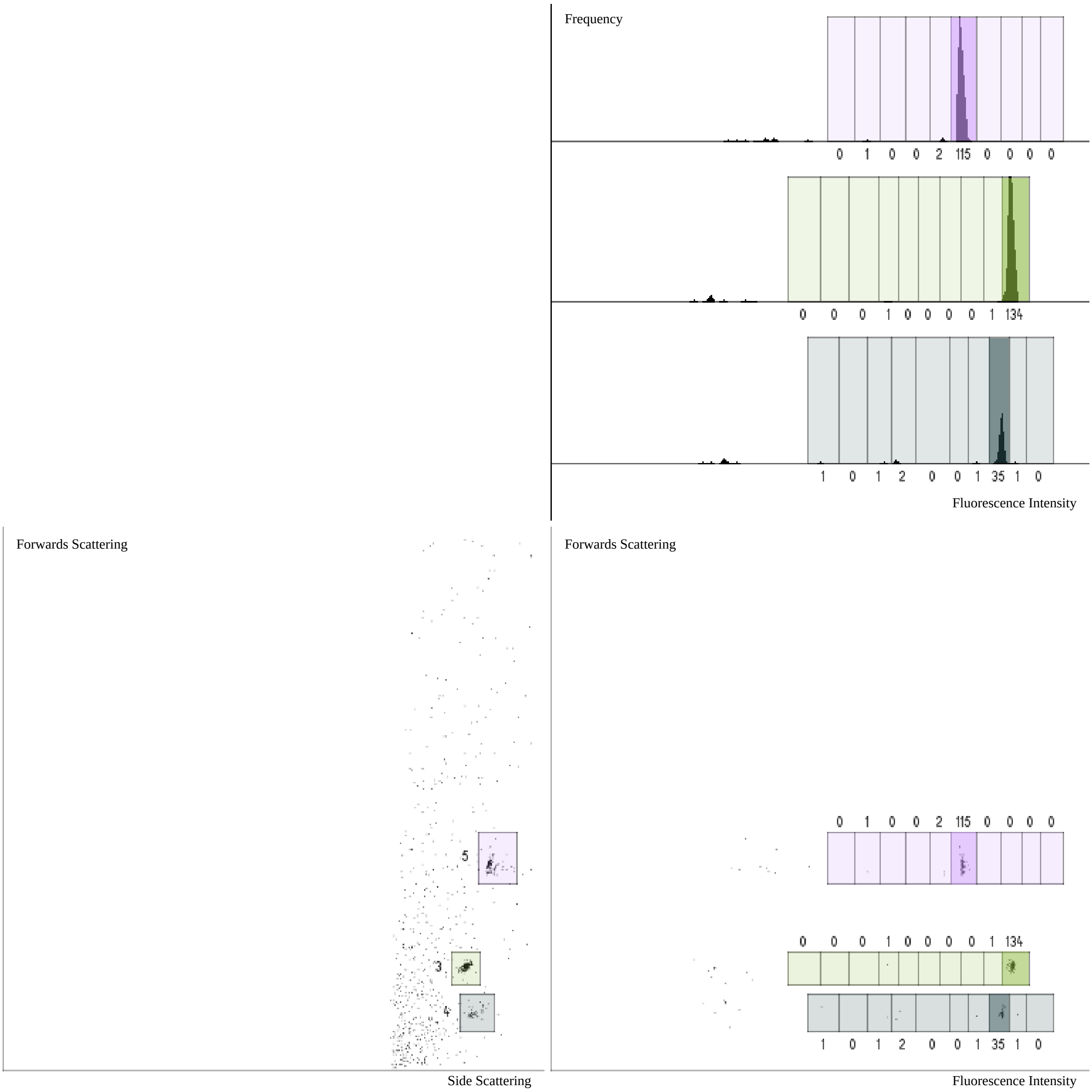

ANNEX 3: TAG DECONVOLUTION - BEAD 395

Passes flow sorting criteria: Yes

Passes tag deconvolution criteria: No

Included in protocol analysis: No

Protocol: N/A

Filename: Bin7_plateA0_C8.fcs

Split 1: Petrol shading

Split 2: Green shading

Split 3: Violet shading

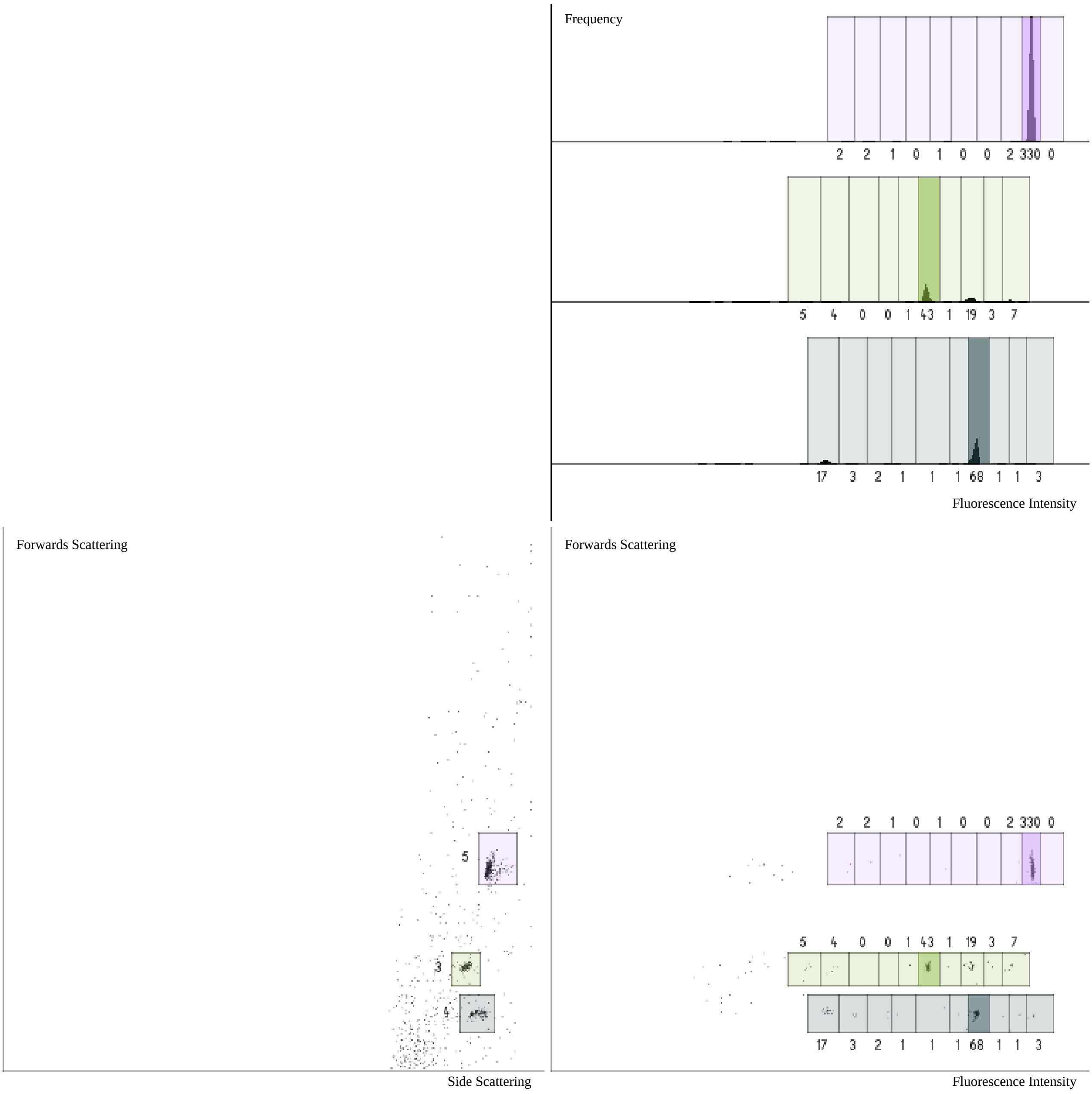

ANNEX 3: TAG DECONVOLUTION - BEAD 396

Passes flow sorting criteria: Yes

Passes tag deconvolution criteria: Yes

Included in protocol analysis: Yes

Protocol: 4, 1, 3, 7

Filename: Bin7_plateA0_C9.fcs

Split 1: Petrol shading

Split 2: Green shading

Split 3: Violet shading

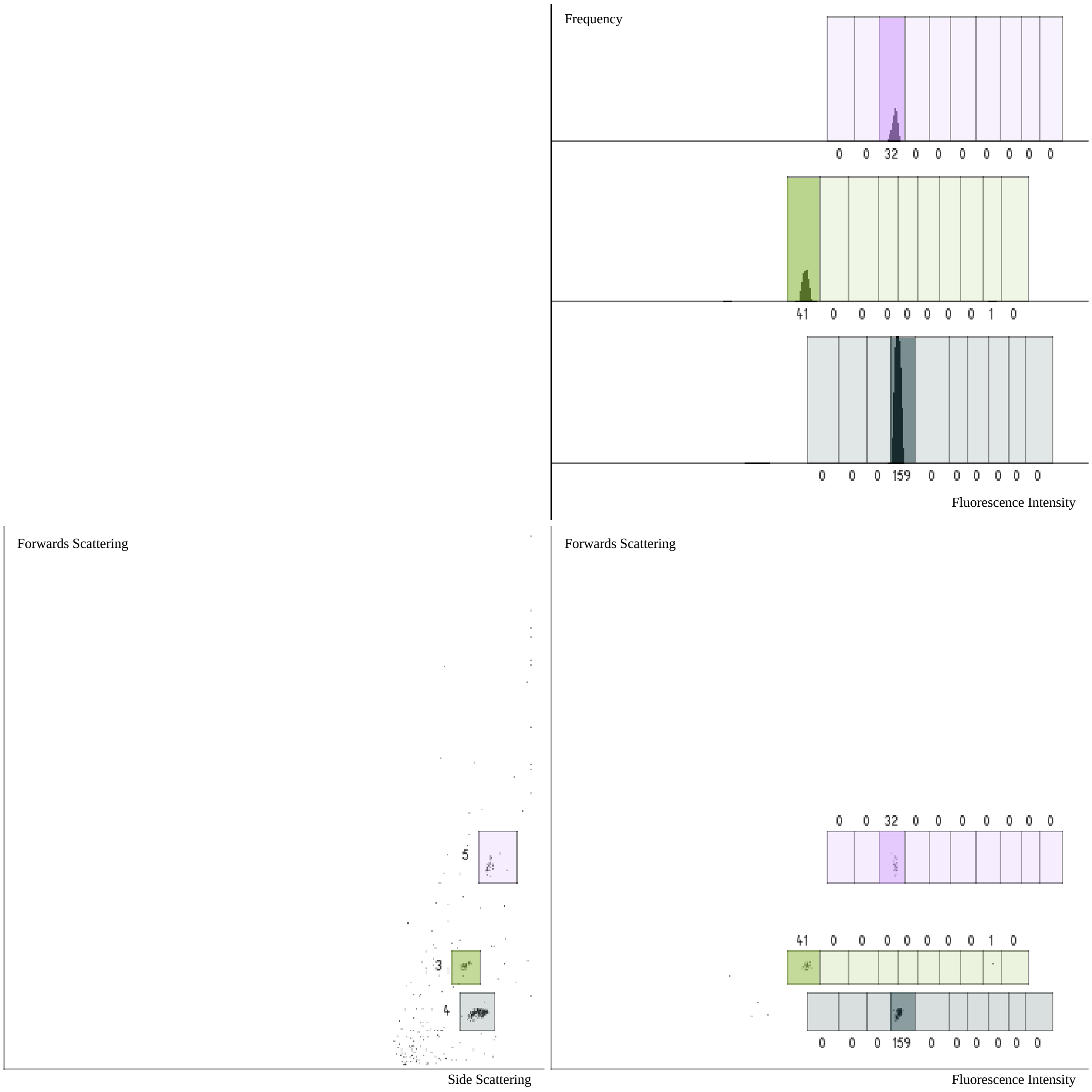

ANNEX 3: TAG DECONVOLUTION - BEAD 397

Passes flow sorting criteria: Yes

Passes tag deconvolution criteria: Yes

Included in protocol analysis: Yes

Protocol: 3, 2, 3, 7

Filename: Bin7_plateA0_C11.fcs

Split 1: Petrol shading

Split 2: Green shading

Split 3: Violet shading

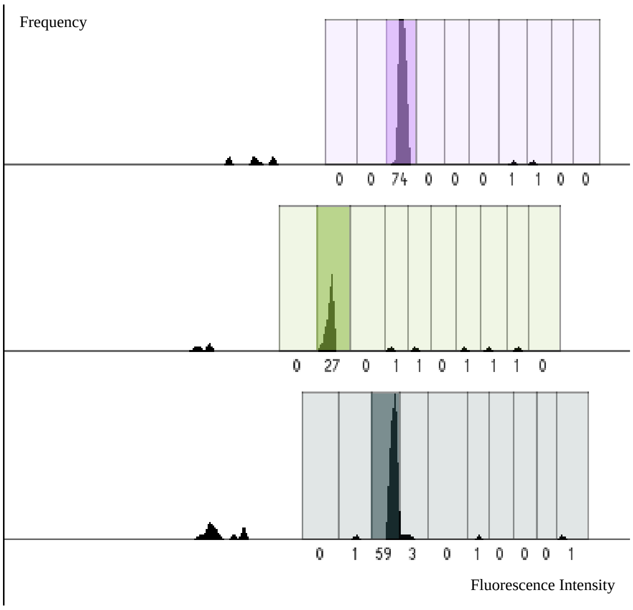

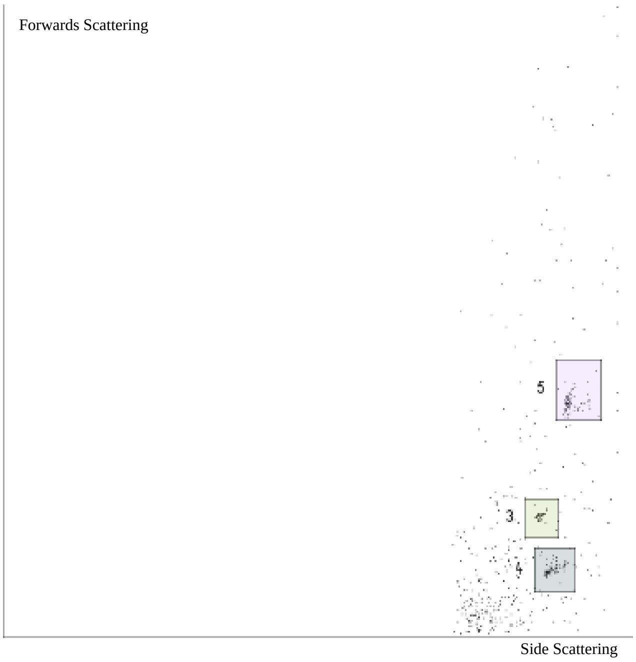

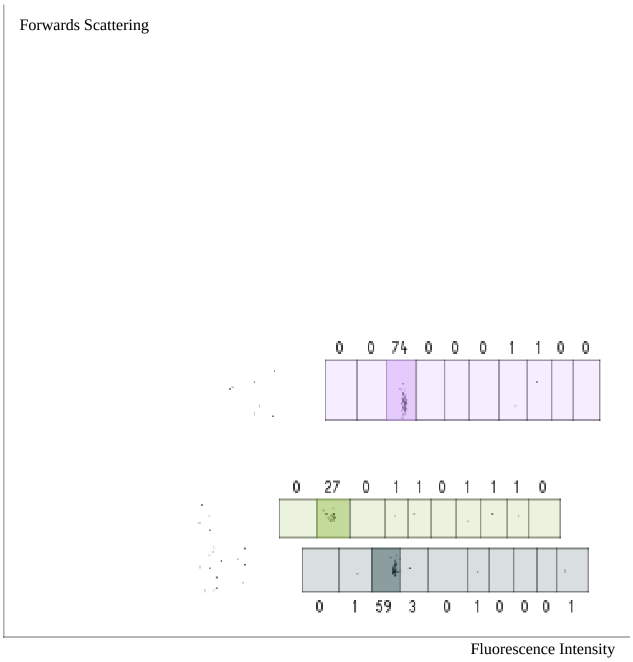

ANNEX 3: TAG DECONVOLUTION - BEAD 398

Passes flow sorting criteria: Yes

Passes tag deconvolution criteria: Yes

Included in protocol analysis: Yes

Protocol: 6, 6, 6, 7

Filename: Bin7_plateA0_C12.fcs

Split 1: Petrol shading

Split 2: Green shading

Split 3: Violet shading

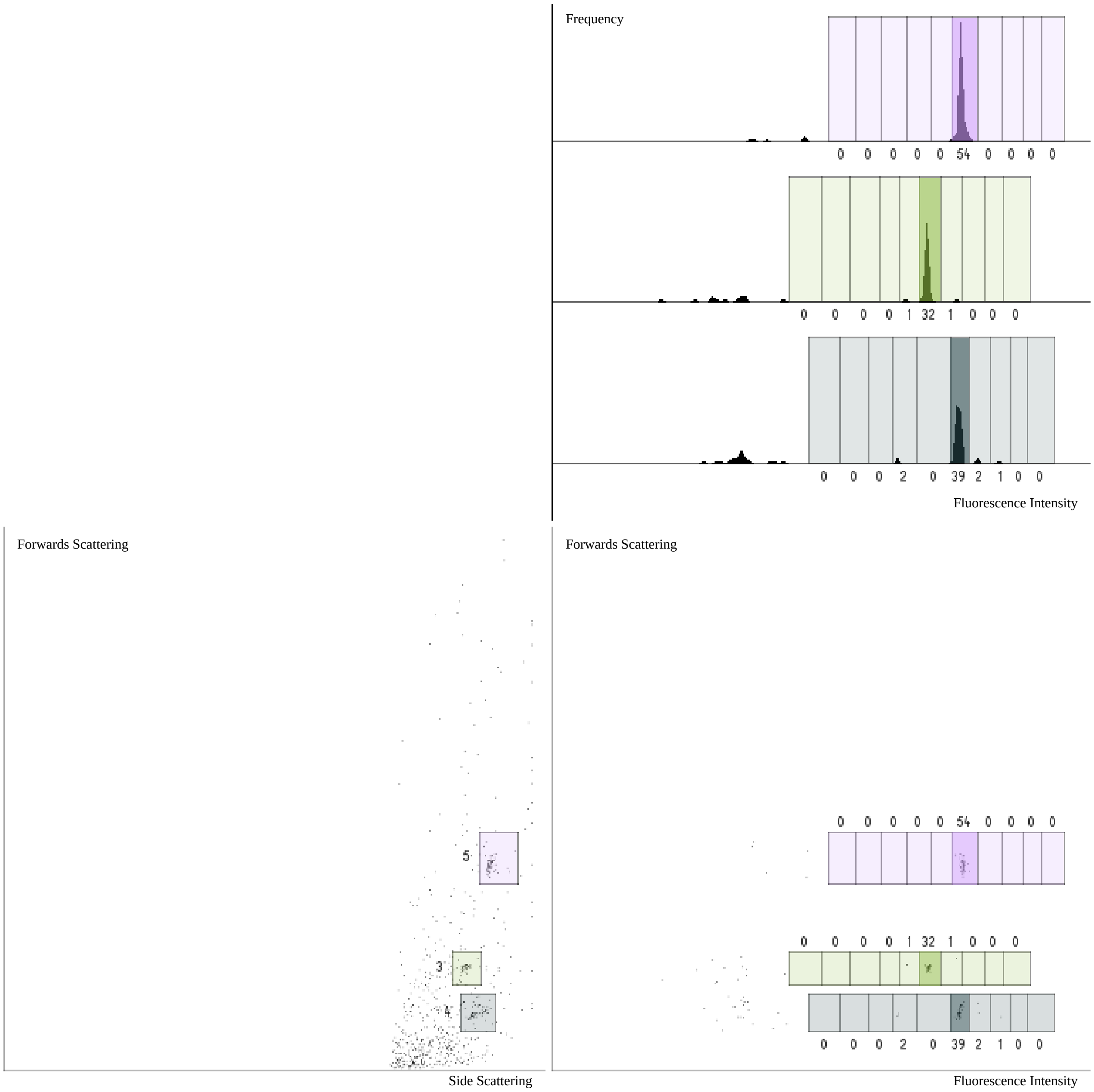

ANNEX 3: TAG DECONVOLUTION - BEAD 399

Passes flow sorting criteria: Yes

Passes tag deconvolution criteria: Yes

Included in protocol analysis: Yes

Protocol: 5, 10, 1, 7

Filename: Bin7_plateA0_D5.fcs

Split 1: Petrol shading

Split 2: Green shading

Split 3: Violet shading

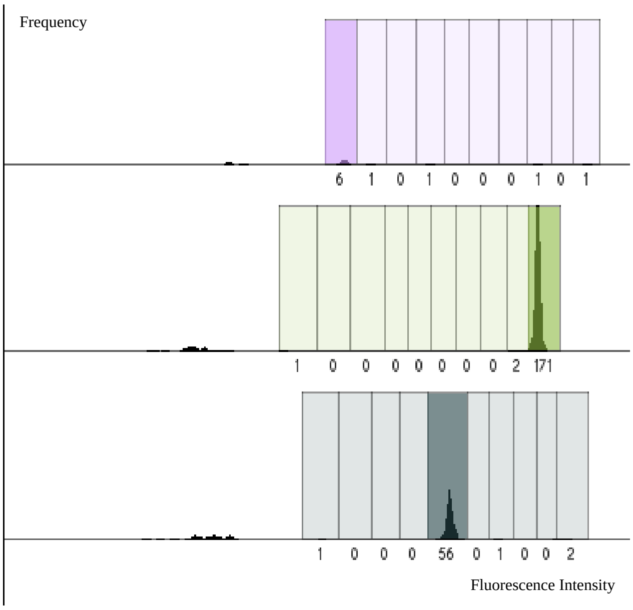

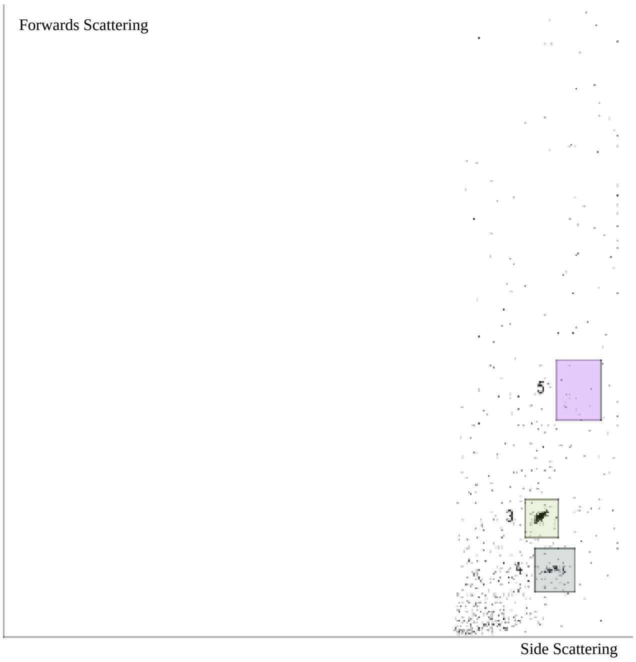

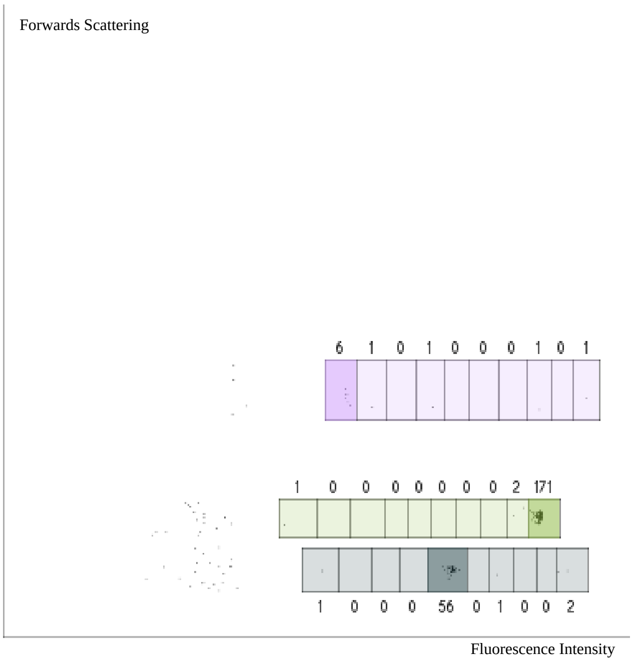

ANNEX 3: TAG DECONVOLUTION - BEAD 400

Passes flow sorting criteria: Yes

Passes tag deconvolution criteria: Yes

Included in protocol analysis: Yes

Protocol: 8, 8, 4, 7

Filename: Bin7_plateA0_D6.fcs

Split 1: Petrol shading

Split 2: Green shading

Split 3: Violet shading

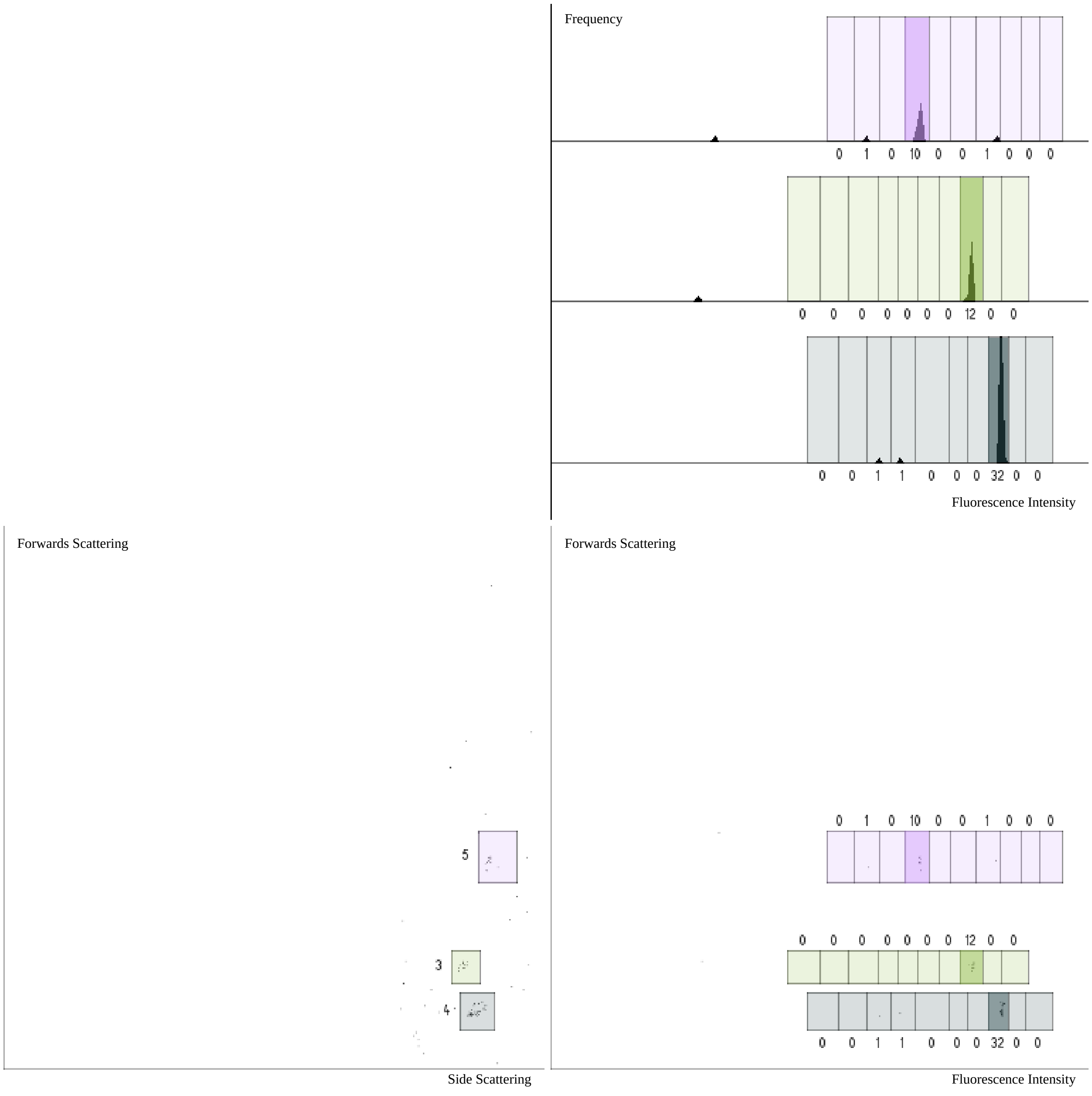

ANNEX 3: TAG DECONVOLUTION - BEAD 401

Passes flow sorting criteria: Yes

Passes tag deconvolution criteria: No

Included in protocol analysis: No

Protocol: N/A

Filename: Bin7_plateA0_D7.fcs

Split 1: Petrol shading

Split 2: Green shading

Split 3: Violet shading

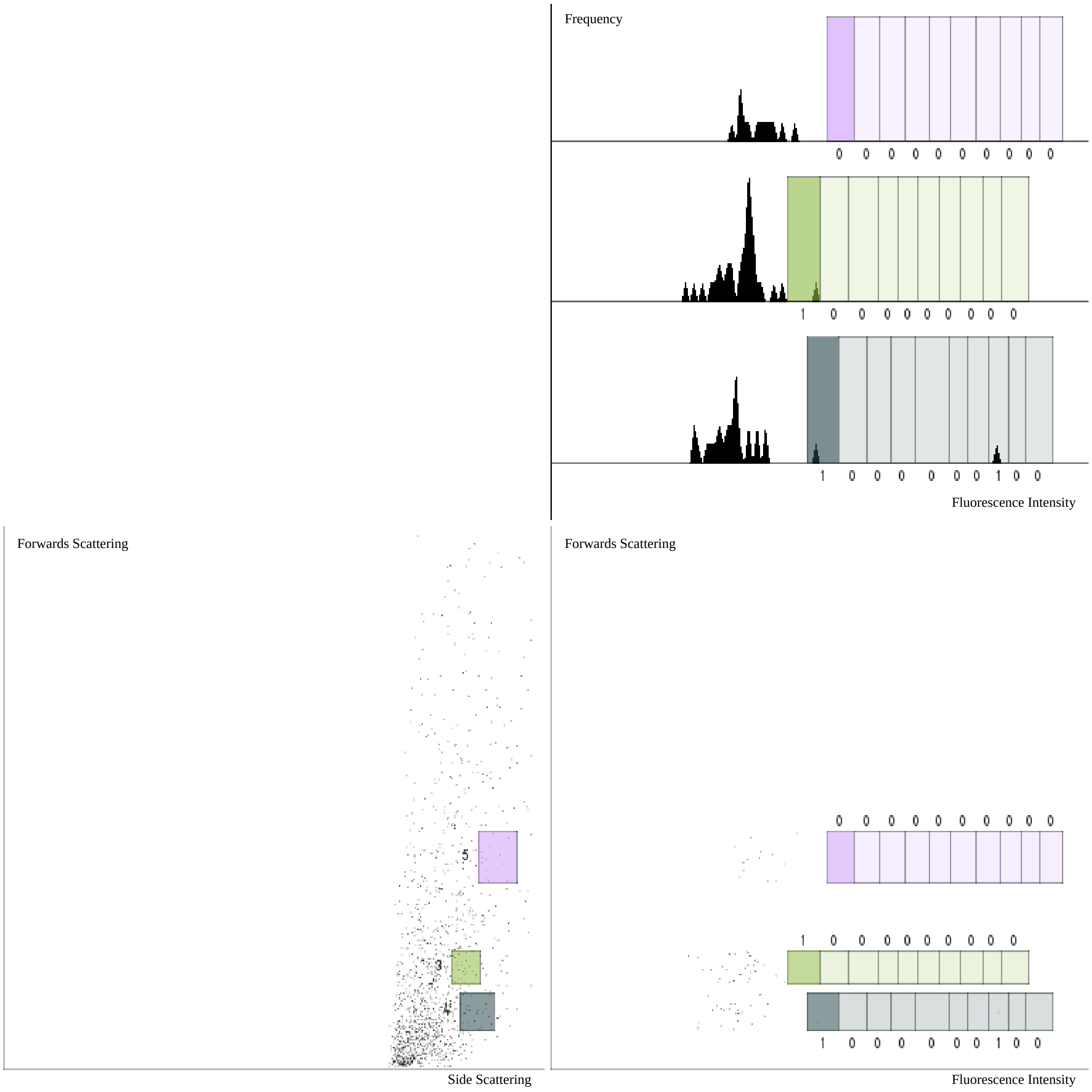

ANNEX 3: TAG DECONVOLUTION - BEAD 402

Passes flow sorting criteria: Yes

Passes tag deconvolution criteria: Yes

Included in protocol analysis: Yes

Protocol: 5, 3, 2, 7

Filename: Bin7_plateA0_D8.fcs

Split 1: Petrol shading

Split 2: Green shading

Split 3: Violet shading

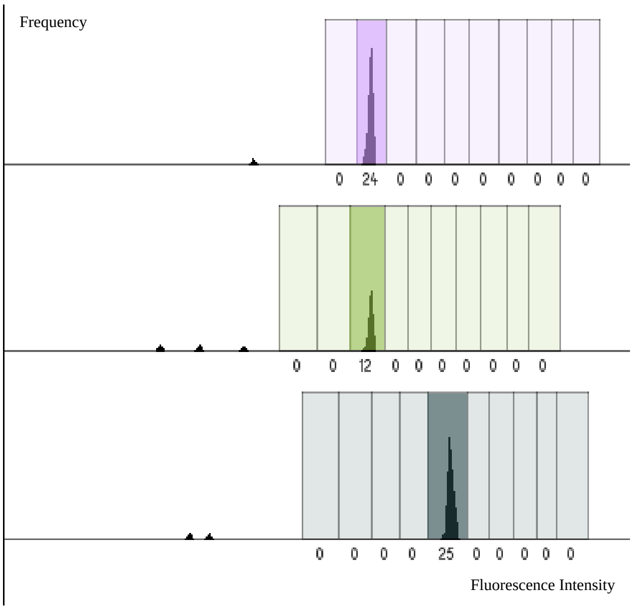

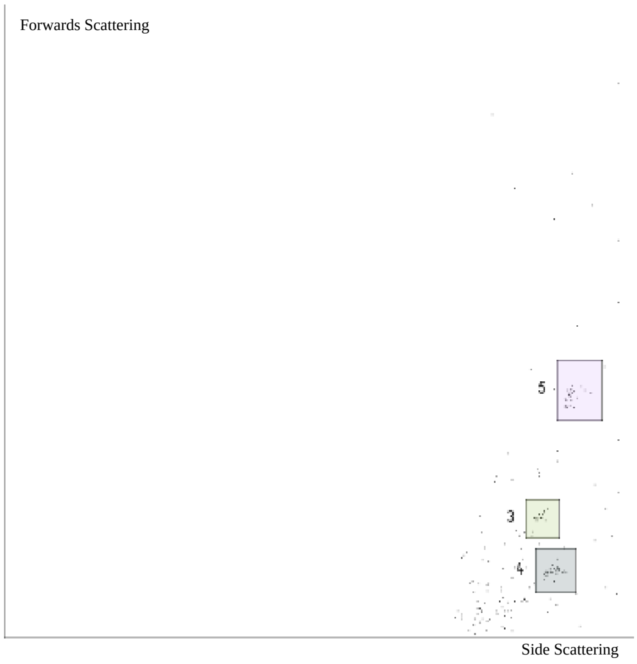

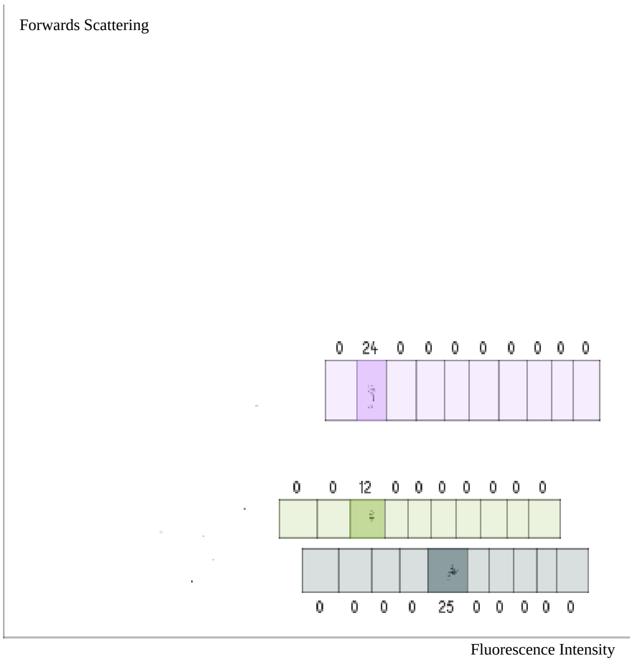

ANNEX 3: TAG DECONVOLUTION - BEAD 403

Passes flow sorting criteria: Yes

Passes tag deconvolution criteria: Yes

Included in protocol analysis: Yes

Protocol: 7, 3, 6, 7

Filename: Bin7_plateA0_D9.fcs

Split 1: Petrol shading

Split 2: Green shading

Split 3: Violet shading

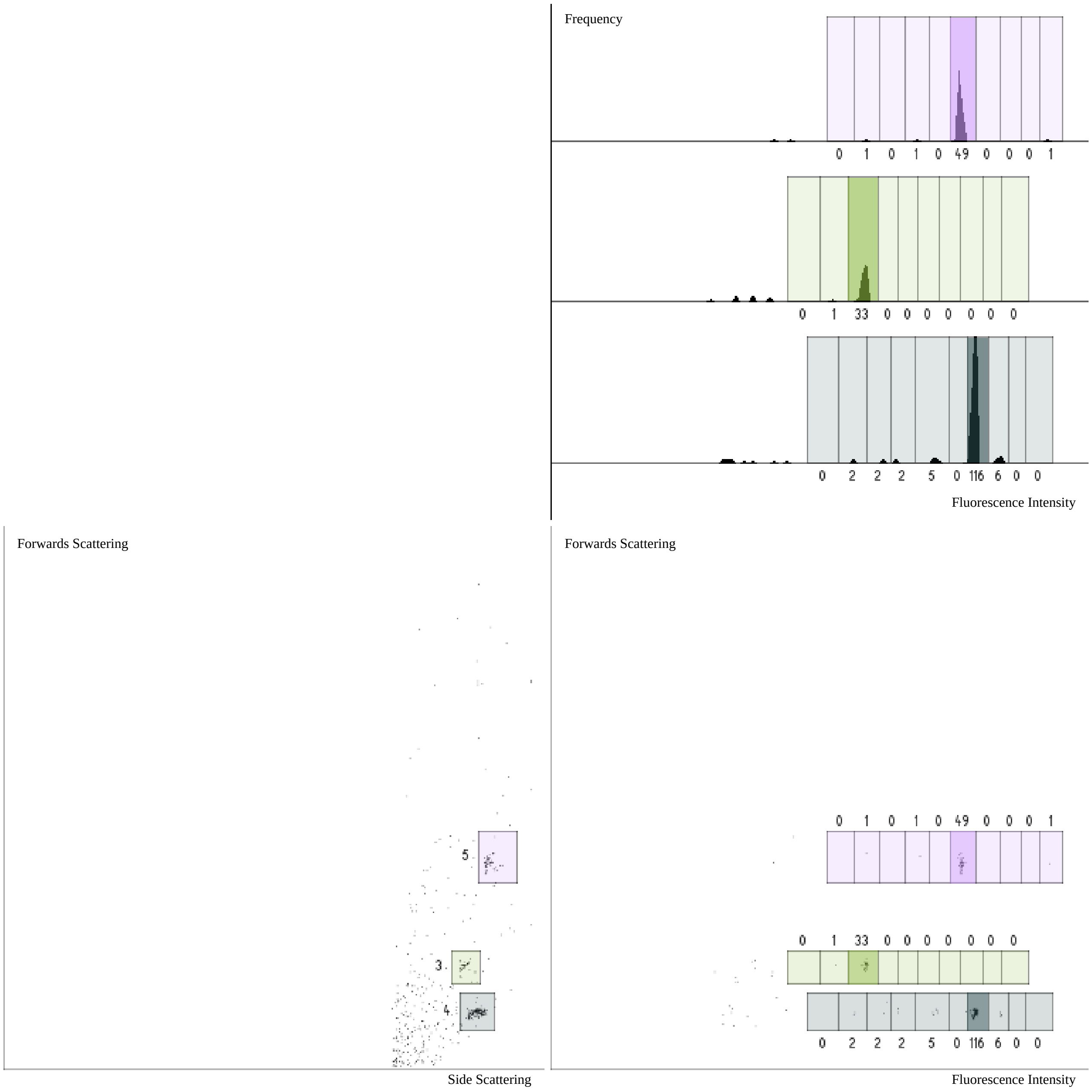

ANNEX 3: TAG DECONVOLUTION - BEAD 404

Passes flow sorting criteria: Yes

Passes tag deconvolution criteria: Yes

Included in protocol analysis: Yes

Protocol: 8, 2, 10, 7

Filename: Bin7_plateA0_D10.fcs

Split 1: Petrol shading

Split 2: Green shading

Split 3: Violet shading

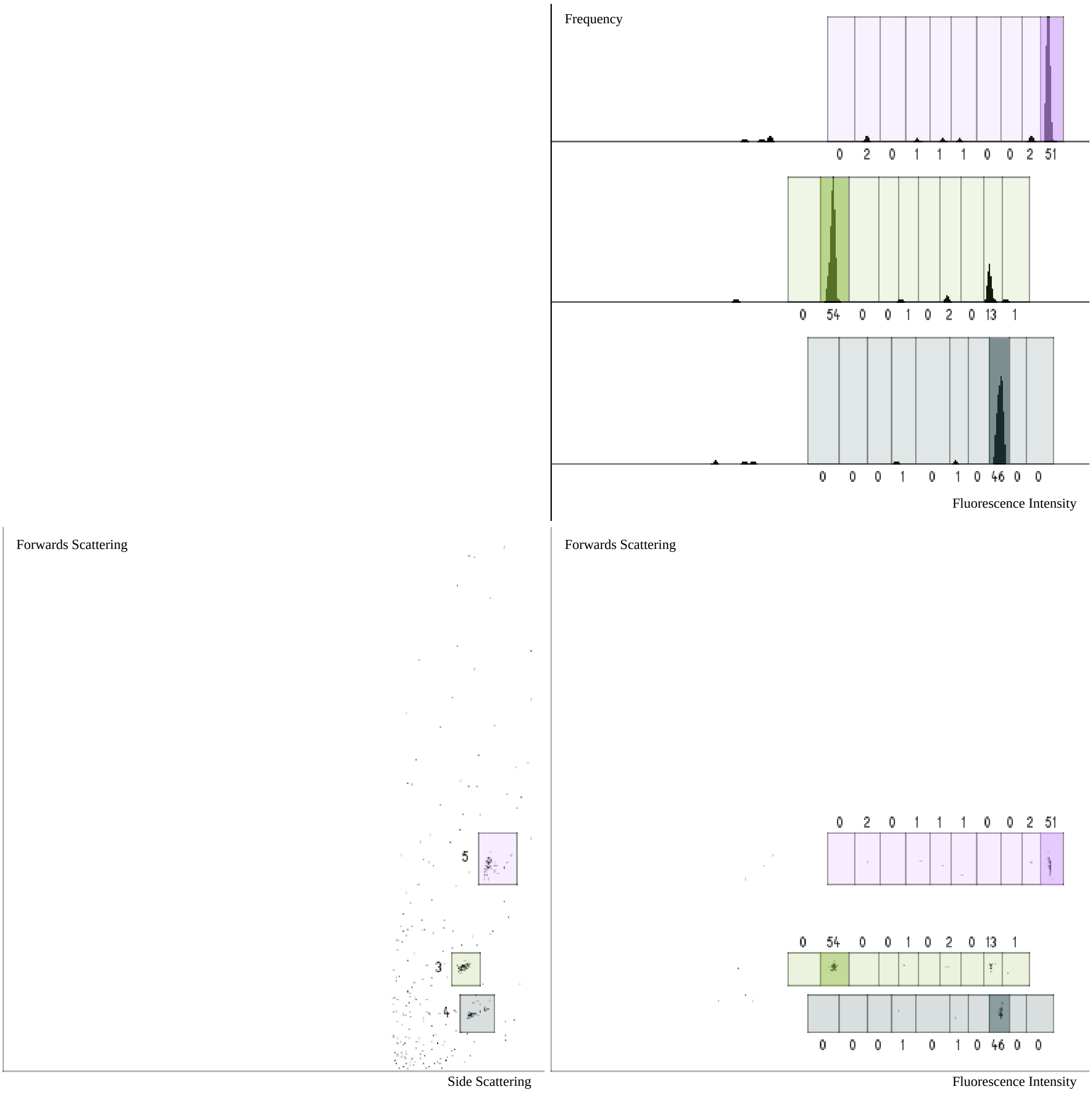

ANNEX 3: TAG DECONVOLUTION - BEAD 405

Passes flow sorting criteria: Yes

Passes tag deconvolution criteria: Yes

Included in protocol analysis: Yes

Protocol: 3, 3, 4, 7

Filename: Bin7_plateA0_D11.fcs

Split 1: Petrol shading

Split 2: Green shading

Split 3: Violet shading

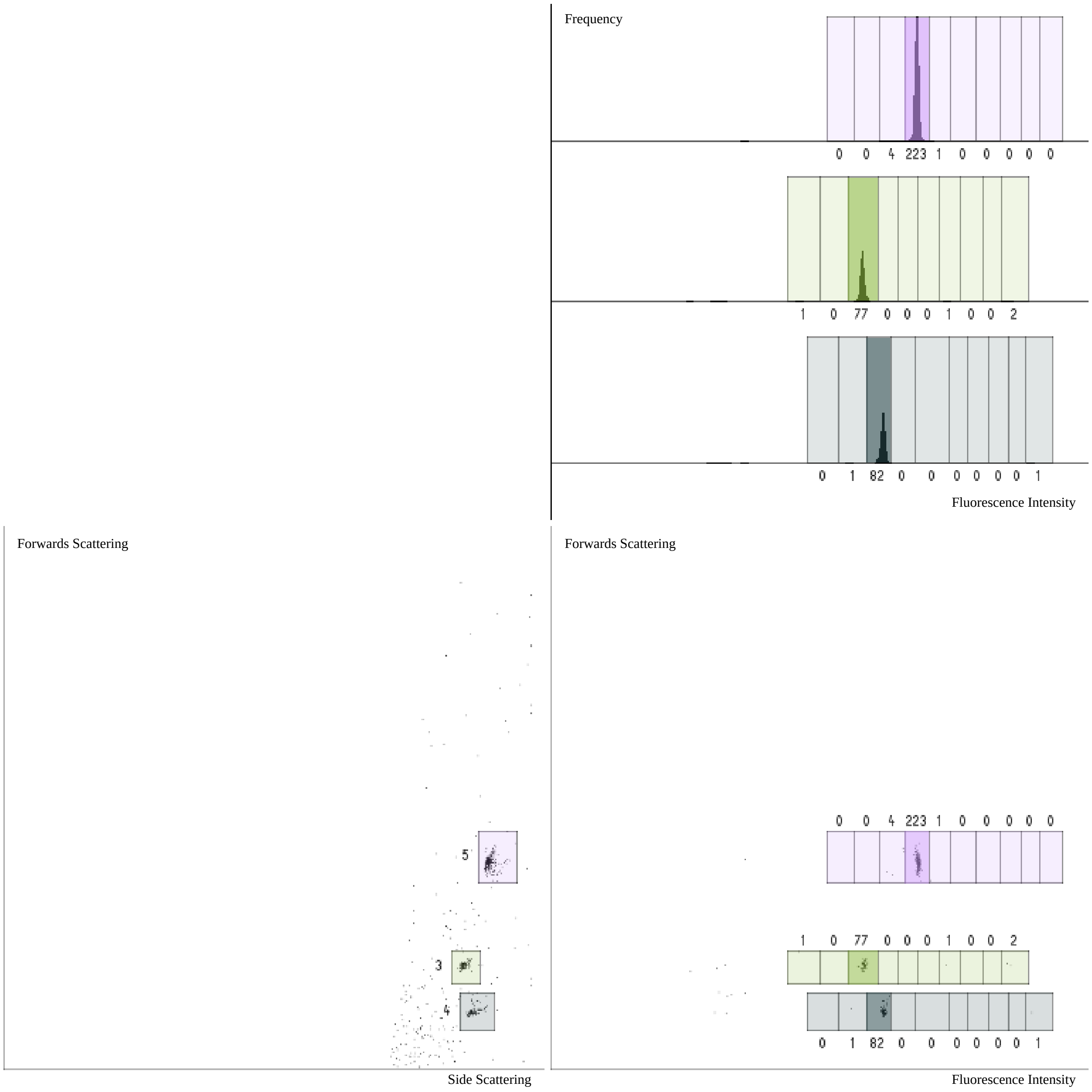

ANNEX 3: TAG DECONVOLUTION - BEAD 406

Passes flow sorting criteria: Yes

Passes tag deconvolution criteria: Yes

Included in protocol analysis: Yes

Protocol: 10, 4, 9, 7

Filename: Bin7_plateA0_D12.fcs

Split 1: Petrol shading

Split 2: Green shading

Split 3: Violet shading

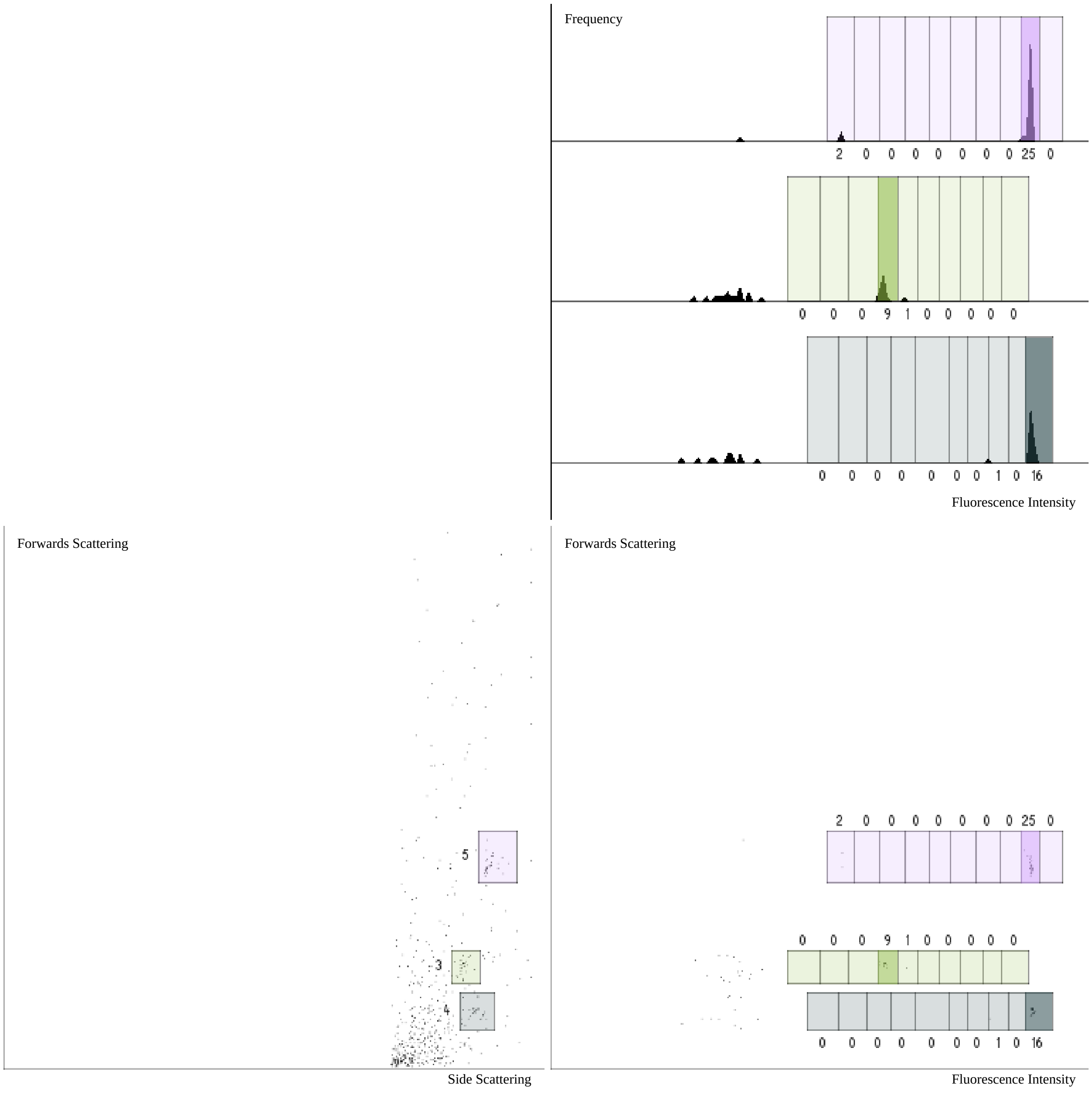

ANNEX 3: TAG DECONVOLUTION - BEAD 407

Passes flow sorting criteria: Yes

Passes tag deconvolution criteria: No

Included in protocol analysis: No

Protocol: N/A

Filename: Bin8_plateA0_F1.fcs

Split 1: Petrol shading

Split 2: Green shading

Split 3: Violet shading

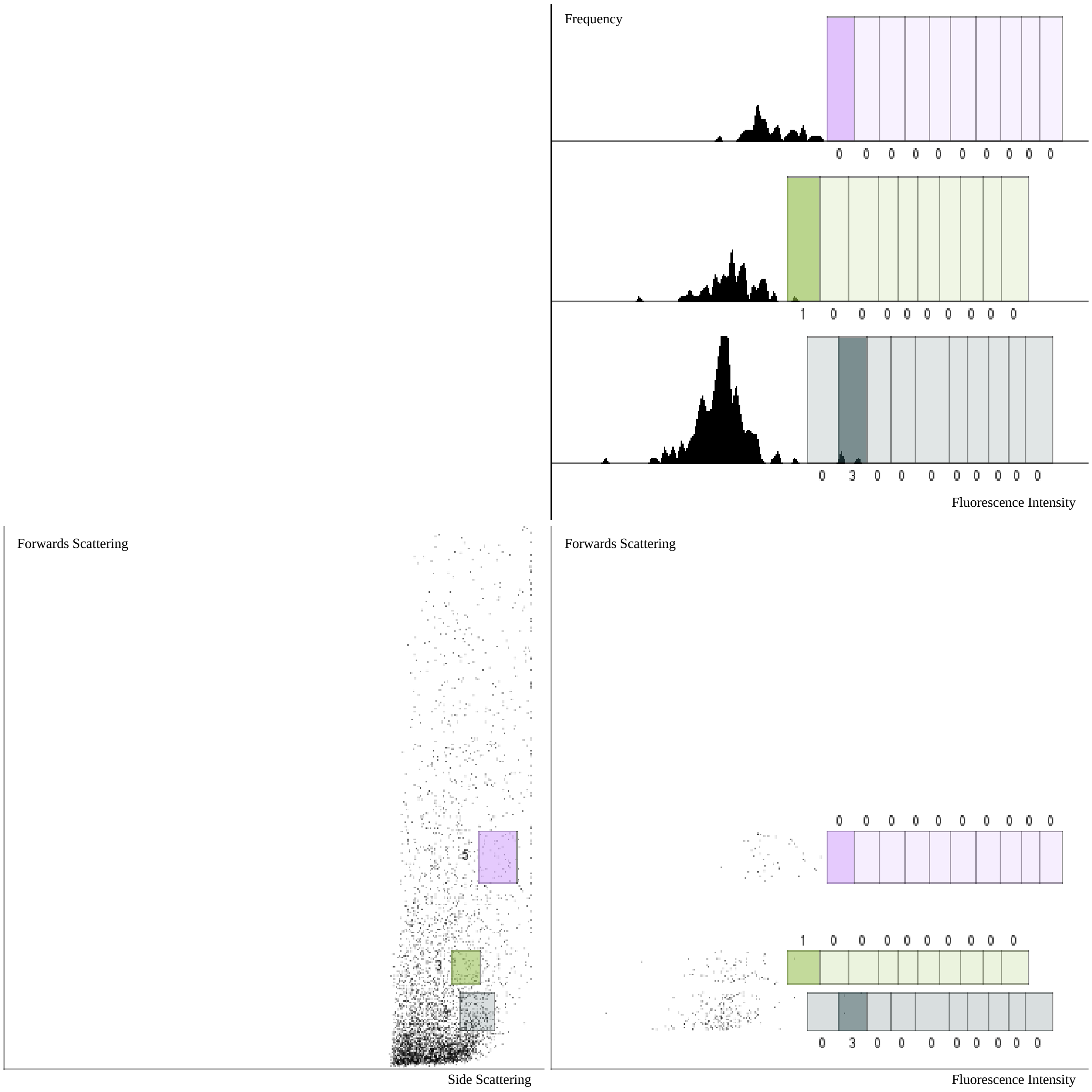

ANNEX 3: TAG DECONVOLUTION - BEAD 408

Passes flow sorting criteria: Yes

Passes tag deconvolution criteria: Yes

Included in protocol analysis: Yes

Protocol: 1, 5, 1, 8

Filename: Bin8_plateA0_F2.fcs

Split 1: Petrol shading

Split 2: Green shading

Split 3: Violet shading

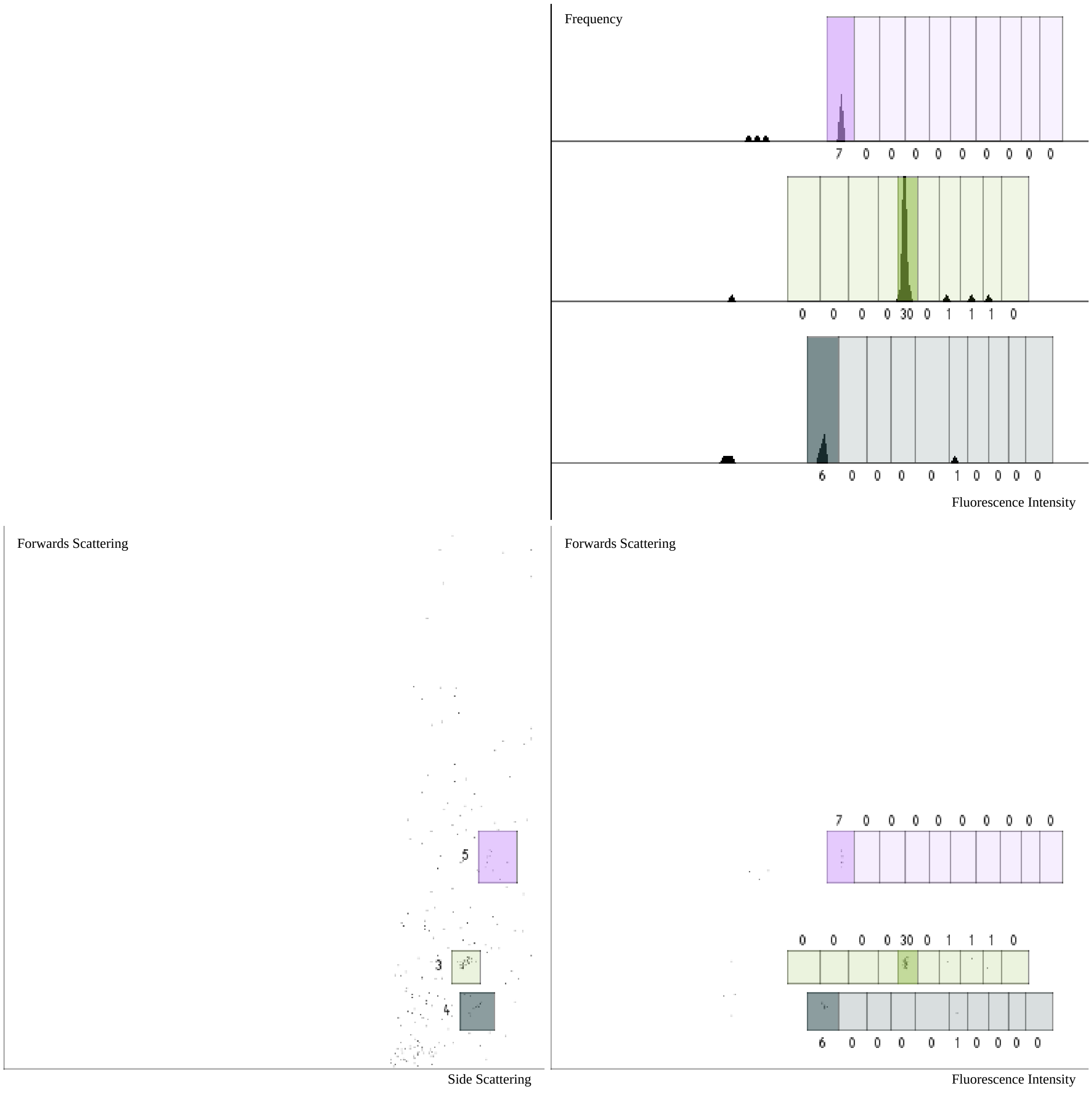

ANNEX 3: TAG DECONVOLUTION - BEAD 409

Passes flow sorting criteria: Yes

Passes tag deconvolution criteria: Yes

Included in protocol analysis: Yes

Protocol: 8, 5, 9, 8

Filename: Bin8_plateA0_F3.fcs

Split 1: Petrol shading

Split 2: Green shading

Split 3: Violet shading

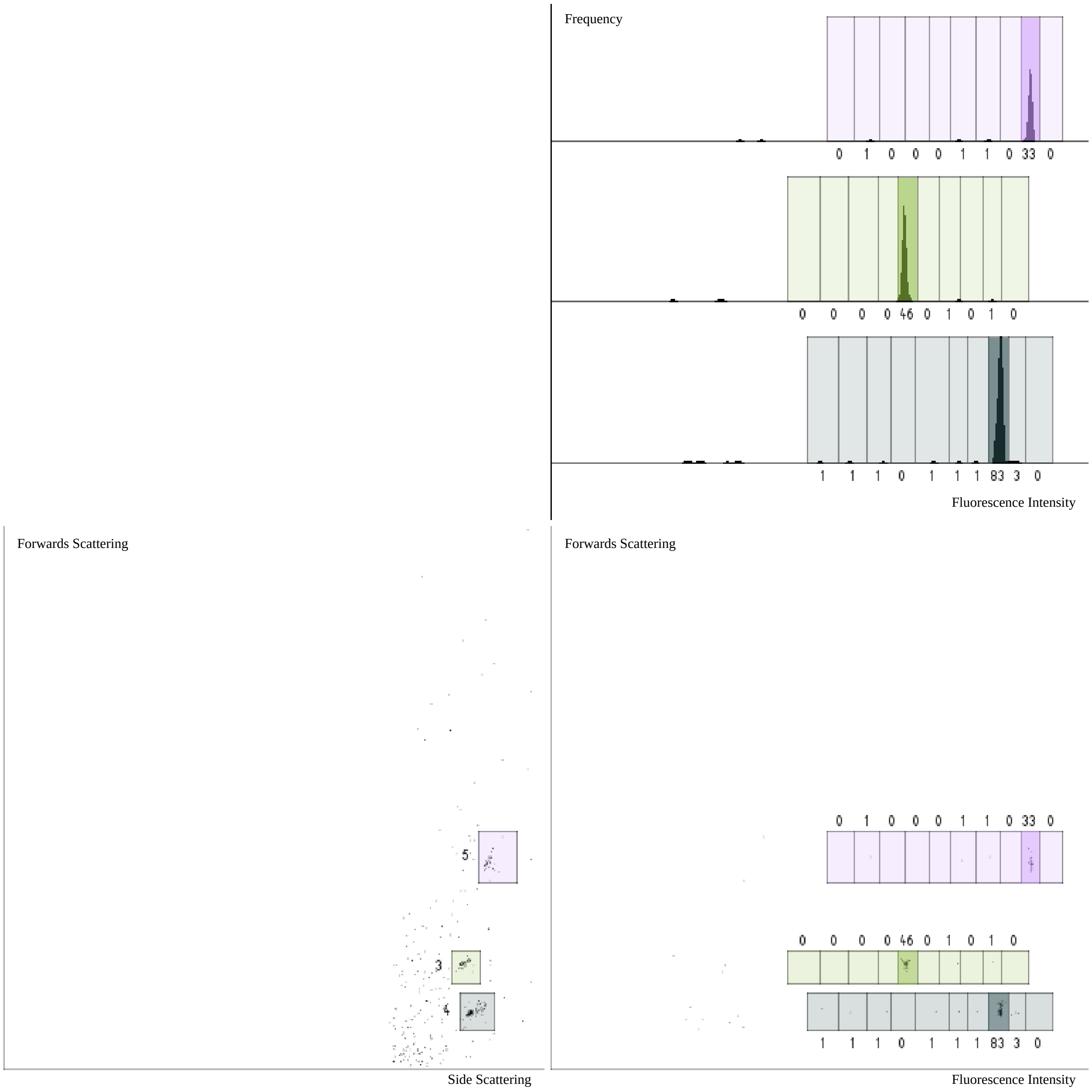

ANNEX 3: TAG DECONVOLUTION - BEAD 410

Passes flow sorting criteria: Yes

Passes tag deconvolution criteria: Yes

Included in protocol analysis: Yes

Protocol: 7, 9, 10, 8

Filename: Bin8_plateA0_F4.fcs

Split 1: Petrol shading

Split 2: Green shading

Split 3: Violet shading

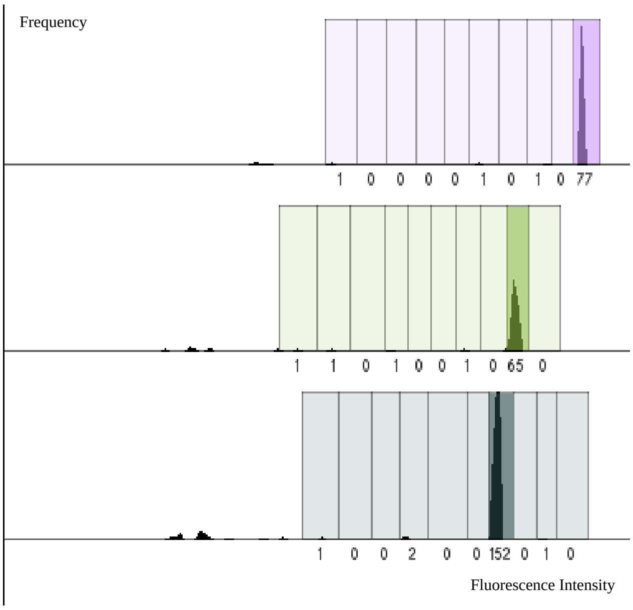

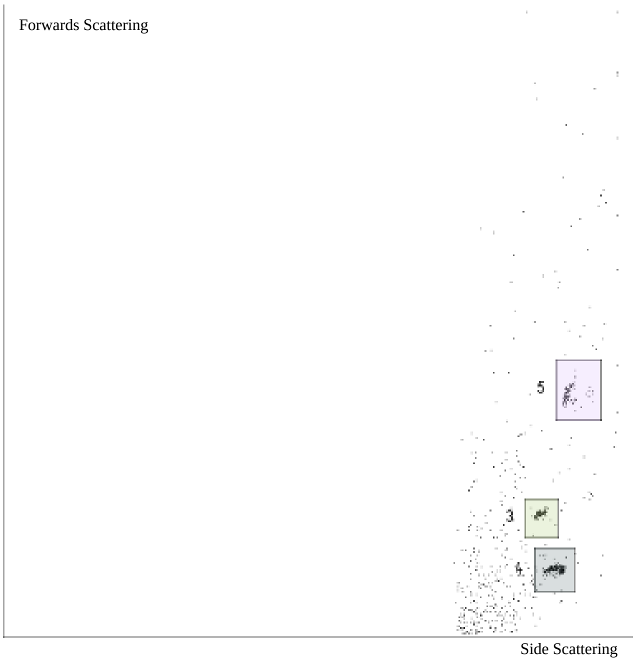

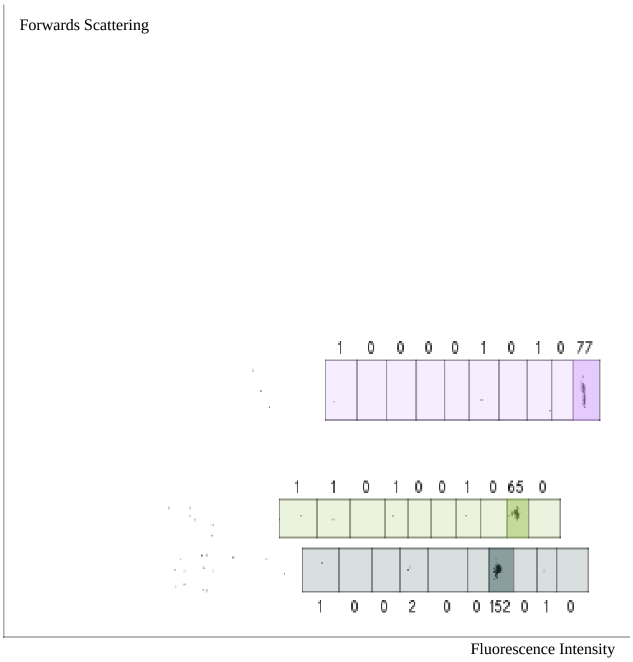

ANNEX 3: TAG DECONVOLUTION - BEAD 411

Passes flow sorting criteria: Yes

Passes tag deconvolution criteria: Yes

Included in protocol analysis: Yes

Protocol: 7, 6, 1, 8

Filename: Bin8_plateA0_F5.fcs

Split 1: Petrol shading

Split 2: Green shading

Split 3: Violet shading

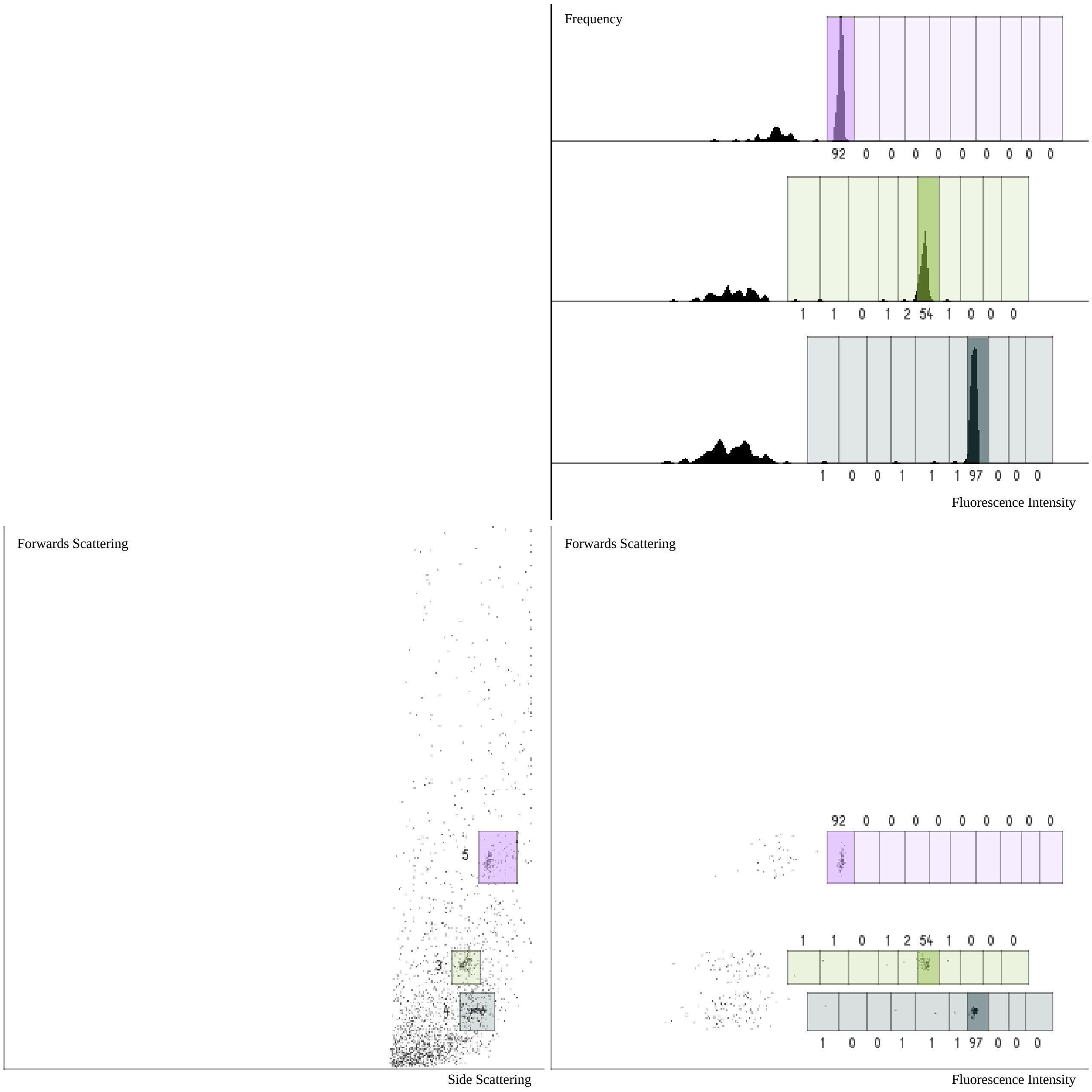

ANNEX 3: TAG DECONVOLUTION - BEAD 412

Passes flow sorting criteria: Yes

Passes tag deconvolution criteria: Yes

Included in protocol analysis: Yes

Protocol: 6, 6, 6, 8

Filename: Bin8_plateA0_F6.fcs

Split 1: Petrol shading

Split 2: Green shading

Split 3: Violet shading

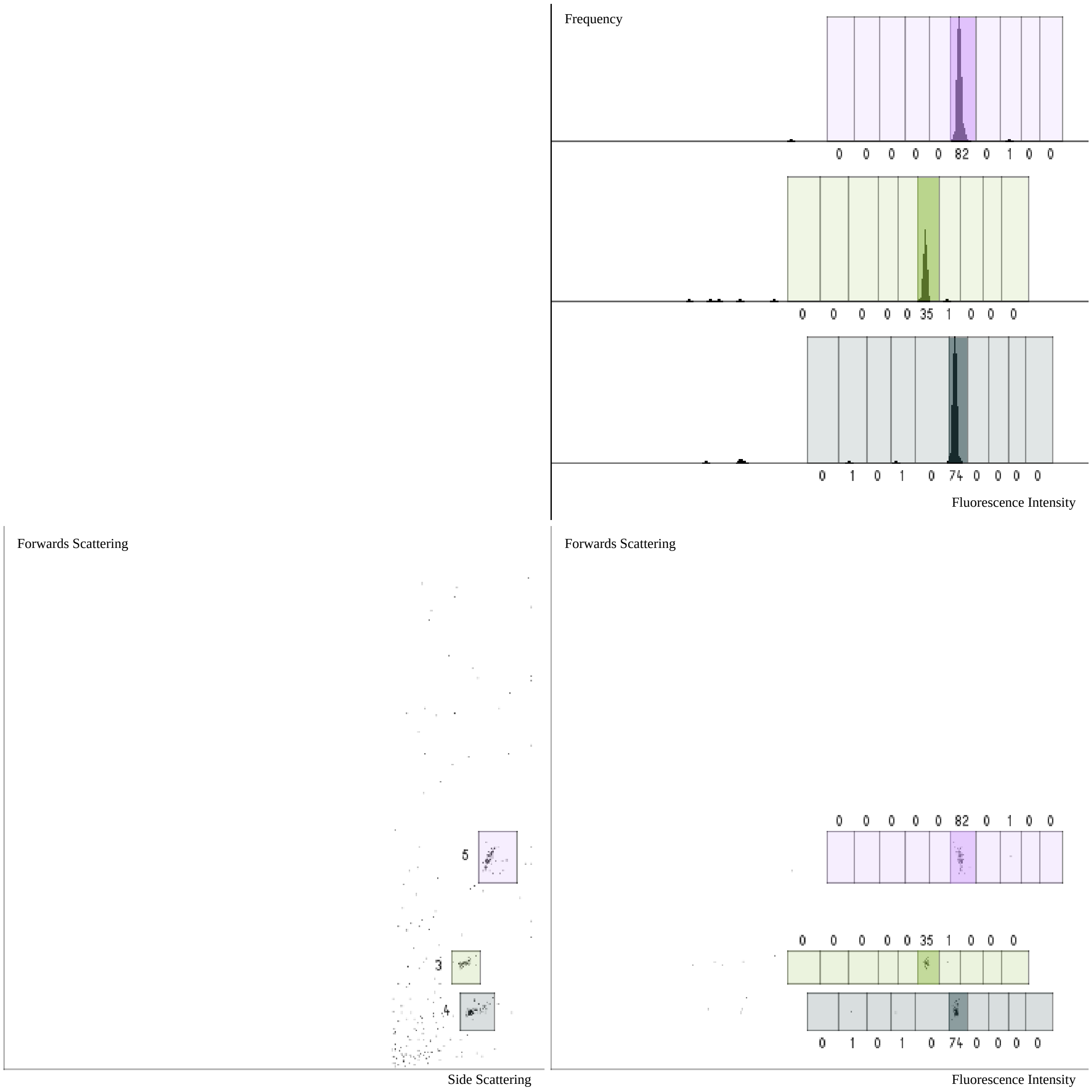

ANNEX 3: TAG DECONVOLUTION - BEAD 413

Passes flow sorting criteria: Yes

Passes tag deconvolution criteria: Yes

Included in protocol analysis: Yes

Protocol: 2, 3, 5, 8

Filename: Bin8_plateA0_F7.fcs

Split 1: Petrol shading

Split 2: Green shading

Split 3: Violet shading

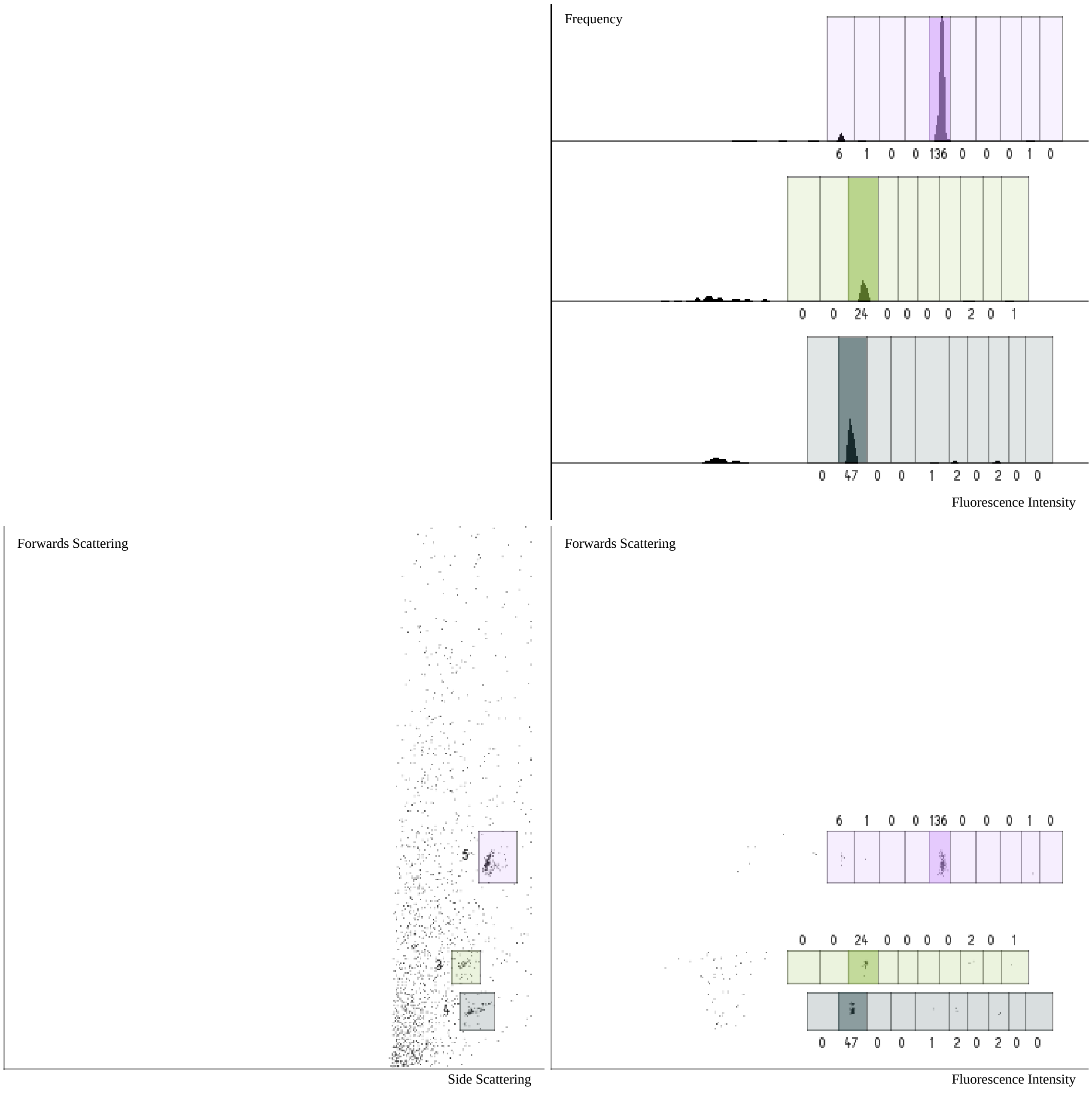

ANNEX 3: TAG DECONVOLUTION - BEAD 414

Passes flow sorting criteria: Yes

Passes tag deconvolution criteria: No

Included in protocol analysis: No

Protocol: N/A

Filename: Bin8_plateA0_F8.fcs

Split 1: Petrol shading

Split 2: Green shading

Split 3: Violet shading

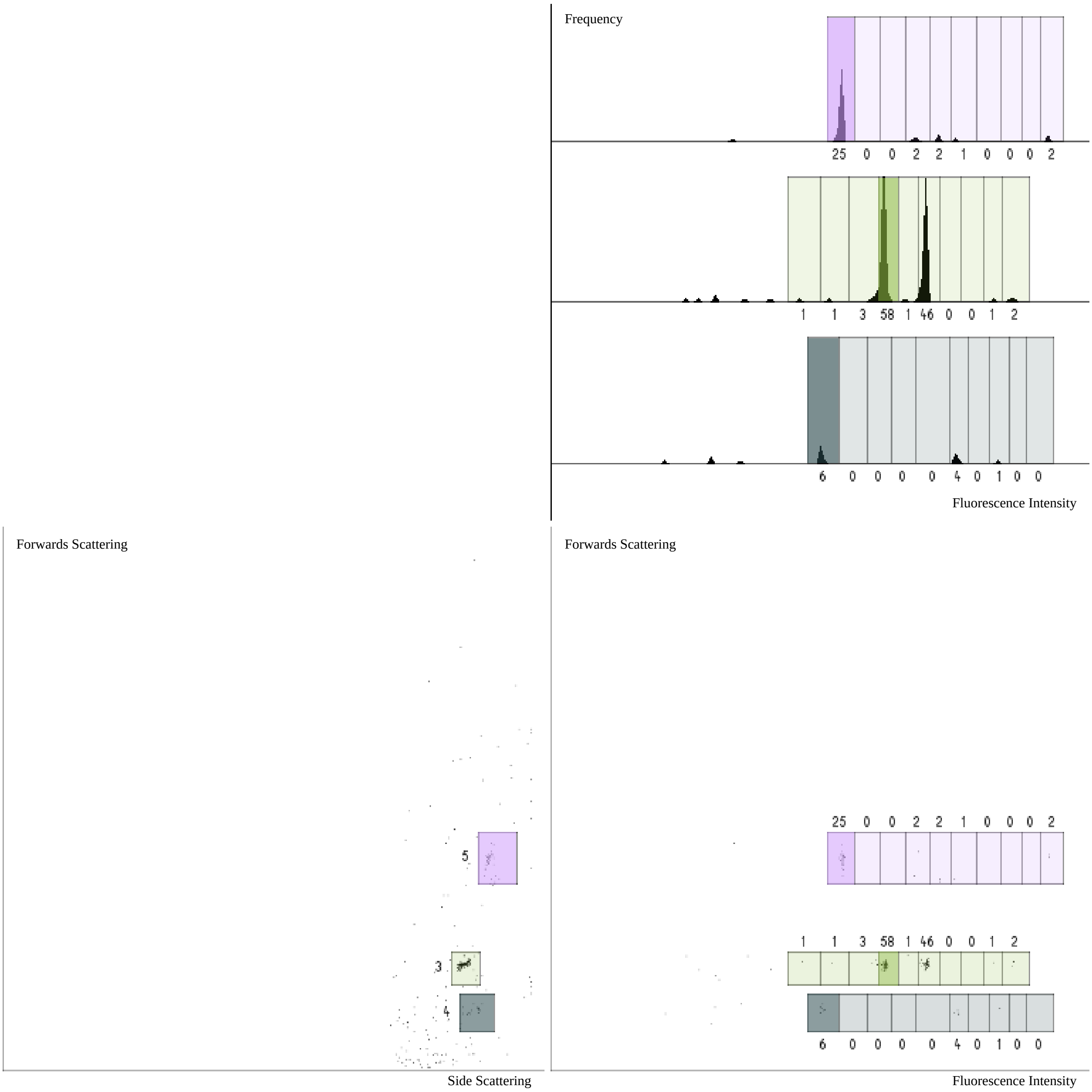

ANNEX 3: TAG DECONVOLUTION - BEAD 415

Passes flow sorting criteria: Yes

Passes tag deconvolution criteria: No

Included in protocol analysis: No

Protocol: N/A

Filename: Bin8_plateA0_F9.fcs

Split 1: Petrol shading

Split 2: Green shading

Split 3: Violet shading

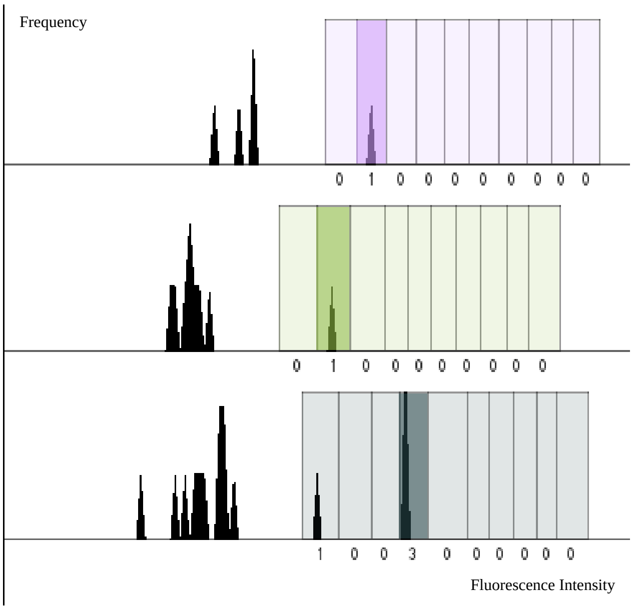

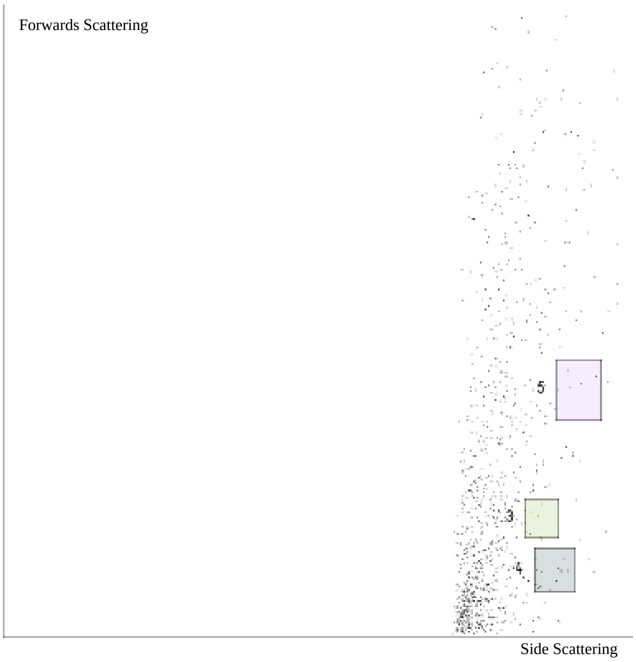

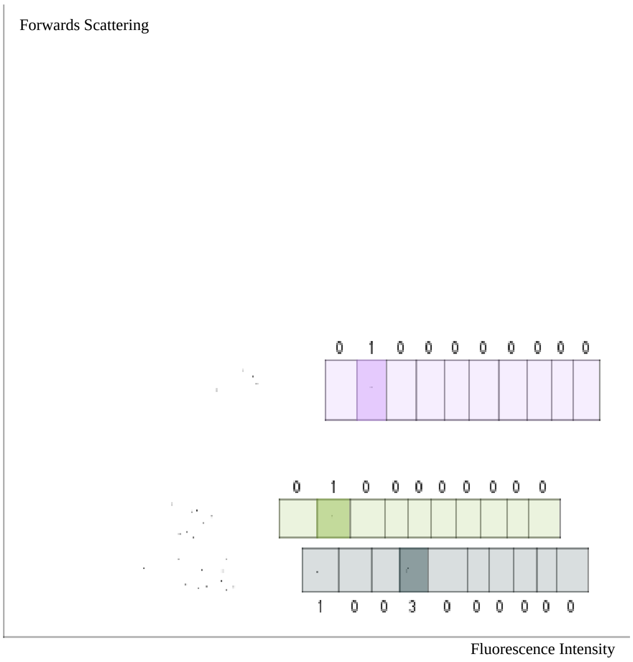

ANNEX 3: TAG DECONVOLUTION - BEAD 416

Passes flow sorting criteria: Yes

Passes tag deconvolution criteria: No

Included in protocol analysis: No

Protocol: N/A

Filename: Bin8_plateA0_F10.fcs

Split 1: Petrol shading

Split 2: Green shading

Split 3: Violet shading

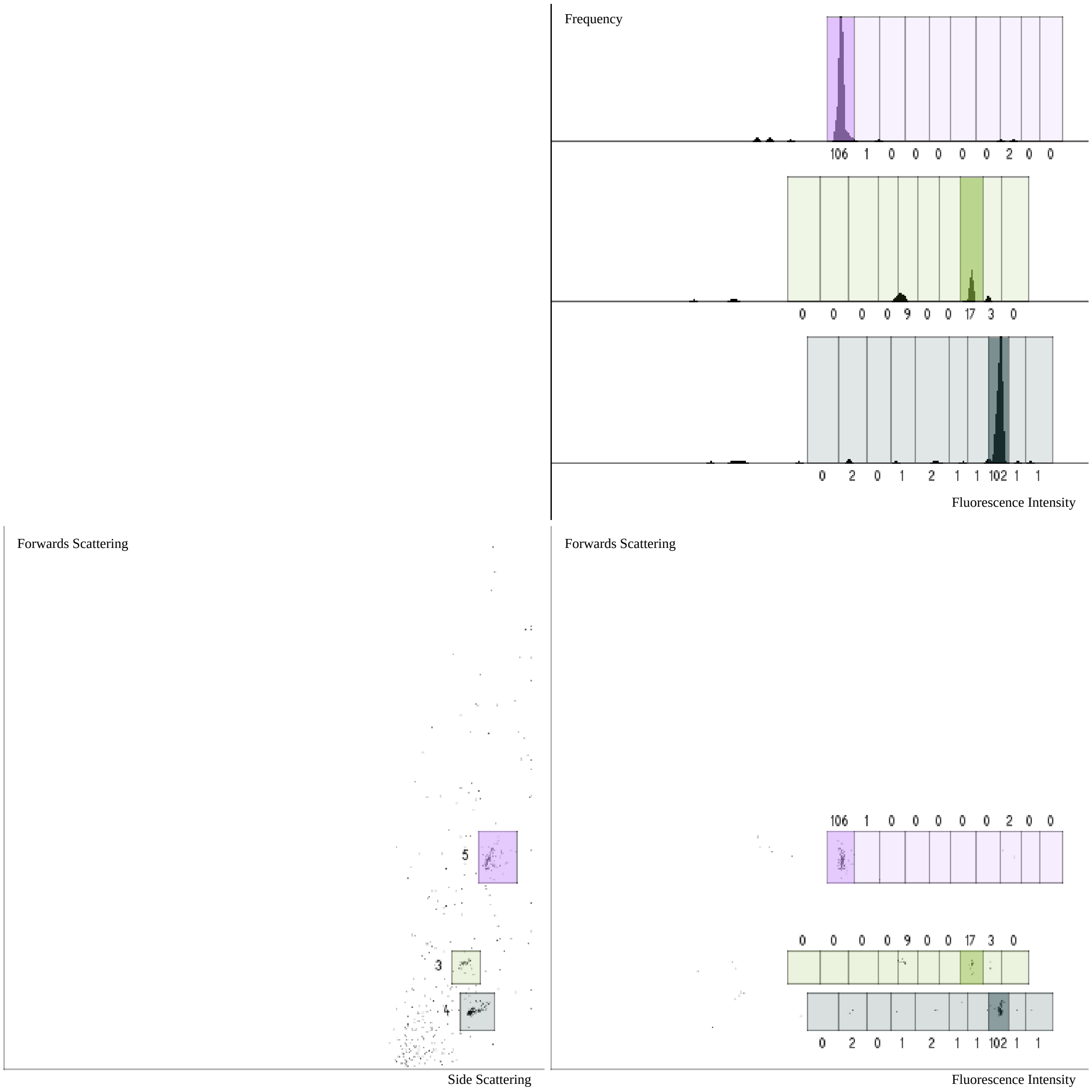

ANNEX 3: TAG DECONVOLUTION - BEAD 417

Passes flow sorting criteria: Yes

Passes tag deconvolution criteria: No

Included in protocol analysis: No

Protocol: N/A

Filename: Bin8_plateA0_F11.fcs

Split 1: Petrol shading

Split 2: Green shading

Split 3: Violet shading

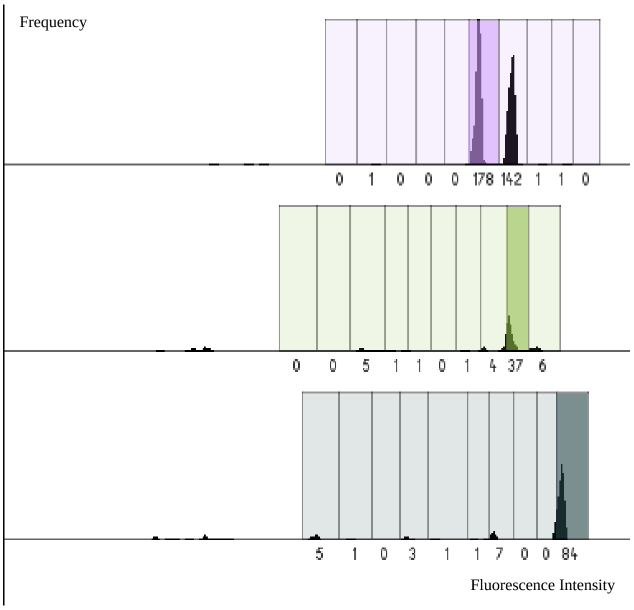

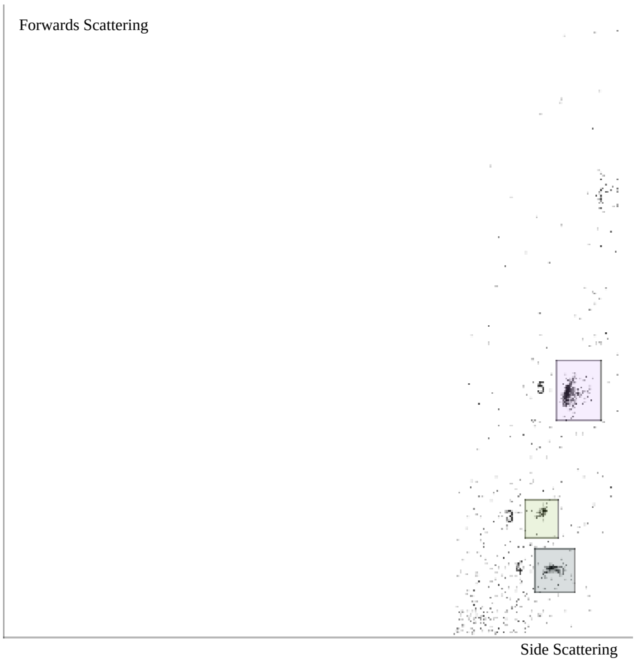

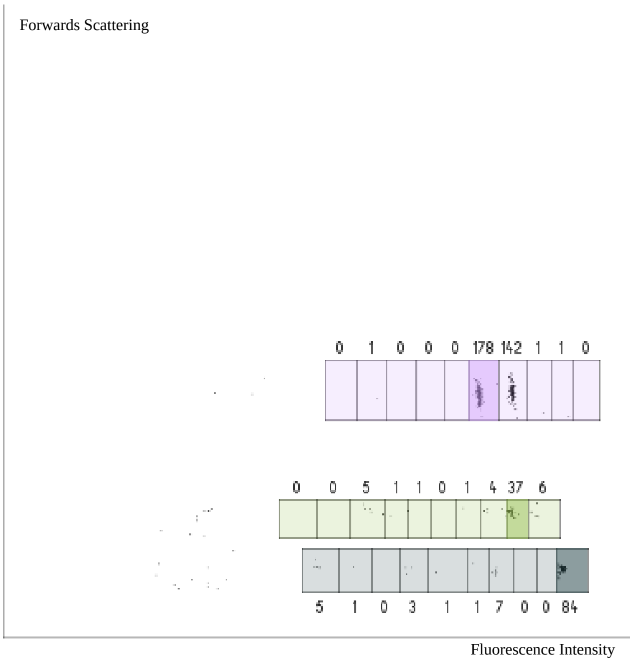

ANNEX 3: TAG DECONVOLUTION - BEAD 418

Passes flow sorting criteria: Yes

Passes tag deconvolution criteria: Yes

Included in protocol analysis: Yes

Protocol: 2, 5, 7, 8

Filename: Bin8_plateA0_F12.fcs

Split 1: Petrol shading

Split 2: Green shading

Split 3: Violet shading

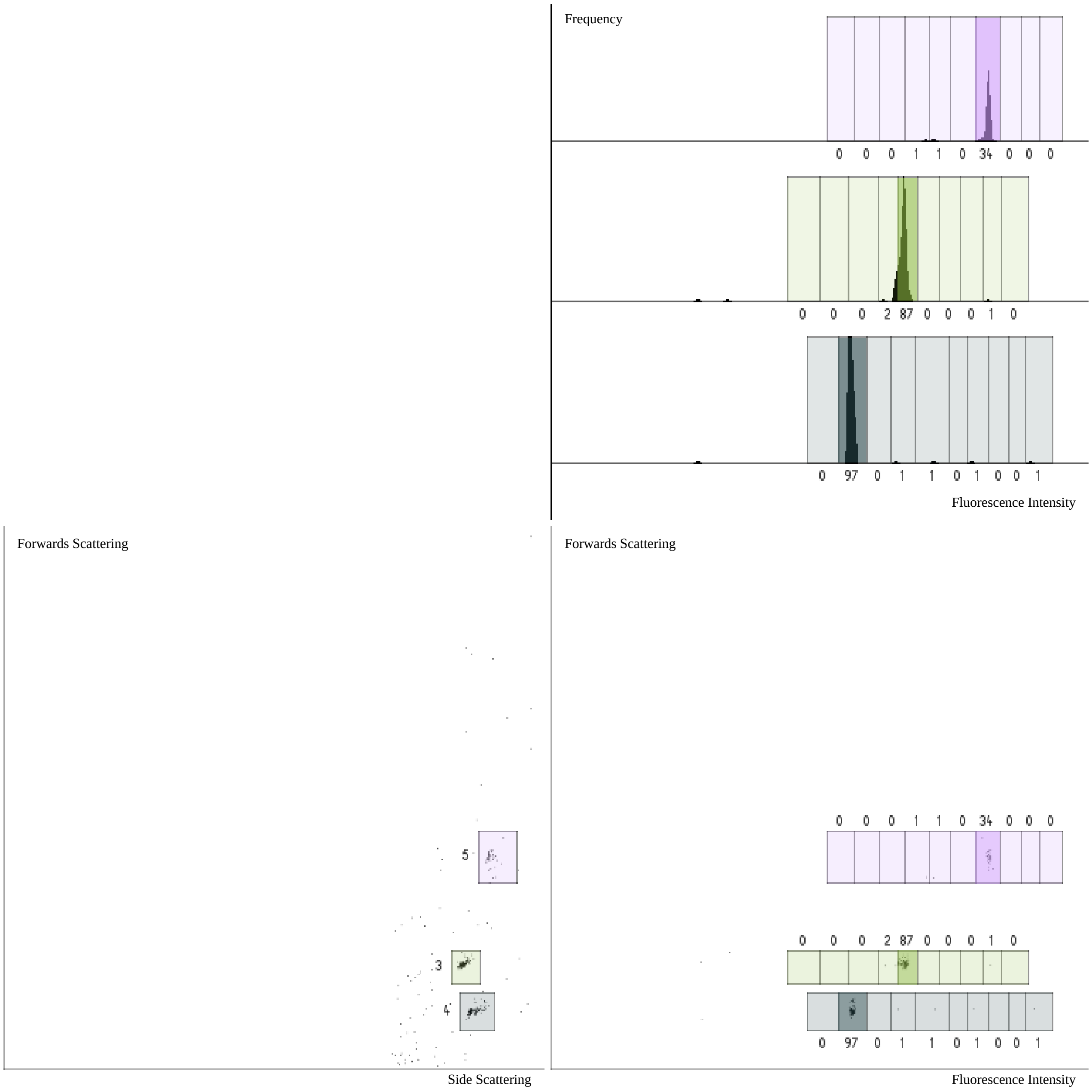

ANNEX 3: TAG DECONVOLUTION - BEAD 419

Passes flow sorting criteria: Yes

Passes tag deconvolution criteria: No

Included in protocol analysis: No

Protocol: N/A

Filename: Bin8_plateA0_G4.fcs

Split 1: Petrol shading

Split 2: Green shading

Split 3: Violet shading

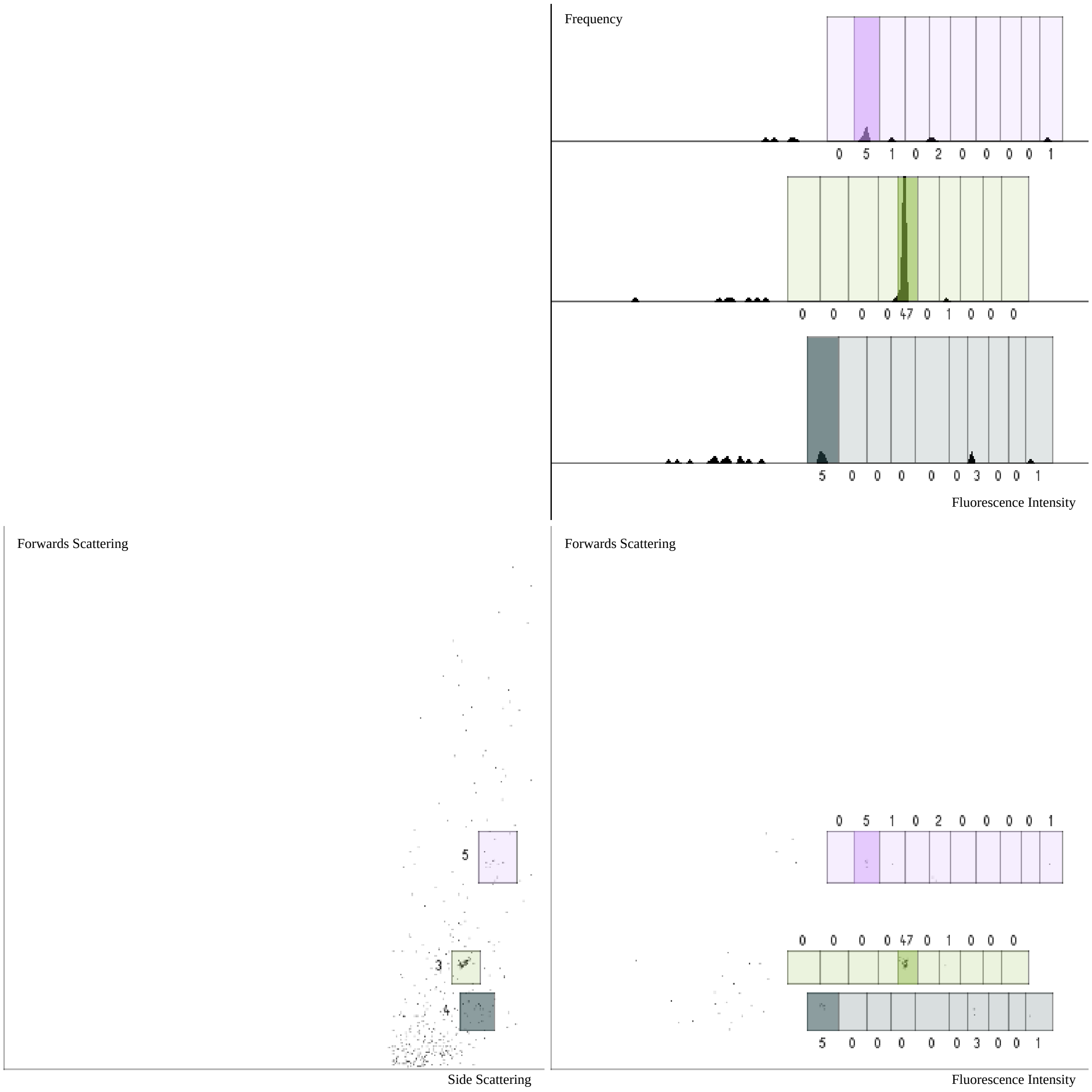

ANNEX 3: TAG DECONVOLUTION - BEAD 420

Passes flow sorting criteria: Yes

Passes tag deconvolution criteria: Yes

Included in protocol analysis: Yes

Protocol: 1, 8, 1, 8

Filename: Bin8_plateA0_G5.fcs

Split 1: Petrol shading

Split 2: Green shading

Split 3: Violet shading

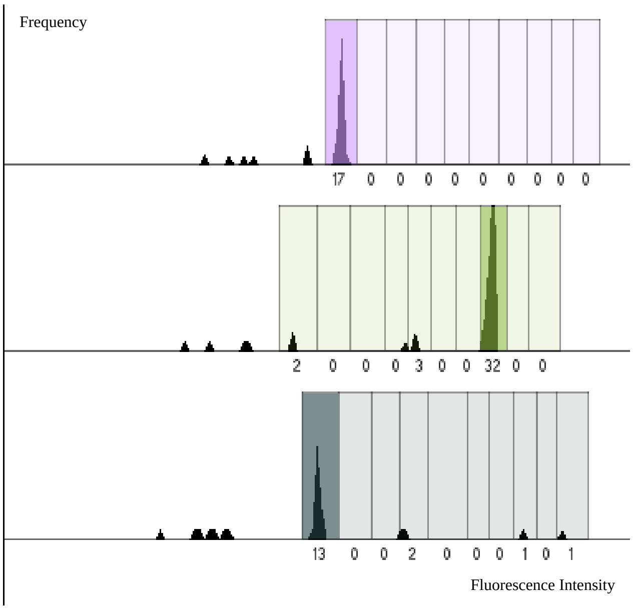

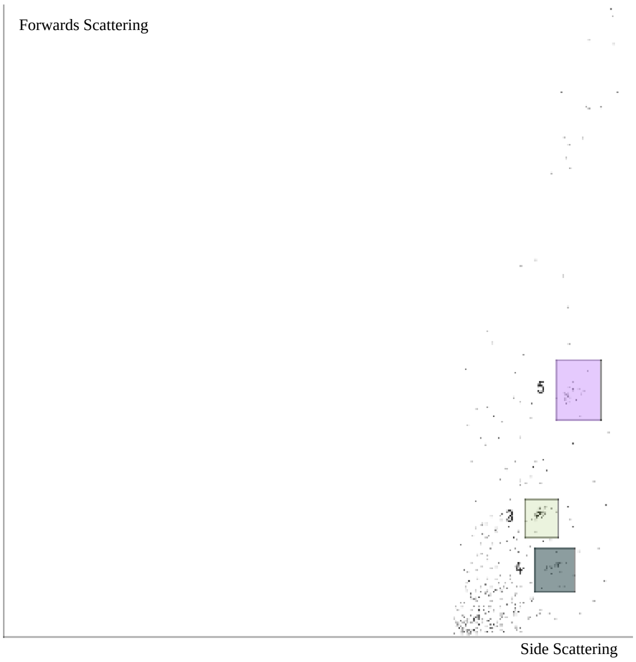

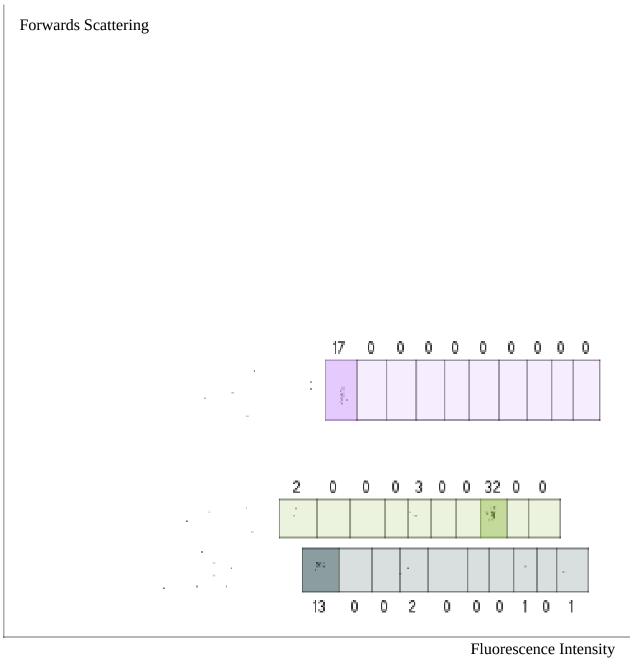

ANNEX 3: TAG DECONVOLUTION - BEAD 421

Passes flow sorting criteria: Yes

Passes tag deconvolution criteria: No

Included in protocol analysis: No

Protocol: N/A

Filename: Bin8_plateA0_G6.fcs

Split 1: Petrol shading

Split 2: Green shading

Split 3: Violet shading

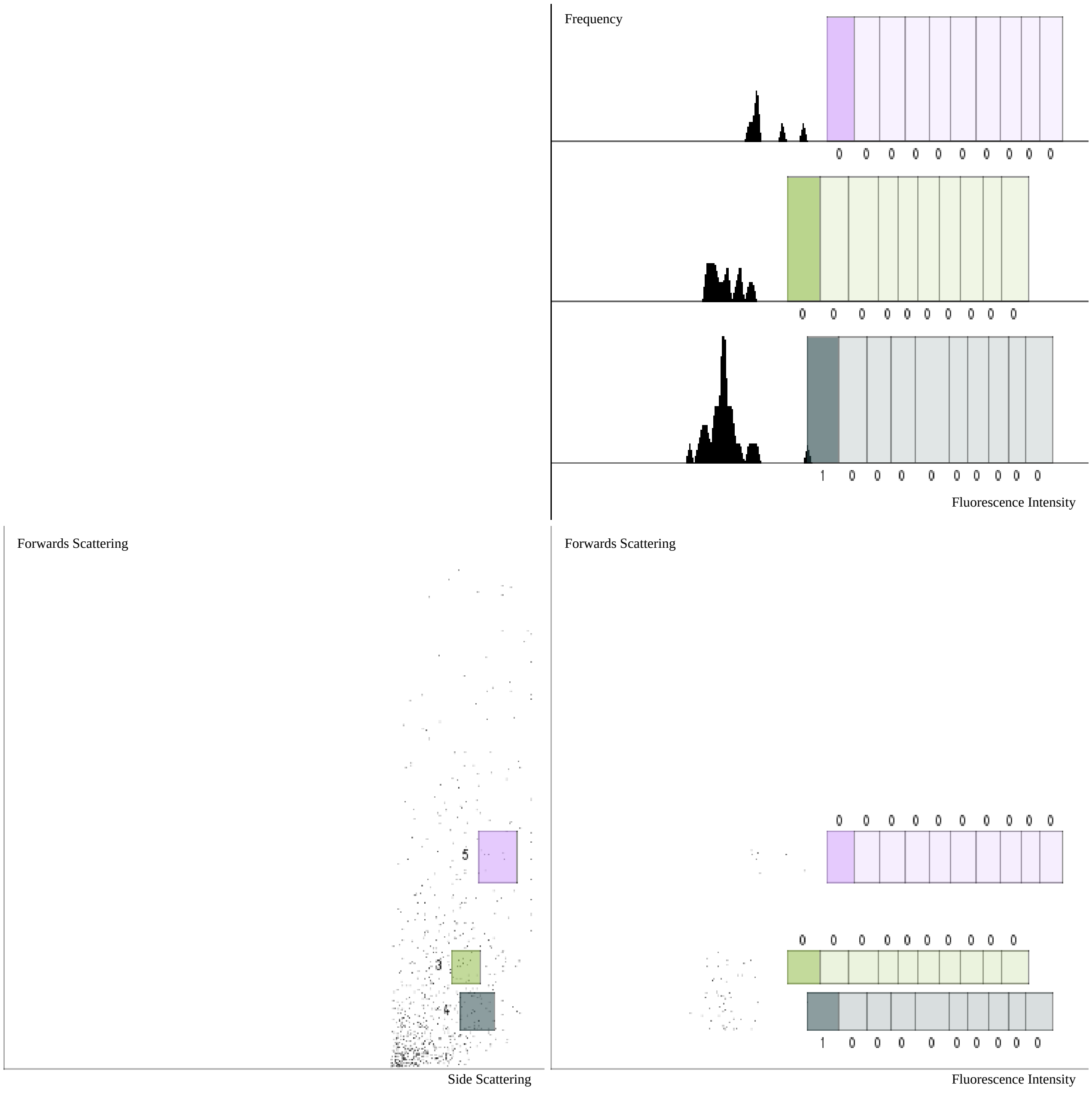

ANNEX 3: TAG DECONVOLUTION - BEAD 422

Passes flow sorting criteria: Yes

Passes tag deconvolution criteria: Yes

Included in protocol analysis: Yes

Protocol: 9, 9, 9, 8

Filename: Bin8_plateA0_G7.fcs

Split 1: Petrol shading

Split 2: Green shading

Split 3: Violet shading

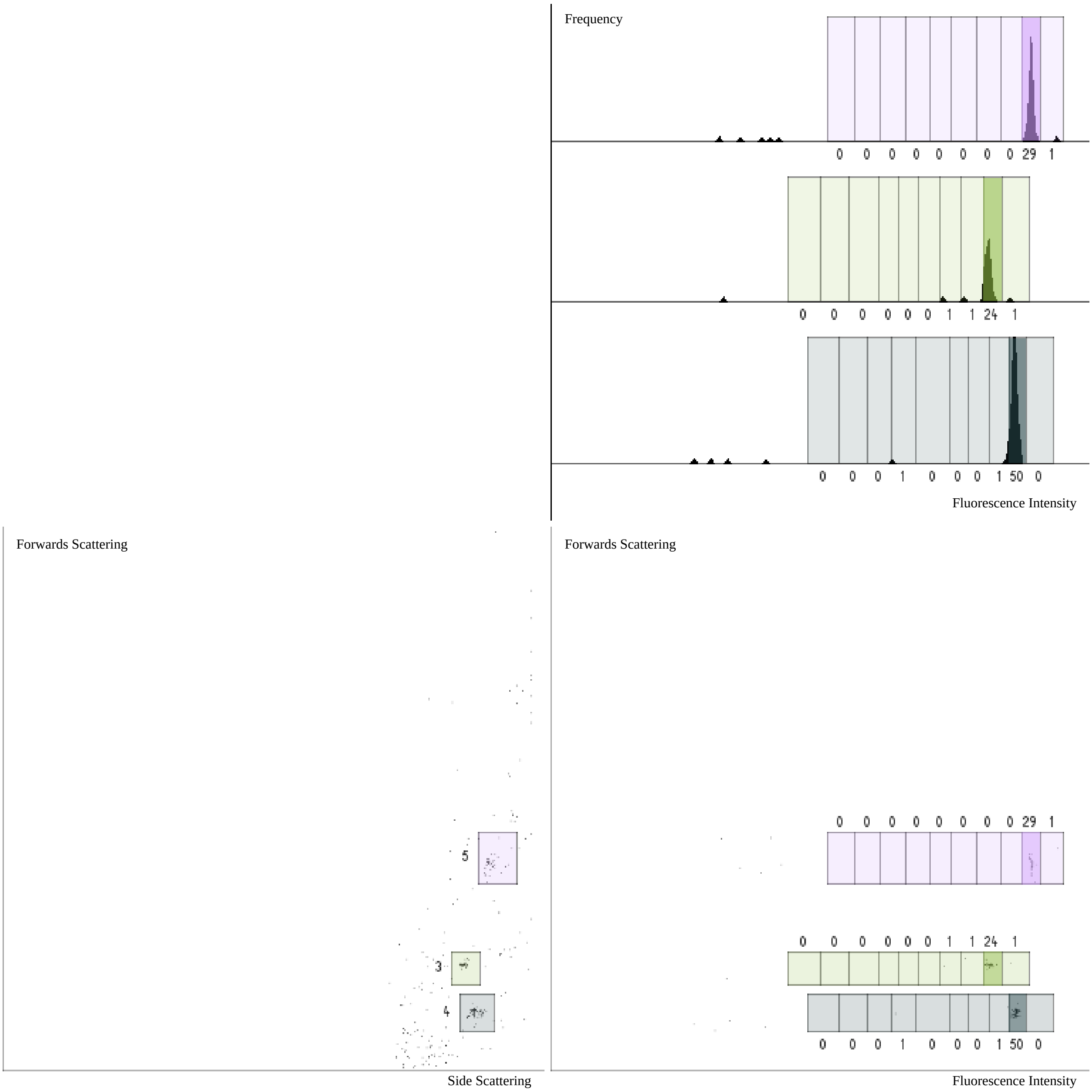

ANNEX 3: TAG DECONVOLUTION - BEAD 423

Passes flow sorting criteria: Yes

Passes tag deconvolution criteria: Yes

Included in protocol analysis: Yes

Protocol: 5, 9, 9, 8

Filename: Bin8_plateA0_G8.fcs

Split 1: Petrol shading

Split 2: Green shading

Split 3: Violet shading

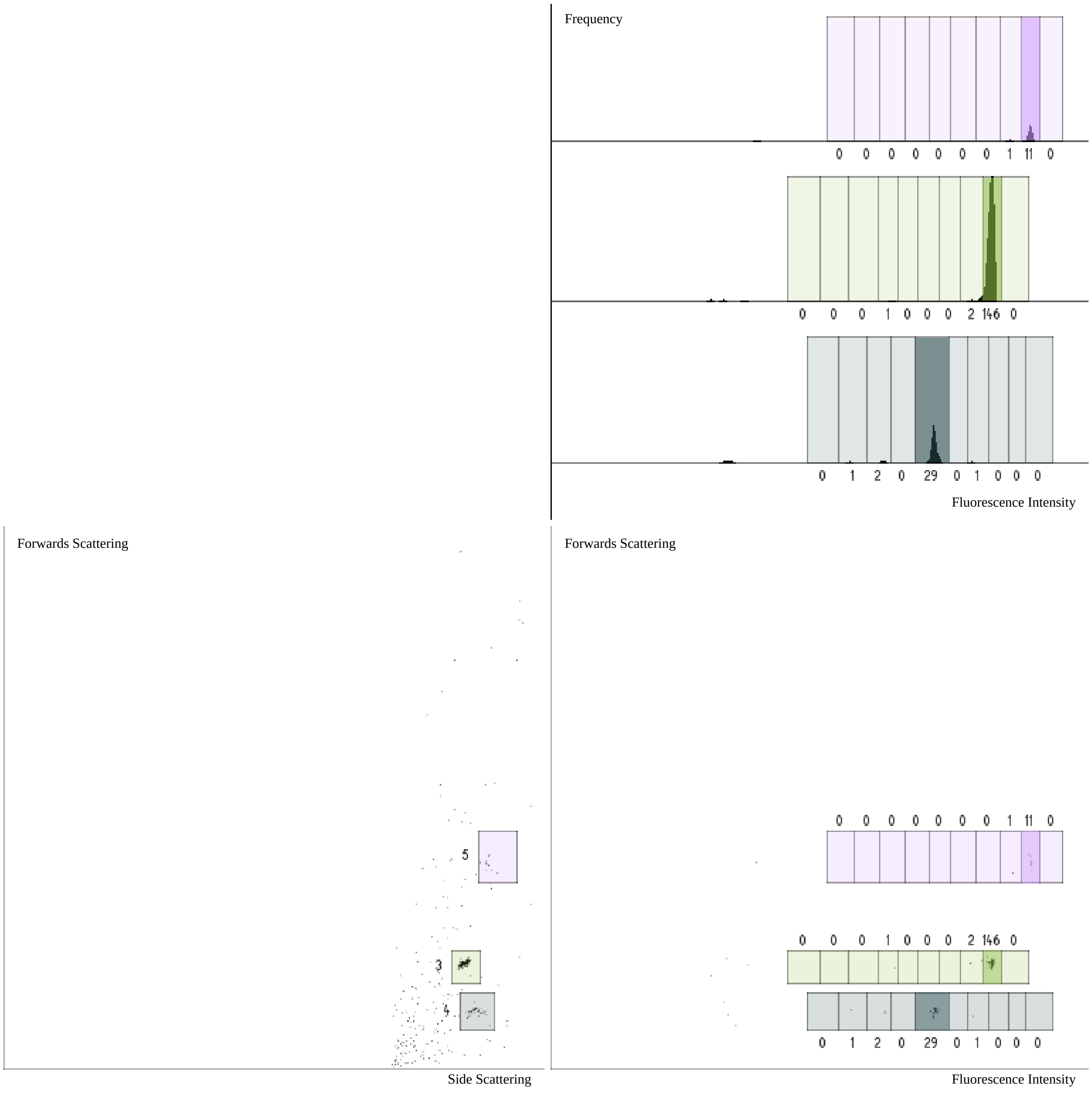

ANNEX 3: TAG DECONVOLUTION - BEAD 424

Passes flow sorting criteria: Yes

Passes tag deconvolution criteria: Yes

Included in protocol analysis: Yes

Protocol: 3, 3, 10, 8

Filename: Bin8_plateA0_G9.fcs

Split 1: Petrol shading

Split 2: Green shading

Split 3: Violet shading

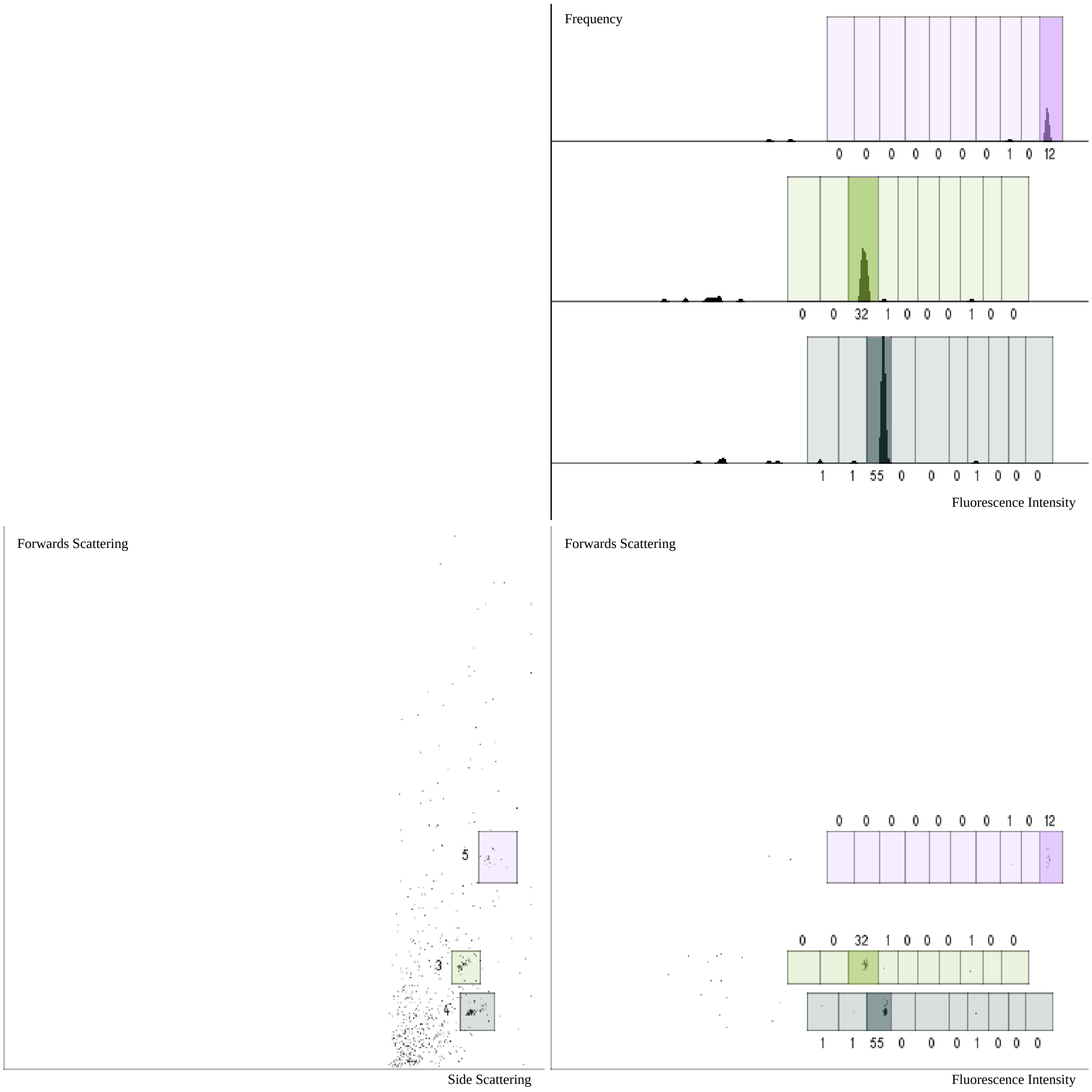

ANNEX 3: TAG DECONVOLUTION - BEAD 425

Passes flow sorting criteria: Yes

Passes tag deconvolution criteria: Yes

Included in protocol analysis: Yes

Protocol: 7, 6, 8, 8

Filename: Bin8_plateA0_G10.fcs

Split 1: Petrol shading

Split 2: Green shading

Split 3: Violet shading

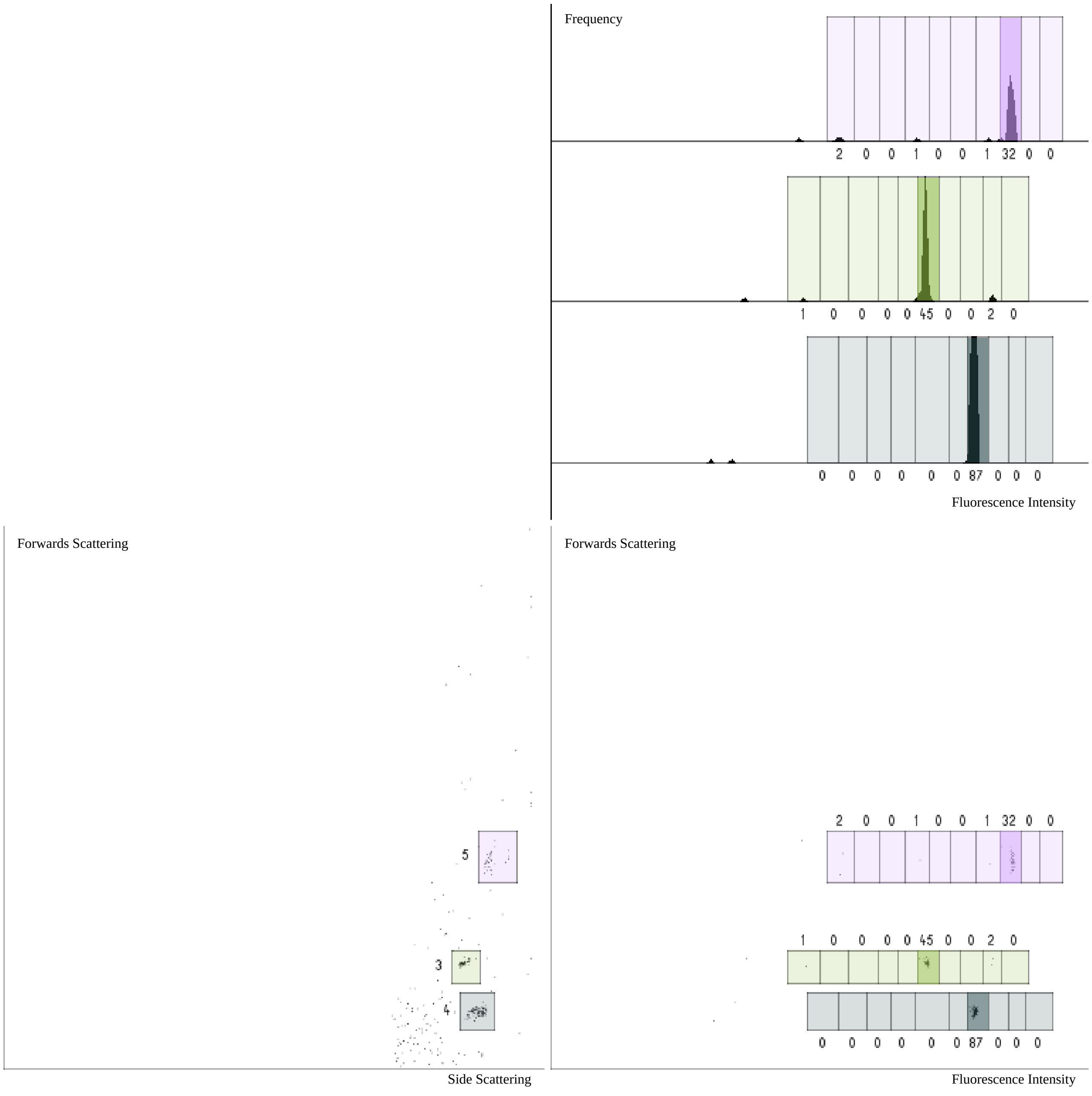

ANNEX 3: TAG DECONVOLUTION - BEAD 426

Passes flow sorting criteria: Yes

Passes tag deconvolution criteria: No

Included in protocol analysis: No

Protocol: N/A

Filename: Bin8_plateA0_G11.fcs

Split 1: Petrol shading

Split 2: Green shading

Split 3: Violet shading

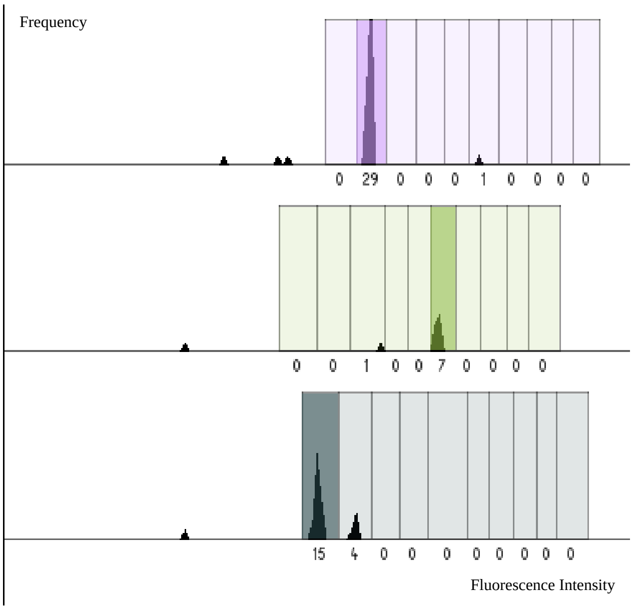

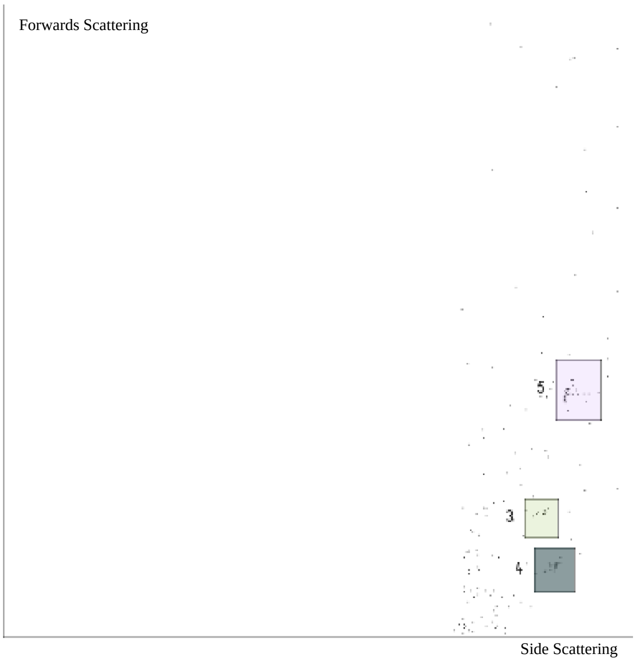

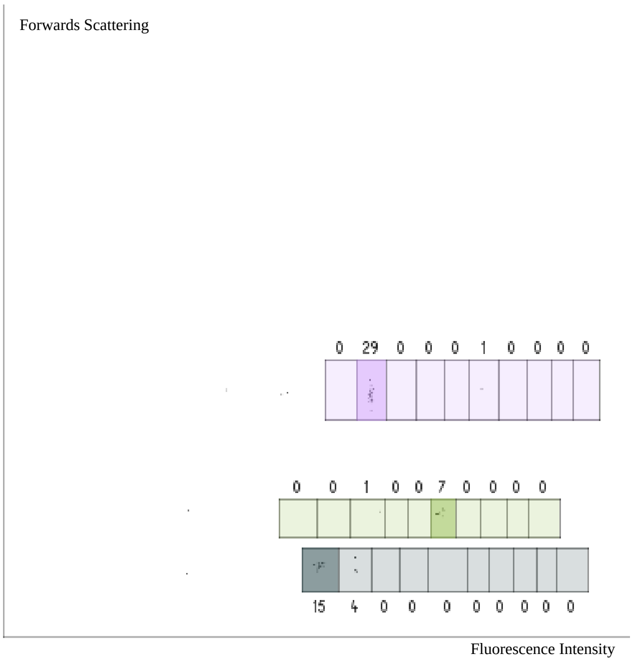

ANNEX 3: TAG DECONVOLUTION - BEAD 427

Passes flow sorting criteria: Yes

Passes tag deconvolution criteria: Yes

Included in protocol analysis: Yes

Protocol: 4, 7, 7, 9

Filename: Bin9_plateA0_D7.fcs

Split 1: Petrol shading

Split 2: Green shading

Split 3: Violet shading

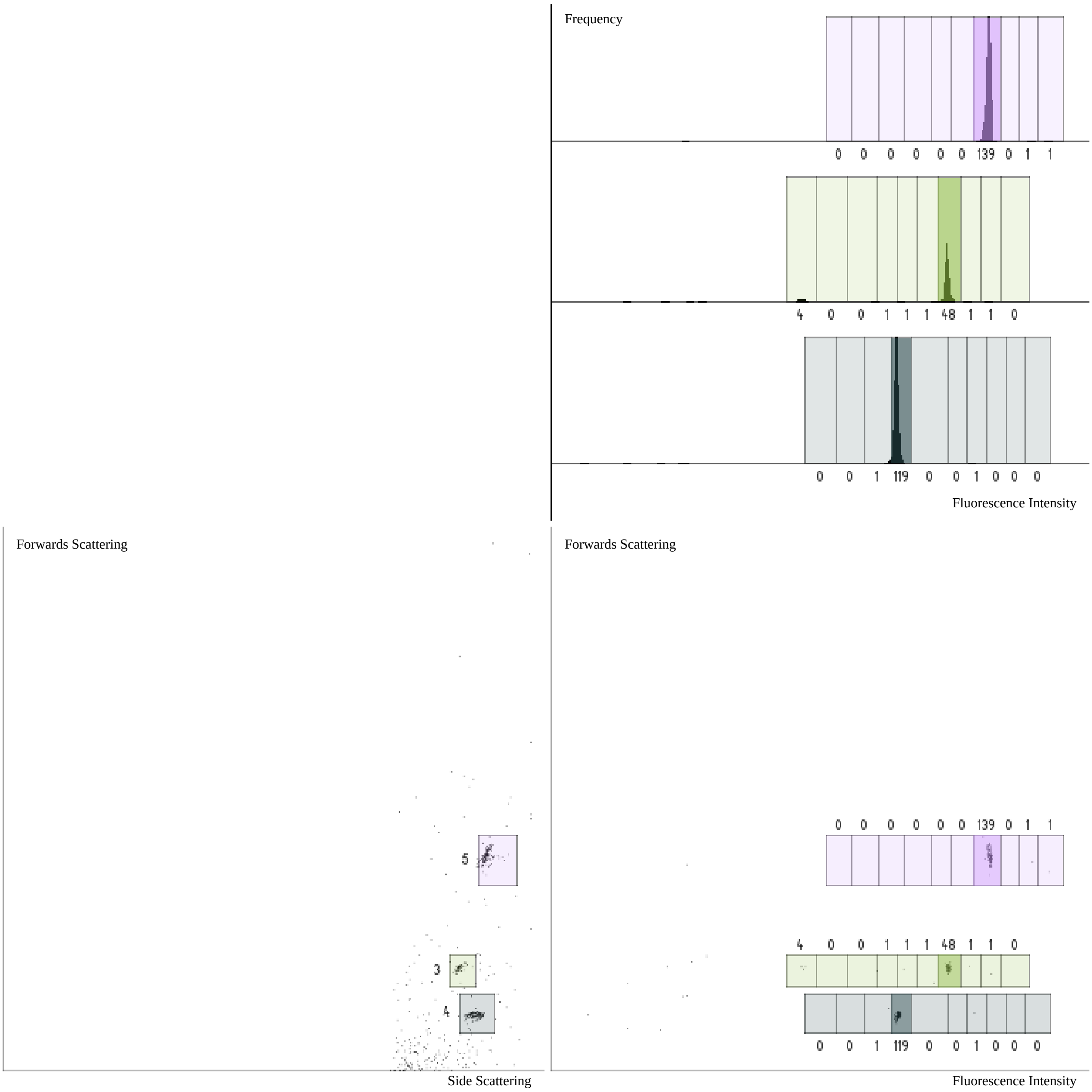

ANNEX 3: TAG DECONVOLUTION - BEAD 428

Passes flow sorting criteria: Yes

Passes tag deconvolution criteria: Yes

Included in protocol analysis: Yes

Protocol: 3, 9, 1, 9

Filename: Bin9_plateA0_A1.fcs

Split 1: Petrol shading

Split 2: Green shading

Split 3: Violet shading

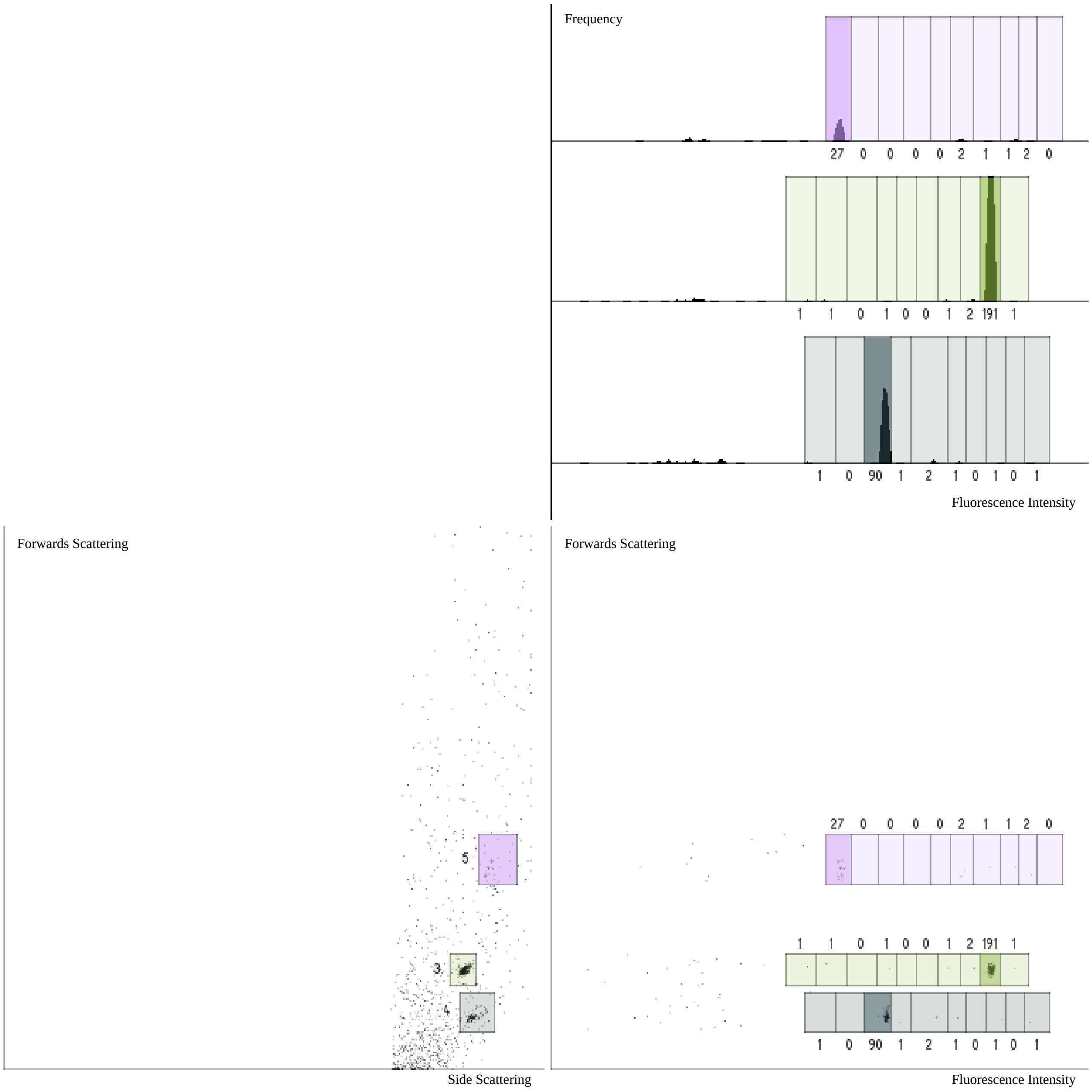

ANNEX 3: TAG DECONVOLUTION - BEAD 429

Passes flow sorting criteria: Yes

Passes tag deconvolution criteria: Yes

Included in protocol analysis: Yes

Protocol: 10, 10, 3, 9

Filename: Bin9_plateA0_A2.fcs

Split 1: Petrol shading

Split 2: Green shading

Split 3: Violet shading

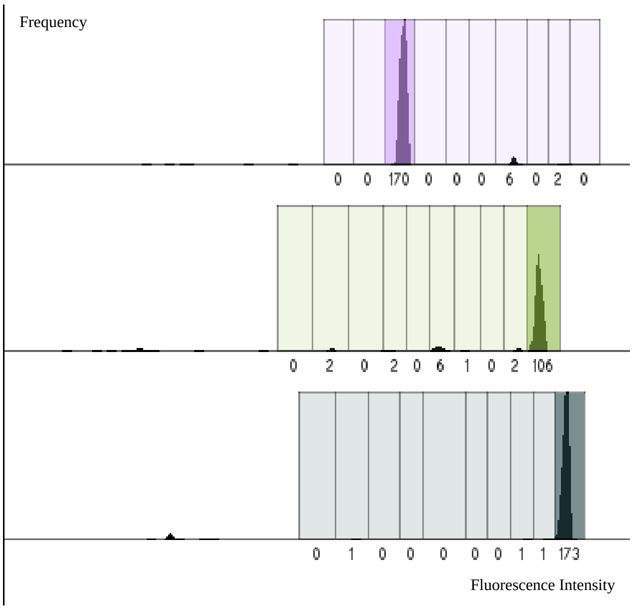

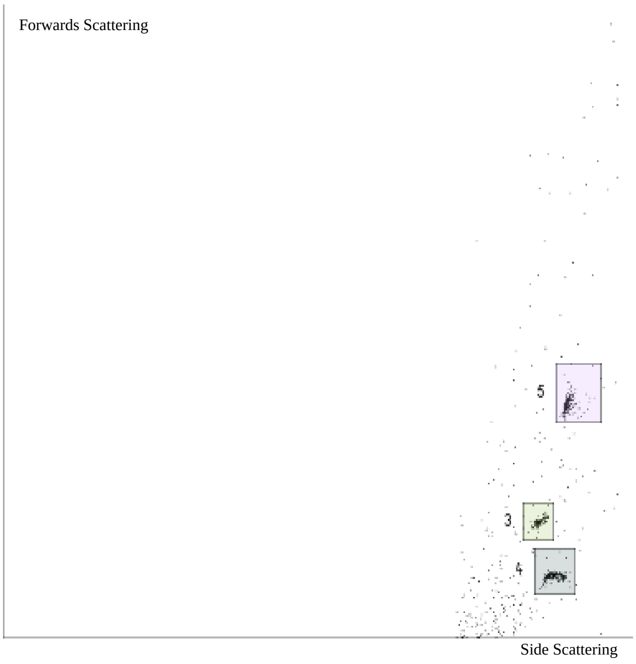

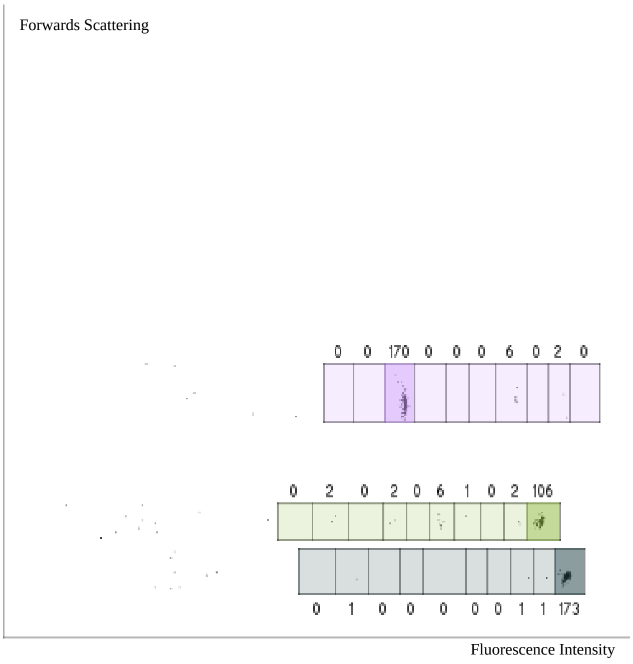

ANNEX 3: TAG DECONVOLUTION - BEAD 430

Passes flow sorting criteria: Yes

Passes tag deconvolution criteria: Yes

Included in protocol analysis: Yes

Protocol: 8, 9, 2, 9

Filename: Bin9_plateA0_A3.fcs

Split 1: Petrol shading

Split 2: Green shading

Split 3: Violet shading

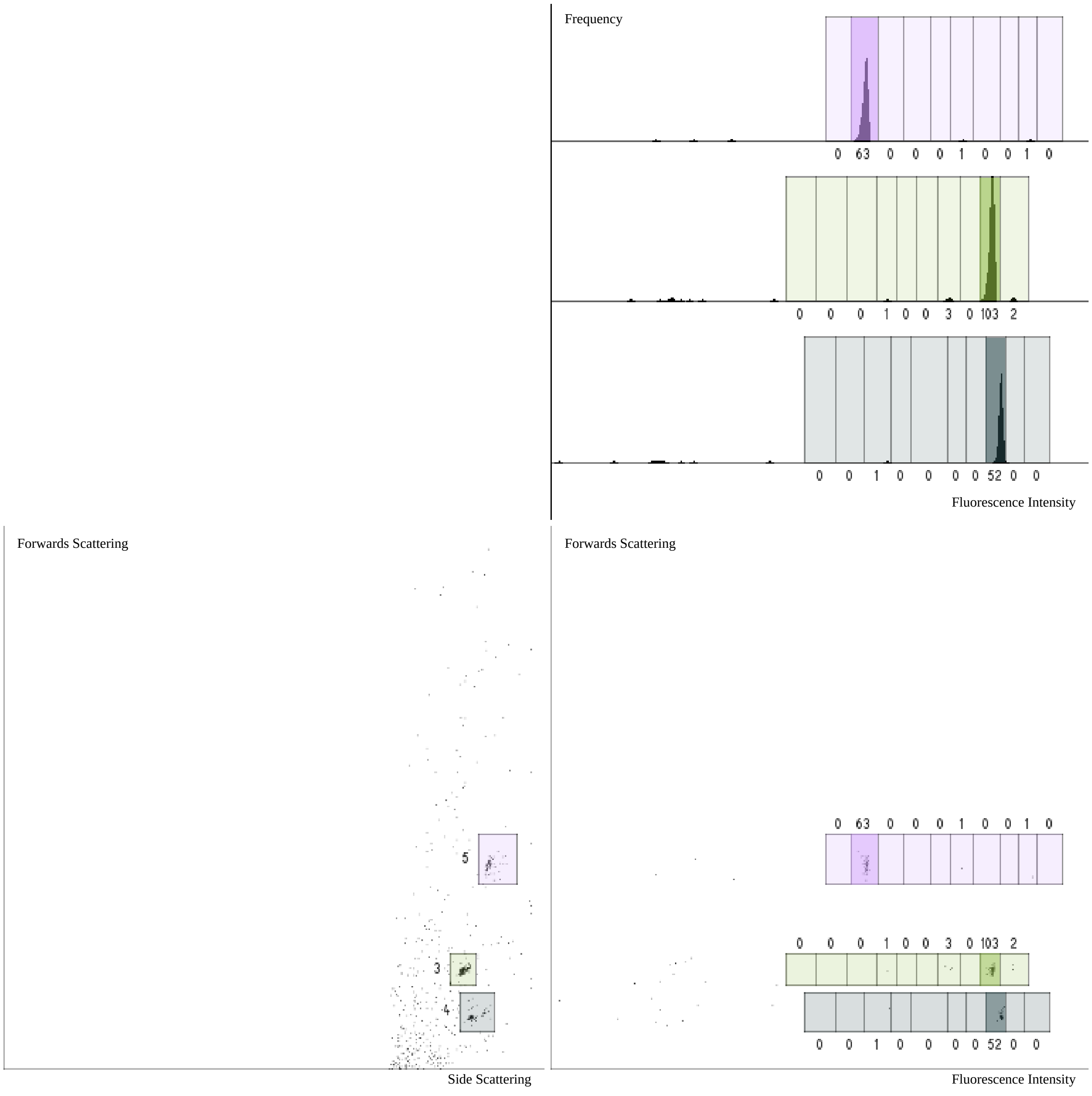

ANNEX 3: TAG DECONVOLUTION - BEAD 431

Passes flow sorting criteria: Yes

Passes tag deconvolution criteria: Yes

Included in protocol analysis: Yes

Protocol: 6, 7, 2, 9

Filename: Bin9_plateA0_A4.fcs

Split 1: Petrol shading

Split 2: Green shading

Split 3: Violet shading

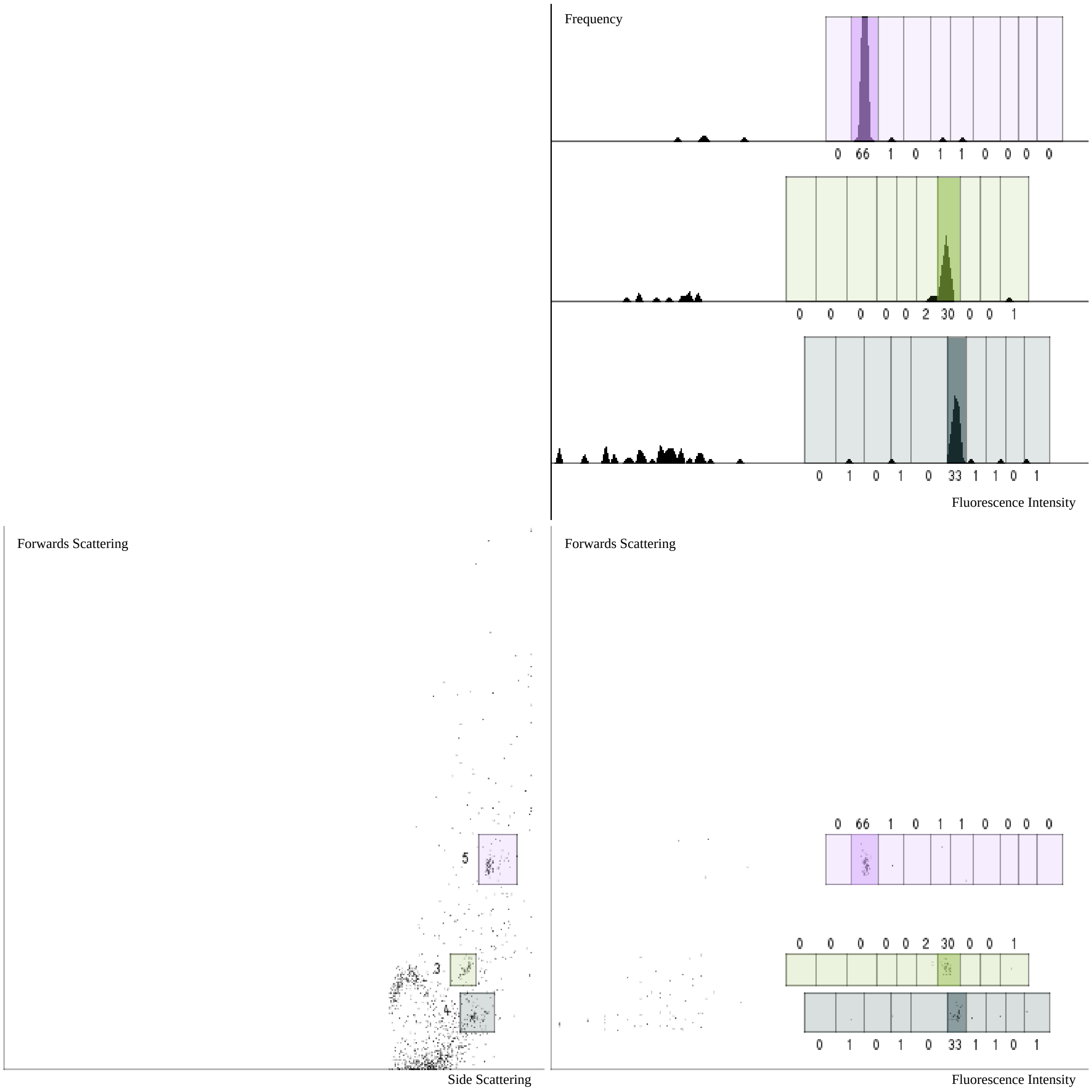

ANNEX 3: TAG DECONVOLUTION - BEAD 432

Passes flow sorting criteria: Yes

Passes tag deconvolution criteria: Yes

Included in protocol analysis: Yes

Protocol: 6, 5, 1, 9

Filename: Bin9_plateA0_A5.fcs

Split 1: Petrol shading

Split 2: Green shading

Split 3: Violet shading

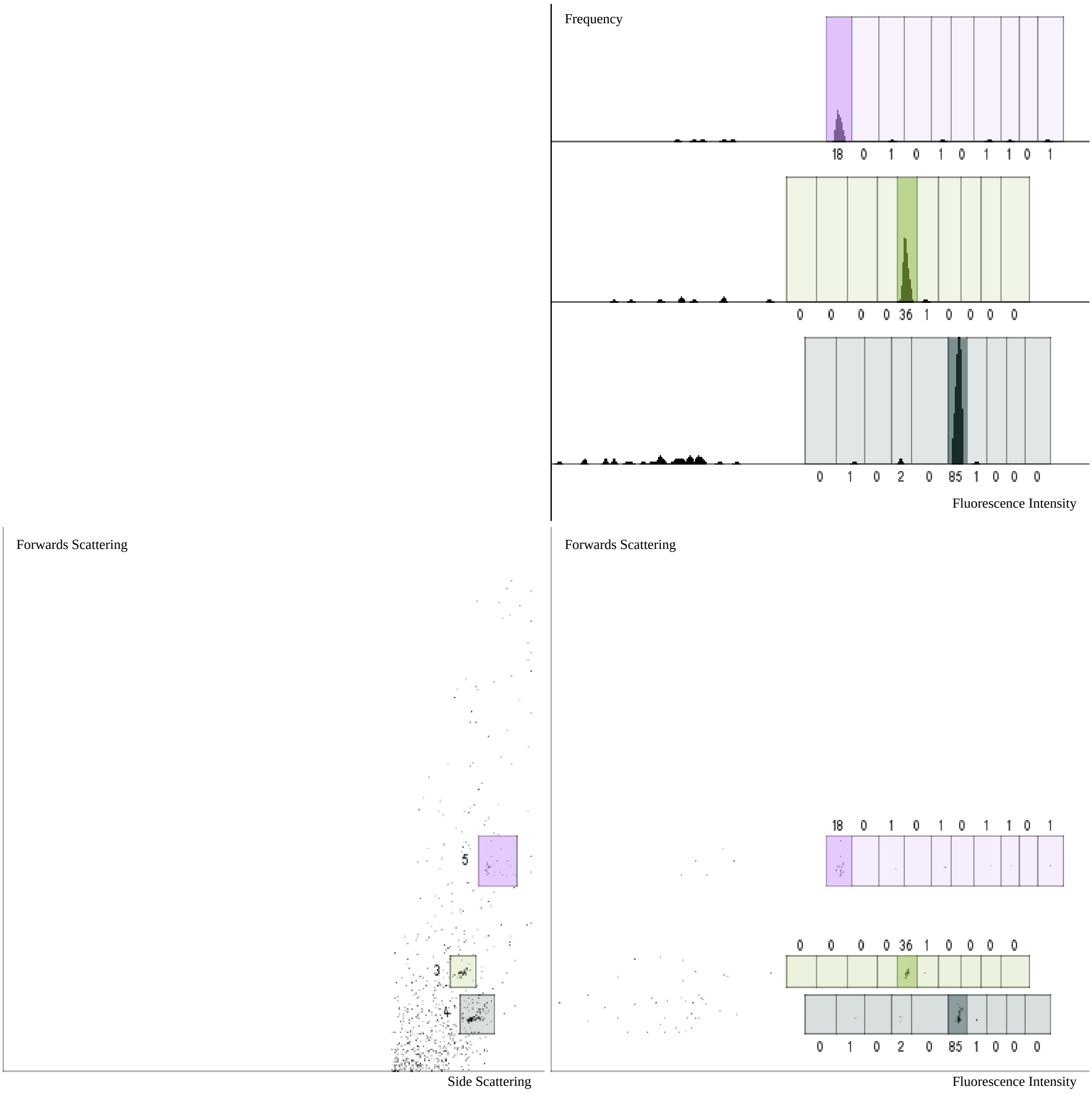

ANNEX 3: TAG DECONVOLUTION - BEAD 433

Passes flow sorting criteria: Yes

Passes tag deconvolution criteria: Yes

Included in protocol analysis: Yes

Protocol: 1, 1, 9, 9

Filename: Bin9_plateA0_A6.fcs

Split 1: Petrol shading

Split 2: Green shading

Split 3: Violet shading

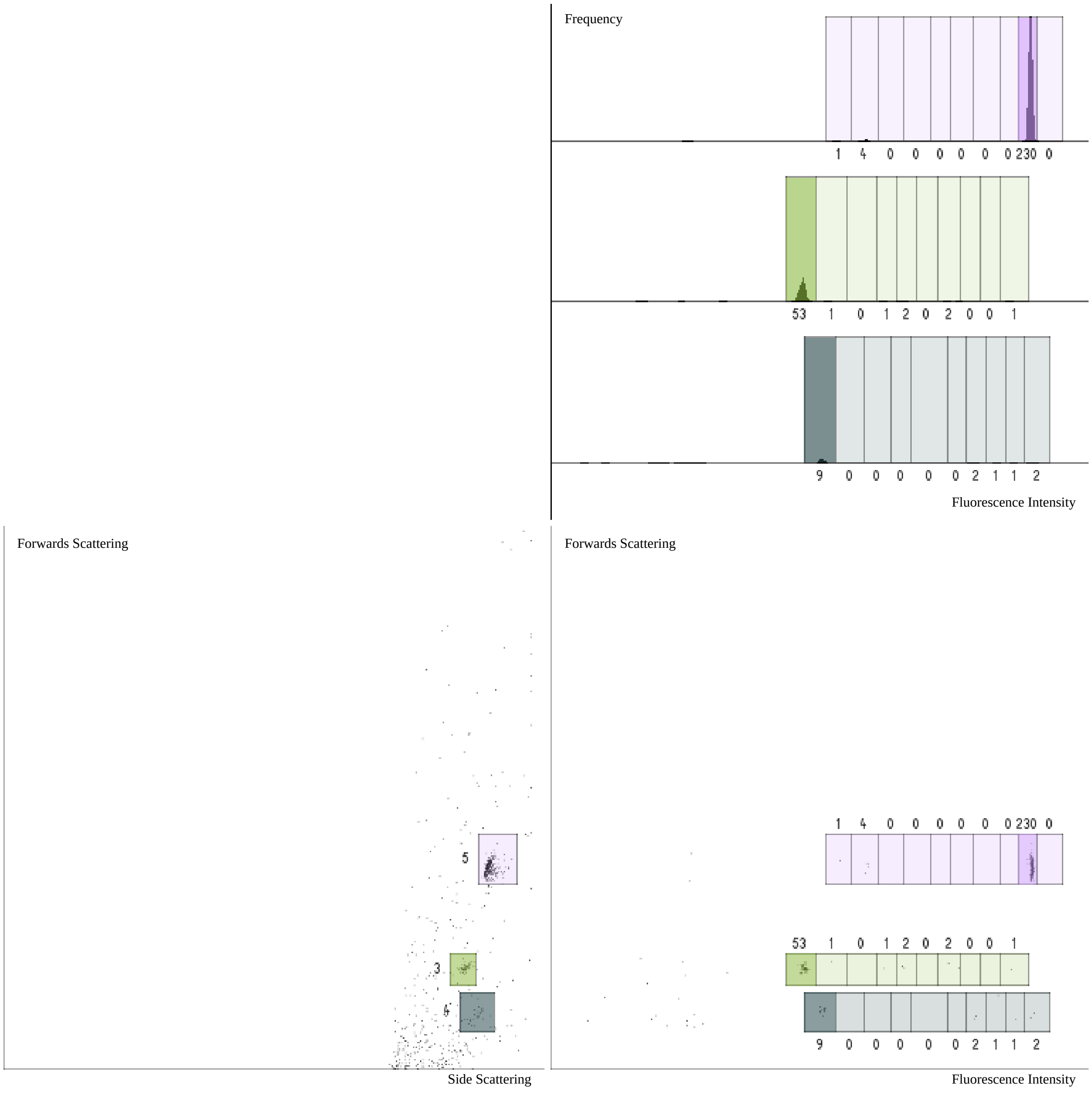

ANNEX 3: TAG DECONVOLUTION - BEAD 434

Passes flow sorting criteria: Yes

Passes tag deconvolution criteria: Yes

Included in protocol analysis: Yes

Protocol: 4, 8, 3, 9

Filename: Bin9_plateA0_A7.fcs

Split 1: Petrol shading

Split 2: Green shading

Split 3: Violet shading

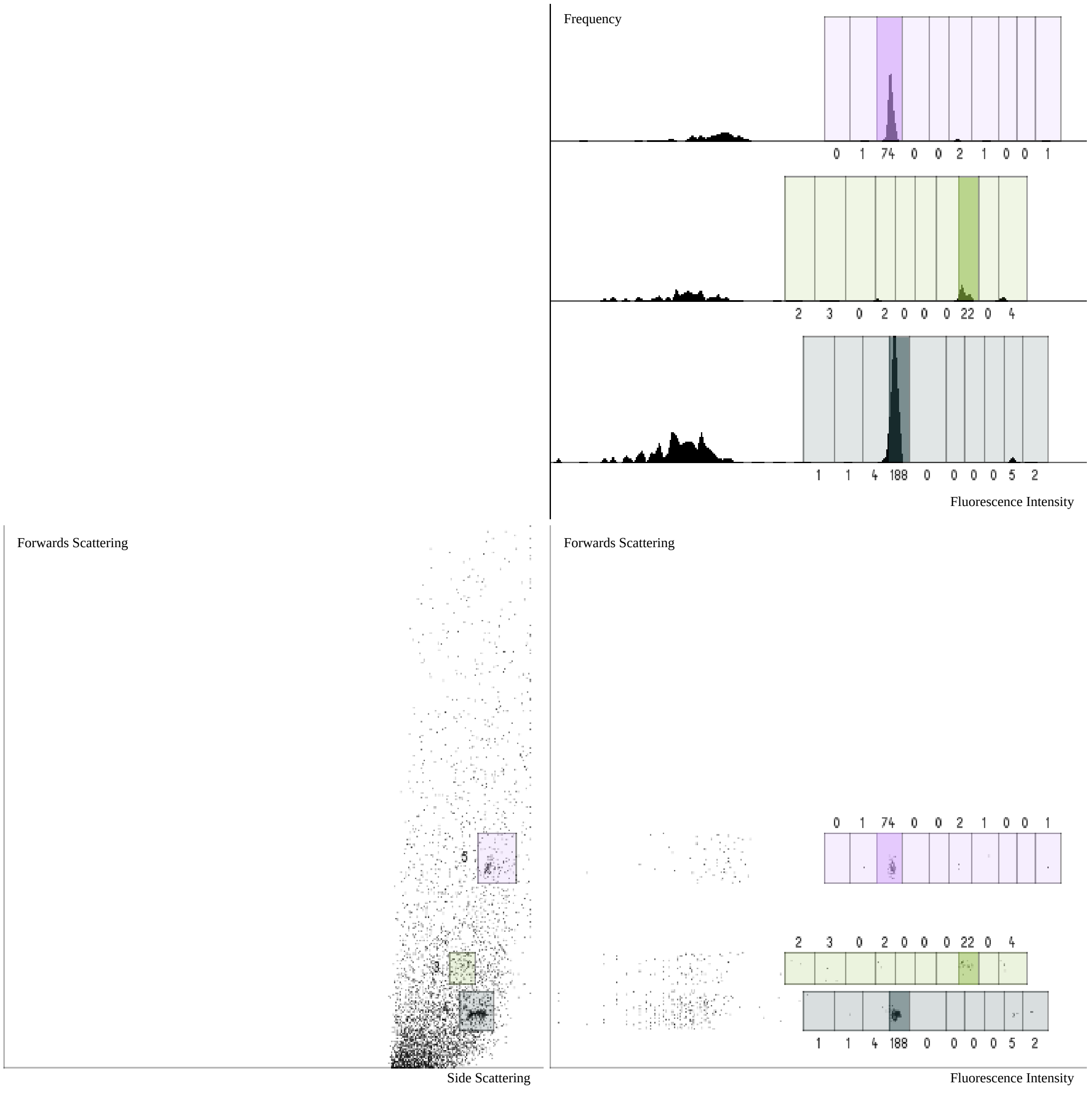

ANNEX 3: TAG DECONVOLUTION - BEAD 435

Passes flow sorting criteria: Yes

Passes tag deconvolution criteria: Yes

Included in protocol analysis: Yes

Protocol: 6, 6, 6, 9

Filename: Bin9_plateA0_A8.fcs

Split 1: Petrol shading

Split 2: Green shading

Split 3: Violet shading

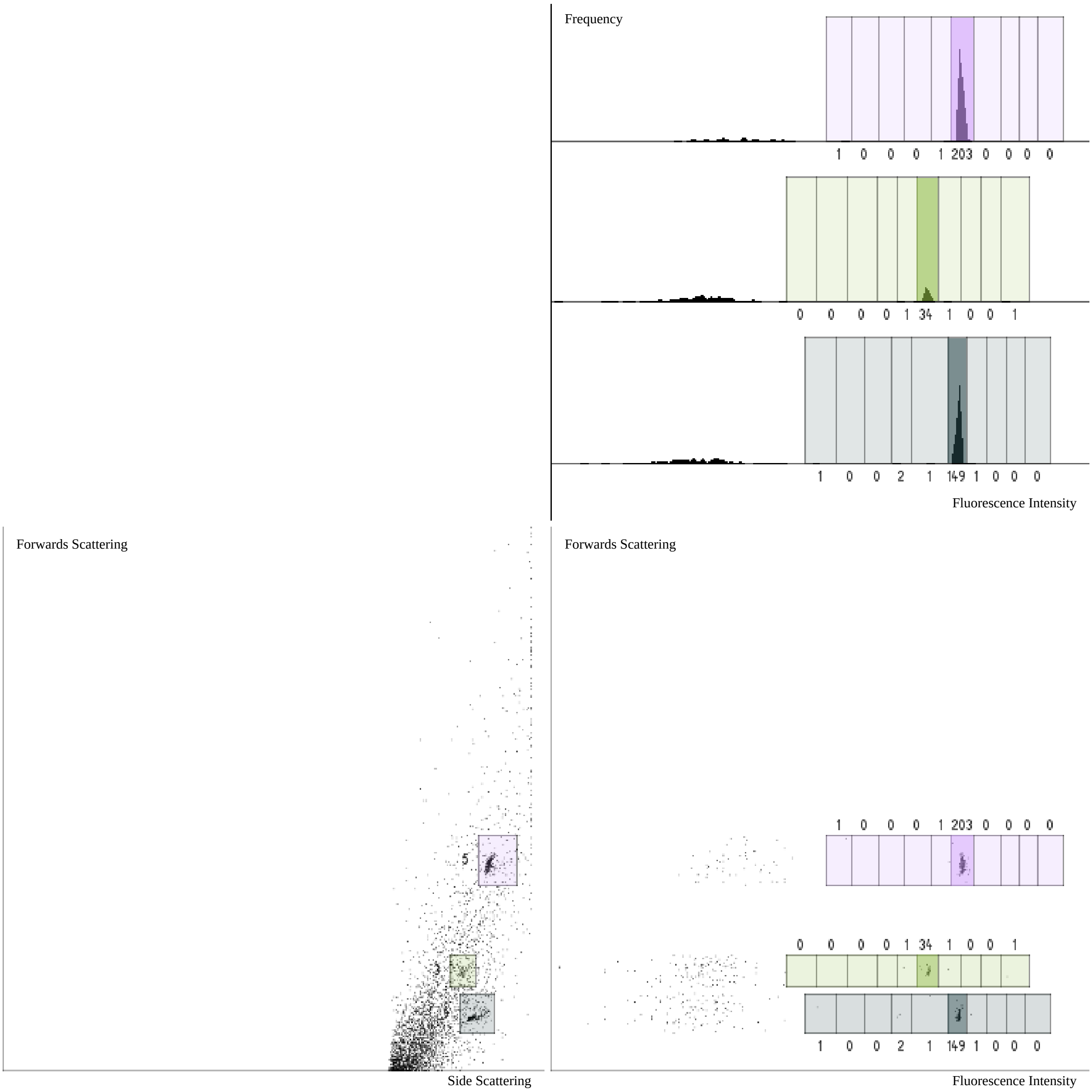

ANNEX 3: TAG DECONVOLUTION - BEAD 436

Passes flow sorting criteria: Yes

Passes tag deconvolution criteria: Yes

Included in protocol analysis: Yes

Protocol: 5, 6, 9, 9

Filename: Bin9_plateA0_A9.fcs

Split 1: Petrol shading

Split 2: Green shading

Split 3: Violet shading

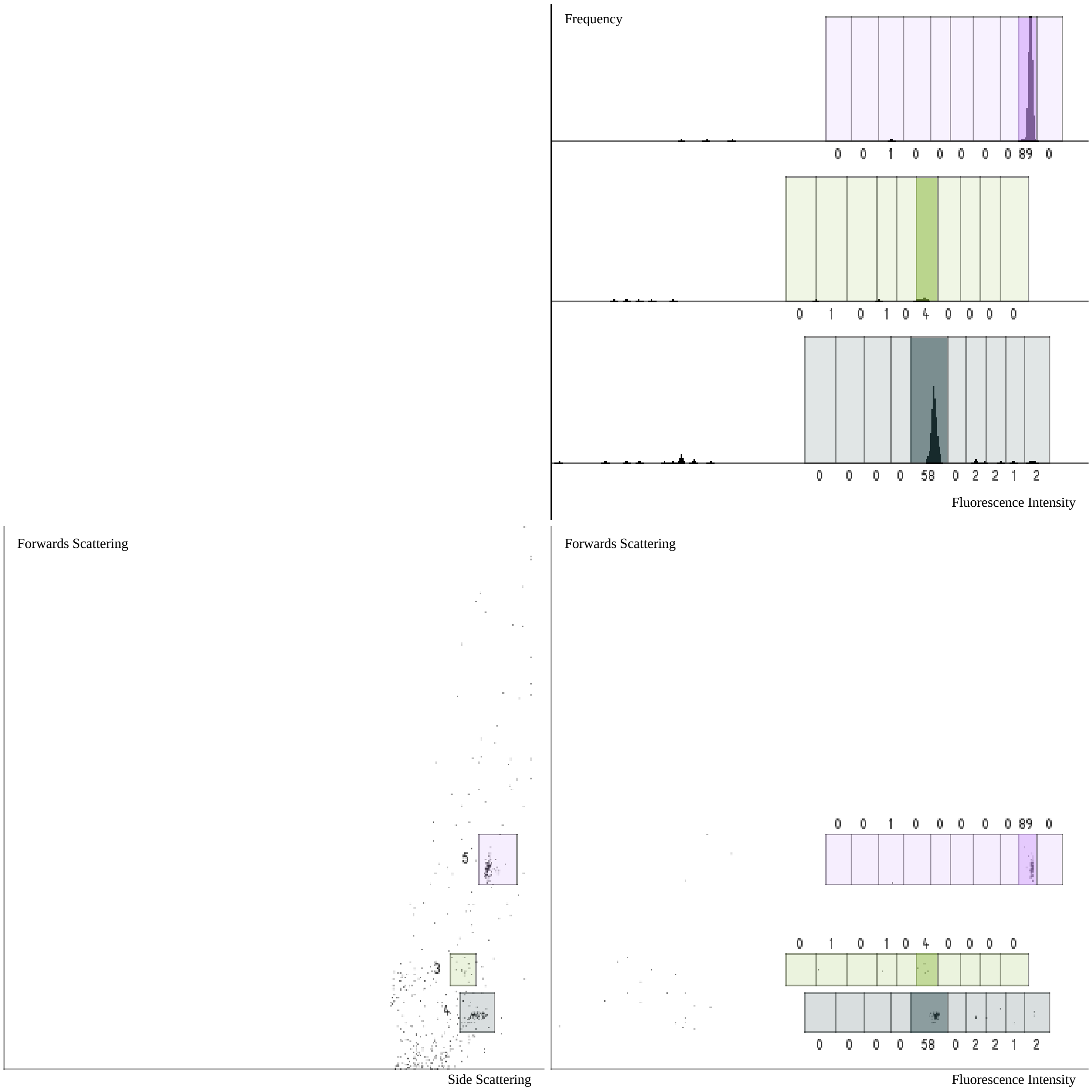

ANNEX 3: TAG DECONVOLUTION - BEAD 437

Passes flow sorting criteria: Yes

Passes tag deconvolution criteria: Yes

Included in protocol analysis: Yes

Protocol: 4, 9, 10, 9

Filename: Bin9_plateA0_A10.fcs

Split 1: Petrol shading

Split 2: Green shading

Split 3: Violet shading

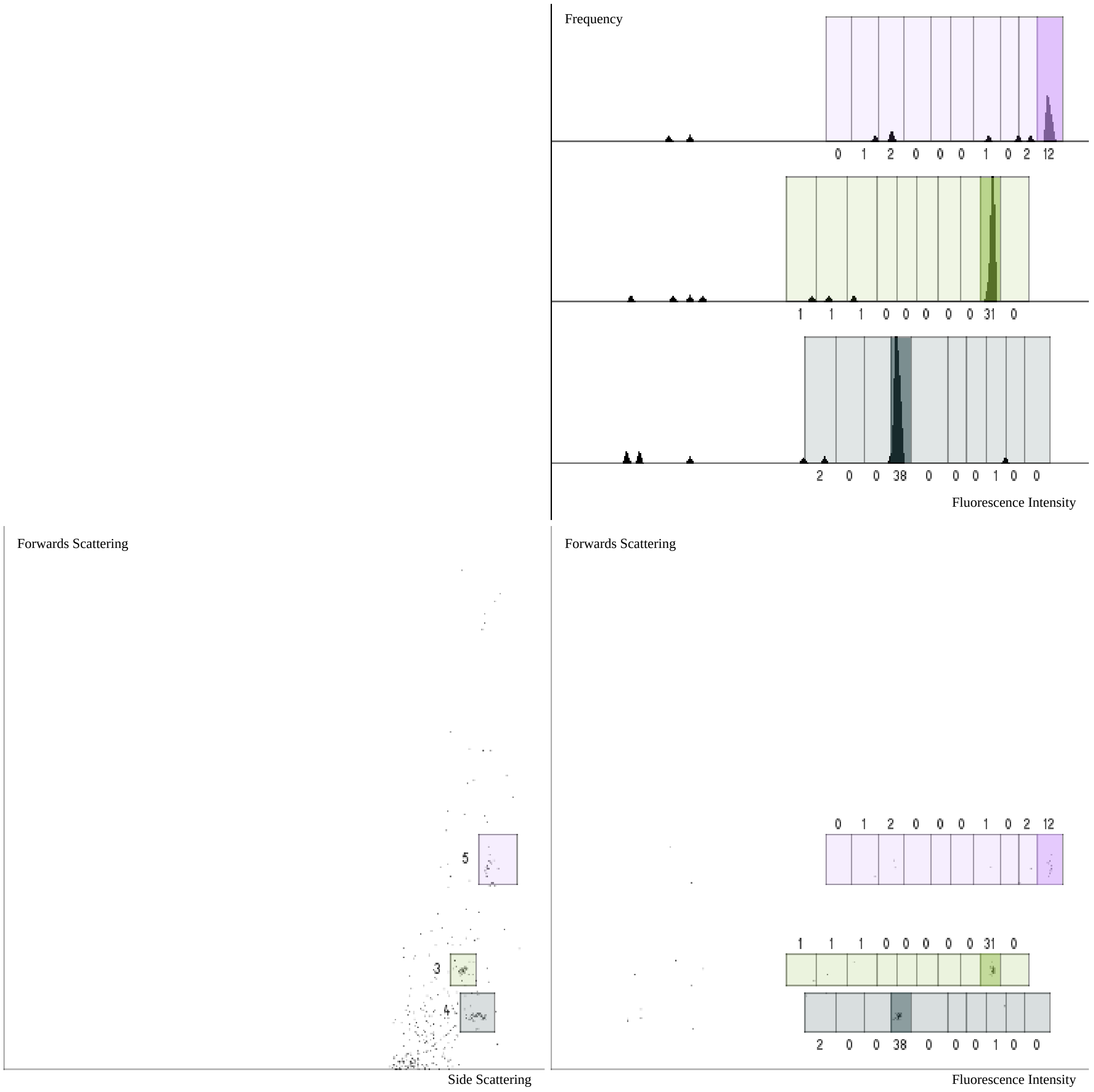

ANNEX 3: TAG DECONVOLUTION - BEAD 438

Passes flow sorting criteria: Yes

Passes tag deconvolution criteria: Yes

Included in protocol analysis: Yes

Protocol: 7, 9, 2, 9

Filename: Bin9_plateA0_A11.fcs

Split 1: Petrol shading

Split 2: Green shading

Split 3: Violet shading

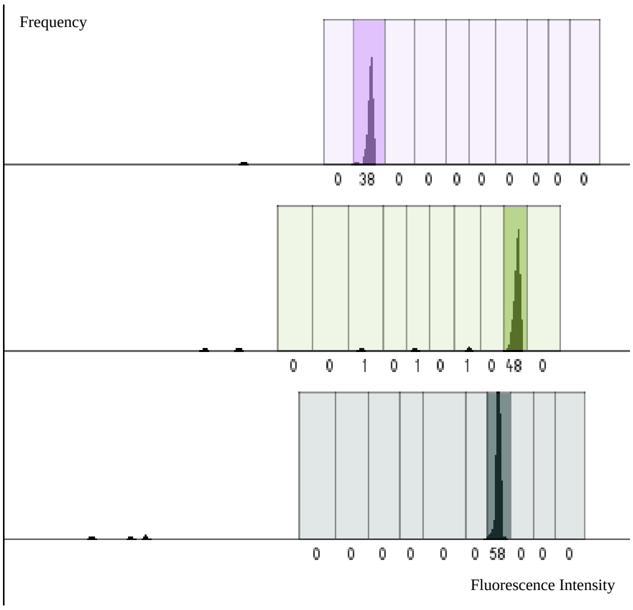

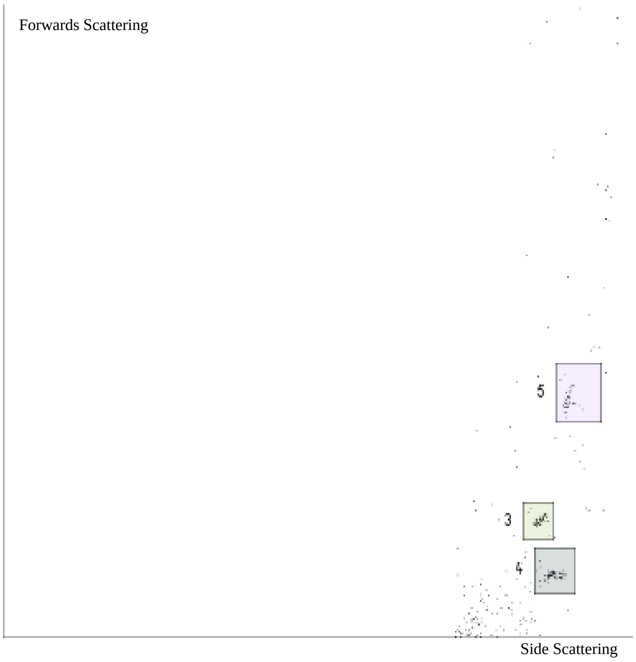

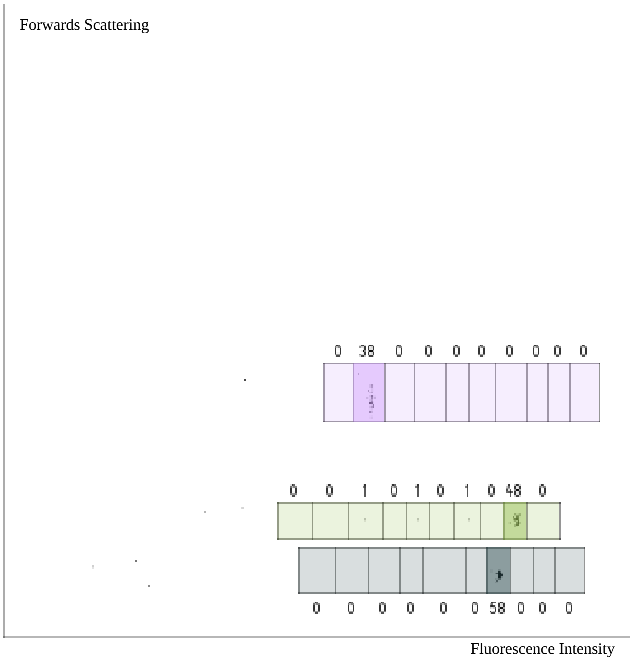

ANNEX 3: TAG DECONVOLUTION - BEAD 439

Passes flow sorting criteria: Yes

Passes tag deconvolution criteria: No

Included in protocol analysis: No

Protocol: N/A

Filename: Bin9_plateA0_B1.fcs

Split 1: Petrol shading

Split 2: Green shading

Split 3: Violet shading

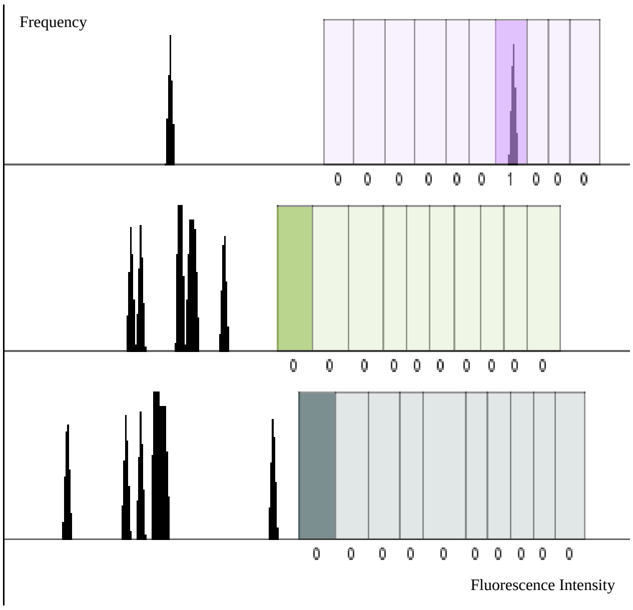

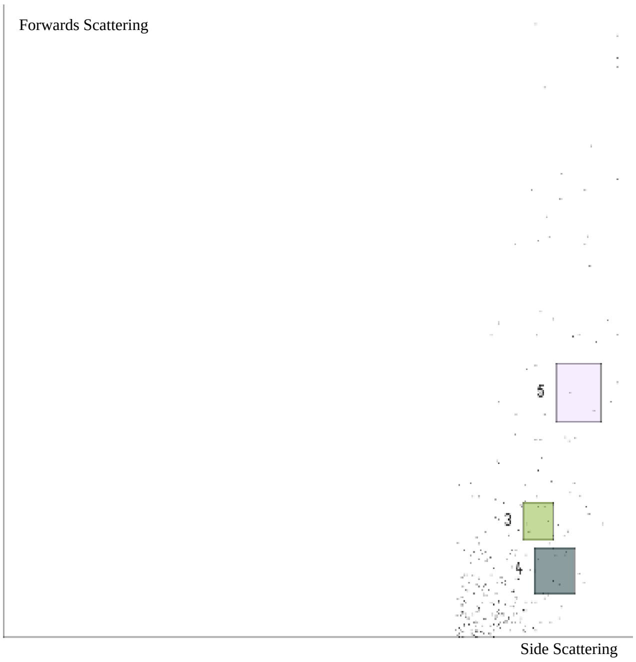

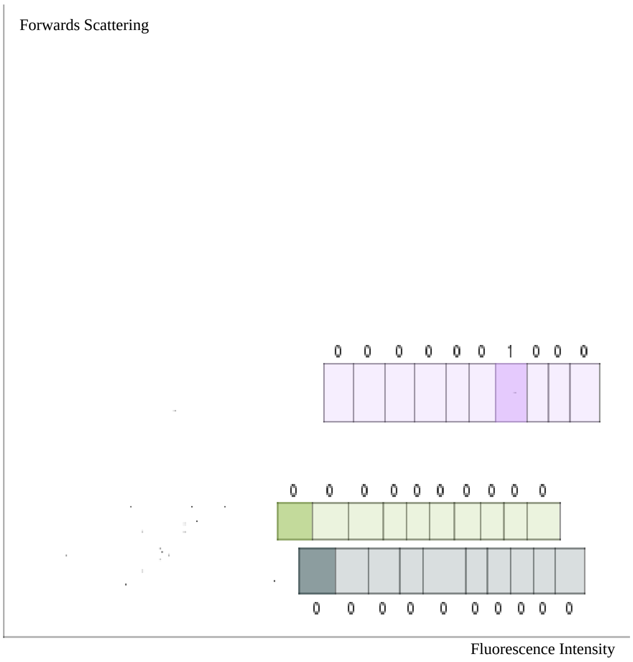

ANNEX 3: TAG DECONVOLUTION - BEAD 440

Passes flow sorting criteria: Yes

Passes tag deconvolution criteria: No

Included in protocol analysis: No

Protocol: N/A

Filename: Bin9_plateA0_B2.fcs

Split 1: Petrol shading

Split 2: Green shading

Split 3: Violet shading

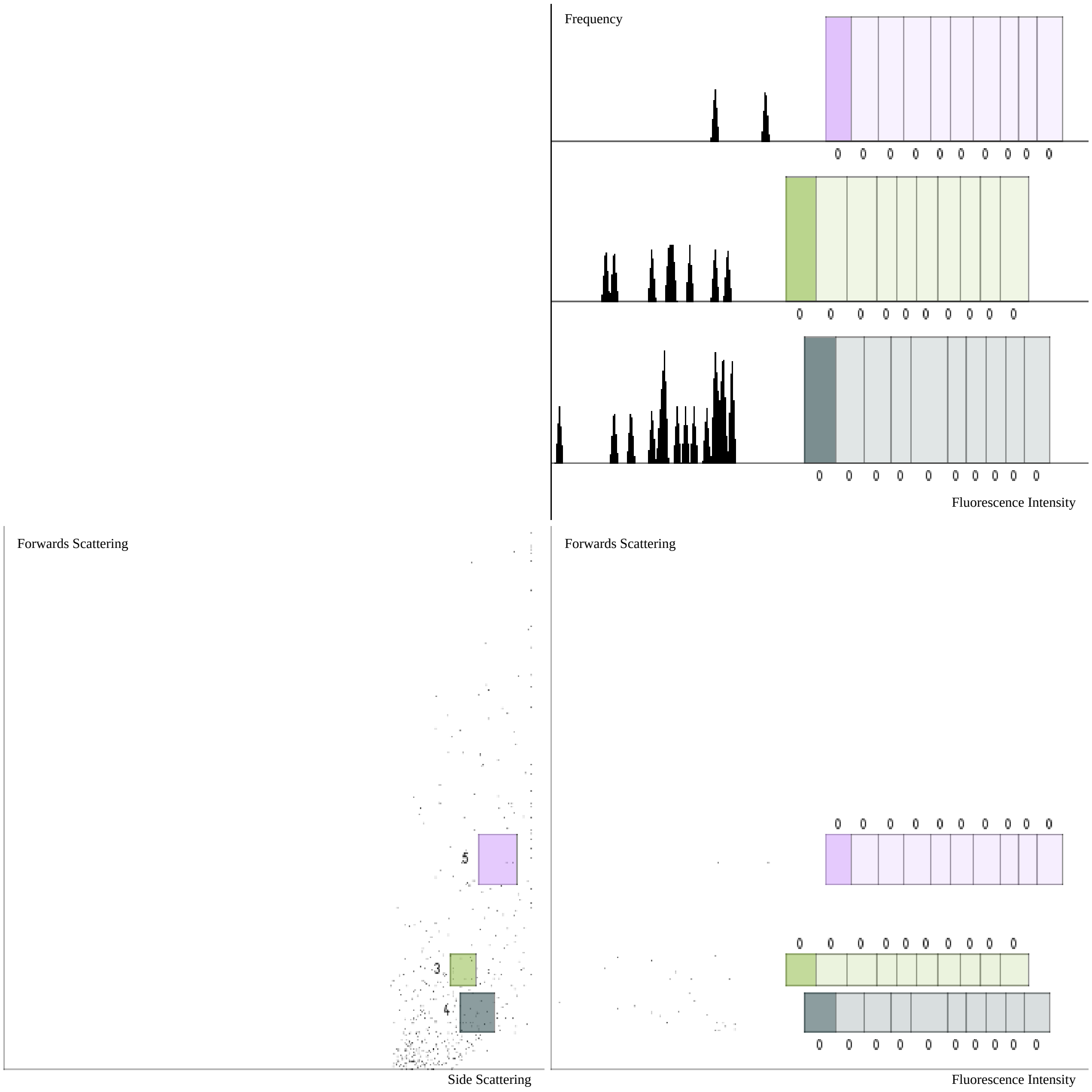

ANNEX 3: TAG DECONVOLUTION - BEAD 441

Passes flow sorting criteria: Yes

Passes tag deconvolution criteria: Yes

Included in protocol analysis: Yes

Protocol: 3, 10, 9, 9

Filename: Bin9_plateA0_B3.fcs

Split 1: Petrol shading

Split 2: Green shading

Split 3: Violet shading

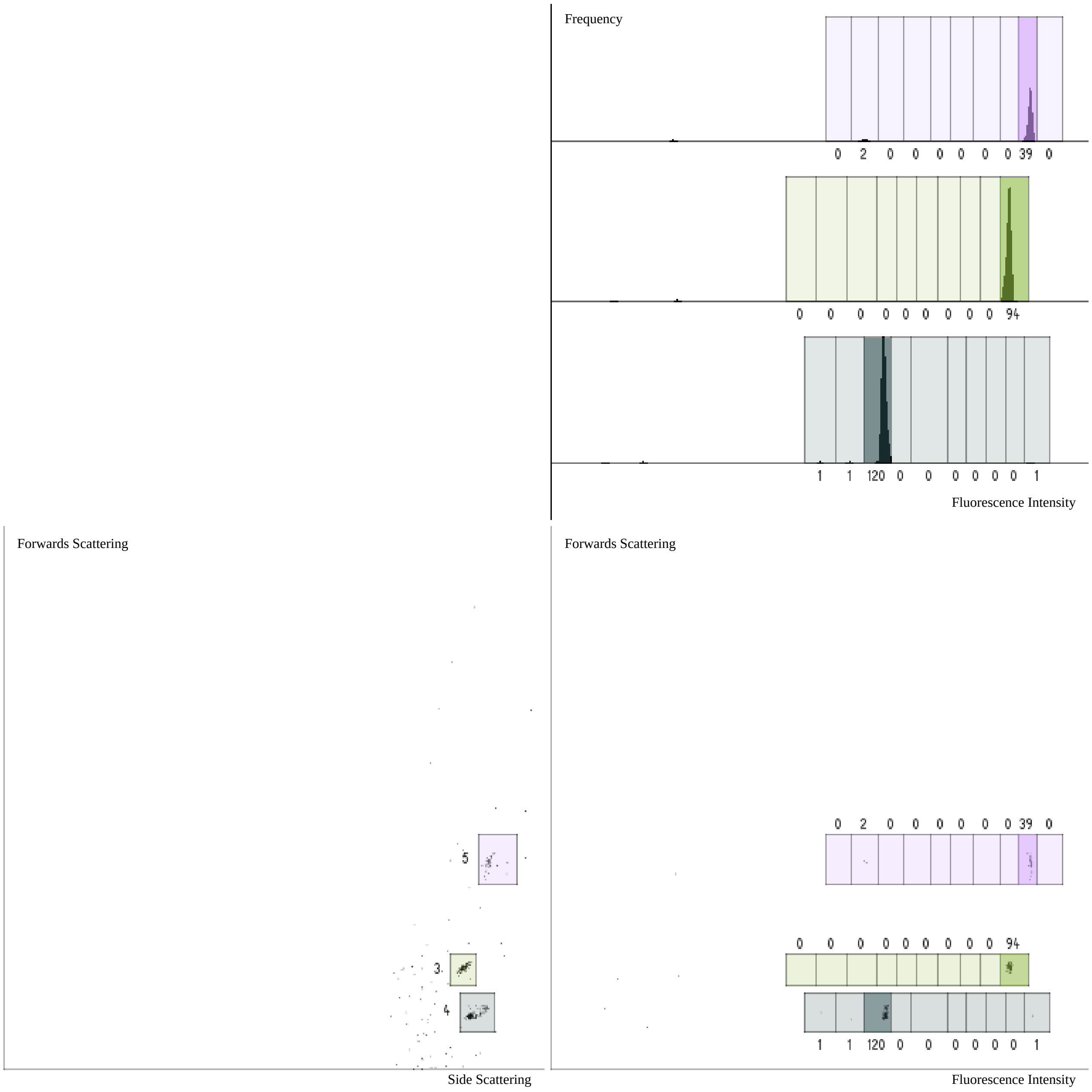

ANNEX 3: TAG DECONVOLUTION - BEAD 442

Passes flow sorting criteria: Yes

Passes tag deconvolution criteria: Yes

Included in protocol analysis: Yes

Protocol: 2, 4, 2, 9

Filename: Bin9_plateA0_B4.fcs

Split 1: Petrol shading

Split 2: Green shading

Split 3: Violet shading

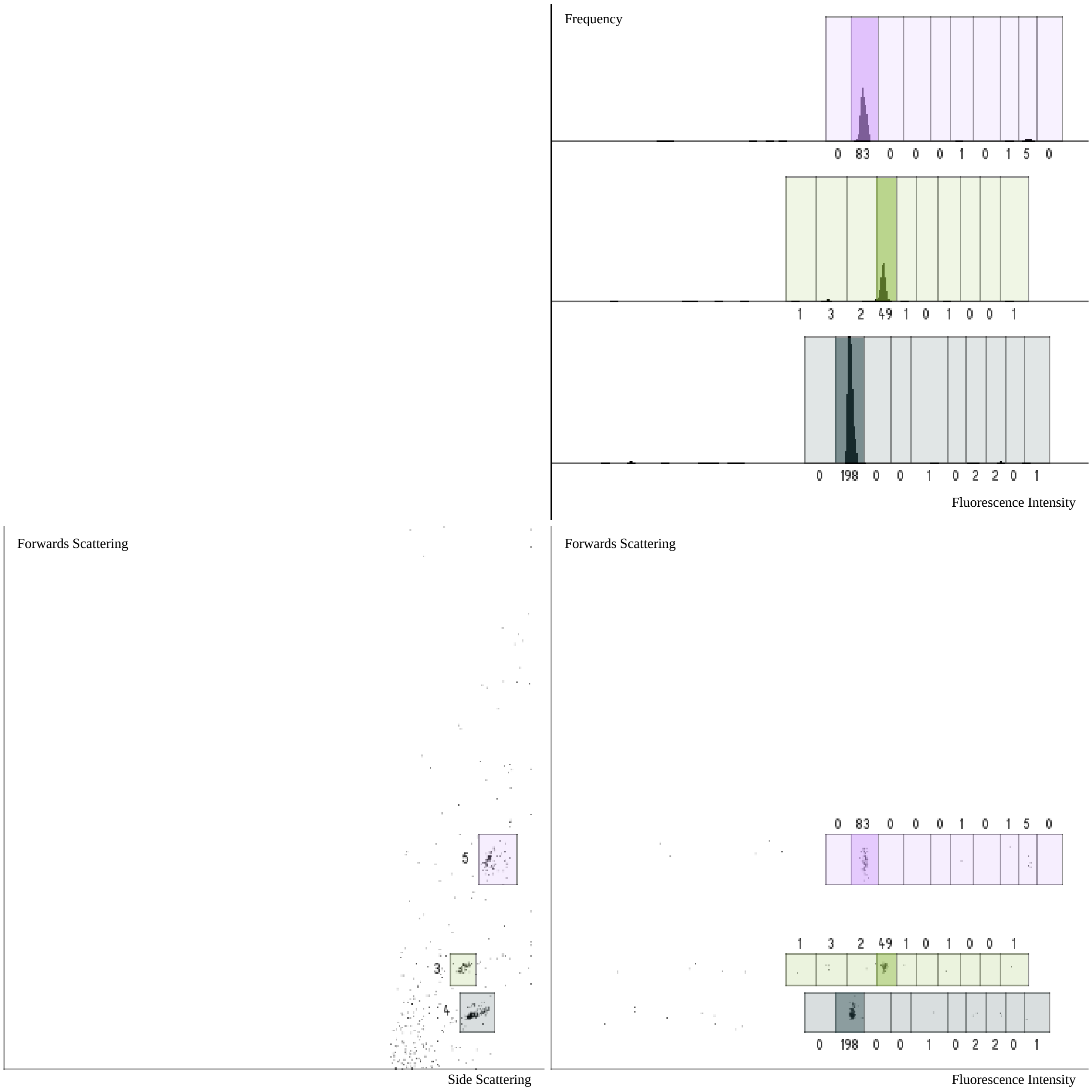

ANNEX 3: TAG DECONVOLUTION - BEAD 443

Passes flow sorting criteria: Yes

Passes tag deconvolution criteria: Yes

Included in protocol analysis: Yes

Protocol: 1, 5, 2, 9

Filename: Bin9_plateA0_B5.fcs

Split 1: Petrol shading

Split 2: Green shading

Split 3: Violet shading

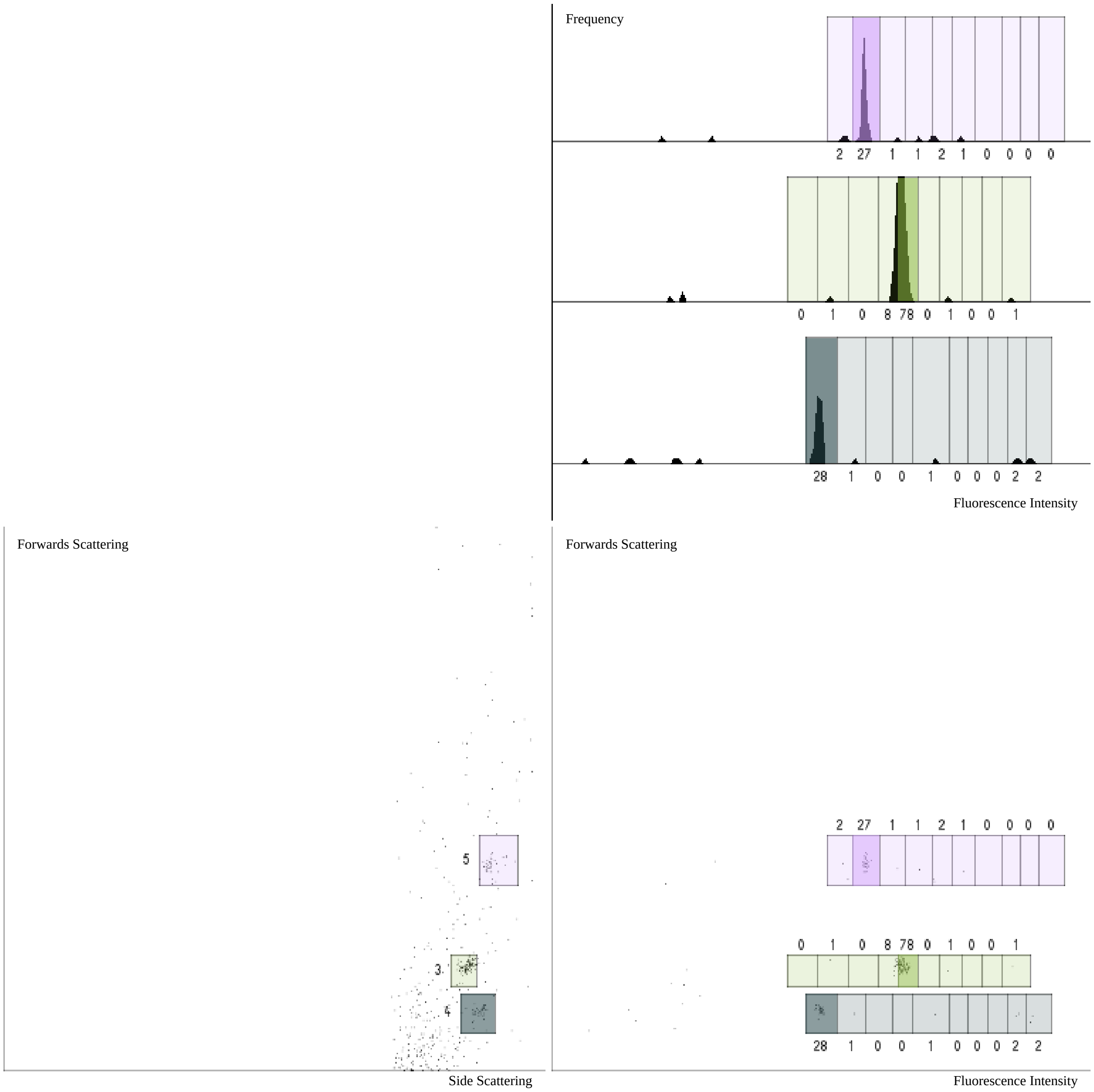

ANNEX 3: TAG DECONVOLUTION - BEAD 444

Passes flow sorting criteria: Yes

Passes tag deconvolution criteria: Yes

Included in protocol analysis: Yes

Protocol: 7, 3, 1, 9

Filename: Bin9_plateA0_B6.fcs

Split 1: Petrol shading

Split 2: Green shading

Split 3: Violet shading

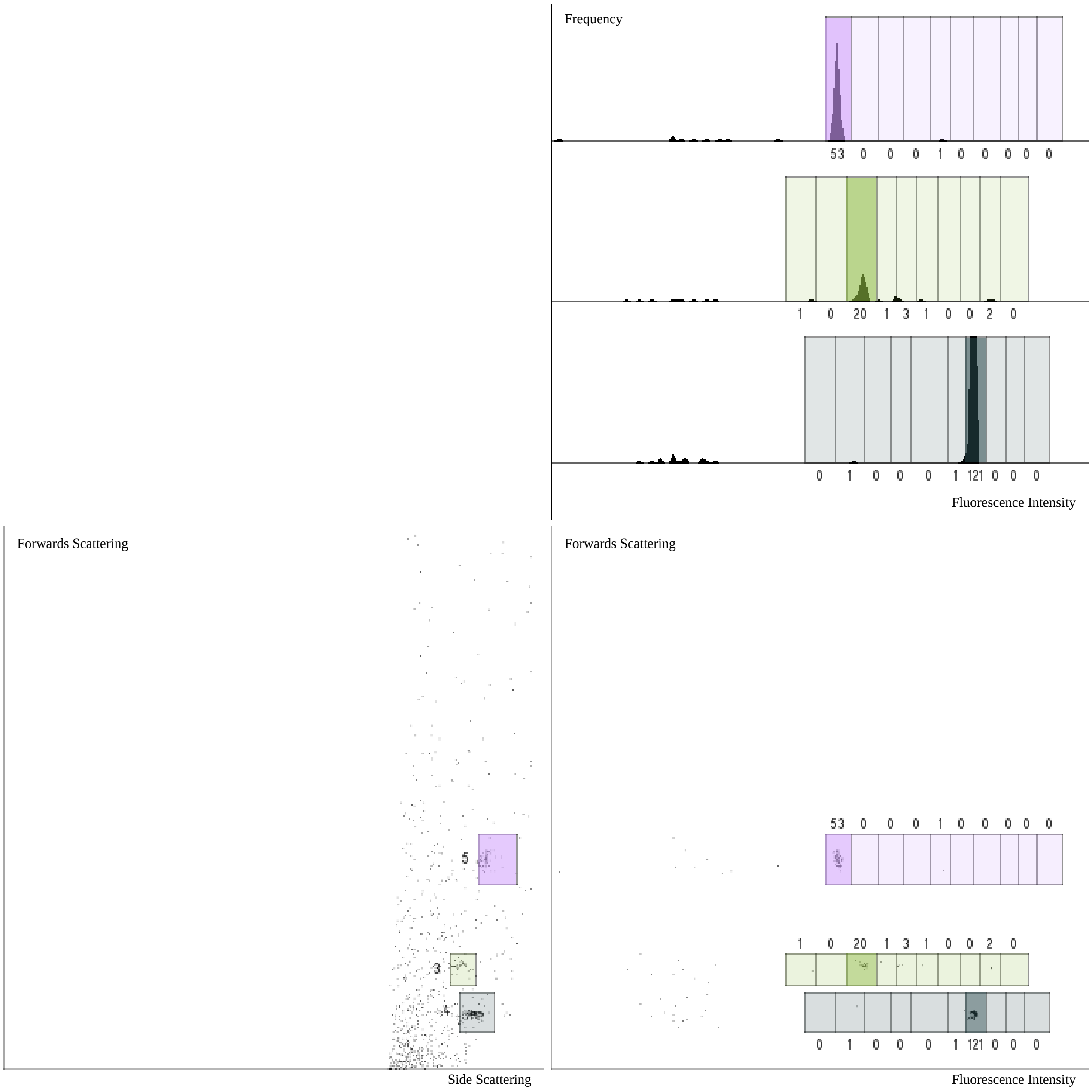

ANNEX 3: TAG DECONVOLUTION - BEAD 445

Passes flow sorting criteria: Yes

Passes tag deconvolution criteria: Yes

Included in protocol analysis: Yes

Protocol: 7, 8, 9, 9

Filename: Bin9_plateA0_B7.fcs

Split 1: Petrol shading

Split 2: Green shading

Split 3: Violet shading

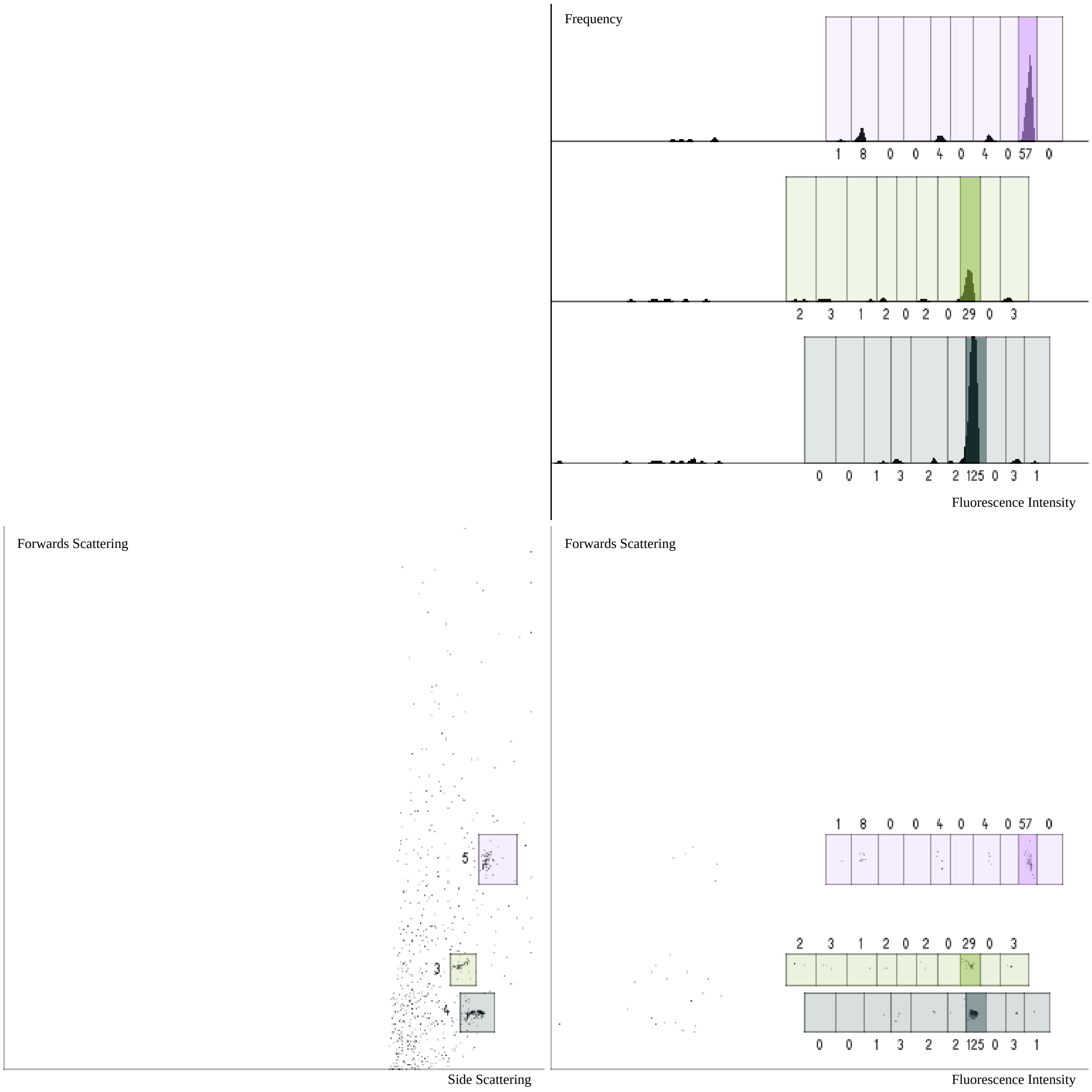

ANNEX 3: TAG DECONVOLUTION - BEAD 446

Passes flow sorting criteria: Yes

Passes tag deconvolution criteria: No

Included in protocol analysis: No

Protocol: N/A

Filename: Bin9_plateA0_B8.fcs

Split 1: Petrol shading

Split 2: Green shading

Split 3: Violet shading

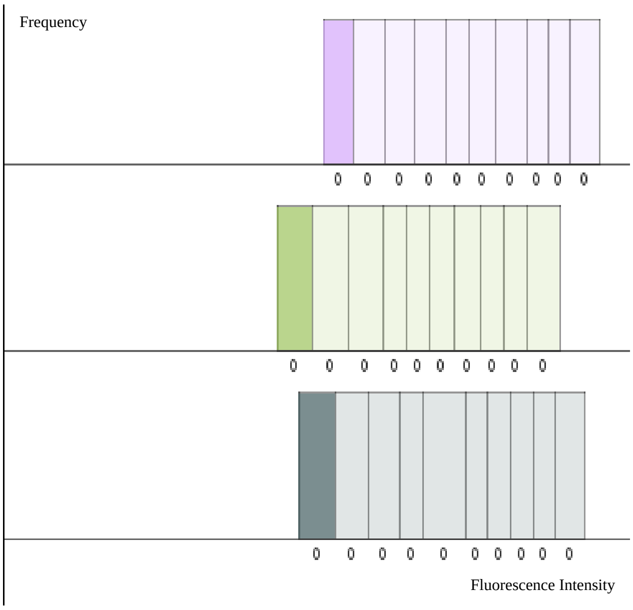

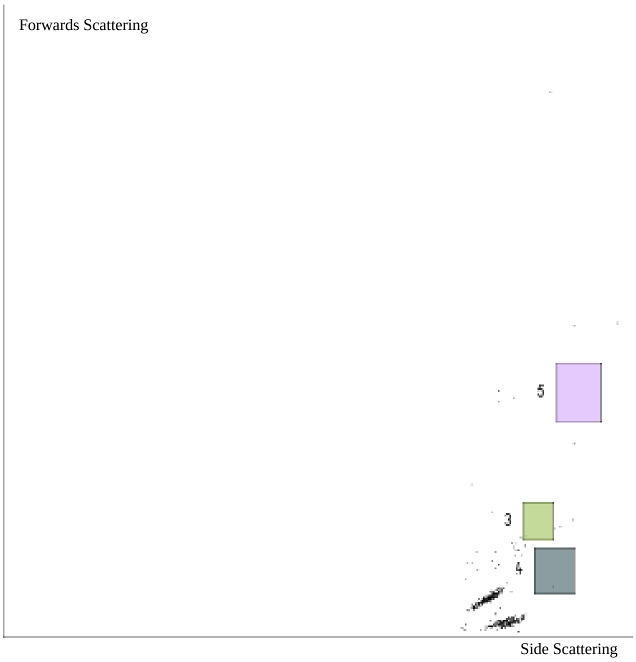

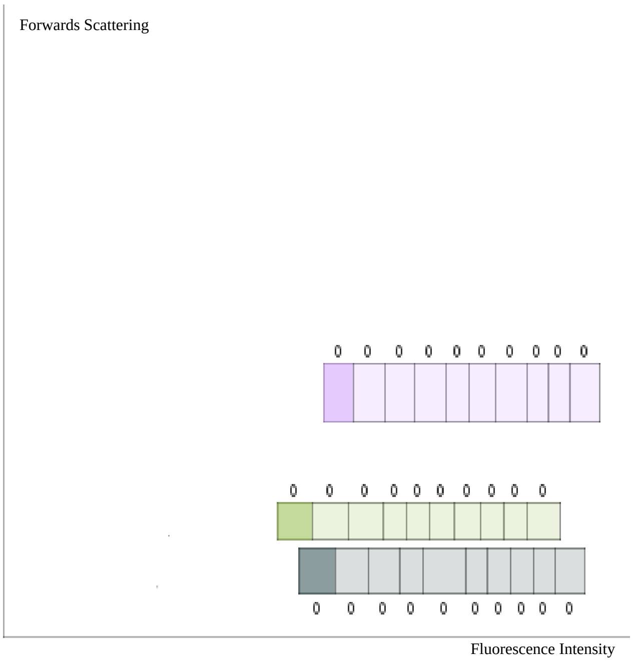

ANNEX 3: TAG DECONVOLUTION - BEAD 447

Passes flow sorting criteria: Yes

Passes tag deconvolution criteria: Yes

Included in protocol analysis: Yes

Protocol: 4, 6, 7, 9

Filename: Bin9_plateA0_B9.fcs

Split 1: Petrol shading

Split 2: Green shading

Split 3: Violet shading

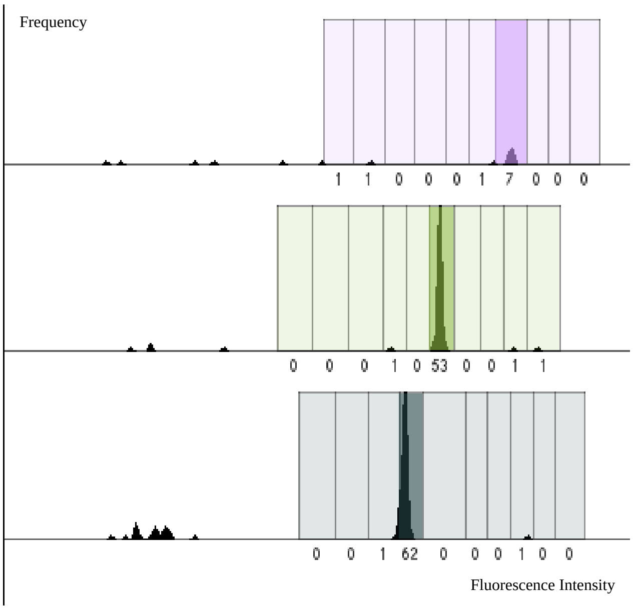

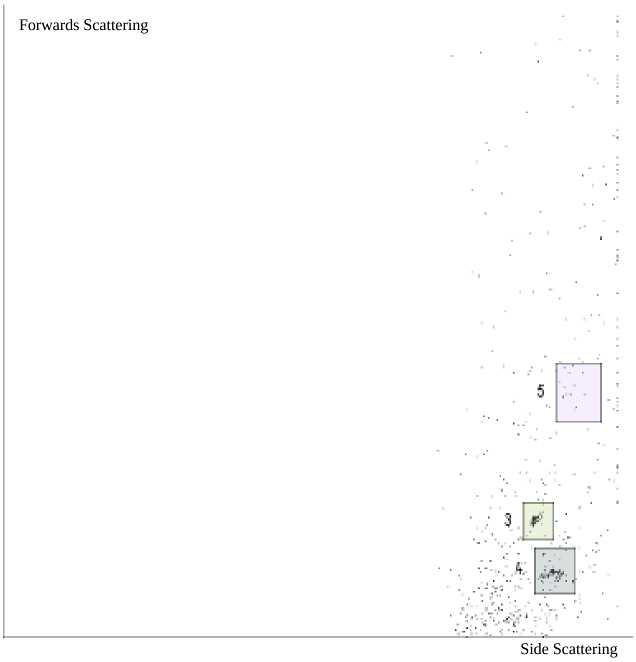

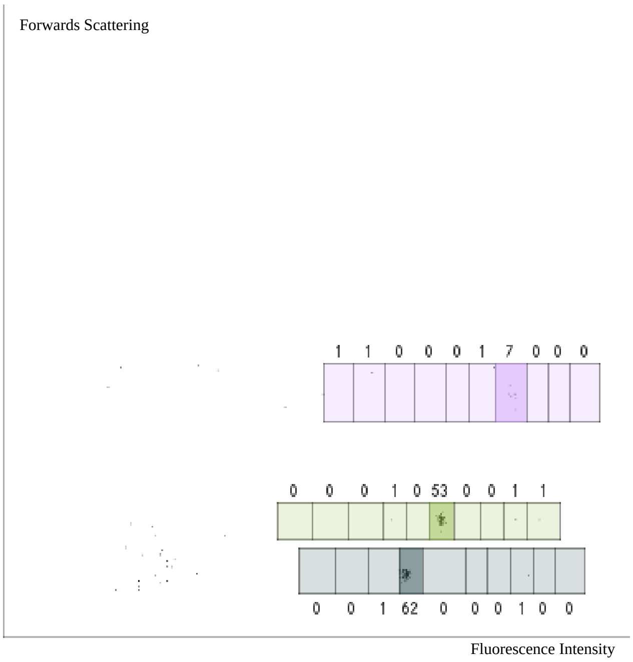

ANNEX 3: TAG DECONVOLUTION - BEAD 448

Passes flow sorting criteria: Yes

Passes tag deconvolution criteria: No

Included in protocol analysis: No

Protocol: N/A

Filename: Bin9_plateA0_B10.fcs

Split 1: Petrol shading

Split 2: Green shading

Split 3: Violet shading

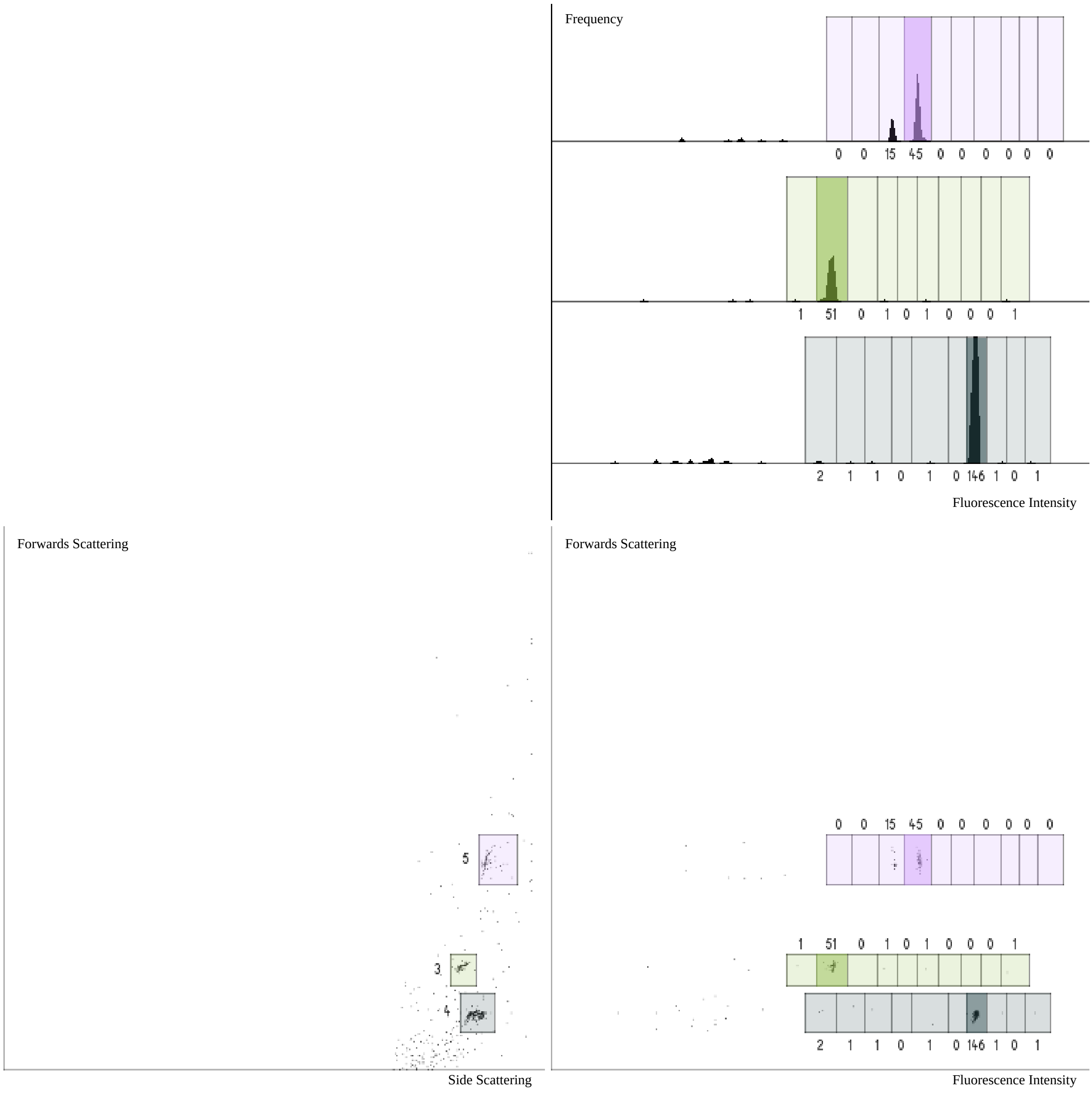

ANNEX 3: TAG DECONVOLUTION - BEAD 449

Passes flow sorting criteria: Yes

Passes tag deconvolution criteria: Yes

Included in protocol analysis: Yes

Protocol: 9, 8, 9, 9

Filename: Bin9_plateA0_B11.fcs

Split 1: Petrol shading

Split 2: Green shading

Split 3: Violet shading

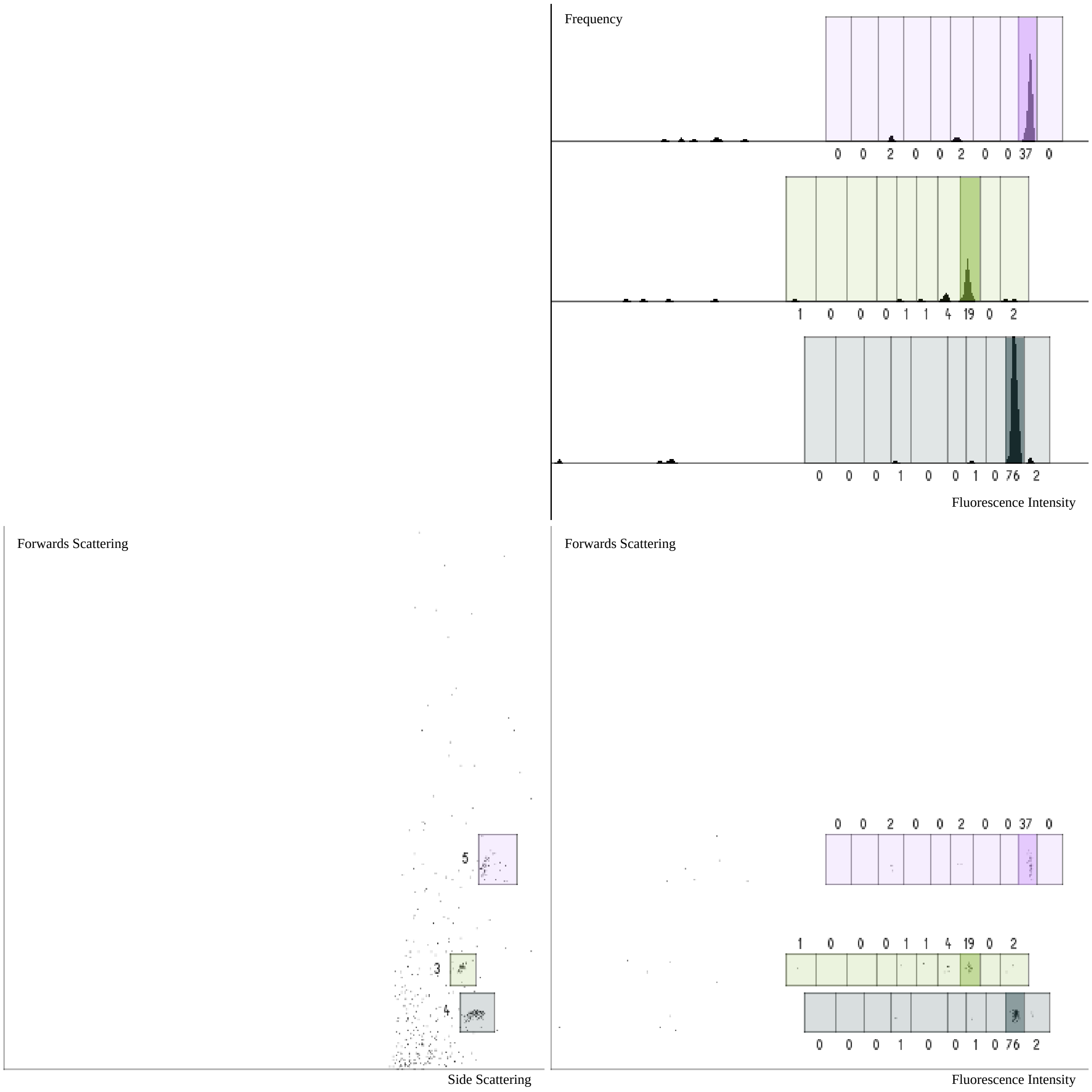

ANNEX 3: TAG DECONVOLUTION - BEAD 450

Passes flow sorting criteria: Yes

Passes tag deconvolution criteria: Yes

Included in protocol analysis: Yes

Protocol: 8, 9, 8, 9

Filename: Bin9_plateA0_B12.fcs

Split 1: Petrol shading

Split 2: Green shading

Split 3: Violet shading

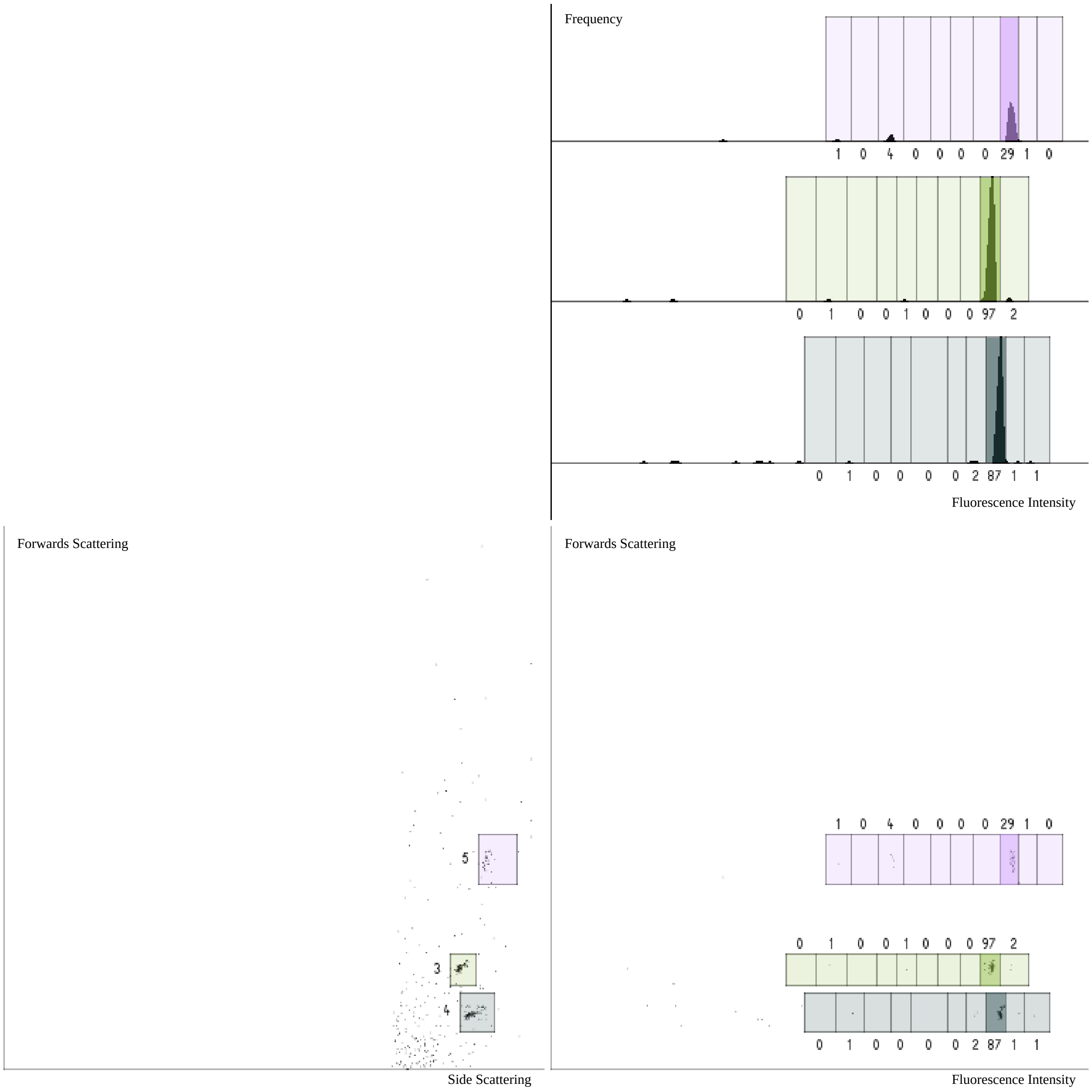

ANNEX 3: TAG DECONVOLUTION - BEAD 451

Passes flow sorting criteria: Yes

Passes tag deconvolution criteria: Yes

Included in protocol analysis: Yes

Protocol: 2, 10, 6, 9

Filename: Bin9_plateA0_C1.fcs

Split 1: Petrol shading

Split 2: Green shading

Split 3: Violet shading

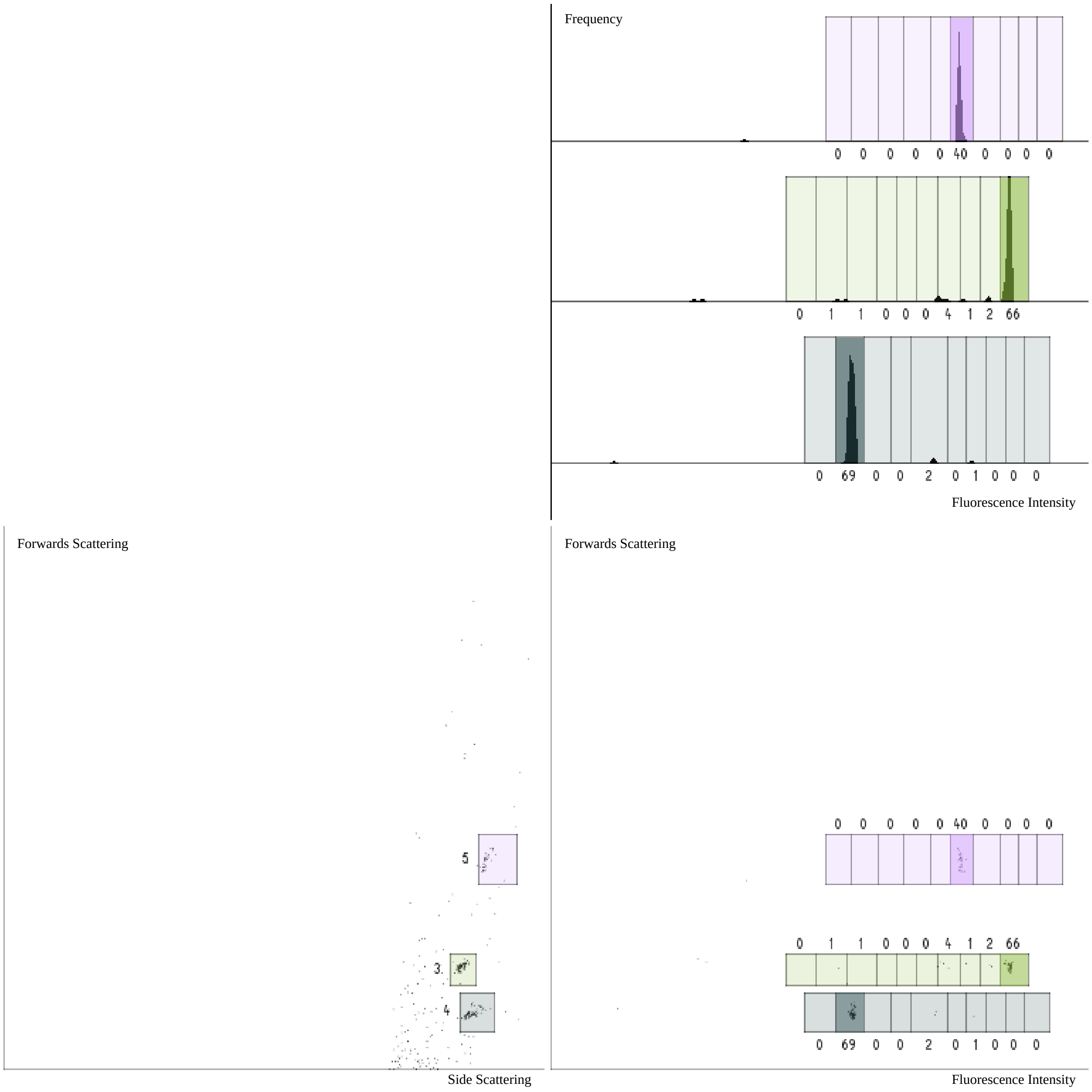

ANNEX 3: TAG DECONVOLUTION - BEAD 452

Passes flow sorting criteria: Yes

Passes tag deconvolution criteria: No

Included in protocol analysis: No

Protocol: N/A

Filename: Bin9_plateA0_C2.fcs

Split 1: Petrol shading

Split 2: Green shading

Split 3: Violet shading

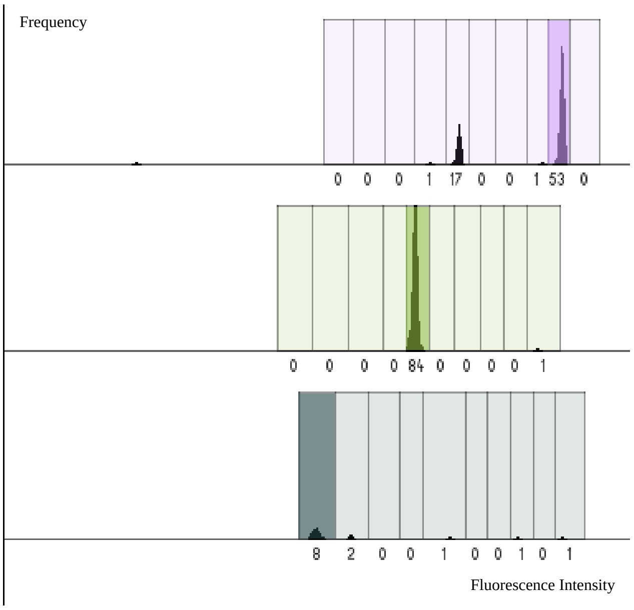

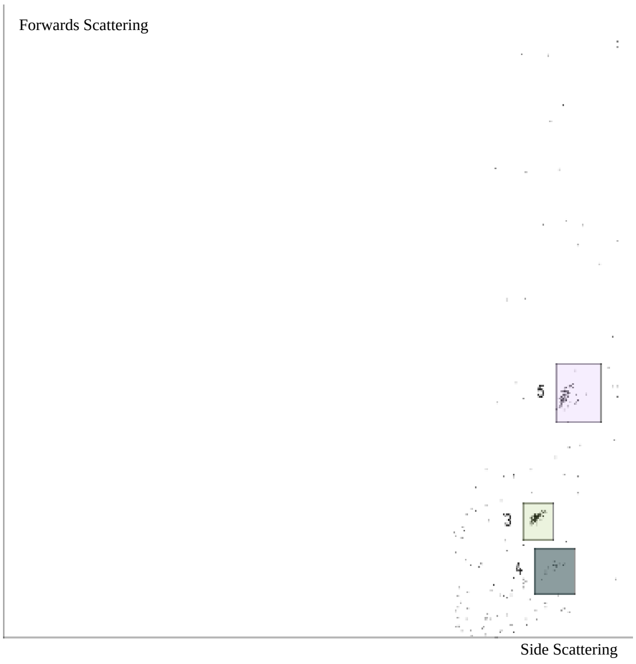

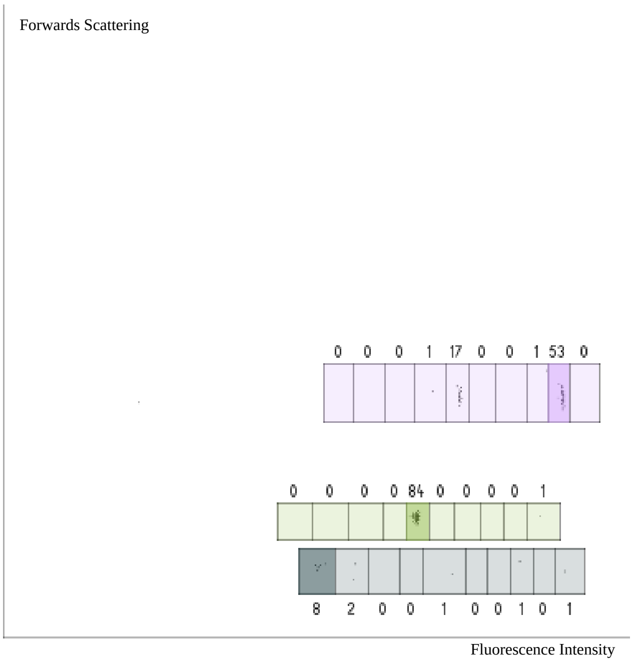

ANNEX 3: TAG DECONVOLUTION - BEAD 453

Passes flow sorting criteria: Yes

Passes tag deconvolution criteria: Yes

Included in protocol analysis: Yes

Protocol: 2, 5, 5, 9

Filename: Bin9_plateA0_C3.fcs

Split 1: Petrol shading

Split 2: Green shading

Split 3: Violet shading

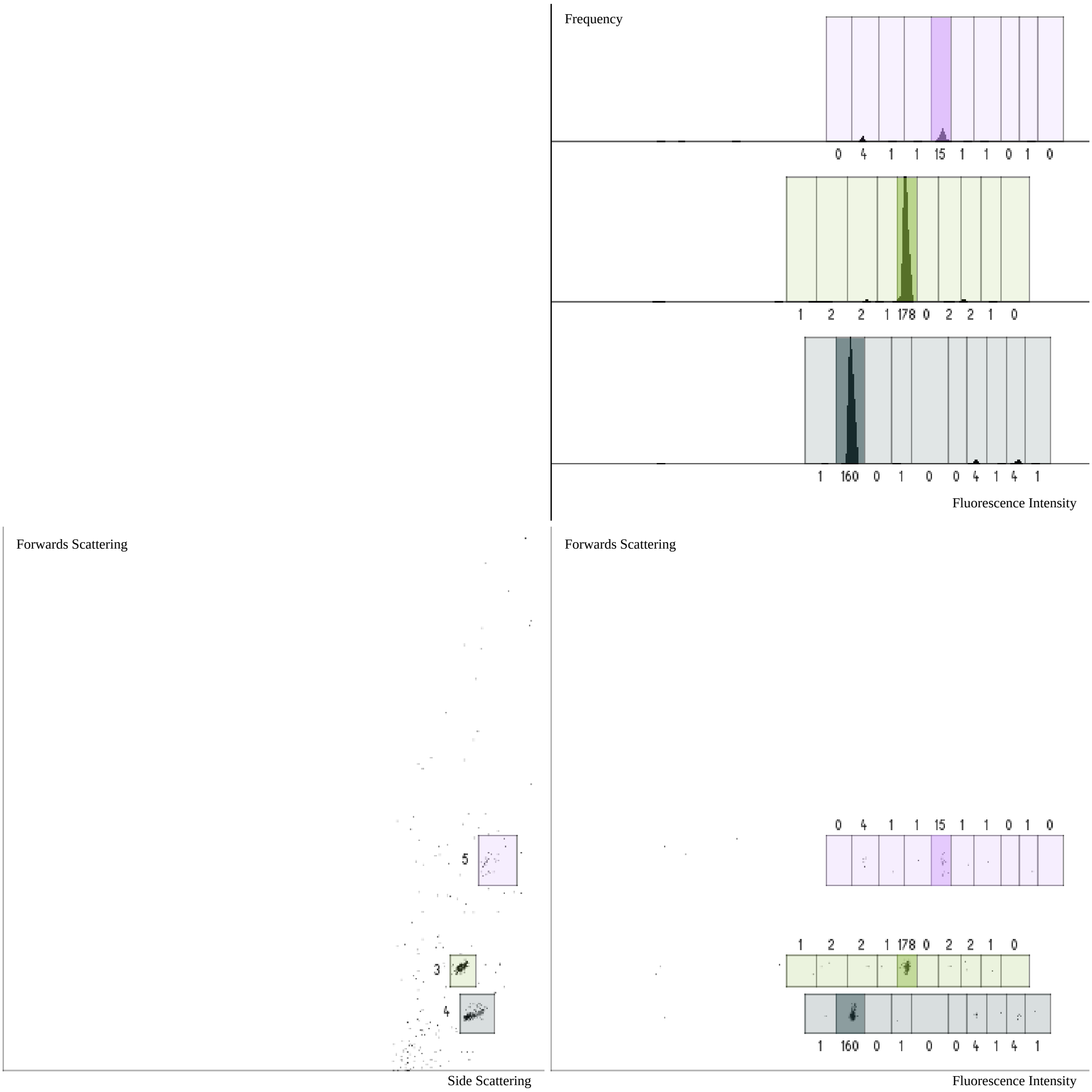

ANNEX 3: TAG DECONVOLUTION - BEAD 454

Passes flow sorting criteria: Yes

Passes tag deconvolution criteria: Yes

Included in protocol analysis: Yes

Protocol: 3, 6, 6, 9

Filename: Bin9_plateA0_C4.fcs

Split 1: Petrol shading

Split 2: Green shading

Split 3: Violet shading

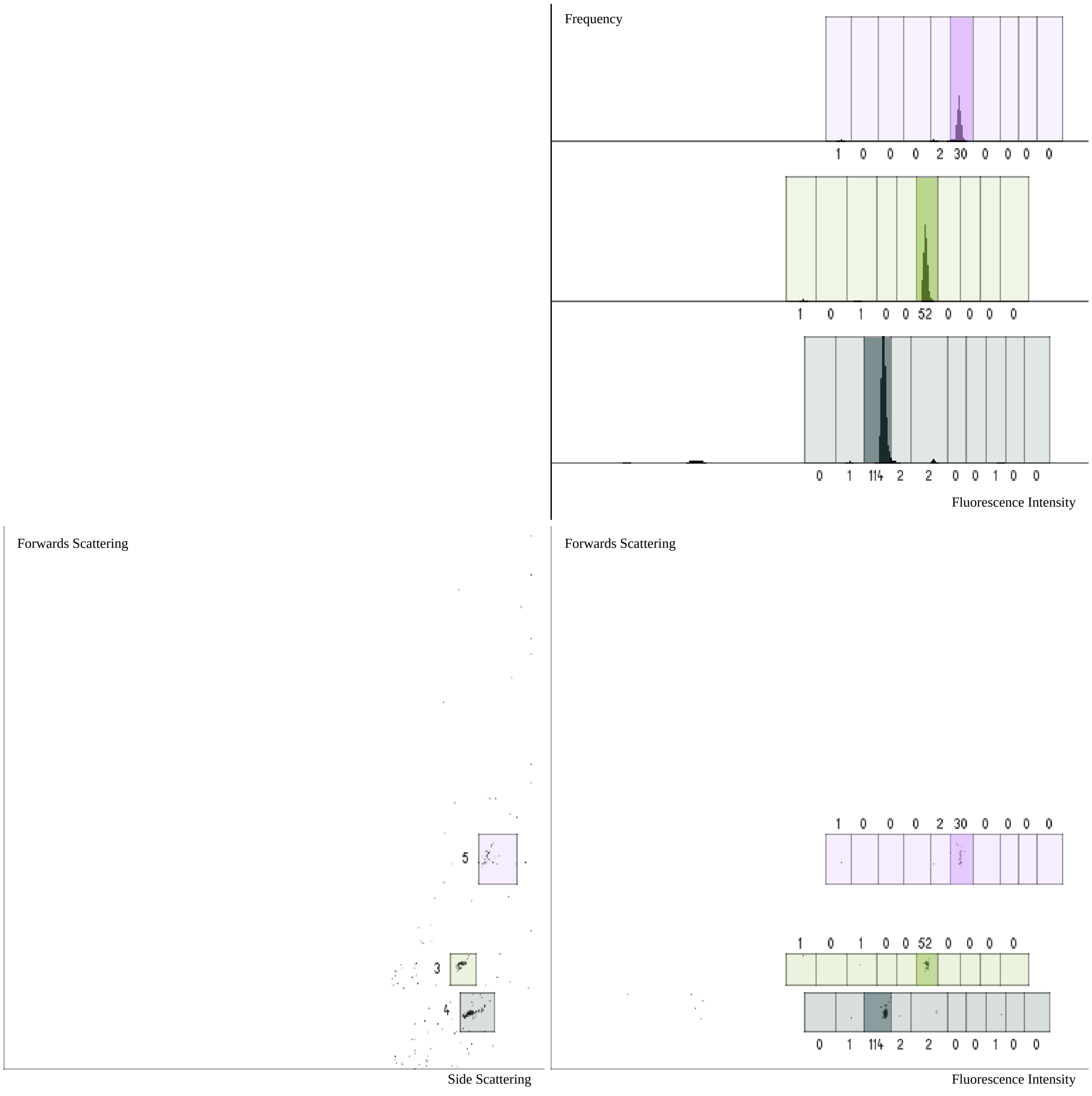

ANNEX 3: TAG DECONVOLUTION - BEAD 455

Passes flow sorting criteria: Yes

Passes tag deconvolution criteria: Yes

Included in protocol analysis: Yes

Protocol: 10, 5, 1, 9

Filename: Bin9_plateA0_C5.fcs

Split 1: Petrol shading

Split 2: Green shading

Split 3: Violet shading

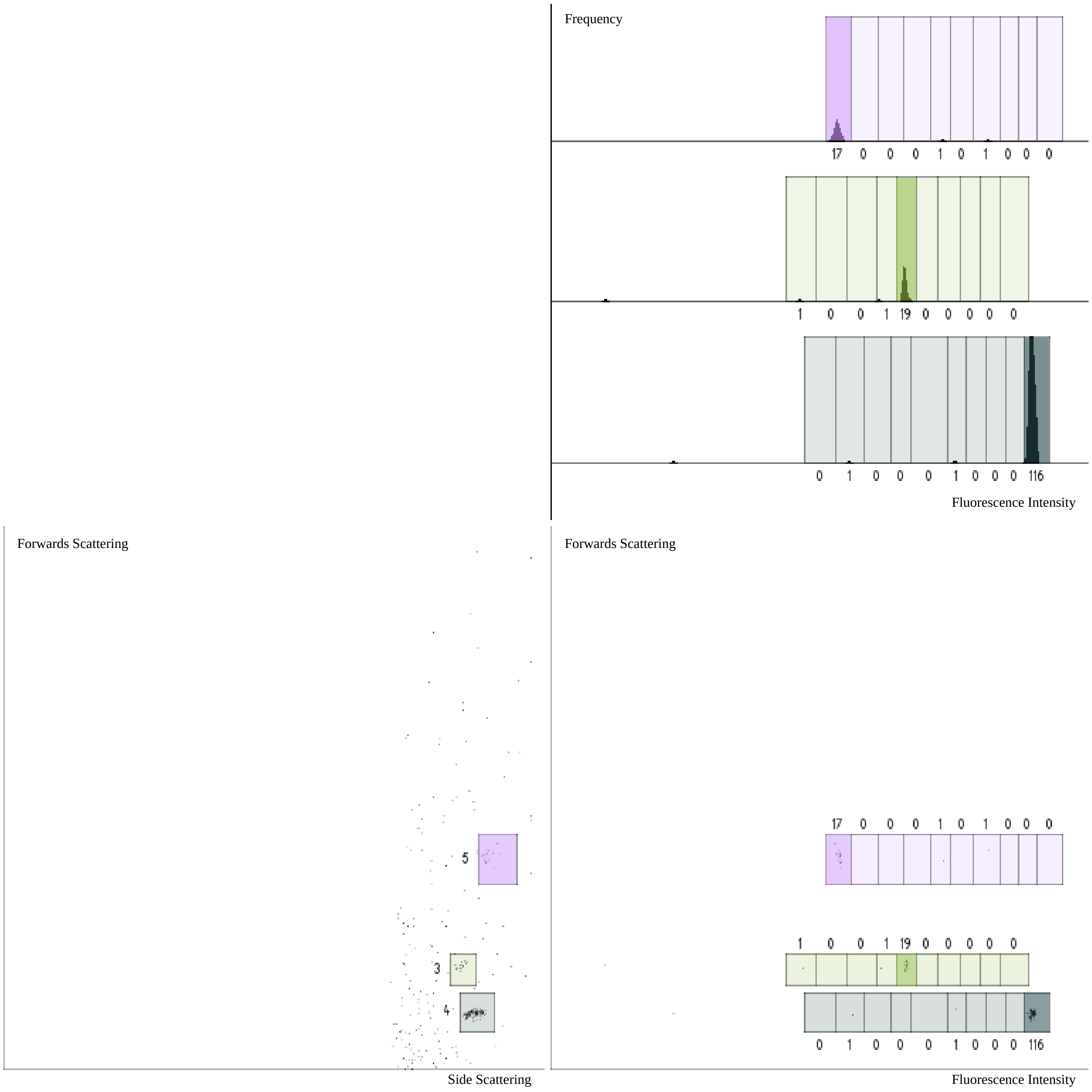

ANNEX 3: TAG DECONVOLUTION - BEAD 456

Passes flow sorting criteria: Yes

Passes tag deconvolution criteria: Yes

Included in protocol analysis: Yes

Protocol: 3, 4, 7, 9

Filename: Bin9_plateA0_C6.fcs

Split 1: Petrol shading

Split 2: Green shading

Split 3: Violet shading

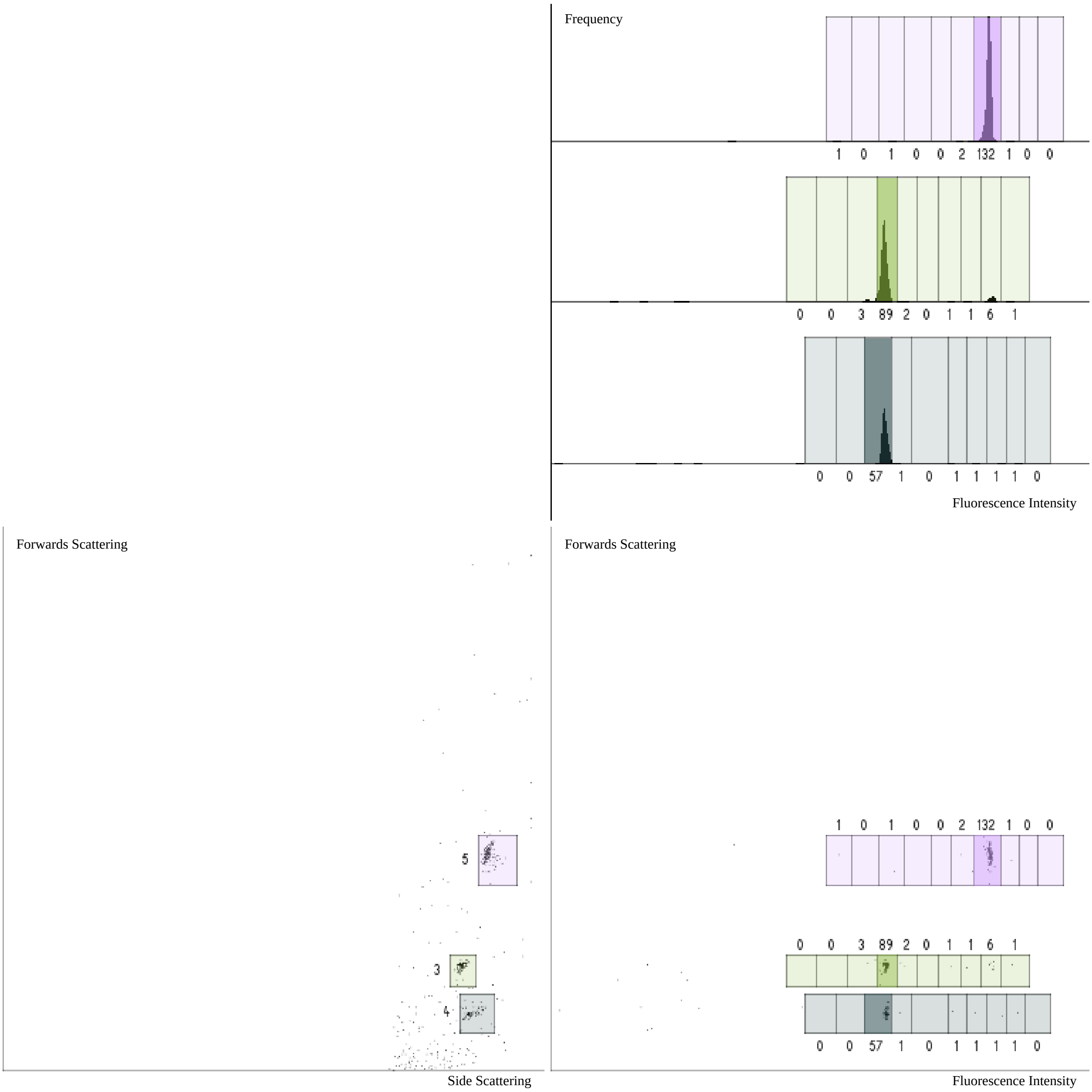

ANNEX 3: TAG DECONVOLUTION - BEAD 457

Passes flow sorting criteria: Yes

Passes tag deconvolution criteria: No

Included in protocol analysis: No

Protocol: N/A

Filename: Bin9_plateA0_C7.fcs

Split 1: Petrol shading

Split 2: Green shading

Split 3: Violet shading

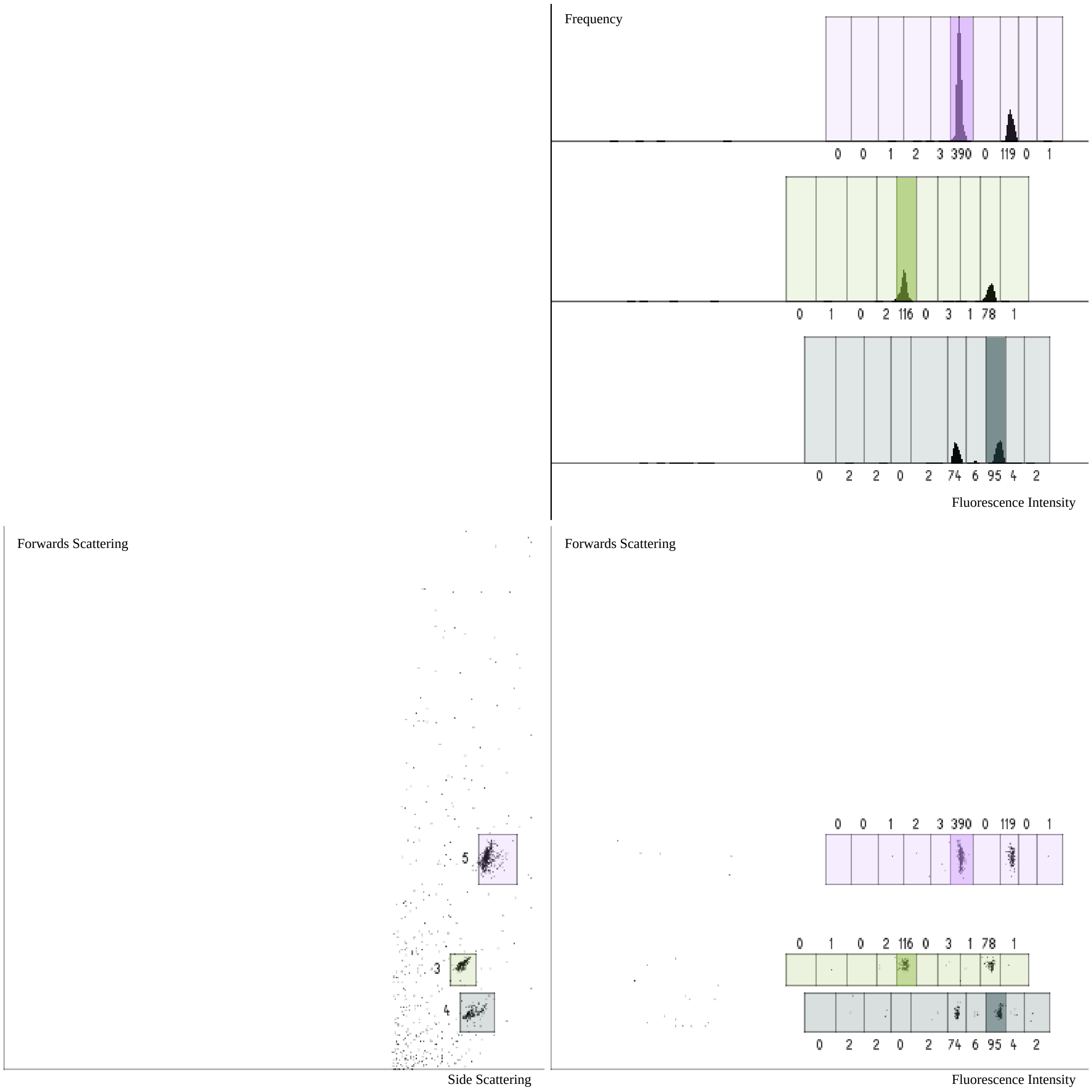

ANNEX 3: TAG DECONVOLUTION - BEAD 458

Passes flow sorting criteria: Yes

Passes tag deconvolution criteria: Yes

Included in protocol analysis: Yes

Protocol: 7, 4, 4, 9

Filename: Bin9_plateA0_C8.fcs

Split 1: Petrol shading

Split 2: Green shading

Split 3: Violet shading

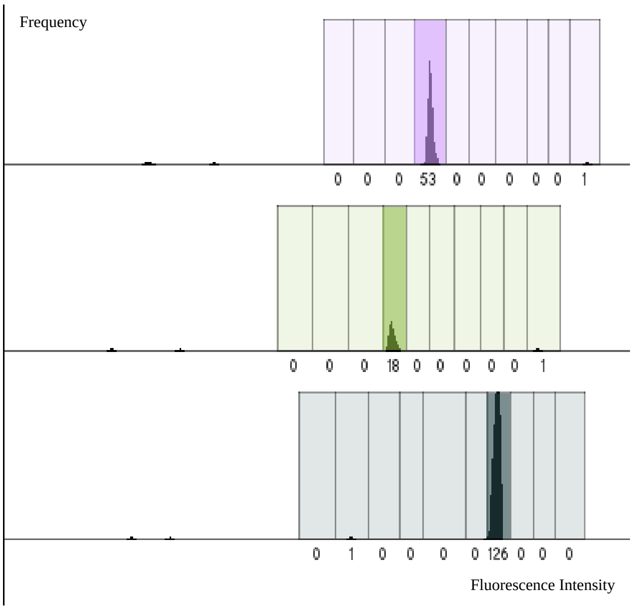

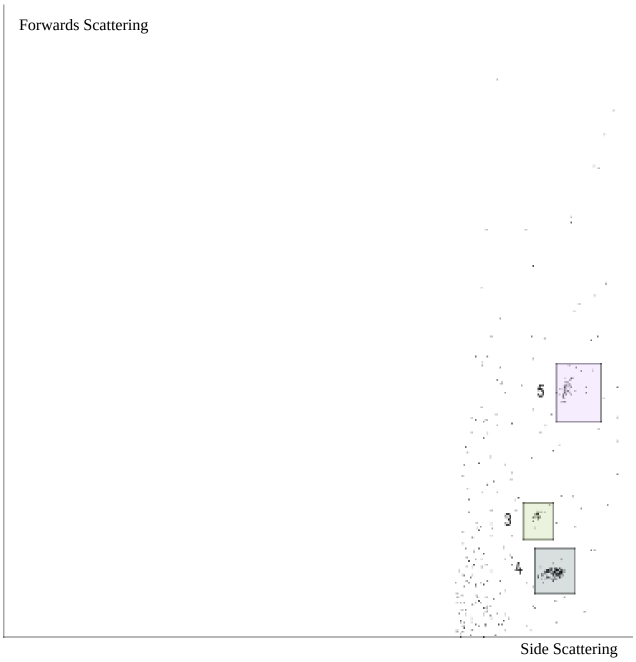

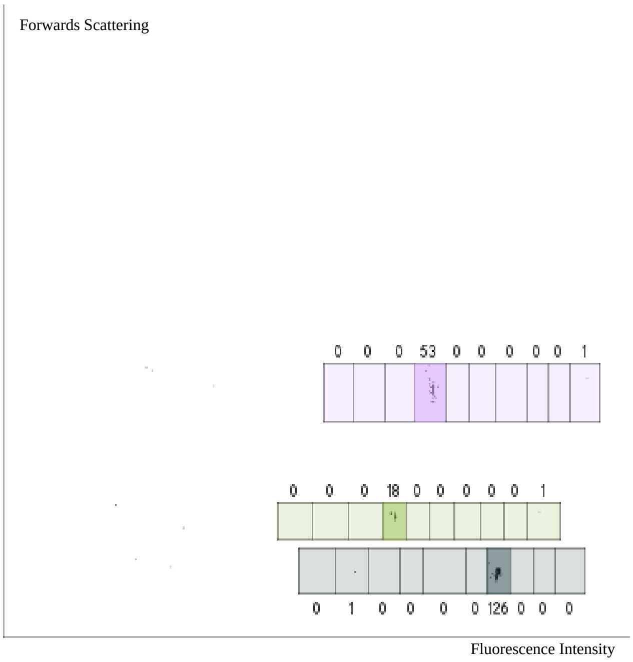

ANNEX 3: TAG DECONVOLUTION - BEAD 459

Passes flow sorting criteria: Yes

Passes tag deconvolution criteria: No

Included in protocol analysis: No

Protocol: N/A

Filename: Bin9_plateA0_C9.fcs

Split 1: Petrol shading

Split 2: Green shading

Split 3: Violet shading

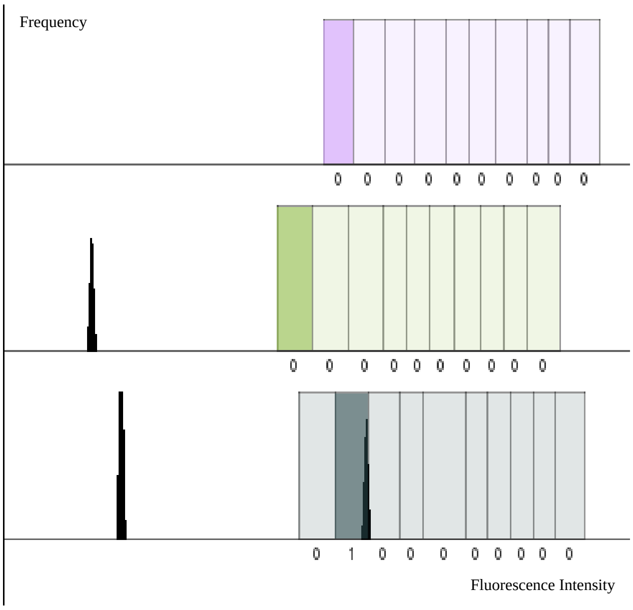

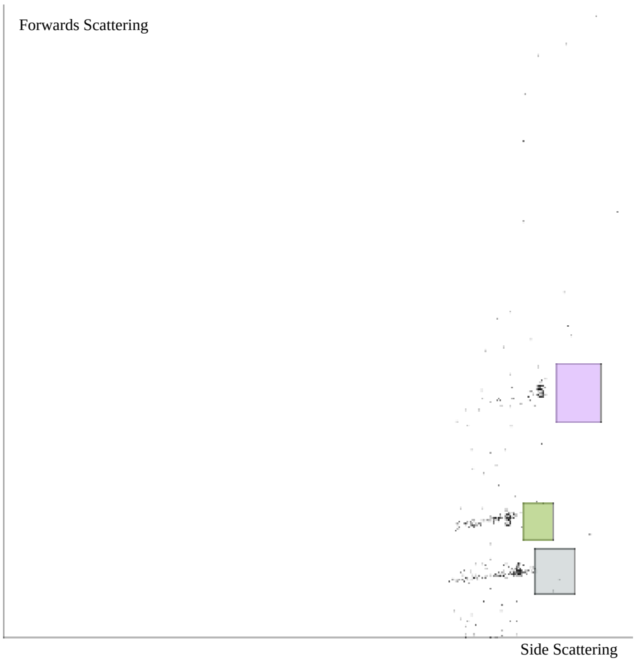

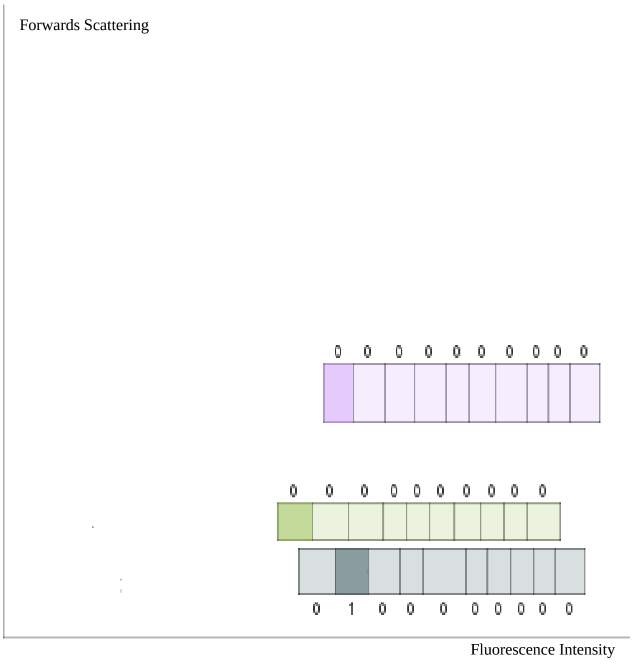

ANNEX 3: TAG DECONVOLUTION - BEAD 460

Passes flow sorting criteria: Yes

Passes tag deconvolution criteria: No

Included in protocol analysis: No

Protocol: N/A

Filename: Bin9_plateA0_C10.fcs

Split 1: Petrol shading

Split 2: Green shading

Split 3: Violet shading

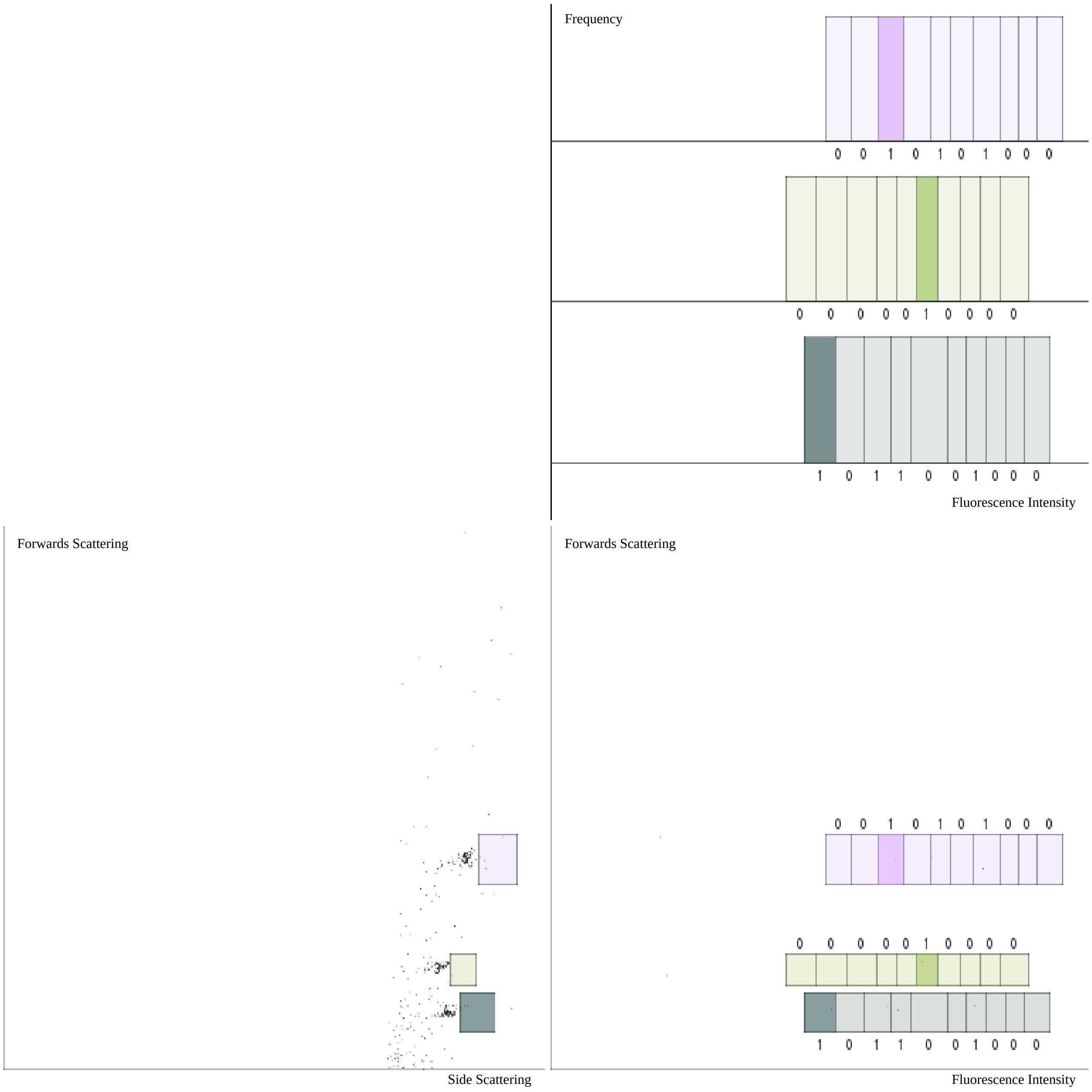

ANNEX 3: TAG DECONVOLUTION - BEAD 461

Passes flow sorting criteria: Yes

Passes tag deconvolution criteria: Yes

Included in protocol analysis: Yes

Protocol: 10, 4, 2, 9

Filename: Bin9_plateA0_C11.fcs

Split 1: Petrol shading

Split 2: Green shading

Split 3: Violet shading

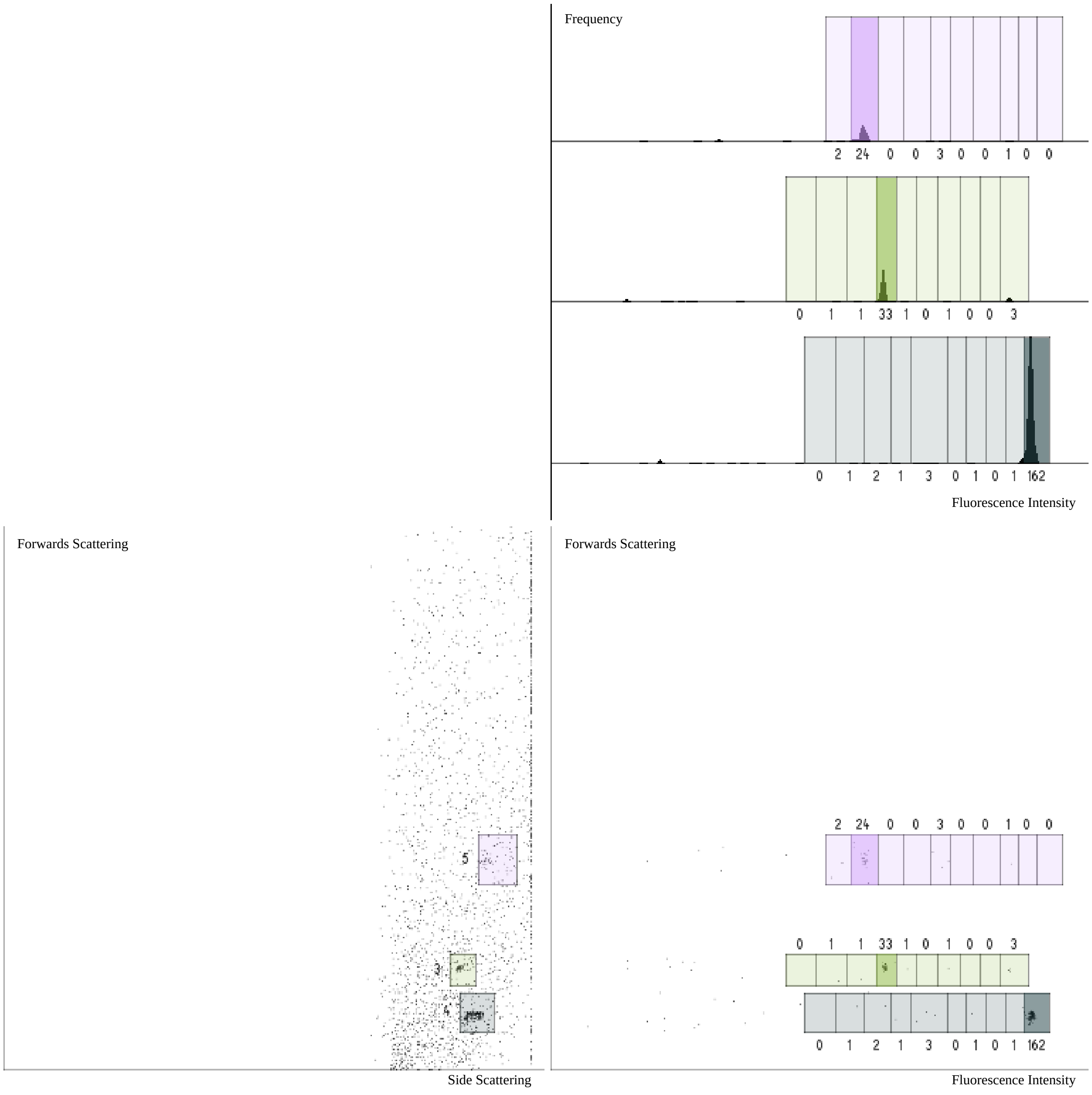

ANNEX 3: TAG DECONVOLUTION - BEAD 462

Passes flow sorting criteria: Yes

Passes tag deconvolution criteria: Yes

Included in protocol analysis: Yes

Protocol: 7, 5, 9, 9

Filename: Bin9_plateA0_C12.fcs

Split 1: Petrol shading

Split 2: Green shading

Split 3: Violet shading

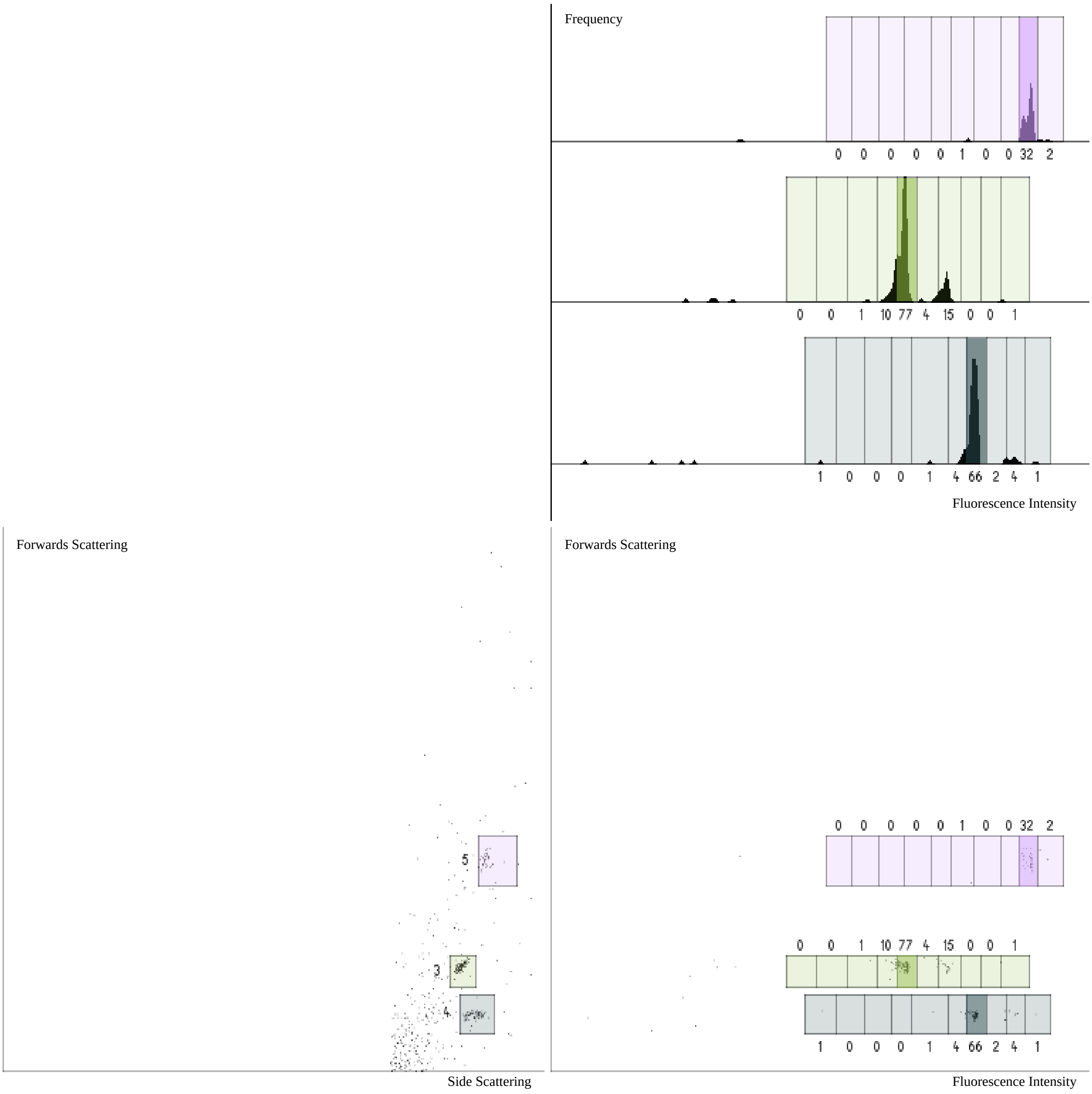

ANNEX 3: TAG DECONVOLUTION - BEAD 463

Passes flow sorting criteria: Yes

Passes tag deconvolution criteria: Yes

Included in protocol analysis: Yes

Protocol: 9, 4, 1, 9

Filename: Bin9_plateA0_D1.fcs

Split 1: Petrol shading

Split 2: Green shading

Split 3: Violet shading

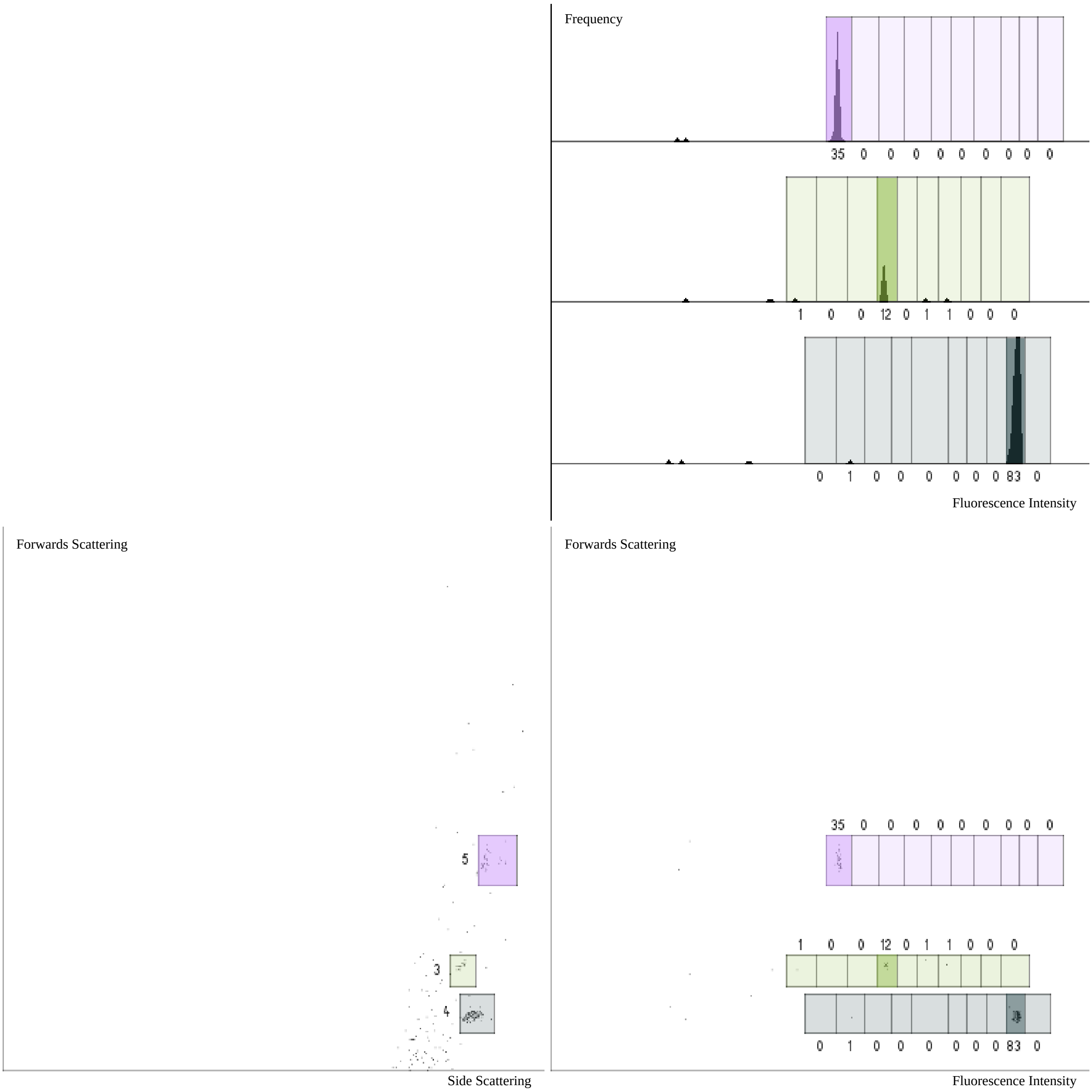

ANNEX 3: TAG DECONVOLUTION - BEAD 464

Passes flow sorting criteria: Yes

Passes tag deconvolution criteria: No

Included in protocol analysis: No

Protocol: N/A

Filename: Bin9_plateA0_D2.fcs

Split 1: Petrol shading

Split 2: Green shading

Split 3: Violet shading

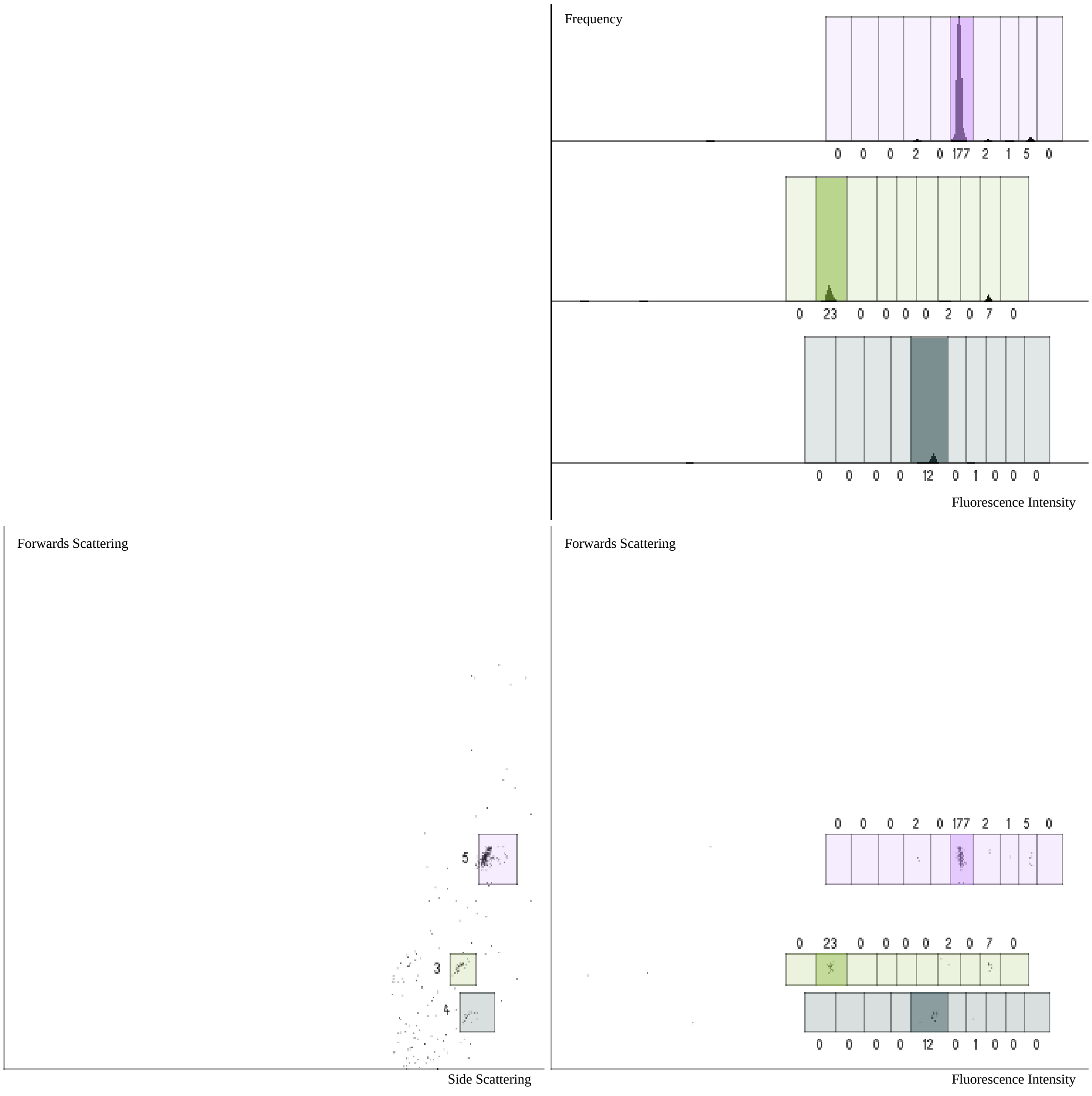

ANNEX 3: TAG DECONVOLUTION - BEAD 465

Passes flow sorting criteria: Yes

Passes tag deconvolution criteria: Yes

Included in protocol analysis: Yes

Protocol: 6, 10, 1, 9

Filename: Bin9_plateA0_D3.fcs

Split 1: Petrol shading

Split 2: Green shading

Split 3: Violet shading

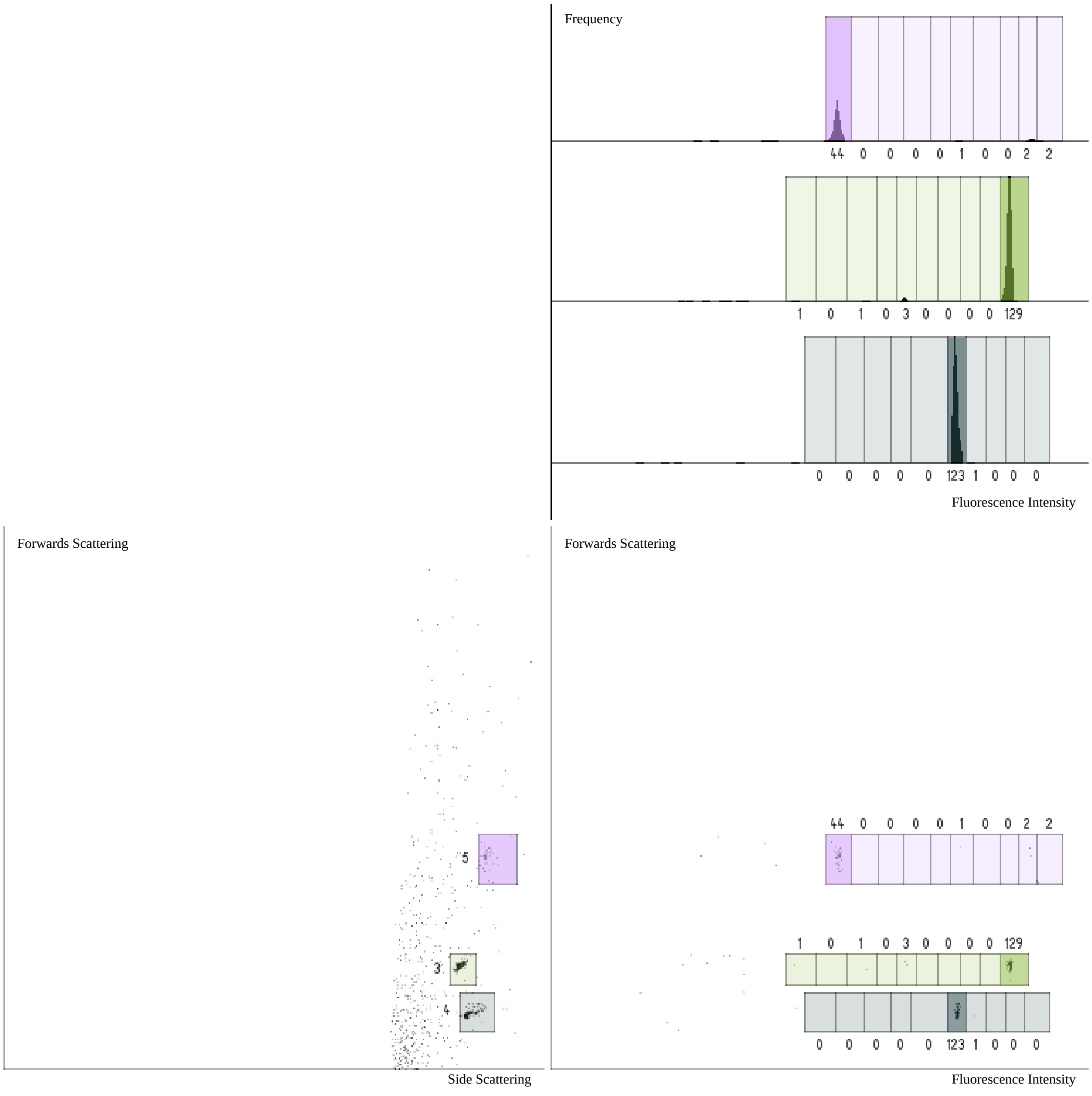

ANNEX 3: TAG DECONVOLUTION - BEAD 466

Passes flow sorting criteria: Yes

Passes tag deconvolution criteria: No

Included in protocol analysis: No

Protocol: N/A

Filename: Bin9_plateA0_D4.fcs

Split 1: Petrol shading

Split 2: Green shading

Split 3: Violet shading

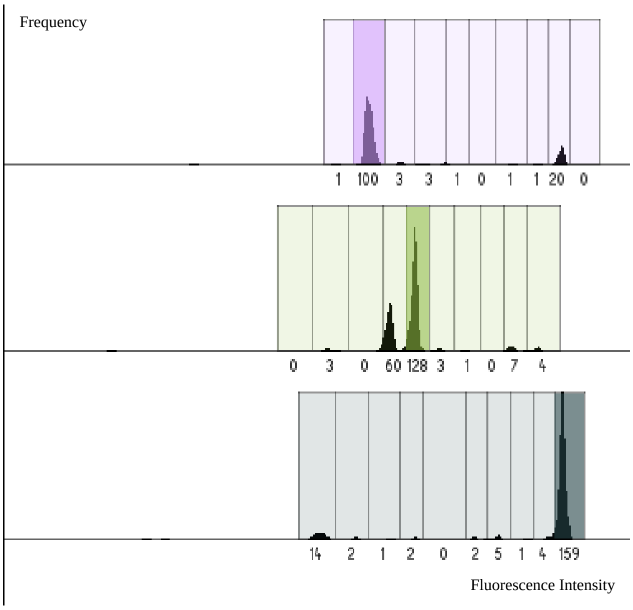

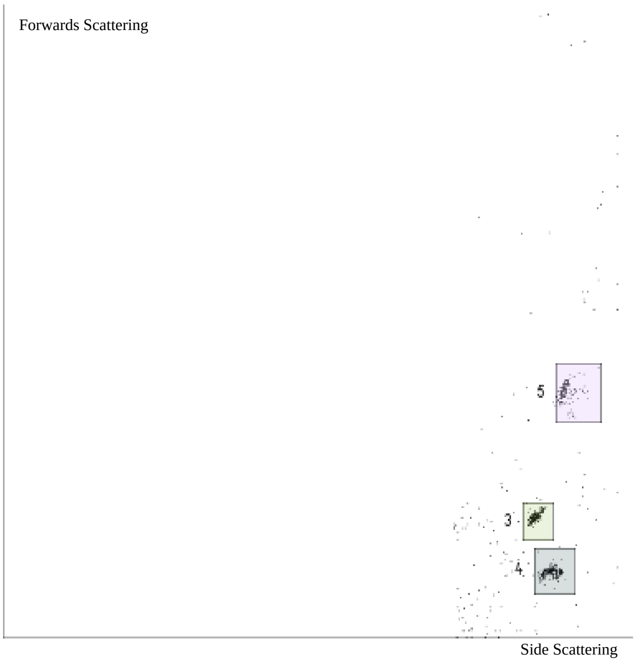

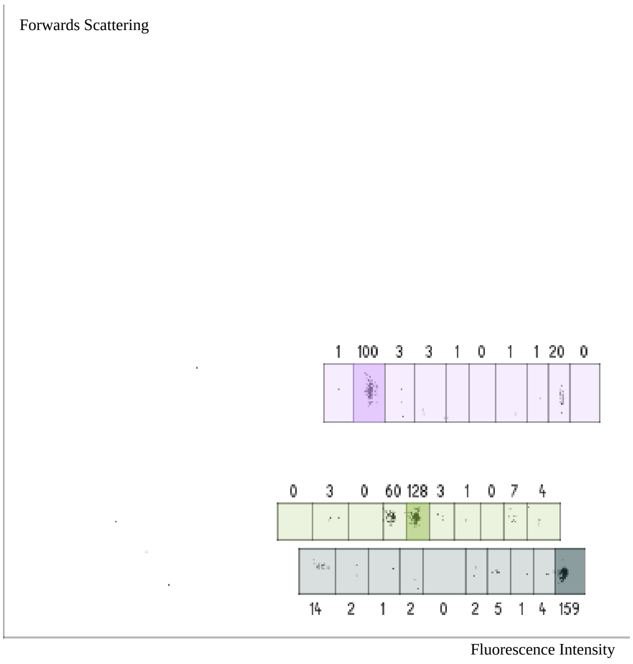

ANNEX 3: TAG DECONVOLUTION - BEAD 467

Passes flow sorting criteria: Yes

Passes tag deconvolution criteria: Yes

Included in protocol analysis: Yes

Protocol: 1, 5, 1, 9

Filename: Bin9_plateA0_D5.fcs

Split 1: Petrol shading

Split 2: Green shading

Split 3: Violet shading

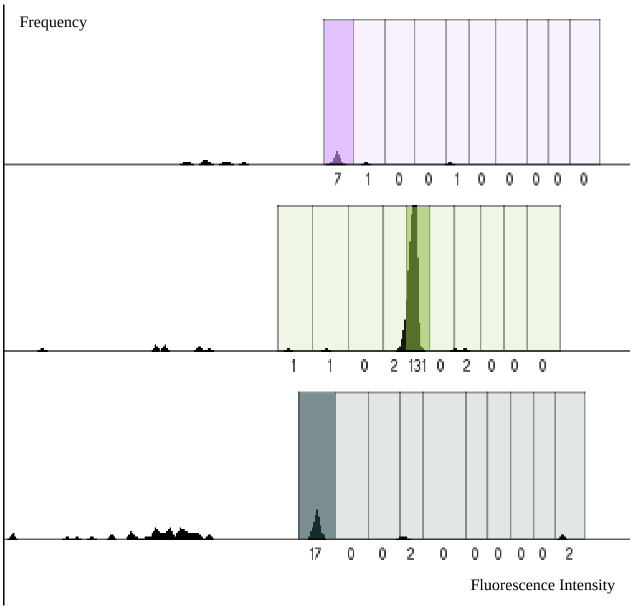

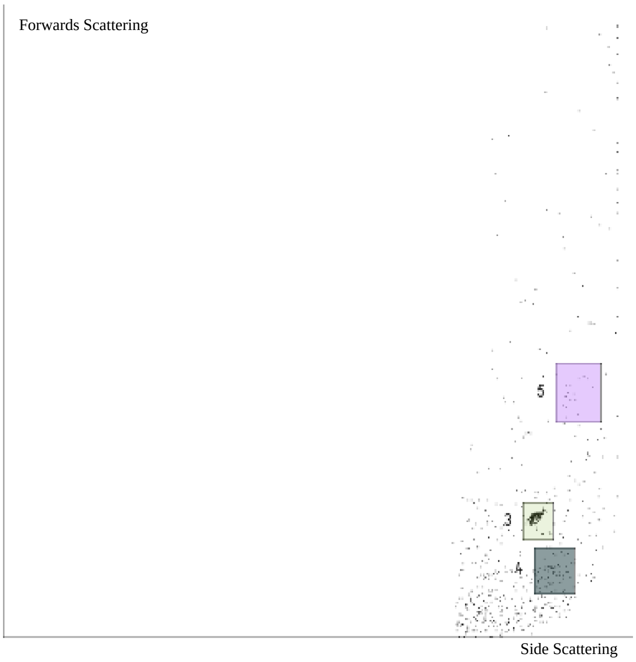

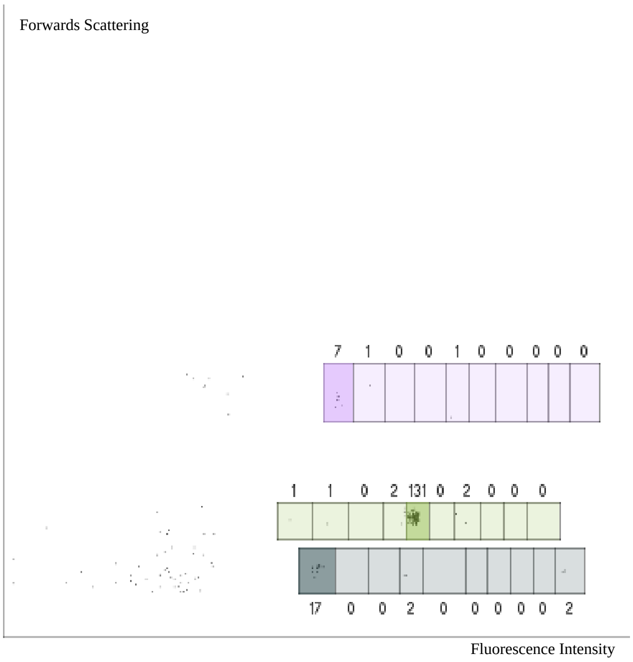

ANNEX 3: TAG DECONVOLUTION - BEAD 468

Passes flow sorting criteria: Yes

Passes tag deconvolution criteria: Yes

Included in protocol analysis: Yes

Protocol: 6, 8, 5, 9

Filename: Bin9_plateA0_D6.fcs

Split 1: Petrol shading

Split 2: Green shading

Split 3: Violet shading

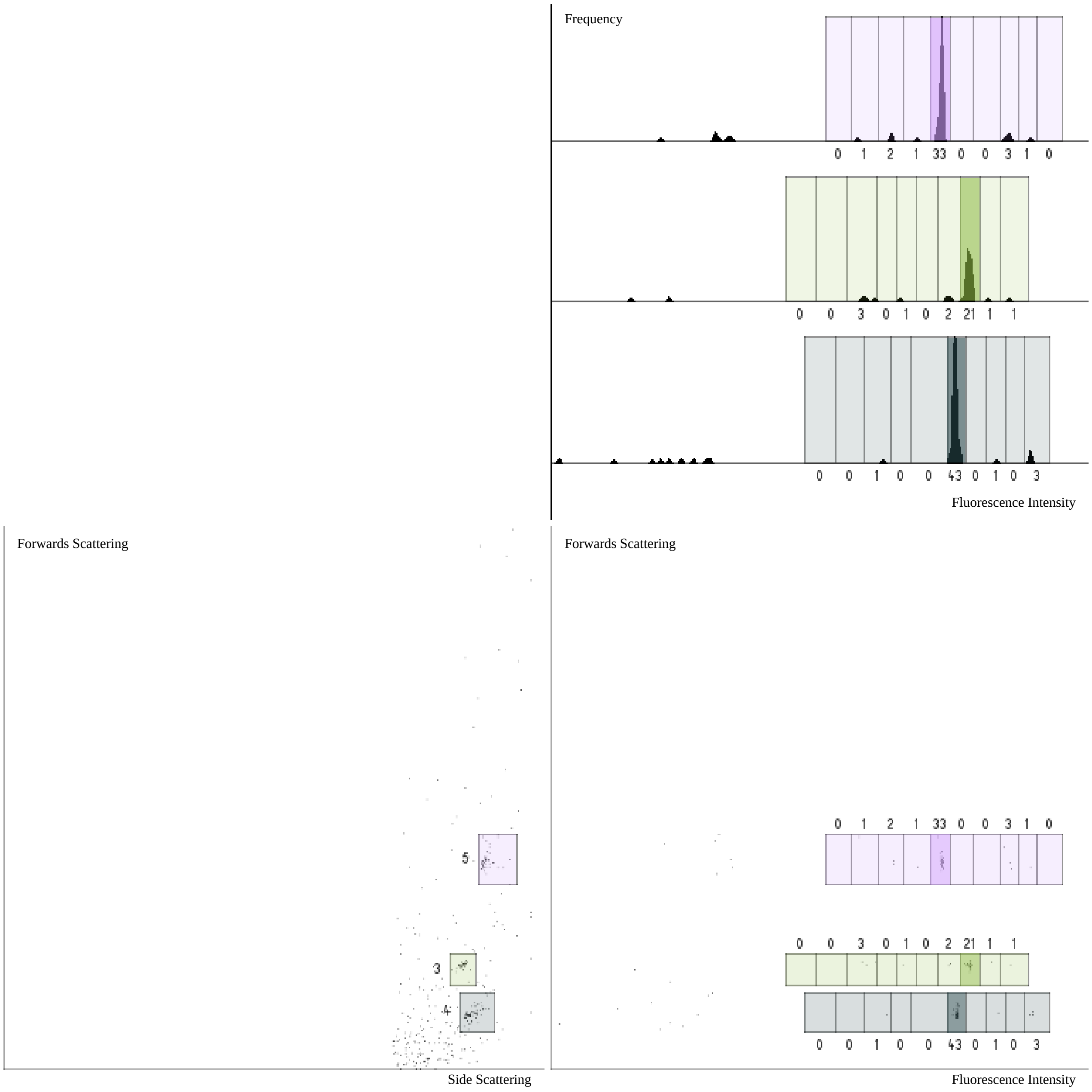

ANNEX 3: TAG DECONVOLUTION - BEAD 469

Passes flow sorting criteria: Yes

Passes tag deconvolution criteria: Yes

Included in protocol analysis: Yes

Protocol: 2, 4, 1, 9

Filename: Bin9_plateA0_H12.fcs

Split 1: Petrol shading

Split 2: Green shading

Split 3: Violet shading

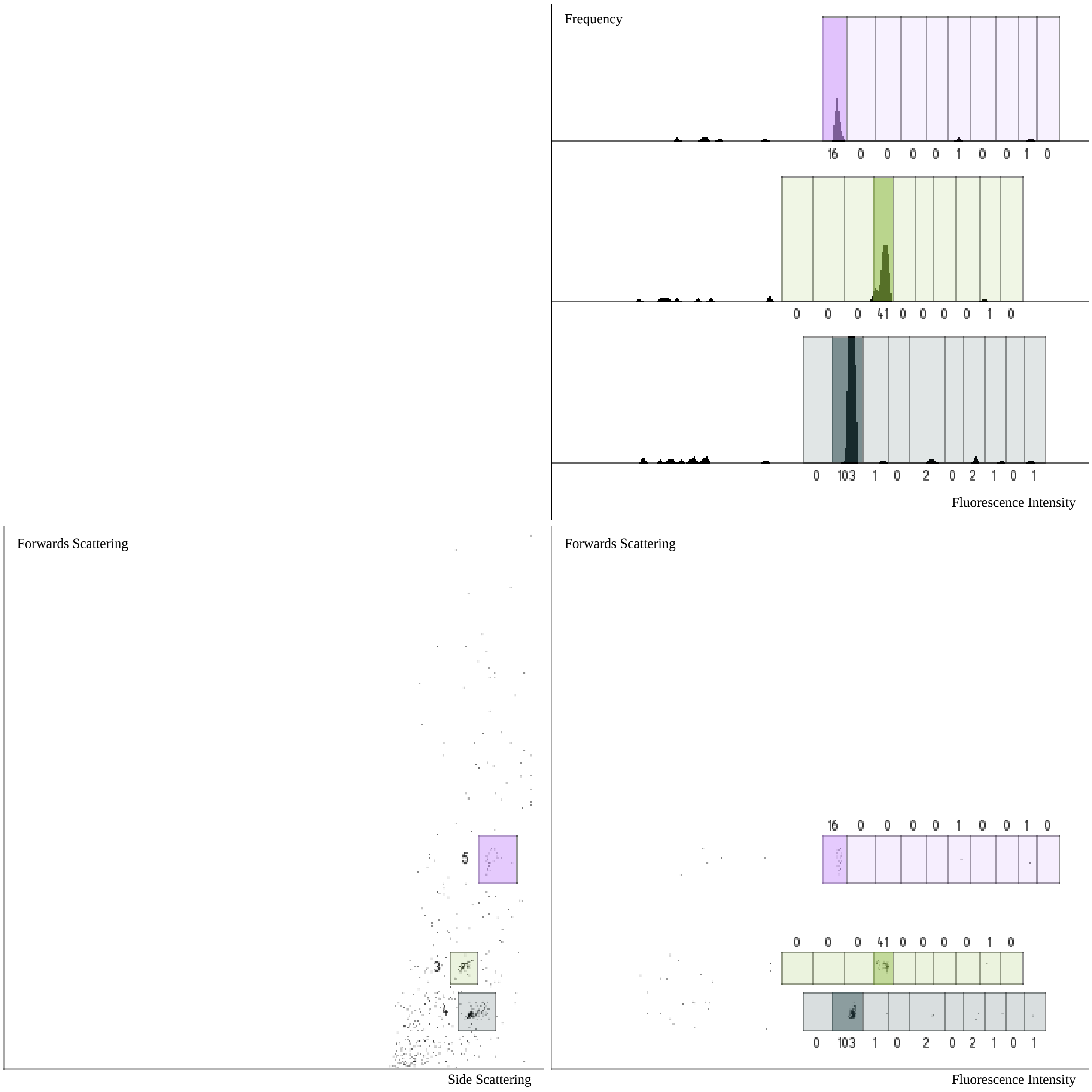

ANNEX 3: TAG DECONVOLUTION - BEAD 470

Passes flow sorting criteria: Yes

Passes tag deconvolution criteria: No

Included in protocol analysis: No

Protocol: N/A

Filename: Bin9_plateA0_D8.fcs

Split 1: Petrol shading

Split 2: Green shading

Split 3: Violet shading

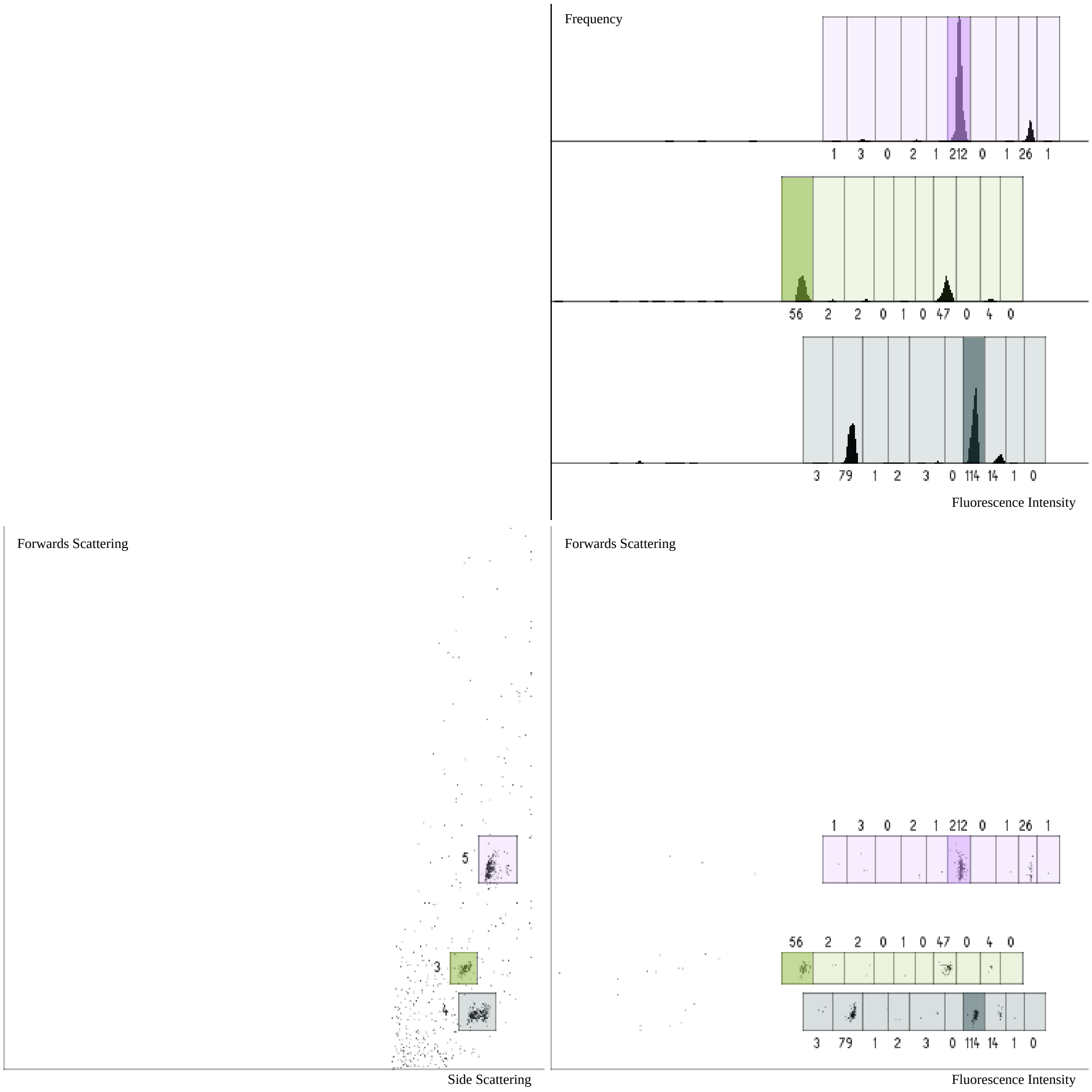

ANNEX 3: TAG DECONVOLUTION - BEAD 471

Passes flow sorting criteria: Yes

Passes tag deconvolution criteria: No

Included in protocol analysis: No

Protocol: N/A

Filename: Bin9_plateA0_D9.fcs

Split 1: Petrol shading

Split 2: Green shading

Split 3: Violet shading

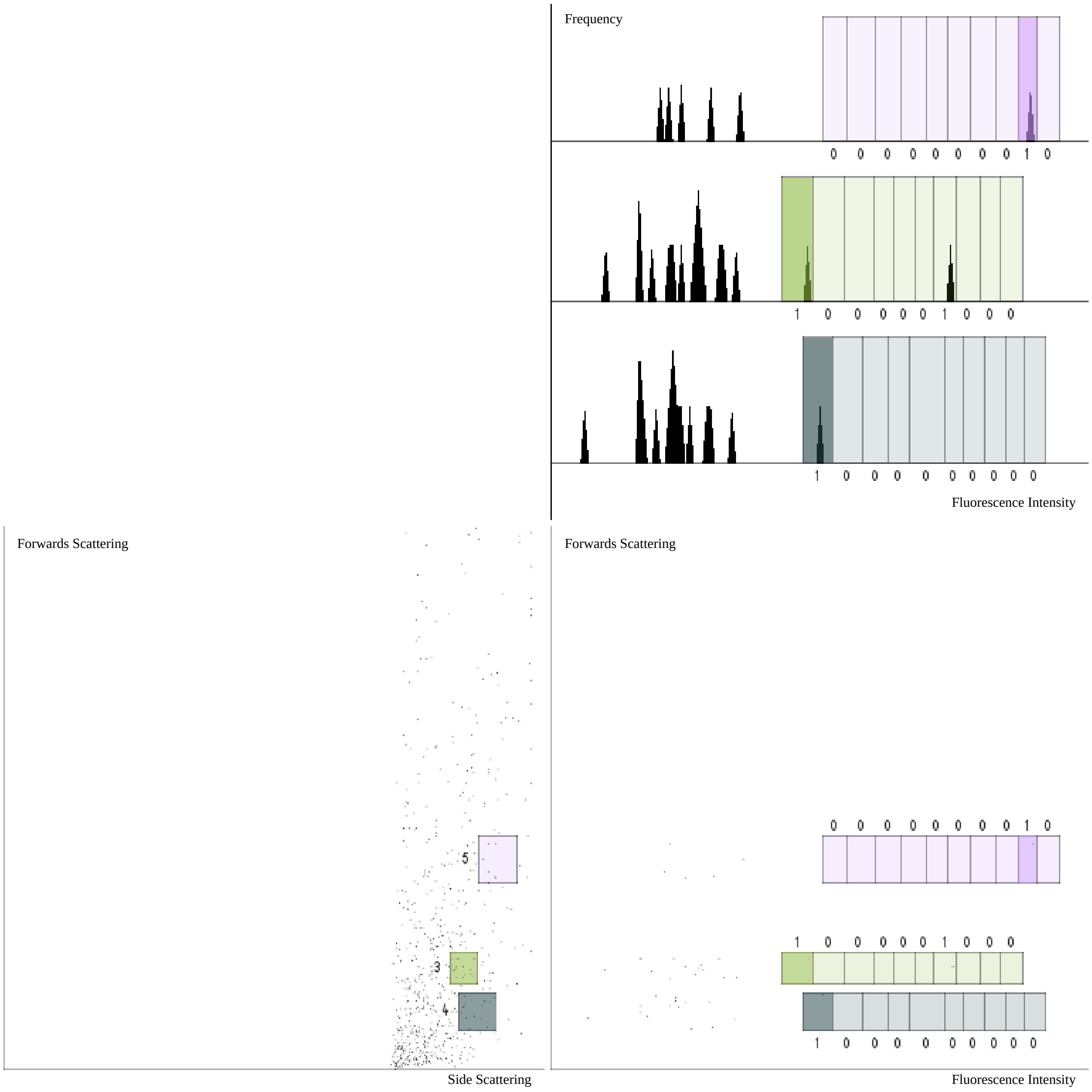

ANNEX 3: TAG DECONVOLUTION - BEAD 472

Passes flow sorting criteria: Yes

Passes tag deconvolution criteria: Yes

Included in protocol analysis: Yes

Protocol: 5, 2, 8, 9

Filename: Bin9_plateA0_D10.fcs

Split 1: Petrol shading

Split 2: Green shading

Split 3: Violet shading

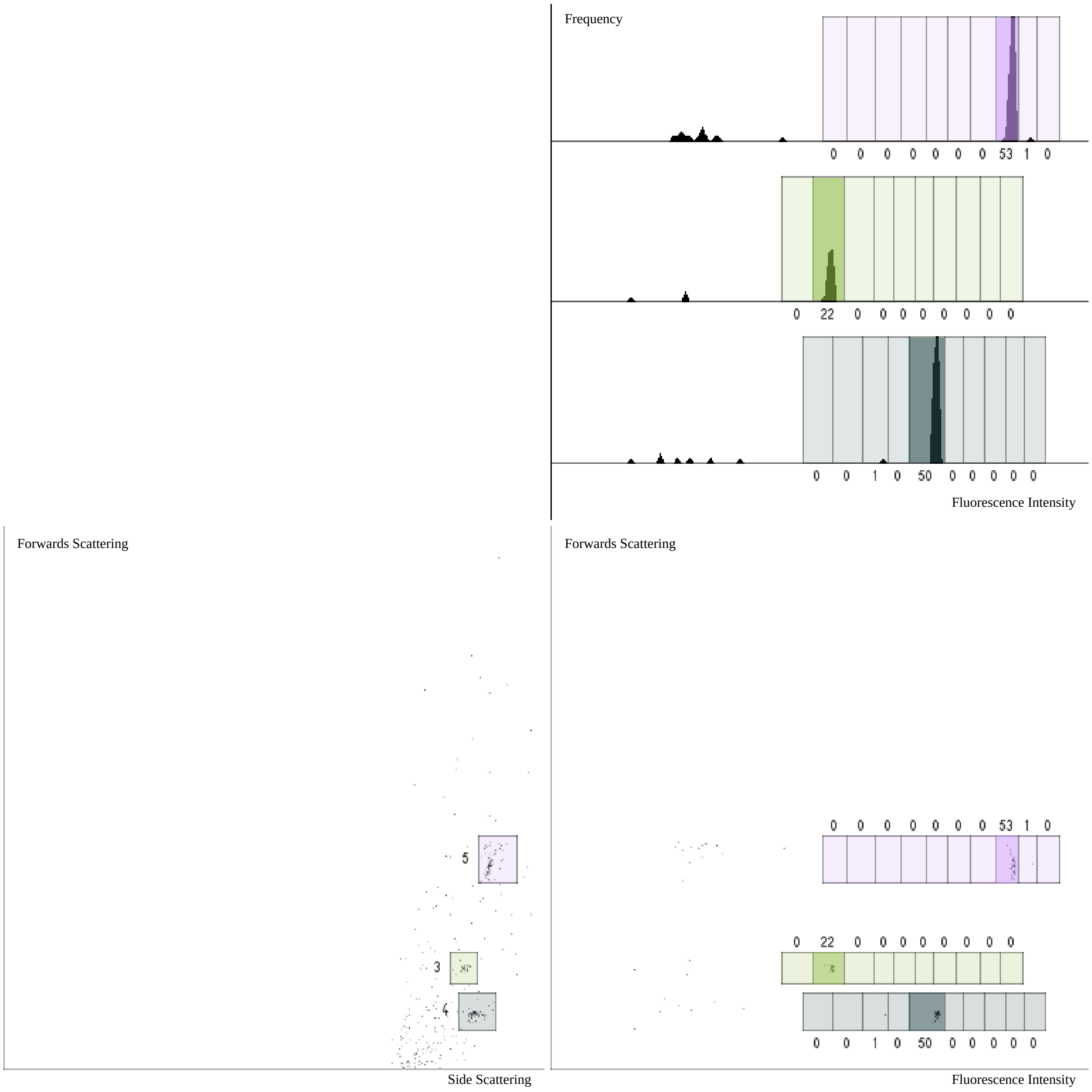

ANNEX 3: TAG DECONVOLUTION - BEAD 473

Passes flow sorting criteria: Yes

Passes tag deconvolution criteria: Yes

Included in protocol analysis: Yes

Protocol: 7, 7, 3, 9

Filename: Bin9_plateA0_D11.fcs

Split 1: Petrol shading

Split 2: Green shading

Split 3: Violet shading

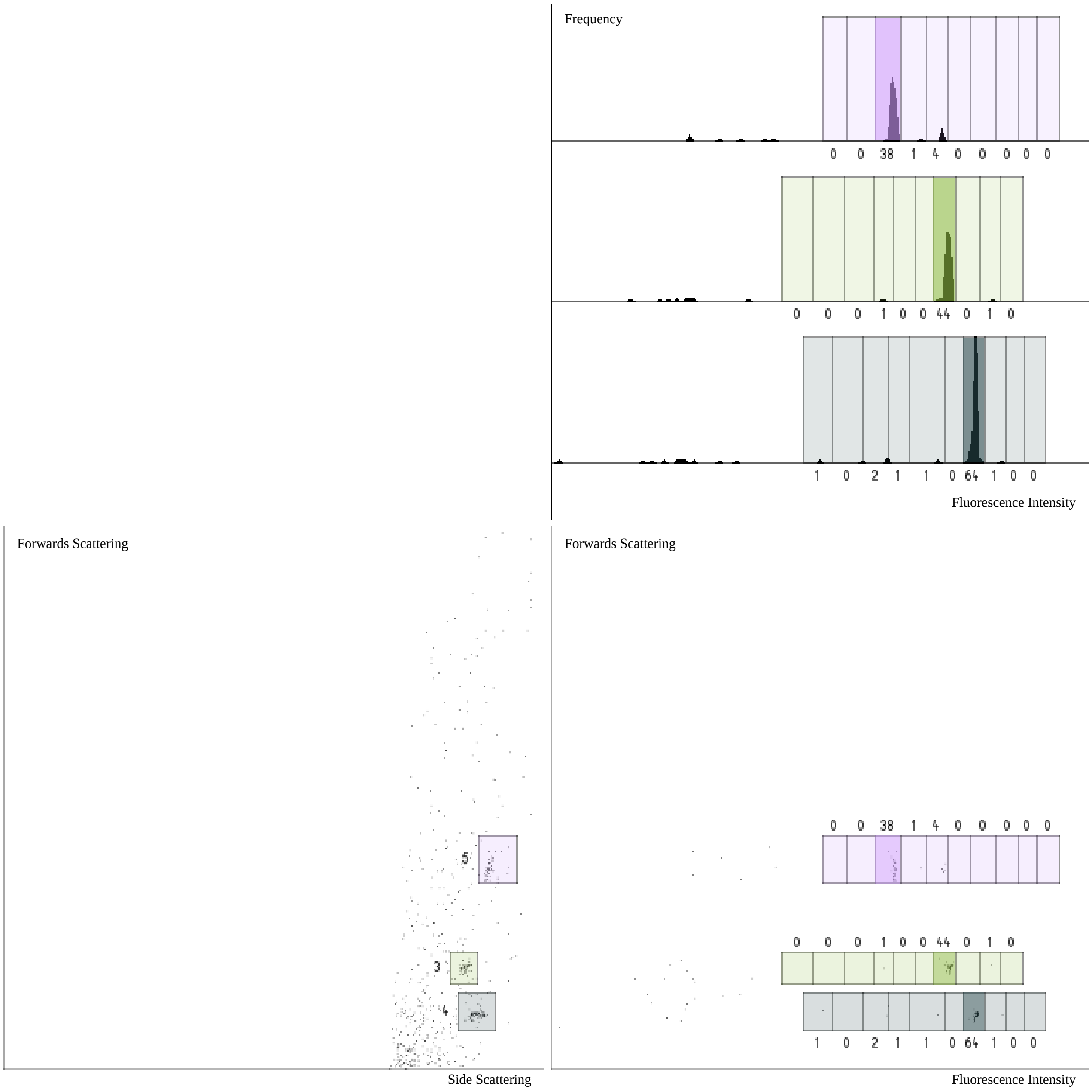

ANNEX 3: TAG DECONVOLUTION - BEAD 474

Passes flow sorting criteria: Yes

Passes tag deconvolution criteria: Yes

Included in protocol analysis: Yes

Protocol: 10, 5, 5, 9

Filename: Bin9_plateA0_D12.fcs

Split 1: Petrol shading

Split 2: Green shading

Split 3: Violet shading

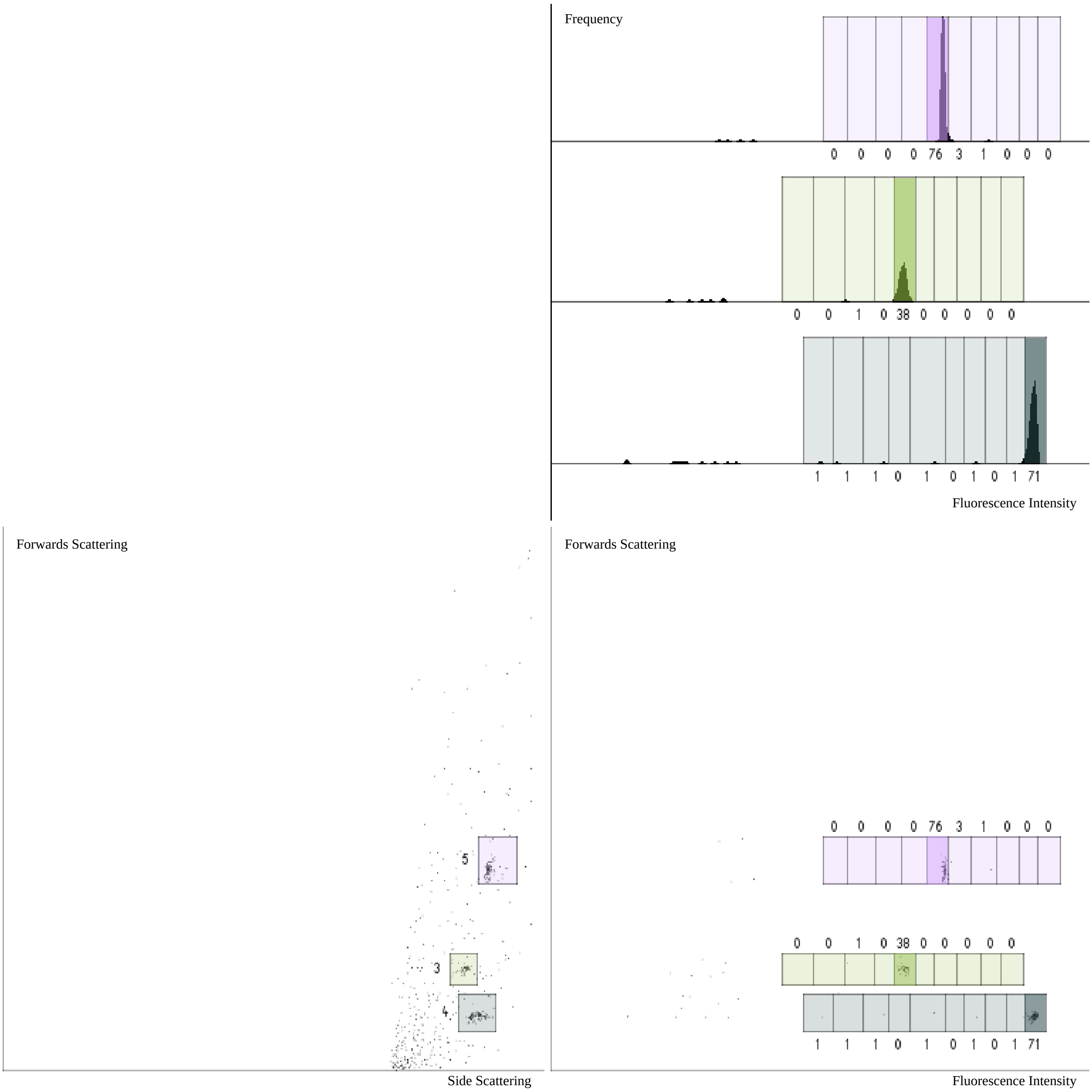

ANNEX 3: TAG DECONVOLUTION - BEAD 475

Passes flow sorting criteria: Yes

Passes tag deconvolution criteria: Yes

Included in protocol analysis: Yes

Protocol: 2, 5, 1, 9

Filename: Bin9_plateA0_E1.fcs

Split 1: Petrol shading

Split 2: Green shading

Split 3: Violet shading

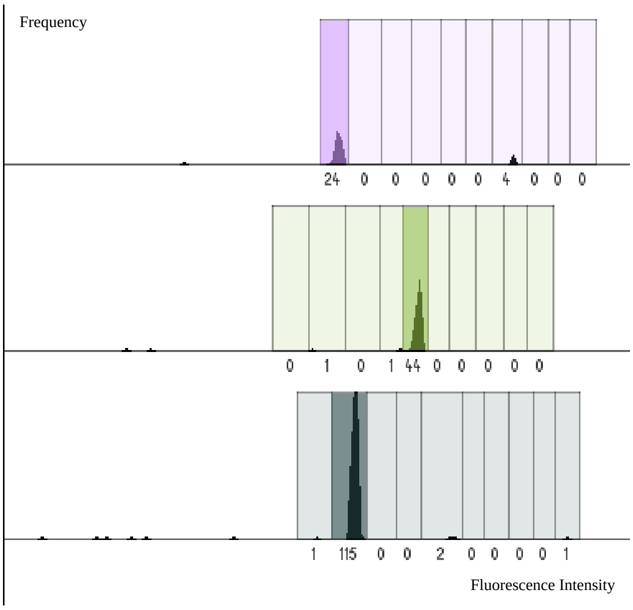

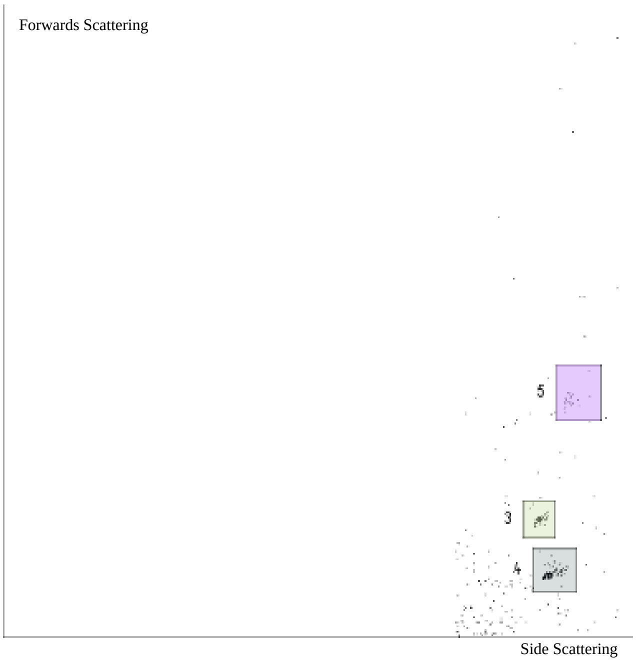

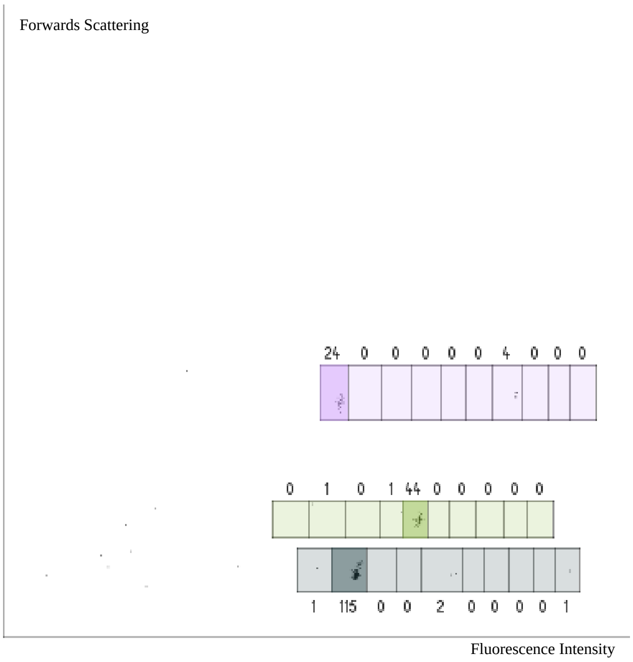

ANNEX 3: TAG DECONVOLUTION - BEAD 476

Passes flow sorting criteria: Yes

Passes tag deconvolution criteria: Yes

Included in protocol analysis: Yes

Protocol: 5, 10, 9, 9

Filename: Bin9_plateA0_E2.fcs

Split 1: Petrol shading

Split 2: Green shading

Split 3: Violet shading

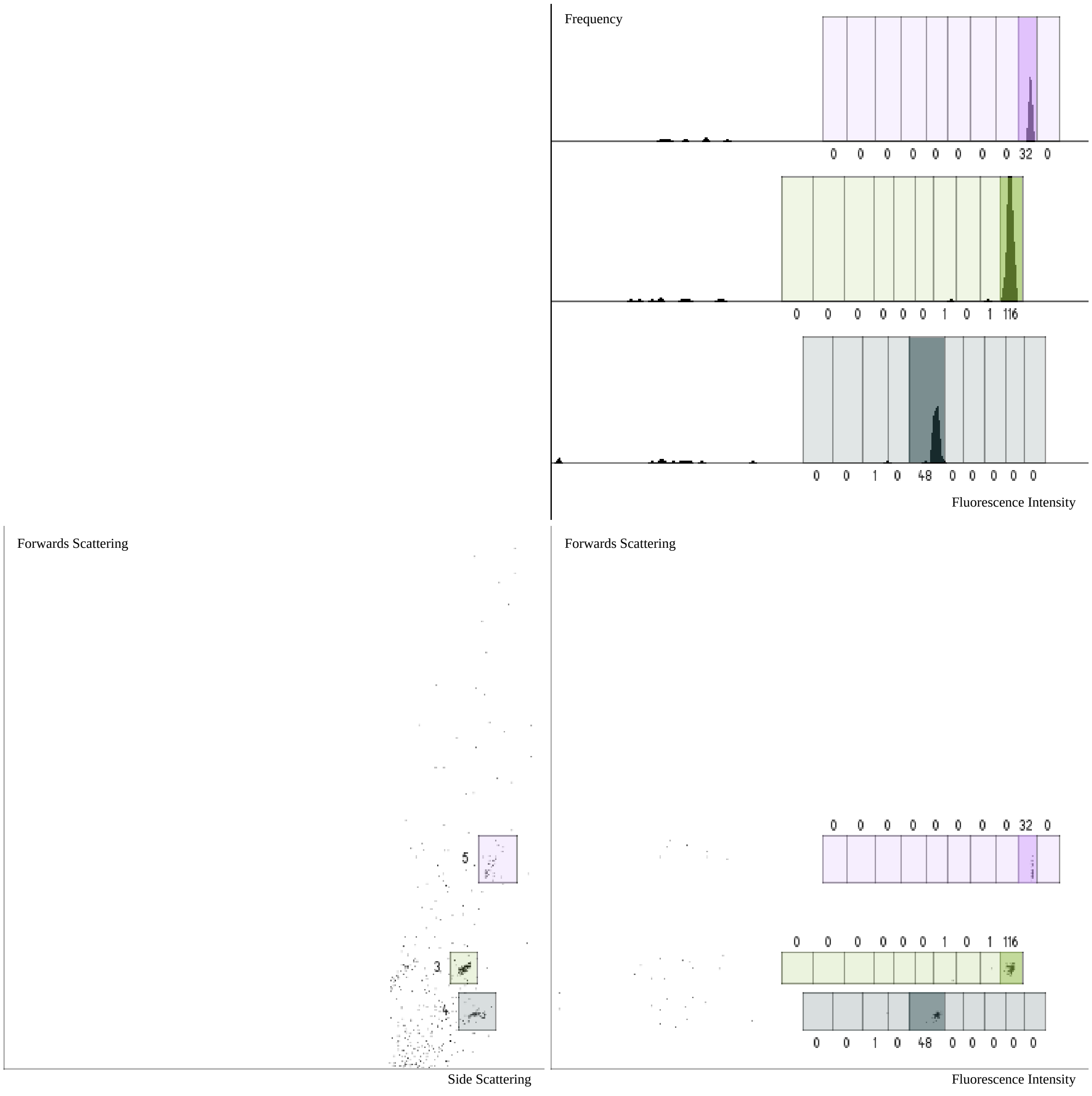

ANNEX 3: TAG DECONVOLUTION - BEAD 477

Passes flow sorting criteria: Yes

Passes tag deconvolution criteria: Yes

Included in protocol analysis: Yes

Protocol: 5, 10, 7, 9

Filename: Bin9_plateA0_E3.fcs

Split 1: Petrol shading

Split 2: Green shading

Split 3: Violet shading

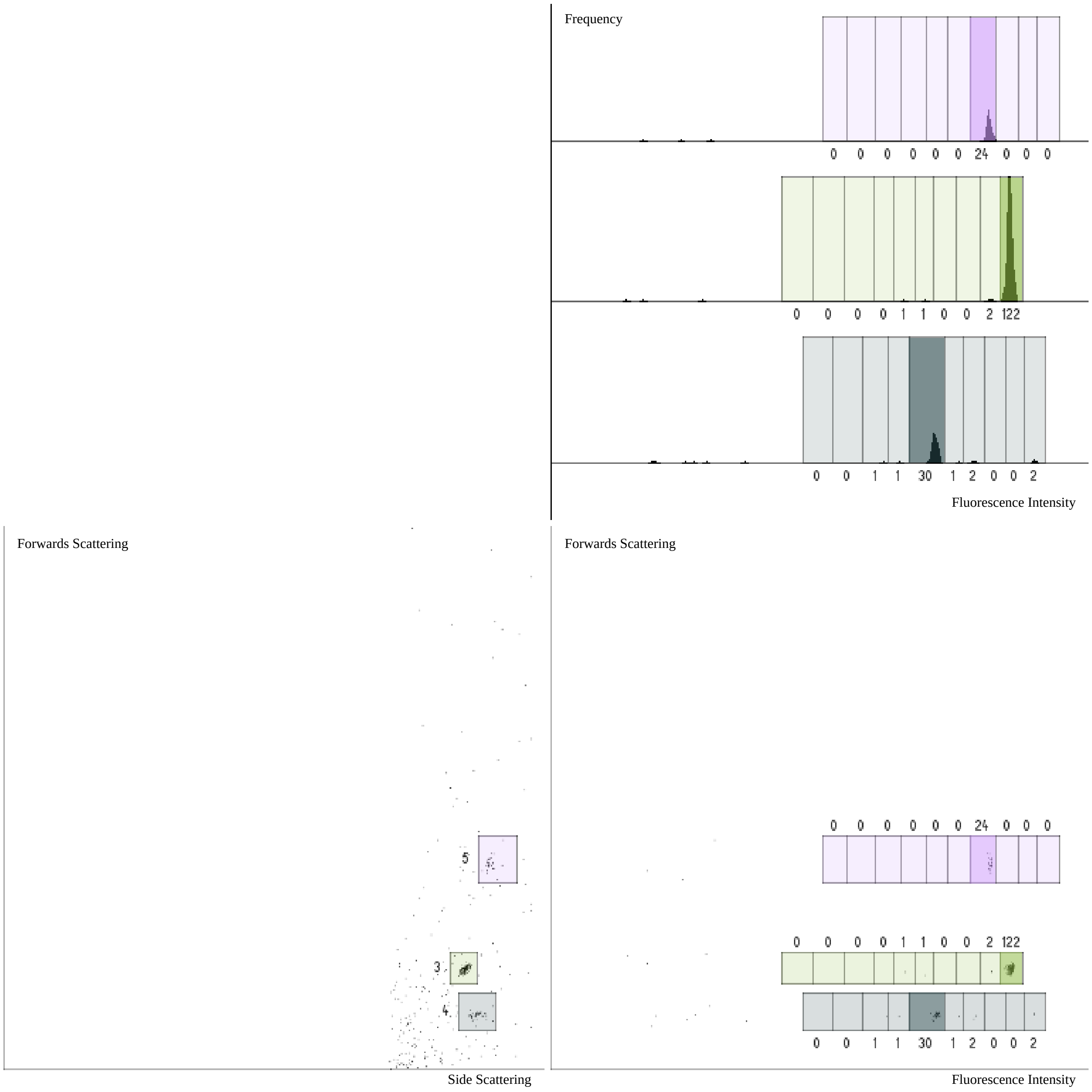

ANNEX 3: TAG DECONVOLUTION - BEAD 478

Passes flow sorting criteria: Yes

Passes tag deconvolution criteria: Yes

Included in protocol analysis: Yes

Protocol: 7, 6, 1, 9

Filename: Bin9_plateA0_E4.fcs

Split 1: Petrol shading

Split 2: Green shading

Split 3: Violet shading

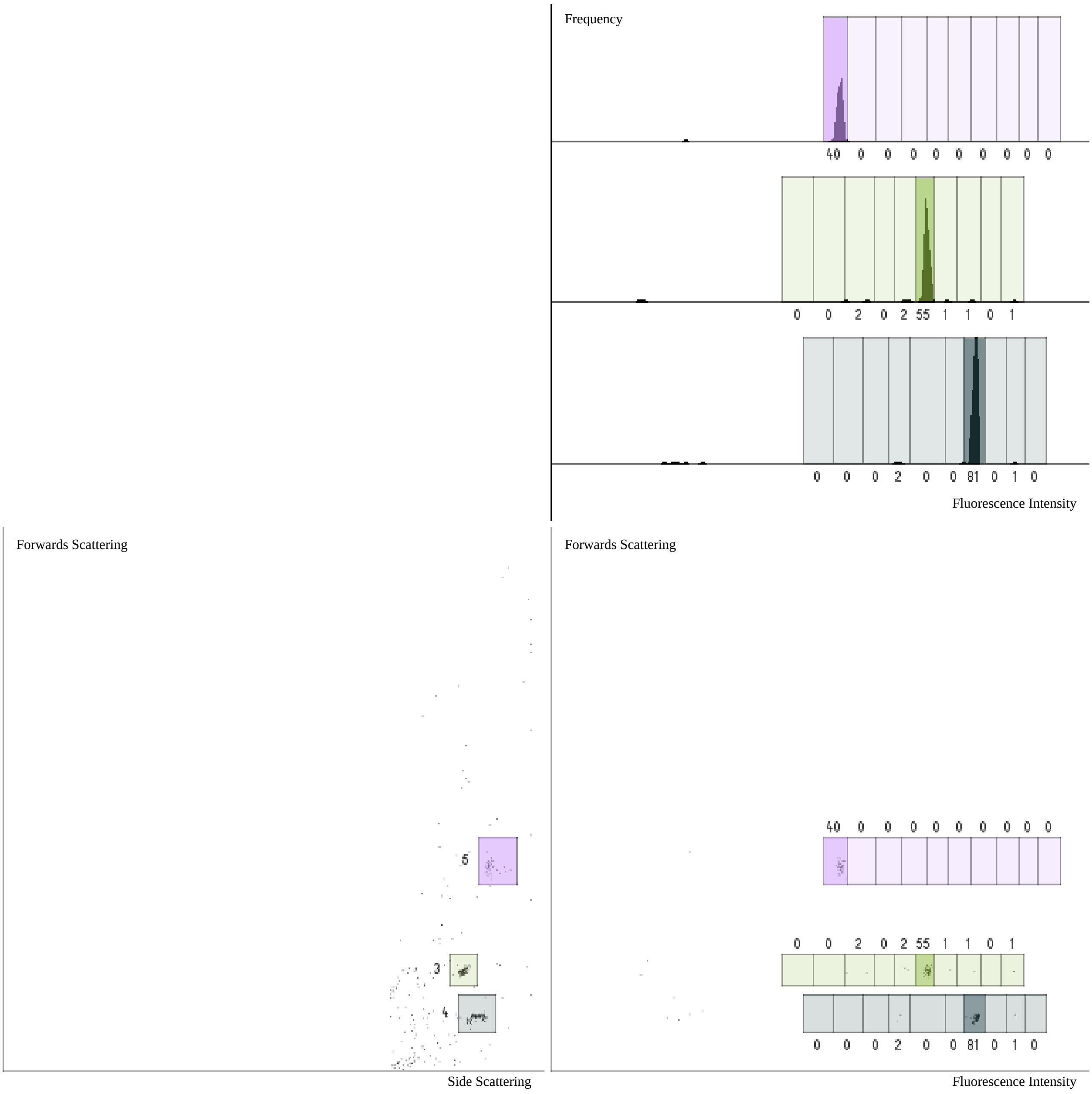

ANNEX 3: TAG DECONVOLUTION - BEAD 479

Passes flow sorting criteria: Yes

Passes tag deconvolution criteria: No

Included in protocol analysis: No

Protocol: N/A

Filename: Bin9_plateA0_E5.fcs

Split 1: Petrol shading

Split 2: Green shading

Split 3: Violet shading

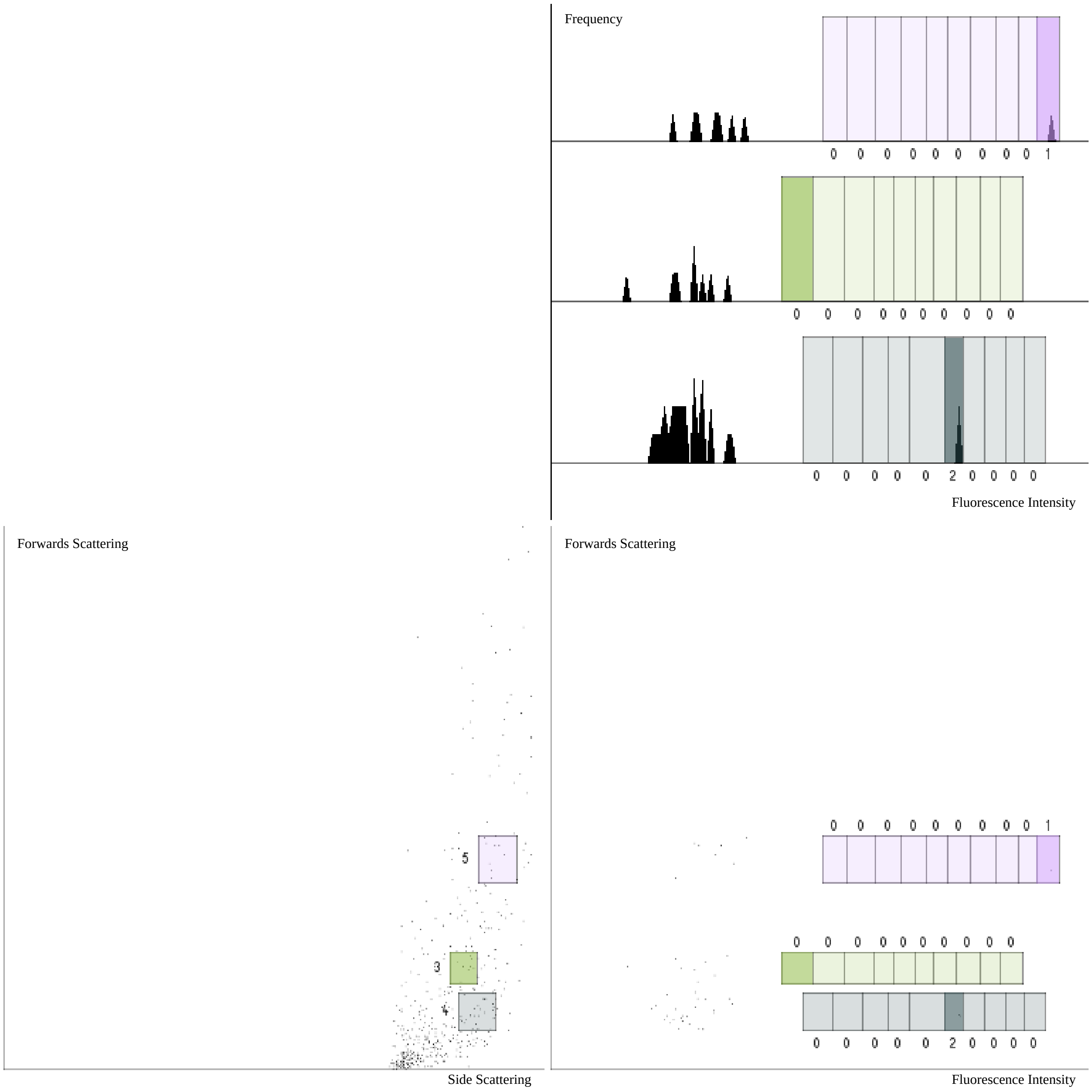

ANNEX 3: TAG DECONVOLUTION - BEAD 480

Passes flow sorting criteria: Yes

Passes tag deconvolution criteria: Yes

Included in protocol analysis: Yes

Protocol: 6, 8, 2, 9

Filename: Bin9_plateA0_E6.fcs

Split 1: Petrol shading

Split 2: Green shading

Split 3: Violet shading

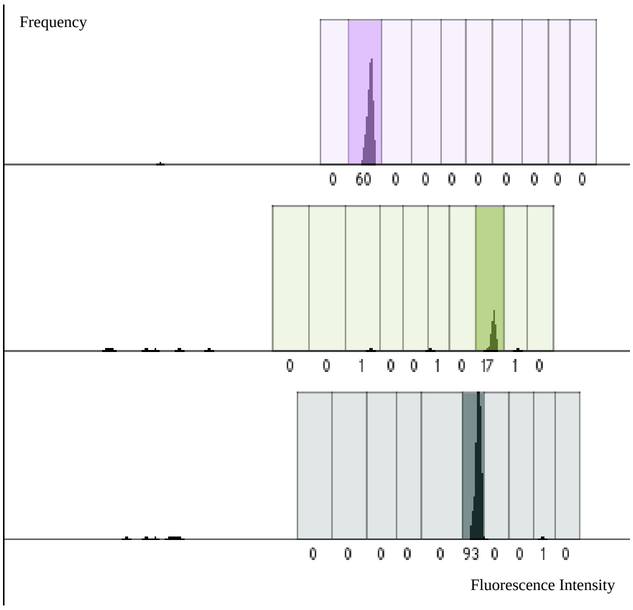

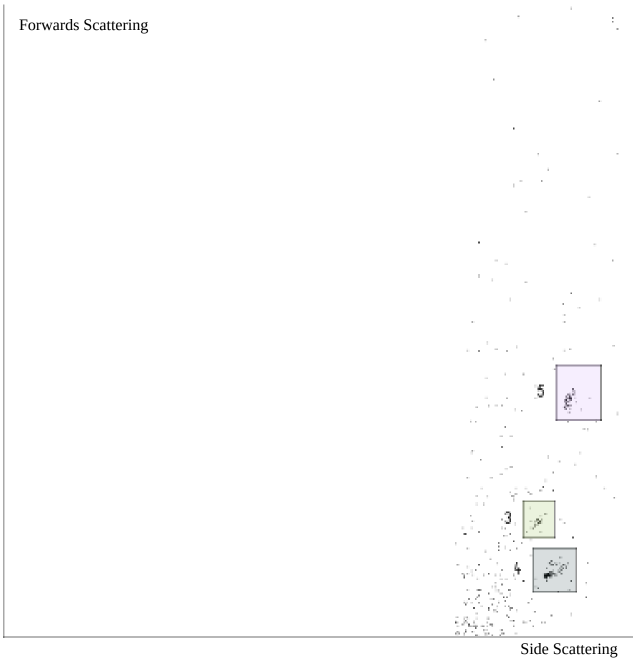

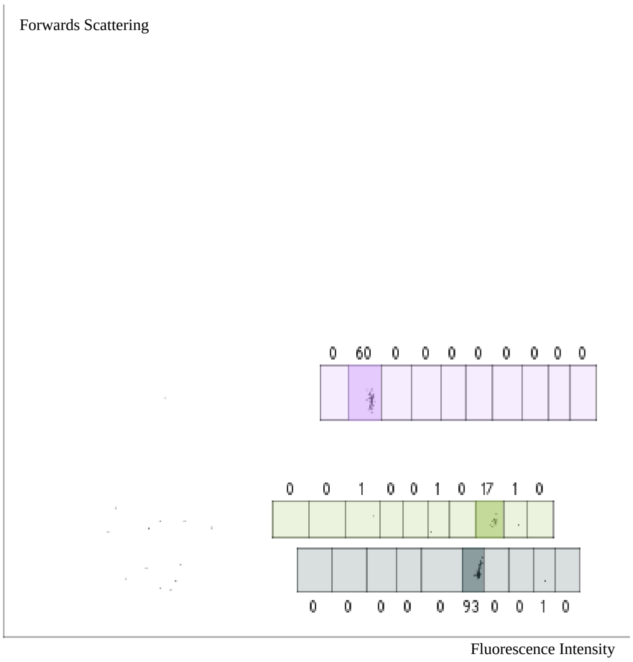

ANNEX 3: TAG DECONVOLUTION - BEAD 481

Passes flow sorting criteria: Yes

Passes tag deconvolution criteria: Yes

Included in protocol analysis: Yes

Protocol: 6, 9, 2, 9

Filename: Bin9_plateA0_E7.fcs

Split 1: Petrol shading

Split 2: Green shading

Split 3: Violet shading

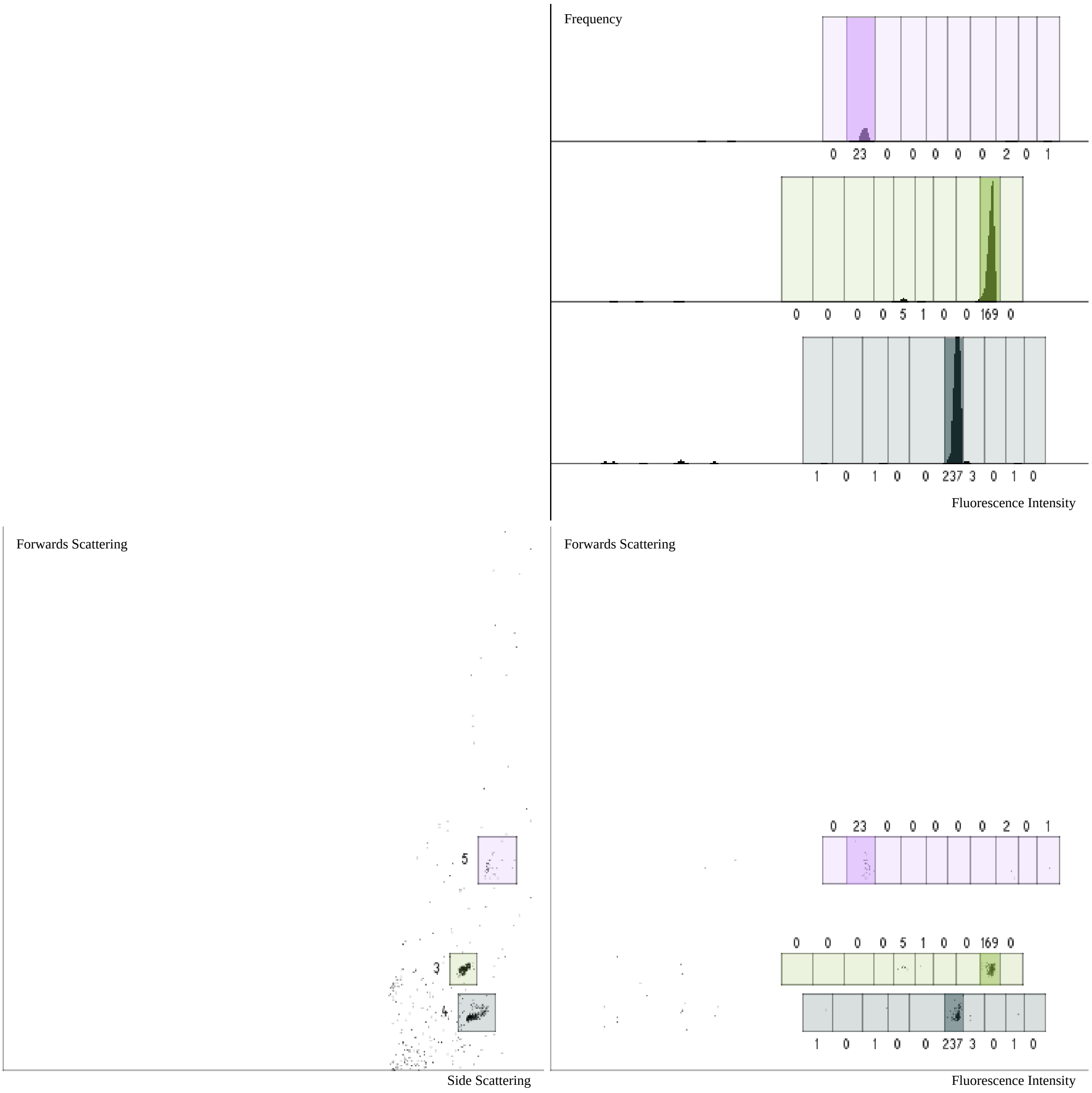

ANNEX 3: TAG DECONVOLUTION - BEAD 482

Passes flow sorting criteria: Yes

Passes tag deconvolution criteria: Yes

Included in protocol analysis: Yes

Protocol: 10, 4, 2, 9

Filename: Bin9_plateA0_E8.fcs

Split 1: Petrol shading

Split 2: Green shading

Split 3: Violet shading

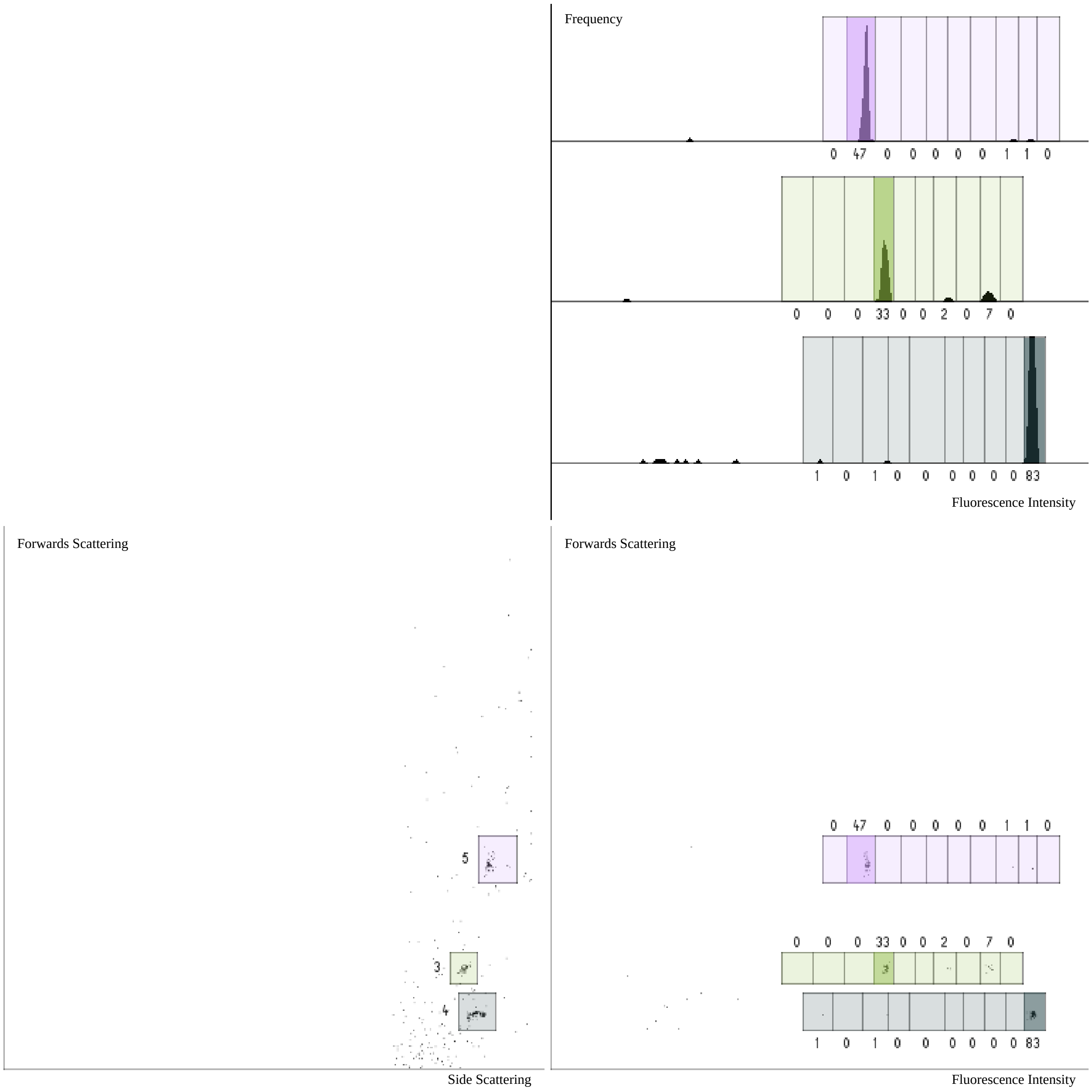

ANNEX 3: TAG DECONVOLUTION - BEAD 483

Passes flow sorting criteria: Yes

Passes tag deconvolution criteria: No

Included in protocol analysis: No

Protocol: N/A

Filename: Bin9_plateA0_E9.fcs

Split 1: Petrol shading

Split 2: Green shading

Split 3: Violet shading

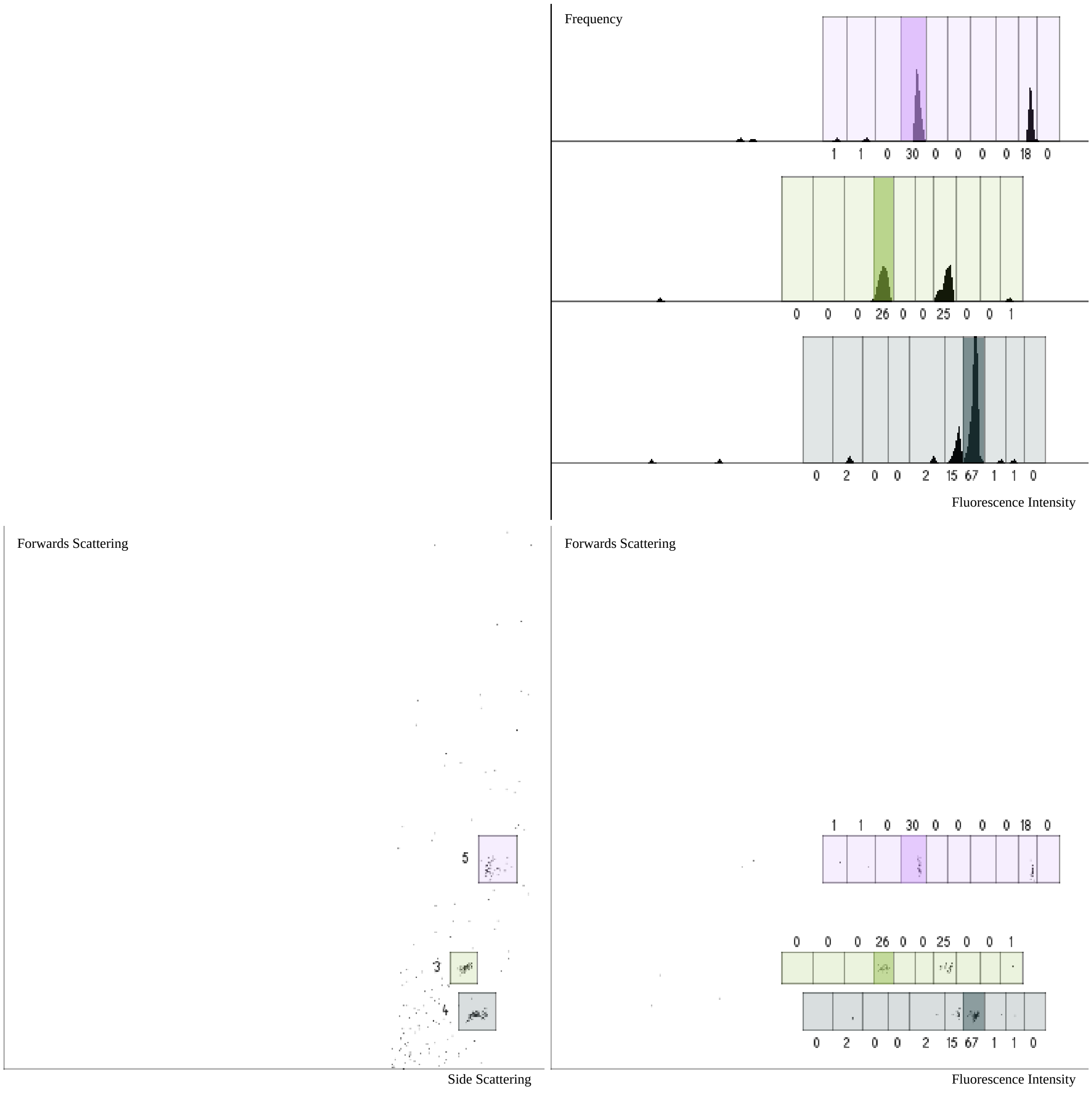

ANNEX 3: TAG DECONVOLUTION - BEAD 484

Passes flow sorting criteria: Yes

Passes tag deconvolution criteria: Yes

Included in protocol analysis: Yes

Protocol: 10, 7, 1, 9

Filename: Bin9_plateA0_E10.fcs

Split 1: Petrol shading

Split 2: Green shading

Split 3: Violet shading

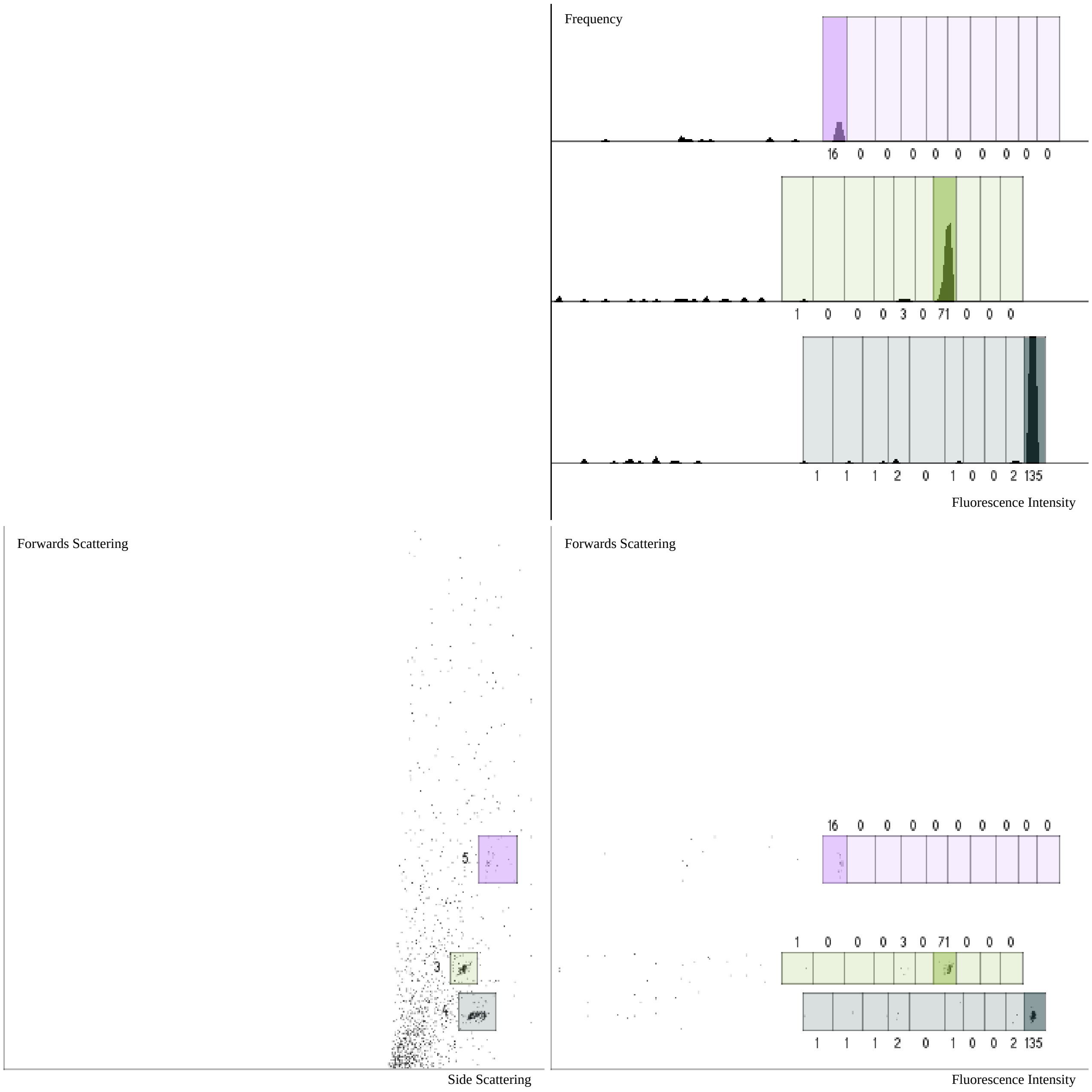

ANNEX 3: TAG DECONVOLUTION - BEAD 485

Passes flow sorting criteria: Yes

Passes tag deconvolution criteria: Yes

Included in protocol analysis: Yes

Protocol: 3, 8, 1, 9

Filename: Bin9_plateA0_E11.fcs

Split 1: Petrol shading

Split 2: Green shading

Split 3: Violet shading

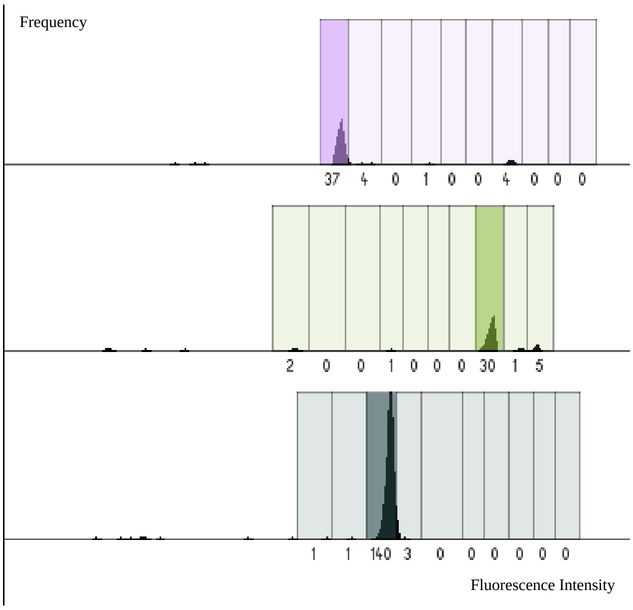

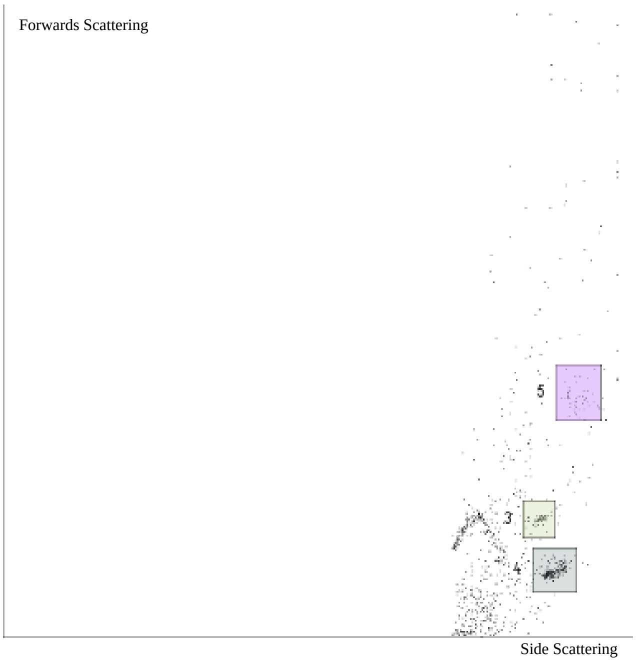

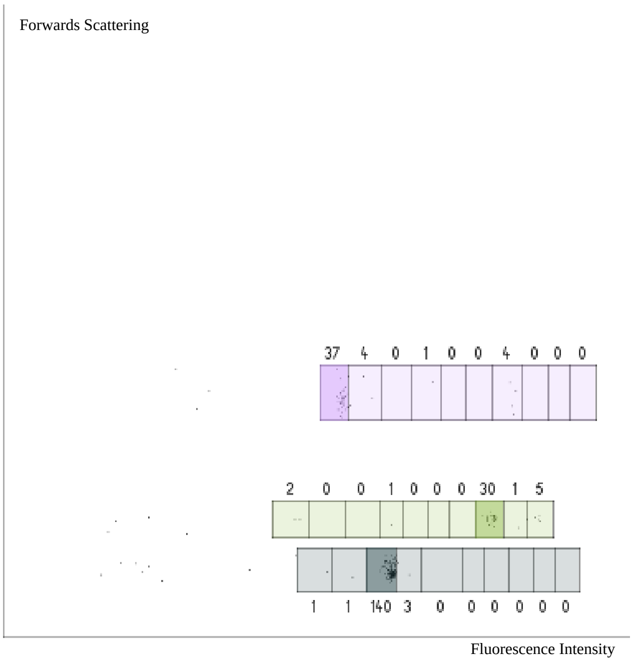

ANNEX 3: TAG DECONVOLUTION - BEAD 486

Passes flow sorting criteria: Yes

Passes tag deconvolution criteria: No

Included in protocol analysis: No

Protocol: N/A

Filename: Bin9_plateA0_E12.fcs

Split 1: Petrol shading

Split 2: Green shading

Split 3: Violet shading

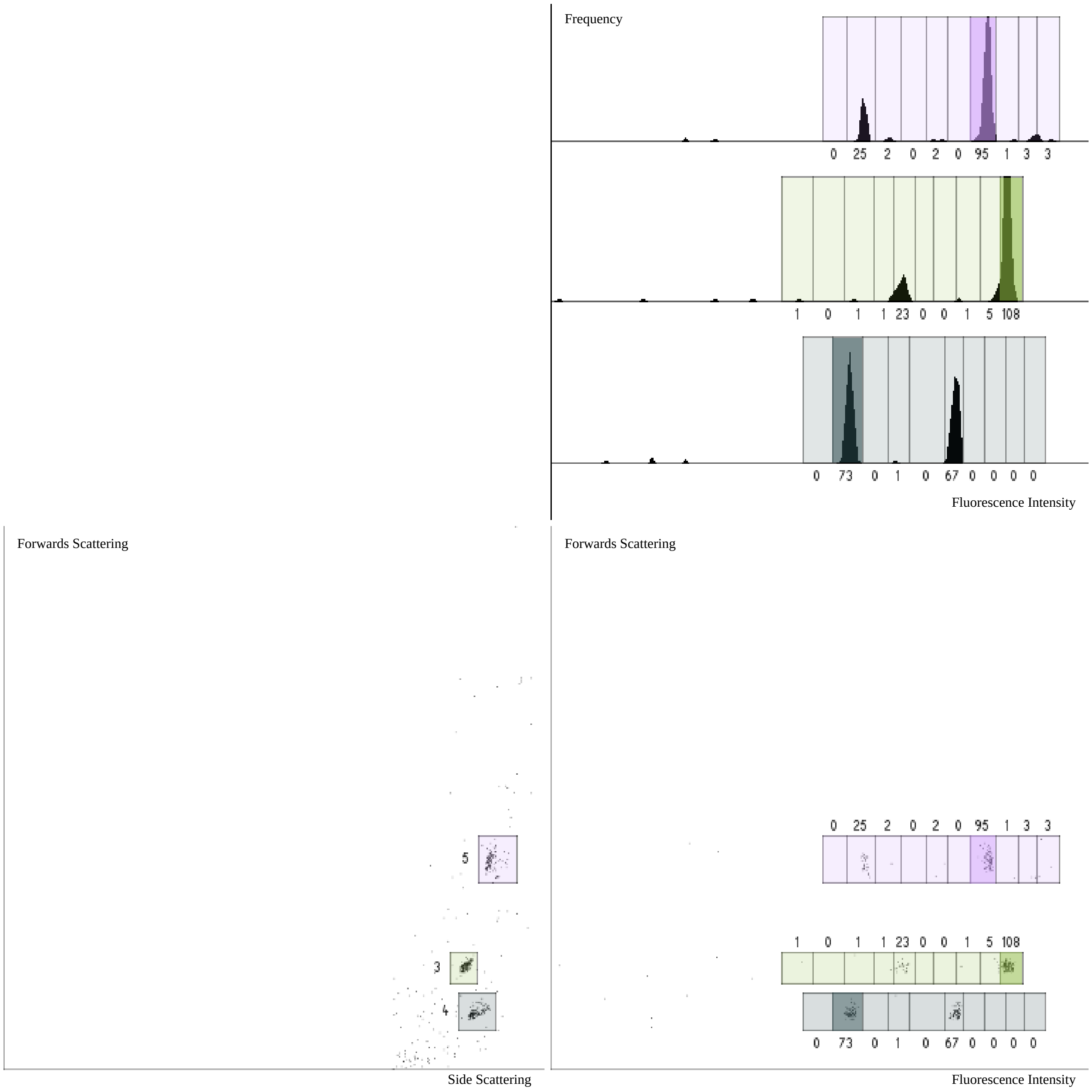

ANNEX 3: TAG DECONVOLUTION - BEAD 487

Passes flow sorting criteria: Yes

Passes tag deconvolution criteria: Yes

Included in protocol analysis: Yes

Protocol: 2, 9, 7, 9

Filename: Bin9_plateA0_F1.fcs

Split 1: Petrol shading

Split 2: Green shading

Split 3: Violet shading

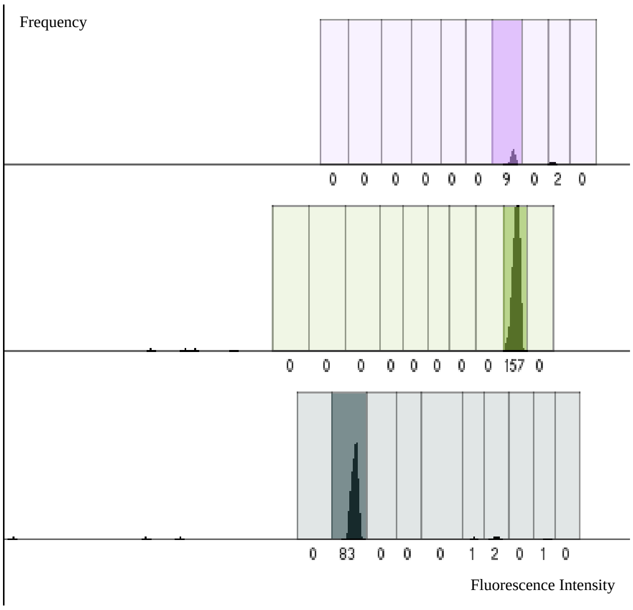

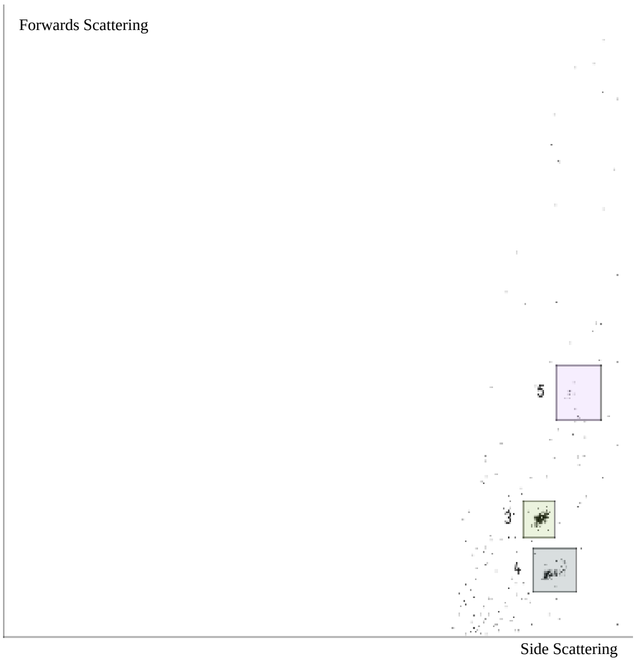

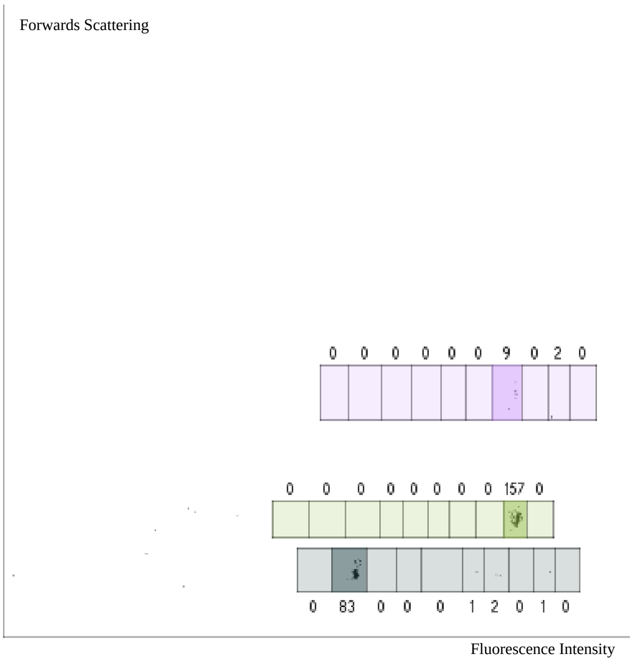

ANNEX 3: TAG DECONVOLUTION - BEAD 488

Passes flow sorting criteria: Yes

Passes tag deconvolution criteria: Yes

Included in protocol analysis: Yes

Protocol: 5, 9, 2, 9

Filename: Bin9_plateA0_F2.fcs

Split 1: Petrol shading

Split 2: Green shading

Split 3: Violet shading

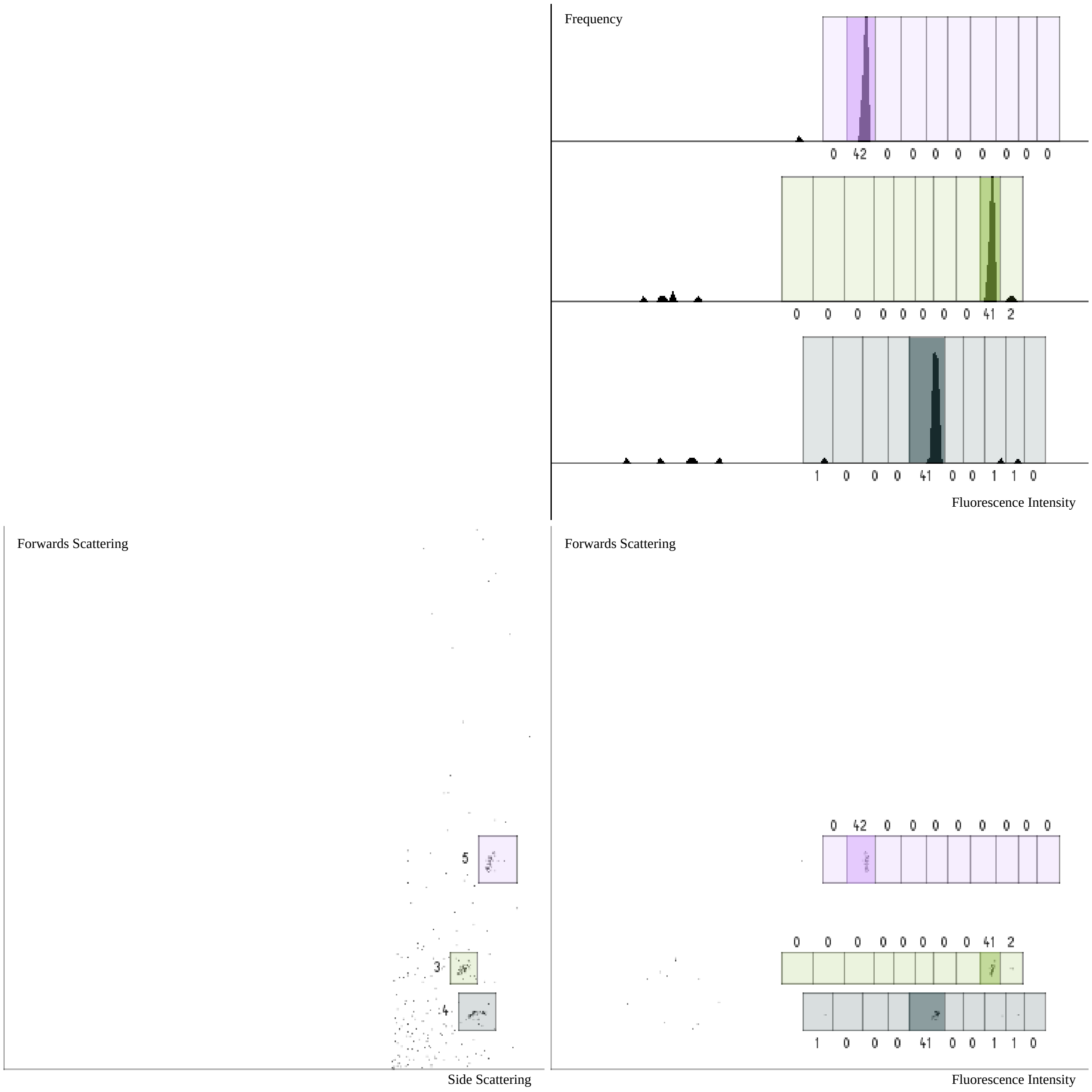

ANNEX 3: TAG DECONVOLUTION - BEAD 489

Passes flow sorting criteria: Yes

Passes tag deconvolution criteria: Yes

Included in protocol analysis: Yes

Protocol: 5, 9, 7, 9

Filename: Bin9_plateA0_F3.fcs

Split 1: Petrol shading

Split 2: Green shading

Split 3: Violet shading

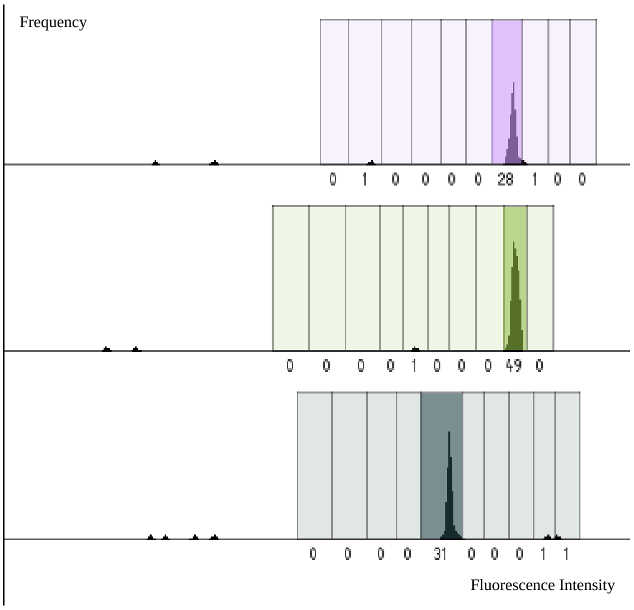

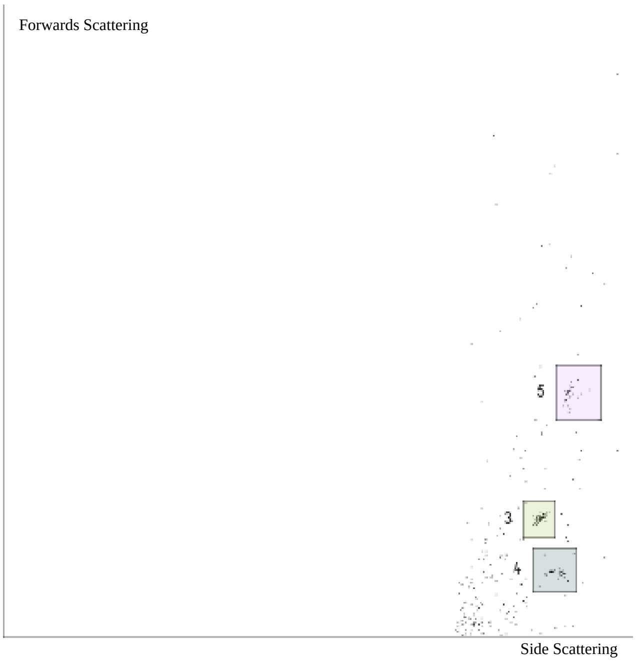

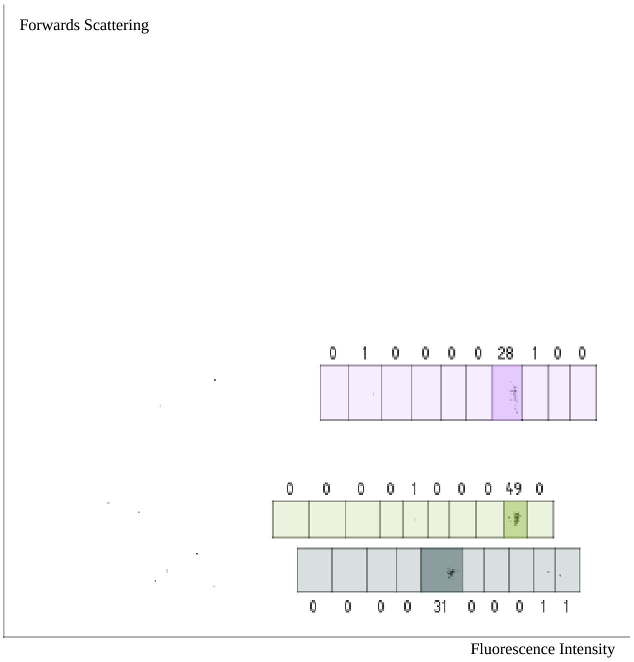

ANNEX 3: TAG DECONVOLUTION - BEAD 490

Passes flow sorting criteria: Yes

Passes tag deconvolution criteria: Yes

Included in protocol analysis: Yes

Protocol: 7, 6, 1, 9

Filename: Bin9_plateA0_F4.fcs

Split 1: Petrol shading

Split 2: Green shading

Split 3: Violet shading

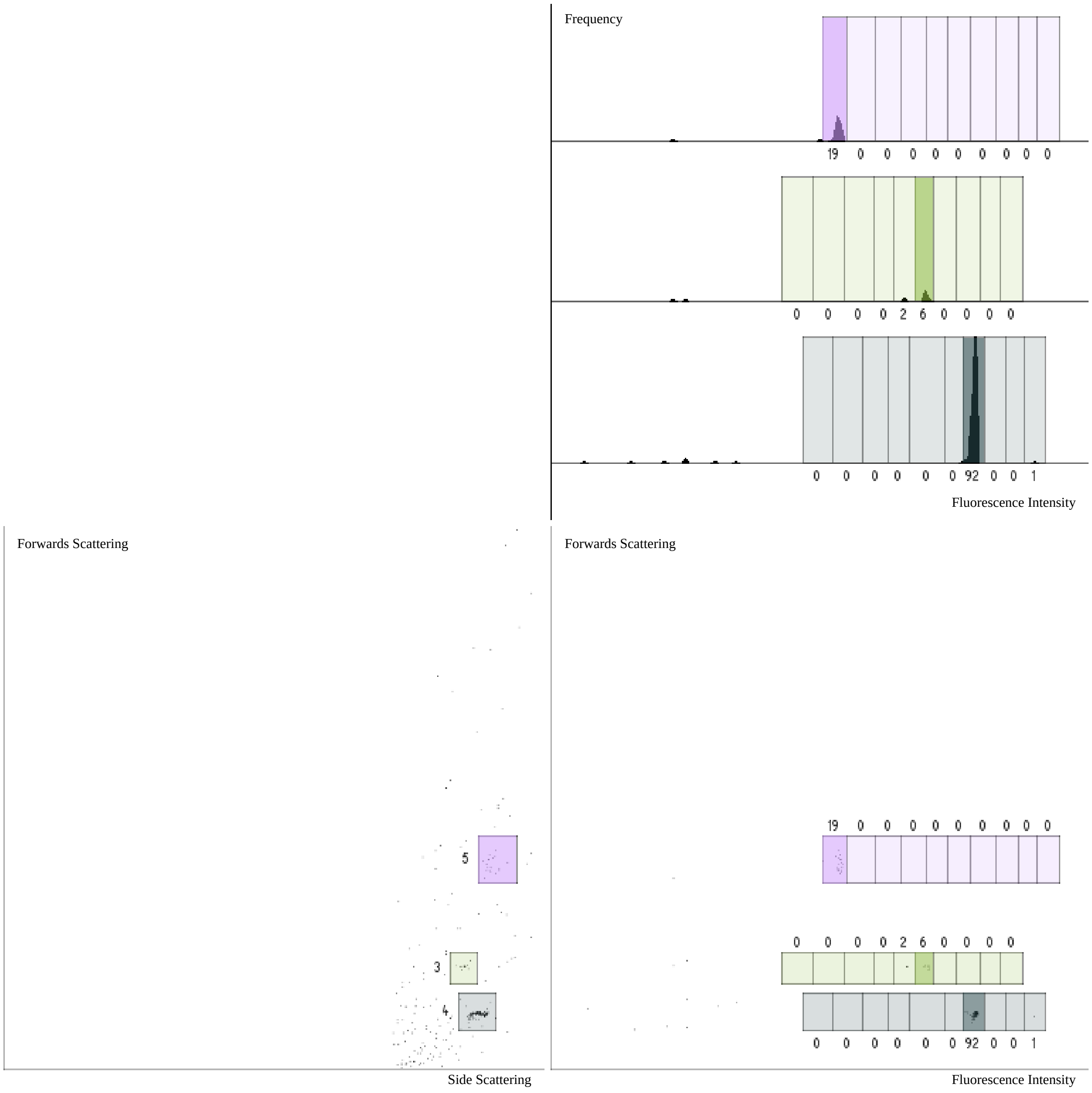

ANNEX 3: TAG DECONVOLUTION - BEAD 491

Passes flow sorting criteria: Yes

Passes tag deconvolution criteria: Yes

Included in protocol analysis: Yes

Protocol: 9, 5, 6, 9

Filename: Bin9_plateA0_F6.fcs

Split 1: Petrol shading

Split 2: Green shading

Split 3: Violet shading

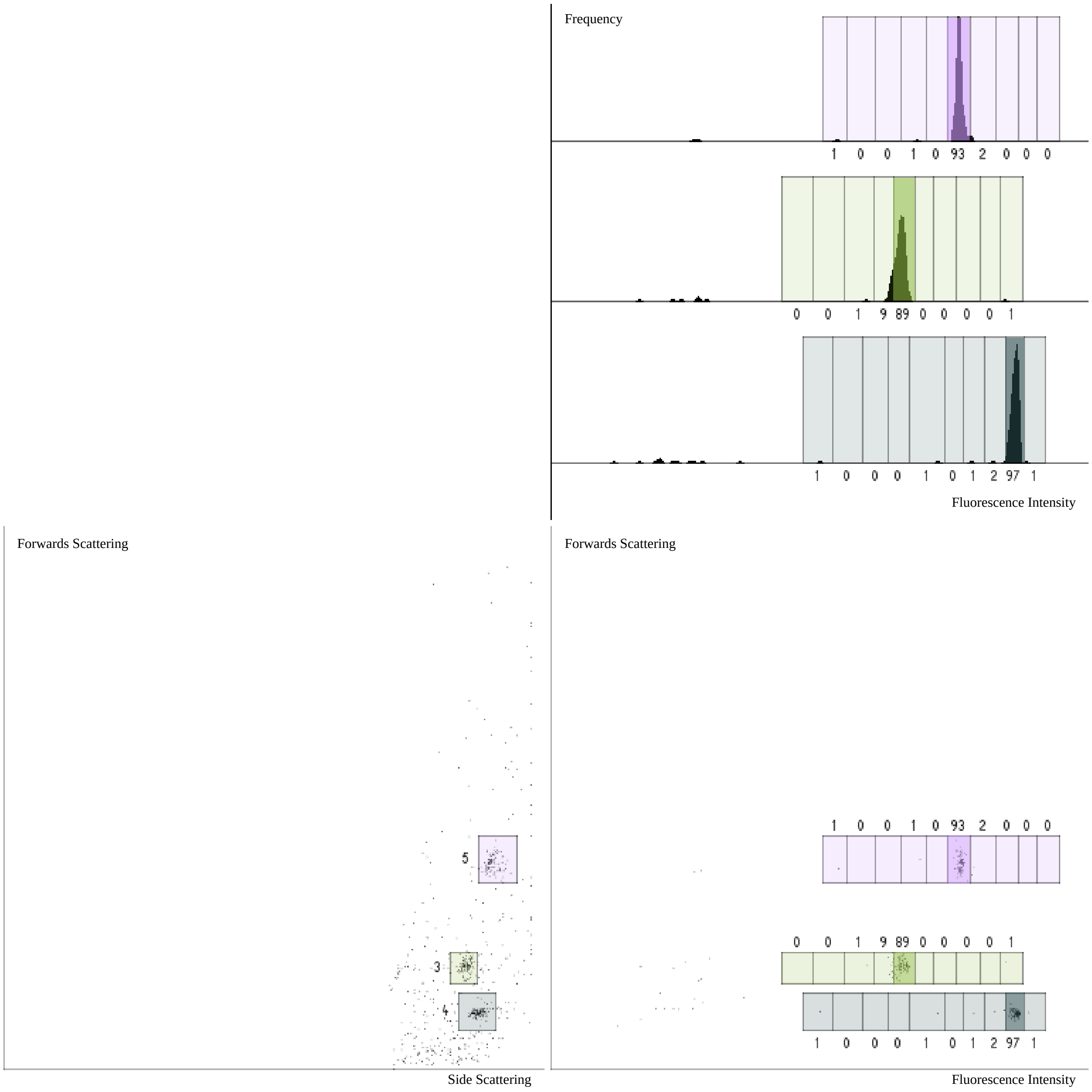

ANNEX 3: TAG DECONVOLUTION - BEAD 492

Passes flow sorting criteria: Yes

Passes tag deconvolution criteria: Yes

Included in protocol analysis: Yes

Protocol: 5, 2, 7, 9

Filename: Bin9_plateA0_F7.fcs

Split 1: Petrol shading

Split 2: Green shading

Split 3: Violet shading

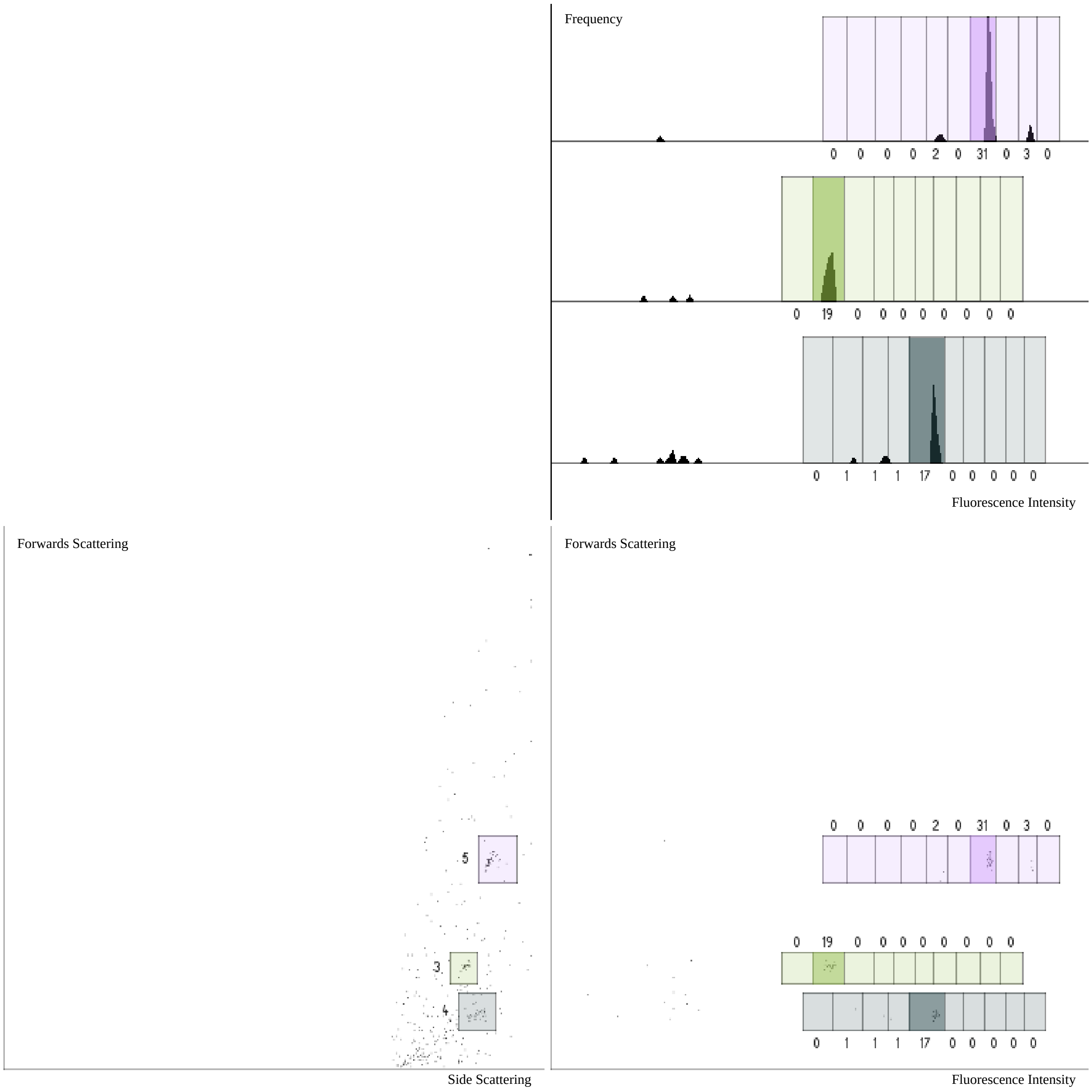

ANNEX 3: TAG DECONVOLUTION - BEAD 493

Passes flow sorting criteria: Yes

Passes tag deconvolution criteria: Yes

Included in protocol analysis: Yes

Protocol: 4, 7, 5, 9

Filename: Bin9_plateA0_F8.fcs

Split 1: Petrol shading

Split 2: Green shading

Split 3: Violet shading

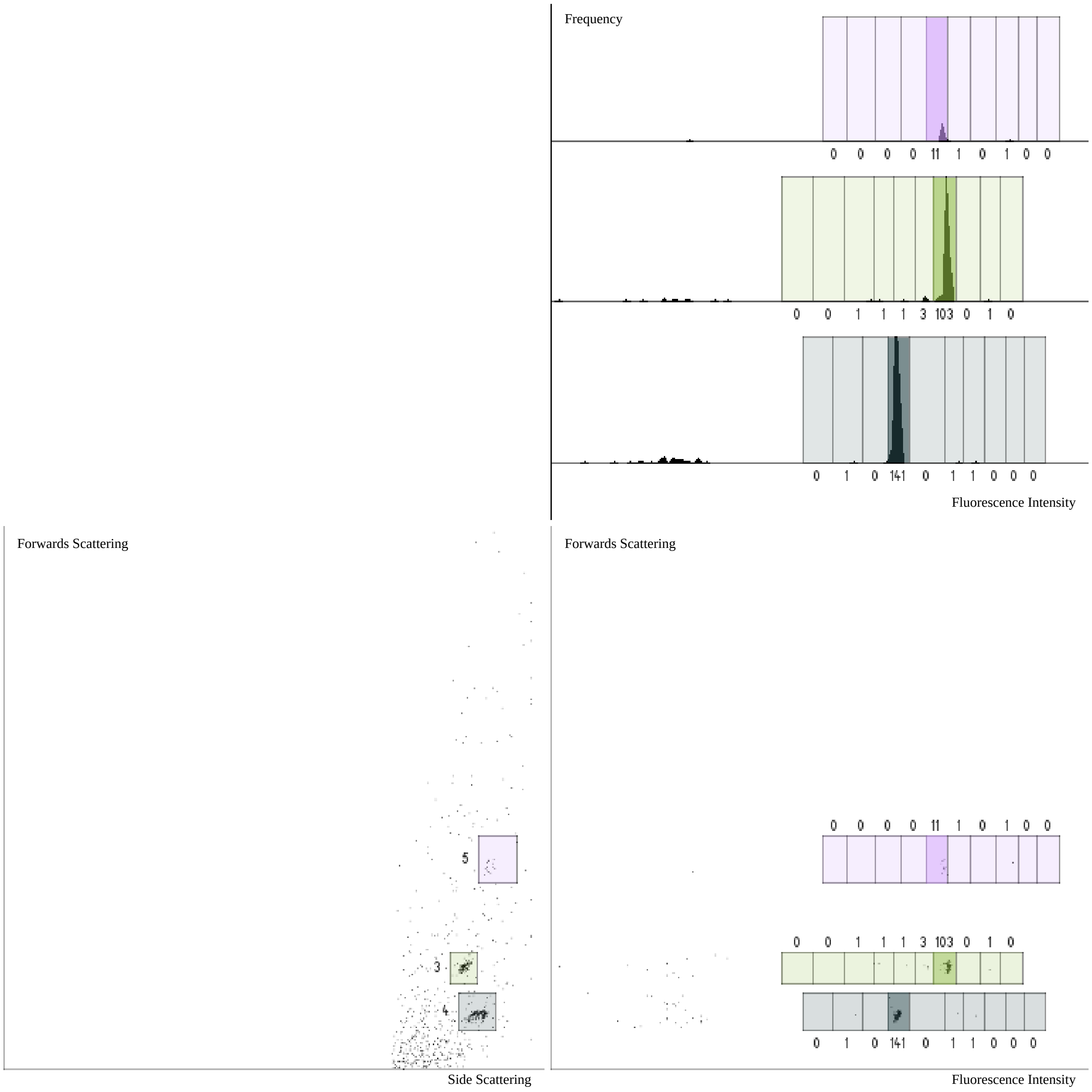

ANNEX 3: TAG DECONVOLUTION - BEAD 494

Passes flow sorting criteria: Yes

Passes tag deconvolution criteria: Yes

Included in protocol analysis: Yes

Protocol: 3, 3, 10, 9

Filename: Bin9_plateA0_F9.fcs

Split 1: Petrol shading

Split 2: Green shading

Split 3: Violet shading

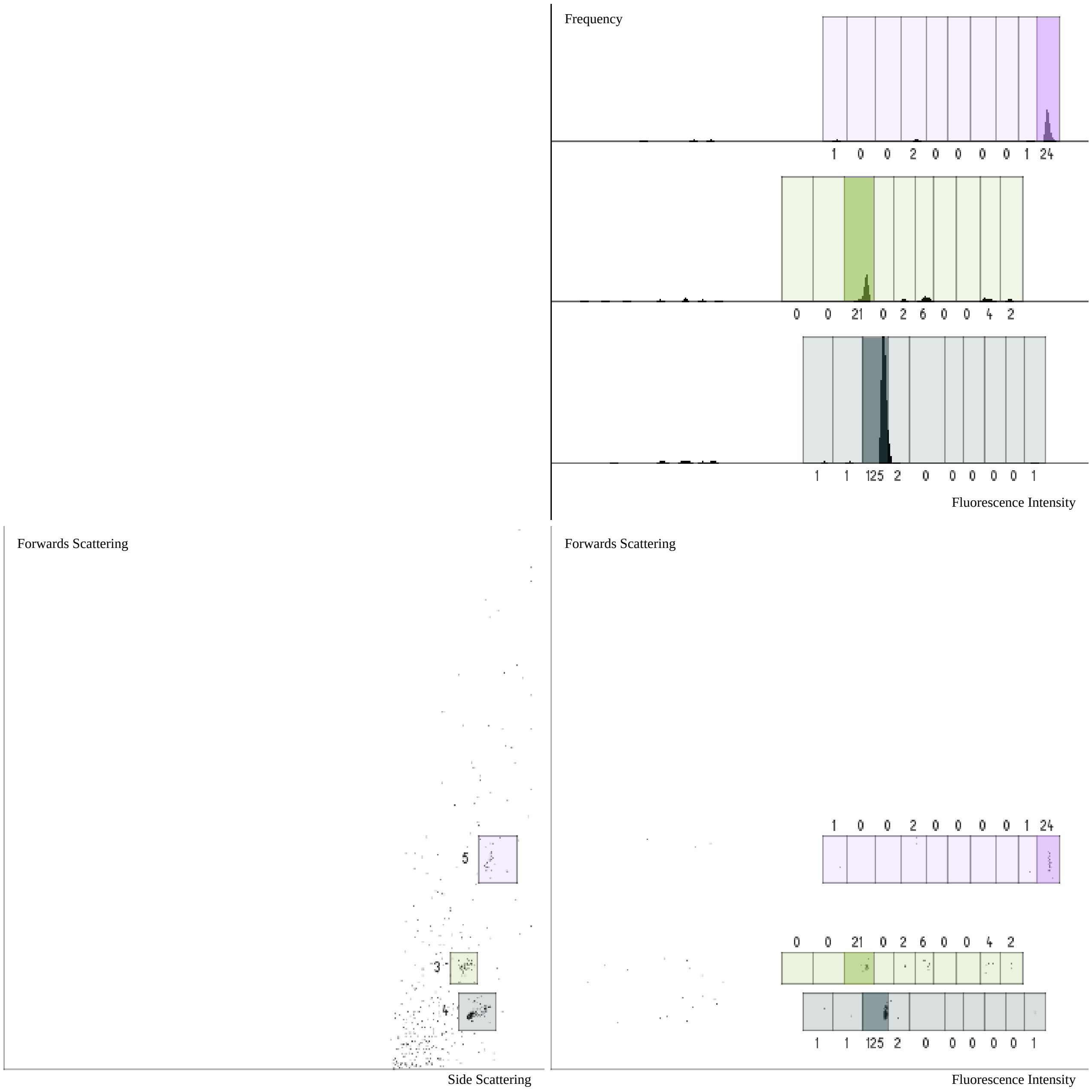

ANNEX 3: TAG DECONVOLUTION - BEAD 495

Passes flow sorting criteria: Yes

Passes tag deconvolution criteria: Yes

Included in protocol analysis: Yes

Protocol: 8, 7, 4, 9

Filename: Bin9_plateA0_F10.fcs

Split 1: Petrol shading

Split 2: Green shading

Split 3: Violet shading

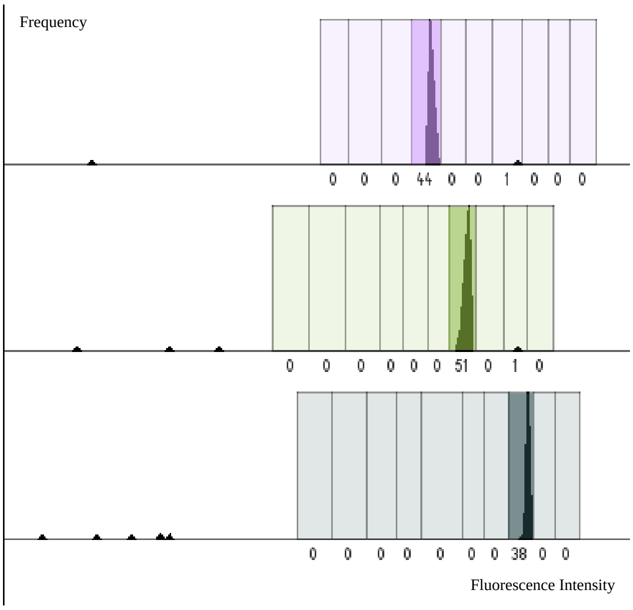

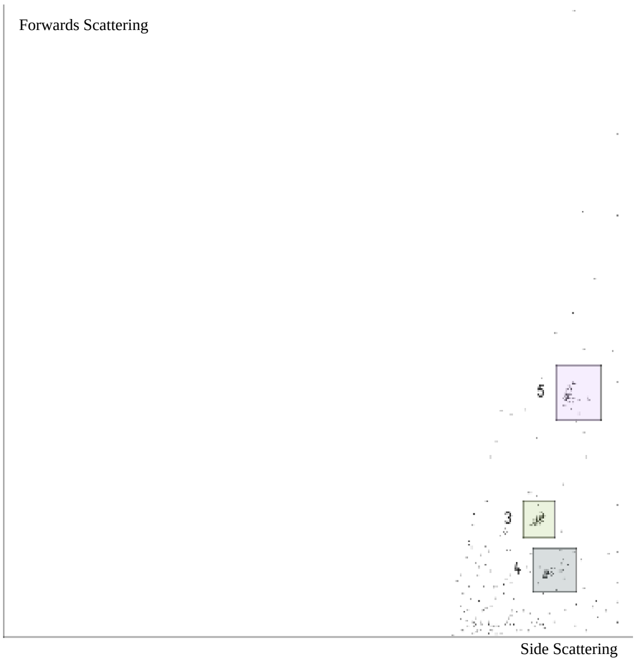

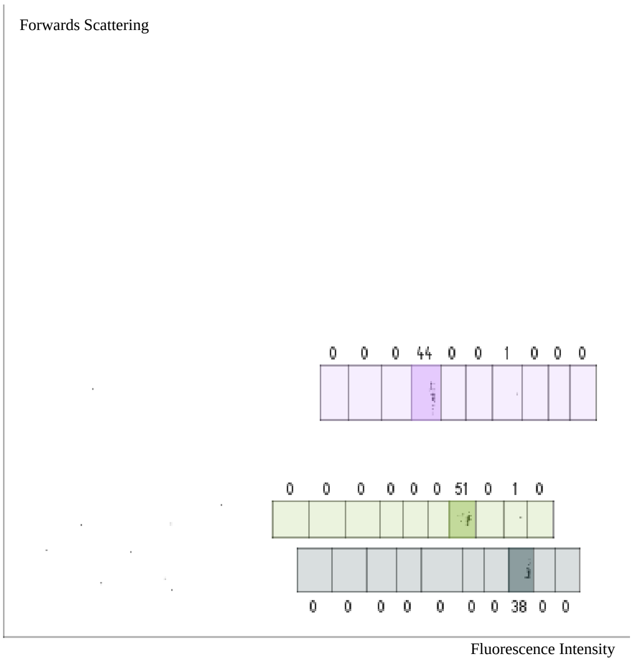

ANNEX 3: TAG DECONVOLUTION - BEAD 496

Passes flow sorting criteria: Yes

Passes tag deconvolution criteria: Yes

Included in protocol analysis: Yes

Protocol: 8, 7, 7, 9

Filename: Bin9_plateA0_F11.fcs

Split 1: Petrol shading

Split 2: Green shading

Split 3: Violet shading

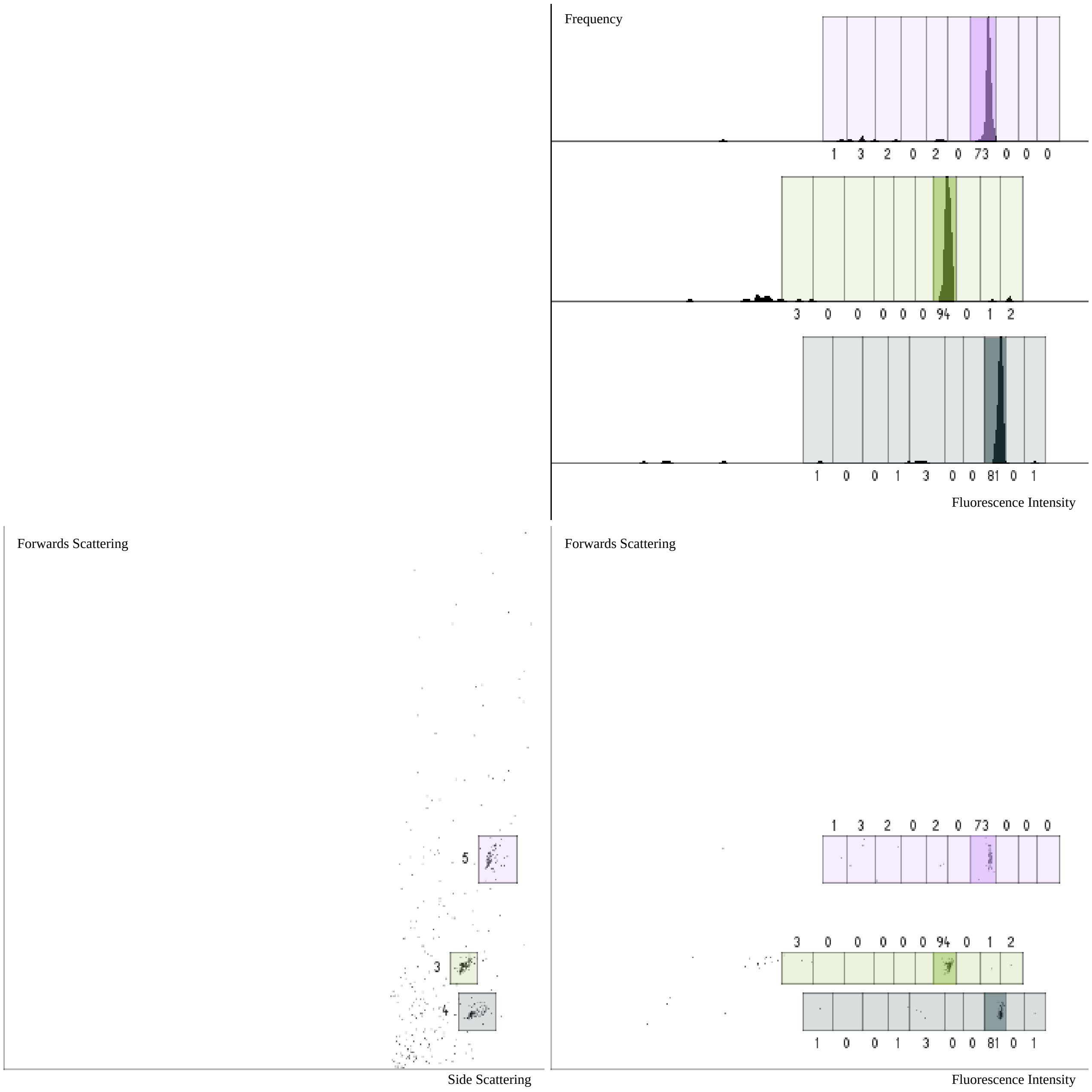

ANNEX 3: TAG DECONVOLUTION - BEAD 497

Passes flow sorting criteria: Yes

Passes tag deconvolution criteria: Yes

Included in protocol analysis: Yes

Protocol: 3, 9, 2, 9

Filename: Bin9_plateA0_F12.fcs

Split 1: Petrol shading

Split 2: Green shading

Split 3: Violet shading

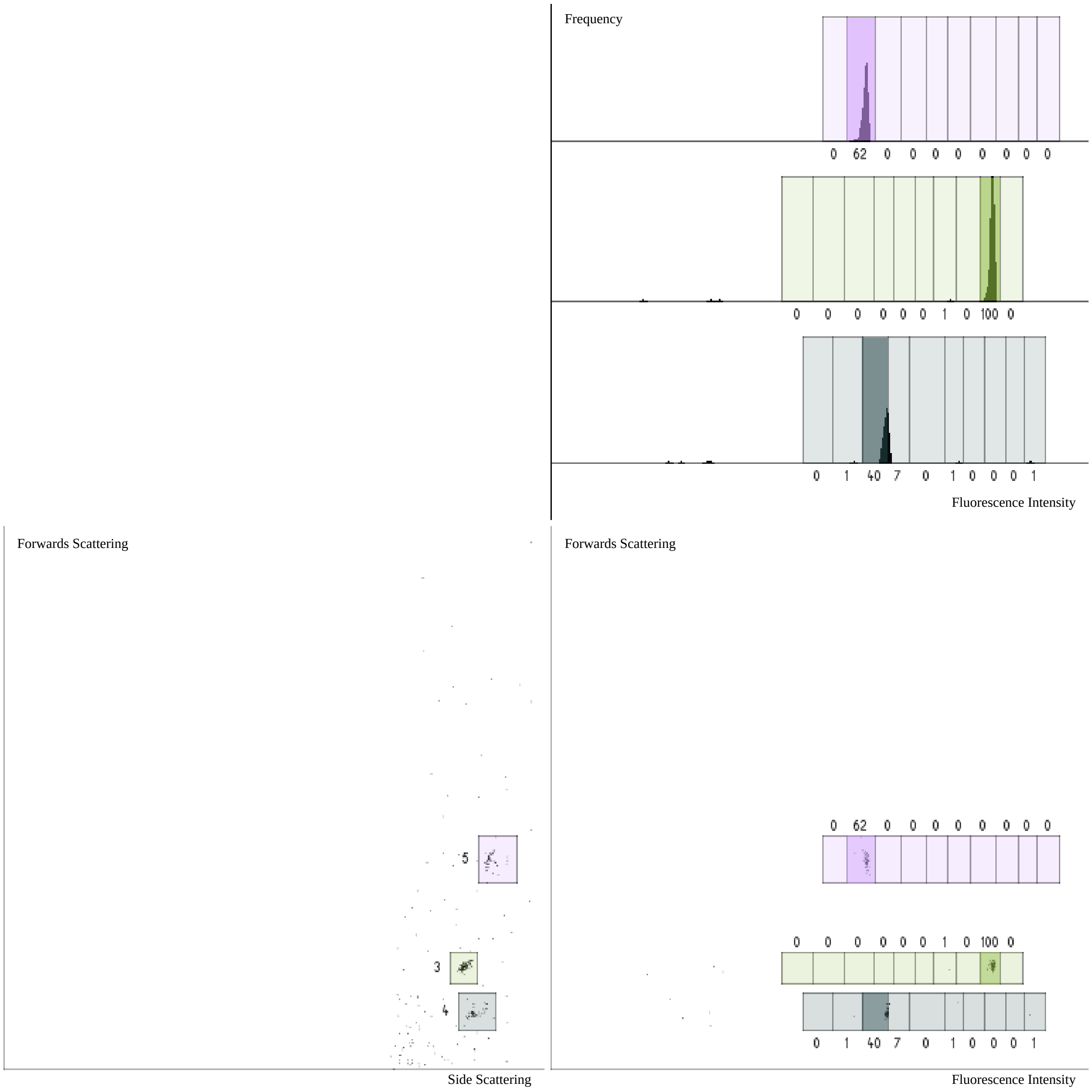

ANNEX 3: TAG DECONVOLUTION - BEAD 498

Passes flow sorting criteria: Yes

Passes tag deconvolution criteria: Yes

Included in protocol analysis: Yes

Protocol: 3, 7, 5, 9

Filename: Bin9_plateA0_G1.fcs

Split 1: Petrol shading

Split 2: Green shading

Split 3: Violet shading

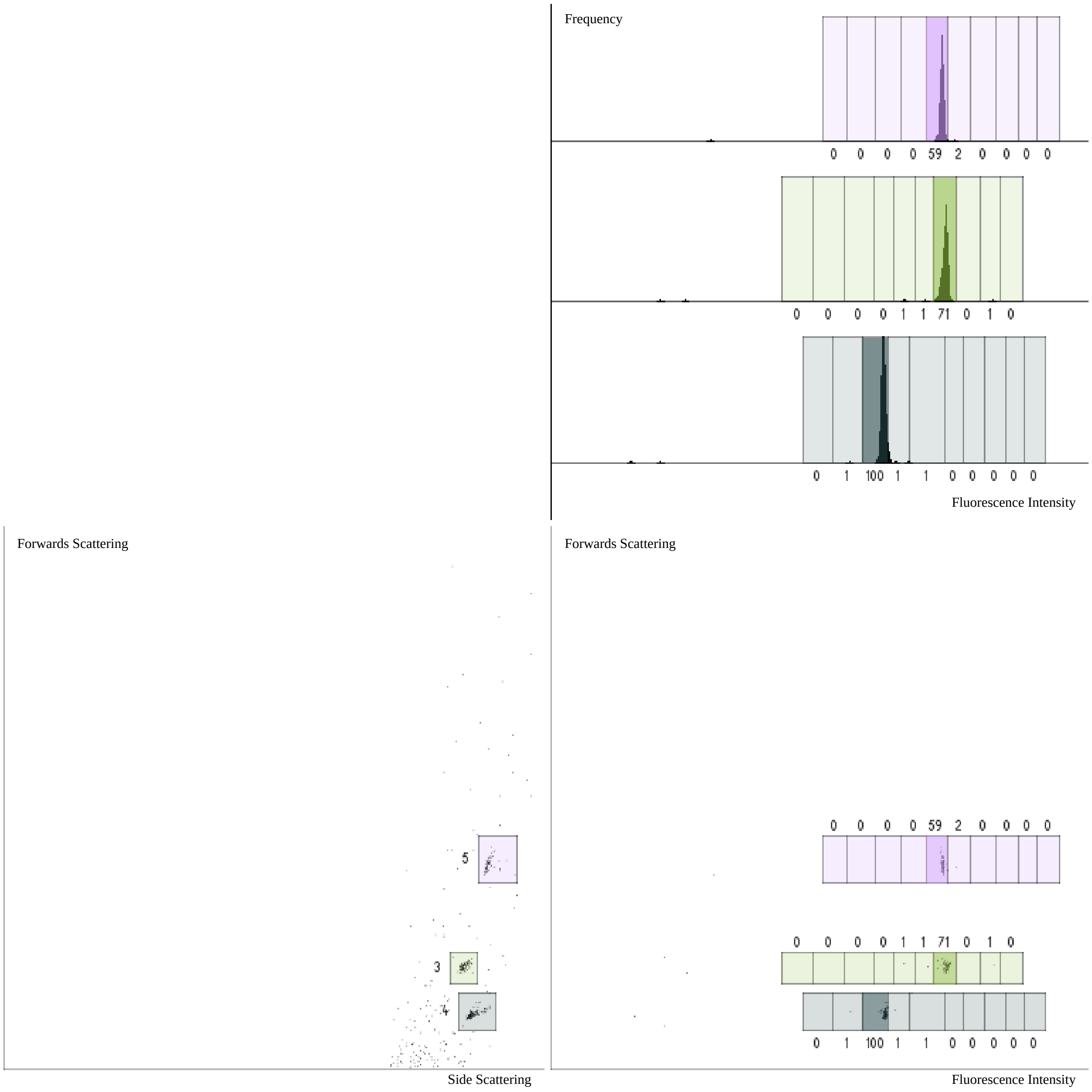

ANNEX 3: TAG DECONVOLUTION - BEAD 499

Passes flow sorting criteria: Yes

Passes tag deconvolution criteria: Yes

Included in protocol analysis: Yes

Protocol: 3, 5, 9, 9

Filename: Bin9_plateA0_G2.fcs

Split 1: Petrol shading

Split 2: Green shading

Split 3: Violet shading

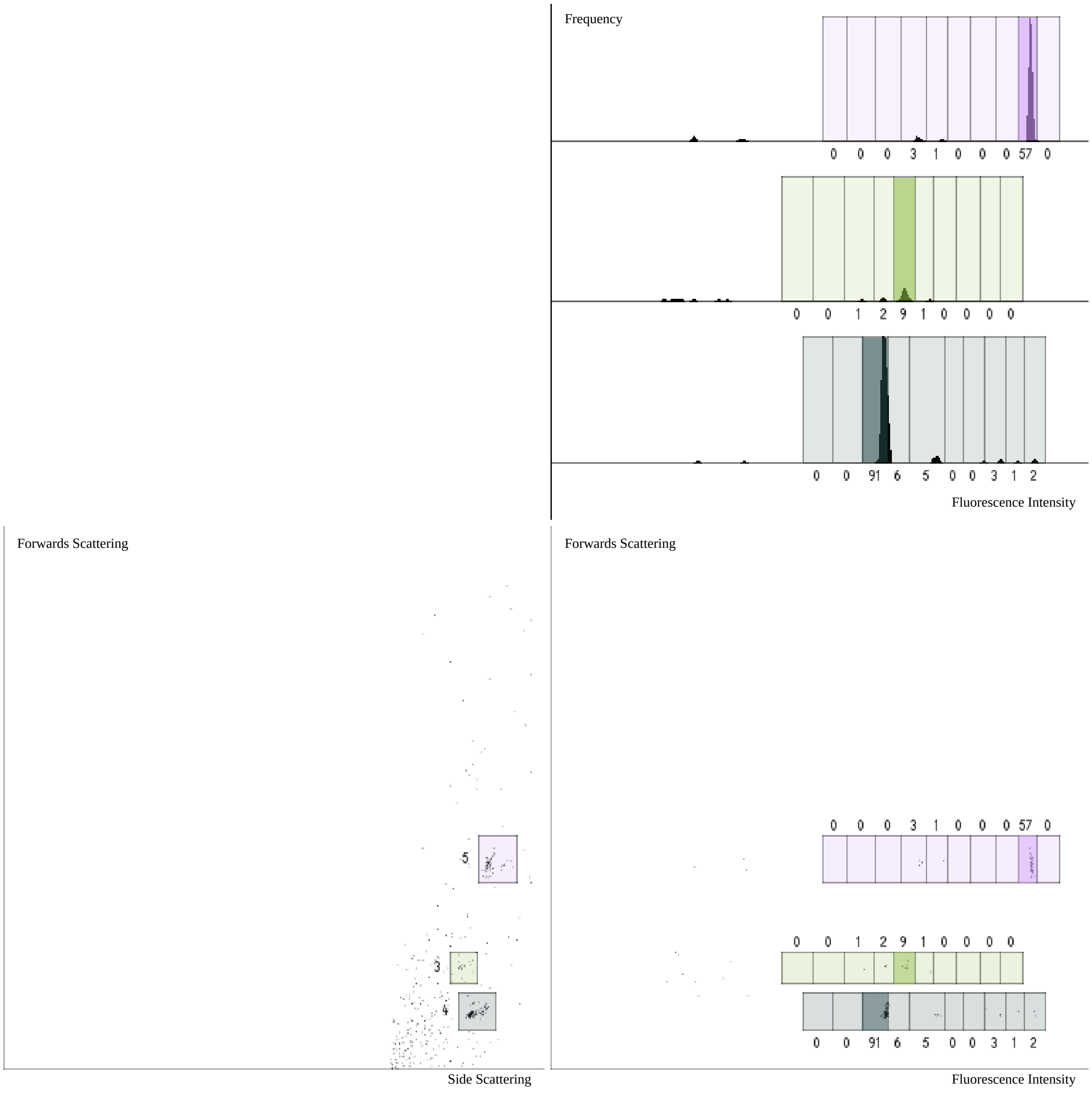

ANNEX 3: TAG DECONVOLUTION - BEAD 500

Passes flow sorting criteria: Yes

Passes tag deconvolution criteria: Yes

Included in protocol analysis: Yes

Protocol: 9, 1, 1, 9

Filename: Bin9_plateA0_G3.fcs

Split 1: Petrol shading

Split 2: Green shading

Split 3: Violet shading

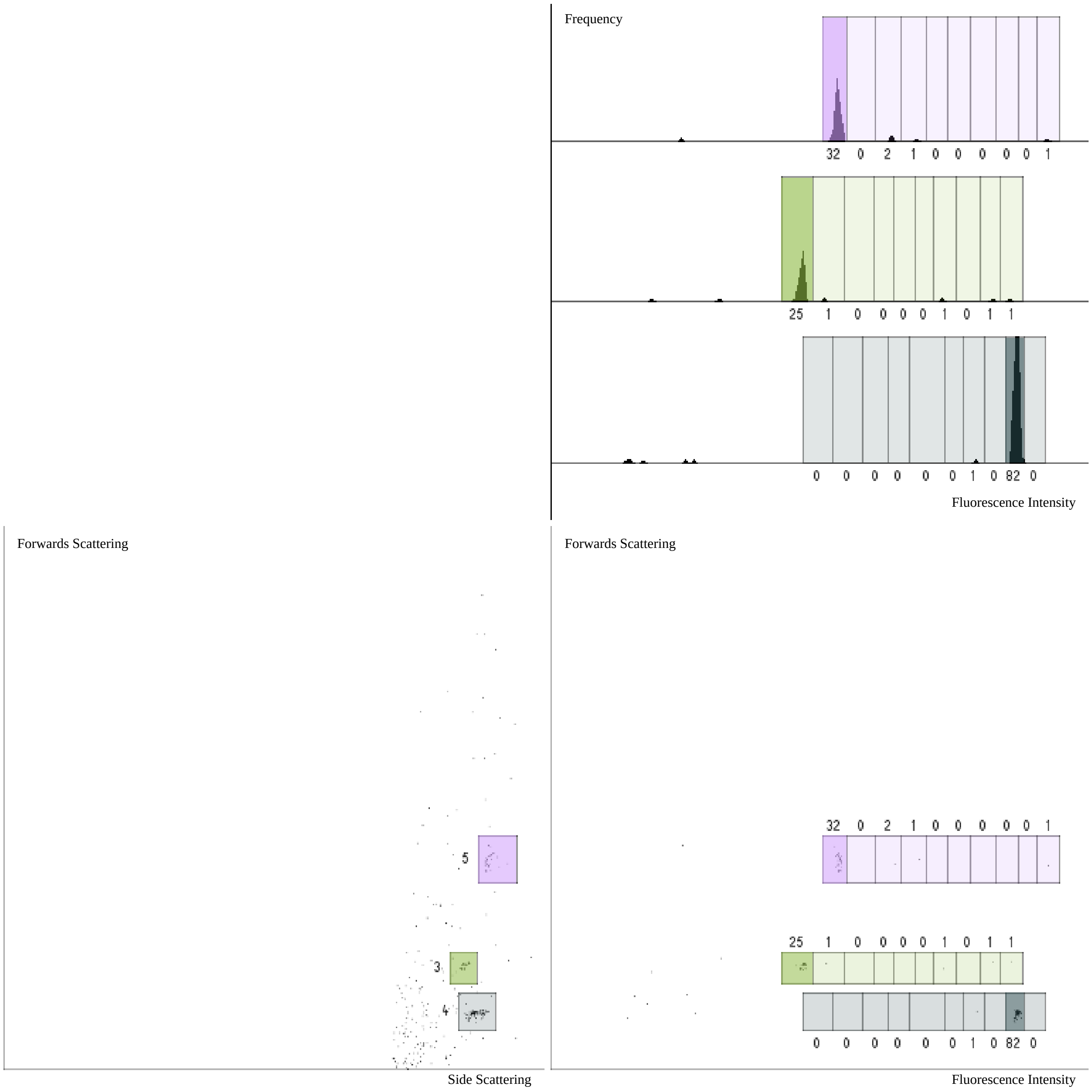

ANNEX 3: TAG DECONVOLUTION - BEAD 501

Passes flow sorting criteria: Yes

Passes tag deconvolution criteria: Yes

Included in protocol analysis: Yes

Protocol: 7, 9, 1, 9

Filename: Bin9_plateA0_G4.fcs

Split 1: Petrol shading

Split 2: Green shading

Split 3: Violet shading

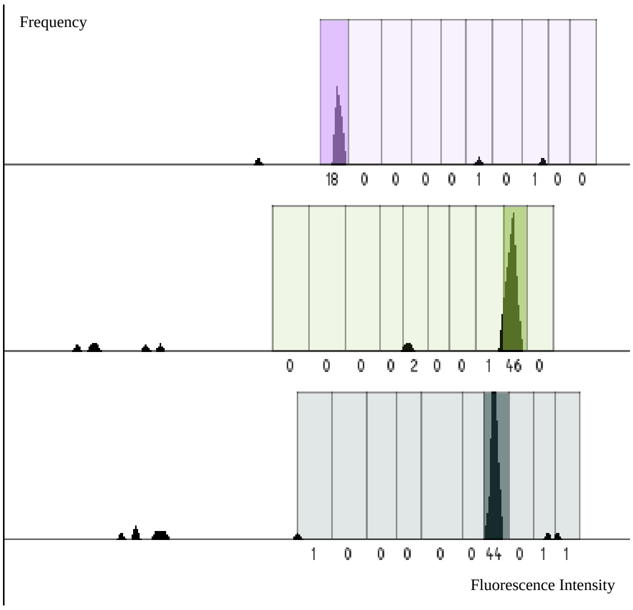

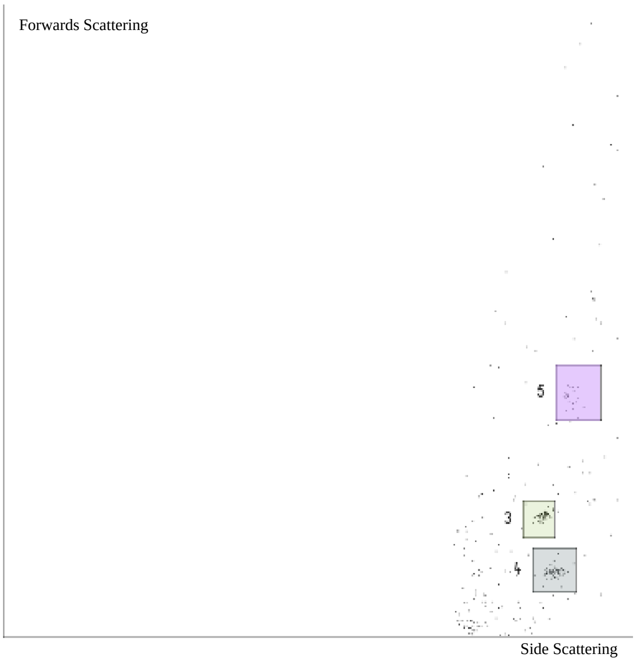

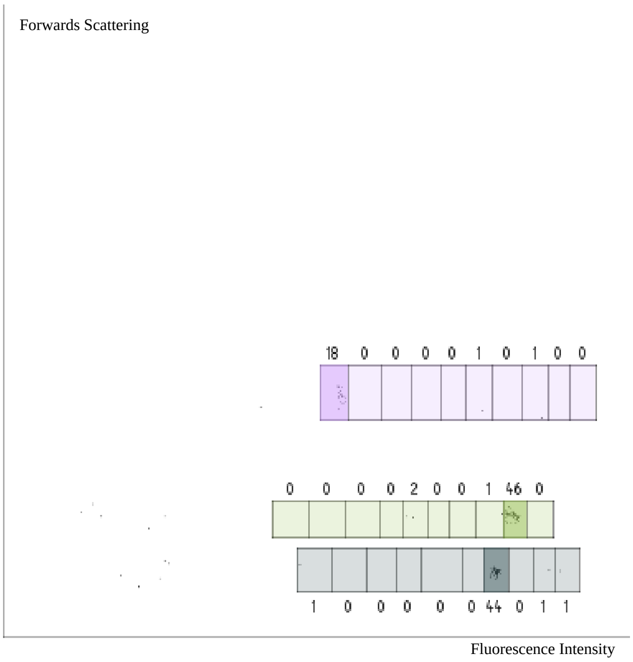

ANNEX 3: TAG DECONVOLUTION - BEAD 502

Passes flow sorting criteria: Yes

Passes tag deconvolution criteria: Yes

Included in protocol analysis: Yes

Protocol: 2, 6, 9, 9

Filename: Bin9_plateA0_G6.fcs

Split 1: Petrol shading

Split 2: Green shading

Split 3: Violet shading

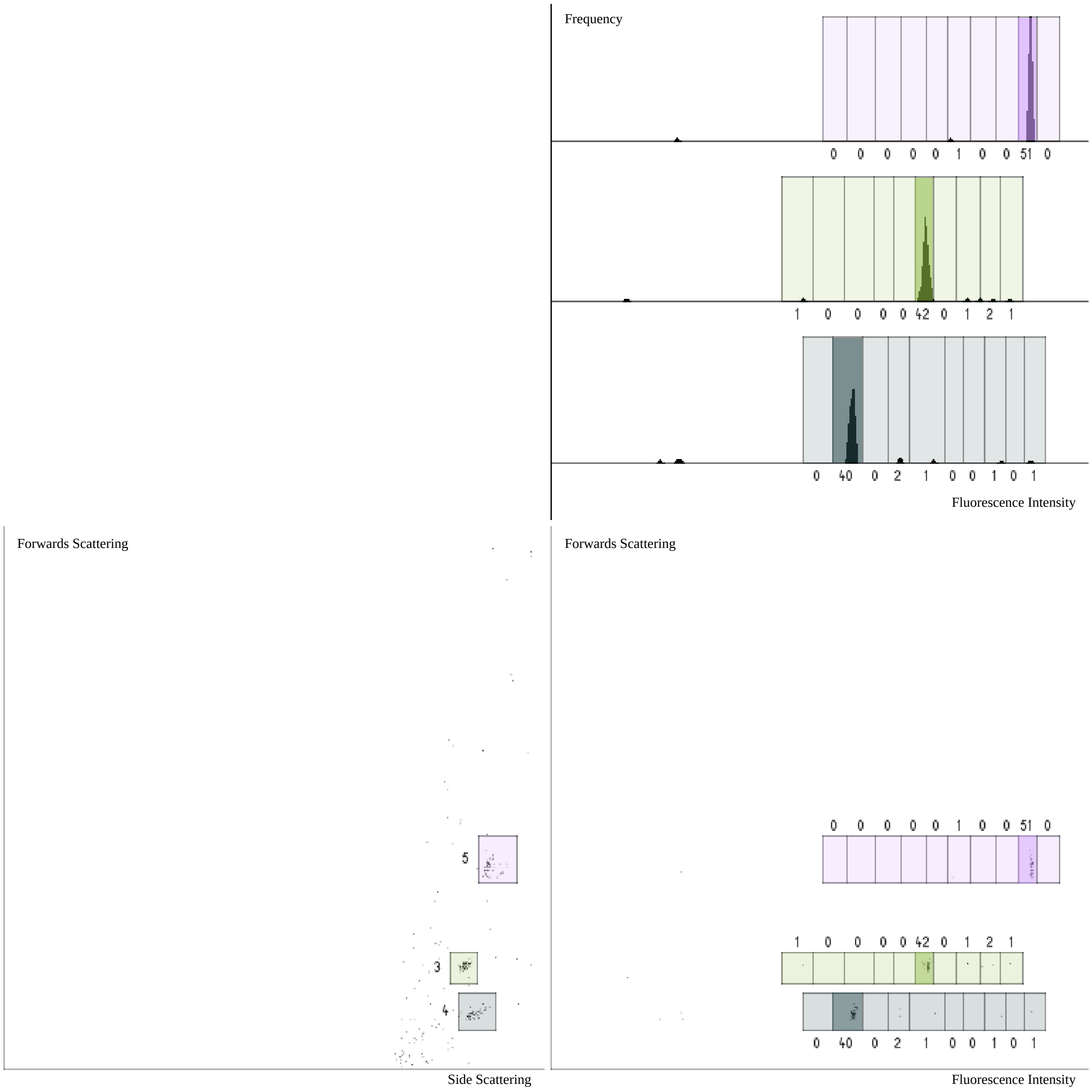

ANNEX 3: TAG DECONVOLUTION - BEAD 503

Passes flow sorting criteria: Yes

Passes tag deconvolution criteria: Yes

Included in protocol analysis: Yes

Protocol: 3, 5, 5, 9

Filename: Bin9_plateA0_G7.fcs

Split 1: Petrol shading

Split 2: Green shading

Split 3: Violet shading

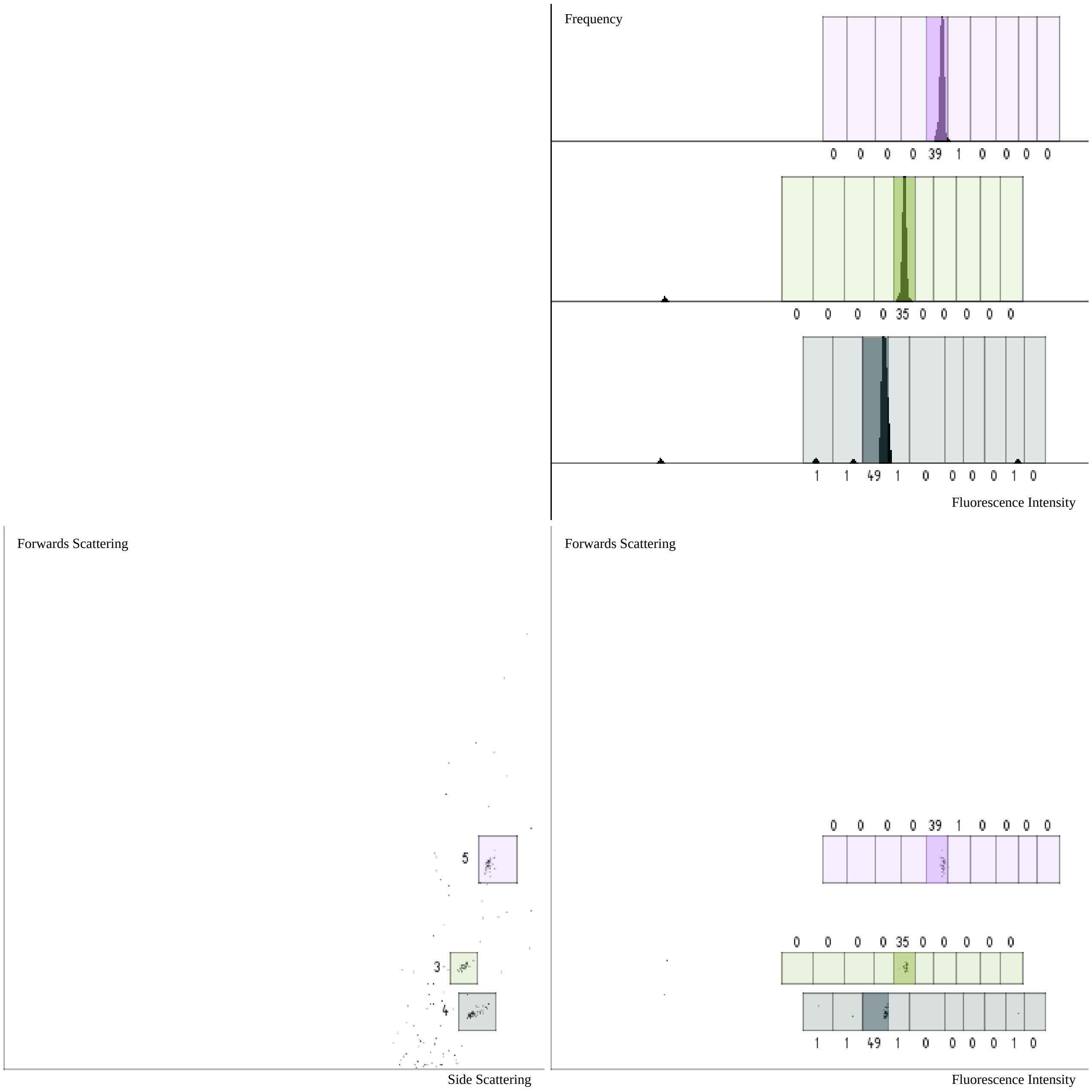

ANNEX 3: TAG DECONVOLUTION - BEAD 504

Passes flow sorting criteria: Yes

Passes tag deconvolution criteria: No

Included in protocol analysis: No

Protocol: N/A

Filename: Bin9_plateA0_G8.fcs

Split 1: Petrol shading

Split 2: Green shading

Split 3: Violet shading

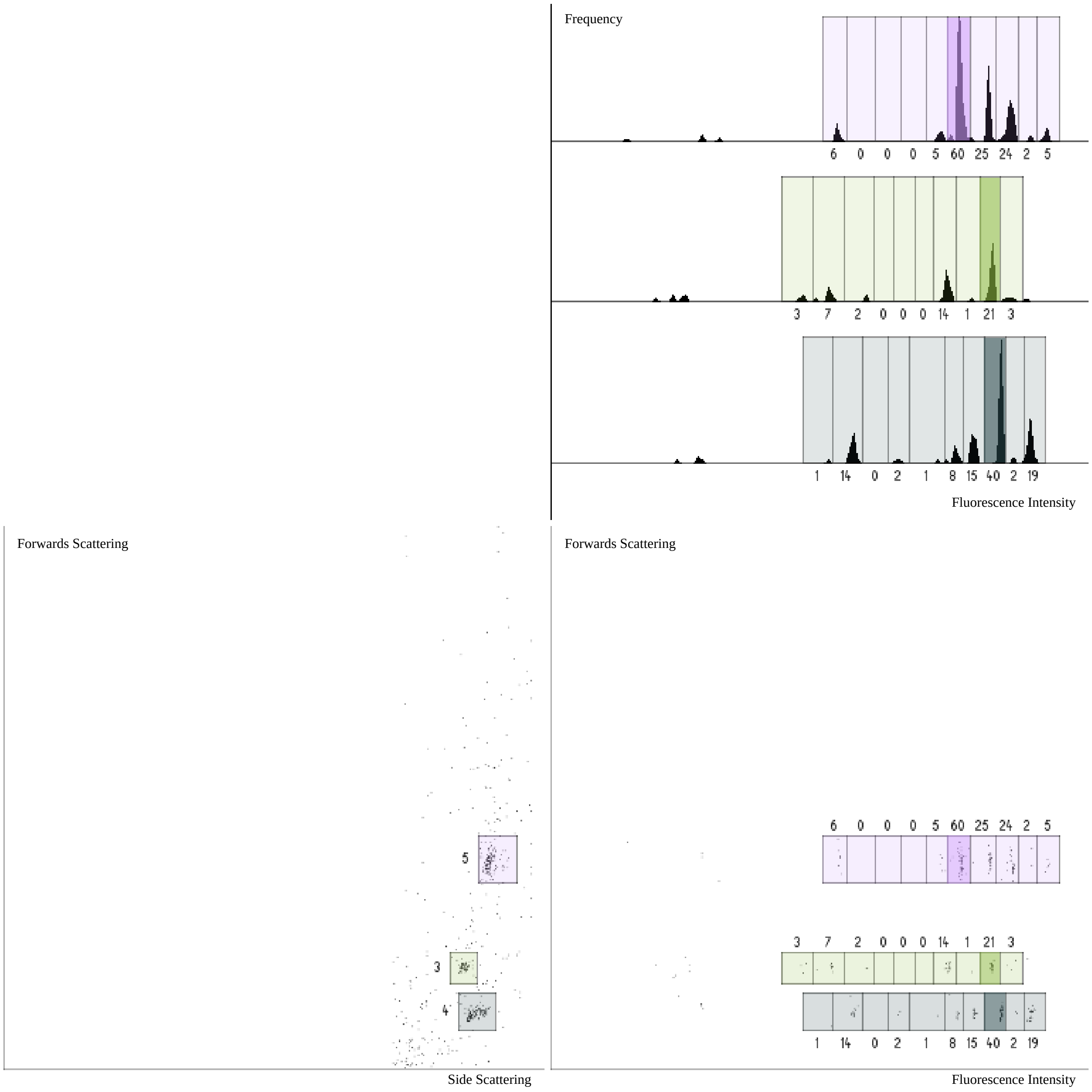

ANNEX 3: TAG DECONVOLUTION - BEAD 505

Passes flow sorting criteria: Yes

Passes tag deconvolution criteria: Yes

Included in protocol analysis: Yes

Protocol: 2, 9, 3, 9

Filename: Bin9_plateA0_G9.fcs

Split 1: Petrol shading

Split 2: Green shading

Split 3: Violet shading

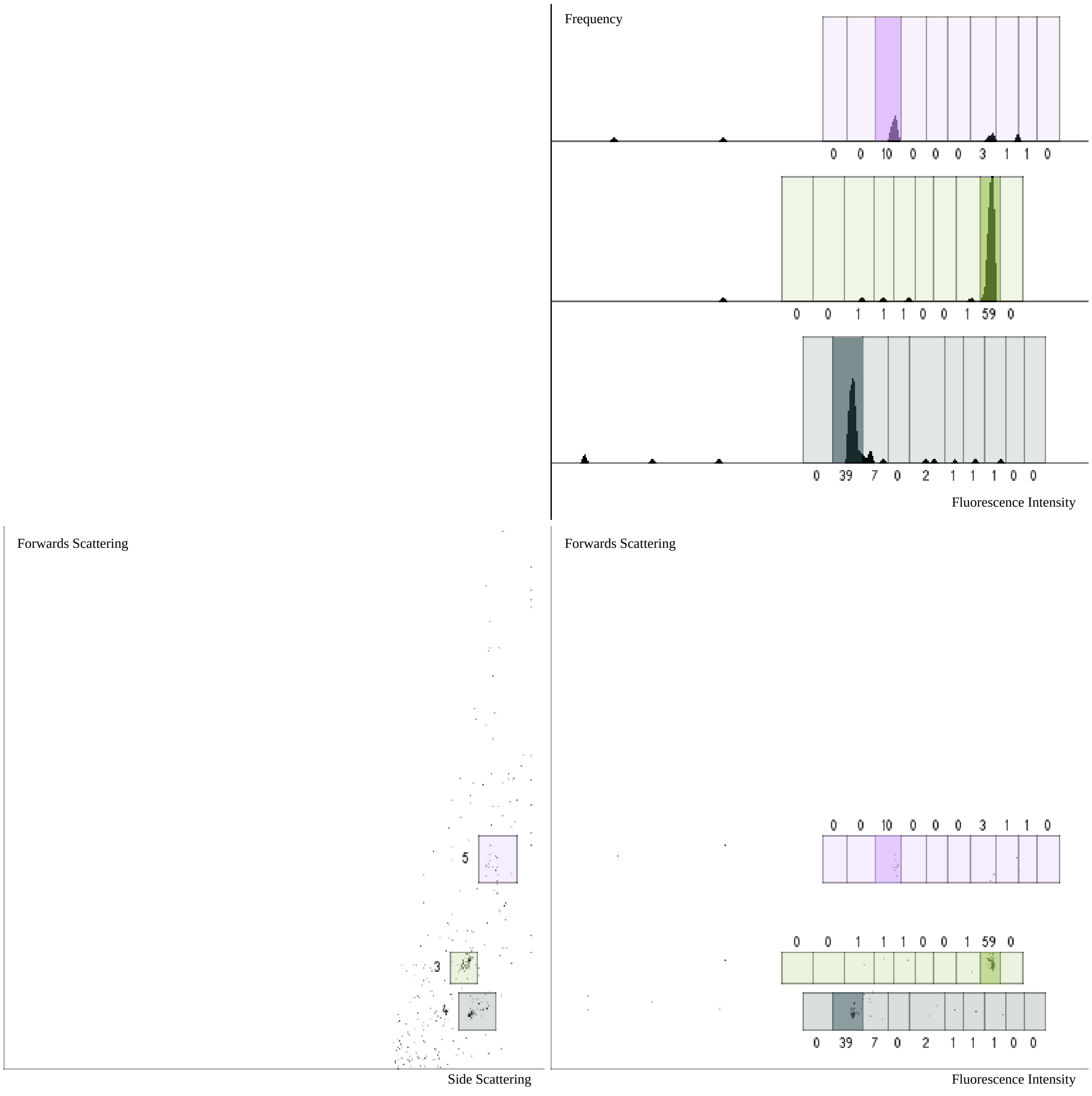

ANNEX 3: TAG DECONVOLUTION - BEAD 506

Passes flow sorting criteria: Yes

Passes tag deconvolution criteria: No

Included in protocol analysis: No

Protocol: N/A

Filename: Bin9_plateA0_G10.fcs

Split 1: Petrol shading

Split 2: Green shading

Split 3: Violet shading

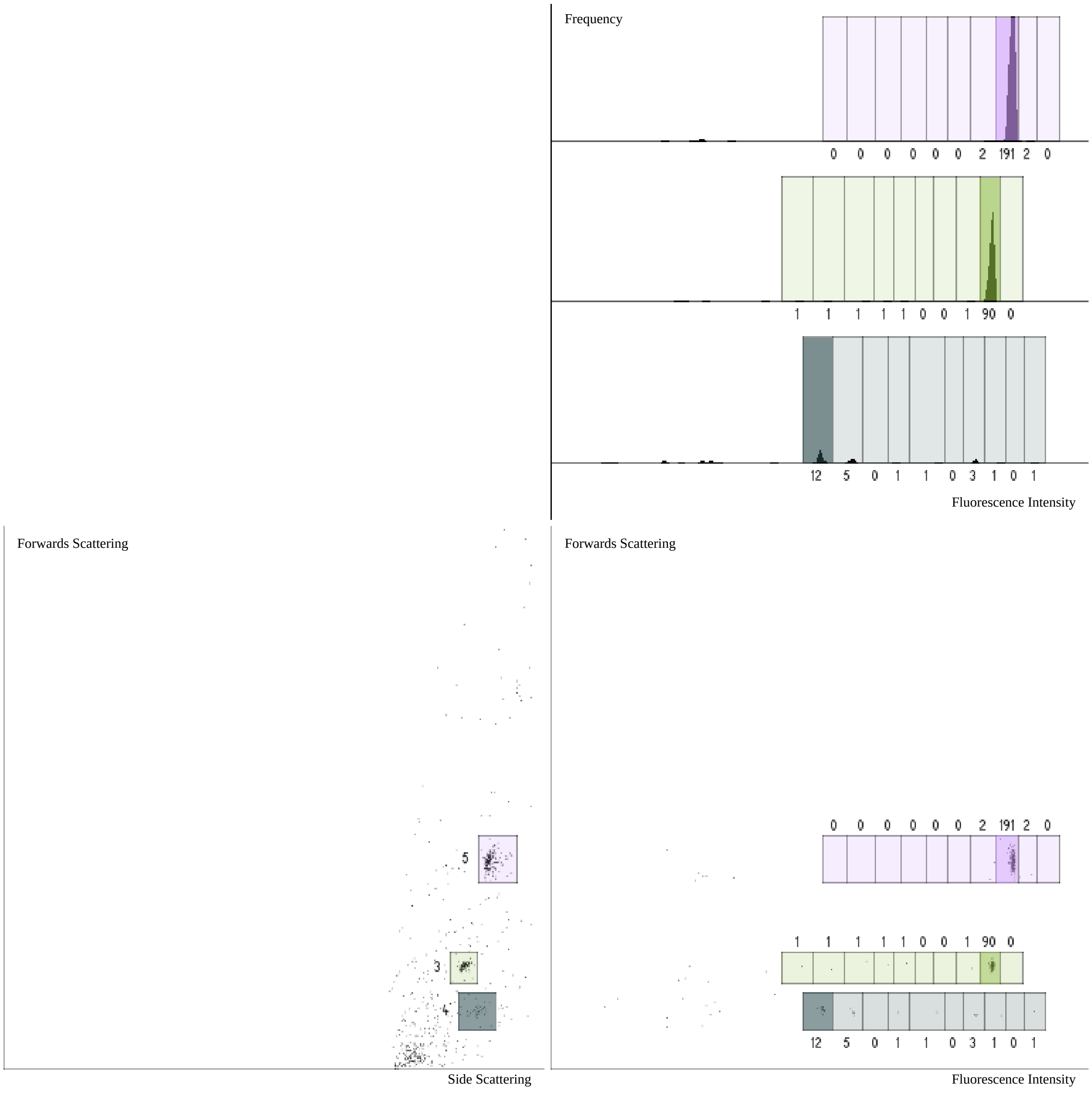

ANNEX 3: TAG DECONVOLUTION - BEAD 507

Passes flow sorting criteria: Yes

Passes tag deconvolution criteria: No

Included in protocol analysis: No

Protocol: N/A

Filename: Bin9_plateA0_G11.fcs

Split 1: Petrol shading

Split 2: Green shading

Split 3: Violet shading

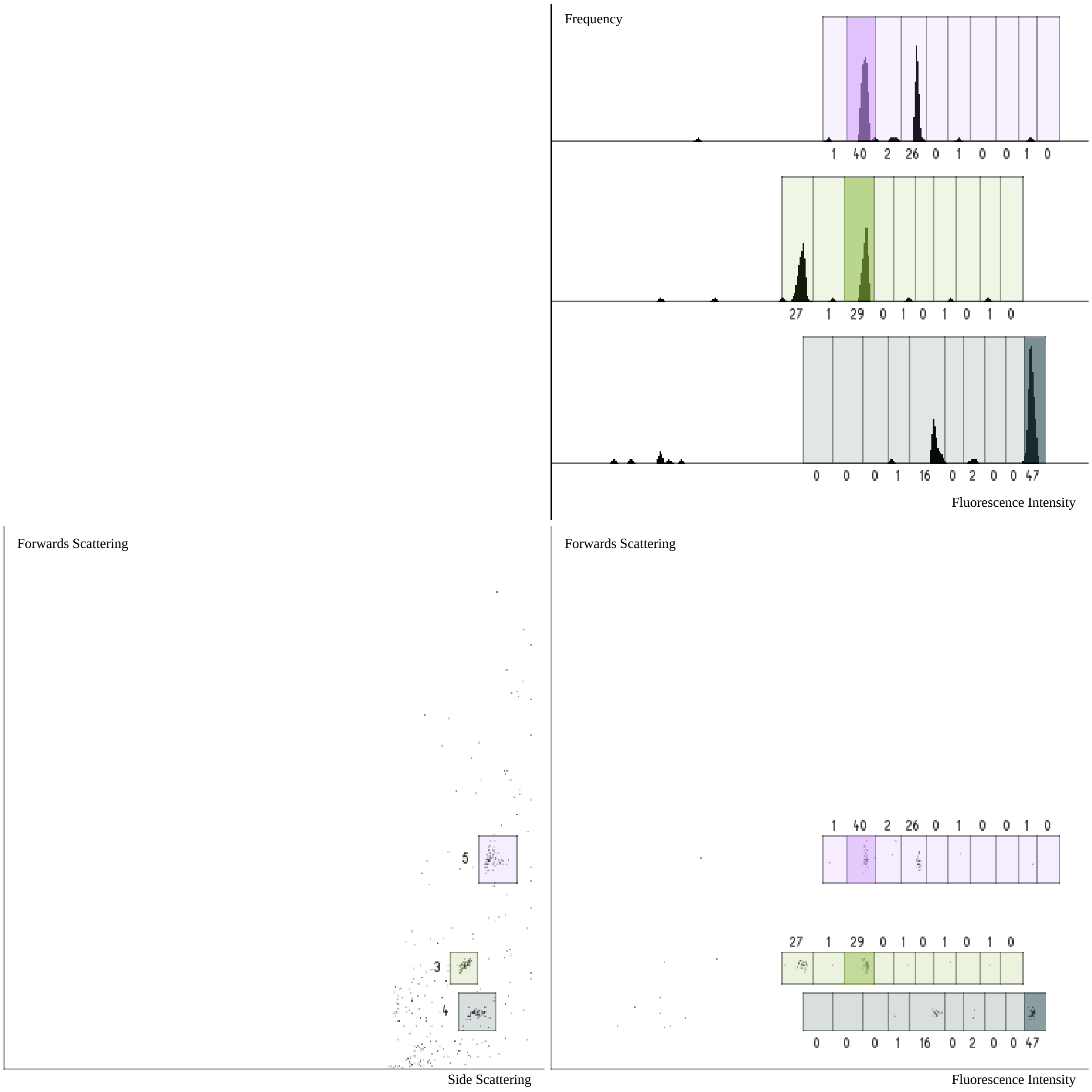

ANNEX 3: TAG DECONVOLUTION - BEAD 508

Passes flow sorting criteria: Yes

Passes tag deconvolution criteria: Yes

Included in protocol analysis: Yes

Protocol: 5, 10, 9, 9

Filename: Bin9_plateA0_G12.fcs

Split 1: Petrol shading

Split 2: Green shading

Split 3: Violet shading

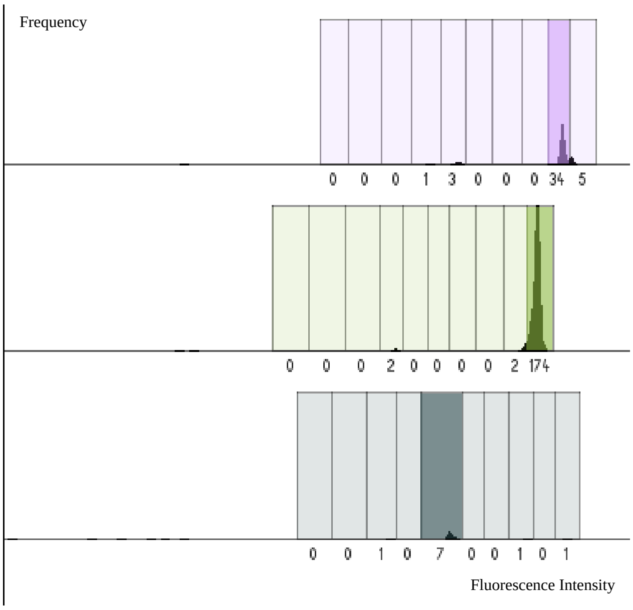

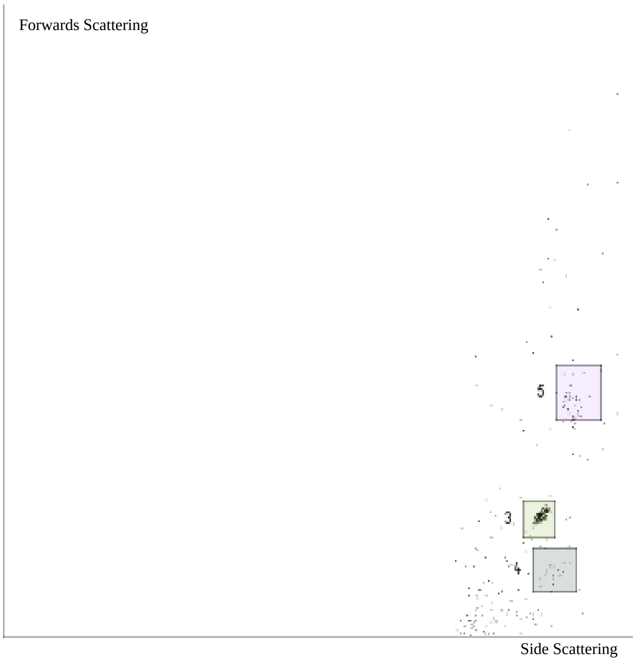

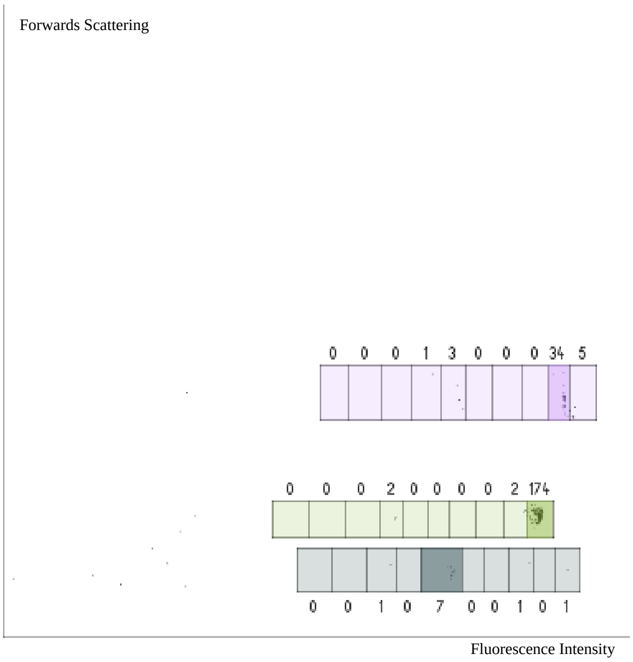

ANNEX 3: TAG DECONVOLUTION - BEAD 509

Passes flow sorting criteria: Yes

Passes tag deconvolution criteria: Yes

Included in protocol analysis: Yes

Protocol: 2, 3, 8, 9

Filename: Bin9_plateA0_H1.fcs

Split 1: Petrol shading

Split 2: Green shading

Split 3: Violet shading

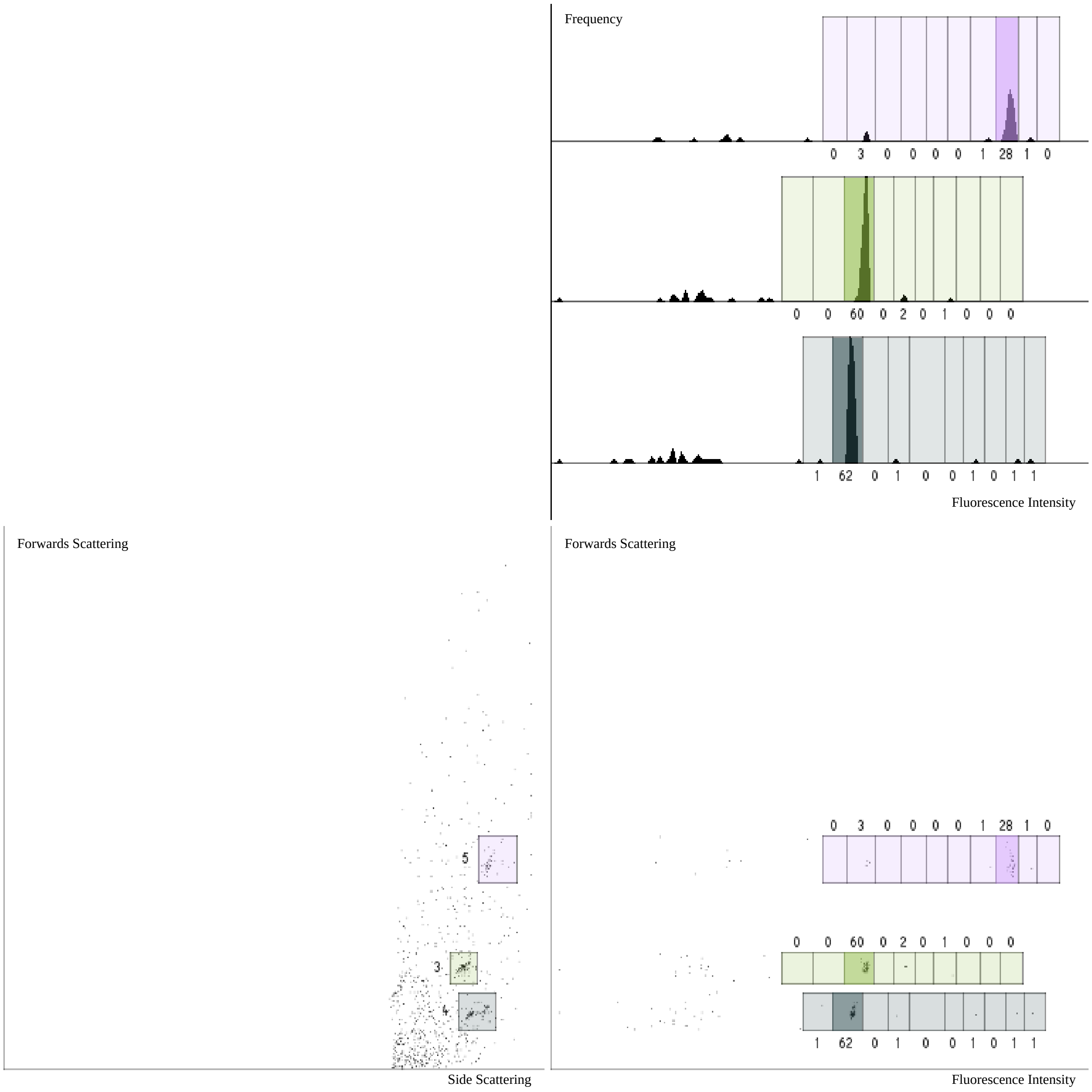

ANNEX 3: TAG DECONVOLUTION - BEAD 510

Passes flow sorting criteria: Yes

Passes tag deconvolution criteria: Yes

Included in protocol analysis: Yes

Protocol: 6, 10, 1, 9

Filename: Bin9_plateA0_H2.fcs

Split 1: Petrol shading

Split 2: Green shading

Split 3: Violet shading

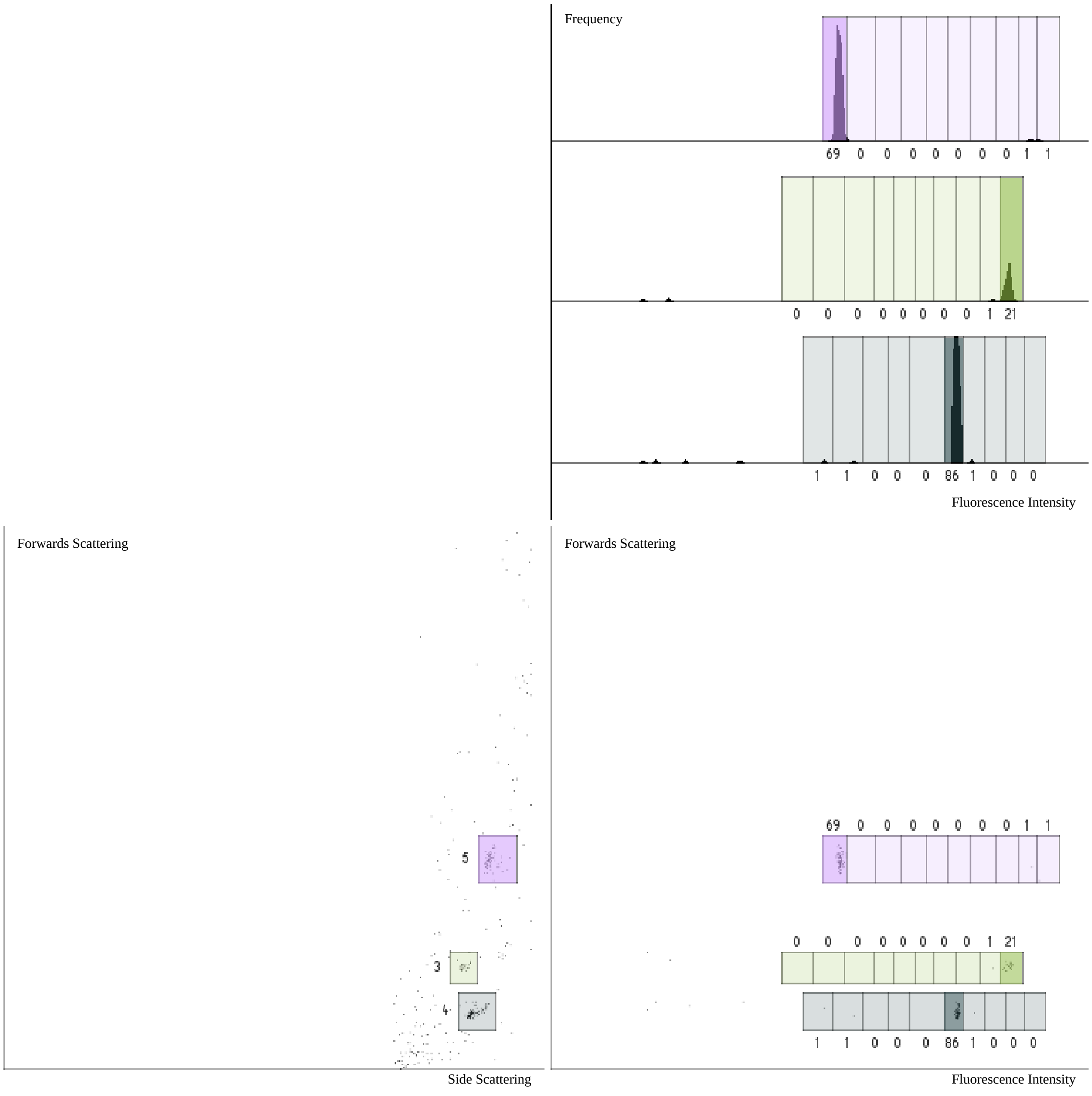

ANNEX 3: TAG DECONVOLUTION - BEAD 511

Passes flow sorting criteria: Yes

Passes tag deconvolution criteria: Yes

Included in protocol analysis: Yes

Protocol: 1, 9, 9, 9

Filename: Bin9_plateA0_H3.fcs

Split 1: Petrol shading

Split 2: Green shading

Split 3: Violet shading

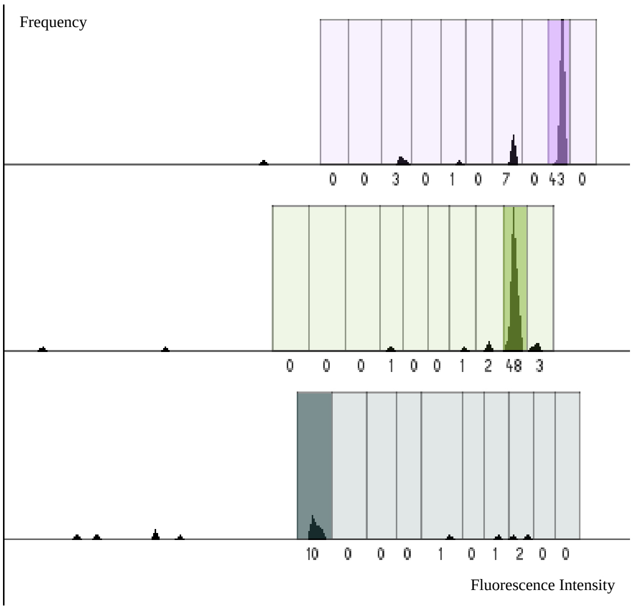

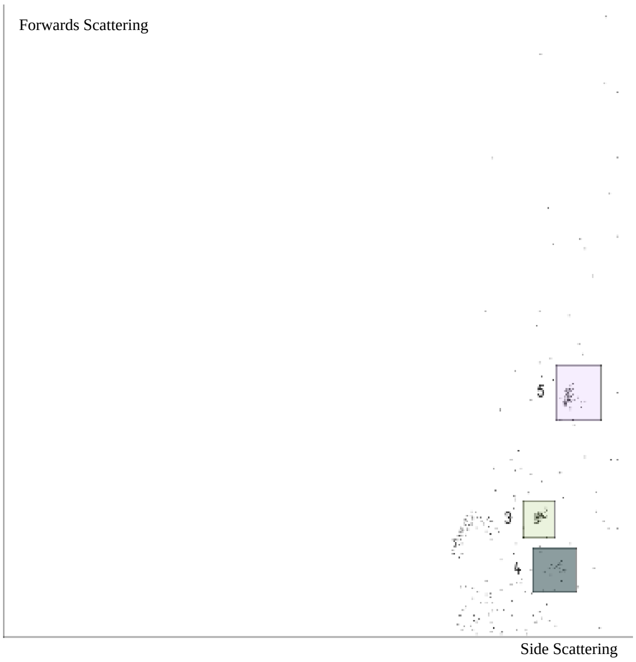

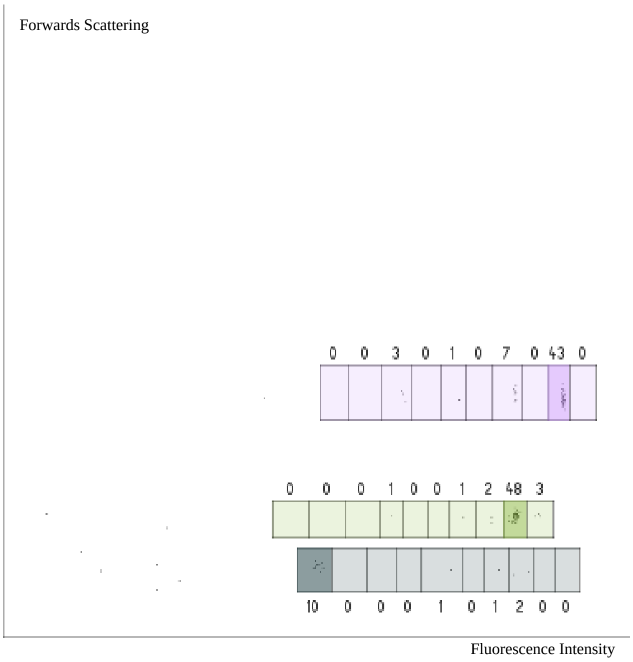

ANNEX 3: TAG DECONVOLUTION - BEAD 512

Passes flow sorting criteria: Yes

Passes tag deconvolution criteria: Yes

Included in protocol analysis: Yes

Protocol: 9, 5, 4, 9

Filename: Bin9_plateA0_H4.fcs

Split 1: Petrol shading

Split 2: Green shading

Split 3: Violet shading

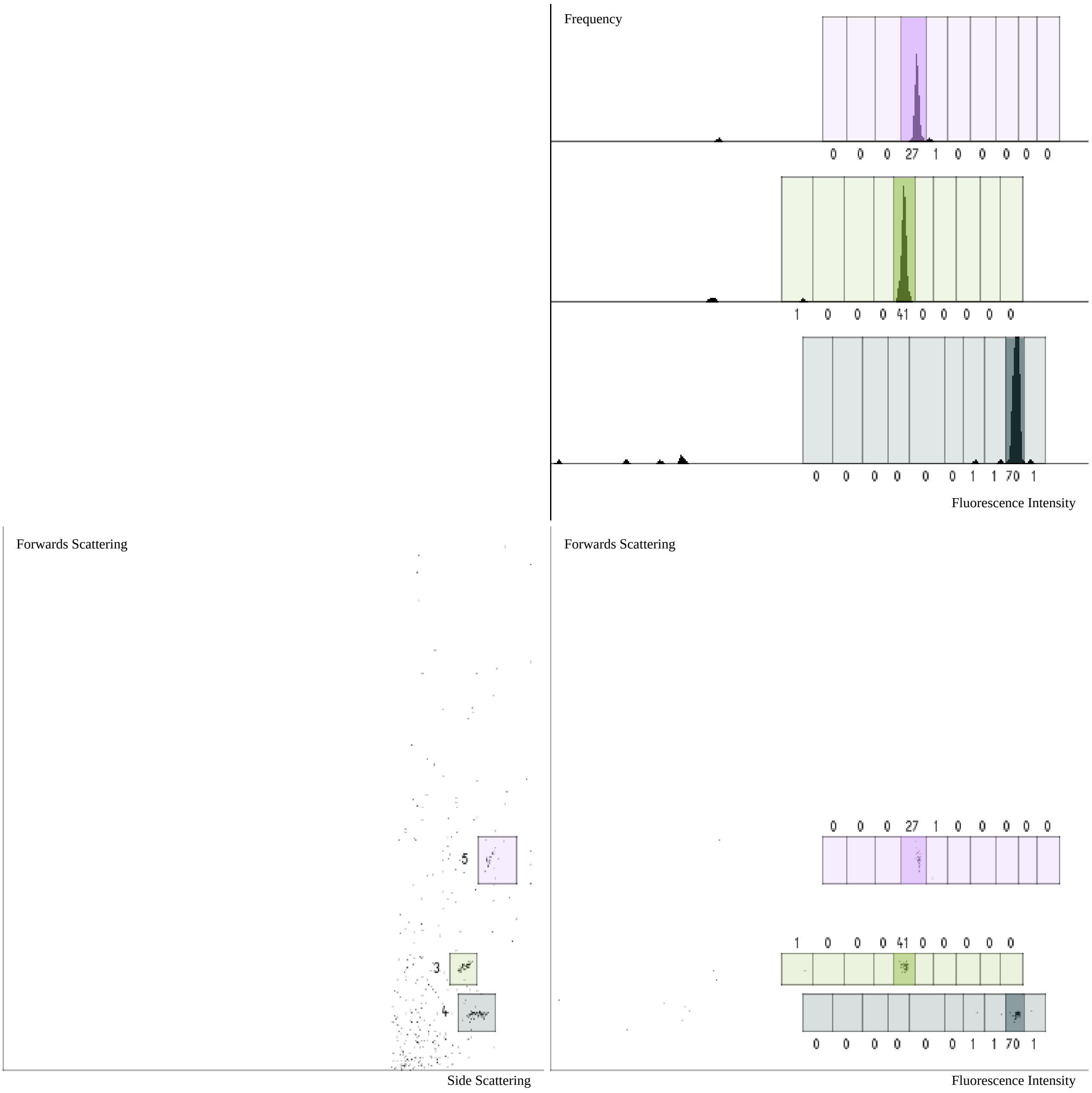

ANNEX 3: TAG DECONVOLUTION - BEAD 513

Passes flow sorting criteria: Yes

Passes tag deconvolution criteria: Yes

Included in protocol analysis: Yes

Protocol: 1, 10, 1, 9

Filename: Bin9_plateA0_H5.fcs

Split 1: Petrol shading

Split 2: Green shading

Split 3: Violet shading

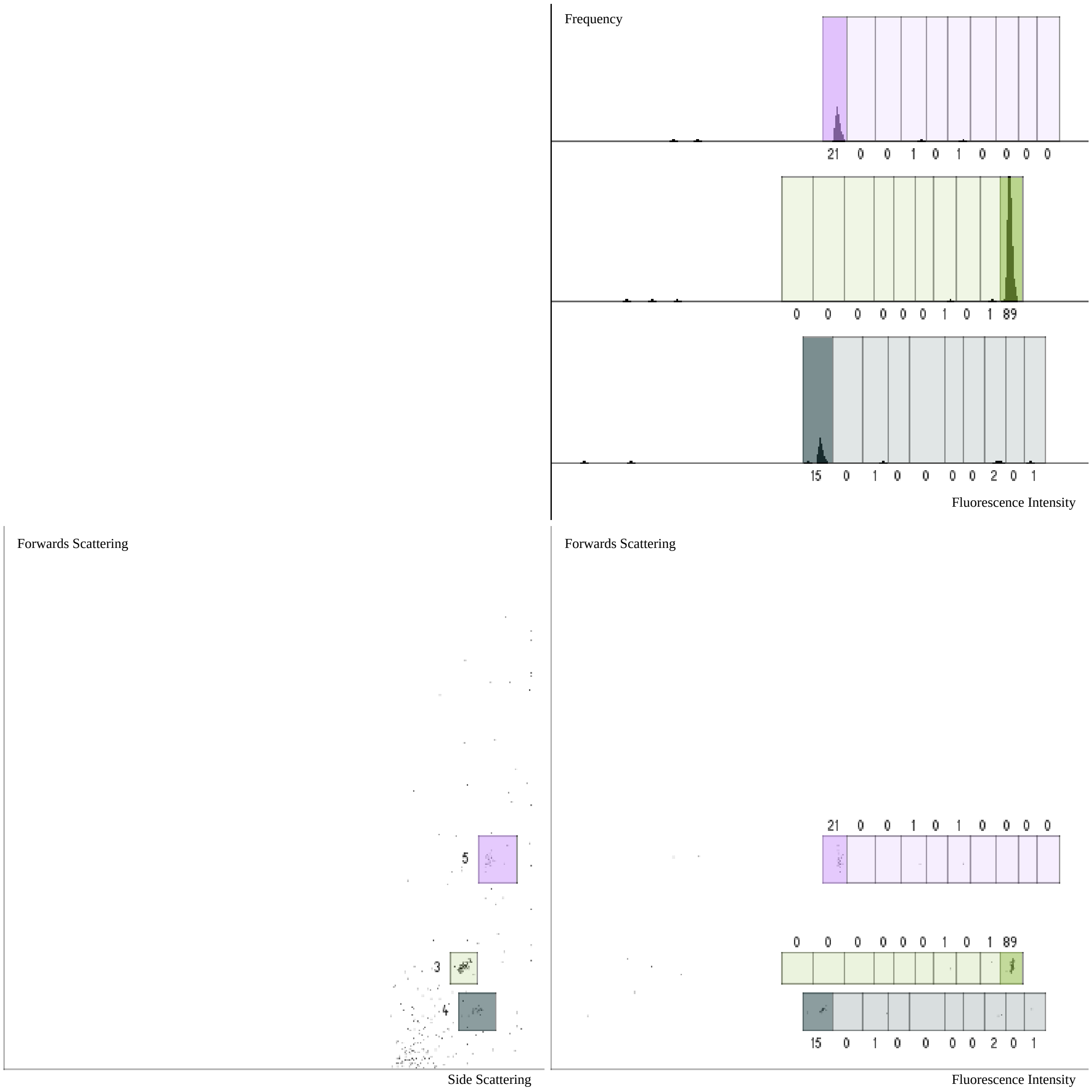

ANNEX 3: TAG DECONVOLUTION - BEAD 514

Passes flow sorting criteria: Yes

Passes tag deconvolution criteria: Yes

Included in protocol analysis: Yes

Protocol: 8, 8, 3, 9

Filename: Bin9_plateA0_H6.fcs

Split 1: Petrol shading

Split 2: Green shading

Split 3: Violet shading

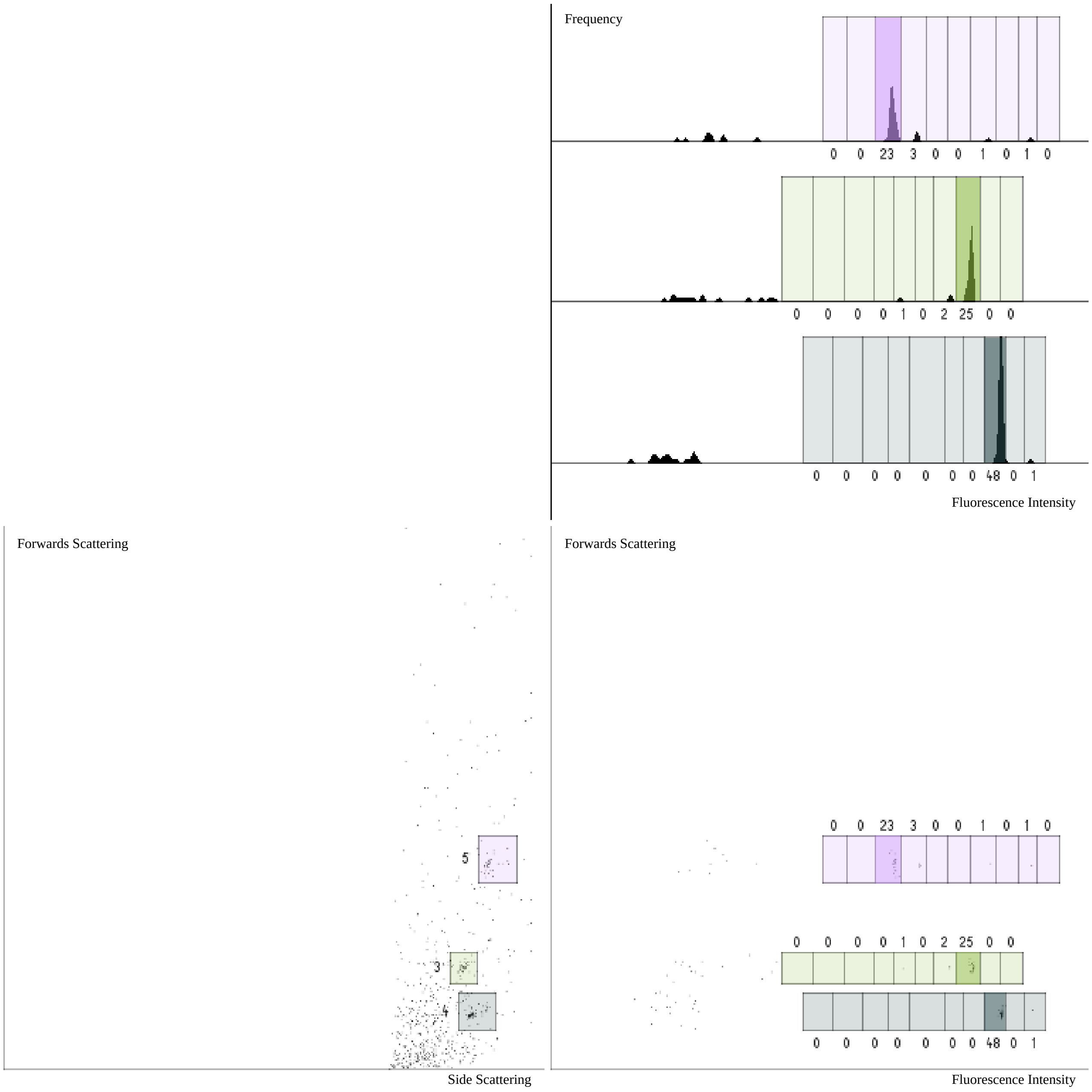

ANNEX 3: TAG DECONVOLUTION - BEAD 515

Passes flow sorting criteria: Yes

Passes tag deconvolution criteria: No

Included in protocol analysis: No

Protocol: N/A

Filename: Bin9_plateA0_H7.fcs

Split 1: Petrol shading

Split 2: Green shading

Split 3: Violet shading

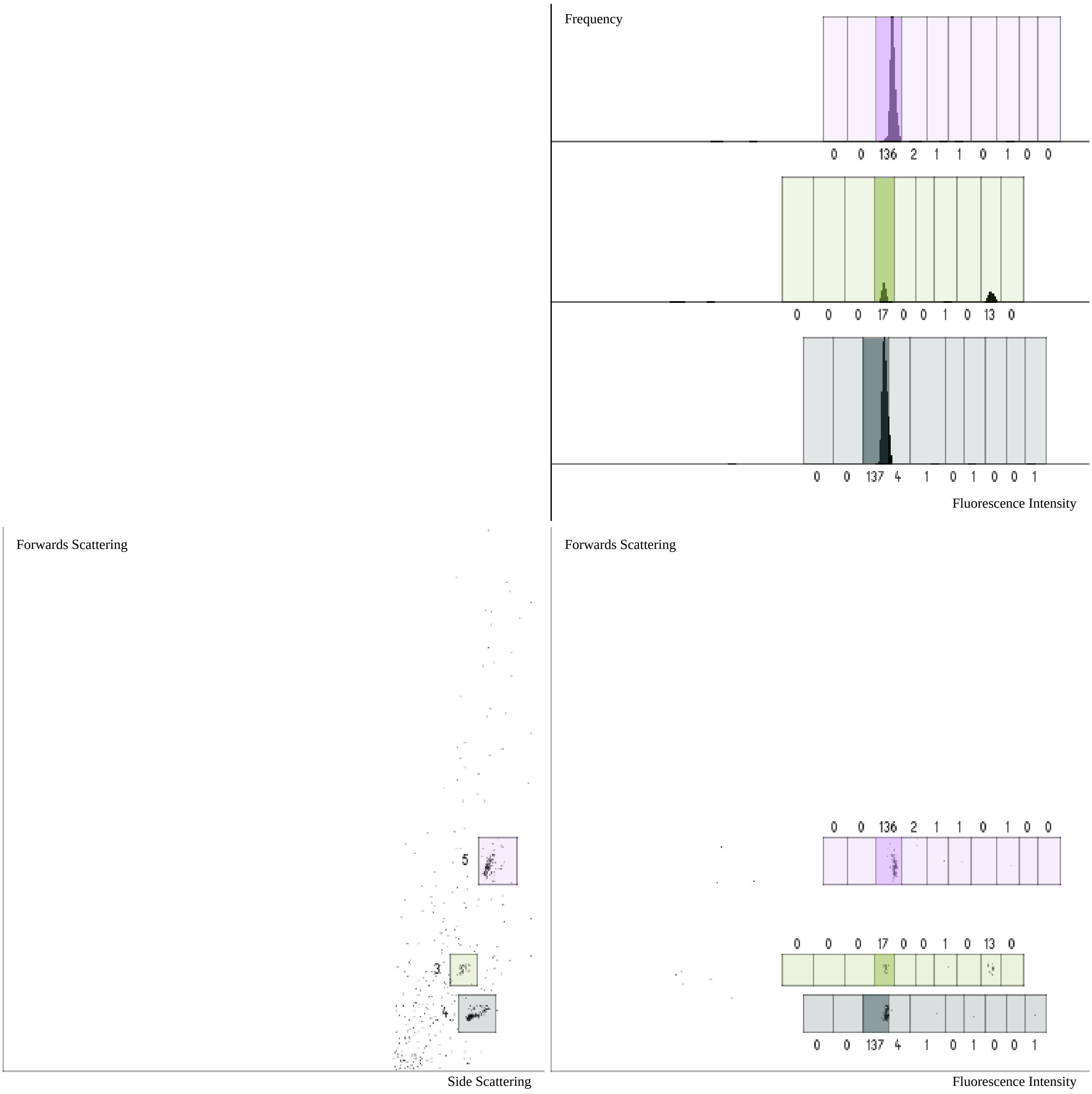

ANNEX 3: TAG DECONVOLUTION - BEAD 516

Passes flow sorting criteria: Yes

Passes tag deconvolution criteria: No

Included in protocol analysis: No

Protocol: N/A

Filename: Bin9_plateA0_H8.fcs

Split 1: Petrol shading

Split 2: Green shading

Split 3: Violet shading

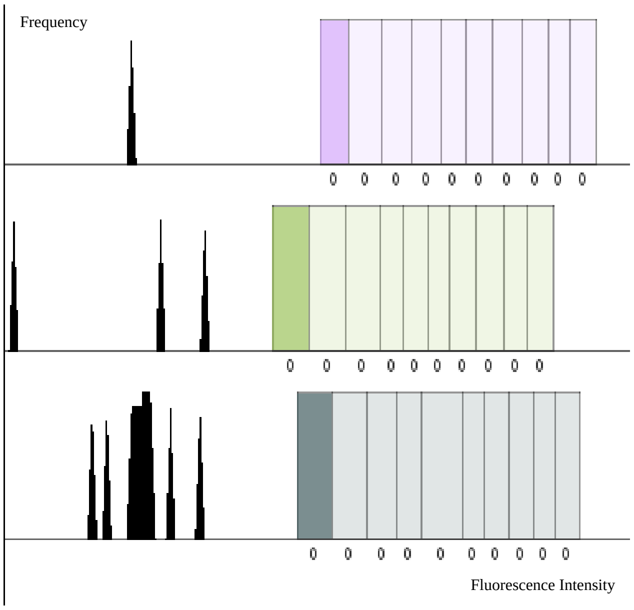

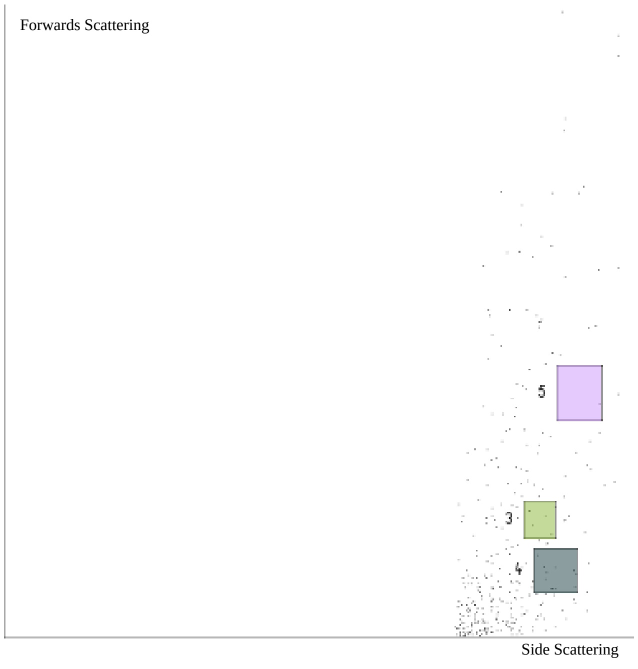

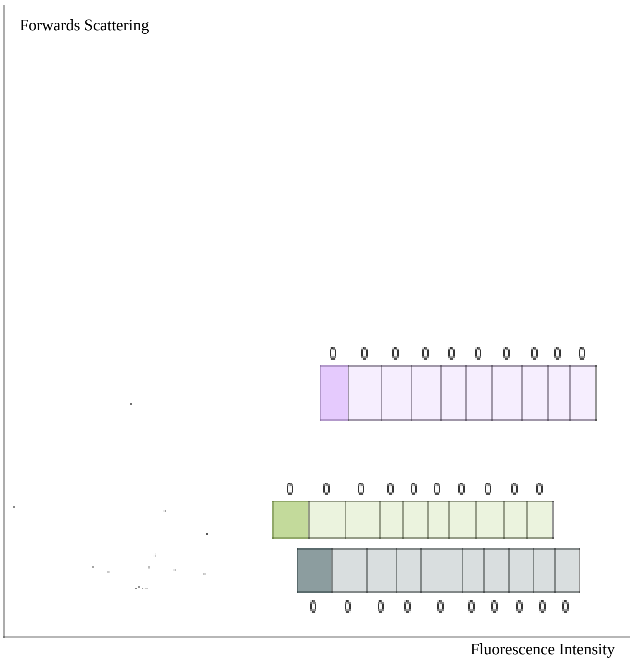

ANNEX 3: TAG DECONVOLUTION - BEAD 517

Passes flow sorting criteria: Yes

Passes tag deconvolution criteria: Yes

Included in protocol analysis: Yes

Protocol: 7, 9, 2, 9

Filename: Bin9_plateA0_H9.fcs

Split 1: Petrol shading

Split 2: Green shading

Split 3: Violet shading

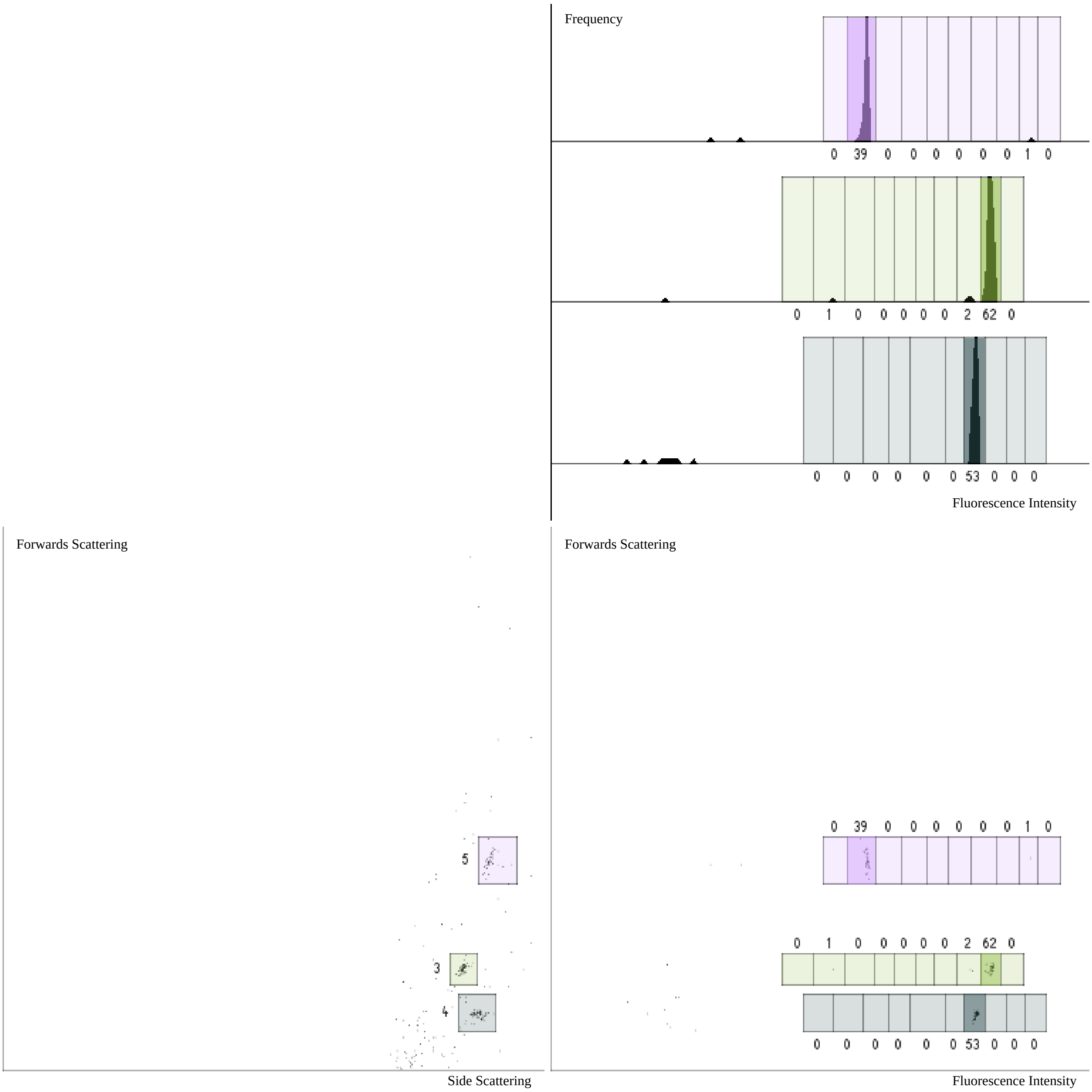

ANNEX 3: TAG DECONVOLUTION - BEAD 518

Passes flow sorting criteria: Yes

Passes tag deconvolution criteria: Yes

Included in protocol analysis: Yes

Protocol: 3, 7, 2, 9

Filename: Bin9_plateA0_H10.fcs

Split 1: Petrol shading

Split 2: Green shading

Split 3: Violet shading

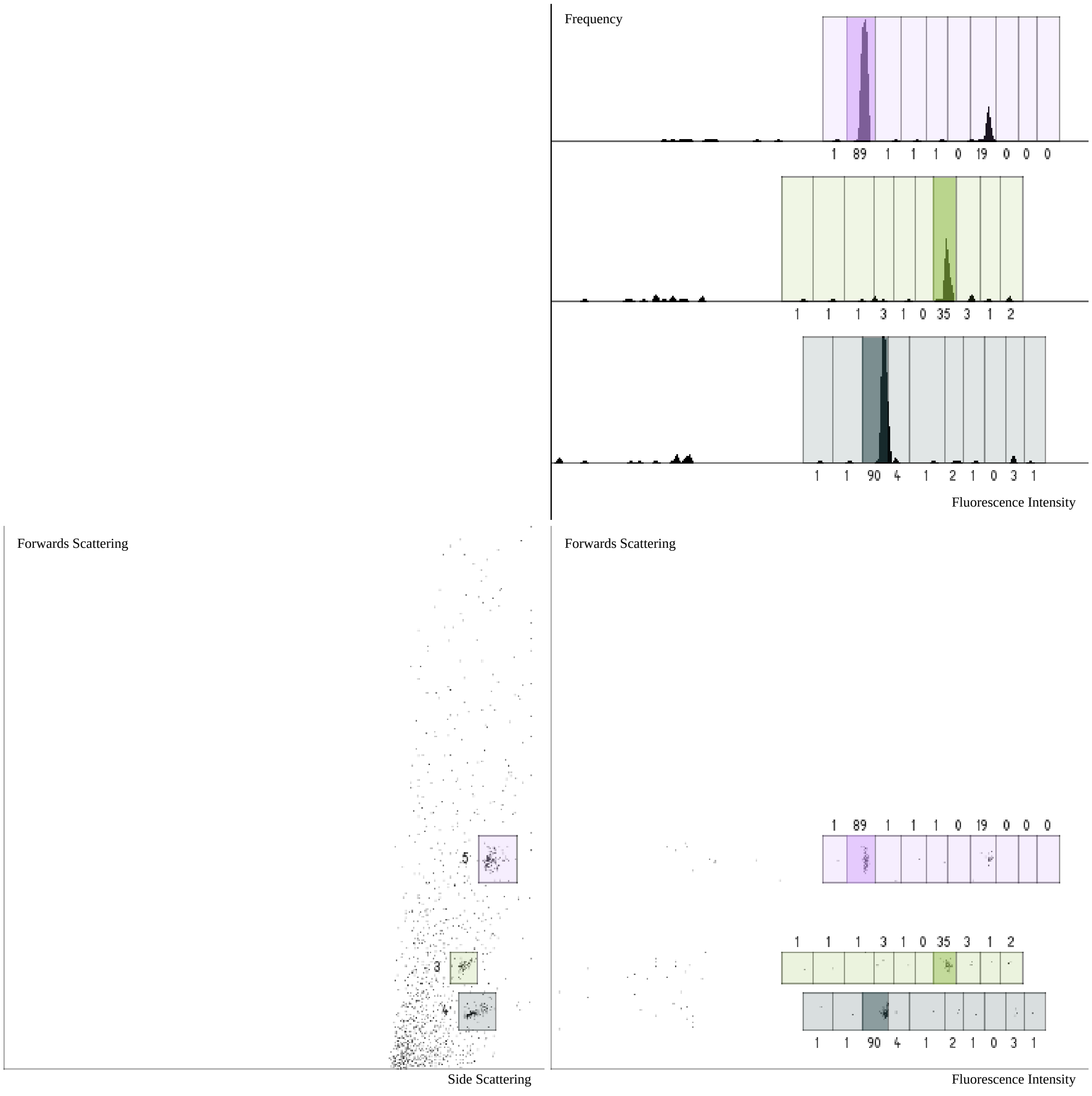

ANNEX 3: TAG DECONVOLUTION - BEAD 519

Passes flow sorting criteria: Yes

Passes tag deconvolution criteria: No

Included in protocol analysis: No

Protocol: N/A

Filename: Bin9_plateA0_H11.fcs

Split 1: Petrol shading

Split 2: Green shading

Split 3: Violet shading

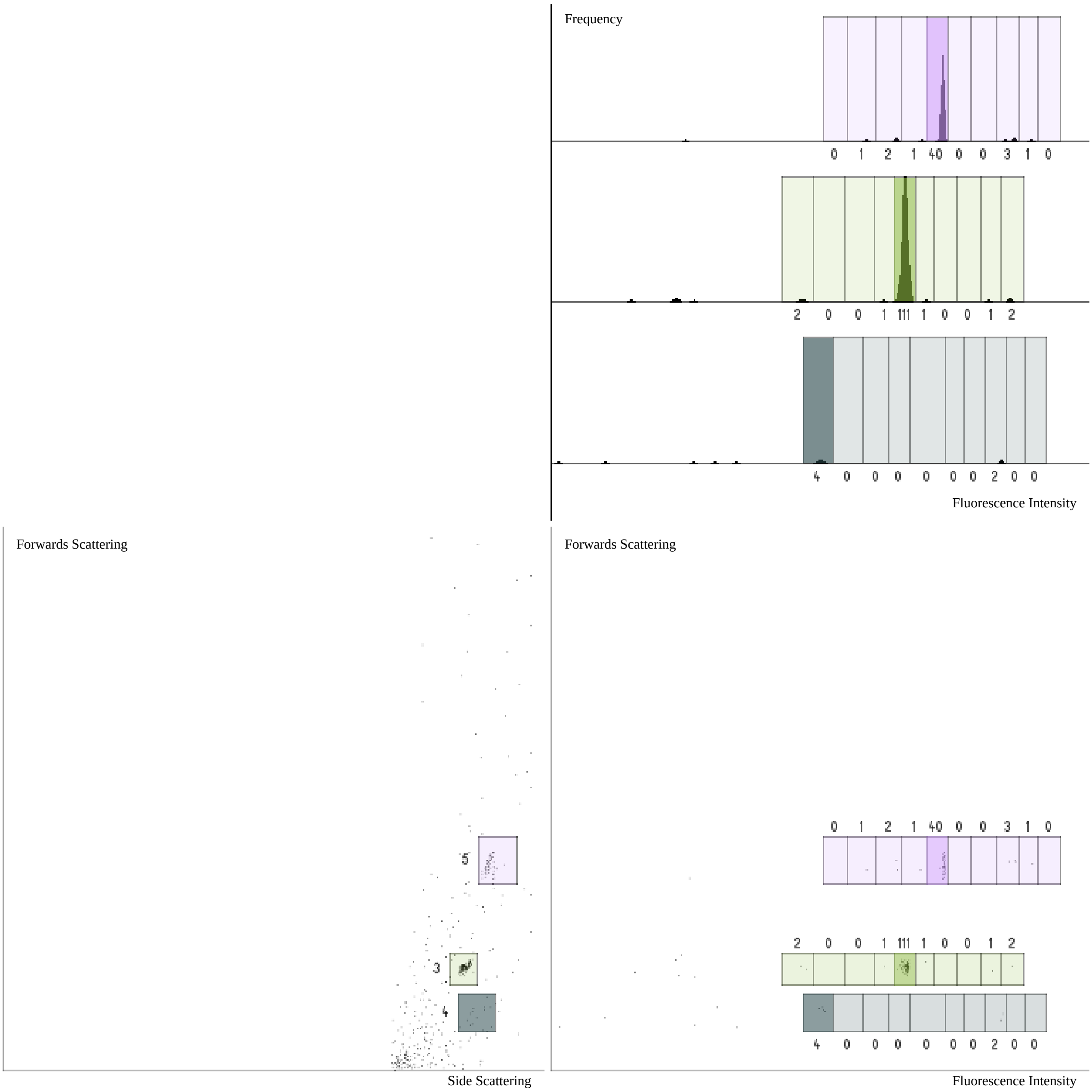

ANNEX 3: TAG DECONVOLUTION - BEAD 520

Passes flow sorting criteria: Yes

Passes tag deconvolution criteria: Yes

Included in protocol analysis: Yes

Protocol: 3, 9, 2, 10

Filename: Bin10_plateA1_D12.fcs

Split 1: Petrol shading

Split 2: Green shading

Split 3: Violet shading

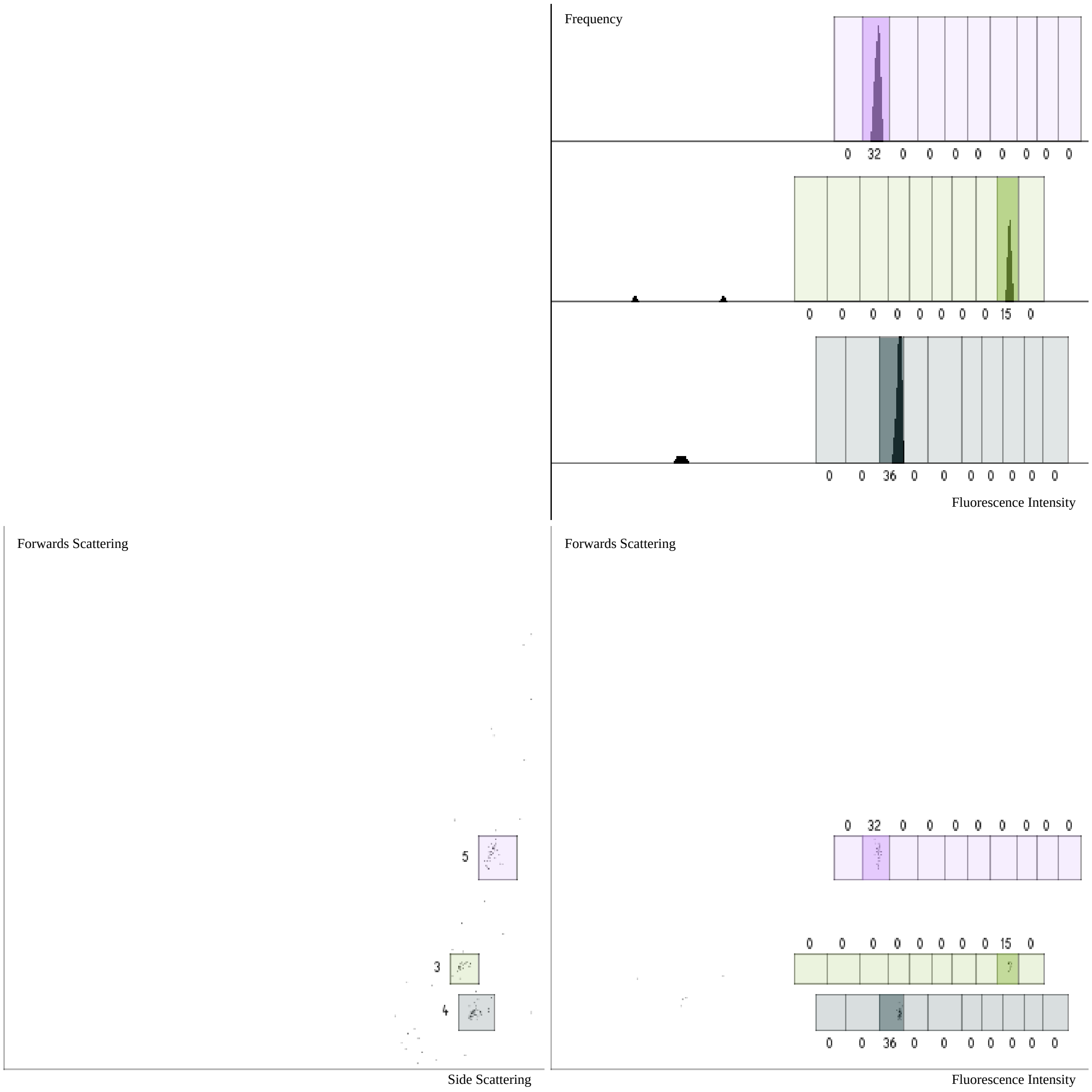

ANNEX 3: TAG DECONVOLUTION - BEAD 521

Passes flow sorting criteria: Yes

Passes tag deconvolution criteria: Yes

Included in protocol analysis: Yes

Protocol: 1, 1, 10, 9

Filename: Bin9_plateB1_A1.fcs

Split 1: Petrol shading

Split 2: Green shading

Split 3: Violet shading

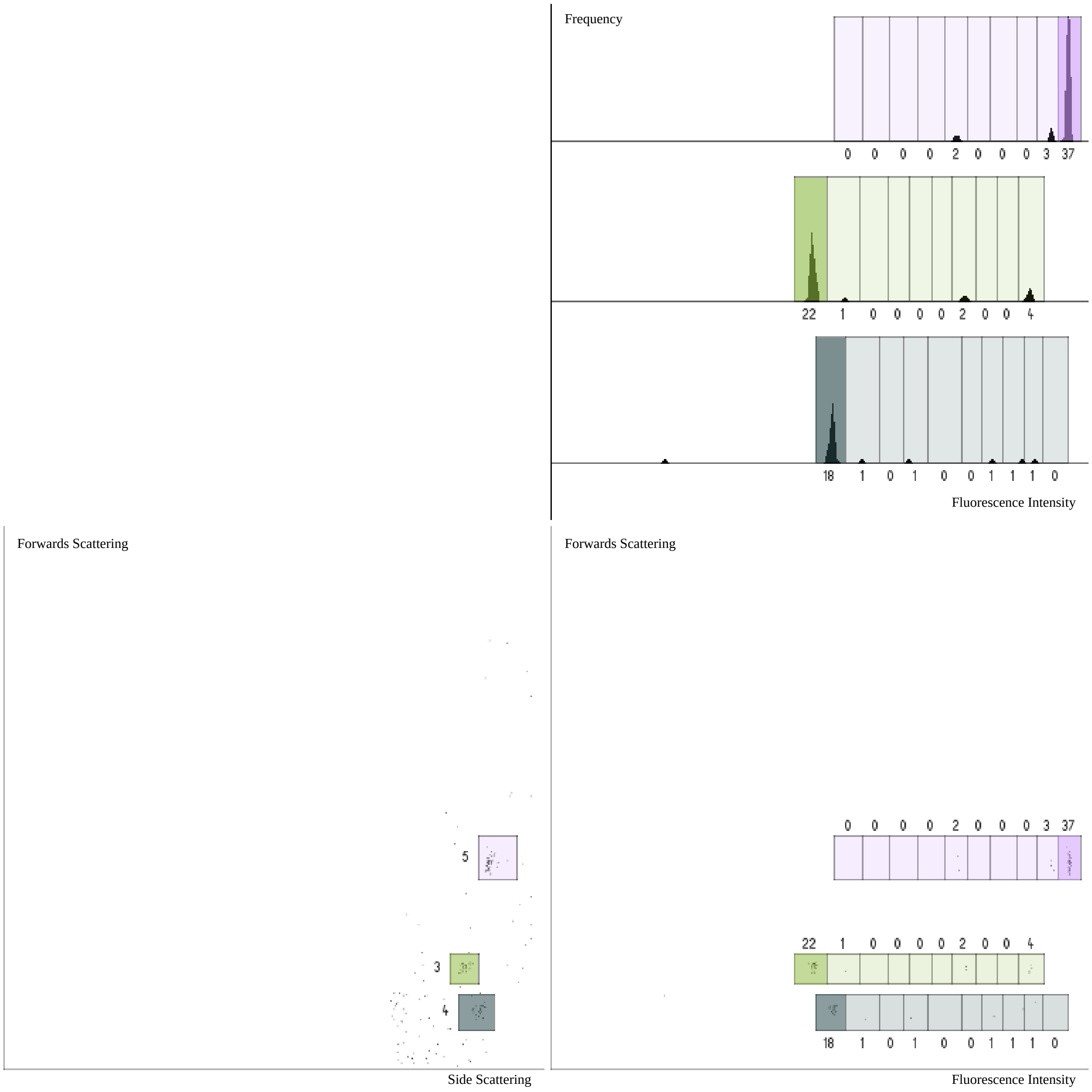

ANNEX 3: TAG DECONVOLUTION - BEAD 522

Passes flow sorting criteria: Yes

Passes tag deconvolution criteria: No

Included in protocol analysis: No

Protocol: N/A

Filename: Bin9_plateB1_A2.fcs

Split 1: Petrol shading

Split 2: Green shading

Split 3: Violet shading

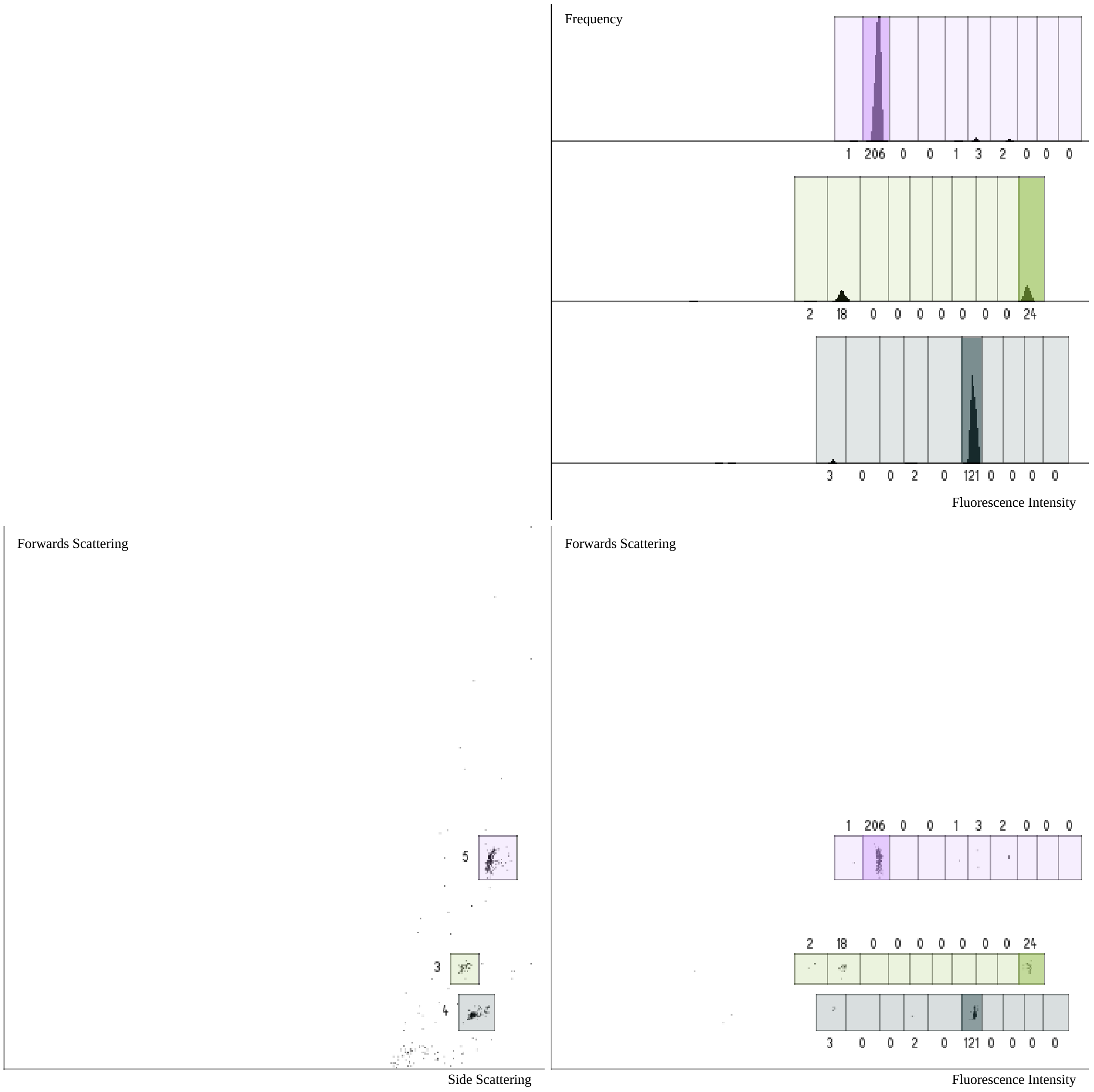

ANNEX 3: TAG DECONVOLUTION - BEAD 523

Passes flow sorting criteria: Yes

Passes tag deconvolution criteria: Yes

Included in protocol analysis: Yes

Protocol: 4, 6, 4, 9

Filename: Bin9_plateB1_A6.fcs

Split 1: Petrol shading

Split 2: Green shading

Split 3: Violet shading

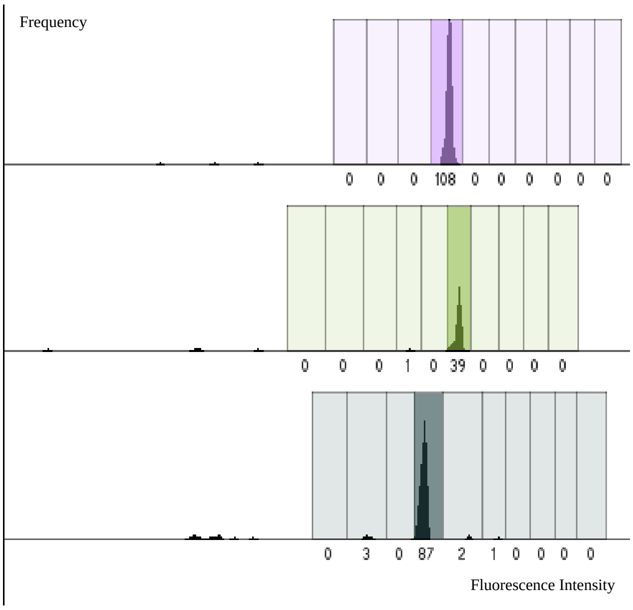

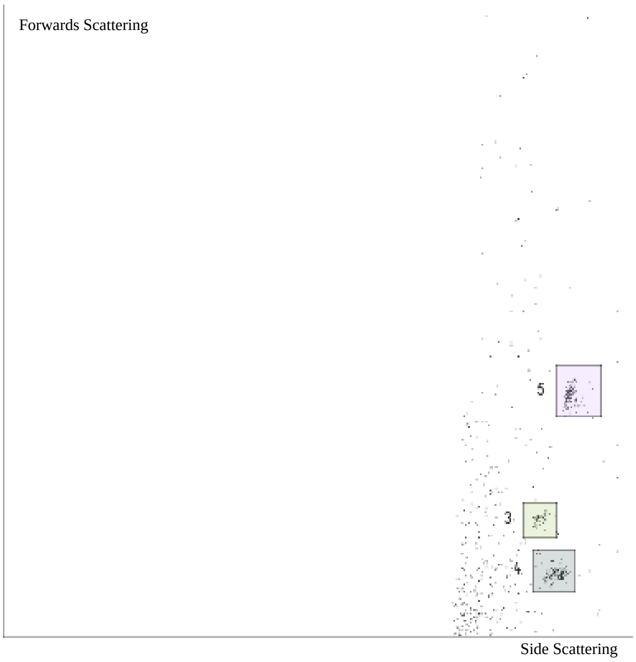

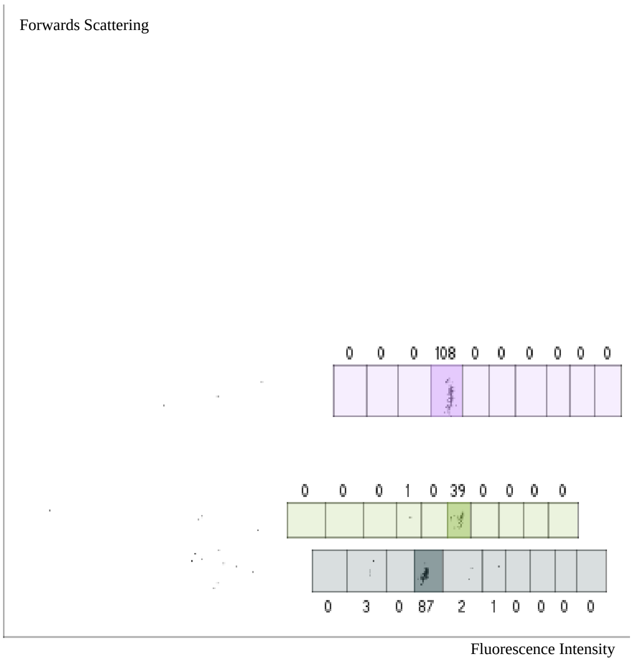

ANNEX 3: TAG DECONVOLUTION - BEAD 524

Passes flow sorting criteria: Yes

Passes tag deconvolution criteria: Yes

Included in protocol analysis: Yes

Protocol: 8, 10, 3, 9

Filename: Bin9_plateB1_A7.fcs

Split 1: Petrol shading

Split 2: Green shading

Split 3: Violet shading

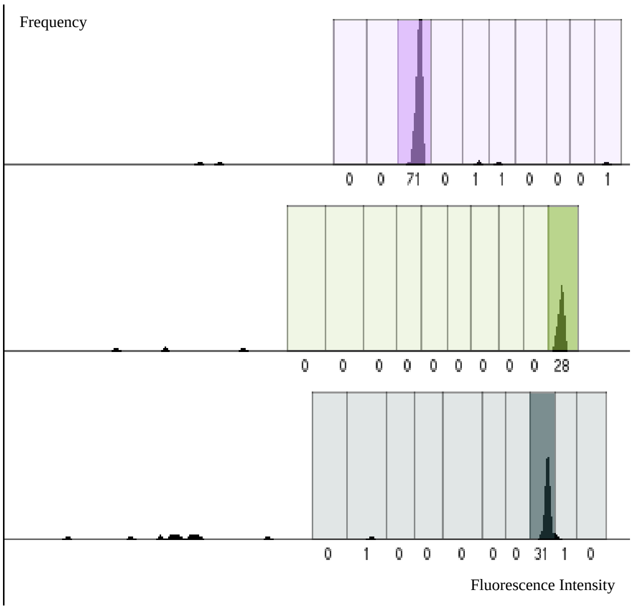

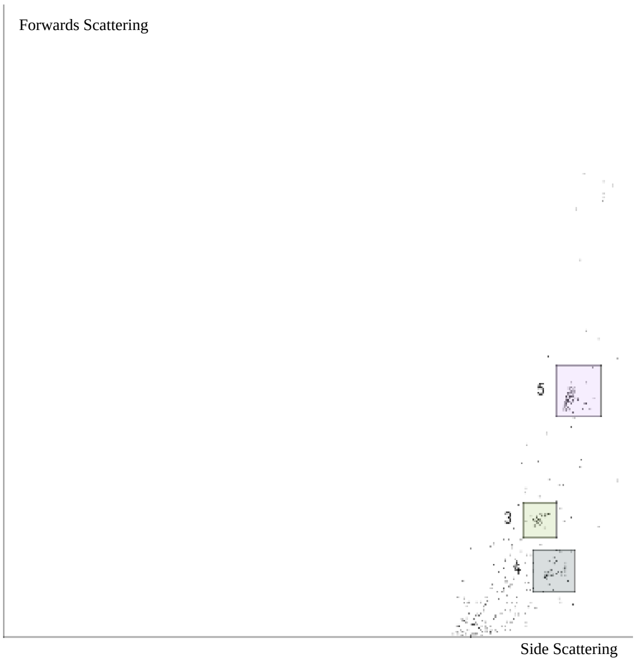

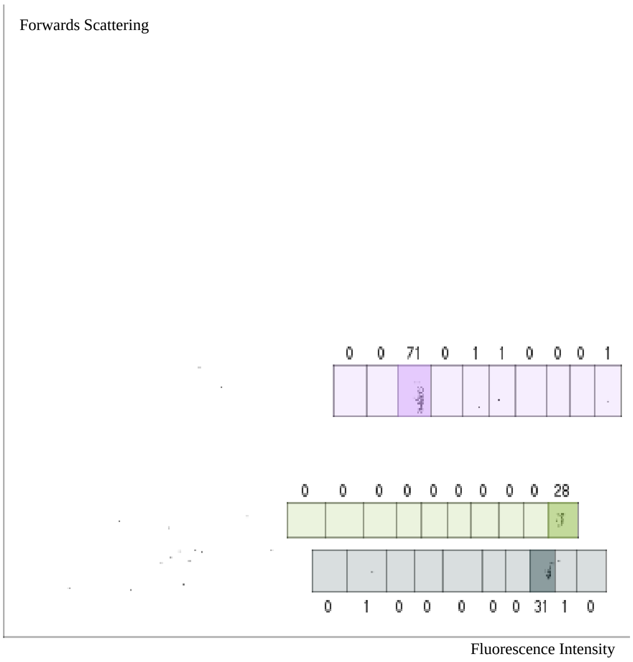

ANNEX 3: TAG DECONVOLUTION - BEAD 525

Passes flow sorting criteria: Yes

Passes tag deconvolution criteria: No

Included in protocol analysis: No

Protocol: N/A

Filename: Bin9_plateB1_A8.fcs

Split 1: Petrol shading

Split 2: Green shading

Split 3: Violet shading

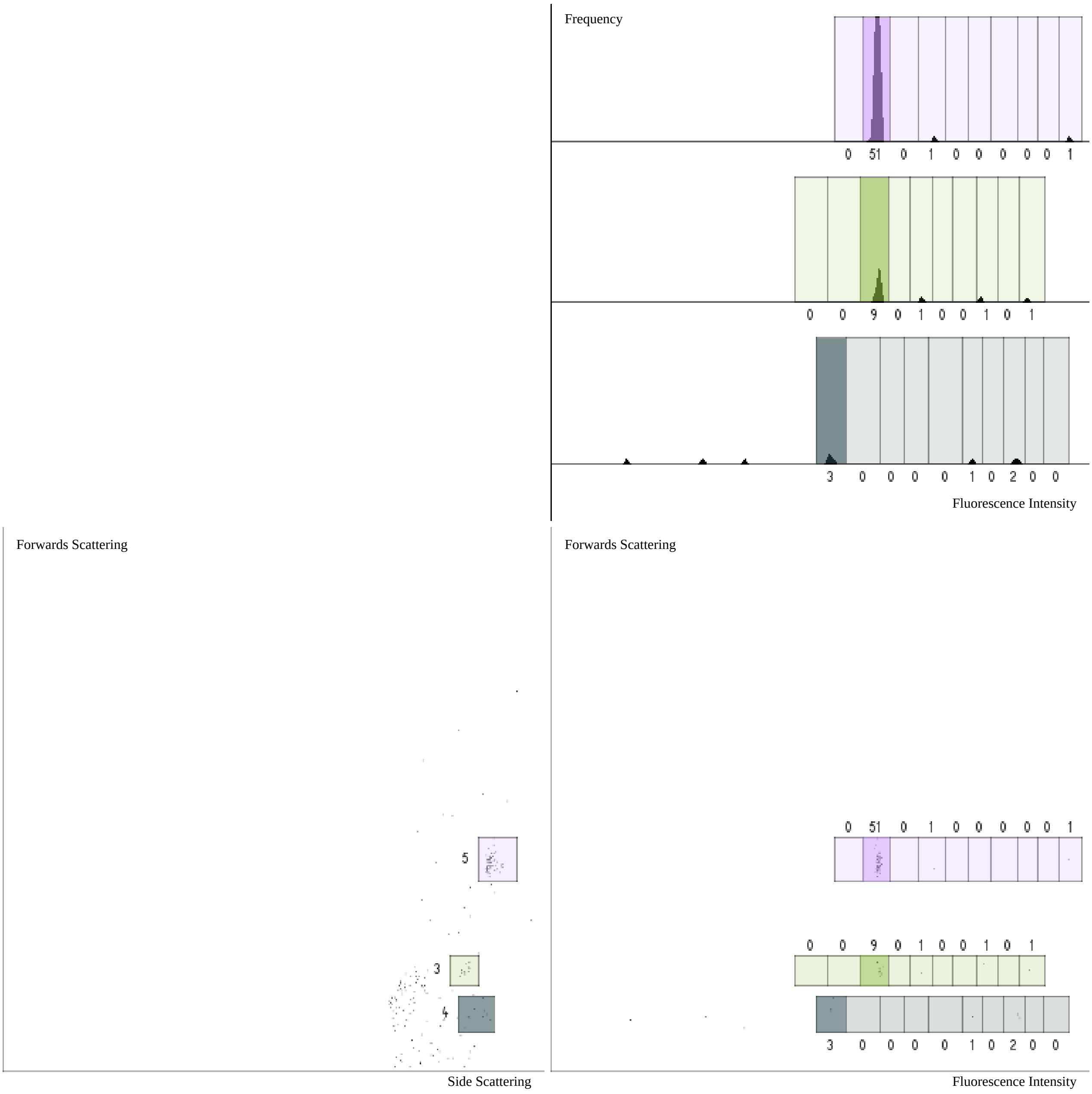

ANNEX 3: TAG DECONVOLUTION - BEAD 526

Passes flow sorting criteria: Yes

Passes tag deconvolution criteria: No

Included in protocol analysis: No

Protocol: N/A

Filename: Bin10_plateA1_C1.fcs

Split 1: Petrol shading

Split 2: Green shading

Split 3: Violet shading

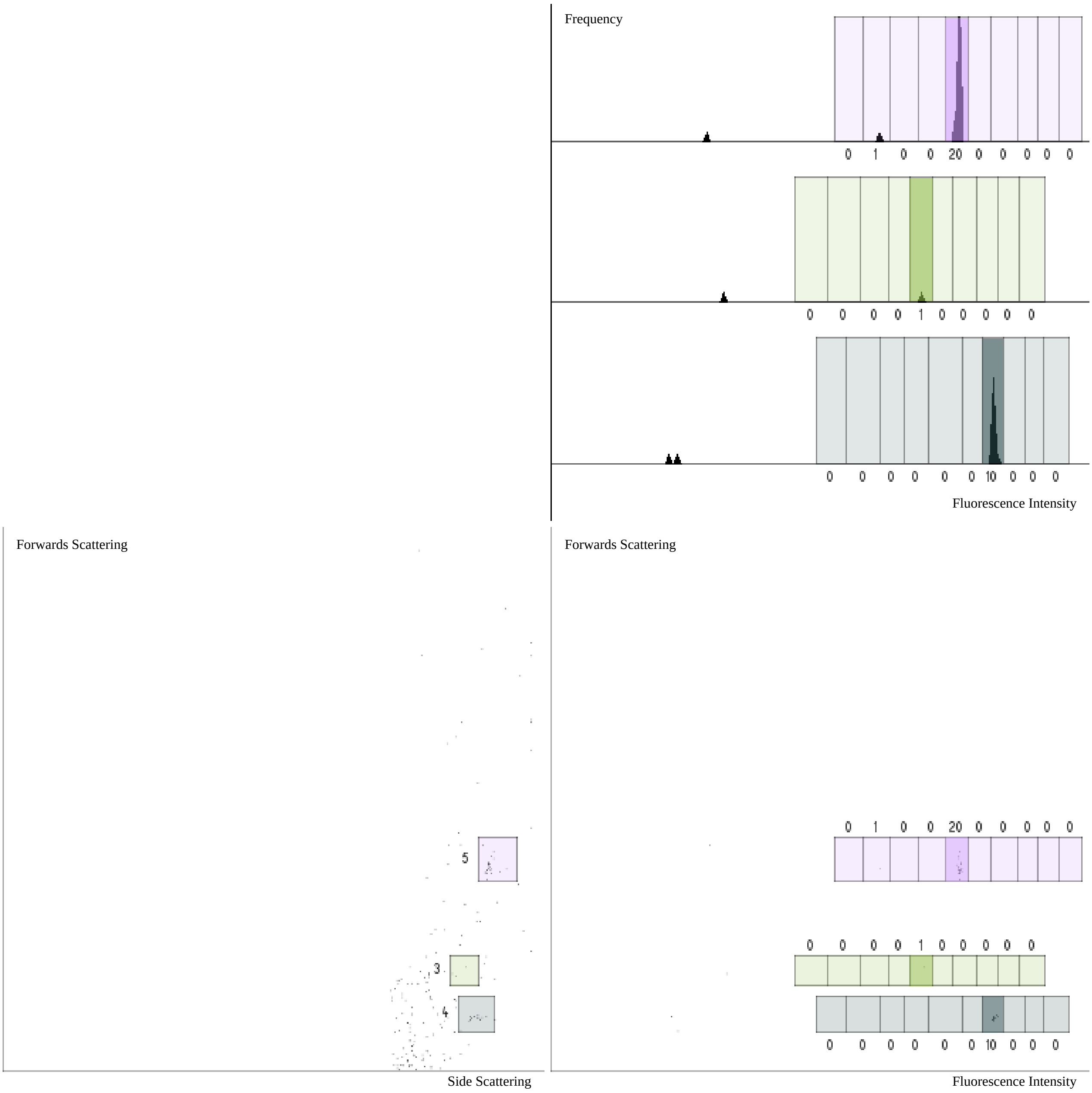

ANNEX 3: TAG DECONVOLUTION - BEAD 527

Passes flow sorting criteria: Yes

Passes tag deconvolution criteria: Yes

Included in protocol analysis: Yes

Protocol: 3, 1, 8, 10

Filename: Bin10_plateA1_C2.fcs

Split 1: Petrol shading

Split 2: Green shading

Split 3: Violet shading

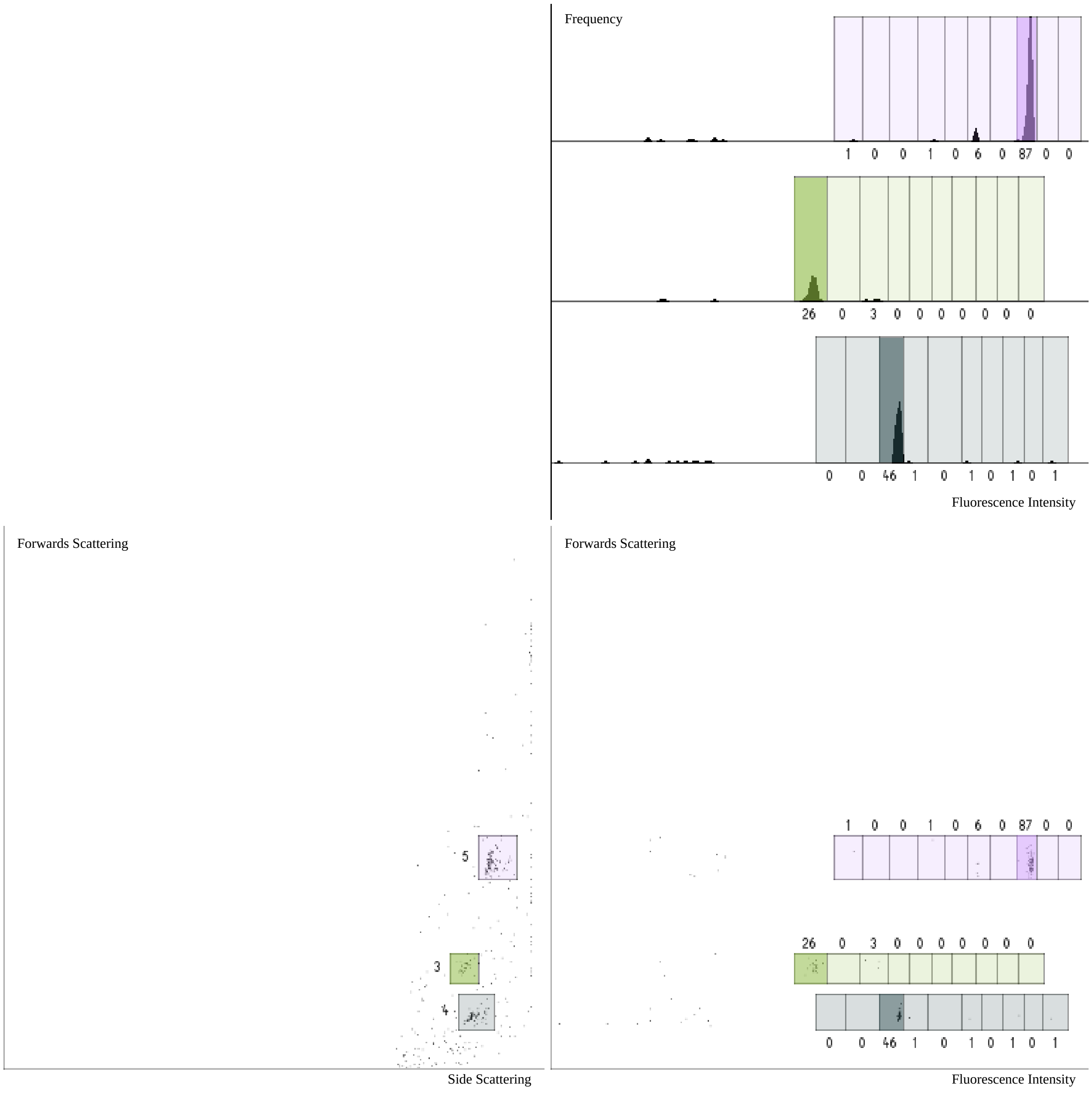

ANNEX 3: TAG DECONVOLUTION - BEAD 528

Passes flow sorting criteria: Yes

Passes tag deconvolution criteria: Yes

Included in protocol analysis: Yes

Protocol: 10, 9, 1, 10

Filename: Bin10_plateA1_C3.fcs

Split 1: Petrol shading

Split 2: Green shading

Split 3: Violet shading

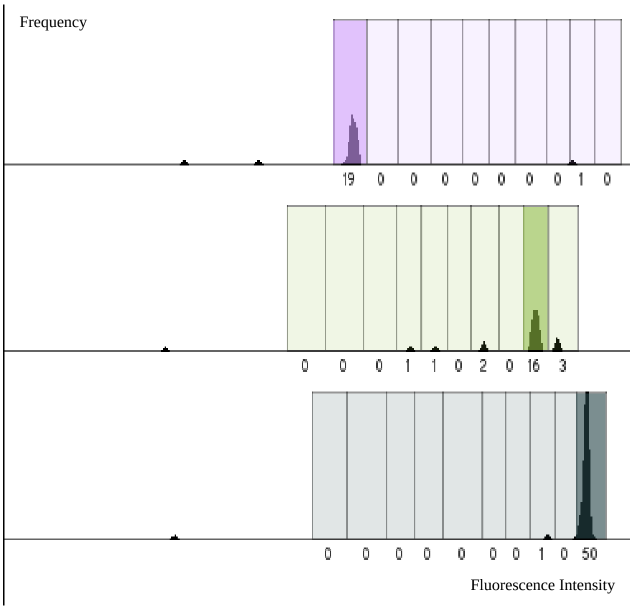

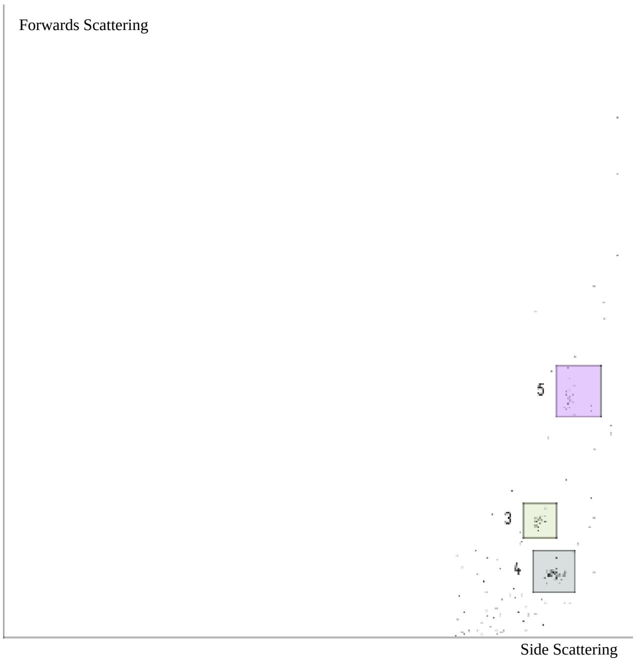

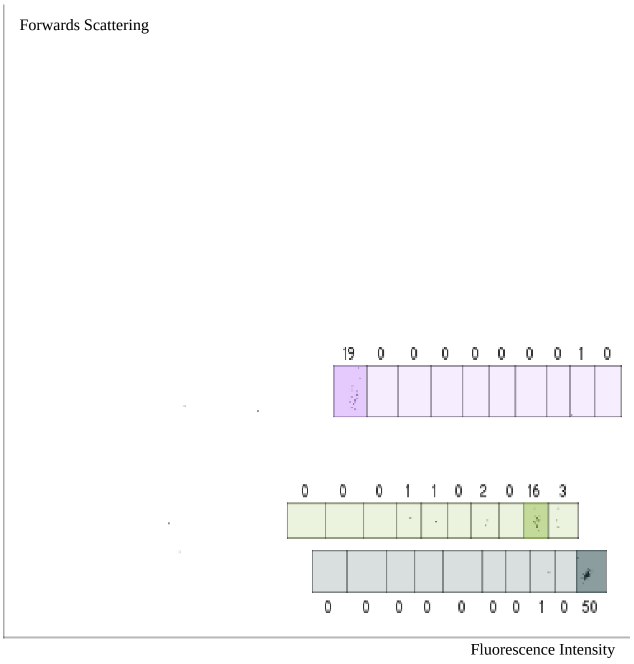

ANNEX 3: TAG DECONVOLUTION - BEAD 529

Passes flow sorting criteria: Yes

Passes tag deconvolution criteria: Yes

Included in protocol analysis: Yes

Protocol: 1, 7, 7, 10

Filename: Bin10_plateA1_C4.fcs

Split 1: Petrol shading

Split 2: Green shading

Split 3: Violet shading

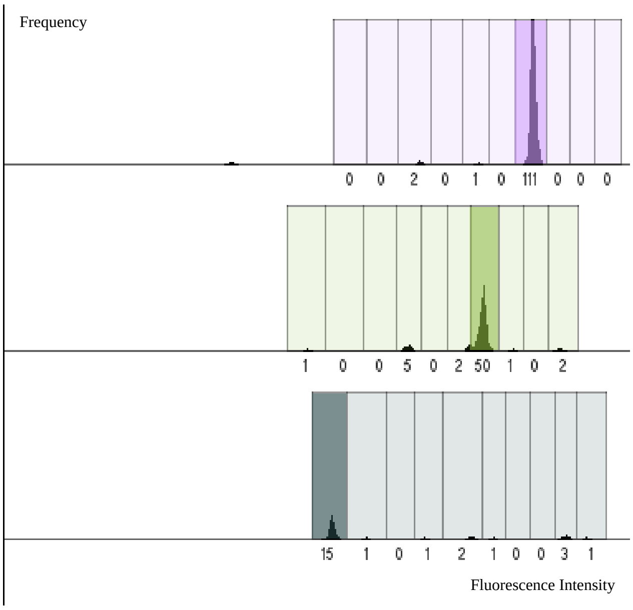

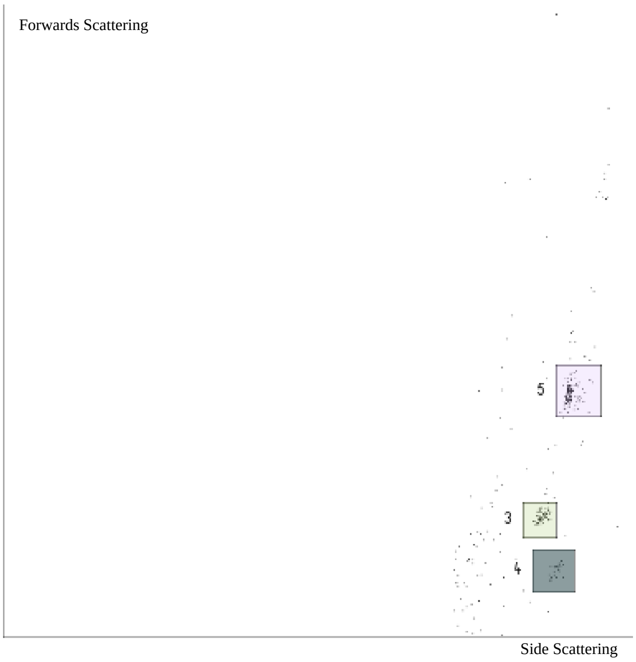

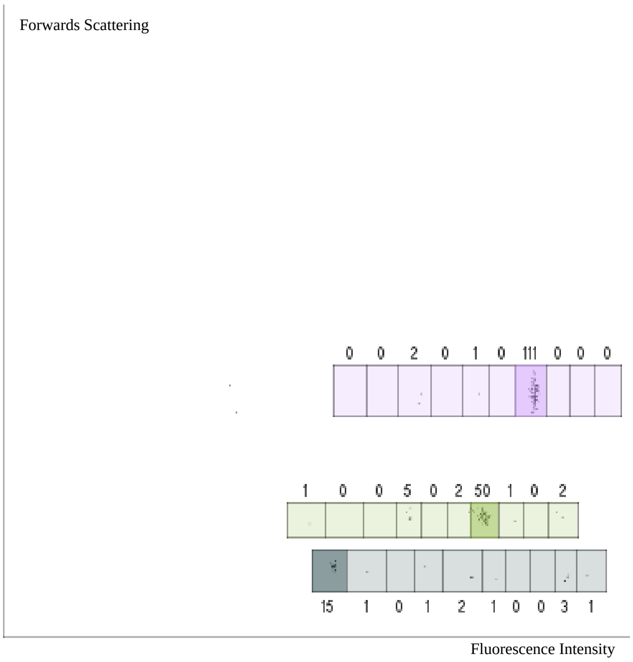

ANNEX 3: TAG DECONVOLUTION - BEAD 530

Passes flow sorting criteria: Yes

Passes tag deconvolution criteria: Yes

Included in protocol analysis: Yes

Protocol: 2, 8, 4, 10

Filename: Bin10_plateA1_C5.fcs

Split 1: Petrol shading

Split 2: Green shading

Split 3: Violet shading

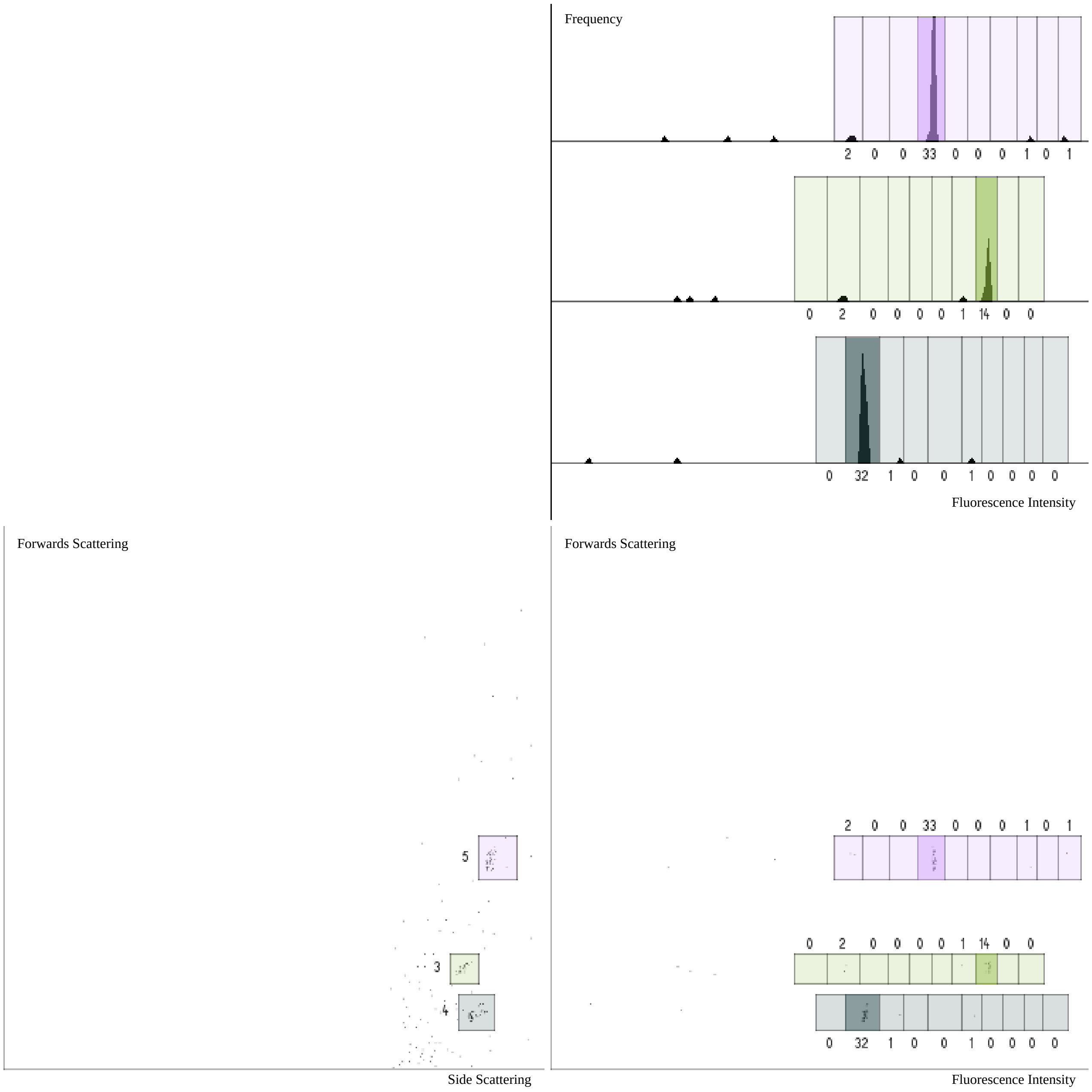

ANNEX 3: TAG DECONVOLUTION - BEAD 531

Passes flow sorting criteria: Yes

Passes tag deconvolution criteria: No

Included in protocol analysis: No

Protocol: N/A

Filename: Bin10_plateA1_C6.fcs

Split 1: Petrol shading

Split 2: Green shading

Split 3: Violet shading

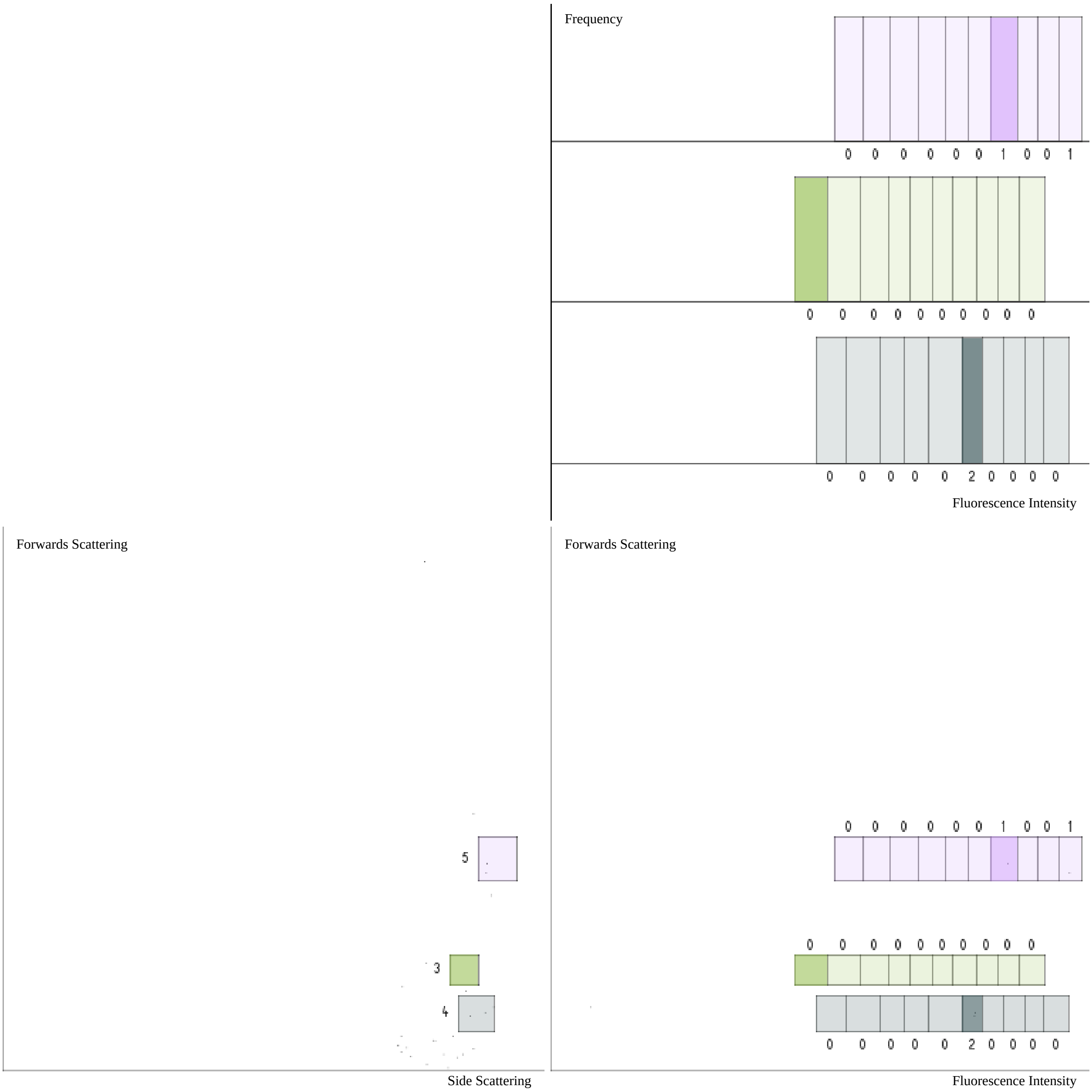

ANNEX 3: TAG DECONVOLUTION - BEAD 532

Passes flow sorting criteria: Yes

Passes tag deconvolution criteria: Yes

Included in protocol analysis: Yes

Protocol: 9, 2, 8, 10

Filename: Bin10_plateA1_C7.fcs

Split 1: Petrol shading

Split 2: Green shading

Split 3: Violet shading

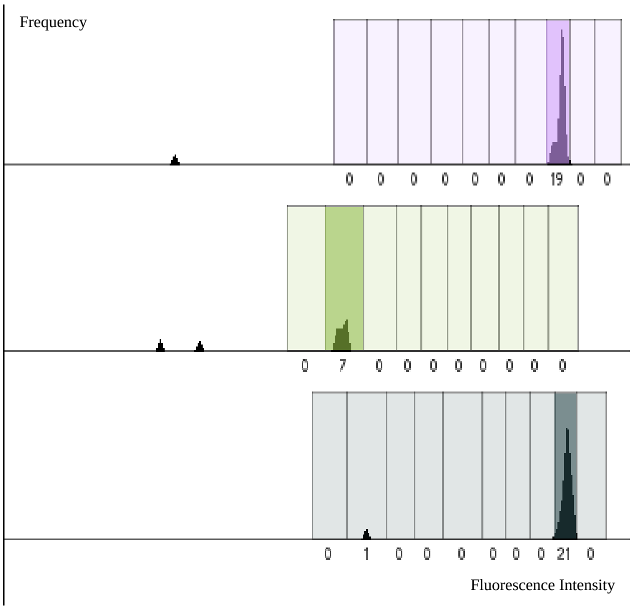

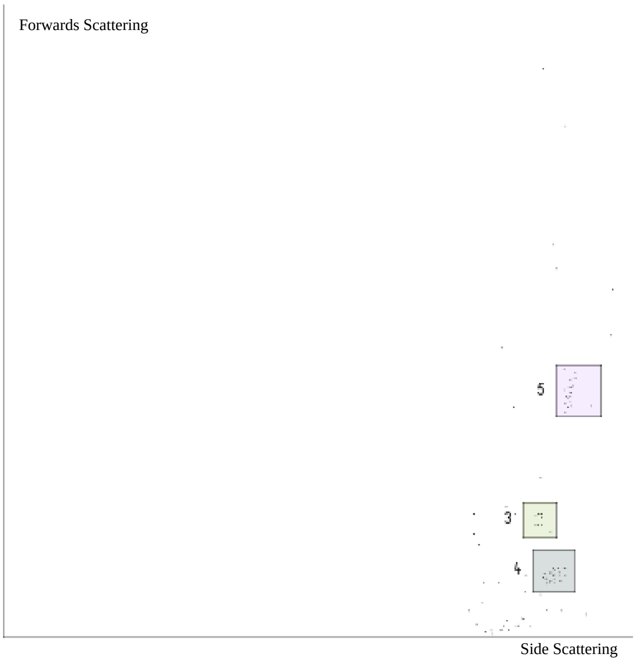

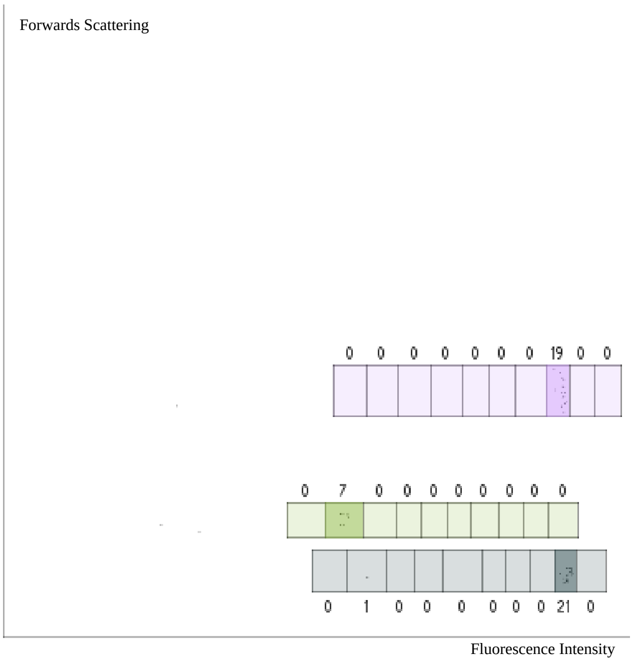

ANNEX 3: TAG DECONVOLUTION - BEAD 533

Passes flow sorting criteria: Yes

Passes tag deconvolution criteria: No

Included in protocol analysis: No

Protocol: N/A

Filename: Bin10_plateA1_C8.fcs

Split 1: Petrol shading

Split 2: Green shading

Split 3: Violet shading

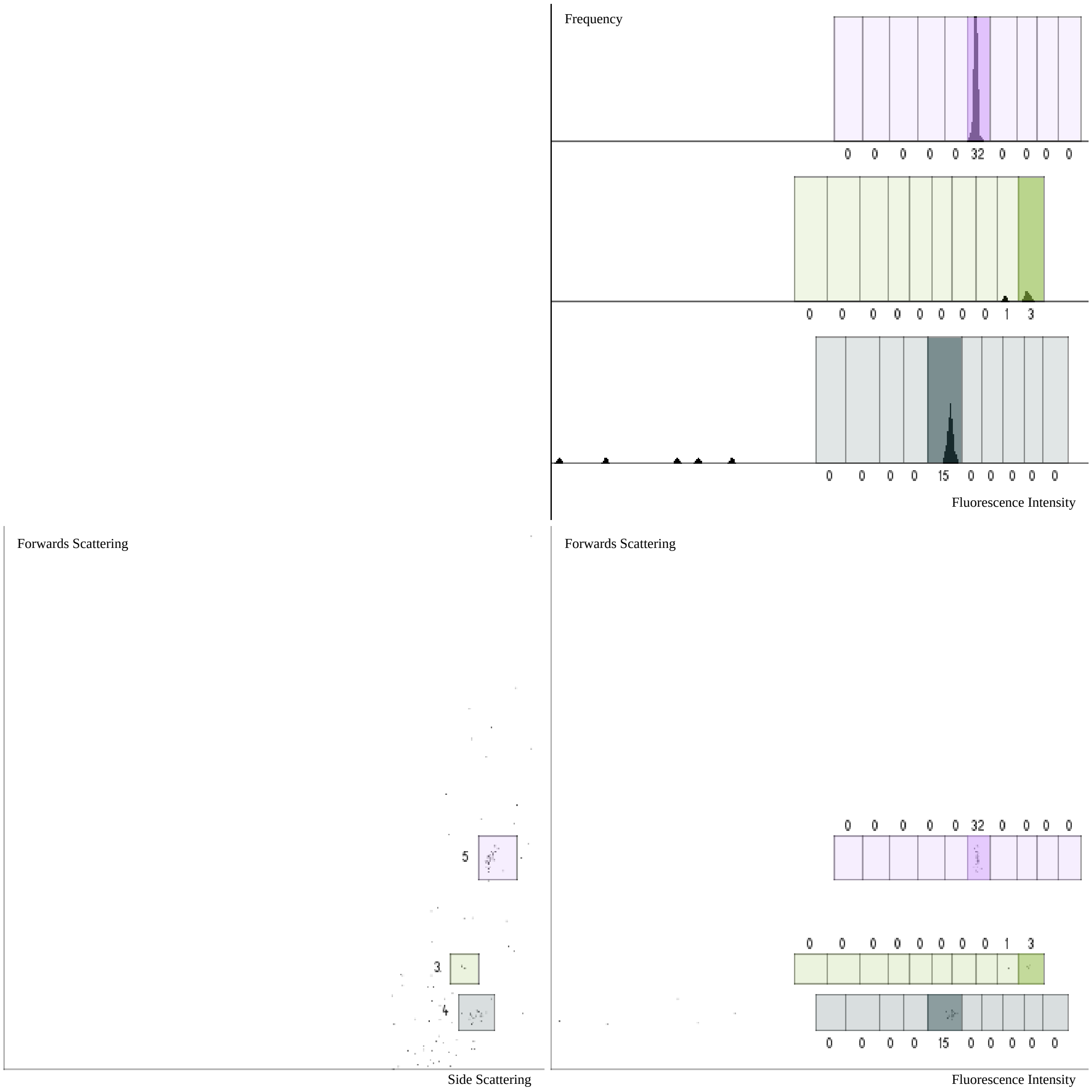

ANNEX 3: TAG DECONVOLUTION - BEAD 534

Passes flow sorting criteria: Yes

Passes tag deconvolution criteria: No

Included in protocol analysis: No

Protocol: N/A

Filename: Bin10_plateA1_C9.fcs

Split 1: Petrol shading

Split 2: Green shading

Split 3: Violet shading

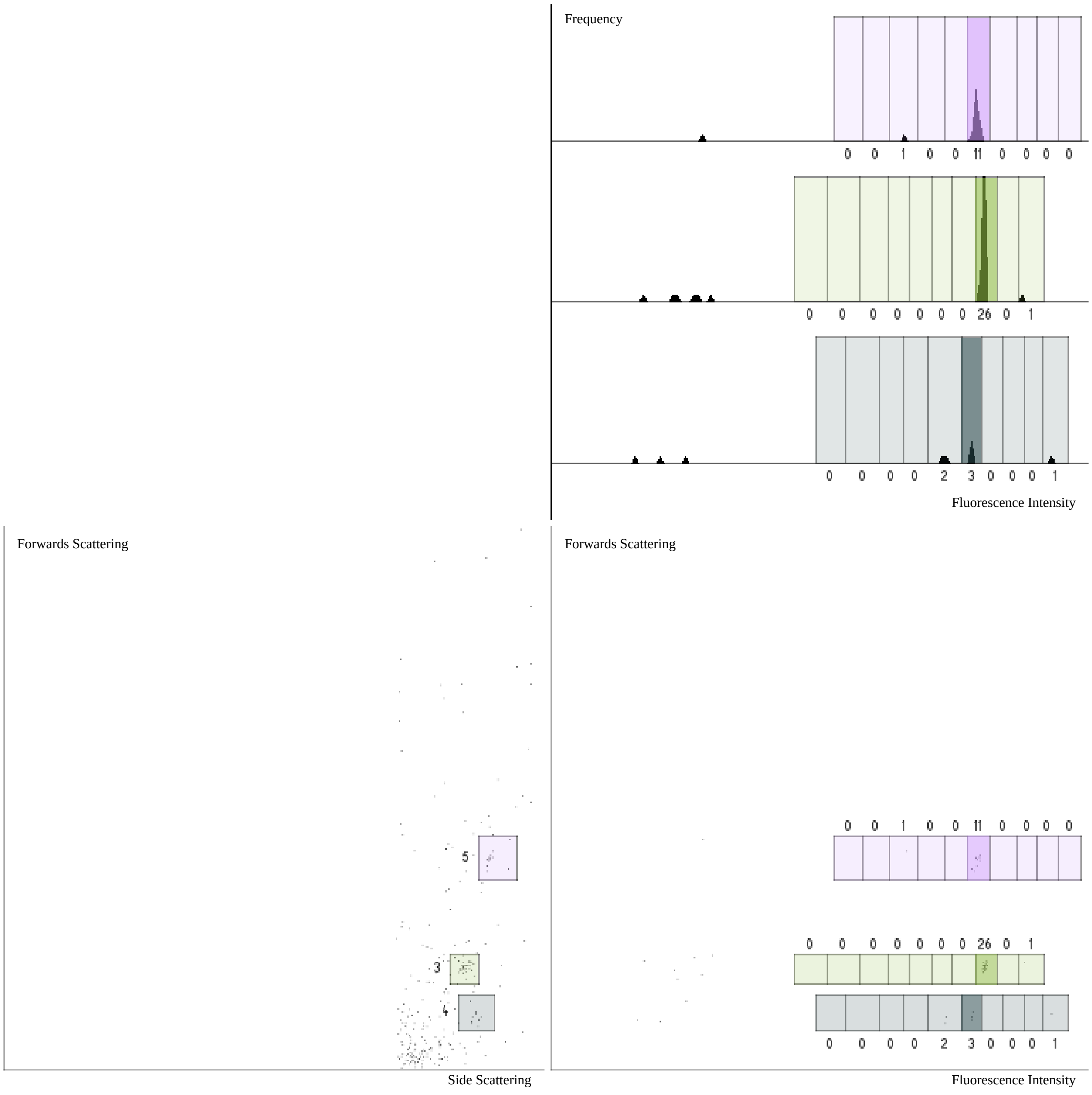

ANNEX 3: TAG DECONVOLUTION - BEAD 535

Passes flow sorting criteria: Yes

Passes tag deconvolution criteria: No

Included in protocol analysis: No

Protocol: N/A

Filename: Bin10_plateA1_C10.fcs

Split 1: Petrol shading

Split 2: Green shading

Split 3: Violet shading

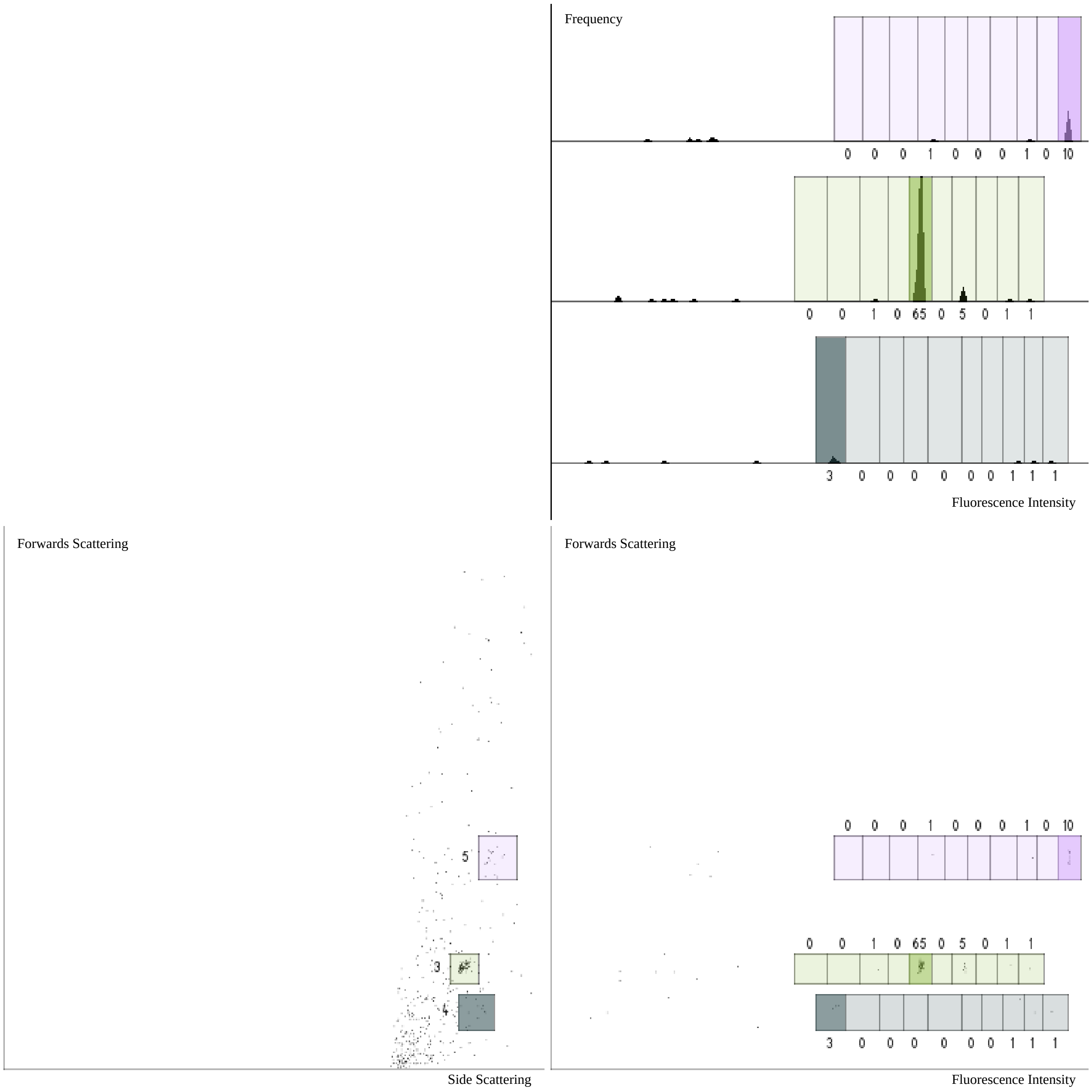

ANNEX 3: TAG DECONVOLUTION - BEAD 536

Passes flow sorting criteria: Yes

Passes tag deconvolution criteria: Yes

Included in protocol analysis: Yes

Protocol: 4, 7, 9, 10

Filename: Bin10_plateA1_D8.fcs

Split 1: Petrol shading

Split 2: Green shading

Split 3: Violet shading

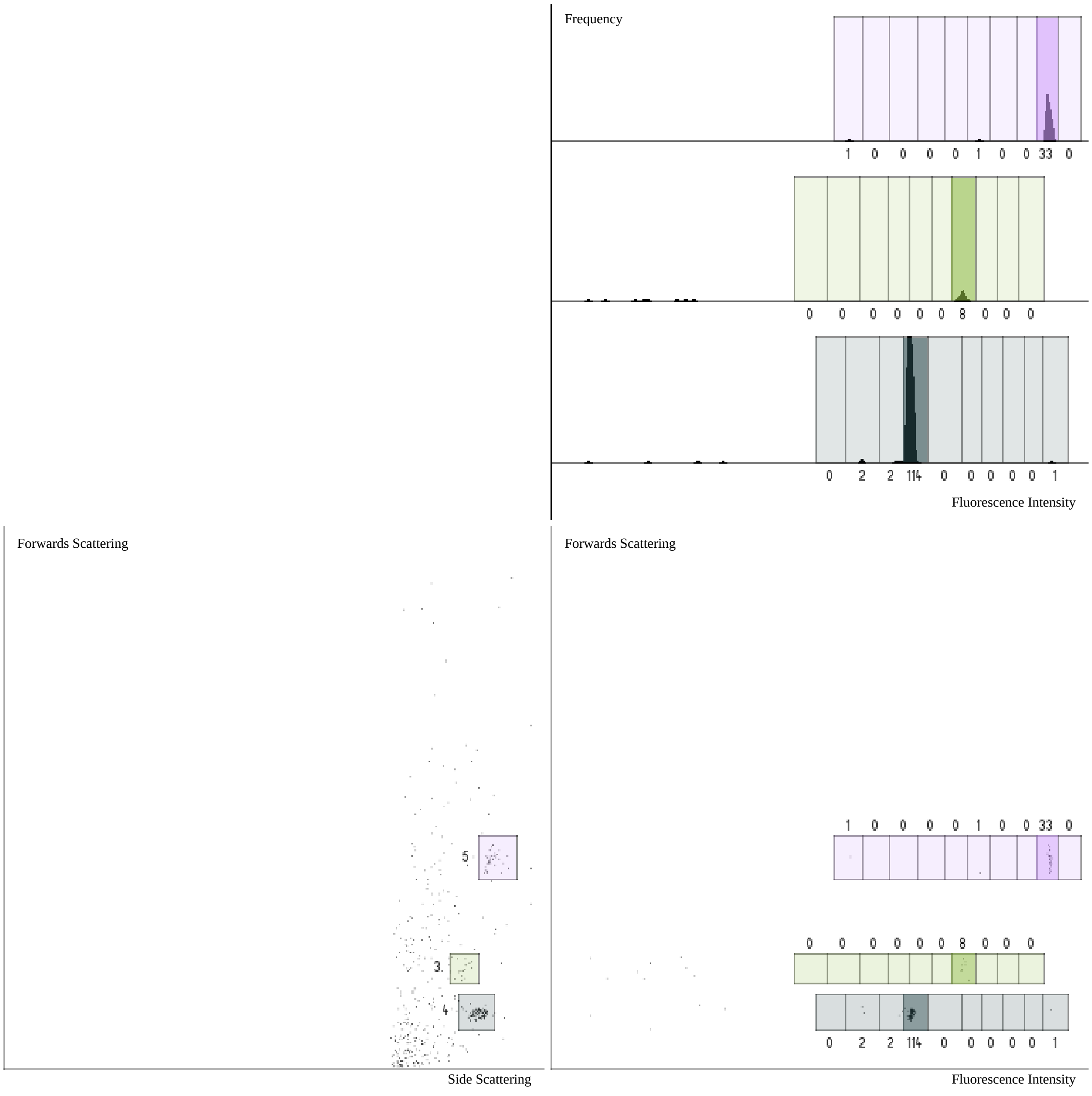

ANNEX 3: TAG DECONVOLUTION - BEAD 537

Passes flow sorting criteria: Yes

Passes tag deconvolution criteria: Yes

Included in protocol analysis: Yes

Protocol: 4, 1, 3, 10

Filename: Bin10_plateA1_D9.fcs

Split 1: Petrol shading

Split 2: Green shading

Split 3: Violet shading

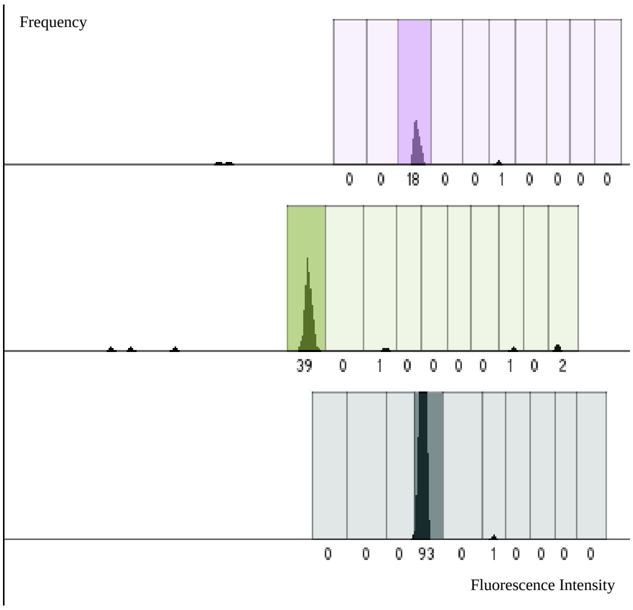

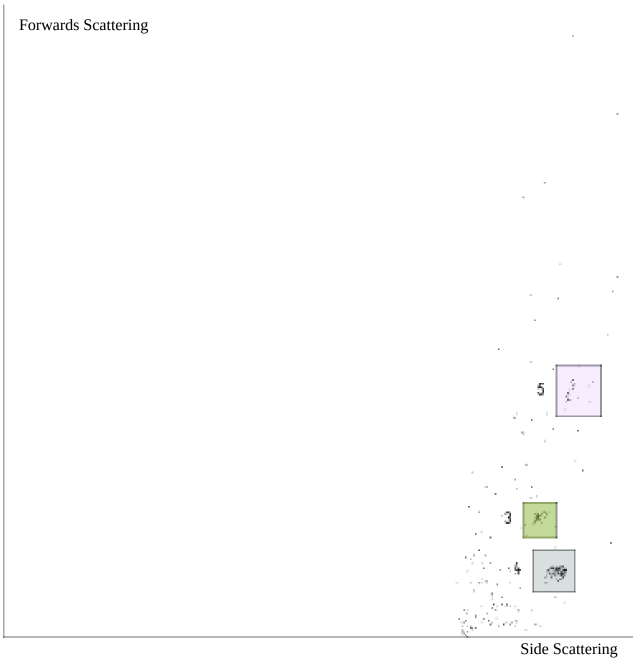

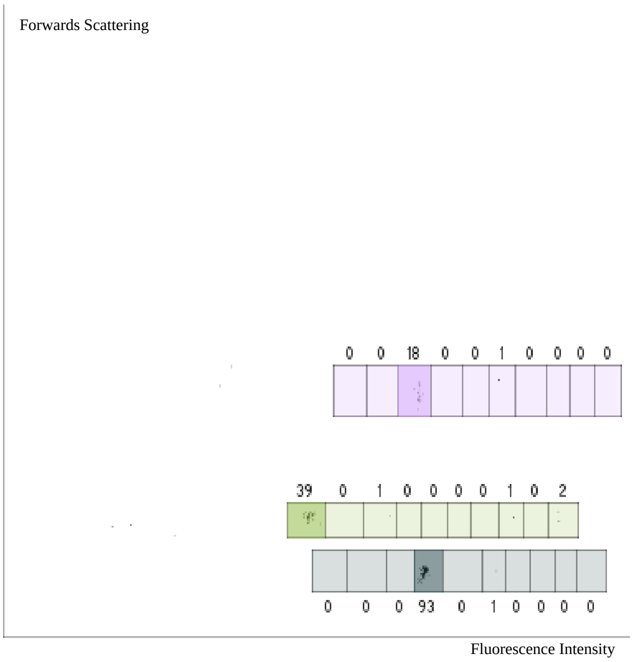

ANNEX 3: TAG DECONVOLUTION - BEAD 538

Passes flow sorting criteria: Yes

Passes tag deconvolution criteria: Yes

Included in protocol analysis: Yes

Protocol: 6, 5, 9, 10

Filename: Bin10_plateA1_D10.fcs

Split 1: Petrol shading

Split 2: Green shading

Split 3: Violet shading

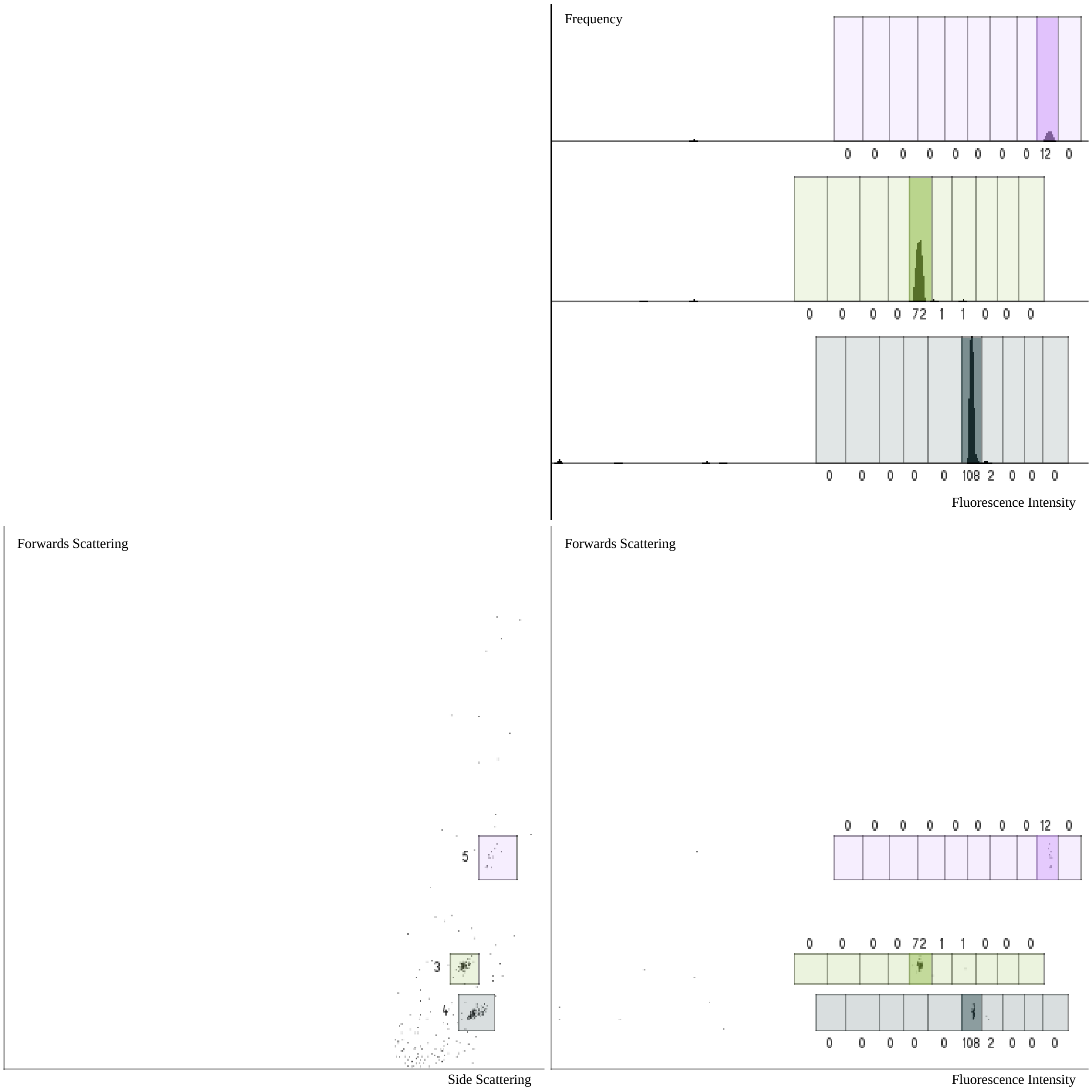

ANNEX 3: TAG DECONVOLUTION - BEAD 539

Passes flow sorting criteria: Yes

Passes tag deconvolution criteria: Yes

Included in protocol analysis: Yes

Protocol: 8, 9, 1, 10

Filename: Bin10_plateA1_D11.fcs

Split 1: Petrol shading

Split 2: Green shading

Split 3: Violet shading

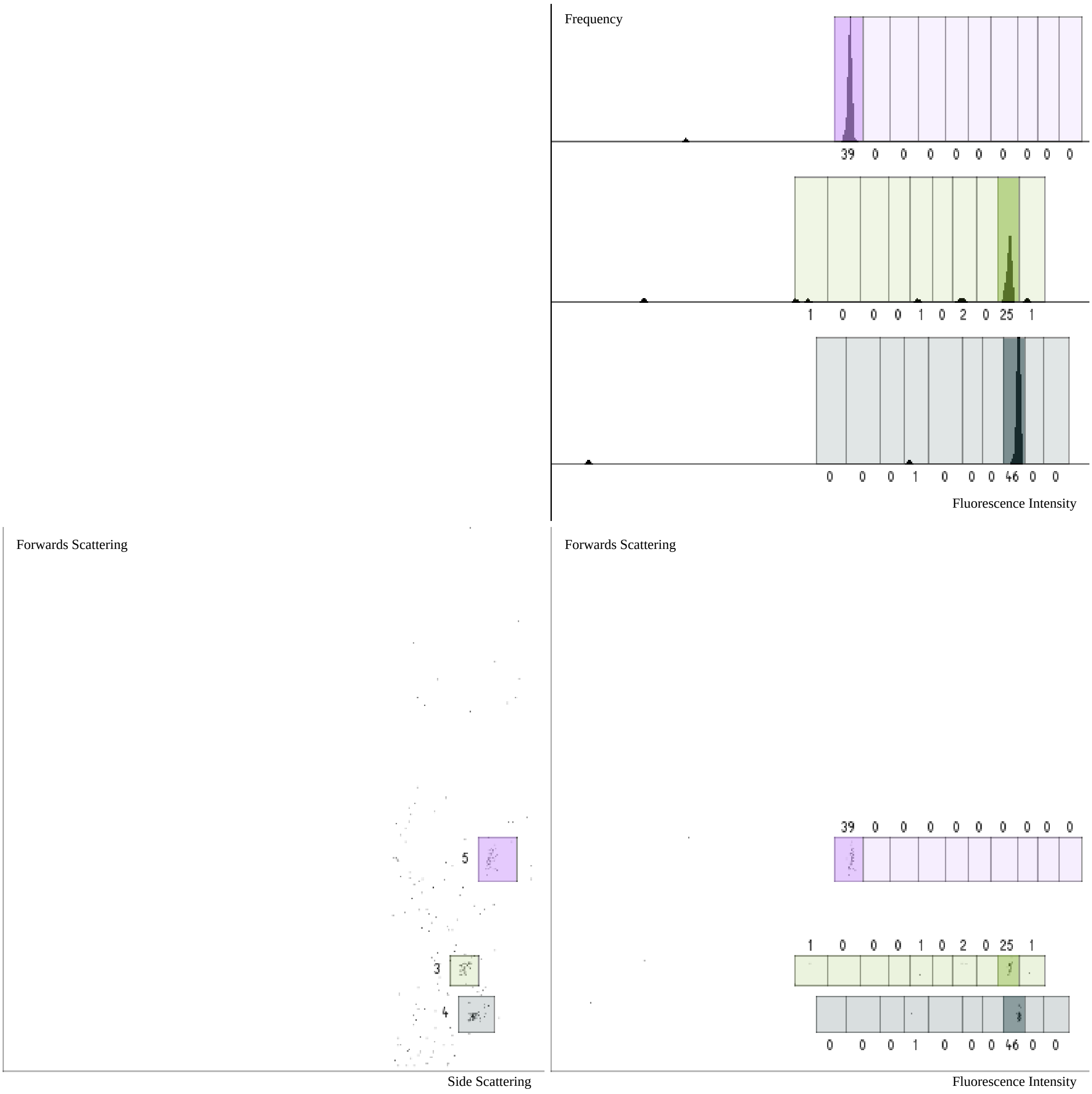

Supplement: Data S3 — Ariadne report for CombiCult screen 3: TH positive neurons from mES cells. (PDF) [file pone.0104301.s018.pdf]
