# Supplementary material for: Directed Differentiation of Embryonic Stem Cells Using a Bead-Based Combinatorial Screening Method
Source: PLoS One. 2014 Sep 24;9(9):e104301. doi: 10.1371/journal.pone.0104301 (PMC4174505; doi:10.1371/journal.pone.0104301)

Ariadne™

Experiment 3 - CombiCult™ screen for Tyrosine Hydroxylase positive neurons using hES

Plasticell

EXECUTIVE SUMMARY

OBJECTIVE

- The objective of this CombiCult™ study was to identify novel serum-free protocols for the differentiation of human embryonic stem cells (hESC) to dopaminergic neurons.

COMBICULT™ EXPERIMENT

- 40 cell culture media were tested in 10,000 combinations
- Approximately 480,000 beads were seeded with hES line Shef6 (Stem Cell Bank, UK)
- The experimental matrix comprised four split-pool cycles, performed on days 2, 9, 16, 23
- 10 media were assayed in each cycle, resulting in an experimental complexity of 10x10x10x10 = 10,000 media combinations
- On completion of cell culture (day 30) all beads were screened using immunocytochemistry assay for the enzyme tyrosine hydroxylase
- 1198 beads were isolated by COPAS

RESULTS

- 367 hits (0.13 % of monomeric beads) had a green fluorescent COPAS sorting value above 136
- The cell culture history of 279 (76 % of hits) could be deduced unambiguously
- The hits were derived from 274 distinct putative differentiation protocols
- Protocols were ranked using bespoke bioinformatics software, Ariadne™ (v1.0)
- The 21 protocols chosen for validation are listed in Table 1

EXECUTIVE SUMMARY CONTINUED

Table 1: The 21 protocols selected for validation as by Ariadne™ (v1.0). Protocols are identified by the series of cell culture media that resulted in differentiation.

Validation number	Protocol	Bead Ids		Basal Medium	Supplements
1	10-4-9-6	226	Split 1 (Day 2)	DMEM/F12	1% BSA, 2mM Glutamine, 1X ITS suppl. 0.5μM retinoic acid
			Split 2 (Day 8)	DMEM/F12	2mM Glutamine, 1X ITS suppl., 0.5μM retinoic acid
			Split 3 (Day 16)	Advanced DMEM	2mM Glutamine
			Split 4 (Day 22)	DMEM/F12	2mM Glutamine, 1X N2 suppl.,
2	10-10-9-6	236	Split 1 (Day 2)	DMEM/F12	1% BSA, 2mM Glutamine, 1X ITS suppl. 0.5μM retinoic acid
			Split 2 (Day 8)	DMEM/F12	2mM Glutamine, 1X ITS suppl., 20ng/mL bFGF, 100ng/mL FGF8b, 200μM ascorbic acid, 10ng/mL BDNF, 1μM purmorphamine
			Split 3 (Day 16)	Advanced DMEM	2mM Glutamine
			Split 4 (Day 22)	DMEM/F12	2mM Glutamine, 1X N2 suppl.,
3	9-7-3-6	191	Split 1 (Day 2)	DMEM/F12	1% BSA, 2mM Glutamine, 1X N2 suppl. 20μM SB431542
			Split 2 (Day 8)	DMEM/F12	2mM Glutamine, 1X ITS suppl., 1X B27 Neuromix, 20ng/mL bFGF, 50ng/mL hr DKK1.
			Split 3 (Day 16)	DMEM/F12	2mM Glutamine, 1X ITS suppl., 100ng/mL FGF8b, 500ng/mL hr Shh.
			Split 4 (Day 22)	DMEM/F12	2mM Glutamine, 1X N2 suppl.,
4	9-7-10-6	194	Split 1 (Day 2)	DMEM/F12	1% BSA, 2mM Glutamine, 1X N2 suppl. 20μM SB431542
			Split 2 (Day 8)	DMEM/F12	2mM Glutamine, 1X ITS suppl., 1X B27 Neuromix, 20ng/mL bFGF, 50ng/mL hr DKK1.
			Split 3 (Day 16)	DMEM/F12	2mM Glutamine, 1X N2 suppl., 20ng/mL bFGF, 20ng/mL EGF, 2ng/mL LIF
			Split 4 (Day 22)	DMEM/F12	2mM Glutamine, 1X N2 suppl.,
5	8-9-7-6	178, 217	Split 1 (Day 2)	DMEM/F12	1% BSA, 2mM Glutamine, 1X N2 suppl. 200ng/mL hr Noggin.
			Split 2 (Day 8)	DMEM/F12	2mM Glutamine, 1X ITS suppl., 100ng/mL FGF8b, 500ng/mL hr Shh.
			Split 3 (Day 16)	DMEM/F12	2mM Glutamine, 1X ITS suppl., 1X B27 neuromix, 20ng/mL bFGF, 50ng/mL hr DKK1.
			Split 4 (Day 22)	DMEM/F12	2mM Glutamine, 1X N2 suppl.,
6	6-6-10-6	180	Split 1 (Day 2)	DMEM/F12	1% BSA, 2mM Glutamine, 1X N2 suppl. 200ng/mL hr Noggin, 20μM SB431542
			Split 2 (Day 8)	DMEM/F12	2mM Glutamine, 1X N2 suppl.
			Split 3 (Day 16)	DMEM/F12	2mM Glutamine, 1X N2 suppl., 20ng/mL bFGF, 20ng/mL EGF, 2ng/mL LIF
			Split 4 (Day 22)	DMEM/F12	2mM Glutamine, 1X N2 suppl.,
7	6-4-10-6	184	Split 1 (Day 2)	DMEM/F12	1% BSA, 2mM Glutamine, 1X N2 suppl. 200ng/mL hr Noggin, 20μM SB431542
			Split 2 (Day 8)	DMEM/F12	2mM Glutamine, 1X ITS suppl., 0.5mM retinoic acid.
			Split 3 (Day 16)	DMEM/F12	2mM Glutamine, 1X N2 suppl., 20ng/mL bFGF, 20ng/mL EGF, 2ng/mL LIF
			Split 4 (Day 22)	DMEM/F12	2mM Glutamine, 1X N2 suppl.
8	3-9-6-6	156	Split 1(Day 2)	Advanced DMEM	2mM Glutamine
			Split 2 (Day 8)	DMEM/F12	2mM Glutamine, 1X ITS suppl., 100ng/mL FGF8b, 500ng/mL hr Shh.
			Split 3 (Day 16)	DMEM/F12	2mM Glutamine, 1X N2 suppl.
			Split 4 (Day 22)	DMEM/F12	2mM Glutamine, 1X N2 suppl.
9	4-4-8-6	234	Split 1 (Day 2)	DMEM/F12	1% BSA, 1X N2, 1X B37 neuromix, 2mM Glutamine
			Split 2 (Day 8)	DMEM/F12	2mM Glutamine, 1X ITS suppl., 0.5μM retinoic acid
			Split 3 (Day 16)	Neurobasal	2mM Glutamine, 1X B37 neuromix, 20ng/mL bFGF

Validation number	Protocol	Bead Ids		Basal Medium	Supplements
			Split 4 (Day 22)	DMEM/F12	2mM Glutamine, 1X N2 suppl.
10	5-2-10-1	18	Split 1 (Day 2)	RHB-A	
			Split 2 (Day 8)	DMEM/F12	2mM Glutamine, 1X ITS suppl., 200ng/mL hr Noggin.
			Split 3 (Day 16)	DMEM/F12	2mM Glutamine, 1X N2 suppl., 20ng/mL bFGF, 20ng/mL EGF, 2ng/mL LIF
			Split 4 (Day 22)	DMEM/F12	2mM Glutamine, 1X N2 suppl., 1mg/mL laminin, 200µM ascorbic acid
11	6-9-1-1	23	Split 1 (Day 2)	DMEM/F12	1% BSA, 2mM Glutamine, 1X N2 suppl. 200ng/mL hr Noggin, 20µM SB431542
			Split 2 (Day 8)	DMEM/F12	2mM Glutamine, 1X ITS suppl., 100ng/mL FGF8b, 500ng/mL hr Shh.
			Split 3 (Day 16)	DMEM/F12	2mM Glutamine, 1X ITS suppl., 20ng/mL bFGF
			Split 4 (Day 22)	DMEM/F12	2mM Glutamine, 1X N2 suppl., 1mg/mL laminin, 200µM ascorbic acid
12	8-10-7-1	15	Split 1 (Day 2)	DMEM/F12	1% BSA, 2mM Glutamine, 1X N2 suppl. 200ng/mL hr Noggin.
			Split 2 (Day 8)	DMEM/F12	2mM Glutamine, 1X ITS suppl., 20ng/mL bFGF, 100ng/mL FGF8b, 200µM ascorbic acid, 10ng/mL BDNF, 1µM purmorphamine
			Split 3 (Day 16)	DMEM/F12	2mM Glutamine, 1X ITS suppl., 1X B27 neuromix, 20ng/mL bFGF, 50ng/mL hr DKK1.
			Split 4 (Day 22)	DMEM/F12	2mM Glutamine, 1X N2 suppl., 1mg/mL laminin, 200µM ascorbic acid
13	4-10-4-1	4, 59	Split 1 (Day 2)	DMEM/F12	1% BSA, 1X N2, 1X B37 neuromix, 2mM Glutamine
			Split 2 (Day 8)	DMEM/F12	2mM Glutamine, 1X ITS suppl., 20ng/mL bFGF, 100ng/mL FGF8b, 200mM ascorbic acid, 10ng/mL BDNF, 1µM purmorphamine
			Split 3 (Day 16)	Neurobasal media	2mM Glutamine, 1X ITS suppl., 20ng/mL BDNF, 10ng/mL GDNF, 0.5mM cAMP, 200µM ascorbic acid, 1ng/mL TGFb
			Split 4 (Day 22)	DMEM/F12	2mM Glutamine, 1X N2 suppl., 1mg/mL laminin, 200µM ascorbic acid
14	6-7-7-5	154	Split 1 (Day 2)	DMEM/F12	1% BSA, 2mM Glutamine, 1X N2 suppl. 200ng/mL hr Noggin, 20µM SB431542
			Split 2 (Day 8)	DMEM/F12	2mM Glutamine, 1X ITS suppl., 1X B27 Neuromix, 20ng/mL bFGF, 50ng/mL hr DKK1.
			Split 3 (Day 16)	DMEM/F12	2mM Glutamine, 1X ITS suppl., 1X B27 neuromix, 20ng/mL bFGF, 50ng/mL hr DKK1.
			Split 4 (Day 22)	RHB-A	
15	7-7-7-4	101	Split 1 (Day 2)	DMEM/F12	1% BSA, 2mM Glutamine, 1X N2 suppl., 20 ng/mL bFGF
			Split 2 (Day 8)	DMEM/F12	2mM Glutamine, 1X ITS suppl., 1X B27 Neuromix, 20ng/mL bFGF, 50ng/mL hr DKK1.
			Split 3 (Day 16)	DMEM/F12	2mM Glutamine, 1X ITS suppl., 1X B27 neuromix, 20ng/mL bFGF, 50ng/mL hr DKK1.
			Split 4 (Day 22)	DMEM/F12	2mM Glutamine, 1X N2 suppl., 0.5µM cAMP, 10ng/mL BDNF
16	10-7-10-4	104	Split 1 (Day 2)	DMEM/F12	1% BSA, 2mM Glutamine, 1X ITS suppl. 0.5µM retinoic acid
			Split 2 (Day 8)	DMEM/F12	2mM Glutamine, 1X ITS suppl., 1X B27 Neuromix, 20ng/mL bFGF, 50ng/mL hr DKK1.
			Split 3 (Day 16)	DMEM/F12	2mM Glutamine, 1X N2 suppl., 20ng/mL bFGF, 20ng/mL EGF, 2ng/mL LIF
			Split 4 (Day 22)	DMEM/F12	2mM Glutamine, 1X N2 suppl., 0.5µM cAMP, 10ng/mL BDNF
17	8-6-3-7	172	Split 1 (Day 2)	DMEM/F12	1% BSA, 2mM Glutamine, 1X N2 suppl. 200ng/mL hr Noggin.
			Split 2 (Day 8)	DMEM/F12	2mM Glutamine, 1X N2 suppl.
			Split 3 (Day 16)	DMEM/F12	2mM Glutamine, 1X ITS suppl., 100ng/mL FGF8b, 500ng/mL hr Shh.
			Split 4 (Day 22)	Neurobasal media	2mM Glutamine, 1X B27 neuromix
18	5-4-9-5	124	Split 1 (Day 2)	RHB-A	
			Split 2 (Day 8)	DMEM/F12	2mM Glutamine, 1X ITS suppl., 0.5µM retinoic acid.
			Split 3 (Day 16)	Advanced DMEM	2mM Glutamine
			Split 4 (Day 22)	RHB-A	
19	2-5-10-4	99, 100	Split 1 (Day 2)	DMEM	10% FBS, 2mM Glutamine, 1X NEAA
			Split 2 (Day 8)	RHB-A	
			Split 3 (Day 16)	DMEM/F12	2mM Glutamine, 1X N2 suppl., 20ng/mL bFGF, 20ng/mL EGF, 2ng/mL LIF

Confidential

Validation number	Protocol	Bead Ids		Basal Medium	Supplements
			Split 4 (Day 22)	DMEM/F12	2mM Glutamine, 1X N2 suppl., 0.5µM cAMP, 10ng/mL BDNF
20	5-6-7-5	134, 136	Split 1 (Day 2)	RHB-A	
			Split 2 (Day 8)	DMEM/F12	2mM Glutamine, 1X N2 suppl.
			Split 3 (Day 16)	DMEM/F12	2mM Glutamine, 1X ITS suppl., 1X B27 neuromix, 20ng/mL bFGF, 50ng/mL hr DKK1.
			Split 4 (Day 22)	RHB-A	
21	7-9-6-4	91, 95	Split 1 (Day 2)	DMEM/F12	1% BSA, 2mM Glutamine, 1X N2 suppl., 20 ng/mL bFGF
			Split 2 (Day 8)	DMEM/F12	2mM Glutamine, 1X ITS suppl., 100ng/mL FGF8b, 500ng/mL hr Shh.
			Split 3 (Day 16)	DMEM/F12	2mM Glutamine, 1X N2 suppl.,
			Split 4 (Day 22)	DMEM/F12	2mM Glutamine, 1X N2 suppl., 0.5µM cAMP, 10ng/mL BDNF

CONTENTS

Executive Summary

Report Nomenclature

1. Introduction

2. Study Setup

3. Split-Pool Experiment
3.1 Cell Culture Media
3.2 Tag Assignment

4. Bead Screening
4.1 Screening Assay
4.2 Flow Sorting of 'Hit' Beads

5. Tag Deconvolution

6. Protocol Analysis
6.1 Dataset Review
6.2 Linkage Analysis
6.3 Fingerprint Analysis
6.4 Hierachical Clustering Dendrogram
6.5 Similarity Matrix

7. Summary and Conclusions

Annex 1: Flow sorting scatter plots

Annex 2: Tag reference set and calibrated selection gates for each tag identification session

Annex 3: Tag identification scatter plots and histograms for each hit

GLOSSARY

Screening matrix refers to the arrangement and identity of media in the split-pool experiment

Experimental complexity is the total number of combinations of media tested, or the number of different protocols tested

Hit is a bead which scores positive in the screening assay and is sorted by COPAS

COPAS is a large particle flow sorter manufactured by Union Bioimetrica Inc.

Deconvolution is the inference of cell culture history by tag analysis

Bin refers to the sum of beads in each final cell culture medium

Session refers to the FACS analysis of tags from a group of hits. Prior to each session a reference tag set was run to calibrate side and forward scatter, and fluorescence intensity gates

Fingerprint anaylsis is a method of finding and comparing hits derived from identical protocols and groups of beads with similar protocols

Probability (of occurrence of a group in the fingerprint analysis) is the probability of that cluster of protocols occurring by chance, assuming beads sample all protocols randomly

Hierarchical clustering is a hierarchy of protocol clusters, represented in a dendrogram

Similarity matrix is a diagrammatical representation of a pair-wise comparison of all protocols

DPBS ++ is Dulbecco's phosphate buffered saline with $CaCl_2$ and $MgCl_2$.

Confidential

1. INTRODUCTION

COMBICULT™ TECHNOLOGY

CombiCult™ is a proprietary bead-based combinatorial technology specifically developed for discovery of novel stem cell differentiation protocols. Stem cells on beads are exposed to multiple combinations of media, containing active agents such as growth factors or small molecules. The optimal combinations for effective differentiation can be deduced reliably, rapidly and cost effectively.

Figure 1: CombiCult™ technology

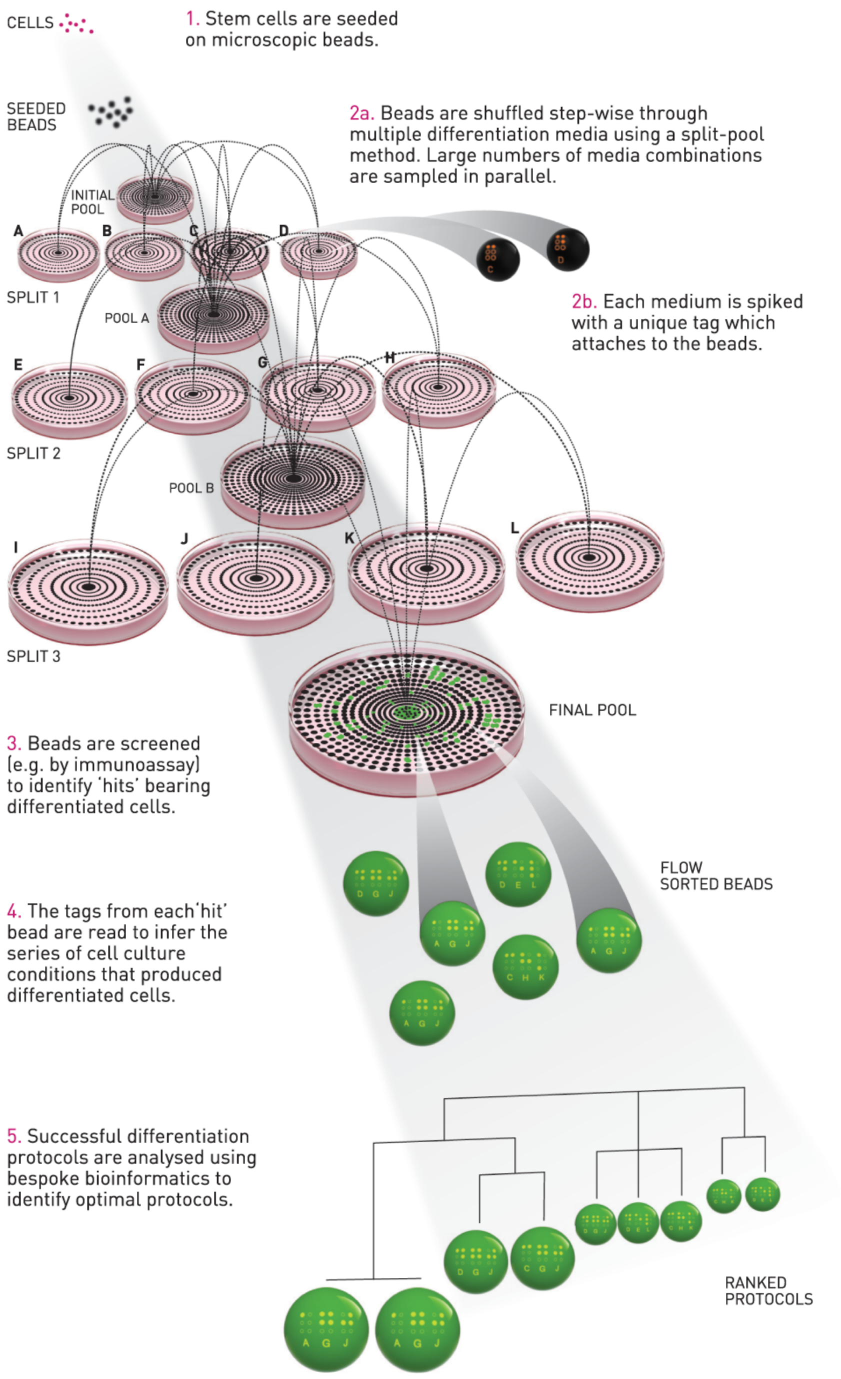

1. INTRODUCTION CONTINUED

OBJECTIVE

The objective of this CombiCult™ study was to identify efficient and/or novel protocols for the differentiation of human embryonic stem cells (hESC) to dopaminergic neurons.

STUDY

The experimental matrix comprised 40 cell culture media distributed over four split-pool cycles (splits) such that a total of 10,000 media combinations (protocols) were tested. Below is a depiction of the experimental design (Figure 2) showing the timing and number of media in each split. On completion of the cell culture phase, on day 30, beads were screened using immunocytochemistry assay to identify differentiated cells.

Figure 2: Schematic diagram of the experimental matrix.

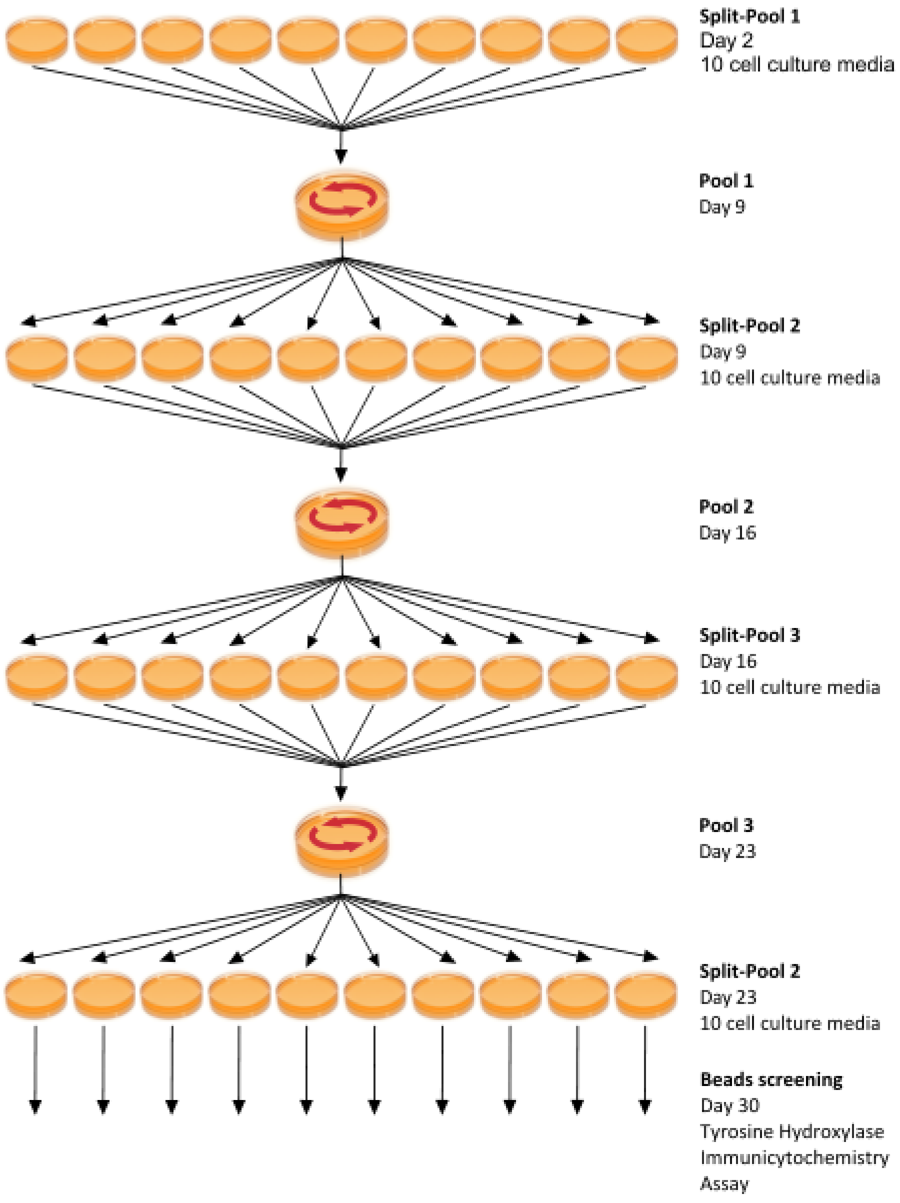

2. STUDY SETUP

OVERVIEW

Study title:	hES neuronal experiment #4
Study start/finish dates:	29-Sep-10 /3-Nov-10

SPLIT-POOL EXPERIMENT

Scientist Name:	Dr Diana Hernandez
Start/Finish Dates:	29-Sep-10/30-Oct-10
Cell Line:	hES Shef6
Split 1 Date:	01-Oct-10 (Day 2)
Split 2 Date:	08-Oct-10 (Day 9)
Split 3 Date:	15-Oct-10 (Day 16)
Split 4 Date:	22-Oct -10 (Day 23)
Differentiation end Date:	29-Oct-10 (Day 30)
Tagging Date - Split 1:	06-Oct-10
Tagging Date - Split 2:	11-Oct-10
Tagging Date - Split 3:	18-Oct-10
Tagging Date - Split 4:	N/A
Number of cell culture media combinations tested:	10,000
Ratio of beads to complexity:	48:1
Total number of beads:	480,000
Ratio of cells to beads during seeding:	90:1
Additional notes:	None

BEAD SCREENING & SORTING

Scientist name:	Dr Diana Hernandez
Start/finish dates:	01-Nov-10/04-Nov-10
Screening assay:	Rb Anti-TH antibody (Millipore) + Alexa Fluor 488 anti-Rb (Invitrogen)
Screening date:	03-Nov-10
Flow sorting instrument:	COPAS PLUS, Union Biometrica
Sorting PMT channel(s):	Green (488/45 nm) optical emission filters
Positive setup control:	N/A
Total number of sorted beads:	1198
Number of brightest 'hits' (green fluorescent COPAS sorting value ≥ 136):	367
Additional notes:	None

TAG DECONVOLUTION

**Bead Digestion**	
Scientist name:	Dr Christopher Johnson
Start/finish dates:	8-Nov-10/22-Nov-10
Additional notes:	None
**Tag Analysis**	
Scientist name:	Dr Christopher Johnson
Start/finish dates:	9-Nov-10/24-Nov-10
Tag analysis flow cytometry instrument:	BD FACSCanto II
Analysis PMT channel(s) optical filter:	BD FACSCanto II FL6 (710/50 nm band pass filter)
Number of analysis sessions:	17
Additional notes:	None

PROTOCOL ANALYSIS & REPORT GENERATION

Software:	Ariadne™ version 1.0
User name:	Dr Christopher Johnson
Date, Time:	11-Jul-11, 16.00

3. SPLIT-POOL EXPERIMENT

3.1 Cell Culture Media

Table 2: Compositions of the cell culture media tested in this study.

Split	Medium ID	Basal Media	Supplements
1	1	KO DMEM	20% KSR, 2mM Glutamine, 1X NEAA, 0.1mM β-mercaptoethanol, 4ng/mL bFGF
1	2	DMEM	10% FBS, 2mM Glutamine, 1X NEAA
1	3	Advanced DMEM	2mM Glutamine
1	4	DMEM/F12	1% BSA, 2mM Glutamine, 1X N2 suppl., 1X B37 neuromix,
1	5	RHB-A	
1	6	DMEM/F12	1% BSA, 2mM Glutamine, 1X N2 suppl. 200ng/mL hr Noggin, 20μM SB431542
1	7	DMEM/F12	1% BSA, 2mM Glutamine, 1X N2 suppl., 20 ng/mL bFGF
1	8	DMEM/F12	1% BSA, 2mM Glutamine, 1X N2 suppl. 200ng/mL hr Noggin.
1	9	DMEM/F12	1% BSA, 2mM Glutamine, 1X N2 suppl. 20μM SB431542
1	10	DMEM/F12	1% BSA, 2mM Glutamine, 1X ITS suppl. 0.5μM retinoic acid
2	1	DMEM/F12	2mM Glutamine, 1X ITS suppl., 5μg/mL fibronectin.
2	2	DMEM/F12	2mM Glutamine, 1X ITS suppl., 200ng/mL hr Noggin.
2	3	DMEM/F12	2mM Glutamine, 1X ITS suppl., 20ng/mL bFGF.
2	4	DMEM/F12	2mM Glutamine, 1X ITS suppl., 0.5μM retinoic acid.
2	5	RHB-A	
2	6	DMEM/F12	2mM Glutamine, 1X N2 suppl.
2	7	DMEM/F12	2mM Glutamine, 1X ITS suppl., 1X B27 Neuromix, 20ng/mL bFGF, 50ng/mL hr DKK1.
2	8	Advanced DMEM	2mM Glutamine
2	9	DMEM/F12	2mM Glutamine, 1X ITS suppl., 100ng/mL FGF8b, 500ng/mL hr Shh.
2	10	DMEM/F12	2mM Glutamine, 1X ITS suppl., 20ng/mL bFGF, 100ng/mL FGF8b, 200μM ascorbic acid, 10ng/mL BDNF, 1μM purmorphamine
3	1	DMEM/F12	2mM Glutamine, 1X ITS suppl., 20ng/mL bFGF
3	2	DMEM/F12	2mM Glutamine, 1X N2 suppl., 1μg/mL laminin, 200μM ascorbic acid, 10ng/mL BDNF
3	3	DMEM/F12	2mM Glutamine, 1X ITS suppl., 100ng/mL FGF8b, 500ng/mL hr Shh.
3	4	Neurobasal media	2mM Glutamine, 1X ITS suppl., 20ng/mL BDNF, 10ng/mL GDNF, 0.5μM cAMP, 200μM ascorbic acid, 1ng/mL TGFβ
3	5	RHB-A	
3	6	DMEM/F12	2mM Glutamine, 1X N2 suppl.,
3	7	DMEM/F12	2mM Glutamine, 1X ITS suppl., 1X B27 neuromix, 20ng/mL bFGF, 50ng/mL hr DKK1.
3	8	Neurobasal media	2mM Glutamine, 1X B27 neuromix, 20ng/mL bFGF.
3	9	Advanced DMEM	2mM Glutamine
3	10	DMEM/F12	2mM Glutamine, 1X N2 suppl., 20ng/mL bFGF, 20ng/mL EGF, 2ng/mL LIF
4	1	DMEM/F12	2mM Glutamine, 1X N2 suppl., 1mg/mL laminin, 200μM ascorbic acid
4	2	DMEM/F12	2mM Glutamine, 1X N2 suppl., 0.5% FBS, 20ng/mL GDNF, 20ng/mL BDNF.
4	3	DMEM/F12	2mM Glutamine, 1X ITS suppl., 200μM ascorbic acid, 10ng/mL BDNF
4	4	DMEM/F12	2mM Glutamine, 1X N2 suppl., 0.5μM cAMP, 10ng/mL BDNF
4	5	RHB-A	

Split	Medium ID	Basal Media	Supplements
4	6	DMEM/F12	2mM Glutamine, 1X N2 suppl.,
4	7	Neurobasal media	2mM Glutamine, 1X B27 neuromix
4	8	DMEM/F12	2mM Glutamine, 1X ITS suppl., 20ng/mL bFGF
4	9	Advanced DMEM	2mM Glutamine
4	10	DMEM/F12	2mM Glutamine, 1X ITS suppl., 10ng/mL NT3

3. SPLIT-POOL

3.1 CELL CULTURE MEDIA CONTINUED

Table 3: Suppliers of basal media and supplements.

Basal Media/Supplement	Supplier	Product Reference
Advanced DMEM	Life Technologies	12491-015
DMEM/F12	Life Technologies	21041-025
FBS	SLI	EU-000-F
Glutamine	Life Technologies	25030-024
NEAA	Life Technologies	11140035
β-mercaptoehtanol	Sigma Aldrich	M7522
KO DMEM	Life Technologies	10829-018
KSR	Life Technologies	10828-028
Neurobasal	Life Technologies	21103-049
RHB-A	Stem Cells Inc.	SCS-SF-NB-01
Ascorbic Acid	Sigma Aldrich	A4403
B27 Neuromix	PAA	F01-002
BSA	Life Technologies	A10008-01
DKK-1	R and D Systems	1765-DK-010
EGF	R and D Systems	2028-EG-200
FGF-8b	R and D Systems	423-F8-025
bFGF	R and D Systems	233-FB-025
Fibronectin	R and D Systems	1918-FN-02M
ITS	Sigma Aldrich	I3146
LIF-ESGRO	Millipore	ESG1106
N2 Supplement	PAA	F005-004
Noggin	R and D Systems	719-NG-050
SB431542	Sigma Aldrich	S4317
Retinoic Acid	Sigma Aldrich	R2625
SHH	R and D Systems	461-SH-025
TGF-β1	R and D Systems	4114-TC-01M
Purmorphamine	Calbiochem	540220
BDNF	R and D Systems	248-BD-005
GDNF	R and D Systems	212-GD-010
Laminin	Sigma Aldrich	L2020
cAMP	Sigma Aldrich	A9501
NT3	R and D Systems	267-N3-005

3. SPLIT-POOL
3.2. TAG ASSIGNMENT

Each cell culture medium (except media in the last split-pool cycle) was spiked with a unique fluorescent tag that attaches to PTC5000 beads. Tag readout from individual beads enables determination of the series of cell culture media to which the bead was exposed. Thirty unique populations of tags were used, which differ in diameter, fluorescence colour and fluorescence intensity (ten gradations). Table 4 shows the tag code, size, fluorescence colour, fluorescence intensity, sample lot number, tag stock solution concentration and volume used, to spike each cell culture medium.

Table 4: The tags used to spike different cell culture media.

Split	Medium ID	Tag code	Tag size	Tag fluorescence colour	Tag fluorescence intensity level	Stock solution concentration (% w/v)	V of stock solution added to media (µL)
1	1	LR01	Large	Red	1	0.5	440
1	2	LR02	Large	Red	2	0.5	440
1	3	LR03	Large	Red	3	0.5	440
1	4	LR04	Large	Red	4	0.5	440
1	5	LR05	Large	Red	5	0.5	440
1	6	LR09	Large	Red	9	0.5	440
1	7	LR06	Large	Red	6	0.5	440
1	8	LR07	Large	Red	7	0.5	440
1	9	LR08	Large	Red	8	0.5	440
1	10	LR10	Large	Red	10	0.5	440
2	1	MR01	Medium	Red	1	0.5	220
2	2	MR02	Medium	Red	2	0.5	220
2	3	MR03	Medium	Red	3	0.5	220
2	4	MR04	Medium	Red	4	0.5	220
2	5	MR05	Medium	Red	5	0.5	220
2	6	MR06	Medium	Red	6	0.5	220
2	7	MR07	Medium	Red	7	0.5	220
2	8	MR08	Medium	Red	8	0.5	220
2	9	MR09	Medium	Red	9	0.5	220
2	10	MR10	Medium	Red	10	0.5	220
3	1	SR01	Small	Red	1	0.5	111
3	2	SR02	Small	Red	2	0.5	111
3	3	SR03	Small	Red	3	0.5	111
3	4	SR04	Small	Red	4	0.5	111
3	5	SR05	Small	Red	5	0.5	111
3	6	SR06	Small	Red	6	0.5	111
3	7	SR07	Small	Red	7	0.5	111
3	8	SR08	Small	Red	8	0.5	111
3	9	SR09	Small	Red	9	0.5	111
3	10	SR10	Small	Red	10	0.5	111
4	1	No tag	-	-	-	-	-
4	2	No tag	-	-	-	-	-
4	3	No tag	-	-	-	-	-
4	4	No tag	-	-	-	-	-
4	5	No tag	-	-	-	-	-
4	6	No tag	-	-	-	-	-
4	7	No tag	-	-	-	-	-
4	8	No tag	-	-	-	-	-
4	9	No tag	-	-	-	-	-
4	10	No tag	-	-	-	-	-

4. BEAD SCREENING

4.1. SCREENING ASSAY

Following completion of the split-pool experiment at day 30, beads were screened using an immunocytochemistry assay for the enzyme tyrosine hydroxylase to identify hits populated with Shef6 hES cells which had differentiated into dopaminergic neurons.

In brief, beads were washed in DPBS, fixed in 4% paraformaldehyde, permeabilised with 0.25% Triton X-100 in PBS for 20 min at 25°C. Following permeabilisation, cells were incubated in blocking solution (0.25% Triton, 1% BSA in PBS) for 30 min at at 25°C and then incubated in Rb anti-tyrosine hydroxylase (Millipore) primary antibody diluted in blocking solution at 4°C overnight. Following primary antibody incubation, beads were washed 3 times in DPBS++, incubated in Alexa Fluor 488 goat anti-rabbit IgG, secondary antibody solution for 2 hours at 25°C, then washed 3 times in DPBS++ and resuspended in PBS before being sorted.

Figure 3: Shows a 'hit' in a background of negative beads (10x objective lens). The image was obtained using a Nikon Eclipse TE2000-S fluorescent microscope with a FITC filter (λex = 485-495 nm, λem = 515-555 nm).

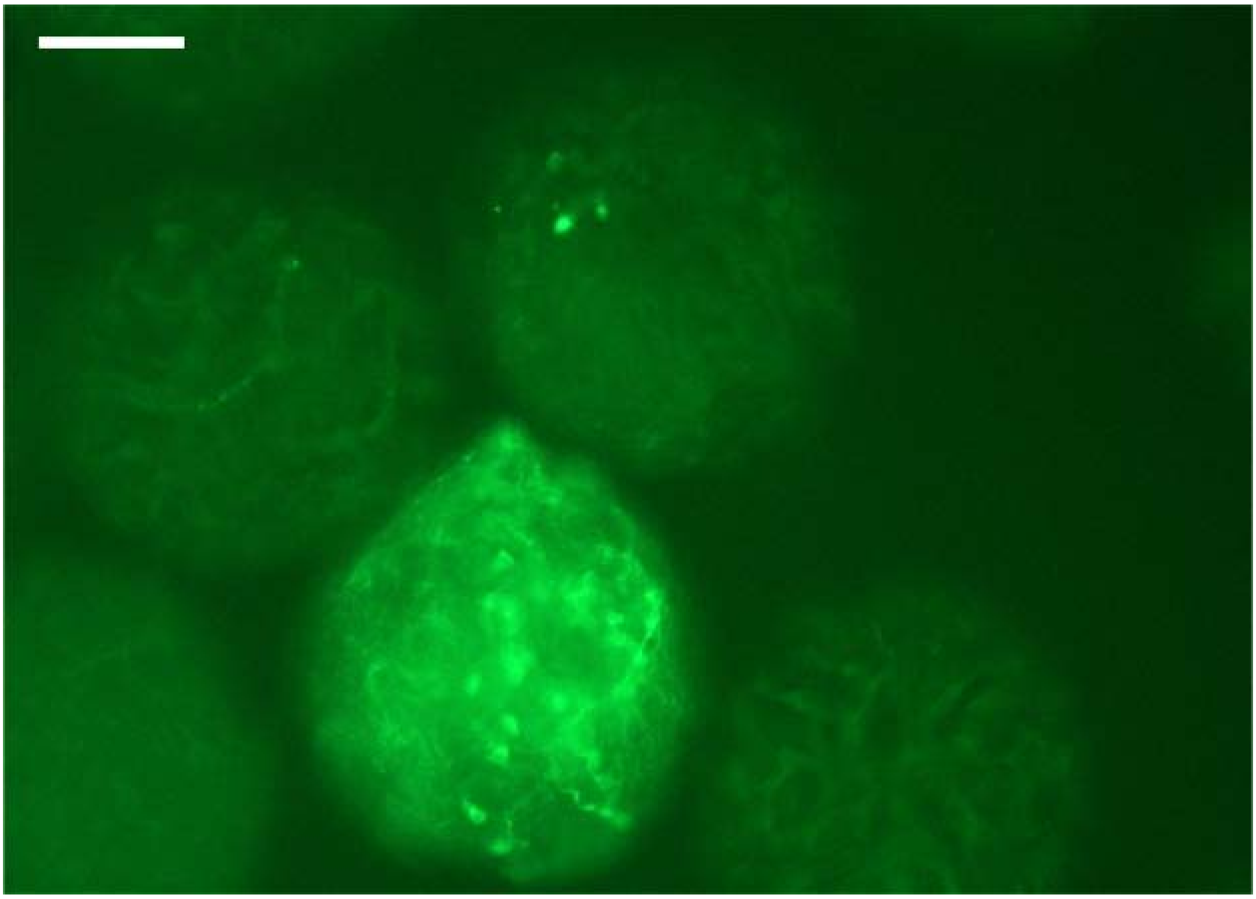

4. BEAD SCREENING

4.2. FLOW SORTING OF ‘HIT’ BEADS

Following the screening assay, beads were sorted using a large particle flow sorter (COPAS PLUS, Union Biometrica; Green PMT 514/23 nm). Beads from each final cell culture medium were labeled as bin 1-10. The data from each bin is contained in one or more flow sorting data files. Annex 1 shows dot plots from each flow sorting data file.
Beads were gated according to size (time of flight [TOF] and optical extinction [ext] values) to exclude bead aggregates that had formed during cell culture. Gated beads were sorted based on their fluorescence properties, as defined by the screening assay, and hits individually dispensed into wells of a 96-well plate.

In figure 4 COPAS data from all bins have been combined on two dot-plots to provide an overview of bead sorting. Figure 4(a) is a dot plot of time of Flight (TOF) vs. optical extinction (ext). Events falling within the gate defining monomeric beads are represented in red: in this experiment 290,314 beads (60 % of the starting number) were sorted. Figure 4(b) is a dot plot of TOF vs. green fluorescence intensity for all sorted beads. Beads with a green fluorescence intensity value above the threshold value (depicted as green dots in Figure 4b) were individually dispensed into 96 well plates.

Once dispensed, the brightest hits with a green fluorescent value above 136 were selected. Table 5 lists the number of positive beads sorted and the brightest ‘hit’ beads selected from each bin. A total of 1198 positive beads were isolated out of which 367 had a green fluorescent value above 136 (Table 5). Tags from the brightest ‘hits’ were subsequently analysed to determine the cell culture history (i.e. differentiation protocol) of each ‘hit’ bead, as detailed in the next section of the report.

Figure 4(a) and (b): COPAS dot plots, showing (a) the gate used to select monomeric beads (red dots) and (b) the threshold fluorescence intensity used to select positive ‘hit’ beads (green dots). The dot plots contain combined data recorded from all bins.

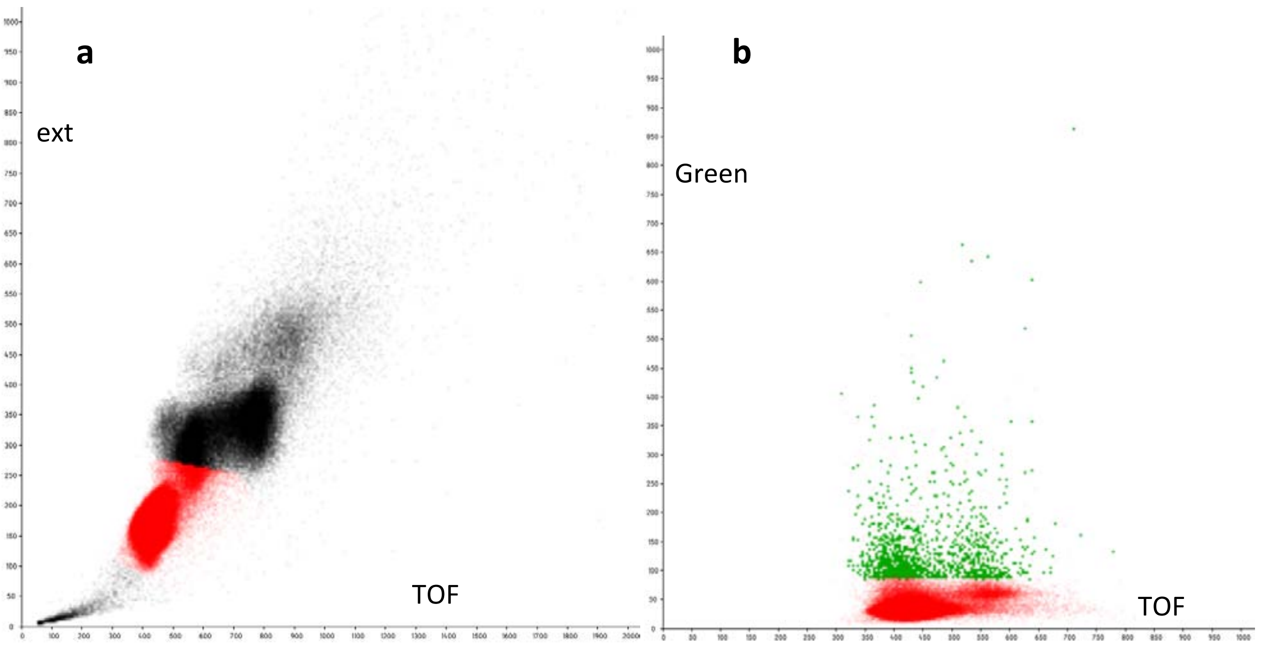

4. BEAD SCREENING

4.2. FLOW SORTING OF 'HITS' CONTINUED

Table 5: Number of positive beads sorted and number of brightest 'hits' (green fluorescent value above 136) selected for tag deconvolution, from each bin.

Bin Number	1	2	3	4	5	6	7	8	9	10	Total
Number of positive beads sorted	210	59	68	151	147	254	40	110	42	117	1198
Number of brightest 'hits' (green fluoresence value above 136)	65	16	15	42	43	98	10	32	19	27	367

Confidential

5. TAG DECONVOLUTION

Following bead sorting, tags from each hit are analysed by flow cytometry and related to the cell culture history. During this process, data is lost or excluded owing to various factors and this is summarized in Table 6.

The flow cytometry data acquisition was performed in a series of sessions and prior to each session a reference tag set was run to calibrate side/forward scatter and fluorescence intensity gates. Dot plots and histograms for each session are shown in Annex 2. The data was loaded into Ariadne™ which automatically identifies tags based on four unique parameter values (forward and side scatter and fluorescence colour and intensity).

Determination of tag identity is based on the number of events (≥ 3) within a gate, and the cluster tightness (Table 7). If two or more clusters of events are identified which mapped to cell media from the same split and/or the signal to noise ratio was too low, an accurate identification is not recorded.

Ariadne™ relates the tag identity to a cell culture medium using the tag assignment (Table 4), allowing it to log the cell culture history of each hit (Table 8).

Table 6. Summary of data attrition owing to various factors.

Bin Number	1	2	3	4	5	6	7	8	9	10	Total
Number brightest 'hits'	65	16	15	42	43	98	10	32	19	27	367
Number of beads lost during digestion and sample preparation	(3)	(4)	(2)	(6)	(9)	(17)	(1)	(4)	(2)	(4)	(52)
Number of beads analysed by flow cytometry	62	12	13	36	34	81	9	28	17	23	315
Number of beads with no tagging data	0	0	0	(1)	0	(6)	0	(1)	0	(3)	(11)
Number of beads with incomplete tagging data	(4)	0	(1)	(4)	(2)	(2)	(1)	0	0	(4)	(18)
Number of beads containing too many tag clusters	(2)	0	(1)	0	0	(3)	0	0	0	(1)	(7)
Number of hits with conclusive tagging data	56	12	11	31	32	70	8	27	17	15	279

Confidential

Table 7. Summary of the minimum, maximum and average number of tags derived from beads in each of the cell culture media. This data demonstrates that the average number of tags for all media is well above the minimum required to link a cell culture medium to a bead (3).

Split	Medium ID	# of 'hit' beads per media	Minimum # of tags	Average # of tags	Maximum # of tags
1	1	22	4	34	139
1	2	18	4	24	48
1	3	30	5	38	160
1	4	34	5	50	176
1	5	24	10	54	289
1	6	26	4	42	134
1	7	21	6	48	166
1	8	37	3	48	171
1	9	34	4	33	84
1	10	33	3	55	271
2	1	15	10	65	202
2	2	25	8	69	152
2	3	25	6	57	154
2	4	32	5	54	186
2	5	21	23	89	300
2	6	29	13	96	523
2	7	46	8	108	459
2	8	28	20	84	204
2	9	32	6	52	144
2	10	26	6	69	164
3	1	19	6	47	222
3	2	23	5	27	69
3	3	25	6	34	110
3	4	33	13	65	238
3	5	22	3	43	147
3	6	27	3	55	230
3	7	49	16	97	371
3	8	22	20	62	155
3	9	28	12	125	375
3	10	31	5	64	240
4	1	56	-	-	-
4	2	12	-	-	-
4	3	11	-	-	-
4	4	31	-	-	-
4	5	32	-	-	-
4	6	72	-	-	-
4	7	8	-	-	-
4	8	27	-	-	-
4	9	17	-	-	-
4	10	14	-	-	-

Table 8. Information for each 'hit': (i) the bead ID; (ii) tag acquisition session number; (iii) flow cytometry tag data file name; (iv) series of cell culture media, i.e. protocol (number of tags upon which assignment of each cell culture media is based are in parentheses, "–" represents unreadable tag data) and (v) whether the bead passes the tag deconvolution.

Bead ID	Tag acquisition session #	Tag data filename	Split 1 media (# of tags)	Split 2 media (# of tags)	Split 3 media (# of tags)	Split 4 media	Pass tag deconvolution criteria
1	1	Bin1_plateA1_D11.fcs	-	9 (67)	4 (82)	1	fail
2	1	Bin1_plateA1_A2.fcs	-	9 (6)	-	1	fail
3	1	Bin1_plateA1_A11.fcs	9 (14)	3 (32)	4 (42)	1	pass
4	1	Bin1_plateA1_A12.fcs	4 (8)	10 (17)	4 (35)	1	pass
5	1	Bin1_plateA1_B1.fcs	8 (15)	6 (33)	6 (56)	1	pass
6	1	Bin1_plateA1_B2.fcs	7 (49)	7 (72)	1 (15)	1	pass
7	1	Bin1_plateA1_B9.fcs	-	-	8 (10)	1	fail
8	1	Bin1_plateA1_C2.fcs	8 (7)	-	2 (6)	1	fail
9	1	Bin1_plateA1_C4.fcs	6 (8)	7 (8)	8 (20)	1	pass
10	1	Bin1_plateA1_C7.fcs	1 (33)	3 (30)	3 (23)	1	pass
11	1	Bin1_plateA1_C8.fcs	9 (36)	6 (92)	7 (144)	1	pass
12	1	Bin1_plateA1_C10.fcs	3 (18)	9 (6)	5 (28)	1	pass
13	2	Bin1_plateA1_F9.fcs	5 (10)	9 (36)	4 (17)	1	pass
14	2	Bin1_plateA1_E3.fcs	1 (139)	6 (523)	4 (211)	1	pass
15	2	Bin1_plateA1_E5.fcs	8 (19)	10 (72)	7 (69)	1	pass
16	2	Bin1_plateA1_E9.fcs	10 (135)	3 (115)	6 (64)	1	pass
17	2	Bin1_plateA1_E11.fcs	6 (4)	4 (8)	5 (5)	1	pass
18	2	Bin1_plateA1_F2.fcs	5 (43)	2 (150)	10 (49)	1	pass
19	3	Bin1_plateA2_F11.fcs	5 (22)	10 (70)	7 (169)	1	pass
20	3	Bin1_plateA2_A5.fcs	9 (28)	9 (31)	2 (8)	1	pass
21	3	Bin1_plateA2_A9.fcs	5 (41)	2 (25)	5 (101)	1	pass
22	3	Bin1_plateA2_A10.fcs	3 (92)	9 (48)	2 (45)	1	pass
23	3	Bin1_plateA2_A11.fcs	6 (39)	9 (41)	1 (222)	1	pass
24	3	Bin1_plateA2_A12.fcs	8 (68)	6 (214)	3 (53)	1	pass
25	3	Bin1_plateA2_B1.fcs	10 (198)	10 (164)	10 (77)	1	pass
26	3	Bin1_plateA2_B3.fcs	4 (52)	8 (64)	4 (70)	1	pass
27	3	Bin1_plateA2_B12.fcs	9 (21)	5 (26)	5 (24)	1	pass
28	3	Bin1_plateA2_C1.fcs	9 (20)	4 (24)	8 (38)	1	pass
29	3	Bin1_plateA2_C2.fcs	10 (42)	6 (90)	7 (44)	1	pass
30	3	Bin1_plateA2_C8.fcs	8 (57)	3 (40)	10 (59)	1	pass
31	3	Bin1_plateA2_D3.fcs	3 (48)	3 (70)	7 (106)	1	pass
32	3	Bin1_plateA2_D4.fcs	5 (58)	2 (66)	1 (19)	1	pass
33	3	Bin1_plateA2_D8.fcs	2 (45)	7 (241)	10 (240)	1	pass
34	3	Bin1_plateA2_D12.fcs	9 (37)	7 (45)	10 (51)	1	pass
35	3	Bin1_plateA2_E2.fcs	5 (35)	2 (76)	6 (44)	1	pass
36	3	Bin1_plateA2_F2.fcs	5 (48)	3 (77)	1 (97)	1	pass
37	3	Bin1_plateA2_F4.fcs	10 (77)	7 (214)	6 (230)	1	pass
38	3	Bin1_plateA2_F7.fcs	-	6 (6)	4 (6)	1	fail
39	3	Bin1_plateA2_F9.fcs	7 (166)	7 (286)	2 (69)	1	pass
40	3	Bin1_plateA2_F10.fcs	3 (10)	2 (27)	8 (45)	1	pass
41	4	Bin3_PlateA3_B5.fcs	3 (26)	5 (41)	6 (13)	3	pass
42	4	Bin1_plateA3_A2.fcs	7 (46)	2 (116)	5 (140)	1	pass
43	4	Bin1_plateA3_A4.fcs	3 (160)	9 (70)	8 (87)	1	pass
44	4	Bin1_plateA3_A5.fcs	4 (155)	4 (186)	5 (69)	1	pass
45	4	Bin1_plateA3_A8.fcs	9 (65)	10 (84)	6 (169)	1	pass
46	4	Bin1_plateA3_A10.fcs	8 (122)	4 (119)	1 (48)	1	pass
47	4	Bin1_plateA3_C2.fcs	8 (20)	10 (117)	3 (48)	1	pass
48	4	Bin1_plateA3_C6.fcs	8 (76)	7 (149)	1 (64)	1	pass
49	4	Bin1_plateA3_C7.fcs	7 (43)	4 (75)	7 (120)	1	pass

Bead ID	Tag acquisition session #	Tag data filename	Split 1 media (# of tags)	Split 2 media (# of tags)	Split 3 media (# of tags)	Split 4 media	Pass tag deconvolution criteria
50	4	Bin1_plateA3_C9.fcs	10 (28)	8 (71)	10 (46)	1	pass
51	4	Bin1_plateA3_C12.fcs	1 (21)	1 (202)	4 (45)	1	pass
52	4	Bin1_plateA3_D1.fcs	4 (33)	2 (62)	9 (255)	1	pass
53	4	Bin1_plateA3_D2.fcs	2 (7)	4 (10)	6 (41)	1	pass
54	4	Bin1_plateA3_D3.fcs	6 (12)	2 (100)	4 (63)	1	pass
55	4	Bin1_plateA3_D6.fcs	4 (41)	8 (30)	8 (63)	1	pass
56	4	Bin1_plateA3_D8.fcs	7 (56)	7 (86)	7 (69)	1	pass
57	4	Bin1_plateA3_D12.fcs	6 (46)	9 (15)	7 (102)	1	pass
58	4	Bin1_plateA3_E2.fcs	-	3 (30)	10 (62)	1	fail
59	4	Bin1_plateA3_E3.fcs	4 (32)	10 (62)	4 (58)	1	pass
60	4	Bin1_plateA3_E6.fcs	7 (30)	6 (49)	2 (32)	1	pass
61	4	Bin1_plateA3_E7.fcs	9 (13)	8 (71)	1 (13)	1	pass
62	4	Bin1_plateA3_F3.fcs	8 (91)	8 (121)	8 (54)	1	pass
63	4	Bin1_plateA3_F4.fcs	7 (13)	3 (18)	9 (45)	1	pass
64	4	Bin2_plateB3_A2.fcs	4 (5)	4 (19)	9 (31)	2	pass
65	4	Bin2_PlateB3_A4.fcs	1 (17)	9 (92)	8 (83)	2	pass
66	4	Bin2_PlateB3_A5.fcs	3 (11)	4 (5)	6 (3)	2	pass
67	4	Bin2_PlateC3_B7.fcs	9 (4)	10 (6)	7 (16)	2	pass
68	4	Bin2_PlateC3_B11.fcs	10 (8)	7 (50)	4 (21)	2	pass
69	4	Bin2_PlateC3_C1.fcs	10 (23)	1 (17)	2 (43)	2	pass
70	4	Bin2_PlateC3_C3.fcs	8 (3)	4 (12)	9 (29)	2	pass
71	4	Bin2_PlateC3_C6.fcs	9 (18)	7 (51)	7 (61)	2	pass
72	4	Bin2_PlateC3_C9.fcs	2 (42)	3 (6)	10 (44)	2	pass
73	4	Bin2_PlateC3_D2.fcs	3 (5)	7 (13)	7 (19)	2	pass
74	4	Bin2_PlateC3_D9.fcs	6 (14)	5 (189)	9 (113)	2	pass
75	4	Bin2_PlateC3_D10.fcs	9 (49)	8 (59)	4 (54)	2	pass
76	4	Bin3_PlateA3_A1.fcs	10 (50)	6 (172)	4 (54)	3	pass
77	5	Bin4_plateA3_H12.fcs	9 (3)	6 (3)	-	4	fail
78	5	Bin3_plateA3_B11.fcs	4 (52)	9 (51)	1 (22)	3	pass
79	5	Bin3_plateA3_C4.fcs	9 (52)	10 (97)	6 (66)	3	pass
80	5	Bin3_plateA3_C5.fcs	1 (10)	5 (101)	7 (75)	3	pass
81	5	Bin3_plateA3_C12.fcs	9 (6)	7 (10)	5 (58)	3	pass
82	5	Bin3_plateA3_D8.fcs	4 (115)	6 (134)	1 (19)	3	pass
83	5	Bin3_plateA3_D9.fcs	7 (109)	7 (169)	3 (40)	3	pass
84	5	Bin3_plateB3_E9.fcs	8 (86)	5 (180)	4 (19)	3	pass
85	5	Bin3_plateB3_F1.fcs	4 (123)	2 (87)	6 (61)	3	pass
86	5	Bin3_plateB3_F6.fcs	4 (176)	10 (158)	10 (78)	3	pass
87	5	Bin3_plateB3_F9.fcs	8 (47)	9 (53)	-	3	fail
88	5	Bin4_plateA3_A8.fcs	4 (19)	2 (28)	3 (39)	4	pass
89	5	Bin4_plateA3_A10.fcs	1 (69)	7 (212)	8 (114)	4	pass
90	5	Bin4_plateA3_A11.fcs	8 (41)	2 (37)	6 (98)	4	pass
91	5	Bin4_plateA3_B1.fcs	7 (48)	9 (9)	6 (4)	4	pass
92	5	Bin4_plateA3_B3.fcs	10 (79)	2 (152)	3 (34)	4	pass
93	5	Bin4_plateA3_B6.fcs	2 (11)	10 (67)	7 (122)	4	pass
94	5	Bin4_plateA3_B8.fcs	9 (34)	7 (99)	7 (279)	4	pass
95	5	Bin4_plateA3_C7.fcs	7 (100)	9 (95)	6 (101)	4	pass
96	5	Bin4_plateA3_C8.fcs	10 (37)	2 (111)	7 (64)	4	pass
97	5	Bin4_plateA3_C9.fcs	-	1 (23)	5 (23)	4	fail
98	5	Bin4_plateA3_D7.fcs	3 (108)	10 (43)	5 (21)	4	pass
99	5	Bin4_plateA3_D9.fcs	2 (37)	5 (51)	10 (40)	4	pass
100	5	Bin4_plateA3_D11.fcs	2 (26)	5 (70)	10 (25)	4	pass
101	5	Bin4_plateA3_E2.fcs	7 (39)	7 (41)	7 (46)	4	pass
102	5	Bin4_plateA3_E4.fcs	1 (5)	10 (13)	10 (5)	4	pass
103	5	Bin4_plateA3_E9.fcs	1 (62)	1 (92)	4 (92)	4	pass
104	5	Bin4_plateA3_E10.fcs	10 (75)	7 (119)	10 (51)	4	pass

Bead ID	Tag acquisition session #	Tag data filename	Split 1 media (# of tags)	Split 2 media (# of tags)	Split 3 media (# of tags)	Split 4 media	Pass tag deconvolution criteria
105	5	Bin4_plateA3_F1.fcs	8 (12)	4 (45)	5 (10)	4	pass
106	5	Bin4_plateA3_G3.fcs	-	-	-	4	fail
107	5	Bin4_plateA3_G10.fcs	1 (4)	-	-	4	fail
108	5	Bin4_plateA3_H1.fcs	3 (7)	3 (29)	8 (32)	4	pass
109	5	Bin4_plateA3_H8.fcs	1 (12)	8 (26)	1 (6)	4	pass
110	6	Bin4_plateA4_D11.fcs	2 (15)	6 (75)	7 (36)	4	pass
111	6	Bin4_plateA4_A2.fcs	9 (58)	9 (29)	7 (98)	4	pass
112	6	Bin4_plateA4_A10.fcs	2 (41)	5 (47)	7 (198)	4	pass
113	6	Bin4_plateA4_A11.fcs	1 (36)	7 (130)	2 (31)	4	pass
114	6	Bin4_plateA4_B2.fcs	7 (8)	5 (40)	9 (88)	4	pass
115	6	Bin4_plateA4_B6.fcs	10 (43)	8 (110)	7 (107)	4	pass
116	6	Bin4_plateA4_B9.fcs	9 (84)	7 (66)	6 (16)	4	pass
117	6	Bin4_plateA4_B10.fcs	-	10 (8)	-	4	fail
118	6	Bin4_plateA4_C1.fcs	4 (26)	6 (29)	6 (30)	4	pass
119	6	Bin4_plateA4_C10.fcs	8 (10)	6 (28)	7 (34)	4	pass
120	6	Bin4_plateA4_C11.fcs	5 (46)	4 (22)	3 (21)	4	pass
121	6	Bin4_plateA4_D9.fcs	5 (30)	9 (31)	6 (25)	4	pass
122	7	Bin7_plateA1_C11.fcs	9 (25)	3 (52)	3 (8)	7	pass
123	7	Bin5_plateA4_A2.fcs	8 (24)	4 (27)	2 (11)	5	pass
124	7	Bin5_plateA4_A3.fcs	5 (21)	4 (34)	9 (159)	5	pass
125	7	Bin5_plateA4_A6.fcs	3 (25)	8 (116)	9 (335)	5	pass
126	7	Bin5_plateA4_A10.fcs	9 (29)	6 (37)	6 (13)	5	pass
127	7	Bin5_plateA4_A11.fcs	-	9 (9)	9 (36)	5	fail
128	7	Bin5_plateA4_B5.fcs	10 (111)	9 (46)	2 (38)	5	pass
129	7	Bin5_plateA4_B6.fcs	3 (16)	7 (36)	7 (47)	5	pass
130	7	Bin5_plateA4_B9.fcs	4 (123)	7 (211)	9 (145)	5	pass
131	7	Bin5_plateA4_B10.fcs	8 (23)	7 (97)	6 (6)	5	pass
132	7	Bin5_plateA4_E7.fcs	5 (40)	9 (44)	5 (40)	5	pass
133	7	Bin5_plateA4_E10.fcs	5 (10)	3 (69)	2 (25)	5	pass
134	7	Bin5_plateA4_F10.fcs	5 (39)	6 (106)	7 (165)	5	pass
135	7	Bin5_plateA4_F11.fcs	3 (12)	4 (65)	9 (84)	5	pass
136	7	Bin5_plateA4_G3.fcs	5 (30)	6 (133)	7 (57)	5	pass
137	7	Bin5_plateA4_G6.fcs	4 (8)	2 (14)	8 (47)	5	pass
138	7	Bin5_plateA4_G7.fcs	3 (54)	9 (9)	6 (40)	5	pass
139	7	Bin5_plateA4_G8.fcs	10 (3)	4 (25)	4 (14)	5	pass
140	7	Bin5_plateA4_H9.fcs	6 (59)	7 (246)	2 (20)	5	pass
141	7	Bin5_plateA5_A1.fcs	10 (271)	4 (44)	2 (17)	5	pass
142	7	Bin5_plateA5_A6.fcs	4 (53)	10 (129)	3 (52)	5	pass
143	7	Bin5_plateA5_B3.fcs	3 (56)	7 (47)	3 (52)	5	pass
144	7	Bin5_plateA5_B9.fcs	1 (26)	6 (135)	7 (84)	5	pass
145	7	Bin5_plateA5_B11.fcs	8 (15)	3 (47)	7 (70)	5	pass
146	7	Bin5_plateA5_C4.fcs	3 (77)	1 (85)	10 (49)	5	pass
147	7	Bin5_plateA5_C7.fcs	6 (106)	1 (58)	3 (56)	5	pass
148	7	Bin5_plateA5_C9.fcs	1 (49)	7 (78)	2 (36)	5	pass
149	7	Bin5_plateA5_C10.fcs	10 (78)	4 (28)	9 (108)	5	pass
150	7	Bin5_plateA5_C12.fcs	10 (14)	5 (83)	8 (33)	5	pass
151	7	Bin5_plateA5_D1.fcs	9 (61)	4 (50)	10 (35)	5	pass
152	7	Bin5_plateA5_D2.fcs	8 (34)	1 (42)	7 (16)	5	pass
153	7	Bin5_plateA5_D6.fcs	1 (4)	6 (55)	9 (180)	5	pass
154	7	Bin5_plateA5_E1.fcs	6 (77)	7 (186)	7 (71)	5	pass
155	7	Bin5_plateA5_E3.fcs	3 (33)	10 (74)	4 (34)	5	pass
156	7	Bin6_plateA5_A1.fcs	3 (24)	9 (13)	6 (16)	6	pass
157	7	Bin6_plateA5_A3.fcs	10 (74)	2 (79)	4 (41)	6	pass
158	7	Bin6_plateA5_A8.fcs	-	-	-	6	fail
159	7	Bin6_plateA5_A11.fcs	5 (289)	4 (123)	2 (15)	6	pass

Confidential

Bead ID	Tag acquisition session #	Tag data filename	Split 1 media (# of tags)	Split 2 media (# of tags)	Split 3 media (# of tags)	Split 4 media	Pass tag deconvolution criteria
160	7	Bin6_plateA5_A12.fcs	4 (12)	2 (8)	3 (28)	6	pass
161	7	Bin6_plateA5_B3.fcs	7 (19)	10 (54)	9 (159)	6	pass
162	7	Bin6_plateA5_B6.fcs	6 (20)	7 (24)	9 (91)	6	pass
163	7	Bin6_plateA5_B9.fcs	8 (24)	8 (87)	3 (22)	6	pass
164	7	Bin6_plateA5_B12.fcs	2 (31)	3 (81)	4 (87)	6	pass
165	7	Bin6_plateA5_C4.fcs	-	6 (8)	-	6	fail
166	7	Bin6_plateA5_C8.fcs	10 (81)	9 (113)	5 (34)	6	pass
167	7	Bin6_plateA5_C9.fcs	6 (86)	5 (63)	8 (69)	6	pass
168	7	Bin7_plateA1_A5.fcs	6 (36)	2 (96)	7 (65)	7	pass
169	7	Bin7_plateA1_A7.fcs	5 (92)	1 (109)	1 (64)	7	pass
170	7	Bin7_plateA1_B1.fcs	10 (10)	3 (7)	7 (20)	7	pass
171	7	Bin7_plateA1_B3.fcs	4 (14)	2 (39)	5 (12)	7	pass
172	7	Bin7_plateA1_B6.fcs	8 (35)	6 (130)	3 (41)	7	pass
173	7	Bin7_plateA1_C3.fcs	5 (69)	5 (189)	10 (95)	7	pass
174	7	Bin7_plateA1_C4.fcs	-	2 (8)	-	7	fail
175	7	Bin7_plateA1_C6.fcs	4 (45)	8 (42)	5 (3)	7	pass
176	8	Bin6_plateA6_H12.fcs	-	-	-	6	fail
177	8	Bin6_plateA5_C12.fcs	10 (58)	8 (184)	5 (81)	6	pass
178	8	Bin6_plateA5_D1.fcs	8 (48)	9 (67)	7 (92)	6	pass
179	8	Bin6_plateA5_D6.fcs	4 (112)	9 (55)	4 (105)	6	pass
180	8	Bin6_plateA5_D7.fcs	6 (46)	6 (49)	10 (29)	6	pass
181	8	Bin6_plateA5_E4.fcs	8 (20)	5 (97)	8 (24)	6	pass
182	8	Bin6_plateA5_E7.fcs	3 (56)	5 (308)	-	6	fail
183	8	Bin6_plateA5_E10.fcs	2 (12)	8 (107)	8 (63)	6	pass
184	8	Bin6_plateA5_F1.fcs	6 (29)	4 (43)	10 (84)	6	pass
185	8	Bin6_plateA5_F4.fcs	10 (30)	6 (65)	10 (134)	6	pass
186	8	Bin6_plateA5_F5.fcs	3 (20)	8 (155)	3 (63)	6	pass
187	8	Bin6_plateA5_F6.fcs	7 (6)	6 (14)	4 (13)	6	pass
188	8	Bin6_plateA5_F7.fcs	4 (31)	6 (68)	9 (100)	6	pass
189	8	Bin6_plateA5_F9.fcs	8 (118)	6 (85)	2 (52)	6	pass
190	8	Bin6_plateA5_F12.fcs	8 (92)	7 (459)	7 (242)	6	pass
191	8	Bin6_plateA5_G2.fcs	9 (16)	7 (121)	3 (54)	6	pass
192	8	Bin6_plateA5_G8.fcs	6 (32)	8 (79)	7 (99)	6	pass
193	8	Bin6_plateA5_G9.fcs	9 (43)	2 (111)	4 (88)	6	pass
194	8	Bin6_plateA5_H3.fcs	9 (17)	7 (47)	10 (27)	6	pass
195	8	Bin6_plateA5_H8.fcs	9 (45)	3 (69)	5 (34)	6	pass
196	8	Bin6_plateA5_H11.fcs	7 (74)	8 (66)	5 (8)	6	pass
197	8	Bin6_plateA6_A4.fcs	8 (51)	10 (85)	7 (33)	6	pass
198	8	Bin6_plateA6_A8.fcs	9 (31)	3 (47)	7 (89)	6	pass
199	8	Bin6_plateA6_A10.fcs	5 (48)	7 (190)	7 (204)	6	pass
200	8	Bin6_plateA6_A12.fcs	7 (49)	10 (28)	1 (14)	6	pass
201	8	Bin6_plateA6_B1.fcs	5 (72)	4 (100)	3 (22)	6	pass
202	8	Bin6_plateA6_C4.fcs	1 (26)	6 (116)	3 (6)	6	pass
203	8	Bin6_plateA6_C6.fcs	6 (41)	8 (65)	2 (40)	6	pass
204	8	Bin6_plateA6_C7.fcs	2 (4)	1 (17)	7 (75)	6	pass
205	8	Bin6_plateA6_C10.fcs	4 (12)	4 (18)	4 (37)	6	pass
206	8	Bin6_plateA6_C11.fcs	3 (25)	3 (51)	1 (26)	6	pass
207	8	Bin6_plateA6_D1.fcs	5 (16)	5 (23)	4 (50)	6	pass
208	8	Bin6_plateA6_D3.fcs	6 (39)	1 (37)	7 (79)	6	pass
209	8	Bin6_plateA6_D5.fcs	2 (7)	1 (21)	4 (18)	6	pass
210	8	Bin6_plateA6_D7.fcs	7 (14)	6 (23)	3 (10)	6	pass
211	8	Bin6_plateA6_E5.fcs	8 (36)	7 (122)	3 (110)	6	pass
212	8	Bin6_plateA6_E6.fcs	1 (13)	8 (20)	5 (3)	6	pass
213	8	Bin6_plateA6_E8.fcs	2 (48)	3 (114)	9 (263)	6	pass
214	8	Bin6_plateA6_E9.fcs	9 (33)	1 (42)	9 (164)	6	pass

Bead ID	Tag acquisition session #	Tag data filename	Split 1 media (# of tags)	Split 2 media (# of tags)	Split 3 media (# of tags)	Split 4 media	Pass tag deconvolution criteria
215	8	Bin6_plateA6_E10.fcs	6 (35)	7 (70)	10 (44)	6	pass
216	8	Bin6_plateA6_F3.fcs	-	-	-	6	fail
217	8	Bin6_plateA6_F8.fcs	8 (46)	9 (110)	7 (90)	6	pass
218	8	Bin6_plateA6_G3.fcs	-	-	-	6	fail
219	8	Bin6_plateA6_G6.fcs	9 (36)	2 (48)	7 (35)	6	pass
220	8	Bin6_plateA6_G9.fcs	6 (69)	6 (68)	-	6	fail
221	8	Bin6_plateA6_G12.fcs	3 (11)	7 (32)	4 (16)	6	pass
222	8	Bin6_plateA6_H1.fcs	4 (45)	8 (98)	-	6	fail
223	8	Bin6_plateA6_H4.fcs	9 (27)	4 (112)	9 (375)	6	pass
224	8	Bin6_plateA6_H7.fcs	6 (16)	10 (53)	4 (76)	6	pass
225	8	Bin6_plateA6_H11.fcs	8 (18)	10 (37)	9 (33)	6	pass
226	9	Bin6_plateA7_E12.fcs	10 (7)	4 (16)	9 (12)	6	pass
227	9	Bin6_plateA7_A2.fcs	9 (10)	6 (13)	10 (7)	6	pass
228	9	Bin6_plateA7_A4.fcs	-	-	-	6	fail
229	9	Bin6_plateA7_A6.fcs	3 (60)	3 (87)	8 (95)	6	pass
230	9	Bin6_plateA7_A11.fcs	6 (134)	5 (98)	10 (60)	6	pass
231	9	Bin6_plateA7_B3.fcs	1 (20)	9 (69)	1 (11)	6	pass
232	9	Bin6_plateA7_B7.fcs	1 (47)	10 (123)	3 (7)	6	pass
233	9	Bin6_plateA7_B9.fcs	7 (29)	9 (26)	7 (99)	6	pass
234	9	Bin6_plateA7_B11.fcs	4 (47)	4 (32)	8 (101)	6	pass
235	9	Bin6_plateA7_C1.fcs	-	-	-	6	fail
236	9	Bin6_plateA7_C3.fcs	10 (6)	10 (10)	9 (17)	6	pass
237	9	Bin6_plateA7_C12.fcs	4 (10)	7 (18)	9 (24)	6	pass
238	9	Bin6_plateA7_D4.fcs	10 (13)	8 (28)	9 (120)	6	pass
239	9	Bin6_plateA7_D8.fcs	3 (5)	7 (42)	9 (26)	6	pass
240	9	Bin6_plateA7_E1.fcs	9 (49)	9 (13)	10 (23)	6	pass
241	9	Bin6_plateA7_E2.fcs	9 (27)	1 (38)	10 (36)	6	pass
242	9	Bin6_plateA7_E5.fcs	9 (19)	5 (40)	2 (14)	6	pass
243	9	Bin6_plateA7_E8.fcs	8 (65)	8 (95)	1 (59)	6	pass
244	9	Bin6_plateA7_E11.fcs	4 (5)	8 (24)	5 (16)	6	pass
245	10	Bin8_plateA1_H9.fcs	8 (20)	10 (11)	3 (10)	8	pass
246	10	Bin8_plateA1_A1.fcs	5 (14)	8 (204)	4 (81)	8	pass
247	10	Bin8_plateA1_A2.fcs	4 (89)	3 (50)	8 (155)	8	pass
248	10	Bin8_plateA1_A4.fcs	2 (8)	8 (59)	2 (21)	8	pass
249	10	Bin8_plateA1_A6.fcs	2 (18)	9 (36)	2 (26)	8	pass
250	10	Bin8_plateA1_A9.fcs	8 (82)	6 (151)	9 (177)	8	pass
251	10	Bin8_plateA1_A11.fcs	9 (7)	6 (35)	2 (12)	8	pass
252	10	Bin8_plateA1_B3.fcs	5 (122)	4 (82)	5 (70)	8	pass
253	10	Bin8_plateA1_B8.fcs	3 (50)	5 (83)	8 (37)	8	pass
254	10	Bin8_plateA1_B10.fcs	5 (48)	1 (127)	2 (30)	8	pass
255	10	Bin8_plateA1_B12.fcs	4 (28)	3 (25)	3 (25)	8	pass
256	10	Bin8_plateA1_C5.fcs	6 (16)	2 (55)	7 (194)	8	pass
257	10	Bin8_plateA1_C9.fcs	-	-	-	8	fail
258	10	Bin8_plateA1_D2.fcs	3 (31)	8 (47)	2 (8)	8	pass
259	10	Bin8_plateA1_D7.fcs	8 (171)	8 (173)	4 (238)	8	pass
260	10	Bin8_plateA1_D9.fcs	8 (15)	7 (44)	1 (6)	8	pass
261	10	Bin8_plateA1_D12.fcs	10 (96)	9 (85)	1 (112)	8	pass
262	10	Bin8_plateA1_F1.fcs	9 (49)	7 (92)	10 (32)	8	pass
263	10	Bin8_plateA1_F2.fcs	1 (23)	3 (154)	7 (371)	8	pass
264	10	Bin8_plateA1_G4.fcs	10 (50)	7 (56)	5 (147)	8	pass
265	10	Bin8_plateA1_G7.fcs	9 (55)	4 (89)	6 (88)	8	pass
266	10	Bin8_plateA1_G8.fcs	3 (35)	2 (71)	10 (97)	8	pass
267	10	Bin8_plateA1_G10.fcs	7 (13)	4 (53)	6 (33)	8	pass
268	10	Bin8_plateA1_H5.fcs	1 (7)	5 (41)	1 (7)	8	pass
269	10	Bin8_plateA1_H7.fcs	5 (41)	1 (73)	1 (72)	8	pass

Bead ID	Tag acquisition session #	Tag data filename	Split 1 media (# of tags)	Split 2 media (# of tags)	Split 3 media (# of tags)	Split 4 media	Pass tag deconvolution criteria
270	11	Bin8_plateA2_A5.fcs	1 (8)	9 (74)	5 (29)	8	pass
271	11	Bin8_plateA2_A3.fcs	10 (20)	6 (46)	4 (42)	8	pass
272	12	Bin9_plateA2_D1.fcs	8 (44)	10 (78)	10 (103)	9	pass
273	12	Bin9_plateA2_A3.fcs	2 (26)	5 (72)	2 (24)	9	pass
274	12	Bin9_plateA2_A7.fcs	3 (39)	6 (83)	3 (12)	9	pass
275	12	Bin9_plateA2_A8.fcs	3 (39)	9 (99)	9 (75)	9	pass
276	12	Bin9_plateA2_A9.fcs	8 (19)	7 (53)	7 (156)	9	pass
277	12	Bin9_plateA2_B3.fcs	3 (15)	7 (77)	10 (125)	9	pass
278	12	Bin9_plateA2_B4.fcs	4 (25)	4 (93)	6 (90)	9	pass
279	12	Bin9_plateA2_B7.fcs	4 (20)	7 (54)	6 (102)	9	pass
280	12	Bin9_plateA2_B11.fcs	10 (19)	9 (144)	4 (59)	9	pass
281	12	Bin9_plateA2_C11.fcs	6 (19)	3 (30)	6 (20)	9	pass
282	13	Bin9_plateA2_F8.fcs	1 (72)	4 (32)	7 (36)	9	pass
283	13	Bin9_plateA2_D5.fcs	8 (51)	7 (137)	10 (65)	9	pass
284	13	Bin9_plateA2_D10.fcs	6 (23)	5 (38)	7 (42)	9	pass
285	13	Bin9_plateA2_D12.fcs	6 (42)	2 (32)	8 (55)	9	pass
286	13	Bin9_plateA2_E7.fcs	4 (96)	10 (101)	7 (123)	9	pass
287	13	Bin9_plateA2_E8.fcs	2 (36)	9 (29)	7 (93)	9	pass
288	13	Bin9_plateA2_F4.fcs	7 (52)	8 (79)	8 (57)	9	pass
289	14	Bin10_plateA2_E1.fcs	3 (5)	-	-	10	fail
290	14	Bin10_plateA2_A1.fcs	3 (28)	4 (68)	10 (111)	10	pass
291	14	Bin10_plateA2_A10.fcs	10 (17)	10 (40)	6 (21)	10	pass
292	14	Bin10_plateA2_B1.fcs	4 (10)	9 (25)	3 (22)	10	pass
293	14	Bin10_plateA2_B2.fcs	8 (12)	7 (79)	4 (67)	10	pass
294	14	Bin10_plateA2_B6.fcs	7 (35)	7 (47)	7 (35)	10	pass
295	14	Bin10_plateA2_B7.fcs	10 (13)	8 (28)	2 (5)	10	pass
296	14	Bin10_plateA2_B11.fcs	8 (3)	-	-	10	fail
297	14	Bin10_plateA2_C6.fcs	-	-	-	10	fail
298	14	Bin10_plateA2_D6.fcs	-	5 (7)	-	10	fail
299	14	Bin10_plateA2_D10.fcs	-	-	-	10	fail
300	15	Bin10_plateA2_H8.fcs	6 (11)	2 (41)	8 (68)	10	pass
301	15	Bin10_plateA2_E7.fcs	1 (39)	8 (130)	4 (44)	10	pass
302	15	Bin10_plateA2_F6.fcs	10 (22)	3 (47)	9 (94)	10	pass
303	15	Bin10_plateA2_F7.fcs	2 (13)	4 (53)	9 (206)	10	pass
304	15	Bin10_plateA2_F9.fcs	6 (93)	7 (227)	4 (122)	10	pass
305	15	Bin10_plateA2_F12.fcs	8 (100)	9 (41)	8 (28)	10	pass
306	15	Bin10_plateA2_G2.fcs	10 (26)	3 (76)	10 (55)	10	pass
307	15	Bin10_plateA2_G6.fcs	4 (61)	2 (88)	6 (31)	10	pass
308	15	Bin10_plateA2_G8.fcs	-	-	5 (23)	10	fail
309	15	Bin10_plateA2_G12.fcs	4 (7)	-	6 (17)	10	fail
310	16	Bin10_plateA3_B4.fcs	5 (13)	-	5 (14)	10	fail
311	16	Bin10_plateA3_A5.fcs	1 (10)	2 (43)	-	10	fail
312	17	Bin8_plateA2_A6.fcs	4 (54)	5 (300)	4 (143)	8	pass
313	17	Bin3_plateA3_A3.fcs	-	6 (6)	2 (4)	3	fail
314	17	Bin4_plateA4_E4.fcs	4 (5)	1 (10)	5 (6)	4	pass
315	17	Bin5_plateA5_C8.fcs	-	7 (15)	8 (16)	5	fail

Confidential

6. PROTOCOL ANALYSIS

6.1 DATASET REVIEW

Once a dataset of protocols has been established, Ariadne™ allows post-acquisition resetting of gates on the COPAS sorting plot to specify a subset of hits for further analysis. For example, if ‘hits were sorted using two antibodies, the user can select those which are positive for both antibodies. No post acquisition flow sort criterion was applied in this study.

Table 9 lists the bead IDs included in the dataset analyzed by Ariadne™ and presented in the following sections of the report. The dataset only includes beads which fall within the (reset) COPAS gates and can be assigned a full cell culture history. Ariadne™ allows a range of analyses to be performed, to select protocols for validation. Analysis methodologies include linkage analysis which identifies frequently occurring media combinations; fingerprint analysis which clusters groups of beads with identical or similar protocols (i.e. protocols with common cell culture media on the same split) and calculates the probability of these clusters occurring randomly; and methods of comparing entire protocols such as hierarchical clustering and a similarity matrix. These results are presented and discussed in the following sections.

Table 9: Dataset Review. The following parameters are shown for each hit: Bead ID, protocol (“–” represents unreadable tag data), whether the bead passed the tag deconvolution, whether it falls within the (reset) COPAS gate and whether it is included in the protocol analysis dataset.

	Protocol				Inclusion Criteria		
Bead ID	Split 1 media	Split 2 media	Split 3 media	Split 4 media	Tag deconvolution	Flow sort criteria	Included in analysis
1	-	9	4	1	fail	pass	No
2	-	9	-	1	fail	pass	No
3	9	3	4	1	pass	pass	Yes
4	4	10	4	1	pass	pass	Yes
5	8	6	6	1	pass	pass	Yes
6	7	7	1	1	pass	pass	Yes
7	-	-	8	1	fail	pass	No
8	8	-	2	1	fail	pass	No
9	6	7	8	1	pass	pass	Yes
10	1	3	3	1	pass	pass	Yes
11	9	6	7	1	pass	pass	Yes
12	3	9	5	1	pass	pass	Yes
13	5	9	4	1	pass	pass	Yes
14	1	6	4	1	pass	pass	Yes
15	8	10	7	1	pass	pass	Yes
16	10	3	6	1	pass	pass	Yes
17	6	4	5	1	pass	pass	Yes
18	5	2	10	1	pass	pass	Yes
19	5	10	7	1	pass	pass	Yes
20	9	9	2	1	pass	pass	Yes
21	5	2	5	1	pass	pass	Yes
22	3	9	2	1	pass	pass	Yes
23	6	9	1	1	pass	pass	Yes
24	8	6	3	1	pass	pass	Yes
25	10	10	10	1	pass	pass	Yes
26	4	8	4	1	pass	pass	Yes
27	9	5	5	1	pass	pass	Yes
28	9	4	8	1	pass	pass	Yes
29	10	6	7	1	pass	pass	Yes
30	8	3	10	1	pass	pass	Yes
31	3	3	7	1	pass	pass	Yes

	Protocol				Inclusion Criteria		
Bead ID	Split 1 media	Split 2 media	Split 3 media	Split 4 media	Tag deconvolution	Flow sort criteria	Included in analysis
32	5	2	1	1	pass	pass	Yes
33	2	7	10	1	pass	pass	Yes
34	9	7	10	1	pass	pass	Yes
35	5	2	6	1	pass	pass	Yes
36	5	3	1	1	pass	pass	Yes
37	10	7	6	1	pass	pass	Yes
38	-	6	4	1	fail	pass	No
39	7	7	2	1	pass	pass	Yes
40	3	2	8	1	pass	pass	Yes
41	3	5	6	3	pass	pass	Yes
42	7	2	5	1	pass	pass	Yes
43	3	9	8	1	pass	pass	Yes
44	4	4	5	1	pass	pass	Yes
45	9	10	6	1	pass	pass	Yes
46	8	4	1	1	pass	pass	Yes
47	8	10	3	1	pass	pass	Yes
48	8	7	1	1	pass	pass	Yes
49	7	4	7	1	pass	pass	Yes
50	10	8	10	1	pass	pass	Yes
51	1	1	4	1	pass	pass	Yes
52	4	2	9	1	pass	pass	Yes
53	2	4	6	1	pass	pass	Yes
54	6	2	4	1	pass	pass	Yes
55	4	8	8	1	pass	pass	Yes
56	7	7	7	1	pass	pass	Yes
57	6	9	7	1	pass	pass	Yes
58	-	3	10	1	fail	pass	No
59	4	10	4	1	pass	pass	Yes
60	7	6	2	1	pass	pass	Yes
61	9	8	1	1	pass	pass	Yes
62	8	8	8	1	pass	pass	Yes
63	7	3	9	1	pass	pass	Yes
64	4	4	9	2	pass	pass	Yes
65	1	9	8	2	pass	pass	Yes
66	3	4	6	2	pass	pass	Yes
67	9	10	7	2	pass	pass	Yes
68	10	7	4	2	pass	pass	Yes
69	10	1	2	2	pass	pass	Yes
70	8	4	9	2	pass	pass	Yes
71	9	7	7	2	pass	pass	Yes
72	2	3	10	2	pass	pass	Yes
73	3	7	7	2	pass	pass	Yes
74	6	5	9	2	pass	pass	Yes
75	9	8	4	2	pass	pass	Yes
76	10	6	4	3	pass	pass	Yes
77	9	6	1	-	fail	pass	No
78	4	9	1	3	pass	pass	Yes
79	9	10	6	3	pass	pass	Yes
80	1	5	7	3	pass	pass	Yes
81	9	7	5	3	pass	pass	Yes
82	4	6	1	3	pass	pass	Yes
83	7	7	3	3	pass	pass	Yes
84	8	5	4	3	pass	pass	Yes
85	4	2	6	3	pass	pass	Yes

	Protocol				Inclusion Criteria		
Bead ID	Split 1 media	Split 2 media	Split 3 media	Split 4 media	Tag deconvolution	Flow sort criteria	Included in analysis
86	4	10	10	3	pass	pass	Yes
87	8	9	-	3	fail	pass	No
88	4	2	3	4	pass	pass	Yes
89	1	7	8	4	pass	pass	Yes
90	8	2	6	4	pass	pass	Yes
91	7	9	6	4	pass	pass	Yes
92	10	2	3	4	pass	pass	Yes
93	2	10	7	4	pass	pass	Yes
94	9	7	7	4	pass	pass	Yes
95	7	9	6	4	pass	pass	Yes
96	10	2	7	4	pass	pass	Yes
97	-	1	5	4	fail	pass	No
98	3	10	5	4	pass	pass	Yes
99	2	5	10	4	pass	pass	Yes
100	2	5	10	4	pass	pass	Yes
101	7	7	7	4	pass	pass	Yes
102	1	10	10	4	pass	pass	Yes
103	1	1	4	4	pass	pass	Yes
104	10	7	10	4	pass	pass	Yes
105	8	4	5	4	pass	pass	Yes
106	-	-	-	4	fail	pass	No
107	1	-	-	4	fail	pass	No
108	3	3	8	4	pass	pass	Yes
109	1	8	1	4	pass	pass	Yes
110	2	6	7	4	pass	pass	Yes
111	9	9	7	4	pass	pass	Yes
112	2	5	7	4	pass	pass	Yes
113	1	7	2	4	pass	pass	Yes
114	7	5	9	4	pass	pass	Yes
115	10	8	7	4	pass	pass	Yes
116	9	7	6	4	pass	pass	Yes
117	-	10	-	4	fail	pass	No
118	4	6	6	4	pass	pass	Yes
119	8	6	7	4	pass	pass	Yes
120	5	4	3	4	pass	pass	Yes
121	5	9	6	4	pass	pass	Yes
122	9	3	3	7	pass	pass	Yes
123	8	4	2	5	pass	pass	Yes
124	5	4	9	5	pass	pass	Yes
125	3	8	9	5	pass	pass	Yes
126	9	6	6	5	pass	pass	Yes
127	-	9	9	5	fail	pass	No
128	10	9	2	5	pass	pass	Yes
129	3	7	7	5	pass	pass	Yes
130	4	7	9	5	pass	pass	Yes
131	8	7	6	5	pass	pass	Yes
132	5	9	5	5	pass	pass	Yes
133	5	3	2	5	pass	pass	Yes
134	5	6	7	5	pass	pass	Yes
135	3	4	9	5	pass	pass	Yes
136	5	6	7	5	pass	pass	Yes
137	4	2	8	5	pass	pass	Yes
138	3	9	6	5	pass	pass	Yes
139	10	4	4	5	pass	pass	Yes

	Protocol				Inclusion Criteria		
Bead ID	Split 1 media	Split 2 media	Split 3 media	Split 4 media	Tag deconvolution	Flow sort criteria	Included in analysis
140	6	7	2	5	pass	pass	Yes
141	10	4	2	5	pass	pass	Yes
142	4	10	3	5	pass	pass	Yes
143	3	7	3	5	pass	pass	Yes
144	1	6	7	5	pass	pass	Yes
145	8	3	7	5	pass	pass	Yes
146	3	1	10	5	pass	pass	Yes
147	6	1	3	5	pass	pass	Yes
148	1	7	2	5	pass	pass	Yes
149	10	4	9	5	pass	pass	Yes
150	10	5	8	5	pass	pass	Yes
151	9	4	10	5	pass	pass	Yes
152	8	1	7	5	pass	pass	Yes
153	1	6	9	5	pass	pass	Yes
154	6	7	7	5	pass	pass	Yes
155	3	10	4	5	pass	pass	Yes
156	3	9	6	6	pass	pass	Yes
157	10	2	4	6	pass	pass	Yes
158	-	-	-	6	fail	pass	No
159	5	4	2	6	pass	pass	Yes
160	4	2	3	6	pass	pass	Yes
161	7	10	9	6	pass	pass	Yes
162	6	7	9	6	pass	pass	Yes
163	8	8	3	6	pass	pass	Yes
164	2	3	4	6	pass	pass	Yes
165	-	6	-	6	fail	pass	No
166	10	9	5	6	pass	pass	Yes
167	6	5	8	6	pass	pass	Yes
168	6	2	7	7	pass	pass	Yes
169	5	1	1	7	pass	pass	Yes
170	10	3	7	7	pass	pass	Yes
171	4	2	5	7	pass	pass	Yes
172	8	6	3	7	pass	pass	Yes
173	5	5	10	7	pass	pass	Yes
174	-	2	-	7	fail	pass	No
175	4	8	5	7	pass	pass	Yes
176	-	-	-	6	fail	pass	No
177	10	8	5	6	pass	pass	Yes
178	8	9	7	6	pass	pass	Yes
179	4	9	4	6	pass	pass	Yes
180	6	6	10	6	pass	pass	Yes
181	8	5	8	6	pass	pass	Yes
182	3	5	-	6	fail	pass	No
183	2	8	8	6	pass	pass	Yes
184	6	4	10	6	pass	pass	Yes
185	10	6	10	6	pass	pass	Yes
186	3	8	3	6	pass	pass	Yes
187	7	6	4	6	pass	pass	Yes
188	4	6	9	6	pass	pass	Yes
189	8	6	2	6	pass	pass	Yes
190	8	7	7	6	pass	pass	Yes
191	9	7	3	6	pass	pass	Yes
192	6	8	7	6	pass	pass	Yes
193	9	2	4	6	pass	pass	Yes

	Protocol				Inclusion Criteria		
Bead ID	Split 1 media	Split 2 media	Split 3 media	Split 4 media	Tag deconvolution	Flow sort criteria	Included in analysis
194	9	7	10	6	pass	pass	Yes
195	9	3	5	6	pass	pass	Yes
196	7	8	5	6	pass	pass	Yes
197	8	10	7	6	pass	pass	Yes
198	9	3	7	6	pass	pass	Yes
199	5	7	7	6	pass	pass	Yes
200	7	10	1	6	pass	pass	Yes
201	5	4	3	6	pass	pass	Yes
202	1	6	3	6	pass	pass	Yes
203	6	8	2	6	pass	pass	Yes
204	2	1	7	6	pass	pass	Yes
205	4	4	4	6	pass	pass	Yes
206	3	3	1	6	pass	pass	Yes
207	5	5	4	6	pass	pass	Yes
208	6	1	7	6	pass	pass	Yes
209	2	1	4	6	pass	pass	Yes
210	7	6	3	6	pass	pass	Yes
211	8	7	3	6	pass	pass	Yes
212	1	8	5	6	pass	pass	Yes
213	2	3	9	6	pass	pass	Yes
214	9	1	9	6	pass	pass	Yes
215	6	7	10	6	pass	pass	Yes
216	-	-	-	6	fail	pass	No
217	8	9	7	6	pass	pass	Yes
218	-	-	-	6	fail	pass	No
219	9	2	7	6	pass	pass	Yes
220	6	6	-	6	fail	pass	No
221	3	7	4	6	pass	pass	Yes
222	4	8	-	6	fail	pass	No
223	9	4	9	6	pass	pass	Yes
224	6	10	4	6	pass	pass	Yes
225	8	10	9	6	pass	pass	Yes
226	10	4	9	6	pass	pass	Yes
227	9	6	10	6	pass	pass	Yes
228	-	-	-	6	fail	pass	No
229	3	3	8	6	pass	pass	Yes
230	6	5	10	6	pass	pass	Yes
231	1	9	1	6	pass	pass	Yes
232	1	10	3	6	pass	pass	Yes
233	7	9	7	6	pass	pass	Yes
234	4	4	8	6	pass	pass	Yes
235	-	-	-	6	fail	pass	No
236	10	10	9	6	pass	pass	Yes
237	4	7	9	6	pass	pass	Yes
238	10	8	9	6	pass	pass	Yes
239	3	7	9	6	pass	pass	Yes
240	9	9	10	6	pass	pass	Yes
241	9	1	10	6	pass	pass	Yes
242	9	5	2	6	pass	pass	Yes
243	8	8	1	6	pass	pass	Yes
244	4	8	5	6	pass	pass	Yes
245	8	10	3	8	pass	pass	Yes
246	5	8	4	8	pass	pass	Yes
247	4	3	8	8	pass	pass	Yes

	Protocol				Inclusion Criteria		
Bead ID	Split 1 media	Split 2 media	Split 3 media	Split 4 media	Tag deconvolution	Flow sort criteria	Included in analysis
248	2	8	2	8	pass	pass	Yes
249	2	9	2	8	pass	pass	Yes
250	8	6	9	8	pass	pass	Yes
251	9	6	2	8	pass	pass	Yes
252	5	4	5	8	pass	pass	Yes
253	3	5	8	8	pass	pass	Yes
254	5	1	2	8	pass	pass	Yes
255	4	3	3	8	pass	pass	Yes
256	6	2	7	8	pass	pass	Yes
257	-	-	-	8	fail	pass	No
258	3	8	2	8	pass	pass	Yes
259	8	8	4	8	pass	pass	Yes
260	8	7	1	8	pass	pass	Yes
261	10	9	1	8	pass	pass	Yes
262	9	7	10	8	pass	pass	Yes
263	1	3	7	8	pass	pass	Yes
264	10	7	5	8	pass	pass	Yes
265	9	4	6	8	pass	pass	Yes
266	3	2	10	8	pass	pass	Yes
267	7	4	6	8	pass	pass	Yes
268	1	5	1	8	pass	pass	Yes
269	5	1	1	8	pass	pass	Yes
270	1	9	5	8	pass	pass	Yes
271	10	6	4	8	pass	pass	Yes
272	8	10	10	9	pass	pass	Yes
273	2	5	2	9	pass	pass	Yes
274	3	6	3	9	pass	pass	Yes
275	3	9	9	9	pass	pass	Yes
276	8	7	7	9	pass	pass	Yes
277	3	7	10	9	pass	pass	Yes
278	4	4	6	9	pass	pass	Yes
279	4	7	6	9	pass	pass	Yes
280	10	9	4	9	pass	pass	Yes
281	6	3	6	9	pass	pass	Yes
282	1	4	7	9	pass	pass	Yes
283	8	7	10	9	pass	pass	Yes
284	6	5	7	9	pass	pass	Yes
285	6	2	8	9	pass	pass	Yes
286	4	10	7	9	pass	pass	Yes
287	2	9	7	9	pass	pass	Yes
288	7	8	8	9	pass	pass	Yes
289	3	-	-	10	fail	pass	No
290	3	4	10	10	pass	pass	Yes
291	10	10	6	10	pass	pass	Yes
292	4	9	3	10	pass	pass	Yes
293	8	7	4	10	pass	pass	Yes
294	7	7	7	10	pass	pass	Yes
295	10	8	2	10	pass	pass	Yes
296	8	-	-	10	fail	pass	No
297	-	-	-	10	fail	pass	No
298	-	5	-	10	fail	pass	No
299	-	-	-	10	fail	pass	No
300	6	2	8	10	pass	pass	Yes
301	1	8	4	10	pass	pass	Yes

	Protocol				Inclusion Criteria		
Bead ID	Split 1 media	Split 2 media	Split 3 media	Split 4 media	Tag deconvolution	Flow sort criteria	Included in analysis
302	10	3	9	10	pass	pass	Yes
303	2	4	9	10	pass	pass	Yes
304	6	7	4	10	pass	pass	Yes
305	8	9	8	10	pass	pass	Yes
306	10	3	10	10	pass	pass	Yes
307	4	2	6	10	pass	pass	Yes
308	-	-	5	10	fail	pass	No
309	4	-	6	10	fail	pass	No
310	5	-	5	10	fail	pass	No
311	1	2	-	10	fail	pass	No
312	4	5	4	8	pass	pass	Yes
313	-	6	2	3	fail	pass	No
314	4	1	5	4	pass	pass	Yes
315	-	7	8	5	fail	pass	No

6. PROTOCOL ANALYSIS

6.2. LINKAGE ANALYSIS

A schematic diagram of the split-pool experiment is shown in Figure 5. Each rectangle corresponds to a cell culture medium and each row of rectangles corresponds to a split. The black upper number within each rectangle is the medium number and the grey number below is the number of beads within the analysis dataset that passed through that medium (the height of each rectangle is proportional to this number). The linkage lines between rectangles depict frequently occurring combinations of media resulting in an overlay of all protocols in the dataset. The opacity of the linkage lines is proportional to the number of protocols that feature a certain media combination (in this dataset the darkest lines correspond to 12 beads). An example of the information the linkage analysis provides is the highly represented transition between cell culture medium 9 (split 3) and medium 6 (split 4), 12 out of the 28 beads which start in medium 9 on split 3 transition to medium 6 on split 4. The high representation of this transition indicates that these media may be relevant for the differentiation of dopaminergic neurons from hES cells.

Figure 5: Schematic diagram showing an overlay of all protocols in the analysis dataset. The darkest line corresponds to 12 beads passing between the 2 connected cell culture media.

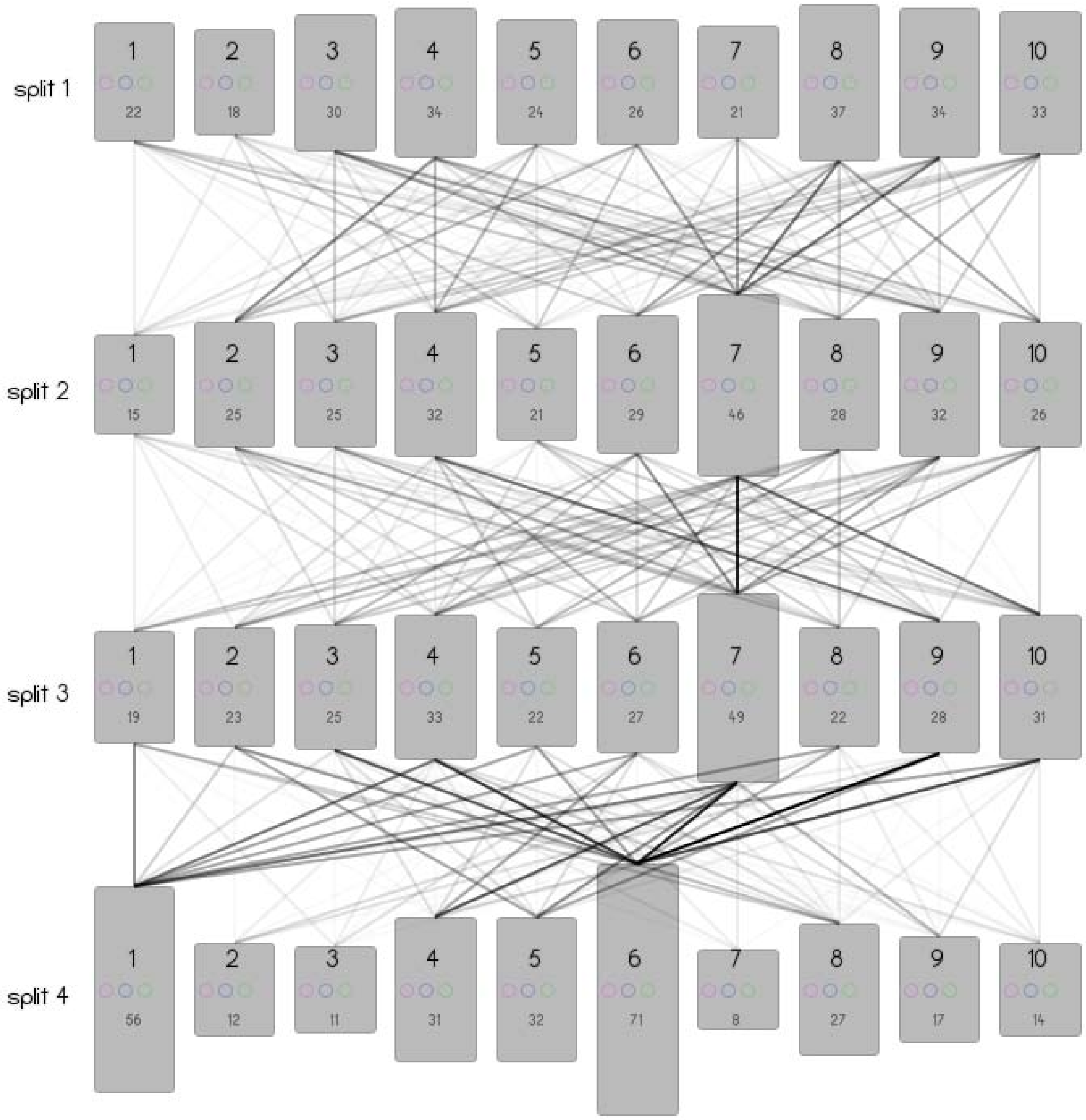

6. PROTOCOL ANALYSIS

6.3. FINGERPRINT ANALYSIS

Fingerprint analysis is a method of finding and comparing hits derived from identical protocols (i.e. four out of four matching cell culture media) and/or groups of beads with similar protocols (i.e. two or three matching media). This analysis method is complementary to linkage analysis which only considers linkages between adjacent splits.

Table 10 details a fingerprint analysis of the dataset. Each row of the table represents a group of hits clustered according to protocol similarity (group number identified in column 1). Column 2 identifies how many cell culture media the group has in common; column 3 how many hits are included in the group; and column 4 the probability of the group occurring by chance. Columns 5 - 8 identify the common media in the groups ("-" represents divergence of media), and column 9 lists the bead IDs included in the group. The table is ordered first by probability value and then by number of media matches.

The two groups which have the lowest probability of occurring by chance are two groups comprising 12 beads sharing two out of four media conditions. Both groups share cell culture media 6 on the final split. The probability of either of these groups occurring by chance is very low (p = 0.00313035). In contrast to the multiple groups clustered around medium 6 (split 4) two sets of groups exist which either feature medium 7 (split 2) or medium 1 (split 4), e.g. groups 3 and 10, respectively.

Therefore three clusters, or types, of protocols for the differentiation of dopaminergic neurons from hES cells have been identified. Two protocol types are dominated by common media on split 4 (medium 1 or 6), and a third protocol type dominated by common media on split 2 (media 7).

Table 10: Fingerprint analysis of protocols. Each row of the table represents a cluster of similar protocols . The second column identifies the number of common cell culture media in a group, the third column the number of hits included in the group, the fourth column the probability of the group occurring by chance, columns 5 – 8 the identity of the common media ("-" represents divergence of media), and the final column lists the beads which yielded the protocols included in the group. The data is sorted first by probability value then by number of media matches.

Group	Matches	Beads	Probability	Split1	Split2	Split3	Split4	BeadIDs
1	2	12	0.00313035	9	-	-	6	191,193,194,195,198,214,219,223,227,240,241,242
2	2	12	0.00313035	-	-	9	6	161,162,188,213,214,223,225,226,236,237,238,239
3	2	11	0.01409913	-	7	7	-	56,71,73,94,101,129,154,190,199,276,294
4	2	11	0.01409913	-	8	-	6	163,177,183,186,192,196,203,212,238,243,244
5	2	11	0.01409913	-	-	7	6	178,190,192,197,198,199,204,208,217,219,233
6	2	10	0.05759115	6	-	-	6	162,167,180,184,192,203,208,215,224,230
7	2	10	0.05759115	8	-	-	6	163,178,181,189,190,197,211,217,225,243
8	2	10	0.05759115	-	7	-	6	162,190,191,194,199,211,215,221,237,239
9	2	10	0.05759115	-	-	4	6	157,164,179,187,193,205,207,209,221,224
10	3	4	0.18085194	5	2	-	1	18,21,32,35
11	3	4	0.18085194	6	-	10	6	180,184,215,230
12	3	4	0.18085194	8	-	7	6	178,190,197,217
13	3	4	0.18085194	9	-	10	6	194,227,240,241
14	3	4	0.18085194	-	8	5	6	177,196,212,244
15	2	9	0.20333147	8	-	7	-	15,119,145,152,178,190,197,217,276
16	2	9	0.20333147	-	-	3	6	160,163,186,191,201,202,210,211,232
17	2	9	0.20333147	-	-	7	4	93,94,96,101,110,111,112,115,119
18	2	9	0.20333147	-	-	10	6	180,184,185,194,215,227,230,240,241
19	2	8	0.55133992	8	7	-	-	48,131,190,211,260,276,283,293
20	2	8	0.55133992	8	-	-	1	5,15,24,30,46,47,48,62
21	2	8	0.55133992	9	7	-	-	34,71,81,94,116,191,194,262
22	2	8	0.55133992	9	-	-	1	3,11,20,27,28,34,45,61
23	2	8	0.55133992	-	2	-	1	18,21,32,35,40,42,52,54
24	2	8	0.55133992	-	4	9	-	64,70,124,135,149,223,226,303

Group	Matches	Beads	Probability	Split1	Split2	Split3	Split4	BeadIDs
25	2	8	0.55133992	-	6	-	6	180,185,187,188,189,202,210,227
26	2	8	0.55133992	-	7	10	-	33,34,104,194,215,262,277,283
27	2	8	0.55133992	-	7	-	1	6,9,33,34,37,39,48,56
28	2	8	0.55133992	-	9	-	6	156,166,178,179,217,231,233,240
29	2	8	0.55133992	-	-	4	1	3,4,13,14,26,51,54,59
30	2	8	0.55133992	-	-	7	1	11,15,19,29,31,49,56,57
31	2	7	0.92770976	3	-	-	5	125,129,135,138,143,146,155
32	2	7	0.92770976	4	2	-	-	52,85,88,137,160,171,307
33	2	7	0.92770976	4	-	-	6	160,179,188,205,234,237,244
34	2	7	0.92770976	5	-	-	1	13,18,19,21,32,35,36
35	2	7	0.92770976	6	-	7	-	57,154,168,192,208,256,284
36	2	7	0.92770976	7	-	-	1	6,39,42,49,56,60,63
37	2	7	0.92770976	9	-	7	-	11,67,71,94,111,198,219
38	2	7	0.92770976	9	-	10	-	34,151,194,227,240,241,262
39	2	7	0.92770976	10	-	-	6	157,166,177,185,226,236,238
40	2	7	0.92770976	-	3	-	1	3,10,16,30,31,36,63
41	2	7	0.92770976	-	4	-	5	123,124,135,139,141,149,151
42	2	7	0.92770976	-	4	-	6	159,184,201,205,223,226,234
43	2	7	0.92770976	-	6	7	-	11,29,110,119,134,136,144
44	2	7	0.92770976	-	7	-	5	129,130,131,140,143,148,154
45	2	7	0.92770976	-	9	-	1	12,13,20,22,23,43,57
46	2	7	0.92770976	-	10	-	1	4,15,19,25,45,47,59
47	2	7	0.92770976	-	10	-	6	161,197,200,224,225,232,236
48	2	7	0.92770976	-	-	1	1	6,23,32,36,46,48,61
49	2	7	0.92770976	-	-	7	5	129,134,136,144,145,152,154
50	3	3	0.9525376	2	5	-	4	99,100,112
51	3	3	0.9525376	2	-	7	4	93,110,112
52	3	3	0.9525376	3	9	-	1	12,22,43
53	3	3	0.9525376	4	-	4	1	4,26,59
54	3	3	0.9525376	7	7	7	-	56,101,294
55	3	3	0.9525376	7	7	-	1	6,39,56
56	3	3	0.9525376	9	7	10	-	34,194,262
57	3	3	0.9525376	10	4	-	5	139,141,149
58	3	3	0.9525376	10	-	9	6	226,236,238
59	3	3	0.9525376	-	4	9	5	124,135,149
60	3	3	0.9525376	-	6	7	5	134,136,144
61	3	3	0.9525376	-	6	10	6	180,185,227
62	3	3	0.9525376	-	7	9	6	162,237,239
63	3	3	0.9525376	-	9	6	4	91,95,121
64	3	3	0.9525376	-	9	7	6	178,217,233
65	3	3	0.9525376	-	10	9	6	161,225,236
66	4	2	0.98003137	2	5	10	4	99,100
67	4	2	0.98003137	4	10	4	1	4,59
68	4	2	0.98003137	5	6	7	5	134,136
69	4	2	0.98003137	7	9	6	4	91,95
70	4	2	0.98003137	8	9	7	6	178,217
71	2	6	0.9997251	3	7	-	-	73,129,143,221,239,277
72	2	6	0.9997251	3	9	-	-	12,22,43,138,156,275
73	2	6	0.9997251	3	-	-	6	156,186,206,221,229,239
74	2	6	0.9997251	4	-	4	-	4,26,59,179,205,312
75	2	6	0.9997251	4	-	-	1	4,26,44,52,55,59
76	2	6	0.9997251	6	7	-	-	9,140,154,162,215,304
77	2	6	0.9997251	7	7	-	-	6,39,56,83,101,294
78	2	6	0.9997251	7	-	-	6	161,187,196,200,210,233
79	2	6	0.9997251	8	6	-	-	5,24,119,172,189,250
80	2	6	0.9997251	8	10	-	-	15,47,197,225,245,272
81	2	6	0.9997251	8	-	3	-	24,47,163,172,211,245
82	2	6	0.9997251	10	-	4	-	68,76,139,157,271,280
83	2	6	0.9997251	-	3	-	6	164,195,198,206,213,229
84	2	6	0.9997251	-	4	-	1	17,28,44,46,49,53
85	2	6	0.9997251	-	6	-	1	5,11,14,24,29,60

Confidential

Group	Matches	Beads	Probability	Split1	Split2	Split3	Split4	BeadIDs
86	2	6	0.9997251	-	7	-	4	89,94,101,104,113,116
87	2	6	0.9997251	-	9	7	-	57,111,178,217,233,287
88	2	6	0.9997251	-	10	7	-	15,19,67,93,197,286
89	2	6	0.9997251	-	-	2	5	123,128,133,140,141,148
90	2	6	0.9997251	-	-	5	1	12,17,21,27,42,44
91	2	6	0.9997251	-	-	5	6	166,177,195,196,212,244
92	2	6	0.9997251	-	-	6	1	5,16,35,37,45,53
93	2	6	0.9997251	-	-	6	4	90,91,95,116,118,121
94	2	6	0.9997251	-	-	8	1	9,28,40,43,55,62
95	2	6	0.9997251	-	-	9	5	124,125,130,135,149,153
96	2	6	0.9997251	-	-	10	1	18,25,30,33,34,50
97	3	2	0.99999999	1	1	4	-	51,103
98	3	2	0.99999999	1	6	-	5	144,153
99	3	2	0.99999999	1	7	2	-	113,148
100	3	2	0.99999999	1	7	-	4	89,113
101	3	2	0.99999999	1	-	3	6	202,232
102	3	2	0.99999999	1	-	4	1	14,51
103	3	2	0.99999999	2	1	-	6	204,209
104	3	2	0.99999999	2	3	-	6	164,213
105	3	2	0.99999999	2	5	10	-	99,100
106	3	2	0.99999999	2	-	2	8	248,249
107	3	2	0.99999999	2	-	4	6	164,209
108	3	2	0.99999999	2	-	10	4	99,100
109	3	2	0.99999999	3	3	8	-	108,229
110	3	2	0.99999999	3	3	-	6	206,229
111	3	2	0.99999999	3	7	7	-	73,129
112	3	2	0.99999999	3	7	-	5	129,143
113	3	2	0.99999999	3	7	-	6	221,239
114	3	2	0.99999999	3	9	6	-	138,156
115	3	2	0.99999999	3	-	8	1	40,43
116	3	2	0.99999999	3	-	9	5	125,135
117	3	2	0.99999999	4	2	3	-	88,160
118	3	2	0.99999999	4	2	6	-	85,307
119	3	2	0.99999999	4	3	-	8	247,255
120	3	2	0.99999999	4	4	-	6	205,234
121	3	2	0.99999999	4	7	9	-	130,237
122	3	2	0.99999999	4	8	5	-	175,244
123	3	2	0.99999999	4	8	-	1	26,55
124	3	2	0.99999999	4	10	4	-	4,59
125	3	2	0.99999999	4	10	-	1	4,59
126	3	2	0.99999999	4	-	1	3	78,82
127	3	2	0.99999999	4	-	4	6	179,205
128	3	2	0.99999999	4	-	5	7	171,175
129	3	2	0.99999999	4	-	6	9	278,279
130	3	2	0.99999999	4	-	9	6	188,237
131	3	2	0.99999999	5	1	1	-	169,269
132	3	2	0.99999999	5	1	-	8	254,269
133	3	2	0.99999999	5	4	3	-	120,201
134	3	2	0.99999999	5	4	-	6	159,201
135	3	2	0.99999999	5	6	7	-	134,136
136	3	2	0.99999999	5	6	-	5	134,136
137	3	2	0.99999999	5	-	1	1	32,36
138	3	2	0.99999999	5	-	7	5	134,136
139	3	2	0.99999999	6	2	7	-	168,256
140	3	2	0.99999999	6	2	8	-	285,300
141	3	2	0.99999999	6	5	-	6	167,230
142	3	2	0.99999999	6	7	-	5	140,154
143	3	2	0.99999999	6	7	-	6	162,215
144	3	2	0.99999999	6	8	-	6	192,203
145	3	2	0.99999999	6	9	-	1	23,57
146	3	2	0.99999999	6	-	7	6	192,208

Group	Matches	Beads	Probability	Split1	Split2	Split3	Split4	BeadIDs
147	3	2	0.99999999	7	6	-	6	187,210
148	3	2	0.99999999	7	9	6	-	91,95
149	3	2	0.99999999	7	9	-	4	91,95
150	3	2	0.99999999	7	10	-	6	161,200
151	3	2	0.99999999	7	-	2	1	39,60
152	3	2	0.99999999	7	-	6	4	91,95
153	3	2	0.99999999	7	-	7	1	49,56
154	3	2	0.99999999	8	6	3	-	24,172
155	3	2	0.99999999	8	6	-	1	5,24
156	3	2	0.99999999	8	7	1	-	48,260
157	3	2	0.99999999	8	7	7	-	190,276
158	3	2	0.99999999	8	7	-	6	190,211
159	3	2	0.99999999	8	7	-	9	276,283
160	3	2	0.99999999	8	8	-	6	163,243
161	3	2	0.99999999	8	9	7	-	178,217
162	3	2	0.99999999	8	9	-	6	178,217
163	3	2	0.99999999	8	10	3	-	47,245
164	3	2	0.99999999	8	10	7	-	15,197
165	3	2	0.99999999	8	10	-	1	15,47
166	3	2	0.99999999	8	10	-	6	197,225
167	3	2	0.99999999	8	-	1	1	46,48
168	3	2	0.99999999	8	-	3	1	24,47
169	3	2	0.99999999	8	-	3	6	163,211
170	3	2	0.99999999	8	-	7	5	145,152
171	3	2	0.99999999	8	-	10	9	272,283
172	3	2	0.99999999	9	1	-	6	214,241
173	3	2	0.99999999	9	2	-	6	193,219
174	3	2	0.99999999	9	3	-	6	195,198
175	3	2	0.99999999	9	7	7	-	71,94
176	3	2	0.99999999	9	7	-	4	94,116
177	3	2	0.99999999	9	7	-	6	191,194
178	3	2	0.99999999	9	10	6	-	45,79
179	3	2	0.99999999	9	-	7	2	67,71
180	3	2	0.99999999	9	-	7	4	94,111
181	3	2	0.99999999	9	-	7	6	198,219
182	3	2	0.99999999	9	-	9	6	214,223
183	3	2	0.99999999	10	2	-	4	92,96
184	3	2	0.99999999	10	3	-	10	302,306
185	3	2	0.99999999	10	4	9	-	149,226
186	3	2	0.99999999	10	6	4	-	76,271
187	3	2	0.99999999	10	8	-	6	177,238
188	3	2	0.99999999	10	-	2	5	128,141
189	3	2	0.99999999	10	-	5	6	166,177
190	3	2	0.99999999	10	-	6	1	16,37
191	3	2	0.99999999	10	-	7	4	96,115
192	3	2	0.99999999	10	-	10	1	25,50
193	3	2	0.99999999	-	1	7	6	204,208
194	3	2	0.99999999	-	2	3	4	88,92
195	3	2	0.99999999	-	2	4	6	157,193
196	3	2	0.99999999	-	2	5	1	21,42
197	3	2	0.99999999	-	4	2	5	123,141
198	3	2	0.99999999	-	4	5	1	17,44
199	3	2	0.99999999	-	4	6	8	265,267
200	3	2	0.99999999	-	4	9	2	64,70
201	3	2	0.99999999	-	4	9	6	223,226
202	3	2	0.99999999	-	5	8	6	167,181
203	3	2	0.99999999	-	5	10	4	99,100
204	3	2	0.99999999	-	6	3	6	202,210
205	3	2	0.99999999	-	6	7	1	11,29
206	3	2	0.99999999	-	6	7	4	110,119
207	3	2	0.99999999	-	7	1	1	6,48

Group	Matches	Beads	Probability	Split1	Split2	Split3	Split4	BeadIDs
208	3	2	0.99999999	-	7	2	5	140,148
209	3	2	0.99999999	-	7	3	6	191,211
210	3	2	0.99999999	-	7	4	10	293,304
211	3	2	0.99999999	-	7	7	2	71,73
212	3	2	0.99999999	-	7	7	4	94,101
213	3	2	0.99999999	-	7	7	5	129,154
214	3	2	0.99999999	-	7	7	6	190,199
215	3	2	0.99999999	-	7	10	1	33,34
216	3	2	0.99999999	-	7	10	6	194,215
217	3	2	0.99999999	-	7	10	9	277,283
218	3	2	0.99999999	-	8	2	8	248,258
219	3	2	0.99999999	-	8	3	6	163,186
220	3	2	0.99999999	-	8	4	8	246,259
221	3	2	0.99999999	-	8	8	1	55,62
222	3	2	0.99999999	-	9	2	1	20,22
223	3	2	0.99999999	-	10	4	1	4,59
224	3	2	0.99999999	-	10	7	1	15,19
225	2	5	0.99999999	1	-	-	4	89,102,103,109,113
226	2	5	0.99999999	2	-	7	-	93,110,112,204,287
227	2	5	0.99999999	2	-	-	4	93,99,100,110,112
228	2	5	0.99999999	2	-	-	6	164,183,204,209,213
229	2	5	0.99999999	3	-	8	-	40,43,108,229,253
230	2	5	0.99999999	3	-	-	1	12,22,31,40,43
231	2	5	0.99999999	4	4	-	-	44,64,205,234,278
232	2	5	0.99999999	4	10	-	-	4,59,86,142,286
233	2	5	0.99999999	4	-	3	-	88,142,160,255,292
234	2	5	0.99999999	4	-	5	-	44,171,175,244,314
235	2	5	0.99999999	4	-	6	-	85,118,278,279,307
236	2	5	0.99999999	4	-	9	-	52,64,130,188,237
237	2	5	0.99999999	5	4	-	-	120,124,159,201,252
238	2	5	0.99999999	5	-	-	5	124,132,133,134,136
239	2	5	0.99999999	6	2	-	-	54,168,256,285,300
240	2	5	0.99999999	6	-	-	1	9,17,23,54,57
241	2	5	0.99999999	7	-	7	-	49,56,101,233,294
242	2	5	0.99999999	9	-	6	-	45,79,116,126,265
243	2	5	0.99999999	10	8	-	-	50,115,177,238,295
244	2	5	0.99999999	10	-	9	-	149,226,236,238,302
245	2	5	0.99999999	10	-	10	-	25,50,104,185,306
246	2	5	0.99999999	10	-	-	1	16,25,29,37,50
247	2	5	0.99999999	10	-	-	5	128,139,141,149,150
248	2	5	0.99999999	-	1	-	6	204,208,209,214,241
249	2	5	0.99999999	-	3	7	-	31,145,170,198,263
250	2	5	0.99999999	-	4	6	-	53,66,265,267,278
251	2	5	0.99999999	-	5	-	6	167,181,207,230,242
252	2	5	0.99999999	-	6	3	-	24,172,202,210,274
253	2	5	0.99999999	-	6	-	5	126,134,136,144,153
254	2	5	0.99999999	-	8	4	-	26,75,246,259,301
255	2	5	0.99999999	-	8	5	-	175,177,196,212,244
256	2	5	0.99999999	-	8	-	1	26,50,55,61,62
257	2	5	0.99999999	-	9	6	-	91,95,121,138,156
258	2	5	0.99999999	-	-	2	8	248,249,251,254,258
259	2	5	0.99999999	-	-	7	9	276,282,284,286,287
260	2	5	0.99999999	-	-	8	6	167,181,183,229,234
261	2	4	0.99999999	1	6	-	-	14,144,153,202
262	2	4	0.99999999	1	-	4	-	14,51,103,301
263	2	4	0.99999999	1	-	7	-	80,144,263,282
264	2	4	0.99999999	1	-	-	6	202,212,231,232
265	2	4	0.99999999	2	5	-	-	99,100,112,273
266	2	4	0.99999999	2	-	10	-	33,72,99,100
267	2	4	0.99999999	3	3	-	-	31,108,206,229
268	2	4	0.99999999	3	-	6	-	41,66,138,156

Group	Matches	Beads	Probability	Split1	Split2	Split3	Split4	BeadIDs
269	2	4	0.99999999	3	-	9	-	125,135,239,275
270	2	4	0.99999999	3	-	10	-	146,266,277,290
271	2	4	0.99999999	4	8	-	-	26,55,175,244
272	2	4	0.99999999	4	-	8	-	55,137,234,247
273	2	4	0.99999999	4	-	-	3	78,82,85,86
274	2	4	0.99999999	5	2	-	-	18,21,32,35
275	2	4	0.99999999	5	-	1	-	32,36,169,269
276	2	4	0.99999999	5	-	7	-	19,134,136,199
277	2	4	0.99999999	5	-	-	6	159,199,201,207
278	2	4	0.99999999	5	-	-	8	246,252,254,269
279	2	4	0.99999999	6	5	-	-	74,167,230,284
280	2	4	0.99999999	6	-	8	-	9,167,285,300
281	2	4	0.99999999	6	-	10	-	180,184,215,230
282	2	4	0.99999999	7	-	-	4	91,95,101,114
283	2	4	0.99999999	8	4	-	-	46,70,105,123
284	2	4	0.99999999	8	8	-	-	62,163,243,259
285	2	4	0.99999999	8	-	1	-	46,48,243,260
286	2	4	0.99999999	8	-	-	5	123,131,145,152
287	2	4	0.99999999	8	-	-	8	245,250,259,260
288	2	4	0.99999999	9	3	-	-	3,122,195,198
289	2	4	0.99999999	9	4	-	-	28,151,223,265
290	2	4	0.99999999	9	6	-	-	11,126,227,251
291	2	4	0.99999999	10	3	-	-	16,170,302,306
292	2	4	0.99999999	10	4	-	-	139,141,149,226
293	2	4	0.99999999	10	6	-	-	29,76,185,271
294	2	4	0.99999999	10	7	-	-	37,68,104,264
295	2	4	0.99999999	10	9	-	-	128,166,261,280
296	2	4	0.99999999	10	-	2	-	69,128,141,295
297	2	4	0.99999999	10	-	7	-	29,96,115,170
298	2	4	0.99999999	10	-	-	4	92,96,104,115
299	2	4	0.99999999	10	-	-	10	291,295,302,306
300	2	4	0.99999999	-	2	6	-	35,85,90,307
301	2	4	0.99999999	-	2	7	-	96,168,219,256
302	2	4	0.99999999	-	2	8	-	40,137,285,300
303	2	4	0.99999999	-	2	-	4	88,90,92,96
304	2	4	0.99999999	-	2	-	6	157,160,193,219
305	2	4	0.99999999	-	4	5	-	17,44,105,252
306	2	4	0.99999999	-	5	8	-	150,167,181,253
307	2	4	0.99999999	-	5	10	-	99,100,173,230
308	2	4	0.99999999	-	5	-	4	99,100,112,114
309	2	4	0.99999999	-	6	4	-	14,76,187,271
310	2	4	0.99999999	-	7	2	-	39,113,140,148
311	2	4	0.99999999	-	7	3	-	83,143,191,211
312	2	4	0.99999999	-	7	4	-	68,221,293,304
313	2	4	0.99999999	-	7	6	-	37,116,131,279
314	2	4	0.99999999	-	7	9	-	130,162,237,239
315	2	4	0.99999999	-	7	-	9	276,277,279,283
316	2	4	0.99999999	-	8	2	-	203,248,258,295
317	2	4	0.99999999	-	8	8	-	55,62,183,288
318	2	4	0.99999999	-	8	-	8	246,248,258,259
319	2	4	0.99999999	-	9	1	-	23,78,231,261
320	2	4	0.99999999	-	9	2	-	20,22,128,249
321	2	4	0.99999999	-	9	5	-	12,132,166,270
322	2	4	0.99999999	-	9	-	4	91,95,111,121
323	2	4	0.99999999	-	10	3	-	47,142,232,245
324	2	4	0.99999999	-	10	4	-	4,59,155,224
325	2	4	0.99999999	-	10	10	-	25,86,102,272
326	2	4	0.99999999	-	-	1	6	200,206,231,243

6. PROTOCOL ANALYSIS
6.4. HIERACHICAL CLUSTERING DENDROGRAM

Hierarchical clustering is a method of grouping protocols according to similarity.

The hierarchical clustering illustrated in figure 6 has two sections. The upper dendrogram depicts the clustering structure and order. The lower coloured array is a graphical representation of the media combinations that comprise each protocol. The y-axis of the dendrogram measures intra-cluster similarity, i.e. horizontal bars closest to the bottom of the dendrogram link identical protocols whilst a horizontal bar half way up the y-axis represents a larger cluster containing beads whose protocols are not identical but do share some similarity. The hierarchical nature of the clustering means that beads are first clustered into small highly similar clusters, which are in turn included in larger clusters. This is repeated until the final cluster is reached (uppermost horizontal bar) which includes all beads in the dataset. Each node at the bottom of the dendrogram (leaf node) corresponds to a protocol. Each row of the coloured array corresponds to a split and each column of the array a protocol, with the columns aligned to the leaf nodes of the dendrogram. The horizontal coloured legend below the array specifies the colour associated with each media number. Three large clusters are labeled A, B and C. Cluster A contains 43 beads, all of which share medium 6 on split 4 and include the majority of the beads contained in groups 2, 5 and 6 of the fingerprint analysis (table 10). Cluster B includes beads which predominantly share medium 1 on the final split and also contains the four beads included in group 10 of the fingerprint analysis which share medium 5 (split 1), medium 2 (split 2) as well as media 1 on the final split. Cluster C contains 27 beads, 21 of which share medium 7 on the second split. The probabilities of these groups occurring by chance are discussed previously in section 6.3.

Figure 6: Dendrogram showing the hierarchical clustering of similar protocols.

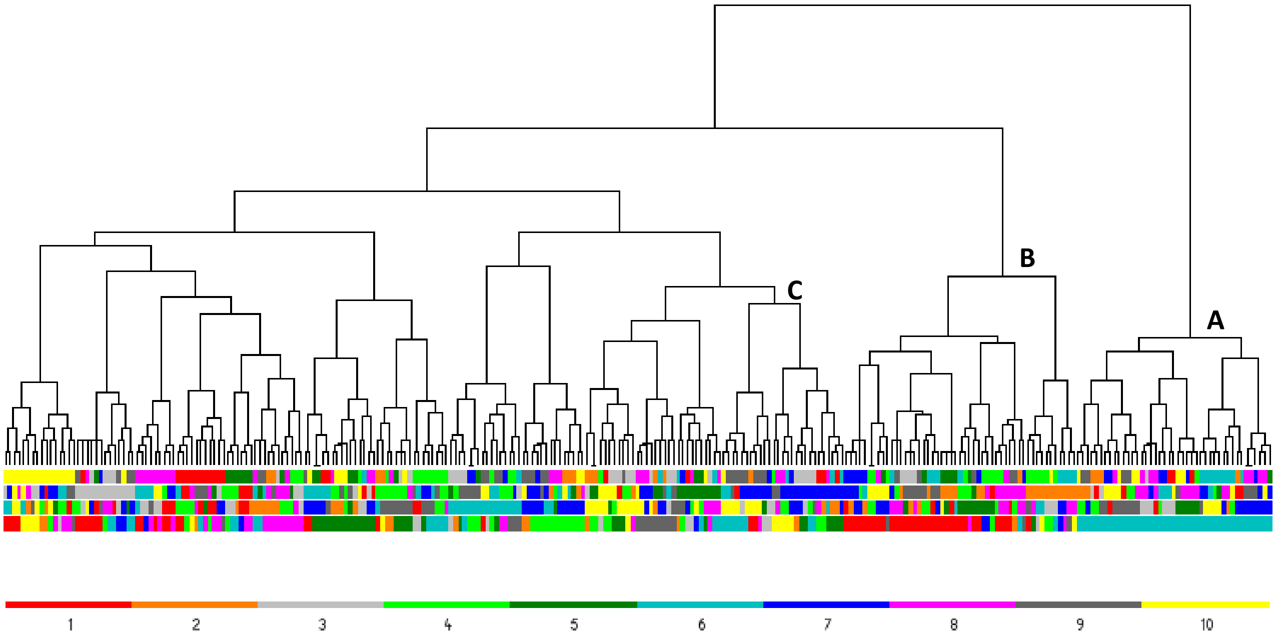

 Confidential

6. PROTOCOL ANALYSIS
6.5. SIMILARITY MATRIX

The similarity matrix is a diagrammatic representation of a pair-wise comparison of all protocols. Each column and each row corresponds to a protocol. The brightness of each cell in the matrix is proportional to the number of cell culture media shared by the two protocols. The brightest cell corresponds to protocols which have common media (i.e. identical protocols) in common, while a black cell corresponds to two protocols with no common media. The diagonal row of cells (from the top left to bottom right) corresponds to beads being compared to themselves. Beads are ordered along both the x- and y-axis according to the hierarchical clustering dendrogram illustrated in figure 6. The coloured arrays on the bottom and right of the matrix illustrate the protocol of each bead. The horizontal coloured legend below the matrix specifies the colour associated with each medium number.

The similarity matrix clearly displays clusters of similar protocols as square regions comprising brightly coloured cells. The size and overall brightness of the square regions are measures of the cluster size and intra-cluster protocol similarity, respectively. Additionally, the similarity matrix provides a global overview of the protocol similarity of beads or clusters to the remaining dataset. For example the brightness of the cells in the columns above cluster A indicate that the beads contained within cluster A also have similarity with other beads in the dataset, including some beads of cluster C. Interestingly, beads in cluster A have little similarity to beads of cluster B.

Figure 7: Similarity matrix comprising a pair-wise comparison of all protocols

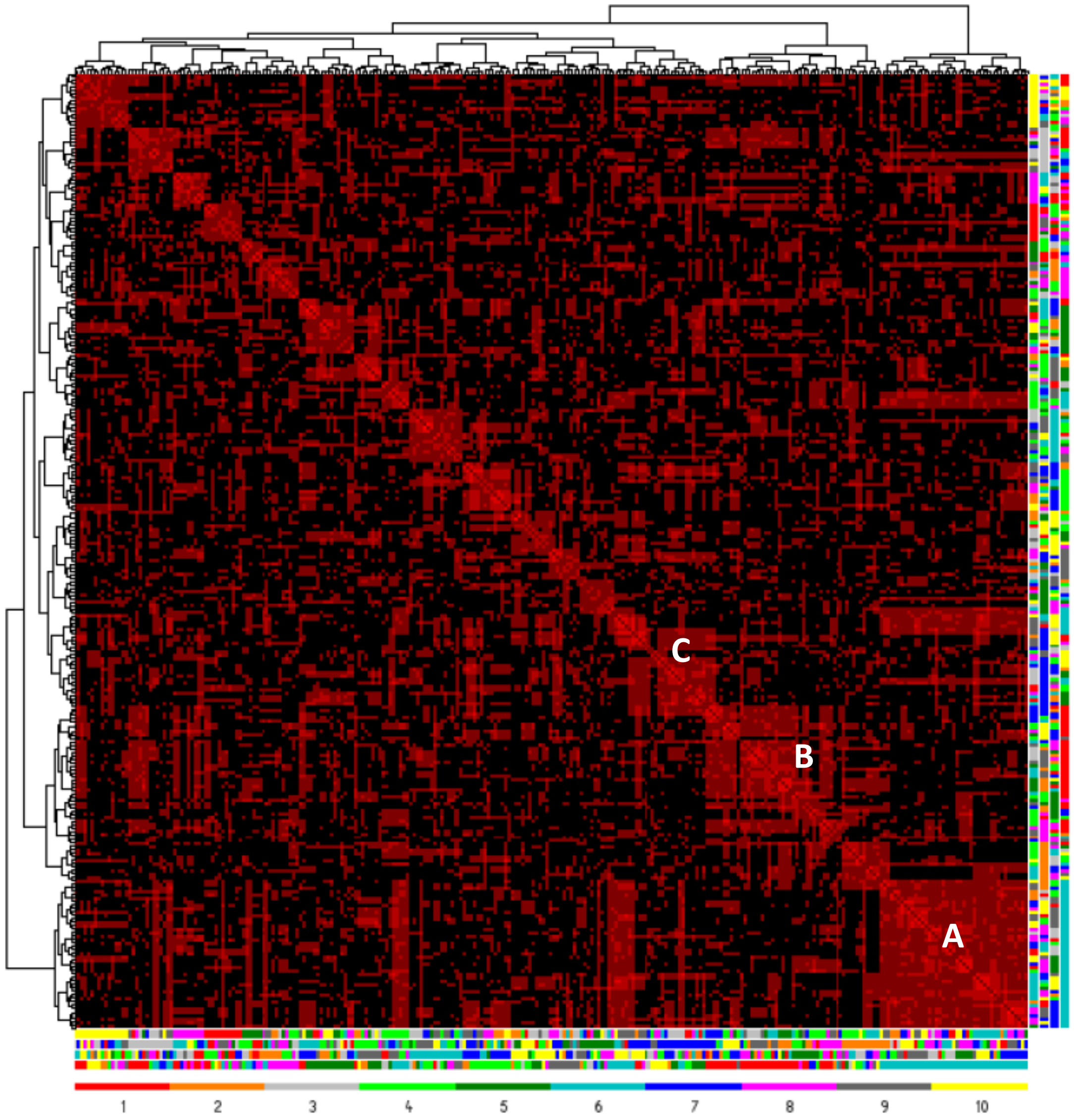

7. SUMMARY AND CONCLUSIONS

A CombiCult™ experiment was performed in which 40 serum-free cell culture media were screened in 10,000 combinations. The 40 media were tested over four split-pool cycles (performed on day 2, day 9, day 16 and day 23 respectively) with 10 media included in each cycle, such that 10,000 media combinations (protocols) are tested. A total of 480,000 beads were used so that on average each protocol was sampled by 48 beads. Following completion of the split-pool experiment (day 30) beads were screened using an immunocytochemistry assay and individual positive beads isolated.

Of the total number of monomeric beads sorted by COPAS 367 (0.13 %) were sorted and verified as brightest 'hits' with a green fluorescence value ≥ 136. The tags from each hit were read to infer the series of cell culture media (protocol) that produced differentiated cells. Of the 367 'hit' beads, tags could be conclusively deconvoluted from 279 (76 %). Putative differentiation protocols were analysed using bespoke bioinformatics software Ariadne™ (v1.0) allowing protocols to be ranked and a subset to be selected for further validation and study. The 21 selected protocols are listed below in Table 11 as well as in the executive summary (Table 1).

The validated protocols numbered 1 - 9 form part of cluster A shown in the hierarchical clustering dendrogram and similarity matrix and share cell culture medium 6 on split 4. The first two validated protocols are members of group 2 in the fingerprint analysis (table 10), and the third and fourth validated protocols are members of group 1 in the fingerprint analysis (table 10). Both groups have a low probability of occurring randomly (p = 0.00313035). The validated protocols numbered 10 - 13 form part of cluster B of the hierarchical clustering dendrogram and similarity matrix and share cell culture medium 1 on split 4. Protocols 14 - 15 have a third general type of protocol. They share cell culture medium 7 on splits 2 and 3 (group 5 in the fingerprint analysis, p = 0.01409913) and is a linkage clearly observed in the pathway analysis diagram (figure 5).

The 21 validated protocols therefore include three general types of protocol for the differentiation of dopaminergic neurons from hES cells. One is dominated by media 6 on split 4, the second is dominated by media 1 on split 4 and the final type dominated by media 7 on split 2.

Table 11: Top 21 protocols determined by Ariadne™ (v1.0). The protocols are identified by the series of cell culture media on each split-pool cycle (split 1 – split 4), e.g. 10-8-5-6.

Validation number	Protocol	Bead Ids		Basal Medium	Supplements
1	10-4-9-6	226	Split 1 (Day 2)	DMEM/F12	1% BSA, 2mM Glutamine, 1X ITS suppl. 0.5μM retinoic acid
			Split 2 (Day 8)	DMEM/F12	2mM Glutamine, 1X ITS suppl., 0.5μM retinoic acid
			Split 3 (Day 16)	Advanced DMEM	2mM Glutamine
			Split 4 (Day 22)	DMEM/F12	2mM Glutamine, 1X N2 suppl.,
2	10-10-9-6	236	Split 1 (Day 2)	DMEM/F12	1% BSA, 2mM Glutamine, 1X ITS suppl. 0.5μM retinoic acid
			Split 2 (Day 8)	DMEM/F12	2mM Glutamine, 1X ITS suppl., 20ng/mL bFGF, 100ng/mL FGF8b, 200μM ascorbic acid, 10ng/mL BDNF, 1μM purmorphamine
			Split 3 (Day 16)	Advanced DMEM	2mM Glutamine
			Split 4 (Day 22)	DMEM/F12	2mM Glutamine, 1X N2 suppl.,
3	9-7-3-6	191	Split 1 (Day 2)	DMEM/F12	1% BSA, 2mM Glutamine, 1X N2 suppl. 20μM SB431542
			Split 2 (Day 8)	DMEM/F12	2mM Glutamine, 1X ITS suppl., 1X B27 Neuromix, 20ng/mL bFGF, 50ng/mL hr DKK1.
			Split 3 (Day 16)	DMEM/F12	2mM Glutamine, 1X ITS suppl., 100ng/mL FGF8b, 500ng/mL hr Shh.
			Split 4 (Day 22)	DMEM/F12	2mM Glutamine, 1X N2 suppl.,
4	9-7-10-6	194	Split 1 (Day 2)	DMEM/F12	1% BSA, 2mM Glutamine, 1X N2 suppl. 20μM SB431542

Validation number	Protocol	Bead Ids		Basal Medium	Supplements
			Split 2 (Day 8)	DMEM/F12	2mM Glutamine, 1X ITS suppl., 1X B27 Neuromix, 20ng/mL bFGF, 50ng/mL hr DKK1.
			Split 3 (Day 16)	DMEM/F12	2mM Glutamine, 1X N2 suppl., 20ng/mL bFGF, 20ng/mL EGF, 2ng/mL LIF
			Split 4 (Day 22)	DMEM/F12	2mM Glutamine, 1X N2 suppl.,
5	8-9-7-6	178, 217	Split 1 (Day 2)	DMEM/F12	1% BSA, 2mM Glutamine, 1X N2 suppl. 200ng/mL hr Noggin.
			Split 2 (Day 8)	DMEM/F12	2mM Glutamine, 1X ITS suppl., 100ng/mL FGF8b, 500ng/mL hr Shh.
			Split 3 (Day 16)	DMEM/F12	2mM Glutamine, 1X ITS suppl., 1X B27 neuromix, 20ng/mL bFGF, 50ng/mL hr DKK1.
			Split 4 (Day 22)	DMEM/F12	2mM Glutamine, 1X N2 suppl.,
6	6-6-10-6	180	Split 1 (Day 2)	DMEM/F12	1% BSA, 2mM Glutamine, 1X N2 suppl. 200ng/mL hr Noggin, 20µM SB431542
			Split 2 (Day 8)	DMEM/F12	2mM Glutamine, 1X N2 suppl.
			Split 3 (Day 16)	DMEM/F12	2mM Glutamine, 1X N2 suppl., 20ng/mL bFGF, 20ng/mL EGF, 2ng/mL LIF
			Split 4 (Day 22)	DMEM/F12	2mM Glutamine, 1X N2 suppl.,
7	6-4-10-6	184	Split 1 (Day 2)	DMEM/F12	1% BSA, 2mM Glutamine, 1X N2 suppl. 200ng/mL hr Noggin, 20µM SB431542
			Split 2 (Day 8)	DMEM/F12	2mM Glutamine, 1X ITS suppl., 0.5mM retinoic acid.
			Split 3 (Day 16)	DMEM/F12	2mM Glutamine, 1X N2 suppl., 20ng/mL bFGF, 20ng/mL EGF, 2ng/mL LIF
			Split 4 (Day 22)	DMEM/F12	2mM Glutamine, 1X N2 suppl.
8	3-9-6-6	156	Split 1(Day 2)	Advanced DMEM	2mM Glutamine
			Split 2 (Day 8)	DMEM/F12	2mM Glutamine, 1X ITS suppl., 100ng/mL FGF8b, 500ng/mL hr Shh.
			Split 3 (Day 16)	DMEM/F12	2mM Glutamine, 1X N2 suppl.
			Split 4 (Day 22)	DMEM/F12	2mM Glutamine, 1X N2 suppl.
9	4-4-8-6	234	Split 1 (Day 2)	DMEM/F12	1% BSA, 1X N2, 1X B37 neuromix, 2mM Glutamine
			Split 2 (Day 8)	DMEM/F12	2mM Glutamine, 1X ITS suppl., 0.5µM retinoic acid
			Split 3 (Day 16)	Neurobasal	2mM Glutamine, 1X B37 neuromix, 20ng/mL bFGF
			Split 4 (Day 22)	DMEM/F12	2mM Glutamine, 1X N2 suppl.
10	5-2-10-1	18	Split 1 (Day 2)	RHB-A	
			Split 2 (Day 8)	DMEM/F12	2mM Glutamine, 1X ITS suppl., 200ng/mL hr Noggin.
			Split 3 (Day 16)	DMEM/F12	2mM Glutamine, 1X N2 suppl., 20ng/mL bFGF, 20ng/mL EGF, 2ng/mL LIF
			Split 4 (Day 22)	DMEM/F12	2mM Glutamine, 1X N2 suppl., 1mg/mL laminin, 200µM ascorbic acid
11	6-9-1-1	23	Split 1 (Day 2)	DMEM/F12	1% BSA, 2mM Glutamine, 1X N2 suppl. 200ng/mL hr Noggin, 20µM SB431542
			Split 2 (Day 8)	DMEM/F12	2mM Glutamine, 1X ITS suppl., 100ng/mL FGF8b, 500ng/mL hr Shh.
			Split 3 (Day 16)	DMEM/F12	2mM Glutamine, 1X ITS suppl., 20ng/mL bFGF
			Split 4 (Day 22)	DMEM/F12	2mM Glutamine, 1X N2 suppl., 1mg/mL laminin, 200µM ascorbic acid
12	8-10-7-1	15	Split 1 (Day 2)	DMEM/F12	1% BSA, 2mM Glutamine, 1X N2 suppl. 200ng/mL hr Noggin.
			Split 2 (Day 8)	DMEM/F12	2mM Glutamine, 1X ITS suppl., 20ng/mL bFGF, 100ng/mL FGF8b, 200µM ascorbic acid, 10ng/mL BDNF, 1µM purmorphamine
			Split 3 (Day 16)	DMEM/F12	2mM Glutamine, 1X ITS suppl., 1X B27 neuromix, 20ng/mL bFGF, 50ng/mL hr DKK1.
			Split 4 (Day 22)	DMEM/F12	2mM Glutamine, 1X N2 suppl., 1mg/mL laminin, 200µM ascorbic acid
13	4-10-4-1	4, 59	Split 1 (Day 2)	DMEM/F12	1% BSA, 1X N2, 1X B37 neuromix, 2mM Glutamine
			Split 2 (Day 8)	DMEM/F12	2mM Glutamine, 1X ITS suppl., 20ng/mL bFGF, 100ng/mL FGF8b, 200mM ascorbic acid, 10ng/mL BDNF, 1µM purmorphamine
			Split 3 (Day 16)	Neurobasal media	2mM Glutamine, 1X ITS suppl., 20ng/mL BDNF, 10ng/mL GDNF, 0.5mM cAMP, 200µM ascorbic acid, 1ng/mL TGFb
			Split 4 (Day 22)	DMEM/F12	2mM Glutamine, 1X N2 suppl., 1mg/mL laminin, 200µM ascorbic acid

Validation number	Protocol	Bead Ids		Basal Medium	Supplements
14	6-7-7-5	154	Split 1 (Day 2)	DMEM/F12	1% BSA, 2mM Glutamine, 1X N2 suppl. 200ng/mL hr Noggin, 20μM SB431542
			Split 2 (Day 8)	DMEM/F12	2mM Glutamine, 1X ITS suppl., 1X B27 Neuromix, 20ng/mL bFGF, 50ng/mL hr DKK1.
			Split 3 (Day 16)	DMEM/F12	2mM Glutamine, 1X ITS suppl., 1X B27 neuromix, 20ng/mL bFGF, 50ng/mL hr DKK1.
			Split 4 (Day 22)	RHB-A	
15	7-7-7-4	101	Split 1 (Day 2)	DMEM/F12	1% BSA, 2mM Glutamine, 1X N2 suppl., 20 ng/mL bFGF
			Split 2 (Day 8)	DMEM/F12	2mM Glutamine, 1X ITS suppl., 1X B27 Neuromix, 20ng/mL bFGF, 50ng/mL hr DKK1.
			Split 3 (Day 16)	DMEM/F12	2mM Glutamine, 1X ITS suppl., 1X B27 neuromix, 20ng/mL bFGF, 50ng/mL hr DKK1.
			Split 4 (Day 22)	DMEM/F12	2mM Glutamine, 1X N2 suppl., 0.5μM cAMP, 10ng/mL BDNF
16	10-7-10-4	104	Split 1 (Day 2)	DMEM/F12	1% BSA, 2mM Glutamine, 1X ITS suppl. 0.5μM retinoic acid
			Split 2 (Day 8)	DMEM/F12	2mM Glutamine, 1X ITS suppl., 1X B27 Neuromix, 20ng/mL bFGF, 50ng/mL hr DKK1.
			Split 3 (Day 16)	DMEM/F12	2mM Glutamine, 1X N2 suppl., 20ng/mL bFGF, 20ng/mL EGF, 2ng/mL LIF
			Split 4 (Day 22)	DMEM/F12	2mM Glutamine, 1X N2 suppl., 0.5μM cAMP, 10ng/mL BDNF
17	8-6-3-7	172	Split 1 (Day 2)	DMEM/F12	1% BSA, 2mM Glutamine, 1X N2 suppl. 200ng/mL hr Noggin.
			Split 2 (Day 8)	DMEM/F12	2mM Glutamine, 1X N2 suppl.
			Split 3 (Day 16)	DMEM/F12	2mM Glutamine, 1X ITS suppl., 100ng/mL FGF8b, 500ng/mL hr Shh.
			Split 4 (Day 22)	Neurobasal media	2mM Glutamine, 1X B27 neuromix
18	5-4-9-5	124	Split 1 (Day 2)	RHB-A	
			Split 2 (Day 8)	DMEM/F12	2mM Glutamine, 1X ITS suppl., 0.5μM retinoic acid.
			Split 3 (Day 16)	Advanced DMEM	2mM Glutamine
			Split 4 (Day 22)	RHB-A	
19	2-5-10-4	99, 100	Split 1 (Day 2)	DMEM	10% FBS, 2mM Glutamine, 1X NEAA
			Split 2 (Day 8)	RHB-A	
			Split 3 (Day 16)	DMEM/F12	2mM Glutamine, 1X N2 suppl., 20ng/mL bFGF, 20ng/mL EGF, 2ng/mL LIF
			Split 4 (Day 22)	DMEM/F12	2mM Glutamine, 1X N2 suppl., 0.5μM cAMP, 10ng/mL BDNF
20	5-6-7-5	134, 136	Split 1 (Day 2)	RHB-A	
			Split 2 (Day 8)	DMEM/F12	2mM Glutamine, 1X N2 suppl.
			Split 3 (Day 16)	DMEM/F12	2mM Glutamine, 1X ITS suppl., 1X B27 neuromix, 20ng/mL bFGF, 50ng/mL hr DKK1.
			Split 4 (Day 22)	RHB-A	
21	7-9-6-4	91, 95	Split 1 (Day 2)	DMEM/F12	1% BSA, 2mM Glutamine, 1X N2 suppl., 20 ng/mL bFGF
			Split 2 (Day 8)	DMEM/F12	2mM Glutamine, 1X ITS suppl., 100ng/mL FGF8b, 500ng/mL hr Shh.
			Split 3 (Day 16)	DMEM/F12	2mM Glutamine, 1X N2 suppl.,
			Split 4 (Day 22)	DMEM/F12	2mM Glutamine, 1X N2 suppl., 0.5μM cAMP, 10ng/mL BDNF

ANNEX 1: FLOW SORTING BIN 1 – File 1 of 1

Bin 1: COPAS scatter plots showing events for medium 1 of the final split-pool cycle (bin 1). In the first scatter plot (time of flight [TOF] vs. optical extinction [ext]), events depicted by a red dot fall within the gate defining monomeric beads and were sorted. The second scatter plot displays the fluorescent values of the sorted beads (TOF vs. green fluorescence intensity). Beads with a red fluorescent intensity above the threshold value are depicted as green dots and were individually dispensed into 96 well plates.

Number of beads individually dispensed: 210

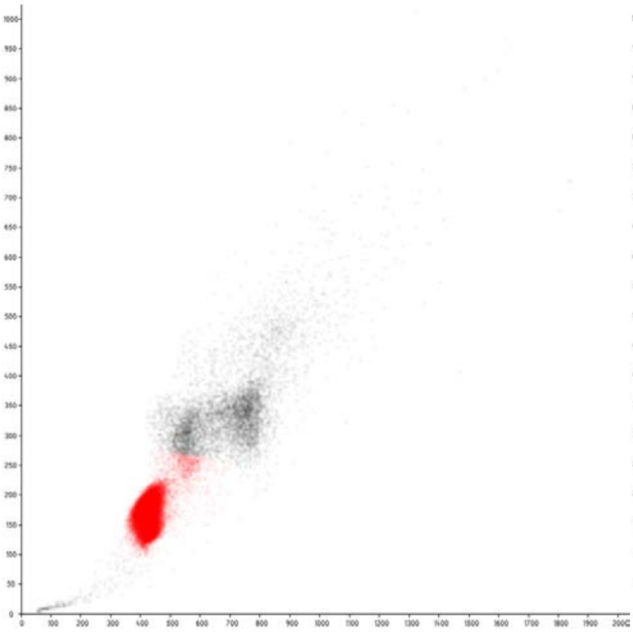

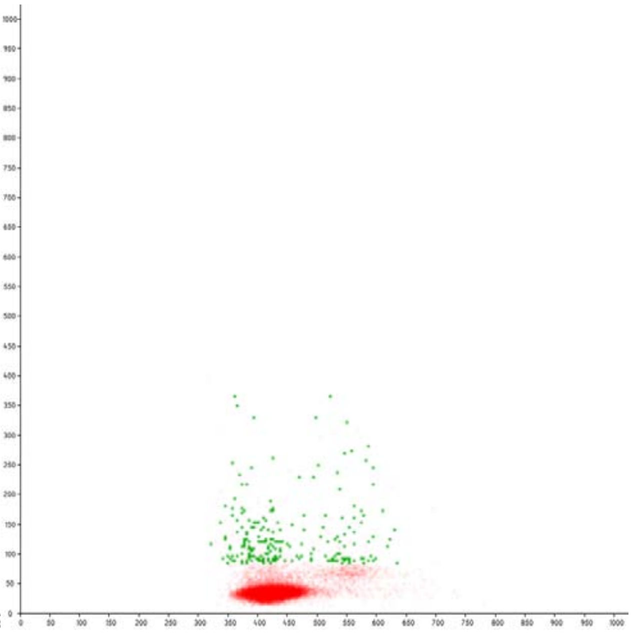

ANNEX 1: FLOW SORTING BIN 2 – File 1 of 3

Bin 2: COPAS scatter plots showing events for medium 2 of the final split-pool cycle (bin 2). In the first scatter plot (time of flight [TOF] vs. optical extinction [ext]), events depicted by a red dot fall within the gate defining monomeric beads and were sorted. The second scatter plot displays the fluorescent values of the sorted beads (TOF vs. green fluorescence intensity). Beads with a red fluorescent intensity above the threshold value are depicted as green dots and were individually dispensed into 96 well plates.

Number of beads individually dispensed: 52

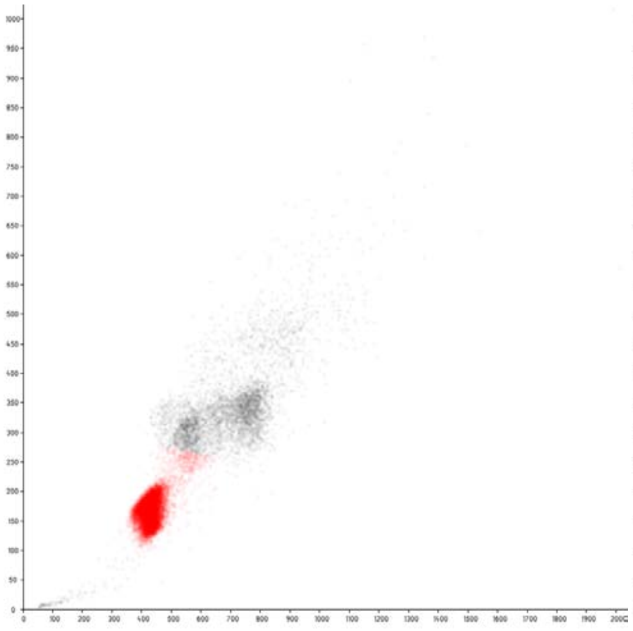

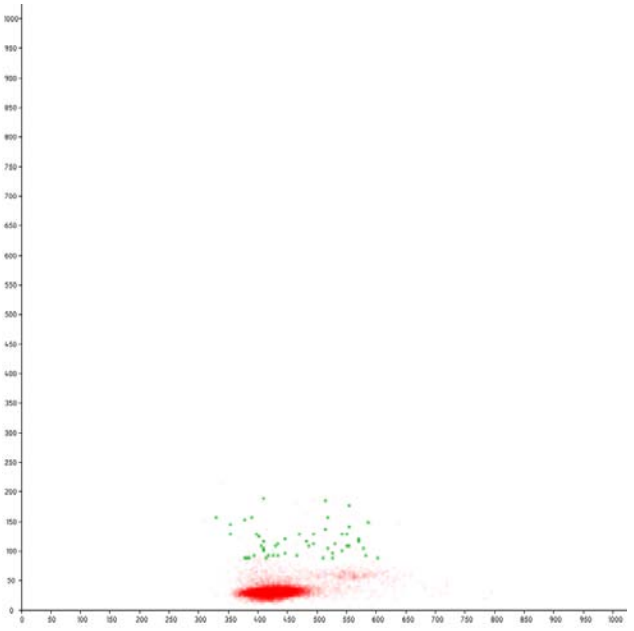

ANNEX 1: FLOW SORTING BIN 2 – File 2 of 3

Bin 2: COPAS scatter plots showing events for medium 2 of the final split-pool cycle (bin 2). In the first scatter plot (time of flight [TOF] vs. optical extinction [ext]), events depicted by a red dot fall within the gate defining monomeric beads and were sorted. The second scatter plot displays the fluorescent values of the sorted beads (TOF vs. green fluorescence intensity). Beads with a red fluorescent intensity above the threshold value are depicted as green dots and were individually dispensed into 96 well plates.

Number of beads individually dispensed: 0

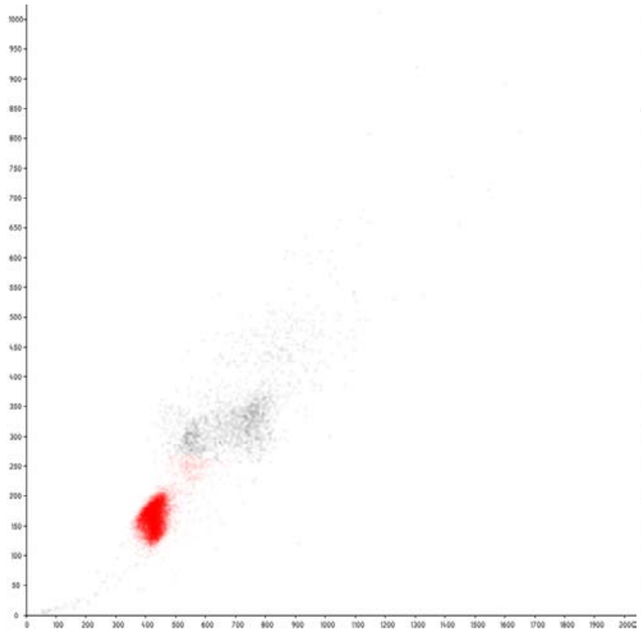

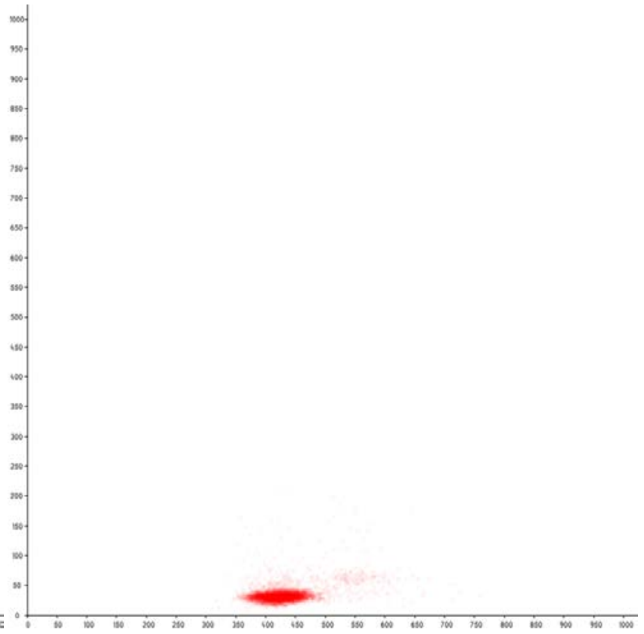

ANNEX 1: FLOW SORTING BIN 2 – File 3 of 3

Bin 2: COPAS scatter plots showing events for medium 2 of the final split-pool cycle (bin 2). In the first scatter plot (time of flight [TOF] vs. optical extinction [ext]), events depicted by a red dot fall within the gate defining monomeric beads and were sorted. The second scatter plot displays the fluorescent values of the sorted beads (TOF vs. green fluorescence intensity). Beads with a red fluorescent intensity above the threshold value are depicted as green dots and were individually dispensed into 96 well plates.

Number of beads individually dispensed: 6

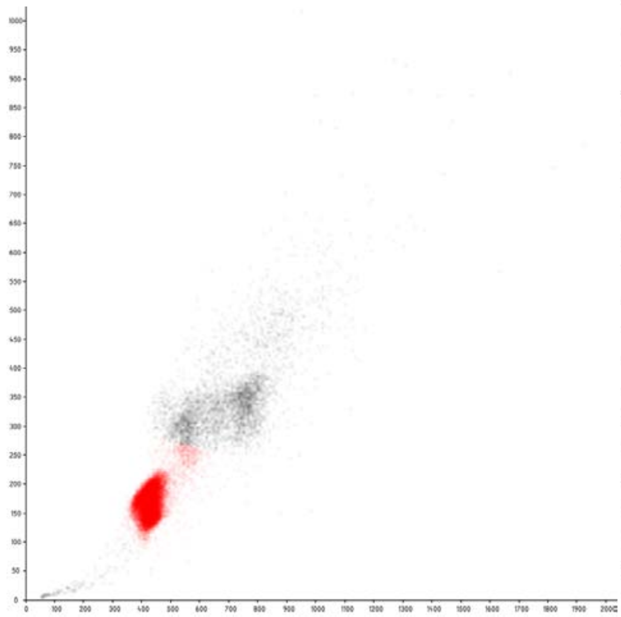

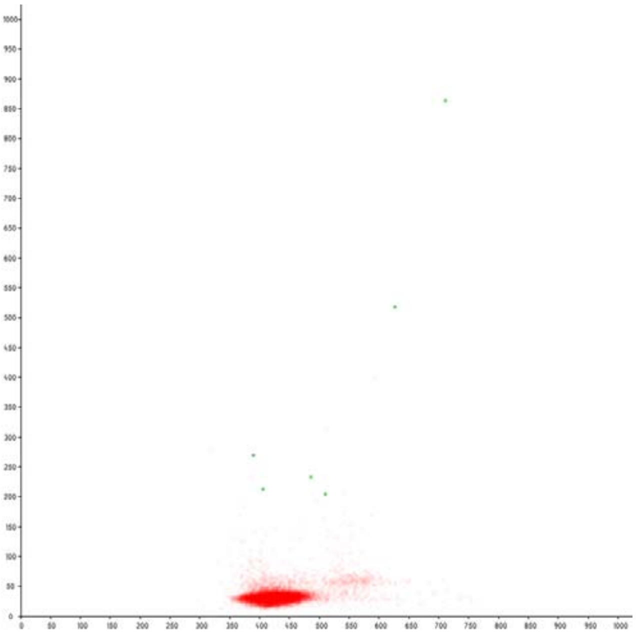

ANNEX 1: FLOW SORTING BIN 3 – File 1 of 2

Bin 3: COPAS scatter plots showing events for medium 3 of the final split-pool cycle (bin 3). In the first scatter plot (time of flight [TOF] vs. optical extinction [ext]), events depicted by a red dot fall within the gate defining monomeric beads and were sorted. The second scatter plot displays the fluorescent values of the sorted beads (TOF vs. green fluorescence intensity). Beads with a red fluorescent intensity above the threshold value are depicted as green dots and were individually dispensed into 96 well plates.

Number of beads individually dispensed: 21

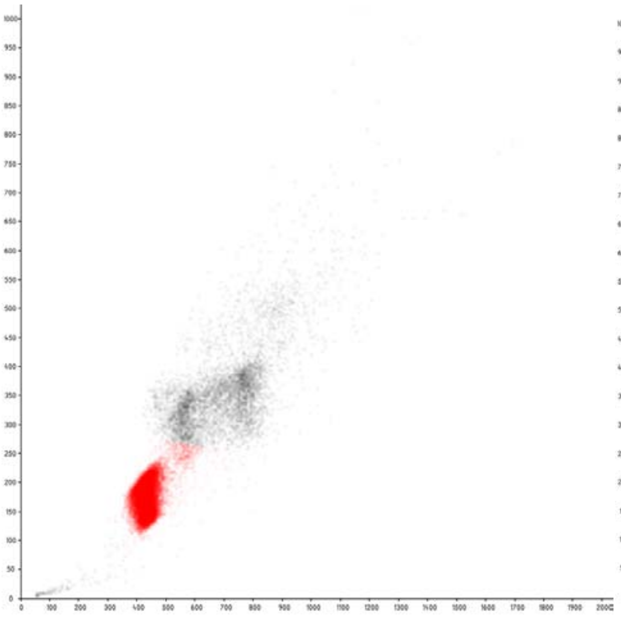

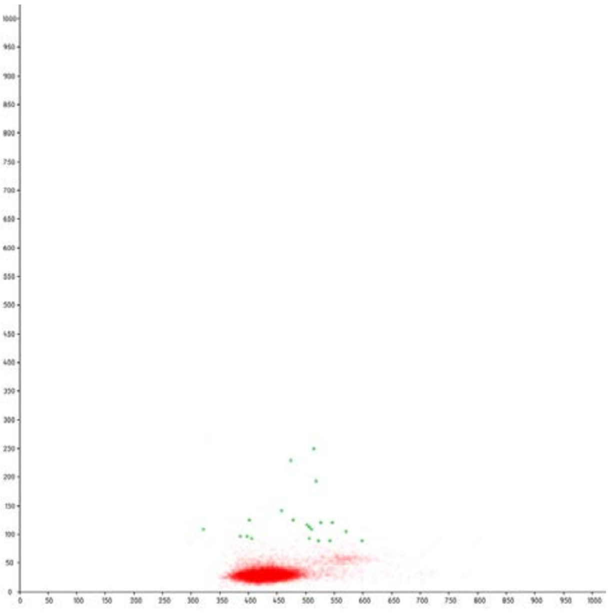

ANNEX 1: FLOW SORTING BIN 3 – File 2 of 2

Bin 3: COPAS scatter plots showing events for medium 3 of the final split-pool cycle (bin 3). In the first scatter plot (time of flight [TOF] vs. optical extinction [ext]), events depicted by a red dot fall within the gate defining monomeric beads and were sorted. The second scatter plot displays the fluorescent values of the sorted beads (TOF vs. green fluorescence intensity). Beads with a red fluorescent intensity above the threshold value are depicted as green dots and were individually dispensed into 96 well plates.

Number of beads individually dispensed: 47

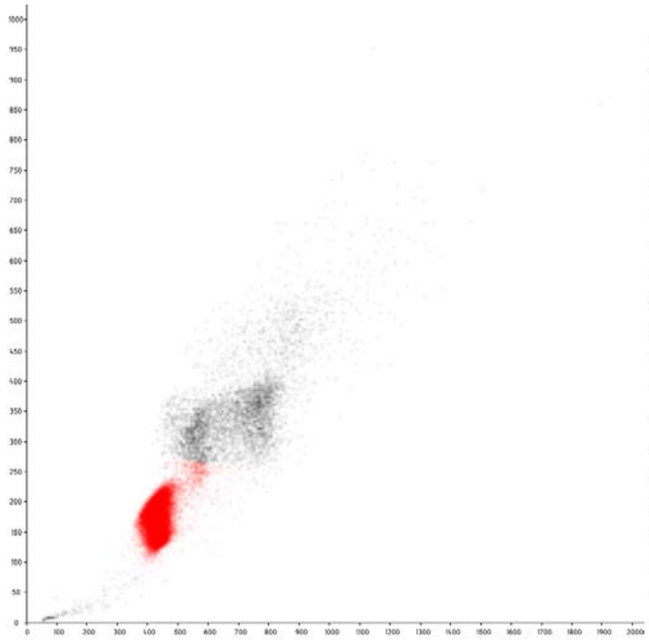

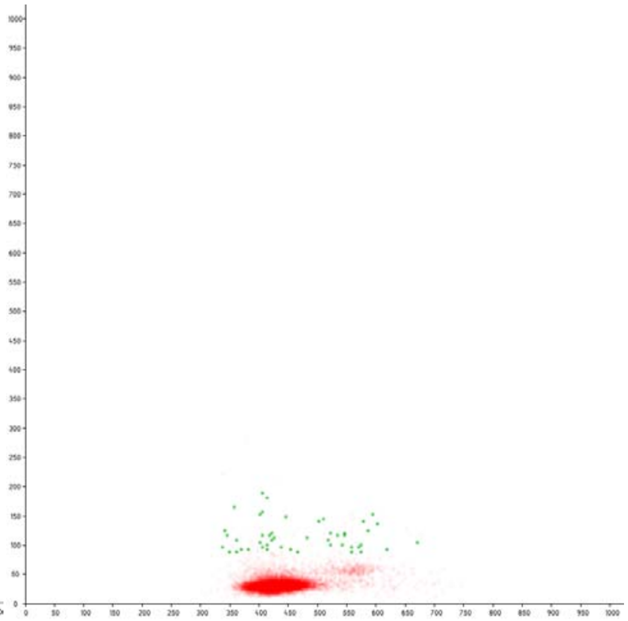

ANNEX 1: FLOW SORTING BIN 4 – File 1 of 1

Bin 4: COPAS scatter plots showing events for medium 4 of the final split-pool cycle (bin 4). In the first scatter plot (time of flight [TOF] vs. optical extinction [ext]), events depicted by a red dot fall within the gate defining monomeric beads and were sorted. The second scatter plot displays the fluorescent values of the sorted beads (TOF vs. green fluorescence intensity). Beads with a red fluorescent intensity above the threshold value are depicted as green dots and were individually dispensed into 96 well plates.

Number of beads individually dispensed: 150

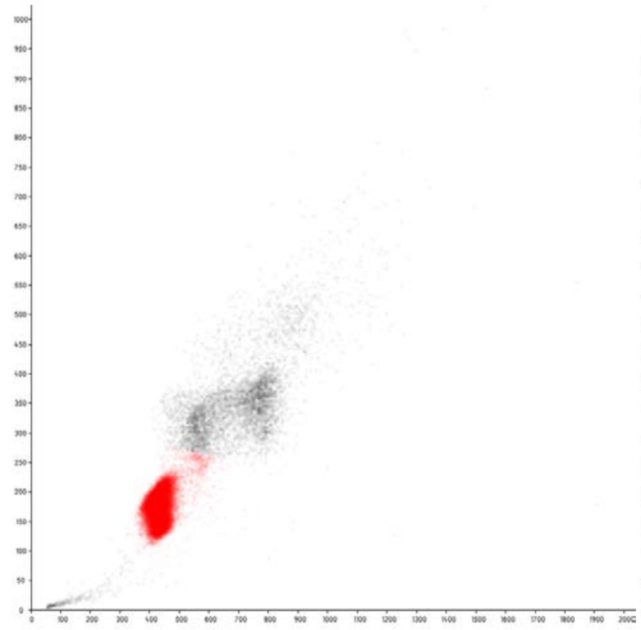

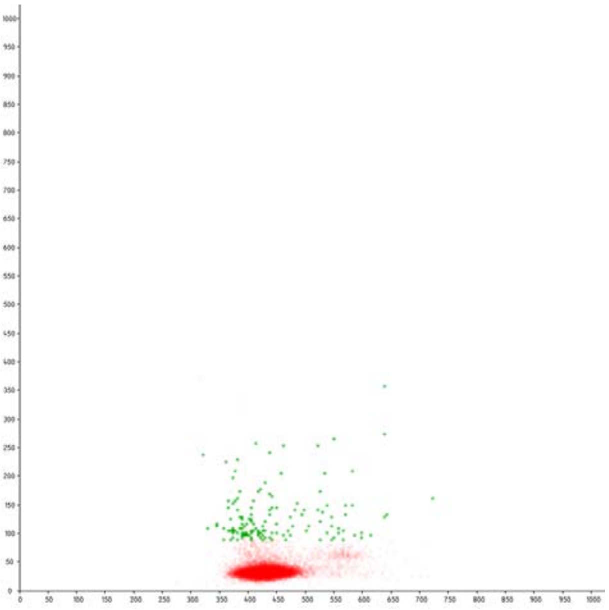

ANNEX 1: FLOW SORTING BIN 5 – File 1 of 1

Bin 5: COPAS scatter plots showing events for medium 5 of the final split-pool cycle (bin 5). In the first scatter plot (time of flight [TOF] vs. optical extinction [ext]), events depicted by a red dot fall within the gate defining monomeric beads and were sorted. The second scatter plot displays the fluorescent values of the sorted beads (TOF vs. green fluorescence intensity). Beads with a red fluorescent intensity above the threshold value are depicted as green dots and were individually dispensed into 96 well plates.

Number of beads individually dispensed: 146

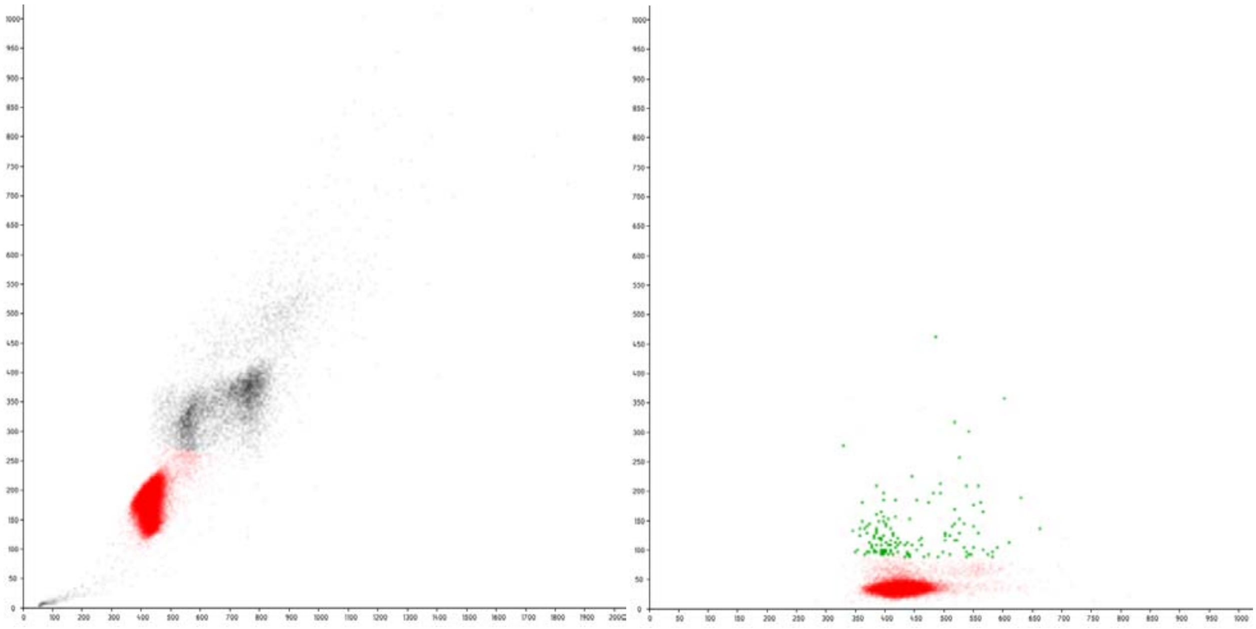

ANNEX 1: FLOW SORTING BIN 6 – File 1 of 1

Bin 6: COPAS scatter plots showing events for medium 6 of the final split-pool cycle (bin 6). In the first scatter plot (time of flight [TOF] vs. optical extinction [ext]), events depicted by a red dot fall within the gate defining monomeric beads and were sorted. The second scatter plot displays the fluorescent values of the sorted beads (TOF vs. green fluorescence intensity). Beads with a red fluorescent intensity above the threshold value are depicted as green dots and were individually dispensed into 96 well plates.

Number of beads individually dispensed: 254

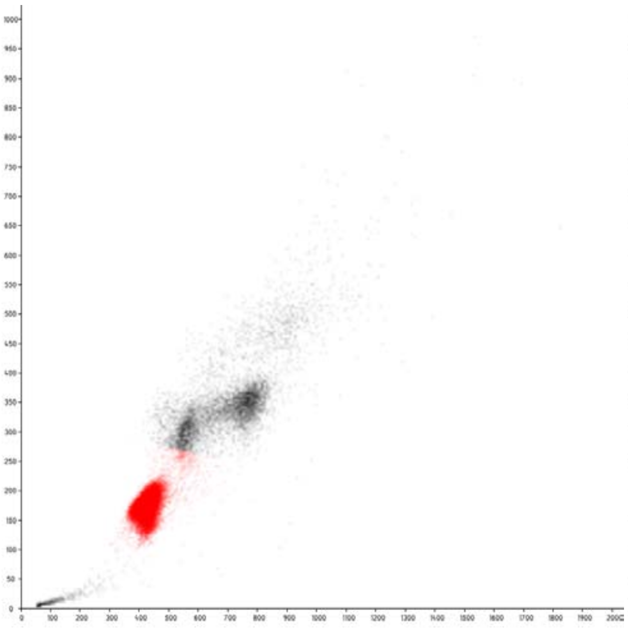

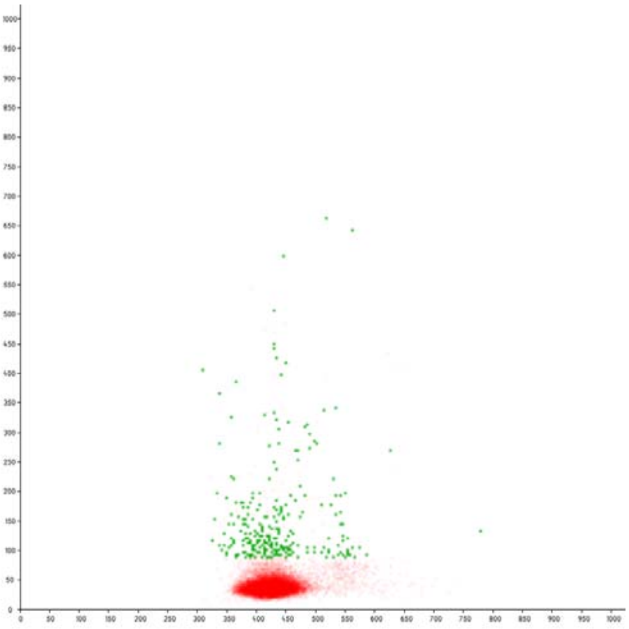

ANNEX 1: FLOW SORTING BIN 7 – File 1 of 1

Bin 7: COPAS scatter plots showing events for medium 7 of the final split-pool cycle (bin 7). In the first scatter plot (time of flight [TOF] vs. optical extinction [ext]), events depicted by a red dot fall within the gate defining monomeric beads and were sorted. The second scatter plot displays the fluorescent values of the sorted beads (TOF vs. green fluorescence intensity). Beads with a red fluorescent intensity above the threshold value are depicted as green dots and were individually dispensed into 96 well plates.

Number of beads individually dispensed: 40

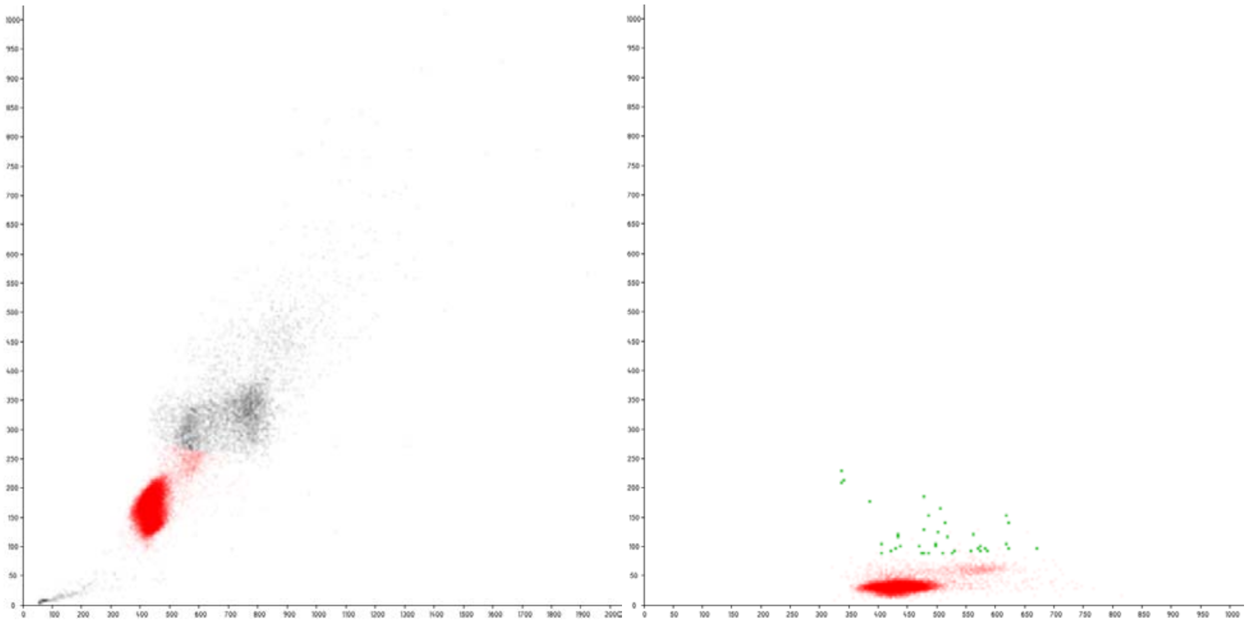

ANNEX 1: FLOW SORTING BIN 8 – File 1 of 1

Bin 8: COPAS scatter plots showing events for medium 8 of the final split-pool cycle (bin 8). In the first scatter plot (time of flight [TOF] vs. optical extinction [ext]), events depicted by a red dot fall within the gate defining monomeric beads and were sorted. The second scatter plot displays the fluorescent values of the sorted beads (TOF vs. green fluorescence intensity). Beads with a red fluorescent intensity above the threshold value are depicted as green dots and were individually dispensed into 96 well plates.

Number of beads individually dispensed: 110

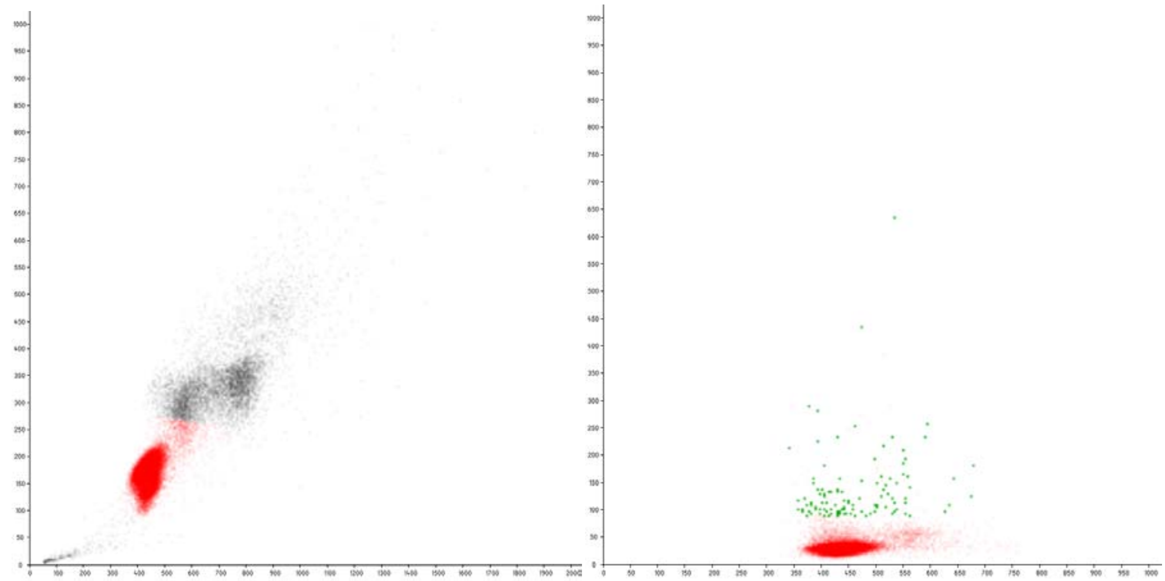

ANNEX 1: FLOW SORTING BIN 9 – File 1 of 1

Bin 9: COPAS scatter plots showing events for medium 9 of the final split-pool cycle (bin 9). In the first scatter plot (time of flight [TOF] vs. optical extinction [ext]), events depicted by a red dot fall within the gate defining monomeric beads and were sorted. The second scatter plot displays the fluorescent values of the sorted beads (TOF vs. green fluorescence intensity). Beads with a red fluorescent intensity above the threshold value are depicted as green dots and were individually dispensed into 96 well plates.

Number of beads individually dispensed: 78

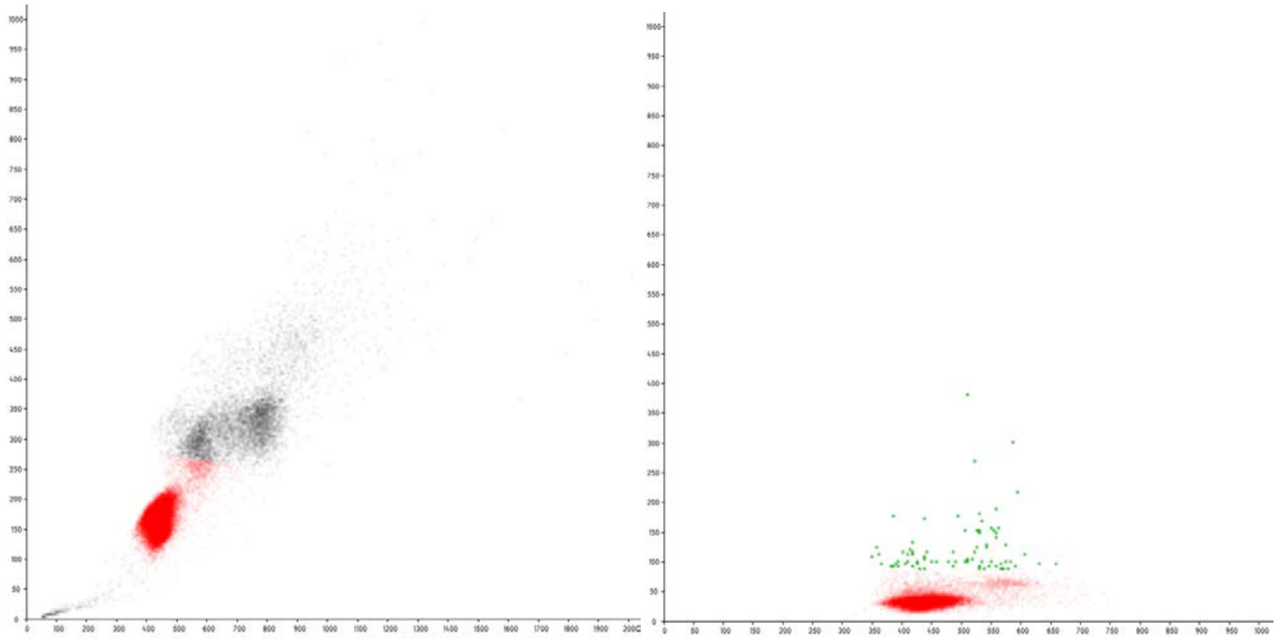

ANNEX 1: FLOW SORTING BIN 10 – File 1 of 1

Bin 10: COPAS scatter plots showing events for medium 10 of the final split-pool cycle (bin 10). In the first scatter plot (time of flight [TOF] vs. optical extinction [ext]), events depicted by a red dot fall within the gate defining monomeric beads and were sorted. The second scatter plot displays the fluorescent values of the sorted beads (TOF vs. green fluorescence intensity). Beads with a red fluorescent intensity above the threshold value are depicted as green dots and were individually dispensed into 96 well plates.

Number of beads individually dispensed: 117

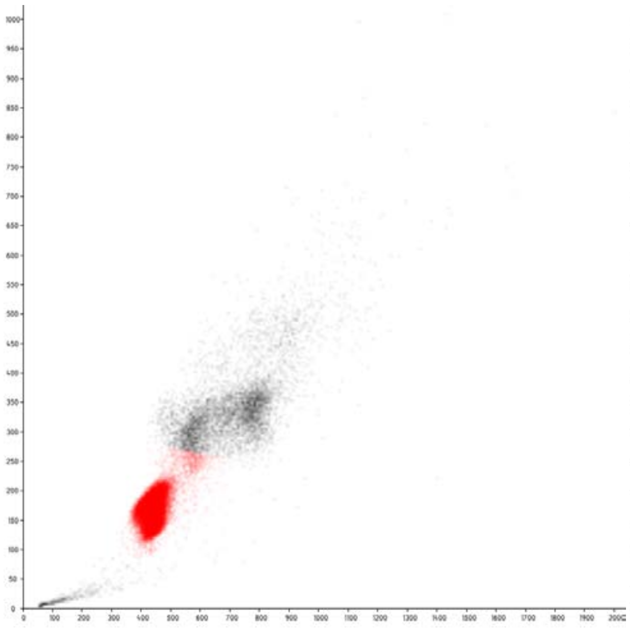

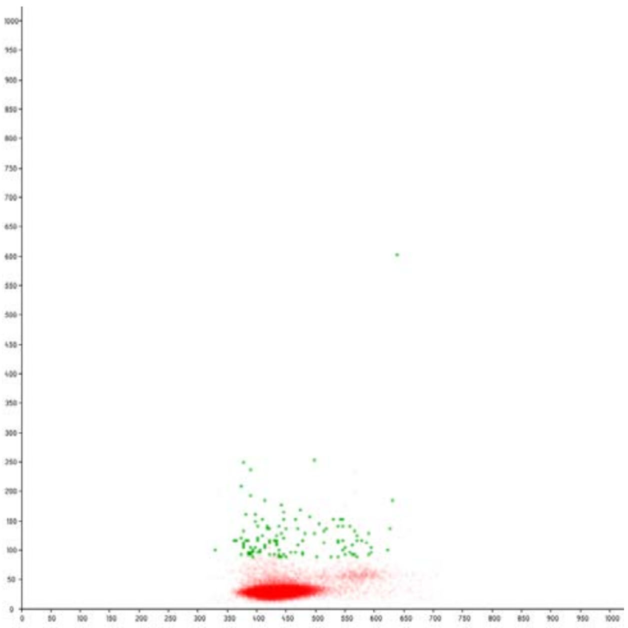

ANNEX 2: TAG REFERENCE SET - TAG DECONVOLUTION SESSION 2

Tag analysis flow cytometry instrument: BD FACSCalibur

Number of gates: 30

Number of beads whose tags were deconvoluted: 6

Number of beads with conclusive tagging data: 6

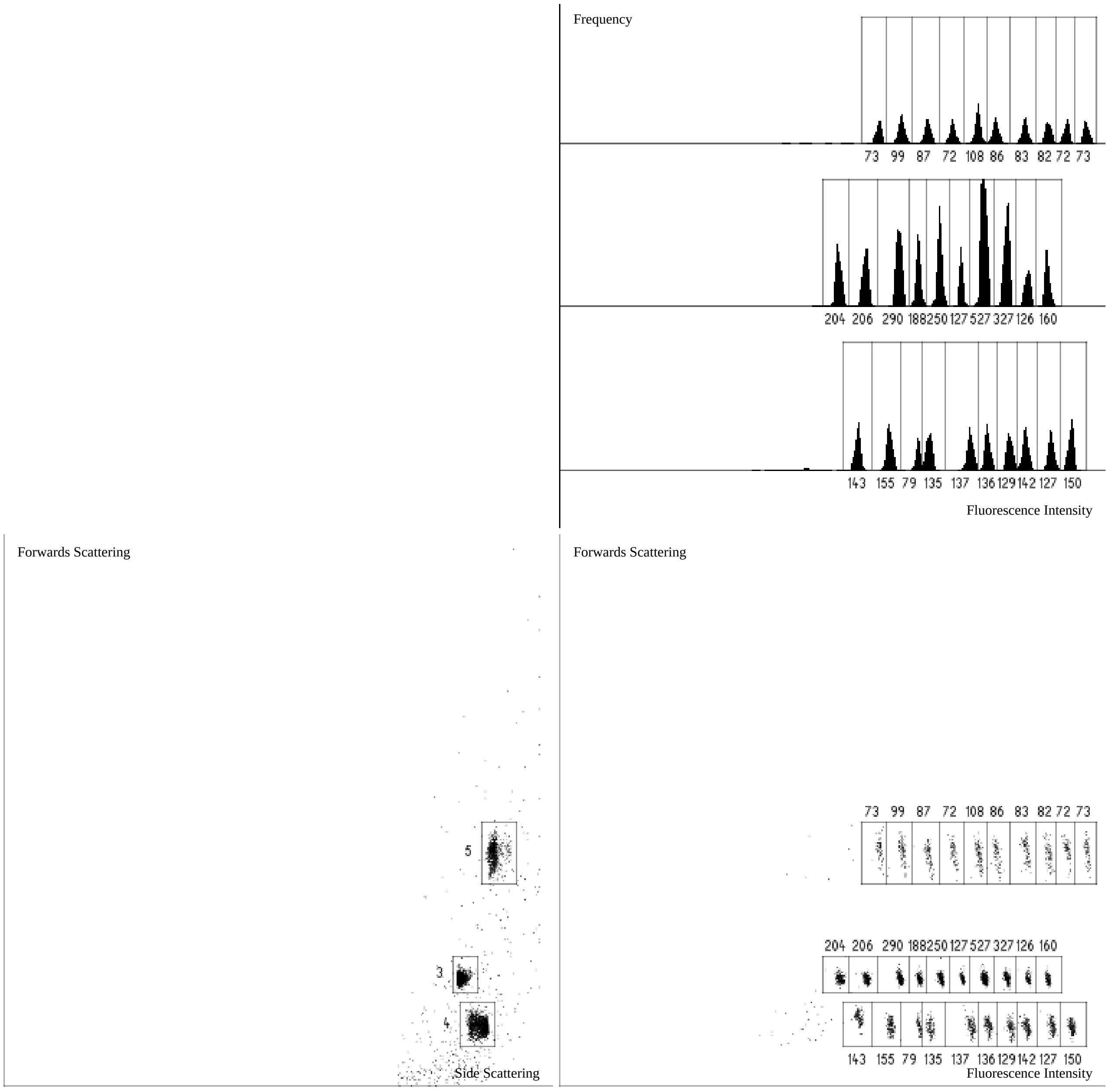

ANNEX 2: TAG REFERENCE SET - TAG DECONVOLUTION SESSION 3

Tag analysis flow cytometry instrument: BD FACSCalibur

Number of gates: 30

Number of beads whose tags were deconvoluted: 22

Number of beads with conclusive tagging data: 21

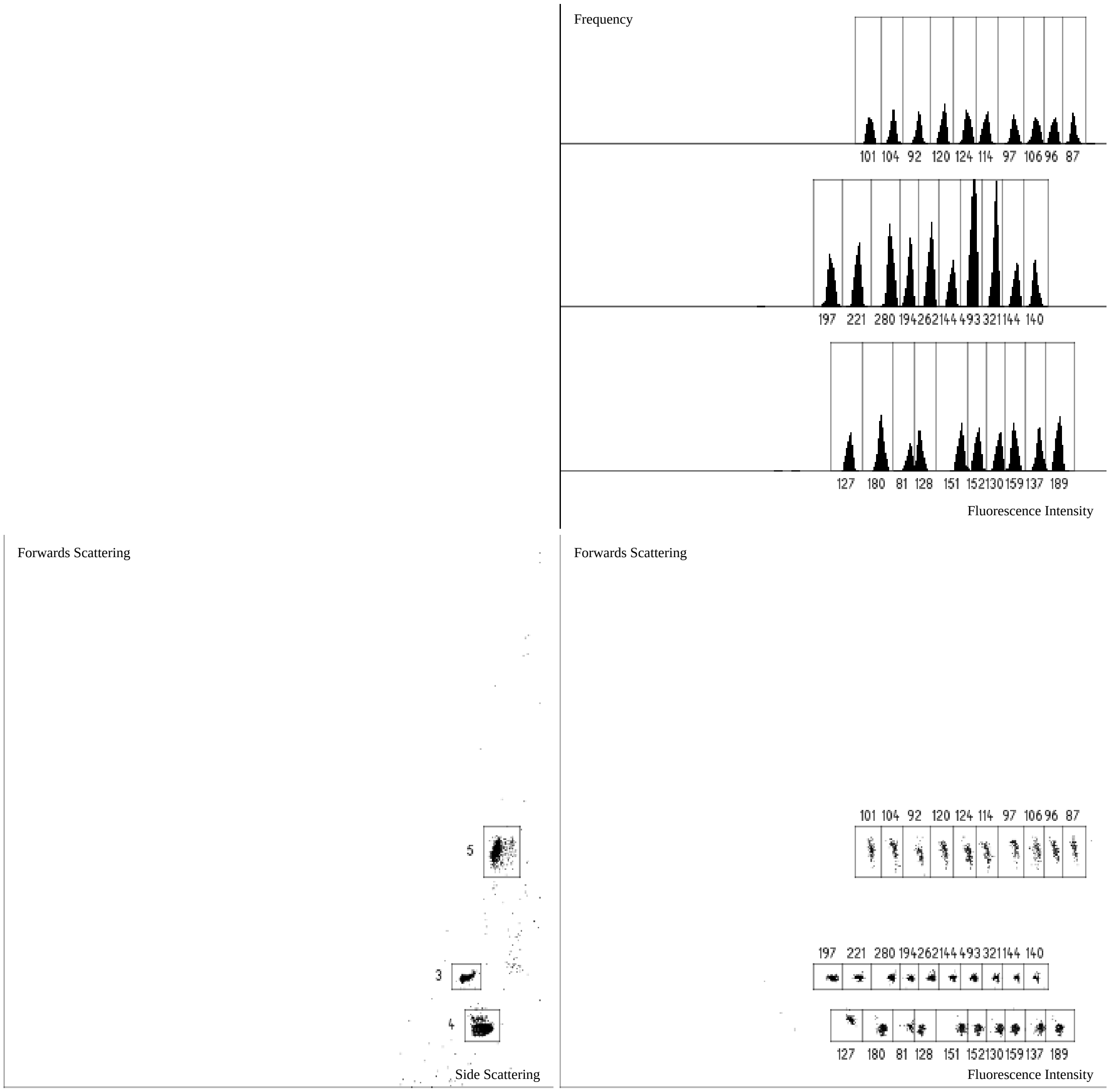

ANNEX 2: TAG REFERENCE SET - TAG DECONVOLUTION SESSION 4

Tag analysis flow cytometry instrument: BD FACSCalibur

Number of gates: 30

Number of beads whose tags were deconvoluted: 36

Number of beads with conclusive tagging data: 35

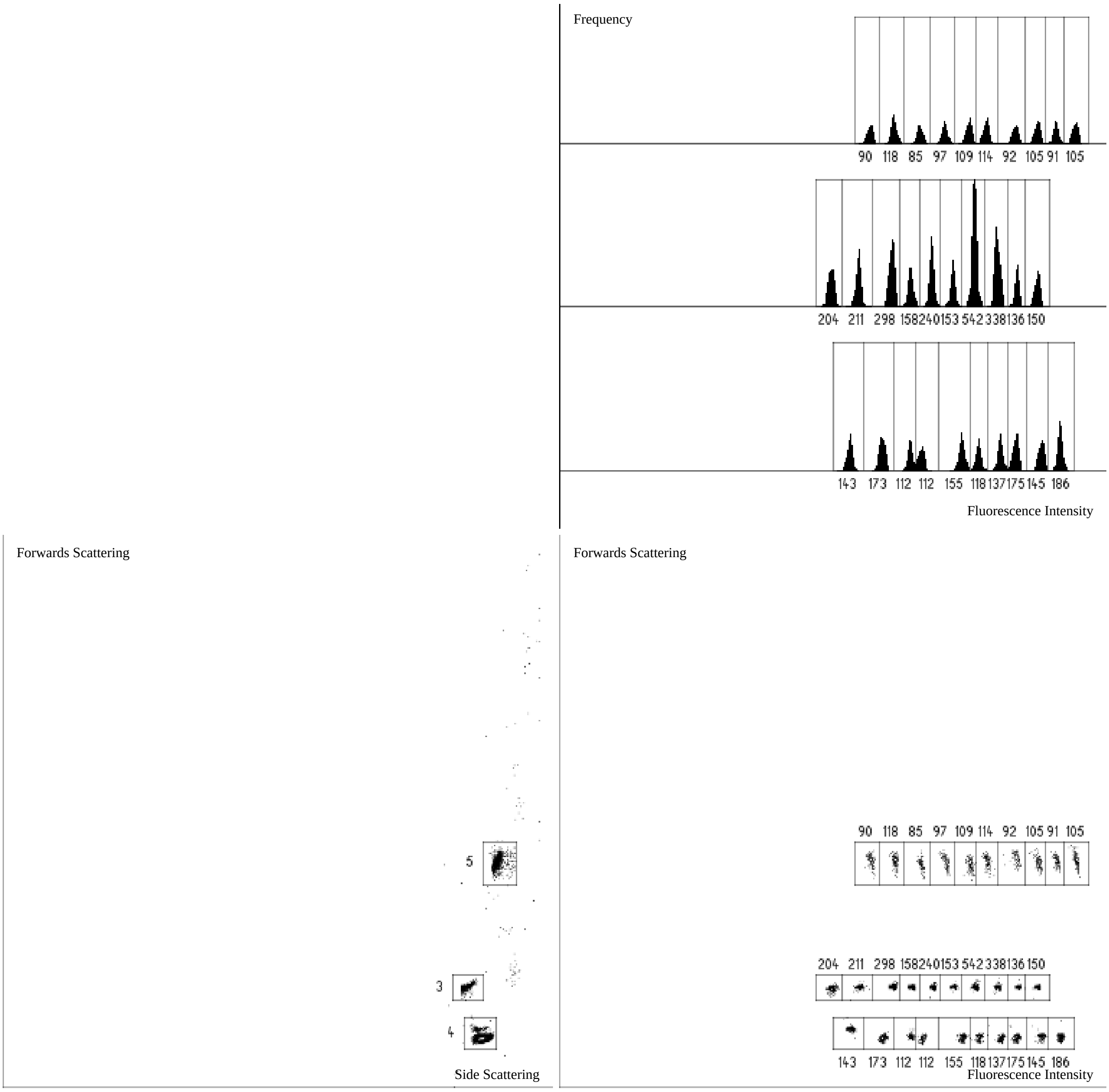

ANNEX 2: TAG REFERENCE SET - TAG DECONVOLUTION SESSION 5

Tag analysis flow cytometry instrument: BD FACSCalibur

Number of gates: 30

Number of beads whose tags were deconvoluted: 33

Number of beads with conclusive tagging data: 28

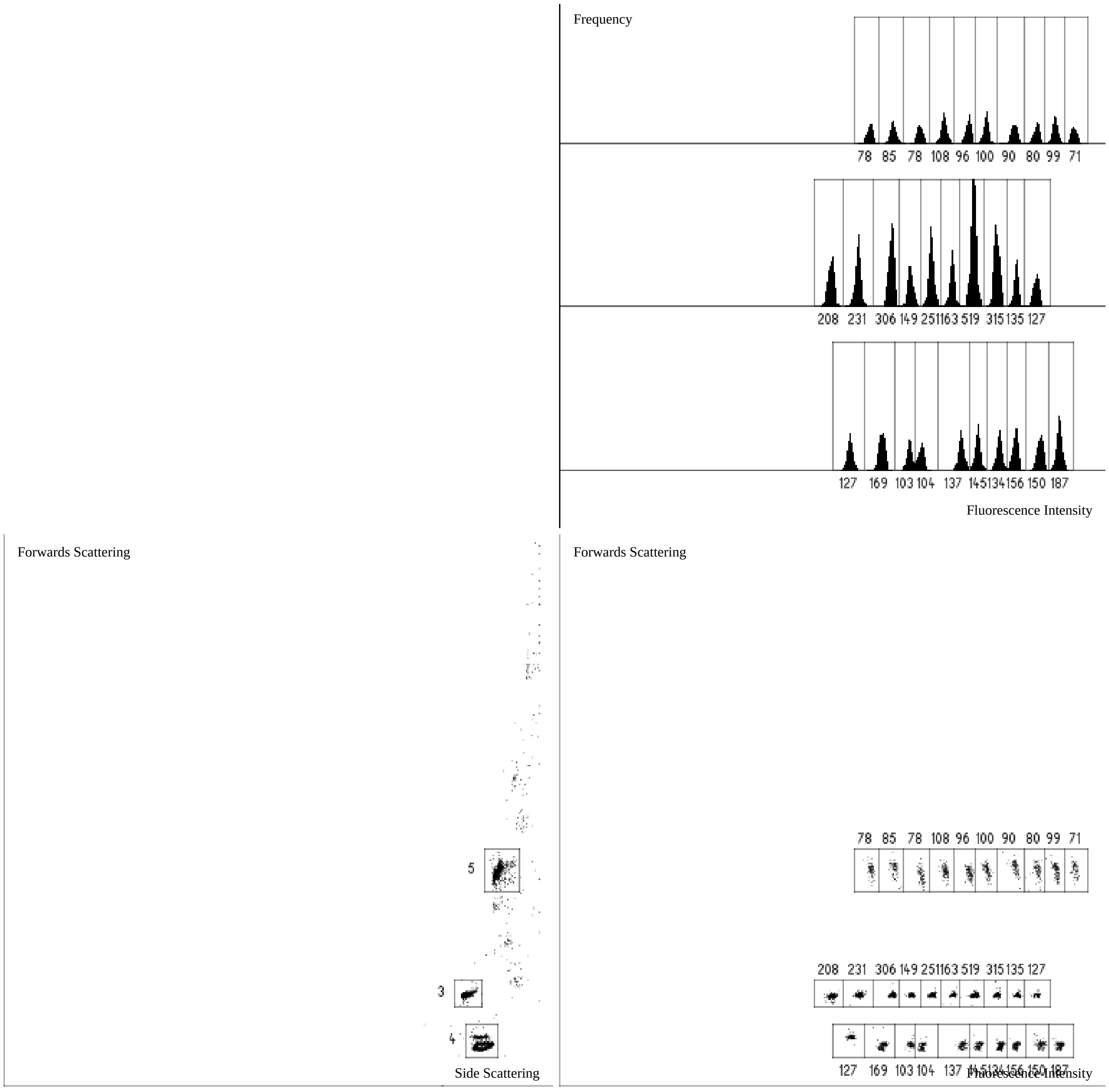

ANNEX 2: TAG REFERENCE SET - TAG DECONVOLUTION SESSION 6

Tag analysis flow cytometry instrument: BD FACSCalibur

Number of gates: 30

Number of beads whose tags were deconvoluted: 12

Number of beads with conclusive tagging data: 11

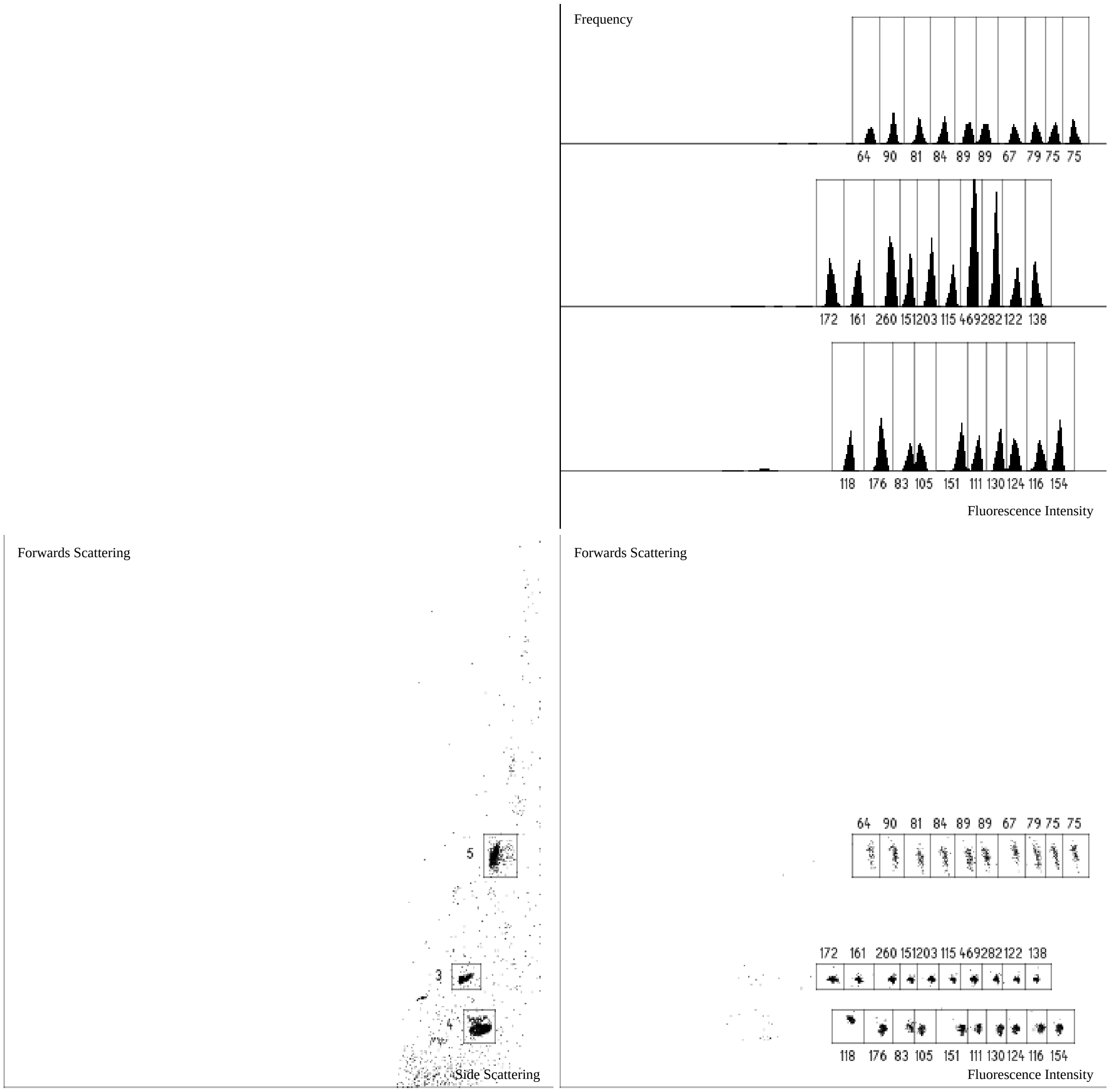

ANNEX 2: TAG REFERENCE SET - TAG DECONVOLUTION SESSION 7

Tag analysis flow cytometry instrument: BD FACSCalibur

Number of gates: 30

Number of beads whose tags were deconvoluted: 54

Number of beads with conclusive tagging data: 50

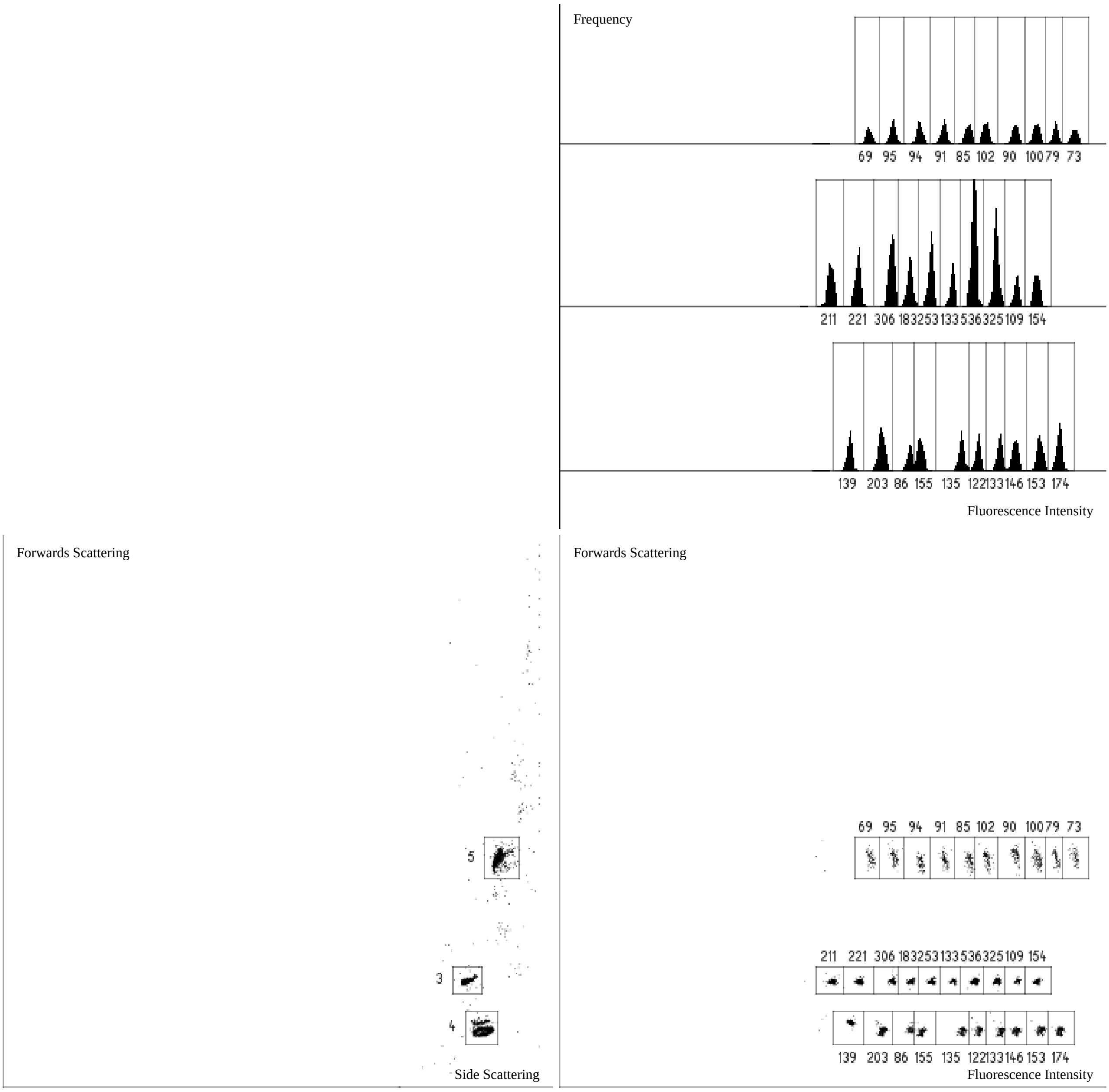

ANNEX 2: TAG REFERENCE SET - TAG DECONVOLUTION SESSION 8

Tag analysis flow cytometry instrument: BD FACSCalibur

Number of gates: 30

Number of beads whose tags were deconvoluted: 50

Number of beads with conclusive tagging data: 44

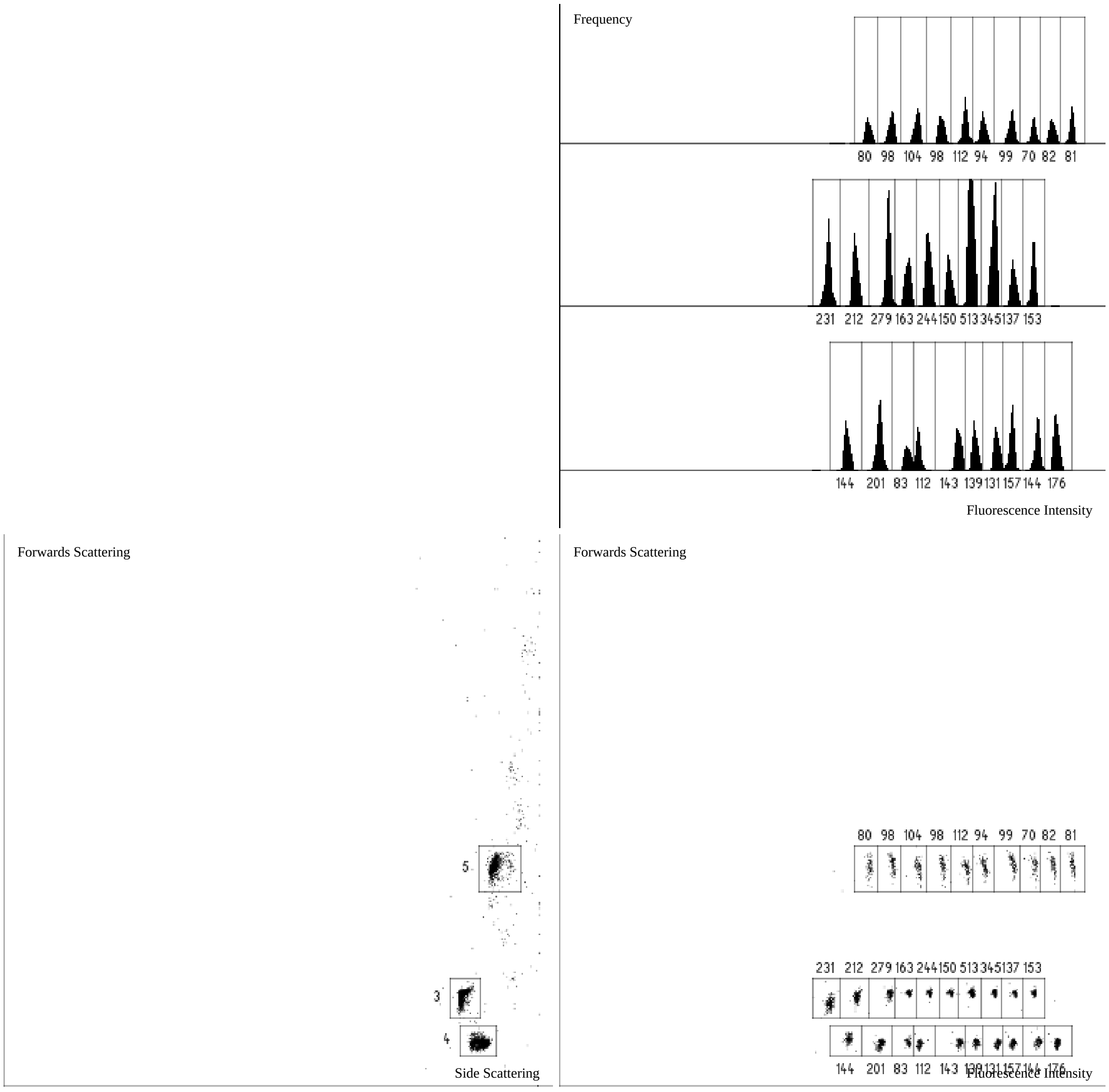

ANNEX 2: TAG REFERENCE SET - TAG DECONVOLUTION SESSION 9

Tag analysis flow cytometry instrument: BD FACSCalibur

Number of gates: 30

Number of beads whose tags were deconvoluted: 19

Number of beads with conclusive tagging data: 17

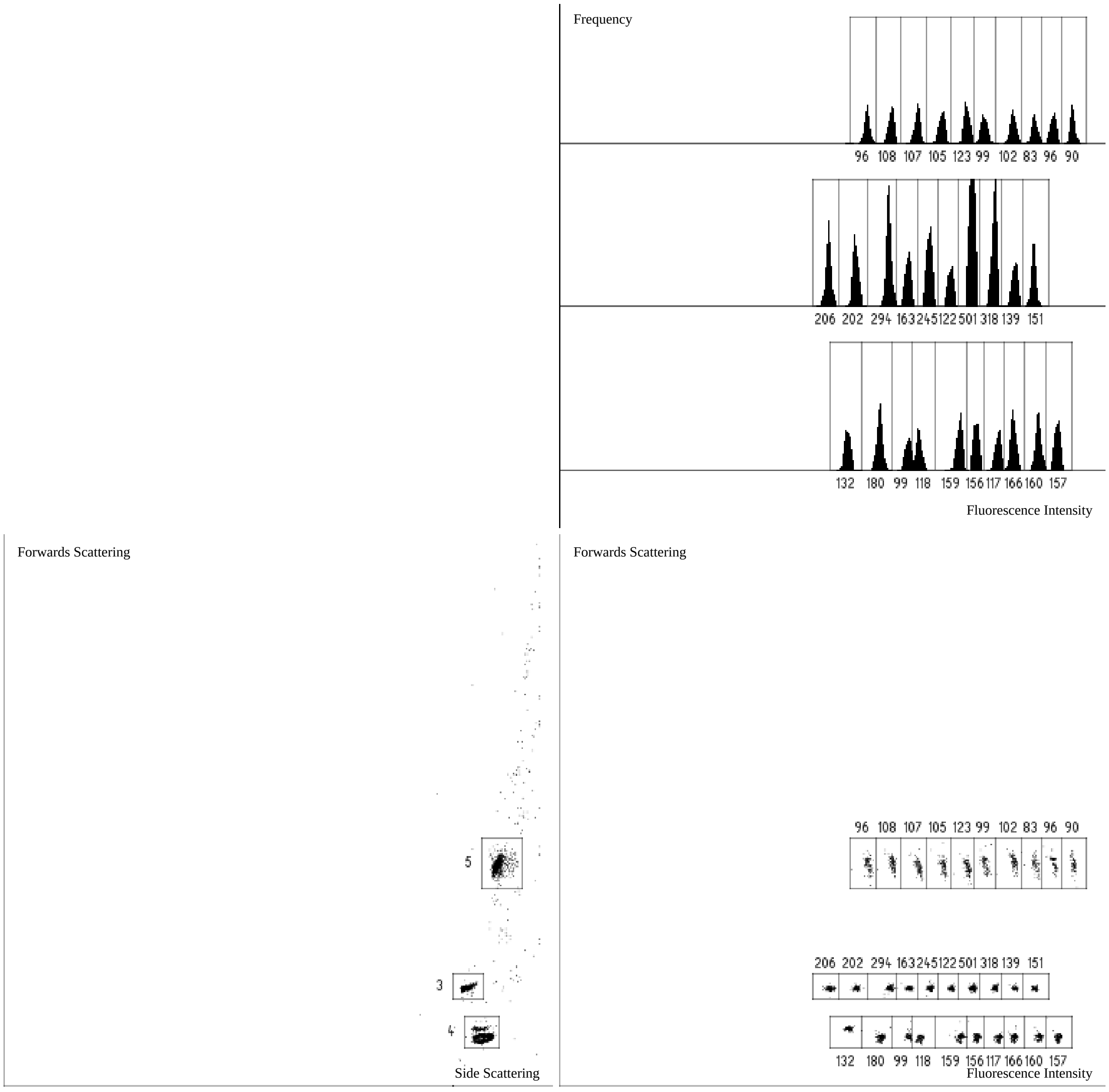

ANNEX 2: TAG REFERENCE SET - TAG DECONVOLUTION SESSION 10

Tag analysis flow cytometry instrument: BD FACSCalibur

Number of gates: 30

Number of beads whose tags were deconvoluted: 25

Number of beads with conclusive tagging data: 24

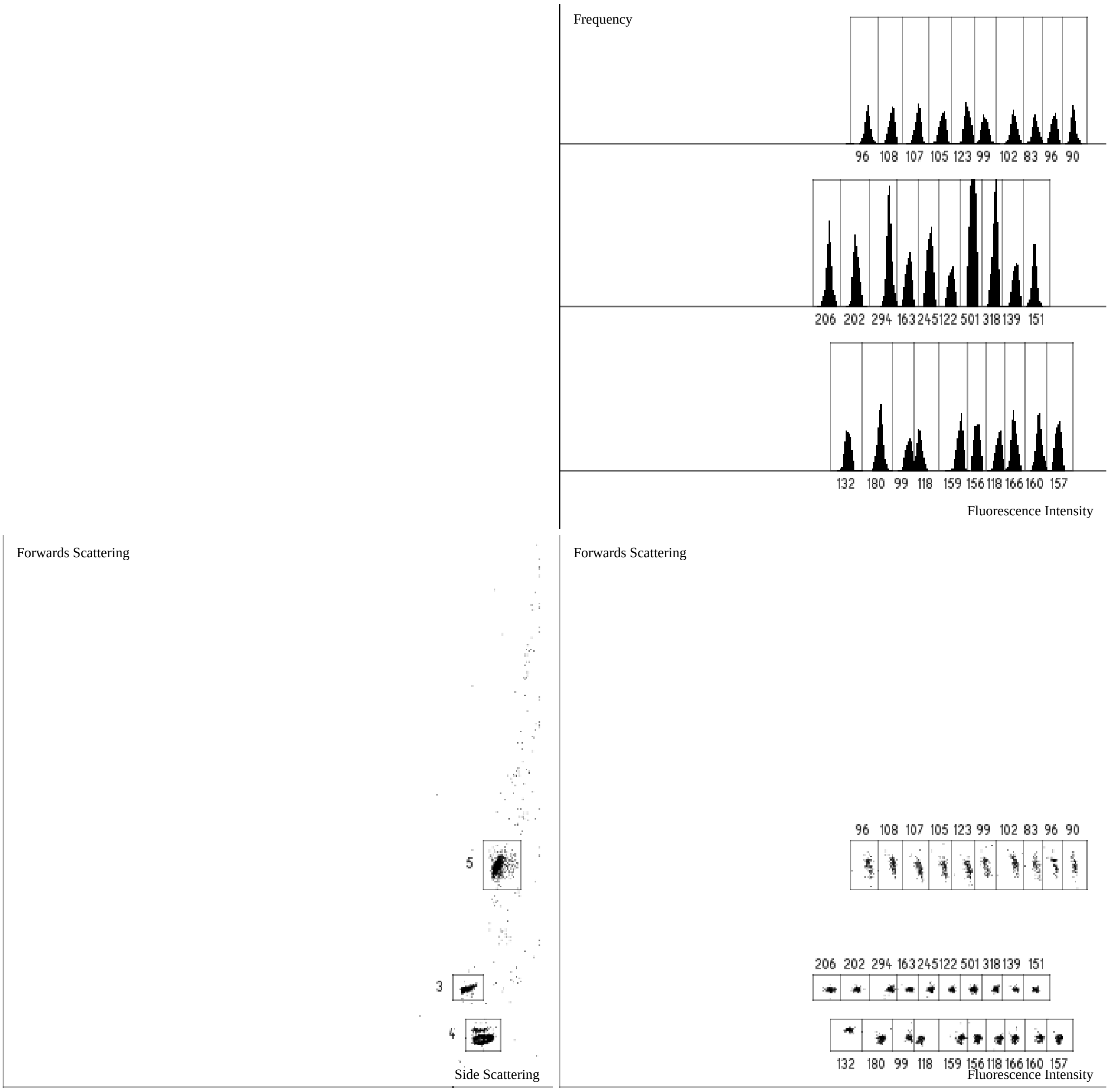

ANNEX 2: TAG REFERENCE SET - TAG DECONVOLUTION SESSION 11

Tag analysis flow cytometry instrument: BD FACSCalibur

Number of gates: 30

Number of beads whose tags were deconvoluted: 2

Number of beads with conclusive tagging data: 2

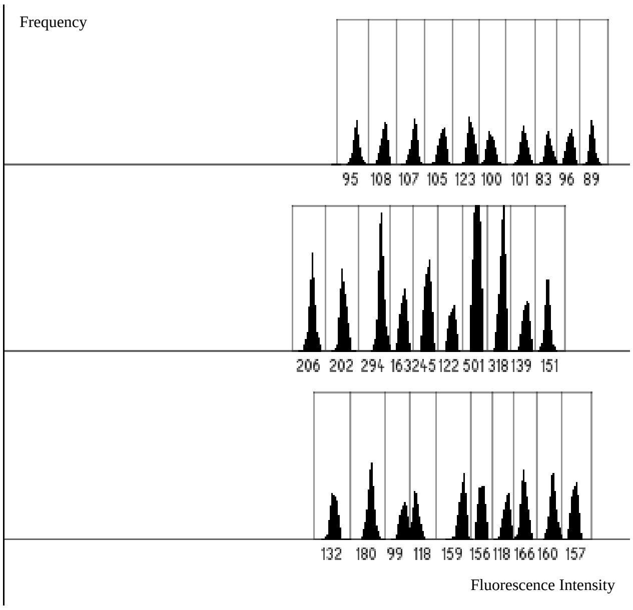

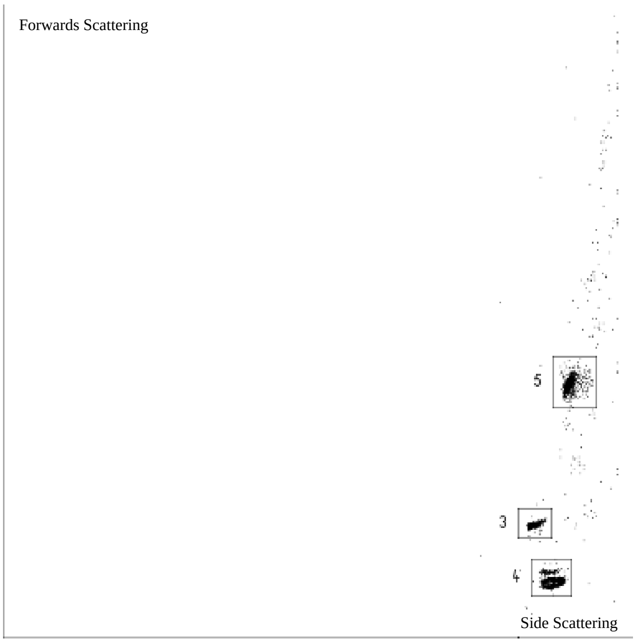

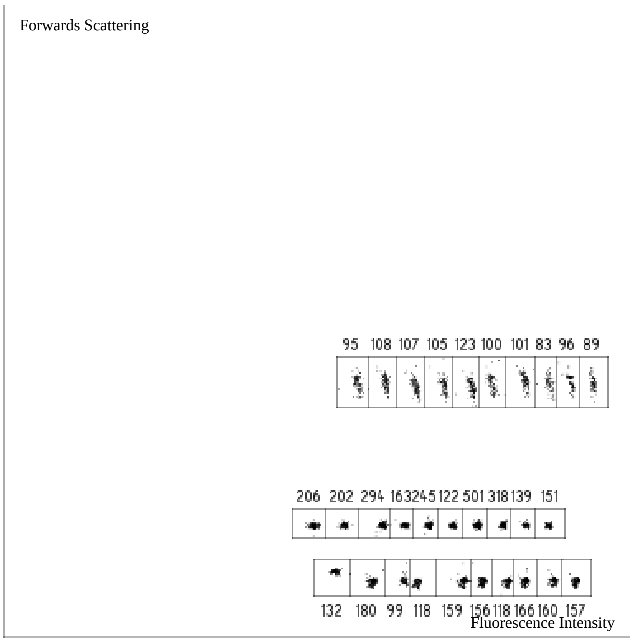

ANNEX 2: TAG REFERENCE SET - TAG DECONVOLUTION SESSION 12

Tag analysis flow cytometry instrument: BD FACSCalibur

Number of gates: 30

Number of beads whose tags were deconvoluted: 10

Number of beads with conclusive tagging data: 10

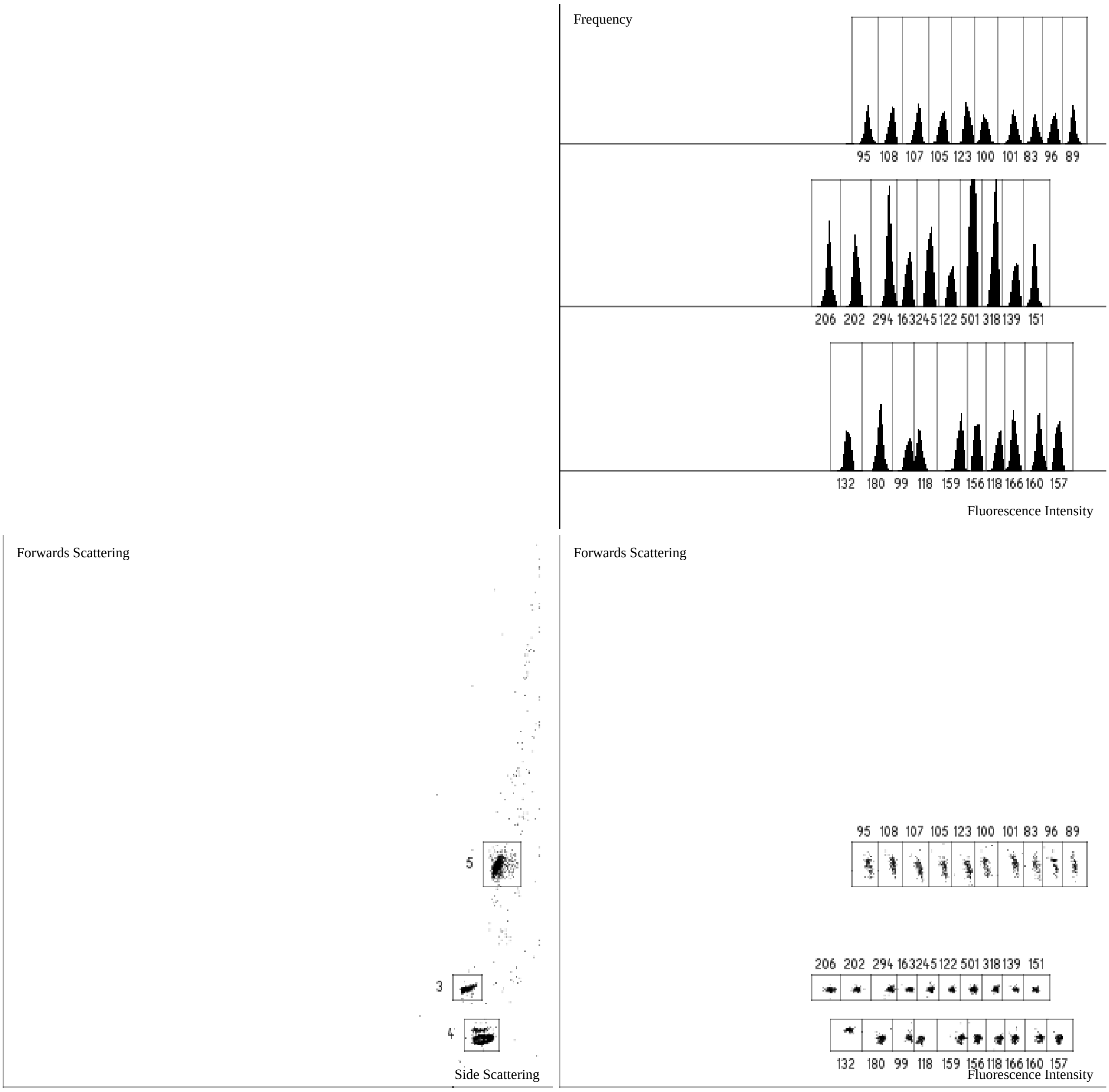

ANNEX 2: TAG REFERENCE SET - TAG DECONVOLUTION SESSION 13

Tag analysis flow cytometry instrument: BD FACSCalibur

Number of gates: 30

Number of beads whose tags were deconvoluted: 7

Number of beads with conclusive tagging data: 7

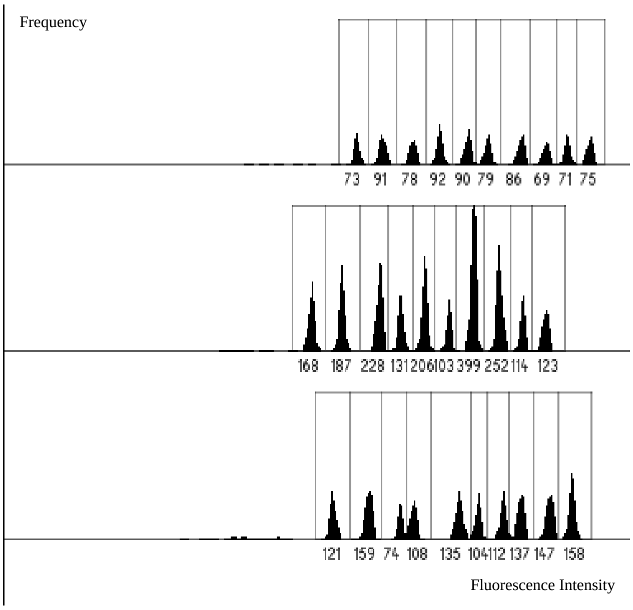

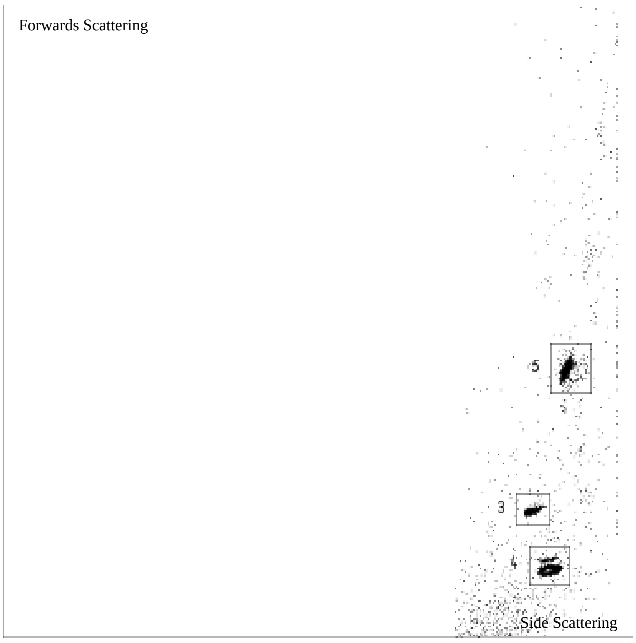

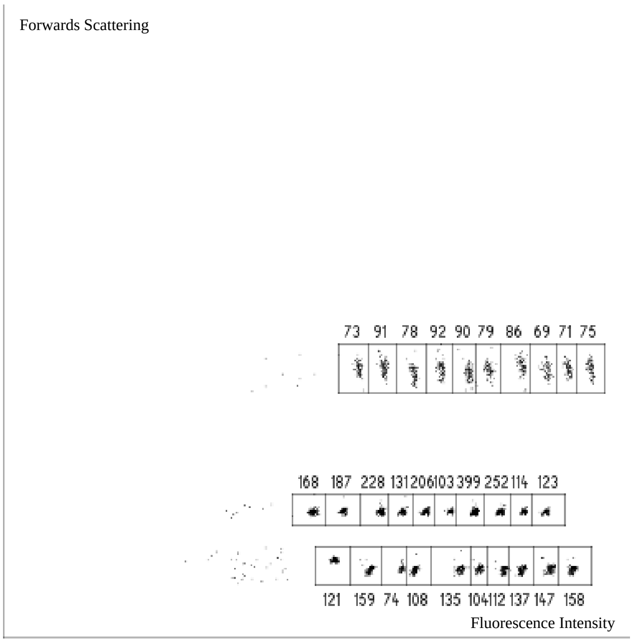

ANNEX 2: TAG REFERENCE SET - TAG DECONVOLUTION SESSION 14

Tag analysis flow cytometry instrument: BD FACSCalibur

Number of gates: 30

Number of beads whose tags were deconvoluted: 11

Number of beads with conclusive tagging data: 6

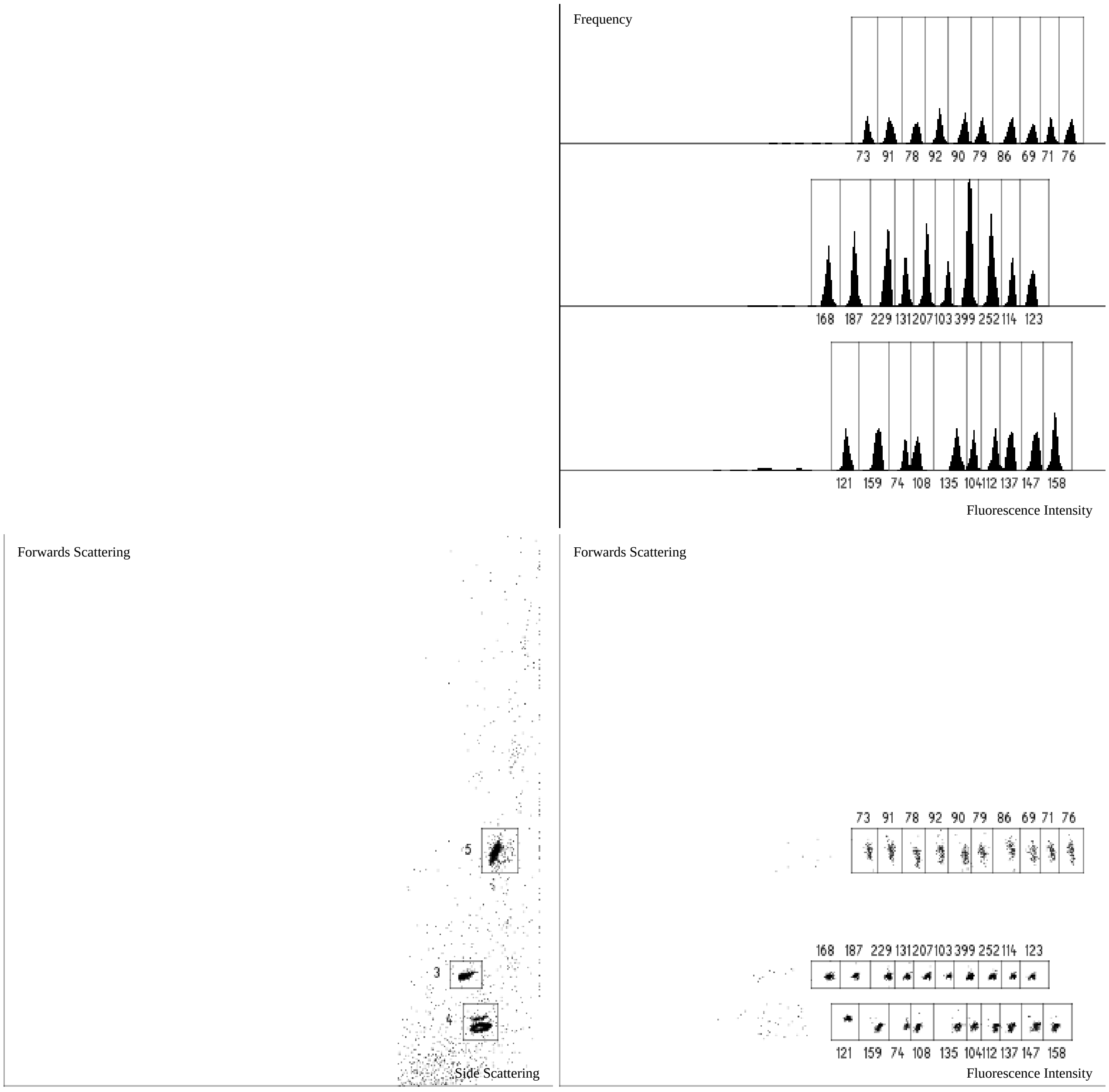

ANNEX 2: TAG REFERENCE SET - TAG DECONVOLUTION SESSION 15

Tag analysis flow cytometry instrument: BD FACSCalibur

Number of gates: 30

Number of beads whose tags were deconvoluted: 10

Number of beads with conclusive tagging data: 8

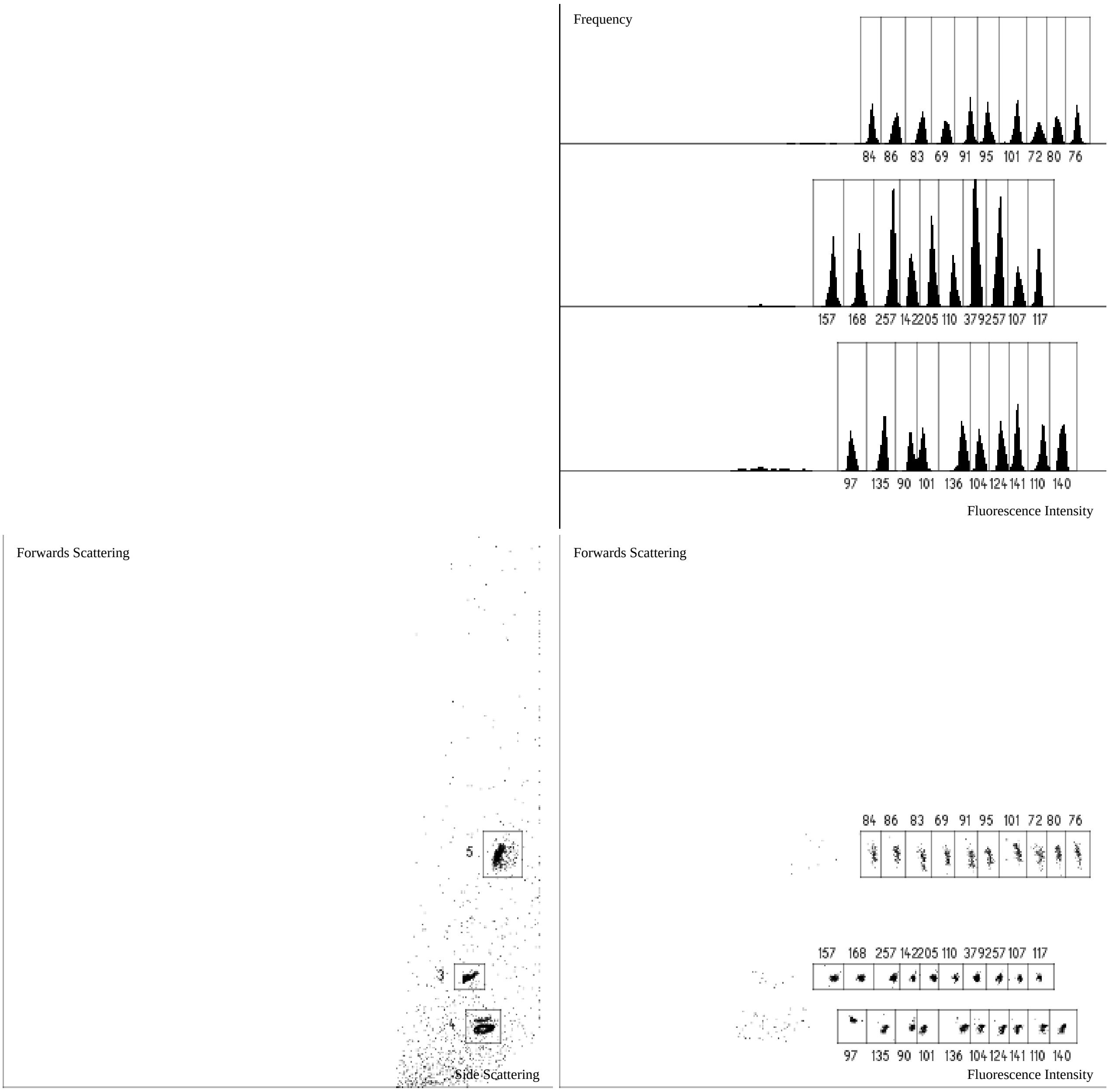

ANNEX 2: TAG REFERENCE SET - TAG DECONVOLUTION SESSION 16

Tag analysis flow cytometry instrument: BD FACSCalibur

Number of gates: 30

Number of beads whose tags were deconvoluted: 2

Number of beads with conclusive tagging data: 0

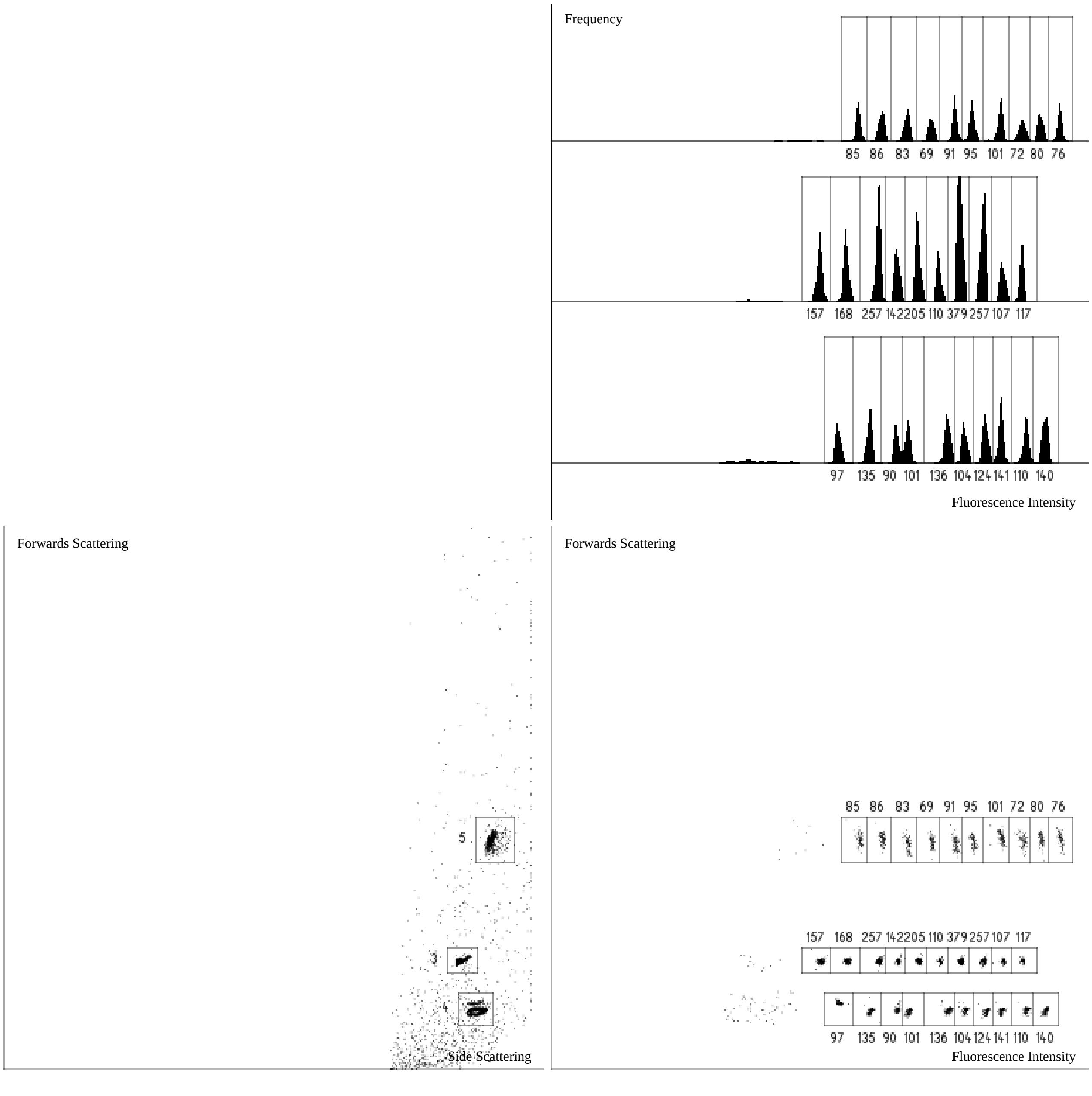

ANNEX 2: TAG REFERENCE SET - TAG DECONVOLUTION SESSION 17

Tag analysis flow cytometry instrument: BD FACSCalibur

Number of gates: 30

Number of beads whose tags were deconvoluted: 4

Number of beads with conclusive tagging data: 2

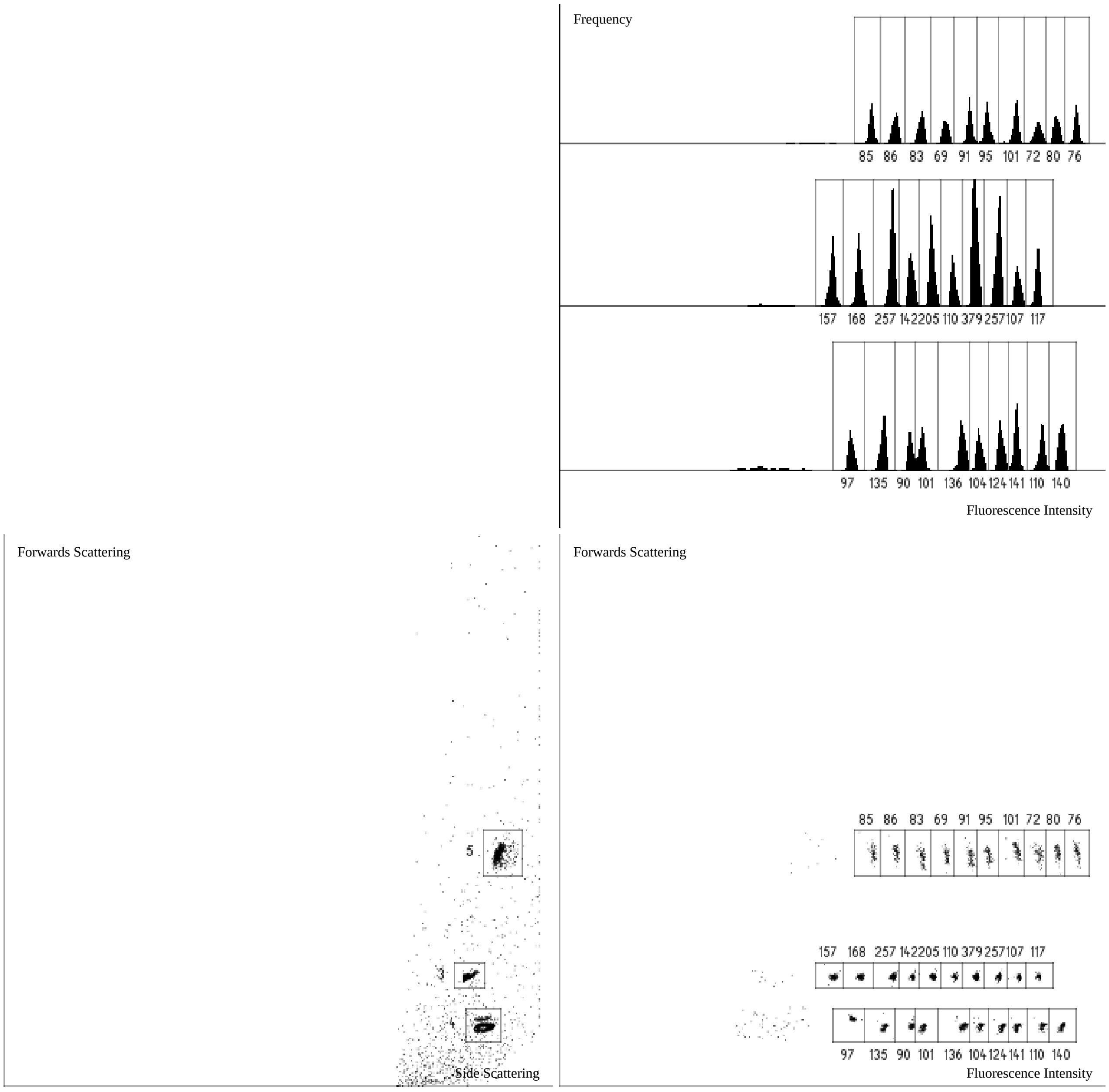

ANNEX 3: TAG DECONVOLUTION - BEAD 1

Passes flow sorting criteria: Yes

Passes tag deconvolution criteria: No

Included in protocol analysis: No

Protocol: N/A

Filename: Bin1_plateA1_D11.fcs

Split 1: Petrol shading

Split 2: Green shading

Split 3: Violet shading

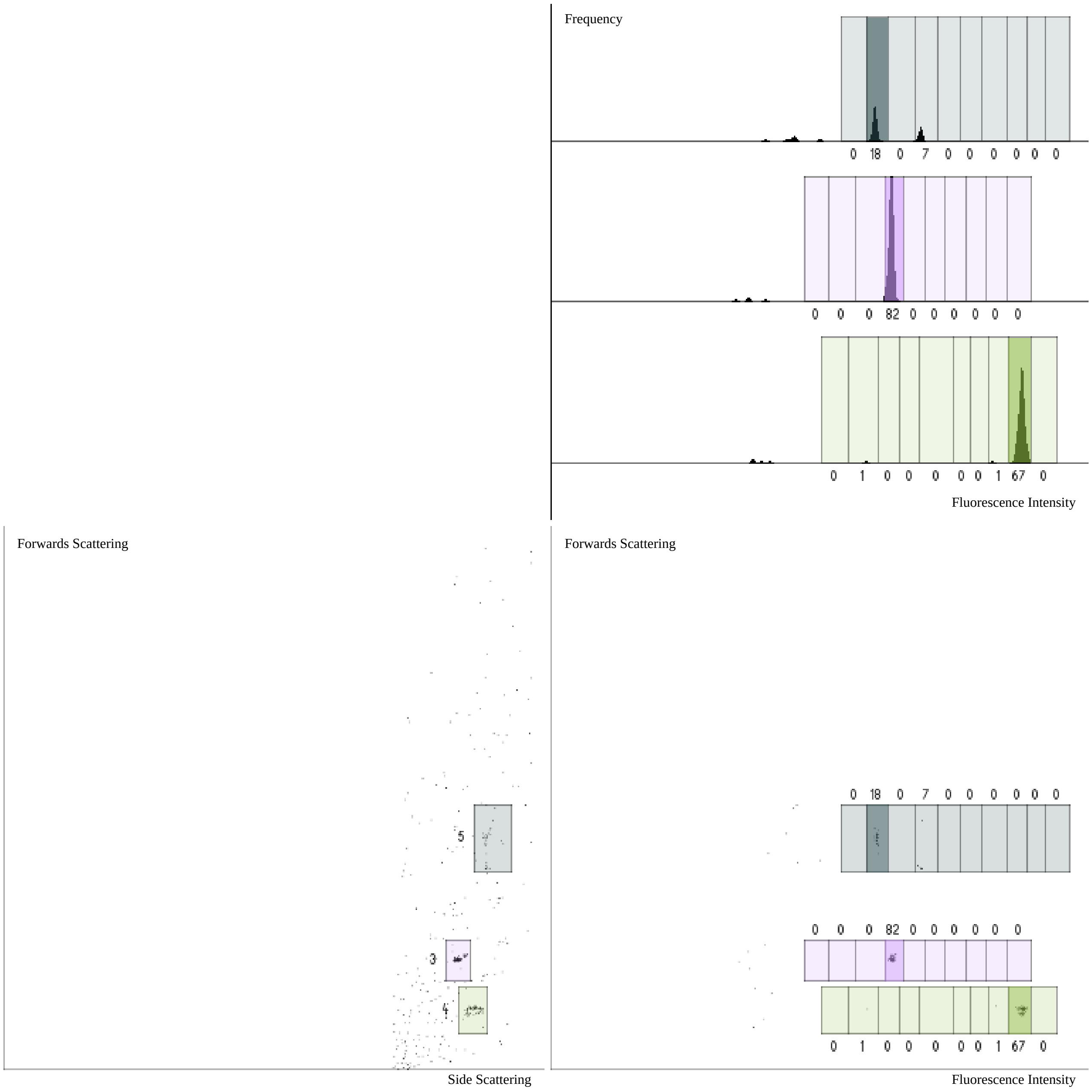

ANNEX 3: TAG DECONVOLUTION - BEAD 2

Passes flow sorting criteria: Yes

Passes tag deconvolution criteria: No

Included in protocol analysis: No

Protocol: N/A

Filename: Bin1_plateA1_A2.fcs

Split 1: Petrol shading

Split 2: Green shading

Split 3: Violet shading

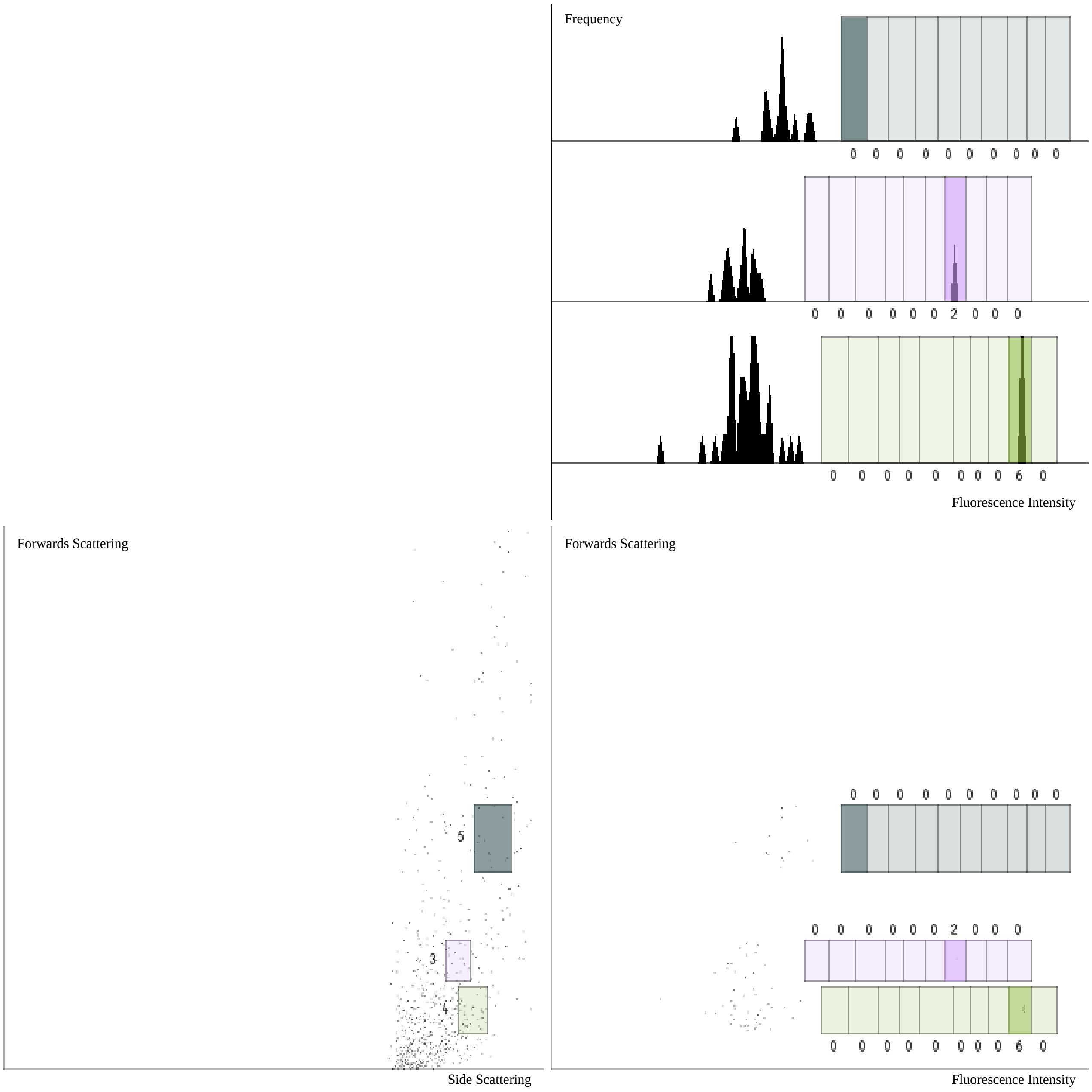

ANNEX 3: TAG DECONVOLUTION - BEAD 3

Passes flow sorting criteria: Yes

Passes tag deconvolution criteria: Yes

Included in protocol analysis: Yes

Protocol: 9, 3, 4, 1

Filename: Bin1_plateA1_A11.fcs

Split 1: Petrol shading

Split 2: Green shading

Split 3: Violet shading

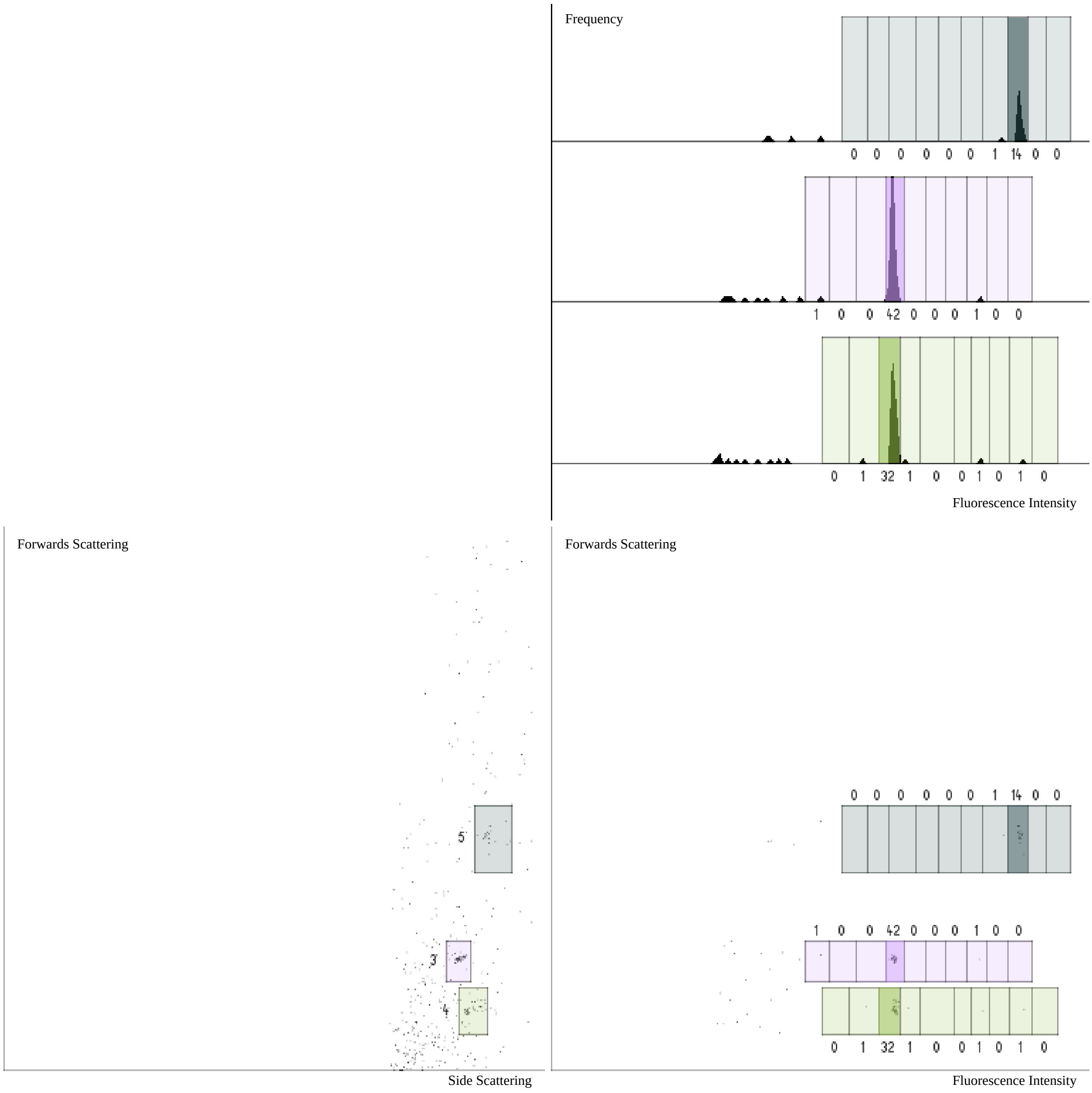

ANNEX 3: TAG DECONVOLUTION - BEAD 4

Passes flow sorting criteria: Yes

Passes tag deconvolution criteria: Yes

Included in protocol analysis: Yes

Protocol: 4, 10, 4, 1

Filename: Bin1_plateA1_A12.fcs

Split 1: Petrol shading

Split 2: Green shading

Split 3: Violet shading

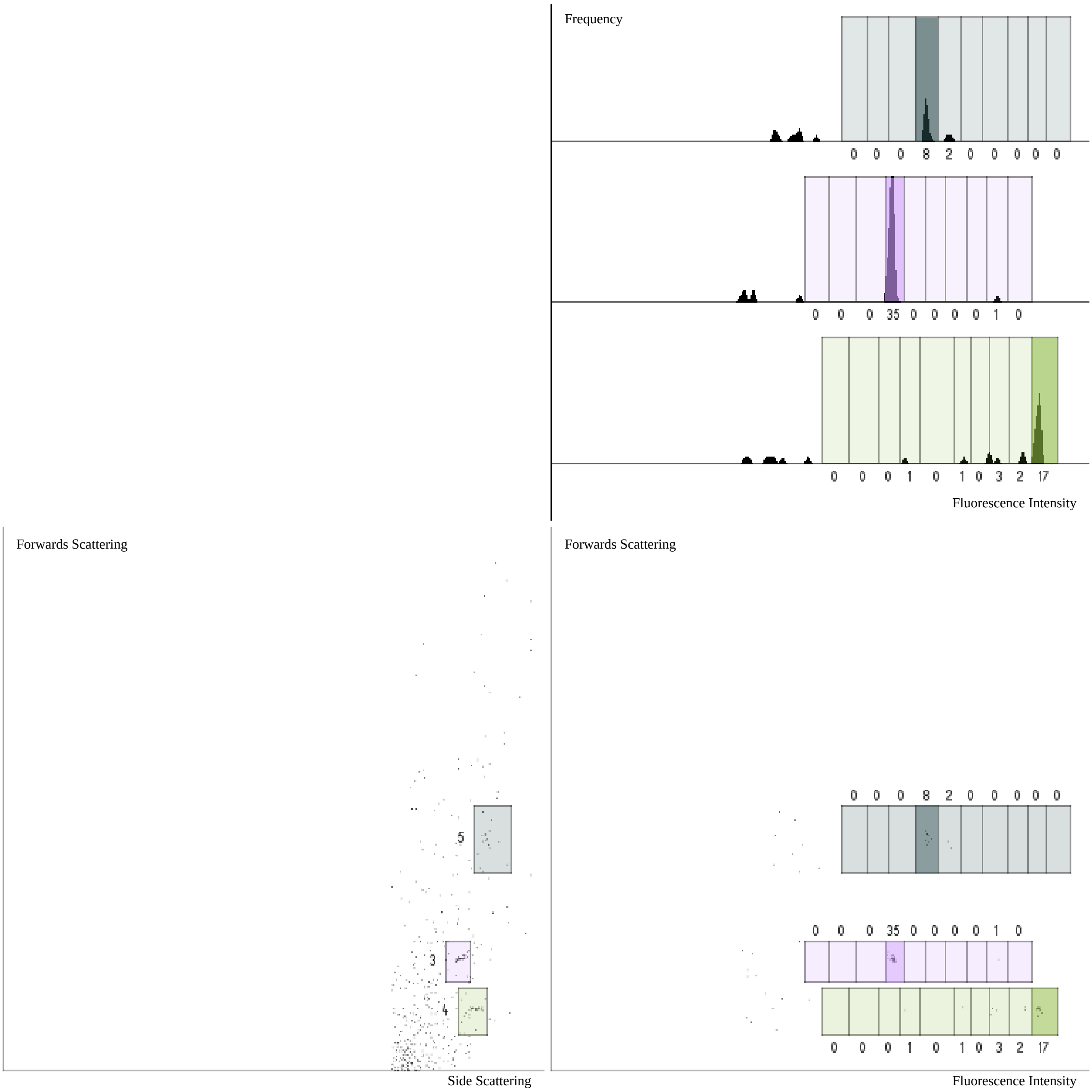

ANNEX 3: TAG DECONVOLUTION - BEAD 5

Passes flow sorting criteria: Yes

Passes tag deconvolution criteria: Yes

Included in protocol analysis: Yes

Protocol: 8, 6, 6, 1

Filename: Bin1_plateA1_B1.fcs

Split 1: Petrol shading

Split 2: Green shading

Split 3: Violet shading

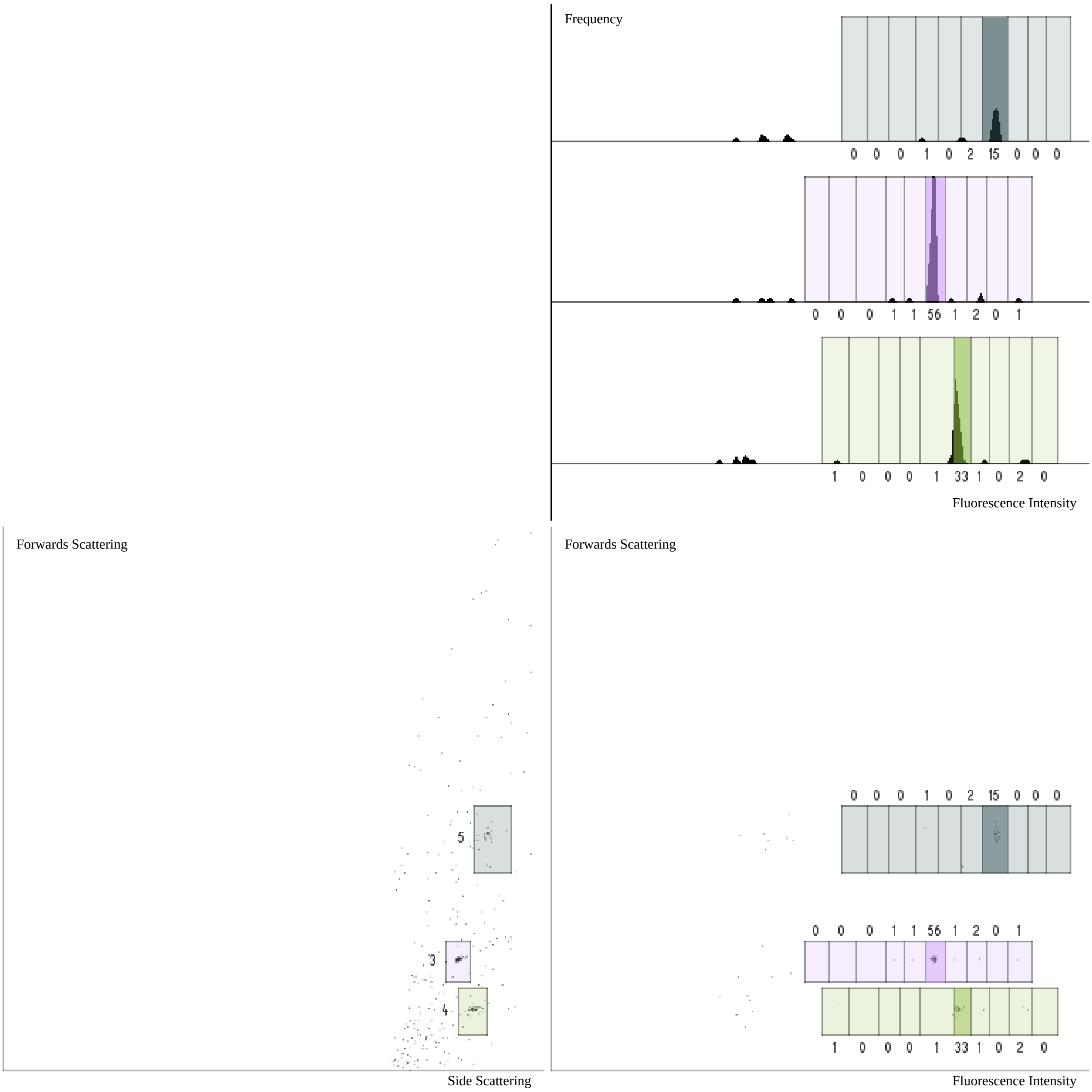

ANNEX 3: TAG DECONVOLUTION - BEAD 6

Passes flow sorting criteria: Yes

Passes tag deconvolution criteria: Yes

Included in protocol analysis: Yes

Protocol: 7, 7, 1, 1

Filename: Bin1_plateA1_B2.fcs

Split 1: Petrol shading

Split 2: Green shading

Split 3: Violet shading

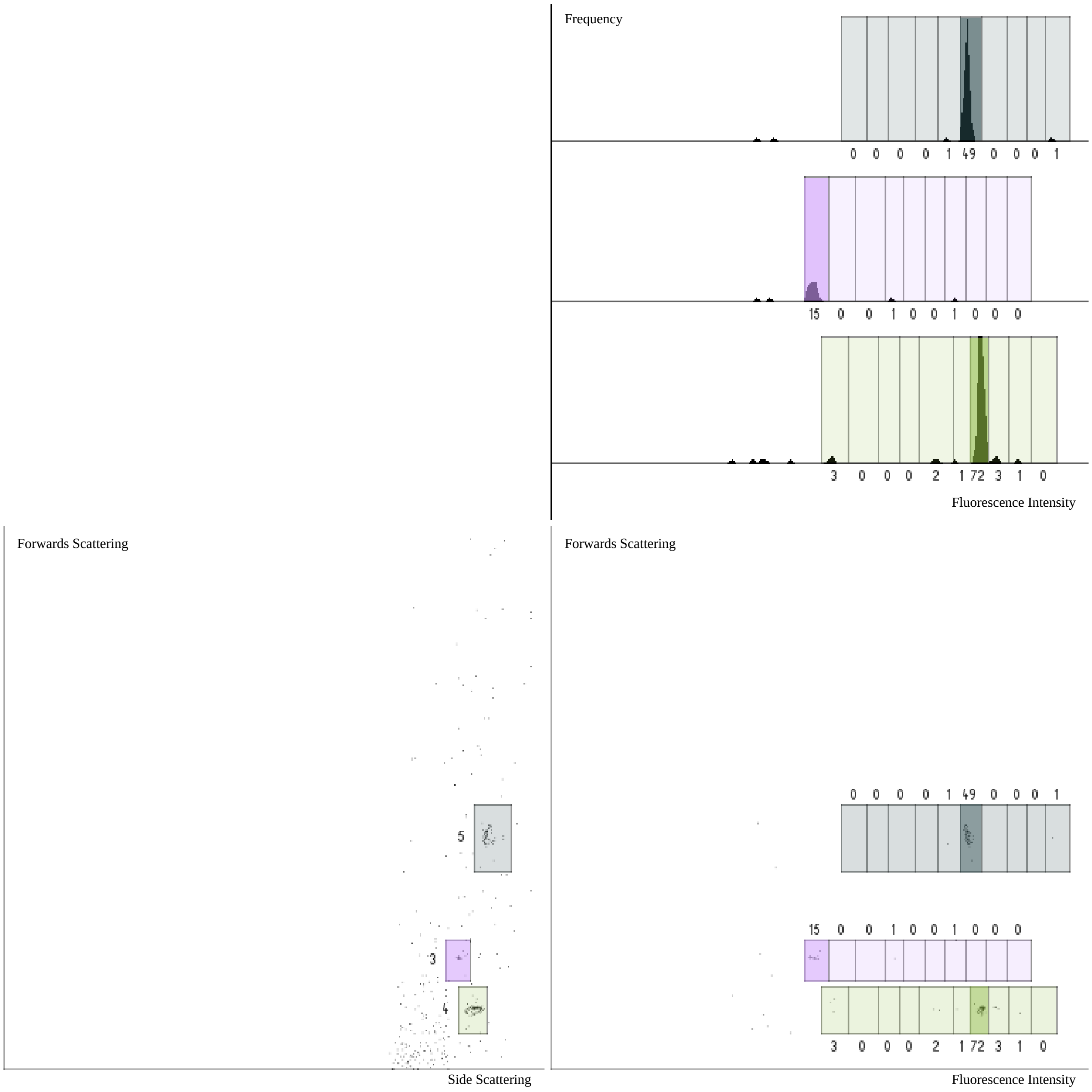

ANNEX 3: TAG DECONVOLUTION - BEAD 7

Passes flow sorting criteria: Yes

Passes tag deconvolution criteria: No

Included in protocol analysis: No

Protocol: N/A

Filename: Bin1_plateA1_B9.fcs

Split 1: Petrol shading

Split 2: Green shading

Split 3: Violet shading

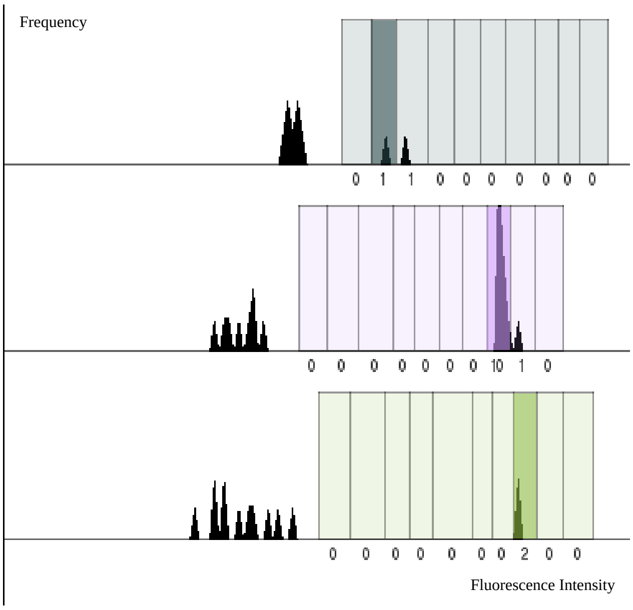

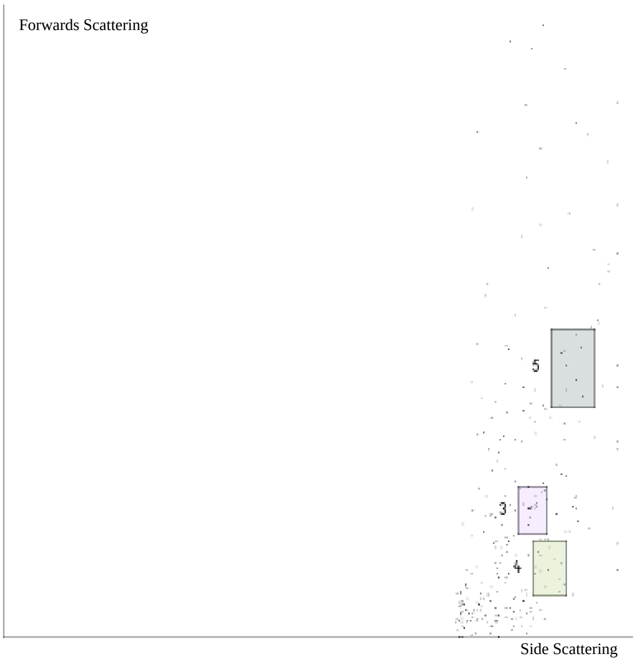

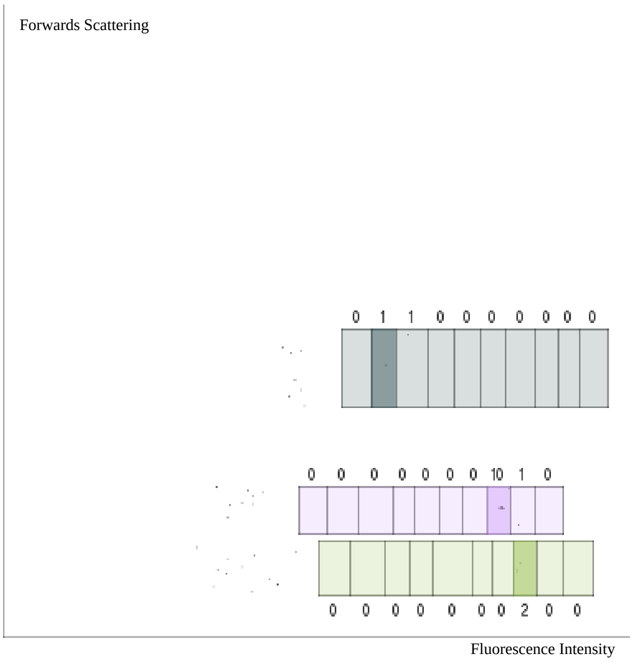

ANNEX 3: TAG DECONVOLUTION - BEAD 8

Passes flow sorting criteria: Yes

Passes tag deconvolution criteria: No

Included in protocol analysis: No

Protocol: N/A

Filename: Bin1_plateA1_C2.fcs

Split 1: Petrol shading

Split 2: Green shading

Split 3: Violet shading

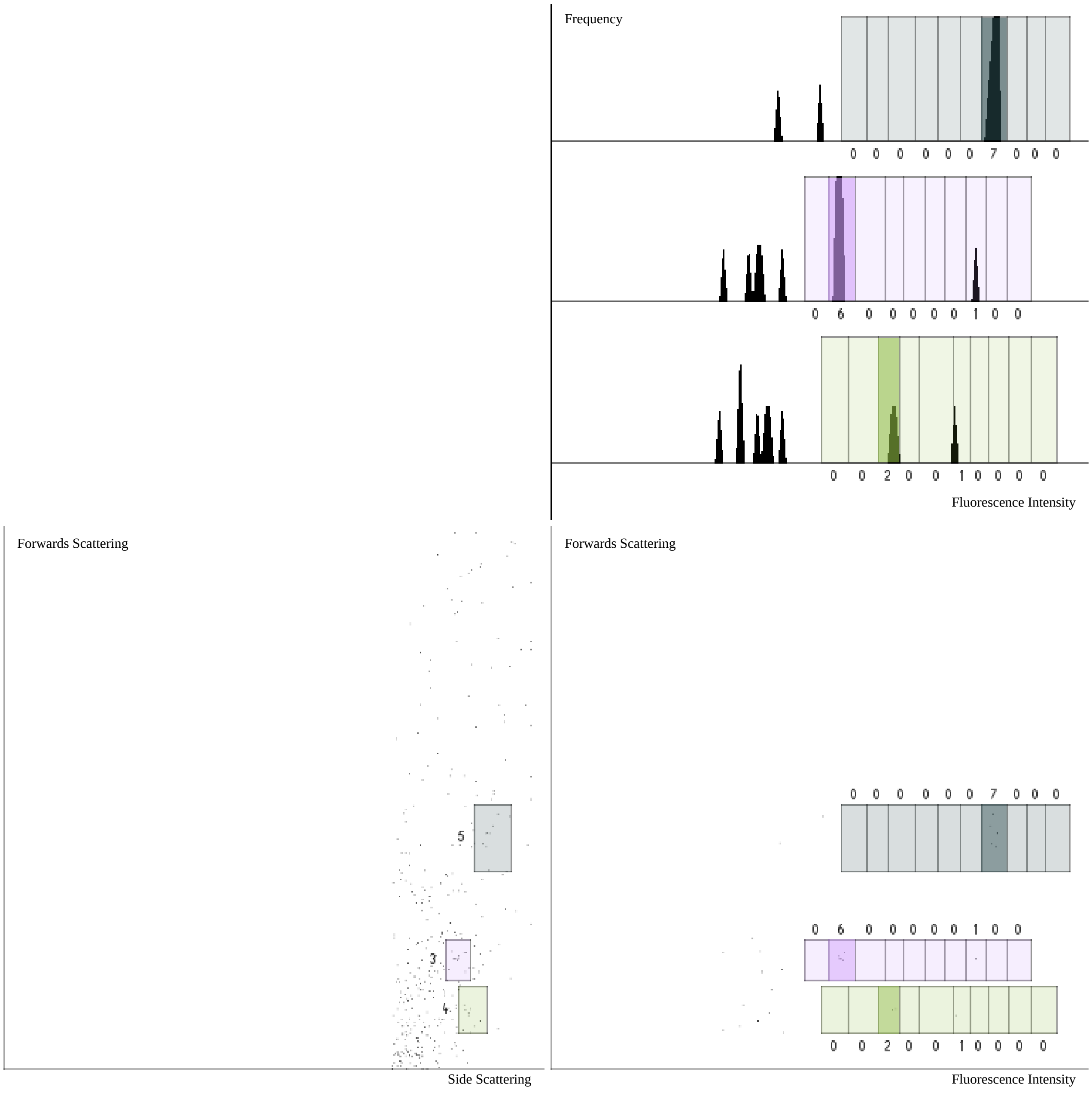

ANNEX 3: TAG DECONVOLUTION - BEAD 9

Passes flow sorting criteria: Yes

Passes tag deconvolution criteria: Yes

Included in protocol analysis: Yes

Protocol: 6, 7, 8, 1

Filename: Bin1_plateA1_C4.fcs

Split 1: Petrol shading

Split 2: Green shading

Split 3: Violet shading

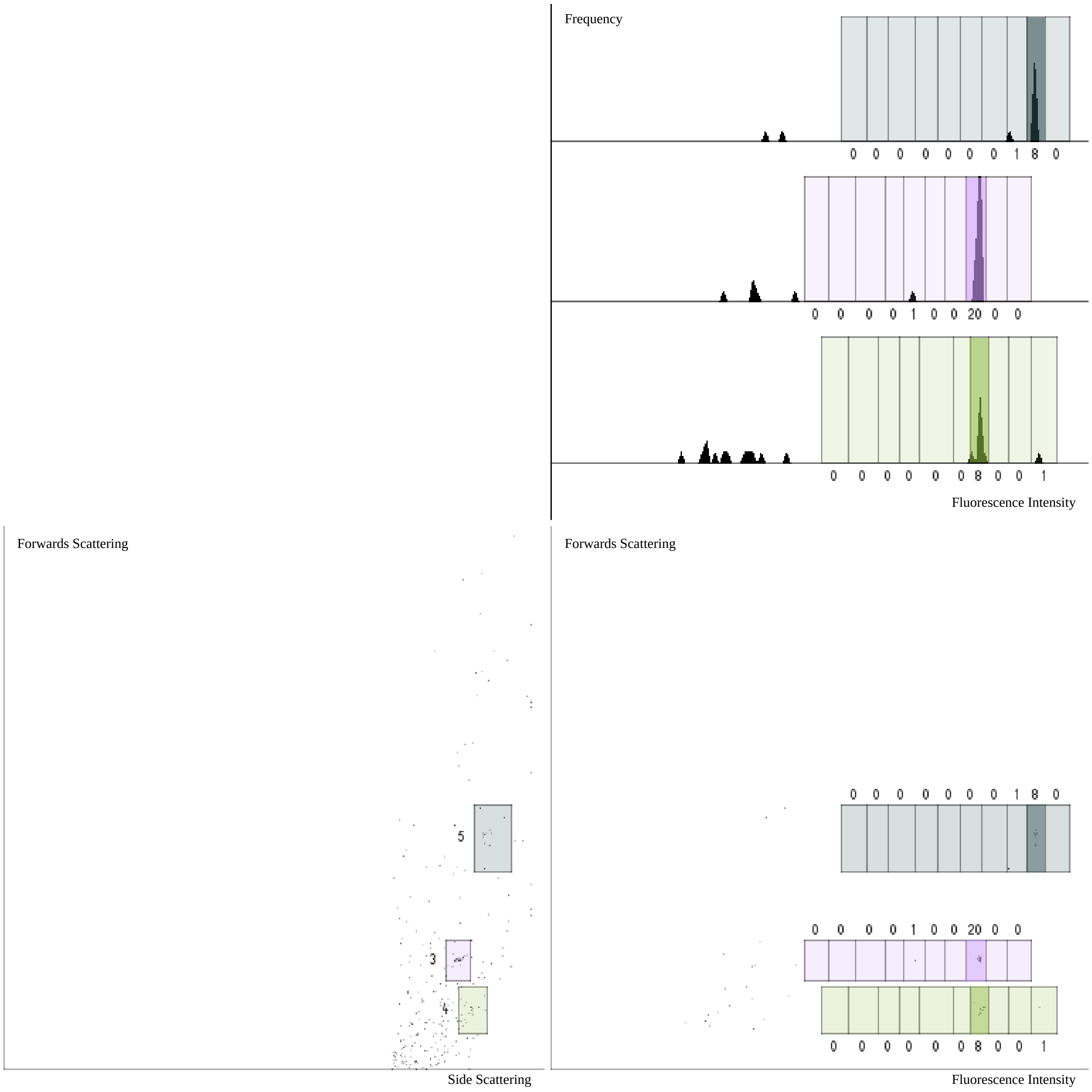

ANNEX 3: TAG DECONVOLUTION - BEAD 10

Passes flow sorting criteria: Yes

Passes tag deconvolution criteria: Yes

Included in protocol analysis: Yes

Protocol: 1, 3, 3, 1

Filename: Bin1_plateA1_C7.fcs

Split 1: Petrol shading

Split 2: Green shading

Split 3: Violet shading

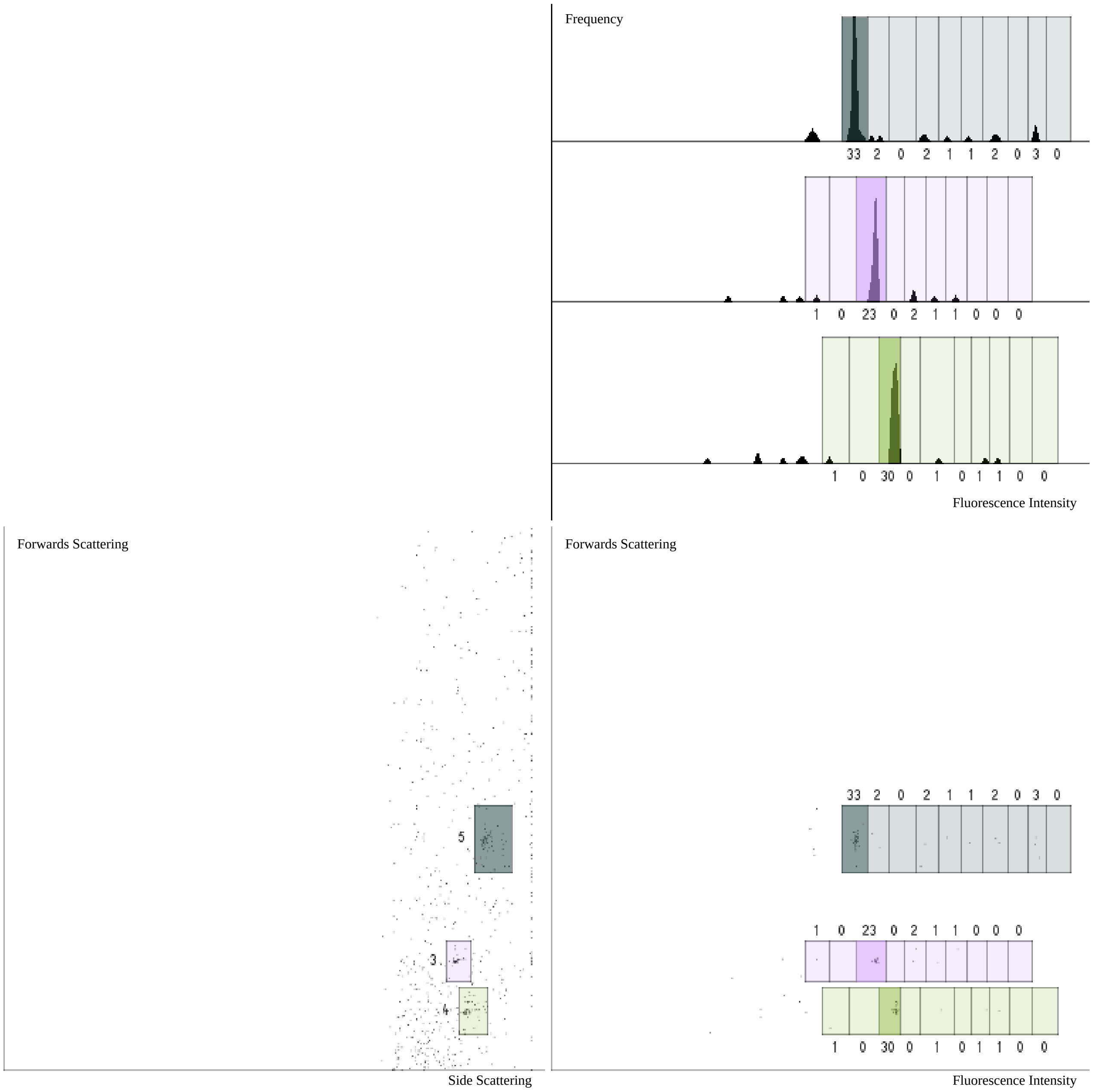

ANNEX 3: TAG DECONVOLUTION - BEAD 11

Passes flow sorting criteria: Yes

Passes tag deconvolution criteria: Yes

Included in protocol analysis: Yes

Protocol: 9, 6, 7, 1

Filename: Bin1_plateA1_C8.fcs

Split 1: Petrol shading

Split 2: Green shading

Split 3: Violet shading

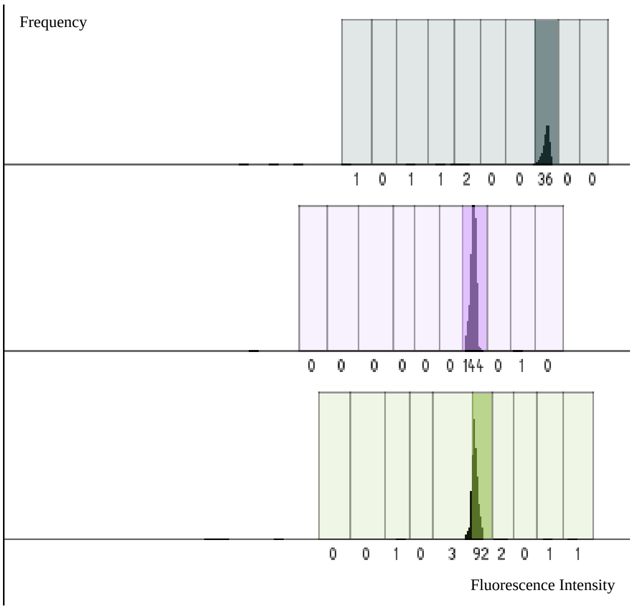

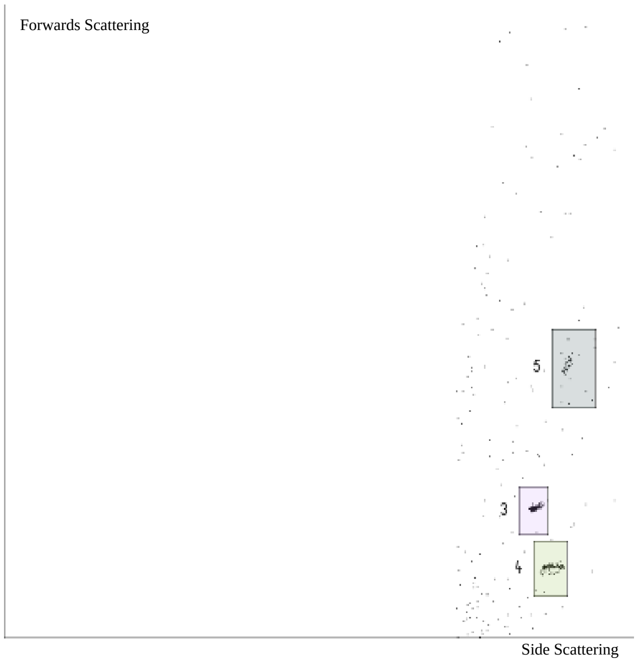

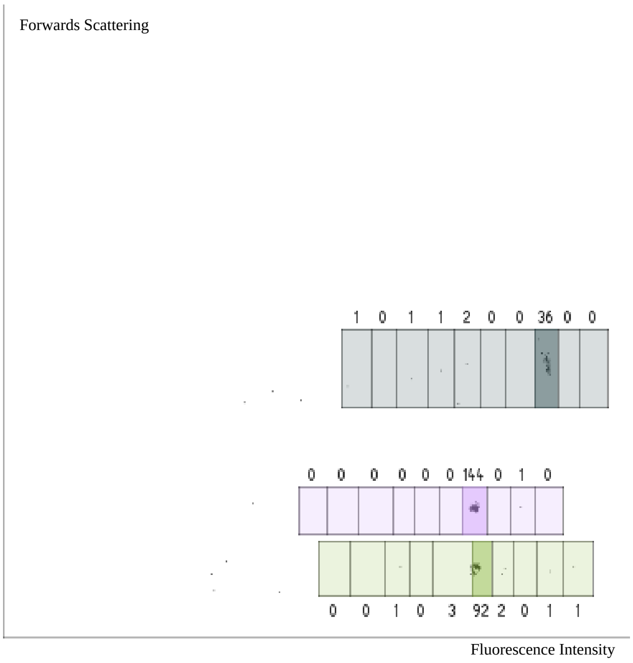

ANNEX 3: TAG DECONVOLUTION - BEAD 12

Passes flow sorting criteria: Yes

Passes tag deconvolution criteria: Yes

Included in protocol analysis: Yes

Protocol: 3, 9, 5, 1

Filename: Bin1_plateA1_C10.fcs

Split 1: Petrol shading

Split 2: Green shading

Split 3: Violet shading

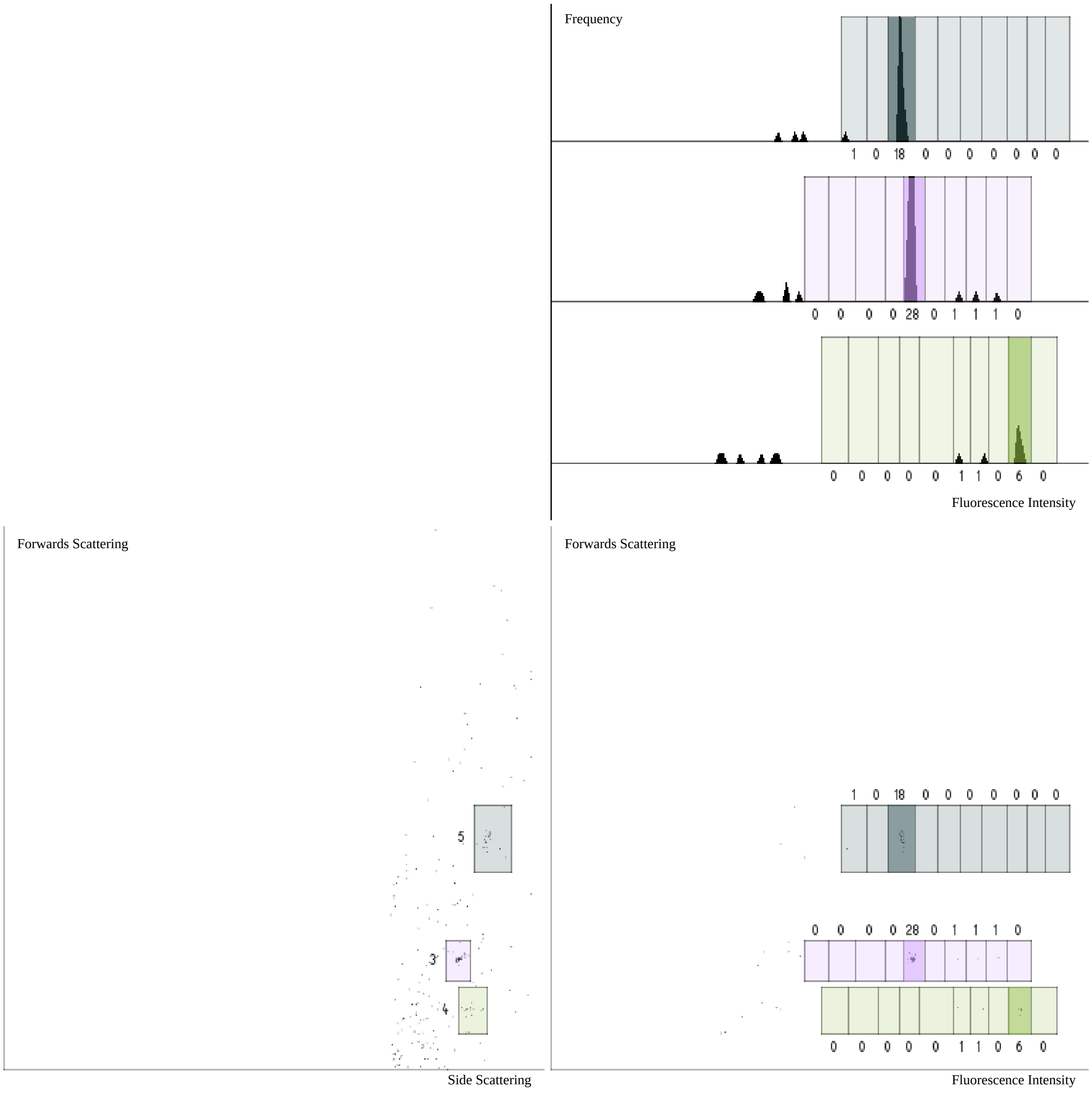

ANNEX 3: TAG DECONVOLUTION - BEAD 13

Passes flow sorting criteria: Yes

Passes tag deconvolution criteria: Yes

Included in protocol analysis: Yes

Protocol: 5, 9, 4, 1

Filename: Bin1_plateA1_F9.fcs

Split 1: Petrol shading

Split 2: Green shading

Split 3: Violet shading

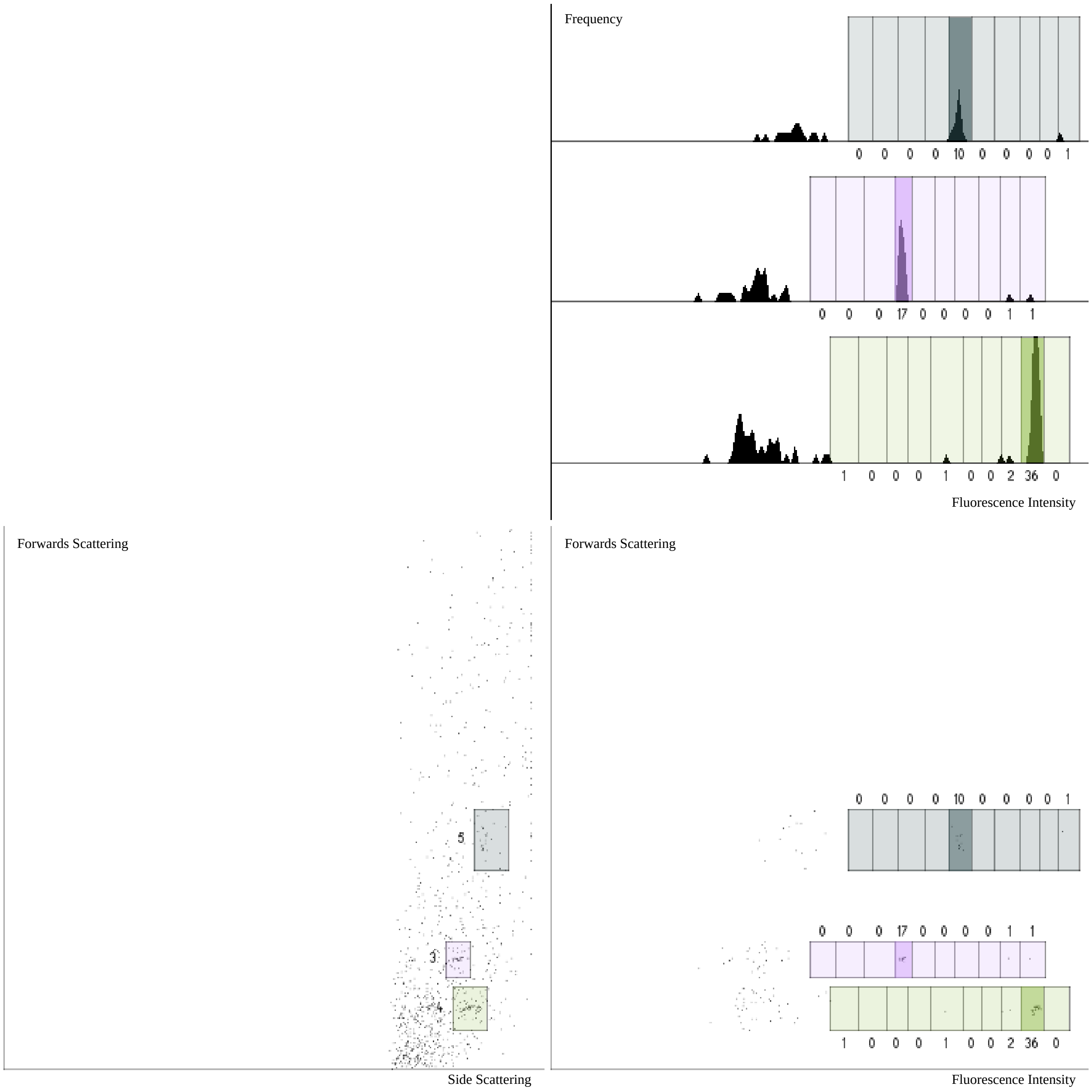

ANNEX 3: TAG DECONVOLUTION - BEAD 14

Passes flow sorting criteria: Yes

Passes tag deconvolution criteria: Yes

Included in protocol analysis: Yes

Protocol: 1, 6, 4, 1

Filename: Bin1_plateA1_E3.fcs

Split 1: Petrol shading

Split 2: Green shading

Split 3: Violet shading

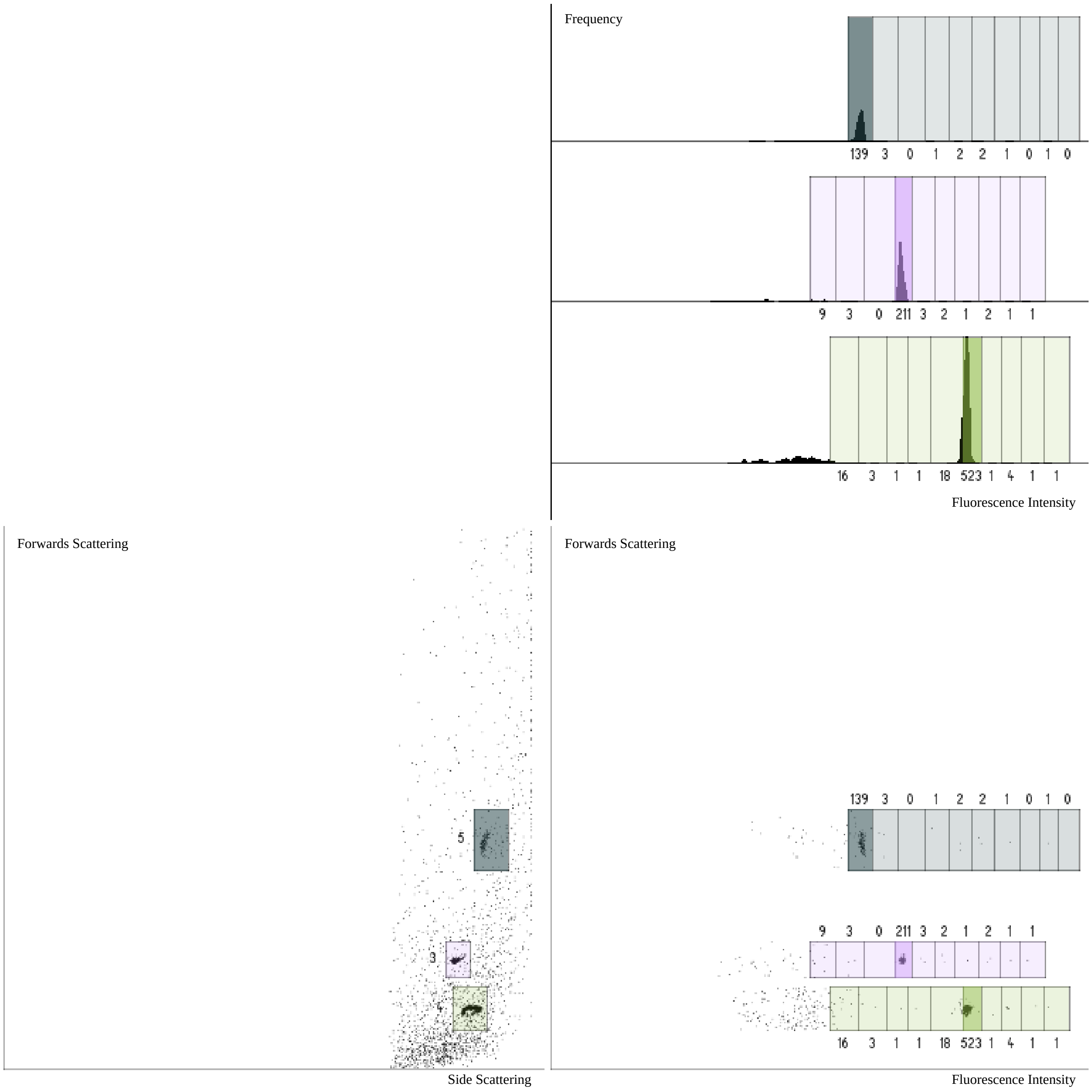

ANNEX 3: TAG DECONVOLUTION - BEAD 15

Passes flow sorting criteria: Yes

Passes tag deconvolution criteria: Yes

Included in protocol analysis: Yes

Protocol: 8, 10, 7, 1

Filename: Bin1_plateA1_E5.fcs

Split 1: Petrol shading

Split 2: Green shading

Split 3: Violet shading

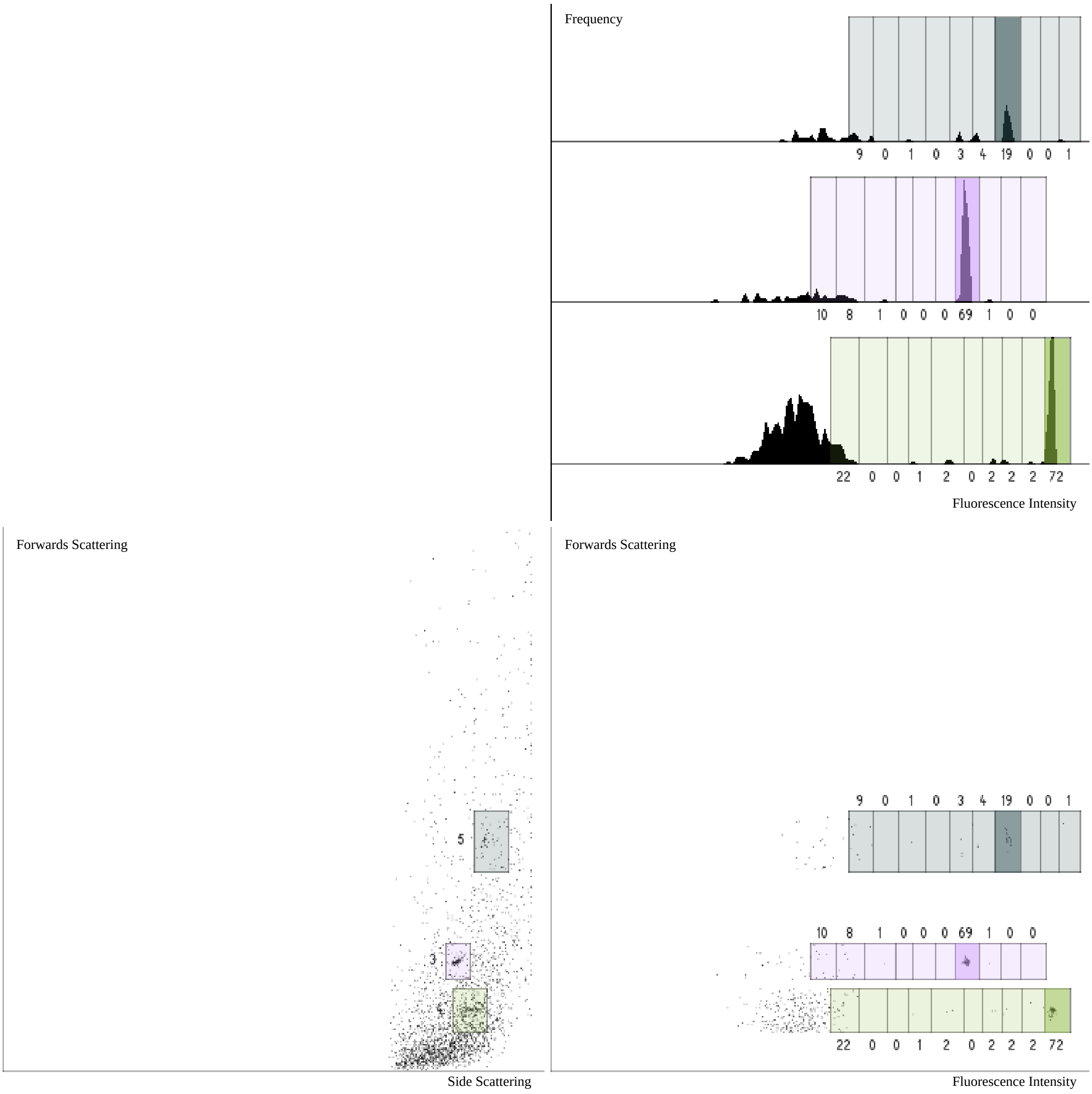

ANNEX 3: TAG DECONVOLUTION - BEAD 16

Passes flow sorting criteria: Yes

Passes tag deconvolution criteria: Yes

Included in protocol analysis: Yes

Protocol: 10, 3, 6, 1

Filename: Bin1_plateA1_E9.fcs

Split 1: Petrol shading

Split 2: Green shading

Split 3: Violet shading

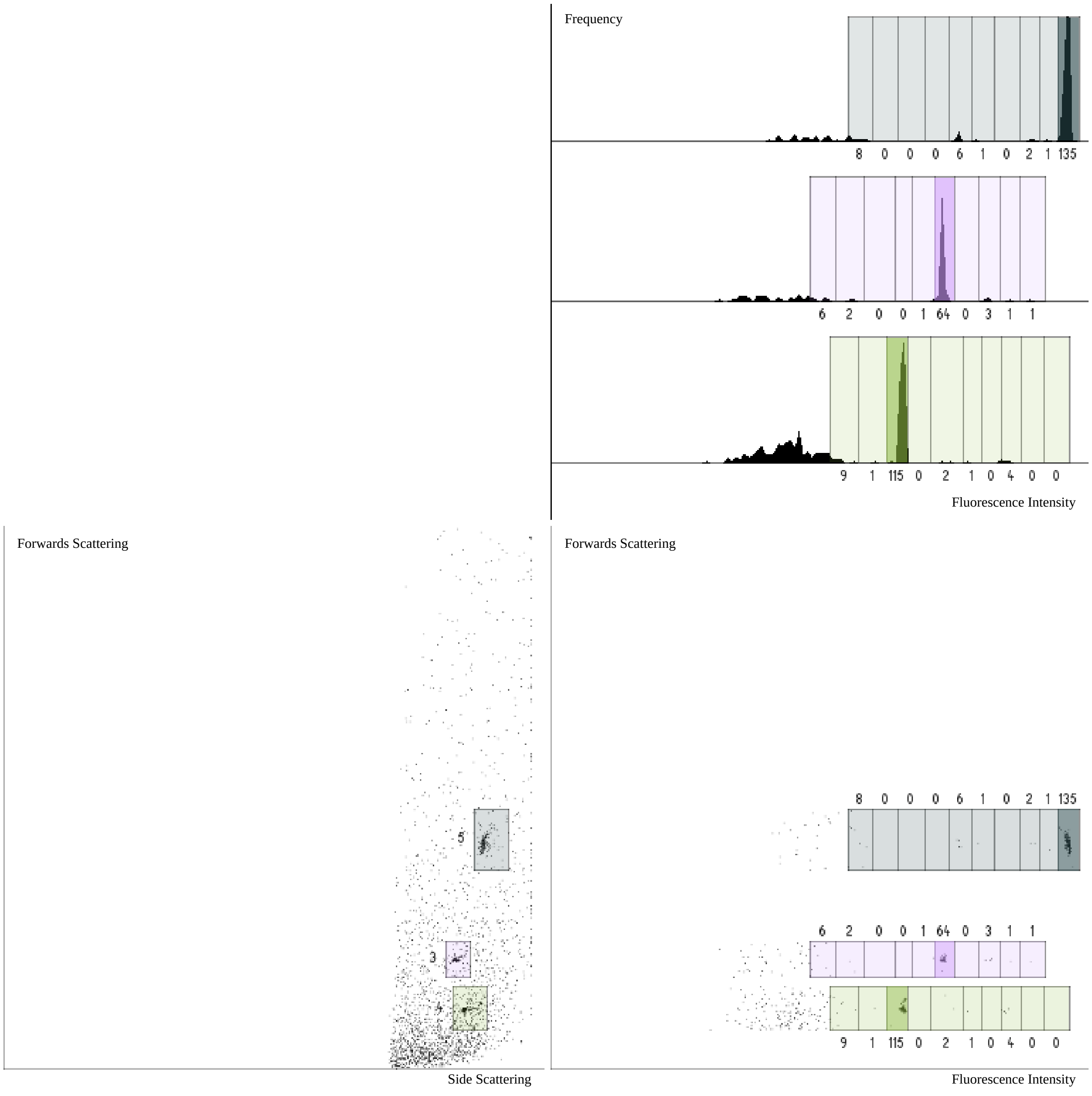

ANNEX 3: TAG DECONVOLUTION - BEAD 17

Passes flow sorting criteria: Yes

Passes tag deconvolution criteria: Yes

Included in protocol analysis: Yes

Protocol: 6, 4, 5, 1

Filename: Bin1_plateA1_E11.fcs

Split 1: Petrol shading

Split 2: Green shading

Split 3: Violet shading

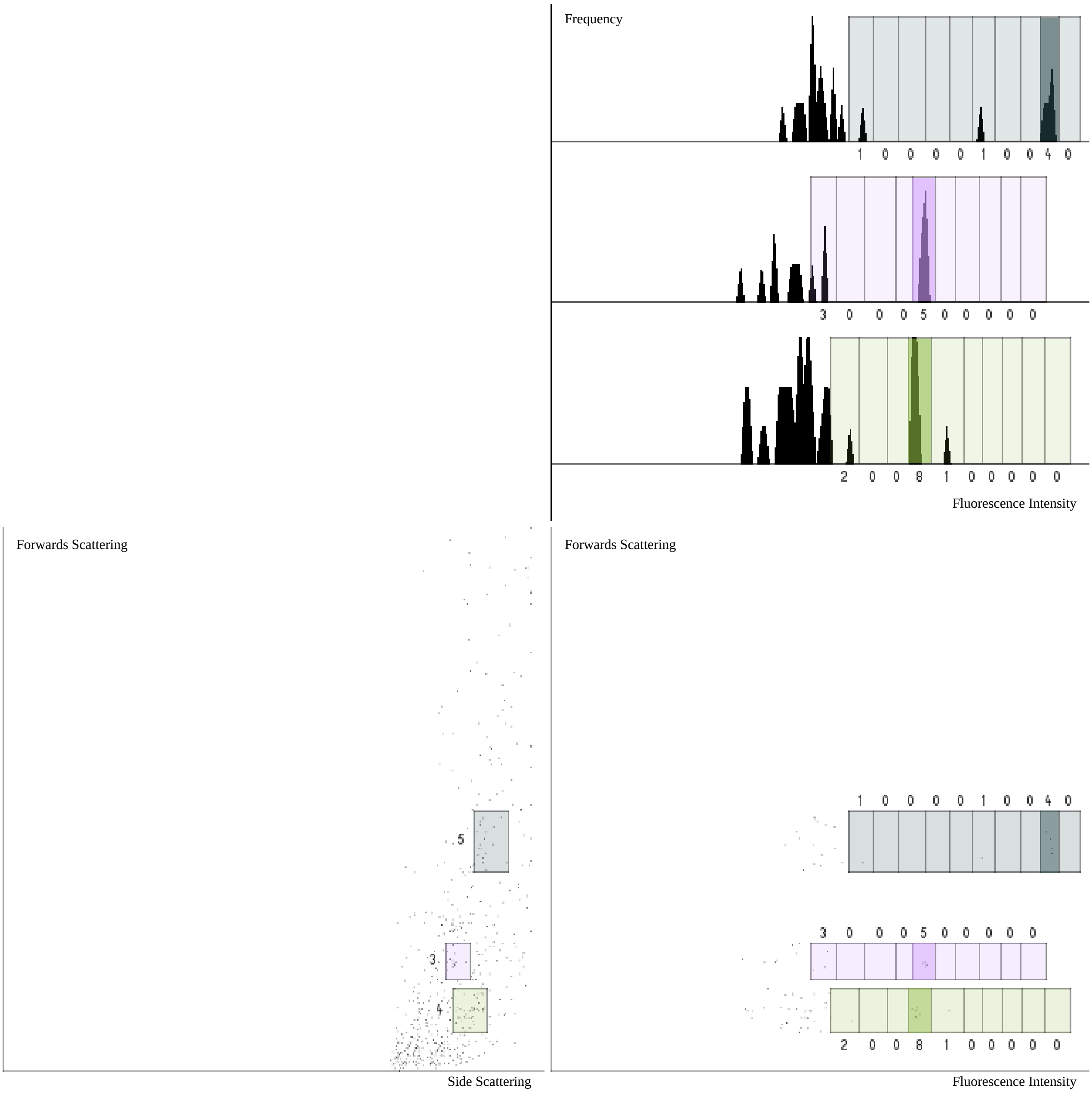

ANNEX 3: TAG DECONVOLUTION - BEAD 18

Passes flow sorting criteria: Yes

Passes tag deconvolution criteria: Yes

Included in protocol analysis: Yes

Protocol: 5, 2, 10, 1

Filename: Bin1_plateA1_F2.fcs

Split 1: Petrol shading

Split 2: Green shading

Split 3: Violet shading

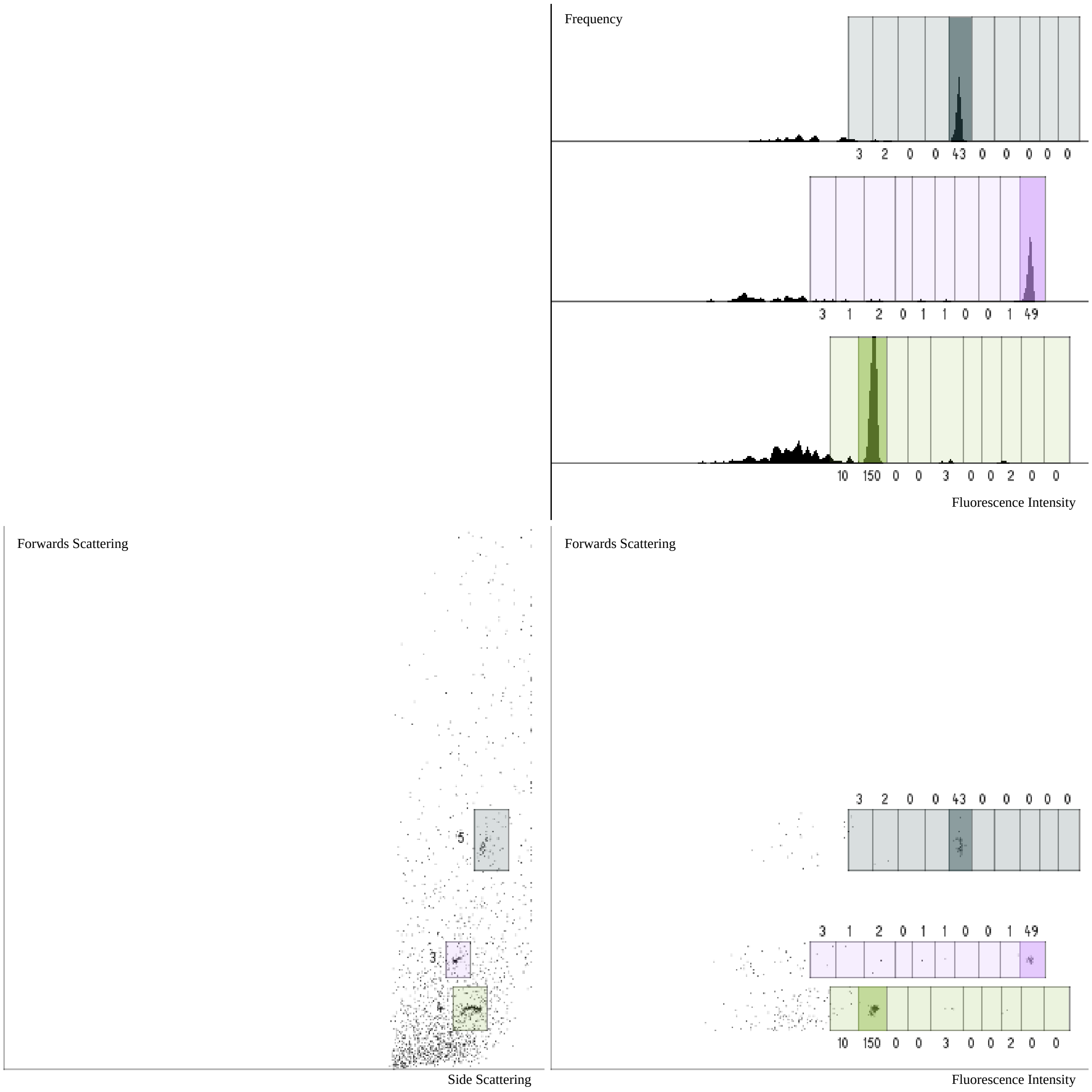

ANNEX 3: TAG DECONVOLUTION - BEAD 19

Passes flow sorting criteria: Yes

Passes tag deconvolution criteria: Yes

Included in protocol analysis: Yes

Protocol: 5, 10, 7, 1

Filename: Bin1_plateA2_F11.fcs

Split 1: Petrol shading

Split 2: Green shading

Split 3: Violet shading

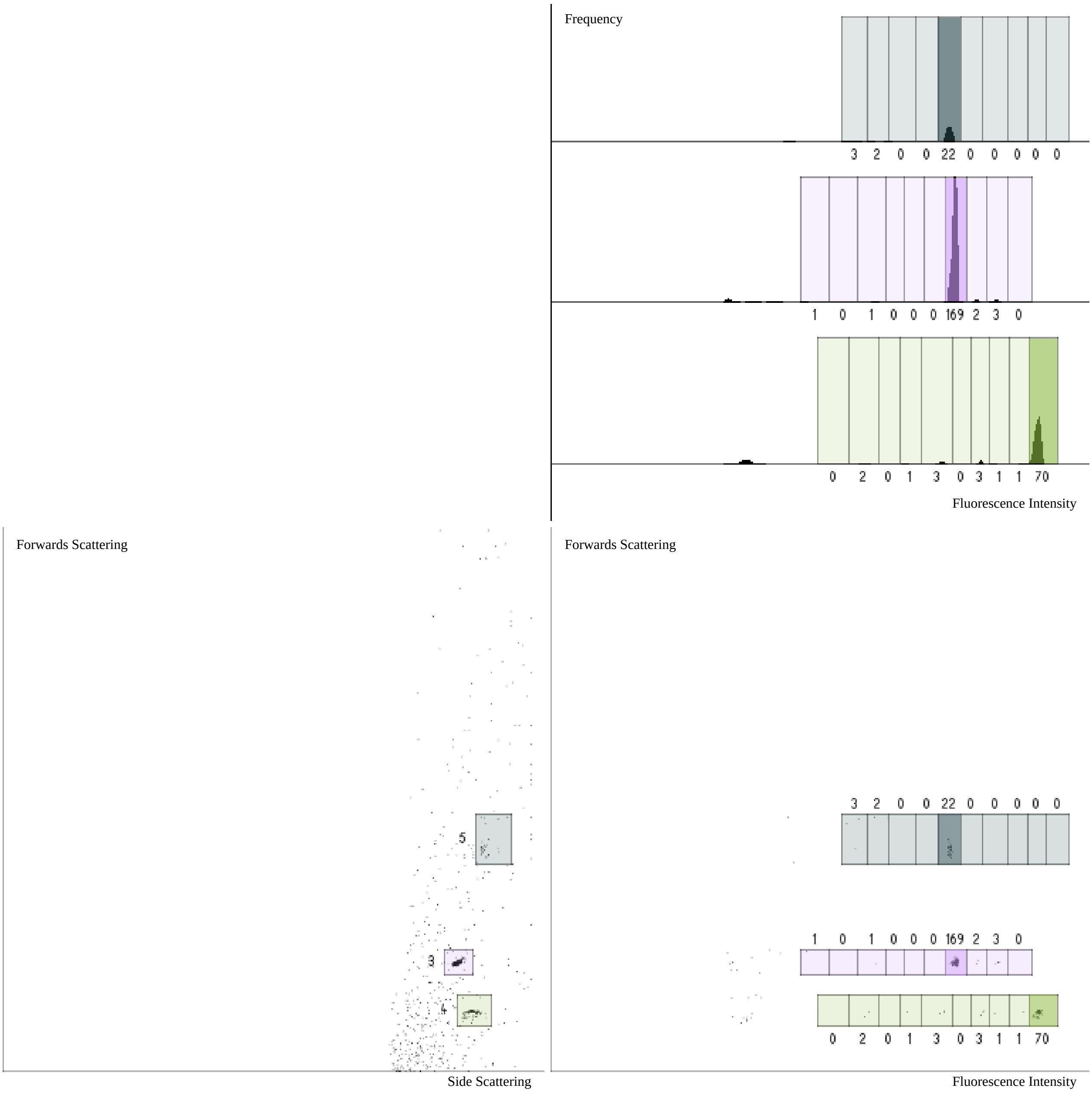

ANNEX 3: TAG DECONVOLUTION - BEAD 20

Passes flow sorting criteria: Yes

Passes tag deconvolution criteria: Yes

Included in protocol analysis: Yes

Protocol: 9, 9, 2, 1

Filename: Bin1_plateA2_A5.fcs

Split 1: Petrol shading

Split 2: Green shading

Split 3: Violet shading

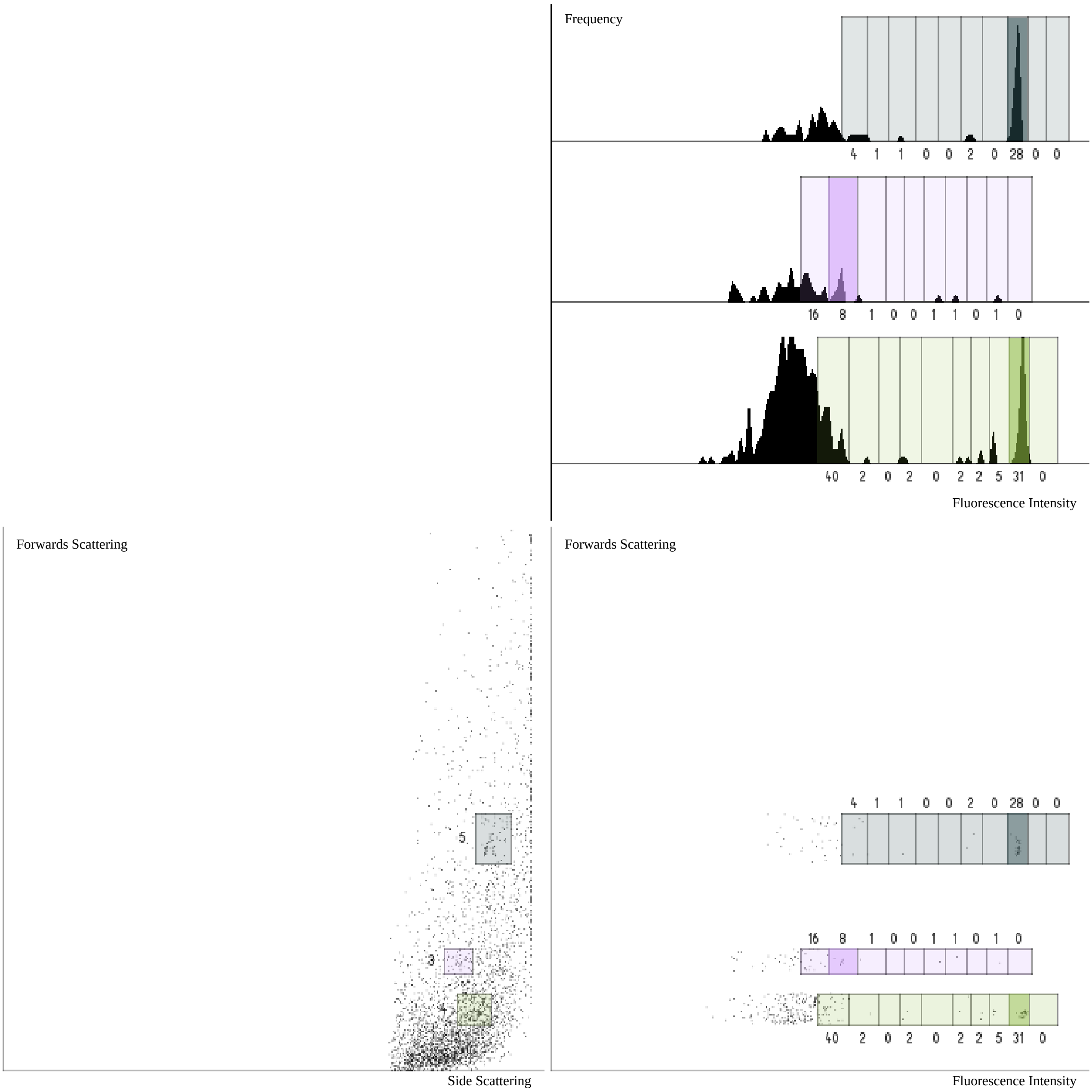

ANNEX 3: TAG DECONVOLUTION - BEAD 21

Passes flow sorting criteria: Yes

Passes tag deconvolution criteria: Yes

Included in protocol analysis: Yes

Protocol: 5, 2, 5, 1

Filename: Bin1_plateA2_A9.fcs

Split 1: Petrol shading

Split 2: Green shading

Split 3: Violet shading

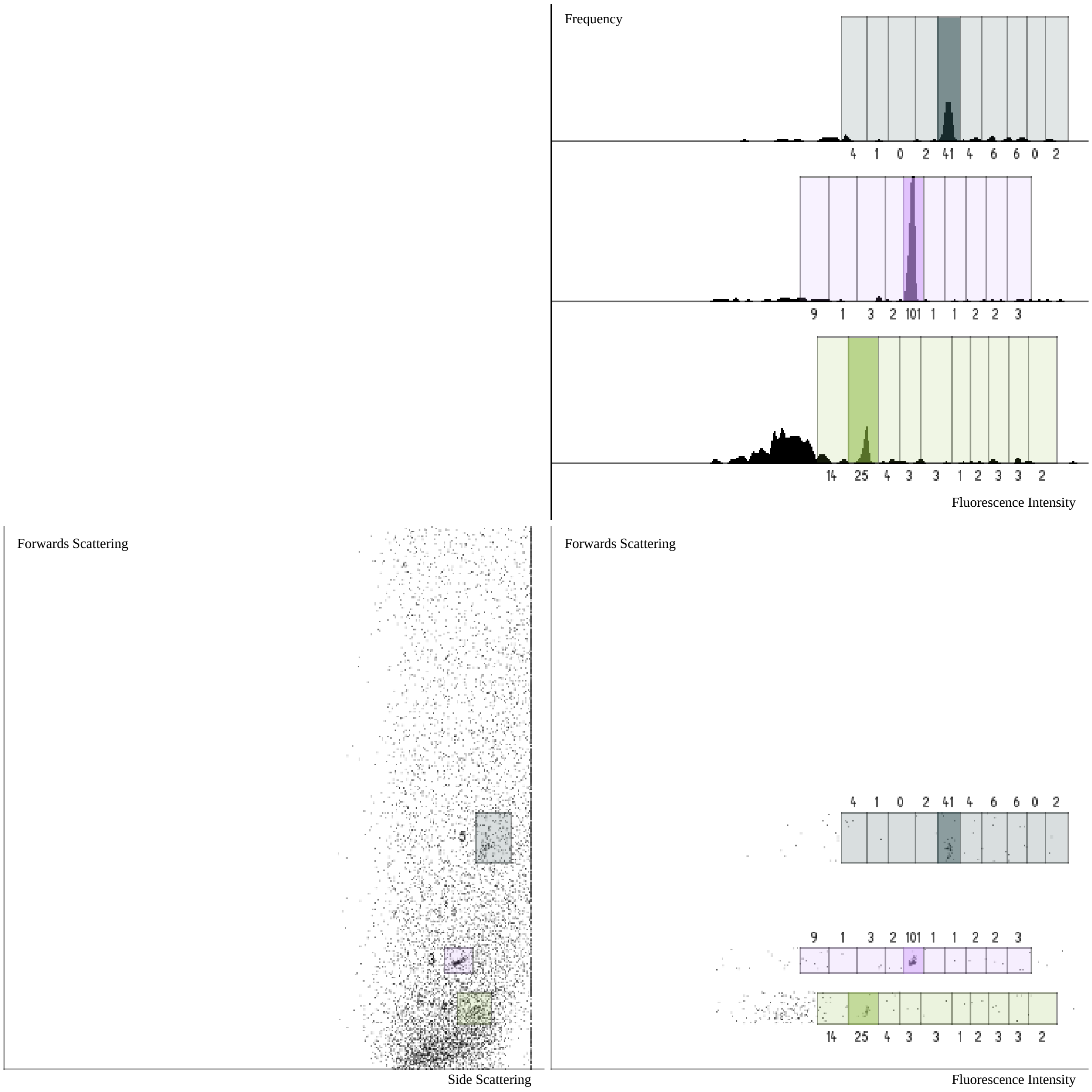

ANNEX 3: TAG DECONVOLUTION - BEAD 22

Passes flow sorting criteria: Yes

Passes tag deconvolution criteria: Yes

Included in protocol analysis: Yes

Protocol: 3, 9, 2, 1

Filename: Bin1_plateA2_A10.fcs

Split 1: Petrol shading

Split 2: Green shading

Split 3: Violet shading

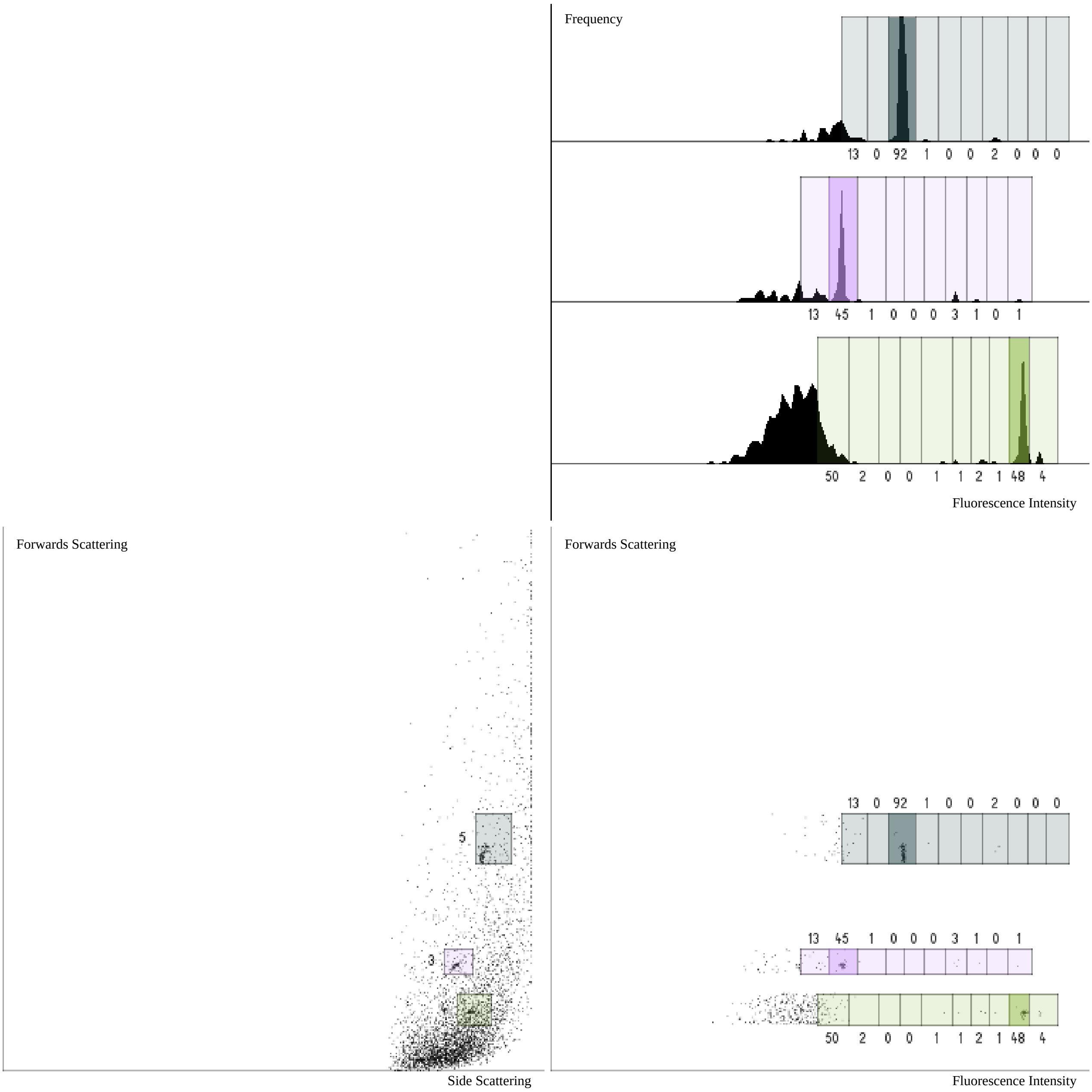

ANNEX 3: TAG DECONVOLUTION - BEAD 23

Passes flow sorting criteria: Yes

Passes tag deconvolution criteria: Yes

Included in protocol analysis: Yes

Protocol: 6, 9, 1, 1

Filename: Bin1_plateA2_A11.fcs

Split 1: Petrol shading

Split 2: Green shading

Split 3: Violet shading

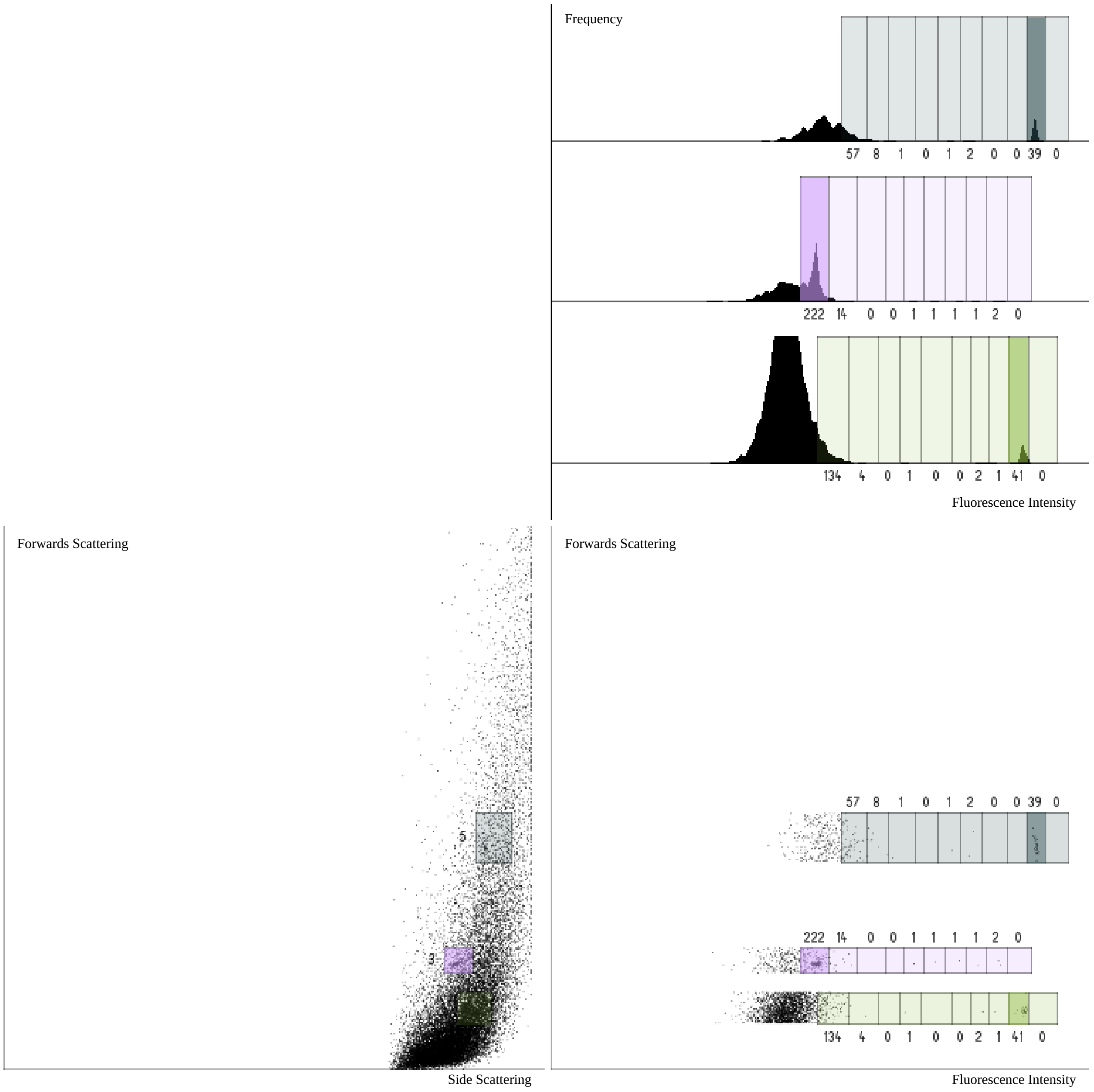

ANNEX 3: TAG DECONVOLUTION - BEAD 24

Passes flow sorting criteria: Yes

Passes tag deconvolution criteria: Yes

Included in protocol analysis: Yes

Protocol: 8, 6, 3, 1

Filename: Bin1_plateA2_A12.fcs

Split 1: Petrol shading

Split 2: Green shading

Split 3: Violet shading

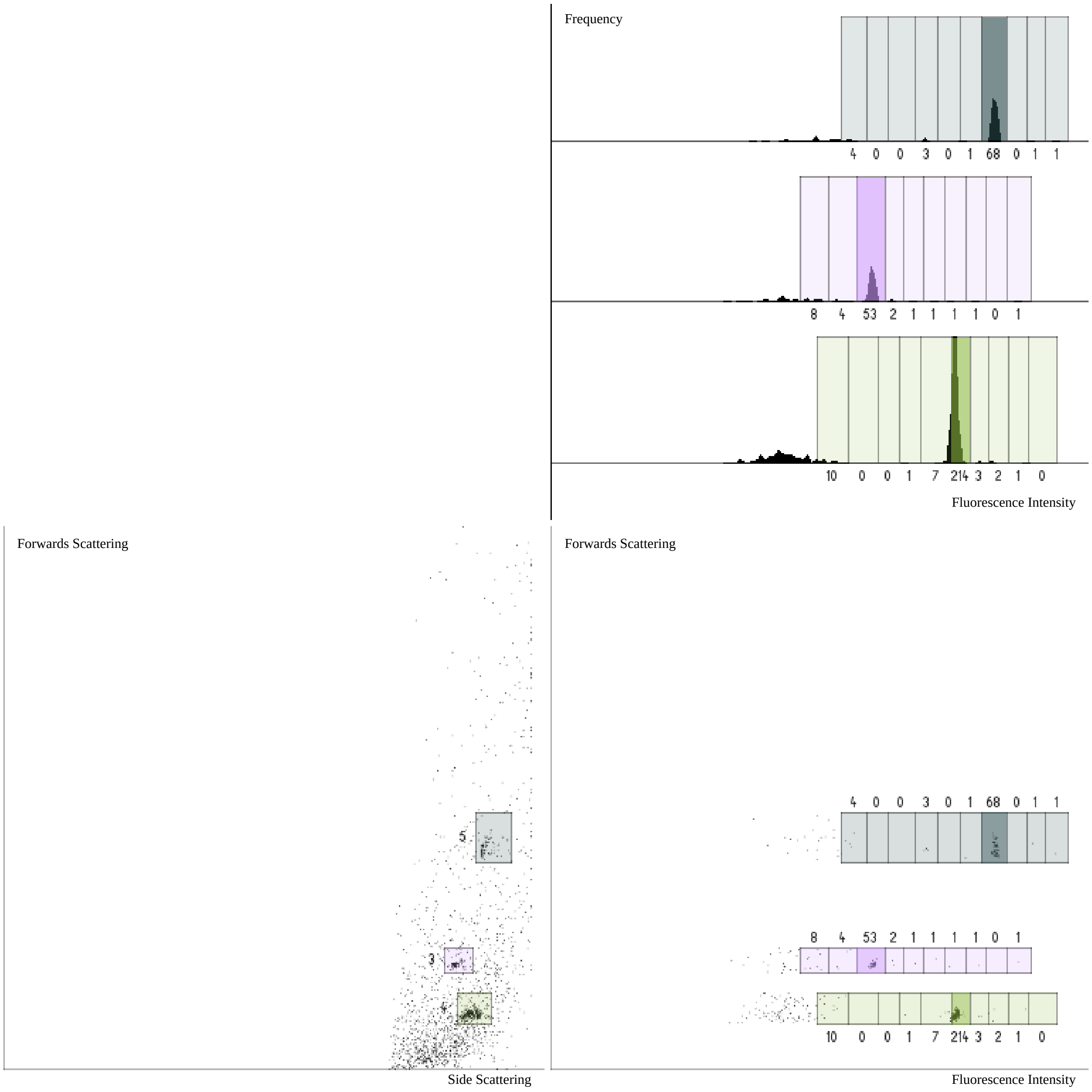

ANNEX 3: TAG DECONVOLUTION - BEAD 25

Passes flow sorting criteria: Yes

Passes tag deconvolution criteria: Yes

Included in protocol analysis: Yes

Protocol: 10, 10, 10, 1

Filename: Bin1_plateA2_B1.fcs

Split 1: Petrol shading

Split 2: Green shading

Split 3: Violet shading

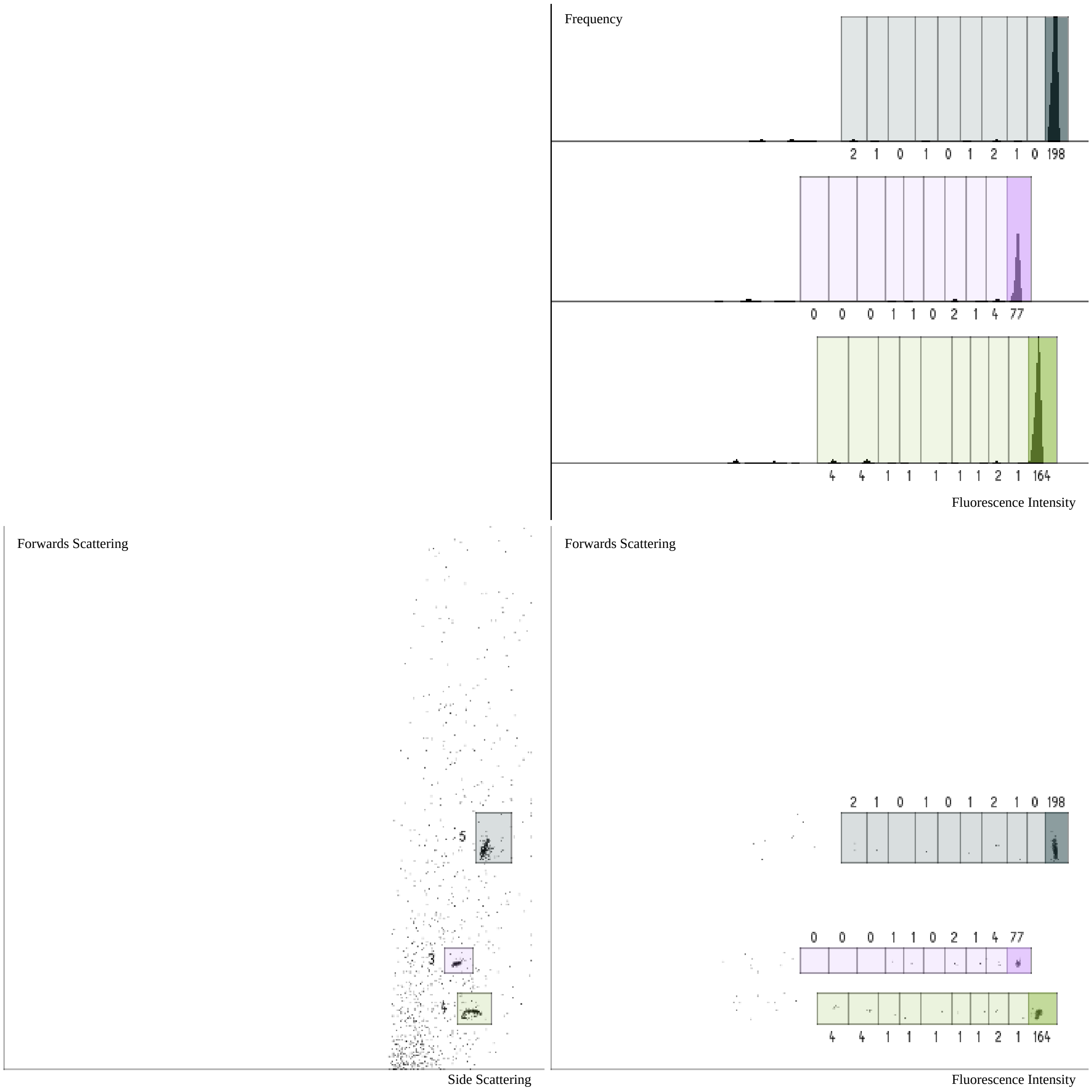

ANNEX 3: TAG DECONVOLUTION - BEAD 26

Passes flow sorting criteria: Yes

Passes tag deconvolution criteria: Yes

Included in protocol analysis: Yes

Protocol: 4, 8, 4, 1

Filename: Bin1_plateA2_B3.fcs

Split 1: Petrol shading

Split 2: Green shading

Split 3: Violet shading

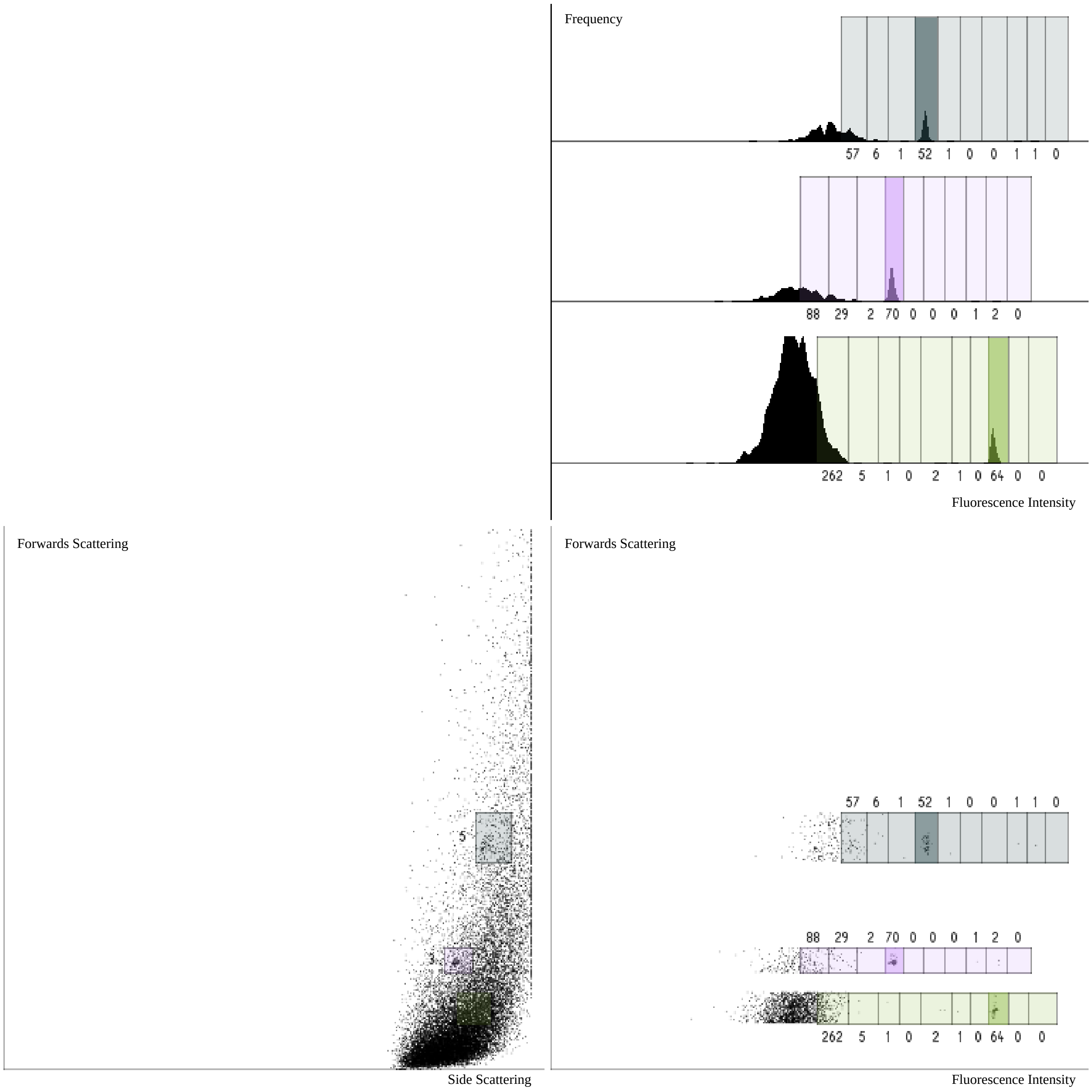

ANNEX 3: TAG DECONVOLUTION - BEAD 27

Passes flow sorting criteria: Yes

Passes tag deconvolution criteria: Yes

Included in protocol analysis: Yes

Protocol: 9, 5, 5, 1

Filename: Bin1_plateA2_B12.fcs

Split 1: Petrol shading

Split 2: Green shading

Split 3: Violet shading

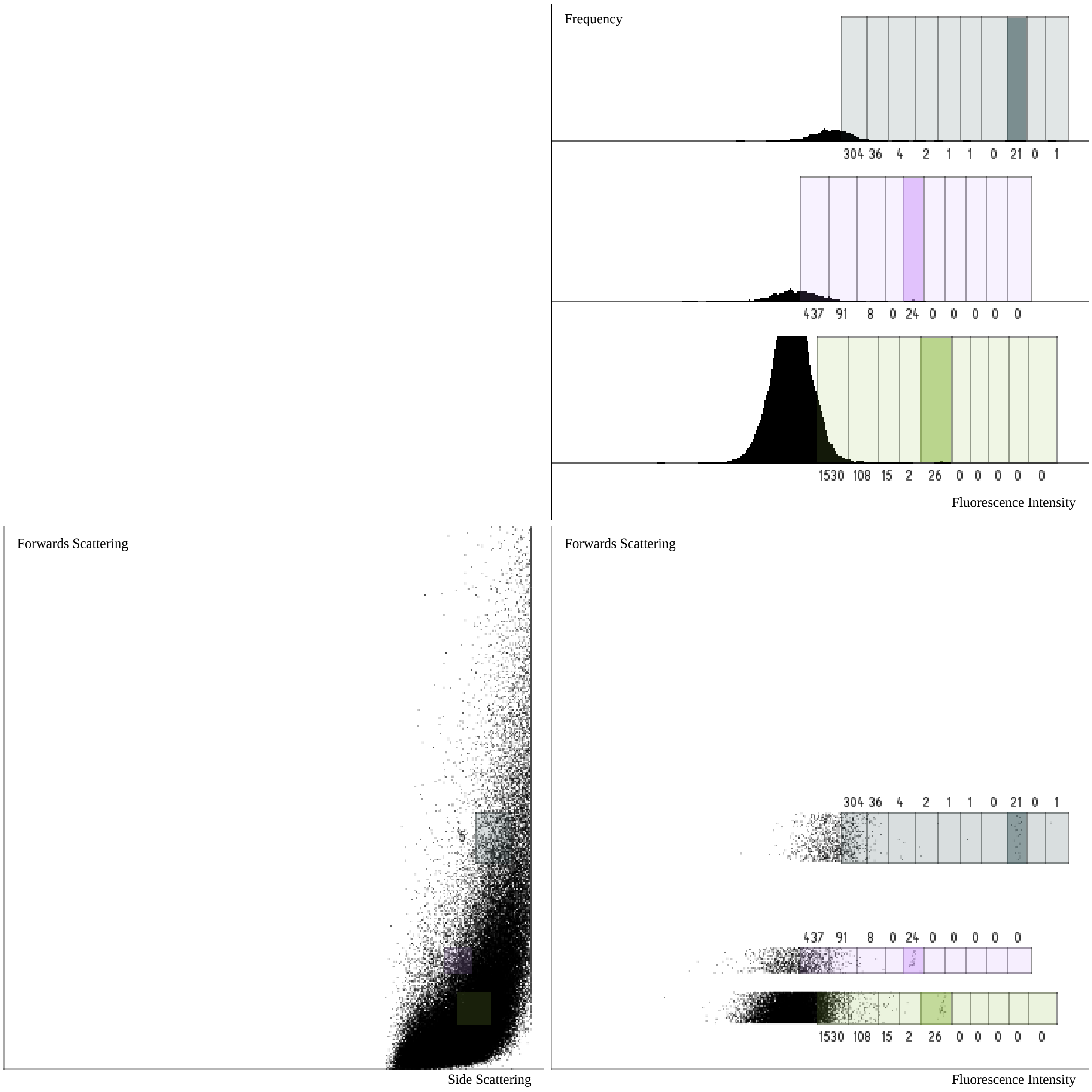

ANNEX 3: TAG DECONVOLUTION - BEAD 28

Passes flow sorting criteria: Yes

Passes tag deconvolution criteria: Yes

Included in protocol analysis: Yes

Protocol: 9, 4, 8, 1

Filename: Bin1_plateA2_C1.fcs

Split 1: Petrol shading

Split 2: Green shading

Split 3: Violet shading

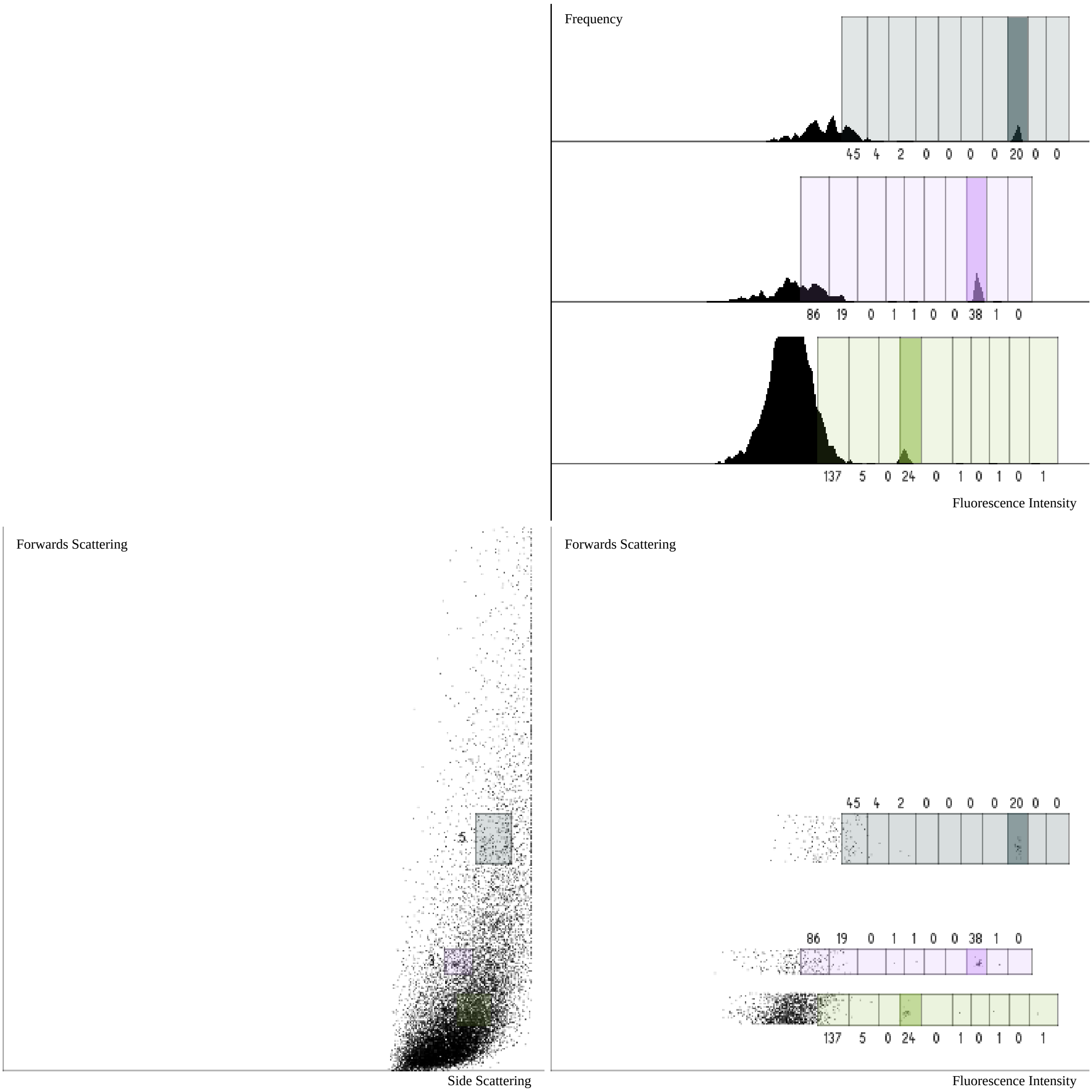

ANNEX 3: TAG DECONVOLUTION - BEAD 29

Passes flow sorting criteria: Yes

Passes tag deconvolution criteria: Yes

Included in protocol analysis: Yes

Protocol: 10, 6, 7, 1

Filename: Bin1_plateA2_C2.fcs

Split 1: Petrol shading

Split 2: Green shading

Split 3: Violet shading

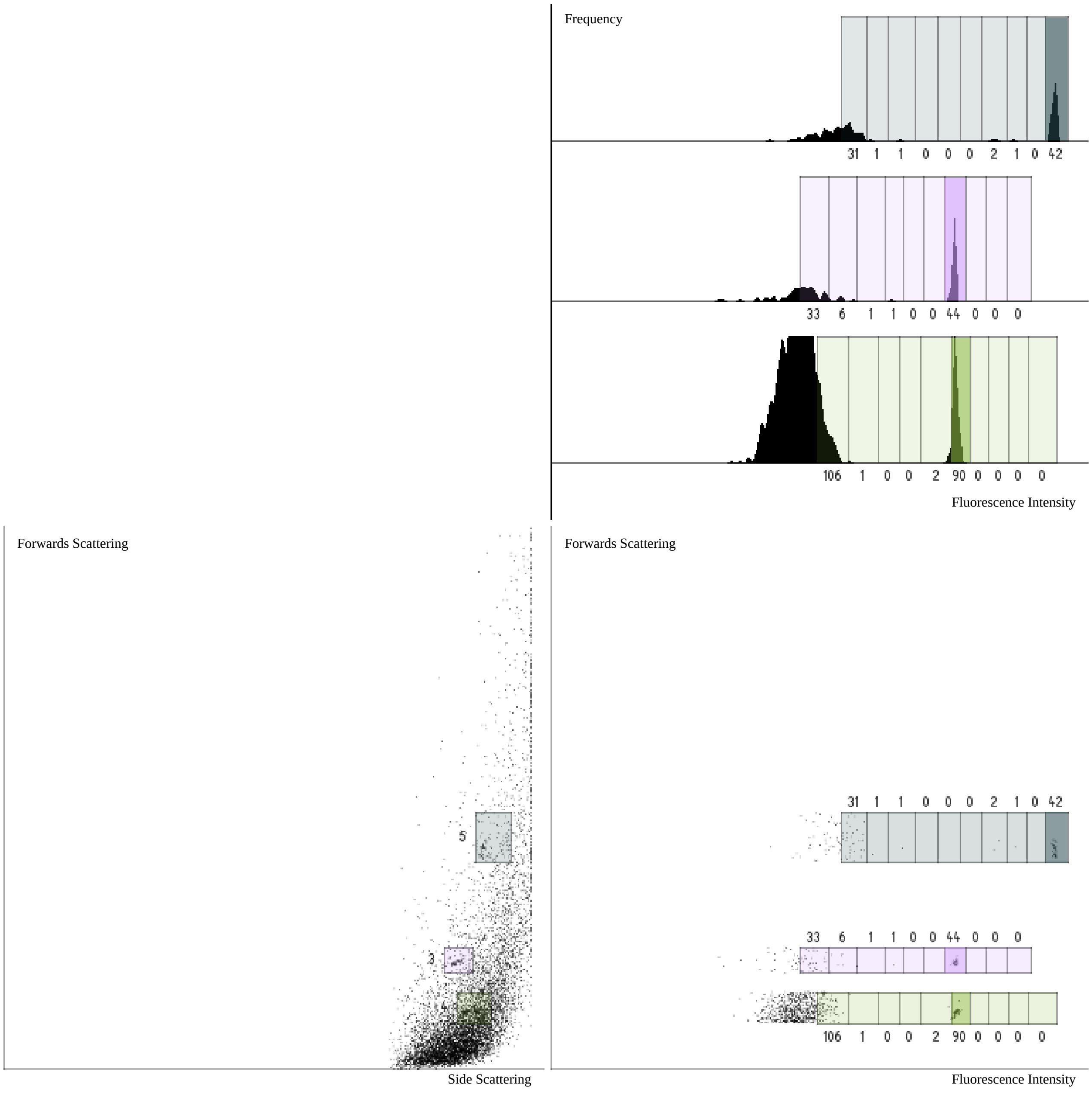

ANNEX 3: TAG DECONVOLUTION - BEAD 30

Passes flow sorting criteria: Yes

Passes tag deconvolution criteria: Yes

Included in protocol analysis: Yes

Protocol: 8, 3, 10, 1

Filename: Bin1_plateA2_C8.fcs

Split 1: Petrol shading

Split 2: Green shading

Split 3: Violet shading

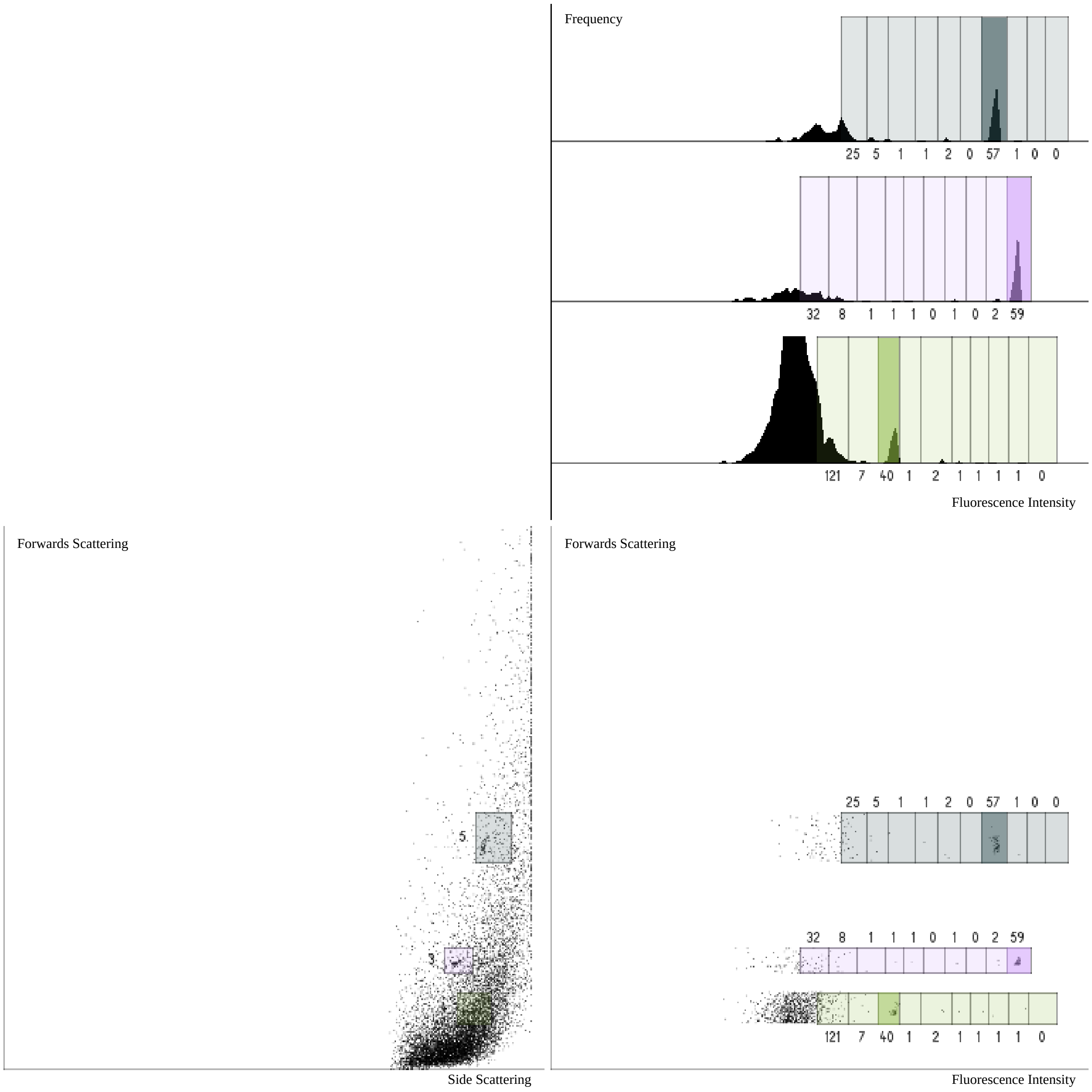

ANNEX 3: TAG DECONVOLUTION - BEAD 31

Passes flow sorting criteria: Yes

Passes tag deconvolution criteria: Yes

Included in protocol analysis: Yes

Protocol: 3, 3, 7, 1

Filename: Bin1_plateA2_D3.fcs

Split 1: Petrol shading

Split 2: Green shading

Split 3: Violet shading

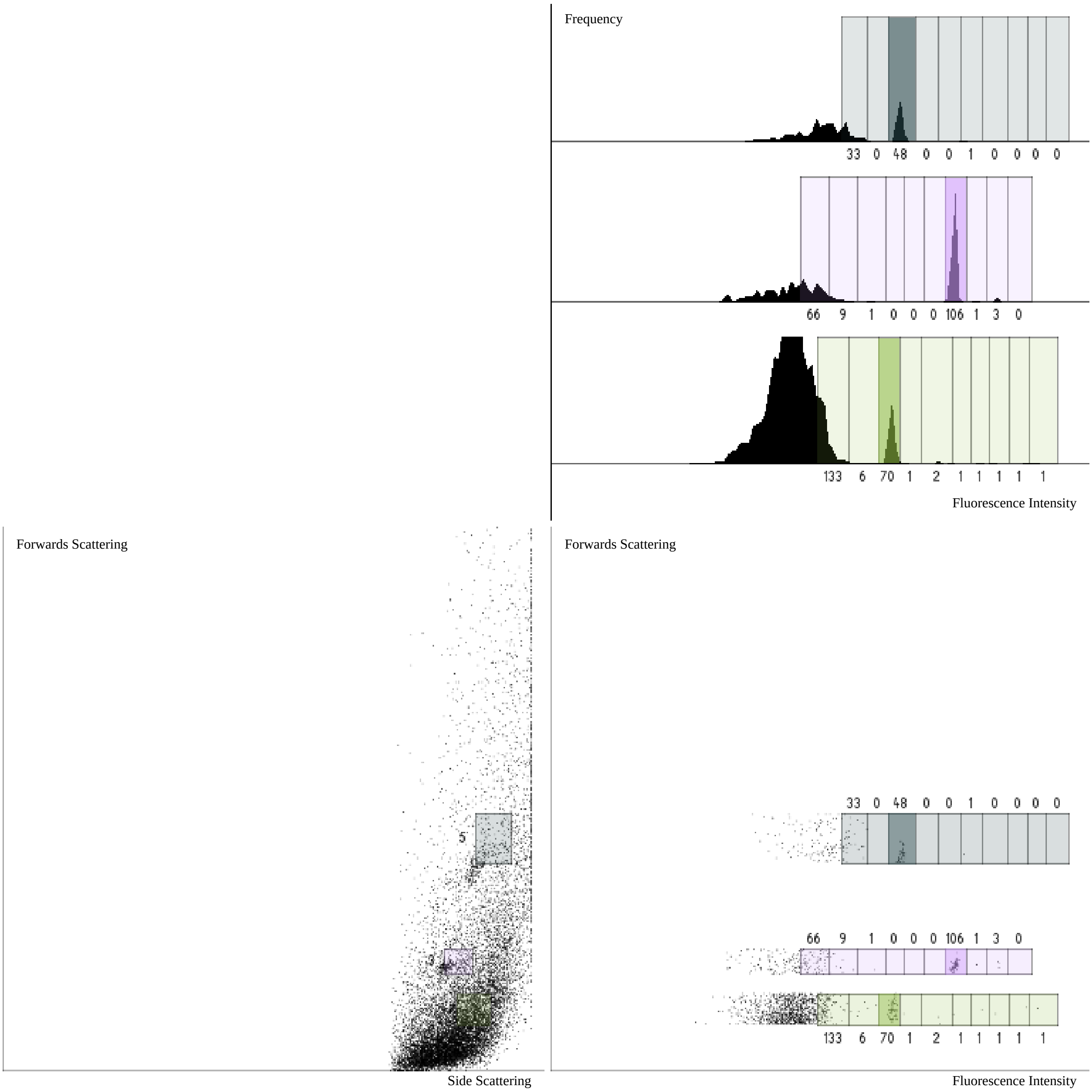

ANNEX 3: TAG DECONVOLUTION - BEAD 32

Passes flow sorting criteria: Yes

Passes tag deconvolution criteria: Yes

Included in protocol analysis: Yes

Protocol: 5, 2, 1, 1

Filename: Bin1_plateA2_D4.fcs

Split 1: Petrol shading

Split 2: Green shading

Split 3: Violet shading

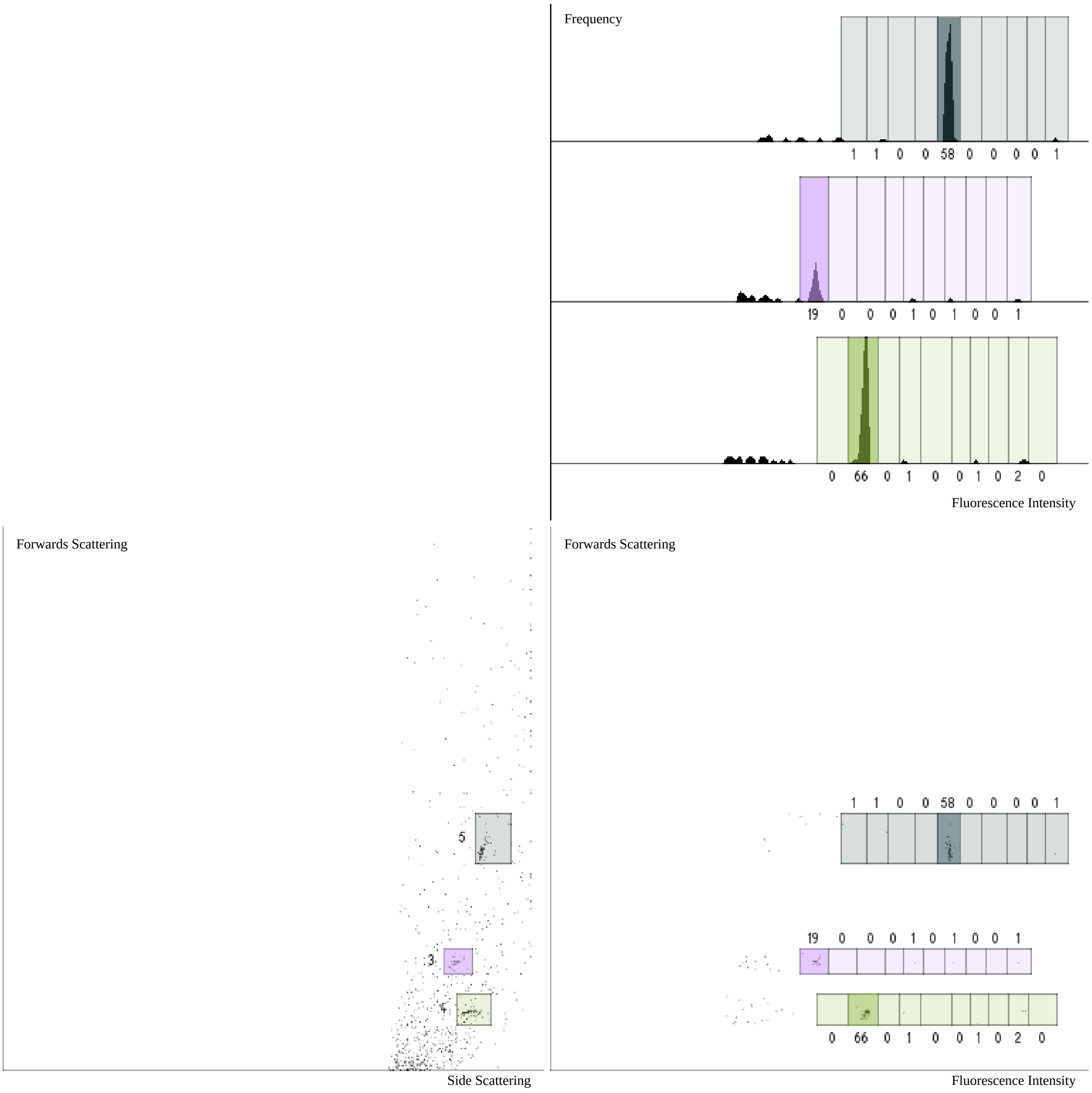

ANNEX 3: TAG DECONVOLUTION - BEAD 33

Passes flow sorting criteria: Yes

Passes tag deconvolution criteria: Yes

Included in protocol analysis: Yes

Protocol: 2, 7, 10, 1

Filename: Bin1_plateA2_D8.fcs

Split 1: Petrol shading

Split 2: Green shading

Split 3: Violet shading

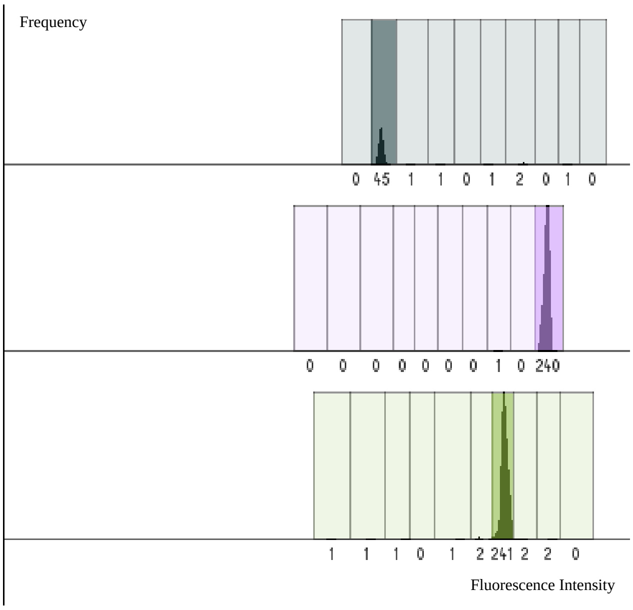

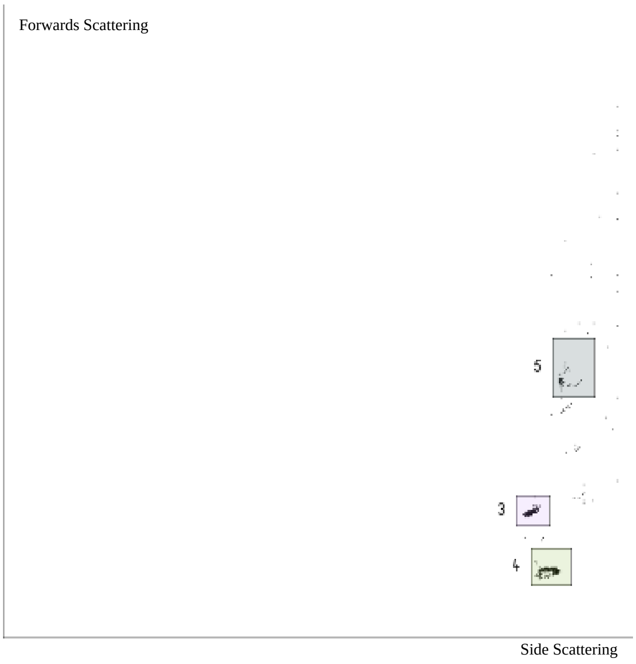

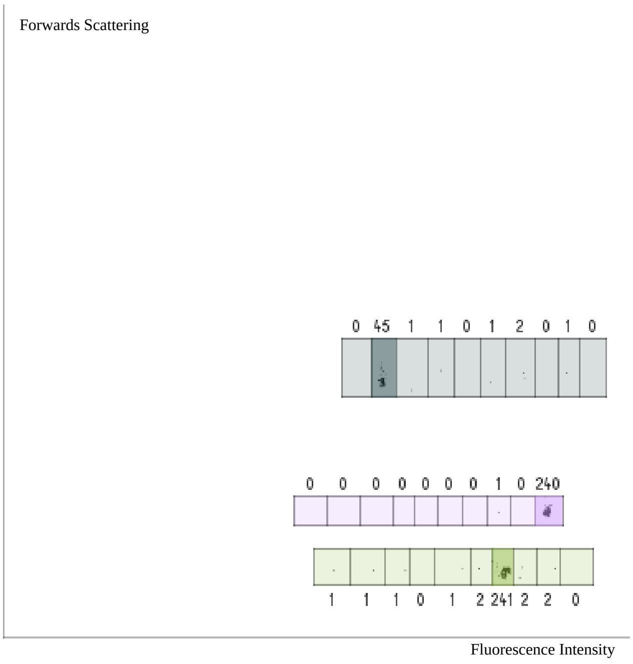

ANNEX 3: TAG DECONVOLUTION - BEAD 34

Passes flow sorting criteria: Yes

Passes tag deconvolution criteria: Yes

Included in protocol analysis: Yes

Protocol: 9, 7, 10, 1

Filename: Bin1_plateA2_D12.fcs

Split 1: Petrol shading

Split 2: Green shading

Split 3: Violet shading

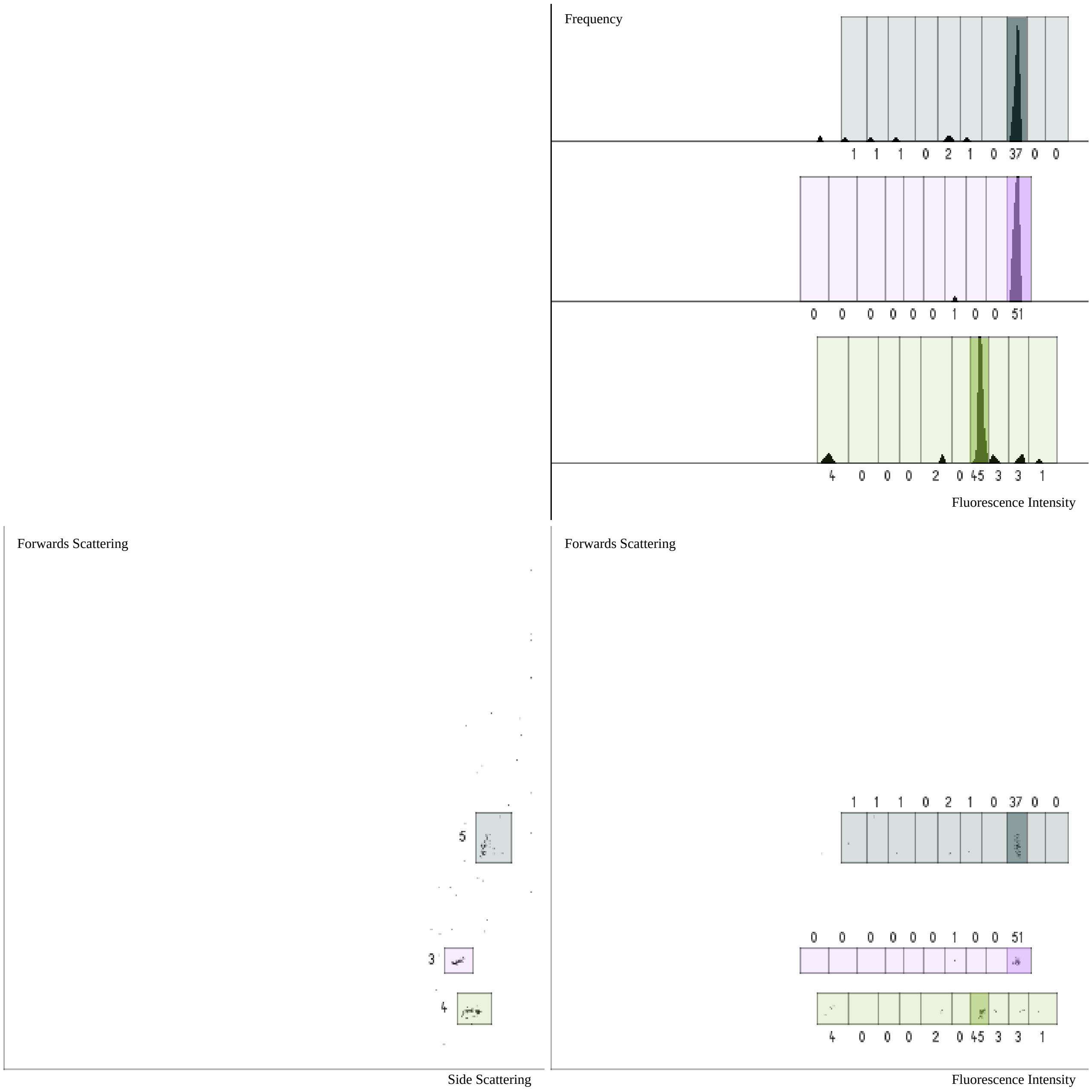

ANNEX 3: TAG DECONVOLUTION - BEAD 35

Passes flow sorting criteria: Yes

Passes tag deconvolution criteria: Yes

Included in protocol analysis: Yes

Protocol: 5, 2, 6, 1

Filename: Bin1_plateA2_E2.fcs

Split 1: Petrol shading

Split 2: Green shading

Split 3: Violet shading

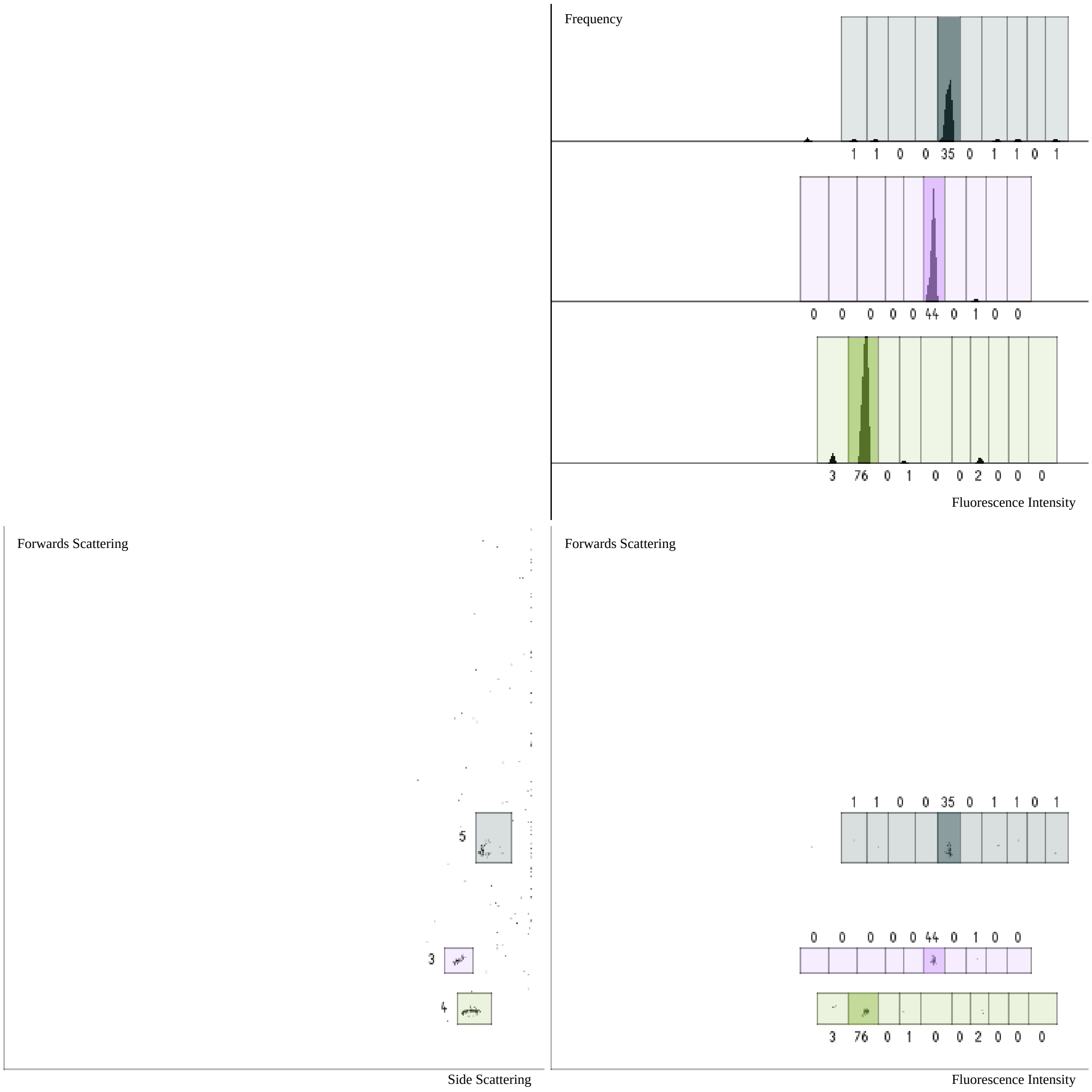

ANNEX 3: TAG DECONVOLUTION - BEAD 36

Passes flow sorting criteria: Yes

Passes tag deconvolution criteria: Yes

Included in protocol analysis: Yes

Protocol: 5, 3, 1, 1

Filename: Bin1_plateA2_F2.fcs

Split 1: Petrol shading

Split 2: Green shading

Split 3: Violet shading

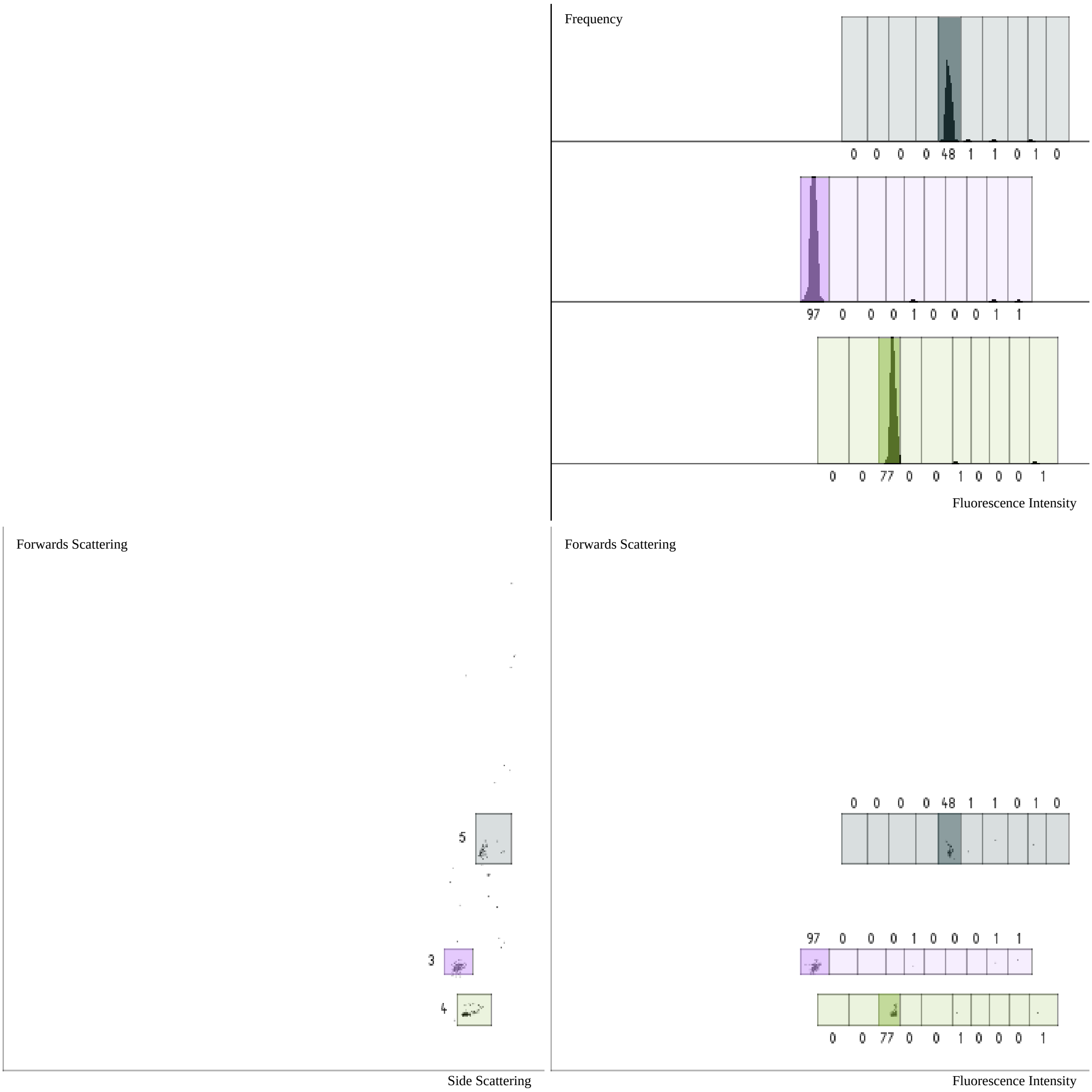

ANNEX 3: TAG DECONVOLUTION - BEAD 37

Passes flow sorting criteria: Yes

Passes tag deconvolution criteria: Yes

Included in protocol analysis: Yes

Protocol: 10, 7, 6, 1

Filename: Bin1_plateA2_F4.fcs

Split 1: Petrol shading

Split 2: Green shading

Split 3: Violet shading

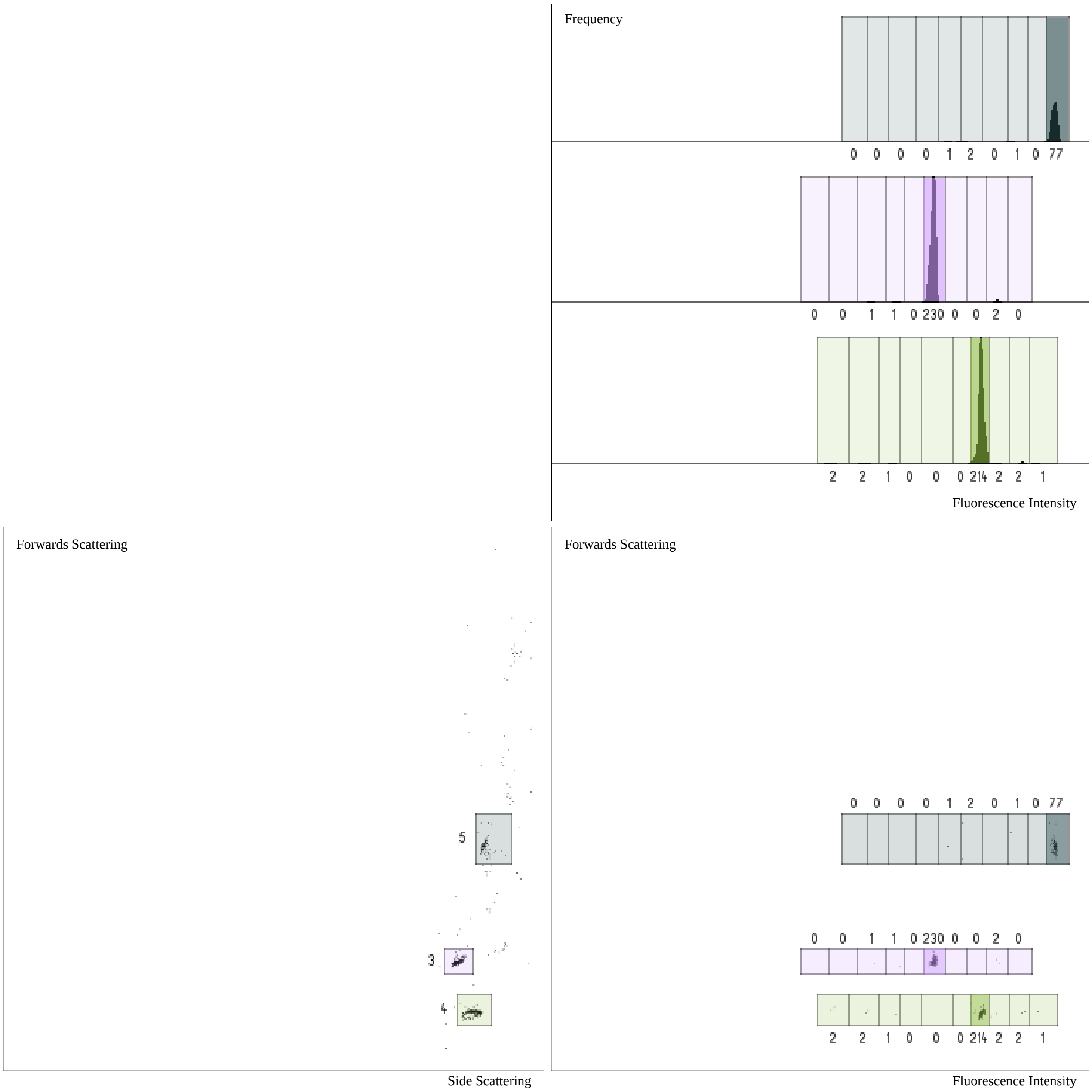

ANNEX 3: TAG DECONVOLUTION - BEAD 38

Passes flow sorting criteria: Yes

Passes tag deconvolution criteria: No

Included in protocol analysis: No

Protocol: N/A

Filename: Bin1_plateA2_F7.fcs

Split 1: Petrol shading

Split 2: Green shading

Split 3: Violet shading

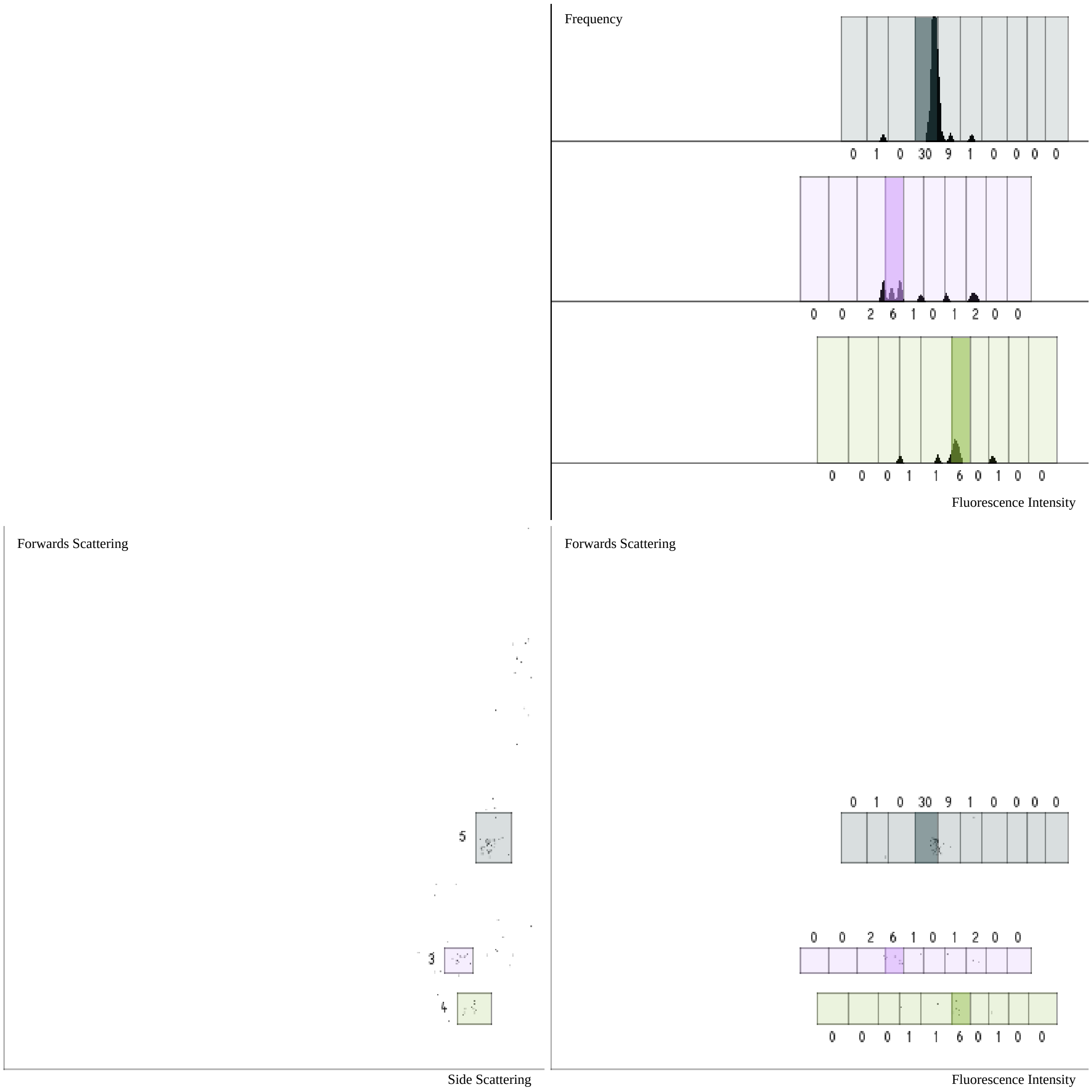

ANNEX 3: TAG DECONVOLUTION - BEAD 39

Passes flow sorting criteria: Yes

Passes tag deconvolution criteria: Yes

Included in protocol analysis: Yes

Protocol: 7, 7, 2, 1

Filename: Bin1_plateA2_F9.fcs

Split 1: Petrol shading

Split 2: Green shading

Split 3: Violet shading

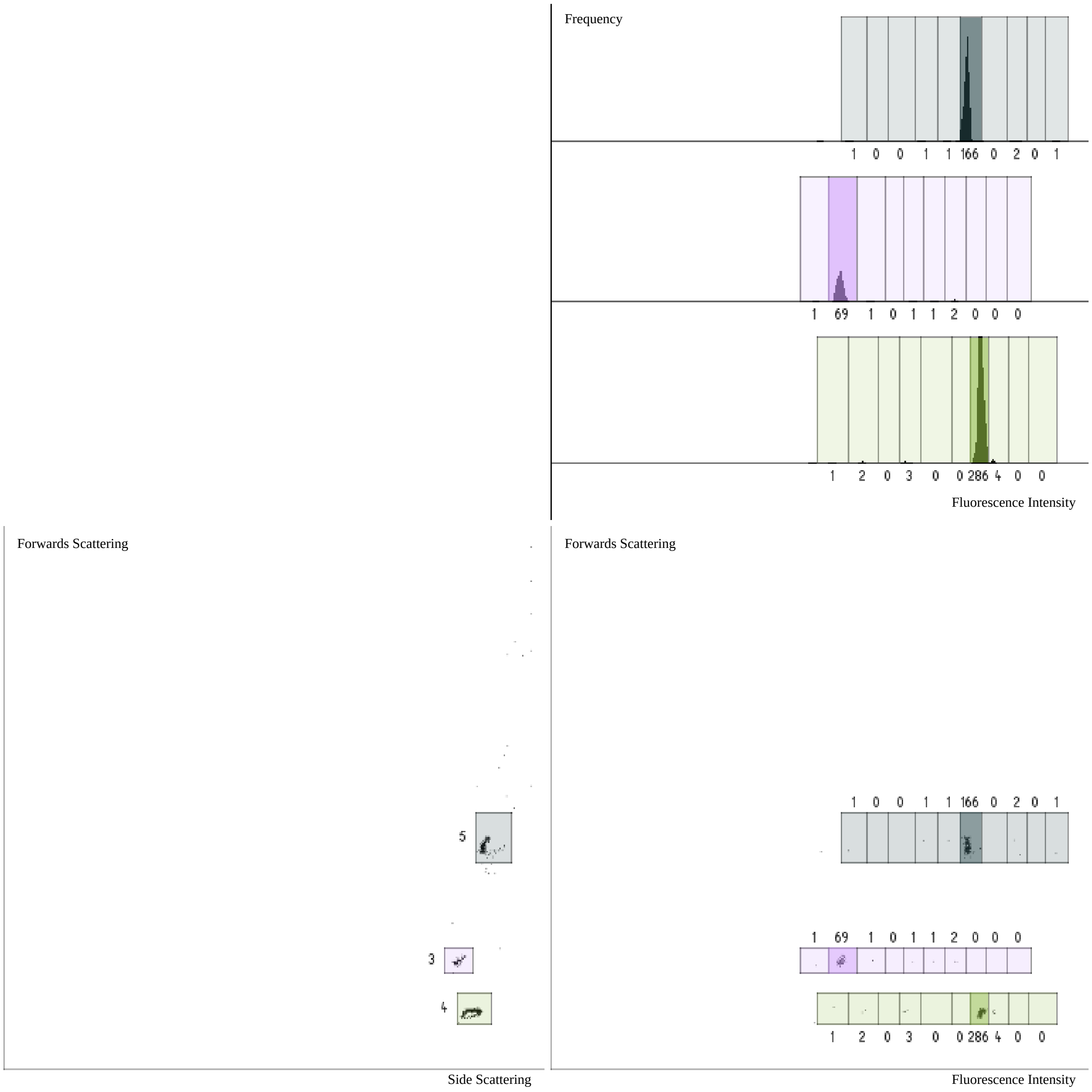

ANNEX 3: TAG DECONVOLUTION - BEAD 40

Passes flow sorting criteria: Yes

Passes tag deconvolution criteria: Yes

Included in protocol analysis: Yes

Protocol: 3, 2, 8, 1

Filename: Bin1_plateA2_F10.fcs

Split 1: Petrol shading

Split 2: Green shading

Split 3: Violet shading

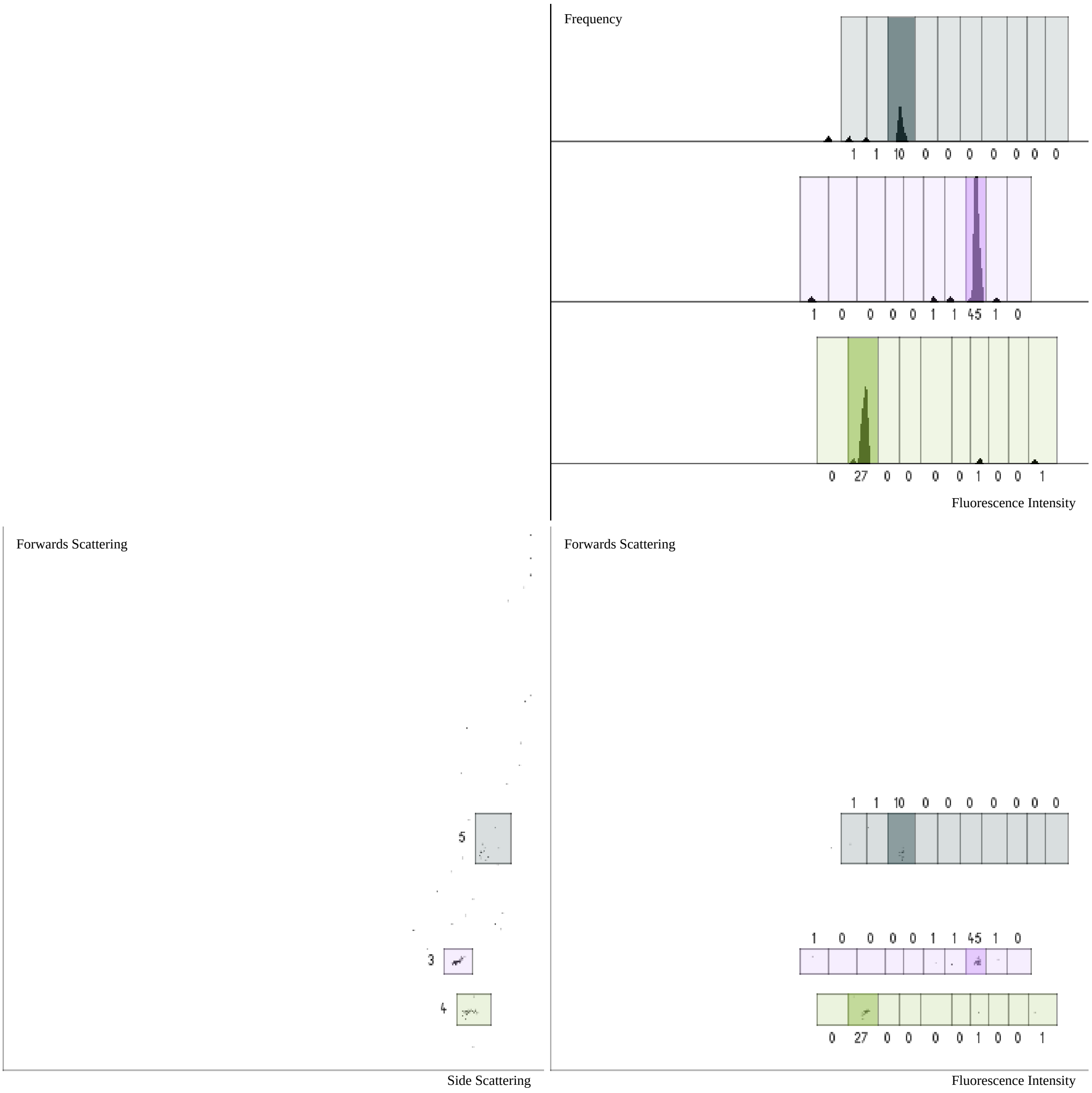

ANNEX 3: TAG DECONVOLUTION - BEAD 41

Passes flow sorting criteria: Yes

Passes tag deconvolution criteria: Yes

Included in protocol analysis: Yes

Protocol: 3, 5, 6, 3

Filename: Bin3_PlateA3_B5.fcs

Split 1: Petrol shading

Split 2: Green shading

Split 3: Violet shading

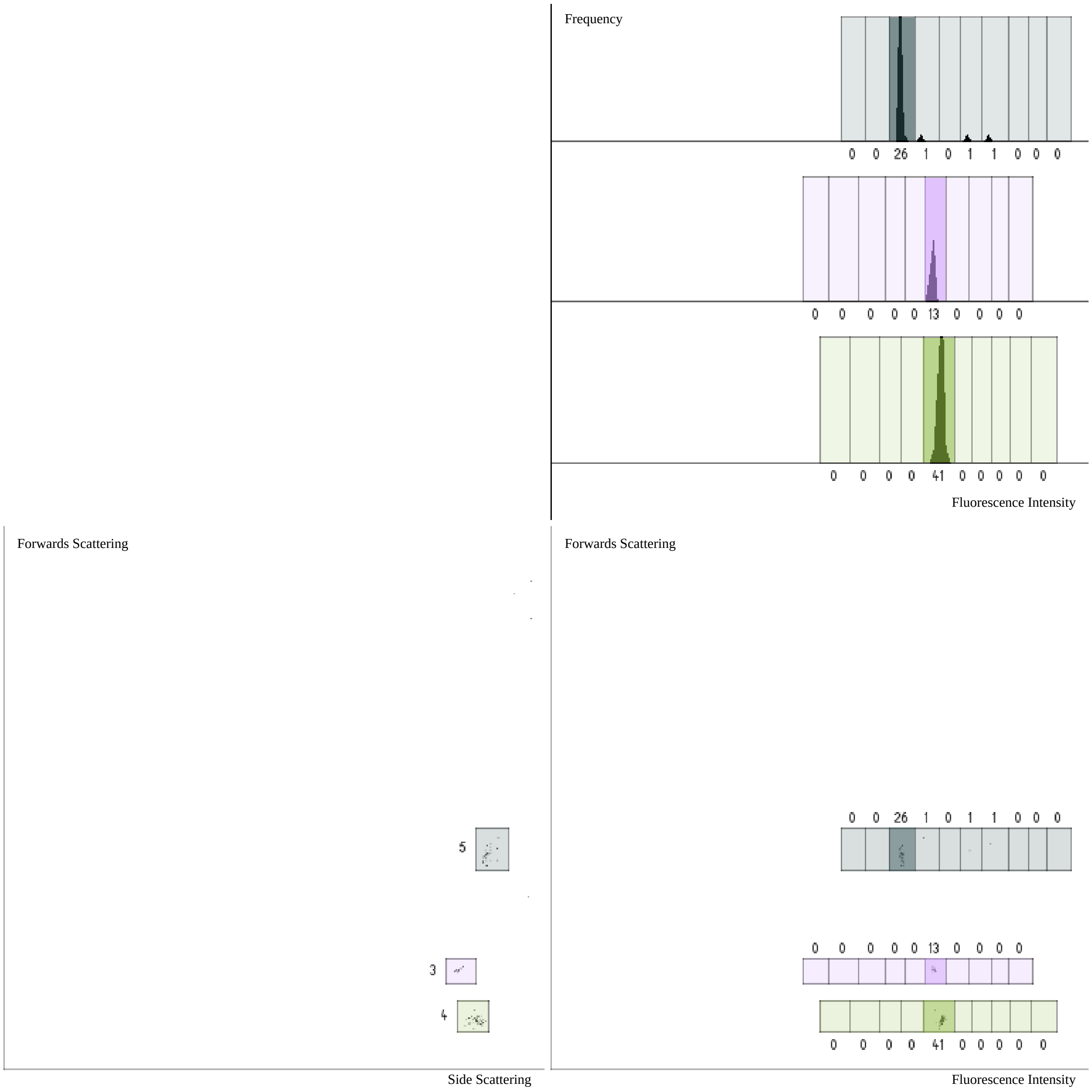

ANNEX 3: TAG DECONVOLUTION - BEAD 42

Passes flow sorting criteria: Yes

Passes tag deconvolution criteria: Yes

Included in protocol analysis: Yes

Protocol: 7, 2, 5, 1

Filename: Bin1_plateA3_A2.fcs

Split 1: Petrol shading

Split 2: Green shading

Split 3: Violet shading

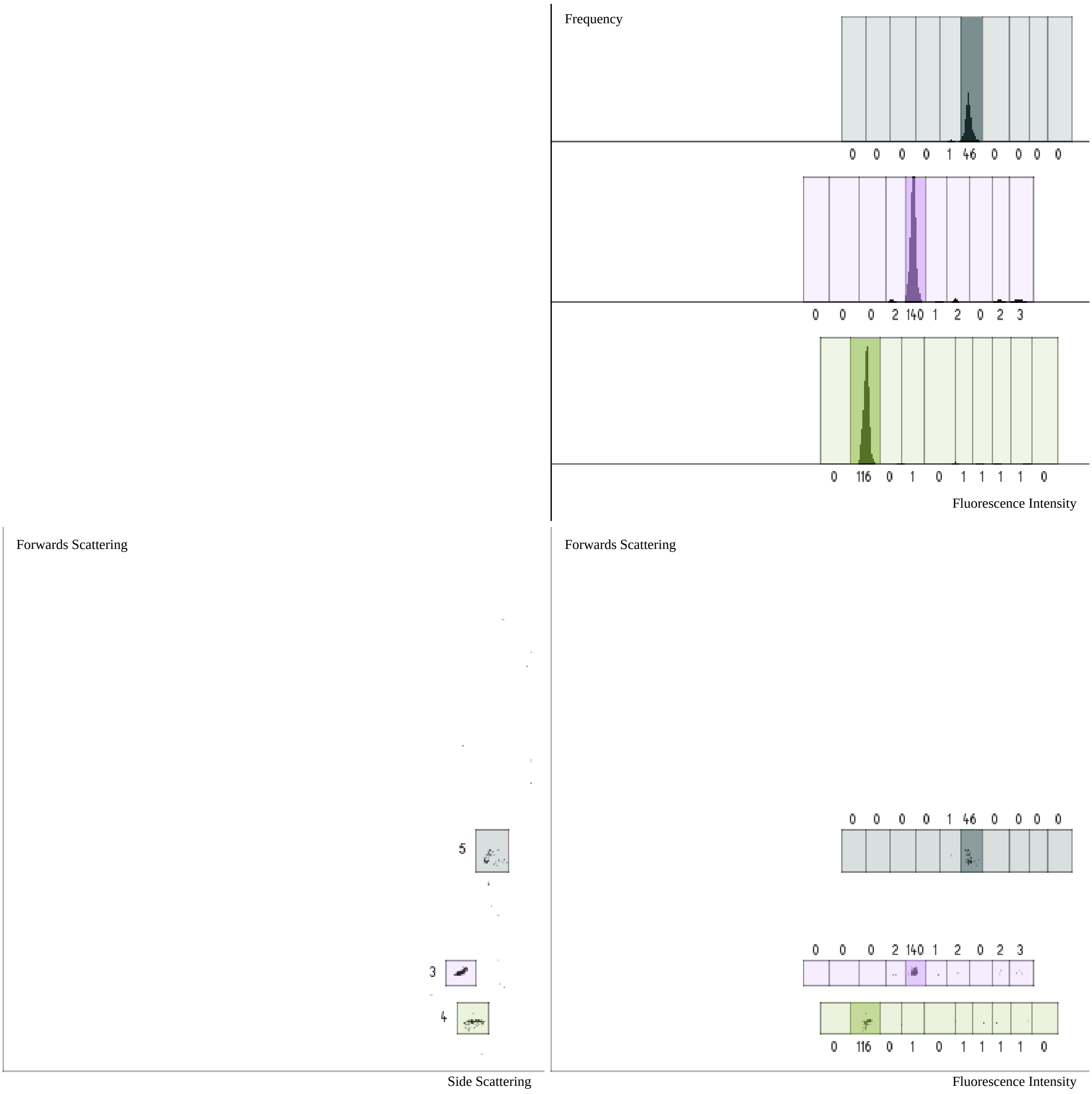

ANNEX 3: TAG DECONVOLUTION - BEAD 43

Passes flow sorting criteria: Yes

Passes tag deconvolution criteria: Yes

Included in protocol analysis: Yes

Protocol: 3, 9, 8, 1

Filename: Bin1_plateA3_A4.fcs

Split 1: Petrol shading

Split 2: Green shading

Split 3: Violet shading

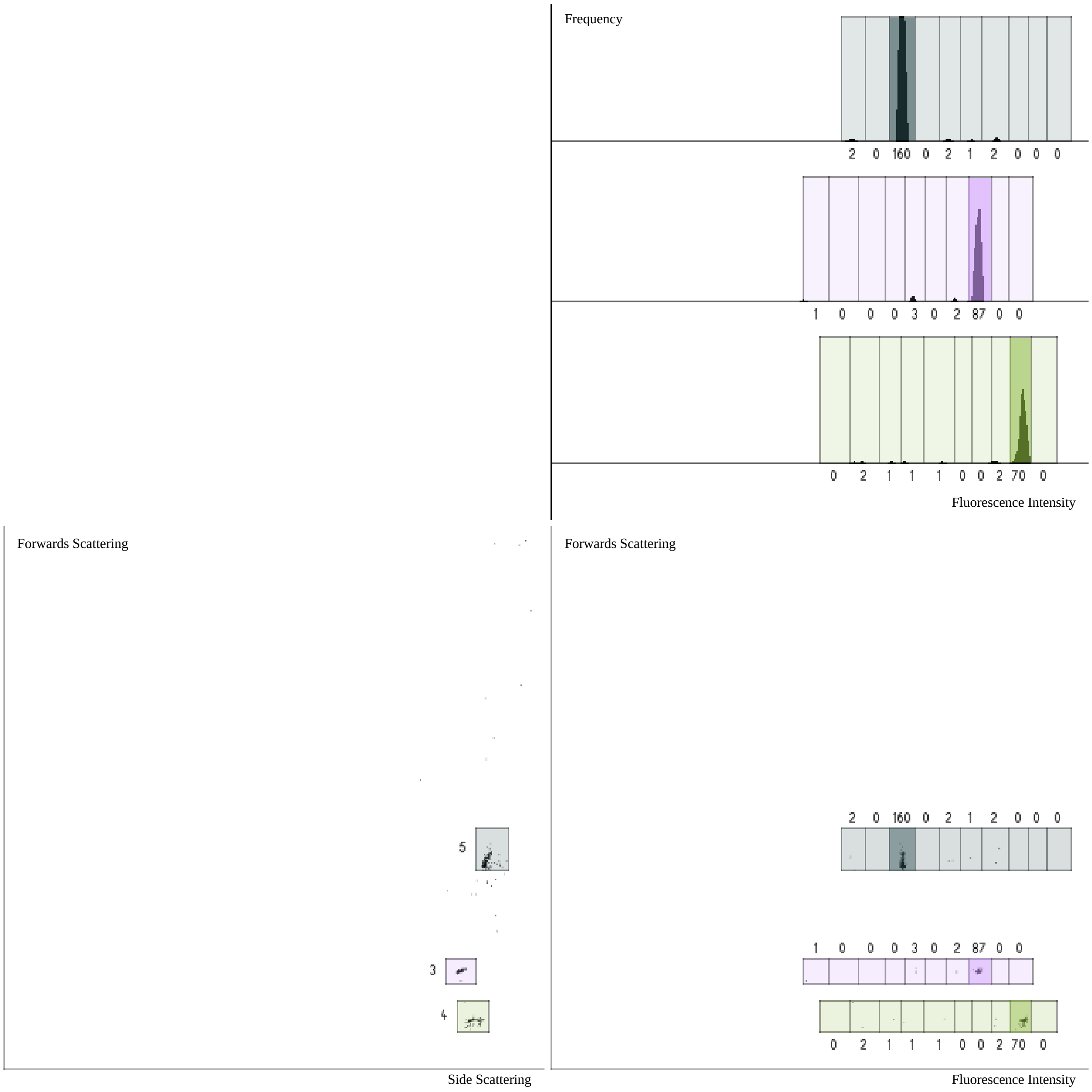

ANNEX 3: TAG DECONVOLUTION - BEAD 44

Passes flow sorting criteria: Yes

Passes tag deconvolution criteria: Yes

Included in protocol analysis: Yes

Protocol: 4, 4, 5, 1

Filename: Bin1_plateA3_A5.fcs

Split 1: Petrol shading

Split 2: Green shading

Split 3: Violet shading

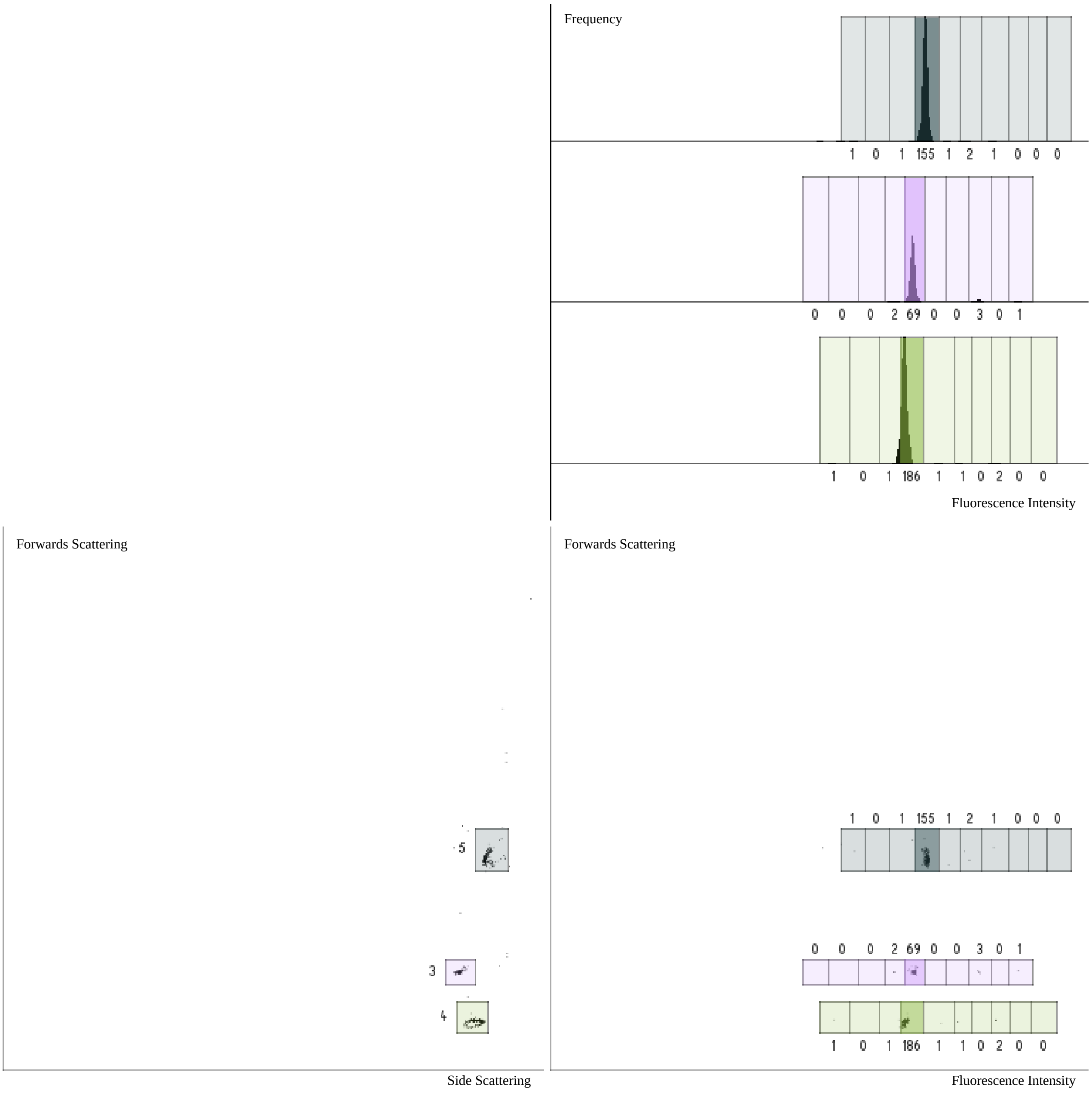

ANNEX 3: TAG DECONVOLUTION - BEAD 45

Passes flow sorting criteria: Yes

Passes tag deconvolution criteria: Yes

Included in protocol analysis: Yes

Protocol: 9, 10, 6, 1

Filename: Bin1_plateA3_A8.fcs

Split 1: Petrol shading

Split 2: Green shading

Split 3: Violet shading

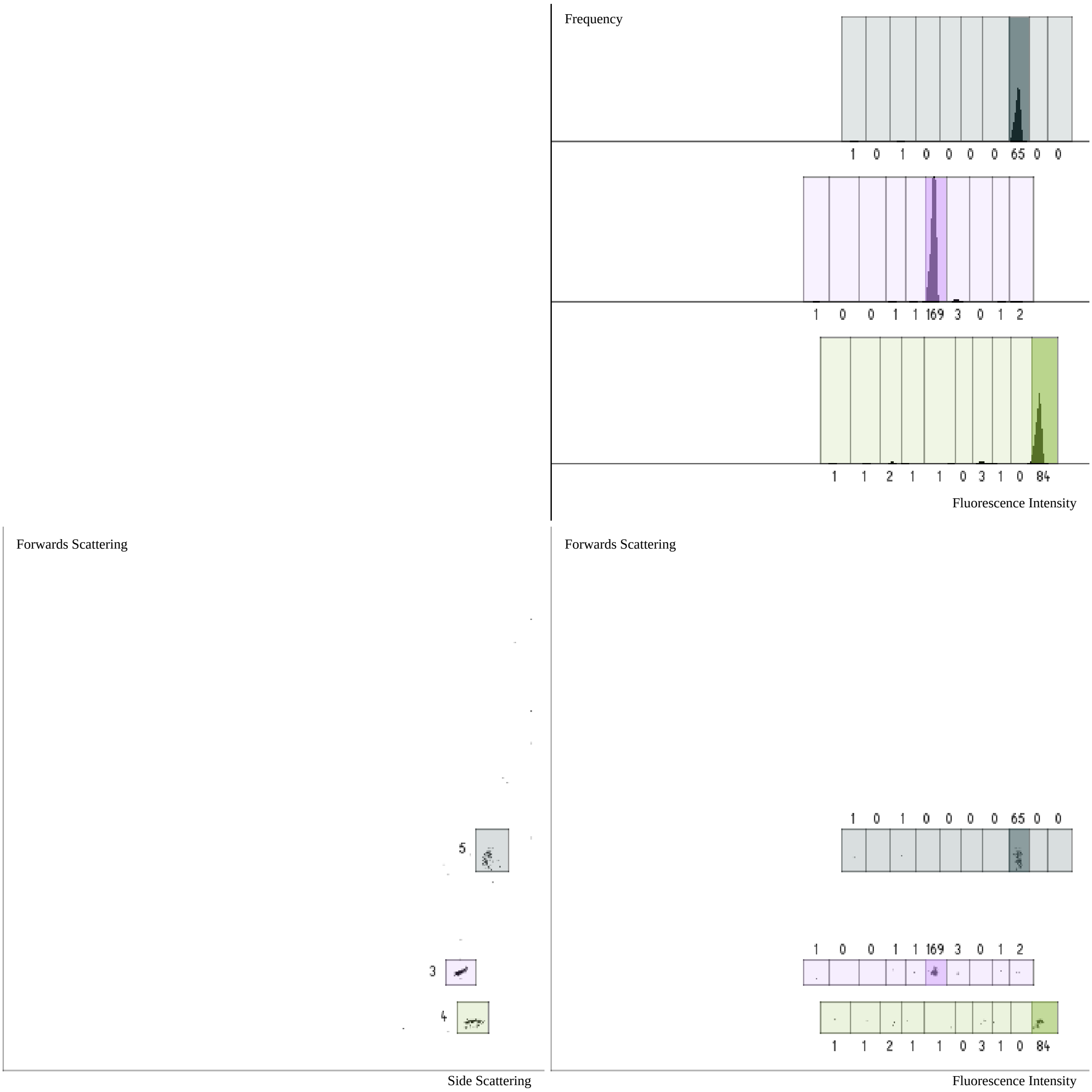

ANNEX 3: TAG DECONVOLUTION - BEAD 46

Passes flow sorting criteria: Yes

Passes tag deconvolution criteria: Yes

Included in protocol analysis: Yes

Protocol: 8, 4, 1, 1

Filename: Bin1_plateA3_A10.fcs

Split 1: Petrol shading

Split 2: Green shading

Split 3: Violet shading

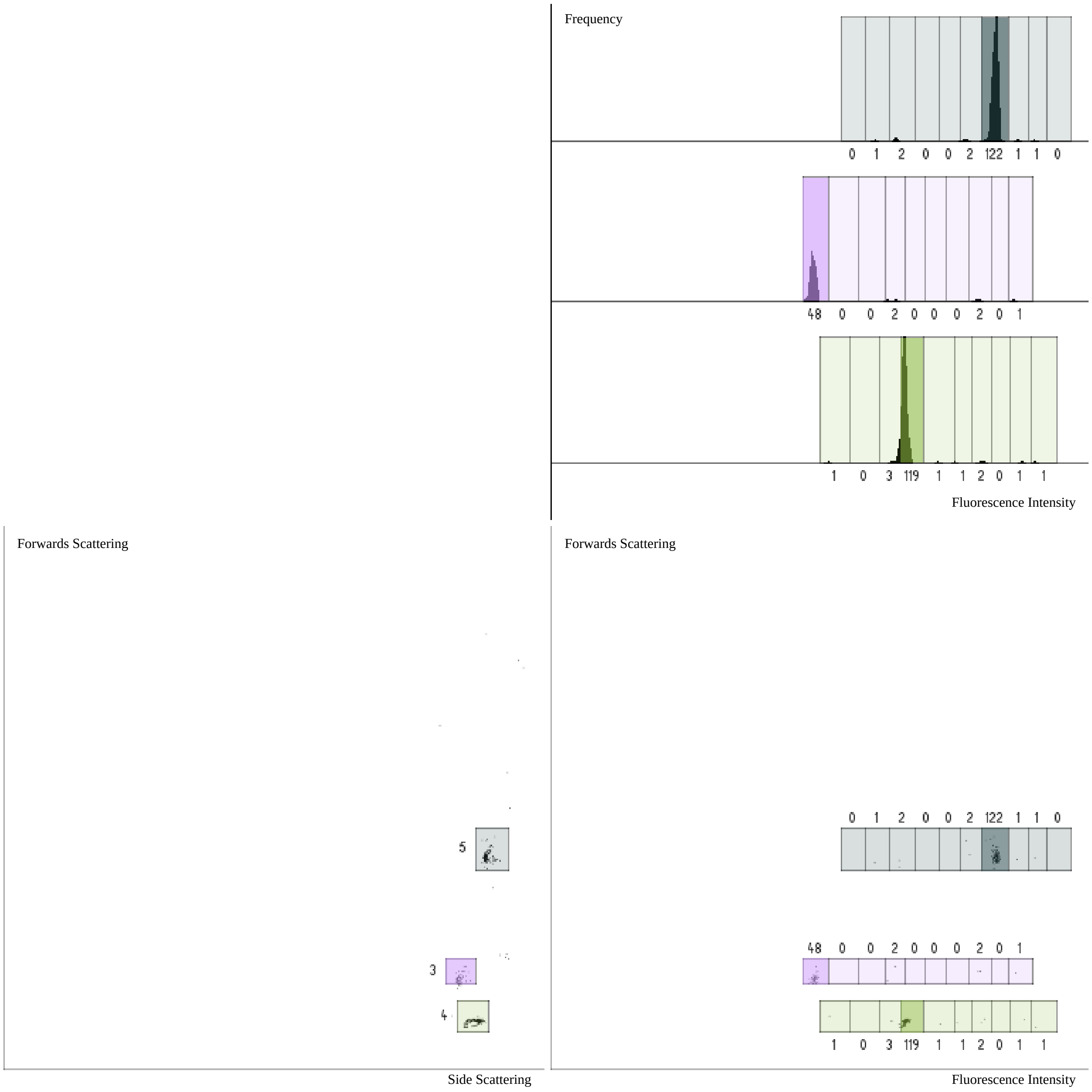

ANNEX 3: TAG DECONVOLUTION - BEAD 47

Passes flow sorting criteria: Yes

Passes tag deconvolution criteria: Yes

Included in protocol analysis: Yes

Protocol: 8, 10, 3, 1

Filename: Bin1_plateA3_C2.fcs

Split 1: Petrol shading

Split 2: Green shading

Split 3: Violet shading

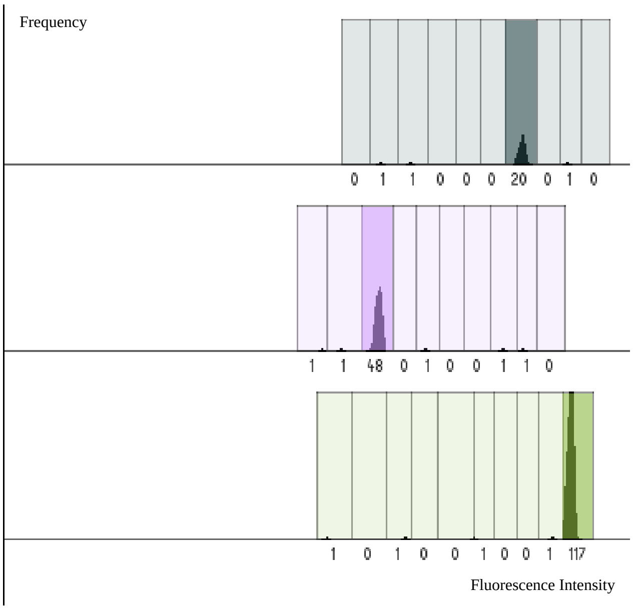

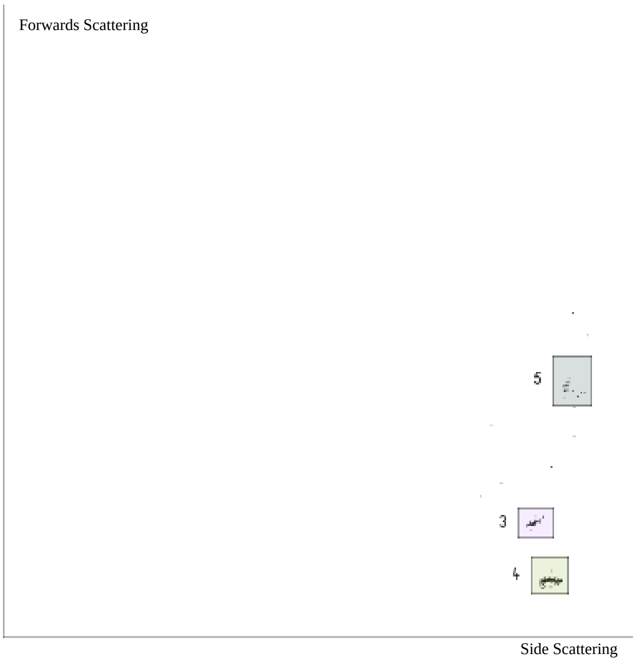

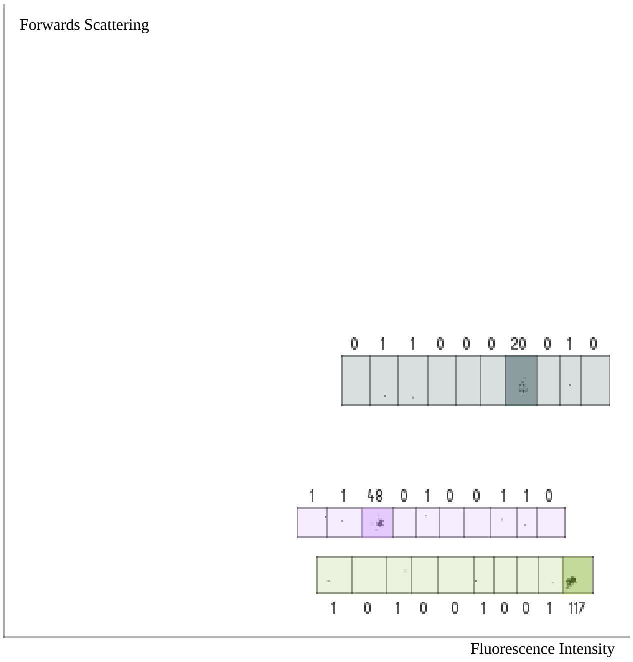

ANNEX 3: TAG DECONVOLUTION - BEAD 48

Passes flow sorting criteria: Yes

Passes tag deconvolution criteria: Yes

Included in protocol analysis: Yes

Protocol: 8, 7, 1, 1

Filename: Bin1_plateA3_C6.fcs

Split 1: Petrol shading

Split 2: Green shading

Split 3: Violet shading

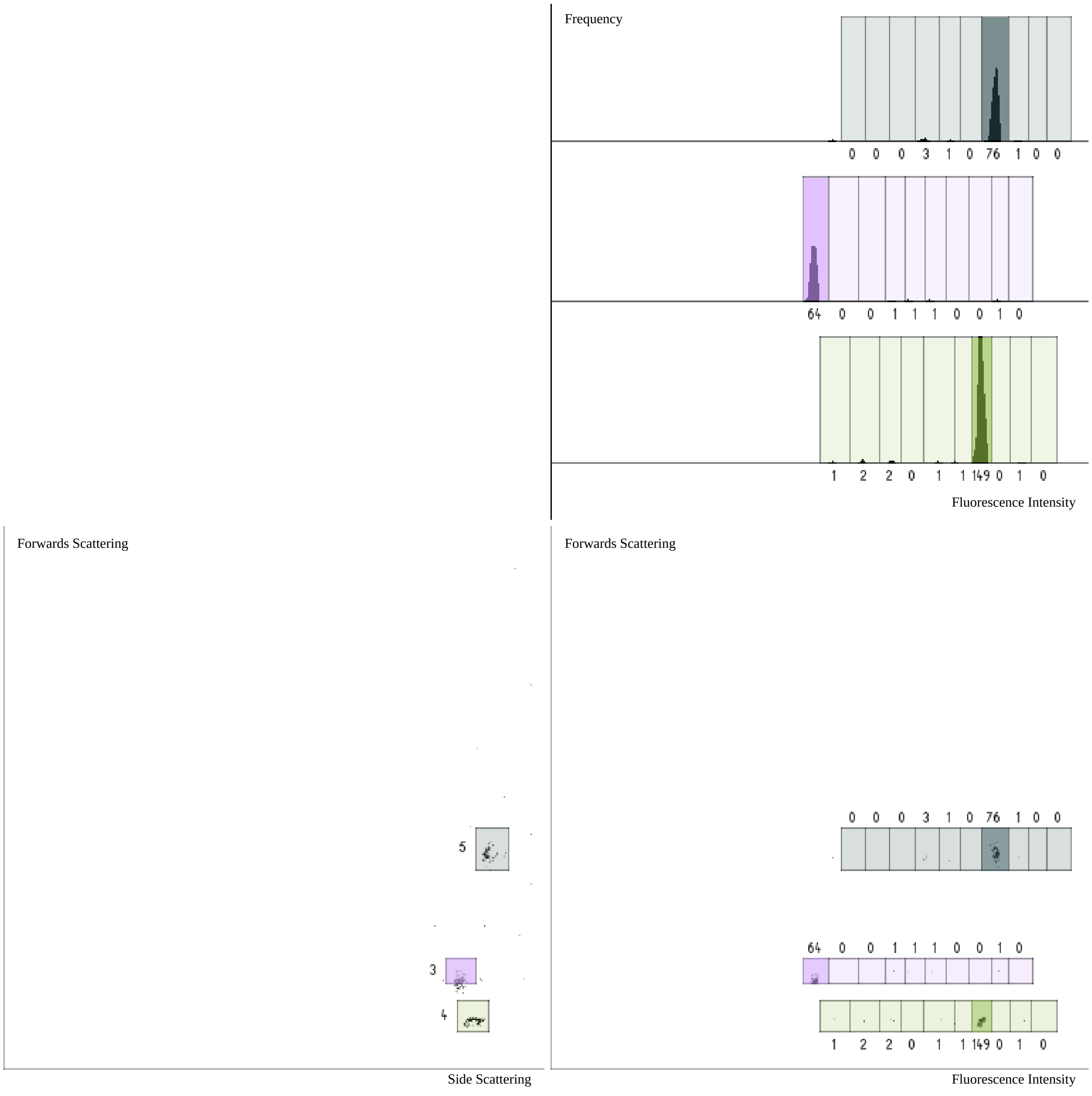

ANNEX 3: TAG DECONVOLUTION - BEAD 49

Passes flow sorting criteria: Yes

Passes tag deconvolution criteria: Yes

Included in protocol analysis: Yes

Protocol: 7, 4, 7, 1

Filename: Bin1_plateA3_C7.fcs

Split 1: Petrol shading

Split 2: Green shading

Split 3: Violet shading

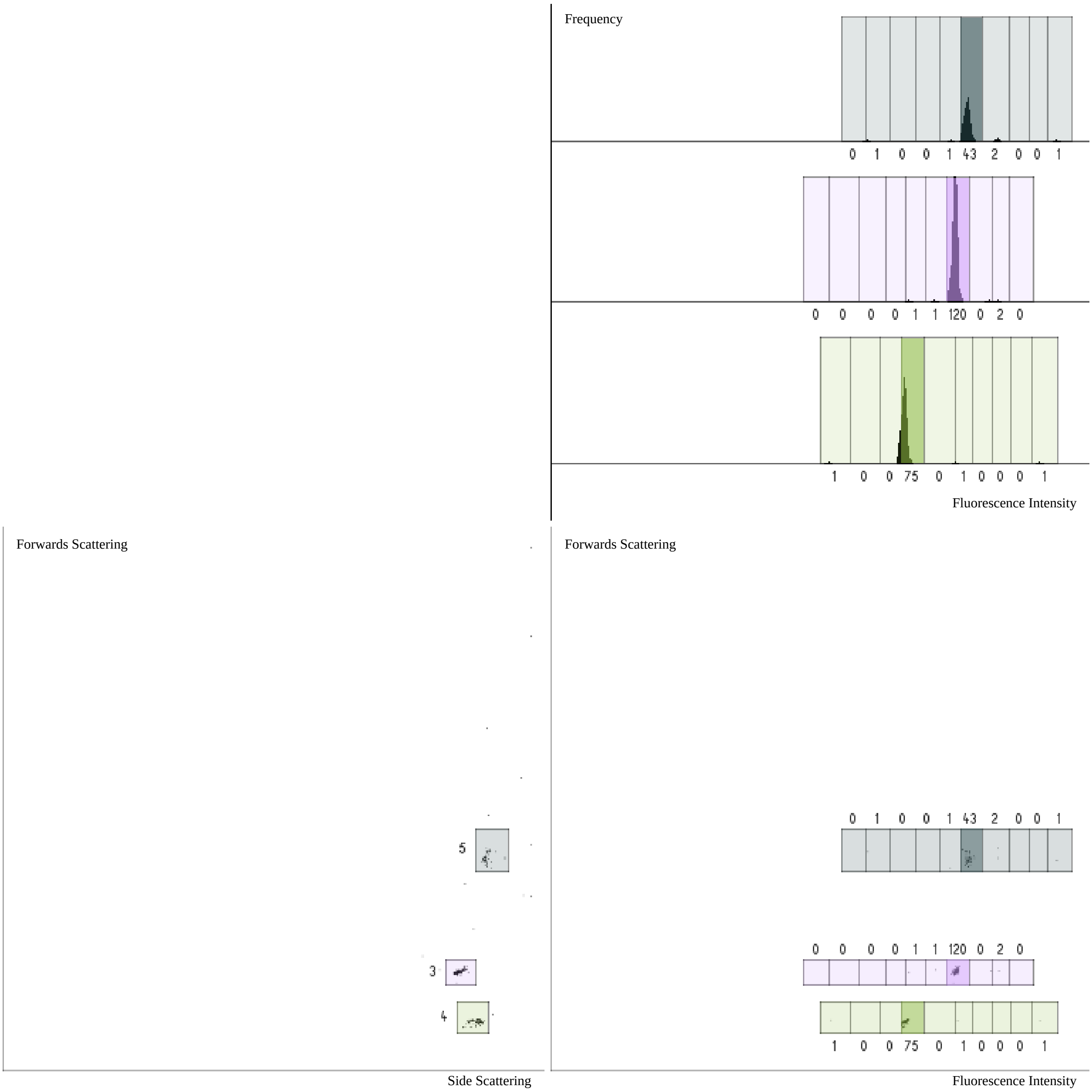

ANNEX 3: TAG DECONVOLUTION - BEAD 50

Passes flow sorting criteria: Yes

Passes tag deconvolution criteria: Yes

Included in protocol analysis: Yes

Protocol: 10, 8, 10, 1

Filename: Bin1_plateA3_C9.fcs

Split 1: Petrol shading

Split 2: Green shading

Split 3: Violet shading

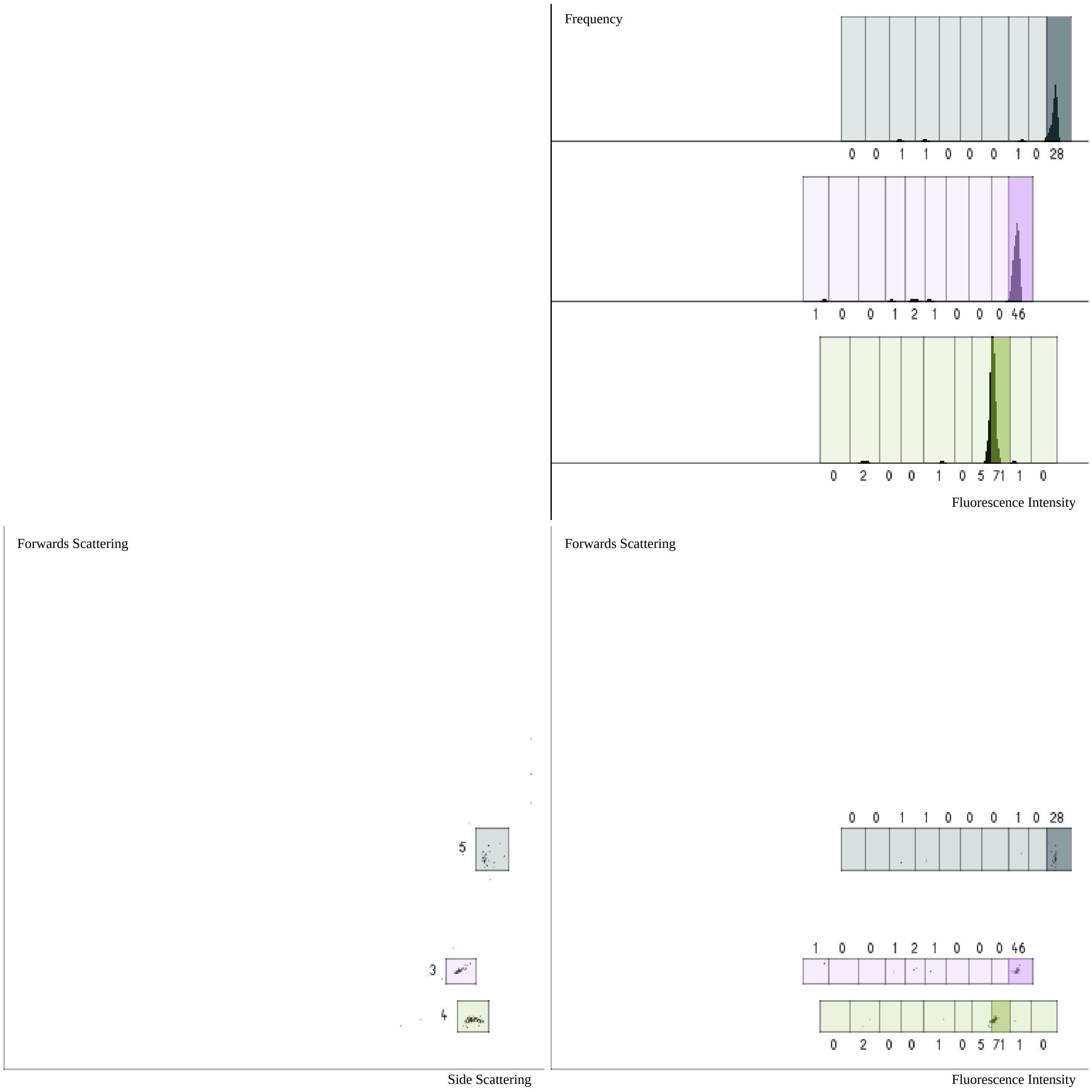

ANNEX 3: TAG DECONVOLUTION - BEAD 51

Passes flow sorting criteria: Yes

Passes tag deconvolution criteria: Yes

Included in protocol analysis: Yes

Protocol: 1, 1, 4, 1

Filename: Bin1_plateA3_C12.fcs

Split 1: Petrol shading

Split 2: Green shading

Split 3: Violet shading

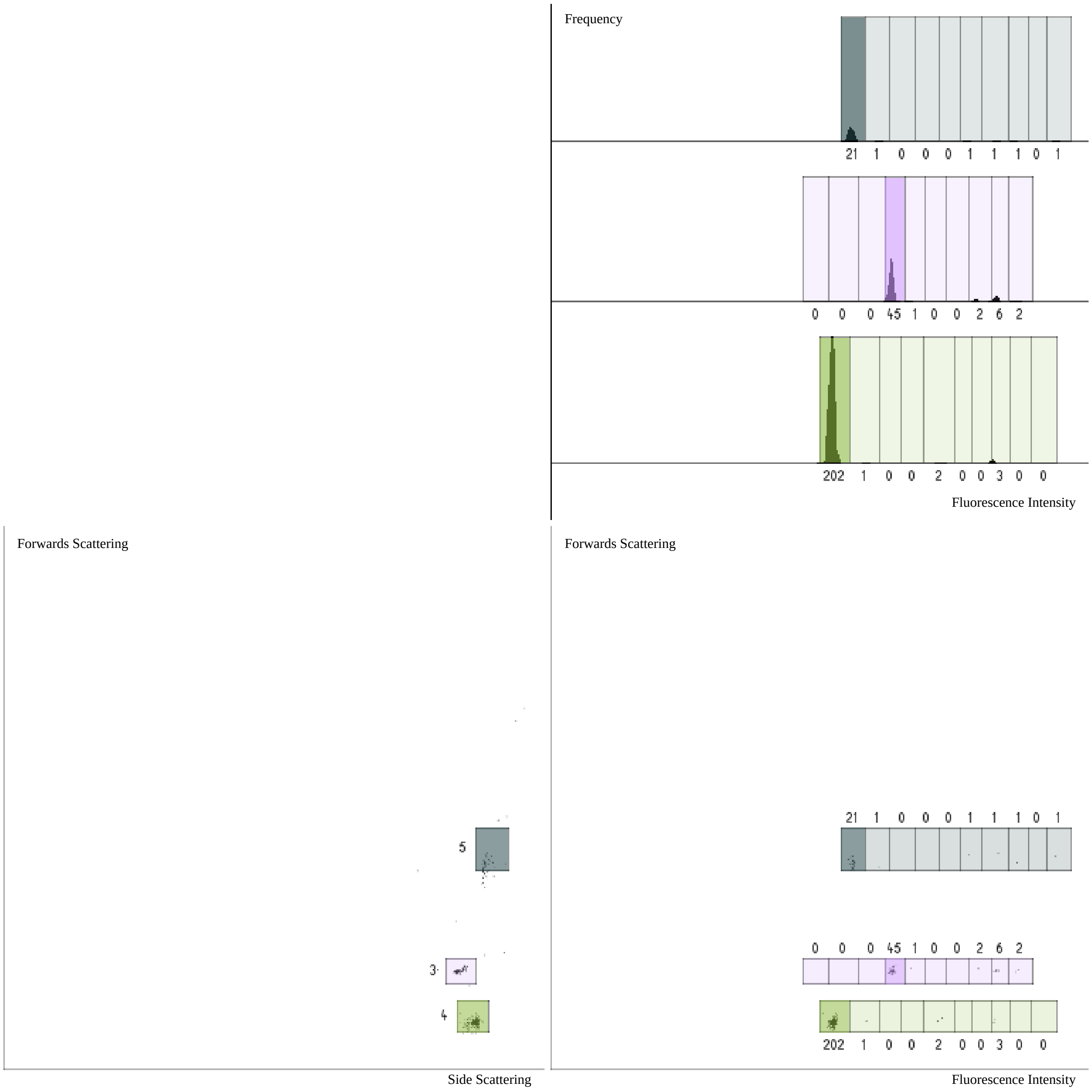

ANNEX 3: TAG DECONVOLUTION - BEAD 52

Passes flow sorting criteria: Yes

Passes tag deconvolution criteria: Yes

Included in protocol analysis: Yes

Protocol: 4, 2, 9, 1

Filename: Bin1_plateA3_D1.fcs

Split 1: Petrol shading

Split 2: Green shading

Split 3: Violet shading

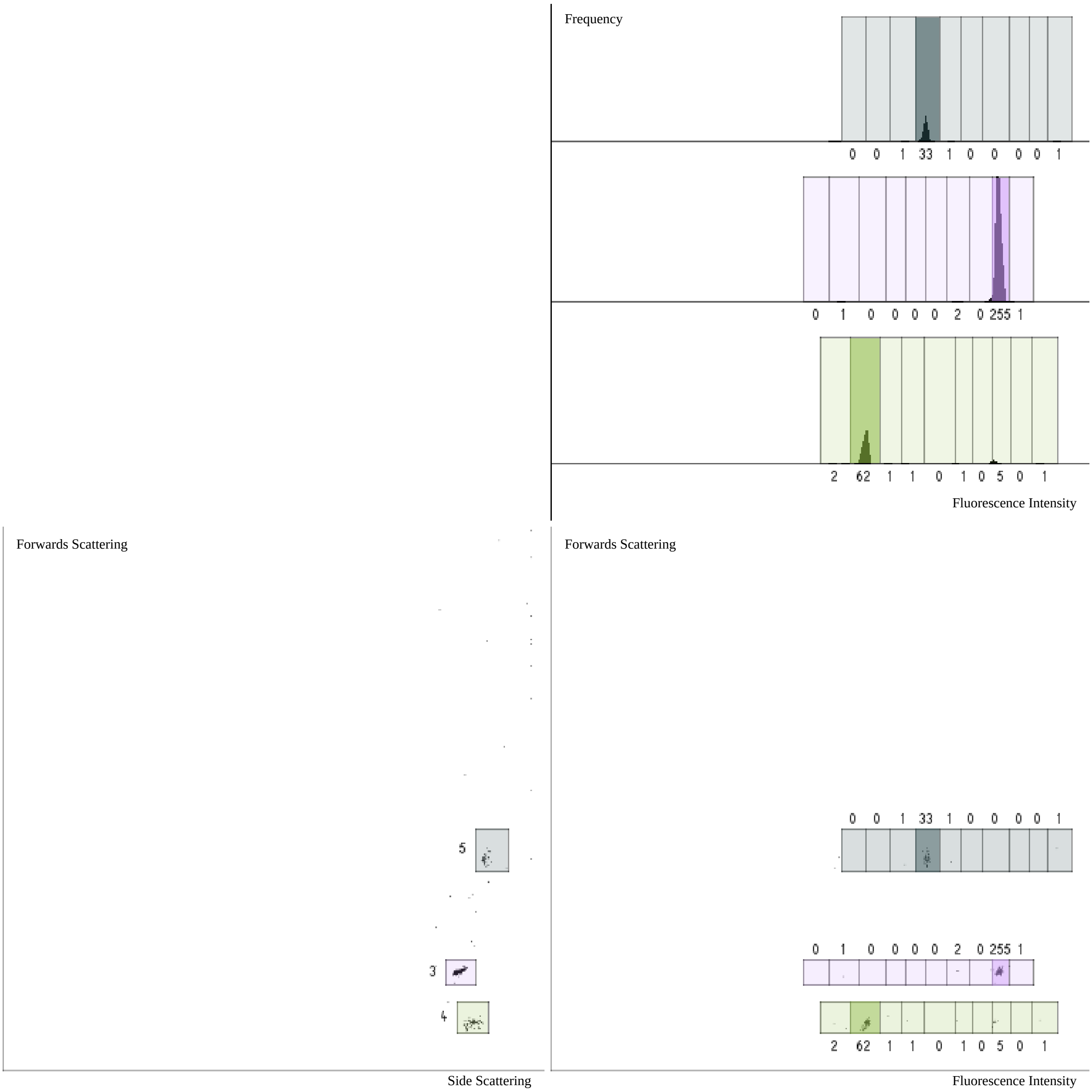

ANNEX 3: TAG DECONVOLUTION - BEAD 53

Passes flow sorting criteria: Yes

Passes tag deconvolution criteria: Yes

Included in protocol analysis: Yes

Protocol: 2, 4, 6, 1

Filename: Bin1_plateA3_D2.fcs

Split 1: Petrol shading

Split 2: Green shading

Split 3: Violet shading

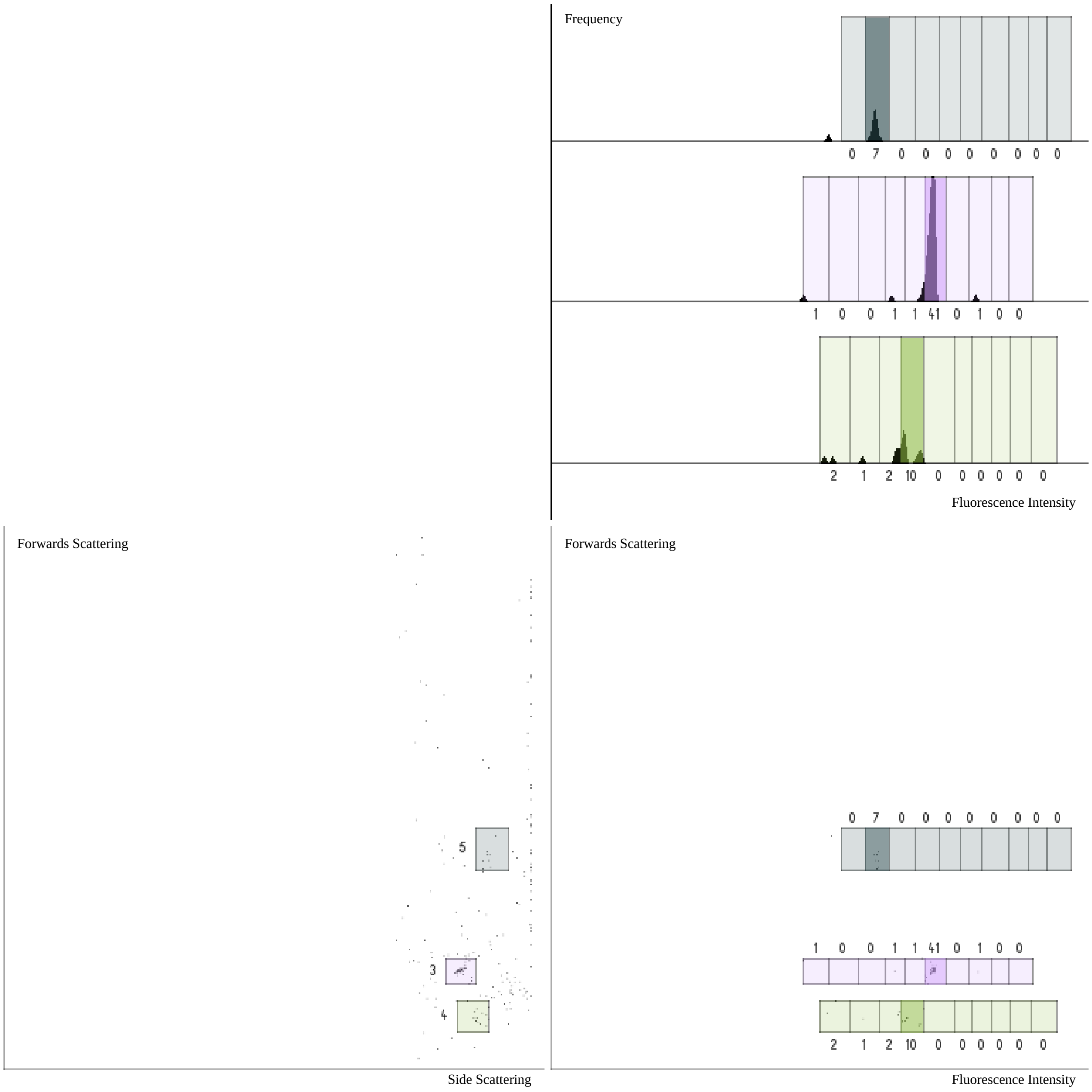

ANNEX 3: TAG DECONVOLUTION - BEAD 54

Passes flow sorting criteria: Yes

Passes tag deconvolution criteria: Yes

Included in protocol analysis: Yes

Protocol: 6, 2, 4, 1

Filename: Bin1_plateA3_D3.fcs

Split 1: Petrol shading

Split 2: Green shading

Split 3: Violet shading

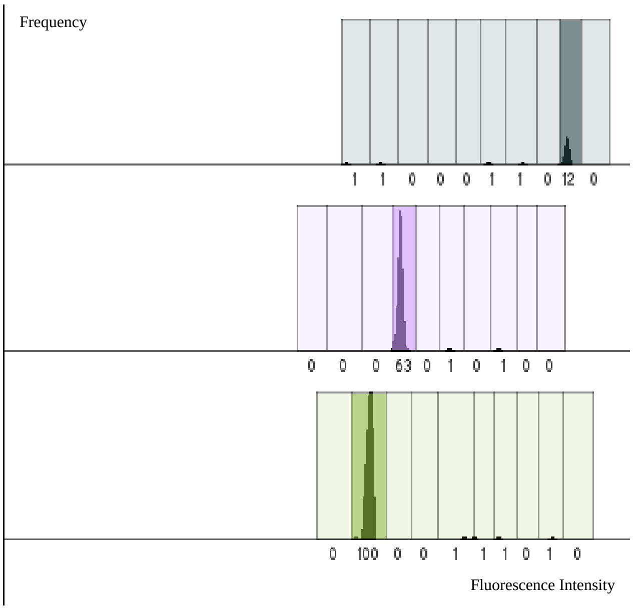

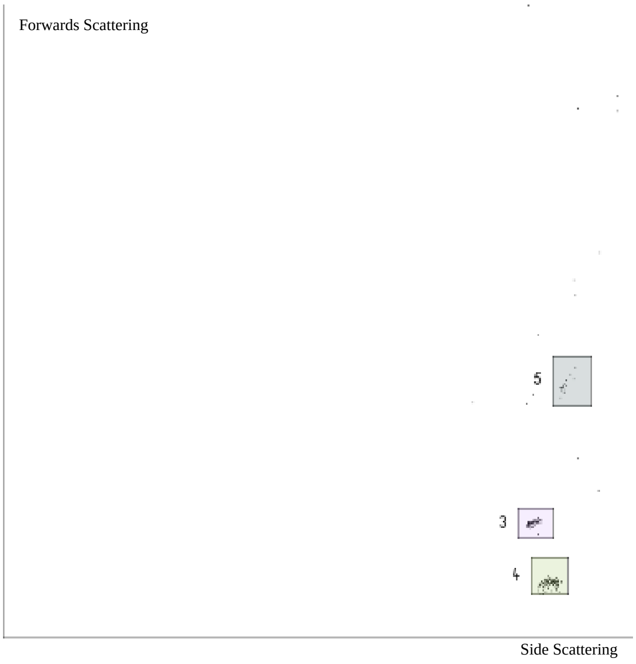

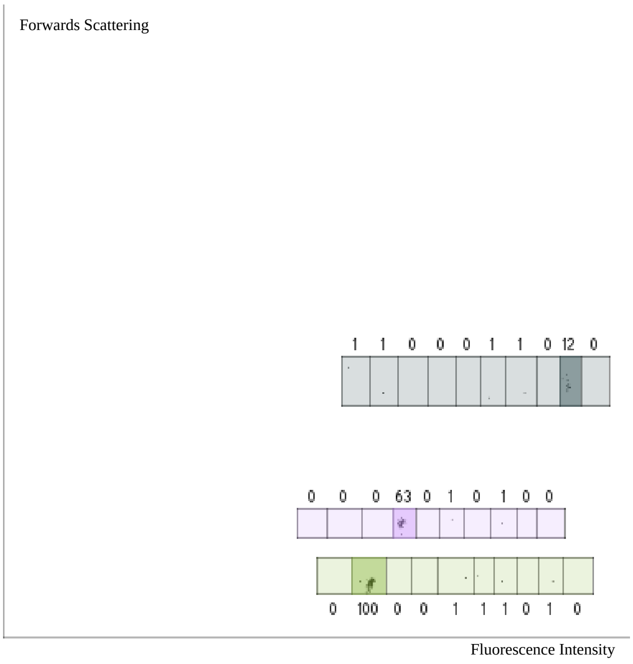

ANNEX 3: TAG DECONVOLUTION - BEAD 55

Passes flow sorting criteria: Yes

Passes tag deconvolution criteria: Yes

Included in protocol analysis: Yes

Protocol: 4, 8, 8, 1

Filename: Bin1_plateA3_D6.fcs

Split 1: Petrol shading

Split 2: Green shading

Split 3: Violet shading

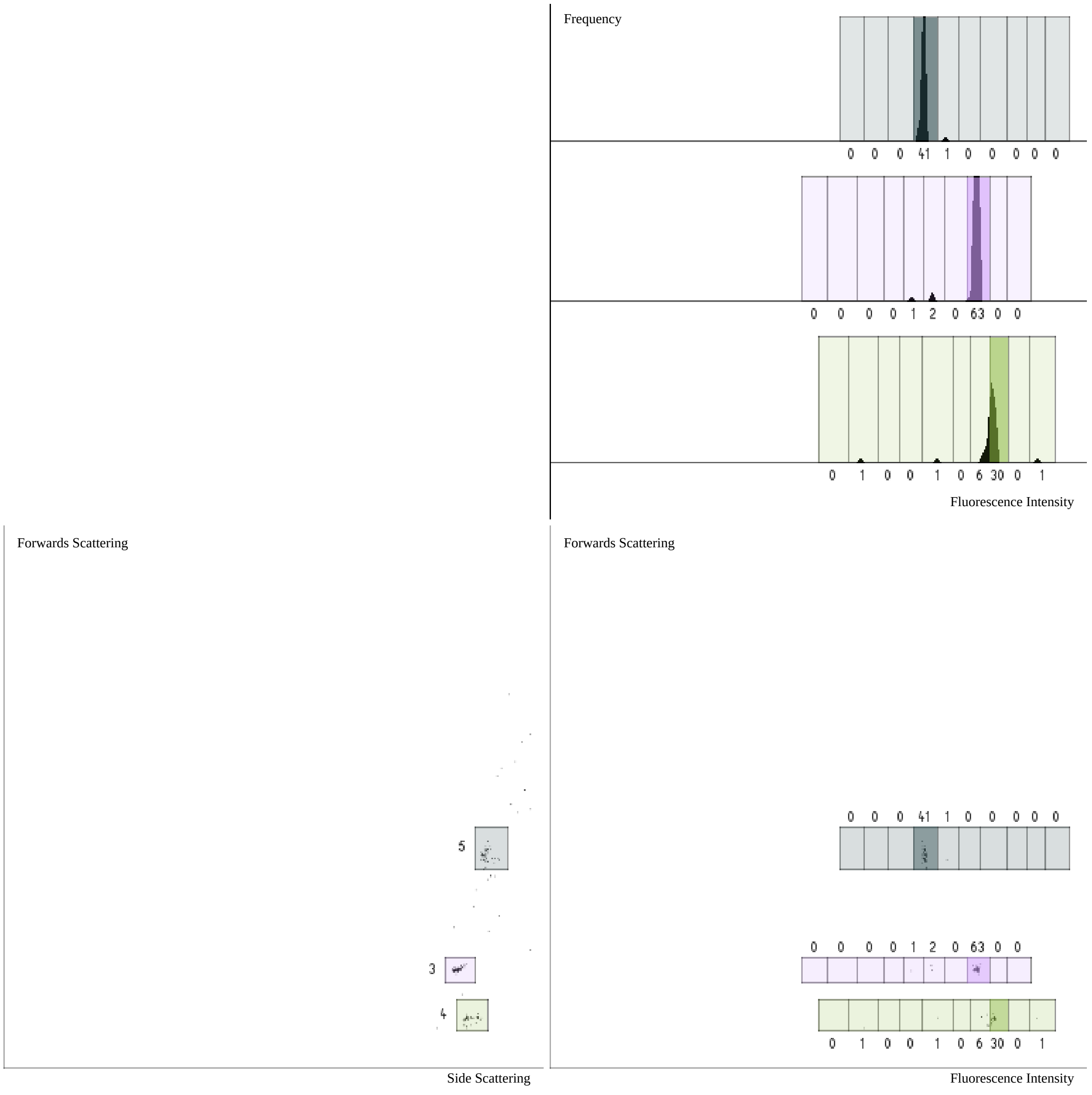

ANNEX 3: TAG DECONVOLUTION - BEAD 56

Passes flow sorting criteria: Yes

Passes tag deconvolution criteria: Yes

Included in protocol analysis: Yes

Protocol: 7, 7, 7, 1

Filename: Bin1_plateA3_D8.fcs

Split 1: Petrol shading

Split 2: Green shading

Split 3: Violet shading

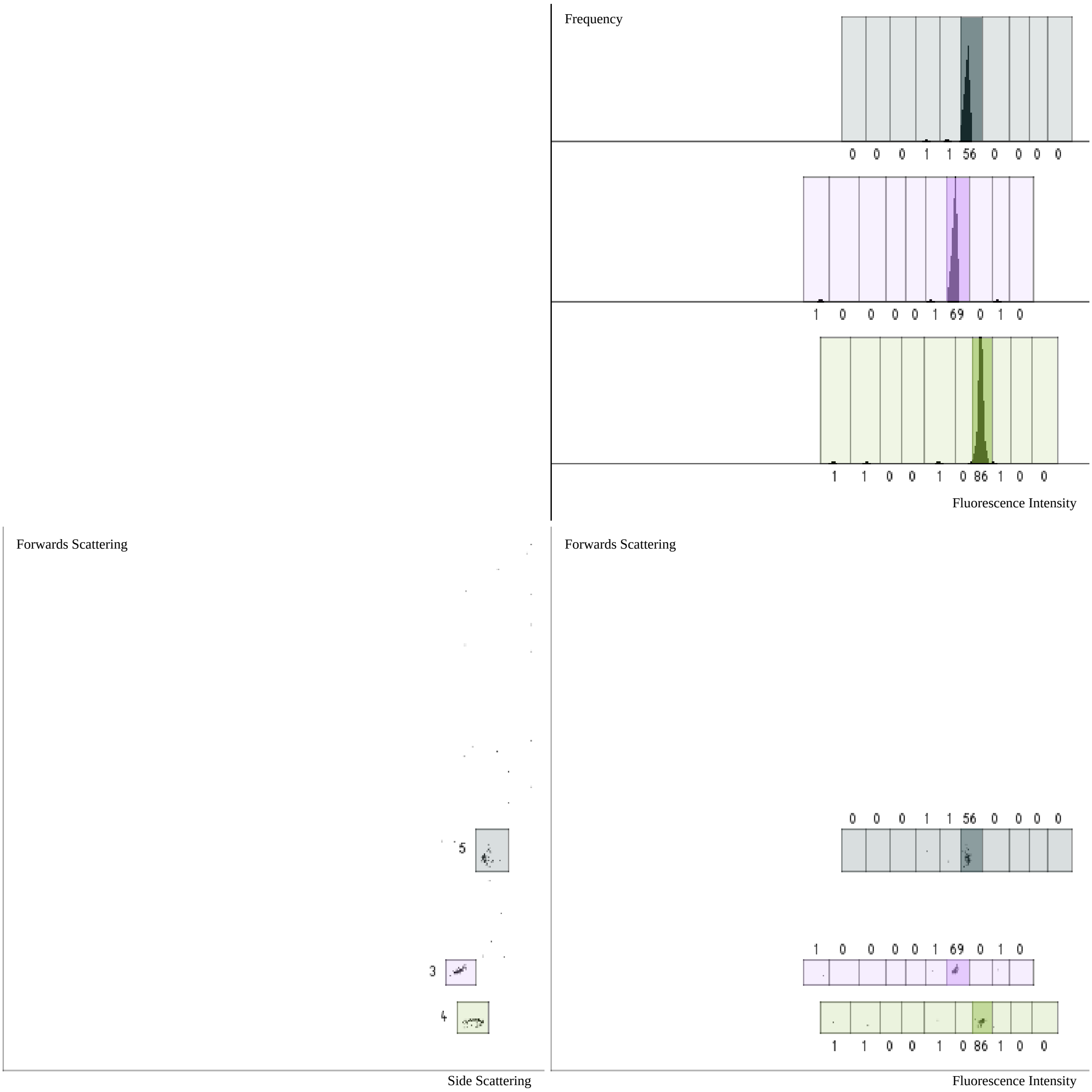

ANNEX 3: TAG DECONVOLUTION - BEAD 57

Passes flow sorting criteria: Yes

Passes tag deconvolution criteria: Yes

Included in protocol analysis: Yes

Protocol: 6, 9, 7, 1

Filename: Bin1_plateA3_D12.fcs

Split 1: Petrol shading

Split 2: Green shading

Split 3: Violet shading

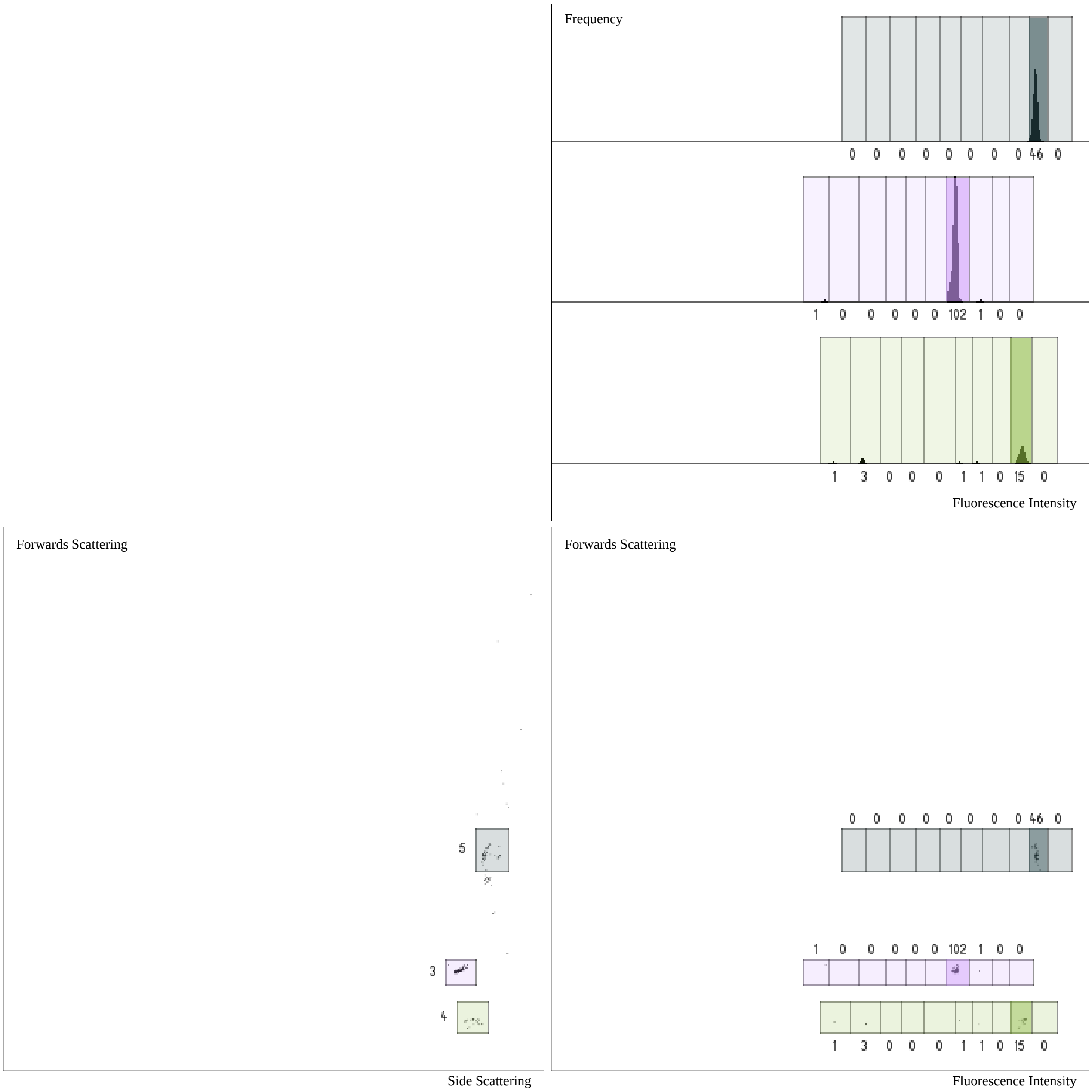

ANNEX 3: TAG DECONVOLUTION - BEAD 58

Passes flow sorting criteria: Yes

Passes tag deconvolution criteria: No

Included in protocol analysis: No

Protocol: N/A

Filename: Bin1_plateA3_E2.fcs

Split 1: Petrol shading

Split 2: Green shading

Split 3: Violet shading

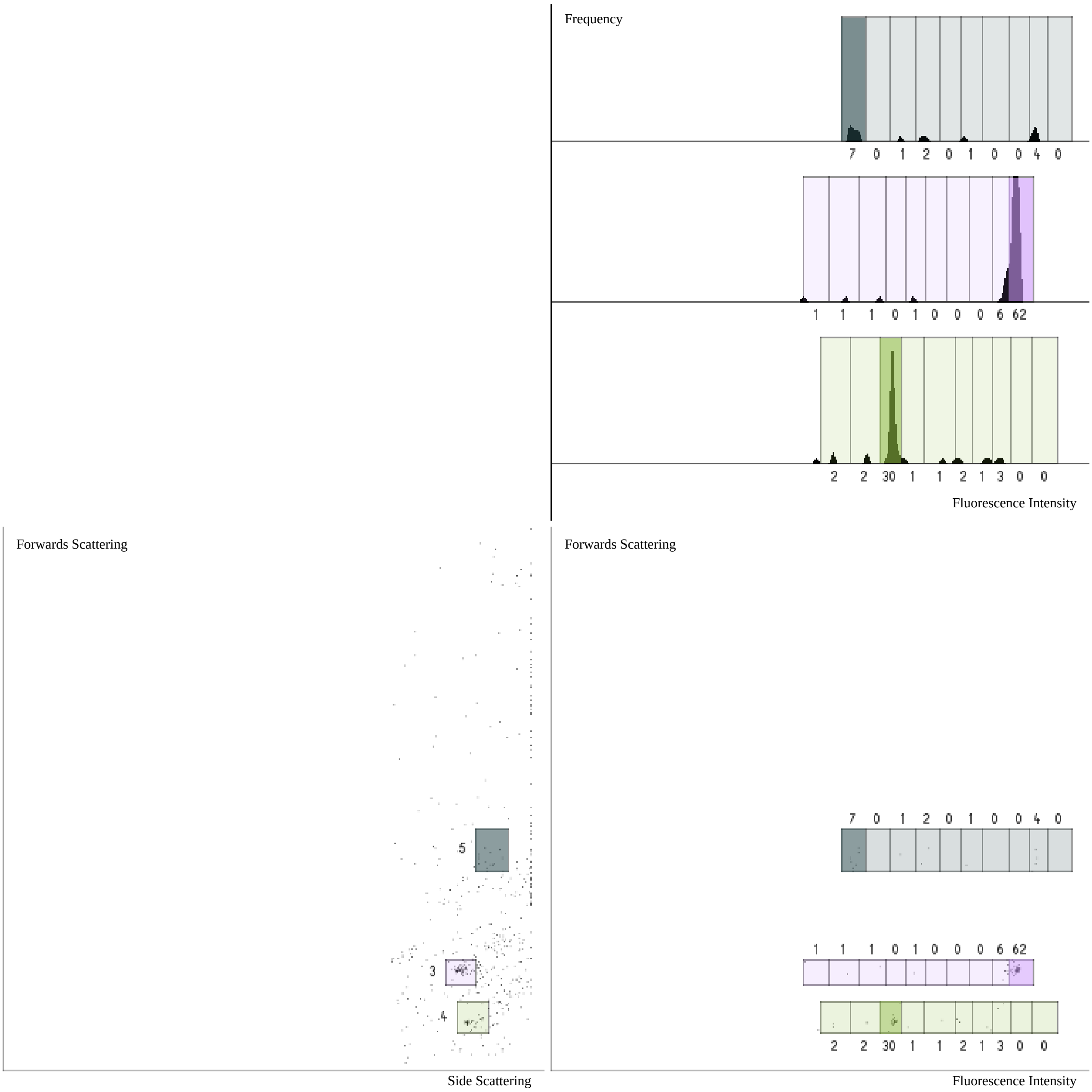

ANNEX 3: TAG DECONVOLUTION - BEAD 59

Passes flow sorting criteria: Yes

Passes tag deconvolution criteria: Yes

Included in protocol analysis: Yes

Protocol: 4, 10, 4, 1

Filename: Bin1_plateA3_E3.fcs

Split 1: Petrol shading

Split 2: Green shading

Split 3: Violet shading

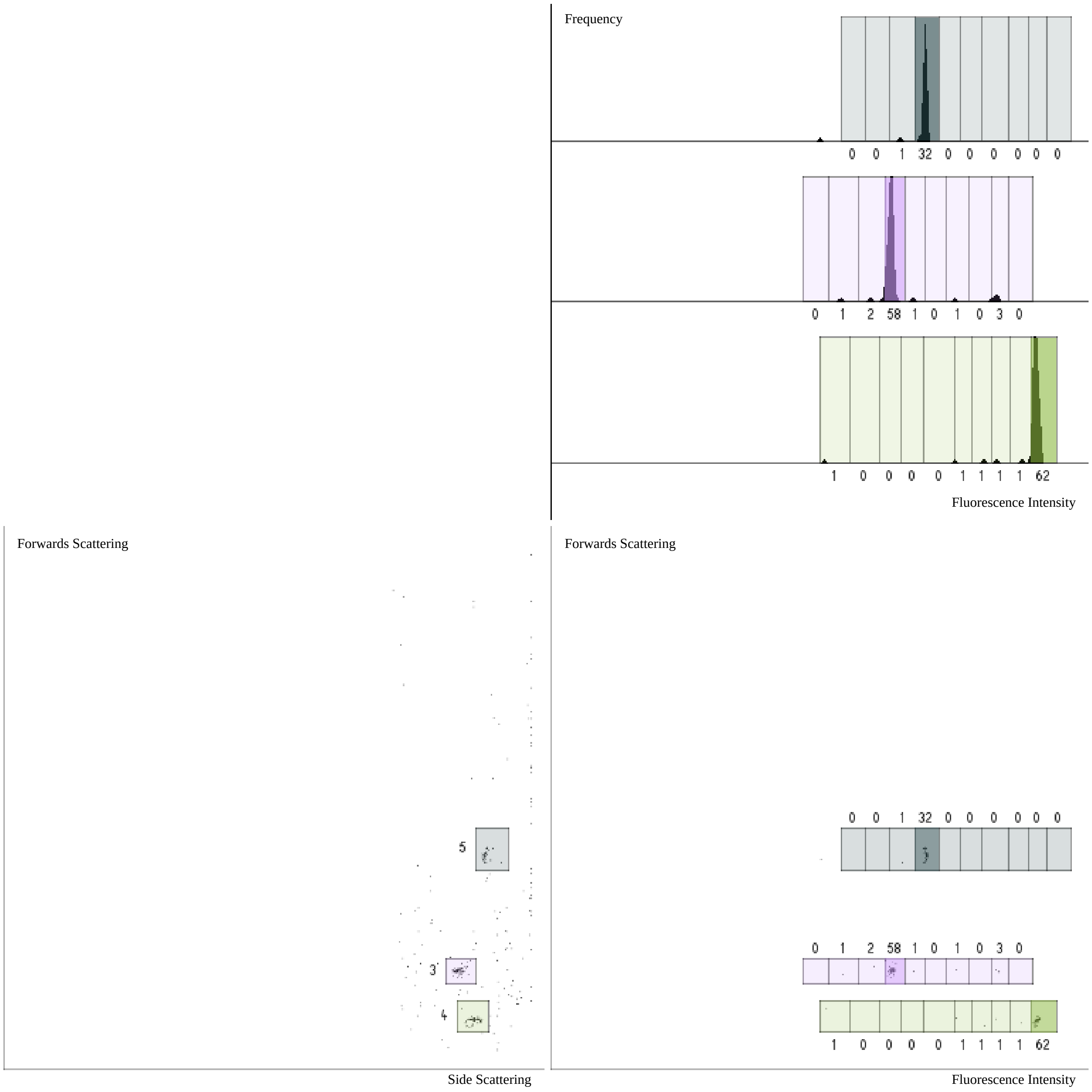

ANNEX 3: TAG DECONVOLUTION - BEAD 60

Passes flow sorting criteria: Yes

Passes tag deconvolution criteria: Yes

Included in protocol analysis: Yes

Protocol: 7, 6, 2, 1

Filename: Bin1_plateA3_E6.fcs

Split 1: Petrol shading

Split 2: Green shading

Split 3: Violet shading

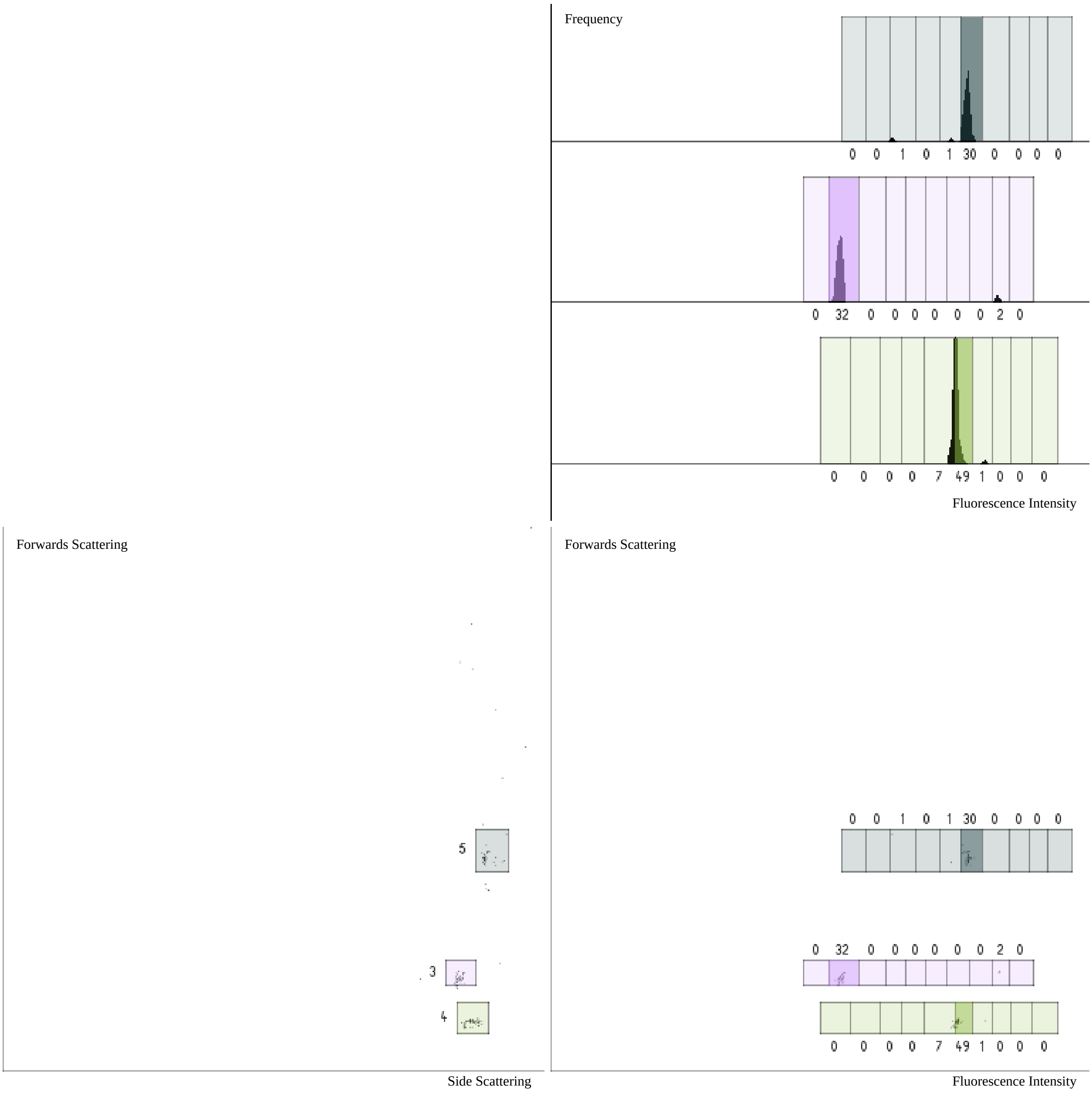

ANNEX 3: TAG DECONVOLUTION - BEAD 61

Passes flow sorting criteria: Yes

Passes tag deconvolution criteria: Yes

Included in protocol analysis: Yes

Protocol: 9, 8, 1, 1

Filename: Bin1_plateA3_E7.fcs

Split 1: Petrol shading

Split 2: Green shading

Split 3: Violet shading

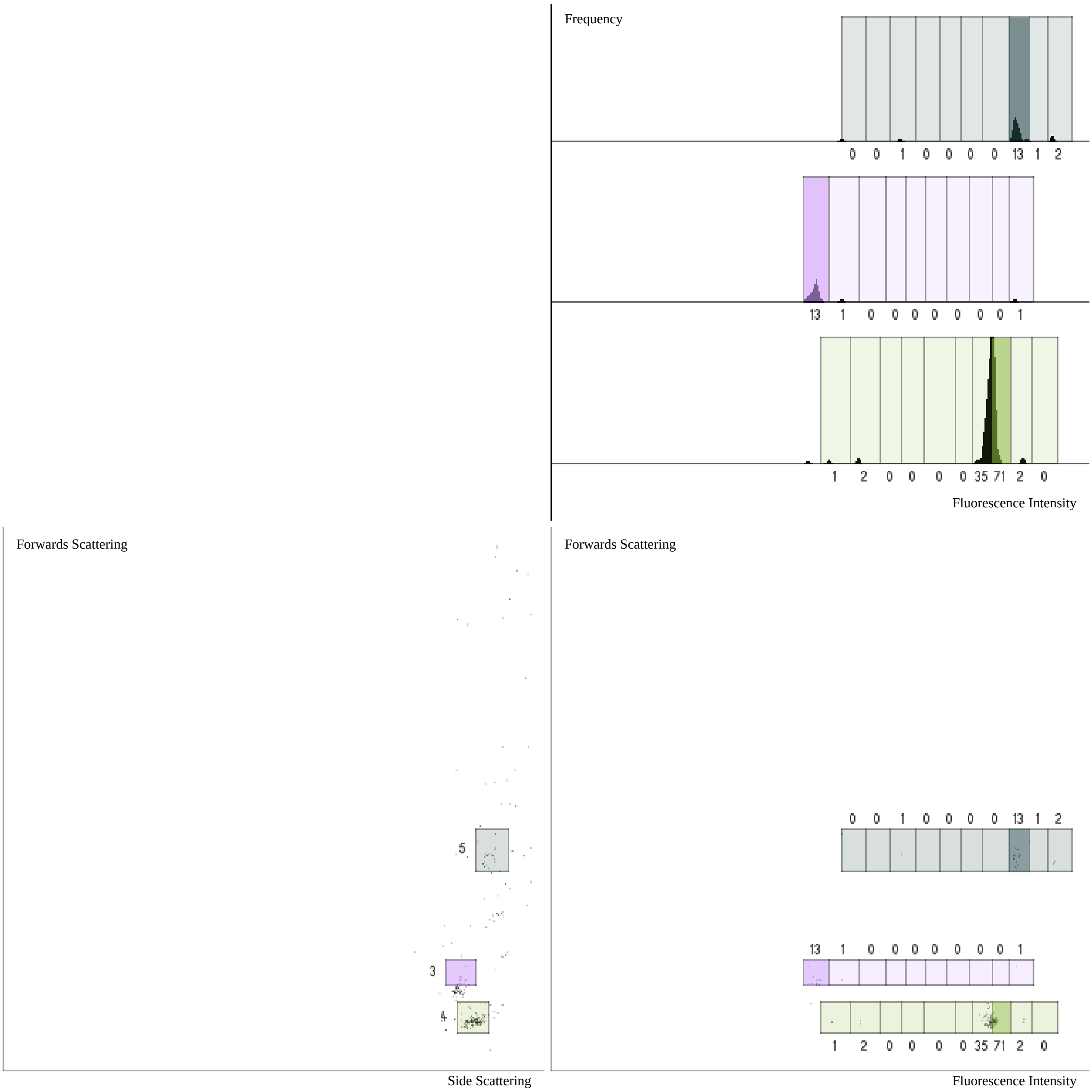

ANNEX 3: TAG DECONVOLUTION - BEAD 62

Passes flow sorting criteria: Yes

Passes tag deconvolution criteria: Yes

Included in protocol analysis: Yes

Protocol: 8, 8, 8, 1

Filename: Bin1_plateA3_F3.fcs

Split 1: Petrol shading

Split 2: Green shading

Split 3: Violet shading

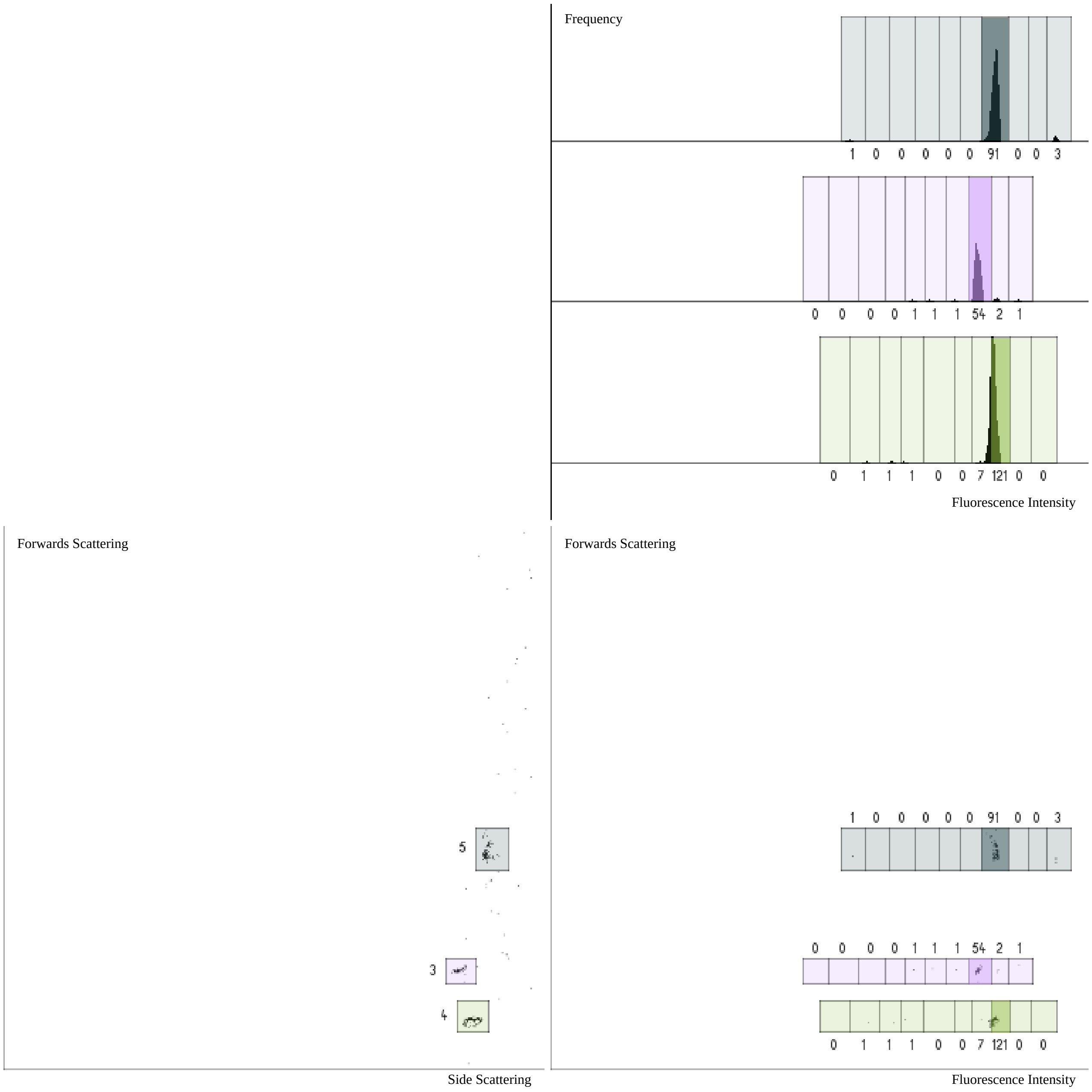

ANNEX 3: TAG DECONVOLUTION - BEAD 63

Passes flow sorting criteria: Yes

Passes tag deconvolution criteria: Yes

Included in protocol analysis: Yes

Protocol: 7, 3, 9, 1

Filename: Bin1_plateA3_F4.fcs

Split 1: Petrol shading

Split 2: Green shading

Split 3: Violet shading

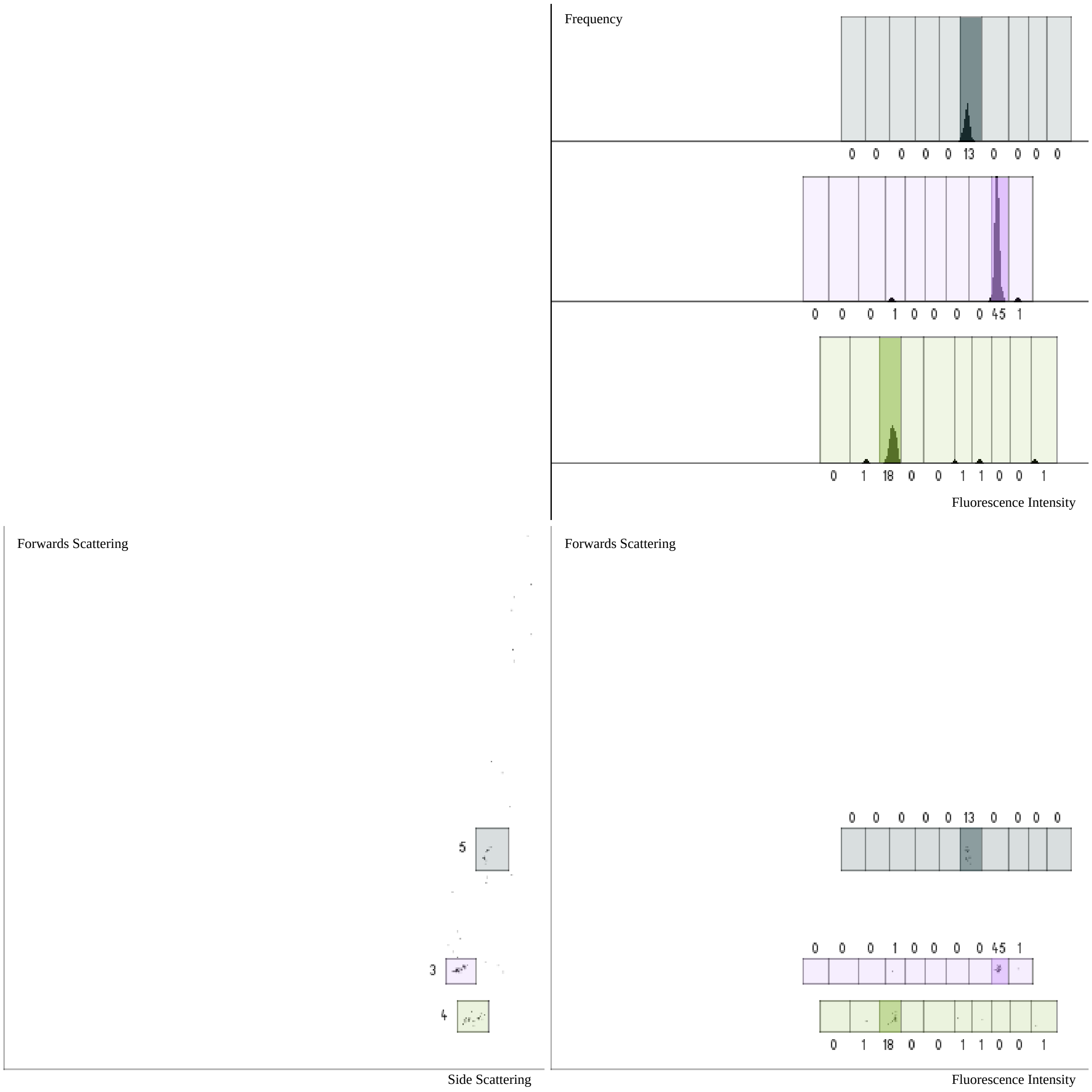

ANNEX 3: TAG DECONVOLUTION - BEAD 64

Passes flow sorting criteria: Yes

Passes tag deconvolution criteria: Yes

Included in protocol analysis: Yes

Protocol: 4, 4, 9, 2

Filename: Bin2_plateB3_A2.fcs

Split 1: Petrol shading

Split 2: Green shading

Split 3: Violet shading

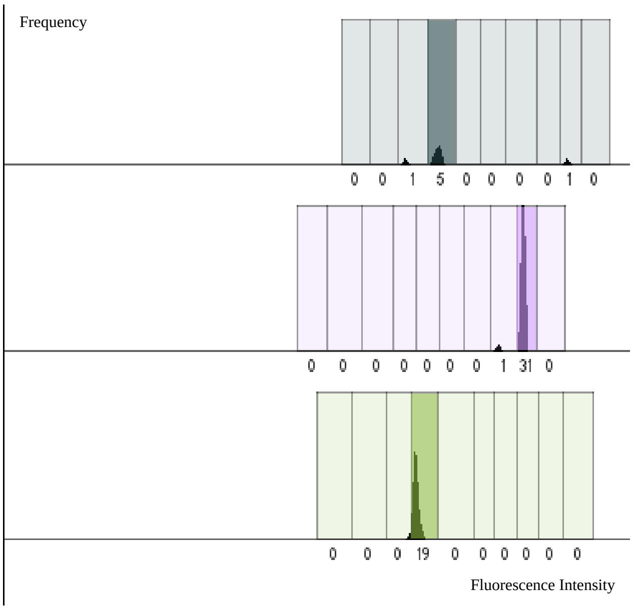

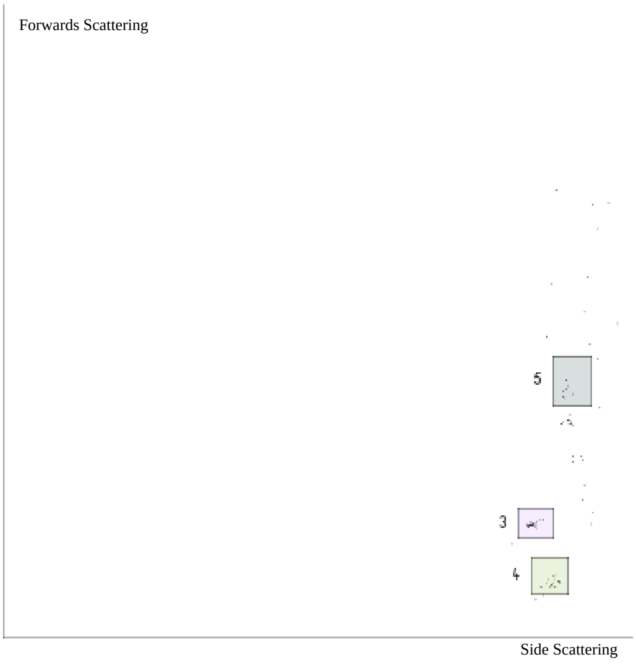

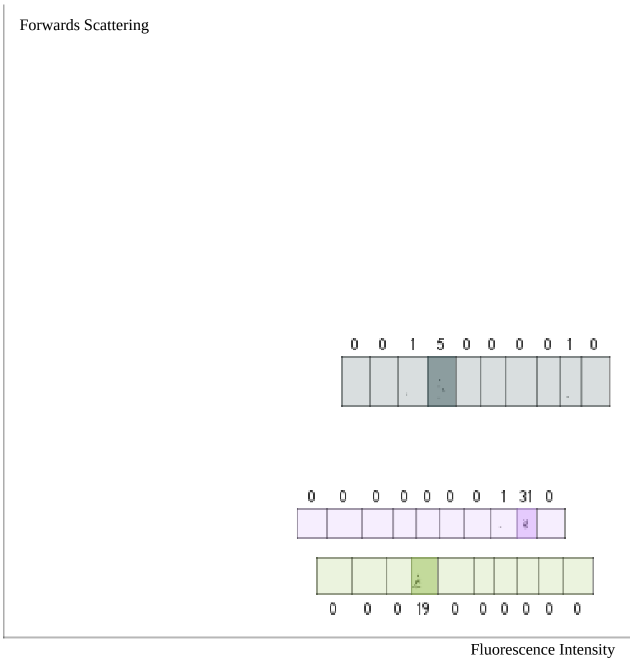

ANNEX 3: TAG DECONVOLUTION - BEAD 65

Passes flow sorting criteria: Yes

Passes tag deconvolution criteria: Yes

Included in protocol analysis: Yes

Protocol: 1, 9, 8, 2

Filename: Bin2_PlateB3_A4.fcs

Split 1: Petrol shading

Split 2: Green shading

Split 3: Violet shading

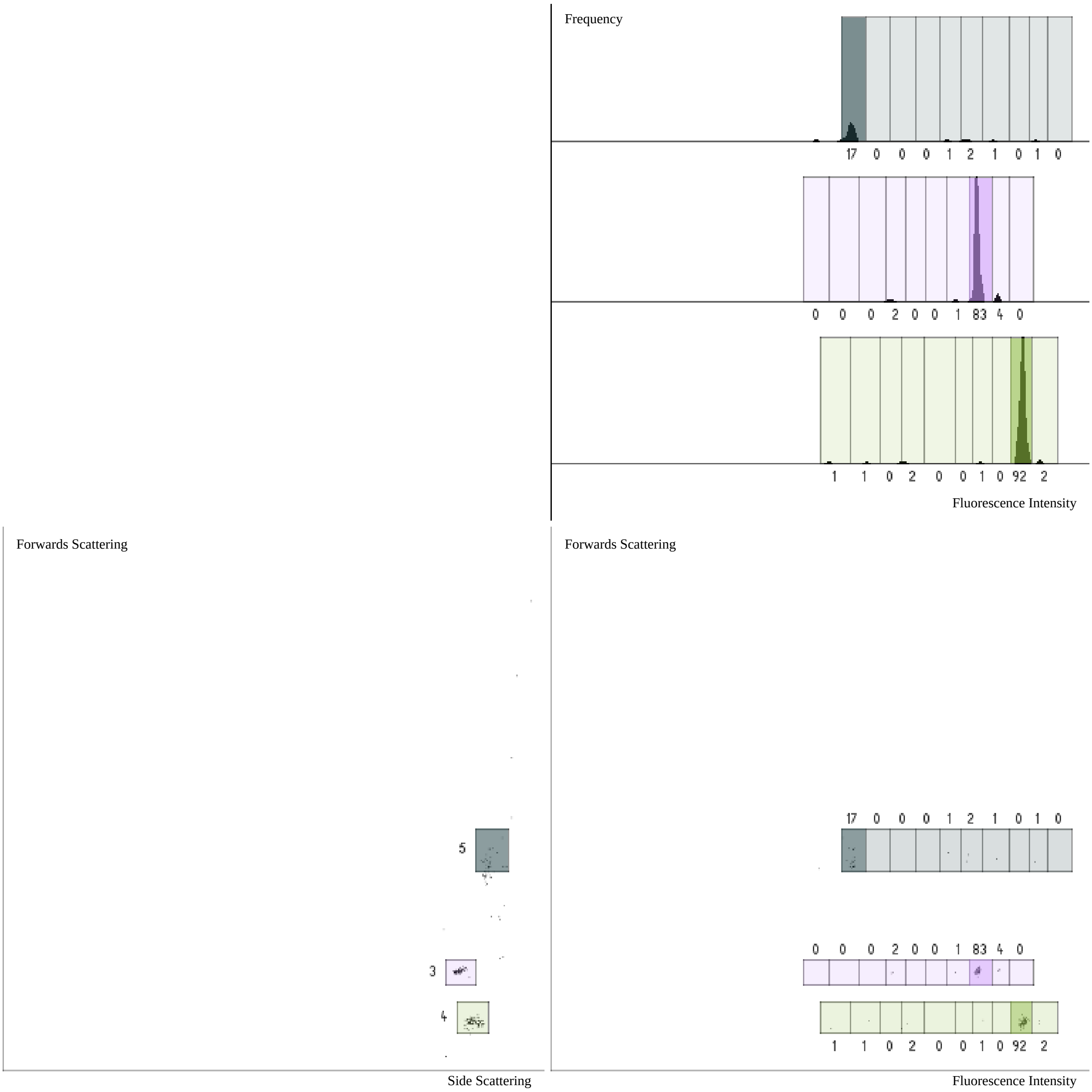

ANNEX 3: TAG DECONVOLUTION - BEAD 66

Passes flow sorting criteria: Yes

Passes tag deconvolution criteria: Yes

Included in protocol analysis: Yes

Protocol: 3, 4, 6, 2

Filename: Bin2_PlateB3_A5.fcs

Split 1: Petrol shading

Split 2: Green shading

Split 3: Violet shading

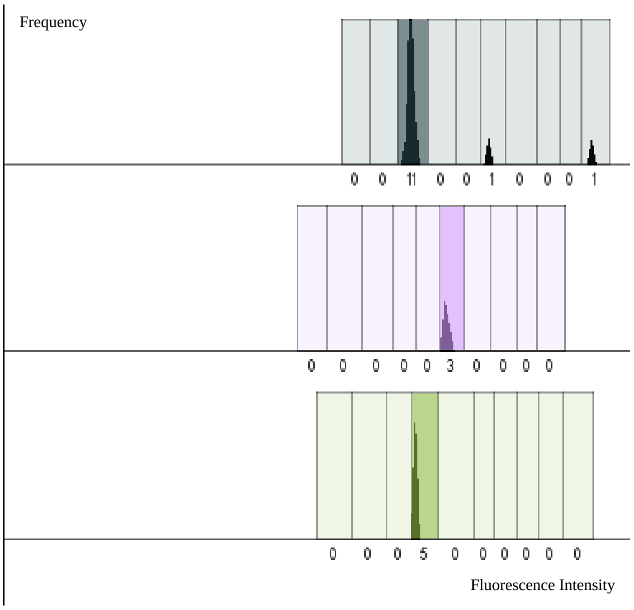

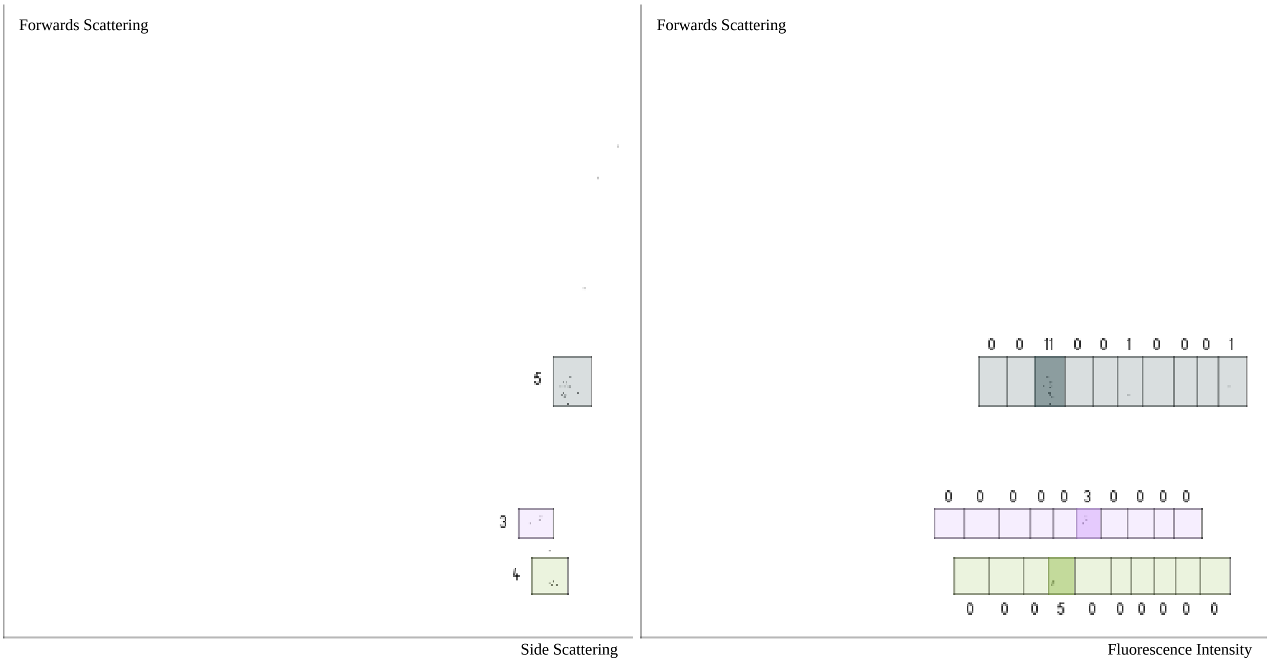

ANNEX 3: TAG DECONVOLUTION - BEAD 67

Passes flow sorting criteria: Yes

Passes tag deconvolution criteria: Yes

Included in protocol analysis: Yes

Protocol: 9, 10, 7, 2

Filename: Bin2_PlateC3_B7.fcs

Split 1: Petrol shading

Split 2: Green shading

Split 3: Violet shading

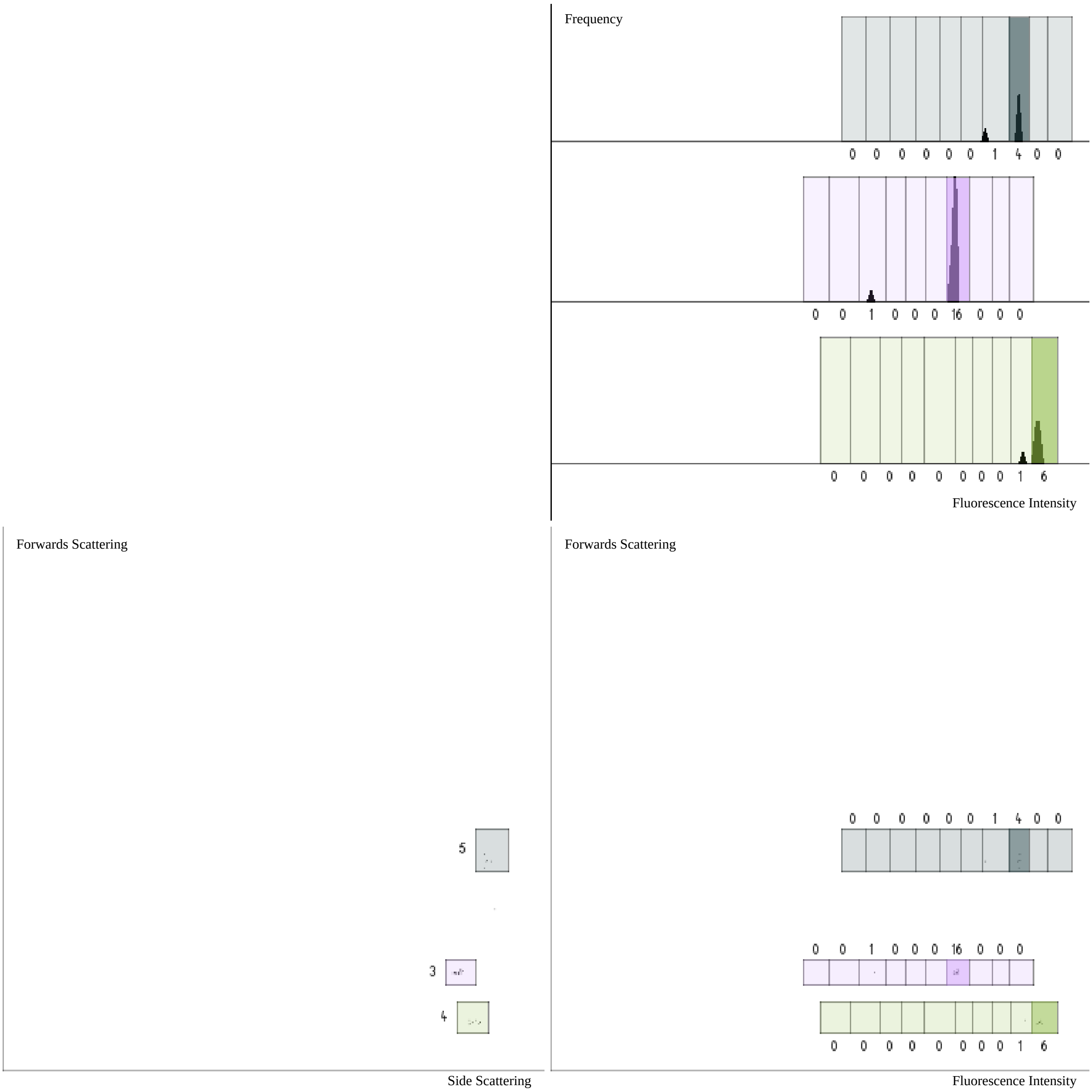

ANNEX 3: TAG DECONVOLUTION - BEAD 68

Passes flow sorting criteria: Yes

Passes tag deconvolution criteria: Yes

Included in protocol analysis: Yes

Protocol: 10, 7, 4, 2

Filename: Bin2_PlateC3_B11.fcs

Split 1: Petrol shading

Split 2: Green shading

Split 3: Violet shading

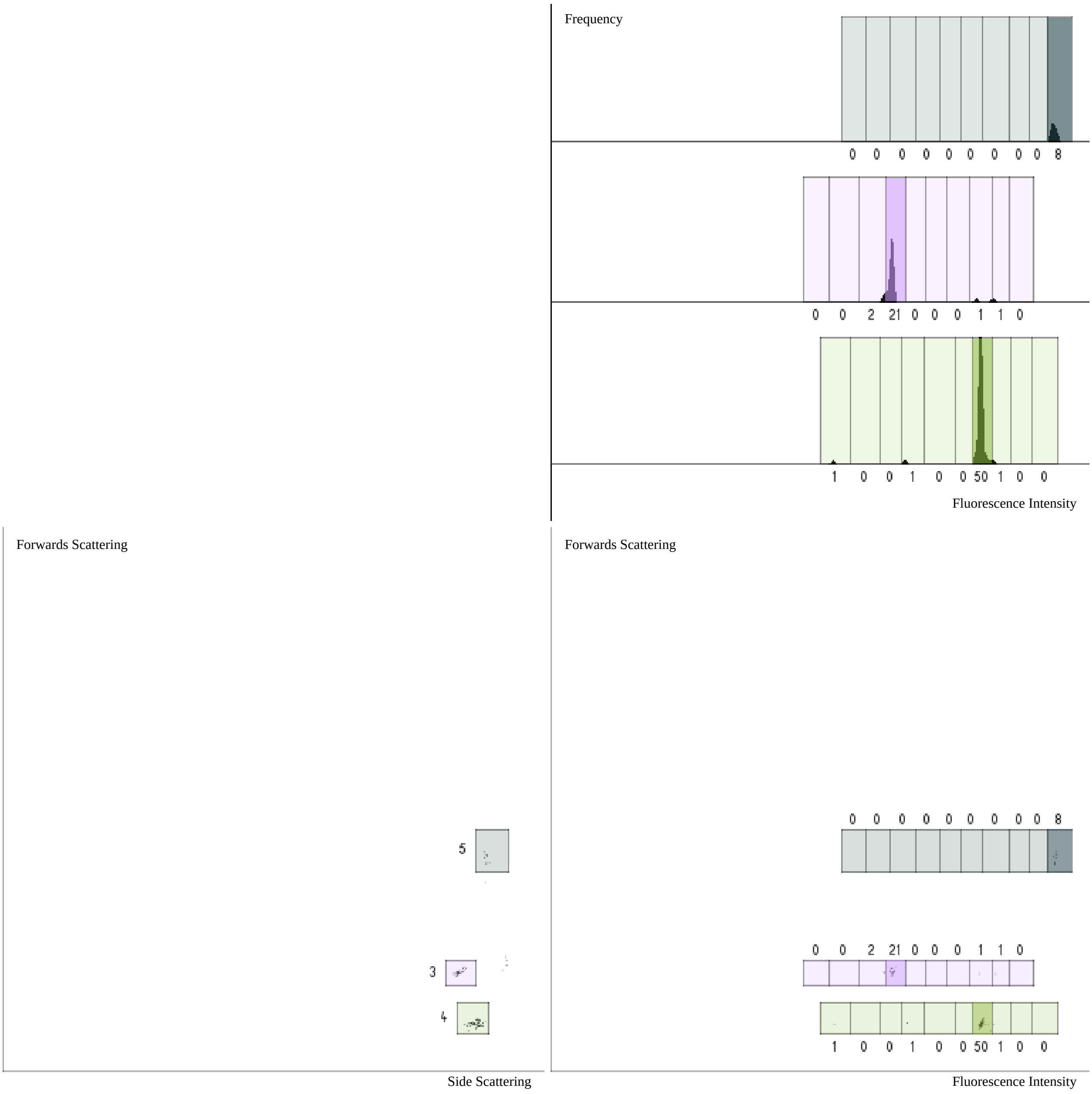

ANNEX 3: TAG DECONVOLUTION - BEAD 69

Passes flow sorting criteria: Yes

Passes tag deconvolution criteria: Yes

Included in protocol analysis: Yes

Protocol: 10, 1, 2, 2

Filename: Bin2_PlateC3_C1.fcs

Split 1: Petrol shading

Split 2: Green shading

Split 3: Violet shading

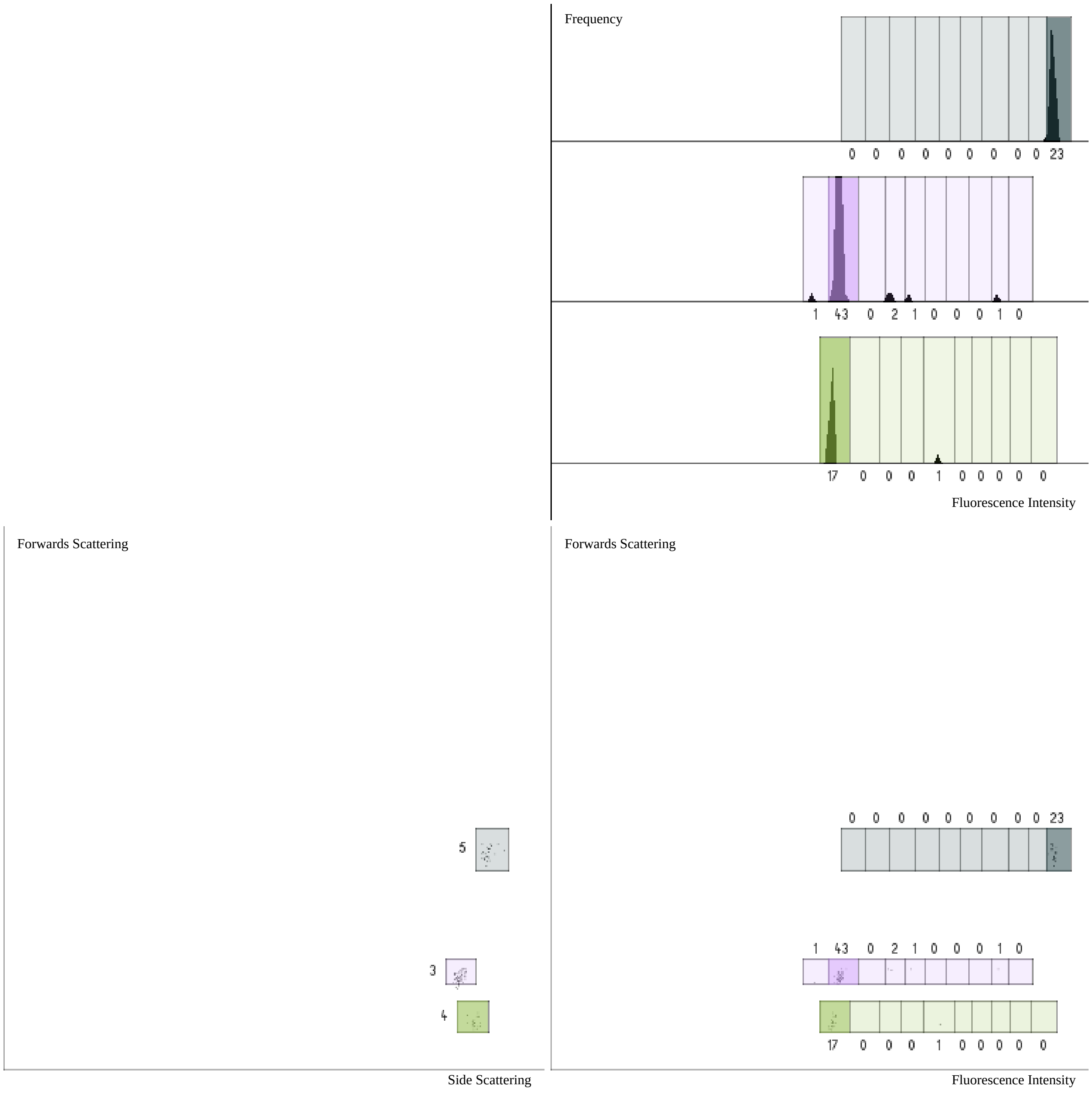

ANNEX 3: TAG DECONVOLUTION - BEAD 70

Passes flow sorting criteria: Yes

Passes tag deconvolution criteria: Yes

Included in protocol analysis: Yes

Protocol: 8, 4, 9, 2

Filename: Bin2_PlateC3_C3.fcs

Split 1: Petrol shading

Split 2: Green shading

Split 3: Violet shading

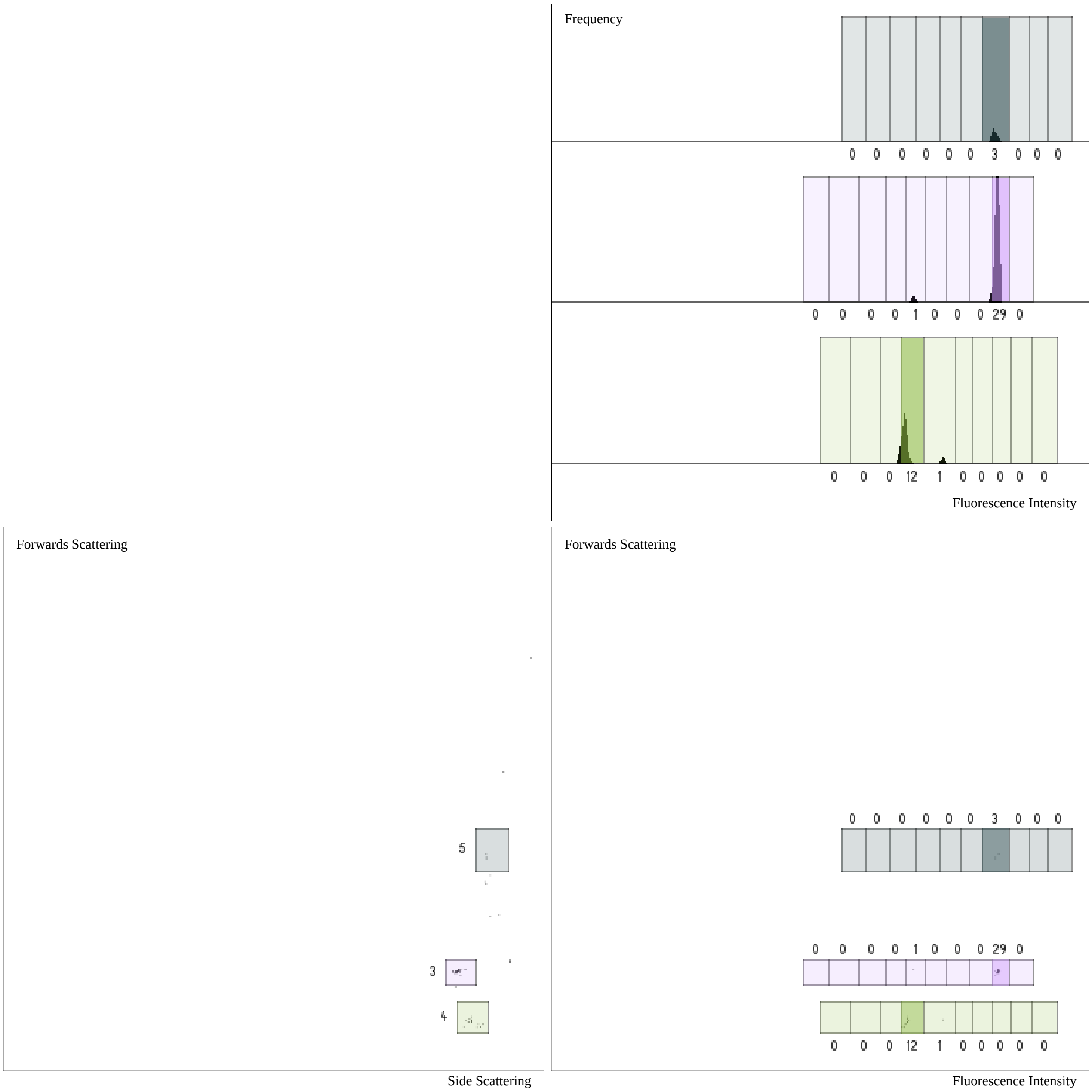

ANNEX 3: TAG DECONVOLUTION - BEAD 71

Passes flow sorting criteria: Yes

Passes tag deconvolution criteria: Yes

Included in protocol analysis: Yes

Protocol: 9, 7, 7, 2

Filename: Bin2_PlateC3_C6.fcs

Split 1: Petrol shading

Split 2: Green shading

Split 3: Violet shading

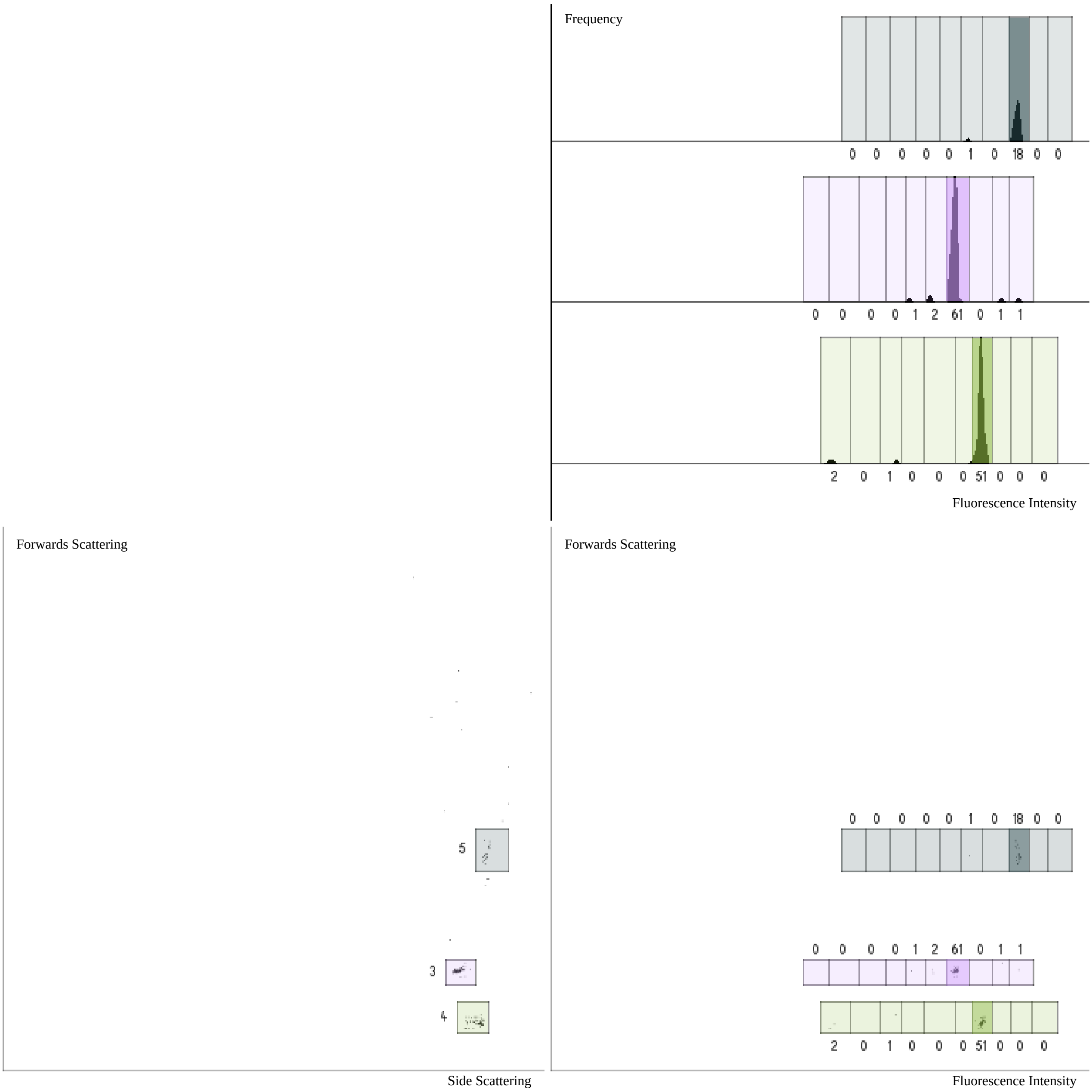

ANNEX 3: TAG DECONVOLUTION - BEAD 72

Passes flow sorting criteria: Yes

Passes tag deconvolution criteria: Yes

Included in protocol analysis: Yes

Protocol: 2, 3, 10, 2

Filename: Bin2_PlateC3_C9.fcs

Split 1: Petrol shading

Split 2: Green shading

Split 3: Violet shading

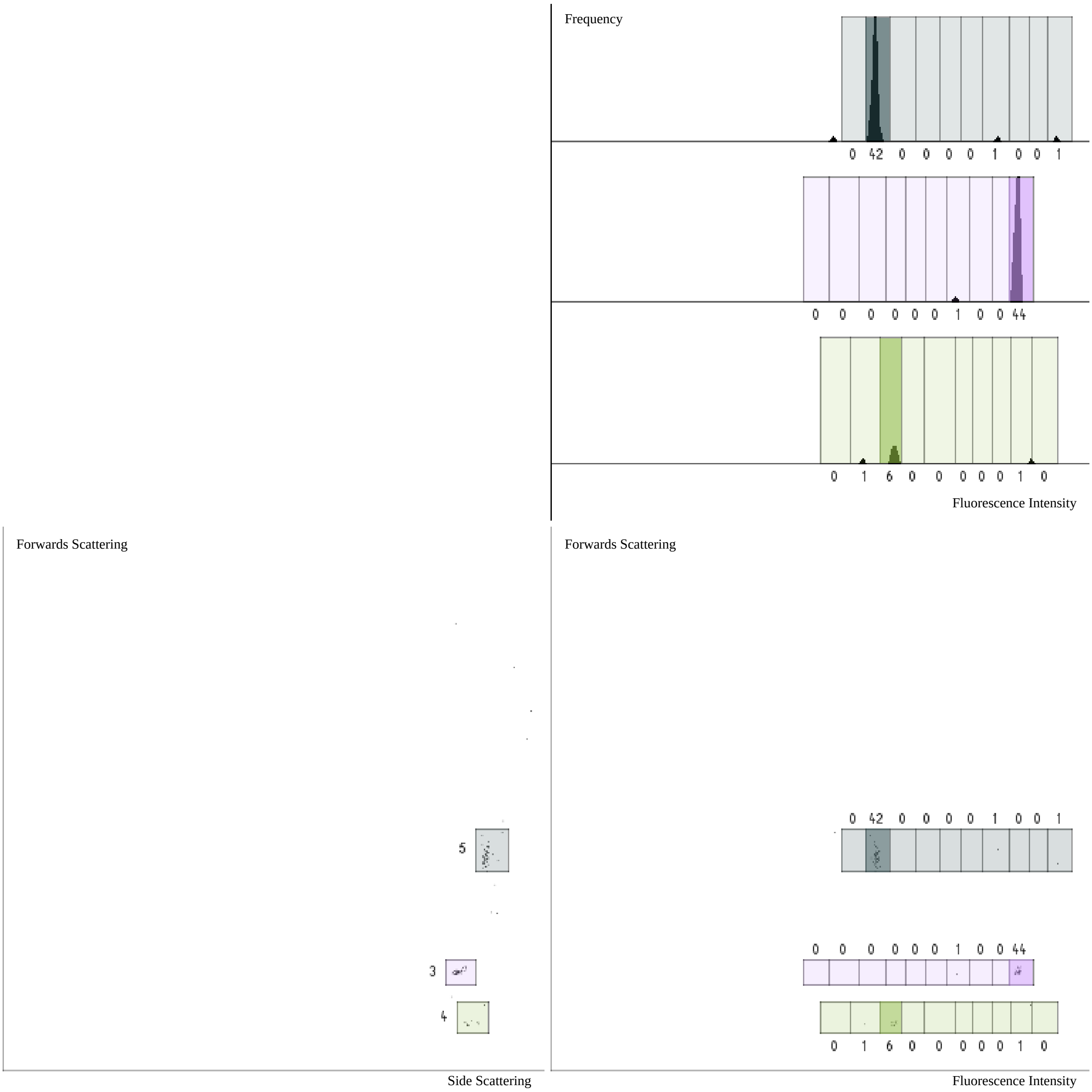

ANNEX 3: TAG DECONVOLUTION - BEAD 73

Passes flow sorting criteria: Yes

Passes tag deconvolution criteria: Yes

Included in protocol analysis: Yes

Protocol: 3, 7, 7, 2

Filename: Bin2_PlateC3_D2.fcs

Split 1: Petrol shading

Split 2: Green shading

Split 3: Violet shading

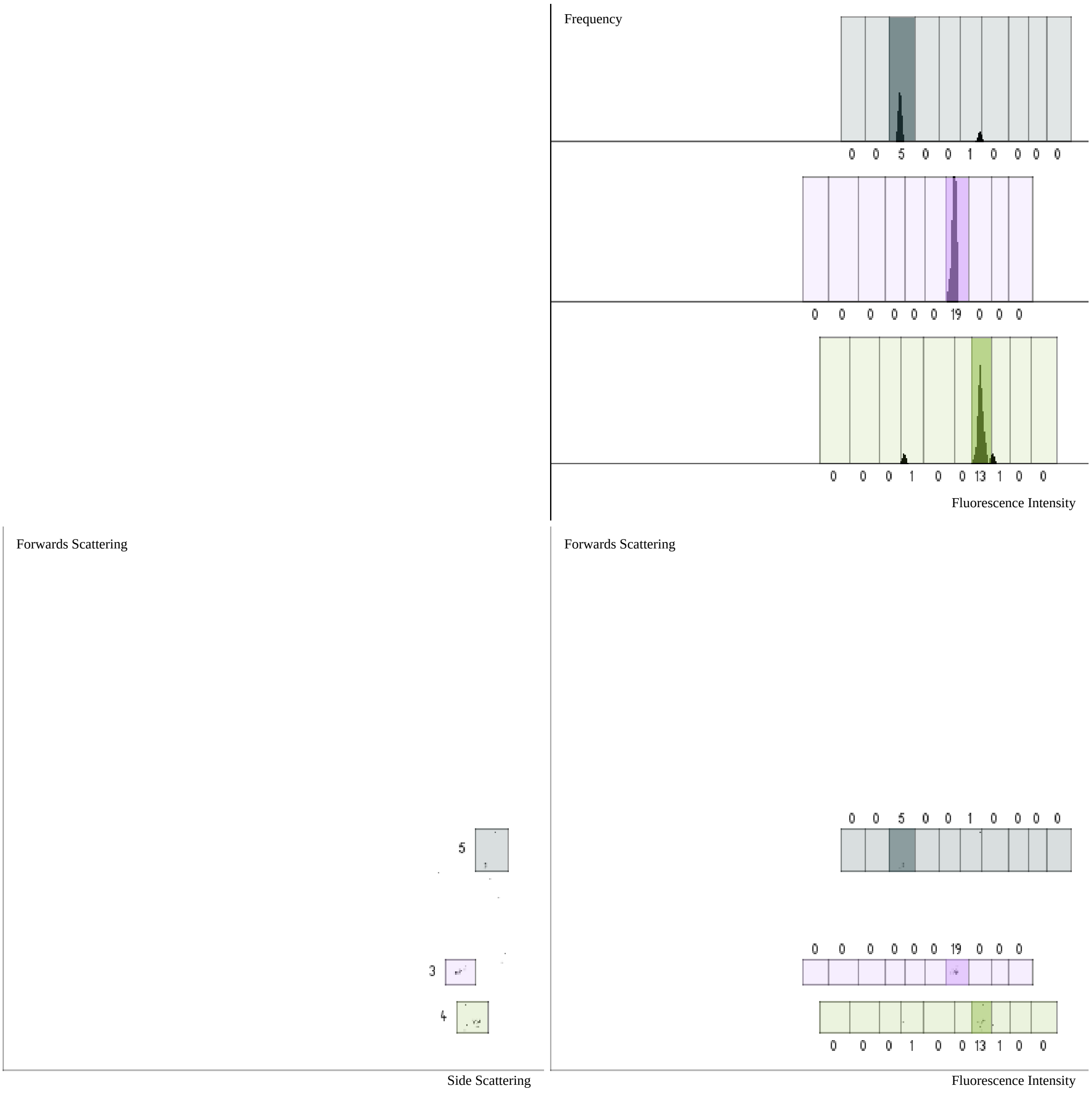

ANNEX 3: TAG DECONVOLUTION - BEAD 74

Passes flow sorting criteria: Yes

Passes tag deconvolution criteria: Yes

Included in protocol analysis: Yes

Protocol: 6, 5, 9, 2

Filename: Bin2_PlateC3_D9.fcs

Split 1: Petrol shading

Split 2: Green shading

Split 3: Violet shading

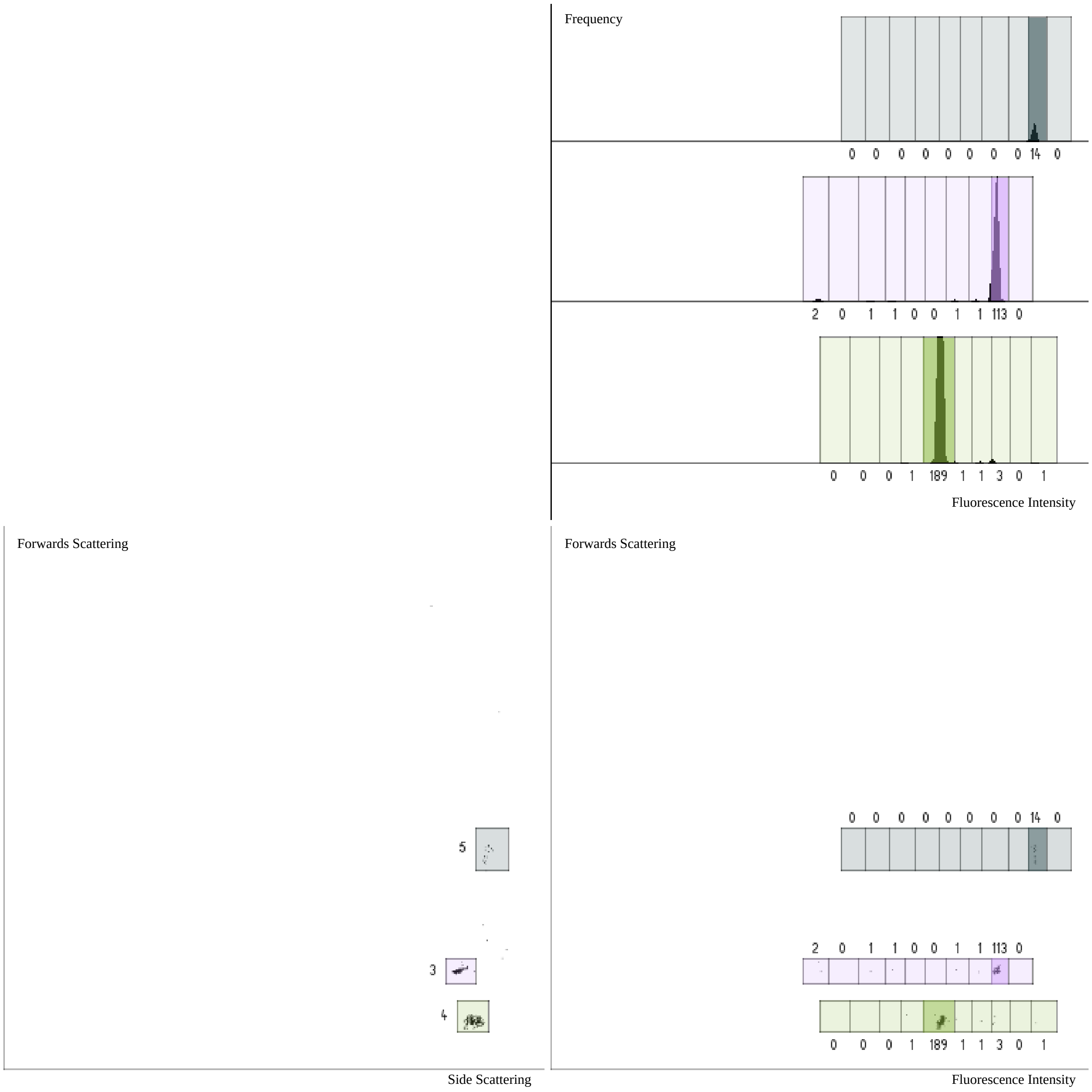

ANNEX 3: TAG DECONVOLUTION - BEAD 75

Passes flow sorting criteria: Yes

Passes tag deconvolution criteria: Yes

Included in protocol analysis: Yes

Protocol: 9, 8, 4, 2

Filename: Bin2_PlateC3_D10.fcs

Split 1: Petrol shading

Split 2: Green shading

Split 3: Violet shading

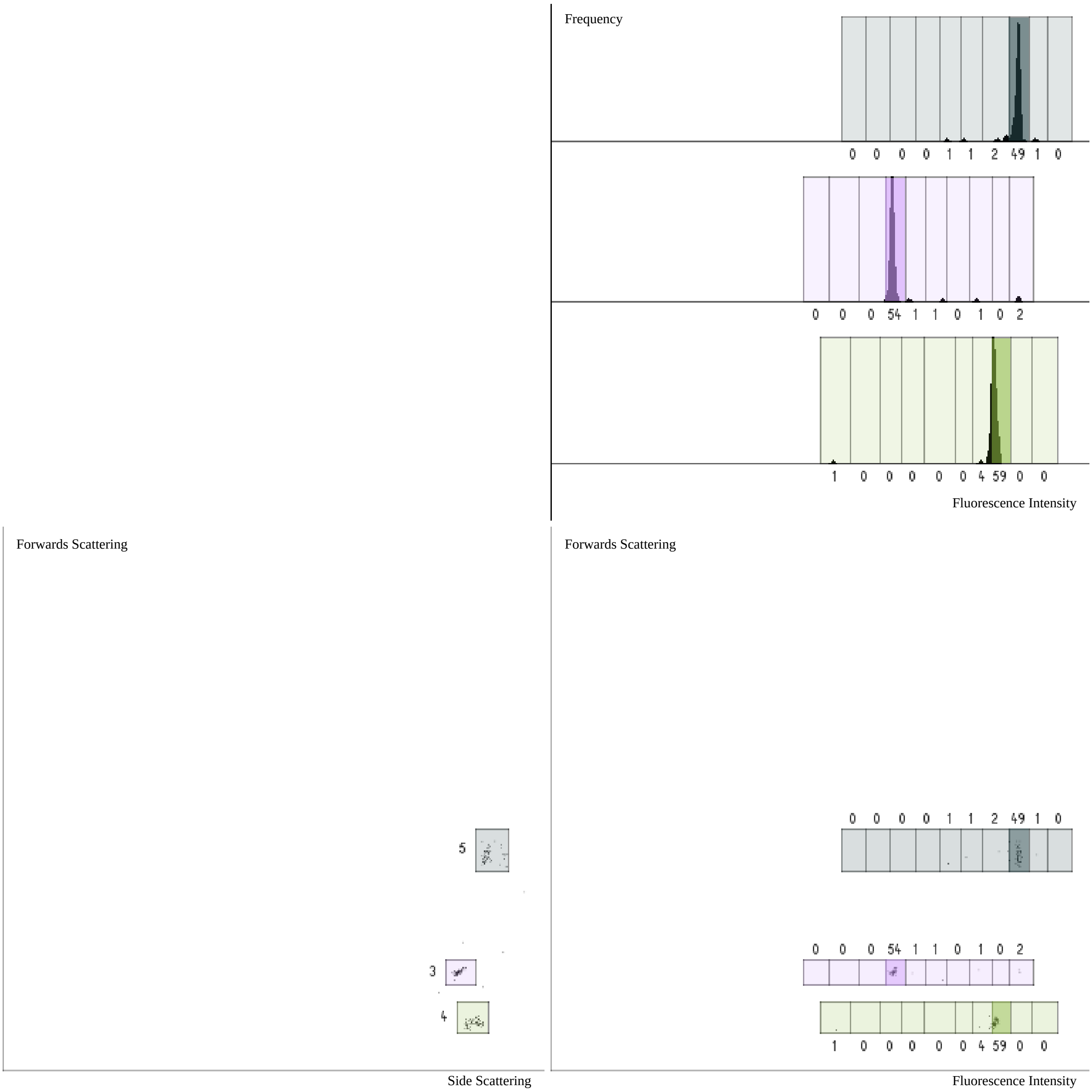

ANNEX 3: TAG DECONVOLUTION - BEAD 76

Passes flow sorting criteria: Yes

Passes tag deconvolution criteria: Yes

Included in protocol analysis: Yes

Protocol: 10, 6, 4, 3

Filename: Bin3_PlateA3_A1.fcs

Split 1: Petrol shading

Split 2: Green shading

Split 3: Violet shading

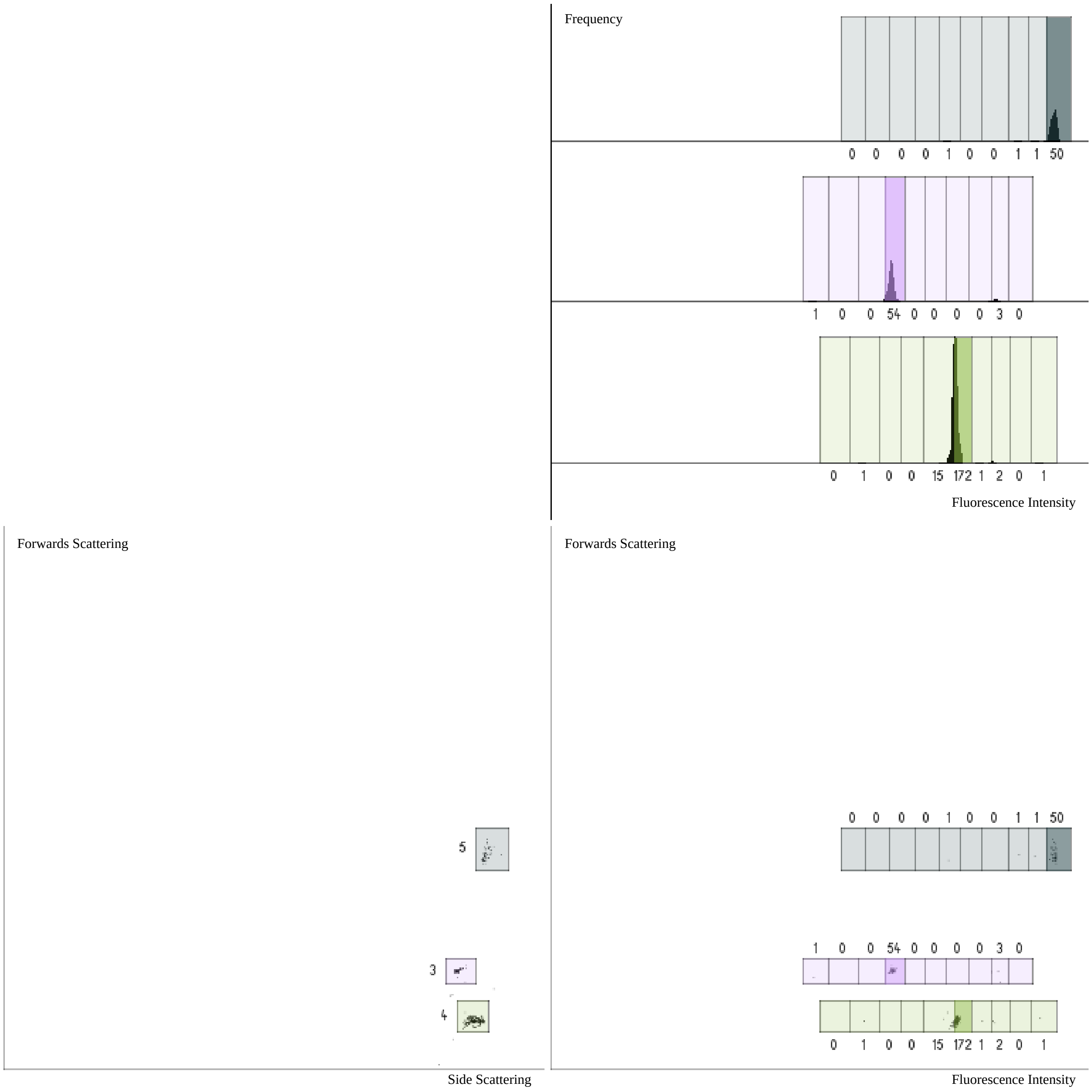

ANNEX 3: TAG DECONVOLUTION - BEAD 77

Passes flow sorting criteria: Yes

Passes tag deconvolution criteria: Yes

Included in protocol analysis: No

Protocol: N/A

Filename: Bin4_plateA3_H12.fcs

Split 1: Petrol shading

Split 2: Green shading

Split 3: Violet shading

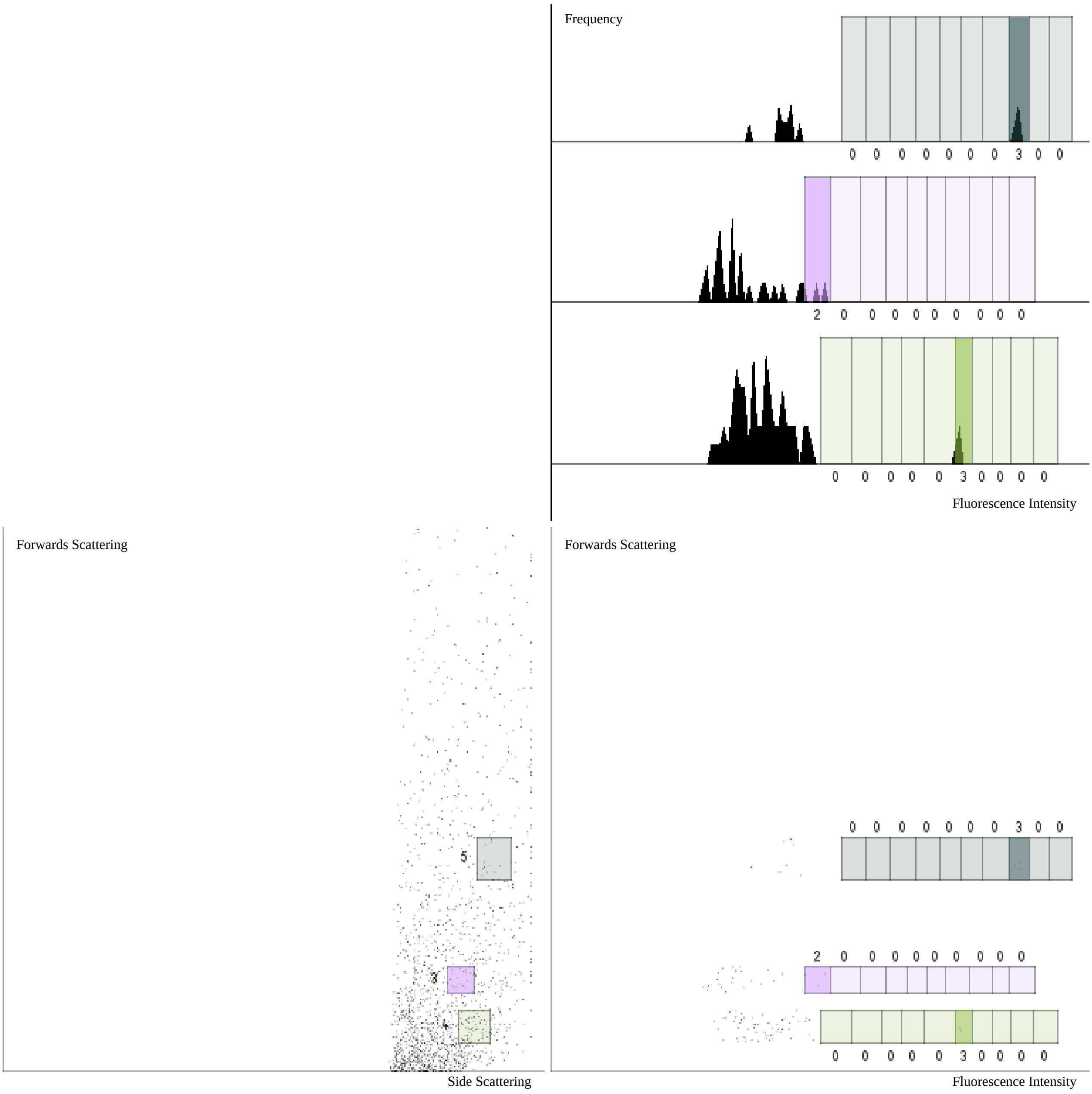

ANNEX 3: TAG DECONVOLUTION - BEAD 78

Passes flow sorting criteria: Yes

Passes tag deconvolution criteria: Yes

Included in protocol analysis: Yes

Protocol: 4, 9, 1, 3

Filename: Bin3_plateA3_B11.fcs

Split 1: Petrol shading

Split 2: Green shading

Split 3: Violet shading

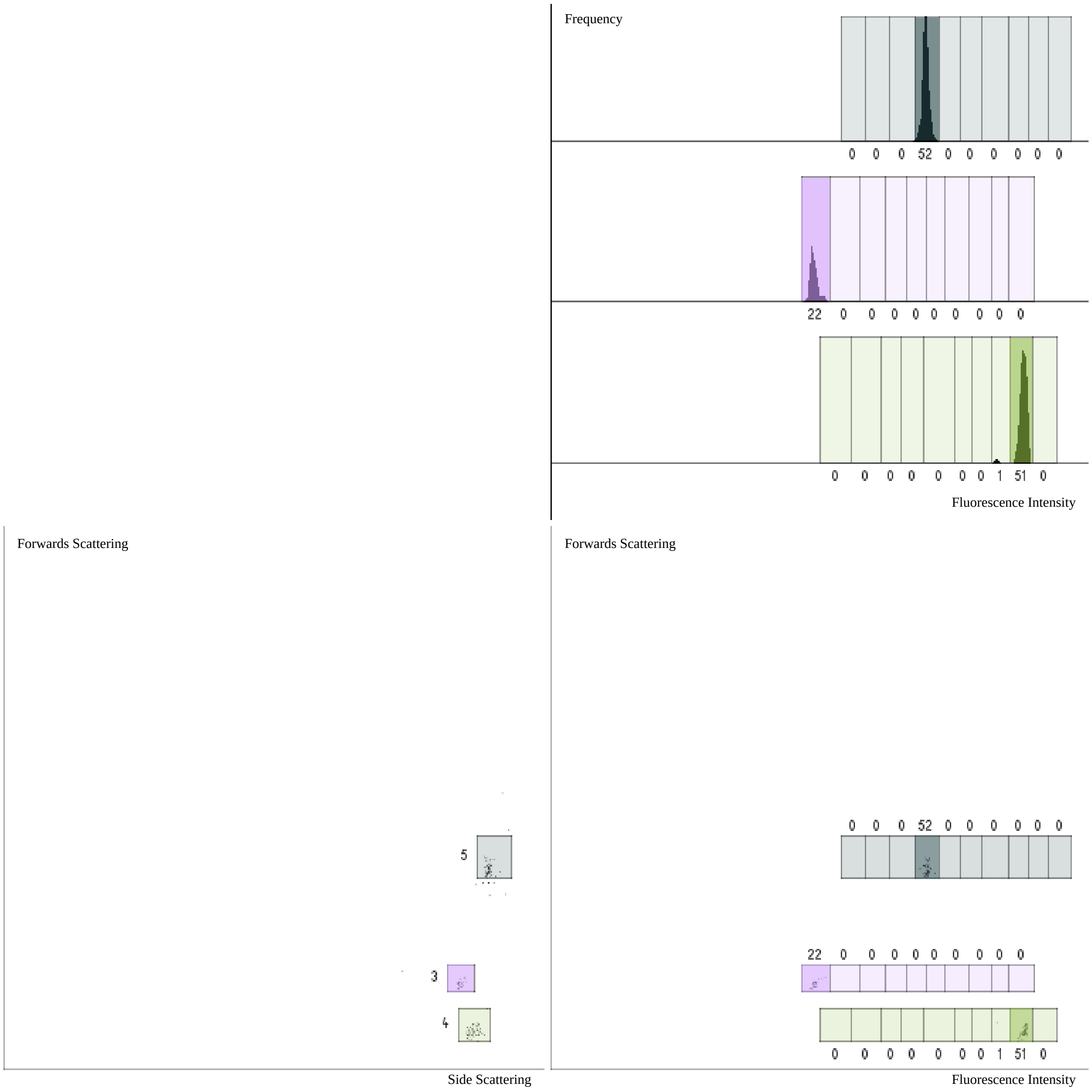

ANNEX 3: TAG DECONVOLUTION - BEAD 79

Passes flow sorting criteria: Yes

Passes tag deconvolution criteria: Yes

Included in protocol analysis: Yes

Protocol: 9, 10, 6, 3

Filename: Bin3_plateA3_C4.fcs

Split 1: Petrol shading

Split 2: Green shading

Split 3: Violet shading

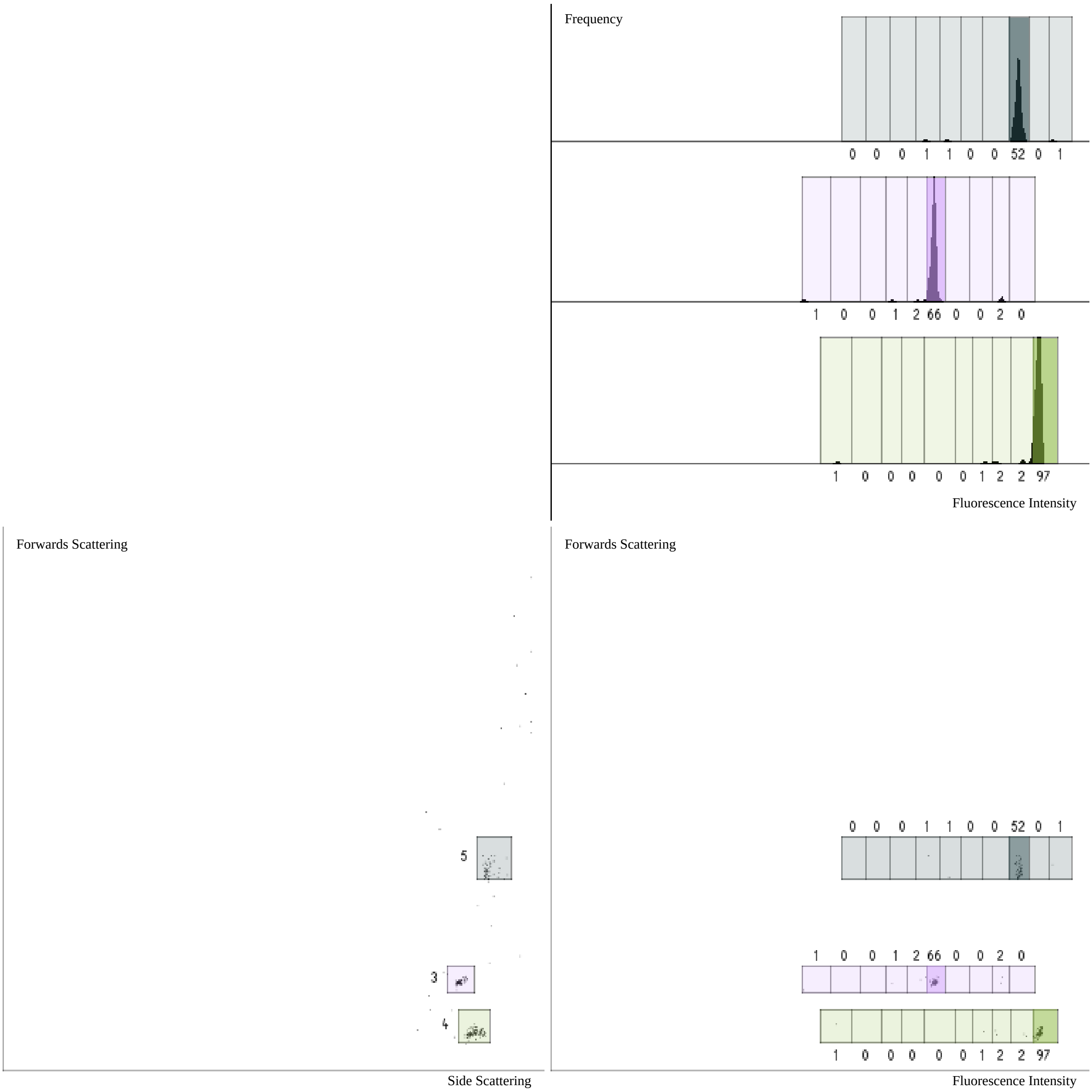

ANNEX 3: TAG DECONVOLUTION - BEAD 80

Passes flow sorting criteria: Yes

Passes tag deconvolution criteria: Yes

Included in protocol analysis: Yes

Protocol: 1, 5, 7, 3

Filename: Bin3_plateA3_C5.fcs

Split 1: Petrol shading

Split 2: Green shading

Split 3: Violet shading

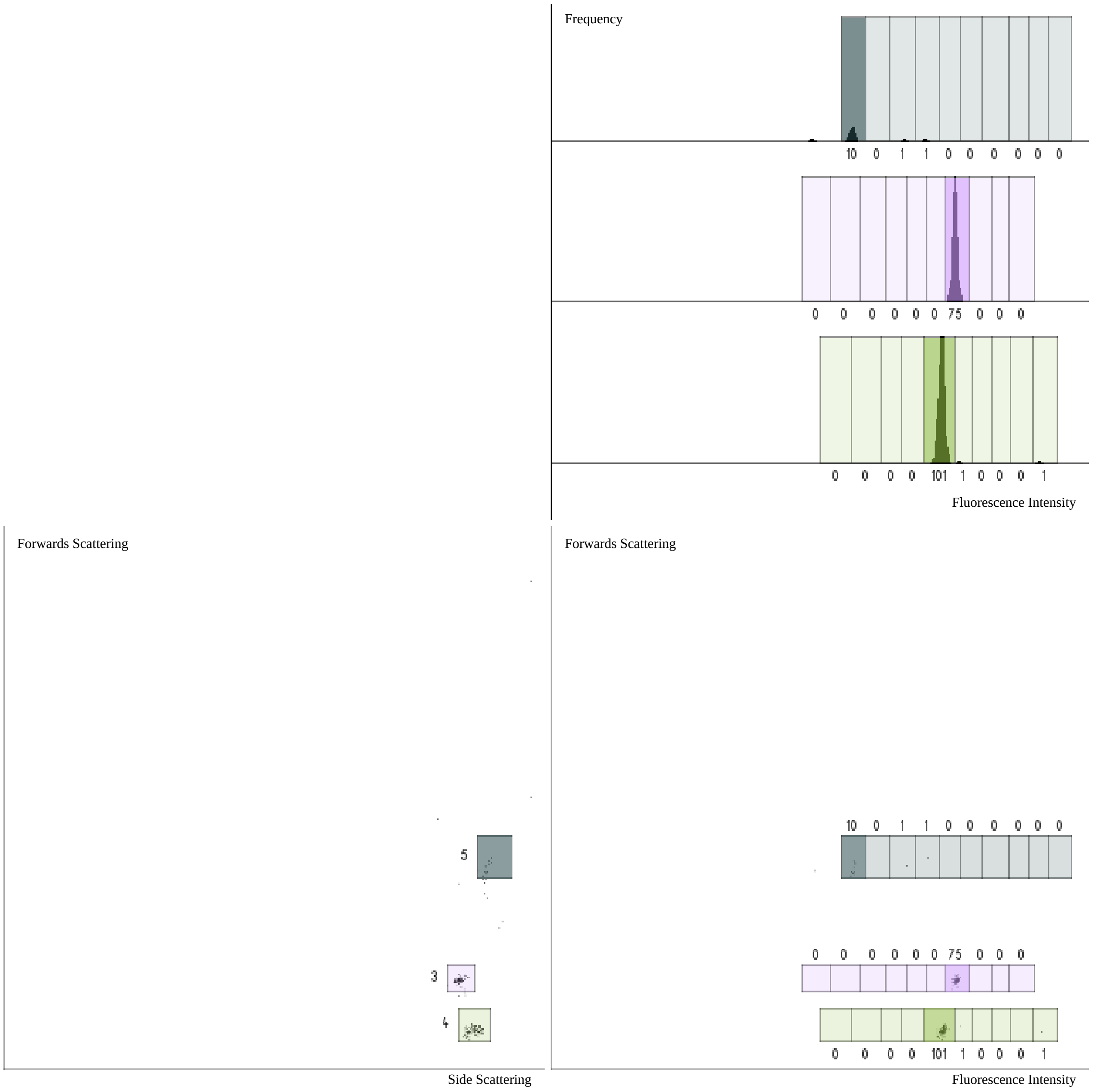

ANNEX 3: TAG DECONVOLUTION - BEAD 81

Passes flow sorting criteria: Yes

Passes tag deconvolution criteria: Yes

Included in protocol analysis: Yes

Protocol: 9, 7, 5, 3

Filename: Bin3_plateA3_C12.fcs

Split 1: Petrol shading

Split 2: Green shading

Split 3: Violet shading

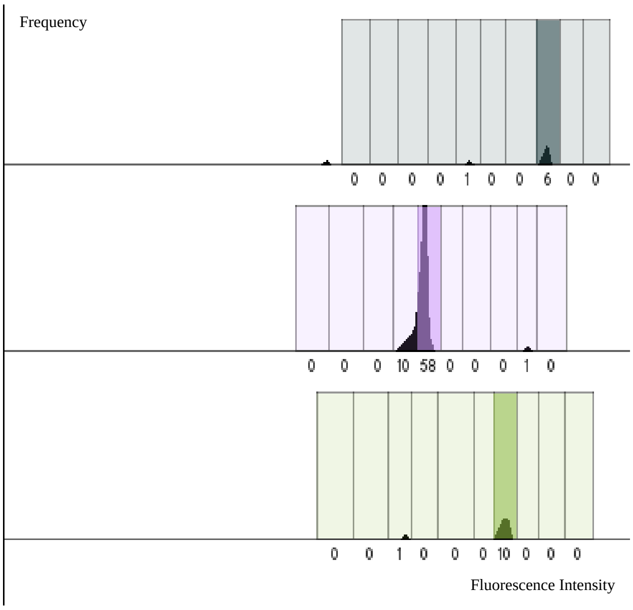

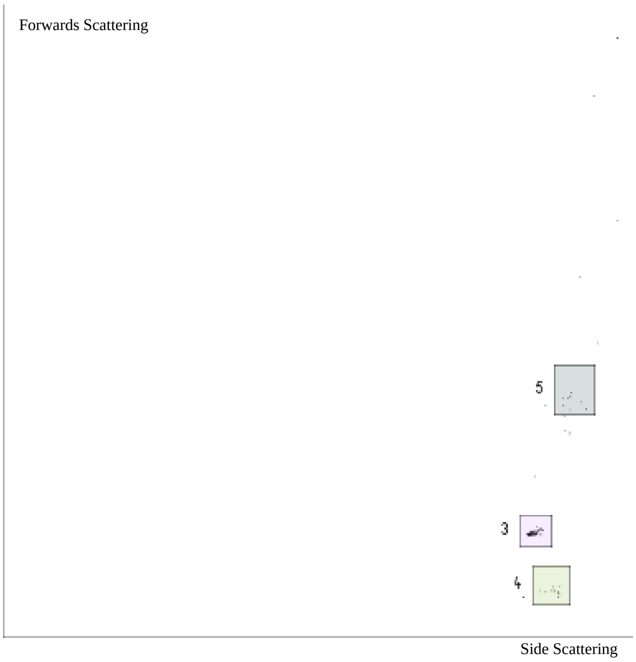

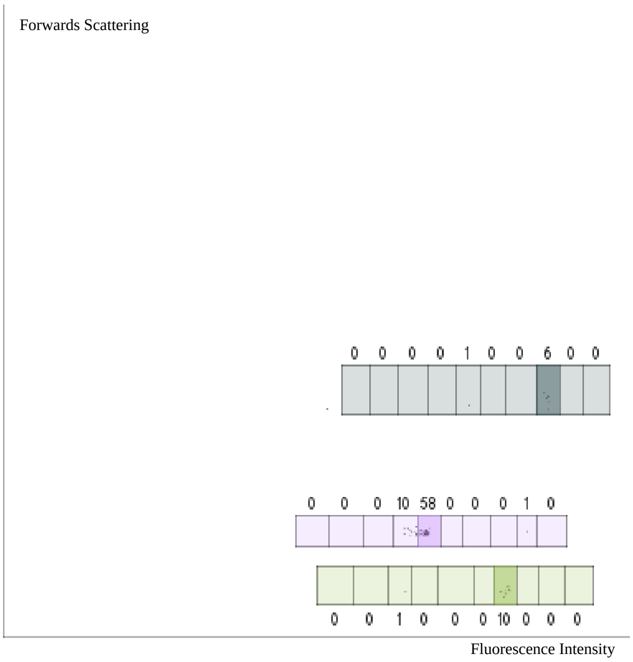

ANNEX 3: TAG DECONVOLUTION - BEAD 82

Passes flow sorting criteria: Yes

Passes tag deconvolution criteria: Yes

Included in protocol analysis: Yes

Protocol: 4, 6, 1, 3

Filename: Bin3_plateA3_D8.fcs

Split 1: Petrol shading

Split 2: Green shading

Split 3: Violet shading

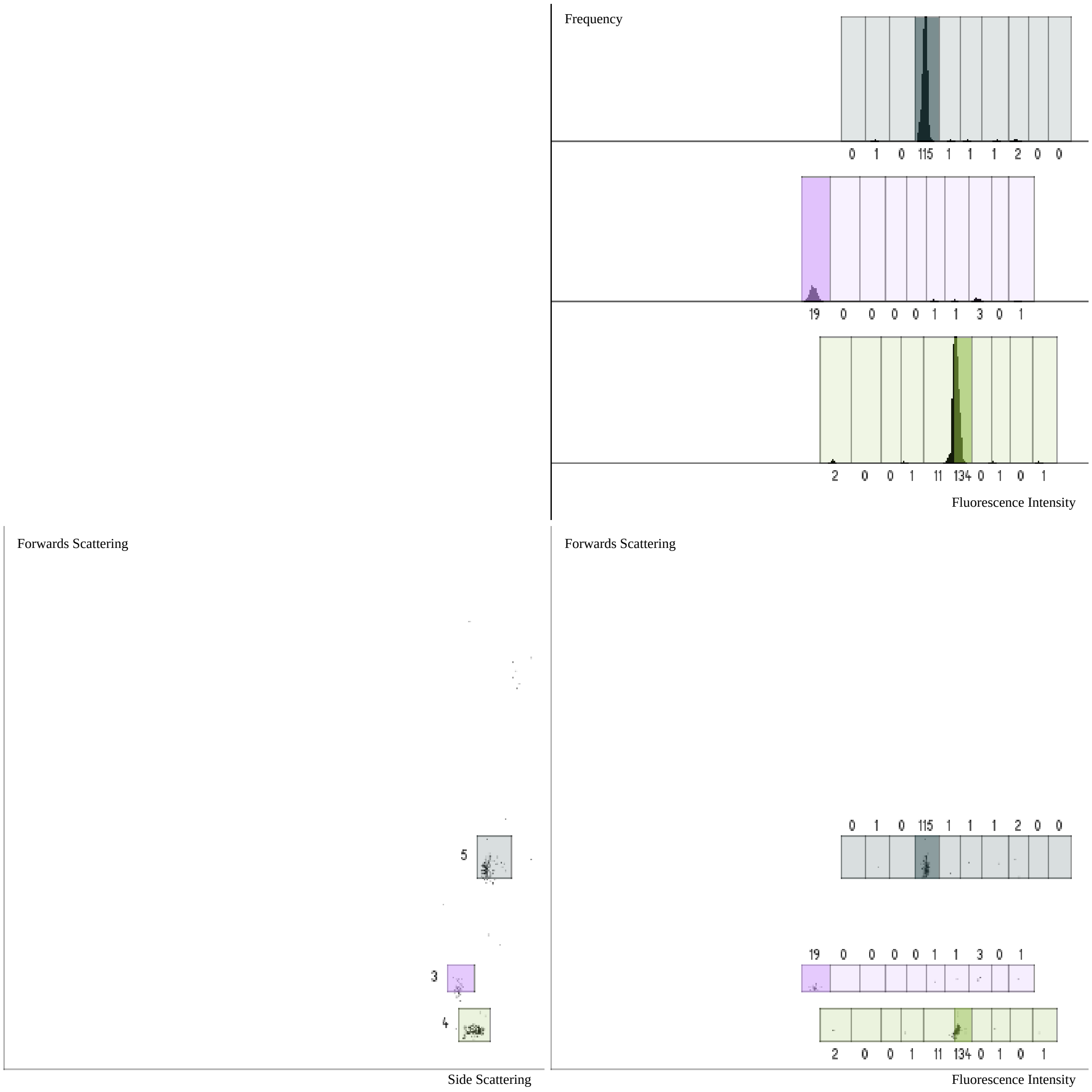

ANNEX 3: TAG DECONVOLUTION - BEAD 83

Passes flow sorting criteria: Yes

Passes tag deconvolution criteria: Yes

Included in protocol analysis: Yes

Protocol: 7, 7, 3, 3

Filename: Bin3_plateA3_D9.fcs

Split 1: Petrol shading

Split 2: Green shading

Split 3: Violet shading

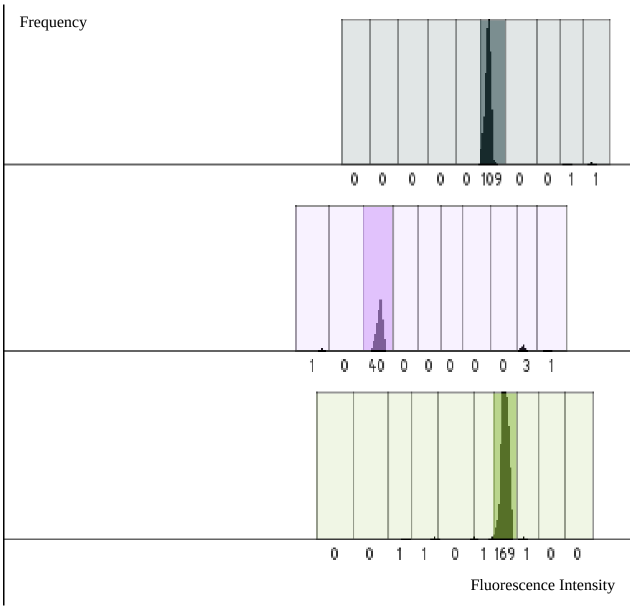

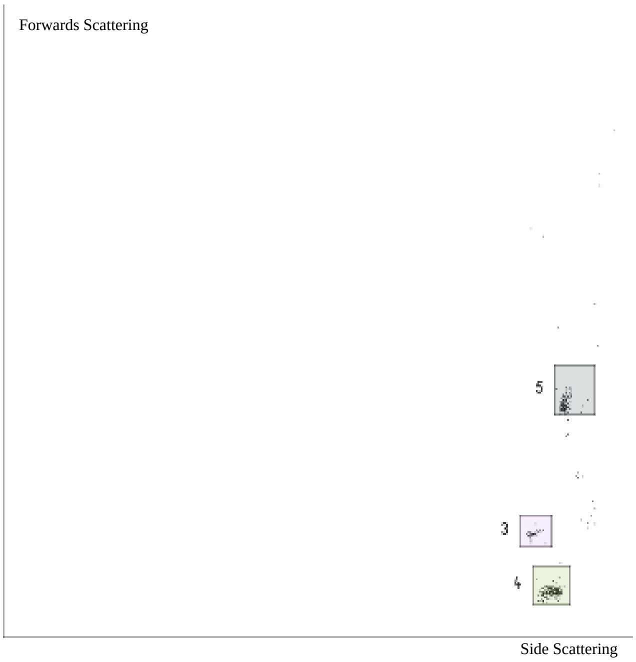

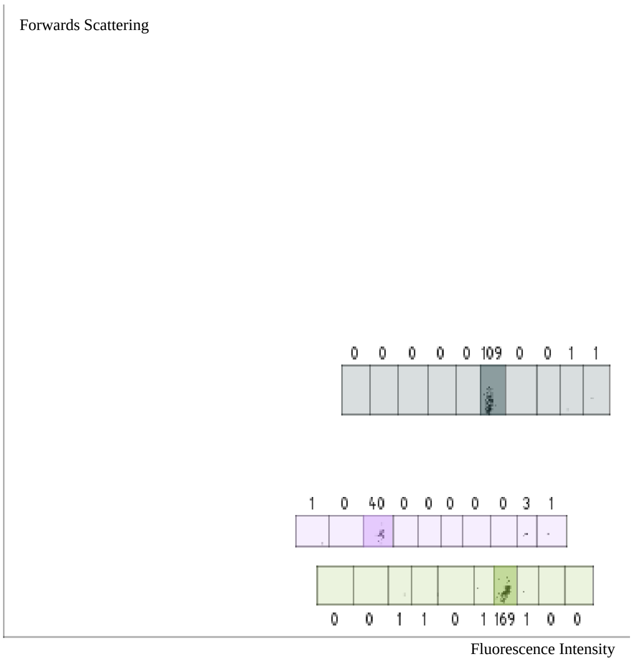

ANNEX 3: TAG DECONVOLUTION - BEAD 84

Passes flow sorting criteria: Yes

Passes tag deconvolution criteria: Yes

Included in protocol analysis: Yes

Protocol: 8, 5, 4, 3

Filename: Bin3_plateB3_E9.fcs

Split 1: Petrol shading

Split 2: Green shading

Split 3: Violet shading

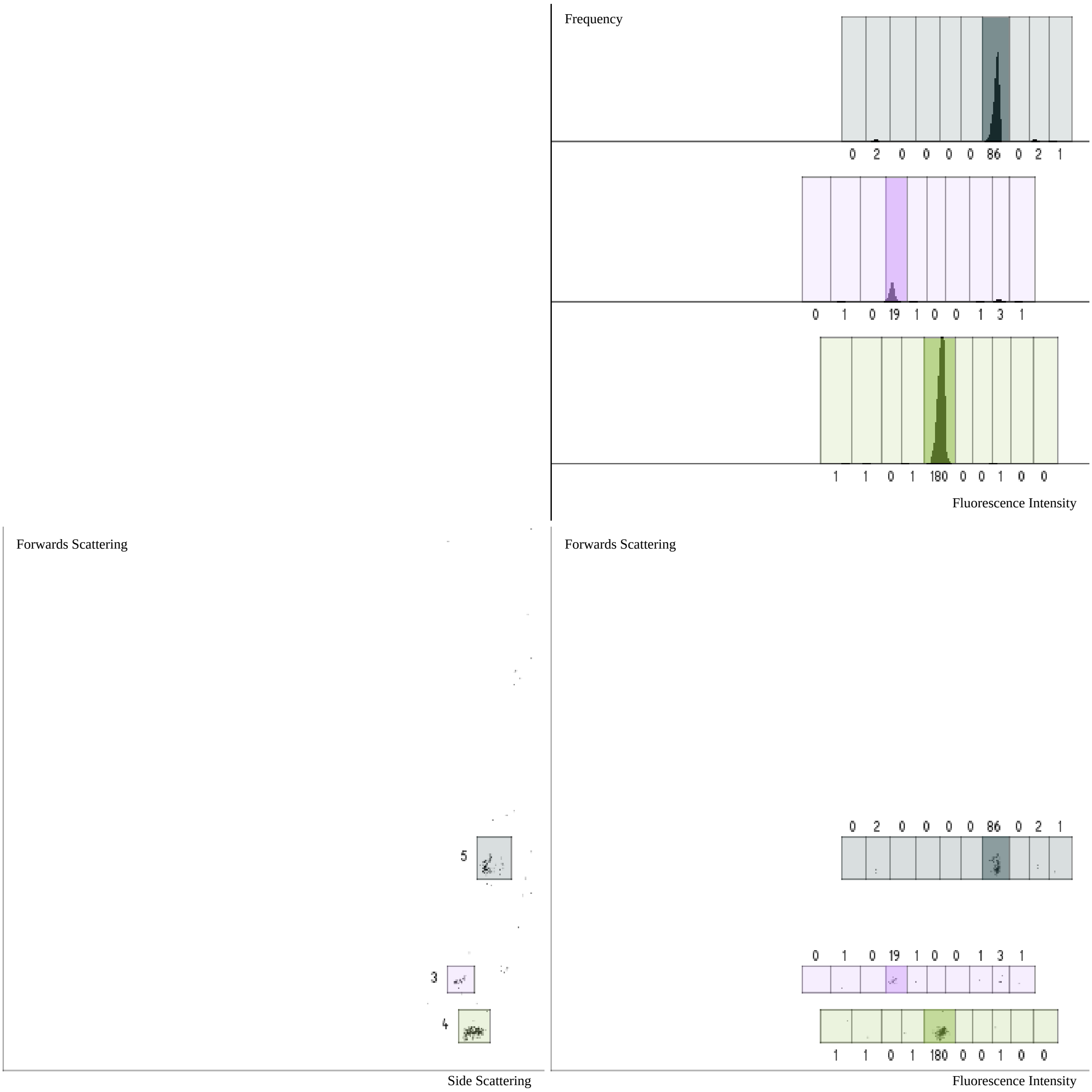

ANNEX 3: TAG DECONVOLUTION - BEAD 85

Passes flow sorting criteria: Yes

Passes tag deconvolution criteria: Yes

Included in protocol analysis: Yes

Protocol: 4, 2, 6, 3

Filename: Bin3_plateB3_F1.fcs

Split 1: Petrol shading

Split 2: Green shading

Split 3: Violet shading

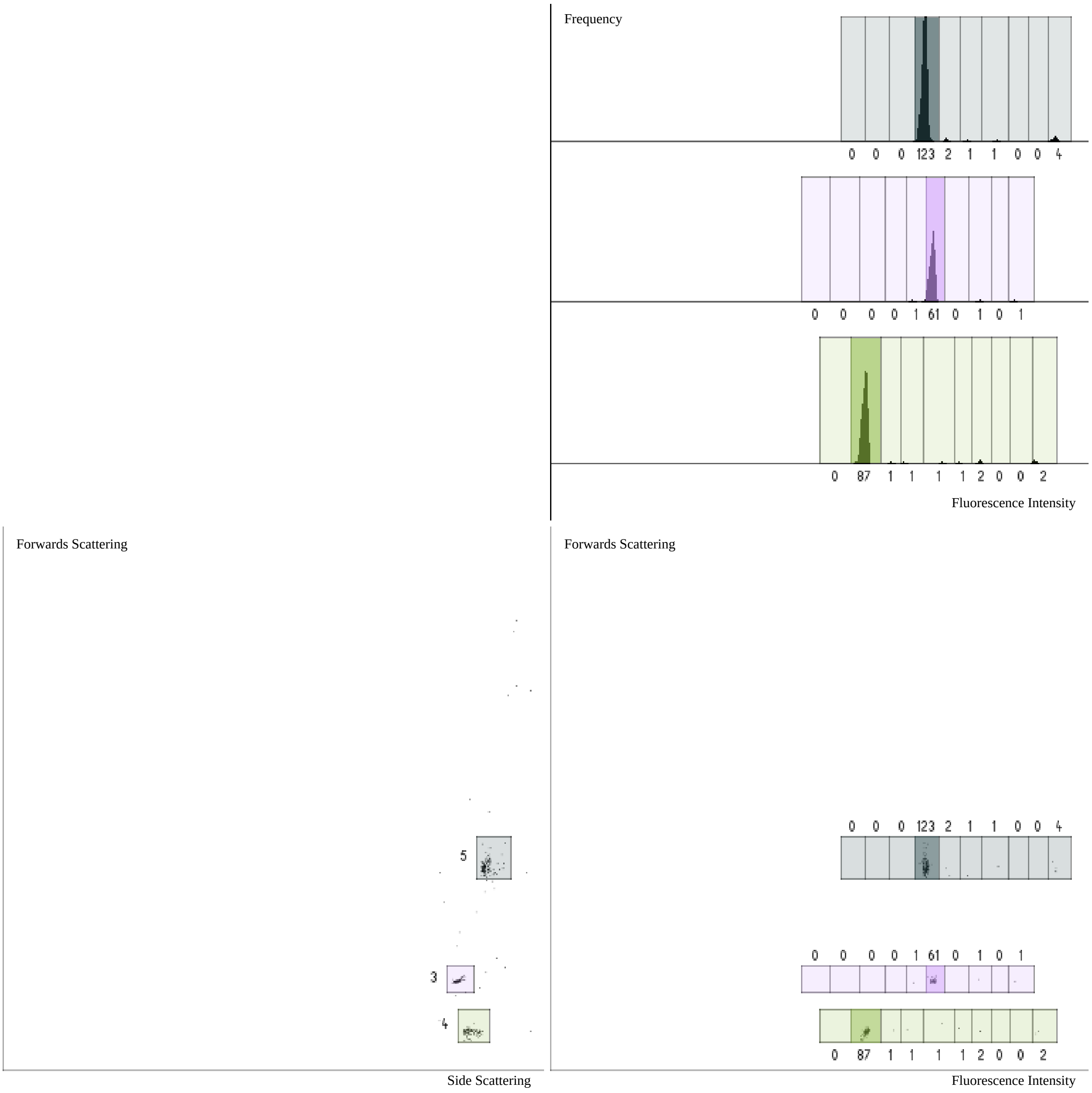

ANNEX 3: TAG DECONVOLUTION - BEAD 86

Passes flow sorting criteria: Yes

Passes tag deconvolution criteria: Yes

Included in protocol analysis: Yes

Protocol: 4, 10, 10, 3

Filename: Bin3_plateB3_F6.fcs

Split 1: Petrol shading

Split 2: Green shading

Split 3: Violet shading

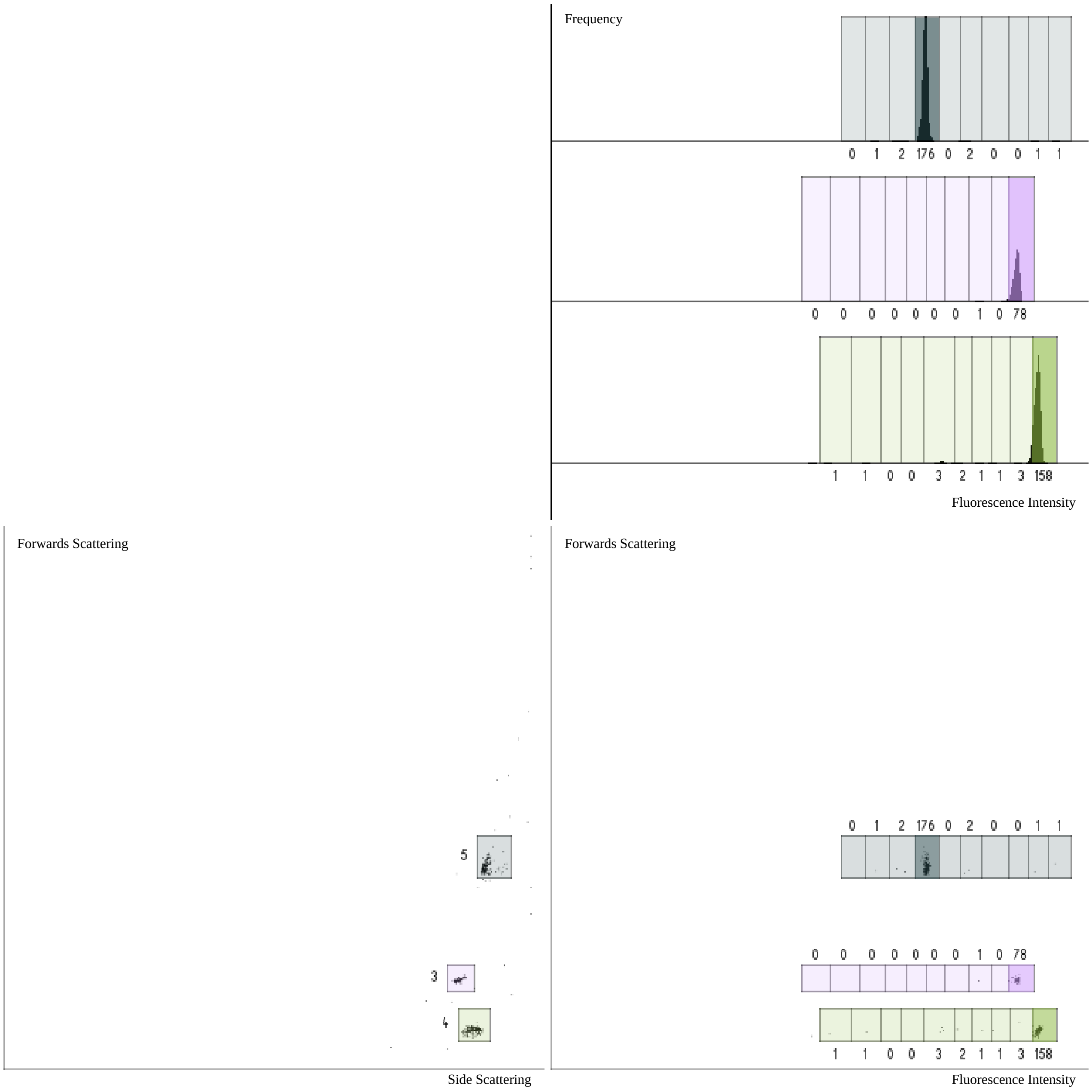

ANNEX 3: TAG DECONVOLUTION - BEAD 87

Passes flow sorting criteria: Yes

Passes tag deconvolution criteria: No

Included in protocol analysis: No

Protocol: N/A

Filename: Bin3_plateB3_F9.fcs

Split 1: Petrol shading

Split 2: Green shading

Split 3: Violet shading

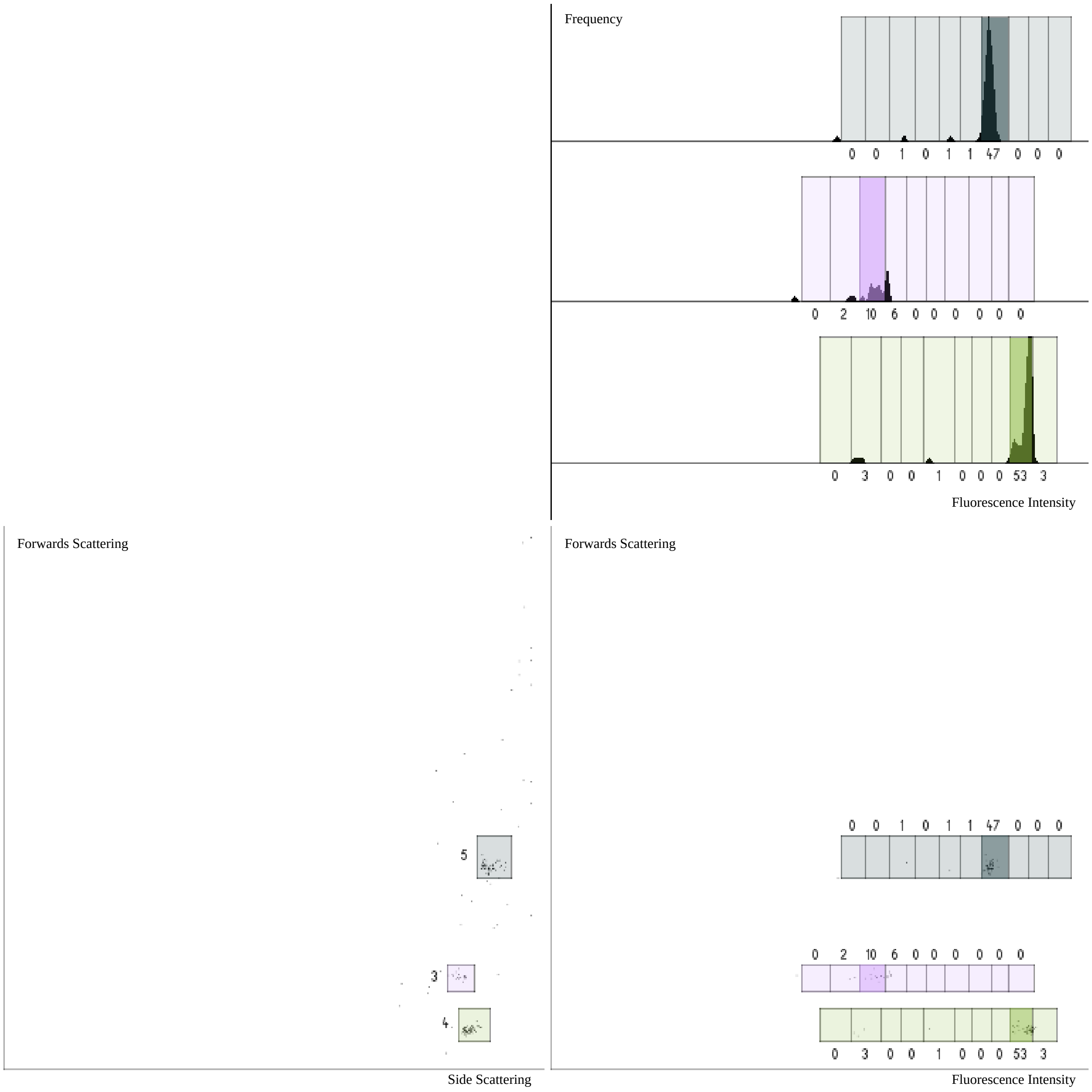

ANNEX 3: TAG DECONVOLUTION - BEAD 88

Passes flow sorting criteria: Yes

Passes tag deconvolution criteria: Yes

Included in protocol analysis: Yes

Protocol: 4, 2, 3, 4

Filename: Bin4_plateA3_A8.fcs

Split 1: Petrol shading

Split 2: Green shading

Split 3: Violet shading

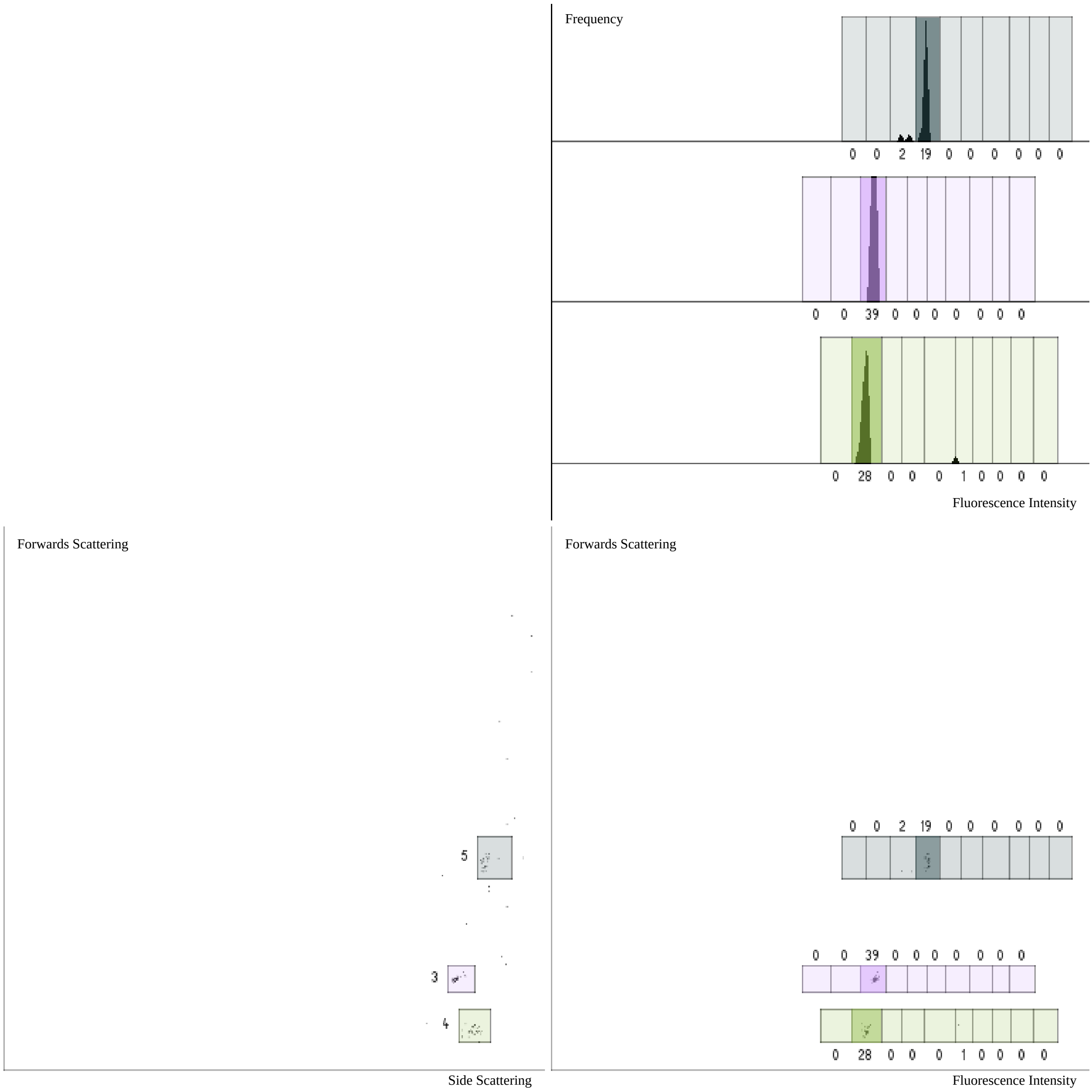

ANNEX 3: TAG DECONVOLUTION - BEAD 89

Passes flow sorting criteria: Yes

Passes tag deconvolution criteria: Yes

Included in protocol analysis: Yes

Protocol: 1, 7, 8, 4

Filename: Bin4_plateA3_A10.fcs

Split 1: Petrol shading

Split 2: Green shading

Split 3: Violet shading

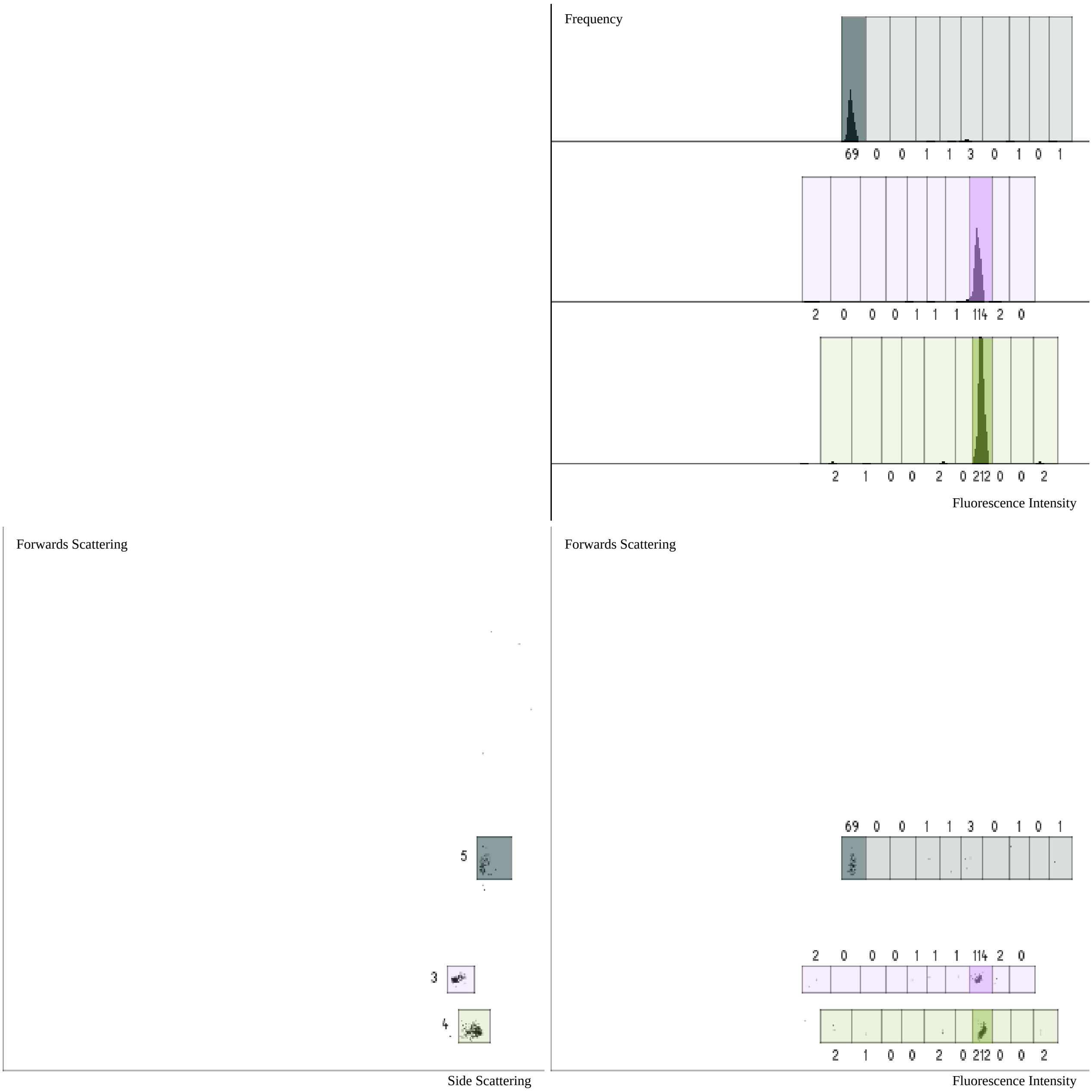

ANNEX 3: TAG DECONVOLUTION - BEAD 90

Passes flow sorting criteria: Yes

Passes tag deconvolution criteria: Yes

Included in protocol analysis: Yes

Protocol: 8, 2, 6, 4

Filename: Bin4_plateA3_A11.fcs

Split 1: Petrol shading

Split 2: Green shading

Split 3: Violet shading

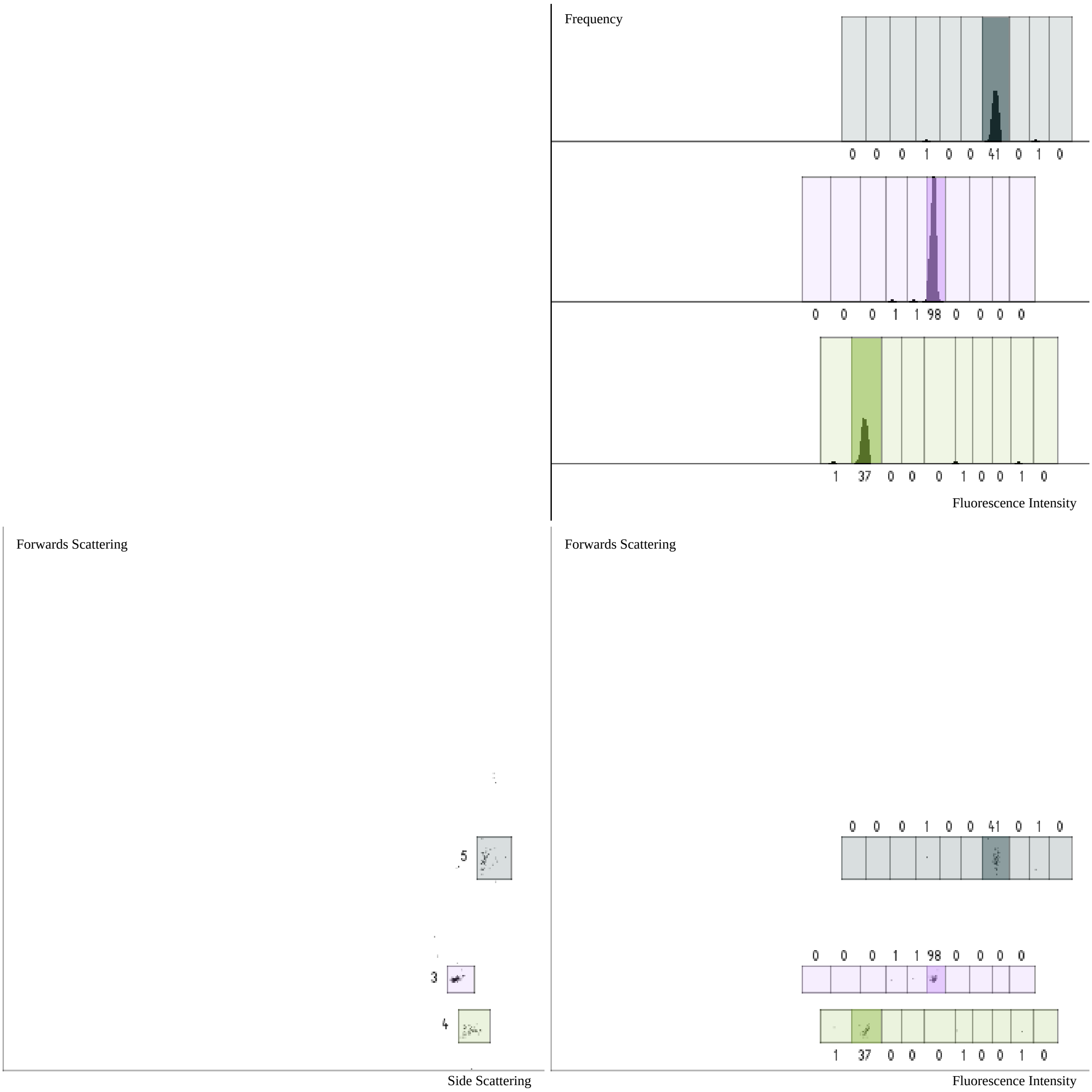

ANNEX 3: TAG DECONVOLUTION - BEAD 91

Passes flow sorting criteria: Yes

Passes tag deconvolution criteria: Yes

Included in protocol analysis: Yes

Protocol: 7, 9, 6, 4

Filename: Bin4_plateA3_B1.fcs

Split 1: Petrol shading

Split 2: Green shading

Split 3: Violet shading

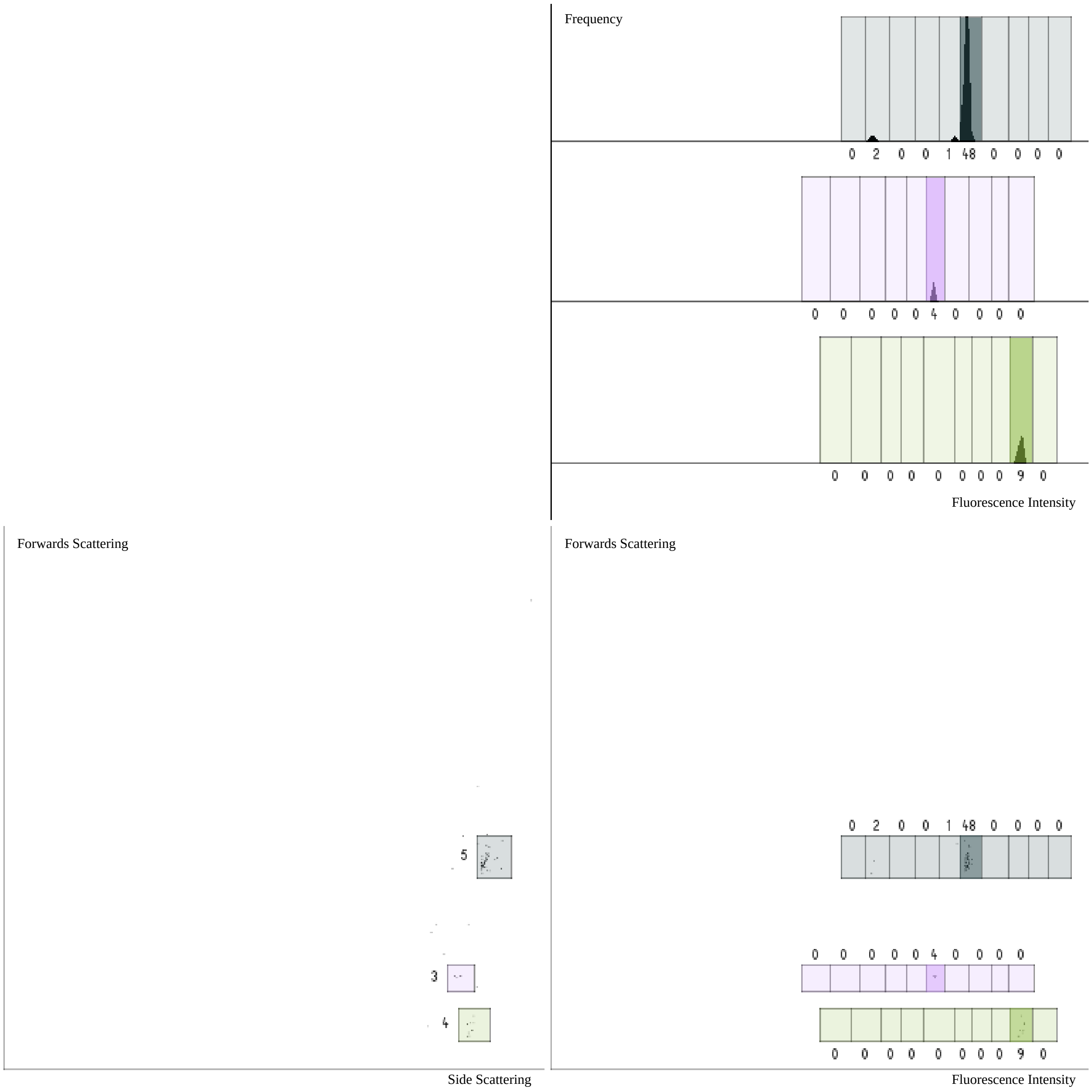

ANNEX 3: TAG DECONVOLUTION - BEAD 92

Passes flow sorting criteria: Yes

Passes tag deconvolution criteria: Yes

Included in protocol analysis: Yes

Protocol: 10, 2, 3, 4

Filename: Bin4_plateA3_B3.fcs

Split 1: Petrol shading

Split 2: Green shading

Split 3: Violet shading

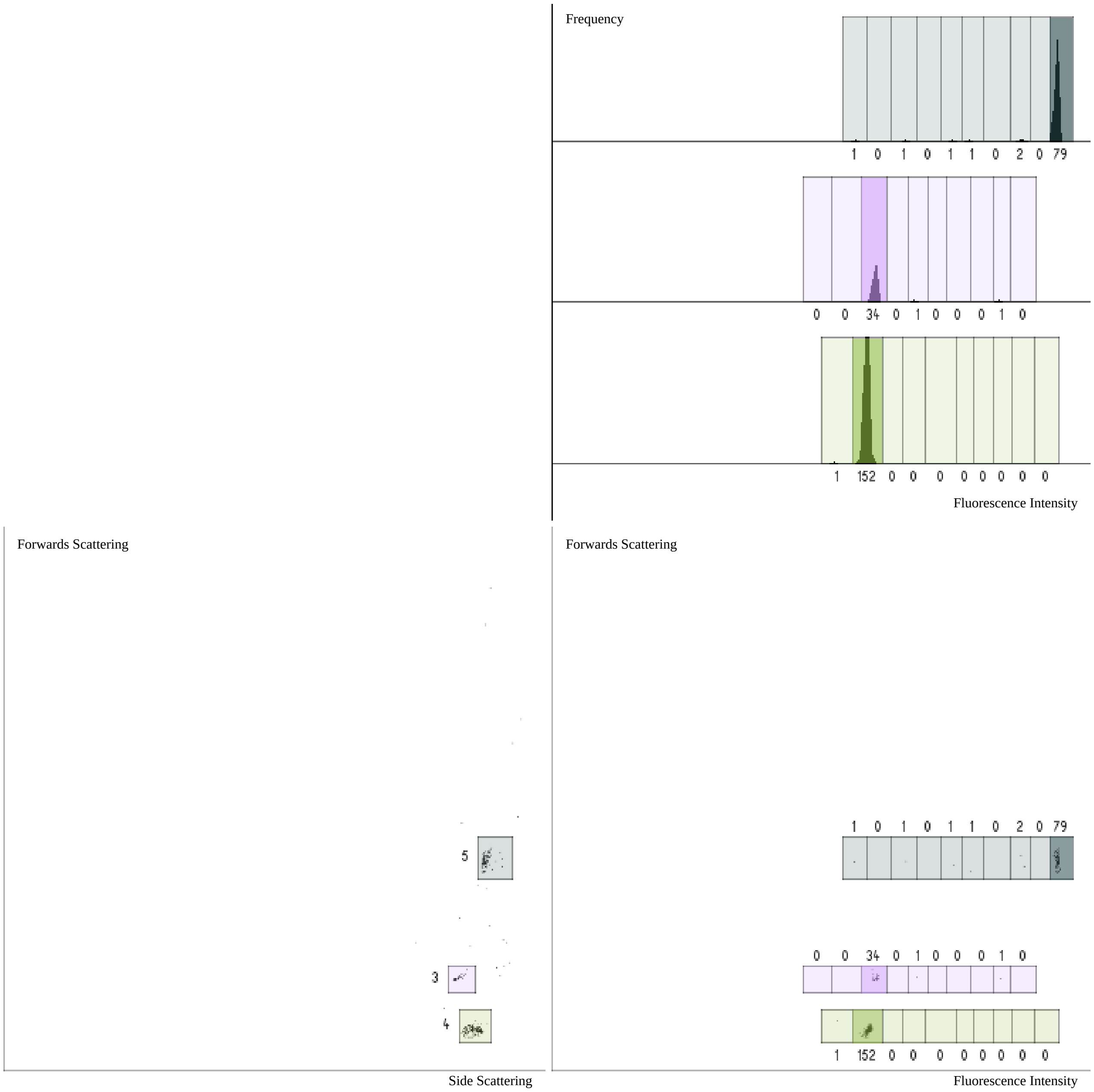

ANNEX 3: TAG DECONVOLUTION - BEAD 93

Passes flow sorting criteria: Yes

Passes tag deconvolution criteria: Yes

Included in protocol analysis: Yes

Protocol: 2, 10, 7, 4

Filename: Bin4_plateA3_B6.fcs

Split 1: Petrol shading

Split 2: Green shading

Split 3: Violet shading

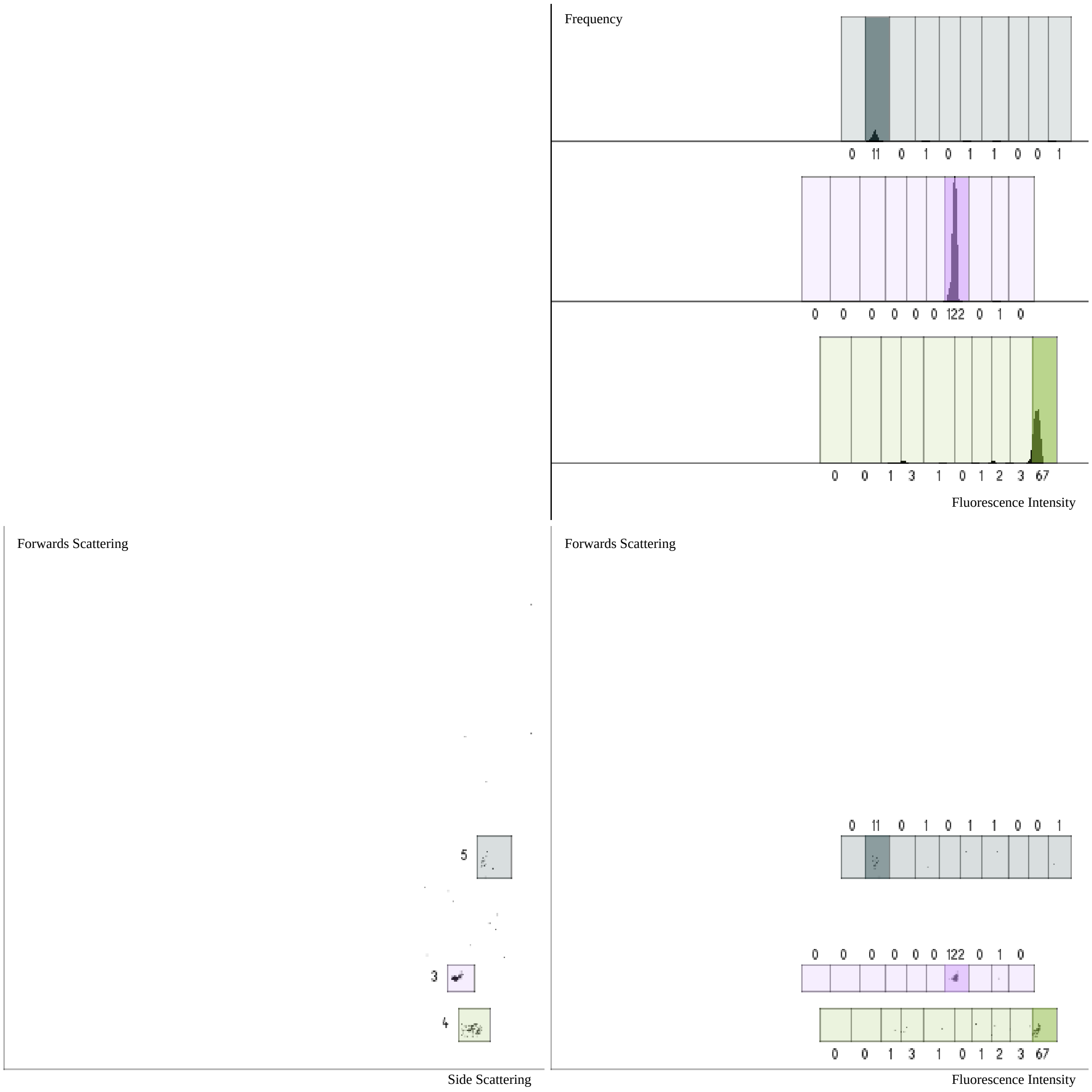

ANNEX 3: TAG DECONVOLUTION - BEAD 94

Passes flow sorting criteria: Yes

Passes tag deconvolution criteria: Yes

Included in protocol analysis: Yes

Protocol: 9, 7, 7, 4

Filename: Bin4_plateA3_B8.fcs

Split 1: Petrol shading

Split 2: Green shading

Split 3: Violet shading

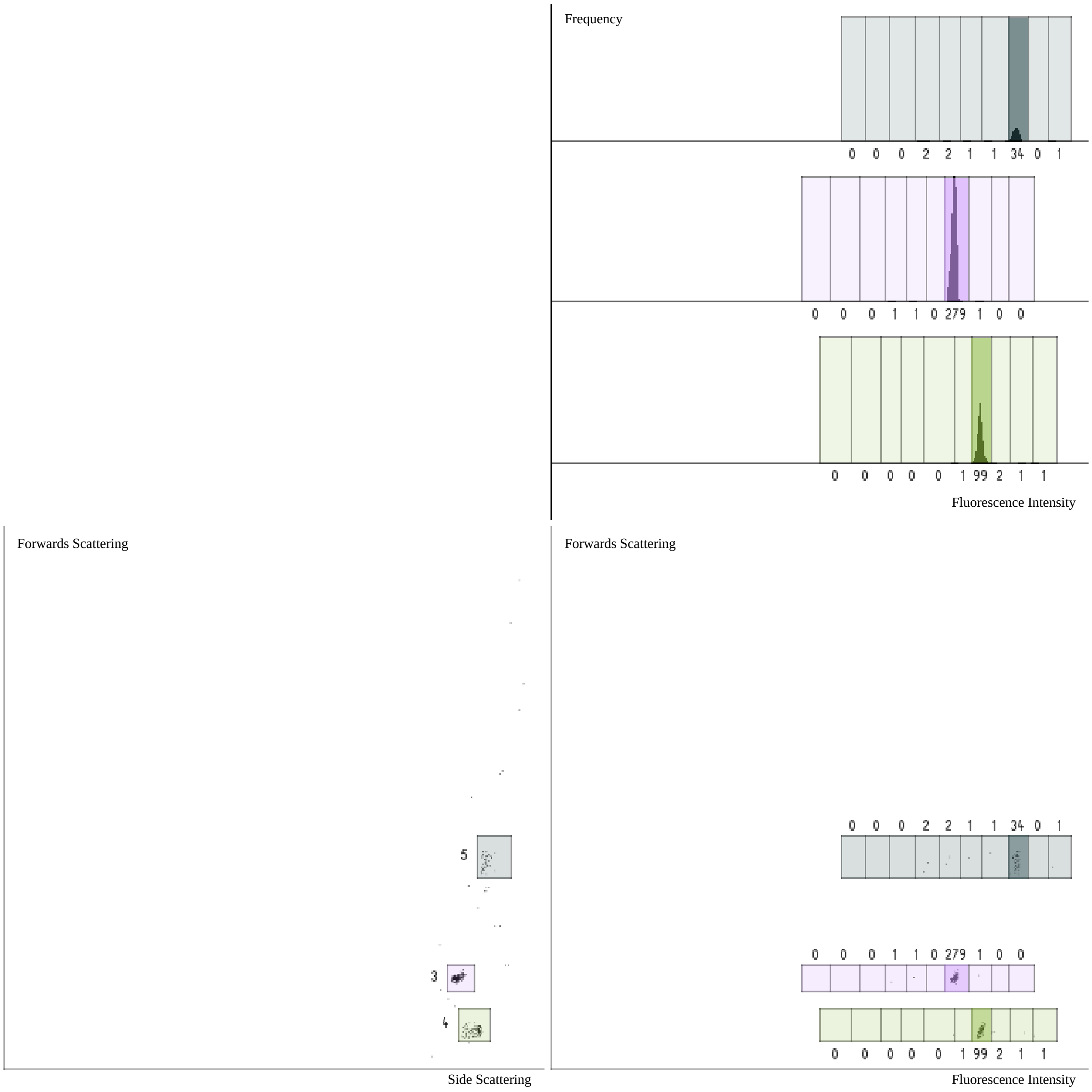

ANNEX 3: TAG DECONVOLUTION - BEAD 95

Passes flow sorting criteria: Yes

Passes tag deconvolution criteria: Yes

Included in protocol analysis: Yes

Protocol: 7, 9, 6, 4

Filename: Bin4_plateA3_C7.fcs

Split 1: Petrol shading

Split 2: Green shading

Split 3: Violet shading

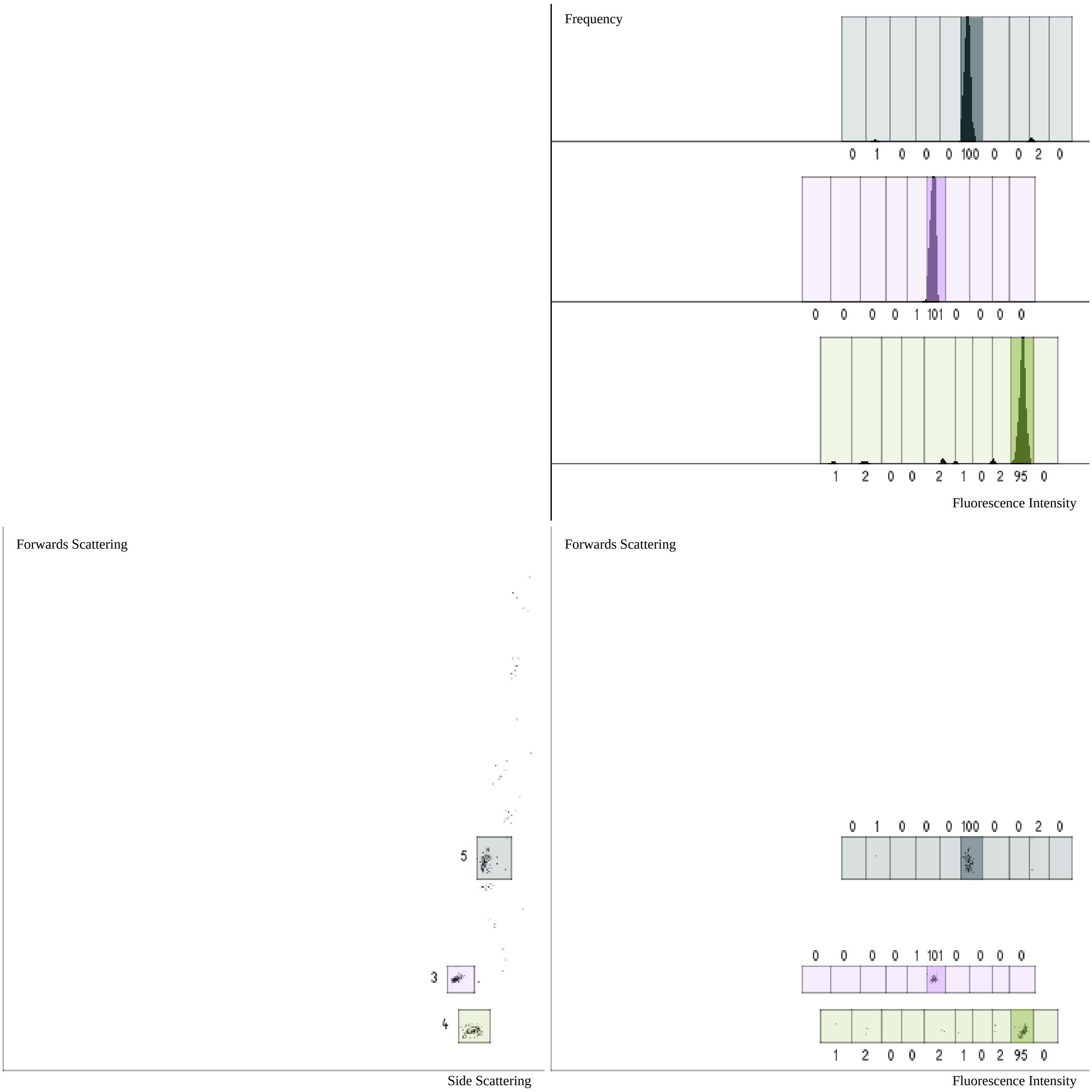

ANNEX 3: TAG DECONVOLUTION - BEAD 96

Passes flow sorting criteria: Yes

Passes tag deconvolution criteria: Yes

Included in protocol analysis: Yes

Protocol: 10, 2, 7, 4

Filename: Bin4_plateA3_C8.fcs

Split 1: Petrol shading

Split 2: Green shading

Split 3: Violet shading

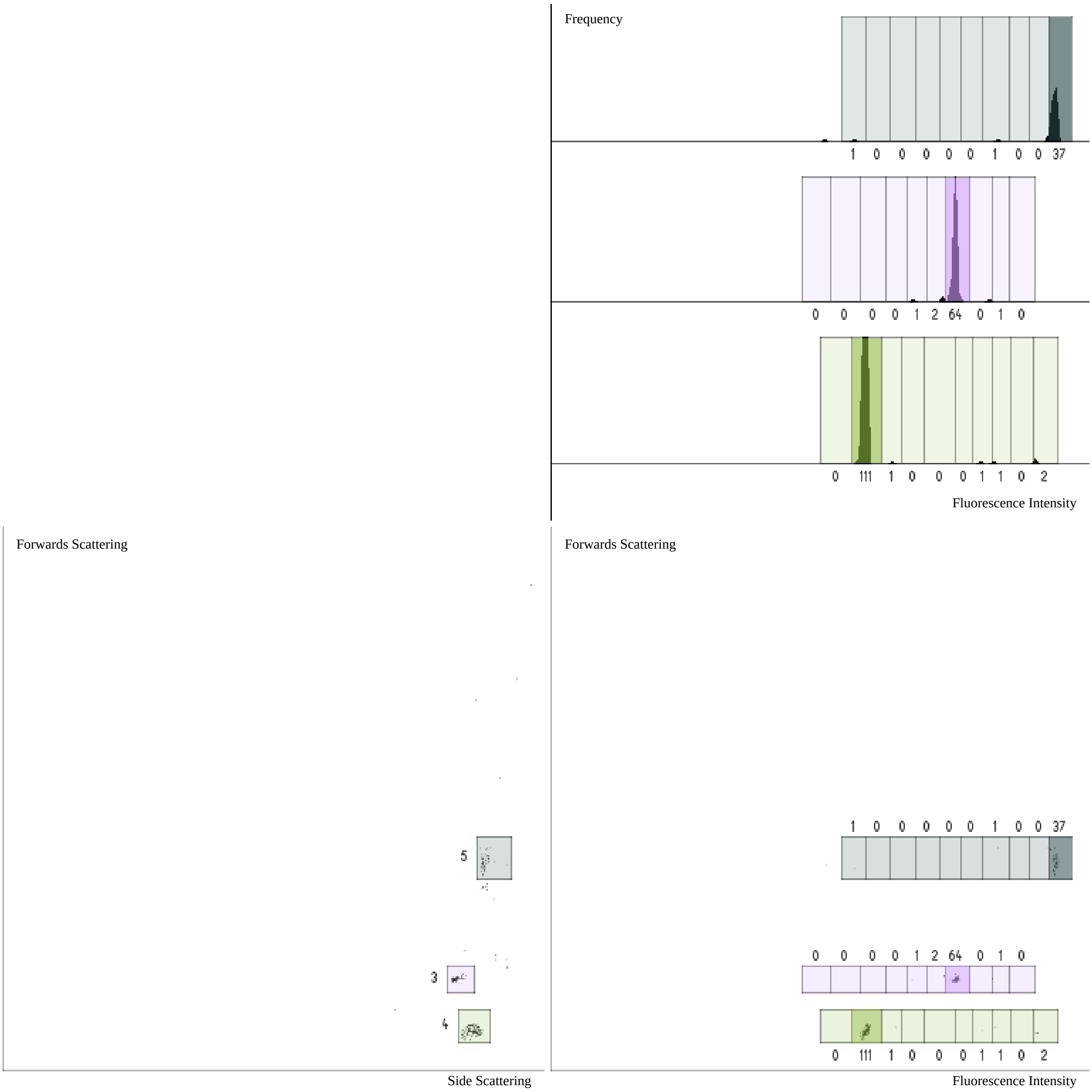

ANNEX 3: TAG DECONVOLUTION - BEAD 97

Passes flow sorting criteria: Yes

Passes tag deconvolution criteria: No

Included in protocol analysis: No

Protocol: N/A

Filename: Bin4_plateA3_C9.fcs

Split 1: Petrol shading

Split 2: Green shading

Split 3: Violet shading

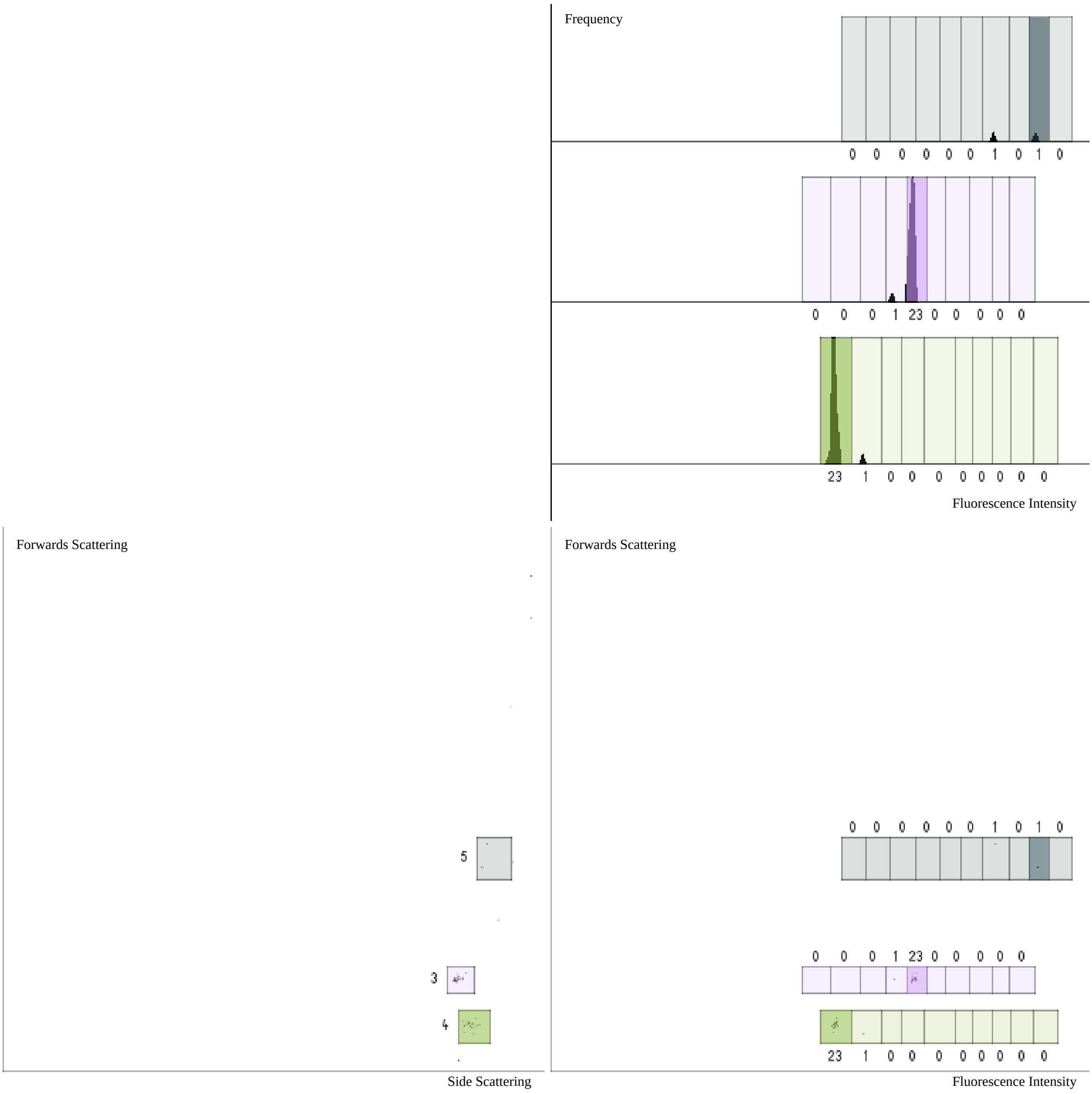

ANNEX 3: TAG DECONVOLUTION - BEAD 98

Passes flow sorting criteria: Yes

Passes tag deconvolution criteria: Yes

Included in protocol analysis: Yes

Protocol: 3, 10, 5, 4

Filename: Bin4_plateA3_D7.fcs

Split 1: Petrol shading

Split 2: Green shading

Split 3: Violet shading

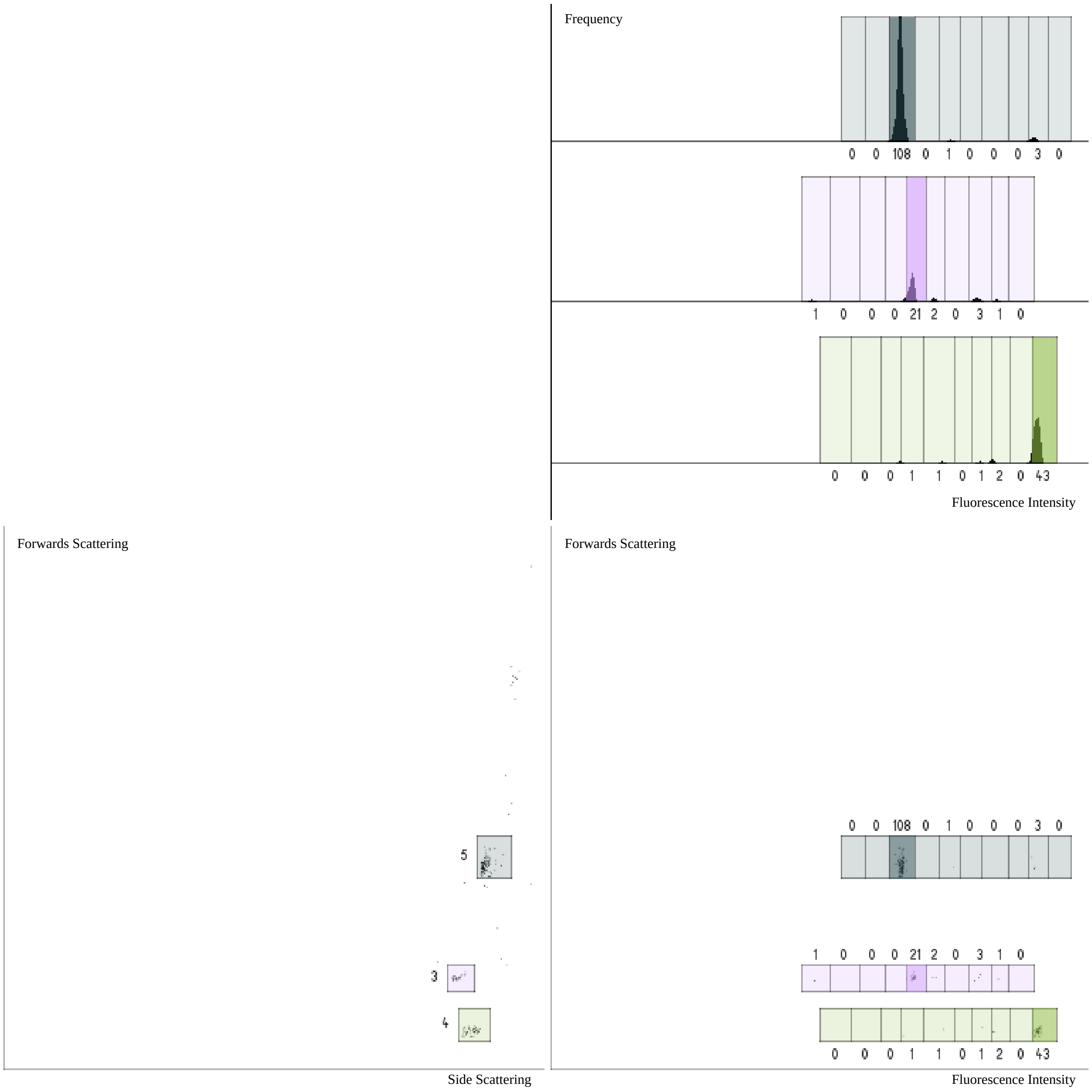

ANNEX 3: TAG DECONVOLUTION - BEAD 99

Passes flow sorting criteria: Yes

Passes tag deconvolution criteria: Yes

Included in protocol analysis: Yes

Protocol: 2, 5, 10, 4

Filename: Bin4_plateA3_D9.fcs

Split 1: Petrol shading

Split 2: Green shading

Split 3: Violet shading

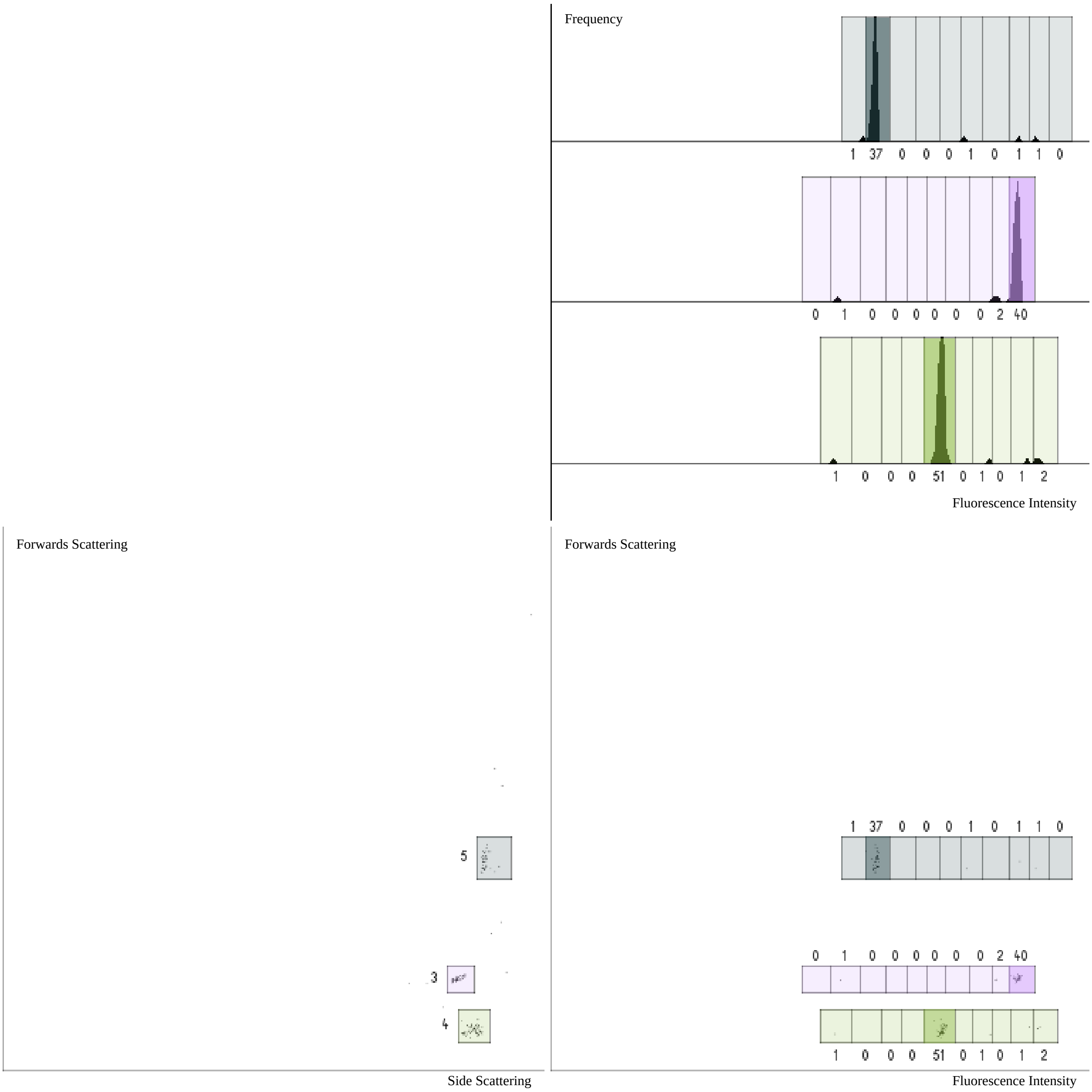

ANNEX 3: TAG DECONVOLUTION - BEAD 100

Passes flow sorting criteria: Yes

Passes tag deconvolution criteria: Yes

Included in protocol analysis: Yes

Protocol: 2, 5, 10, 4

Filename: Bin4_plateA3_D11.fcs

Split 1: Petrol shading

Split 2: Green shading

Split 3: Violet shading

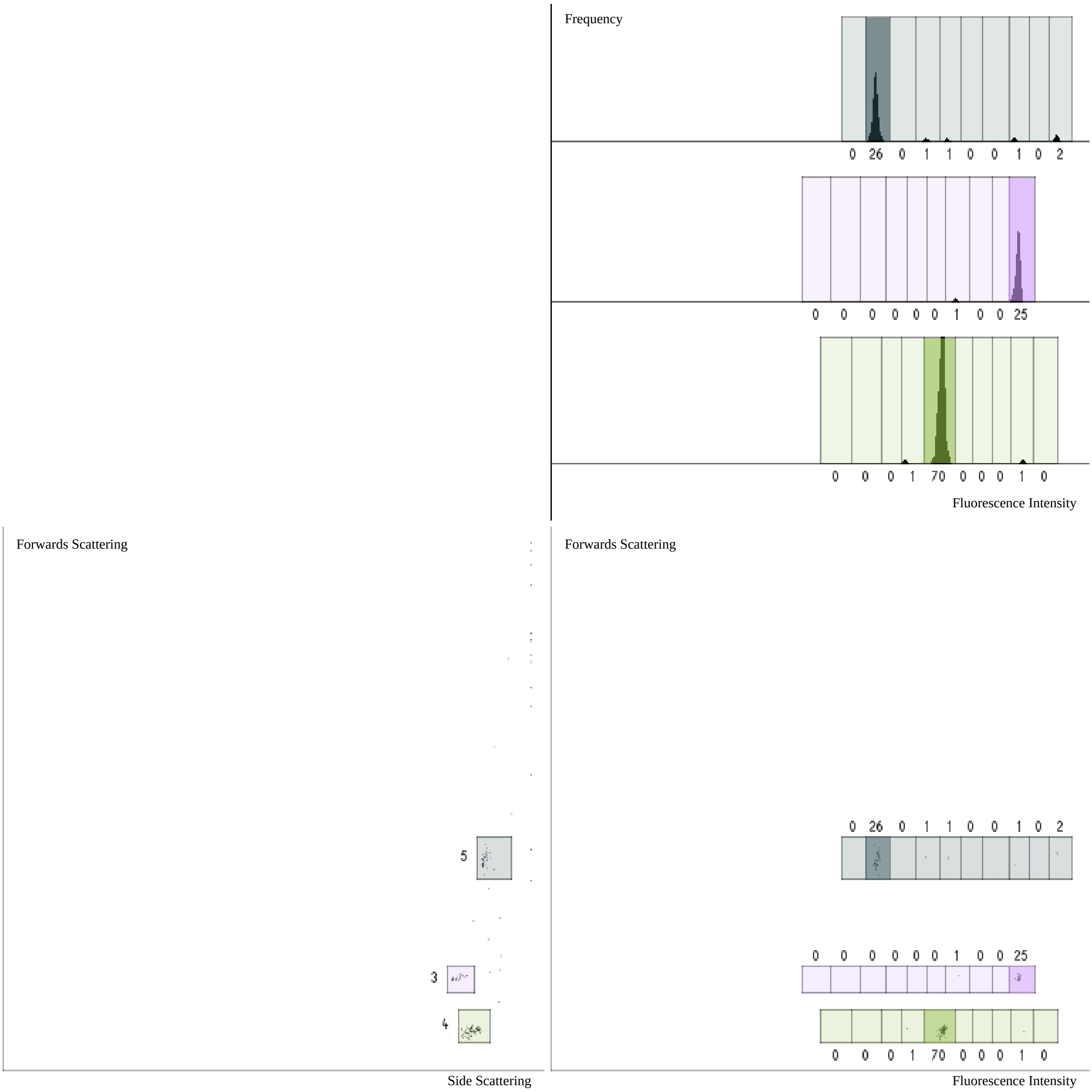

ANNEX 3: TAG DECONVOLUTION - BEAD 101

Passes flow sorting criteria: Yes

Passes tag deconvolution criteria: Yes

Included in protocol analysis: Yes

Protocol: 7, 7, 7, 4

Filename: Bin4_plateA3_E2.fcs

Split 1: Petrol shading

Split 2: Green shading

Split 3: Violet shading

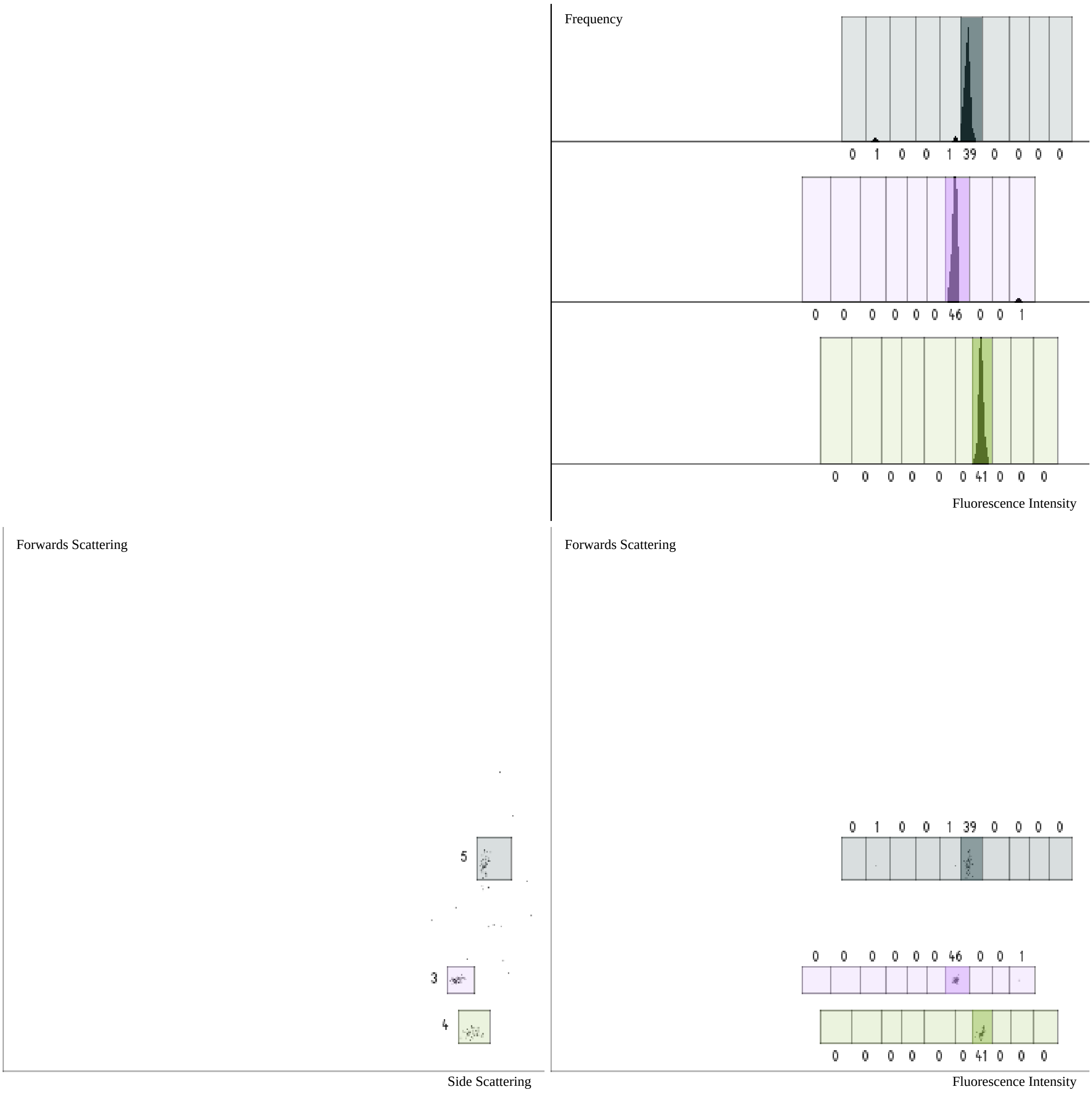

ANNEX 3: TAG DECONVOLUTION - BEAD 102

Passes flow sorting criteria: Yes

Passes tag deconvolution criteria: Yes

Included in protocol analysis: Yes

Protocol: 1, 10, 10, 4

Filename: Bin4_plateA3_E4.fcs

Split 1: Petrol shading

Split 2: Green shading

Split 3: Violet shading

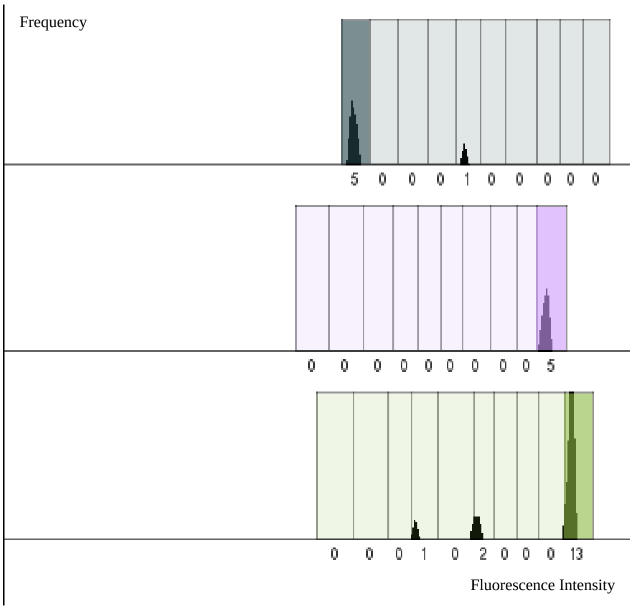

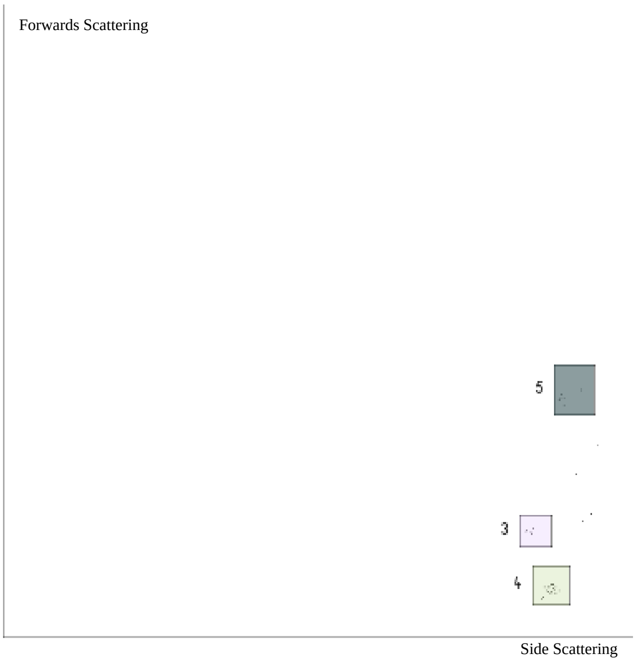

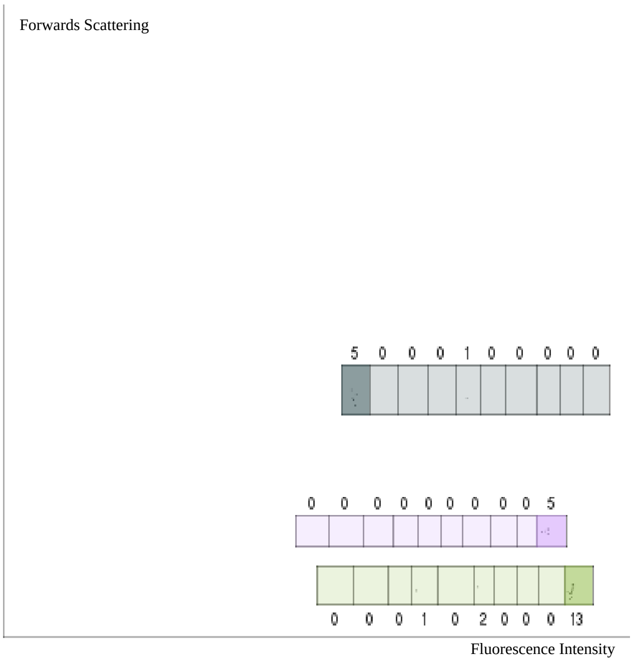

ANNEX 3: TAG DECONVOLUTION - BEAD 103

Passes flow sorting criteria: Yes

Passes tag deconvolution criteria: Yes

Included in protocol analysis: Yes

Protocol: 1, 1, 4, 4

Filename: Bin4_plateA3_E9.fcs

Split 1: Petrol shading

Split 2: Green shading

Split 3: Violet shading

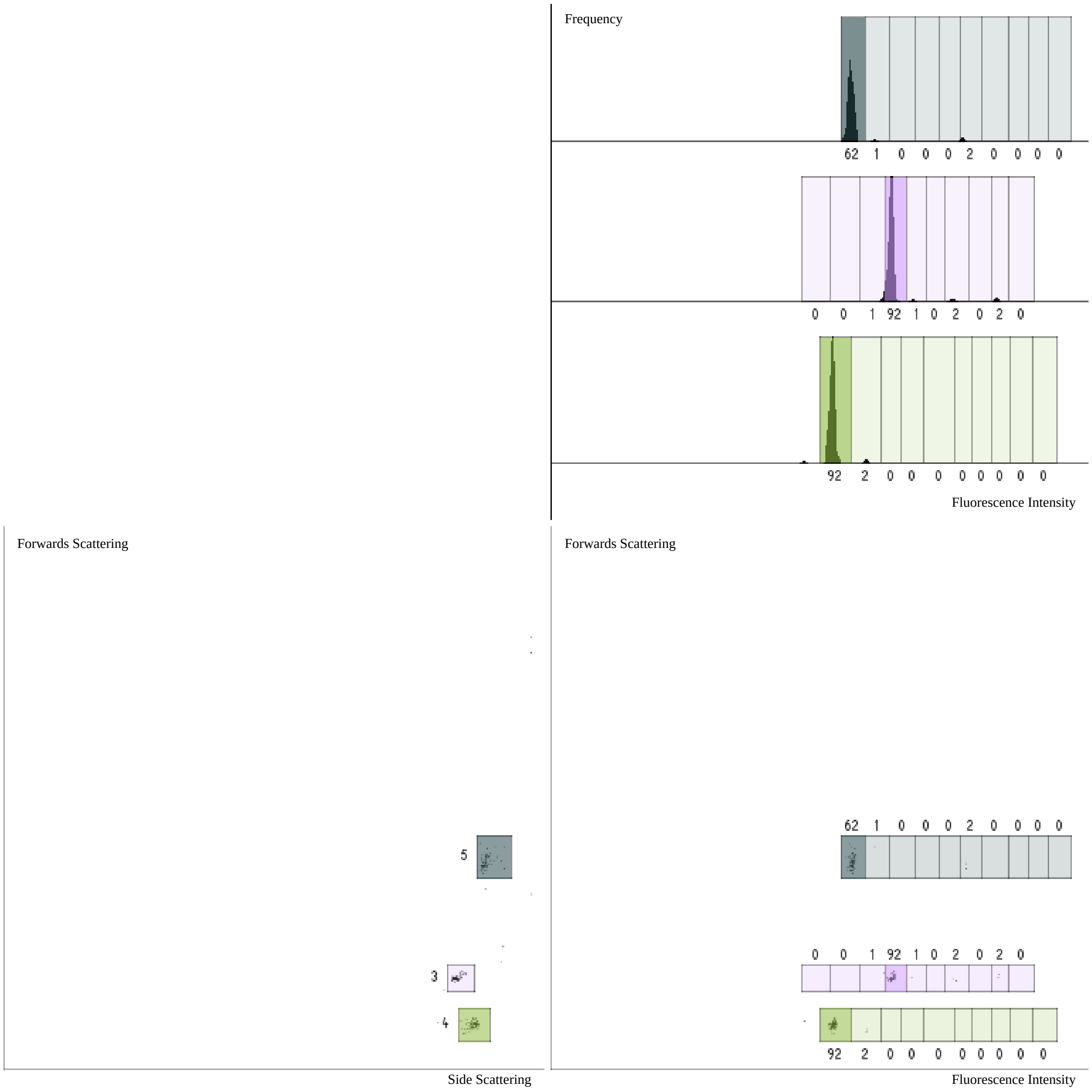

ANNEX 3: TAG DECONVOLUTION - BEAD 104

Passes flow sorting criteria: Yes

Passes tag deconvolution criteria: Yes

Included in protocol analysis: Yes

Protocol: 10, 7, 10, 4

Filename: Bin4_plateA3_E10.fcs

Split 1: Petrol shading

Split 2: Green shading

Split 3: Violet shading

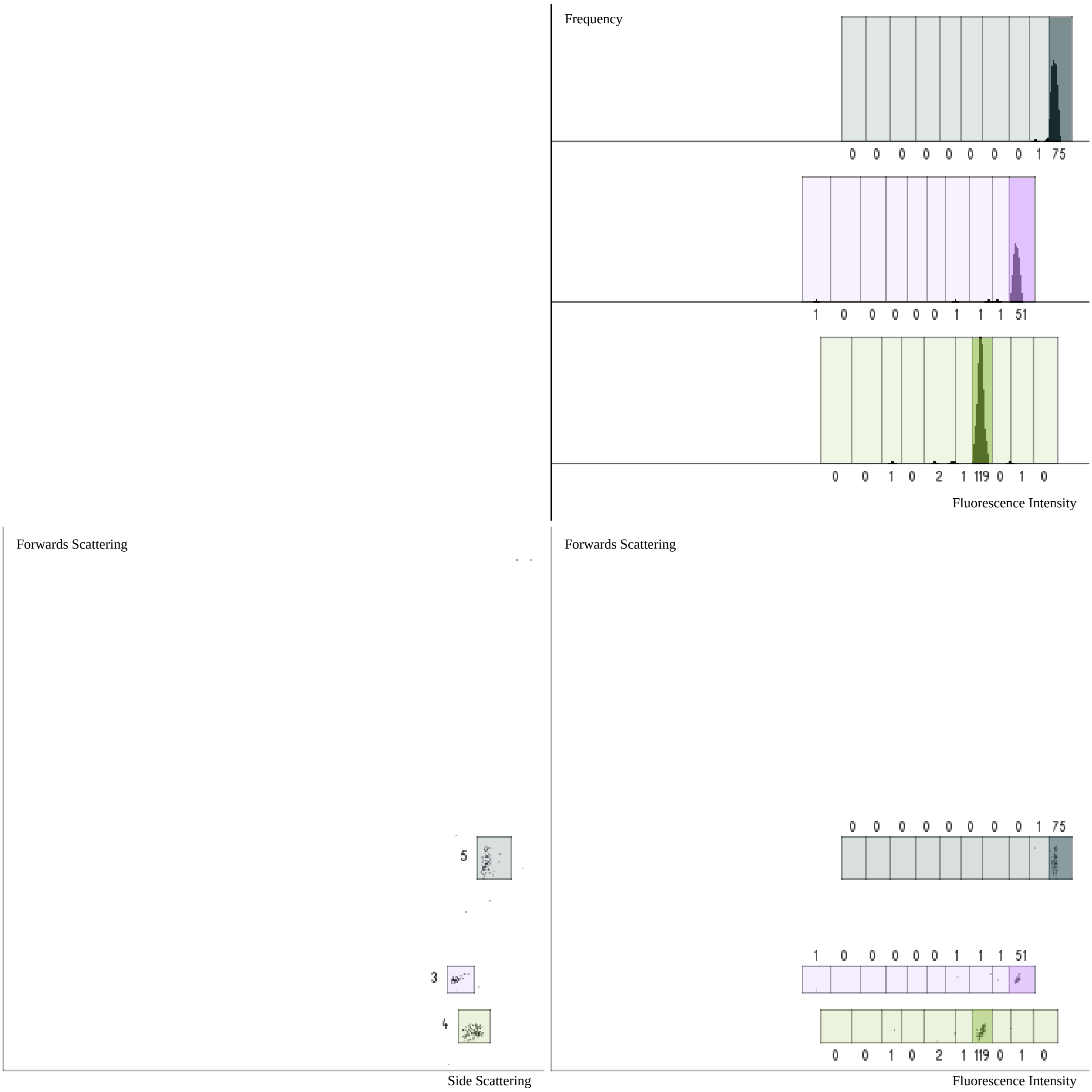

ANNEX 3: TAG DECONVOLUTION - BEAD 105

Passes flow sorting criteria: Yes

Passes tag deconvolution criteria: Yes

Included in protocol analysis: Yes

Protocol: 8, 4, 5, 4

Filename: Bin4_plateA3_F1.fcs

Split 1: Petrol shading

Split 2: Green shading

Split 3: Violet shading

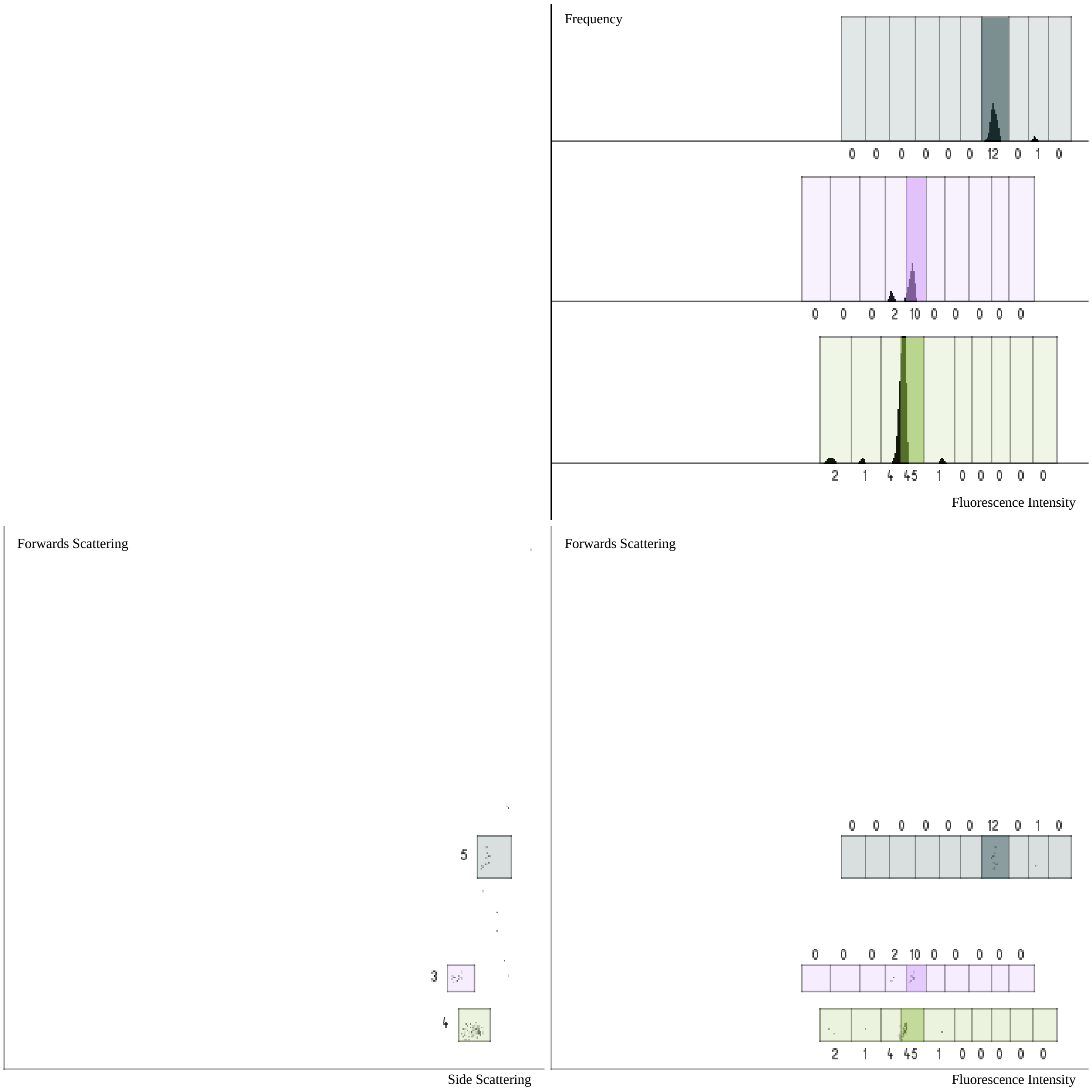

ANNEX 3: TAG DECONVOLUTION - BEAD 106

Passes flow sorting criteria: Yes

Passes tag deconvolution criteria: No

Included in protocol analysis: No

Protocol: N/A

Filename: Bin4_plateA3_G3.fcs

Split 1: Petrol shading

Split 2: Green shading

Split 3: Violet shading

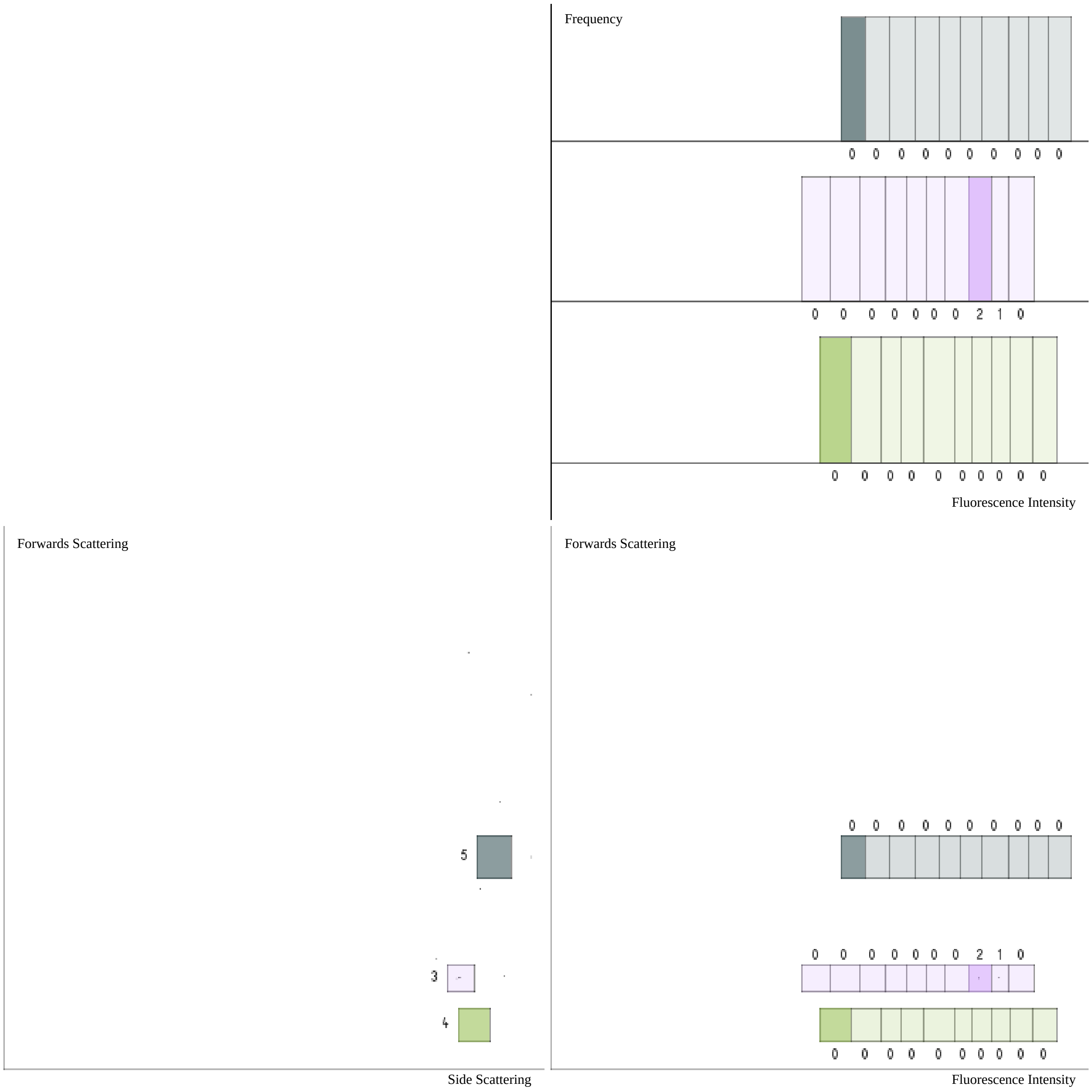

ANNEX 3: TAG DECONVOLUTION - BEAD 107

Passes flow sorting criteria: Yes

Passes tag deconvolution criteria: No

Included in protocol analysis: No

Protocol: N/A

Filename: Bin4_plateA3_G10.fcs

Split 1: Petrol shading

Split 2: Green shading

Split 3: Violet shading

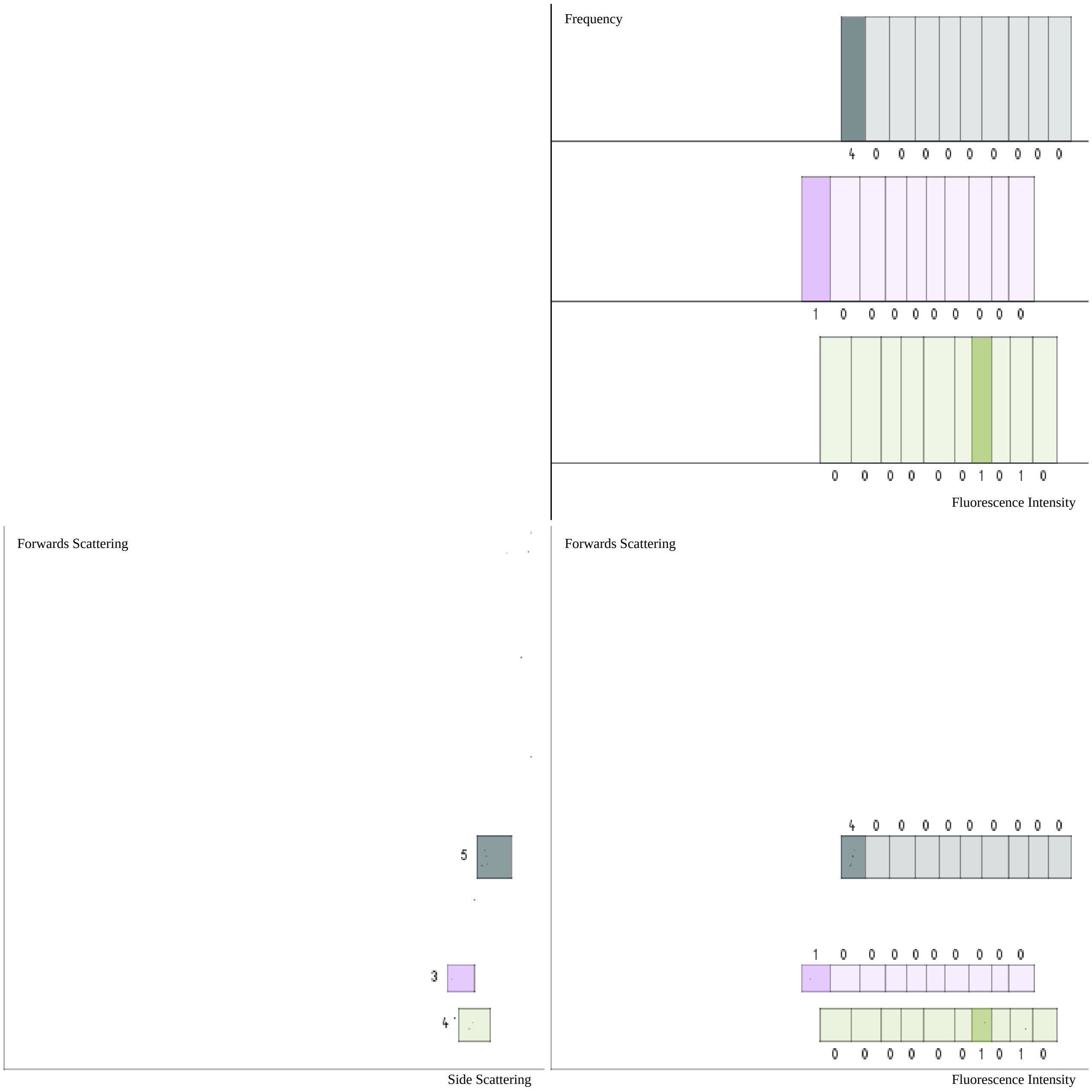

ANNEX 3: TAG DECONVOLUTION - BEAD 108

Passes flow sorting criteria: Yes

Passes tag deconvolution criteria: Yes

Included in protocol analysis: Yes

Protocol: 3, 3, 8, 4

Filename: Bin4_plateA3_H1.fcs

Split 1: Petrol shading

Split 2: Green shading

Split 3: Violet shading

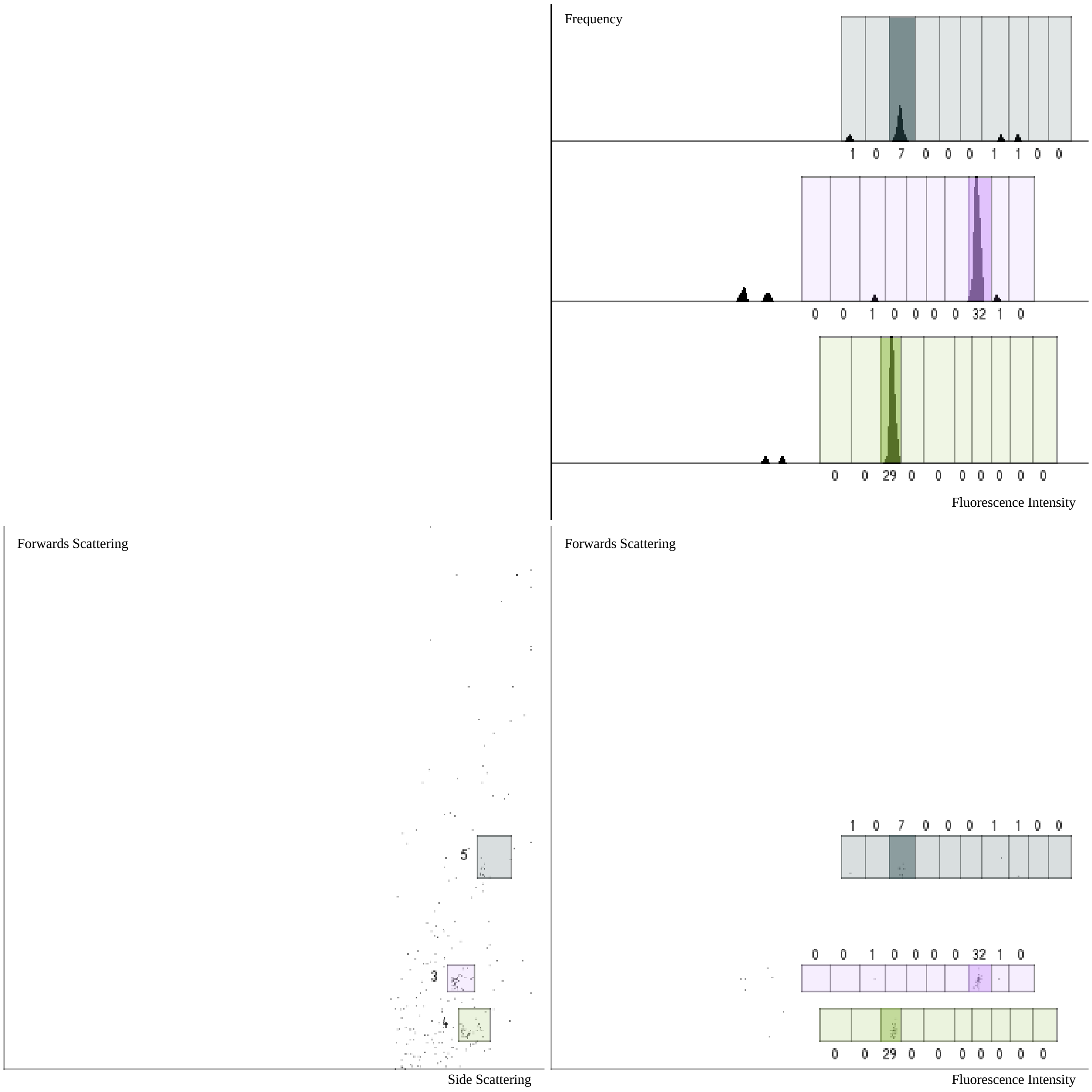

ANNEX 3: TAG DECONVOLUTION - BEAD 109

Passes flow sorting criteria: Yes

Passes tag deconvolution criteria: Yes

Included in protocol analysis: Yes

Protocol: 1, 8, 1, 4

Filename: Bin4_plateA3_H8.fcs

Split 1: Petrol shading

Split 2: Green shading

Split 3: Violet shading

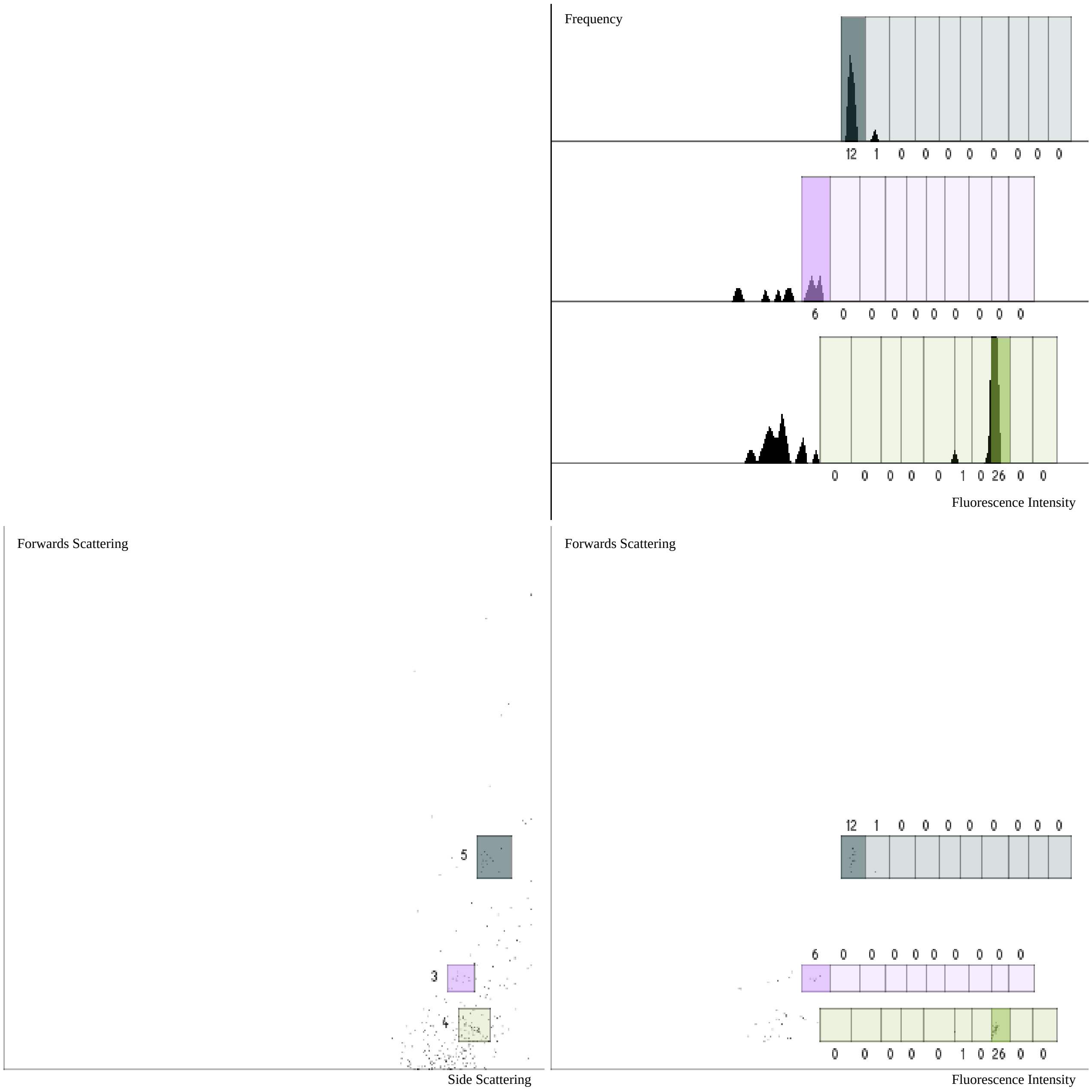

ANNEX 3: TAG DECONVOLUTION - BEAD 110

Passes flow sorting criteria: Yes

Passes tag deconvolution criteria: Yes

Included in protocol analysis: Yes

Protocol: 2, 6, 7, 4

Filename: Bin4_plateA4_D11.fcs

Split 1: Petrol shading

Split 2: Green shading

Split 3: Violet shading

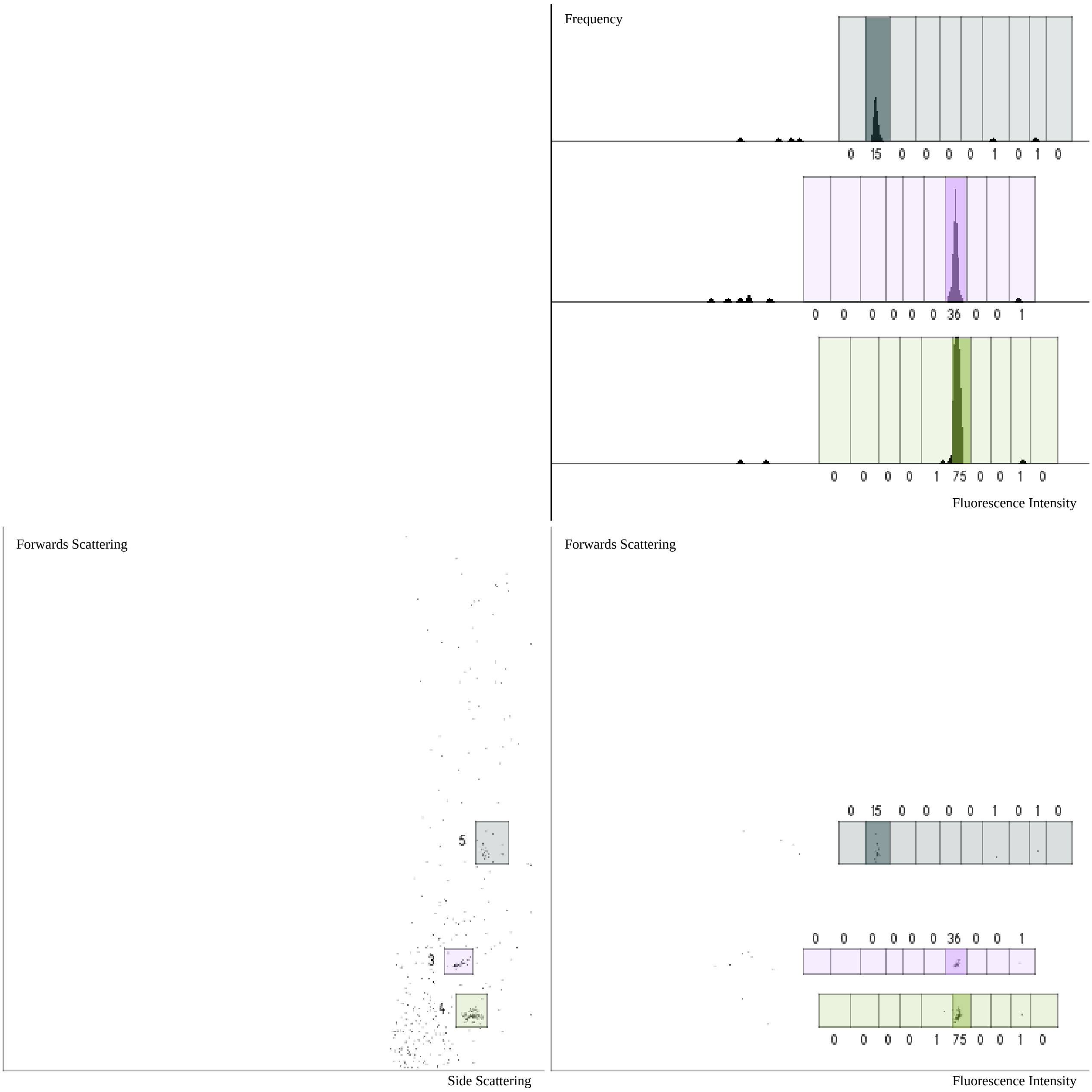

ANNEX 3: TAG DECONVOLUTION - BEAD 111

Passes flow sorting criteria: Yes

Passes tag deconvolution criteria: Yes

Included in protocol analysis: Yes

Protocol: 9, 9, 7, 4

Filename: Bin4_plateA4_A2.fcs

Split 1: Petrol shading

Split 2: Green shading

Split 3: Violet shading

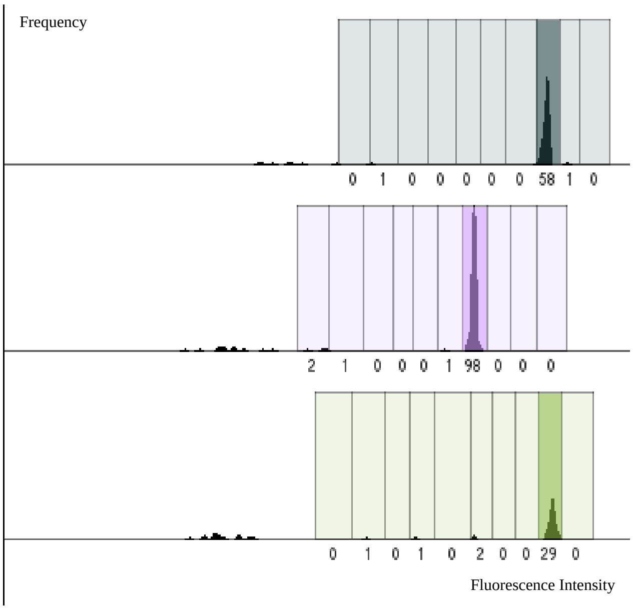

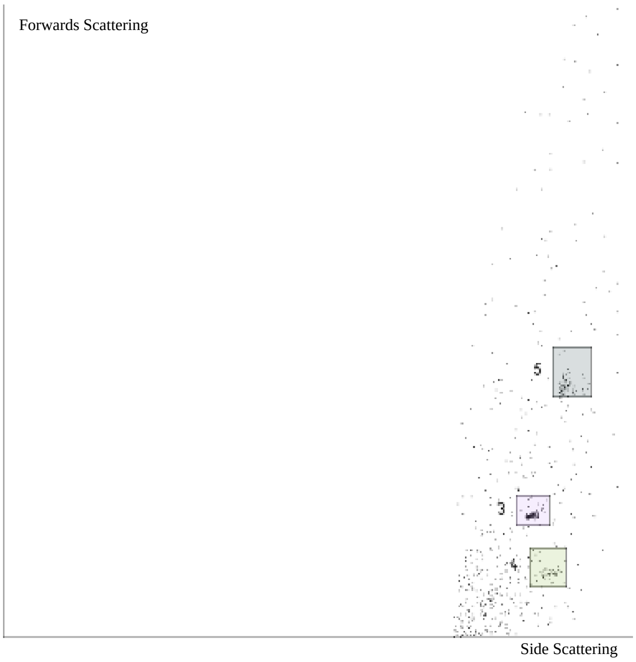

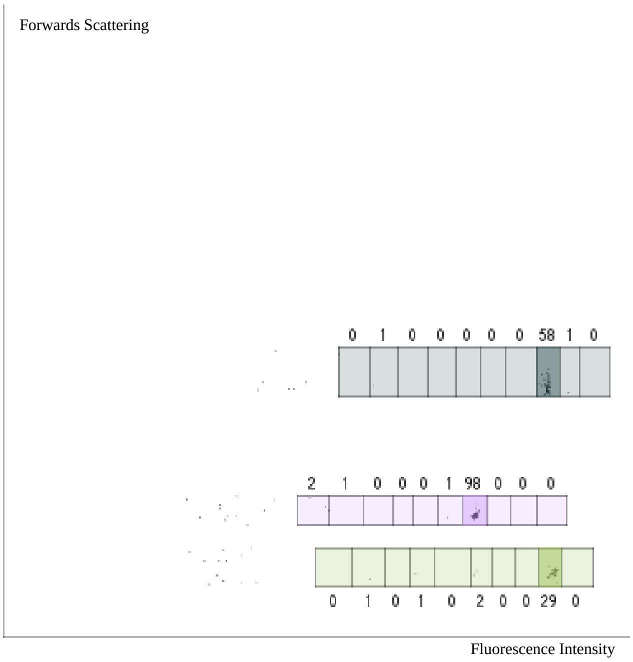

ANNEX 3: TAG DECONVOLUTION - BEAD 112

Passes flow sorting criteria: Yes

Passes tag deconvolution criteria: Yes

Included in protocol analysis: Yes

Protocol: 2, 5, 7, 4

Filename: Bin4_plateA4_A10.fcs

Split 1: Petrol shading

Split 2: Green shading

Split 3: Violet shading

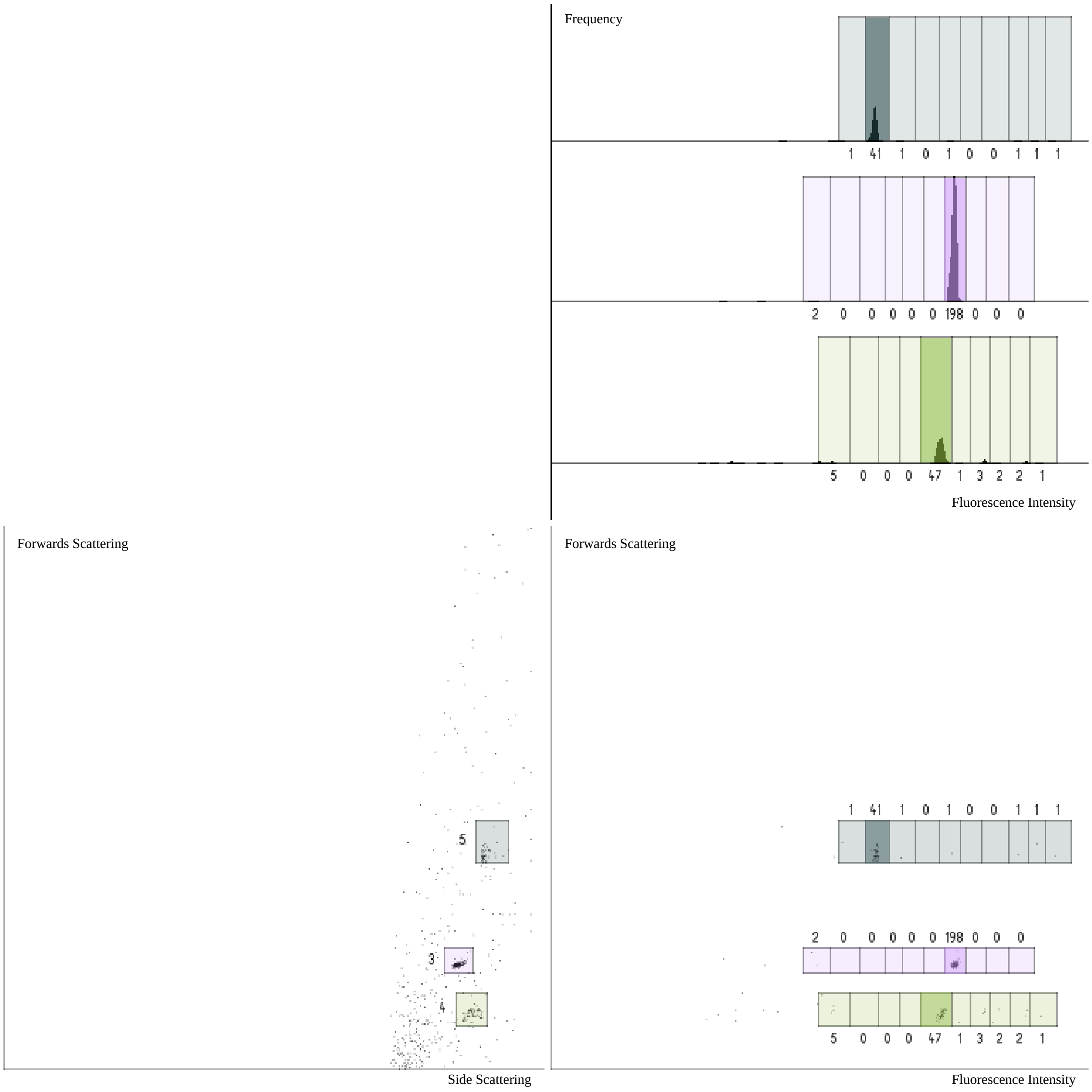

ANNEX 3: TAG DECONVOLUTION - BEAD 113

Passes flow sorting criteria: Yes

Passes tag deconvolution criteria: Yes

Included in protocol analysis: Yes

Protocol: 1, 7, 2, 4

Filename: Bin4_plateA4_A11.fcs

Split 1: Petrol shading

Split 2: Green shading

Split 3: Violet shading

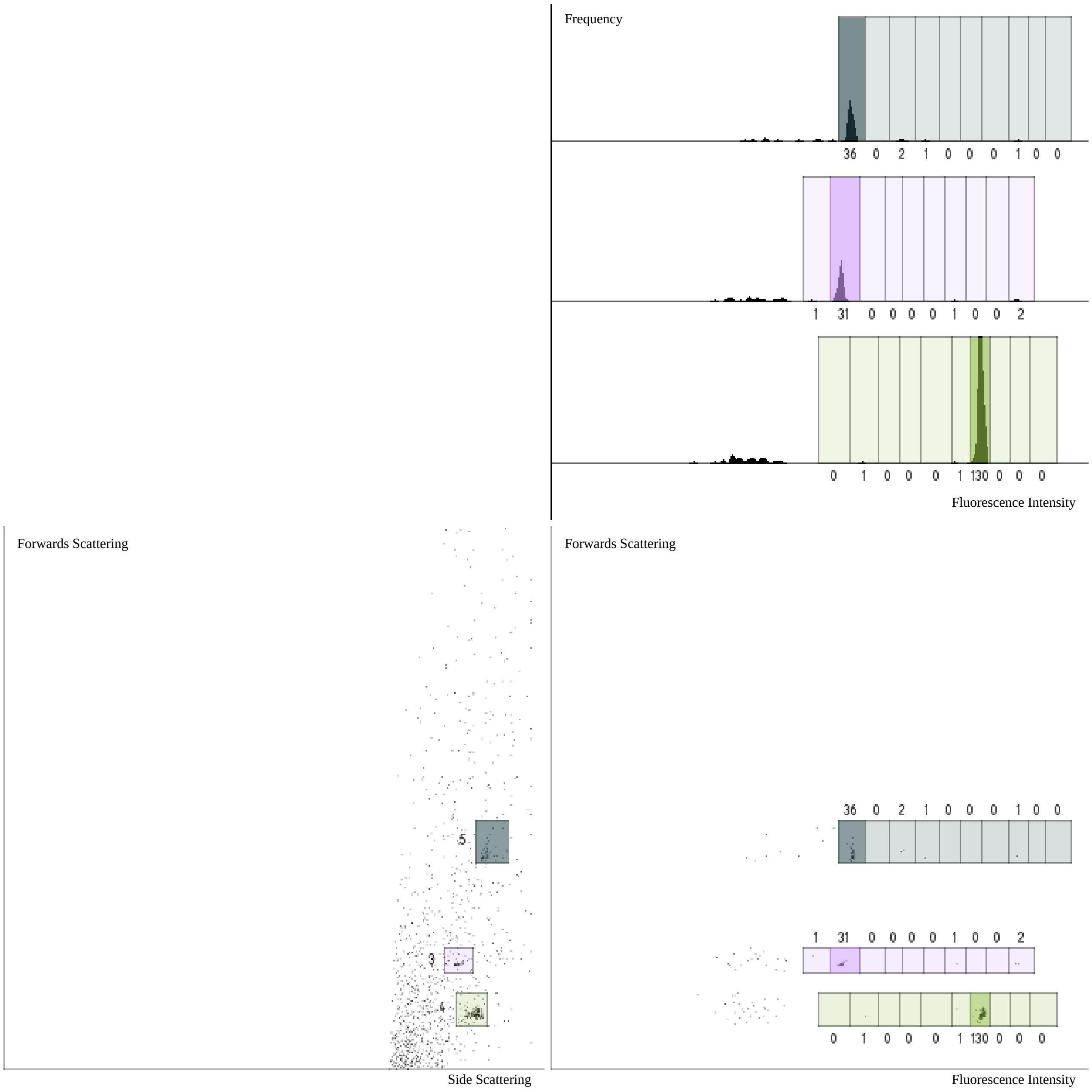

ANNEX 3: TAG DECONVOLUTION - BEAD 114

Passes flow sorting criteria: Yes

Passes tag deconvolution criteria: Yes

Included in protocol analysis: Yes

Protocol: 7, 5, 9, 4

Filename: Bin4_plateA4_B2.fcs

Split 1: Petrol shading

Split 2: Green shading

Split 3: Violet shading

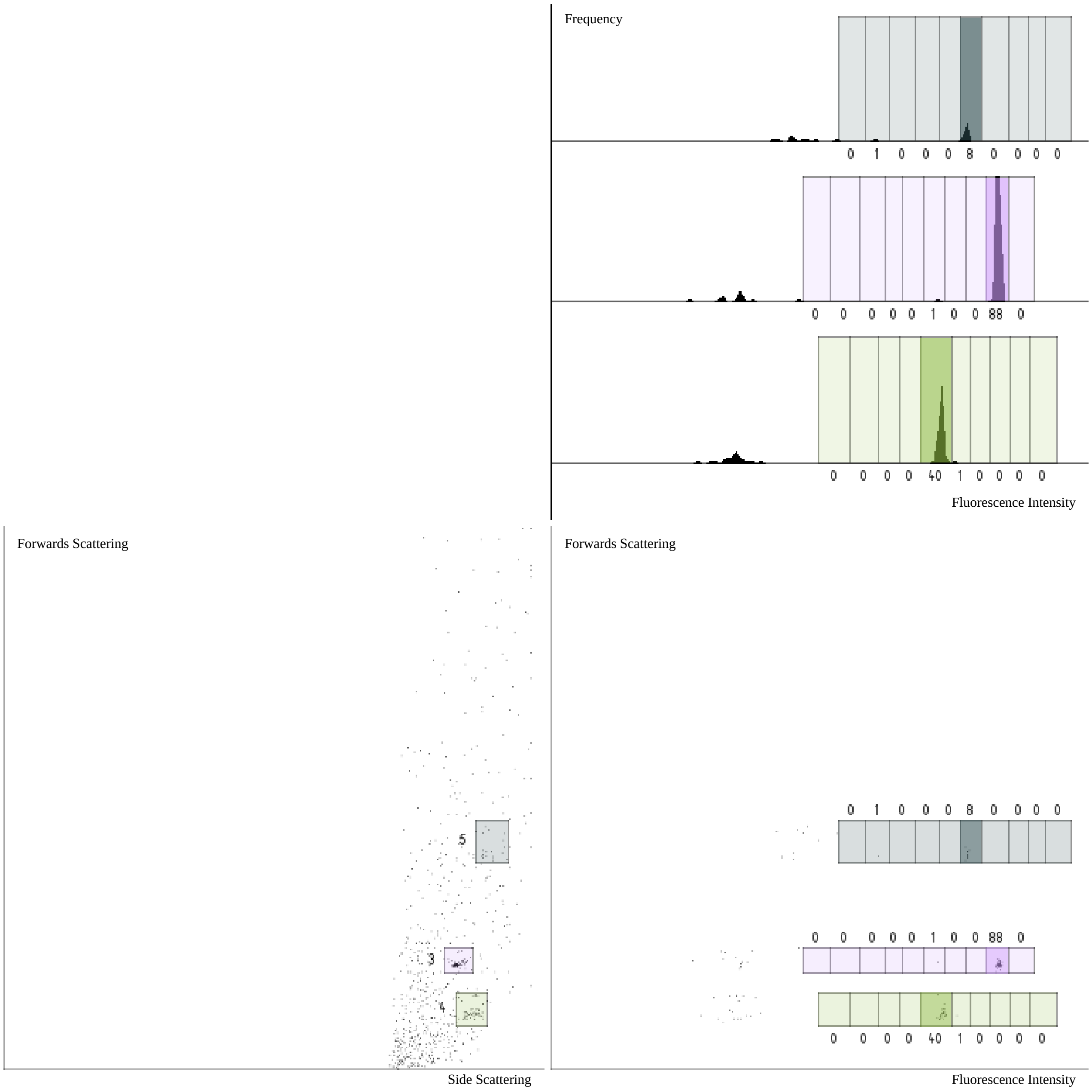

ANNEX 3: TAG DECONVOLUTION - BEAD 115

Passes flow sorting criteria: Yes

Passes tag deconvolution criteria: Yes

Included in protocol analysis: Yes

Protocol: 10, 8, 7, 4

Filename: Bin4_plateA4_B6.fcs

Split 1: Petrol shading

Split 2: Green shading

Split 3: Violet shading

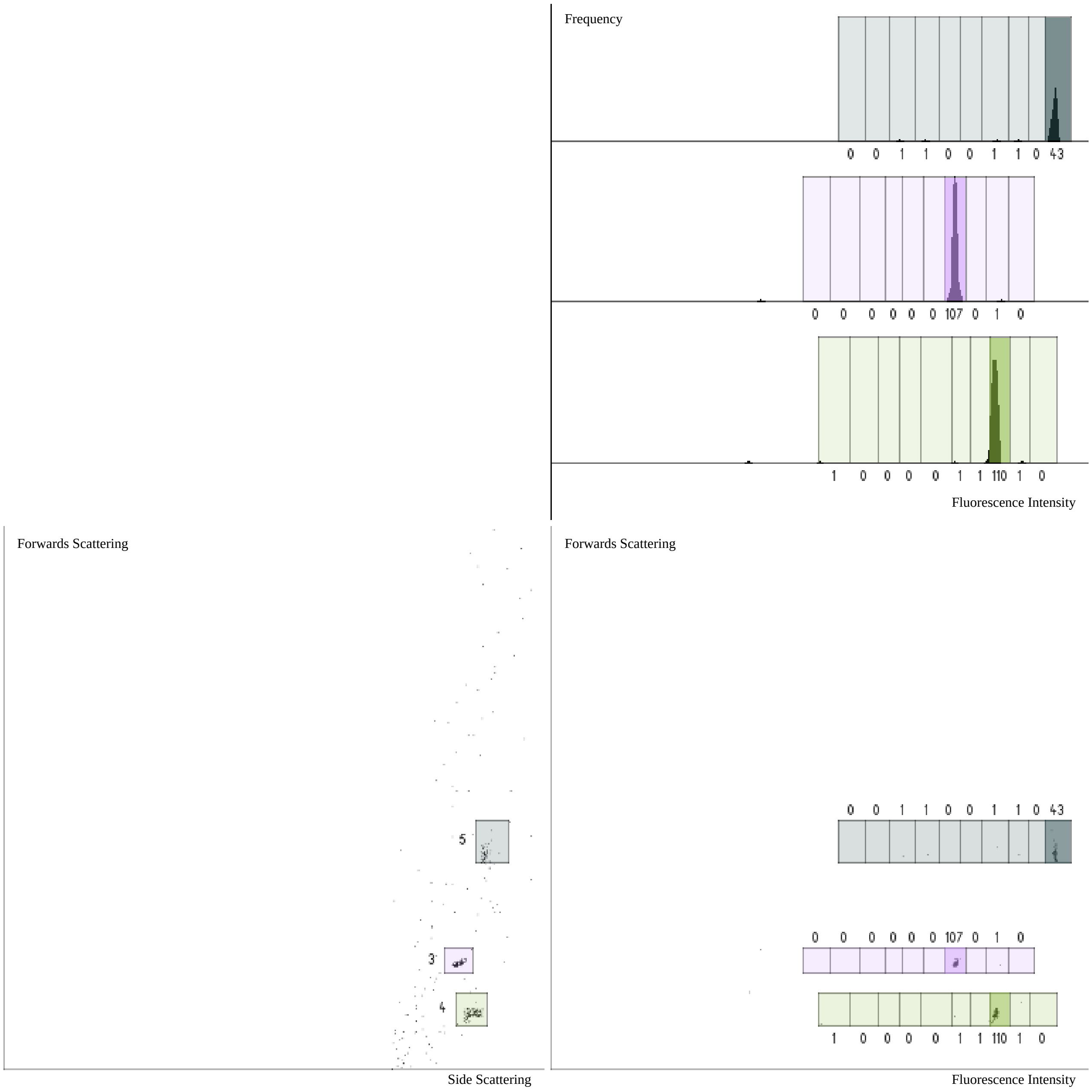

ANNEX 3: TAG DECONVOLUTION - BEAD 116

Passes flow sorting criteria: Yes

Passes tag deconvolution criteria: Yes

Included in protocol analysis: Yes

Protocol: 9, 7, 6, 4

Filename: Bin4_plateA4_B9.fcs

Split 1: Petrol shading

Split 2: Green shading

Split 3: Violet shading

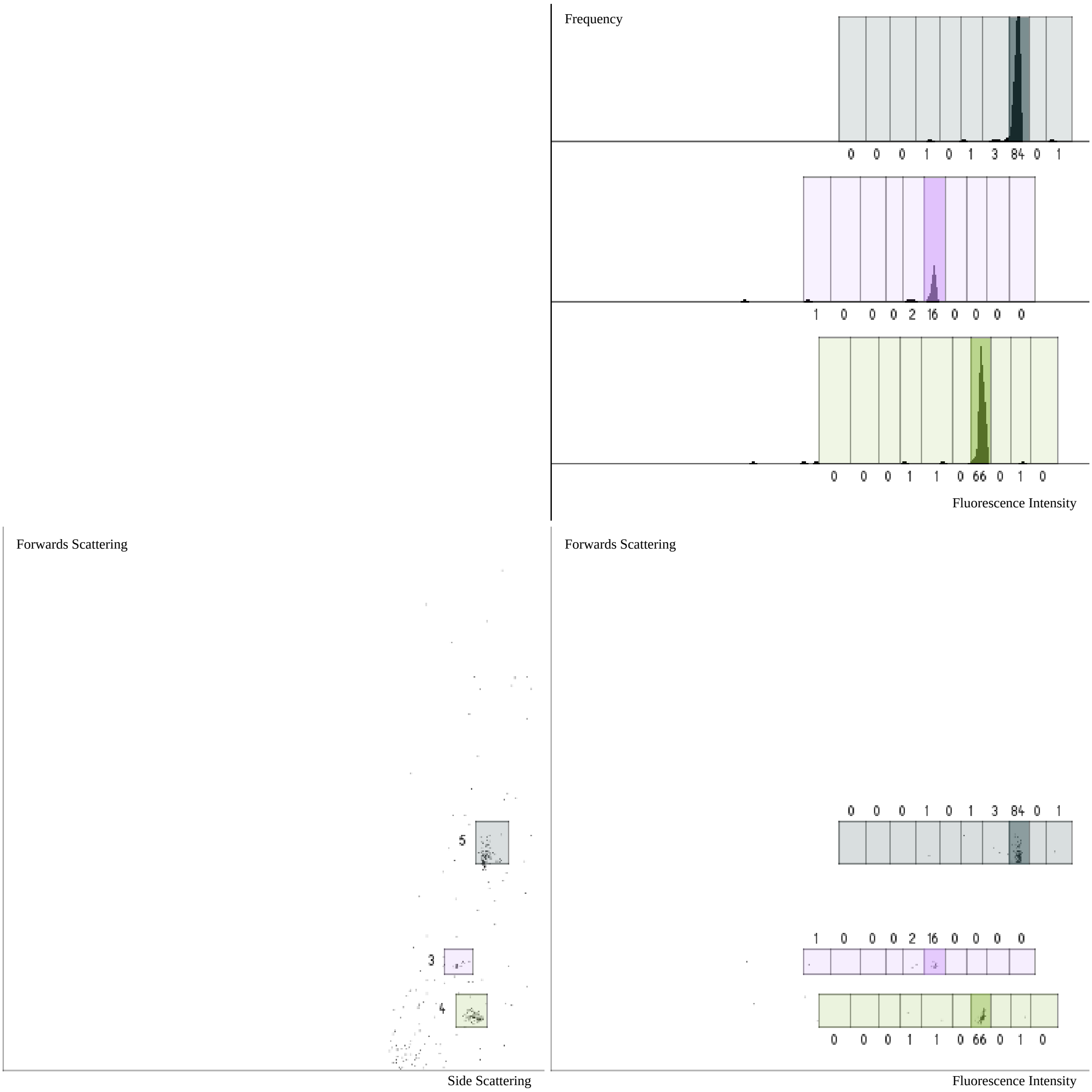

ANNEX 3: TAG DECONVOLUTION - BEAD 117

Passes flow sorting criteria: Yes

Passes tag deconvolution criteria: No

Included in protocol analysis: No

Protocol: N/A

Filename: Bin4_plateA4_B10.fcs

Split 1: Petrol shading

Split 2: Green shading

Split 3: Violet shading

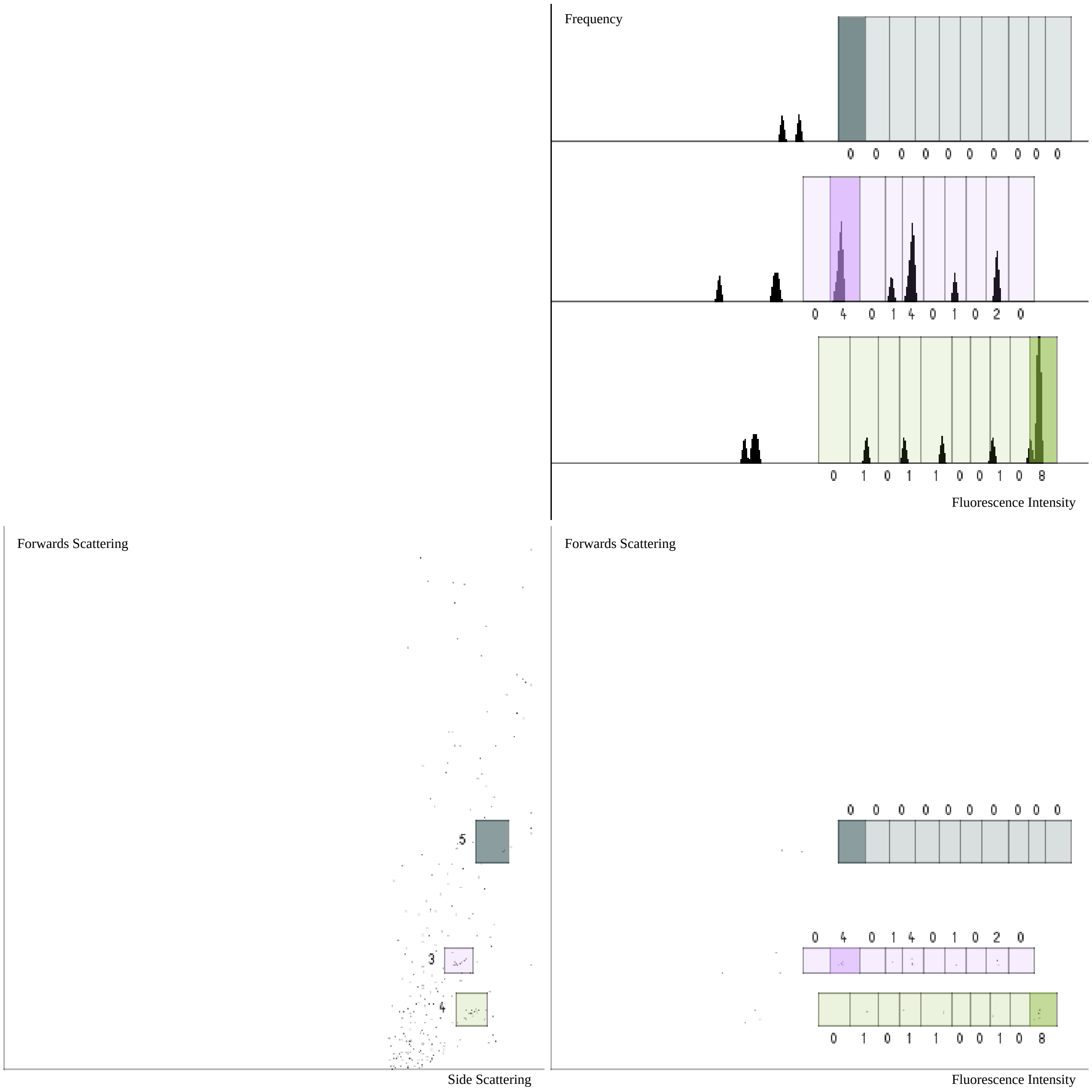

ANNEX 3: TAG DECONVOLUTION - BEAD 118

Passes flow sorting criteria: Yes

Passes tag deconvolution criteria: Yes

Included in protocol analysis: Yes

Protocol: 4, 6, 6, 4

Filename: Bin4_plateA4_C1.fcs

Split 1: Petrol shading

Split 2: Green shading

Split 3: Violet shading

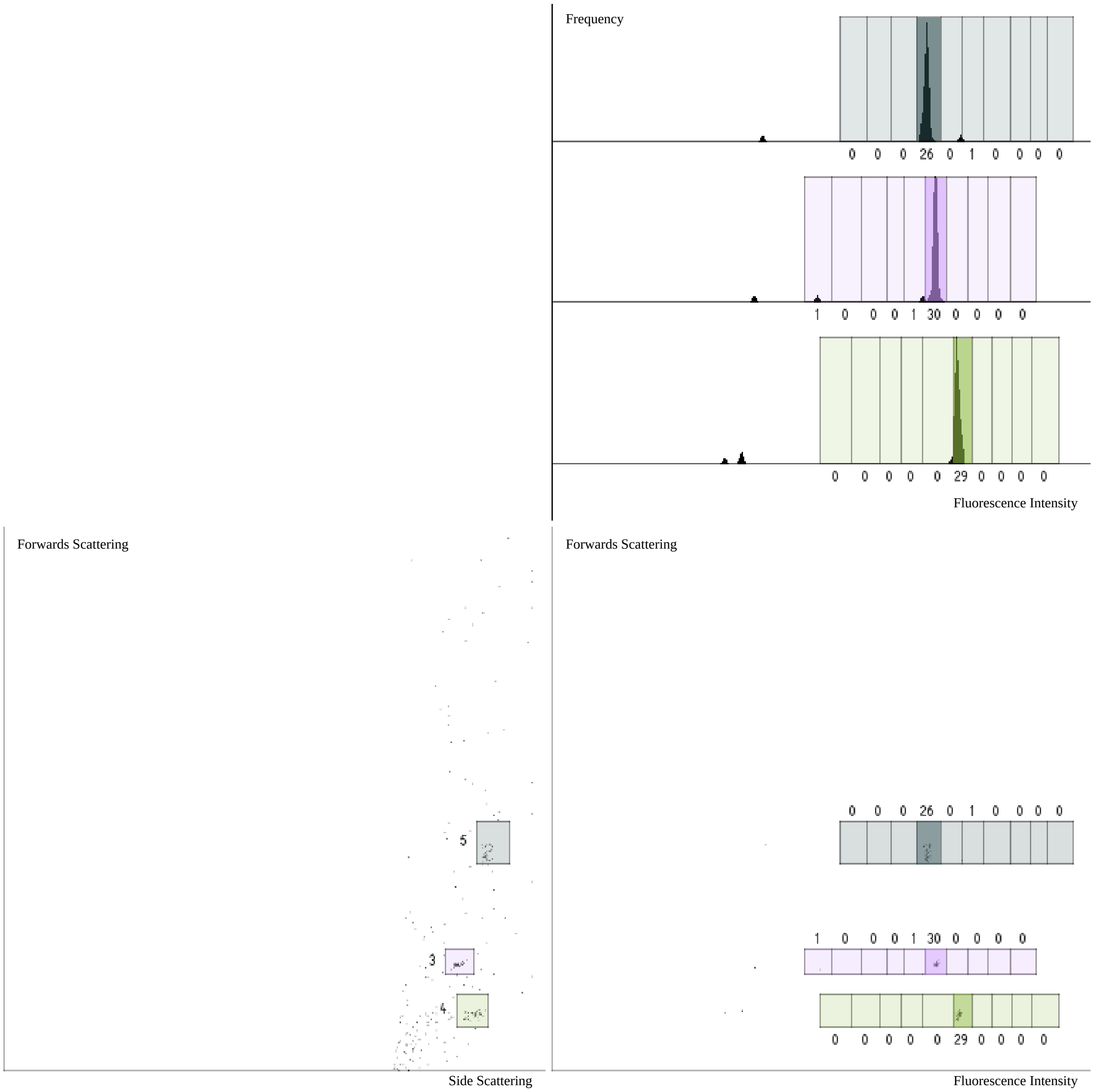

ANNEX 3: TAG DECONVOLUTION - BEAD 119

Passes flow sorting criteria: Yes

Passes tag deconvolution criteria: Yes

Included in protocol analysis: Yes

Protocol: 8, 6, 7, 4

Filename: Bin4_plateA4_C10.fcs

Split 1: Petrol shading

Split 2: Green shading

Split 3: Violet shading

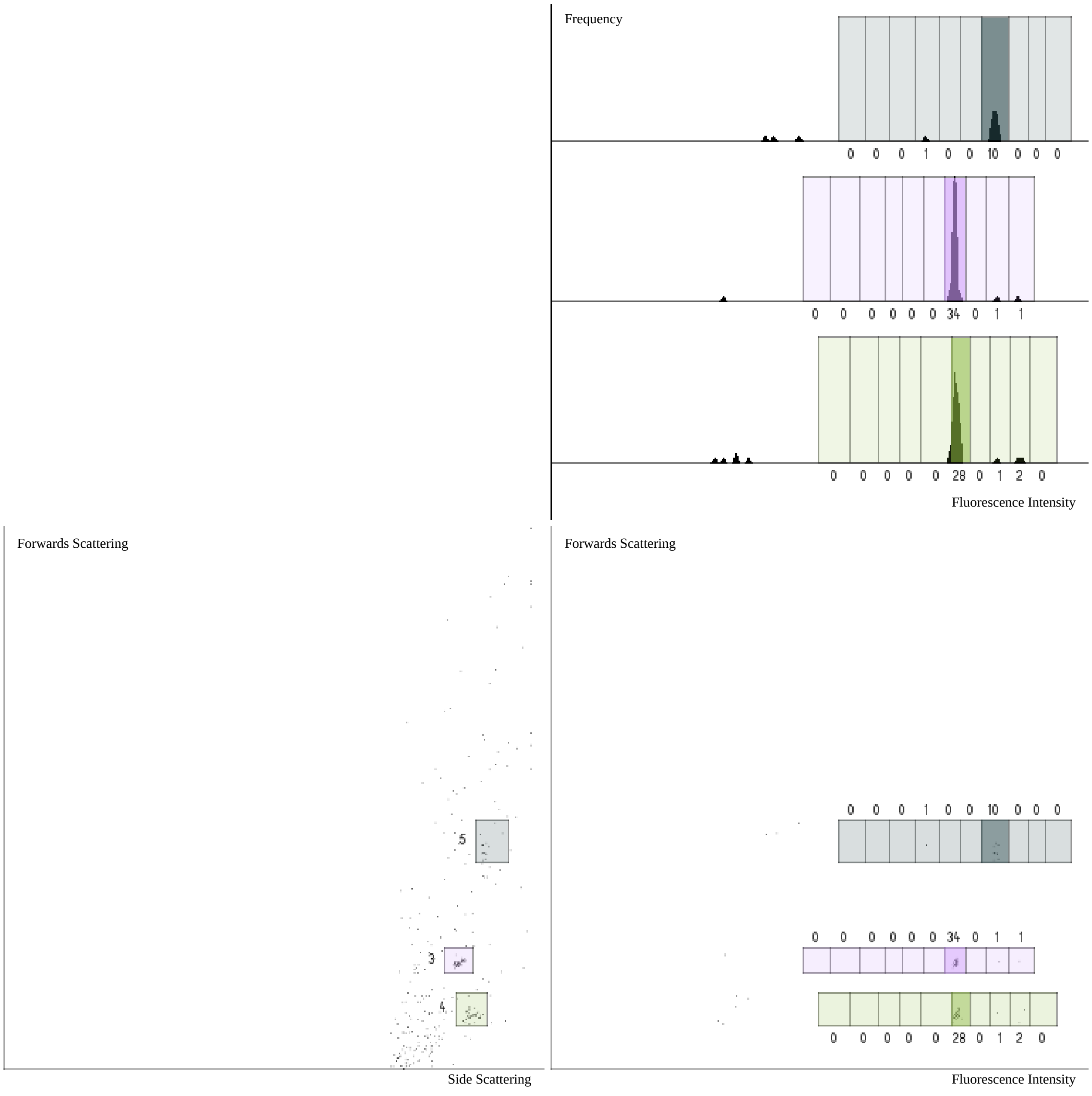

ANNEX 3: TAG DECONVOLUTION - BEAD 120

Passes flow sorting criteria: Yes

Passes tag deconvolution criteria: Yes

Included in protocol analysis: Yes

Protocol: 5, 4, 3, 4

Filename: Bin4_plateA4_C11.fcs

Split 1: Petrol shading

Split 2: Green shading

Split 3: Violet shading

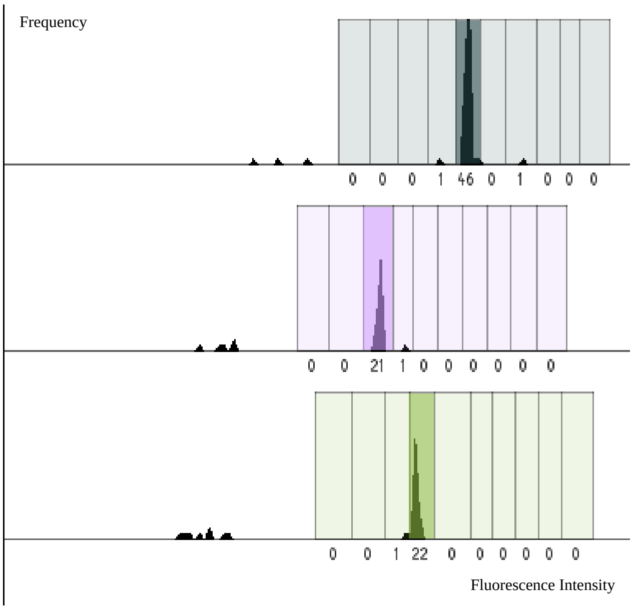

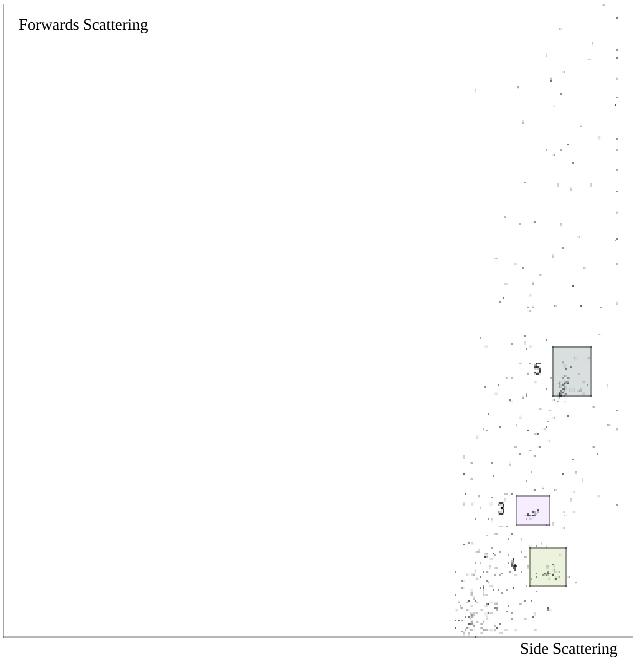

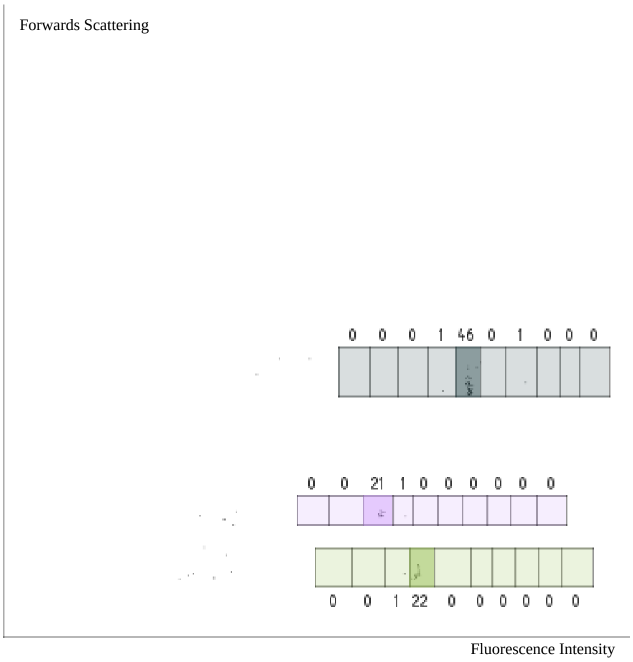

ANNEX 3: TAG DECONVOLUTION - BEAD 121

Passes flow sorting criteria: Yes

Passes tag deconvolution criteria: Yes

Included in protocol analysis: Yes

Protocol: 5, 9, 6, 4

Filename: Bin4_plateA4_D9.fcs

Split 1: Petrol shading

Split 2: Green shading

Split 3: Violet shading

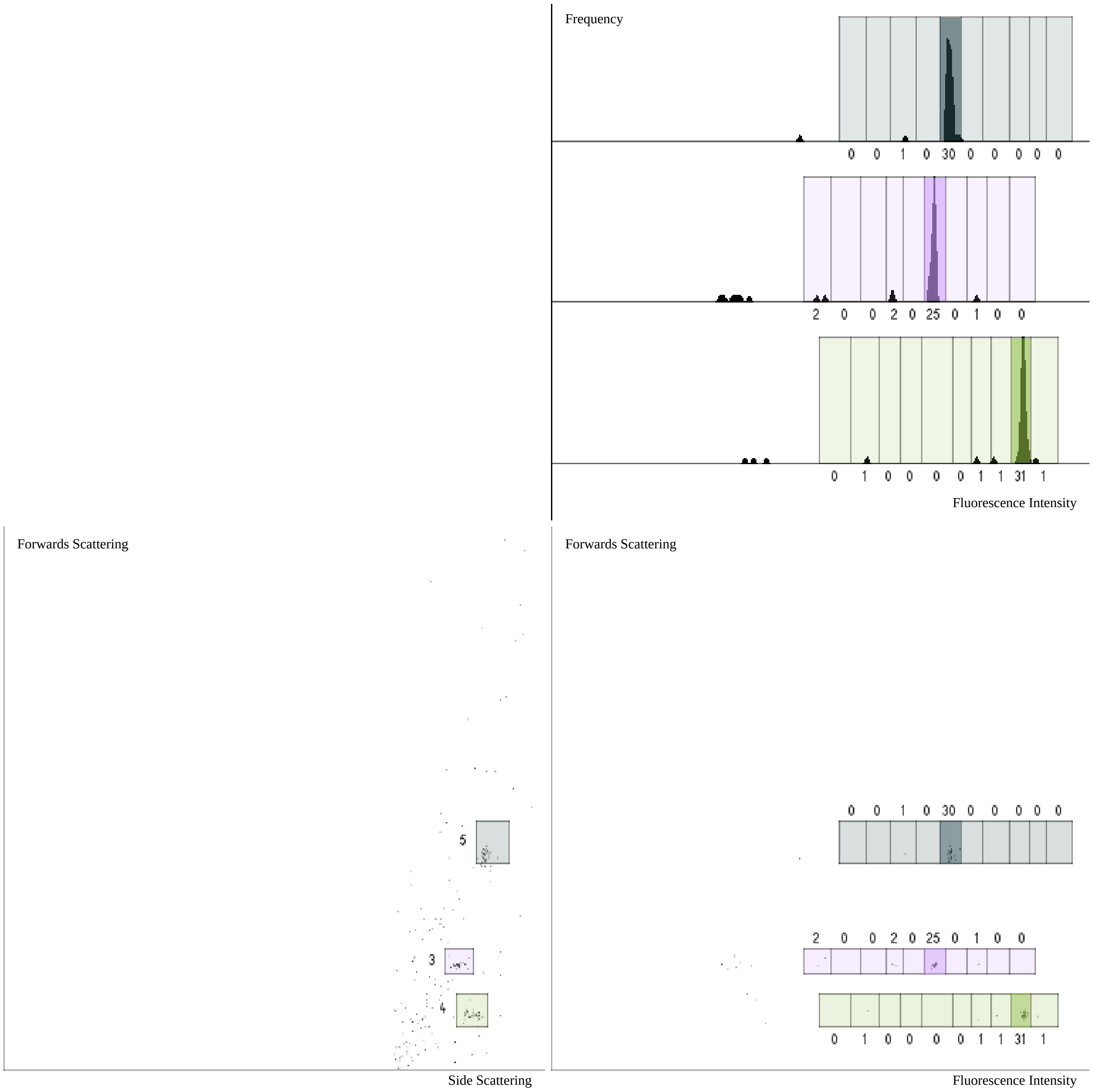

ANNEX 3: TAG DECONVOLUTION - BEAD 122

Passes flow sorting criteria: Yes

Passes tag deconvolution criteria: Yes

Included in protocol analysis: Yes

Protocol: 9, 3, 3, 7

Filename: Bin7_plateA1_C11.fcs

Split 1: Petrol shading

Split 2: Green shading

Split 3: Violet shading

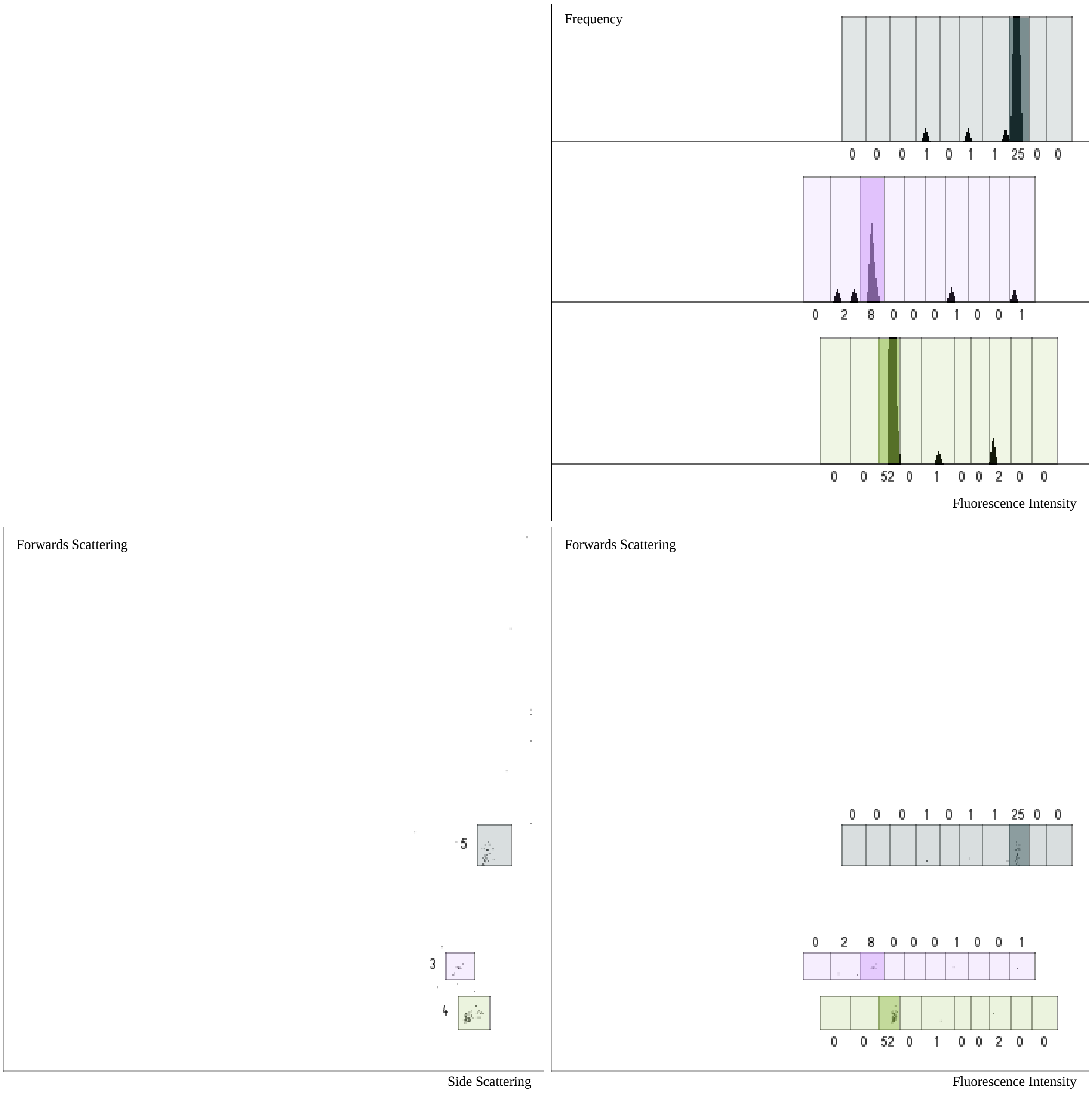

ANNEX 3: TAG DECONVOLUTION - BEAD 123

Passes flow sorting criteria: Yes

Passes tag deconvolution criteria: Yes

Included in protocol analysis: Yes

Protocol: 8, 4, 2, 5

Filename: Bin5_plateA4_A2.fcs

Split 1: Petrol shading

Split 2: Green shading

Split 3: Violet shading

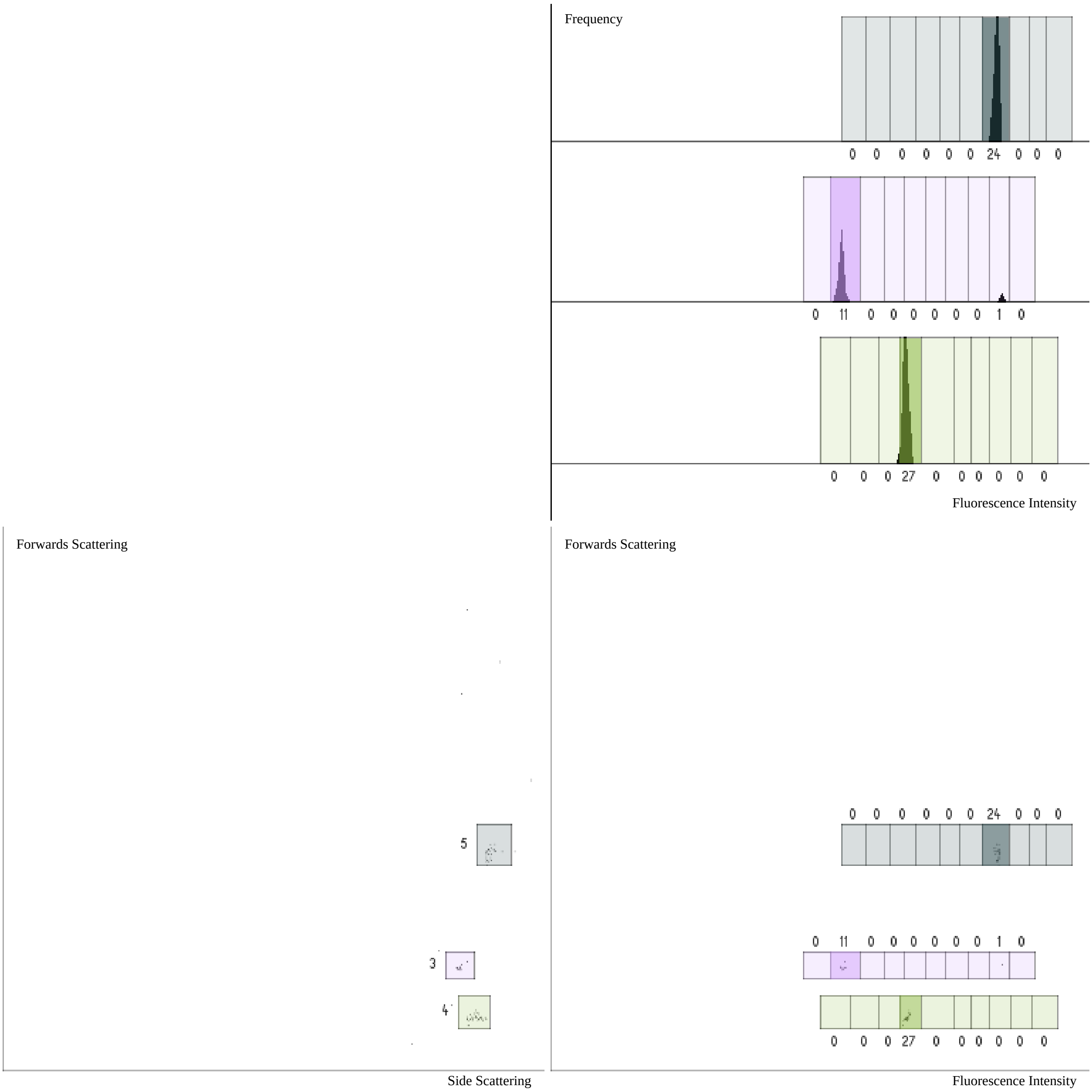

ANNEX 3: TAG DECONVOLUTION - BEAD 124

Passes flow sorting criteria: Yes

Passes tag deconvolution criteria: Yes

Included in protocol analysis: Yes

Protocol: 5, 4, 9, 5

Filename: Bin5_plateA4_A3.fcs

Split 1: Petrol shading

Split 2: Green shading

Split 3: Violet shading

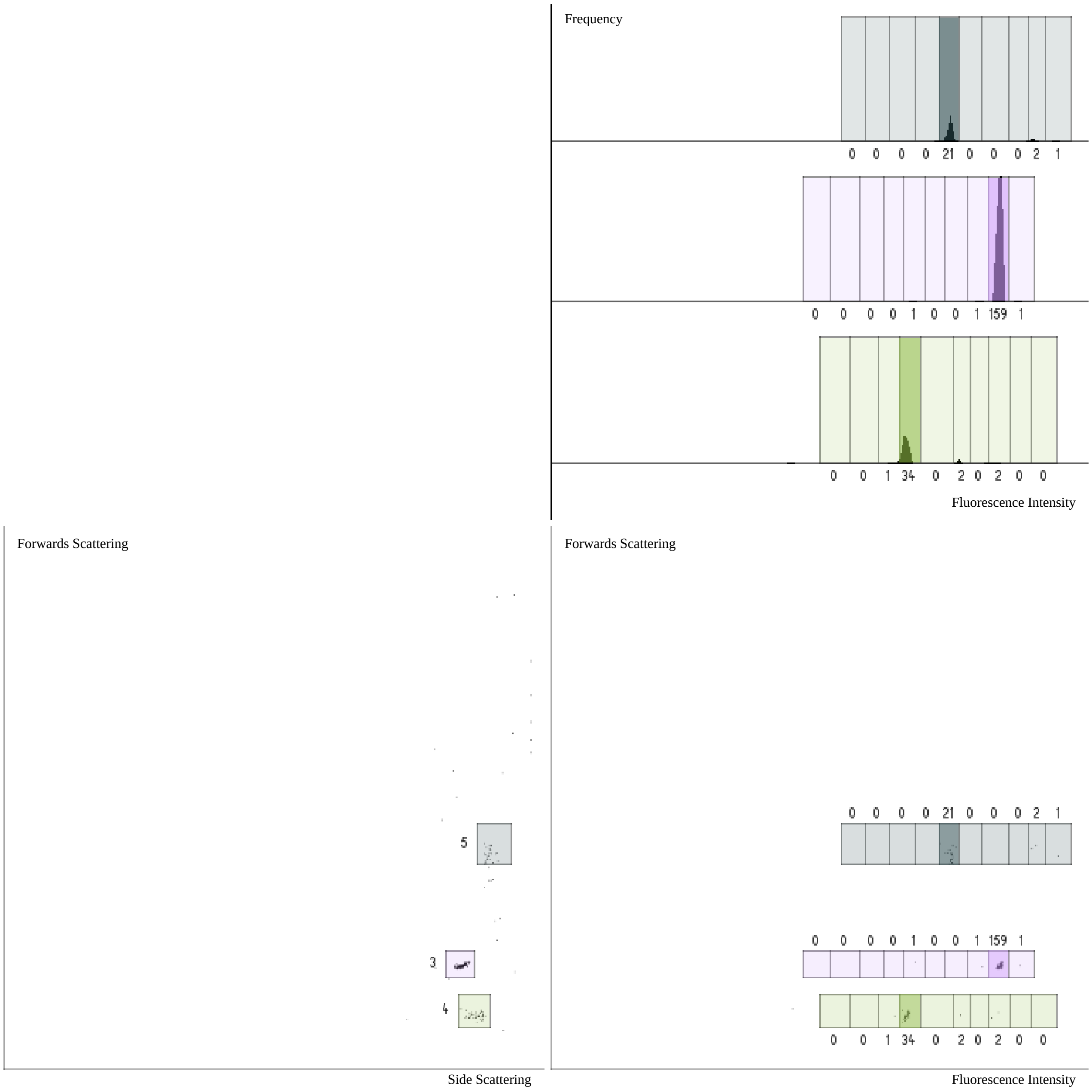

ANNEX 3: TAG DECONVOLUTION - BEAD 125

Passes flow sorting criteria: Yes

Passes tag deconvolution criteria: Yes

Included in protocol analysis: Yes

Protocol: 3, 8, 9, 5

Filename: Bin5_plateA4_A6.fcs

Split 1: Petrol shading

Split 2: Green shading

Split 3: Violet shading

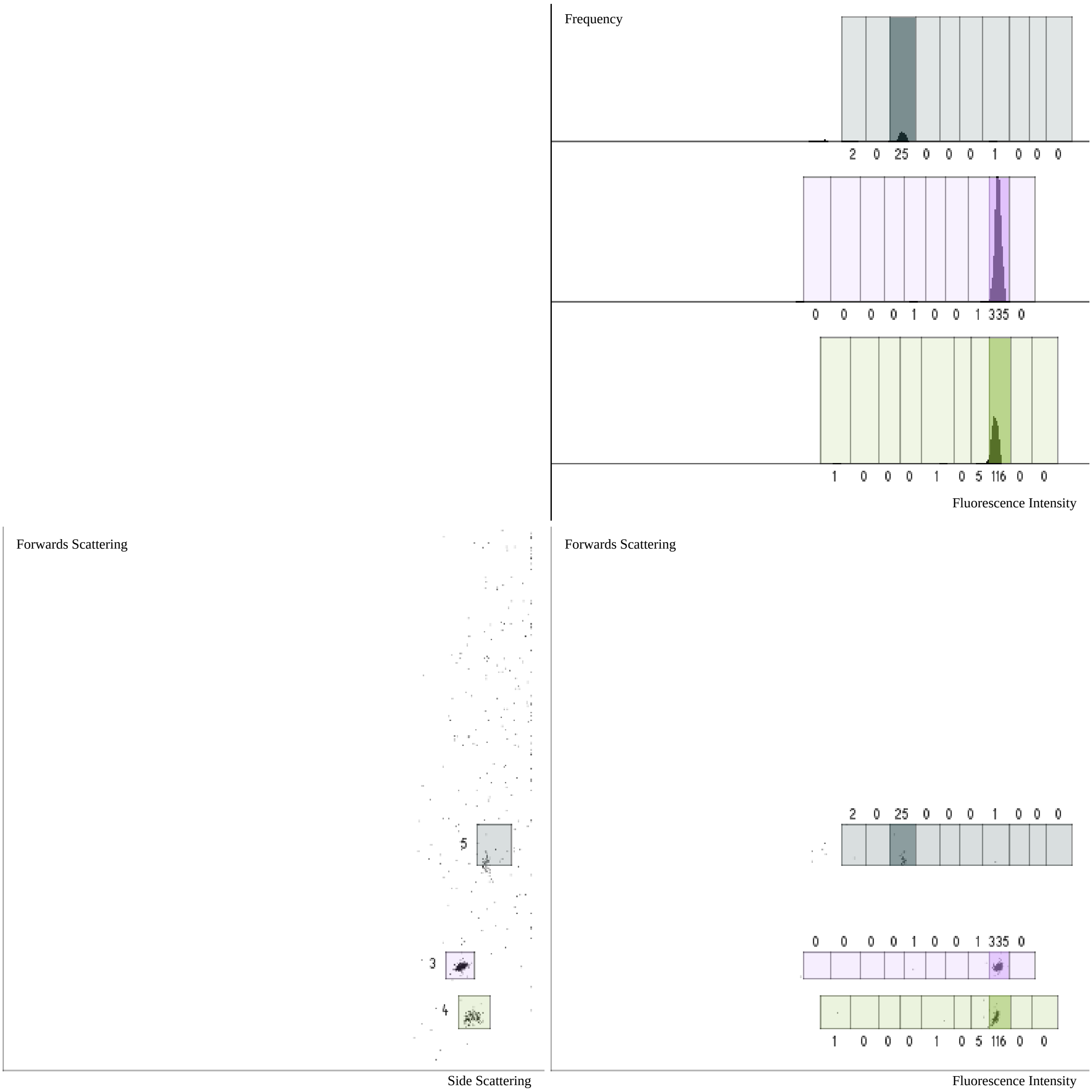

ANNEX 3: TAG DECONVOLUTION - BEAD 126

Passes flow sorting criteria: Yes

Passes tag deconvolution criteria: Yes

Included in protocol analysis: Yes

Protocol: 9, 6, 6, 5

Filename: Bin5_plateA4_A10.fcs

Split 1: Petrol shading

Split 2: Green shading

Split 3: Violet shading

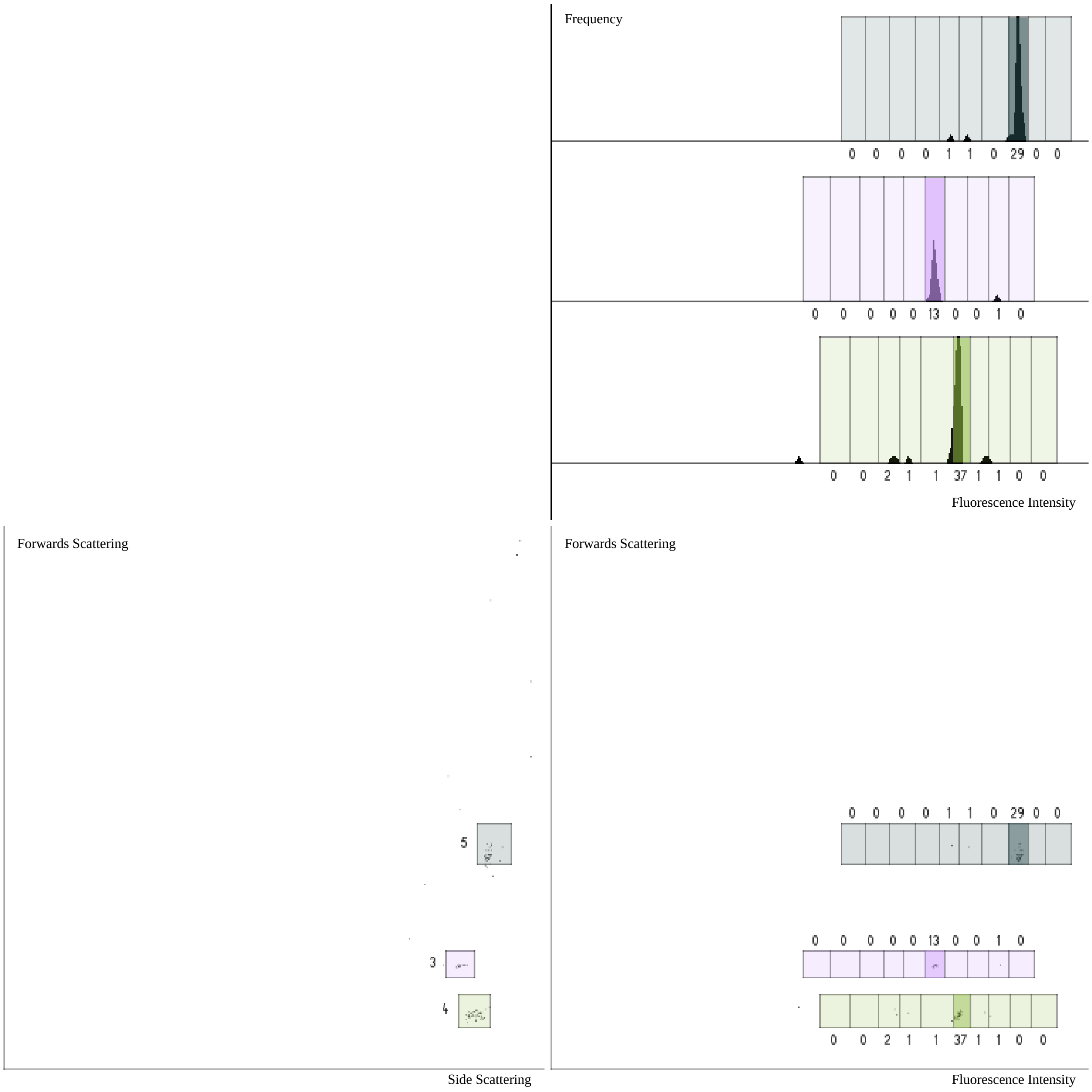

ANNEX 3: TAG DECONVOLUTION - BEAD 127

Passes flow sorting criteria: Yes

Passes tag deconvolution criteria: No

Included in protocol analysis: No

Protocol: N/A

Filename: Bin5_plateA4_A11.fcs

Split 1: Petrol shading

Split 2: Green shading

Split 3: Violet shading

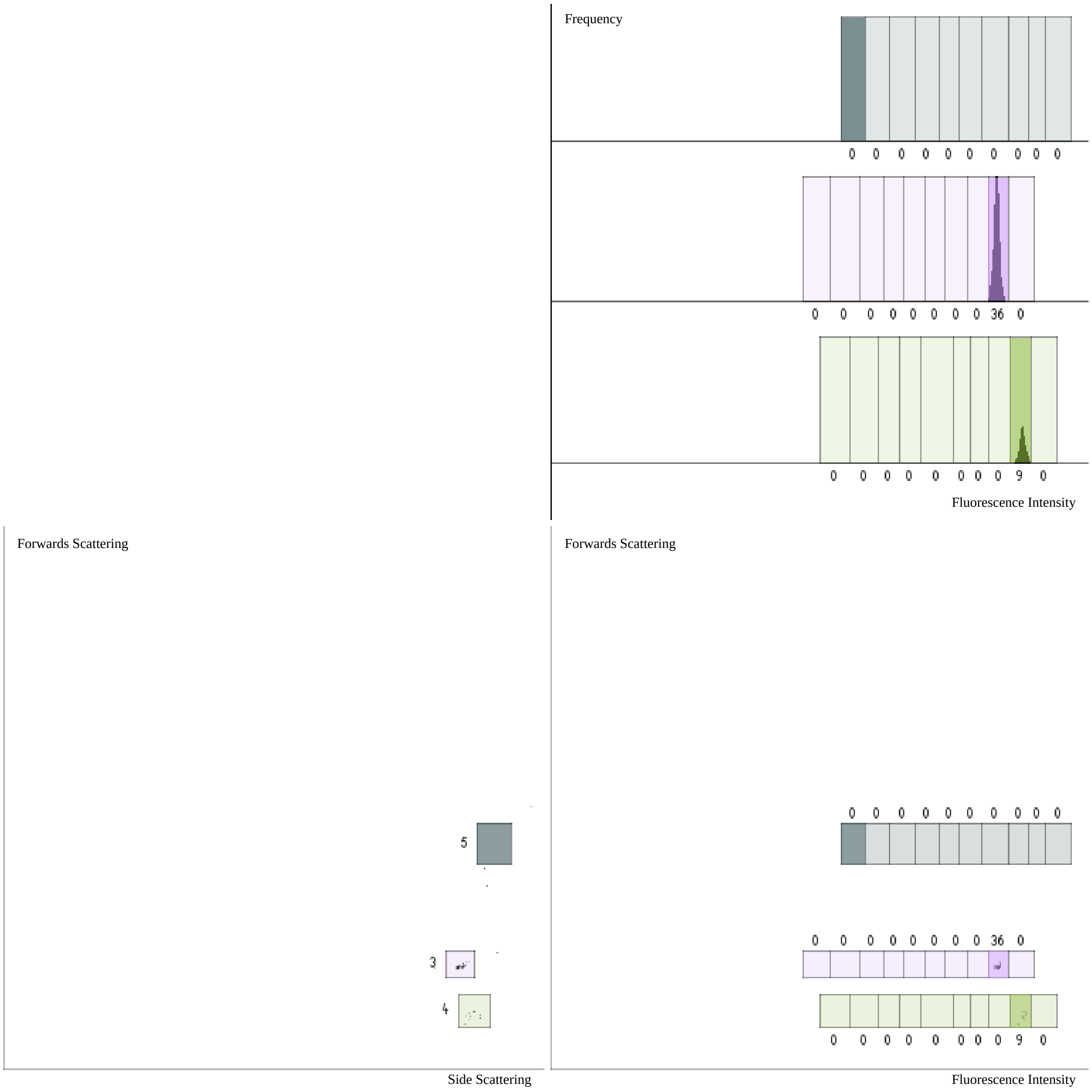

ANNEX 3: TAG DECONVOLUTION - BEAD 128

Passes flow sorting criteria: Yes

Passes tag deconvolution criteria: Yes

Included in protocol analysis: Yes

Protocol: 10, 9, 2, 5

Filename: Bin5_plateA4_B5.fcs

Split 1: Petrol shading

Split 2: Green shading

Split 3: Violet shading

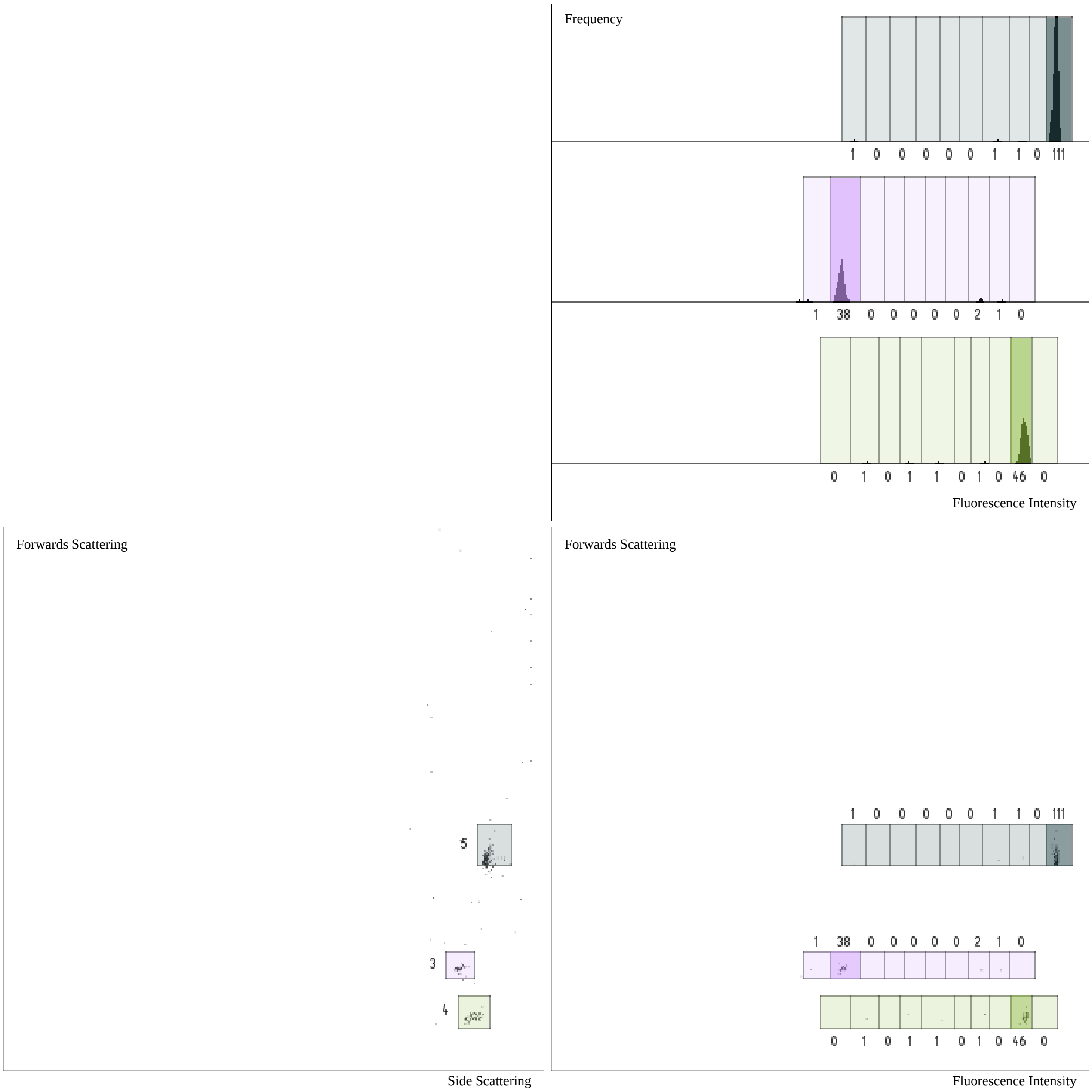

ANNEX 3: TAG DECONVOLUTION - BEAD 129

Passes flow sorting criteria: Yes

Passes tag deconvolution criteria: Yes

Included in protocol analysis: Yes

Protocol: 3, 7, 7, 5

Filename: Bin5_plateA4_B6.fcs

Split 1: Petrol shading

Split 2: Green shading

Split 3: Violet shading

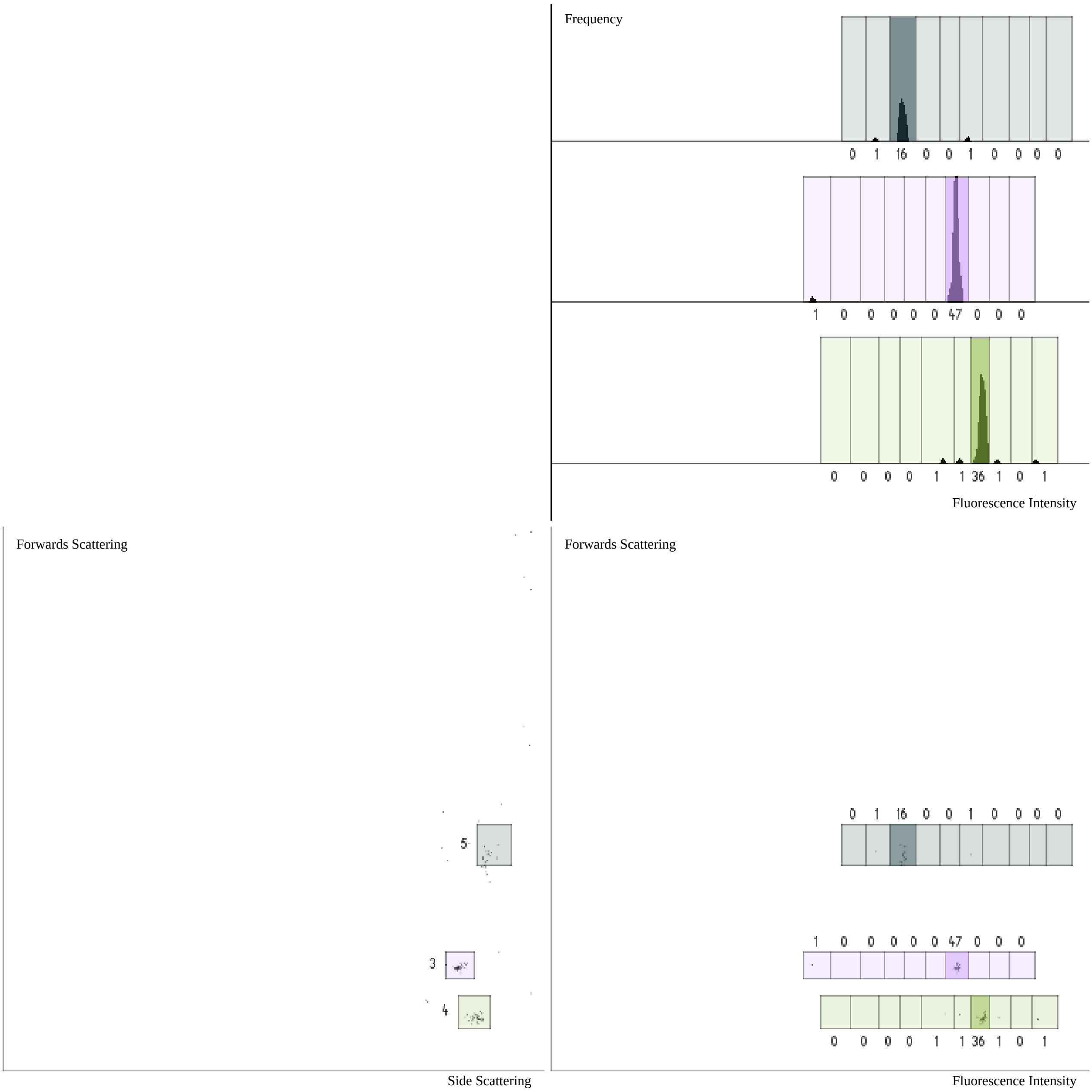

ANNEX 3: TAG DECONVOLUTION - BEAD 130

Passes flow sorting criteria: Yes

Passes tag deconvolution criteria: Yes

Included in protocol analysis: Yes

Protocol: 4, 7, 9, 5

Filename: Bin5_plateA4_B9.fcs

Split 1: Petrol shading

Split 2: Green shading

Split 3: Violet shading

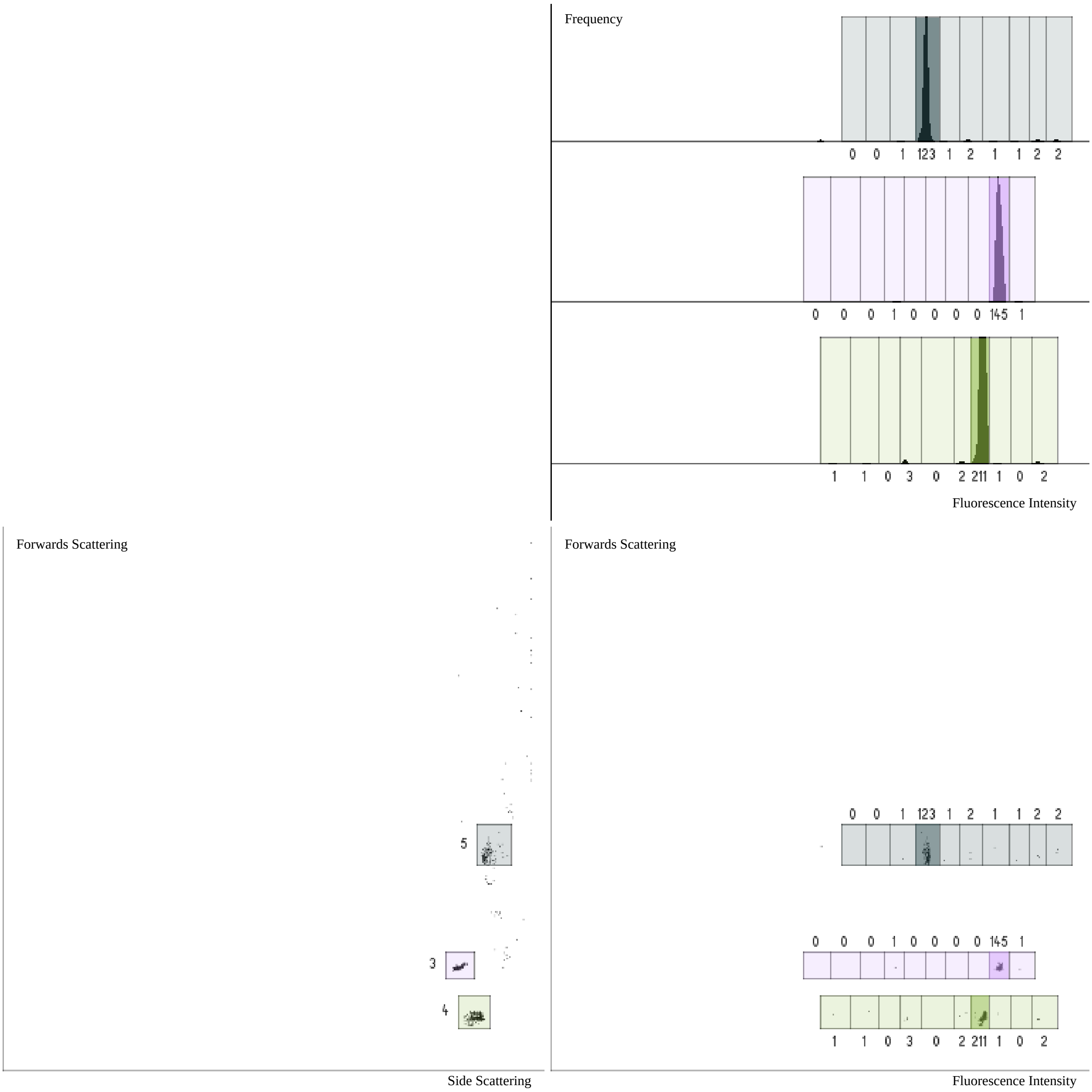

ANNEX 3: TAG DECONVOLUTION - BEAD 131

Passes flow sorting criteria: Yes

Passes tag deconvolution criteria: Yes

Included in protocol analysis: Yes

Protocol: 8, 7, 6, 5

Filename: Bin5_plateA4_B10.fcs

Split 1: Petrol shading

Split 2: Green shading

Split 3: Violet shading

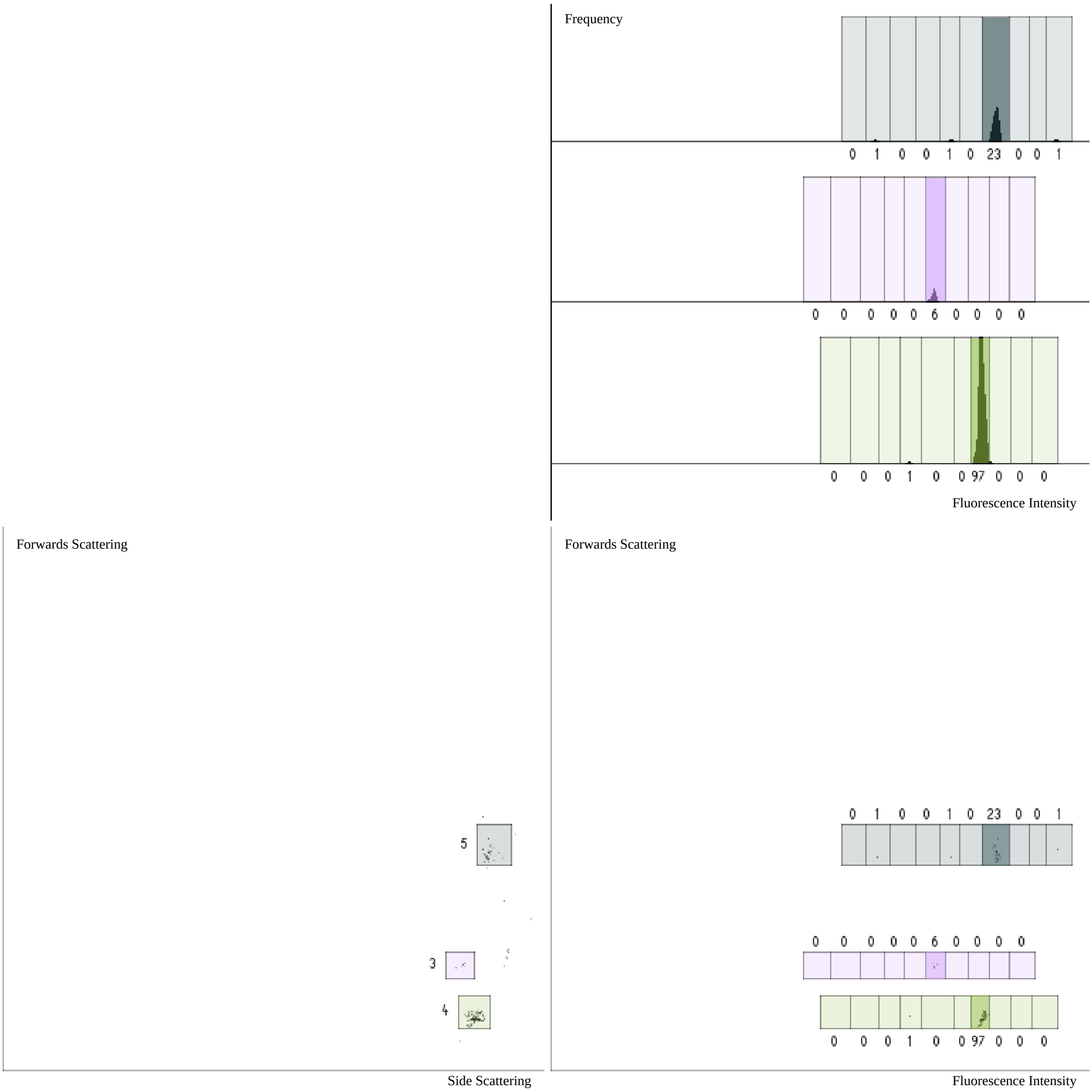

ANNEX 3: TAG DECONVOLUTION - BEAD 132

Passes flow sorting criteria: Yes

Passes tag deconvolution criteria: Yes

Included in protocol analysis: Yes

Protocol: 5, 9, 5, 5

Filename: Bin5_plateA4_E7.fcs

Split 1: Petrol shading

Split 2: Green shading

Split 3: Violet shading

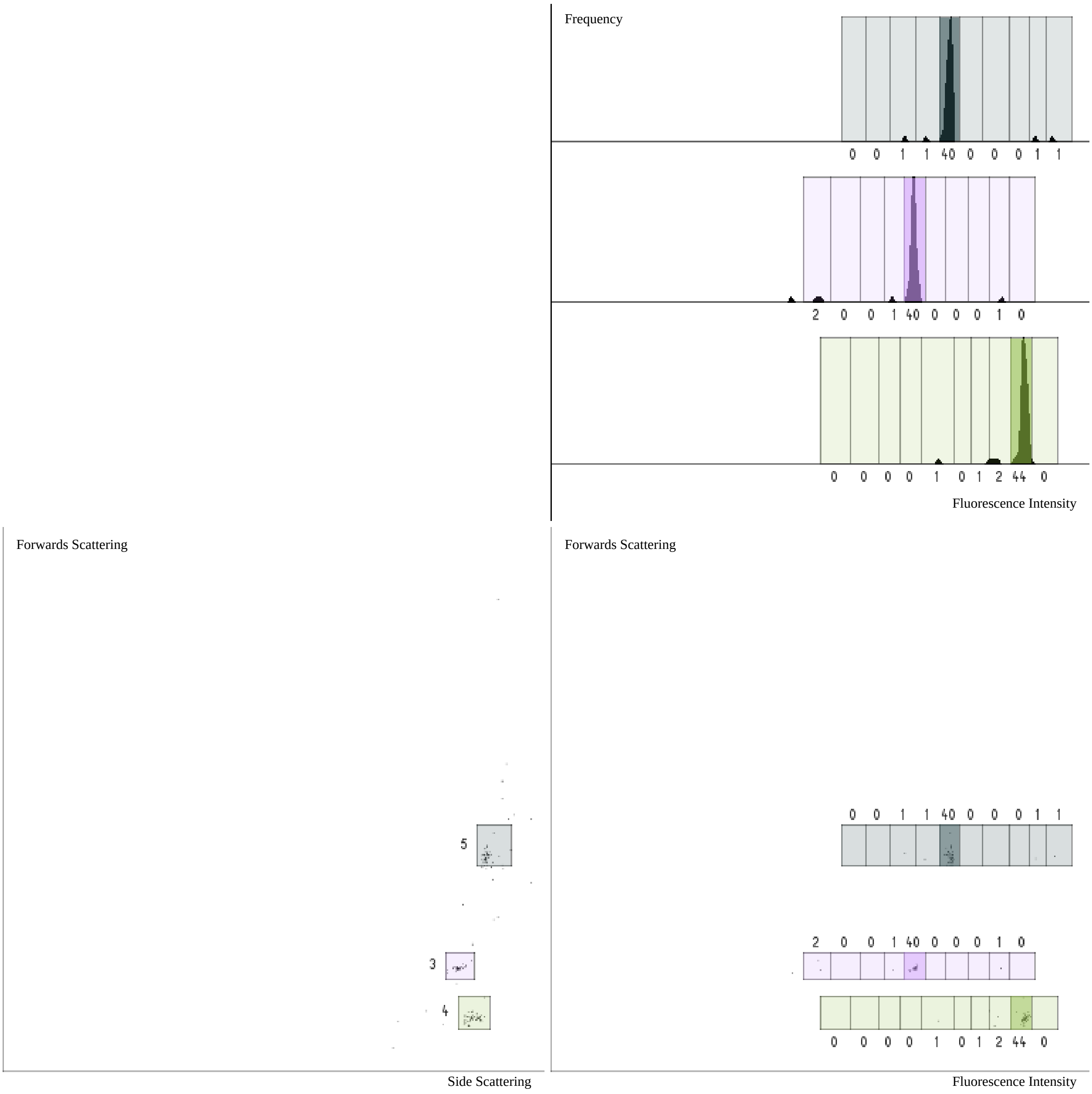

ANNEX 3: TAG DECONVOLUTION - BEAD 133

Passes flow sorting criteria: Yes

Passes tag deconvolution criteria: Yes

Included in protocol analysis: Yes

Protocol: 5, 3, 2, 5

Filename: Bin5_plateA4_E10.fcs

Split 1: Petrol shading

Split 2: Green shading

Split 3: Violet shading

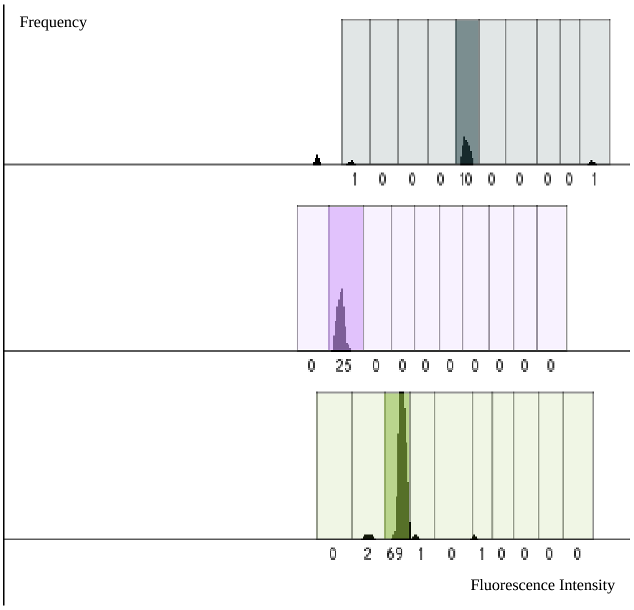

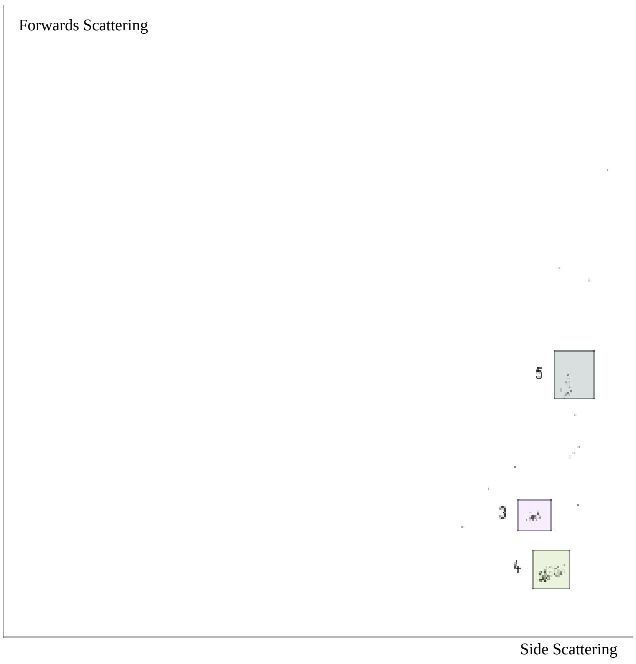

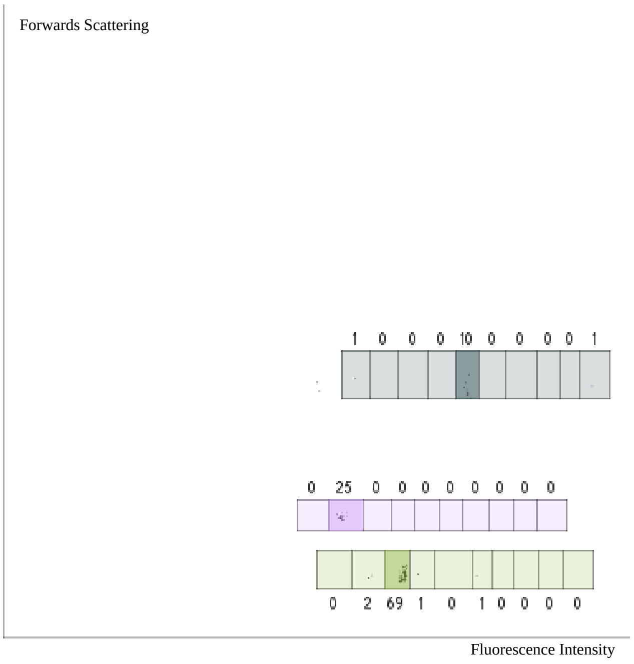

ANNEX 3: TAG DECONVOLUTION - BEAD 134

Passes flow sorting criteria: Yes

Passes tag deconvolution criteria: Yes

Included in protocol analysis: Yes

Protocol: 5, 6, 7, 5

Filename: Bin5_plateA4_F10.fcs

Split 1: Petrol shading

Split 2: Green shading

Split 3: Violet shading

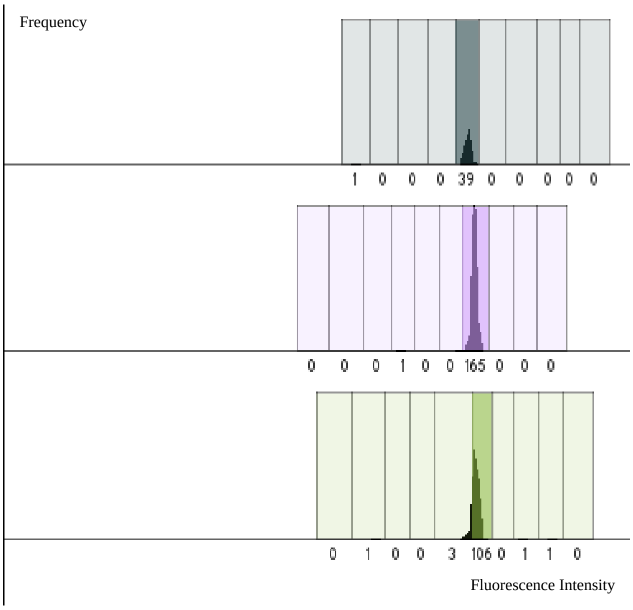

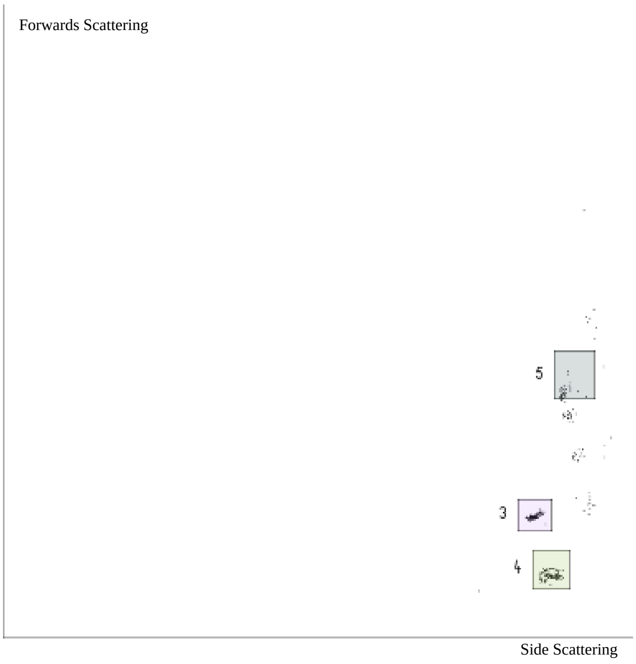

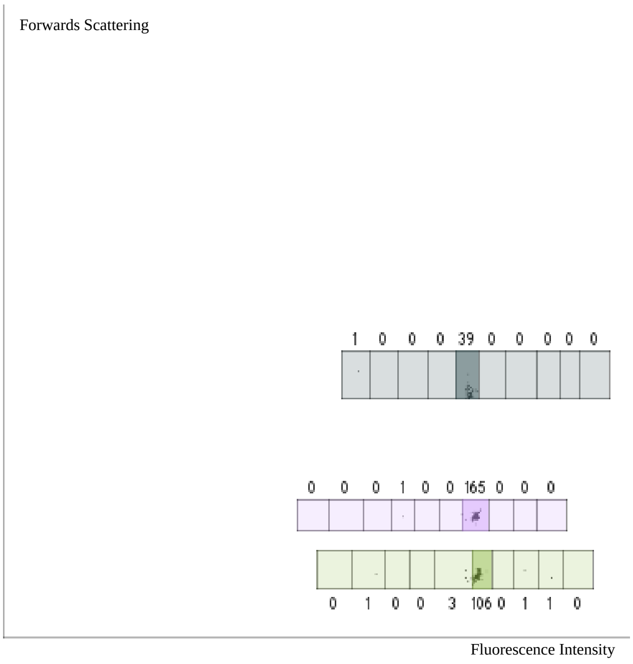

ANNEX 3: TAG DECONVOLUTION - BEAD 135

Passes flow sorting criteria: Yes

Passes tag deconvolution criteria: Yes

Included in protocol analysis: Yes

Protocol: 3, 4, 9, 5

Filename: Bin5_plateA4_F11.fcs

Split 1: Petrol shading

Split 2: Green shading

Split 3: Violet shading

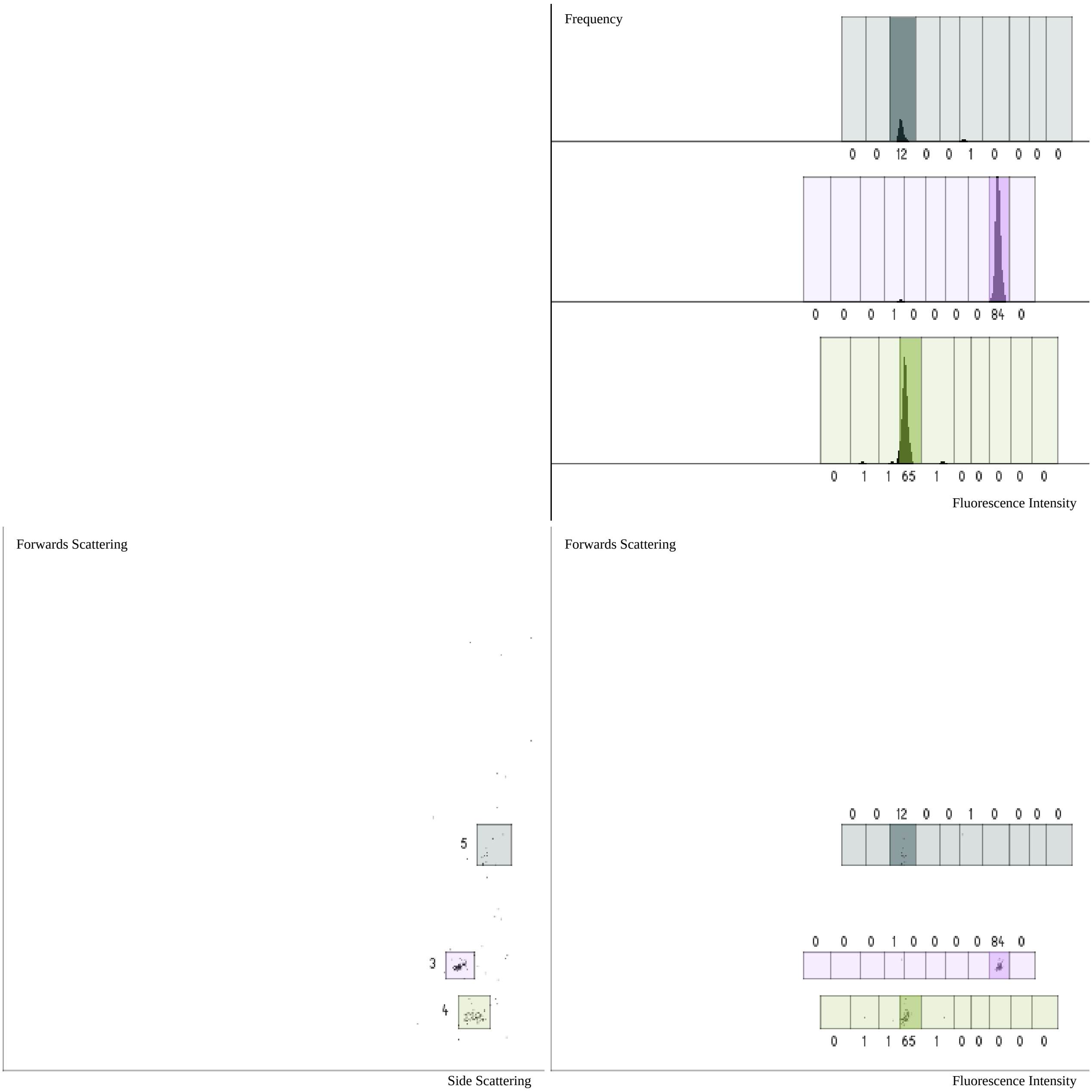

ANNEX 3: TAG DECONVOLUTION - BEAD 136

Passes flow sorting criteria: Yes

Passes tag deconvolution criteria: Yes

Included in protocol analysis: Yes

Protocol: 5, 6, 7, 5

Filename: Bin5_plateA4_G3.fcs

Split 1: Petrol shading

Split 2: Green shading

Split 3: Violet shading

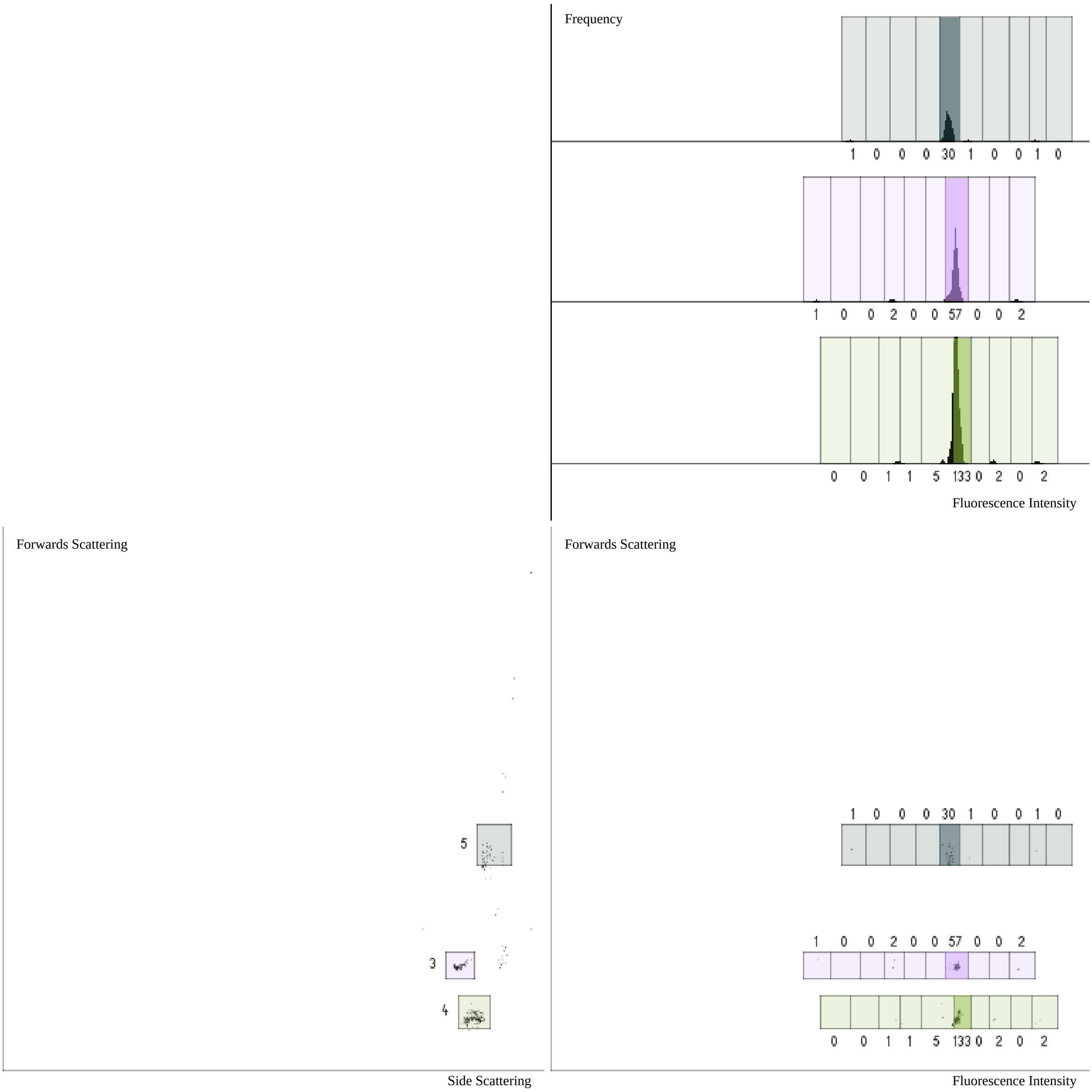

ANNEX 3: TAG DECONVOLUTION - BEAD 137

Passes flow sorting criteria: Yes

Passes tag deconvolution criteria: Yes

Included in protocol analysis: Yes

Protocol: 4, 2, 8, 5

Filename: Bin5_plateA4_G6.fcs

Split 1: Petrol shading

Split 2: Green shading

Split 3: Violet shading

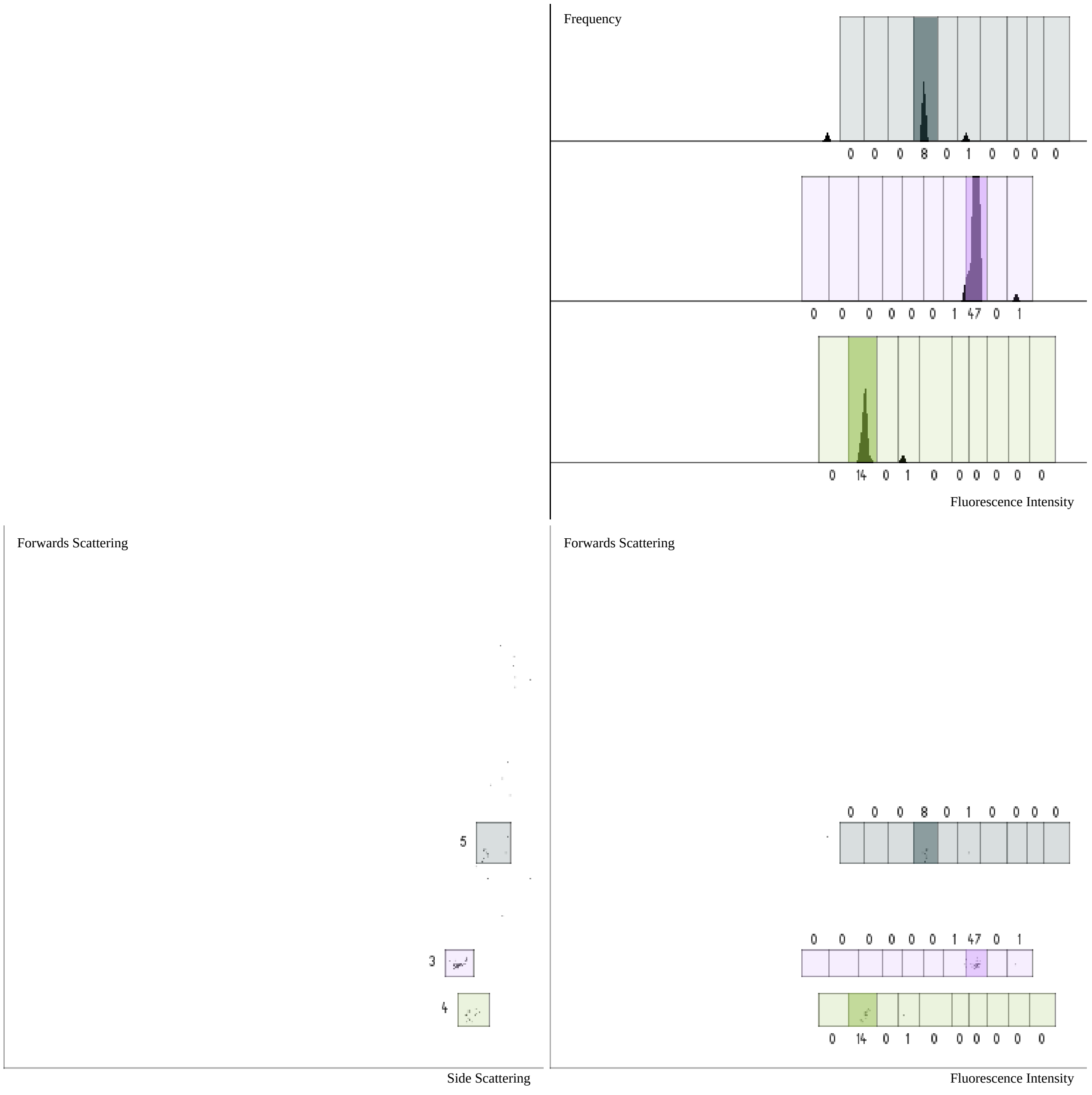

ANNEX 3: TAG DECONVOLUTION - BEAD 138

Passes flow sorting criteria: Yes

Passes tag deconvolution criteria: Yes

Included in protocol analysis: Yes

Protocol: 3, 9, 6, 5

Filename: Bin5_plateA4_G7.fcs

Split 1: Petrol shading

Split 2: Green shading

Split 3: Violet shading

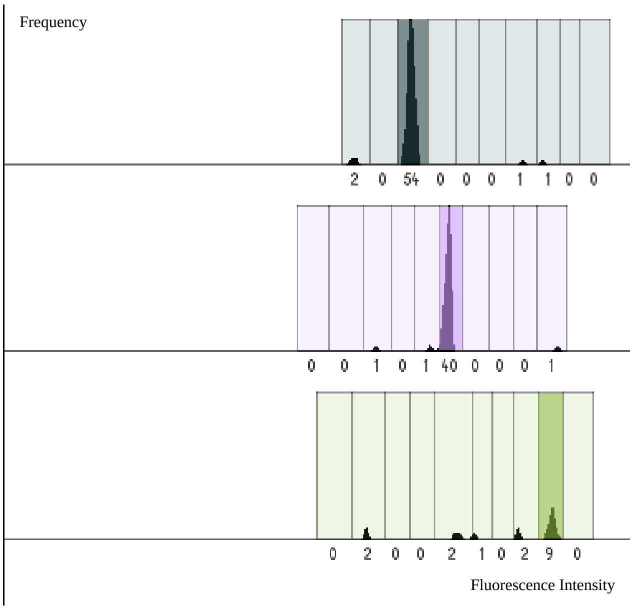

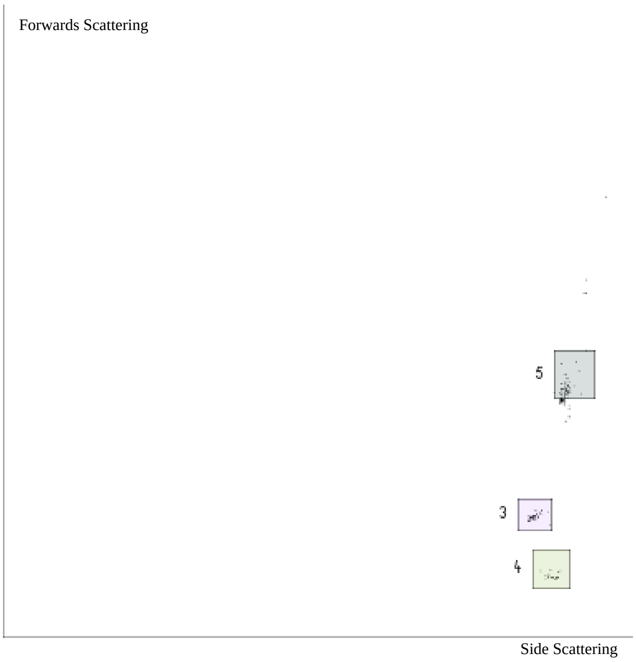

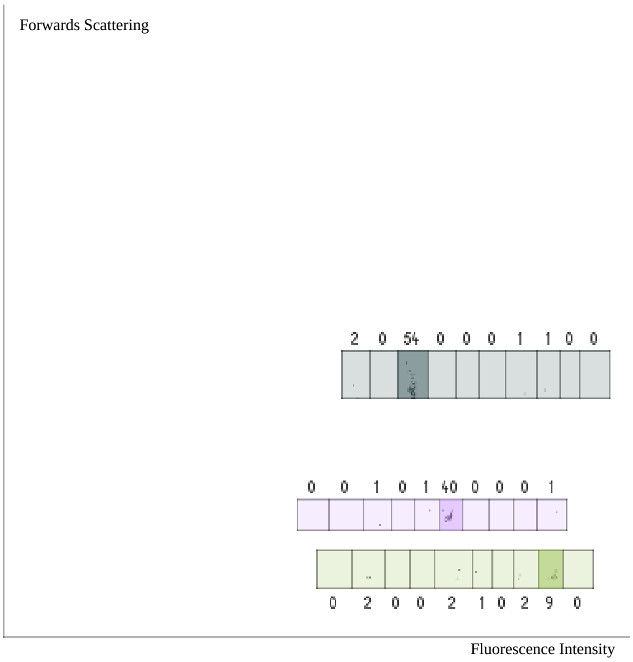

ANNEX 3: TAG DECONVOLUTION - BEAD 139

Passes flow sorting criteria: Yes

Passes tag deconvolution criteria: Yes

Included in protocol analysis: Yes

Protocol: 10, 4, 4, 5

Filename: Bin5_plateA4_G8.fcs

Split 1: Petrol shading

Split 2: Green shading

Split 3: Violet shading

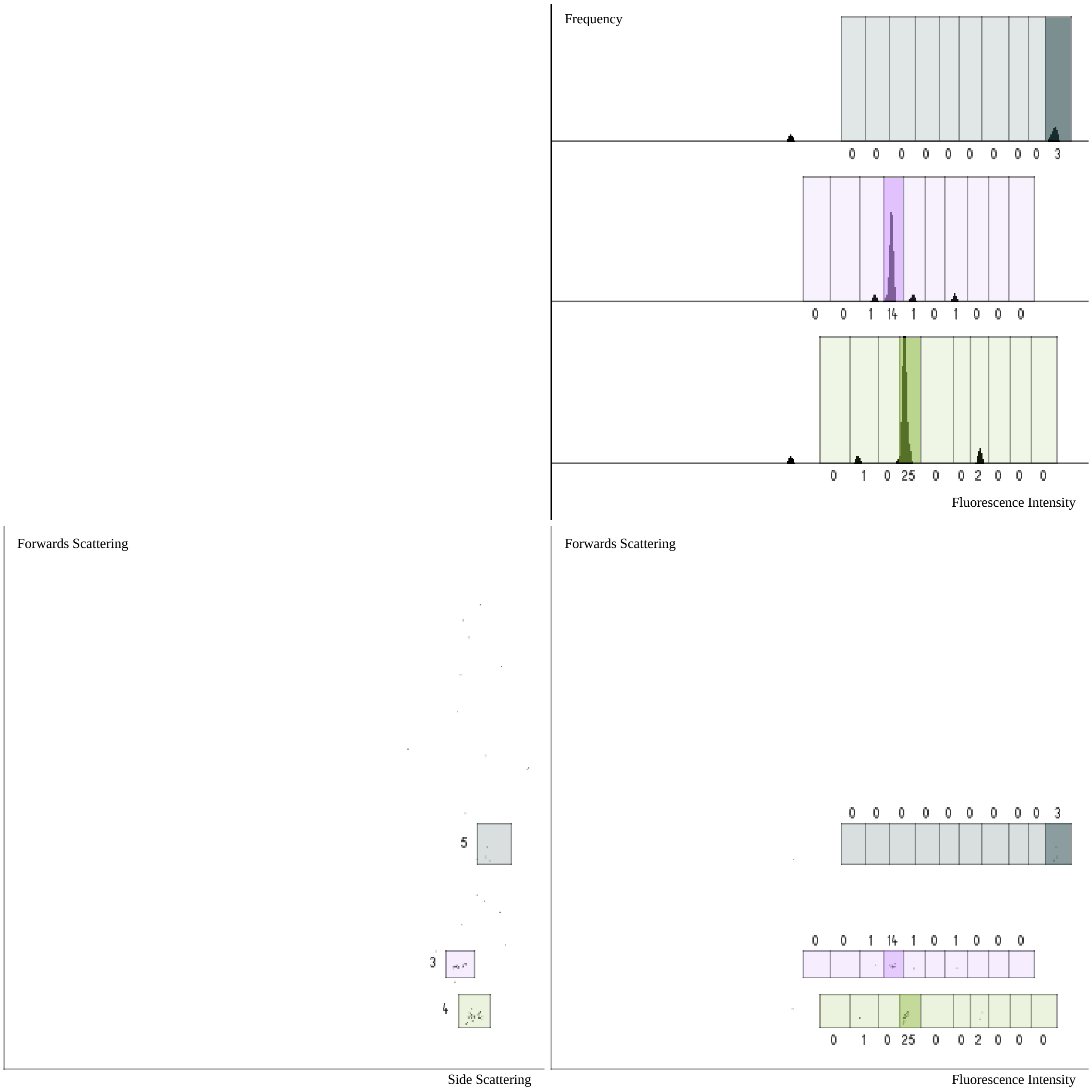

ANNEX 3: TAG DECONVOLUTION - BEAD 140

Passes flow sorting criteria: Yes

Passes tag deconvolution criteria: Yes

Included in protocol analysis: Yes

Protocol: 6, 7, 2, 5

Filename: Bin5_plateA4_H9.fcs

Split 1: Petrol shading

Split 2: Green shading

Split 3: Violet shading

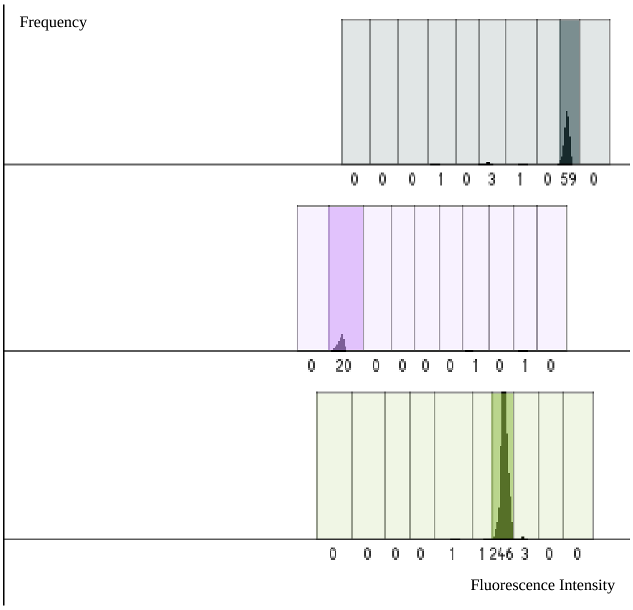

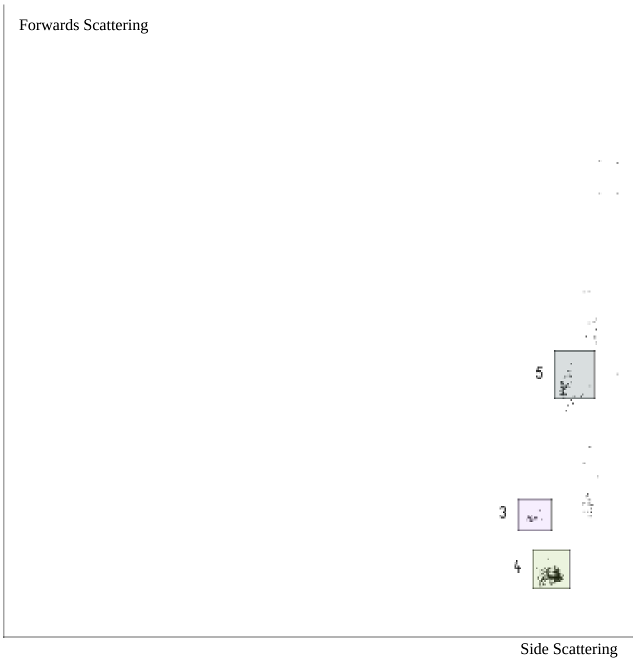

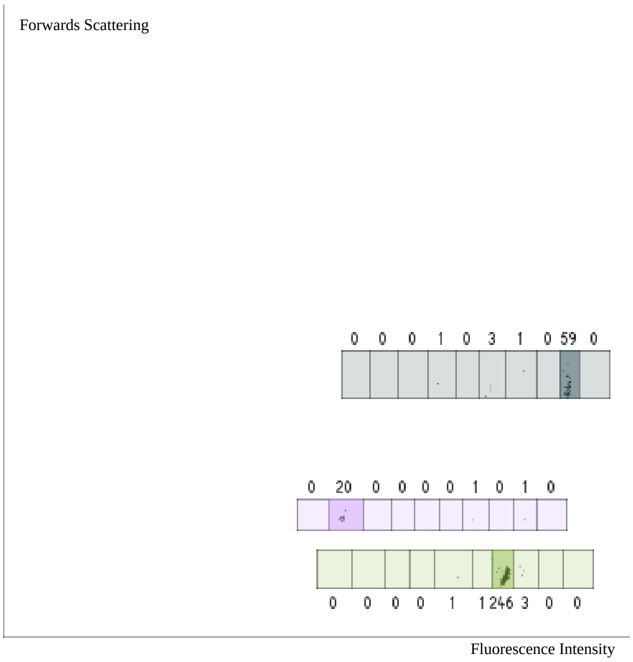

ANNEX 3: TAG DECONVOLUTION - BEAD 141

Passes flow sorting criteria: Yes

Passes tag deconvolution criteria: Yes

Included in protocol analysis: Yes

Protocol: 10, 4, 2, 5

Filename: Bin5_plateA5_A1.fcs

Split 1: Petrol shading

Split 2: Green shading

Split 3: Violet shading

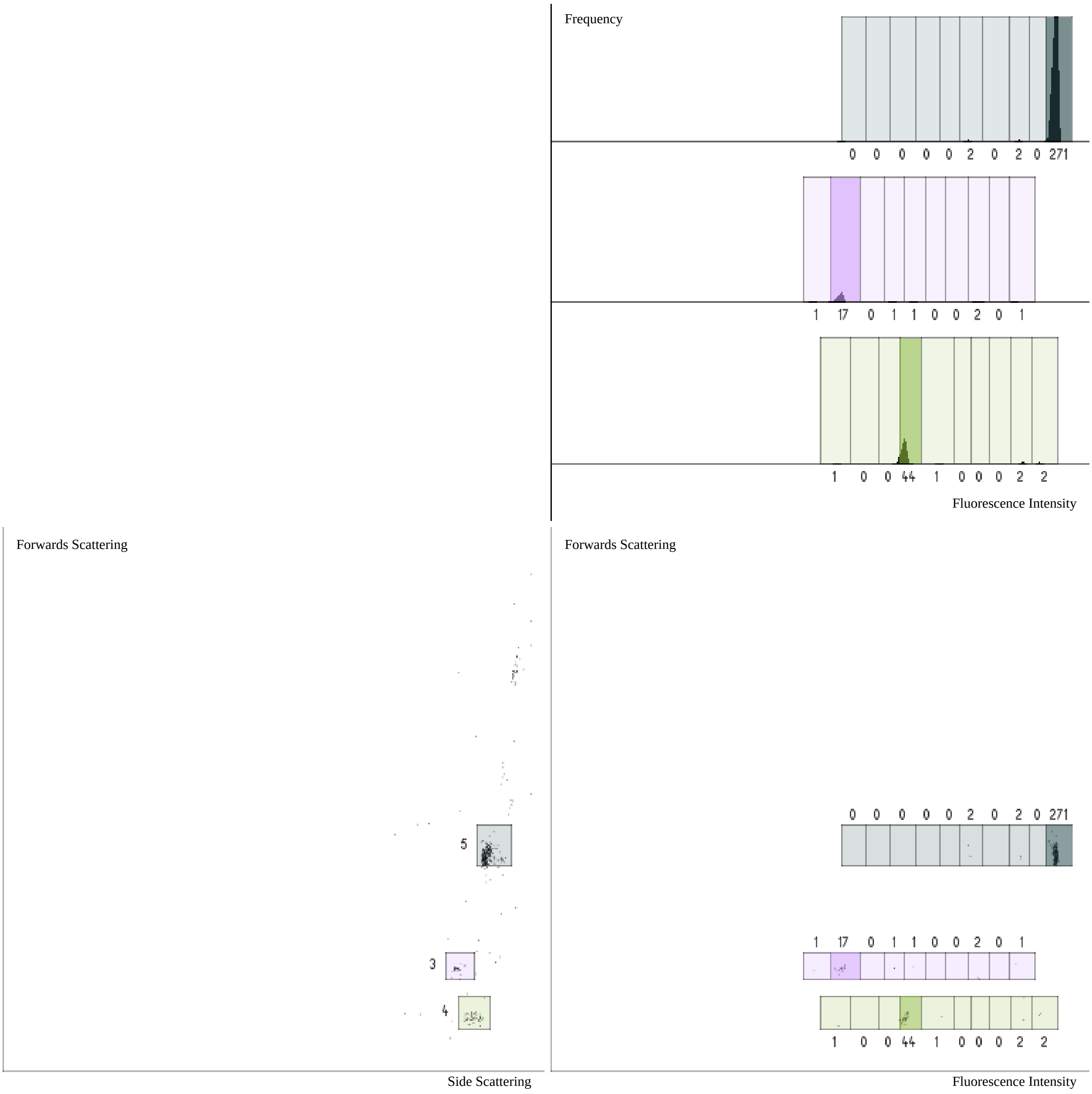

ANNEX 3: TAG DECONVOLUTION - BEAD 142

Passes flow sorting criteria: Yes

Passes tag deconvolution criteria: Yes

Included in protocol analysis: Yes

Protocol: 4, 10, 3, 5

Filename: Bin5_plateA5_A6.fcs

Split 1: Petrol shading

Split 2: Green shading

Split 3: Violet shading

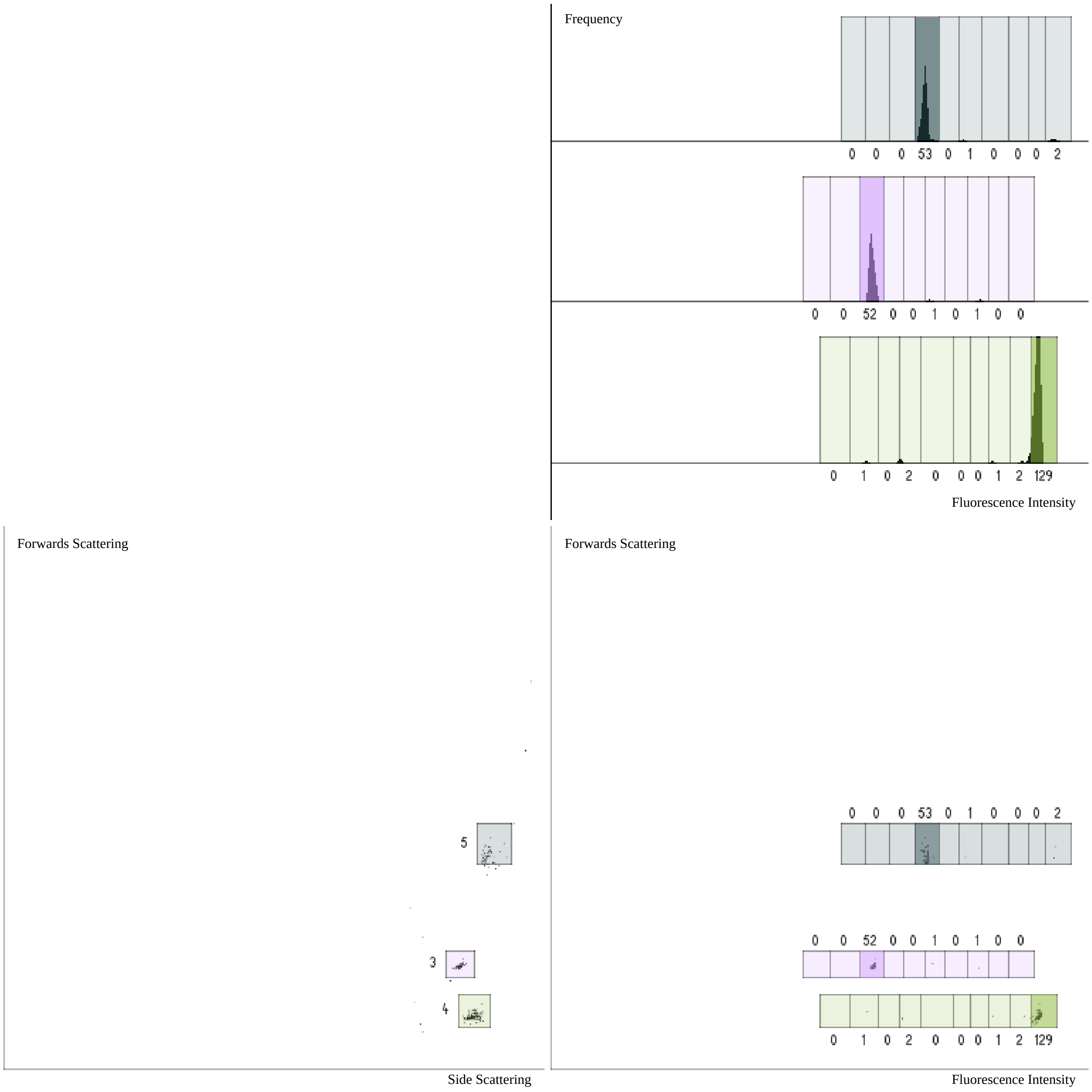

ANNEX 3: TAG DECONVOLUTION - BEAD 143

Passes flow sorting criteria: Yes

Passes tag deconvolution criteria: Yes

Included in protocol analysis: Yes

Protocol: 3, 7, 3, 5

Filename: Bin5_plateA5_B3.fcs

Split 1: Petrol shading

Split 2: Green shading

Split 3: Violet shading

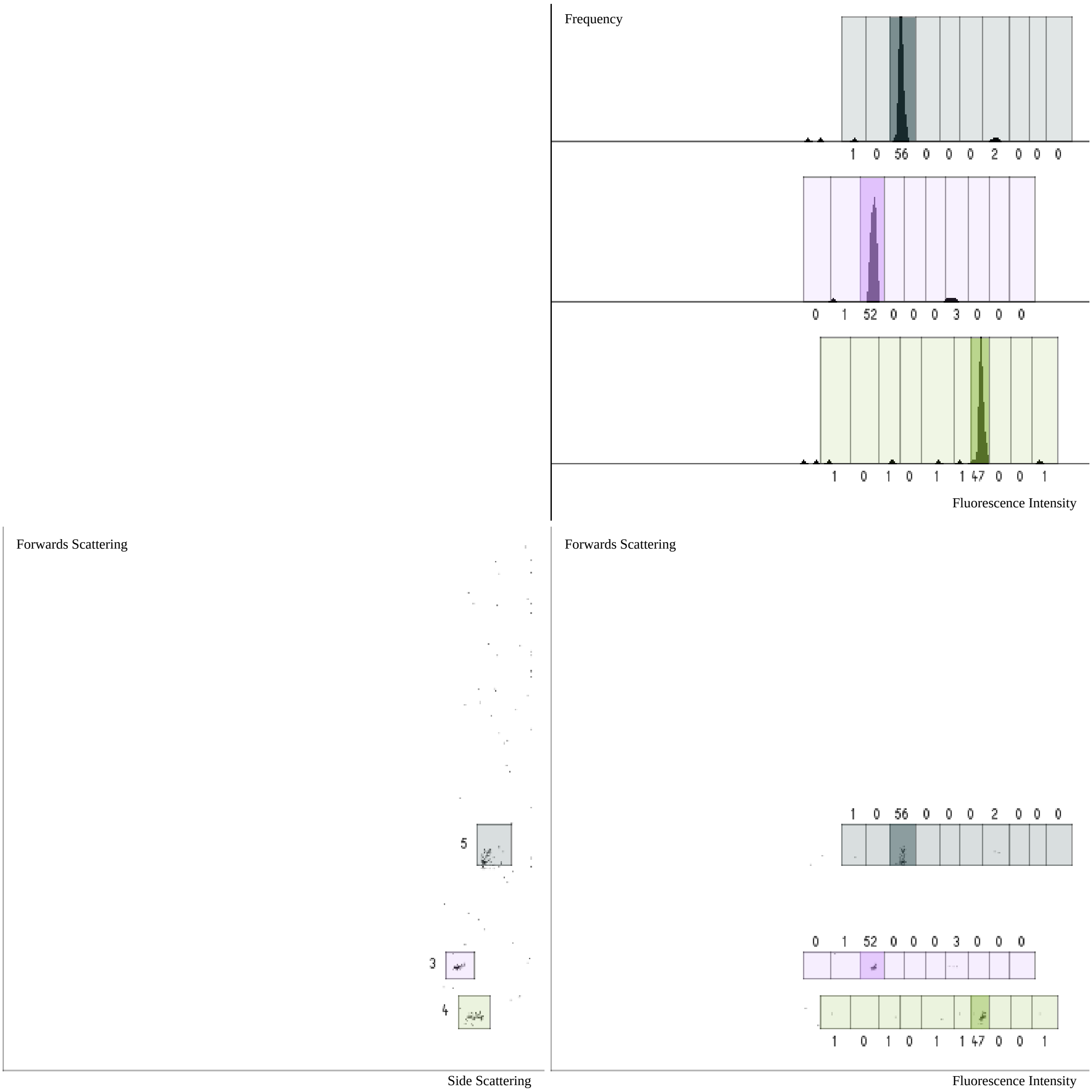

ANNEX 3: TAG DECONVOLUTION - BEAD 144

Passes flow sorting criteria: Yes

Passes tag deconvolution criteria: Yes

Included in protocol analysis: Yes

Protocol: 1, 6, 7, 5

Filename: Bin5_plateA5_B9.fcs

Split 1: Petrol shading

Split 2: Green shading

Split 3: Violet shading

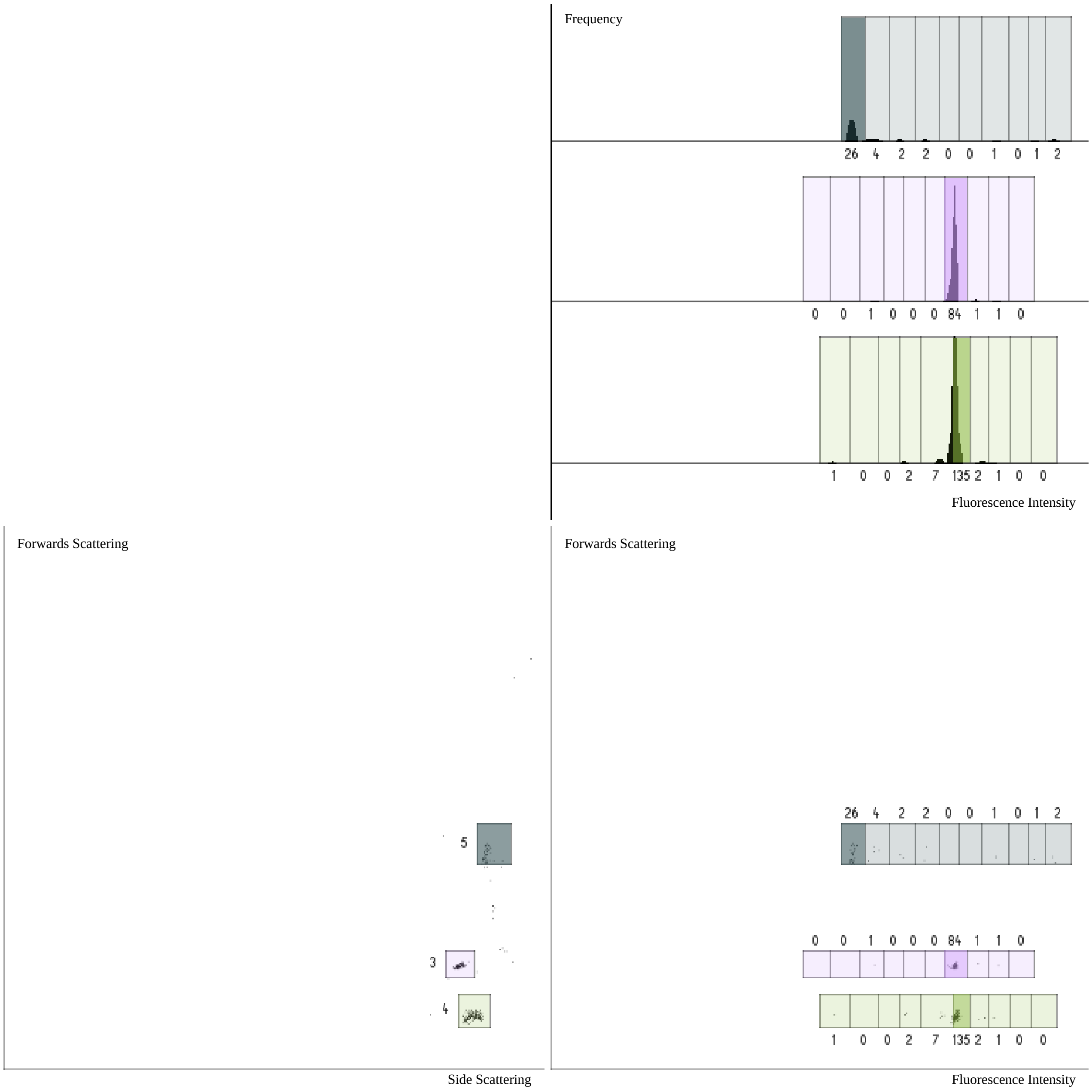

ANNEX 3: TAG DECONVOLUTION - BEAD 145

Passes flow sorting criteria: Yes

Passes tag deconvolution criteria: Yes

Included in protocol analysis: Yes

Protocol: 8, 3, 7, 5

Filename: Bin5_plateA5_B11.fcs

Split 1: Petrol shading

Split 2: Green shading

Split 3: Violet shading

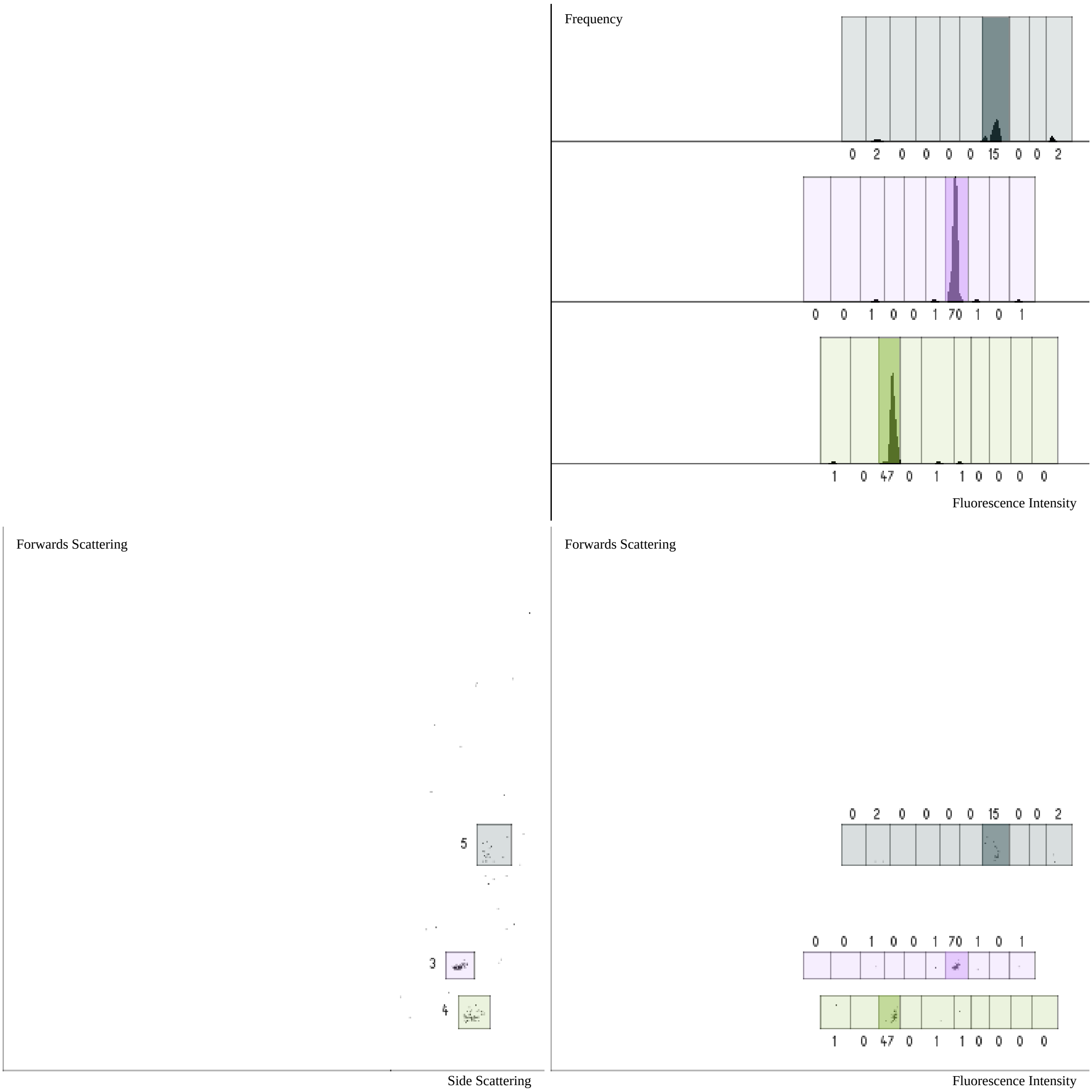

ANNEX 3: TAG DECONVOLUTION - BEAD 146

Passes flow sorting criteria: Yes

Passes tag deconvolution criteria: Yes

Included in protocol analysis: Yes

Protocol: 3, 1, 10, 5

Filename: Bin5_plateA5_C4.fcs

Split 1: Petrol shading

Split 2: Green shading

Split 3: Violet shading

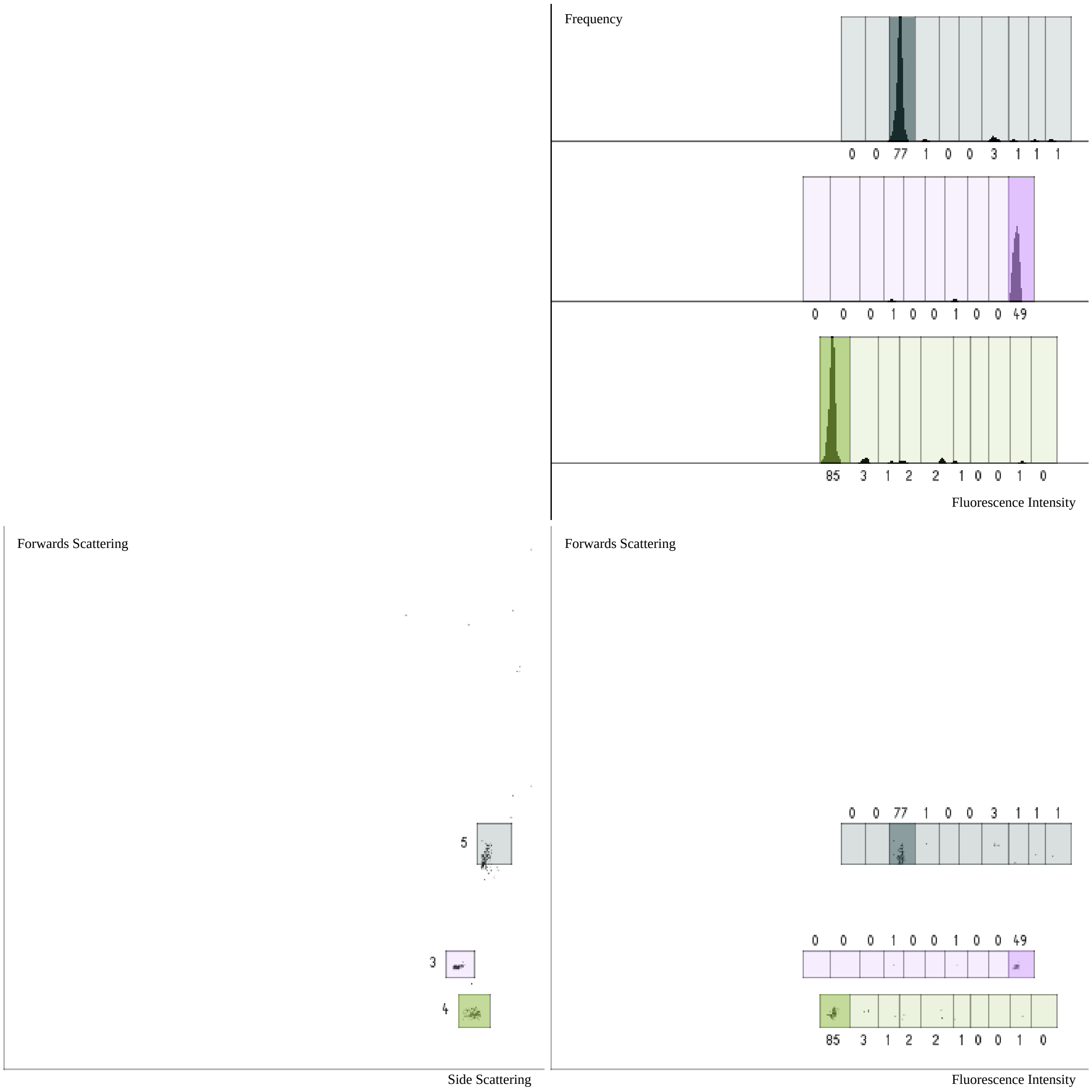

ANNEX 3: TAG DECONVOLUTION - BEAD 147

Passes flow sorting criteria: Yes

Passes tag deconvolution criteria: Yes

Included in protocol analysis: Yes

Protocol: 6, 1, 3, 5

Filename: Bin5_plateA5_C7.fcs

Split 1: Petrol shading

Split 2: Green shading

Split 3: Violet shading

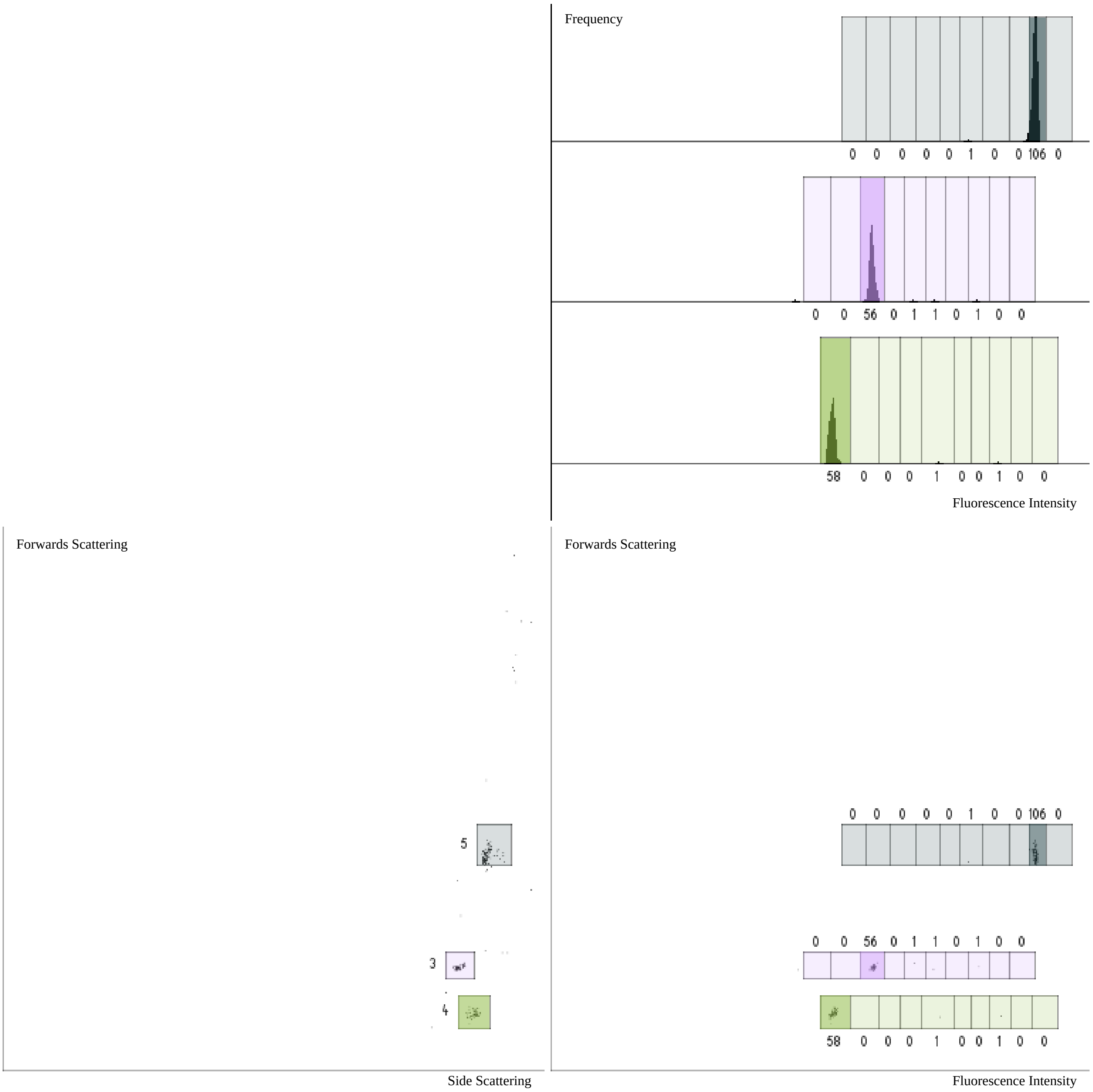

ANNEX 3: TAG DECONVOLUTION - BEAD 148

Passes flow sorting criteria: Yes

Passes tag deconvolution criteria: Yes

Included in protocol analysis: Yes

Protocol: 1, 7, 2, 5

Filename: Bin5_plateA5_C9.fcs

Split 1: Petrol shading

Split 2: Green shading

Split 3: Violet shading

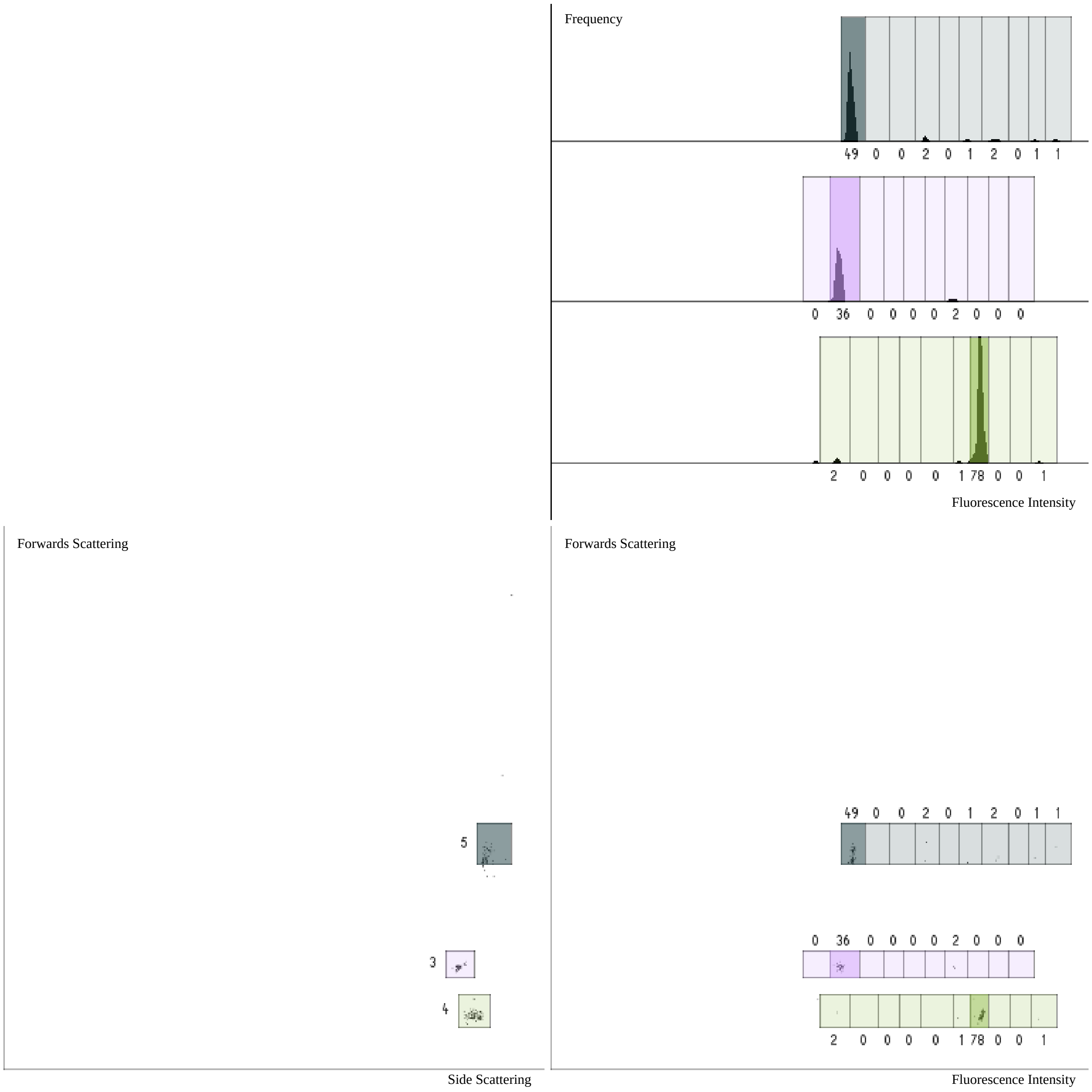

ANNEX 3: TAG DECONVOLUTION - BEAD 149

Passes flow sorting criteria: Yes

Passes tag deconvolution criteria: Yes

Included in protocol analysis: Yes

Protocol: 10, 4, 9, 5

Filename: Bin5_plateA5_C10.fcs

Split 1: Petrol shading

Split 2: Green shading

Split 3: Violet shading

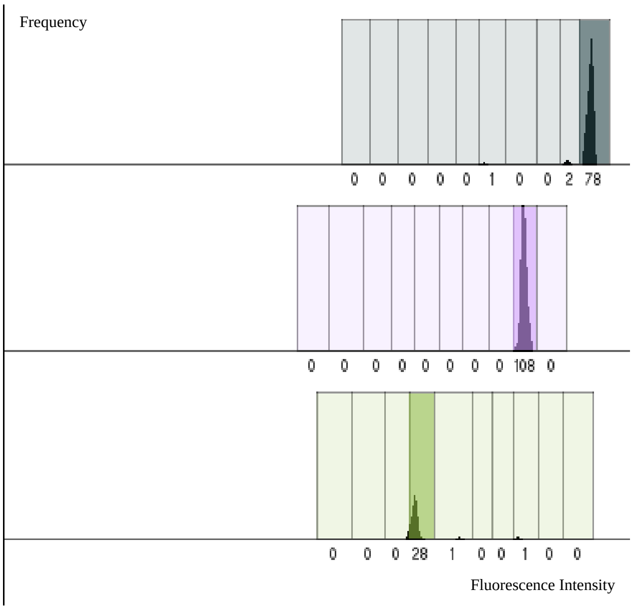

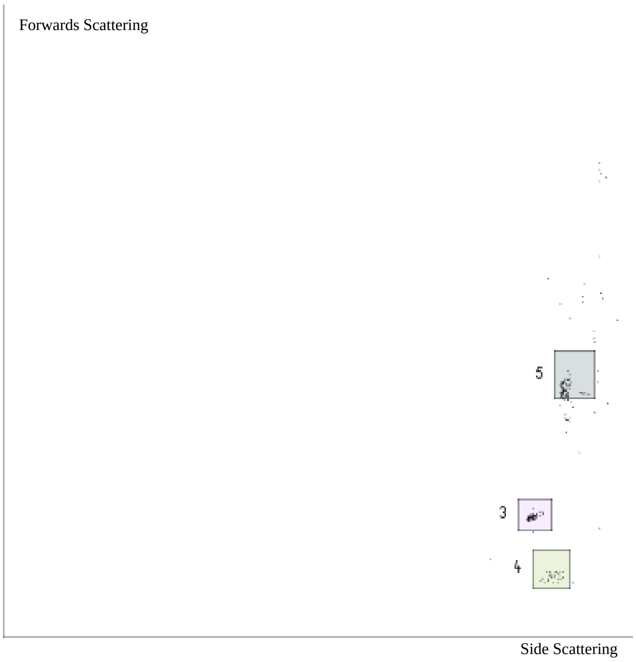

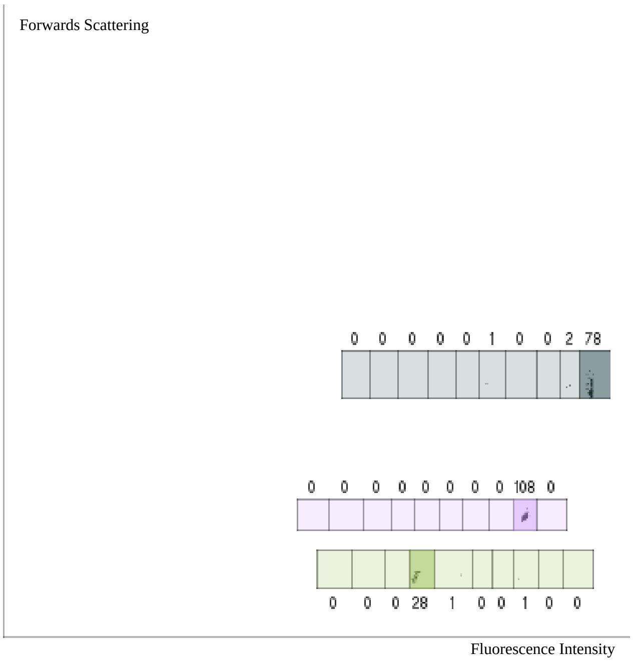

ANNEX 3: TAG DECONVOLUTION - BEAD 150

Passes flow sorting criteria: Yes

Passes tag deconvolution criteria: Yes

Included in protocol analysis: Yes

Protocol: 10, 5, 8, 5

Filename: Bin5_plateA5_C12.fcs

Split 1: Petrol shading

Split 2: Green shading

Split 3: Violet shading

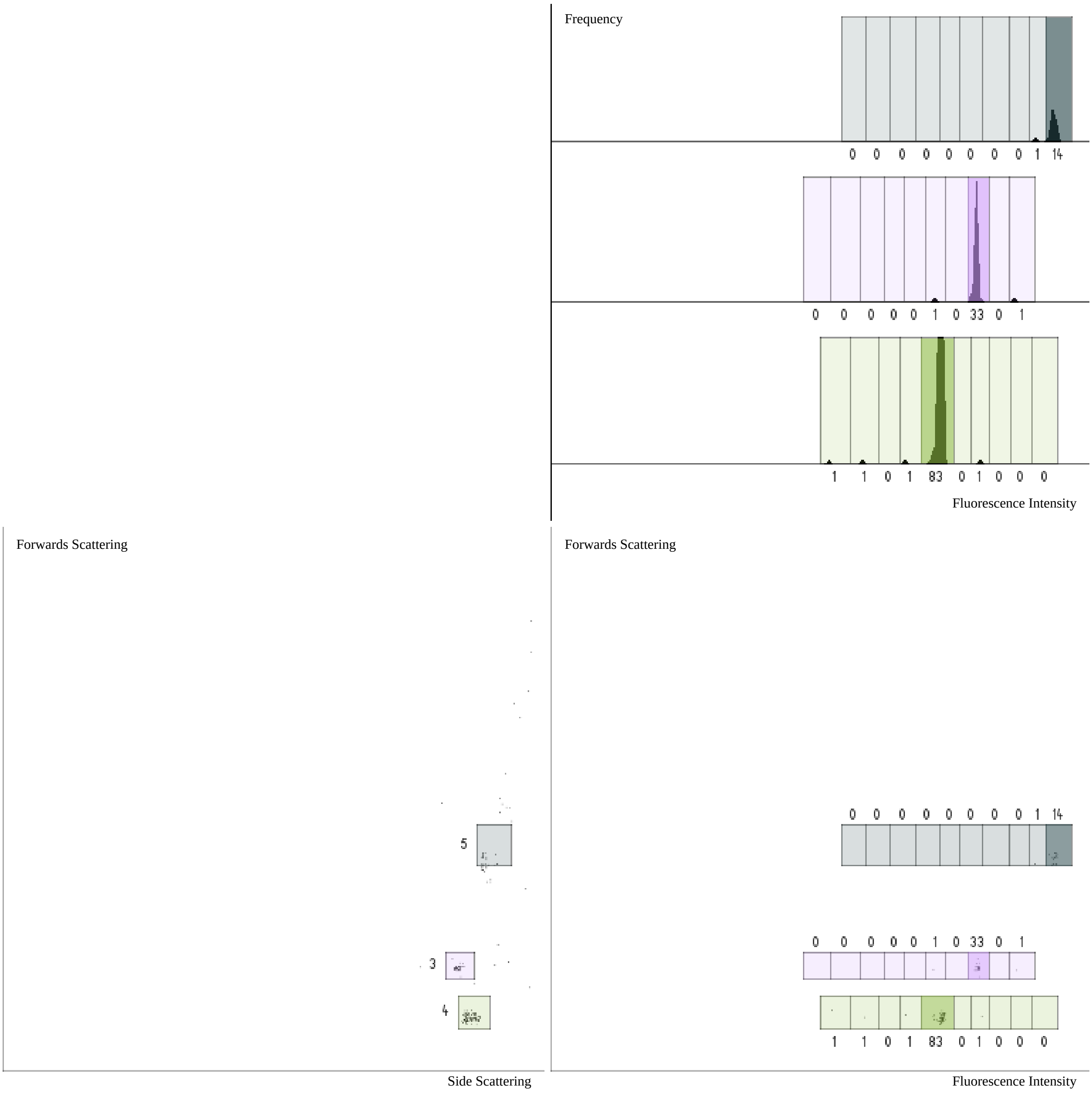

ANNEX 3: TAG DECONVOLUTION - BEAD 151

Passes flow sorting criteria: Yes

Passes tag deconvolution criteria: Yes

Included in protocol analysis: Yes

Protocol: 9, 4, 10, 5

Filename: Bin5_plateA5_D1.fcs

Split 1: Petrol shading

Split 2: Green shading

Split 3: Violet shading

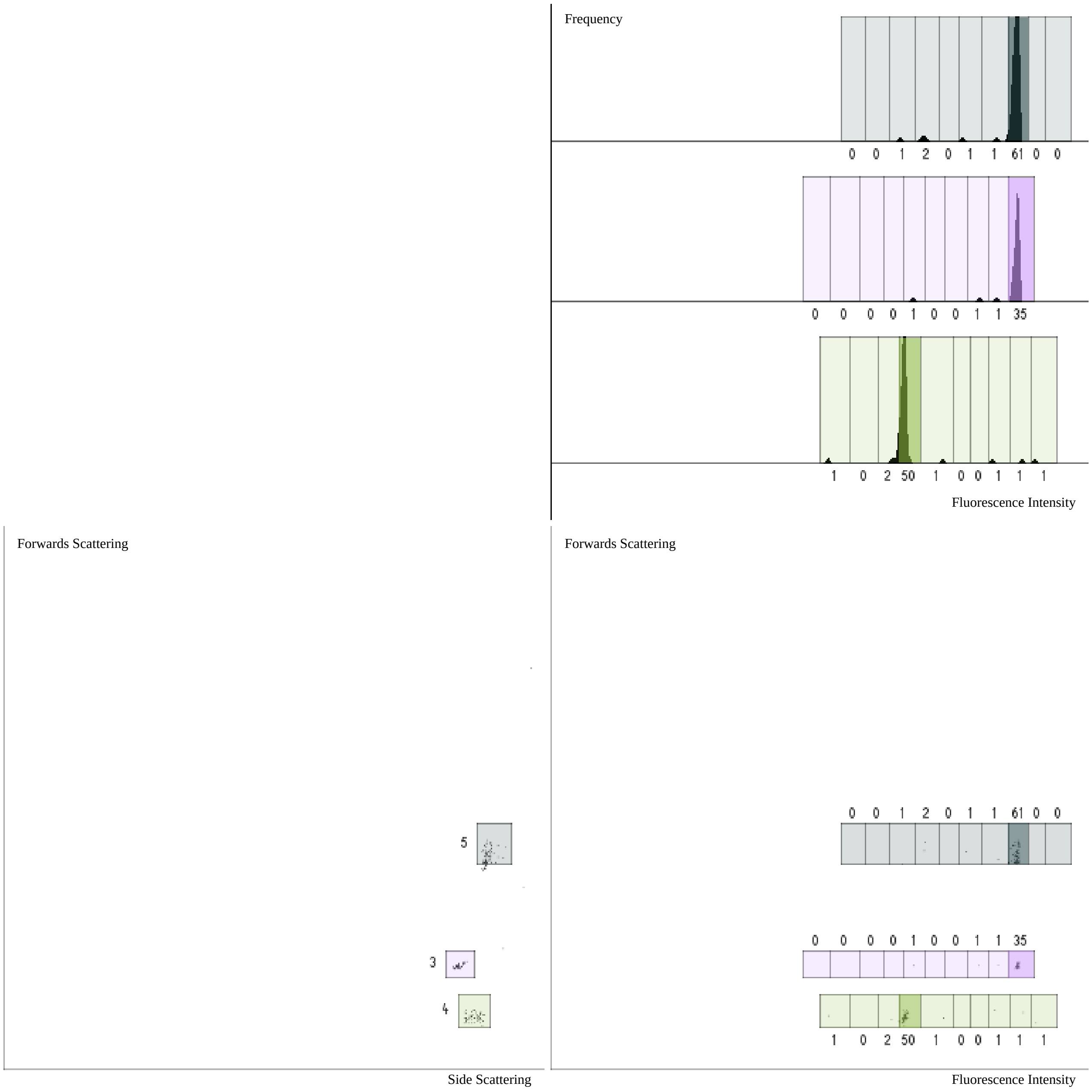

ANNEX 3: TAG DECONVOLUTION - BEAD 152

Passes flow sorting criteria: Yes

Passes tag deconvolution criteria: Yes

Included in protocol analysis: Yes

Protocol: 8, 1, 7, 5

Filename: Bin5_plateA5_D2.fcs

Split 1: Petrol shading

Split 2: Green shading

Split 3: Violet shading

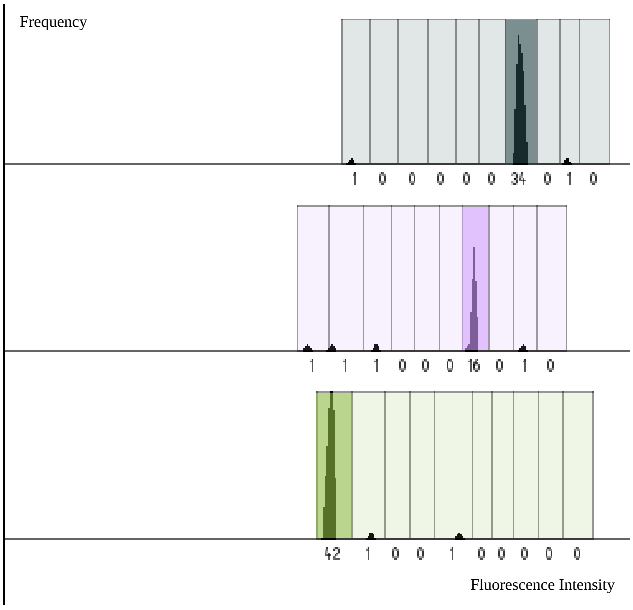

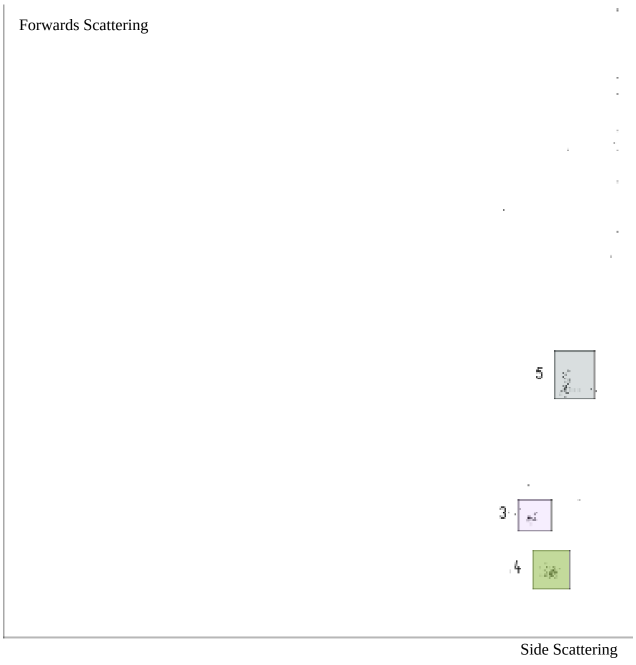

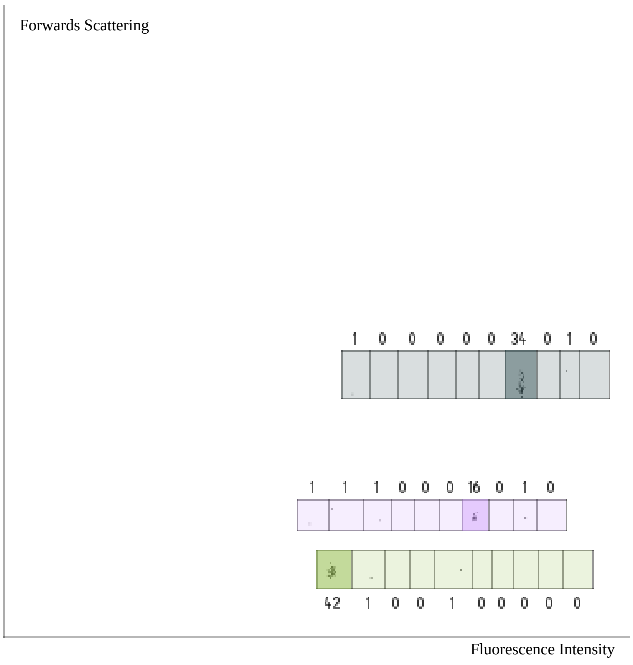

ANNEX 3: TAG DECONVOLUTION - BEAD 153

Passes flow sorting criteria: Yes

Passes tag deconvolution criteria: Yes

Included in protocol analysis: Yes

Protocol: 1, 6, 9, 5

Filename: Bin5_plateA5_D6.fcs

Split 1: Petrol shading

Split 2: Green shading

Split 3: Violet shading

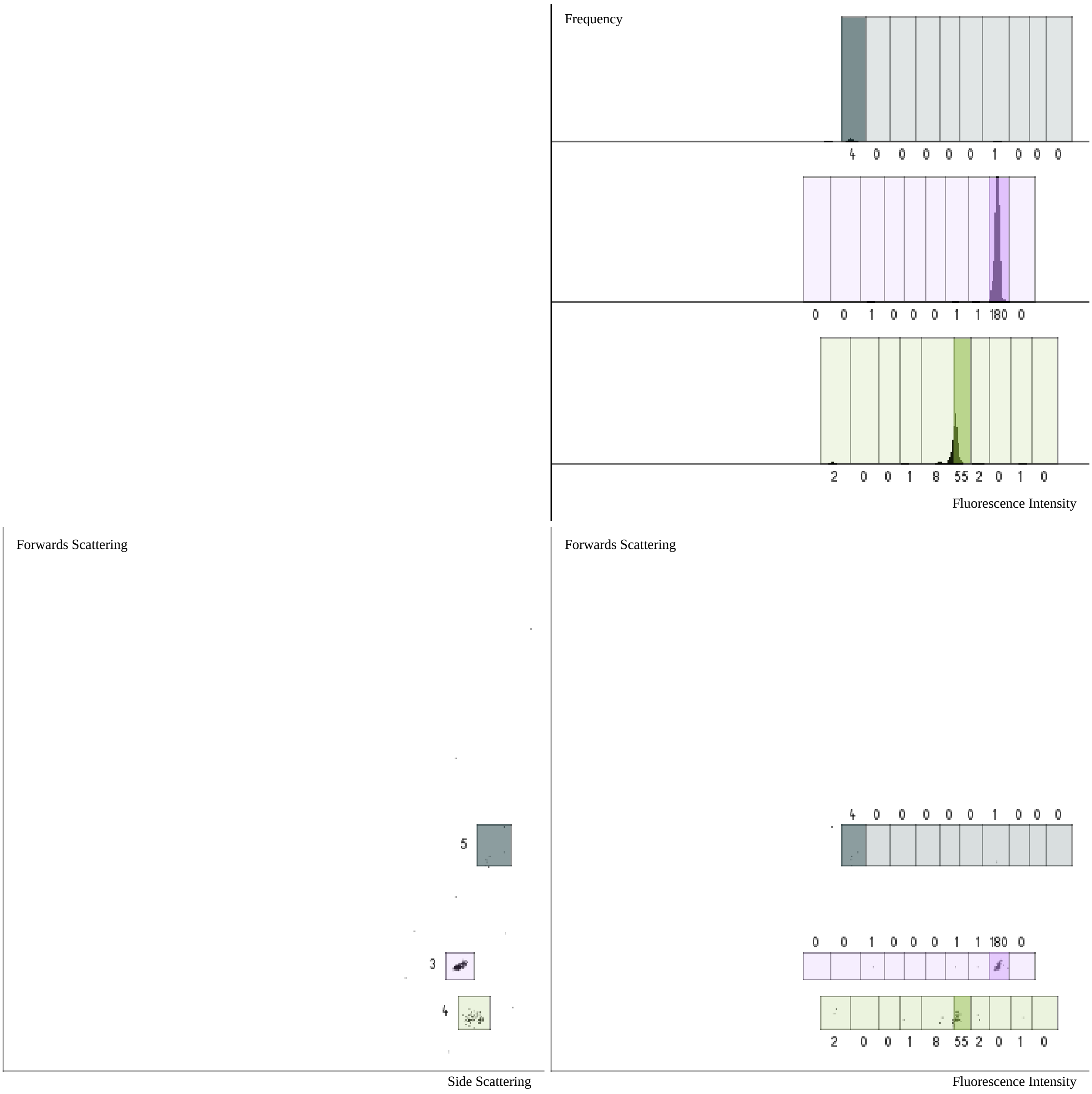

ANNEX 3: TAG DECONVOLUTION - BEAD 154

Passes flow sorting criteria: Yes

Passes tag deconvolution criteria: Yes

Included in protocol analysis: Yes

Protocol: 6, 7, 7, 5

Filename: Bin5_plateA5_E1.fcs

Split 1: Petrol shading

Split 2: Green shading

Split 3: Violet shading

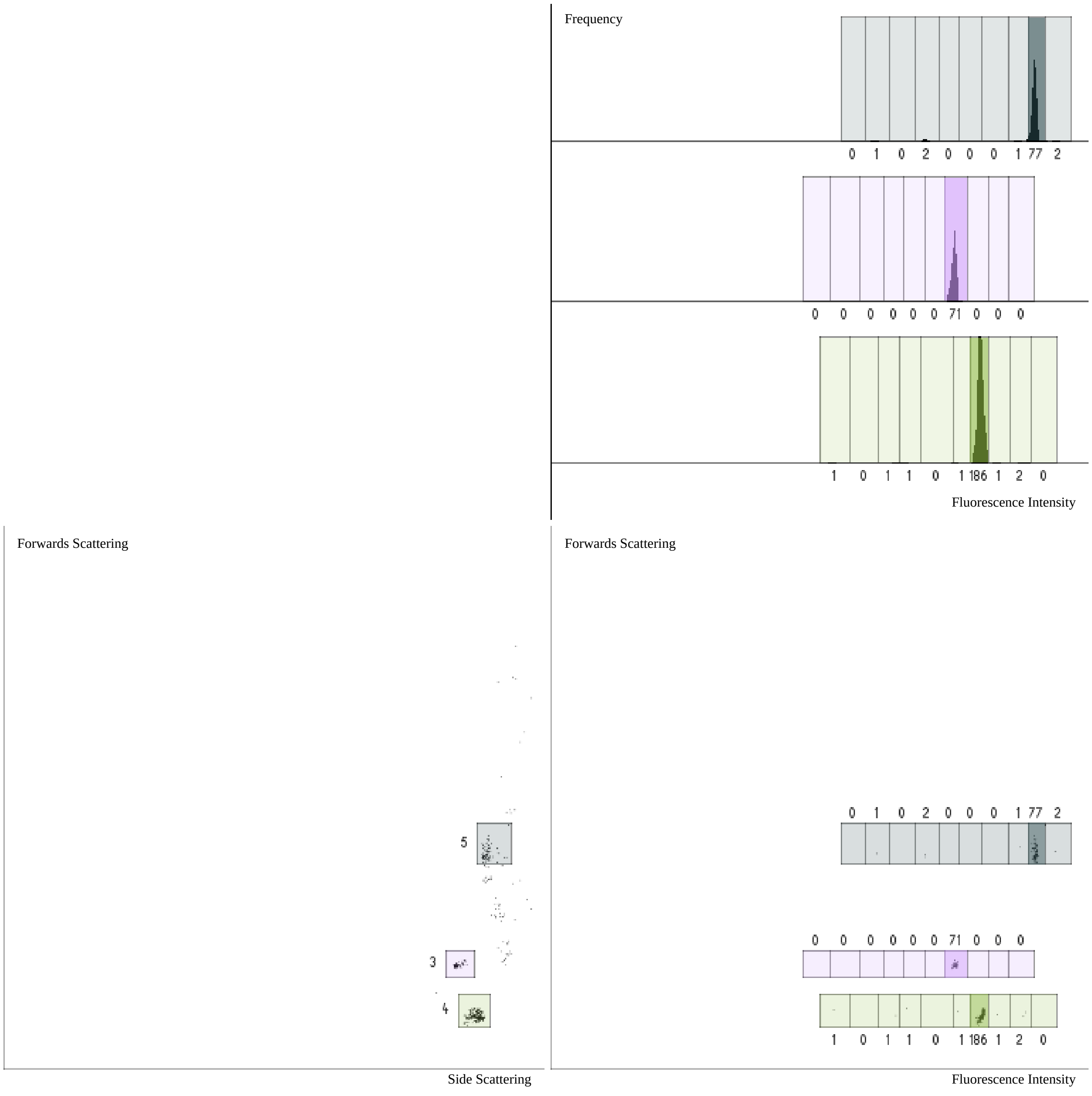

ANNEX 3: TAG DECONVOLUTION - BEAD 155

Passes flow sorting criteria: Yes

Passes tag deconvolution criteria: Yes

Included in protocol analysis: Yes

Protocol: 3, 10, 4, 5

Filename: Bin5_plateA5_E3.fcs

Split 1: Petrol shading

Split 2: Green shading

Split 3: Violet shading

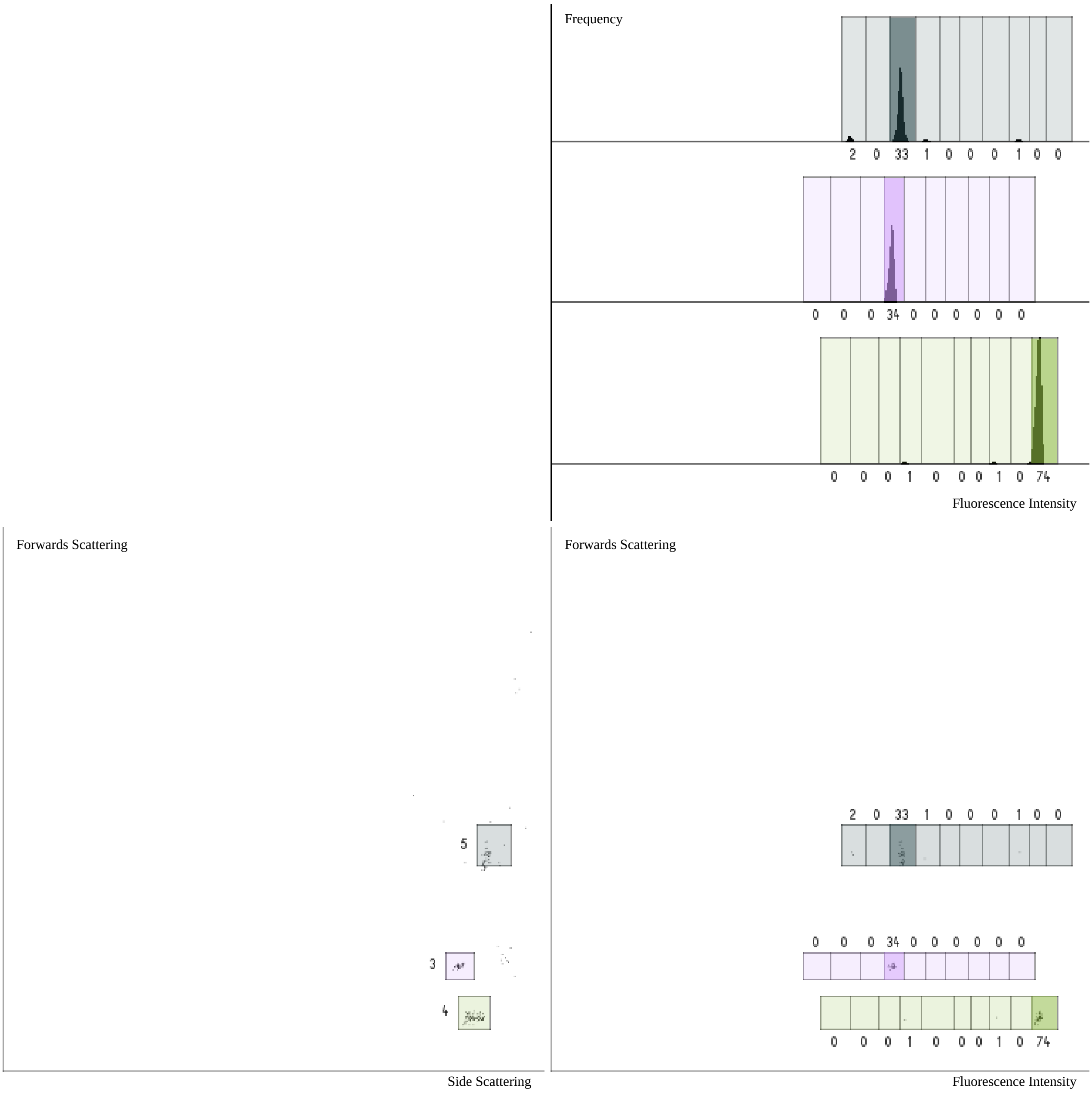

ANNEX 3: TAG DECONVOLUTION - BEAD 156

Passes flow sorting criteria: Yes

Passes tag deconvolution criteria: Yes

Included in protocol analysis: Yes

Protocol: 3, 9, 6, 6

Filename: Bin6_plateA5_A1.fcs

Split 1: Petrol shading

Split 2: Green shading

Split 3: Violet shading

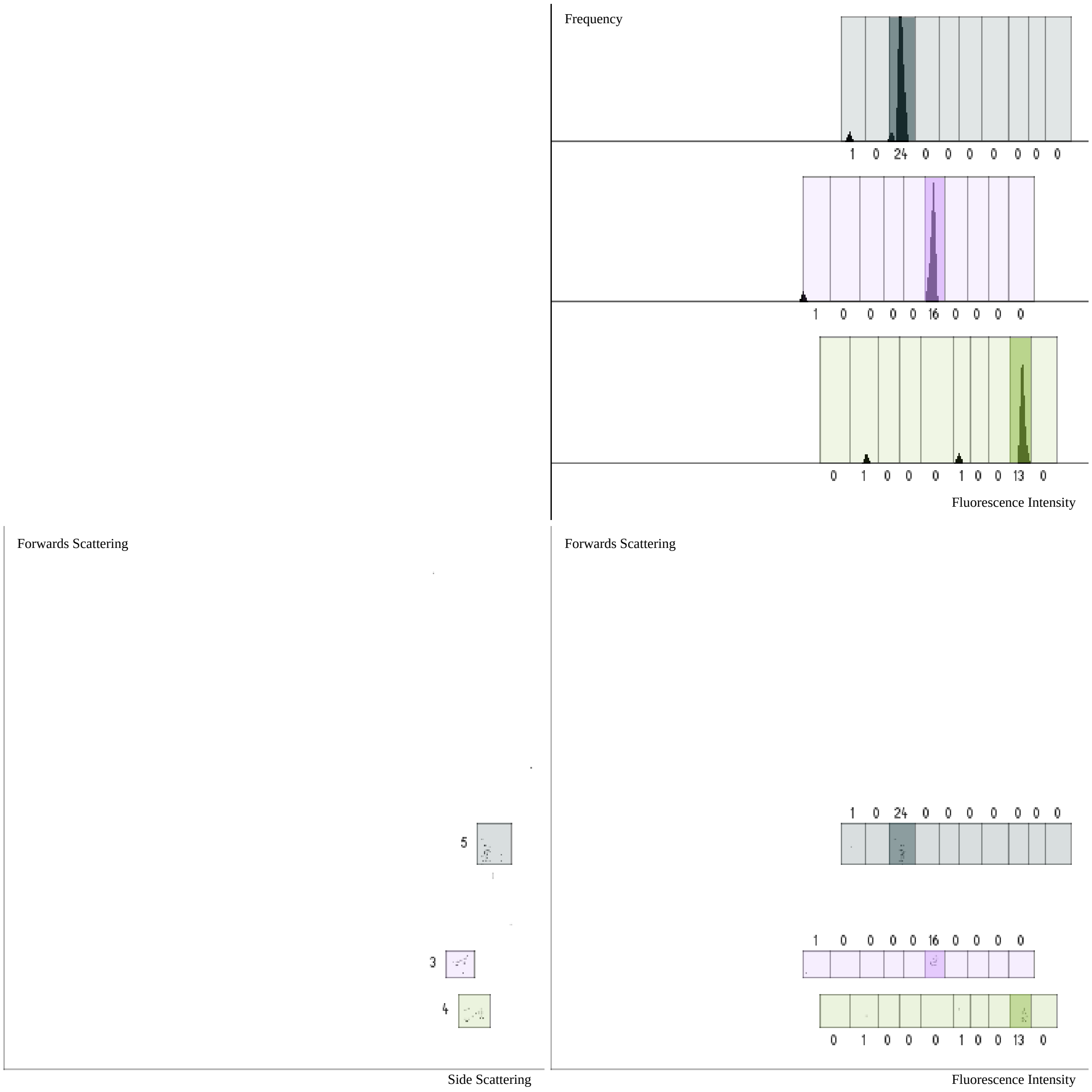

ANNEX 3: TAG DECONVOLUTION - BEAD 157

Passes flow sorting criteria: Yes

Passes tag deconvolution criteria: Yes

Included in protocol analysis: Yes

Protocol: 10, 2, 4, 6

Filename: Bin6_plateA5_A3.fcs

Split 1: Petrol shading

Split 2: Green shading

Split 3: Violet shading

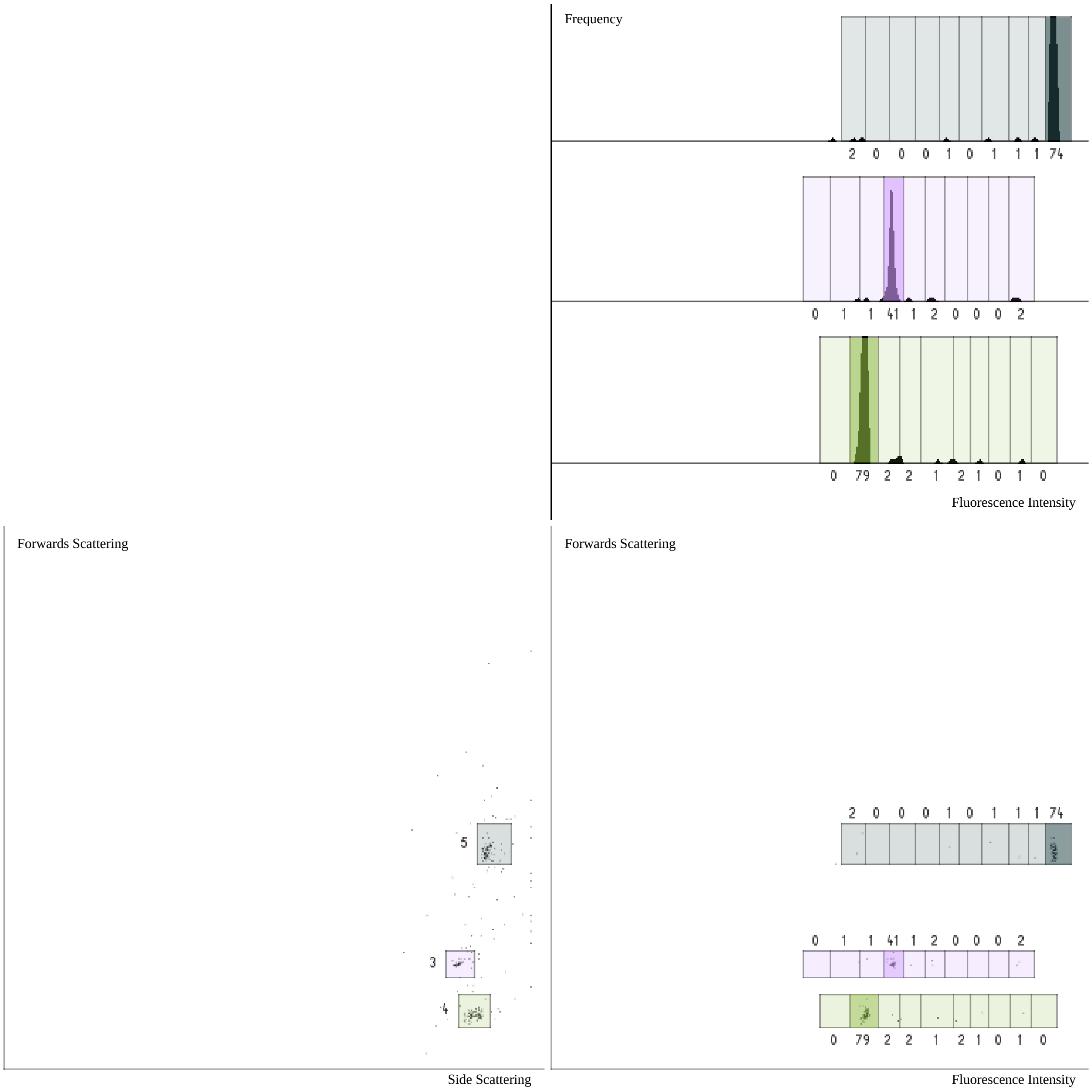

ANNEX 3: TAG DECONVOLUTION - BEAD 158

Passes flow sorting criteria: Yes

Passes tag deconvolution criteria: No

Included in protocol analysis: No

Protocol: N/A

Filename: Bin6_plateA5_A8.fcs

Split 1: Petrol shading

Split 2: Green shading

Split 3: Violet shading

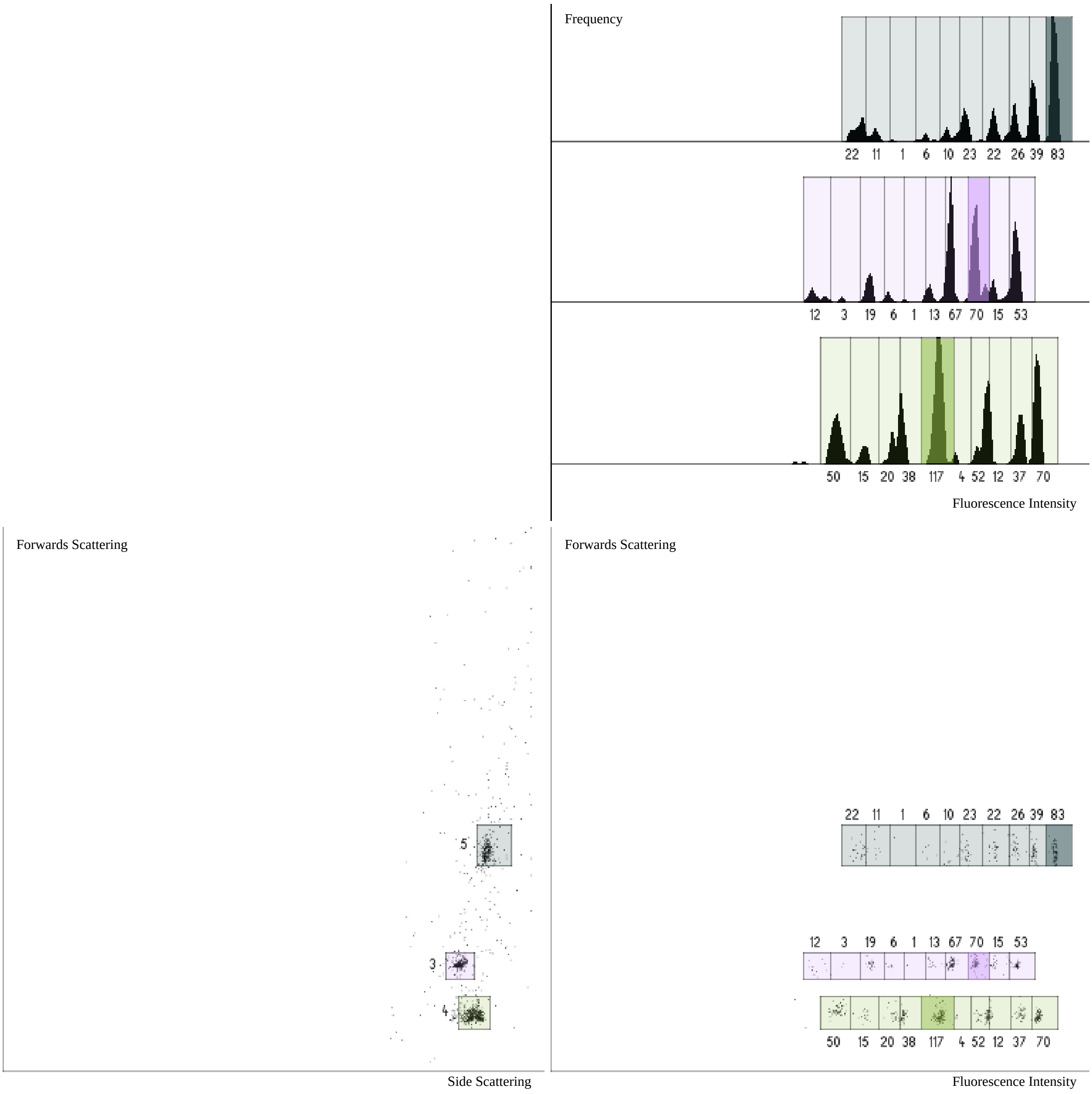

ANNEX 3: TAG DECONVOLUTION - BEAD 159

Passes flow sorting criteria: Yes

Passes tag deconvolution criteria: Yes

Included in protocol analysis: Yes

Protocol: 5, 4, 2, 6

Filename: Bin6_plateA5_A11.fcs

Split 1: Petrol shading

Split 2: Green shading

Split 3: Violet shading

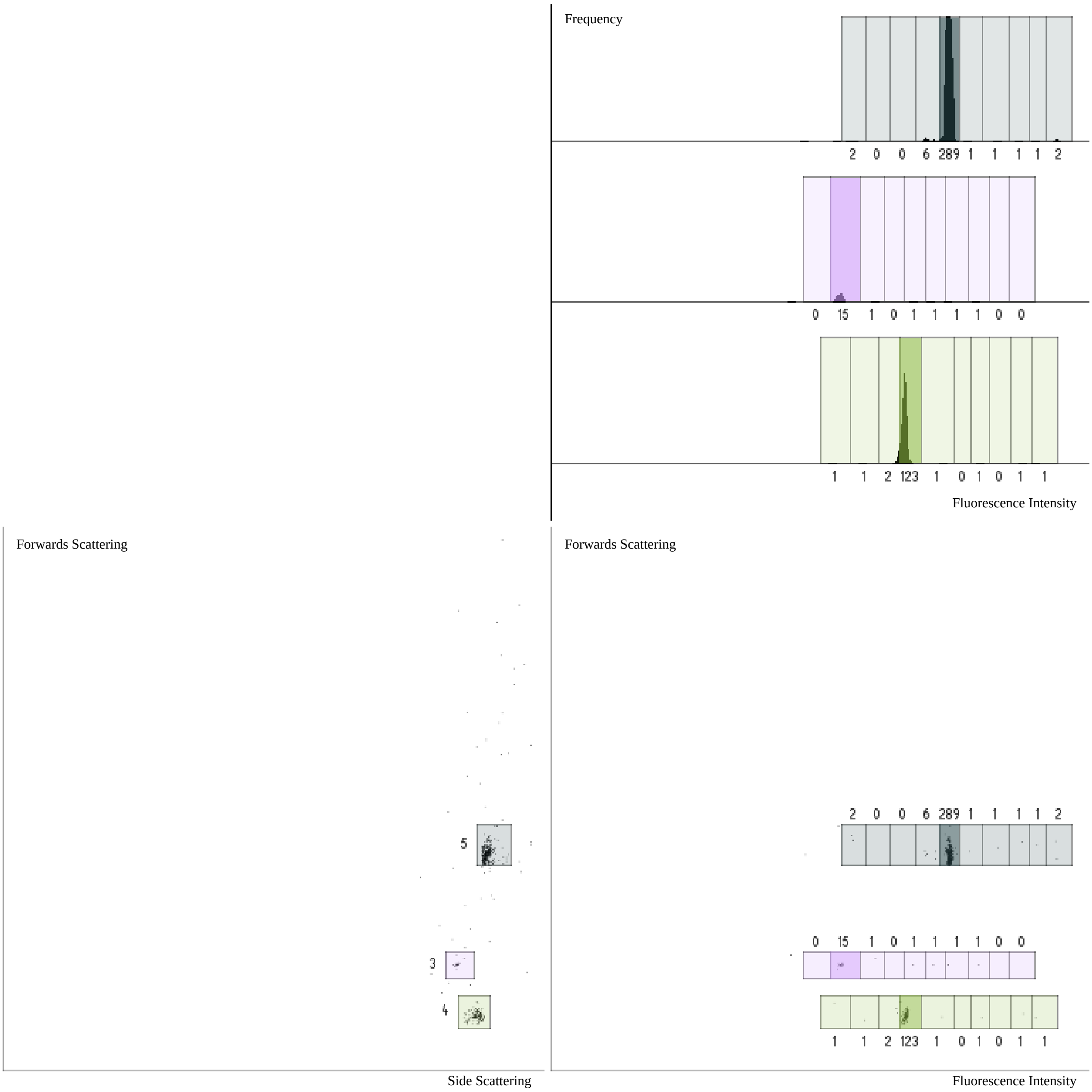

ANNEX 3: TAG DECONVOLUTION - BEAD 160

Passes flow sorting criteria: Yes

Passes tag deconvolution criteria: Yes

Included in protocol analysis: Yes

Protocol: 4, 2, 3, 6

Filename: Bin6_plateA5_A12.fcs

Split 1: Petrol shading

Split 2: Green shading

Split 3: Violet shading

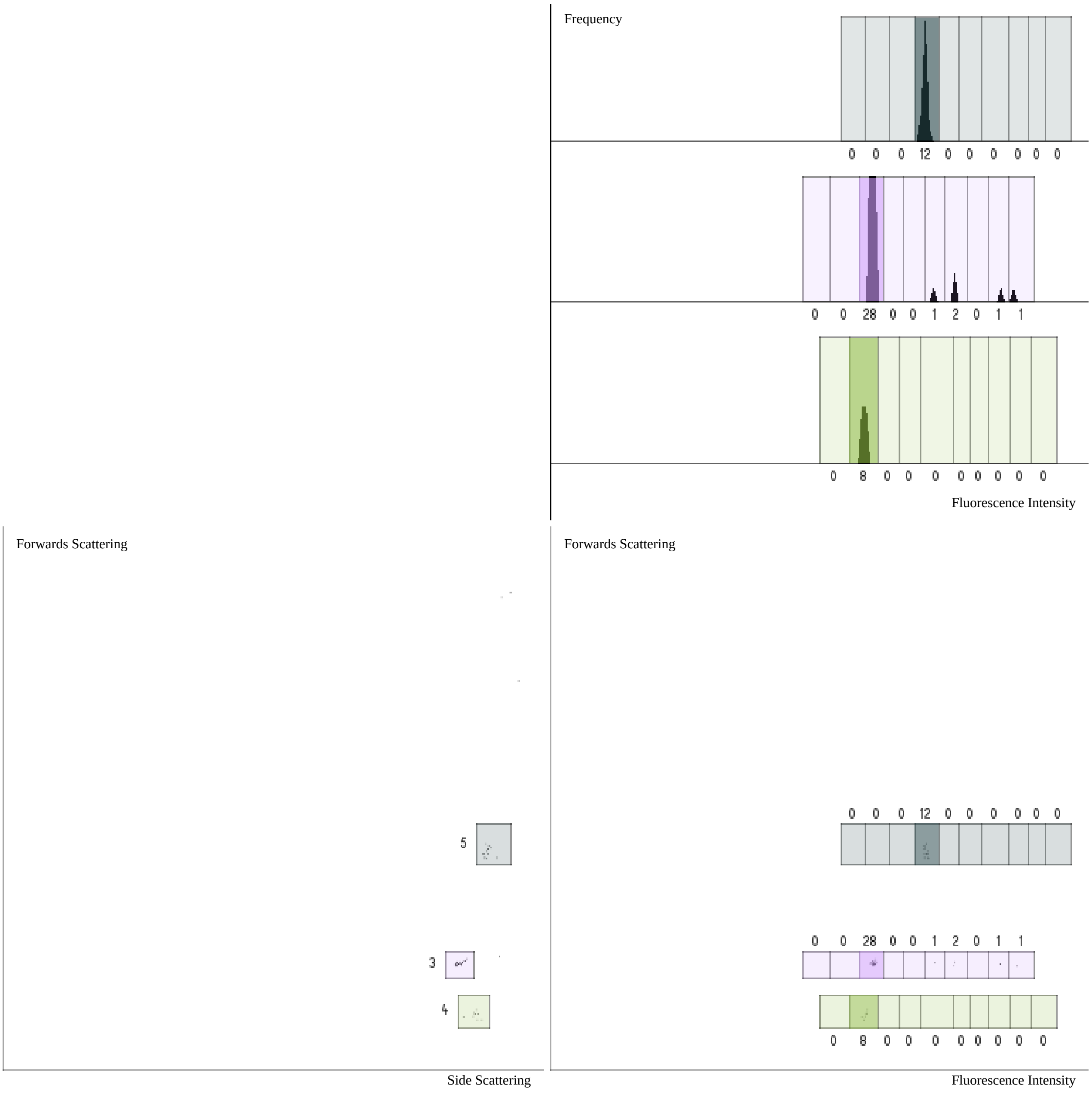

ANNEX 3: TAG DECONVOLUTION - BEAD 161

Passes flow sorting criteria: Yes

Passes tag deconvolution criteria: Yes

Included in protocol analysis: Yes

Protocol: 7, 10, 9, 6

Filename: Bin6_plateA5_B3.fcs

Split 1: Petrol shading

Split 2: Green shading

Split 3: Violet shading

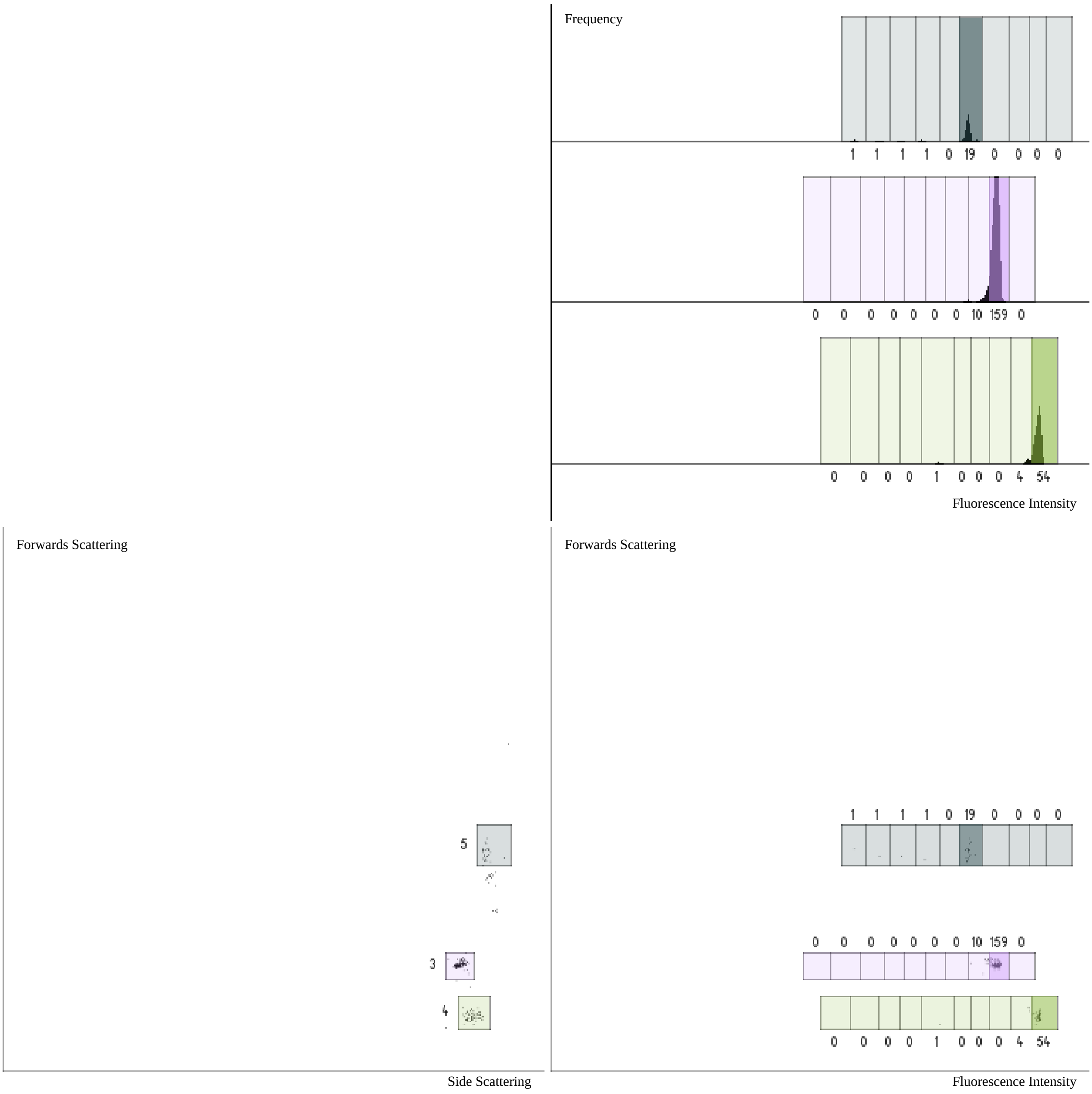

ANNEX 3: TAG DECONVOLUTION - BEAD 162

Passes flow sorting criteria: Yes

Passes tag deconvolution criteria: Yes

Included in protocol analysis: Yes

Protocol: 6, 7, 9, 6

Filename: Bin6_plateA5_B6.fcs

Split 1: Petrol shading

Split 2: Green shading

Split 3: Violet shading

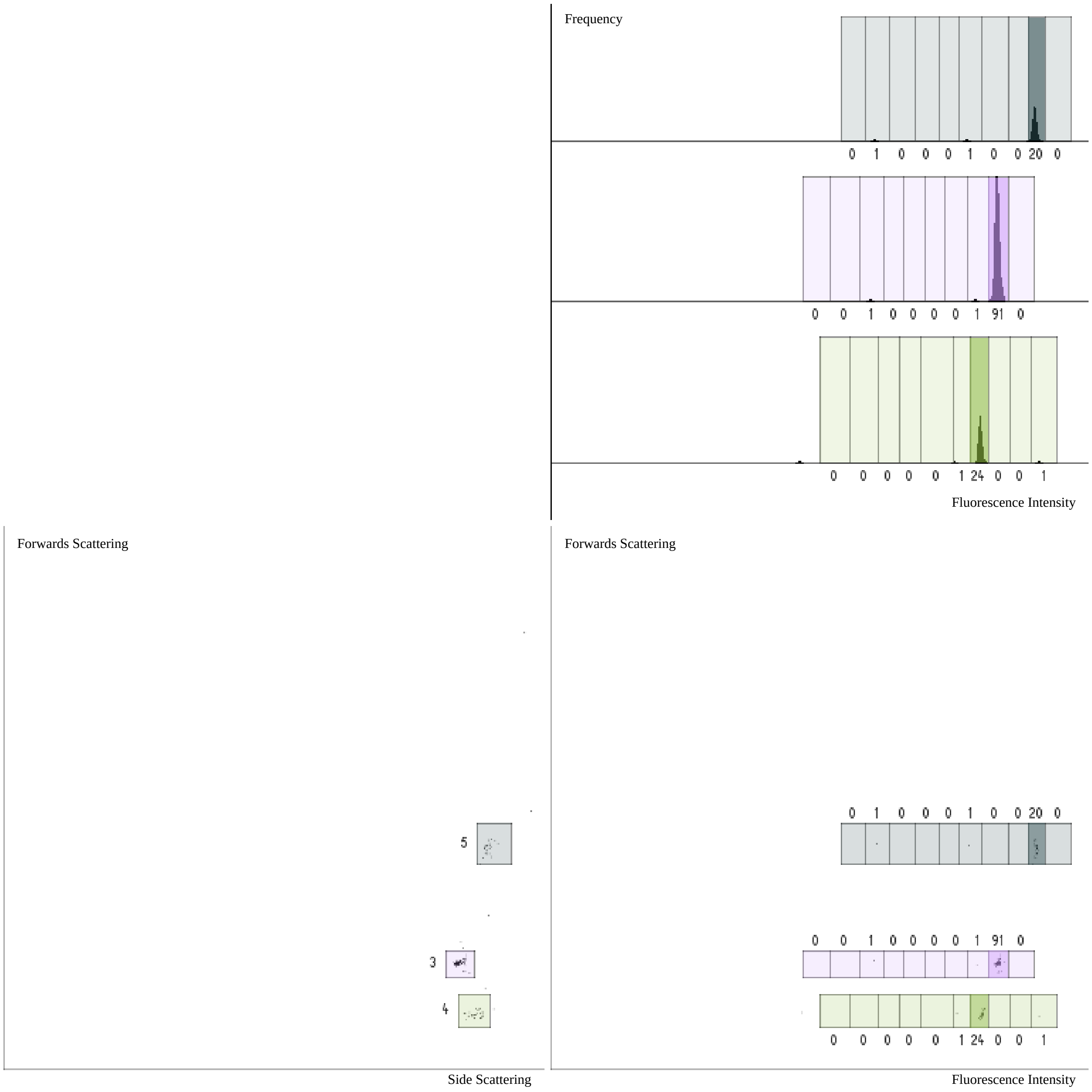

ANNEX 3: TAG DECONVOLUTION - BEAD 163

Passes flow sorting criteria: Yes

Passes tag deconvolution criteria: Yes

Included in protocol analysis: Yes

Protocol: 8, 8, 3, 6

Filename: Bin6_plateA5_B9.fcs

Split 1: Petrol shading

Split 2: Green shading

Split 3: Violet shading

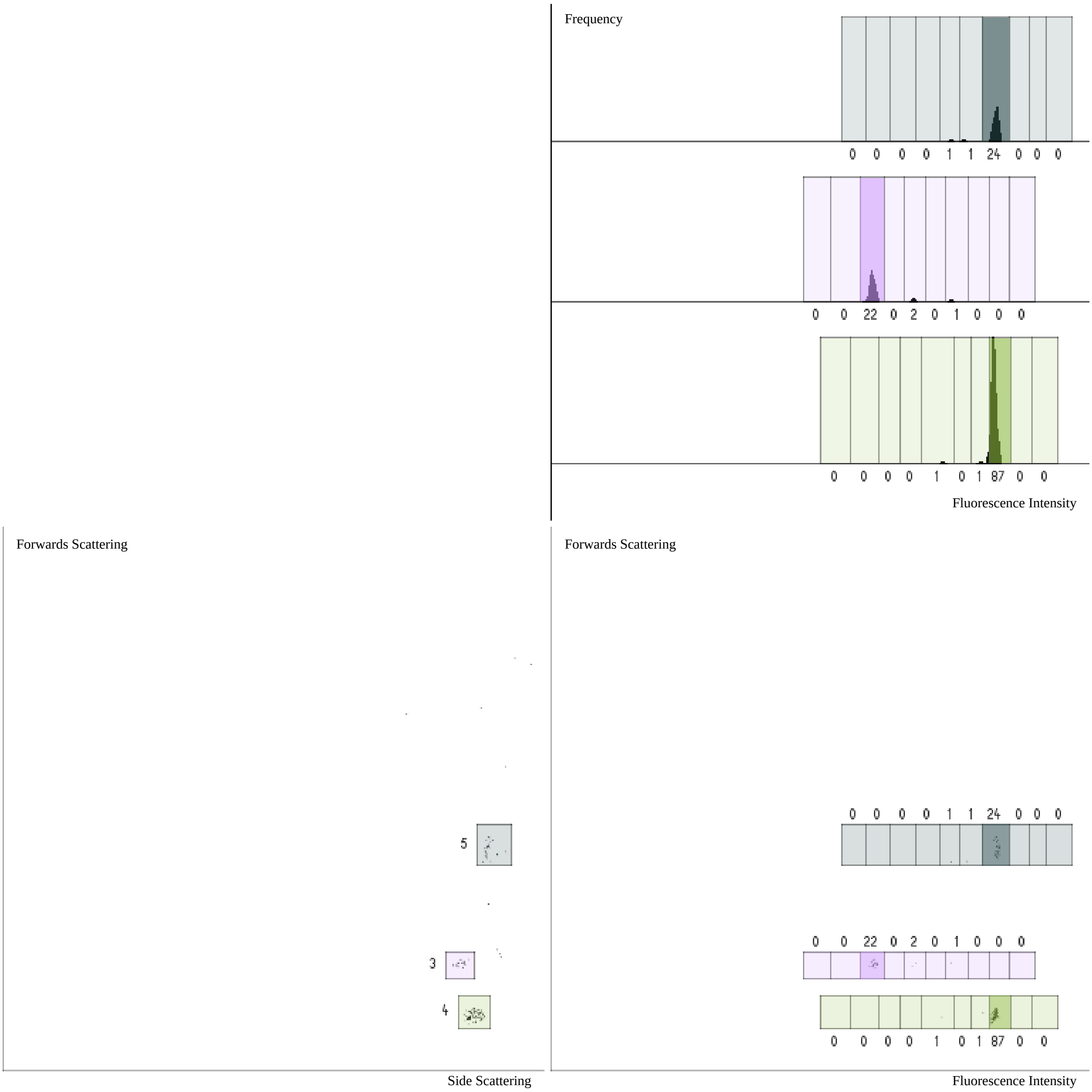

ANNEX 3: TAG DECONVOLUTION - BEAD 164

Passes flow sorting criteria: Yes

Passes tag deconvolution criteria: Yes

Included in protocol analysis: Yes

Protocol: 2, 3, 4, 6

Filename: Bin6_plateA5_B12.fcs

Split 1: Petrol shading

Split 2: Green shading

Split 3: Violet shading

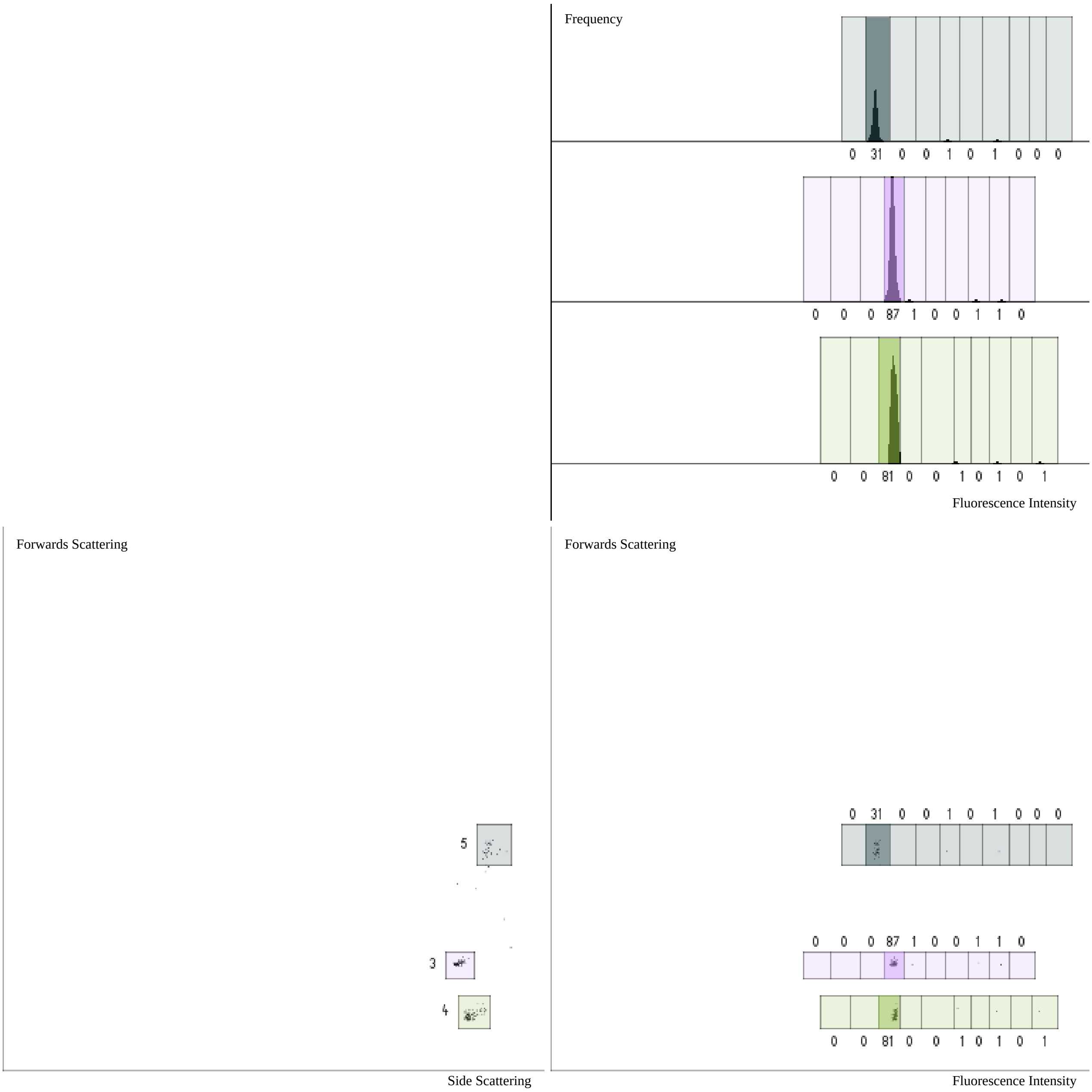

ANNEX 3: TAG DECONVOLUTION - BEAD 165

Passes flow sorting criteria: Yes

Passes tag deconvolution criteria: No

Included in protocol analysis: No

Protocol: N/A

Filename: Bin6_plateA5_C4.fcs

Split 1: Petrol shading

Split 2: Green shading

Split 3: Violet shading

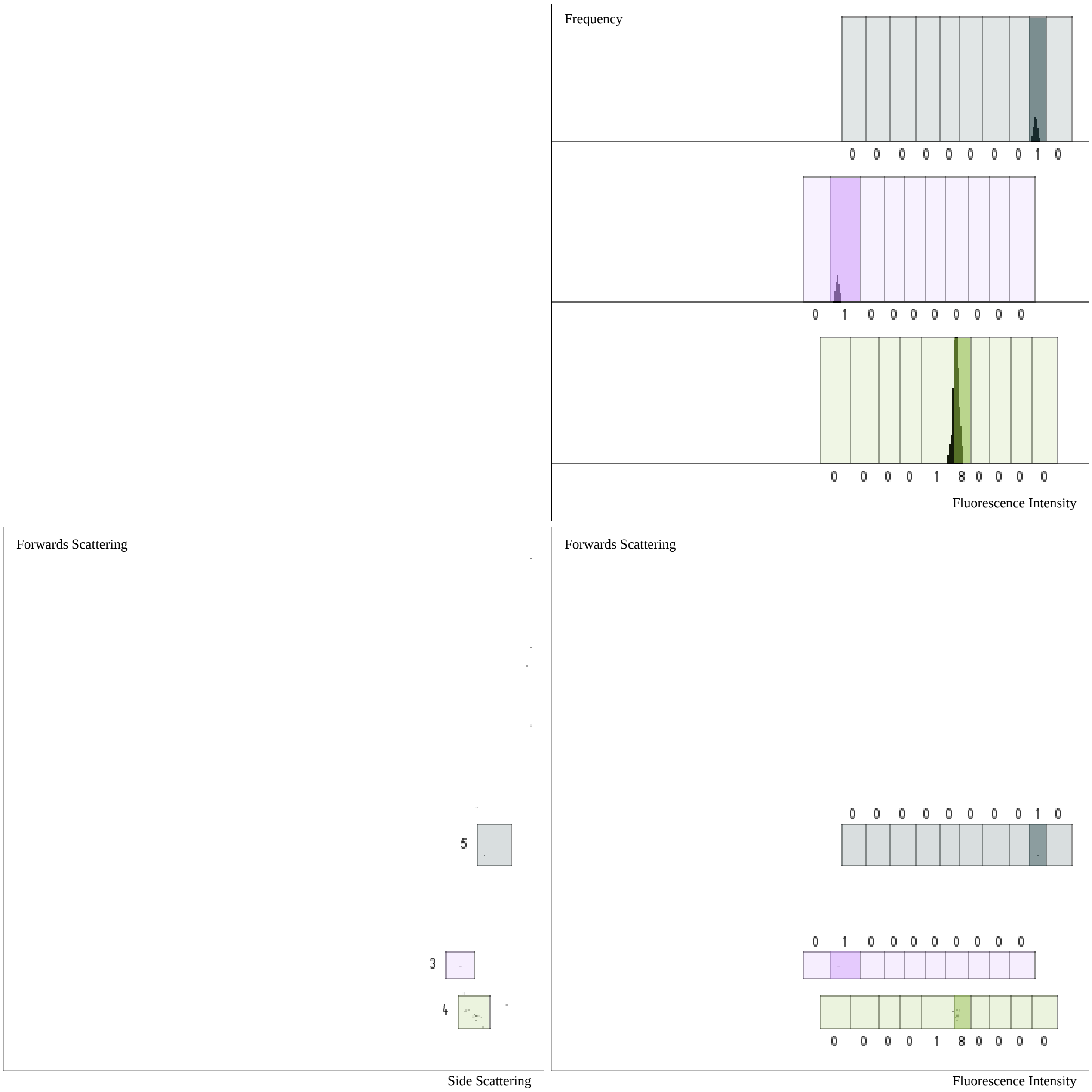

ANNEX 3: TAG DECONVOLUTION - BEAD 166

Passes flow sorting criteria: Yes

Passes tag deconvolution criteria: Yes

Included in protocol analysis: Yes

Protocol: 10, 9, 5, 6

Filename: Bin6_plateA5_C8.fcs

Split 1: Petrol shading

Split 2: Green shading

Split 3: Violet shading

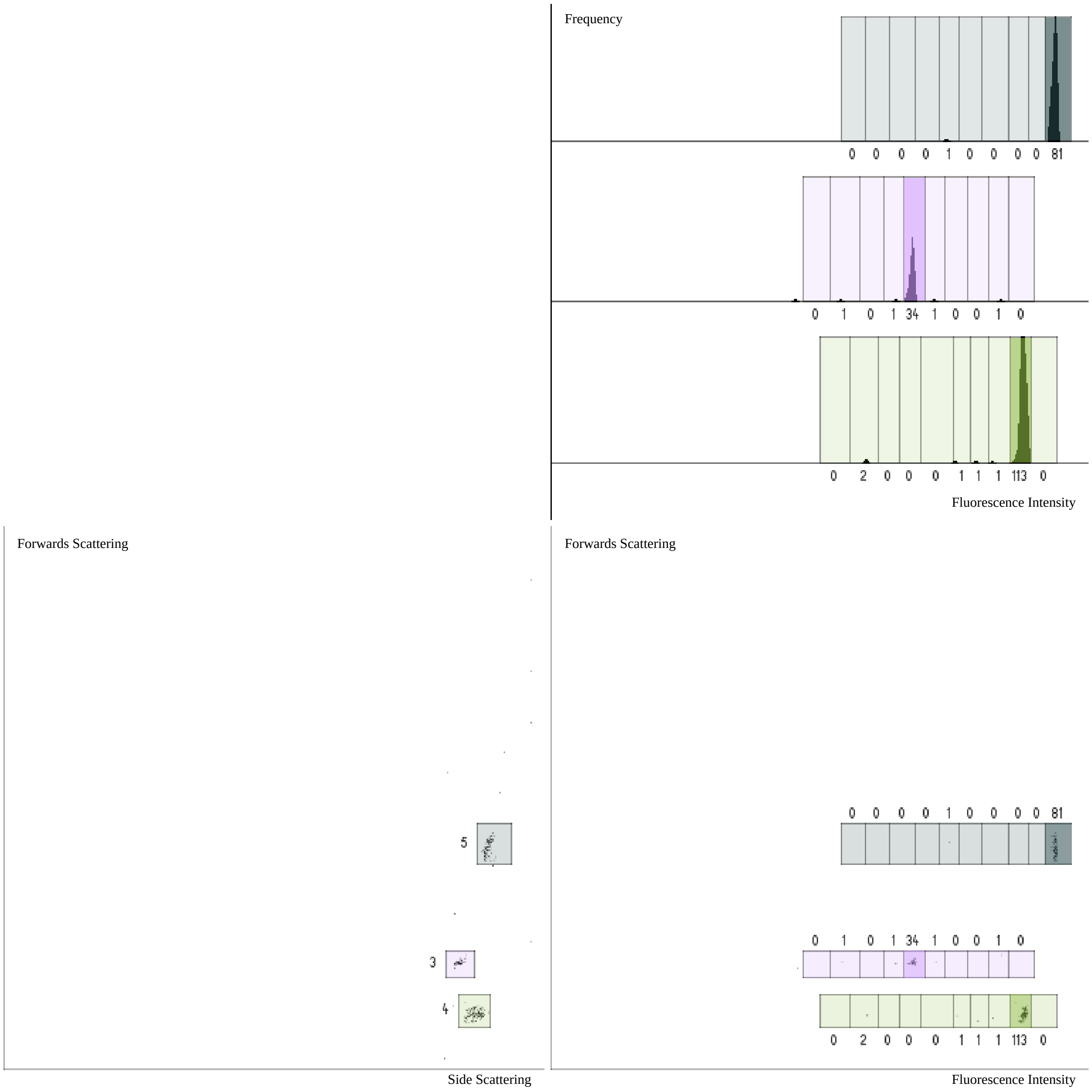

ANNEX 3: TAG DECONVOLUTION - BEAD 167

Passes flow sorting criteria: Yes

Passes tag deconvolution criteria: Yes

Included in protocol analysis: Yes

Protocol: 6, 5, 8, 6

Filename: Bin6_plateA5_C9.fcs

Split 1: Petrol shading

Split 2: Green shading

Split 3: Violet shading

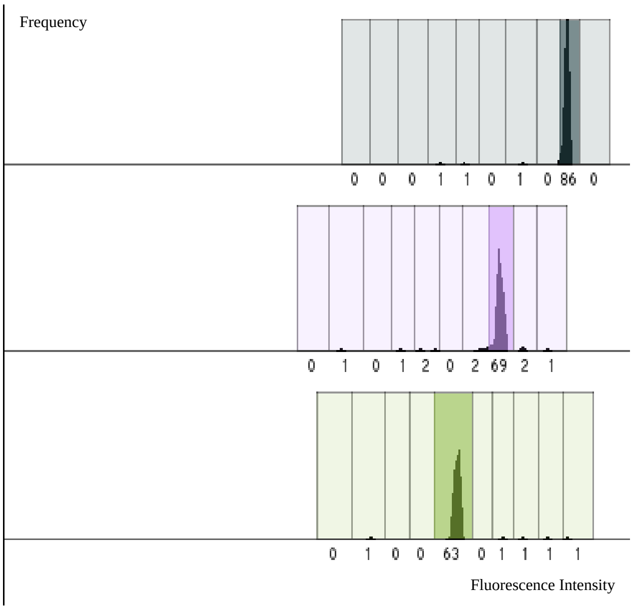

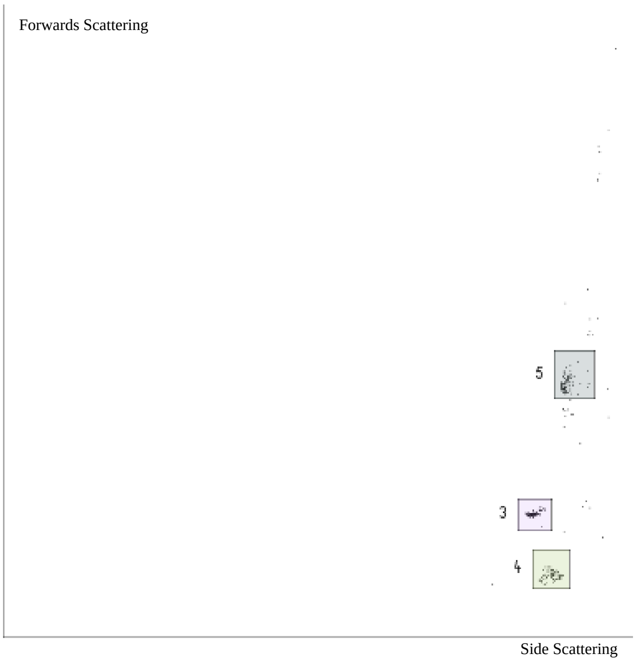

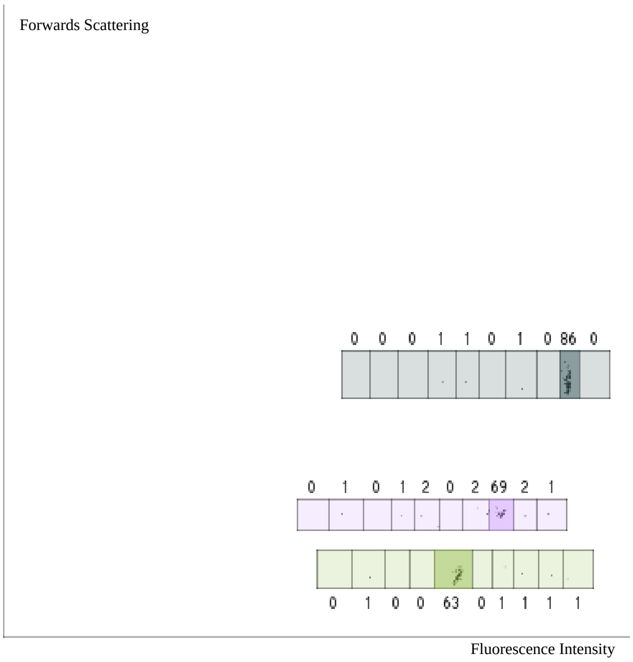

ANNEX 3: TAG DECONVOLUTION - BEAD 168

Passes flow sorting criteria: Yes

Passes tag deconvolution criteria: Yes

Included in protocol analysis: Yes

Protocol: 6, 2, 7, 7

Filename: Bin7_plateA1_A5.fcs

Split 1: Petrol shading

Split 2: Green shading

Split 3: Violet shading

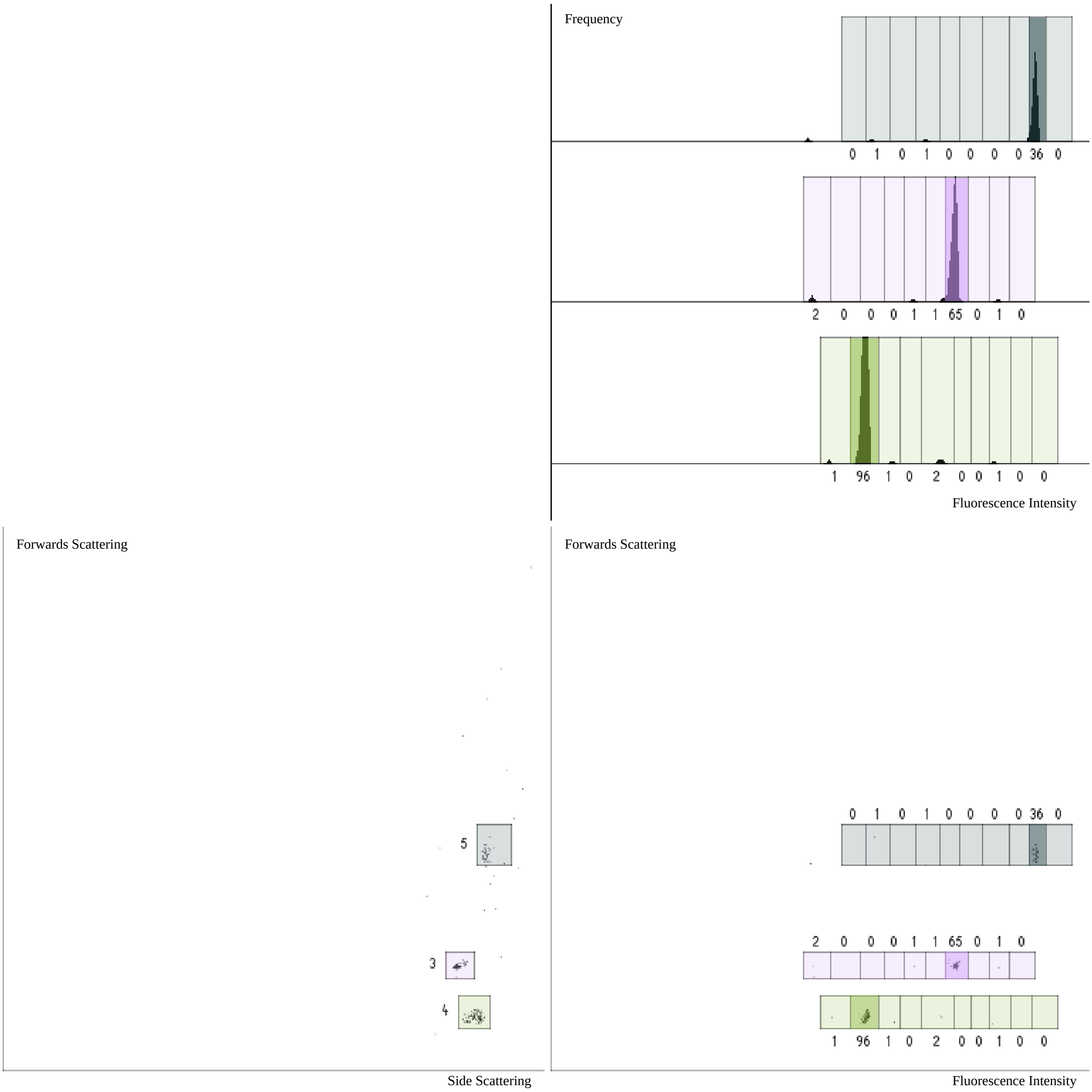

ANNEX 3: TAG DECONVOLUTION - BEAD 169

Passes flow sorting criteria: Yes

Passes tag deconvolution criteria: Yes

Included in protocol analysis: Yes

Protocol: 5, 1, 1, 7

Filename: Bin7_plateA1_A7.fcs

Split 1: Petrol shading

Split 2: Green shading

Split 3: Violet shading

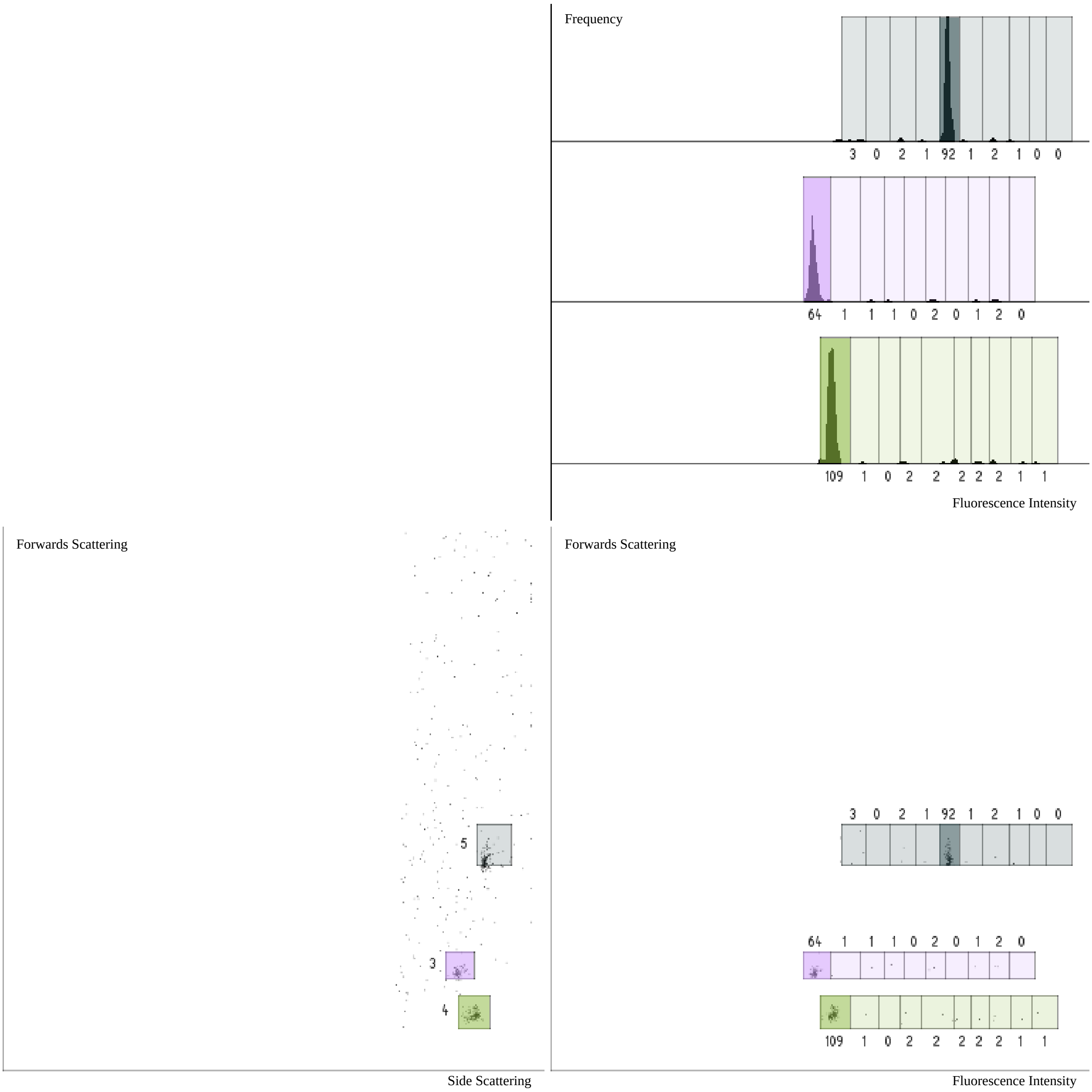

ANNEX 3: TAG DECONVOLUTION - BEAD 170

Passes flow sorting criteria: Yes

Passes tag deconvolution criteria: Yes

Included in protocol analysis: Yes

Protocol: 10, 3, 7, 7

Filename: Bin7_plateA1_B1.fcs

Split 1: Petrol shading

Split 2: Green shading

Split 3: Violet shading

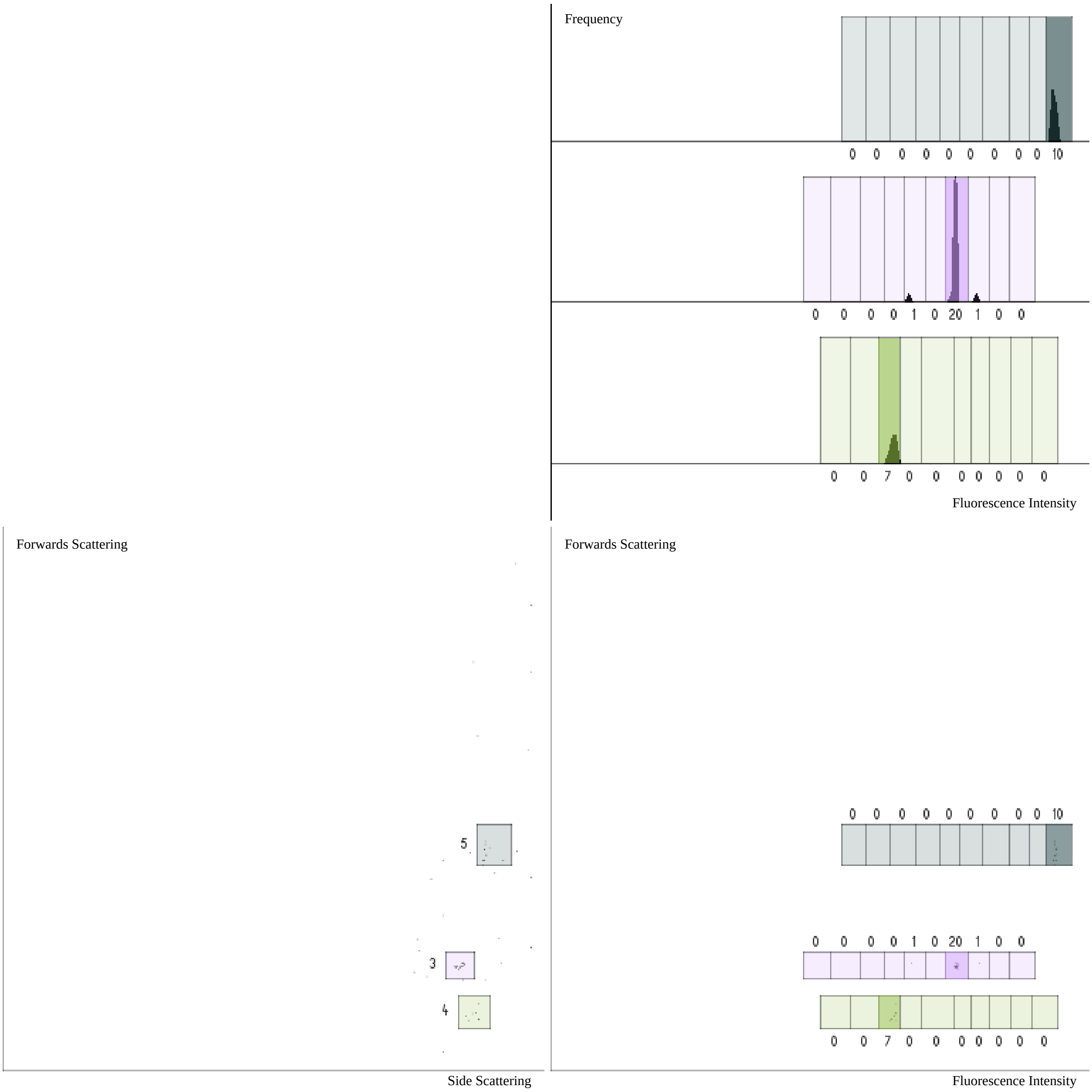

ANNEX 3: TAG DECONVOLUTION - BEAD 171

Passes flow sorting criteria: Yes

Passes tag deconvolution criteria: Yes

Included in protocol analysis: Yes

Protocol: 4, 2, 5, 7

Filename: Bin7_plateA1_B3.fcs

Split 1: Petrol shading

Split 2: Green shading

Split 3: Violet shading

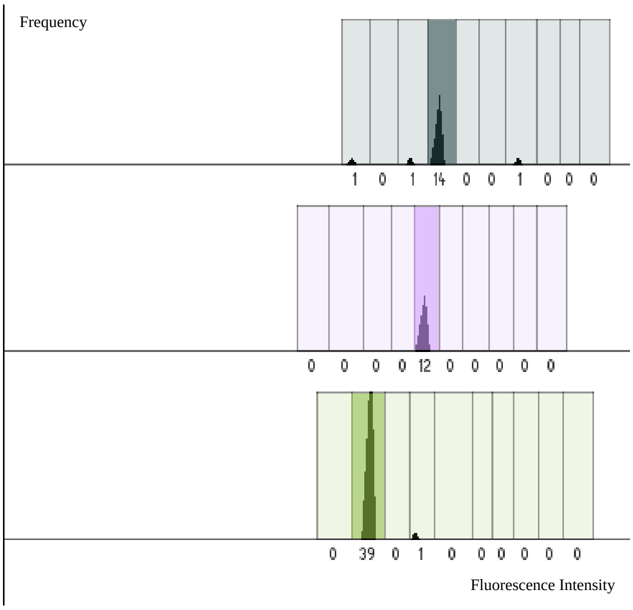

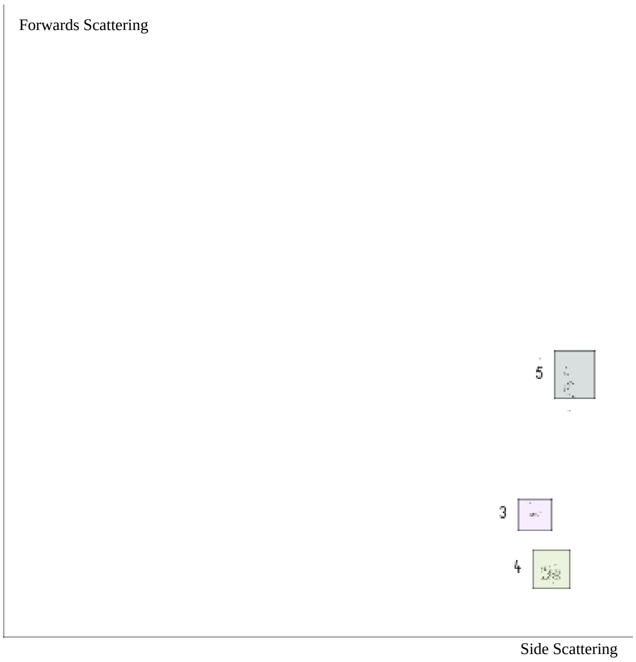

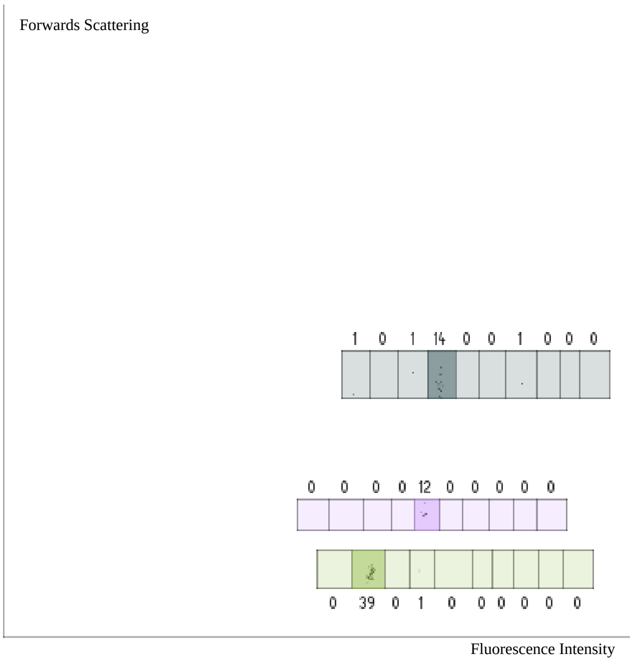

ANNEX 3: TAG DECONVOLUTION - BEAD 172

Passes flow sorting criteria: Yes

Passes tag deconvolution criteria: Yes

Included in protocol analysis: Yes

Protocol: 8, 6, 3, 7

Filename: Bin7_plateA1_B6.fcs

Split 1: Petrol shading

Split 2: Green shading

Split 3: Violet shading

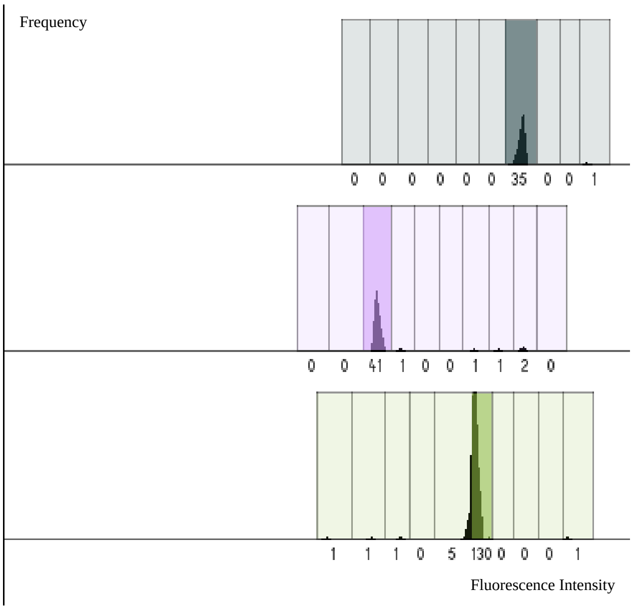

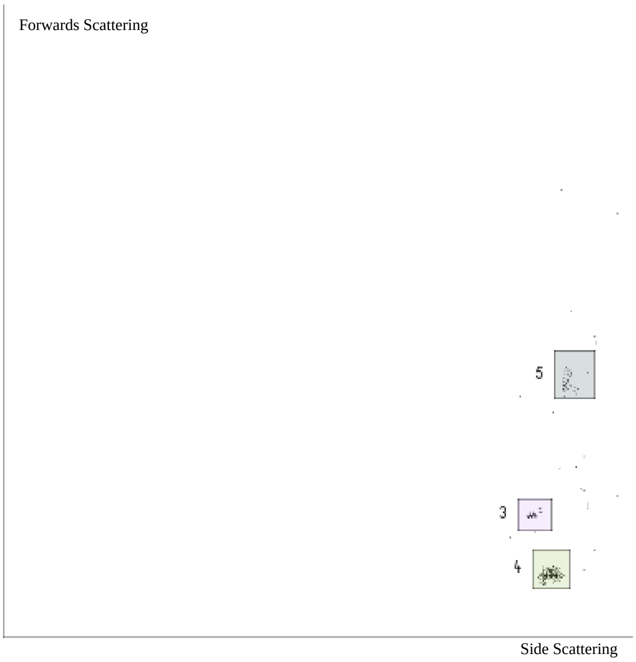

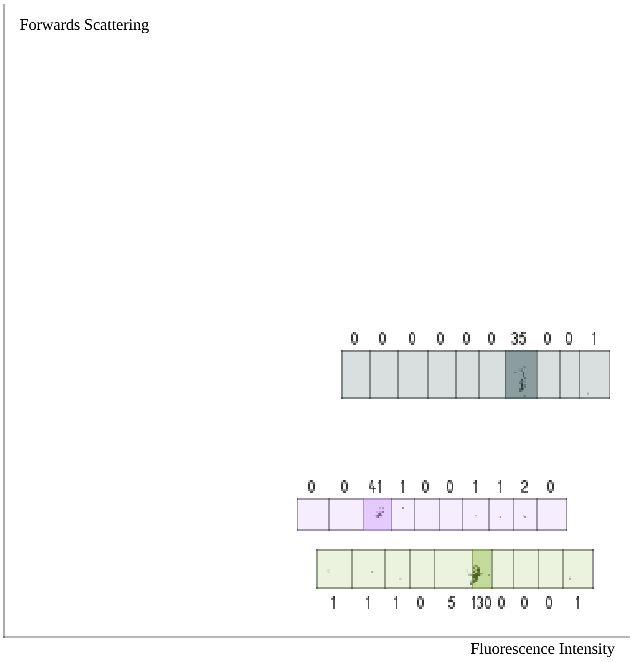

ANNEX 3: TAG DECONVOLUTION - BEAD 173

Passes flow sorting criteria: Yes

Passes tag deconvolution criteria: Yes

Included in protocol analysis: Yes

Protocol: 5, 5, 10, 7

Filename: Bin7_plateA1_C3.fcs

Split 1: Petrol shading

Split 2: Green shading

Split 3: Violet shading

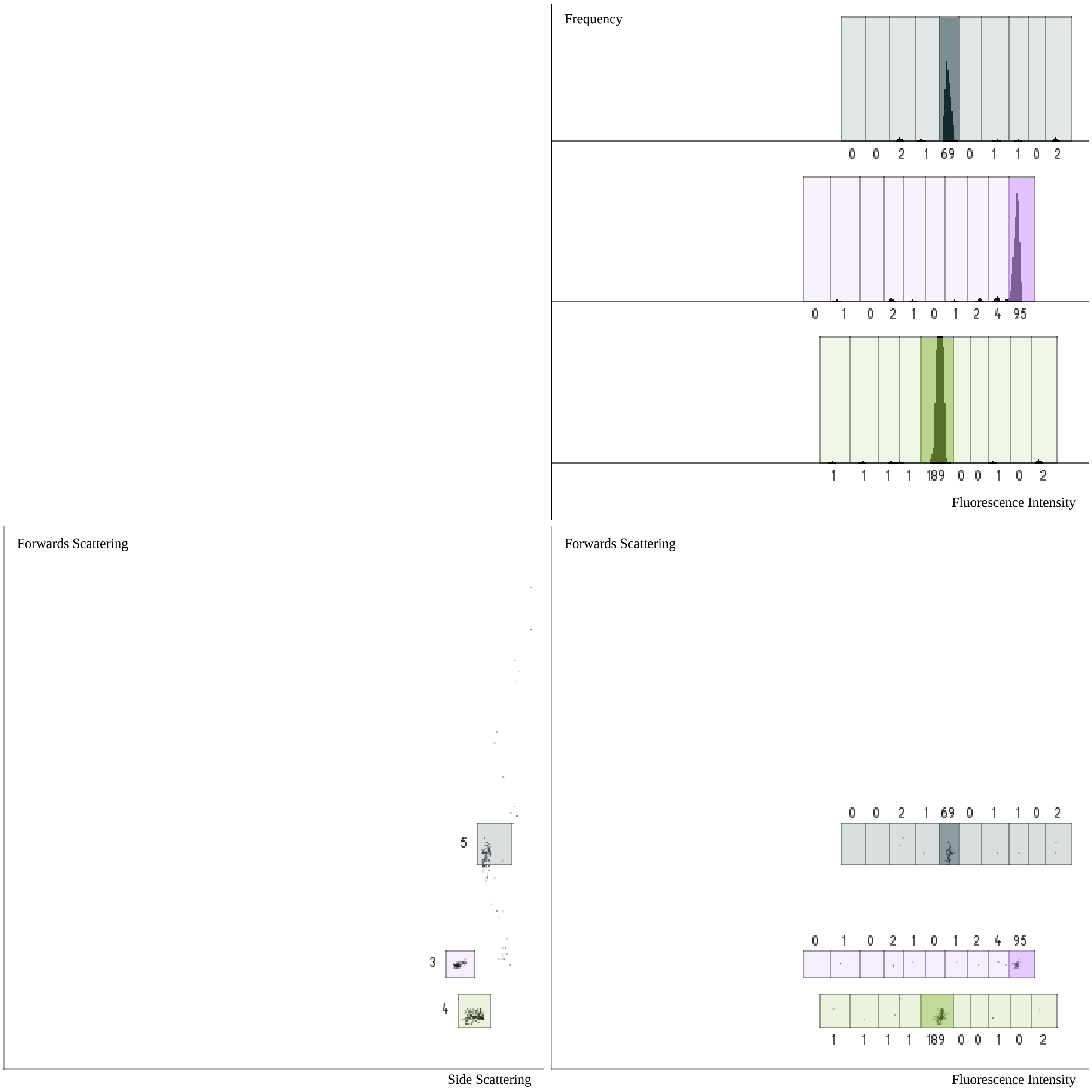

ANNEX 3: TAG DECONVOLUTION - BEAD 174

Passes flow sorting criteria: Yes

Passes tag deconvolution criteria: No

Included in protocol analysis: No

Protocol: N/A

Filename: Bin7_plateA1_C4.fcs

Split 1: Petrol shading

Split 2: Green shading

Split 3: Violet shading

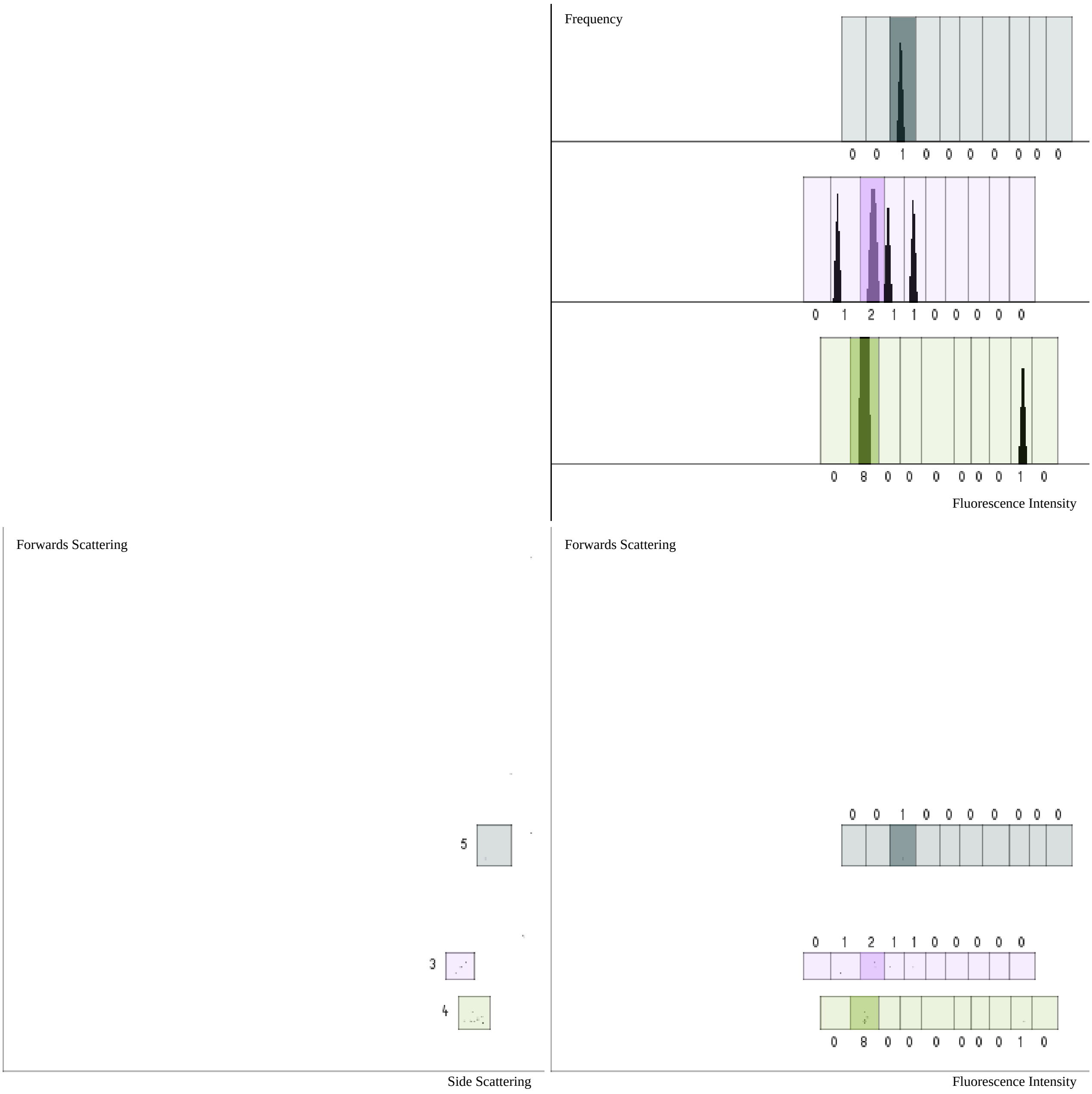

ANNEX 3: TAG DECONVOLUTION - BEAD 175

Passes flow sorting criteria: Yes

Passes tag deconvolution criteria: Yes

Included in protocol analysis: Yes

Protocol: 4, 8, 5, 7

Filename: Bin7_plateA1_C6.fcs

Split 1: Petrol shading

Split 2: Green shading

Split 3: Violet shading

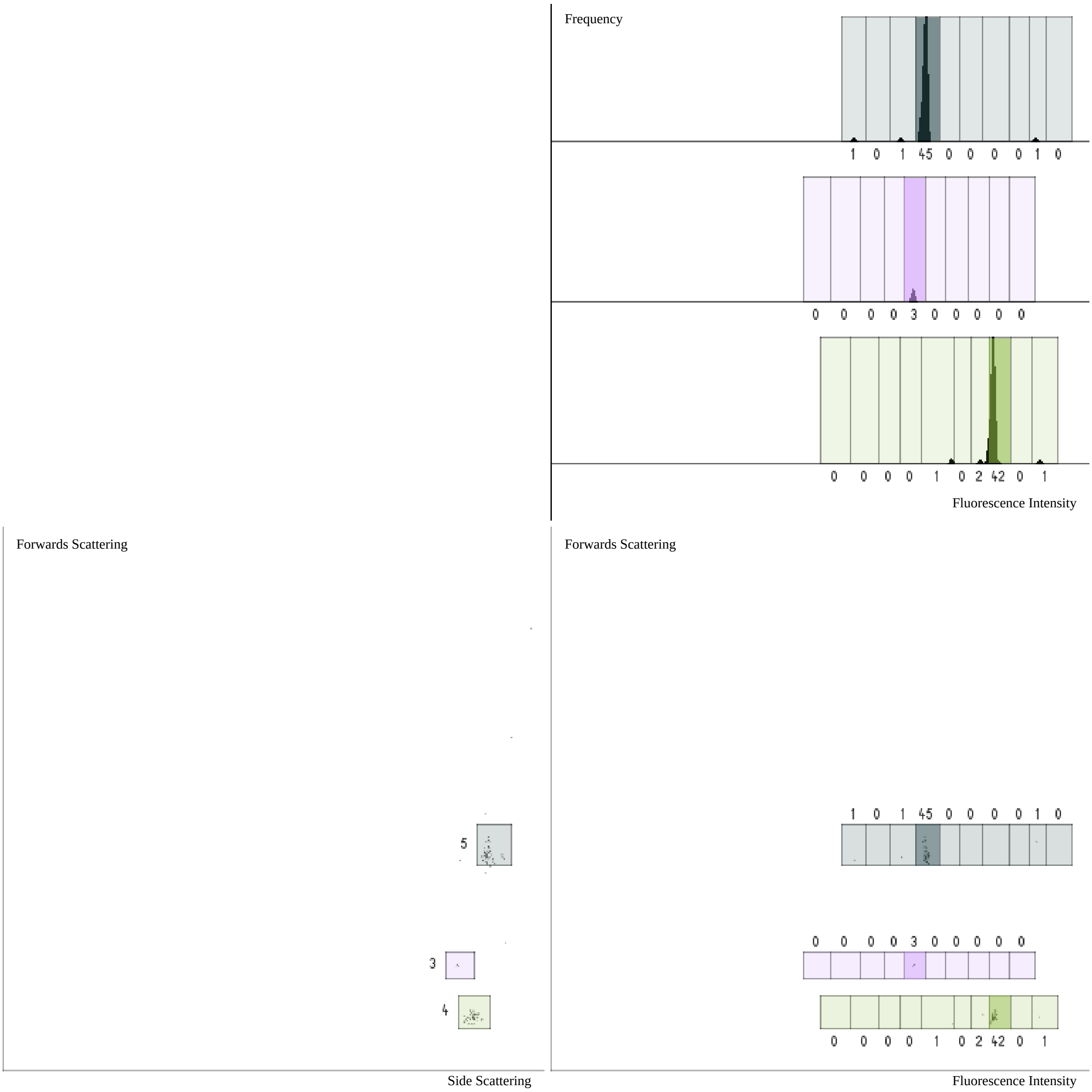

ANNEX 3: TAG DECONVOLUTION - BEAD 176

Passes flow sorting criteria: Yes

Passes tag deconvolution criteria: No

Included in protocol analysis: No

Protocol: N/A

Filename: Bin6_plateA6_H12.fcs

Split 1: Petrol shading

Split 2: Green shading

Split 3: Violet shading

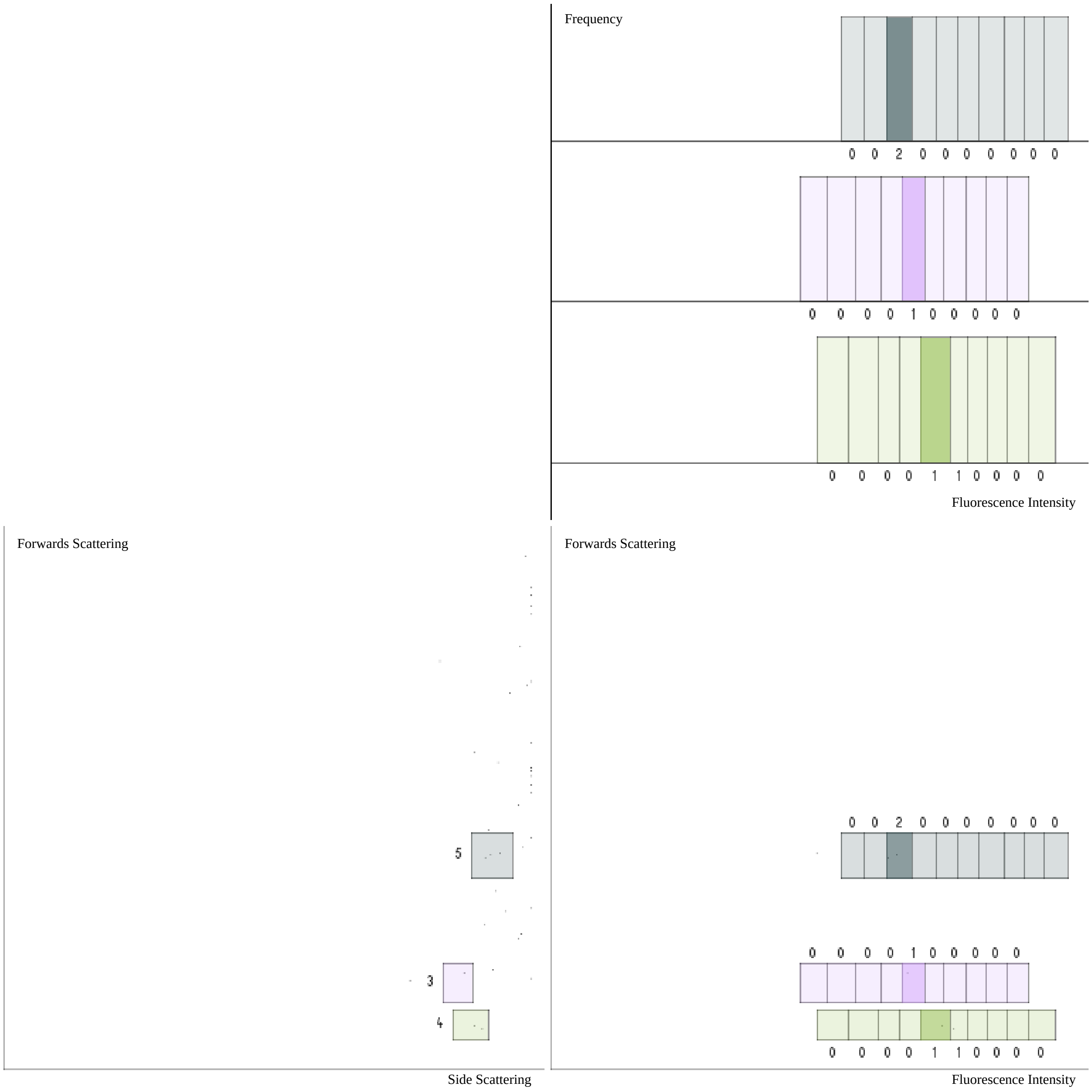

ANNEX 3: TAG DECONVOLUTION - BEAD 177

Passes flow sorting criteria: Yes

Passes tag deconvolution criteria: Yes

Included in protocol analysis: Yes

Protocol: 10, 8, 5, 6

Filename: Bin6_plateA5_C12.fcs

Split 1: Petrol shading

Split 2: Green shading

Split 3: Violet shading

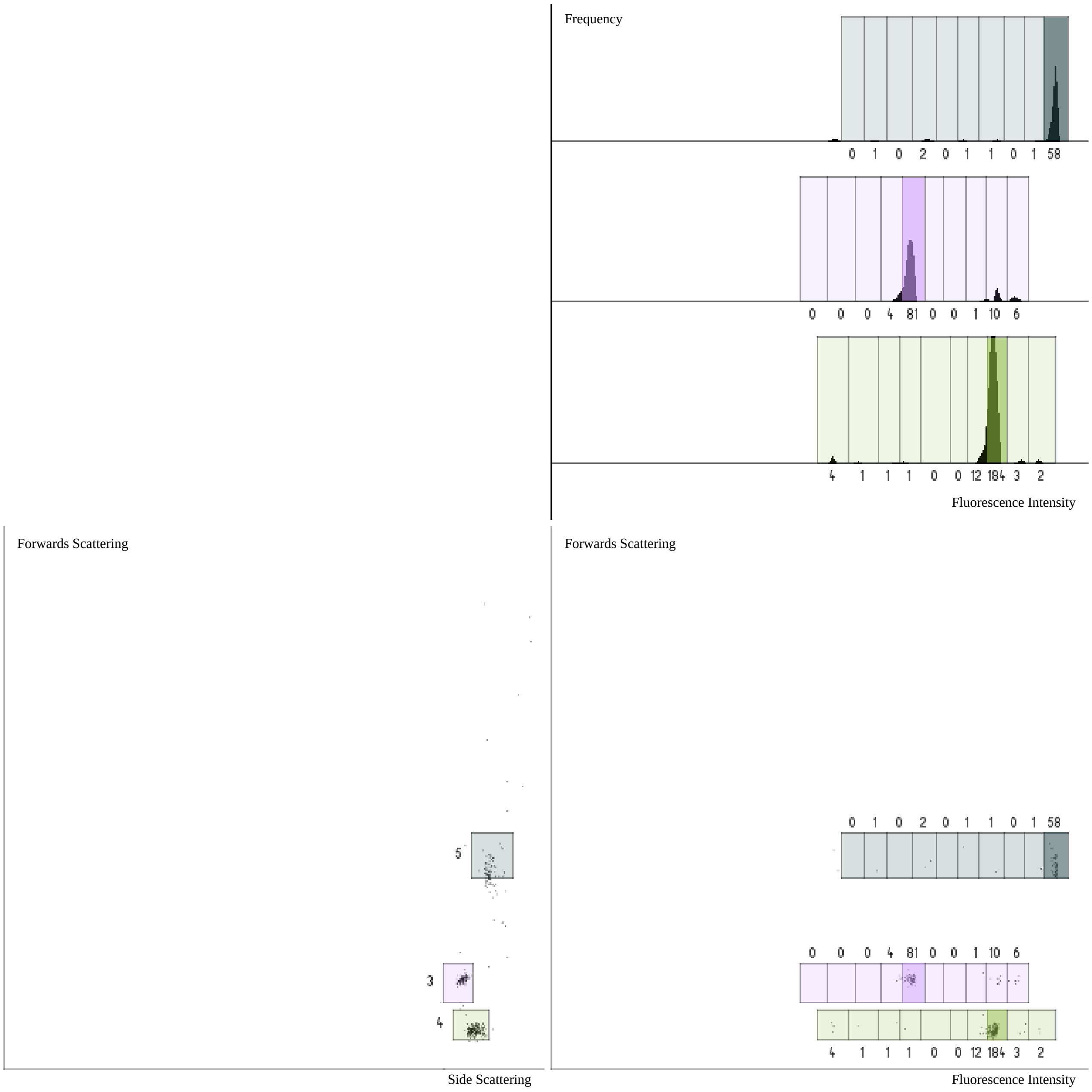

ANNEX 3: TAG DECONVOLUTION - BEAD 178

Passes flow sorting criteria: Yes

Passes tag deconvolution criteria: Yes

Included in protocol analysis: Yes

Protocol: 8, 9, 7, 6

Filename: Bin6_plateA5_D1.fcs

Split 1: Petrol shading

Split 2: Green shading

Split 3: Violet shading

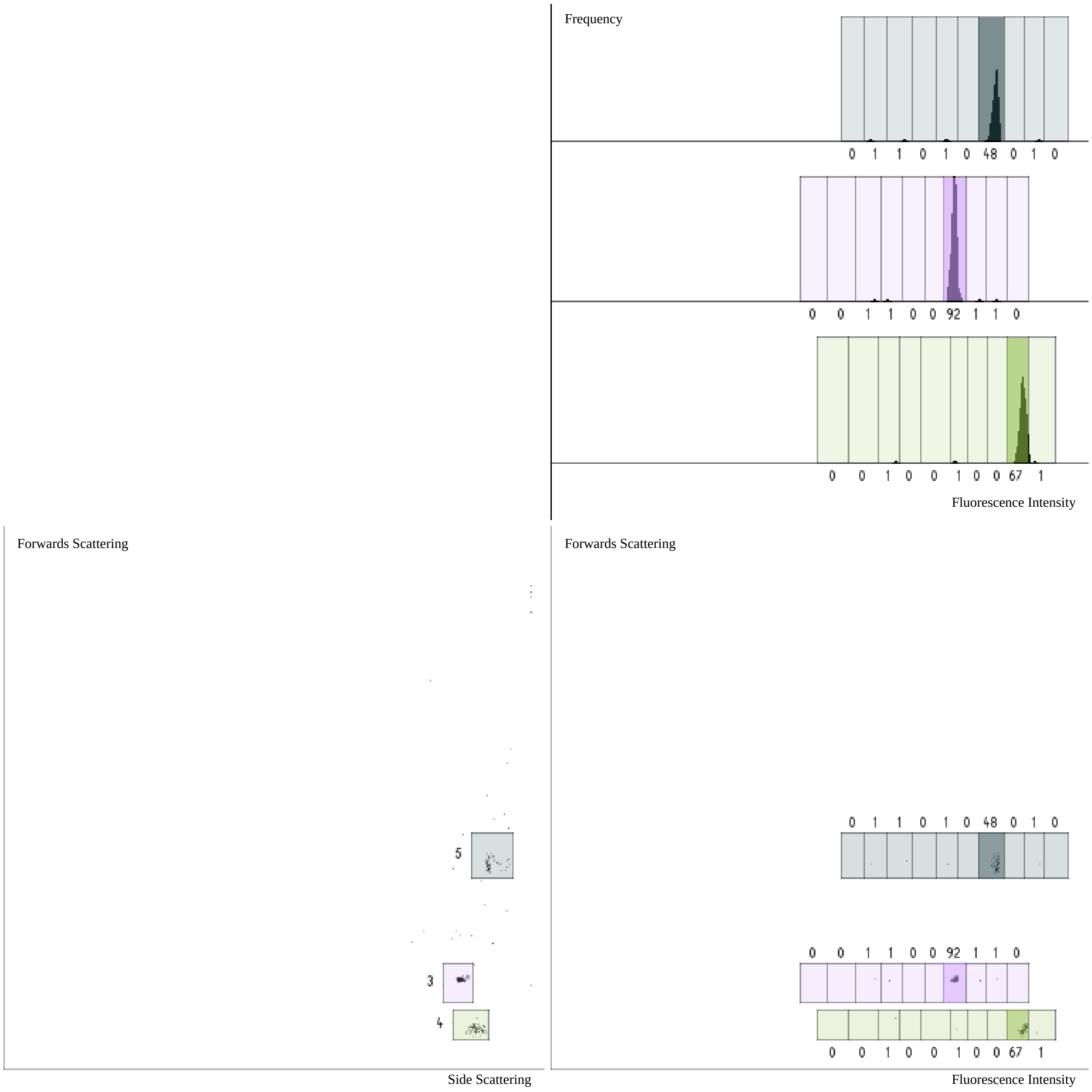

ANNEX 3: TAG DECONVOLUTION - BEAD 179

Passes flow sorting criteria: Yes

Passes tag deconvolution criteria: Yes

Included in protocol analysis: Yes

Protocol: 4, 9, 4, 6

Filename: Bin6_plateA5_D6.fcs

Split 1: Petrol shading

Split 2: Green shading

Split 3: Violet shading

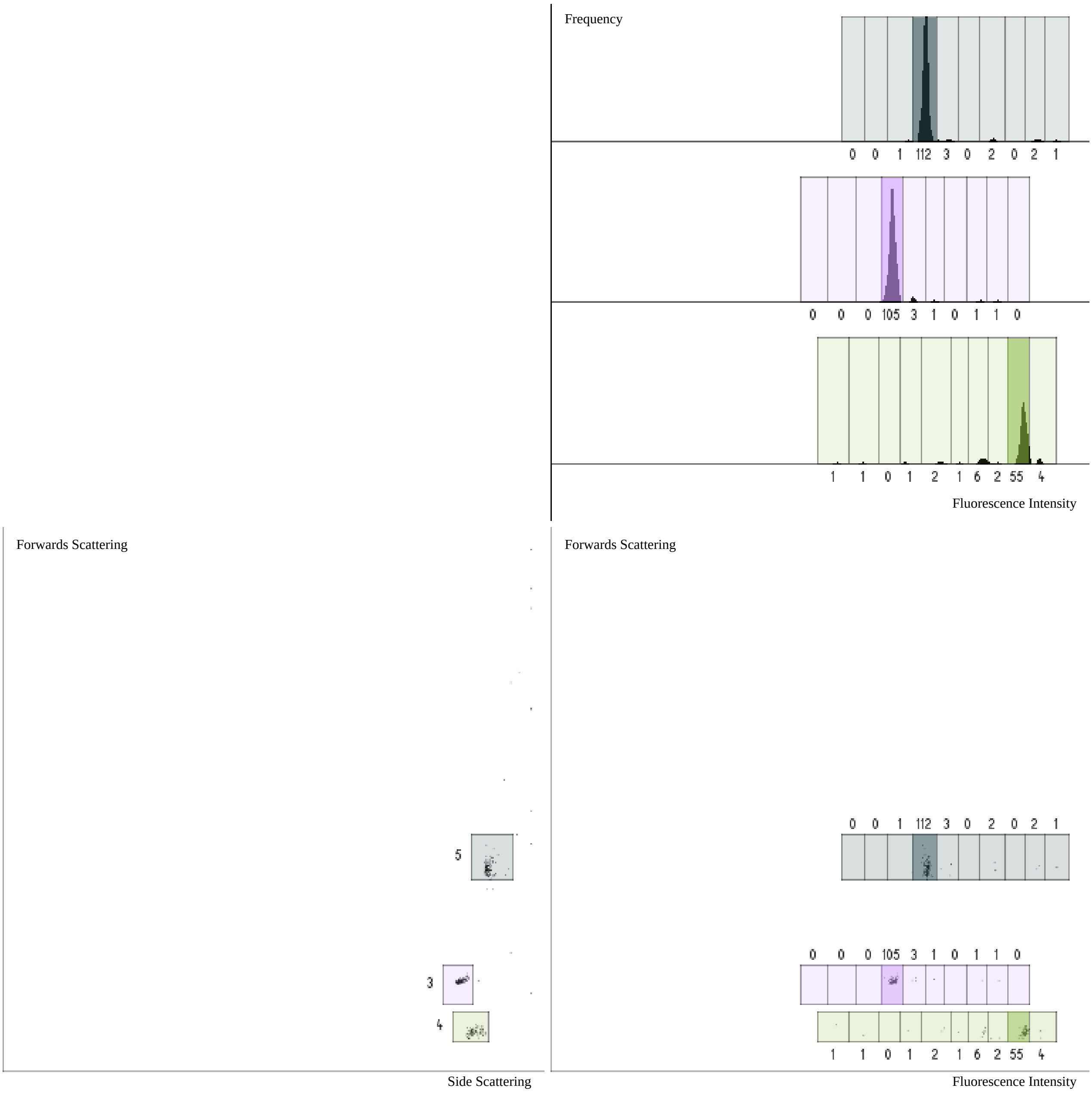

ANNEX 3: TAG DECONVOLUTION - BEAD 180

Passes flow sorting criteria: Yes

Passes tag deconvolution criteria: Yes

Included in protocol analysis: Yes

Protocol: 6, 6, 10, 6

Filename: Bin6_plateA5_D7.fcs

Split 1: Petrol shading

Split 2: Green shading

Split 3: Violet shading

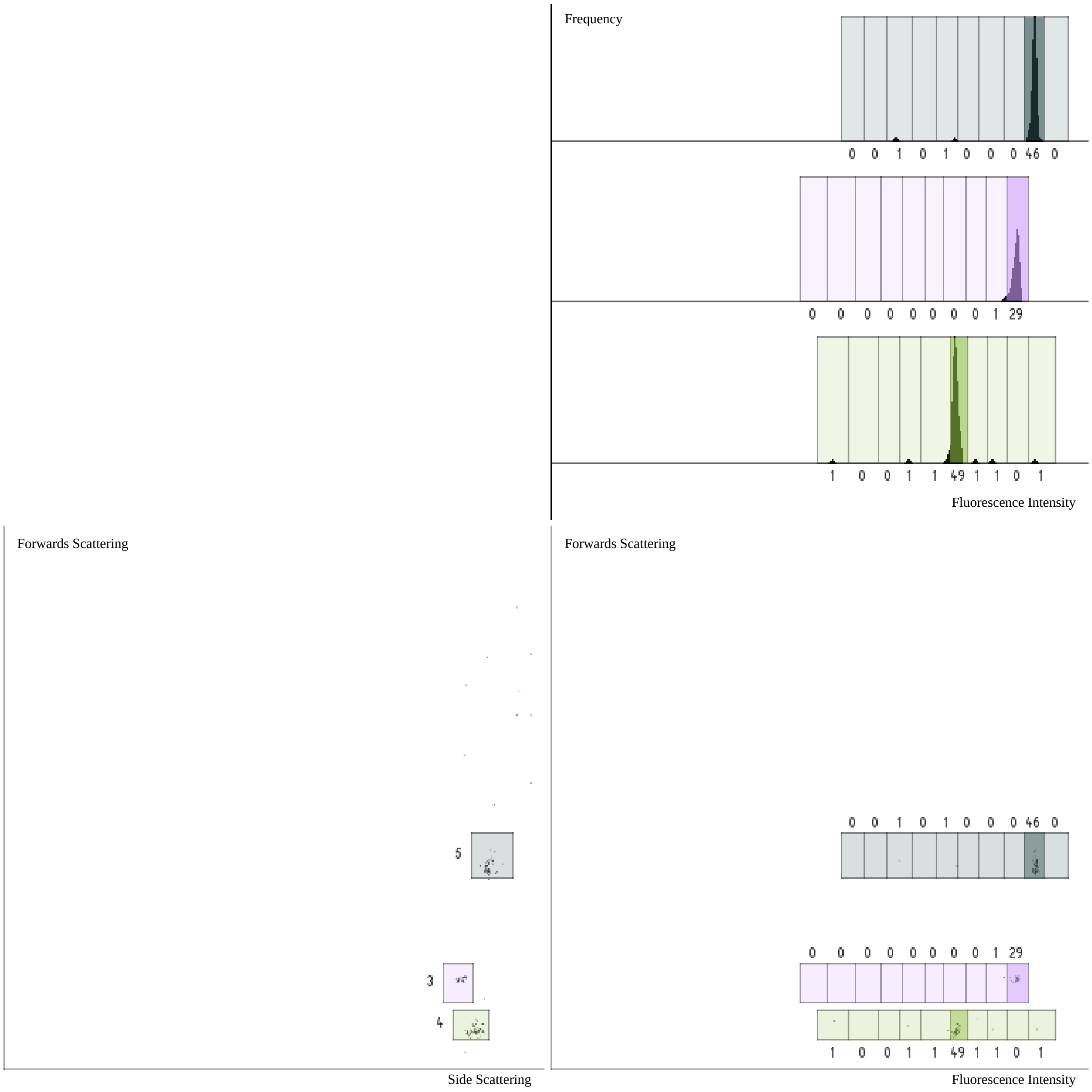

ANNEX 3: TAG DECONVOLUTION - BEAD 181

Passes flow sorting criteria: Yes

Passes tag deconvolution criteria: Yes

Included in protocol analysis: Yes

Protocol: 8, 5, 8, 6

Filename: Bin6_plateA5_E4.fcs

Split 1: Petrol shading

Split 2: Green shading

Split 3: Violet shading

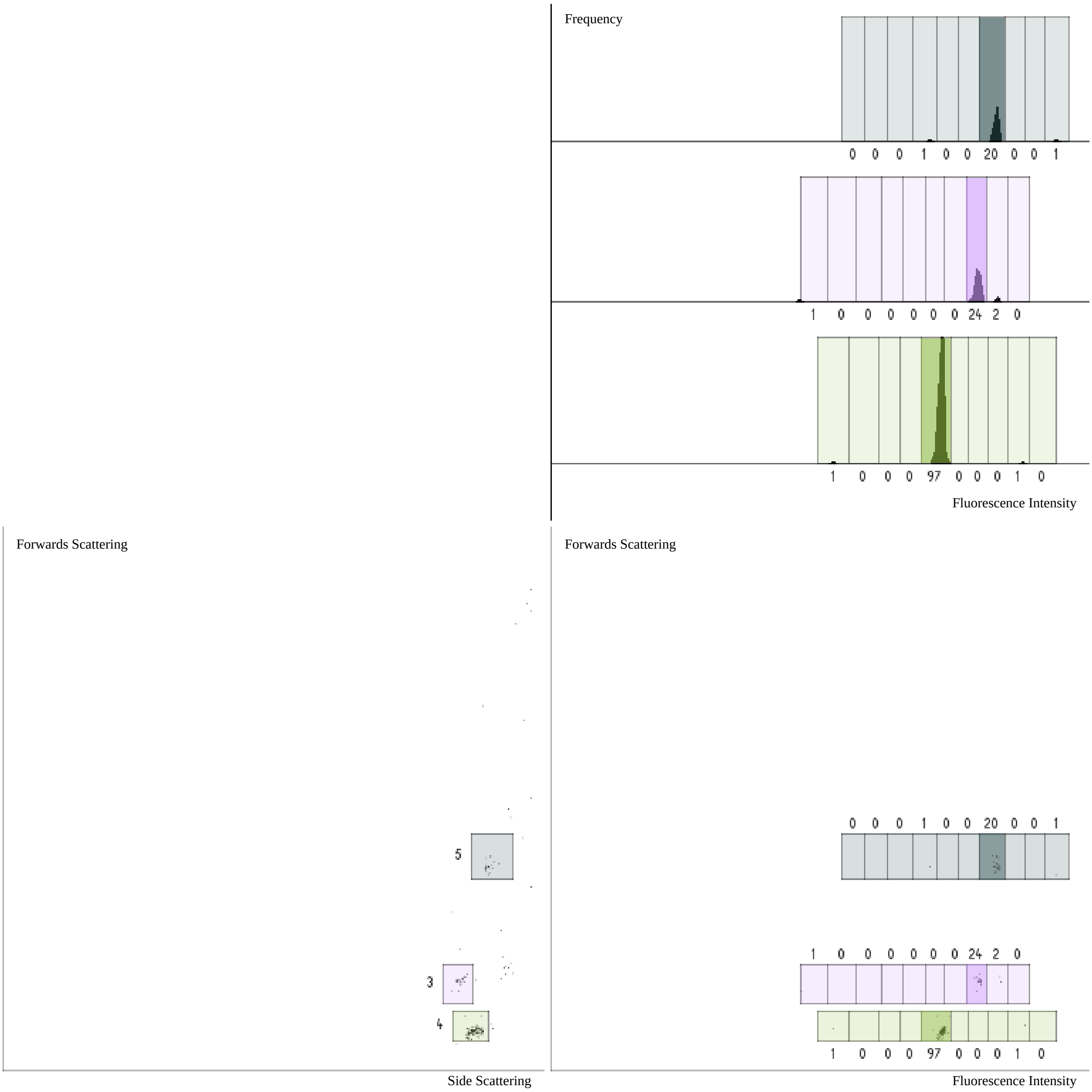

ANNEX 3: TAG DECONVOLUTION - BEAD 182

Passes flow sorting criteria: Yes

Passes tag deconvolution criteria: No

Included in protocol analysis: No

Protocol: N/A

Filename: Bin6_plateA5_E7.fcs

Split 1: Petrol shading

Split 2: Green shading

Split 3: Violet shading

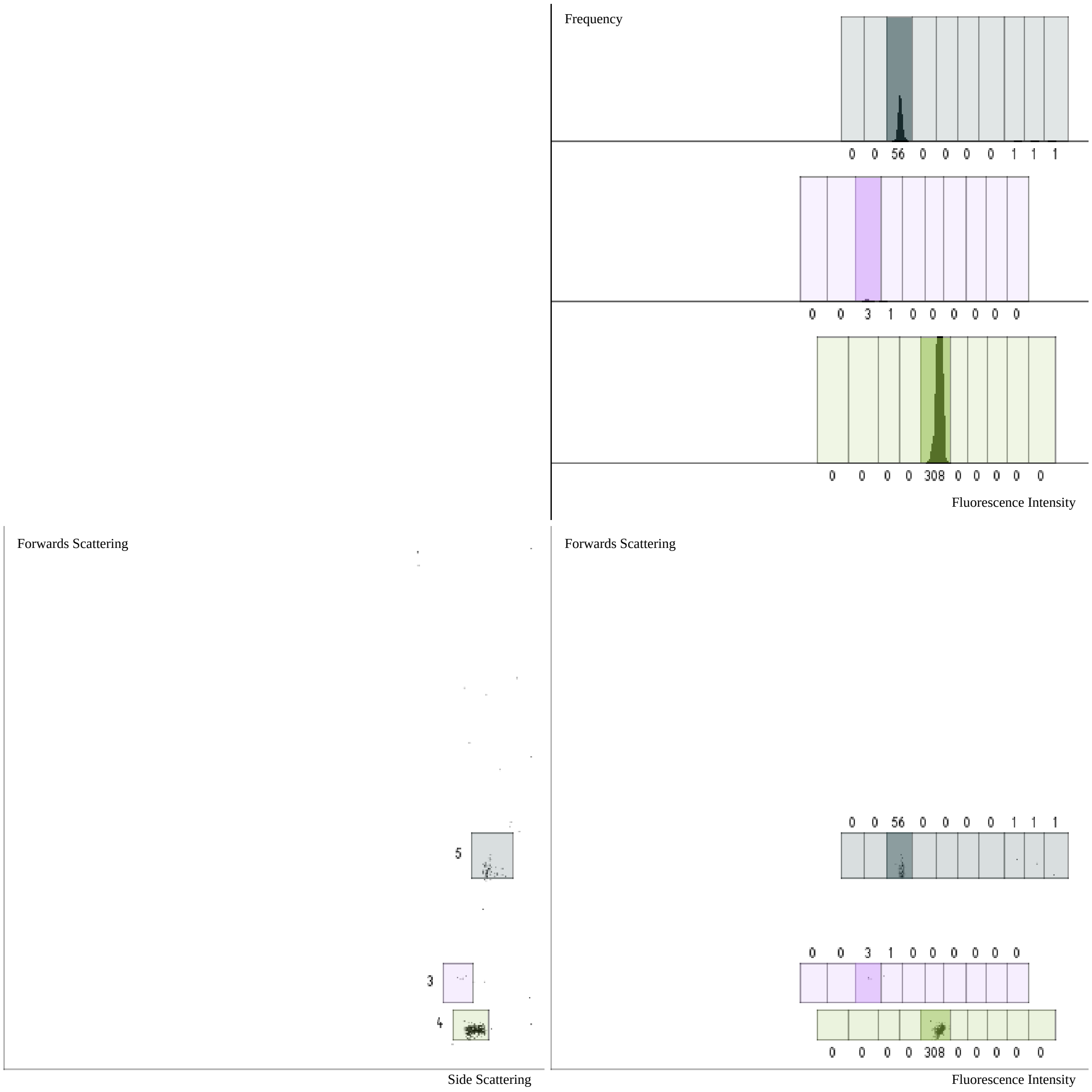

ANNEX 3: TAG DECONVOLUTION - BEAD 183

Passes flow sorting criteria: Yes

Passes tag deconvolution criteria: Yes

Included in protocol analysis: Yes

Protocol: 2, 8, 8, 6

Filename: Bin6_plateA5_E10.fcs

Split 1: Petrol shading

Split 2: Green shading

Split 3: Violet shading

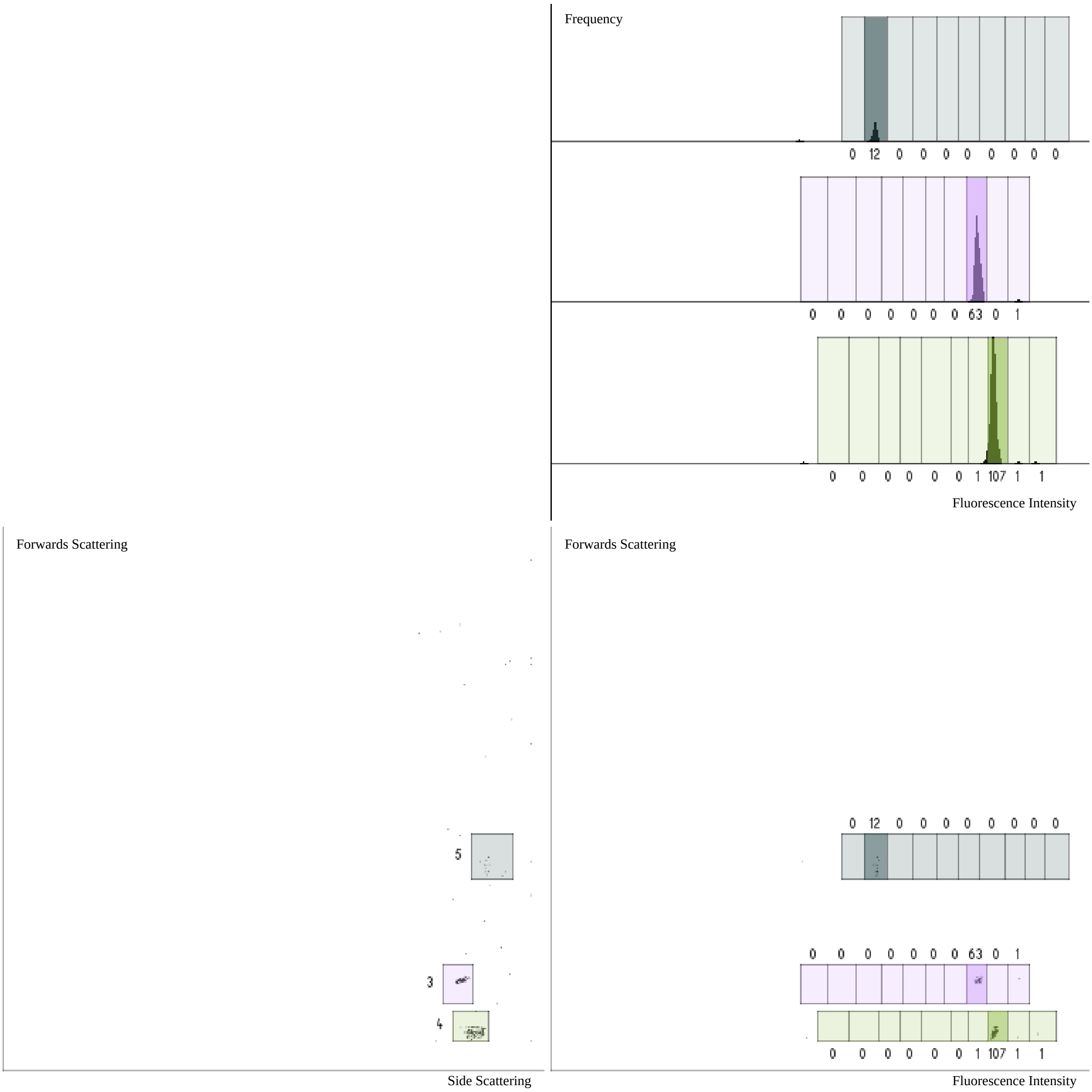

ANNEX 3: TAG DECONVOLUTION - BEAD 184

Passes flow sorting criteria: Yes

Passes tag deconvolution criteria: Yes

Included in protocol analysis: Yes

Protocol: 6, 4, 10, 6

Filename: Bin6_plateA5_F1.fcs

Split 1: Petrol shading

Split 2: Green shading

Split 3: Violet shading

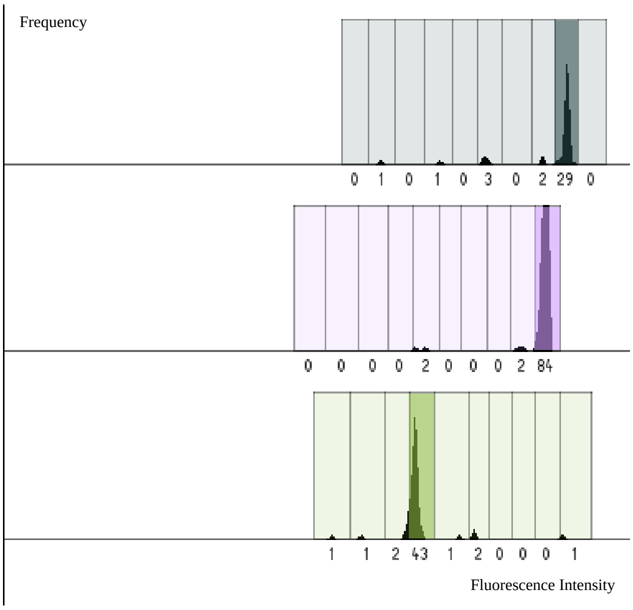

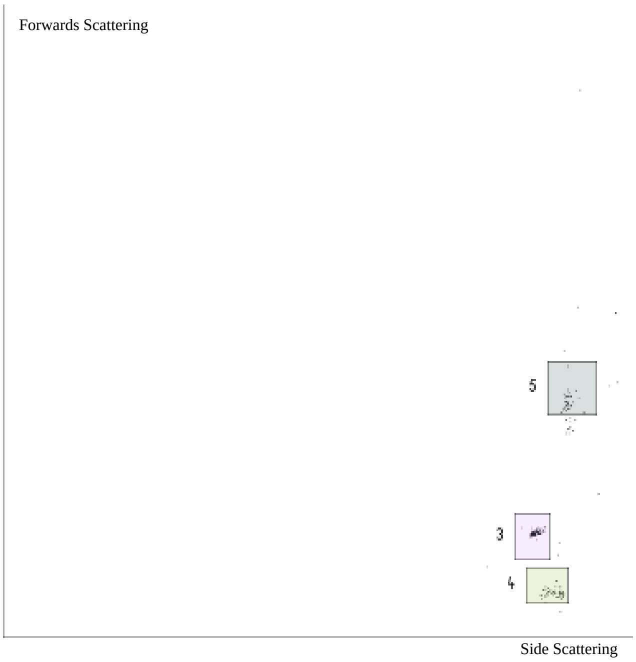

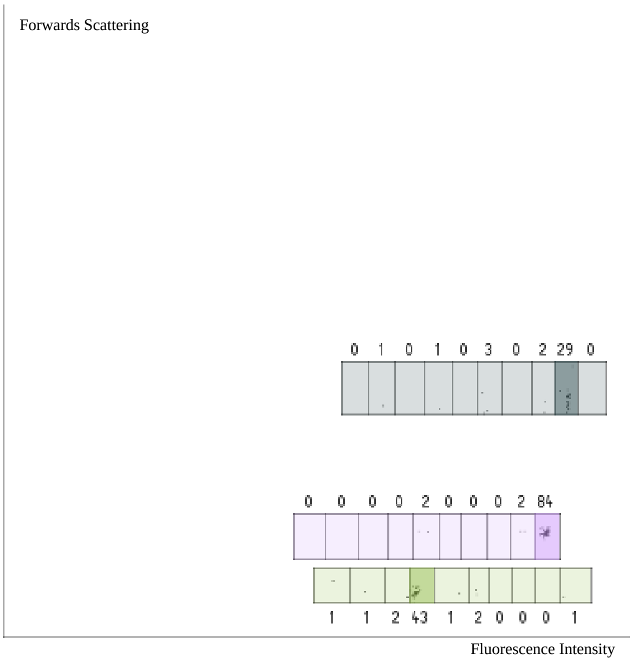

ANNEX 3: TAG DECONVOLUTION - BEAD 185

Passes flow sorting criteria: Yes

Passes tag deconvolution criteria: Yes

Included in protocol analysis: Yes

Protocol: 10, 6, 10, 6

Filename: Bin6_plateA5_F4.fcs

Split 1: Petrol shading

Split 2: Green shading

Split 3: Violet shading

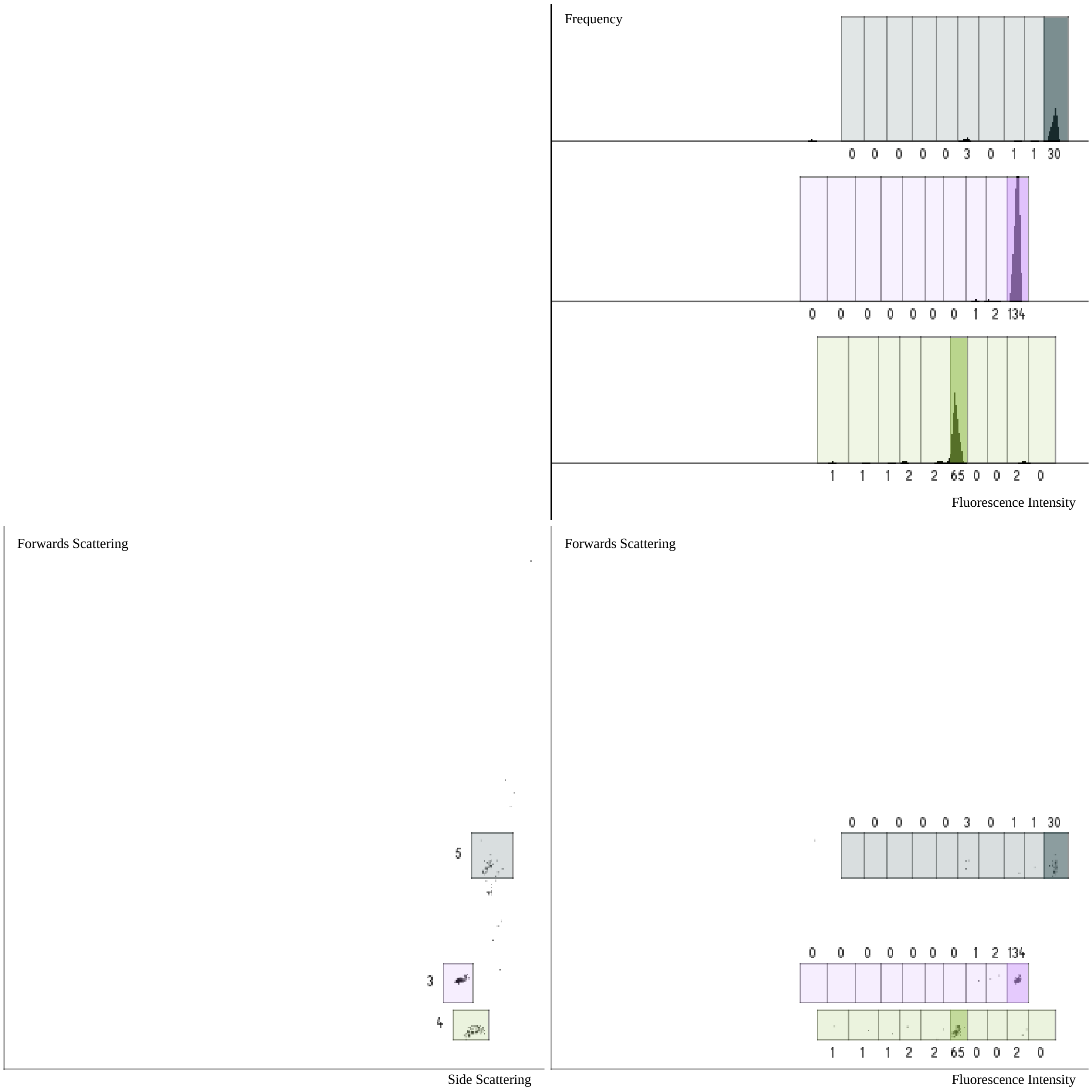

ANNEX 3: TAG DECONVOLUTION - BEAD 186

Passes flow sorting criteria: Yes

Passes tag deconvolution criteria: Yes

Included in protocol analysis: Yes

Protocol: 3, 8, 3, 6

Filename: Bin6_plateA5_F5.fcs

Split 1: Petrol shading

Split 2: Green shading

Split 3: Violet shading

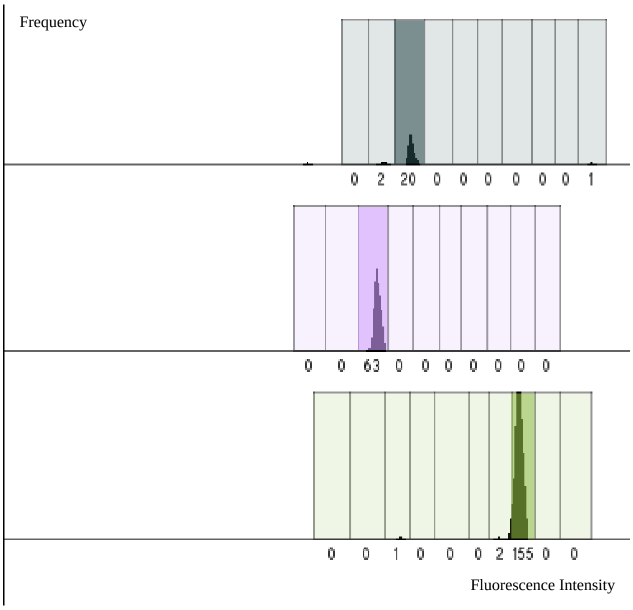

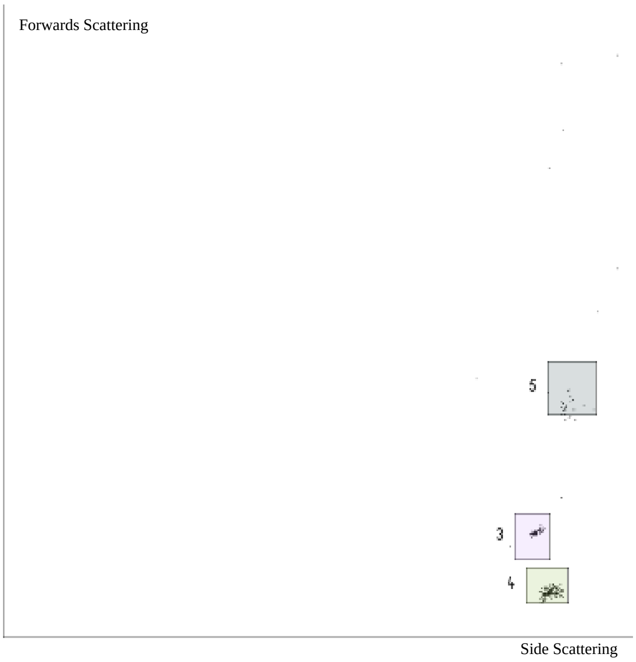

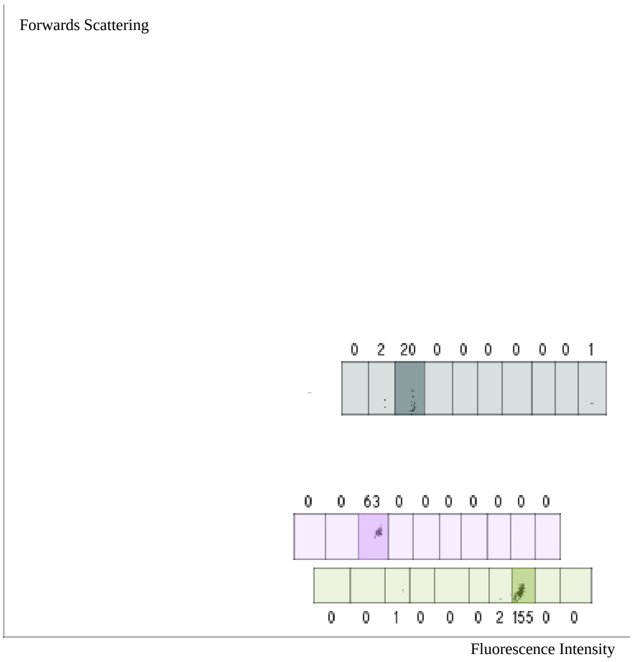

ANNEX 3: TAG DECONVOLUTION - BEAD 187

Passes flow sorting criteria: Yes

Passes tag deconvolution criteria: Yes

Included in protocol analysis: Yes

Protocol: 7, 6, 4, 6

Filename: Bin6_plateA5_F6.fcs

Split 1: Petrol shading

Split 2: Green shading

Split 3: Violet shading

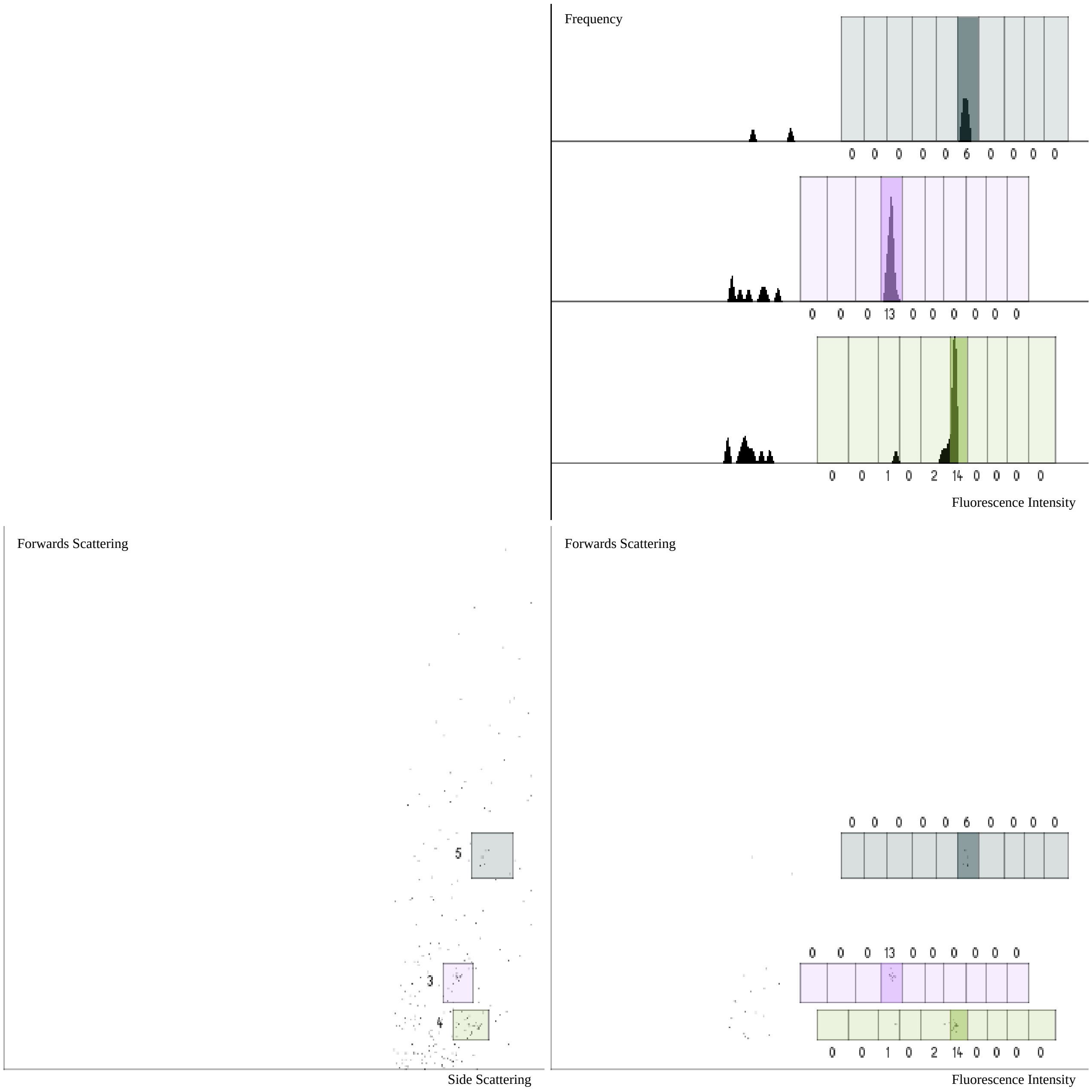

ANNEX 3: TAG DECONVOLUTION - BEAD 188

Passes flow sorting criteria: Yes

Passes tag deconvolution criteria: Yes

Included in protocol analysis: Yes

Protocol: 4, 6, 9, 6

Filename: Bin6_plateA5_F7.fcs

Split 1: Petrol shading

Split 2: Green shading

Split 3: Violet shading

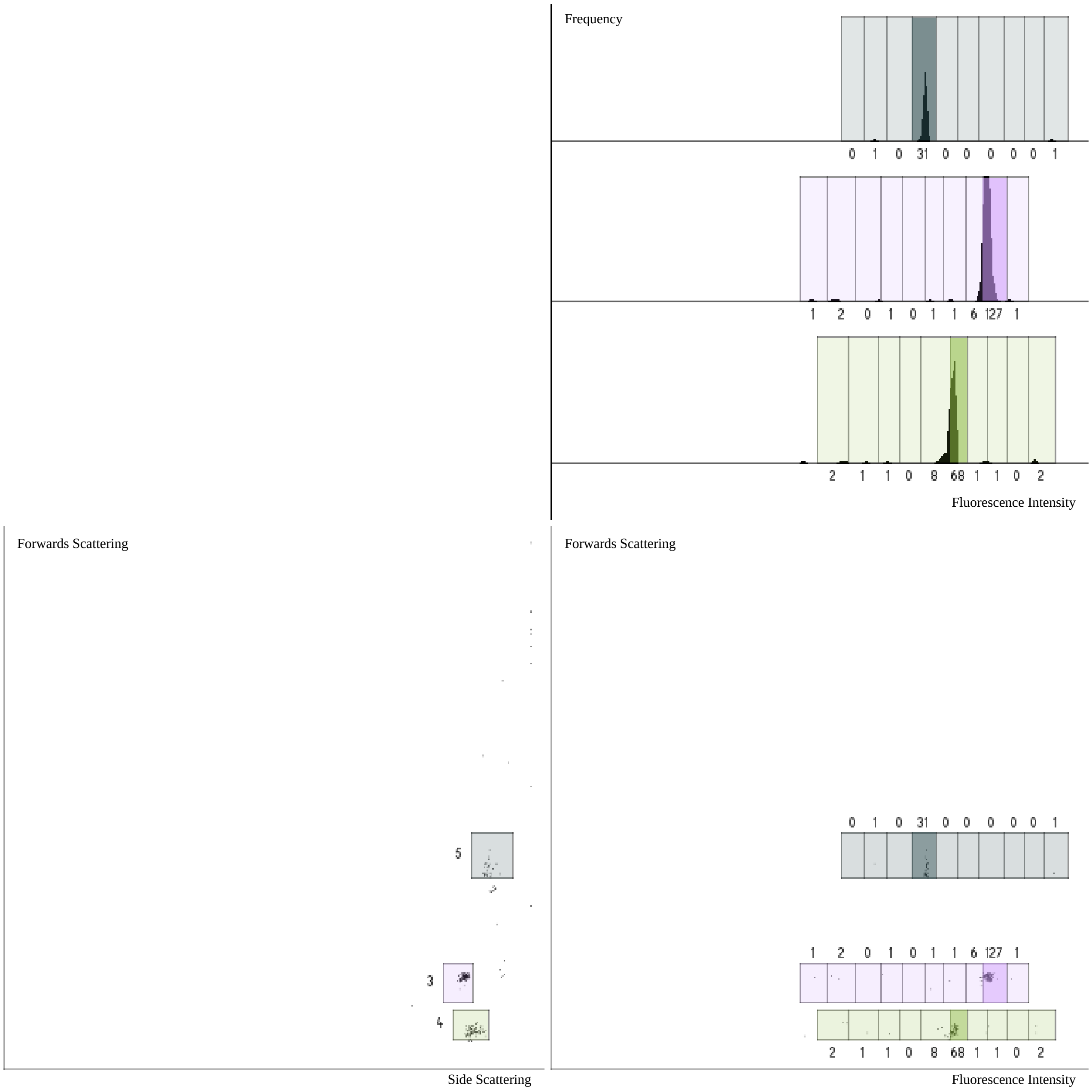

ANNEX 3: TAG DECONVOLUTION - BEAD 189

Passes flow sorting criteria: Yes

Passes tag deconvolution criteria: Yes

Included in protocol analysis: Yes

Protocol: 8, 6, 2, 6

Filename: Bin6_plateA5_F9.fcs

Split 1: Petrol shading

Split 2: Green shading

Split 3: Violet shading

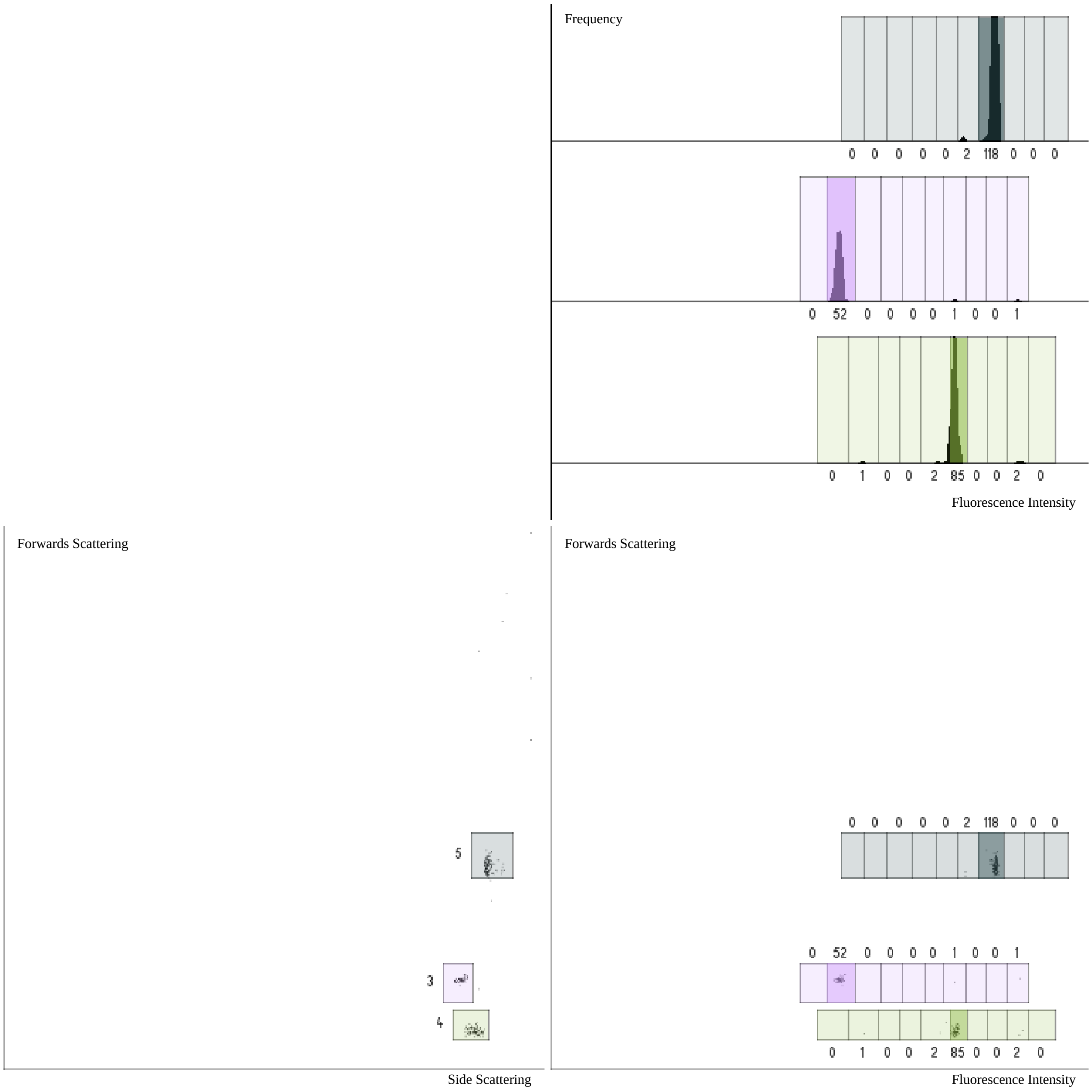

ANNEX 3: TAG DECONVOLUTION - BEAD 190

Passes flow sorting criteria: Yes

Passes tag deconvolution criteria: Yes

Included in protocol analysis: Yes

Protocol: 8, 7, 7, 6

Filename: Bin6_plateA5_F12.fcs

Split 1: Petrol shading

Split 2: Green shading

Split 3: Violet shading

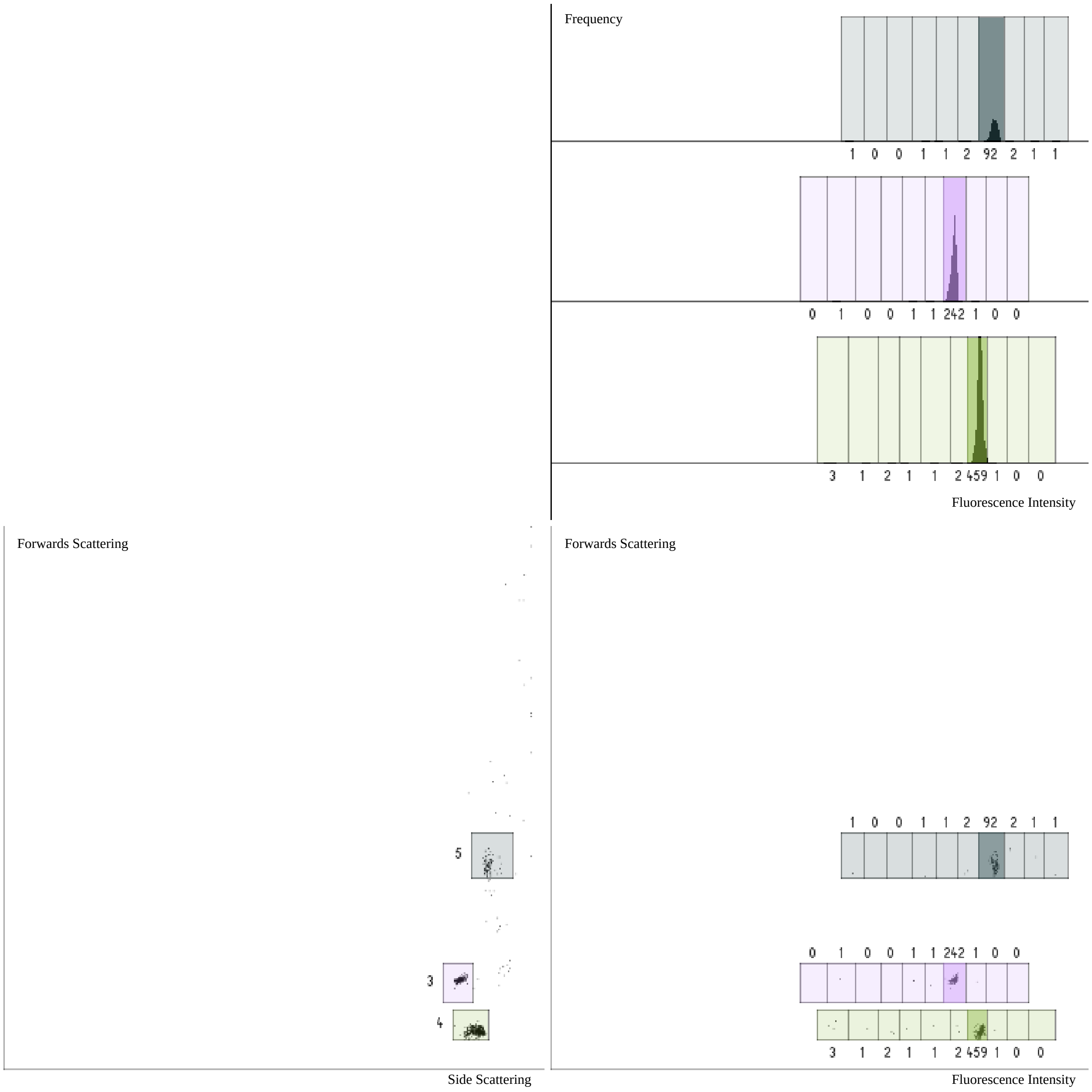

ANNEX 3: TAG DECONVOLUTION - BEAD 191

Passes flow sorting criteria: Yes

Passes tag deconvolution criteria: Yes

Included in protocol analysis: Yes

Protocol: 9, 7, 3, 6

Filename: Bin6_plateA5_G2.fcs

Split 1: Petrol shading

Split 2: Green shading

Split 3: Violet shading

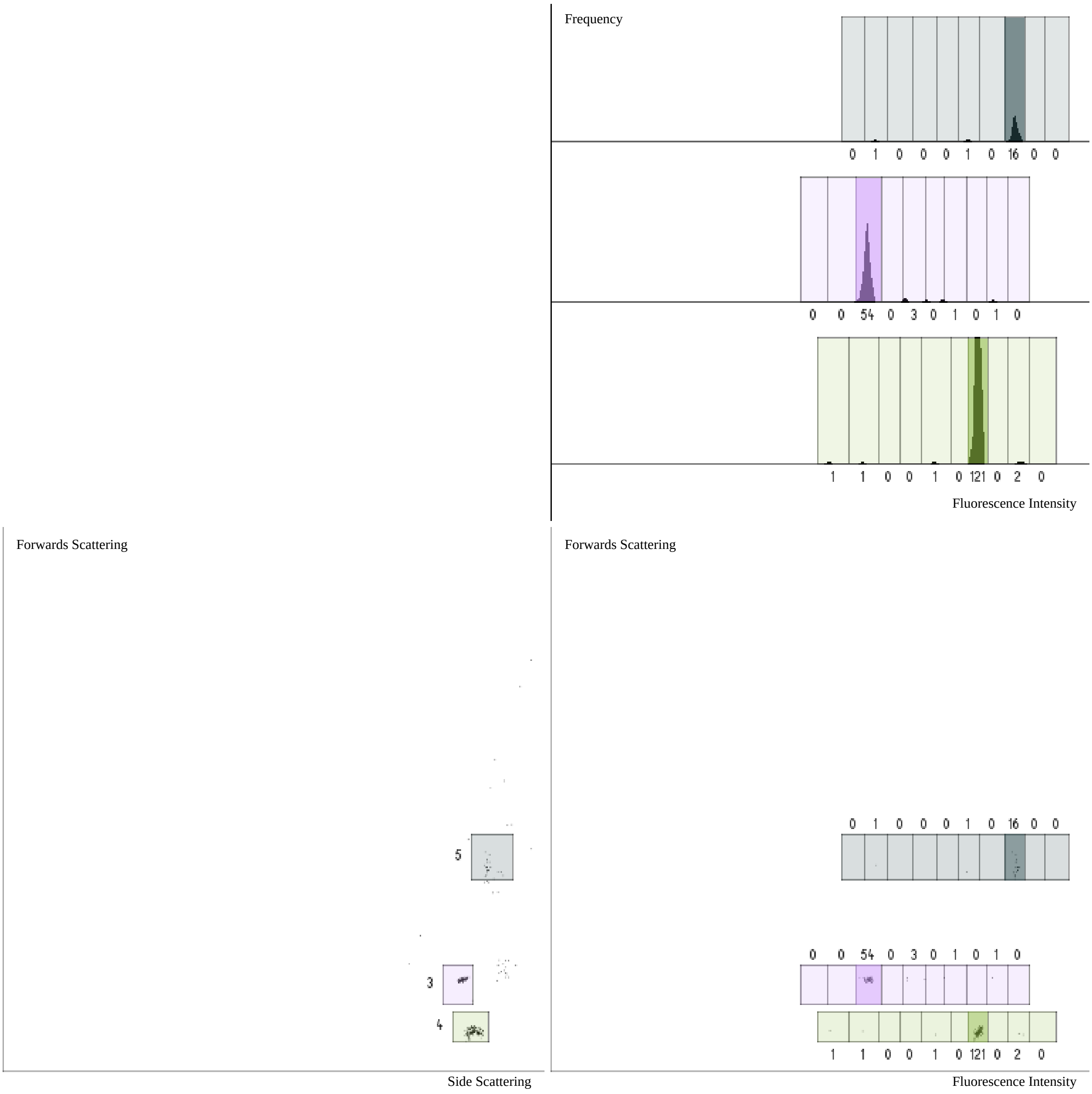

ANNEX 3: TAG DECONVOLUTION - BEAD 192

Passes flow sorting criteria: Yes

Passes tag deconvolution criteria: Yes

Included in protocol analysis: Yes

Protocol: 6, 8, 7, 6

Filename: Bin6_plateA5_G8.fcs

Split 1: Petrol shading

Split 2: Green shading

Split 3: Violet shading

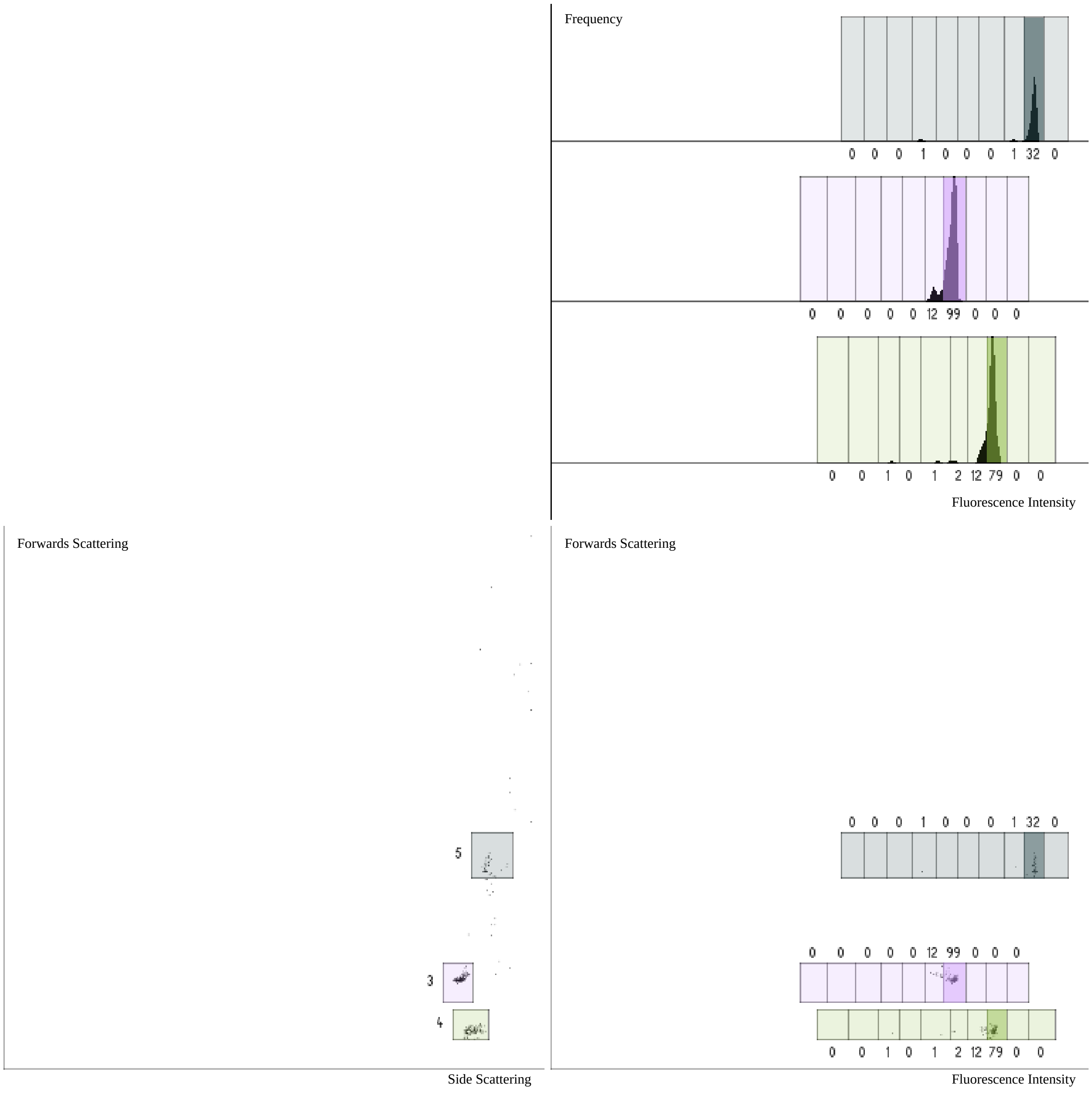

ANNEX 3: TAG DECONVOLUTION - BEAD 193

Passes flow sorting criteria: Yes

Passes tag deconvolution criteria: Yes

Included in protocol analysis: Yes

Protocol: 9, 2, 4, 6

Filename: Bin6_plateA5_G9.fcs

Split 1: Petrol shading

Split 2: Green shading

Split 3: Violet shading

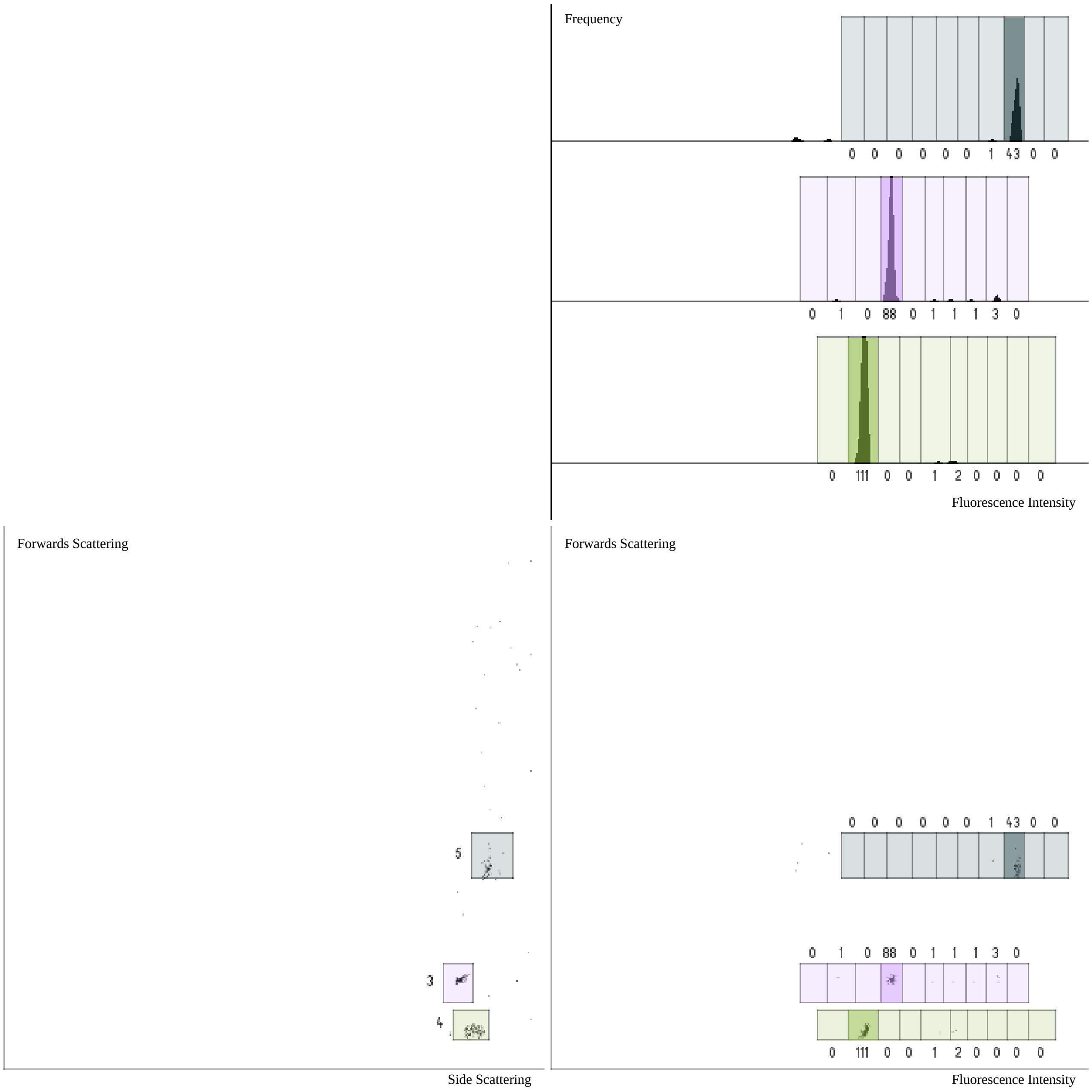

ANNEX 3: TAG DECONVOLUTION - BEAD 194

Passes flow sorting criteria: Yes

Passes tag deconvolution criteria: Yes

Included in protocol analysis: Yes

Protocol: 9, 7, 10, 6

Filename: Bin6_plateA5_H3.fcs

Split 1: Petrol shading

Split 2: Green shading

Split 3: Violet shading

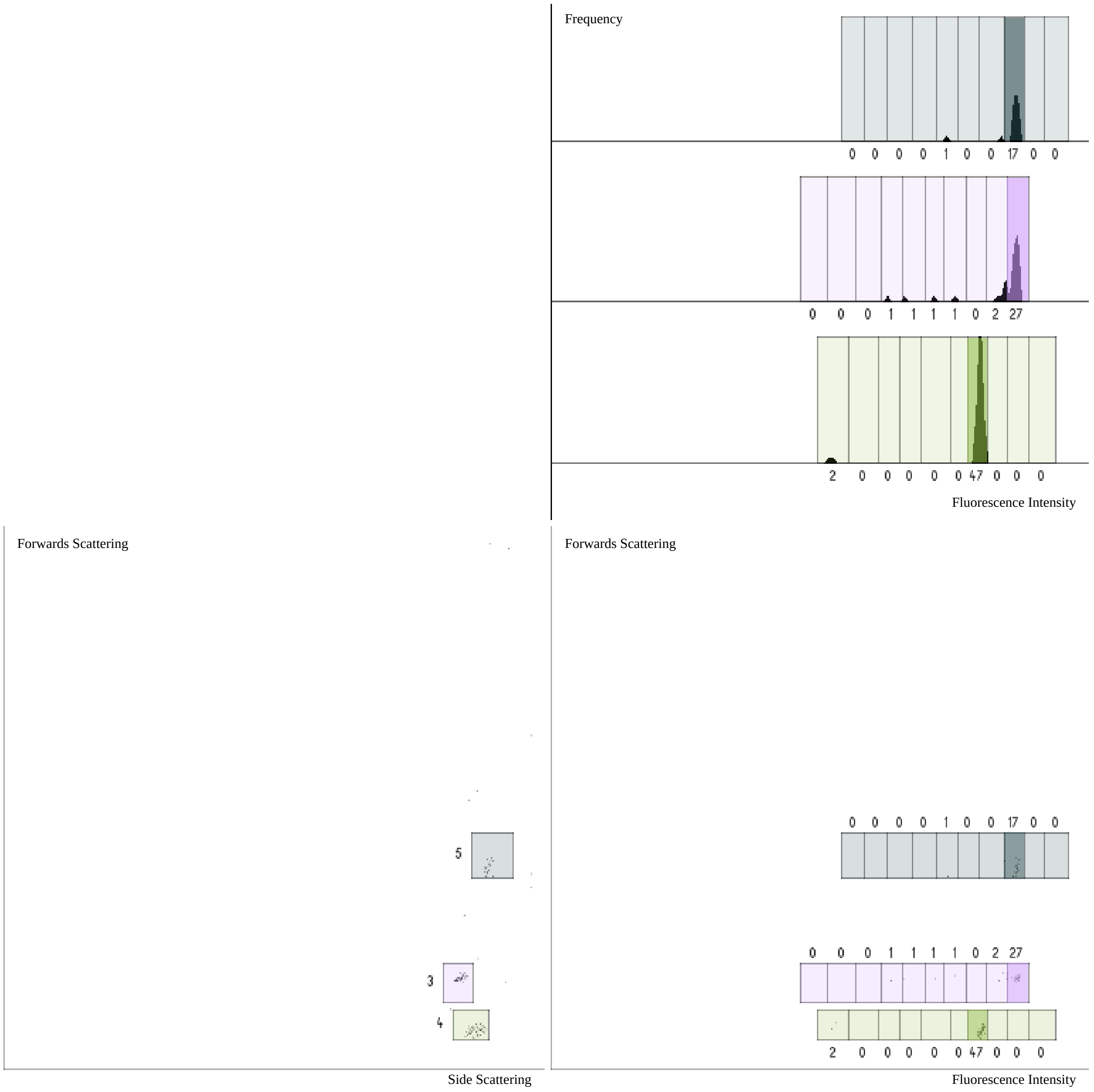

ANNEX 3: TAG DECONVOLUTION - BEAD 195

Passes flow sorting criteria: Yes

Passes tag deconvolution criteria: Yes

Included in protocol analysis: Yes

Protocol: 9, 3, 5, 6

Filename: Bin6_plateA5_H8.fcs

Split 1: Petrol shading

Split 2: Green shading

Split 3: Violet shading

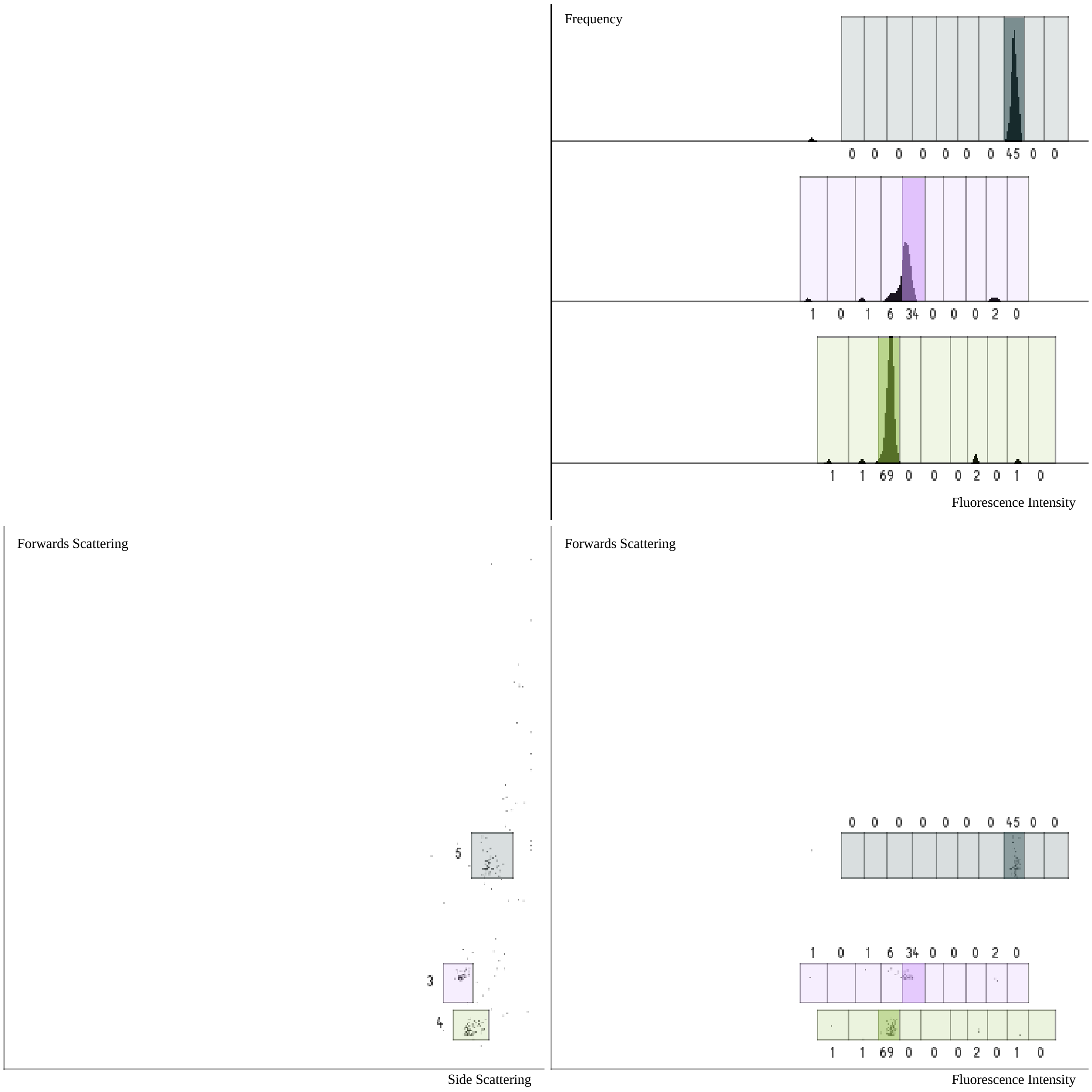

ANNEX 3: TAG DECONVOLUTION - BEAD 196

Passes flow sorting criteria: Yes

Passes tag deconvolution criteria: Yes

Included in protocol analysis: Yes

Protocol: 7, 8, 5, 6

Filename: Bin6_plateA5_H11.fcs

Split 1: Petrol shading

Split 2: Green shading

Split 3: Violet shading

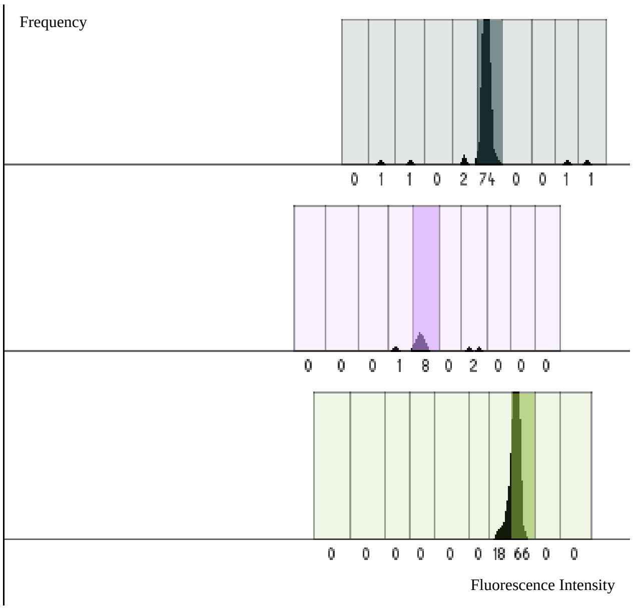

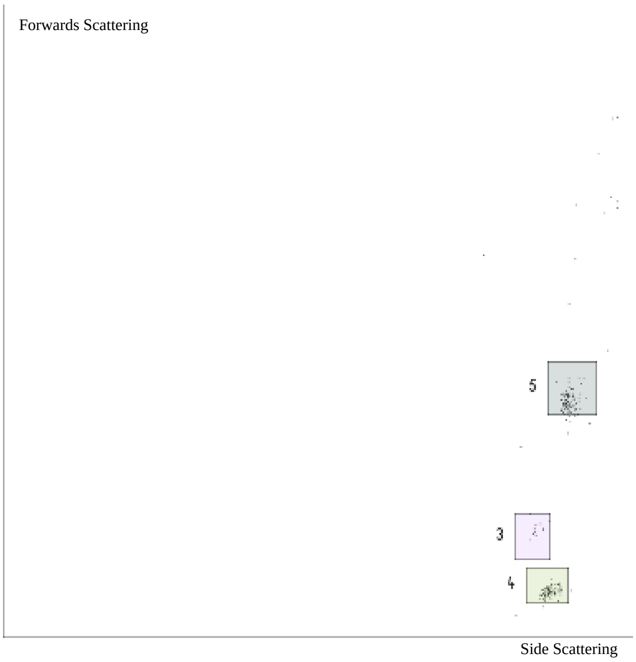

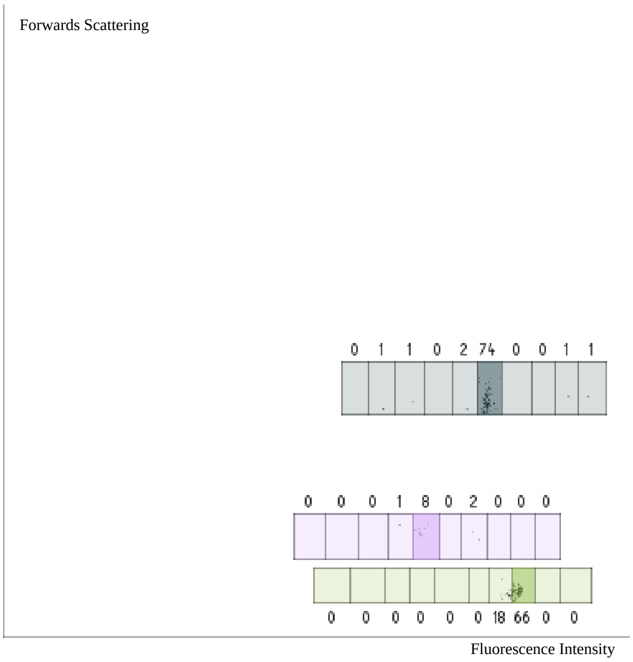

ANNEX 3: TAG DECONVOLUTION - BEAD 197

Passes flow sorting criteria: Yes

Passes tag deconvolution criteria: Yes

Included in protocol analysis: Yes

Protocol: 8, 10, 7, 6

Filename: Bin6_plateA6_A4.fcs

Split 1: Petrol shading

Split 2: Green shading

Split 3: Violet shading

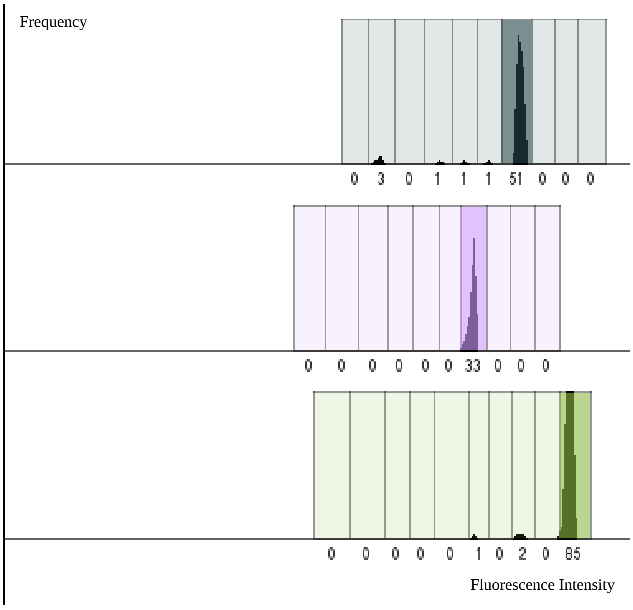

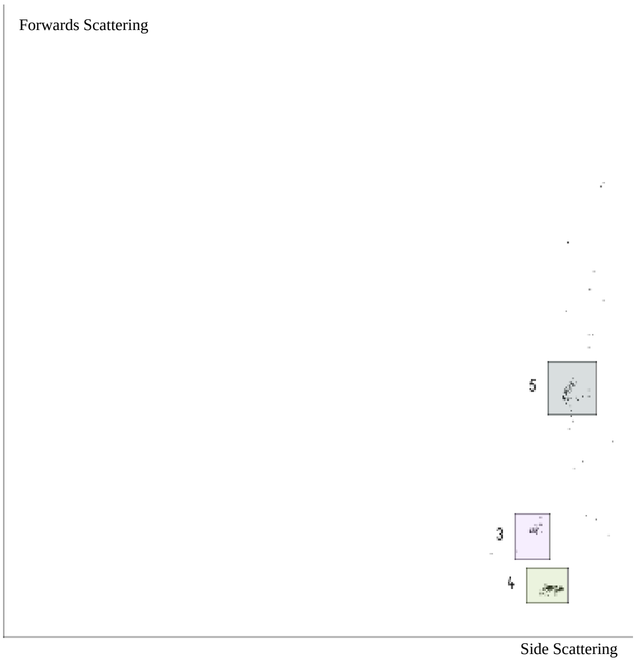

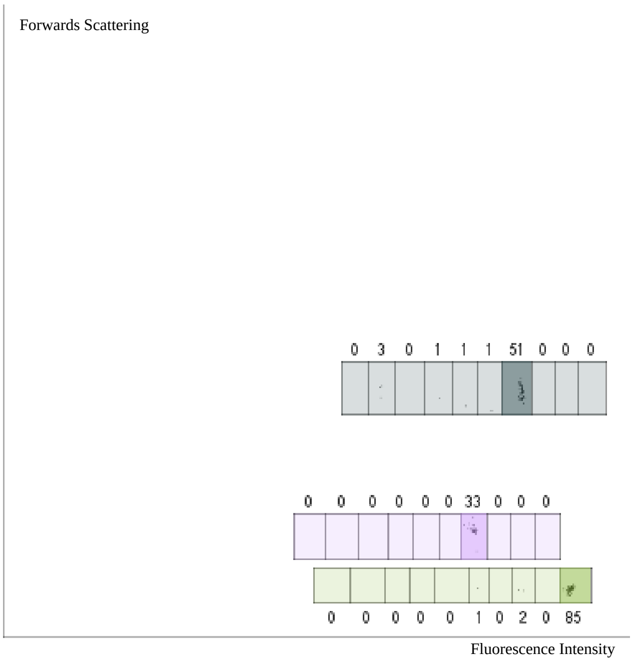

ANNEX 3: TAG DECONVOLUTION - BEAD 198

Passes flow sorting criteria: Yes

Passes tag deconvolution criteria: Yes

Included in protocol analysis: Yes

Protocol: 9, 3, 7, 6

Filename: Bin6_plateA6_A8.fcs

Split 1: Petrol shading

Split 2: Green shading

Split 3: Violet shading

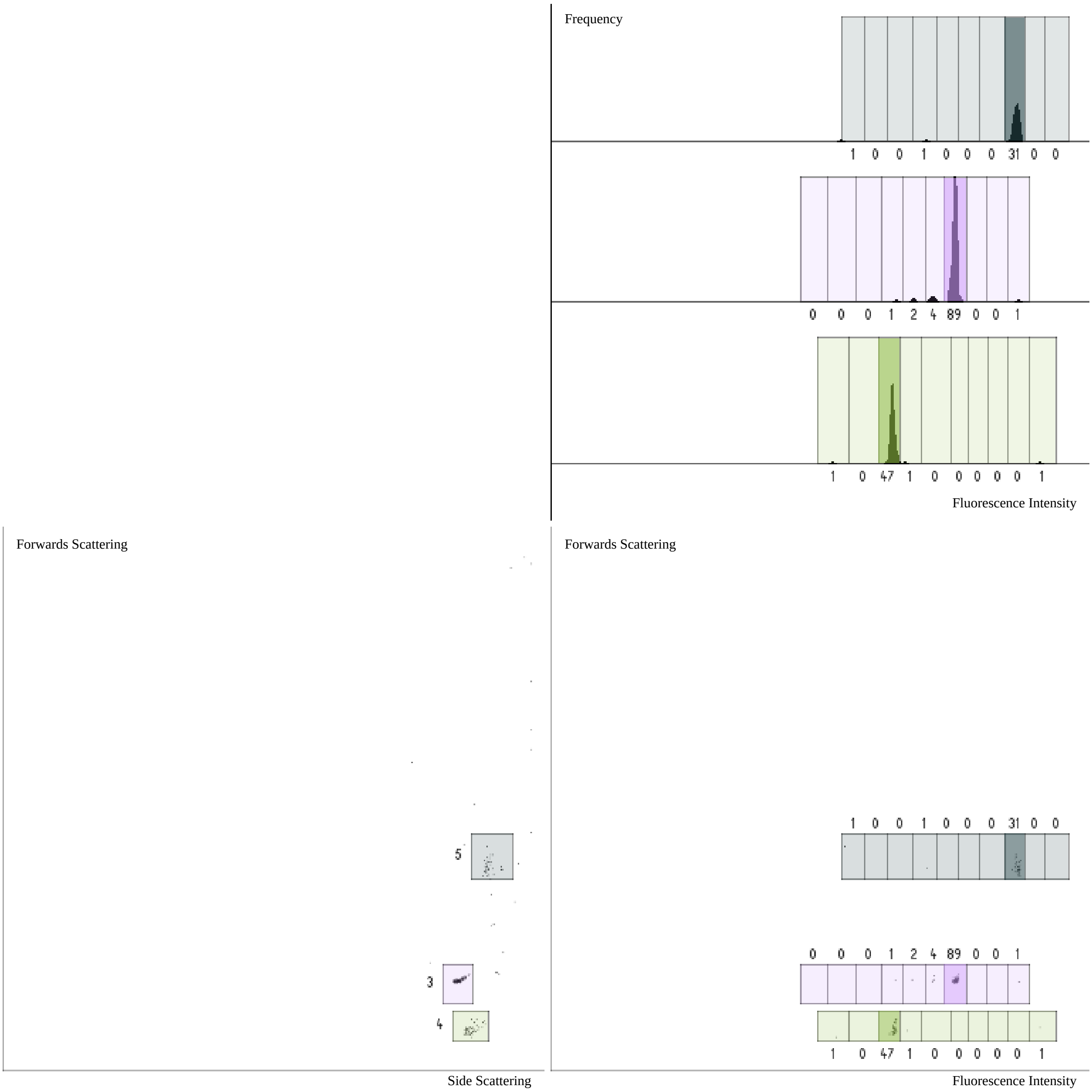

ANNEX 3: TAG DECONVOLUTION - BEAD 199

Passes flow sorting criteria: Yes

Passes tag deconvolution criteria: Yes

Included in protocol analysis: Yes

Protocol: 5, 7, 7, 6

Filename: Bin6_plateA6_A10.fcs

Split 1: Petrol shading

Split 2: Green shading

Split 3: Violet shading

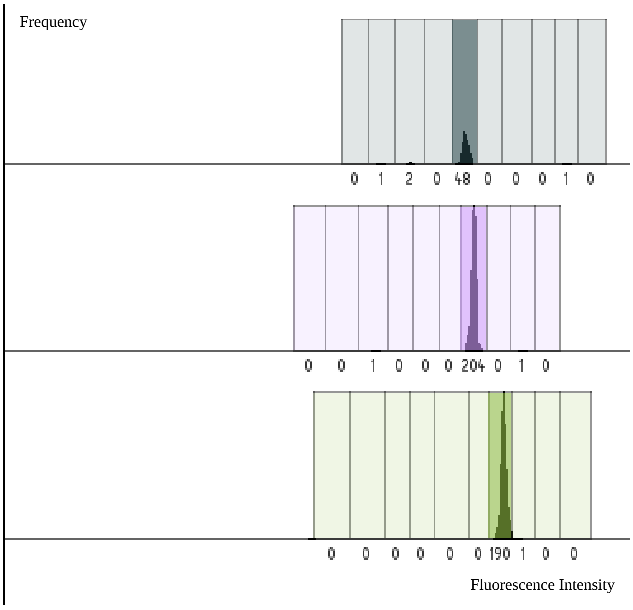

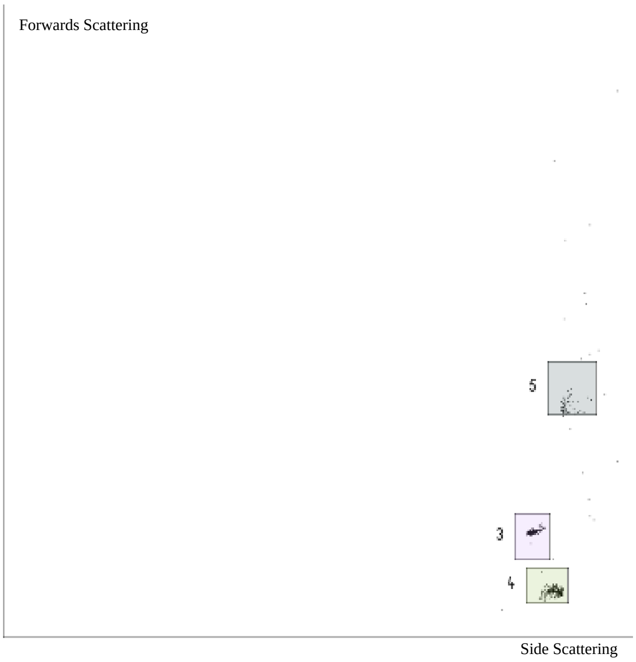

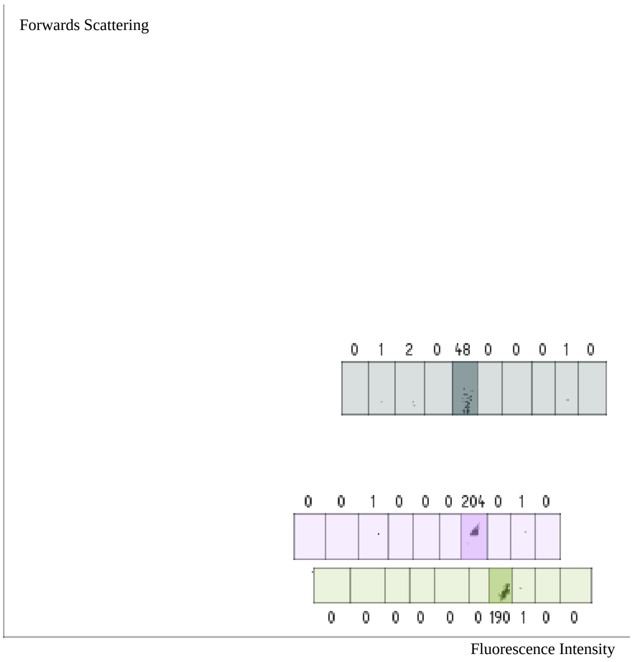

ANNEX 3: TAG DECONVOLUTION - BEAD 200

Passes flow sorting criteria: Yes

Passes tag deconvolution criteria: Yes

Included in protocol analysis: Yes

Protocol: 7, 10, 1, 6

Filename: Bin6_plateA6_A12.fcs

Split 1: Petrol shading

Split 2: Green shading

Split 3: Violet shading

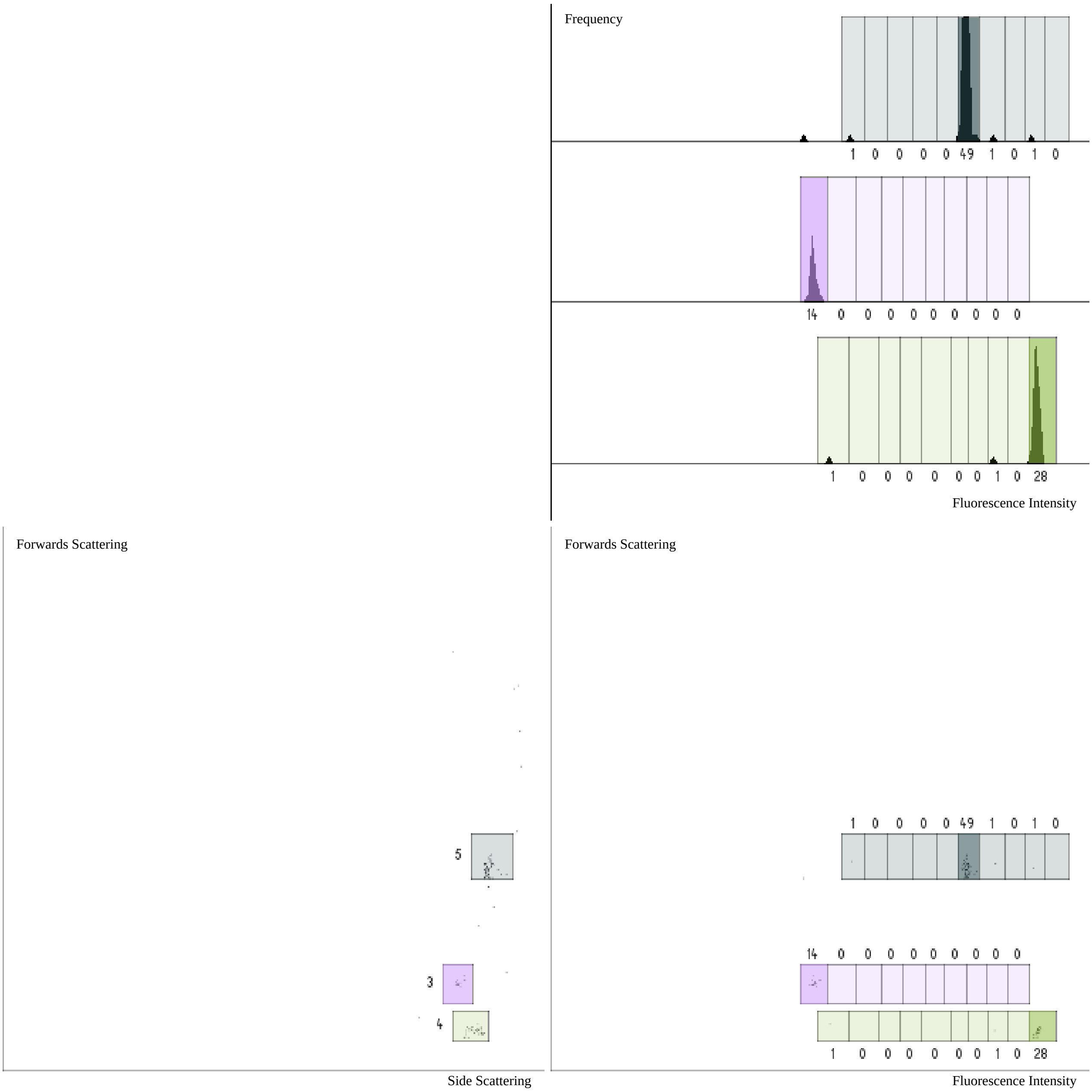

ANNEX 3: TAG DECONVOLUTION - BEAD 201

Passes flow sorting criteria: Yes

Passes tag deconvolution criteria: Yes

Included in protocol analysis: Yes

Protocol: 5, 4, 3, 6

Filename: Bin6_plateA6_B1.fcs

Split 1: Petrol shading

Split 2: Green shading

Split 3: Violet shading

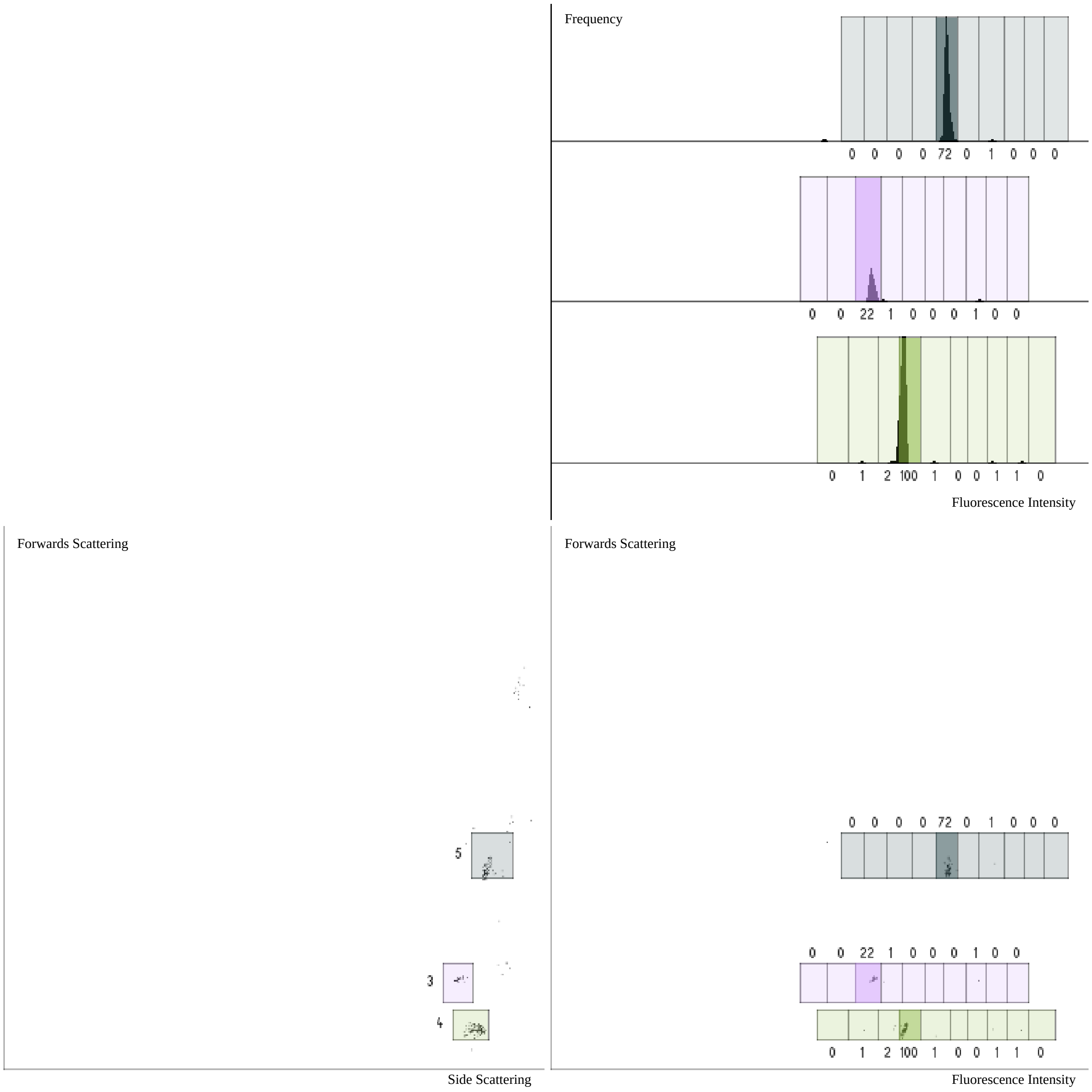

ANNEX 3: TAG DECONVOLUTION - BEAD 202

Passes flow sorting criteria: Yes

Passes tag deconvolution criteria: Yes

Included in protocol analysis: Yes

Protocol: 1, 6, 3, 6

Filename: Bin6_plateA6_C4.fcs

Split 1: Petrol shading

Split 2: Green shading

Split 3: Violet shading

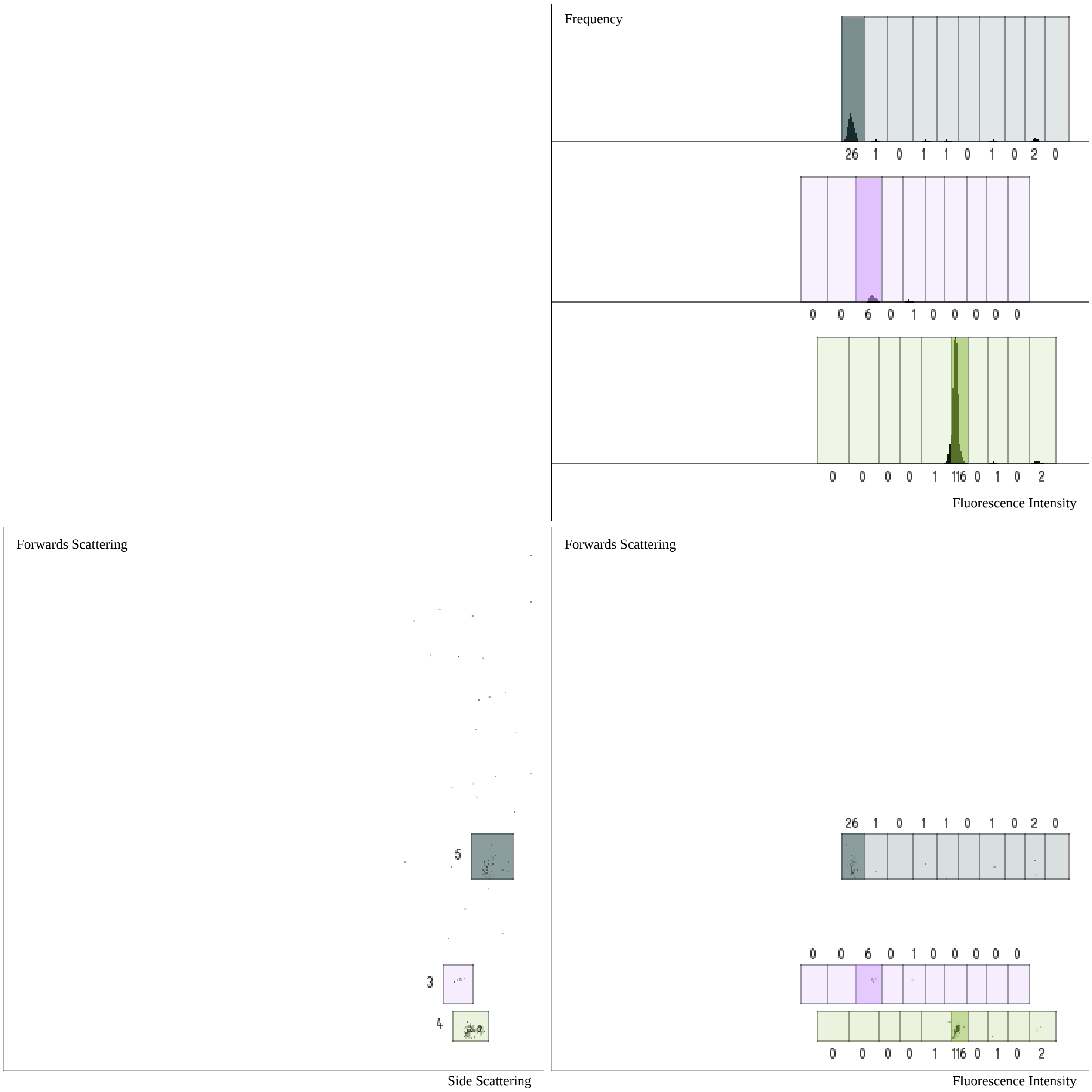

ANNEX 3: TAG DECONVOLUTION - BEAD 203

Passes flow sorting criteria: Yes

Passes tag deconvolution criteria: Yes

Included in protocol analysis: Yes

Protocol: 6, 8, 2, 6

Filename: Bin6_plateA6_C6.fcs

Split 1: Petrol shading

Split 2: Green shading

Split 3: Violet shading

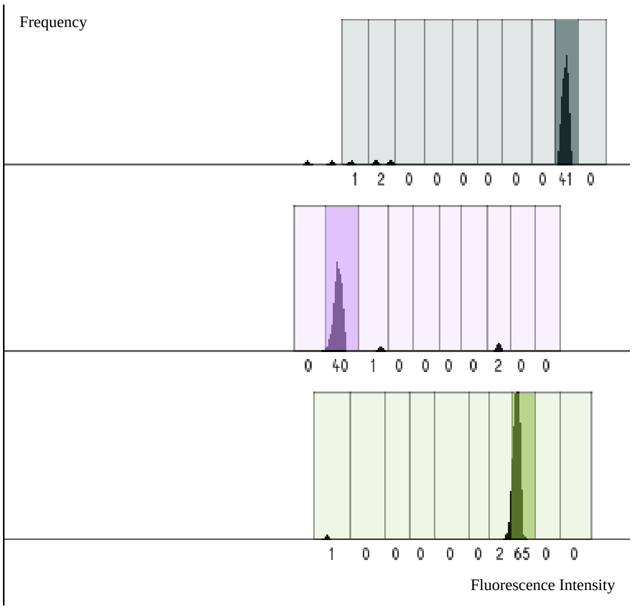

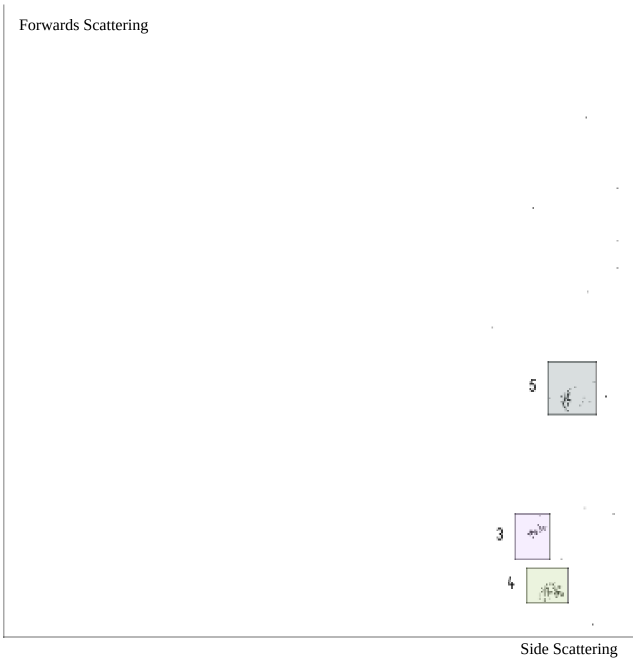

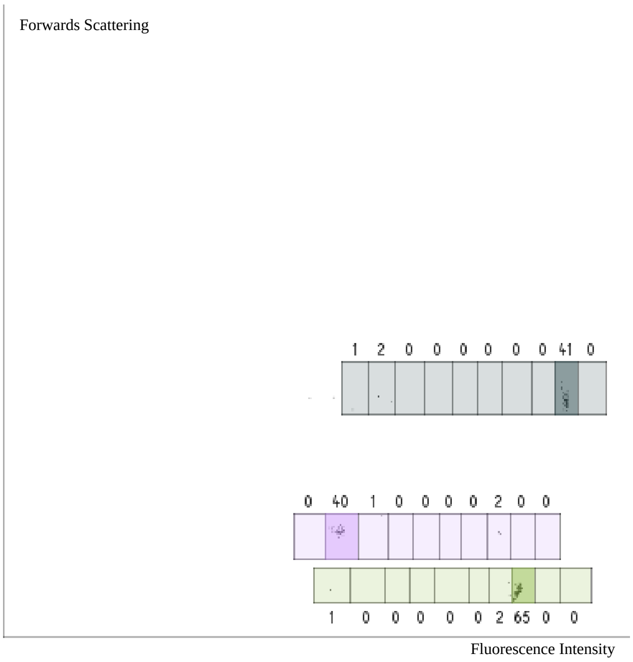

ANNEX 3: TAG DECONVOLUTION - BEAD 204

Passes flow sorting criteria: Yes

Passes tag deconvolution criteria: Yes

Included in protocol analysis: Yes

Protocol: 2, 1, 7, 6

Filename: Bin6_plateA6_C7.fcs

Split 1: Petrol shading

Split 2: Green shading

Split 3: Violet shading

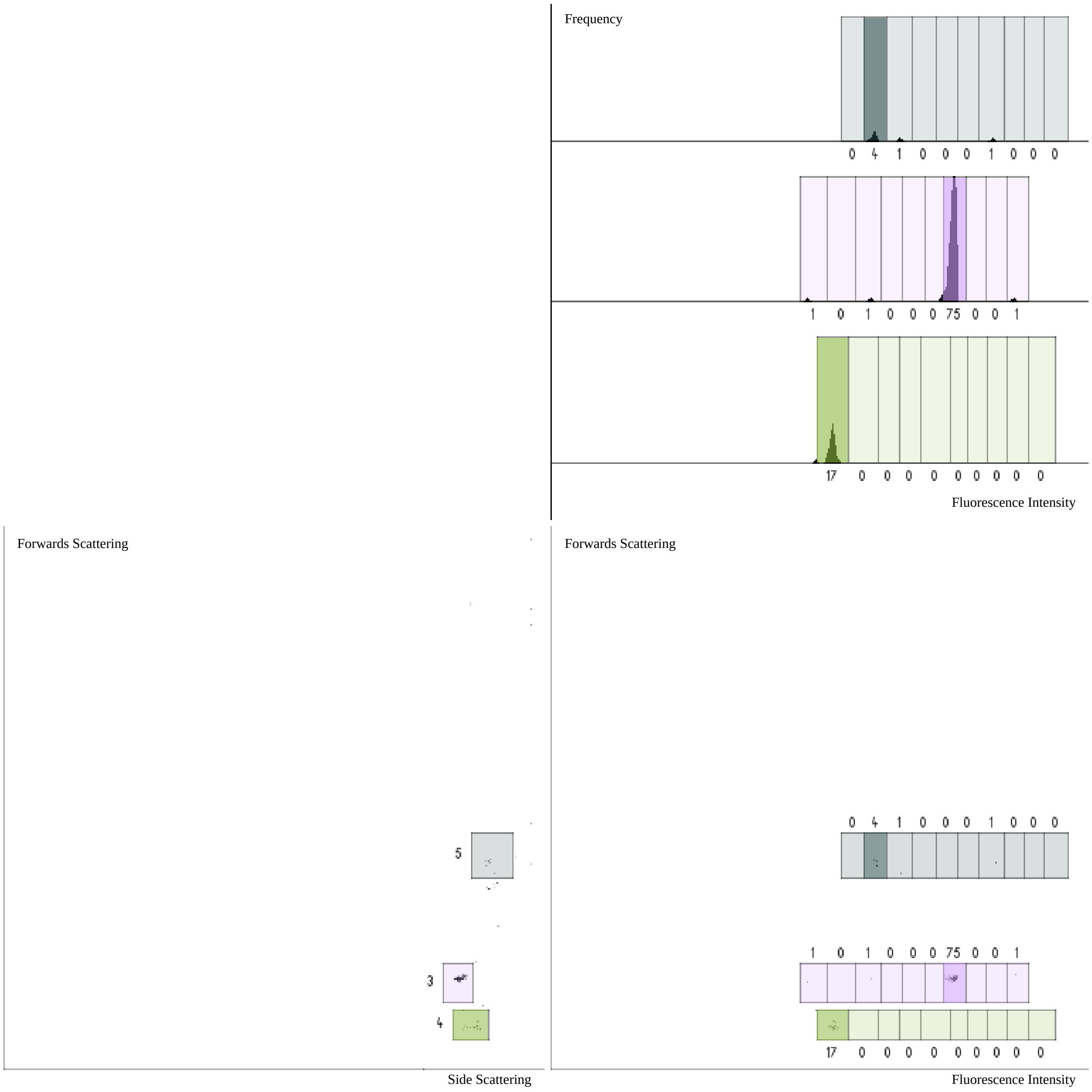

ANNEX 3: TAG DECONVOLUTION - BEAD 205

Passes flow sorting criteria: Yes

Passes tag deconvolution criteria: Yes

Included in protocol analysis: Yes

Protocol: 4, 4, 4, 6

Filename: Bin6_plateA6_C10.fcs

Split 1: Petrol shading

Split 2: Green shading

Split 3: Violet shading

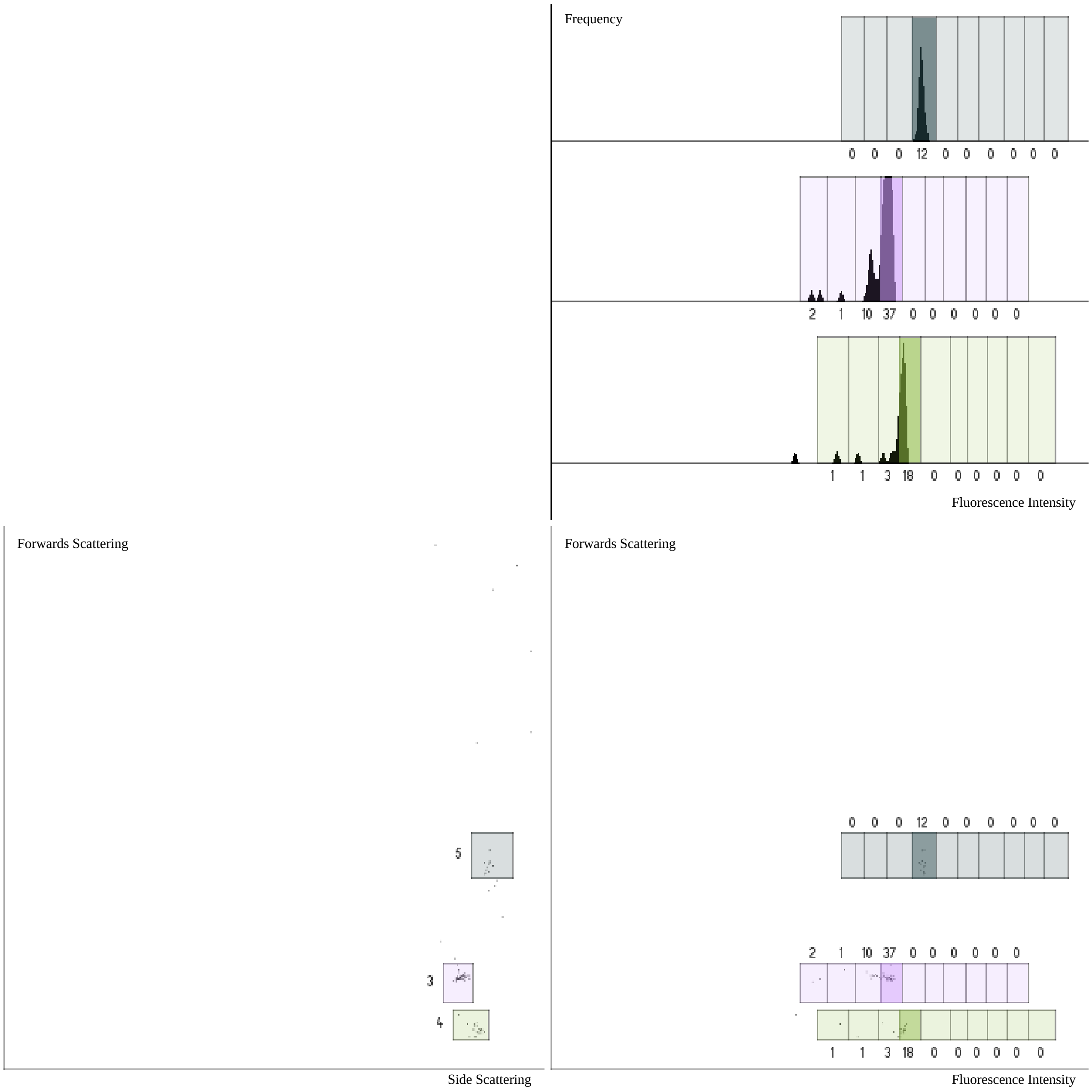

ANNEX 3: TAG DECONVOLUTION - BEAD 206

Passes flow sorting criteria: Yes

Passes tag deconvolution criteria: Yes

Included in protocol analysis: Yes

Protocol: 3, 3, 1, 6

Filename: Bin6_plateA6_C11.fcs

Split 1: Petrol shading

Split 2: Green shading

Split 3: Violet shading

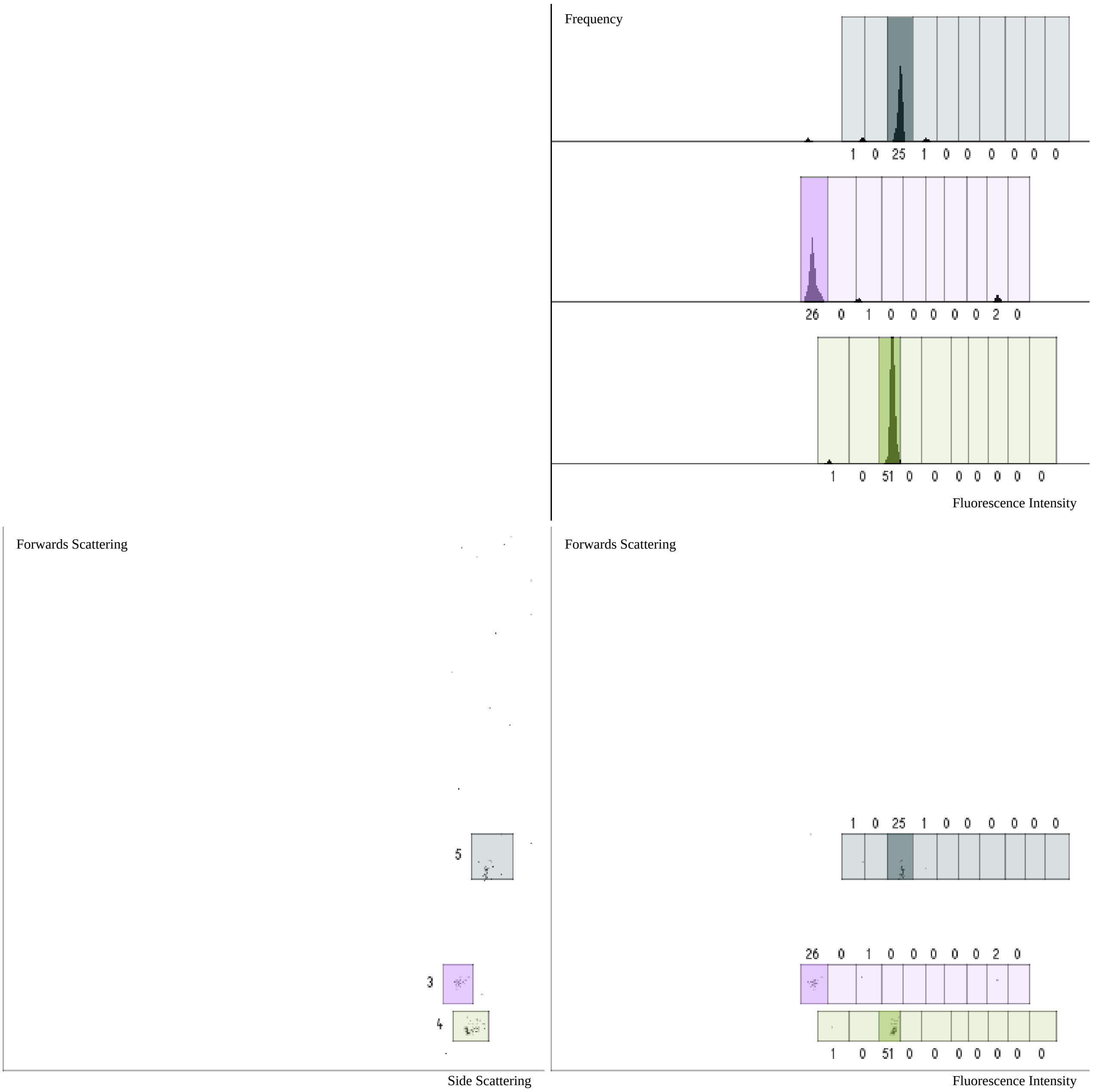

ANNEX 3: TAG DECONVOLUTION - BEAD 207

Passes flow sorting criteria: Yes

Passes tag deconvolution criteria: Yes

Included in protocol analysis: Yes

Protocol: 5, 5, 4, 6

Filename: Bin6_plateA6_D1.fcs

Split 1: Petrol shading

Split 2: Green shading

Split 3: Violet shading

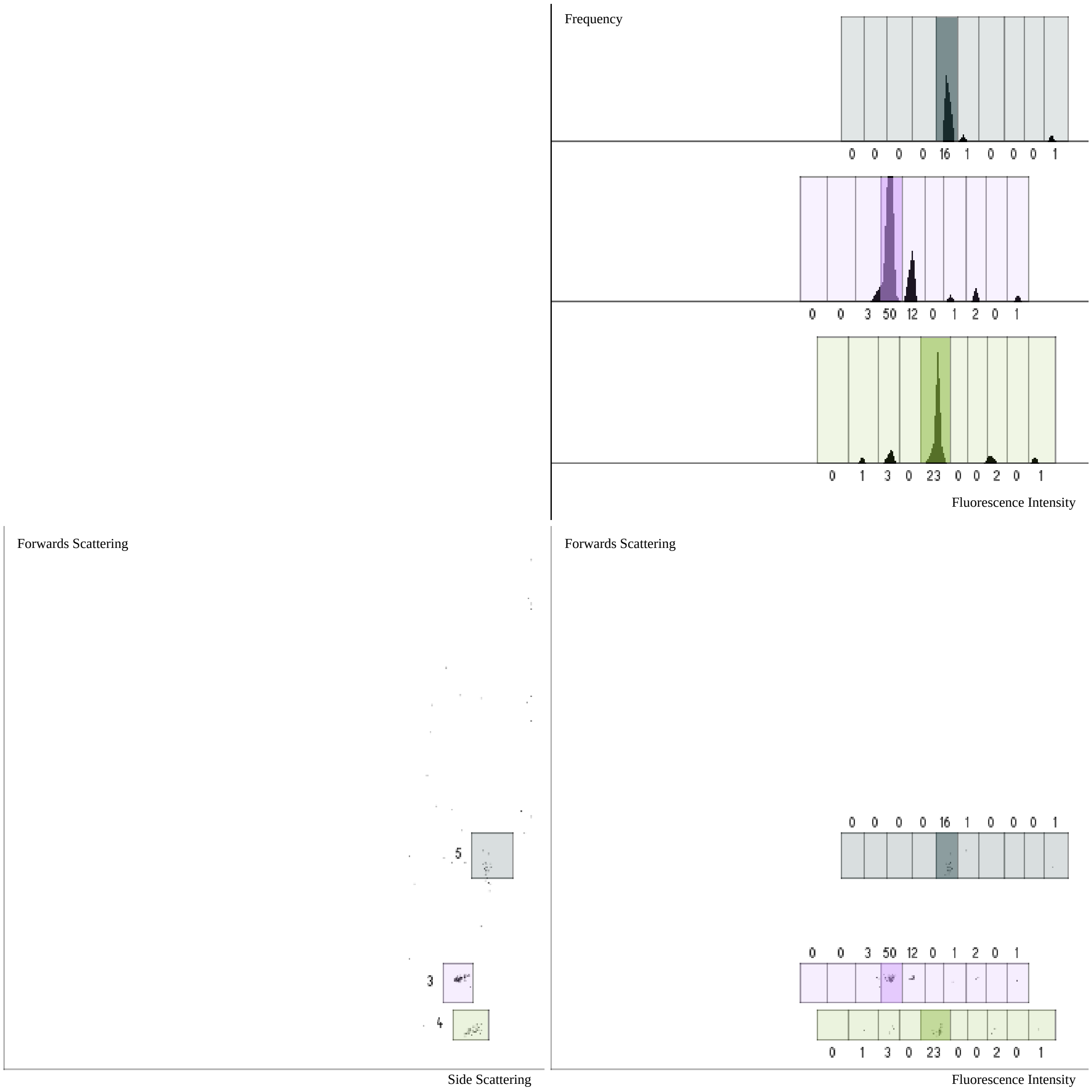

ANNEX 3: TAG DECONVOLUTION - BEAD 208

Passes flow sorting criteria: Yes

Passes tag deconvolution criteria: Yes

Included in protocol analysis: Yes

Protocol: 6, 1, 7, 6

Filename: Bin6_plateA6_D3.fcs

Split 1: Petrol shading

Split 2: Green shading

Split 3: Violet shading

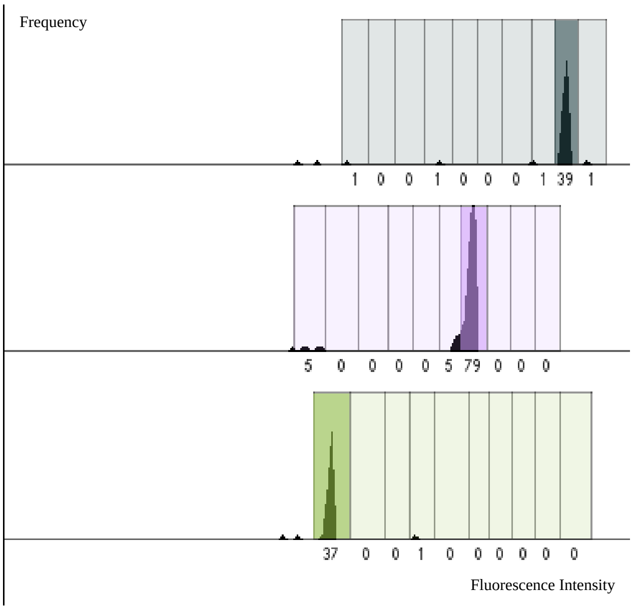

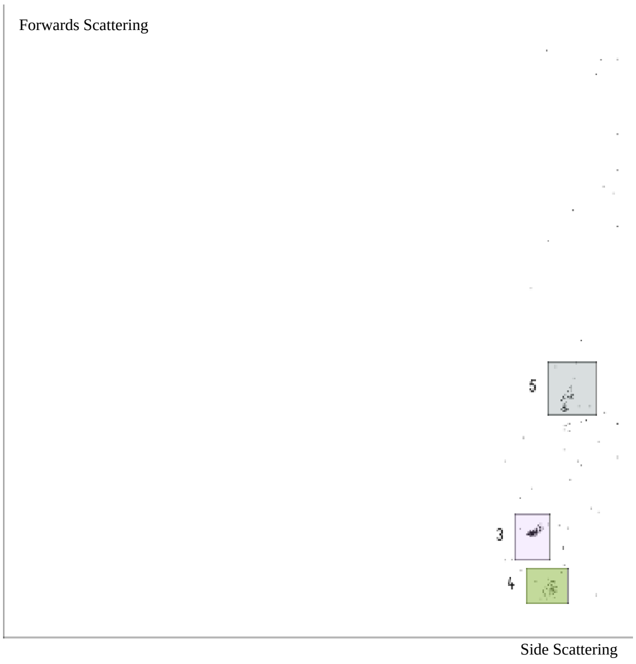

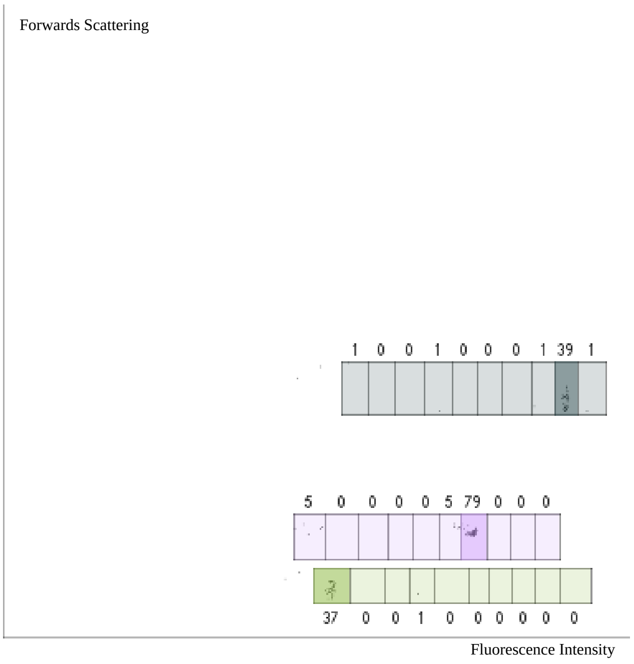

ANNEX 3: TAG DECONVOLUTION - BEAD 209

Passes flow sorting criteria: Yes

Passes tag deconvolution criteria: Yes

Included in protocol analysis: Yes

Protocol: 2, 1, 4, 6

Filename: Bin6_plateA6_D5.fcs

Split 1: Petrol shading

Split 2: Green shading

Split 3: Violet shading

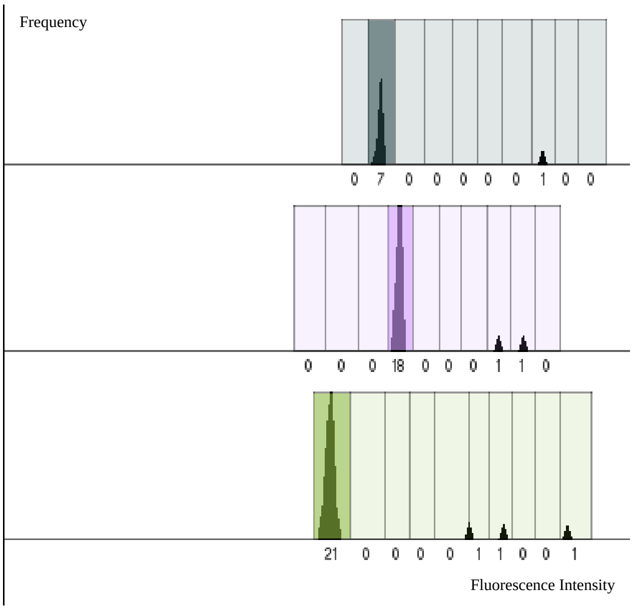

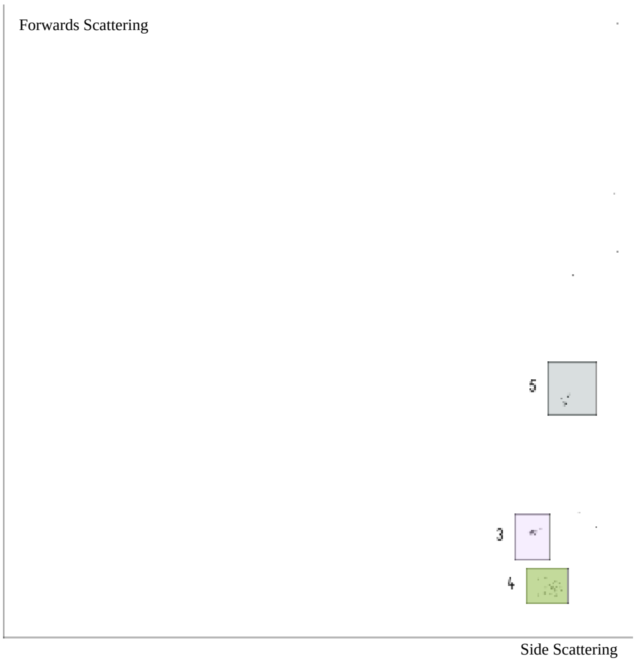

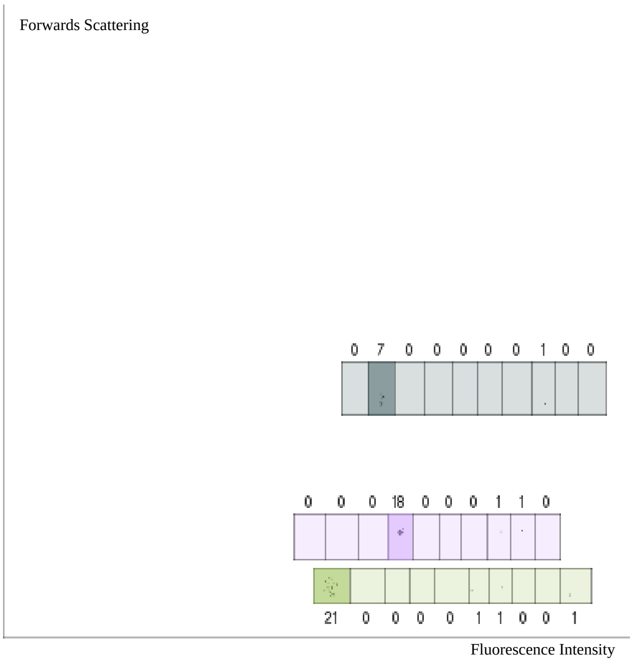

ANNEX 3: TAG DECONVOLUTION - BEAD 210

Passes flow sorting criteria: Yes

Passes tag deconvolution criteria: Yes

Included in protocol analysis: Yes

Protocol: 7, 6, 3, 6

Filename: Bin6_plateA6_D7.fcs

Split 1: Petrol shading

Split 2: Green shading

Split 3: Violet shading

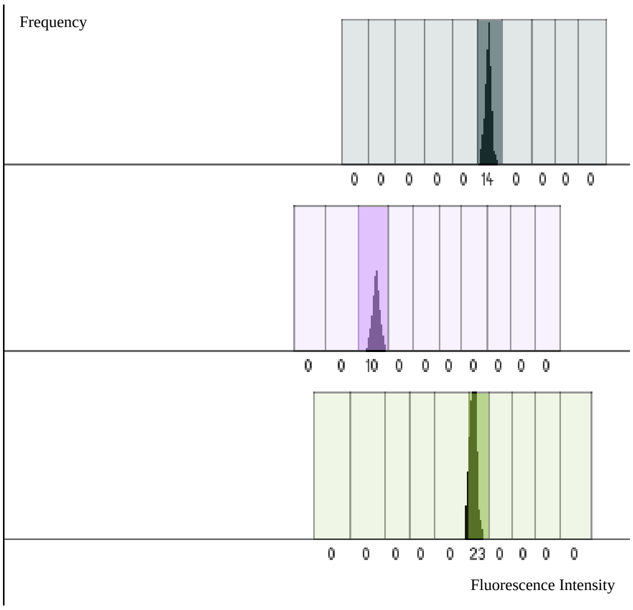

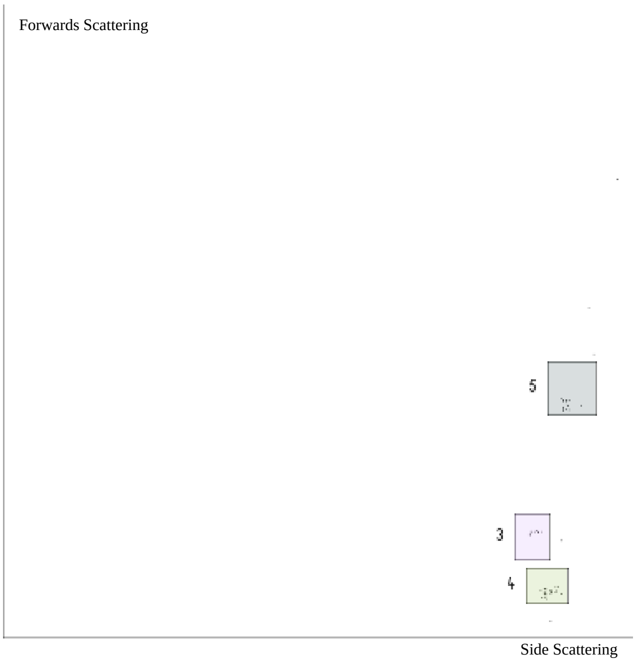

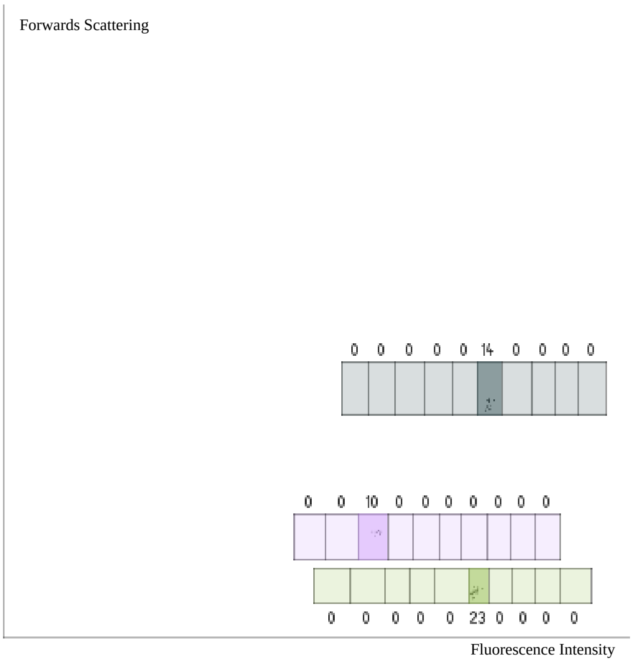

ANNEX 3: TAG DECONVOLUTION - BEAD 211

Passes flow sorting criteria: Yes

Passes tag deconvolution criteria: Yes

Included in protocol analysis: Yes

Protocol: 8, 7, 3, 6

Filename: Bin6_plateA6_E5.fcs

Split 1: Petrol shading

Split 2: Green shading

Split 3: Violet shading

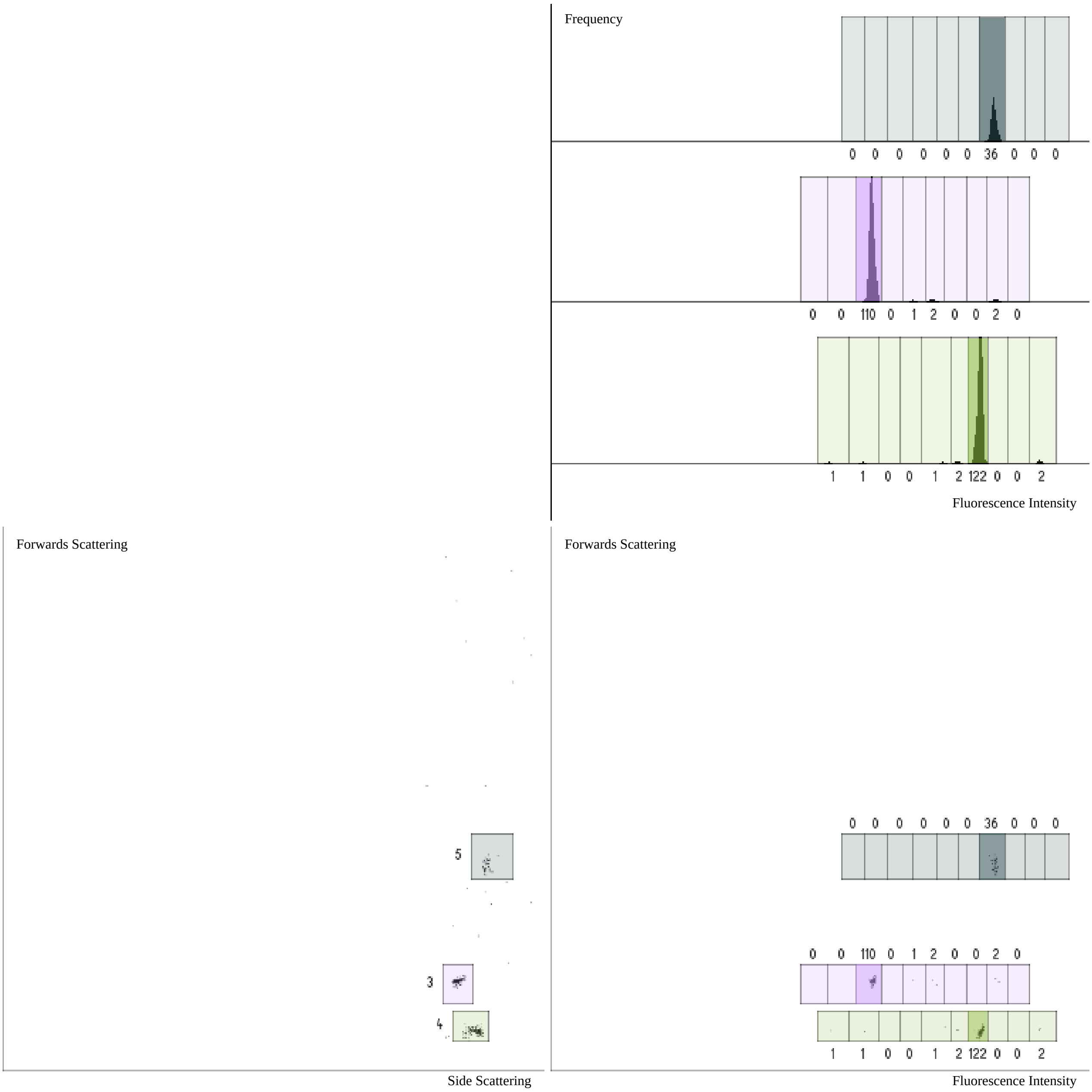

ANNEX 3: TAG DECONVOLUTION - BEAD 212

Passes flow sorting criteria: Yes

Passes tag deconvolution criteria: Yes

Included in protocol analysis: Yes

Protocol: 1, 8, 5, 6

Filename: Bin6_plateA6_E6.fcs

Split 1: Petrol shading

Split 2: Green shading

Split 3: Violet shading

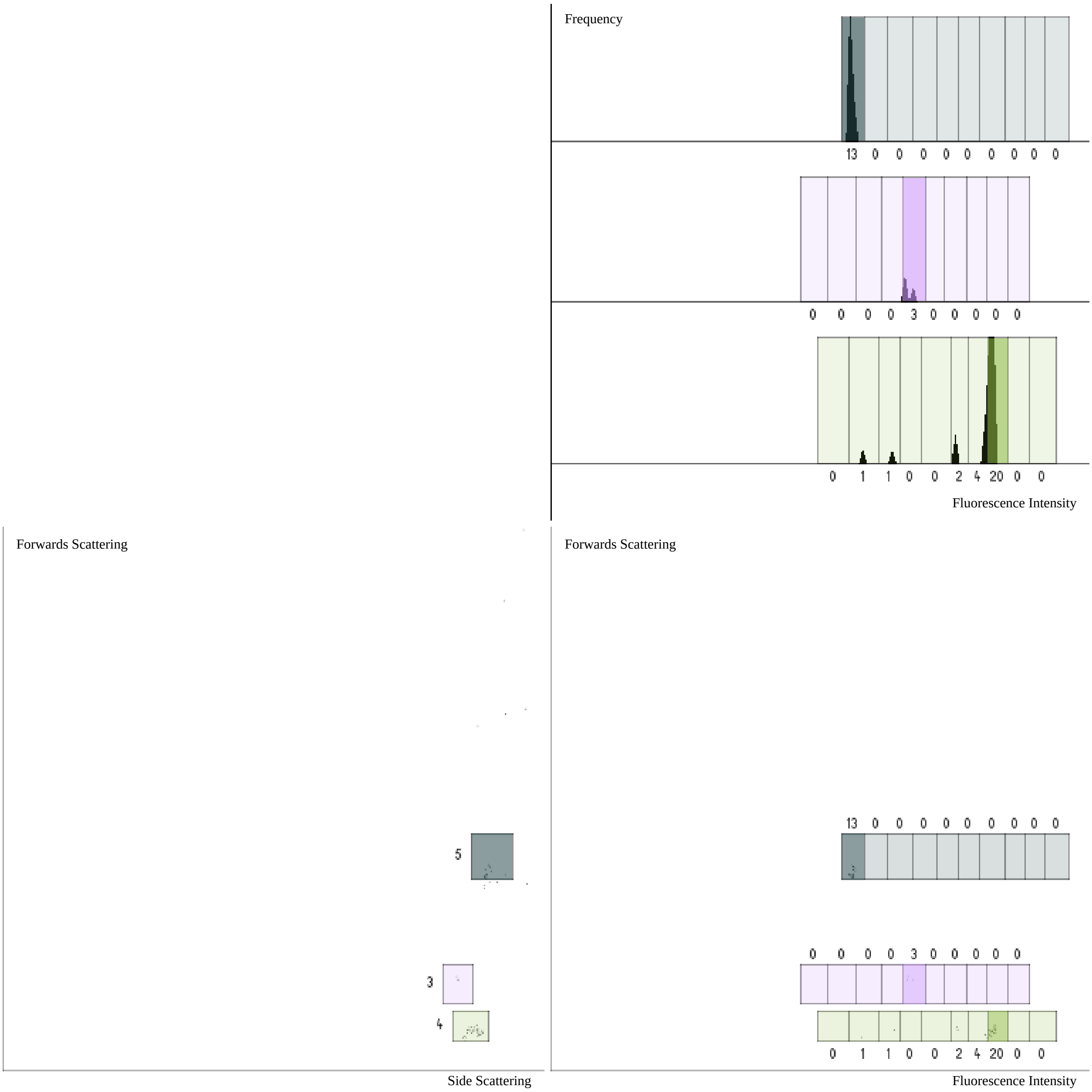

ANNEX 3: TAG DECONVOLUTION - BEAD 213

Passes flow sorting criteria: Yes

Passes tag deconvolution criteria: Yes

Included in protocol analysis: Yes

Protocol: 2, 3, 9, 6

Filename: Bin6_plateA6_E8.fcs

Split 1: Petrol shading

Split 2: Green shading

Split 3: Violet shading

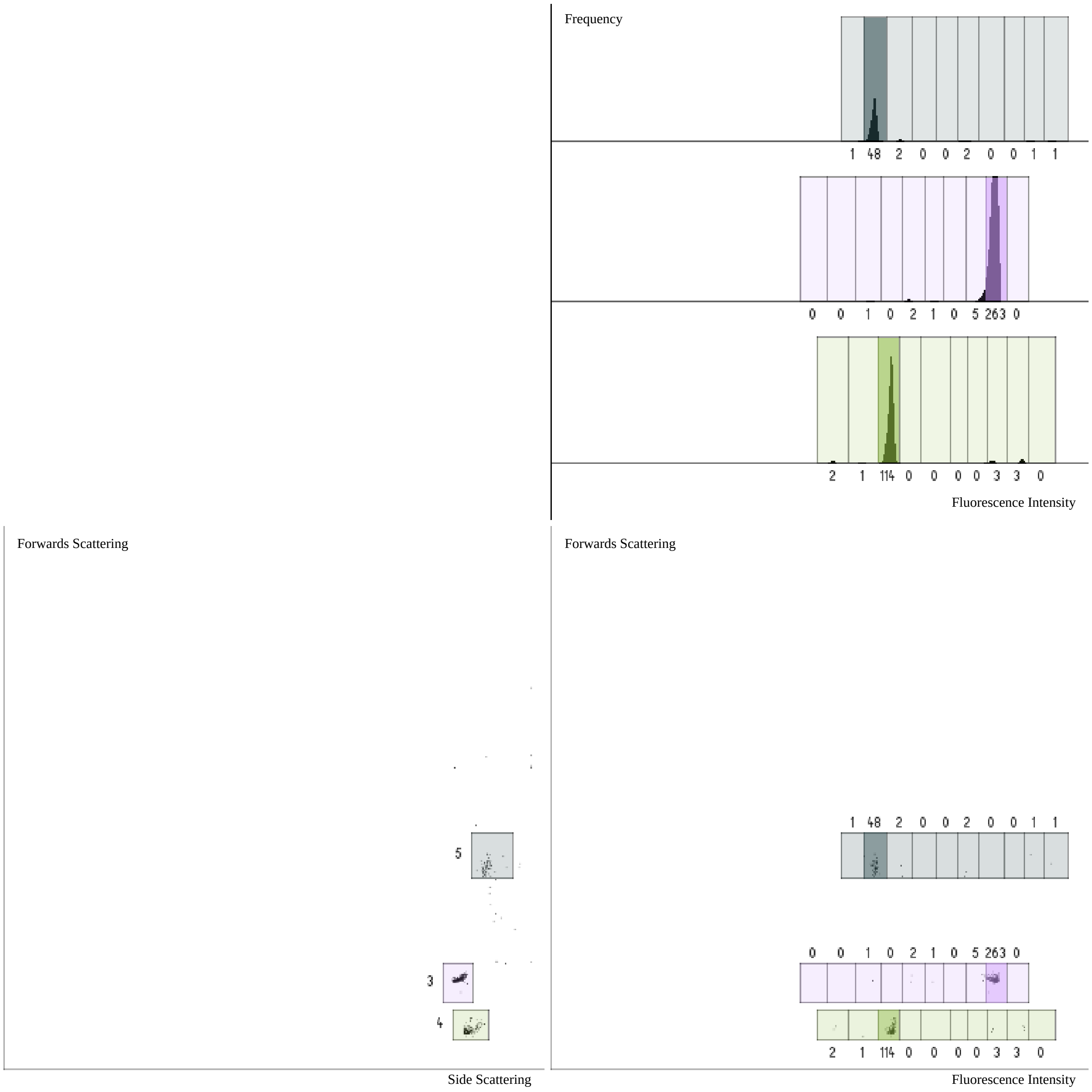

ANNEX 3: TAG DECONVOLUTION - BEAD 214

Passes flow sorting criteria: Yes

Passes tag deconvolution criteria: Yes

Included in protocol analysis: Yes

Protocol: 9, 1, 9, 6

Filename: Bin6_plateA6_E9.fcs

Split 1: Petrol shading

Split 2: Green shading

Split 3: Violet shading

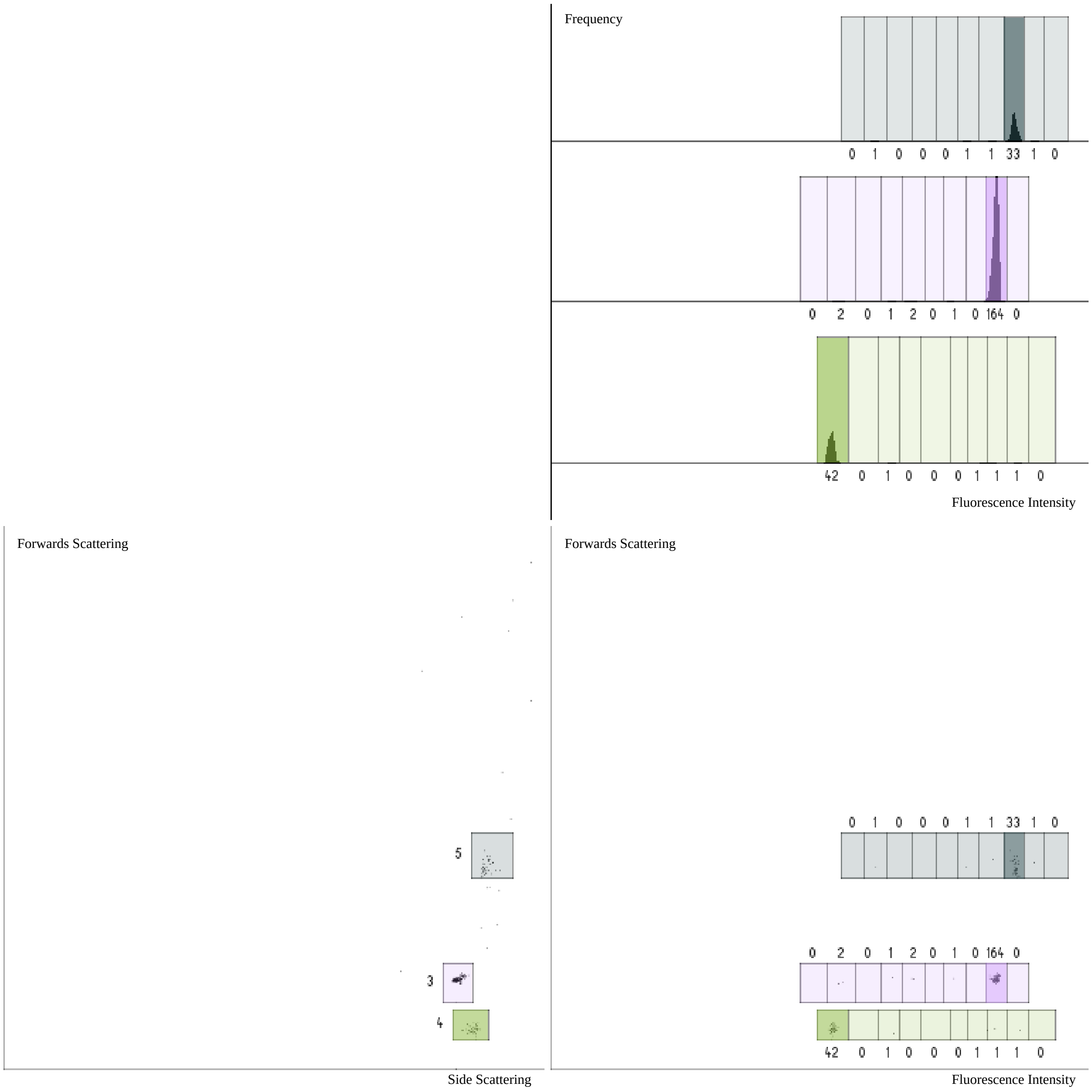

ANNEX 3: TAG DECONVOLUTION - BEAD 215

Passes flow sorting criteria: Yes

Passes tag deconvolution criteria: Yes

Included in protocol analysis: Yes

Protocol: 6, 7, 10, 6

Filename: Bin6_plateA6_E10.fcs

Split 1: Petrol shading

Split 2: Green shading

Split 3: Violet shading

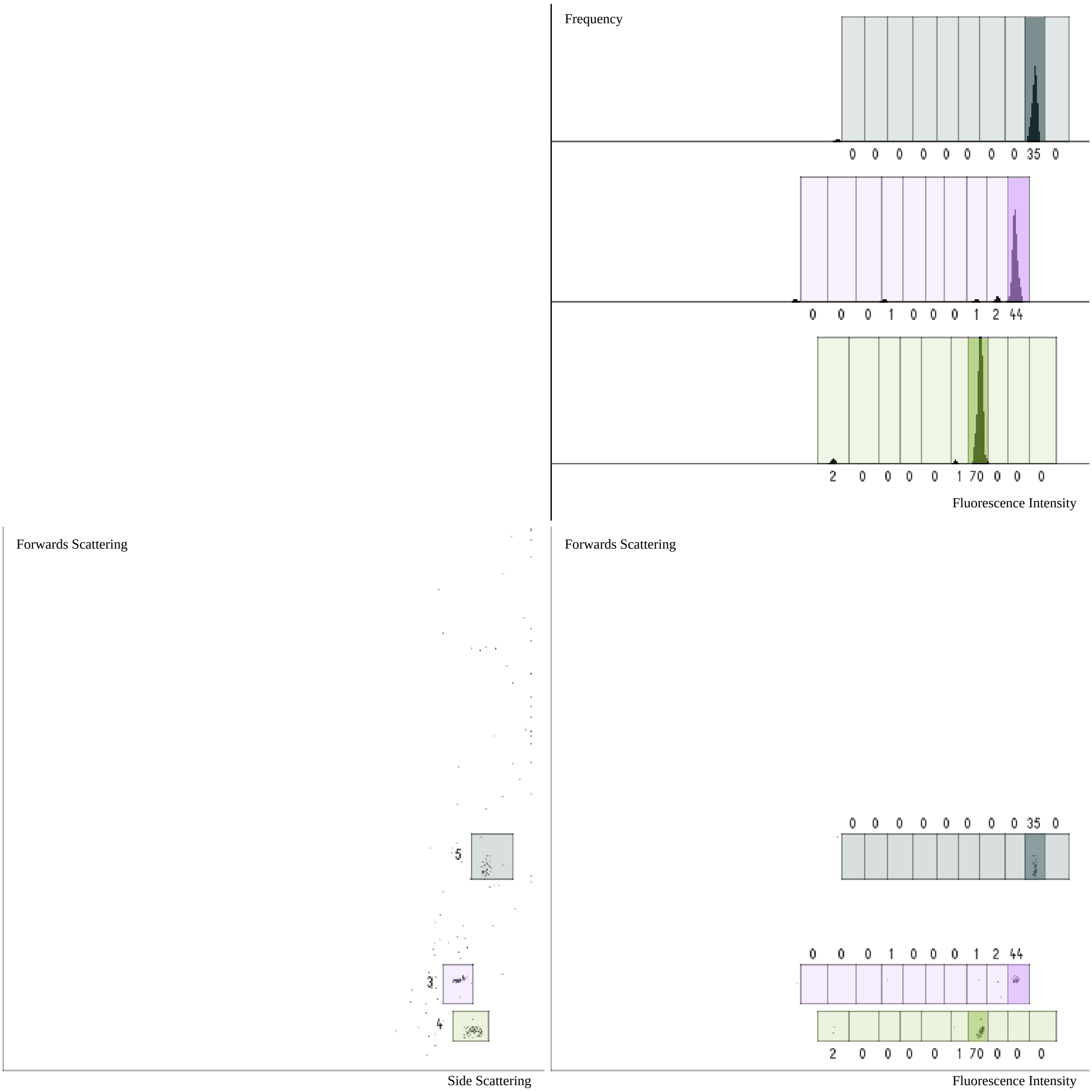

ANNEX 3: TAG DECONVOLUTION - BEAD 216

Passes flow sorting criteria: Yes

Passes tag deconvolution criteria: No

Included in protocol analysis: No

Protocol: N/A

Filename: Bin6_plateA6_F3.fcs

Split 1: Petrol shading

Split 2: Green shading

Split 3: Violet shading

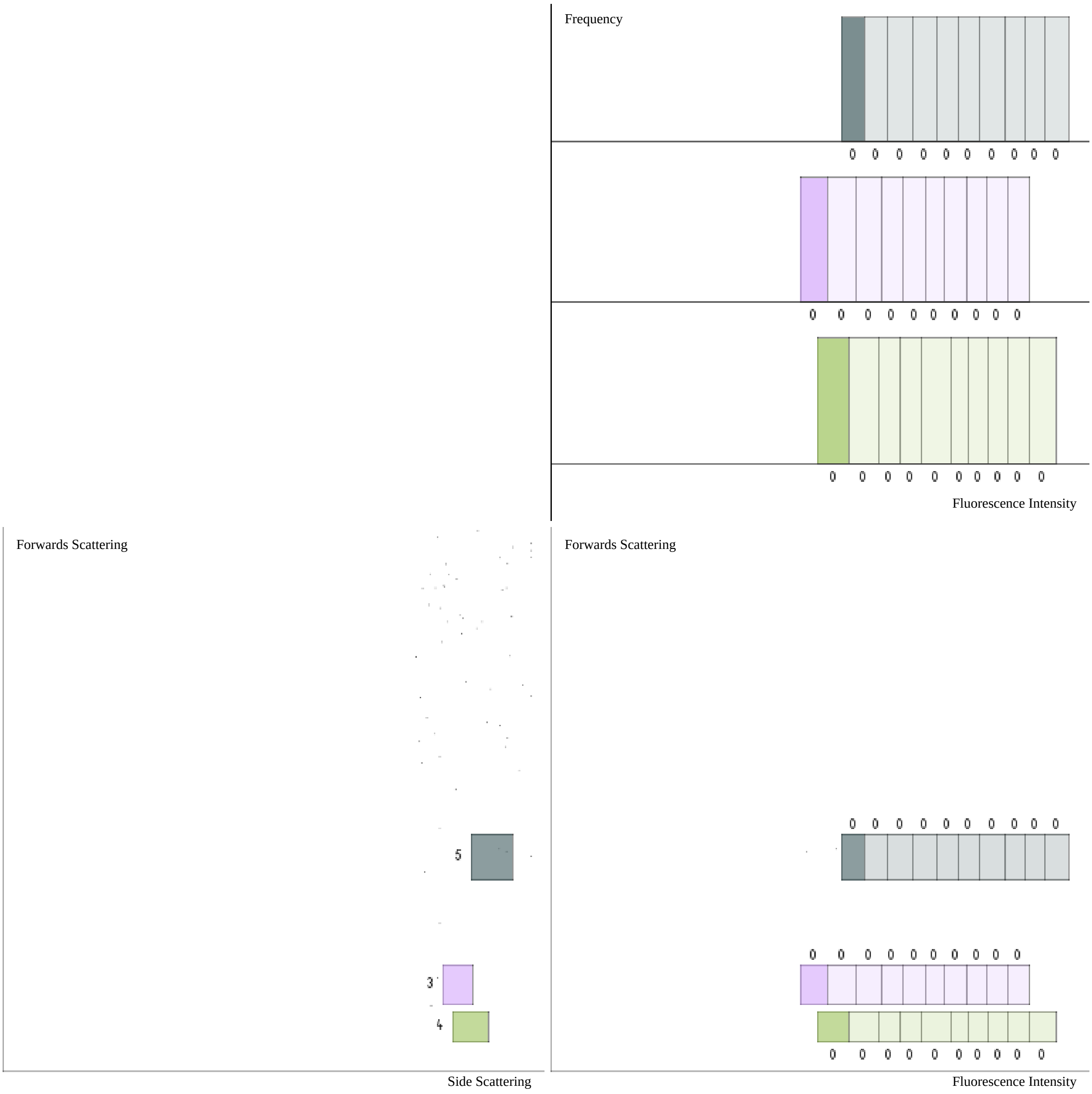

ANNEX 3: TAG DECONVOLUTION - BEAD 217

Passes flow sorting criteria: Yes

Passes tag deconvolution criteria: Yes

Included in protocol analysis: Yes

Protocol: 8, 9, 7, 6

Filename: Bin6_plateA6_F8.fcs

Split 1: Petrol shading

Split 2: Green shading

Split 3: Violet shading

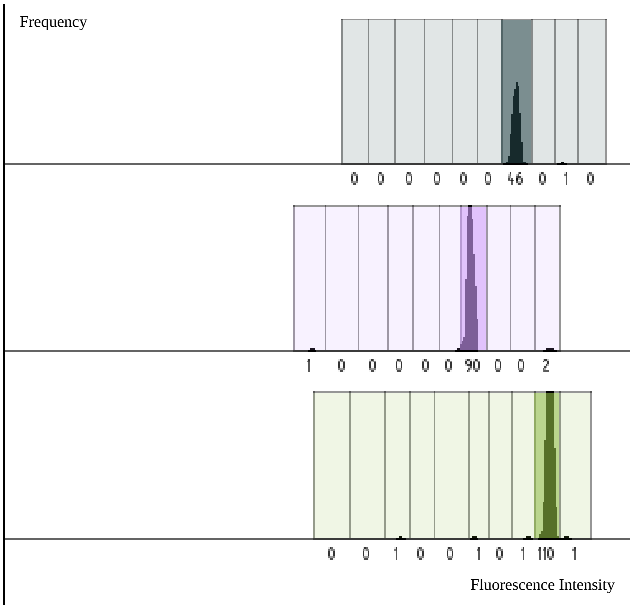

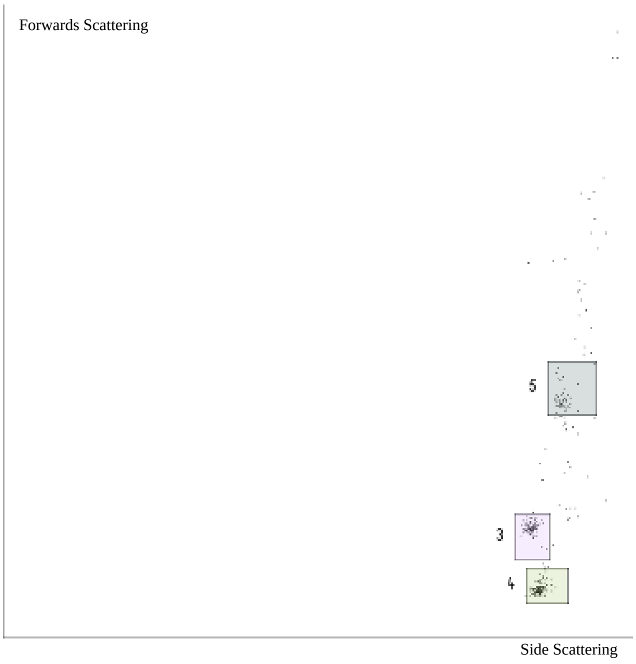

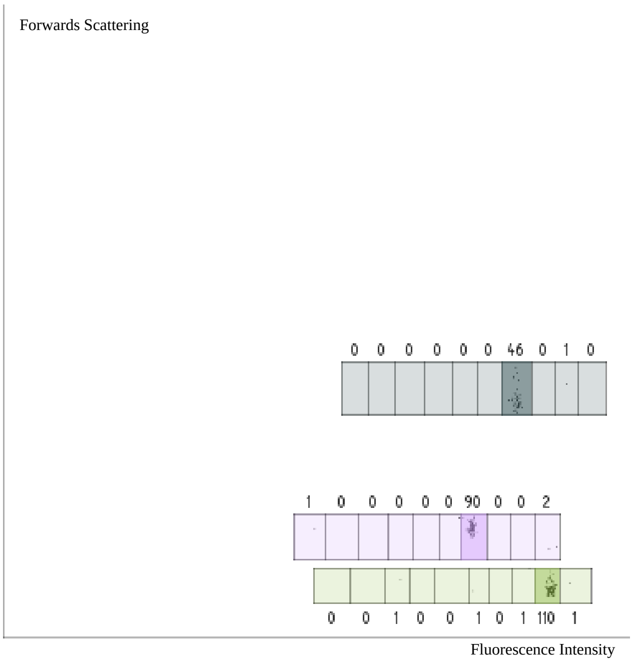

ANNEX 3: TAG DECONVOLUTION - BEAD 218

Passes flow sorting criteria: Yes

Passes tag deconvolution criteria: No

Included in protocol analysis: No

Protocol: N/A

Filename: Bin6_plateA6_G3.fcs

Split 1: Petrol shading

Split 2: Green shading

Split 3: Violet shading

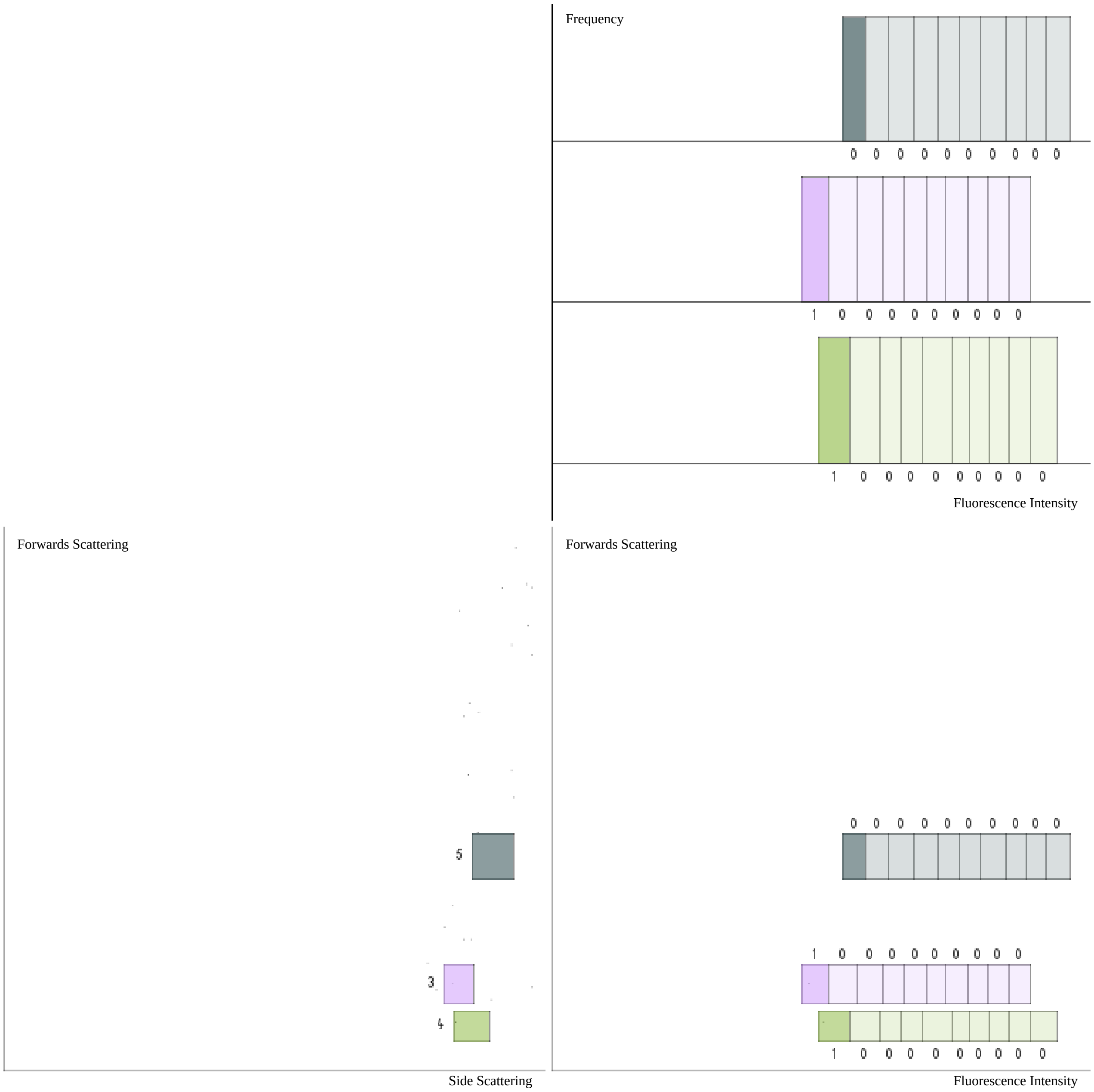

ANNEX 3: TAG DECONVOLUTION - BEAD 219

Passes flow sorting criteria: Yes

Passes tag deconvolution criteria: Yes

Included in protocol analysis: Yes

Protocol: 9, 2, 7, 6

Filename: Bin6_plateA6_G6.fcs

Split 1: Petrol shading

Split 2: Green shading

Split 3: Violet shading

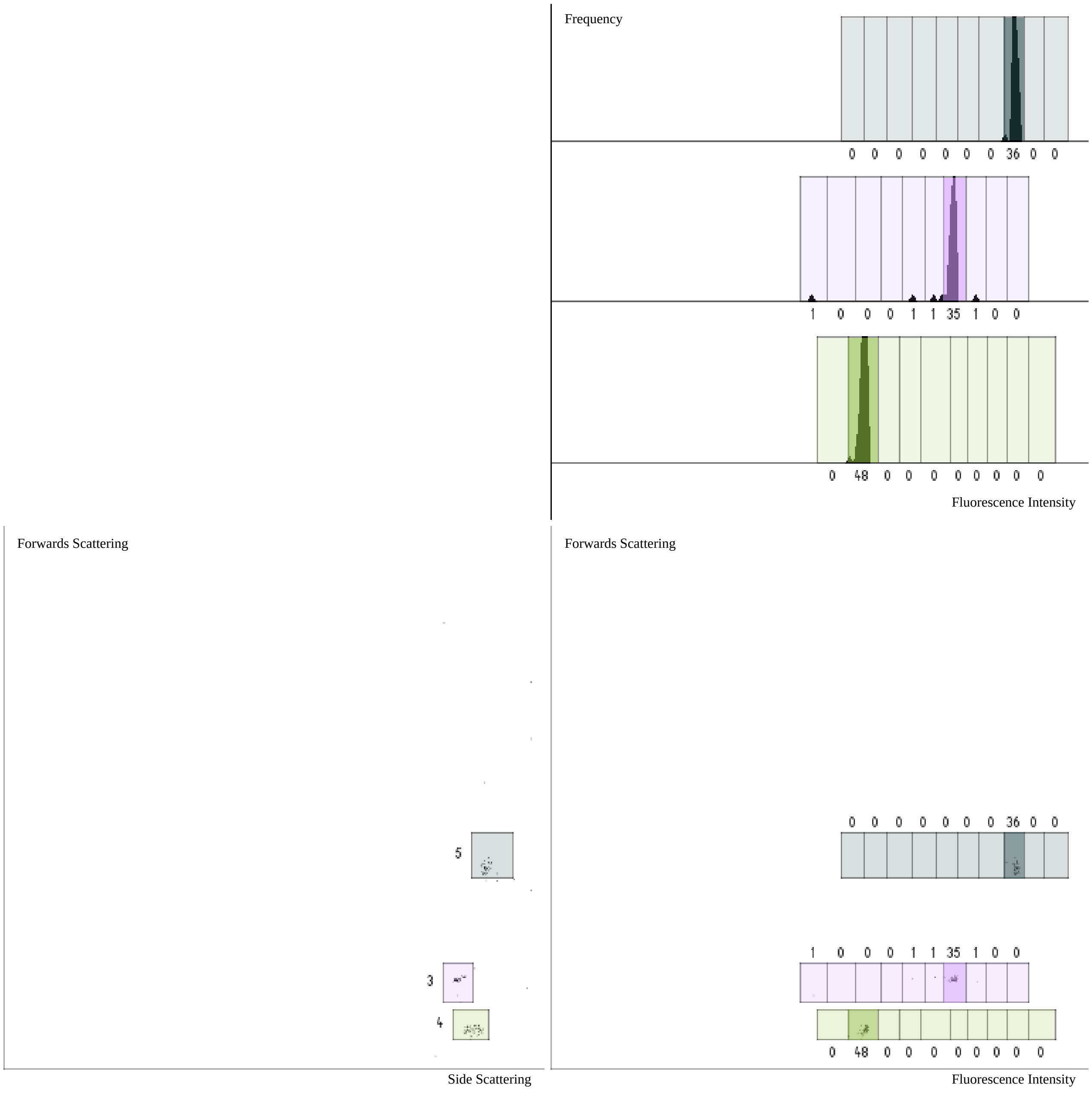

ANNEX 3: TAG DECONVOLUTION - BEAD 220

Passes flow sorting criteria: Yes

Passes tag deconvolution criteria: No

Included in protocol analysis: No

Protocol: N/A

Filename: Bin6_plateA6_G9.fcs

Split 1: Petrol shading

Split 2: Green shading

Split 3: Violet shading

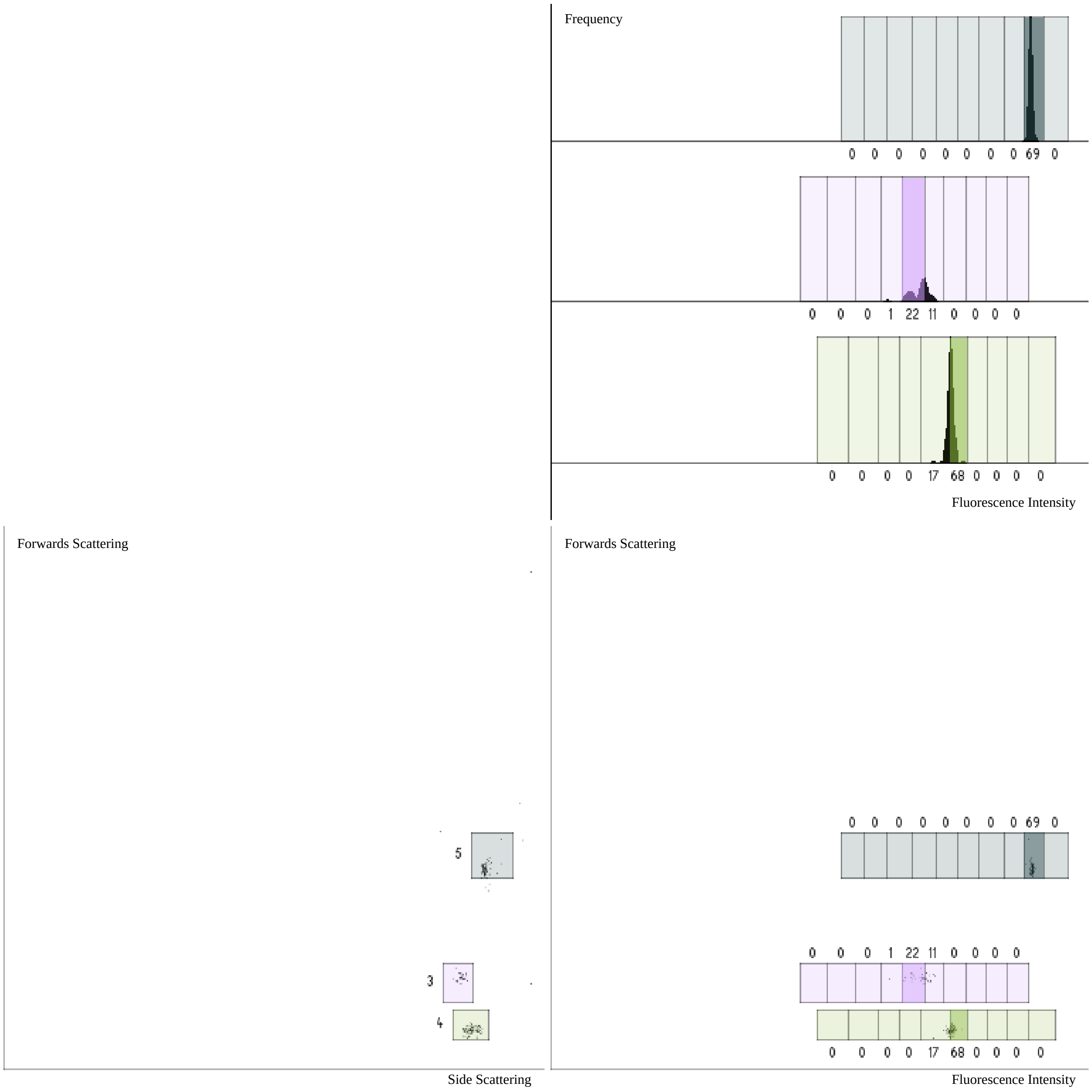

ANNEX 3: TAG DECONVOLUTION - BEAD 221

Passes flow sorting criteria: Yes

Passes tag deconvolution criteria: Yes

Included in protocol analysis: Yes

Protocol: 3, 7, 4, 6

Filename: Bin6_plateA6_G12.fcs

Split 1: Petrol shading

Split 2: Green shading

Split 3: Violet shading

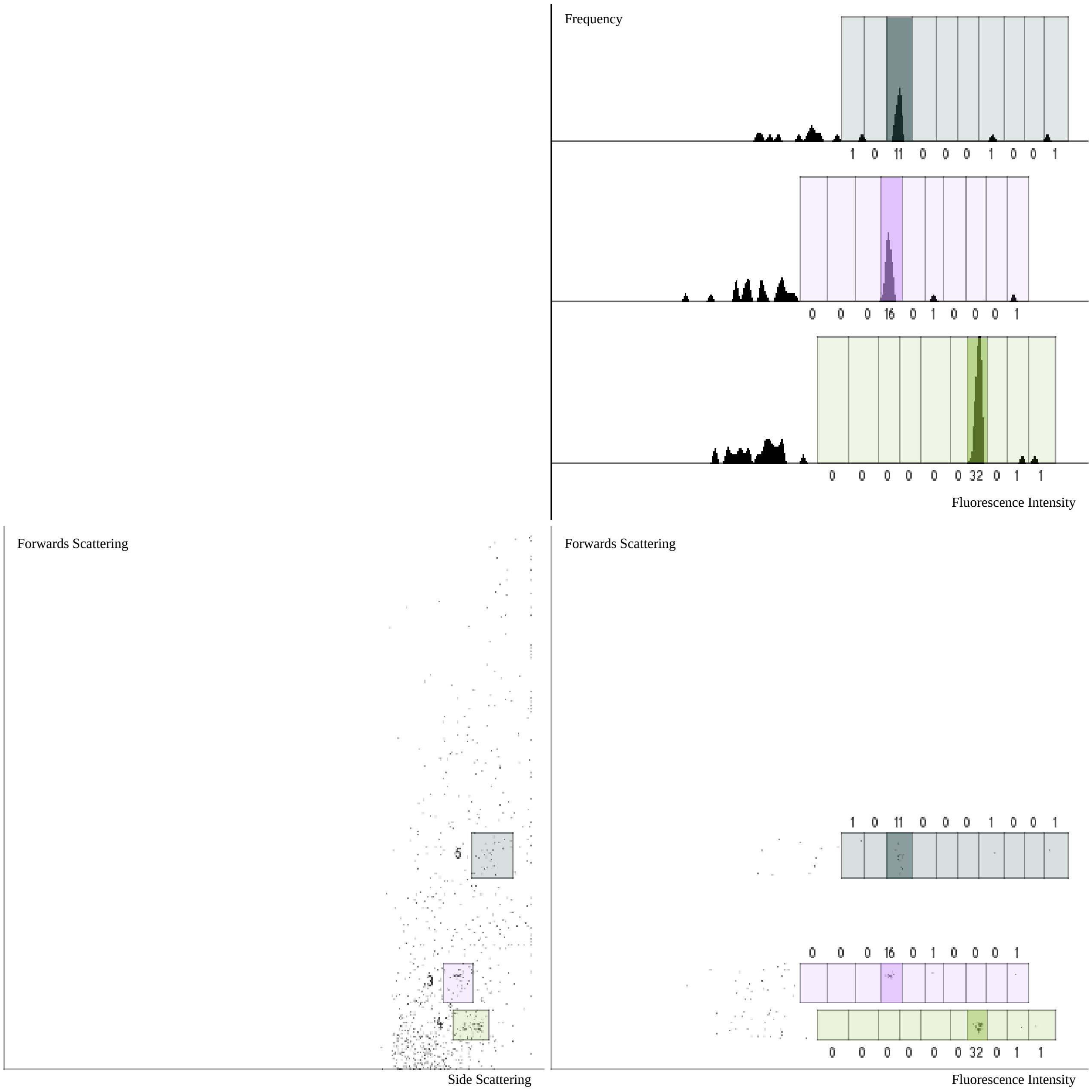

ANNEX 3: TAG DECONVOLUTION - BEAD 222

Passes flow sorting criteria: Yes

Passes tag deconvolution criteria: No

Included in protocol analysis: No

Protocol: N/A

Filename: Bin6_plateA6_H1.fcs

Split 1: Petrol shading

Split 2: Green shading

Split 3: Violet shading

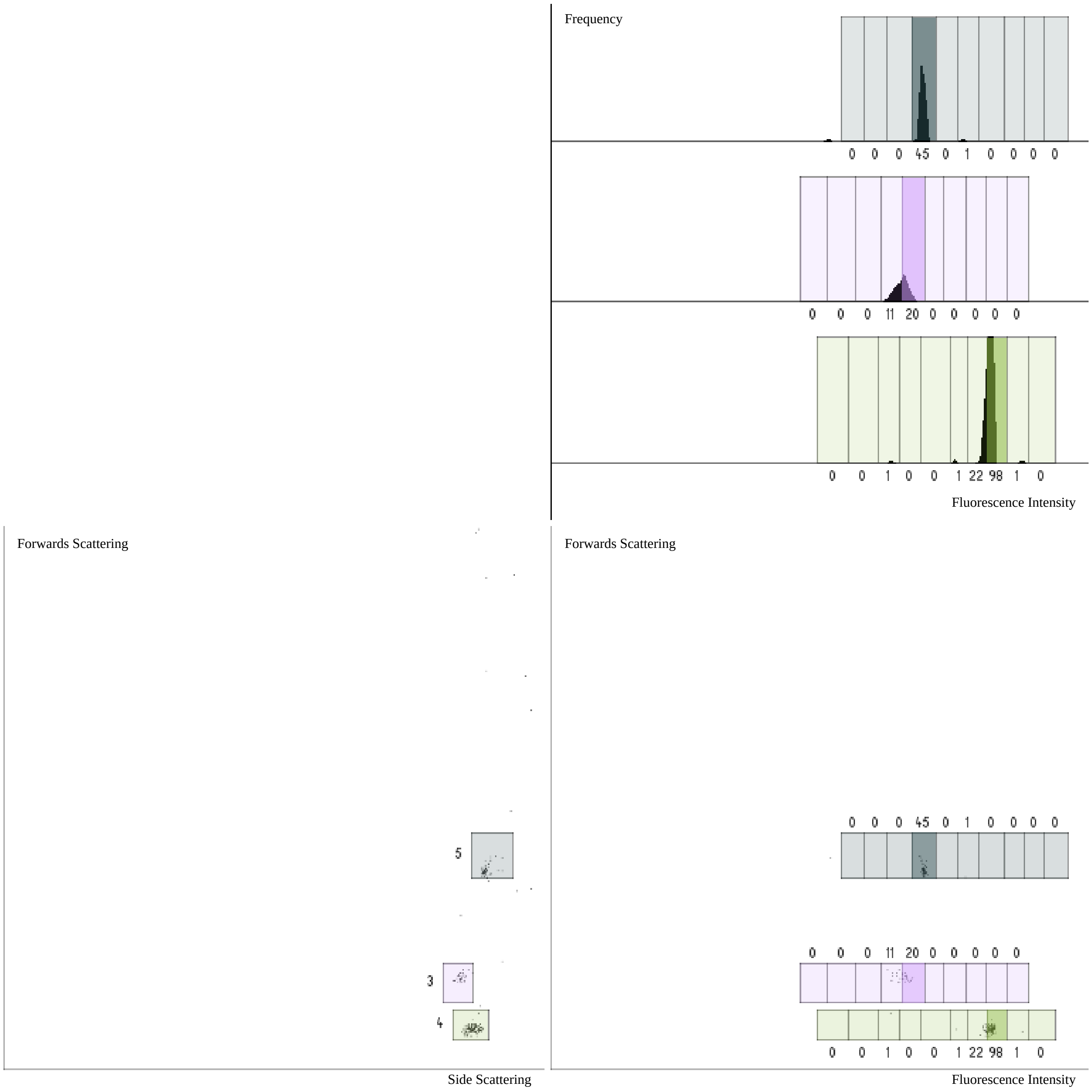

ANNEX 3: TAG DECONVOLUTION - BEAD 223

Passes flow sorting criteria: Yes

Passes tag deconvolution criteria: Yes

Included in protocol analysis: Yes

Protocol: 9, 4, 9, 6

Filename: Bin6_plateA6_H4.fcs

Split 1: Petrol shading

Split 2: Green shading

Split 3: Violet shading

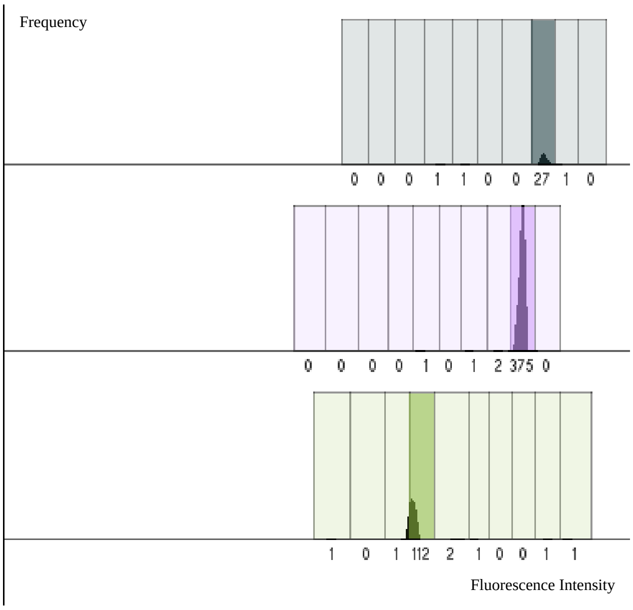

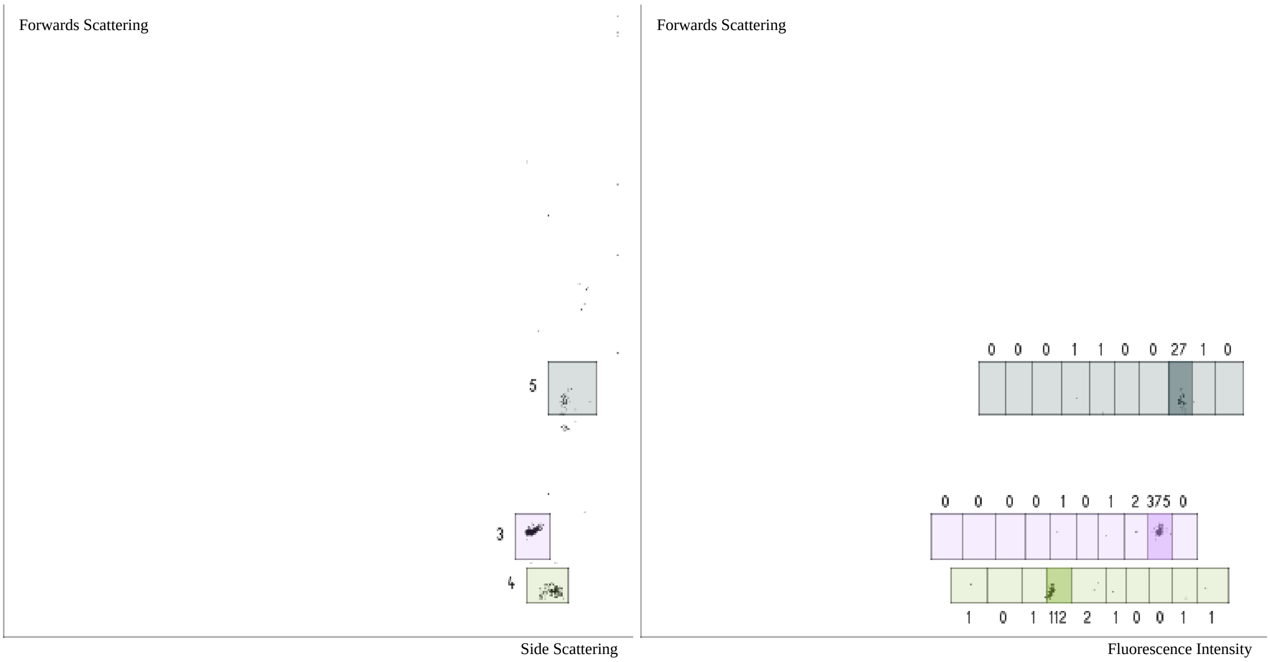

ANNEX 3: TAG DECONVOLUTION - BEAD 224

Passes flow sorting criteria: Yes

Passes tag deconvolution criteria: Yes

Included in protocol analysis: Yes

Protocol: 6, 10, 4, 6

Filename: Bin6_plateA6_H7.fcs

Split 1: Petrol shading

Split 2: Green shading

Split 3: Violet shading

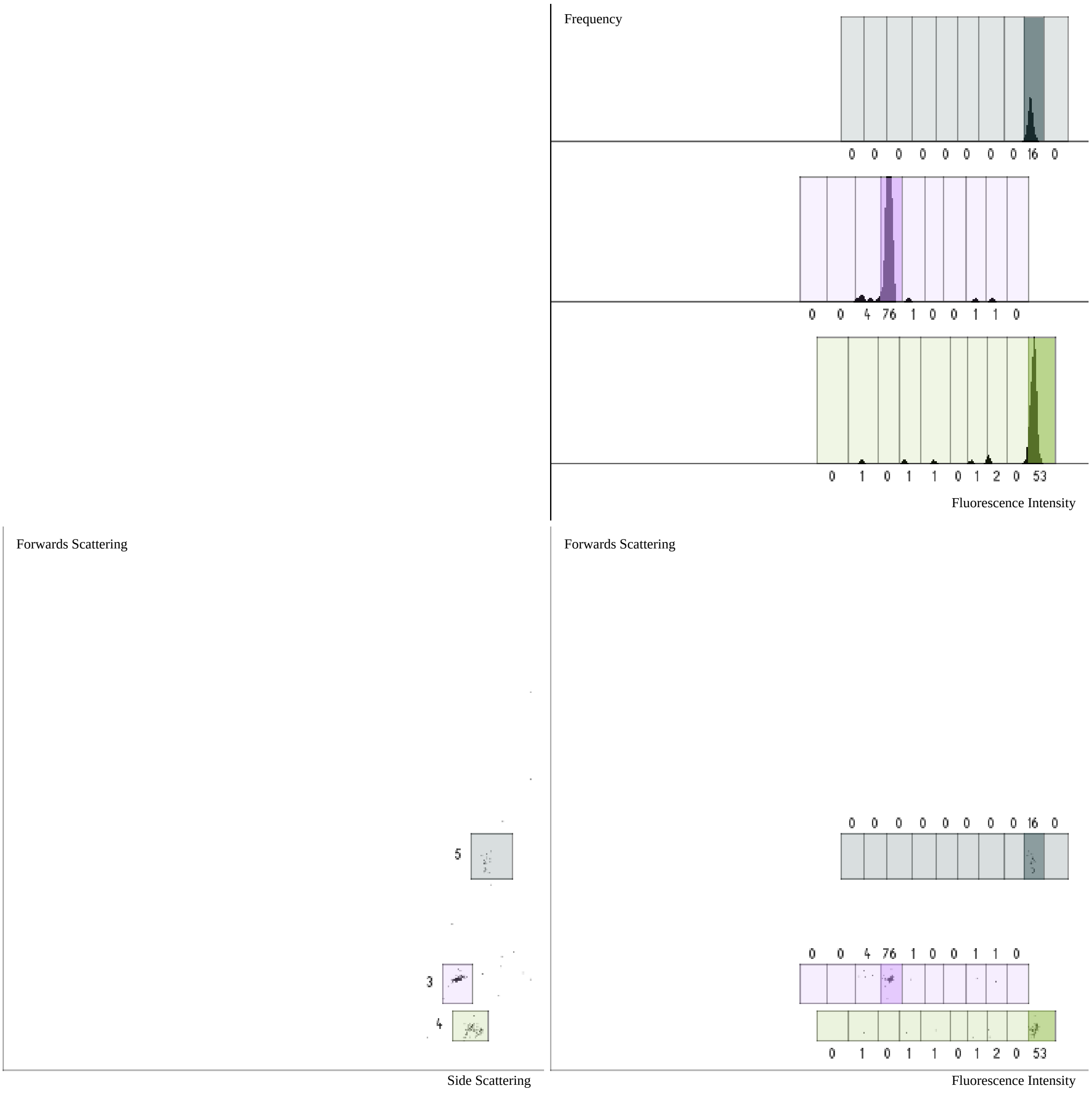

ANNEX 3: TAG DECONVOLUTION - BEAD 225

Passes flow sorting criteria: Yes

Passes tag deconvolution criteria: Yes

Included in protocol analysis: Yes

Protocol: 8, 10, 9, 6

Filename: Bin6_plateA6_H11.fcs

Split 1: Petrol shading

Split 2: Green shading

Split 3: Violet shading

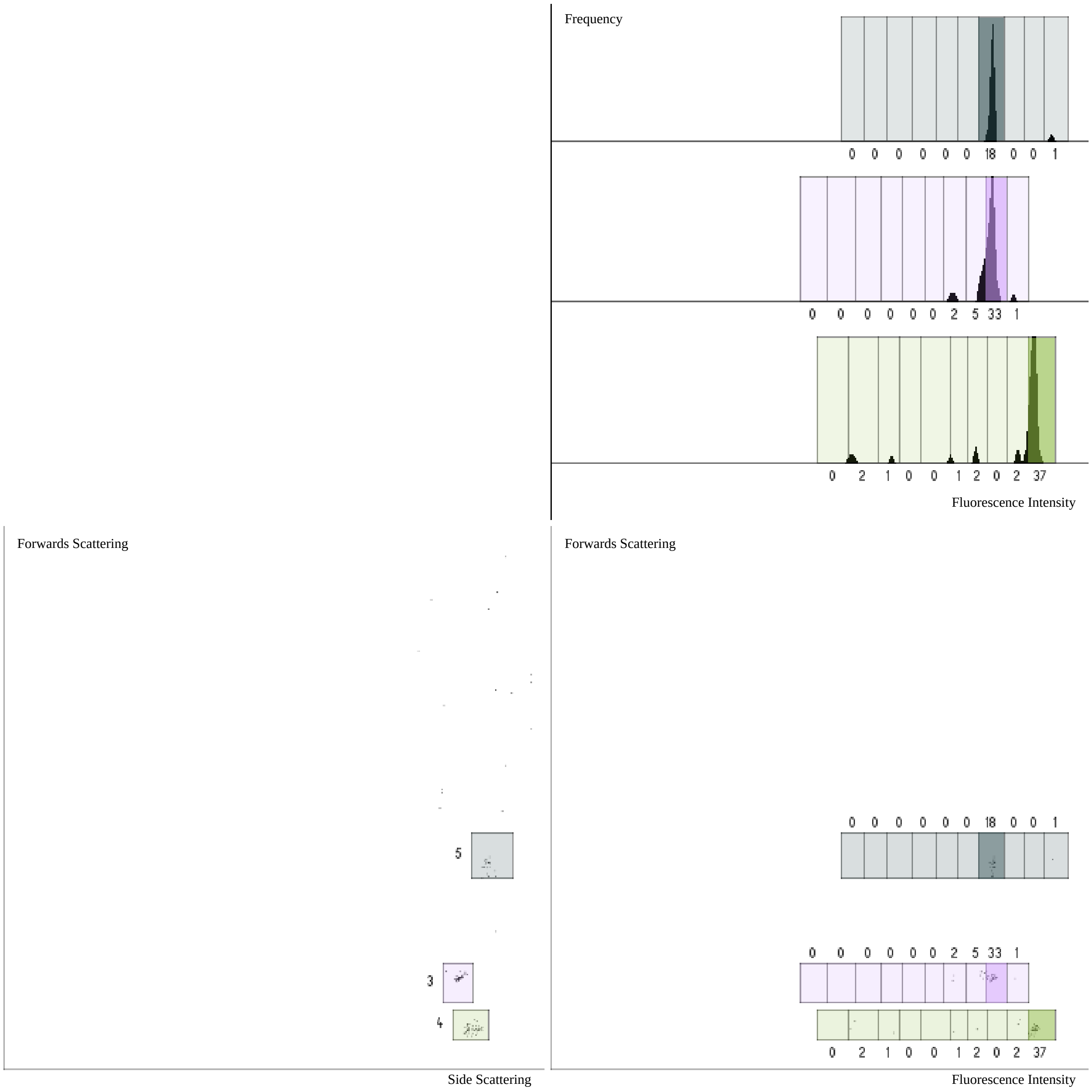

ANNEX 3: TAG DECONVOLUTION - BEAD 226

Passes flow sorting criteria: Yes

Passes tag deconvolution criteria: Yes

Included in protocol analysis: Yes

Protocol: 10, 4, 9, 6

Filename: Bin6_plateA7_E12.fcs

Split 1: Petrol shading

Split 2: Green shading

Split 3: Violet shading

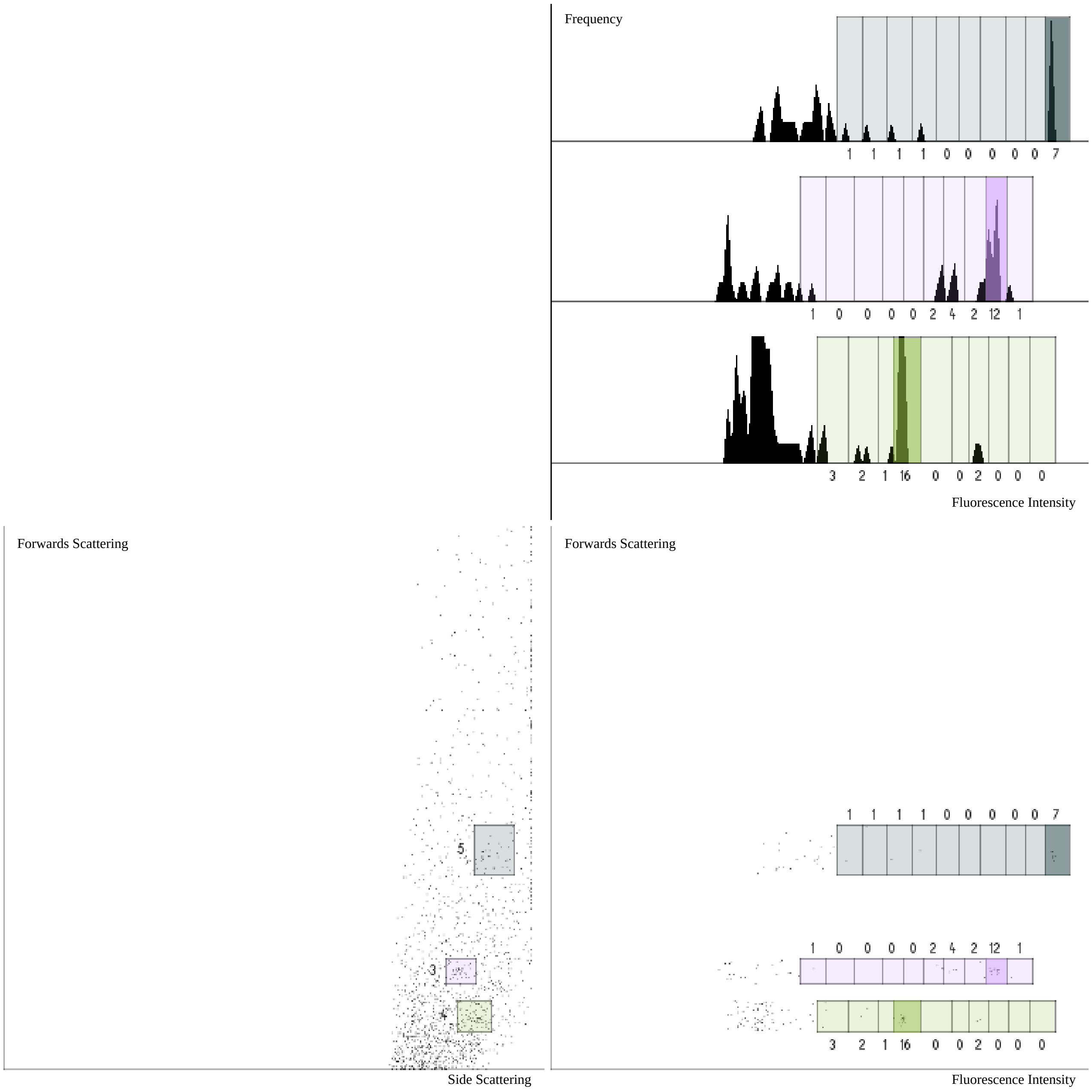

ANNEX 3: TAG DECONVOLUTION - BEAD 227

Passes flow sorting criteria: Yes

Passes tag deconvolution criteria: Yes

Included in protocol analysis: Yes

Protocol: 9, 6, 10, 6

Filename: Bin6_plateA7_A2.fcs

Split 1: Petrol shading

Split 2: Green shading

Split 3: Violet shading

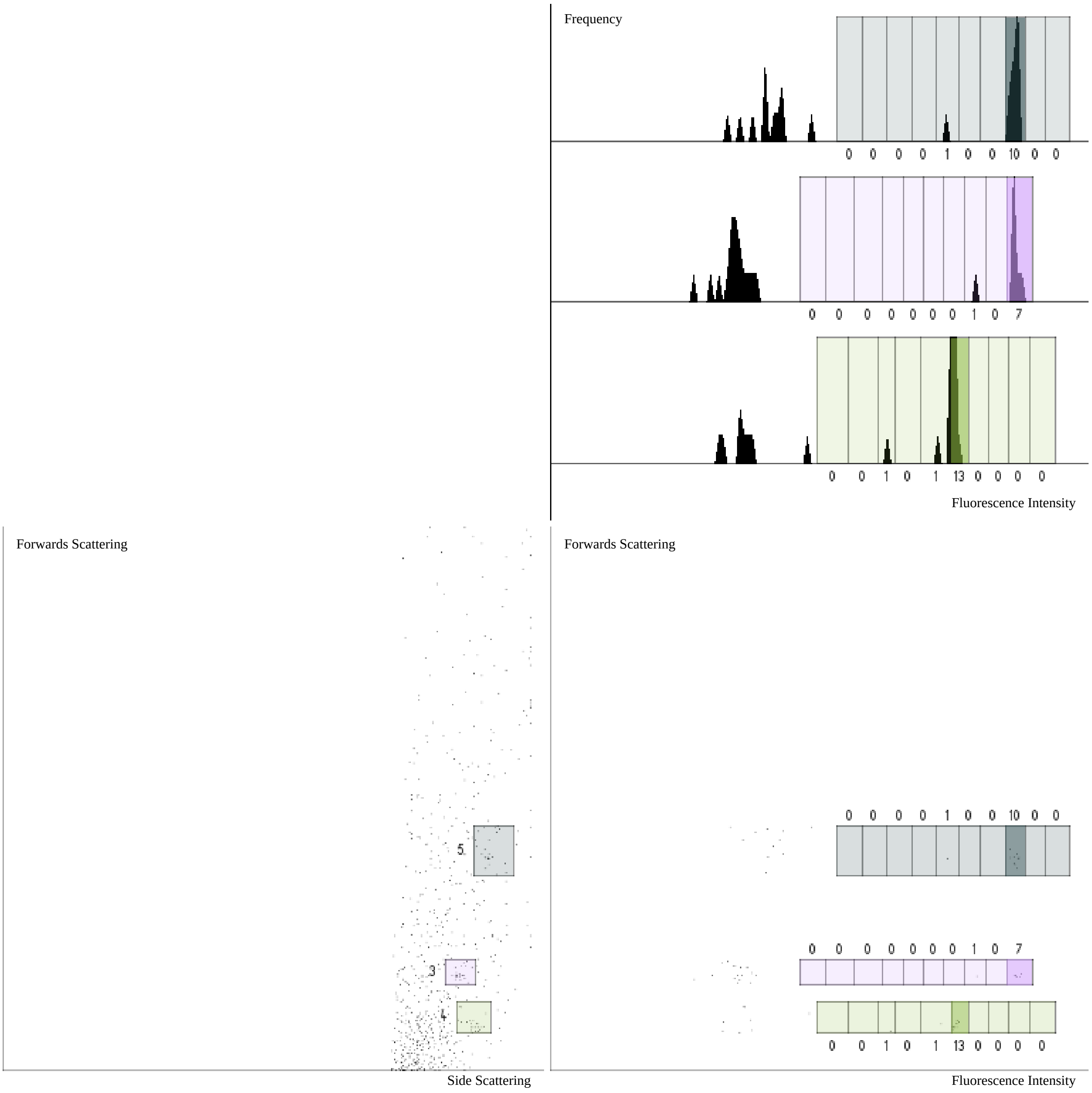

ANNEX 3: TAG DECONVOLUTION - BEAD 228

Passes flow sorting criteria: Yes

Passes tag deconvolution criteria: No

Included in protocol analysis: No

Protocol: N/A

Filename: Bin6_plateA7_A4.fcs

Split 1: Petrol shading

Split 2: Green shading

Split 3: Violet shading

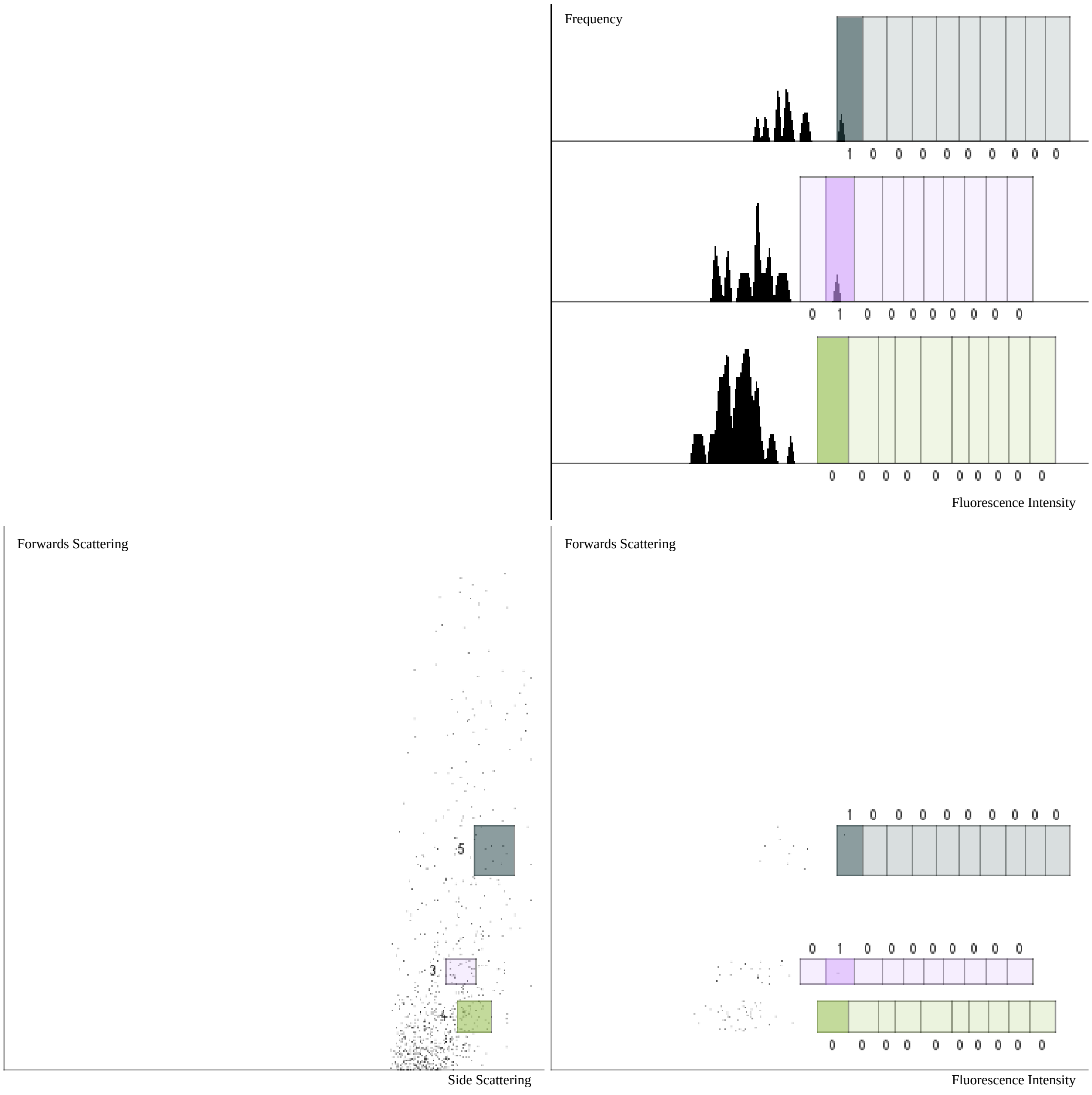

ANNEX 3: TAG DECONVOLUTION - BEAD 229

Passes flow sorting criteria: Yes

Passes tag deconvolution criteria: Yes

Included in protocol analysis: Yes

Protocol: 3, 3, 8, 6

Filename: Bin6_plateA7_A6.fcs

Split 1: Petrol shading

Split 2: Green shading

Split 3: Violet shading

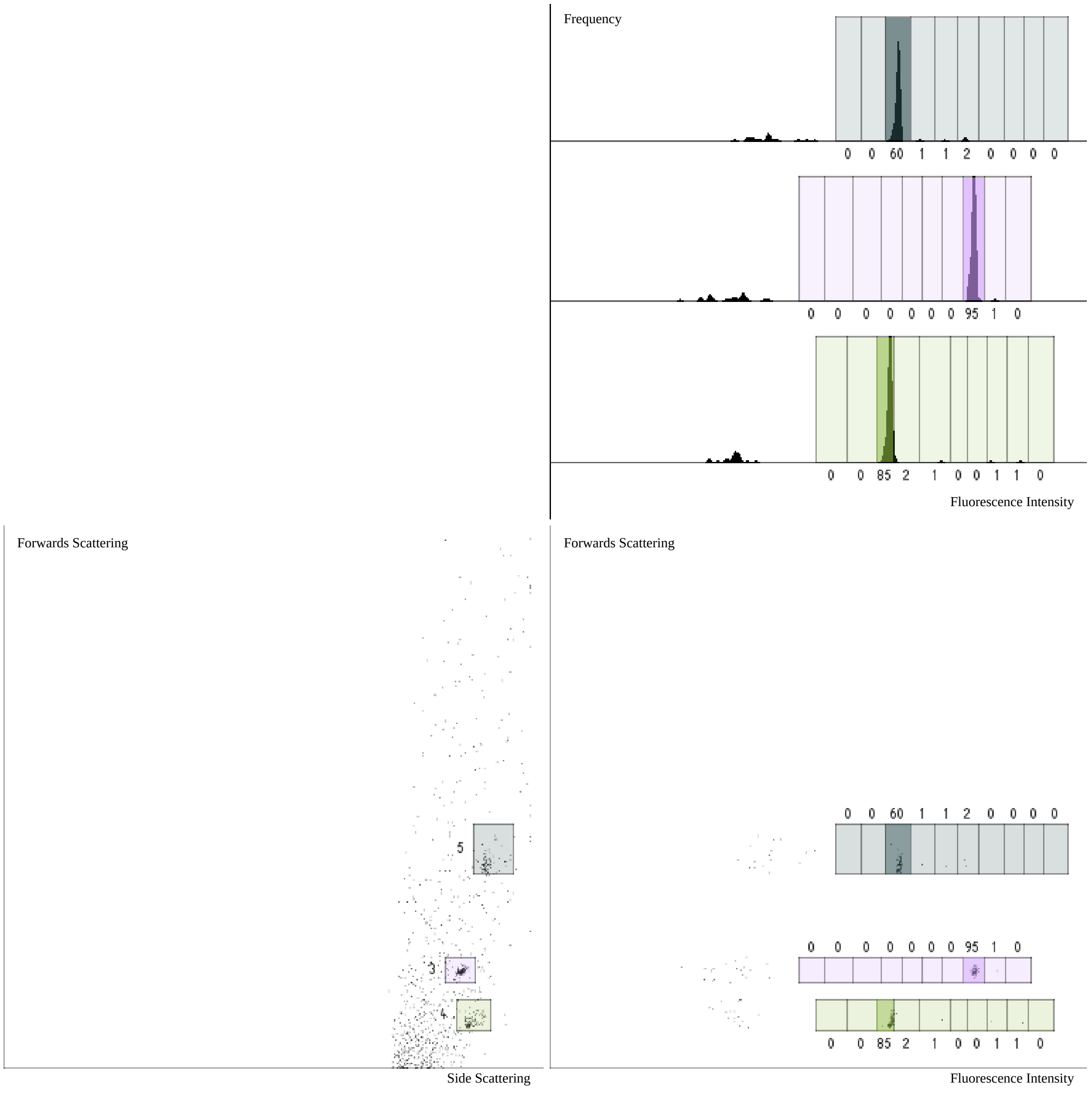

ANNEX 3: TAG DECONVOLUTION - BEAD 230

Passes flow sorting criteria: Yes

Passes tag deconvolution criteria: Yes

Included in protocol analysis: Yes

Protocol: 6, 5, 10, 6

Filename: Bin6_plateA7_A11.fcs

Split 1: Petrol shading

Split 2: Green shading

Split 3: Violet shading

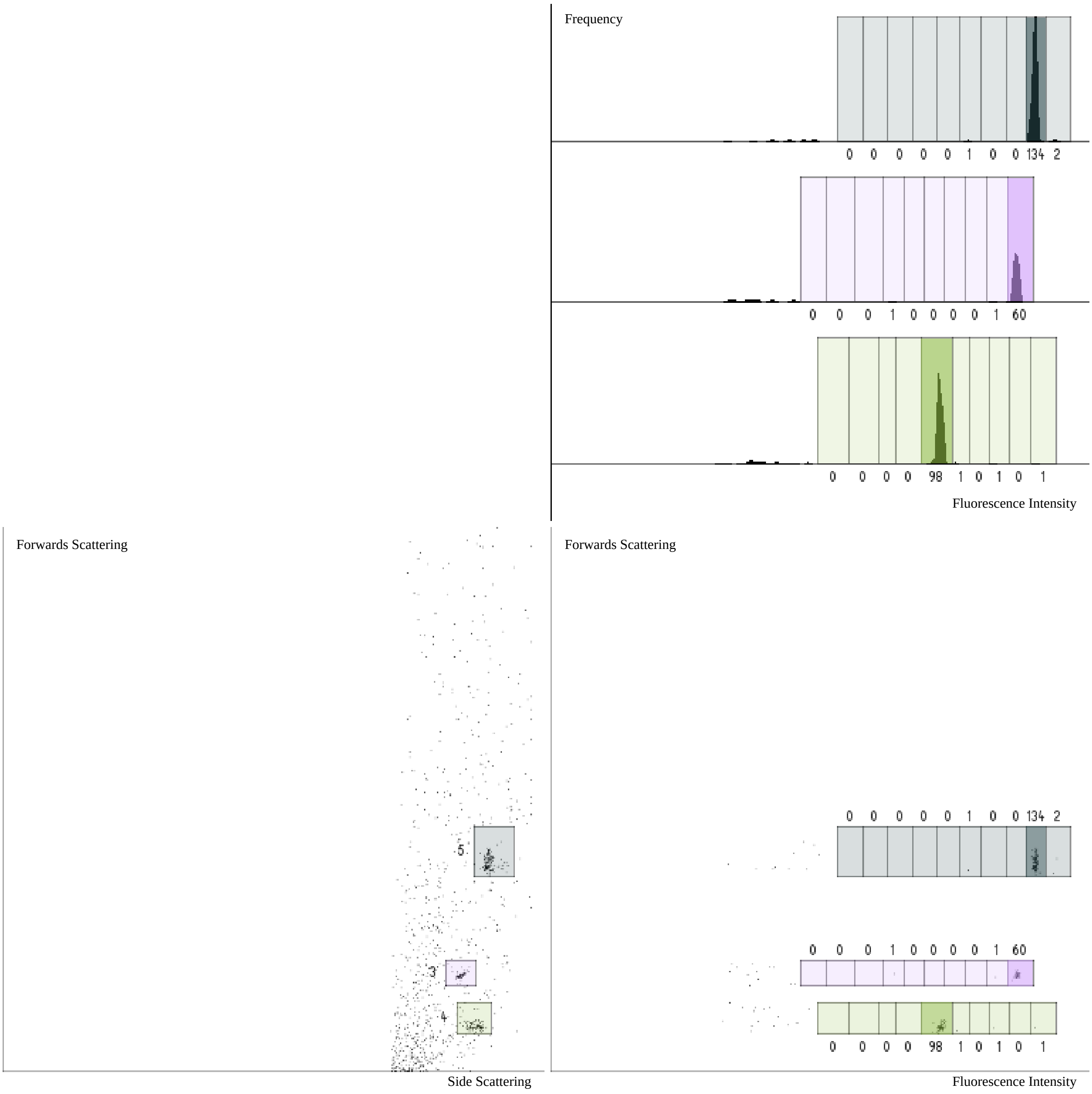

ANNEX 3: TAG DECONVOLUTION - BEAD 231

Passes flow sorting criteria: Yes

Passes tag deconvolution criteria: Yes

Included in protocol analysis: Yes

Protocol: 1, 9, 1, 6

Filename: Bin6_plateA7_B3.fcs

Split 1: Petrol shading

Split 2: Green shading

Split 3: Violet shading

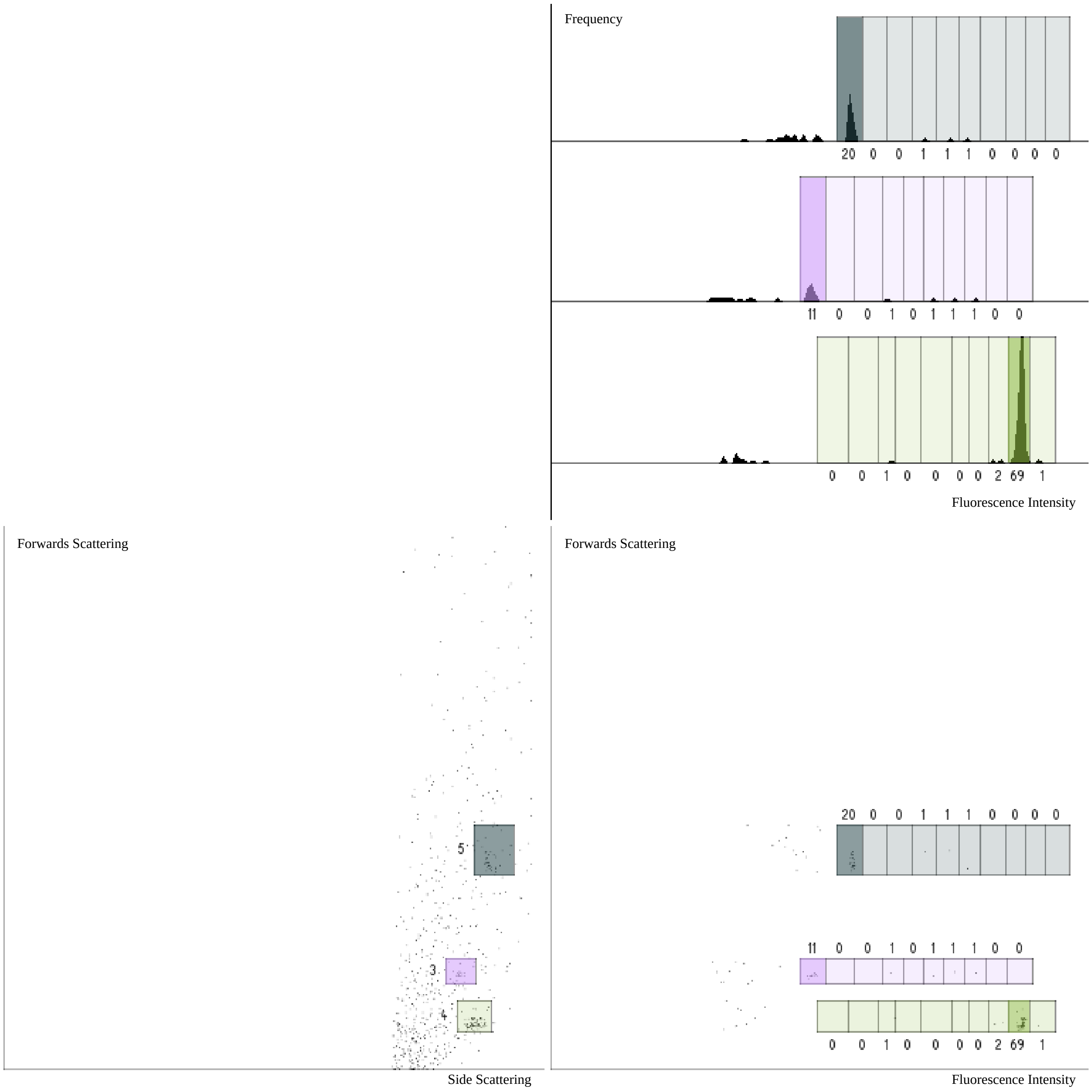

ANNEX 3: TAG DECONVOLUTION - BEAD 232

Passes flow sorting criteria: Yes

Passes tag deconvolution criteria: Yes

Included in protocol analysis: Yes

Protocol: 1, 10, 3, 6

Filename: Bin6_plateA7_B7.fcs

Split 1: Petrol shading

Split 2: Green shading

Split 3: Violet shading

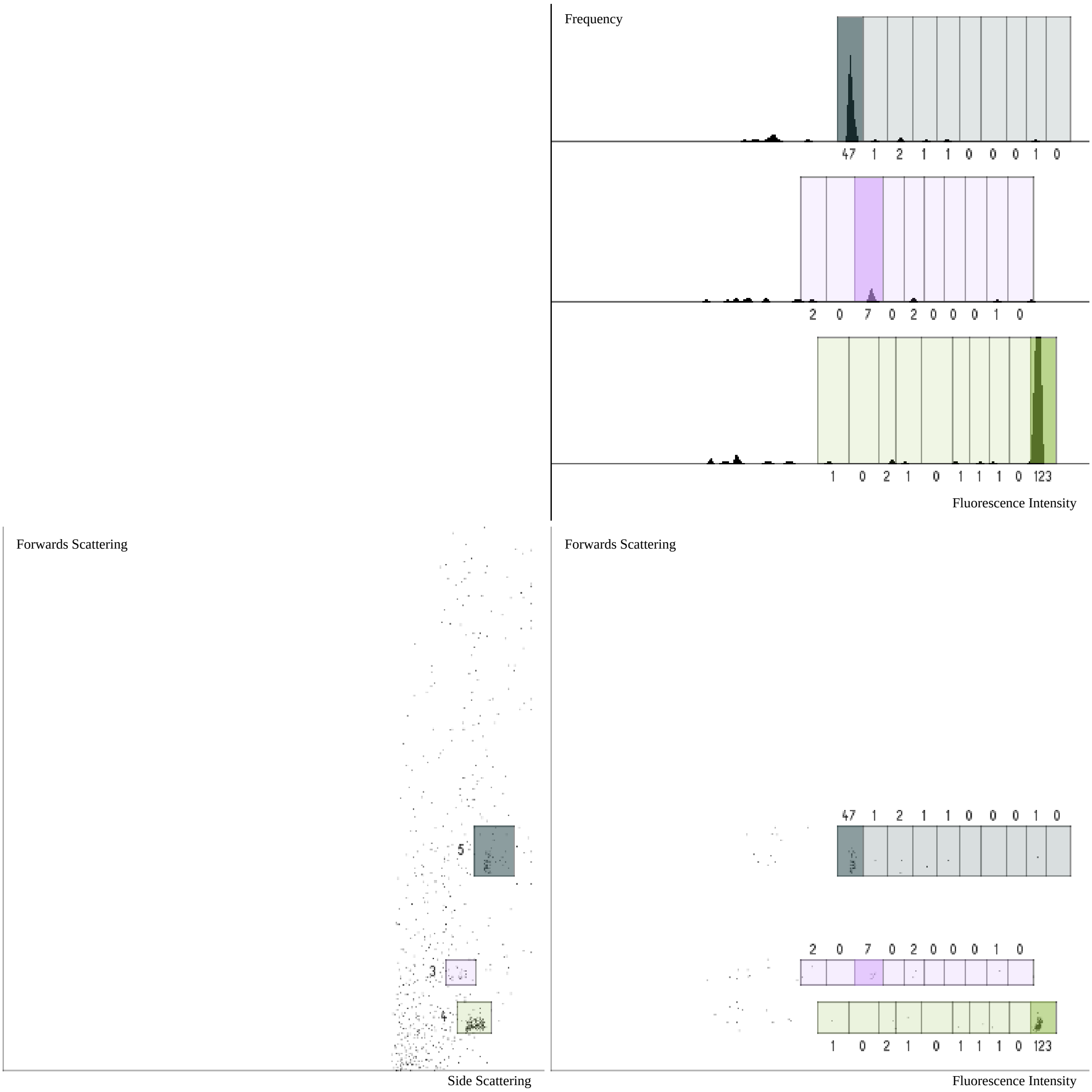

ANNEX 3: TAG DECONVOLUTION - BEAD 233

Passes flow sorting criteria: Yes

Passes tag deconvolution criteria: Yes

Included in protocol analysis: Yes

Protocol: 7, 9, 7, 6

Filename: Bin6_plateA7_B9.fcs

Split 1: Petrol shading

Split 2: Green shading

Split 3: Violet shading

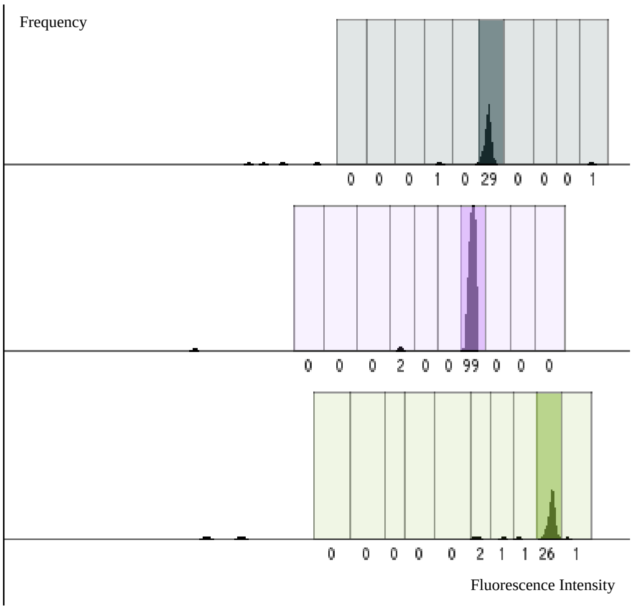

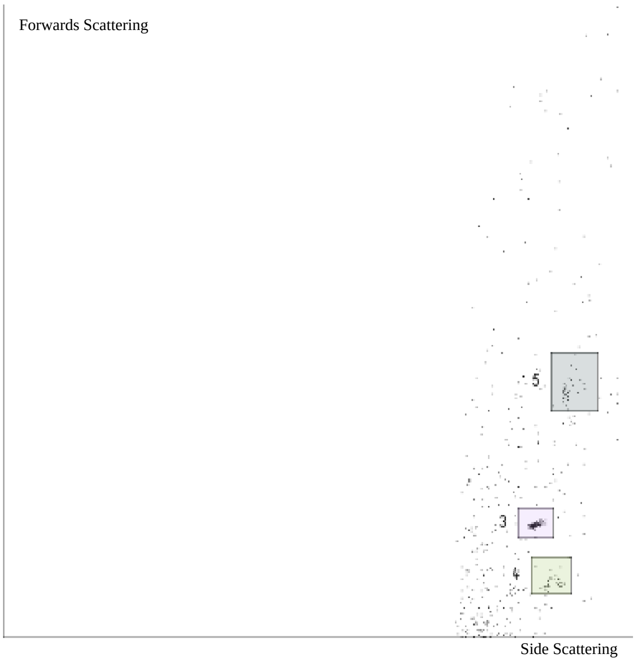

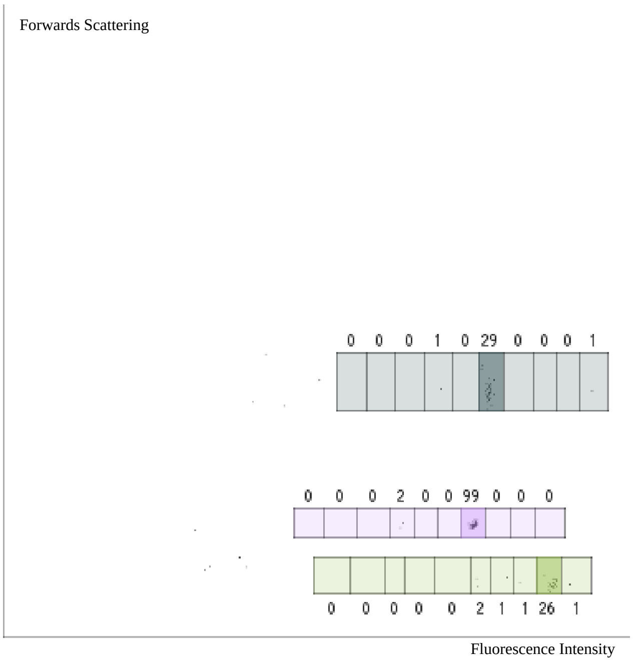

ANNEX 3: TAG DECONVOLUTION - BEAD 234

Passes flow sorting criteria: Yes

Passes tag deconvolution criteria: Yes

Included in protocol analysis: Yes

Protocol: 4, 4, 8, 6

Filename: Bin6_plateA7_B11.fcs

Split 1: Petrol shading

Split 2: Green shading

Split 3: Violet shading

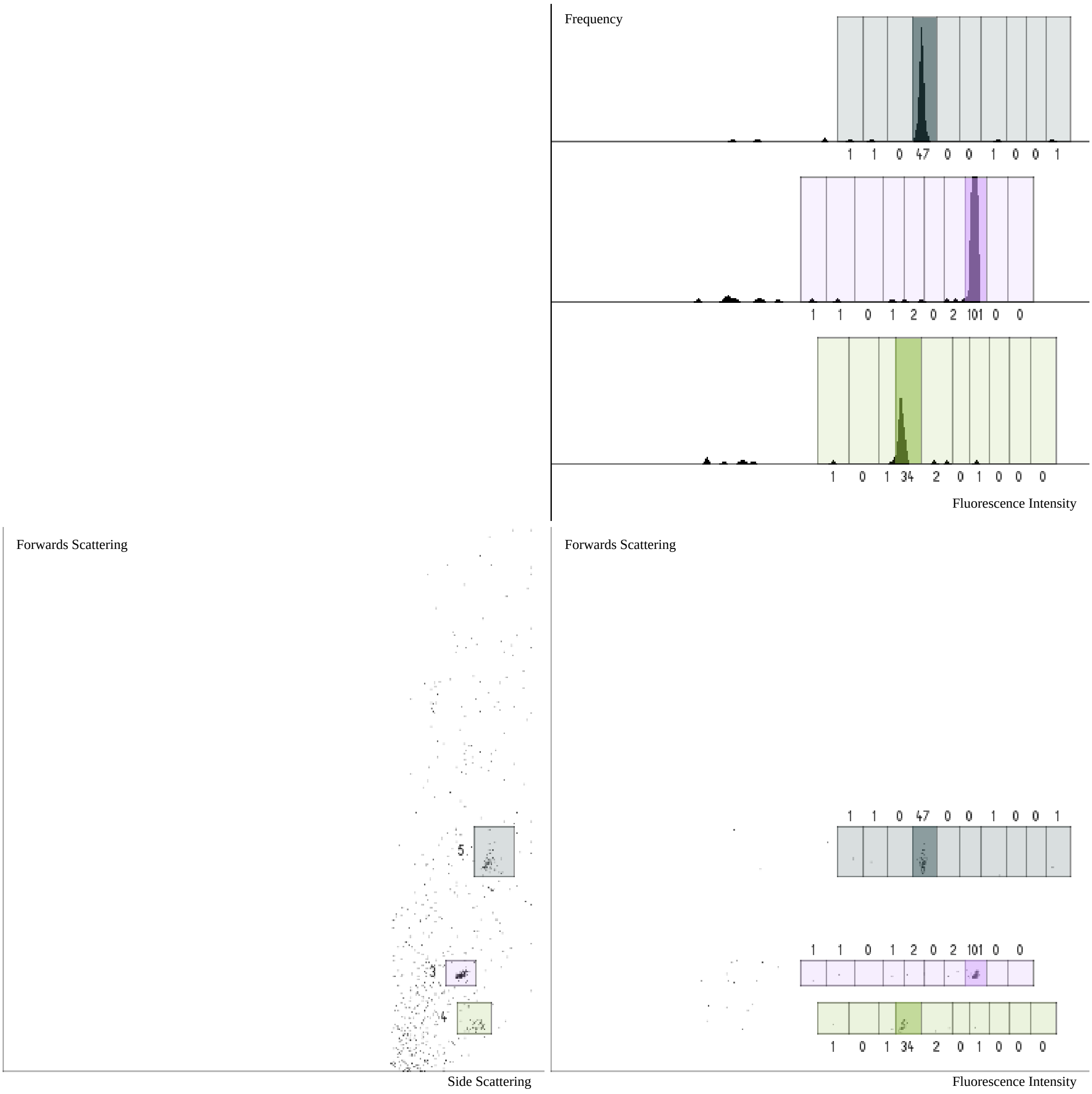

ANNEX 3: TAG DECONVOLUTION - BEAD 235

Passes flow sorting criteria: Yes

Passes tag deconvolution criteria: No

Included in protocol analysis: No

Protocol: N/A

Filename: Bin6_plateA7_C1.fcs

Split 1: Petrol shading

Split 2: Green shading

Split 3: Violet shading

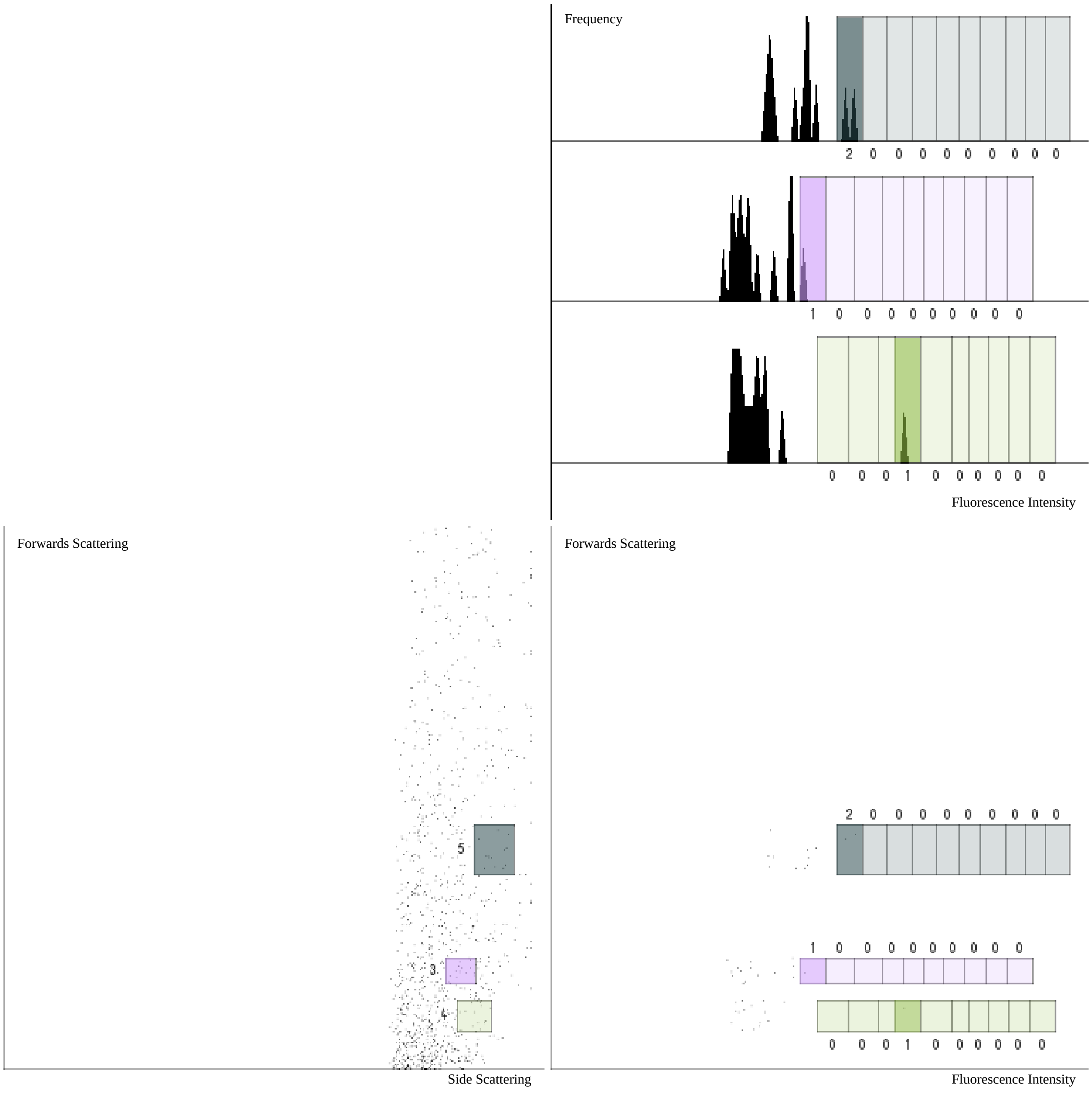

ANNEX 3: TAG DECONVOLUTION - BEAD 236

Passes flow sorting criteria: Yes

Passes tag deconvolution criteria: Yes

Included in protocol analysis: Yes

Protocol: 10, 10, 9, 6

Filename: Bin6_plateA7_C3.fcs

Split 1: Petrol shading

Split 2: Green shading

Split 3: Violet shading

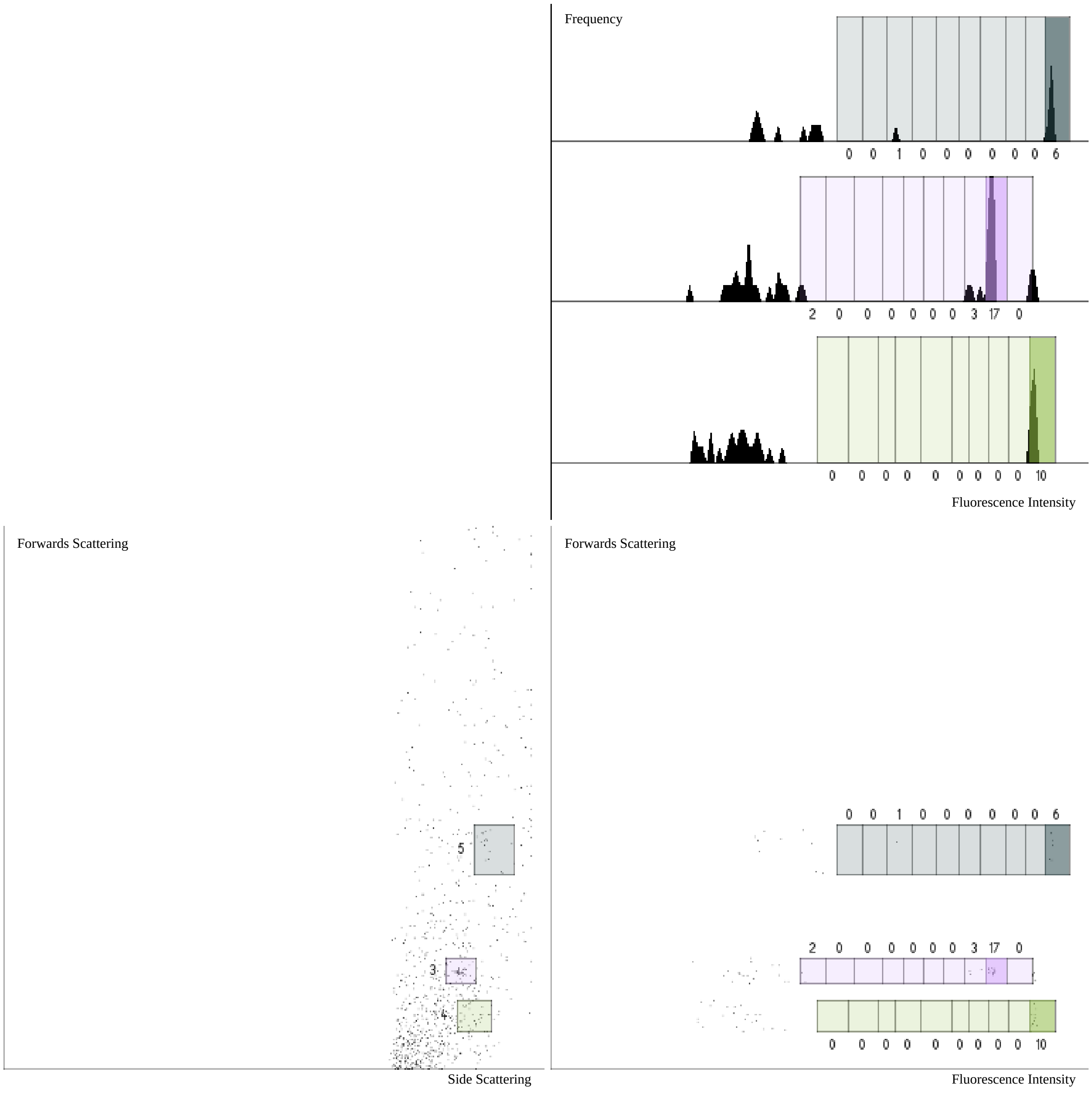

ANNEX 3: TAG DECONVOLUTION - BEAD 237

Passes flow sorting criteria: Yes

Passes tag deconvolution criteria: Yes

Included in protocol analysis: Yes

Protocol: 4, 7, 9, 6

Filename: Bin6_plateA7_C12.fcs

Split 1: Petrol shading

Split 2: Green shading

Split 3: Violet shading

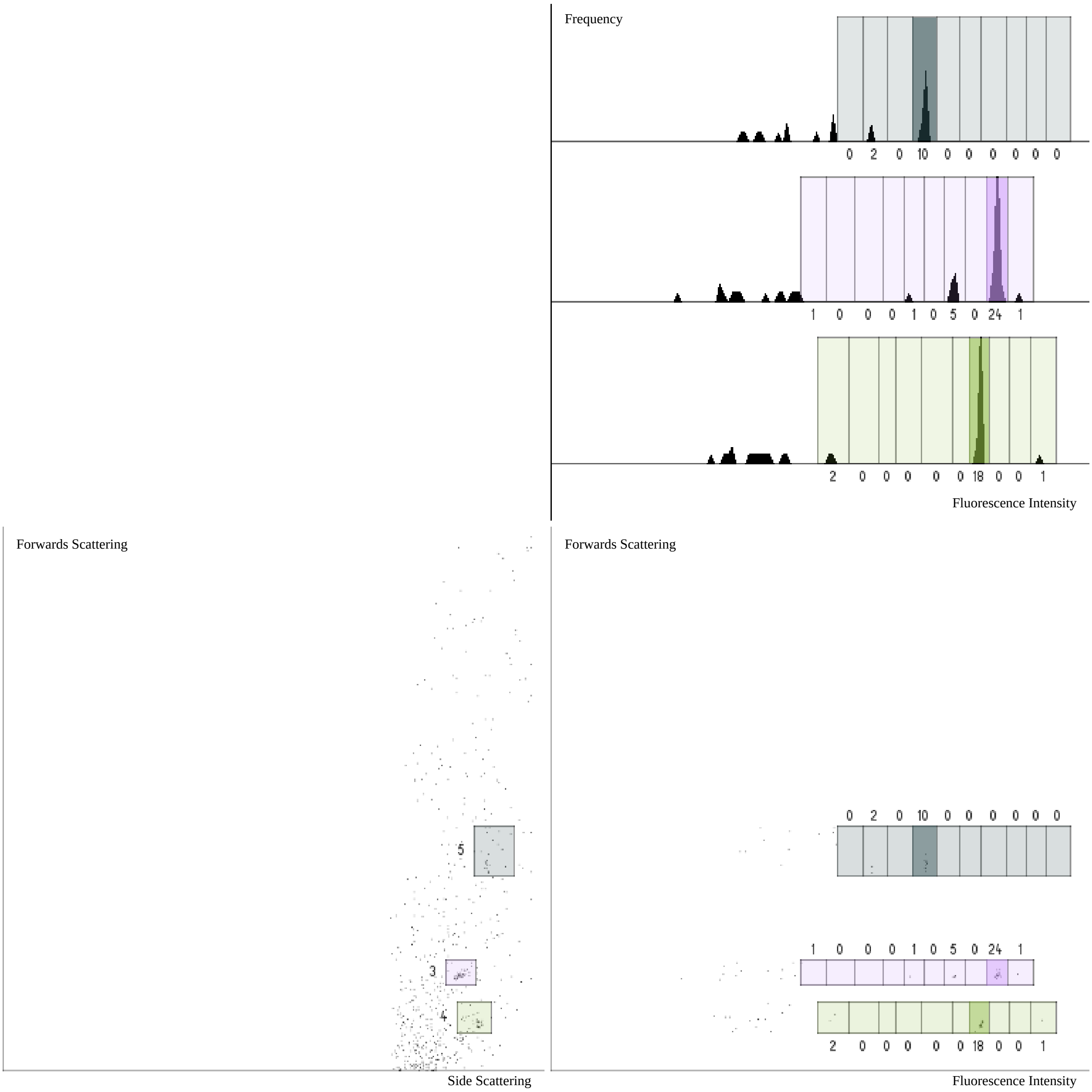

ANNEX 3: TAG DECONVOLUTION - BEAD 238

Passes flow sorting criteria: Yes

Passes tag deconvolution criteria: Yes

Included in protocol analysis: Yes

Protocol: 10, 8, 9, 6

Filename: Bin6_plateA7_D4.fcs

Split 1: Petrol shading

Split 2: Green shading

Split 3: Violet shading

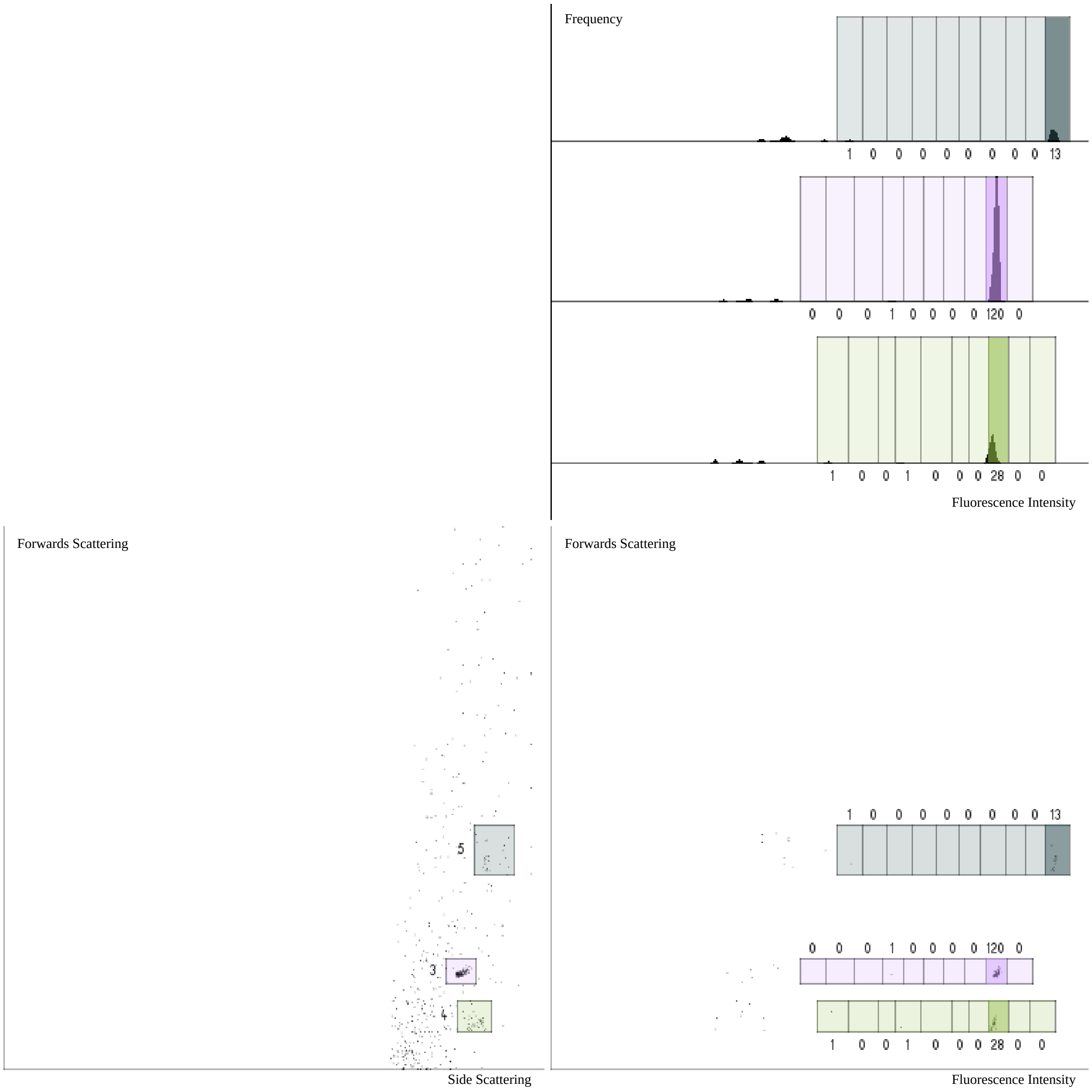

ANNEX 3: TAG DECONVOLUTION - BEAD 239

Passes flow sorting criteria: Yes

Passes tag deconvolution criteria: Yes

Included in protocol analysis: Yes

Protocol: 3, 7, 9, 6

Filename: Bin6_plateA7_D8.fcs

Split 1: Petrol shading

Split 2: Green shading

Split 3: Violet shading

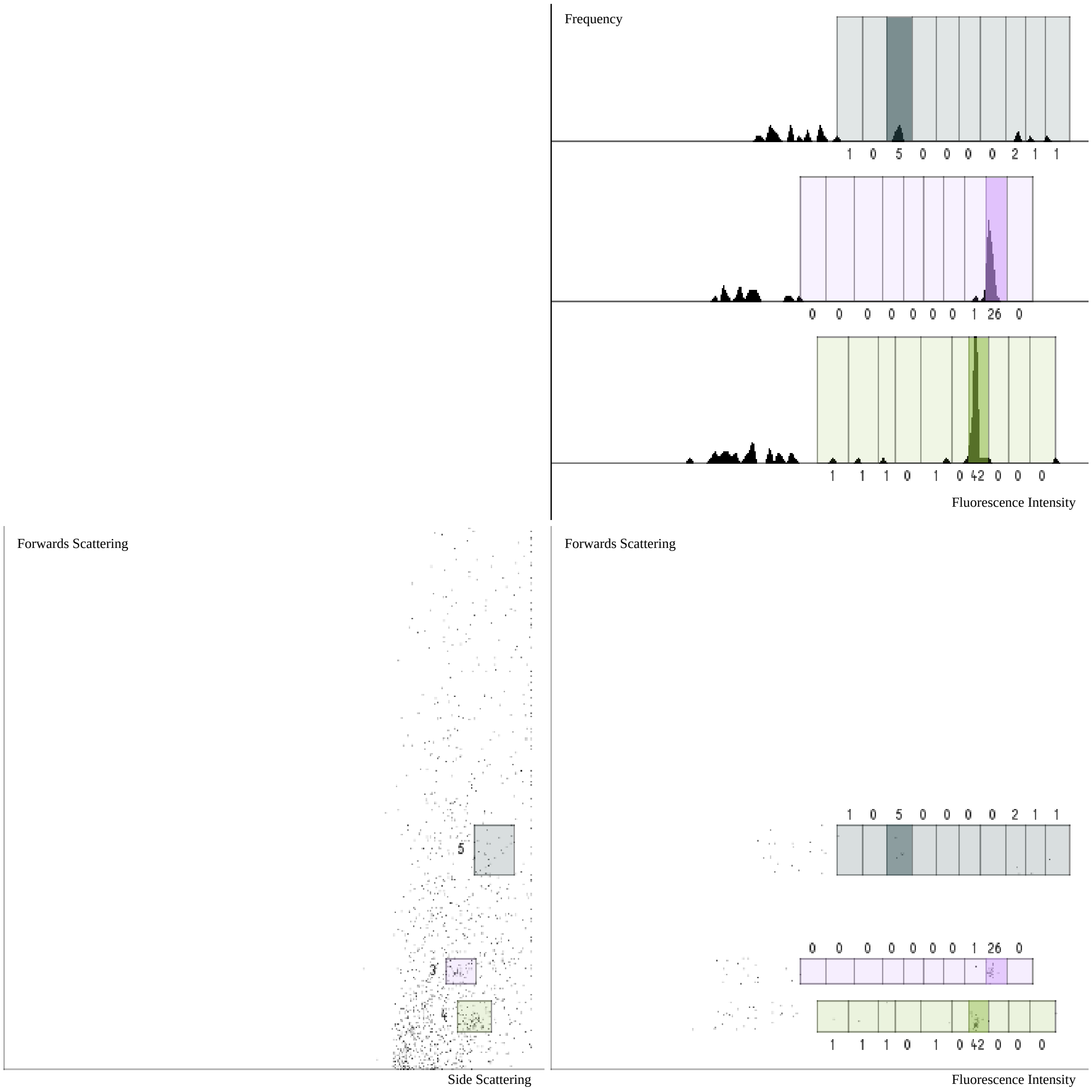

ANNEX 3: TAG DECONVOLUTION - BEAD 240

Passes flow sorting criteria: Yes

Passes tag deconvolution criteria: Yes

Included in protocol analysis: Yes

Protocol: 9, 9, 10, 6

Filename: Bin6_plateA7_E1.fcs

Split 1: Petrol shading

Split 2: Green shading

Split 3: Violet shading

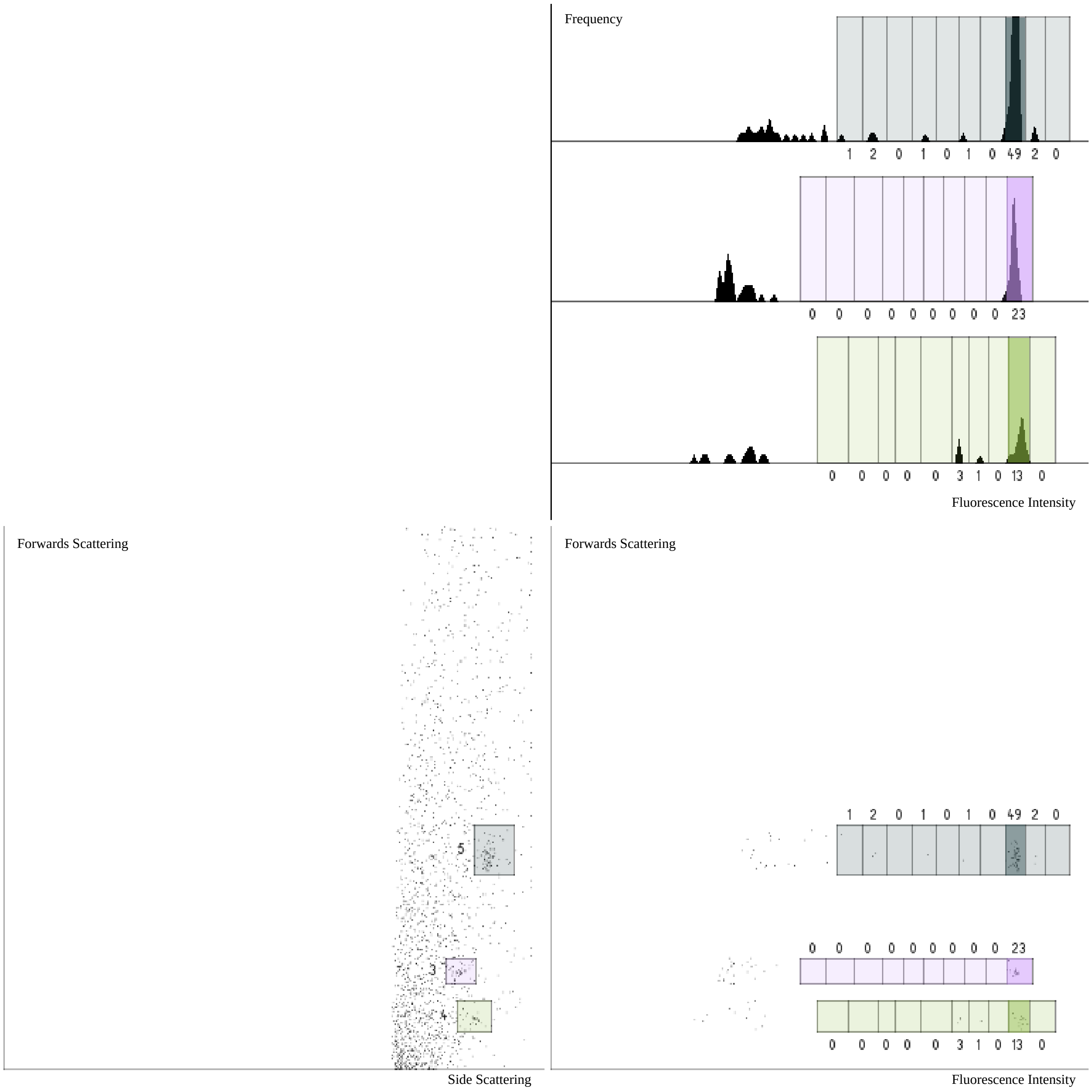

ANNEX 3: TAG DECONVOLUTION - BEAD 241

Passes flow sorting criteria: Yes

Passes tag deconvolution criteria: Yes

Included in protocol analysis: Yes

Protocol: 9, 1, 10, 6

Filename: Bin6_plateA7_E2.fcs

Split 1: Petrol shading

Split 2: Green shading

Split 3: Violet shading

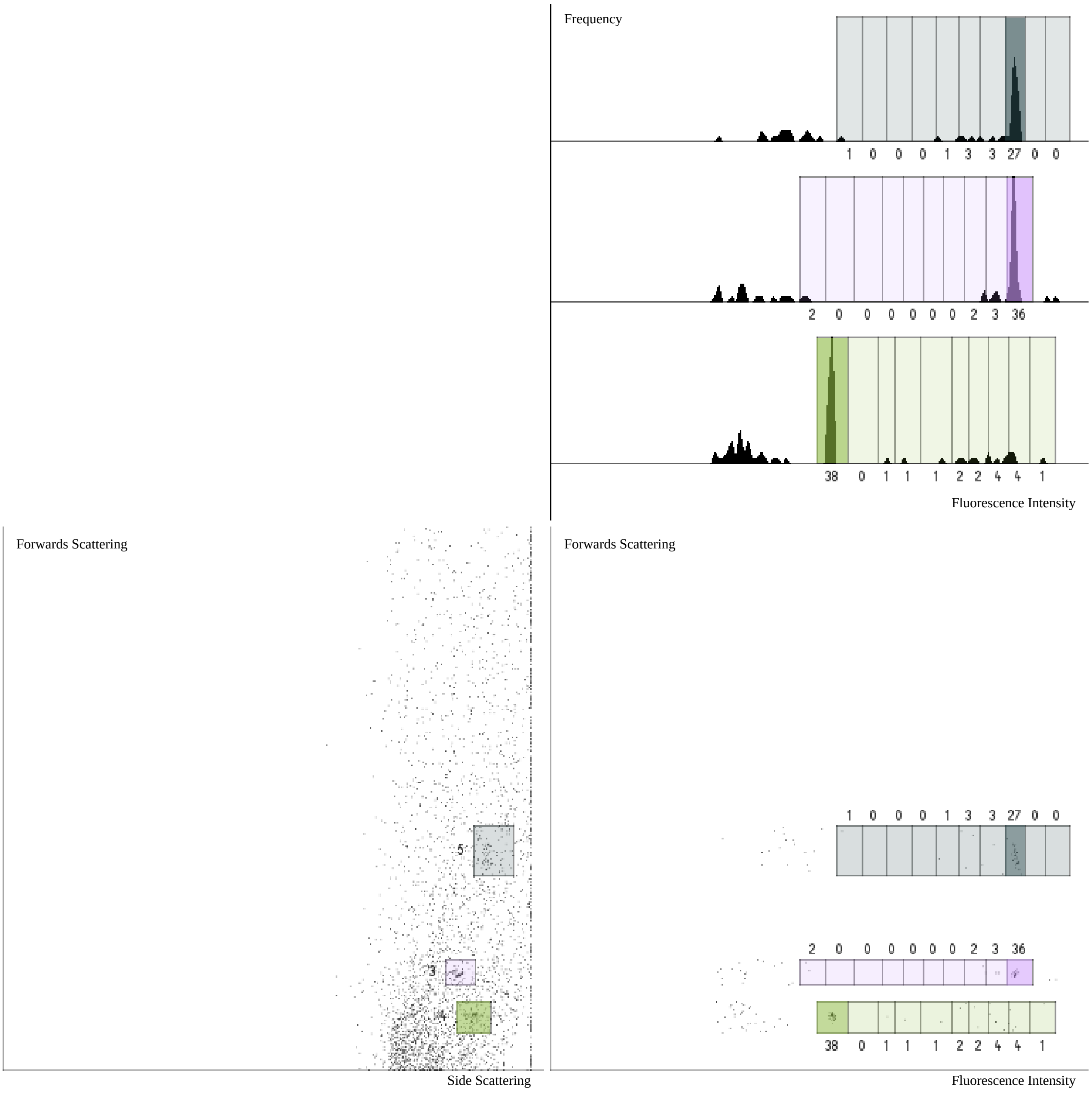

ANNEX 3: TAG DECONVOLUTION - BEAD 242

Passes flow sorting criteria: Yes

Passes tag deconvolution criteria: Yes

Included in protocol analysis: Yes

Protocol: 9, 5, 2, 6

Filename: Bin6_plateA7_E5.fcs

Split 1: Petrol shading

Split 2: Green shading

Split 3: Violet shading

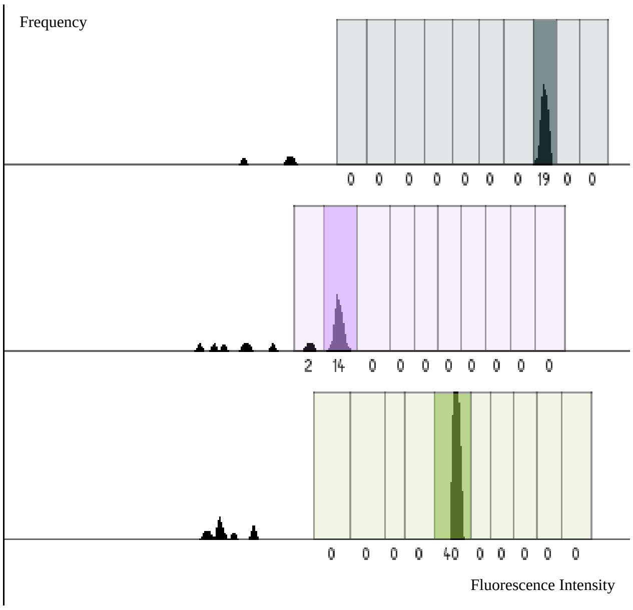

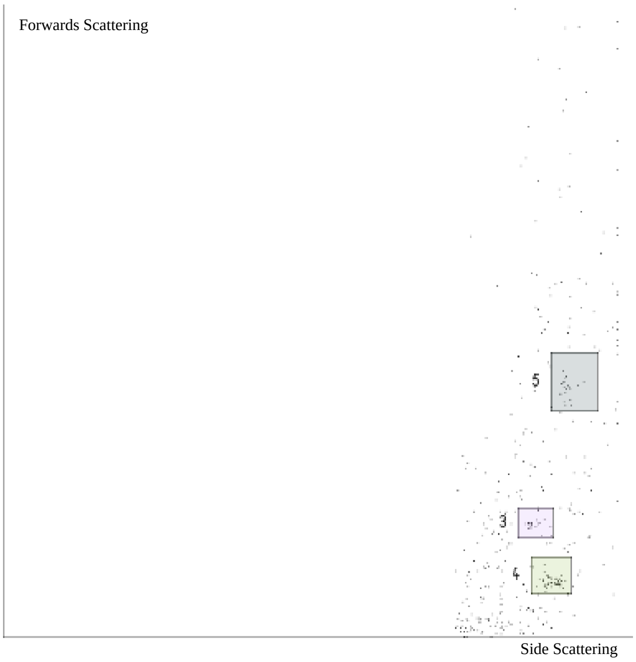

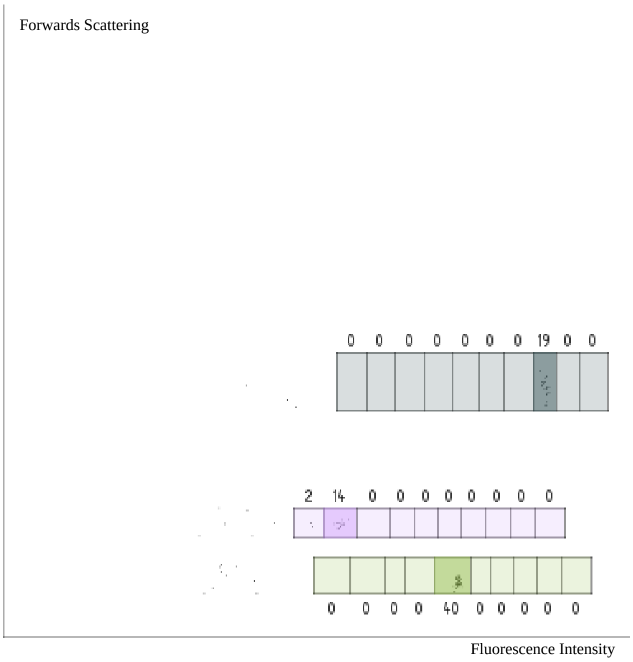

ANNEX 3: TAG DECONVOLUTION - BEAD 243

Passes flow sorting criteria: Yes

Passes tag deconvolution criteria: Yes

Included in protocol analysis: Yes

Protocol: 8, 8, 1, 6

Filename: Bin6_plateA7_E8.fcs

Split 1: Petrol shading

Split 2: Green shading

Split 3: Violet shading

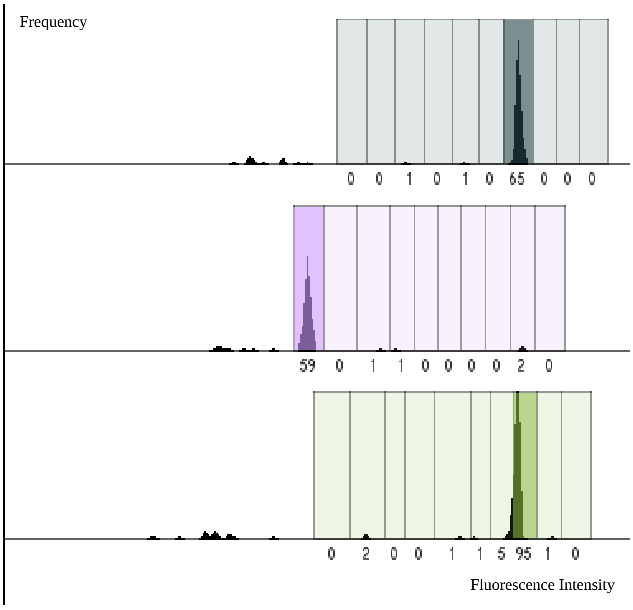

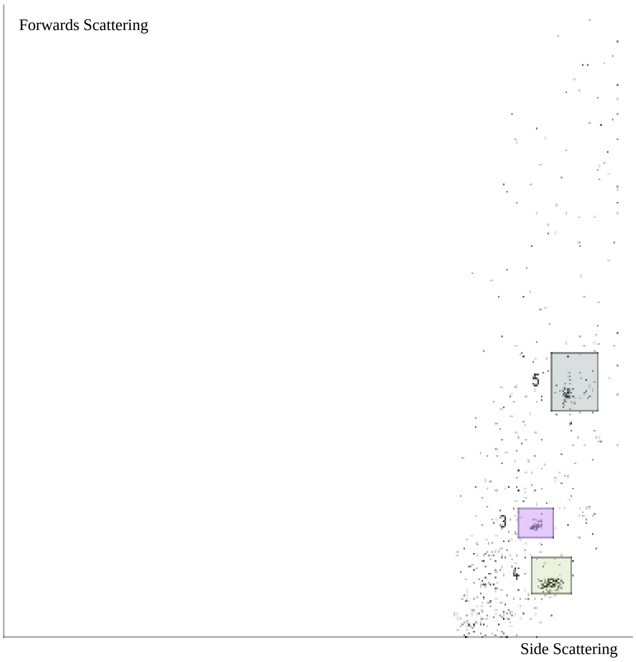

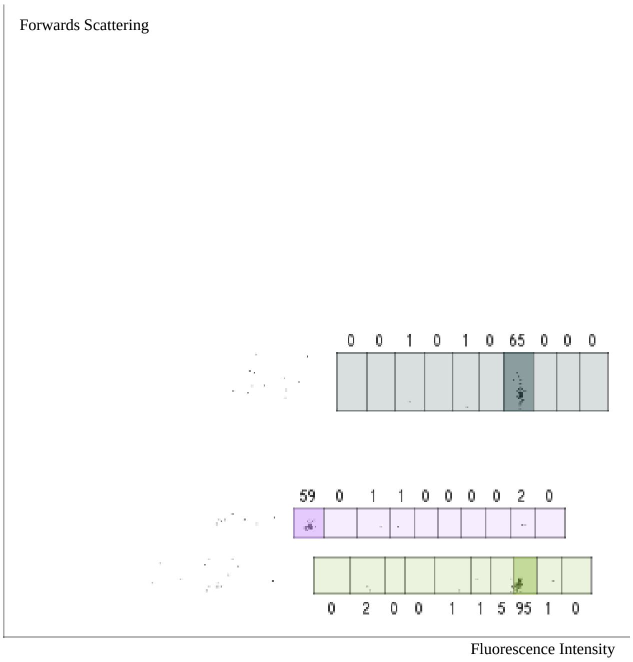

ANNEX 3: TAG DECONVOLUTION - BEAD 244

Passes flow sorting criteria: Yes

Passes tag deconvolution criteria: Yes

Included in protocol analysis: Yes

Protocol: 4, 8, 5, 6

Filename: Bin6_plateA7_E11.fcs

Split 1: Petrol shading

Split 2: Green shading

Split 3: Violet shading

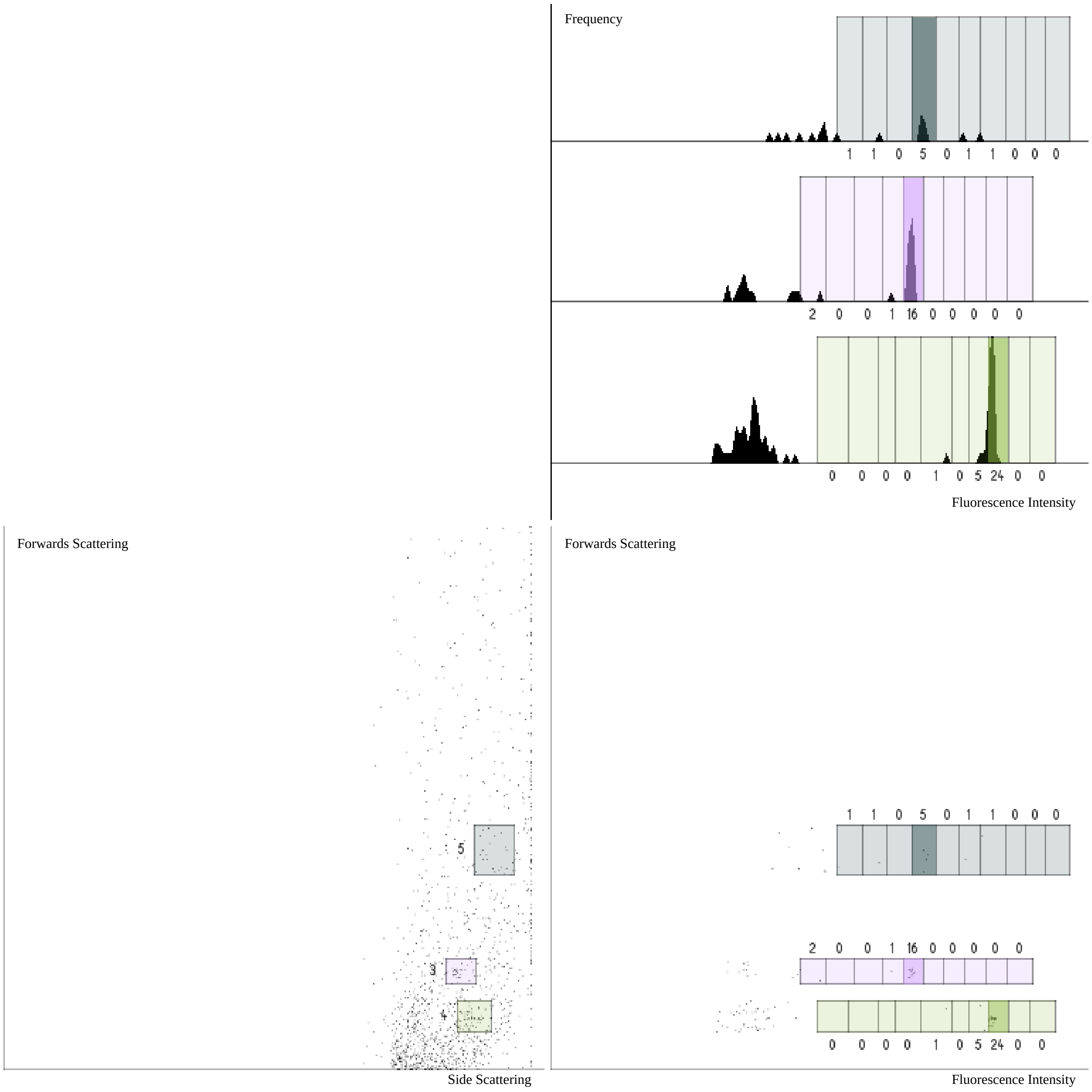

ANNEX 3: TAG DECONVOLUTION - BEAD 245

Passes flow sorting criteria: Yes

Passes tag deconvolution criteria: Yes

Included in protocol analysis: Yes

Protocol: 8, 10, 3, 8

Filename: Bin8_plateA1_H9.fcs

Split 1: Petrol shading

Split 2: Green shading

Split 3: Violet shading

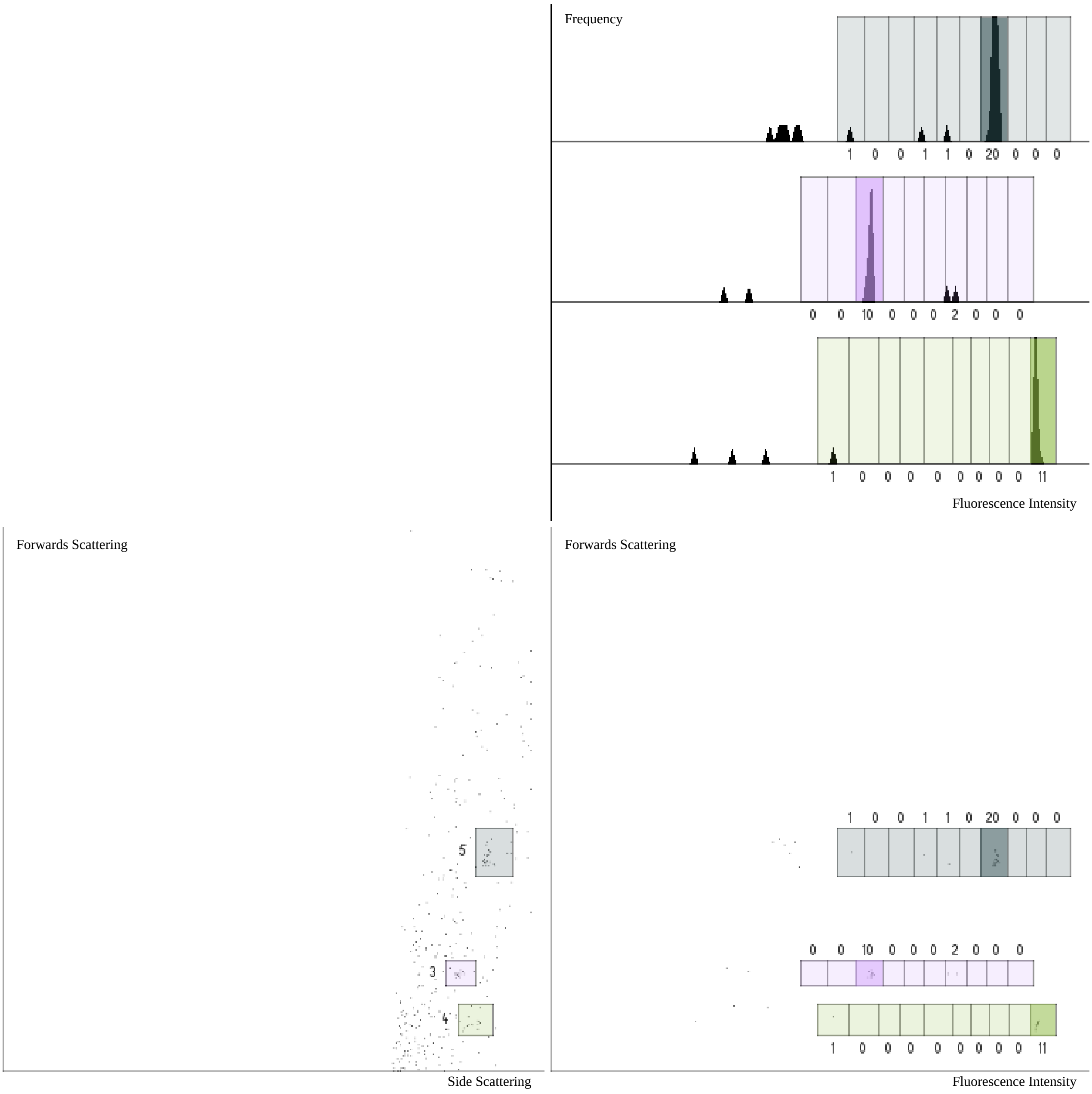

ANNEX 3: TAG DECONVOLUTION - BEAD 246

Passes flow sorting criteria: Yes

Passes tag deconvolution criteria: Yes

Included in protocol analysis: Yes

Protocol: 5, 8, 4, 8

Filename: Bin8_plateA1_A1.fcs

Split 1: Petrol shading

Split 2: Green shading

Split 3: Violet shading

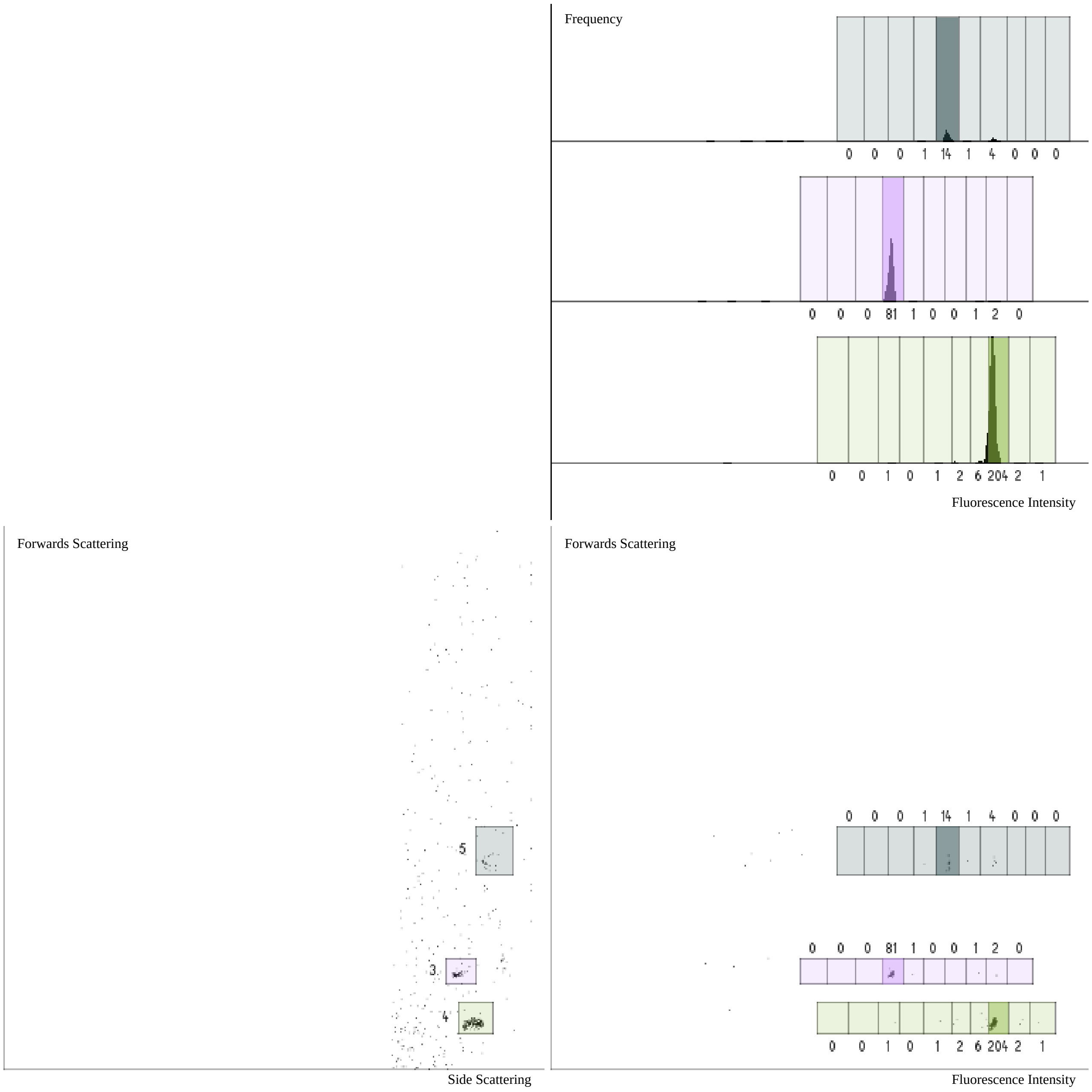

ANNEX 3: TAG DECONVOLUTION - BEAD 247

Passes flow sorting criteria: Yes

Passes tag deconvolution criteria: Yes

Included in protocol analysis: Yes

Protocol: 4, 3, 8, 8

Filename: Bin8_plateA1_A2.fcs

Split 1: Petrol shading

Split 2: Green shading

Split 3: Violet shading

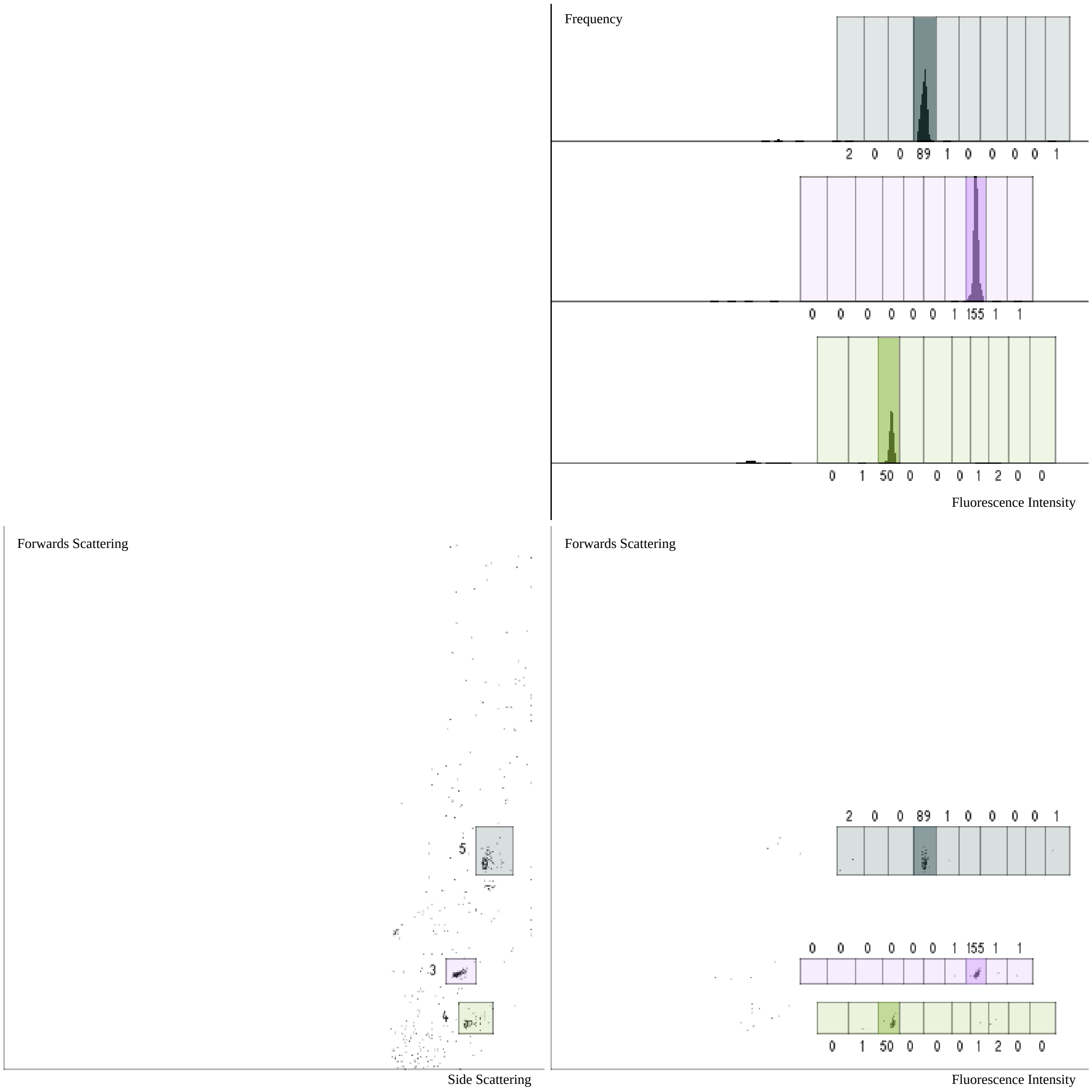

ANNEX 3: TAG DECONVOLUTION - BEAD 248

Passes flow sorting criteria: Yes

Passes tag deconvolution criteria: Yes

Included in protocol analysis: Yes

Protocol: 2, 8, 2, 8

Filename: Bin8_plateA1_A4.fcs

Split 1: Petrol shading

Split 2: Green shading

Split 3: Violet shading

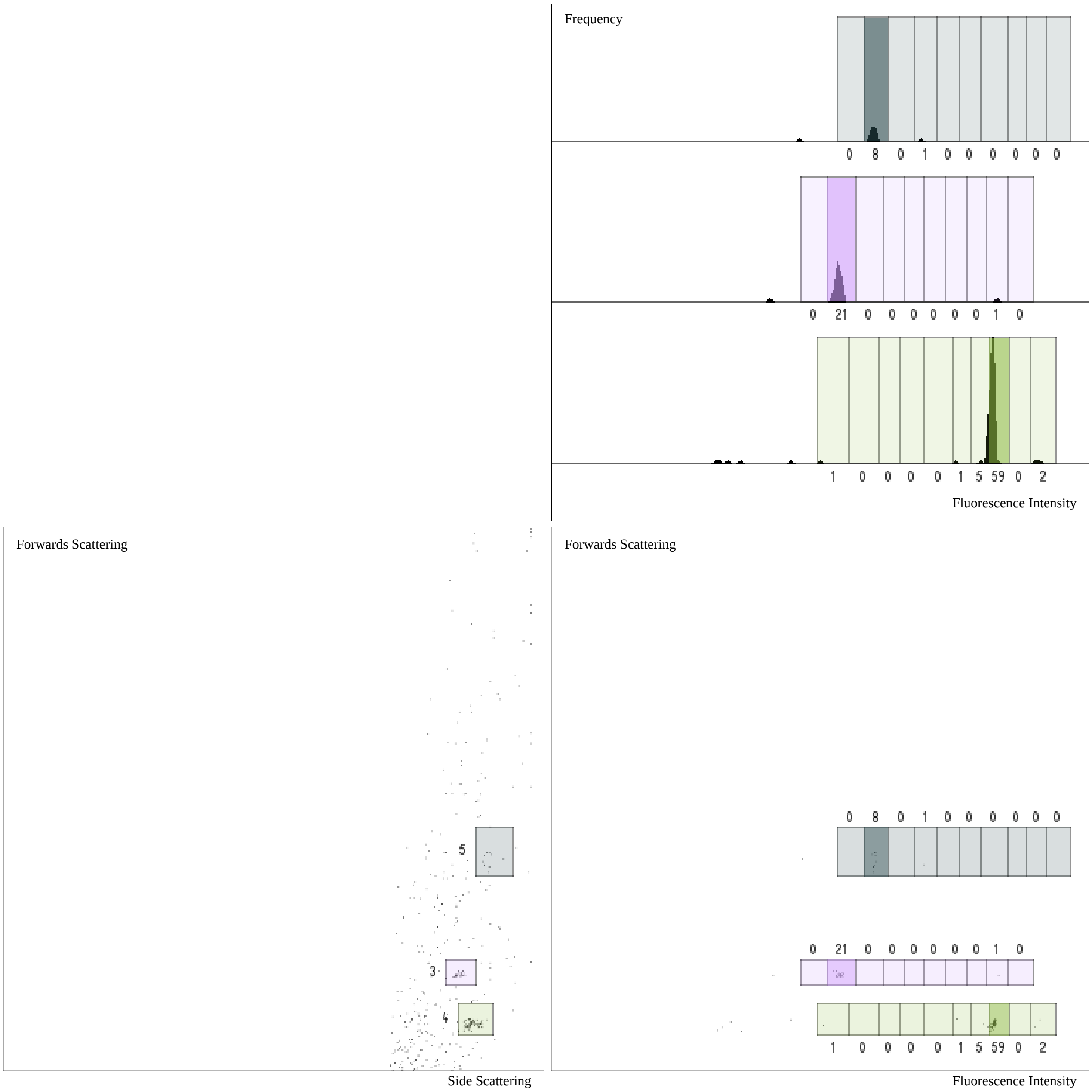

ANNEX 3: TAG DECONVOLUTION - BEAD 249

Passes flow sorting criteria: Yes

Passes tag deconvolution criteria: Yes

Included in protocol analysis: Yes

Protocol: 2, 9, 2, 8

Filename: Bin8_plateA1_A6.fcs

Split 1: Petrol shading

Split 2: Green shading

Split 3: Violet shading

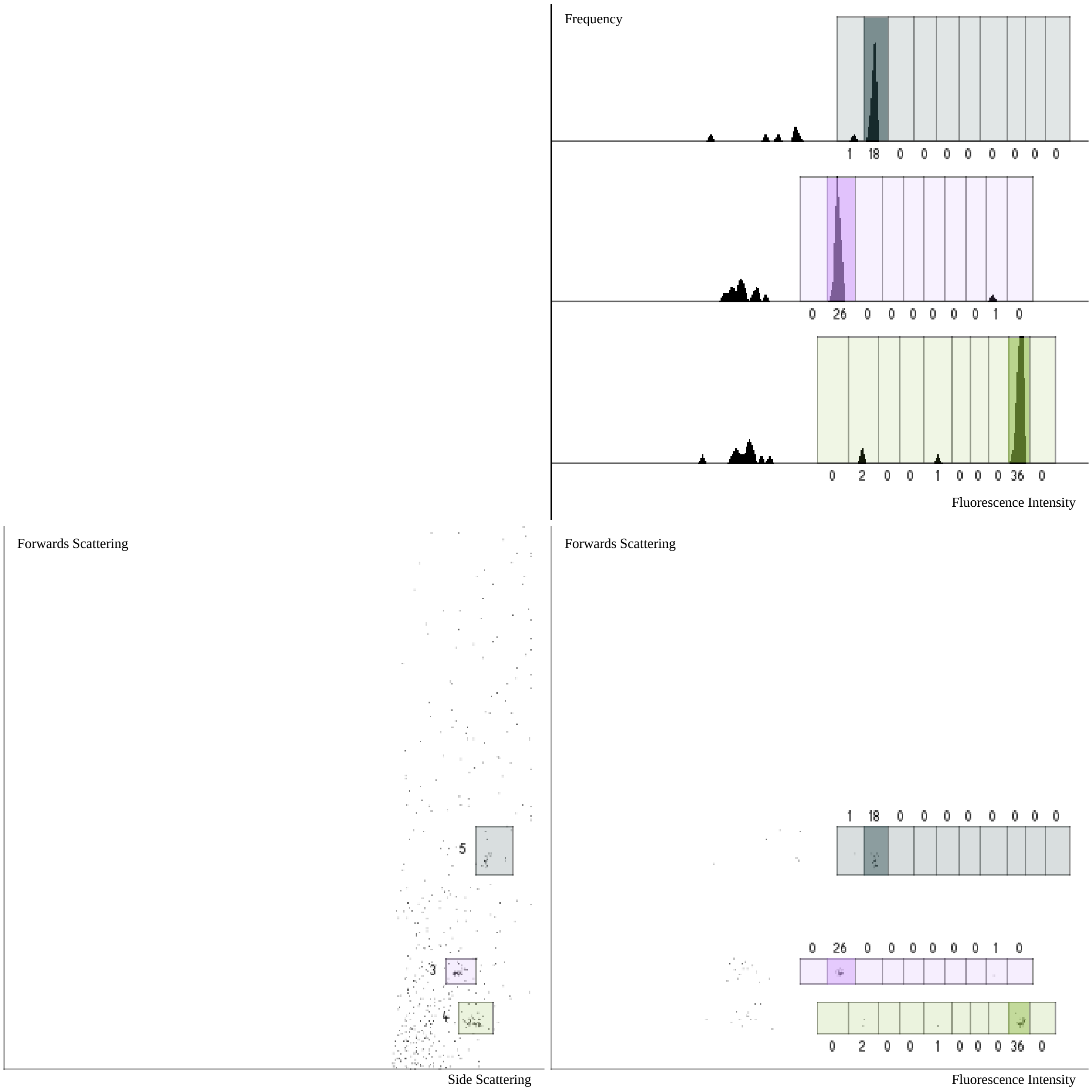

ANNEX 3: TAG DECONVOLUTION - BEAD 250

Passes flow sorting criteria: Yes

Passes tag deconvolution criteria: Yes

Included in protocol analysis: Yes

Protocol: 8, 6, 9, 8

Filename: Bin8_plateA1_A9.fcs

Split 1: Petrol shading

Split 2: Green shading

Split 3: Violet shading

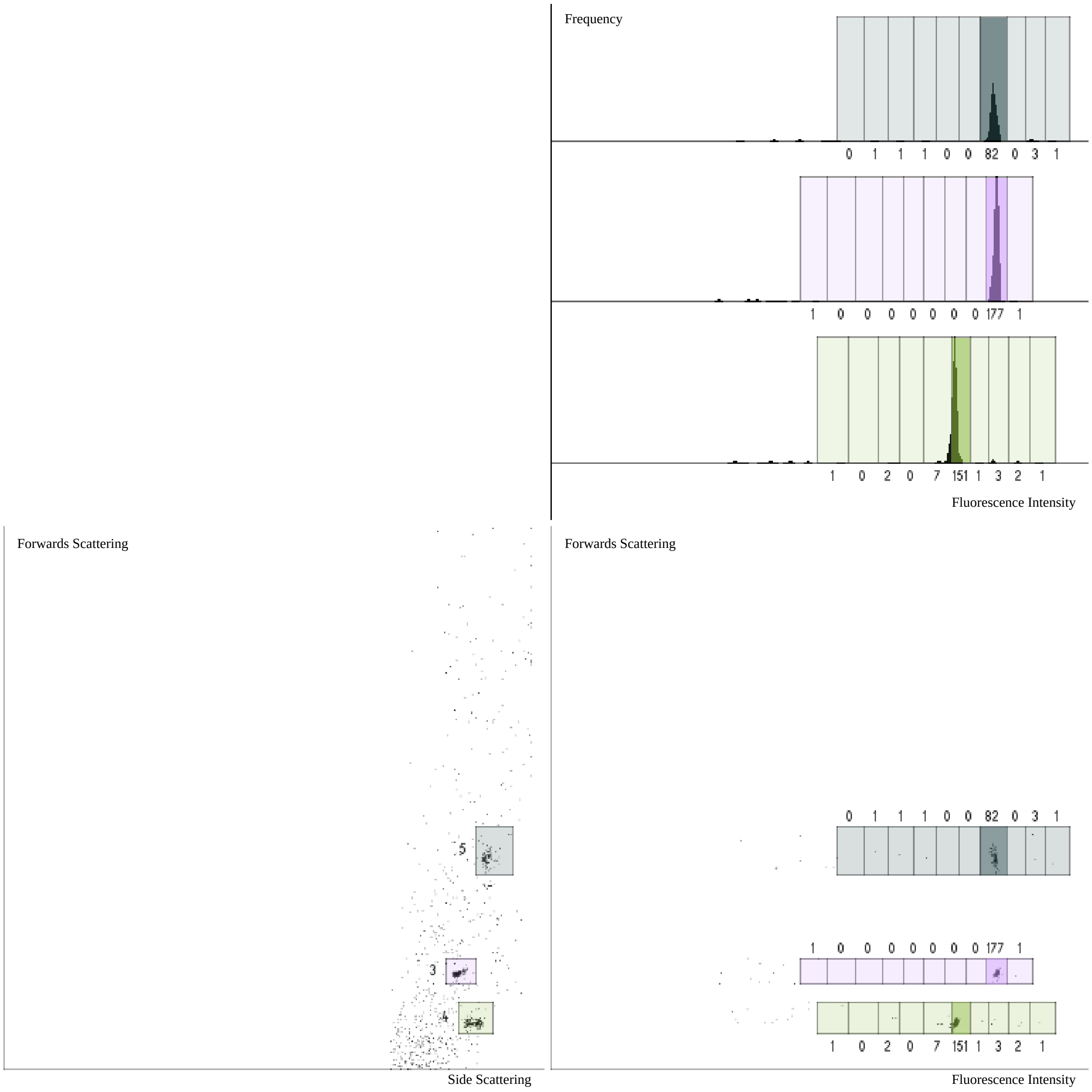

ANNEX 3: TAG DECONVOLUTION - BEAD 251

Passes flow sorting criteria: Yes

Passes tag deconvolution criteria: Yes

Included in protocol analysis: Yes

Protocol: 9, 6, 2, 8

Filename: Bin8_plateA1_A11.fcs

Split 1: Petrol shading

Split 2: Green shading

Split 3: Violet shading

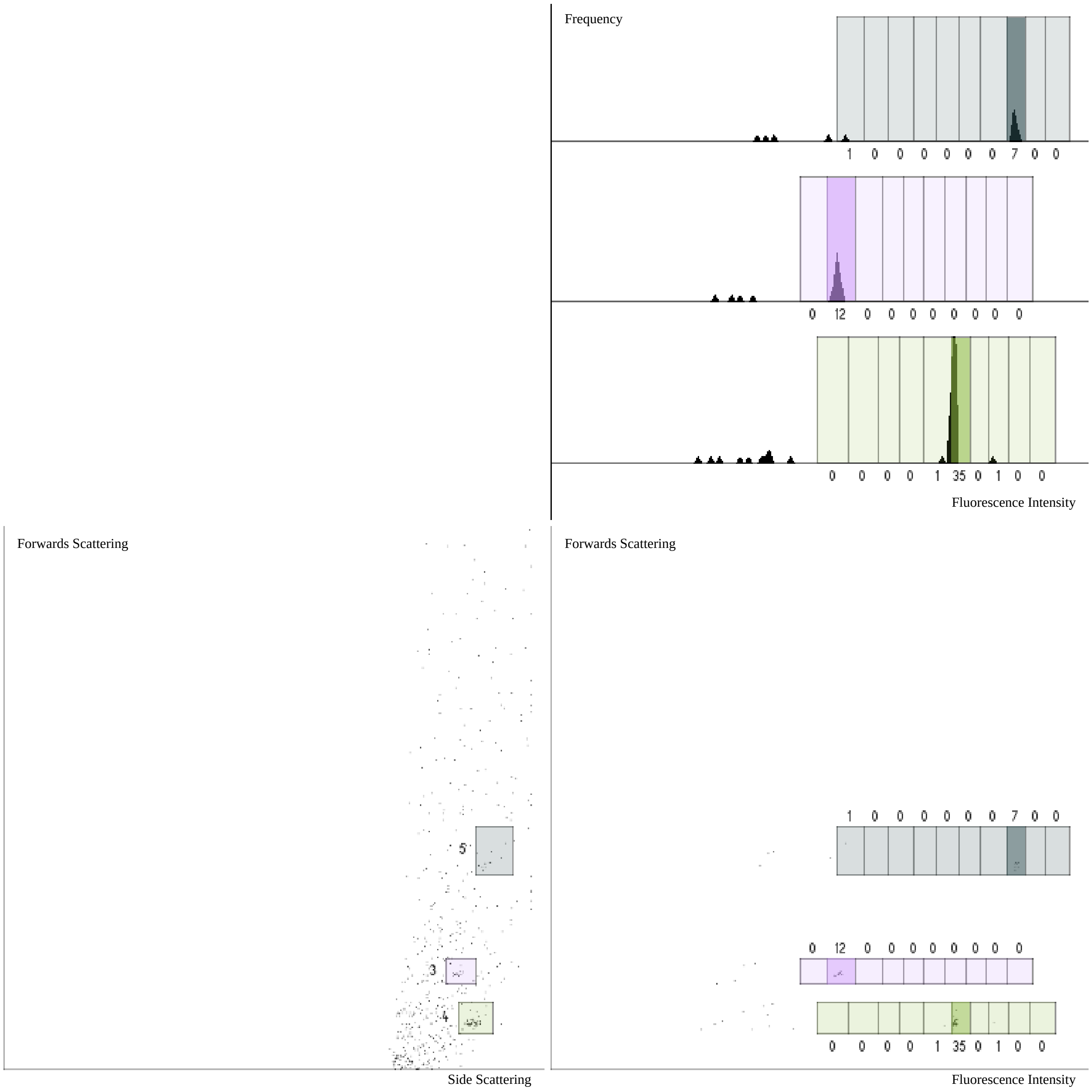

ANNEX 3: TAG DECONVOLUTION - BEAD 252

Passes flow sorting criteria: Yes

Passes tag deconvolution criteria: Yes

Included in protocol analysis: Yes

Protocol: 5, 4, 5, 8

Filename: Bin8_plateA1_B3.fcs

Split 1: Petrol shading

Split 2: Green shading

Split 3: Violet shading

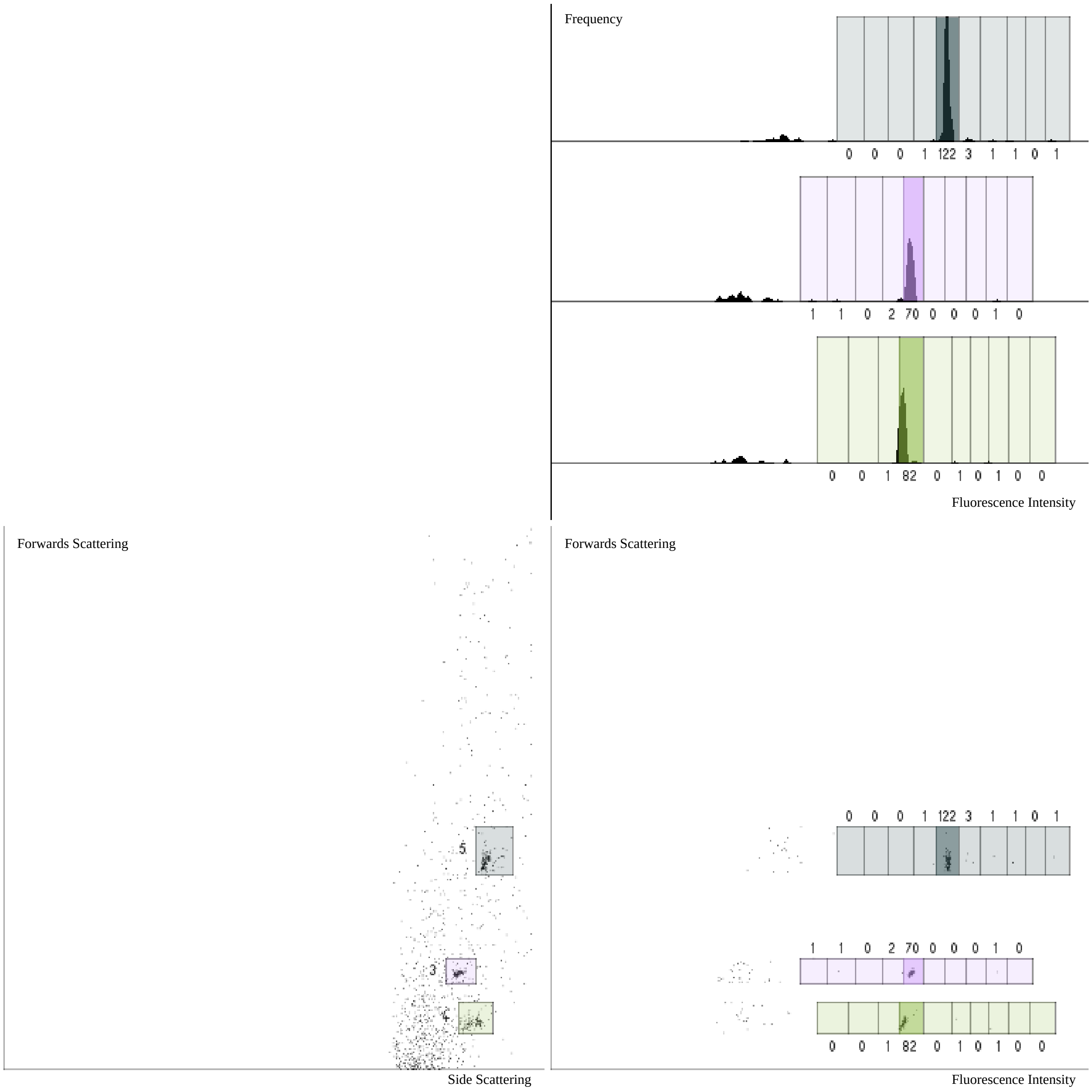

ANNEX 3: TAG DECONVOLUTION - BEAD 253

Passes flow sorting criteria: Yes

Passes tag deconvolution criteria: Yes

Included in protocol analysis: Yes

Protocol: 3, 5, 8, 8

Filename: Bin8_plateA1_B8.fcs

Split 1: Petrol shading

Split 2: Green shading

Split 3: Violet shading

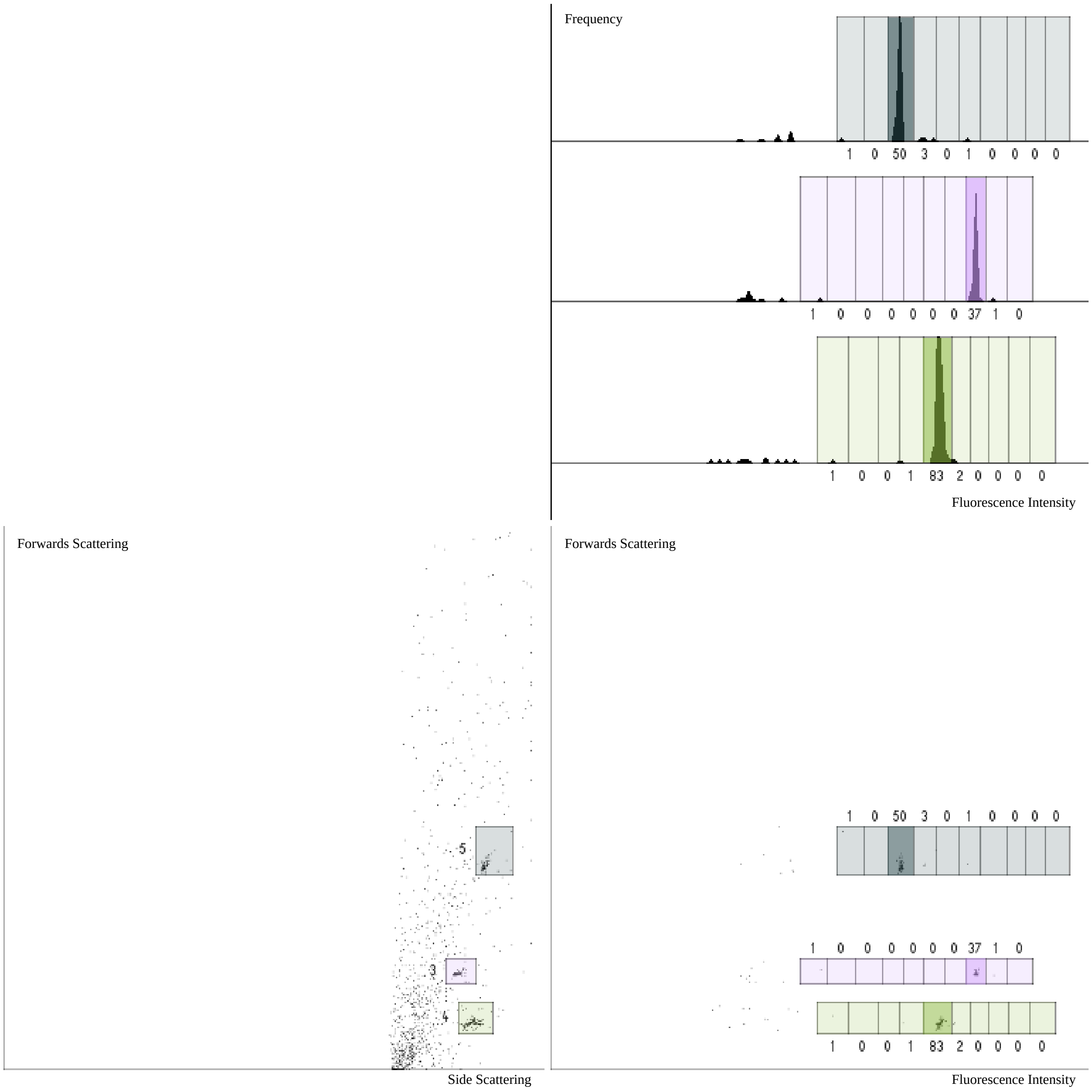

ANNEX 3: TAG DECONVOLUTION - BEAD 254

Passes flow sorting criteria: Yes

Passes tag deconvolution criteria: Yes

Included in protocol analysis: Yes

Protocol: 5, 1, 2, 8

Filename: Bin8_plateA1_B10.fcs

Split 1: Petrol shading

Split 2: Green shading

Split 3: Violet shading

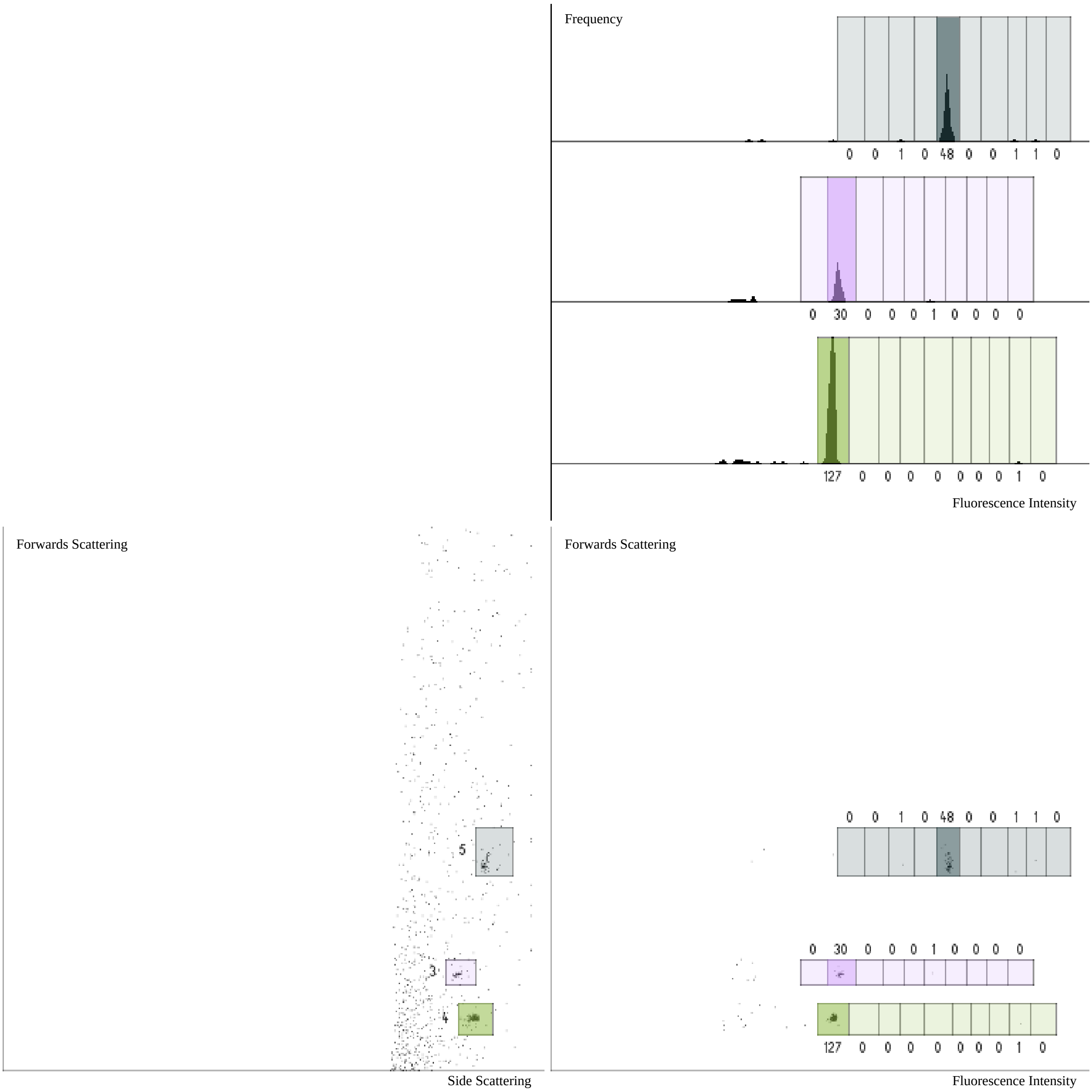

ANNEX 3: TAG DECONVOLUTION - BEAD 255

Passes flow sorting criteria: Yes

Passes tag deconvolution criteria: Yes

Included in protocol analysis: Yes

Protocol: 4, 3, 3, 8

Filename: Bin8_plateA1_B12.fcs

Split 1: Petrol shading

Split 2: Green shading

Split 3: Violet shading

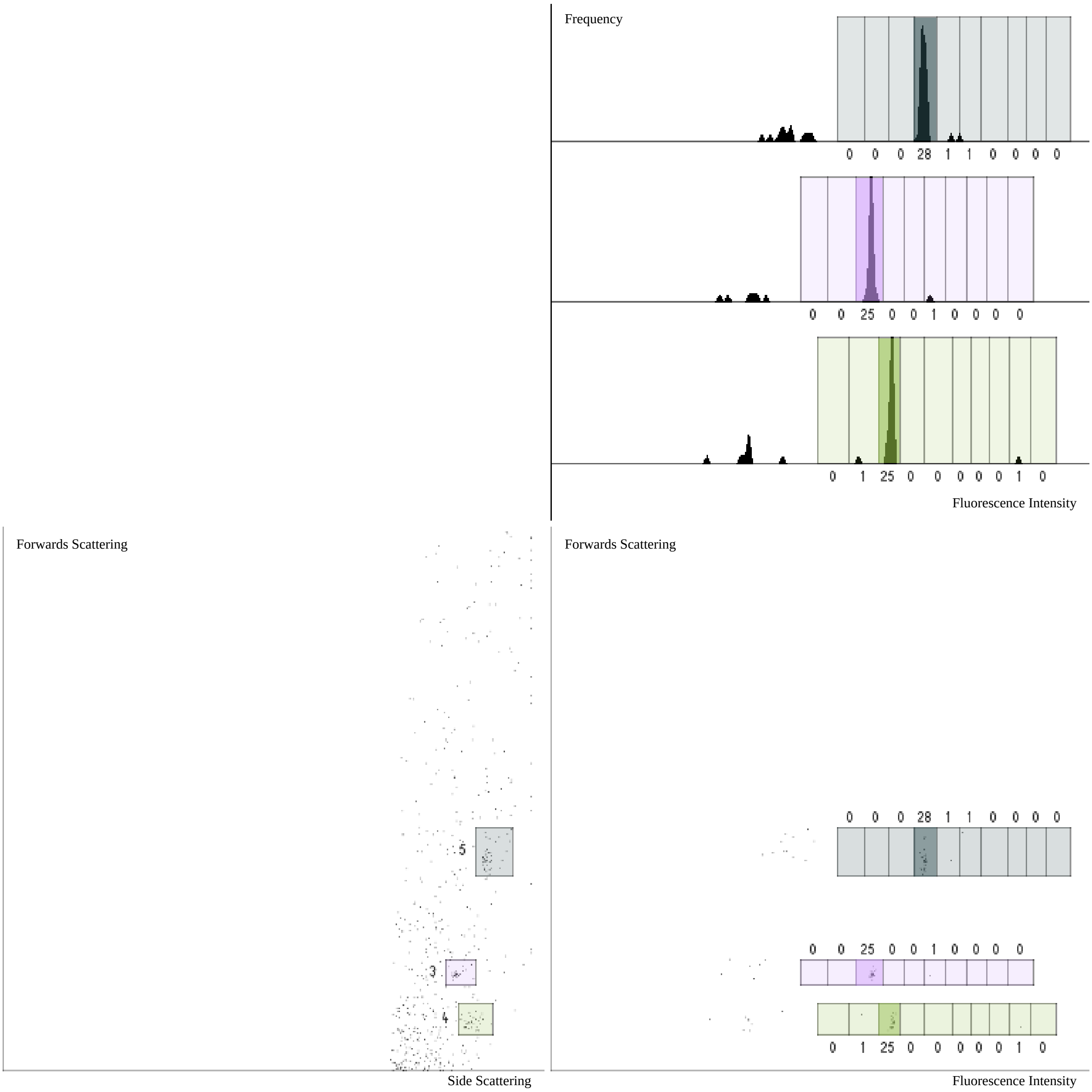

ANNEX 3: TAG DECONVOLUTION - BEAD 256

Passes flow sorting criteria: Yes

Passes tag deconvolution criteria: Yes

Included in protocol analysis: Yes

Protocol: 6, 2, 7, 8

Filename: Bin8_plateA1_C5.fcs

Split 1: Petrol shading

Split 2: Green shading

Split 3: Violet shading

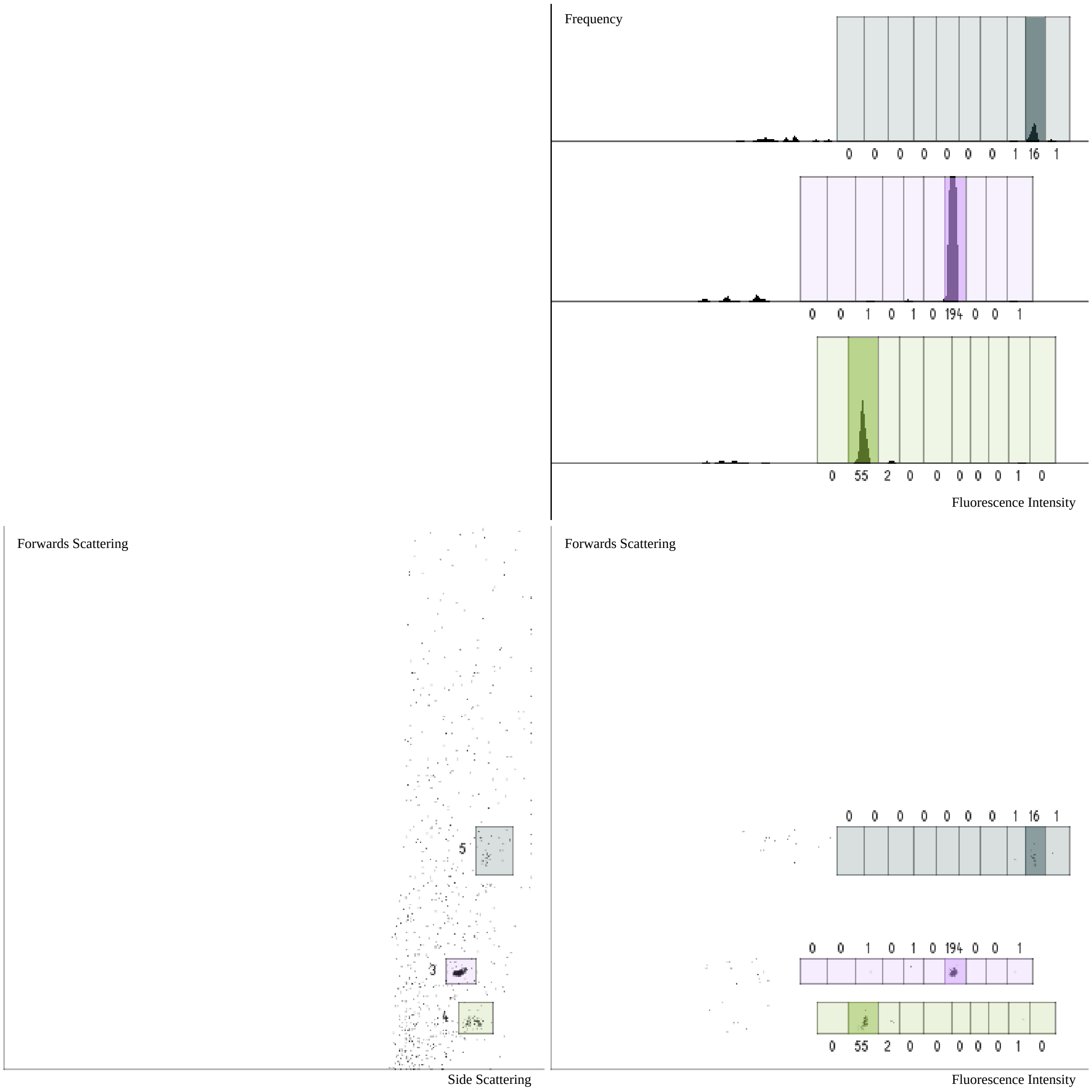

ANNEX 3: TAG DECONVOLUTION - BEAD 257

Passes flow sorting criteria: Yes

Passes tag deconvolution criteria: No

Included in protocol analysis: No

Protocol: N/A

Filename: Bin8_plateA1_C9.fcs

Split 1: Petrol shading

Split 2: Green shading

Split 3: Violet shading

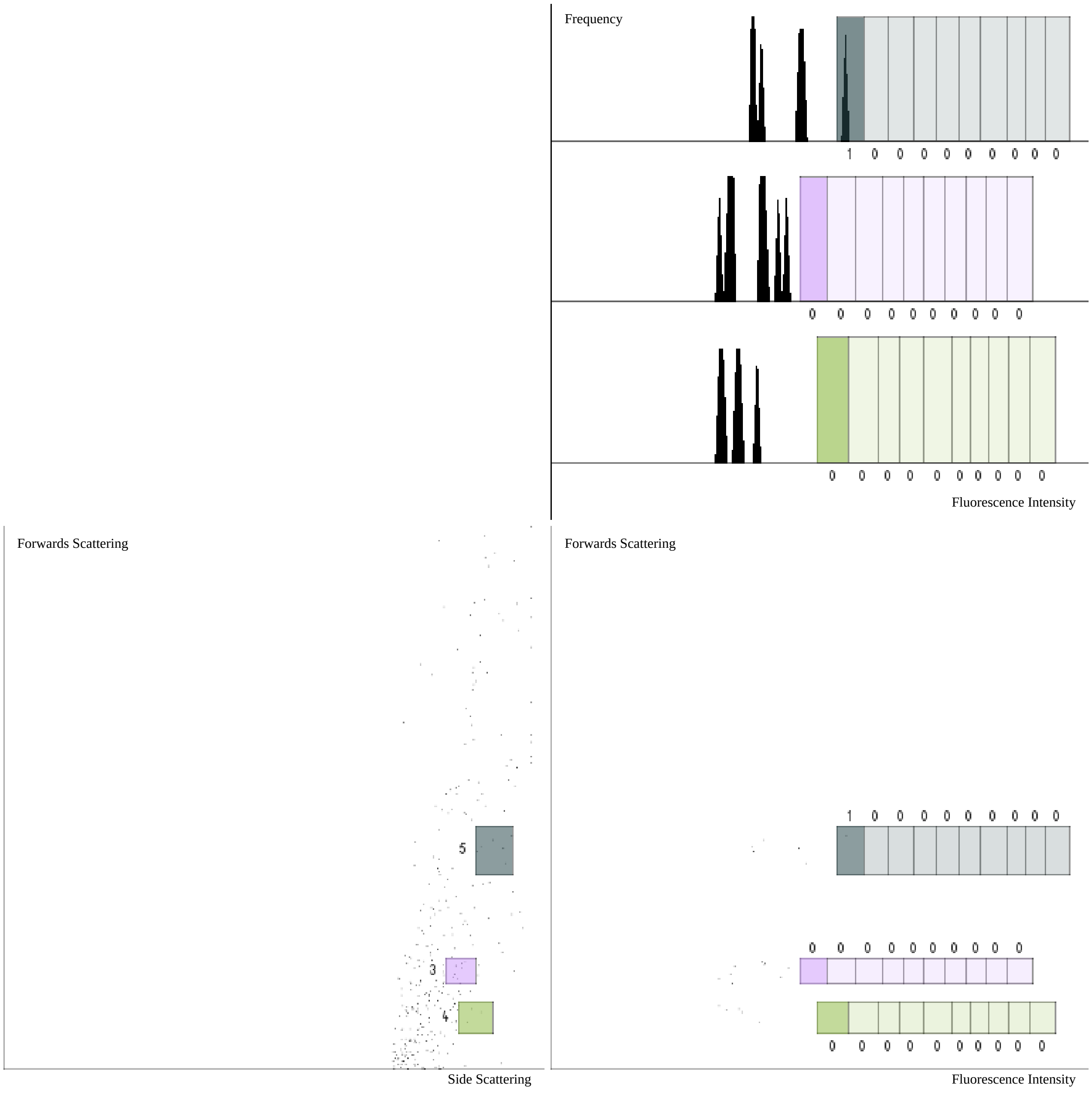

ANNEX 3: TAG DECONVOLUTION - BEAD 258

Passes flow sorting criteria: Yes

Passes tag deconvolution criteria: Yes

Included in protocol analysis: Yes

Protocol: 3, 8, 2, 8

Filename: Bin8_plateA1_D2.fcs

Split 1: Petrol shading

Split 2: Green shading

Split 3: Violet shading

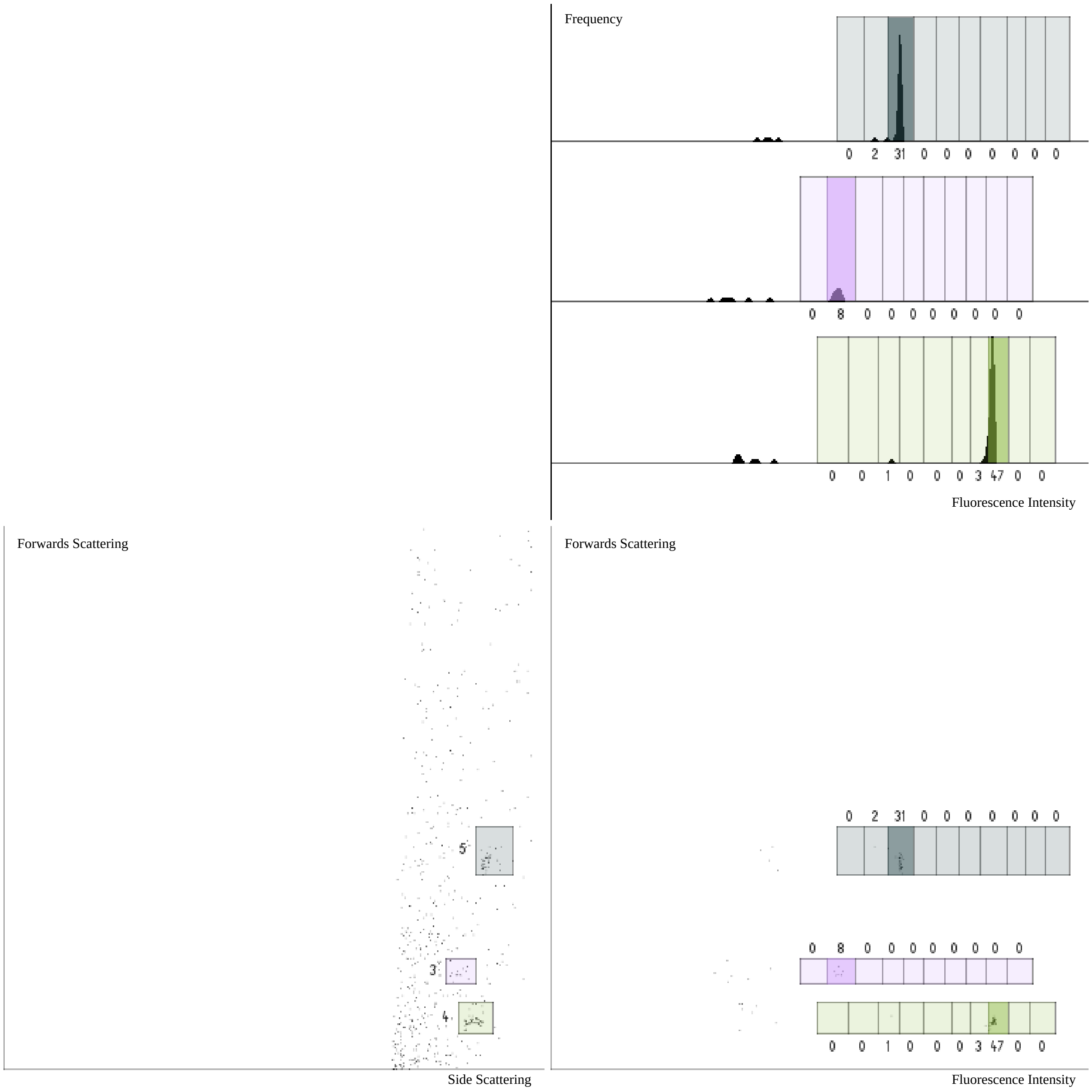

ANNEX 3: TAG DECONVOLUTION - BEAD 259

Passes flow sorting criteria: Yes

Passes tag deconvolution criteria: Yes

Included in protocol analysis: Yes

Protocol: 8, 8, 4, 8

Filename: Bin8_plateA1_D7.fcs

Split 1: Petrol shading

Split 2: Green shading

Split 3: Violet shading

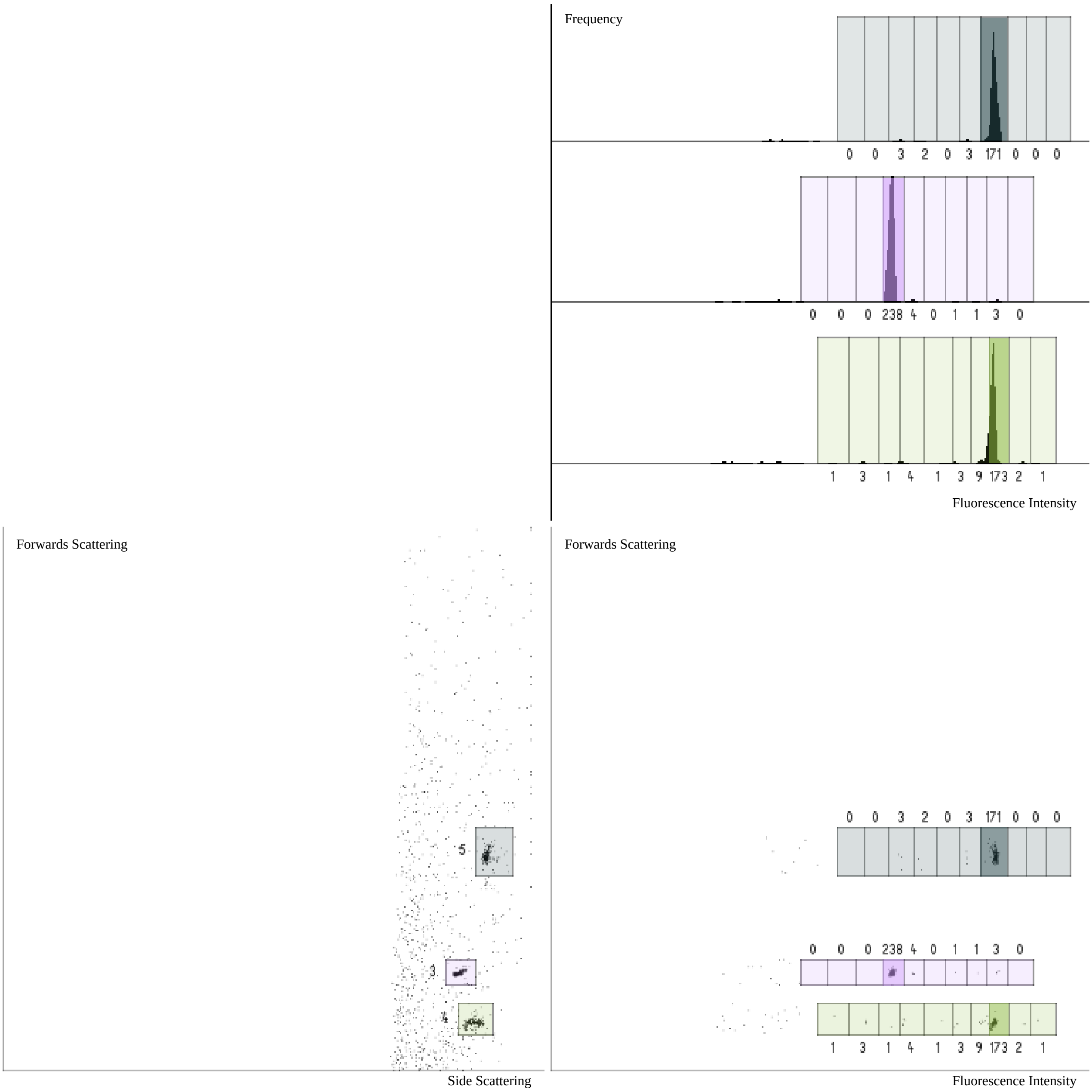

ANNEX 3: TAG DECONVOLUTION - BEAD 260

Passes flow sorting criteria: Yes

Passes tag deconvolution criteria: Yes

Included in protocol analysis: Yes

Protocol: 8, 7, 1, 8

Filename: Bin8_plateA1_D9.fcs

Split 1: Petrol shading

Split 2: Green shading

Split 3: Violet shading

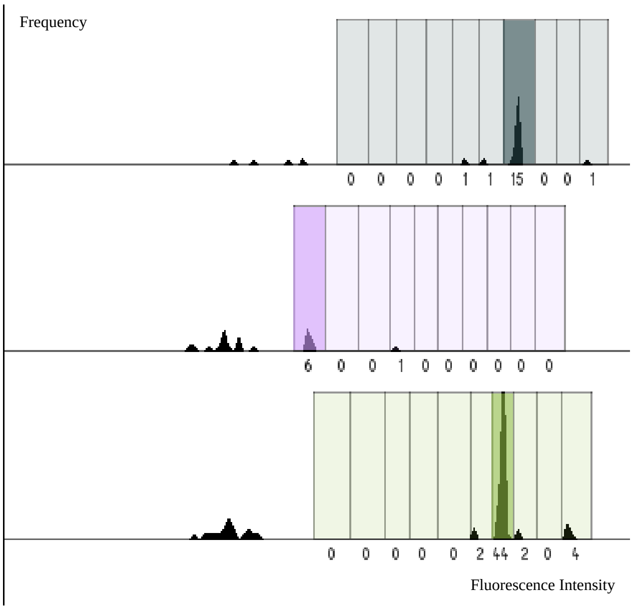

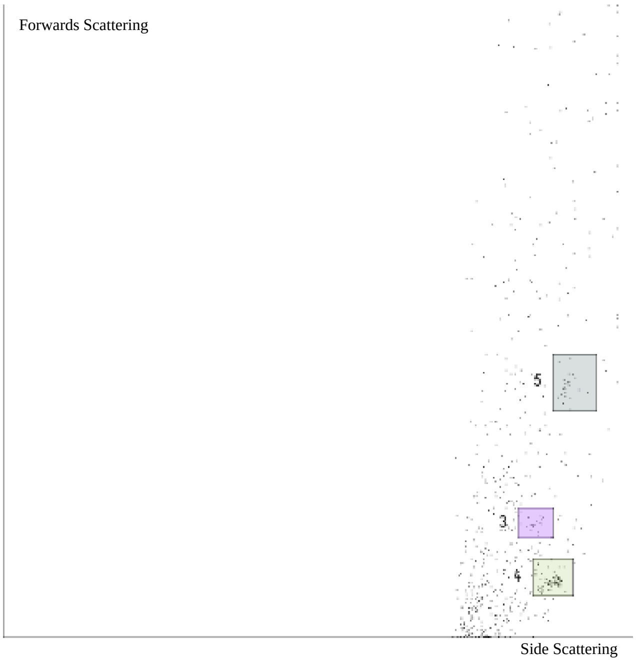

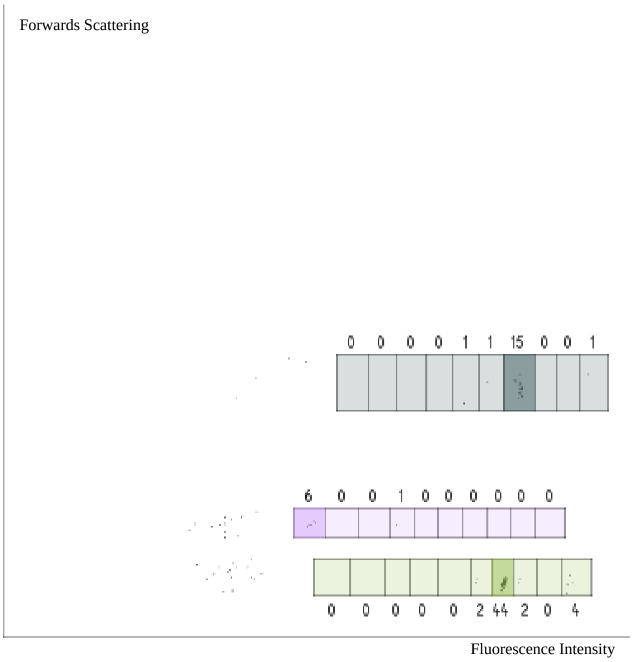

ANNEX 3: TAG DECONVOLUTION - BEAD 261

Passes flow sorting criteria: Yes

Passes tag deconvolution criteria: Yes

Included in protocol analysis: Yes

Protocol: 10, 9, 1, 8

Filename: Bin8_plateA1_D12.fcs

Split 1: Petrol shading

Split 2: Green shading

Split 3: Violet shading

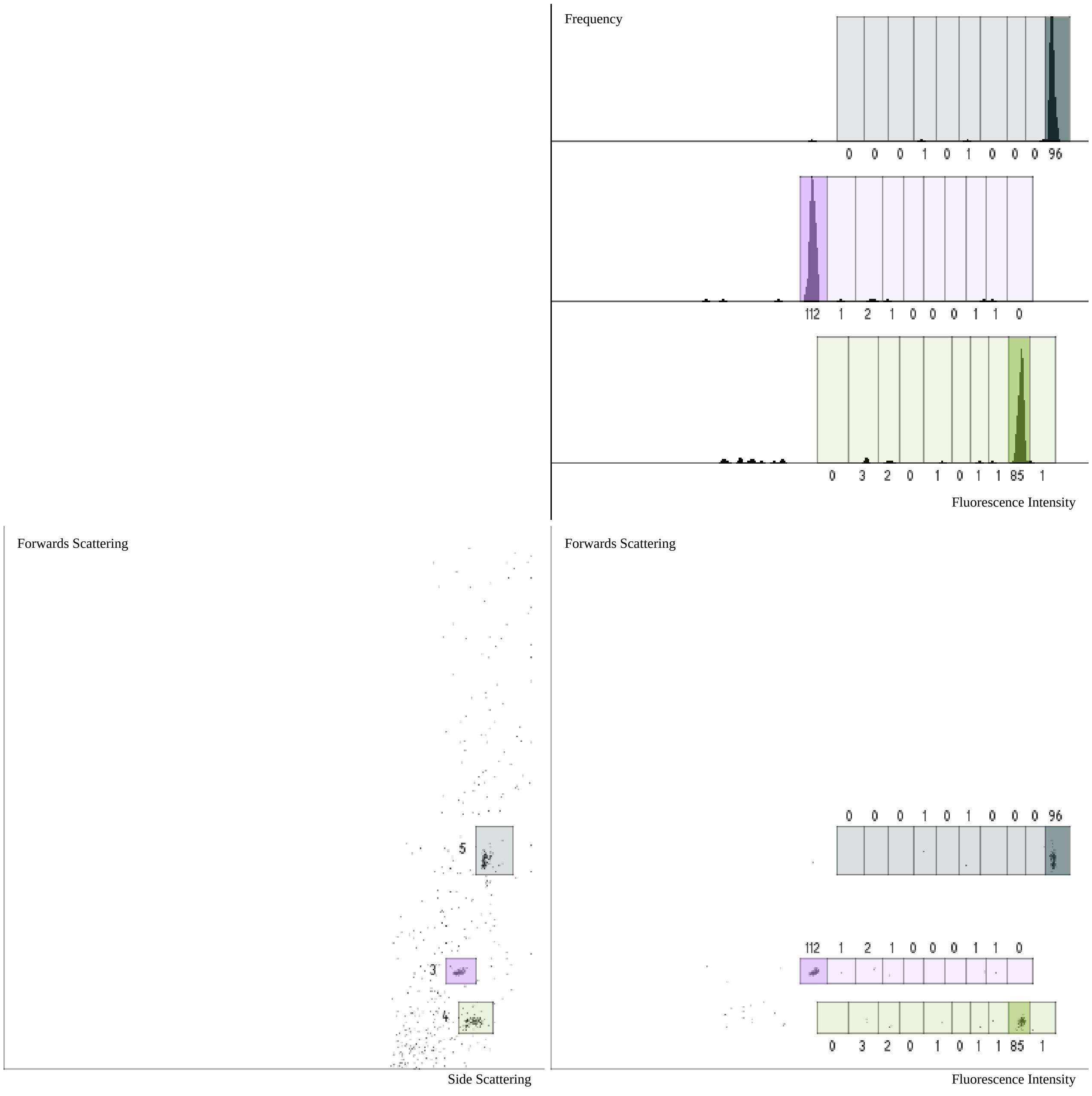

ANNEX 3: TAG DECONVOLUTION - BEAD 262

Passes flow sorting criteria: Yes

Passes tag deconvolution criteria: Yes

Included in protocol analysis: Yes

Protocol: 9, 7, 10, 8

Filename: Bin8_plateA1_F1.fcs

Split 1: Petrol shading

Split 2: Green shading

Split 3: Violet shading

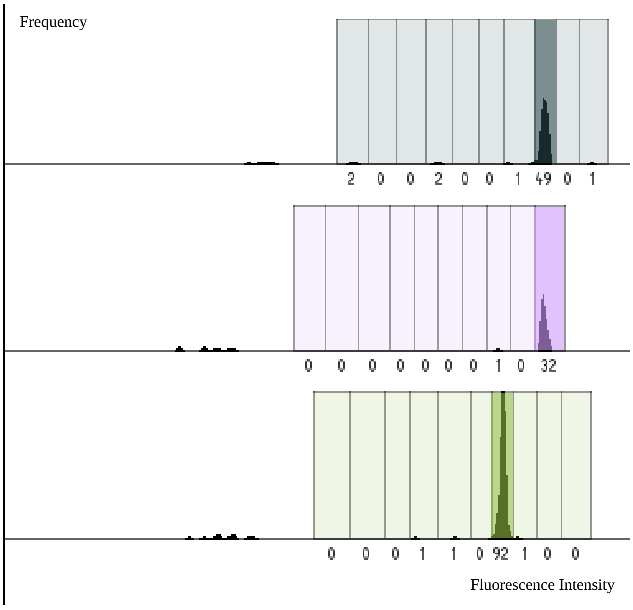

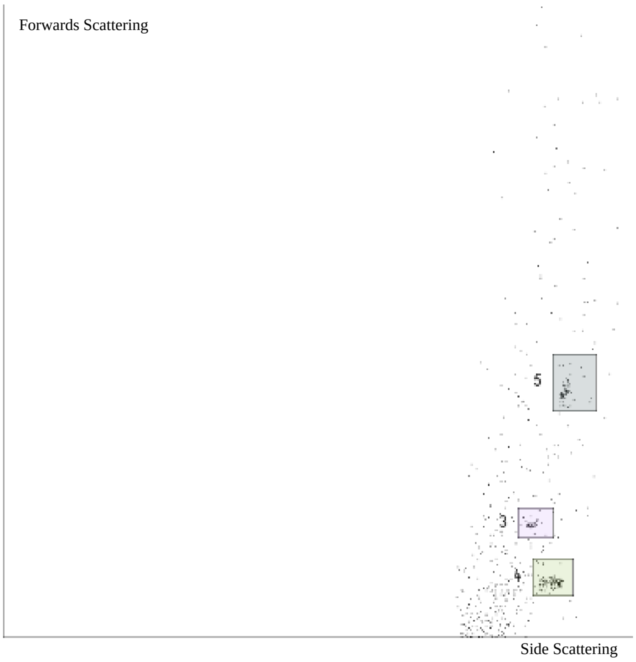

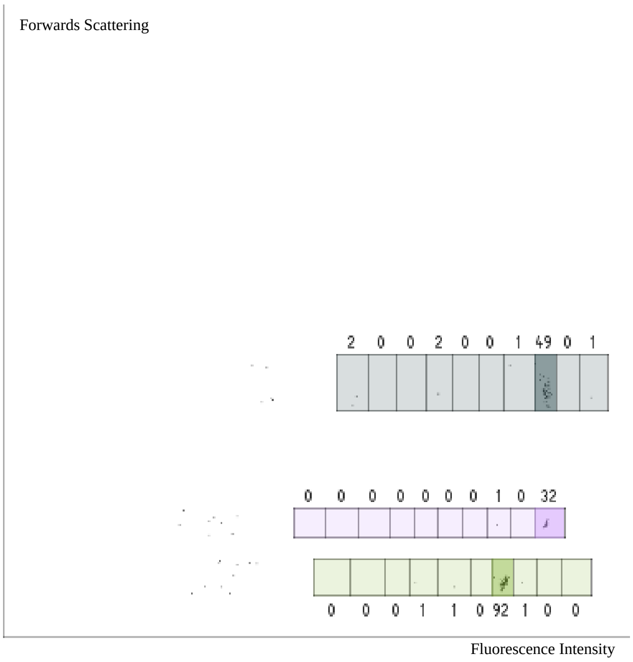

ANNEX 3: TAG DECONVOLUTION - BEAD 263

Passes flow sorting criteria: Yes

Passes tag deconvolution criteria: Yes

Included in protocol analysis: Yes

Protocol: 1, 3, 7, 8

Filename: Bin8_plateA1_F2.fcs

Split 1: Petrol shading

Split 2: Green shading

Split 3: Violet shading

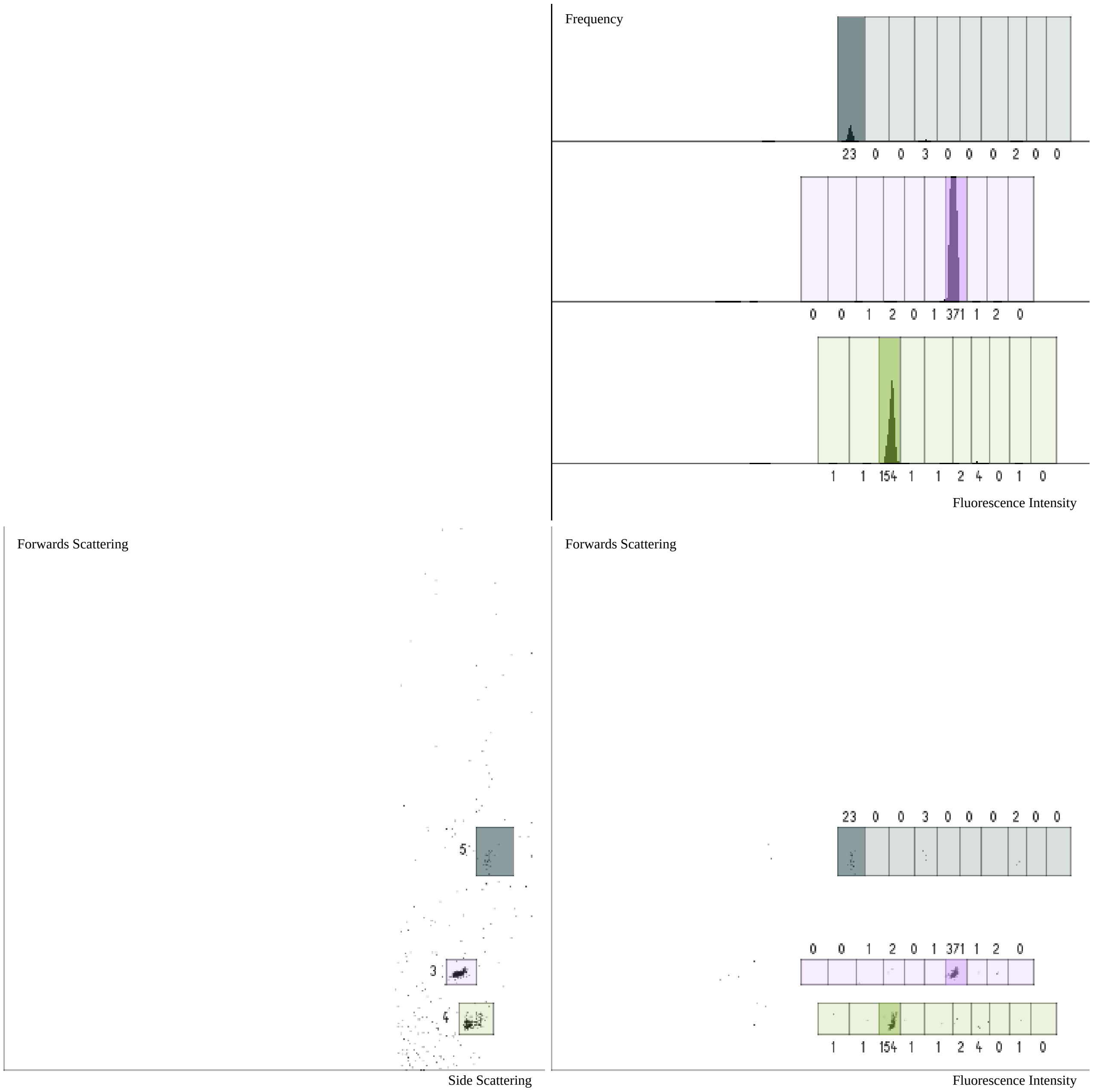

ANNEX 3: TAG DECONVOLUTION - BEAD 264

Passes flow sorting criteria: Yes

Passes tag deconvolution criteria: Yes

Included in protocol analysis: Yes

Protocol: 10, 7, 5, 8

Filename: Bin8_plateA1_G4.fcs

Split 1: Petrol shading

Split 2: Green shading

Split 3: Violet shading

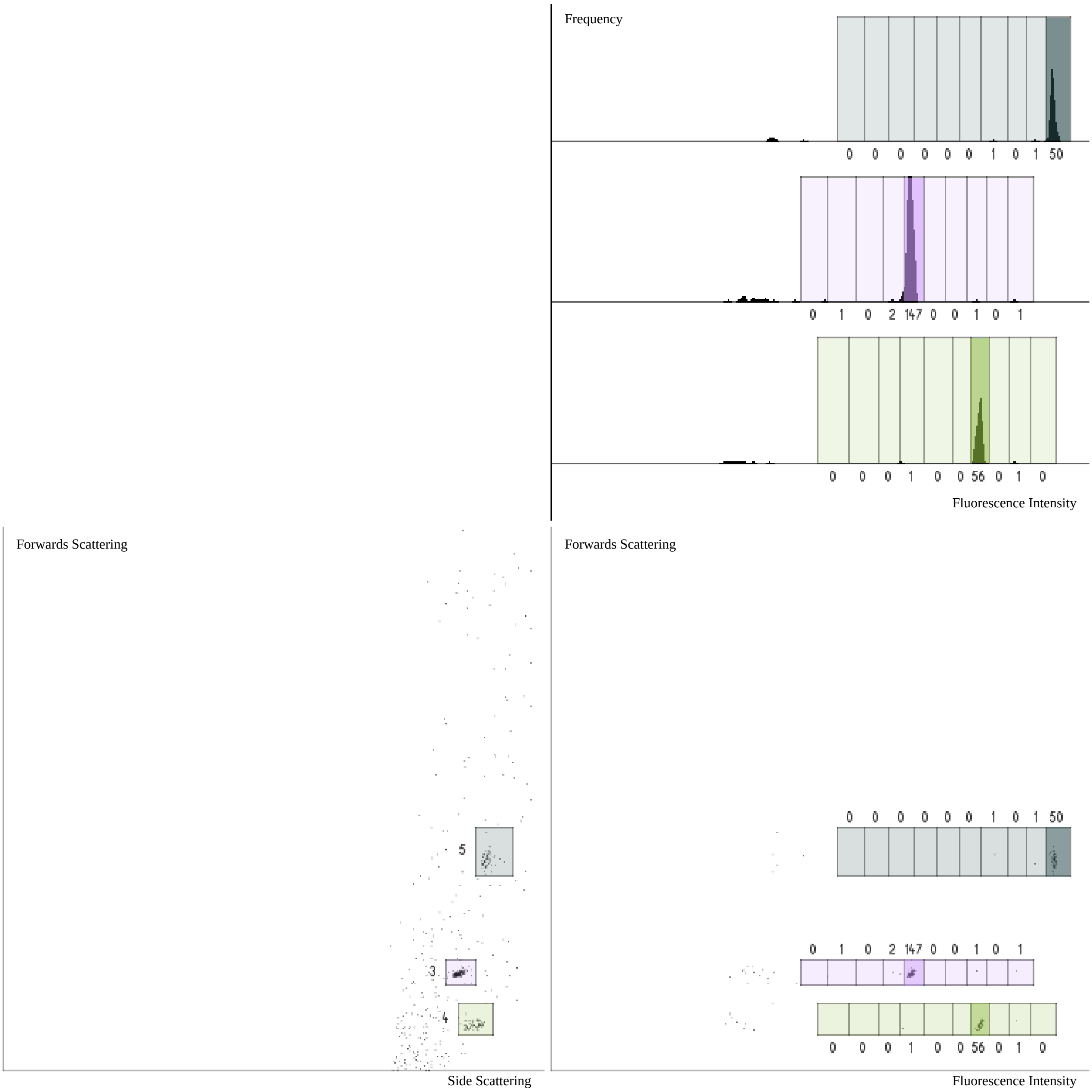

ANNEX 3: TAG DECONVOLUTION - BEAD 265

Passes flow sorting criteria: Yes

Passes tag deconvolution criteria: Yes

Included in protocol analysis: Yes

Protocol: 9, 4, 6, 8

Filename: Bin8_plateA1_G7.fcs

Split 1: Petrol shading

Split 2: Green shading

Split 3: Violet shading

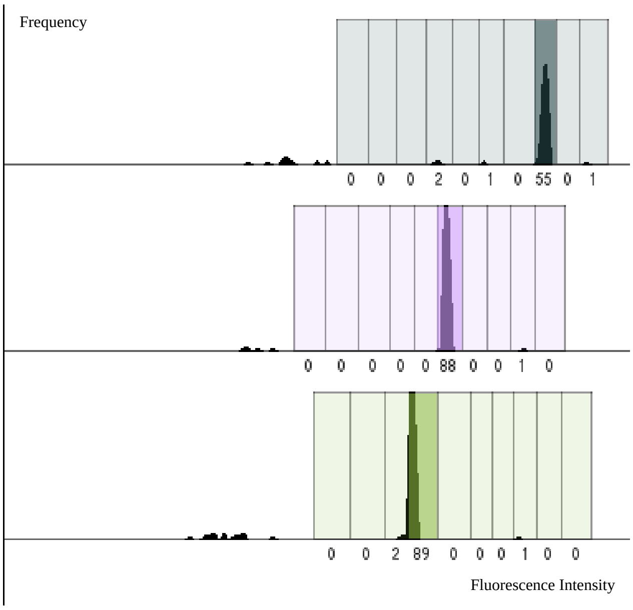

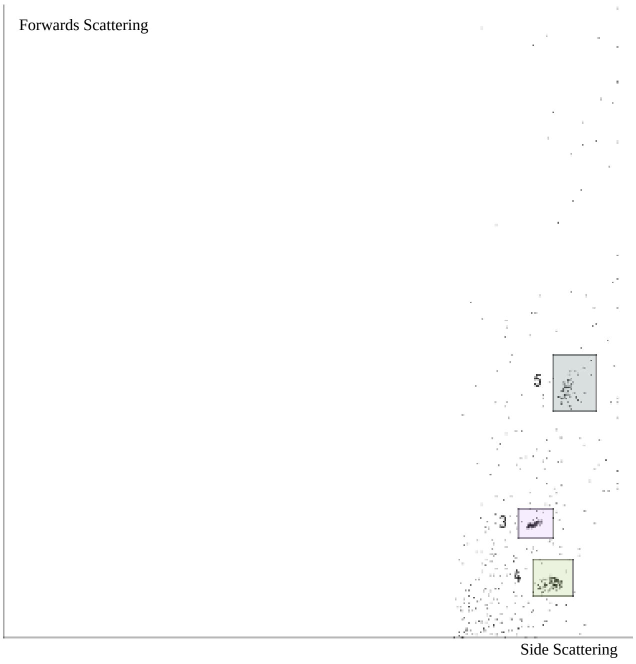

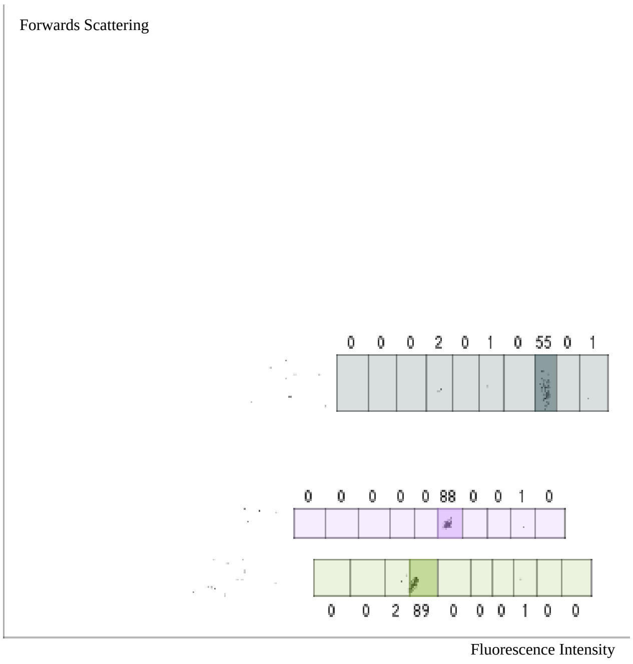

ANNEX 3: TAG DECONVOLUTION - BEAD 266

Passes flow sorting criteria: Yes

Passes tag deconvolution criteria: Yes

Included in protocol analysis: Yes

Protocol: 3, 2, 10, 8

Filename: Bin8_plateA1_G8.fcs

Split 1: Petrol shading

Split 2: Green shading

Split 3: Violet shading

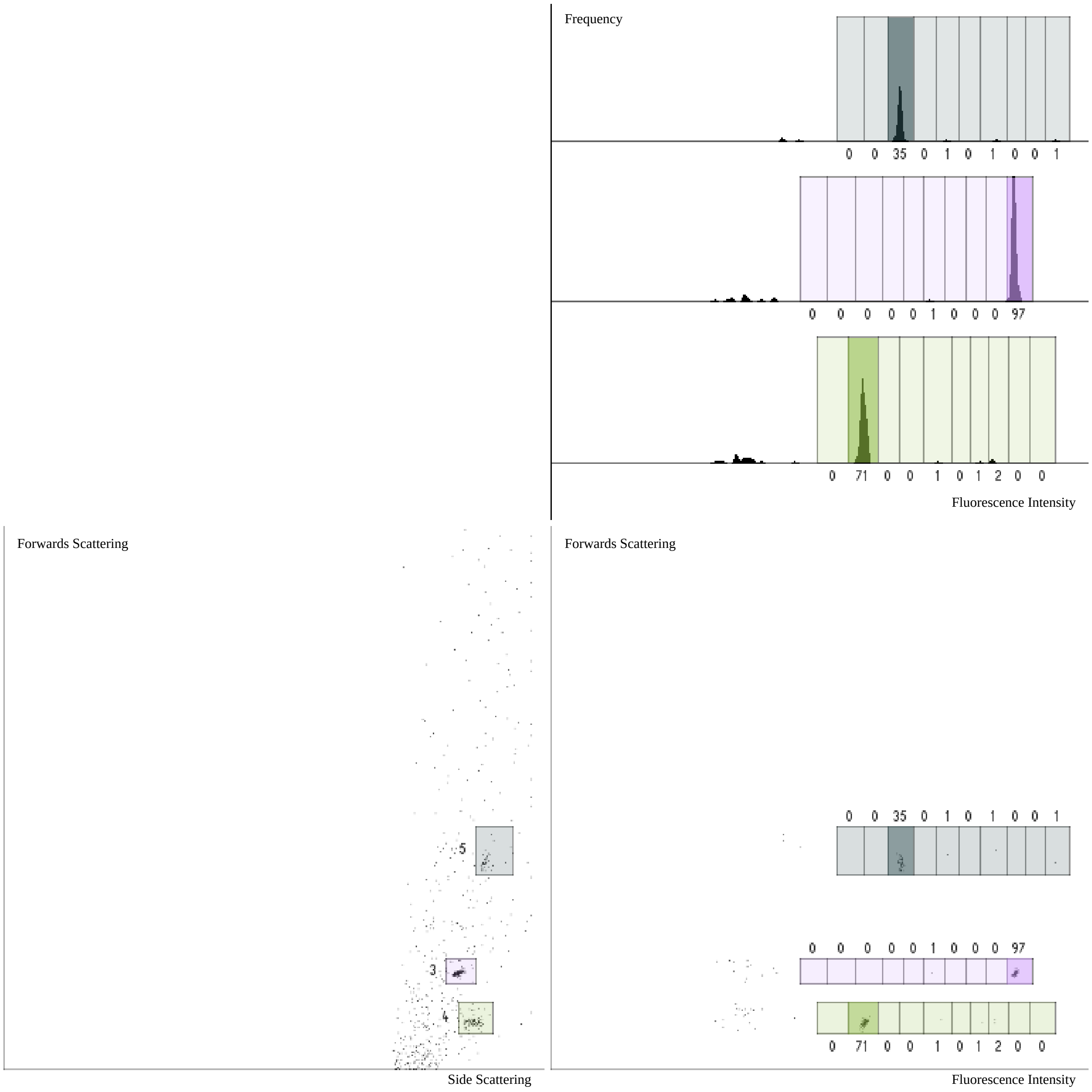

ANNEX 3: TAG DECONVOLUTION - BEAD 267

Passes flow sorting criteria: Yes

Passes tag deconvolution criteria: Yes

Included in protocol analysis: Yes

Protocol: 7, 4, 6, 8

Filename: Bin8_plateA1_G10.fcs

Split 1: Petrol shading

Split 2: Green shading

Split 3: Violet shading

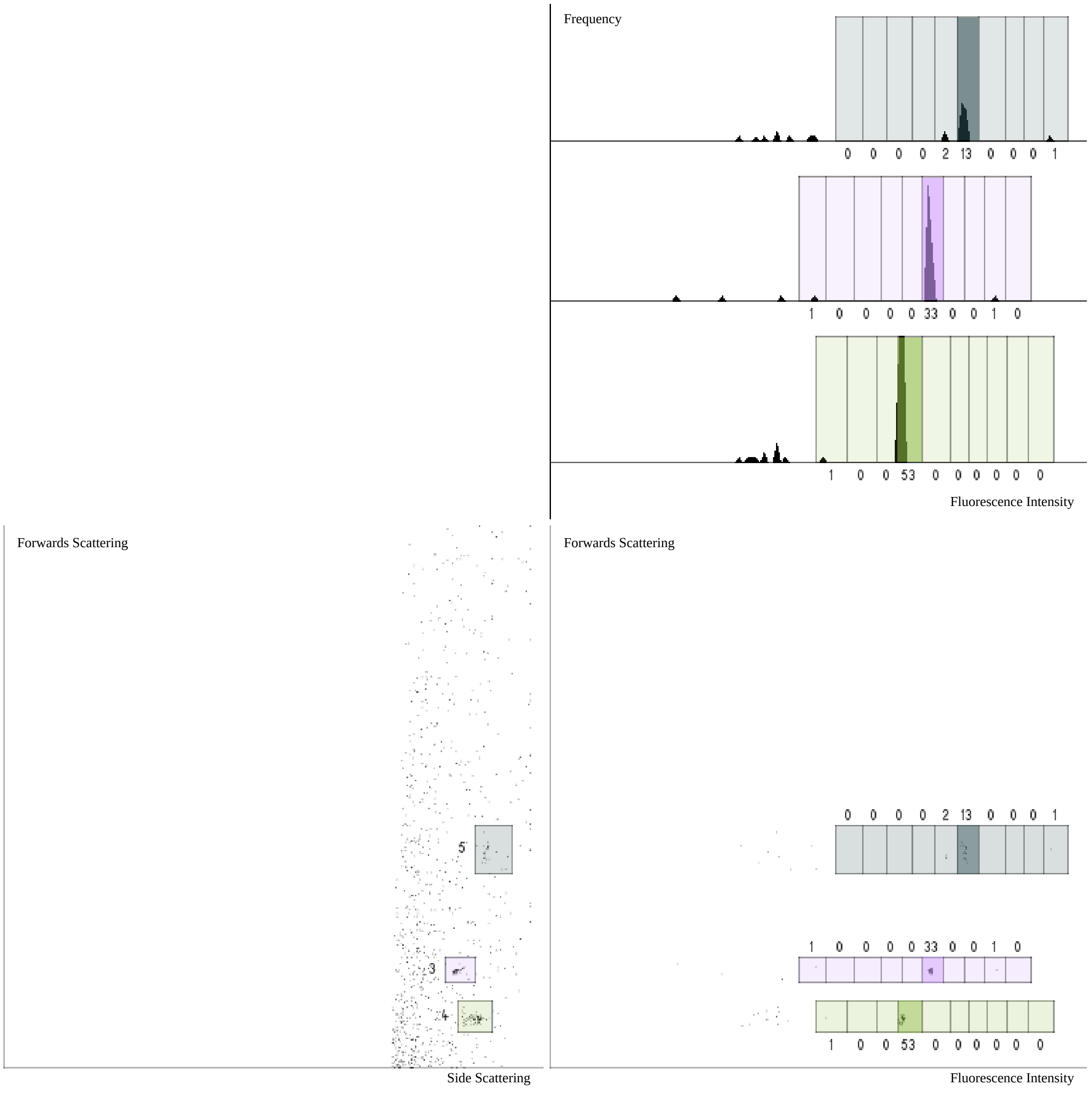

ANNEX 3: TAG DECONVOLUTION - BEAD 268

Passes flow sorting criteria: Yes

Passes tag deconvolution criteria: Yes

Included in protocol analysis: Yes

Protocol: 1, 5, 1, 8

Filename: Bin8_plateA1_H5.fcs

Split 1: Petrol shading

Split 2: Green shading

Split 3: Violet shading

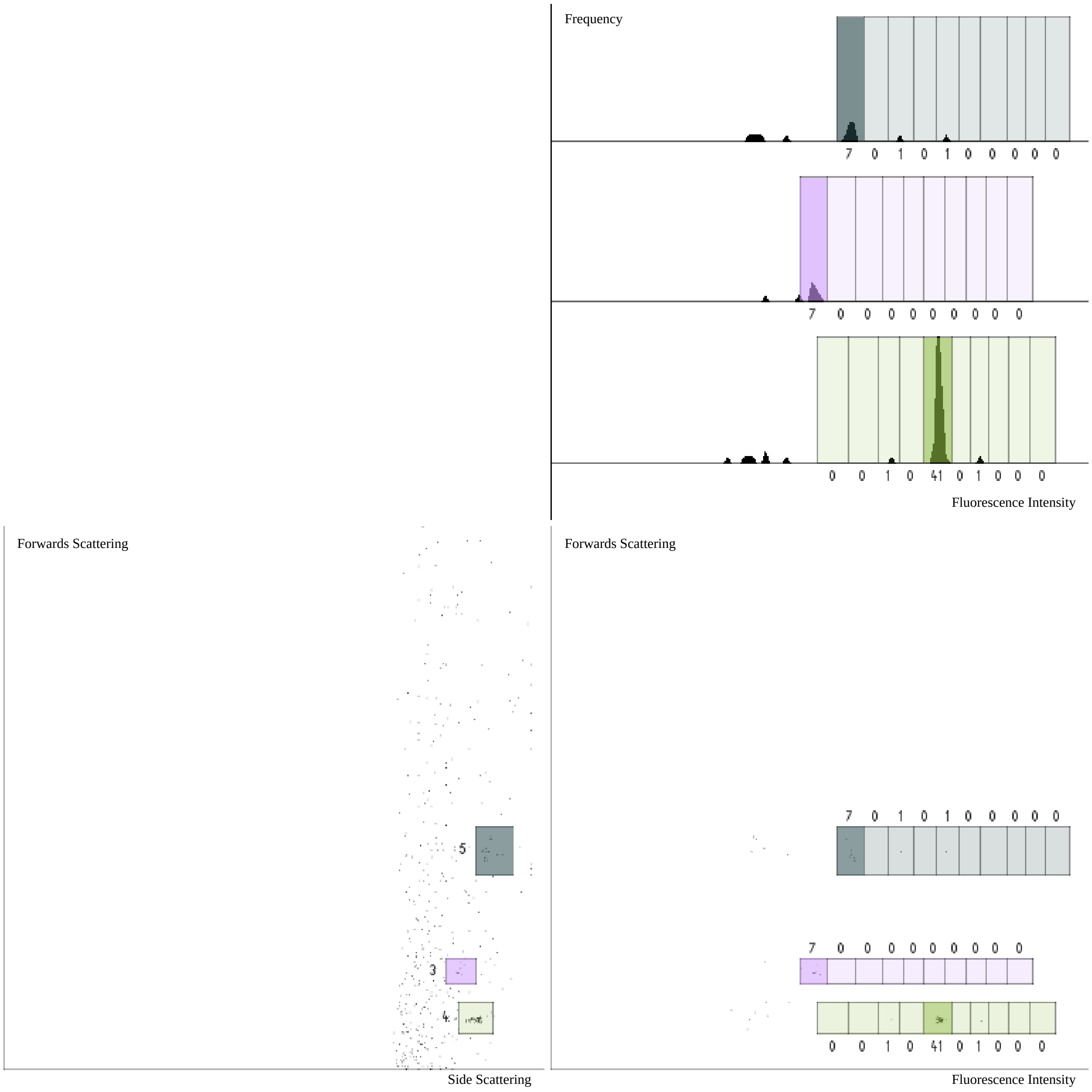

ANNEX 3: TAG DECONVOLUTION - BEAD 269

Passes flow sorting criteria: Yes

Passes tag deconvolution criteria: Yes

Included in protocol analysis: Yes

Protocol: 5, 1, 1, 8

Filename: Bin8_plateA1_H7.fcs

Split 1: Petrol shading

Split 2: Green shading

Split 3: Violet shading

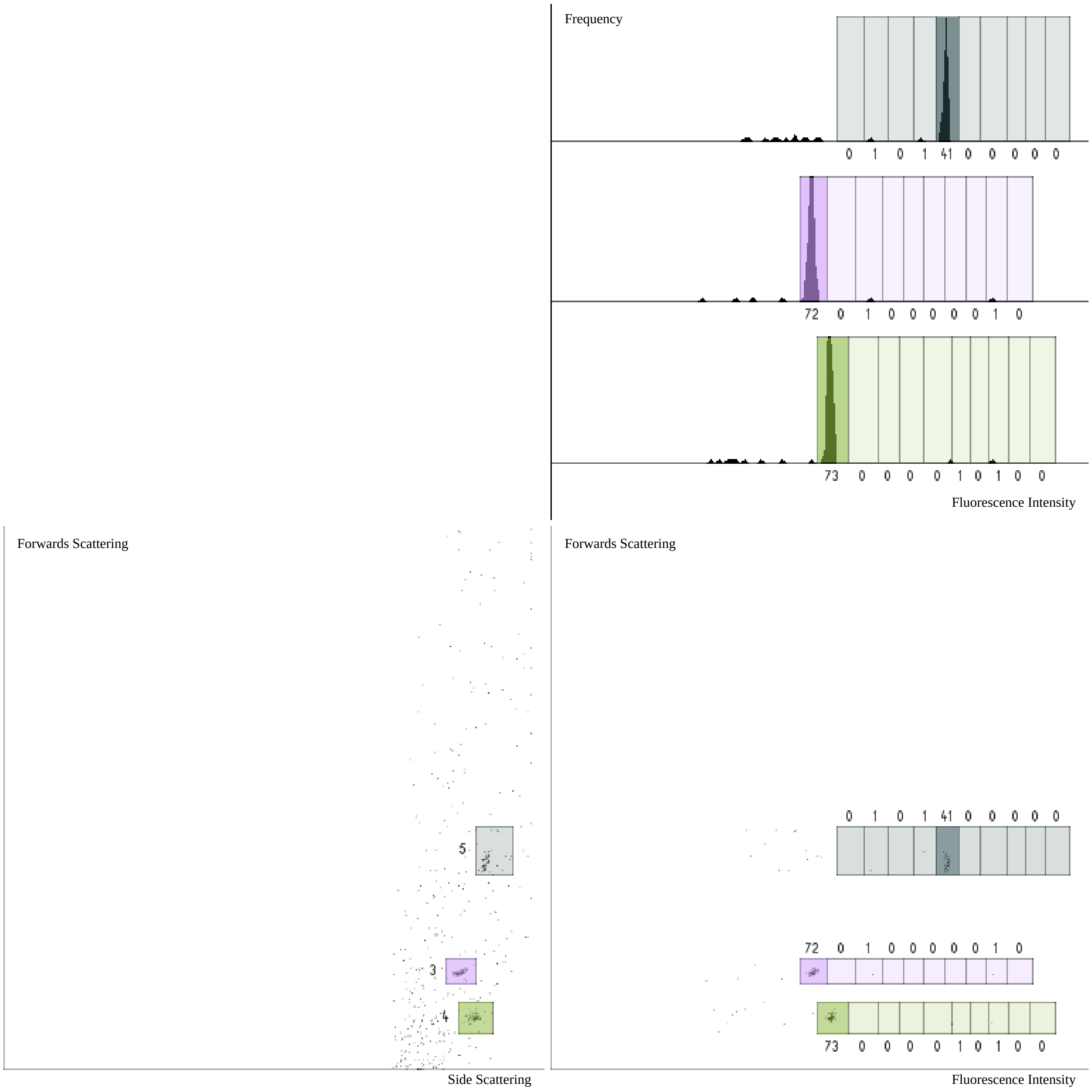

ANNEX 3: TAG DECONVOLUTION - BEAD 270

Passes flow sorting criteria: Yes

Passes tag deconvolution criteria: Yes

Included in protocol analysis: Yes

Protocol: 1, 9, 5, 8

Filename: Bin8_plateA2_A5.fcs

Split 1: Petrol shading

Split 2: Green shading

Split 3: Violet shading

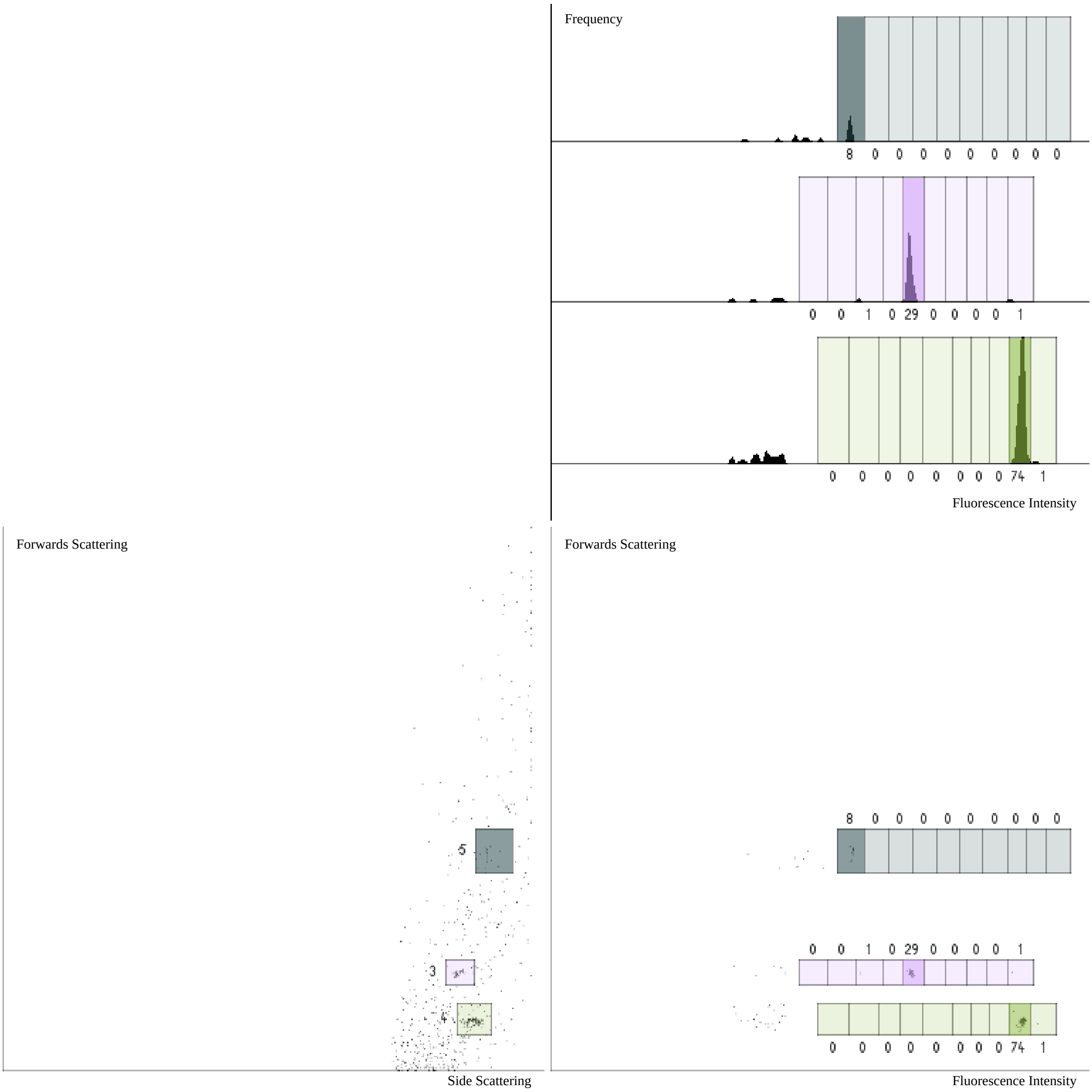

ANNEX 3: TAG DECONVOLUTION - BEAD 271

Passes flow sorting criteria: Yes

Passes tag deconvolution criteria: Yes

Included in protocol analysis: Yes

Protocol: 10, 6, 4, 8

Filename: Bin8_plateA2_A3.fcs

Split 1: Petrol shading

Split 2: Green shading

Split 3: Violet shading

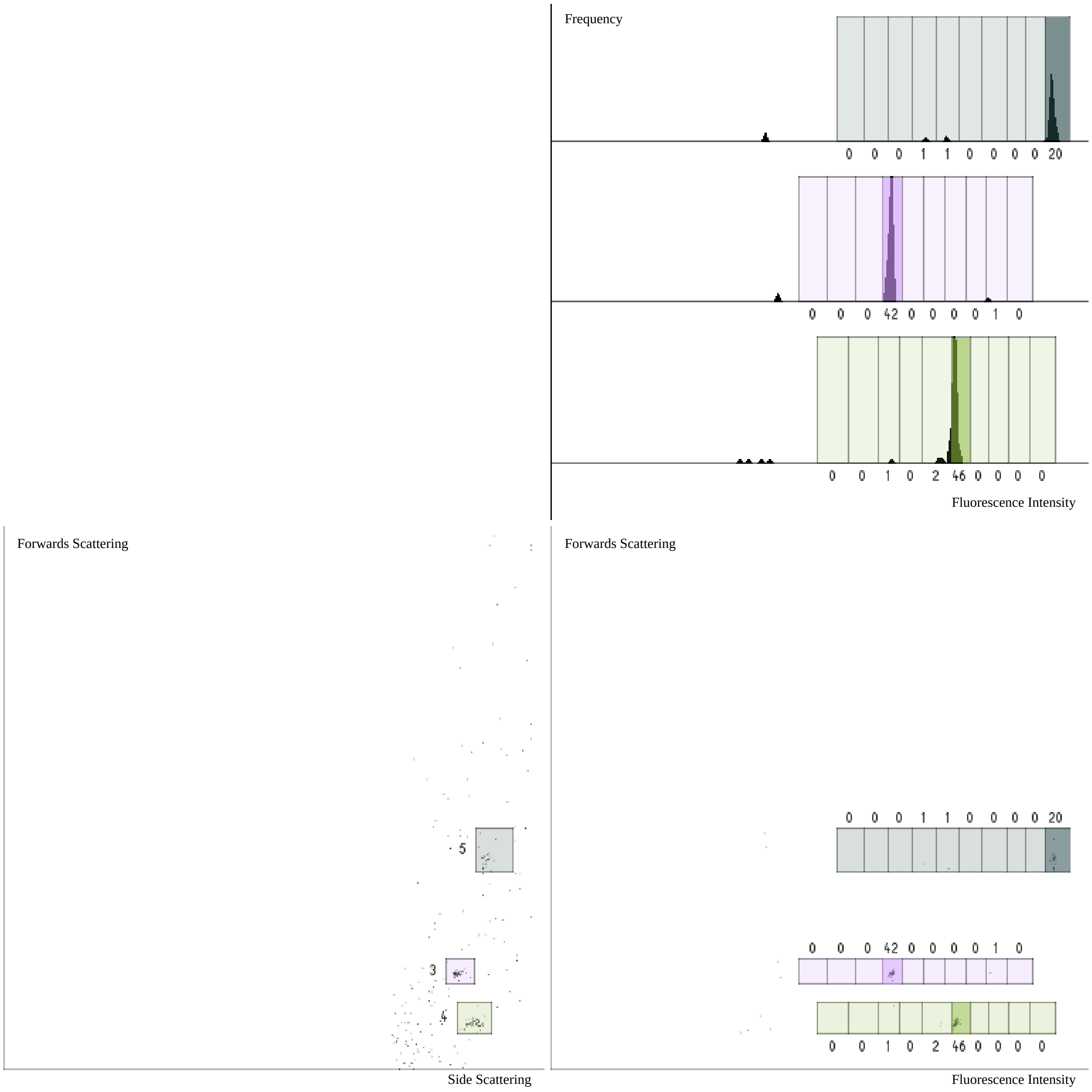

ANNEX 3: TAG DECONVOLUTION - BEAD 272

Passes flow sorting criteria: Yes

Passes tag deconvolution criteria: Yes

Included in protocol analysis: Yes

Protocol: 8, 10, 10, 9

Filename: Bin9_plateA2_D1.fcs

Split 1: Petrol shading

Split 2: Green shading

Split 3: Violet shading

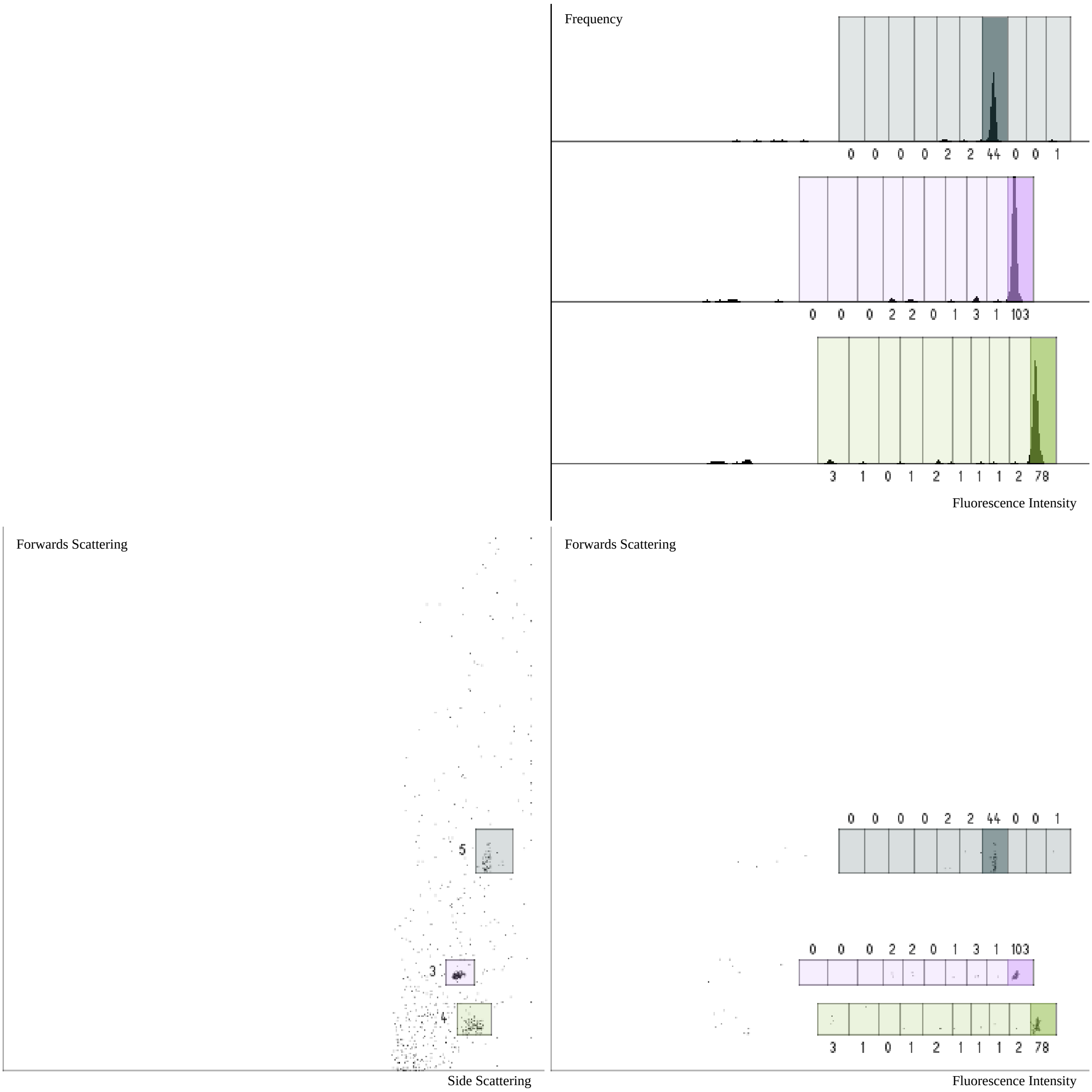

ANNEX 3: TAG DECONVOLUTION - BEAD 273

Passes flow sorting criteria: Yes

Passes tag deconvolution criteria: Yes

Included in protocol analysis: Yes

Protocol: 2, 5, 2, 9

Filename: Bin9_plateA2_A3.fcs

Split 1: Petrol shading

Split 2: Green shading

Split 3: Violet shading

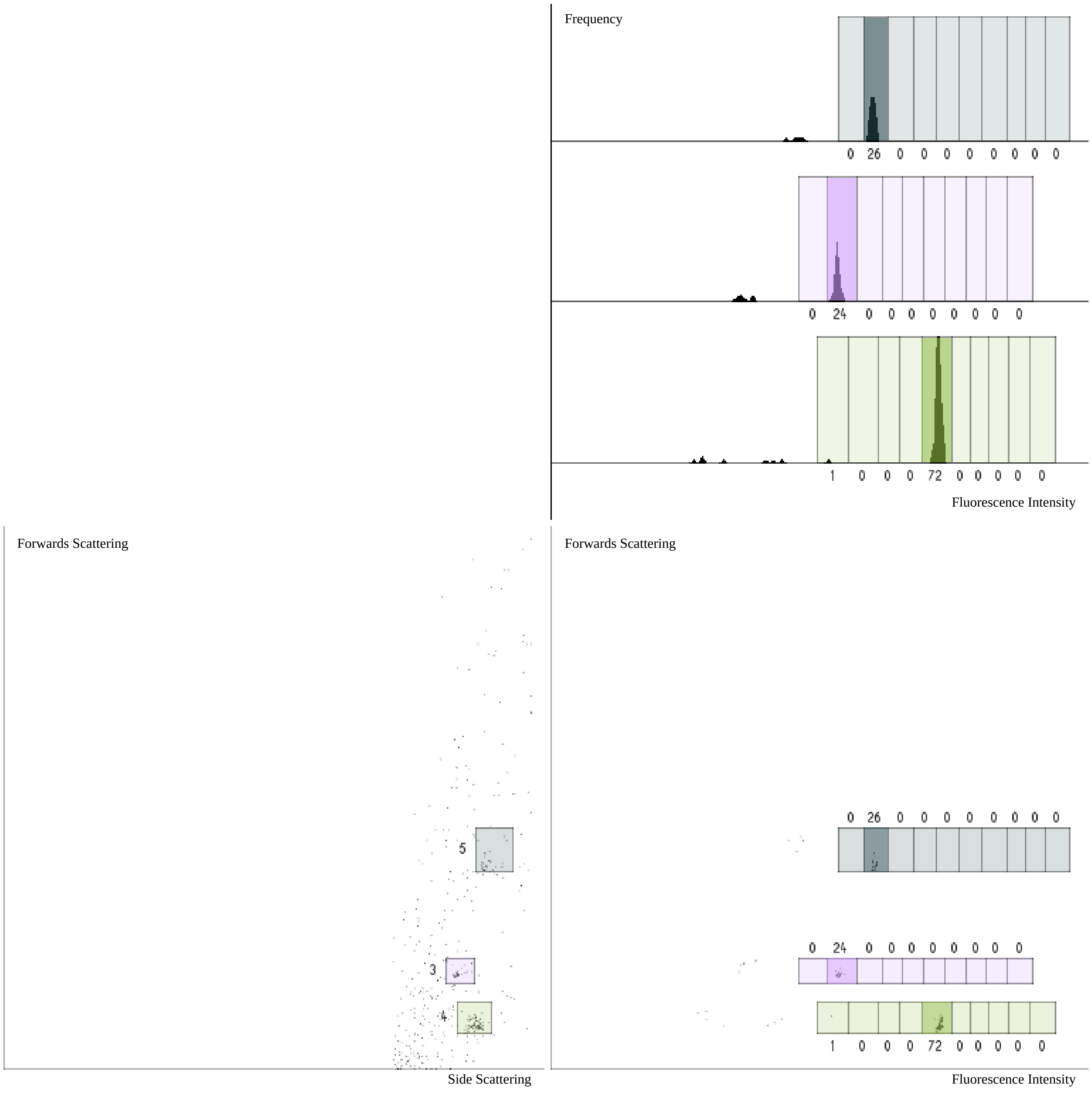

ANNEX 3: TAG DECONVOLUTION - BEAD 274

Passes flow sorting criteria: Yes

Passes tag deconvolution criteria: Yes

Included in protocol analysis: Yes

Protocol: 3, 6, 3, 9

Filename: Bin9_plateA2_A7.fcs

Split 1: Petrol shading

Split 2: Green shading

Split 3: Violet shading

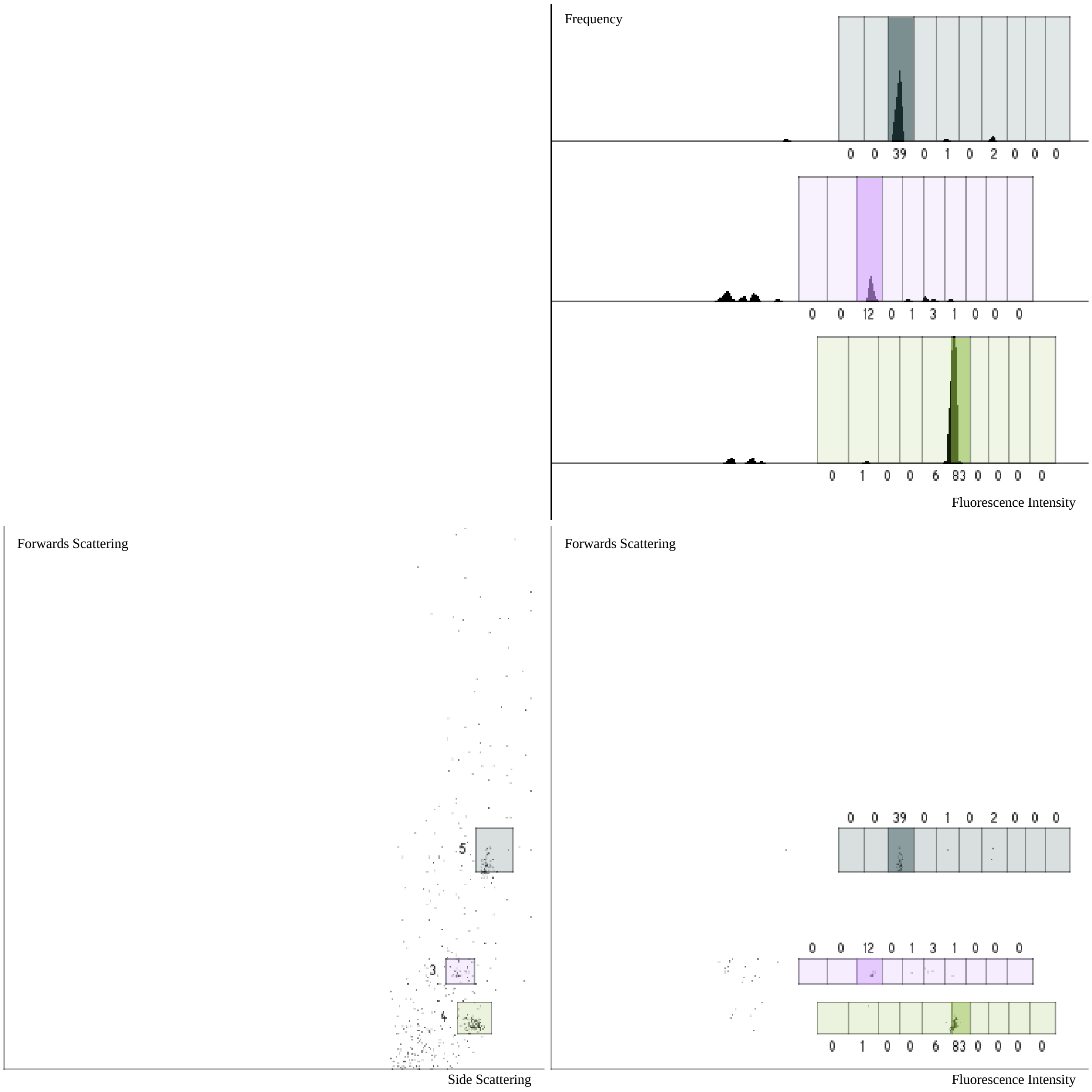

ANNEX 3: TAG DECONVOLUTION - BEAD 275

Passes flow sorting criteria: Yes

Passes tag deconvolution criteria: Yes

Included in protocol analysis: Yes

Protocol: 3, 9, 9, 9

Filename: Bin9_plateA2_A8.fcs

Split 1: Petrol shading

Split 2: Green shading

Split 3: Violet shading

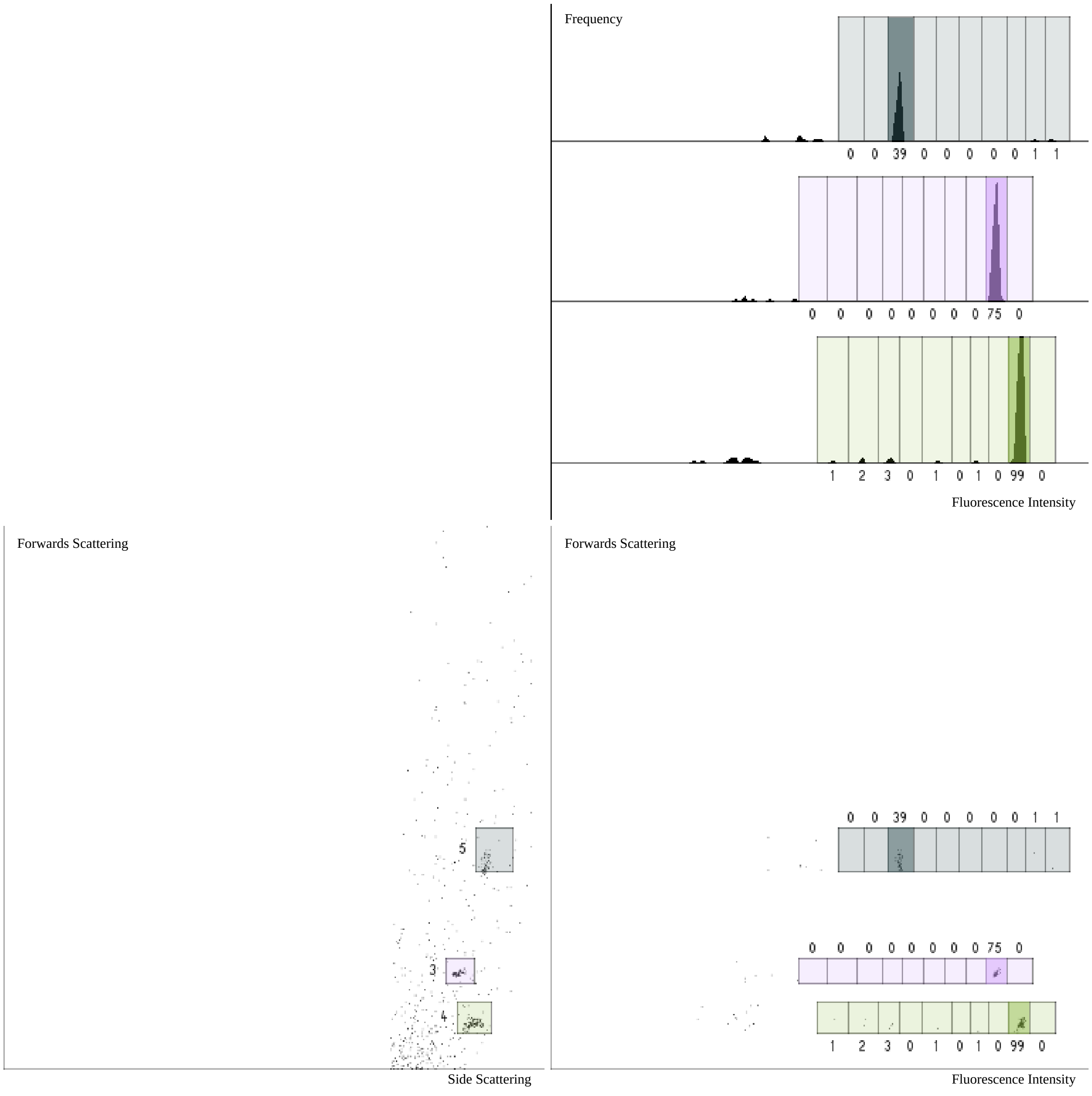

ANNEX 3: TAG DECONVOLUTION - BEAD 276

Passes flow sorting criteria: Yes

Passes tag deconvolution criteria: Yes

Included in protocol analysis: Yes

Protocol: 8, 7, 7, 9

Filename: Bin9_plateA2_A9.fcs

Split 1: Petrol shading

Split 2: Green shading

Split 3: Violet shading

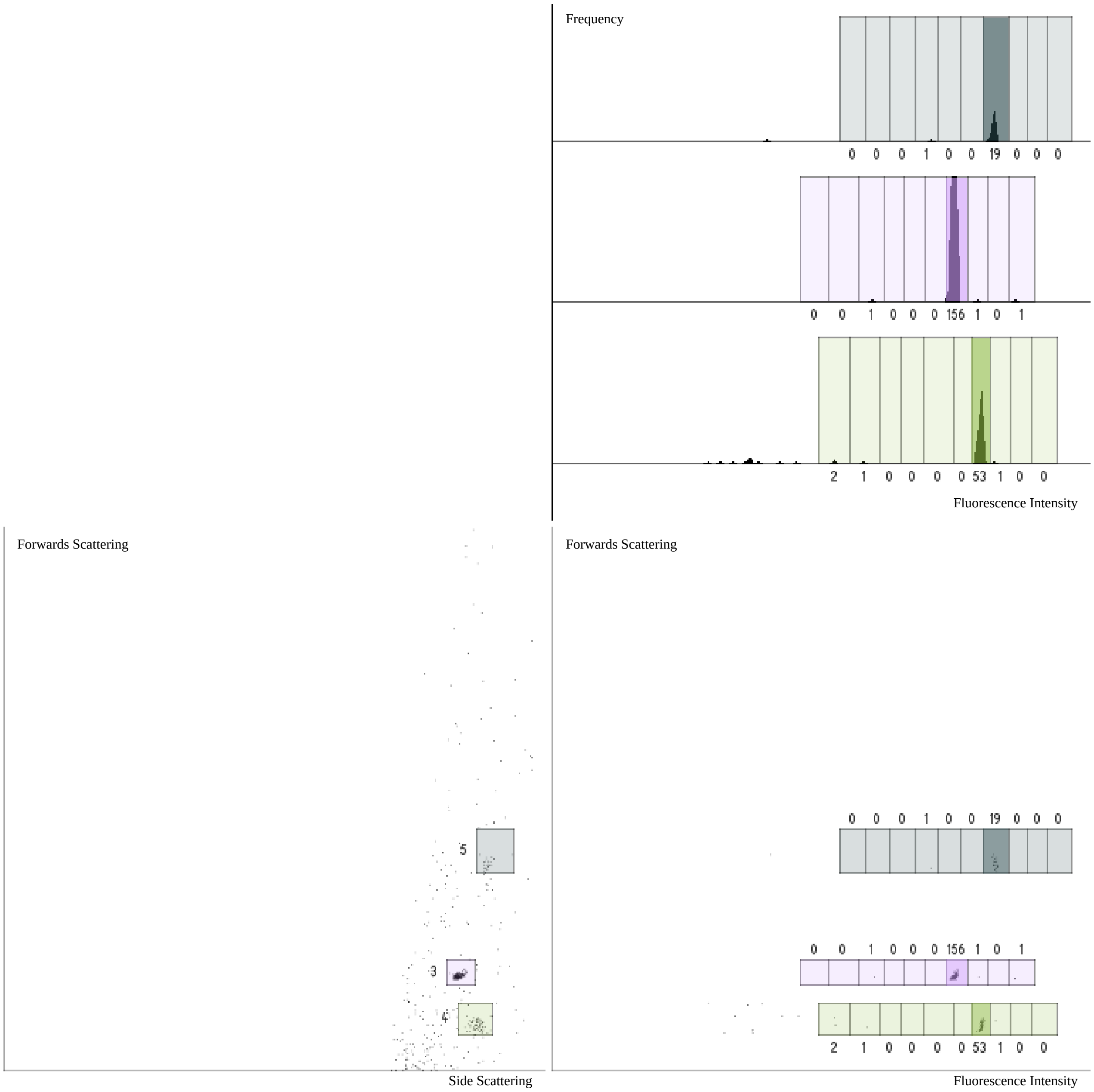

ANNEX 3: TAG DECONVOLUTION - BEAD 277

Passes flow sorting criteria: Yes

Passes tag deconvolution criteria: Yes

Included in protocol analysis: Yes

Protocol: 3, 7, 10, 9

Filename: Bin9_plateA2_B3.fcs

Split 1: Petrol shading

Split 2: Green shading

Split 3: Violet shading

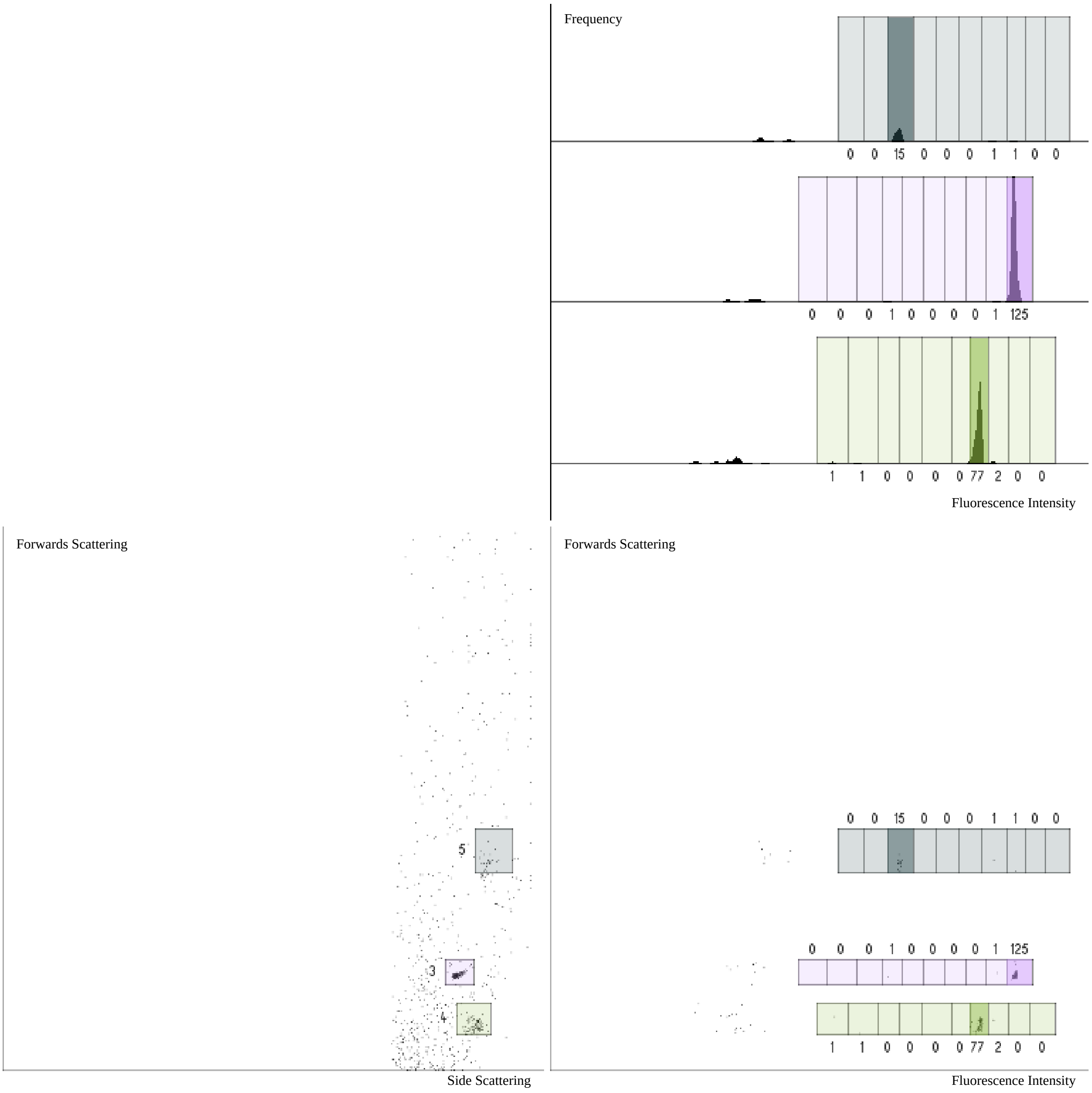

ANNEX 3: TAG DECONVOLUTION - BEAD 278

Passes flow sorting criteria: Yes

Passes tag deconvolution criteria: Yes

Included in protocol analysis: Yes

Protocol: 4, 4, 6, 9

Filename: Bin9_plateA2_B4.fcs

Split 1: Petrol shading

Split 2: Green shading

Split 3: Violet shading

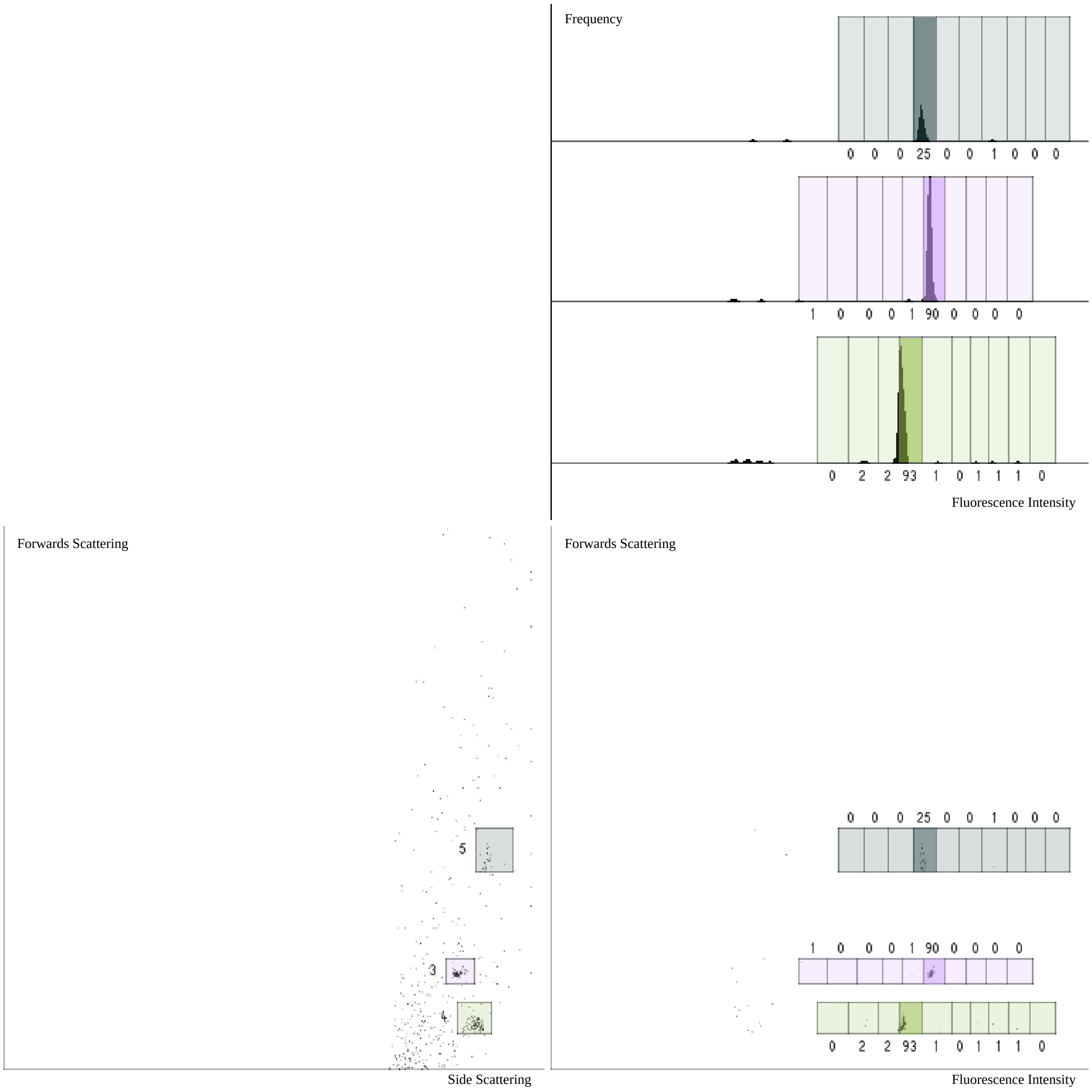

ANNEX 3: TAG DECONVOLUTION - BEAD 279

Passes flow sorting criteria: Yes

Passes tag deconvolution criteria: Yes

Included in protocol analysis: Yes

Protocol: 4, 7, 6, 9

Filename: Bin9_plateA2_B7.fcs

Split 1: Petrol shading

Split 2: Green shading

Split 3: Violet shading

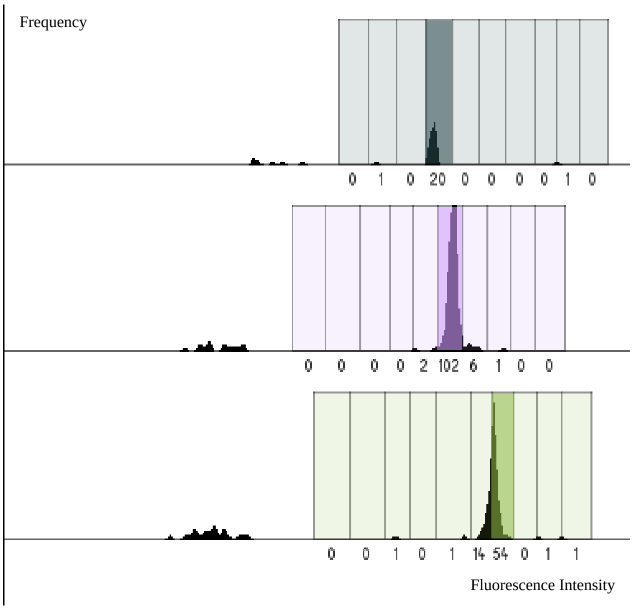

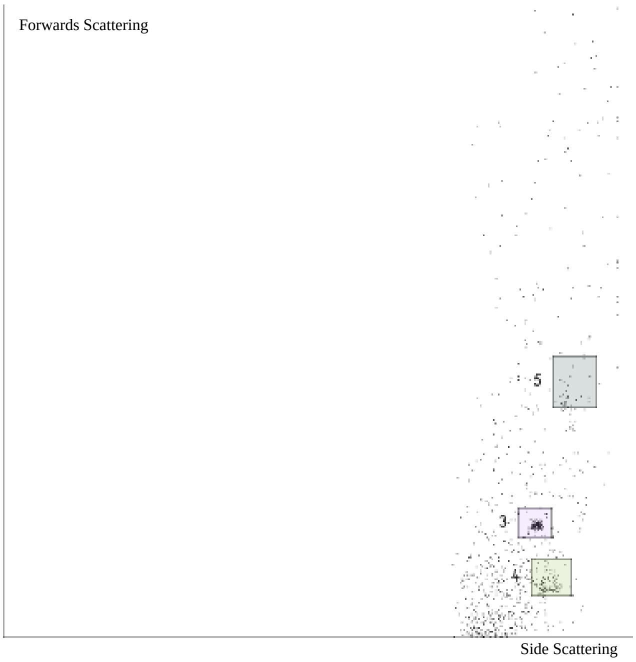

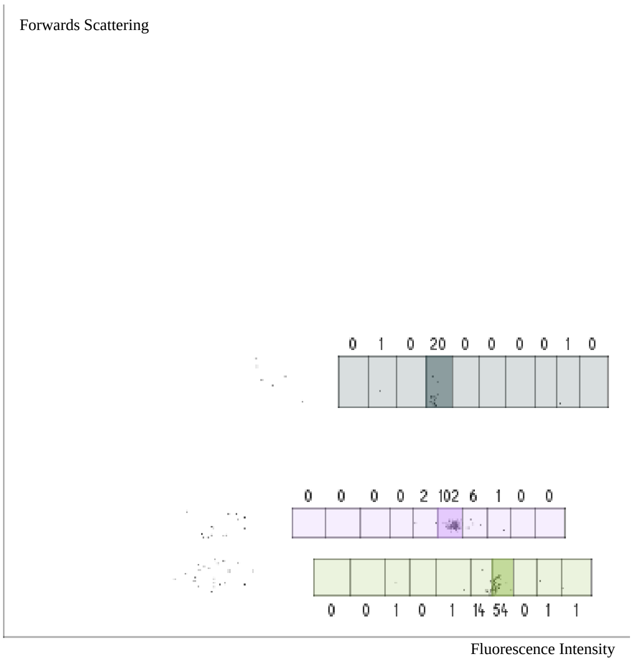

ANNEX 3: TAG DECONVOLUTION - BEAD 280

Passes flow sorting criteria: Yes

Passes tag deconvolution criteria: Yes

Included in protocol analysis: Yes

Protocol: 10, 9, 4, 9

Filename: Bin9_plateA2_B11.fcs

Split 1: Petrol shading

Split 2: Green shading

Split 3: Violet shading

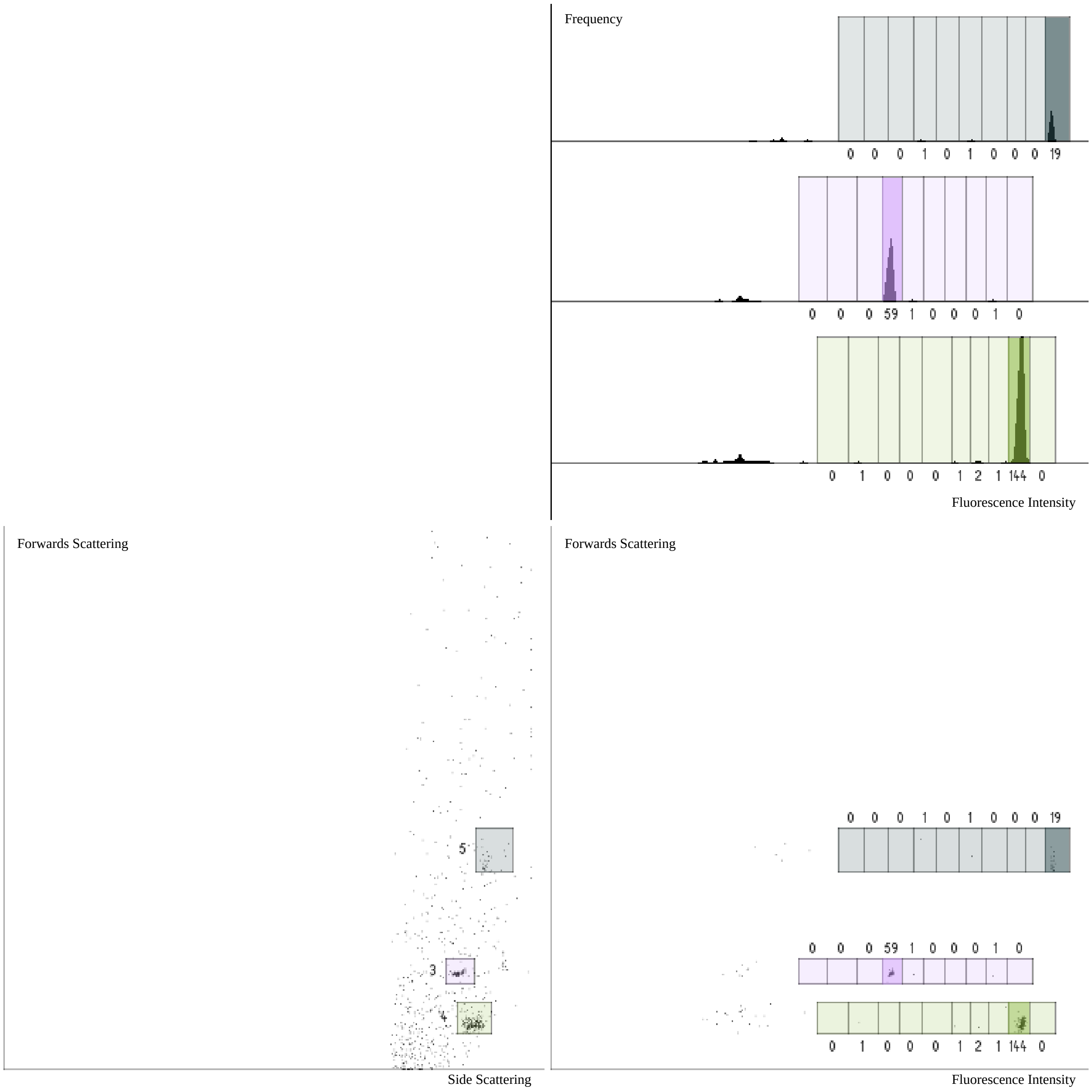

ANNEX 3: TAG DECONVOLUTION - BEAD 281

Passes flow sorting criteria: Yes

Passes tag deconvolution criteria: Yes

Included in protocol analysis: Yes

Protocol: 6, 3, 6, 9

Filename: Bin9_plateA2_C11.fcs

Split 1: Petrol shading

Split 2: Green shading

Split 3: Violet shading

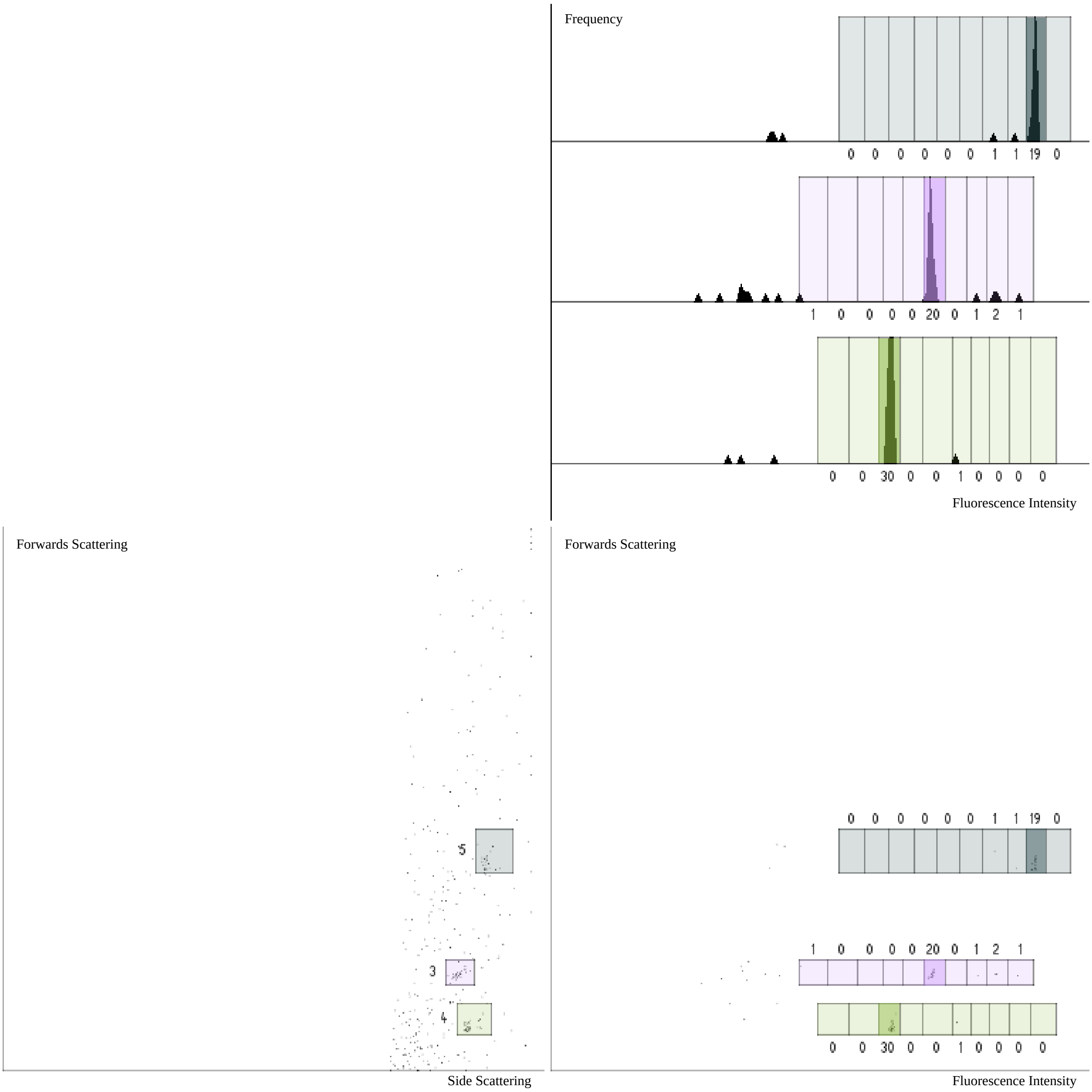

ANNEX 3: TAG DECONVOLUTION - BEAD 282

Passes flow sorting criteria: Yes

Passes tag deconvolution criteria: Yes

Included in protocol analysis: Yes

Protocol: 1, 4, 7, 9

Filename: Bin9_plateA2_F8.fcs

Split 1: Petrol shading

Split 2: Green shading

Split 3: Violet shading

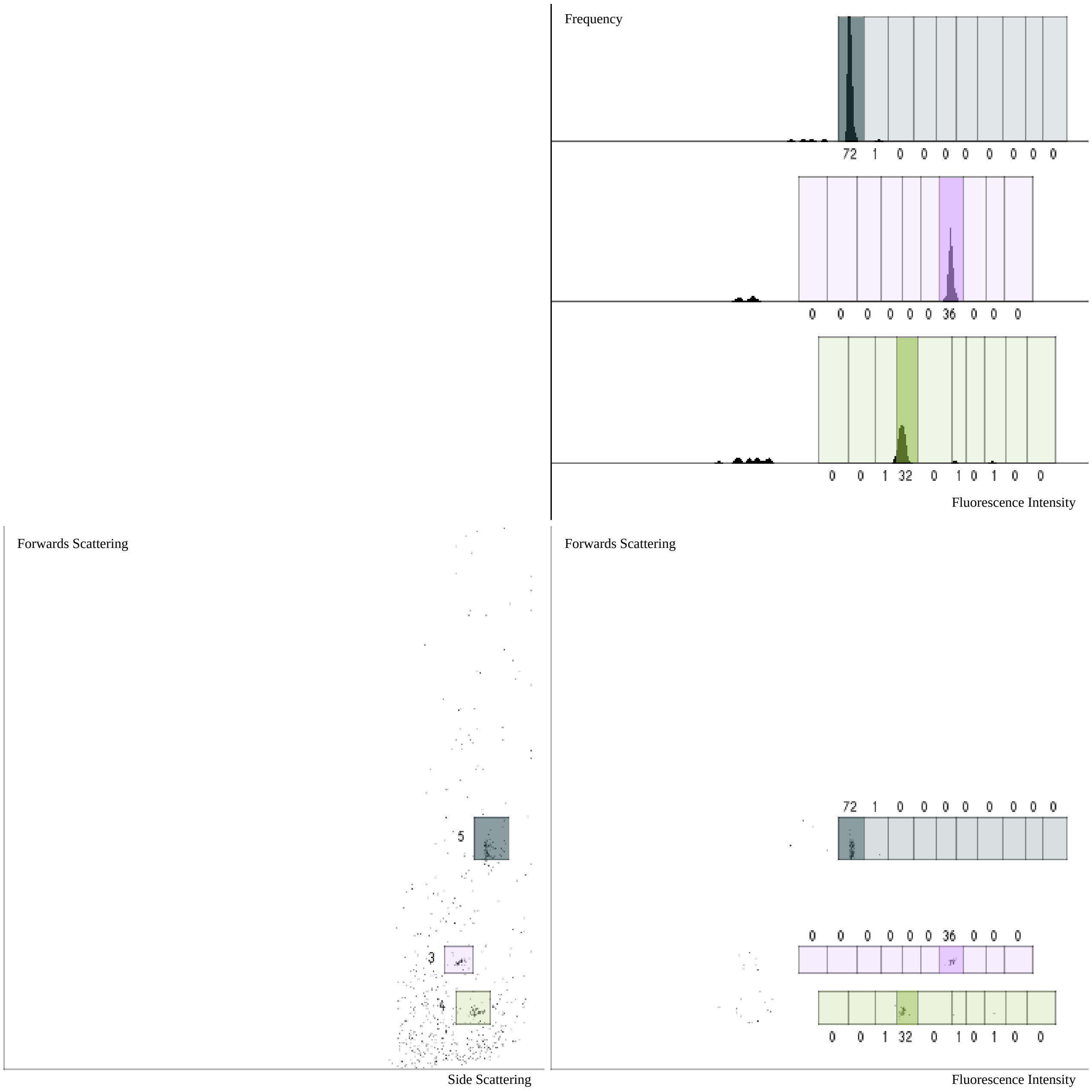

ANNEX 3: TAG DECONVOLUTION - BEAD 283

Passes flow sorting criteria: Yes

Passes tag deconvolution criteria: Yes

Included in protocol analysis: Yes

Protocol: 8, 7, 10, 9

Filename: Bin9_plateA2_D5.fcs

Split 1: Petrol shading

Split 2: Green shading

Split 3: Violet shading

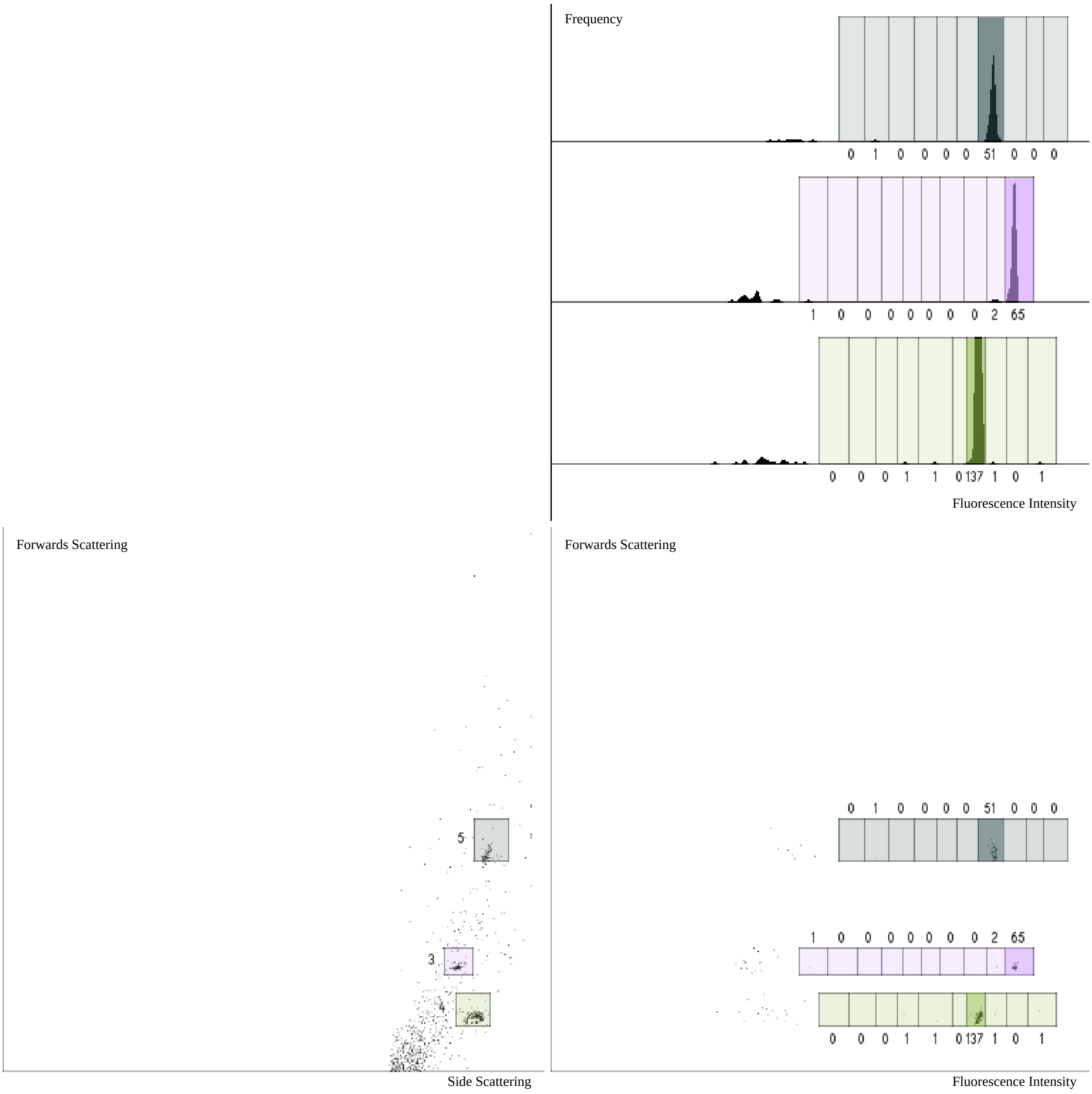

ANNEX 3: TAG DECONVOLUTION - BEAD 284

Passes flow sorting criteria: Yes

Passes tag deconvolution criteria: Yes

Included in protocol analysis: Yes

Protocol: 6, 5, 7, 9

Filename: Bin9_plateA2_D10.fcs

Split 1: Petrol shading

Split 2: Green shading

Split 3: Violet shading

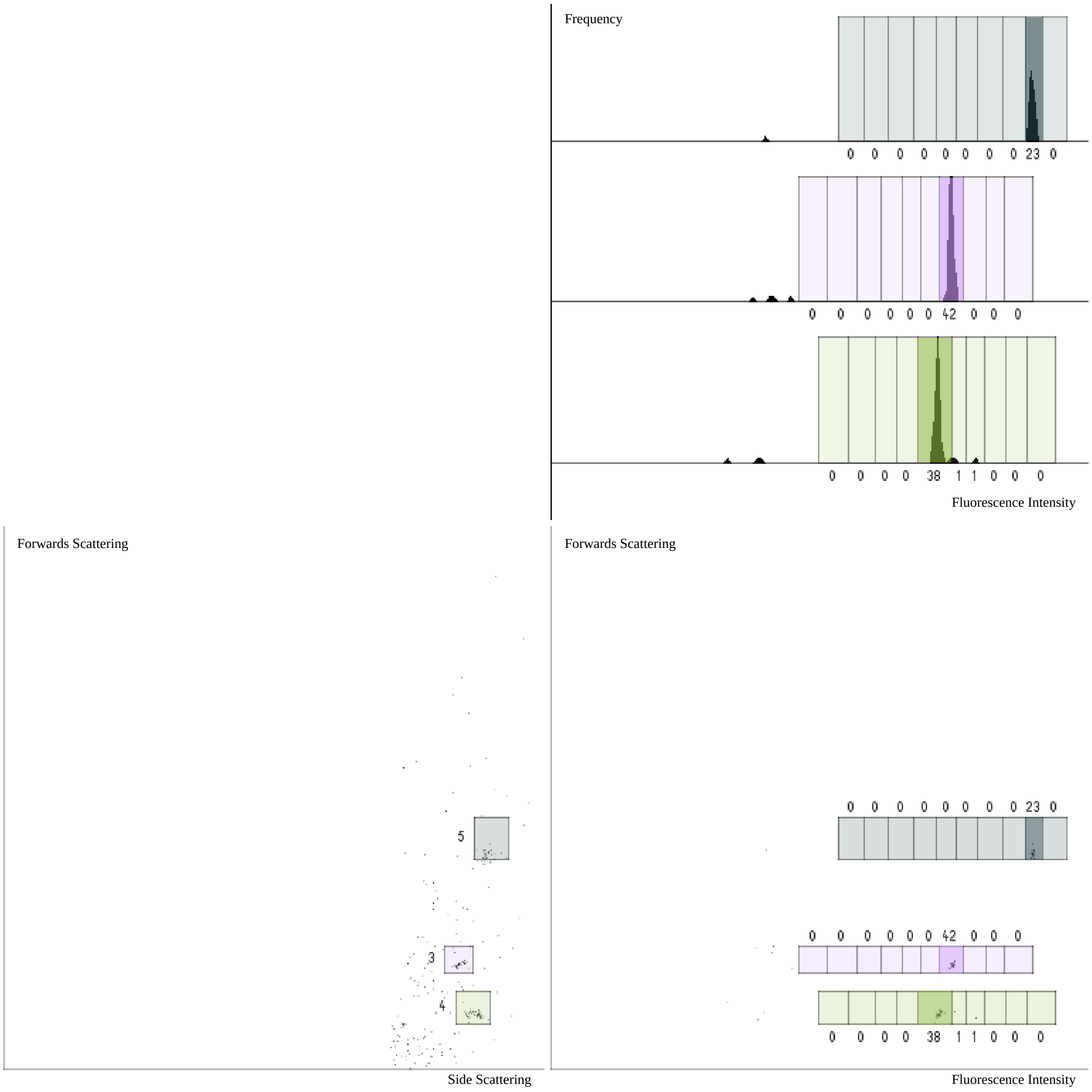

ANNEX 3: TAG DECONVOLUTION - BEAD 285

Passes flow sorting criteria: Yes

Passes tag deconvolution criteria: Yes

Included in protocol analysis: Yes

Protocol: 6, 2, 8, 9

Filename: Bin9_plateA2_D12.fcs

Split 1: Petrol shading

Split 2: Green shading

Split 3: Violet shading

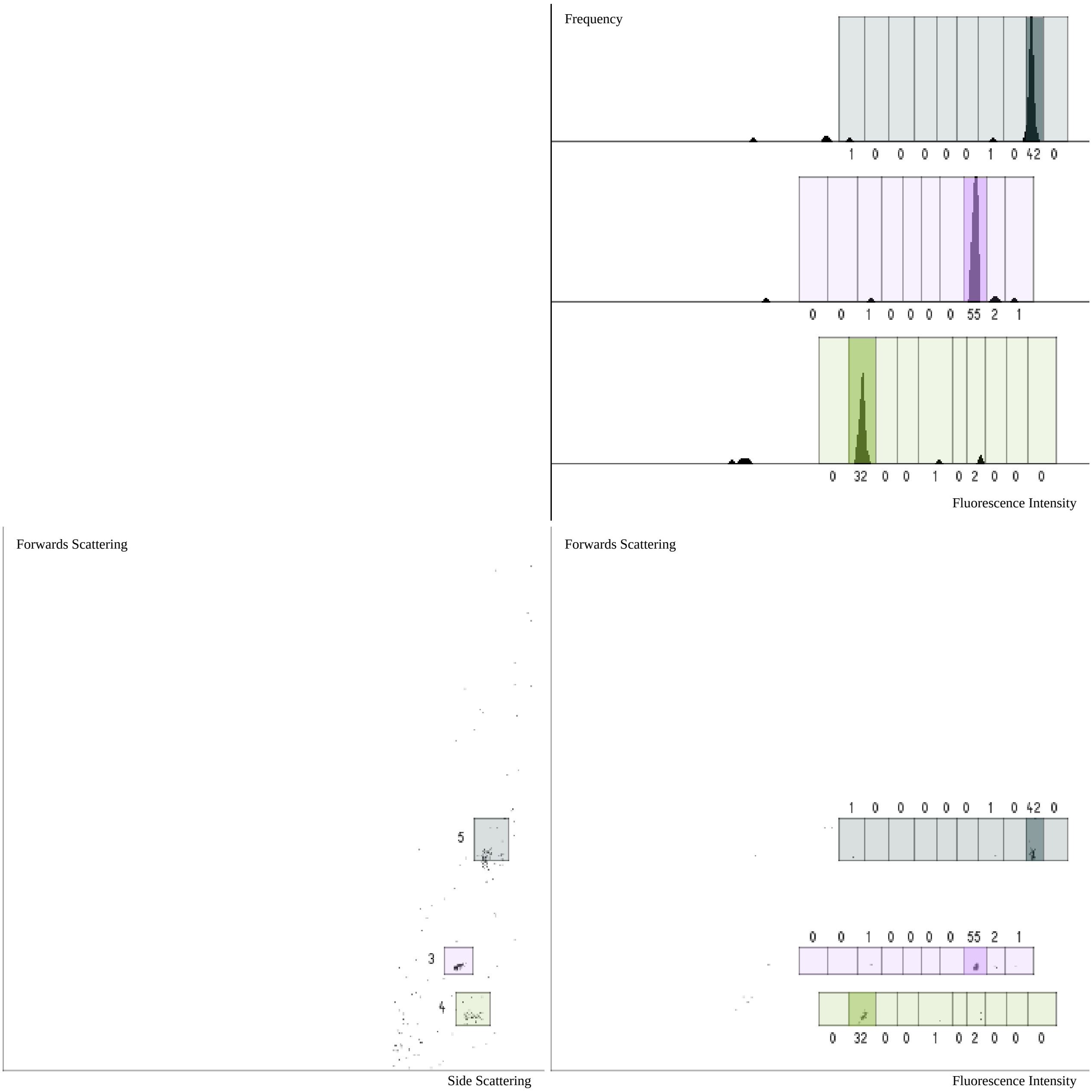

ANNEX 3: TAG DECONVOLUTION - BEAD 286

Passes flow sorting criteria: Yes

Passes tag deconvolution criteria: Yes

Included in protocol analysis: Yes

Protocol: 4, 10, 7, 9

Filename: Bin9_plateA2_E7.fcs

Split 1: Petrol shading

Split 2: Green shading

Split 3: Violet shading

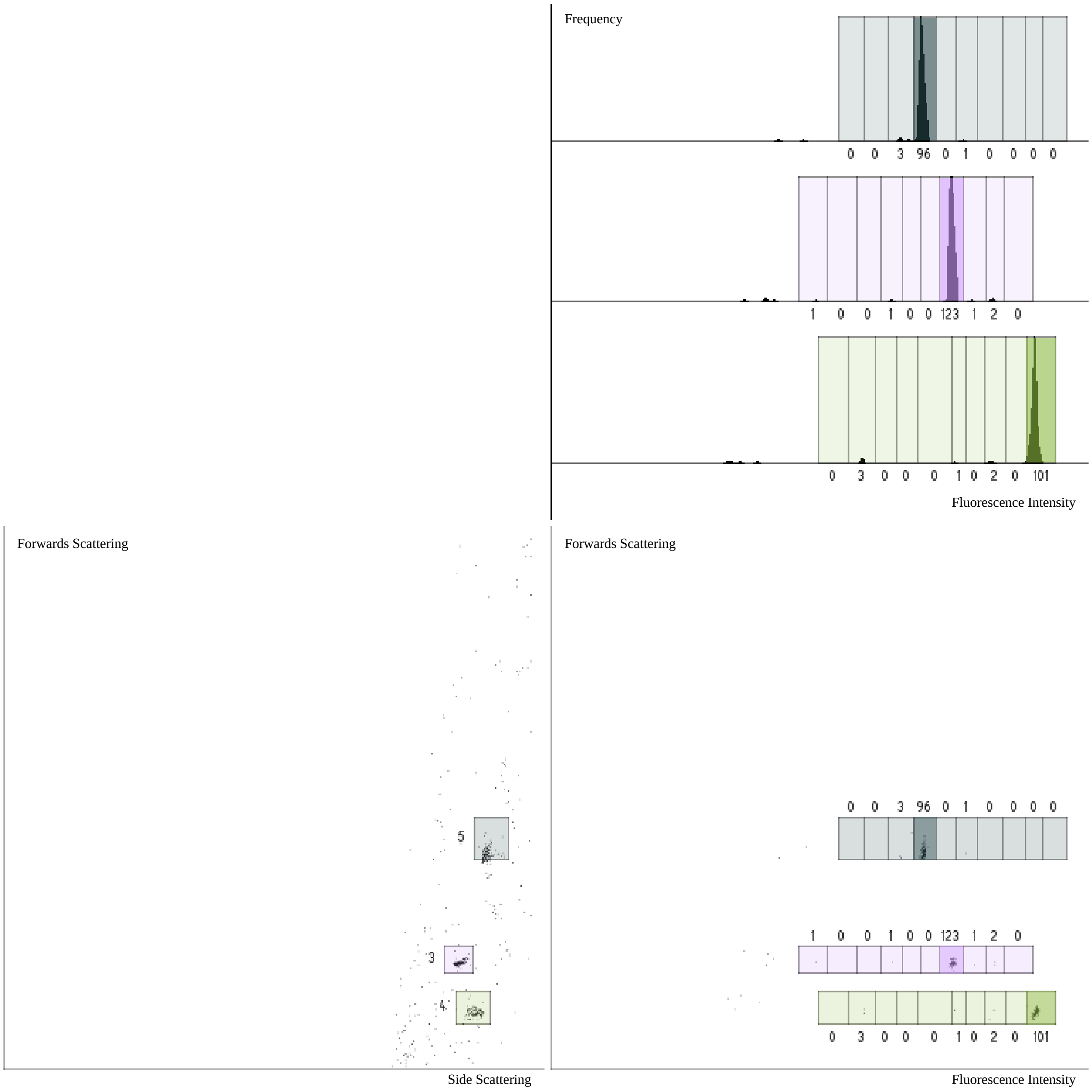

ANNEX 3: TAG DECONVOLUTION - BEAD 287

Passes flow sorting criteria: Yes

Passes tag deconvolution criteria: Yes

Included in protocol analysis: Yes

Protocol: 2, 9, 7, 9

Filename: Bin9_plateA2_E8.fcs

Split 1: Petrol shading

Split 2: Green shading

Split 3: Violet shading

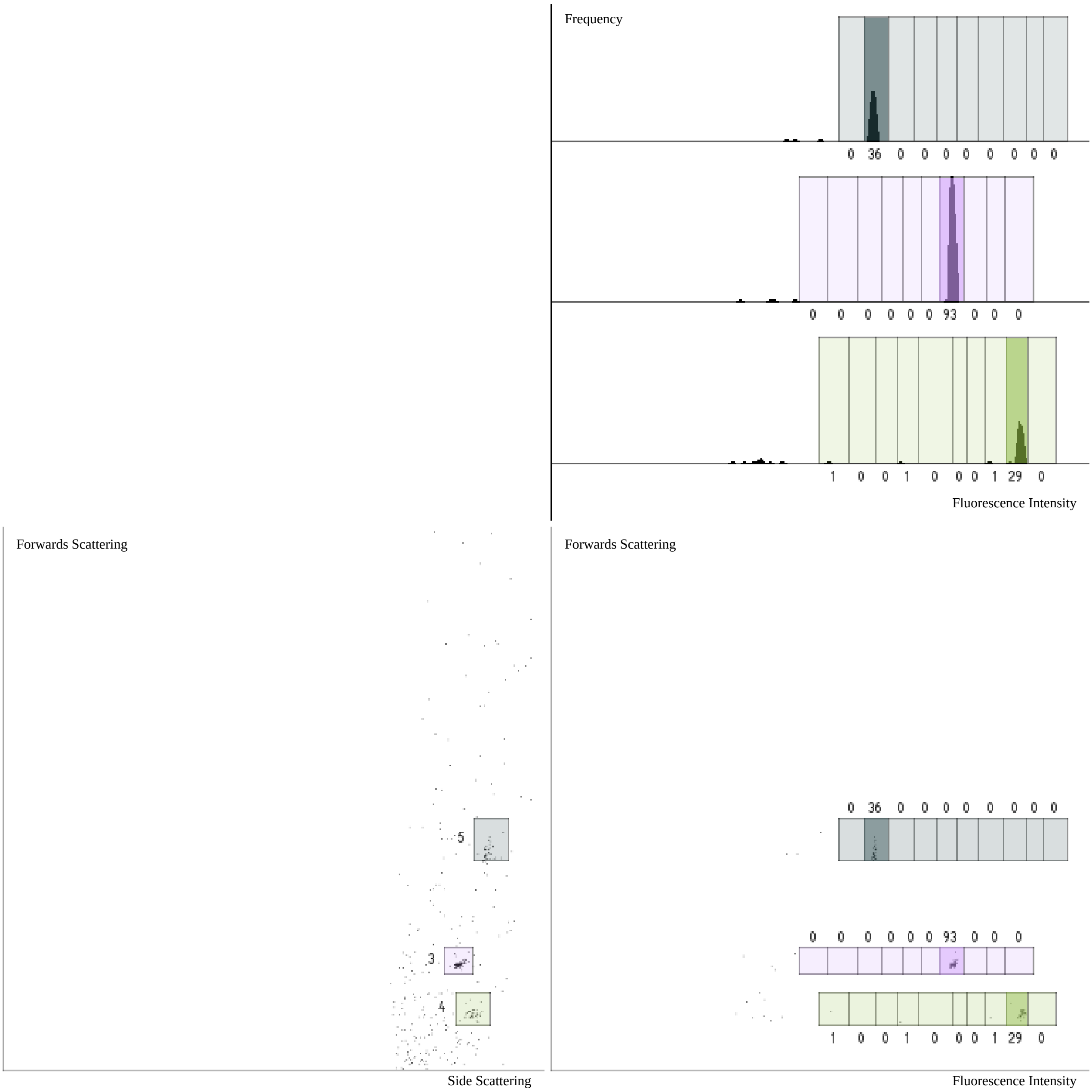

ANNEX 3: TAG DECONVOLUTION - BEAD 288

Passes flow sorting criteria: Yes

Passes tag deconvolution criteria: Yes

Included in protocol analysis: Yes

Protocol: 7, 8, 8, 9

Filename: Bin9_plateA2_F4.fcs

Split 1: Petrol shading

Split 2: Green shading

Split 3: Violet shading

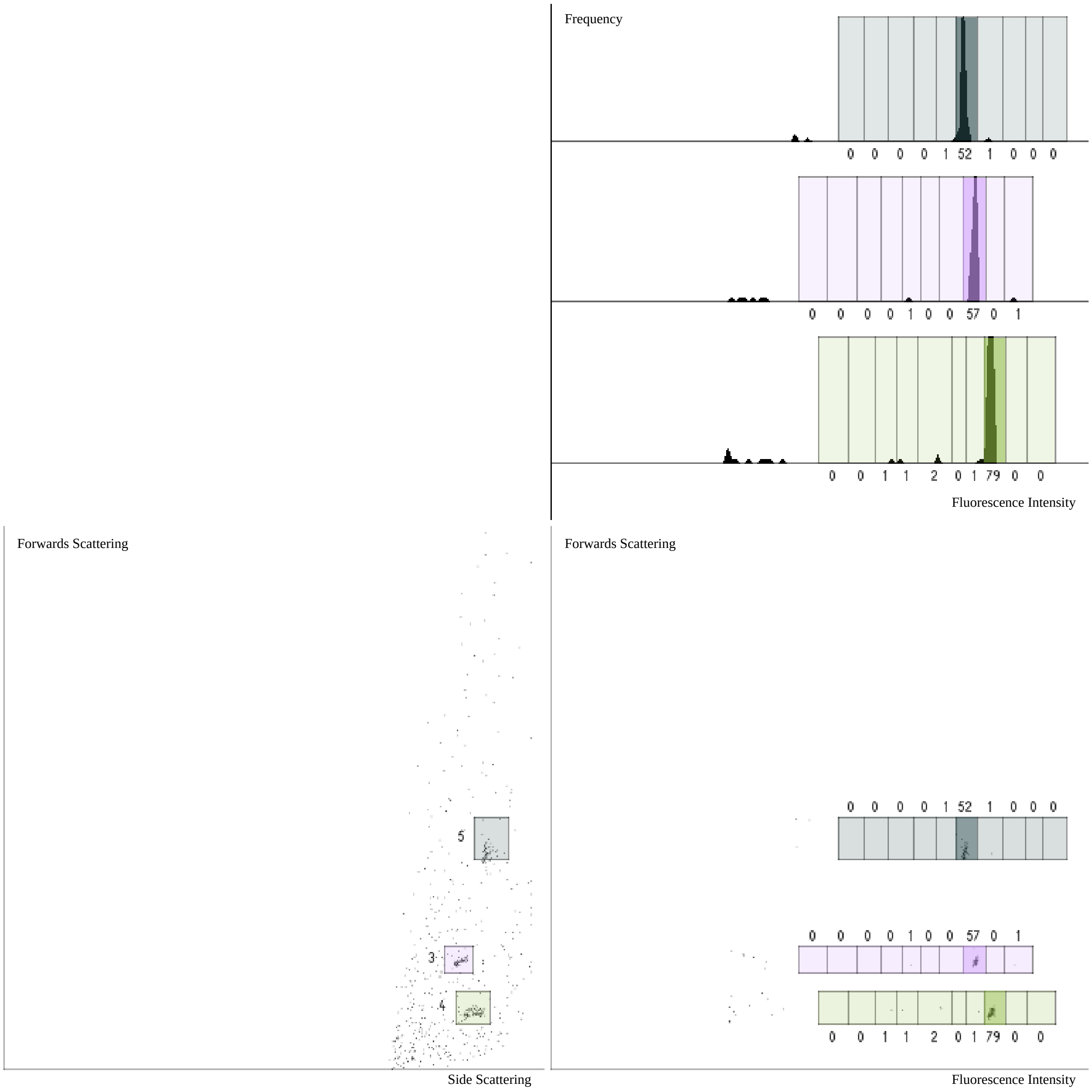

ANNEX 3: TAG DECONVOLUTION - BEAD 289

Passes flow sorting criteria: Yes

Passes tag deconvolution criteria: No

Included in protocol analysis: No

Protocol: N/A

Filename: Bin10_plateA2_E1.fcs

Split 1: Petrol shading

Split 2: Green shading

Split 3: Violet shading

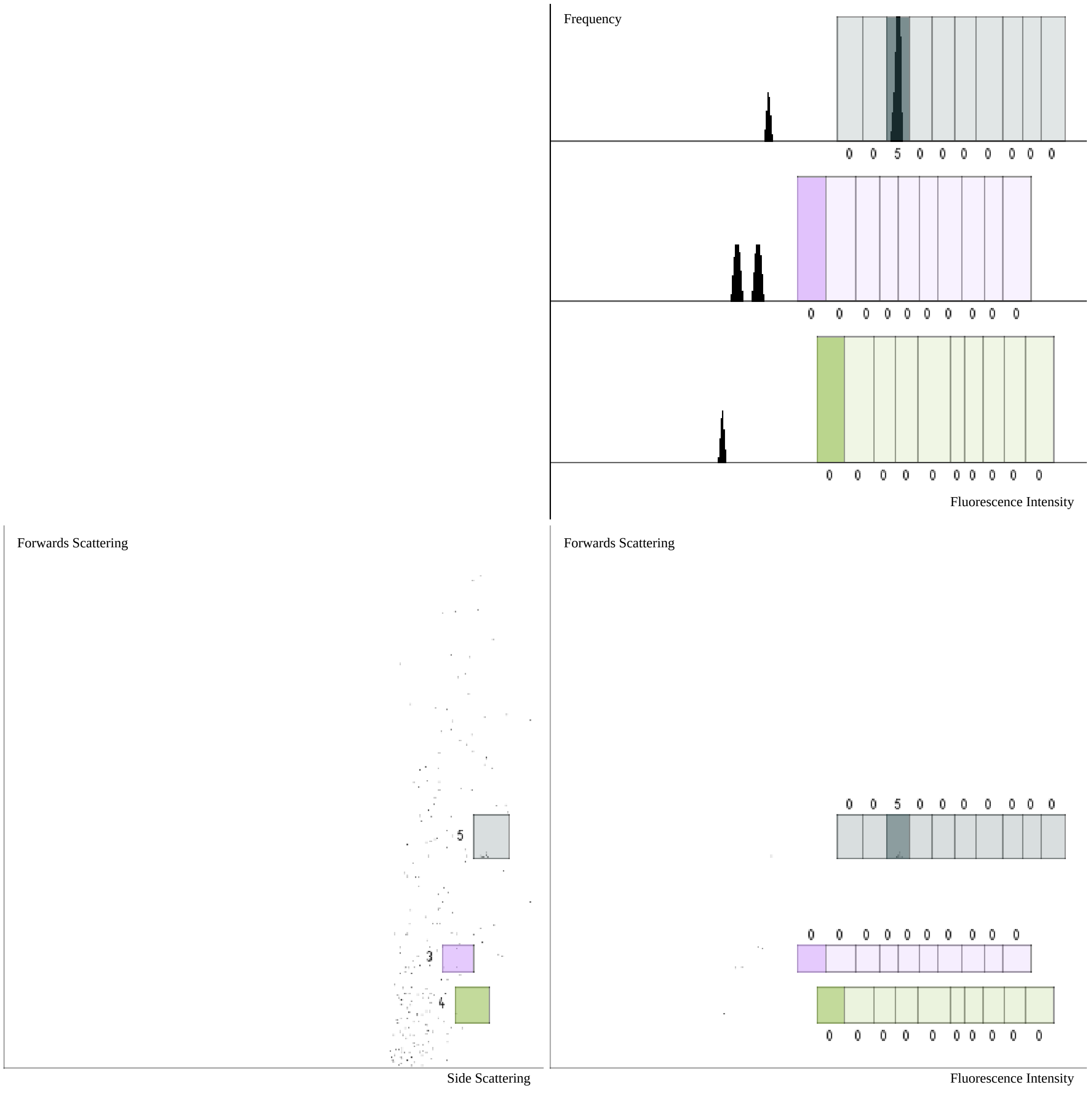

ANNEX 3: TAG DECONVOLUTION - BEAD 290

Passes flow sorting criteria: Yes

Passes tag deconvolution criteria: Yes

Included in protocol analysis: Yes

Protocol: 3, 4, 10, 10

Filename: Bin10_plateA2_A1.fcs

Split 1: Petrol shading

Split 2: Green shading

Split 3: Violet shading

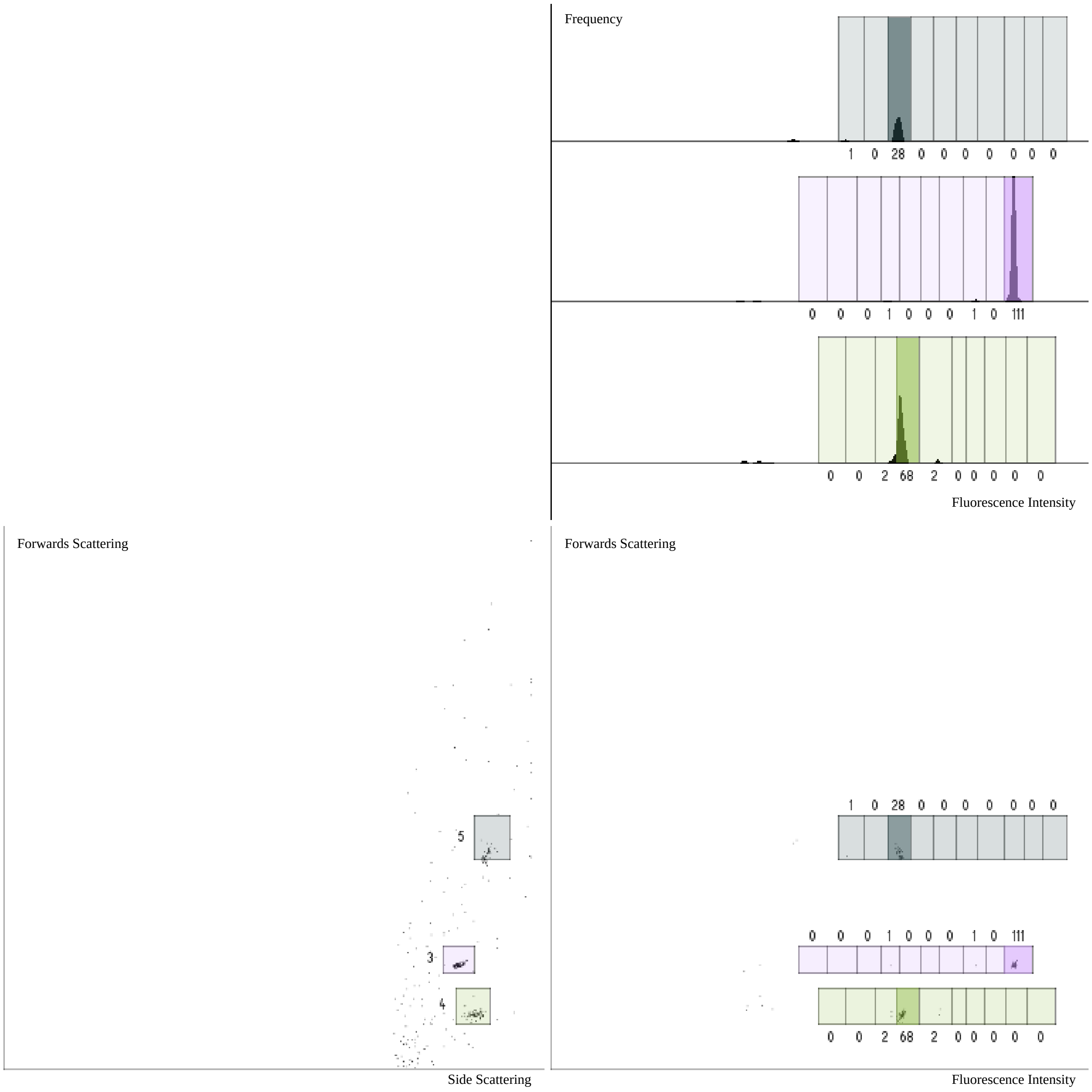

ANNEX 3: TAG DECONVOLUTION - BEAD 291

Passes flow sorting criteria: Yes

Passes tag deconvolution criteria: Yes

Included in protocol analysis: Yes

Protocol: 10, 10, 6, 10

Filename: Bin10_plateA2_A10.fcs

Split 1: Petrol shading

Split 2: Green shading

Split 3: Violet shading

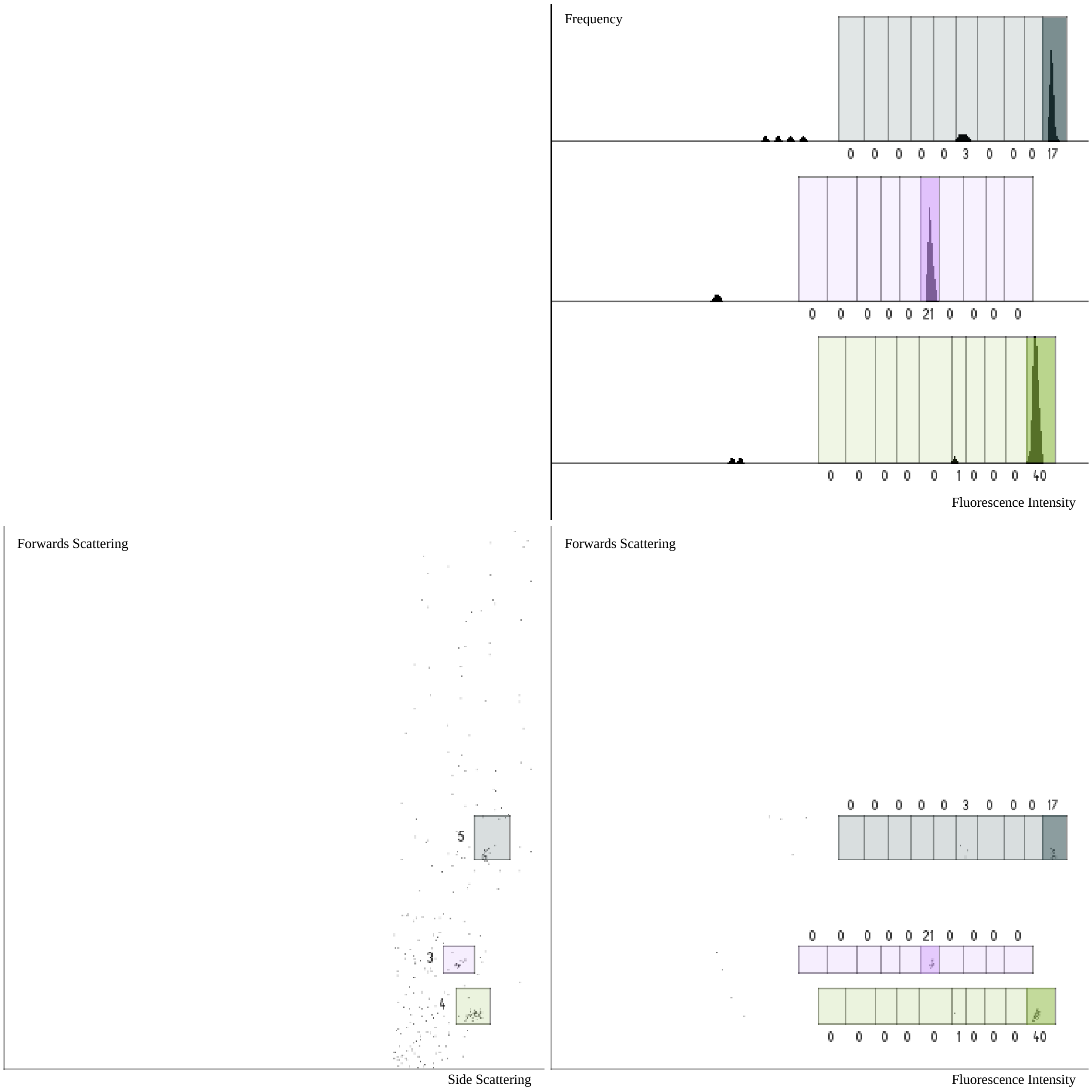

ANNEX 3: TAG DECONVOLUTION - BEAD 292

Passes flow sorting criteria: Yes

Passes tag deconvolution criteria: Yes

Included in protocol analysis: Yes

Protocol: 4, 9, 3, 10

Filename: Bin10_plateA2_B1.fcs

Split 1: Petrol shading

Split 2: Green shading

Split 3: Violet shading

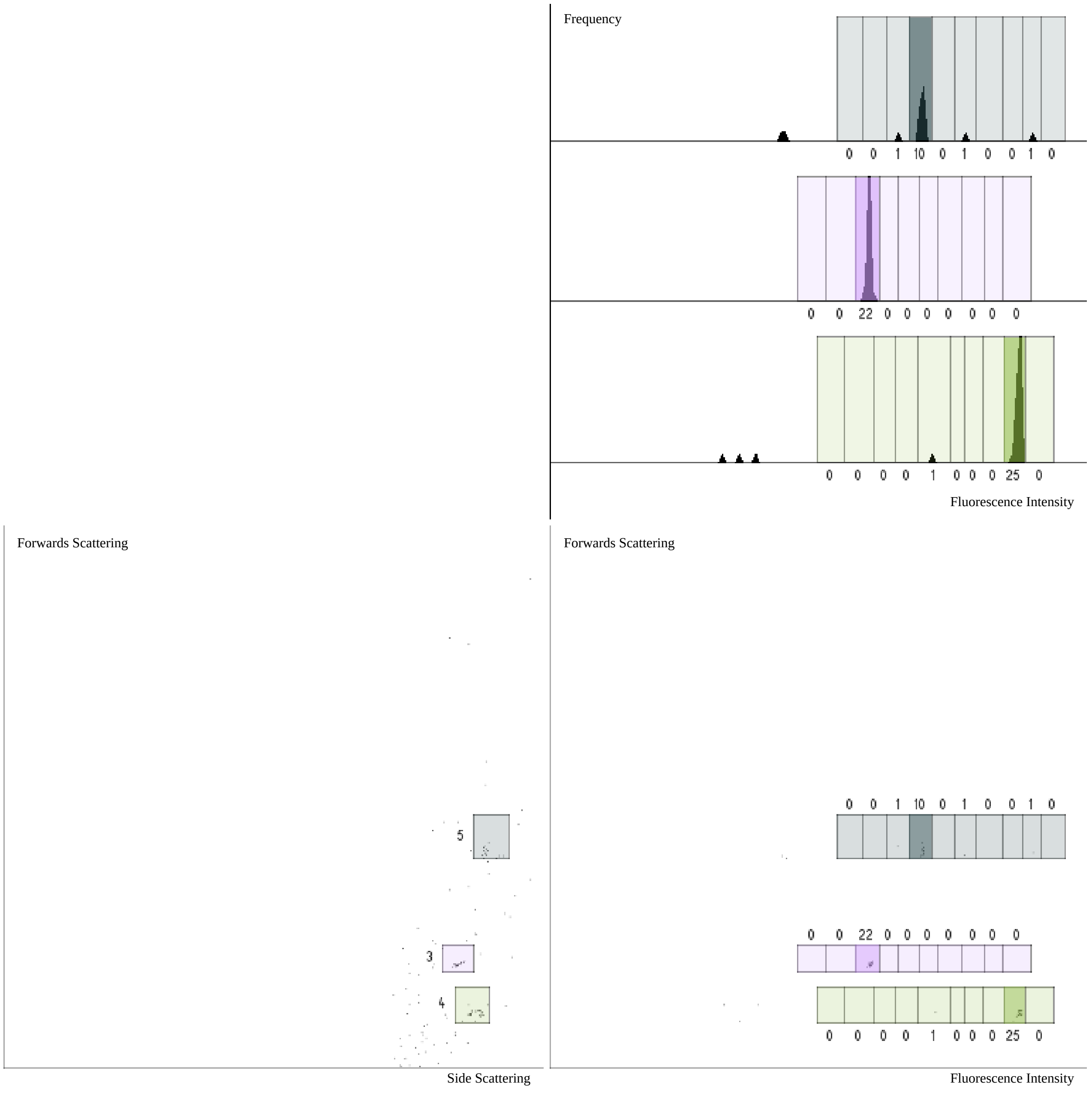

ANNEX 3: TAG DECONVOLUTION - BEAD 293

Passes flow sorting criteria: Yes

Passes tag deconvolution criteria: Yes

Included in protocol analysis: Yes

Protocol: 8, 7, 4, 10

Filename: Bin10_plateA2_B2.fcs

Split 1: Petrol shading

Split 2: Green shading

Split 3: Violet shading

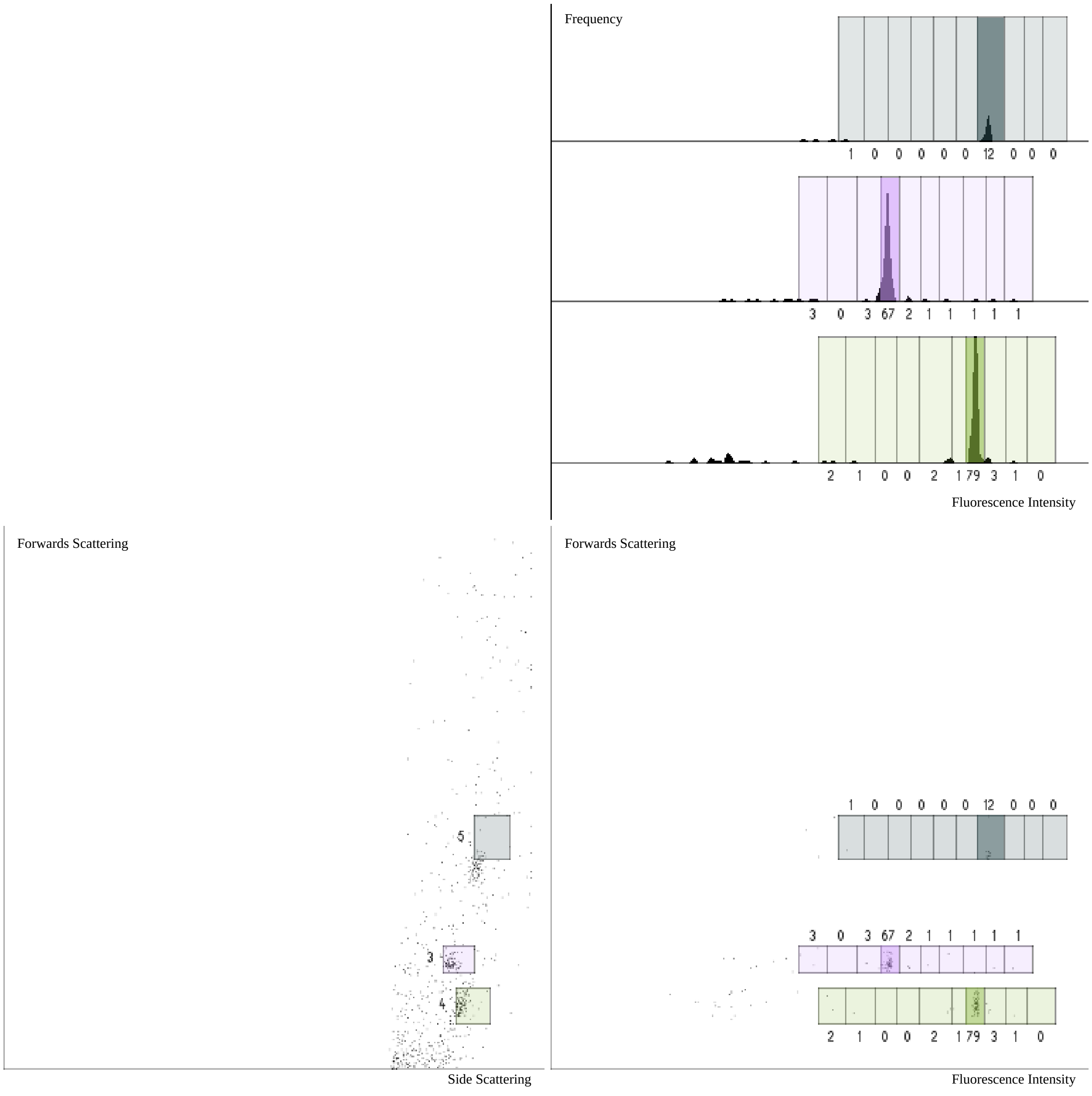

ANNEX 3: TAG DECONVOLUTION - BEAD 294

Passes flow sorting criteria: Yes

Passes tag deconvolution criteria: Yes

Included in protocol analysis: Yes

Protocol: 7, 7, 7, 10

Filename: Bin10_plateA2_B6.fcs

Split 1: Petrol shading

Split 2: Green shading

Split 3: Violet shading

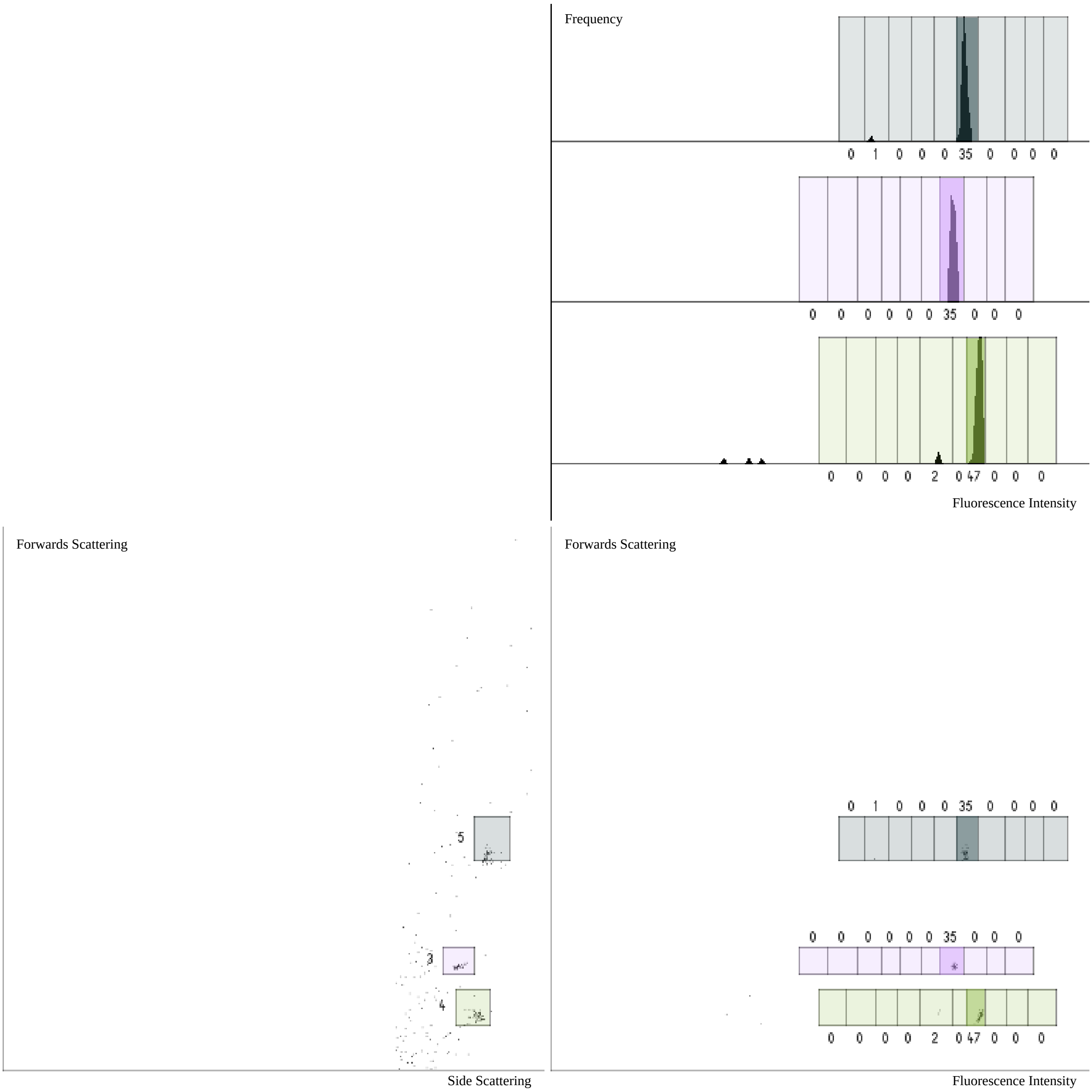

ANNEX 3: TAG DECONVOLUTION - BEAD 295

Passes flow sorting criteria: Yes

Passes tag deconvolution criteria: Yes

Included in protocol analysis: Yes

Protocol: 10, 8, 2, 10

Filename: Bin10_plateA2_B7.fcs

Split 1: Petrol shading

Split 2: Green shading

Split 3: Violet shading

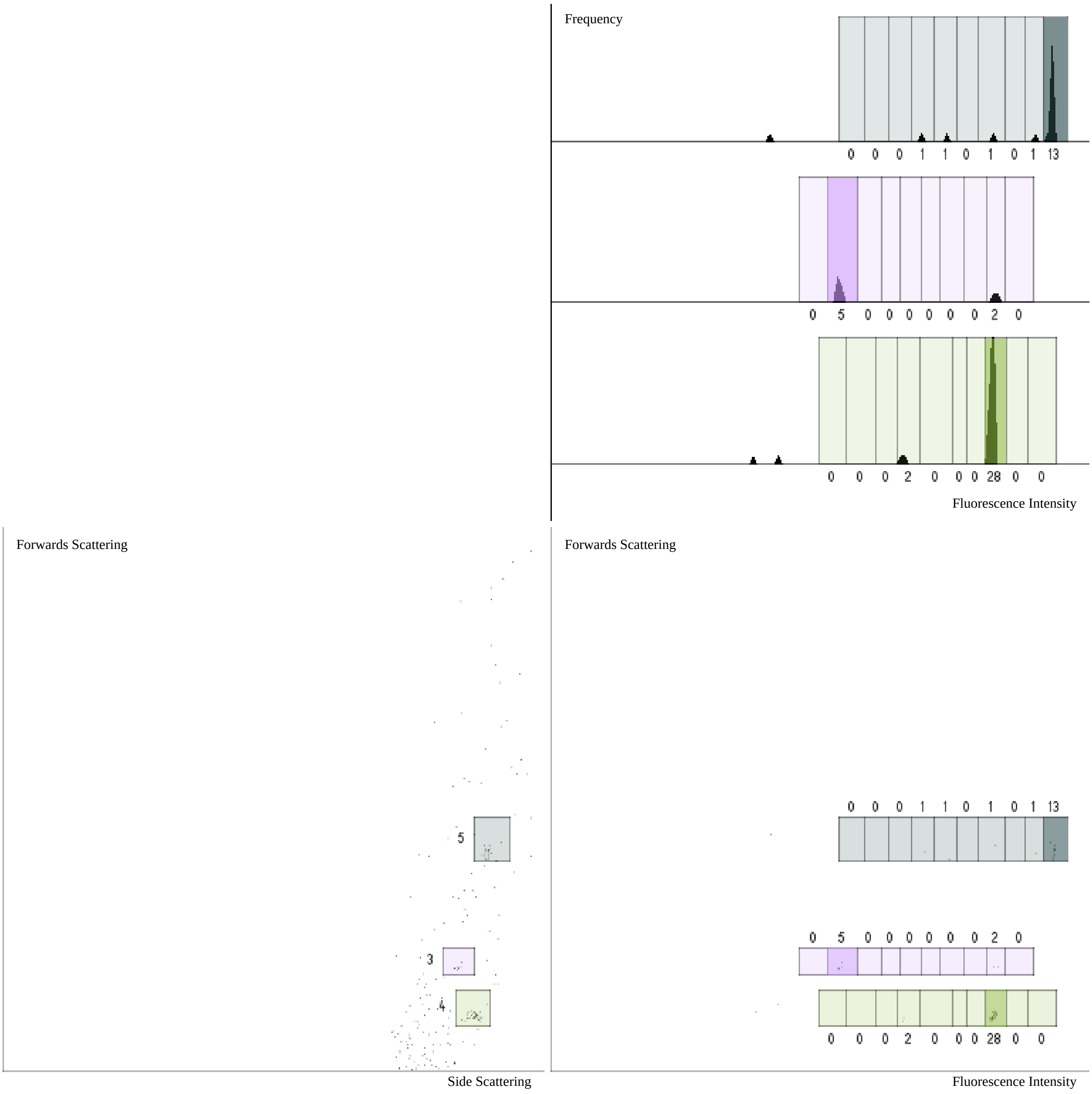

ANNEX 3: TAG DECONVOLUTION - BEAD 296

Passes flow sorting criteria: Yes

Passes tag deconvolution criteria: No

Included in protocol analysis: No

Protocol: N/A

Filename: Bin10_plateA2_B11.fcs

Split 1: Petrol shading

Split 2: Green shading

Split 3: Violet shading

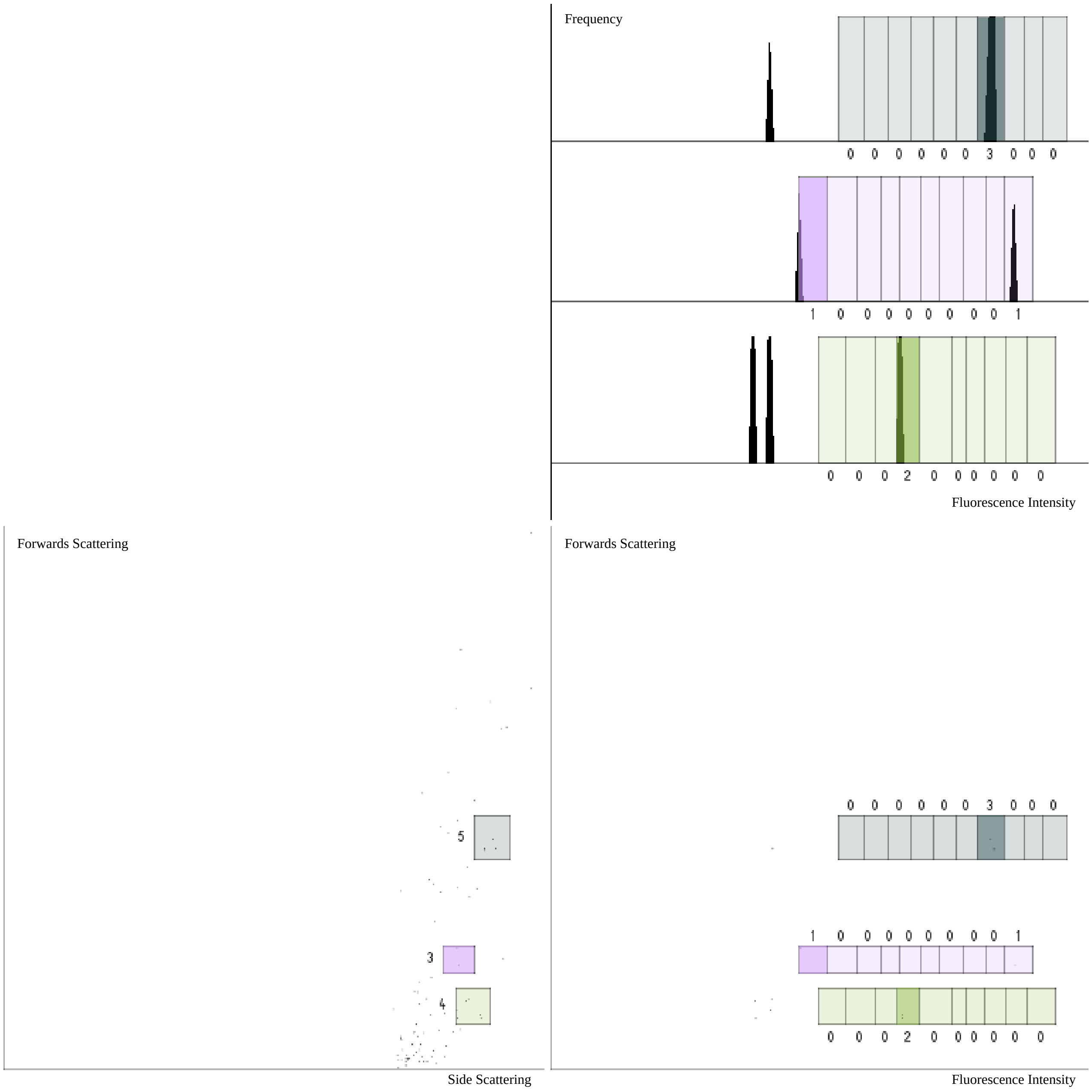

ANNEX 3: TAG DECONVOLUTION - BEAD 297

Passes flow sorting criteria: Yes

Passes tag deconvolution criteria: No

Included in protocol analysis: No

Protocol: N/A

Filename: Bin10_plateA2_C6.fcs

Split 1: Petrol shading

Split 2: Green shading

Split 3: Violet shading

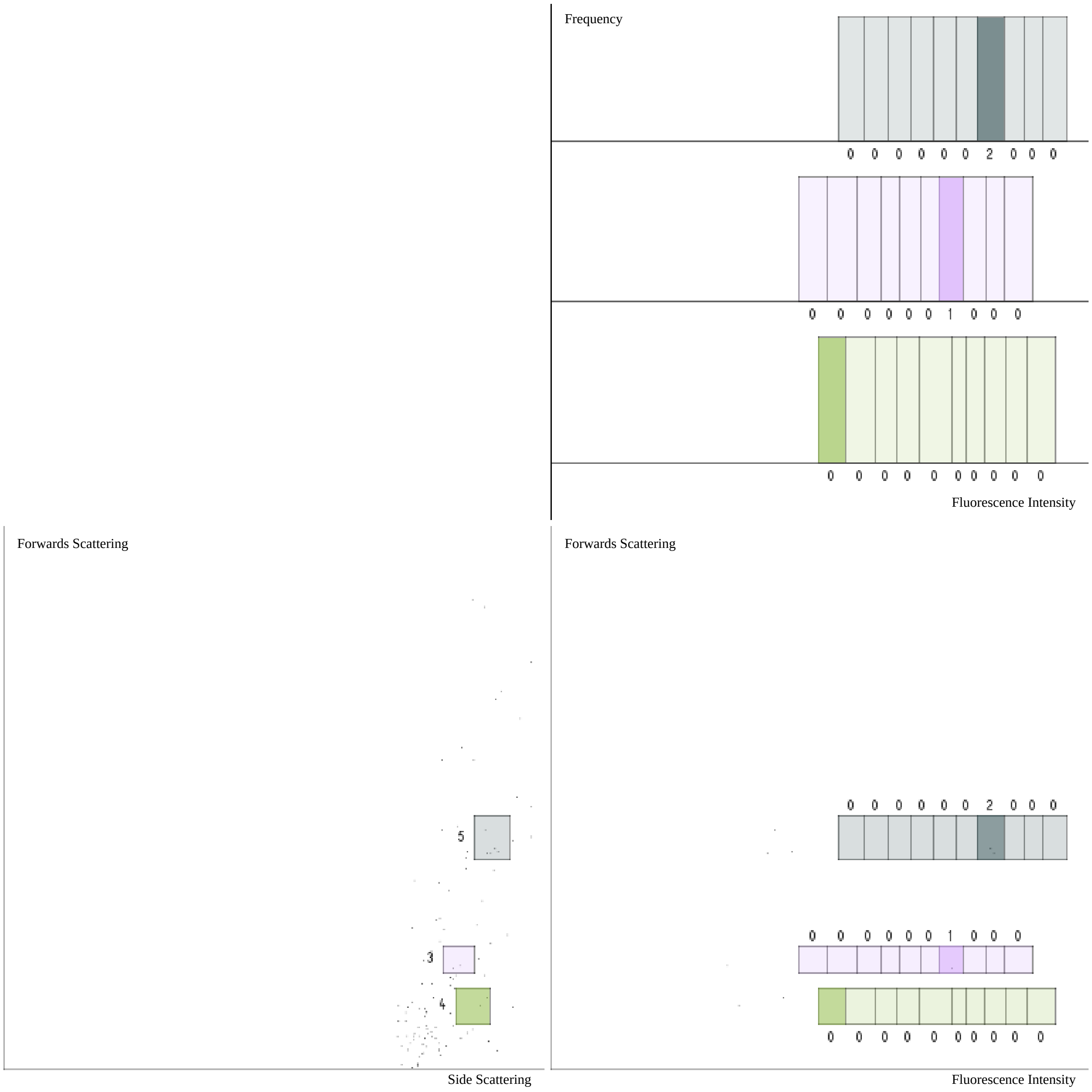

ANNEX 3: TAG DECONVOLUTION - BEAD 298

Passes flow sorting criteria: Yes

Passes tag deconvolution criteria: No

Included in protocol analysis: No

Protocol: N/A

Filename: Bin10_plateA2_D6.fcs

Split 1: Petrol shading

Split 2: Green shading

Split 3: Violet shading

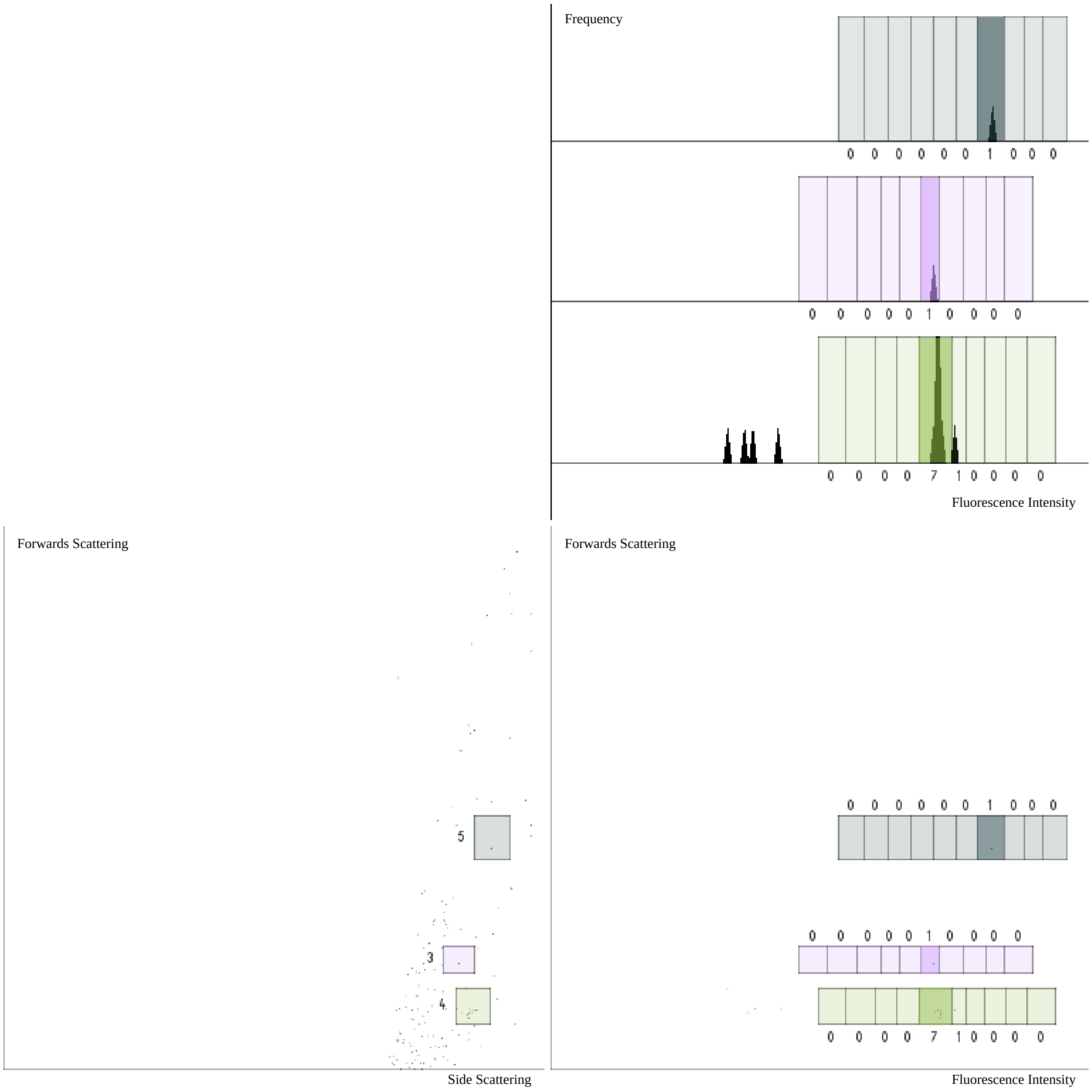

ANNEX 3: TAG DECONVOLUTION - BEAD 299

Passes flow sorting criteria: Yes

Passes tag deconvolution criteria: No

Included in protocol analysis: No

Protocol: N/A

Filename: Bin10_plateA2_D10.fcs

Split 1: Petrol shading

Split 2: Green shading

Split 3: Violet shading

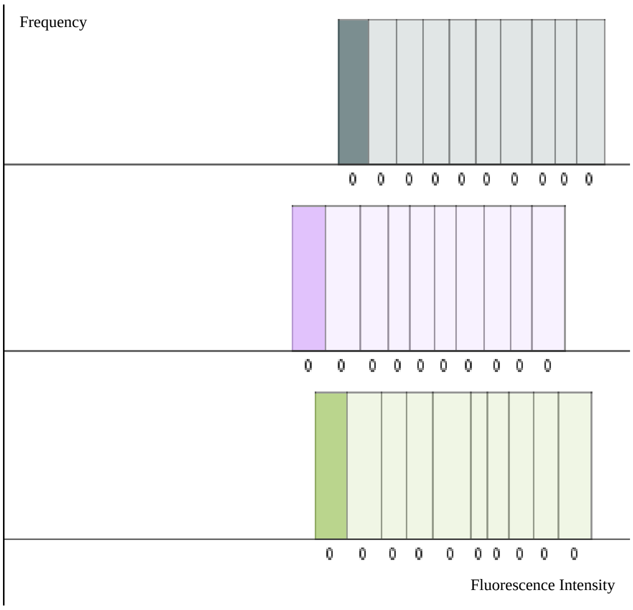

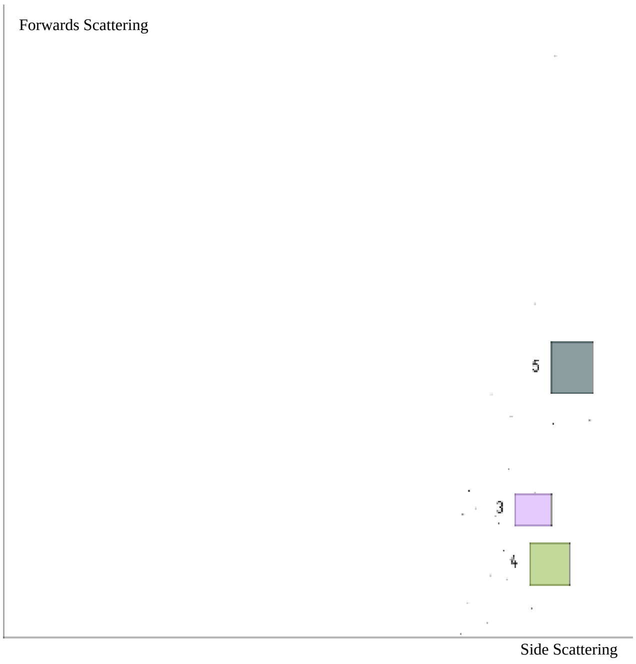

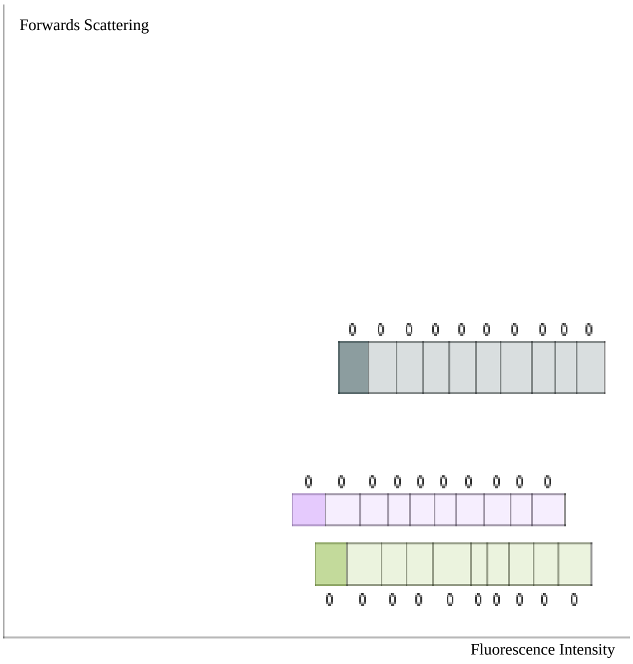

ANNEX 3: TAG DECONVOLUTION - BEAD 300

Passes flow sorting criteria: Yes

Passes tag deconvolution criteria: Yes

Included in protocol analysis: Yes

Protocol: 6, 2, 8, 10

Filename: Bin10_plateA2_H8.fcs

Split 1: Petrol shading

Split 2: Green shading

Split 3: Violet shading

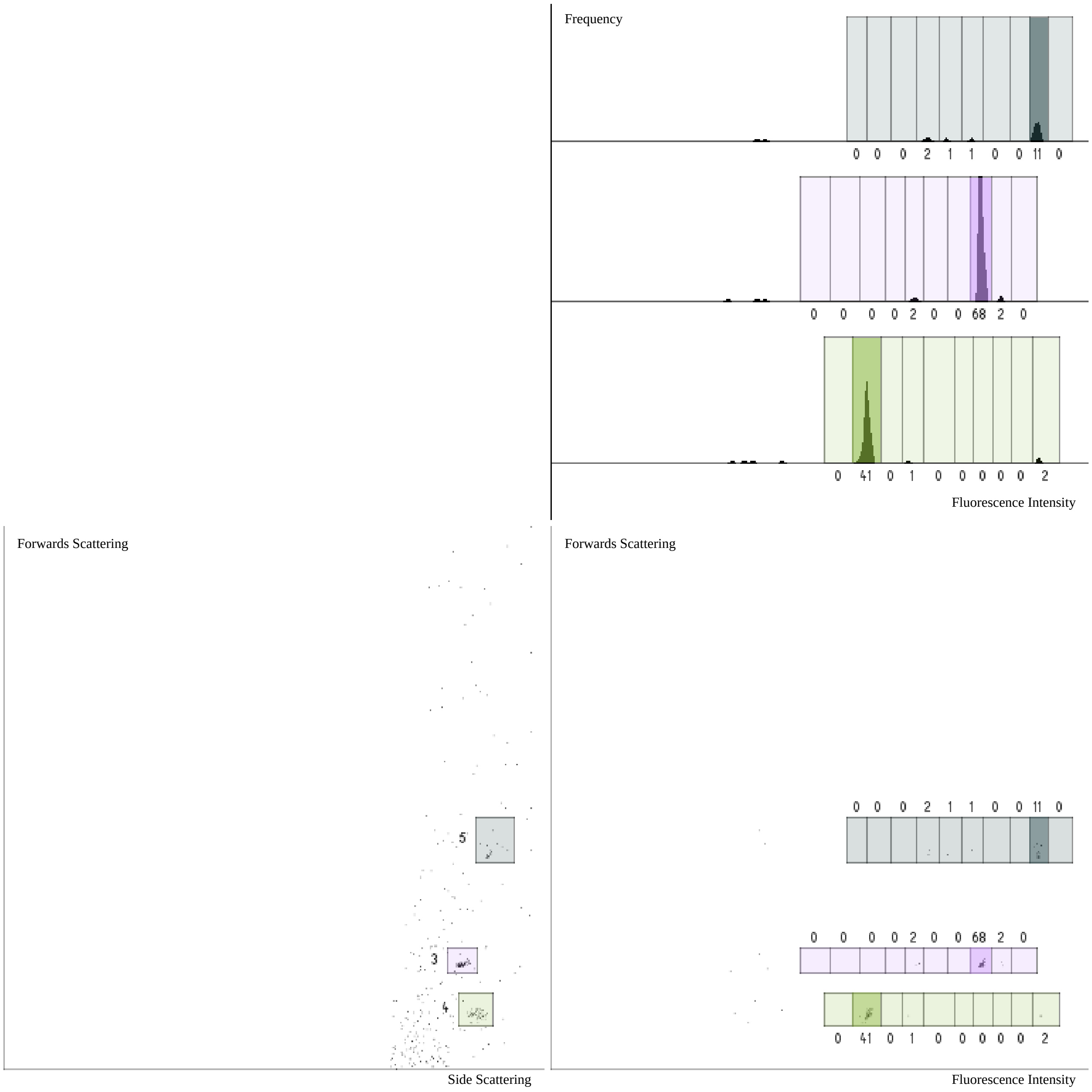

ANNEX 3: TAG DECONVOLUTION - BEAD 301

Passes flow sorting criteria: Yes

Passes tag deconvolution criteria: Yes

Included in protocol analysis: Yes

Protocol: 1, 8, 4, 10

Filename: Bin10_plateA2_E7.fcs

Split 1: Petrol shading

Split 2: Green shading

Split 3: Violet shading

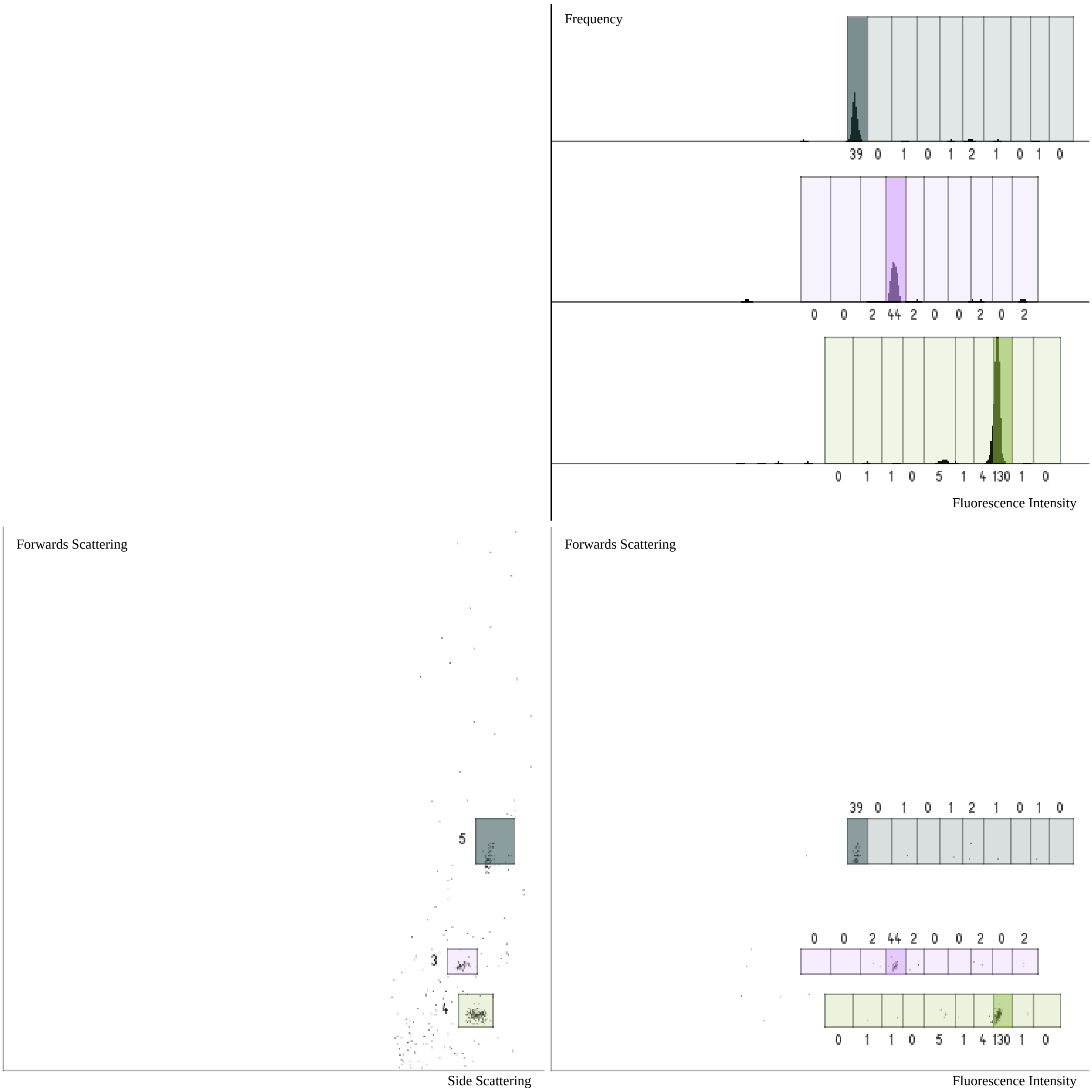

ANNEX 3: TAG DECONVOLUTION - BEAD 302

Passes flow sorting criteria: Yes

Passes tag deconvolution criteria: Yes

Included in protocol analysis: Yes

Protocol: 10, 3, 9, 10

Filename: Bin10_plateA2_F6.fcs

Split 1: Petrol shading

Split 2: Green shading

Split 3: Violet shading

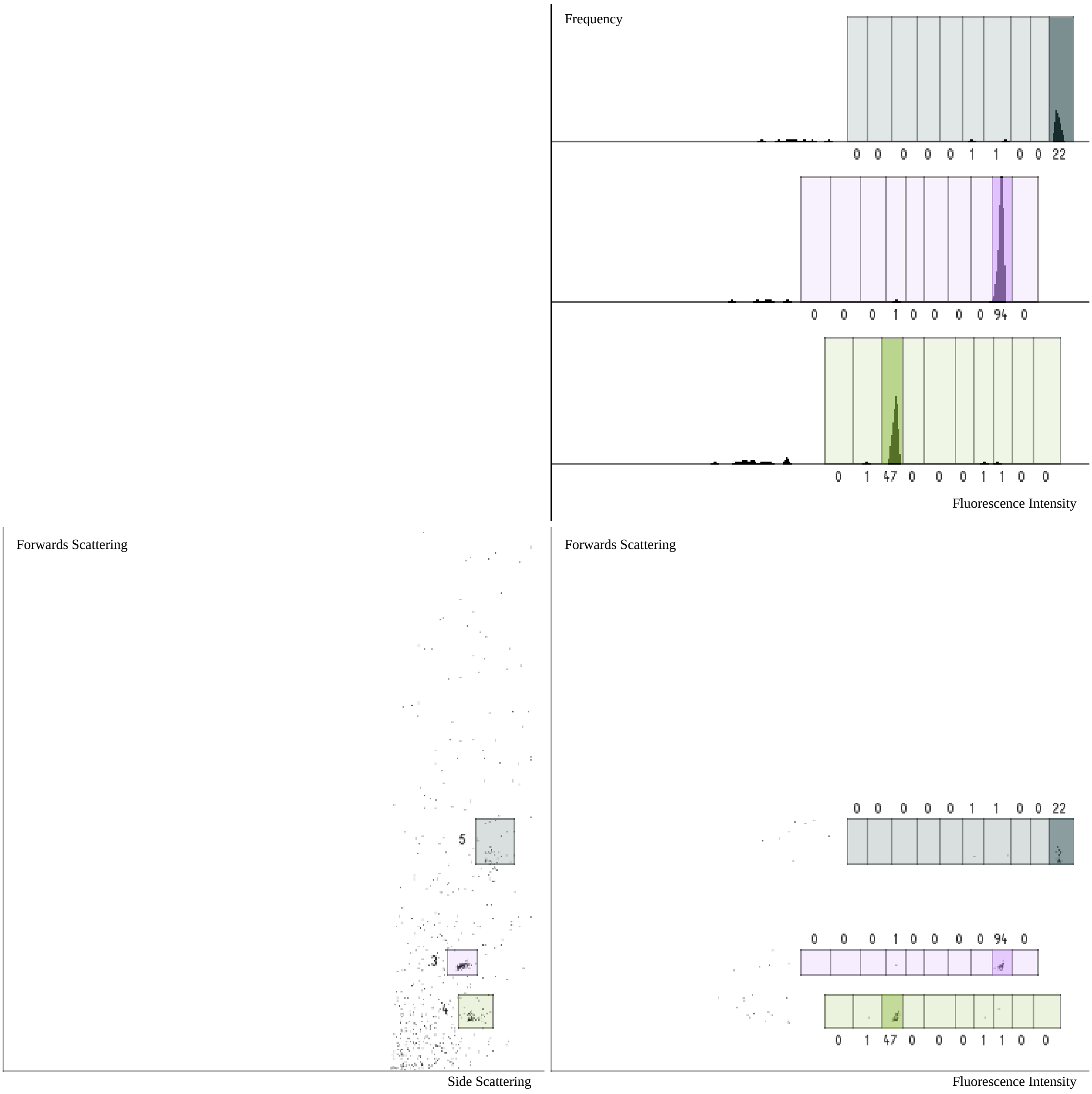

ANNEX 3: TAG DECONVOLUTION - BEAD 303

Passes flow sorting criteria: Yes

Passes tag deconvolution criteria: Yes

Included in protocol analysis: Yes

Protocol: 2, 4, 9, 10

Filename: Bin10_plateA2_F7.fcs

Split 1: Petrol shading

Split 2: Green shading

Split 3: Violet shading

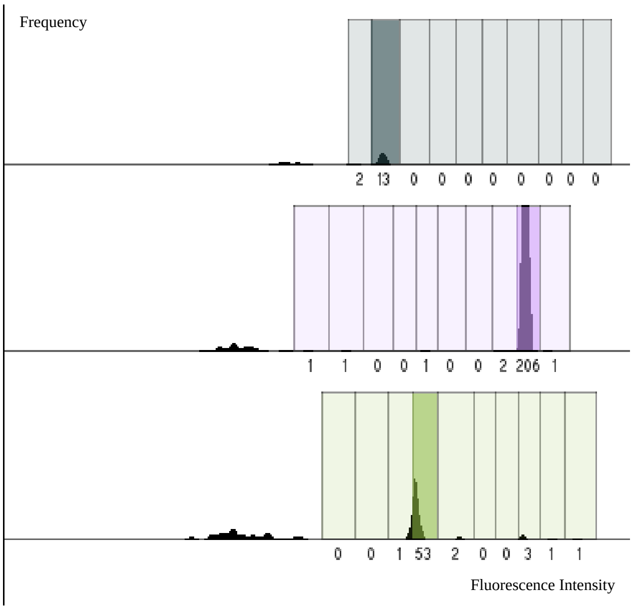

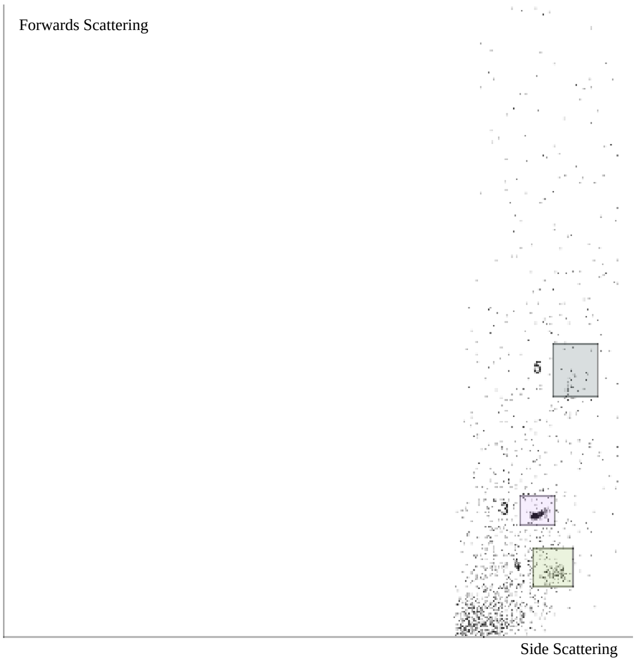

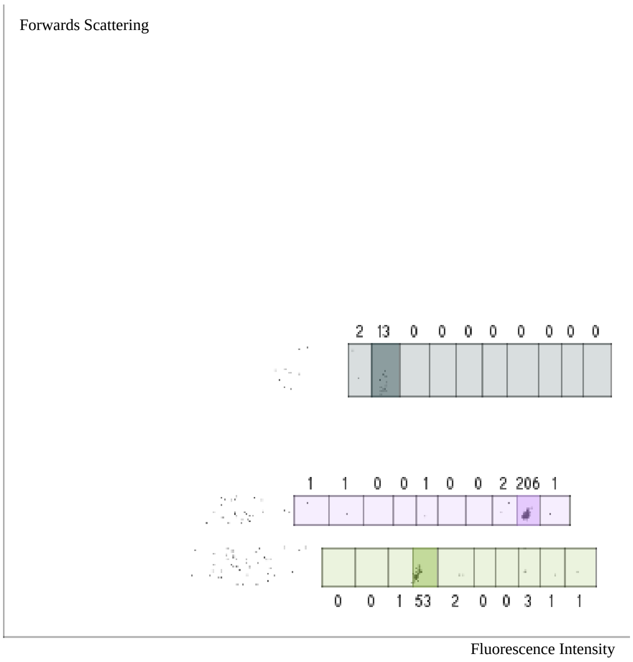

ANNEX 3: TAG DECONVOLUTION - BEAD 304

Passes flow sorting criteria: Yes

Passes tag deconvolution criteria: Yes

Included in protocol analysis: Yes

Protocol: 6, 7, 4, 10

Filename: Bin10_plateA2_F9.fcs

Split 1: Petrol shading

Split 2: Green shading

Split 3: Violet shading

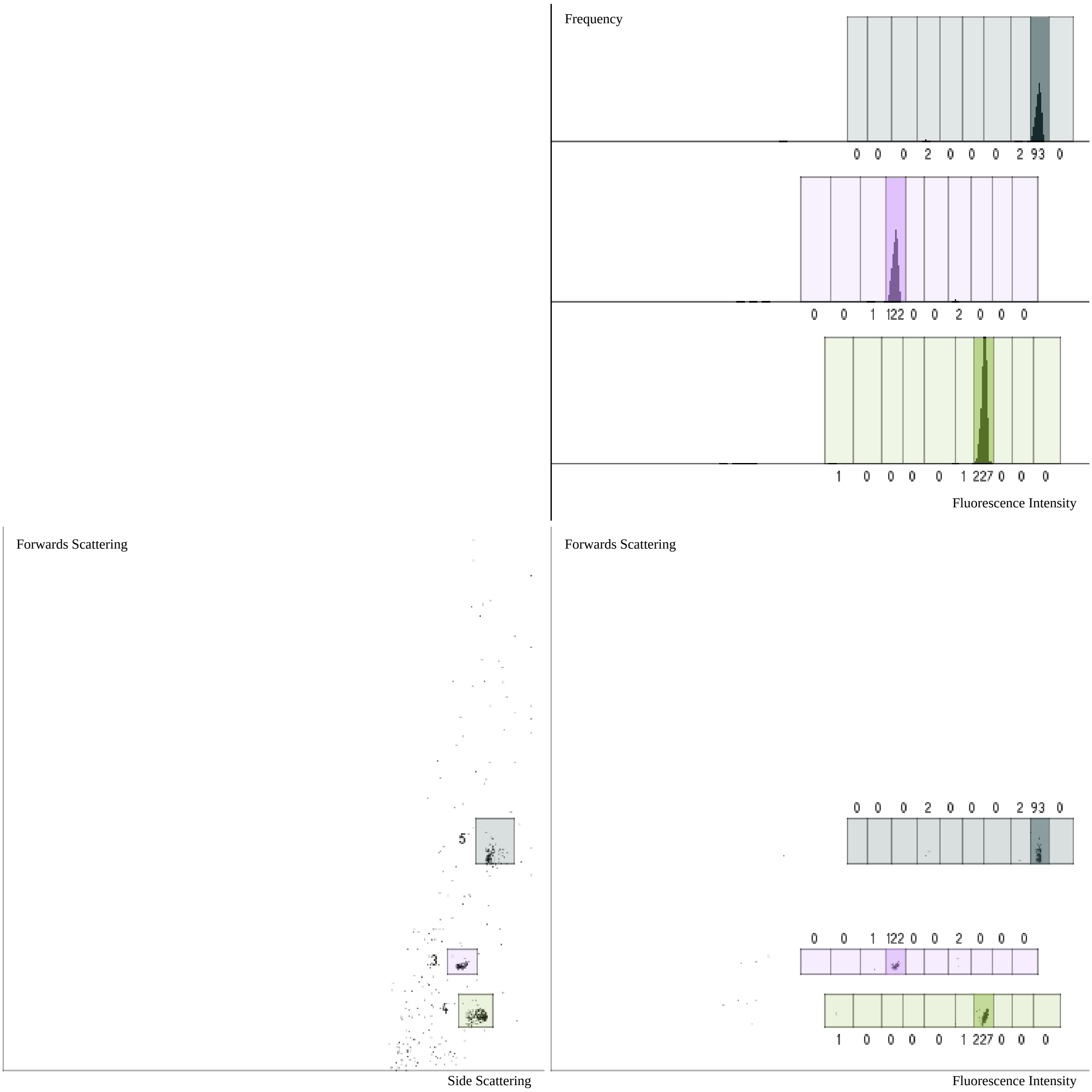

ANNEX 3: TAG DECONVOLUTION - BEAD 305

Passes flow sorting criteria: Yes

Passes tag deconvolution criteria: Yes

Included in protocol analysis: Yes

Protocol: 8, 9, 8, 10

Filename: Bin10_plateA2_F12.fcs

Split 1: Petrol shading

Split 2: Green shading

Split 3: Violet shading

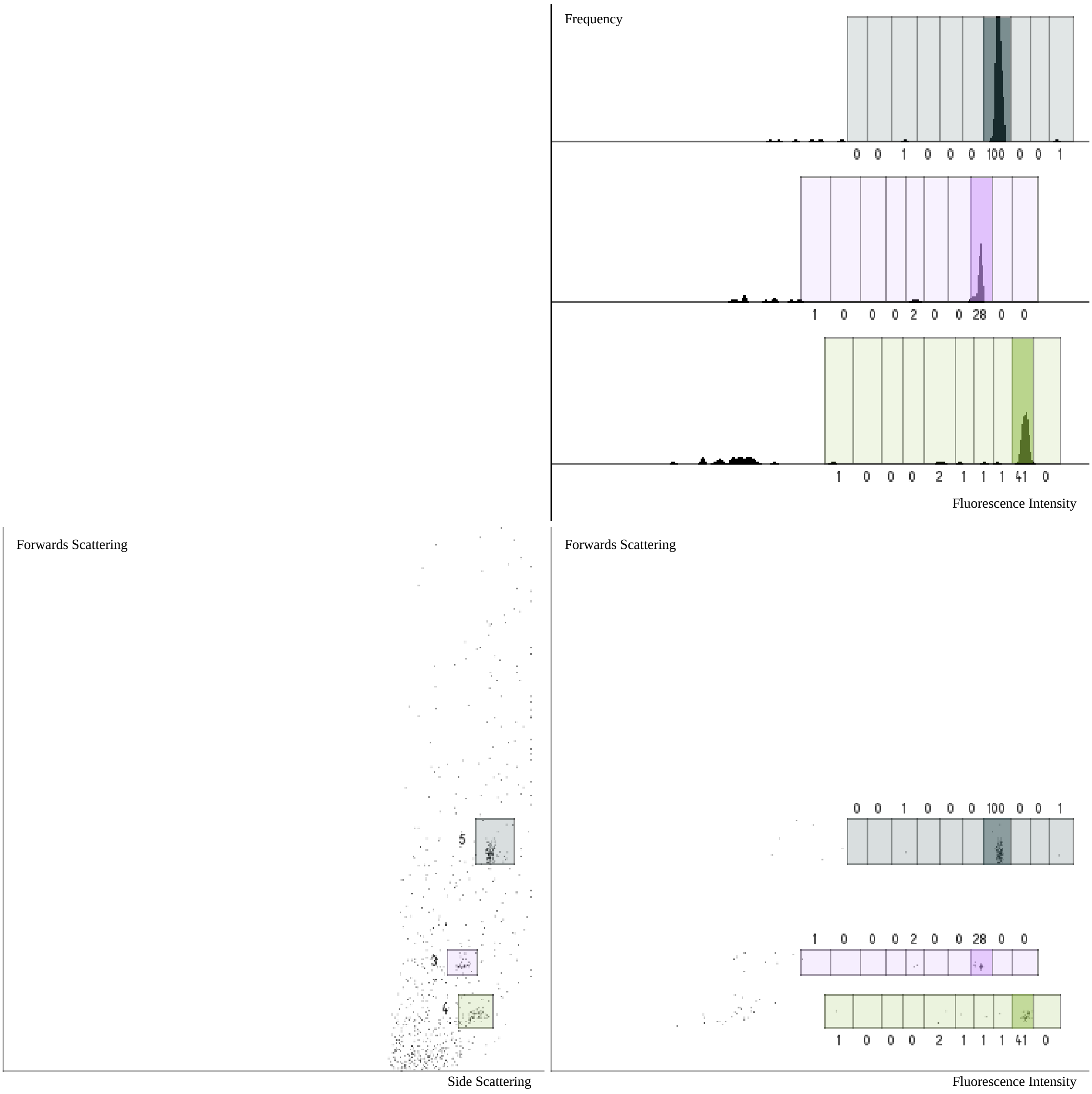

ANNEX 3: TAG DECONVOLUTION - BEAD 306

Passes flow sorting criteria: Yes

Passes tag deconvolution criteria: Yes

Included in protocol analysis: Yes

Protocol: 10, 3, 10, 10

Filename: Bin10_plateA2_G2.fcs

Split 1: Petrol shading

Split 2: Green shading

Split 3: Violet shading

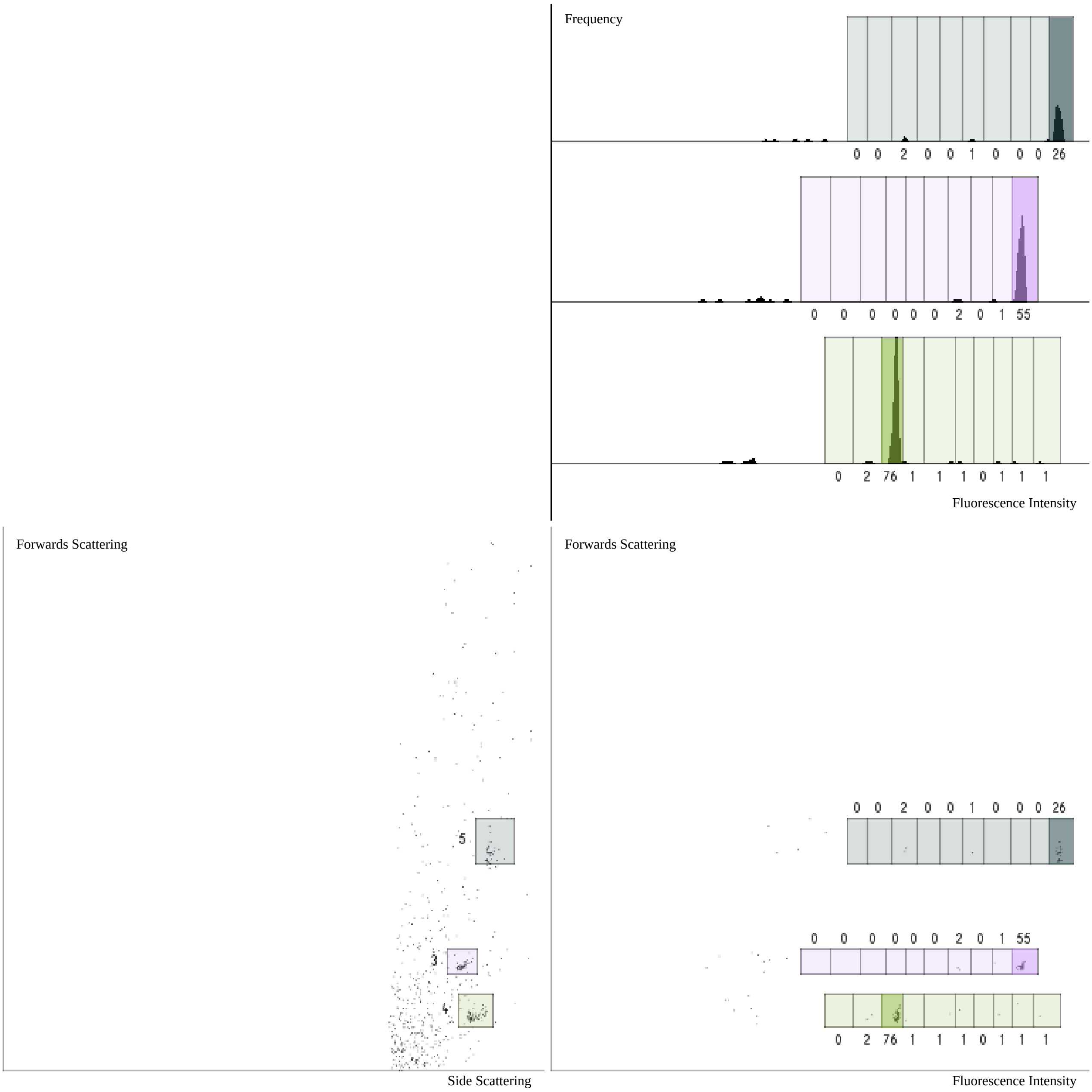

ANNEX 3: TAG DECONVOLUTION - BEAD 307

Passes flow sorting criteria: Yes

Passes tag deconvolution criteria: Yes

Included in protocol analysis: Yes

Protocol: 4, 2, 6, 10

Filename: Bin10_plateA2_G6.fcs

Split 1: Petrol shading

Split 2: Green shading

Split 3: Violet shading

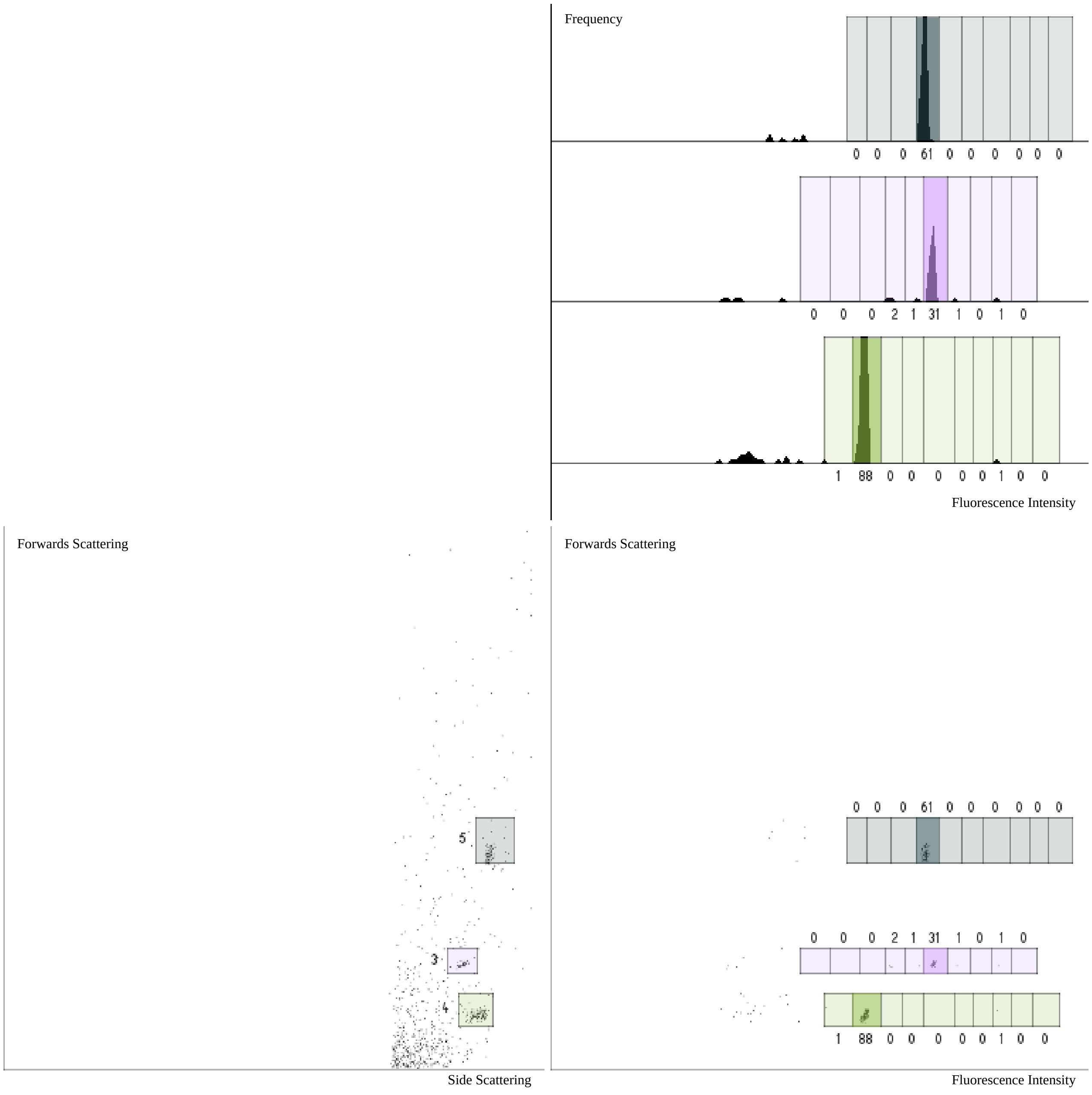

ANNEX 3: TAG DECONVOLUTION - BEAD 308

Passes flow sorting criteria: Yes

Passes tag deconvolution criteria: No

Included in protocol analysis: No

Protocol: N/A

Filename: Bin10_plateA2_G8.fcs

Split 1: Petrol shading

Split 2: Green shading

Split 3: Violet shading

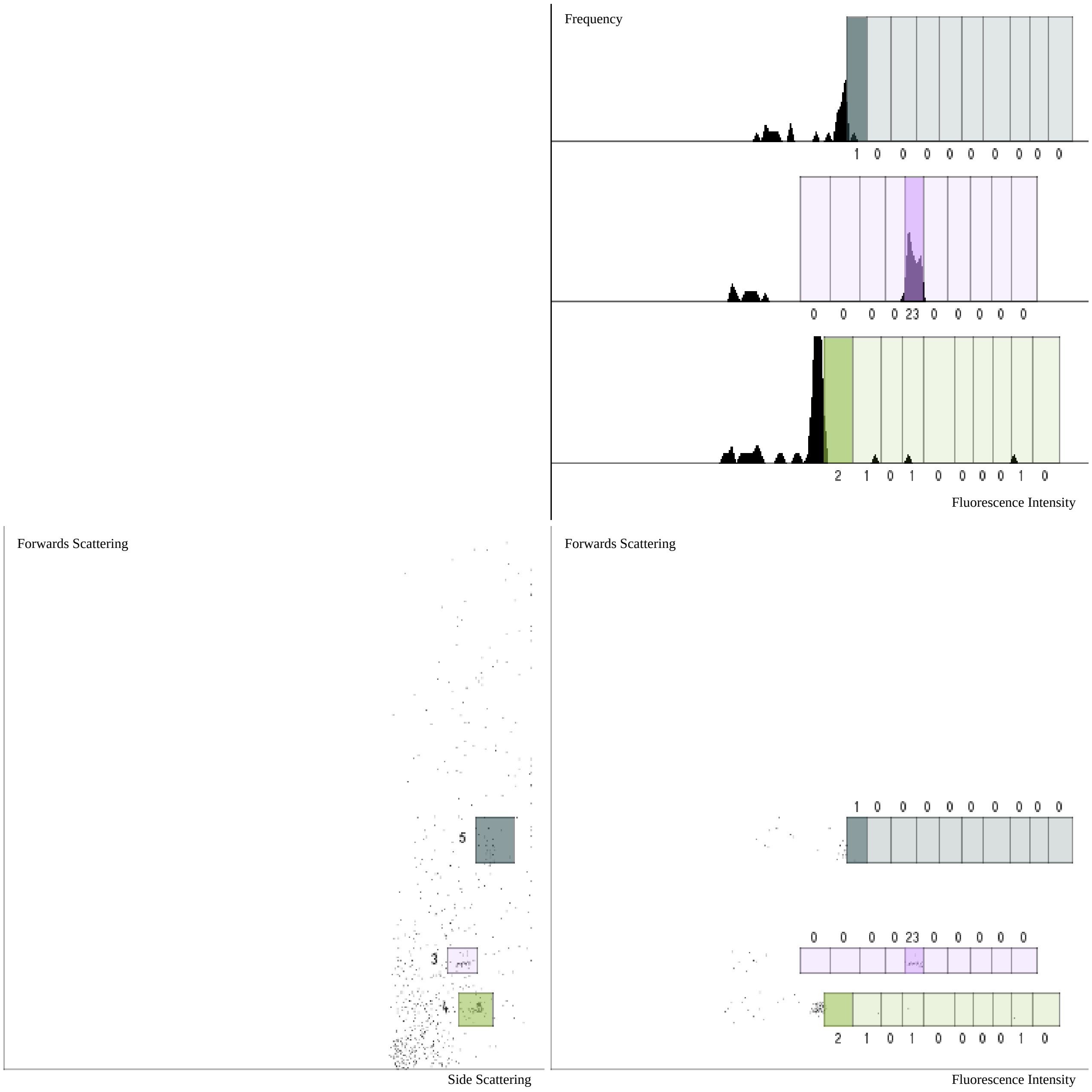

ANNEX 3: TAG DECONVOLUTION - BEAD 309

Passes flow sorting criteria: Yes

Passes tag deconvolution criteria: No

Included in protocol analysis: No

Protocol: N/A

Filename: Bin10_plateA2_G12.fcs

Split 1: Petrol shading

Split 2: Green shading

Split 3: Violet shading

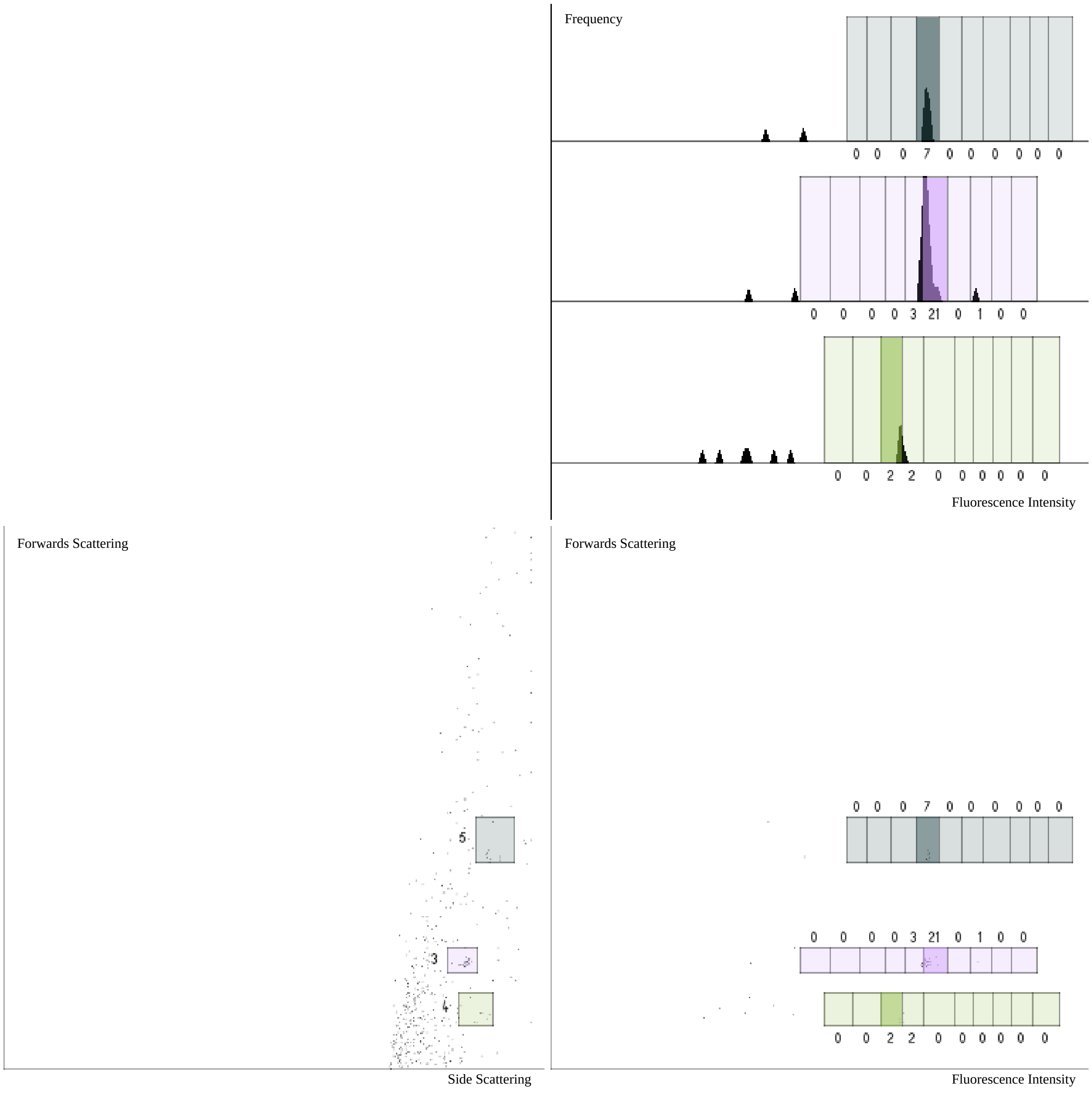

ANNEX 3: TAG DECONVOLUTION - BEAD 310

Passes flow sorting criteria: Yes

Passes tag deconvolution criteria: Yes

Included in protocol analysis: No

Protocol: N/A

Filename: Bin10_plateA3_B4.fcs

Split 1: Petrol shading

Split 2: Green shading

Split 3: Violet shading

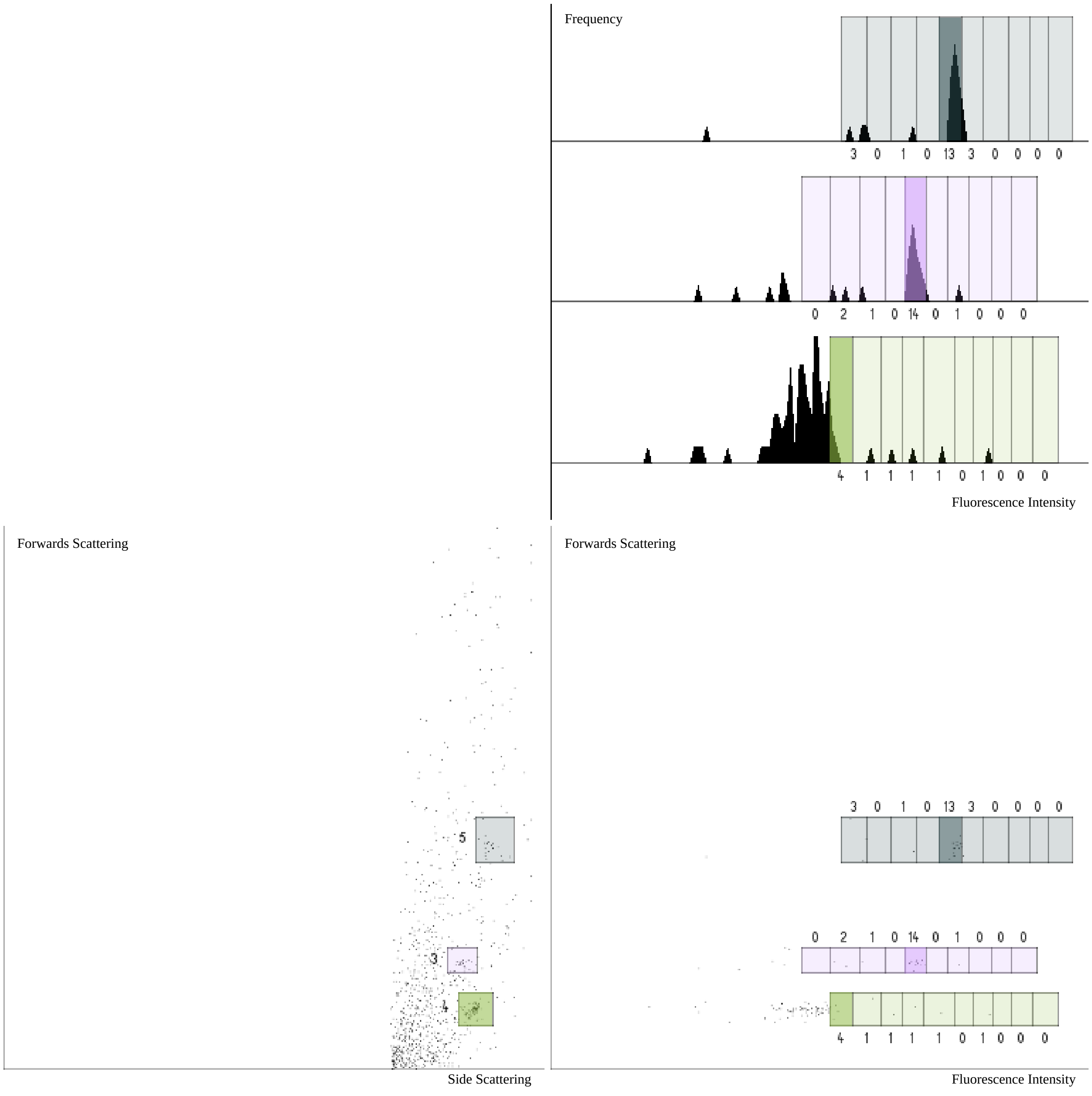

ANNEX 3: TAG DECONVOLUTION - BEAD 311

Passes flow sorting criteria: Yes

Passes tag deconvolution criteria: No

Included in protocol analysis: No

Protocol: N/A

Filename: Bin10_plateA3_A5.fcs

Split 1: Petrol shading

Split 2: Green shading

Split 3: Violet shading

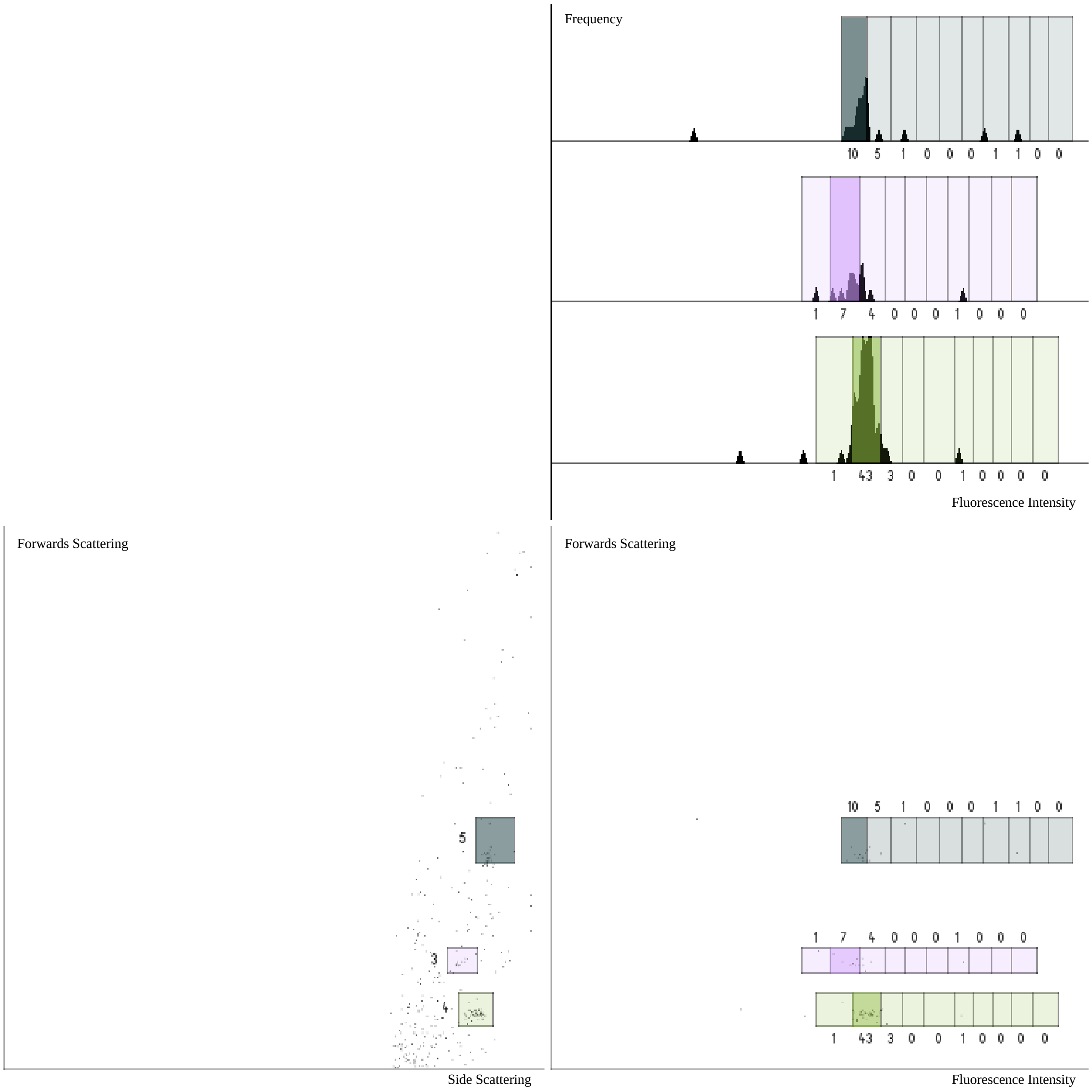

ANNEX 3: TAG DECONVOLUTION - BEAD 312

Passes flow sorting criteria: Yes

Passes tag deconvolution criteria: Yes

Included in protocol analysis: Yes

Protocol: 4, 5, 4, 8

Filename: Bin8_plateA2_A6.fcs

Split 1: Petrol shading

Split 2: Green shading

Split 3: Violet shading

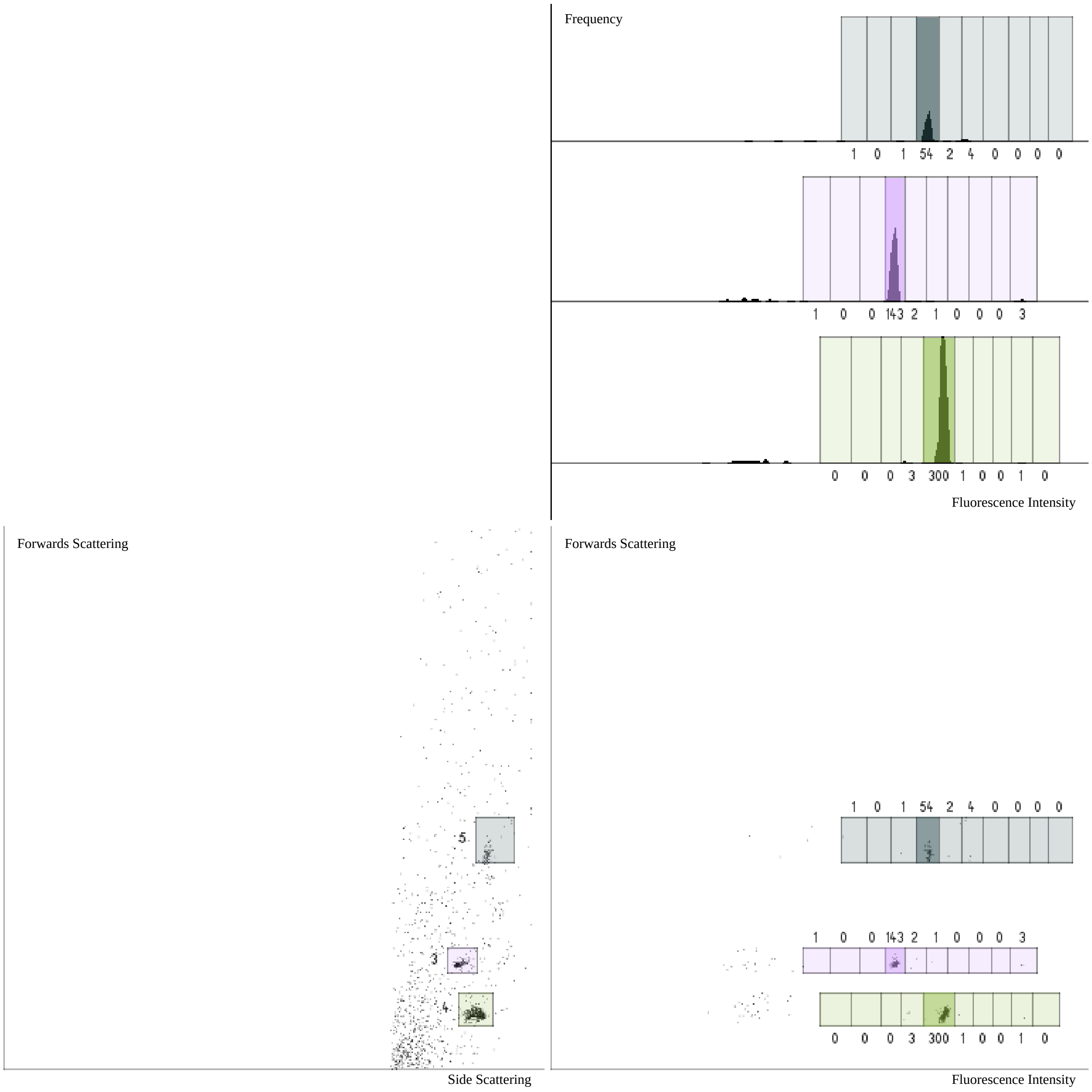

ANNEX 3: TAG DECONVOLUTION - BEAD 313

Passes flow sorting criteria: Yes

Passes tag deconvolution criteria: No

Included in protocol analysis: No

Protocol: N/A

Filename: Bin3_plateA3_A3.fcs

Split 1: Petrol shading

Split 2: Green shading

Split 3: Violet shading

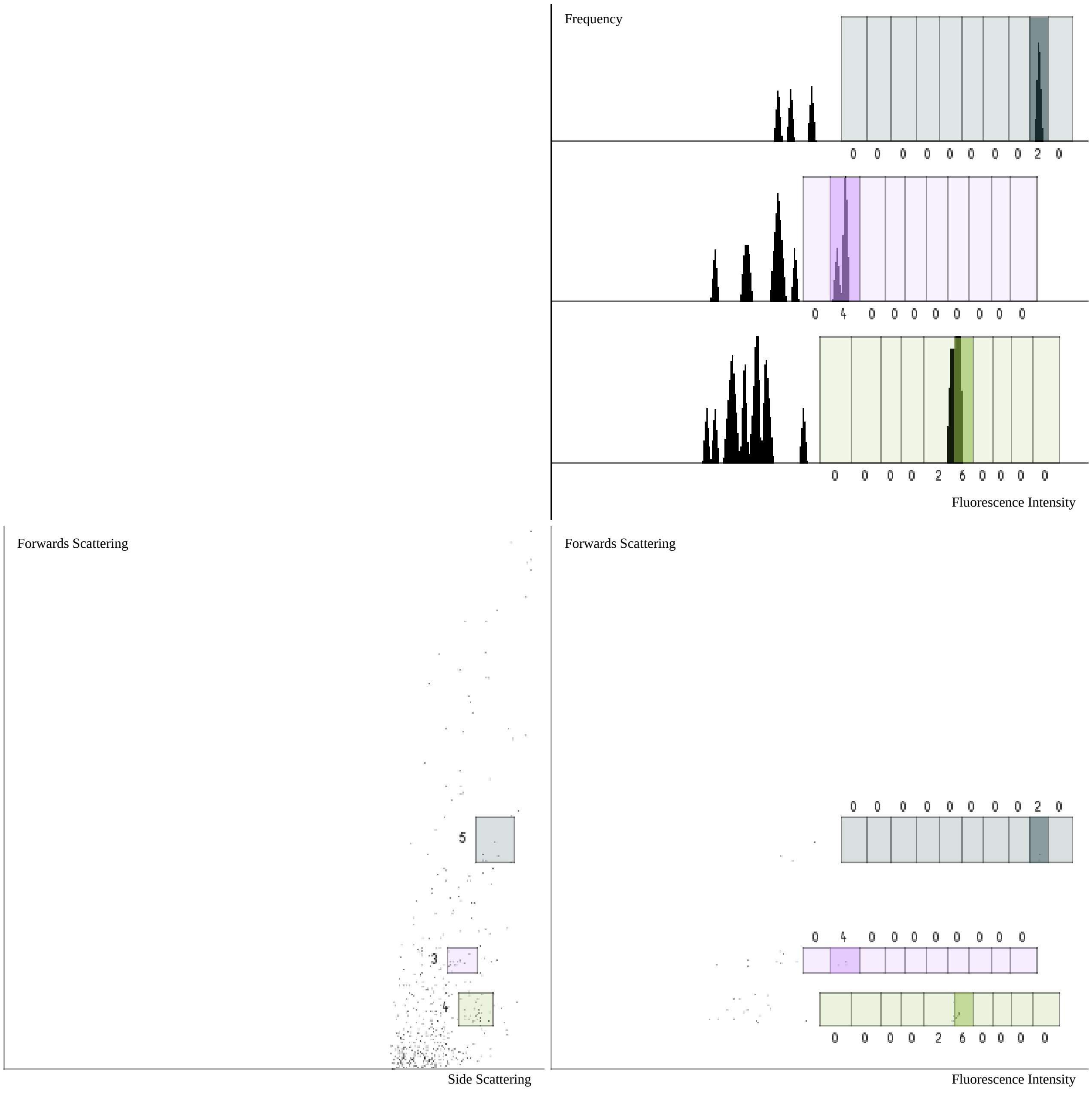

ANNEX 3: TAG DECONVOLUTION - BEAD 314

Passes flow sorting criteria: Yes

Passes tag deconvolution criteria: Yes

Included in protocol analysis: Yes

Protocol: 4, 1, 5, 4

Filename: Bin4_plateA4_E4.fcs

Split 1: Petrol shading

Split 2: Green shading

Split 3: Violet shading

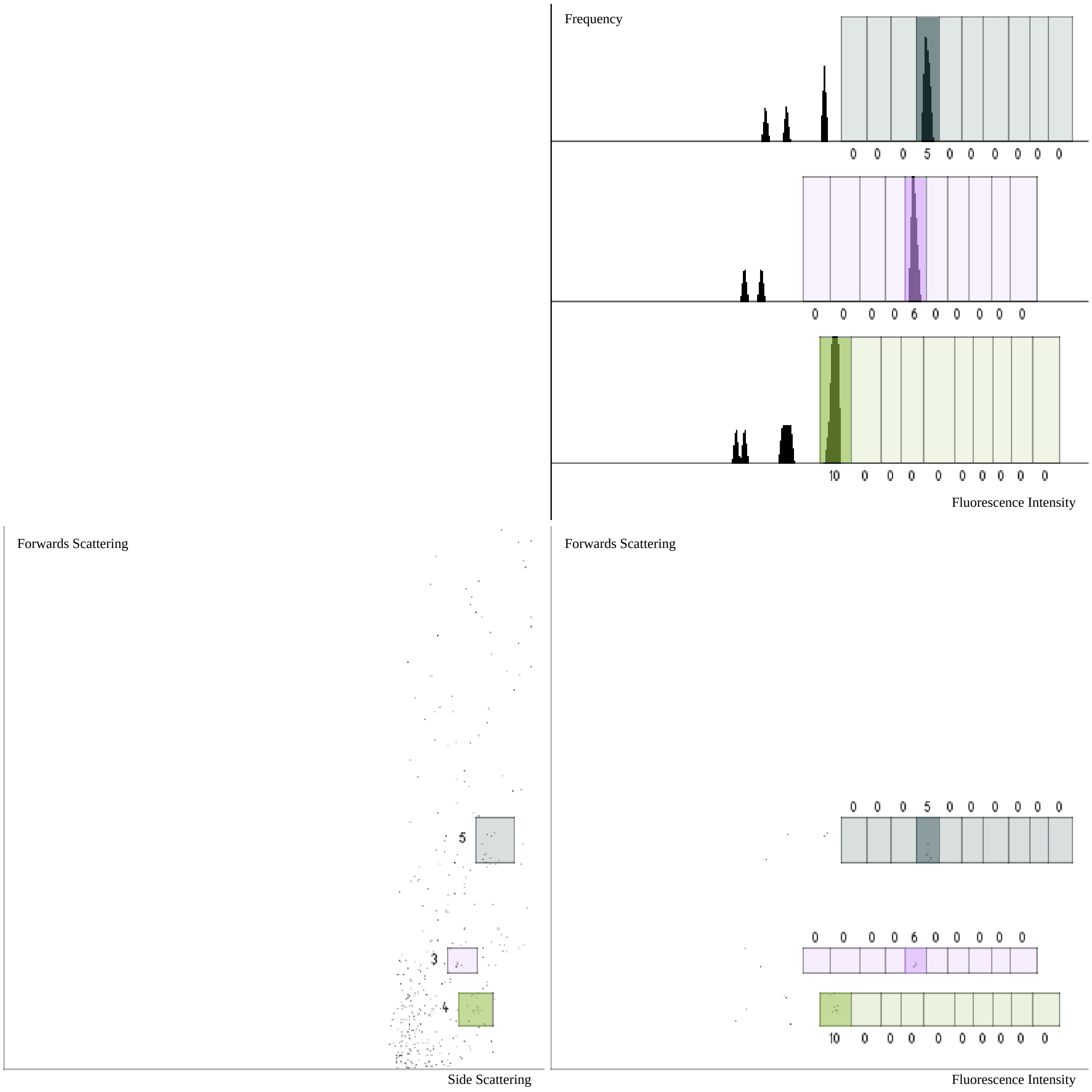

ANNEX 3: TAG DECONVOLUTION - BEAD 315

Passes flow sorting criteria: Yes

Passes tag deconvolution criteria: No

Included in protocol analysis: No

Protocol: N/A

Filename: Bin5_plateA5_C8.fcs

Split 1: Petrol shading

Split 2: Green shading

Split 3: Violet shading

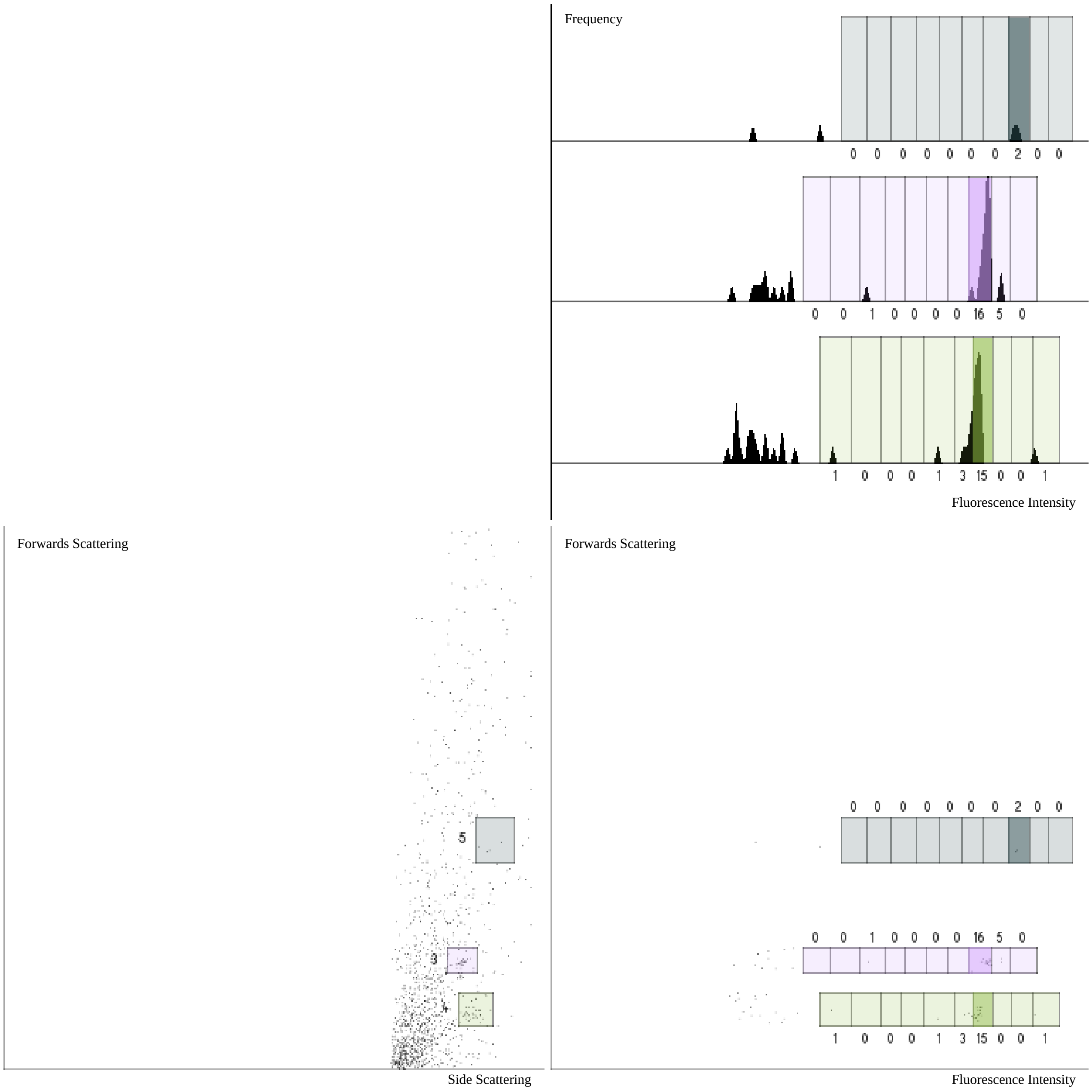

Supplement: Data S4 — Ariadne report for CombiCult screen 4: TH positive neurons from hES cells. (PDF) [file pone.0104301.s019.pdf]
